# Circulating proteomic panels for risk stratification of intracranial aneurysm and its rupture

**DOI:** 10.15252/emmm.202114713

**Published:** 2022-01-03

**Authors:** Yueting Xiong, Yongtao Zheng, Yan Yan, Jun Yao, Hebin Liu, Fenglin Shen, Siyuan Kong, Shuang Yang, Guoquan Yan, Huanhuan Zhao, Xinwen Zhou, Jia Hu, Bin Zhou, Tao Jin, Huali Shen, Bing Leng, Pengyuan Yang, Xiaohui Liu

**Affiliations:** ^1^ The Fifth People's Hospital of Shanghai Shanghai Key Laboratory of Medical Epigenetics The International Co‐laboratory of Medical Epigenetics and Metabolism, Ministry of Science and Technology Institutes of Biomedical Sciences Fudan University Shanghai China; ^2^ Department of Neurosurgery Ruijin Hospital School of Medicine Shanghai Jiaotong University Shanghai China; ^3^ Huashan Hospital Fudan University Shanghai China; ^4^ Shanghai Omicsolution Co., Ltd. Shanghai China

**Keywords:** biomarker discovery, intracranial aneurysm, mass spectrometry, serum proteome profiling, Biomarkers, Neuroscience, Vascular Biology & Angiogenesis

## Abstract

The prevalence of intracranial aneurysm (IA) is increasing, and the consequences of its rupture are severe. This study aimed to reveal specific, sensitive, and non‐invasive biomarkers for diagnosis and classification of ruptured and unruptured IA, to benefit the development of novel treatment strategies and therapeutics altering the course of the disease. We first assembled an extensive candidate biomarker bank of IA, comprising up to 717 proteins, based on altered proteins discovered in the current tissue and serum proteomic analysis, as well as from previous studies. Mass spectrometry assays for hundreds of biomarkers were efficiently designed using our proposed deep learning‐based method, termed DeepPRM. A total of 113 potential markers were further quantitated in serum cohort I (*n* = 212) & II (*n* = 32). Combined with a machine‐learning‐based pipeline, we built two sets of biomarker combinations (P6 & P8) to accurately distinguish IA from healthy controls (accuracy: 87.50%) or classify IA rupture patients (accuracy: 91.67%) upon evaluation in the external validation set (*n* = 32). This extensive circulating biomarker development study provides valuable knowledge about IA biomarkers.

## Graphical abstract

This study constructed a comprehensive mass spectrometry‐based proteomics strategy for serum protein biomarker discovery for intracranial aneurysm (IA).

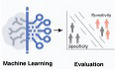

**The paper explained**

**Problem**

Intracranial aneurysm (IA) is a common cerebrovascular disorder with steadily increasing prevalence, whose rupturing consequences are severe. Patients are typically diagnosed when the aneurysm becomes large or ruptures with an extremely severe headache. The development of disease‐modifying therapeutics is hampered by the lack of specific tests for early detection of IA.

**Results**

We constructed a comprehensive mass spectrometry‐based proteomics strategy for serum protein biomarker discovery for intracranial aneurysm (IA). An extensive serum candidate biomarker bank of IA was constructed based on the altered protein expressions discovered in current tissue and serum proteomic analysis and previous studies, comprising up to 717 proteins. Mass spectrometry assays for hundreds of biomarkers were efficiently designed using our proposed deep learning‐based method, termed DeepPRM. A total of 113 potential markers were further quantitated in a large serum cohort (*n* = 212) and an additional validation set (*n* = 32). Machine learning successfully classified IA from healthy controls based on a six‐protein biomarker combination with accuracy of 87.50%, while the classification of ruptured and unruptured IA based on an eight‐protein model reached a remarkable accuracy of 91.67%.

**Impact**

These findings provide valuable knowledge on serum biomarkers associated with IA formation or rupture, and shed light on the pathogenesis and diagnosis of IA. Furthermore, this study improves our understanding of IA biology and identifies pathways to be further investigated for the development of novel treatment strategies.

## Introduction

Intracranial aneurysm (IA) is a complex, multifactorial cerebrovascular disorder, commonly occurring in 2–3% of the general population (Vlak *et al*, 2011). Approximately 1% of IAs rupture per year (Rinkel *et al*, 1998), leading to aneurysmal subarachnoid hemorrhage (aSAH) (Macdonald & Schweizer, 2017), with one‐third of patients dying and another third remaining dependent for daily life activities (Nieuwkamp *et al*, 2009; Bakker *et al*, 2020). Despite the available neuroimaging modalities, most patients are not diagnosed in time due to the asymptomatic nature of IA. Furthermore, the patients at risk of IA rupture and those who subsequently develop cerebral vasospasm cannot be predicted to date. Therefore, once the disease is confirmed by CT or MRI, clinicians are confronted with the dilemma of choosing preventive treatments, each with inherent risks of complications, or conservative management, which leaves patients at a small but definite risk of aneurysm rupture (Etminan & Rinkel, 2017). Factors such as hypertension, cigarette smoking, alcohol consumption, female sex, and lipid accumulation have been reported to be associated with an increased risk of harboring an IA (Chalouhi *et al*, 2013; Hussain *et al*, 2015). However, to the best of our knowledge, no biomarker has satisfactorily addressed these challenges. In this regard, it is of immense value to develop biological signatures that assist in the early diagnosis of IA and the prediction or classification of IA rupture (Baker *et al*, 1995; Mack *et al*, 2002).

Changes in human plasma or serum proteins have long been recognized as indicators of pathophysiological changes caused by various diseases. There are more than 100 FDA‐cleared or FDA‐approved clinical plasma or serum proteins categorized as abundant, functional plasma/serum proteins (50%), followed by tissue leakage markers without a dedicated function in the circulation (25%), and signal molecules including receptor ligands, immunoglobulins, and aberrant secretions (Anderson, 2010; Geyer *et al*, 2017). Previous attempts to comparatively analyze specific samples revealed that aberrant gene and protein expression may drive structural alterations of vasculature found in IA, and hundreds of proteins and genes were reported to be relevant to IA formation (Peters *et al*, 2001; Shi *et al*, 2009; Pera *et al*, 2010). However, only few of them were further validated in serum for further clinical trials. Therefore, we hypothesize that a system‐wide analysis of IA, linking changes in circulation levels and dysregulation in the diseased organs, as well as the previously reported proteins, would provide informative knowledge for deciphering the molecular mechanisms underlying these events and contribute to a comprehensive serum biomarker survey for the classification of IA and IA rupture.

Mass spectrometry (MS)‐based proteomics approaches have made significant advances in biomedical and clinical research (Altelaar *et al*, 2013; Aebersold & Mann, 2016; Budayeva & Kirkpatrick, 2020; Wu *et al*, 2020) and offered a powerful pipeline for unbiased biomarker discovery and targeted validation, avoiding the restrictions of antibodies (Shen *et al*, 2020; Shu *et al*, 2020). For example, multiplex quantitative proteomics revealed characteristic protein changes in the sera of severe COVID‐19 patients, which might be used in the selection of potential blood biomarkers for severity evaluation (Messner *et al*, 2020; Demichev *et al*, 2021; Geyer *et al*, 2021). For a cancer biomarker study, reproducible quantification assays were developed for 1000 cancer‐related proteins using the targeted proteomic strategy (Hüttenhain *et al*, 2012; Zhang *et al*, 2019).

In an attempt to develop circulation markers for IA and IA rupture, we employed a MS‐based proteomic approach to profile the proteome of IA serum and tissue and build a comprehensive knowledge bank of IA altered proteins by connecting the results of the current and previous studies. MS assays were developed to extensively validate the serum detectable proteins in cohort I (Dataset EV1), containing 212 serum samples from healthy controls (normal controls, NC), patients with unruptured IA (UR), and ruptured IA (R) using a targeted proteomics strategy named DeepPRM. To identify potential biomarkers for the accurate classification of different samples, we developed a machine‐learning‐based pipeline, resulting in a six‐protein based biomarker combinations to accurately distinguish IA, as well as an eight‐protein based panel to classify IA rupture. Both proteomic models trained on the training set (75% of cohort I) demonstrated a comparably high patient stratification performance when applied to the internal validation set (25% of cohort I) and the external validation cohort II (*n* = 32) (Dataset EV2). Some identified biomarkers were further examined for their serum levels via the enzyme‐linked immunosorbent assay (ELISA), whose findings were in accordance with the proteomic results. These striking discoveries provide valuable knowledge about serum biomarkers associated with IA formation or rupture and might shed light on the pathogenesis and diagnosis of IA to ultimately achieve an overall improvement in patient survival rates.

## Results

### Proteomics analysis

**Figure 1:**
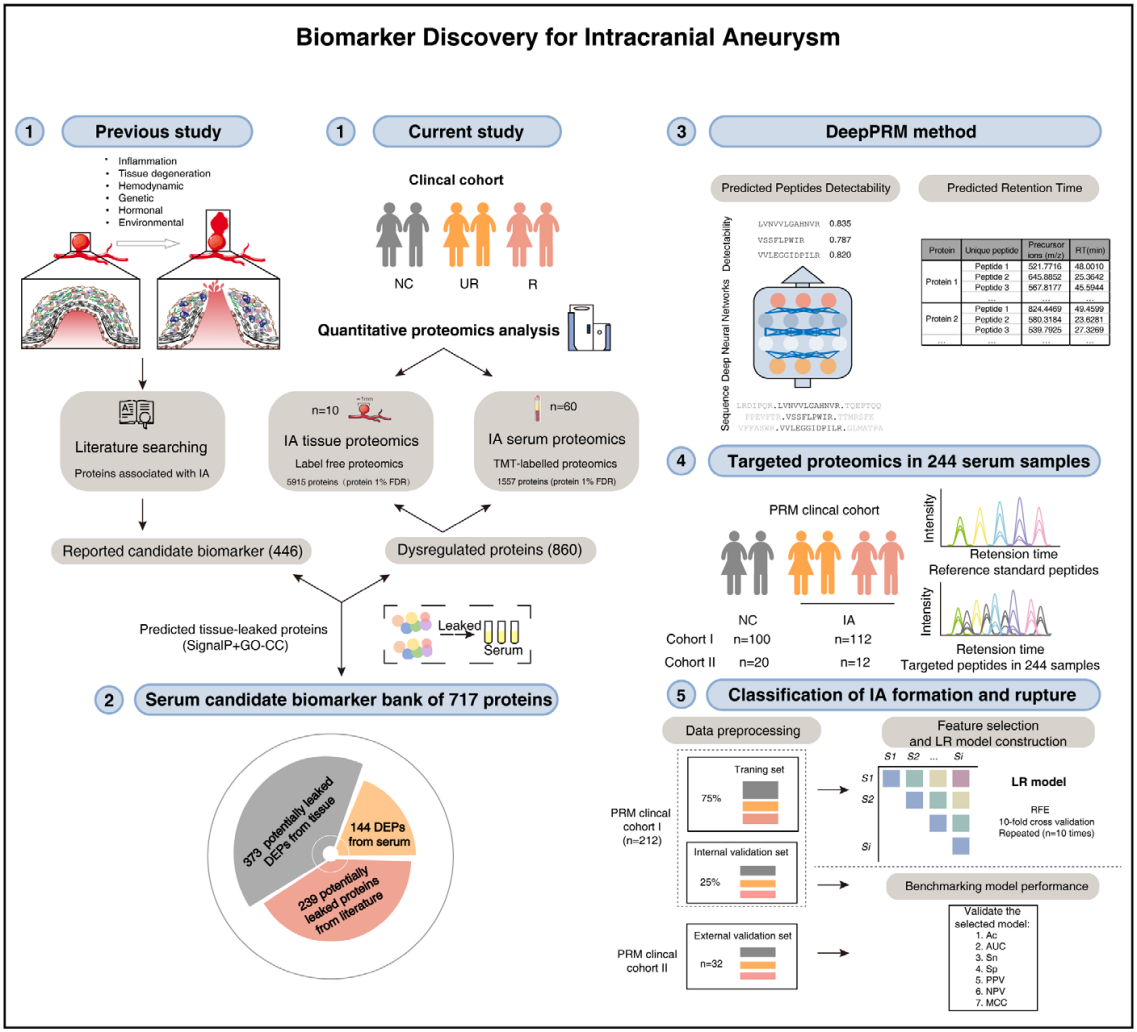
Framework for biomarker discovery in intracranial aneurysms disease For a comprehensive survey of potential protein biomarkers of IA, the altered proteins reported in previous studies were summarized, and the high‐throughput MS‐based quantitative proteomics technology was applied to profile tissue and serum samples from a large clinical cohort of IA and healthy controls, resulting in 446 reported candidate biomarkers and 860 DEPs found in the current proteomic study ①. A comprehensive serum protein candidate biomarker bank (SPCBB) was built, consisting of potential tissue‐leaked DEPs, serum DEPs, and reported IA candidate signatures that may be detected in the serum, amounting to 717 proteins ②. An instrument‐specific deep learning‐based DeepPRM strategy was proposed for the high‐efficiency development of PRM assays of numerous candidate proteins in SPCBB ③. To further validate the proteins, the developed PRM assays were used to quantitate the observed peptides in a large cohort of serum samples under strict quality control ④. The machine‐learning approach was used to measure the predictive power of quantitated peptides in the serum of the normal controls (NC), unruptured IA (UR), and ruptured IA (R) group ⑤.

**Figure 2:**
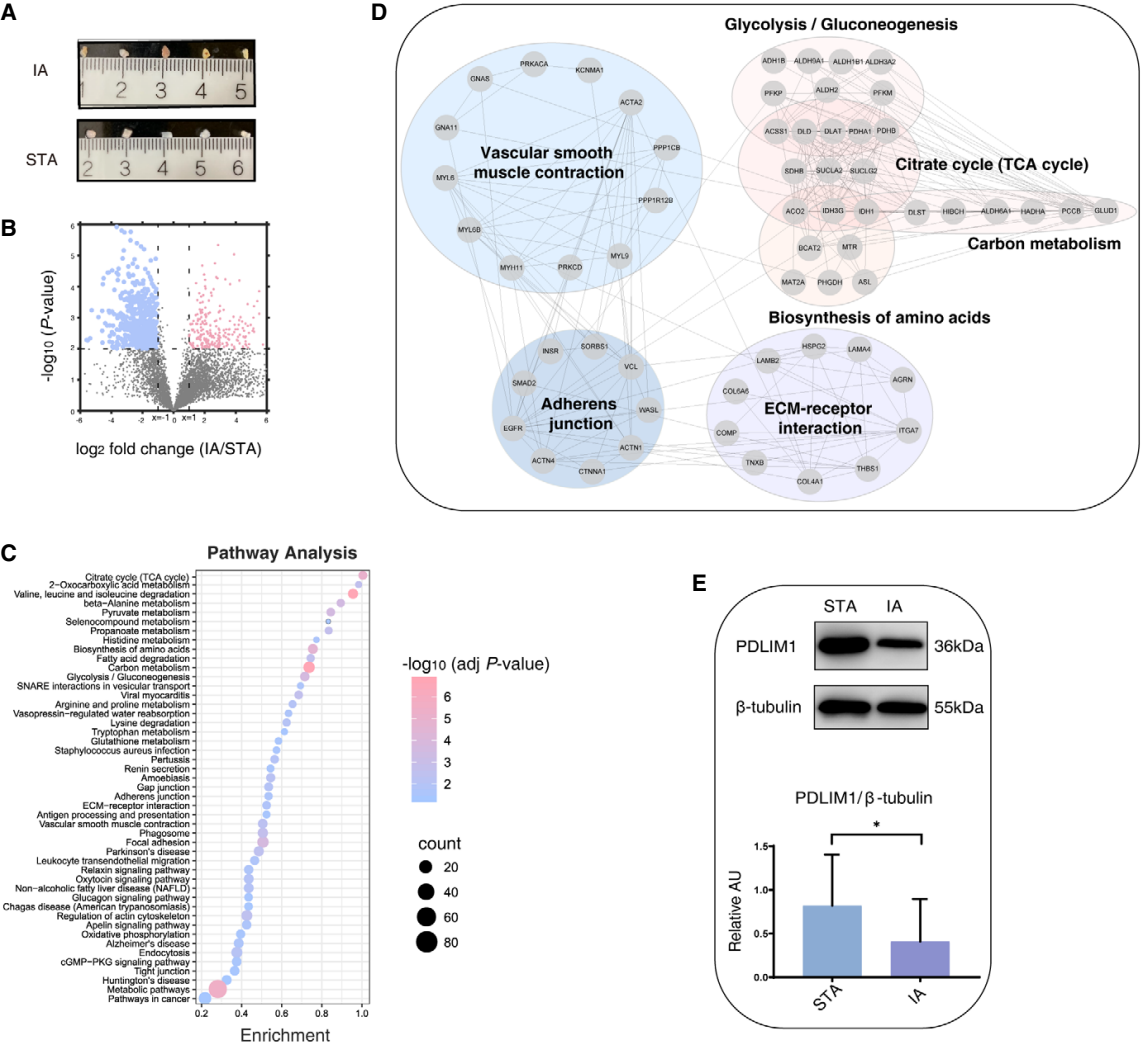
IA tissue proteome remodeling due to IA formation and rupture Five pairs of IA and STA tissues surgically excised from intracranial aneurysm patients.Differentially expressed proteins between IA group and STA group, as determined by plotting Student’s *t*‐test (*P*‐value < 0.05, two‐sided) *P*‐values versus the log2 fold change (IA/STA) are represented on volcano plots. Proteins with significant change in expression levels are indicated by pink (upregulated) and blue (downregulated) dots. The dashed line (x = ±1) represents the cutoff of the log2 fold change in protein levels, and dashed line (y = 2) represents the cutoff of the −log10 *t*‐test *P*‐value.Significantly altered top ranked pathways in IA tissues compared with STA tissues.Interaction network of tissue downregulated proteins in IA group compared to STA group.Western blot validation of selected protein (PDLIM1) differentially expressed between STA and IA. Downregulation of PDLIM1 in IA tissue as compared to STA tissue was confirmed. Immunoblots showing expression differences of PDLIM1 in STA and IA specimens are shown in the left panel using β‐tubulin as the loading control. The semi‐quantitative densitometric measurement of blot bands is summarized in the lower panel. Error bars indicate standard deviations (three biological replicates for each group, two‐tailed Student’s *t*‐test, * *P*‐value < 0.05). Source data are available online for this figure.

The overall design of this study is shown in Fig 1. During the discovery stage, samples weighing approximately 0.83–2 mg from five pairs of IA tissues and matched superficial temporal artery (STA) tissues from IA patients were analyzed by LC‐MS/MS‐based label‐free quantitation (LFQ). The STA tissues obtained intraoperatively were set as the control tissue samples (Fig 2A). A total of 5,915 proteins were identified, with 5,677 proteins quantified at the protein FDR < 1%. The reproducibility and correlation coefficient of the LFQ intensities within and between the two groups are shown in Appendix Fig S1A and B. In total, 724 differentially expressed proteins (DEPs) were found in IA tissues compared to matched STA tissues, which accounted for 12.2% of the total quantified proteome. Of these, 497 proteins (68.6%) were downregulated, and 227 proteins (31.4%) were upregulated in IA samples (Fig 2B; Dataset EV3). Multivariate principal component analysis (PCA) and unsupervised hierarchical clustering analysis effectively distinguished the IA groups from STA groups with high confidence (Appendix Fig S1C and D). Additionally, functional pathway annotation and enrichment analysis by the Kyoto Encyclopedia of Genes and Genomes (KEGG) analysis showed that upregulated proteins were overrepresented in the complement and coagulation cascades, cell adhesion molecules (CAMs), fluid shear stress, and atherosclerosis, while the downregulated proteins were enriched in the smooth muscle cells (SMCs) contraction pathway, extracellular matrix (ECM)–receptor interaction pathway, tricarboxylic acid (TCA) cycle pathway, and metabolism‐associated pathways (Fig 2C and D; Appendix Fig S1E). Of these, 14 proteins comprising up to 11.7% of the proteins in the SMCs contraction pathway and 10 proteins accounting to 12.3% of the proteins in the ECM–receptor interaction pathway were markedly lower expressed in IA tissues. These results were consistent with the previous reports of the prominent features of activated inflammation, and the inhibition of muscle formation, development, and contraction‐related functions in IA. Besides, 11 of 30 proteins involved in the TCA cycle were downregulated with an average of 74% reduction by detailed analysis of individual proteins. What’s more, an additional 29 significantly changed proteins were largely involved in metabolism‐associated pathways including amino acid, carbon, and glycol metabolism pathways. These data suggested fundamental differences between IA tissues and STA tissues that possibly reflected differing energy interventions of IA. Proteins crucial for organizing the cytoskeleton and its maintenance, such as PDZ and LIM domain protein 1 (PDLIM1), were significantly downregulated in the IA group. We further validated its expression in IA tissue specimens by immunoblotting (Fig 2E).

**Figure 3:**
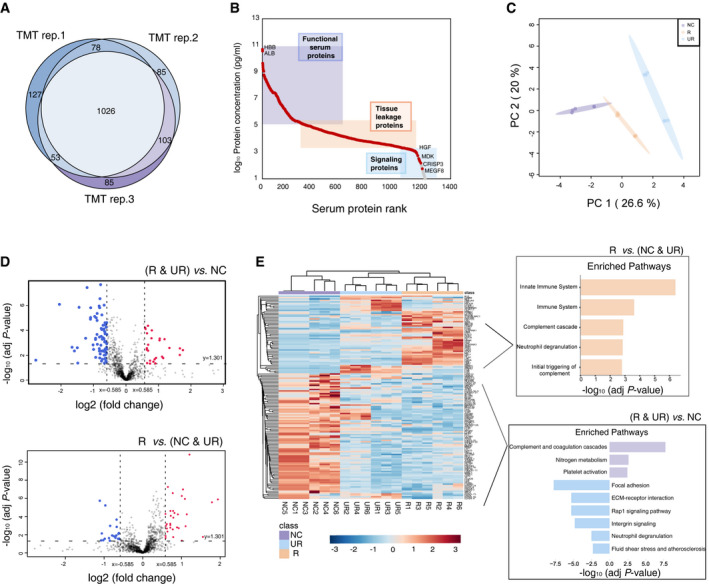
Remodeled serum proteome due to IA formation and rupture Venn diagram of common and “exclusive” proteins identified from serum proteins from triplicate TMT experiment.Concentration range of serum proteins identified in this study according to the plasma proteome database in May 2017 (http://www.plasmaproteomedatabase.org/). Colored rectangles categorize the entire abundance range into three classes representing functional serum proteins (purple), tissue leakage proteins (orange), signaling proteins (blue).PCA score plot of R, UR, and NC based on the triplicate serum TMT data.Volcano plots of the results of Student’s *t*‐test comparing the (R & UR) versus NC group or R versus (NC & UR) group. Proteins with significant change in expression levels are indicated by red (upregulated) and blue (downregulated) dots. The dashed line (x = ±0.585) represents the cutoff of the log2 fold change in protein levels, and dashed line (y = 1.301) represents the cutoff of the ‐log10 *t*‐test *P*‐value.Multigroup heatmap with dendrogram of differential expressed proteins levels incorporating UR, R, and NC groups. Enriched pathways based on differentially expressed proteins between IA group (R & UR) versus NC group, or R group compared to control group (NC & UR). Source data are available online for this figure.

Sixty serum samples applied for the proteome analysis were separated to three groups (NC, UR, and R groups) with 20 age‐ and sex‐matched individuals in each group. High abundant protein depletion and a tandem mass tag‐labeled (TMT‐labeled) proteomic strategy (Fig 1) were used for the relatively quantitation of serum proteome. Two pooled biological replicates of each group and three technical replicates were performed, which resulted in a total of 1,557 proteins identified with protein FDR < 1% (Fig 3A). The concentration of the detected proteins spanned 11 orders of magnitude from the lowest of 4.3 pg/ml (MEGF8) to almost 50 mg/ml (HBB & ALB) according to the plasma proteome database (Nanjappa *et al*, 2014) (Fig 3B). The PCA using all the detected proteins clearly separated the NC, UR, and R groups (Fig 3C). We then performed two comparisons between the three groups: first, IA (R & UR) versus NC, aiming to find the altered proteins in the serum samples of IA patients. A total of 103 DEPs were found in IA group (Benjamini–Hochberg‐adjusted *P*‐value < 0.05) with 26 proteins having increased expression (> 1.50‐fold) and 77 proteins showing lower expression (< 0.667‐fold); the second is R versus the control group (UR & NC), to identify potential biomarkers for predicting the rupture of IA which resulted in 53 DEPs, including 32 upregulated and 21 downregulated proteins (Fig 3D, Dataset EV4). Unsupervised heatmap clustering analysis of the dysregulated proteins of IA showed not only the proteomic diversity between samples from NC and IA, but also the variations within the IA of UR and R group (Fig 3E). The most enriched pathways of the dysregulated proteins in IA are illustrated in Fig 3E. Notably, proteins positively related to ruptured IA were significantly enriched in the innate immune system and neutrophil degranulation, which indicated an extraordinary activation of the inflammation system (Fig 3E).

### Construction of serum candidate circulating biomarker bank for IA

To take full advantage of data generated from preceding study of IA, we assembled a protein candidate biomarker bank (PCBB) by pooling the results from this study and the reported biomarkers of IA in the literature together. The articles and reviews in English published in public databases between 2000 and 2020 were summarized, and a total of 446 genes or proteins have been addressed to be related with IA from different biological specimens (Dataset EV5). Combined with the 860 DEPs found in present study, 1241 proteins were deposited in the PCBB with 65 proteins overlapped in current and previous study (Appendix Fig S2A, Dataset EV6).

**Figure 4:**
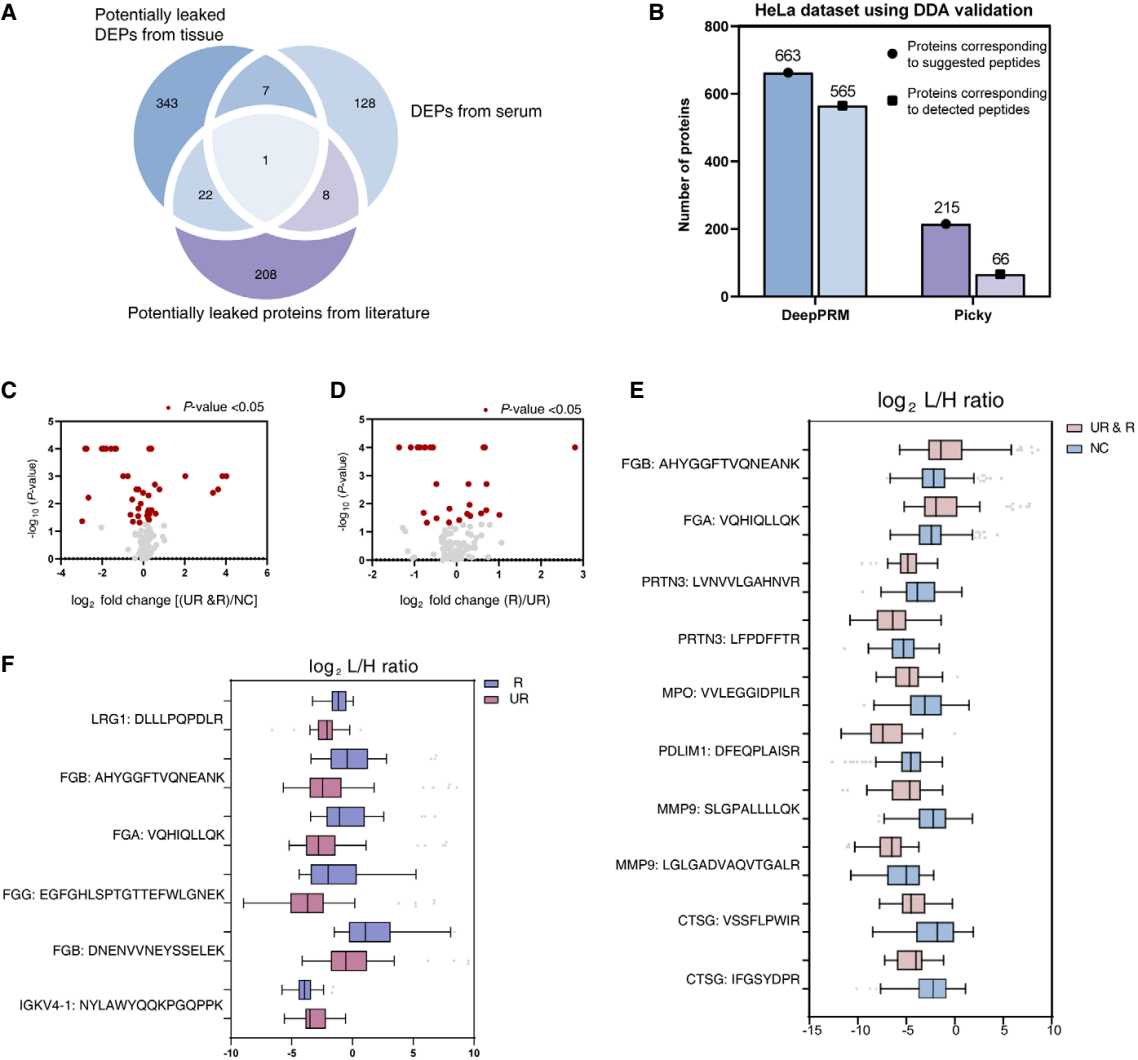
Targeted proteomics of IA candidate biomarker bank AOverlap of serum candidate proteins related to IA.BBar graph showing the number of proteins corresponding to suggested peptides by DeepPRM and Picky methods, and proteins corresponding to detected peptides from DDA validation.C, DVolcano analyses showing log2 fold change of (UR & R)/NC (C), R/ UR (D) of peptides according to the *P*‐value. Proteins meeting the indicated statistical cutoff criteria (Mann−Whitney *U*‐test, *P*‐value < 0.05) are colored in red.E, FTen peptide relative quantification (log2 L/H ratio) between (R & UR) (*n* = 72) and NC group (*n* = 80) (E), and six peptide relative quantification (log2 L/H ratio) between R (*n* = 35) and UR group (*n* = 37) (F). For each protein, 1–2 proteotypic peptides were monitored. The light peptide (corresponding to the endogenous peptide present in serum) and the heavy peptide (which corresponds to the synthetic peptide spiked‐in serum) were monitored, and the light/heavy ratio for each of the 12 peptides was obtained by Skyline. Box plots represent the median and interquartile range, whiskers represent the 1–99 percentile, and outliers are represented by empty circles. Source data are available online for this figure.

The PCBB was further prioritized by selecting detectable proteins in the serum. Apart from serum‐altered proteins, we investigated and found out that a total of 373 DEPs discovered in tissue and 239 reported proteins were predicted to be leaked or secreted into the serum (Datasets EV3 and EV5) according to the cellular components and signal peptide prediction information. As a result, the IA‐related Serum Protein Candidate Biomarker Bank (SPCBB) was built with 717 proteins (Fig 4A, Dataset EV7). These proteins were further validated in serum‐targeted proteomic analysis.

### DeepPRM for SPCBB MS assay development

The development of targeted assays, such as parallel reaction monitoring (PRM) and selected reaction monitoring (SRM) assays, is tedious and typically requires peptide selection, synthesis, and MS analysis (Zauber *et al*, 2018). Further, without the prior knowledge of the peptides’ detectability, scientists face difficulties regarding peptide selection or the decision to synthesize hundreds and thousands of peptides, which is costly (Kusebauch *et al*, 2016). To validate hundreds of candidate biomarkers in SPCBB, we developed the DeepPRM algorithm to aid the large‐scale assay development by predicting the peptide detectability and retention time, which are critical parameters for MS assay development. The DeepPRM is based on an instrument‐specific model constructed on a given LC‐MS/MS platform using the deep learning approach, as previously described (Yang *et al*, 2020). To validate the performance of DeepPRM, we first compared the DeepPRM with a well‐known online PRM and SRM method designer‐Picky (Zauber *et al*, 2018), which suggests targeted peptides and their predicted RT of given protein datasets from serum and Hela cell digests. The detection of the proposed peptides was further validated by MS acquisition. Consequently, DeepPRM achieves more targeted peptides with corresponding RT information, and the detection rate (detected peptides/suggested peptides) of DeepPRM (85.23%) is far larger than that obtained by Picky (30.70%) (Fig 4B, Appendix Fig S2B–D, Datasets [Link], [Link], [Link]). This indicates that our newly developed DeepPRM provides an informative targeted peptide selection with high efficiency, especially for large‐scale candidate proteins, which is in line with the objectives for the extensive protein biomarker validation in this study.

We then employed the DeepPRM for MS assay development of 717 SPCBB proteins, and 1254 unique peptides were suggested with good detectability (≥ 0.5). Among these, 367 peptides were further observed in the serum digestion mixtures, with 113 peptides (corresponding to 100 proteins) detected with high confidence by manually checking (Appendix Fig S3, Datasets EV11 and EV12). These peptides were further quantified in cohort I serum samples [*n* = 212, within R (*n* = 55), UR (*n* = 57), and NC (*n* = 100)] (Dataset EV1) and cohort II serum samples [*n* = 32, within R (*n* = 6), UR (*n* = 6) and NC (*n* = 20)] (Dataset EV2).

### DeepPRM‐based quantification of biomarker candidates in serum of IA patients

Rigorous experimental controls were set to monitor the variation introduced by batch effects for the large‐scale sample analysis and the subsequent statistical analysis by setting quality control samples and spiking‐in iRT peptides (Appendix Fig S4A and B). The raw data were uploaded to Skyline to perform automatic PRM peak integration, detect interferences, and extract single transition intensities. A two‐step normalization of the quantification results of the peptides among 212 samples was carried out before further statistical analysis to account for the variability in instrument performance within each batch and between batches (Appendix Fig S4C and D). The orthogonal partial least‐squares discrimination analysis (OPLS‐DA), based on the normalized peak area of the 113 peptides corresponding to 100 protein candidates, identified a significant spatial separation of three groups NC, UR, and R (Appendix Fig S4E and F). Further, the variable importance in projection (VIP) of each feature was calculated (Dataset EV13).

To preselect potential proteomic signatures for IA and IA rupture, the Mann–Whitney U‐test was employed to analyze the variables that revealed 42 significantly changed peptides (36 proteins) in IA patients (UR & R), and 26 altered peptides (24 proteins) clearly distinguished the R group from the UR group (*P* < 0.05) (Fig 4C and D; Appendix Fig S4G and H).

Based on the PRM quantitation results of the first 60 serum samples, 12 stable isotope‐labeled peptides (of proteins whose expression levels changed significantly) were spiked into the remaining 152 serum samples as reference peptides. According to the relatively quantified amounts of these peptides, 10 peptides indicated marked change (*P* < 0.001) between the IA (UR & R) groups and NC groups. The quantitation results (log_2_ (L/H ratio)) are listed in Fig 4E. Furthermore, six peptides corresponding to five proteins (leucine‐rich alpha‐2‐glycoprotein (LRG1), fibrinogen alpha chain (FGA), fibrinogen beta chain (FGB), and fibrinogen gamma chain (FGG)) showed significantly higher expression in the R group than in UR group (*P* < 0.001), while the immunoglobulin kappa variable 4‐1(IGKV4‐1) was found in lower abundance in the R group (*P* < 0.001) (Fig 4F). The quality of PRM data of the above peptides is illustrated in Appendix Fig S5.

### Machine‐learning‐based selection of biomarker combinations for classification of IA cases

**Figure 5:**
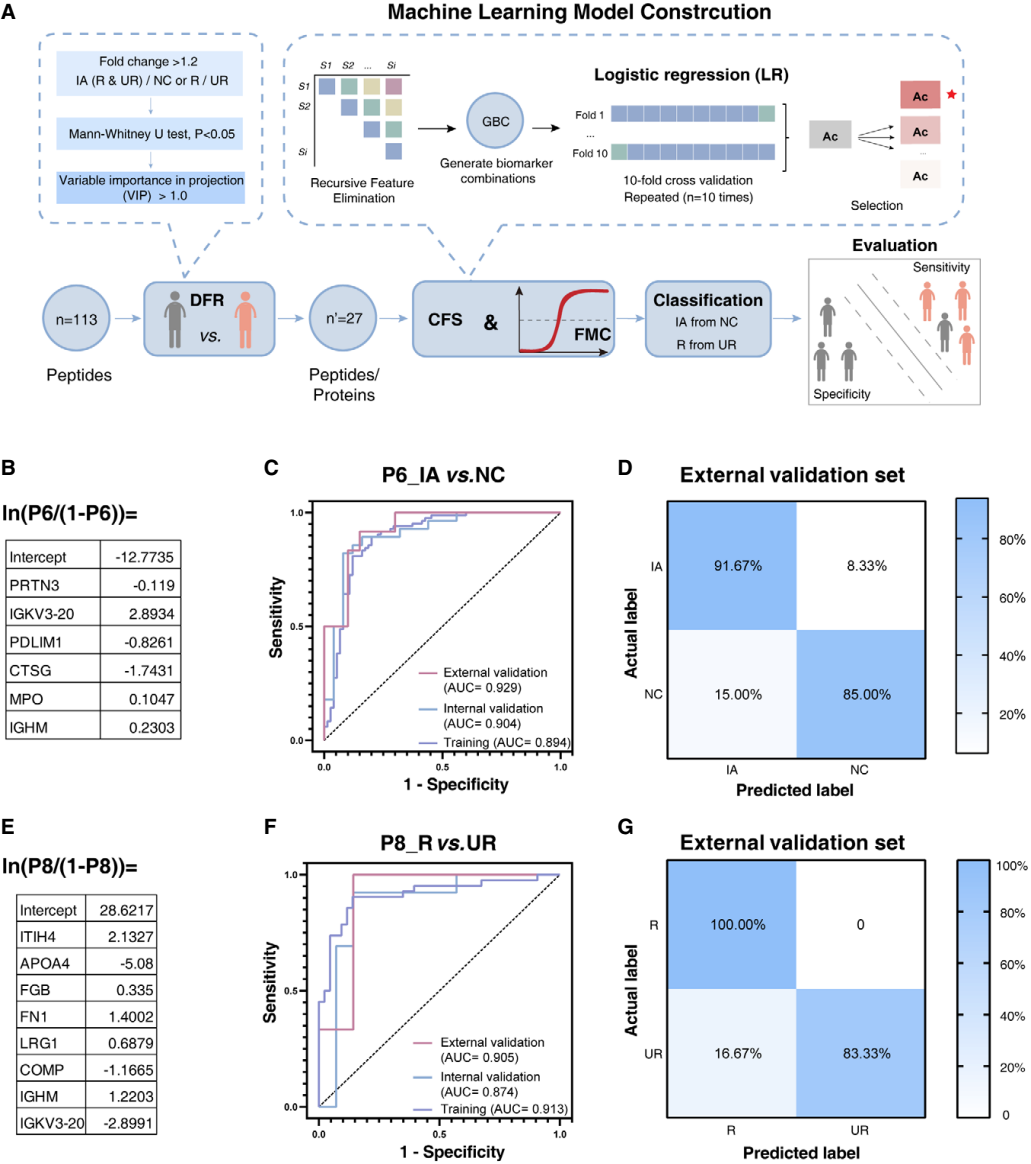
Identification of potential biomarker combinations for classification of IA different outcomes from healthy controls using machine‐learning method AWorkflow of data processing and machine‐learning model construction.B–GLogistic regression (LR) model for the classification of IA and NC (B) or R and UR (E). Receiver operating characteristic (ROC) curve of the LR‐based model in IA versus NC (C) or R versus UR (F) in training, internal validation, and external validation set. Confusion matrix showing the model performance for classifying IA and NC (D) or R and UR (G) in the external validation set. Source data are available online for this figure.

We investigated the possibility of discriminating patients with ruptured or unruptured IA from healthy controls based on the molecular signatures of serum proteins. On the basis of the serum proteomic data of PRM cohort I (*n* = 212), we developed a computational pipeline including differential feature reservation (DFR), candidate feature selection, and final model construction (CFS & FMC) for identifying potential biomarker combinations to classify IA cases (Fig 5A). In the DFR step, 32 peptides corresponding to 27 proteins were identified as highly ranked DEPs (fold change (FC) > 1.2, *P*‐value < 0.05 and VIP > 1.0). For proteins associated with multiple peptides, we selected the best peptide based on the area under the curve (AUC) and PRM raw data, to ensure that each peptide corresponded to a single protein (Kim *et al*, 2021) which resulted in remaining 27 peptides (Appendix Fig S6A). Subsequently, the combined dataset (cohort I) was randomly divided into a training and an internal validation set with a ratio of three to one (Datasets EV14 and EV15). In the CFS & FMC step, logistic regression was used for model building, and recursive feature elimination (RFE) with cross‐validation (10‐fold CV, repeated 10 times) was performed to select the optimal biomarker combination on the training set (75% of cohort I), based on the highest average accuracy (Ac) (Fig 5A).

We sought to utilize the above machine‐learning strategy to classify different clinical outcomes of IA (e.g., IA versus NC, or R versus UR) based on the significant features with more economical combinations of the molecules. For the classification of IA (R & UR) patients and healthy controls, we identified a compact biomarker combination containing six proteins (dubbed P6) (Fig 5B), including CTSG, PDLIM1, myeloblastin (PRTN3), myeloperoxidase (MPO), immunoglobulin heavy constant mu (IGHM), and immunoglobulin kappa variable 3–20 (IGKV3‐20). This model reached an AUC of 0.894 (95% CI = 0.836–0.937) based on the receiver operating characteristic curve (ROC) analysis and an Ac of 83.65% in the training set (Fig 5C; Table 1; Dataset EV16).

**Table 1:** Performance of circulating protein panel P6 in differential diagnosis of IA from healthy controls and P8 in classification of IA rupture.

Classification	Dataset	AUC (95% CI)	Sn	Sp	Ac	PPV	NPV	MCC
P6 (IA versus NC)	Training	0.894 (0.836–0.937)	85.71%	81.33%	83.65%	83.72%	83.56%	0.672
	Internal validation	0.904 (0.792–0.968)	85.71%	88.00%	86.79%	88.89%	84.62%	0.736
	External validation	0.929 (0.780–0.990)	91.67%	85.00%	87.50%	78.57%	94.44%	0.748
P8 (R versus UR)	Training	0.913 (0.832–0.963)	88.10%	86.05%	87.06%	86.05%	88.10%	0.741
	Internal validation	0.874 (0.689–0.969)	84.62%	85.71%	85.19%	84.62%	85.71%	0.703
	External validation	0.905 (0.616–0.996)	100.00%	83.33%	91.67%	85.71%	100.00%	0.845

We then tested the P6 model on the internal validation set (25% of cohort I), resulting in a promising AUC of 0.904 (95% CI = 0.792–0.968) with high sensitivity and specificity for distinguishing different IA groups (R & UR) from healthy controls (Fig 5B; Table 1). To evaluate the reliability of the machine‐learning strategy, confusion matrices were compiled, and the results demonstrated that different samples could be correctly classified with a high Ac of 86.79% (Appendix Fig S6B; Table 1).

To validate the Ac of the machine‐learning‐based classification of IA cases, we further collected 32 serum samples of a new cohort (II) as the external validation set. The AUC values were calculated as 0.929 (95% CI = 0.780–0.990) for distinguishing IA cases from NC (Fig 5C; Table 1). Accordingly, the corresponding confusion matrix results also demonstrated that P6 exhibited a promising Ac of 87.50% on the independent cohort (Fig 5D; Table 1; Dataset EV16).

Finally, we plotted the learning curves of P6 model (Appendix Fig S6C). The curves of the training and internal validation sets are getting flattened, and the accuracy becomes stable and larger than 0.8, indicating a good fit of the model (Emmert‐Streib & Dehmer, 2019).

### Biomarker combinations for classification of ruptured and unruptured IA

The potential IA rupture is a complex and challenging condition that confuses clinicians regarding the choice of treatments. To classify UR and R outcomes, we identified an eight‐protein combination (P8) containing inter‐alpha‐trypsin inhibitor heavy‐chain H4 (ITIH4), apolipoprotein A‐IV (APOA4), FGG, fibronectin (FN1), LRG1, cartilage oligomeric matrix protein (COMP), IGHM, and IGKV3D‐20, with an AUC of 0.913 (95% CI = 0.832–0.963) and an Ac of 87.06% in the training set, and an AUC of 0.874 (95% CI = 0.689–0.969) and an Ac of 85.19% in the internal validation set to distinguish ruptured from unruptured IA patients (Fig 5E and F; Table 1; Appendix Fig S6D; Dataset EV16). It also yielded an excellent classification Ac of 91.67% and a high AUC value of 0.905 in the external validation set, which further demonstrates the robustness of the model (Fig 5F and G; Table 1). The learning curves of the P8 model also illustrated that the model fits well (Appendix Fig S6E).

We further validated two low abundant proteins PRTN3 (tissue original) and CTSG (serum original) using ELISA in an additional cohort (III) that included 40 R and 40 UR patients plus 40 healthy controls, showing high concordance with the PRM results (Appendix Fig S7A–D; Dataset EV17).

In summary, these data strongly support the potential clinical value of serum proteomics‐derived panels for the identification of IA and determination of its rupture.

## Discussion

The disruption of internal elastic lamina and subsequent mechanical overload and shift in tensile forces are critical for the formation, progression, and rupture of IA. However, the molecular mechanisms involved still remain poorly recognized, which hampers the clinical management of IA. Currently, the golden standard diagnosis of IA is digital subtraction angiography (DSA), with no molecular diagnostic model existing in the clinic to date. There are few reports of IA‐related circulation signatures that could also discriminate ruptured and unruptured IA. Herein, we performed a systematic proteomic analysis of the diseased organs and serum samples, developed a comprehensive biomarker discovery strategy, and identified six‐ and eight‐protein‐based biomarker combinations with potential clinical utility for classifying IA and its rupture.

There are several notable findings. First, the proteome data of IA tissue and serum reiterate that the main pathophysiological processes involved in IA development, such as SMC contraction and adhesion, are inhibited, and inflammatory reactions are activated, as previously reported (Etminan & Rinkel, 2017). Proteins related to these pathways dramatically alter their expression levels. In particular, serum samples collected from patients with ruptured IA demonstrate a distinct enhancement of the immune system and related pathways. Furthermore, the data highlight profound differences in proteins involved in key energy metabolic pathways, such as the TCA cycle, which are markedly downregulated, indicating different energy mediation of IA patients.

For the biomarker discovery of IA, scientists investigated the disease using various strategies and perspectives, thus generating different results. To make full use of this precious data and construct a comprehensive survey of the IA processes, we assembled results from previous studies and the present study to form the most extensive protein biomarker bank reported to date. We then refined it by predicting the likelihood that identified proteins are detected in serum samples for downstream clinical applications. A total of 1,241 proteins were reviewed, with 717 proteins collected in the SPCBB having various functions. The candidate biomarker bank may provide an informative reference for further IA studies.

Considering the costs and workload of the unbiased validation of 717 proteins in the SPCBB, we proposed a highly efficient and time‐saving PRM assay approach (DeepPRM). Due to the different conditions used in the pretreatment of samples, the types of instruments, as well as variable instrumental settings in different laboratories, the peptide detection, and RTs differ across studies. We therefore built an instrument‐specific model based on deep learning the spectra generated in a given LC‐MS platform and made a prediction of the peptide detectability and RT. Based on the analysis of the HeLa cell line and serum datasets, the DeepPRM strategy achieved better performance than the Picky‐derived results from the global model. Furthermore, the algorithm used by DeepPRM was based on the deep learning of peptide spectra, thus indicating the potential to be widely used without the restriction of actual specimens from human and mouse models. In this study, we used DeepPRM and our rapid serum sample preparation (RSP) method (Shen *et al*, 2021), to construct a highly efficient PRM assay developing strategy and validate the candidate peptides in serum samples.

For further large‐scale analysis of serum samples, we elaborated the experimental conditions to eliminate differences caused by instrument performance and set strict quality control criteria for the data acquisition and analysis. Accordingly, two biomarker combinations were identified as biomarkers using the LR machine‐learning strategy. These biomarkers could classify the different outcomes of IA cases with high Ac, AUC, sensitivity, and specificity. Further, the alternations of these proteins provide valuable insight into the pathogenesis of IA. Moreover, the Ac of these biomarkers to distinguish IA formation and rupture were further validated via proteomics and ELISA using the serum samples from 25% of cohort I and two additional cohorts (II & III) of IA and NC, respectively. These results confirm that the altered serum protein levels identified in this study indeed reflect the pathophysiological changes in response to IA. Therefore, these proteins show potential for further development as clinical biomarkers. In this respect, the DeepPRM and our developed comprehensive serum biomarker screening strategy are expected to benefit flexible biomarkers discovery studies.

Notably, some of these proteins were reported to be strongly relevant to cardiovascular or cerebrovascular disease. For instance, two of the biomarker candidates derived from immunoglobulins, IGHM and IGKV3‐20 are important mediators of the inflammatory responses which are reported to be the shared pathological mechanisms in a variety of vascular diseases, including atherosclerosis, abdominal aortic aneurysm, and arteritis (Chyatte *et al*, 1999; Chalouhi *et al*, 2012). The markedly increased levels of two acute‐phase proteins (APPs) including FGG and ITIH4 are usually reported to occur in response to inflammation or tissue injury (Gabay & Kushner, 1999). FN1 and COMP are mapped in the focal adhesion pathway, which is known to maintain cerebrovascular integrity (Liu *et al*, 2012; Xu *et al*, 2019). The four regulators LRG1, MPO, CTSG, and PRTN3 are all involved in the neutrophil degranulation pathway. Notably, MPO, CTSG, and PRTN3 are all neutrophil‐related proteins, of which MPO has been revealed to be correlated with the severity and outcome of vascular disease (Schindhelm *et al*, 2009; Gounis *et al*, 2014). Further, CTSG has been reported to induce early atherosclerotic lesion formation (Ortega‐Gomez *et al*, 2016), while PRTN3 is possibly involved in neuroinflammation caused by excess neutrophils entering the brain (Kwon *et al*, 2013). Nevertheless, there is little information available regarding the role of PDLIM1 in cardiovascular or cerebrovascular disease. A previous study reported that PDLIM1 could interact with and stabilize the E‐cadherin/β‐catenin complex in cancer (Chen *et al*, 2016). Interestingly, VE‐cadherin, a unique adhesion factor of endothelial cells, directly or indirectly participates in intercellular signals to determine the stability of cell connections (Giannotta *et al*, 2013). Therefore, we considered that PDLIM1 might play a crucial role in regulating cell adhesion between endothelial cells and maintaining the endothelial cell barrier.

Our study has several limitations. First, there are a limited number of patients, especially for the discovery of tissue samples due to special IA treatment strategies and technical complexity. However, our goal in this step was to generate candidate biomarkers and validate them in large cohort of serum samples; therefore, more cases in the discovery stages would not have yielded a different result. Another possible drawback of this work is that different therapeutic strategies used during the treatment of different patients might affect the results, although the protein‐level changes uncovered here are consistent within different cohorts. Finally, the detailed roles of the biomarker proteins in the pathogenesis of IA require further investigation on potential therapeutic targets, such as PDLIM1 and CTSG, which must be further elucidated or experimentally validated.

In summary, we outlined a comprehensive protein candidate biomarker discovery pipeline for IA that takes full advantage of data from the present and previous studies using our newly developed DeepPRM method. Our study provides a highly valuable proteomics resource for the research community to better understand IA‐associated response, sheds light on the pathogenesis of IA formation and rupture, and identifies a serial of valuable biomarker candidates that may assist in the clinical decision‐making process, thus leading to appropriate diagnosis and effective treatment of IA. We further envisioned the widespread application of the extensive serum biomarker strategy to other proteomic signature discovery studies.

## Materials and Methods

### Study approval

The study was conducted in accordance with the guidelines of the Declaration of Helsinki and the Department of Health and Human Services Belmont Report, with the approval of the Research Ethics Committee from the Huashan Hospital, Fudan University. Written informed consent was obtained from all patients or their legal representatives prior to their participation in the study.

### Patients and specimens

In total, 244 blood samples of IA patients and healthy controls were collected between September 2017 and October 2019 which were categorized as follows: NC (*n* = 120), IA (*n* = 124), UR (*n* = 63), and R (*n* = 61) (Datasets EV1 and EV2). Three‐dimensional rotational DSA images of all patients were observed by Sante DICOM Viewer Free software (Philips Allua Xper, the Netherlands) and labeled with size, width, and neck diameter of aneurysm. Blood samples were collected after overnight fasting under routine clinical blood guidance using Vacutainer tubes (Becton Dickinson, Franklin Lakes, NJ, USA) with no anticoagulant. The blood samples were centrifuged at 1,500 *g* for 10 min at 4°C after clotting at room temperature. Serum samples were immediately aliquoted in sterile centrifuge tubes and were stored at −80°C for future analysis. Each aliquoted serum was only analyzed once without any freeze–thaw cycles. The decision of clipping an IA was made by two neurosurgeons and one neuro‐intervention specialist. The control vessel specimen was taken from the STA. All tissues samples were immediately transferred to liquid nitrogen and placed in sterile centrifuge tubes and stored at −80°C for future analysis.

### Sample pretreatment of quantitative proteomics analysis

Five pairs of IA tissues and matched STA tissues from patients with IA were prepared using a commercial sample preparation kit (iST kit, PreOmics GmbH), according to the manufacturer’s instructions. The serum samples were subjected to immunoaffinity depletion for removal of the top 12 high abundance proteins. Protein concentration was then determined by BCA assay. A total of 100 µg of proteins from each group were then digested, followed by TMT labeling according to the manufacturer’s instructors, and high pH RPLC separation. Two biological replicates and three technical replicates were performed.

### Liquid chromatography tandem mass spectrometry (LC‐MS/MS) analysis

The protein samples from trace IA and STA tissue were analyzed on an UltiMate 3000 nanosystem (Thermo Fisher Scientific, USA) connected to an Orbitrap Exploris 480 MS in combination with Field Asymmetric Waveform Ion Mobility Spectrometry (FAIMS). The peptides were separated on a 75 μm × 25 cm long column (2 μm id) at a flow rate of 300 nl/min for 150 min. The data of TMT‐labeled serum peptides were acquired on an easyNano system (Thermo Fisher Scientific, USA) with a 75 μm × 25 cm long column (2 μm id) connected to Orbitrap Fusion Tribrid Mass Spectrometer (MS) (Thermo Fisher Scientific, USA) by a 120 min LC separation.

### DeepPRM method

The list of 717 proteins in SPCBB was submitted to the previously developed instrument‐specific model for predicting unique peptides and their detectability and iRT information (Yang *et al*, 2020). Suggested peptides with the probability of detectability > 0.5 were selected and further monitored in digested serum samples using an Orbitrap Exploris™ 480 MS (Thermo Fisher Scientific, MA, USA) coupled to an EASY‐NanoLC 1200 system (Thermo Fisher Scientific, MA, USA) with a 75 μm × 50 cm long column (2 μm id) with 60‐min acquisition. Chromatographic conditions were as follows: 65‐min gradient at a flow rate of 200 nl/min starting with 94% A (0.1% FA), followed by 35% B (80% ACN, 0.1%FA) at 45 min, followed by a step increase to 50% B until 54 min, and climbing to 100% B at a flow rate of 300 nl/min for the last 11 min. For all PRM runs, scheduled injections with a 3‐min elution window were used. A blank was set between samples to avoid carryover. Eighteen stable isotope‐labeled (SIL), targeted peptides [> 98% purity], and iRT standards were spiked into the digested serum as quantity control.

The identified peptides meet the criterion of three aspects (proteotypic peptides, charge state, and transition selection) were checked manually and used for further PRM quantitation in large cohort of serum samples (*n* = 244).

Peptide selection: For each protein, preferably two, at least one proteotypic peptides (PTPs) were selected from DeepPRM method based on the following criteria: (i) unique to a particular protein; (ii) peptides of 7–25 amino acids in length; (iii) peptides of mass ≤ 6,000 Da and detectability > 0.5; and (iv) without of methionine, cysteine, or other post‐translational modification sites. For proteins without eligible peptides were removed for further large‐scale quantification. Charge state selection: the peptide charge state picked up for PRM analysis was selected based on the following criteria: (i) the *m/z* of targeted peptide was selected within the optimal MS scan range of 350–1,250; (ii) when two charge states of a peptide could be detected simultaneously, we selected the better one based on the manually checking the mass spectrum signal response, such as the signal‐to‐noise ratio, the transitions, the peak shape, and the peak area (Appendix Fig S3). Transition selection: the transitions were selected based on the rank of intensity identified in the spectral libraries. For each peptide, at least top 3 fragment ions of the spectral library were monitored excluding those short fragments ions (y1, y2, y3 and b1, b2, b3).

### Statistical analysis

The raw files were searched against the human Swiss‐Prot database (20,379 entries with 11 iRT peptides) by Proteome Discovery (PD, Thermo Scientific, USA) using the MASCOT search engine. The false discovery rate (FDR) of protein identification was set to < 1%. Data statistical analysis was performed with MetaboAnalyst 4.0 (Chong *et al*, 2018). After missing value imputations and data normalization, significance was assessed using Student’s *t*‐test to identify differentially expressed proteins in the IA tissue proteome and the IA serum proteome. For data visualization, volcano plots, heatmaps, and Venn diagrams were constructed using an online platform (http://www.bioinformatics.com.cn). The home‐made MATLAB script was used for GO annotation and pathway enrichment analyses. IPA (Ingenuity Pathway Analysis, Ingenuity Systems) tools were used to analyze the functions and interactions of the evaluable proteins obtained from the tissue and serum samples. GO‐CC term and signalP (Petersen *et al*, 2011) were used for predicting leaked or secreted proteins from tissue.

Acquired DeepPRM raw data were analyzed using the open‐source Skyline‐daily software for transition identification and peak area integration. The peak area of targeted peptides was exported from Skyline into an Excel report spreadsheet and transformed to log10 format, which was more closely conformed to normal distribution and better suitable for the statistical modeling assumptions for downstream analysis. We modified the normalization step that was established by Ruedi Aebersold (Huettenhain *et al*, 2019) in two steps: the first normalization was a longitudinal correction, while the second normalization step was transverse correction, all conducted to remove systematic variations caused by the instrument performance and batch effects. The precise relative quantification of each endogenous peptide was calculated as follows: C_endogenous_ = C_SIS_ × peak area _endogenous_/peak area _SIS_. The Mann–Whitney *U*‐test was used to obtain variables with significant differences between the (R & UR) versus NC groups, and the R versus UR groups (*P*‐value < 0.001).

### Machine‐learning strategy

For the identification of different types of biomarker combinations, we first classified the proteomic datasets of R, UR, and NC into different groups: (i) For the classification of IA and NC, the R and UR groups were combined and set as the IA group. (ii) For the classification of the R and UR groups.

In the DFR step, we compared the PRM quantitation results of peptides in UR, R, and NC groups and reserved high potential DEPs as a candidate reservoir (FC > 1.2, *P* < 0.05, and VIP > 1). To avoid overfitting, the number of proteins in a combination must be significantly smaller than the sample size (Shu *et al*, 2020). Thus, in the CFS & FMC step, we used binary logistic regression to construct the models, and RFE with CV (10‐fold, repeated 10 times) was further implemented to remove features of low importance and select the best subsets of biomarker combinations based on the highest average Ac.

The combined dataset (cohort I) was randomly divided into a training set and internal validation set with a ratio of three (training set) to one (the internal validation set). The newly enrolled cohort II (*n* = 32) was set as external validation set. The internal and external validation sets were only used to evaluate the performance, but not for model training. To evaluate the accuracy of the LR model, true‐positive (TP), true‐negative (TN), false‐positive (FP), and false‐negative (FN) numbers were counted. Then, six measurements including sensitivity (Sn), specificity (Sp), accuracy (Ac), positive predictive value (PPV), negative predictive value (NPV), and Mathew correlation coefficient (MCC) were calculated, as shown in Table 1.

To evaluate whether the model was overfitting, we plotted a learning curve of the P6/P8 model based on the accuracy of the training and internal validation sets using R package (version 4.6‐14) (Appendix Fig S6C and E). The ROC curve was illustrated using MedCalc (version 19.5.6) and GraphPad Prism (version 8.0.2).

### Enzyme‐linked immunosorbent assay (ELISA)

The serum levels of two candidate biomarkers were further determined in an additional cohort (III) that included 40 R and 40 UR patients plus 40 healthy controls using the following ELISA kits: polymorphonuclear leukocyte serine protease 3 (PRTN3) (#LS‐F57404, LifeSpan BioSciences, WA, USA) and Cathepsin G (CTSG) (#E4639‐100, Biovision, CA, USA).

### Western blotting

The tissue level of PDLIM1 was further determined using Western blotting analysis. IAs and matched STA tissues were extracted with RIPA lysis buffer (Thermo Fisher Scientific, USA) and quantified by the BCA Protein Assay (Thermo Fisher Scientific, USA). Briefly, 20–25 μg of purified protein was separated by SDS–PAGE and electrotransferred onto a PVDF membrane. The membranes were blocked for 1 h at room temperature in TBST (20 mM Tris–Cl, 140 mM NaCl, pH 7.5, 0.05% Tween‐20) containing 5% nonfat dry milk and then incubated with rabbit polyclonal anti‐PDLIM1 antibody (Affinity Biosciences, OH, USA) and rabbit polyclonal anti‐tubulin beta antibody (Affinity Biosciences, OH, USA) overnight at 4°C. After washing three times in TBST, membranes were incubated with secondary antibody (goat anti‐rabbit IgG (H + L) HRP, Affinity Biosciences, OH, USA) for 1 h at room temperature. Blots were visualized using an ECL detection system and proteins were quantified using a ImageQuant LAS 4000 mini (GE Healthcare, Piscataway, NJ, USA). The expression of PDLIM1 was evaluated by densitometric analysis. Ratios of densitometric measurements of target proteins relative to β‐tubulin were compared between the IA and STA.

## Author contributions

XL conceived and designed the research. YX performed the experiments and data analysis. YZ, YY, JH, BZ, TJ, and BL coordinated samples and clinical data collection. JY, FS, SK, SY, GY, HZ, XZ, and HS performed mass spectrometry analyses. HL performed machine‐learning analysis. XL and PY provided technical support and manuscript preparation. All authors were involved in the design of this work.

## Conflict of interest

The authors declare that they have no conflict of interest.

## For more information

- pyyangteam.fudan.edu.cn/

## Supporting information



Appendix

Dataset EV1

Dataset EV2

Dataset EV3

Dataset EV4

Dataset EV5

Dataset EV6

Dataset EV7

Dataset EV8

Dataset EV9

Dataset EV10

Dataset EV11

Dataset EV12

Dataset EV13

Dataset EV14

Dataset EV15

Dataset EV16

Dataset EV17

Dataset EV18

Source Data for Figure 2

Source Data for Figure 3

Source Data for Figure 4

Source Data for Figure 5

## Data Availability

The mass spectrometry proteomics data have been deposited to the ProteomeXchange Consortium via the iProX partner repository (Ma *et al*, 2019) with the dataset identifier PXD024615 (http://proteomecentral.proteomexchange.org/cgi/GetDataset?ID=PXD024615). The DeepPRM algorithm is available at https://deepdia.omicsolution.com/ (Yang *et al*, 2020).
