# Supplementary material for: Circulating proteomic panels for risk stratification of intracranial aneurysm and its rupture
Source: EMBO Mol Med. 2022 Jan 3;14(2):e14713. doi: 10.15252/emmm.202114713 (PMC8819334; doi:10.15252/emmm.202114713)
Supplement: Supplementary file 1 — Appendix [file EMMM-14-e14713-s020.pdf]

Circulating proteomic panels for risk stratification of intracranial aneurysm and its rupture

Yueting Xiong[1#], Yongtao Zheng[2#], Yan Yan[3#], Jun Yao[1], Hebin Liu[4], Fenglin Shen[1], Siyuan Kong[1], Shuang Yang[1], Guoquan Yan[1], Huanhuan Zhao[1], Xinwen Zhou[1], Jia Hu[3], Bin Zhou[3], Tao Jin[3], Bing Leng[3, *], Pengyuan Yang[1, &], Xiaohui Liu[1, *]

1. The Fifth People's Hospital of Shanghai, Fudan university, and the Shanghai Key Laboratory of Medical Epigenetics, the International Co-laboratory of Medical Epigenetics and Metabolism, Ministry of Science and Technology, and Institutes of Biomedical Sciences, Fudan University, Shanghai 200032, China
2. Department of neurosurgery, Ruijin Hospital, Shanghai jiaotong University, Shanghai, 200010, China
3. Huashan Hospital, Fudan University, Shanghai 200032, China
4. Shanghai Omicsolution Co., Ltd., Shanghai 200000, China

[#]: These authors contributed equally: Yueting Xiong, Yongtao Zheng, Yan, Yan

*: To whom correspondence should be addressed. E-mail: lengbing99999@126.com; liuxiaohui@fudan.edu.cn

& : This study is dedicated to the memory of Professor Pengyuan Yang (1949-2021)

Fax: +86 2154237482; Phone: +86 2154237482

Table of Content:

- **Appendix Figures S1-7**

Appendix Figures

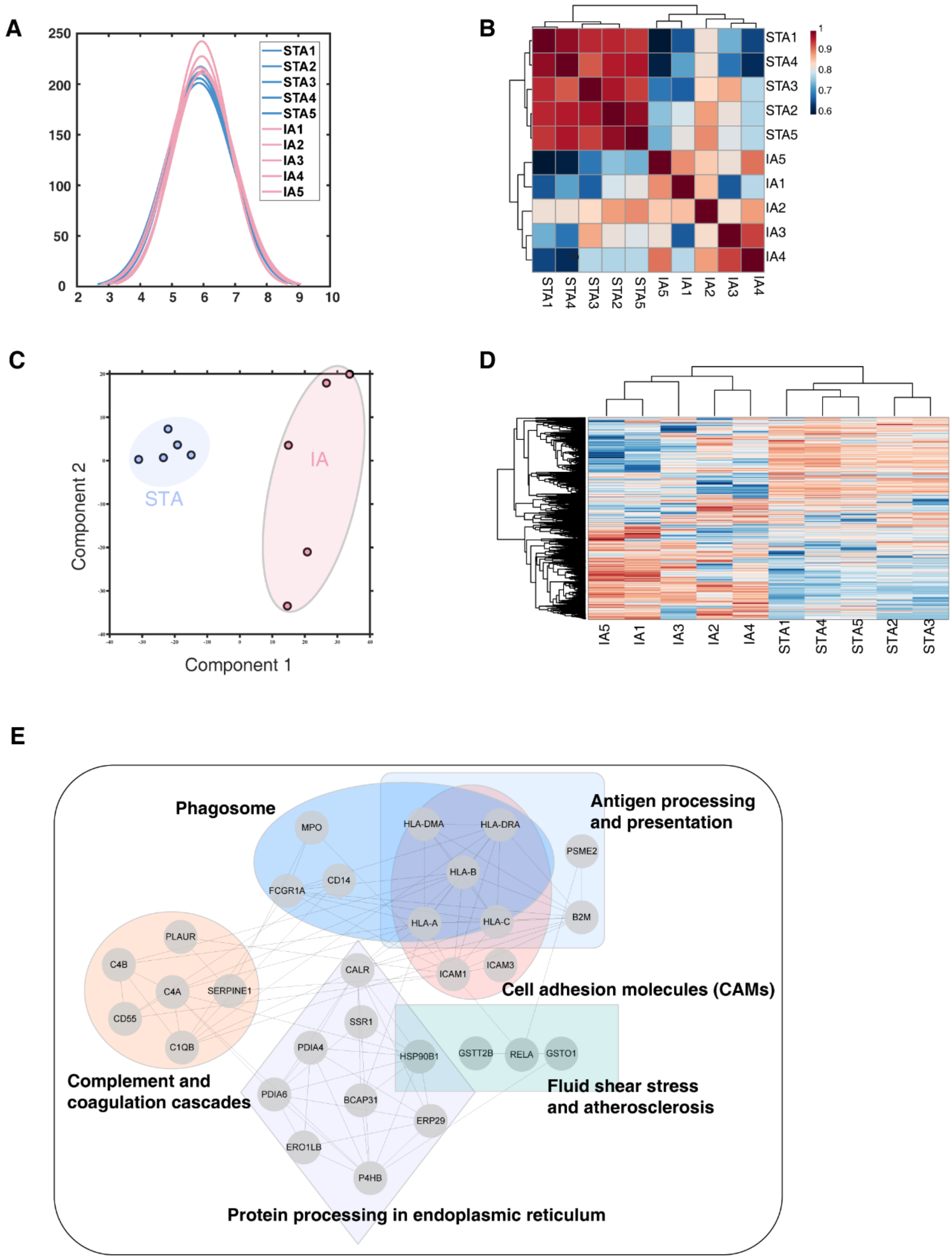

Appendix Figure S1. IA tissue proteome remodeling due to IA formation and rupture.

A) The normal distribution of the proteome abundance of the ten samples (5 IAs and 5 STAs).

B) Heat map of Pearson correlation coefficients derived from the comparison of the proteome abundance of the 10-patient samples in IA tissues and STA tissues.

C) PCA score plot of IA and STA group based on the unbiased label free proteomics data.

D) Heatmap of the differential expressed proteins identified in IA tissue and STA tissue. Values for each protein (rows) and for each surgically excised sample (columns) are colored based on the protein abundance.

E) Interaction network of tissue upregulated proteins in IA group compared to STA group. Different pathways were colored differentially such as complement and coagulation cascades (orange), protein processing in endoplasmic reticulum (purple), fluid shear stress and atherosclerosis (green), cell adhesion molecules (CAMs) (pink), protein processing in endoplasmic reticulum (blue) and phagosome (dark blue).

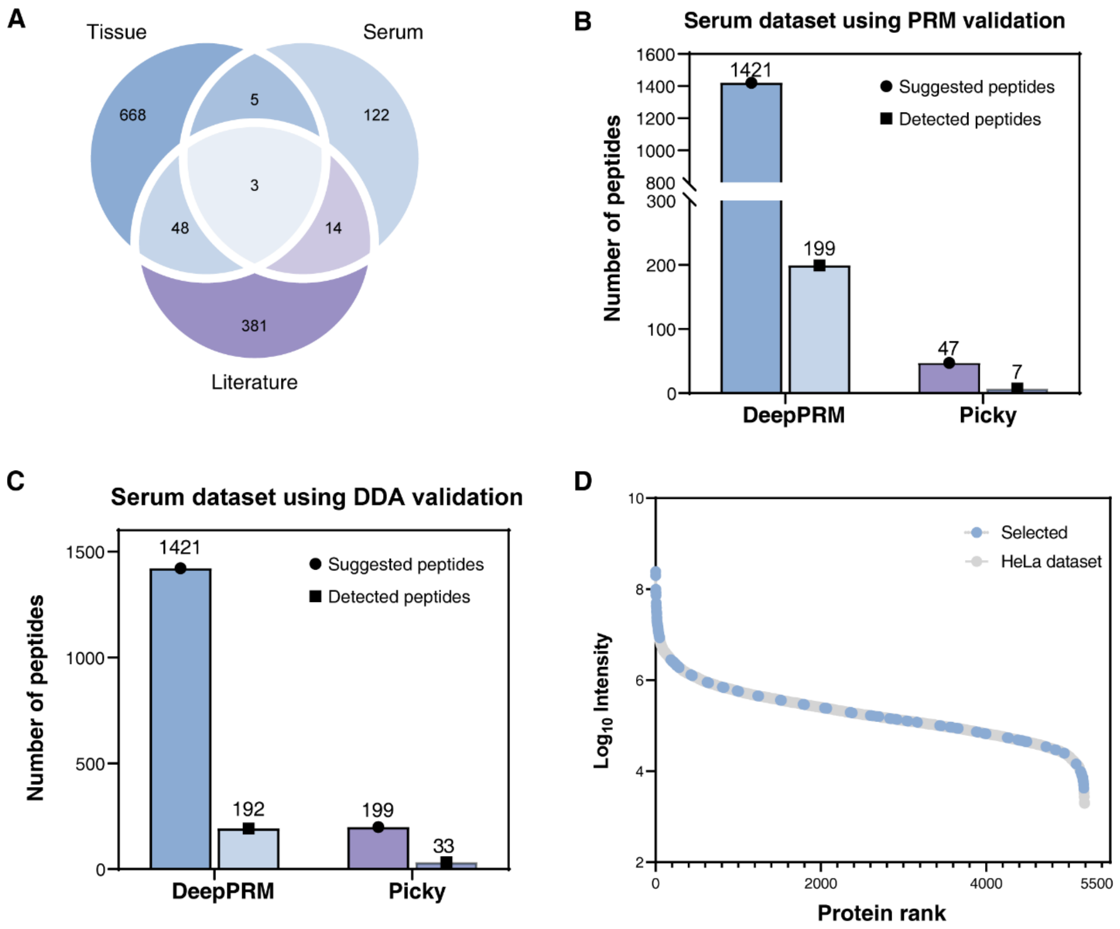

Appendix Figure S2. Developed DeepPRM method for IA candidate proteins biomarker bank.

A) Overlap of candidate proteins related to IA from IA tissue proteome, IA serum proteome and previous studies.

B, C) The histogram of the number of suggested peptides by DeepPRM and Picky methods, and detected peptides from PRM (**B**) or DDA (**C**) validation.

D) Intensity range of proteins from public HeLa DDA dataset. The red dots represented the randomly selected 686 proteins for further peptide selection.

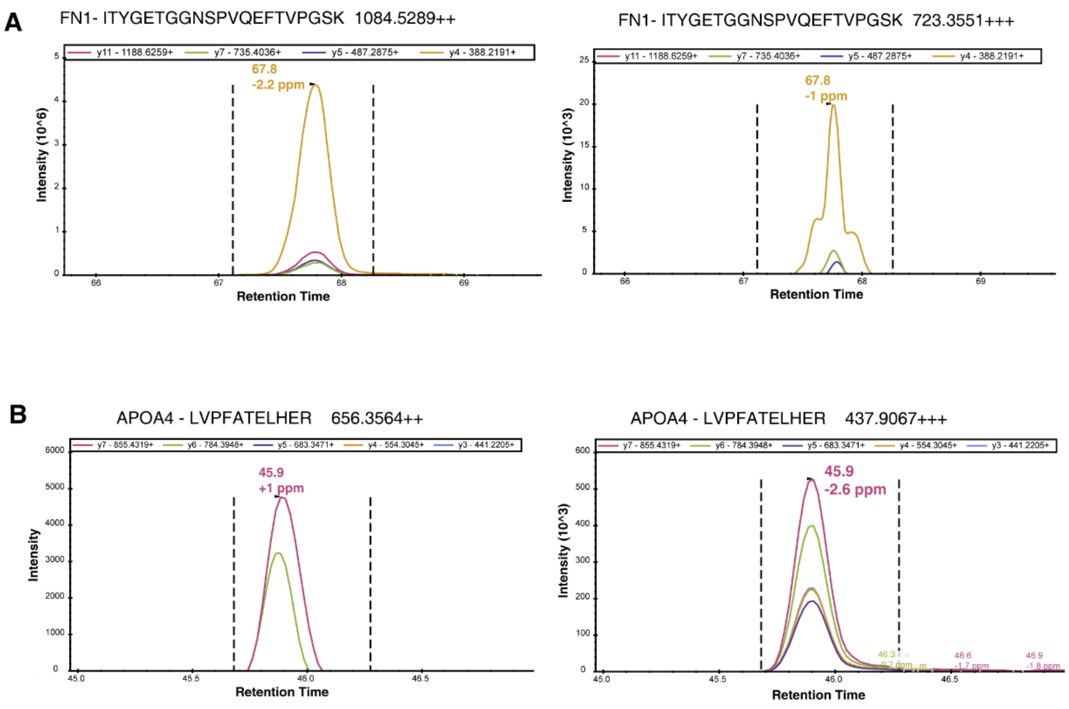

Appendix Figure S3. The comparation of same peptide with charge state of 2 and 3.

A) Skyline display of ion pattern and chromatograph of serum peptide (ITYGETGGNSPVQEFTVPGSK) of fibronectin protein with charge state of 2 and 3.

B) Skyline display of ion pattern and chromatograph of serum peptide (LVPFATELHER) of apolipoprotein A-IV protein with charge state of 2 and 3.

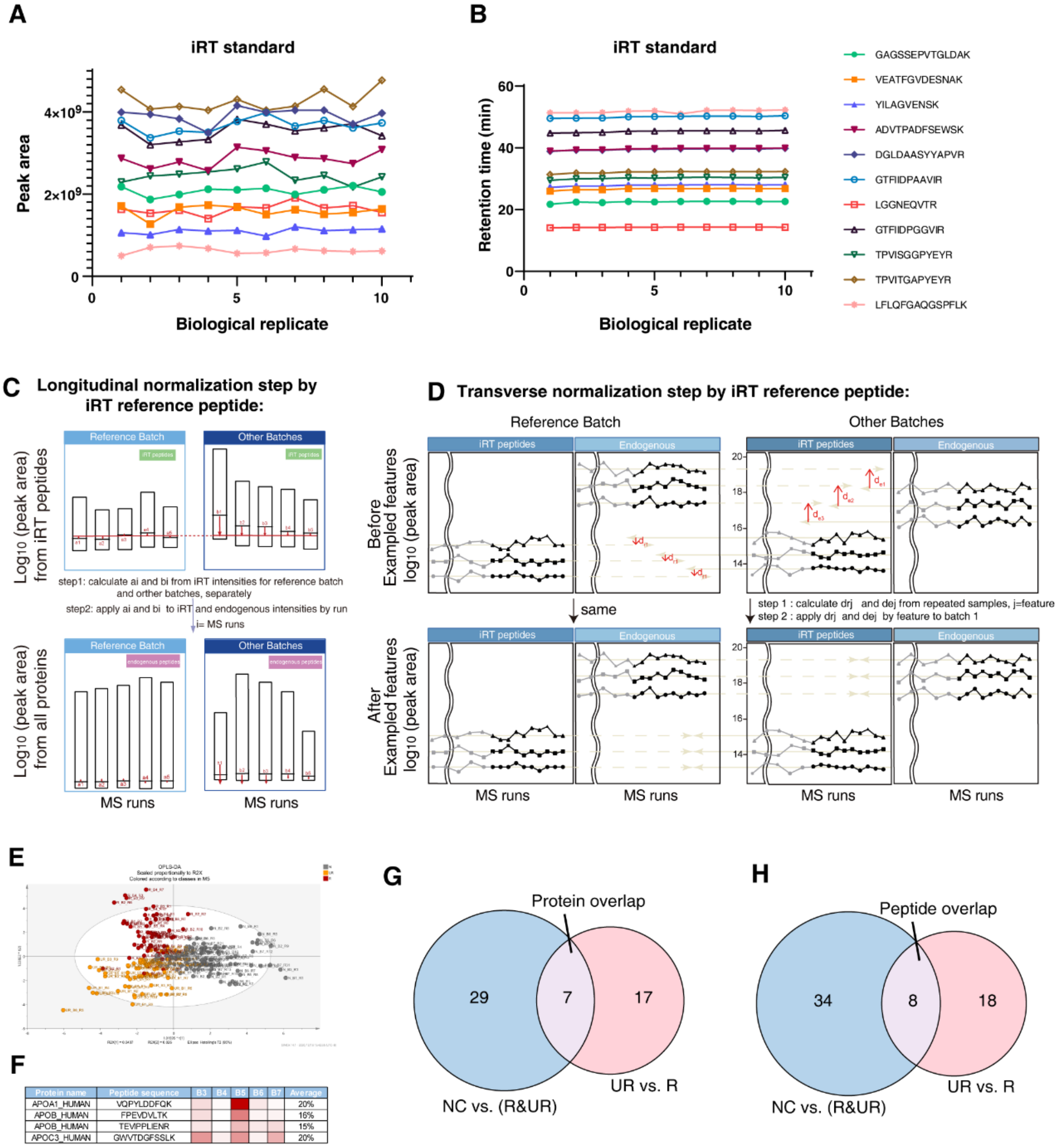

Appendix Figure S4. SPCBB quantification in large-scale IA serum samples by DeepPRM method.

A-B) The peak area (**A**) and retention time (**B**) of iRT samples among biological replicate for the evaluation of DeepPRM-MS method in large-scale serum sample validation analysis.

C) The longitudinal normalization step by iRT reference peptide of the DeepPRM data.

D) The transverse normalization step by iRT reference peptide of the DeepPRM data.

E) OPLS-DA of the three groups of patients by normalized DeepPRM data.

F) The average CV of peak area of four heavy peptides corresponding to 4 non-differential proteins.

G-H) The overlap of significantly changed proteins (**G**) or peptides (**H**) (P-value < 0.05) quantified in the (R & UR) group *vs.* NC group and the R group *vs.* UR group. The quantitation results of the 113 peptides using statistical analysis (Mann-Whitney U test).

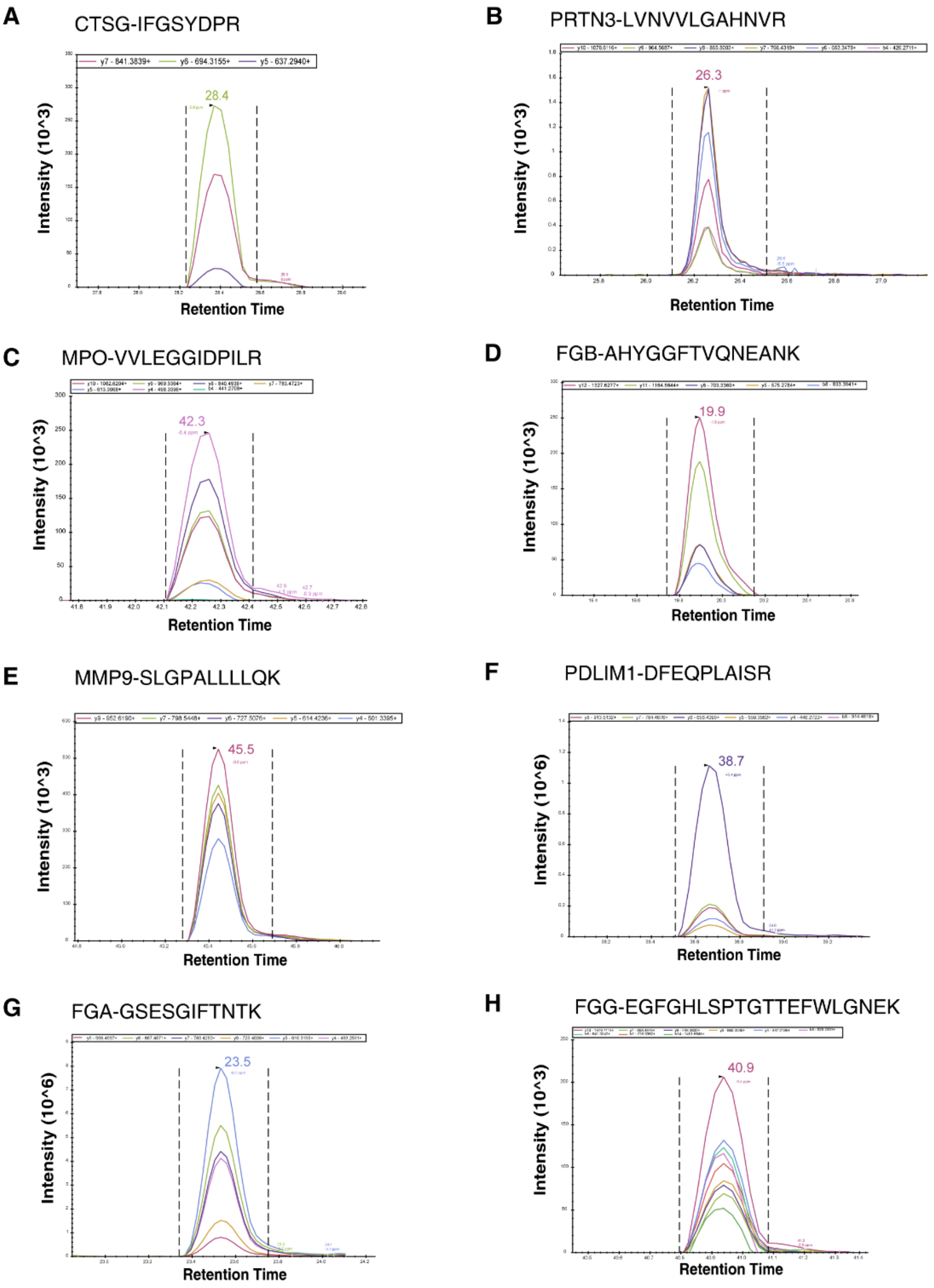

Appendix Figure S5. Skyline display of ion pattern and chromatograph of 8 serum peptides.

A) Serum peptide IFGSYDPR of CTSG protein.

B) Serum peptide LVNVVLGAHNVR of PRTN3 protein.

C) Serum peptide VVLEGGIDPILR of MPO protein.

D) Serum peptide AHYGGFTVQNEANK of FGB protein.

E) Serum peptide SLGPALLLLQK of MMP9 protein.

F) Serum peptide DFEQPLAISR of PDLIM1 protein.

G) Serum peptide GSESGIFTNTK of FGA protein.

H) Serum peptide EGFGHLSPTGTTEFWLGNEK of FGG protein.

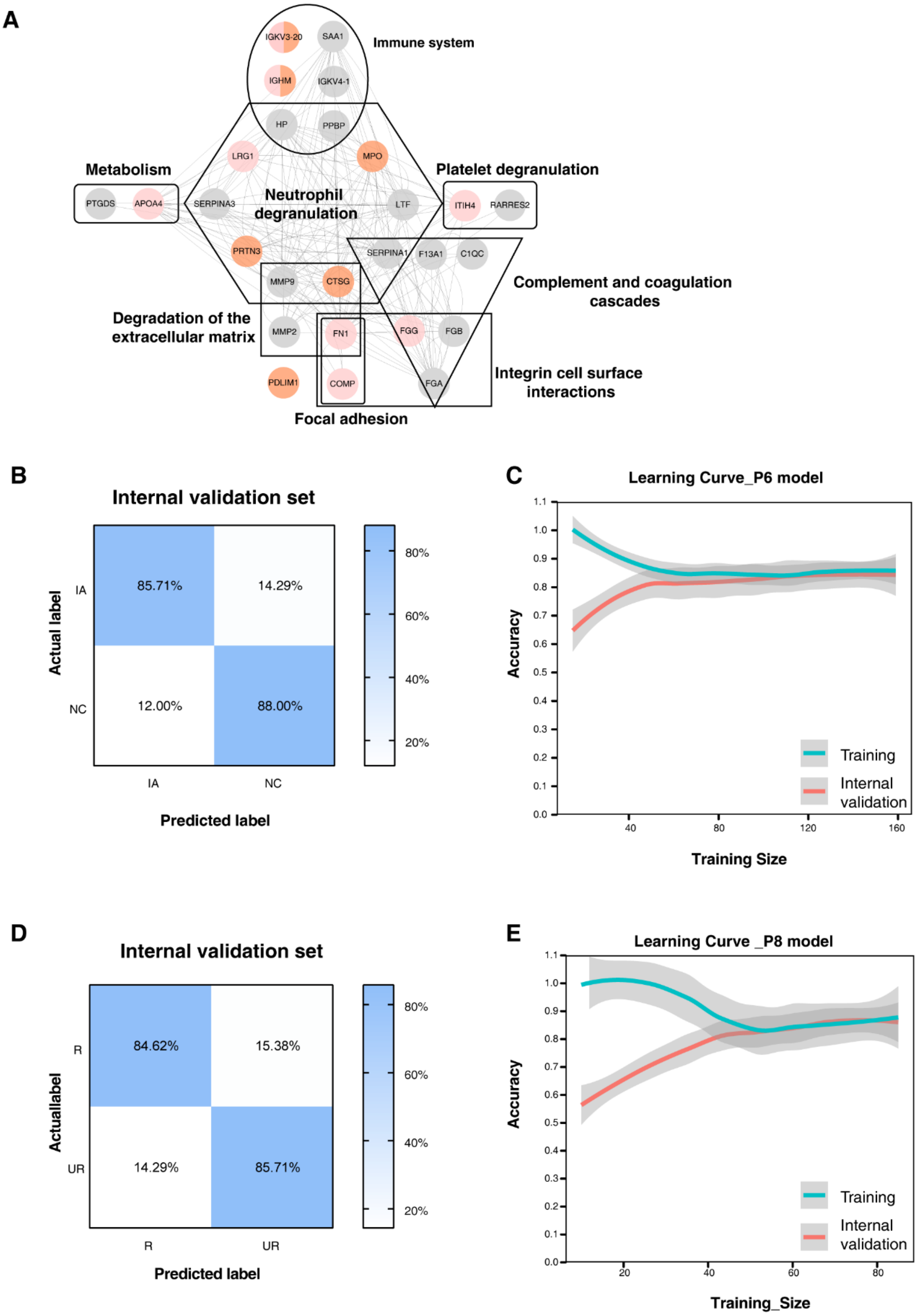

Appendix Figure S6: Machine learning strategy for the classification of IA cases and healthy controls.

A) A serum protein regulatory network based on 27 highly ranked proteins associated with IA.

B, D) Confusion matrix showing the model performance for classifying IA and NC (**B**) or R and UR (**D**) in the internal validation set.

C, E) The learning curve showing the fitting situation of P6 model (**C**) and P8 model (**E**) based on the accuracy of the training set and the internal validation set.

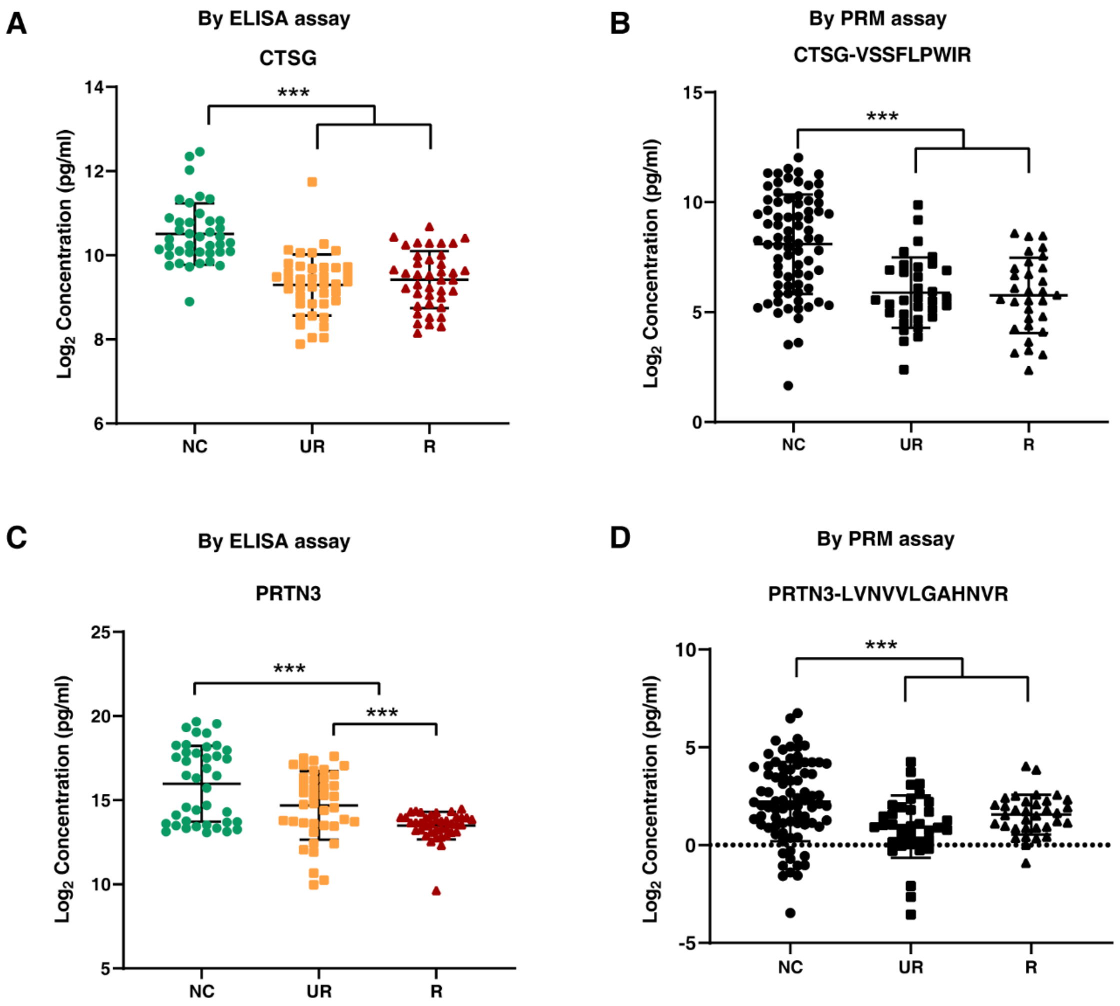

Appendix Figure S7: Serological Validation of IA Biomarkers.

A-D) Serum levels of CTSG and PRTN3 were detected via ELISA in cohort III containing 40 R patients, 40 UR patients and 40 healthy individuals (**A, C**) or PRM in cohort I of 212 serum samples (100 NC, 57 UR, 55 R) (**B, D**). Data were analyzed by Mann- Whitney U test. * $P < 0.05$; ** $P < 0.01$; *** $P < 0.001$.
