# Supplementary material for: Circulating proteomic panels for risk stratification of intracranial aneurysm and its rupture
Source: EMBO Mol Med. 2022 Jan 3;14(2):e14713. doi: 10.15252/emmm.202114713 (PMC8819334; doi:10.15252/emmm.202114713)

Source Data For Figure 2A

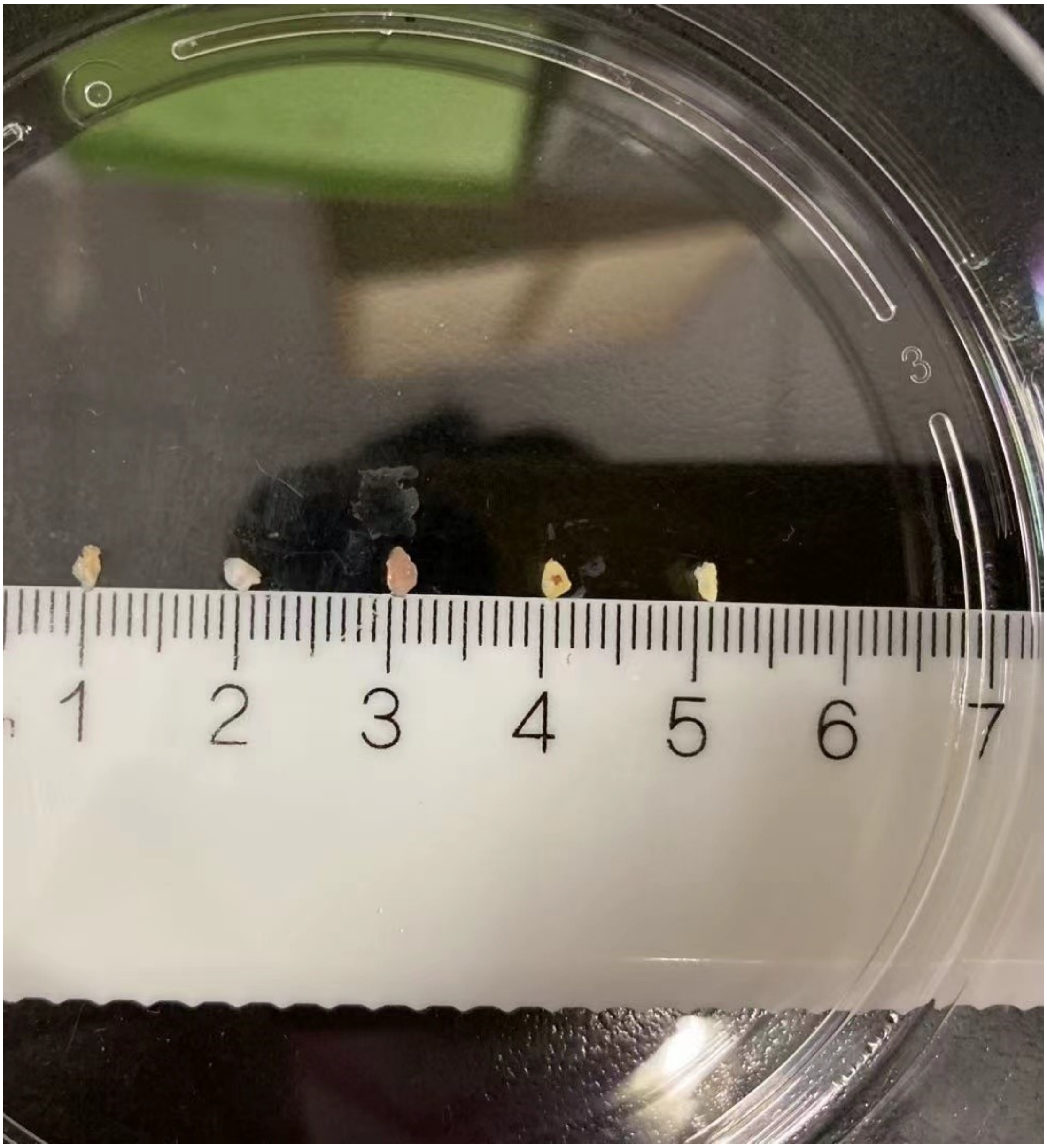

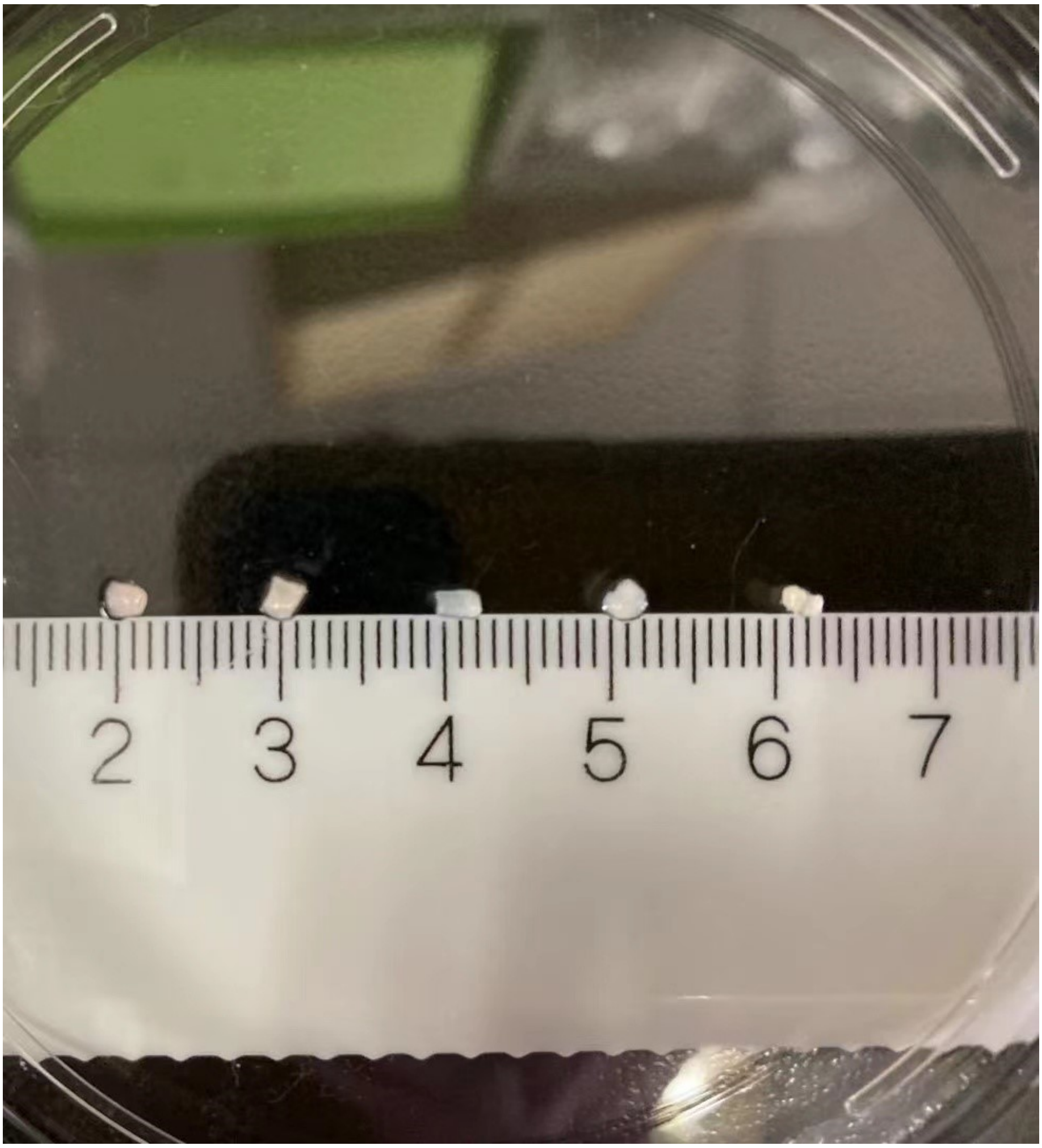
2 3 4 5 6 7

SourceDataForFigure2B							
y expressed	Accession	Uniprot ID	Gene Symbol	Foldchange of IA/STA	Log2(FC)	P-value	Predicted as leakage proteins
1	Q9Y2A7	NCKP1_HUMAN	NCKAP1	0.400826969	-1.318948515	0.005567	Yes
2	P35221	CTNA1_HUMAN	CTNNA1	0.348759776	-1.519694436	0.000326	
3	P06396	GELS_HUMAN	GSN	0.343586163	-1.54125616	0.000636	Yes
4	P14649	MYL6B_HUMAN	MYL6B	0.342751106	-1.544766772	0.0081	Yes
5	P24844	MYL9_HUMAN	MYL9	0.316465315	-1.65988071	0.008604	
6	O60237	MYPT2_HUMAN	PPP1R12B	0.311996321	-1.680399076	0.00382	
7	Q676U5	A16L1_HUMAN	ATG16L1	0.280095655	-1.83600849	0.003406	
8	O43294	TGFI1_HUMAN	TGFB1I1	0.275092456	-1.862011519	0.008193	
9	P09493	TPM1_HUMAN	TPM1	0.273986765	-1.867821892	0.001631	
10	P18206	VINC_HUMAN	VCL	0.265637791	-1.912467689	0.001149	Yes
11	P17661	DESM_HUMAN	DES	0.234398658	-2.092963786	5.09E-06	Yes
12	P12814	ACTN1_HUMAN	ACTN1	0.195691504	-2.353346969	0.00691	Yes
13	P62736	ACTA_HUMAN	ACTA2	0.184132316	-2.441185245	7.43E-05	Yes
14	Q02045	MYL5_HUMAN	MYL5	0.175850549	-2.507578258	1.32E-05	
15	O00401	WASL_HUMAN	WASL	0.129509601	-2.948869039	0.002474	Yes
16	O43707	ACTN4_HUMAN	ACTN4	0.109344836	-3.193043007	7.06E-05	Yes
17	P35609	ACTN2_HUMAN	ACTN2	0.075242696	-3.732304644	0.008447	Yes
18	P35749	MYH11_HUMAN	MYH11	0.051759378	-4.272035917	0.001783	Yes
19	Q9HBL0	TENS1_HUMAN	TNS1	0.024265337	-5.364959311	0.004613	
20	O00151	PDLI1_HUMAN	PDLIM1	0.344334247	-1.53811842	0.001333	Yes
21	O60725	ICMT_HUMAN	ICMT	0.343152459	-1.5430784	0.004152	Yes
22	O75110	ATP9A_HUMAN	ATP9A	0.336345846	-1.571982655	0.008468	Yes
23	A6NMZ7	CO6A6_HUMAN	COL6A6	0.263938928	-1.921723946	0.003662	Yes
24	Q7Z4I7	LIMS2_HUMAN	LIMS2	0.246668228	-2.019356194	0.009928	
25	P39060	COIA1_HUMAN	COL18A1	0.242066952	-2.046521963	0.002156	Yes
26	P39059	COFA1_HUMAN	COL15A1	0.194937726	-2.358914776	4E-06	Yes
27	Q9Y6F6	IRAG1_HUMAN	IRAG1	0.192820676	-2.374668338	0.009074	Yes
28	P60981	DEST_HUMAN	DSTN	0.19082886	-2.389648721	0.000276	Yes
29	Q05707	COEA1_HUMAN	COL14A1	0.160394854	-2.64030024	0.000262	Yes
30	O43293	DAPK3_HUMAN	DAPK3	0.13721218	-2.865519545	0.000163	
31	O75112	LDB3_HUMAN	LDB3	0.131630691	-2.925432188	0.001666	
32	Q5TD97	FHL5_HUMAN	FHL5	0.102427496	-3.287325052	0.002132	
33	Q8IXS6	PALM2_HUMAN	PALM2	0.067459341	-3.889837975	0.009244	
34	Q86UY8	NT5D3_HUMAN	NT5DC3	0.042768113	-4.547320634	8.37E-05	
35	Q96IZ0	PAWR_HUMAN	PAWR	0.35669503	-1.48723698	0.001941	
36	Q8NDA8	MROH1_HUMAN	MROH1	0.208428464	-2.262375784	0.000162	
37	Q9NYN1	RASLC_HUMAN	RASL12	0.091953311	-3.442954659	0.000945	
38	Q13884	SNTB1_HUMAN	SNTB1	0.086958229	-3.523533638	0.00737	
39	Q9BQI4	CCDC3_HUMAN	CCDC3	0.057785688	-4.113143979	0.005948	Yes
40	Q13424	SNTA1_HUMAN	SNTA1	0.055833556	-4.162723742	0.0006	
41	Q9NZQ3	SPN90_HUMAN	NCKIPSD	0.439065358	-1.187492385	0.003621	
42	Q15796	SMAD2_HUMAN	SMAD2	0.400399941	-1.320486333	0.005288	
43	P20339	RAB5A_HUMAN	RAB5A	0.388559062	-1.363794188	0.001321	Yes
44	Q8TDZ2	MICA1_HUMAN	MICAL1	0.327079613	-1.612286258	0.006078	Yes
45	Q9UQR1	ZN148_HUMAN	ZNF148	0.270156938	-1.888130364	0.00871	Yes
46	Q53SF7	COBL1_HUMAN	COBLL1	0.163642751	-2.611378403	0.001768	Yes
47	O95631	NET1_HUMAN	NTN1	0.119327866	-3.06699711	0.005661	Yes
48	Q9NZN4	EHD2_HUMAN	EHD2	0.095663627	-3.3858857	0.000232	Yes
49	Q8IVI9	NOSTN_HUMAN	NOSTRIN	0.053148236	-4.233834372	3.55E-05	
50	Q8N3P4	VPS8_HUMAN	VPS8	0.309693688	-1.691086118	0.009048	
51	Q12792	TWF1_HUMAN	TWF1	0.268813593	-1.895322001	6.26E-06	
52	P09525	ANXA4_HUMAN	ANXA4	0.259670689	-1.945244917	6.33E-05	Yes
53	Q16832	DDR2_HUMAN	DDR2	0.25019877	-1.9988534	0.000139	Yes
54	Q9NRY4	RHG35_HUMAN	ARHGAP35	0.229943644	-2.120647773	0.002309	
55	P10301	RRAS_HUMAN	RRAS	0.18528118	-2.432211746	0.00276	Yes
56	P21333	FLNA_HUMAN	FLNA	0.134365756	-2.895762594	4.15E-05	Yes
57	O94875	SRBS2_HUMAN	SORBS2	0.125919622	-2.989424975	0.000428	
58	O43157	PLXB1_HUMAN	PLXNB1	0.106133527	-3.236047632	0.006332	Yes
59	Q8NF91	SYNE1_HUMAN	SYNE1	0.157986427	-2.662127474	0.000263	Yes
60	P21291	CSRP1_HUMAN	CSRP1	0.153160607	-2.706882813	0.000403	Yes
61	Q6XZF7	DNMBP_HUMAN	DNMBP	0.148405615	-2.752382417	0.000515	Yes
62	Q14315	FLNC_HUMAN	FLNC	0.133403132	-2.906135555	2.25E-05	
63	Q9H492	MLP3A_HUMAN	MAP1LC3A	0.348487992	-1.520819149	0.001137	
64	P42166	LAP2A_HUMAN	TMPO	0.341688537	-1.549246244	0.008455	
65	Q3YEC7	RABL6_HUMAN	RABL6	0.315522552	-1.66418497	0.002521	
66	Q8IVF7	FMNL3_HUMAN	FMNL3	0.312992583	-1.675799623	0.003213	Yes
67	Q96PE2	ARHGH_HUMAN	ARHGEF17	0.311727761	-1.681641454	0.003297	
68	Q92629	SGCD_HUMAN	SGCD	0.294005942	-1.76608278	0.000323	Yes
69	Q9HCE6	ARGAL_HUMAN	ARHGEF10L	0.292375078	-1.774107754	0.00187	
70	Q5VT25	MRCKA_HUMAN	CDC42BPA	0.263460758	-1.924340001	0.000818	Yes
71	Q16585	SGCB_HUMAN	SGCB	0.236543278	-2.079823931	0.004196	
72	O43556	SGCE_HUMAN	SGCE	0.222190957	-2.170127992	8.13E-05	Yes
73	P51911	CNN1_HUMAN	CNN1	0.187280367	-2.416728428	0.004314	
74	Q7Z7B0	FLIP1_HUMAN	FILIP1	0.182561652	-2.453544342	0.005471	
75	Q9BR76	COR1B_HUMAN	CORO1B	0.154626572	-2.693139836	0.000525	Yes
76	P28906	CD34_HUMAN	CD34	0.145412997	-2.781771869	0.00021	Yes
77	Q9Y281	COF2_HUMAN	CFL2	0.119444674	-3.065585573	0.003966	Yes
78	Q13425	SNTB2_HUMAN	SNTB2	0.105038404	-3.251011199	0.001125	
79	Q9BR39	JPH2_HUMAN	JPH2	0.083167088	-3.58784347	6.16E-05	Yes
80	Q9Y4J8	DTNA_HUMAN	DTNA	0.064730786	-3.949404176	3.02E-05	

81	Q9UM07	PADI4_HUMAN	PADI4	0.434753726	-1.201729702	0.007018	
82	Q9H857	NT5D2_HUMAN	NT5DC2	0.351581811	-1.508067659	0.003193	
83	Q8NB37	GALD1_HUMAN	GATD1	0.349865247	-1.515128731	0.000757	Yes
84	Q68EM7	RHG17_HUMAN	ARHGAP17	0.343761866	-1.540518582	0.009111	
85	Q6NZI2	CAVN1_HUMAN	CAVIN1	0.220739232	-2.179585031	0.00036	Yes
86	Q96Q05	TPPC9_HUMAN	TRAPPC9	0.146728589	-2.768778093	0.004714	Yes
87	Q6SZW1	SARM1_HUMAN	SARM1	0.07004766	-3.835519335	0.000765	
88	Q15124	PGM5_HUMAN	PGM5	0.014561841	-6.101663448	0.001482	
89	P09619	PGFRB_HUMAN	PDGFRB	0.461857006	-1.114481843	0.00041	Yes
90	O43639	NCK2_HUMAN	NCK2	0.447475104	-1.160120678	0.000715	Yes
91	P42025	ACTY_HUMAN	ACTR1B	0.398288595	-1.328113926	0.000303	Yes
92	O00291	HIP1_HUMAN	HIP1	0.320590021	-1.641198577	3.62E-05	Yes
93	Q96RF0	SNX18_HUMAN	SNX18	0.320101408	-1.643399074	0.000135	Yes
94	Q5VZK9	CARL1_HUMAN	CARMIL1	0.31520591	-1.66563351	0.000735	Yes
95	Q9UKS6	PACN3_HUMAN	PACSIN3	0.277798076	-1.847891488	0.000118	Yes
96	P00533	EGFR_HUMAN	EGFR	0.252576222	-1.98520927	0.000298	Yes
97	Q9Y5X1	SNX9_HUMAN	SNX9	0.18797408	-2.411394354	0.000208	Yes
98	Q99972	MYOC_HUMAN	MYOC	0.141513475	-2.820988661	0.002493	Yes
99	P09960	LKHA4_HUMAN	LTA4H	0.392764243	-1.348264502	0.003234	Yes
100	Q9NVD7	PARVA_HUMAN	PARVA	0.361480276	-1.468011166	0.001736	
101	P07996	TSP1_HUMAN	THBS1	0.347459292	-1.525084132	0.000415	Yes
102	O75382	TRIM3_HUMAN	TRIM3	0.248702917	-2.007504667	0.004874	Yes
103	P26022	PTX3_HUMAN	PTX3	0.15504984	-2.68919606	0.000575	Yes
104	P98160	PGBM_HUMAN	HSPG2	0.149402797	-2.742720938	0.007029	Yes
105	Q7Z7G0	TARSH_HUMAN	ABI3BP	0.103283177	-3.275322813	1.24E-05	Yes
106	Q96AC1	FERM2_HUMAN	FERMT2	0.067178845	-3.895849194	0.001805	
107	Q96DB5	RMD1_HUMAN	RMDN1	0.461367427	-1.116011944	0.000367	
108	Q5T5P2	SKT_HUMAN	KIAA1217	0.366306254	-1.448877762	0.008907	
109	P22105	TENX_HUMAN	TNXB	0.336911514	-1.569558361	0.001754	Yes
110	Q6QEF8	CORO6_HUMAN	CORO6	0.325683085	-1.618459303	0.001826	
111	O00468	AGRIN_HUMAN	AGRN	0.252350173	-1.986501016	0.000791	Yes
112	Q13332	PTPRS_HUMAN	PTPRS	0.170150513	-2.555116597	0.001684	Yes
113	Q6P0A1	F180B_HUMAN	FAM180B	0.155743342	-2.682757607	0.001676	Yes
114	P02462	CO4A1_HUMAN	COL4A1	0.095206922	-3.392789724	0.004714	Yes
115	Q9HB63	NET4_HUMAN	NTN4	0.09481182	-3.398789264	0.00072	Yes
116	Q9HCB6	SPON1_HUMAN	SPON1	0.084761806	-3.560441855	0.004574	Yes
117	Q96AM1	MRGRF_HUMAN	MRGPRF	0.084205221	-3.569946501	0.00418	
118	Q13683	ITA7_HUMAN	ITGA7	0.082085437	-3.606729906	0.001031	
119	O43813	LANC1_HUMAN	LANCL1	0.079151384	-3.659241616	1.19E-06	
120	Q8IVN8	SBSPO_HUMAN	SBSPON	0.015314871	-6.028922967	0.005437	Yes
121	Q99720	SGMR1_HUMAN	SIGMAR1	0.351548838	-1.508202969	0.006959	Yes
122	Q86SQ0	PHLB2_HUMAN	PHLDB2	0.317529587	-1.65503707	0.009242	
123	Q9Y5A9	YTHD2_HUMAN	YTHDF2	0.26467033	-1.917731619	0.003299	
124	Q8N474	SFRP1_HUMAN	SFRP1	0.06559172	-3.930342473	0.008525	Yes
125	Q9UKG1	DP13A_HUMAN	APPL1	0.384345803	-1.37952318	8.07E-05	Yes
126	O00292	LFTY2_HUMAN	LEFTY2	0.359460462	-1.476095002	0.005885	Yes
127	Q9BXJ8	TACAN_HUMAN	TMEM120A	0.357200985	-1.485192035	0.004773	
128	P55268	LAMB2_HUMAN	LAMB2	0.343992372	-1.539551523	0.00753	Yes
129	Q5JT25	RAB41_HUMAN	RAB41	0.324653066	-1.623029262	0.000653	Yes
130	O60645	EXOC3_HUMAN	EXOC3	0.317320521	-1.65598727	0.002182	Yes
131	Q9H4A4	AMPB_HUMAN	RNPEP	0.316659686	-1.658994885	0.000808	Yes
132	Q9H3U1	UN45A_HUMAN	UNC45A	0.248125848	-2.010856063	0.004164	Yes
133	O14791	APOL1_HUMAN	APOL1	0.178167	-2.488697948	0.001784	Yes
134	P08651	NFIC_HUMAN	NFIC	0.17496321	-2.514876504	0.002161	
135	Q86UP2	KTN1_HUMAN	KTN1	0.172558267	-2.534844503	0.002179	Yes
136	Q9BX10	GTPB2_HUMAN	GTPBP2	0.165149823	-2.598152675	0.000231	Yes
137	Q93084	AT2A3_HUMAN	ATP2A3	0.148295723	-2.753451106	0.000349	Yes
138	P50895	BCAM_HUMAN	BCAM	0.145850831	-2.777434489	0.001048	Yes
139	O60499	STX10_HUMAN	STX10	0.139239742	-2.844357048	0.001884	Yes
140	O76024	WFS1_HUMAN	WFS1	0.131642852	-2.925298911	0.0006	Yes
141	P17152	TMM11_HUMAN	TMEM11	0.127795866	-2.968086925	0.002244	
142	Q9BZE9	ASPC1_HUMAN	ASPSCR1	0.12380527	-3.013855368	0.004084	
143	P24593	IBP5_HUMAN	IGFBP5	0.113031361	-3.145204989	0.002402	Yes
144	Q6UXV4	MIC27_HUMAN	APOOL	0.099736913	-3.325728635	0.005112	Yes
145	P13521	SCG2_HUMAN	SCG2	0.097037706	-3.365310747	0.001539	Yes
146	P48449	LSS_HUMAN	LSS	0.41546817	-1.267190141	0.00926	Yes
147	P08133	ANXA6_HUMAN	ANXA6	0.338748044	-1.561715477	0.000251	Yes
148	O95183	VAMP5_HUMAN	VAMP5	0.31637743	-1.660281414	0.001625	Yes
149	Q9UBY9	HSPB7_HUMAN	HSPB7	0.294794267	-1.762219625	0.002788	
150	O94851	MICA2_HUMAN	MICAL2	0.264797041	-1.917041096	0.000195	
151	Q96AG3	S2546_HUMAN	SLC25A46	0.244810849	-2.030260601	0.001457	
152	O76062	ERG24_HUMAN	TM7SF2	0.23787351	-2.071733477	0.003737	Yes
153	O00182	LEG9_HUMAN	LGALS9	0.236259504	-2.081555729	0.000756	Yes
154	O75751	S22A3_HUMAN	SLC22A3	0.220729022	-2.179651761	0.004758	
155	P49747	COMP_HUMAN	COMP	0.214224034	-2.22280775	0.004405	Yes
156	Q9HCH5	SYTL2_HUMAN	SYTL2	0.20117181	-2.31349994	0.00888	
157	P43121	MUC18_HUMAN	MCAM	0.186081272	-2.425995229	0.002786	Yes
158	Q92599	SEPT8_HUMAN	SEPTIN8	0.468916179	-1.092598039	0.001353	
159	Q92843	B2CL2_HUMAN	BCL2L2	0.413097687	-1.275445113	9.82E-05	
160	Q9BTE6	AASD1_HUMAN	AARSD1	0.369454339	-1.436532021	0.003521	
161	Q15035	TRAM2_HUMAN	TRAM2	0.279618694	-1.83846728	0.001382	Yes
162	Q8N3D4	EH1L1_HUMAN	EHBP1L1	0.260819084	-1.938878658	0.006482	

163	O00339	MATN2_HUMAN	MATN2	0.163130854	-2.615898417	0.005441	Yes
164	Q9P0V9	SEP10_HUMAN	SEPTIN10	0.108503515	-3.204186314	6.86E-06	
165	P78356	PI42B_HUMAN	PIP4K2B	0.460256927	-1.11948866	0.000118	Yes
166	Q9P291	ARMX1_HUMAN	ARMCX1	0.348517447	-1.520697217	0.004154	
167	Q99969	RARR2_HUMAN	RARRES2	0.214522667	-2.220797998	0.002598	Yes
168	Q92575	UBXN4_HUMAN	UBXN4	0.177280275	-2.495896073	0.000264	Yes
169	Q9Y646	CBPQ_HUMAN	CPQ	0.169891615	-2.557313441	0.001294	Yes
170	Q9ULV4	COR1C_HUMAN	CORO1C	0.163372447	-2.613763407	7.48E-05	
171	Q7KYR7	BT2A1_HUMAN	BTN2A1	0.113492399	-3.139332414	0.001597	Yes
172	P19075	TSN8_HUMAN	TSPAN8	0.035190436	-4.828672792	0.000962	Yes
173	Q6L8Q7	PDE12_HUMAN	PDE12	0.388898824	-1.362533221	0.003255	
174	Q9BWH2	FUND2_HUMAN	FUNDC2	0.376756146	-1.408297048	0.002195	
175	Q7Z5L7	PODN_HUMAN	PODN	0.287391033	-1.798913049	0.000429	Yes
176	Q15848	ADIPO_HUMAN	ADIPOQ	0.286725936	-1.802255685	0.002287	Yes
177	Q99707	METH_HUMAN	MTR	0.284207521	-1.814983361	0.007123	
178	O60493	SNX3_HUMAN	SNX3	0.228502145	-2.129720384	0.001164	Yes
179	O43414	ERI3_HUMAN	ERI3	0.215482191	-2.214359454	0.001362	
180	O75339	CILP1_HUMAN	CILP	0.209354428	-2.255980665	0.001269	Yes
181	P54821	PRRX1_HUMAN	PRRX1	0.189994513	-2.395970337	0.003431	
182	Q5TGL8	PXDC1_HUMAN	PXDC1	0.16703559	-2.581772566	0.000595	
183	Q9NR12	PDLI7_HUMAN	PDLIM7	0.150305678	-2.734028582	0.001887	
184	Q8WWA0	ITLN1_HUMAN	ITLN1	0.102078475	-3.292249411	0.000163	Yes
185	P35070	BTC_HUMAN	BTC	0.459431682	-1.122077747	0.009449	Yes
186	Q01973	ROR1_HUMAN	ROR1	0.298349209	-1.744926141	0.00667	Yes
187	P32004	L1CAM_HUMAN	L1CAM	0.047609493	-4.392606938	0.001518	Yes
188	Q13510	ASAH1_HUMAN	ASAH1	0.453374251	-1.141225639	0.006324	Yes
189	Q99714	HCD2_HUMAN	HSD17B10	0.380616974	-1.393588192	0.004256	
190	P43155	CACP_HUMAN	CRAT	0.341226619	-1.551197899	0.004142	Yes
191	P09110	THIK_HUMAN	ACAA1	0.301115449	-1.731611364	0.003376	Yes
192	Q16836	HCDH_HUMAN	HADH	0.180038229	-2.473624818	0.000534	
193	Q06136	KDSR_HUMAN	KDSR	0.146897874	-2.767114581	0.002253	Yes
194	P40939	ECHA_HUMAN	HADHA	0.065049948	-3.942308287	0.006274	
195	O75874	IDHC_HUMAN	IDH1	0.055429031	-4.173214392	3.51E-05	Yes
196	Q9H1E5	TMX4_HUMAN	TMX4	0.418841251	-1.255524558	0.006044	Yes
197	P09417	DHPR_HUMAN	QDPR	0.360616906	-1.471461062	0.008032	Yes
198	Q8N436	CPXM2_HUMAN	CPXM2	0.296636528	-1.753231831	0.007839	Yes
199	Q8N0X4	CLYBL_HUMAN	CLYBL	0.288341668	-1.794148761	0.00055	
200	Q86YB7	ECHD2_HUMAN	ECHDC2	0.172210165	-2.53775779	0.005858	
201	Q96Q06	PLIN4_HUMAN	PLIN4	0.166821918	-2.583619246	0.007051	
202	O95562	SFT2B_HUMAN	SFT2D2	0.162675065	-2.619934969	1.83E-05	Yes
203	P15088	CBPA3_HUMAN	CPA3	0.021707374	-5.525670949	0.005405	Yes
204	Q96QR8	PURB_HUMAN	PURB	0.267433265	-1.902749166	0.003016	
205	Q13439	GOGA4_HUMAN	GOLGA4	0.410849446	-1.283318275	0.001292	Yes
206	Q15043	S39AE_HUMAN	SLC39A14	0.314623073	-1.668303618	0.000121	Yes
207	O75131	CPNE3_HUMAN	CPNE3	0.28793855	-1.796167142	0.00034	Yes
208	P51687	SUOX_HUMAN	SUOX	0.189269529	-2.401485928	0.007091	
209	P27216	ANX13_HUMAN	ANXA13	0.123066925	-3.022485015	0.001919	Yes
210	Q6VEQ5	WASH2_HUMAN	WASH2P	0.369388745	-1.436788185	0.008147	
211	Q04760	LGUL_HUMAN	GLO1	0.285578983	-1.808038285	0.002582	Yes
212	P04424	ARLY_HUMAN	ASL	0.268211514	-1.898556923	0.006354	Yes
213	O14880	MGST3_HUMAN	MGST3	0.233259609	-2.099991583	0.002536	Yes
214	Q969Z3	MARC2_HUMAN	MTARC2	0.115918638	-3.108815547	0.003557	
215	P11217	PYGM_HUMAN	PYGM	0.057443025	-4.121724475	0.000682	Yes
216	Q16881	TRXR1_HUMAN	TXNRD1	0.456953884	-1.129879519	0.005428	Yes
217	Q9NNW7	TRXR2_HUMAN	TXNRD2	0.426471397	-1.229479109	0.006622	
218	P21912	SDHB_HUMAN	SDHB	0.403146534	-1.310623777	0.0085	
219	Q99798	ACON_HUMAN	ACO2	0.377194537	-1.406619315	0.001605	
220	Q3ZCQ8	TIM50_HUMAN	TIMM50	0.357178262	-1.485283813	0.007009	
221	P09622	DLDH_HUMAN	DLD	0.342689345	-1.545026758	0.002809	
222	P07919	QCR6_HUMAN	UQCRH	0.328688496	-1.605207132	0.002204	
223	P51553	IDH3G_HUMAN	IDH3G	0.326639065	-1.61423075	0.008401	
224	P05166	PCCB_HUMAN	PCCB	0.326534207	-1.614693963	0.005851	
225	P36957	ODO2_HUMAN	DLST	0.321599471	-1.636663061	0.004936	
226	P06576	ATPB_HUMAN	ATP5F1B	0.314193432	-1.670275072	0.008803	Yes
227	P11177	ODPB_HUMAN	PDHB	0.308999883	-1.694321803	0.00243	
228	Q96EY8	MMAB_HUMAN	MMAB	0.304003311	-1.717841059	0.002566	
229	P28331	NDUS1_HUMAN	NDUFS1	0.286966596	-1.801045284	0.002954	
230	P31930	QCR1_HUMAN	UQCRC1	0.28239678	-1.824204458	0.000803	
231	Q86TU7	SETD3_HUMAN	SETD3	0.267624756	-1.901716519	0.000668	
232	P00367	DHE3_HUMAN	GLUD1	0.263290546	-1.925272378	0.000197	
233	P10515	ODP2_HUMAN	DLAT	0.213347033	-2.228726048	0.000266	
234	Q8N5M1	ATPF2_HUMAN	ATPAF2	0.209810846	-2.252838836	0.000324	
235	P08559	ODPA_HUMAN	PDHA1	0.201796389	-2.309027736	0.000188	
236	Q96I99	SUCB2_HUMAN	SUCLG2	0.176435796	-2.502784806	0.005206	
237	P49753	ACOT2_HUMAN	ACOT2	0.173711855	-2.525231881	7.71E-05	
238	Q9P2R7	SUCB1_HUMAN	SUCLA2	0.158239392	-2.659819309	0.00023	Yes
239	O95299	NDUAA_HUMAN	NDUFA10	0.116841641	-3.09737357	0.004666	
240	Q9BZQ8	NIBA1_HUMAN	NIBAN1	0.08618448	-3.5364281	2.65E-05	Yes
241	P49321	NASP_HUMAN	NASP	0.079363384	-3.655382655	3.59E-05	
242	Q15120	PDK3_HUMAN	PDK3	0.042106637	-4.569808525	0.006387	
243	P49189	AL9A1_HUMAN	ALDH9A1	0.466549122	-1.099899108	0.000223	Yes
244	Q9C0E8	LNP_HUMAN	LNPK	0.408326948	-1.292203311	0.000634	Yes

245	Q9NUB1	ACS2L_HUMAN	ACSS1	0.327331367	-1.61117624	0.001492	
246	Q92890	UFD1_HUMAN	UFD1	0.303278161	-1.721286482	0.001891	Yes
247	P51648	AL3A2_HUMAN	ALDH3A2	0.2852721	-1.80958944	0.003214	Yes
248	O95841	ANGL1_HUMAN	ANGPTL1	0.278554028	-1.843970917	0.000787	Yes
249	Q9Y3D6	FIS1_HUMAN	FIS1	0.245874541	-2.024005736	0.008553	Yes
250	P31937	3HIDH_HUMAN	HIBADH	0.240455757	-2.056156625	0.000723	
251	P55809	SCOT1_HUMAN	OXCT1	0.239752199	-2.060384046	0.000466	
252	P50995	ANX11_HUMAN	ANXA11	0.236088421	-2.082600807	0.005944	Yes
253	Q9UJ70	NAGK_HUMAN	NAGK	0.231390075	-2.111601108	0.000516	Yes
254	Q01813	PFKAP_HUMAN	PFKP	0.224221264	-2.157004991	0.000655	Yes
255	P42126	ECI1_HUMAN	ECI1	0.212393641	-2.235187524	0.003208	
256	O15382	BCAT2_HUMAN	BCAT2	0.185794521	-2.42822014	0.001409	
257	Q02252	MMSA_HUMAN	ALDH6A1	0.162482084	-2.621647443	0.000539	
258	Q9NVH6	TMLH_HUMAN	TMLHE	0.148183527	-2.754543015	0.002679	
259	P08237	PFKAM_HUMAN	PFKM	0.146465238	-2.771369803	3.24E-06	
260	P05091	ALDH2_HUMAN	ALDH2	0.122798253	-3.025638063	0.000927	Yes
261	Q6NVY1	HIBCH_HUMAN	HIBCH	0.116184352	-3.105512314	0.000149	
262	P00325	ADH1B_HUMAN	ADH1B	0.089584733	-3.480603307	0.002492	
263	Q8WUY3	PRUN2_HUMAN	PRUNE2	0.081527018	-3.616577944	0.007162	
264	P30837	AL1B1_HUMAN	ALDH1B1	0.04154129	-4.589310158	0.000604	
265	P09172	DOPO_HUMAN	DBH	0.020746897	-5.590960597	0.005234	Yes
266	Q8NFW8	NEUA_HUMAN	CMAS	0.392151056	-1.350518608	0.008905	
267	A1A4S6	RHG10_HUMAN	ARHGAP10	0.369671491	-1.435684309	0.006916	
268	P35612	ADDB_HUMAN	ADD2	0.292857609	-1.771728715	0.000168	
269	Q93034	CUL5_HUMAN	CUL5	0.260745535	-1.939285549	0.005862	
270	P24310	CX7A1_HUMAN	COX7A1	0.235736478	-2.084753073	0.006122	
271	Q9NQE9	HINT3_HUMAN	HINT3	0.211218235	-2.243193704	0.001704	
272	Q9Y6C2	EMIL1_HUMAN	EMILIN1	0.146164833	-2.774331852	0.002109	Yes
273	Q9BQ24	ZFY21_HUMAN	ZFYVE21	0.124942725	-3.000661198	0.000239	
274	Q14938	NFIX_HUMAN	NFIX	0.098763382	-3.339879954	1.57E-06	
275	Q6YN16	HSDL2_HUMAN	HSDL2	0.094084068	-3.409905748	0.000198	
276	Q9BY89	K1671_HUMAN	KIAA1671	0.093936781	-3.412166027	0.001826	
277	Q9NRG7	D39U1_HUMAN	SDR39U1	0.058104355	-4.105209897	0.001771	
278	Q96JJ3	ELMO2_HUMAN	ELMO2	0.468790527	-1.092984678	0.003072	
279	O00635	TRI38_HUMAN	TRIM38	0.245240852	-2.027728775	0.002285	
280	P62140	PP1B_HUMAN	PPP1CB	0.184473534	-2.438514244	0.001237	Yes
281	O00629	IMA3_HUMAN	KPNA4	0.438225434	-1.190254877	0.001663	
282	Q9UL54	TAOK2_HUMAN	TAOK2	0.374366121	-1.417478214	0.00608	
283	Q13107	UBP4_HUMAN	USP4	0.36827314	-1.441151918	0.004573	
284	O00505	IMA4_HUMAN	KPNA3	0.350190231	-1.513789254	4.87E-05	
285	O60282	KIF5C_HUMAN	KIF5C	0.326322897	-1.615627873	0.007931	
286	P07196	NFL_HUMAN	NEFL	0.235673225	-2.085140234	0.001218	
287	Q9UK99	FBX3_HUMAN	FBXO3	0.21105831	-2.244286459	0.008901	
288	Q13043	STK4_HUMAN	STK4	0.148225646	-2.754133012	1.72E-06	
289	O14958	CASQ2_HUMAN	CASQ2	0.026009579	-5.264813159	0.000577	Yes
290	P17987	TCPA_HUMAN	TCP1	0.444324215	-1.170315327	0.001376	Yes
291	Q9H4B7	TBB1_HUMAN	TUBB1	0.376663166	-1.408653135	0.002467	Yes
292	Q14152	EIF3A_HUMAN	EIF3A	0.360561423	-1.471683047	0.000309	
293	Q5JSL3	DOC11_HUMAN	DOCK11	0.313513632	-1.673399921	0.008662	
294	P40227	TCPZ_HUMAN	CCT6A	0.307257039	-1.702482034	0.000314	Yes
295	P50990	TCPQ_HUMAN	CCT8	0.29754163	-1.748836559	0.000489	Yes
296	Q9BTW9	TBCD_HUMAN	TBCD	0.276180177	-1.856318319	0.002234	
297	Q9H2J4	PDCL3_HUMAN	PDCL3	0.240998232	-2.052905534	0.004149	Yes
298	B5ME19	EIFCL_HUMAN	EIF3CL	0.24025686	-2.05735047	0.002352	
299	Q9BQJ4	TMM47_HUMAN	TMEM47	0.235215194	-2.087946837	0.000841	
300	Q9Y2T2	AP3M1_HUMAN	AP3M1	0.232604385	-2.104049802	0.002264	Yes
301	Q9BVA1	TBB2B_HUMAN	TUBB2B	0.156587263	-2.674961224	0.000554	
302	P60510	PP4C_HUMAN	PPP4C	0.447736577	-1.159277915	0.008824	
303	Q9H2G2	SLK_HUMAN	SLK	0.374100699	-1.418501434	0.002017	Yes
304	Q8IXM6	NRM_HUMAN	NRM	0.252592647	-1.985115451	0.004509	
305	Q93062	RBPMS_HUMAN	RBPMS	0.198572096	-2.332265187	2.34E-05	
306	Q6UWY5	OLFL1_HUMAN	OLFML1	0.177223742	-2.496356208	0.00139	Yes
307	Q96N67	DOCK7_HUMAN	DOCK7	0.445706744	-1.165833303	0.000776	
308	P62068	UBP46_HUMAN	USP46	0.406204014	-1.299723598	0.004605	
309	Q9UMR2	DD19B_HUMAN	DDX19B	0.355519212	-1.492000572	0.002805	Yes
310	Q9BTV4	TMM43_HUMAN	TMEM43	0.313461462	-1.67364001	0.000567	Yes
311	A9UHW6	MI4GD_HUMAN	MIF4GD	0.254417632	-1.974729438	0.000698	Yes
312	Q4L180	FIL1L_HUMAN	FILIP1L	0.169895843	-2.557277542	0.002275	
313	Q8TAF3	WDR48_HUMAN	WDR48	0.160555323	-2.638857599	0.003088	
314	Q9Y4F5	C170B_HUMAN	CEP170B	0.132790154	-2.912779915	0.003901	
315	P40123	CAP2_HUMAN	CAP2	0.071604366	-3.803808623	0.000128	
316	O95260	ATE1_HUMAN	ATE1	0.064702748	-3.950029195	0.000638	
317	Q12816	TROP_HUMAN	TRO	0.283747353	-1.817321163	0.004073	
318	Q7Z3D6	GLUCM_HUMAN	DGLUCY	0.281364203	-1.829489303	0.00454	
319	Q86TV6	TTC7B_HUMAN	TTC7B	0.257115269	-1.959512805	0.004703	
320	Q6ZMZ3	SYNE3_HUMAN	SYNE3	0.25629478	-1.964123998	0.00098	Yes
321	Q13075	BIRC1_HUMAN	NAIP	0.256193265	-1.964695548	8.15E-05	
322	A1L390	PKHG3_HUMAN	PLEKHG3	0.208297267	-2.263284182	0.001926	
323	Q9UPT6	JIP3_HUMAN	MAPK8IP3	0.126527349	-2.982478841	0.000427	Yes
324	Q9Y2J2	E41L3_HUMAN	EPB41L3	0.104931405	-3.252481572	0.008731	
325	Q9NUJ3	T11L1_HUMAN	TCP11L1	0.294723197	-1.762567478	0.001295	
326	Q7RTP6	MICA3_HUMAN	MICAL3	0.277283719	-1.850565187	0.002432	Yes

327	O75534	CSDE1_HUMAN	CSDE1	0.24360513	-2.037383581	0.005623	Yes
328	O94856	NFASC_HUMAN	NFASC	0.204182256	-2.292070599	0.004429	Yes
329	Q5TBA9	FRY_HUMAN	FRY	0.19713728	-2.342727472	0.000748	
330	Q96GK7	FAH2A_HUMAN	FAHD2A	0.192007848	-2.380762818	0.005046	
331	Q6UWP7	LCLT1_HUMAN	LCLAT1	0.181408791	-2.462683727	0.004072	Yes
332	P46736	BRCC3_HUMAN	BRCC3	0.443892644	-1.171717294	0.005841	
333	Q9H8L6	MMRN2_HUMAN	MMRN2	0.422373132	-1.24341003	0.000235	Yes
334	Q9BUL8	PDC10_HUMAN	PDCD10	0.402035001	-1.314606988	0.00056	Yes
335	Q5HYK3	COQ5_HUMAN	COQ5	0.259270847	-1.947468101	0.005799	
336	Q5VSL9	STRP1_HUMAN	STRIP1	0.240587489	-2.055366472	0.003194	Yes
337	Q9P289	STK26_HUMAN	STK26	0.220133807	-2.183547372	0.000253	Yes
338	Q13033	STRN3_HUMAN	STRN3	0.194422961	-2.362729487	0.001657	Yes
339	Q6P1X6	CH082_HUMAN	C8orf82	0.176967023	-2.498447546	0.006687	
340	O76070	SYUG_HUMAN	SNCG	0.161264393	-2.632500171	0.000209	Yes
341	Q9NRL3	STRN4_HUMAN	STRN4	0.138820989	-2.848702386	0.002045	
342	Q63ZY3	KANK2_HUMAN	KANK2	0.133745657	-2.902436055	0.00028	
343	Q14BN4	SLMAP_HUMAN	SLMAP	0.131781389	-2.923781456	0.000271	Yes
344	Q99689	FEZ1_HUMAN	FEZ1	0.112042865	-3.157877318	0.000266	Yes
345	Q02790	FKBP4_HUMAN	FKBP4	0.39703152	-1.33267455	0.000832	Yes
346	Q96FJ2	DYL2_HUMAN	DYNLL2	0.324987225	-1.621545088	0.001479	
347	Q9NTI5	PDS5B_HUMAN	PDS5B	0.278623361	-1.843611868	0.009741	
348	O75694	NU155_HUMAN	NUP155	0.261877799	-1.933034339	0.001611	
349	Q9UQ13	SHOC2_HUMAN	SHOC2	0.253587491	-1.979444516	0.00022	
350	Q9NS87	KIF15_HUMAN	KIF15	0.236191616	-2.08197034	0.000948	
351	Q8TDX7	NEK7_HUMAN	NEK7	0.236124883	-2.082378015	0.000404	
352	Q8TD57	DYH3_HUMAN	DNAH3	0.231251074	-2.112468026	0.004392	
353	P51808	DYLT3_HUMAN	DYNLT3	0.224415746	-2.15575419	0.001566	
354	O43237	DC1L2_HUMAN	DYNC1LI2	0.222437931	-2.16852527	0.004022	
355	Q8NFH4	NUP37_HUMAN	NUP37	0.195123196	-2.357542803	0.00904	
356	Q9NPY3	C1QR1_HUMAN	CD93	0.117940486	-3.083869051	0.00124	Yes
357	O14576	DC1I1_HUMAN	DYNC1I1	0.058256187	-4.101444918	0.00392	
358	Q9UKV8	AGO2_HUMAN	AGO2	0.451449754	-1.147362668	1.27E-05	Yes
359	Q92608	DOCK2_HUMAN	DOCK2	0.351964827	-1.506496833	0.005921	Yes
360	Q7KZF4	SND1_HUMAN	SND1	0.338140466	-1.564305416	0.00126	Yes
361	P60660	MYL6_HUMAN	MYL6	0.272240037	-1.877048843	0.000797	Yes
362	O14744	ANM5_HUMAN	PRMT5	0.232140446	-2.106930189	0.007728	Yes
363	P62834	RAP1A_HUMAN	RAP1A	0.22998777	-2.120370951	0.002777	Yes
364	O15294	OGT1_HUMAN	OGT	0.171246514	-2.545853474	0.000789	
365	Q9BX66	SRBS1_HUMAN	SORBS1	0.138377974	-2.85331377	0.000419	
366	Q01518	CAP1_HUMAN	CAP1	0.461144619	-1.116708831	0.001469	Yes
367	Q99536	VAT1_HUMAN	VAT1	0.44545358	-1.166652995	0.006134	Yes
368	Q13618	CUL3_HUMAN	CUL3	0.419786159	-1.252273496	0.000442	Yes
369	O95817	BAG3_HUMAN	BAG3	0.389399846	-1.360675782	0.008705	
370	Q9UBF6	RBX2_HUMAN	RNF7	0.350217504	-1.513676902	0.001316	
371	Q05086	UBE3A_HUMAN	UBE3A	0.306027346	-1.708267521	0.009916	
372	P07355	ANXA2_HUMAN	ANXA2	0.293278817	-1.769655221	0.001428	Yes
373	P46934	NEDD4_HUMAN	NEDD4	0.28182961	-1.8271049	0.009298	Yes
374	P00387	NB5R3_HUMAN	CYB5R3	0.246911434	-2.017934448	0.002986	Yes
375	Q15139	KPCD1_HUMAN	PRKD1	0.176613504	-2.501332436	0.002949	Yes
376	P11216	PYGB_HUMAN	PYGB	0.134132692	-2.898267185	3.86E-05	Yes
377	Q05655	KPCD_HUMAN	PRKCD	0.041570317	-4.588302451	0.002643	Yes
378	Q14432	PDE3A_HUMAN	PDE3A	0.277446437	-1.849718821	0.002062	
379	Q9UMS6	SYNP2_HUMAN	SYNPO2	0.144503337	-2.790825282	0.004956	
380	Q12791	KCMA1_HUMAN	KCNMA1	0.080037074	-3.643187758	0.002418	
381	O75380	NDUS6_HUMAN	NDUFS6	0.424324938	-1.236758624	0.007081	
382	P56556	NDUA6_HUMAN	NDUFA6	0.393461232	-1.345706602	0.007569	
383	P49821	NDUV1_HUMAN	NDUFV1	0.290565877	-1.783062806	0.00902	
384	O94826	TOM70_HUMAN	TOMM70	0.170184202	-2.554830976	0.004318	Yes
385	P12235	ADT1_HUMAN	SLC25A4	0.16834435	-2.570512794	0.006175	
386	Q14318	FKBP8_HUMAN	FKBP8	0.105119046	-3.249904006	0.000822	Yes
387	Q9NSD9	SYFB_HUMAN	FARSB	0.221986367	-2.171457017	0.001581	
388	P05026	AT1B1_HUMAN	ATP1B1	0.483104839	-1.04959179	0.000354	Yes
389	Q8N122	RPTOR_HUMAN	RPTOR	0.322695767	-1.631753441	0.003338	
390	Q86TI0	TBCD1_HUMAN	TBC1D1	0.321380662	-1.637644974	0.003484	
391	Q9Y4B6	DCAF1_HUMAN	DCAF1	0.284594418	-1.813020729	0.004448	
392	P55042	RAD_HUMAN	RRAD	0.278109136	-1.846276956	0.000917	
393	P78527	PRKDC_HUMAN	PRKDC	0.233432221	-2.09892438	0.001129	
394	Q6DD88	ATLA3_HUMAN	ATL3	0.144607307	-2.789787644	0.001504	Yes
395	Q9NY47	CA2D2_HUMAN	CACNA2D2	0.471617868	-1.084309717	0.006702	Yes
396	P62879	GBB2_HUMAN	GNB2	0.412936887	-1.276006797	0.005734	Yes
397	P61163	ACTZ_HUMAN	ACTR1A	0.412815372	-1.276431401	0.001422	Yes
398	P08754	GNAI3_HUMAN	GNAI3	0.408329256	-1.292195159	0.00583	Yes
399	P04899	GNAI2_HUMAN	GNAI2	0.377200396	-1.406596906	0.004854	Yes
400	O00451	GFRA2_HUMAN	GFRA2	0.363495603	-1.459990183	0.006968	Yes
401	O43741	AAKB2_HUMAN	PRKAB2	0.353959287	-1.498344667	0.000451	
402	O14939	PLD2_HUMAN	PLD2	0.329498166	-1.60165766	0.000604	Yes
403	P62873	GBB1_HUMAN	GNB1	0.32848017	-1.606121814	0.003516	Yes
404	P10415	BCL2_HUMAN	BCL2	0.314077157	-1.670809078	0.002285	Yes
405	O75935	DCTN3_HUMAN	DCTN3	0.267922197	-1.900113983	0.003675	
406	P17612	KAPCA_HUMAN	PRKACA	0.264254128	-1.920002086	0.005128	Yes
407	Q9UBI6	GBG12_HUMAN	GNG12	0.233743748	-2.097000317	0.00096	Yes
408	P13861	KAP2_HUMAN	PRKAR2A	0.230591735	-2.116587293	0.000297	Yes

409	P29992	GNA11_HUMAN	GNA11	0.195035513	-2.358191257	6.79E-05	Yes
410	P63167	DYL1_HUMAN	DYNLL1	0.177414883	-2.494801052	0.005823	
411	Q5JWF2	GNAS1_HUMAN	GNAS	0.167141879	-2.580854834	0.001065	Yes
412	Q15172	2A5A_HUMAN	PPP2R5A	0.149430311	-2.742455277	0.000631	
413	Q5BJF6	ODFP2_HUMAN	ODF2	0.129068049	-2.953796193	0.001474	
414	P06213	INSR_HUMAN	INSR	0.123989712	-3.011707676	0.007678	Yes
415	P63096	GNAI1_HUMAN	GNAI1	0.079905918	-3.64555384	0.009842	Yes
416	Q96CV9	OPTN_HUMAN	OPTN	0.044255203	-4.498009118	5.39E-05	Yes
417	Q9UGJ0	AAKG2_HUMAN	PRKAG2	0.012631783	-6.306797932	3.09E-06	Yes
418	Q99873	ANM1_HUMAN	PRMT1	0.406326146	-1.299289894	0.002358	
419	Q9NR30	DDX21_HUMAN	DDX21	0.258715021	-1.950564276	0.005154	
420	O60264	SMCA5_HUMAN	SMARCA5	0.233854067	-2.096319577	0.006007	
421	Q9UKM9	RALY_HUMAN	RALY	0.308791812	-1.695293596	0.001928	
422	Q96EY5	MB12A_HUMAN	MVB12A	0.274494096	-1.865152978	0.003429	Yes
423	Q9NSY0	NRBP2_HUMAN	NRBP2	0.172546447	-2.53494333	0.000707	
424	Q9UDY4	DNJB4_HUMAN	DNAJB4	0.133547903	-2.904570776	0.001505	
425	P63244	RACK1_HUMAN	RACK1	0.478768145	-1.062600931	0.000856	Yes
426	P61353	RL27_HUMAN	RPL27	0.449306408	-1.154228458	0.000148	Yes
427	P61247	RS3A_HUMAN	RPS3A	0.440137584	-1.183973525	0.002278	Yes
428	P18124	RL7_HUMAN	RPL7	0.364619386	-1.455536825	0.001778	
429	P61513	RL37A_HUMAN	RPL37A	0.330402842	-1.597701998	0.004407	Yes
430	P47813	IF1AX_HUMAN	EIF1AX	0.303831351	-1.718657352	0.003446	
431	O15371	EIF3D_HUMAN	EIF3D	0.295939145	-1.756627555	0.003472	
432	P62888	RL30_HUMAN	RPL30	0.230381492	-2.117903272	0.000364	Yes
433	Q13347	EIF3I_HUMAN	EIF3I	0.216457867	-2.207841857	0.000488	Yes
434	Q9H008	LHPP_HUMAN	LHPP	0.137867216	-2.85864866	0.007862	
435	P62166	NCS1_HUMAN	NCS1	0.314415191	-1.669257169	0.002926	Yes
436	Q96JB5	CK5P3_HUMAN	CDK5RAP3	0.256776629	-1.961414197	0.003175	
437	Q5JPE7	NOMO2_HUMAN	NOMO2	0.17288297	-2.532132337	0.001165	Yes
438	O95980	RECK_HUMAN	RECK	0.483893097	-1.047239737	0.006483	Yes
439	Q9BQ69	MACD1_HUMAN	MACROD1	0.400780756	-1.319114859	0.000216	
440	Q8N1G4	LRC47_HUMAN	LRRC47	0.376562173	-1.409040011	0.000186	
441	Q92973	TNPO1_HUMAN	TNPO1	0.314857074	-1.667231015	0.001644	Yes
442	O75355	ENTP3_HUMAN	ENTPD3	0.28368977	-1.817613966	2.71E-05	
443	Q9NRF8	PYRG2_HUMAN	CTPS2	0.242138659	-2.046094661	0.000203	
444	Q8NDH3	PEPL1_HUMAN	NPEPL1	0.219350722	-2.188688638	0.000388	
445	P54098	DPOG1_HUMAN	POLG	0.213953708	-2.224629413	0.006687	
446	P17812	PYRG1_HUMAN	CTPS1	0.174677843	-2.517231472	0.000321	
447	O43390	HNRPR_HUMAN	HNRNPR	0.456358283	-1.131761177	0.001444	Yes
448	Q15717	ELAV1_HUMAN	ELAVL1	0.4146693	-1.269966854	0.003247	Yes
449	Q8WUA2	PPIL4_HUMAN	PPIL4	0.413050603	-1.275609556	0.006957	
450	Q9Y3I0	RTCB_HUMAN	RTCB	0.368000445	-1.442220583	6.01E-05	Yes
451	P27144	KAD4_HUMAN	AK4	0.326724736	-1.613852411	0.009203	
452	Q9UHX1	PUF60_HUMAN	PUF60	0.282110821	-1.825666089	0.001971	
453	P08621	RU17_HUMAN	SNRNP70	0.275297632	-1.860935895	0.006912	
454	P30876	RPB2_HUMAN	POLR2B	0.230524037	-2.117010905	0.009816	
455	O75323	NIPS2_HUMAN	NIPSNAP2	0.19979501	-2.323407542	0.00516	
456	O75746	CMC1_HUMAN	SLC25A12	0.194805769	-2.359891689	0.004105	
457	P78524	DEN2B_HUMAN	DENND2B	0.161350956	-2.631725968	0.007684	
458	A1IGU5	ARH37_HUMAN	ARHGEF37	0.16052596	-2.639121466	0.000773	
459	Q6NY19	KANK3_HUMAN	KANK3	0.080503969	-3.634796282	0.006128	
460	P41252	SYIC_HUMAN	IARS1	0.466039456	-1.101475992	0.006279	Yes
461	Q9H2U1	DHX36_HUMAN	DHX36	0.354697534	-1.495338797	0.007523	Yes
462	Q17RN3	FA98C_HUMAN	FAM98C	0.352289045	-1.505168482	0.000713	
463	Q9P2J5	SYLC_HUMAN	LARS1	0.290176999	-1.784994928	0.005715	Yes
464	Q9NSE4	SYIM_HUMAN	IARS2	0.233186117	-2.100446194	0.008377	
465	P12081	HARS1_HUMAN	HARS1	0.180060311	-2.47344788	8.87E-05	
466	Q9UGM6	SYWM_HUMAN	WARS2	0.159653985	-2.64697953	0.003698	
467	Q8IX01	SUGP2_HUMAN	SUGP2	0.494760003	-1.015199218	0.004579	
468	P12956	XRCC6_HUMAN	XRCC6	0.463433367	-1.109566173	0.005229	Yes
469	Q02153	GCYB1_HUMAN	GUCY1B1	0.460117546	-1.119925623	0.000371	
470	O60524	NEMF_HUMAN	NEMF	0.445936899	-1.165088513	0.000762	
471	Q9NXR7	BABA2_HUMAN	BABAM2	0.445771936	-1.165622301	0.001498	
472	P31153	METK2_HUMAN	MAT2A	0.368158544	-1.441600912	0.003102	
473	O14787	TNPO2_HUMAN	TNPO2	0.325939067	-1.617325809	0.009623	
474	O15484	CAN5_HUMAN	CAPN5	0.277637974	-1.848723188	0.000223	Yes
475	Q9NZJ9	NUDT4_HUMAN	NUDT4	0.215023408	-2.217434372	0.002202	
476	Q9GZZ1	NAA50_HUMAN	NAA50	0.164652333	-2.602505144	4.27E-05	Yes
477	Q9BQI9	NRIP2_HUMAN	NRIP2	0.139294329	-2.843791567	4.76E-05	
478	Q6PGP7	TTC37_HUMAN	TTC37	0.081455242	-3.617848637	0.000837	
479	Q9NYL2	M3K20_HUMAN	MAP3K20	0.069990336	-3.836700461	0.003376	
480	Q9BS92	NPS3B_HUMAN	NIPSNAP3B	0.064206253	-3.961142384	0.002917	
481	P25789	PSA4_HUMAN	PSMA4	0.433082608	-1.207285859	0.000484	Yes
482	Q8N1F7	NUP93_HUMAN	NUP93	0.339273366	-1.559479918	0.000733	
483	O95757	HS74L_HUMAN	HSPA4L	0.296775415	-1.752556509	0.008328	
484	Q15008	PSMD6_HUMAN	PSMD6	0.239780403	-2.060214344	0.000762	Yes
485	P25788	PSA3_HUMAN	PSMA3	0.152458606	-2.7135105	0.007152	Yes
486	Q7L014	DDX46_HUMAN	DDX46	0.415913115	-1.265645916	0.009219	
487	Q16363	LAMA4_HUMAN	LAMA4	0.369286593	-1.437187208	0.000902	Yes
488	Q9Y4F1	FARP1_HUMAN	FARP1	0.362073766	-1.465644443	1.44E-05	
489	O14936	CSKP_HUMAN	CASK	0.268049061	-1.899431013	0.000384	Yes
490	Q9BXJ5	C1QT2_HUMAN	C1QTNF2	0.212835159	-2.232191603	0.000702	Yes

491	Q7Z3J2	VP35L_HUMAN	VPS35L	0.199274442	-2.327171408	0.002547	
492	Q9BZG1	RAB34_HUMAN	RAB34	0.198413821	-2.333415572	0.000233	Yes
493	Q93052	LPP_HUMAN	LPP	0.182837948	-2.451362564	0.006573	
494	Q9H902	REEP1_HUMAN	REEP1	0.173807792	-2.524435331	0.000679	Yes
495	O43175	SERA_HUMAN	PHGDH	0.155811197	-2.682129179	0.000142	Yes
496	Q9P0L2	MARK1_HUMAN	MARK1	0.112131807	-3.156732533	1.8E-06	
497	Q9P2K5	MYEF2_HUMAN	MYEF2	0.084867483	-3.5586443	4.11E-05	
498	Q03405	UPAR_HUMAN	PLAUR	36.05587057	5.172162271	0.000429	Yes
499	P17813	EGLN_HUMAN	ENG	9.335813418	3.222775728	0.000563	Yes
500	Q15904	VAS1_HUMAN	ATP6AP1	6.326864653	2.661490734	1.78E-05	Yes
501	O43252	PAPS1_HUMAN	PAPSS1	4.165518536	2.058496098	0.000116	
502	P50281	MMP14_HUMAN	MMP14	2.43321364	1.282862997	0.001269	Yes
503	P19440	GGT1_HUMAN	GGT1	90.43263413	6.498771582	0.00545	Yes
504	Q9BV19	CA050_HUMAN	C1orf50	18.2605643	4.190659444	0.001705	
505	A6QL63	BTBDB_HUMAN	BTBD11	16.83335333	4.073250696	0.005245	
506	Q9NSY1	BMP2K_HUMAN	BMP2K	7.713139837	2.947318267	0.005315	
507	Q66K79	CBPZ_HUMAN	CPZ	5.652291765	2.498835938	0.007526	Yes
508	Q9Y289	SC5A6_HUMAN	SLC5A6	4.302320773	2.105115093	0.006479	
509	Q9NVA4	T184C_HUMAN	TMEM184C	2.13385646	1.093463133	0.007081	
510	P05109	S10A8_HUMAN	S100A8	35.1801687	5.136690496	0.000996	Yes
511	Q8TF64	GIPC3_HUMAN	GIPC3	14.48295992	3.856284575	0.000939	
512	P19971	TYPH_HUMAN	TYMP	7.2406569	2.85612059	0.00951	
513	P55786	PSA_HUMAN	NPEPPS	4.7530935	2.248866782	0.006843	Yes
514	Q15126	PMVK_HUMAN	PMVK	3.450161627	1.786663948	0.00749	Yes
515	Q969T9	WBP2_HUMAN	WBP2	2.829274484	1.500432148	0.004659	
516	P05121	PAI1_HUMAN	SERPINE1	45.564863	5.509849825	0.000746	Yes
517	O75781	PALM_HUMAN	PALM	7.910447759	2.983759359	0.000417	
518	Q9NZ52	GGA3_HUMAN	GGA3	7.796604351	2.962845925	0.000632	Yes
519	Q14677	EPN4_HUMAN	CLINT1	5.471224209	2.451863678	0.000195	Yes
520	O75379	VAMP4_HUMAN	VAMP4	5.417496095	2.437626208	0.006859	Yes
521	O75154	RFIP3_HUMAN	RAB11FIP3	5.052607109	2.337028	0.003835	Yes
522	P52209	6PGD_HUMAN	PGD	4.082851101	2.029576954	9.71E-05	Yes
523	Q9NUQ9	CYRIB_HUMAN	CYRIB	3.683396935	1.881036875	0.001482	Yes
524	O00299	CLIC1_HUMAN	CLIC1	3.661775148	1.872543205	0.000315	Yes
525	Q9UEU0	VTI1B_HUMAN	VTI1B	3.586905494	1.842739734	0.000558	Yes
526	P49768	PSN1_HUMAN	PSEN1	3.370200215	1.752834301	0.003521	Yes
527	Q92542	NICA_HUMAN	NCSTN	2.976331887	1.573535408	0.001103	Yes
528	Q3T906	GNPTA_HUMAN	GNPTAB	2.481662958	1.311307192	0.009449	Yes
529	P20338	RAB4A_HUMAN	RAB4A	2.37192128	1.24605613	0.009143	Yes
530	Q9NY33	DPP3_HUMAN	DPP3	2.235362109	1.160508554	0.00771	Yes
531	Q9Y3P9	RBGP1_HUMAN	RABGAP1	34.02380847	5.088472734	0.002644	
532	Q9BPZ3	PAIP2_HUMAN	PAIP2	28.81641758	4.848819088	0.003104	
533	Q9P1F3	ABRAL_HUMAN	ABRACL	22.54983276	4.495044829	0.00411	
534	Q9H773	DCTP1_HUMAN	DCTPP1	18.64143508	4.220441023	0.001206	
535	Q15800	MSMO1_HUMAN	MSMO1	3.293494022	1.719618932	0.000331	Yes
536	Q96AG4	LRC59_HUMAN	LRRC59	2.552857792	1.352113174	0.002873	Yes
537	P27707	DCK_HUMAN	DCK	14.83186323	3.89062794	9.14E-06	
538	P54687	BCAT1_HUMAN	BCAT1	7.489597026	2.904888097	0.002262	
539	P20933	ASPG_HUMAN	AGA	3.948751726	1.981396663	0.004671	Yes
540	P17174	AATC_HUMAN	GOT1	2.896533947	1.534327573	0.000773	Yes
541	P17677	NEUM_HUMAN	GAP43	35.163717	5.136015674	0.002253	
542	Q9P2A4	ABI3_HUMAN	ABI3	13.47695103	3.752422239	0.007545	
543	Q9HB40	RISC_HUMAN	SCPEP1	10.66448803	3.414742802	0.006424	Yes
544	Q5SW79	CE170_HUMAN	CEP170	7.367978261	2.881268805	0.00368	
545	O94760	DDAH1_HUMAN	DDAH1	6.721229828	2.748725237	4.99E-05	Yes
546	P50225	ST1A1_HUMAN	SULT1A1	6.718934767	2.748232523	0.001234	
547	O95372	LYPA2_HUMAN	LYPLA2	6.370742447	2.671461514	0.008418	Yes
548	Q8IXM2	BAP18_HUMAN	BAP18	3.961931206	1.98620383	0.008683	
549	P53367	ARFP1_HUMAN	ARFIP1	3.170558692	1.664737084	0.004927	Yes
550	Q9Y5S2	MRCKB_HUMAN	CDC42BPB	2.88656378	1.529353103	0.000274	Yes
551	Q6WKZ4	RFIP1_HUMAN	RAB11FIP1	21.25712602	4.409874651	0.001335	
552	Q9NX08	COMD8_HUMAN	COMMD8	12.04779846	3.590697637	0.002336	
553	P15374	UCHL3_HUMAN	UCHL3	8.869798565	3.148901341	0.006853	
554	P78417	GSTO1_HUMAN	GSTO1	7.677126383	2.940566398	0.00249	Yes
555	P0CG30	GSTT2_HUMAN	GSTT2B	5.811712336	2.538963295	0.003891	Yes
556	P09936	UCHL1_HUMAN	UCHL1	5.619909739	2.490546959	0.006042	Yes
557	Q8NBI6	XXLT1_HUMAN	XXYLT1	5.113535245	2.354321044	0.002547	Yes
558	P53985	MOT1_HUMAN	SLC16A1	4.826691083	2.271034495	0.000281	Yes
559	Q9H4A6	GOLP3_HUMAN	GOLPH3	4.225334662	2.079065613	0.005602	Yes
560	Q8TEW8	PAR3L_HUMAN	PARD3B	3.149231851	1.654999975	0.000164	
561	Q86X83	COMD2_HUMAN	COMMD2	2.39768181	1.261640215	0.002448	
562	P31997	CEAM8_HUMAN	CEACAM8	112.1513459	6.809303124	0.004001	Yes
563	Q9Y3L5	RAP2C_HUMAN	RAP2C	6.698329263	2.743801295	0.0019	Yes
564	P35613	BASI_HUMAN	BSG	3.850286323	1.944965734	0.003342	Yes
565	P18085	ARF4_HUMAN	ARF4	3.528372255	1.819002778	0.003587	Yes
566	Q96CW1	AP2M1_HUMAN	AP2M1	2.047051258	1.033547228	0.00361	Yes
567	O43914	TYOBP_HUMAN	TYROBP	24.98732181	4.643124374	0.004043	Yes
568	O60711	LPXN_HUMAN	LPXN	18.81994168	4.234190253	6.37E-05	
569	P13796	PLSL_HUMAN	LCP1	15.82910672	3.984507937	0.002376	Yes
570	P12314	FCGR1_HUMAN	FCGR1A	12.31258641	3.622061944	0.001282	Yes
571	P10321	HLAC_HUMAN	HLA-C	10.06295213	3.3309817	5.79E-05	Yes
572	Q01628	IFM3_HUMAN	IFITM3	8.034511119	3.006210242	0.004389	

573	P61769	B2MG_HUMAN	B2M	7.776963553	2.959206978	0.003062	Yes
574	P05362	ICAM1_HUMAN	ICAM1	7.330775718	2.873965868	0.00465	Yes
575	P01889	HLAB_HUMAN	HLA-B	7.090982665	2.825985569	5.44E-05	Yes
576	P80217	IN35_HUMAN	IFI35	5.043891744	2.334537312	0.00068	
577	P04439	HLAA_HUMAN	HLA-A	4.709040722	2.235433199	0.005335	Yes
578	Q16658	FSCN1_HUMAN	FSCN1	3.702442502	1.888477331	0.002393	Yes
579	Q16864	VATF_HUMAN	ATP6V1F	3.62582684	1.858310027	0.001352	Yes
580	Q99439	CNN2_HUMAN	CNN2	3.570555852	1.836148685	0.00931	Yes
581	P27797	CALR_HUMAN	CALR	3.229597482	1.691354367	0.001109	Yes
582	O00764	PDXK_HUMAN	PDXK	2.378076204	1.249794946	0.007933	Yes
583	O95445	APOM_HUMAN	APOM	27.39973622	4.776090099	0.001832	Yes
584	Q8WUH6	TM263_HUMAN	TMEM263	22.24809011	4.475609588	0.009515	
585	Q9Y3Q8	T22D4_HUMAN	TSC22D4	6.320322408	2.659998154	0.001307	
586	P55209	NP1L1_HUMAN	NAP1L1	3.449507605	1.786390441	0.000958	
587	O43790	KRT86_HUMAN	KRT86	3.35499159	1.74630915	0.000631	Yes
588	P51572	BAP31_HUMAN	BCAP31	2.11917467	1.083502505	0.001587	Yes
589	Q9Y3L3	3BP1_HUMAN	SH3BP1	36.21788746	5.178630493	0.00285	
590	Q6P589	TP8L2_HUMAN	TNFAIP8L2	27.19292528	4.765159453	0.000364	
591	Q8IUI8	CRLF3_HUMAN	CRLF3	14.95095248	3.902165492	0.0029	
592	Q9NR99	MXRA5_HUMAN	MXRA5	10.62649451	3.409593852	0.008069	Yes
593	O14657	TOR1B_HUMAN	TOR1B	7.475521945	2.902174313	0.005008	Yes
594	Q13077	TRAF1_HUMAN	TRAF1	5.505807414	2.460954147	0.004175	
595	P30260	CDC27_HUMAN	CDC27	5.437357342	2.442905645	0.003831	
596	Q92805	GOGA1_HUMAN	GOLGA1	18.96869622	4.245548616	0.006724	Yes
597	P0C0L5	CO4B_HUMAN	C4B	13.81581587	3.788248856	0.003531	Yes
598	P10124	SRGN_HUMAN	SRGN	11.48363043	3.521506901	0.003431	Yes
599	Q01459	DIAC_HUMAN	CTBS	10.31704819	3.366958355	0.004231	Yes
600	Q9GZX9	TWSG1_HUMAN	TWSG1	6.899084927	2.78640502	0.005691	Yes
601	Q13641	TPBG_HUMAN	TPBG	4.301639057	2.104886476	0.00159	Yes
602	O75976	CBPD_HUMAN	CPD	3.594430742	1.845763306	2.11E-05	Yes
603	Q6QNY0	BL1S3_HUMAN	BLOC1S3	2.999394676	1.584671372	0.004274	
604	Q9BRR9	RHG09_HUMAN	ARHGAP9	27.51805466	4.78230658	0.009135	Yes
605	Q3ZCW2	LEGL_HUMAN	LGALSL	11.07319785	3.469000016	0.007407	
606	Q15025	TNIP1_HUMAN	TNIP1	9.384409595	3.230265983	0.002547	
607	Q13093	PAFA_HUMAN	PLA2G7	5.14631972	2.363541089	0.001768	Yes
608	Q9ULQ1	TPC1_HUMAN	TPCN1	4.545784722	2.184529363	0.008451	
609	Q96DF8	ESS2_HUMAN	ESS2	3.598467888	1.847382784	0.000677	
610	P02008	HBAZ_HUMAN	HBZ	31.17506713	4.962320761	0.001721	Yes
611	Q11201	SIA4A_HUMAN	ST3GAL1	23.15710036	4.533382711	0.002633	Yes
612	P32942	ICAM3_HUMAN	ICAM3	17.55434992	4.133756665	0.005456	Yes
613	O95466	FMNL1_HUMAN	FMNL1	7.982287097	2.996802169	0.001077	Yes
614	P55957	BID_HUMAN	BID	7.29026104	2.865970474	4.58E-06	
615	P58546	MTPN_HUMAN	MTPN	7.427255215	2.892829153	0.006725	
616	Q8TD55	PKHO2_HUMAN	PLEKHO2	5.968145367	2.577282676	0.000121	Yes
617	Q8N1S5	S39AB_HUMAN	SLC39A11	4.284393353	2.099090941	0.008145	Yes
618	Q96HH9	GRM2B_HUMAN	GRAMD2B	4.082612686	2.029492707	6.05E-05	
619	Q9ULI3	HEG1_HUMAN	HEG1	4.062497059	2.022366768	0.001252	Yes
620	P47224	MSS4_HUMAN	RABIF	3.984369563	1.994351468	0.005194	
621	Q9BUH6	PAXX_HUMAN	PAXX	9.364403381	3.227187081	0.003744	
622	Q9H488	OFUT1_HUMAN	POFUT1	6.3364971	2.66368552	0.006722	Yes
623	Q5U3C3	TM164_HUMAN	TMEM164	3.764172969	1.912332923	0.000189	
624	P80303	NUCB2_HUMAN	NUCB2	3.395497586	1.763623007	0.002031	Yes
625	Q9BV40	VAMP8_HUMAN	VAMP8	65.11469841	6.024911336	0.001216	Yes
626	O43665	RGS10_HUMAN	RGS10	24.80557273	4.632592362	0.00587	
627	A0MZ66	SHOT1_HUMAN	SHTN1	24.72538641	4.627921163	0.001555	
628	O43805	SSNA1_HUMAN	SSNA1	17.70102166	4.145760727	0.004159	
629	Q04206	TF65_HUMAN	RELA	12.43778687	3.636657895	8.31E-05	
630	P28067	DMA_HUMAN	HLA-DMA	5.245708926	2.391137757	0.003403	Yes
631	P01903	DRA_HUMAN	HLA-DRA	4.640446346	2.214263579	0.003278	Yes
632	O15155	BET1_HUMAN	BET1	4.077357115	2.027634321	0.000813	Yes
633	P28065	PSB9_HUMAN	PSMB9	3.373469275	1.754233022	0.001625	Yes
634	O00499	BIN1_HUMAN	BIN1	3.222907142	1.688362622	0.000538	
635	Q99961	SH3G1_HUMAN	SH3GL1	2.902487599	1.537289903	0.002581	
636	Q9UL46	PSME2_HUMAN	PSME2	2.835376565	1.503540352	0.00488	Yes
637	P52594	AGFG1_HUMAN	AGFG1	2.686915215	1.425950798	0.000832	
638	P52907	CAZA1_HUMAN	CAPZA1	2.290103499	1.195412801	0.002423	Yes
639	Q9UQ88	CD11A_HUMAN	CDK11A	2.148927721	1.103616959	0.001663	
640	Q9BVK6	TMED9_HUMAN	TMED9	2.12144402	1.08504661	0.006714	Yes
641	Q13951	PEBB_HUMAN	CBFB	22.04620224	4.462458248	0.008622	
642	P54105	ICLN_HUMAN	CLNS1A	4.393455093	2.135355948	0.00785	
643	P17947	SPI1_HUMAN	SPI1	4.2060289	2.072458763	0.00077	
644	Q9NR97	TLR8_HUMAN	TLR8	11.41403864	3.512737447	0.004784	Yes
645	Q9BT09	CNPY3_HUMAN	CNPY3	6.487307439	2.697619811	0.003277	Yes
646	Q68CQ7	GL8D1_HUMAN	GLT8D1	4.950467252	2.307564701	0.008526	Yes
647	Q6P4E1	GOLM2_HUMAN	GOLM2	4.090277857	2.03219885	0.008254	Yes
648	Q86YB8	ERO1B_HUMAN	ERO1B	3.920603886	1.971075888	4.06E-05	Yes
649	Q8WU79	SMAP2_HUMAN	SMAP2	3.587385747	1.842932885	0.007829	
650	O75506	HSBP1_HUMAN	HSBP1	2.96068496	1.565930984	0.001244	
651	Q9ULZ3	ASC_HUMAN	PYCARD	86.59079097	6.436141696	0.007221	Yes
652	P24158	PRTN3_HUMAN	PRTN3	53.75145173	5.748231815	0.007418	Yes
653	P05164	PERM_HUMAN	MPO	24.19629841	4.596714454	0.004406	Yes
654	Q99615	DNJC7_HUMAN	DNAJC7	7.699235064	2.944715118	0.003884	Yes

655	P28676	GRAN_HUMAN	GCA	5.9709122	2.577951354	0.007369	Yes
656	P43307	SSRA_HUMAN	SSR1	4.405121348	2.139181762	0.002788	Yes
657	P61011	SRP54_HUMAN	SRP54	4.300913739	2.104643196	0.004806	
658	Q8TDP1	RNH2C_HUMAN	RNASEH2C	2.747194189	1.457958893	0.001574	
659	Q96RD7	PANX1_HUMAN	PANX1	2.318064028	1.212920416	0.001572	Yes
660	P0C0L4	CO4A_HUMAN	C4A	19.81752852	4.308705148	0.000961	Yes
661	Q8NBJ4	GOLM1_HUMAN	GOLM1	19.19454618	4.262624546	0.00357	Yes
662	P01033	TIMP1_HUMAN	TIMP1	15.18216586	3.924305713	0.0099	Yes
663	P02746	C1QB_HUMAN	C1QB	14.86374366	3.893725621	0.008663	Yes
664	O43765	SGTA_HUMAN	SGTA	4.686448381	2.228494992	0.009029	
665	Q15084	PDIA6_HUMAN	PDIA6	4.244180318	2.085485952	0.00095	Yes
666	Q14766	LTBP1_HUMAN	LTBP1	2.885998565	1.529070583	0.001575	Yes
667	P07237	PDIA1_HUMAN	P4HB	2.686467689	1.425710486	0.009972	Yes
668	P14625	ENPL_HUMAN	HSP90B1	2.316289626	1.211815657	0.002544	Yes
669	Q9UPI3	FLVC2_HUMAN	FLVCR2	45.44452088	5.506034458	0.00014	
670	Q9NZK5	ADA2_HUMAN	ADA2	41.79518138	5.385264717	0.000293	Yes
671	Q9NRG1	PRDC1_HUMAN	PRTFDC1	15.21262261	3.927196986	0.001699	
672	Q96BP2	CHCH1_HUMAN	CHCHD1	10.50018425	3.392342739	0.002383	
673	Q9BRT3	MIEN1_HUMAN	MIEN1	7.922861639	2.986021608	0.007571	
674	Q96E11	RRFM_HUMAN	MRRF	7.848676125	2.972449328	0.000604	
675	Q96AY3	FKB10_HUMAN	FKBP10	7.599186071	2.925844904	0.008754	Yes
676	Q92665	RT31_HUMAN	MRPS31	5.078166466	2.344307688	0.007562	
677	Q86U28	ISCA2_HUMAN	ISCA2	3.99436051	1.997964549	0.001629	
678	P07332	FES_HUMAN	FES	3.662395627	1.872787645	0.006638	Yes
679	P19623	SPEE_HUMAN	SRM	2.65322035	1.407744497	0.009856	
680	Q6IA17	SIGIR_HUMAN	SIGIRR	28.95757935	4.855869103	0.00715	
681	Q9BU89	DOHH_HUMAN	DOHH	25.49307805	4.672033671	0.007705	
682	P15104	GLNA_HUMAN	GLUL	21.89904603	4.452796119	0.002186	Yes
683	P08571	CD14_HUMAN	CD14	10.01504902	3.324097578	0.003103	Yes
684	O95400	CD2B2_HUMAN	CD2BP2	7.79527563	2.962600036	0.006244	
685	Q01844	EWS_HUMAN	EWSR1	7.092439626	2.826281965	0.00774	
686	P11171	EPB41_HUMAN	EPB41	4.003195889	2.001152213	0.005219	Yes
687	Q99757	THIOM_HUMAN	TXN2	3.377787586	1.756078606	0.000713	
688	Q9P2I0	CPSF2_HUMAN	CPSF2	3.282880038	1.714962032	0.003975	
689	P22307	NLTP_HUMAN	SCP2	3.013895998	1.591629634	0.003028	
690	P31943	HNRH1_HUMAN	HNRNPH1	2.926251838	1.549053935	0.008318	
691	P23246	SFPQ_HUMAN	SFPQ	2.459626952	1.298439521	0.009739	
692	Q9UJ68	MSRA_HUMAN	MSRA	2.041372364	1.029539366	0.006411	Yes
693	P11274	BCR_HUMAN	BCR	17.41830866	4.122532638	0.00235	Yes
694	Q9H3U7	SMOC2_HUMAN	SMOC2	6.870850455	2.780488683	0.008567	Yes
695	Q96KP4	CNDP2_HUMAN	CNDP2	5.333339207	2.415039088	0.000156	Yes
696	Q96EP5	DAZP1_HUMAN	DAZAP1	5.14835834	2.364112472	0.003091	
697	P60174	TPIS_HUMAN	TPI1	4.87086583	2.284178244	0.009486	Yes
698	P06132	DCUP_HUMAN	UROD	4.546998747	2.184914607	0.002849	
699	Q8TEX9	IPO4_HUMAN	IPO4	4.295559934	2.102846201	0.001534	
700	Q9GZR7	DDX24_HUMAN	DDX24	4.292316093	2.101756322	0.000891	
701	Q9UKD2	MRT4_HUMAN	MRTO4	3.897194739	1.962436024	4.09E-05	
702	P30040	ERP29_HUMAN	ERP29	3.600696091	1.848275837	0.009858	Yes
703	Q96HQ2	C2AIL_HUMAN	CDKN2AIPNL	3.508708748	1.810940197	0.006715	
704	Q96BQ5	CC127_HUMAN	CCDC127	3.418732131	1.773461387	0.000209	
705	Q9Y315	DEOC_HUMAN	DERA	2.000616719	1.0004448	0.003958	Yes
706	O15321	TM9S1_HUMAN	TM9SF1	11.90991683	3.574091433	0.003472	Yes
707	Q9NRN7	ADPPT_HUMAN	AASDHPPT	11.87121258	3.569395401	0.005147	Yes
708	Q9NWM8	FKB14_HUMAN	FKBP14	11.86136204	3.568197779	0.002417	Yes
709	O15305	PMM2_HUMAN	PMM2	9.431920269	3.237551523	0.003987	
710	O15431	COPT1_HUMAN	SLC31A1	4.738400626	2.244400182	0.000739	
711	P80511	S10AC_HUMAN	S100A12	73.80859632	6.205716948	0.002013	Yes
712	Q96MW1	CCD43_HUMAN	CCDC43	4.619035458	2.207591621	0.005741	
713	P49792	RBP2_HUMAN	RANBP2	2.726410623	1.447002862	7.75E-05	
714	P51659	DHB4_HUMAN	HSD17B4	2.389982777	1.257000222	0.006654	
715	P09326	CD48_HUMAN	CD48	24.73313479	4.6283732	0.007201	Yes
716	P15289	ARSA_HUMAN	ARSA	9.474693259	3.244079237	0.008668	Yes
717	Q53H96	P5CR3_HUMAN	PYCR3	7.510570539	2.908922506	0.000666	
718	Q04756	HGFA_HUMAN	HGFAC	6.080321833	2.604147688	0.004446	Yes
719	O75787	RENR_HUMAN	ATP6AP2	4.150722734	2.053362564	0.00922	Yes
720	P08174	DAF_HUMAN	CD55	4.12265903	2.043575146	0.000473	Yes
721	P13667	PDIA4_HUMAN	PDIA4	3.447493228	1.785547718	0.00259	Yes
722	Q08379	GOGA2_HUMAN	GOLGA2	3.339561899	1.739658855	0.004171	Yes
723	P13693	TCTP_HUMAN	TPT1	3.324363574	1.733078173	0.005222	Yes
724	Q9NYB9	ABI2_HUMAN	ABI2	2.904636726	1.538357741	0.007954	

SourceDataForFigure2C							
#term ID	term description	observed g	backgroun	strength	false discovery rate	matching p	matching proteins in your network (labels)
hsa00020	Citrate cycle (TCA cycle)	11	30	1	9.47E-06	9606.ENSI	DLD,ACO2,IDH3G,DLAT,PDHB,DLST,SDHB,SUCLA2,PDHA1,IDH1,SUCLG2
hsa01210	2-Oxocarboxylic acid met	6	17	0.98	0.0025	9606.ENSI	ACO2,IDH3G,BCAT2,GOT1,IDH1,BCAT1
hsa00280	Valine, leucine and isoleu	16	48	0.95	1.67E-07	9606.ENSI	HSD17B10,OXCT1,DLD,ALDH2,HIBADH,BCAT2,ACAA1,ALDH3A2,ALDH9A1,HIBCH,ALDH1B1,HADHA,PCCB,BCAT1,ALDH6A1,HADH
hsa00410	beta-Alanine metabolism	9	31	0.89	0.00029	9606.ENSI	ALDH2,CNDP2,ALDH3A2,ALDH9A1,HIBCH,SRM,ALDH1B1,HADHA,ALDH6A1
hsa00620	Pyruvate metabolism	10	39	0.84	0.00026	9606.ENSI	DLD,ALDH2,DLAT,PDHB,ACSS1,ALDH3A2,ALDH9A1,GLO1,ALDH1B1,PDHA1
hsa00640	Propanoate metabolism	8	32	0.83	0.002	9606.ENSI	DLD,ACSS1,HIBCH,SUCLA2,HADHA,PCCB,SUCLG2,ALDH6A1
hsa00450	Selenocompound metabol	4	16	0.83	0.0355	9606.ENSI	PAPSS1,MTR,TXNRD2,TXNRD1
hsa00340	Histidine metabolism	5	23	0.77	0.024	9606.ENSI	ALDH2,CNDP2,ALDH3A2,ALDH9A1,ALDH1B1
hsa01230	Biosynthesis of amino aci	15	72	0.75	2.33E-05	9606.ENSI	ACO2,IDH3G,PYCRL,TPI1,MAT2A,ASL,GLUL,BCAT2,PFKM,MTR,PHGDH,GOT1,PFKP,IDH1,BCAT1
hsa00071	Fatty acid degradation	9	44	0.74	0.0023	9606.ENSI	ALDH2,ECI1,ADH1B,ACAA1,ALDH3A2,ALDH9A1,ALDH1B1,HADHA,HADH
hsa01200	Carbon metabolism	23	116	0.73	1.67E-07	9606.ENSI	DLD,ACO2,IDH3G,TPI1,PGD,GLUD1,DLAT,PDHB,ACSS1,DLST,PFKM,HIBCH,PHGDH,GOT1,SDHB,SUCLA2,PDHA1,HADHA,PFKP,IDH1,PCCB,SUCLG2,ALDH6A1
hsa00010	Glycolysis / Gluconeogen	13	68	0.71	0.00023	9606.ENSI	DLD,TPI1,ALDH2,DLAT,ADH1B,PDHB,ACSS1,PFKM,ALDH3A2,ALDH9A1,ALDH1B1,PDHA1,PFKP
hsa04130	SNARE interactions in ve	6	33	0.69	0.0217	9606.ENSI	BET1,VAMP4,VAMP8,VAMP5,VTI1B,STX10
hsa05416	Viral myocarditis	10	56	0.68	0.0024	9606.ENSI	ICAM1,BID,SGCD,CD55,HLA-DMA,HLA-C,SGCB,HLA-DRA,HLA-A,HLA-B
hsa00330	Arginine and proline meta	8	48	0.65	0.0103	9606.ENSI	PYCRL,ALDH2,CNDP2,ALDH3A2,ALDH9A1,GOT1,SRM,ALDH1B1
hsa04962	Vasopressin-regulated wa	7	44	0.63	0.021	9606.ENSI	DYNC1LI2,RAB5A,PRKACA,DYNC1I1,GNAS,DYNLL1,DYNLL2
hsa00310	Lysine degradation	9	59	0.62	0.0096	9606.ENSI	ALDH2,SETD3,TMLHE,DLST,ALDH3A2,ALDH9A1,ALDH1B1,HADHA,HADH
hsa00380	Tryptophan metabolism	6	40	0.61	0.038	9606.ENSI	ALDH2,ALDH3A2,ALDH9A1,ALDH1B1,HADHA,HADH
hsa00480	Glutathione metabolism	7	50	0.58	0.0314	9606.ENSI	PGD,GSTT2B,MGST3,GSTO1,SRM,GGT1,IDH1
hsa05150	Staphylococcus aureus inf	7	51	0.57	0.0336	9606.ENSI	ICAM1,C1QB,FCGR1A,HLA-DMA,HLA-DRA,C4A,C4B
hsa05133	Pertussis	10	74	0.56	0.0103	9606.ENSI	PYCARD,CFL2,CD14,GNAI2,C1QB,GNAI1,GNAI3,RELA,C4A,C4B
hsa05146	Amoebiasis	12	94	0.54	0.0064	9606.ENSI	GNA11,VCL,LAMA4,ACTN4,RAB5A,CD14,PRKACA,GNAS,COL4A1,ACTN1,RELA,LAMB2
hsa04924	Renin secretion	8	63	0.54	0.0305	9606.ENSI	GUCY1B3,KCNMA1,PRKACA,GNAI2,GNAI1,PDE3A,GNAI3,GNAS
hsa04540	Gap junction	11	87	0.53	0.0101	9606.ENSI	GNA11,TUBB1,TUBB2B,PDGFRB,GUCY1B3,EGFR,PRKACA,GNAI2,GNAI1,GNAI3,GNAS
hsa04520	Adherens junction	9	71	0.53	0.021	9606.ENSI	VCL,WASL,ACTN4,SMAD2,EGFR,INSR,CTNNA1,SORBS1,ACTN1
hsa04512	ECM-receptor interaction	10	81	0.52	0.0166	9606.ENSI	COMP,LAMA4,THBS1,COL6A6,HSPG2,COL4A1,AGRN,LAMB2,TNXB,ITGA7
hsa04612	Antigen processing and pr	8	66	0.52	0.0354	9606.ENSI	PSME2,CALR,HLA-DMA,HLA-C,HLA-DRA,HLA-A,HLA-B,B2M
hsa04510	Focal adhesion	23	197	0.5	0.0002	9606.ENSI	VCL,COMP,LAMA4,ACTN4,THBS1,PDGFRB,EGFR,MYL9,FLNC,PARVA,COL6A6,RAP1A,FLNA,COL4A1,ACTN1,PPP1CB,BCL2,MYL5,ARHGAP35,LAMB2,TNXB,ITGA7,PPP1R12B
hsa04145	Phagosome	17	145	0.5	0.002	9606.ENSI	TUBB1,COMP,MPO,DYNC1LI2,TUBB2B,THBS1,RAB5A,CD14,DYNC1I1,CALR,FCGR1A,ATP6AP1,HLA-DMA,HLA-C,HLA-DRA,HLA-A,HLA-B
hsa04270	Vascular smooth muscle c	14	119	0.5	0.0049	9606.ENSI	GNA11,GUCY1B3,MYL9,KCNMA1,PRKACA,GNAS,PRKCD,PPP1CB,MYH11,ACTA2,MRVI1,MYL6,MYL6B,PPP1R12B
hsa05012	Parkinson's disease	16	142	0.48	0.0033	9606.ENSI	UQCRC1,NDUFA10,ATP5B,NDUFS6,SLC25A4,UCHL1,COX7A1,UQCRH,PRKACA,GNAI2,NDUFV1,GNAI1,GNAI3,SDHB,NDUFS1,NDUFA6
hsa04670	Leukocyte transendothelia	12	112	0.46	0.0172	9606.ENSI	VCL,ACTN4,ICAM1,MYL9,CTNNA1,GNAI2,GNAI1,RAP1A,GNAI3,ACTN1,MYL5,ARHGAP35
hsa04921	Oxytocin signaling pathw	15	149	0.43	0.0103	9606.ENSI	PRKAB2,GUCY1B3,EGFR,MYL9,PRKAG2,PRKACA,GNAI2,GNAI1,GNAI3,GNAS,PPP1CB,CACNA2D2,MYL6,MYL6B,PPP1R12B
hsa04932	Non-alcoholic fatty liver c	15	149	0.43	0.0103	9606.ENSI	UQCRC1,NDUFA10,PRKAB2,NDUFS6,PRKAG2,COX7A1,INSR,UQCRH,BID,NDUFV1,SDHB,RELA,ADIPOQ,NDUFS1,NDUFA6
hsa04926	Relaxin signaling pathway	13	130	0.43	0.0191	9606.ENSI	SMAD2,EGFR,GNB2,PRKACA,GNAI2,GNAI1,GNAI3,GNG12,GNAS,COL4A1,RELA,ACTA2,GNB1
hsa04922	Glucagon signaling pathw	10	100	0.43	0.0391	9606.ENSI	PYGM,PYGB,PRKAB2,PPP4C,PRKAG2,PDHB,PRKACA,GNAS,PDHA1,PRMT1
hsa05142	Chagas disease (Americar	10	101	0.43	0.0408	9606.ENSI	GNA11,SERPINE1,SMAD2,GNAI2,C1QB,CALR,GNAI1,GNAI3,GNAS,RELA
hsa04810	Regulation of actin cytosk	20	205	0.42	0.0033	9606.ENSI	VCL,WASL,RRAS,ACTN4,PDGFRB,EGFR,MYL9,ABI2,CFL2,CD14,NCKAP1,GNG12,GSN,ACTN1,PPP1CB,MYL5,ARHGAP35,ITGA7,PPP1R12B,PIP4K2B
hsa04371	Apelin signaling pathway	13	133	0.42	0.021	9606.ENSI	SERPINE1,RRAS,PRKAB2,SMAD2,PRKAG2,GNB2,PRKACA,GNAI2,GNAI1,GNAI3,GNG12,ACTA2,G

hsa00190	Oxidative phosphorylatio	12	131	0.39	0.0368	9606.ENS	UQCRC1,NDUFA10,ATP5B,NDUFS6,COX7A1,UQCRH,NDUFV1,LHPP,ATP6AP1,SDHB,NDUFS1,ND
hsa05010	Alzheimer's disease	15	168	0.38	0.0217	9606.ENS	HSD17B10,UQCRC1,NDUFA10,ATP5B,NDUFS6,COX7A1,NCSTN,UQCRH,BID,NDUFV1,PSEN1,ATP2A3,SDHB,NDUFS1,NDUFA6
hsa04144	Endocytosis	21	242	0.37	0.0079	9606.ENS	WASL,SNX3,SMAD2,RAB11FIP3,PLD2,CAPZA1,EHD2,SH3GL1,RAB5A,EGFR,AP2M1,BIN1,MVB12A,RAB11FIP1,RAB4A,SMAP2,HLA-C,HLA-A,KIF5C,HLA-B,NEDD4
hsa04022	cGMP-PKG signaling pat	14	160	0.37	0.0314	9606.ENS	GNA11,GUCY1B3,MYL9,SLC25A4,KCNMA1,INSR,GNAI2,GNAI1,PDE3A,ATP2A3,ATP1B1,GNAI3,PPP1CB,MRVI1
hsa04530	Tight junction	14	167	0.36	0.038	9606.ENS	WASL,ACTN4,PRKAB2,MYL9,PRKAG2,ARHGAP17,PRKACA,RAP2C,RAP1A,ACTN1,MYH11,NEDD4,MYL6,MYL6B
hsa05016	Huntington's disease	15	193	0.32	0.0483	9606.ENS	UQCRC1,NDUFA10,DNAH3,ATP5B,NDUFS6,SLC25A4,AP2M1,COX7A1,UQCRH,NDUFV1,HIP1,SDHB,POLR2B,NDUFS1,NDUFA6
hsa01100	Metabolic pathways	87	1250	0.27	3.33E-06	9606.ENS	PYGM,HSD17B10,UQCRC1,DLD,ACO2,PYGB,IDH3G,PYCRL,LTA4H,TPI1,CMAS,ACOT2,UROD,NDUFA10,MSMO1,ALDH2,ATP5B,PLD2,PAPSS1,HIBADH,POLG,PMM2,PGD,NDUFS6,PLA2G7,GLUD1,TM7SF2,DLAT,QDPR,DCK,COQ5,PDXK,MAT2A,ADH1B,ASL,PDHB,GLUL,UQCRH,LCLAT1,ACSS1,NDUFV1,DCTPP1,BCAT2,CNDP2,ACAA1,DLST,PFKM,ALDH3A2,ALDH9A1,HIBCH,MTR,PMVK,PHGDH,ATP6AP1,GOT1,SCP2,CTPS1,SDHB,SRM,ALDH1B1,SUCLA2,PDHA1,HADHA,PFKP,POLR2B,ASAH1,DBH,SUOX,AK4,TYMP,LSS,CECR1,GGT1,KDSR,IDH1,NDUFS1,CTPS2,NDUFA6,PCCB,SUCLG2,HSD17B4,ST3GAL1,BCAT1,MMAB,ALDH6A1,HADH,NAGK
hsa05200	Pathways in cancer	31	515	0.21	0.0483	9606.ENS	GNA11,SPI1,LAMA4,PDGFRB,SMAD2,EGFR,APPL1,GSTT2B,HSP90B1,BCR,CTNNA1,GNB2,PRKACA,GNAI2,BID,GNAI1,MGST3,GSTO1,GNAI3,GNG12,GNAS,STK4,TRAF1,COL4A1,BCL2,TXNRD2,RELA,LAMB2,TXNRD1,DAPK3,GNB1

SourceDataForFigure2D												
#node1	node2	node1 stri	node2 stri	neighborhc	gene fusio	phylogenet	homology	coexpressi	experimen	database a	automated	combined score
ACO2	DLD	9606.ENSI	9606.ENSI	0.051	0	0	0	0.153	0	0	0.773	0.802
ACO2	BCAT2	9606.ENSI	9606.ENSI	0	0	0	0	0.082	0.267	0	0.195	0.411
ACO2	ASL	9606.ENSI	9606.ENSI	0.097	0	0	0	0	0	0	0.377	0.413
ACO2	PFKP	9606.ENSI	9606.ENSI	0	0	0	0	0.083	0.062	0	0.391	0.43
ACO2	ALDH2	9606.ENSI	9606.ENSI	0.044	0	0	0	0.062	0.055	0	0.443	0.465
ACO2	PFKM	9606.ENSI	9606.ENSI	0	0	0	0	0.063	0.062	0	0.444	0.469
ACO2	HADHA	9606.ENSI	9606.ENSI	0	0	0	0	0.14	0.072	0	0.396	0.476
ACO2	SUCLG2	9606.ENSI	9606.ENSI	0.079	0	0	0	0.171	0.134	0	0.415	0.562
ACO2	GLUD1	9606.ENSI	9606.ENSI	0	0	0	0	0.096	0.092	0	0.593	0.637
ACO2	PDHA1	9606.ENSI	9606.ENSI	0	0	0	0	0.132	0	0	0.662	0.694
ACO2	DLAT	9606.ENSI	9606.ENSI	0.06	0	0	0	0.156	0	0	0.647	0.695
ACO2	SDHB	9606.ENSI	9606.ENSI	0.077	0	0	0	0.281	0	0	0.781	0.842
ACO2	SUCLA2	9606.ENSI	9606.ENSI	0.079	0	0	0	0.44	0.134	0	0.781	0.889
ACO2	DLST	9606.ENSI	9606.ENSI	0.06	0	0	0	0.554	0	0	0.782	0.9
ACO2	PDHB	9606.ENSI	9606.ENSI	0.042	0	0	0	0.806	0.055	0	0.74	0.948
ACO2	IDH1	9606.ENSI	9606.ENSI	0.135	0	0	0	0.129	0.08	0.9	0.752	0.979
ACO2	IDH3G	9606.ENSI	9606.ENSI	0.11	0	0.245	0	0.685	0.695	0.9	0.759	0.998
ACSS1	ALDH2	9606.ENSI	9606.ENSI	0.043	0	0	0	0.148	0.085	0.9	0.383	0.945
ACSS1	DLAT	9606.ENSI	9606.ENSI	0.054	0	0	0	0.078	0	0.9	0.136	0.914
ACSS1	PDHB	9606.ENSI	9606.ENSI	0.072	0	0	0	0.064	0	0.8	0.302	0.862
ACSS1	ALDH1B1	9606.ENSI	9606.ENSI	0.043	0	0	0	0.148	0.085	0.65	0.284	0.779
ACSS1	ALDH9A1	9606.ENSI	9606.ENSI	0.043	0	0	0	0.174	0.085	0.65	0.335	0.801
ACSS1	SUCLG2	9606.ENSI	9606.ENSI	0.046	0	0	0	0.142	0	0.8	0.115	0.835
ACSS1	SUCLA2	9606.ENSI	9606.ENSI	0.046	0	0	0	0.133	0	0.8	0.13	0.837
ACSS1	PDHA1	9606.ENSI	9606.ENSI	0	0	0	0	0.113	0	0.8	0.25	0.855
ACSS1	ALDH6A1	9606.ENSI	9606.ENSI	0	0	0	0	0.117	0	0.9	0.127	0.916
ACSS1	PCCB	9606.ENSI	9606.ENSI	0.071	0	0	0	0.063	0	0.9	0.196	0.92
ACSS1	ALDH3A2	9606.ENSI	9606.ENSI	0.043	0	0	0	0.136	0.08	0.9	0.452	0.95
ACTA2	VCL	9606.ENSI	9606.ENSI	0	0	0	0	0.063	0.382	0.9	0.551	0.97
ACTA2	WASL	9606.ENSI	9606.ENSI	0	0	0	0	0	0.715	0	0.403	0.822
ACTA2	ACTN4	9606.ENSI	9606.ENSI	0	0	0	0	0.087	0.381	0	0.36	0.607
ACTA2	SMAD2	9606.ENSI	9606.ENSI	0	0	0	0	0	0.133	0	0.473	0.524
ACTA2	EGFR	9606.ENSI	9606.ENSI	0	0	0	0	0.06	0.393	0	0.314	0.574
ACTA2	MYL9	9606.ENSI	9606.ENSI	0	0	0	0	0.356	0.291	0.9	0.519	0.975
ACTA2	SORBS1	9606.ENSI	9606.ENSI	0	0	0	0	0.087	0	0.9	0.058	0.906
ACTA2	COL4A1	9606.ENSI	9606.ENSI	0	0	0	0	0.155	0	0	0.459	0.524
ACTA2	ACTN1	9606.ENSI	9606.ENSI	0	0	0	0	0.087	0.381	0	0.455	0.665
ACTA2	PRKCD	9606.ENSI	9606.ENSI	0	0	0	0	0	0.403	0	0.132	0.46
ACTA2	MYH11	9606.ENSI	9606.ENSI	0	0	0	0	0.339	0.568	0.9	0.784	0.993
ACTA2	MYL6B	9606.ENSI	9606.ENSI	0	0	0	0	0.063	0.188	0.9	0.301	0.939
ACTA2	MYL6	9606.ENSI	9606.ENSI	0	0	0	0	0.063	0.188	0.9	0.325	0.941
ACTN1	VCL	9606.ENSI	9606.ENSI	0	0	0	0	0.273	0.974	0.9	0.77	0.999
ACTN1	ACTN4	9606.ENSI	9606.ENSI	0	0	0	0.982	0.107	0.997	0.9	0.725	0.999
ACTN1	THBS1	9606.ENSI	9606.ENSI	0	0	0	0	0.098	0	0.9	0.186	0.92
ACTN1	MYL9	9606.ENSI	9606.ENSI	0	0	0	0	0.177	0.132	0	0.37	0.511
ACTN1	CTNNA1	9606.ENSI	9606.ENSI	0	0	0	0	0.082	0.689	0.9	0.364	0.979
ACTN1	MYL6	9606.ENSI	9606.ENSI	0	0	0	0	0.118	0.132	0	0.321	0.435
ACTN1	MYH11	9606.ENSI	9606.ENSI	0	0	0	0	0.17	0.186	0	0.254	0.452
ACTN1	ITGA7	9606.ENSI	9606.ENSI	0	0	0	0	0.064	0.16	0.6	0.263	0.737
ACTN4	VCL	9606.ENSI	9606.ENSI	0	0	0	0	0.114	0.96	0.9	0.734	0.998
ACTN4	WASL	9606.ENSI	9606.ENSI	0	0	0	0	0	0.157	0	0.355	0.433
ACTN4	MYH11	9606.ENSI	9606.ENSI	0	0	0	0	0.17	0.186	0	0.187	0.403
ACTN4	MYL6	9606.ENSI	9606.ENSI	0	0	0	0	0.124	0.132	0	0.357	0.469
ACTN4	EGFR	9606.ENSI	9606.ENSI	0	0	0	0	0.088	0.393	0	0.25	0.549
ACTN4	ITGA7	9606.ENSI	9606.ENSI	0	0	0	0	0.064	0.16	0.6	0.143	0.694
ACTN4	THBS1	9606.ENSI	9606.ENSI	0	0	0	0	0.061	0	0.9	0.116	0.909
ACTN4	CTNNA1	9606.ENSI	9606.ENSI	0	0	0	0	0.07	0.381	0.8	0.352	0.915
ADH1B	ALDH2	9606.ENSI	9606.ENSI	0.048	0	0	0	0.14	0	0.9	0.924	0.993
ADH1B	ALDH9A1	9606.ENSI	9606.ENSI	0.048	0	0	0	0.063	0	0.65	0.427	0.797
ADH1B	ALDH1B1	9606.ENSI	9606.ENSI	0.048	0	0	0	0.063	0	0.65	0.587	0.854
ADH1B	ALDH3A2	9606.ENSI	9606.ENSI	0.048	0	0	0	0.063	0	0.9	0.492	0.948
AGRN	LAMA4	9606.ENSI	9606.ENSI	0	0	0	0.539	0.059	0.136	0.9	0.264	0.921
AGRN	EGFR	9606.ENSI	9606.ENSI	0	0	0	0	0.119	0.262	0	0.329	0.526
AGRN	HSPG2	9606.ENSI	9606.ENSI	0	0	0	0.581	0.19	0	0.9	0.909	0.947
AGRN	ITGA7	9606.ENSI	9606.ENSI	0	0	0	0	0.098	0	0.9	0.27	0.928
AGRN	LAMB2	9606.ENSI	9606.ENSI	0	0	0	0.54	0.161	0.185	0.9	0.138	0.929
ALDH1B1	ALDH2	9606.ENSI	9606.ENSI	0	0	0.449	0.976	0.06	0	0.8	0.734	0.809
ALDH1B1	GLUD1	9606.ENSI	9606.ENSI	0.049	0	0	0	0.099	0.164	0.6	0.251	0.746
ALDH1B1	ALDH3A2	9606.ENSI	9606.ENSI	0	0	0	0.639	0.063	0	0.8	0.776	0.858
ALDH1B1	PCCB	9606.ENSI	9606.ENSI	0	0	0	0	0.077	0	0.6	0.092	0.635
ALDH1B1	ALDH6A1	9606.ENSI	9606.ENSI	0	0	0.344	0.76	0.16	0	0.9	0.663	0.931
ALDH2	PCCB	9606.ENSI	9606.ENSI	0	0	0	0	0.077	0	0.6	0.092	0.635
ALDH2	GLUD1	9606.ENSI	9606.ENSI	0.049	0	0	0	0.099	0.164	0.6	0.319	0.769
ALDH2	ALDH3A2	9606.ENSI	9606.ENSI	0	0	0	0.632	0.063	0	0.8	0.755	0.858
ALDH2	ALDH6A1	9606.ENSI	9606.ENSI	0	0	0.344	0.741	0.19	0.295	0.9	0.652	0.952
ALDH3A2	GLUD1	9606.ENSI	9606.ENSI	0.049	0	0	0	0.112	0.138	0.6	0.251	0.742
ALDH3A2	PCCB	9606.ENSI	9606.ENSI	0	0.077	0	0	0.064	0	0.6	0.092	0.644
ALDH3A2	ALDH6A1	9606.ENSI	9606.ENSI	0	0	0	0.593	0.077	0	0.9	0.61	0.927
ALDH6A1	GLUD1	9606.ENSI	9606.ENSI	0	0	0	0	0.122	0	0	0.467	0.512
ALDH6A1	DLAT	9606.ENSI	9606.ENSI	0	0	0	0	0.092	0	0.8	0.209	0.843
ALDH6A1	BCAT2	9606.ENSI	9606.ENSI	0	0	0	0	0.06	0	0	0.439	0.45
ALDH6A1	ALDH9A1	9606.ENSI	9606.ENSI	0	0	0.362	0.765	0.16	0	0.9	0.717	0.932
ALDH6A1	HIBCH	9606.ENSI	9606.ENSI	0	0	0	0	0.319	0.406	0	0.726	0.879
ALDH6A1	HADHA	9606.ENSI	9606.ENSI	0	0	0	0	0.153	0	0	0.576	0.625
ALDH6A1	PCCB	9606.ENSI	9606.ENSI	0	0	0	0	0.308	0	0.9	0.502	0.962
ALDH9A1	IDH3G	9606.ENSI	9606.ENSI	0.055	0	0	0	0.054	0.123	0	0.354	0.426

ALDH9A1	GLUD1	9606.ENSI	9606.ENSI	0.049	0	0	0	0.217	0.164	0.6	0.21	0.767
ALDH9A1	PCCB	9606.ENSI	9606.ENSI	0	0	0	0	0.077	0	0.6	0.158	0.662
ASL	IDH3G	9606.ENSI	9606.ENSI	0.068	0	0	0	0.088	0	0.8	0.199	0.845
ASL	GLUD1	9606.ENSI	9606.ENSI	0.042	0	0	0	0.061	0	0	0.407	0.42
ASL	SDHB	9606.ENSI	9606.ENSI	0.043	0	0	0	0.063	0	0.8	0.196	0.836
ASL	IDH1	9606.ENSI	9606.ENSI	0.042	0	0	0	0.063	0	0.8	0.239	0.845
BCAT2	DLD	9606.ENSI	9606.ENSI	0	0	0	0	0.104	0	0.9	0.54	0.955
BCAT2	IDH3G	9606.ENSI	9606.ENSI	0.135	0	0	0	0.081	0.084	0	0.285	0.41
BCAT2	GLUD1	9606.ENSI	9606.ENSI	0.162	0	0	0	0.133	0	0	0.491	0.598
BCAT2	PDHB	9606.ENSI	9606.ENSI	0.147	0	0	0	0.063	0.127	0	0.318	0.461
BCAT2	DLST	9606.ENSI	9606.ENSI	0.046	0	0	0	0.324	0.081	0	0.173	0.444
BCAT2	SDHB	9606.ENSI	9606.ENSI	0	0	0	0	0.428	0	0	0.092	0.458
COL4A1	LAMA4	9606.ENSI	9606.ENSI	0	0	0	0	0.202	0	0	0.483	0.57
COL4A1	THBS1	9606.ENSI	9606.ENSI	0	0	0	0	0.115	0.333	0	0.399	0.614
COL4A1	EGFR	9606.ENSI	9606.ENSI	0	0	0	0	0.23	0	0	0.262	0.408
COL4A1	COL6A6	9606.ENSI	9606.ENSI	0	0	0	0.554	0.062	0	0.9	0.296	0.913
COL4A1	HSPG2	9606.ENSI	9606.ENSI	0	0	0	0	0.242	0.157	0	0.656	0.761
COL4A1	LAMB2	9606.ENSI	9606.ENSI	0	0	0	0	0.258	0	0	0.266	0.433
COL4A1	ITGA7	9606.ENSI	9606.ENSI	0	0	0	0	0.116	0	0.6	0.319	0.738
COL6A6	ITGA7	9606.ENSI	9606.ENSI	0	0	0	0	0.052	0	0.6	0.208	0.673
COMP	HSPG2	9606.ENSI	9606.ENSI	0	0	0	0	0.061	0	0	0.399	0.411
COMP	ITGA7	9606.ENSI	9606.ENSI	0	0	0	0	0	0.173	0.6	0.422	0.792
CTNNA1	VCL	9606.ENSI	9606.ENSI	0	0	0	0.58	0.085	0.974	0.9	0.643	0.998
CTNNA1	EGFR	9606.ENSI	9606.ENSI	0	0	0	0	0.077	0.513	0.9	0.272	0.962
CTNNA1	ITGA7	9606.ENSI	9606.ENSI	0	0	0	0	0.062	0	0	0.538	0.548
DLAT	DLD	9606.ENSI	9606.ENSI	0.076	0.513	0.359	0	0.61	0.919	0.9	0.921	0.999
DLAT	IDH3G	9606.ENSI	9606.ENSI	0.046	0	0	0	0.103	0.156	0	0.508	0.597
DLAT	GLUD1	9606.ENSI	9606.ENSI	0.11	0	0	0	0.173	0	0	0.329	0.463
DLAT	PFKM	9606.ENSI	9606.ENSI	0.074	0	0	0	0.081	0	0	0.363	0.411
DLAT	IDH1	9606.ENSI	9606.ENSI	0	0	0	0	0.149	0	0	0.496	0.552
DLAT	SDHB	9606.ENSI	9606.ENSI	0.167	0	0	0	0.301	0.416	0	0.424	0.778
DLAT	SUCLG2	9606.ENSI	9606.ENSI	0.081	0	0	0	0.262	0.083	0.8	0.293	0.896
DLAT	SUCLA2	9606.ENSI	9606.ENSI	0.081	0	0	0	0.363	0.128	0.8	0.56	0.946
DLAT	DLST	9606.ENSI	9606.ENSI	0	0	0.413	0.696	0.455	0.841	0.9	0.836	0.993
DLAT	PDHB	9606.ENSI	9606.ENSI	0.111	0.001	0.433	0	0.899	0.962	0.9	0.905	0.999
DLAT	PDHA1	9606.ENSI	9606.ENSI	0	0	0.432	0	0.747	0.884	0.9	0.833	0.999
DLD	PFKM	9606.ENSI	9606.ENSI	0.07	0	0	0	0.088	0	0	0.357	0.407
DLD	IDH1	9606.ENSI	9606.ENSI	0	0	0	0	0.117	0	0	0.463	0.506
DLD	PHGDH	9606.ENSI	9606.ENSI	0	0	0	0	0.062	0	0	0.558	0.568
DLD	IDH3G	9606.ENSI	9606.ENSI	0.077	0	0	0	0.198	0.262	0	0.425	0.644
DLD	SUCLG2	9606.ENSI	9606.ENSI	0.081	0	0	0	0.245	0.416	0.36	0.174	0.747
DLD	SDHB	9606.ENSI	9606.ENSI	0.167	0	0	0	0.266	0.458	0	0.57	0.838
DLD	GLUD1	9606.ENSI	9606.ENSI	0.086	0	0	0	0.268	0.05	0.8	0.434	0.915
DLD	PCCB	9606.ENSI	9606.ENSI	0.044	0	0	0	0.063	0	0.9	0.179	0.916
DLD	SUCLA2	9606.ENSI	9606.ENSI	0.081	0	0	0	0.871	0.05	0	0.427	0.927
DLD	PDHA1	9606.ENSI	9606.ENSI	0	0	0.208	0	0.445	0.815	0.9	0.788	0.997
DLD	DLST	9606.ENSI	9606.ENSI	0.076	0	0.398	0	0.846	0.886	0.9	0.852	0.999
DLD	PDHB	9606.ENSI	9606.ENSI	0.111	0	0.225	0	0.864	0.807	0.9	0.823	0.999
DLST	IDH3G	9606.ENSI	9606.ENSI	0.046	0	0	0	0.137	0.156	0	0.594	0.68
DLST	GLUD1	9606.ENSI	9606.ENSI	0.11	0	0	0	0.144	0	0	0.342	0.455
DLST	PDHB	9606.ENSI	9606.ENSI	0.111	0.043	0.291	0	0.322	0.382	0.9	0.579	0.986
DLST	IDH1	9606.ENSI	9606.ENSI	0	0	0	0	0.145	0	0	0.628	0.668
DLST	SDHB	9606.ENSI	9606.ENSI	0.167	0	0	0	0.868	0	0	0.467	0.936
DLST	SUCLG2	9606.ENSI	9606.ENSI	0.081	0	0	0	0.245	0.083	0.9	0.226	0.941
DLST	PDHA1	9606.ENSI	9606.ENSI	0	0	0.266	0	0.168	0.132	0.9	0.469	0.966
DLST	SUCLA2	9606.ENSI	9606.ENSI	0.081	0	0	0	0.677	0.419	0.9	0.702	0.993
EGFR	GNA11	9606.ENSI	9606.ENSI	0	0	0	0	0.089	0.064	0	0.484	0.521
EGFR	VCL	9606.ENSI	9606.ENSI	0	0	0	0	0.082	0	0	0.505	0.526
EGFR	WASL	9606.ENSI	9606.ENSI	0	0	0	0	0.076	0.393	0.9	0.402	0.961
EGFR	THBS1	9606.ENSI	9606.ENSI	0	0	0	0	0.088	0	0	0.48	0.506
EGFR	SMAD2	9606.ENSI	9606.ENSI	0	0	0	0	0	0	0	0.591	0.591
EGFR	PRKCD	9606.ENSI	9606.ENSI	0	0	0	0.552	0	0.379	0	0.399	0.482
EGFR	PFKP	9606.ENSI	9606.ENSI	0	0	0	0	0	0.171	0	0.46	0.534
EGFR	SUCLG2	9606.ENSI	9606.ENSI	0	0	0	0	0	0	0	0.558	0.558
EGFR	GNAS	9606.ENSI	9606.ENSI	0	0	0	0	0	0.308	0	0.477	0.623
EGFR	HSPG2	9606.ENSI	9606.ENSI	0	0	0	0	0.154	0.33	0	0.433	0.651
EGFR	IDH1	9606.ENSI	9606.ENSI	0	0	0	0	0	0	0	0.726	0.726
GLUD1	IDH3G	9606.ENSI	9606.ENSI	0.111	0	0	0	0.087	0	0.8	0.364	0.883
GLUD1	PHGDH	9606.ENSI	9606.ENSI	0.131	0	0	0	0.066	0	0	0.336	0.414
GLUD1	SUCLG2	9606.ENSI	9606.ENSI	0.071	0	0	0	0.233	0	0	0.244	0.414
GLUD1	SDHB	9606.ENSI	9606.ENSI	0	0	0	0	0.219	0	0	0.287	0.42
GLUD1	PFKP	9606.ENSI	9606.ENSI	0.045	0	0	0	0.063	0.057	0	0.425	0.45
GLUD1	PFKM	9606.ENSI	9606.ENSI	0.045	0	0	0	0.063	0.057	0	0.479	0.502
GLUD1	PDHB	9606.ENSI	9606.ENSI	0.167	0	0	0	0.231	0	0	0.381	0.569
GLUD1	PDHA1	9606.ENSI	9606.ENSI	0	0	0	0	0.172	0	0	0.568	0.627
GLUD1	IDH1	9606.ENSI	9606.ENSI	0	0	0	0	0.108	0.183	0.8	0.604	0.934
GNA11	IDH1	9606.ENSI	9606.ENSI	0	0	0	0	0	0	0	0.493	0.493
GNA11	GNAS	9606.ENSI	9606.ENSI	0	0	0	0.877	0.062	0.387	0.54	0.793	0.74
GNA11	PRKACA	9606.ENSI	9606.ENSI	0	0	0	0	0.064	0.173	0.9	0.231	0.932
GNAS	PRKACA	9606.ENSI	9606.ENSI	0	0	0	0	0.082	0.173	0.9	0.272	0.937
GNAS	IDH1	9606.ENSI	9606.ENSI	0	0	0	0	0	0	0	0.569	0.569
HADHA	HIBCH	9606.ENSI	9606.ENSI	0	0	0	0.577	0.05	0	0.9	0.369	0.915
HADHA	SDHB	9606.ENSI	9606.ENSI	0	0	0	0	0.163	0	0	0.32	0.407
HIBCH	SUCLA2	9606.ENSI	9606.ENSI	0.042	0	0	0	0.198	0	0	0.302	0.417
HIBCH	PCCB	9606.ENSI	9606.ENSI	0.072	0	0	0	0.152	0	0	0.421	0.505
HSPG2	VCL	9606.ENSI	9606.ENSI	0	0	0	0	0.204	0	0	0.619	0.684
HSPG2	LAMA4	9606.ENSI	9606.ENSI	0	0	0	0.543	0.118	0.136	0.9	0.304	0.927
HSPG2	THBS1	9606.ENSI	9606.ENSI	0	0	0	0	0.126	0	0	0.49	0.535

HSPG2	LAMB2	9606.ENSI	9606.ENSI	0	0	0	0.546	0.231	0.185	0.9	0.452	0.945
IDH1	IDH3G	9606.ENSI	9606.ENSI	0.06	0	0	0	0.079	0.872	0.9	0.681	0.995
IDH1	PDHB	9606.ENSI	9606.ENSI	0	0	0	0	0.113	0	0	0.49	0.529
IDH1	PFKM	9606.ENSI	9606.ENSI	0.111	0	0	0	0.062	0	0	0.511	0.557
IDH1	PHGDH	9606.ENSI	9606.ENSI	0.044	0	0	0	0.052	0	0	0.522	0.529
IDH1	SDHB	9606.ENSI	9606.ENSI	0.126	0	0	0	0.17	0	0	0.674	0.742
IDH1	SUCLA2	9606.ENSI	9606.ENSI	0.135	0	0	0	0.217	0	0	0.599	0.705
IDH1	PDHA1	9606.ENSI	9606.ENSI	0	0	0	0	0.085	0	0	0.506	0.529
IDH1	PFKP	9606.ENSI	9606.ENSI	0.111	0	0	0	0.062	0	0	0.359	0.418
IDH1	SUCLG2	9606.ENSI	9606.ENSI	0.135	0	0	0	0.184	0	0	0.559	0.662
IDH3G	PFKM	9606.ENSI	9606.ENSI	0.045	0	0	0	0.071	0	0	0.481	0.499
IDH3G	PFKP	9606.ENSI	9606.ENSI	0.045	0	0	0	0.064	0	0	0.493	0.507
IDH3G	PDHA1	9606.ENSI	9606.ENSI	0.053	0	0	0	0.155	0	0	0.465	0.535
IDH3G	PDHB	9606.ENSI	9606.ENSI	0	0	0	0	0.145	0.05	0	0.492	0.551
IDH3G	SUCLG2	9606.ENSI	9606.ENSI	0.082	0	0	0	0.171	0.178	0	0.522	0.661
IDH3G	SUCLA2	9606.ENSI	9606.ENSI	0.082	0	0	0	0.225	0.178	0	0.638	0.76
IDH3G	SDHB	9606.ENSI	9606.ENSI	0.185	0	0	0	0.433	0	0	0.628	0.813
INSR	SMAD2	9606.ENSI	9606.ENSI	0	0	0	0	0.053	0.417	0	0.192	0.515
INSR	PRKCD	9606.ENSI	9606.ENSI	0	0	0	0.553	0.062	0.398	0	0.085	0.423
INSR	SORBS1	9606.ENSI	9606.ENSI	0	0	0	0	0.07	0.393	0.9	0.517	0.969
ITGA7	LAMA4	9606.ENSI	9606.ENSI	0	0	0	0	0.062	0.157	0.9	0.409	0.947
ITGA7	THBS1	9606.ENSI	9606.ENSI	0	0	0	0	0	0.173	0.6	0.115	0.682
ITGA7	LAMB2	9606.ENSI	9606.ENSI	0	0	0	0	0.108	0	0.9	0.173	0.919
ITGA7	TNXB	9606.ENSI	9606.ENSI	0	0	0	0	0.125	0	0.6	0.145	0.674
ITGA7	MYL6B	9606.ENSI	9606.ENSI	0	0	0	0	0.063	0	0	0.528	0.538
KCNMA1	VCL	9606.ENSI	9606.ENSI	0	0	0	0	0.138	0.268	0	0.282	0.507
KCNMA1	PRKACA	9606.ENSI	9606.ENSI	0	0	0	0	0.072	0.361	0.8	0.058	0.873
LAMA4	THBS1	9606.ENSI	9606.ENSI	0	0	0	0	0.089	0.129	0	0.399	0.481
LAMA4	LAMB2	9606.ENSI	9606.ENSI	0	0	0	0.547	0.207	0.195	0.9	0.472	0.944
MAT2A	SDHB	9606.ENSI	9606.ENSI	0.046	0	0	0	0	0.413	0	0.179	0.5
MAT2A	MTR	9606.ENSI	9606.ENSI	0.135	0	0	0	0.064	0	0.9	0.663	0.969
MTR	PCCB	9606.ENSI	9606.ENSI	0	0	0	0	0	0	0	0.609	0.609
MYH11	VCL	9606.ENSI	9606.ENSI	0	0	0	0	0.063	0.17	0.9	0.456	0.952
MYH11	MYL9	9606.ENSI	9606.ENSI	0	0	0	0	0.202	0.316	0.9	0.72	0.982
MYH11	SORBS1	9606.ENSI	9606.ENSI	0	0	0	0	0.108	0	0.9	0.093	0.912
MYH11	PPP1CB	9606.ENSI	9606.ENSI	0	0	0	0	0.059	0.158	0.9	0.051	0.914
MYH11	PPP1R12B	9606.ENSI	9606.ENSI	0	0	0	0	0.135	0.157	0.9	0.354	0.946
MYH11	MYL6B	9606.ENSI	9606.ENSI	0	0	0	0	0	0.327	0.9	0.696	0.977
MYH11	MYL6	9606.ENSI	9606.ENSI	0	0	0	0	0	0.382	0.9	0.68	0.978
MYL6	VCL	9606.ENSI	9606.ENSI	0	0	0	0	0.047	0	0.9	0.32	0.929
MYL6	MYL9	9606.ENSI	9606.ENSI	0	0	0.229	0.74	0.098	0	0.9	0.633	0.925
MYL6	PHGDH	9606.ENSI	9606.ENSI	0	0	0	0	0	0.421	0	0.05	0.427
MYL6	SORBS1	9606.ENSI	9606.ENSI	0	0	0	0	0.063	0	0.9	0.05	0.903
MYL6	PPP1CB	9606.ENSI	9606.ENSI	0	0	0	0	0.048	0.401	0.9	0.057	0.939
MYL6	PPP1R12B	9606.ENSI	9606.ENSI	0	0	0	0	0	0.105	0.9	0.161	0.918
MYL6	MYL6B	9606.ENSI	9606.ENSI	0	0	0.403	0.978	0	0.387	0.9	0.641	0.937
MYL6B	VCL	9606.ENSI	9606.ENSI	0	0	0	0	0	0	0.9	0.25	0.921
MYL6B	MYL9	9606.ENSI	9606.ENSI	0	0	0	0.725	0.129	0	0.9	0.406	0.918
MYL6B	SORBS1	9606.ENSI	9606.ENSI	0	0	0	0	0.063	0	0.9	0.05	0.903
MYL6B	PPP1CB	9606.ENSI	9606.ENSI	0	0	0	0	0.064	0.187	0.6	0.064	0.676
MYL6B	PPP1R12B	9606.ENSI	9606.ENSI	0	0	0	0	0	0.105	0.6	0.309	0.731
MYL9	VCL	9606.ENSI	9606.ENSI	0	0	0	0	0.123	0	0.9	0.434	0.946
MYL9	SORBS1	9606.ENSI	9606.ENSI	0	0	0	0	0.062	0	0.9	0.257	0.924
MYL9	PPP1CB	9606.ENSI	9606.ENSI	0	0	0	0	0	0.187	0.9	0.427	0.949
MYL9	PPP1R12B	9606.ENSI	9606.ENSI	0	0	0	0	0	0.264	0.9	0.495	0.959
PCCB	PDHB	9606.ENSI	9606.ENSI	0.078	0	0	0	0.313	0	0	0.268	0.496
PDHA1	PDHB	9606.ENSI	9606.ENSI	0	0.843	0.448	0	0.977	0.996	0.9	0.952	0.999
PDHA1	PFKM	9606.ENSI	9606.ENSI	0.059	0	0	0	0.063	0.185	0	0.587	0.663
PDHA1	SDHB	9606.ENSI	9606.ENSI	0	0	0	0	0.129	0.419	0	0.37	0.653
PDHA1	SUCLA2	9606.ENSI	9606.ENSI	0	0	0	0	0.309	0.055	0	0.651	0.752
PDHA1	PFKP	9606.ENSI	9606.ENSI	0.059	0	0	0	0.063	0.185	0	0.481	0.577
PDHB	SDHB	9606.ENSI	9606.ENSI	0	0	0	0	0.253	0	0	0.361	0.502
PDHB	SUCLG2	9606.ENSI	9606.ENSI	0	0	0	0	0.186	0	0	0.466	0.547
PDHB	PFKP	9606.ENSI	9606.ENSI	0.069	0	0	0	0.062	0.05	0	0.538	0.565
PDHB	PFKM	9606.ENSI	9606.ENSI	0.069	0	0	0	0.062	0.05	0	0.573	0.598
PDHB	SUCLA2	9606.ENSI	9606.ENSI	0	0	0	0	0.682	0.05	0	0.708	0.904
PFKM	SDHB	9606.ENSI	9606.ENSI	0	0	0	0	0.072	0	0	0.396	0.416
PFKM	PFKP	9606.ENSI	9606.ENSI	0	0	0.448	0.975	0.061	0.945	0.8	0.909	0.989
PPP1CB	PPP1R12B	9606.ENSI	9606.ENSI	0	0	0	0	0	0.866	0.9	0.554	0.993
SDHB	SUCLG2	9606.ENSI	9606.ENSI	0.185	0	0	0	0.263	0	0.9	0.346	0.955
SDHB	SUCLA2	9606.ENSI	9606.ENSI	0.185	0	0	0	0.256	0.416	0.9	0.655	0.985
SMAD2	THBS1	9606.ENSI	9606.ENSI	0	0	0	0	0	0	0	0.54	0.54
SORBS1	VCL	9606.ENSI	9606.ENSI	0	0	0	0	0.064	0.596	0.9	0.704	0.987
SUCLA2	SUCLG2	9606.ENSI	9606.ENSI	0	0	0.448	0.929	0.082	0.064	0.8	0.862	0.829
VCL	WASL	9606.ENSI	9606.ENSI	0	0	0	0	0	0	0.8	0.657	0.928

Source Data For Figure 2E

NO.1

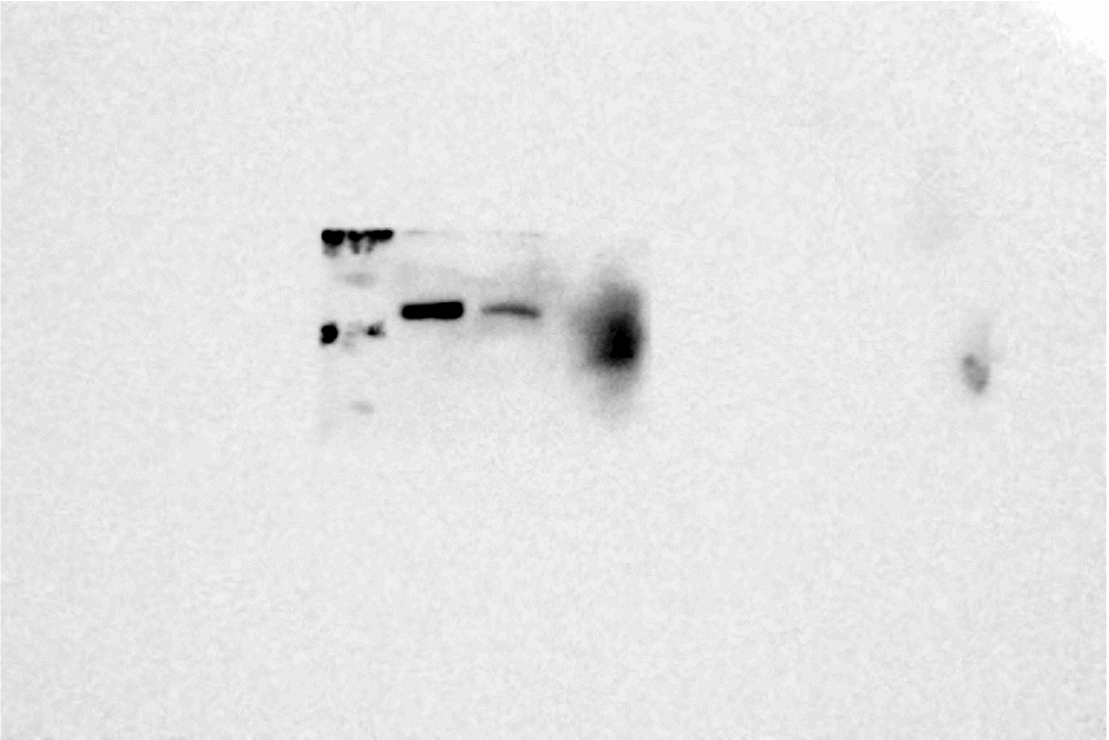

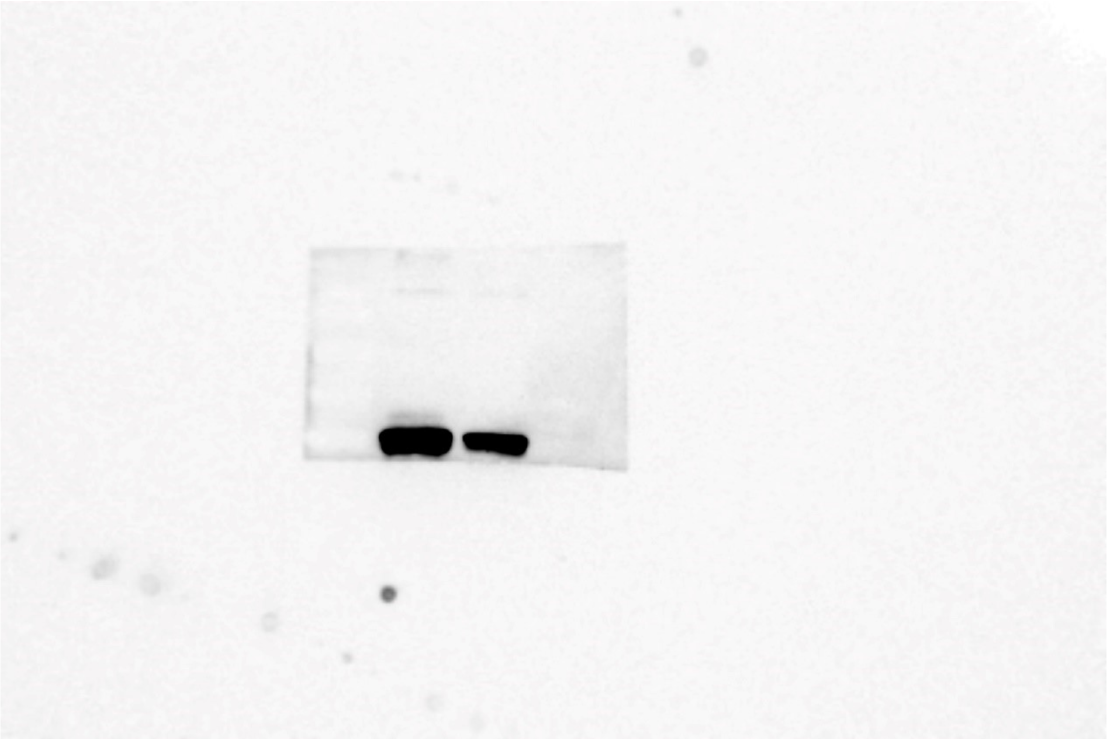

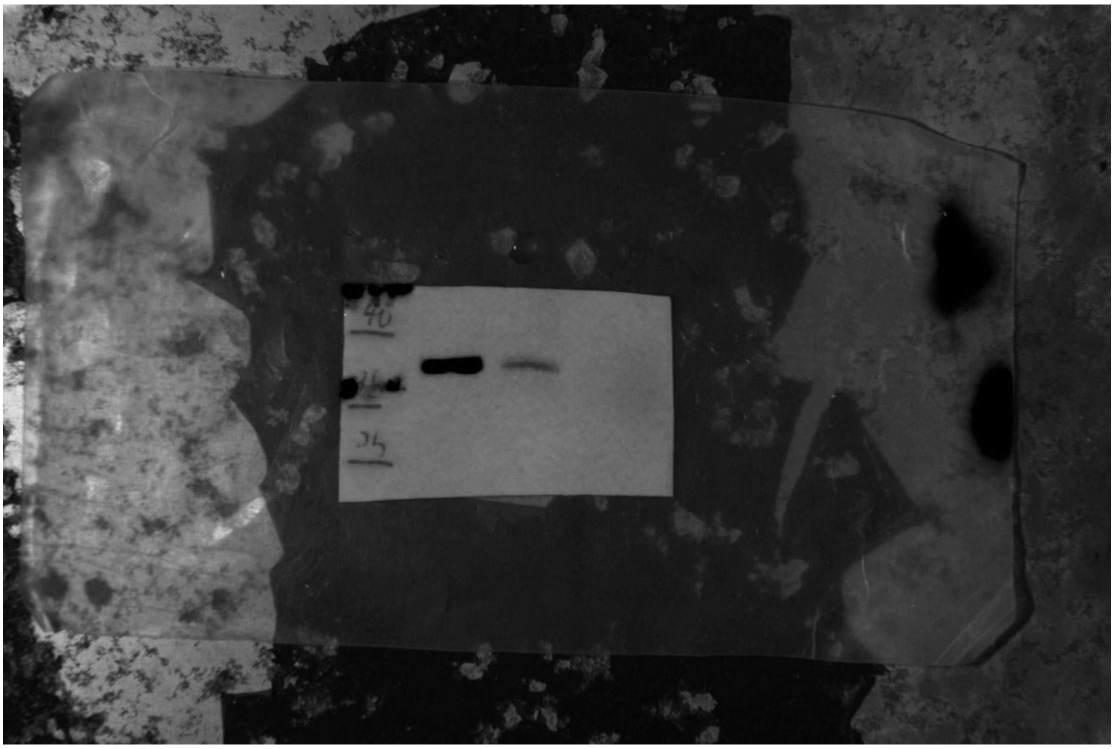
40

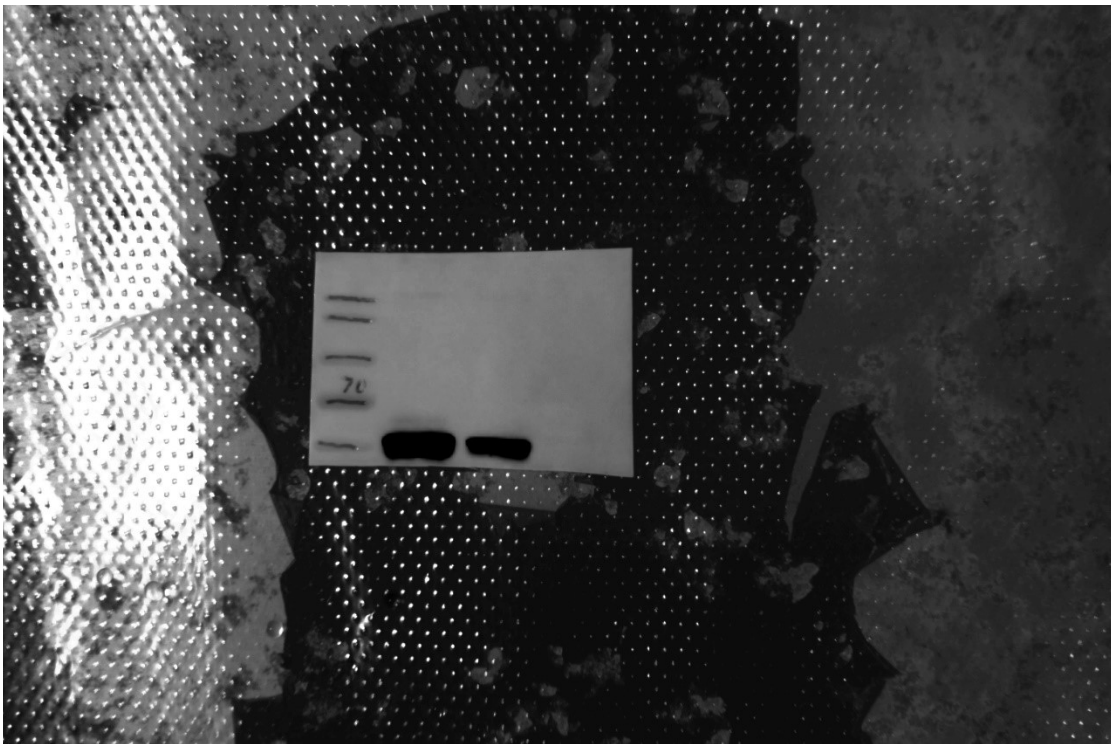
70

NO.2

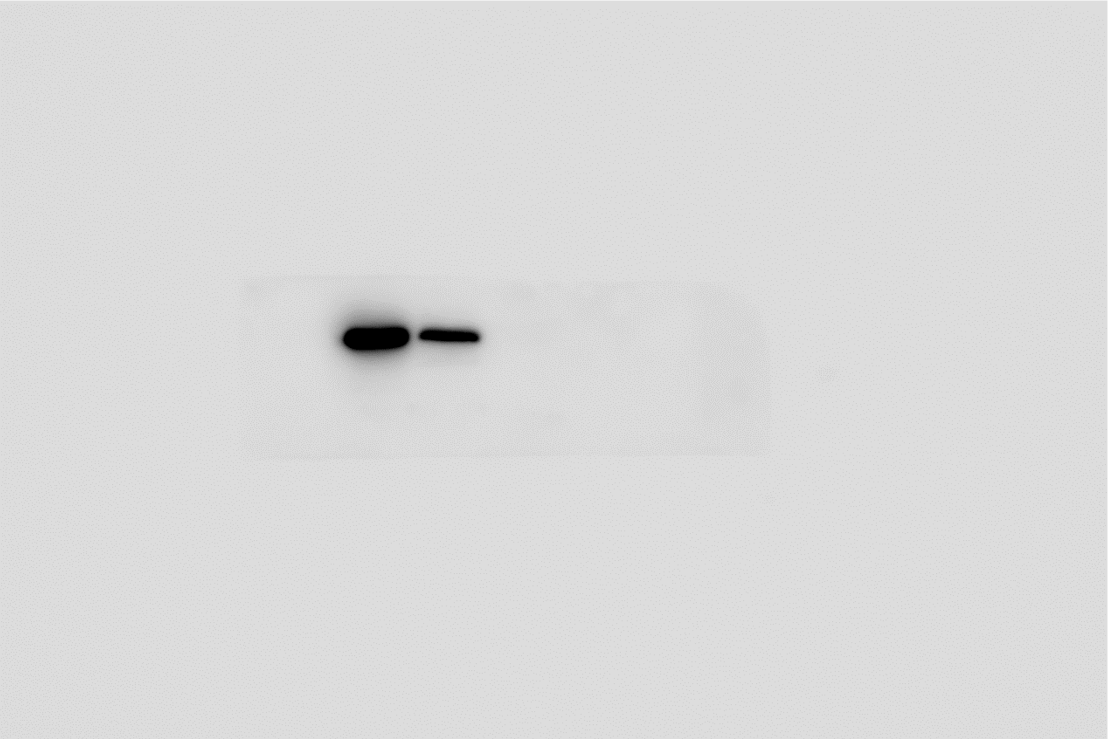

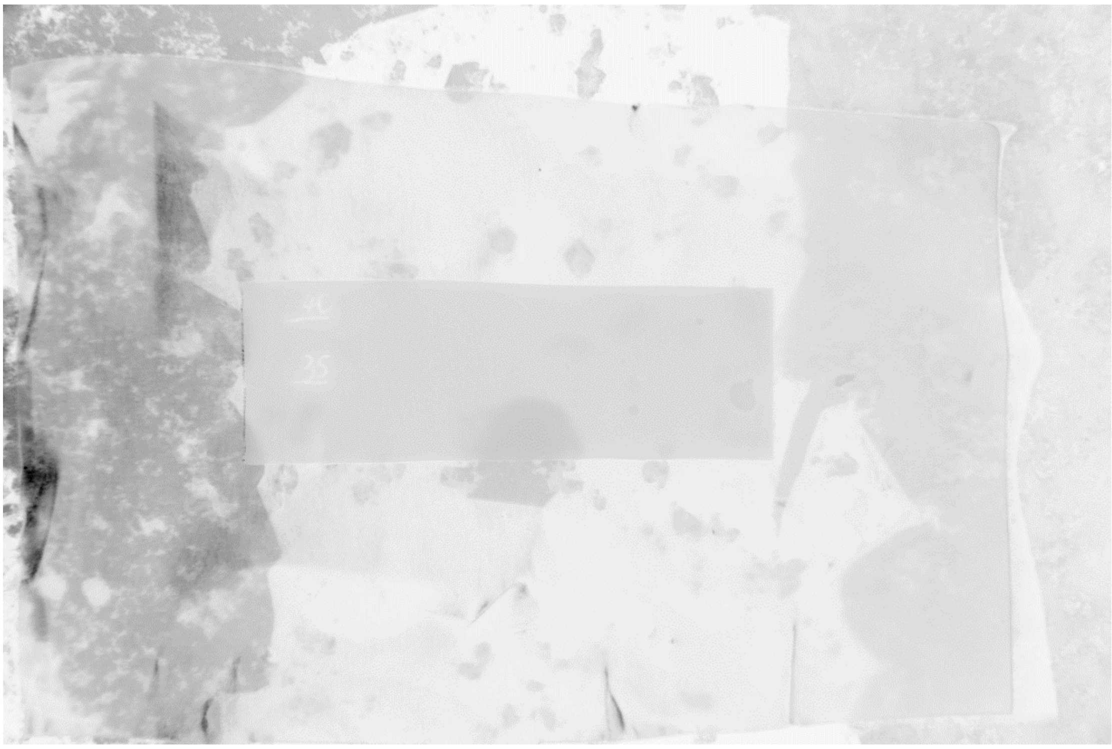

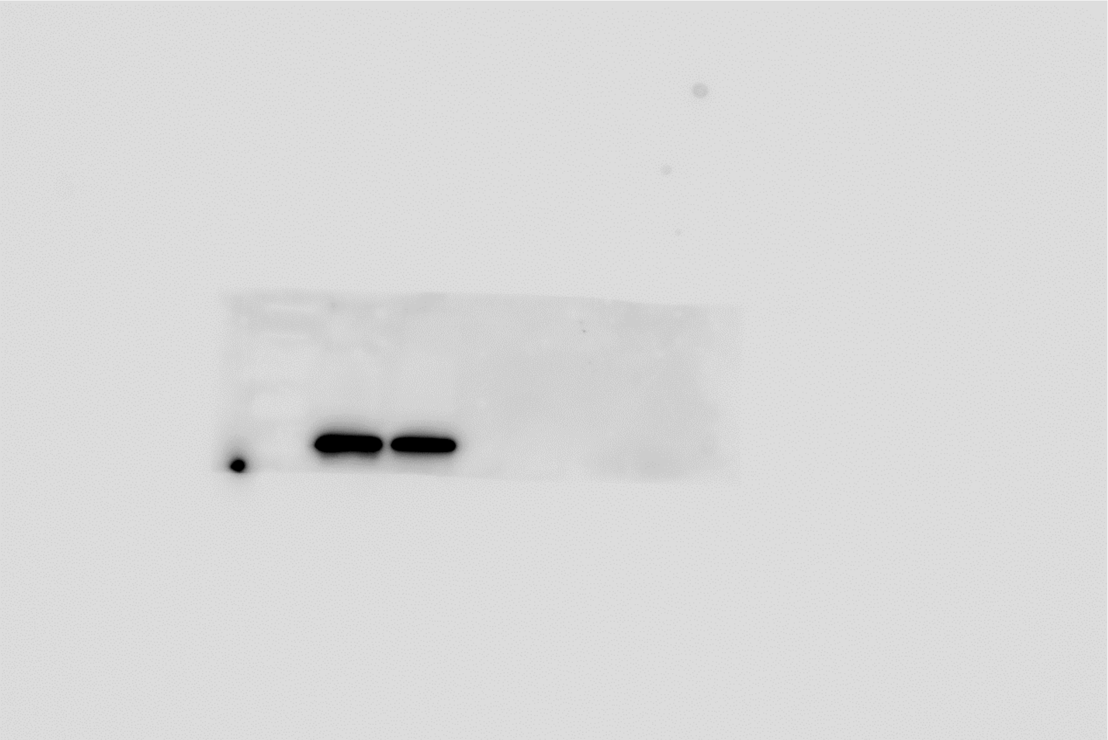

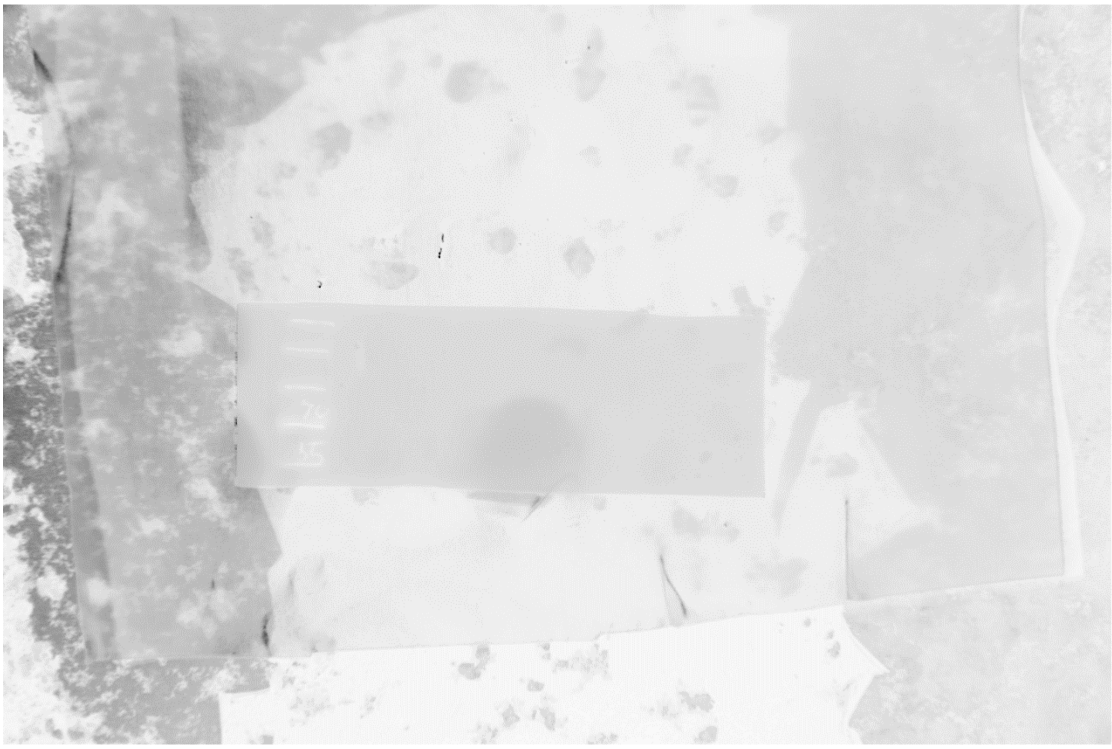

NO.3

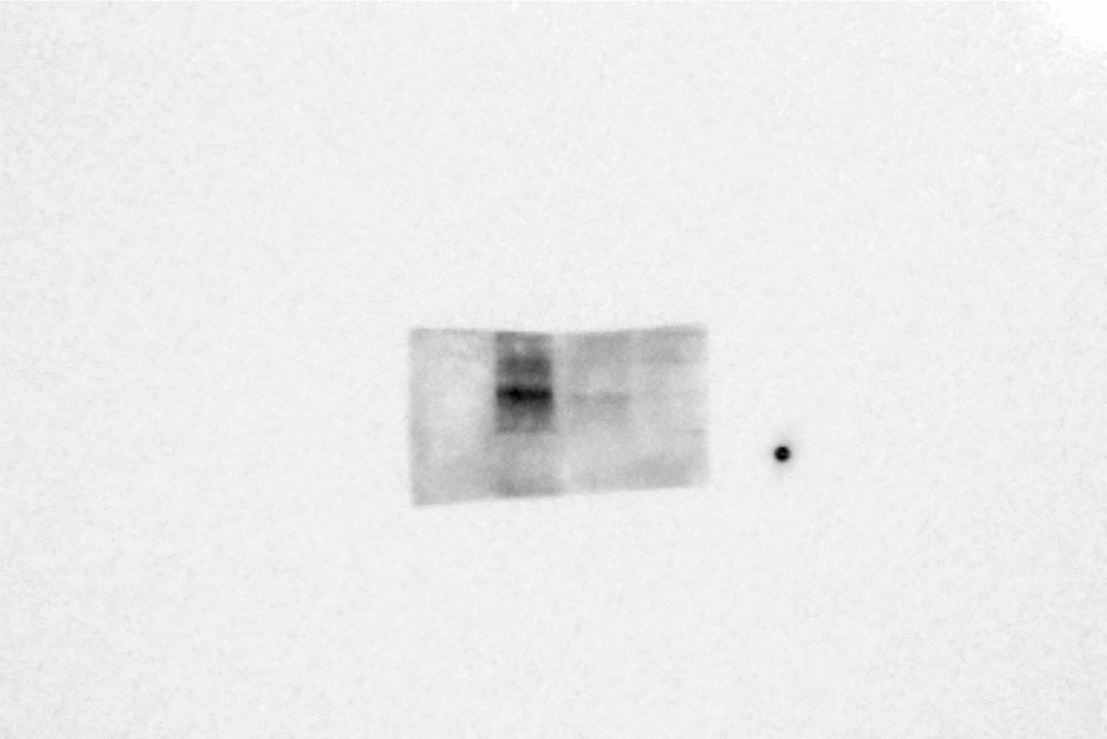

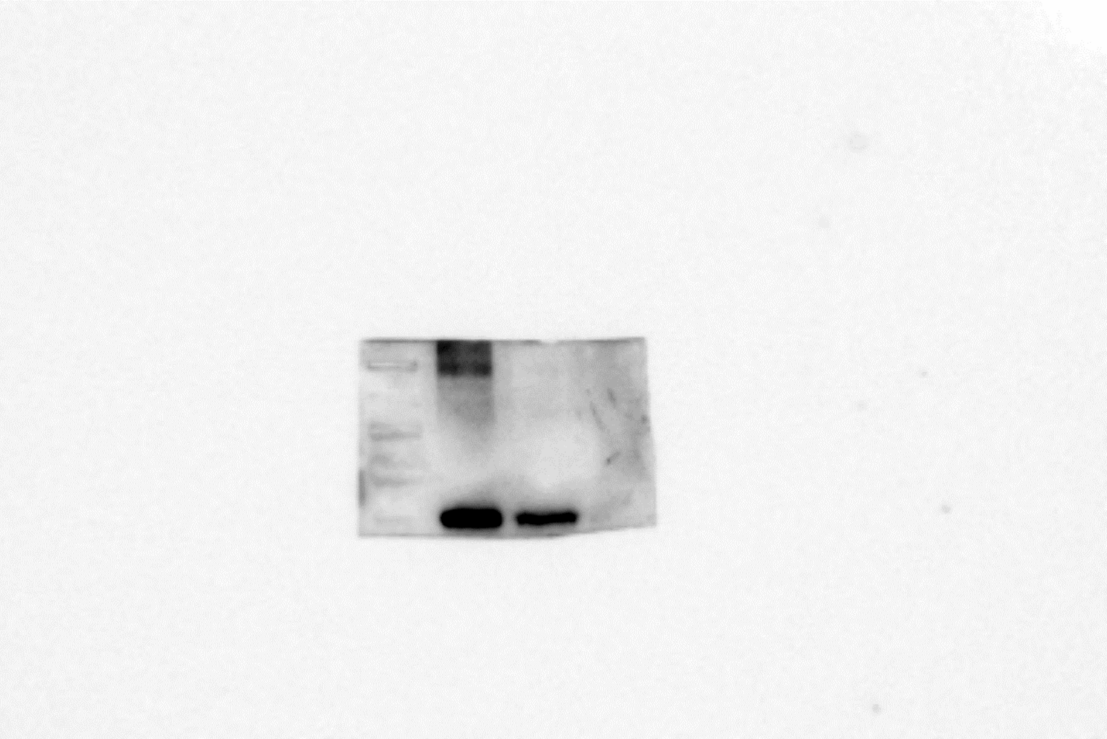

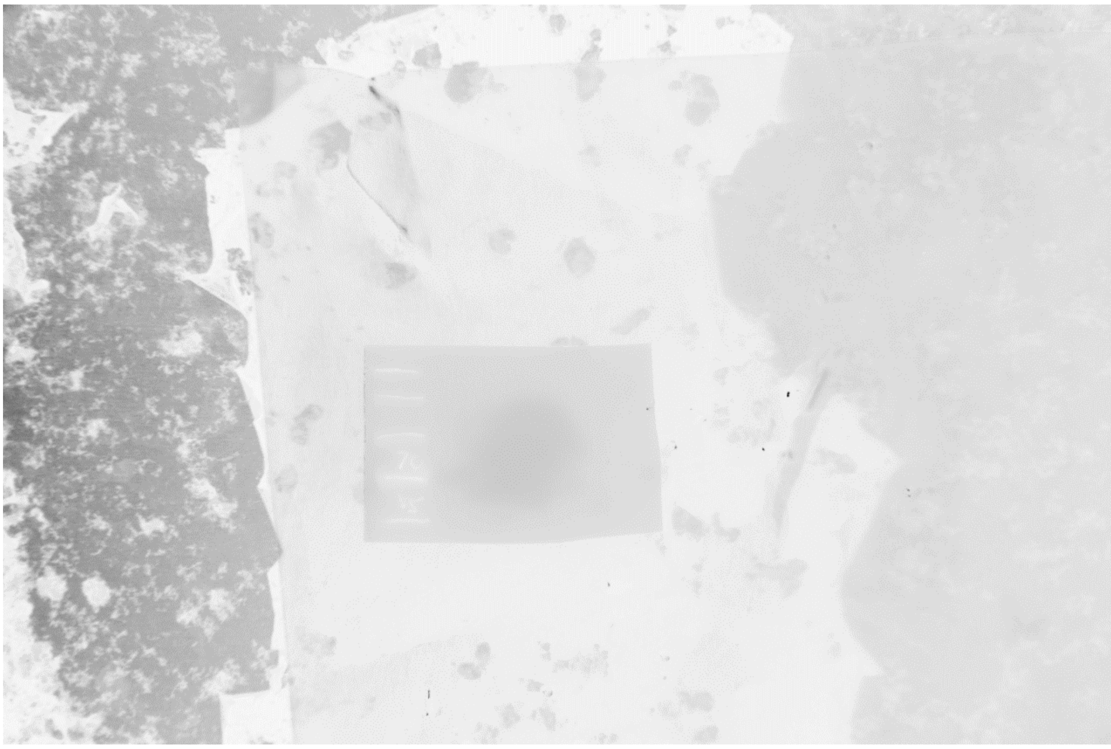

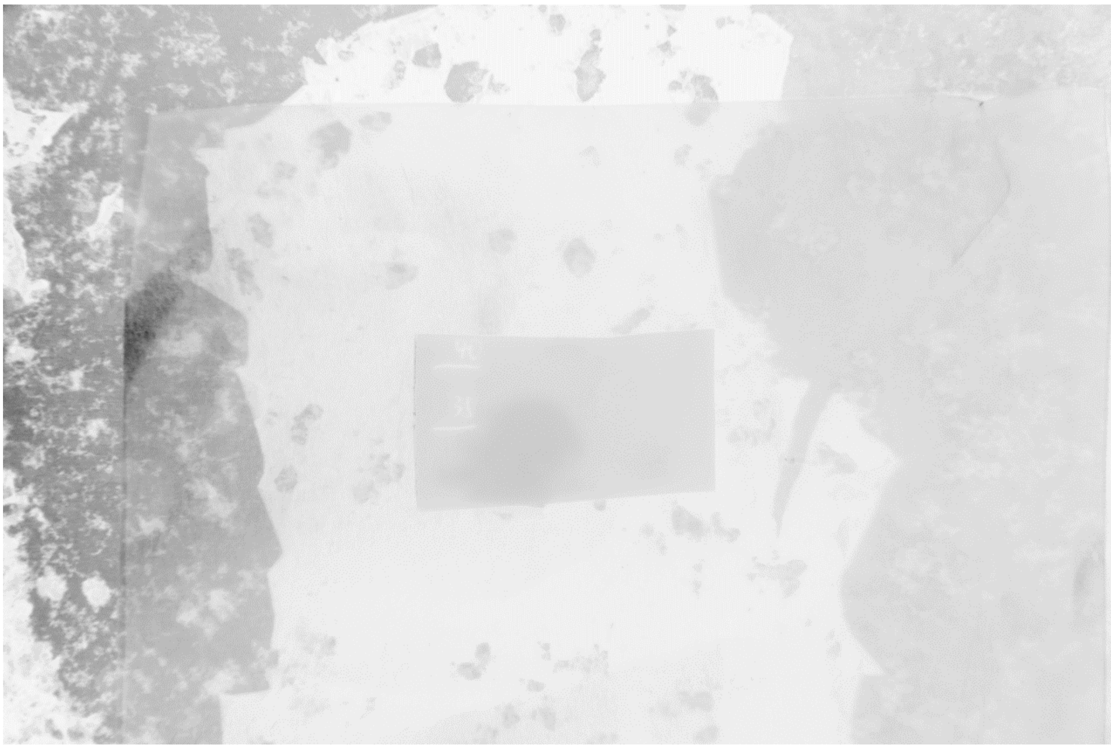

Supplement: Supplementary file 20 — Source Data for Figure 2 [file EMMM-14-e14713-s012.pdf]
