# Supplementary material for: Circulating proteomic panels for risk stratification of intracranial aneurysm and its rupture
Source: EMBO Mol Med. 2022 Jan 3;14(2):e14713. doi: 10.15252/emmm.202114713 (PMC8819334; doi:10.15252/emmm.202114713)
Supplement: Supplementary file 21 — Source Data for Figure 3 [file EMMM-14-e14713-s019.pdf]

SourceDataForFigure3A		
**TMT rep.1**	**TMT rep.2**	**TMT rep.3**
P01024	P01024	P01024
P04114	P04114	P04114
P01023	P01023	P01023
P00450	P00450	P00450
P02774	P02774	P02774
P02768	P02768	P02768
P0C0L5	P02790	P02790
P02790	P0C0L5	P0C0L5
P0C0L4	P0C0L4	P0C0L4
P00738	P02647	P02647
P02647	P00738	P08603
P08603	P01008	P00738
P01042	P08603	P01008
P19823	P01009	P01042
P01008	P01042	P01009
P01009	P19823	P19823
P00751	P00734	P00751
P00734	P00751	P00734
P00747	P00747	P00747
P19827	P19827	P19827
Q14624	Q14624	Q14624
P01031	P10643	P10643
P04003	P01031	P01031
P10643	P04003	P06396
P05155	P00739	P02760
P06396	P05155	P04003
P02760	P13671	P13671
P02787	P02760	P00739
P00739	P06396	P02787
P02751	P02787	P05155
P13671	P02765	P06727
P05156	P06727	P02751
P06727	P02751	P02765
P02765	P05156	P05156
P03952	P09871	P03952
P09871	P06681	P10909
O75882	P03952	P09871
P10909	P10909	P06681
P02748	P01019	O75882
P06681	P43652	P07358
P07358	P02748	P43652
P07357	O75882	P01019
P01019	P07358	P05546
P43652	P07357	P02748
P05546	P04217	P01876
P02649	P02749	P07357
P04275	P04275	P04217
P02749	P05546	P02649
P04217	P01876	P04275
P07996	P04196	P04196
P20742	P02649	P02749
P04004	P04004	P01871
P01871	P07996	P01834
P04196	P01834	P04004
P01834	P01871	P20742
P00736	P20742	P07996

P02656	P68871	P00736
P02763	P00736	P02763
P01876	P02763	P0DOX6
P68871	P02656	P68871
P04264	Q06033	P02656
P05090	P04264	P05090
Q06033	P08697	P08697
P0DOX6	Q96PD5	P04264
P08697	P05090	P12259
P12259	P0DOX6	Q96PD5
P02753	P12259	P0DOX7
Q96PD5	P02753	P02753
P0DOY2	P0DOX7	P27169
P27169	P27169	P0DOY2
P0DOX7	P0DOY2	Q06033
P01857	P36955	P07225
P36955	P07225	P36955
P07225	P0DOX5	P0DOX5
P51884	P35858	P51884
P02671	P51884	P35858
A0M8Q6	P35527	P05452
P08519	P05452	P22792
P02652	P08519	A0M8Q6
P22792	P02671	P08519
P05452	P22792	P80108
P35858	A0M8Q6	P02750
P02750	P02750	P02671
P80108	P80108	P02747
P35527	P00742	P35527
Q08380	P07360	P02652
P26927	P26927	P26927
P00742	P02747	P07360
P02747	Q08380	P00742
P48740	P48740	B9A064
P27918	P27918	P48740
P02746	P02652	Q96KN2
P07360	Q96KN2	P27918
P35908	P19652	P0DJI8
P01859	B9A064	Q08380
Q14520	P35908	Q9UGM5
P0DOX8	Q14520	Q14520
P08185	Q9UGM5	P01861
P01860	P01861	P19652
Q9UGM5	P08185	P01859
P19652	P02042	P35908
Q96KN2	P02746	P01860
P0DJI9	P01859	P02746
P0DJI8	P0DJI8	P02042
P01861	Q16610	P02655
P23142	P01860	P0DJI9
P02042	P23142	P23142
P01011	P01011	Q16610
O43866	O43866	P08185
P13645	P15169	P02766
Q04756	Q04756	P15169
P02655	P0DJI9	O43866
P15169	P02655	P01011
P00740	P13645	P13645

P02675	P49747	P00740
Q16610	P02766	P69905
P02654	P00740	P25311
P60709	P69905	Q04756
P02766	P25311	P02654
P49747	P02679	P02675
P69905	P60709	P02679
P02775	Q96IY4	P11226
Q12805	P43251	P43251
P25311	P02675	P60709
P11226	P02775	P49747
P0DOX2	P02654	P03951
O95445	P03951	P08571
Q92954	P0DOX2	Q12805
Q96IY4	Q92954	O95445
Q03591	P22105	Q96IY4
P02679	O95445	P0DOX2
Q02985	P11021	P02775
P03951	P13796	Q02985
Q13790	P11226	O00391
P08571	Q03591	P04070
P04070	O75636	P11021
O14791	O00391	Q03591
P13796	P08571	O75636
P11021	O14791	P35542
P17936	P35542	P63261
O00391	P17936	O14791
O75636	Q9NZP8	P05543
O00187	P05543	P22352
P22105	Q12805	Q9NZP8
Q07954	P63261	P13796
P05543	Q13790	P22105
P35542	P04070	P17936
P43251	Q02985	Q92954
P20851	O00187	O00187
P22352	P06276	P02743
P02743	P20851	P01591
P06276	P24821	P14151
P01591	P02743	P20851
P14151	Q9UK55	P06276
Q86VB7	Q86VB7	O00533
P24821	P01591	Q86VB7
Q9NZP8	Q07954	P24821
P36980	P98160	Q13790
O00533	P22352	Q9NQ79
Q9NQ79	Q9Y6R7	P00746
P19320	P14151	P19320
Q9Y6R7	O00533	P98160
Q9UK55	Q9NQ79	P36980
P00746	P00746	Q07954
P04278	P04278	P04278
P00488	P09172	P09486
P98160	P19320	Q9UK55
Q13201	P36980	P00488
P22891	Q9Y490	P40197
Q76LX8	Q15166	Q9Y6R7
P35443	P09486	Q9Y490
Q9BXR6	P68363	P35443

Q14766	Q15582	Q15166
Q92496	Q9BWP8	Q9BWP8
P00915	P22891	Q13201
Q15166	Q76LX8	Q14515
P05362	Q9BXR6	Q14766
Q15582	P61626	Q9BXR6
P68363	P00488	P00915
P61626	P00915	Q15582
P09172	P13591	P13591
Q2TV78	P35443	Q2TV78
Q9UHG3	P40197	Q76LX8
Q15113	Q13201	P04180
Q71U36	P35555	P07359
P35555	P07359	Q71U36
Q9Y490	P63267	P35555
P09486	Q14515	P22891
P01034	P01034	P68366
P05154	Q15113	Q92496
P63267	Q2TV78	Q9UNW1
P00748	P04180	P02776
P21333	P00748	Q15113
Q15485	Q9UHG3	Q15485
P18206	P02776	P63267
P04180	Q14766	Q9UHG3
A1L4H1	Q15485	P05362
P13591	P22897	P09172
P14780	Q9UNW1	P21333
Q7Z7M0	Q8NBP7	P14625
P02776	P14543	P12111
P08253	P21333	P10720
Q14515	P08253	Q92820
P05062	P14780	O14786
P40197	P14625	P18428
P14543	Q7Z7M0	P14780
Q9UNW1	P33908	P33908
P14625	Q92496	P05154
Q92820	P10720	Q14126
Q9BWP8	P05362	P08253
P22897	P18065	P07195
P49908	P02533	P02533
P10720	P08779	P15144
P07359	P05062	Q13103
P33908	P43121	P18206
P05556	P07195	P01034
Q16706	Q92820	A1L4H1
Q14126	Q14126	P05062
Q9Y5Y7	P05556	Q6YHK3
Q9HDC9	Q16706	P33151
Q6YHK3	P05154	Q7Z7M0
P18428	P15144	Q9HDC9
P04075	O14786	Q16706
Q01459	Q9Y5Y7	P22897
P07195	P12111	P00748
P43121	P49908	P48668
P00338	Q6EMK4	P61626
P33151	Q13103	P04259
P12111	Q13822	P08779
P12955	P18206	Q8NBP7

Q12860	P00338	P18065
P02533	P33151	P11717
P08779	Q6YHK3	P43121
Q13103	P04259	Q13822
P15144	P12955	P00338
P48668	P55058	Q9Y5Y7
P55058	Q7Z794	Q9ULI3
P05067	Q9HDC9	P14543
Q9NPY3	Q12860	P04040
P07737	P04040	P04075
P05160	P04075	P11142
P29622	P18428	P54289
O14786	P08294	P12955
Q13822	P55056	Q12860
P11717	A1L4H1	P01033
Q8NBP7	P11717	P12830
P13647	P29622	Q9NPY3
P23470	P02538	P13647
P24043	P04406	P07737
P55290	P05067	P55056
P55056	Q9NPY3	P01344
P54289	Q9ULI3	P05067
P18065	P07602	P55058
Q99784	P01344	P05556
Q6EMK4	P02745	Q6EMK4
P12830	Q04721	P23470
P01344	Q01459	P04406
Q16270	P13647	P02745
P02745	P13646	P40926
Q9ULI3	P07333	P07339
P07333	P54289	Q04721
P07602	P07339	P08294
P04040	Q12913	Q92859
P09960	P23470	P07333
P07942	P61769	Q01459
P11142	P07942	P61769
P08294	Q99784	Q12913
Q12913	P01033	P12109
Q9H4A9	P13797	P13598
P54108	P11142	P55290
P04745	P32119	Q8IZF2
P55103	Q8IZF2	P37802
P37802	P37802	P29622
Q86YZ3	P12830	P04746
Q9BTY2	Q15063	Q86YZ3
P07237	P09960	P07602
Q6UXB8	P40926	P05106
P80188	Q16270	Q99784
P05106	P04745	P04745
P01033	P54108	P16109
P16070	P13598	P05160
P08709	P07237	Q16270
P04746	Q86YZ3	P80188
P13637	P05160	Q9UM47
P78509	P17301	Q9Y6Z7
P61769	P80188	P07942
P24593	P10586	P46531
Q9NZK5	Q9UM47	Q9BTY2

Q04721	P11047	P32119
Q8IZF2	Q92859	P07998
Q9NPH3	P07998	P41222
P04406	P39060	P10586
P05534	Q9H4A9	P54802
P10124	P04066	Q6UX71
P06310	P13637	Q13093
P13797	P41222	O95497
Q15063	P16109	P11597
P13598	P04746	P02741
P07998	Q9NPH3	Q15063
P41222	Q9Y6Z7	P10124
Q9UM47	P07737	P24593
Q13740	P00558	P78509
P54802	P08709	P49908
Q9Y6Z7	Q7Z7G0	P11047
Q16853	P12109	P08709
Q9H4G4	Q9BTY2	Q6UXB8
A0A075B6P5	P55103	P10721
A0A075B6S2	P78509	Q16853
P10721	P12821	Q9NZK5
P23528	Q9NZK5	P0CG38
Q9NPR2	Q9UJJ9	Q9UJJ9
P0CG38	O95497	Q9NPH3
Q9UJJ9	P02741	P07237
Q9HBR0	Q9H4G4	P32942
P10586	P23528	Q9UBG0
P09972	P55290	Q9H4A9
P16109	Q6UXB8	Q9NPR2
Q9Y5C1	P05106	P13637
P11047	Q9HBR0	P01833
P63104	P05534	P13797
P12821	Q16853	Q5XKE5
Q8WZ75	P24043	P01780
P12109	P16070	P24043
P07339	Q13093	P09960
P20023	P01833	P23528
Q5XKE5	P24593	Q13740
P40926	Q9NZ08	P78385
P08238	P01780	P16930
P01833	P10124	P02452
Q92859	Q5XKE5	Q14118
Q86SQ4	P19022	P24592
P78385	Q9NPR2	P01764
P01780	Q13740	Q9BY67
O95980	P78385	P08195
P19022	Q9BY67	P19022
P05164	Q04695	P06733
Q9BY67	P11597	P0DP24
P02741	P80723	P11279
P00441	P06310	P17813
P00558	P14314	A0A075B6P5
Q13093	Q6UX71	P06310
P07477	P31146	Q9HBR0
Q8TER0	Q9UEW3	P08514
P16930	Q15848	P09972
A0A075B6K5	Q8TER0	Q7Z7G0
P69892	P20023	P00558

Q9NZ08	P00441	P0DP03
P01880	Q14118	P17301
O95497	A0A075B6P5	P03950
P11279	P11279	Q9H4G4
P03950	O15031	P37837
Q04695	Q8WWZ8	Q04695
Q6UX71	P16930	P30101
Q9H8L6	P54802	P0DOX3
P07911	P10721	P39060
O95479	P08195	Q86U17
O60462	P30508	Q12841
O43493	A0A075B6S2	Q86UD1
P04439	P69892	Q15848
Q14118	P03950	O95479
P15151	P24592	P35916
P67936	P09972	O43493
O15031	P17174	P55103
P28827	O95479	P63104
Q9NZT1	Q6UY14	P12821
P39060	Q9NY15	P04066
P11597	Q07075	P30501
Q02818	P0CG38	P15151
P17813	O75144	Q86SQ4
P81605	P67936	P16070
P80723	P30101	P13727
Q15848	P46531	P11362
Q7Z7G0	O43493	P05534
Q13885	Q12841	Q8IXL6
O15394	Q8TDJ6	P10451
P46531	P32004	P05107
P35916	P78417	P00441
Q07075	O60462	P25774
P16035	P25774	P32004
Q9Y646	O76013	O15031
P35590	P01892	P67936
P40189	O15394	Q6UY14
P17301	Q02818	Q99969
Q9BUN1	P37837	Q02487
Q6UY14	P05107	P17174
P78417	Q92823	Q8WWZ8
Q13423	P17813	Q8TDJ6
Q9NY97	P30464	P52566
A0A0C4DH38	P07477	Q6UVK1
Q8TDJ6	P61916	Q9HBB8
Q09160	Q9Y5C1	P16035
Q15323	Q13423	P14314
Q9NY15	Q29940	Q9NZ08
Q9BYE9	P08514	Q9Y5C1
P61916	Q5VY43	P49746
P10451	P20848	O76013
P24592	P22692	P10645
Q86YW5	P11362	P00533
Q86WI1	P02452	Q86TH1
P07437	O43157	P14618
P01892	P15151	P40189
P49746	P07911	O00584
P14618	P0DP03	P69892
P07196	P0DP24	P06312

P08195	Q8WZ75	O00602
P34931	O95980	O60462
Q9H1U4	Q6UVK1	Q5VY43
Q13449	P15924	Q9BYE9
Q92823	Q9Y646	P19367
P11362	P10451	P54108
Q99969	Q02487	P31146
Q99650	P06733	P01892
Q12841	Q9NY97	Q9UEW3
P08514	P35590	Q9Y646
P06312	P00533	Q8TER0
P0DP03	P49746	P10619
Q86UD1	Q15323	P78417
O76013	Q9H1U4	Q8NBJ4
P02452	O00592	P00390
P31146	A0A0C4DH38	Q9BUN1
P32119	Q13885	Q10588
O00584	P08567	Q13423
P17174	P06312	P07437
Q15828	P63104	P07858
P06732	Q12884	P10599
O75144	Q99969	A0A0B4J1X5
O76011	A0A0B4J1X5	Q13477
Q9Y4G6	Q86WI1	P27797
Q8IXL6	P11150	P32754
P32754	P19367	Q4LDE5
Q13477	P28906	P07477
P61981	P49913	P01619
O00602	Q86U17	O15394
P60900	P35442	P28827
A0A0A0MS15	Q10588	Q13449
P36222	P10619	P07196
P30101	Q8IXL6	P23083
P14314	P13727	P05164
P30508	P35579	Q99650
Q4LDE5	Q15828	O94985
O43157	P00451	Q01518
P04066	Q86UD1	Q9NY15
A0A0B4J1X5	P0DMV8	P28799
Q86U17	Q16394	P26038
P07478	P32942	P16284
P15924	Q13477	P22692
P06733	Q12907	Q15828
P26038	P10645	Q13508
P62937	O76011	O43157
P29401	Q99650	P07911
O43505	P01137	P78386
P25774	P54764	O00592
P19367	P81605	P80723
P10599	Q9UBG0	A0A0A0MS15
Q12884	P26038	Q9Y4L1
P00367	Q8NBJ4	P01825
Q9NTU7	Q9UQM7	P12110
P08567	P10599	Q96S96
P35442	P18669	Q7Z3B1
Q5VY43	O00602	P12814
P52566	P14618	Q15323
P02788	P36222	P30464

P25786	Q7Z3B1	Q9NQ38
Q9UEW3	Q01518	Q8WU03
Q9Y4L1	A0A075B6K4	P0DMV8
Q12907	P01880	Q8WZ75
P12814	P01700	Q9UBX1
P06702	Q9UBQ6	P25788
P28799	P05164	Q9UBQ6
P23471	P40189	P78504
O94985	P07196	Q7Z7M8
Q07507	P05109	Q9H1U4
P49913	P04350	P01700
P00533	P35916	Q13332
P50895	P06732	Q9UBR2
A0A075B6K4	P32754	Q12907
Q9UBG0	Q86YW5	Q5SYB0
P13727	P52566	P07148
Q02487	P28827	P61916
P10645	P05186	P06702
P32004	Q9Y4L1	Q9HCB6
Q8WWZ8	O43852	A0A0C4DH38
P05019	O95428	Q86UX7
P20848	P50895	P15291
Q13421	P25786	Q9NTU7
P10619	P49641	P81605
Q9NQ38	Q9H8L6	P02788
P62258	O00584	P60900
Q10588	P29401	O95980
P07858	P78386	P10646
Q86TH1	P07148	Q07075
P34096	Q9HBB8	P49913
P0DP24	P28799	P35590
Q13508	O76009	P35579
P30479	P06702	A0A0C4DH25
P04222	P60900	P20023
Q8IWV2	P27797	P05109
P01137	P15291	Q13885
P30464	Q9UBP4	P19021
P11150	Q9BYE9	P52209
P31946	P42785	P06732
Q8WU03	Q15262	Q8NDA2
P01619	P60660	Q16394
O14818	Q9NSB4	P23141
Q99972	P10646	Q12884
P46777	Q13508	Q9HCU0
P13489	P13489	P08581
P08174	Q13509	Q07507
P00918	Q8NBS9	Q9UQM7
P00451	P52272	Q9UBP4
P27797	P00390	P29401
O60814	P13611	Q15262
P15291	P07686	P15924
P01782	O14498	P46777
Q9NSB4	Q92520	A0A075B6K5
Q15262	P13473	P25786
P07686	Q96RD9	P36222
O00299	Q9NTU7	P49641
Q96EE4	P25788	P11150
O75874	Q07507	Q9NZT1

P22692	Q14525	A0A075B6R2
P04083	P07478	P08833
Q99983	Q8IWV2	A0A0C4DH29
Q7Z3B1	P08174	P06576
Q8NDA2	P34096	P10153
Q9UBP4	Q8N149	P07738
Q8NBJ4	P13667	P07988
P08581	O00151	Q9NSB4
P09619	Q14956	P34096
Q5SYB0	P50395	O75874
P23381	Q96HD1	P18669
P07148	P01619	O43852
Q13557	P07988	P69849
Q9HBB8	P08833	O43505
O00461	Q9UBR2	P13489
P60660	Q86SQ4	Q92520
Q96RD9	P35900	Q86WI1
Q13509	Q9UQP3	P00451
A0A0C4DH29	A0A075B6K5	P20848
Q8N3T6	Q13332	P14209
A0A0J9YXX1	Q9NZT1	Q99623
P05109	Q9UBX1	Q92854
Q8TDL5	P23471	O75144
P0DMV8	P09668	P31150
P01130	P51693	O00299
O75356	A0A0A0MS15	P61224
Q99623	P09382	P04430
Q8NBS9	Q13421	Q96HD1
P08311	P09619	Q9Y4G6
Q06481	Q8IZP9	P00918
Q9UQP3	Q6Q788	P07478
P52272	Q6P179	Q10471
Q13332	P62258	O00151
Q99941	Q99972	P07686
Q9BS26	Q7Z7M8	O60814
Q04917	Q86UN3	Q9UI15
Q6UVK1	A0A0J9YXX1	P06753
P42785	P02788	O76009
P69849	Q96S96	A0A0B4J1X8
P50395	O94985	P13667
Q92520	P19021	Q86YW5
P78386	P16035	P28906
Q6P179	O75015	P00505
Q8WUA8	Q10471	A0A0C4DH68
P62942	P62937	Q6UWP8
O00592	Q9BUN1	P20774
P52209	Q8WU03	Q99972
P01742	Q4LDE5	Q8NI99
Q86UX7	P00918	P56199
Q93063	O75874	P09668
P09668	Q8TDL5	P23284
P10153	Q5D862	P01137
Q8IUL8	Q5SYB0	P02144
Q9UBR2	Q8NI99	P01782
Q8IZP9	O43707	Q13421
P18669	P23083	Q02818
Q92854	Q8NDA2	P62942
Q02413	Q8WUA8	Q92823

Q9HCU0	Q92854	P08174
P21926	P23141	Q9H8L6
Q14697	Q86UX7	Q96KG7
P16284	P27348	P50895
P23083	O60814	O95236
P36269	Q8IUL8	P09619
P14923	P04899	P62873
P13667	P14209	P54764
P32942	P20061	P25815
P01040	P07858	Q8IWV2
Q9UBX1	P06576	P60660
A0A075B6H9	O00468	Q14314
Q86UN3	P01599	A0A075B6I9
Q5T2D2	Q99983	P04899
Q9HCN6	P52209	P01130
P0DP01	Q8IUC1	P68104
O00468	A0A0C4DH25	Q96EE4
O75023	P61224	O43707
P00491	P01742	P50395
P28072	Q9Y4D7	P01594
P01599	P21926	P21926
P13611	O95897	Q9UNN8
A0A0C4DH25	P31150	P31946
P01825	P13929	P80511
Q10471	P10153	Q6P179
Q9HCB6	Q03154	Q3ZCW2
P08575	P61981	O00468
P0CG48	Q96EE4	Q14767
P07900	Q9NX62	P01742
Q8NI99	Q14112	O00451
P10646	P08581	P05019
O14798	P23284	Q8N6C8
A0A0C4DH68	Q9HCU0	P50552
P20061	P49257	P52272
P01624	O95831	P60174
P01700	P02144	P13611
P25788	Q9P232	P08575
P58166	P31946	Q13228
Q8N6C8	Q96KG7	P24298
Q9UNN8	P14923	P05186
P02144	A0A0C4DH29	P23471
P55268	P13688	A0A0J9YXX1
P58546	P62873	Q96RD9
O00151	Q9BS26	P62258
P01709	A0A0C4DH68	P09622
O75083	P56199	Q9HBW9
O43852	A0A0B4J1Y9	P61981
P37840	P12110	Q6Q788
P01594	P08575	Q13275
P35579	P01594	Q86UN3
P31151	Q9HCB6	Q8IWL1
Q16394	P69849	Q03154
Q02978	P01130	Q3LXA3
Q96H15	P08311	Q13509
Q08ET2	Q6UWP8	Q8IUC1
P06576	P08319	P62937
P36871	Q86Y46	P30530
P16150	P00367	Q9BS26

Q8IUC1	P62942	P49736
P07738	Q93063	P11215
P08670	Q99941	Q14162
P28906	O43505	P23381
P56199	P52799	P08311
Q9UBX5	Q14532	P00491
P60174	P60174	P14923
P00390	P27105	Q99983
Q6Q788	P01825	O75083
Q16627	P00505	A0A0C4DH72
O00462	Q8N8Z6	A0A075B6K4
P19256	P12814	P31151
P37837	P04430	P13987
Q96S96	Q14162	P27348
P27930	Q96AP7	P02461
P54764	P58335	Q8N3T6
P24298	Q96J42	P27930
P00505	P16150	O00462
Q86X29	O75976	Q14697
P13987	Q3LXA3	P09493
Q7Z7M8	Q15404	O95428
P23141	O00462	Q13231
Q9HCL0	A0A075B6R2	Q8N149
P49720	Q9UNN8	Q8IZP9
P20618	P06753	Q01469
Q96HD1	O00299	P01709
Q14956	Q99623	Q8IUL8
P05186	Q14314	Q9HCL0
O95236	O75326	Q16627
P08319	Q8TDY8	P11177
P24387	Q9UI15	P20061
A0A0B4J1V6	Q9NQ38	P30043
Q8N1N4	Q13449	P51693
Q06830	P23468	Q6UXK5
P51693	P05019	A0A0C4DH67
P58335	Q13867	P13929
P07711	O00451	Q9NSC7
Q12794	Q86TH1	Q5T2D2
P78504	A0A0B4J1X8	P58335
Q96AP7	Q14697	P25787
P06865	O00461	Q16620
Q9HC84	P03973	A0A0B4J1Y9
P05451	P07711	P52799
P34932	Q86TY3	Q8WUA8
O95274	Q9P121	Q9UBX5
P13535	Q9HBW9	Q96QR1
P00352	Q14767	P08670
O75015	Q99497	P17900
Q8WZA1	Q13228	Q99436
O00757	Q15223	Q9P232
P17900	P00491	P42785
A0A0B4J1Y8	P58166	P13535
O95831	P07738	Q8WUJ3
P49257	O15335	O60641
Q14112	Q3ZCW2	Q9NS71
P61224	Q6PCE3	P01599
P07988	Q58EX2	A0A075B6H9
Q9H6X2	O60641	Q96AP7

P80511	Q8N6C8	Q9Y240
Q99715	Q9HCL0	O14498
P09382	O60449	Q86X29
Q12866	Q9UBX5	Q99497
Q9NS98	O14818	P49257
O95810	Q02809	P17931
Q5JRA6	Q06830	Q9Y251
Q9UIB8	P37840	P58546
P14207	P16284	P13688
P07585	P58546	O95274
P00568	P01040	Q93063
P25815	Q12794	Q92484
Q15303	P20062	Q9H4B7
Q16851	P13535	Q58EX2
Q14767	P14207	P09603
Q8NFT8	P01624	Q02978
P05107	O95236	O95810
Q9HD89	P39748	O15335
Q8IUC0	P18850	A0A075B6K6
Q9P232	Q16851	P16150
P22061	P19256	P49720
P02786	P28838	Q9H2X0
P23284	Q14623	Q02413
P01601	Q01469	P15311
O14793	Q8NHL6	Q14956
O95998	A0A075B6I1	P61764
O15335	P80511	P35968
Q08554	O95810	P29279
P39748	P20774	Q15404
P13224	P05060	Q03167
O95428	P13987	P23468
P11215	Q92896	P15090
Q01518	Q8IXJ6	P09467
Q9H4B7	Q15293	P02795
P28070	P40925	P01624
O15204	O75356	O43405
Q93070	P09467	Q7Z5L0
O60667	Q9NS71	P0DJD7
Q8N149	P36269	Q9UQP3
A0A075B6I9	Q86X29	P01601
P53634	P22894	P03973
P55000	P25815	O15145
P23468	O15204	Q86VD1
Q96RI9	Q03167	P22061
P08833	P00352	O14818
Q6UWP8	Q86UW7	Q96J42
P00480	P49720	P27105
A0A0C4DH67	P25787	P61978
P49641	Q8TAQ9	P01127
P59666	Q96FE7	P00352
Q9NR99	P15311	Q9GZT8
Q03154	Q16620	P06737
P13497	Q9H6X2	Q9NY97
Q03181	Q8N3T6	P07108
Q9Y251	P30043	Q96H15
P52565	Q06481	P22894
Q8TDY8	A0A075B6K6	O94769
Q8WWA0	P36871	P18850

Q9P2X0	Q08554	Q14847
O43895	Q8IWL1	Q8NBS9
Q7Z4R8	P27930	O75023
Q13867	P27487	Q8IXJ6
P06744	Q7Z5L0	P15531
P35247	P17405	P22392
P20774	P24387	Q86UW7
Q3LXA3	Q08ET2	Q15942
Q86UW7	P52565	Q6E0U4
P30043	P17931	P24387
O75976	Q9NSC7	P62328
A0A075B6I1	Q16627	Q8TDL5
Q03167	Q9BU40	P29122
P08648	P08670	A0A075B6I1
P05783	P11215	P28072
Q9HBW9	P51149	P21709
P31150	Q12866	Q96RI9
P06737	Q8WUJ3	Q12866
Q6UXH0	P07900	Q8WZA1
Q8N8Z6	A6NMY6	P21741
P45880	P46777	Q99426
A0A0A0MT36	Q14847	A0A075B6I0
P09603	Q16787	Q92896
P01701	P09211	Q8TAQ9
P09467	P68104	Q16787
Q01469	P00325	P13473
P18850	P60953	Q14623
Q8IWL1	O14960	P19256
Q9GZT8	P61764	P07951
Q8N335	P24298	Q13867
P25787	P30530	O75976
Q8TF66	P13497	P51149
P03973	P53801	P15088
P40925	P48637	Q8N8Z6
P21741	Q9UKU6	Q9Y4D7
P09622	Q13231	P0CG48
A0A0B4J1Y9	Q9H4B7	Q92187
Q8NHL6	P04424	Q9UMX5
Q7RTS7	Q93070	P20700
P04211	Q8N6Q3	P39748
Q9NQ76	P28062	P05060
O00560	O95967	P28062
Q68CJ9	O43278	Q16851
A0A075B6K6	Q9NZD4	Q06830
P15090	P13798	Q14393
P20062	P0DJD7	Q68CJ9
Q9H8J5	Q99436	P20062
P00325	Q9HCN6	Q04446
Q12882	Q9UK23	P36269
Q92484	P06744	P35247
A0A075B6I0	Q86SF2	P29966
P09493	P09622	Q08554
Q9UBQ6	Q15465	P28838
Q9C075	P40121	Q9P121
Q7Z5L0	O43895	Q93070
Q13308	P21709	P59666
P13521	P26447	P35030
Q14508	P02461	Q9UIB8

Q9Y240	Q16769	P00325
Q13630	P15090	P10912
A0A0C4DH72	P55000	Q5D862
P21709	P50552	Q8WWA0
P61160	A0A0C4DH72	Q96BZ4
P21695	Q5T2D2	O14960
Q16620	P55268	O43278
P08123	P62328	Q9BRK5
Q99542	P17900	O95831
Q9BRK5	P15531	P06744
P08473	P41271	Q02763
P07384	P04054	O75015
Q58EX2	P09913	A6NMY6
Q99497	Q9P2X0	P31431
P68104	P29279	Q86TY3
P62491	P01706	P98095
O15143	Q92484	P55268
Q9NZD4	P01709	P62805
P15531	Q96RI9	P19440
P23467	P04083	P17405
Q02809	Q9GZT8	P62826
P61764	P98095	P01701
Q99426	P29122	O95897
P14770	P22392	P13224
Q8TAQ9	P59666	Q92764
P31431	Q8N5C7	Q14112
P27105	Q05707	Q06141
P14209	Q9BRK5	P52790
P01706	P06737	Q99715
Q96J42	Q02413	P06865
Q9NS71	Q9NS98	P58166
Q9UMX5	P78504	P04054
P10912	Q8TF66	Q96KK5
Q9UKU6	P0CG48	A0A0B4J1Y8
P09326	P01701	Q9BU40
P19021	Q99426	Q8NHL6
Q9HAT2	Q5JRA6	Q9UK23
P55287	P00568	Q8NFL0
O14498	O95274	Q15223
P13639	P51124	A8MVU1
Q13637	Q9UIB8	Q6PCE3
Q5D862	Q92743	O43895
P28066	O94769	P08493
P28838	P01127	Q16769
Q99436	Q6UXK5	O60279
Q92896	Q04760	O14798
O60641	Q96H15	A0A075B6Y3
A0A0A0MRZ8	P09326	P28066
P48960	P20701	P63313
O60279	Q9HD89	P40429
Q9NSC7	P20933	P00995
Q9BYJ0	Q8WWA0	Q9BYJ0
Q9BXS4	Q9C0C4	P00367
Q96KG7	P02730	P08254
P12110	Q13308	Q9NX62
P16152	A0A075B6H9	P48960
Q15746	P21695	P04083
Q9BXP2	P28072	O15144

P55285	Q14019	P00568
P15311	O15144	Q5JRA6
P40429	P10912	Q9P2E9
Q9ULC0	Q15303	Q8TDY8
Q9BQ51	P08493	P53634
O14960	Q8NFL0	P01714
Q86VP6	Q08257	P39059
Q08495	A0A0B4J1V6	Q9C0C4
Q6UXK5	Q03181	Q8TD31
Q9UI15	P40429	P48163
P62328	O15145	Q68BL7
Q9BU40	P07451	P01706
P35052	Q6E0U4	Q13642
P15088	Q9Y4E6	Q9H6X2
Q14847	Q96QR1	Q99941
A0A0B4J1U7	P61160	Q9HD89
Q9UKX3	Q9BXP2	P36871
Q9BUD6	Q96PD2	P20618
Q8NCC3	Q86VD1	Q15293
P61158	P30740	P55285
Q9H0U4	P00995	P15529
Q15465	Q8IZM9	Q08495
P22894	P08254	Q16143
Q16769	P31431	Q92743
P35968	O95633	P55157
P05121	Q13404	Q13404
P26447	Q14393	P09326
Q6UX06	Q8N1N4	P60842
P01714	P39059	P07711
O15144	Q9UMX5	Q02747
Q06141	A0A075B6I0	P07585
P62826	P13521	P30740
Q9NYU2	P01111	Q8WWQ8
P02795	O43405	P20933
Q9H4F8	Q13275	Q01524
Q6ZRP7	Q13418	P08567
Q9Y279	Q9ULV4	P13497
Q9C0C4	P48960	P25789
Q9ULV4	Q13835	O15204
O43598	Q96NZ9	Q6ZRP7
Q9NT99	Q99536	Q86Y46
Q8IZM9	Q08722	Q8IZM9
Q17RQ9	Q68BL7	O75594
P09471	Q02952	P53801
O15145	Q96RW7	P14770
Q15293	Q9Y240	Q01995
P43304	Q9UKX5	P21695
P61106	Q68CJ9	Q8NCL4
Q8IVW4	P01714	P08648
P20700	O43280	O60234
P07108	P31949	P23467
Q9NR34	P23467	P28482
Q9BWV1	P19440	Q9NT99
Q01973	P04626	Q9UKU6
Q16787	P78324	Q13232
Q96FE7	P05451	O14793
P48539	P48506	Q9H0U4
P48723	Q6UXH0	O95998

Q96PD2	Q6ZRP7	Q13835
O43278	Q01524	Q9GZM5
Q14019	P16112	P40121
P52790	Q9NR34	Q14141
Q86SF2	Q8NCC3	P55196
Q86Z14	Q9Y251	P52565
Q13217	P48061	Q9Y279
Q9H299	Q9GZM5	P20701
Q15223	Q15691	O75326
O95897	Q92187	Q9Y4E6
Q96KK5	P01717	Q8NCC3
P31949	P62805	P34932
P61978	A0A0B4J1V0	P07900
Q08174	P07108	A0A075B6J9
P13686	P35030	Q99439
Q9NUQ9	P50281	P48061
P50552	P21741	Q9UMF0
P61088	P06703	Q8WXD2
P0DPA2	Q9Y2E5	A0A0A0MRZ8
Q08830	P06280	P09471
O00244	P23229	P05121
Q08257	P55145	Q08174
O75326	A0A0B4J1U7	P55899
O00194	P55285	O75223
P48509	P62826	P09382
P04424	Q9Y6N6	Q86U42
Q02747	Q53RD9	Q96B86
O75563	Q02747	A0A0A0MT36
Q9HC57	Q7Z6G8	P22314
P04085	P22061	P35670
Q6IPM2	A0A0A0MRZ8	Q05315
P13473	P55196	P22626
Q96QK1	Q8IUK5	Q15303
Q9UJV3	Q9BYJ0	P60981
P04062	Q7Z7L1	Q8IZW8
P13942	O75223	P23280
Q8NCL4	P14384	Q96FE7
Q86VD1	O95998	Q6UXM1
P30530	Q96QK1	Q9Y2E5
P41271	P35754	Q86Z14
Q9P121	P52790	P47756
P21589	Q9UL25	P61158
P55899	P53634	O95633
Q8NFY4	P23280	P09211
O43280	P04156	P31949
Q6P2E9	P23381	Q9HC84
P31994	P55157	P08118
Q969E1	A0A0A0MT36	Q14574
Q86SR1	Q9NTN9	Q01973
P29279	O00757	Q9H299
P55196	P07307	Q8N1N4
Q32MZ4	Q9UMF0	Q14667
P15085	P62917	P04424
P02730	Q86VP6	P55287
P20933	O60279	Q13630
A6NGU5	Q14141	P07307
Q13231	Q5T749	P61019
Q5T749	P22455	Q7Z5N4

O94769	P63313	P40925
P17931	Q8WZA1	P05451
P13798	P53396	Q969E1
P00995	P13639	Q9NPG4
Q9H0X4	P35247	A0A0B4J1U7
Q9H251	P07585	Q02809
P04156	P55899	P48723
Q6UXD5	Q9UBS4	P27487
P07307	P16233	Q8IVW4
Q9Y2T3	P28066	Q15691
Q13555	P61158	Q03181
Q96EG1	O00194	P49454
Q01524	Q96CM4	Q13308
Q05315	Q9HC57	Q96PD2
P39059	P31151	P00387
Q14162	P15088	P48735
P02461	P06865	P13798
P27487	P48735	P01717
Q86SJ6	Q8NCL4	P50281
P53801	P25789	P26572
Q9H2M3	Q6IPM2	Q9UBU7
Q9NP84	O14793	Q8N5C7
P14384	A0A0G2JS06	Q06481
O75223	A0A075B6Y3	Q8IUC0
Q7Z5N4	O60664	P22455
O14983	Q92692	Q08345
P61204	P22314	Q6UX06
Q9Y274	Q96E52	O75534
Q13228	P13942	Q92692
Q9UKX5	Q9BUD6	Q8NFY4
P60842	Q01995	P01040
P78324	P12429	O94973
P0DJD7	Q02978	Q96E52
P07451	P02795	Q9NYU2
Q6U841	Q14574	Q6IPM2
Q5TCZ1	O14798	Q9HBI1
Q9NRN5	Q9UJV3	Q08257
P23280	P22413	Q9BXS4
Q9Y4D7	P05089	P16152
Q04760	Q05315	Q86VP6
P04626	Q9UL03	P63220
Q4KMG0	Q86Z14	P49247
Q69YW2	P17050	Q00532
Q8NFL0	O75594	P13521
Q9P2E9	Q02763	Q8IUK5
Q96NZ9	P08473	Q14203
Q68BL7	Q00532	P49767
P09913	P34932	P16403
P13501	Q969E1	P01160
O15511	Q14667	P07451
P48436	Q06141	P17050
Q01995	Q2UY09	P00480
P08887	O15467	P02786
A6NMY6	O75822	O76061
P63313	Q96CG8	P63010
P48637	Q8IVW4	Q9NR99
Q05682	P15085	P28074
P81172	P55287	P35754

P26572	Q9Y624	A0A0B4J1V2
Q9HBW1	Q13217	A0A1B0GTC6
P35030	P02786	Q9BV35
P51149	A0A075B6I9	Q7Z7L1
P54760	Q07065	Q9BUD6
Q6IBS0	P14210	O75356
O15230	P54725	P20810
P48735	Q9UJU6	Q96NZ9
O00451	P35670	Q9UP79
Q6UXM1	Q9Y279	A0A0G2JS06
P62873	P13686	Q9Y624
Q9BTV5	Q92876	P21291
P78552	Q8IZW8	Q5TFQ8
Q9NX62	P00387	Q9Y376
P16581	Q99542	Q15375
Q99536	P56202	Q96RW7
Q9HC38	P00480	P19971
Q10472	P16581	P14207
P35237	Q08495	P16581
P17927	Q5PSV4	Q9UL25
P28074	P19971	P08123
Q96EN8	P53004	Q99536
P30041	P04062	O43280
A0A1B0GUS4	P54760	Q9H4M9
P48681	Q7Z4R8	Q9Y2Y8
Q99674	Q15836	P22304
Q02156	P48723	Q96IS3
Q9UJW2	P20618	Q9ULC0
P26006	P59998	Q7Z2D5
P49721	P20810	P40306
A0A0B4J1V0	P04085	Q8IZQ1
Q02952	Q96KK5	A0A0A0MT89
Q13835	P20138	P35367
Q8NI35	A0A087WSY6	P20160
P01236	A7E2Y1	P98172
A0A075B6Y3	P13224	Q9H4F8
P63010	P78552	Q8NI35
Q9HBI1	P05023	A0A0B4J1V0
Q14623	Q9H3S1	O94923
P78423	Q8IZQ1	O15240
Q2UY09	Q9Y6N7	Q13042
P40306	P26572	P26447
Q53RD9	Q6UXM1	Q12894
Q7Z7L1	P17655	Q2UY09
Q8NEN0	P15907	P45880
P10768	O75023	Q9UKX5
P83731	P11177	O14773
Q9NZN5	P28074	Q9NQW7
P13284	P14317	Q9H1Z8
Q9UPZ9	Q4KMG0	Q9BQ51
Q14574	Q17RQ9	A0A0B4J1V6
Q15375	P22304	O60883
Q02763	A0A1B0GTC6	Q8N465
P15529	Q6P4E1	O60832
Q8WUJ3	O76061	Q5TCZ1
P48061	Q08830	P62491
P13716	P84085	Q96CM4
Q8WVQ1	P13501	P13639

Q9BZR8	P35367	P08319
Q14141	P34059	P48506
O60832	Q8TEU7	P16871
Q8NB25	P63010	A5YM72
P35670	Q04446	P21399
Q13275	Q9UPZ9	O95866
Q5U5X0	O60234	P51124
Q15643	Q9UJX2	P50876
P35613	Q9H8J5	P06858
P14317	Q9HBW1	Q17RQ9
O60234	Q96BZ4	Q9GZX9
P30086	A0A0C4DH43	P53396
P49454	Q96CX2	Q8TF21
P19971	Q02156	Q8NHQ9
P58215	Q13683	Q12797
Q92692	P09471	A6NDG6
Q9H1B5	P42338	Q14019
O43556	Q9NP84	P35613
O95202	P49247	Q8NFT8
Q9HC56	P21291	Q9BZR6
Q06418	Q12864	P22413
P00492	P62491	Q9UPN3
P05387	P09601	O00194
P35754	P14770	P42126
O14594	O75069	Q9HAT2
Q7Z6G8	Q9BZR8	Q9UNH6
P26022	Q9H0U4	Q9UPZ9
Q8IX15	P45880	Q5U5X0
P49767	P20930	P43304
Q8N967	P09603	Q9H5L6
Q6UXH9	P16152	Q96CX2
P22314	O95782	Q8NHP8
Q9GZR7	O95866	Q6UWL2
O75051	Q8IVU3	P05089
P05089	P48681	P05026
P09601	O94856	O43768
Q8IZW8	P16871	P04156
Q96BZ4	Q9BXJ4	Q9Y6N6
Q86U42	Q8NHQ9	P04216
P98172	Q9H2X3	P13501
Q14203	Q9HC56	P04626
P05060	P0C7U1	Q9NS62
Q92743	Q9BQ51	Q86SF2
A0A0C4DH43	P08118	Q9ULV4
Q05193	O15143	Q93034
Q96CX2	P31153	Q96DT5
P37173	Q14508	P31153
P21583	Q9NQ76	P09913
Q96PL1	O14773	Q05586
P05026	O43768	Q99523
Q8NHP8	Q99435	A6NMZ7
P25789	Q9H9E3	P07384
P06703	Q8NFT8	P45877
Q92626	Q7Z2D5	P0C7W6
P05023	Q05682	P15907
Q8N5C7	P22626	P01225
P16112	Q86U42	Q13418
P10253	P15086	P26022

P60981	Q8N2S1	P63167
Q99538	Q9H4A4	Q9H8J5
Q7Z2D5	Q6UX06	P54760
Q14314	Q9UP79	O15143
O95967	P98164	Q9H9E3
Q86TY3	Q01638	Q9BPX5
P27361	P60981	Q08830
O60341	Q8NB25	P22735
P48059	Q9HD15	Q9BX84
Q15404	O75083	P21589
Q9UBX7	Q8NEN0	Q9UBX7
Q9BUJ0	Q8NHP8	Q9UBC9
Q8IUX7	Q8WWQ8	O14745
P14210	P21399	P30086
P56202	Q06828	Q9BUJ0
Q8WXD2	P61978	Q9HD15
O75382	Q9H299	P98161
Q6V0I7	Q5SQ64	Q9Y6Y8
Q9HD15	Q9HAT2	O95071
P07814	P05387	Q15283
Q6ZR08	Q15149	P48681
P42126	P02545	Q05682
P15086	Q9NT99	P17655
P98161	P30041	O75368
Q8WWQ8	P14920	P12429
Q96RW7	Q15375	Q86UP2
Q709C8	Q9Y6Y8	Q02952
Q14667	Q8TDD5	P02545
Q9Y4W6	Q9BWS9	Q96EP1
Q86UP2	Q9NR99	P68431
P15153	O14594	Q9BQS8
Q8N2S1	P35052	Q14789
Q14191	Q7Z6J4	O95210
Q14789	Q9UBX7	Q07065
Q07065	A0A0B4J1Y8	P06703
P53004	P21589	Q9Y274
A6NMZ7	P52888	P48637
Q8NFI4	Q9BXS4	Q8NFI4
P46783	P49736	P49721
P11177	Q9NRN5	P60953
Q3V6T2	Q9UNN5	P48059
A4UGR9	P07384	Q8TEU7
A6NEC2	Q16891	Q9NU22
Q08722	P47756	O94986
Q15413	P04179	P35052
Q9Y4G8	O75368	P52888
P98095	Q86UP2	Q9NTI5
P48506	P35237	Q86YW9
Q8WW22	Q92614	Q9NQ76
B2RXH8	O43556	P13942
P20810	P68431	Q8WY91
Q15691	P21810	Q13724
Q15942	Q9Y274	Q15058
Q8TF21	A6NMZ7	P29083
P14927	P42766	A0A0C4DH34
P52888	Q5U5X0	Q9H1B5
Q9NTI5	Q92626	Q6KC79
Q8IYJ0	Q99715	Q9UKW4

Q9NRC6	Q12797	Q9UJX2
Q8WY91	O95202	P63218
P14174	Q8WW22	Q96EN8
Q13724	P06756	Q9BRS2
Q13126	Q15942	Q8NB25
P49247	Q9NPG4	Q8IY85
Q8NA56	Q9NU22	Q6KB66
Q93050	P05026	Q6W2J9
Q13085	Q9HBI1	A6NDX5
Q6KC79	P09110	Q7Z6G8
Q7Z7M9	Q9NTI5	Q6Q0C0
Q6PGP7	P31994	Q7Z4Q2
Q8IY85	Q8IYJ0	Q68DL7
Q8IZX4	Q8WY91	Q9H0X4
Q8NET8	Q13724	O00461
Q6Q0C0	Q15058	Q4G0X9
Q9H9E3	O75683	O14986
P23458	Q4G163	Q53RD9
Q7Z4Q2	P29083	Q14159
O15240	A0A0C4DH34	Q7Z5M8
Q494V2	Q9H1B5	Q7Z418
Q8IYU4	Q6KC79	P98174
O14986	A6NHR9	Q9NR34
Q14159	A5YKK6	Q9UGM3
Q9UMF0	Q5T1H1	Q5VWQ8
Q9NY47	P49454	P04062
Q7Z418	Q8IY85	P05981
P08254	Q8WXD2	Q7KZ85
P55145	Q8N4C7	P19086
Q8WVM7	Q8IZX4	P16112
Q7KZ85	P15586	Q9H3S1
Q9BPW4	Q6KB66	Q5T124
Q8IV08	Q8NET8	O43933
P20908	A6NDX5	Q8IVU3
P55157	P50748	P16233
Q13105	Q6Q0C0	Q9ULD2
Q7Z4N2	P0C7W6	Q6P4Q7
A2RTY3	Q2TAC6	P48436
Q5T124	Q86YP4	Q8N967
P0C7X5	P29317	Q5PSV4
Q68BL8	Q494V2	Q9Y315
P11684	Q4G0X9	Q02156
Q96EH3	Q96L50	Q9BTV5
Q8IVU3	O14986	P0C7U1
P08118	Q14159	P22303
Q16288	Q7Z418	Q96SM3
Q5VTJ3	Q9UGM3	P26992
P51124	Q5VWQ8	P21583
Q96GX5	Q5TCZ1	P0DPA2
Q6ZMJ2	Q7KZ85	Q8IWA4
Q9UNH6	O60341	O75150
A8MVW5	Q8IV08	
Q9UK23	P19086	
Q9NNX6	Q7LGC8	
O00206	A2RTY3	
Q5PSV4	Q9UIW2	
Q6ZNQ3	P11684	
P22304	O43933	

Q6E0U4	Q8IWK6
P01718	Q8IXT1
Q9NX70	Q5VTJ3
P52799	Q9BZQ6
Q9UK05	Q6ZMJ2
P55774	Q9NP70
Q8NGL6	P20160
O76061	Q0VD83
P63129	Q10472
Q14393	Q9BTV5
	Q9BV57
	Q8WVQ1
	Q9Y580
	Q5JS37
	Q8IYS5
	Q9UBC9
	Q9P2E9
	Q8IWA4

SourceDataForFigure3B					
**Common elements in TMT result and plasma proteome database :**					
**Accession**	**Gene Symbol**	**Estimated pg/ml**	**concentration Log10**	**rank**	**type**
P68871	HBB	50014999984	10.69910027	1	transporter
P02768	ALB	37315000004	10.57188345	2	transporter
P04217	A1BG	4527559996	9.655864214	3	other
P02787	TF	3268888889	9.514400159	4	transporter
P55056	APOC4	2305000001	9.36267093	5	transporter
P01023	A2M	1648090910	9.216981164	6	transporter
P00738	HP	1427500000	9.154576117	7	peptidase
P01009	SERPINA1	1269155555	9.103514855	8	other
P02647	APOA1	1242983332	9.094465305	9	transporter
P01024	C3	1176779231	9.070694995	10	peptidase
P02671	FGA	1138911804	9.056490094	11	other
P19652	ORM2	610000000	8.785329835	12	other
P04114	APOB	563369874.6	8.75079362	13	transporter
P02790	HPX	516618999.5	8.713170374	14	transporter
P02765	AHSG	462333333	8.664955206	16	other
P02763	ORM1	462297545.9	8.664921588	17	other
P02675	FGB	349333333.4	8.543240028	19	other
P02679	FGG	348543199.9	8.542256614	20	other
P02652	APOA2	327921624.7	8.515770057	21	transporter
P02766	TTR	324414333.3	8.511100034	22	transporter
P01861	IGHG4	319999999.8	8.505149978	23	other
P0C0L4	C4A	273333333.5	8.436692598	24	other
P08603	CFH	270949999.8	8.432889155	25	other
P00450	CP	261899999.7	8.418135498	26	enzyme
P06396	GSN	258471000	8.412411823	27	other
P02774	GC	255872000.1	8.408022764	28	transporter
P01011	SERPINA3	248999999.9	8.396199347	29	other
P05155	SERPING1	219250000	8.340939602	30	other
P01042	KNG1	210749999.9	8.323767583	31	other
P02753	RBP4	180499999.9	8.256477206	32	other
P02788	LTF	180135000	8.255598104	33	peptidase
P00751	CFB	175315000	8.243819076	34	peptidase
P01871	IGHM	169000000.2	8.227886705	35	transmembrane receptor
Q9BPW4	APOL4	164000000	8.214843848	36	other
P05154	SERPINA5	160698571.4	8.206012016	37	other
P04004	VTN	150542769.4	8.177659901	38	other
P01008	SERPINC1	143520833.2	8.156914947	39	enzyme
Q6EMK4	VASN	130041999.9	8.11408364	40	other
P10909	CLU	124499909	8.095169034	41	other
P43652	AFM	122535000	8.088260155	42	transporter
P02749	APOH	112120800	8.049686188	43	transporter
P02751	FN1	105282937.6	8.022357994	44	enzyme
P06727	APOA4	97837199.97	7.990504015	45	transporter
P02656	APOC3	97398749.94	7.988553383	46	transporter
P0C0L5	C4B	96999999.94	7.986771734	47	other
P04196	HRG	91866666.77	7.963157959	48	other
P01031	C5	88750000.06	7.948168362	49	cytokine
P00747	PLG	86603000.08	7.937532937	50	peptidase
P05090	APOD	81999999.93	7.913813852	51	transporter
P08519	LPA	70000700.01	7.845102383	52	other
P00734	F2	68353127.26	7.834758389	53	peptidase
P02655	APOC2	67500000.03	7.829303773	54	transporter
P25311	AZGP1	65820166.72	7.818358978	55	transporter
P05019	IGF1	62903215.01	7.798672843	56	growth factor

P08697	SERPINF2	61158714.31	7.786458347	57	other
Q03591	CFHR1	57000000.04	7.755874856	58	other
P36980	CFHR2	57000000.04	7.755874856	59	other
P00736	C1R	56859999.95	7.754806855	60	peptidase
P05546	SERPIND1	53382000.01	7.727394841	61	other
P08185	SERPINA6	47066666.67	7.672713442	62	other
P10643	C7	46919999.95	7.671358003	63	other
P27169	PON1	46174999.99	7.664406904	64	phosphatase
Q14624	ITIH4	41999999.96	7.62324929	65	other
P07357	C8A	41200000	7.614897216	66	other
P69905	HBA1	41000000.03	7.612783857	67	#N/A
P00739	HPR	40699999.98	7.609594409	68	peptidase
P02748	C9	39825999.96	7.600166689	69	other
P02760	AMBP	39500000.03	7.596597096	70	transporter
P13671	C6	35925000.02	7.555396777	72	other
P02743	APCS	35020399.98	7.544321102	73	other
P03952	KLKB1	34200000	7.534026106	74	peptidase
P05452	CLEC3B	33100000.02	7.519827994	75	other
P09871	C1S	33040000.03	7.519040039	76	peptidase
P02042	HBD	30000000.02	7.477121255	77	transporter
P35542	SAA4	30000000.02	7.477121255	78	transporter
P69892	HBG2	30000000.02	7.477121255	79	other
Q9NSB4	KRT82	27999999.98	7.447158031	80	other
P04264	KRT1	27999999.98	7.447158031	81	other
Q8N1N4	KRT78	27999999.98	7.447158031	84	other
P35908	KRT2	27999999.98	7.447158031	85	other
Q86Y46	KRT73	27999999.98	7.447158031	86	other
P08670	VIM	27999999.98	7.447158031	89	other
P78386	KRT85	27999999.98	7.447158031	90	other
P06744	GPI	27574999.99	7.440515521	93	enzyme
P02649	APOE	26473749.98	7.422815463	94	transporter
P05156	CFI	26251424.99	7.419152883	95	peptidase
P01019	AGT	25705124.99	7.41001972	96	growth factor
P02654	APOC1	24649083.33	7.391800773	97	transporter
P19827	ITIH1	24000000.02	7.380211242	98	other
P15169	CPN1	23573333.32	7.372420997	99	peptidase
P06681	C2	23000000	7.361727836	100	peptidase
P05362	ICAM1	22929600.02	7.360396479	101	transmembrane receptor
P19823	ITIH2	21000000.01	7.322219295	102	other
P00748	F12	18186473.08	7.259748484	103	peptidase
P07225	PROS1	17425000.01	7.241172787	104	other
P23142	FBLN1	16809999.98	7.225567713	105	other
P35858	IGFALS	16499999.99	7.217483944	106	other
P27918	CFP	16332500.01	7.213052667	107	other
P20742	PZP	16150000.01	7.208172527	108	other
P22352	GPX3	15866666.67	7.200485698	109	enzyme
P35527	KRT9	15000000	7.176091259	110	other
P40197	GP5	14484500	7.160903508	111	other
Q96PD5	PGLYRP2	14000000.01	7.146128036	113	transmembrane receptor
P00488	F13A1	12988666.65	7.113564571	114	enzyme
P41222	PTGDS	12000000	7.079181246	115	enzyme
P29622	SERPINA4	11599999.99	7.064457989	116	other
P04003	C4BPA	11000000	7.041392685	117	other
P05543	SERPINA7	10433333.34	7.018423083	118	transporter
P05160	F13B	9189999.992	6.963315511	119	enzyme
P18428	LBP	9106666.661	6.95935944	120	transporter
P53634	CTSC	7800000.006	6.892094603	121	peptidase
P14618	PKM	7501250.004	6.87513364	122	kinase

Q13790	APOF	7366672.672	6.867271373	123	transporter
P00742	F10	7333333.332	6.865301426	124	peptidase
P0DJI8	SAA1	6967249.996	6.843061394	125	transporter
P12259	F5	6319624.993	6.800691308	126	other
O43866	CD5L	5600000	6.748188027	127	transmembrane receptor
P01591	IGJ	5600000	6.748188027	128	other
P36955	SERPINF1	5569999.998	6.745855195	129	other
P0DJI9	SAA2	5549999.998	6.744292983	130	other
P01344	IGF2	5139020.002	6.710880308	131	growth factor
Q08380	LGALS3BP	4770000	6.678518379	132	transmembrane receptor
P07998	RNASE1	4700000.001	6.672097858	133	enzyme
Q07954	LRP1	4423333.332	6.645749668	134	transmembrane receptor
P04275	VWF	4067999.998	6.609380944	135	other
Q15848	ADIPOQ	4006666.667	6.602783213	136	other
P51884	LUM	3999999.997	6.602059991	137	other
P61769	B2M	3973249.999	6.599145892	138	transmembrane receptor
P22792	CPN2	3900000	6.591064607	139	peptidase
O14791	APOL1	3550000	6.550228353	141	transporter
P02775	PPBP	3515724.998	6.546014897	142	cytokine
P12830	CDH1	3504999.998	6.544688022	143	other
Q96IY4	CPB2	3440000.7	6.536558531	144	peptidase
P22692	IGFBP4	3199999.998	6.505149978	145	other
P49908	SEPP1	2905000.002	6.463146137	147	other
P00740	F9	2900000.001	6.462397998	148	peptidase
O00187	MASP2	2799999.998	6.447158031	149	peptidase
P07358	C8B	2799999.998	6.447158031	150	other
P05106	ITGB3	2760499.997	6.440987751	151	transmembrane receptor
P02750	LRG1	2699999.999	6.431363764	152	other
P03951	F11	2431500.001	6.385874274	153	peptidase
P00746	CFD	2396000.002	6.379486814	154	peptidase
Q9UBX7	KLK11	2200000.001	6.342422681	155	peptidase
P02741	CRP	2135624.999	6.329524996	156	other
P01034	CST3	2073333.333	6.31666913	157	other
Q06033	ITIH3	2000000.002	6.301029996	158	other
P17936	IGFBP3	1967935.712	6.294010907	159	other
P06702	S100A9	1900000	6.278753601	160	other
P22891	PROZ	1822666.666	6.260707251	161	peptidase
P62328	TMSB4X	1800000	6.255272505	163	other
P24593	IGFBP5	1699999.999	6.230448921	166	other
Q14520	HABP2	1680999.998	6.225567713	167	peptidase
P04070	PROC	1593375.001	6.202317999	168	peptidase
P10153	RNASE2	1500000	6.176091259	169	enzyme
O95445	APOM	1500000	6.176091259	170	transporter
P11597	CETP	1284666.668	6.108790456	172	enzyme
P02786	TFRC	1253816.668	6.098234039	173	transporter
P02745	C1QA	1200000	6.079181246	174	other
P08571	CD14	1159999.999	6.064457989	175	transmembrane receptor
P07360	C8G	1100000	6.041392685	176	transporter
P24592	IGFBP6	1100000	6.041392685	177	other
Q9UK55	SERPINA10	1086666.666	6.036096345	178	other
P05556	ITGB1	1055500.001	6.023458238	179	transmembrane receptor
O75636	FCN3	1000000	6	180	other
O00602	FCN1	949999.9994	5.977723605	181	other
P02746	C1QB	930000.001	5.968482949	182	other
P02747	C1QC	909999.9993	5.959041392	183	other
P13987	CD59	909999.9993	5.959041392	184	other
P49747	COMP	810499.9997	5.908753019	185	other
Q13103	SPP2	800000	5.903089987	186	other

Q16610	ECM1	769999.9997	5.886490725	187	transporter
P63261	ACTG1	749999.9993	5.875061263	189	other
P08118	MSMB	710000.0005	5.851258349	193	other
O75882	ATRN	660000.0007	5.819543936	194	other
P14151	SELL	628285.7143	5.798157185	195	transmembrane receptor
P00441	SOD1	599999.9995	5.77815125	196	enzyme
P00915	CA1	590000.0005	5.770852012	197	enzyme
Q99969	RARRES2	570000.0004	5.755874856	198	transmembrane receptor
P20851	C4BPB	530000.0005	5.72427587	200	other
P24821	TNC	520999.9996	5.716837723	201	other
P32119	PRDX2	509999.9999	5.707570176	202	enzyme
P35442	THBS2	509999.9999	5.707570176	203	other
P43251	BTD	490000	5.69019608	204	enzyme
Q16270	IGFBP7	479999.9996	5.681241237	205	transporter
P80108	GPLD1	460000.0003	5.662757832	206	enzyme
P21333	FLNA	410000.0003	5.612783857	210	other
Q6UXB8	PI16	375499.9997	5.574609941	211	other
P07478	PRSS2	369999.9999	5.568201724	212	peptidase
P49913	CAMP	369999.9999	5.568201724	213	other
Q01459	CTBS	360000.0002	5.556302501	214	enzyme
Q02747	GUCA2A	349999.9997	5.544068044	215	other
Q96S96	PEBP4	349999.9997	5.544068044	216	other
P67936	TPM4	340000	5.531478917	217	other
P06753	TPM3	340000	5.531478917	218	other
Q9Y490	TLN1	340000	5.531478917	219	other
P03950	ANG	340000	5.531478917	220	enzyme
P07951	TPM2	340000	5.531478917	221	other
Q04756	HGFAC	319999.9998	5.505149978	222	peptidase
Q9Y6R7	FCGBP	300000.0002	5.477121255	223	other
P08709	F7	289833.3336	5.462148332	224	peptidase
P07996	THBS1	287124.9999	5.458071008	225	other
Q9UGM5	FETUB	269999.9999	5.431363764	226	other
P05109	S100A8	269999.9999	5.431363764	227	other
Q15828	CST6	269999.9999	5.431363764	229	other
P28799	GRN	269999.9999	5.431363764	230	growth factor
P18065	IGFBP2	265000	5.423245874	231	other
Q12805	EFEMP1	260000	5.414973348	232	enzyme
Q92954	PRG4	260000	5.414973348	233	other
Q9NZP8	C1RL	260000	5.414973348	234	peptidase
P08253	MMP2	254171.4286	5.40512673	235	peptidase
Q9NZD4	AHSP	250000.0002	5.397940009	236	other
Q92820	GGH	250000.0002	5.397940009	237	peptidase
P07477	PRSS1	234500	5.370142847	238	peptidase
Q96KN2	CNDP1	230000	5.361727836	239	peptidase
P61916	NPC2	230000	5.361727836	240	transporter
Q9H8L6	MMRN2	230000	5.361727836	241	other
P10720	PF4V1	230000	5.361727836	242	cytokine
P10451	SPP1	224906.6667	5.352002329	243	cytokine
P04180	LCAT	220000.0001	5.342422681	244	enzyme
P04040	CAT	220000.0001	5.342422681	245	enzyme
P04075	ALDOA	210000.0001	5.322219295	246	enzyme
Q04695	KRT17	210000.0001	5.322219295	247	other
O76013	KRT36	210000.0001	5.322219295	248	other
P13645	KRT10	210000.0001	5.322219295	249	other
P14923	JUP	210000.0001	5.322219295	250	other
P35900	KRT20	210000.0001	5.322219295	251	other
Q14525	KRT33B	210000.0001	5.322219295	252	other
P08779	KRT16	210000.0001	5.322219295	253	other

Q15323	KRT31	210000.0001	5.322219295	254	other
Q9NQ38	SPINK5	210000.0001	5.322219295	255	other
Q92520	FAM3C	210000.0001	5.322219295	257	cytokine
P02533	KRT14	210000.0001	5.322219295	259	other
P13646	KRT13	210000.0001	5.322219295	260	other
P80188	LCN2	208499.9999	5.319106059	261	transporter
P60174	TPI1	200000.0002	5.301029996	263	enzyme
P19320	VCAM1	195999.9998	5.292256071	264	transmembrane receptor
P02452	COL1A1	195144.9999	5.290357428	265	other
Q86YZ3	HRNR	190000	5.278753601	266	other
P30086	PEBP1	190000	5.278753601	267	other
P04278	SHBG	180499.9999	5.256477206	268	other
P40189	IL6ST	180433.3332	5.256316772	269	transmembrane receptor
P31946	YWHAB	180000	5.255272505	270	other
Q04917	YWHAH	180000	5.255272505	271	transcription regulator
P61981	YWHAG	180000	5.255272505	274	other
P62258	YWHAE	180000	5.255272505	275	other
P63104	YWHAZ	180000	5.255272505	276	enzyme
P27348	YWHAQ	180000	5.255272505	277	other
Q16627	CCL14	174250.0001	5.241172787	278	cytokine
P06276	BCHE	169999.9999	5.230448921	279	enzyme
P21583	KITLG	165029.5001	5.217561584	281	growth factor
O75368	SH3BGRL	160000.0001	5.204119983	283	other
P48740	MASP1	154500.0001	5.188928484	284	peptidase
P13688	CEACAM1	150250.0001	5.176814481	285	transporter
P13797	PLS3	150000	5.176091259	286	other
P63313	TMSB10	150000	5.176091259	287	other
P13796	LCP1	150000	5.176091259	288	other
O00391	QSOX1	150000	5.176091259	289	enzyme
Q02985	CFHR3	140000.0001	5.146128036	290	other
P62937	PPIA	140000.0001	5.146128036	292	enzyme
P23528	CFL1	140000.0001	5.146128036	293	other
Q15582	TGFBI	140000.0001	5.146128036	294	other
P30043	BLVRB	140000.0001	5.146128036	295	enzyme
P05062	ALDOB	129999.9999	5.113943352	297	enzyme
P09668	CTSH	129999.9999	5.113943352	300	peptidase
P07737	PFN1	129999.9999	5.113943352	302	other
P81605	DCD	129999.9999	5.113943352	303	other
P00995	SPINK1	125500.0001	5.098643726	304	other
P12814	ACTN1	120000	5.079181246	305	transcription regulator
P55290	CDH13	120000	5.079181246	306	other
P00352	ALDH1A1	120000	5.079181246	307	enzyme
P98160	HSPG2	120000	5.079181246	308	enzyme
P37802	TAGLN2	120000	5.079181246	309	other
O43707	ACTN4	120000	5.079181246	310	transcription regulator
P08294	SOD3	117999.9999	5.071882007	312	enzyme
P09493	TPM1	114666.6667	5.059437188	313	other
P10721	KIT	114333.3332	5.058172865	314	transmembrane receptor
P00451	F8	112707.5001	5.051952817	315	other
Q9UNN8	PROCR	111233.3333	5.046234952	316	other
Q15113	PCOLCE	110000	5.041392685	317	other
P09172	DBH	110000	5.041392685	318	enzyme
P08567	PLEK	110000	5.041392685	319	other
P55000	SLURP1	110000	5.041392685	320	cytokine
P26927	MST1	109666.6666	5.040074643	322	growth factor
P05164	MPO	103500	5.01494035	324	enzyme
P32942	ICAM3	101800	5.007747778	325	transmembrane receptor
P27797	CALR	100000	5	326	transcription regulator

P35555	FBN1	100000	5	327	other
P11021	HSPA5	100000	5	328	enzyme
P0DMV8	HSPA1A	100000	5	329	enzyme
P11142	HSPA8	100000	5	330	enzyme
P12111	COL6A3	100000	5	331	other
P00558	PGK1	99000.00009	4.995635195	333	kinase
O15467	CCL16	98916.66669	4.995269473	334	cytokine
P16109	SELP	98128.57135	4.991795476	335	transmembrane receptor
P16070	CD44	95999.99999	4.982271233	337	other
Q86VB7	CD163	94000.00009	4.973127854	338	transmembrane receptor
P02144	MB	93700.00002	4.971739591	339	transporter
P35443	THBS4	91999.99993	4.963787827	340	other
Q9H4G4	GLIPR2	91999.99993	4.963787827	341	other
P08493	MGP	91999.99993	4.963787827	342	other
P09972	ALDOC	91333.33331	4.960629308	343	enzyme
P68366	TUBA4A	90999.99993	4.959041392	345	other
P04406	GAPDH	90999.99993	4.959041392	346	enzyme
Q6Q788	APOA5	90250.00009	4.955447211	348	transporter
P18206	VCL	89999.99991	4.954242509	349	enzyme
O75083	WDR1	89999.99991	4.954242509	350	other
P17900	GM2A	89999.99991	4.954242509	351	enzyme
P29401	TKT	89000.00007	4.949390007	352	enzyme
P14625	HSP90B1	87999.99997	4.944482672	353	other
P07900	HSP90AA1	87999.99997	4.944482672	354	enzyme
P08238	HSP90AB1	87999.99997	4.944482672	355	enzyme
Q13228	SELENBP1	87000.00008	4.939519253	356	other
P11226	MBL2	87000.00008	4.939519253	357	other
P10645	CHGA	86333.33326	4.936178509	358	other
P07195	LDHB	85999.99995	4.934498451	359	enzyme
P00338	LDHA	85999.99995	4.934498451	360	enzyme
P06732	CKM	85000.00006	4.929418926	361	kinase
P01033	TIMP1	82723.74991	4.917630213	362	cytokine
Q16853	AOC3	82333.33342	4.915575699	363	enzyme
Q8NBP7	PCSK9	81999.99993	4.913813852	364	peptidase
P09382	LGALS1	81000.00002	4.908485019	365	other
P02776	PF4	76374.99996	4.882951223	367	cytokine
O15204	ADAMDEC1	75999.99995	4.880813592	368	peptidase
O60814	HIST1H2BK	74999.99993	4.875061263	369	other
P00325	ADH1B	74000.00005	4.86923172	370	enzyme
P19022	CDH2	72999.99998	4.86332286	374	other
P22105	TNXB	70000	4.84509804	375	other
P41271	NBL1	70000	4.84509804	377	other
P12955	PEPD	70000	4.84509804	378	peptidase
Q9H299	SH3BGRL3	68000.00005	4.832508913	380	other
P09486	SPARC	66000.00007	4.819543936	381	other
P07339	CTSD	65966.66659	4.819324539	382	peptidase
Q14767	LTBP2	64000	4.806179974	384	other
P55058	PLTP	64000	4.806179974	385	enzyme
P12821	ACE	63699.99995	4.804139432	387	peptidase
Q15166	PON3	63499.99996	4.802773725	388	enzyme
P16035	TIMP2	63349.99997	4.801746619	389	other
P08887	IL6R	63140.00006	4.800304578	390	transmembrane receptor
O75015	FCGR3B	62999.99993	4.799340549	393	transmembrane receptor
P08833	IGFBP1	62490.00005	4.795810525	394	other
Q969E1	LEAP2	61999.99993	4.792391689	395	other
P68104	EEF1A1	61000	4.785329835	396	translation regulator
P14780	MMP9	60610	4.782544284	397	peptidase
Q06830	PRDX1	59999.99995	4.77815125	398	enzyme

O00151	PDLIM1	59000.00005	4.770852012	399	transcription regulator
Q92496	CFHR4	59000.00005	4.770852012	401	transporter
P36222	CHI3L1	57303.33339	4.758179886	402	enzyme
P35579	MYH9	57000.00004	4.755874856	403	enzyme
P11684	SCGB1A1	56000	4.748188027	406	cytokine
Q99784	OLFM1	56000	4.748188027	407	other
P16930	FAH	56000	4.748188027	408	enzyme
P17174	GOT1	56000	4.748188027	409	enzyme
O95497	VNN1	56000	4.748188027	410	enzyme
P06733	ENO1	54999.99994	4.740362689	411	enzyme
P00367	GLUD1	54999.99994	4.740362689	412	enzyme
P13929	ENO3	54999.99994	4.740362689	413	enzyme
Q9UHG3	PCYOX1	54000.00002	4.73239376	414	enzyme
Q14515	SPARCL1	53000.00005	4.72427587	415	other
P61626	LYZ	50999.99999	4.707570176	418	enzyme
P07108	DBI	50000.49997	4.698974347	421	other
P11047	LAMC1	49999.99996	4.698970004	422	other
P37840	SNCA	49000	4.69019608	423	enzyme
P04216	THY1	49000	4.69019608	424	other
P18669	PGAM1	47999.99996	4.681241237	425	phosphatase
O15240	VGF	47999.99996	4.681241237	427	growth factor
P26038	MSN	47000.00001	4.672097858	430	other
P09467	FBP1	47000.00001	4.672097858	431	phosphatase
P05451	REG1A	47000.00001	4.672097858	432	growth factor
O00757	FBP2	47000.00001	4.672097858	433	phosphatase
P15311	EZR	47000.00001	4.672097858	434	other
P28827	PTPRM	47000.00001	4.672097858	435	phosphatase
P23141	CES1	46000.00003	4.662757832	436	enzyme
Q14766	LTBP1	46000.00003	4.662757832	437	other
P33151	CDH5	46000.00003	4.662757832	438	other
P15090	FABP4	41999.99996	4.62324929	442	transporter
P00918	CA2	41999.99996	4.62324929	444	enzyme
Q14112	NID2	41999.99996	4.62324929	445	other
P14543	NID1	41999.99996	4.62324929	446	other
P05186	ALPL	41000.00003	4.612783857	447	phosphatase
P31146	CORO1A	41000.00003	4.612783857	448	other
Q01469	FABP5	39999.99997	4.602059991	449	transporter
Q13822	ENPP2	39000	4.591064607	452	enzyme
P40925	MDH1	39000	4.591064607	453	enzyme
P29279	CTGF	38000.00003	4.579783597	454	growth factor
P61158	ACTR3	38000.00003	4.579783597	456	other
P13501	CCL5	37100.00003	4.56937391	457	cytokine
P13598	ICAM2	36999.99999	4.568201724	459	other
P15144	ANPEP	36000.00002	4.556302501	460	peptidase
P15529	CD46	34999.99997	4.544068044	462	transmembrane receptor
P10646	TFPI	34999.99997	4.544068044	463	other
P10586	PTPRF	34999.99997	4.544068044	465	phosphatase
P39060	COL18A1	34999.99997	4.544068044	466	other
Q8WWA0	ITLN1	34580	4.538824989	467	other
Q14118	DAG1	34000	4.531478917	468	transmembrane receptor
P35754	GLRX	34000	4.531478917	469	enzyme
Q01524	DEFA6	34000	4.531478917	470	other
P07602	PSAP	33000.00001	4.51851394	471	enzyme
P78417	GSTO1	33000.00001	4.51851394	472	enzyme
Q15942	ZYX	33000.00001	4.51851394	473	other
P13591	NCAM1	33000.00001	4.51851394	474	other
P07858	CTSB	32399.99998	4.51054501	475	peptidase
Q86UX7	FERMT3	31999.99998	4.505149978	476	enzyme

P30101	PDIA3	31999.99998	4.505149978	478	peptidase
P80511	S100A12	31999.99998	4.505149978	479	other
Q9BY67	CADM1	31999.99998	4.505149978	480	other
P09211	GSTP1	31999.99998	4.505149978	481	enzyme
P55774	CCL18	31000.00001	4.491361694	482	cytokine
P16403	HIST1H1C	30000.00002	4.477121255	484	other
Q13508	ART3	30000.00002	4.477121255	486	enzyme
P13639	EEF2	30000.00002	4.477121255	487	translation regulator
Q03154	ACY1	30000.00002	4.477121255	488	peptidase
P22894	MMP8	29563.33332	4.4707534	489	peptidase
Q96FE7	PIK3IP1	29000.00001	4.462397998	490	other
P31150	GDI1	29000.00001	4.462397998	491	other
P50395	GDI2	29000.00001	4.462397998	492	other
Q9HDC9	APMAP	29000.00001	4.462397998	493	enzyme
P52209	PGD	29000.00001	4.462397998	494	enzyme
P06858	LPL	27999.99998	4.447158031	497	enzyme
P06703	S100A6	27999.99998	4.447158031	500	transporter
P04439	HLA-A	27999.99998	4.447158031	501	other
Q9BU40	CHRDL1	27999.99998	4.447158031	503	other
Q14574	DSC3	26999.99999	4.431363764	505	other
Q13509	TUBB3	26999.99999	4.431363764	506	other
Q15485	FCN2	26999.99999	4.431363764	507	other
Q01518	CAP1	26999.99999	4.431363764	508	other
P31151	S100A7	26999.99999	4.431363764	509	other
Q9NQ79	CRTAC1	26999.99999	4.431363764	510	other
Q9H4B7	TUBB1	26999.99999	4.431363764	511	other
O75594	PGLYRP1	26000	4.414973348	517	transmembrane receptor
P11717	IGF2R	26000	4.414973348	518	transmembrane receptor
P09110	ACAA1	26000	4.414973348	519	enzyme
P12109	COL6A1	26000	4.414973348	521	other
P08254	MMP3	25949.99999	4.414137362	522	peptidase
P09960	LTA4H	25000.00002	4.397940009	523	enzyme
P43121	MCAM	25000.00002	4.397940009	524	other
P01833	PIGR	25000.00002	4.397940009	525	transporter
P30041	PRDX6	25000.00002	4.397940009	526	enzyme
Q8N6C8	LILRA3	25000.00002	4.397940009	527	other
P17813	ENG	24966.66667	4.397360563	528	transmembrane receptor
Q13740	ALCAM	24200	4.383815366	529	other
P07333	CSF1R	24000.00002	4.380211242	532	kinase
P01137	TGFB1	23809.99998	4.376759395	533	growth factor
P15151	PVR	23000	4.361727836	534	other
P07237	P4HB	23000	4.361727836	535	enzyme
O00584	RNASET2	23000	4.361727836	536	enzyme
Q99497	PARK7	23000	4.361727836	537	enzyme
Q9HD89	RETN	22849.99998	4.358886204	539	other
P03973	SLPI	22499.99999	4.352182518	540	other
O00299	CLIC1	22000.00001	4.342422681	545	ion channel
P23284	PPIB	21000.00001	4.322219295	546	enzyme
Q16851	UGP2	21000.00001	4.322219295	547	enzyme
Q9ULV4	CORO1C	21000.00001	4.322219295	549	other
P50281	MMP14	21000.00001	4.322219295	550	peptidase
Q6E0U4	DMKN	20000.00002	4.301029996	553	other
P47756	CAPZB	20000.00002	4.301029996	554	other
P25787	PSMA2	20000.00002	4.301029996	555	peptidase
Q06141	REG3A	20000.00002	4.301029996	556	enzyme
Q10588	BST1	20000.00002	4.301029996	557	enzyme
P15924	DSP	20000.00002	4.301029996	558	other
P05060	CHGB	20000.00002	4.301029996	559	other

P05121	SERPINE1	19460	4.289142836	561	other
P24043	LAMA2	19000	4.278753601	562	other
Q6YHK3	CD109	19000	4.278753601	563	other
Q96EE4	CCDC126	18000	4.255272505	565	other
P05067	APP	18000	4.255272505	567	other
P31949	S100A11	18000	4.255272505	568	other
P58546	MTPN	18000	4.255272505	570	transcription regulator
P51693	APLP1	18000	4.255272505	571	other
P07359	GP1BA	18000	4.255272505	572	transmembrane receptor
Q02818	NUCB1	18000	4.255272505	573	other
P60900	PSMA6	16999.99999	4.230448921	577	peptidase
P13497	BMP1	16999.99999	4.230448921	578	peptidase
P36871	PGM1	16999.99999	4.230448921	580	enzyme
Q13418	ILK	16999.99999	4.230448921	581	kinase
P16581	SELE	16108	4.207041621	583	transmembrane receptor
P24298	GPT	16000.00001	4.204119983	584	enzyme
Q9UNW1	MINPP1	16000.00001	4.204119983	585	phosphatase
P13647	KRT5	16000.00001	4.204119983	586	other
P78324	SIRPA	16000.00001	4.204119983	587	phosphatase
P20774	OGN	16000.00001	4.204119983	588	growth factor
Q92823	NRCAM	16000.00001	4.204119983	589	other
Q13449	LSAMP	16000.00001	4.204119983	590	other
P48059	LIMS1	16000.00001	4.204119983	591	other
Q15063	POSTN	16000.00001	4.204119983	592	other
P01236	PRL	16000.00001	4.204119983	593	cytokine
P08123	COL1A2	15000	4.176091259	594	other
P14314	PRKCSH	15000	4.176091259	595	enzyme
P40926	MDH2	15000	4.176091259	596	enzyme
P20908	COL5A1	15000	4.176091259	597	other
Q9UBP4	DKK3	15000	4.176091259	599	cytokine
P50552	VASP	15000	4.176091259	600	other
P28838	LAP3	15000	4.176091259	602	peptidase
Q10471	GALNT2	15000	4.176091259	603	enzyme
P37837	TALDO1	15000	4.176091259	604	enzyme
P54289	CACNA2D1	15000	4.176091259	605	ion channel
Q8N2S1	LTBP4	15000	4.176091259	606	growth factor
P62942	FKBP1A	14000.00001	4.146128036	607	enzyme
Q9NS71	GKN1	14000.00001	4.146128036	608	growth factor
P30740	SERPINB1	14000.00001	4.146128036	609	other
O00533	CHL1	14000.00001	4.146128036	611	other
Q8WXD2	SCG3	14000.00001	4.146128036	614	other
Q12860	CNTN1	14000.00001	4.146128036	615	enzyme
P60660	MYL6	14000.00001	4.146128036	618	enzyme
Q02413	DSG1	14000.00001	4.146128036	619	other
P08514	ITGA2B	12999.99999	4.113943352	624	transmembrane receptor
P10768	ESD	12999.99999	4.113943352	626	enzyme
P32754	HPD	12999.99999	4.113943352	627	enzyme
P61160	ACTR2	12999.99999	4.113943352	628	other
P55103	INHBC	12999.99999	4.113943352	629	growth factor
O14786	NRP1	12999.99999	4.113943352	630	transmembrane receptor
Q9UBR2	CTSZ	12000	4.079181246	632	peptidase
Q9Y5C1	ANGPTL3	12000	4.079181246	633	growth factor
P08319	ADH4	12000	4.079181246	634	enzyme
O95633	FSTL3	12000	4.079181246	635	other
Q8IZF2	GPR116	12000	4.079181246	636	G-protein coupled receptor
Q13231	CHIT1	12000	4.079181246	641	enzyme
P08195	SLC3A2	12000	4.079181246	642	transporter
P04626	ERBB2	11000	4.041392685	646	kinase

P00505	GOT2	11000	4.041392685	647	enzyme
P52565	ARHGDIA	11000	4.041392685	648	other
P58166	INHBE	11000	4.041392685	649	growth factor
Q12907	LMAN2	11000	4.041392685	650	transporter
P34932	HSPA4	11000	4.041392685	652	other
Q7Z7G0	ABI3BP	11000	4.041392685	653	other
P25788	PSMA3	11000	4.041392685	654	peptidase
Q08257	CRYZ	11000	4.041392685	655	enzyme
P25789	PSMA4	11000	4.041392685	656	peptidase
P48637	GSS	11000	4.041392685	657	enzyme
P22392	NME2	11000	4.041392685	658	kinase
Q02809	PLOD1	11000	4.041392685	659	enzyme
Q9BXR6	CFHR5	11000	4.041392685	660	other
P35968	KDR	10637.5	4.026839573	661	kinase
Q92876	KLK6	10449.99999	4.01911629	662	peptidase
P52566	ARHGDIB	10000	4	664	enzyme
Q8NBS9	TXNDC5	10000	4	668	enzyme
Q05682	CALD1	10000	4	670	other
P00533	EGFR	9966.666676	3.998549934	671	kinase
Q12913	PTPRJ	9900.000009	3.995635195	672	phosphatase
P07738	BPGM	9800.000007	3.991226076	674	phosphatase
Q14847	LASP1	9800.000007	3.991226076	675	transporter
P20933	AGA	9599.999999	3.982271233	680	enzyme
P01040	CSTA	9599.999999	3.982271233	681	other
Q14508	WFDC2	9499.999994	3.977723605	683	other
O14818	PSMA7	9400.000009	3.973127854	684	peptidase
Q8NBJ4	GOLM1	9199.999993	3.963787827	685	other
P00491	PNP	9199.999993	3.963787827	686	enzyme
Q15404	RSU1	8900.000007	3.949390007	691	other
P39059	COL15A1	8799.999997	3.944482672	693	other
P22314	UBA1	8500.000006	3.929418926	696	enzyme
P10599	TXN	8399.999999	3.924279286	697	enzyme
P25786	PSMA1	8399.999999	3.924279286	698	peptidase
Q03167	TGFBR3	8299.999993	3.919078092	701	kinase
Q9NZT1	CALML5	8100.000002	3.908485019	703	other
O15143	ARPC1B	8100.000002	3.908485019	705	other
Q3LXA3	DAK	8000	3.903089987	707	kinase
P52799	EFNB2	8000	3.903089987	709	kinase
O94985	CLSTN1	7899.999995	3.897627091	712	other
O95810	SDPR	7899.999995	3.897627091	714	other
Q02763	TEK	7800.000006	3.892094603	715	kinase
Q9NZK5	CECR1	7699.999997	3.886490725	716	enzyme
P28066	PSMA5	7699.999997	3.886490725	717	peptidase
P21291	CSRP1	7599.999995	3.880813592	718	other
Q14314	FGL2	7499.999993	3.875061263	722	peptidase
P02545	LMNA	7499.999993	3.875061263	724	other
Q15149	PLEC	7499.999993	3.875061263	725	other
P25815	S100P	7299.999998	3.86332286	730	other
P80723	BASP1	7299.999998	3.86332286	732	transcription regulator
P13473	LAMP2	7299.999998	3.86332286	734	enzyme
P49721	PSMB2	7199.999993	3.857332496	735	peptidase
Q13201	MMRN1	7199.999993	3.857332496	736	other
P27930	IL1R2	7100.000005	3.851258349	738	transmembrane receptor
P04179	SOD2	7100.000005	3.851258349	739	enzyme
P16112	ACAN	7100.000005	3.851258349	740	other
Q01638	IL1RL1	7000	3.84509804	742	transmembrane receptor
P20618	PSMB1	7000	3.84509804	743	peptidase
P51124	GZMM	6900.000004	3.838849091	746	peptidase

Q9UJJ9	GNPTG	6800.000005	3.832508913	748	enzyme
Q86U17	SERPINA11	6700.000005	3.826074803	750	other
O75874	IDH1	6600.000007	3.819543936	752	enzyme
Q9ULI3	HEG1	6600.000007	3.819543936	754	other
P48163	ME1	6600.000007	3.819543936	755	enzyme
Q9Y4L1	HYOU1	6600.000007	3.819543936	756	other
P16284	PECAM1	6600.000007	3.819543936	758	other
P13611	VCAN	6600.000007	3.819543936	759	other
Q9H4A9	DPEP2	6500.000005	3.812913357	762	peptidase
P55145	MANF	6500.000005	3.812913357	763	other
Q14697	GANAB	6400	3.806179974	765	enzyme
P13667	PDIA4	6400	3.806179974	766	enzyme
Q8WWZ8	OIT3	6400	3.806179974	768	other
Q13332	PTPRS	6400	3.806179974	771	phosphatase
P21399	ACO1	6299.999993	3.799340549	773	enzyme
Q9Y5Y7	LYVE1	6199.999993	3.792391689	774	transmembrane receptor
P06576	ATP5B	6199.999993	3.792391689	775	transporter
P04083	ANXA1	6199.999993	3.792391689	776	enzyme
P15531	NME1	6100	3.785329835	781	kinase
P13716	ALAD	6100	3.785329835	785	enzyme
P27487	DPP4	5999.999995	3.77815125	789	peptidase
P23381	WARS	5999.999995	3.77815125	790	enzyme
P04424	ASL	5900.000005	3.770852012	792	enzyme
Q04760	GLO1	5900.000005	3.770852012	798	enzyme
P61224	RAP1B	5900.000005	3.770852012	799	enzyme
Q9NY97	B3GNT2	5800.000006	3.763427994	802	enzyme
P98172	EFNB1	5800.000006	3.763427994	804	other
A5YKK6	CNOT1	5700.000004	3.755874856	805	other
P01225	FSHB	5700.000004	3.755874856	806	other
Q13642	FHL1	5600	3.748188027	808	other
P22897	MRC1	5600	3.748188027	810	transmembrane receptor
P33908	MAN1A1	5600	3.748188027	812	enzyme
Q9P121	NTM	5499.999994	3.740362689	814	other
P34096	RNASE4	5499.999994	3.740362689	816	enzyme
P06737	PYGL	5400.000002	3.73239376	817	enzyme
P09603	CSF1	5126.399999	3.70981249	822	cytokine
P28070	PSMB4	5099.999999	3.707570176	825	peptidase
Q9NZ08	ERAP1	5099.999999	3.707570176	826	peptidase
P25774	CTSS	5099.999999	3.707570176	830	peptidase
P00480	OTC	4999.999996	3.698970004	832	enzyme
P28074	PSMB5	4999.999996	3.698970004	833	peptidase
Q3ZCW2	LGALSL	4900	3.69019608	841	other
P02730	SLC4A1	4900	3.69019608	842	transporter
P23470	PTPRG	4900	3.69019608	844	phosphatase
O95479	H6PD	4900	3.69019608	845	enzyme
P07307	ASGR2	4799.999996	3.681241237	846	transmembrane receptor
Q9NPY3	CD93	4799.999996	3.681241237	849	other
Q99436	PSMB7	4799.999996	3.681241237	851	peptidase
P07451	CA3	4799.999996	3.681241237	852	enzyme
P02795	MT2A	4700.000001	3.672097858	854	other
Q9Y6Z7	COLEC10	4700.000001	3.672097858	855	other
Q9NPH3	IL1RAP	4700.000001	3.672097858	860	transmembrane receptor
Q04446	GBE1	4600.000003	3.662757832	861	enzyme
Q6UXH0	C19orf80	4600.000003	3.662757832	862	other
P62826	RAN	4600.000003	3.662757832	863	enzyme
Q5D862	FLG2	4600.000003	3.662757832	864	other
Q96AP7	ESAM	4600.000003	3.662757832	868	other
O15144	ARPC2	4500.000002	3.653212514	869	other

Q6UWP8	SBSN	4500.000002	3.653212514	870	other
Q9UBX5	FBLN5	4500.000002	3.653212514	872	other
Q9BS26	ERP44	4500.000002	3.653212514	873	enzyme
Q9HBB8	CDHR5	4399.999995	3.643452676	874	other
P42126	ECI1	4399.999995	3.643452676	875	enzyme
Q6IBS0	TWF2	4399.999995	3.643452676	876	kinase
Q92859	NEO1	4199.999996	3.62324929	881	transcription regulator
P22626	HNRNPA2B1	4100.000003	3.612783857	889	other
P35916	FLT4	4100.000003	3.612783857	890	transmembrane receptor
P05783	KRT18	3999.999997	3.602059991	894	other
Q7Z3B1	NEGR1	3999.999997	3.602059991	898	other
P46777	RPL5	3999.999997	3.602059991	899	other
P60842	EIF4A1	3999.999997	3.602059991	900	translation regulator
P09622	DLD	3900	3.591064607	902	enzyme
P21695	GPD1	3900	3.591064607	903	enzyme
O95998	IL18BP	3900	3.591064607	904	other
P04066	FUCA1	3900	3.591064607	906	enzyme
O00468	AGRN	3900	3.591064607	909	other
Q16706	MAN2A1	3900	3.591064607	910	enzyme
P22061	PCMT1	3900	3.591064607	911	enzyme
P08311	CTSG	3699.999999	3.568201724	915	peptidase
P61088	UBE2N	3699.999999	3.568201724	916	enzyme
P35237	SERPINB6	3600.000002	3.556302501	918	other
P04054	PLA2G1B	3600.000002	3.556302501	919	enzyme
Q12841	FSTL1	3600.000002	3.556302501	920	other
P21926	CD9	3600.000002	3.556302501	923	other
Q13835	PKP1	3600.000002	3.556302501	925	other
O43157	PLXNB1	3499.999997	3.544068044	929	transmembrane receptor
Q9BRK5	SDF4	3499.999997	3.544068044	932	other
P27105	STOM	3499.999997	3.544068044	934	other
P00390	GSR	3499.999997	3.544068044	935	enzyme
P28072	PSMB6	3499.999997	3.544068044	937	peptidase
P42785	PRCP	3499.999997	3.544068044	939	peptidase
O14798	TNFRSF10C	3400	3.531478917	945	transmembrane receptor
P02461	COL3A1	3400	3.531478917	948	other
Q13867	BLMH	3300.000001	3.51851394	950	peptidase
Q9HBR0	SLC38A10	3300.000001	3.51851394	951	other
P07148	FABP1	3300.000001	3.51851394	952	transporter
Q8TDL5	BPIFB1	3300.000001	3.51851394	954	other
O43505	B3GNT1	3300.000001	3.51851394	955	enzyme
Q15691	MAPRE1	3300.000001	3.51851394	957	other
P26447	S100A4	3199.999998	3.505149978	958	other
P07384	CAPN1	3100.000001	3.491361694	960	peptidase
Q9HBI1	PARVB	3100.000001	3.491361694	962	other
O15031	PLXNB2	3100.000001	3.491361694	963	transmembrane receptor
Q99439	CNN2	3100.000001	3.491361694	968	other
Q9BYE9	CDHR2	2900.000001	3.462397998	974	other
Q04721	NOTCH2	2799.999998	3.447158031	980	transcription regulator
Q9HBW9	ELTD1	2799.999998	3.447158031	981	G-protein coupled receptor
Q8N6Q3	CD177	2699.999999	3.431363764	985	other
Q02487	DSC2	2699.999999	3.431363764	986	other
Q14126	DSG2	2699.999999	3.431363764	987	other
P49720	PSMB3	2699.999999	3.431363764	989	peptidase
P21709	EPHA1	2699.999999	3.431363764	990	kinase
P00492	HPRT1	2600	3.414973348	999	enzyme
P07942	LAMB1	2600	3.414973348	1000	other
P08581	MET	2500.000002	3.397940009	1002	kinase
P62873	GNB1	2500.000002	3.397940009	1004	enzyme

Q8IYS5	OSCAR	2500.000002	3.397940009	1005	other
P59998	ARPC4	2500.000002	3.397940009	1007	other
P55268	LAMB2	2500.000002	3.397940009	1008	enzyme
P30530	AXL	2500.000002	3.397940009	1010	kinase
Q9Y279	VSIG4	2400.000002	3.380211242	1014	other
Q08830	FGL1	2300	3.361727836	1020	other
Q16620	NTRK2	2300	3.361727836	1025	kinase
Q08ET2	SIGLEC14	2200.000001	3.342422681	1027	other
O75144	ICOSLG	2200.000001	3.342422681	1030	other
O43768	ENSA	2100.000001	3.322219295	1034	transporter
Q07075	ENPEP	2100.000001	3.322219295	1036	peptidase
P17655	CAPN2	2000.000002	3.301029996	1040	peptidase
P20023	CR2	2000.000002	3.301029996	1041	transmembrane receptor
P49454	CENPF	2000.000002	3.301029996	1042	other
P10619	CTSA	2000.000002	3.301029996	1043	peptidase
P08575	PTPRC	2000.000002	3.301029996	1046	phosphatase
P22455	FGFR4	2000.000002	3.301029996	1048	kinase
Q08495	DMTN	2000.000002	3.301029996	1050	other
P16233	PNLIP	2000.000002	3.301029996	1052	enzyme
Q12884	FAP	2000.000002	3.301029996	1053	peptidase
P11362	FGFR1	2000.000002	3.301029996	1055	kinase
Q99650	OSMR	1949.999998	3.290034611	1057	transmembrane receptor
O15145	ARPC3	1800	3.255272505	1068	other
Q06481	APLP2	1800	3.255272505	1075	other
O95980	RECK	1800	3.255272505	1076	other
Q9H4M9	EHD1	1800	3.255272505	1077	other
O00462	MANBA	1699.999999	3.230448921	1080	enzyme
P46783	RPS10	1699.999999	3.230448921	1082	other
O14773	TPP1	1699.999999	3.230448921	1085	peptidase
Q15262	PTPRK	1699.999999	3.230448921	1088	phosphatase
P11279	LAMP1	1600.000001	3.204119983	1091	other
Q9NYU2	UGGT1	1600.000001	3.204119983	1094	enzyme
Q6UX71	PLXDC2	1600.000001	3.204119983	1098	other
P12429	ANXA3	1600.000001	3.204119983	1100	enzyme
P48506	GCLC	1500	3.176091259	1102	enzyme
Q13557	CAMK2D	1500	3.176091259	1104	kinase
P60981	DSTN	1500	3.176091259	1105	other
P07686	HEXB	1500	3.176091259	1108	enzyme
P61978	HNRNPK	1400.000001	3.146128036	1113	transcription regulator
P07911	UMOD	1400.000001	3.146128036	1115	other
O95831	AIFM1	1400.000001	3.146128036	1116	enzyme
P54802	NAGLU	1400.000001	3.146128036	1117	enzyme
Q13232	NME3	1400.000001	3.146128036	1118	kinase
P37173	TGFBR2	1299.999999	3.113943352	1120	kinase
Q9BTY2	FUCA2	1299.999999	3.113943352	1121	enzyme
O60664	PLIN3	1299.999999	3.113943352	1122	other
Q76LX8	ADAMTS13	1200	3.079181246	1128	peptidase
P20700	LMNB1	1200	3.079181246	1131	other
P52888	THOP1	1200	3.079181246	1134	peptidase
P53396	ACLY	1100	3.041392685	1135	enzyme
Q6P179	ERAP2	980.0000007	2.991226076	1142	peptidase
Q15223	PVRL1	919.9999993	2.963787827	1143	other
P20930	FLG	819.9999993	2.913813852	1146	other
Q4LDE5	SVEP1	540.0000002	2.73239376	1154	other
P01127	PDGFB	319.9999998	2.505149978	1162	growth factor
P08473	MME	250.0000002	2.397940009	1166	peptidase
P14210	HGF	205.4999999	2.312811826	1169	growth factor
P21741	MDK	150	2.176091259	1177	growth factor

P01160	NPPA	56	1.748188027	1188	other
P54108	CRISP3	6.299999993	0.799340549	1212	other
Q7Z7M0	MEGF8	4.300000004	0.633468456	1213	other

SourceDataForFigure3C		
	PC1	PC2
NC1	-5.9957	1.1722
NC2	-10.049	0.5214
NC3	-5.9957	1.1722
NC4	-10.629	-0.00188
NC5	-5.9957	1.1722
NC6	-10.765	-0.69184
R1	-0.93771	-0.80681
R2	2.7253	-7.966
R3	-0.7458	-1.326
R4	2.9869	-7.8253
R5	-1.0709	-1.6216
R6	2.8239	-8.0036
UR1	9.0366	-3.2794
UR2	5.1233	11.002
UR3	9.3312	-3.2096
UR4	4.8029	11.101
UR5	9.9129	-3.0828
UR6	5.4413	11.673

SourceDataForFigure3D									
NO.	Uniprot Accession Number	Uniprot ID	Gene Symbol	Foldchange of (R & UR)/NC	Log2[FC((R & UR)/NC)]	P.ajusted [(R & UR)/NC]	Foldchange of R /(UR & N)	log2[FC(R /(UR & N))]	P.ajusted [R /(UR & N)]
103	A2RTY3	HEAT9_HUMAN	HEATR9	0.1461	-2.7755	0.0248			
102	Q8NI35	INADL_HUMAN	PATJ	0.3163	-1.6605	0.0023			
101	P13224	GP1BB_HUMAN	GP1BB	0.3327	-1.5879	0.0045			
100	Q8NHQ9	DDX55_HUMAN	DDX55	0.3541	-1.4979	0.0025			
99	Q8IWA4	MFN1_HUMAN	MFN1	0.3594	-1.4765	0.0413			
98	P05106	ITB3_HUMAN	ITGB3	0.3673	-1.4448	0.0000			
97	P0DOX6	IGM_HUMAN	#N/A	0.4227	-1.2422	0.0004			
96	P08514	ITA2B_HUMAN	ITGA2B	0.4371	-1.1941	0.0000			
95	O43866	CD5L_HUMAN	CD5L	0.4685	-1.0938	0.0000			
94	Q86YW5	TRML1_HUMAN	TREML1	0.4694	-1.0910	0.0000			
93	P21926	CD9_HUMAN	CD9	0.4706	-1.0876	0.0000			
92	A0A0B4J1X5	HV374_HUMAN	IGHV3-74	0.4951	-1.0141	0.0015			
91	P53801	PTTG_HUMAN	PTTG1IP	0.5010	-0.9972	0.0034			
90	Q6UX06	OLFM4_HUMAN	OLFM4	0.5128	-0.9637	0.0000			
89	P08311	CATG_HUMAN	CTSG	0.5221	-0.9376	0.0005			
88	P01871	IGHM_HUMAN	IGHM	0.5225	-0.9366	0.0004			
87	P02751	FINC_HUMAN	FN1	0.5241	-0.9321	0.0148			
86	P07996	TSP1_HUMAN	THBS1	0.5295	-0.9172	0.0000			
85	Q9Y490	TLN1_HUMAN	TLN1	0.5386	-0.8928	0.0002			
84	P01619	KV320_HUMAN	IGKV3-20	0.5399	-0.8892	0.0000			
83	P61224	RAP1B_HUMAN	RAP1B	0.5405	-0.8875	0.0000			
82	Q15404	RSU1_HUMAN	RSU1	0.5428	-0.8816	0.0013			
81	A0A0A0MRZ8	KVD11_HUMAN	IGKV3D-11	0.5643	-0.8256	0.0000			
80	P06727	APOA4_HUMAN	APOA4	0.5659	-0.8214	0.0029	0.4631	-1.1106	0.0000
79	P60709	ACTB_HUMAN	ACTB	0.5671	-0.8182	0.0046			
78	P04430	KV116_HUMAN	IGKV1-16	0.5678	-0.8167	0.0000			
77	P01591	IGJ_HUMAN	JCHAIN	0.5682	-0.8155	0.0003			
76	P02788	TRFL_HUMAN	LTF	0.5706	-0.8095	0.0000			
75	Q14141	SEPT6_HUMAN	SEPTIN6	0.5710	-0.8084	0.0044			
74	P63261	ACTG_HUMAN	ACTG1	0.5713	-0.8076	0.0164			
73	Q96CM4	NXNL1_HUMAN	NXNL1	0.5740	-0.8010	0.0000			
72	A0A075B6P5	KV228_HUMAN	IGKV2-28	0.5767	-0.7942	0.0079			
71	P02787	TRFE_HUMAN	TF	0.5815	-0.7822	0.0000			
70	A0A0A0MT36	KVD21_HUMAN	IGKV6D-21	0.5853	-0.7727	0.0016	0.6423	-0.6386	0.0284
69	O43852	CALU_HUMAN	CALU	0.5902	-0.7608	0.0001			
68	P01780	HV307_HUMAN	IGHV3-7	0.5905	-0.7600	0.0003			
67	O95810	CAVN2_HUMAN	CAVIN2	0.5933	-0.7532	0.0296			
66	P10720	PF4V_HUMAN	PF4V1	0.5936	-0.7524	0.0046			
65	P0DOX7	IGK_HUMAN	#N/A	0.5967	-0.7449	0.0001			
64	P27105	STOM_HUMAN	STOM	0.5980	-0.7418	0.0000			
63	Q9UL25	RAB21_HUMAN	RAB21	0.6028	-0.7303	0.0005			
62	P0DP03	HVC05_HUMAN	IGHV3-30-5	0.6035	-0.7286	0.0211			
61	O95236	APOL3_HUMAN	APOL3	0.6117	-0.7091	0.0044	0.5573	-0.8434	0.0011
60	P00488	F13A_HUMAN	F13A1	0.6131	-0.7057	0.0000			
59	P21333	FLNA_HUMAN	FLNA	0.6141	-0.7035	0.0004	0.6573	-0.6054	0.0272
58	P02775	CXCL7_HUMAN	PPBP	0.6173	-0.6960	0.0001			
57	P06312	KV401_HUMAN	IGKV4-1	0.6186	-0.6928	0.0047			
56	Q9H6X2	ANTR1_HUMAN	ANTXR1	0.6250	-0.6781	0.0005	0.6467	-0.6288	0.0081
55	P01709	LV208_HUMAN	IGLV2-8	0.6251	-0.6778	0.0001			
54	P40197	GPV_HUMAN	GP5	0.6270	-0.6735	0.0000			
53	Q05315	LEG10_HUMAN	CLC	0.6278	-0.6716	0.0098	0.5115	-0.9671	0.0000
52	Q5VTJ3	KLD7A_HUMAN	KLHDC7A	0.6301	-0.6663	0.0001			
51	O60641	AP180_HUMAN	SNAP91	0.6303	-0.6660	0.0434			
50	Q7Z5L0	VMO1_HUMAN	VMO1	0.6346	-0.6561	0.0009			
49	Q9UMX5	NENF_HUMAN	NENF	0.6362	-0.6524	0.0007			
48	P01860	IGHG3_HUMAN	IGHG3	0.6380	-0.6485	0.0001			
47	Q9HBI1	PARVB_HUMAN	PARVB	0.6385	-0.6472	0.0188			
46	Q86UX7	URP2_HUMAN	FERMT3	0.6390	-0.6462	0.0000			
45	P22894	MMP8_HUMAN	MMP8	0.6401	-0.6436	0.0002			

44	Q8IXJ6	SIR2_HUMAN	SIRT2	0.6416	-0.6402	0.0000			
43	P00748	FA12_HUMAN	F12	0.6418	-0.6398	0.0186			
42	A0A075B6K4	LV310_HUMAN	IGLV3-10	0.6422	-0.6390	0.0044			
41	Q9HCN6	GPVI_HUMAN	GP6	0.6423	-0.6387	0.0008			
40	Q9Y6Y8	S23IP_HUMAN	SEC23IP	0.6438	-0.6354	0.0034			
39	P01782	HV309_HUMAN	IGHV3-9	0.6454	-0.6317	0.0057			
38	O43707	ACTN4_HUMAN	ACTN4	0.6468	-0.6287	0.0289			
37	Q14766	LTBP1_HUMAN	LTBP1	0.6485	-0.6248	0.0000			
36	Q12797	ASPH_HUMAN	ASPH	0.6487	-0.6244	0.0094			
35	P09471	GNAO_HUMAN	GNAO1	0.6502	-0.6212	0.0263			
34	Q8NDA2	HMCN2_HUMAN	HMCN2	0.6517	-0.6178	0.0008			
33	P63267	ACTH_HUMAN	ACTG2	0.6581	-0.6036	0.0064			
32	A0A1B0GTC6	CC085_HUMAN	C3orf85	0.6583	-0.6032	0.0001			
31	Q96KG7	MEG10_HUMAN	MEGF10	0.6603	-0.5989	0.0033			
30	P05160	F13B_HUMAN	F13B	0.6604	-0.5985	0.0000			
29	P01701	LV151_HUMAN	IGLV1-51	0.6605	-0.5983	0.0023			
28	A0A0C4DH25	KVD20_HUMAN	IGKV3D-20	0.6621	-0.5948	0.0002			
27	Q9NP84	TNR12_HUMAN	TNFRSF12A	0.6630	-0.5929	0.0012			
26	Q03181	PPARD_HUMAN	PPARD	1.5007	0.5856	0.0001			
25	Q15485	FCN2_HUMAN	FCN2	1.5164	0.6006	0.0001			
24	Q06141	REG3A_HUMAN	REG3A	1.5597	0.6412	0.0035			
23	P00915	CAH1_HUMAN	CA1	1.5619	0.6433	0.0027			
22	P69892	HBG2_HUMAN	HBG2	1.5667	0.6477	0.0002			
21	Q9Y279	VSIG4_HUMAN	VSIG4	1.5710	0.6517	0.0440	1.8754	0.9072	0.0001
20	P00918	CAH2_HUMAN	CA2	1.5989	0.6771	0.0000			
19	P02750	A2GL_HUMAN	LRG1	1.6025	0.6803	0.0043	1.7211	0.7834	0.0000
18	P48681	NEST_HUMAN	NES	1.6043	0.6820	0.0014			
17	Q86YZ3	HORN_HUMAN	HRNR	1.6709	0.7406	0.0273	1.7255	0.7870	0.0188
16	Q5D862	FILA2_HUMAN	FLG2	1.6725	0.7420	0.0471			
15	Q8WU03	GLYL2_HUMAN	GLYATL2	1.7235	0.7854	0.0004			
14	P00740	FA9_HUMAN	F9	1.7356	0.7954	0.0001	1.6368	0.7109	0.0009
13	P00738	HPT_HUMAN	HP	1.7935	0.8428	0.0324	2.3076	1.2064	0.0000
12	Q9P2E9	RRBP1_HUMAN	RRBP1	1.8351	0.8759	0.0214			
11	Q9H9E3	COG4_HUMAN	COG4	1.8732	0.9055	0.0471			
10	P02671	FIBA_HUMAN	FGA	1.8981	0.9245	0.0163			
9	Q9UPZ9	CILK1_HUMAN	CILK1	2.0472	1.0336	0.0006			
8	Q9UBX7	KLK11_HUMAN	KLK11	2.0544	1.0387	0.0005			
7	Q9H4F8	SMOC1_HUMAN	SMOC1	2.1598	1.1109	0.0131			
6	P05154	IPSP_HUMAN	SERPINA5	2.2129	1.1460	0.0169			
5	Q86Z14	KLOTB_HUMAN	KLB	2.2757	1.1863	0.0005			
4	Q13093	PAFA_HUMAN	PLA2G7	2.5270	1.3374	0.0026			
3	P02741	CRP_HUMAN	CRP	3.1529	1.6567	0.0095	2.9353	1.5535	0.0177
2	P02679	FIBG_HUMAN	FGG	3.1593	1.6596	0.0028			
1	P02675	FIBB_HUMAN	FGB	3.3697	1.7526	0.0039			
156	P06727	APOA4_HUMAN	APOA4				0.4631	-1.1106	0.0000
155	O00151	PDLI1_HUMAN	PDLIM1				0.4745	-1.0755	0.0425
154	O43768	ENSA_HUMAN	ENSA				0.4801	-1.0586	0.0074
153	Q8WW22	DNJA4_HUMAN	DNAJA4				0.4931	-1.0199	0.0408
152	P08567	PLEK_HUMAN	PLEK				0.5060	-0.9829	0.0283
151	Q05315	LEG10_HUMAN	CLC				0.5115	-0.9671	0.0000
150	P51124	GRAM_HUMAN	GZMM				0.5458	-0.8734	0.0240
149	Q96KK5	H2A1H_HUMAN	H2AC12				0.5544	-0.8511	0.0465
148	O95236	APOL3_HUMAN	APOL3				0.5573	-0.8434	0.0011
147	P62491	RB11A_HUMAN	RAB11A				0.5776	-0.7920	0.0123
146	P06753	TPM3_HUMAN	TPM3				0.5940	-0.7515	0.0146
145	Q9UNN8	EPCR_HUMAN	PROCR				0.6125	-0.7072	0.0220
144	A0A075B6R2	HV404_HUMAN	IGHV4-4				0.6131	-0.7059	0.0157
143	P01825	HV459_HUMAN	IGHV4-59				0.6222	-0.6846	0.0123
142	Q15691	MARE1_HUMAN	MAPRE1				0.6252	-0.6777	0.0002
141	P23083	HV102_HUMAN	IGHV1-2				0.6265	-0.6746	0.0014
140	A0A0A0MT36	KVD21_HUMAN	IGKV6D-21				0.6423	-0.6386	0.0284

139	Q9H6X2	ANTR1_HUMAN	ANTXR1	0.6467	-0.6288	0.0081
138	A0A0B4J1V6	HV373_HUMAN	IGHV3-73	0.6507	-0.6200	0.0067
137	P21333	FLNA_HUMAN	FLNA	0.6573	-0.6054	0.0272
136	O95866	G6B_HUMAN	MPIG6B	0.6580	-0.6038	0.0304
135	P08519	APOA_HUMAN	LPA	1.5008	0.5858	0.0001
134	Q02985	FHR3_HUMAN	CFHR3	1.5055	0.5902	0.0141
133	P04746	AMYP_HUMAN	AMY2A	1.5060	0.5908	0.0233
132	P01009	A1AT_HUMAN	SERPINA1	1.5133	0.5977	0.0011
131	P81605	DCD_HUMAN	DCD	1.5368	0.6200	0.0007
130	Q99436	PSB7_HUMAN	PSMB7	1.5380	0.6211	0.0021
129	O75356	ENTP5_HUMAN	ENTPD5	1.5557	0.6376	0.0046
128	Q8WWA0	ITLN1_HUMAN	ITLN1	1.5673	0.6483	0.0000
127	Q7KZ85	SPT6H_HUMAN	SUPT6H	1.6340	0.7084	0.0006
126	P04745	AMY1A_HUMAN	AMY1A	1.6364	0.7106	0.0017
125	P00740	FA9_HUMAN	F9	1.6368	0.7109	0.0009
124	P02144	MYG_HUMAN	MB	1.6398	0.7135	0.0000
123	P10645	CMGA_HUMAN	CHGA	1.6494	0.7220	0.0001
122	P01011	AACT_HUMAN	SERPINA3	1.7095	0.7736	0.0026
121	P68871	HBB_HUMAN	HBB	1.7101	0.7741	0.0000
120	P02750	A2GL_HUMAN	LRG1	1.7211	0.7834	0.0000
119	Q86YZ3	HORN_HUMAN	HRNR	1.7255	0.7870	0.0188
118	P07451	CAH3_HUMAN	CA3	1.7288	0.7898	0.0001
117	O00602	FCN1_HUMAN	FCN1	1.7636	0.8186	0.0000
116	P06732	KCRM_HUMAN	CKM	1.8449	0.8836	0.0016
115	Q9Y279	VSIG4_HUMAN	VSIG4	1.8754	0.9072	0.0001
114	Q9NSC7	SIA7A_HUMAN	ST6GALNAC1	1.9034	0.9286	0.0019
113	P02042	HBD_HUMAN	HBD	2.0107	1.0077	0.0000
112	Q969E1	LEAP2_HUMAN	LEAP2	2.0123	1.0088	0.0459
111	P69905	HBA_HUMAN	HBA1	2.0545	1.0388	0.0000
110	P26022	PTX3_HUMAN	PTX3	2.1264	1.0884	0.0000
109	Q08830	FGL1_HUMAN	FGL1	2.1532	1.1065	0.0012
108	Q6IPM2	IQCE_HUMAN	IQCE	2.1986	1.1366	0.0001
107	P00738	HPT_HUMAN	HP	2.3076	1.2064	0.0000
106	P02741	CRP_HUMAN	CRP	2.9353	1.5535	0.0177
105	P0DJI9	SAA2_HUMAN	SAA2	3.4499	1.7865	0.0000
104	P0DJI8	SAA1_HUMAN	SAA1	3.8381	1.9404	0.0000

SourceDataForFigure3E																			
sample	N-1	N-2	N-3	N-4	N-5	N-6	UR1	UR2	UR3	UR4	UR5	UR6	R1	R2	R3	R4	R5	R6
group	N	N	N	N	N	N	UR	UR	UR	UR	UR	UR	R	R	R	R	R	R
P01024	1	1.020016	1	1.00926	1	1.025731	1.338406	1.12269	1.336507	1.126479	1.335405	1.125667	1.055885	1.252489	1.03507	1.25733	1.042922	1.261161
P04114	1	0.795446	1	0.82193	1	0.82501	1.955772	1.134346	1.932931	1.140767	1.961634	1.135864	0.678163	1.226278	0.677023	1.255558	0.670156	1.257412
P01023	1	0.900913	1	0.92874	1	0.927949	0.951954	0.610638	0.969156	0.618592	0.952208	0.610135	0.729063	1.149428	0.727811	1.163253	0.734425	1.152233
P00450	1	1.119552	1	1.129909	1	1.156592	1.033395	1.051513	1.04714	1.069955	1.035804	1.061443	1.194653	1.277458	1.18177	1.28625	1.200194	1.278296
P02774	1	1.167755	1	1.152836	1	1.186299	1.049813	1.044862	1.042974	1.052663	1.053217	1.039295	1.107871	1.047908	1.096145	1.041078	1.09458	1.042982
P02768	1	0.52146	1	0.53932	1	0.546441	0.153223	0.191143	0.159518	0.198761	0.164302	0.194677	0.21204	0.20971	0.211533	0.21711	0.216952	0.214479
P0C0L5	1	1.013376	1	0.958473	1	0.949424	1.545452	1.309224	1.603712	1.307718	1.569195	1.324449	0.734814	1.196061	0.695121	1.27365	0.780085	1.285531
P02790	1	0.990065	1	0.988645	1	0.998973	1.072036	1.027219	1.080321	1.038544	1.060785	1.034023	1.145685	1.079636	1.12335	1.09762	1.128206	1.086033
P0C0L4	1	0.899508	1	0.723528	1	0.780697	1.758569	1.136338	1.656875	1.154883	1.681671	1.199112	0.650311	1.147251	0.735997	1.160242	0.690172	1.137985
P00738	1	0.959089	1	0.941048	1	0.975066	1.185186	1.200268	1.182715	1.217815	1.170357	1.204525	2.348551	2.516842	2.343913	2.567647	2.348356	2.580799
P02647	1	0.871385	1	0.875833	1	0.880851	1.397368	0.734762	1.399129	0.737056	1.408876	0.734015	0.515133	1.089467	0.513145	1.101449	0.519703	1.112415
P08603	1	0.927155	1	0.921901	1	0.948519	0.683106	0.792688	0.670424	0.798916	0.678836	0.797279	0.829722	0.890096	0.814552	0.882167	0.833432	0.893186
P01042	1	1.00805	1	1.038117	1	1.049048	1.010906	0.983814	1.007643	0.993456	1.008459	0.983025	1.004109	1.021238	1.003516	1.029621	1.031166	1.033829
P19823	1	0.958782	1	0.957698	1	0.979851	1.039845	1.054645	1.03613	1.061803	1.030184	1.05972	1.121948	1.009303	1.119005	1.015372	1.116736	1.022335
P01008	1	1.221001	1	1.224792	1	1.24703	1.550239	1.31832	1.567502	1.319055	1.573616	1.317878	1.321919	1.475816	1.305329	1.458216	1.31096	1.50636
P01009	1	1.071923	1	1.129688	1	1.138349	0.811339	0.796763	0.80641	0.795199	0.781367	0.786803	1.405716	1.354104	1.360389	1.375325	1.407036	1.385817
P00751	1	1.183498	1	1.168087	1	1.186038	1.166345	1.134668	1.166388	1.14341	1.178992	1.135478	1.318638	1.227918	1.305978	1.221418	1.312371	1.234282
P00734	1	1.053491	1	1.046757	1	1.053317	1.089888	1.061321	1.063007	1.064206	1.061549	1.073265	1.056363	1.023946	1.06562	1.022632	1.069977	1.030923
P00747	1	1.007173	1	1	1	1.004257	0.948667	0.967361	0.94869	0.973485	0.93081	0.967503	1	0.959615	1.001148	0.947467	1.018568	0.944046
P19827	1	1.274697	1	1.239283	1	1.267502	1.091983	1.050835	1.08451	1.056414	1.089665	1.050328	1.206401	1.083369	1.173396	1.068919	1.21627	1.08138
Q14624	1	1.019719	1	1.057684	1	1.052192	1.389571	1.203722	1.39727	1.221224	1.422936	1.218539	1.187485	1.340693	1.191407	1.344558	1.194805	1.35683
P01031	1	0.93459	1	0.948675	1	0.958667	1.198251	1.135607	1.191769	1.146631	1.206263	1.148812	1.229055	1.219647	1.222547	1.245723	1.222743	1.259025
P04003	1	0.869708	1	0.877705	1	0.925284	0.975946	1.069683	0.965077	1.076329	0.977774	1.083402	0.972885	1.11488	0.9358	1.096466	0.979893	1.125431
P10643	1	0.988179	1	1.005859	1	1.001635	1.099551	0.965322	1.084362	0.974268	1.072852	0.952659	1.29737	1.309458	1.30706	1.317478	1.296718	1.300325
P05155	1	0.906114	1	0.890132	1	0.900339	1.286336	1.173081	1.257345	1.167284	1.266416	1.177279	1.242333	1.419539	1.202617	1.372318	1.247574	1.44672
P06396	1	1.104281	1	1.076154	1	1.101739	0.887089	0.872144	0.890937	0.872221	0.875903	0.873368	0.840892	0.773544	0.835	0.774352	0.820173	0.784061
P02760	1	0.958015	1	0.977991	1	0.998401	0.969553	1.05343	0.969474	1.066643	0.97954	1.058975	1.162565	1.054232	1.175478	1.073077	1.166667	1.06161
P02787	1	0.916815	1	0.900219	1	0.898008	0.554163	0.565608	0.560205	0.579994	0.557611	0.575405	0.595896	0.592739	0.593143	0.594992	0.598126	0.59364
P00739	1	1.526273	1	1.457977	1	1.615633	0.84429	0.911833	0.797446	0.89828	0.826222	0.90899	1.138922	1.055832	1.106091	1.069156	1.175855	1.108785
P02751	1	0.542065	1	0.55161	1	0.555282	0.342611	0.66267	0.368016	0.676133	0.354846	0.660808	0.212306	0.473553	0.235284	0.484077	0.233484	0.491238
P13671	1	1.031338	1	1.028568	1	1.034364	0.983979	1.074647	0.964715	1.074485	0.982137	1.082693	1.303956	1.241305	1.281327	1.25058	1.30197	1.264146
P05156	1	1.274138	1	1.295129	1	1.294844	1.14734	1.11802	1.122805	1.130207	1.130778	1.130755	1.169339	1.343477	1.185575	1.331411	1.173908	1.320593
P06727	1	1.118947	1	1.04868	1	1.117724	0.822461	0.822847	0.825824	0.818771	0.830787	0.827397	0.386396	0.473209	0.372854	0.464455	0.386767	0.481276
P02765	1	0.981152	1	0.94599	1	0.957464	1.002326	0.956934	0.984519	0.955258	0.994696	0.959266	0.928894	0.85463	0.893223	0.859152	0.912379	0.889423
P03952	1	0.927103	1	0.920423	1	0.936239	0.879095	1.057318	0.880578	1.059102	0.882739	1.056328	1.079364	0.976748	1.073738	0.989979	1.071634	0.978749
P09871	1	0.929791	1	0.982137	1	0.975555	0.997669	1.043835	1.002834	1.044016	0.993469	1.048174	1.156533	1.210281	1.164328	1.234603	1.14866	1.235473
O75882	1	0.763295	1	0.750415	1	0.775069	0.956028	1.015652	0.968729	1.027642	0.958008	1.031323	0.965542	1.022233	0.966433	1.049152	0.979896	1.057224
P10909	1	1.024057	1	0.980549	1	0.991329	0.685869	0.888014	0.691736	0.894914	0.696566	0.892625	0.803832	0.813943	0.780998	0.811612	0.79818	0.818329
P02748	1	0.975243	1	0.978479	1	1.015149	1.227623	1.151542	1.236084	1.177147	1.229415	1.175229	1.408014	1.436811	1.374082	1.450385	1.352106	1.418086
P06681	1	1.077089	1	1.071998	1	1.124132	1.076434	1.067155	1.074467	1.081437	1.077119	1.06959	1.264261	1.226956	1.257973	1.241474	1.281824	1.254978
P07358	1	1.20386	1	1.145206	1	1.213551	0.941869	1.067572	0.942572	1.073941	0.945542	1.052503	1.195148	1.155165	1.171532	1.164906	1.181604	1.155769
P07357	1	0.983719	1	0.996511	1	1.009832	1.013451	1.002595	0.995079	1.023371	1.011339	1.014123	1.090558	1.079696	1.105595	1.081227	1.09181	1.080014
P01019	1	0.997669	1	1.016519	1	1.040794	1.157269	1.087481	1.159148	1.095336	1.158526	1.102647	1.217366	1.637338	1.213902	1.614655	1.229906	1.644421
P43652	1	0.97491	1	0.981554	1	1.006654	0.934199	1.010514	0.926813	1.020789	0.94563	1.019798	1.136062	0.916759	1.109452	0.926018	1.132979	0.92927
P05546	1	0.906845	1	0.899268	1	0.932907	1.321389	1.098668	1.303391	1.095969	1.337187	1.124163	0.730398	0.869089	0.729846	0.862337	0.726294	0.888159
P02649	1	0.945853	1	0.943392	1	0.944991	1.144113	0.95565	1.134984	0.969238	1.123917	0.970122	0.665971	0.907325	0.671748	0.923874	0.685605	0.905213
P04275	1	0.797305	1	0.801658	1	0.78951	0.610549	0.656876	0.620033	0.679246	0.621199	0.670361	0.497664	1.190695	0.516377	1.163997	0.490761	1.220793

P02749	1	0.970329	1	0.87557	1	0.909814	1.0297	0.96588	1.030966	0.948161	1.018386	0.959276	1.215883	0.993321	1.087293	0.975932	1.162979	0.97183
P04217	1	0.890738	1	0.9415	1	0.947567	1.170317	1.06975	1.171148	1.074063	1.170694	1.071026	0.893917	1.089596	0.896055	1.109691	0.892804	1.102099
P07996	1	0.987111	1	0.995757	1	1.010692	0.408751	0.599157	0.414677	0.594075	0.413994	0.601969	0.57632	0.604937	0.588663	0.624554	0.587346	0.630956
P20742	1	1.038303	1	1.083297	1	1.053353	1.037199	1.425056	1.066431	1.50565	1.019681	1.38271	1.4244	1.665155	1.375244	1.751762	1.396842	1.721714
P04004	1	1.17675	1	1.167773	1	1.199507	1.031678	1.043054	1.017424	1.061518	1.046171	1.059955	1.036508	1.082838	1.008548	1.05922	1.024667	1.105831
P01871	1	0.579626	1	0.605234	1	0.584919	0.346631	0.519162	0.344754	0.518322	0.347641	0.521822	0.422408	0.47186	0.410382	0.475468	0.417033	0.464659
P04196	1	1.462323	1	1.473109	1	1.443162	1.264759	1.104413	1.253334	1.090636	1.239422	1.101976	1.192118	1.070086	1.199344	1.117281	1.186201	1.105245
P01834	1	0.523427	1	0.590133	1	0.594948	0.611393	0.585306	0.646772	0.620532	0.633333	0.616179	0.357621	0.611477	0.393049	0.692456	0.427147	0.669153
P00736	1	1.049191	1	1.017539	1	0.993059	1.038075	1.013313	1.014661	1.03928	1.001444	1.019884	1.195251	1.235048	1.198919	1.225409	1.148799	1.206383
P02656	1	1.213917	1	1.214607	1	1.152333	0.898541	0.844242	0.907261	0.857468	0.915571	0.869282	1.083866	0.941786	1.083487	0.957485	1.064388	0.946954
P02763	1	0.719216	1	0.711547	1	0.721129	1.011096	0.880924	1.006487	0.876569	1.004077	0.881127	0.673677	2.184517	0.662591	2.07825	0.671161	2.060281
P01876	1	0.873283	1	0.815418	1	0.792635	0.580852	0.780747	0.577773	0.789231	0.566798	0.783337	0.609827	1.039502	0.574223	1.012576	0.584455	0.991706
P68871	1	1.150983	1	1.165727	1	1.195697	1.038108	0.957467	1.096175	1.038483	1.073244	1.002324	1.846346	1.724277	1.85822	1.66036	1.89164	1.682665
P04264	1	1.015276	1	1.027571	1	1.01141	1.45658	1.895245	1.440846	1.863931	1.416015	1.832207	0.870144	3.255781	0.851658	3.17408	0.851155	3.192036
P05090	1	0.691336	1	0.670047	1	0.670798	0.836314	0.784689	0.844946	0.801946	0.790895	0.77888	0.870132	0.854316	0.868449	0.858468	0.852698	0.841736
Q06033	1	1.140283	1	1.115814	1	1.110575	1.081559	1.056742	1.07332	1.074941	1.046969	1.059654	1.233693	1.565615	1.217186	1.577829	1.234209	1.612948
P0DOX6	1	0.675751	1	0.842501	1	0.773411	0.31745	0.473075	0.140789	0.490983	0.311422	0.48788	0.424928	0.416485	0.467445	0.329251	0.416549	0.43003
P08697	1	0.96782	1	0.968447	1	1.014245	1.230178	1.316085	1.227442	1.340518	1.198801	1.293333	1.133397	1.297586	1.103606	1.30721	1.063271	1.269936
P12259	1	0.822965	1	0.8313	1	0.837427	0.893554	0.996673	0.900082	0.999594	0.908561	0.991727	1.078147	1.084857	1.063355	1.092738	1.09081	1.092264
P02753	1	0.986819	1	0.990597	1	1.021322	0.949169	1.080509	0.923599	1.061218	0.932141	1.057086	0.882773	0.748765	0.860987	0.764562	0.865296	0.754071
Q96PD5	1	1.034071	1	1.070067	1	1.01031	0.954337	0.978669	0.966126	0.980463	0.954356	0.969682	0.992783	0.919434	1.012708	0.931372	1.010457	0.945121
P0DOY2	1	0.678331	1	0.644209	1	0.654264	0.721939	0.650144	0.708088	0.649097	0.693207	0.622012	0.475072	0.689637	0.462478	0.665269	0.441941	0.691102
P27169	1	0.980823	1	0.996117	1	0.971717	0.81882	0.911009	0.794407	0.900631	0.814064	0.924434	0.88249	0.864822	0.85662	0.874304	0.852604	0.85425
P0DOX7	1	0.816487	1	0.920667	1	0.886112	0.549361	0.575872	0.586485	0.62072	0.610517	0.653026	0.402959	0.677365	0.458373	0.703162	0.479144	0.736798
P01857	1	0.840973					2.001138	0.69581					0.434682	0.867599				
P36955	1	0.831263	1	0.855353	1	0.865652	0.961102	1.019933	0.971405	1.011033	0.975707	1.029823	1.051902	0.976983	1.02092	1.004981	1.080281	1.01357
P07225	1	0.838677	1	0.840661	1	0.825527	0.803537	0.88157	0.779622	0.903746	0.771636	0.898517	0.891415	0.950749	0.898432	0.951273	0.914049	0.966923
P51884	1	0.911042	1	0.892711	1	0.922405	0.984522	0.87884	0.977693	0.888766	0.983258	0.875402	0.861691	0.892516	0.857292	0.898868	0.857546	0.899944
P02671	1	0.931087	1	0.898166	1	0.902388	1.97402	2.28732	2.292207	3.346948	2.306141	2.991829	1.578665	0.94865	1.659313	0.996659	1.657436	1.005959
A0M8Q6	1	1.049981	1	0.957122	1	1.010817	1.147606	1.249922	0.896509	1.079595	1.049608	1.001382	1.036914	1.425912	1.085716	1.117783	1.099732	1.016687
P08519	1	0.962795	1	0.95629	1	0.939048	0.814906	1.100607	0.791672	1.112086	0.830531	1.138388	1.543205	1.310936	1.513064	1.314072	1.536122	1.3317
P02652	1	0.888673	1	0.895626	1	0.922331	0.700439	0.756599	0.707254	0.773087	0.710959	0.782527	0.888508	0.867805	0.894192	0.876845	0.901288	0.873968
P22792	1	0.924325	1	0.93599	1	0.95537	0.96265	1.012018	0.950199	1.021893	0.958499	1.033637	1.013521	1.099544	0.990315	1.114435	1.021244	1.130372
P05452	1	0.775462	1	0.72228	1	0.734575	1.061066	0.920862	1.093816	0.917581	1.1377	0.892068	0.778321	0.82697	0.692401	0.837509	0.716622	0.840217
P35858	1	1.038144	1	1.093597	1	0.989668	0.982445	1.057355	0.998683	1.086052	0.979355	1.089933	0.963706	0.920364	0.975451	0.948342	0.989874	0.968098
P02750	1	0.900696	1	0.934602	1	0.910999	1.330553	1.284881	1.348697	1.251529	1.308932	1.283314	1.707072	2.233023	1.582833	2.08459	1.661307	2.134453
P80108	1	0.830217	1	0.799633	1	0.796048	0.775294	0.831991	0.799094	0.8412	0.781963	0.848316	0.82112	0.828048	0.791016	0.828912	0.809274	0.832889
P35527	1	1.146397	1	1.105634	1	1.143147	1.762813	2.007752	1.572065	1.872634	1.593405	1.860926	1.076468	3.813703	1.050106	3.449715	1.037474	3.386605
Q08380	1	0.805697	1	0.904691	1	0.852465	1.357471	0.999358	1.385247	0.982636	1.380164	1.001083	0.9636	1.21951	0.964386	1.253642	0.958074	1.230283
P26927	1	1.029127	1	0.996703	1	1.031125	1.047056	1.042794	1.052799	1.047272	1.0658	1.06298	1.125365	1.112361	1.15228	1.155793	1.130249	1.140729
P00742	1	0.975727	1	1.019608	1	1.011971	1.20038	1.062563	1.107971	1.075001	1.090996	1.074099	1.132281	1.163164	1.098486	1.141512	1.15137	1.182153
P02747	1	0.759876	1	0.722191	1	0.702237	0.987291	0.960431	0.99349	0.993486	0.958626	0.967679	0.827321	0.822194	0.832682	0.829268	0.81358	0.827189
P48740	1	0.891416	1	0.930071	1	0.885615	0.992403	1.140945	1.00038	1.138685	0.980544	1.139614	1.110348	1.11233	1.087663	1.087796	1.094773	1.122014
P27918	1	0.990693	1	0.980435	1	0.994789	1.024049	1.186127	1.025389	1.195707	1.026018	1.239463	1.260224	1.078539	1.233588	1.058788	1.267877	1.113607
P02746	1	1.022348	1	1.037509	1	1.044592	0.94282	0.999896	0.922172	0.994551	0.926183	0.990859	0.897723	1.075339	0.913382	1.132521	0.935852	1.118498
P07360	1	1.017787	1	1.018827	1	1.100257	1.265574	1.119935	1.235148	1.120002	1.239179	1.121551	1.052821	1.236052	1.086399	1.229668	1.061648	1.265865
P35908	1	0.989271	1	0.936704	1	0.95865	1.360665	1.335325	1.272302	1.287525	1.327212	1.296579	0.948834	3.22486	0.905234	3.115727	0.881753	2.819812
P01859	1	0.867333	1	0.796444	1	0.865244	1.013022	0.643565	1.294331	0.647636	1.649217	0.644618	0.388764	0.870423	0.378494	0.867221	0.382421	0.949185
Q14520	1	1.004918	1	0.99827	1	1.016855	1.004136	1.007715	0.985431	1.009046	1.01127	0.994061	1.019315	1.101345	0.994106	1.113304	0.979222	1.122635
P0DOX8	1	0.910419					1.043705	0.951264					0.565191	1.133278				

P08185	1	0.595854	1	0.63962	1	0.651097	2.367995	1.367581	2.334792	1.324926	2.39243	1.336295	0.426044	1.639261	0.423139	1.509407	0.43799	1.628361
P01860	1	0.635882	1	0.741284	1	0.901093	0.505511	0.602826	0.561799	0.658931	0.568624	0.681683	0.54077	0.574556	0.556182	0.598521	0.584964	0.650989
Q9UGM5	1	0.943018	1	1.014311	1	0.983935	1.041439	0.939765	1.016306	0.975417	1.036434	0.949517	0.776175	0.76842	0.777722	0.794076	0.842041	0.798014
P19652	1	0.535752	1	0.601266	1	0.583896	1.110212	0.693353	1.104744	0.706752	1.097075	0.694991	0.457939	1.991757	0.445161	2.062126	0.453477	1.995621
Q96KN2	1	1.078637	1	1.080742	1	1.07181	0.774513	0.960513	0.761976	0.947815	0.761907	0.953371	1.126994	0.972965	1.084865	0.987758	1.102683	0.974115
P0DJI9	1	0.926613	1	0.994724	1	0.907865	0.947868	1.205679	1.050557	1.179748	1.074542	1.223297	2.494948	4.07053	2.171229	5.155656	2.097839	5.208179
P0DJI8	1	1.0648	1	1.112602	1	1.201896	1.246946	0.923406	1.233835	0.849895	1.213968	0.882523	2.34153	5.469264	3.041428	5.683633	2.737289	4.823365
P01861	1	0.971337	1	0.936535	1	0.94769	1.42604	0.687326	1.506495	0.754645	1.605725	0.759543	0.593253	1.04449	0.616065	1.112378	0.601364	1.137075
P23142	1	0.711692	1	0.773944	1	0.714836	0.764365	0.793169	0.734329	0.816632	0.773241	0.793213	0.577443	0.696708	0.624008	0.729608	0.625492	0.713213
P02042	1	1.033863	1	0.996246	1	0.954446	0.963083	1.044935	0.854391	1.043365	0.85379	1.054268	2.175563	1.69861	2.396549	1.689229	2.010299	1.631802
P01011	1	1.190944	1	1.249758	1	1.201493	1.357309	1.330906	1.340574	1.340594	1.34351	1.364453	1.355649	2.570803	1.436916	2.70136	1.433438	2.837989
O43866	1	0.942612	1	0.922261	1	0.96083	0.340926	0.523876	0.334841	0.544721	0.326631	0.542099	0.565565	0.477215	0.540954	0.468011	0.555371	0.498134
P13645	1	0.786021	1	0.852613	1	0.842774	1.435876	1.261904	1.379617	1.233043	1.41228	1.260859	0.689461	2.394361	0.724237	2.352902	0.702219	2.454228
Q04756	1	0.939495	1	1.036947	1	0.93325	1.118205	1.066207	1.129714	1.069234	1.113678	1.04292	1.096155	1.023033	1.135659	1.117266	1.141107	1.063201
P02655	1	0.876085	1	0.885681	1	0.88185	0.91976	0.890107	0.946237	0.885409	0.928295	0.874993	0.95924	0.928736	0.905313	0.900104	0.911009	0.905887
P15169	1	0.818156	1	0.820951	1	0.848257	0.981452	1.032239	0.948737	1.039204	0.944373	1.030736	0.909948	1.111214	0.969125	1.097027	0.997879	1.096907
P00740	1	0.797411	1	0.837349	1	0.835666	1.454505	1.477462	1.423159	1.437241	1.41649	1.412225	1.978858	1.951795	1.782458	1.862052	1.808022	1.841823
P02675	1	0.858443	1	0.983512	1	1.018604	4.668588	6.317659	4.113068	6.06468	4.363779	5.55252	2.260754	1.288007	2.401845	1.290197	2.421984	1.290367
Q16610	1	1.02348	1	1.000587	1	0.991916	1.415068	1.349866	1.41232	1.350593	1.412408	1.311007	1.195753	1.192793	1.203443	1.196984	1.221932	1.172297
P02654	1	0.87684	1	0.854123	1	0.861889	0.730478	0.778732	0.722959	0.771032	0.717761	0.776196	0.88525	0.837796	0.860213	0.831692	0.875236	0.828613
P60709	1	1.501168	1	2.237007	1	1.958721	0.859918	0.957871	0.870084	1.077979	0.570491	1.037501	0.829871	0.917777	0.928036	0.824903	0.846999	0.567352
P02766	1	1.756091	1	1.637392	1	1.782463	1.266229	1.182232	1.243361	1.177006	1.202771	1.153789	1.097059	1.203604	1.045181	1.064513	1.053755	1.073491
P49747	1	1.029675	1	1.012642	1	1.017008	0.859202	0.787869	0.874778	0.831529	0.845832	0.80212	0.719014	0.622676	0.660079	0.674627	0.724209	0.663329
P69905	1	0.910275	1	0.884759	1	0.924344	1.059826	0.924916	1.018685	0.936863	1.028998	0.941982	2.205106	1.734029	2.154556	1.737684	2.1502	1.724633
P02775	1	1.401845	1	1.411136	1	1.253483	0.644405	0.738026	0.634318	0.743549	0.63892	0.727923	0.830609	0.816805	0.822108	0.836804	0.838437	0.815328
Q12805	1	0.939155	1	0.895685	1	0.917391	1.019272	0.965156	0.988449	0.987767	1.027116	0.96448	0.841515	0.865034	0.838869	0.838376	0.811116	0.855025
P25311	1	0.965542	1	1.00174	1	1.023065	3.877244	1.312584	4.184026	1.342829	4.045864	1.320782	0.590722	1.464773	0.579111	1.463216	0.584557	1.50514
P11226	1	1.064659	1	1.008035	1	1.111338	1.144704	0.922782	1.120617	0.943383	1.192698	0.942689	1.02203	0.926713	0.983676	0.907596	0.957839	0.97822
P0DOX2	1	0.904582	1	0.886542	1	0.909084	0.583203	0.777887	0.614211	0.782057	0.639696	0.783522	0.696905	0.913812	0.712473	0.903748	0.659477	0.940192
O95445	1	0.978583	1	0.948386	1	0.993877	0.957103	0.950452	0.94001	0.950286	0.914876	0.940809	1.070978	0.944138	1.06293	0.899507	1.071765	0.931535
Q92954	1	0.869416	1	0.85305	1	0.853047	0.907568	0.99509	0.925294	0.978291	0.943547	1.006739	1.104801	1.069126	1.055056	1.077513	1.088988	1.051415
Q96IY4	1	1.015869	1	1.004443	1	1.00105	1.040738	1.13142	1.024002	1.116341	1.001611	1.109267	1.262756	1.019004	1.235741	1.053566	1.160834	0.984879
Q03591	1	0.826111	1	0.783808	1	0.900604	0.621562	0.771621	0.595043	0.772721	0.729063	0.801525	0.758544	0.844488	0.747056	0.807227	0.766899	0.919634
P02679	1	1.074529	1	0.903579	1	0.837235	4.27329	5.233908	4.279387	5.226605	4.008218	5.279949	2.324283	1.313536	2.290466	1.28837	2.169932	1.356074
Q02985	1	0.752777	1	0.664152	1	0.746466	0.512068	0.927074	0.484004	0.91773	0.522085	0.940683	1.139611	1.166025	1.118827	1.240892	1.121418	1.218076
P03951	1	0.846103	1	0.88228	1	0.931775	1.056879	1.11687	1.095429	1.137203	1.112359	1.147589	1.251025	1.243943	1.230456	1.3022	1.283737	1.341655
Q13790	1	0.954209	1	0.980722	1	1.009356	0.68371	0.738048	0.709099	0.748188	0.692383	0.765317	0.768312	0.829291	0.719868	0.908757	0.777611	0.887243
P08571	1	1.094106	1	1.025979	1	1.062202	1.231941	1.038742	1.277197	1.022229	1.195607	0.994663	1.367012	1.306285	1.348327	1.29118	1.363403	1.213811
P04070	1	0.959674	1	0.943578	1	0.952627	0.93848	1.096703	0.8852	1.118006	0.89322	1.092411	0.949325	1.133023	0.957931	1.15717	0.918374	1.105607
O14791	1	0.868167	1	0.83344	1	0.872101	0.850493	0.924343	0.817631	0.928091	0.807805	0.911712	0.882444	0.946012	0.905393	0.990802	0.899811	0.967782
P13796	1	0.953811	1	0.916216	1	0.899712	1.111638	0.983552	1.08546	0.985675	1.029075	0.961002	0.897129	1.099328	0.860067	1.071952	0.902576	1.042173
P11021	1	1.035821	1	0.99593	1	1.111088	0.93145	1.004484	0.918293	1.008372	0.925766	1.020319	0.935213	0.965189	0.893525	0.938423	0.937946	0.948041
P17936	1	1.253331	1	1.070803	1	0.989903	0.863429	1.088178	0.889036	1.101392	0.881081	1.100886	1.061443	0.881236	1.005777	0.897294	1.025535	0.840053
O00391	1	1.037449	1	1.027679	1	1.108077	1.088607	1.003195	1.059675	1.00908	1.100927	1.020864	1.098978	1.099974	1.055319	1.032964	1.085525	1.05604
O75636	1	0.967453	1	0.850987	1	0.872927	1.028885	1.218691	1.000384	1.254807	0.93291	1.214818	1.118106	1.17511	1.02709	1.16752	1.040068	1.133049
O00187	1	0.990936	1	0.967701	1	1.016393	0.947412	1.167799	0.946005	1.173211	0.927919	1.161457	1.073224	1.140329	1.035133	1.181716	1.072462	1.166836
P22105	1	0.984707	1	0.983943	1	0.998117	1.006396	0.961406	1.018268	0.97279	1.023385	0.964731	1.153163	0.998398	1.072868	0.949917	1.079818	0.94683
Q07954	1	0.912374	1	0.916952	1	0.986771	0.870846	0.899815	0.873677	0.907847	0.863059	0.918004	1.046371	0.997084	1.093367	0.970329	1.047611	1.007376
P05543	1	0.982928	1	0.987977	1	1.018503	2.804667	1.032566	2.908875	1.034175	2.704078	1.015531	0.657455	1.827437	0.642596	1.797667	0.670873	1.81869
P35542	1	1.087459	1	1.109865	1	1.125339	0.866038	0.935013	0.850887	0.929854	0.840312	0.944952	1.330582	1.139614	1.351978	1.199861	1.370218	1.198675

P43251	1	1.013844	1	0.999941	1	0.909067	1.070009	1.10147	1.118926	1.071252	0.993569	1.035859	1.300067	1.124543	1.158122	1.117736	1.063526	1.042437
P20851	1	0.959796	1	0.932791	1	0.926501	0.795501	0.93139	0.786331	0.929546	0.775382	0.937422	0.995234	1.155415	0.975934	1.180356	0.94965	1.183488
P22352	1	0.990318	1	0.92645	1	0.954876	0.964596	0.989306	0.965963	1.020818	0.928435	0.992105	1.05155	1.062856	1.033311	1.117904	1.034698	1.09261
P02743	1	0.885559	1	0.926233	1	0.885696	4.934572	0.914403	4.873736	0.920425	4.709537	0.898087	0.427918	1.737321	0.570456	1.583409	0.517728	1.672198
P06276	1	0.693655	1	0.64571	1	0.724333	1.51661	1.019603	1.48465	1.039725	1.498852	1.049474	0.655897	1.426147	0.675741	1.400186	0.634361	1.434947
P01591	1	0.73013	1	0.693298	1	0.690377	0.385705	0.535493	0.375624	0.519885	0.411413	0.530539	0.575354	0.554202	0.552387	0.51023	0.599048	0.549421
P14151	1	1.064705	1	1.126217	1	1.162742	0.968727	1.104088	0.974726	1.106502	1.013021	1.138901	1.209783	1.282914	1.200856	1.285688	1.229182	1.30843
Q86VB7	1	1.062522	1	1.021111	1	1.023163	1.134688	1.173514	1.130904	1.199125	1.154957	1.145721	1.799926	1.224969	1.815754	1.183567	1.609917	1.16937
P24821	1	0.901874	1	0.973814	1	0.94479	1.088076	1.139029	1.120164	1.126047	1.038454	1.17491	1.271421	1.380269	1.246843	1.310094	1.271312	1.391088
Q9NZP8	1	1.249275	1	1.238815	1	1.287403	1.021462	1.27222	1.012042	1.264238	1.025141	1.269259	1.475237	1.339113	1.36704	1.251984	1.372285	1.292671
P36980	1	1.011269	1	0.975544	1	0.987701	0.981524	0.900424	0.974803	0.908276	1.018306	0.9548	0.767639	1.128025	0.854771	1.041476	0.819822	1.150248
O00533	1	1.22225	1	1.04991	1	1.058259	0.971991	0.98258	0.94792	0.960393	0.993932	0.967615	1.288667	1.139656	1.199205	1.116497	1.221452	1.098452
Q9NQ79	1	1.033393	1	1.050901	1	1.111076	1.028229	0.796699	1.032006	0.823843	1.033262	0.820321	0.812869	0.913816	0.840362	0.87382	0.851856	0.924248
P19320	1	0.946807	1	0.995366	1	0.983615	1.00018	0.963231	1	1.001769	0.974924	0.989171	1.229102	1.059694	1.227709	1.075229	1.274396	1.092988
Q9Y6R7	1	1.040117	1	1.021319	1	1.089422	1.026012	0.92152	1.045758	0.923875	0.990841	0.962666	0.886996	0.900444	0.905262	0.893013	0.921889	0.910699
Q9UK55	1	0.872183	1	0.911444	1	0.956569	1.062102	1.169886	1.080277	1.177635	1.098973	1.183628	1.326069	1.249041	1.295666	1.248834	1.337661	1.248972
P00746	1	1.351701	1	1.232227	1	1.261791	1.25901	0.995248	1.153315	0.947224	1.230603	0.980812	0.949478	0.926509	0.939588	0.891475	0.903424	0.869808
P04278	1	0.925887	1	0.916574	1	0.996715	1.084408	1.059008	1.111071	1.055858	1.132851	1.072602	1.066261	1.211505	1.043367	1.168483	1.08197	1.140948
P00488	1	0.974863	1	1.162922	1	1.307803	0.671317	0.712457	0.668059	0.708203	0.68091	0.723735	0.551806	0.742072	0.520215	0.74452	0.614701	0.943681
P98160	1	0.91747	1	0.927121	1	0.91496	0.953604	0.900084	0.948623	0.908911	0.94306	0.909591	0.871277	0.857473	0.896923	0.921693	0.919919	0.870322
Q13201	1	0.95371	1	0.927873	1	1.013168	0.689554	0.77887	0.692591	0.781872	0.697312	0.792697	0.784964	0.797808	0.768948	0.811246	0.814571	0.808686
P22891	1	1.272423	1	1.414268	1	1.318861	1.048864	1.07829	1.041373	1.068771	1.062401	1.053219	1.240131	0.864588	1.235673	0.84144	1.278409	0.878059
Q76LX8	1	1.037421	1	1.082046	1	0.969306	0.865022	0.988845	0.884396	0.994273	0.856205	0.997939	0.78766	0.852321	0.794083	0.899728	0.726311	0.859764
P35443	1	1.105716	1	1.077708	1	0.929771	0.800022	0.729433	0.905138	0.791231	0.855874	0.806625	0.792561	0.697133	0.775427	0.741074	0.752172	0.697108
Q9BXR6	1	0.858649	1	0.83821	1	0.934585	0.832736	0.948917	0.802983	0.972726	0.819474	0.966456	0.960593	1.168882	0.941606	1.176017	0.925719	1.137595
Q14766	1	1.077923	1	1.102384	1	1.121336	0.598866	0.722968	0.561993	0.753093	0.592771	0.737752	0.712688	0.819492	0.691134	0.804533	0.7172	0.835896
Q92496	1	0.86007	1	0.867495	1	0.864892	0.868995	1.099914	0.833091	1.150438	0.830265	1.160254	0.945885	1.164567	0.953963	1.126192	0.982135	1.132826
P00915	1	0.954143	1	1.147694	1	1.035554	2.10224	1.670132	2.167077	1.685622	1.950496	1.612073	1.096113	1.828418	1.192392	1.930196	1.160433	1.724307
Q15166	1	0.994223	1	1.017146	1	0.94987	0.68776	0.734667	0.733946	0.745987	0.733738	0.741237	0.649568	0.88183	0.697894	0.883615	0.64824	0.886772
P05362	1	1.135273	1	1.064823	1	1.005425	1.11865	1.092521	1.162572	1.085949	1.159571	1.081651	0.955943	1.137392	0.933129	1.101377	0.960896	1.08712
Q15582	1	0.718631	1	0.790132	1	0.752394	1.180924	0.893803	1.327695	0.914972	1.25117	0.932411	0.768434	1.172754	0.798421	1.242601	0.832829	1.171172
P68363	1	0.958415	1	1.255609			0.367522	0.720248	1.353002	0.873832			0.457657	0.712932	0.812315	0.806027		
P61626	1	1.009319	1	0.902999	1	0.925985	1.013418	0.995133	1.032744	0.995303	1.024303	0.94693	0.8971	0.689084	0.900713	0.697717	0.884948	0.691512
P09172	1	0.950546	1	0.983887	1	0.978763	1.428347	1.067175	1.377807	1.08293	1.443335	1.076921	1.150165	0.876427	1.165566	0.916982	1.196409	0.926659
Q2TV78	1	0.90251	1	0.927525	1	0.887803	4.7658	1.478966	4.55917	1.370558	4.301997	1.342537	0.789572	2.363711	0.74507	2.203257	0.796654	2.118303
Q9UHG3	1	0.698183	1	0.758161	1	0.764804	1.095935	1.029047	1.150194	1.082796	1.119191	1.051838	0.704998	1.265972	0.785536	1.24712	0.752157	1.236456
Q15113	1	0.80294	1	0.875518	1	0.7767	1.110436	0.895746	1.110365	0.903084	1.121356	0.89609	0.703634	0.697424	0.709489	0.732099	0.700234	0.720696
Q71U36	1				1	1.075423					0.813031	0.887637					0.57385	1.002985
P35555	1	0.879738	1	0.875348	1	0.965746	0.897592	0.947382	0.846956	0.932768	0.931117	0.952574	1.028287	1.01352	1.058093	0.986783	1.049436	0.974337
Q9Y490	1	1.118925	1	1.057338	1	1.043408	0.459234	0.949675	0.436872	0.837782	0.461413	0.852473	0.554884	0.536903	0.489965	0.444466	0.53021	0.514311
P09486	1	1.145482	1	1.204355	1	1.211928	0.854042	0.792591	0.867974	0.772603	0.846065	0.783815	0.899641	1.027238	0.88555	1.010166	0.964918	1.010917
P01034	1	1.28483	1	1.354892	1	1.378087	1.093266	1.00543	1.107535	1.00703	1.14243	0.996803	1.097128	1.023813	1.242682	1.035002	1.207405	1.007725
P05154	1	0.964378	1	0.945355	1	0.974942	3.955778	1.960359	4.286404	2.25628	4.230821	2.277266	1.157216	1.645868	1.168785	1.695128	1.074255	1.736252
P63267	1	1.518223	1	1.427637	1	1.588278	0.655253	0.864159	0.604336	0.809656	0.689254	0.870243	0.879291	1.095931	0.793194	1.05372	0.864432	1.147969
P00748	1	0.61211	1	0.63333	1	0.646568	0.393867	0.414329	0.448314	0.456951	0.43173	0.469177	0.668063	0.61388	0.681339	0.649574	0.704079	0.635425
P21333	1	1.049432	1	1.034618	1	1.144684	0.558743	1.015151	0.575064	0.947164	0.575495	0.902168	0.60414	0.568676	0.636215	0.544727	0.601271	0.532861
Q15485	1	1.224907	1	1.111531	1	1.1971	1.464787	1.67107	1.477999	1.717042	1.477444	1.708434	2.032785	1.724039	1.975594	1.766583	1.904952	1.749755
P18206	1	0.893396	1	0.919439	1	0.896115	0.964459	0.91965	0.946068	0.90189	0.912352	0.924034	0.863739	1.007701	0.852755	0.974973	0.858287	0.923477
P04180	1	0.599823	1	0.691568	1	0.599224	0.766082	0.857472	0.81183	0.876574	0.819389	0.860523	0.731805	0.869488	0.706626	0.915394	0.730237	0.947336
A1L4H1	1	0.940392	1	0.866123	1	0.959401	0.935765	1.029072	0.949059	1.058974	0.936025	1.092941	0.993308	0.990698	0.951838	1.025252	0.977506	1.013685

P13591	1	0.978368	1	0.981952	1	0.958573	1.057536	1.067268	1.0851	1.056858	1.13247	1.063757	1.015375	0.969886	0.998469	0.981041	0.981865	1.049421
P14780	1	1.384742	1	1.372061	1	1.37794	0.720297	0.84239	0.671405	0.897828	0.709838	0.872036	1.236985	1.170818	1.208244	1.239578	1.225788	1.224962
Q7Z7M0	1	1.061475	1	1.106222	1	1.172287	1.073278	1.022954	1.078527	1.046614	1.10827	1.087368	1.145348	1.044111	1.209687	1.041239	1.245717	1.115109
P02776	1	1.034261	1	1.104109	1	1.015537	1.560003	1.135003	1.556078	1.232241	1.581325	1.176037	1.375002	0.763899	1.379397	0.681152	1.360217	0.643716
P08253	1	1.003375	1	1.030179	1	1.088677	0.932656	0.858715	0.923735	0.848672	0.944287	0.8552	0.865491	0.853235	0.81371	0.821424	0.843124	0.908813
Q14515	1	1.166512	1	1.093171	1	1.10479	1.02016	0.93575	1.083633	0.946577	1.040919	0.89744	0.998633	1.186106	0.95915	1.105621	1.02429	1.194343
P05062	1	1.129476	1	1.074887	1	1.154593	0.975436	1.348676	0.967351	1.500023	0.938969	1.512336	0.877031	1.097947	0.833215	0.993302	0.9866	1.095715
P40197	1	1.110052	1	1.116937	1	1.096588	0.597421	0.659785	0.654112	0.646743	0.672869	0.694615	0.758758	0.710142	0.698818	0.706319	0.776214	0.70497
P14543	1	1.081604	1	1.033302	1	0.874102	0.852423	0.909596	0.860687	0.896944	0.795432	0.945722	1.052549	0.948978	1.092481	0.953742	1.128639	0.949905
Q9UNW1	1	0.9758	1	1.020886	1	1.086527	0.896079	0.92673	0.859716	1.014684	0.846956	0.994595	1.130978	0.969809	1.106193	0.988596	1.131963	1.005728
P14625	1	0.916954	1	0.936676	1	0.991952	0.842373	1.014066	0.831865	1.01265	0.871123	0.995042	0.790948	1.170125	0.839343	1.174936	0.88034	1.152045
Q92820	1	0.90612	1	0.949262	1	0.960459	1.466676	0.990279	1.462628	1.067253	1.590493	1.06992	0.887577	1.062595	0.986177	1.113822	0.955953	1.143913
Q9BWP8	1	1.134503	1	1.080925	1	1.167602	0.865196	1.039483	0.785888	1.051568	0.852985	1.021615	1.094471	1.09326	0.998334	1.008169	1.04232	1.131006
P22897	1	0.863043	1	0.985633	1	0.943211	1.208701	1.069989	1.230439	1.090923	1.291156	1.091851	1.250863	1.258905	1.34847	1.265899	1.314591	1.26974
P49908	1	1.065295	1	1.069997	1	0.79199	0.804472	0.933923	0.814434	0.963704	0.786971	0.941435	1.032199	0.994343	1.043778	1.008657	1.077633	0.922759
P10720	1	2.07044	1	1.759113	1	1.73484	0.825048	1.002063	1.115166	1.116261	0.783831	0.991053	0.841895	0.687488	0.903289	0.905397	0.858298	0.579937
P07359	1	1.098252	1	0.990585	1	1.139404	0.815381	0.895544	0.840478	0.909737	0.814345	0.891079	1.084173	1.005265	1.038518	0.963708	1.050312	0.968156
P33908	1	1.23136	1	1.18826	1	1.232171	1.130437	1.16965	1.142707	1.163551	1.126977	1.104104	1.521053	1.454075	1.488001	1.420624	1.47131	1.424908
P05556	1	0.851042	1	0.926124	1	0.900741	0.743039	0.780488	0.758827	0.812277	0.797109	0.776537	0.89154	0.824705	0.931358	0.837711	0.862644	0.849127
Q16706	1	0.96827	1	1.022877	1	1.072791	0.913122	0.947216	0.903095	1.007587	0.980875	0.973801	0.951745	1.054609	0.988005	1.07061	0.991891	1.070071
Q14126	1	1.232932	1	1.136825	1	1.179166	0.857798	0.909504	0.85354	0.919502	0.853578	0.920106	0.884323	0.864334	0.895646	0.834451	0.94596	0.832163
Q9Y5Y7	1	0.853618	1	0.858402	1	0.890343	1.220531	0.923719	1.170207	0.955229	1.262104	0.9561	1.410817	1.076705	1.448987	1.07406	1.471695	1.093408
Q9HDC9	1	0.791624	1	0.786935	1	0.78414	0.911856	0.896306	0.930547	0.924571	0.917799	0.921747	0.92609	0.925798	0.922964	0.899463	0.990763	0.940795
Q6YHK3	1	0.932721	1	0.915446	1	1.119721	1.122724	0.913065	1.52782	0.946031	1.172122	0.926563	0.849089	1.038063	0.853951	1.191167	0.963955	1.125632
P18428	1	0.76542	1	0.789902	1	0.838577	0.914703	1.132071	0.9771	1.039851	0.983358	1.021253	0.950346	1.35968	0.941273	1.411937	0.994952	1.385789
P04075	1	1.17354	1	1.252019	1	1.133148	0.796527	0.892462	0.870578	0.91422	0.936542	0.960878	1.129235	1.05835	1.220348	1.059463	1.131109	1.12303
Q01459	1	1.221486	1	1.152908	1	1.218541	1.007131	1.023936	0.962344	1.068756	0.90195	1.04459	1.070634	1.123846	1.152024	1.129269	1.222693	1.125017
P07195	1	0.990459	1	0.94687	1	0.970175	1.434225	0.907038	1.543114	0.909426	1.54021	0.905946	0.911346	1.111565	0.738967	1.117416	0.784807	1.199475
P43121	1	1.216682	1	1.076614	1	1.111724	1.167399	0.960248	1.210761	0.915505	1.149832	0.93602	1.135708	1.120388	1.154197	1.132054	1.134832	1.204217
P00338	1	0.813819	1	0.851656	1	0.839527	1.108825	0.964932	1.093036	0.918125	1.145762	0.950838	1.032821	1.109623	0.943322	1.052887	0.975543	1.122087
P33151	1	1.104803	1	0.969451	1	1.029304	0.857667	0.991598	0.707125	0.939386	0.736017	0.930228	0.784093	0.797103	0.800536	0.734046	0.767442	0.798697
P12111	1	0.960964	1	0.940663	1	0.918579	1.190821	0.903717	1.184666	0.90694	1.298329	0.919614	0.770062	0.955684	0.869643	0.937963	0.855164	0.908903
P12955	1	1.416369	1	1.14962	1	1.233545	1.155466	0.979746	1.120162	0.991822	1.161155	0.984021	0.97811	0.881743	1.043839	0.820402	1.080622	0.915849
Q12860	1	0.917708	1	0.939238	1	0.897319	1.068095	0.943013	1.11312	1.008992	1.119179	1.014915	0.944815	1.094342	1.053569	1.103572	1.038482	1.118403
P02533	1	1.107889	1	1.288123	1	1.301431	1.135054	1.359969	1.146195	1.329664	1.197612	1.523163	0.857192	2.336151	0.870025	2.547434	0.917225	2.929142
P08779	1	1.462256	1	1.427838	1	1.305249	1.570761	1.194084	1.52168	0.983634	1.388846	1.065467	0.556454	2.517508	0.479066	2.022546	0.505959	2.330466
Q13103	1	0.800974	1	0.901143	1	0.899903	0.985095	1.031583	1.06286	1.066647	1.017397	1.04112	0.915682	0.907522	0.998017	0.925395	1.001406	0.887844
P15144	1	1.056576	1	1.012596	1	1.043945	1.226925	1.306656	1.229665	1.32764	1.206347	1.337187	1.256153	1.142674	1.138939	1.065372	1.152864	1.045628
P48668	1	1.191107			1	0.263916	1.367372	0.822877			0.307875	0.406259	0.569945	1.80625			0.236899	0.598322
P55058	1	0.719292	1	0.841926	1	0.835726	1.42631	0.890929	1.310736	0.881019	1.408162	0.933625	0.680905	1.17321	0.615996	1.221628	0.604296	1.251007
P05067	1	1.070455	1	1.101536	1	1.157776	0.747018	0.734396	0.774987	0.795897	0.752431	0.773845	0.813038	0.938446	0.871655	0.953358	0.802049	0.974208
Q9NPY3	1	1.036155	1	1.15109	1	1.091778	1.141746	0.881007	1.121723	0.918204	1.029889	0.902337	0.923896	0.930721	0.869424	0.964777	0.831015	0.915095
P07737	1	0.979224	1	1.091147	1	0.987383	0.549766	1.143766	0.757672	1.196216	0.58277	1.157322	0.776246	0.786668	0.812953	0.82467	0.789587	0.770185
P05160	1	0.904548	1	0.974512	1	0.927196	0.821747	0.785311	0.751108	0.783837	0.677418	0.734248	0.586678	0.62917	0.57301	0.598016	0.578954	0.546757
P29622	1	1.004884	1	1.024002	1	1.041249	1.772852	1.158515	1.626271	1.158708	1.713486	1.194885	0.74304	1.284173	0.750931	1.265359	0.777155	1.238818
O14786	1	1.196676	1	1.272941	1	1.200143	1.166524	1.035988	1.106883	1.047953	1.04466	1.061319	1.120056	1.155348	1.19985	1.145819	1.114278	1.173022
Q13822	1	1.063727	1	1.176636	1	1.15068	1.062536	0.950049	0.993772	0.97436	1.05593	0.972643	1.145629	1.037946	1.043916	1.024124	1.078768	1.06559
P11717	1	0.910092	1	0.830592	1	0.861115	0.956402	0.997565	0.951408	1.002034	0.986901	0.962902	1.202261	0.999801	1.156702	1.010766	1.152126	0.994689
Q8NBP7	1	0.785522	1	0.813603	1	0.89502	0.948534	0.994301	0.968067	0.972196	0.93049	0.936807	1.150233	0.995014	1.062385	1.053715	1.022841	1.087984
P13647	1	0.950331	1	0.912143	1	0.790109	1.16742	1.44356	1.132298	1.344236	1.11348	1.331461	0.924034	1.989913	0.8242	1.800949	0.824153	1.959346

P23470	1	0.88917	1	0.881909	1	0.905954	1.147006	1.011606	1.083766	1.048084	1.089205	1.016377	1.06065	0.964791	1.040381	0.994546	1.022152	1.012249
P24043	1	1.075081	1	1.067266	1	1.034635	1.10179	0.978091	1.096197	0.964322	1.042348	0.907272	1.020119	1.00452	1.032586	1.060185	1.04693	1.069172
P55290	1	1.194141	1	1.024394	1	0.994485	0.98413	0.934291	0.945075	0.944234	1.139678	0.970379	0.911144	1.064372	0.840115	0.912717	0.801107	1.033481
P55056	1	1.114998	1	1.247851	1	1.147495	1.165297	1.078175	1.175482	1.09349	1.123388	1.067669	1.243325	1.098383	1.215474	1.189779	1.182167	1.070829
P54289	1	1.028891	1	0.935971	1	0.879205	0.858633	0.838156	0.83842	0.809721	0.816739	0.799247	1.04618	0.999005	1.083181	0.982519	1.030588	0.954554
P18065	1	1.256886	1	1.218186	1	1.177972	0.842477	0.754049	0.862284	0.7878	0.809748	0.779043	1.228495	1.406252	1.210337	1.294599	1.217166	1.349012
Q99784	1	0.835144	1	0.80802	1	0.90744	0.924405	0.963054	0.871772	0.94853	0.863993	0.937188	1.107372	1.149839	1.118509	1.135322	1.135706	1.147654
Q6EMK4	1	0.83131	1	0.936057	1	0.800327	1.032571	0.947147	1.049069	0.989885	0.949984	0.975172	0.997642	0.917136	0.940218	0.908404	0.988099	0.900931
P12830	1	1.012515	1	1.065097	1	1.035857	0.977946	0.991634	0.968494	0.98201	1.00712	0.99064	1.014739	0.992342	1.059383	0.881381	1.109332	0.815971
P01344	1	0.86289	1	0.868155	1	0.968436	0.888834	1.097071	0.875775	1.085202	0.863902	1.124904	0.969353	0.786452	0.881292	0.791369	0.98058	0.802363
Q16270	1	1.035857	1	1.109047	1	1.085806	0.996482	1.005155	0.992321	1.105503	1.025202	1.14954	1.224412	1.039147	1.197933	1.059196	1.333338	1.13597
P02745	1	0.804199	1	0.824315	1	0.782426	0.924792	1.064957	0.935521	1.060019	0.950753	1.071122	1.088379	0.606937	1.126471	0.599528	1.112545	0.653644
Q9ULI3	1	0.996461	1	0.966278	1	0.886417	1.032469	1.074142	0.966932	1.031964	0.934135	1.024468	1.119539	1.023099	1.082608	1.026132	1.011838	0.980255
P07333	1	1.034156	1	0.997455	1	1.105563	1.302983	0.936297	1.45864	1.072275	1.405344	1.098638	1.146061	1.128362	1.206867	1.060315	1.330242	1.153334
P07602	1	0.839841	1	0.883718	1	0.870532	0.734002	0.862634	0.777399	0.892435	0.719679	0.892762	0.953271	0.964987	0.975319	0.966233	0.969866	0.960072
P04040	1	0.916796	1	0.950086	1	1.081932	1.509333	1.13825	1.467385	1.222094	1.479452	1.21647	1.090728	1.346	1.196593	1.390678	1.169535	1.485615
P09960	1	0.925578	1	0.941774	1	0.928458	1.002359	1.074093	1.019571	1.146929	0.9977	1.054311	1.099829	1.184911	1.120296	1.218064	1.171365	1.177956
P07942	1	1.123389	1	1.044375	1	1.189294	1.051495	0.949799	0.996491	1.02073	1.077021	0.973921	1.026684	1.057869	1.047107	1.150036	1.090481	1.176941
P11142	1	1.161718	1	1.199733	1	1.232278	0.834327	1.039811	0.872689	1.021412	0.751436	0.982773	0.952373	0.921264	0.987217	1.003654	1.034187	0.890374
P08294	1	1.373415	1	1.310945	1	1.429646	1.079108	1.408167	1.101232	1.43884	1.503127	1.448484	0.985336	0.950463	0.920364	0.935411	0.973094	0.928806
Q12913	1	0.933777	1	1.005268	1	1.116781	1.08608	1.087308	1.147257	1.028963	1.125929	1.077012	1.112181	1.093086	1.037921	1.062789	1.126896	1.080636
Q9H4A9	1	0.913662	1	0.957975	1	0.966216	1.182222	1.012317	1.211927	1.014813	1.20604	1.019445	1.051388	1.096315	1.011519	1.101289	1.059671	1.071145
P54108	1	0.978853	1	0.940182	1	0.957085	1.8789	1.306974	1.81709	1.340816	1.875838	1.411181	1.052613	1.552074	1.00199	1.37801	1.225062	1.459475
P04745	1	0.699919	1	0.941042	1	0.778887	0.8423	1.132665	1.067445	1.311227	0.908763	1.028266	2.007235	1.315292	1.880469	1.248828	1.724611	1.163467
P55103	1	0.981037	1	1.012005	1	1.066602	0.694145	0.965628	0.754802	1.051989	0.624186	0.961123	0.985394	0.984703	1.033226	1.093171	0.977592	0.957501
P37802	1	0.821335	1	0.950305	1	0.917435	0.402413	2.516012	0.468967	2.544573	0.711035	2.256501	0.501247	0.420443	0.552654	0.511124	0.574163	0.650274
Q86YZ3	1	1.197012	1	1.076134	1	1.159224	1.587313	1.757083	1.213427	1.541376	1.351137	1.792937	1.308377	2.932518	1.412324	2.360841	1.489444	3.716404
Q9BTY2	1	0.822416	1	0.807853	1	0.810448	1.079993	1.07775	1.061786	1.064462	1.062907	1.052304	1.116648	1.070027	1.037905	1.064274	1.083491	1.032258
P07237	1	0.98697	1	0.988467	1	0.951201	0.847531	0.917086	0.800205	0.944902	0.78641	0.935099	1.286455	1.087942	1.229379	1.043988	1.275756	1.027418
Q6UXB8	1	0.956091	1	0.935038	1	0.962022	0.951996	0.965589	0.972245	0.981664	0.931365	0.955146	0.865573	0.709026	0.80459	0.769326	0.852099	0.774939
P80188	1	0.836385	1	1.011545	1	1.084359	0.798837	0.71527	0.863228	0.774822	0.884892	0.882757	0.659889	0.70907	0.67421	0.886196	0.814879	0.919205
P05106	1	1.128819	1	1.278386	1	1.273488	0.278853	0.479554	0.306025	0.53887	0.332637	0.502964	0.500303	0.377208	0.515378	0.437165	0.447089	0.416508
P01033	1	0.968578	1	1.06252	1	1.0673	0.974469	0.85872	0.927236	0.872553	0.95929	0.888034	0.88116	1.1569	0.931674	1.166	1.004051	1.143142
P16070	1	0.905723	1	0.955933	1	0.876136	1.134704	0.982349	1.159186	1.022362	1.224576	0.991051	1.059862	1.013956	1.263496	1.163212	1.203203	1.119177
P08709	1	1.073045	1	1.077009	1	1.10951	1.234095	1.060955	1.012945	1.076061	1.232166	1.107853	0.908518	1.077637	0.929655	1.091566	0.867453	1.135699
P04746	1	0.601316	1	0.608721	1	0.624913	0.746294	0.97378	0.849878	0.952868	0.7897	1.090873	1.670841	0.960523	1.238354	0.962217	1.678206	1.022509
P13637	1	0.792008	1	1.024663	1	0.94585	1.202932	0.777552	1.130377	0.83718	1.080132	0.89348	0.768991	0.524022	0.782382	0.689696	0.874322	0.845747
P78509	1	0.870796	1	0.818266	1	0.818159	0.98472	0.978335	0.943157	1.033423	0.861332	0.991847	1.038982	0.952889	0.969431	0.921271	0.968208	0.913776
P61769	1	1.114391	1	1.052494	1	1.051129	1.19585	1.266214	1.154366	1.272003	1.150682	1.266573	1.577374	1.20576	1.474688	1.175065	1.443262	1.181263
P24593	1	1.21477	1	1.265967	1	1.261444	0.818185	0.97906	0.811012	0.955336	0.936812	1.048686	1.044359	0.851046	0.972659	0.921488	0.946984	0.861231
Q9NZK5	1	1.012093	1	1.327906	1	1.040839	1.227431	1.314292	1.2469	1.18954	1.301031	1.297674	1.660545	1.315873	1.60016	1.294392	1.697957	1.314127
Q04721	1	0.975354	1	1.410009	1	1.373665	0.883731	0.95947	0.94964	0.961674	0.987721	0.945588	0.929946	0.960661	1.069745	1.046606	1.047538	1.045388
Q8IZF2	1	1.079409	1	0.984729	1	0.97018	1.010419	1.098595	1.055933	1.079704	0.990469	1.051462	1.311663	1.28422	1.414238	1.28713	1.358302	1.216026
Q9NPH3	1	0.859754	1	0.910571	1	0.763498	0.931838	0.973542	0.908982	0.999724	0.872512	1.008719	1.157388	1.110711	1.102637	1.07596	1.180331	1.17673
P04406	1	1.192944	1	1.141852	1	1.116759	0.82678	1.011615	0.82707	1.053742	0.788376	1.07876	1.558289	1.285773	1.326358	1.226971	1.54758	1.292024
P05534	1	0.638597	1	0.739052	1	0.657677	0.618963	0.782661	0.889964	0.939724	1.04445	1.047765	0.693567	0.822867	0.72736	0.825114	0.783841	0.873476
P10124	1	1.508378	1	1.549318	1	1.425223	0.919612	1.19752	0.960511	1.208925	0.864282	1.091005	1.369547	1.422392	1.425147	1.455134	1.366045	1.423753
P06310	1	0.537936	1	0.442952	1	0.433847	0.451551	0.636446	0.406261	0.659176	0.569824	0.70659	0.46128	0.611231	0.389332	0.55262	0.458704	0.495233
P13797	1	0.765004	1	1.003063	1	0.65774	0.973805	0.895962	1.059985	0.822994	0.882824	0.864283	0.950485	0.94421	0.694484	1.031368	0.841464	0.92874
Q15063	1	1.127054	1	1.049182	1	1.108582	0.919943	0.789395	0.868013	0.798412	0.952401	0.809611	0.697034	0.764506	0.672112	0.687929	0.724124	0.739965

P13598	1	1.268054	1	1.130985	1	1.280149	1.467718	1.140963	1.281739	1.089228	1.282576	1.156344	1.540768	1.305216	1.340102	1.159909	1.333259	1.243502
P07998	1	1.033132	1	0.988485	1	1.123159	0.983466	1.057493	1.016634	1.075634	1.010872	1.148291	1.457526	0.975106	1.428197	0.98755	1.542909	1.056782
P41222	1	1.400954	1	1.337381	1	1.418618	1.204577	0.980705	1.213842	0.995408	1.177592	1.007327	0.943227	0.95703	0.838441	0.917845	0.941104	0.922017
Q9UM47	1	0.790408	1	0.860659	1	0.936368	0.789563	0.799851	0.742546	0.829189	0.793066	0.8226	0.985081	0.713858	0.992267	0.737094	0.962805	0.748657
Q13740	1	1.048979	1	0.937832	1	1.00569	0.989677	1.011764	1.071326	0.998944	1.094996	1.048812	1.100961	1.142312	1.054533	0.994133	1.10455	1.093811
P54802	1	1.012163	1	0.934746	1	0.974321	1.182185	0.926566	1.146556	1.11201	1.102109	1.009086	0.798805	0.844932	0.974728	0.897137	0.858119	0.852269
Q9Y6Z7	1	1.018688	1	1.042904	1	1.034089	0.822297	1.045484	0.844974	1.071684	0.801572	1.015422	0.987807	0.953778	0.913067	1.02679	0.873926	1.007517
Q16853	1	1.06838	1	0.976169	1	1.134568	1.081796	1.051947	1.008805	1.06954	1.083916	1.042821	1.229321	1.176004	1.09533	1.154184	1.115456	1.261167
Q9H4G4	1	0.855834	1	0.877799	1	0.975319	0.867288	0.971923	1.011447	1.016417	1.008598	1.077718	0.971647	0.757561	1.044679	0.81188	1.092978	0.846842
A0A075B(	1	1.021568	1	1.575135	1	1.535198	0.802448	1.149411	0.369634	0.812793	0.363992	0.665456	0.693387	0.891808	0.713859	0.717852	0.697165	0.728675
A0A075B(	1	0.493182	1	0.67899			0.856461	0.823703	1.116047	0.662044			0.65571	0.727425	0.480737	0.696844		
P10721	1	1.239943	1	1.050904	1	1.096578	1.007483	1.101739	0.969357	1.136361	0.968191	1.112823	1.358625	1.115483	1.305511	1.082833	1.202459	1.086752
P23528	1	1.097161	1	1.019627	1	0.807617	0.621961	1.369672	0.54637	1.215819	0.534438	1.333947	0.884929	0.793592	0.790559	0.668769	0.829545	0.703479
Q9NPR2	1	1.226178	1	1.086281	1	1.02743	0.940015	1.158111	0.948757	1.193119	0.920434	1.11552	1.699385	1.114344	1.757374	1.136255	1.692873	0.990194
P0CG38	1	1.784114	1	0.968835	1	1.372107	1.304097	0.95964	2.462262	1.326918	2.224347	1.408236	0.754529	1.121794	0.827296	1.906553	0.982532	2.207642
Q9UJJ9	1	1.034412	1	0.885096	1	0.91763	1.0863	0.856174	1.024951	1.042754	1.170033	1.098849	0.801504	0.824648	0.935805	0.949774	0.997025	0.970545
Q9HBR0	1	1.080906	1	1.192039	1	0.999799	1.05357	1.012083	1.1151	1.042362	1.009279	1.035439	0.96447	0.975293	0.979878	0.940374	0.947694	0.924866
P10586	1	1.037958	1	0.970203	1	1.006854	1.070648	1.035096	1.016593	0.959263	1.087948	0.977873	1.012569	1.12043	0.982941	1.123929	0.931445	1.134825
P09972	1	1.133694	1	0.991568	1	0.926848	0.916935	1.030159	0.702103	0.922167	0.7911	0.980947	0.961916	1.021535	0.953636	1.027806	0.936638	0.879289
P16109	1	0.880413	1	0.827295	1	0.868048	0.795264	1.188925	0.722419	1.206995	0.756683	1.207057	1.119508	0.994989	1.158044	0.967081	1.071824	1.018444
Q9Y5C1	1	1.070863	1	1.110067	1	0.969542	0.908393	0.968476	0.901975	0.986958	0.918492	0.965738	1.020835	1.029173	1.040865	1.023496	0.958129	1.056685
P11047	1	0.948315	1	0.932681	1	1.006797	0.971886	0.90834	0.97729	0.95081	0.977475	0.93909	0.899428	1.082518	1.042432	1.148286	0.996679	1.167957
P63104	1	1.211905	1	1.191587	1	1.105234	0.685188	0.885936	0.645059	0.79432	0.722602	0.794487	1.157892	0.931526	0.93109	0.852143	1.127138	0.914707
P12821	1	0.867083	1	0.973847	1	1.044888	1.374535	0.990326	1.458034	0.965657	1.326068	1.042898	1.05347	1.137639	1.021645	1.097261	1.105955	1.013882
Q8WZ75	1	1.113866	1	0.976132	1	0.923438	1.128745	0.988391	1.0351	0.970137	1.134742	1.05005	1.077993	0.972461	0.8984	0.95203	1.039536	1.150844
P12109	1	1.193035	1	1.121174	1	1.049184	1.128351	0.891477	0.970815	0.94214	0.943656	0.925105	1.130079	0.926237	1.141263	1.026829	1.173911	0.930794
P07339	1	1.039986	1	0.972742	1	0.952437	1.025514	1.06153	1.029448	1.034871	1.036206	1.034285	1.429412	1.206644	1.444485	1.254347	1.41539	1.1646
P20023	1	1.070735	1	0.954593	1	0.919487	1.069967	1.242872	1.071481	1.214884	1.016253	1.290128	1.168866	0.882237	1.25359	0.836533	1.161963	0.986096
Q5XKE5	1	1.45245	1	1.606227	1	2.1494	1.115405	0.856423	1.403851	0.787572	1.341238	0.766606	0.992466	4.50548	1.07152	4.80528	1.159725	5.401668
P40926	1	1.08874	1	1.202271	1	1.163206	1.118468	0.874649	0.967285	0.799166	0.962172	0.816032	0.858277	0.773888	0.980714	0.72712	0.805654	0.612727
P08238	1	0.978787					0.976081	1.280712					1.210351	1.157909				
P01833	1	0.904522		0.84432	1	0.717238	0.619047	0.729324	0.564924	0.752174	0.409667	0.71026	0.565611	0.715561	0.685525	0.813178	0.577946	0.74905
Q92859	1	0.887959	1	0.952111	1	0.903756	1.071172	0.999359	1.044488	1.027803	1.028048	0.965625	1.057505	0.97445	1.083036	1.054297	1.080438	1.021274
Q86SQ4	1	0.891052	1	0.785613	1	0.684711	0.904225	0.982769	0.892329	0.938113	0.916154	0.894541	1.022785	0.99188	1.017063	0.989935	0.946828	1.063719
P78385	1	1.053685	1	1.084096	1	0.852236	0.996033	17.57112	0.936815	14.95054	0.999312	14.14265	0.8172	0.491172	0.738536	0.592101	0.833187	0.666794
P01780	1	0.877889	1	0.912723	1	0.871131	0.605423	0.63332	0.600866	0.624287	0.567455	0.612879	0.376404	0.718484	0.431835	0.742933	0.416586	0.70108
O95980	1	0.96473	1	1.046715	1	0.916807	0.955652	1.012355	0.928493	0.952886	0.92104	0.937456	1.282618	1.096957	1.183088	1.011644	1.077173	0.984182
P19022	1	0.997076	1	1.097015	1	1.060951	1.092038	0.986008	1.108075	1.029173	1.096848	1.036695	1.272638	1.068246	1.244705	1.130653	1.333499	1.064972
P05164	1	0.953362	1	1.19568	1	1.039816	0.53046	0.83797	0.738684	0.869005	0.675496	0.86186	0.655601	0.620319	0.749497	0.731849	0.795352	0.697312
Q9BY67	1	0.834206	1	1.170294	1	0.935385	1.184791	1.000502	1.234224	0.936267	1.125946	0.949366	1.012341	1.122737	1.022216	1.165794	0.900518	1.211607
P02741	1	0.987026	1	0.819954	1	1.050714	2.594066	1.753185	1.926669	1.938881	2.324828	1.817836	1.711962	5.936954	1.741466	7.491187	1.771511	7.479353
P00441	1	1.196416	1	1.039613	1	1.23977	0.980196	1.561596	0.938656	1.467989	0.925607	1.502892	1.607716	1.224883	1.444115	1.299161	1.603354	1.263194
P00558	1	0.968264	1	1.130709	1	1.174158	0.796969	0.969017	0.840034	1.087301	0.767529	1.009481	0.883782	0.655777	1.195953	0.969098	0.949134	0.886148
Q13093	1	0.932078	1	1.050549	1	0.949152	4.112422	2.507938	4.556599	2.762194	5.011645	2.621622	1.262736	1.708529	1.593385	1.936694	1.638046	1.869667
P07477	1	0.626555	1	0.485517	1	0.619881	1.463809	0.944967	1.39642	0.979206	1.431232	0.950689	0.762894	1.363107	0.674537	1.418151	0.757591	1.371633
Q8TER0	1	1.134588	1	1.001627	1	0.937584	0.960232	0.962767	0.895088	0.991339	0.855522	1.067786	0.945506	0.789188	0.916888	0.953569	0.95397	0.883467
P16930	1	1.042278	1	0.856754	1	0.907322	0.851367	1.129693	0.939276	1.153568	0.882292	1.054559	1.074396	0.988138	1.068406	0.976464	0.957992	0.973433
A0A075B(	1	0.691256	1	0.465258	1	0.57641	0.876951	0.807019	0.756773	0.825932	0.764739	0.813895	0.61311	0.716364	0.386896	0.809224	0.479338	0.742108
P69892	1	1.071917	1	1.293823	1	1.099361	1.397085	1.700646	1.480084	1.683584	1.529523	1.72775	1.762808	2.139852	1.70221	2.061879	1.756333	2.206863
Q9NZ08	1	0.931993	1	1.102401	1	0.958269	0.983808	1.017188	1.027582	1.059463	0.991504	0.993835	1.128965	1.258931	1.298414	1.220428	1.203018	1.201681

P01880	1	0.886252	1	0.861777			0.761319	0.648647	0.687715	0.651423			0.536526	0.72186	0.643345	0.673285		
O95497	1	1.229093	1	1.14889	1	1.140254	1.427587	1.265634	1.509614	1.246897	1.427123	1.284173	1.380649	1.677361	1.250886	1.366774	1.416349	1.424643
P11279	1	1.026496	1	0.785324	1	1.174507	0.914486	0.953048	0.834832	0.947918	0.850539	0.90249	1.110491	0.973566	1.095197	0.911104	1.060471	0.957995
P03950	1	0.933505	1	1.07167	1	1.113048	1.206232	1.40469	1.1076	1.298824	1.146105	1.384426	1.602736	1.237233	1.749435	1.242555	1.905418	1.315861
Q04695	1	0.876047	1	1.045436	1	1.006829	0.665069	0.70106	0.948802	0.712155	0.877602	0.79913	0.59397	1.563403	0.574523	1.742216	0.685329	1.354555
Q6UX71	1	0.944413	1	1.016009	1	0.884803	0.886388	0.891163	0.898214	0.959242	0.882646	1.001983	1.020772	0.92936	1.070059	1.010144	1.152049	0.97397
Q9H8L6	1	0.987277	1	1.171508	1	1.114085	1.739921	0.782542	1.019903	0.842265	0.680516	0.768063	0.654531	1.028308	0.993371	0.920676	0.725783	0.973036
P07911	1	1.064279	1	0.933567	1	0.857712	0.94568	0.973405	1.018671	1.033177	0.921399	0.978851	1.077732	1.023242	1.08567	1.08012	1.17247	1.179411
O95479	1	1.251136	1	0.975585	1	1.169687	1.301845	1.146237	1.261925	1.172037	1.359921	1.182397	1.194063	1.417226	1.220004	1.342393	1.244278	1.414986
O60462	1	0.931273	1	0.984563	1	1.035673	1.11785	1.057845	1.060389	1.038452	1.062703	0.986883	1.089095	0.911853	1.083769	1.038226	1.089734	1.021623
O43493	1	0.720941	1	0.756777	1	0.809263	1.043091	1.010135	0.901473	0.984041	0.914896	0.9747	1.038166	0.80971	0.999279	0.842479	0.980937	0.828453
P04439	1																	
Q14118	1	1.018913	1	1.04003	1	1.090093	0.90672	0.84586	0.899615	0.755768	0.965751	0.771286	0.945826	0.96547	0.86381	0.849898	0.885737	0.879819
P15151	1	0.661348	1	0.67762	1	0.729182	0.90184	1.032722	0.88601	1.044009	0.824267	1.043082	1.259092	1.079941	1.221717	1.157506	1.294831	1.176827
P67936	1	1.110556	1	1.261476	1	1.445995	0.716253	2.666923	0.739004	2.464629	0.75636	2.218457	0.953528	0.763236	0.81543	0.774379	1.023897	0.826604
O15031	1	0.974276	1	1.23804	1	1.217894	1.002175	1.093645	1.011321	1.008675	1.011958	0.960048	1.452342	1.081426	1.309919	0.956386	1.260293	0.986717
P28827	1	1.339801	1	0.853175	1	0.859459	1.221949	1.108897	1.203058	1.044324	1.354921	1.099977	1.377967	1.187719	1.115327	1.136072	1.214435	1.213485
Q9NZT1	1	1.521991	1	1.293351	1	0.947975	1.192782	1.094798	0.857782	1.055516	1.075806	1.164667	0.945337	1.175759	0.96086	0.950792	0.777609	1.268878
P39060	1	0.948353	1	0.775376	1	0.875887	1.167153	0.988354	1.068621	0.902373	1.097511	0.955994	0.92391	1.035302	0.839089	0.878241	0.684883	1.030352
P11597	1	0.727464	1	0.933757	1	0.857857	1.064518	0.964648	1.176371	1.00441	1.171829	1	0.609901	0.811002	0.583761	0.818287	0.583737	0.790943
Q02818	1	1.138495	1	1.0741	1	1.05011	0.833937	1.063201	0.930578	1.074286	0.874297	1.02995	1.570213	1.512873	1.357739	1.389302	1.526653	1.465268
P17813	1	0.994902	1	0.893019	1	1.085406	0.948426	0.852051	0.818155	0.903326	0.930115	0.971029	0.817977	0.974736	0.884775	0.932698	0.963213	0.996978
P81605	1	1.024372	1	1.01373	1	0.967309	1.054819	1.21979	1.107246	1.223257	1.123519	1.27868	1.243425	1.964688	1.192279	2.053511	1.35502	1.991226
P80723	1	1.25857	1	1.135833	1	1.174701	1.048847	1.04207	0.992741	1.11677	0.9664	1.066517	1.043163	1.010968	1.038889	0.943485	1.058256	1.001194
Q15848	1	0.677479	1	0.711918	1	0.816776	1.085251	0.949274	1.232386	1.105282	1.20429	0.896976	0.905301	1.660912	0.969645	2.333564	0.847488	1.607623
Q7Z7G0	1	0.897188	1	0.951575	1	0.91606	1.090293	1.008301	0.944958	1.058727	1.020928	1.000288	1.031748	0.995226	1.011718	1.043048	1.042819	1.087953
Q13885	1	1.949631	1	1.726941	1	2.205641	1.373045	0.765515	1.290663	0.929772	1.441856	1.147235	1.197781	0.767321	1.770081	1.355045	1.764466	1.143919
O15394	1	0.894377	1	0.889386	1	0.980609	1.420551	1.206111	1.43395	1.185543	1.392977	1.149477	0.747177	1.484232	0.82458	1.364363	0.849201	1.371631
P46531	1	1.35387	1	1.247684	1	1.27767	0.919713	1.014562	0.940695	0.961163	0.8979	1.030385	1.062407	0.885646	1.199527	0.862103	1.108584	0.970326
P35916	1	0.830234	1	0.837256	1	0.934926	0.974704	1.225823	1.0124	1.233489	1.041432	1.198499	1.231551	1.115465	1.231603	1.113022	1.15928	1.107766
Q07075	1	0.968535	1	1.053981	1	1.108034	1.035039	1.175543	1.048676	1.155531	1.091543	1.212304	0.96502	1.011036	1.084764	1.056024	1.096333	0.955003
P16035	1	0.889508	1	0.777753	1	0.81221	0.815175	0.832032	0.832068	0.912423	0.851304	0.848644	0.976114	0.7997	0.967786	0.848678	0.994061	0.836864
Q9Y646	1	1.109007	1	1.035228	1	1.241188	1.41583	0.971604	1.165844	0.932044	1.201695	0.931804	0.772905	0.981237	0.918349	1.057765	0.871378	1.101407
P35590	1	1.063033	1	0.995013	1	1.009913	1.236788	1.10839	1.263269	1.08226	1.094099	1.0839	1.184181	1.009335	1.192997	0.971622	1.230998	1.005292
P40189	1	0.912546	1	1.015901	1	0.917741	1.002766	1.115675	1.055325	1.107081	1.025815	1.056879	1.248385	1.104213	1.121212	1.096913	1.091916	1.069228
P17301	1	0.94272	1	1.008817	1	1.082387	0.934454	0.876654	0.977908	0.747143	0.962227	0.841435	0.930482	1.00464	0.852202	0.956498	0.891044	0.976204
Q9BUN1	1	0.873577	1	0.970819	1	0.814574	0.928587	0.864354	0.876987	0.900417	0.82566	0.839922	0.86528	1.072533	0.964547	0.996367	1.018015	1.019556
Q6UY14	1	0.90425	1	0.983137	1	0.795504	0.727424	0.856554	0.859712	1.007994	0.711941	0.880994	0.707105	0.752659	0.80797	0.903525	0.766059	0.695573
P78417	1	1.135283	1	1.173882	1	1.068881	1.141904	1.071208	1.165499	1.124581	1.107566	1.090761	1.190134	1.193718	1.098419	1.151165	1.107527	1.154368
Q13423	1	1.14639	1	1.639782	1	1.298676	1.06127	0.804703	0.979216	0.816971	1.092152	0.848541	1.291213	0.958945	1.221355	0.81708	1.022252	0.814359
Q9NY97	1	0.966204	1	1.03555	1	1.054556	1.087164	0.993306	1.158726	1.0573	1.179722	1.034809	1.070343	1.073194	1.181887	1.117634	1.213581	1.17521
A0A0C4D	1	1.160462	1	0.944359	1	1.230132	0.808914	0.701165	0.783097	0.878944	0.797956	0.804408	0.489097	0.824226	0.879702	0.815076	0.949033	0.626716
Q8TDJ6	1	1.634561	1	1.547586	1	1.694022	1.011501	0.769689	1.124071	0.948262	1.45383	1.361368	0.799651	0.795404		1.15291		0.910676
Q09160	1																	
Q15323	1	0.443197	1		1		0.937204	9.625756			0.804645	20.90783	1.037181	0.768376			0.867749	0.645393
Q9NY15	1	1.009485	1	0.932927	1	1.10143	0.970108	0.973488	1.103823	1.063753	0.918774	1.038461	0.994483	0.991441	1.051798	1.249852	1.144132	0.991416
Q9BYE9	1	1.098193	1	1.092646	1	1.279998	0.871916	1.259201	0.781379	1.082925	1.009434	1.29912	1.067181	0.983895	1.162995	0.943731	1.116942	1.053598
P61916	1	0.808529	1	0.762855	1	0.808221	1.041531	1.059768	1.021656	1.127187	0.97977	1.045104	1.17449	0.864964	1.160326	0.911175	1.115878	0.849434
P10451	1	1.310817	1	1.147953	1	1.319958	1.409294	1.179121	1.373772	1.349147	1.490587	1.260967	1.662604	1.491269	1.558647	1.37346	1.64843	1.370409
P24592	1	0.986685	1	1.043698	1	1.007083	1.146746	0.993765	1.08108	1.018553	1.012647	0.969249	1.003935	0.983573	1.032255	1.154495	1.02987	1.003762

Q86YW5	1	1.017052	1	1.033192	1	1.029271	0.391519	0.534717	0.367479	0.607818	0.562689	0.628515	0.344564	0.409257	0.463712	0.439323	0.607063	0.639135
Q86WI1	1	1.026053	1	1.077567	1	1.056247	1.103389	1.073766	1.04222	1.078188	1.019043	0.915924	0.897371	1.023192	0.993594	0.895105	0.993057	0.924529
P07437	1	1.750559			1	1.560909	1.802248	0.854478			1.459313	0.92388	1.00251	0.646133			1.07282	0.663625
P01892	1	0.897561	1	0.908776	1	0.830192	0.986559	0.872034	0.688391	0.860121	0.662849	0.797554	0.791319	0.878387	0.752088	0.764213	0.752933	0.896833
P49746	1	0.726677	1	0.836385	1	1.137485	0.648669	0.913349	0.699859	0.920179	0.763919	0.932027	0.981696	0.604757	0.878557	0.615887	0.771852	0.686936
P14618	1	0.955612	1	1.075137	1	1.160997	0.813598	0.773283	0.734989	0.761607	0.785061	0.786592	0.782392	0.818099	0.828799	0.857854	0.802158	0.837627
P07196	1	1.71351	1	1.326685	1	1.895105	1.607306	1.060619	1.128576	0.763087	1.218085	0.71246	0.769058	0.877855	1.00502	0.958381	0.717828	1.221181
P08195	1	0.953988	1	0.960488	1	1.110818	1.023977	0.944111	1.275188	0.947685	1.166808	0.976603	0.8295	0.834464	0.764688	0.883708	0.754485	0.915618
P34931	1																	
Q9H1U4	1	1.049893	1	0.888809	1	0.980572	0.821583	1.034585	0.766611	1.0796	0.805148	1.071201	1.199234	1.093813	1.222261	1.003984	1.160961	1.074426
Q13449	1	1.0403	1	1.266613	1	0.995031	0.953082	1.039588	0.941415	1.075251	0.976582	1.007219	1.36037	0.87951	1.177868	0.922012	1.175439	0.942245
Q92823	1	1.352717	1	0.964884	1	1.001554	1.023152	0.971666	0.834233	0.970924	0.79372	1.063536	1.032211	1.092159	1.053675	0.834883	0.972789	0.862605
P11362	1	0.966338	1	1.033766	1	1.012222	0.895212	0.991584	1.034106	1.029544	0.950014	1.028336	1.052349	1.034184	1.03791	1.070187	1.021881	1.056621
Q99969	1	1.416251	1	1.280416	1	1.236266	1.054877	1.027375	0.90842	1.117076	0.897681	1.073685	0.898541	0.906755	1.004997	0.980725	0.943977	0.991273
Q99650	1	1.232518	1	1.14905	1	1.152262	1.20675	1.110555	1.157279	1.06925	1.264598	1.122035	1.312971	1.240452	1.223836	1.286259	1.23665	1.389164
Q12841	1	0.976753	1	0.989961	1	1.006734	0.840618	1.00475	0.819397	0.948055	0.926798	1.014917	1.24586	1.052695	1.253971	1.045412	1.325915	1.096948
P08514	1	0.937286	1	0.883447	1	0.918763	0.359441	0.484387	0.387868	0.533041	0.35753	0.492415	0.428097	0.401258	0.469671	0.467686	0.400806	0.485512
P06312	1	0.692952	1	0.538018	1	0.560311	0.438278	0.631996	0.45138	0.628159	0.469181	0.675081	0.477537	0.513608	0.395256	0.513517	0.513144	0.560599
P0DP03	1	0.521706	1	0.779744	1	0.391296	0.538939	0.578182	0.561996	0.584361	0.593935	0.561888	0.394116	0.438957	0.352435	0.507583	0.349226	0.531011
Q86UD1	1	1.192446	1	1.178854	1	1.16796	0.822804	0.863344	0.79986	0.913345	0.838011	0.925746	1.113122	0.953885	1.064603	0.890294	1.146737	0.968133
O76013	1	1.128823	1	0.991321	1	0.848435	1.326195	10.45844	1.1809	12.22417	1.151021	12.07206	0.945349	0.875501	1.002179	0.934464	1.114503	0.714314
P02452	1	1.027094	1	1.242046	1	1.011128	1.076809	1.208064	0.908758	1.205972	1.055735	1.240899	0.940029	0.839718	0.907351	1.012017	0.974171	0.979041
P31146	1	1.117586	1	0.909403	1	0.927677	0.601766	0.814586	0.642619	0.943804	0.69979	0.944311	0.986405	0.488247	0.956155	0.871193	1.0159	0.837914
P32119	1	1.215933	1	1.109878	1	1.138217	1.038245	1.100316	1.034916	1.09024	1.032279	1.103024	1.782484	1.160467	1.668381	1.131003	1.629478	1.158098
O00584	1	1.307777	1	1.338841	1	1.296491	1.095285	1.037814	1.117576	0.989552	1.03472	0.950549	1.43911	1.156904	1.55932	1.141178	1.470638	1.215759
P17174	1	1.139574	1	0.736979	1	0.821699	1.183881	1.052576	1.079751	1.088779	1.0009	1.064106	1.17739	1.070156	0.963348	1.0872	1.083847	0.983423
Q15828	1	1.357702	1	1.190537	1	1.242805	1.310597	1.161905	1.204492	1.14367	1.368303	1.184181	1.026717	0.88541	1.002835	1.016212	1.04303	0.96
P06732	1	0.938443	1	0.929079	1	0.847727	0.691336	0.50768	0.760755	0.572106	0.80218	0.533097	1.443807	1.611624	1.332564	1.59572	1.241734	1.5355
O75144	1	1.157424	1	1.232342	1	1.16423	1.194879	0.977517	1.087349	0.939297	1.021019	0.993099	0.855684	1.182926	1.115132	1.007294	1.207684	1.04816
O76011	1	0.36704	1				1.393562	11.58491					0.988471	0.592614				
Q9Y4G6	1	0.785822			1	0.879823	1.328394	0.630094			0.558901	1.637285	0.321999	0.752299			1.415186	1.246576
Q8IXL6	1	1.208607	1	1.350835	1	1.368118	0.973875	0.980591	1.003342	0.988738	0.942343	0.978162	1.233868	1.065547	1.369078	1.176525	1.275931	1.201551
P32754	1	1.31783	1	1.621723	1	1.493585	1.080634	1.527447	1.112827	1.643167	1.060045	1.600996	1.118342	1.045097	1.2616	1.118411	1.209242	1.044293
Q13477	1	1.145339	1	1.091079	1	1.2039	0.854898	0.985473	0.998293	1.101572	0.992068	1.040019	0.775715	0.896362	0.55885	0.798069	1.060473	0.879788
P61981	1	1.15283	1	1.060625	1	0.860101	0.77526	0.853282	0.861498	0.892405	0.554969	0.778073	1.02441	0.76756	0.677553	0.729576	0.827033	0.72765
O00602	1	1.048842	1	1.050058	1	1.045165	1.109054	0.872806	1.091146	0.858503	1.320655	0.858329	1.51054	2.036435	1.566322	2.109943	1.471759	1.942681
P60900	1	0.844526	1	0.645472	1	0.62976	1.191342	1.197722	1.054556	1.179588	1.006828	1.130623	1.45834	1.280559	1.439128	1.219167	1.512192	1.379348
A0A0A0M	1	0.805732	1	0.856995	1	0.663562	0.512715	0.656894	0.808766	0.668847	0.643395	0.653323	0.423515	0.762063	0.645615	0.883642	0.489458	0.791358
P36222	1	0.736637	1	1.158003	1	1.33309	1.740607	1.062612	1.669535	1.146515	1.670608	1.17628	1.685921	0.554669	1.660376	0.654964	1.51996	0.525174
P30101	1	0.932308	1	0.789778	1	0.913142	0.83745	0.99018	0.937339	1.025346	0.940584	1.000483	1.083101	0.940348	1.071526	1.018329	1.094412	1.036763
P14314	1	0.851133	1	0.869857	1	0.838606	0.891747	0.866658	0.910607	0.968282	0.927772	0.961074	0.987196	0.977776	0.972302	1.052909	1.067241	1.122012
P30508	1		1	1.087556					1.400531	1.29276					0.919885	1.124227		
Q4LDE5	1	1.051332	1	0.922637	1	0.925805	0.734335	0.695869	0.743289	0.687533	0.62793	0.628047	0.849642	0.929617	0.84311	0.895381	0.825992	0.841111
O43157	1	1.078545	1	1.115915	1	1.299275	0.946978	0.995142	1.102609	1.056712	1.144049	1.111787	0.862532	1.070201	1.005941	1.003294	1.055599	1.060795
P04066	1	1.168669	1	1.021558	1	0.952875	1.125413	1.087096	1.140547	1.070441	1.126433	0.981366	1.093977	1.083448	1.195623	1.104652	1.052991	1.015505
A0A0B4J1	1	0.79346	1	0.709179	1	0.757724	0.408292	0.520911	0.307636	0.48113	0.356911	0.473701	0.373906	0.60656	0.246052	0.623956	0.293005	0.794378
Q86U17	1	0.942308	1	1.072958	1	1.018119	1.1108	1.046813	1.196333	1.100872	1.161696	1.028137	1.160179	1.00657	1.195323	1.094627	1.15883	1.011571
P07478	1	0.944509	1	0.784958	1	0.902995	1.190011	0.977627	1.048346	0.97096	1.145006	1.050319	1.277677	1.050401	1.173248	0.999854	1.287098	0.970534
P15924	1	1.141096	1	1.130373	1	1.277917	1.247315	1.884443	1.164291	1.719733	1.124296	1.796213	0.985389	2.38557	0.918728	2.484754	0.901043	2.059765
P06733	1	1.210751	1	1.271351	1	1.401684	0.888233	1.084466	0.742249	1.063711	0.79274	1.108611	1.051635	1.002785	1.121688	1.000146	1.262555	1.040808

P26038	1	0.891593	1	0.98061	1	0.90162	0.933393	0.939683	0.80977	0.994353	0.868336	0.983792	1.023085	0.938559	1.018632	0.910735	0.995903	0.898176
P62937	1	1.391368	1	1.278766	1	1.355808	0.671962	1.566333	0.731484	1.505042	0.852805	1.456732	1.05591	0.712345	0.977885	0.910766	1.077262	1.013788
P29401	1	1.088286	1	1.238148	1	1.178389	1.416206	0.872804	1.549418	0.939206	1.254351	0.86406	0.933853	0.815137	1.028872	0.894678	1.053299	0.863466
O43505	1	1.000722	1	0.824501	1	0.97759	0.959125	0.986883	0.901502	0.948375	0.860863	0.979212	1.022147	0.887902	1.346051	1.063318	1.317857	1.008402
P25774	1	1.18002	1	1.05035	1	1.242732	1.031763	1.063758	1.008229	1.073435	1.049341	1.064156	1.144759	0.884006	1.075388	0.921355	1.097538	0.937663
P19367	1	0.997819	1	0.97847	1	1.134923	1.269206	0.749953	1.166	0.78976	1.174619	0.839681	1.021182	0.959734	0.98968	0.675292	0.96729	0.739505
P10599	1	1.254924	1	1.157299	1	0.858568	0.99982	1.025554	0.996899	1.156142	0.720462	1.168882	0.891216	0.92463	0.948425	1.055677	0.813661	1.111049
Q12884	1	0.990727	1	0.932189	1	1.000117	0.808701	1.030999	1.083555	1.218687	1.031635	1.055214	0.757042	0.939115	0.727414	1.171063	0.648384	1.027476
P00367	1	1.392318	1	1.449917	1	0.90968	1.575451	0.989192	1.732806	1.403816	1.459551	1.273094	0.78947	1.168898	1.074562	1.227912	1.011467	1.028064
Q9NTU7	1	0.976699	1	1.128765	1	0.832396	0.971082	1.012614	1.205283	1.076712	0.758435	0.910668	1.147031	0.823913	1.167701	0.968354	1.102687	0.843376
P08567	1	1.191306	1	1.361709	1	1.012981	0.536802	1.457568	0.678203	1.22398	0.347062	1.572233	0.628806	0.345293	0.566487	0.439432	0.615913	0.454665
P35442	1	1.311197	1	0.969842			1.348708	1.26858	0.640258	0.706614			1.758254	1.07884	0.871673	0.463873		
Q5VY43	1	1.149405	1	1.208626	1	1.240338	1.047352	0.965814	1.144485	1.05279	1.34693	1.03517	0.993865	0.965891	0.968035	1.094909	0.972536	1.18134
P52566	1	1.079257	1	1.310336	1	1.166251	0.743586	1.280713	0.787821	1.0858	0.811098	1.079712	0.977295	0.981188	0.999804	1.038057	0.920074	1.028989
P02788	1	0.966292	1	0.785965	1	0.928518	0.552521	0.582594	0.629432	0.636542	0.654874	0.616904	0.429866	0.564821	0.473302	0.571372	0.507353	0.595861
P25786	1	0.964112	1	0.930623	1	0.927562	1.018614	1.048104	1.115972	1.180096	1.067774	1.1192	1.294559	1.284227	1.291019	1.20658	1.282193	1.276475
Q9UEW3	1	1.035372	1	1.064226	1	0.943927	0.837557	1.114241	0.668085	1.085734	0.731478	1.093506	1.385557	1.031824	1.380172	0.844096	1.399375	0.822187
Q9Y4L1	1	1.084409	1	0.931861	1	1.002464	0.876405	0.990221	0.921075	0.964198	0.867381	0.977213	1.053139	0.883545	1.055269	0.898073	1.055135	0.866107
Q12907	1	0.750079	1	0.736187	1	0.86007	0.941569	0.956316	1.047112	1.028085	1.047688	1.010519	1.040041	0.791966	1.000732	0.927514	1.091786	0.851891
P12814	1	1.219113	1	1.155664	1	1.176477	0.804919	0.916659	0.91324	0.948055	0.66452	0.959657	0.781744	0.89414	0.783208	1.073048	1.165709	0.942547
P06702	1	1.407917	1	1.62495	1	1.436705	1.17296	1.144748	1.399762	1.057773	1.273392	1.067342	1.178475	1.264831	1.084399	1.196796	1.010752	1.185607
P28799	1	1.121922	1	0.757745	1	0.984345	0.995932	1.059529	0.937881	1.069022	1.04694	1.101969	1.357465	1.201124	1.367341	1.101919	1.372356	1.16171
P23471	1	1.08379	1	0.929374	1	0.846909	1.276075	1.093861	1.241668	1.073952	1.215182	1.053017	0.896912	1.026529	0.847428	1.008899	0.858535	1.06419
O94985	1	0.866002	1	0.909981	1	1.088532	0.858382	0.93217	0.913204	0.912288	1.032232	0.952144	0.91322	0.882576	0.82848	0.850531	0.936904	0.930973
Q07507	1	0.704018	1	0.977319	1	0.792823	1.248528	0.773308	1.326496	0.885582	1.108785	0.90846	0.731141	0.640726	0.911877	0.819802	1.010606	0.803245
P49913	1	1.03857	1	1.352027	1	1.307403	0.980518	0.991539	0.943619	1.014775	0.961727	1.019581	0.812662	0.847734	0.817526	0.837999	0.809736	0.810135
P00533	1	1.172157	1	0.891069	1	1.156129	0.996912	1.029296	1.01558	0.972503	1.015805	0.992757	1.136112	1.07438	1.081343	0.998627	1.121697	1.143524
P50895	1	1.083731	1	1.170902	1	1.143422	0.948065	0.965402	0.848148	1.018388	1.067844	1.065404	1.35269	1.057405	1.438319	1.054979	1.35775	1.204456
A0A075B(	1	0.748764	1	0.642124	1	0.750646	0.695929	0.631668	0.752375	0.566803	0.707851	0.555372	0.491757	0.532479	0.335736	0.550672	0.512157	0.625299
Q9UBG0	1	0.973753	1	0.942221	1	1.016039	1.148337	0.850044	1.090639	0.975099	1.072393	0.995022	1.009131	0.975029	0.987551	1.015635	0.954455	1.075452
P13727	1	0.825099	1	0.83914	1	0.896623	1.437971	0.922543	1.249191	0.96677	1.258236	0.934548	0.947645	1.085294	0.885963	1.120155	0.841854	1.039215
Q02487	1	0.934159	1	1.092403	1	1.227633	1.114562	1.068155	0.997915	1.066434	1.085419	0.941233	1.096536	0.824952	0.958005	0.88431	0.996261	0.924352
P10645	1	0.993867	1	1.091196	1	0.968187	0.878939	0.874671	0.789001	0.910316	0.778506	0.847708	1.73262	1.384115	1.661202	1.315741	1.651057	1.26795
P32004	1	0.997128	1	0.953695	1	1.018956	0.995631	0.86662	0.952111	0.862722	0.958735	0.906516	1.095738	1.020835	1.119989	0.957171	1.176832	0.89621
Q8WWZ8	1	0.909854	1	0.825491	1	0.936509	0.954325	0.958612	0.933876	1.02101	0.961342	0.976919	0.981355	1.005701	1.022328	1.047063	1.025569	1.025835
P05019	1	0.9085	1	1.360009	1	1.300088	0.863373	1.076422	1.118288	1.368794	0.899128	1.203377	0.905915	0.847473	1.091929	1.093886	1.074176	0.987347
P20848	1	0.458988	1	0.242161	1	0.44644	2.118769	0.783417	3.25487	0.754994	2.38414	0.81953	0.869104	1.275674	0.863752	1.237339	0.919562	1.238066
Q13421	1	0.704845	1	0.827445	1	0.965354	1.23273	0.820709	1.149967	0.927138	1.268686	0.990091	1.067811	0.940897	1.122102	1.059211	1.135706	1.151607
P10619	1	1.225212	1	1.043714	1	1.189451	0.636098	0.826398	1.063333	0.961491	1.049458	1.037269	0.914482	0.875735	0.978594	0.975849	0.994156	1.1258
Q9NQ38	1	0.969987	1	0.882155	1	1.029461	0.859565	1.022288	0.924678	1.055392	0.938526	0.975297	1.151239	0.818705	1.076273	0.938838	1.120854	0.773128
P62258	1	1.366769	1	1.574995	1	1.505177	1.113492	1.031655	1.100346	0.999558	1.046379	0.983439	1.1878	1.315352	1.374748	1.447361	1.360408	2.889799
Q10588	1	1.638901	1	1.686721	1	1.532033	1.183569	1.137624	1.1638	1.156044	1.065019	1.155725	1.447661	1.472745	1.381068	1.393621	1.446967	1.498846
P07858	1	0.876923	1	0.808527	1	0.76159	0.871425	0.971894	0.826498	1.008208	0.903532	0.942498	1.583517	0.963998	1.600553	0.949241	1.569896	0.949857
Q86TH1	1	0.724495	1	0.926757	1	0.72925	0.819267	0.970553	1.025726	1.086966	0.808238	0.865701	1.219875	1.070077	1.26387	1.01899	1.077079	0.962827
P34096	1	0.792115	1	0.956006	1	0.779626	1.114366	1.149321	1.220961	1.146524	1.115637	1.170782	1.182296	1.172263	1.268024	1.106971	1.133912	1.163658
P0DP24	1	1.136737	1	1.288966	1	2.137079	0.859676	2.247545	0.914464	1.9999	0.942764	2.273507	0.948519	1.115664	0.919743	1.225021	1.088548	1.154085
Q13508	1	1.087015	1	1.081977	1	1.093195	1.068348	0.985803	1.104335	0.956319	1.060143	0.960884	0.946609	1.034533	0.874084	0.952645	0.985508	0.907402
P30479	1																	
P04222	1	0.825611					0.6007	0.732759					0.626079	0.504462				
Q8IWV2	1	0.938281	1	0.917434	1	1.115965	1.001574	0.867079	1.055854	1.012266	1.148879	0.94612	1.113124	1.049608	1.124352	1.247428	1.02771	1.183187

P01137	1	1.28099	1	0.955709	1	0.995282	0.599823	0.87077	0.593474	0.825055	0.597446	0.780661	0.712767	0.840331	0.710018	0.829222	0.754094	0.834532
P30464	1	0.968585	1	1.112385	1	1.026609	1.250945	1.465356	1.07917	1.524684	0.974184	1.09195	1.281187	1.018506	1.399198	0.833658	0.92853	1.103303
P11150	1	0.739808	1	0.869727	1	0.938433	0.97211	1.038833	1.026227	1.132258	1.043464	1.140177	0.625042	0.749043	0.666379	0.722052	0.65532	0.60856
P31946	1	0.976729	1	1.799447	1	0.863467	0.759764	0.964104	0.721129	0.730706	0.704862	0.889558	1.077901	1.040547	1.008925	1.01109	1.058945	0.905039
Q8WU03	1	0.713269	1	0.686722	1	0.747805	2.019969	1.422768	2.020241	1.428191	2.008725	1.438758	1.251163	1.512746	1.261746	1.512352	1.261122	1.5266
P01619	1	0.966988	1	0.838208	1	0.916655	0.457181	0.469977	0.865151	0.564368	0.571653	0.505406	0.485084	0.495456	0.505901	0.541522	0.527563	0.486859
O14818	1	1.041599	1	1.080501	1	0.868902	1.181852	1.079512	1.30452	1.111221	1.067213	1.143481	1.16835	1.190477	1.218523	1.336304	1.166551	1.176799
Q99972	1	0.722643	1	0.615353	1	0.708046	0.955356	0.818269	1.006347	0.821404	0.920685	0.770846	0.846498	0.751245	0.753849	0.645864	0.714877	0.620121
P46777	1	0.898568	1	0.774134	1	1.017197	1.154613	0.786516	0.857679		1.259245	1.118589	0.981082	1.212971	0.753906	0.901087	0.828008	1.028285
P13489	1	1.147746	1	1.096792	1	1.199438	0.914523	0.941979	0.941845	0.97759	0.991906	1.076453	1.095577	0.817463	1.090645	0.785915	1.1364	0.808157
P08174	1	0.767963	1	0.893313	1	0.957948	0.949803	0.97498	0.96744	0.965179	1.073491	1.013327	1.168772	0.940541	1.005502	0.920802	1.133929	1.006256
P00918	1	1.1765	1	1.133387	1	1.08652	1.996355	1.703877	2.267846	1.601885	2.342039	1.870139	1.60451	1.687747	1.436117	1.595548	1.687506	1.618655
P00451	1	0.93121	1	0.943112	1	0.920733	1.008481	1.052462	1.020141	0.922393	0.994503	0.949636	1.252527	1.211054	1.188265	1.271992	1.164245	1.265086
P27797	1	0.753788	1	0.722638	1	0.744868	0.974009	1	0.975487	1.07908	0.962009	1.013015	1.188192	0.995007	1.2279	1.050992	1.064863	0.934682
O60814	1	0.756976	1	1.056024	1	0.89354	0.867343	3.579589	0.818098	3.301952	0.759406	3.278922	0.664548	0.83738	0.667937	0.845483	0.685949	0.921144
P15291	1	0.91201	1	0.80264	1	0.934103	0.90435	0.956681	0.864097	0.806048	1.054463	0.856275	1.188032	1.05983	0.962681	0.981362	0.873985	0.980925
P01782	1	1.265824			1	1.386828	0.488764	0.726793			0.594987	0.781863	0.752024	0.513341			1.178745	0.778583
Q9NSB4	1	1.114213	1	0.600689	1	0.114386	1.343745	13.72013	2.542511	27.18996	2.957449	23.16	0.932792	0.840313	1.098324	0.747178	0.59283	0.861146
Q15262	1	1.154341	1	0.977723	1	1.240318	1.095726	1.131147	1.159835	1.087197	1.219221	1.077863	2.013788	1.140182	1.533189	1.219124	1.510923	1.302613
P07686	1	1.0495	1	0.97087	1	1.066654	1.069645	1.024644	1.073325	0.951451	1.042726	0.992565	1.495963	1.330786	1.509318	1.239844	1.405624	1.230344
O00299	1	0.91801	1	0.984559	1	1.148502	0.816146	1.083007	1.029548	1.162217	0.922074	1.105564	0.992153	0.854708	1.027155	1.087484	0.854787	0.929948
Q96EE4	1	0.902144	1	0.953419	1	0.994364	0.571047	0.794569	1.00969	0.781585	0.928812	0.776676	0.93199	0.743119	0.903503	1.093183	1.095498	1.00856
O75874	1	1.081672	1	0.931335	1	0.893363	1.096569	1.067413	0.949943	1.070958	0.902676	1.095591	1.010164	0.863486	0.828332	0.833027	0.818976	0.790098
P22692	1	1.62858	1	1.186872	1	1.337246	1.091972	0.934076	0.929436	0.959654	0.865072	0.970681	1.050426	1.026457	1.045867	0.816105	1.003447	0.906415
P04083	1	1.649467	1	0.839099	1	0.691426	0.926115	1.058153	1.109601	0.81966	0.610561	0.78159	0.977247	1.313372	1.160541	1.626969	0.753639	1.007691
Q99983	1	0.599955	1	0.534192	1	0.616272	0.826969	0.946646	0.823699	0.954272	0.814683	0.95296	1.140025	1.035232	1.017161	1.015007	1.026463	1.072517
Q7Z3B1	1	0.927676	1	0.876201	1	1.014131	1.363619	0.981603	1.155436	0.95392	1.181865	1.010208	1.26409	1.210228	1.04458	0.947203	1.185102	1.091358
Q8NDA2	1	1.571195	1	0.920121	1	1.002547	0.895566	0.750508	0.763179	0.630292	0.764465	0.772592	0.843875	0.737931	0.761603	0.608526	0.740959	0.572169
Q9UBP4	1	1.400924	1	0.964046	1	1.035793	1.283115	1.041636	0.798833	0.856282	1.20404	0.96386	1.280112	1.380363	1.148022	0.926995	1.036803	1.113423
Q8NBJ4	1	1.102274	1	1.075015	1	1.096381	1.0952	0.935572	1.094912	1.034055	1.111723	0.942071	1.57943	1.399901	1.629036	1.416391	1.538015	1.394318
P08581	1	0.934467	1	0.879099	1	0.979975	1.171485	0.928537	1.112591	0.878337	1.024178	0.862273	0.900798	1.025241	0.907922	1.03067	0.920457	0.91211
P09619	1	0.850705	1	1.187869	1	0.940639	1.104163	1.067928	1.329076	0.989691	1.15716	0.937257	1.30229	1.282185	1.311316	1.089259	1.343195	1.17448
Q5SYB0	1	0.691864	1	0.70921	1	0.73178	0.868178	0.954015	0.895668	0.951684	0.874948	0.952706	0.721838	0.731545	0.720884	0.731808	0.759967	0.761612
P23381	1	1.466191	1	1.402702	1	1.189138	1.180053	1.065536	1.508229	1.020715	1.267851	1.031623	1.233677	1.561133	1.040924	1.111781	0.99403	1.629956
P07148	1	1.398708	1	1.379364	1	1.299159	1.441487	1.302824	1.280495	1.277583	1.218804	1.301676	1.368193	1.108868	1.064598	1.143805	1.246451	1.088834
Q13557	1	1.05898					1.239488	0.631388					0.651828	0.554639				
Q9HBB8	1	0.909844	1	1.07988	1	0.991971	0.831846	1.005889	0.900939	1.053561	0.909023	1.075767	1.010121	0.861908	1.021995	0.862464	1.014016	0.920399
O00461	1	0.586497	1	0.46077	1	0.661003	0.944199	1.1579	0.869586	1.076715	0.909497	1.085214	1.171709	0.94959	1.245314	1.012163	1.317696	0.911687
P60660	1	1.173734	1	1.250205	1	1.208638	0.749342	0.992745	0.790507	1.039078	0.815793	1.016555	0.932303	0.939896	0.914793	0.8877	0.952718	0.983072
Q96RD9	1	0.807706	1	0.882705	1	0.838735	0.877131	1.093906	0.952621	1.087074	0.783317	1.09511	1.124981	1.02	1.062014	1.169022	1.150242	1.174981
Q13509	1	1.24956	1	1.242261	1	0.971134	1.218446	0.800645	1.257963	0.921428	1.002931	1.048393	1.041826	0.963042	1.019931	0.919776	0.912622	0.967677
A0A0C4D	1	0.813144	1	0.881667	1	1.170686	0.901354	0.728924	1.075885	0.866804	1.065389	0.949371	0.599539	0.859862	1.035627	1.252936	0.875945	1.08934
Q8N3T6	1	0.975347	1	0.839173	1	0.879656	1.066458	0.940147	1.034889	0.983041	1.116558	0.979712	1.036946	1.015078	1.098719	0.988315	0.899247	0.938525
A0A0J9Y	1	1.200929	1	1.23965	1	1.230954	1.142087	0.895196	1.161749	0.887951	1.175409	0.895174	0.436318	1.313377	0.454552	1.368957	0.427093	1.323609
P05109	1	1.433279	1	1.342007	1	1.454221	1.051171	1.156108	1.009148	1.107212	1.033484	1.081977	1.297851	1.235392	1.250336	1.098905	1.196062	1.158856
Q8TDL5	1	0.768741	1	0.702754	1	0.659175	0.981551	0.971679	0.8216	0.887275	0.771615	0.912185	0.673261	1.01509	0.65114	0.970624	0.596796	1.029879
P0DMV8	1	1.421868	1	1.015375	1	1.496337	0.974183	1.062192	0.679422	0.97797	1.018114	1.077902	0.920025	1.309301	0.975218	0.857161	0.972635	1.389584
P01130	1	1.022632	1	0.798433	1	1.042478	1.066205	1.139455	1.080633	1.13675	1.172326	1.075616	1.212641	1.156019	1.223537	1.321609	1.250181	1.211857
O75356	1	1.416701	1	0.702865	1	0.7683	0.743968	0.957402	0.773545	0.73254	0.829475	0.976244	1.684242	1.427931	1.451471	1.156747	1.494552	1.107459
Q99623	1	1.240891	1	1.536487	1	2.067432	1.531119	1.362271	1.928174	1.139556	2.286457	1.128332	2.097178	1.542507	1.313061	1.380453	2.210447	1.019419

Q8NBS9	1	1.190683	1	1.034896	1	1.464024	1.281042	1.276406	0.964629	1.095365	1.096005	1.096774	1.168053	1.256085	1.15905	1.189125	0.950486	0.910331
P08311	1	1.193107	1	0.891779	1	0.622488	0.431357	0.654197	0.396755	0.588776	0.366556	0.534385	0.637657	0.627311	0.575915	0.65371	0.385628	0.395244
Q06481	1	0.927061	1	1.122299	1	1.408672	0.595038	0.746639	0.653842	0.849627	0.926088	0.925561	0.842267	0.765349	0.8049	0.770873	0.961532	0.78185
Q9UQP3	1	0.750159	1	0.936451	1	0.791213	0.898385	0.965832	0.915997	1.050714	0.828808	0.957473	1.013038	0.957741	0.980036	1.010477	0.954466	0.850466
P52272	1	1.002214	1	0.659532	1	0.853981	1.850394	0.766416	0.909512	0.688179	1.21764	1.063251	1.423256	1.225192	1.152964	0.592003	1.579261	0.753436
Q13332	1	0.952057	1	0.869216	1	0.948831	1.056596	0.78138	0.9792	0.961164	1.140197	1.041963	0.590446	0.666701	0.855146	0.79014	0.881407	0.925886
Q99941	1	1.239404	1	1.120202	1	1.218958	1.008277	1.176312	0.887353	0.976025	1.022796	1.151263	1.36203	1.404046	1.144993	1.115712	1.409205	1.727239
Q9BS26	1	0.956474	1	0.990259	1	0.933033	0.987578	1.019975	1.001318	0.971932	0.972142	0.926525	0.90029	0.816936	0.958773	0.790656	0.941203	0.758526
Q04917	1	1.410901					0.860398	1.078869					1.091196	0.829458				
Q6UVK1	1	0.979187	1	1.045791	1	0.894011	1.025493	0.938262	0.961035	0.888164	0.914171	0.883811	1.163115	1.021024	1.051554	0.908445	1.023939	1.026839
P42785	1	0.950708	1	1.046473	1	1.10445	1.119456	1.133604	1.190415	1.086719	1.123508	1.1504	1.191067	1.048239	1.192222	1.037814	1.489095	1.203581
P69849	1	1.109251	1	1.228763	1	1.180772	0.960506	0.978497	1.012987	1.037041	1.063091	1.0345	1.306433	0.982613	1.244143	1.139626	1.364993	1.150758
P50395	1	1.42584	1	1.135337	1	1.092107	0.961916	1.141922	1.049198	1.103827	0.981754	0.884528	1.06898	0.998004	1.241655	1.006613	1.184383	1.123346
Q92520	1	1.204323	1	1.271661	1	1.056133	1.083885	0.911885	0.948875	0.832361	0.871448	0.901256	0.928279	0.767846	0.828137	0.765591	0.941117	0.847332
P78386	1	2.336128	1	1.377627	1	1.214689	0.585948	23.63047	0.999534	11.57224	1.11786	14.93689	1.794488	1.691177	0.729138	1.102654	1.07148	0.884908
Q6P179	1	0.798208	1	0.889261	1	0.7065	1.466872	1.131521	1.590512	1.25667	1.423181	1.256785	1.01269	1.374765	0.955568	1.52328	1.05618	1.534473
Q8WUA8	1	1.756644	1	1.57403	1	1.329555	0.912562	1.03277	1.047166	1.039146	1.113981	1.221061	0.955139	1.701959	1.165351	1.562192	1.148582	1.764757
P62942	1	1.359709	1	1.525017	1	1.414572	0.809934	0.986469	0.920672	1.304333	0.684105	1.165496	0.939193	0.854031	0.855969	0.941886	0.88246	0.699532
O00592	1	0.817272	1	0.787265	1	0.891296	0.864593	0.945527	0.839882	0.973923	0.805297	0.939877	1.02236	1.05568	1.165363	0.936749	1.220298	0.949515
P52209	1	0.87747	1	1.136262	1	1.239989	0.926751	1.189246	0.940214	1.096902	0.824674	1.031493	1.229909	1.18131	1.031487	1.136533	0.942608	0.813294
P01742	1	0.901052	1	0.708972	1	0.646848	1.051988	0.655654	0.764088	0.582361	0.729857	0.574879	0.394901	0.98854	0.264424	0.811335	0.279013	0.831185
Q86UX7	1	1.128261	1	1.086518	1	1.180478	0.596342	0.869581	0.697339	0.687465	0.784515	0.811486	0.718881	0.891419	0.57117	0.607979	0.672636	0.6554
Q93063	1	1.010487	1	0.829786	1	0.884993	1.198737	0.929067	1.142247	0.973571	1.106748	0.975888	1.021995	0.967406	1.026546	0.958298	1.182337	1.027525
P09668	1	1.468979	1	1.34297	1	1.183577	1.074466	1.11163	0.991866	1.021922	1.028835	1.095486	1.601811	1.348689	1.482645	1.294065	1.481527	1.218124
P10153	1	1.035628	1	1.265912	1	1.180604	1.188997	0.856473	1.463538	0.962496	1.287598	0.994657	0.99744	1.192327	0.945769	0.918024	0.895874	1.096012
Q8IUL8	1	1.047393	1	0.982431	1	1.175252	0.925565	0.948501	0.994701	0.942666	1.19387	0.962959	0.712258	0.713878	0.799765	0.829973	0.850924	0.828421
Q9UBR2	1	0.768018	1	0.612538	1	0.867223	1.155104	1.025565	1.188386	1.005474	1.101463	1.002385	1.516701	1.297756	1.723312	1.196987	1.61035	1.138573
Q8IZP9	1	1.741841	1	2.249526	1	1.61325	1.392761	1.180889	0.94495	1.026274	1.520663	1.001756	1.190685	1.099917	0.939703	1.231994	0.572401	0.972049
P18669	1	1.057926	1	1.343665	1	1.00954	0.895999	1.088661	0.980703	0.956909	0.873221	1.024982	1.60076	1.002532	1.603834	1.018721	1.655771	1.059944
Q92854	1	0.841787	1	0.798617	1	0.851881	0.692081	0.817785	0.775946	0.80207	0.645228	0.755062	0.883354	0.580896	0.818074	0.660706	0.901287	0.747971
Q02413	1	1.161335	1	1.086785	1	1.187418	1.160909	1.031966	0.974059	1.109547	0.982423	1.207831	0.775529	1.415374	0.968311	1.89925	0.953381	1.474787
Q9HCU0	1	0.882076	1	1.092713	1	0.93504	0.900031	0.859631	0.793875	0.700284	0.776258	0.787739	0.868354	0.901292	0.770804	0.731441	0.697113	0.82472
P21926	1	1.385991	1	1.347338	1	1.253888	0.400712	0.61816	0.510756	0.659604	0.599476	0.698173	0.543339	0.535527	0.479578	0.686594	0.55699	0.592569
Q14697	1	0.93192	1	0.833428	1	0.854397	0.79349	0.889042	0.846336	0.882777	0.856863	0.880479	0.998994	0.963577	0.926643	0.953958	1.045942	1.001554
P16284	1	1.458011	1	1.413192	1	1.469478	0.984595	0.879424	0.943222	0.933021	0.980079	0.942529	0.907025	0.787746	0.963305	0.723893	1.010231	0.876524
P23083	1	0.788053	1	1.070581	1	0.978782	0.873297	0.716585	1.058662	0.932303	1.061827	0.868998	0.422266	0.526276	0.826182	0.548768	0.505674	0.665046
P36269	1	0.910917	1	1.269839	1	0.814889	1.048399	0.952787	0.955246	1.048457	0.842644	0.986429	0.96197	1.279677	0.954318	1.313844	1.159814	1.131682
P14923	1	1.408358	1	1.145203	1	1.346845	1.348305	4.389843	1.312537	2.456217	1.381243	2.437084	0.752694	2.358503	0.742235	1.768075	0.925822	1.836638
P13667	1	0.857035	1	0.944289	1	1.077344	1.000654	1.015744	1.12256	1.006449	1.03911	0.957891	0.98575	1.07756	1.030211	1.140375	1.048191	1.177932
P32942	1	0.668162	1	1.009046	1	0.807747	0.966303	0.985353	1.056182	0.984045	1.082276	1.014492	1.290261	1.033915	1.38589	1.029591	1.257834	1.063025
P01040	1	1.171107	1	0.820277	1	0.886477	2.434902	0.981547	2.402117	1.168759	2.215935	1.02853	0.809652	1.595868	0.591124	1.496708	0.584558	1.412465
Q9UBX1	1	0.705133	1	0.780755	1	0.904882	0.942355	0.820133	1.019349	0.838351	1.026096	0.835702	0.724006	0.723012	0.844886	0.897662	0.840931	0.856089
A0A075B[illegible]	1	0.326985	1	0.315303	1	0.344696	0.431031	0.50856	0.44611	0.609351	0.427895	0.451163	0.322776	0.489338	0.402384	0.519609	0.324694	0.48777
Q86UN3	1	0.935084	1	0.899698	1	1.026507	0.962111	1.064742	1.171152	1.10468	1.056355	1.059829	0.842188	0.968389	0.845346	0.98521	0.820492	1.036642
Q5T2D2	1	1.006587	1	0.927467	1	0.878743	0.955255	0.931606	1.189776	0.975964	1.123115	0.936328	1.028135	1.097184	0.874455	1.096211	0.90301	1.131085
Q9HCN6	1	0.896819	1	0.996913			0.454637	0.906341	0.591758	0.916494			0.546111	0.671558	0.599746	0.501924		
P0DP01	1	1.295022					0.920571	0.662261					0.725673	0.863389				
O00468	1	1.034008	1	1.219699	1	1.360821	1.066425	0.99145	1.113928	0.953935	1.179255	1.050504	1.226343	0.866063	1.256483	0.944564	1.407041	1.107036
O75023	1	1.139934	1	1.166713	1	1.331692	0.963779	1.034867	1.02582	0.956721	1.03984	0.932984	1.208	1.159124	1.155723	1.004087	1.032553	0.985377
P00491	1	1.47104	1	1.275394	1	1.517534	1.109152	1.119817	1.487464	1.15502	1.143441	1.200349	1.332416	1.27391	1.174618	1.275854	1.290596	1.387349

P28072	1	0.72617	1	1.061295	1	0.802252	0.998704	1.071931	1.173015	1.088467	1.131182	1.185366	1.15855	1.114462	1.217022	1.230837	1.178933	1.191593
P01599	1	0.642474	1	0.662905	1	0.705127	2.486169	1.504749	0.83461	0.982169	0.72328	0.666953	1.120278	3.315348	0.632617	0.945923	0.653502	0.575775
P13611	1	0.884082	1	1.105465	1	1.180171	1.283457	1.125658	1.172686	1.162913	1.257931	1.169366	1.145257	1.280415	1.088153	1.184641	1.166	1.202059
A0A0C4D	1	1.012264	1	0.957802	1	0.979644	0.562794	0.549047	0.644736	0.778989	0.74804	0.92914	0.551287	0.654634	0.601783	0.67626	0.728361	0.846226
P01825	1	0.79146	1	0.761492	1	0.652942	1.002296	0.727472	0.769001	0.57494	0.910485	0.601113	0.371577	0.674517	0.374653	0.5951	0.369814	0.630887
Q10471	1	0.830027	1	0.82189	1	0.796657	0.964018	0.888013	0.90547	0.889548	0.915792	0.865238	1.054461	0.951485	1.082495	1.045818	1.099952	1.082173
Q9HCB6	1	0.87155	1	1.03174	1	0.87562	1.20924	1.290884	1.20396	1.387722	1.182482	1.367875	1.280956	1.104862	1.268764	1.048619	1.287806	1.150666
P08575	1	1.074194	1	0.954814	1	1.032506	1.113165	1.008071	1.152617	1.018405	1.151966	0.999384	1.043011	1.345639	1.133235	1.326174	1.122674	1.431334
P0CG48	1	1.21192	1	1.192756	1	1.010219	1.848278	1.486413	2.426411	1.671943	2.018564	1.519017	0.731603	1.404727	0.887942	1.457809	1.03824	1.526375
P07900	1	0.966499	1	0.996875	1	1.212585	0.808408	0.941952	1.091787	1.078054	1.050661	0.994406	0.930371	1.082195	0.991797	1.074726	1.106887	1.197349
Q8NI99	1	0.973324	1	0.825962	1	0.922542	1.019098	1.389868	1.141152	1.352469	1.328072	1.292759	1.044692	1.240123	1.367814	1.044718	1.104856	1.024905
P10646	1	0.871904	1	0.915759	1	0.973779	1.031054	0.97184	1.042521	1.161451	1.011499	1.127606	0.933918	1.04887	0.861526	0.908846	1.084541	0.948462
O14798	1	1.338891	1	0.950793	1	0.910588	1.371422	1.29645	1.024396	0.945419	1.359529	1.098036	1.648021	1.583463	0.893136	1.099858	1.235446	1.566919
A0A0C4D	1	0.423667	1	0.30792	1	0.309995	0.584779	0.59746	0.288076	0.463474	0.288735	0.493783	0.462291	0.436507	0.277534	0.373542	0.304929	0.368235
P20061	1	0.64138	1	0.862872	1	0.898815	1.382168	0.959167	1.129118	0.98889	1.034409	0.992654	0.868534	1.752378	0.933223	1.232849	0.989677	0.984497
P01624	1	0.845888	1	0.981767	1	1.013257	0.644664	0.753114	0.798806	0.616981	0.796956	0.627128	0.6892	0.802689	0.595721	0.654359	0.66505	0.673914
P01700	1	0.430241	1	0.420433	1	0.425382	1.074713	0.688395	1.020125	0.693856	1.005	0.765193	0.482454	0.710259	0.550718	0.76725	0.557859	0.720891
P25788	1	1.03103	1	0.894326	1	0.909235	1.140946	1.116609	1.028755	1.154238	0.992064	1.175159	1.443475	1.383031	1.421075	1.366651	1.506161	1.363307
P58166	1	0.972353	1	0.961635	1	0.893576	0.867905	0.795232	0.84693	0.793493	0.97267	0.845075	0.99961	1.010618	0.973586	0.97611	0.874709	0.973045
Q8N6C8	1	1.198825	1	2.78959	1	1.981787	1.359557	3.302391	1.339442	2.957056	1.580139	4.258539	1.730153	2.758838	1.891658	2.058322	1.894382	2.93175
Q9UNN8	1	0.817012	1	0.763884	1	0.947612	1.830454	1.102932	1.882866	1.270999	1.913817	1.07953	0.655446	0.710422	0.693615	0.929459	0.642768	0.740845
P02144	1	1.393948	1	1.169255	1	0.908045	0.988011	1.035704	0.923155	1.023775	1.063926	1.036919	1.70345	1.796273	1.901607	1.527902	1.753397	1.391998
P55268	1	1.136067	1	1.130464	1	1.097251	1.030003	0.96467	1.061568	1.001587	0.979462	0.815859	1.200953	0.932608	0.886216	1.026967	0.962217	1.049129
P58546	1	1.653342	1	1.416812	1	1.374995	0.686993	1.50971	0.85262	1.572546	0.960029	1.617554	0.956845	0.876873	0.958376	0.810198	0.991867	1.025612
O00151	1	1.561249	1	1.055449	1	1.051086	0.484475	2.175662	0.527969	1.555966	0.413161	1.666158	0.509186	0.321469	0.720749	0.407058	0.567804	0.578583
P01709	1	0.859454	1	0.931022	1	0.95421	0.561238	0.631297	0.597345	0.537505	0.681293	0.622482	0.550011	0.725184	0.474723	0.707878	0.608296	0.830433
O75083	1	1.110696	1	1.235479	1	1.093146	0.802406	0.844973	0.785245	0.972047	0.684388	0.91583	0.85445	1.007991	0.883407	0.956459	0.94006	0.955555
O43852	1	0.916077	1	1.203528	1	0.988152	0.525178	0.647811	0.480147	0.611218	0.473393	0.61003	0.703583	0.74461	0.639164	0.693777	0.630681	0.774164
P37840	1	1.142375	1	1.268931			0.630593	0.764047	0.759153	0.811039			0.608019	0.710865	0.802506	0.996716		
P01594	1	1.029482	1	1.10814	1	1.05552	0.889013	0.754334	0.808744	0.614769	1.010109	0.719299	0.525055	0.951572	0.43105	0.720043	0.790245	0.883857
P35579	1	1.112618	1	1.268935	1	1.310272	0.89572	1.095393	0.861317	1.08355	0.821217	1.075935	0.924078	0.99687	0.985622	1.105585	0.96157	1.058663
P31151	1	1.213628	1	1.083925	1	1.156324	1.631807	1.106937	1.424453	1.084566	1.379837	1.054367	1.205621	1.104595	1.260907	0.938764	1.287889	1.081073
Q16394	1	0.888592	1	0.868749	1	0.795546	1.037756	0.907353	1.09264	1.014798	0.994577	0.939781	1.134787	1.017126	1.051293	1.074986	0.993292	1.026257
Q02978	1	0.878547	1	0.905964	1	0.96627	0.822029	0.461104	1.240173	0.711039	1.189338	0.708701	0.946339	0.817226	1.201791	1.291813	1.111772	1.328463
Q96H15	1	1.116053	1	1.866813	1	0.968231	1.18749	1.008627	1.108794	1.073309	1.367663	1.107543	1.762039	0.972447	1.943864	1.098442	1.36883	1.034721
Q08ET2	1	0.87359	1	0.657727			1.282762	1.034448	0.997119	0.981142			1.125855	0.711308	1.068691	0.712744		
P06576	1	0.977569	1	0.990991	1	0.785783	1.068258	0.952716	1.303441	0.910849	1.257751	0.920477	1.047442	0.571723	1.085449	0.468496	0.915654	0.528316
P36871	1	0.972825	1	0.684472	1	0.743888	0.878776	0.938278	0.745195	0.927666	0.642216	0.783217	0.807937	0.870875	0.794907	0.69432	0.774461	0.656572
P16150	1	1.363234	1	1.217687	1	1.113032	0.977252	1.04174	0.996827	1.073285	1.058707	1.064281	1.245554	1.355363	1.077733	1.317234	1.133955	1.250896
Q8IUC1	1	1.474006	1	0.799636	1	0.841053	1.445786	4.896762	1.060757	7.655901	1.13024	18.82231	2.361945	1.598278	1.387943	0.955421	1.775655	1.011547
P07738	1	1.10233	1	1.144235	1	1.254057	1.329658	1.143089	1.093403	1.052088	1.247806	1.2448	1.24755	1.184115	1.215802	1.186535	1.335363	1.24017
P08670	1	1.16628	1	1.082993	1	1.487173	0.87753	1.005642	0.831456	0.887527	0.942992	1.004603	0.856827	0.883604	0.806593	0.910095	1.018665	1.126042
P28906	1	1.259978	1	1.108914	1	0.982866	0.820884	0.868603	0.901263	0.960098	0.736929	0.711183	0.947226	0.9878	0.920188	0.933684	1.101901	0.842666
P56199	1	0.962891	1	1.121057	1	1.116588	1.113402	1.015687	0.832364	0.886581	1.007763	0.945622	1.129031	1.139227	1.170692	0.994194	1.227939	1.082928
Q9UBX5	1	1.251526	1	0.913871	1	1.109615	0.671029	0.940433	0.763502	0.848249	0.843772	0.911391	0.786434	0.702377	0.835354	0.80404	0.829768	0.84024
P60174	1	1.009917	1	1.173751	1	1.014786	1.257562	0.926551	1.399392	1.195252	1.181652	1.116005	0.985347	0.980749	1.161626	0.884873	1.21371	0.931608
P00390	1	0.976489	1	1.178132	1	0.958776	1.249342	0.966534	1.299175	1.049277	1.211735	0.991054	1.043202	0.939437	0.948016	0.985011	0.937668	0.988372
Q6Q788	1	1.118944	1	1.040791	1	1.05411	0.847039	0.967318	0.978277	0.837768	0.919172	0.919441	0.916326	1.311334	1.196711	1.645827	1.128205	1.637228
Q16627	1	1.174938	1	1.068773	1	1.118137	1.073646	0.974692	1.023912	1.059835	0.957928	0.944185	1.161879	0.967056	1.145269	0.977255	1.193663	1.031485
O00462	1	1.129319	1	1.699861	1	1.197884	1.160453	0.872942	1.207995	0.98255	1.025818	1.067046	0.926089	1.232704	1.163888	1.186708	1.18797	1.280503

P19256	1	1.47022	1	1.302966	1	1.392918	1.234677	1.13626	1.137165	0.983061	1.016378	1.162869	1.239395	1.432379	1.045904	1.047354	1.180801	1.240058
P37837	1	0.954894	1	1.095649	1	0.993404	0.969317	1.016031	0.976621	0.998798	0.962079	1.003449	0.982447	1.076839	0.946309	1.100384	0.957835	1.142115
Q96S96	1	0.703759	1	0.773162	1	0.703202	0.855187	0.773424	0.962284	0.779503	0.899464	0.809881	0.756053	0.623417	0.773296	0.639611	0.813003	0.684377
P27930	1	1.138438	1	0.921633	1	0.741469	1.297443	1.030253	1.161087	1.063222	0.959819	0.940429	1.222062	1.10995	1.22095	1.310138	1.301434	1.015977
P54764	1	0.935718	1	1.146714	1	0.955936	1.030121	0.986061	1.014306	0.997652	1.052055	0.967713	1.258757	0.91958	1.222662	0.944841	1.03019	0.832534
P24298	1	1.188684	1	1.291836	1	1.219105	1.116219	1.124261	1.017743	1.197176	1.072703	1.025604	1.014492	1.065645	1.099491	1.08781	0.987103	1.040858
P00505	1	1.723867	1	1.332792	1	1.92031	3.563804	1.286495	3.260085	1.336569	2.711735	1.145784	1.753159	1.368422	1.054169	1.238986	1.338736	1.291116
Q86X29	1	0.929225	1	0.731384	1	0.793553	1.131933	1.166811	0.999052	1.056697	0.827413	1.058967	1.090075	1.22347	1.158164	1.148894	1.209046	1.273667
P13987	1	0.791672	1	1.177678	1	1.196741	1.10765	1.160723	1.152839	1.022	1.090317	1.155955	1.181275	1.139657	1.202535	1.190213	1.165081	1.125752
Q7Z7M8	1	0.901683	1	1.081737	1	1.191742	1.085288	0.934936	1.120022	0.89532	1.06251	0.955724	0.948188	0.981391	1.138465	0.90123	0.889905	1.003529
P23141	1	1.064929	1	1.248576	1	1.007028	1.15267	1.045747	1.20753	1.281188	1.116466	1.239808	0.853215	1.047516	1.192734	1.253935	1.224904	1.1954
Q9HCL0	1	0.844327	1	0.94942	1	1.022573	0.903497	1.073641	0.924028	1.114541	0.964615	1.084013	0.780158	0.908251	0.892987	0.860309	0.753761	0.972107
P49720	1	0.837352	1	0.925274	1	0.914879	1.114064	1.11807	1.060977	0.912031	1.015572	1.073123	0.892917	1.463466	1.115835	1.534568	1.153473	1.257226
P20618	1	1.325787	1	1.164744	1	1.14257	1.092626	1.045153	1.03544	1.054198	1.028126	1.094074	1.266005	1.338017	1.342001	1.109832	1.333085	1.306556
Q96HD1	1	1.303072	1	1.196996	1	1.266243	1.226169	1.069911	0.805049	0.997483	1.093736	1.031245	1.079995	1.182316	1.122939	0.980784	1.155833	1.121043
Q14956	1	1.096421	1	1.342515	1	1.102392	0.956579	0.959296	1.030441	1.041797	1.015327	1.015735	1.109505	1.022409	1.244222	1.031299	1.118205	0.939423
P05186	1	1.03566	1	1.043345	1	1.196808	1.31423	1.165673	1.074062	1.242091	1.050618	1.092463	1.344455	1.227675	1.34755	1.211326	1.289697	1.161889
O95236	1	0.727629	1	0.835856	1	0.887575	0.691087	0.884954	0.617291	0.858635	0.493527	0.8273	0.37956	0.407095	0.513391	0.401091	0.510466	0.481299
P08319	1	0.843723	1	0.990584	1	0.908562	0.760534	1.13778	1.019264	1.165629	1.067982	1.094693	0.765605	0.564435	0.829261	0.800566	0.796518	0.82437
P24387	1	0.923696	1	0.928449	1	1.01143	0.915941	0.897392	0.946447	0.835013	0.869609	0.906661	0.927408	0.966829	0.879487	0.863796	1.00802	0.951532
A0A0B4J1	1	0.784708	1	0.762065	1	0.498788	0.866934	0.790478	1.130687	0.960956	1.048981	0.847462	0.523032	0.713457	0.527545	0.475497	0.566145	0.611934
Q8N1N4	1	0.811042	1	1.115406	1	0.837172	1.088173	0.916993	1.493809	1.119909	1.43151	1.364051	0.711766	2.548437	1.186784	1.61588	1.073618	1.940149
Q06830	1	1.162414	1	1.167829	1	1.174125	1.057384	1.033385	1.06389	1.142124	1.086503	1.155751	1.363311	1.31501	1.315655	1.23983	1.303147	1.221005
P51693	1	0.916855	1	0.610101	1	0.829007	0.853393	0.823937	0.650461	0.833664	0.759695	1.001977	0.772548	0.763764	0.619205	0.721366	0.949979	0.870961
P58335	1	0.858751	1	0.832099	1	0.770489	0.847897	0.642447	0.769476	0.561182	0.992433	0.608574	0.564462	0.679361	0.588568	0.603033	0.652301	0.64218
P07711	1	1.383385	1	1.323761	1	1.142729	1.206478	1.171568	1.254843	1.129349	1.127499	1.103469	1.362098	0.940559	1.503706	1.068203	1.448115	0.982896
Q12794	1	1.173733	1	1.071686			0.659639	0.809023	0.912598	0.726558			0.866095	1.018909	0.72335	0.846923		
P78504	1	1.15996	1	1.296972	1	1.05804	0.884394	0.991063	0.940524	0.989949	0.999128	0.972732	1.162723	0.998507	1.312103	1.118657	1.099579	1.04862
Q96AP7	1	0.720197	1	0.904749	1	0.957824	0.789914	0.913837	0.819161	0.930952	0.817218	1.062551	1.013523	0.457247	1.008603	0.340703	1.073487	0.601549
P06865	1	1.170149	1	1.134687	1	1.251734	1.007603	1.049205	0.980198	0.98098	0.978251	0.969074	1.289812	1.226299	1.270374	1.14626	1.300326	1.052456
Q9HC84	1	1.329121			1	1.247276	0.770984	0.996045			0.669018	0.888118	1.15951	1.033265			0.97661	0.870457
P05451	1	0.732235	1	0.637066	1	0.59431	1.406405	0.879186	0.680611	0.528413	1.054512	0.824023	0.821549	1.073236	0.640593	0.572272	0.914858	0.986996
P34932	1	1.355802	1	0.970702	1	0.989866	1.152931	1.019911	1.107785	1.062231	0.988751	1.117465	1.209076	1.240253	1.217624	1.068215	1.338511	1.123281
O95274	1	1.463447	1	0.931949	1	1.168969	1.05228	1.169998	1.07833	1.133743	1.092383	1.112411	1.371372	1.171118	1.200208	0.954099	1.171879	0.947219
P13535	1	4.091261	1	2.881113	1	2.519571	2.52786	1.702164	1.707915	0.620182	1.768904	1.085713	3.088959	2.058665	2.2057	4.286708	2.03795	4.077197
P00352	1	0.959666	1	1.09807	1	1.067652	1.122957	1.059763	1.008192	0.9581	1.149734	1.063617	1.222373	1.153885	0.65818	0.861187	0.804348	0.859856
O75015	1	1.009309	1	1.091365	1	1.06945	1.553538	1.408239	1.473684	1.435013	1.475213	1.510829	1.72105	1.617263	1.864059	1.506768	1.671096	1.621623
Q8WZA1	1	1.3058	1	1.096639	1	1.18158	1.145282	1.055001	1.06851	0.953402	1.016072	0.972837	1.416307	1.017893	0.885118	0.89266	1.070528	0.906104
O00757	1	0.685377	1	0.789711			1.11452	1.111343	0.893693	1.152883			0.860292	0.730816	0.853986	0.652542		
P17900	1	0.946933	1	1.036904	1	0.921956	0.756572	0.881872	0.815223	0.944418	0.683194	0.841172	0.899749	0.824113	1.020109	0.841203	0.89661	0.839018
A0A0B4J1	1	0.51728	1	0.505028	1	0.419892	0.601813	0.684966	0.544602	0.71534	0.527859	0.687521	0.431965	0.862817	0.430898	0.427658	0.406097	0.442048
O95831	1	1.018824	1	0.899481	1	1.056609	0.734709	0.914474	0.654473	1.000217	0.792249	0.759735	0.707045	0.919001	0.737583	1.14946	1.151946	0.932179
P49257	1	1.034853	1	0.971336	1	1.002327	0.923756	0.845481	0.820128	0.786593	0.996118	0.854631	0.765862	0.7412	0.746021	0.711621	0.629336	0.818777
Q14112	1	1.283492	1	1.034247	1	1.208861	0.893271	0.885771	0.665544	0.758264	1.00319	0.85284	1.104026	1.00616	0.775899	0.684233	0.792527	0.785936
P61224	1	1.047994	1	0.812621	1	0.858961	0.467717	0.705859	0.476302	0.641518	0.437366	0.602252	0.487322	0.515022	0.522436	0.641356	0.497846	0.505233
P07988	1	0.622293	1	0.609716	1	0.70945	1.093294	0.816101	0.961397	0.829072	1.097893	0.897484	0.914147	0.937583	0.698887	0.862367	0.85452	0.991682
Q9H6X2	1	0.919637	1	1.154462	1	0.931855	0.904384	0.622926	0.828982	0.651319	0.833816	0.674937	0.440903	0.621552	0.520739	0.62453	0.533941	0.623022
P80511	1	1.826904	1	1.997785	1	1.856699	0.675181	1.189698	0.62038	1.166428	0.617282	1.164939	1.52716	1.78732	1.525749	1.711826	1.542621	1.67447
Q99715	1	1.164093	1	1.235423	1	1.305615	0.797728	0.815362	0.820718	0.908574	0.746273	0.816342	0.887114	0.756645	1.191902	0.854442	0.908482	0.783642
P09382	1	1.336657	1	1.194235	1	1.03527	0.895505	0.851632	0.816488	0.801913	0.890396	0.927181	0.858001	0.793881	0.644416	0.780588	0.767482	0.736731

Q12866	1	1.102046	1	1.150335	1	1.084596	1.126125	1.094596	1.186329	1.11041	1.309528	1.170485	1.809878	1.349496	1.729472	1.282374	1.768056	1.38578
Q9NS98	1	1.266195	1	1.829089			1.042069	0.992413	1.149448	1.010483			1.566464	0.893936	1.251513	1.168474		
O95810	1	0.796728	1	1.0819	1	0.748246	0.287605	1.154329	0.484439	0.993426	0.309911	1.238539	0.4699	0.393992	0.45064	0.568767	0.333067	0.427657
Q5JRA6	1	1.040232	1	1.457397	1	1.061397	1.014439	1.018871	0.908588	1.342241	0.990724	0.99182	1.106269	1.581768	1.774863	1.875718	1.313538	1.191454
Q9UIB8	1	1.112002	1	0.787397	1	1.42828	1.029953	1.197119	1.105289	0.97269	1.130767	0.945985	1.026965	1.003943	0.920736	1.580876	0.898097	0.871748
P14207	1	0.901949	1	0.982847	1	1.405257	1.197115	1.034258	1.080196	1.114171	1.062202	1.104727	1.350472	1.04292	1.480274	1.060517	1.451054	1.02263
P07585	1	1.24142	1	1.499108	1	1.55174	1.015892	1.045161	0.995979	1.076083	0.995461	0.961826	1.299128	1.115867	1.351122	1.063308	1.345754	0.98253
P00568	1	1.278308	1	1.032659	1	1.275459	1.047441	1.434623	0.948066	1.690365	0.940956	1.514464	1.455938	1.003498	1.684656	0.825425	1.516701	0.942475
P25815	1	1.129691	1	1.164968	1	0.905943	0.867386	1.053646	0.808836	1.052112	0.723725	0.915455	1.241603	1.179457	1.183064	1.083286	1.130154	1.095649
Q15303	1	0.729795	1	0.594603	1	0.734665	0.980016	0.964747	0.851496	0.998541	0.720057	1.076038	1.135595	1.147264	1.468251	0.924095	1.425556	0.984245
Q16851	1	0.909402	1	0.846977	1	0.893347	1.039055	1.029794	1.116139	0.994734	1.012067	1.007256	1.074605	1.390309	0.996471	1.172076	1.079597	0.992013
Q14767	1	1.109515	1	0.903547	1	0.954846	1.077795	1.047794	0.888652	0.866316	0.887499	0.90317	1.442709	0.935025	1.07804	0.995645	1.136579	0.867086
Q8NFT8	1	1.055847	1	0.528343	1	0.53833	1.283545	0.889301	0.770949	0.908746	0.693784	0.852038	1.074044	1.291159	1.118909	0.847313	1.085201	0.749264
P05107	1	0.916818	1	1.006985	1	0.978776	0.833253	0.879042	0.835971	0.830595	0.864939	0.943504	0.99468	0.865257	0.861951	0.828802	1.000147	0.914355
Q9HD89	1	1.365757	1	0.990741	1	1.180941	1.121242	1.00124	1.201948	0.974733	1.185086	0.995756	1.159098	1.19304	0.915628	1.23211	0.991109	2.351145
Q8IUC0	1				1													
Q9P232	1	0.975588	1	0.952618	1	1.021218	0.969867	0.991539	0.999783	0.988172	1.001559	1.031226	1.324596	1.083278	1.234751	1.095892	1.254278	1.183734
P22061	1	1.322172	1	1.201165	1	1.236301	0.878329	0.567416	0.812384	1.085688	0.812486	1.066748	0.858802	1.069152	1.177331	0.920457	0.731651	1.231889
P02786	1	1.162361	1	0.947481	1	0.848937	1.273649	0.911594	0.676189	0.787684	0.916218	0.871526	1.003576	1.080771	0.78498	1.209457	0.719019	1.057078
P23284	1	0.767252	1	0.845092	1	0.755223	0.965278	1.288108	1.029566	1.601511	0.936394	1.374794	0.814932	1.117124	0.865981	1.067296	0.759392	1.032233
P01601	1	1.061889			1	0.48185	0.862134	0.805457			0.884701	0.900787	0.649671	0.727511			1.081712	0.874096
O14793	1	0.880503	1	0.87436	1	1.009245	0.864103	0.875973	0.895415	0.977701	1.064435	0.955717	0.781187	0.729966	0.589572	0.70295	0.822181	0.916552
O95998	1	1.18752	1	1.235064	1	1.27391	1.031692	1.081312	1.11051	1.108073	0.991865	1.018815	1.566947	1.077506	1.439514	1.05517	1.558855	1.097992
O15335	1	0.747522	1	0.949163	1	0.892673	0.973334	0.957987	0.960404	0.839228	0.915542	0.872578	0.769625	0.629249	0.59969	0.639693	0.734925	0.728637
Q08554	1	1.819677	1	1.182273	1	1.242644	0.961885	1.153553	1.023503	0.876827	1.114092	1.018103	1.134848	1.72272	1.000093	1.244228	1.028862	1.169398
P39748	1	0.926891	1	0.877164	1	0.961527	1.567981	1.523771	1.583437	1.505821	1.535153	1.642909	1.219172	1.096458	1.177203	1.254119	1.394646	1.313934
P13224	1	0.732271	1	0.912552	1	0.928291	0.169967	0.429276	0.410098	0.601505	0.176329	0.453072	0.1295	0.335156	0.271174	0.568029	0.047711	0.339377
O95428	1	1.17223	1	1.120692	1	1.010008	0.79603	0.917923	0.824829	0.921588	0.898818	0.852717	1.226647	0.835972	1.138065	0.850444	1.04457	0.955439
P11215	1	1.123093	1	0.86469	1	1.450119	0.692225	0.699465	0.977743	0.807	1.004119	0.939066	0.839247	0.886521	0.796548	0.821018	0.999375	1.040558
Q01518	1	1.153592	1	1.178645	1	1.25097	0.487623	2.30462	0.591714	2.397874	0.557426	2.715561	0.747298	0.611644	0.860328	0.7066	0.720165	0.616023
Q9H4B7	1	0.793603	1	1.190653	1	1.262844	0.429849	0.58886	0.675362	0.703056	0.714909	0.624826	0.503469	0.372737	0.814047	0.785483	1.833192	0.688517
P28070	1	0.975434					1.183673	1.039203					1.070826	1.544666				
O15204	1	1.005186	1	0.925098	1	1.168891	0.6527	0.938369	0.549327	0.876897	0.590896	1.150066	0.841568	0.867525	0.734046	0.864237	0.927205	1
Q93070	1	1.257132	1	1.271375	1	1.290142	1.115013	0.946724	1.174061	0.992969	1.10737	1.046497	1.376617	1.169188	1.028809	1.25887	1.101777	1.183738
O60667	1	0.430105					0.464749	0.688813					0.355452	0.519176				
Q8N149	1	0.935787	1	1.270763	1	1.236306	0.887931	0.846448	1.078785	1.101456	0.765986	1.213366	0.969503	0.828832	0.851239	1.182113	1.08148	1.039771
A0A075B(	1	0.666036	1	1.044971	1	0.411178	0.483177	0.768928	0.567217	0.754831	0.524393	0.624032	0.546159	0.640463	0.522063	0.66907	0.609711	0.651816
P53634	1	1.121397	1	0.853959	1	1.028945	1.132256	0.833808	0.91583	0.955444	1.21774	1.108071	1.045403	1.087064	0.96967	1.030793	1.120562	1.036728
P55000	1	1.533313	1	1.390674			0.957125	1.098967	0.950657	0.790942			1.527618	1.131822	0.879813	0.795055		
P23468	1	1.045335	1	0.969554	1	1.009721	0.964295	0.960119	1.153402	0.983859	1.157756	1.093973	0.90687	1.073234	0.951984	1.025721	1.029416	1.061303
Q96RI9	1	0.982099	1	1.076354	1	1.002839	5.978122	1.045337	5.13602	1.042759	6.175519	1.023775	0.346618	1.709547	0.29636	1.596084	0.357815	1.709294
P08833	1	1.190652	1	1.509999	1	1.368717	1.137195	0.923437	1.338975	0.974546	1.285883	0.907702	0.724235	0.93785	1.01468	1.12129	0.97448	1.020262
Q6UWP8	1	1.025682	1	0.961105	1	0.876259	0.744556	0.916013	0.893451	0.939598	0.829408	0.893185	0.888879	1.026158	0.790474	0.850325	0.881928	0.863973
P00480	1	1.670152	1	1.494659	1	1.460297	0.89311	2.310331	1.052245	1.3743	0.989898	1.522832	0.79992	1.449455	1.072677	1.26435	0.994706	1.186038
A0A0C4D	1	0.978077			1	1.787813	1.03286	1.192103			1.030746	0.613394	1.052891	0.927566			1.152038	1.068999
P49641	1	0.752143	1	0.893663	1	0.943056	0.936497	0.718489	0.994939	0.866717	1.01905	0.96182	0.523332	0.833122	0.60743	0.804035	0.817748	0.887077
P59666	1	0.778864	1	1.318688	1	1.365513	0.668434	0.959828	0.868201	1.192275	0.877737	1.241019	0.84995	0.70399	1.069209	1.079896	1.093565	1.115454
Q9NR99	1	0.733339	1	0.739982	1	0.569832	0.937331	0.975519	1.112142	1.060476	0.979875	1.077591	1.009866	1.124156	1.116516	1.181013	1.091209	1.159143
Q03154	1	1.099673	1	1.098647	1	0.872303	1.223681	1.173772	1.274325	1.273115	1.378843	1.062515	1.387782	1.449454	1.321904	1.396072	1.46894	1.839209
P13497	1	1.293131	1	1.123962	1	1.035615	0.921527	0.985236	1.021451	1.055134	0.969066	0.937859	0.975786	0.851538	0.944848	0.969165	0.984492	0.820966

Q03181	1	0.958016	1	1.028139	1	1.045389	1.537625	2.024754	1.520403	2.036457	1.558258	1.959315	1.505305	1.390346	1.469319	1.297429	1.46412	1.28152
Q9Y251	1	0.943925	1	0.926215	1	0.810352	0.756476	0.724859	0.692611	0.76891	0.76327	0.746651	0.634421	0.864117	0.684339	0.774301	0.841606	0.955202
P52565	1	1.092389	1	1.171048	1	1.161099	0.849053	1.156387	0.932827	1.191479	0.935359	1.095975	0.798423	0.857203	1.051794	0.954967	0.803501	0.913786
Q8TDY8	1	1.436475	1	1.275642	1	1.222917	1.032475	0.957325	1.000441	0.935134	1.233577	1.029121	1.203934	0.991553	0.96238	1.248152	0.926336	1.870896
Q8WWA0	1	0.933114	1	1.071614	1	0.953145	1.118151	0.996904	1.064581	1.049503	1.080723	0.966152	1.594276	1.697499	1.476893	1.564741	1.497152	1.571678
Q9P2X0	1	0.812444	1	0.877029			0.925204	0.950263	0.797366	1.017207			0.695387	0.658001	0.824844	0.725926		
O43895	1	1.113402	1	1.022816	1	1.076389	1.54147	1.167049	1.381678	1.113419	1.753871	1.28002	1.31671	1.224203	1.331793	1.01067	1.331353	1.130191
Q7Z4R8	1	1.95962	1	1.595796			0.872106	0.766414	1.040596	0.879801			0.867515	1.135973	1.00218	1.112505		
Q13867	1	0.709254	1	0.830482	1	0.892423	0.887369	0.834877	0.953934	0.944229	1.012953	0.780349	0.591037	0.564431	0.729839	0.523809	0.583158	0.582023
P06744	1	0.934968	1	1.065757	1	1	1.527212	1.093638	1.180718	1.014266	1.446236	1.11346	0.864586	1.393929	1.056721	1.023201	0.890692	1.244056
P35247	1	1.84253	1	1.349189	1	1.256336	0.861986	0.884179	0.777926	0.941035	1.038213	0.857102	0.915622	1.017921	0.755373	0.997962	0.879088	1.05116
P20774	1	0.865341	1	1.088538	1	1.004755	1.091508	1.039467	1.128194	0.975939	1.070427	1.051769	1.227019	0.964783	1.068306	0.979877	1.257456	1.039733
Q3LXA3	1	1.331249	1	0.850962	1	1.058618	1.028177	1.176881	0.84856	1.220411	0.894087	1.140619	1.067	0.704175	1.251036	0.633813	1.113691	0.723968
Q86UW7	1	0.683797	1	0.740334	1	0.78519	1.35195	0.929355	1.428055	1.002766	1.375021	1.014064	0.928747	0.456171	0.859232	0.468865	0.896154	0.413073
P30043	1	1.045252	1	1.171547	1	1.269986	0.975199	1.061725	0.996695	1.237079	0.996282	1.231693	1.554161	0.962479	1.601507	1.123371	1.458345	0.968272
O75976	1	0.862074	1	0.842909	1	0.936231	0.948778	0.844706	1.092441	0.998549	0.89181	0.923211	1.009877	0.965237	0.992722	1.055361	0.800677	1.059336
A0A075B(	1	0.377074	1	0.269765	1	0.579427	0.60466	0.626539	0.610794	0.530092	0.685683	0.677902	0.622621	0.604334	0.43948	0.46449	0.592791	0.62508
Q03167	1	0.532329	1	0.640306	1	0.787652	0.775522	0.664908	0.770814	0.847944	0.792673	0.788552	0.582837	0.660705	0.740366	0.855488	0.720438	0.773195
P08648	1	0.711052			1	1.136281	0.930191	0.865024			0.922075	0.942237	1.015132	1.050249			1.01699	0.883864
P05783	1	1.873898					1.394058	1.276585					1.465155	1.551388				
Q9HBW9	1	1.015099	1	1.02453	1	1.077561	1.213891	1.058003	1.377277	1.121353	1.320526	1.166465	1.180411	1.307734	1.16684	1.42349	1.130039	1.438019
P31150	1	1.033095	1	1.113434	1	0.974685	0.992924	0.934319	0.888506	0.927054	0.991356	0.948477	1.201227	0.933237	1.192667	0.961983	1.045839	1.001501
P06737	1	1.03393	1	1.082755	1	1.114701	0.893154	1.082764	0.86161	1.047704	1.019296	1.018019	1.253945	1.284322	1.120703	1.256087	1.096206	1.229725
Q6UXH0	1	1.029689	1	0.883428			0.758938	0.974786	0.554396	0.965594			0.839699	0.781751	0.960928	0.754128		
Q8N8Z6	1	0.886757	1	1.058586	1	0.937382	0.943433	0.972372	0.939711	1.043799	0.969248	0.888049	1.141449	0.826875	1.218614	0.996497	1.043657	0.804965
P45880	1	1.466612	1	1.277982	1	0.99289	1.043072	0.732528	1.463207	0.371128	1.30017	0.43929	0.828371	0.572246	0.591427	0.414833	0.788672	0.335401
A0A0A0M	1	1.223422	1	1.149614	1	1.732858	0.852962	0.708987	0.869768	0.749549	0.838503	0.886873	0.549691	0.567885	0.457242	0.53696	0.968555	0.728128
P09603	1	0.774728	1	1.145486	1	0.940235	1.357363	1.042726	1.577213	1.138172	1.090903	1.010534	1.043905	1.145791	1.115935	1.239872	1.192887	1.320945
P01701	1	0.743358	1	0.769872	1	0.852022	0.821906	0.506918	0.806378	0.60757	0.790607	0.619417	0.500469	0.562431	0.490807	0.636847	0.512644	0.594445
P09467	1	1.77375	1	1.468002	1	1.460873	0.976853	1.107794	1.490413	1.26658	1.659504	1.168812	0.854905	0.954601	1.242859	1.284203	0.887029	0.918155
Q01469	1	0.858503	1	0.653192	1	0.536244	0.794499	1.325785	0.976979	1.285917	0.844345	1.298422	0.890082	1.07457	1.095987	0.571744	0.914266	0.682507
P18850	1	1.18855	1	0.688729	1	0.905269	0.941366	1.057944	0.760148	0.974274	0.851925	1.031775	1.476353	1.180854	1.282857	1.246781	1.409467	1.260549
Q8IWL1	1	1.927191	1	1.012266	1	0.92503	0.958539	0.943251	0.924063	0.850434	1.040502	1.000209	0.612547	0.957897	0.915104	0.995381	1.070524	1.173495
Q9GZT8	1	0.683668	1	1.386268	1	0.764468	0.93584	0.952064	0.86739	0.922748	0.894094	1.000301	1.013694	0.877145	1.10029	0.928807	0.888859	0.795681
Q8N335	1	1.62986					1.626606	0.797621					1.003873	1.121862				
P25787	1	1.129861	1	1.41235	1	1.107851	1.291315	1.0502	1.465433	1.451932	1.082464	1.185759	1.371228	1.321898	1.546683	1.509726	1.383638	1.297
Q8TF66	1	0.595326	1	0.588447			0.976083	22.36804	1.471603	27.0263			1.570149	0.219371	1.379386	0.456121		
P03973	1	0.657511	1	0.594985	1	0.591738	1.020927	1.068272	1.027689	1.207255	0.959888	1.083034	1.018529	0.804342	0.957281	0.795943	0.990973	0.633314
P40925	1	1.361055	1	0.931013	1	1.126145	1.497449	0.936802	1.044696	0.924103	1.094084	0.942132	0.936012	0.946947	0.865185	0.870848	0.986179	0.80118
P21741	1		1	0.351829	1		7.881504	13.78516	4.200194	6.452787	7.717663	13.23008	6.890749	0.425983	3.69251	0.616699	7.157712	0.514794
P09622	1	0.969122	1	0.906374	1	0.87106	0.856814	1.07175	0.994906	1.122528	1.004841	1.075905	1.06323	0.858332	1.011431	1.023193	1.106598	0.850608
A0A0B4J1	1	0.801821	1	0.733519	1	0.660091	0.79635	0.777523	0.587859	0.802262	0.594284	0.776863	0.659761	0.933423	0.580179	0.764107	0.603931	0.799092
Q8NHL6	1	0.295083	1	1.692154	1	1.461838	1.39224	1.011462	1.137987	1.217717	1.110578	1.007408	0.802506	0.9181	1.301098	1.360481	1.243459	1.146625
Q7RTS7	1	1.723881					1.301979	1.023933					1.069153	1.422102				
P04211	1	0.851228					0.628039	0.442674					0.428113	0.59605				
Q9NQ76	1	1.632242	1	1.272885	1	1.127813	0.670543	0.789872	0.952737	0.921422	1.164314	0.956613	0.909378	0.931523	0.984549	0.978293	0.90551	0.973974
O00560	1	2.27175					1.269617	1.211948					1.089715	0.912851				
Q68CJ9	1	1.242595	1	1.120916	1	1.601314	1.342876	1.311731	1.031955	1.017227	1.247367	1.044751	1.313852	1.191051	1.24136	0.900541	1.568989	1.178302
A0A075B(	1	0.368752	1	0.419184	1	0.521363	0.206526	0.572301	0.32176	0.602695	0.287087	0.682688	0.270413	0.456261	0.382902	0.512746	0.408722	0.48039
P15090	1	1.084489	1	1.296643	1	1.29759	0.850742	0.773167	1.166098	1.0188	1.084955	0.900196	1.281185	0.655023	1.88065	0.435109	1.69017	0.797763

P20062	1	1.125458	1	1.014086	1	1.001454	1.298724	1.216867	1.508485	1.093291	1.329257	1.154932	1.391122	1.515954	1.267784	1.466557	1.282797	1.603509
Q9H8J5	1	2.221307	1	1.222402	1	1.477278	1.607067	1.083528	1.563383	1.136302	1.175102	0.999864	0.993119	1.210859	0.999734	1.224692	1.006234	1.014557
P00325	1	0.813766	1	0.908167	1	0.805906	0.736235	0.96623	0.845103	1.018104	0.865169	1.103948	0.828812	0.689156	0.852724	0.77396	0.965168	0.795085
Q12882	1	1.210816					1.064721	1.015503					1.124733	1.33054				
Q92484	1	1.525289	1	0.723632	1	0.934877	1.479284	1.020473	1.237418	1.004367	1.423759	1.076246	1.568434	1.385602	1.244099	1.085322	1.289221	1.242831
A0A075B(	1	0.474261	1	0.478462	1	0.473825	0.745926	0.63091	0.711422	0.732391	0.817515	0.693181	0.528354	0.56758	0.582391	0.591003	0.652514	0.624776
P09493	1	0.776285			1	0.808575	1.510348	1.057078			0.563474	1.121805	1.119837	1.095767			0.835051	0.44452
Q9UBQ6	1	1.280829	1	1.136771	1	1.096839	1.163535	0.932655	0.981501	0.973346	1.038684	0.978705	1.032962	0.815657	0.999163	0.99025	1.065744	1.075655
Q9C075	1	1.06098					1.971314	1.094728					0.882819	2.250173				
Q7Z5L0	1	1.79412	1	1.343132	1	1.277674	0.818839	0.912425	0.65506	0.819815	0.662744	0.817349	0.814109	0.9125	0.999907	0.750524	0.879805	0.763053
Q13308	1	1.375092	1	1.187495	1	1.054146	1.054822	0.904944	0.936582	0.993272	1.00739	1.020625	1.327171	1.141907	1.125448	0.993288	1.062859	1.106306
P13521	1	1.161828	1	1.219073	1	1.042304	1.127223	1.462342	1.106084	1.200023	1.146475	1.18167	0.94601	1.026337	1.128805	0.770181	1.323662	0.850319
Q14508	1	1.068359	1	1.150584			1.011306	1.061228	0.855405	1.048871			1.436989	1.350981	1.029743	1.02666		
Q9Y240	1	0.746245	1	0.836983	1	0.902828	0.980911	0.939221	1.148974	1.021921	1.041167	1.038342	1.018027	0.81548	0.894419	0.864758	0.944297	0.922911
Q13630	1	1.689034			1	1.358613	0.787109	0.876216			0.806774	0.969523	1.534399	1.04373			1.507124	0.956816
A0A0C4D	1	1.058349	1	1.438287	1	1.660971	1.850045	1.057462	1.179847	1.089392	1.342025	1.01381	0.756329	0.815252	1.672173	1.134817	1.355037	1.283456
P21709	1	1.598735	1	1.370602	1	1.292935	1.12429	1.05952	0.885495	0.948239	0.999192	0.910968	0.861941	0.945382	0.714783	0.85517	0.922309	0.887965
P61160	1	1.194452	1	1.433551			0.92629	0.938728	1.065045	0.912393			0.918533	0.942708	0.806413	0.941201		
P21695	1	1.25232	1	0.914404	1	0.87994	1.269948	1.117671	1.07569	0.988734	1.079232	1.030206	1.099063	0.977264	1.013107	0.94816	1.035503	0.897107
Q16620	1	1.597255	1	1.335718	1	1.274384	1.054052	0.905878	1.239036	1.112264	1.283316	1.023566	1.183724	0.970666	1.056069	1.167532	1.152159	1.12958
P08123	1	1.261647			1	1.159266	0.940857	1.113206			0.803644	1.232831	1.004884	0.954319			1.09251	0.978169
Q99542	1	1.502275	1	1.304095			1.245401	1.062225	0.932174	0.889242			1.509494	1.318501	1.623668	1.165886		
Q9BRK5	1	1.43392	1	1.812381	1	1.902735	1.586266	1.137289	1.216111	1.127355	1.366659	1.300597	1.223417	0.94222	1.199533	1.02967	1.337413	1.26559
P08473	1	0.927627	1	1.29915			0.781361	1.211996	0.762996	1.71093			0.409171	1.00404	1.193688	1.124825		
P07384	1	1.068744	1	0.844777	1	0.865439	0.977843	0.930469	1.253193	0.845827	1.265527	1.016452	0.98645	0.972339	0.753357	0.834574	0.986839	1.219231
Q58EX2	1	1.258134	1	0.701969	1	1.187362	0.988653	1.052519	1.507349	1.397939	1.148615	1.052748	1.240686	0.604724	1.618843	0.849796	1.006288	0.752097
Q99497	1	1.196346	1	1.38499	1	1.317707	0.881177	1.611895	0.896066	1.29555	1.102399	1.892285	1.417647	0.917051	1.349225	0.95815	1.482303	0.936702
P68104	1	0.713204	1	0.721037	1	0.66951	1.472711	1.258939	3.033791	1.273536	3.933117	1.137384	0.595259	0.972078	0.573263	1.722335	0.679239	1.99086
P62491	1	1.173623	1	1.161615	1	1.331774	0.668513	1.338583	0.656781	1.317138	0.612294	1.808769	0.57013	0.635737	0.661103	0.667896	0.544304	0.601572
O15143	1	1.118861	1	1.144093	1	1.191881	0.996086	0.87836	0.752671	0.906526	0.834408	0.956284	0.87169	0.758574	1.063091	0.551342	1.043592	0.609351
Q9NZD4	1	1.059727	1	0.920906			0.904965	1.064969	1.11255	1.158039			1.314886	1.028845	1.692479	1.213953		
P15531	1	1.659033	1	0.668866	1	0.593524	0.848478	1.141177	1.013925	1.160512	0.90712	1.170456	1.69226	1.115788	1.4507	0.87912	1.476209	0.805209
P23467	1	1.379189	1	1.16123	1	0.705243	0.900552	1.02326	1.118884	0.956743	0.824973	0.75944	1.328531	0.950693	1.198764	0.763056	1.124524	0.699747
Q02809	1	1.113459	1	0.711989	1	0.803327	0.824703	0.881866	0.927207	0.888437	0.861145	0.971865	1.354067	1.223305	1.338585	1.007264	1.573245	1.076562
P61764	1	1.021169	1	0.977722	1	0.95707	1.140165	0.916302	0.928554	0.782724	1.067001	0.909854	0.832268	0.669599	0.971797	0.83529	0.888418	0.805135
Q99426	1	1.651055	1	1.702418	1	1.571707	0.708281	2.122947	0.638221	2.105582	0.522143	2.399377	0.997221	0.636619	0.976406	0.647961	0.764435	0.656673
P14770	1	1.098824	1	0.823167	1	1.374011	0.693087	0.681501	0.236493	0.511986	1.257184	0.999414	0.696791	0.528894	0.230931	0.211399	0.944795	1.055484
Q8TAQ9	1	0.873245	1	0.785193	1	0.820102	0.91071	1.1357	0.923003	1.17244	0.926267	1.169003	1.268121	1.113585	1.366583	1.07647	1.300145	1.149384
P31431	1	1.076511	1	0.987317	1	0.93848	0.843157	0.876841	0.790336	0.986277	0.809114	0.875336	0.738881	0.891052	1.151616	1.100036	0.79838	0.966222
P27105	1	1.168691	1	1.056279	1	0.866685	0.676069	0.666188	0.599113	0.67354	0.642944	0.58027	0.728746	0.686035	0.655981	0.625577	0.474305	0.614732
P14209	1	1.011652	1	0.964662	1	1.163071	1.009062	0.895949	0.875895	0.84538	0.855605	0.822211	1.011608	1.055884	0.85482	0.794852	0.947973	0.824295
P01706	1	0.774843	1	0.991129	1	1.146363	0.819944	0.770182	0.708776	0.765725	0.774625	0.79915	0.82616	1.074463	0.804657	0.94892	0.854086	0.877946
Q96J42	1	1.363751	1	1.253228	1	1.28815	1.012412	1.065179	1.289905	0.990987	0.938855	0.835797	0.924776	1.387482	1.045785	1.245005	0.954072	0.999958
Q9NS71	1	1.049111	1	1.109803	1	1.276706	1.398593	1.133312	1.399662	0.955526	1.461445	1.109036	1.556341	1.617757	1.370304	1.576973	1.473239	1.821081
Q9UMX5	1	1.443433	1	1.244648	1	1.269747	0.88066	0.607609	0.880795	0.717034	0.929837	0.85298	0.594175	0.889911	0.535114	0.839812	0.731139	0.794898
P10912	1	1.030204	1	1.016606	1	0.95735	1.162238	1.177775	1.237623	0.997475	1.14687	0.952432	0.876237	1.187331	0.864039	1.092094	1.014737	1.149842
Q9UKU6	1	0.886823	1	0.760151	1	0.600928	1.128705	0.963749	1.00158	0.984534	1.091524	0.946424	0.948626	0.985256	1.030234	0.920047	0.802725	0.998879
P09326	1	1.180246	1	1.194798	1	0.911306	0.967455	1.027128	0.985647	1.120639	1.024843	1.098476	1.223094	1.264027	1.235762	1.249626	1.106584	0.892033
P19021	1	1.095293	1	1.102194	1	0.964142	1.092352	0.997491	0.908776	0.944663	1.047573	0.964581	1.073123	0.899184	1.059706	1.000177	1.051437	0.974557
Q9HAT2	1	0.752751	1	0.862466	1	0.838127	0.929343	0.885729	0.992802	1.042691	1.1112	1.011283	0.905321	0.807392	0.87037	0.974167	0.770421	0.990836

P55287	1	0.618747	1	0.683556	1	0.837795	0.579452	0.962944	0.781233	0.894405	0.948971	0.785773	0.84142	0.78874	0.731112	0.916721	0.670238	0.642624
O14498	1	1.51184	1	1.102437	1	1.189992	0.99237	0.88776	0.879401	0.833243	0.908187	0.855544	0.978973	1.027006	0.938326	0.912366	0.907445	0.925293
P13639	1	0.818239	1	1.387895	1	1.602563	0.874101	1.119383	0.873335	1.144993	1.027655	1.031858	0.8768	0.861407	1.065214	0.86226	0.911619	0.769494
Q13637	1	2.098946					0.847645	1.152079					1.286705	1.016981				
Q5D862	1	0.644963	1	0.792705	1	0.693882	1.626201	1.604596	1.228272	1.526433	1.516179	1.368334	0.740753	1.888575	0.828517	2.91694	0.933384	1.900598
P28066	1	1.052358	1	0.871534	1	1.031643	0.8946	1.117888	0.710144	1.06829	1.137331	1.188847	1.540522	1.119693	1.289672	0.847895	1.697929	1.028327
P28838	1	1.115213	1	1.00084	1	1.14075	1.176138	1.362174	1.119443	1.828103	1.201484	1.661703	1.13515	1.2305	1.365069	1.539194	1.408711	1.665169
Q99436	1	0.799897	1	0.666694	1	0.89035	0.793728	1.074795	0.554394	0.910926	0.833605	1.122762	1.286621	1.466723	1.168107	1.177492	1.417005	1.516456
Q92896	1	1.264714	1	1.260018	1	1.26351	0.797861	0.896288	0.724963	0.989663	0.822098	0.923713	1.005773	0.905935	0.982733	0.972841	1.059213	0.864716
O60641	1	1.830766	1	1.785853	1	1.38432	1.190406	0.672785	1.173902	0.728886	1.412911	0.513302	0.940923	0.56839	0.996344	0.524294	1.132704	0.603379
A0A0A0M	1	1.015516	1	0.959564	1	0.81146	0.492355	0.576648	0.616758	0.6617	0.499382	0.546648	0.534318	0.573999	0.620644	0.662933	0.534203	0.523333
P48960	1	0.679622	1	0.928455	1	0.845376	0.825996	1.059642	0.992702	1.084201	0.982356	1.126778	1.183063	0.902393	1.142067	1.067516	1.166485	1.199222
O60279	1	0.956124	1	2.278101	1	0.936384	1.030048	0.88698	1.379028	0.946715	0.957964	0.950884	1.087825	0.982048	1.133317	1.088494	1.093817	0.821971
Q9NSC7	1	1.91892	1	2.104733	1	2.383611	1.356223	1.670298	1.113681	1.411527	1.397725	1.42778	3.868426	2.570601	2.529098	2.459864	2.9682	2.125682
Q9BYJ0	1	0.951969	1	0.719933	1	1.008308	0.884297	0.730828	0.911043	0.77853	0.909278	0.725079	0.748483	0.907128	0.531717	0.777588	0.610801	0.977878
Q9BXS4	1	0.939174	1	1.111302	1	1.031784	0.837772	0.781026	0.787231	0.668928	0.762348	0.790128	0.826937	1.083906	0.725285	0.902292	0.688282	0.901673
Q96KG7	1	0.898761	1	0.997391	1	1.410519	0.522053	0.777708	0.783751	0.793126	0.765616	0.865681	0.646941	0.464981	0.907136	0.663107	0.868355	0.663919
P12110	1	0.936908	1	0.988714	1	1.055568	1.079849	0.964127	1.214182	1.034225	1.144803	0.966394	0.93182	0.874126	1.005347	1.003812	0.986004	1.082564
P16152	1	1.36418	1	1.201082	1	1.290932	0.990978	1.086644	1.099181	1.08114	0.988998	0.880345	1.205987	1.168839	0.858449	1.125513	1.069707	0.924065
Q15746	1	1.281643					0.862929	1.750563					0.937206	0.833703				
Q9BXP2	1	0.679712	1	0.662206			1.542688	1.029616	1.600312	1.036103			0.537001	1.023349	0.747807	1.147667		
P55285	1	0.770778	1	0.848269	1	0.928567	0.58353	0.899912	0.562932	0.848389	0.596022	0.856182	0.639403	0.540579	0.637542	0.554456	0.634405	0.579077
P15311	1	1.08214	1	0.983327	1	0.985949	1.03754	0.990239	0.885899	0.965406	0.867205	0.98295	1.101439	1.045989	1.022809	0.924625	1.07941	1.024866
P40429	1		1		1													
Q9ULC0	1	1.564784			1	1.353484	1.085513	1.161581			1.094428	1.359761	1.249111	1.1614			1.559796	1.279632
Q9BQ51	1	1.301054	1	1.398952	1	1.339174	1.222362	1.154911	1.517127	1.265837	1.128012	1.043486	0.845926	1.216934	0.938489	1.253135	0.843178	1.007156
O14960	1	0.659662	1	0.674197	1	0.644542	0.95185	0.957416	0.941542	0.849694	0.995613	0.929309	0.671693	0.669439	0.593526	0.643646	0.632349	0.665949
Q86VP6	1	1.150244	1	1.027323	1	0.846046	1.107618	1.18523	1.116526	1.112015	0.797787	1.097506	1.540951		0.995875	0.385702	1.471994	0.703382
Q08495	1	0.975312	1	1.032119	1	1.273068	0.654811	1.019037	0.460875	0.999783	0.646766	0.991509	0.936611	0.655212	1.170454	0.578852	1.108086	0.785024
Q6UXK5	1	1.108912	1	1.612147	1	1.427519	0.812089	0.855714	0.68243	0.779329	0.722365	0.922316	0.863771	0.853602	0.948111	0.786123	1.105522	0.946277
Q9UI15	1	1.326602	1	1.362871	1	1.467417	1.145078	0.594807	1.109449	0.528523	1.339204	0.668406	0.793201	0.700962	0.878387	0.638225	0.930319	0.598097
P62328	1	1.371187	1	1.395186	1	1.431369	0.385644	1.923629	0.420246	1.770579	0.35583	1.868492	1.002922	1.2888	0.98617	1.386893	0.908736	1.393101
Q9BU40	1	1.102553	1	0.767255	1	0.766523	1.365786	1.184553	0.990708	0.701278	0.815258	0.63512	1.171737	1.040697	0.958508	0.614023	0.77927	0.869416
P35052	1	0.824786	1	0.719749	1	0.780128	1.068273	1.091964	1.213678	0.937715	1.393397	1.109794	1.048817	0.949013	1.252972	1.047012	1.226352	1.314486
P15088	1	0.482543	1	0.566715	1	0.486004	1.28686	0.919167	1.325374	0.966768	1.245763	0.931457	0.828834	0.789566	0.776155	0.877785	0.797641	0.834317
Q14847	1	0.876189	1	0.946753	1	0.863579	0.855062	1.336469	0.741344	1.201574	0.797907	1.342025	1.302359	0.86003	1.112495	0.838802	1.02198	0.813823
A0A0B4J1	1	0.752092	1	0.695645	1	0.689848	0.774641	0.543151	0.603055	0.677592	0.672642	0.529152	0.679315	0.695533	0.693197	0.679787	0.595908	0.620453
Q9UKX3	1	1.156126					1.031104	1.043504					0.667752	0.975515				
Q9BUD6	1	0.680625	1	0.727025	1	0.950816	2.112532	0.891244	1.244886	0.752344	1.894466	0.833036	0.586469	1.411539	0.603325	1.09527	0.758333	1.268047
Q8NCC3	1	1.220197	1	1.63617	1	1.257149	1.135576	1.027762	1.046334	1.063488	1.293587	1.095352	1.284882	1.067641	1.33418	0.949913	1.482713	0.981641
P61158	1	0.870078	1	0.770226	1	1.13593	0.787706	0.654731	0.894835	0.663097	0.812386	0.676168	0.844127	0.565765	0.685049	0.693988	0.639601	0.612651
Q9H0U4	1	1.361612	1	1.302109	1	1.377472	0.742074	1.210324	0.632712	1.2639	0.611167	1.044603	0.900222	1.085503	1.070393	1.1286	1.108814	1.045983
Q15465	1	0.999279	1	1.082463			0.868968	0.87234	0.945709	0.845129			1.094093	1.045498	0.887884	0.875104		
P22894	1	1.390225	1	1.233802	1	1.279566	0.636089	0.78056	0.712303	0.833544	0.620658	0.787902	0.714105	0.90438	0.682072	0.955017	0.674899	0.933464
Q16769	1	1.013139	1	1.189914	1	1.15674	1.043239	1.344113	1.182777	1.329284	1.006664	1.14682	1.377539	2.121419	1.330398	1.052771	1.404843	2.098896
P35968	1	1.126818			1	0.973823	1.296161	1.090178			1.339207	1.174357	0.857549	1.180593			0.904362	1.266608
P05121	1	1.048932			1	0.724106	1.504723	0.83322			1.072935	0.64164	0.48388	1.578584			0.391411	1.060514
P26447	1	1.273966	1	1.373405	1	1.248886	0.82537	1.05696	0.837555	1.200094	0.825994	1.07336	1.509821	0.96507	1.387996	1.009208	1.323375	1.021098
Q6UX06	1	1.276646	1	1.188861	1	1.551805	0.611602	0.468831	0.734652	0.72004	0.624557	0.654054	0.50196	0.606857	0.651174	0.682971	0.592129	0.667827
P01714	1	0.425415	1	0.986077	1	0.471194	0.698885	0.531133	0.828171	0.671228	0.688982	0.537082	0.367064	0.332862	0.797639	0.551344	0.398395	0.361025

O15144	1	0.880983	1	1.36319	1	1.314761	0.915299	0.842724	1.113771	0.891336	1.030722	0.836162	0.722524	0.730941	0.96344	1.054522	0.841317	1.072181
Q06141	1	0.872441	1	0.859099	1	0.744969	1.52565	1.02778	1.555181	1.230487	1.616655	1.15109	1.612385	1.811216	1.818984	1.280758	1.66031	1.540667
P62826	1	0.95607	1	1.376509	1	1.499128	0.931703	1.046575	0.992459	1.372411	0.964776	1.464364	0.970805	1.00726	1.586172	1.191147	1.491851	1.149065
Q9NYU2	1	0.567306			1	1.094488	0.939598	0.692813			0.915119	0.989552	0.699015	0.928898			1.037538	1.07111
P02795	1	0.371122	1	0.958601	1	0.647795	0.738513	1.58852	0.799507	1.5639	0.652105	1.535589	1.876376	0.857209	2.072089	1.102237	1.926329	0.92814
Q9H4F8	1	0.795337			1	0.872078	2.59623	3.22297			2.844614	3.723004	1.938972	0.849372			2.369867	0.923157
Q6ZRP7	1	1.0745	1	1.062837	1	1.102833	0.880841	1.129687	0.89534	1.174894	0.914853	1.124511	1.051373	0.923804	0.958834	0.78397	1.02878	0.867358
Q9Y279	1	1.006795	1	1.143963	1	1.119081	1.201783	1.283973	1.318673	1.334469	1.272976	1.272311	2.377051	1.488211	2.446026	1.589633	2.874863	1.97298
Q9C0C4	1	1.200508	1	1.085992	1	1.111855	1.065398	0.978111	0.970797	0.965351	0.922541	0.988857	0.998041	1.111197	0.923694	0.843054	1.16665	0.954868
Q9ULV4	1	1.725811	1	1.328164	1	1.61979	0.738368	3.763037	0.559109	3.170811	0.564351	2.876213	0.832943	1.580841	0.474146	1.161151	0.579496	1.455066
O43598	1	0.864954					1.28552	1.593545					0.740502	1.206304				
Q9NT99	1	1.591072	1	1.011208	1	1.297096	1.081801	0.911248	1.198749	1.064516	1.325458	1.091606	1.002045	1.229725	1.069099	1.276901	1	1.633614
Q8IZM9	1	0.633697	1	0.574805	1	0.618181	1.137068	1.048122	1.14487	1.041976	1.148983	1.069595	0.775303	1.415056	0.773949	1.382681	0.746977	1.71229
Q17RQ9	1	1.006643	1	1.077951	1	1.072967	0.806958	0.921345	0.735522	0.900022	0.834836	0.833688	0.986482	0.737992	0.89766	0.689996	0.891672	0.703694
P09471	1	1.399649	1	2.086879	1	1.018835	1.153668	0.666234	1.038107		0.819251	0.696265	1.120025	0.662316		0.578322		0.6065
O15145	1	1.358516	1	1.199105	1	1.171319	0.967311	0.961068	0.970349	0.996815	1.22851	0.986566	1.058299	0.975014	1.130427	0.943565	1.058399	1.094001
Q15293	1	0.824417	1	0.949948	1	0.871621	0.696544	0.904647	0.836205	1.045121	0.874926	0.895461	0.900634	0.689334	1.027502	0.811294	0.900355	0.887031
P43304	1	1.304895			1	1.615421	1.331752	0.955461			1.846427	1.20784	0.992192	0.813596			1.646284	1.165468
P61106	1	1.275062					0.565011	1.290485					0.710588	0.841324				
Q8IVW4	1	0.645041	1	0.595895	1	0.639274	1.215778	0.939573	1.099035	0.87055	1.173293	0.941298	1.082073	1.176541	0.928716	1.05779	1.146685	1.10684
P20700	1	0.244717			1	0.314229	1.656221	1.036506			1.382734	1.099131	0.469634	0.805372			0.413757	0.789828
P07108	1	1.325903	1	1.245212	1	1.013941	0.880374	1.253988	0.74825	1.185021	0.818962	1.28116	1.139309	0.93369	1.117368	0.83598	0.992426	0.814334
Q9NR34	1	0.977634	1	1.080514	1	1.043003	1.399756	1.091161	0.965777	1.157381	1.583959	1.093242	0.973857	1.230901	1.506326	1.108245	1.130476	1.067271
Q9BWV1	1	0.914908					0.653071	0.646829					0.579341	0.588109				
Q01973	1	0.705634			1	0.977269	0.888677	0.912965			0.882274	0.758842	0.814917	0.538342			0.873168	0.676462
Q16787	1	0.87385	1	0.927795	1	0.854363	1.056032	1.04117	0.725974	0.92299	0.648436	0.791414	0.936517	1.0659	1.333468	0.797712	1.241088	0.84762
Q96FE7	1	1.414608	1	0.825467	1	1.187043	1.458642	1.196397	1.301088	1.067999	1.290661	1.060082	1.381336	1.214522	1.408497	1.035538	1.182922	1.158239
P48539	1	0.963918					0.906324	0.743527					0.743362	0.51446				
P48723	1	1.143699	1	1.158348	1	0.88473	1.156018	0.8255	1.058576	0.815136	0.944888	0.659659	0.947191	1.033207	1.099072	1.041367	1.001023	0.889478
Q96PD2	1	1.486107	1	1.11409	1	1.178167	1.204754	1.135814	1.144149	0.92399	0.88458	1.005939	1.700231	1.231334	1.258654	1.190873	1.403941	1.036612
O43278	1	1.034894	1	1.234347	1	1.190108	1.075627	1.126976	1.166992	1.066913	1.245666	1.025545	1.114681	0.864381	1.158918	1.080783	1.116345	1.060442
Q14019	1	0.685252	1	1.138773	1	1.101561	0.605546	1.438807	0.651335	1.597658	0.49281	1.603473	0.725478	0.509093	0.790157	0.599459	0.637101	0.590298
P52790	1	0.884225	1	0.984582	1	1.07147	0.808699	0.879126	0.682229	1.026886	0.912229	1.039908	1.210422	0.780848	1.173178	0.863335	1.235834	0.977876
Q86SF2	1	0.586103	1	0.967723	1	1.363775	1.319645	1.050394	1.012095	0.79066	1.106253	1.15793	0.785552	0.866794	0.98997	0.770849	0.873488	0.868351
Q86Z14	1	0.525737	1	0.485284	1	0.472505	1.883216	1.850171	1.966613	1.897748	1.921333	1.859332	2.204686	1.188752	2.114843	1.250015	2.204757	1.163222
Q13217	1	0.956354	1	0.844575			0.688965	0.958052	0.680243	0.790496			1.202649	1.032279	0.938662	1.103478		
Q9H299	1	0.618591	1	0.711376	1	1.057154	0.366077	1.582274	0.364256	1.379576	0.522965	1.474253	0.738161	0.409231	0.705099	0.621925	0.751646	0.5726
Q15223	1	1.034716	1	0.912637	1	1.104911	1.275116	1.140927	1.394483	1.089038	1.316599	1.148372	0.788389	1.113835	0.679296	1.212475	0.812618	1.284212
O95897	1	0.922067	1	0.879422	1	0.829917	1.020977	1.045766	0.99222	0.917139	0.9331	0.873255	1.168332	1.009628	0.935733	0.973527	0.879714	0.999221
Q96KK5	1	0.562647	1	1.442878	1	1.923132	0.943612	1.100715	1.010449	0.602249	1.020567	0.833102	0.457382	0.648674	0.145217	0.778333	0.47533	0.911131
P31949	1	0.675225	1	0.852249	1	0.879852	0.730798	0.845038	0.737347	0.941751	0.770573	0.932731	0.97504	0.938759	0.843111	0.957714	0.889922	1.035155
P61978	1	0.926547	1	1.204087	1	0.969384	1.32565	0.670747	1.835716	1.133342	0.869275	0.569891	0.876277	1.406059	1.025285	1.382522	0.896995	0.887242
Q08174	1	1.032823			1	1.546673	0.86953	0.968163			0.878698	0.993713	0.991569	0.719237			0.704749	0.782946
P13686	1	1.76455	1	2.16501			0.875405	0.991795	1.20172	0.997718			1.130845	1.465805	1.123073	1.649711		
Q9NUQ9	1	0.935033					1.405185	1.079278					1.280303	1.182112				
P50552	1	1.640595	1	1.408193	1	1.138972	0.904067	1.693914	1.114675	1.215167	0.662256	1.596841	0.854068	0.907708	1.03052	0.815542	0.556603	0.663183
P61088	1	1.809878					0.851572	1.663385					0.781058	1.139577				
P0DPA2	1	1.163364			1	0.881848	1.222538	4.16573			1.10074	4.617903	0.994092	1.790276			1.183081	1.855659
Q08830	1	0.659793	1	0.781849	1	0.779162	0.761652	0.6208	0.862113	0.894501	0.67622	0.842982	1.122827	3.007189	1.184801	1.987412	1.193242	2.017315
O00244	1	0.967106					1.685656	1.172541					1.536862	2.280553				

Q08257	1	1.23756	1	1.003124	1	1.127086	0.726616	1.034312	0.819504	1.03685	0.764533	0.966814	0.879829	1.177208	0.830969	0.955379	0.867879	1.341826
O75326	1	0.99334	1	1.449143	1	1.406914	1.265981	1.103547	1.047097	1.131591	1.061553	1.028835	1.874207	1.319079	1.830623	1.152661	1.783544	1.258571
O00194	1	0.87869	1	0.709373	1	0.541912	0.717105	1.2522	0.470471	1.085233	0.398123	1.208613	0.481626	0.674357	0.539915	0.565552	0.409463	0.514072
P48509	1	1.32288					0.427242	0.649537					0.57478	0.536955				
P04424	1	1.333282	1	0.962825	1	0.814406	1.634636	1.27405	1.377705	0.921084	1.075549	1.430771	1.287273	1.244098	0.970619	0.87811	1.220824	1.209214
Q02747	1	1.143254	1	1.240924	1	1.149655	0.917116	1.081307	1.273445	1.244796	0.968014	1.283407	1.189823	0.847164	1.291835	0.975895	1.155607	0.894703
O75563	1	1.606199					0.55385	1.061178					0.771996	0.746347				
Q9HC57	1	0.61551	1	0.210409			0.66908	0.880479	0.449501	0.76831			0.775415	0.705941	0.868929	0.543355		
P04085	1	0.983629	1	1.266282			0.762975	0.740132	0.7756	0.85035			0.883227	1.346895	0.949228	1.061153		
Q6IPM2	1	1.663674	1	1.160589	1	1.059834	1.120516	0.945353	1.179328	1.365861	1.211425	1.397398	1.73224	3.854091	1.832305	2.183341	2.63281	2.974453
P13473	1	1.187376	1	1.123784	1	1.240696	1.030551	0.962056	0.966386	0.991789	1.058977	1.179769	1.014197	0.90603	1.104626	1.069238	1.288324	1.198835
Q96QK1	1	0.734635	1	0.766646			0.974433	0.846671	1.049903	0.962596			1.356807	1.118995	1.158564	0.927011		
Q9UJV3	1	0.263994	1	0.271547			0.366158	0.646242	0.35519	0.628007			0.443867	0.225152	0.36958	0.298993		
P04062	1	0.842001	1	0.690962	1	0.545116	0.924334	0.994727	0.810466	0.954734	0.830311	0.8875	1.15508	1.041447	1.075105	0.907755	0.931486	0.793582
P13942	1	0.750979	1	0.666971	1	0.38994	0.882222	1.281402	0.757948	0.981692	0.797532	0.888362	1.101472	0.828743	0.783135	0.781038	0.702506	0.642839
Q8NCL4	1	0.934747	1	0.99799	1	1.049353	1.019471	1.06563	0.986851	1.047586	1.013912	0.995692	1.158328	1.264134	1.045794	1.066545	1.117287	1.132401
Q86VD1	1	0.462799	1	0.348131	1	0.378124	6.140502	0.920485	5.199692	0.920319	6.299196	0.947418	0.493524	1.28147	0.274623	0.937915		1.218251
P30530	1	0.89686	1	0.672112	1	0.586059	1.019066	0.937913	1.153959	0.996241	1.249442	1.051278	1.322952	0.802112	1.142613	1.018525	1.112439	0.897333
P41271	1	1.056252	1	0.81783			0.979812	1.072066	1.09178	1.512337			1.083165	1.082853	0.921562	1.126915		
Q9P121	1	0.517269	1	0.943227	1	1.050968	0.789394	0.714582	1.051596	0.980835	1.010497	0.959822	0.663	0.724524	1.179613	0.879597	1.5076	0.916138
P21589	1	0.878701	1	1.302828	1	1.750282	0.855197	1.571413	0.863719	1.225367	0.857933	1.604495	1.227202	1.04288	0.763162	1.056753	1.225764	1.701117
P55899	1	1.159	1	1.227639	1	1.009976	1.420622	1.059481	1.550589	1.094429	1.19531	1.195585	0.972833	0.857527	0.807339	1.033938	0.887638	0.996165
Q8NFY4	1	1.108775			1	1.201068	0.992909	1.063072			0.893052	1.072625	1.148817	0.913523			1.224931	1.013
O43280	1	0.577079	1	1.01544	1	1.076533	0.911262	0.695947	1.127462	1.091441	1.118242	0.808589	0.562316	0.748128	0.75519	0.830398	0.780134	0.921102
Q6P2E9	1	0.749704					0.833205	0.497946					0.581584	0.796451				
P31994	1	0.627197	1	0.714885			1.368584	1.051898	1.262607	1.015781			1.112206	1.231123	1.114258	1.224451		
Q969E1	1	0.298077	1	0.313647	1	0.263748	0.46184	1.040503	0.456309	1.127571	0.456791	1.103523	1.811339	0.898874	1.883228	0.954204	1.920501	0.868256
Q86SR1	1	1.211105					1.073125	1.099896					1.3012	1.234581				
P29279	1	0.876683	1	0.859453	1	0.734403	0.771828	0.848211	0.650923	0.79075	0.739019	0.876664	0.845693	0.88682	0.898302	0.885147	0.897267	0.853858
P55196	1	0.912437	1	1.017855	1	0.989799	1.494608	1.214649	1.561227	1.24781	1.561352	1.360397	1.288457	1.296323	1.134739	1.43108	1.263062	1.467436
Q32MZ4	1	1.718822					1.644512	0.876408					1.110512	0.632631				
P15085	1	1.150344	1	1.643669			1.083087	1.125136	1.2116	0.77525			1.498356	1.190605	1.23896	1.10452		
P02730	1	1.025491	1	0.990708			0.810121	0.639216	0.635921	0.841114			0.86423	0.5674	1.047476	0.914665		
P20933	1	0.766276	1	0.745977	1	0.77203	0.944962	1.035142	0.993804	1.07616	0.969751	1.030006	1.353558	1.009062	1.423374	1.151186	1.42748	1.163465
A6NGU5	1	0.702697					1.108549	1.087963					1.086992	1.144017				
Q13231	1	0.730675	1	0.779797	1	0.840047	0.999058	1.002151	0.927198	0.890334	1.021609	0.890153	0.76005	0.709276	0.748156	0.742278	0.828225	0.747259
Q5T749	1	1.219045	1	1.332909			1.244106	1.597045	1.28725	1.222476			1.011718	1.62065	1.03102	1.591394		
O94769	1	1.169469	1	1.01247	1	1.119613	0.901605	0.946722	0.971126	0.958209	1.004024	1.008812	1.181404	0.993046	0.947847	0.806909	1.162472	0.880252
P17931	1	0.856021	1	1.119496	1	0.934388	0.872445	1.020556	1.070652	1.152934	1.026094	1.036957	0.941739	0.588297	1.106897	1.146542	1.127956	0.948204
P13798	1	2.835692	1	1.065913	1	1.244198	1.07518	1.270083	1.0961	1.217068	1.235883	1.180997	1.553146	1.301323	1.799252	1.246153	2.141449	1.129446
P00995	1	0.860206	1	0.74555	1	0.715768	0.834338	0.922724	0.996419	1.113219	0.998322	1.077143	1.405203	0.857127	1.443664	0.931995	1.297436	0.884742
Q9H0X4	1	1.012473			1	0.850271	1.219818	0.993035			0.975036	0.99622	1.017907	0.89068			1.119691	0.958834
Q9H251	1	1.025897					0.788329	1.088527					0.885149	0.594006				
P04156	1	1.030386	1	1.630286	1	1.522614	1.794799	1.083764	1.531522	1.145429	1.433943	1.023301	0.90685	1.090293	1.071988	0.928223	1.067722	0.988981
Q6UXD5	1	1.479527					0.790545	0.99427					1.576783	0.866582				
P07307	1	1.173892	1	1.131415	1	1.316361	0.749011	0.975685	0.690887	0.907923	0.748205	0.828901	1.270016	1.295211	1.143348	1.154314	0.976403	1.038739
Q9Y2T3	1	0.996636					1.623002	1.215799					0.553317	0.984103				
Q13555	1	1.327205					1.192996	0.925555					0.96806	0.832036				
Q96EG1	1	1.060994					0.887839	0.921255					0.600983	1.178453				
Q01524	1	1.431072	1	1.056023	1	1.226421	0.76051	0.67186	0.658767	0.648729	0.718546	0.772136	1.3744	0.497273	1.241981	0.465261	1.472142	0.638357

Q05315	1	0.898075	1	0.95412	1	0.982805	0.764805	0.862887	0.801728	0.892379	0.813123	0.883315	0.364074	0.505381	0.383834	0.564107	0.379446	0.538986
P39059	1	0.645447	1	0.674652	1	0.600122	0.994449	0.930597	1.080402	0.925208	0.76895	0.822447	0.656357	0.797262	0.664895	0.745026	0.476459	0.689399
Q14162	1	1.206239	1	1.078173	1	1.601736	1.112995	1.104739	0.932572	1.162356	0.938073	1.185957	1.232601	1.168746	0.606046	0.900618	0.745841	0.812683
P02461	1	1.001365	1	0.896522	1	1.278209	0.760988	0.891786	1.052087	0.961191	0.855108	0.962561	0.949041	0.772947	0.861685	0.860107	1.01078	1.001112
P27487	1	1.047288	1	0.787767	1	0.689992	0.625195	0.629135	0.739045	0.684673	0.762363	0.742394	0.712956	0.58646	0.619951	0.648395	0.558453	0.5905
Q86SJ6	1	1.403544					1.613383	3.741096					1.984432	1.734516				
P53801	1	1.037898	1	1.500085	1	2.068575	0.283326	0.647555	0.781623	0.72914	0.720977	0.767179	0.565444	0.408709	0.775382	0.793677	0.696494	0.78279
Q9H2M3	1	1.28747					1.038338	1.154498					1.132309	1.682291				
Q9NP84	1	1.153377	1	1.272131			0.644637	0.730089	0.614077	0.774302			1.072829	0.633382	0.881934	0.586973		
P14384	1	0.865296	1	0.825023			0.687688	0.823431	1.106282	1.040949			1.134489	0.787877	1.318005	0.990785		
O75223	1	1.561972	1	0.907176	1	0.985792	0.575215	0.930761	1.105085	1.081021	0.951273	1.086626	1.106244	1.071384	1.294176	1.34593	1.33064	1.319731
Q7Z5N4	1	1.041179			1	0.956191	1.058569	0.929327			0.866236	1.034002	0.928124	0.85039			0.962368	0.691203
O14983	1	2.058556					1.51905	1.26222					1.330425	1.016793				
P61204	1	0.823922					0.91098	0.956882					0.641643	0.799801				
Q9Y274	1	0.996303	1	1.169196	1	1.09432	0.627124	0.783256	0.77635	0.90855	0.829096	0.87198	0.927097	0.745957	0.969976	0.825009	0.90341	0.837389
Q13228	1	1.055518	1	1.07366	1	1.054037	1.202982	1.167473	1.248476	1.023158	1.18889	1.13232	1.265654	0.991049	0.981427	1.065198	1.176257	0.866681
Q9UKX5	1	1.169602	1	0.854696	1	0.824292	0.84461	0.781263	0.935584	0.920211	0.82877	0.982928	0.895381	0.776692	0.893673	0.820024	1.081124	0.823269
P60842	1	1.293148			1	1.140417	1.277701	2.106342			1.076644	1.839197	1.184703	0.876078			0.998003	0.873737
P78324	1	1.191366	1	1.095341			0.677727	0.73518	1.09413	0.983394			0.715455	0.818143	0.875258	0.746068		
P0DJD7	1	1.302051	1	1.292074	1	0.773815	1.098196	1.038704	1.165813	1.075074	1.044592	0.950439	1.317409	1.482399	1.45576	1.470445	1.316059	1.60454
P07451	1	0.721705	1	0.892724	1	0.797572	0.885445	0.98635	0.893052	0.977306	0.993807	0.9867	2.081427	1.810143	1.554572	1.101033	1.515513	1.36083
Q6U841	1	0.391665					3.404861	0.746501					0.05186	0.94296				
Q5TCZ1	1	0.5876	1	0.663061	1	0.621292	0.815588	0.945011	0.895338	0.95839	0.92218	1.02582	1.182813	0.890665	1.099408	0.938659	1.149953	0.987496
Q9NRN5	1	0.531921	1	1.192415			0.939976	0.957441	1.049763	0.859523			0.701194	0.690687	1.040681	0.889799		
P23280	1	0.588358	1	1.182766	1	1.474433	0.744155	0.663059	1.423208	0.725777	1.190211	0.840992	0.843126	0.73366	0.755424	0.856954	1.208833	1.099698
Q9Y4D7	1	0.973828	1	0.917617	1	0.919808	1.007693	1.036927	1.093654	0.96806	1.159793	0.944439	1.257489	1.126912	1.178664	1.079563	1.188908	1.137945
Q04760	1	0.966491	1	0.822071			1.510045	1.286726	1.06539	1.112007			1.597812	1.029137	1.07149	0.98044		
P04626	1	1.324563	1	0.999135	1	1.596741	1.195173	0.920743	1.060868	1.128185	1.042604	0.892829	1.182289	1.145168	1.35264	1.134213	1.066203	1.110105
Q4KMG0	1	1.110237	1	1.035479			0.957595	1.028172	1.053465	0.873849			0.895904	0.810942	0.850788	0.958639		
Q69YW2	1	1.64401					1.322022	1.31763					0.942191	0.911903				
Q8NFL0	1	0.307664	1	1.173851	1	0.377785	0.866518	0.984661	1.396361	1.244976	1.002184	1.004469	0.626125	0.795847	1.210641	1.343434	0.745417	0.856068
Q9P2E9	1	0.761801	1	0.794997	1	1.177428	1.024201	2.370577	1.227173	3.480084	0.997509	3.346639	1.401588	1.497779	1.880638	1.927693	1.500021	1.650079
Q96NZ9	1	0.783954	1	1.063014	1	1.028928	1.203425	1.185416	1.448928	1.091094	1.433112	0.827166	1.277711	1.251902	1.135862	1.602084	0.80989	1.409814
Q68BL7	1	1.181818	1	1.139173	1	1.038586	0.967467	0.963198	0.989096	0.974533	0.952893	1.01079	1.008892	0.905919	1.071027	0.871338	0.867371	0.891308
P09913	1		1	2.371925	1	2.253379			0.978955	1.75329	1.152106	1.870287			3.179369	1.096346	3.161724	1.27482
P13501	1	0.954616	1	0.96336	1	1.069993	0.838079	0.893375	0.744309	0.890741	0.786124	0.912164	0.909181	1.075604	0.998928	1.079505	1.054624	1.138273
O15511	1	1.141715					0.817672	0.892837					0.729823	0.781942				
P48436	1	0.462069			1	0.43267	0.589077	0.820953			0.669463	0.828091	0.818103	0.610786			0.922455	0.706683
Q01995	1	0.668832	1	0.961011	1	1.242863	1.467913	0.826707	1.94363	0.795254	1.484338	0.748734	2.243424	1.308303	1.235164	1.032658	2.021761	1.123096
P08887	1	0.913626					0.970393	0.948064					1.602606	1.115703				
A6NMY6	1	1.022795	1	1.105341	1	1.301518	1.027914	1.134067	1.420382	1.519703	1.361512	1.154857	1.037466	1.602446	0.854814	2.252425	0.851588	1.845932
P63313	1	0.888844	1	0.751759	1	1.053498	0.975171	1.020222	0.627013	0.933889	0.952906	1.260894	1.284456	1.236399	1.040355	0.743536	1.568463	1.386402
P48637	1	1.394582	1	1.526297	1	1.762066	1.552677	1.02659	1.918127	1.169797	1.633814	1.056715	0.804769	1.050911	1.061524	1.163047	1.018474	0.983262
Q05682	1	1.054383	1	1.115463	1	0.984179	0.863422	1.803553	0.682149	1.328815	0.451269	1.148594	0.881092	1.016184	0.845145	0.852577	0.781905	0.55496
P81172	1	1.326855					1.066242	0.757329					1.061125	1.018764				
P26572	1	0.941582	1	0.76799	1	0.957203	1.003358	0.888808	0.900835	0.876851	0.930498	0.960948	0.866918	0.942103	0.738727	0.839161	0.839474	0.980541
Q9HBW1	1	1.32528	1	1.083622			1.134722	0.878269	0.909371	1.050412			1.076595	1.15253	1.226587	1.041733		
P35030	1	1.174093	1	1.323845	1	1.014713	1.062485	1.094258	1.073162	0.858125	0.946496	1.009473	1.033623	1.318377	1.098398	1.196094	1.166972	1.299021
P51149	1	1.32186	1	1.466513	1	1.157818	1.38351	1.031126	0.964192	1.42	0.859354	0.907989	0.929862	1.088669	0.665263	0.702278	0.883666	0.887552
P54760	1	1.023014	1	1.305651	1	1.395181	1.306421	1.243272	1.134395	1.02544	1.26128	1.120511	1.525129	1.243005	1.292643	2.214994	1.484921	1.747598

Q6IBS0	1	0.752104					0.828369	0.926554					0.694732	0.668333				
O15230	1	1.261494					1.048936	1.012222					1.441877	1.451068				
P48735	1	0.763456	1	1.134823	1	1.349541	0.7199	0.915495	1.225168	1.103594	1.276916	1.115094	1.250834	1.171363	1.554707	1.516358	1.943728	1.206244
O00451	1	1.179268	1	1.312673	1	1.319076	1.059037	1.115085	1.284122	0.9732	1.124099	0.990532	1.322468	1.066228	1.036654	1.492417	1.169638	1.553489
Q6UXM1	1	1.201918	1	1.179548	1	1.135228	1.592791	1.653955	1.507638	1.644147	1.565352	1.57073	1.25541	1.066534	1.165639	1.087008	1.168778	1.022123
P62873	1	1.20884	1	1.160019	1	1.159736	0.824043	0.874672	0.643566	0.779463	0.724046	0.908351	1.116668	0.748836	0.77293	0.616855	0.96994	0.783721
Q9BTV5	1	1.629739	1	1.506861	1	0.626994	1.176715	1.729984	1.551259	1.799394	1.210717	1.614384	1.512035	1.313065	1.373769	1.121294	1.503998	1.470869
P78552	1	0.731994	1	0.820934			0.916003	0.997004	1.05526	1.05792			1.059422	0.966021	1.244209	1.057533		
Q9NX62	1	1.090753	1	1.05286	1	0.869375	0.983705	0.826306	0.979551	0.914465	0.937601	0.814292	1.057002	1.140894	0.906693	1.024747	0.880198	1.179514
P16581	1	0.959696	1	1.226856	1	0.839819	1.062728	1.064259	1.2486	1.188503	1.069957	1.059823	1.228524	1.045728	1.531949	1.553395	1.103168	1.143277
Q99536	1	1.103163	1	0.927692	1	1.0907	1.442861	0.829279	2.048443	0.924734	1.225989	0.928125	0.771879	0.95901	0.726114	1.16729	0.992814	1.086336
Q9HC38	1	1.038087					1.181272	1.104843					1.287383	1.061299				
Q10472	1	1.119596	1	1.806002			0.847406	0.81063	1.321411	1.349564			0.779181	0.826065	1.380852	1.496937		
P35237	1	0.998901	1	1.597655			1.276614	1.258385	1.845662	1.262726			0.595521	1.099214	0.851465	0.845641		
P17927	1	1.61764					1.262534	0.912299					1.132338	0.886331				
P28074	1	1.558123	1	0.851079	1	0.760939	1.253944	1.364897	1.151437	1.068095	1.115729	1.011146	1.440137	1.829364	1.048602	1.474794	1.270474	1.472524
Q96EN8	1	1.072534			1	0.647254	1.594591	1.106779			1.425629	1.12236	1.015072	0.116378			0.945224	0.188731
P30041	1	1.182342	1	1.654059			0.953785	1.069267	1.053049	1.137888			0.944343	1.035018	1.090179	1.110149		
A0A1B0G	1	0.986174					0.763245	1.347011					0.974295	1.225791				
P48681	1	0.483807	1	0.570188	1		1.416323	1.602127	1.29693	1.639875			1.191725	1.296613	1.273645	1.577616		
Q99674	1	0.541331					1.584645	1.053213					0.470772	1.453353				
Q02156	1	2.071623	1	2.089938	1	1.489461	1.719821	1.856651	1.612801	1.908542	1.407382	1.582294	0.884214	1.488597	0.989066	1.526986	0.947493	1.260188
Q9UJW2	1	2.560673					1.100534	1.005827					1.381331	1.318288				
P26006	1	1.02645					1.04105	1.042979					1.091019	0.945687				
P49721	1	1.647778			1	1.057524	0.925273	0.972378			1.17607	0.70034	1.349081	1.052284			0.594765	1.161155
A0A0B4J1	1	0.416511	1	0.672588	1	0.528338	0.675362	0.617918	0.931632	0.637838	0.909684	0.677566	0.390159	0.425596	0.625132	0.568668	0.533636	0.666473
Q02952	1	0.903255	1	1.124375	1	1.07606	1.634769	1.040912	0.898661	0.843491	0.652774	0.87355	0.508168	1.267926	0.719783	1.023193	1.11729	0.938393
Q13835	1	1.557375	1	1.151318	1	1.330446	1.195202	2.725952	1.267884	2.257795	1.212154	2.501378	0.975531	1.843006	0.53415	1.391893	0.618135	1.704641
Q8NI35	1	0.421178			1	0.406749	0.087165	0.183641			0.098779	0.19859	0.203316	0.204108			0.200723	0.252442
P01236	1	0.614362					1.268404	2.384522					2.203461	1.559607				
A0A075B[illegible]	1	0.598225	1	0.342069	1	0.477364	0.873563	1.252045	1.408475	1.60251	1.302143	1.511706	0.838236	0.852926	0.897359	1.048997	0.862212	0.972536
P63010	1	1.950082	1	2.000789	1	1.498923	1.238148	1.126638	1.259295	1.207981	1.185292	1.033043	1.212636	0.671661	1.072902	0.658253	1.163927	0.7235
Q9HBI1	1	0.93487	1	0.837713	1	1.129126	0.482591	1.057161	0.457733	1.103579	0.451495	1.285461	0.483614	0.492594	0.507462	0.518775	0.655707	0.503234
Q14623	1	0.878408	1	1.008562	1	1.865375	0.990581	0.975697	0.827959	0.944357	1.161165	0.952401	1.155982	0.59401	0.900736	0.620499	0.905035	0.85584
P78423	1	1.246204					0.998272	0.832503					0.955746	1.005868				
Q2UY09	1	1.116508	1	1.027247	1	1.112755	1.290912	1.083357	1.184136	1.045673	1.19164	1.051416	0.950859	0.970302	0.922208	0.867524	0.921266	0.871916
P40306	1	0.895464			1	0.894801	1.120197	1.213912			0.942247	1.249581	1.364152	1.230355			1.378497	1.072989
Q53RD9	1	0.976296	1	0.894622	1	1.127104	0.981956	0.859557	1.135767	0.890367	1.193459	0.847766	0.900787	0.980766	0.835889	0.772503	0.845223	0.822897
Q7Z7L1	1	1.293841	1	1.141817	1	0.938209	1.177217	1.137301	1.043368	1.118028	0.943705	1.089623	1.507525	0.845434	1.371241	1.276463	1.478887	1.367462
Q8NEN0	1	0.910576	1	0.98534			1.20838	0.785341	1.133299	0.67499			0.929471	0.862754	1.100014	0.869295		
P10768	1	0.824332					1.130329	1.132535					1.328399	0.941039				
P83731	1	0.719815					2.711024	1.015365					0.676656	0.691887				
Q9NZN5	1	0.633225					1.515111	1.839537					1.94163	1.543572				
P13284	1	1.269038					0.929794	1.266237					2.100165	1.272323				
Q9UPZ9	1	0.600799	1	0.655111	1	0.639844	1.753291	1.611065	1.712758	1.771532	1.788407	1.821984	1.076028	2.142658	1.342033	2.495008	1.183335	2.427435
Q14574	1	1.24837	1	1.469847	1	1.582345	1.006293	0.990276	0.979968	1.058453	1.119494	1.026321	0.979332	0.816755	1.045527	1.146221	0.960982	0.938385
Q15375	1	0.963633	1	1.843249	1	1.207167	0.896453	0.866804	1.194687	1.035564	0.807418	1.111186	1.348894	0.908332	1.66927	1.080908	1.459817	1.123286
Q02763	1	1.266533	1	0.955664	1	1.805566	1.075753	1.16875	1.09495	1.04929	1.744851	1.053304	1.420956	0.9378	1.563179	0.870858	1.141313	1.406772
P15529	1	0.751936			1	0.952262	1.131674	1.136094			1.317978	1.091719	1.137279	0.739302			1.195185	0.85728
Q8WUJ3	1	0.979748	1	0.894265	1	1.181695	1.512614	0.964826	1.139479	1.019671	1.0798	1.070128	1.051074	0.910984	1.118904	0.949686	1.136697	0.957238

P48061	1	1.192951	1	1.219416	1	0.994524	1.409479	0.795721	0.785975	0.962626	0.810287	0.808447	0.83406	0.779554	0.535009	0.815203	0.644097	0.756328
P13716	1	1.706273					0.762841	0.596067					0.76232	0.466829				
Q8WVQ1	1	1.193947	1	1.073421			1.075309	1.041625	0.901429	0.889758			1.011301	1.417642	0.912171	1.013386		
Q9BZR8	1	1.142721	1	0.966608			1.111516	1.660484	1.199441	1.954608			1.405192	1.106213	1.466162	1.26391		
Q14141	1	1.5789	1	1.010881	1	1.136603	0.930995	0.398076	0.936119	0.449021	0.874586	0.662629	0.57674	0.615066	0.446093	0.553462	0.700189	0.873473
O60832	1	0.894234			1	0.963076	0.932181	1.01826			0.934172	1.001927	1.084521	1.085388			1.131235	1.089005
Q8NB25	1	0.784268	1	0.813821	1	0.713859	1.00797	1.225041	1.005188	1.194506	0.94969	1.167518	1.42469	1.152184	1.347128	1.224158	1.360916	1.21883
P35670	1	0.7186	1	0.749162	1	0.873654	1.212653	1.296656	1.397657	1.547384	1.293533	1.386711	0.925748	0.971895	1.015013	1.245931	1.085786	1.157515
Q13275	1	1.101521	1	0.61376	1	0.992129	1.198674	1.03804	1.240875	1.05967	1.075073	1.035523	0.899325	0.617344	0.814997	0.628889	1.123909	0.888655
Q5U5X0	1		1		1													
Q15643	1	0.73379					0.71551	0.772665					0.560791	0.716784				
P35613	1	0.92251			1	0.943195	0.841437	0.856231			0.761347	0.910941	0.623895	0.830359			1.038569	0.934048
P14317	1	1.008463	1	1.00273			0.684238	1.190672	0.649281	1.115378			1.025043	0.935551	0.830928	0.89748		
O60234	1	1.096339	1	1.033031	1	0.980342	0.619	1.208523	0.610414	1.028958	0.602894	1.109892	0.915671	1.011154	0.813491	1.259609	0.78551	1.060173
P30086	1	1.123427			1	1.758448	0.916161	1.379633			1.627532	1.212075	1.190656	0.909534			1.221435	1.419311
P49454	1		1		1	1.43001					0.743	0.723147					0.777222	0.552668
P19971	1	0.763006	1	0.777208	1	1.138633	0.785428	0.808785	0.752351	0.942643	1.278256	0.976047	0.857363	0.68157	0.850831	0.812141	1.390006	1.251006
P58215	1	1.203649					0.779307	0.721773					0.850077	1.509787				
Q92692	1	0.986985	1	0.637632	1	0.40566	1.096009	0.893182	1.127587	0.996459	1.098491	0.939778	0.722906	0.81996	0.832935	0.867926	0.785148	0.875257
Q9H1B5	1	1.113577	1	2.151991	1	1.1839	0.846632	0.594756	1.252005	1.065601	1.142888	0.912365	0.60235	0.930232	0.945707	1.070653	0.642163	0.93339
O43556	1	0.92238	1	1.318186			0.843273	0.912533	1.218928	1.028922			0.899394	0.772305	1.043117	0.782758		
O95202	1	0.883872	1	1.105723			1.54401	1.383677	1.401949	1.210748			1.279829	1.379425	1.283243	1.098151		
Q9HC56	1	0.685078	1	1.085119			0.720271	0.646807	0.802076	1.120142			0.79162	0.638707	1.097099	0.9634		
Q06418	1	0.898988					1.161784	1.065641					1.110036	1.082525				
P00492	1	1.220638					1.479138	1.366243					2.000892	2.098322				
P05387	1	1.094689	1	0.987071			1.254928	1.596457	1.506932	1.448649			0.918806	1.391798	1.132951	1.068943		
P35754	1	1.802818	1	1.285152	1	1.163069	1.295512	1.496224	0.68924	0.96011	0.646962	0.952302	1.627754	1.097144	1.250272	0.929871	1.233791	0.917041
O14594	1	0.719473	1	1.05832			1.050857	0.916103	1.010901	1.287724			0.889824	0.977956	1.017481	0.893654		
Q7Z6G8	1		1		1													
P26022	1	1.133294			1	0.836782	0.760675	1.029286			1.092335	0.886454	1.941	2.099			2.328896	2.62755
Q8IX15	1	1.091567					1.950231	1.949554					1.890377	1.667927				
P49767	1	0.621924			1	0.496625	0.490729	0.620582			0.414772	0.614475	0.706038	0.505465			0.62299	0.504027
Q8N967	1	1.152851			1	0.687706	1.659199	1.139202			1.946717	1.464538	0.787921	1.158925			0.980964	1.513666
Q6UXH9	1	0.763604					1.03432	1.063142					0.733728	0.892747				
P22314	1	0.991548	1	0.807952	1	1.344066	0.783693	0.806684	0.677776	0.947252	1.031744	0.968711	0.979054	0.702932	0.810019	0.792065	1.037603	0.964264
Q9GZR7	1	0.684326					1.006601	0.951112					0.60574	0.799468				
O75051	1	0.802284					0.808151	0.990919					1.197953	0.897291				
P05089	1	1.073474	1	0.987793	1	0.881951	0.84842	1.063683	0.881183	1.04508	0.843554	1.018768	1.031927	1.205861	0.768906	1.360004	0.70704	1.167453
P09601	1	1.050893	1	1.106854			0.920006	0.908973	1.141226	1.01906			0.824061	0.852208	1.116447	1.117406		
Q8IZW8	1	0.788605	1	0.756916	1	0.497589	1.398633	1.838574	1.355	1.719707	1.410735	1.962545	1.040525	1.053097	1.010906	1.059843	1.026525	1.156064
Q96BZ4	1	1.328087	1	1.07848	1	1.380531	0.861919	1.041664	0.827848	0.946015	0.936117	1.093124	0.996981	0.873931	0.932448	0.743822	0.797901	0.784716
Q86U42	1		1		1													
P98172	1	0.744128			1	0.876266	0.769579	0.814536			0.799378	0.805113	0.814201	1.033969			0.968835	0.849877
Q14203	1	1.055909			1	0.978169	1.266964	0.844622			1.239496	0.79357	0.683752	0.806477			0.695306	0.788637
P05060	1	1.911755	1	1.240633	1	2.246543	0.721674	1.23054	0.805871	1.261573	1.070858	1.255995	1.311796	1.379481	1.053868	1.023089	1.502927	1.120278
Q92743	1	0.996462	1	0.945204	1	0.960826	0.674635	0.943836	0.619105	0.797339	0.637648	0.990112	1.323164	1.313274	1.086935	0.981766	1.093812	1.070081
A0A0C4D	1	0.997306	1	1.008459			1.327281	0.662076	1.241915	0.584359			0.341142	1.09218	0.301646	1.071467		
Q05193	1	0.781355					0.87576	1.153985					1.224252	1.457882				
Q96CX2	1	0.772868	1	0.817003	1	0.691131	0.93485	0.595195	0.967938	0.757265	0.882305	0.555223	0.629661	0.772624	0.642561	0.658463	0.491173	0.698459
P37173	1	1.36162					1.373594	0.895867					0.871306	1.014068				

P21583	1	0.788491			1	0.946981	0.873851	0.683867			0.786196	0.499921	0.828675	0.675741			0.958048	0.6546
Q96PL1	1	1.328478					1.247718	1.166676					0.966941	1.368215				
P05026	1	0.842501	1	1.00671	1	0.838599	1.187527	1.152266	1.096498	0.983414	1.145441	0.946481	1.322307	1.209299	1.099534	1.030615	1.057893	1.012696
Q8NHP8	1	1.3271	1	1.296203	1	1.289104	1.273431	1.041696	1.153367	0.642476	1.099507	1.458228	1.467359	1.143824	1.593176	0.9395	1.382325	1.379303
P25789	1	0.771576	1	1.216708	1	1.064869	1.234236	1.19288	1.257114	1.013213	1.091745	1.180149	1.415561	1.438791	1.43577	1.577856	1.451735	1.474348
P06703	1	1.229681	1	1.171201	1	1.336496	1.024472	1.085591	1.055156	1.144357	1.115595	1.175237	1.523501	0.886992	1.622657	0.947193	1.671008	1.004466
Q92626	1	1.124411	1	1.061824			1.210672	1.388076	1.153011	1.237259			1.128522	1.149512	1.219115	1.11854		
P05023	1	0.946783	1	1.013324			1.236038	0.58808	1.000341	0.723213			0.578688	0.902121	0.60244	0.671594		
Q8N5C7	1	1.696894	1	1.125044	1	1.112857	1.703951	1.543611	1.753218	1.738087	1.900488	1.815247	1.359557	1.245496	1.255262	1.262919	1.456208	1.399122
P16112	1	1.322911	1	0.88504	1	0.847843	2.431287	1.169666	1.039478	1.047689	1.045541	1.245222	0.83809	1.386729	1.371873	0.989021	1.307812	1.038297
P10253	1	1.368017					1.117973	1.01233					1.057156	1.006323				
P60981	1		1		1													
Q99538	1	1.22991					0.807815	1.068045					1.012043	0.978175				
Q7Z2D5	1	0.795926	1	0.985455	1	0.802395	1.518043	1.296559	1.342496	1.19933	1.45187	1.35441	1.369681	0.790126	1.51657	0.77511	1.28786	0.903455
Q14314	1	1.111846	1	1.134393	1	1.233953	1.117236	0.9299	1.220451	0.926125	1.107878	0.934123	1.071285	0.843728	1.025044	1.00206	1.111689	1.090857
O95967	1	0.87619	1	0.839869			0.940626	1.00575	1.132242	1.210946			0.687802	0.89601	0.875384	0.939437		
Q86TY3	1	0.867825	1	0.993662	1	0.786069	1.048723	1.052956	0.943593	1.01921	0.80074	0.897422	1.126865	1.730813	1.037355	1.146988	0.97151	1.061732
P27361	1																	
O60341	1	0.956509	1	1.735065			0.718126	0.566653	0.961228	0.931843			0.700522	0.813646	1.259463	0.919526		
P48059	1				1	1.232734					0.324614	0.681824					0.41817	0.362506
Q15404	1	2.04223	1	1.088908	1	1.153264	0.624029	0.945896	0.424435	0.815097	0.652307	0.774033	0.555953	0.928175	0.551394	0.561425	0.725608	0.722895
Q9UBX7	1	0.855088	1	0.836036	1	0.890524	1.966513	1.79208	1.923285	1.758714	1.874507	1.781834	1.463191	2.934514	1.366827	2.880779	1.378703	2.925499
Q9BUJ0	1	1.415428			1	1.221589	0.708553	0.985158			0.541479	0.973883	0.954545	0.956624			0.996589	1.042348
Q8IUX7	1	1.543507					1.501967	1.350324					1.470908	0.96848				
P14210	1	0.88515	1	0.854204			0.88785	1.024911	0.871498	1.05013			0.989362	0.867493	0.893474	0.719585		
P56202	1	1.203696	1	0.67407			0.821385	0.93546	0.602126	0.728997			1.128449	0.848748	0.707607	0.642745		
Q8WXD2	1	0.665429	1	0.698452	1	0.700527	1.578735	0.956205	1.076008	1.012463	1	0.967767	0.51562	1.04145	0.985026	0.945134	1.037069	0.863343
O75382	1	1.388072					0.670465	0.776242					0.894152	1.573107				
Q6V0I7	1	1.031491					0.9259	1.014712					0.511431	0.86187				
Q9HD15	1	0.600379	1	0.650672	1	0.717816	1.140272	0.61149	1.114991	0.66684	1.143159	0.598552	0.752709	0.885615	0.712764	1.036115	0.697854	1.006417
P07814	1	0.728128					0.864335	0.912854					1.085509	0.861805				
Q6ZR08	1																	
P42126	1	0.424965			1	0.36814	1.413766	1.305107			1.734184	1.346749	0.374084	1.169826				1.060009
P15086	1	1.058145	1	1.491646			1.670244	0.839295	1.605706	1.103425			1.136759	0.339945	1.156451	0.559477		
P98161	1	0.715457			1	0.893099	0.99753	0.652382			0.668361	0.714382	0.74631	0.832189			0.498251	0.625671
Q8WWQ8	1	1.261199	1	1.487296	1	1.29347	0.839957	1.037482	0.961176	1.377514	0.943708	0.883344	0.893971	0.671861	0.612177	0.629436	1.062623	0.911583
Q96RW7	1	0.910314	1	1.235974	1	1.502648	1.080978	1.009878	1.00182	1.164792	1.047146	1.24157	1.10636	0.804272	1.308239	0.639266	1.544868	1.017323
Q709C8	1	1.314251					1.538488	1.453624					1.111155	1.041281				
Q14667	1	0.85686	1	0.842947	1	0.723277	1.486642	1.427845	1.609491	1.512319	1.560639	1.327719	0.685367	1.311194	1.033266	1.382941	0.761957	1.373482
Q9Y4W6	1	2.208353					0.948837	1.061516					1.34947	1.080328				
Q86UP2	1	0.985144	1	1.021116	1	0.977325	1.603732	0.837704	1.573467	0.861297	1.629504	0.885092	0.741485	2.519078	0.667399	2.240133	0.817232	2.093259
P15153	1	0.98863					0.668228	0.898569					0.673956	0.324408				
Q8N2S1	1	1.129823	1	0.77487			0.995132	1.092884	0.930368	0.845149			1.333562	1.091614	1.017065	0.516493		
Q14191	1	1.274716					2.940044	1.227257					1.059208	1.952503				
Q14789	1	0.565182			1	1.12247	1.154795	0.950425			1.089921	1.019098	1.072151	0.98034			1.131672	1.120734
Q07065	1	1.357729	1	1.092027	1	1.133477	1.056735	1.085305	1.020099	1.159422	1.040524	1.185914	0.862638	0.874131	0.740054	0.909906	0.798761	0.899784
P53004	1	1.243922	1	1.034668			1.302783	1.193372	0.999639	1.175906			1.148847	0.911862	1.061125	0.85214		
A6NMZ7	1	1.189102	1	1.102111	1	1.070623	1.098604	0.833172	0.88577	0.737556	0.919881	0.654389	0.671936	1.014214	0.638827	0.923526	0.610898	0.834102
Q8NFI4	1	0.808388			1	1.185967	0.56849	0.981205			0.965967	1.163557	0.902777	0.760645			1.173855	1.913105
P46783	1	1.888672					1.276492	1.069435					1.151007	0.848859				

P11177	1		1	1.301214	1	1.683443	1.496284		1.262261	1.057996	1.132071	0.844366	0.720648		0.976236	0.920268	1.142695	0.863957
Q3V6T2	1	0.828106					0.755821	0.796221					0.564819	0.330938				
A4UGR9	1	0.650137					1.212934	1.063295					1.035652	0.57159				
A6NEC2	1	1.551363					1.437863	0.849941					1.603959	1.14226				
Q08722	1	0.767423	1	0.811463			0.629764	0.873858	0.623395	1.026618			0.850666	0.779628	0.76164	0.648694		
Q15413	1	1.946048					1.144255	1.654814					1.531429	1.443289				
Q9Y4G8	1	0.455778					1.699084	1.246979					0.625439	0.796663				
P98095	1	2.293135	1	0.980187	1	1.013148	0.631155	0.923815	0.70617	1.056818	0.942107	0.705454	0.852548	0.90839	1.058752	0.974129	0.703548	1.009591
P48506	1	1.413963	1	1.433327	1	1.287694	1.352573	1.164903	1.394613	1.269225	0.839626	0.85634	1.603863	1.557051	1.630994	1.403481	1.126426	0.897585
Q8WW22	1	0.499428	1	1.41287			2.535965	1.068817	2.086132	1.016515			0.143013	0.989805	0.626856	0.38561		
B2RXH8	1	0.779454					2.050746	1.711623					1.909456	1.339063				
P20810	1	3.158832	1	1.415904	1	1.399995	1.643075	0.956828	1.514038	1.100184	1.461397	1.134974	0.744404	1.263512	1.144372	1.262781	1.161881	1.430613
Q15691	1	0.965253	1	1.170577	1	0.929844	0.975433	1.387483	0.84542	1.312446	0.820517	1.273208	0.68842	0.549787	0.723852	0.674722	0.6881	0.538768
Q15942	1	1.111162	1	0.862215	1	0.742035	0.568655	1.433937	0.559277	1.603993	0.510778	1.364876	0.824561	0.690635	0.651081	0.665293	0.889022	0.638181
Q8TF21	1	3.197253			1	5.061252	1.318503	1.08724			0.877863	0.685752	1.269444	1.700503			1.38599	2.197655
P14927	1	0.797265					1.317033	1.032193					0.759106	0.670295				
P52888	1	0.896791	1	0.842405	1	1.063519	0.851085	0.918161	0.954509	1.020833	0.992501	1.069893	1.039282	1.022384	0.86028	1.075412	1.148623	1.146923
Q9NTI5	1	1.257515	1	1.090692	1	1.235991	1.04757	1.335476	0.969429	1.31461	1.039396	1.369467	1.473013	1.20604	1.634147	1.370014	1.712022	1.345373
Q8IYJ0	1	0.630822	1	0.431363			0.989961	1.018755	1.095647	0.895224			0.980478	0.739199	1.001278	0.583984		
Q9NRC6	1	0.797188					2.264283	1.919775					1.439823	1.885034				
Q8WY91	1	0.608156	1	0.635291	1	0.95183	27.18982	1.322536	16.36578	0.946694	15.21619	1.23415		1.757509		1.178665	1.07281	1.738446
P14174	1	1.162012					0.868076	1.037312					1.035858	0.431578				
Q13724	1	1.420888	1	1.1354	1	1.442209	0.850825	0.725159	0.679921	0.801882	0.681598	0.872229	0.933052	0.870056	0.883855	0.850912	0.910921	0.942085
Q13126	1	1.436406					0.936046	0.91854					1.056018	0.839501				
P49247	1	0.951048	1	0.808995	1	0.742669	0.855981	1.187075	0.960013	1.155176	1.019451	1.256395	1.345195	0.336065	1.702878	0.496632	1.750844	0.450242
Q8NA56	1	0.675607					1.023275	1.255553					1.073352	0.952256				
Q93050	1	0.741575					1.12677	1.0395					0.724361	1.170658				
Q13085	1	3.425862					2.261086	1.244957					0.97855	1.986071				
Q6KC79	1	0.736161	1	1.738292	1	1.212793	1.043755	1.186473	1.106423	1.248444	1.094589	1.199919	1.330324	1.310442	1.370906	1.527709	1.292073	1.54071
Q7Z7M9	1	1.217793					0.902509	0.922787					1.051232	0.732483				
Q6PGP7	1	0.880657					0.481759	0.598222					0.778145	0.587814				
Q8IY85	1		1		1													
Q8IZX4	1	1.185631	1	0.965142			0.484441	0.763603	1.014513	0.907012			1.49396	0.618886	1.012315	1.156295		
Q8NET8	1	1.388118	1	1.870311			1.01997	0.931175	1.054296	1.031793			1.051621	1.018564	0.936315	0.86264		
Q6Q0C0	1	1.4507	1	1.503448	1	0.739189	0.95956	0.806464	1.038246	0.866942	0.890033	0.811403	0.831224	0.567561	0.803091	0.643471	0.804841	0.572822
Q9H9E3	1	0.524437	1	0.67638	1	0.707976	2.994595	1.18625	2.761667	1.233632	2.862798	1.301866	0.778342	1.382318	0.964941	1.508128	0.931041	1.485183
P23458	1	1.205746					1.315081	1.22155					0.836674	0.494018				
Q7Z4Q2	1	0.516531			1	0.713243	1.25208	0.917766			1.336171	0.912793	0.817239	0.649649			0.786035	0.728311
O15240	1	2.69033			1	2.464538	1.230486	1.423255			1.358215	1.448239	1.145768	0.49568			0.895378	0.865759
Q494V2	1	1.406955	1	1.627527			2.260114	1.840766	2.17497	1.79655			1.366798	1.867557	1.546915	1.983051		
Q8IYU4	1	0.756772					1.033372	1.284028					1.310929	0.993436				
O14986	1	0.77767	1	0.87121	1	0.797258	1.003667	0.681082	1.390884	0.745418	0.996503	0.667661	0.736334	0.755288	0.677246	0.920255	0.764315	0.692795
Q14159	1	0.447899	1	0.61843	1	0.703818	2.28294	1.651496	2.398195	1.683079	2.051272	1.49881	0.677063	1.163747	0.595867	1.223739	0.105202	1.092412
Q9UMF0	1	0.751914	1	1.097294	1	1.197262	0.875262	0.717968	0.942242	0.845415	1.042874	0.820651	0.635557	1.610039	0.79673	0.751832	0.77614	0.825083
Q9NY47	1	1.390486					1.528316	0.789679					1.310407	1.157482				
Q7Z418	1		1		1													
P08254	1	0.79217	1	0.670806	1	0.799938	0.762581	1.127439	0.811968	1.186851	0.826083	1.197063	0.825737	1.066877	0.901354	1.052804	0.843429	1.046853
P55145	1	1.138978	1	1.333636			0.810879	1.59181	0.564597	1.566832			0.945077	0.909643	0.952886	0.72988		
Q8WVM7	1	0.511509					0.676181	0.943546					1.012758	0.814569				
Q7KZ85	1	0.805113	1	0.938714	1	1.159691	0.795296	0.715454	0.911304	1.095162	0.853915	1.032579	1.409961	1.095548	1.800437	1.496229	1.763977	1.493485

Q9BPW4	1	0.866205					1.407804	1.187955					1.563466	1.152291				
Q8IV08	1	0.993584	1	0.979426			0.742701	0.864052	0.973797	0.935031			0.678765	0.679854	0.793332	0.795701		
P20908	1	0.927164					1.053987	1.457541					1.292034	1.266071				
P55157	1	1.265132	1	1.961369	1	1.687023	0.838101	1.072891	0.864176	1.097357	0.823908	0.963559	0.944577	0.880433	1.234677	0.924101	0.914851	0.911963
Q13105	1	0.7241					0.554983	1.166602					0.969873	0.202799				
Q7Z4N2	1	0.904929					2.619778	1.175681					1.167041	1.534864				
A2RTY3	1	14.95653	1	14.10325			0.808085	0.628993	0.430243	0.764496			0.882208	0.903346	0.255282	0.58782		
Q5T124	1	0.975493			1	0.991494	2.915132	1.812438			3.401962	1.969196	0.601971	1.590589			0.484067	1.671679
P0C7X5	1	1.442213					1.208368	2.04883					4.215213	4.140761				
Q68BL8	1	1.120921					0.821178	0.577038					0.787163	0.828133				
P11684	1	0.718213	1	0.738342			1.033174	1.20461	1.054746	1.21358			1.214372	0.699563	1.205019	0.709244		
Q96EH3	1	1.01611					0.89433	1.209824					1.430427	1.201866				
Q8IVU3	1	1.648716	1	1.563238	1	1.053004	0.83065	1.05689	0.905074	1.044503	0.602269	1.16351	1.496462	1.164935	1.677741	1.208259	1.6127	1.392399
P08118	1	0.996611	1	1.24488	1	0.826166	1.148515	1.365396	1.046866	1.116986	1.087837	0.659342	1.61157	1.082299	0.820615	0.578833	0.908164	0.709782
Q16288	1	0.624384					0.499179	1.026705					0.752369	0.792799				
Q5VTJ3	1	1.426349	1	0.954734			0.609169	0.759756	0.563667	0.808301			0.668453	0.53674	0.644829	0.793421		
P51124	1	0.537167	1	0.591114	1	0.645544	1.331266	1.279857	1.487768	1.228458	1.4524	1.174537	0.736262	0.432163	0.730412	0.407944	0.805591	0.257862
Q96GX5	1	0.705366					1.642472	1.174101					0.645515	0.181994				
Q6ZMJ2	1	0.64723	1	0.915054			0.481307	0.778661	0.920613	1.001868			0.513295	0.623605	0.832245	0.910963		
Q9UNH6	1	0.962434			1	0.941123	0.934457	1.042884			0.991757	1.273786	1.204133	1.114279			1.293517	1.201588
A8MVW5	1	1.167088					0.993073	1.137686					1.476266	1.269368				
Q9UK23	1	1.082556	1	0.931628	1	0.694994	1.197922	1.253773	1.170194	0.938988	1.097438	1.107271	1.371668	1.1109	1.046684	0.965374	1.171977	1.060631
Q9NNX6	1	1.240089					1.073992	0.804143					0.707258	1.056468				
O00206	1	0.867661					1.670182	0.877589					0.377209	0.838232				
Q5PSV4	1	0.577395	1	0.475434	1	0.358567	0.930381	1.139947	0.991489	1.149074	1.135195	1.186014	1.059539	1.180145	1.211842	1.214019	1.17113	1.180989
Q6ZNQ3	1	0.676322					1.816914	1.43349					1.472376	1.918175				
P22304	1	0.820333	1	1.2573	1	1.113277	1.055413	0.928431	1.1962	1.04872	1.090871	0.954926	0.903418	1.000199	0.926143	1.021894	0.900314	0.859894
Q6E0U4	1	0.925724	1	0.831645	1	0.88153	0.69948	0.802313	0.624471	0.822551	0.643939	0.809329	0.805176	0.772631	0.923534	0.775146	0.897532	0.830513
P01718	1	0.62568					0.86965	0.831504					0.485635	0.697819				
Q9NX70	1	0.434382					1.990317	0.703512					0.407993	0.837113				
P52799	1	0.817765	1	0.752394	1	0.861883	1.03886	1.003091	0.840812	0.960088	0.972232	1.00258	1.201733	0.975253	1.04115	0.93731	1.108828	0.992818
Q9UK05	1	1.012003					0.899516	0.999927					0.947691	0.807112				
P55774	1	0.793343					1.09489	1.174376					0.805669	0.759109				
Q8NGL6	1	1.18218					1.690188	2.082029					1.199671	1.570983				
O76061	1	0.890678	1	1.045789	1	0.908601	1.155027	0.989087	0.735458	0.959655	0.814789	1.059979	0.977339	1.144891	0.976307	0.869344	1.097617	0.987029
P63129	1	1.210343					6.033289	1.064409					0.18202	1.218109				
Q14393	1	0.711229	1	1.143168	1	0.996797	1.23442	0.861384	1.135522	0.975962	0.970888	0.877869	0.769204	0.885289	1.316087	1.164866	0.90522	0.888861
P0DOX5			1	0.752794	1	0.801417			2.37297	0.744526	2.557839	0.718435			0.401452	0.933671	0.422727	0.926124
B9A064			1	0.875626	1	0.909033			1.027743	0.979112	1.076543	0.963096			0.492614	1.093763	0.602924	1.133221
P63261			1	2.386594	1	2.615483			0.632792	0.806697	0.975339	0.870061			1.032567	0.954073	1.253396	0.969575
P04259			1	1.321296	1	0.909283			1.556771	0.579847	0.473546	0.294065			0.210567	2.261786	0.286532	0.994994
Q7Z794			1	0.783497					1.178879	0.999336					0.695705	1.485107		
P02538			1	1.190695					1.948805	0.833101					0.12301	2.437721		
P13646			1															
Q29940			1	0.624709					0.911212	0.745904					0.444133	1.287609		
Q9UQM7			1	1.192365	1	1.632605			1.110853	0.551814	1.122568	0.590417			0.872921	0.715393	0.838571	0.655757
P04350			1	1.44999					1.448182	0.939136					1.045245	0.821603		
O76009			1		1	0.65016					1.047313	6.859023					0.90185	0.665669
Q14525			1															
P35900			1															

O43707	1	1.586111	1	2.400336	0.591269	0.832482	0.706206	0.934769	1.000346	1.076172	1.175619	1.158291
P27348	1	0.977141	1	1.148748	2.638212	0.64575	0.768103	0.602619	0.809752	0.566147	0.529485	0.604444
P04899	1	1.099115	1	1.233801	0.764985	0.63625	1.114933	0.447927	0.900582	0.859518	1.043176	0.903547
P13929	1	1.192572	1	1.191871	0.874225	0.846848	0.750674	0.799355	0.817371	1.112814	0.981281	1.086328
P13688	1	1.057844	1	0.681868	1.180644	0.872675	1.164691	1.293931	0.92375	0.87501	1.110462	1.086513
Q86Y46	1	1.308054	1	1.989355	1.082036	1.298455	0.976552	0.780727	0.793253	3.079991	0.900215	2.390305
Q14532	1	0.895792			1.24177	6.533278			0.899439	0.960702		
P04430	1	0.880207	1	0.988373	0.62293	0.702116	0.450498	0.566676	0.482979	0.65377	0.563013	0.574753
A0A075B6R2	1	0.559754	1	0.641419	0.737101	0.713008	0.836842	0.789502	0.257539	0.556322	0.261545	0.614362
P06753	1	1.525904	1	1.759155	1.303518	2.160737	1.08857	2.198504	0.551112	0.689624	0.874456	0.911138
A0A0B4J1X8	1	0.906123	1	0.899883	0.61844	0.884738	0.693629	0.939616	0.740343	0.595179	0.80761	0.614773
Q3ZCW2	1	1.102258	1	0.791162	0.601575	1.449547	0.579483	1.527431	1.435637	1.357448	0.916976	0.914962
Q6PCE3	1	0.823614	1	0.857196	1.193099	1.185874	1.393961	1.110203	0.769328	0.639192	0.8691	0.67884
O60449	1	1.60658			1.011658	1.125776			1.405807	1.082661		
Q8IXJ6	1	1.269891	1	1.329467	0.7885	0.648601	0.866476	0.663872	0.737441	0.736122	0.745672	0.736443
P17405	1	0.986124	1	0.864745	1.483837	1.244171	1.235797	1.018226	1.283759	1.221696	0.993397	1.060165
P09211	1	1.053566	1	1.19786	0.827178	1.115376	0.442851	1.111311	0.862933	0.947928	1.103238	0.995221
P60953	1	0.87095	1	0.94173	0.538817	1.056632	0.903311	0.992219	0.792618	0.511446	0.653919	0.65249
Q8N6Q3	1	0.812346			0.504736	0.701148			2.061354	1.03084		
P28062	1	0.851413	1	1.094147	1.088255	0.831661	1.231185	0.922226	0.709439	0.986143	0.882074	1.100931
P40121	1	1.094667	1	1.353932	0.809855	0.872947	0.796086	0.968878	1.105008	1.81193	1.007117	1.408423
P04054	1	1.030936	1	1.185725	0.774055	0.995477	0.863444	0.942974	1.208341	0.911242	1.229936	0.875558
P29122	1	0.836796	1	0.689131	0.659074	0.976815	0.72541	0.816247	1.004836	0.817058	0.826835	0.863602
P22392	1	1.572567	1	0.832443	0.998175	1.157114	1.080413	1.179818	1.493808	1.100545	1.432439	1.306997
Q05707	1	0.950913			0.808785	1.081209			0.781958	0.614482		
P01127	1	0.769147	1	1.126646	1.083048	0.787408	1.033897	0.777229	0.690708	0.954991	0.996465	1.009799
P20701	1	0.91156	1	1.115301	1.05333	0.948632	1.146178	0.933475	1.22832	1.018164	1.357251	1.123006
P08493	1	0.500863	1	0.517898	1.053978	0.938348	1.184634	0.976191	0.970676	0.84148	0.909991	0.788597
Q9Y4E6	1	0.890599	1	0.690341	1.161853	0.686078	1.012351	0.502538	0.946256	0.92036	0.757351	0.905703
Q96QR1	1	0.76723	1	1.700027	1.0861	0.780852	1.176167	0.794493	0.697822	0.594951	0.839718	0.975249
P30740	1	1.211301	1	1.145205	1.547303	1.17535	1.370272	0.982536	0.996256	1.548542	1.120594	1.295585
O95633	1	0.992537	1	0.628259	0.852077	0.975734	0.957341	0.88268	1.210109	1.010189	1.187735	0.946584
Q13404	1	1.276385	1	1.214651	0.952954	1.34982	0.967472	1.337866	1.310363	1.288999	1.28486	1.373862
P01111	1	0.677354			0.846964	0.635471			0.558666	0.335022		
O43405	1	0.861635	1	1.080309	0.682212	0.907746	0.798671	0.870109	0.867252	0.610129	1.096877	0.651166
Q13418	1	0.76438	1	0.745482	0.601553	0.736096	0.404108	0.697226	0.853409	0.975469	0.574489	0.525996
P19440	1	1.342417	1	0.974676	1.120866	1.374426	1.051111	1.015579	0.7201	1.005925	0.531474	0.985435
Q9GZM5	1	1.073887	1	1.080738	1.220521	1.183577	1.227682	1.105296	1.244597	1.33282	1.21687	1.25811
Q92187	1	1.432027	1	1.368094	0.952061	0.886156	0.878433	0.827381	0.918384	0.838395	0.826595	0.852644
P01717	1	0.883712	1	0.992655	1.039853	0.878361	0.876947	0.92011	0.664637	0.728941	0.708149	0.824357
P62805	1	0.975415	1	0.724089	1.249925	2.795675	1.8376	2.856749	0.865751	1.107698	0.646783	0.952324
P50281	1	0.841106	1	0.762735	1.040645	0.845322	0.928861	0.744558	0.940132	1.070501	0.925577	0.996645
Q9Y2E5	1	1.240068	1	0.971381	0.931538	1.088136	0.839458	0.889791	0.523779	1.042648	1.257846	0.968044
P06280	1	1.457242			0.944549	0.640151			0.830076	1.17741		
P23229	1	1.022487			0.711314	0.72001			0.597226	0.595479		
Q9Y6N6	1	1.263372	1	1.168699	0.964164	1.054296	1.124369	0.97921	1.113654	0.937687	1.200891	0.979549
Q8IUK5	1	1.640672	1	0.788224	1.650771	1.303485	0.961202	0.702266	1.234009	1.398092	0.896583	1.886799
Q9UL25	1	1.342548	1	1.522992	0.585123	0.816028	0.553026	0.994913	0.735922	0.541738	0.877844	0.699929
Q9NTN9	1	1.621283			1.211477	0.953836			0.989435	1.176433		
P62917	1	1.116649			1.164911	1.056303			1.173357	1.019924		

P22455	1	1.015037	1	1.162435	1.335567	1.152405	1.232832	1.122835	1.560412	0.559314	1.687827	0.492798
P53396	1	1.414364	1	1.286514	0.823585	0.978634	0.852102	0.972736	1.039117	1.057236	1.019328	0.777556
Q9UBS4	1	1.414673			0.54411	0.726033			1.004236	1.232248		
P16233	1	1.152648	1	1.470997	1.450532	1.146109	2.393497	1.865179	1.704955	1.542368	2.323738	2.381643
Q96CM4	1	0.977153	1	0.926817	0.558953	0.67847	0.486914	0.682376	0.541414	0.588385	0.47001	0.603476
A0A0G2JS06	1	0.325793	1	0.327109	0.55219	0.533658	0.529458	0.503432	0.323107	0.368709	0.291849	0.324795
O60664	1	0.859749			0.54815	0.863128			0.775223	0.717863		
Q96E52	1		1									
P12429	1	0.691776	1	0.771324	0.561456	0.905791	1.298397	0.907408	0.626631	0.682685	0.942355	1.402891
P22413	1	1.091366	1	1.239499	0.942761	1.084633	0.926099	1.135831	1.080396	1.14864	1.194999	1.245433
Q9UL03	1	1.266061			1.77804	1.788461			1.293712	1.2041		
P17050	1	0.940293	1	0.94592	0.931298	0.804947	0.766262	0.781106	0.878569	1.308658	0.96824	0.840083
O75594	1	1.776696	1	1.500473	1.04929	0.748314	0.899636	0.801339	1.089435	0.943196	0.88109	0.823068
Q00532	1	1.041379	1	1.215662	0.937394	0.964107	1.024921	1.029311	1.357265	1.318608	1.435401	1.457462
O15467	1	1.275741			1.534053	0.760749			0.5875	0.859101		
O75822	1	0.711578			4.372778	1.088905			0.79894	1.433271		
Q96CG8	1	1.188286			0.812769	0.847438			0.990198	0.611674		
Q9Y624	1	0.777305	1	1.107457	0.812215	0.830642	0.918794	0.748881	0.642751	0.558329	0.632246	0.487677
P54725	1	0.72573			0.773178	1.290788			0.973688	0.782728		
Q9UJU6	1	1.006872			0.721515	1.503404			1.048518	0.807348		
Q92876	1	1.14272			0.857003	0.916905			0.724999	0.73563		
P00387	1	1.438383	1	0.701993	0.876963	0.916141	0.928177	0.564486	1.170418	0.855949	0.875963	0.94512
Q15836	1											
P59998	1	0.891391			0.748484	0.856914			0.896779	0.699622		
P20138	1	1.370191			1.276826	1.107103			1.331539	1.045439		
A0A087WSY6	1	0.592263			1.094231	1.518058			1.66139	1.967233		
A7E2Y1	1	0.99382			1.987461	1.376792			1.016599	1.322592		
Q9H3S1	1		1	1.020625		1.122067	1.029507	0.779259		0.909272	0.857828	1.161535
Q8IZQ1	1	0.61549	1	0.735631	1.400356	1.354148	1.364006	1.280788	2.152216	1.102585	1.164414	1.090689
Q9Y6N7	1	0.739299			0.879793	0.871282			1.037913	0.676325		
P17655	1	1.23608	1	1.146634	0.792193	1.099562	0.87936	0.999979	1.141995	1.149176	0.994645	0.961149
P15907	1	1.677885	1	0.96823	0.686333	0.985353	0.645705	0.748292	1.166776	1.056682	0.924547	0.956251
A0A1B0GTC6	1	0.953835	1	0.968163	0.677468	0.67043	0.704796	0.699762	0.534554	0.52242	0.680857	0.893826
Q6P4E1	1	0.935911			1.145421	1.129326			1.386037	1.281709		
P84085	1	0.667096			1.067182	1.149093			0.632974	0.822879		
P35367	1	0.939509	1	0.848396	1.463015	2.026145	1.536751	2.071643	1.372148	1.23536	1.499127	1.325087
P34059	1	1.779815			1.097068	1.11843			1.445849	1.404288		
Q8TEU7	1	0.344779	1	0.373166	0.756711	0.731352	1.008335	0.966394	1.410194	1.07209	1.081809	0.982582
Q04446	1	1.373707	1	1.326042	1.398309	1.54963	1.383087	1.142855	1.008016	1.519076	1.094278	1.201798
Q9UJX2	1	2.32975	1	2.376562	0.354193	1.372133	0.419741	1.450081	2.004212	0.386591	2.108282	0.34227
Q13683	1	0.904106			0.971867	1.163005			1.074788	0.991177		
P42338	1	0.660366			0.491987	0.695181			0.604754	0.544064		
P21291	1	1.375879	1	1.73487	0.800555	1.060855	0.967066	1.433257	1.118145	0.875175	1.108307	0.959609
Q12864	1	1.689644			1.185569	1.344327			1.331732	1.476725		
O75069	1	1.118124			1.087025	0.56328			0.443614	0.714116		
P20930	1	0.707875			1.57203	1.273622			1.072136	1.648258		
O95782	1	0.532758			1.416617	0.895338			0.867396	1.508799		
O95866	1	1.470508	1	1.640313	0.620503	1.233735	0.785225	1.266926	0.518799	0.62089	0.569621	0.762543
O94856	1	2.117897			1.079971	0.941584			1.124175	1.044139		
P16871	1	0.566871	1	0.923648	0.940705	0.73279	1.276232	0.875358	0.963366	0.807281	1.108573	1.207509

Q9BXJ4	1	3.214771			1.138898	1.371871			2.118819	1.681889		
Q8NHQ9	1	0.490989	1	0.467939	0.251803	0.335838	0.087864	0.173142	0.267756	0.312765	0.201158	0.183541
Q9H2X3	1	1.49989			0.966073	1.136394			1.174799	1.084974		
P0C7U1	1	1.697833	1	1.314505	1.763685	1.060314	1.607621	0.940603	0.888962	1.303472	0.805922	0.981989
P31153	1	1.111627	1	0.907353	1.522412	0.942276	1.540929	1.051752	1.005286	1.105739	0.999895	1.034527
O14773	1	1.013886	1	1.160312	1.080884	0.951094	1.196698	0.854989	1.095719	0.857287	1.15471	1.099433
O43768	1	0.409304	1	0.362875	0.982302	0.79087	0.892507	0.716618	0.267512	0.293454	0.239044	0.270834
Q99435	1	0.906199			1.104751	1.09302			0.835027	0.83557		
P22626	1	1.241318	1	0.886643	0.749636	1.076188	0.615352	1.120249	0.776606	0.989993	0.770532	0.877754
Q9H4A4	1	1.23688			1.246235	0.974147			0.617307	0.987162		
Q9UP79	1	1.258011	1	1.078146	1.108996	0.899241	1.02799	0.824755	0.97773	0.729856	0.664206	0.746874
P98164	1	0.693588			1.104862	0.819701			0.599371	0.916122		
Q01638	1	1.996787			1.12481	0.5618			1.351156	1.974753		
P21399	1	0.535096	1	0.933358	0.525934	0.909323	0.666806	1.019577	0.677692	0.440568	0.94721	0.585135
Q06828	1	1.167347			1.279552	0.948809			0.976759	1.078555		
Q5SQ64	1	1.201438			1.66926	1.19222			1.08875	1.238778		
Q15149	1	1.72364			0.775515	1.08945			1.146544	1.035017		
P02545	1	2.98628	1	0.703442	2.392736	2.338262	0.715959	0.852322	2.929759	2.336531	0.82328	0.75096
P14920	1											
Q9Y6Y8	1	1.076982	1	1.041784	0.441902	0.707019	0.404092	0.66966	0.741983	0.901845	0.752097	0.858906
Q8TDD5	1	0.588586			3.721529	0.737865			0.128896	1.173658		
Q9BWS9	1	1.073106			0.975098	1.138712			1.07628	1.076769		
Q7Z6J4	1	0.479303			1.921172	1.538015			0.890332	1.445173		
P49736	1	1.36784	1	1.239851	1.816834	0.998846	1.37046	0.589491	0.784884	0.94546	0.488433	0.710041
Q9UNN5	1	0.603465			0.736299	0.897579			1.737009	1.338528		
Q16891	1	0.761044			0.640301	0.623104			0.725219	0.559767		
P47756	1	1.319481	1	1.310288	0.98191	1.04885	0.721775	0.88682	0.973692	0.811184	0.824216	0.911953
P04179	1	0.750032			0.173381	1.102347			0.684781	0.483137		
O75368	1	1.003956	1	1.309727	1.098844	1.128498	0.969042	0.683086	0.891884	1.010457	0.621387	1.302193
Q92614	1	0.878112			0.671796	0.733889			0.668344	0.640194		
P68431	1	0.741733	1	0.69652	1.96851	5.508723	1.916935	4.223946	0.973852	1.154706	1.089773	1.078783
P21810	1	1.112333			0.799155	0.897416			0.747886	0.96152		
P42766	1											
Q12797	1	1.148552	1	1.586515	0.696218	0.749822	1.066027	1.051242	0.655374	0.407433	0.82004	0.530292
P06756	1	0.617888			0.765423	0.67469			0.600601	0.550635		
Q9NPG4	1	1.43391	1	1.136565	0.942751	1.183636	0.963333	1.047402	1.029849	0.831427	0.658622	0.847456
Q9NU22	1	0.720872	1	0.640831	0.961915	1.119643	0.990249	1.162529	1.126979	1.150448	1.139945	1.170049
P09110	1	1.044231			2.440139	1.317162			1.348249	1.318045		
Q15058	1	0.761282	1	0.310526	1.740742	1.311163	1.901224	1.597073	0.672631	0.75744	0.657161	0.956009
O75683	1	0.762474			2.401157	1.218187			0.652063	1.125642		
Q4G163	1	0.500796			0.650707	0.911182			0.957598	0.925648		
P29083	1	0.820626	1	0.998616	1.537373	1.709393	1.494466	1.901982	1.547931	1.38111	1.681492	1.366764
A0A0C4DH34	1	0.599143	1	0.57326	0.895258	0.798944	1.089711	0.859871	0.628318	1.210561	0.666091	1.360964
A6NHR9	1	0.786628			0.288116	0.978357			0.172663	0.158838		
A5YKK6	1	0.974798			1.094571	1.110131			1.181099	1.124794		
Q5T1H1	1	0.773326			1.004458	0.981811			1.116678	0.969633		
Q8N4C7	1	0.511703			1.390009	0.485345			0.398307	0.695311		
P15586	1	0.953773			0.928988	0.882761			1.055798	1.042723		
Q6KB66	1	1.357039	1	0.649458	1.945154	0.861362	1.290631	1.492177		2.944077	1.201633	2.198739
A6NDX5	1	0.539934	1	0.54185	5.032092	1.089081	5.308829	1.099926	0.28459	1.628571	0.280851	1.499498

P50748	1											
P0C7W6	1	0.763995	1	0.842807	1.007447	1.06184	1.230903	1.123282	0.817825	0.904516	0.93985	0.970858
Q2TAC6	1	1.110055			5.118563	1.379885			0.557039	1.357163		
Q86YP4	1	2.325454			1.452678	1.198325			1.302629	0.468966		
P29317	1	0.979944			0.817341	0.799018			0.645515	0.881656		
Q4G0X9	1	0.535031	1	0.414861	0.871172	0.815763	0.911614	0.669467	0.591242	0.550413	0.431812	0.46151
Q96L50	1	0.749694			5.807937	1.20196			0.182563	0.972455		
Q9UGM3	1	0.96748	1	0.917188	0.74071	0.977562	0.815726	0.980277	0.814866	0.877494	0.828354	0.917438
Q5VWQ8	1	1.36612	1	2.186801	1.894992	0.977725	1.348288	1.309354	0.617141	1.138632	1.147921	0.719191
P19086	1	1.839358	1	1.776821	0.831253	0.981188	0.696786	0.934496	0.99755	1.062668	0.976806	1.057666
Q7LGC8	1	1.104667			0.973771	1.130358			0.806931	1.141212		
Q9UIW2	1	0.66072			1.262328	0.958985			0.7988	1.037983		
O43933	1	0.715041	1	0.717682	0.337054	0.993123	0.178412	1.040495	1.214209	0.804335	1.144174	0.773656
Q8IWK6	1	0.852516			0.879664	1.00715			1.237693	1.12406		
Q8IXT1	1	1.419728			0.181579	0.565768			0.513595	0.599186		
Q9BZQ6	1	1.080704			1.874613	2.105207			1.061045	1.668629		
Q9NP70	1	1.185167			1.263643	1.064012			1.26765	1.32642		
P20160	1	0.816899	1	0.866325	1.408075	1.435012	1.467397	1.370561	0.762742	0.820391	0.580571	0.955007
Q0VD83	1	0.975698			0.729659	0.789686			0.58432	0.887088		
Q9BV57	1	0.484546			0.855087	0.943354			1.068421	0.976653		
Q9Y580	1	1.419992			1.370251	0.93103			1.032432	1.320451		
Q5JS37	1	0.919364			1.96299	0.677845				0.937786		
Q8IYS5	1	1.272441			1.340735	0.978303			1.19378	0.789239		
Q9UBC9	1	1.383878	1	1.086625	0.78535	1.130127	0.648572	1.105284	1.077258	0.865819	1.059277	0.877589
Q8IWA4	1	0.325157	1	0.314729	0.16954	0.426642	0.04481	0.444709	0.070147	0.076728	0.066687	0.05827
P68366			1	0.935604			0.409683	0.642001			0.472768	0.634747
P01764			1	0.946533			0.686107	0.821163			0.650104	0.82328
P0DOX3			1	0.944077			0.737412	0.693293			0.684623	0.749079
P30501			1	0.940316			1.247308	1.141154			0.84548	1.032412
Q9H2X0			1	1.246985			1.003363	1.048139			1.422752	1.627174
P07951			1	0.71169			0.627747	0.649817			1.647628	1.270885
P29966			1	1.369996			0.864858	0.877449			0.849079	1.003357
Q92764			1	1.096438			1.184405	6.354861			0.913719	0.855709
A8MVU1			1	1.263695			1.02838	1.11594			0.861932	1.149704
Q8TD31			1	0.950643			0.309043	0.620684			0.478466	0.389147
P48163			1	1.111845			0.751496	0.957547			0.870022	0.195537
Q13642			1	0.966003			0.659961	2.174462			0.50932	0.67652
Q16143			1	1.032624			0.468894	0.994459			0.776063	0.620077
P28482			1	1.309162			0.775423	0.858351			0.853059	0.712329
Q13232			1	0.944019			0.970432	1.014224			1.18806	0.848452
A0A075B6J9			1	1.407165			1.153484	0.605291			0.591393	0.897199
Q99439			1	1.34303			0.412511	1.640245			0.454281	0.533098
Q96B86			1	1.543592			0.902047	0.820888			0.680207	0.822633
P61019			1	1.429845			1.097738	1.357678			0.583561	0.787299
Q9UBU7			1	0.939117			0.843164	0.946825			1.373858	0.982627
Q08345			1	1.793399			1.822822	1.157374			0.97256	1.421432
O75534			1	0.515391			1.025835	0.895698			0.911567	1.405302
O94973			1	0.483458			1.921056	0.838787			0.5522	1.797385
P63220			1									
P16403			1	1.21242			0.878887	1.940421			0.923432	0.959665

P01160	1	0.681967	0.950409	0.815258	1.193095	1.04635
A0A0B4J1V2	1	0.468422	0.86057	0.698929	0.256271	0.670431
Q9BV35	1	0.953345	1.14666	1.045953	0.980553	1.039834
Q5TFQ8	1	1.330811	1.094458	0.896356	0.804988	0.912782
Q9Y376	1	0.860796	0.879406	0.975283	1.023482	1.090911
Q9H4M9	1	1.490853	0.897559	0.761628	1.048905	0.992506
Q9Y2Y8	1	1.038537	1.432887	0.905282	0.732815	0.839072
Q96IS3	1	0.987711	1.04784	1.191271	2.155023	1.111213
A0A0A0MT89	1	0.511967	0.562838	0.613962	0.601254	0.640755
O94923	1	0.816517	1.065408	0.905273	0.713177	0.882325
Q13042	1	0.185643	1.313804	1.149327	0.984925	1.295396
Q12894	1	0.730927	1.325923	0.888145	1.341885	1.124798
Q9NQW7	1	0.866581	0.848064	1.017457	0.796082	0.909931
Q9H1Z8	1	1.025989	0.657485	1.208746	1.690368	1.211991
O60883	1	1.833606	1.199123	1.336386	1.260183	1.237404
Q8N465	1	2.232191	1.064476	1.083147	1.657092	1.740876
A5YM72	1	1.144253	1.263363	1.499957	1.722884	0.787755
P50876	1	2.224263	3.892972	1.99083	1.617285	2.843866
P06858	1	1.012048	0.917958	1.068627	0.795627	1.003145
Q9GZX9	1	1.03419	1.071735	0.948451	1.162312	0.978782
A6NDG6	1	0.620671	0.963227	0.950319	0.955028	0.589516
Q9BZR6	1	1.545883	1.003421	1.007359	0.77691	0.710893
Q9UPN3	1	0.717479	0.519562	1.254682	1.95961	0.401772
Q9H5L6	1	0.473252	1.572921	1.060025	0.67614	1.201207
Q6UWL2	1	0.783592	0.735264	0.838595	0.930433	0.786687
P04216	1	1.232871	1.549364	0.971217	1.081556	1.076122
Q9NS62	1	1.059598	0.844032	1.101543	1.32105	0.991713
Q93034	1	2.523454	1.568126	1.061613	0.97809	0.264079
Q96DT5	1	0.412735	2.539646	1.22941	0.447813	1.674278
Q05586	1	0.975592	1.169238	0.897654	1.143947	1.310139
Q99523	1	1.192163	0.787862	0.880675	0.753598	0.580245
P45877	1	0.749542	1.189813	1.05797	0.929443	1.07068
P01225	1	0.514807	0.563171	0.736744	0.421731	0.663864
P63167	1	0.958805	0.662366	2.067546	0.812853	0.756309
Q9BPX5	1	0.969473	0.744429	0.775104	0.980401	0.76603
P22735	1	0.765687	1.102293	1.242417	0.8016	1.590199
Q9BX84	1	1.423959	0.742521	1.059251	1.488189	1.306853
O14745	1	1.074189	0.722445	1.290203	0.927692	1.007121
O95071	1					
Q15283	1	0.555375	2.299556	1.210551	0.541133	1.098108
Q96EP1	1	0.736557	1.682067	1.939183	1.160457	1.146064
Q9BQS8	1	0.901089	1.483073	1.213658	0.745299	0.819284
O95210	1	0.743487	0.748501	0.919839	0.968828	0.81006
O94986	1	0.554195	1.36363	0.983226	0.808789	0.736429
Q86YW9	1	1.159839	0.675317	0.994365	0.912034	0.954838
Q9UKW4	1	0.986941	0.817904	0.859949	0.826404	0.686484
P63218	1	0.905948	0.593595	0.691657	0.856043	0.734147
Q9BRS2	1	0.519413	2.146971	0.734562	0.248662	1.34416
Q6W2J9	1	0.295175	0.708724	0.807884	0.852666	0.616382
Q68DL7	1	0.874218	1.401021	1.104478	0.735951	1.416339

Q7Z5M8	1	0.72701	0.57116	0.381468	0.325467	0.344573
P98174	1	0.624079	0.816741	0.998077	1.023772	0.940533
P05981	1	1.010597	3.466307	0.696346	0.360854	0.889707
Q9ULD2	1	1.222054	1.321061	1.054835	1.198796	1.310214
Q6P4Q7	1	0.511501	0.274739	0.399001	0.362802	0.363405
Q9Y315	1	1.25648	1.164217	0.976036	0.916078	1.184302
P22303	1	3.327308	1.37885	0.835641	1.029448	1.983896
Q96SM3	1	1.346473	0.835253	1.037974	1.184254	0.919799
P26992	1	0.596259	1.05407	0.913051	1.198042	0.84355
O75150	1	0.82944	1.13592	1.173785	0.969691	1.347145
