# Supplementary material for: Circulating proteomic panels for risk stratification of intracranial aneurysm and its rupture
Source: EMBO Mol Med. 2022 Jan 3;14(2):e14713. doi: 10.15252/emmm.202114713 (PMC8819334; doi:10.15252/emmm.202114713)
Supplement: Supplementary file 22 — Source Data for Figure 4 [file EMMM-14-e14713-s014.pdf]

SourceDataForFigure4A

Tissue

No.	Uniprot Accession Number	Uniprot ID	Gene Symbol
1	Q9Y2A7	NCKP1 HUMAN	NCKAP1
3	P06396	GELS HUMAN	GSN
4	P14649	MYL6B HUMAN	MYL6B
10	P18206	VINC HUMAN	VCL
11	P17661	DESM HUMAN	DES
12	P12814	ACTN1 HUMAN	ACTN1
13	P62736	ACTA HUMAN	ACTA2
15	O00401	WASL HUMAN	WASL
16	O43707	ACTN4 HUMAN	ACTN4
17	P35609	ACTN2 HUMAN	ACTN2
18	P35749	MYH11 HUMAN	MYH11
20	O00151	PDLI1 HUMAN	PDLIM1
21	O60725	ICMT HUMAN	ICMT
22	O75110	ATP9A HUMAN	ATP9A
23	A6NMZ7	CO6A6 HUMAN	COL6A6
25	P39060	COIA1 HUMAN	COL18A1
26	P39059	COFA1 HUMAN	COL15A1
27	Q9Y6F6	IRAG1 HUMAN	IRAG1
28	P60981	DEST HUMAN	DSTN
29	Q05707	COEA1 HUMAN	COL14A1
39	Q9BQI4	CCDC3 HUMAN	CCDC3
43	P20339	RAB5A HUMAN	RAB5A
44	Q8TDZ2	MICA1 HUMAN	MICAL1
45	Q9UQR1	ZN148 HUMAN	ZNF148
46	Q53SF7	COBL1 HUMAN	COBLL1
47	O95631	NET1 HUMAN	NTN1
48	Q9NZN4	EHD2 HUMAN	EHD2
52	P09525	ANXA4 HUMAN	ANXA4
53	Q16832	DDR2 HUMAN	DDR2
55	P10301	RRAS HUMAN	RRAS
56	P21333	FLNA HUMAN	FLNA
58	O43157	PLXB1 HUMAN	PLXNB1
59	Q8NF91	SYNE1 HUMAN	SYNE1
60	P21291	CSRP1 HUMAN	CSRP1
61	Q6XZF7	DNMBP HUMAN	DNMBP
66	Q8IVF7	FMNL3 HUMAN	FMNL3
68	Q92629	SGCD HUMAN	SGCD
70	Q5VT25	MRCKA HUMAN	CDC42BPA
72	O43556	SGCE HUMAN	SGCE
75	Q9BR76	COR1B HUMAN	CORO1B
76	P28906	CD34 HUMAN	CD34
77	Q9Y281	COF2 HUMAN	CFL2
79	Q9BR39	JPH2 HUMAN	JPH2
83	Q8NB37	GALD1 HUMAN	GATD1
85	Q6NZI2	CAVN1 HUMAN	CAVIN1
86	Q96Q05	TPPC9 HUMAN	TRAPPC9
89	P09619	PGFRB HUMAN	PDGFRB
90	O43639	NCK2 HUMAN	NCK2
91	P42025	ACTY HUMAN	ACTR1B
92	O00291	HIP1 HUMAN	HIP1
93	Q96RF0	SNX18 HUMAN	SNX18
94	Q5VZK9	CARL1 HUMAN	CARMIL1
95	Q9UKS6	PACN3 HUMAN	PACSIN3
96	P00533	EGFR HUMAN	EGFR
97	Q9Y5X1	SNX9 HUMAN	SNX9
98	Q99972	MYOC HUMAN	MYOC

Serum

NO.	Uniprot Accession Number	Uniprot ID	Gene Symbol
1	P02675	FIBB HUMAN	FGB
2	P02679	FIBG HUMAN	FGG
3	P02741	CRP HUMAN	CRP
4	Q13093	PAFA HUMAN	PLA2G7
5	Q86Z14	KLOTB HUMAN	KLB
6	P05154	IPSP HUMAN	SERPINA5
7	Q9H4F8	SMOC1 HUMAN	SMOC1
8	Q9UBX7	KLK11 HUMAN	KLK11
9	Q9UPZ9	CILK1 HUMAN	CILK1
10	P02671	FIBA HUMAN	FGA
11	Q9H9E3	COG4 HUMAN	COG4
12	Q9P2E9	RRBP1 HUMAN	RRBP1
13	P00738	HPT HUMAN	HP
14	P00740	FA9 HUMAN	F9
15	Q8WU03	GLYL2 HUMAN	GLYATL2
16	Q5D862	FILA2 HUMAN	FLG2
17	Q86YZ3	HORN HUMAN	HRNR
18	P48681	NEST HUMAN	NES
19	P02750	A2GL HUMAN	LRG1
20	P00918	CAH2 HUMAN	CA2
21	Q9Y279	VSIG4 HUMAN	VSIG4
22	P69892	HBG2 HUMAN	HBG2
23	P00915	CAH1 HUMAN	CA1
24	Q06141	REG3A HUMAN	REG3A
25	Q15485	FCN2 HUMAN	FCN2
26	Q03181	PPARD HUMAN	PPARD
27	Q9NP84	TNR12 HUMAN	TNFRSF12A
28	A0A0C4DH25	KVD20 HUMAN	IGKV3D-20
29	P01701	LV151 HUMAN	IGLV1-51
30	P05160	F13B HUMAN	F13B
31	Q96KG7	MEG10 HUMAN	MEGF10
32	A0A1B0GTC6	CC085 HUMAN	C3orf85
33	P63267	ACTH HUMAN	ACTG2
34	Q8NDA2	HMCN2 HUMAN	HMCN2
35	P09471	GNAO HUMAN	GNAO1
36	Q12797	ASPH HUMAN	ASPH
37	Q14766	LTBP1 HUMAN	LTBP1
38	O43707	ACTN4 HUMAN	ACTN4
39	P01782	HV309 HUMAN	IGHV3-9
40	Q9Y6Y8	S23IP HUMAN	SEC23IP
41	Q9HCN6	GPVI HUMAN	GP6
42	A0A075B6K4	LV310 HUMAN	IGLV3-10
43	P00748	FA12 HUMAN	F12
44	Q8IXJ6	SIR2 HUMAN	SIRT2
45	P22894	MMP8 HUMAN	MMP8
46	Q86UX7	URP2 HUMAN	FERMT3
47	Q9HBI1	PARVB HUMAN	PARVB
48	P01860	IGHG3 HUMAN	IGHG3
49	Q9UMX5	NENF HUMAN	NENF
50	Q7Z5L0	VMO1 HUMAN	VMO1
51	O60641	AP180 HUMAN	SNAP91
52	Q5VTJ3	KLD7A HUMAN	KLHDC7A
53	Q05315	LEG10 HUMAN	CLC
54	P40197	GPV HUMAN	GP5
55	P01709	LV208 HUMAN	IGLV2-8
56	Q9H6X2	ANTR1 HUMAN	ANTXR1

Literature

No.	Uniprot Accession Number	Gene Symbol	Uniprot ID
1	P05164	MPO	PERM HUMAN
4	P24821	TNC	TENA HUMAN
5	P02649	APOE	APOE HUMAN
7	P13500	CCL2	CCL2 HUMAN
9	P01019	AGT	ANGT HUMAN
10	P16581	SELE	LYAM2 HUMAN
14	Q07325	CXCL9	CXCL9 HUMAN
15	P02778	CXCL10	CXL10 HUMAN
16	P51671	CCL11	CCL11 HUMAN
17	P10145	CXCL8	IL8 HUMAN
18	Q16552	IL17A	IL17 HUMAN
19	P14091	CTSE	CATE HUMAN
20	P05231	IL6	IL6 HUMAN
21	O00206	TLR4	TLR4 HUMAN
22	P01024	C3	CO3 HUMAN
23	P01031	C5	CO5 HUMAN
25	P01135	TGFA	TGFA HUMAN
26	P03956	MMP1	MMP1 HUMAN
27	P08254	MMP3	MMP3 HUMAN
28	P09237	MMP7	MMP7 HUMAN
29	P09238	MMP10	MMP10 HUMAN
30	P39900	MMP12	MMP12 HUMAN
31	P08253	MMP2	MMP2 HUMAN
32	P14780	MMP9	MMP9 HUMAN
33	P15692	VEGFA	VEGFA HUMAN
34	P19320	VCAM1	VCAM1 HUMAN
35	P05362	ICAM1	ICAM1 HUMAN
36	Q12762	ECRP	Q12762 HUMAN
38	P11678	EPX	PERE HUMAN
39	Q9HD89	RETN	RETN HUMAN
42	P13224	GP1BB	GP1BB HUMAN
43	B3VMW0	#N/A	B3VMW0 HUMAN
44	P59666	DEFA3	DEF3 HUMAN
45	B2MV14	LTF	B2MV14 HUMAN
48	L7UUZ7	ITGB3	L7UUZ7 HUMAN
49	P08514	ITGA2B	ITA2B HUMAN
51	P12724	RNASE3	ECP HUMAN
53	P08697	SERPINF2	A2AP HUMAN
56	P01889	HLAB	HLAB HUMAN
57	P55058	PLTP	PLTP HUMAN
59	P02765	AHSG	FETUA HUMAN
61	C5IWY0	HLAA	C5IWY0 HUMAN
62	Q07507	DPT	DERM HUMAN
63	Q5HYE3	DKFZp686H1812	Q5HYE3 HUMAN
65	Q6UWY5	OLFML1	OLFL1 HUMAN
66	Q9BXN1	ASPN	ASPN HUMAN
68	P41222	PTGDS	PTGDS HUMAN
69	Q9NRN5	OLFML3	OLFL3 HUMAN
74	P48061	CXCL12	SDF1 HUMAN
77	P55268	LAMB2	LAMB2 HUMAN
78	P05106	ITGB3	ITB3 HUMAN
79	Q96HF1	SFRP2	SFRP2 HUMAN
80	P15088	CPA3	CBPA3 HUMAN
81	P02671	FGA	FIBA HUMAN
83	P20160	AZU1	CAP7 HUMAN
85	P59665	DEFA1	DEF1 HUMAN

99	P09960	LKHA4_HUMAN	LTA4H
101	P07996	TSP1_HUMAN	THBS1
102	O75382	TRIM3_HUMAN	TRIM3
103	P26022	PTX3_HUMAN	PTX3
104	P98160	PGBM_HUMAN	HSPG2
105	Q7Z7G0	TARSH_HUMAN	ABI3BP
109	P22105	TENX_HUMAN	TNXB
111	O00468	AGRIN_HUMAN	AGRN
112	Q13332	PTPRS_HUMAN	PTPRS
113	Q6P0A1	F180B_HUMAN	FAM180B
114	P02462	CO4A1_HUMAN	COL4A1
115	Q9HB63	NET4_HUMAN	NTN4
116	Q9HCB6	SPON1_HUMAN	SPON1
120	Q8IVN8	SBSPO_HUMAN	SBSPON
121	Q99720	SGMR1_HUMAN	SIGMAR1
124	Q8N474	SFRP1_HUMAN	SFRP1
125	Q9UKG1	DP13A_HUMAN	APPL1
126	O00292	LFTY2_HUMAN	LEFTY2
128	P55268	LAMB2_HUMAN	LAMB2
129	Q5JT25	RAB41_HUMAN	RAB41
130	O60645	EXOC3_HUMAN	EXOC3
131	Q9H4A4	AMPB_HUMAN	RNPEP
132	Q9H3U1	UN45A_HUMAN	UNC45A
133	O14791	APOL1_HUMAN	APOL1
135	Q86UP2	KTN1_HUMAN	KTN1
136	Q9BX10	GTPB2_HUMAN	GTPBP2
137	Q93084	AT2A3_HUMAN	ATP2A3
138	P50895	BCAM_HUMAN	BCAM
139	O60499	STX10_HUMAN	STX10
140	O76024	WFS1_HUMAN	WFS1
143	P24593	IBP5_HUMAN	IGFBP5
144	Q6UXV4	MIC27_HUMAN	APOOL
145	P13521	SCG2_HUMAN	SCG2
146	P48449	LSS_HUMAN	LSS
147	P08133	ANXA6_HUMAN	ANXA6
148	O95183	VAMP5_HUMAN	VAMP5
152	O76062	ERG24_HUMAN	TM7SF2
153	O00182	LEG9_HUMAN	LGALS9
155	P49747	COMP_HUMAN	COMP
157	P43121	MUC18_HUMAN	MCAM
161	Q15035	TRAM2_HUMAN	TRAM2
163	O00339	MATN2_HUMAN	MATN2
165	P78356	PI42B_HUMAN	PIP4K2B
167	Q99969	RARR2_HUMAN	RARRES2
168	Q92575	UBXN4_HUMAN	UBXN4
169	Q9Y646	CBPQ_HUMAN	CPQ
171	Q7KYR7	BT2A1_HUMAN	BTN2A1
172	P19075	TSN8_HUMAN	TSPAN8
175	Q7Z5L7	PODN_HUMAN	PODN
176	Q15848	ADIPO_HUMAN	ADIPOQ
178	O60493	SNX3_HUMAN	SNX3
180	O75339	CILP1_HUMAN	CILP
184	Q8WWA0	ITLN1_HUMAN	ITLN1
185	P35070	BTC_HUMAN	BTC
186	Q01973	ROR1_HUMAN	ROR1
187	P32004	L1CAM_HUMAN	L1CAM
188	Q13510	ASAH1_HUMAN	ASAH1
190	P43155	CACP_HUMAN	CRAT
191	P09110	THIK_HUMAN	ACAA1

57	P06312	KV401_HUMAN	IGKV4-1
58	P02775	CXCL7_HUMAN	PPBP
59	P21333	FLNA_HUMAN	FLNA
60	P00488	F13A_HUMAN	F13A1
61	O95236	APOL3_HUMAN	APOL3
62	P0DP03	HVC05_HUMAN	IGHV3-30-5
63	Q9UL25	RAB21_HUMAN	RAB21
64	P27105	STOM_HUMAN	STOM
65	P0DOX7	IGK_HUMAN	#N/A
66	P10720	PF4V_HUMAN	PF4V1
67	O95810	CAVN2_HUMAN	CAVIN2
68	P01780	HV307_HUMAN	IGHV3-7
69	O43852	CALU_HUMAN	CALU
70	A0A0A0MT36	KVD21_HUMAN	IGKV6D-21
71	P02787	TRFE_HUMAN	TF
72	A0A075B6P5	KV228_HUMAN	IGKV2-28
73	Q96CM4	NXNL1_HUMAN	NXNL1
74	P63261	ACTG_HUMAN	ACTG1
75	Q14141	SEPT6_HUMAN	SEPTIN6
76	P02788	TRFL_HUMAN	LTF
77	P01591	IGJ_HUMAN	JCHAIN
78	P04430	KV116_HUMAN	IGKV1-16
79	P60709	ACTB_HUMAN	ACTB
80	P06727	APOA4_HUMAN	APOA4
81	A0A0A0MRZ8	KVD11_HUMAN	IGKV3D-11
82	Q15404	RSU1_HUMAN	RSU1
83	P61224	RAP1B_HUMAN	RAP1B
84	P01619	KV320_HUMAN	IGKV3-20
85	Q9Y490	TLN1_HUMAN	TLN1
86	P07996	TSP1_HUMAN	THBS1
87	P02751	FINC_HUMAN	FN1
88	P01871	IGHM_HUMAN	IGHM
89	P08311	CATG_HUMAN	CTSG
90	Q6UX06	OLFM4_HUMAN	OLFM4
91	P53801	PTTG_HUMAN	PTTG1IP
92	A0A0B4J1X5	HV374_HUMAN	IGHV3-74
93	P21926	CD9_HUMAN	CD9
94	Q86YW5	TRML1_HUMAN	TREML1
95	O43866	CD5L_HUMAN	CD5L
96	P08514	ITA2B_HUMAN	ITGA2B
97	P0DOX6	IGM_HUMAN	#N/A
98	P05106	ITB3_HUMAN	ITGB3
99	Q8IWA4	MFN1_HUMAN	MFN1
100	Q8NHQ9	DDX55_HUMAN	DDX55
101	P13224	GP1BB_HUMAN	GP1BB
102	Q8NI35	INADL_HUMAN	PATJ
103	A2RTY3	HEAT9_HUMAN	HEATR9
104	P0DJI8	SAA1_HUMAN	SAA1
105	P0DJI9	SAA2_HUMAN	SAA2
106	P02741	CRP_HUMAN	CRP
107	P00738	HPT_HUMAN	HP
108	Q6IPM2	IQCE_HUMAN	IQCE
109	Q08830	FGL1_HUMAN	FGL1
110	P26022	PTX3_HUMAN	PTX3
111	P69905	HBA_HUMAN	HBA1
112	Q969E1	LEAP2_HUMAN	LEAP2
113	P02042	HBD_HUMAN	HBD
114	Q9NSC7	SIA7A_HUMAN	ST6GALNAC1
115	Q9Y279	VSIG4_HUMAN	VSIG4

88	Q13201	MMRN1	MMRN1_HUMAN
89	P02675	FGB	FIBB_HUMAN
90	P23219	PTGS1	PGH1_HUMAN
95	P02775	PPBP	CXCL7_HUMAN
97	P06702	S100A9	S10A9_HUMAN
98	P11215	ITGAM	ITAM_HUMAN
99	Q9BXX0	EMILIN2	EMIL2_HUMAN
100	P08311	CTSG	CATG_HUMAN
101	Q15063	POSTN	POSTN_HUMAN
102	Q96IY4	CPB2	CBPB2_HUMAN
103	P04003	C4BPA	C4BPA_HUMAN
104	P01023	A2M	A2MG_HUMAN
108	P0C0L5	C4B	CO4B_HUMAN
109	P00450	CP	CERU_HUMAN
111	P00736	C1R	C1R_HUMAN
112	Q8IUX7	AEBP1	AEBP1_HUMAN
123	P12111	COL6A3	CO6A3_HUMAN
130	Q2UY09	COL28A1	COSA1_HUMAN
133	O14958	CASQ2	CASQ2_HUMAN
143	P12110	COL6A2	CO6A2_HUMAN
145	P07585	DCN	PGS2_HUMAN
146	P08603	CFH	CFAH_HUMAN
147	P02746	C1QB	C1QB_HUMAN
148	P35443	THBS4	TSP4_HUMAN
149	P02763	ORM1	A1AG1_HUMAN
150	P35542	SAA4	SAA4_HUMAN
151	P07858	CTSB	CATB_HUMAN
152	P80188	LCN2	NGAL_HUMAN
153	P35354	PTGS2	PGH2_HUMAN
154	P55083	MFAP4	MFAP4_HUMAN
155	Q9BYF1	ACE2	ACE2_HUMAN
156	P04085	PDGFA	PDGFA_HUMAN
157	P01033	TIMP1	TIMP1_HUMAN
158	P02751	FN1	FINC_HUMAN
159	P02461	COL3A1	CO3A1_HUMAN
160	P02452	COL1A1	CO1A1_HUMAN
161	P08123	COL1A2	CO1A2_HUMAN
162	P12109	COL6A1	CO6A1_HUMAN
163	P15502	ELN	ELN_HUMAN
164	P35625	TIMP3	TIMP3_HUMAN
165	P09486	SPARC	SPRC_HUMAN
166	Q14515	SPARCL1	SPRL1_HUMAN
167	P29279	CCN2	CCN2_HUMAN
168	Q15582	TGFBI	BGH3_HUMAN
169	P07339	CTSD	CATD_HUMAN
170	P00751	CFB	CFAB_HUMAN
171	P02747	C1QC	C1QC_HUMAN
172	P02745	C1QA	C1QA_HUMAN
173	P00746	CFD	CFAD_HUMAN
174	Q13093	PLA2G7	PAFA_HUMAN
175	P10147	CCL3	CCL3_HUMAN
176	P13501	CCL5	CCL5_HUMAN
177	P09341	CXCL1	GROA_HUMAN
178	P80162	CXCL6	CXCL6_HUMAN
179	P16871	IL7R	IL7RA_HUMAN
180	Q13651	IL10RA	I10R1_HUMAN
181	Q08334	IL10RB	I10R2_HUMAN
182	P78552	IL13RA1	I13R1_HUMAN
183	P15260	IFNGR1	INGR1_HUMAN

193	Q06136	KDSR_HUMAN	KDSR
195	O75874	IDHC_HUMAN	IDH1
196	Q9H1E5	TMX4_HUMAN	TMX4
197	P09417	DHPR_HUMAN	QDPR
198	Q8N436	CPXM2_HUMAN	CPXM2
202	O95562	SFT2B_HUMAN	SFT2D2
203	P15088	CBPA3_HUMAN	CPA3
205	Q13439	GOGA4_HUMAN	GOLGA4
206	Q15043	S39AE_HUMAN	SLC39A14
207	O75131	CPNE3_HUMAN	CPNE3
209	P27216	ANX13_HUMAN	ANXA13
211	Q04760	LGUL_HUMAN	GLO1
212	P04424	ARLY_HUMAN	ASL
213	O14880	MGST3_HUMAN	MGST3
215	P11217	PYGM_HUMAN	PYGM
216	Q16881	TRXR1_HUMAN	TXNRD1
226	P06576	ATPB_HUMAN	ATP5F1B
238	Q9P2R7	SUCB1_HUMAN	SUCLA2
240	Q9BZQ8	NIBA1_HUMAN	NIBAN1
243	P49189	AL9A1_HUMAN	ALDH9A1
244	Q9C0E8	LNP_HUMAN	LNPK
246	Q92890	UFD1_HUMAN	UFD1
247	P51648	AL3A2_HUMAN	ALDH3A2
248	O95841	ANGL1_HUMAN	ANGPTL1
249	Q9Y3D6	FIS1_HUMAN	FIS1
252	P50995	ANX11_HUMAN	ANXA11
253	Q9UJ70	NAGK_HUMAN	NAGK
254	Q01813	PFKAP_HUMAN	PFKP
260	P05091	ALDH2_HUMAN	ALDH2
265	P09172	DOPO_HUMAN	DBH
272	Q9Y6C2	EMIL1_HUMAN	EMILIN1
280	P62140	PP1B_HUMAN	PPP1CB
289	O14958	CASQ2_HUMAN	CASQ2
290	P17987	TCPA_HUMAN	TCP1
291	Q9H4B7	TBB1_HUMAN	TUBB1
294	P40227	TCPZ_HUMAN	CCT6A
295	P50990	TCPQ_HUMAN	CCT8
297	Q9H2J4	PDCL3_HUMAN	PDCL3
300	Q9Y2T2	AP3M1_HUMAN	AP3M1
303	Q9H2G2	SLK_HUMAN	SLK
306	Q6UWY5	OLFL1_HUMAN	OLFML1
309	Q9UMR2	DD19B_HUMAN	DDX19B
310	Q9BTV4	TMM43_HUMAN	TMEM43
311	A9UHW6	MI4GD_HUMAN	MIF4GD
320	Q6ZMZ3	SYNE3_HUMAN	SYNE3
323	Q9UPT6	JIP3_HUMAN	MAPK8IP3
326	Q7RTP6	MICA3_HUMAN	MICAL3
327	O75534	CSDE1_HUMAN	CSDE1
328	O94856	NFASC_HUMAN	NFASC
331	Q6UWP7	LCLT1_HUMAN	LCLAT1
333	Q9H8L6	MMRN2_HUMAN	MMRN2
334	Q9BUL8	PDC10_HUMAN	PDCD10
336	Q5VSL9	STRP1_HUMAN	STRIP1
337	Q9P289	STK26_HUMAN	STK26
338	Q13033	STRN3_HUMAN	STRN3
340	O76070	SYUG_HUMAN	SNCG
343	Q14BN4	SLMAP_HUMAN	SLMAP
344	Q99689	FEZ1_HUMAN	FEZ1
345	Q02790	FKBP4_HUMAN	FKBP4

116	P06732	KCRM_HUMAN	CKM
117	O00602	FCN1_HUMAN	FCN1
118	P07451	CAH3_HUMAN	CA3
119	Q86YZ3	HORN_HUMAN	HRNR
120	P02750	A2GL_HUMAN	LRG1
121	P68871	HBB_HUMAN	HBB
122	P01011	AACT_HUMAN	SERPINA3
123	P10645	CMGA_HUMAN	CHGA
124	P02144	MYG_HUMAN	MB
125	P00740	FA9_HUMAN	F9
126	P04745	AMY1A_HUMAN	AMY1A
127	Q7KZ85	SPT6H_HUMAN	SUPT6H
128	Q8WWA0	ITLN1_HUMAN	ITLN1
129	O75356	ENTP5_HUMAN	ENTPD5
130	Q99436	PSB7_HUMAN	PSMB7
131	P81605	DCD_HUMAN	DCD
132	P01009	A1AT_HUMAN	SERPINA1
133	P04746	AMYP_HUMAN	AMY2A
134	Q02985	FHR3_HUMAN	CFHR3
135	P08519	APOA_HUMAN	LPA
136	O95866	G6B_HUMAN	MPIG6B
137	P21333	FLNA_HUMAN	FLNA
138	A0A0B4J1V6	HV373_HUMAN	IGHV3-73
139	Q9H6X2	ANTR1_HUMAN	ANTXR1
140	A0A0A0MT36	KVD21_HUMAN	IGKV6D-21
141	P23083	HV102_HUMAN	IGHV1-2
142	Q15691	MARE1_HUMAN	MAPRE1
143	P01825	HV459_HUMAN	IGHV4-59
144	A0A075B6R2	HV404_HUMAN	IGHV4-4
145	Q9UNN8	EPCR_HUMAN	PROCR
146	P06753	TPM3_HUMAN	TPM3
147	P62491	RB11A_HUMAN	RAB11A
148	O95236	APOL3_HUMAN	APOL3
149	Q96KK5	H2A1H_HUMAN	H2AC12
150	P51124	GRAM_HUMAN	GZMM
151	Q05315	LEG10_HUMAN	CLC
152	P08567	PLEK_HUMAN	PLEK
153	Q8WW22	DNJA4_HUMAN	DNAJA4
154	O43768	ENSA_HUMAN	ENSA
155	O00151	PDLI1_HUMAN	PDLIM1
156	P06727	APOA4_HUMAN	APOA4

184	P38484	IFNGR2	INGR2_HUMAN
185	P01137	TGFB1	TGFB1_HUMAN
186	Q99731	CCL19	CCL19_HUMAN
187	O00585	CCL21	CCL21_HUMAN
188	O95715	CXCL14	CXL14_HUMAN
189	P08571	CD14	CD14_HUMAN
190	P34810	CD68	CD68_HUMAN
191	O95711	LY86	LY86_HUMAN
192	Q9Y6Y9	LY96	LY96_HUMAN
193	O75509	TNFRSF21	TNR21_HUMAN
194	P25116	F2R	PAR1_HUMAN
195	P10124	SRGN	SRGN_HUMAN
196	O00300	TNFRSF11B	TR11B_HUMAN
197	P12544	GZMA	GRAA_HUMAN
198	Q92956	TNFRSF14	TNR14_HUMAN
199	P20718	GZMH	GRAH_HUMAN
200	Q969K3	RNF34	RNF34_HUMAN
201	P05107	ITGB2	ITB2_HUMAN
202	O75578	ITGA10	ITA10_HUMAN
203	Q9UKX5	ITGA11	ITA11_HUMAN
204	P06756	ITGAV	ITAV_HUMAN
205	P16144	ITGB4	ITB4_HUMAN
206	P26006	ITGA3	ITA3_HUMAN
207	P10451	SPP1	OSTP_HUMAN
208	P00533	EGFR	EGFR_HUMAN
209	P12107	COL11A1	COBA1_HUMAN
210	P05997	COL5A2	CO5A2_HUMAN
211	P20908	COL5A1	CO5A1_HUMAN
212	P53420	COL4A4	CO4A4_HUMAN
213	Q14031	COL4A6	CO4A6_HUMAN
214	Q9Y6N6	LAMC3	LAMC3_HUMAN
215	P07942	LAMB1	LAMB1_HUMAN
216	Q16787	LAMA3	LAMA3_HUMAN
217	Q16363	LAMA4	LAMA4_HUMAN
218	Q4ZHG4	FNDC1	FNDC1_HUMAN
219	P78509	RELN	RELN_HUMAN
220	P35442	THBS2	TSP2_HUMAN
221	Q02487	DSC2	DSC2_HUMAN
222	P12314	FCGR1A	FCGR1_HUMAN
223	O15533	TAPBP	TPSN_HUMAN
224	P0C0L4	C4A	CO4A_HUMAN
225	P06681	C2	CO2_HUMAN
226	P04234	CD3D	CD3D_HUMAN
227	Q9BX59	TAPBPL	TPSNR_HUMAN
228	P43489	TNFRSF4	TNR4_HUMAN
229	P32241	VIPR1	VIPR1_HUMAN
230	P23229	ITGA6	ITA6_HUMAN
231	Q9UHC6	CNTNAP2	CNTP2_HUMAN
232	Q9BQT9	CLSTN3	CSTN3_HUMAN
233	P78539	SRPX	SRPX_HUMAN
234	Q13444	ADAM15	ADA15_HUMAN
235	P21583	KITLG	SCF_HUMAN
236	O94856	NFASC	NFASC_HUMAN
237	Q9ULC0	EMCN	MUCEN_HUMAN
238	P28906	CD34	CD34_HUMAN
239	P33151	CDH5	CADH5_HUMAN
240	P55107	GDF10	GDF10_HUMAN
241	Q9BU40	CHRDL1	CRDL1_HUMAN
242	P05019	IGF1	IGF1_HUMAN

356	Q9NPY3	C1QR1 HUMAN	CD93
358	Q9UKV8	AGO2 HUMAN	AGO2
359	Q92608	DOCK2 HUMAN	DOCK2
360	Q7KZF4	SND1 HUMAN	SND1
361	P60660	MYL6 HUMAN	MYL6
362	O14744	ANM5 HUMAN	PRMT5
363	P62834	RAP1A HUMAN	RAP1A
366	Q01518	CAP1 HUMAN	CAP1
367	Q99536	VAT1 HUMAN	VAT1
368	Q13618	CUL3 HUMAN	CUL3
372	P07355	ANXA2 HUMAN	ANXA2
373	P46934	NEDD4 HUMAN	NEDD4
374	P00387	NB5R3 HUMAN	CYB5R3
375	Q15139	KPCD1 HUMAN	PRKD1
376	P11216	PYGB HUMAN	PYGB
377	Q05655	KPCD HUMAN	PRKCD
384	O94826	TOM70 HUMAN	TOMM70
386	Q14318	FKBP8 HUMAN	FKBP8
388	P05026	AT1B1 HUMAN	ATP1B1
394	Q6DD88	ATLA3 HUMAN	ATL3
395	Q9NY47	CA2D2 HUMAN	CACNA2D2
396	P62879	GBB2 HUMAN	GNB2
397	P61163	ACTZ HUMAN	ACTR1A
398	P08754	GNAI3 HUMAN	GNAI3
399	P04899	GNAI2 HUMAN	GNAI2
400	O00451	GFRA2 HUMAN	GFRA2
402	O14939	PLD2 HUMAN	PLD2
403	P62873	GBB1 HUMAN	GNB1
404	P10415	BCL2 HUMAN	BCL2
406	P17612	KAPCA HUMAN	PRKACA
407	Q9UBI6	GBG12 HUMAN	GNG12
408	P13861	KAP2 HUMAN	PRKAR2A
409	P29992	GNA11 HUMAN	GNA11
411	Q5JWF2	GNAS1 HUMAN	GNAS
414	P06213	INSR HUMAN	INSR
415	P63096	GNAI1 HUMAN	GNAI1
416	Q96CV9	OPTN HUMAN	OPTN
417	Q9UGJ0	AAKG2 HUMAN	PRKAG2
422	Q96EY5	MB12A HUMAN	MVB12A
425	P63244	RACK1 HUMAN	RACK1
426	P61353	RL27 HUMAN	RPL27
427	P61247	RS3A HUMAN	RPS3A
429	P61513	RL37A HUMAN	RPL37A
432	P62888	RL30 HUMAN	RPL30
433	Q13347	EIF3I HUMAN	EIF3I
435	P62166	NCS1 HUMAN	NCS1
437	Q5JPE7	NOMO2 HUMAN	NOMO2
438	O95980	RECK HUMAN	RECK
441	Q92973	TNPO1 HUMAN	TNPO1
447	O43390	HNRPR HUMAN	HNRNPR
448	Q15717	ELAV1 HUMAN	ELAVL1
450	Q9Y3I0	RTCB HUMAN	RTCB
460	P41252	SYIC HUMAN	IARS1
461	Q9H2U1	DHX36 HUMAN	DHX36
463	Q9P2J5	SYLC HUMAN	LARS1
468	P12956	XRCC6 HUMAN	XRCC6
474	O15484	CAN5 HUMAN	CAPN5
476	Q9GZZ1	NAA50 HUMAN	NAA50
481	P25789	PSA4 HUMAN	PSMA4

243	O75015	FCGR3B	FCG3B HUMAN
244	P10619	CTSA	PPGB HUMAN
245	P07711	CTSL	CATL1 HUMAN
246	P25774	CTSS	CATS HUMAN
247	P53634	CTSC	CATC HUMAN
248	Q99542	MMP19	MMP19 HUMAN
249	Q9Y251	HPSE	HPSE HUMAN
250	Q03405	PLAUR	UPAR HUMAN
251	P29400	COL4A5	CO4A5 HUMAN
252	Q05707	COL14A1	COEA1 HUMAN
253	Q96P44	COL21A1	COLA1 HUMAN
254	Q9BXR5	TLR10	TLR10 HUMAN
255	P50281	MMP14	MMP14 HUMAN
256	P12318	FCGR2A	FCG2A HUMAN
257	P01732	CD8A	CD8A HUMAN
258	P32248	CCR7	CCR7 HUMAN
259	P26842	CD27	CD27 HUMAN
260	P07766	CD3E	CD3E HUMAN
261	P98095	FBLN2	FBLN2 HUMAN
262	P17813	ENG	EGLN HUMAN
263	P00750	PLAT	TPA HUMAN
264	Q9NS62	THSD1	THSD1 HUMAN
265	P25101	EDNRA	EDNRA HUMAN
266	Q03167	TGFBR3	TGBR3 HUMAN
267	Q9UKY0	PRND	PRND HUMAN
268	Q8N6Y2	LRRC17	LRC17 HUMAN
269	P10721	KIT	KIT HUMAN
270	P78423	CX3CL1	X3CL1 HUMAN
271	P13598	ICAM2	ICAM2 HUMAN
272	O14625	CXCL11	CXL11 HUMAN
279	Q86VB7	CD163	C163A HUMAN
282	P09601	HMOX1	HMOX1 HUMAN
348	Q6UWP8	SBSN	SBSN HUMAN
349	P49913	CAMP	CAMP HUMAN
350	Q14624	ITIH4	ITIH4 HUMAN
351	P19827	ITIH1	ITIH1 HUMAN
352	Q8NI99	ANGPTL6	ANGL6 HUMAN
353	Q13508	ART3	NAR3 HUMAN
354	P48740	MASP1	MASP1 HUMAN
355	Q99784	OLFM1	NOE1 HUMAN
356	P20851	C4BPB	C4BPB HUMAN
357	Q9Y6Z7	COLEC10	COL10 HUMAN
358	P20742	PZP	PZP HUMAN
359	Q9UBP4	DKK3	DKK3 HUMAN
360	P37837	TALDO1	TALDO HUMAN
361	Q9UGM3	DMBT1	DMBT1 HUMAN
362	Q8IZF2	ADGRF5	AGRF5 HUMAN
363	P24592	IGFBP6	IBP6 HUMAN
364	P16278	GLB1	BGAL HUMAN
365	P13473	LAMP2	LAMP2 HUMAN
366	P08709	F7	FA7 HUMAN
367	P07225	PROS1	PROS HUMAN
368	P05556	ITGB1	ITB1 HUMAN
369	P05090	APOD	APOD HUMAN
370	P05062	ALDOB	ALDOB HUMAN
371	P04070	PROC	PROC HUMAN
372	P00739	HPR	HPTR HUMAN
373	O95497	VNN1	VNN1 HUMAN
374	O60279	SUSD5	SUSD5 HUMAN

484	Q15008	PSMD6 HUMAN	PSMD6
485	P25788	PSA3 HUMAN	PSMA3
487	Q16363	LAMA4 HUMAN	LAMA4
489	O14936	CSKP HUMAN	CASK
490	Q9BXJ5	C1QT2 HUMAN	C1QTNF2
492	Q9BZG1	RAB34 HUMAN	RAB34
494	Q9H902	REEP1 HUMAN	REEP1
495	O43175	SERA HUMAN	PHGDH
498	Q03405	UPAR HUMAN	PLAUR
499	P17813	EGLN HUMAN	ENG
500	Q15904	VAS1 HUMAN	ATP6AP1
502	P50281	MMP14 HUMAN	MMP14
503	P19440	GGT1 HUMAN	GGT1
507	Q66K79	CBPZ HUMAN	CPZ
510	P05109	S10A8 HUMAN	S100A8
513	P55786	PSA HUMAN	NPEPPS
514	Q15126	PMVK HUMAN	PMVK
516	P05121	PAI1 HUMAN	SERPINE1
518	Q9NZ52	GGA3 HUMAN	GGA3
519	Q14677	EPN4 HUMAN	CLINT1
520	O75379	VAMP4 HUMAN	VAMP4
521	O75154	RFIP3 HUMAN	RAB11FIP3
522	P52209	6PGD HUMAN	PGD
523	Q9NUQ9	CYRIB HUMAN	CYRIB
524	O00299	CLIC1 HUMAN	CLIC1
525	Q9UEU0	VTI1B HUMAN	VTI1B
526	P49768	PSN1 HUMAN	PSEN1
527	Q92542	NICA HUMAN	NCSTN
528	Q3T906	GNPTA HUMAN	GNPTAB
529	P20338	RAB4A HUMAN	RAB4A
530	Q9NY33	DPP3 HUMAN	DPP3
535	Q15800	MSMO1 HUMAN	MSMO1
536	Q96AG4	LRC59 HUMAN	LRRC59
539	P20933	ASPG HUMAN	AGA
540	P17174	AATC HUMAN	GOT1
543	Q9HB40	RISC HUMAN	SCPEP1
545	O94760	DDAH1 HUMAN	DDAH1
547	O95372	LYPA2 HUMAN	LYPLA2
549	P53367	ARFP1 HUMAN	ARFIP1
550	Q9Y5S2	MRCKB HUMAN	CDC42BPB
554	P78417	GSTO1 HUMAN	GSTO1
555	P0CG30	GSTT2 HUMAN	GSTT2B
556	P09936	UCHL1 HUMAN	UCHL1
557	Q8NBI6	XXLT1 HUMAN	XXYLT1
558	P53985	MOT1 HUMAN	SLC16A1
559	Q9H4A6	GOLP3 HUMAN	GOLPH3
562	P31997	CEAM8 HUMAN	CEACAM8
563	Q9Y3L5	RAP2C HUMAN	RAP2C
564	P35613	BASI HUMAN	BSG
565	P18085	ARF4 HUMAN	ARF4
566	Q96CW1	AP2M1 HUMAN	AP2M1
567	O43914	TYOBP HUMAN	TYROBP
569	P13796	PLSL HUMAN	LCP1
570	P12314	FCGR1 HUMAN	FCGR1A
571	P10321	HLAC HUMAN	HLA-C
573	P61769	B2MG HUMAN	B2M
574	P05362	ICAM1 HUMAN	ICAM1
575	P01889	HLAB HUMAN	HLA-B
577	P04439	HLAA HUMAN	HLA-A

375	P01344	IGF2	IGF2 HUMAN
376	P09668	CTSH	CATH HUMAN
377	O75022	LILRB3	LIRB3 HUMAN
378	A0A0J9YXX1	IGHV5101	HV5X1 HUMAN
379	A0A0B4J1Y8	IGLV949	LV949 HUMAN
380	A0A075B6I0	IGLV861	LV861 HUMAN

578	Q16658	FSCN1 HUMAN	FSCN1
579	Q16864	VATF HUMAN	ATP6V1F
580	Q99439	CNN2 HUMAN	CNN2
581	P27797	CALR HUMAN	CALR
582	O00764	PDXK HUMAN	PDXK
583	O95445	APOM HUMAN	APOM
587	O43790	KRT86 HUMAN	KRT86
588	P51572	BAP31 HUMAN	BCAP31
592	Q9NR99	MXRA5 HUMAN	MXRA5
593	O14657	TOR1B HUMAN	TOR1B
596	Q92805	GOGA1 HUMAN	GOLGA1
597	P0C0L5	CO4B HUMAN	C4B
598	P10124	SRGN HUMAN	SRGN
599	Q01459	DIAC HUMAN	CTBS
600	Q9GZX9	TWSG1 HUMAN	TWSG1
601	Q13641	TPBG HUMAN	TPBG
602	O75976	CBPD HUMAN	CPD
604	Q9BRR9	RHG09 HUMAN	ARHGAP9
607	Q13093	PAFA HUMAN	PLA2G7
610	P02008	HBAZ HUMAN	HBZ
611	Q11201	SIA4A HUMAN	ST3GAL1
612	P32942	ICAM3 HUMAN	ICAM3
613	O95466	FMNL1 HUMAN	FMNL1
616	Q8TD55	PKHO2 HUMAN	PLEKHO2
617	Q8N1S5	S39AB HUMAN	SLC39A11
619	Q9ULI3	HEG1 HUMAN	HEG1
622	Q9H488	OFUT1 HUMAN	POFUT1
624	P80303	NUCB2 HUMAN	NUCB2
625	Q9BV40	VAMP8 HUMAN	VAMP8
630	P28067	DMA HUMAN	HLA-DMA
631	P01903	DRA HUMAN	HLA-DRA
632	O15155	BET1 HUMAN	BET1
633	P28065	PSB9 HUMAN	PSMB9
636	Q9UL46	PSME2 HUMAN	PSME2
638	P52907	CAZA1 HUMAN	CAPZA1
640	Q9BVK6	TMED9 HUMAN	TMED9
644	Q9NR97	TLR8 HUMAN	TLR8
645	Q9BT09	CNPY3 HUMAN	CNPY3
646	Q68CQ7	GL8D1 HUMAN	GLT8D1
647	Q6P4E1	GOLM2 HUMAN	GOLM2
648	Q86YB8	ERO1B HUMAN	ERO1B
651	Q9ULZ3	ASC HUMAN	PYCARD
652	P24158	PRTN3 HUMAN	PRTN3
653	P05164	PERM HUMAN	MPO
654	Q99615	DNJC7 HUMAN	DNAJC7
655	P28676	GRAN HUMAN	GCA
656	P43307	SSRA HUMAN	SSR1
659	Q96RD7	PANX1 HUMAN	PANX1
660	P0C0L4	CO4A HUMAN	C4A
661	Q8NBJ4	GOLM1 HUMAN	GOLM1
662	P01033	TIMP1 HUMAN	TIMP1
663	P02746	C1QB HUMAN	C1QB
665	Q15084	PDIA6 HUMAN	PDIA6
666	Q14766	LTBP1 HUMAN	LTBP1
667	P07237	PDIA1 HUMAN	P4HB
668	P14625	ENPL HUMAN	HSP90B1
670	Q9NZK5	ADA2 HUMAN	ADA2
675	Q96AY3	FKB10 HUMAN	FKBP10
678	P07332	FES HUMAN	FES

682	P15104	GLNA_HUMAN	GLUL
683	P08571	CD14_HUMAN	CD14
686	P11171	EPB41_HUMAN	EPB41
692	Q9UJ68	MSRA_HUMAN	MSRA
693	P11274	BCR_HUMAN	BCR
694	Q9H3U7	SMOC2_HUMAN	SMOC2
695	Q96KP4	CNDP2_HUMAN	CNDP2
697	P60174	TPIS_HUMAN	TPI1
702	P30040	ERP29_HUMAN	ERP29
705	Q9Y315	DEOC_HUMAN	DERA
706	O15321	TM9S1_HUMAN	TM9SF1
707	Q9NRN7	ADPPT_HUMAN	AASDHPPT
708	Q9NWM8	FKB14_HUMAN	FKBP14
711	P80511	S10AC_HUMAN	S100A12
715	P09326	CD48_HUMAN	CD48
716	P15289	ARSA_HUMAN	ARSA
718	Q04756	HGFA_HUMAN	HGFAC
719	O75787	RENR_HUMAN	ATP6AP2
720	P08174	DAF_HUMAN	CD55
721	P13667	PDIA4_HUMAN	PDIA4
722	Q08379	GOGA2_HUMAN	GOLGA2
723	P13693	TCTP_HUMAN	TPT1

SourceDataForFigure4B: The number of suggested peptides of HeLa dataset by Picky method and peptides detected by DDA analysis.									
**Compound**	**Formula**	**Precursor (m/z)**	**Precursor Charge (z)**	**t start (min)**	**t stop (min)**	**Collision Energy (%)**	**Maximum Injection Time (ms)**	**Charge (by Search Engine): Sequest HT**	**Detected by DDA**
P07910	IVGCSVHK	450.24	2	6.12	15.56	28	30		
P33316	LSEHATAPTR	541.78	2	6.12	15.56	30	50		
P25705	LTDADAMK	432.71	2	9.76	20.08	20	30	2	Yes
P83881	DSLYAQGK	441.22	2	10.28	20.72	28	30	2	Yes
P29401	ISSDLDGHPVPK	421.89	3	14.04	25.31	23	30	3	Yes
P35637	EFSGNPIK	446.23	2	16.05	27.73	20	30		
Q15149	EAIAELER	465.75	2	20.50	33.04	20	30		
P26038	SGYLAGDK	405.70	2	23.23	36.25	28	30	2	Yes
P13010	EGLEIVK	394.23	2	24.78	38.06	20	30		
P35613	SSEHINEGETAMLVCK	601.94	3	26.16	39.65	28	30	3	Yes
P06748	VTLATLK	373.24	2	27.68	41.42	20	30		
P62249	TLLVADPR	442.76	2	27.98	41.77	28	30	2	Yes
P78417	LLPDDPYEK	545.28	2	28.06	41.86	23	30	2	Yes
P04075	QLLLTADDR	522.79	2	34.78	49.55	35	30	2	Yes
Q9Y2Z9	EQIMAFASK	512.76	2	35.93	50.85	23	150		
O75369	GLEELVK	394.23	2	36.53	51.52	23	30		
P11387	TYNASITLQQQLK	754.41	2	38.79	53.96	23	50		
P52789	DVVALIR	393.25	2	48.23	63.46	20	100		
P05783	TVQSLEIDLDSMR	753.88	2	51.10	66.43	28	50	2	Yes
Q9Y2W1	GFYPWGQYNR	644.30	2	53.65	69.21	28	50		
P30048	TSLTNLLCSGSSQAK	783.89	2	53.98	69.56	28	30		
P26447	ELPSFLGK	445.75	2	54.44	70.05	28	30	2	Yes
Q01082	SWHNVYCVINNQEMGFYK	763.34	3	61.27	77.21	20	30	3	Yes
P07237	THILLFLPK	541.34	2	63.52	79.49	23	30	2	Yes
O43390	GPPPDSVYSGVQPGIGTEVFV	1254.13	2	64.30	80.27	23	30	3	
P08238	ALLFIPR	415.27	2	66.23	82.20	25	30	2	Yes
P06733	AVEHINK	405.73	2	69.62	85.60	23	30	2	Yes
Q16881	GFHVLGPNAGEVTQGFAAA	1141.62	2	76.63	92.81	23	30	3	
P12814	IDQLEGDHQLIQEALIFDNK	780.07	3	78.06	94.26	20	30	3	Yes
Q14974	MELITILEK	545.31	2	78.66	94.87	20	30		
P10809	TLNDELEIIEGMK	752.88	2	80.19	96.40	20	30	2	Yes
P04843	FPLFGGWK	476.26	2	80.22	96.43	23	30	2	Yes
O75369	IPYLPITNFNQNWQDGK	1024.52	2	83.65	99.83	28	30	3	

P26639	TPYQIACGISQGLADNTVIA[illegible]	1161.09	2	86.02	102.14	23	30	3	
P11388	YIFTMLSSLAR	651.35	2	86.57	102.67	20	100	2	Yes
P50395	FVSISDLLVPK	609.36	2	96.58	112.16	20	30	2	Yes
Q14974	VQHQDALQISDVVMASLLR	708.05	3	97.90	113.39	28	30	4	
P08195	LLTSFLPAQLLR	686.42	2	100.20	115.50	20	30	2	Yes
Q14204	ILDDDTIITTLENLK	858.97	2	103.48	118.49	20	30		
P07602	EIVDSYLPVILDIIK	865.50	2	104.14	119.08	28	30		
P31949	TEFLSFMNTELAAFTK	925.46	2	107.85	122.40	20	30	2	Yes
O75643	LIGLSATLPNYEDVATFLR	1047.07	2	109.98	124.28	20	30		
P26358	PEPSPSPR	433.72	2	6.75	16.34	35	200		
P18846	TDDPQLK	408.71	2	10.12	20.53	20	200		
Q6XZF7	NPNELSVSANQK	650.83	2	11.16	21.80	28	200	2	Yes
P52701	ELAETIK	402.23	2	12.09	22.94	20	100	2	Yes
Q8N4H5	IEGLAPK	364.22	2	12.86	23.88	20	50	2	Yes
Q6IA86	TLLASACK	432.24	2	13.85	25.08	35	100	2	Yes
P49257	NDYEFCAK	523.72	2	16.85	28.70	20	30		
O75844	DIQEDSGMEPR	638.78	2	18.08	30.16	28	50	2	Yes
Q9UNS2	DGMVSFHDNPEK	688.30	2	20.09	32.55	28	30	3	
O94874	SVFMSSTTSASGTGR	738.34	2	20.76	33.34	28	50	2	Yes
Q9UNZ2	LGAAPEEESAYVAGEK	810.89	2	22.93	35.89	23	30	2	Yes
P49721	NLADCLR	431.22	2	23.89	37.02	25	30	2	Yes
Q9H936	GIAGLYK	361.22	2	24.16	37.32	20	50		
Q6YN16	GTYLASK	370.20	2	31.82	46.18	28	30		
Q96FV9	ILMGNEELTR	588.31	2	33.96	48.63	28	150		
P30622	YGLFAPVHK	516.29	2	34.21	48.91	25	200		
P26358	AIGLEIK	372.24	2	35.61	50.50	23	200		
Q9Y277	GYGFGMVK	429.71	2	37.00	52.03	20	30	2	Yes
Q96FJ2	DIAAYIK	397.23	2	39.55	54.74	20	30	2	Yes
Q8WUQ7	DMTTITEDEISK	691.82	2	43.28	58.44	28	150		
Q13561	LTPVLLAK	427.79	2	45.50	60.62	23	30	2	Yes
Q92542	SGAGVPAVILR	520.32	2	47.06	62.22	20	100	2	Yes
O15160	VVLGEFGVR	488.28	2	51.13	66.46	30	50		
Q8N4H5	VTPFILK	409.26	2	53.32	68.84	23	50		
P23634	EGDFGCTVMELR	707.31	2	54.44	70.04	28	50	2	Yes
Q08379	AGMQLNLEELQK	687.36	2	54.85	70.47	28	150	2	Yes
O15305	TIYFFGDK	495.75	2	55.33	70.99	28	50	2	Yes
P52701	YQLEIPENFTTR	755.88	2	61.26	77.20	20	100	2	Yes
Q9NZM1	NANPEWNQVVNLQIK	883.96	2	62.97	78.95	20	100		

Q9UHG3	SNLISGSVMYIEEK	785.40	2	65.41	81.38	28	30		
Q9BVV7	YIFVEIESYPR	708.36	2	68.14	84.11	28	200		
Q5JTH9	AFMDIMSAQASSGSTSVLR	979.97	2	69.10	85.07	20	100		
Q9H4A4	AFFPCFDTPAVK	700.34	2	71.70	87.74	20	30	2	Yes
O14617	ALDLLYGMVSK	605.33	2	75.54	91.70	20	50		
Q86U86	NQPDYYEVVSQPIDLMK	1019.99	2	76.66	92.84	28	200		
P43686	LQQELEFLEVQEEYIK	1019.52	2	84.29	100.46	20	30		
Q99459	LVLPAPQISDAELQEVVK	975.05	2	87.22	103.30	23	50	3	
O95453	VMDIPYLNLEGPDLQPK	971.50	2	88.48	104.51	20	100	3	
O94874	TYDLPGNFLTQALTQR	919.48	2	91.96	107.83	25	50		
Q9Y4R8	FNSVAGHFFFPLLQR	593.65	3	92.16	108.02	20	200		
Q92552	IESEGLLSLTTQLVK	815.97	2	92.65	108.48	25	50	3	
P02656	GWVTDGFSSLK	598.80	2	93.93	109.68	23	30		
Q5JTH9	SWLLPVIR	492.31	2	97.03	112.57	30	100	2	Yes
Q9NY61	ALLTTNQLPQPDVFPLFK	1021.57	2	101.39	116.59	23	100		
Q96JB5	DNTYLVELSSLLVR	811.44	2	105.83	120.60	28	50		
Q5VYK3	DIALVQQLFEALCK	824.44	2	107.64	122.22	20	100		
Q14657	DADADAGGGADGGDGR	688.77	2	6.12	15.56	28	150		
O60566	EIQTTQQER	566.78	2	6.12	15.56	28	200		
O95139	MVHGVYK	417.22	2	6.33	15.82	20	100		
Q7Z6K5	LEAGTVTK	409.73	2	9.60	19.88	23	50		
Q9H3H5	VLGPIHER	460.77	2	9.69	19.99	35	100	2	Yes
P50750	NPATTNQTEFER	704.33	2	10.51	21.00	23	100	2	Yes
Q92747	SVQVSTLK	431.26	2	12.56	23.51	20	30		
Q92797	MADSEIPR	459.72	2	13.77	24.99	28	100		
Q8WVV9	VPNGSNPYTLK	595.31	2	18.35	30.48	23	50		
P46100	SVPVTVDDDDDDNDPENR	1008.92	2	18.99	31.25	23	150		
Q9NWV8	EEEMSWK	469.70	2	19.85	32.27	28	100		
Q9H9Y6	NLPSGPSLK	456.76	2	20.41	32.93	20	200		
Q02127	LPEDQAVINR	577.81	2	20.65	33.22	25	150	2	Yes
Q92747	TQIALSPNNHEVHIYK	466.24	4	24.08	37.24	23	30		
Q12983	EVESILK	409.24	2	26.43	39.97	20	100		
P53384	ILVLSGK	365.25	2	30.22	44.34	35	50	2	Yes
Q86U38	EVETQALALSTNR	716.38	2	32.30	46.73	23	100		
P78537	SIELDMR	432.22	2	34.19	48.89	28	30		
P49006	LSGLSFK	376.22	2	36.09	51.03	20	30		
Q14181	VLGCPEALTGSYK	697.85	2	37.33	52.40	23	150	2	Yes
Q9Y696	EMTGIWR	446.72	2	38.19	53.33	28	30		

O60566	LLPEEDLDVK	585.82	2	42.04	57.20	23	200	2	Yes
Q7Z4W1	GALDMLTK	424.73	2	44.91	60.04	20	30	2	Yes
Q53FA7	MAGAIPLVTAGSQK	672.37	2	47.05	62.21	23	50		
Q9UPN9	AVALYFEDK	528.27	2	47.41	62.59	28	150	2	Yes
Q6P1Q0	NLMSYVVTK	527.78	2	49.06	64.33	28	150	2	Yes
Q709C8	TVPLLLAESK	535.83	2	55.66	71.34	23	200		
P57076	EAEAQLWWAAK	651.83	2	57.39	73.17	20	200		
Q6P1N0	TLLEALEQR	536.80	2	57.96	73.78	30	50	2	Yes
Q969Y2	DLALAAEALR	521.80	2	59.70	75.60	30	100		
Q96SZ5	SLLTQLR	415.76	2	61.58	77.53	25	50		
Q969U7	CIPEIDDSEFCIR	827.37	2	62.69	78.66	23	100		
Q13303	QFNLTPPICEQAEYHMFQR	803.37	3	65.54	81.51	28	100		
O00566	SPVFSDEDSDLDFDISK	958.43	2	70.60	86.61	23	100		
P17706	FSYMAIIEGAK	615.32	2	72.73	88.81	20	150		
Q9UHY1	VIFITEYMSSGSLK	787.91	2	79.04	95.25	20	100		
O75381	SEINSLK	395.72	2	80.43	96.64	20	100		
Q96DB5	LAAFWLMK	490.28	2	84.07	100.24	28	50	2	Yes
Q969E8	WLGGAVEDYFMR	722.34	2	85.25	101.39	28	200	2	Yes
Q02818	YLQEVIDVLETDGHFR	967.49	2	85.97	102.09	28	30	3	
Q96KN7	SQLEDVSILQMTLK	802.93	2	86.04	102.16	23	110		
P16422	TYWIIIELK	589.84	2	93.14	108.94	28	30		
Q9BSL1	TILEENIQDQDVLLLIK	999.06	2	94.35	110.08	23	100		
Q9NP81	SADLPAIISTWQELR	850.45	2	96.62	112.20	35	50	3	
P52564	TVDCPFTVTFYGALFR	947.46	2	103.31	118.33	20	100		
Q8IVS2	SLGIENPVCEVSNYLFPDCR	1185.05	2	103.34	118.36	25	150	3	
Q9H9Y6	ELFFLPLGFALK	697.91	2	104.73	119.61	28	200		
Q9H892	LLEALVSFLDFSDK	798.93	2	106.30	121.02	28	150		
Q9Y3Z3	VGNIIDTMITDAFLK	825.94	2	106.76	121.43	28	30		
Q9BV73	ATASSPTQQDGR	609.79	2	6.12	15.56	28	200		
O14787	DSQSPNTATQR	602.78	2	6.12	15.56	23	100		
O95235	QEDQGCVR	496.22	2	6.12	15.56	20	150		
O15126	EMQNLSQHGR	600.28	2	6.12	15.56	28	50		
Q8N1G0	GPSAHAHLYSQHPSFQTQQA	602.04	4	9.72	20.02	30	150		
Q63ZY3	FLGHPTAGR	478.26	2	9.82	20.15	28	30		
O00255	GLGTGQGAVSGPPR	627.33	2	12.48	23.42	23	200	2	Yes
P02144	HPGDFGADAQGAMNK	758.34	2	15.06	26.54	28	100		
Q9NRG7	WNETFQK	476.73	2	17.18	29.09	25	150		
O94915	LINQVNTIK	521.82	2	19.94	32.38	20	200		

Q8N4Q0	TEPVGTVLK	472.28	2	20.39	32.91	23	150		
Q9BRZ2	AGWYDEEAR	548.74	2	20.70	33.27	20	150		
P42702	GTDYEVCIENR	678.30	2	25.80	39.23	25	110		
Q8IWZ8	EWAEQLTK	502.76	2	26.15	39.65	20	150		
Q5T200	GNIETTSEDGQVFSPK	854.90	2	27.19	40.85	25	200		
Q86TP1	CSQISLSQSTTASLSK	849.42	2	29.75	43.81	28	150		
Q9H2X9	LAWEGNETVTTR	688.84	2	29.89	43.96	28	110		
Q8IWJ2	ELMCQIEASAK	640.30	2	33.41	48.00	28	100		
O75122	QTEDVAEVLNR	637.32	2	33.98	48.64	35	200		
Q53GG5	INLESEPQDGNYFEHK	640.29	3	34.66	49.41	23	100		
Q9C0B5	SFHFDPLSSGSR	668.82	2	44.14	59.29	30	150		
Q8IWA5	IFDDSPCPFTAK	699.32	2	44.45	59.58	20	50		
Q96JY6	SFQSLACSPGLPAADR	838.91	2	44.55	59.68	23	30		
Q9UP83	ASFWTNMEK	557.26	2	46.55	61.68	28	200		
Q9Y2D4	NNMFAQFR	514.24	2	47.46	62.65	35	100		
Q96FV2	NISNQLSIGTDISAQHPELR	731.38	3	49.12	64.39	23	50	3	Yes
P42858	LLLQINPER	548.33	2	54.36	69.96	25	200		
Q8NG31	ILAMTPESIYSNPSIQGCK	1055.02	2	60.67	76.60	28	200		
Q8TDB6	FGGPEMYGYPDPSYLK	910.91	2	61.44	77.39	28	50		
Q9Y6A5	SFSDLFK	422.22	2	61.49	77.44	20	100		
Q9H9Q2	CIPYSVLLK	546.81	2	62.69	78.66	23	50		
Q9BYX2	YALAIFK	413.25	2	62.85	78.82	20	100		
Q969Z3	IFGLDIK	403.24	2	68.58	84.55	23	50		
Q9NYV6	FIPLITVK	465.80	2	70.69	86.70	20	110		
Q9NZJ6	ILDVGCGGGLLTEPLGR	863.96	2	77.77	93.96	25	100		
Q9UIF9	QLPAFQEGIMSWSPK	859.93	2	79.72	95.93	28	200		
O95235	EELLQVVEAMK	644.84	2	80.39	96.60	28	150		
O75122	SLLVAGAAQYDCFFQHLR	1048.52	2	82.02	98.22	23	200		
Q96BJ8	LVPLASDMIFAR	666.87	2	82.85	99.04	28	100		
Q15800	WYFLLAR	484.77	2	85.23	101.37	28	100	2	Yes
Q9BYX2	YDEYGFLTVPDYEVEDLK	1098.01	2	85.91	102.03	20	100	3	
Q8TDB6	DSLPGYESFGTIVITYSMK	1054.52	2	97.38	112.90	20	50		
Q7Z4H7	EFLGLSPFSLIK	675.89	2	99.67	115.01	23	150		
Q8WUH2	ELISLYPFLLPTSSSFTR	1036.06	2	103.88	118.84	28	100		
Q9NYV6	YVPSTPWFLMPILVEK	960.52	2	103.92	118.88	28	110		
O95427	EVTLPFLFTPFK	719.90	2	104.18	119.11	28	100		
Q9HB40	DLAMVASDMMVLLK	768.90	2	105.62	120.41	28	50		
Q9BY41	DEASGFCYLNDAVLGILR	1006.99	2	107.85	122.40	23	200		

O94915	EDVLSGFVYFIVR	772.41	2	107.85	122.40	20	200		
Q14746	ILNSQSSK	438.74	2	5.33	14.57	30	100		
Q5VW38	HLQEASATDGK	578.78	2	6.12	15.56	23	50	2	Yes
Q32NC0	GLHDSCPGQAR	599.28	2	6.12	15.56	28	110		
O95249	LCTSYSHSSTR	649.79	2	6.12	15.56	35	100		
Q9C0I1	AVSVNEGYK	483.75	2	9.33	19.55	23	200		
P49588	ALNEALK	379.72	2	11.17	21.81	28	30		
Q6P1X6	ACSGDGGVSYTQGQSPEPR	976.92	2	11.41	22.11	25	50		
P49588	GLEVTDDSPK	530.76	2	15.56	27.15	30	30	2	Yes
O00257	YMENGMQAVK	585.77	2	15.68	27.30	28	110		
Q9ULD2	SDLNADK	381.69	2	16.38	28.13	20	200		
P07858	ICEPGYSPTYK	657.81	2	19.19	31.48	20	30	2	Yes
P55884	GTYLATFHQR	597.31	2	20.09	32.55	28	30	3	
P35659	EPFTIAQGK	495.77	2	25.21	38.55	20	30	2	Yes
P53396	EAGVFVPR	437.74	2	25.88	39.33	20	30	2	Yes
P56182	VGAEELTADQNLK	694.36	2	26.18	39.68	25	50	2	Yes
Q9UKB3	DLMLEESDK	540.25	2	32.17	46.59	28	110		
Q9BRP1	LLHVFACACPGCSTGGAR	644.96	3	33.28	47.85	20	150		
P57740	EADLDVATITK	588.31	2	37.58	52.67	20	100		
Q96E22	AAQDFCQLVAQK	689.84	2	37.63	52.73	20	110		
P35659	VYENYPTYDLTER	831.89	2	39.14	54.33	20	30	2	Yes
O43464	EPSFPDVQHGVLIHK	567.97	3	39.54	54.73	20	50		
Q13769	LMAEIQDLK	530.79	2	43.49	58.65	23	100		
Q5VW38	AGITAAINLAK	521.82	2	45.81	60.93	23	50		
O75347	LEAAYLDLQR	596.32	2	46.75	61.89	20	30	2	Yes
Q8IUR7	YTQEYICQIFSHCCK	1018.94	2	54.04	69.62	28	50		
Q12986	NVETHTGSLIEQLTTEK	950.49	2	54.20	69.79	28	200		
P78318	NEDLEEIASTDLK	738.86	2	54.25	69.85	23	100	2	Yes
P36873	LNIDSIIQR	536.31	2	58.02	73.84	20	50	2	Yes
Q9NRX5	GVVPCNILVGYK	659.86	2	60.48	76.40	20	100		
Q8IUR7	DMGIVDILHK	570.81	2	63.16	79.13	25	50		
Q12907	GYYFGASAGTGDLSDNHDII	910.43	3	63.48	79.45	23	30	3	Yes
Q96GW9	LGPETGLLFPR	600.34	2	67.67	83.65	20	50		
P51610	ENQWFDVGVIK	667.84	2	69.74	85.72	20	50	2	Yes
O43847	ELAALWGIETR	629.84	2	73.12	89.21	28	100		
Q96QE3	YFPLLLK	447.28	2	77.69	93.89	20	110		
Q8N442	GVIANVALFDGVVSK	744.92	2	79.03	95.24	20	150		
P25445	YITTIAGVMTLSQVK	812.95	2	79.51	95.72	28	50		

Q8NCE2	DGGFTLEDICMLR	763.85	2	85.93	102.05	28	150		
Q86WQ0	DFYPEDNNSLQTFPIPAAEVI	917.11	3	86.01	102.13	28	50	3	Yes
P00533	DNIGSQYLLNWCVQIAK	1011.51	2	86.26	102.37	25	150		
Q9BRX5	LELPLWLAK	541.83	2	87.84	103.90	20	100	2	Yes
Q9UPW5	LVLPCLQLLR	612.88	2	93.44	109.23	23	110		
P15848	ELIHISDWLPTLVK	832.47	2	95.20	110.87	23	150		
O60294	GYVQDPFAALLVPGAAR	872.97	2	97.34	112.86	20	110		
Q8N1F8	EPWEELFSIGLR	738.38	2	103.67	118.66	25	200		
Q9ULD2	FEALTVVIQHLLSER	585.33	3	104.05	119.00	28	200		
Q96JI7	NVEELIPALDLLCSAIR	963.52	2	104.28	119.21	28	200		
Q9Y5T5	FPEILDLAPFCTLK	832.44	2	106.28	121.00	28	150		
Q8IY45	VSQLLDLCLWCFMK	906.95	2	107.85	122.40	25	200		
Q9UDY8	APLVDVYELTNLLR	808.46	2	110.10	124.38	28	100		

SourceDataForFigure4B: The number of suggested peptides of HeLa dataset by DeepPRM method and peptides detected by DDA analysis.								
StrippedSequence	iRT	Accession	protein name	PrecursorMz	Precursor Charge	Detectability	Charge (by Search Engine): Sequest HT	Detected by DDA
ALNSASTSLPTSCPGSEPVPTHQQGQPALELK	46.06047	Q9Y6A5	TACC3_HUMAN	1651.820262	2	0.784355938	3	
ALNSASTSLPTSCPGSEPVPTHQQGQPALELK	46.06047	Q9Y6A5	TACC3_HUMAN	1101.549449	3	0.784355938	3	Yes
AMTLSPQEEVAAGQMASSSR	42.24765	Q9Y6A5	TACC3_HUMAN	1025.980796	2	0.741617799		
AMTLSPQEEVAAGQMASSSR	42.24765	Q9Y6A5	TACC3_HUMAN	684.3231387	3	0.741617799		
AQAEALALQASLR	45.55912	Q9Y6A5	TACC3_HUMAN	671.3784325	2	0.771613061		
AQAEALALQASLR	45.55912	Q9Y6A5	TACC3_HUMAN	447.9215633	3	0.771613061		
DPAEVLGTGAEVDYLEQFGTSSFK	127.8232	Q9Y6A5	TACC3_HUMAN	1280.608662	2	0.741547823		
DPAEVLGTGAEVDYLEQFGTSSFK	127.8232	Q9Y6A5	TACC3_HUMAN	854.0750493	3	0.741547823		
DQLTTDLNSMEK	33.61954	Q9Y6A5	TACC3_HUMAN	697.8275805	2	0.805745304		
DQLTTDLNSMEK	33.61954	Q9Y6A5	TACC3_HUMAN	465.5543287	3	0.805745304		
FEEVVYQAMEEVQK	68.78257	Q9Y6A5	TACC3_HUMAN	864.9116405	2	0.820465863		
FEEVVYQAMEEVQK	68.78257	Q9Y6A5	TACC3_HUMAN	576.943702	3	0.820465863		
GSYHLDWDK	11.29826	Q9Y6A5	TACC3_HUMAN	560.7570825	2	0.67088747		
GSYHLDWDK	11.29826	Q9Y6A5	TACC3_HUMAN	374.1739967	3	0.67088747		
LEAPFTQDDTLGLENSHPVWTQK	69.24026	Q9Y6A5	TACC3_HUMAN	1313.643375	2	0.789589047	3	
LEAPFTQDDTLGLENSHPVWTQK	69.24026	Q9Y6A5	TACC3_HUMAN	876.0981913	3	0.789589047	3	Yes
LQLANEEIAQVR	35.37946	Q9Y6A5	TACC3_HUMAN	692.3837145	2	0.726007104		
LQLANEEIAQVR	35.37946	Q9Y6A5	TACC3_HUMAN	461.9250847	3	0.726007104		
MDDPNFIPFGGDTK	70.44232	Q9Y6A5	TACC3_HUMAN	777.351223	2	0.734464049		
MDDPNFIPFGGDTK	70.44232	Q9Y6A5	TACC3_HUMAN	518.5700903	3	0.734464049		
NTENCDFLFSPPEVTGR	76.55728	Q9Y6A5	TACC3_HUMAN	991.949821	2	0.755636394		
NTENCDFLFSPPEVTGR	76.55728	Q9Y6A5	TACC3_HUMAN	661.6358223	3	0.755636394		
SGCSEAQPPESPETR	-19.96138	Q9Y6A5	TACC3_HUMAN	816.3524825	2	0.755123496		
SGCSEAQPPESPETR	-19.96138	Q9Y6A5	TACC3_HUMAN	544.57093	3	0.755123496		
VSGSPEQAVEENLSSYSLDR	62.66575	Q9Y6A5	TACC3_HUMAN	1084.011656	2	0.766401708		
VSGSPEQAVEENLSSYSLDR	62.66575	Q9Y6A5	TACC3_HUMAN	723.0103787	3	0.766401708		
VTFQTPLR	26.86573	Q9Y6A5	TACC3_HUMAN	481.277459	2	0.703995109		
VTFQTPLR	26.86573	Q9Y6A5	TACC3_HUMAN	321.187581	3	0.703995109		
VTPASETLEDPCR	15.10699	Q9Y6A5	TACC3_HUMAN	737.846305	2	0.64084655	2	Yes
VTPASETLEDPCR	15.10699	Q9Y6A5	TACC3_HUMAN	492.2334783	3	0.64084655	2	
AGSDGESIGNCPFSQR	17.15689	Q9Y696	CLIC4_HUMAN	841.365924	2	0.697907448	2	Yes
AGSDGESIGNCPFSQR	17.15689	Q9Y696	CLIC4_HUMAN	561.2465577	3	0.697907448	2	
DEFTNTCPSDK	-7.779972	Q9Y696	CLIC4_HUMAN	657.2698985	2	0.681300282	2	Yes
DEFTNTCPSDK	-7.779972	Q9Y696	CLIC4_HUMAN	438.515874	3	0.681300282	2	
FLDGNEMTLADCNLLPK	84.12822	Q9Y696	CLIC4_HUMAN	975.969168	2	0.913477182		
FLDGNEMTLADCNLLPK	84.12822	Q9Y696	CLIC4_HUMAN	650.9820537	3	0.913477182		
GVVFSVTTVDLK	64.50734	Q9Y696	CLIC4_HUMAN	632.8615525	2	0.802670956		
GVVFSVTTVDLK	64.50734	Q9Y696	CLIC4_HUMAN	422.2436433	3	0.802670956		
HPESNTAGMDIFAK	21.61301	Q9Y696	CLIC4_HUMAN	759.3568395	2	0.661852956	2	Yes
HPESNTAGMDIFAK	21.61301	Q9Y696	CLIC4_HUMAN	506.5738347	3	0.661852956	2	
IEEFLEEVLCPPK	95.51234	Q9Y696	CLIC4_HUMAN	801.9083735	2	0.785008073		
IEEFLEEVLCPPK	95.51234	Q9Y696	CLIC4_HUMAN	534.941524	3	0.785008073		
KPADLQNLAPGTHPPFITFNSEVK	63.12082	Q9Y696	CLIC4_HUMAN	1311.190295	2	0.663235545	4	
KPADLQNLAPGTHPPFITFNSEVK	63.12082	Q9Y696	CLIC4_HUMAN	874.4628047	3	0.663235545	4	
LDEYLNSPLPDEIDENSMEDIK	87.93132	Q9Y696	CLIC4_HUMAN	1290.089443	2	0.760510206	3	
LDEYLNSPLPDEIDENSMEDIK	87.93132	Q9Y696	CLIC4_HUMAN	860.39557	3	0.760510206	3	Yes
YLTNAYSR	-9.562222	Q9Y696	CLIC4_HUMAN	494.248889	2	0.704847038	2	Yes
YLTNAYSR	-9.562222	Q9Y696	CLIC4_HUMAN	329.835201	3	0.704847038	2	
ALVNVEWNICQDCK	60.9427	Q9Y5T5	UBP16_HUMAN	874.9089135	2	0.688586652		
ALVNVEWNICQDCK	60.9427	Q9Y5T5	UBP16_HUMAN	583.6085507	3	0.688586652		
ENPPMNSPCQITVK	28.28144	Q9Y5T5	UBP16_HUMAN	807.884907	2	0.630263925		
ENPPMNSPCQITVK	28.28144	Q9Y5T5	UBP16_HUMAN	538.9258797	3	0.630263925		
EVFNTDECSIQHCLYQFTR	62.23759	Q9Y5T5	UBP16_HUMAN	1224.041912	2	0.604115129		
EVFNTDECSIQHCLYQFTR	62.23759	Q9Y5T5	UBP16_HUMAN	816.363883	3	0.604115129		
FQQAGFNLR	23.56018	Q9Y5T5	UBP16_HUMAN	540.7834395	2	0.669878125		
FQQAGFNLR	23.56018	Q9Y5T5	UBP16_HUMAN	360.8582347	3	0.669878125		
GQWFHISDTHVQAVPTTK	32.05234	Q9Y5T5	UBP16_HUMAN	1026.519064	2	0.805784464		
GQWFHISDTHVQAVPTTK	32.05234	Q9Y5T5	UBP16_HUMAN	684.681984	3	0.805784464		
GYQQQDSQELLR	14.45791	Q9Y5T5	UBP16_HUMAN	732.858057	2	0.781821609		
GYQQQDSQELLR	14.45791	Q9Y5T5	UBP16_HUMAN	488.9079797	3	0.781821609		
MIESVTDNQK	-5.523529	Q9Y5T5	UBP16_HUMAN	582.7824445	2	0.62094605		
MIESVTDNQK	-5.523529	Q9Y5T5	UBP16_HUMAN	388.8575713	3	0.62094605		
NINMDNDLEVLTSSPTR	67.19112	Q9Y5T5	UBP16_HUMAN	959.9629275	2	0.831426382		
NINMDNDLEVLTSSPTR	67.19112	Q9Y5T5	UBP16_HUMAN	640.3112267	3	0.831426382		
NLNLNAALHPDEINIEILNDSHTPGTK	72.20506	Q9Y5T5	UBP16_HUMAN	1477.254882	2	0.603878736		
NLNLNAALHPDEINIEILNDSHTPGTK	72.20506	Q9Y5T5	UBP16_HUMAN	985.1725293	3	0.603878736		
SMPSFVDR	17.60208	Q9Y5T5	UBP16_HUMAN	469.724201	2	0.648299992		
SMPSFVDR	17.60208	Q9Y5T5	UBP16_HUMAN	313.485409	3	0.648299992		
SNHISQEGVMHK	-34.38102	Q9Y5T5	UBP16_HUMAN	683.8307915	2	0.661106706		
SNHISQEGVMHK	-34.38102	Q9Y5T5	UBP16_HUMAN	456.223136	3	0.661106706		
TANSHLSNLVLHGDIPQDFEMESK	56.53962	Q9Y5T5	UBP16_HUMAN	1341.64559	2	0.639859915		
TANSHLSNLVLHGDIPQDFEMESK	56.53962	Q9Y5T5	UBP16_HUMAN	894.766335	3	0.639859915		
TVPIDDSSETLEPVCR	42.66312	Q9Y5T5	UBP16_HUMAN	909.4334725	2	0.647306561		
TVPIDDSSETLEPVCR	42.66312	Q9Y5T5	UBP16_HUMAN	606.6249233	3	0.647306561		
VLHLNDICTIDHPEDSEYEAEMSLQGEVNIK	72.66748	Q9Y5T5	UBP16_HUMAN	1799.837986	2	0.720417202		
VLHLNDICTIDHPEDSEYEAEMSLQGEVNIK	72.66748	Q9Y5T5	UBP16_HUMAN	1200.227932	3	0.720417202		
VLNSQAYLLFYER	78.74234	Q9Y5T5	UBP16_HUMAN	808.428113	2	0.628218949		
VLNSQAYLLFYER	78.74234	Q9Y5T5	UBP16_HUMAN	539.288017	3	0.628218949		
VLYSLYGVVEHSGTMR	57.55863	Q9Y5T5	UBP16_HUMAN	905.9619945	2	0.751098514		
VLYSLYGVVEHSGTMR	57.55863	Q9Y5T5	UBP16_HUMAN	604.3106047	3	0.751098514		
YLLDGMR	27.78542	Q9Y5T5	UBP16_HUMAN	434.223833	2	0.726853907		
YLLDGMR	27.78542	Q9Y5T5	UBP16_HUMAN	289.818497	3	0.726853907		
APGMNTIDQGMAALK	48.17893	Q9Y5A9	YTHD2_HUMAN	759.3767185	2	0.870891333		
APGMNTIDQGMAALK	48.17893	Q9Y5A9	YTHD2_HUMAN	506.5870873	3	0.870891333		
HTTSIFDDFSHYEK	35.87793	Q9Y5A9	YTHD2_HUMAN	863.8895535	2	0.876164317	3	
HTTSIFDDFSHYEK	35.87793	Q9Y5A9	YTHD2_HUMAN	576.2623107	3	0.876164317	3	Yes
LGSTEVASNVPK	2.155155	Q9Y5A9	YTHD2_HUMAN	601.3253335	2	0.842514157		
LGSTEVASNVPK	2.155155	Q9Y5A9	YTHD2_HUMAN	401.2194973	3	0.842514157		
SAVDYNTCAGVWSQDK	33.78315	Q9Y5A9	YTHD2_HUMAN	900.897053	2	0.729439616		
SAVDYNTCAGVWSQDK	33.78315	Q9Y5A9	YTHD2_HUMAN	600.933977	3	0.729439616		
AVGSGSITSNIVASNSLPPATIAPPKPASWADIAS	82.29144	Q9Y5A9	YTHD2_HUMAN	2057.61333	2	0.646173775		
AVGSGSITSNIVASNSLPPATIAPPKPASWADIAS	82.29144	Q9Y5A9	YTHD2_HUMAN	1372.078162	3	0.646173775		
YNIWCSTEHGNK	-0.876606	Q9Y5A9	YTHD2_HUMAN	754.833527	2	0.760176957	3	
YNIWCSTEHGNK	-0.876606	Q9Y5A9	YTHD2_HUMAN	503.558293	3	0.760176957	3	Yes
DCGGAAQLAGPAAEADPLGR	53.22333	Q9Y508	RN114_HUMAN	948.947614	2	0.66939801		
DCGGAAQLAGPAAEADPLGR	53.22333	Q9Y508	RN114_HUMAN	632.9676843	3	0.66939801		
FSYDTFVDYDVDEEDMMNQVLQR	117.7625	Q9Y508	RN114_HUMAN	1430.110381	2	0.659346879		
FSYDTFVDYDVDEEDMMNQVLQR	117.7625	Q9Y508	RN114_HUMAN	953.742862	3	0.659346879		

NFDQEGLVEHCK	4.415398	Q9Y508	RN114_HUMAN	738.3333645	2	0.778277636	3	
NFDQEGLVEHCK	4.415398	Q9Y508	RN114_HUMAN	492.5581847	3	0.778277636	3	Yes
YQNYIMEGVK	28.01649	Q9Y508	RN114_HUMAN	622.8031715	2	0.767508268		
YQNYIMEGVK	28.01649	Q9Y508	RN114_HUMAN	415.538056	3	0.767508268		
YTFPCPYCPEK	39.71072	Q9Y508	RN114_HUMAN	731.313047	2	0.750749052		
YTFPCPYCPEK	39.71072	Q9Y508	RN114_HUMAN	487.877973	3	0.750749052		
ALLLLQR	40.89122	Q9Y4R8	TELO2_HUMAN	413.779637	2	0.725389302		
ALLLLQR	40.89122	Q9Y4R8	TELO2_HUMAN	276.189033	3	0.725389302		
AMEAVLTGLVEAALGPEVLSR	154.8126	Q9Y4R8	TELO2_HUMAN	1063.580472	2	0.679920137		
AMEAVLTGLVEAALGPEVLSR	154.8126	Q9Y4R8	TELO2_HUMAN	709.3895893	3	0.679920137		
AVLICLAQLGEPELR	92.8363	Q9Y4R8	TELO2_HUMAN	841.4694645	2	0.68942219		
AVLICLAQLGEPELR	92.8363	Q9Y4R8	TELO2_HUMAN	561.3155847	3	0.68942219		
DCVEALTTSEDIER	42.79036	Q9Y4R8	TELO2_HUMAN	819.370341	2	0.749195874		
DCVEALTTSEDIER	42.79036	Q9Y4R8	TELO2_HUMAN	546.5828357	3	0.749195874		
DELLASMMAGVK	80.8903	Q9Y4R8	TELO2_HUMAN	632.817973	2	0.766096294		
DELLASMMAGVK	80.8903	Q9Y4R8	TELO2_HUMAN	422.2145903	3	0.766096294		
ELLETWGSSSAIR	54.14107	Q9Y4R8	TELO2_HUMAN	724.873179	2	0.724747062	2	Yes
ELLETWGSSSAIR	54.14107	Q9Y4R8	TELO2_HUMAN	483.5847277	3	0.724747062	2	
FNSVAGHFFFPLLQR	88.3136	Q9Y4R8	TELO2_HUMAN	890.4706555	2	0.743904829		
FNSVAGHFFFPLLQR	88.3136	Q9Y4R8	TELO2_HUMAN	593.9830453	3	0.743904829		
LAALTQGSYLHQR	13.95971	Q9Y4R8	TELO2_HUMAN	729.3971525	2	0.814087987		
LAALTQGSYLHQR	13.95971	Q9Y4R8	TELO2_HUMAN	486.60071	3	0.814087987		
LAVLMEAQCR	30.5135	Q9Y4R8	TELO2_HUMAN	595.8052005	2	0.693480909		
LAVLMEAQCR	30.5135	Q9Y4R8	TELO2_HUMAN	397.5394087	3	0.693480909		
LGMIVAEVVSAR	64.75415	Q9Y4R8	TELO2_HUMAN	622.855747	2	0.737648368		
LGMIVAEVVSAR	64.75415	Q9Y4R8	TELO2_HUMAN	415.5731063	3	0.737648368		
LLEDLMDELLEAR	148.9077	Q9Y4R8	TELO2_HUMAN	780.403455	2	0.826336026	3	
LLEDLMDELLEAR	148.9077	Q9Y4R8	TELO2_HUMAN	520.6049117	3	0.826336026	3	Yes
LLFLQSR	42.17339	Q9Y4R8	TELO2_HUMAN	438.769269	2	0.729254782		
LLFLQSR	42.17339	Q9Y4R8	TELO2_HUMAN	292.8487877	3	0.729254782		
LLGNLVVK	36.79348	Q9Y4R8	TELO2_HUMAN	428.2872945	2	0.767723024	2	Yes
LLGNLVVK	36.79348	Q9Y4R8	TELO2_HUMAN	285.8608047	3	0.767723024	2	
LQQENLAEFFPQNYFR	100.0419	Q9Y4R8	TELO2_HUMAN	1022.500334	2	0.857957721		
LQQENLAEFFPQNYFR	100.0419	Q9Y4R8	TELO2_HUMAN	682.002831	3	0.857957721		
LSPAWLELLPHGR	77.93655	Q9Y4R8	TELO2_HUMAN	744.920267	2	0.736416757	3	
LSPAWLELLPHGR	77.93655	Q9Y4R8	TELO2_HUMAN	496.949453	3	0.736416757	3	Yes
LTTPMLQSLLGHLAMDSQR	104.2135	Q9Y4R8	TELO2_HUMAN	1056.551192	2	0.750386596		
LTTPMLQSLLGHLAMDSQR	104.2135	Q9Y4R8	TELO2_HUMAN	704.7034027	3	0.750386596		
RPLLLQVLK	41.05382	Q9Y4R8	TELO2_HUMAN	540.3691505	2	0.691899896		
RPLLLQVLK	41.05382	Q9Y4R8	TELO2_HUMAN	360.582042	3	0.691899896		
VLLHLEEK	5.624699	Q9Y4R8	TELO2_HUMAN	490.792941	2	0.811572194		
VLLHLEEK	5.624699	Q9Y4R8	TELO2_HUMAN	327.5312357	3	0.811572194		
VVALPDHLGNR	16.96999	Q9Y4R8	TELO2_HUMAN	595.836203	2	0.782243013		
VVALPDHLGNR	16.96999	Q9Y4R8	TELO2_HUMAN	397.560077	3	0.782243013		
YLGEMEPPALPR	55.24596	Q9Y4R8	TELO2_HUMAN	686.8506575	2	0.809407115		
YLGEMEPPALPR	55.24596	Q9Y4R8	TELO2_HUMAN	458.23638	3	0.809407115		
ANIALWPPVR	64.45277	Q9Y4C2	TCAF1_HUMAN	568.832932	2	0.785176814		
ANIALWPPVR	64.45277	Q9Y4C2	TCAF1_HUMAN	379.5578963	3	0.785176814		
DSLGVYCIDAYNETMTEK	68.98553	Q9Y4C2	TCAF1_HUMAN	1054.961729	2	0.687151194		
DSLGVYCIDAYNETMTEK	68.98553	Q9Y4C2	TCAF1_HUMAN	703.6437607	3	0.687151194		
EVATSLAYLPEWK	76.60044	Q9Y4C2	TCAF1_HUMAN	753.896119	2	0.83793658		
EVATSLAYLPEWK	76.60044	Q9Y4C2	TCAF1_HUMAN	502.9333543	3	0.83793658		
GDTSFFK	12.66564	Q9Y4C2	TCAF1_HUMAN	401.19306	2	0.709519684		
GDTSFFK	12.66564	Q9Y4C2	TCAF1_HUMAN	267.7979817	3	0.709519684		
GLWGPVHELGR	35.14643	Q9Y4C2	TCAF1_HUMAN	610.830921	2	0.835570931	3	
GLWGPVHELGR	35.14643	Q9Y4C2	TCAF1_HUMAN	407.5565557	3	0.835570931	3	Yes
IPVLVSCEDDLSDDR	55.92334	Q9Y4C2	TCAF1_HUMAN	866.90709	2	0.68591392		
IPVLVSCEDDLSDDR	55.92334	Q9Y4C2	TCAF1_HUMAN	578.2740017	3	0.68591392		
IQENPGPWGELATDNIILTVPTANLR	127.7259	Q9Y4C2	TCAF1_HUMAN	1416.748705	2	0.751031101		
IQENPGPWGELATDNIILTVPTANLR	127.7259	Q9Y4C2	TCAF1_HUMAN	944.8350783	3	0.751031101		
IQIGCHTDDLTR	3.342319	Q9Y4C2	TCAF1_HUMAN	714.8491825	2	0.727133155		
IQIGCHTDDLTR	3.342319	Q9Y4C2	TCAF1_HUMAN	476.9020633	3	0.727133155		
LGAEPFPLR	46.32761	Q9Y4C2	TCAF1_HUMAN	500.2852835	2	0.737506628	2	Yes
LGAEPFPLR	46.32761	Q9Y4C2	TCAF1_HUMAN	333.859464	3	0.737506628	2	
LGETTLEEWK	37.47961	Q9Y4C2	TCAF1_HUMAN	603.30661	2	0.841700315		
LGETTLEEWK	37.47961	Q9Y4C2	TCAF1_HUMAN	402.5403483	3	0.841700315		
LGPFLLNAVR	72.04482	Q9Y4C2	TCAF1_HUMAN	550.3353075	2	0.700805366	2	Yes
LGPFLLNAVR	72.04482	Q9Y4C2	TCAF1_HUMAN	367.2261467	3	0.700805366	2	
LWDEVMQAVAR	72.09525	Q9Y4C2	TCAF1_HUMAN	659.335179	2	0.780781269		
LWDEVMQAVAR	72.09525	Q9Y4C2	TCAF1_HUMAN	439.8927277	3	0.780781269		
LYLLTQMPH	56.46712	Q9Y4C2	TCAF1_HUMAN	558.3000725	2	0.726051807		
LYLLTQMPH	56.46712	Q9Y4C2	TCAF1_HUMAN	372.53599	3	0.726051807		
NQTNLPTENVDK	-3.652519	Q9Y4C2	TCAF1_HUMAN	686.8393365	2	0.641450047	2	Yes
NQTNLPTENVDK	-3.652519	Q9Y4C2	TCAF1_HUMAN	458.2288327	3	0.641450047	2	
STLAEFQVIMGR	71.80572	Q9Y4C2	TCAF1_HUMAN	676.3561115	2	0.795545399		
STLAEFQVIMGR	71.80572	Q9Y4C2	TCAF1_HUMAN	451.240016	3	0.795545399		
TLENPEPLLR	36.68777	Q9Y4C2	TCAF1_HUMAN	591.330419	2	0.778259158		
TLENPEPLLR	36.68777	Q9Y4C2	TCAF1_HUMAN	394.556221	3	0.778259158		
ADIPLPEGEASPPAPPLK	67.20378	Q9Y487	VPP2_HUMAN	899.9834585	2	0.821507573		
ADIPLPEGEASPPAPPLK	67.20378	Q9Y487	VPP2_HUMAN	600.324914	3	0.821507573		
DLNQNVSSFQR	23.92023	Q9Y487	VPP2_HUMAN	654.3211135	2	0.827629328		
DLNQNVSSFQR	23.92023	Q9Y487	VPP2_HUMAN	436.5500173	3	0.827629328		
ESGATIPSFMNIIPTK	93.87265	Q9Y487	VPP2_HUMAN	853.4456545	2	0.821490765	2	Yes
ESGATIPSFMNIIPTK	93.87265	Q9Y487	VPP2_HUMAN	569.2997113	3	0.821490765	2	
FTEGFQNIVDAYGVGSYR	86.01006	Q9Y487	VPP2_HUMAN	1011.98197	2	0.805115938		
FTEGFQNIVDAYGVGSYR	86.01006	Q9Y487	VPP2_HUMAN	674.9905883	3	0.805115938		
GPYPLGIDPIWNLATNR	116.3102	Q9Y487	VPP2_HUMAN	949.0025125	2	0.771593928		
GPYPLGIDPIWNLATNR	116.3102	Q9Y487	VPP2_HUMAN	633.0042833	3	0.771593928		
HNSILQLDPSIPGVFR	83.5849	Q9Y487	VPP2_HUMAN	896.989409	2	0.776935697		
HNSILQLDPSIPGVFR	83.5849	Q9Y487	VPP2_HUMAN	598.328881	3	0.776935697		
ILVYLVQEINR	78.3988	Q9Y487	VPP2_HUMAN	680.403914	2	0.61461997		
ILVYLVQEINR	78.3988	Q9Y487	VPP2_HUMAN	453.938551	3	0.61461997		
IQDLYTVLHK	30.84378	Q9Y487	VPP2_HUMAN	615.348608	2	0.822713017	3	
IQDLYTVLHK	30.84378	Q9Y487	VPP2_HUMAN	410.568347	3	0.822713017	3	Yes
LGFVSGLINQGK	62.16331	Q9Y487	VPP2_HUMAN	616.8540615	2	0.837990522	2	Yes
LGFVSGLINQGK	62.16331	Q9Y487	VPP2_HUMAN	411.5719827	3	0.837990522	2	
LNQSQEIMR	-5.909069	Q9Y487	VPP2_HUMAN	559.7853215	2	0.704674661	2	Yes
LNQSQEIMR	-5.909069	Q9Y487	VPP2_HUMAN	373.526156	3	0.704674661	2	
MVLWNDSVVR	53.58796	Q9Y487	VPP2_HUMAN	609.819164	2	0.612067699		
MVLWNDSVVR	53.58796	Q9Y487	VPP2_HUMAN	406.882051	3	0.612067699		
NLLELIEYTHMLR	119.6078	Q9Y487	VPP2_HUMAN	822.9430775	2	0.829770446	3	

NLLELIEYTHMLR	119.6078	Q9Y487	VPP2 HUMAN	548.96466	3	0.829770446	3	Yes
NVEFEPTYEEFPSLESDSLLDYSCMQR	122.0404	Q9Y487	VPP2 HUMAN	1643.216105	2	0.675118506		
NVEFEPTYEEFPSLESDSLLDYSCMQR	122.0404	Q9Y487	VPP2 HUMAN	1095.813345	3	0.675118506		
QVLEMQEQLQK	24.88129	Q9Y487	VPP2 HUMAN	687.3588515	2	0.755161226		
QVLEMQEQLQK	24.88129	Q9Y487	VPP2 HUMAN	458.575176	3	0.755161226		
SVNLFGSGWNVSAMYSSSHPPAEHK	60.25534	Q9Y487	VPP2 HUMAN	1345.127363	2	0.604433894		
SVNLFGSGWNVSAMYSSSHPPAEHK	60.25534	Q9Y487	VPP2 HUMAN	897.0875167	3	0.604433894		
TSGLYTGQEYVQR	17.68174	Q9Y487	VPP2 HUMAN	751.3682535	2	0.878201663		
TSGLYTGQEYVQR	17.68174	Q9Y487	VPP2 HUMAN	501.2481107	3	0.878201663		
WLVFSAETSR	50.94872	Q9Y487	VPP2 HUMAN	598.3094865	2	0.729683578		
WLVFSAETSR	50.94872	Q9Y487	VPP2 HUMAN	399.2089327	3	0.729683578		
ADDYIEITGAGGK	35.30177	Q9Y3Z3	SAMH1 HUMAN	655.317702	2	0.752305269		
ADDYIEITGAGGK	35.30177	Q9Y3Z3	SAMH1 HUMAN	437.2144097	3	0.752305269		
DCHHLGIQNNFDYK	15.33736	Q9Y3Z3	SAMH1 HUMAN	880.8946475	2	0.646038949		
DCHHLGIQNNFDYK	15.33736	Q9Y3Z3	SAMH1 HUMAN	587.59904	3	0.646038949		
EDYESLPK	6.9062	Q9Y3Z3	SAMH1 HUMAN	490.7327415	2	0.620632887		
EDYESLPK	6.9062	Q9Y3Z3	SAMH1 HUMAN	327.4911027	3	0.620632887		
EQIVGPLESPVEDSLWPYK	106.4463	Q9Y3Z3	SAMH1 HUMAN	1093.554972	2	0.715240061		
EQIVGPLESPVEDSLWPYK	106.4463	Q9Y3Z3	SAMH1 HUMAN	729.3725893	3	0.715240061		
EVGNLYDMFHTR	53.86732	Q9Y3Z3	SAMH1 HUMAN	741.3462705	2	0.830911994		
EVGNLYDMFHTR	53.86732	Q9Y3Z3	SAMH1 HUMAN	494.5667887	3	0.830911994		
FENLGVSSLGER	38.4967	Q9Y3Z3	SAMH1 HUMAN	654.3336895	2	0.791512847		
FENLGVSSLGER	38.4967	Q9Y3Z3	SAMH1 HUMAN	436.5584013	3	0.791512847		
FIPLARPEVK	22.50917	Q9Y3Z3	SAMH1 HUMAN	585.35624	2	0.730188012		
FIPLARPEVK	22.50917	Q9Y3Z3	SAMH1 HUMAN	390.573435	3	0.730188012		
GGFEEPVLLK	48.4692	Q9Y3Z3	SAMH1 HUMAN	544.803506	2	0.812036991		
GGFEEPVLLK	48.4692	Q9Y3Z3	SAMH1 HUMAN	363.538279	3	0.812036991		
IIDTPQFQR	23.38787	Q9Y3Z3	SAMH1 HUMAN	559.304205	2	0.671719134		
IIDTPQFQR	23.38787	Q9Y3Z3	SAMH1 HUMAN	373.2054117	3	0.671719134		
ISTAIDDMEAYTK	39.03025	Q9Y3Z3	SAMH1 HUMAN	729.345602	2	0.829426944		
ISTAIDDMEAYTK	39.03025	Q9Y3Z3	SAMH1 HUMAN	486.566343	3	0.829426944		
LLSYIQR	21.08845	Q9Y3Z3	SAMH1 HUMAN	446.766722	2	0.662922025		
LLSYIQR	21.08845	Q9Y3Z3	SAMH1 HUMAN	298.180423	3	0.662922025		
LTDNIFLEILYSTDPK	131.4487	Q9Y3Z3	SAMH1 HUMAN	941.496394	2	0.702965617		
LTDNIFLEILYSTDPK	131.4487	Q9Y3Z3	SAMH1 HUMAN	628.0002043	3	0.702965617		
LVQIHVDTMK	13.58638	Q9Y3Z3	SAMH1 HUMAN	592.3293655	2	0.672983348		
LVQIHVDTMK	13.58638	Q9Y3Z3	SAMH1 HUMAN	395.2221853	3	0.672983348		
NFTKPQDGDVIAPLITPQK	52.78365	Q9Y3Z3	SAMH1 HUMAN	1041.565679	2	0.7180264		
NFTKPQDGDVIAPLITPQK	52.78365	Q9Y3Z3	SAMH1 HUMAN	694.713061	3	0.7180264		
NPIDHVSFYCK	19.35779	Q9Y3Z3	SAMH1 HUMAN	690.3248065	2	0.759210706		
NPIDHVSFYCK	19.35779	Q9Y3Z3	SAMH1 HUMAN	460.5524793	3	0.759210706		
NQVSQLLPEK	22.5827	Q9Y3Z3	SAMH1 HUMAN	578.322594	2	0.822886765		
NQVSQLLPEK	22.5827	Q9Y3Z3	SAMH1 HUMAN	385.8843377	3	0.822886765		
SFLYEIVSNK	63.96301	Q9Y3Z3	SAMH1 HUMAN	600.319515	2	0.814299703		
SFLYEIVSNK	63.96301	Q9Y3Z3	SAMH1 HUMAN	400.5489517	3	0.814299703		
TPSNTPSAEADWSPGLELHPDYK	53.37183	Q9Y3Z3	SAMH1 HUMAN	1256.58552	2	0.711147428		
TPSNTPSAEADWSPGLELHPDYK	53.37183	Q9Y3Z3	SAMH1 HUMAN	838.0596217	3	0.711147428		
TWGPEQVCSFLR	71.56648	Q9Y3Z3	SAMH1 HUMAN	740.356643	2	0.758858085		
TWGPEQVCSFLR	71.56648	Q9Y3Z3	SAMH1 HUMAN	493.907037	3	0.758858085		
VCEVDNELR	-0.858898	Q9Y3Z3	SAMH1 HUMAN	567.2669615	2	0.753876328		
VCEVDNELR	-0.858898	Q9Y3Z3	SAMH1 HUMAN	378.513916	3	0.753876328		
VGNIIDTMITDAFLK	130.986	Q9Y3Z3	SAMH1 HUMAN	825.942748	2	0.82007277		
VGNIIDTMITDAFLK	130.986	Q9Y3Z3	SAMH1 HUMAN	550.9644403	3	0.82007277		
VINDPIHGHIELHPLLVR	46.61062	Q9Y3Z3	SAMH1 HUMAN	1036.592365	2	0.73497951	5	
VINDPIHGHIELHPLLVR	46.61062	Q9Y3Z3	SAMH1 HUMAN	691.397518	3	0.73497951	5	
YVGETQPTGQIK	-5.72089	Q9Y3Z3	SAMH1 HUMAN	660.8438875	2	0.823490024		
YVGETQPTGQIK	-5.72089	Q9Y3Z3	SAMH1 HUMAN	440.8985333	3	0.823490024		
AFDLFNPNFK	80.99509	Q9Y3D9	RT23 HUMAN	606.8065795	2	0.831058323	2	Yes
AFDLFNPNFK	80.99509	Q9Y3D9	RT23 HUMAN	404.8736613	3	0.831058323	2	
ALLAEGVILR	59.28667	Q9Y3D9	RT23 HUMAN	527.8351405	2	0.887302041	2	Yes
ALLAEGVILR	59.28667	Q9Y3D9	RT23 HUMAN	352.2260353	3	0.887302041	2	
APIQDIWYHEDR	36.7056	Q9Y3D9	RT23 HUMAN	771.870967	2	0.866650105	3	
APIQDIWYHEDR	36.7056	Q9Y3D9	RT23 HUMAN	514.9165863	3	0.866650105	3	Yes
EKPLWFDVYDAFPPLR	119.5748	Q9Y3D9	RT23 HUMAN	997.0150885	2	0.734285235	3	
EKPLWFDVYDAFPPLR	119.5748	Q9Y3D9	RT23 HUMAN	665.0126673	3	0.734285235	3	Yes
EVPQDQHLEAPADQSK	-4.402573	Q9Y3D9	RT23 HUMAN	896.429579	2	0.857894421		
EVPQDQHLEAPADQSK	-4.402573	Q9Y3D9	RT23 HUMAN	597.955661	3	0.857894421		
FYSVYGSGQR	10.87228	Q9Y3D9	RT23 HUMAN	582.278178	2	0.748336673		
FYSVYGSGQR	10.87228	Q9Y3D9	RT23 HUMAN	388.5213937	3	0.748336673		
LETVGSIFSR	44.72898	Q9Y3D9	RT23 HUMAN	554.804037	2	0.851895809	2	Yes
LETVGSIFSR	44.72898	Q9Y3D9	RT23 HUMAN	370.2052997	3	0.851895809	2	
DYVFYLAVGNYR	93.6508	Q9Y3D6	FIS1 HUMAN	740.36752	2	0.718471587		
DYVFYLAVGNYR	93.6508	Q9Y3D6	FIS1 HUMAN	493.9142883	3	0.718471587		
GIVLLEELLPK	108.9636	Q9Y3D6	FIS1 HUMAN	612.384663	2	0.801601529		
GIVLLEELLPK	108.9636	Q9Y3D6	FIS1 HUMAN	408.5923837	3	0.801601529		
GLLQTEPQNNQAK	-0.292862	Q9Y3D6	FIS1 HUMAN	720.876254	2	0.777242184		
GLLQTEPQNNQAK	-0.292862	Q9Y3D6	FIS1 HUMAN	480.920111	3	0.777242184		
MEAVLNELVSVEDLLK	146.2435	Q9Y3D6	FIS1 HUMAN	901.484976	2	0.603080869		
MEAVLNELVSVEDLLK	146.2435	Q9Y3D6	FIS1 HUMAN	601.3259257	3	0.603080869		
HTLDGAACLLNSNK	23.4905	Q9Y3A3	PHOCN HUMAN	757.3755635	2	0.787309885	3	
HTLDGAACLLNSNK	23.4905	Q9Y3A3	PHOCN HUMAN	505.252984	3	0.787309885	3	Yes
IFSHAYFHHR	-25.72474	Q9Y3A3	PHOCN HUMAN	657.828708	2	0.725940585		
IFSHAYFHHR	-25.72474	Q9Y3A3	PHOCN HUMAN	438.8884137	3	0.725940585		
ILEPPEGQDEGVWK	46.35715	Q9Y3A3	PHOCN HUMAN	798.8993945	2	0.883223116	2	Yes
ILEPPEGQDEGVWK	46.35715	Q9Y3A3	PHOCN HUMAN	532.935538	3	0.883223116	2	
QIFDEYENETFLCHR	53.93655	Q9Y3A3	PHOCN HUMAN	1000.944535	2	0.640470862		
QIFDEYENETFLCHR	53.93655	Q9Y3A3	PHOCN HUMAN	667.632298	3	0.640470862		
GTYADDCLVQR	10.40285	Q9Y324	FCF1 HUMAN	649.296247	2	0.770789266	2	Yes
GTYADDCLVQR	10.40285	Q9Y324	FCF1 HUMAN	433.2001063	3	0.770789266	2	
IPGVPIMYISNHR	54.74777	Q9Y324	FCF1 HUMAN	748.906298	2	0.708796799		
IPGVPIMYISNHR	54.74777	Q9Y324	FCF1 HUMAN	499.606807	3	0.708796799		
AAPHSGPLVSWR	17.08524	Q9Y2Z9	COQ6 HUMAN	639.3416525	2	0.695321977		
AAPHSGPLVSWR	17.08524	Q9Y2Z9	COQ6 HUMAN	426.56371	3	0.695321977		
DLGSVSHLTGYETER	31.68499	Q9Y2Z9	COQ6 HUMAN	832.4002855	2	0.886552691		
DLGSVSHLTGYETER	31.68499	Q9Y2Z9	COQ6 HUMAN	555.2694653	3	0.886552691		
HNTALLAATDLLK	50.90754	Q9Y2Z9	COQ6 HUMAN	690.896458	2	0.833545387		
HNTALLAATDLLK	50.90754	Q9Y2Z9	COQ6 HUMAN	460.9335803	3	0.833545387		
ILLLEAGPK	43.79777	Q9Y2Z9	COQ6 HUMAN	477.3056845	2	0.839267969	2	Yes
ILLLEAGPK	43.79777	Q9Y2Z9	COQ6 HUMAN	318.5397313	3	0.839267969	2	
LLIGADGHNSGVR	4.878635	Q9Y2Z9	COQ6 HUMAN	654.855124	2	0.728472292		
LLIGADGHNSGVR	4.878635	Q9Y2Z9	COQ6 HUMAN	436.9060243	3	0.728472292		

LYSTSASPLVLLR	73.37252	Q9Y2Z9	COQ6_HUMAN	710.4144785	2	0.860887289		
LYSTSASPLVLLR	73.37252	Q9Y2Z9	COQ6_HUMAN	473.945594	3	0.860887289		
TWGLQATNAVSPLK	56.70276	Q9Y2Z9	COQ6_HUMAN	743.40719	2	0.857352912		
TWGLQATNAVSPLK	56.70276	Q9Y2Z9	COQ6_HUMAN	495.940735	3	0.857352912		
VALIGDAAHR	-4.180058	Q9Y2Z9	COQ6_HUMAN	511.7912645	2	0.842878819		
VALIGDAAHR	-4.180058	Q9Y2Z9	COQ6_HUMAN	341.530118	3	0.842878819		
VLFPLGLGHAAEYVRPR	62.31017	Q9Y2Z9	COQ6_HUMAN	948.03669	2	0.676423252		
VLFPLGLGHAAEYVRPR	62.31017	Q9Y2Z9	COQ6_HUMAN	632.3604017	3	0.676423252		
VSSISPGSATLLSSFGAWDHICNMR	95.7726	Q9Y2Z9	COQ6_HUMAN	1347.144703	2	0.707707882		
VSSISPGSATLLSSFGAWDHICNMR	95.7726	Q9Y2Z9	COQ6_HUMAN	898.4324103	3	0.707707882		
ASAVSELSPR	2.206005	Q9Y2W1	TR150_HUMAN	508.7727365	2	0.790519476		
ASAVSELSPR	2.206005	Q9Y2W1	TR150_HUMAN	339.517766	3	0.790519476		
ASESSKPWPDATYGTGSASR	14.83751	Q9Y2W1	TR150_HUMAN	1027.974877	2	0.716111422		
ASESSKPWPDATYGTGSASR	14.83751	Q9Y2W1	TR150_HUMAN	685.6525263	3	0.716111422		
DSRPSQAAGDNQGDEAK	-36.11312	Q9Y2W1	TR150_HUMAN	873.388442	2	0.639611781		
DSRPSQAAGDNQGDEAK	-36.11312	Q9Y2W1	TR150_HUMAN	582.594903	3	0.639611781		
EQTFSGGTSQDTK	-25.9875	Q9Y2W1	TR150_HUMAN	693.3131525	2	0.664999068	2	Yes
EQTFSGGTSQDTK	-25.9875	Q9Y2W1	TR150_HUMAN	462.54471	3	0.664999068	2	
FSGEEGEIEDDESGTENR	12.71386	Q9Y2W1	TR150_HUMAN	1000.404151	2	0.613665402	2	Yes
FSGEEGEIEDDESGTENR	12.71386	Q9Y2W1	TR150_HUMAN	667.2720423	3	0.613665402	2	
GFYPWGQYNR	47.03719	Q9Y2W1	TR150_HUMAN	644.299445	2	0.825081706		
GFYPWGQYNR	47.03719	Q9Y2W1	TR150_HUMAN	429.868905	3	0.825081706		
GSFSDTGLGDGK	9.335564	Q9Y2W1	TR150_HUMAN	570.7625665	2	0.840583444	2	Yes
GSFSDTGLGDGK	9.335564	Q9Y2W1	TR150_HUMAN	380.8443193	3	0.840583444	2	
IDISPSTFR	37.9627	Q9Y2W1	TR150_HUMAN	518.277655	2	0.819505692	2	Yes
IDISPSTFR	37.9627	Q9Y2W1	TR150_HUMAN	345.8543783	3	0.819505692	2	
SIFQHIQSAQSQR	7.779007	Q9Y2W1	TR150_HUMAN	765.395145	2	0.808760047	2	Yes
SIFQHIQSAQSQR	7.779007	Q9Y2W1	TR150_HUMAN	510.5993717	3	0.808760047	2	
SNWQNYR	-12.02669	Q9Y2W1	TR150_HUMAN	484.2232095	2	0.619388282	2	Yes
SNWQNYR	-12.02669	Q9Y2W1	TR150_HUMAN	323.1514147	3	0.619388282	2	
SPSELFAQHIVTIVHHVK	47.8204	Q9Y2W1	TR150_HUMAN	1021.563273	2	0.745192289	3	
SPSELFAQHIVTIVHHVK	47.8204	Q9Y2W1	TR150_HUMAN	681.3781237	3	0.745192289	3	Yes
TEELEEESFPER	23.92267	Q9Y2W1	TR150_HUMAN	747.8339165	2	0.656063318	2	Yes
TEELEEESFPER	23.92267	Q9Y2W1	TR150_HUMAN	498.891886	3	0.656063318	2	
WEGLVYAPPGK	45.05689	Q9Y2W1	TR150_HUMAN	608.822226	2	0.729491591	2	Yes
WEGLVYAPPGK	45.05689	Q9Y2W1	TR150_HUMAN	406.2174257	3	0.729491591	2	
AAMVQFDR	10.4939	Q9Y2V7	COG6_HUMAN	469.232194	2	0.789779186		
AAMVQFDR	10.4939	Q9Y2V7	COG6_HUMAN	313.1574043	3	0.789779186		
ALSTFFVENSLR	69.05768	Q9Y2V7	COG6_HUMAN	692.3675325	2	0.82185328		
ALSTFFVENSLR	69.05768	Q9Y2V7	COG6_HUMAN	461.9142967	3	0.82185328		
AQVADAFLSK	25.91124	Q9Y2V7	COG6_HUMAN	525.2854805	2	0.676142097		
AQVADAFLSK	25.91124	Q9Y2V7	COG6_HUMAN	350.526262	3	0.676142097		
AYGEVYAAVMNPINEYK	74.35193	Q9Y2V7	COG6_HUMAN	966.4645625	2	0.627282679		
AYGEVYAAVMNPINEYK	74.35193	Q9Y2V7	COG6_HUMAN	644.64565	3	0.627282679		
EGPITEDFFK	58.23595	Q9Y2V7	COG6_HUMAN	591.788053	2	0.790394306		
EGPITEDFFK	58.23595	Q9Y2V7	COG6_HUMAN	394.8613103	3	0.790394306		
EQTQDLIVK	10.04316	Q9Y2V7	COG6_HUMAN	537.2960455	2	0.671749294		
EQTQDLIVK	10.04316	Q9Y2V7	COG6_HUMAN	358.5333053	3	0.671749294		
EVLASHDSSVVPLDAR	28.25459	Q9Y2V7	COG6_HUMAN	847.9395815	2	0.842644334		
EVLASHDSSVVPLDAR	28.25459	Q9Y2V7	COG6_HUMAN	565.628996	3	0.842644334		
FQLTSDEMSLLR	72.53793	Q9Y2V7	COG6_HUMAN	720.364133	2	0.829039812	2	Yes
FQLTSDEMSLLR	72.53793	Q9Y2V7	COG6_HUMAN	480.578697	3	0.829039812	2	
FYHHTISGIVGNSATALLTTIEEMHLLSK	114.7807	Q9Y2V7	COG6_HUMAN	1592.329532	2	0.676890314		
FYHHTISGIVGNSATALLTTIEEMHLLSK	114.7807	Q9Y2V7	COG6_HUMAN	1061.888963	3	0.676890314		
GFIDALTR	51.71339	Q9Y2V7	COG6_HUMAN	446.748534	2	0.673489869	2	Yes
GFIDALTR	51.71339	Q9Y2V7	COG6_HUMAN	298.1682977	3	0.673489869	2	
HVTTQGVEENIQEVVGHITEGVCRPLK	68.67896	Q9Y2V7	COG6_HUMAN	1515.277836	2	0.623006165		
HVTTQGVEENIQEVVGHITEGVCRPLK	68.67896	Q9Y2V7	COG6_HUMAN	1010.521166	3	0.623006165		
IEQVIVAEPGAVLLYK	82.9044	Q9Y2V7	COG6_HUMAN	871.509108	2	0.770798147		
IEQVIVAEPGAVLLYK	82.9044	Q9Y2V7	COG6_HUMAN	581.3420137	3	0.770798147		
IFFNSLSLHASK	44.25426	Q9Y2V7	COG6_HUMAN	682.3726175	2	0.78019166		
IFFNSLSLHASK	44.25426	Q9Y2V7	COG6_HUMAN	455.25102	3	0.78019166		
SLAINEEFVSIFK	98.13966	Q9Y2V7	COG6_HUMAN	748.9039475	2	0.794138551	2	Yes
SLAINEEFVSIFK	98.13966	Q9Y2V7	COG6_HUMAN	499.60524	3	0.794138551	2	
TLTQESCDVSPVLTQAMEALQDRPVLYK	106.7651	Q9Y2V7	COG6_HUMAN	1596.799948	2	0.696075737		
TLTQESCDVSPVLTQAMEALQDRPVLYK	106.7651	Q9Y2V7	COG6_HUMAN	1064.86924	3	0.696075737		
TNQQTAGLEIMEQMALLQETAYER	129.7115	Q9Y2V7	COG6_HUMAN	1384.665663	2	0.693961978		
TNQQTAGLEIMEQMALLQETAYER	129.7115	Q9Y2V7	COG6_HUMAN	923.4463837	3	0.693961978		
TTLALFEFTDR	81.02699	Q9Y2V7	COG6_HUMAN	657.3409845	2	0.815803051		
TTLALFEFTDR	81.02699	Q9Y2V7	COG6_HUMAN	438.5632647	3	0.815803051		
VELPPPDLGPSSALNQTLMLLR	128.005	Q9Y2V7	COG6_HUMAN	1181.14651	2	0.702620447		
VELPPPDLGPSSALNQTLMLLR	128.005	Q9Y2V7	COG6_HUMAN	787.766948	3	0.702620447		
VGLSYIYNTVQQHKPEQGSLANMPNLDSVTLK	76.5177	Q9Y2V7	COG6_HUMAN	1772.909402	2	0.700319767		
VGLSYIYNTVQQHKPEQGSLANMPNLDSVTLK	76.5177	Q9Y2V7	COG6_HUMAN	1182.275543	3	0.700319767		
YTLDEFGTAR	34.74293	Q9Y2V7	COG6_HUMAN	586.783298	2	0.749699831	2	Yes
YTLDEFGTAR	34.74293	Q9Y2V7	COG6_HUMAN	391.524807	3	0.749699831	2	
YVGDMLAWLHQATASEK	74.64977	Q9Y2V7	COG6_HUMAN	960.4701885	2	0.816074133	3	
YVGDMLAWLHQATASEK	74.64977	Q9Y2V7	COG6_HUMAN	640.6494007	3	0.816074133	3	Yes
AADVENVPPVISTPHHYLISIYR	64.90896	Q9Y2T2	AP3M1_HUMAN	1296.185003	2	0.739111304	4	
AADVENVPPVISTPHHYLISIYR	64.90896	Q9Y2T2	AP3M1_HUMAN	864.459277	3	0.739111304	4	
FDITIGPK	41.74378	Q9Y2T2	AP3M1_HUMAN	445.753285	2	0.772890866	2	Yes
FDITIGPK	41.74378	Q9Y2T2	AP3M1_HUMAN	297.5047983	3	0.772890866	2	
GLVNLQSGAPKPEENPSLNIQFK	62.43401	Q9Y2T2	AP3M1_HUMAN	1240.661369	2	0.805718958	3	
GLVNLQSGAPKPEENPSLNIQFK	62.43401	Q9Y2T2	AP3M1_HUMAN	827.443521	3	0.805718958	3	Yes
LLDDVSFHPCIR	46.26883	Q9Y2T2	AP3M1_HUMAN	736.3722925	2	0.667716563	3	
LLDDVSFHPCIR	46.26883	Q9Y2T2	AP3M1_HUMAN	491.2508033	3	0.667716563	3	Yes
LSGMPDLSLSFMNPR	94.11383	Q9Y2T2	AP3M1_HUMAN	832.910924	2	0.642699897		
LSGMPDLSLSFMNPR	94.11383	Q9Y2T2	AP3M1_HUMAN	555.609891	3	0.642699897		
SVVNSITGSSNVGDTLPTGQLSNIPWR	95.4232	Q9Y2T2	AP3M1_HUMAN	1400.217768	2	0.78018713		
SVVNSITGSSNVGDTLPTGQLSNIPWR	95.4232	Q9Y2T2	AP3M1_HUMAN	933.8144537	3	0.78018713		
TIEGITVTVHMPK	40.71458	Q9Y2T2	AP3M1_HUMAN	713.3926945	2	0.707327247	3	
TIEGITVTVHMPK	40.71458	Q9Y2T2	AP3M1_HUMAN	475.9310713	3	0.707327247	3	Yes
VADTFQDYFGECSEAAIK	71.40758	Q9Y2T2	AP3M1_HUMAN	1025.957308	2	0.817043483		
VADTFQDYFGECSEAAIK	71.40758	Q9Y2T2	AP3M1_HUMAN	684.3074803	3	0.817043483		
VLSFIPPDGNFR	69.8164	Q9Y2T2	AP3M1_HUMAN	681.364793	2	0.804019094		
VLSFIPPDGNFR	69.8164	Q9Y2T2	AP3M1_HUMAN	454.579137	3	0.804019094		
VLTWDVGK	32.80245	Q9Y2T2	AP3M1_HUMAN	459.2587345	2	0.791185498		
VLTWDVGK	32.80245	Q9Y2T2	AP3M1_HUMAN	306.5084313	3	0.791185498		
VSSQNLVAIPVYVK	63.90597	Q9Y2T2	AP3M1_HUMAN	758.9408605	2	0.831731915	2	Yes
VSSQNLVAIPVYVK	63.90597	Q9Y2T2	AP3M1_HUMAN	506.2965153	3	0.831731915	2	
VVLNMNLTPTQGSYTFDPVTK	85.34406	Q9Y2T2	AP3M1_HUMAN	1163.093939	2	0.731930554		

VVLNMNLTPTQGSYTFDPVTK	85.34406	Q9Y2T2	AP3M1_HUMAN	775.731901	3	0.731930554		
YTNNEAYFDVVEEIDAIIDK	142.7217	Q9Y2T2	AP3M1_HUMAN	1181.060811	2	0.638649762		
YTNNEAYFDVVEEIDAIIDK	142.7217	Q9Y2T2	AP3M1_HUMAN	787.7098153	3	0.638649762		
FLDELEDEAK	38.08993	Q9Y2R0	COA3_HUMAN	604.7882495	2	0.769281745	2	Yes
FLDELEDEAK	38.08993	Q9Y2R0	COA3_HUMAN	403.528108	3	0.769281745	2	
LTPEQLHSMR	-4.144783	Q9Y2R0	COA3_HUMAN	606.3142465	2	0.673485935	3	
LTPEQLHSMR	-4.144783	Q9Y2R0	COA3_HUMAN	404.5454393	3	0.673485935	3	Yes
AYIDELWEMALSK	120.8394	Q9Y2D4	EXC6B_HUMAN	784.8874365	2	0.77501905		
AYIDELWEMALSK	120.8394	Q9Y2D4	EXC6B_HUMAN	523.5942327	3	0.77501905		
EIESTDTACIGPTLR	36.34129	Q9Y2D4	EXC6B_HUMAN	831.9043515	2	0.763933897		
EIESTDTACIGPTLR	36.34129	Q9Y2D4	EXC6B_HUMAN	554.9388427	3	0.763933897		
ETFENYYR	12.79108	Q9Y2D4	EXC6B_HUMAN	561.249086	2	0.781668127		
ETFENYYR	12.79108	Q9Y2D4	EXC6B_HUMAN	374.501999	3	0.781668127		
FPFSEFVPK	69.15579	Q9Y2D4	EXC6B_HUMAN	549.2874915	2	0.775520682		
FPFSEFVPK	69.15579	Q9Y2D4	EXC6B_HUMAN	366.5276027	3	0.775520682		
FSEDLHLSSTEVDDMIR	58.19935	Q9Y2D4	EXC6B_HUMAN	997.4627605	2	0.830919147		
FSEDLHLSSTEVDDMIR	58.19935	Q9Y2D4	EXC6B_HUMAN	665.3111153	3	0.830919147		
HAAEEEIYTNLNQK	10.21265	Q9Y2D4	EXC6B_HUMAN	830.402828	2	0.770234346		
HAAEEEIYTNLNQK	10.21265	Q9Y2D4	EXC6B_HUMAN	553.937827	3	0.770234346		
IDQFLQLADYDWMTGDLGNK	126.5481	Q9Y2D4	EXC6B_HUMAN	1172.052271	2	0.676360548		
IDQFLQLADYDWMTGDLGNK	126.5481	Q9Y2D4	EXC6B_HUMAN	781.7041223	3	0.676360548		
LMLCLPVLEMYSK	113.7757	Q9Y2D4	EXC6B_HUMAN	798.914086	2	0.674812615		
LMLCLPVLEMYSK	113.7757	Q9Y2D4	EXC6B_HUMAN	532.9453323	3	0.674812615		
LVLQPPSNMHETLDGYR	42.37754	Q9Y2D4	EXC6B_HUMAN	985.4941945	2	0.878731132		
LVLQPPSNMHETLDGYR	42.37754	Q9Y2D4	EXC6B_HUMAN	657.3320713	3	0.878731132		
LYGTTTFK	5.829269	Q9Y2D4	EXC6B_HUMAN	465.7507385	2	0.735892653	2	Yes
LYGTTTFK	5.829269	Q9Y2D4	EXC6B_HUMAN	310.836434	3	0.735892653	2	
MAEAESLETAAEHER	10.41666	Q9Y2D4	EXC6B_HUMAN	837.3759575	2	0.836026967		
MAEAESLETAAEHER	10.41666	Q9Y2D4	EXC6B_HUMAN	558.58658	3	0.836026967		
NILDSDNYSPIPVTSEEMYK	77.48096	Q9Y2D4	EXC6B_HUMAN	1158.041563	2	0.741269588		
NILDSDNYSPIPVTSEEMYK	77.48096	Q9Y2D4	EXC6B_HUMAN	772.3636503	3	0.741269588		
NLDNIVLQQPR	41.16856	Q9Y2D4	EXC6B_HUMAN	655.3653245	2	0.894948363		
NLDNIVLQQPR	41.16856	Q9Y2D4	EXC6B_HUMAN	437.246158	3	0.894948363		
SCKYLEEFITNITNVLPETVHTTK	102.7554	Q9Y2D4	EXC6B_HUMAN	1419.22367	2	0.768784523		
SCKYLEEFITNITNVLPETVHTTK	102.7554	Q9Y2D4	EXC6B_HUMAN	946.485055	3	0.768784523		
STFAVFTHLPGK	43.44713	Q9Y2D4	EXC6B_HUMAN	652.854062	2	0.785622656		
STFAVFTHLPGK	43.44713	Q9Y2D4	EXC6B_HUMAN	435.571983	3	0.785622656		
SVYDGEEHGR	-31.04831	Q9Y2D4	EXC6B_HUMAN	574.7525285	2	0.661869466		
SVYDGEEHGR	-31.04831	Q9Y2D4	EXC6B_HUMAN	383.504294	3	0.661869466		
THSSYCSDPNLVLDLK	42.4048	Q9Y2D4	EXC6B_HUMAN	924.9440025	2	0.639749408		
THSSYCSDPNLVLDLK	42.4048	Q9Y2D4	EXC6B_HUMAN	616.9652767	3	0.639749408		
TLEHLEHTYLPQVSHYR	23.46965	Q9Y2D4	EXC6B_HUMAN	1062.037611	2	0.864869952		
TLEHLEHTYLPQVSHYR	23.46965	Q9Y2D4	EXC6B_HUMAN	708.3610157	3	0.864869952		
TLSNSLQNVIK	37.2766	Q9Y2D4	EXC6B_HUMAN	608.8489755	2	0.806685984		
TLSNSLQNVIK	37.2766	Q9Y2D4	EXC6B_HUMAN	406.2352587	3	0.806685984		
VMVDNIPK	13.00195	Q9Y2D4	EXC6B_HUMAN	458.2525945	2	0.627781153		
VMVDNIPK	13.00195	Q9Y2D4	EXC6B_HUMAN	305.8376713	3	0.627781153		
VNPVTALTLLEK	83.00784	Q9Y2D4	EXC6B_HUMAN	649.390477	2	0.857728839		
VNPVTALTLLEK	83.00784	Q9Y2D4	EXC6B_HUMAN	433.2629263	3	0.857728839		
VVGQFPFQDIELEK	80.24857	Q9Y2D4	EXC6B_HUMAN	824.9332375	2	0.854927778		
VVGQFPFQDIELEK	80.24857	Q9Y2D4	EXC6B_HUMAN	550.2914333	3	0.854927778		
AGGLIYQLDSYAPR	65.68116	Q9Y296	TPPC4_HUMAN	762.3968135	2	0.879515409		
AGGLIYQLDSYAPR	65.68116	Q9Y296	TPPC4_HUMAN	508.600484	3	0.879515409		
EVLEYLGNPANYPVSIR	85.27365	Q9Y296	TPPC4_HUMAN	967.50508	2	0.831603229		
EVLEYLGNPANYPVSIR	85.27365	Q9Y296	TPPC4_HUMAN	645.3393283	3	0.831603229		
FVVLADPR	35.1884	Q9Y296	TPPC4_HUMAN	458.7667265	2	0.801171005		
FVVLADPR	35.1884	Q9Y296	TPPC4_HUMAN	306.180426	3	0.801171005		
IYEIYSDFALK	72.64703	Q9Y296	TPPC4_HUMAN	681.353551	2	0.796450138		
IYEIYSDFALK	72.64703	Q9Y296	TPPC4_HUMAN	454.5716423	3	0.796450138		
LHCYQTLTGIK	6.168865	Q9Y296	TPPC4_HUMAN	667.3508255	2	0.812622905		
LHCYQTLTGIK	6.168865	Q9Y296	TPPC4_HUMAN	445.236492	3	0.812622905		
NPFYSLEMPIR	77.5432	Q9Y296	TPPC4_HUMAN	683.8453745	2	0.817593038	2	Yes
NPFYSLEMPIR	77.5432	Q9Y296	TPPC4_HUMAN	456.232858	3	0.817593038	2	
TFSYPLDLLLK	112.3461	Q9Y296	TPPC4_HUMAN	655.374291	2	0.788677335		
TFSYPLDLLLK	112.3461	Q9Y296	TPPC4_HUMAN	437.2521357	3	0.788677335		
VLVAFGQR	25.1869	Q9Y296	TPPC4_HUMAN	445.266894	2	0.790595055	2	Yes
VLVAFGQR	25.1869	Q9Y296	TPPC4_HUMAN	297.1805377	3	0.790595055	2	
AADFQLHTHVNDGTEFGGSIYQK	37.38812	Q9Y277	VDAC3_HUMAN	1268.09913	2	0.744331062	4	
AADFQLHTHVNDGTEFGGSIYQK	37.38812	Q9Y277	VDAC3_HUMAN	845.7353617	3	0.744331062	4	
GYGFGMVK	24.34583	Q9Y277	VDAC3_HUMAN	429.7131015	2	0.723889172	2	Yes
GYGFGMVK	24.34583	Q9Y277	VDAC3_HUMAN	286.8113427	3	0.723889172	2	
IETSINLAWTAGSNNTR	58.65834	Q9Y277	VDAC3_HUMAN	924.466495	2	0.658499479		
IETSINLAWTAGSNNTR	58.65834	Q9Y277	VDAC3_HUMAN	616.6469383	3	0.658499479		
LSQNNFALGYK	29.23994	Q9Y277	VDAC3_HUMAN	627.828039	2	0.833220363	2	Yes
LSQNNFALGYK	29.23994	Q9Y277	VDAC3_HUMAN	418.8879677	3	0.833220363	2	
LTLDTIFVPNTGK	79.79625	Q9Y277	VDAC3_HUMAN	709.8986665	2	0.787816465	2	Yes
LTLDTIFVPNTGK	79.79625	Q9Y277	VDAC3_HUMAN	473.6017193	3	0.787816465	2	
SCSGVEFSTSGHAYTDTGK	5.610382	Q9Y277	VDAC3_HUMAN	995.926539	2	0.725918412	3	
SCSGVEFSTSGHAYTDTGK	5.610382	Q9Y277	VDAC3_HUMAN	664.2869677	3	0.725918412	3	Yes
VCNYGLTFTQK	33.23521	Q9Y277	VDAC3_HUMAN	665.827183	2	0.683513284	2	Yes
VCNYGLTFTQK	33.23521	Q9Y277	VDAC3_HUMAN	444.2207303	3	0.683513284	2	
VNNASLIGLGYTQTLRPGVK	57.0201	Q9Y277	VDAC3_HUMAN	1051.092391	2	0.718382299	3	
VNNASLIGLGYTQTLRPGVK	57.0201	Q9Y277	VDAC3_HUMAN	701.064202	3	0.718382299	3	Yes
WNTDNTLGTEISWENK	57.63953	Q9Y277	VDAC3_HUMAN	954.4426855	2	0.837708473	3	
WNTDNTLGTEISWENK	57.63953	Q9Y277	VDAC3_HUMAN	636.6310653	3	0.837708473	3	Yes
ACPHMATCGNVLFEGR	25.33658	Q9UPW5	CBPC1_HUMAN	910.406016	2	0.611552417		
ACPHMATCGNVLFEGR	25.33658	Q9UPW5	CBPC1_HUMAN	607.2732857	3	0.611552417		
DCSLPLTVLTCAK	78.5193	Q9UPW5	CBPC1_HUMAN	739.3736445	2	0.665995061		
DCSLPLTVLTCAK	78.5193	Q9UPW5	CBPC1_HUMAN	493.2517047	3	0.665995061		
ESPPHEDLMVQIHSILAK	59.95374	Q9UPW5	CBPC1_HUMAN	1022.530781	2	0.788085878		
ESPPHEDLMVQIHSILAK	59.95374	Q9UPW5	CBPC1_HUMAN	682.023129	3	0.788085878		
ETVWHTNDNATSCDVVEDTGYR	31.41389	Q9UPW5	CBPC1_HUMAN	1285.048977	2	0.663889408		
ETVWHTNDNATSCDVVEDTGYR	31.41389	Q9UPW5	CBPC1_HUMAN	857.0352593	3	0.663889408		
GLLQYLAAVK	83.53323	Q9UPW5	CBPC1_HUMAN	538.329687	2	0.791784406		
GLLQYLAAVK	83.53323	Q9UPW5	CBPC1_HUMAN	359.2223997	3	0.791784406		
GPIVVPTAGEETSGNSGNLR	37.78988	Q9UPW5	CBPC1_HUMAN	977.995617	2	0.710959613		
GPIVVPTAGEETSGNSGNLR	37.78988	Q9UPW5	CBPC1_HUMAN	652.3330197	3	0.710959613		
GSTGMEILLSTLENTK	94.15062	Q9UPW5	CBPC1_HUMAN	847.4380265	2	0.740909278		
GSTGMEILLSTLENTK	94.15062	Q9UPW5	CBPC1_HUMAN	565.294626	3	0.740909278		
GTLEYLMSNNPTAQSLR	65.37595	Q9UPW5	CBPC1_HUMAN	947.970552	2	0.772771835		
GTLEYLMSNNPTAQSLR	65.37595	Q9UPW5	CBPC1_HUMAN	632.3163097	3	0.772771835		

ILHLAQSQEK	-22.74821	Q9UPW5	CBPC1_HUMAN	583.8305865	2	0.812939286		
ILHLAQSQEK	-22.74821	Q9UPW5	CBPC1_HUMAN	389.5563327	3	0.812939286		
ILYNTSQECLAVR	33.72836	Q9UPW5	CBPC1_HUMAN	783.90141	2	0.761706948		
ILYNTSQECLAVR	33.72836	Q9UPW5	CBPC1_HUMAN	522.9368817	3	0.761706948		
INGALNITLNLVK	78.45277	Q9UPW5	CBPC1_HUMAN	691.922475	2	0.710614562		
INGALNITLNLVK	78.45277	Q9UPW5	CBPC1_HUMAN	461.6175917	3	0.710614562		
ITLQNIPSQTAPGFTAEMK	65.84002	Q9UPW5	CBPC1_HUMAN	1024.030615	2	0.859128237		
ITLQNIPSQTAPGFTAEMK	65.84002	Q9UPW5	CBPC1_HUMAN	683.0230183	3	0.859128237		
IVGLLAQLEK	65.30925	Q9UPW5	CBPC1_HUMAN	542.3427985	2	0.80474925		
IVGLLAQLEK	65.30925	Q9UPW5	CBPC1_HUMAN	361.8978073	3	0.80474925		
LESAHNPQQIYFR	16.30779	Q9UPW5	CBPC1_HUMAN	801.905341	2	0.830730796		
LESAHNPQQIYFR	16.30779	Q9UPW5	CBPC1_HUMAN	534.9395023	3	0.830730796		
LIHQSDIIDR	0.080894	Q9UPW5	CBPC1_HUMAN	605.333493	2	0.752612174		
LIHQSDIIDR	0.080894	Q9UPW5	CBPC1_HUMAN	403.8916037	3	0.752612174		
LTSPLEYNLPSSLLDFENDLIESSCK	150.8769	Q9UPW5	CBPC1_HUMAN	1492.726438	2	0.654037297		
LTSPLEYNLPSSLLDFENDLIESSCK	150.8769	Q9UPW5	CBPC1_HUMAN	995.4869	3	0.654037297		
LVLPCLQLLR	93.31186	Q9UPW5	CBPC1_HUMAN	612.8790255	2	0.641433299		
LVLPCLQLLR	93.31186	Q9UPW5	CBPC1_HUMAN	408.9219587	3	0.641433299		
MGTDICYYK	19.90629	Q9UPW5	CBPC1_HUMAN	575.749551	2	0.680858314		
MGTDICYYK	19.90629	Q9UPW5	CBPC1_HUMAN	384.1689757	3	0.680858314		
NRPYVFLSAR	18.4917	Q9UPW5	CBPC1_HUMAN	611.838741	2	0.602306187		
NRPYVFLSAR	18.4917	Q9UPW5	CBPC1_HUMAN	408.2284357	3	0.602306187		
NVFMYGCSIK	37.27238	Q9UPW5	CBPC1_HUMAN	609.7864715	2	0.741748989		
NVFMYGCSIK	37.27238	Q9UPW5	CBPC1_HUMAN	406.860256	3	0.741748989		
SVPEYSEVAYPDYFGHIPPPFK	86.77342	Q9UPW5	CBPC1_HUMAN	1270.113177	2	0.842080712		
SVPEYSEVAYPDYFGHIPPPFK	86.77342	Q9UPW5	CBPC1_HUMAN	847.0780593	3	0.842080712		
SYTMESTLCGCDQGK	17.5704	Q9UPW5	CBPC1_HUMAN	868.8504005	2	0.695239246		
SYTMESTLCGCDQGK	17.5704	Q9UPW5	CBPC1_HUMAN	579.569542	3	0.695239246		
SYYTITFTVNFPHK	67.99164	Q9UPW5	CBPC1_HUMAN	859.433396	2	0.65064162		
SYYTITFTVNFPHK	67.99164	Q9UPW5	CBPC1_HUMAN	573.291539	3	0.65064162		
TISSVHGLNNDIVK	12.66826	Q9UPW5	CBPC1_HUMAN	748.907553	2	0.630836904		
TISSVHGLNNDIVK	12.66826	Q9UPW5	CBPC1_HUMAN	499.6076437	3	0.630836904		
TLDPLVNTSSLIMR	78.63449	Q9UPW5	CBPC1_HUMAN	780.4272645	2	0.764673293		
TLDPLVNTSSLIMR	78.63449	Q9UPW5	CBPC1_HUMAN	520.6207847	3	0.764673293		
TLQQQPGDQNR	-31.45769	Q9UPW5	CBPC1_HUMAN	642.8187395	2	0.784505248		
TLQQQPGDQNR	-31.45769	Q9UPW5	CBPC1_HUMAN	428.881768	3	0.784505248		
VALDTLAALLK	106.1558	Q9UPW5	CBPC1_HUMAN	564.355906	2	0.830083013		
VALDTLAALLK	106.1558	Q9UPW5	CBPC1_HUMAN	376.5732123	3	0.830083013		
VHPGETNASWVMK	9.719646	Q9UPW5	CBPC1_HUMAN	728.3566425	2	0.738824248		
VHPGETNASWVMK	9.719646	Q9UPW5	CBPC1_HUMAN	485.9070367	3	0.738824248		
VTSPTTYVLDEDEPR	41.21369	Q9UPW5	CBPC1_HUMAN	861.4156015	2	0.832709074		
VTSPTTYVLDEDEPR	41.21369	Q9UPW5	CBPC1_HUMAN	574.6130093	3	0.832709074		
VVYDLDNPNYTIPEEGDILK	85.38251	Q9UPW5	CBPC1_HUMAN	1154.073721	2	0.812527835		
VVYDLDNPNYTIPEEGDILK	85.38251	Q9UPW5	CBPC1_HUMAN	769.7184223	3	0.812527835		
VYSANSVNSVSLGK	18.91621	Q9UPW5	CBPC1_HUMAN	712.873174	2	0.635898292		
VYSANSVNSVSLGK	18.91621	Q9UPW5	CBPC1_HUMAN	475.5847243	3	0.635898292		
AVALYFEDK	36.38102	Q9UPN9	TRI33_HUMAN	528.274577	2	0.79265976	2	Yes
AVALYFEDK	36.38102	Q9UPN9	TRI33_HUMAN	352.518993	3	0.79265976	2	
DIGKPEVEYDCDNLQHSK	10.69077	Q9UPN9	TRI33_HUMAN	1073.98967	2	0.731429517	4	
DIGKPEVEYDCDNLQHSK	10.69077	Q9UPN9	TRI33_HUMAN	716.329055	3	0.731429517	4	
GAIENLLAK	36.4371	Q9UPN9	TRI33_HUMAN	464.777291	2	0.705604613		
GAIENLLAK	36.4371	Q9UPN9	TRI33_HUMAN	310.187469	3	0.705604613		
HSGPQYSMMQPHLQR	2.188908	Q9UPN9	TRI33_HUMAN	898.920143	2	0.743131399		
HSGPQYSMMQPHLQR	2.188908	Q9UPN9	TRI33_HUMAN	599.616037	3	0.743131399		
HSQHYQIPDDFVADVR	43.5643	Q9UPN9	TRI33_HUMAN	963.9588335	2	0.76517278	3	
HSQHYQIPDDFVADVR	43.5643	Q9UPN9	TRI33_HUMAN	642.975164	3	0.76517278	3	Yes
HVMNFTNWAIASGSSTALLYSK	81.10606	Q9UPN9	TRI33_HUMAN	1199.597179	2	0.667624712		
HVMNFTNWAIASGSSTALLYSK	81.10606	Q9UPN9	TRI33_HUMAN	800.0673943	3	0.667624712		
IEPADMNESCK	-6.265343	Q9UPN9	TRI33_HUMAN	647.276669	2	0.77860719		
IEPADMNESCK	-6.265343	Q9UPN9	TRI33_HUMAN	431.853721	3	0.77860719		
LISVQTMQR	16.27384	Q9UPN9	TRI33_HUMAN	538.300608	2	0.736001432		
LISVQTMQR	16.27384	Q9UPN9	TRI33_HUMAN	359.2030137	3	0.736001432		
LLQQQNDITGLSR	30.57199	Q9UPN9	TRI33_HUMAN	743.4051785	2	0.890705943		
LLQQQNDITGLSR	30.57199	Q9UPN9	TRI33_HUMAN	495.939394	3	0.890705943		
LQHMQQQVYAQK	-23.40034	Q9UPN9	TRI33_HUMAN	751.3831885	2	0.807166576		
LQHMQQQVYAQK	-23.40034	Q9UPN9	TRI33_HUMAN	501.2580673	3	0.807166576		
LTEIYSDR	1.718426	Q9UPN9	TRI33_HUMAN	498.7540085	2	0.725166559		
LTEIYSDR	1.718426	Q9UPN9	TRI33_HUMAN	332.838614	3	0.725166559		
MQQPPAPVPTTTTTTQQHPR	6.439888	Q9UPN9	TRI33_HUMAN	1109.058229	2	0.770214856		
MQQPPAPVPTTTTTTQQHPR	6.439888	Q9UPN9	TRI33_HUMAN	739.708094	3	0.770214856		
VVNLGNLVIESKPAPGYTPNVVVGQVPPGTNHIS	80.94614	Q9UPN9	TRI33_HUMAN	1855.007769	2	0.699115276		
VVNLGNLVIESKPAPGYTPNVVVGQVPPGTNHIS	80.94614	Q9UPN9	TRI33_HUMAN	1237.007787	3	0.699115276		
NYVHFAATQVQNR	9.264744	Q9UPN9	TRI33_HUMAN	774.3898585	2	0.646791041	2	Yes
NYVHFAATQVQNR	9.264744	Q9UPN9	TRI33_HUMAN	516.5958473	3	0.646791041	2	
QAAPQMLQQQPPR	12.75079	Q9UPN9	TRI33_HUMAN	746.8886425	2	0.73386997		
QAAPQMLQQQPPR	12.75079	Q9UPN9	TRI33_HUMAN	498.2617033	3	0.73386997		
SLLQQLENVTK	70.56654	Q9UPN9	TRI33_HUMAN	636.8620835	2	0.847435713	2	Yes
SLLQQLENVTK	70.56654	Q9UPN9	TRI33_HUMAN	424.910664	3	0.847435713	2	
TFAPLPEFEQEEDDGEVTEDSDEDFIQPR	95.56615	Q9UPN9	TRI33_HUMAN	1692.731694	2	0.613273203		
TFAPLPEFEQEEDDGEVTEDSDEDFIQPR	95.56615	Q9UPN9	TRI33_HUMAN	1128.823738	3	0.613273203		
TPGQINLAQLR	38.27864	Q9UPN9	TRI33_HUMAN	605.849311	2	0.85869658	2	Yes
TPGQINLAQLR	38.27864	Q9UPN9	TRI33_HUMAN	404.2354823	3	0.85869658	2	
VVQVYADTQEINLK	43.17664	Q9UPN9	TRI33_HUMAN	810.43614	2	0.738352656		
VVQVYADTQEINLK	43.17664	Q9UPN9	TRI33_HUMAN	540.6267017	3	0.738352656		
YQFLEEAFQNQK	55.58844	Q9UPN9	TRI33_HUMAN	772.8731755	2	0.853784621		
YQFLEEAFQNQK	55.58844	Q9UPN9	TRI33_HUMAN	515.5847253	3	0.853784621		
ASFWTNMEK	33.25981	Q9UP83	COG5_HUMAN	557.2558655	2	0.838324904		
ASFWTNMEK	33.25981	Q9UP83	COG5_HUMAN	371.839852	3	0.838324904		
DSLQPYEAAYLSK	52.13795	Q9UP83	COG5_HUMAN	742.867554	2	0.851921678	2	Yes
DSLQPYEAAYLSK	52.13795	Q9UP83	COG5_HUMAN	495.5809777	3	0.851921678	2	
EFAPVYPIMVQLLQK	121.5708	Q9UP83	COG5_HUMAN	888.492404	2	0.778416276		
EFAPVYPIMVQLLQK	121.5708	Q9UP83	COG5_HUMAN	592.664211	3	0.778416276		
ELLQDGCYSDFLNEDFDVK	106.7356	Q9UP83	COG5_HUMAN	1154.010268	2	0.796333492		
ELLQDGCYSDFLNEDFDVK	106.7356	Q9UP83	COG5_HUMAN	769.6761203	3	0.796333492		
FSQWLDDHPSEK	15.68958	Q9UP83	COG5_HUMAN	744.841879	2	0.843611062		
FSQWLDDHPSEK	15.68958	Q9UP83	COG5_HUMAN	496.8971943	3	0.843611062		
GALEAYVQSVR	38.51771	Q9UP83	COG5_HUMAN	596.8202145	2	0.831188202		
GALEAYVQSVR	38.51771	Q9UP83	COG5_HUMAN	398.2160847	3	0.831188202		
HEDLLAQATGIESLEGVLQMMQTR	140.2997	Q9UP83	COG5_HUMAN	1335.66547	2	0.803823471		
HEDLLAQATGIESLEGVLQMMQTR	140.2997	Q9UP83	COG5_HUMAN	890.7795883	3	0.803823471		
HFECLDFVFDNTEAIAQR	80.02333	Q9UP83	COG5_HUMAN	1106.510578	2	0.803040445		

HFECLDFVFDNTEAIAQR	80.02333	Q9UP83	COG5_HUMAN	738.00966	3	0.803040445		
IGALQGAVDR	9.531067	Q9UP83	COG5_HUMAN	500.2832725	2	0.805809081		
IGALQGAVDR	9.531067	Q9UP83	COG5_HUMAN	333.8581233	3	0.805809081		
LAADFAQMELAVGPFCR	98.94278	Q9UP83	COG5_HUMAN	948.461314	2	0.617200673		
LAADFAQMELAVGPFCR	98.94278	Q9UP83	COG5_HUMAN	632.6434843	3	0.617200673		
LAQGISQLDR	11.04462	Q9UP83	COG5_HUMAN	550.8071115	2	0.815293789	2	Yes
LAQGISQLDR	11.04462	Q9UP83	COG5_HUMAN	367.5406827	3	0.815293789	2	
LFDPINLVFPPGGR	124.0523	Q9UP83	COG5_HUMAN	771.427925	2	0.771889806		
LFDPINLVFPPGGR	124.0523	Q9UP83	COG5_HUMAN	514.621225	3	0.771889806		
LMDHIYAVCGQVQHLQK	28.93344	Q9UP83	COG5_HUMAN	1020.511866	2	0.746219158		
LMDHIYAVCGQVQHLQK	28.93344	Q9UP83	COG5_HUMAN	680.6771853	3	0.746219158		
LQVACDLLR	43.00293	Q9UP83	COG5_HUMAN	544.300608	2	0.689631402		
LQVACDLLR	43.00293	Q9UP83	COG5_HUMAN	363.2030137	3	0.689631402		
LYNDLWK	36.38699	Q9UP83	COG5_HUMAN	476.2509045	2	0.610688448		
LYNDLWK	36.38699	Q9UP83	COG5_HUMAN	317.8365447	3	0.610688448		
NPPSSDELDGIIK	41.44963	Q9UP83	COG5_HUMAN	692.851912	2	0.803471625		
NPPSSDELDGIIK	41.44963	Q9UP83	COG5_HUMAN	462.2372163	3	0.803471625		
NVAVVNSLYK	24.83702	Q9UP83	COG5_HUMAN	553.8144	2	0.735213578		
NVAVVNSLYK	24.83702	Q9UP83	COG5_HUMAN	369.5455417	3	0.735213578		
SAADDINPAPANMEGGGGSVAVAGLGAR	62.65672	Q9UP83	COG5_HUMAN	1263.106633	2	0.822563529		
SAADDINPAPANMEGGGGSVAVAGLGAR	62.65672	Q9UP83	COG5_HUMAN	842.4070303	3	0.822563529		
SEQLLSTQGDASQVIGPLTEGQR	65.72387	Q9UP83	COG5_HUMAN	1207.612075	2	0.844792724		
SEQLLSTQGDASQVIGPLTEGQR	65.72387	Q9UP83	COG5_HUMAN	805.410658	3	0.844792724		
TIASELNVAAVDTNLTLAVSK	88.78394	Q9UP83	COG5_HUMAN	1065.586808	2	0.710598528		
TIASELNVAAVDTNLTLAVSK	88.78394	Q9UP83	COG5_HUMAN	710.7271467	3	0.710598528		
TYTSQSIHQAVIAEQLAK	39.35295	Q9UP83	COG5_HUMAN	994.5265495	2	0.80832386		
TYTSQSIHQAVIAEQLAK	39.35295	Q9UP83	COG5_HUMAN	663.3536413	3	0.80832386		
VLTQPSQSAVR	-12.04562	Q9UP83	COG5_HUMAN	593.3334935	2	0.631886125	2	Yes
VLTQPSQSAVR	-12.04562	Q9UP83	COG5_HUMAN	395.891604	3	0.631886125	2	
ASSSILIDESEPTTNIQIR	58.86817	Q9UNZ2	NSF1C_HUMAN	1037.537314	2	0.740636945		
ASSSILIDESEPTTNIQIR	58.86817	Q9UNZ2	NSF1C_HUMAN	692.0274843	3	0.740636945		
EANLLNAVIVQR	67.42959	Q9UNZ2	NSF1C_HUMAN	670.3887995	2	0.826447785		
EANLLNAVIVQR	67.42959	Q9UNZ2	NSF1C_HUMAN	447.261808	3	0.826447785		
EFVAVTGAEEDR	21.95522	Q9UNZ2	NSF1C_HUMAN	661.8153305	2	0.767575979	2	Yes
EFVAVTGAEEDR	21.95522	Q9UNZ2	NSF1C_HUMAN	441.546162	3	0.767575979	2	
ELADESQTLK	-0.657509	Q9UNZ2	NSF1C_HUMAN	567.288417	2	0.646102011		
ELADESQTLK	-0.657509	Q9UNZ2	NSF1C_HUMAN	378.5282197	3	0.646102011		
LAHGGQVNLDMEDHR	-3.129951	Q9UNZ2	NSF1C_HUMAN	846.4001015	2	0.727988482		
LAHGGQVNLDMEDHR	-3.129951	Q9UNZ2	NSF1C_HUMAN	564.602676	3	0.727988482		
LGAAPEEESAYVAGEK	18.6476	Q9UNZ2	NSF1C_HUMAN	810.891762	2	0.861216068	2	Yes
LGAAPEEESAYVAGEK	18.6476	Q9UNZ2	NSF1C_HUMAN	540.9304497	3	0.861216068	2	
LGSTAPQVLSTSSPAQQAENEAK	34.49314	Q9UNZ2	NSF1C_HUMAN	1157.580243	2	0.825110495	3	
LGSTAPQVLSTSSPAQQAENEAK	34.49314	Q9UNZ2	NSF1C_HUMAN	772.0561033	3	0.825110495	3	Yes
SGFSLDNGELR	33.43282	Q9UNZ2	NSF1C_HUMAN	597.7916575	2	0.676204801	2	Yes
SGFSLDNGELR	33.43282	Q9UNZ2	NSF1C_HUMAN	398.8637133	3	0.676204801	2	
SGQQIVGPPR	-5.088608	Q9UNZ2	NSF1C_HUMAN	519.788722	2	0.794118047	2	Yes
SGQQIVGPPR	-5.088608	Q9UNZ2	NSF1C_HUMAN	346.8617563	3	0.794118047	2	
SPNELVDDLFK	75.27197	Q9UNZ2	NSF1C_HUMAN	638.825166	2	0.797216713		
SPNELVDDLFK	75.27197	Q9UNZ2	NSF1C_HUMAN	426.2193857	3	0.797216713		
SYQDPSNAQFLESIR	54.86081	Q9UNZ2	NSF1C_HUMAN	877.9213845	2	0.801326275	2	Yes
SYQDPSNAQFLESIR	54.86081	Q9UNZ2	NSF1C_HUMAN	585.6168647	3	0.801326275	2	
AMDQEITVNPQFVQK	47.70379	Q9UNS2	CSN3_HUMAN	874.4383615	2	0.817414045		
AMDQEITVNPQFVQK	47.70379	Q9UNS2	CSN3_HUMAN	583.2948493	3	0.817414045		
DGMVSFHDNPEK	9.223801	Q9UNS2	CSN3_HUMAN	688.3015325	2	0.800860763	3	
DGMVSFHDNPEK	9.223801	Q9UNS2	CSN3_HUMAN	459.20363	3	0.800860763	3	Yes
FIKPLSNAYHELAQVYSTNNPSELR	63.21262	Q9UNS2	CSN3_HUMAN	1446.238495	2	0.746016204	4	
FIKPLSNAYHELAQVYSTNNPSELR	63.21262	Q9UNS2	CSN3_HUMAN	964.494938	3	0.746016204	4	
QLSAQGQMTQLCELINK	70.29998	Q9UNS2	CSN3_HUMAN	981.4933425	2	0.633037269	3	
QLSAQGQMTQLCELINK	70.29998	Q9UNS2	CSN3_HUMAN	654.6648367	3	0.633037269	3	Yes
TFLTLSLQDMASR	85.32558	Q9UNS2	CSN3_HUMAN	741.885233	2	0.815524042		
TFLTLSLQDMASR	85.32558	Q9UNS2	CSN3_HUMAN	494.926097	3	0.815524042		
VQLSGPQEAEK	-6.363029	Q9UNS2	CSN3_HUMAN	593.309684	2	0.837526202		
VQLSGPQEAEK	-6.363029	Q9UNS2	CSN3_HUMAN	395.875731	3	0.837526202		
YATDTFAGLCHQLTNALVER	78.37186	Q9UNS2	CSN3_HUMAN	1140.558056	2	0.796032488	3	
YATDTFAGLCHQLTNALVER	78.37186	Q9UNS2	CSN3_HUMAN	760.7079787	3	0.796032488	3	Yes
YNNPAMLHNIDQEMLK	45.78531	Q9UNS2	CSN3_HUMAN	965.9616725	2	0.836534441	3	
YNNPAMLHNIDQEMLK	45.78531	Q9UNS2	CSN3_HUMAN	644.31039	3	0.836534441	3	Yes
YVLHMIEDGEIFASINQK	72.41902	Q9UNS2	CSN3_HUMAN	1054.03061	2	0.674809515		
YVLHMIEDGEIFASINQK	72.41902	Q9UNS2	CSN3_HUMAN	703.0230147	3	0.674809515		
IQENALPLMLAEPPQNLIAQSQSGTGK	105.3033	Q9UMR2	DD19B_HUMAN	1424.747848	2	0.824518323	3	
IQENALPLMLAEPPQNLIAQSQSGTGK	105.3033	Q9UMR2	DD19B_HUMAN	950.1678403	3	0.824518323	3	Yes
TAAFVLAMLSQVEPANK	112.758	Q9UMR2	DD19B_HUMAN	895.480029	2	0.818379343		
TAAFVLAMLSQVEPANK	112.758	Q9UMR2	DD19B_HUMAN	597.3226277	3	0.818379343		
GPATLVAPASVITIVK	83.09889	Q9ULX9	MAFF_HUMAN	768.972166	2	0.807460845	2	Yes
GPATLVAPASVITIVK	83.09889	Q9ULX9	MAFF_HUMAN	512.9840523	3	0.807460845	2	
DEFGELEGTRPNK	11.43533	Q9ULJ3	ZBT21_HUMAN	746.3578935	2	0.789559007		
DEFGELEGTRPNK	11.43533	Q9ULJ3	ZBT21_HUMAN	497.9078707	3	0.789559007		
EAMDDKPGVSGQLPK	5.541264	Q9ULJ3	ZBT21_HUMAN	786.3908795	2	0.631946206		
EAMDDKPGVSGQLPK	5.541264	Q9ULJ3	ZBT21_HUMAN	524.596528	3	0.631946206		
EEPVEEAEEEAPEASTAPK	27.59935	Q9ULJ3	ZBT21_HUMAN	1021.458396	2	0.635485291		
EEPVEEAEEEAPEASTAPK	27.59935	Q9ULJ3	ZBT21_HUMAN	681.3082053	3	0.635485291		
GKPGFQGQSSSQAQQVIK	-8.944382	Q9ULJ3	ZBT21_HUMAN	937.9901385	2	0.810074091		
GKPGFQGQSSSQAQQVIK	-8.944382	Q9ULJ3	ZBT21_HUMAN	625.6627007	3	0.810074091		
IQPLEPDSPTGLSENPTPATEK	48.67423	Q9ULJ3	ZBT21_HUMAN	1161.079544	2	0.728311896		
IQPLEPDSPTGLSENPTPATEK	48.67423	Q9ULJ3	ZBT21_HUMAN	774.388971	3	0.728311896		
LFVPQESDTLFYHAPPLSAITFK	107.0875	Q9ULJ3	ZBT21_HUMAN	1311.186685	2	0.828021646	3	
LFVPQESDTLFYHAPPLSAITFK	107.0875	Q9ULJ3	ZBT21_HUMAN	874.4603983	3	0.828021646	3	Yes
LGLVIPSSGSGSGNQSIDR	50.6865	Q9ULJ3	ZBT21_HUMAN	922.479602	2	0.77133286		
LGLVIPSSGSGSGNQSIDR	50.6865	Q9ULJ3	ZBT21_HUMAN	615.322343	3	0.77133286		
LSSLLEQGSHER	0.198235	Q9ULJ3	ZBT21_HUMAN	678.349871	2	0.771116853		
LSSLLEQGSHER	0.198235	Q9ULJ3	ZBT21_HUMAN	452.569189	3	0.771116853		
LWSHFQSHMSQASEESAHK	-9.986401	Q9ULJ3	ZBT21_HUMAN	1114.00345	2	0.663703799		
LWSHFQSHMSQASEESAHK	-9.986401	Q9ULJ3	ZBT21_HUMAN	743.0049083	3	0.663703799		
NAVLPSKPLQDR	1.637806	Q9ULJ3	ZBT21_HUMAN	669.3809745	2	0.768026769		
NAVLPSKPLQDR	1.637806	Q9ULJ3	ZBT21_HUMAN	446.5899247	3	0.768026769		
NLLYYSK	14.39326	Q9ULJ3	ZBT21_HUMAN	450.7454505	2	0.608162999		
NLLYYSK	14.39326	Q9ULJ3	ZBT21_HUMAN	300.8329087	3	0.608162999		
NVLAASSEYFQSLFTNK	95.47606	Q9ULJ3	ZBT21_HUMAN	959.981442	2	0.891873121		
NVLAASSEYFQSLFTNK	95.47606	Q9ULJ3	ZBT21_HUMAN	640.3235697	3	0.891873121		
SLEHSGSLDDPNR	-20.21046	Q9ULJ3	ZBT21_HUMAN	713.832042	2	0.773216784		
SLEHSGSLDDPNR	-20.21046	Q9ULJ3	ZBT21_HUMAN	476.2239697	3	0.773216784		

SLSMDSQVPVYSPSIDLK	75.26292	Q9ULJ3	ZBT21_HUMAN	983.4960675	2	0.719098389		
SLSMDSQVPVYSPSIDLK	75.26292	Q9ULJ3	ZBT21_HUMAN	655.9999867	3	0.719098389		
SSQGSSSVSSDAPGNVLCALSQK	55.4004	Q9ULJ3	ZBT21_HUMAN	1133.534977	2	0.665133119		
SSQGSSSVSSDAPGNVLCALSQK	55.4004	Q9ULJ3	ZBT21_HUMAN	756.025926	3	0.665133119		
SVIVCQSR	-20.03171	Q9ULJ3	ZBT21_HUMAN	474.7507505	2	0.71664691		
SVIVCQSR	-20.03171	Q9ULJ3	ZBT21_HUMAN	316.836442	3	0.71664691		
TAFSLWSHEQTHN	26.19556	Q9ULJ3	ZBT21_HUMAN	779.358228	2	0.626774013		
TAFSLWSHEQTHN	26.19556	Q9ULJ3	ZBT21_HUMAN	519.9080937	3	0.626774013		
TALDDRPQVLQPHR	0.248718	Q9ULJ3	ZBT21_HUMAN	823.442627	2	0.767781019		
TALDDRPQVLQPHR	0.248718	Q9ULJ3	ZBT21_HUMAN	549.297693	3	0.767781019		
TEPSSPLSDPSDIIR	57.68857	Q9ULJ3	ZBT21_HUMAN	807.40504	2	0.766745329		
TEPSSPLSDPSDIIR	57.68857	Q9ULJ3	ZBT21_HUMAN	538.6059683	3	0.766745329		
TPQAPFPTCPNR	17.65513	Q9ULJ3	ZBT21_HUMAN	693.335711	2	0.778422952		
TPQAPFPTCPNR	17.65513	Q9ULJ3	ZBT21_HUMAN	462.559749	3	0.778422952		
TVSQNQPDVSHTSRPSPSIAVK	-3.149803	Q9ULJ3	ZBT21_HUMAN	1168.104219	2	0.642250419		
TVSQNQPDVSHTSRPSPSIAVK	-3.149803	Q9ULJ3	ZBT21_HUMAN	779.0720873	3	0.642250419		
VFVEDDENSSQK	-6.745426	Q9ULJ3	ZBT21_HUMAN	698.8155265	2	0.705584109		
VFVEDDENSSQK	-6.745426	Q9ULJ3	ZBT21_HUMAN	466.2129593	3	0.705584109		
VNEHGSPVSEDNFEEGSSPTLLDADFPDSDLNK	74.04399	Q9ULJ3	ZBT21_HUMAN	1781.29298	2	0.628361046		
VNEHGSPVSEDNFEEGSSPTLLDADFPDSDLNK	74.04399	Q9ULJ3	ZBT21_HUMAN	1187.864595	3	0.628361046		
VTVGDAATTAAASSSSVTR	17.60796	Q9ULJ3	ZBT21_HUMAN	876.4426865	2	0.816402316		
VTVGDAATTAAASSSSVTR	17.60796	Q9ULJ3	ZBT21_HUMAN	584.631066	3	0.816402316		
AYEASLSEIK	21.46336	Q9ULD2	MTUS1_HUMAN	555.788048	2	0.768945396		
AYEASLSEIK	21.46336	Q9ULD2	MTUS1_HUMAN	370.861307	3	0.768945396		
DLGTQNHTSELILSSPPGQK	40.70333	Q9ULD2	MTUS1_HUMAN	1061.542932	2	0.696137011		
DLGTQNHTSELILSSPPGQK	40.70333	Q9ULD2	MTUS1_HUMAN	708.0312293	3	0.696137011		
DSICQCPALVGTEKPK	19.23217	Q9ULD2	MTUS1_HUMAN	901.942954	2	0.775088549		
DSICQCPALVGTEKPK	19.23217	Q9ULD2	MTUS1_HUMAN	601.6312443	3	0.775088549		
DTYIEEAEK	5.296768	Q9ULD2	MTUS1_HUMAN	549.2540385	2	0.678827941		
DTYIEEAEK	5.296768	Q9ULD2	MTUS1_HUMAN	366.5053007	3	0.678827941		
EAPVNLCKPSLGK	12.98901	Q9ULD2	MTUS1_HUMAN	706.882493	2	0.700885892		
EAPVNLCKPSLGK	12.98901	Q9ULD2	MTUS1_HUMAN	471.590937	3	0.700885892		
FEALTVVIQHLLSER	111.4045	Q9ULD2	MTUS1_HUMAN	877.9941605	2	0.754596233		
FEALTVVIQHLLSER	111.4045	Q9ULD2	MTUS1_HUMAN	585.665382	3	0.754596233		
GELVTASTTCEK	-11.13188	Q9ULD2	MTUS1_HUMAN	648.311567	2	0.734803557		
GELVTASTTCEK	-11.13188	Q9ULD2	MTUS1_HUMAN	432.543653	3	0.734803557		
IEDELQTFFTSDKDGNTHAYNPK	51.7303	Q9ULD2	MTUS1_HUMAN	1335.620092	2	0.768524528		
IEDELQTFFTSDKDGNTHAYNPK	51.7303	Q9ULD2	MTUS1_HUMAN	890.749336	3	0.768524528		
LEIEASHSEK	-26.17178	Q9ULD2	MTUS1_HUMAN	571.7885835	2	0.671887875		
LEIEASHSEK	-26.17178	Q9ULD2	MTUS1_HUMAN	381.5283307	3	0.671887875		
LSMENEELLWK	64.48896	Q9ULD2	MTUS1_HUMAN	696.347951	2	0.705394804		
LSMENEELLWK	64.48896	Q9ULD2	MTUS1_HUMAN	464.567909	3	0.705394804		
LVDNNTALVDK	10.91312	Q9ULD2	MTUS1_HUMAN	601.3253335	2	0.820824146		
LVDNNTALVDK	10.91312	Q9ULD2	MTUS1_HUMAN	401.2194973	3	0.820824146		
MESAECLEMTYVPNIDR	70.49537	Q9ULD2	MTUS1_HUMAN	1029.453021	2	0.647342324		
MESAECLEMTYVPNIDR	70.49537	Q9ULD2	MTUS1_HUMAN	686.6379553	3	0.647342324		
MQLQEQFDNLNAAHETSK	35.07222	Q9ULD2	MTUS1_HUMAN	1052.492385	2	0.842707396		
MQLQEQFDNLNAAHETSK	35.07222	Q9ULD2	MTUS1_HUMAN	701.9975313	3	0.842707396		
NELQTVYEAFVQQHQAEK	70.35851	Q9ULD2	MTUS1_HUMAN	1081.529821	2	0.821022034		
NELQTVYEAFVQQHQAEK	70.35851	Q9ULD2	MTUS1_HUMAN	721.355822	3	0.821022034		
NISKPDSCGLR	-26.39549	Q9ULD2	MTUS1_HUMAN	623.81461	2	0.684934855		
NISKPDSCGLR	-26.39549	Q9ULD2	MTUS1_HUMAN	416.2123483	3	0.684934855		
NLFTALNAVEK	63.90412	Q9ULD2	MTUS1_HUMAN	610.338244	2	0.741963029		
NLFTALNAVEK	63.90412	Q9ULD2	MTUS1_HUMAN	407.2281043	3	0.741963029		
NPQIMYLEQELESLK	96.45084	Q9ULD2	MTUS1_HUMAN	917.9669465	2	0.61985153		
NPQIMYLEQELESLK	96.45084	Q9ULD2	MTUS1_HUMAN	612.313906	3	0.61985153		
NSGSFPSPSISPR	27.07188	Q9ULD2	MTUS1_HUMAN	666.8313135	2	0.687122881		
NSGSFPSPSISPR	27.07188	Q9ULD2	MTUS1_HUMAN	444.8901507	3	0.687122881		
PNDNLNCAGYCDALELNQTFDMTVDK	92.32785	Q9ULD2	MTUS1_HUMAN	1509.649883	2	0.658729792		
PNDNLNCAGYCDALELNQTFDMTVDK	92.32785	Q9ULD2	MTUS1_HUMAN	1006.769197	3	0.658729792		
QLSTEQAVLQESLEK	46.9118	Q9ULD2	MTUS1_HUMAN	851.947073	2	0.66427511		
QLSTEQAVLQESLEK	46.9118	Q9ULD2	MTUS1_HUMAN	568.300657	3	0.66427511		
SEAQVLNPEHK	-20.09137	Q9ULD2	MTUS1_HUMAN	626.3205825	2	0.706864834		
SEAQVLNPEHK	-20.09137	Q9ULD2	MTUS1_HUMAN	417.8829967	3	0.706864834		
SLCIQPQTAPDALPPEK	47.97334	Q9ULD2	MTUS1_HUMAN	932.977851	2	0.838218451		
SLCIQPQTAPDALPPEK	47.97334	Q9ULD2	MTUS1_HUMAN	622.3211757	3	0.838218451		
SPTSSAIPLQSPR	22.11088	Q9ULD2	MTUS1_HUMAN	670.8626145	2	0.703525305		
SPTSSAIPLQSPR	22.11088	Q9ULD2	MTUS1_HUMAN	447.5776847	3	0.703525305		
TGSTPSIASTHSELSTYSNNSGNAAVIK	29.86029	Q9ULD2	MTUS1_HUMAN	1397.678668	2	0.797347307		
TGSTPSIASTHSELSTYSNNSGNAAVIK	29.86029	Q9ULD2	MTUS1_HUMAN	932.1217203	3	0.797347307		
TLELTQYK	18.58144	Q9ULD2	MTUS1_HUMAN	498.2745775	2	0.790107906		
TLELTQYK	18.58144	Q9ULD2	MTUS1_HUMAN	332.5189933	3	0.790107906		
TLSQELVNLR	43.04292	Q9ULD2	MTUS1_HUMAN	586.8358685	2	0.817848086		
TLSQELVNLR	43.04292	Q9ULD2	MTUS1_HUMAN	391.559854	3	0.817848086		
TMCMSTPVLEPTK	39.65357	Q9ULD2	MTUS1_HUMAN	747.8542305	2	0.630148113		
TMCMSTPVLEPTK	39.65357	Q9ULD2	MTUS1_HUMAN	498.9054287	3	0.630148113		
VGPPVSCLR	12.05231	Q9ULD2	MTUS1_HUMAN	492.7689435	2	0.639694929		
VGPPVSCLR	12.05231	Q9ULD2	MTUS1_HUMAN	328.8485707	3	0.639694929		
VGSSFGLTWDANDMVISTDK	88.39482	Q9ULD2	MTUS1_HUMAN	1072.004793	2	0.638307691		
VGSSFGLTWDANDMVISTDK	88.39482	Q9ULD2	MTUS1_HUMAN	715.0058037	3	0.638307691		
VTETEDTQMVSK	-11.14685	Q9ULD2	MTUS1_HUMAN	684.3221315	2	0.625130653		
VTETEDTQMVSK	-11.14685	Q9ULD2	MTUS1_HUMAN	456.550696	3	0.625130653		
VTFSVSPIEATEK	49.02689	Q9ULD2	MTUS1_HUMAN	704.372481	2	0.79818666		
VTFSVSPIEATEK	49.02689	Q9ULD2	MTUS1_HUMAN	469.9175957	3	0.79818666		
VTSEYTDGSQQR	-26.33986	Q9ULD2	MTUS1_HUMAN	685.813315	2	0.775238037		
VTSEYTDGSQQR	-26.33986	Q9ULD2	MTUS1_HUMAN	457.5448183	3	0.775238037		
DFQAISDVIGNK	65.38126	Q9UKL0	RCOR1_HUMAN	653.8360655	2	0.804057837		
DFQAISDVIGNK	65.38126	Q9UKL0	RCOR1_HUMAN	436.226652	3	0.804057837		
DNLGMLVWSPNQNLSEAK	93.19821	Q9UKL0	RCOR1_HUMAN	1008.496938	2	0.805108547		
DNLGMLVWSPNQNLSEAK	93.19821	Q9UKL0	RCOR1_HUMAN	672.6672333	3	0.805108547		
EVPPTETVPQVK	20.94512	Q9UKL0	RCOR1_HUMAN	662.361917	2	0.810964942		
EVPPTETVPQVK	20.94512	Q9UKL0	RCOR1_HUMAN	441.910553	3	0.810964942		
FNIDEVLQEWEAEHGK	95.98987	Q9UKL0	RCOR1_HUMAN	972.4608785	2	0.781008303		
FNIDEVLQEWEAEHGK	95.98987	Q9UKL0	RCOR1_HUMAN	648.643194	3	0.781008303		
GMFLSQEDVEAVSANATAATTVLR	92.64896	Q9UKL0	RCOR1_HUMAN	1241.118678	2	0.780312657		
GMFLSQEDVEAVSANATAATTVLR	92.64896	Q9UKL0	RCOR1_HUMAN	827.7483937	3	0.780312657		
LDGGIEPYR	14.6783	Q9UKL0	RCOR1_HUMAN	510.262001	2	0.765487254		
LDGGIEPYR	14.6783	Q9UKL0	RCOR1_HUMAN	340.510609	3	0.765487254		
MPEEEDEAPVLDVR	44.35385	Q9UKL0	RCOR1_HUMAN	814.8778015	2	0.861476541	2	Yes
MPEEEDEAPVLDVR	44.35385	Q9UKL0	RCOR1_HUMAN	543.5878093	3	0.861476541	2	
VGPQYQAVVPDFDPAK	60.41574	Q9UKL0	RCOR1_HUMAN	865.94159	2	0.858479977		

VGPQYQAVVPDFDPAK	60.41574	Q9UKL0	RCOR1 HUMAN	577.630335	3	0.858479977		
WTTEEQLLAVQAIR	76.44775	Q9UKL0	RCOR1 HUMAN	829.4495865	2	0.836621225	2	Yes
WTTEEQLLAVQAIR	76.44775	Q9UKL0	RCOR1 HUMAN	553.3023327	3	0.836621225	2	
DAPSELLR	26.8251	Q9UKB3	DJC12 HUMAN	450.743448	2	0.756091714		
DAPSELLR	26.8251	Q9UKB3	DJC12 HUMAN	300.8315737	3	0.756091714		
DLMLEESDK	26.53876	Q9UKB3	DJC12 HUMAN	540.2504455	2	0.735438943		
DLMLEESDK	26.53876	Q9UKB3	DJC12 HUMAN	360.5029053	3	0.735438943		
SQMSMPFQQWEALNDSVK	86.98146	Q9UKB3	DJC12 HUMAN	1063.488256	2	0.692670703		
SQMSMPFQQWEALNDSVK	86.98146	Q9UKB3	DJC12 HUMAN	709.3281123	3	0.692670703		
AALHDPGFPSYCQSLK	33.29557	Q9UIF9	BAZ2A HUMAN	895.930699	2	0.833254099		
AALHDPGFPSYCQSLK	33.29557	Q9UIF9	BAZ2A HUMAN	597.6230743	3	0.833254099		
AGDPGEMPQSPTGLGQPK	29.86752	Q9UIF9	BAZ2A HUMAN	883.923076	2	0.888598919		
AGDPGEMPQSPTGLGQPK	29.86752	Q9UIF9	BAZ2A HUMAN	589.6179923	3	0.888598919		
ALLSTPNGAPEGTTTEISYEITPR	67.67879	Q9UIF9	BAZ2A HUMAN	1259.637754	2	0.661709309		
ALLSTPNGAPEGTTTEISYEITPR	67.67879	Q9UIF9	BAZ2A HUMAN	840.094444	3	0.661709309		
ASPVTSPAAAFPTASPANK	39.04555	Q9UIF9	BAZ2A HUMAN	892.9630575	2	0.709618628		
ASPVTSPAAAFPTASPANK	39.04555	Q9UIF9	BAZ2A HUMAN	595.6446467	3	0.709618628		
AVSLGQDR	-21.17491	Q9UIF9	BAZ2A HUMAN	423.2279655	2	0.721338332		
AVSLGQDR	-21.17491	Q9UIF9	BAZ2A HUMAN	282.4879187	3	0.721338332		
DGEVDATASSIPELER	52.31304	Q9UIF9	BAZ2A HUMAN	844.902862	2	0.882453978		
DGEVDATASSIPELER	52.31304	Q9UIF9	BAZ2A HUMAN	563.6045163	3	0.882453978		
DNVSEILR	32.89562	Q9UIF9	BAZ2A HUMAN	473.2541795	2	0.652520537		
DNVSEILR	32.89562	Q9UIF9	BAZ2A HUMAN	315.838728	3	0.652520537		
DPEMLDAMLK	73.22015	Q9UIF9	BAZ2A HUMAN	581.7783165	2	0.777769208	2	Yes
DPEMLDAMLK	73.22015	Q9UIF9	BAZ2A HUMAN	388.1881527	3	0.777769208	2	
DTPEGLQWVQLSAEEIPSR	97.65633	Q9UIF9	BAZ2A HUMAN	1078.037482	2	0.65892446	3	
DTPEGLQWVQLSAEEIPSR	97.65633	Q9UIF9	BAZ2A HUMAN	719.0275963	3	0.65892446	3	Yes
DVSSFLETTADVEEITGEGLTASGSGDVMR	130.133	Q9UIF9	BAZ2A HUMAN	1537.211886	2	0.692958355		
DVSSFLETTADVEEITGEGLTASGSGDVMR	130.133	Q9UIF9	BAZ2A HUMAN	1025.143866	3	0.692958355		
EDLAYCEHLSDSQEDITWR	48.90047	Q9UIF9	BAZ2A HUMAN	1184.013874	2	0.707382977	3	
EDLAYCEHLSDSQEDITWR	48.90047	Q9UIF9	BAZ2A HUMAN	789.678524	3	0.707382977	3	Yes
EPLWPTHEVVLEK	46.64056	Q9UIF9	BAZ2A HUMAN	788.9226725	2	0.78957355		
EPLWPTHEVVLEK	46.64056	Q9UIF9	BAZ2A HUMAN	526.28439	3	0.78957355		
QDSEQPQAQLQPEAQLHAPAQPQPQLQLQSH	50.79541	Q9UIF9	BAZ2A HUMAN	1948.986092	2	0.756125271		
QDSEQPQAQLQPEAQLHAPAQPQPQLQLQSH	50.79541	Q9UIF9	BAZ2A HUMAN	1299.660003	3	0.756125271		
GWTCPSPDSTR	1.730747	Q9UIF9	BAZ2A HUMAN	632.2753185	2	0.754749417		
GWTCPSPDSTR	1.730747	Q9UIF9	BAZ2A HUMAN	421.8528207	3	0.754749417		
IMEETSGMEEEEEEESIAAVPGR	58.49046	Q9UIF9	BAZ2A HUMAN	1276.554915	2	0.720023394		
IMEETSGMEEEEEEESIAAVPGR	58.49046	Q9UIF9	BAZ2A HUMAN	851.3725517	3	0.720023394		
KPTEDMCLTDHQPLPDFSR	38.60995	Q9UIF9	BAZ2A HUMAN	1144.028278	2	0.773879528		
KPTEDMCLTDHQPLPDFSR	38.60995	Q9UIF9	BAZ2A HUMAN	763.02146	3	0.773879528		
LAALEQNVER	8.425331	Q9UIF9	BAZ2A HUMAN	571.8123935	2	0.848855078	2	Yes
LAALEQNVER	8.425331	Q9UIF9	BAZ2A HUMAN	381.544204	3	0.848855078	2	
LEAQETLNEEDK	0.848217	Q9UIF9	BAZ2A HUMAN	709.8364595	2	0.658280909		
LEAQETLNEEDK	0.848217	Q9UIF9	BAZ2A HUMAN	473.560248	3	0.658280909		
LPLQHGWR	2.981461	Q9UIF9	BAZ2A HUMAN	503.7832425	2	0.737891555		
LPLQHGWR	2.981461	Q9UIF9	BAZ2A HUMAN	336.1914367	3	0.737891555		
MELAGSNTTASSPAR	-1.389915	Q9UIF9	BAZ2A HUMAN	746.8572035	2	0.67460072		
MELAGSNTTASSPAR	-1.389915	Q9UIF9	BAZ2A HUMAN	498.240744	3	0.67460072		
MPVGDFFEER	58.72637	Q9UIF9	BAZ2A HUMAN	613.779705	2	0.755120277		
MPVGDFFEER	58.72637	Q9UIF9	BAZ2A HUMAN	409.5224117	3	0.755120277		
NPMDFSTMR	31.03349	Q9UIF9	BAZ2A HUMAN	549.7395255	2	0.733278394		
NPMDFSTMR	31.03349	Q9UIF9	BAZ2A HUMAN	366.8289587	3	0.733278394		
SEVEMEGPEECLGR	31.43421	Q9UIF9	BAZ2A HUMAN	811.3458115	2	0.711137533		
SEVEMEGPEECLGR	31.43421	Q9UIF9	BAZ2A HUMAN	541.2331493	3	0.711137533		
SGYSLNFSEGDGR	32.19865	Q9UIF9	BAZ2A HUMAN	694.8080315	2	0.629408419		
SGYSLNFSEGDGR	32.19865	Q9UIF9	BAZ2A HUMAN	463.541296	3	0.629408419		
TQPFQAQPPQQK	-1.343536	Q9UIF9	BAZ2A HUMAN	699.3627835	2	0.682527363		
TQPFQAQPPQQK	-1.343536	Q9UIF9	BAZ2A HUMAN	466.5777973	3	0.682527363		
TTNPLDLAVMR	63.54005	Q9UIF9	BAZ2A HUMAN	615.8297295	2	0.787501574		
TTNPLDLAVMR	63.54005	Q9UIF9	BAZ2A HUMAN	410.8890947	3	0.787501574		
VAAHASLNPALFSMK	43.13081	Q9UIF9	BAZ2A HUMAN	778.916867	2	0.797887087		
VAAHASLNPALFSMK	43.13081	Q9UIF9	BAZ2A HUMAN	519.613853	3	0.797887087		
VIMSDLQIR	42.0776	Q9UIF9	BAZ2A HUMAN	537.802983	2	0.639502883		
VIMSDLQIR	42.0776	Q9UIF9	BAZ2A HUMAN	358.8712637	3	0.639502883		
WEEFYQGK	19.54391	Q9UIF9	BAZ2A HUMAN	543.7487265	2	0.651404321		
WEEFYQGK	19.54391	Q9UIF9	BAZ2A HUMAN	362.8350927	3	0.651404321		
WQGETWYYGPCGK	45.6949	Q9UIF9	BAZ2A HUMAN	816.3515495	2	0.614127576		
WQGETWYYGPCGK	45.6949	Q9UIF9	BAZ2A HUMAN	544.570308	3	0.614127576		
GIPNMLLSEEETES	81.82715	Q9UI30	TR112 HUMAN	774.859077	2	0.613359392		
GIPNMLLSEEETES	81.82715	Q9UI30	TR112 HUMAN	516.9086597	3	0.613359392		
GPVEGYEENEEFLR	43.41407	Q9UI30	TR112 HUMAN	834.381561	2	0.733214796	2	Yes
GPVEGYEENEEFLR	43.41407	Q9UI30	TR112 HUMAN	556.5903157	3	0.733214796	2	
ICPVEFNPNFVAR	65.64579	Q9UI30	TR112 HUMAN	781.893392	2	0.756855667	3	
ICPVEFNPNFVAR	65.64579	Q9UI30	TR112 HUMAN	521.598203	3	0.756855667	3	Yes
LLTHNLLSSHVR	3.517723	Q9UI30	TR112 HUMAN	695.402241	2	0.802401245	3	
LLTHNLLSSHVR	3.517723	Q9UI30	TR112 HUMAN	463.9374357	3	0.802401245	3	Yes
TMHHLLLEVEVIEGTLQCPESGR	75.67828	Q9UI30	TR112 HUMAN	1324.66273	2	0.608952165		
TMHHLLLEVEVIEGTLQCPESGR	75.67828	Q9UI30	TR112 HUMAN	883.4444283	3	0.608952165		
VEWSAFLEAADNLR	100.8703	Q9UI30	TR112 HUMAN	810.9050105	2	0.837893963	3	
VEWSAFLEAADNLR	100.8703	Q9UI30	TR112 HUMAN	540.939282	3	0.837893963	3	Yes
FNVTGTPEQYVPYSTTR	54.49574	Q9UI09	NDUAC HUMAN	980.476521	2	0.847604752		
FNVTGTPEQYVPYSTTR	54.49574	Q9UI09	NDUAC HUMAN	653.9869557	3	0.847604752		
IQEWIPPSTPYK	56.18179	Q9UI09	NDUAC HUMAN	729.8855545	2	0.771973968		
IQEWIPPSTPYK	56.18179	Q9UI09	NDUAC HUMAN	486.9263113	3	0.771973968		
NTFWDVDGSMVPPEWHR	78.17323	Q9UI09	NDUAC HUMAN	1036.968348	2	0.740079463	3	
NTFWDVDGSMVPPEWHR	78.17323	Q9UI09	NDUAC HUMAN	691.6481737	3	0.740079463	3	Yes
VGTLVGEDK	-9.623699	Q9UI09	NDUAC HUMAN	459.2511065	2	0.616369963		
VGTLVGEDK	-9.623699	Q9UI09	NDUAC HUMAN	306.503346	3	0.616369963		
AVFDNLIQLEHLNIVK	95.63252	Q9UHY1	NRBP HUMAN	933.528367	2	0.836838603	3	
AVFDNLIQLEHLNIVK	95.63252	Q9UHY1	NRBP HUMAN	622.6881863	3	0.836838603	3	Yes
ELLFHPALFEVPSLK	95.16516	Q9UHY1	NRBP HUMAN	870.490722	2	0.757546425		
ELLFHPALFEVPSLK	95.16516	Q9UHY1	NRBP HUMAN	580.6630897	3	0.757546425		
EPVQTLYSQSPALELDK	57.52568	Q9UHY1	NRBP HUMAN	959.494383	2	0.725605309		
EPVQTLYSQSPALELDK	57.52568	Q9UHY1	NRBP HUMAN	639.9988637	3	0.725605309		
IGSVAPDTINNHVK	0.672401	Q9UHY1	NRBP HUMAN	732.894446	2	0.787936389		
IGSVAPDTINNHVK	0.672401	Q9UHY1	NRBP HUMAN	488.932239	3	0.787936389		
LLAAHCIVGHQHMIPENALEEITK	50.76306	Q9UHY1	NRBP HUMAN	1362.70219	2	0.715166628		
LLAAHCIVGHQHMIPENALEEITK	50.76306	Q9UHY1	NRBP HUMAN	908.804068	3	0.715166628		
LTSLLEETLNK	52.78265	Q9UHY1	NRBP HUMAN	630.8564665	2	0.86984539		
LTSLLEETLNK	52.78265	Q9UHY1	NRBP HUMAN	420.9069193	3	0.86984539		

NMDTSAVLAEIPAGPGR	66.71276	Q9UHY1	NRBP_HUMAN	849.9281605	2	0.773789585		
NMDTSAVLAEIPAGPGR	66.71276	Q9UHY1	NRBP_HUMAN	566.9547153	3	0.773789585		
VIFITEYMSSGSLK	78.95229	Q9UHY1	NRBP_HUMAN	787.9109115	2	0.745072901		
VIFITEYMSSGSLK	78.95229	Q9UHY1	NRBP_HUMAN	525.6098827	3	0.745072901		
VVLMQCNIESVEEGVK	54.88093	Q9UHY1	NRBP_HUMAN	917.458436	2	0.675289929		
VVLMQCNIESVEEGVK	54.88093	Q9UHY1	NRBP_HUMAN	611.974899	3	0.675289929		
AAVHTYGIASVPNQPLK	30.14751	Q9UHI6	DDX20_HUMAN	883.483956	2	0.853156686		
AAVHTYGIASVPNQPLK	30.14751	Q9UHI6	DDX20_HUMAN	589.3252457	3	0.853156686		
AQHLADILSSK	15.77666	Q9UHI6	DDX20_HUMAN	591.8280435	2	0.823564529	2	Yes
AQHLADILSSK	15.77666	Q9UHI6	DDX20_HUMAN	394.8879707	3	0.823564529	2	
AWQEYYAAASHSYYWNAQR	49.3521	Q9UHI6	DDX20_HUMAN	1183.025223	2	0.689100504	3	
AWQEYYAAASHSYYWNAQR	49.3521	Q9UHI6	DDX20_HUMAN	789.0194237	3	0.689100504	3	Yes
EALPVSLPQIPCLSSFK	114.8198	Q9UHI6	DDX20_HUMAN	943.508786	2	0.761126399		
EALPVSLPQIPCLSSFK	114.8198	Q9UHI6	DDX20_HUMAN	629.341799	3	0.761126399		
EGLEKPVEIIR	26.90338	Q9UHI6	DDX20_HUMAN	641.872451	2	0.711110532		
EGLEKPVEIIR	26.90338	Q9UHI6	DDX20_HUMAN	428.250909	3	0.711110532		
EIAVQIHSVITAIGIK	75.56627	Q9UHI6	DDX20_HUMAN	846.506904	2	0.66029948		
EIAVQIHSVITAIGIK	75.56627	Q9UHI6	DDX20_HUMAN	564.6738777	3	0.66029948		
GFPAECISGNMNQNQR	28.62986	Q9UHI6	DDX20_HUMAN	911.902151	2	0.748612106		
GFPAECISGNMNQNQR	28.62986	Q9UHI6	DDX20_HUMAN	608.270709	3	0.748612106		
IHQPYTLTFAELVEDYEHYIK	104.1529	Q9UHI6	DDX20_HUMAN	1305.150291	2	0.787848473	4	
IHQPYTLTFAELVEDYEHYIK	104.1529	Q9UHI6	DDX20_HUMAN	870.4361357	3	0.787848473	4	
IPFNQALVFSNLHSR	61.17261	Q9UHI6	DDX20_HUMAN	871.971019	2	0.667989433	3	
IPFNQALVFSNLHSR	61.17261	Q9UHI6	DDX20_HUMAN	581.6499543	3	0.667989433	3	Yes
ISLEQPPNGSDTPNPEK	21.44726	Q9UHI6	DDX20_HUMAN	911.945061	2	0.642138362		
ISLEQPPNGSDTPNPEK	21.44726	Q9UHI6	DDX20_HUMAN	608.2993157	3	0.642138362		
LFILDEADK	53.41146	Q9UHI6	DDX20_HUMAN	532.2876885	2	0.763132215		
LFILDEADK	53.41146	Q9UHI6	DDX20_HUMAN	355.1944007	3	0.763132215		
LNSSDPSLIGLK	43.39734	Q9UHI6	DDX20_HUMAN	622.348808	2	0.770834088	2	Yes
LNSSDPSLIGLK	43.39734	Q9UHI6	DDX20_HUMAN	415.235147	3	0.770834088	2	
LQTEAQEDDWYDCHR	18.70277	Q9UHI6	DDX20_HUMAN	983.405774	2	0.815883338	2	Yes
LQTEAQEDDWYDCHR	18.70277	Q9UHI6	DDX20_HUMAN	655.939791	3	0.815883338	2	
LSFSDTYQDYEEYWR	81.33434	Q9UHI6	DDX20_HUMAN	1001.429231	2	0.7463727		
LSFSDTYQDYEEYWR	81.33434	Q9UHI6	DDX20_HUMAN	667.955429	3	0.7463727		
SYLEGSSDNQLK	5.168308	Q9UHI6	DDX20_HUMAN	670.8206075	2	0.783225536		
SYLEGSSDNQLK	5.168308	Q9UHI6	DDX20_HUMAN	447.54968	3	0.783225536		
TGDVLLAEPADFESLLLSRPVLEGLR	142.3247	Q9UHI6	DDX20_HUMAN	1405.769106	2	0.632582843		
TGDVLLAEPADFESLLLSRPVLEGLR	142.3247	Q9UHI6	DDX20_HUMAN	937.5153453	3	0.632582843		
TQHLQELFSR	16.91954	Q9UHI6	DDX20_HUMAN	629.831118	2	0.748068333		
TQHLQELFSR	16.91954	Q9UHI6	DDX20_HUMAN	420.2233537	3	0.748068333		
VLISTDLTSR	31.0713	Q9UHI6	DDX20_HUMAN	552.8171445	2	0.812886953		
VLISTDLTSR	31.0713	Q9UHI6	DDX20_HUMAN	368.8807047	3	0.812886953		
VVNSYPLAHK	-17.00735	Q9UHI6	DDX20_HUMAN	564.3145675	2	0.788151503		
VVNSYPLAHK	-17.00735	Q9UHI6	DDX20_HUMAN	376.5456533	3	0.788151503		
YQESPGIQMK	5.105476	Q9UHI6	DDX20_HUMAN	590.787526	2	0.801289916		
YQESPGIQMK	5.105476	Q9UHI6	DDX20_HUMAN	394.1942923	3	0.801289916		
FLNEMIAPVMR	68.43211	Q9UHG3	PCYOX_HUMAN	660.844325	2	0.814213455	2	Yes
FLNEMIAPVMR	68.43211	Q9UHG3	PCYOX_HUMAN	440.898825	3	0.814213455	2	
GELNTSIFSSRPIDK	34.77336	Q9UHG3	PCYOX_HUMAN	832.4366745	2	0.79146421		
GELNTSIFSSRPIDK	34.77336	Q9UHG3	PCYOX_HUMAN	555.2937247	3	0.79146421		
IAIIGAGIGGTSAAYYLR	82.47626	Q9UHG3	PCYOX_HUMAN	883.9941525	2	0.776348829	3	
IAIIGAGIGGTSAAYYLR	82.47626	Q9UHG3	PCYOX_HUMAN	589.6653767	3	0.776348829	3	Yes
IFSQETLTK	8.775902	Q9UHG3	PCYOX_HUMAN	533.7931385	2	0.66538167	2	Yes
IFSQETLTK	8.775902	Q9UHG3	PCYOX_HUMAN	356.198034	3	0.66538167	2	
KPWLAYPHYKPPEK	6.3172	Q9UHG3	PCYOX_HUMAN	877.475398	2	0.781865954	4	
KPWLAYPHYKPPEK	6.3172	Q9UHG3	PCYOX_HUMAN	585.3195403	3	0.781865954	4	
LATMMVQGQEYEAGGSVIHPLNLHMK	57.5963	Q9UHG3	PCYOX_HUMAN	1427.706611	2	0.832356572		
LATMMVQGQEYEAGGSVIHPLNLHMK	57.5963	Q9UHG3	PCYOX_HUMAN	952.1403487	3	0.832356572		
LFLSYDYAVK	60.3672	Q9UHG3	PCYOX_HUMAN	609.8244295	2	0.768687487		
LFLSYDYAVK	60.3672	Q9UHG3	PCYOX_HUMAN	406.8855613	3	0.768687487		
LLHALGGDDFLGMLNR	81.74133	Q9UHG3	PCYOX_HUMAN	871.456888	2	0.783311784		
LLHALGGDDFLGMLNR	81.74133	Q9UHG3	PCYOX_HUMAN	581.3072003	3	0.783311784		
LVCSGLLQASK	24.51395	Q9UHG3	PCYOX_HUMAN	588.3268225	2	0.773104072	2	Yes
LVCSGLLQASK	24.51395	Q9UHG3	PCYOX_HUMAN	392.5538233	3	0.773104072	2	
MYEVVYQIGTETR	50.17149	Q9UHG3	PCYOX_HUMAN	794.8879645	2	0.826095343	2	Yes
MYEVVYQIGTETR	50.17149	Q9UHG3	PCYOX_HUMAN	530.2612513	3	0.826095343	2	
SDFYDIVLVATPLNR	117.7083	Q9UHG3	PCYOX_HUMAN	861.957239	2	0.670204043		
SDFYDIVLVATPLNR	117.7083	Q9UHG3	PCYOX_HUMAN	574.974101	3	0.670204043		
SNLISGSVMYIEEK	60.04493	Q9UHG3	PCYOX_HUMAN	785.395625	2	0.783320427		
SNLISGSVMYIEEK	60.04493	Q9UHG3	PCYOX_HUMAN	523.933025	3	0.783320427		
TLLETLQK	29.34449	Q9UHG3	PCYOX_HUMAN	473.2849495	2	0.686866164	2	Yes
TLLETLQK	29.34449	Q9UHG3	PCYOX_HUMAN	315.8592413	3	0.686866164	2	
WNGHTDMIDQDGLYEK	31.14171	Q9UHG3	PCYOX_HUMAN	961.423435	2	0.68290019		
WNGHTDMIDQDGLYEK	31.14171	Q9UHG3	PCYOX_HUMAN	641.2848983	3	0.68290019		
YQSHDYAFSSVEK	1.279427	Q9UHG3	PCYOX_HUMAN	780.852435	2	0.742635369		
YQSHDYAFSSVEK	1.279427	Q9UHG3	PCYOX_HUMAN	520.9042317	3	0.742635369		
DQDTLIQHGIHDGLTVHLVIK	57.67085	Q9UHD9	UBQL2_HUMAN	1176.637699	2	0.69694674		
DQDTLIQHGIHDGLTVHLVIK	57.67085	Q9UHD9	UBQL2_HUMAN	784.761074	3	0.69694674		
FQQQLEQLNAMGFLNR	83.97464	Q9UHD9	UBQL2_HUMAN	968.989084	2	0.814072013	3	
FQQQLEQLNAMGFLNR	83.97464	Q9UHD9	UBQL2_HUMAN	646.3286643	3	0.814072013	3	Yes
GPAAAQGSAAAPAEPK	-19.73581	Q9UHD9	UBQL2_HUMAN	697.3576975	2	0.720041692	2	Yes
GPAAAQGSAAAPAEPK	-19.73581	Q9UHD9	UBQL2_HUMAN	465.2410733	3	0.720041692	2	
NPEISHLLNNPDIMR	51.52491	Q9UHD9	UBQL2_HUMAN	881.949426	2	0.835307002	3	
NPEISHLLNNPDIMR	51.52491	Q9UHD9	UBQL2_HUMAN	588.3022257	3	0.835307002	3	Yes
NQDLALSNLESIPGGYNALR	85.93335	Q9UHD9	UBQL2_HUMAN	1073.050919	2	0.754882574		
NQDLALSNLESIPGGYNALR	85.93335	Q9UHD9	UBQL2_HUMAN	715.7032207	3	0.754882574		
PQLPAFLQQMQNPDTLSAMSNPR	100.5136	Q9UHD9	UBQL2_HUMAN	1292.636515	2	0.825127125		
PQLPAFLQQMQNPDTLSAMSNPR	100.5136	Q9UHD9	UBQL2_HUMAN	862.0936183	3	0.825127125		
SQTDQLVLIFAGK	84.78116	Q9UHD9	UBQL2_HUMAN	710.396291	2	0.679288447	2	Yes
SQTDQLVLIFAGK	84.78116	Q9UHD9	UBQL2_HUMAN	473.933469	3	0.679288447	2	
ASSVGVLSPQK	4.246323	Q9UGU5	HMGX4_HUMAN	536.804037	2	0.801083922		
ASSVGVLSPQK	4.246323	Q9UGU5	HMGX4_HUMAN	358.2052997	3	0.801083922		
DEQGALLLGHELQSFLK	106.2261	Q9UGU5	HMGX4_HUMAN	949.5050895	2	0.718059719	3	
DEQGALLLGHELQSFLK	106.2261	Q9UGU5	HMGX4_HUMAN	633.3393347	3	0.718059719	3	Yes
DSELYFLGTDTHK	46.82985	Q9UGU5	HMGX4_HUMAN	763.3626405	2	0.874033332		
DSELYFLGTDTHK	46.82985	Q9UGU5	HMGX4_HUMAN	509.2443687	3	0.874033332		
EDCFDGDHTFEDIGLAAGR	60.28616	Q9UGU5	HMGX4_HUMAN	1062.95055	2	0.664144099		
EDCFDGDHTFEDIGLAAGR	60.28616	Q9UGU5	HMGX4_HUMAN	708.9696417	3	0.664144099		
GSSSVDEESFQYPSQQATVK	31.4479	Q9UGU5	HMGX4_HUMAN	1087.498382	2	0.713976204		
GSSSVDEESFQYPSQQATVK	31.4479	Q9UGU5	HMGX4_HUMAN	725.334863	3	0.713976204		
MKPLYVNTETLTLR	43.24467	Q9UGU5	HMGX4_HUMAN	839.9640105	2	0.726013124		

MKPLYVNTETLTLR	43.24467	Q9UGU5	HMGX4_HUMAN	560.3119487	3	0.726013124		
SPPTTMLLPASPAK	47.50644	Q9UGU5	HMGX4_HUMAN	705.8872445	2	0.781779289		
SPPTTMLLPASPAK	47.50644	Q9UGU5	HMGX4_HUMAN	470.927438	3	0.781779289		
SSPQSTDTAMDLLK	47.51924	Q9UGU5	HMGX4_HUMAN	747.3617875	2	0.735845804		
SSPQSTDTAMDLLK	47.51924	Q9UGU5	HMGX4_HUMAN	498.5771333	3	0.735845804		
VSGSSGELPLEDGGSHK	5.28437	Q9UGU5	HMGX4_HUMAN	828.3977465	2	0.706570268		
VSGSSGELPLEDGGSHK	5.28437	Q9UGU5	HMGX4_HUMAN	552.601106	3	0.706570268		
VTIVADHPGIDFGELSK	58.57458	Q9UGU5	HMGX4_HUMAN	899.473258	2	0.847557008		
VTIVADHPGIDFGELSK	58.57458	Q9UGU5	HMGX4_HUMAN	599.9847803	3	0.847557008		
LATSEMSAFHQPGPCEDPSCLTHGDYYDNLSLAS	72.09017	Q9UGP4	LIMD1_HUMAN	2106.41753	2	0.64922756		
LATSEMSAFHQPGPCEDPSCLTHGDYYDNLSLAS	72.09017	Q9UGP4	LIMD1_HUMAN	1404.614295	3	0.64922756		
FIEDLNMYEASK	54.60661	Q9UGP4	LIMD1_HUMAN	730.3428615	2	0.874269545	2	Yes
FIEDLNMYEASK	54.60661	Q9UGP4	LIMD1_HUMAN	487.2311827	3	0.874269545	2	
GAGNNPEFEETR	-13.48069	Q9UGP4	LIMD1_HUMAN	660.794929	2	0.840641022		
GAGNNPEFEETR	-13.48069	Q9UGP4	LIMD1_HUMAN	440.8658943	3	0.840641022		
IHLQQQQQLLQEETLPR	38.14826	Q9UGP4	LIMD1_HUMAN	1115.601117	2	0.806737661	3	
IHLQQQQQLLQEETLPR	38.14826	Q9UGP4	LIMD1_HUMAN	744.0700193	3	0.806737661	3	Yes
LPCQPLVPGPELRPSAAELK	57.13031	Q9UGP4	LIMD1_HUMAN	1086.596456	2	0.746641576		
LPCQPLVPGPELRPSAAELK	57.13031	Q9UGP4	LIMD1_HUMAN	724.733579	3	0.746641576		
LSPTSLVHPVMSTLPELSCK	78.16479	Q9UGP4	LIMD1_HUMAN	1098.574332	2	0.777771056	3	
LSPTSLVHPVMSTLPELSCK	78.16479	Q9UGP4	LIMD1_HUMAN	732.7188293	3	0.777771056	3	Yes
VDGAAKPPLAASTGAPGAVTTLAAGQPPYPPQE	66.92004	Q9UGP4	LIMD1_HUMAN	1799.94738	2	0.769652426		
VDGAAKPPLAASTGAPGAVTTLAAGQPPYPPQE	66.92004	Q9UGP4	LIMD1_HUMAN	1200.300861	3	0.769652426		
SSEGSLGGQNSGIGGR	-11.42194	Q9UGP4	LIMD1_HUMAN	731.848224	2	0.82320857	2	Yes
SSEGSLGGQNSGIGGR	-11.42194	Q9UGP4	LIMD1_HUMAN	488.2347577	3	0.82320857	2	
SSEKPTGLWSTASSQR	9.286118	Q9UGP4	LIMD1_HUMAN	861.4268385	2	0.852182388		
SSEKPTGLWSTASSQR	9.286118	Q9UGP4	LIMD1_HUMAN	574.6205007	3	0.852182388		
SSSAPSSSPAGLDGSQQGAVPGLGPKPGCTDLGT	58.95438	Q9UGP4	LIMD1_HUMAN	1893.418521	2	0.723143697		
SSSAPSSSPAGLDGSQQGAVPGLGPKPGCTDLGT	58.95438	Q9UGP4	LIMD1_HUMAN	1262.614956	3	0.723143697		
VFCEEDFLYSGFQQSADR	83.6073	Q9UGP4	LIMD1_HUMAN	1099.478939	2	0.768253505		
VFCEEDFLYSGFQQSADR	83.6073	Q9UGP4	LIMD1_HUMAN	733.3219007	3	0.768253505		
YDDLGLEASK	22.83774	Q9UGP4	LIMD1_HUMAN	555.7698555	2	0.783687592		
YDDLGLEASK	22.83774	Q9UGP4	LIMD1_HUMAN	370.8491787	3	0.783687592		
AGVSNIVAVHAAVTGLSVEEVVR	79.85928	Q9UGM6	SYWM_HUMAN	1139.132248	2	0.68557173		
AGVSNIVAVHAAVTGLSVEEVVR	79.85928	Q9UGM6	SYWM_HUMAN	759.7574403	3	0.68557173		
AVTDFTSEVTYDPAGR	51.99864	Q9UGM6	SYWM_HUMAN	864.907944	2	0.765414417		
AVTDFTSEVTYDPAGR	51.99864	Q9UGM6	SYWM_HUMAN	576.9412377	3	0.765414417		
ITDSPEEIVQK	17.33135	Q9UGM6	SYWM_HUMAN	629.830449	2	0.693999648		
ITDSPEEIVQK	17.33135	Q9UGM6	SYWM_HUMAN	420.2229077	3	0.693999648		
LAVADAVIEK	28.71431	Q9UGM6	SYWM_HUMAN	514.803506	2	0.815612495	2	Yes
LAVADAVIEK	28.71431	Q9UGM6	SYWM_HUMAN	343.538279	3	0.815612495	2	
LDKDHLEK	-50	Q9UGM6	SYWM_HUMAN	499.26983	2	0.600818396		
LDKDHLEK	-50	Q9UGM6	SYWM_HUMAN	333.182495	3	0.600818396		
STHVPVGEDQVQHMELVQDLAQGFNK	76.44627	Q9UGM6	SYWM_HUMAN	1453.709255	2	0.767576635	3	
STHVPVGEDQVQHMELVQDLAQGFNK	76.44627	Q9UGM6	SYWM_HUMAN	969.4754447	3	0.767576635	3	Yes
VFSGIQPTGILHLGNYLGAIESWVR	139.7903	Q9UGM6	SYWM_HUMAN	1364.235032	2	0.721924245		
VFSGIQPTGILHLGNYLGAIESWVR	139.7903	Q9UGM6	SYWM_HUMAN	909.825963	3	0.721924245		
YGEFFPVPESILTSMK	120.954	Q9UGM6	SYWM_HUMAN	922.961133	2	0.812374234		
YGEFFPVPESILTSMK	120.954	Q9UGM6	SYWM_HUMAN	615.6433637	3	0.812374234		
MCLEANIPLEK	50.3935	Q9UFW8	CGBP1_HUMAN	659.3312475	2	0.758760691		
MCLEANIPLEK	50.3935	Q9UFW8	CGBP1_HUMAN	439.8901067	3	0.758760691		
TALYVTPLDR	38.91659	Q9UFW8	CGBP1_HUMAN	574.8196835	2	0.833066523	2	Yes
TALYVTPLDR	38.91659	Q9UFW8	CGBP1_HUMAN	383.549064	3	0.833066523	2	
VSVIQDFVK	50.8487	Q9UFW8	CGBP1_HUMAN	517.7982235	2	0.709918678		
VSVIQDFVK	50.8487	Q9UFW8	CGBP1_HUMAN	345.5347573	3	0.709918678		
VTEFGGELHEDGGK	2.478416	Q9UFW8	CGBP1_HUMAN	737.8446195	2	0.757648826	3	
VTEFGGELHEDGGK	2.478416	Q9UFW8	CGBP1_HUMAN	492.2323547	3	0.757648826	3	Yes
ALTDPIQGTEYSAESLVR	69.67886	Q9UDY8	MALT1_HUMAN	975.4949145	2	0.828529477		
ALTDPIQGTEYSAESLVR	69.67886	Q9UDY8	MALT1_HUMAN	650.6658847	3	0.828529477		
APLVDVYELTNLLR	135.0216	Q9UDY8	MALT1_HUMAN	808.456875	2	0.786956608		
APLVDVYELTNLLR	135.0216	Q9UDY8	MALT1_HUMAN	539.3071917	3	0.786956608		
ATGHPFVQYQWFK	44.09998	Q9UDY8	MALT1_HUMAN	804.902071	2	0.783024073	3	
ATGHPFVQYQWFK	44.09998	Q9UDY8	MALT1_HUMAN	536.9373223	3	0.783024073	3	Yes
AVLAGQFVK	26.17365	Q9UDY8	MALT1_HUMAN	466.782377	2	0.713515222	2	Yes
AVLAGQFVK	26.17365	Q9UDY8	MALT1_HUMAN	311.524193	3	0.713515222	2	
GCTVTELSDFLQAMEHTEVLQLLSPPGIK	165.6834	Q9UDY8	MALT1_HUMAN	1607.312696	2	0.633213162		
GCTVTELSDFLQAMEHTEVLQLLSPPGIK	165.6834	Q9UDY8	MALT1_HUMAN	1071.877739	3	0.633213162		
GTPEETGSYLVSK	16.79173	Q9UDY8	MALT1_HUMAN	684.338634	2	0.813384056	2	Yes
GTPEETGSYLVSK	16.79173	Q9UDY8	MALT1_HUMAN	456.5616977	3	0.813384056	2	
ITVLLDEVAEDMGK	88.44997	Q9UDY8	MALT1_HUMAN	766.898006	2	0.762889743		
ITVLLDEVAEDMGK	88.44997	Q9UDY8	MALT1_HUMAN	511.601279	3	0.762889743		
LQICVEPTSQK	21.29352	Q9UDY8	MALT1_HUMAN	651.8402945	2	0.772374272		
LQICVEPTSQK	21.29352	Q9UDY8	MALT1_HUMAN	434.896138	3	0.772374272		
LSCLDLEQCSLK	44.18059	Q9UDY8	MALT1_HUMAN	733.355451	2	0.714232147		
LSCLDLEQCSLK	44.18059	Q9UDY8	MALT1_HUMAN	489.2395757	3	0.714232147		
LSELLDQAPEGR	33.31631	Q9UDY8	MALT1_HUMAN	664.3467975	2	0.776189089	2	Yes
LSELLDQAPEGR	33.31631	Q9UDY8	MALT1_HUMAN	443.2338067	3	0.776189089	2	
NDYDDTIPILDALK	99.81635	Q9UDY8	MALT1_HUMAN	803.404505	2	0.813426495		
NDYDDTIPILDALK	99.81635	Q9UDY8	MALT1_HUMAN	535.938945	3	0.813426495		
NELPLTHETK	-16.02648	Q9UDY8	MALT1_HUMAN	591.3122265	2	0.765056849		
NELPLTHETK	-16.02648	Q9UDY8	MALT1_HUMAN	394.5440927	3	0.765056849		
SENCLCVQNILK	49.17546	Q9UDY8	MALT1_HUMAN	739.3610675	2	0.69017607		
SENCLCVQNILK	49.17546	Q9UDY8	MALT1_HUMAN	493.24332	3	0.69017607		
SNVPVETTDEIPFSFSDR	79.80695	Q9UDY8	MALT1_HUMAN	1020.482008	2	0.848206222		
SNVPVETTDEIPFSFSDR	79.80695	Q9UDY8	MALT1_HUMAN	680.65728	3	0.848206222		
TDEAVECTEDELNNLGHPDNK	27.88776	Q9UDY8	MALT1_HUMAN	1200.516982	2	0.676021457	3	
TDEAVECTEDELNNLGHPDNK	27.88776	Q9UDY8	MALT1_HUMAN	800.680596	3	0.676021457	3	Yes
TPDAFISSFAHHASCHFSR	35.08025	Q9UDY8	MALT1_HUMAN	1087.995429	2	0.675699055		
TPDAFISSFAHHASCHFSR	35.08025	Q9UDY8	MALT1_HUMAN	725.6662273	3	0.675699055		
VALLIGNMNYR	56.05471	Q9UDY8	MALT1_HUMAN	632.3480845	2	0.886580706		
VALLIGNMNYR	56.05471	Q9UDY8	MALT1_HUMAN	421.9013313	3	0.886580706		
VLEPEGSPSLCLLK	64.39864	Q9UDY8	MALT1_HUMAN	771.4163655	2	0.78499347	2	Yes
VLEPEGSPSLCLLK	64.39864	Q9UDY8	MALT1_HUMAN	514.6135187	3	0.78499347	2	
VVSLLDLTEYEMR	95.93909	Q9UDY8	MALT1_HUMAN	784.4059935	2	0.888770461	3	
VVSLLDLTEYEMR	95.93909	Q9UDY8	MALT1_HUMAN	523.2732707	3	0.888770461	3	Yes
AHIHMLLEGLR	29.54068	Q9UBK9	UXT_HUMAN	645.361531	2	0.685328066		
AHIHMLLEGLR	29.54068	Q9UBK9	UXT_HUMAN	430.5769623	3	0.685328066		
ELQGLQNFPEKPHH	11.61694	Q9UBK9	UXT_HUMAN	837.4239025	2	0.800833464		
ELQGLQNFPEKPHH	11.61694	Q9UBK9	UXT_HUMAN	558.6185433	3	0.800833464		
SSLLTELSNSLTK	75.38609	Q9UBK9	UXT_HUMAN	696.883212	2	0.837467074		
SSLLTELSNSLTK	75.38609	Q9UBK9	UXT_HUMAN	464.9247497	3	0.837467074		

YETFISDVLQR	68.78488	Q9UBK9	UXT_HUMAN	685.8517115	2	0.827373981		
YETFISDVLQR	68.78488	Q9UBK9	UXT_HUMAN	457.570416	3	0.827373981		
AADTEEMDIEMDSGDEA	46.44917	Q9UBB5	MBD2_HUMAN	914.840953	2	0.744871497		
AADTEEMDIEMDSGDEA	46.44917	Q9UBB5	MBD2_HUMAN	610.2299103	3	0.744871497		
AFIVTDEDIR	40.22025	Q9UBB5	MBD2_HUMAN	589.806777	2	0.833344996		
AFIVTDEDIR	40.22025	Q9UBB5	MBD2_HUMAN	393.5404597	3	0.833344996		
GKPDLNTTLPIR	25.81201	Q9UBB5	MBD2_HUMAN	662.8833505	2	0.826250196	2	Yes
GKPDLNTTLPIR	25.81201	Q9UBB5	MBD2_HUMAN	442.2581753	3	0.826250196	2	
QGVGPGSNDETLLSAVASALHTSSAPITGQVSAA'	121.6801	Q9UBB5	MBD2_HUMAN	1860.458508	2	0.730979502		
QGVGPGSNDETLLSAVASALHTSSAPITGQVSAA'	121.6801	Q9UBB5	MBD2_HUMAN	1240.641613	3	0.730979502		
LQGLSASDVTEQIIK	58.32456	Q9UBB5	MBD2_HUMAN	801.4414265	2	0.810291409	2	Yes
LQGLSASDVTEQIIK	58.32456	Q9UBB5	MBD2_HUMAN	534.630226	3	0.810291409	2	
NPAVWLNTSQPLCK	50.32251	Q9UBB5	MBD2_HUMAN	814.4172315	2	0.815055966		
NPAVWLNTSQPLCK	50.32251	Q9UBB5	MBD2_HUMAN	543.2807627	3	0.815055966		
SDVYYFSPSGK	29.46569	Q9UBB5	MBD2_HUMAN	625.2909505	2	0.824192643	2	Yes
SDVYYFSPSGK	29.46569	Q9UBB5	MBD2_HUMAN	417.1965753	3	0.824192643	2	
YLGNTVDLSSFDFR	83.28476	Q9UBB5	MBD2_HUMAN	817.397014	2	0.721022666	2	Yes
YLGNTVDLSSFDFR	83.28476	Q9UBB5	MBD2_HUMAN	545.2672843	3	0.721022666	2	
ELQSQIQEAR	-6.215591	Q9P2X0	DPM3_HUMAN	601.312758	2	0.763668835	2	Yes
ELQSQIQEAR	-6.215591	Q9P2X0	DPM3_HUMAN	401.2111137	3	0.763668835	2	
VATFHDCEDAAR	-21.21286	Q9P2X0	DPM3_HUMAN	696.3046075	2	0.610141158	2	Yes
VATFHDCEDAAR	-21.21286	Q9P2X0	DPM3_HUMAN	464.5390133	3	0.610141158	2	
AFCADQLDVFLQK	80.15683	Q9P2N5	RBM27_HUMAN	777.8852335	2	0.715175748		
AFCADQLDVFLQK	80.15683	Q9P2N5	RBM27_HUMAN	518.9260973	3	0.715175748		
ALTVGGFIEEEK	52.1011	Q9P2N5	RBM27_HUMAN	646.840817	2	0.811674118		
ALTVGGFIEEEK	52.1011	Q9P2N5	RBM27_HUMAN	431.563153	3	0.811674118		
AMSGLEGPLTK	24.79311	Q9P2N5	RBM27_HUMAN	552.2924485	2	0.848024309	2	Yes
AMSGLEGPLTK	24.79311	Q9P2N5	RBM27_HUMAN	368.5309073	3	0.848024309	2	
DQPGTSAVPNLASVGTR	46.12032	Q9P2N5	RBM27_HUMAN	835.429382	2	0.742952108		
DQPGTSAVPNLASVGTR	46.12032	Q9P2N5	RBM27_HUMAN	557.288863	3	0.742952108		
EDLLQHFSTANQGPK	31.61547	Q9P2N5	RBM27_HUMAN	842.91865	2	0.729862571		
EDLLQHFSTANQGPK	31.61547	Q9P2N5	RBM27_HUMAN	562.2817083	3	0.729862571		
ELLDTELDLHK	43.35398	Q9P2N5	RBM27_HUMAN	663.3515485	2	0.833981514		
ELLDTELDLHK	43.35398	Q9P2N5	RBM27_HUMAN	442.5703073	3	0.833981514		
FGTIVNIQVAFK	81.08458	Q9P2N5	RBM27_HUMAN	668.885362	2	0.6925897		
FGTIVNIQVAFK	81.08458	Q9P2N5	RBM27_HUMAN	446.2595163	3	0.6925897		
GDPEAALIQYLTNEEAR	108.5664	Q9P2N5	RBM27_HUMAN	945.466157	2	0.838092148	3	
GDPEAALIQYLTNEEAR	108.5664	Q9P2N5	RBM27_HUMAN	630.646713	3	0.838092148	3	Yes
LFESLYTK	36.88933	Q9P2N5	RBM27_HUMAN	500.77167	2	0.695177436		
LFESLYTK	36.88933	Q9P2N5	RBM27_HUMAN	334.1837217	3	0.695177436		
LGILPVGR	42.62767	Q9P2N5	RBM27_HUMAN	412.771812	2	0.769163132		
LGILPVGR	42.62767	Q9P2N5	RBM27_HUMAN	275.5171497	3	0.769163132		
LLEPICDADPSALANYVVALVK	154.8301	Q9P2N5	RBM27_HUMAN	1186.133064	2	0.601227999		
LLEPICDADPSALANYVVALVK	154.8301	Q9P2N5	RBM27_HUMAN	791.0913173	3	0.601227999		
LPPPLPQNLLYTVSER	96.52734	Q9P2N5	RBM27_HUMAN	919.0150885	2	0.775581956		
LPPPLPQNLLYTVSER	96.52734	Q9P2N5	RBM27_HUMAN	613.0126673	3	0.775581956		
LPVPQGHGQPPPSVVLPIPRPPITQSSLINSR	79.18875	Q9P2N5	RBM27_HUMAN	1689.954616	2	0.689282537		
LPVPQGHGQPPPSVVLPIPRPPITQSSLINSR	79.18875	Q9P2N5	RBM27_HUMAN	1126.972352	3	0.689282537		
LQISWHKPK	-5.600307	Q9P2N5	RBM27_HUMAN	568.832932	2	0.768896699		
LQISWHKPK	-5.600307	Q9P2N5	RBM27_HUMAN	379.5578963	3	0.768896699		
LQLGTPPPLLAAR	65.28795	Q9P2N5	RBM27_HUMAN	673.9119115	2	0.797471404		
LQLGTPPPLLAAR	65.28795	Q9P2N5	RBM27_HUMAN	449.6105493	3	0.797471404		
LSQLQVEAAR	9.802044	Q9P2N5	RBM27_HUMAN	557.8149365	2	0.78657037	2	Yes
LSQLQVEAAR	9.802044	Q9P2N5	RBM27_HUMAN	372.212566	3	0.78657037	2	
LSSGEDTTELR	-5.67556	Q9P2N5	RBM27_HUMAN	604.2942305	2	0.759865761	2	Yes
LSSGEDTTELR	-5.67556	Q9P2N5	RBM27_HUMAN	403.198762	3	0.759865761	2	
MLIEDVDALK	57.97975	Q9P2N5	RBM27_HUMAN	573.807931	2	0.601521373		
MLIEDVDALK	57.97975	Q9P2N5	RBM27_HUMAN	382.8745623	3	0.601521373		
MMSKPQTSGAYVLNK	4.153431	Q9P2N5	RBM27_HUMAN	827.9187455	2	0.749197602		
MMSKPQTSGAYVLNK	4.153431	Q9P2N5	RBM27_HUMAN	552.281772	3	0.749197602		
NYLPLLEPVKPEPKPLVQEK	55.72988	Q9P2N5	RBM27_HUMAN	1166.170306	2	0.794885695		
NYLPLLEPVKPEPKPLVQEK	55.72988	Q9P2N5	RBM27_HUMAN	777.7828123	3	0.794885695		
TQTQRPNLIGLTSGDMDVNPR	46.5045	Q9P2N5	RBM27_HUMAN	1157.084973	2	0.687761188		
TQTQRPNLIGLTSGDMDVNPR	46.5045	Q9P2N5	RBM27_HUMAN	771.7259233	3	0.687761188		
DLEASHQHSSPNEQLK	-25.08176	Q9P2B4	CT2NL_HUMAN	910.432652	2	0.660244584		
DLEASHQHSSPNEQLK	-25.08176	Q9P2B4	CT2NL_HUMAN	607.291043	3	0.660244584		
DLSPTLIDNSAAK	43.35588	Q9P2B4	CT2NL_HUMAN	672.854455	2	0.847082138		
DLSPTLIDNSAAK	43.35588	Q9P2B4	CT2NL_HUMAN	448.9055783	3	0.847082138		
DLVIEALK	56.01728	Q9P2B4	CT2NL_HUMAN	450.774217	2	0.666210473		
DLVIEALK	56.01728	Q9P2B4	CT2NL_HUMAN	300.8520863	3	0.666210473		
ELTAGNNVENQVPPR	15.96868	Q9P2B4	CT2NL_HUMAN	819.416274	2	0.780924499		
ELTAGNNVENQVPPR	15.96868	Q9P2B4	CT2NL_HUMAN	546.6134577	3	0.780924499		
FQSQADQDQQASGLQSPPSR	7.830536	Q9P2B4	CT2NL_HUMAN	1088.007245	2	0.778403163		
FQSQADQDQQASGLQSPPSR	7.830536	Q9P2B4	CT2NL_HUMAN	725.674105	3	0.778403163		
FTSQQGPIKPVSPNSSPFGTDYR	43.83462	Q9P2B4	CT2NL_HUMAN	1255.619698	2	0.763167918		
FTSQQGPIKPVSPNSSPFGTDYR	43.83462	Q9P2B4	CT2NL_HUMAN	837.41574	3	0.763167918		
GLQTEAQVEK	-17.39045	Q9P2B4	CT2NL_HUMAN	551.7911275	2	0.672155321		
GLQTEAQVEK	-17.39045	Q9P2B4	CT2NL_HUMAN	368.1966933	3	0.672155321		
GTATEPLMLMSVFCQTESFPAER	130.5298	Q9P2B4	CT2NL_HUMAN	1301.603493	2	0.660857379		
GTATEPLMLMSVFCQTESFPAER	130.5298	Q9P2B4	CT2NL_HUMAN	868.0716033	3	0.660857379		
HAQDTAEGDDVTYMLEK	25.63794	Q9P2B4	CT2NL_HUMAN	961.926008	2	0.690077662		
HAQDTAEGDDVTYMLEK	25.63794	Q9P2B4	CT2NL_HUMAN	641.619947	3	0.690077662		
KPGLTPSPSATTPLTK	7.829262	Q9P2B4	CT2NL_HUMAN	798.4543375	2	0.845240355		
KPGLTPSPSATTPLTK	7.829262	Q9P2B4	CT2NL_HUMAN	532.6388333	3	0.845240355		
LLGSSASSPGYQSSYQVGINQR	40.17101	Q9P2B4	CT2NL_HUMAN	1150.069836	2	0.761772215		
LLGSSASSPGYQSSYQVGINQR	40.17101	Q9P2B4	CT2NL_HUMAN	767.0491653	3	0.761772215		
LTQQLEFEK	18.34002	Q9P2B4	CT2NL_HUMAN	568.3038705	2	0.833992422		
LTQQLEFEK	18.34002	Q9P2B4	CT2NL_HUMAN	379.2051887	3	0.833992422		
MLSQLAAAESR	19.19805	Q9P2B4	CT2NL_HUMAN	588.806254	2	0.769652069		
MLSQLAAAESR	19.19805	Q9P2B4	CT2NL_HUMAN	392.8734443	3	0.769652069		
MTNTGLPGPATPAYSYAK	42.62506	Q9P2B4	CT2NL_HUMAN	920.4514605	2	0.741514564		
MTNTGLPGPATPAYSYAK	42.62506	Q9P2B4	CT2NL_HUMAN	613.9702487	3	0.741514564		
VILDLEEER	37.37653	Q9P2B4	CT2NL_HUMAN	558.301327	2	0.7612288		
VILDLEEER	37.37653	Q9P2B4	CT2NL_HUMAN	372.5368263	3	0.7612288		
VSSPLSPLSPGIK	49.50887	Q9P2B4	CT2NL_HUMAN	641.374826	2	0.807029426		
VSSPLSPLSPGIK	49.50887	Q9P2B4	CT2NL_HUMAN	427.919159	3	0.807029426		
YNISDPLMALQR	72.99387	Q9P2B4	CT2NL_HUMAN	710.8668385	2	0.836388528	2	Yes
YNISDPLMALQR	72.99387	Q9P2B4	CT2NL_HUMAN	474.2471673	3	0.836388528	2	
ADVLSGENAYMCAK	31.10432	Q9P275	UBP36_HUMAN	764.8427035	2	0.734474123		
ADVLSGENAYMCAK	31.10432	Q9P275	UBP36_HUMAN	510.2310773	3	0.734474123		
ALELFVK	46.71992	Q9P275	UBP36_HUMAN	410.2529205	2	0.703601897	2	Yes

ALELFVK	46.71992	Q9P275	UBP36_HUMAN	273.8378887	3	0.703601897	2	
ATGNDLRPPPPSPSSDLTHPMK	22.7752	Q9P275	UBP36_HUMAN	1158.076616	2	0.694196284		
ATGNDLRPPPPSPSSDLTHPMK	22.7752	Q9P275	UBP36_HUMAN	772.3870187	3	0.694196284		
DVVLSTSPK	8.022926	Q9P275	UBP36_HUMAN	473.266756	2	0.775867701		
DVVLSTSPK	8.022926	Q9P275	UBP36_HUMAN	315.8471123	3	0.775867701		
EGQAQLPAVR	7.677246	Q9P275	UBP36_HUMAN	534.7940045	2	0.684118032		
EGQAQLPAVR	7.677246	Q9P275	UBP36_HUMAN	356.865278	3	0.684118032		
ESDVVQELLK	56.26452	Q9P275	UBP36_HUMAN	580.3144345	2	0.781139493	2	Yes
ESDVVQELLK	56.26452	Q9P275	UBP36_HUMAN	387.2122313	3	0.781139493	2	
FGNQEDAHEFLR	15.56693	Q9P275	UBP36_HUMAN	731.839671	2	0.855340779	3	
FGNQEDAHEFLR	15.56693	Q9P275	UBP36_HUMAN	488.2290557	3	0.855340779	3	Yes
GAEGLGEEGGLHQDPLR	18.90489	Q9P275	UBP36_HUMAN	867.924464	2	0.740622044	3	
GAEGLGEEGGLHQDPLR	18.90489	Q9P275	UBP36_HUMAN	578.952251	3	0.740622044	3	Yes
LQPPFSPHPTLLSSTPKPPGTSEPR	46.7119	Q9P275	UBP36_HUMAN	1334.708852	2	0.712318182		
LQPPFSPHPTLLSSTPKPPGTSEPR	46.7119	Q9P275	UBP36_HUMAN	890.1418427	3	0.712318182		
LSQTPTHMPTILDDPGK	36.03389	Q9P275	UBP36_HUMAN	925.970026	2	0.804962277		
LSQTPTHMPTILDDPGK	36.03389	Q9P275	UBP36_HUMAN	617.6492923	3	0.804962277		
MGLSQAPPVSWNGER	48.228	Q9P275	UBP36_HUMAN	814.896663	2	0.761765778		
MGLSQAPPVSWNGER	48.228	Q9P275	UBP36_HUMAN	543.6003837	3	0.761765778		
MSAVSQDAIEDSR	13.12152	Q9P275	UBP36_HUMAN	704.822829	2	0.802829385	2	Yes
MSAVSQDAIEDSR	13.12152	Q9P275	UBP36_HUMAN	470.2178277	3	0.802829385	2	
NFWSVTHPAK	14.93029	Q9P275	UBP36_HUMAN	593.8043715	2	0.712580621		
NFWSVTHPAK	14.93029	Q9P275	UBP36_HUMAN	396.2055227	3	0.712580621		
RPEDTAASALQEGQTQR	-5.139378	Q9P275	UBP36_HUMAN	929.45666	2	0.778678238		
RPEDTAASALQEGQTQR	-5.139378	Q9P275	UBP36_HUMAN	619.973715	3	0.778678238		
SFSYQLEALK	47.89079	Q9P275	UBP36_HUMAN	593.3116905	2	0.774026692	2	Yes
SFSYQLEALK	47.89079	Q9P275	UBP36_HUMAN	395.8770687	3	0.774026692	2	
SPVLSNTTTEPASTMSPPPAK	31.57642	Q9P275	UBP36_HUMAN	1057.028269	2	0.772600532		
SPVLSNTTTEPASTMSPPPAK	31.57642	Q9P275	UBP36_HUMAN	705.021454	3	0.772600532		
TAQGLPGTSNSNSSR	-23.21037	Q9P275	UBP36_HUMAN	738.856049	2	0.617851138		
TAQGLPGTSNSNSSR	-23.21037	Q9P275	UBP36_HUMAN	492.906641	3	0.617851138		
TETVVDDWDEEFDR	62.61948	Q9P275	UBP36_HUMAN	878.371395	2	0.800385714	2	Yes
TETVVDDWDEEFDR	62.61948	Q9P275	UBP36_HUMAN	585.9168717	3	0.800385714	2	
TSHPVVASTWPVHR	6.216568	Q9P275	UBP36_HUMAN	787.4158805	2	0.840615392	4	
TSHPVVASTWPVHR	6.216568	Q9P275	UBP36_HUMAN	525.279862	3	0.840615392	4	
TSNVLTLSLK	44.33723	Q9P275	UBP36_HUMAN	538.3220625	2	0.834524572		
TSNVLTLSLK	44.33723	Q9P275	UBP36_HUMAN	359.2173167	3	0.834524572		
VLFPTER	20.74168	Q9P275	UBP36_HUMAN	431.245627	2	0.626381934		
VLFPTER	20.74168	Q9P275	UBP36_HUMAN	287.8330263	3	0.626381934		
VLTWDGK	12.71774	Q9P275	UBP36_HUMAN	409.7245275	2	0.623746037		
VLTWDGK	12.71774	Q9P275	UBP36_HUMAN	273.4856267	3	0.623746037		
VVLNQQAYVLFYLR	101.6984	Q9P275	UBP36_HUMAN	863.4885055	2	0.649672329		
VVLNQQAYVLFYLR	101.6984	Q9P275	UBP36_HUMAN	575.9949453	3	0.649672329		
YTIDAMQK	6.069756	Q9P275	UBP36_HUMAN	485.2396805	2	0.670244634		
YTIDAMQK	6.069756	Q9P275	UBP36_HUMAN	323.829062	3	0.670244634		
YVLLNPK	18.992	Q9P275	UBP36_HUMAN	423.7583655	2	0.649199128	2	Yes
YVLLNPK	18.992	Q9P275	UBP36_HUMAN	282.8415187	3	0.649199128	2	
AACILTSQTLMR	41.35015	Q9P265	DIP2B_HUMAN	682.855422	2	0.812084198		
AACILTSQTLMR	41.35015	Q9P265	DIP2B_HUMAN	455.5728897	3	0.812084198		
AQLAELELELSEGDITQK	88.81883	Q9P265	DIP2B_HUMAN	994.015684	2	0.845149398		
AQLAELELELSEGDITQK	88.81883	Q9P265	DIP2B_HUMAN	663.0130643	3	0.845149398		
DAGLWHGMFANVMNK	73.66259	Q9P265	DIP2B_HUMAN	845.895609	2	0.633627236		
DAGLWHGMFANVMNK	73.66259	Q9P265	DIP2B_HUMAN	564.2663477	3	0.633627236		
DIGLSPR	10.81191	Q9P265	DIP2B_HUMAN	379.2143265	2	0.658224881		
DIGLSPR	10.81191	Q9P265	DIP2B_HUMAN	253.1454927	3	0.658224881		
DLGQIEENDLVR	50.55463	Q9P265	DIP2B_HUMAN	700.8549865	2	0.856778979		
DLGQIEENDLVR	50.55463	Q9P265	DIP2B_HUMAN	467.5725993	3	0.856778979		
DLHWAMMAHR	27.5156	Q9P265	DIP2B_HUMAN	634.295334	2	0.645362735		
DLHWAMMAHR	27.5156	Q9P265	DIP2B_HUMAN	423.1994977	3	0.645362735		
DWQPHISPAGTEPAYIEYK	54.34072	Q9P265	DIP2B_HUMAN	1101.529285	2	0.764575303	3	
DWQPHISPAGTEPAYIEYK	54.34072	Q9P265	DIP2B_HUMAN	734.688798	3	0.764575303	3	Yes
EFFVDDSEEIVEVPQPDPNQPKPEGR	71.30176	Q9P265	DIP2B_HUMAN	1499.209806	2	0.72589612	3	
EFFVDDSEEIVEVPQPDPNQPKPEGR	71.30176	Q9P265	DIP2B_HUMAN	999.8091457	3	0.72589612	3	Yes
GAPQSLLLSESGK	34.39476	Q9P265	DIP2B_HUMAN	643.8517155	2	0.831868947	2	Yes
GAPQSLLLSESGK	34.39476	Q9P265	DIP2B_HUMAN	429.5704187	3	0.831868947	2	
GLEPSPAAVAALPPEVR	66.24463	Q9P265	DIP2B_HUMAN	837.465236	2	0.819165707		
GLEPSPAAVAALPPEVR	66.24463	Q9P265	DIP2B_HUMAN	558.646099	3	0.819165707		
GLGNQVEVLK	22.07214	Q9P265	DIP2B_HUMAN	528.80658	2	0.823541164		
GLGNQVEVLK	22.07214	Q9P265	DIP2B_HUMAN	352.8736617	3	0.823541164		
[illegible]GDSHLGEIWVNSPHTASGYYTIYDSETLQADHF[illegible]	71.87679	Q9P265	DIP2B_HUMAN	2168.00486	2	0.766916096		
[illegible]GDSHLGEIWVNSPHTASGYYTIYDSETLQADHF[illegible]	71.87679	Q9P265	DIP2B_HUMAN	1445.672515	3	0.766916096		
GTSGSLADVFANTR	47.96014	Q9P265	DIP2B_HUMAN	698.347329	2	0.769022822	2	Yes
GTSGSLADVFANTR	47.96014	Q9P265	DIP2B_HUMAN	465.9008277	3	0.769022822	2	
GTTVCTASCLQLHK	3.57793	Q9P265	DIP2B_HUMAN	788.3850755	2	0.638800263		
GTTVCTASCLQLHK	3.57793	Q9P265	DIP2B_HUMAN	525.925992	3	0.638800263		
HQFLAEILQWR	80.44321	Q9P265	DIP2B_HUMAN	720.89151	2	0.652120948		
HQFLAEILQWR	80.44321	Q9P265	DIP2B_HUMAN	480.9302817	3	0.652120948		
IAVFSVSVFYDER	88.52895	Q9P265	DIP2B_HUMAN	766.393743	2	0.717102408		
IAVFSVSVFYDER	88.52895	Q9P265	DIP2B_HUMAN	511.2651037	3	0.717102408		
LLSPYSPQTQETDSAVQK	32.05163	Q9P265	DIP2B_HUMAN	996.500197	2	0.809278727	3	
LLSPYSPQTQETDSAVQK	32.05163	Q9P265	DIP2B_HUMAN	664.6694063	3	0.809278727	3	Yes
LSFGDAAQTLWAR	80.74651	Q9P265	DIP2B_HUMAN	718.3706075	2	0.875763416	2	Yes
LSFGDAAQTLWAR	80.74651	Q9P265	DIP2B_HUMAN	479.24968	3	0.875763416	2	
MDGLLMVSGR	51.34108	Q9P265	DIP2B_HUMAN	539.7733685	2	0.720860481	2	Yes
MDGLLMVSGR	51.34108	Q9P265	DIP2B_HUMAN	360.184854	3	0.720860481	2	
MHTISVPYSVMK	33.55693	Q9P265	DIP2B_HUMAN	696.8548855	2	0.759691834		
MHTISVPYSVMK	33.55693	Q9P265	DIP2B_HUMAN	464.9058653	3	0.759691834		
NEPVLKPGDR	-23.212	Q9P265	DIP2B_HUMAN	562.807111	2	0.636601567		
NEPVLKPGDR	-23.212	Q9P265	DIP2B_HUMAN	375.5406823	3	0.636601567		
NTFEVIPVNSAGSPVGDVPFIR	107.0335	Q9P265	DIP2B_HUMAN	1158.105699	2	0.719055474		
NTFEVIPVNSAGSPVGDVPFIR	107.0335	Q9P265	DIP2B_HUMAN	772.4064073	3	0.719055474		
RHNADDIVATGLAVESIK	38.41657	Q9P265	DIP2B_HUMAN	955.01107	2	0.846380949		
RHNADDIVATGLAVESIK	38.41657	Q9P265	DIP2B_HUMAN	637.0099883	3	0.846380949		
RPGVPGAPLPGR	7.174747	Q9P265	DIP2B_HUMAN	587.3467385	2	0.777339935		
RPGVPGAPLPGR	7.174747	Q9P265	DIP2B_HUMAN	391.900434	3	0.777339935		
SDIHTEAVQAALAK	20.37119	Q9P265	DIP2B_HUMAN	727.3864545	2	0.60911715	3	
SDIHTEAVQAALAK	20.37119	Q9P265	DIP2B_HUMAN	485.2602447	3	0.60911715	3	Yes
SSLMDTADGVPVSSR	37.51595	Q9P265	DIP2B_HUMAN	761.364861	2	0.78109175		
SSLMDTADGVPVSSR	37.51595	Q9P265	DIP2B_HUMAN	507.9125157	3	0.78109175		
STFVQSPADACTPPDTSSASEDEGSLR	40.92651	Q9P265	DIP2B_HUMAN	1406.614882	2	0.649023116		
STFVQSPADACTPPDTSSASEDEGSLR	40.92651	Q9P265	DIP2B_HUMAN	938.079196	3	0.649023116		

TCPLSWVQR	38.30914	Q9P265	DIP2B_HUMAN	573.7904075	2	0.742221832		
TCPLSWVQR	38.30914	Q9P265	DIP2B_HUMAN	382.86288	3	0.742221832		
TDEIGEICVSSR	29.40645	Q9P265	DIP2B_HUMAN	683.3199225	2	0.654847383	2	Yes
TDEIGEICVSSR	29.40645	Q9P265	DIP2B_HUMAN	455.8825567	3	0.654847383	2	
TGGMMYFGLAGVTK	74.4477	Q9P265	DIP2B_HUMAN	716.852344	2	0.739905894		
TGGMMYFGLAGVTK	74.4477	Q9P265	DIP2B_HUMAN	478.2375043	3	0.739905894		
TGYLGFVR	35.6834	Q9P265	DIP2B_HUMAN	456.7510725	2	0.623767257	2	Yes
TGYLGFVR	35.6834	Q9P265	DIP2B_HUMAN	304.8366567	3	0.623767257	2	
TPLGGIHISQTK	5.838127	Q9P265	DIP2B_HUMAN	626.356969	2	0.77979219		
TPLGGIHISQTK	5.838127	Q9P265	DIP2B_HUMAN	417.9072543	3	0.77979219		
TWPTIIDTDDLPR	77.88387	Q9P265	DIP2B_HUMAN	771.8941125	2	0.856225967		
TWPTIIDTDDLPR	77.88387	Q9P265	DIP2B_HUMAN	514.9320167	3	0.856225967		
VALQQSFSK	2.186348	Q9P265	DIP2B_HUMAN	504.280198	2	0.73538661		
VALQQSFSK	2.186348	Q9P265	DIP2B_HUMAN	336.5227403	3	0.73538661		
VVIVNPETK	6.865929	Q9P265	DIP2B_HUMAN	499.7982235	2	0.747482061	2	Yes
VVIVNPETK	6.865929	Q9P265	DIP2B_HUMAN	333.5347573	3	0.747482061	2	
YHPIDIETSVSR	17.72311	Q9P265	DIP2B_HUMAN	708.8600675	2	0.667830765	3	
YHPIDIETSVSR	17.72311	Q9P265	DIP2B_HUMAN	472.90932	3	0.667830765	3	Yes
YLSKPPK	-28.54283	Q9P265	DIP2B_HUMAN	416.7505405	2	0.626368821		
YLSKPPK	-28.54283	Q9P265	DIP2B_HUMAN	278.1696353	3	0.626368821		
AAEHEVPLQER	-20.98271	Q9P260	RELCH_HUMAN	639.8260325	2	0.815296471		
AAEHEVPLQER	-20.98271	Q9P260	RELCH_HUMAN	426.88663	3	0.815296471		
AGSISTLDSLDFAR	63.76231	Q9P260	RELCH_HUMAN	726.8706365	2	0.825594842		
AGSISTLDSLDFAR	63.76231	Q9P260	RELCH_HUMAN	484.916366	3	0.825594842		
ALNFLVNEFLLK	117.3089	Q9P260	RELCH_HUMAN	710.9141185	2	0.79460299		
ALNFLVNEFLLK	117.3089	Q9P260	RELCH_HUMAN	474.2786873	3	0.79460299		
DEFVIPHLHK	25.27249	Q9P260	RELCH_HUMAN	617.833129	2	0.749366403		
DEFVIPHLHK	25.27249	Q9P260	RELCH_HUMAN	412.2246943	3	0.749366403		
DFGNHQVTGK	-27.08478	Q9P260	RELCH_HUMAN	551.7679865	2	0.725147903		
DFGNHQVTGK	-27.08478	Q9P260	RELCH_HUMAN	368.181266	3	0.725147903		
DQYLLTALELHTELLESGR	114.7609	Q9P260	RELCH_HUMAN	1101.076603	2	0.656557381	3	
DQYLLTALELHTELLESGR	114.7609	Q9P260	RELCH_HUMAN	734.3870103	3	0.656557381	3	Yes
DYFSNPGNFER	43.65701	Q9P260	RELCH_HUMAN	673.29456	2	0.85534519		
DYFSNPGNFER	43.65701	Q9P260	RELCH_HUMAN	449.1989817	3	0.85534519		
EPSTASGGGQLNR	-27.52044	Q9P260	RELCH_HUMAN	637.3107465	2	0.683651149		
EPSTASGGGQLNR	-27.52044	Q9P260	RELCH_HUMAN	425.2097727	3	0.683651149		
FPRPMSPLQDVSTIIGSR	82.77634	Q9P260	RELCH_HUMAN	1001.033491	2	0.644242883		
FPRPMSPLQDVSTIIGSR	82.77634	Q9P260	RELCH_HUMAN	667.6916023	3	0.644242883		
IATIPAFGTIMETVIQR	129.9108	Q9P260	RELCH_HUMAN	931.0167795	2	0.837421775		
IATIPAFGTIMETVIQR	129.9108	Q9P260	RELCH_HUMAN	621.0137947	3	0.837421775		
INVTSTACVHEFSR	16.30746	Q9P260	RELCH_HUMAN	810.8941205	2	0.708865523		
INVTSTACVHEFSR	16.30746	Q9P260	RELCH_HUMAN	540.932022	3	0.708865523		
ISLLNSEK	13.13557	Q9P260	RELCH_HUMAN	452.261473	2	0.726669192	2	Yes
ISLLNSEK	13.13557	Q9P260	RELCH_HUMAN	301.8435903	3	0.726669192	2	
LALVNNLQIVDSK	61.66229	Q9P260	RELCH_HUMAN	713.9173895	2	0.848749459		
LALVNNLQIVDSK	61.66229	Q9P260	RELCH_HUMAN	476.280868	3	0.848749459		
LHGEVPQIEVTR	15.55505	Q9P260	RELCH_HUMAN	689.3784325	2	0.85485363		
LHGEVPQIEVTR	15.55505	Q9P260	RELCH_HUMAN	459.9215633	3	0.85485363		
LLVAESCGALAPYLPK	73.42387	Q9P260	RELCH_HUMAN	851.466386	2	0.765860975		
LLVAESCGALAPYLPK	73.42387	Q9P260	RELCH_HUMAN	567.980199	3	0.765860975		
LSEENIDSSAGNGVLTK	26.0927	Q9P260	RELCH_HUMAN	867.431786	2	0.804335475		
LSEENIDSSAGNGVLTK	26.0927	Q9P260	RELCH_HUMAN	578.623799	3	0.804335475		
LSIDAIAAQLLR	113.1673	Q9P260	RELCH_HUMAN	642.3882685	2	0.786198378		
LSIDAIAAQLLR	113.1673	Q9P260	RELCH_HUMAN	428.5947873	3	0.786198378		
LSPAFHQALLSFCR	52.2039	Q9P260	RELCH_HUMAN	823.9277665	2	0.709826648		
LSPAFHQALLSFCR	52.2039	Q9P260	RELCH_HUMAN	549.6211193	3	0.709826648		
MGQLTTSGAMLANVFQR	79.37053	Q9P260	RELCH_HUMAN	912.9589385	2	0.690146685		
MGQLTTSGAMLANVFQR	79.37053	Q9P260	RELCH_HUMAN	608.975234	3	0.690146685		
MQLASFLEDPQYQDQHSLHTEIIK	71.93637	Q9P260	RELCH_HUMAN	1436.203269	2	0.672313809		
MQLASFLEDPQYQDQHSLHTEIIK	71.93637	Q9P260	RELCH_HUMAN	957.8047877	3	0.672313809		
NTDIHLSISDEADSTIPK	45.2435	Q9P260	RELCH_HUMAN	978.4820075	2	0.745288014		
NTDIHLSISDEADSTIPK	45.2435	Q9P260	RELCH_HUMAN	652.65728	3	0.745288014		
SSPEIQEPIKPLEK	25.30755	Q9P260	RELCH_HUMAN	797.938519	2	0.851704478		
SSPEIQEPIKPLEK	25.30755	Q9P260	RELCH_HUMAN	532.2949543	3	0.851704478		
TDMEHLSPEHEVILSSMIK	60.48213	Q9P260	RELCH_HUMAN	1098.537946	2	0.72189343		
TDMEHLSPEHEVILSSMIK	60.48213	Q9P260	RELCH_HUMAN	732.694572	3	0.72189343		
TVQEPQGSMSIAASLVSEDTK	64.20757	Q9P260	RELCH_HUMAN	1089.533915	2	0.706173062		
TVQEPQGSMSIAASLVSEDTK	64.20757	Q9P260	RELCH_HUMAN	726.691885	3	0.706173062		
VAPALVTLSSDPEFSVR	76.46483	Q9P260	RELCH_HUMAN	894.4810825	2	0.762902856		
VAPALVTLSSDPEFSVR	76.46483	Q9P260	RELCH_HUMAN	596.6566633	3	0.762902856		
VAVLEFELR	67.44659	Q9P260	RELCH_HUMAN	538.311498	2	0.803939939		
VAVLEFELR	67.44659	Q9P260	RELCH_HUMAN	359.2102737	3	0.803939939		
VEAELLPQCWEQINHK	57.6401	Q9P260	RELCH_HUMAN	997.494199	2	0.644970417	3	
VEAELLPQCWEQINHK	57.6401	Q9P260	RELCH_HUMAN	665.3320743	3	0.644970417	3	Yes
VGPNAEPR	-29.306	Q9P260	RELCH_HUMAN	420.222683	2	0.607143104		
VGPNAEPR	-29.306	Q9P260	RELCH_HUMAN	280.484397	3	0.607143104		
VKPQFQEILR	26.36524	Q9P260	RELCH_HUMAN	629.369879	2	0.833678365	3	
VKPQFQEILR	26.36524	Q9P260	RELCH_HUMAN	419.915861	3	0.833678365	3	Yes
WSLMEQIR	51.31984	Q9P260	RELCH_HUMAN	531.7742255	2	0.738683462		
WSLMEQIR	51.31984	Q9P260	RELCH_HUMAN	354.852092	3	0.738683462		
AGGAAVVITEPEHTK	0.571125	Q9P258	RCC2_HUMAN	740.39428	2	0.811711729	3	
AGGAAVVITEPEHTK	0.571125	Q9P258	RCC2_HUMAN	493.9321283	3	0.811711729	3	Yes
AVQDLCGWR	25.39342	Q9P258	RCC2_HUMAN	552.7669325	2	0.725043893	2	Yes
AVQDLCGWR	25.39342	Q9P258	RCC2_HUMAN	368.84723	3	0.725043893	2	
DGQILPVPNVVVR	78.79025	Q9P258	RCC2_HUMAN	703.412275	2	0.828117192	2	Yes
DGQILPVPNVVVR	78.79025	Q9P258	RCC2_HUMAN	469.2774583	3	0.828117192	2	
GNLYSFGCPEYGQLGHNSDGK	42.99833	Q9P258	RCC2_HUMAN	1150.506015	2	0.759526491	3	
GNLYSFGCPEYGQLGHNSDGK	42.99833	Q9P258	RCC2_HUMAN	767.3399513	3	0.759526491	3	Yes
IEYDCELVPR	35.90738	Q9P258	RCC2_HUMAN	647.311365	2	0.832261682	2	Yes
IEYDCELVPR	35.90738	Q9P258	RCC2_HUMAN	431.8768517	3	0.832261682	2	
LFDFPGR	47.48524	Q9P258	RCC2_HUMAN	426.2246945	2	0.684317052	2	Yes
LFDFPGR	47.48524	Q9P258	RCC2_HUMAN	284.485738	3	0.684317052	2	
LIEGLSHEVIVSAACGR	49.99735	Q9P258	RCC2_HUMAN	905.9781845	2	0.7333076	3	
LIEGLSHEVIVSAACGR	49.99735	Q9P258	RCC2_HUMAN	604.321398	3	0.7333076	3	Yes
LPEYNPR	-3.962448	Q9P258	RCC2_HUMAN	444.732879	2	0.601859391		
LPEYNPR	-3.962448	Q9P258	RCC2_HUMAN	296.8245277	3	0.601859391		
NHTLALTETGSVFAFGENK	57.41279	Q9P258	RCC2_HUMAN	1018.508361	2	0.664141059	3	
NHTLALTETGSVFAFGENK	57.41279	Q9P258	RCC2_HUMAN	679.3415153	3	0.664141059	3	Yes
NLGQNLWGPHR	21.69711	Q9P258	RCC2_HUMAN	646.3369015	2	0.815840483	3	
NLGQNLWGPHR	21.69711	Q9P258	RCC2_HUMAN	431.2272093	3	0.815840483	3	Yes
SSIIVAADESTISWGPSPTFGELGYGDHKPK	79.49385	Q9P258	RCC2_HUMAN	1623.801858	2	0.637581706		

SSIIVAADESTISWGPSPTFGELGYGDHKPK	79.49385	Q9P258	RCC2_HUMAN	1082.870514	3	0.637581706		
TLDGIFSEQVAMGYSHSLVIAR	84.59685	Q9P258	RCC2_HUMAN	1197.610287	2	0.663383901	3	
TLDGIFSEQVAMGYSHSLVIAR	84.59685	Q9P258	RCC2_HUMAN	798.7427997	3	0.663383901	3	Yes
TVVSGSCAAHSLLITTEGK	31.44819	Q9P258	RCC2_HUMAN	965.9993145	2	0.684904814	3	
TVVSGSCAAHSLLITTEGK	31.44819	Q9P258	RCC2_HUMAN	644.3354847	3	0.684904814	3	Yes
VFSWGFGGYGR	66.42473	Q9P258	RCC2_HUMAN	616.7965425	2	0.770302474	2	Yes
VFSWGFGGYGR	66.42473	Q9P258	RCC2_HUMAN	411.5336367	3	0.770302474	2	
AAEGAEAQGFSGLFAAYTDHPPPFR	85.74458	Q9P253	VPS18_HUMAN	1304.117323	2	0.784388304		
AAEGAEAQGFSGLFAAYTDHPPPFR	85.74458	Q9P253	VPS18_HUMAN	869.7474903	3	0.784388304		
ASIHELLASHGDTEHMVYFAVIMQDYER	93.11397	Q9P253	VPS18_HUMAN	1631.768977	2	0.67397207		
ASIHELLASHGDTEHMVYFAVIMQDYER	93.11397	Q9P253	VPS18_HUMAN	1088.181926	3	0.67397207		
DSSTGQLWAYTER	42.53752	Q9P253	VPS18_HUMAN	757.3500645	2	0.847875655		
DSSTGQLWAYTER	42.53752	Q9P253	VPS18_HUMAN	505.2359847	3	0.847875655		
EFPSNLGYSELAFYTPK	89.1936	Q9P253	VPS18_HUMAN	981.978364	2	0.765376866		
EFPSNLGYSELAFYTPK	89.1936	Q9P253	VPS18_HUMAN	654.9881843	3	0.765376866		
ERPDCLDTVLAR	25.31361	Q9P253	VPS18_HUMAN	722.864832	2	0.744269192		
ERPDCLDTVLAR	25.31361	Q9P253	VPS18_HUMAN	482.2458297	3	0.744269192		
GQLVESVGWNK	29.79346	Q9P253	VPS18_HUMAN	608.8202185	2	0.764135182		
GQLVESVGWNK	29.79346	Q9P253	VPS18_HUMAN	406.2160873	3	0.764135182		
GRPDSLLAYLEQAGASPHR	61.38255	Q9P253	VPS18_HUMAN	1019.527415	2	0.631110072	4	
GRPDSLLAYLEQAGASPHR	61.38255	Q9P253	VPS18_HUMAN	680.0208847	3	0.631110072	4	
IDFTPSER	13.1725	Q9P253	VPS18_HUMAN	482.7409055	2	0.659519494		
IDFTPSER	13.1725	Q9P253	VPS18_HUMAN	322.163212	3	0.659519494		
IEDVLPFFPDFVTIDHFK	136.214	Q9P253	VPS18_HUMAN	1090.059698	2	0.655624092		
IEDVLPFFPDFVTIDHFK	136.214	Q9P253	VPS18_HUMAN	727.0424067	3	0.655624092		
LGALQGDPEALTLYR	67.78174	Q9P253	VPS18_HUMAN	808.9363075	2	0.901651442	2	Yes
LGALQGDPEALTLYR	67.78174	Q9P253	VPS18_HUMAN	539.6268133	3	0.901651442	2	
MFLDHTGSHLLIALSSTEVLYVNR	97.00275	Q9P253	VPS18_HUMAN	1358.710532	2	0.601326704		
MFLDHTGSHLLIALSSTEVLYVNR	97.00275	Q9P253	VPS18_HUMAN	906.142963	3	0.601326704		
SAVLQPGCPSVGIPHSGYVNAQLEK	51.17321	Q9P253	VPS18_HUMAN	1304.663583	2	0.767598629	3	
SAVLQPGCPSVGIPHSGYVNAQLEK	51.17321	Q9P253	VPS18_HUMAN	870.1116633	3	0.767598629	3	Yes
SIDRPFIDPQR	21.51488	Q9P253	VPS18_HUMAN	672.3574995	2	0.732268453	3	
SIDRPFIDPQR	21.51488	Q9P253	VPS18_HUMAN	448.5742747	3	0.732268453	3	Yes
TYLDMNR	6.208157	Q9P253	VPS18_HUMAN	456.716372	2	0.758248568	2	Yes
TYLDMNR	6.208157	Q9P253	VPS18_HUMAN	304.813523	3	0.758248568	2	
VEAVCTLTGQVVLR	55.98655	Q9P253	VPS18_HUMAN	772.927433	2	0.71198833		
VEAVCTLTGQVVLR	55.98655	Q9P253	VPS18_HUMAN	515.620897	3	0.71198833		
VVAYHCQHEAYEEALAVLAR	46.27834	Q9P253	VPS18_HUMAN	1165.073868	2	0.669493914		
VVAYHCQHEAYEEALAVLAR	46.27834	Q9P253	VPS18_HUMAN	777.0518537	3	0.669493914		
AVGFVSEDEYLEIQGITR	86.20508	Q9P121	NTRI_HUMAN	1013.510565	2	0.740264058		
AVGFVSEDEYLEIQGITR	86.20508	Q9P121	NTRI_HUMAN	676.0096513	3	0.740264058		
EQSGDYECSASNDVAAPVVR	30.89411	Q9P121	NTRI_HUMAN	1077.474384	2	0.804619193		
EQSGDYECSASNDVAAPVVR	30.89411	Q9P121	NTRI_HUMAN	718.6521977	3	0.804619193		
GTLQCEASAVPSAEFQWYK	66.14043	Q9P121	NTRI_HUMAN	1086.5075	2	0.607473731		
GTLQCEASAVPSAEFQWYK	66.14043	Q9P121	NTRI_HUMAN	724.6742747	3	0.607473731		
LIFFNVSEHDYGNYTCVASNK	63.66882	Q9P121	NTRI_HUMAN	1239.573897	2	0.650448442		
LIFFNVSEHDYGNYTCVASNK	63.66882	Q9P121	NTRI_HUMAN	826.7185393	3	0.650448442		
VHLIVQVSPK	13.66771	Q9P121	NTRI_HUMAN	560.348415	2	0.858550787		
VHLIVQVSPK	13.66771	Q9P121	NTRI_HUMAN	373.9015517	3	0.858550787		
VTVNYPPYISEAK	42.65852	Q9P121	NTRI_HUMAN	740.8882895	2	0.799205363		
VTVNYPPYISEAK	42.65852	Q9P121	NTRI_HUMAN	494.261468	3	0.799205363		
AASPSELTCPPGWEWEDDAWSYDINR	105.9005	Q9NZM1	MYOF_HUMAN	1526.656879	2	0.68246299	3	
AASPSELTCPPGWEWEDDAWSYDINR	105.9005	Q9NZM1	MYOF_HUMAN	1018.107194	3	0.68246299	3	Yes
ACGDVLVTAELILR	110.4058	Q9NZM1	MYOF_HUMAN	765.421983	2	0.750126123		
ACGDVLVTAELILR	110.4058	Q9NZM1	MYOF_HUMAN	510.6172637	3	0.750126123		
ADCLMGEFK	31.51563	Q9NZM1	MYOF_HUMAN	535.736452	2	0.774246275	2	Yes
ADCLMGEFK	31.51563	Q9NZM1	MYOF_HUMAN	357.4935763	3	0.774246275	2	
AEDIPQMDDAFSQTVK	58.40225	Q9NZM1	MYOF_HUMAN	897.914916	2	0.872943878		
AEDIPQMDDAFSQTVK	58.40225	Q9NZM1	MYOF_HUMAN	598.9458857	3	0.872943878		
AMEELQDQEGWEYASLIGWK	116.0416	Q9NZM1	MYOF_HUMAN	1192.049728	2	0.763944089		
AMEELQDQEGWEYASLIGWK	116.0416	Q9NZM1	MYOF_HUMAN	795.0357603	3	0.763944089		
ANVTVLDTQIR	32.49998	Q9NZM1	MYOF_HUMAN	615.346601	2	0.876673937	2	Yes
ANVTVLDTQIR	32.49998	Q9NZM1	MYOF_HUMAN	410.567009	3	0.876673937	2	
ASLLSAPPCR	20.64359	Q9NZM1	MYOF_HUMAN	536.2849575	2	0.748059332	2	Yes
ASLLSAPPCR	20.64359	Q9NZM1	MYOF_HUMAN	357.8592467	3	0.748059332	2	
DFETIGQNK	12.65018	Q9NZM1	MYOF_HUMAN	526.25692	2	0.796402931	2	Yes
DFETIGQNK	12.65018	Q9NZM1	MYOF_HUMAN	351.1738883	3	0.796402931	2	
DGSNLPILPPQR	55.77435	Q9NZM1	MYOF_HUMAN	653.859875	2	0.806896627	2	Yes
DGSNLPILPPQR	55.77435	Q9NZM1	MYOF_HUMAN	436.242525	3	0.806896627	2	
DHYIPNTLNPVFGR	57.92509	Q9NZM1	MYOF_HUMAN	821.920991	2	0.767617881	3	
DHYIPNTLNPVFGR	57.92509	Q9NZM1	MYOF_HUMAN	548.283269	3	0.767617881	3	Yes
DIVIEMEDTKPLLASK	62.38828	Q9NZM1	MYOF_HUMAN	901.4849765	2	0.791453242		
DIVIEMEDTKPLLASK	62.38828	Q9NZM1	MYOF_HUMAN	601.325926	3	0.791453242		
DNDSDDVESNLLLPAGIALR	116.6273	Q9NZM1	MYOF_HUMAN	1064.032396	2	0.620614231		
DNDSDDVESNLLLPAGIALR	116.6273	Q9NZM1	MYOF_HUMAN	709.690872	3	0.620614231		
DQLRPTQLLQNVAR	48.47997	Q9NZM1	MYOF_HUMAN	826.466102	2	0.794521987		
DQLRPTQLLQNVAR	48.47997	Q9NZM1	MYOF_HUMAN	551.313343	3	0.794521987		
DVILDEK	11.88081	Q9NZM1	MYOF_HUMAN	416.2270995	2	0.645877719		
DVILDEK	11.88081	Q9NZM1	MYOF_HUMAN	277.8206747	3	0.645877719		
DYSLDEFEANK	44.01447	Q9NZM1	MYOF_HUMAN	665.7940585	2	0.83523345	2	Yes
DYSLDEFEANK	44.01447	Q9NZM1	MYOF_HUMAN	444.1986473	3	0.83523345	2	
EDIVPQLK	25.72446	Q9NZM1	MYOF_HUMAN	471.269299	2	0.629168391	2	Yes
EDIVPQLK	25.72446	Q9NZM1	MYOF_HUMAN	314.5154743	3	0.629168391	2	
EELYMPPLVIK	80.47107	Q9NZM1	MYOF_HUMAN	666.3681515	2	0.782957196	2	Yes
EELYMPPLVIK	80.47107	Q9NZM1	MYOF_HUMAN	444.581376	3	0.782957196	2	
ELPDSVPQECTVR	26.05082	Q9NZM1	MYOF_HUMAN	765.3674045	2	0.819595158	2	Yes
ELPDSVPQECTVR	26.05082	Q9NZM1	MYOF_HUMAN	510.580878	3	0.819595158	2	
EYTGFPDPYDELNTGK	65.76101	Q9NZM1	MYOF_HUMAN	923.4130545	2	0.780852377		
EYTGFPDPYDELNTGK	65.76101	Q9NZM1	MYOF_HUMAN	615.9446447	3	0.780852377		
FDTTCKPLASTTQYSR	10.1955	Q9NZM1	MYOF_HUMAN	938.4494535	2	0.80871284		
FDTTCKPLASTTQYSR	10.1955	Q9NZM1	MYOF_HUMAN	625.9689107	3	0.80871284		
FGKPDPIVSVIFK	63.89124	Q9NZM1	MYOF_HUMAN	723.921944	2	0.834299147	2	Yes
FGKPDPIVSVIFK	63.89124	Q9NZM1	MYOF_HUMAN	482.950571	3	0.834299147	2	
FYASSGEHEK	-30.42572	Q9NZM1	MYOF_HUMAN	577.759822	2	0.603086114		
FYASSGEHEK	-30.42572	Q9NZM1	MYOF_HUMAN	385.5091563	3	0.603086114		
GFPQPILSEDGSR	44.22886	Q9NZM1	MYOF_HUMAN	701.852247	2	0.740158916	2	Yes
GFPQPILSEDGSR	44.22886	Q9NZM1	MYOF_HUMAN	468.2374397	3	0.740158916	2	
GIPLDFSSSLGIIVK	113.2582	Q9NZM1	MYOF_HUMAN	773.4485225	2	0.75292325		
GIPLDFSSSLGIIVK	113.2582	Q9NZM1	MYOF_HUMAN	515.96829	3	0.75292325		
GLELQPQDNNGLCDPYIK	60.55646	Q9NZM1	MYOF_HUMAN	1037.499674	2	0.695175946		
GLELQPQDNNGLCDPYIK	60.55646	Q9NZM1	MYOF_HUMAN	692.002391	3	0.695175946		

GPVGTVSEAQLAR	17.94575	Q9NZM1	MYOF_HUMAN	642.849508	2	0.850207448	2	Yes
GPVGTVSEAQLAR	17.94575	Q9NZM1	MYOF_HUMAN	428.9022803	3	0.850207448	2	
GWEWEGEWIVDPER	96.80542	Q9NZM1	MYOF_HUMAN	894.405375	2	0.696743727	2	Yes
GWEWEGEWIVDPER	96.80542	Q9NZM1	MYOF_HUMAN	596.6061917	3	0.696743727	2	
GWEYGITIPPDHKPK	33.73612	Q9NZM1	MYOF_HUMAN	869.4521245	2	0.824629843		
GWEYGITIPPDHKPK	33.73612	Q9NZM1	MYOF_HUMAN	579.9706913	3	0.824629843		
GWIPGNEENK	13.70656	Q9NZM1	MYOF_HUMAN	572.2756435	2	0.684919059	2	Yes
GWIPGNEENK	13.70656	Q9NZM1	MYOF_HUMAN	381.8530373	3	0.684919059	2	
GWWPCYAEK	45.53137	Q9NZM1	MYOF_HUMAN	598.763854	2	0.707628965		
GWWPCYAEK	45.53137	Q9NZM1	MYOF_HUMAN	399.5118443	3	0.707628965		
HSATTVFGANTPIVSCNFDR	47.93986	Q9NZM1	MYOF_HUMAN	1097.521477	2	0.662671506	3	
HSATTVFGANTPIVSCNFDR	47.93986	Q9NZM1	MYOF_HUMAN	732.016926	3	0.662671506	3	Yes
IDVGFVYDEPGHAVMR	56.29836	Q9NZM1	MYOF_HUMAN	902.938524	2	0.734213769	3	
IDVGFVYDEPGHAVMR	56.29836	Q9NZM1	MYOF_HUMAN	602.2949577	3	0.734213769	3	Yes
ILHQHLGAPEER	-25.59879	Q9NZM1	MYOF_HUMAN	700.376224	2	0.785170555		
ILHQHLGAPEER	-25.59879	Q9NZM1	MYOF_HUMAN	467.2534243	3	0.785170555		
ILVELATFLEK	101.9982	Q9NZM1	MYOF_HUMAN	638.3821205	2	0.80245465	2	Yes
ILVELATFLEK	101.9982	Q9NZM1	MYOF_HUMAN	425.924022	3	0.80245465	2	
IPAHQVLYSTSGENASGK	-1.315628	Q9NZM1	MYOF_HUMAN	929.9688665	2	0.793925345	3	
IPAHQVLYSTSGENASGK	-1.315628	Q9NZM1	MYOF_HUMAN	620.315186	3	0.793925345	3	Yes
IPANQLAELWLK	86.03061	Q9NZM1	MYOF_HUMAN	698.4039185	2	0.881327748	2	Yes
IPANQLAELWLK	86.03061	Q9NZM1	MYOF_HUMAN	465.938554	3	0.881327748	2	
ISVYDYDTFTR	51.98228	Q9NZM1	MYOF_HUMAN	690.328065	2	0.90012008	2	Yes
ISVYDYDTFTR	51.98228	Q9NZM1	MYOF_HUMAN	460.5546517	3	0.90012008	2	
IYPLPDDPSVPAPPR	64.75692	Q9NZM1	MYOF_HUMAN	817.4334	2	0.786847889	2	Yes
IYPLPDDPSVPAPPR	64.75692	Q9NZM1	MYOF_HUMAN	545.2915417	3	0.786847889	2	
KPVVGQCTIER	-27.3478	Q9NZM1	MYOF_HUMAN	643.848454	2	0.793411016	2	Yes
KPVVGQCTIER	-27.3478	Q9NZM1	MYOF_HUMAN	429.5682443	3	0.793411016	2	
LALHILR	18.64803	Q9NZM1	MYOF_HUMAN	418.279804	2	0.739471078		
LALHILR	18.64803	Q9NZM1	MYOF_HUMAN	279.1891443	3	0.739471078		
LDAVNTLLAMAER	105.5828	Q9NZM1	MYOF_HUMAN	708.8799505	2	0.871015787	2	Yes
LDAVNTLLAMAER	105.5828	Q9NZM1	MYOF_HUMAN	472.9225753	3	0.871015787	2	
LDMIPDLK	51.47404	Q9NZM1	MYOF_HUMAN	472.7602525	2	0.635592103	2	Yes
LDMIPDLK	51.47404	Q9NZM1	MYOF_HUMAN	315.5094433	3	0.635592103	2	
LEGALGADTTEDGDEK	12.69585	Q9NZM1	MYOF_HUMAN	810.865946	2	0.621548116	2	Yes
LEGALGADTTEDGDEK	12.69585	Q9NZM1	MYOF_HUMAN	540.913239	3	0.621548116	2	
LEPISNDDLLVVEK	64.33472	Q9NZM1	MYOF_HUMAN	792.4305265	2	0.767296851	2	Yes
LEPISNDDLLVVEK	64.33472	Q9NZM1	MYOF_HUMAN	528.6229593	3	0.767296851	2	
LIDEVIEDTR	39.16355	Q9NZM1	MYOF_HUMAN	601.8173415	2	0.761122346	2	Yes
LIDEVIEDTR	39.16355	Q9NZM1	MYOF_HUMAN	401.5475027	3	0.761122346	2	
LIGTATVALK	32.35348	Q9NZM1	MYOF_HUMAN	493.816417	2	0.797595024	2	Yes
LIGTATVALK	32.35348	Q9NZM1	MYOF_HUMAN	329.5468863	3	0.797595024	2	
LIIQIWDNDK	70.3315	Q9NZM1	MYOF_HUMAN	629.346069	2	0.605619907		
LIIQIWDNDK	70.3315	Q9NZM1	MYOF_HUMAN	419.8999877	3	0.605619907		
LISLLNEK	37.52668	Q9NZM1	MYOF_HUMAN	465.287491	2	0.726546884	2	Yes
LISLLNEK	37.52668	Q9NZM1	MYOF_HUMAN	310.5276023	3	0.726546884	2	
LLWHPVMNGDK	26.86866	Q9NZM1	MYOF_HUMAN	655.340264	2	0.633486867		
LLWHPVMNGDK	26.86866	Q9NZM1	MYOF_HUMAN	437.229451	3	0.633486867		
LNSEMDITPK	13.85757	Q9NZM1	MYOF_HUMAN	574.2873625	2	0.778932154		
LNSEMDITPK	13.85757	Q9NZM1	MYOF_HUMAN	383.1941833	3	0.778932154		
LQMWVDVFPK	87.94186	Q9NZM1	MYOF_HUMAN	631.8342835	2	0.714196444	2	Yes
LQMWVDVFPK	87.94186	Q9NZM1	MYOF_HUMAN	421.5587973	3	0.714196444	2	
LQTNIEALK	20.44442	Q9NZM1	MYOF_HUMAN	515.3011305	2	0.861821294	2	Yes
LQTNIEALK	20.44442	Q9NZM1	MYOF_HUMAN	343.8700287	3	0.861821294	2	
LTIYDWDR	51.09098	Q9NZM1	MYOF_HUMAN	541.269826	2	0.825745821		
LTIYDWDR	51.09098	Q9NZM1	MYOF_HUMAN	361.1824923	3	0.825745821		
MAPSETHGAAAIFK	14.11022	Q9NZM1	MYOF_HUMAN	715.8590185	2	0.610738099	3	
MAPSETHGAAAIFK	14.11022	Q9NZM1	MYOF_HUMAN	477.5752873	3	0.610738099	3	Yes
MLSNKPQDFQIR	16.57214	Q9NZM1	MYOF_HUMAN	738.885567	2	0.82046628		
MLSNKPQDFQIR	16.57214	Q9NZM1	MYOF_HUMAN	492.9263197	3	0.82046628		
MYELSCYLPQEK	55.88072	Q9NZM1	MYOF_HUMAN	780.857818	2	0.850194514		
MYELSCYLPQEK	55.88072	Q9NZM1	MYOF_HUMAN	520.9078203	3	0.850194514		
NANPEWNQVVNLQIK	64.51096	Q9NZM1	MYOF_HUMAN	883.963391	2	0.803778052		
NANPEWNQVVNLQIK	64.51096	Q9NZM1	MYOF_HUMAN	589.644869	3	0.803778052		
NDVVGTTYLHLSK	31.26688	Q9NZM1	MYOF_HUMAN	723.883543	2	0.865392923		
NDVVGTTYLHLSK	31.26688	Q9NZM1	MYOF_HUMAN	482.9249703	3	0.865392923		
SDENEDPSVVGEFK	32.31813	Q9NZM1	MYOF_HUMAN	776.344648	2	0.675170064		
SDENEDPSVVGEFK	32.31813	Q9NZM1	MYOF_HUMAN	517.8990403	3	0.675170064		
SITGEEMSDIYVK	40.56847	Q9NZM1	MYOF_HUMAN	736.3534265	2	0.80878967	2	Yes
SITGEEMSDIYVK	40.56847	Q9NZM1	MYOF_HUMAN	491.238226	3	0.80878967	2	
SLDGEGNFNWR	37.47718	Q9NZM1	MYOF_HUMAN	647.7947315	2	0.741016209		
SLDGEGNFNWR	37.47718	Q9NZM1	MYOF_HUMAN	432.199096	3	0.741016209		
SLGPPGPPFNITPR	63.88264	Q9NZM1	MYOF_HUMAN	725.396625	2	0.759879649	2	Yes
SLGPPGPPFNITPR	63.88264	Q9NZM1	MYOF_HUMAN	483.9336917	3	0.759879649	2	
SLLTEADAGHTEFTDEVYQNESR	47.89309	Q9NZM1	MYOF_HUMAN	1306.591531	2	0.794409394	3	
SLLTEADAGHTEFTDEVYQNESR	47.89309	Q9NZM1	MYOF_HUMAN	871.3969623	3	0.794409394	3	Yes
SLSQIHEAAVR	-10.4757	Q9NZM1	MYOF_HUMAN	605.8311175	2	0.767199278	3	
SLSQIHEAAVR	-10.4757	Q9NZM1	MYOF_HUMAN	404.2233533	3	0.767199278	3	Yes
STLAEIEDWLDK	97.01764	Q9NZM1	MYOF_HUMAN	710.354288	2	0.821876824	2	Yes
STLAEIEDWLDK	97.01764	Q9NZM1	MYOF_HUMAN	473.905467	3	0.821876824	2	
TLHSTFQPNISQGK	1.132977	Q9NZM1	MYOF_HUMAN	779.4051785	2	0.813577831	3	
TLHSTFQPNISQGK	1.132977	Q9NZM1	MYOF_HUMAN	519.939394	3	0.813577831	3	Yes
TPNFPSSVLFMK	75.03522	Q9NZM1	MYOF_HUMAN	684.3555795	2	0.768227577	2	Yes
TPNFPSSVLFMK	75.03522	Q9NZM1	MYOF_HUMAN	456.5729947	3	0.768227577	2	
VEMTLEILNEK	65.08763	Q9NZM1	MYOF_HUMAN	659.850327	2	0.726351321	2	Yes
VEMTLEILNEK	65.08763	Q9NZM1	MYOF_HUMAN	440.2361597	3	0.726351321	2	
VGETIIDLENR	43.29841	Q9NZM1	MYOF_HUMAN	629.8360655	2	0.825266063	2	Yes
VGETIIDLENR	43.29841	Q9NZM1	MYOF_HUMAN	420.226652	3	0.825266063	2	
VIIWNTK	26.78574	Q9NZM1	MYOF_HUMAN	437.2638195	2	0.708200395		
VIIWNTK	26.78574	Q9NZM1	MYOF_HUMAN	291.8451547	3	0.708200395		
VIMELFDNDQVGK	66.24896	Q9NZM1	MYOF_HUMAN	754.3772405	2	0.855050385	2	Yes
VIMELFDNDQVGK	66.24896	Q9NZM1	MYOF_HUMAN	503.254102	3	0.855050385	2	
VIVESASNIPK	15.48476	Q9NZM1	MYOF_HUMAN	578.832794	2	0.811355054	2	Yes
VIVESASNIPK	15.48476	Q9NZM1	MYOF_HUMAN	386.224471	3	0.811355054	2	
VNIWLGLSAVEK	82.20822	Q9NZM1	MYOF_HUMAN	664.8828185	2	0.71417129	2	Yes
VNIWLGLSAVEK	82.20822	Q9NZM1	MYOF_HUMAN	443.591154	3	0.71417129	2	
VSMFVLGTGDEPPPER	63.64415	Q9NZM1	MYOF_HUMAN	865.9250865	2	0.783726752		
VSMFVLGTGDEPPPER	63.64415	Q9NZM1	MYOF_HUMAN	577.6193327	3	0.783726752		
VYIYHLR	5.552856	Q9NZM1	MYOF_HUMAN	482.27471	2	0.71080631	2	Yes
VYIYHLR	5.552856	Q9NZM1	MYOF_HUMAN	321.852415	3	0.71080631	2	
WGTSGLVGR	20.00175	Q9NZM1	MYOF_HUMAN	466.751608	2	0.604982376		

WGTSGLVGR	20.00175	Q9NZM1	MYOF_HUMAN	311.5036803	3	0.604982376		
WLLLNDPEDTSSGSK	57.19693	Q9NZM1	MYOF_HUMAN	831.40504	2	0.722302675	2	Yes
WLLLNDPEDTSSGSK	57.19693	Q9NZM1	MYOF_HUMAN	554.6059683	3	0.722302675	2	
YTLPLTEGK	31.91533	Q9NZM1	MYOF_HUMAN	511.2824025	2	0.752076805	2	Yes
YTLPLTEGK	31.91533	Q9NZM1	MYOF_HUMAN	341.1908767	3	0.752076805	2	
AARPLISSAVYVK	20.97529	Q9NZJ6	COQ3_HUMAN	687.9093635	2	0.753250837		
AARPLISSAVYVK	20.97529	Q9NZJ6	COQ3_HUMAN	458.942184	3	0.753250837		
GETEELQANACTNPAVHEK	-2.915279	Q9NZJ6	COQ3_HUMAN	1049.479475	2	0.729624152		
GETEELQANACTNPAVHEK	-2.915279	Q9NZJ6	COQ3_HUMAN	699.9889247	3	0.729624152		
ILDVGCGGGLLTEPLGR	78.86115	Q9NZJ6	COQ3_HUMAN	863.962004	2	0.781984091		
ILDVGCGGGLLTEPLGR	78.86115	Q9NZJ6	COQ3_HUMAN	576.310611	3	0.781984091		
LGASVIGIDPVDENIK	70.2461	Q9NZJ6	COQ3_HUMAN	820.449251	2	0.819401324		
LGASVIGIDPVDENIK	70.2461	Q9NZJ6	COQ3_HUMAN	547.302109	3	0.819401324		
LGSSGGWFLR	49.53188	Q9NZJ6	COQ3_HUMAN	540.2858145	2	0.707010984		
LGSSGGWFLR	49.53188	Q9NZJ6	COQ3_HUMAN	360.5264847	3	0.707010984		
LYSTSQTTVDSGEVK	3.372246	Q9NZJ6	COQ3_HUMAN	807.8970445	2	0.859013855		
LYSTSQTTVDSGEVK	3.372246	Q9NZJ6	COQ3_HUMAN	538.9339713	3	0.859013855		
NQLSGTLQIKPGVFNEYR	53.3052	Q9NZJ6	COQ3_HUMAN	1032.547816	2	0.813401937		
NQLSGTLQIKPGVFNEYR	53.3052	Q9NZJ6	COQ3_HUMAN	688.701152	3	0.813401937		
TIFSCLNR	31.74034	Q9NZJ6	COQ3_HUMAN	505.7585755	2	0.760315061		
TIFSCLNR	31.74034	Q9NZJ6	COQ3_HUMAN	337.5083253	3	0.760315061		
VQEHPASAEFVLK	17.18917	Q9NZJ6	COQ3_HUMAN	727.88609	2	0.811752975		
VQEHPASAEFVLK	17.18917	Q9NZJ6	COQ3_HUMAN	485.593335	3	0.811752975		
WWDEQGVYAPLHSMNDLR	68.8634	Q9NZJ6	COQ3_HUMAN	1109.013283	2	0.704515994		
WWDEQGVYAPLHSMNDLR	68.8634	Q9NZJ6	COQ3_HUMAN	739.67813	3	0.704515994		
AISVHSTPEGCSSACK	-25.72555	Q9NZI8	IF2B1_HUMAN	845.880353	2	0.78090626		
AISVHSTPEGCSSACK	-25.72555	Q9NZI8	IF2B1_HUMAN	564.256177	3	0.78090626		
DQTPDENDQVIVK	17.16397	Q9NZI8	IF2B1_HUMAN	750.863009	2	0.799515903	2	Yes
DQTPDENDQVIVK	17.16397	Q9NZI8	IF2B1_HUMAN	500.911281	3	0.799515903	2	
IIGHFYASQMAQR	18.25579	Q9NZI8	IF2B1_HUMAN	761.3857305	2	0.837271929		
IIGHFYASQMAQR	18.25579	Q9NZI8	IF2B1_HUMAN	507.9264287	3	0.837271929		
ISYSGQFLVK	40.13371	Q9NZI8	IF2B1_HUMAN	571.316776	2	0.797670722	2	Yes
ISYSGQFLVK	40.13371	Q9NZI8	IF2B1_HUMAN	381.2137923	3	0.797670722	2	
ITISSLQDLTLYNPER	89.37259	Q9NZI8	IF2B1_HUMAN	931.9970925	2	0.819527626	3	
ITISSLQDLTLYNPER	89.37259	Q9NZI8	IF2B1_HUMAN	621.6673367	3	0.819527626	3	Yes
LLVPTQYVGAIIGK	87.28198	Q9NZI8	IF2B1_HUMAN	736.4483225	2	0.821109593	2	Yes
LLVPTQYVGAIIGK	87.28198	Q9NZI8	IF2B1_HUMAN	491.30149	3	0.821109593	2	
LYIGNLNESVTPADLEK	67.8762	Q9NZI8	IF2B1_HUMAN	938.4891	2	0.878014326		
LYIGNLNESVTPADLEK	67.8762	Q9NZI8	IF2B1_HUMAN	625.9953417	3	0.878014326		
MILEIMHK	29.79046	Q9NZI8	IF2B1_HUMAN	507.7779225	2	0.747767627	2	Yes
MILEIMHK	29.79046	Q9NZI8	IF2B1_HUMAN	338.8545567	3	0.747767627	2	
SGYAFVDCPDEHWAMK	47.53854	Q9NZI8	IF2B1_HUMAN	956.903824	2	0.904069901	3	
SGYAFVDCPDEHWAMK	47.53854	Q9NZI8	IF2B1_HUMAN	638.2718243	3	0.904069901	3	Yes
TVNELQNLTAAEVVVPR	76.69616	Q9NZI8	IF2B1_HUMAN	927.010539	2	0.829920769	3	
TVNELQNLTAAEVVVPR	76.69616	Q9NZI8	IF2B1_HUMAN	618.3429677	3	0.829920769	3	Yes
VSYIPDEQIAQGPENGR	35.82337	Q9NZI8	IF2B1_HUMAN	936.958499	2	0.717761159		
VSYIPDEQIAQGPENGR	35.82337	Q9NZI8	IF2B1_HUMAN	624.974941	3	0.717761159		
FSHGTMPLR	-11.2546	Q9NZ01	TECR_HUMAN	523.266568	2	0.66390264	2	Yes
FSHGTMPLR	-11.2546	Q9NZ01	TECR_HUMAN	349.1803203	3	0.66390264	2	
HYEVEILDAK	24.87327	Q9NZ01	TECR_HUMAN	608.8145975	2	0.73825109	3	
HYEVEILDAK	24.87327	Q9NZ01	TECR_HUMAN	406.21234	3	0.73825109	3	Yes
LLETLFVHR	45.07352	Q9NZ01	TECR_HUMAN	564.332765	2	0.788353026	3	
LLETLFVHR	45.07352	Q9NZ01	TECR_HUMAN	376.557785	3	0.788353026	3	Yes
LPVGTTATLYFR	60.40921	Q9NZ01	TECR_HUMAN	669.8749905	2	0.714590907	2	Yes
LPVGTTATLYFR	60.40921	Q9NZ01	TECR_HUMAN	446.9192687	3	0.714590907	2	
VEPHATIAEIK	2.240326	Q9NZ01	TECR_HUMAN	604.3382445	2	0.725814104	2	Yes
VEPHATIAEIK	2.240326	Q9NZ01	TECR_HUMAN	403.2281047	3	0.725814104	2	
VPFIYGHK	7.173038	Q9NZ01	TECR_HUMAN	480.769265	2	0.738772511		
VPFIYGHK	7.173038	Q9NZ01	TECR_HUMAN	320.848785	3	0.738772511		
DLINIFDK	78.28741	Q9NYV6	RRN3_HUMAN	489.2692985	2	0.658921003		
DLINIFDK	78.28741	Q9NYV6	RRN3_HUMAN	326.515474	3	0.658921003		
EGDVDVSDSDDEDDNLPANFDTCHR	41.72659	Q9NYV6	RRN3_HUMAN	1419.057927	2	0.677418768		
EGDVDVSDSDDEDDNLPANFDTCHR	41.72659	Q9NYV6	RRN3_HUMAN	946.3745597	3	0.677418768		
EGLQYLQSLNFER	72.75672	Q9NYV6	RRN3_HUMAN	798.9050065	2	0.787284553		
EGLQYLQSLNFER	72.75672	Q9NYV6	RRN3_HUMAN	532.9392793	3	0.787284553		
FGGTVTEVLLK	54.79929	Q9NYV6	RRN3_HUMAN	582.3377135	2	0.85605967		
FGGTVTEVLLK	54.79929	Q9NYV6	RRN3_HUMAN	388.561084	3	0.85605967		
GETNDFELLK	43.3623	Q9NYV6	RRN3_HUMAN	583.2909595	2	0.822551429		
GETNDFELLK	43.3623	Q9NYV6	RRN3_HUMAN	389.1965813	3	0.822551429		
GEVPQNDTVIGITPSSFDTHFR	66.07997	Q9NYV6	RRN3_HUMAN	1209.090778	2	0.719456434		
GEVPQNDTVIGITPSSFDTHFR	66.07997	Q9NYV6	RRN3_HUMAN	806.39646	3	0.719456434		
LDQMVHPVAER	0.819778	Q9NYV6	RRN3_HUMAN	647.8328035	2	0.790968895		
LDQMVHPVAER	0.819778	Q9NYV6	RRN3_HUMAN	432.2244773	3	0.790968895		
LGFAEAFLEHLWK	112.8237	Q9NYV6	RRN3_HUMAN	780.9146505	2	0.79327029		
LGFAEAFLEHLWK	112.8237	Q9NYV6	RRN3_HUMAN	520.9457087	3	0.79327029		
TLECYVHNLLR	46.40452	Q9NYV6	RRN3_HUMAN	709.367006	2	0.816109896		
TLECYVHNLLR	46.40452	Q9NYV6	RRN3_HUMAN	473.247279	3	0.816109896		
YVPSTPWFLMPILVEK	139.8149	Q9NYV6	RRN3_HUMAN	960.521161	2	0.621085405		
YVPSTPWFLMPILVEK	139.8149	Q9NYV6	RRN3_HUMAN	640.6833823	3	0.621085405		
AEAGLETESPVR	7.544453	Q9NYL4	FKB11_HUMAN	629.817873	2	0.656692564		
AEAGLETESPVR	7.544453	Q9NYL4	FKB11_HUMAN	420.2145237	3	0.656692564		
DPLVIELGQK	58.53925	Q9NYL4	FKB11_HUMAN	556.322063	2	0.785100281		
DPLVIELGQK	58.53925	Q9NYL4	FKB11_HUMAN	371.217317	3	0.785100281		
LQVETLVEPPEPCAEPAAFGDTLHIHYTGSLVDG	84.64706	Q9NYL4	FKB11_HUMAN	1960.462733	2	0.701176643		
LQVETLVEPPEPCAEPAAFGDTLHIHYTGSLVDG	84.64706	Q9NYL4	FKB11_HUMAN	1307.311097	3	0.701176643		
AVQLAIQTLFTNSDGNPGSR	85.37915	Q9NYL2	M3K20_HUMAN	1045.037817	2	0.631957889		
AVQLAIQTLFTNSDGNPGSR	85.37915	Q9NYL2	M3K20_HUMAN	697.0278193	3	0.631957889		
DLHQPNTIPGMPLHPETDSR	37.67879	Q9NYL2	M3K20_HUMAN	1128.047859	2	0.655330181		
DLHQPNTIPGMPLHPETDSR	37.67879	Q9NYL2	M3K20_HUMAN	752.3678473	3	0.655330181		
DSGFSSGNTDTSSER	-15.92935	Q9NYL2	M3K20_HUMAN	773.816786	2	0.747904062		
DSGFSSGNTDTSSER	-15.92935	Q9NYL2	M3K20_HUMAN	516.213799	3	0.747904062		
DVTFNTNLPDAEILK	83.08067	Q9NYL2	M3K20_HUMAN	845.4388835	2	0.825087309		
DVTFNTNLPDAEILK	83.08067	Q9NYL2	M3K20_HUMAN	563.961864	3	0.825087309		
EAEILSVLSHR	46.62184	Q9NYL2	M3K20_HUMAN	627.3466	2	0.772402227	2	Yes
EAEILSVLSHR	46.62184	Q9NYL2	M3K20_HUMAN	418.5670083	3	0.772402227	2	
GDSSAEMSVYASLFK	80.36313	Q9NYL2	M3K20_HUMAN	796.3696075	2	0.70257622		
GDSSAEMSVYASLFK	80.36313	Q9NYL2	M3K20_HUMAN	531.2490133	3	0.70257622		
GLEGLQVAWLVVEK	109.9826	Q9NYL2	M3K20_HUMAN	770.9408655	2	0.748761654		
GLEGLQVAWLVVEK	109.9826	Q9NYL2	M3K20_HUMAN	514.2965187	3	0.748761654		
GMHYLHMEAPVK	8.943439	Q9NYL2	M3K20_HUMAN	706.8448525	2	0.868790746		
GMHYLHMEAPVK	8.943439	Q9NYL2	M3K20_HUMAN	471.5658433	3	0.868790746		

GSISLNSSPR	-3.132938	Q9NYL2	M3K20_HUMAN	509.2703605	2	0.775635123		
GSISLNSSPR	-3.132938	Q9NYL2	M3K20_HUMAN	339.8495153	3	0.775635123		
LLLLEEEDLK	61.09486	Q9NYL2	M3K20_HUMAN	607.84811	2	0.856638551		
LLLLEEEDLK	61.09486	Q9NYL2	M3K20_HUMAN	405.568015	3	0.856638551		
LTIPSSCPR	10.48049	Q9NYL2	M3K20_HUMAN	515.771683	2	0.745578229	2	Yes
LTIPSSCPR	10.48049	Q9NYL2	M3K20_HUMAN	344.1837303	3	0.745578229	2	
MTKPPFVMEK	13.63393	Q9NYL2	M3K20_HUMAN	604.3148695	2	0.75761795		
MTKPPFVMEK	13.63393	Q9NYL2	M3K20_HUMAN	403.2125213	3	0.75761795		
NFSSLHLNSR	4.296673	Q9NYL2	M3K20_HUMAN	587.802359	2	0.772274852		
NFSSLHLNSR	4.296673	Q9NYL2	M3K20_HUMAN	392.204181	3	0.772274852		
NVVIAADGVLK	39.41396	Q9NYL2	M3K20_HUMAN	549.830055	2	0.798853397		
NVVIAADGVLK	39.41396	Q9NYL2	M3K20_HUMAN	366.8893117	3	0.798853397		
SDSSADCQWLDTLR	59.39801	Q9NYL2	M3K20_HUMAN	827.36285	2	0.849739671		
SDSSADCQWLDTLR	59.39801	Q9NYL2	M3K20_HUMAN	551.911175	3	0.849739671		
SEEMDMDHIMTWATDVAK	79.95102	Q9NYL2	M3K20_HUMAN	1055.450483	2	0.755907774		
SEEMDMDHIMTWATDVAK	79.95102	Q9NYL2	M3K20_HUMAN	703.9695967	3	0.755907774		
SFAELLHQCWEADAK	57.91001	Q9NYL2	M3K20_HUMAN	902.9203355	2	0.613763154		
SFAELLHQCWEADAK	57.91001	Q9NYL2	M3K20_HUMAN	602.282832	3	0.613763154		
SQSNPILGSPFFSHFDGQDSYAAAVR	83.43344	Q9NYL2	M3K20_HUMAN	1399.662625	2	0.712907374	3	
SQSNPILGSPFFSHFDGQDSYAAAVR	83.43344	Q9NYL2	M3K20_HUMAN	933.444358	3	0.712907374	3	Yes
SQTVECTVTYESDVR	17.38468	Q9NYL2	M3K20_HUMAN	887.4021685	2	0.742532909		
SQTVECTVTYESDVR	17.38468	Q9NYL2	M3K20_HUMAN	591.9373873	3	0.742532909		
SSSPTQYGLTK	-2.713661	Q9NYL2	M3K20_HUMAN	584.796405	2	0.812447548		
SSSPTQYGLTK	-2.713661	Q9NYL2	M3K20_HUMAN	390.2002117	3	0.812447548		
VSQSALNPHQSPDFK	-4.719944	Q9NYL2	M3K20_HUMAN	827.913367	2	0.711181462	3	
VSQSALNPHQSPDFK	-4.719944	Q9NYL2	M3K20_HUMAN	552.2781863	3	0.711181462	3	Yes
YQQITPVNQSR	-0.466572	Q9NYL2	M3K20_HUMAN	667.347128	2	0.724393964	2	Yes
YQQITPVNQSR	-0.466572	Q9NYL2	M3K20_HUMAN	445.234027	3	0.724393964	2	
AEGEWEDQEALDYFSDK	73.77203	Q9NYF8	BCLF1_HUMAN	1016.426894	2	0.801705599		
AEGEWEDQEALDYFSDK	73.77203	Q9NYF8	BCLF1_HUMAN	677.953871	3	0.801705599		
DDGVDYWAK	37.98186	Q9NYF8	BCLF1_HUMAN	534.735816	2	0.753752768		
DDGVDYWAK	37.98186	Q9NYF8	BCLF1_HUMAN	356.8264857	3	0.753752768		
DLFDYSPPLHK	55.06246	Q9NYF8	BCLF1_HUMAN	666.335697	2	0.812789083		
DLFDYSPPLHK	55.06246	Q9NYF8	BCLF1_HUMAN	444.5597397	3	0.812789083		
DTFEHDPSESIDEFNK	36.25717	Q9NYF8	BCLF1_HUMAN	955.408508	2	0.750586808		
DTFEHDPSESIDEFNK	36.25717	Q9NYF8	BCLF1_HUMAN	637.274947	3	0.750586808		
GVFAGTNTGPNNSNTTFQK	18.11057	Q9NYF8	BCLF1_HUMAN	977.96686	2	0.78276217		
GVFAGTNTGPNNSNTTFQK	18.11057	Q9NYF8	BCLF1_HUMAN	652.3138483	3	0.78276217		
IDISPSTLR	32.24947	Q9NYF8	BCLF1_HUMAN	501.28548	2	0.743085623	2	Yes
IDISPSTLR	32.24947	Q9NYF8	BCLF1_HUMAN	334.5262617	3	0.743085623	2	
LLASTLVHSVK	18.02782	Q9NYF8	BCLF1_HUMAN	584.3589795	2	0.817770004	3	
LLASTLVHSVK	18.02782	Q9NYF8	BCLF1_HUMAN	389.9085947	3	0.817770004	3	Yes
MAPVPLDDSNRPASLTK	29.73675	Q9NYF8	BCLF1_HUMAN	906.470192	2	0.757302284		
MAPVPLDDSNRPASLTK	29.73675	Q9NYF8	BCLF1_HUMAN	604.649403	3	0.757302284		
SPHSPSPIATPPSQSSSCSDAPMLSTVHSAK	28.03828	Q9NYF8	BCLF1_HUMAN	1574.737882	2	0.642438352		
SPHSPSPIATPPSQSSSCSDAPMLSTVHSAK	28.03828	Q9NYF8	BCLF1_HUMAN	1050.161196	3	0.642438352		
SSATSGDIWPGLSAYDNSPR	68.77371	Q9NYF8	BCLF1_HUMAN	1040.982702	2	0.803964734		
SSATSGDIWPGLSAYDNSPR	68.77371	Q9NYF8	BCLF1_HUMAN	694.3244093	3	0.803964734		
SSFYPDGGDQETAK	3.171413	Q9NYF8	BCLF1_HUMAN	751.326255	2	0.803215146	2	Yes
SSFYPDGGDQETAK	3.171413	Q9NYF8	BCLF1_HUMAN	501.2201117	3	0.803215146	2	
STSESFIQHIVSLVHHVK	68.35463	Q9NYF8	BCLF1_HUMAN	1024.550362	2	0.706410229	4	
STSESFIQHIVSLVHHVK	68.35463	Q9NYF8	BCLF1_HUMAN	683.3695163	3	0.706410229	4	
ALLTTNQLPQPDVFPLFK	117.8546	Q9NY61	AATF_HUMAN	1021.570233	2	0.885926247		
ALLTTNQLPQPDVFPLFK	117.8546	Q9NY61	AATF_HUMAN	681.3827637	3	0.885926247		
DLDEEIFDDDDFYHQLLR	110.6631	Q9NY61	AATF_HUMAN	1149.514032	2	0.775107622		
DLDEEIFDDDDFYHQLLR	110.6631	Q9NY61	AATF_HUMAN	766.6786297	3	0.775107622		
FADFTVYR	40.03429	Q9NY61	AATF_HUMAN	509.753812	2	0.70623368		
FADFTVYR	40.03429	Q9NY61	AATF_HUMAN	340.1718163	3	0.70623368		
FDEGEDGEGDFLVVGSIR	82.78525	Q9NY61	AATF_HUMAN	970.947801	2	0.722266078		
FDEGEDGEGDFLVVGSIR	82.78525	Q9NY61	AATF_HUMAN	647.6344757	3	0.722266078		
LASASLLDTDK	21.90118	Q9NY61	AATF_HUMAN	567.3066095	2	0.766227245	2	Yes
LASASLLDTDK	21.90118	Q9NY61	AATF_HUMAN	378.540348	3	0.766227245	2	
LLSFMAPIDHTTMNDDAR	61.85377	Q9NY61	AATF_HUMAN	1024.482974	2	0.659742177		
LLSFMAPIDHTTMNDDAR	61.85377	Q9NY61	AATF_HUMAN	683.324591	3	0.659742177		
NQIALWDQLLEGR	107.1297	Q9NY61	AATF_HUMAN	778.415546	2	0.846898317		
NQIALWDQLLEGR	107.1297	Q9NY61	AATF_HUMAN	519.279639	3	0.846898317		
NSEDDGVVMTFSSVK	53.97583	Q9NY61	AATF_HUMAN	807.869976	2	0.775232792		
NSEDDGVVMTFSSVK	53.97583	Q9NY61	AATF_HUMAN	538.9159257	3	0.775232792		
SILTQIDHILMDK	86.3286	Q9NY61	AATF_HUMAN	763.916533	2	0.816334665	3	
SILTQIDHILMDK	86.3286	Q9NY61	AATF_HUMAN	509.6136303	3	0.816334665	3	Yes
SLFGQLHPPDEGHGD	40.94755	Q9NY61	AATF_HUMAN	803.368793	2	0.735545158		
SLFGQLHPPDEGHGD	40.94755	Q9NY61	AATF_HUMAN	535.915137	3	0.735545158		
SLVGLQEELLFQYPDTR	106.7665	Q9NY61	AATF_HUMAN	1004.523475	2	0.78868109	3	
SLVGLQEELLFQYPDTR	106.7665	Q9NY61	AATF_HUMAN	670.0182583	3	0.78868109	3	Yes
TPGFSVQSISDFEK	59.09519	Q9NY61	AATF_HUMAN	771.3782945	2	0.792241335	2	Yes
TPGFSVQSISDFEK	59.09519	Q9NY61	AATF_HUMAN	514.588138	3	0.792241335	2	
VLGKPEPAAQPVPESLPGEPEILPQAPANAHLK	60.22771	Q9NY61	AATF_HUMAN	1697.922647	2	0.879494786	4	
VLGKPEPAAQPVPESLPGEPEILPQAPANAHLK	60.22771	Q9NY61	AATF_HUMAN	1132.284373	3	0.879494786	4	
YLVDGTKPNAGSEEISSEDDELVEEK	39.53709	Q9NY61	AATF_HUMAN	1427.162182	2	0.736469269		
YLVDGTKPNAGSEEISSEDDELVEEK	39.53709	Q9NY61	AATF_HUMAN	951.7773963	3	0.736469269		
ASAQQENSSTCIGSAIK	6.859962	Q9NXV6	CARF_HUMAN	876.415614	2	0.807258904		
ASAQQENSSTCIGSAIK	6.859962	Q9NXV6	CARF_HUMAN	584.6130177	3	0.807258904		
ATLDVFFVPLK	104.8051	Q9NXV6	CARF_HUMAN	625.363731	2	0.699731648	2	Yes
ATLDVFFVPLK	104.8051	Q9NXV6	CARF_HUMAN	417.2450957	3	0.699731648	2	
ELADLPQNK	8.339436	Q9NXV6	CARF_HUMAN	514.2751125	2	0.717232227		
ELADLPQNK	8.339436	Q9NXV6	CARF_HUMAN	343.1860167	3	0.717232227		
ILSMAEGIK	28.69606	Q9NXV6	CARF_HUMAN	481.273527	2	0.692127705	2	Yes
ILSMAEGIK	28.69606	Q9NXV6	CARF_HUMAN	321.1849597	3	0.692127705	2	
LVAVGGFSPNVNHGELLNAAIEALK	96.06059	Q9NXV6	CARF_HUMAN	1267.192837	2	0.757192969	3	
LVAVGGFSPNVNHGELLNAAIEALK	96.06059	Q9NXV6	CARF_HUMAN	845.131166	3	0.757192969	3	Yes
NAGDLAPAGGAASASTDEAADAESGTR	20.58368	Q9NXV6	CARF_HUMAN	1217.04221	2	0.638957083	3	
NAGDLAPAGGAASASTDEAADAESGTR	20.58368	Q9NXV6	CARF_HUMAN	811.697415	3	0.638957083	3	Yes
NSSSSGTSLLTPK	12.08725	Q9NXV6	CARF_HUMAN	639.8309795	2	0.638265252	2	Yes
NSSSSGTSLLTPK	12.08725	Q9NXV6	CARF_HUMAN	426.889928	3	0.638265252	2	
SSSSTNTSLLTSK	2.155697	Q9NXV6	CARF_HUMAN	656.833719	2	0.74446094		
SSSSTNTSLLTSK	2.155697	Q9NXV6	CARF_HUMAN	438.2250877	3	0.74446094		
STSLASVSQLASK	32.02756	Q9NXV6	CARF_HUMAN	639.8491725	2	0.754654586	2	Yes
STSLASVSQLASK	32.02756	Q9NXV6	CARF_HUMAN	426.9020567	3	0.754654586	2	
STSQVAASLLASK	34.28727	Q9NXV6	CARF_HUMAN	631.8517155	2	0.80058825		
STSQVAASLLASK	34.28727	Q9NXV6	CARF_HUMAN	421.5704187	3	0.80058825		
SVSSQSSSSVSSQVTTAGSGK	-14.77005	Q9NXV6	CARF_HUMAN	979.469628	2	0.649321914		

SVSSQSSSSVSSQVTTAGSGK	-14.77005	Q9NXV6	CARF HUMAN	653.3156937	3	0.649321914		
SVYLGTGCGK	-3.991673	Q9NXV6	CARF HUMAN	521.255862	2	0.71944052		
SVYLGTGCGK	-3.991673	Q9NXV6	CARF HUMAN	347.8398497	3	0.71944052		
VTDAPTYTTR	-19.11592	Q9NXV6	CARF HUMAN	562.7832985	2	0.834959269	2	Yes
VTDAPTYTTR	-19.11592	Q9NXV6	CARF HUMAN	375.5248073	3	0.834959269	2	
GLMEPLLPPK	61.03358	Q9NXS2	QPCTL HUMAN	547.818102	2	0.778896034	2	Yes
GLMEPLLPPK	61.03358	Q9NXS2	QPCTL HUMAN	365.5480097	3	0.778896034	2	
HLAQLMESIPHSPGPTR	27.78195	Q9NXS2	QPCTL HUMAN	935.9838015	2	0.813215494		
HLAQLMESIPHSPGPTR	27.78195	Q9NXS2	QPCTL HUMAN	624.3251427	3	0.813215494		
LNLLQSHPQEVMYFQPGEPFGSVEDDHIPFLR	106.6854	Q9NXS2	QPCTL HUMAN	1870.414856	2	0.846897602	4	
LNLLQSHPQEVMYFQPGEPFGSVEDDHIPFLR	106.6854	Q9NXS2	QPCTL HUMAN	1247.279179	3	0.846897602	4	
TPGSPGNLQVR	0.522446	Q9NXS2	QPCTL HUMAN	563.304736	2	0.690370679		
TPGSPGNLQVR	0.522446	Q9NXS2	QPCTL HUMAN	375.8724323	3	0.690370679		
VPLIGSLPEAR	54.13401	Q9NXS2	QPCTL HUMAN	576.3433295	2	0.762972474	2	Yes
VPLIGSLPEAR	54.13401	Q9NXS2	QPCTL HUMAN	384.564828	3	0.762972474	2	
VVGQLDPQR	-3.60038	Q9NXS2	QPCTL HUMAN	506.2832725	2	0.726773798	2	Yes
VVGQLDPQR	-3.60038	Q9NXS2	QPCTL HUMAN	337.8581233	3	0.726773798	2	
AFLEDMK	22.56248	Q9NXG2	THUM1 HUMAN	427.2103955	2	0.799378514	2	Yes
AFLEDMK	22.56248	Q9NXG2	THUM1 HUMAN	285.142872	3	0.799378514	2	
FQSVESGANNVVFIR	50.81796	Q9NXG2	THUM1 HUMAN	833.9315595	2	0.87003088	2	Yes
FQSVESGANNVVFIR	50.81796	Q9NXG2	THUM1 HUMAN	556.2903147	3	0.87003088	2	
GTFQIVYK	29.03814	Q9NXG2	THUM1 HUMAN	478.2665555	2	0.685698688	2	Yes
GTFQIVYK	29.03814	Q9NXG2	THUM1 HUMAN	319.180312	3	0.685698688	2	
LVHHILQDMYK	12.07652	Q9NXG2	THUM1 HUMAN	698.874467	2	0.840459168		
LVHHILQDMYK	12.07652	Q9NXG2	THUM1 HUMAN	466.252253	3	0.840459168		
MLPISGTCK	14.16704	Q9NXG2	THUM1 HUMAN	503.7571875	2	0.76890254		
MLPISGTCK	14.16704	Q9NXG2	THUM1 HUMAN	336.1740667	3	0.76890254		
VPENTEELGQTKPTSNPQVVNEGGAKPELASQA'	30.59085	Q9NXG2	THUM1 HUMAN	2203.571306	2	0.675192773	4	
VPENTEELGQTKPTSNPQVVNEGGAKPELASQA'	30.59085	Q9NXG2	THUM1 HUMAN	1469.383479	3	0.675192773	4	
TLGIEPEK	2.821774	Q9NXG2	THUM1 HUMAN	443.7481995	2	0.801883578		
TLGIEPEK	2.821774	Q9NXG2	THUM1 HUMAN	296.1680747	3	0.801883578		
VDLTNPQYTVVVEIIK	102.6106	Q9NXG2	THUM1 HUMAN	916.0147545	2	0.662689507		
VDLTNPQYTVVVEIIK	102.6106	Q9NXG2	THUM1 HUMAN	611.0124447	3	0.662689507		
YAETFLEPWFK	91.2215	Q9NXG2	THUM1 HUMAN	715.853723	2	0.852097511	2	Yes
YAETFLEPWFK	91.2215	Q9NXG2	THUM1 HUMAN	477.571757	3	0.852097511	2	
AAAVALFNLDIR	87.26039	Q9NXC5	MIO HUMAN	637.367336	2	0.744838595		
AAAVALFNLDIR	87.26039	Q9NXC5	MIO HUMAN	425.247499	3	0.744838595		
AIQILNEGASSEK	23.45324	Q9NXC5	MIO HUMAN	680.3599045	2	0.807964563		
AIQILNEGASSEK	23.45324	Q9NXC5	MIO HUMAN	453.9092113	3	0.807964563		
AVQGVTVDPYFHDR	28.38679	Q9NXC5	MIO HUMAN	802.3973495	2	0.769175053		
AVQGVTVDPYFHDR	28.38679	Q9NXC5	MIO HUMAN	535.267508	3	0.769175053		
DGVDLMESYVDR	67.84329	Q9NXC5	MIO HUMAN	699.8144685	2	0.810844779		
DGVDLMESYVDR	67.84329	Q9NXC5	MIO HUMAN	466.8789207	3	0.810844779		
EAGNLEGILLTGLTK	103.1971	Q9NXC5	MIO HUMAN	764.9332375	2	0.796550751		
EAGNLEGILLTGLTK	103.1971	Q9NXC5	MIO HUMAN	510.2914333	3	0.796550751		
FEKPVLTLTEQPKPLTK	35.66257	Q9NXC5	MIO HUMAN	985.0726085	2	0.812534451		
FEKPVLTLTEQPKPLTK	35.66257	Q9NXC5	MIO HUMAN	657.051014	3	0.812534451		
FLSDTQLNR	16.28381	Q9NXC5	MIO HUMAN	547.2860115	2	0.787351727		
FLSDTQLNR	16.28381	Q9NXC5	MIO HUMAN	365.1932827	3	0.787351727		
GDLNLNVVAMALSGYTDEK	111.8668	Q9NXC5	MIO HUMAN	1005.4966	2	0.773723066		
GDLNLNVVAMALSGYTDEK	111.8668	Q9NXC5	MIO HUMAN	670.667008	3	0.773723066		
GFSQYGVSGSPTK	9.006466	Q9NXC5	MIO HUMAN	657.8204115	2	0.75824362		
GFSQYGVSGSPTK	9.006466	Q9NXC5	MIO HUMAN	438.8828827	3	0.75824362		
GSLVYAGIK	20.40472	Q9NXC5	MIO HUMAN	454.266555	2	0.740370154		
GSLVYAGIK	20.40472	Q9NXC5	MIO HUMAN	303.1803117	3	0.740370154		
GTDVDVGPFLNSLVQEGEWER	121.9897	Q9NXC5	MIO HUMAN	1174.064228	2	0.756035089		
GTDVDVGPFLNSLVQEGEWER	121.9897	Q9NXC5	MIO HUMAN	783.045427	3	0.756035089		
HGGHAGHMLSWFR	13.80174	Q9NXC5	MIO HUMAN	746.854936	2	0.705835104		
HGGHAGHMLSWFR	13.80174	Q9NXC5	MIO HUMAN	498.2392323	3	0.705835104		
LLLAGMHR	6.896175	Q9NXC5	MIO HUMAN	455.7687465	2	0.609791934		
LLLAGMHR	6.896175	Q9NXC5	MIO HUMAN	304.1817727	3	0.609791934		
LSEDSAATLLSINSDTPYMK	78.64409	Q9NXC5	MIO HUMAN	1078.525554	2	0.738005757		
LSEDSAATLLSINSDTPYMK	78.64409	Q9NXC5	MIO HUMAN	719.3529773	3	0.738005757		
LYDMQHTPTPIGDETEPTIIER	56.32402	Q9NXC5	MIO HUMAN	1278.618507	2	0.863751829	3	
LYDMQHTPTPIGDETEPTIIER	56.32402	Q9NXC5	MIO HUMAN	852.7482797	3	0.863751829	3	Yes
NLLDAWR	53.7255	Q9NXC5	MIO HUMAN	444.2408755	2	0.771014571		
NLLDAWR	53.7255	Q9NXC5	MIO HUMAN	296.4965253	3	0.771014571		
SSLGMVESSR	-0.996544	Q9NXC5	MIO HUMAN	526.756229	2	0.781913161		
SSLGMVESSR	-0.996544	Q9NXC5	MIO HUMAN	351.506761	3	0.781913161		
TGDVQTASYCMLQGSPLDVLK	90.48857	Q9NXC5	MIO HUMAN	1142.053957	2	0.680597186		
TGDVQTASYCMLQGSPLDVLK	90.48857	Q9NXC5	MIO HUMAN	761.7052463	3	0.680597186		
TGLLATLTR	42.87097	Q9NXC5	MIO HUMAN	473.2905665	2	0.744075954		
TGLLATLTR	42.87097	Q9NXC5	MIO HUMAN	315.862986	3	0.744075954		
TMSDFTVFER	54.0098	Q9NXC5	MIO HUMAN	616.7849875	2	0.783441126		
TMSDFTVFER	54.0098	Q9NXC5	MIO HUMAN	411.5259333	3	0.783441126		
VASFYEGQVAIWDLR	88.26074	Q9NXC5	MIO HUMAN	877.4495815	2	0.838784873		
VASFYEGQVAIWDLR	88.26074	Q9NXC5	MIO HUMAN	585.3023293	3	0.838784873		
VQYWIENYR	42.63805	Q9NXC5	MIO HUMAN	635.8149275	2	0.844572008		
VQYWIENYR	42.63805	Q9NXC5	MIO HUMAN	424.21256	3	0.844572008		
VTSCPGCRKPLPR	-28.15437	Q9NXC5	MIO HUMAN	764.3983195	2	0.734821558		
VTSCPGCRKPLPR	-28.15437	Q9NXC5	MIO HUMAN	509.9348213	3	0.734821558		
VVLTSLGQDHNSK	-0.186401	Q9NXC5	MIO HUMAN	699.3733465	2	0.782520711		
VVLTSLGQDHNSK	-0.186401	Q9NXC5	MIO HUMAN	466.5848393	3	0.782520711		
YGLDTEQVWR	45.01121	Q9NXC5	MIO HUMAN	633.809847	2	0.816367745		
YGLDTEQVWR	45.01121	Q9NXC5	MIO HUMAN	422.8758397	3	0.816367745		
YTPDIVPMEK	38.34344	Q9NXC5	MIO HUMAN	596.800102	2	0.83144176		
YTPDIVPMEK	38.34344	Q9NXC5	MIO HUMAN	398.2026763	3	0.83144176		
DMFAFMGSLDTK	97.98233	Q9NWV8	BABA1 HUMAN	681.8076055	2	0.771660686	2	Yes
DMFAFMGSLDTK	97.98233	Q9NWV8	BABA1 HUMAN	454.8743453	3	0.771660686	2	
SEGEGEAASADDGSLNTSGAGPK	-3.332325	Q9NWV8	BABA1 HUMAN	1053.957083	2	0.676135004		
SEGEGEAASADDGSLNTSGAGPK	-3.332325	Q9NWV8	BABA1 HUMAN	702.9739967	3	0.676135004		
SWQVPPPAPEVQIR	62.23051	Q9NWV8	BABA1 HUMAN	802.433739	2	0.879310727		
SWQVPPPAPEVQIR	62.23051	Q9NWV8	BABA1 HUMAN	535.2917677	3	0.879310727		
TELPVTENVQTIPPPYVVR	74.98131	Q9NWV8	BABA1 HUMAN	1076.586607	2	0.830843389		
TELPVTENVQTIPPPYVVR	74.98131	Q9NWV8	BABA1 HUMAN	718.0603463	3	0.830843389		
TNALNVSQK	-21.4974	Q9NWV8	BABA1 HUMAN	487.7674545	2	0.73826617		
TNALNVSQK	-21.4974	Q9NWV8	BABA1 HUMAN	325.5142447	3	0.73826617		
EGIIHTLIVDNR	38.34909	Q9NVQ4	FAIM1 HUMAN	690.386257	2	0.66360122		
EGIIHTLIVDNR	38.34909	Q9NVQ4	FAIM1 HUMAN	460.5934463	3	0.66360122		
LETAGEFVDDGTETHFSIGNHDCYIK	51.32504	Q9NVQ4	FAIM1 HUMAN	1478.159256	2	0.768984139		
LETAGEFVDDGTETHFSIGNHDCYIK	51.32504	Q9NVQ4	FAIM1 HUMAN	985.7754453	3	0.768984139		

TTNTWVLHMDGENFR	49.49173	Q9NVQ4	FAIM1 HUMAN	910.9234095	2	0.803899288		
TTNTWVLHMDGENFR	49.49173	Q9NVQ4	FAIM1 HUMAN	607.6182147	3	0.803899288		
GVVPCNILVGYK	54.97346	Q9NRX5	SERC1 HUMAN	659.863568	2	0.780003846		
GVVPCNILVGYK	54.97346	Q9NRX5	SERC1 HUMAN	440.244987	3	0.780003846		
LTLTSDESTLIEDGGAR	49.5062	Q9NRX5	SERC1 HUMAN	889.4448945	2	0.809707284		
LTLTSDESTLIEDGGAR	49.5062	Q9NRX5	SERC1 HUMAN	593.2992047	3	0.809707284		
SDGSLEDGDDVHR	-22.57738	Q9NRX5	SERC1 HUMAN	701.2980325	2	0.679588377	2	Yes
SDGSLEDGDDVHR	-22.57738	Q9NRX5	SERC1 HUMAN	467.8679633	3	0.679588377	2	
AARPPWEPPK	-1.40588	Q9NRR4	RNC HUMAN	574.8147395	2	0.743389785		
AARPPWEPPK	-1.40588	Q9NRR4	RNC HUMAN	383.545768	3	0.743389785		
ALVPEEEIANMLQWEELEWQK	144.5216	Q9NRR4	RNC HUMAN	1293.133796	2	0.753439188		
ALVPEEEIANMLQWEELEWQK	144.5216	Q9NRR4	RNC HUMAN	862.425139	3	0.753439188		
DILELYDWNLK	115.9286	Q9NRR4	RNC HUMAN	711.3697365	2	0.753117561		
DILELYDWNLK	115.9286	Q9NRR4	RNC HUMAN	474.5824327	3	0.753117561		
EFILNQDWNDPK	59.66475	Q9NRR4	RNC HUMAN	759.865354	2	0.775094271		
EFILNQDWNDPK	59.66475	Q9NRR4	RNC HUMAN	506.9128443	3	0.775094271		
EVTVELSSQGFWK	66.2402	Q9NRR4	RNC HUMAN	755.38338	2	0.720918238		
EVTVELSSQGFWK	66.2402	Q9NRR4	RNC HUMAN	503.9248617	3	0.720918238		
EVWLNYPLHPLQLQEPNTDR	74.75402	Q9NRR4	RNC HUMAN	1231.627326	2	0.778104603		
EVWLNYPLHPLQLQEPNTDR	74.75402	Q9NRR4	RNC HUMAN	821.4208253	3	0.778104603		
FLPDGGK	-6.380993	Q9NRR4	RNC HUMAN	367.1981455	2	0.627284884		
FLPDGGK	-6.380993	Q9NRR4	RNC HUMAN	245.1347053	3	0.627284884		
FMLYAHGPDLCR	35.74249	Q9NRR4	RNC HUMAN	740.3477595	2	0.804154396		
FMLYAHGPDLCR	35.74249	Q9NRR4	RNC HUMAN	493.9011147	3	0.804154396		
GINTLINIMSR	71.42596	Q9NRR4	RNC HUMAN	616.345546	2	0.769070864		
GINTLINIMSR	71.42596	Q9NRR4	RNC HUMAN	411.2329723	3	0.769070864		
GMIVTNPGTKPSSVR	-0.301239	Q9NRR4	RNC HUMAN	772.4172315	2	0.782154322		
GMIVTNPGTKPSSVR	-0.301239	Q9NRR4	RNC HUMAN	515.2807627	3	0.782154322		
GPLFEDSPPCCPR	34.29276	Q9NRR4	RNC HUMAN	766.337593	2	0.650242329		
GPLFEDSPPCCPR	34.29276	Q9NRR4	RNC HUMAN	511.2276703	3	0.650242329		
GPSIQQAEMGAAMDALEK	66.10251	Q9NRR4	RNC HUMAN	923.9378685	2	0.824959636		
GPSIQQAEMGAAMDALEK	66.10251	Q9NRR4	RNC HUMAN	616.2945207	3	0.824959636		
HLLANSPK	-28.70709	Q9NRR4	RNC HUMAN	440.2565255	2	0.691550732		
HLLANSPK	-28.70709	Q9NRR4	RNC HUMAN	293.840292	3	0.691550732		
HLPPYPLPK	21.24127	Q9NRR4	RNC HUMAN	531.311297	2	0.726193905		
HLPPYPLPK	21.24127	Q9NRR4	RNC HUMAN	354.543473	3	0.726193905		
LHDELWYNDPGQMNDGPLCK	55.78086	Q9NRR4	RNC HUMAN	1201.53118	2	0.626285434		
LHDELWYNDPGQMNDGPLCK	55.78086	Q9NRR4	RNC HUMAN	801.356728	3	0.626285434		
LIGYTFQDR	33.63007	Q9NRR4	RNC HUMAN	556.790926	2	0.769540846		
LIGYTFQDR	33.63007	Q9NRR4	RNC HUMAN	371.5298923	3	0.769540846		
LLFNDPDLR	52.1523	Q9NRR4	RNC HUMAN	551.7987545	2	0.788366079		
LLFNDPDLR	52.1523	Q9NRR4	RNC HUMAN	368.201778	3	0.788366079		
NTDSWAPPLEIVNHR	60.77625	Q9NRR4	RNC HUMAN	874.939916	2	0.82163465		
NTDSWAPPLEIVNHR	60.77625	Q9NRR4	RNC HUMAN	583.629219	3	0.82163465		
SQLQQCCLTLR	21.28494	Q9NRR4	RNC HUMAN	703.8481285	2	0.732036948		
SQLQQCCLTLR	21.28494	Q9NRR4	RNC HUMAN	469.5680273	3	0.732036948		
SYGLSVVPEPAGCTPELPGEIIK	96.03377	Q9NRR4	RNC HUMAN	1207.120154	2	0.768400848		
SYGLSVVPEPAGCTPELPGEIIK	96.03377	Q9NRR4	RNC HUMAN	805.0827107	3	0.768400848		
TAIVQNQHLAMLAK	28.02406	Q9NRR4	RNC HUMAN	769.4301425	2	0.830731571		
TAIVQNQHLAMLAK	28.02406	Q9NRR4	RNC HUMAN	513.28937	3	0.830731571		
TLQTVGPSHAR	-27.56473	Q9NRR4	RNC HUMAN	583.818011	2	0.752107143		
TLQTVGPSHAR	-27.56473	Q9NRR4	RNC HUMAN	389.547949	3	0.752107143		
TVGFNHLTLGHNQR	-0.387772	Q9NRR4	RNC HUMAN	797.416412	2	0.835844934		
TVGFNHLTLGHNQR	-0.387772	Q9NRR4	RNC HUMAN	531.946883	3	0.835844934		
TYTVAVYFK	45.81565	Q9NRR4	RNC HUMAN	546.292766	2	0.619571507		
TYTVAVYFK	45.81565	Q9NRR4	RNC HUMAN	364.531119	3	0.619571507		
VAEELGMQEYAITNDK	45.19646	Q9NRR4	RNC HUMAN	905.9305615	2	0.812278032		
VAEELGMQEYAITNDK	45.19646	Q9NRR4	RNC HUMAN	604.2896493	3	0.812278032		
YNFPQMAHQK	1.769215	Q9NRR4	RNC HUMAN	632.301135	2	0.78962034		
YNFPQMAHQK	1.769215	Q9NRR4	RNC HUMAN	421.8700317	3	0.78962034		
AEWLLAVR	57.71704	Q9NRN7	ADPPT HUMAN	479.280001	2	0.733408213	2	Yes
AEWLLAVR	57.71704	Q9NRN7	ADPPT HUMAN	319.8559423	3	0.733408213	2	
AIGVGLGFELQR	72.01655	Q9NRN7	ADPPT HUMAN	630.3595115	2	0.81062746		
AIGVGLGFELQR	72.01655	Q9NRN7	ADPPT HUMAN	420.575616	3	0.81062746		
DEWTQLDMFYR	91.45206	Q9NRN7	ADPPT HUMAN	752.332829	2	0.761813581		
DEWTQLDMFYR	91.45206	Q9NRN7	ADPPT HUMAN	501.891161	3	0.761813581		
GSIPEFFHIMK	63.08509	Q9NRN7	ADPPT HUMAN	653.33719	2	0.831171155	3	
GSIPEFFHIMK	63.08509	Q9NRN7	ADPPT HUMAN	435.8940683	3	0.831171155	3	Yes
IDEHHFVAVALR	20.37839	Q9NRN7	ADPPT HUMAN	703.881142	2	0.787418425	3	
IDEHHFVAVALR	20.37839	Q9NRN7	ADPPT HUMAN	469.5900363	3	0.787418425	3	Yes
IGQFVFAR	37.97739	Q9NRN7	ADPPT HUMAN	469.266894	2	0.694294155	2	Yes
IGQFVFAR	37.97739	Q9NRN7	ADPPT HUMAN	313.1805377	3	0.694294155	2	
LFLDGEEEK	22.64008	Q9NRN7	ADPPT HUMAN	540.266953	2	0.777294993		
LFLDGEEEK	22.64008	Q9NRN7	ADPPT HUMAN	360.5139103	3	0.777294993		
LNIPWNHIR	35.14917	Q9NRN7	ADPPT HUMAN	581.8281805	2	0.840388417		
LNIPWNHIR	35.14917	Q9NRN7	ADPPT HUMAN	388.2213953	3	0.840388417		
AFIPLPSAVVQAVFGR	137.6595	Q9NRG7	D39U1 HUMAN	836.483232	2	0.691998482		
AFIPLPSAVVQAVFGR	137.6595	Q9NRG7	D39U1 HUMAN	557.9914297	3	0.691998482		
AIMLLEGQK	33.3242	Q9NRG7	D39U1 HUMAN	501.786802	2	0.800705194		
AIMLLEGQK	33.3242	Q9NRG7	D39U1 HUMAN	334.8604763	3	0.800705194		
GGGAMGHMLLPFR	49.35789	Q9NRG7	D39U1 HUMAN	672.339742	2	0.712209344		
GGGAMGHMLLPFR	49.35789	Q9NRG7	D39U1 HUMAN	448.5624363	3	0.712209344		
LETTQLLAK	21.06979	Q9NRG7	D39U1 HUMAN	508.8035065	2	0.772307754		
LETTQLLAK	21.06979	Q9NRG7	D39U1 HUMAN	339.5382793	3	0.772307754		
TLATGYQYSFPELGAALK	87.96017	Q9NRG7	D39U1 HUMAN	965.5020075	2	0.834194064		
TLATGYQYSFPELGAALK	87.96017	Q9NRG7	D39U1 HUMAN	644.0039467	3	0.834194064		
EALPDGVNISK	24.86336	Q9NRF9	DPOE3 HUMAN	571.8067765	2	0.804976106	2	Yes
EALPDGVNISK	24.86336	Q9NRF9	DPOE3 HUMAN	381.5404593	3	0.804976106	2	
TLNASDVLSAMEEMEFQR	117.854	Q9NRF9	DPOE3 HUMAN	1035.977721	2	0.759431005		
TLNASDVLSAMEEMEFQR	117.854	Q9NRF9	DPOE3 HUMAN	690.9877557	3	0.759431005		
ATTVPDFQETRPR	9.376587	Q9NR46	SHLB2 HUMAN	759.389529	2	0.753736734	3	
ATTVPDFQETRPR	9.376587	Q9NR46	SHLB2 HUMAN	506.5956277	3	0.753736734	3	Yes
DFIHTASISFLTPLR	104.3301	Q9NR46	SHLB2 HUMAN	859.4677785	2	0.764846861	3	
DFIHTASISFLTPLR	104.3301	Q9NR46	SHLB2 HUMAN	573.3144607	3	0.764846861	3	Yes
LASDAGIFFTR	55.52339	Q9NR46	SHLB2 HUMAN	599.317312	2	0.811753094	2	Yes
LASDAGIFFTR	55.52339	Q9NR46	SHLB2 HUMAN	399.8808163	3	0.811753094	2	
LLLEGISSTHVNHLR	32.48405	Q9NR46	SHLB2 HUMAN	844.9763015	2	0.86920476	3	
LLLEGISSTHVNHLR	32.48405	Q9NR46	SHLB2 HUMAN	563.653476	3	0.86920476	3	Yes
NFLEGDWK	38.61435	Q9NR46	SHLB2 HUMAN	504.743448	2	0.834554672	2	Yes
NFLEGDWK	38.61435	Q9NR46	SHLB2 HUMAN	336.8315737	3	0.834554672	2	
SQTTYYAQCYR	0.730545	Q9NR46	SHLB2 HUMAN	720.8147955	2	0.601138353	2	Yes

SQTTYYAQCYR	0.730545	Q9NR46	SHLB2 HUMAN	480.8791387	3	0.601138353	2	
TELDAHFENLLAR	57.62255	Q9NR46	SHLB2 HUMAN	764.8919035	2	0.784613371	3	
TELDAHFENLLAR	57.62255	Q9NR46	SHLB2 HUMAN	510.2638773	3	0.784613371	3	Yes
VTNGELLAQYMADAASELGPTTPYGK	113.2676	Q9NR46	SHLB2 HUMAN	1349.157992	2	0.717324317		
VTNGELLAQYMADAASELGPTTPYGK	113.2676	Q9NR46	SHLB2 HUMAN	899.774603	3	0.717324317		
ALLANQDSGEVQQDPK	10.13619	Q9NP81	SYSM HUMAN	856.926672	2	0.858950973		
ALLANQDSGEVQQDPK	10.13619	Q9NP81	SYSM HUMAN	571.6203897	3	0.858950973		
DGSVLVPPALQSYLGTDR	102.3845	Q9NP81	SYSM HUMAN	944.4947175	2	0.750784278		
DGSVLVPPALQSYLGTDR	102.3845	Q9NP81	SYSM HUMAN	629.9990867	3	0.750784278		
DLNLAGTAEVGLAGYFMDHTVAFR	122.9567	Q9NP81	SYSM HUMAN	1284.631752	2	0.630846977		
DLNLAGTAEVGLAGYFMDHTVAFR	122.9567	Q9NP81	SYSM HUMAN	856.7571093	3	0.630846977		
EAQLEEQFYLQALK	78.91956	Q9NP81	SYSM HUMAN	855.4414225	2	0.76066637		
EAQLEEQFYLQALK	78.91956	Q9NP81	SYSM HUMAN	570.6302233	3	0.76066637		
EGYSALPQLDIER	59.70453	Q9NP81	SYSM HUMAN	745.8784575	2	0.869048297		
EGYSALPQLDIER	59.70453	Q9NP81	SYSM HUMAN	497.5882467	3	0.869048297		
FCACPEEAAHALELR	40.08829	Q9NP81	SYSM HUMAN	887.4065385	2	0.607185066		
FCACPEEAAHALELR	40.08829	Q9NP81	SYSM HUMAN	591.9403007	3	0.607185066		
FDIEAWMPGR	90.97435	Q9NP81	SYSM HUMAN	611.29024	2	0.702016592		
FDIEAWMPGR	90.97435	Q9NP81	SYSM HUMAN	407.8627683	3	0.702016592		
FGEVTSASNCTDFQSR	22.51154	Q9NP81	SYSM HUMAN	903.392139	2	0.827811003	2	Yes
FGEVTSASNCTDFQSR	22.51154	Q9NP81	SYSM HUMAN	602.5973677	3	0.827811003	2	
GAGALLQHGLVNFTFNK	63.10508	Q9NP81	SYSM HUMAN	893.9841275	2	0.692433774	3	
GAGALLQHGLVNFTFNK	63.10508	Q9NP81	SYSM HUMAN	596.32536	3	0.692433774	3	Yes
GFTPMTVPDLLR	87.60318	Q9NP81	SYSM HUMAN	673.86103	2	0.74157244		
GFTPMTVPDLLR	87.60318	Q9NP81	SYSM HUMAN	449.5766283	3	0.74157244		
ITAPTHVPLQYIGPNQPR	46.1212	Q9NP81	SYSM HUMAN	1001.54762	2	0.779238999	3	
ITAPTHVPLQYIGPNQPR	46.1212	Q9NP81	SYSM HUMAN	668.0343547	3	0.779238999	3	Yes
LLIALLESNQQK	69.34791	Q9NP81	SYSM HUMAN	685.406658	2	0.773556948		
LLIALLESNQQK	69.34791	Q9NP81	SYSM HUMAN	457.2737137	3	0.773556948		
LPNQTHPDVPVGDESQAR	6.615913	Q9NP81	SYSM HUMAN	980.4801345	2	0.684037447		
LPNQTHPDVPVGDESQAR	6.615913	Q9NP81	SYSM HUMAN	653.9893647	3	0.684037447		
NLLYEYAR	32.23996	Q9NP81	SYSM HUMAN	521.272364	2	0.821382523	2	Yes
NLLYEYAR	32.23996	Q9NP81	SYSM HUMAN	347.850851	3	0.821382523	2	
SADLPAIISTWQELR	112.2742	Q9NP81	SYSM HUMAN	850.454868	2	0.839921594	3	
SADLPAIISTWQELR	112.2742	Q9NP81	SYSM HUMAN	567.3058537	3	0.839921594	3	Yes
VLDMPTQELGLPAYR	76.98998	Q9NP81	SYSM HUMAN	851.945818	2	0.889811039		
VLDMPTQELGLPAYR	76.98998	Q9NP81	SYSM HUMAN	568.2998203	3	0.889811039		
VLHMVGDKPVFSFQPR	40.33449	Q9NP81	SYSM HUMAN	928.9961805	2	0.735409737	4	
VLHMVGDKPVFSFQPR	40.33449	Q9NP81	SYSM HUMAN	619.6667287	3	0.735409737	4	
AGPNGTLFVADAYK	46.88792	Q9HDC9	APMAP HUMAN	712.3649865	2	0.643332362		
AGPNGTLFVADAYK	46.88792	Q9HDC9	APMAP HUMAN	475.2459327	3	0.643332362		
DYLLLVMEGTDDGR	104.3198	Q9HDC9	APMAP HUMAN	798.882883	2	0.738393068		
DYLLLVMEGTDDGR	104.3198	Q9HDC9	APMAP HUMAN	532.9245303	3	0.738393068		
EPPLLLGVLHPNTK	63.15704	Q9HDC9	APMAP HUMAN	764.4488575	2	0.601661444	3	
EPPLLLGVLHPNTK	63.15704	Q9HDC9	APMAP HUMAN	509.9685133	3	0.601661444	3	Yes
GLFEVNPWK	65.76212	Q9HDC9	APMAP HUMAN	545.2905655	2	0.82128191		
GLFEVNPWK	65.76212	Q9HDC9	APMAP HUMAN	363.8629853	3	0.82128191		
IYFTDSSSK	7.934677	Q9HDC9	APMAP HUMAN	524.253841	2	0.609747112	2	Yes
IYFTDSSSK	7.934677	Q9HDC9	APMAP HUMAN	349.8385023	3	0.609747112	2	
LFENQLVGPESIAHIGDVMFTGTADGR	112.7416	Q9HDC9	APMAP HUMAN	1437.708723	2	0.735621631	3	
LFENQLVGPESIAHIGDVMFTGTADGR	112.7416	Q9HDC9	APMAP HUMAN	958.8084237	3	0.735621631	3	Yes
LFSQETVMK	18.02191	Q9HDC9	APMAP HUMAN	541.7817165	2	0.77883023	2	Yes
LFSQETVMK	18.02191	Q9HDC9	APMAP HUMAN	361.5237527	3	0.77883023	2	
LLEYDTVTR	23.86602	Q9HDC9	APMAP HUMAN	555.296041	2	0.809258223	2	Yes
LLEYDTVTR	23.86602	Q9HDC9	APMAP HUMAN	370.5333023	3	0.809258223	2	
LLLSSETPIEGK	39.22121	Q9HDC9	APMAP HUMAN	643.8642915	2	0.851571321	2	Yes
LLLSSETPIEGK	39.22121	Q9HDC9	APMAP HUMAN	429.5788027	3	0.851571321	2	
NMSFVNDLTVTQDGR	59.25226	Q9HDC9	APMAP HUMAN	848.9021425	2	0.788837433		
NMSFVNDLTVTQDGR	59.25226	Q9HDC9	APMAP HUMAN	566.2707033	3	0.788837433		
RPLRPQVVTDDDGQAPEAK	-9.316402	Q9HDC9	APMAP HUMAN	1046.543267	2	0.617507637	3	
RPLRPQVVTDDDGQAPEAK	-9.316402	Q9HDC9	APMAP HUMAN	698.0314527	3	0.617507637	3	Yes
SLHDPDGLVATYISEVHEHDGHLYLGSFR	76.39735	Q9HDC9	APMAP HUMAN	1632.789612	2	0.725784481	4	
SLHDPDGLVATYISEVHEHDGHLYLGSFR	76.39735	Q9HDC9	APMAP HUMAN	1088.86235	3	0.725784481	4	
VLLDQLR	35.52835	Q9HDC9	APMAP HUMAN	428.7667265	2	0.723516047	2	Yes
VLLDQLR	35.52835	Q9HDC9	APMAP HUMAN	286.180426	3	0.723516047	2	
YSLVLELSDSGAFR	86.16042	Q9HDC9	APMAP HUMAN	778.901932	2	0.766039371		
YSLVLELSDSGAFR	86.16042	Q9HDC9	APMAP HUMAN	519.6038963	3	0.766039371		
DLVTQQLPHLMPSNCGLEEK	67.06915	Q9HCY8	S10AE HUMAN	1155.067403	2	0.675763369		
DLVTQQLPHLMPSNCGLEEK	67.06915	Q9HCY8	S10AE HUMAN	770.3808767	3	0.675763369		
ETLTPSELR	15.60088	Q9HCY8	S10AE HUMAN	523.280395	2	0.716182053		
ETLTPSELR	15.60088	Q9HCY8	S10AE HUMAN	349.1895383	3	0.716182053		
NFHQYSVEGGK	-25.40872	Q9HCY8	S10AE HUMAN	633.299646	2	0.67345506		
NFHQYSVEGGK	-25.40872	Q9HCY8	S10AE HUMAN	422.5357057	3	0.67345506		
SANAEDAQEFSDVER	19.69466	Q9HCY8	S10AE HUMAN	834.361361	2	0.697072387		
SANAEDAQEFSDVER	19.69466	Q9HCY8	S10AE HUMAN	556.576849	3	0.697072387		
SFWELIGEAAK	90.06024	Q9HCY8	S10AE HUMAN	625.8249695	2	0.824798703		
SFWELIGEAAK	90.06024	Q9HCY8	S10AE HUMAN	417.552588	3	0.824798703		
DETLQPFFEGLLPR	131.3985	Q9HCG8	CWC22 HUMAN	831.430862	2	0.745703816		
DETLQPFFEGLLPR	131.3985	Q9HCG8	CWC22 HUMAN	554.623183	3	0.745703816		
DQEMHIDLENK	12.94452	Q9HCG8	CWC22 HUMAN	686.31464	2	0.776842952		
DQEMHIDLENK	12.94452	Q9HCG8	CWC22 HUMAN	457.879035	3	0.776842952		
DYFDYSR	30.26933	Q9HCG8	CWC22 HUMAN	483.204147	2	0.619143784		
DYFDYSR	30.26933	Q9HCG8	CWC22 HUMAN	322.4720397	3	0.619143784		
EYMESFEGIFK	73.4668	Q9HCG8	CWC22 HUMAN	690.313573	2	0.804041266		
EYMESFEGIFK	73.4668	Q9HCG8	CWC22 HUMAN	460.5449903	3	0.804041266		
FFGLLAGR	61.28885	Q9HCG8	CWC22 HUMAN	440.756162	2	0.630126953	2	Yes
FFGLLAGR	61.28885	Q9HCG8	CWC22 HUMAN	294.173383	3	0.630126953	2	
FPQIGELILK	83.45349	Q9HCG8	CWC22 HUMAN	579.3506235	2	0.813849807		
FPQIGELILK	83.45349	Q9HCG8	CWC22 HUMAN	386.5696907	3	0.813849807		
IFFQELCEYMGLPK	116.1498	Q9HCG8	CWC22 HUMAN	887.931322	2	0.650144935		
IFFQELCEYMGLPK	116.1498	Q9HCG8	CWC22 HUMAN	592.2901563	3	0.650144935		
LSEETTTSSSR	-30.09926	Q9HCG8	CWC22 HUMAN	599.2838625	2	0.667652369		
LSEETTTSSSR	-30.09926	Q9HCG8	CWC22 HUMAN	399.8585167	3	0.667652369		
MDPNFMENEEK	23.09607	Q9HCG8	CWC22 HUMAN	692.281951	2	0.81464684	2	Yes
MDPNFMENEEK	23.09607	Q9HCG8	CWC22 HUMAN	461.8572423	3	0.81464684	2	
MEFPESQTK	7.222004	Q9HCG8	CWC22 HUMAN	548.753156	2	0.662178993	2	Yes
MEFPESQTK	7.222004	Q9HCG8	CWC22 HUMAN	366.171379	3	0.662178993	2	
MMQEQITDK	-4.833893	Q9HCG8	CWC22 HUMAN	562.2602915	2	0.666196465		
MMQEQITDK	-4.833893	Q9HCG8	CWC22 HUMAN	375.176136	3	0.666196465		
NILHESEIDK	-6.117218	Q9HCG8	CWC22 HUMAN	599.309683	2	0.740530372		
NILHESEIDK	-6.117218	Q9HCG8	CWC22 HUMAN	399.8757303	3	0.740530372		

TGGAYIPPAK	1.096577	Q9HCG8	CWC22 HUMAN	487.7694625	2	0.621115088		
TGGAYIPPAK	1.096577	Q9HCG8	CWC22 HUMAN	325.5155833	3	0.621115088		
VIVAQKPDVEQNK	-17.39278	Q9HCG8	CWC22 HUMAN	734.412472	2	0.755040109		
VIVAQKPDVEQNK	-17.39278	Q9HCG8	CWC22 HUMAN	489.9442563	3	0.755040109		
DKPITFTQHQMPLFR	42.41944	Q9HC62	SENP2 HUMAN	929.9858135	2	0.82643646		
DKPITFTQHQMPLFR	42.41944	Q9HC62	SENP2 HUMAN	620.326484	3	0.82643646		
EISNALGHGPQDEILSSAFK	58.06122	Q9HC62	SENP2 HUMAN	1057.032199	2	0.653592169		
EISNALGHGPQDEILSSAFK	58.06122	Q9HC62	SENP2 HUMAN	705.0240743	3	0.653592169		
GNPESSLMWK	31.47079	Q9HC62	SENP2 HUMAN	574.774422	2	0.748487473		
GNPESSLMWK	31.47079	Q9HC62	SENP2 HUMAN	383.5188897	3	0.748487473		
GVNLFEQEIILVPIHR	98.42188	Q9HC62	SENP2 HUMAN	939.0363595	2	0.831164598		
GVNLFEQEIILVPIHR	98.42188	Q9HC62	SENP2 HUMAN	626.3601813	3	0.831164598		
GWGEEQNHGVK	-28.85845	Q9HC62	SENP2 HUMAN	620.78945	2	0.70619154		
GWGEEQNHGVK	-28.85845	Q9HC62	SENP2 HUMAN	414.195575	3	0.70619154		
GYQLEPDLSEEVSAR	49.85593	Q9HC62	SENP2 HUMAN	846.907943	2	0.761169732		
GYQLEPDLSEEVSAR	49.85593	Q9HC62	SENP2 HUMAN	564.941237	3	0.761169732		
ICEILLQYLQDESK	102.3654	Q9HC62	SENP2 HUMAN	876.44839	2	0.605202913		
ICEILLQYLQDESK	102.3654	Q9HC62	SENP2 HUMAN	584.6348683	3	0.605202913		
ILGTIFR	43.89761	Q9HC62	SENP2 HUMAN	410.2585375	2	0.659859717		
ILGTIFR	43.89761	Q9HC62	SENP2 HUMAN	273.8416333	3	0.659859717		
ITDTETMVGIR	30.54947	Q9HC62	SENP2 HUMAN	618.319195	2	0.732431233		
ITDTETMVGIR	30.54947	Q9HC62	SENP2 HUMAN	412.5487383	3	0.732431233		
LDCFIHQVK	10.34065	Q9HC62	SENP2 HUMAN	580.300608	2	0.772776246		
LDCFIHQVK	10.34065	Q9HC62	SENP2 HUMAN	387.2030137	3	0.772776246		
NVAPSGEVFSNSSSCELTGSGSWNNMLK	73.52238	Q9HC62	SENP2 HUMAN	1480.164017	2	0.67918992		
NVAPSGEVFSNSSSCELTGSGSWNNMLK	73.52238	Q9HC62	SENP2 HUMAN	987.111953	3	0.67918992		
PQEQAVTEMISEESGK	35.83746	Q9HC62	SENP2 HUMAN	881.912373	2	0.871332109		
PQEQAVTEMISEESGK	35.83746	Q9HC62	SENP2 HUMAN	588.2775237	3	0.871332109		
SDSTLFSTVDTDEIPAK	62.15829	Q9HC62	SENP2 HUMAN	913.4392775	2	0.853720307		
SDSTLFSTVDTDEIPAK	62.15829	Q9HC62	SENP2 HUMAN	609.29546	3	0.853720307		
TDDLLELTEDMEK	86.21524	Q9HC62	SENP2 HUMAN	776.3589105	2	0.813151717		
TDDLLELTEDMEK	86.21524	Q9HC62	SENP2 HUMAN	517.9085487	3	0.813151717		
VLPSFGFTLNSEGCNR	73.06461	Q9HC62	SENP2 HUMAN	899.4336095	2	0.667566538		
VLPSFGFTLNSEGCNR	73.06461	Q9HC62	SENP2 HUMAN	599.958348	3	0.667566538		
YLDSMGQK	-6.960888	Q9HC62	SENP2 HUMAN	471.22403	2	0.703956306		
YLDSMGQK	-6.960888	Q9HC62	SENP2 HUMAN	314.485295	3	0.703956306		
APSTWEESGLR	16.53377	Q9HC36	MRM3 HUMAN	616.799483	2	0.801953793	2	Yes
APSTWEESGLR	16.53377	Q9HC36	MRM3 HUMAN	411.535597	3	0.801953793	2	
ASDHGWVCDQR	-22.57368	Q9HC36	MRM3 HUMAN	665.783842	2	0.803042054		
ASDHGWVCDQR	-22.57368	Q9HC36	MRM3 HUMAN	444.1918363	3	0.803042054		
DPGNLGTILR	50.07165	Q9HC36	MRM3 HUMAN	528.2963795	2	0.892587543	2	Yes
DPGNLGTILR	50.07165	Q9HC36	MRM3 HUMAN	352.533528	3	0.892587543	2	
DWSDLVTPQGIMGIFAKPDHVK	106.5355	Q9HC36	MRM3 HUMAN	1227.628485	2	0.655508578		
DWSDLVTPQGIMGIFAKPDHVK	106.5355	Q9HC36	MRM3 HUMAN	818.7549313	3	0.655508578		
GCVDAWEPK	10.47137	Q9HC36	MRM3 HUMAN	531.240216	2	0.769681334		
GCVDAWEPK	10.47137	Q9HC36	MRM3 HUMAN	354.4960857	3	0.769681334		
TQLQHSLPLLLICDNLR	81.65706	Q9HC36	MRM3 HUMAN	1017.562416	2	0.646941841	3	
TQLQHSLPLLLICDNLR	81.65706	Q9HC36	MRM3 HUMAN	678.7108857	3	0.646941841	3	Yes
VVFPSGEVVEQK	32.3135	Q9HC36	MRM3 HUMAN	659.356634	2	0.83979857	2	Yes
VVFPSGEVVEQK	32.3135	Q9HC36	MRM3 HUMAN	439.907031	3	0.83979857	2	
VYVADNCGLYAQAEMSNK	43.70263	Q9HC36	MRM3 HUMAN	1016.959323	2	0.754953325		
VYVADNCGLYAQAEMSNK	43.70263	Q9HC36	MRM3 HUMAN	678.3088237	3	0.754953325		
AGHMVPSDQGDMALK	5.099625	Q9HB40	RISC HUMAN	778.863975	2	0.727083802		
AGHMVPSDQGDMALK	5.099625	Q9HB40	RISC HUMAN	519.5785917	3	0.727083802		
DALSQLMNGPIR	77.00791	Q9HB40	RISC HUMAN	657.8459105	2	0.750544906		
DALSQLMNGPIR	77.00791	Q9HB40	RISC HUMAN	438.899882	3	0.750544906		
DLAMVASDMMVLLK	141.6381	Q9HB40	RISC HUMAN	768.8958975	2	0.700628877		
DLAMVASDMMVLLK	141.6381	Q9HB40	RISC HUMAN	512.9332067	3	0.700628877		
EATELWGK	12.8013	Q9HB40	RISC HUMAN	467.237999	2	0.61239028		
EATELWGK	12.8013	Q9HB40	RISC HUMAN	311.827941	3	0.61239028		
MAAGIGLELYK	56.53274	Q9HB40	RISC HUMAN	583.318462	2	0.791674614		
MAAGIGLELYK	56.53274	Q9HB40	RISC HUMAN	389.2149163	3	0.791674614		
VAEQVLNAVNK	14.72586	Q9HB40	RISC HUMAN	592.8358685	2	0.826282382	2	Yes
VAEQVLNAVNK	14.72586	Q9HB40	RISC HUMAN	395.559854	3	0.826282382	2	
EFMLCHDEEHEK	-12.61889	Q9HAZ1	CLK4 HUMAN	802.3299645	2	0.735547125		
EFMLCHDEEHEK	-12.61889	Q9HAZ1	CLK4 HUMAN	535.2225847	3	0.735547125		
ENSFLPFQIDHIR	68.88135	Q9HAZ1	CLK4 HUMAN	808.4155455	2	0.736228406		
ENSFLPFQIDHIR	68.88135	Q9HAZ1	CLK4 HUMAN	539.2796387	3	0.736228406		
ILGPIPQHMIQK	32.37646	Q9HAZ1	CLK4 HUMAN	687.9004895	2	0.816205502		
ILGPIPQHMIQK	32.37646	Q9HAZ1	CLK4 HUMAN	458.936268	3	0.816205502		
ITLDEALQHPFFDLLK	109.5924	Q9HAZ1	CLK4 HUMAN	950.514926	2	0.798836708		
ITLDEALQHPFFDLLK	109.5924	Q9HAZ1	CLK4 HUMAN	634.012559	3	0.798836708		
LTHTDLKPENILFVK	43.1701	Q9HAZ1	CLK4 HUMAN	884.504361	2	0.854562104		
LTHTDLKPENILFVK	43.1701	Q9HAZ1	CLK4 HUMAN	590.0055157	3	0.854562104		
MLEYDPTQR	14.69514	Q9HAZ1	CLK4 HUMAN	576.771876	2	0.75826025		
MLEYDPTQR	14.69514	Q9HAZ1	CLK4 HUMAN	384.8505257	3	0.75826025		
SEIQVLEHLNSTDPNSVFR	71.03517	Q9HAZ1	CLK4 HUMAN	1093.04838	2	0.677586496		
SEIQVLEHLNSTDPNSVFR	71.03517	Q9HAZ1	CLK4 HUMAN	729.0348617	3	0.677586496		
SIEDDEEGHLICQSGDVLR	41.2784	Q9HAZ1	CLK4 HUMAN	1086.497864	2	0.714278042		
SIEDDEEGHLICQSGDVLR	41.2784	Q9HAZ1	CLK4 HUMAN	724.6678507	3	0.714278042		
VVECIDHGMDGMHVAVK	21.74345	Q9HAZ1	CLK4 HUMAN	948.942431	2	0.729711533		
VVECIDHGMDGMHVAVK	21.74345	Q9HAZ1	CLK4 HUMAN	632.964229	3	0.729711533		
YFHHNQLDWDEHSSAGR	-1.952332	Q9HAZ1	CLK4 HUMAN	1049.959896	2	0.828428209		
YFHHNQLDWDEHSSAGR	-1.952332	Q9HAZ1	CLK4 HUMAN	700.3092053	3	0.828428209		
AAGYNFYGTER	17.47621	Q9H9Y6	RPA2 HUMAN	624.7863675	2	0.841997623	2	Yes
AAGYNFYGTER	17.47621	Q9H9Y6	RPA2 HUMAN	416.8601867	3	0.841997623	2	
AALQELTR	6.625015	Q9H9Y6	RPA2 HUMAN	451.259266	2	0.731523454		
AALQELTR	6.625015	Q9H9Y6	RPA2 HUMAN	301.1754523	3	0.731523454		
DALLAHGTSFLLHDR	44.24671	Q9H9Y6	RPA2 HUMAN	833.4395525	2	0.805099607	4	
DALLAHGTSFLLHDR	44.24671	Q9H9Y6	RPA2 HUMAN	555.96231	3	0.805099607	4	
GPGYTQYGVSMHCVR	15.19073	Q9H9Y6	RPA2 HUMAN	856.3881405	2	0.614812434		
GPGYTQYGVSMHCVR	15.19073	Q9H9Y6	RPA2 HUMAN	571.2613687	3	0.614812434		
HLTDPSYGIPR	14.98493	Q9H9Y6	RPA2 HUMAN	628.325664	2	0.796309114		
HLTDPSYGIPR	14.98493	Q9H9Y6	RPA2 HUMAN	419.2197177	3	0.796309114		
ISFTILDAVISPPTVPK	124.6153	Q9H9Y6	RPA2 HUMAN	899.5222195	2	0.798058629		
ISFTILDAVISPPTVPK	124.6153	Q9H9Y6	RPA2 HUMAN	600.0174213	3	0.798058629		
IVMEEGCSTQK	-19.54169	Q9H9Y6	RPA2 HUMAN	641.2948625	2	0.660936177	2	Yes
IVMEEGCSTQK	-19.54169	Q9H9Y6	RPA2 HUMAN	427.86585	3	0.660936177	2	
LDDDGLPFIGAK	71.01159	Q9H9Y6	RPA2 HUMAN	630.8277095	2	0.809397042	2	Yes
LDDDGLPFIGAK	71.01159	Q9H9Y6	RPA2 HUMAN	420.887748	3	0.809397042	2	
LTADINWAVNGISK	64.79289	Q9H9Y6	RPA2 HUMAN	751.4046465	2	0.80954212		

LTADINWAVNGISK	64.79289	Q9H9Y6	RPA2_HUMAN	501.2723727	3	0.80954212		
LVRPVQNLALGK	19.8338	Q9H9Y6	RPA2_HUMAN	654.412078	2	0.725680053		
LVRPVQNLALGK	19.8338	Q9H9Y6	RPA2_HUMAN	436.6106603	3	0.725680053		
MTIGMLIESMAGK	97.83571	Q9H9Y6	RPA2_HUMAN	691.3485835	2	0.652184069	2	Yes
MTIGMLIESMAGK	97.83571	Q9H9Y6	RPA2_HUMAN	461.2349973	3	0.652184069	2	
NLPSGPSLK	3.758423	Q9H9Y6	RPA2_HUMAN	456.7616405	2	0.758496404		
NLPSGPSLK	3.758423	Q9H9Y6	RPA2_HUMAN	304.843702	3	0.758496404		
NSVSQMLR	3.308189	Q9H9Y6	RPA2_HUMAN	467.742925	2	0.771718621		
NSVSQMLR	3.308189	Q9H9Y6	RPA2_HUMAN	312.1645583	3	0.771718621		
QVLNYLGECFR	61.12389	Q9H9Y6	RPA2_HUMAN	699.8459065	2	0.606775463		
QVLNYLGECFR	61.12389	Q9H9Y6	RPA2_HUMAN	466.8998793	3	0.606775463		
SDTIDTVSVPYVFR	82.42926	Q9H9Y6	RPA2_HUMAN	799.907215	2	0.786612213	2	Yes
SDTIDTVSVPYVFR	82.42926	Q9H9Y6	RPA2_HUMAN	533.6074183	3	0.786612213	2	
SEFIDLSEK	36.58413	Q9H9Y6	RPA2_HUMAN	534.2669525	2	0.682992458		
SEFIDLSEK	36.58413	Q9H9Y6	RPA2_HUMAN	356.51391	3	0.682992458		
TGLGLLQDSGLCVVADK	76.69847	Q9H9Y6	RPA2_HUMAN	873.459294	2	0.798874974		
TGLGLLQDSGLCVVADK	76.69847	Q9H9Y6	RPA2_HUMAN	582.6421377	3	0.798874974		
YFVAELAAMNIK	80.7204	Q9H9Y6	RPA2_HUMAN	685.3634005	2	0.796557426		
YFVAELAAMNIK	80.7204	Q9H9Y6	RPA2_HUMAN	457.2448753	3	0.796557426		
DAAHPSEATFSCDCVADALILR	73.02842	Q9H9Q4	NHEJ1_HUMAN	1210.055024	2	0.663189709	3	
DAAHPSEATFSCDCVADALILR	73.02842	Q9H9Q4	NHEJ1_HUMAN	807.0392907	3	0.663189709	3	Yes
DLEIQDYQESGATLIR	69.23203	Q9H9Q4	NHEJ1_HUMAN	925.9607075	2	0.864404202		
DLEIQDYQESGATLIR	69.23203	Q9H9Q4	NHEJ1_HUMAN	617.64308	3	0.864404202		
ELATLLHMK	32.10735	Q9H9Q4	NHEJ1_HUMAN	528.3000765	2	0.663600445		
ELATLLHMK	32.10735	Q9H9Q4	NHEJ1_HUMAN	352.5359927	3	0.663600445		
ESTGTSGPLQRPQLSK	-2.40572	Q9H9Q4	NHEJ1_HUMAN	843.445032	2	0.733776867		
ESTGTSGPLQRPQLSK	-2.40572	Q9H9Q4	NHEJ1_HUMAN	562.6326297	3	0.733776867		
TEPFEENSFLEQFMIEK	116.5683	Q9H9Q4	NHEJ1_HUMAN	1059.490987	2	0.682067811		
TEPFEENSFLEQFMIEK	116.5683	Q9H9Q4	NHEJ1_HUMAN	706.6632663	3	0.682067811		
ATASSSAQEMEQQLAER	23.81054	Q9H9Q2	CSN7B_HUMAN	918.9238035	2	0.809147537	3	
ATASSSAQEMEQQLAER	23.81054	Q9H9Q2	CSN7B_HUMAN	612.9518107	3	0.809147537	3	Yes
ESLPELSTAQQNK	21.77461	Q9H9Q2	CSN7B_HUMAN	722.868094	2	0.836736441		
ESLPELSTAQQNK	21.77461	Q9H9Q2	CSN7B_HUMAN	482.2480043	3	0.836736441		
HLTIVSLASR	21.75455	Q9H9Q2	CSN7B_HUMAN	548.8278465	2	0.700542569		
HLTIVSLASR	21.75455	Q9H9Q2	CSN7B_HUMAN	366.2211727	3	0.700542569		
TQQQVEAEVTNIK	12.76453	Q9H9Q2	CSN7B_HUMAN	744.3891945	2	0.829417646	2	Yes
TQQQVEAEVTNIK	12.76453	Q9H9Q2	CSN7B_HUMAN	496.5954047	3	0.829417646	2	
DFLITQMTGK	56.8426	Q9H9J2	RM44_HUMAN	577.300274	2	0.813337326	2	Yes
DFLITQMTGK	56.8426	Q9H9J2	RM44_HUMAN	385.202791	3	0.813337326	2	
EAVLLNLK	38.86988	Q9H9J2	RM44_HUMAN	450.282209	2	0.609025598		
EAVLLNLK	38.86988	Q9H9J2	RM44_HUMAN	300.524081	3	0.609025598		
IINPMGLLVEELK	125.2836	Q9H9J2	RM44_HUMAN	734.926369	2	0.77949208		
IINPMGLLVEELK	125.2836	Q9H9J2	RM44_HUMAN	490.2868543	3	0.77949208		
LIAEGPGETVLVAEEEAAR	64.30099	Q9H9J2	RM44_HUMAN	977.510569	2	0.858296275	3	
LIAEGPGETVLVAEEEAAR	64.30099	Q9H9J2	RM44_HUMAN	652.0096543	3	0.858296275	3	Yes
LQENFSLDLLK	74.67079	Q9H9J2	RM44_HUMAN	660.3644585	2	0.721787572	2	Yes
LQENFSLDLLK	74.67079	Q9H9J2	RM44_HUMAN	440.578914	3	0.721787572	2	
LYGFTENR	14.95023	Q9H9J2	RM44_HUMAN	500.2488935	2	0.771420062		
LYGFTENR	14.95023	Q9H9J2	RM44_HUMAN	333.835204	3	0.771420062		
NLVDFLTGEEVVCHVAR	83.31737	Q9H9J2	RM44_HUMAN	979.494199	2	0.841049612	3	
NLVDFLTGEEVVCHVAR	83.31737	Q9H9J2	RM44_HUMAN	653.3320743	3	0.841049612	3	Yes
SEKPNWDYHAEIQAFGHR	25.16683	Q9H9J2	RM44_HUMAN	1093.014671	2	0.735517144		
SEKPNWDYHAEIQAFGHR	25.16683	Q9H9J2	RM44_HUMAN	729.012389	3	0.735517144		
TAFVNSCYIK	24.72102	Q9H9J2	RM44_HUMAN	601.7978935	2	0.784393549	2	Yes
TAFVNSCYIK	24.72102	Q9H9J2	RM44_HUMAN	401.5345373	3	0.784393549	2	
AILHSIDCCSSDDTK	2.263237	Q9H981	ARP8_HUMAN	861.377643	2	0.716519892		
AILHSIDCCSSDDTK	2.263237	Q9H981	ARP8_HUMAN	574.5877037	3	0.716519892		
AQEFLQHR	-20.42403	Q9H981	ARP8_HUMAN	514.7677895	2	0.744396091		
AQEFLQHR	-20.42403	Q9H981	ARP8_HUMAN	343.514468	3	0.744396091		
ATDTLPASIPHVIAR	44.26225	Q9H981	ARP8_HUMAN	781.4390215	2	0.75698483		
ATDTLPASIPHVIAR	44.26225	Q9H981	ARP8_HUMAN	521.2952893	3	0.75698483		
ETFCHLDQDISGLQDHEFQIR	55.58045	Q9H981	ARP8_HUMAN	1294.595902	2	0.733529508	4	
ETFCHLDQDISGLQDHEFQIR	55.58045	Q9H981	ARP8_HUMAN	863.399876	3	0.733529508	4	
HPDSPALLYQFR	42.50854	Q9H981	ARP8_HUMAN	722.3731455	2	0.712144375		
HPDSPALLYQFR	42.50854	Q9H981	ARP8_HUMAN	481.9180387	3	0.712144375		
LCLAYGGSDVSR	24.22418	Q9H981	ARP8_HUMAN	649.314439	2	0.603932202		
LCLAYGGSDVSR	24.22418	Q9H981	ARP8_HUMAN	433.2122343	3	0.603932202		
LHSQEVDLGSAQGDGLMAGNDSEEALTALMSR	88.47685	Q9H981	ARP8_HUMAN	1651.767368	2	0.674222052		
LHSQEVDLGSAQGDGLMAGNDSEEALTALMSR	88.47685	Q9H981	ARP8_HUMAN	1101.514187	3	0.674222052		
LQAPMALFYPATFGIVGQK	122.1258	Q9H981	ARP8_HUMAN	1026.553525	2	0.662401259		
LQAPMALFYPATFGIVGQK	122.1258	Q9H981	ARP8_HUMAN	684.7049583	3	0.662401259		
MDCLLLQHLK	37.78976	Q9H981	ARP8_HUMAN	635.8365005	2	0.776549578		
MDCLLLQHLK	37.78976	Q9H981	ARP8_HUMAN	424.226942	3	0.776549578		
MVDQAIWSK	26.67632	Q9H981	ARP8_HUMAN	539.2740585	2	0.625615835		
MVDQAIWSK	26.67632	Q9H981	ARP8_HUMAN	359.8519807	3	0.625615835		
PAILDHCSGNK	-26.23291	Q9H981	ARP8_HUMAN	606.2960535	2	0.656821549		
PAILDHCSGNK	-26.23291	Q9H981	ARP8_HUMAN	404.5333107	3	0.656821549		
SASKPIGFEGDLR	19.59759	Q9H981	ARP8_HUMAN	688.8626145	2	0.788095534		
SASKPIGFEGDLR	19.59759	Q9H981	ARP8_HUMAN	459.5776847	3	0.788095534		
TAISLFEGK	37.8808	Q9H981	ARP8_HUMAN	483.269299	2	0.668405175		
TAISLFEGK	37.8808	Q9H981	ARP8_HUMAN	322.5154743	3	0.668405175		
YLEIPLK	45.97971	Q9H981	ARP8_HUMAN	438.2660235	2	0.731522262		
YLEIPLK	45.97971	Q9H981	ARP8_HUMAN	292.5132907	3	0.731522262		
HDAVYGPADTMVQYMELFNK	94.11526	Q9H944	MED20_HUMAN	1165.035563	2	0.642823219	3	
HDAVYGPADTMVQYMELFNK	94.11526	Q9H944	MED20_HUMAN	777.0263167	3	0.642823219	3	Yes
SVQQTVELLTR	43.38304	Q9H944	MED20_HUMAN	637.3597085	2	0.754253983	2	Yes
SVQQTVELLTR	43.38304	Q9H944	MED20_HUMAN	425.242414	3	0.754253983	2	
VGTVTMGPSAR	-2.85577	Q9H944	MED20_HUMAN	538.2824155	2	0.845136464	2	Yes
VGTVTMGPSAR	-2.85577	Q9H944	MED20_HUMAN	359.1908853	3	0.845136464	2	
GVNEDTYSGILDCAR	45.91716	Q9H936	GHC1_HUMAN	835.378496	2	0.692017674	2	Yes
GVNEDTYSGILDCAR	45.91716	Q9H936	GHC1_HUMAN	557.254939	3	0.692017674	2	
ILAAQGQLSAQGGAQPSVEAPAAPRPTATQLTR	47.57993	Q9H936	GHC1_HUMAN	1628.874022	2	0.680836022	3	
ILAAQGQLSAQGGAQPSVEAPAAPRPTATQLTR	47.57993	Q9H936	GHC1_HUMAN	1086.251956	3	0.680836022	3	Yes
SEGYFGMYR	31.71902	Q9H936	GHC1_HUMAN	555.240207	2	0.821106851		
SEGYFGMYR	31.71902	Q9H936	GHC1_HUMAN	370.4960797	3	0.821106851		
VYTSMSDCLIK	36.83324	Q9H936	GHC1_HUMAN	658.815426	2	0.833227098	2	Yes
VYTSMSDCLIK	36.83324	Q9H936	GHC1_HUMAN	439.5462257	3	0.833227098	2	
ALVDCEWALK	51.29552	Q9H892	TTC12_HUMAN	602.805723	2	0.774596334		
ALVDCEWALK	51.29552	Q9H892	TTC12_HUMAN	402.2064237	3	0.774596334		
GNEAFAEGNYETAILR	53.02713	Q9H892	TTC12_HUMAN	877.921385	2	0.885340571		
GNEAFAEGNYETAILR	53.02713	Q9H892	TTC12_HUMAN	585.616865	3	0.885340571		

GYLNQVDLQEK	27.67544	Q9H892	TTC12_HUMAN	653.8360615	2	0.861425519		
GYLNQVDLQEK	27.67544	Q9H892	TTC12_HUMAN	436.2266493	3	0.861425519		
ILEINPK	11.85548	Q9H892	TTC12_HUMAN	413.755827	2	0.667874396		
ILEINPK	11.85548	Q9H892	TTC12_HUMAN	276.1731597	3	0.667874396		
LHGLEILNSTMK	37.60486	Q9H892	TTC12_HUMAN	678.371761	2	0.80344373		
LHGLEILNSTMK	37.60486	Q9H892	TTC12_HUMAN	452.5837823	3	0.80344373		
LLAALLSSK	51.01134	Q9H892	TTC12_HUMAN	458.297859	2	0.651448905		
LLAALLSSK	51.01134	Q9H892	TTC12_HUMAN	305.8678477	3	0.651448905		
LLLMEEDQEEDECR	38.88216	Q9H892	TTC12_HUMAN	904.8880405	2	0.651951194		
LLLMEEDQEEDECR	38.88216	Q9H892	TTC12_HUMAN	603.5946353	3	0.651951194		
NVDEISNLIQEMNSDDPVVQQK	114.2548	Q9H892	TTC12_HUMAN	1258.103224	2	0.789655447		
NVDEISNLIQEMNSDDPVVQQK	114.2548	Q9H892	TTC12_HUMAN	839.071424	3	0.789655447		
SAEEINSEAFLASVEK	62.31129	Q9H892	TTC12_HUMAN	862.4234295	2	0.791880429		
SAEEINSEAFLASVEK	62.31129	Q9H892	TTC12_HUMAN	575.2848947	3	0.791880429		
SLIINHLDLTR	42.10327	Q9H892	TTC12_HUMAN	647.8780675	2	0.831548393		
SLIINHLDLTR	42.10327	Q9H892	TTC12_HUMAN	432.2546533	3	0.831548393		
TAVQVNAGIALGK	34.08517	Q9H892	TTC12_HUMAN	621.364794	2	0.759731293		
TAVQVNAGIALGK	34.08517	Q9H892	TTC12_HUMAN	414.5791377	3	0.759731293		
TDLLQVLLK	96.33963	Q9H892	TTC12_HUMAN	521.829524	2	0.760763049		
TDLLQVLLK	96.33963	Q9H892	TTC12_HUMAN	348.222291	3	0.760763049		
AFQNTATACAPVSHYR	3.75349	Q9H814	PHAX_HUMAN	897.423573	2	0.830511987	3	
AFQNTATACAPVSHYR	3.75349	Q9H814	PHAX_HUMAN	598.6183237	3	0.830511987	3	Yes
AVESVDSSEESFSDSDDDSCLWK	57.82218	Q9H814	PHAX_HUMAN	1297.522125	2	0.661000609		
AVESVDSSEESFSDSDDDSCLWK	57.82218	Q9H814	PHAX_HUMAN	865.3506917	3	0.661000609		
DIFYIENQK	46.76767	Q9H814	PHAX_HUMAN	585.2960405	2	0.819997549	2	Yes
DIFYIENQK	46.76767	Q9H814	PHAX_HUMAN	390.533302	3	0.819997549	2	
ELDEYMHGGK	-5.938553	Q9H814	PHAX_HUMAN	589.761508	2	0.683995306		
ELDEYMHGGK	-5.938553	Q9H814	PHAX_HUMAN	393.5102803	3	0.683995306		
ETFASDTNEALASLDESQEGHAEAK	42.46215	Q9H814	PHAX_HUMAN	1325.591732	2	0.839135885		
ETFASDTNEALASLDESQEGHAEAK	42.46215	Q9H814	PHAX_HUMAN	884.063763	3	0.839135885		
QSETYNYLLAK	33.45958	Q9H814	PHAX_HUMAN	665.3384325	2	0.741861224		
QSETYNYLLAK	33.45958	Q9H814	PHAX_HUMAN	443.8948967	3	0.741861224		
SLNFQEDDDTSR	12.12546	Q9H814	PHAX_HUMAN	713.808233	2	0.73766315	2	Yes
SLNFQEDDDTSR	12.12546	Q9H814	PHAX_HUMAN	476.208097	3	0.73766315	2	
TPGGVFLNLLK	82.13832	Q9H814	PHAX_HUMAN	579.848248	2	0.686186671	2	Yes
TPGGVFLNLLK	82.13832	Q9H814	PHAX_HUMAN	386.9014403	3	0.686186671	2	
YEITAEDSQEK	-4.224907	Q9H814	PHAX_HUMAN	656.7993415	2	0.60080111	2	Yes
YEITAEDSQEK	-4.224907	Q9H814	PHAX_HUMAN	438.2021693	3	0.60080111	2	
AGHSLLMDAR	-8.32016	Q9H7H0	MET17_HUMAN	535.774757	2	0.806841195		
AGHSLLMDAR	-8.32016	Q9H7H0	MET17_HUMAN	357.519113	3	0.806841195		
ALAALVPGVTQVDNK	56.08118	Q9H7H0	MET17_HUMAN	748.4281225	2	0.802696645		
ALAALVPGVTQVDNK	56.08118	Q9H7H0	MET17_HUMAN	499.2880233	3	0.802696645		
FLENPDLSQTEEK	31.79712	Q9H7H0	MET17_HUMAN	775.373209	2	0.812482834		
FLENPDLSQTEEK	31.79712	Q9H7H0	MET17_HUMAN	517.2514143	3	0.812482834		
GAVLHALR	-17.78725	Q9H7H0	MET17_HUMAN	418.759236	2	0.690166295		
GAVLHALR	-17.78725	Q9H7H0	MET17_HUMAN	279.5087657	3	0.690166295		
GGSESGEPYIPGVFFR	86.43251	Q9H7H0	MET17_HUMAN	849.910289	2	0.659372687	2	Yes
GGSESGEPYIPGVFFR	86.43251	Q9H7H0	MET17_HUMAN	566.942801	3	0.659372687	2	
HLPVEPEELQR	14.26188	Q9H7H0	MET17_HUMAN	673.8573325	2	0.822135329		
HLPVEPEELQR	14.26188	Q9H7H0	MET17_HUMAN	449.5741633	3	0.822135329		
LDGGFAAVSR	20.86976	Q9H7H0	MET17_HUMAN	496.7621725	2	0.748018622		
LDGGFAAVSR	20.86976	Q9H7H0	MET17_HUMAN	331.5107233	3	0.748018622		
NPAFQPQTLMDFGSGTGSVTWAAHSIWGQSLR	115.6625	Q9H7H0	MET17_HUMAN	1724.331131	2	0.727141619		
NPAFQPQTLMDFGSGTGSVTWAAHSIWGQSLR	115.6625	Q9H7H0	MET17_HUMAN	1149.890029	3	0.727141619		
SAAMLVLAEK	38.72653	Q9H7H0	MET17_HUMAN	516.792084	2	0.808623195		
SAAMLVLAEK	38.72653	Q9H7H0	MET17_HUMAN	344.8639977	3	0.808623195		
WCPGLGVAPQAR	36.1411	Q9H7H0	MET17_HUMAN	656.335514	2	0.779630542		
WCPGLGVAPQAR	36.1411	Q9H7H0	MET17_HUMAN	437.892951	3	0.779630542		
EYNALVAQGVR	24.00938	Q9H7C9	AAMDC_HUMAN	610.325664	2	0.724598289	2	Yes
EYNALVAQGVR	24.00938	Q9H7C9	AAMDC_HUMAN	407.2197177	3	0.724598289	2	
VLQTEQAVK	-20.09409	Q9H7C9	AAMDC_HUMAN	508.293306	2	0.758895993		
VLQTEQAVK	-20.09409	Q9H7C9	AAMDC_HUMAN	339.1981457	3	0.758895993		
VPSSTVEYLK	17.37126	Q9H7C9	AAMDC_HUMAN	561.806241	2	0.786783159		
VPSSTVEYLK	17.37126	Q9H7C9	AAMDC_HUMAN	374.8734357	3	0.786783159		
APAEGVLTLR	31.23362	Q9H6S3	ES8L2_HUMAN	513.801298	2	0.742218792		
APAEGVLTLR	31.23362	Q9H6S3	ES8L2_HUMAN	342.8701403	3	0.742218792		
ARPPSEGEFIDCFQK	33.1961	Q9H6S3	ES8L2_HUMAN	890.9203355	2	0.846705079		
ARPPSEGEFIDCFQK	33.1961	Q9H6S3	ES8L2_HUMAN	594.282832	3	0.846705079		
DELMQHMDEVNDELIR	62.43356	Q9H6S3	ES8L2_HUMAN	993.9489635	2	0.773803413		
DELMQHMDEVNDELIR	62.43356	Q9H6S3	ES8L2_HUMAN	662.968584	3	0.773803413		
DEVLEVLEDGR	70.7318	Q9H6S3	ES8L2_HUMAN	637.3177055	2	0.794778883		
DEVLEVLEDGR	70.7318	Q9H6S3	ES8L2_HUMAN	425.214412	3	0.794778883		
EMSLWESLGESWMR	117.34	Q9H6S3	ES8L2_HUMAN	870.8901885	2	0.80091542		
EMSLWESLGESWMR	117.34	Q9H6S3	ES8L2_HUMAN	580.9294007	3	0.80091542		
EPQVPLYVPK	44.57507	Q9H6S3	ES8L2_HUMAN	585.3324265	2	0.782870114		
EPQVPLYVPK	44.57507	Q9H6S3	ES8L2_HUMAN	390.5575593	3	0.782870114		
THSGWEPPVDVLQEAPWEVEGLASAPIEEVSPVSI	141.0042	Q9H6S3	ES8L2_HUMAN	1922.447411	2	0.732047021		
THSGWEPPVDVLQEAPWEVEGLASAPIEEVSPVSI	141.0042	Q9H6S3	ES8L2_HUMAN	1281.967549	3	0.732047021		
ILYDFTAR	42.98326	Q9H6S3	ES8L2_HUMAN	499.769462	2	0.696166694		
ILYDFTAR	42.98326	Q9H6S3	ES8L2_HUMAN	333.515583	3	0.696166694		
IVENLGILTGPQLFSLNK	110.348	Q9H6S3	ES8L2_HUMAN	978.562407	2	0.765537798	3	
IVENLGILTGPQLFSLNK	110.348	Q9H6S3	ES8L2_HUMAN	652.7108797	3	0.765537798	3	Yes
IWTQEMLLQVNDQSLR	83.22356	Q9H6S3	ES8L2_HUMAN	987.509849	2	0.781424761		
IWTQEMLLQVNDQSLR	83.22356	Q9H6S3	ES8L2_HUMAN	658.675841	3	0.781424761		
LAINLLAK	47.58542	Q9H6S3	ES8L2_HUMAN	428.2872945	2	0.717677772	2	Yes
LAINLLAK	47.58542	Q9H6S3	ES8L2_HUMAN	285.8608047	3	0.717677772	2	
LLDIESQEELEDFPLPTVQR	110.9884	Q9H6S3	ES8L2_HUMAN	1186.105562	2	0.772201836		
LLDIESQEELEDFPLPTVQR	110.9884	Q9H6S3	ES8L2_HUMAN	791.0729827	3	0.772201836		
SQPVSQPLTYESGPDEVR	31.26119	Q9H6S3	ES8L2_HUMAN	994.982171	2	0.80513978		
SQPVSQPLTYESGPDEVR	31.26119	Q9H6S3	ES8L2_HUMAN	663.657389	3	0.80513978		
SQTVLNQLR	13.71626	Q9H6S3	ES8L2_HUMAN	529.801829	2	0.733642101	2	Yes
SQTVLNQLR	13.71626	Q9H6S3	ES8L2_HUMAN	353.537161	3	0.733642101	2	
VGPQVPLSEPGFR	48.79816	Q9H6S3	ES8L2_HUMAN	691.8755255	2	0.877515435	2	Yes
VGPQVPLSEPGFR	48.79816	Q9H6S3	ES8L2_HUMAN	461.586292	3	0.877515435	2	
VYSQLTMQK	2.548935	Q9H6S3	ES8L2_HUMAN	549.287162	2	0.839094102		
VYSQLTMQK	2.548935	Q9H6S3	ES8L2_HUMAN	366.527383	3	0.839094102		
YSNSNVIMHETSQYHVQHLATFIMDK	54.06946	Q9H6S3	ES8L2_HUMAN	1547.23203	2	0.775569379		
YSNSNVIMHETSQYHVQHLATFIMDK	54.06946	Q9H6S3	ES8L2_HUMAN	1031.823962	3	0.775569379		
AALVAGMYPNLVHVDR	58.65142	Q9H6S0	YTDC2_HUMAN	863.4594265	2	0.780545831		
AALVAGMYPNLVHVDR	58.65142	Q9H6S0	YTDC2_HUMAN	575.9755593	3	0.780545831		
ALPTDWLIYDEMTR	106.7017	Q9H6S0	YTDC2_HUMAN	862.4221755	2	0.830551624		

ALPTDWLIYDEMTR	106.7017	Q9H6S0	YTDC2 HUMAN	575.2840587	3	0.830551624		
APEPPPALIVR	40.46043	Q9H6S0	YTDC2 HUMAN	580.3458725	2	0.700823188	2	Yes
APEPPPALIVR	40.46043	Q9H6S0	YTDC2 HUMAN	387.23319	3	0.700823188	2	
APSKPWSQVDEATIR	22.73509	Q9H6S0	YTDC2 HUMAN	842.9368425	2	0.83786577		
APSKPWSQVDEATIR	22.73509	Q9H6S0	YTDC2 HUMAN	562.2938367	3	0.83786577		
ASNGWMALDWAK	66.94457	Q9H6S0	YTDC2 HUMAN	675.3195285	2	0.674968183		
ASNGWMALDWAK	66.94457	Q9H6S0	YTDC2 HUMAN	450.5489607	3	0.674968183		
DGQELEPLVGEQLLQLWER	159.948	Q9H6S0	YTDC2 HUMAN	1126.582057	2	0.780401587		
DGQELEPLVGEQLLQLWER	159.948	Q9H6S0	YTDC2 HUMAN	751.3906463	3	0.780401587		
DPFVLPTQASQK	46.03404	Q9H6S0	YTDC2 HUMAN	665.854259	2	0.775695503		
DPFVLPTQASQK	46.03404	Q9H6S0	YTDC2 HUMAN	444.238781	3	0.775695503		
DVNCLEPWLIK	86.93115	Q9H6S0	YTDC2 HUMAN	693.8584865	2	0.704243362	2	Yes
DVNCLEPWLIK	86.93115	Q9H6S0	YTDC2 HUMAN	462.908266	3	0.704243362	2	
EMDACLSDIWLHK	64.50829	Q9H6S0	YTDC2 HUMAN	809.374175	2	0.794081926		
EMDACLSDIWLHK	64.50829	Q9H6S0	YTDC2 HUMAN	539.918725	3	0.794081926		
EMEFPSSLTSTER	48.99986	Q9H6S0	YTDC2 HUMAN	757.346137	2	0.817229867		
EMEFPSSLTSTER	48.99986	Q9H6S0	YTDC2 HUMAN	505.2333663	3	0.817229867		
EMFLEDILR	92.54532	Q9H6S0	YTDC2 HUMAN	583.300273	2	0.828121722	2	Yes
EMFLEDILR	92.54532	Q9H6S0	YTDC2 HUMAN	389.2027903	3	0.828121722	2	
ENLVLTGPK	19.547	Q9H6S0	YTDC2 HUMAN	485.7825735	2	0.750682354	2	Yes
ENLVLTGPK	19.547	Q9H6S0	YTDC2 HUMAN	324.1909907	3	0.750682354	2	
FHPASVLSQPQYK	19.07212	Q9H6S0	YTDC2 HUMAN	751.394078	2	0.827484608		
FHPASVLSQPQYK	19.07212	Q9H6S0	YTDC2 HUMAN	501.265327	3	0.827484608		
FQNMLEFQTPELLR	95.64909	Q9H6S0	YTDC2 HUMAN	883.4512715	2	0.682103813		
FQNMLEFQTPELLR	95.64909	Q9H6S0	YTDC2 HUMAN	589.303456	3	0.682103813		
FTAGAFSDHMALLR	54.57051	Q9H6S0	YTDC2 HUMAN	768.8855675	2	0.802696586		
FTAGAFSDHMALLR	54.57051	Q9H6S0	YTDC2 HUMAN	512.92632	3	0.802696586		
GFASQVEQLISMGANVHSK	82.61316	Q9H6S0	YTDC2 HUMAN	1002.004931	2	0.704596221		
GFASQVEQLISMGANVHSK	82.61316	Q9H6S0	YTDC2 HUMAN	668.3392287	3	0.704596221		
GIWSTTPSNER	14.3773	Q9H6S0	YTDC2 HUMAN	624.3049325	2	0.776106358	2	Yes
GIWSTTPSNER	14.3773	Q9H6S0	YTDC2 HUMAN	416.53923	3	0.776106358	2	
GNVFAVEAENR	19.43745	Q9H6S0	YTDC2 HUMAN	603.29965	2	0.714148045		
GNVFAVEAENR	19.43745	Q9H6S0	YTDC2 HUMAN	402.5357083	3	0.714148045		
HSETSATALMVAAGR	20.46434	Q9H6S0	YTDC2 HUMAN	751.375564	2	0.817408204		
HSETSATALMVAAGR	20.46434	Q9H6S0	YTDC2 HUMAN	501.2529843	3	0.817408204		
IAVNIALER	38.05798	Q9H6S0	YTDC2 HUMAN	499.80384	2	0.677667618		
IAVNIALER	38.05798	Q9H6S0	YTDC2 HUMAN	333.5385017	3	0.677667618		
IGQTIGYQIR	23.57918	Q9H6S0	YTDC2 HUMAN	574.8253005	2	0.823670268	2	Yes
IGQTIGYQIR	23.57918	Q9H6S0	YTDC2 HUMAN	383.5528087	3	0.823670268	2	
LASNALQEPSSFR	29.29582	Q9H6S0	YTDC2 HUMAN	710.365521	2	0.845095396		
LASNALQEPSSFR	29.29582	Q9H6S0	YTDC2 HUMAN	473.9129557	3	0.845095396		
LLAPVNCPIADFLMK	107.2922	Q9H6S0	YTDC2 HUMAN	851.457511	2	0.786960483		
LLAPVNCPIADFLMK	107.2922	Q9H6S0	YTDC2 HUMAN	567.9742823	3	0.786960483		
LSQSLGLVSK	19.83741	Q9H6S0	YTDC2 HUMAN	516.308955	2	0.747871876	2	Yes
LSQSLGLVSK	19.83741	Q9H6S0	YTDC2 HUMAN	344.5419117	3	0.747871876	2	
MPLQELCLHTK	29.95956	Q9H6S0	YTDC2 HUMAN	685.352515	2	0.693529129		
MPLQELCLHTK	29.95956	Q9H6S0	YTDC2 HUMAN	457.2376183	3	0.693529129		
NFLSQATMEIIIGMR	133.965	Q9H6S0	YTDC2 HUMAN	862.4476745	2	0.61897409		
NFLSQATMEIIIGMR	133.965	Q9H6S0	YTDC2 HUMAN	575.301058	3	0.61897409		
NLEISQQK	-14.39061	Q9H6S0	YTDC2 HUMAN	480.262005	2	0.743433654		
NLEISQQK	-14.39061	Q9H6S0	YTDC2 HUMAN	320.5106117	3	0.743433654		
SFDALNFVTMLK	115.9543	Q9H6S0	YTDC2 HUMAN	693.360862	2	0.755447209		
SFDALNFVTMLK	115.9543	Q9H6S0	YTDC2 HUMAN	462.5765163	3	0.755447209		
SPSPALHPPQK	-25.88379	Q9H6S0	YTDC2 HUMAN	579.817479	2	0.651190758		
SPSPALHPPQK	-25.88379	Q9H6S0	YTDC2 HUMAN	386.8809277	3	0.651190758		
SQDWGSAGLGGVFK	60.48052	Q9H6S0	YTDC2 HUMAN	704.846965	2	0.819238663	2	Yes
SQDWGSAGLGGVFK	60.48052	Q9H6S0	YTDC2 HUMAN	470.2339183	3	0.819238663	2	
SSADTEFSDECTTAER	3.462696	Q9H6S0	YTDC2 HUMAN	903.3607015	2	0.802158654	2	Yes
SSADTEFSDECTTAER	3.462696	Q9H6S0	YTDC2 HUMAN	602.5764093	3	0.802158654	2	
TIDAMDTWEDLTELGYHLADLPVEPHLGK	122.6004	Q9H6S0	YTDC2 HUMAN	1640.298095	2	0.733748078		
TIDAMDTWEDLTELGYHLADLPVEPHLGK	122.6004	Q9H6S0	YTDC2 HUMAN	1093.868005	3	0.733748078		
TLMAGDSTLSTVTHVIVDEVHER	68.09671	Q9H6S0	YTDC2 HUMAN	1255.631953	2	0.827304363	4	
TLMAGDSTLSTVTHVIVDEVHER	68.09671	Q9H6S0	YTDC2 HUMAN	837.42391	3	0.827304363	4	
TQLLGQLR	23.95831	Q9H6S0	YTDC2 HUMAN	464.7829085	2	0.806883454		
TQLLGQLR	23.95831	Q9H6S0	YTDC2 HUMAN	310.191214	3	0.806883454		
TTQIPQFLLDDCFK	97.92674	Q9H6S0	YTDC2 HUMAN	863.4300055	2	0.723949969		
TTQIPQFLLDDCFK	97.92674	Q9H6S0	YTDC2 HUMAN	575.9559453	3	0.723949969		
TVLNVTDEYDLLDDGGDAVFSQLTEK	132.2848	Q9H6S0	YTDC2 HUMAN	1429.185462	2	0.751603961		
TVLNVTDEYDLLDDGGDAVFSQLTEK	132.2848	Q9H6S0	YTDC2 HUMAN	953.1262493	3	0.751603961		
VVLIVGETGSGK	30.7655	Q9H6S0	YTDC2 HUMAN	579.84062	2	0.823062062		
VVLIVGETGSGK	30.7655	Q9H6S0	YTDC2 HUMAN	386.896355	3	0.823062062		
YQVFMLHSNMQTSDQK	31.6326	Q9H6S0	YTDC2 HUMAN	978.9513055	2	0.725152612		
YQVFMLHSNMQTSDQK	31.6326	Q9H6S0	YTDC2 HUMAN	652.9701453	3	0.725152612		
AGGLQDSDTEDECWSDTEAVPR	46.92106	Q9H6H4	REEP4 HUMAN	1219.506615	2	0.814113438		
AGGLQDSDTEDECWSDTEAVPR	46.92106	Q9H6H4	REEP4 HUMAN	813.3403513	3	0.814113438		
EIDAYIVQAK	30.92605	Q9H6H4	REEP4 HUMAN	575.311691	2	0.723166466	2	Yes
EIDAYIVQAK	30.92605	Q9H6H4	REEP4 HUMAN	383.877069	3	0.723166466	2	
GLNIAASAAVQAATK	45.5724	Q9H6H4	REEP4 HUMAN	693.39154	2	0.791185677		
GLNIAASAAVQAATK	45.5724	Q9H6H4	REEP4 HUMAN	462.5969683	3	0.791185677		
SFSMQDLR	26.4157	Q9H6H4	REEP4 HUMAN	492.234933	2	0.733997822		
SFSMQDLR	26.4157	Q9H6H4	REEP4 HUMAN	328.4925637	3	0.733997822		
SISDAPAPAYHDPLYLEDQVSHR	46.46458	Q9H6H4	REEP4 HUMAN	1291.120057	2	0.713478923	3	
SISDAPAPAYHDPLYLEDQVSHR	46.46458	Q9H6H4	REEP4 HUMAN	861.0826463	3	0.713478923	3	Yes
SYETVLSFGK	46.17902	Q9H6H4	REEP4 HUMAN	565.790591	2	0.784320712	2	Yes
SYETVLSFGK	46.17902	Q9H6H4	REEP4 HUMAN	377.529669	3	0.784320712	2	
AEFGPPGPGAGSR	10.04779	Q9H4A4	AMPB HUMAN	600.2943685	2	0.741583884	2	Yes
AEFGPPGPGAGSR	10.04779	Q9H4A4	AMPB HUMAN	400.5321873	3	0.741583884	2	
AFELLHLHLDLR	66.08595	Q9H4A4	AMPB HUMAN	738.920267	2	0.852565765	4	
AFELLHLHLDLR	66.08595	Q9H4A4	AMPB HUMAN	492.949453	3	0.852565765	4	
AFFPCFDTPAVK	73.02761	Q9H4A4	AMPB HUMAN	700.3399305	2	0.778504312	2	Yes
AFFPCFDTPAVK	73.02761	Q9H4A4	AMPB HUMAN	467.2292287	3	0.778504312	2	
AIEAVAISPWK	58.68547	Q9H4A4	AMPB HUMAN	592.83788	2	0.752658188	2	Yes
AIEAVAISPWK	58.68547	Q9H4A4	AMPB HUMAN	395.561195	3	0.752658188	2	
ETFASTASQLHSNVVNYVQQIVAPK	85.30566	Q9H4A4	AMPB HUMAN	1366.206669	2	0.82017225	3	
ETFASTASQLHSNVVNYVQQIVAPK	85.30566	Q9H4A4	AMPB HUMAN	911.1403873	3	0.82017225	3	Yes
GLSGTAVLDLR	46.91387	Q9H4A4	AMPB HUMAN	551.317312	2	0.766776085	2	Yes
GLSGTAVLDLR	46.91387	Q9H4A4	AMPB HUMAN	367.8808163	3	0.766776085	2	
IEPGVDPDDTYNETPYEK	38.0191	Q9H4A4	AMPB HUMAN	1041.463473	2	0.732589722	3	
IEPGVDPDDTYNETPYEK	38.0191	Q9H4A4	AMPB HUMAN	694.6449233	3	0.732589722	3	Yes
KPFVYTQGQAVLNR	20.18365	Q9H4A4	AMPB HUMAN	810.9470095	2	0.768995464		
KPFVYTQGQAVLNR	20.18365	Q9H4A4	AMPB HUMAN	540.9672813	3	0.768995464		

LDSHPCLEVTAAALR	41.56599	Q9H4A4	AMPB_HUMAN	826.925421	2	0.804526091	3	
LDSHPCLEVTAAALR	41.56599	Q9H4A4	AMPB_HUMAN	551.6195557	3	0.804526091	3	Yes
LFGPYVWGR	66.24654	Q9H4A4	AMPB_HUMAN	547.7932715	2	0.653331816	2	Yes
LFGPYVWGR	66.24654	Q9H4A4	AMPB_HUMAN	365.531456	3	0.653331816	2	
LGDTYPSISNAR	19.98564	Q9H4A4	AMPB_HUMAN	647.3258605	2	0.83917743		
LGDTYPSISNAR	19.98564	Q9H4A4	AMPB_HUMAN	431.8865153	3	0.83917743		
LQVLLTYR	45.2626	Q9H4A4	AMPB_HUMAN	503.3087545	2	0.764766753	2	Yes
LQVLLTYR	45.2626	Q9H4A4	AMPB_HUMAN	335.8751113	3	0.764766753	2	
RPLHSAQAVDVASASNFR	6.447746	Q9H4A4	AMPB_HUMAN	963.5012035	2	0.793433547	4	
RPLHSAQAVDVASASNFR	6.447746	Q9H4A4	AMPB_HUMAN	642.6700773	3	0.793433547	4	
TYQLVYFLDK	74.95933	Q9H4A4	AMPB_HUMAN	645.342987	2	0.765798807	2	Yes
TYQLVYFLDK	74.95933	Q9H4A4	AMPB_HUMAN	430.5645997	3	0.765798807	2	
VDIIPGFEFDR	87.55275	Q9H4A4	AMPB_HUMAN	654.3357015	2	0.631360352	2	Yes
VDIIPGFEFDR	87.55275	Q9H4A4	AMPB_HUMAN	436.5597427	3	0.631360352	2	
VGEGPGVCWLAPEQTAGK	55.019	Q9H4A4	AMPB_HUMAN	928.4545435	2	0.794033527	2	Yes
VGEGPGVCWLAPEQTAGK	55.019	Q9H4A4	AMPB_HUMAN	619.3056373	3	0.794033527	2	
VWAEPCLIDAAK	54.65827	Q9H4A4	AMPB_HUMAN	686.850662	2	0.761642754	2	Yes
VWAEPCLIDAAK	54.65827	Q9H4A4	AMPB_HUMAN	458.236383	3	0.761642754	2	
YSALIEVPDGFTAVMSASTWEK	126.5483	Q9H4A4	AMPB_HUMAN	1201.583404	2	0.633996785		
YSALIEVPDGFTAVMSASTWEK	126.5483	Q9H4A4	AMPB_HUMAN	801.391544	3	0.633996785		
YTLPLYHAMMGGSEVAQTLAK	69.895	Q9H4A4	AMPB_HUMAN	1141.071949	2	0.616954267		
YTLPLYHAMMGGSEVAQTLAK	69.895	Q9H4A4	AMPB_HUMAN	761.050574	3	0.616954267		
ACHPFLLGPPLVR	55.3956	Q9H496	IFG15_HUMAN	738.9113885	2	0.728894591		
ACHPFLLGPPLVR	55.3956	Q9H496	IFG15_HUMAN	492.943534	3	0.728894591		
ADYCPECYPDNPANR	17.85808	Q9H496	IFG15_HUMAN	921.3650585	2	0.728686213		
ADYCPECYPDNPANR	17.85808	Q9H496	IFG15_HUMAN	614.579314	3	0.728686213		
SLVLPWSFPLEWAPQNLTR	157.2194	Q9H496	IFG15_HUMAN	1127.605138	2	0.755178988		
SLVLPWSFPLEWAPQNLTR	157.2194	Q9H496	IFG15_HUMAN	752.0727	3	0.755178988		
VAGFNPALQLILTR	99.17255	Q9H496	IFG15_HUMAN	756.949025	2	0.797478318	2	Yes
VAGFNPALQLILTR	99.17255	Q9H496	IFG15_HUMAN	504.968625	3	0.797478318	2	
SLSFLGTFILK	109.6146	Q9H3H5	GPT_HUMAN	613.3637305	2	0.673790038	2	Yes
SLSFLGTFILK	109.6146	Q9H3H5	GPT_HUMAN	409.2450953	3	0.673790038	2	
AEDASGEAAAMLNNMR	47.79758	Q9H2X9	S12A5_HUMAN	825.8647025	2	0.762777328		
AEDASGEAAAMLNNMR	47.79758	Q9H2X9	S12A5_HUMAN	550.91241	3	0.762777328		
DFFSMKPEWENLNQSNVR	77.32722	Q9H2X9	S12A5_HUMAN	1121.023851	2	0.8011446		
DFFSMKPEWENLNQSNVR	77.32722	Q9H2X9	S12A5_HUMAN	747.6851753	3	0.8011446		
DGVSHLIQSGGLGGLQHNTVLVGWPR	77.10355	Q9H2X9	S12A5_HUMAN	1349.215167	2	0.683134198		
DGVSHLIQSGGLGGLQHNTVLVGWPR	77.10355	Q9H2X9	S12A5_HUMAN	899.8127197	3	0.683134198		
DLTTFLYHLR	78.06563	Q9H2X9	S12A5_HUMAN	639.8462325	2	0.607144892		
DLTTFLYHLR	78.06563	Q9H2X9	S12A5_HUMAN	426.9000967	3	0.607144892		
ENLWSSYLTK	62.49335	Q9H2X9	S12A5_HUMAN	620.814597	2	0.786376655		
ENLWSSYLTK	62.49335	Q9H2X9	S12A5_HUMAN	414.2123397	3	0.786376655		
ESSPFINSTDTEK	16.52629	Q9H2X9	S12A5_HUMAN	727.836459	2	0.808394313		
ESSPFINSTDTEK	16.52629	Q9H2X9	S12A5_HUMAN	485.5602477	3	0.808394313		
ETTAGHLALLVTK	33.06093	Q9H2X9	S12A5_HUMAN	677.391009	2	0.763261437		
ETTAGHLALLVTK	33.06093	Q9H2X9	S12A5_HUMAN	451.9299477	3	0.763261437		
FLNATCDEYFTR	46.82864	Q9H2X9	S12A5_HUMAN	768.843561	2	0.825752139		
FLNATCDEYFTR	46.82864	Q9H2X9	S12A5_HUMAN	512.8983157	3	0.825752139		
GFCQVVISSNLR	47.37447	Q9H2X9	S12A5_HUMAN	690.359185	2	0.743376374		
GFCQVVISSNLR	47.37447	Q9H2X9	S12A5_HUMAN	460.5753983	3	0.743376374		
GLTIVGSVLEGTFLENHPQAQR	90.82069	Q9H2X9	S12A5_HUMAN	1183.627331	2	0.835814834		
GLTIVGSVLEGTFLENHPQAQR	90.82069	Q9H2X9	S12A5_HUMAN	789.4208287	3	0.835814834		
ITAEVEVVEMHESDISAYTYEK	61.89359	Q9H2X9	S12A5_HUMAN	1272.097068	2	0.796948314		
ITAEVEVVEMHESDISAYTYEK	61.89359	Q9H2X9	S12A5_HUMAN	848.4006533	3	0.796948314		
LAWEGNETVTTR	24.18416	Q9H2X9	S12A5_HUMAN	688.8444225	2	0.812101662		
LAWEGNETVTTR	24.18416	Q9H2X9	S12A5_HUMAN	459.5655567	3	0.812101662		
LWGLFCSSR	66.12129	Q9H2X9	S12A5_HUMAN	563.279675	2	0.603241503		
LWGLFCSSR	66.12129	Q9H2X9	S12A5_HUMAN	375.855725	3	0.603241503		
NNVTEIQGIPGAASGLIK	66.02285	Q9H2X9	S12A5_HUMAN	891.491982	2	0.897157669		
NNVTEIQGIPGAASGLIK	66.02285	Q9H2X9	S12A5_HUMAN	594.6639297	3	0.897157669		
NVSMFPGNPER	24.18671	Q9H2X9	S12A5_HUMAN	624.296053	2	0.735995591		
NVSMFPGNPER	24.18671	Q9H2X9	S12A5_HUMAN	416.5333103	3	0.735995591		
SAFDPPNFPICLLGNR	112.9058	Q9H2X9	S12A5_HUMAN	909.454345	2	0.622716546		
SAFDPPNFPICLLGNR	112.9058	Q9H2X9	S12A5_HUMAN	606.6388383	3	0.622716546		
VDQDQNVVHPQLLSLTSQLK	68.42177	Q9H2X9	S12A5_HUMAN	1131.608606	2	0.814605653		
VDQDQNVVHPQLLSLTSQLK	68.42177	Q9H2X9	S12A5_HUMAN	754.741679	3	0.814605653		
FEDEYSEYLK	38.97963	Q9H2H8	PPIL3_HUMAN	661.793523	2	0.747116327		
FEDEYSEYLK	38.97963	Q9H2H8	PPIL3_HUMAN	441.5316237	3	0.747116327		
GFMVQTGDPTGTGR	23.93797	Q9H2H8	PPIL3_HUMAN	712.3359085	2	0.76698333	2	Yes
GFMVQTGDPTGTGR	23.93797	Q9H2H8	PPIL3_HUMAN	475.2265473	3	0.76698333	2	
VIDGLETLDELEK	74.49932	Q9H2H8	PPIL3_HUMAN	737.388328	2	0.873675823	2	Yes
VIDGLETLDELEK	74.49932	Q9H2H8	PPIL3_HUMAN	491.9281603	3	0.873675823	2	
AMAGDTSLSENYAFAGMYHVFDQHVDEAVPR	88.70602	Q9H1Z4	WDR13_HUMAN	1714.769706	2	0.71096468		
AMAGDTSLSENYAFAGMYHVFDQHVDEAVPR	88.70602	Q9H1Z4	WDR13_HUMAN	1143.515745	3	0.71096468		
AVYEDRPPGSVVPTSAAEASR	22.14848	Q9H1Z4	WDR13_HUMAN	1080.040552	2	0.714419127		
AVYEDRPPGSVVPTSAAEASR	22.14848	Q9H1Z4	WDR13_HUMAN	720.362976	3	0.714419127		
GSYQLQAQMNR	0.971992	Q9H1Z4	WDR13_HUMAN	648.312231	2	0.776950896		
GSYQLQAQMNR	0.971992	Q9H1Z4	WDR13_HUMAN	432.5440957	3	0.776950896		
LVVHEGSPVTSISAR	16.86262	Q9H1Z4	WDR13_HUMAN	776.428653	2	0.852301776		
LVVHEGSPVTSISAR	16.86262	Q9H1Z4	WDR13_HUMAN	517.9550437	3	0.852301776		
MEDFEDDPR	7.826687	Q9H1Z4	WDR13_HUMAN	577.227502	2	0.652559638		
MEDFEDDPR	7.826687	Q9H1Z4	WDR13_HUMAN	385.1542763	3	0.652559638		
SFPIEQSSHPVR	4.427505	Q9H1Z4	WDR13_HUMAN	692.3549565	2	0.751923382		
SFPIEQSSHPVR	4.427505	Q9H1Z4	WDR13_HUMAN	461.9059127	3	0.751923382		
TPTFPQFR	35.1733	Q9H1Z4	WDR13_HUMAN	497.261809	2	0.68985194		
TPTFPQFR	35.1733	Q9H1Z4	WDR13_HUMAN	331.8438143	3	0.68985194		
VLALSFDAPGR	55.81214	Q9H1Z4	WDR13_HUMAN	573.3198545	2	0.745416284	2	Yes
VLALSFDAPGR	55.81214	Q9H1Z4	WDR13_HUMAN	382.549178	3	0.745416284	2	
VVDNEGTLQLK	17.80949	Q9H1Z4	WDR13_HUMAN	608.333159	2	0.88711071		
VVDNEGTLQLK	17.80949	Q9H1Z4	WDR13_HUMAN	405.891381	3	0.88711071		
YGPLSEPGSAR	2.924126	Q9H1Z4	WDR13_HUMAN	567.2834645	2	0.679277718		
YGPLSEPGSAR	2.924126	Q9H1Z4	WDR13_HUMAN	378.524918	3	0.679277718		
GDTYELQVR	14.70342	Q9H0U3	MAGT1_HUMAN	540.7701905	2	0.661477864		
GDTYELQVR	14.70342	Q9H0U3	MAGT1_HUMAN	360.849402	3	0.661477864		
GFSAEQIAR	7.5037	Q9H0U3	MAGT1_HUMAN	489.7543475	2	0.688652456	2	Yes
GFSAEQIAR	7.5037	Q9H0U3	MAGT1_HUMAN	326.83884	3	0.688652456	2	
SNMEFLFNK	52.22791	Q9H0U3	MAGT1_HUMAN	565.271515	2	0.799025118		
SNMEFLFNK	52.22791	Q9H0U3	MAGT1_HUMAN	377.1836183	3	0.799025118		
VSQLMEWTNK	32.24117	Q9H0U3	MAGT1_HUMAN	618.3086295	2	0.791739702	2	Yes
VSQLMEWTNK	32.24117	Q9H0U3	MAGT1_HUMAN	412.5416947	3	0.791739702	2	
AFMEAAEITDEDNSIAAMYQAVGELPQANR	123.9523	Q9H0H5	RGAP1_HUMAN	1628.250819	2	0.698605478		

AFMEAAEITDEDNSIAAMYQAVGELPQANR	123.9523	Q9H0H5	RGAP1 HUMAN	1085.836487	3	0.698605478		
GLTETGLYR	14.54706	Q9H0H5	RGAP1 HUMAN	505.2698265	2	0.779228866		
GLTETGLYR	14.54706	Q9H0H5	RGAP1 HUMAN	337.1824927	3	0.779228866		
LSTIDESGSILSDISFDK	92.81473	Q9H0H5	RGAP1 HUMAN	963.9813085	2	0.72069025		
LSTIDESGSILSDISFDK	92.81473	Q9H0H5	RGAP1 HUMAN	642.9901473	3	0.72069025		
NLFEQLVR	64.56876	Q9H0H5	RGAP1 HUMAN	509.78819	2	0.762630045		
NLFEQLVR	64.56876	Q9H0H5	RGAP1 HUMAN	340.194735	3	0.762630045		
SALAFLNR	38.33271	Q9H0H5	RGAP1 HUMAN	446.2565255	2	0.762965798	2	Yes
SALAFLNR	38.33271	Q9H0H5	RGAP1 HUMAN	297.840292	3	0.762965798	2	
SIGSAVDQGNESIVAK	21.60501	Q9H0H5	RGAP1 HUMAN	787.9052075	2	0.846559465	2	Yes
SIGSAVDQGNESIVAK	21.60501	Q9H0H5	RGAP1 HUMAN	525.60608	3	0.846559465	2	
TDESLDWDSSLVK	60.24155	Q9H0H5	RGAP1 HUMAN	747.852109	2	0.80785042	2	Yes
TDESLDWDSSLVK	60.24155	Q9H0H5	RGAP1 HUMAN	498.9040143	3	0.80785042	2	
TTVTVPNDGGPIEAVSTIETVPYWTR	109.4623	Q9H0H5	RGAP1 HUMAN	1402.211618	2	0.709125757		
TTVTVPNDGGPIEAVSTIETVPYWTR	109.4623	Q9H0H5	RGAP1 HUMAN	935.1436867	3	0.709125757		
TVIKPESCVPCGK	-6.281609	Q9H0H5	RGAP1 HUMAN	737.8738115	2	0.603737235		
TVIKPESCVPCGK	-6.281609	Q9H0H5	RGAP1 HUMAN	492.251816	3	0.603737235		
VDDIHAICSLLK	63.71788	Q9H0H5	RGAP1 HUMAN	692.3692185	2	0.830724001		
VDDIHAICSLLK	63.71788	Q9H0H5	RGAP1 HUMAN	461.9154207	3	0.830724001		
VEILSEGNEVQFIQLAK	91.15285	Q9H0H5	RGAP1 HUMAN	959.020572	2	0.799298823		
VEILSEGNEVQFIQLAK	91.15285	Q9H0H5	RGAP1 HUMAN	639.6829897	3	0.799298823		
VSLLGPVTTPEHQLLK	56.11932	Q9H0H5	RGAP1 HUMAN	866.5043615	2	0.882751465	3	
VSLLGPVTTPEHQLLK	56.11932	Q9H0H5	RGAP1 HUMAN	578.005516	3	0.882751465	3	Yes
GLAPQNKPELQK	-21.85579	Q9H098	F107B HUMAN	661.8755255	2	0.762433887	3	
GLAPQNKPELQK	-21.85579	Q9H098	F107B HUMAN	441.586292	3	0.762433887	3	Yes
LEQLELEK	16.01044	Q9H098	F107B HUMAN	501.2798635	2	0.780019879	2	Yes
LEQLELEK	16.01044	Q9H098	F107B HUMAN	334.5225173	3	0.780019879	2	
LQEEQENAPEFVK	19.94804	Q9H098	F107B HUMAN	780.8812015	2	0.759432495		
LQEEQENAPEFVK	19.94804	Q9H098	F107B HUMAN	520.9234093	3	0.759432495		
SDLEIELLK	61.80488	Q9H098	F107B HUMAN	530.3007955	2	0.77627182		
SDLEIELLK	61.80488	Q9H098	F107B HUMAN	353.8698053	3	0.77627182		
ADILQVGLR	44.48732	Q9H074	PAIP1 HUMAN	492.7960155	2	0.750543952	2	Yes
ADILQVGLR	44.48732	Q9H074	PAIP1 HUMAN	328.8666187	3	0.750543952	2	
APGFLQPPPLR	53.04079	Q9H074	PAIP1 HUMAN	596.84604	2	0.828037322		
APGFLQPPPLR	53.04079	Q9H074	PAIP1 HUMAN	398.2333017	3	0.828037322		
ATPENDPNYFMNEPTFYTSDGVPFTAADPDYQE	102.8828	Q9H074	PAIP1 HUMAN	2000.855076	2	0.725418746		
ATPENDPNYFMNEPTFYTSDGVPFTAADPDYQE	102.8828	Q9H074	PAIP1 HUMAN	1334.239326	3	0.725418746		
ELLNALFSNPMDDNLICAVK	126.4728	Q9H074	PAIP1 HUMAN	1139.06687	2	0.706386983		
ELLNALFSNPMDDNLICAVK	126.4728	Q9H074	PAIP1 HUMAN	759.713855	3	0.706386983		
IENVVLDANCSR	25.38006	Q9H074	PAIP1 HUMAN	695.3437315	2	0.70387423	2	Yes
IENVVLDANCSR	25.38006	Q9H074	PAIP1 HUMAN	463.8984293	3	0.70387423	2	
LTGSVLEDAWK	48.27062	Q9H074	PAIP1 HUMAN	609.822427	2	0.834936559	2	Yes
LTGSVLEDAWK	48.27062	Q9H074	PAIP1 HUMAN	406.8842263	3	0.834936559	2	
MDMEEIIQR	47.75668	Q9H074	PAIP1 HUMAN	582.7735655	2	0.765530229	2	Yes
MDMEEIIQR	47.75668	Q9H074	PAIP1 HUMAN	388.851652	3	0.765530229	2	
DWCWELER	66.76241	Q9C0I1	MTMRC HUMAN	597.256397	2	0.784916639		
DWCWELER	66.76241	Q9C0I1	MTMRC HUMAN	398.506873	3	0.784916639		
EFYDSWHSK	9.65873	Q9C0I1	MTMRC HUMAN	599.7623645	2	0.678703308		
EFYDSWHSK	9.65873	Q9C0I1	MTMRC HUMAN	400.177518	3	0.678703308		
EQDDGILQIQK	29.99779	Q9C0I1	MTMRC HUMAN	643.8335235	2	0.815577924		
EQDDGILQIQK	29.99779	Q9C0I1	MTMRC HUMAN	429.5582907	3	0.815577924		
EVTLHLLPGEQLLCEASTVLK	101.7617	Q9C0I1	MTMRC HUMAN	1175.638518	2	0.690434754	3	
EVTLHLLPGEQLLCEASTVLK	101.7617	Q9C0I1	MTMRC HUMAN	784.0949537	3	0.690434754	3	Yes
HHSQQAPQAEAPCLLR	5.890942	Q9C0I1	MTMRC HUMAN	921.955576	2	0.68638283		
HHSQQAPQAEAPCLLR	5.890942	Q9C0I1	MTMRC HUMAN	614.9729923	3	0.68638283		
IAFLGDDESALDNDETQFK	70.33861	Q9C0I1	MTMRC HUMAN	1064.49003	2	0.907622278		
IAFLGDDESALDNDETQFK	70.33861	Q9C0I1	MTMRC HUMAN	709.9959617	3	0.907622278		
IGFQSLIQK	46.77887	Q9C0I1	MTMRC HUMAN	517.306216	2	0.817571938		
IGFQSLIQK	46.77887	Q9C0I1	MTMRC HUMAN	345.2067523	3	0.817571938		
LFLFSYATAAQNNTVTDPK	83.71332	Q9C0I1	MTMRC HUMAN	1051.034207	2	0.734312832		
LFLFSYATAAQNNTVTDPK	83.71332	Q9C0I1	MTMRC HUMAN	701.025413	3	0.734312832		
LIIHCKDLR	-20.78157	Q9C0I1	MTMRC HUMAN	584.3375245	2	0.6017856		
LIIHCKDLR	-20.78157	Q9C0I1	MTMRC HUMAN	389.8942913	3	0.6017856		
LINSSDELQDNFR	35.70654	Q9C0I1	MTMRC HUMAN	775.8764495	2	0.823397875	2	Yes
LINSSDELQDNFR	35.70654	Q9C0I1	MTMRC HUMAN	517.586908	3	0.823397875	2	
LLEMMEEVQSLQEK	78.57559	Q9C0I1	MTMRC HUMAN	853.921155	2	0.872362256		
LLEMMEEVQSLQEK	78.57559	Q9C0I1	MTMRC HUMAN	569.6167117	3	0.872362256		
LPAYFVVPTPLPEENVQR	94.5081	Q9C0I1	MTMRC HUMAN	1035.057485	2	0.731924713	3	
LPAYFVVPTPLPEENVQR	94.5081	Q9C0I1	MTMRC HUMAN	690.374265	3	0.731924713	3	Yes
LSSLFPFALLQR	120.3409	Q9C0I1	MTMRC HUMAN	696.406461	2	0.600248337		
LSSLFPFALLQR	120.3409	Q9C0I1	MTMRC HUMAN	464.6069157	3	0.600248337		
NPLYVEKPK	-17.30088	Q9C0I1	MTMRC HUMAN	544.3114935	2	0.77927351		
NPLYVEKPK	-17.30088	Q9C0I1	MTMRC HUMAN	363.2102707	3	0.77927351		
SFLDGIYK	50.79778	Q9C0I1	MTMRC HUMAN	471.7507375	2	0.778365254		
SFLDGIYK	50.79778	Q9C0I1	MTMRC HUMAN	314.8364333	3	0.778365254		
STDYHGLLLPHIEGPEIK	56.5429	Q9C0I1	MTMRC HUMAN	1010.031467	2	0.803649247	4	
STDYHGLLLPHIEGPEIK	56.5429	Q9C0I1	MTMRC HUMAN	673.690253	3	0.803649247	4	
TEDLSSNFLSLQEIQTAYSK	102.5389	Q9C0I1	MTMRC HUMAN	1137.560982	2	0.762908816		
TEDLSSNFLSLQEIQTAYSK	102.5389	Q9C0I1	MTMRC HUMAN	758.7099297	3	0.762908816		
TIHRPPYEIVK	-1.633274	Q9C0I1	MTMRC HUMAN	676.8884315	2	0.764594674		
TIHRPPYEIVK	-1.633274	Q9C0I1	MTMRC HUMAN	451.594896	3	0.764594674		
VIGENDITLHCVDQIYGVFDEK	87.56439	Q9C0I1	MTMRC HUMAN	1282.621049	2	0.742979109		
VIGENDITLHCVDQIYGVFDEK	87.56439	Q9C0I1	MTMRC HUMAN	855.416641	3	0.742979109		
WFSLLESSSWLDIIR	159.2575	Q9C0I1	MTMRC HUMAN	926.486167	2	0.671867132		
WFSLLESSSWLDIIR	159.2575	Q9C0I1	MTMRC HUMAN	617.9933863	3	0.671867132		
WIPEAQILGGGQVATLSK	79.39711	Q9C0I1	MTMRC HUMAN	934.5180005	2	0.734669566	3	
WIPEAQILGGGQVATLSK	79.39711	Q9C0I1	MTMRC HUMAN	623.347942	3	0.734669566	3	Yes
ALVEMQDVVAELLR	129.671	Q9C0H2	TTYH3 HUMAN	793.43509	2	0.794426978		
ALVEMQDVVAELLR	129.671	Q9C0H2	TTYH3 HUMAN	529.2926683	3	0.794426978		
TVPWEQPATK	15.85807	Q9C0H2	TTYH3 HUMAN	578.804038	2	0.823710382		
TVPWEQPATK	15.85807	Q9C0H2	TTYH3 HUMAN	386.2053003	3	0.823710382		
VWDTAVGLNHTAEPSLQTLER	58.80835	Q9C0H2	TTYH3 HUMAN	1169.095863	2	0.687026978		
VWDTAVGLNHTAEPSLQTLER	58.80835	Q9C0H2	TTYH3 HUMAN	779.7331837	3	0.687026978		
AQPGVSETEEVALQPLLTPK	76.83723	Q9C0B5	ZDHC5 HUMAN	1054.068252	2	0.781916976	3	
AQPGVSETEEVALQPLLTPK	76.83723	Q9C0B5	ZDHC5 HUMAN	703.0481093	3	0.781916976	3	Yes
DSPPTPTMYK	13.24296	Q9C0B5	ZDHC5 HUMAN	568.768802	2	0.717201829		
DSPPTPTMYK	13.24296	Q9C0B5	ZDHC5 HUMAN	379.515143	3	0.717201829		
EEEPGLGDSGIQSTPGSGHAPR	12.62069	Q9C0B5	ZDHC5 HUMAN	1089.507078	2	0.750003338	3	
EEEPGLGDSGIQSTPGSGHAPR	12.62069	Q9C0B5	ZDHC5 HUMAN	726.6739933	3	0.750003338	3	Yes
GSLEITESQSADAEPPPPPKPDLSR	35.25716	Q9C0B5	ZDHC5 HUMAN	1309.651396	2	0.717228174		
GSLEITESQSADAEPPPPPKPDLSR	35.25716	Q9C0B5	ZDHC5 HUMAN	873.436872	3	0.717228174		

GVGSPEPGPTAPYLGR	37.56205	Q9C0B5	ZDHC5_HUMAN	777.899725	2	0.787409127		
GVGSPEPGPTAPYLGR	37.56205	Q9C0B5	ZDHC5_HUMAN	518.9357583	3	0.787409127		
IMDNGIQGELR	29.66292	Q9C0B5	ZDHC5_HUMAN	623.316986	2	0.620218992		
IMDNGIQGELR	29.66292	Q9C0B5	ZDHC5_HUMAN	415.880599	3	0.620218992		
SAQGTGFELGQLQSIR	60.8065	Q9C0B5	ZDHC5_HUMAN	846.43975	2	0.810528994		
SAQGTGFELGQLQSIR	60.8065	Q9C0B5	ZDHC5_HUMAN	564.6291083	3	0.810528994		
SEPSLEPESFR	28.92984	Q9C0B5	ZDHC5_HUMAN	639.3045975	2	0.809176266		
SEPSLEPESFR	28.92984	Q9C0B5	ZDHC5_HUMAN	426.5390067	3	0.809176266		
SFHFDPLSSGSR	30.1288	Q9C0B5	ZDHC5_HUMAN	668.8182065	2	0.700119197		
SFHFDPLSSGSR	30.1288	Q9C0B5	ZDHC5_HUMAN	446.214746	3	0.700119197		
SLGSASPGPGQPPLSSPTR	28.90498	Q9C0B5	ZDHC5_HUMAN	896.9635885	2	0.743627429		
SLGSASPGPGQPPLSSPTR	28.90498	Q9C0B5	ZDHC5_HUMAN	598.3116673	3	0.743627429		
THLGLATNEDSSLLAK	30.11801	Q9C0B5	ZDHC5_HUMAN	835.4419575	2	0.740538061	3	
THLGLATNEDSSLLAK	30.11801	Q9C0B5	ZDHC5_HUMAN	557.2972467	3	0.740538061	3	Yes
ADAEEGIFEIK	50.14173	Q9BYX2	TBD2A_HUMAN	611.304067	2	0.736026227		
ADAEEGIFEIK	50.14173	Q9BYX2	TBD2A_HUMAN	407.8719863	3	0.736026227		
ASSAYLAAAEDKDR	-5.486469	Q9BYX2	TBD2A_HUMAN	734.357889	2	0.72381717		
ASSAYLAAAEDKDR	-5.486469	Q9BYX2	TBD2A_HUMAN	489.9078677	3	0.72381717		
DFLSQQGK	3.444801	Q9BYX2	TBD2A_HUMAN	461.7356235	2	0.624616265		
DFLSQQGK	3.444801	Q9BYX2	TBD2A_HUMAN	308.1596907	3	0.624616265		
EQQVQELQQHVQLLMDK	58.12923	Q9BYX2	TBD2A_HUMAN	1047.536596	2	0.764394641	3	
EQQVQELQQHVQLLMDK	58.12923	Q9BYX2	TBD2A_HUMAN	698.6936723	3	0.764394641	3	Yes
HFTCPTSSFPDK	7.325481	Q9BYX2	TBD2A_HUMAN	712.319726	2	0.710787177		
HFTCPTSSFPDK	7.325481	Q9BYX2	TBD2A_HUMAN	475.215759	3	0.710787177		
HLGTEIQNTMHNIR	2.132343	Q9BYX2	TBD2A_HUMAN	832.420837	2	0.721210539		
HLGTEIQNTMHNIR	2.132343	Q9BYX2	TBD2A_HUMAN	555.2831663	3	0.721210539		
LMNIAFNDMNPFR	88.54356	Q9BYX2	TBD2A_HUMAN	791.879427	2	0.751059711		
LMNIAFNDMNPFR	88.54356	Q9BYX2	TBD2A_HUMAN	528.2555597	3	0.751059711		
LQEALGDEASECSELLR	51.97974	Q9BYX2	TBD2A_HUMAN	960.454936	2	0.831404686		
LQEALGDEASECSELLR	51.97974	Q9BYX2	TBD2A_HUMAN	640.6392323	3	0.831404686		
SHHLLGLEAVDRPLR	14.39943	Q9BYX2	TBD2A_HUMAN	856.9819185	2	0.742476702	4	
SHHLLGLEAVDRPLR	14.39943	Q9BYX2	TBD2A_HUMAN	571.6572207	3	0.742476702	4	
TAQDANPLDSIDLSSAVFDCK	100.2944	Q9BYX2	TBD2A_HUMAN	1134.028997	2	0.79946357		
TAQDANPLDSIDLSSAVFDCK	100.2944	Q9BYX2	TBD2A_HUMAN	756.3552727	3	0.79946357		
TQNCFLNSEIHQVTK	25.94235	Q9BYX2	TBD2A_HUMAN	909.944342	2	0.783434868		
TQNCFLNSEIHQVTK	25.94235	Q9BYX2	TBD2A_HUMAN	606.965503	3	0.783434868		
VAALEQQVLMLTK	68.02442	Q9BYX2	TBD2A_HUMAN	722.4161695	2	0.837625325		
VAALEQQVLMLTK	68.02442	Q9BYX2	TBD2A_HUMAN	481.9467213	3	0.837625325		
VLQDLLSEK	40.91619	Q9BYX2	TBD2A_HUMAN	522.800963	2	0.779609025		
VLQDLLSEK	40.91619	Q9BYX2	TBD2A_HUMAN	348.869917	3	0.779609025		
VQHLHTPGCYQELLSR	20.02401	Q9BYX2	TBD2A_HUMAN	969.4867045	2	0.74566102		
VQHLHTPGCYQELLSR	20.02401	Q9BYX2	TBD2A_HUMAN	646.6604113	3	0.74566102		
VTQDFTHPPDQSPLRPDAANR	18.10194	Q9BYX2	TBD2A_HUMAN	1181.580912	2	0.790342569		
VTQDFTHPPDQSPLRPDAANR	18.10194	Q9BYX2	TBD2A_HUMAN	788.0565493	3	0.790342569		
VWDAFLYEGTK	68.88517	Q9BYX2	TBD2A_HUMAN	664.830248	2	0.795564651		
VWDAFLYEGTK	68.88517	Q9BYX2	TBD2A_HUMAN	443.556107	3	0.795564651		
WAALGDLVPSAELK	85.584	Q9BYX2	TBD2A_HUMAN	735.4041155	2	0.819867671	2	Yes
WAALGDLVPSAELK	85.584	Q9BYX2	TBD2A_HUMAN	490.605352	3	0.819867671	2	
WFFYDER	50.9846	Q9BYX2	TBD2A_HUMAN	531.7381615	2	0.7687819		
WFFYDER	50.9846	Q9BYX2	TBD2A_HUMAN	354.8280493	3	0.7687819		
YDEYGFLTVPDYEVEDLK	106.0887	Q9BYX2	TBD2A_HUMAN	1098.007512	2	0.829950809	3	
YDEYGFLTVPDYEVEDLK	106.0887	Q9BYX2	TBD2A_HUMAN	732.3409493	3	0.829950809	3	Yes
LLPQGLAVYASPENK	56.29241	Q9BYD2	RM09_HUMAN	800.441225	2	0.816060185	3	
LLPQGLAVYASPENK	56.29241	Q9BYD2	RM09_HUMAN	533.963425	3	0.816060185	3	Yes
NLGVVVAPHTLK	21.34093	Q9BYD2	RM09_HUMAN	624.377704	2	0.776821971		
NLGVVVAPHTLK	21.34093	Q9BYD2	RM09_HUMAN	416.5877443	3	0.776821971		
VPMSVVNFEKPK	26.06303	Q9BYD2	RM09_HUMAN	687.876679	2	0.696553111	3	
VPMSVVNFEKPK	26.06303	Q9BYD2	RM09_HUMAN	458.9203943	3	0.696553111	3	Yes
WELNPEIVAR	57.79805	Q9BYD2	RM09_HUMAN	613.830586	2	0.796090961	2	Yes
WELNPEIVAR	57.79805	Q9BYD2	RM09_HUMAN	409.5563323	3	0.796090961	2	
YWLAQQAAK	14.16564	Q9BYD2	RM09_HUMAN	539.7881865	2	0.801764131	2	Yes
YWLAQQAAK	14.16564	Q9BYD2	RM09_HUMAN	360.1947327	3	0.801764131	2	
AGAVGAHLPASGLDIFGDLK	78.80748	Q9BY50	SC11C_HUMAN	955.013082	2	0.765154541		
AGAVGAHLPASGLDIFGDLK	78.80748	Q9BY50	SC11C_HUMAN	637.0113297	3	0.765154541		
GDLLFLTNFR	91.9064	Q9BY50	SC11C_HUMAN	598.3276795	2	0.779845297	2	Yes
GDLLFLTNFR	91.9064	Q9BY50	SC11C_HUMAN	399.2210613	3	0.779845297	2	
GLIVLTGSESPIVVVLSGSMEPAFHR	123.3099	Q9BY50	SC11C_HUMAN	1348.228562	2	0.663911223		
GLIVLTGSESPIVVVLSGSMEPAFHR	123.3099	Q9BY50	SC11C_HUMAN	899.154983	3	0.663911223		
ASMVHSLIEAYALHK	45.28781	Q9BY41	HDAC8_HUMAN	835.440702	2	0.778756857		
ASMVHSLIEAYALHK	45.28781	Q9BY41	HDAC8_HUMAN	557.2964097	3	0.778756857		
EVYQAFNPK	12.33398	Q9BY41	HDAC8_HUMAN	548.277651	2	0.754205048		
EVYQAFNPK	12.33398	Q9BY41	HDAC8_HUMAN	365.8543757	3	0.754205048		
FSPGFFPGTGDVSDVGLGK	86.2874	Q9BY41	HDAC8_HUMAN	942.4628905	2	0.824153006		
FSPGFFPGTGDVSDVGLGK	86.2874	Q9BY41	HDAC8_HUMAN	628.6445353	3	0.824153006		
IQQILNYIK	49.38107	Q9BY41	HDAC8_HUMAN	566.8404185	2	0.714996278		
IQQILNYIK	49.38107	Q9BY41	HDAC8_HUMAN	378.229554	3	0.714996278		
VAINWSGGWHHAK	10.75647	Q9BY41	HDAC8_HUMAN	731.8711085	2	0.646669865		
VAINWSGGWHHAK	10.75647	Q9BY41	HDAC8_HUMAN	488.250014	3	0.646669865		
VASMEEMATFHTDAYLQHLQK	51.1143	Q9BY41	HDAC8_HUMAN	1225.578131	2	0.848575473		
VASMEEMATFHTDAYLQHLQK	51.1143	Q9BY41	HDAC8_HUMAN	817.3880287	3	0.848575473		
YYQICESVLK	36.36749	Q9BY41	HDAC8_HUMAN	651.824104	2	0.781635642		
YYQICESVLK	36.36749	Q9BY41	HDAC8_HUMAN	434.8853443	3	0.781635642		
YYSVNVPIQDGIQDEK	57.11832	Q9BY41	HDAC8_HUMAN	934.457796	2	0.827864528		
YYSVNVPIQDGIQDEK	57.11832	Q9BY41	HDAC8_HUMAN	623.3078057	3	0.827864528		
IDLPEYQGEPDEISIQK	65.40163	Q9BY32	ITPA_HUMAN	987.4892975	2	0.915902436	3	
IDLPEYQGEPDEISIQK	65.40163	Q9BY32	ITPA_HUMAN	658.66214	3	0.915902436	3	Yes
LEEVVQILGDK	63.53612	Q9BY32	ITPA_HUMAN	621.8511845	2	0.621259689		
LEEVVQILGDK	63.53612	Q9BY32	ITPA_HUMAN	414.903398	3	0.621259689		
LKPEGLHQLLAGFEDK	51.07739	Q9BY32	ITPA_HUMAN	897.991618	2	0.884828746		
LKPEGLHQLLAGFEDK	51.07739	Q9BY32	ITPA_HUMAN	598.9970203	3	0.884828746		
DMVTELFDPLVQGEVQHR	111.0116	Q9BXV9	GON7_HUMAN	1057.023321	2	0.688299537	3	
DMVTELFDPLVQGEVQHR	111.0116	Q9BXV9	GON7_HUMAN	705.0181557	3	0.688299537	3	Yes
MELLGEYVGQEGKPQK	37.76291	Q9BXV9	GON7_HUMAN	903.4592895	2	0.815404773		
MELLGEYVGQEGKPQK	37.76291	Q9BXV9	GON7_HUMAN	602.6421347	3	0.815404773		
AQHTLPQDPAIVQSSLGSASASPFQPHVPYSPFR	77.04797	Q9BX40	LS14B_HUMAN	1809.410962	2	0.751865208	4	
AQHTLPQDPAIVQSSLGSASASPFQPHVPYSPFR	77.04797	Q9BX40	LS14B_HUMAN	1206.609916	3	0.751865208	4	
DITVCEPPK	14.14352	Q9BX40	LS14B_HUMAN	529.763524	2	0.781362414		
DITVCEPPK	14.14352	Q9BX40	LS14B_HUMAN	353.5116243	3	0.781362414		
DLAVVTQSAEAPAEEDLLGPNCYYDK	94.93849	Q9BX40	LS14B_HUMAN	1434.666378	2	0.635458589	3	
DLAVVTQSAEAPAEEDLLGPNCYYDK	94.93849	Q9BX40	LS14B_HUMAN	956.7801933	3	0.635458589	3	Yes
EEIYEYIIFR	87.57535	Q9BX40	LS14B_HUMAN	687.8511755	2	0.726356924		

EEIYEYIIFR	87.57535	Q9BX40	LS14B_HUMAN	458.903392	3	0.726356924		
FEGDFDFESANAQFNR	63.25803	Q9BX40	LS14B_HUMAN	947.406103	2	0.826152384		
FEGDFDFESANAQFNR	63.25803	Q9BX40	LS14B_HUMAN	631.9400103	3	0.826152384		
LNTETFGVSGR	17.11782	Q9BX40	LS14B_HUMAN	590.802026	2	0.784142911	2	Yes
LNTETFGVSGR	17.11782	Q9BX40	LS14B_HUMAN	394.203959	3	0.784142911	2	
SFFDNISSELK	75.66204	Q9BX40	LS14B_HUMAN	643.8173405	2	0.787938356	2	Yes
SFFDNISSELK	75.66204	Q9BX40	LS14B_HUMAN	429.547502	3	0.787938356	2	
SPMVEQAVQTGSADNLNAK	26.62855	Q9BX40	LS14B_HUMAN	980.476203	2	0.76253897	3	
SPMVEQAVQTGSADNLNAK	26.62855	Q9BX40	LS14B_HUMAN	653.9867437	3	0.76253897	3	Yes
TASDVVQPAAVQAQGQVNDENR	23.7162	Q9BX40	LS14B_HUMAN	1149.060009	2	0.728360593	3	
TASDVVQPAAVQAQGQVNDENR	23.7162	Q9BX40	LS14B_HUMAN	766.3759477	3	0.728360593	3	Yes
GMGPMGPGPQNYGSGMR	28.56956	Q9BWW4	SSBP3_HUMAN	847.366355	2	0.748362601		
GMGPMGPGPQNYGSGMR	28.56956	Q9BWW4	SSBP3_HUMAN	565.246845	3	0.748362601		
MGNQPPGGVPGTQPLLPNSMDPTR	67.49196	Q9BWW4	SSBP3_HUMAN	1231.102309	2	0.680849493		
MGNQPPGGVPGTQPLLPNSMDPTR	67.49196	Q9BWW4	SSBP3_HUMAN	821.070814	3	0.680849493		
NSPNNISGISNPPGTPR	20.41173	Q9BWW4	SSBP3_HUMAN	861.4324545	2	0.797388077		
NSPNNISGISNPPGTPR	20.41173	Q9BWW4	SSBP3_HUMAN	574.6242447	3	0.797388077		
ACVQVLDPK	14.70104	Q9BWS9	CHID1_HUMAN	515.274059	2	0.652706623		
ACVQVLDPK	14.70104	Q9BWS9	CHID1_HUMAN	343.851981	3	0.652706623		
AESVVLEHR	-17.7908	Q9BWS9	CHID1_HUMAN	520.280729	2	0.759357154		
AESVVLEHR	-17.7908	Q9BWS9	CHID1_HUMAN	347.189761	3	0.759357154		
EMFEVTGLHDVDQGWMR	70.91811	Q9BWS9	CHID1_HUMAN	1025.462042	2	0.746947289		
EMFEVTGLHDVDQGWMR	70.91811	Q9BWS9	CHID1_HUMAN	683.977303	3	0.746947289		
FTQISPVWLQLK	89.71503	Q9BWS9	CHID1_HUMAN	730.419569	2	0.800087333	2	Yes
FTQISPVWLQLK	89.71503	Q9BWS9	CHID1_HUMAN	487.282321	3	0.800087333	2	
GLVVTDLK	23.51374	Q9BWS9	CHID1_HUMAN	422.76111	2	0.737045884		
GLVVTDLK	23.51374	Q9BWS9	CHID1_HUMAN	282.1766817	3	0.737045884		
ILLGLNFYGMDYATSK	103.2259	Q9BWS9	CHID1_HUMAN	903.461296	2	0.7280671		
ILLGLNFYGMDYATSK	103.2259	Q9BWS9	CHID1_HUMAN	602.6434723	3	0.7280671		
LLFEDWTYDDFR	105.188	Q9BWS9	CHID1_HUMAN	810.373008	2	0.867460012		
LLFEDWTYDDFR	105.188	Q9BWS9	CHID1_HUMAN	540.5846137	3	0.867460012		
MVWDSQASEHFFEYK	57.3899	Q9BWS9	CHID1_HUMAN	952.4201635	2	0.815100312	3	
MVWDSQASEHFFEYK	57.3899	Q9BWS9	CHID1_HUMAN	635.2827173	3	0.815100312	3	Yes
NQHFDGFVVEVWNQLLSQK	124.7913	Q9BWS9	CHID1_HUMAN	1144.577109	2	0.749872208		
NQHFDGFVVEVWNQLLSQK	124.7913	Q9BWS9	CHID1_HUMAN	763.3873473	3	0.749872208		
NVLDSEDEIEELSK	58.06586	Q9BWS9	CHID1_HUMAN	810.3865125	2	0.876639724		
NVLDSEDEIEELSK	58.06586	Q9BWS9	CHID1_HUMAN	540.5936167	3	0.876639724		
SQFSDKPVQDR	-26.74094	Q9BWS9	CHID1_HUMAN	653.8234895	2	0.67215246		
SQFSDKPVQDR	-26.74094	Q9BWS9	CHID1_HUMAN	436.218268	3	0.67215246		
VGLIHMLTHLAEALHQAR	88.25354	Q9BWS9	CHID1_HUMAN	1005.557469	2	0.816557527		
VGLIHMLTHLAEALHQAR	88.25354	Q9BWS9	CHID1_HUMAN	670.7075873	3	0.816557527		
DQEQVELEGESSAPPR	20.5685	Q9BW61	DDA1_HUMAN	885.9112185	2	0.745852113	2	Yes
DQEQVELEGESSAPPR	20.5685	Q9BW61	DDA1_HUMAN	590.9434207	3	0.745852113	2	
EYPSEQIIVTEK	31.98312	Q9BW61	DDA1_HUMAN	718.369934	2	0.779064894	2	Yes
EYPSEQIIVTEK	31.98312	Q9BW61	DDA1_HUMAN	479.249231	3	0.779064894	2	
GLPVYNK	-5.021633	Q9BW61	DDA1_HUMAN	395.7270655	2	0.626244605		
GLPVYNK	-5.021633	Q9BW61	DDA1_HUMAN	264.1539853	3	0.626244605		
FYIEGSEPGK	16.87291	Q9BVV7	TIM21_HUMAN	563.774941	2	0.730516851	2	Yes
FYIEGSEPGK	16.87291	Q9BVV7	TIM21_HUMAN	376.1859023	3	0.730516851	2	
LLLPYIVLNK	91.16011	Q9BVV7	TIM21_HUMAN	593.3844615	2	0.707226396		
LLLPYIVLNK	91.16011	Q9BVV7	TIM21_HUMAN	395.9255827	3	0.707226396		
SHPEVIGVFGESVK	37.96658	Q9BVV7	TIM21_HUMAN	742.891372	2	0.652627468	2	Yes
SHPEVIGVFGESVK	37.96658	Q9BVV7	TIM21_HUMAN	495.5968563	3	0.652627468	2	
TIWTQGPSPR	16.91789	Q9BVV7	TIM21_HUMAN	571.8018295	2	0.793977022		
TIWTQGPSPR	16.91789	Q9BVV7	TIM21_HUMAN	381.5371613	3	0.793977022		
YIFVEIESYPR	70.08543	Q9BVV7	TIM21_HUMAN	708.36445	2	0.767998099		
YIFVEIESYPR	70.08543	Q9BVV7	TIM21_HUMAN	472.5789083	3	0.767998099		
ALTALGLAVPR	60.87808	Q9BVQ7	SPA5L_HUMAN	541.3405905	2	0.799837351	2	Yes
ALTALGLAVPR	60.87808	Q9BVQ7	SPA5L_HUMAN	361.2296687	3	0.799837351	2	
DGADGFVQLDPLCASPGAAVGASR	95.88339	Q9BVQ7	SPA5L_HUMAN	1166.055881	2	0.654348612		
DGADGFVQLDPLCASPGAAVGASR	95.88339	Q9BVQ7	SPA5L_HUMAN	777.706529	3	0.654348612		
EVVIGTPTLK	34.28143	Q9BVQ7	SPA5L_HUMAN	528.8191565	2	0.728047788	2	Yes
EVVIGTPTLK	34.28143	Q9BVQ7	SPA5L_HUMAN	352.882046	3	0.728047788	2	
EVVVVGATNRPDALDPALR	45.35644	Q9BVQ7	SPA5L_HUMAN	996.54782	2	0.689289212	3	
EVVVVGATNRPDALDPALR	45.35644	Q9BVQ7	SPA5L_HUMAN	664.701155	3	0.689289212	3	Yes
FPWEFVR	67.99238	Q9BVQ7	SPA5L_HUMAN	490.753619	2	0.664701462		
FPWEFVR	67.99238	Q9BVQ7	SPA5L_HUMAN	327.505021	3	0.664701462		
GPSLLFLDEMDALCPQR	116.7102	Q9BVQ7	SPA5L_HUMAN	981.4771605	2	0.792090178		
GPSLLFLDEMDALCPQR	116.7102	Q9BVQ7	SPA5L_HUMAN	654.6540487	3	0.792090178		
GVLLAGPPGVGK	38.02856	Q9BVQ7	SPA5L_HUMAN	532.827316	2	0.843364537	2	Yes
GVLLAGPPGVGK	38.02856	Q9BVQ7	SPA5L_HUMAN	355.5541523	3	0.843364537	2	
GVLLYGPPGCAK	37.32868	Q9BVQ7	SPA5L_HUMAN	616.3293615	2	0.778215766	2	Yes
GVLLYGPPGCAK	37.32868	Q9BVQ7	SPA5L_HUMAN	411.2221827	3	0.778215766	2	
IIYIPPPDHK	22.31271	Q9BVQ7	SPA5L_HUMAN	596.8404185	2	0.74487561		
IIYIPPPDHK	22.31271	Q9BVQ7	SPA5L_HUMAN	398.229554	3	0.74487561		
ISLPDGGSCLCTAWPR	72.33422	Q9BVQ7	SPA5L_HUMAN	895.4221885	2	0.758797765		
ISLPDGGSCLCTAWPR	72.33422	Q9BVQ7	SPA5L_HUMAN	597.2840673	3	0.758797765		
LGPAALHALGAR	17.00134	Q9BVQ7	SPA5L_HUMAN	573.841289	2	0.719193697	3	
LGPAALHALGAR	17.00134	Q9BVQ7	SPA5L_HUMAN	382.896801	3	0.719193697	3	Yes
LLLVPCPPLR	70.99638	Q9BVQ7	SPA5L_HUMAN	589.3604685	2	0.732790947	2	Yes
LLLVPCPPLR	70.99638	Q9BVQ7	SPA5L_HUMAN	393.2429207	3	0.732790947	2	
LLPLDAR	24.83429	Q9BVQ7	SPA5L_HUMAN	399.2481695	2	0.615689218		
LLPLDAR	24.83429	Q9BVQ7	SPA5L_HUMAN	266.501388	3	0.615689218		
NQDNPVIDEIDFLEAFK	139.7495	Q9BVQ7	SPA5L_HUMAN	1003.989469	2	0.78982383		
NQDNPVIDEIDFLEAFK	139.7495	Q9BVQ7	SPA5L_HUMAN	669.662254	3	0.78982383		
NTAAVLEAAQELLR	97.95549	Q9BVQ7	SPA5L_HUMAN	749.915379	2	0.837192893	3	
NTAAVLEAAQELLR	97.95549	Q9BVQ7	SPA5L_HUMAN	500.2795277	3	0.837192893	3	Yes
SSQQEFQEVFNR	35.34165	Q9BVQ7	SPA5L_HUMAN	749.8502355	2	0.860224962	2	Yes
SSQQEFQEVFNR	35.34165	Q9BVQ7	SPA5L_HUMAN	500.2360987	3	0.860224962	2	
SVIGLMDIKPVDWEEIGGLEDVK	117.6851	Q9BVQ7	SPA5L_HUMAN	1271.659647	2	0.734673738		
SVIGLMDIKPVDWEEIGGLEDVK	117.6851	Q9BVQ7	SPA5L_HUMAN	848.1090393	3	0.734673738		
SVMIIAATNRPDVLDTALLRPGR	71.44057	Q9BVQ7	SPA5L_HUMAN	1240.194857	2	0.663162053		
SVMIIAATNRPDVLDTALLRPGR	71.44057	Q9BVQ7	SPA5L_HUMAN	827.132513	3	0.663162053		
TVKPSLSCKDLALYENLFK	63.1236	Q9BVQ7	SPA5L_HUMAN	1113.596117	2	0.783409178		
TVKPSLSCKDLALYENLFK	63.1236	Q9BVQ7	SPA5L_HUMAN	742.7333527	3	0.783409178		
VAVWPVLR	57.91692	Q9BVQ7	SPA5L_HUMAN	470.2929115	2	0.816747069	2	Yes
VAVWPVLR	57.91692	Q9BVQ7	SPA5L_HUMAN	313.8645493	3	0.816747069	2	
VLSVLLNELDGVGLK	107.0616	Q9BVQ7	SPA5L_HUMAN	784.9670795	2	0.854455948		
VLSVLLNELDGVGLK	107.0616	Q9BVQ7	SPA5L_HUMAN	523.647328	3	0.854455948		
VSLGGEPPSEAQPQPEVPLGGLSEAADSLR	93.26096	Q9BVQ7	SPA5L_HUMAN	1494.252006	2	0.803255975		
VSLGGEPPSEAQPQPEVPLGGLSEAADSLR	93.26096	Q9BVQ7	SPA5L_HUMAN	996.5039453	3	0.803255975		

VVAQVLTLLDGASGDR	86.92046	Q9BVQ7	SPA5L HUMAN	807.4470435	2	0.793585718		
VVAQVLTLLDGASGDR	86.92046	Q9BVQ7	SPA5L HUMAN	538.6339707	3	0.793585718		
FSPDSTLLATCSADQTCK	47.02655	Q9BVC4	LST8 HUMAN	1001.448798	2	0.671269		
FSPDSTLLATCSADQTCK	47.02655	Q9BVC4	LST8 HUMAN	667.9684733	3	0.671269		
FWQAHSGICTR	-0.108704	Q9BVC4	LST8 HUMAN	681.8227705	2	0.726677239		
FWQAHSGICTR	-0.108704	Q9BVC4	LST8 HUMAN	454.8844553	3	0.726677239		
LWCVETGEIK	40.76826	Q9BVC4	LST8 HUMAN	617.8110055	2	0.787428081		
LWCVETGEIK	40.76826	Q9BVC4	LST8 HUMAN	412.2099453	3	0.787428081		
NIASVGFHEDGR	1.161079	Q9BVC4	LST8 HUMAN	651.3158315	2	0.653021276		
NIASVGFHEDGR	1.161079	Q9BVC4	LST8 HUMAN	434.546496	3	0.653021276		
SMIAAAGYQHIR	10.05629	Q9BVC4	LST8 HUMAN	659.3407915	2	0.800358951		
SMIAAAGYQHIR	10.05629	Q9BVC4	LST8 HUMAN	439.8964693	3	0.800358951		
TSNFSLMTELSIK	75.17326	Q9BVC4	LST8 HUMAN	735.8796155	2	0.631780922		
TSNFSLMTELSIK	75.17326	Q9BVC4	LST8 HUMAN	490.922352	3	0.631780922		
TVQHQDSQVNALEVTPDR	14.40172	Q9BVC4	LST8 HUMAN	1019.003974	2	0.762575746		
TVQHQDSQVNALEVTPDR	14.40172	Q9BVC4	LST8 HUMAN	679.6719243	3	0.762575746		
WMYTGGEDCTAR	17.66845	Q9BVC4	LST8 HUMAN	723.7930145	2	0.66388464		
WMYTGGEDCTAR	17.66845	Q9BVC4	LST8 HUMAN	482.864618	3	0.66388464		
AALELLSLDLK	93.54948	Q9BV73	CP250 HUMAN	593.358645	2	0.820753515		
AALELLSLDLK	93.54948	Q9BV73	CP250 HUMAN	395.9083717	3	0.820753515		
AAQAGSLEISK	1.092262	Q9BV73	CP250 HUMAN	537.7936695	2	0.712521851		
AAQAGSLEISK	1.092262	Q9BV73	CP250 HUMAN	358.8650547	3	0.712521851		
AAQLQLR	-4.293983	Q9BV73	CP250 HUMAN	400.243419	2	0.725094497		
AAQLQLR	-4.293983	Q9BV73	CP250 HUMAN	267.1648877	3	0.725094497		
ADALQGALEQAHMTLK	57.02659	Q9BV73	CP250 HUMAN	848.938529	2	0.811206102		
ADALQGALEQAHMTLK	57.02659	Q9BV73	CP250 HUMAN	566.294961	3	0.811206102		
AEALQEALGK	19.43634	Q9BV73	CP250 HUMAN	515.282938	2	0.766786993		
AEALQEALGK	19.43634	Q9BV73	CP250 HUMAN	343.8579003	3	0.766786993		
AEEAGAELQADLR	26.63905	Q9BV73	CP250 HUMAN	686.839337	2	0.865449965		
AEEAGAELQADLR	26.63905	Q9BV73	CP250 HUMAN	458.228833	3	0.865449965		
AEQSIAELSSSENTLK	36.68531	Q9BV73	CP250 HUMAN	853.9263365	2	0.780020237		
AEQSIAELSSSENTLK	36.68531	Q9BV73	CP250 HUMAN	569.620166	3	0.780020237		
ALLALQQQCAEQAQEHEVETR	32.41276	Q9BV73	CP250 HUMAN	1226.598445	2	0.748237729		
ALLALQQQCAEQAQEHEVETR	32.41276	Q9BV73	CP250 HUMAN	818.0682383	3	0.748237729		
ALQDSWLQAQAVLK	71.96584	Q9BV73	CP250 HUMAN	785.933572	2	0.896558881		
ALQDSWLQAQAVLK	71.96584	Q9BV73	CP250 HUMAN	524.2916563	3	0.896558881		
ALQENLALLTQTLAER	89.34575	Q9BV73	CP250 HUMAN	892.4998075	2	0.826628447		
ALQENLALLTQTLAER	89.34575	Q9BV73	CP250 HUMAN	595.3358133	3	0.826628447		
AQALQEQGELK	-8.643513	Q9BV73	CP250 HUMAN	607.822959	2	0.824032903		
AQALQEQGELK	-8.643513	Q9BV73	CP250 HUMAN	405.5512477	3	0.824032903		
AQASAAGILEEDLR	47.61178	Q9BV73	CP250 HUMAN	722.3760865	2	0.849273682		
AQASAAGILEEDLR	47.61178	Q9BV73	CP250 HUMAN	481.9199993	3	0.849273682		
AQDLALSLAQTK	38.2247	Q9BV73	CP250 HUMAN	629.854259	2	0.831937313		
AQDLALSLAQTK	38.2247	Q9BV73	CP250 HUMAN	420.238781	3	0.831937313		
ASLTLSLMEK	49.41423	Q9BV73	CP250 HUMAN	546.8026485	2	0.778707206		
ASLTLSLMEK	49.41423	Q9BV73	CP250 HUMAN	364.8710407	3	0.778707206		
ASLWAQEAK	12.07964	Q9BV73	CP250 HUMAN	502.264548	2	0.758311212		
ASLWAQEAK	12.07964	Q9BV73	CP250 HUMAN	335.1789737	3	0.758311212		
DDLAALQEESSSLLQDK	83.9285	Q9BV73	CP250 HUMAN	931.4554585	2	0.783487082		
DDLAALQEESSSLLQDK	83.9285	Q9BV73	CP250 HUMAN	621.3062473	3	0.783487082		
DQELEALQQEQQQAQGQEER	32.12609	Q9BV73	CP250 HUMAN	1193.049839	2	0.740685999		
DQELEALQQEQQQAQGQEER	32.12609	Q9BV73	CP250 HUMAN	795.702501	3	0.740685999		
DQLEQQLQGLHR	29.62645	Q9BV73	CP250 HUMAN	732.881871	2	0.676166058		
DQLEQQLQGLHR	29.62645	Q9BV73	CP250 HUMAN	488.9238557	3	0.676166058		
DTLAGQTVDLQGEVDSLSK	69.43938	Q9BV73	CP250 HUMAN	988.495116	2	0.779036582		
DTLAGQTVDLQGEVDSLSK	69.43938	Q9BV73	CP250 HUMAN	659.3326857	3	0.779036582		
EADFLAQEAQLLEELEASHITEQQLR	145.9837	Q9BV73	CP250 HUMAN	1506.252006	2	0.680042267		
EADFLAQEAQLLEELEASHITEQQLR	145.9837	Q9BV73	CP250 HUMAN	1004.503946	3	0.680042267		
EALESSHLEGELLR	34.34515	Q9BV73	CP250 HUMAN	791.90775	2	0.81192106		
EALESSHLEGELLR	34.34515	Q9BV73	CP250 HUMAN	528.2744417	3	0.81192106		
EEQQEELHLAVR	15.45107	Q9BV73	CP250 HUMAN	740.873711	2	0.736320794		
EEQQEELHLAVR	15.45107	Q9BV73	CP250 HUMAN	494.251749	3	0.736320794		
ELLESSLFEAQQQNSVIEVTK	90.40773	Q9BV73	CP250 HUMAN	1196.616293	2	0.769096196		
ELLESSLFEAQQQNSVIEVTK	90.40773	Q9BV73	CP250 HUMAN	798.080137	3	0.769096196		
ELQLTLAQK	33.36279	Q9BV73	CP250 HUMAN	522.308956	2	0.772916853		
ELQLTLAQK	33.36279	Q9BV73	CP250 HUMAN	348.5419123	3	0.772916853		
ELSAQMELLR	49.63036	Q9BV73	CP250 HUMAN	595.3164545	2	0.787415504		
ELSAQMELLR	49.63036	Q9BV73	CP250 HUMAN	397.213578	3	0.787415504		
EPAQLLLLLAK	99.27643	Q9BV73	CP250 HUMAN	604.8848305	2	0.721194088		
EPAQLLLLLAK	99.27643	Q9BV73	CP250 HUMAN	403.5924953	3	0.721194088		
EQEILELR	33.82423	Q9BV73	CP250 HUMAN	515.2829375	2	0.687239766		
EQEILELR	33.82423	Q9BV73	CP250 HUMAN	343.8579	3	0.687239766		
EQEIVVLQQQLQEAR	56.29151	Q9BV73	CP250 HUMAN	905.9870645	2	0.799830019		
EQEIVVLQQQLQEAR	56.29151	Q9BV73	CP250 HUMAN	604.327318	3	0.799830019		
EQETTGILQTQLQEAQR	46.63338	Q9BV73	CP250 HUMAN	987.000901	2	0.807880163		
EQETTGILQTQLQEAQR	46.63338	Q9BV73	CP250 HUMAN	658.3365423	3	0.807880163		
EQHLLEQAELSR	11.07428	Q9BV73	CP250 HUMAN	726.8762535	2	0.758018017		
EQHLLEQAELSR	11.07428	Q9BV73	CP250 HUMAN	484.9201107	3	0.758018017		
EQIEELQR	0.22728	Q9BV73	CP250 HUMAN	522.7701945	2	0.710949123		
EQIEELQR	0.22728	Q9BV73	CP250 HUMAN	348.8494047	3	0.710949123		
EQQTEMEAIQAQR	9.189968	Q9BV73	CP250 HUMAN	781.3679365	2	0.772098601		
EQQTEMEAIQAQR	9.189968	Q9BV73	CP250 HUMAN	521.2478993	3	0.772098601		
EQSLQSQLDEAQR	13.08495	Q9BV73	CP250 HUMAN	766.3715325	2	0.751173437	3	
EQSLQSQLDEAQR	13.08495	Q9BV73	CP250 HUMAN	511.2502967	3	0.751173437	3	Yes
ESQWQMEQEFFK	70.59226	Q9BV73	CP250 HUMAN	808.8566725	2	0.843809009		
ESQWQMEQEFFK	70.59226	Q9BV73	CP250 HUMAN	539.5737233	3	0.843809009		
ETVEILETNHTELMEHEASLSR	48.67013	Q9BV73	CP250 HUMAN	1284.616499	2	0.704673469		
ETVEILETNHTELMEHEASLSR	48.67013	Q9BV73	CP250 HUMAN	856.7469407	3	0.704673469		
EVVLLQAQLTLER	78.68361	Q9BV73	CP250 HUMAN	756.4437725	2	0.827269375		
EVVLLQAQLTLER	78.68361	Q9BV73	CP250 HUMAN	504.63179	3	0.827269375		
GEQGVQLGEVSGVEAEPSPDGMEK	53.04987	Q9BV73	CP250 HUMAN	1215.061026	2	0.697411895		
GEQGVQLGEVSGVEAEPSPDGMEK	53.04987	Q9BV73	CP250 HUMAN	810.3766253	3	0.697411895		
GPLLTALSAEAVASALHK	90.43784	Q9BV73	CP250 HUMAN	874.999443	2	0.740732849		
GPLLTALSAEAVASALHK	90.43784	Q9BV73	CP250 HUMAN	583.6689037	3	0.740732849		
GQIQELEK	-9.474884	Q9BV73	CP250 HUMAN	472.756556	2	0.73240453		
GQIQELEK	-9.474884	Q9BV73	CP250 HUMAN	315.506979	3	0.73240453		
GQLEVQIQTVTQAK	37.14854	Q9BV73	CP250 HUMAN	771.9284875	2	0.836752176		
GQLEVQIQTVTQAK	37.14854	Q9BV73	CP250 HUMAN	514.9549333	3	0.836752176		
HQQEAATTQLEQLHQEAK	-3.01379	Q9BV73	CP250 HUMAN	1045.51725	2	0.724995494		
HQQEAATTQLEQLHQEAK	-3.01379	Q9BV73	CP250 HUMAN	697.3474413	3	0.724995494		
ILEEDLEQIK	42.52228	Q9BV73	CP250 HUMAN	615.335367	2	0.839065433		

ILEEDLEQIK	42.52228	Q9BV73	CP250_HUMAN	410.5595197	3	0.839065433		
IQEGEIQDQDLR	12.90465	Q9BV73	CP250_HUMAN	722.357894	2	0.742484152		
IQEGEIQDQDLR	12.90465	Q9BV73	CP250_HUMAN	481.907871	3	0.742484152		
IQVLEDQR	4.201828	Q9BV73	CP250_HUMAN	500.77528	2	0.82831347		
IQVLEDQR	4.201828	Q9BV73	CP250_HUMAN	334.1861283	3	0.82831347		
LDESLTQSLTSPGPVLLHPSPSTTQAASR	70.88235	Q9BV73	CP250_HUMAN	1495.775648	2	0.600370765		
LDESLTQSLTSPGPVLLHPSPSTTQAASR	70.88235	Q9BV73	CP250_HUMAN	997.519707	3	0.600370765		
LEATGGPIPQR	6.862492	Q9BV73	CP250_HUMAN	569.814937	2	0.793384314		
LEATGGPIPQR	6.862492	Q9BV73	CP250_HUMAN	380.2125663	3	0.793384314		
LEHLQQAVAR	-15.81729	Q9BV73	CP250_HUMAN	582.8283785	2	0.613725781		
LEHLQQAVAR	-15.81729	Q9BV73	CP250_HUMAN	388.888194	3	0.613725781		
LHSPGATSTAELGSR	-13.35569	Q9BV73	CP250_HUMAN	742.3791605	2	0.812745571		
LHSPGATSTAELGSR	-13.35569	Q9BV73	CP250_HUMAN	495.255382	3	0.812745571		
LLVLQEADSIR	50.72702	Q9BV73	CP250_HUMAN	628.864626	2	0.861910105		
LLVLQEADSIR	50.72702	Q9BV73	CP250_HUMAN	419.5790257	3	0.861910105		
LQDELELTR	29.31299	Q9BV73	CP250_HUMAN	558.798952	2	0.807992697		
LQDELELTR	29.31299	Q9BV73	CP250_HUMAN	372.8685763	3	0.807992697		
LQEMLMGLEAK	57.4198	Q9BV73	CP250_HUMAN	631.828341	2	0.747144103		
LQEMLMGLEAK	57.4198	Q9BV73	CP250_HUMAN	421.5548357	3	0.747144103		
LSALNEALALDK	55.31548	Q9BV73	CP250_HUMAN	629.3566335	2	0.892744422		
LSALNEALALDK	55.31548	Q9BV73	CP250_HUMAN	419.9070307	3	0.892744422		
LSGSLLTCCLR	41.23639	Q9BV73	CP250_HUMAN	640.3290395	2	0.63200438		
LSGSLLTCCLR	41.23639	Q9BV73	CP250_HUMAN	427.221968	3	0.63200438		
LSLQQVIK	36.93051	Q9BV73	CP250_HUMAN	464.795484	2	0.724323273		
LSLQQVIK	36.93051	Q9BV73	CP250_HUMAN	310.1995977	3	0.724323273		
LTVDWSR	25.26152	Q9BV73	CP250_HUMAN	438.7328835	2	0.610722542		
LTVDWSR	25.26152	Q9BV73	CP250_HUMAN	292.8245307	3	0.610722542		
NALQVDLAEAEK	37.39532	Q9BV73	CP250_HUMAN	650.841348	2	0.859166384		
NALQVDLAEAEK	37.39532	Q9BV73	CP250_HUMAN	434.2301737	3	0.859166384		
NQEVDLQQEQIQELEK	46.85083	Q9BV73	CP250_HUMAN	985.987458	2	0.857370079		
NQEVDLQQEQIQELEK	46.85083	Q9BV73	CP250_HUMAN	657.6609137	3	0.857370079		
NVLEHQLLELEK	45.19405	Q9BV73	CP250_HUMAN	732.907022	2	0.851371765		
NVLEHQLLELEK	45.19405	Q9BV73	CP250_HUMAN	488.940623	3	0.851371765		
QDLAEQLQGLSSAK	49.52688	Q9BV73	CP250_HUMAN	744.389194	2	0.763391018		
QDLAEQLQGLSSAK	49.52688	Q9BV73	CP250_HUMAN	496.5954043	3	0.763391018		
QQLEVLEQEAWR	55.3958	Q9BV73	CP250_HUMAN	764.891904	2	0.725584984		
QQLEVLEQEAWR	55.3958	Q9BV73	CP250_HUMAN	510.2638777	3	0.725584984		
RHFLEMK	-23.93357	Q9BV73	CP250_HUMAN	480.7583785	2	0.608562648		
RHFLEMK	-23.93357	Q9BV73	CP250_HUMAN	320.8415273	3	0.608562648		
SQELIQLK	21.89983	Q9BV73	CP250_HUMAN	479.7825735	2	0.769985557		
SQELIQLK	21.89983	Q9BV73	CP250_HUMAN	320.1909907	3	0.769985557		
SQELQAQSSQIHDLESHSTVLAR	28.17444	Q9BV73	CP250_HUMAN	1282.639154	2	0.835759759		
SQELQAQSSQIHDLESHSTVLAR	28.17444	Q9BV73	CP250_HUMAN	855.428711	3	0.835759759		
SQLVAQDDSQR	-24.28954	Q9BV73	CP250_HUMAN	623.805297	2	0.765041113		
SQLVAQDDSQR	-24.28954	Q9BV73	CP250_HUMAN	416.2061397	3	0.765041113		
SQVHTELQDLQR	3.852081	Q9BV73	CP250_HUMAN	727.3738785	2	0.799726665		
SQVHTELQDLQR	3.852081	Q9BV73	CP250_HUMAN	485.2518607	3	0.799726665		
SVLEHLPMAVQER	39.16548	Q9BV73	CP250_HUMAN	754.8986745	2	0.858411431		
SVLEHLPMAVQER	39.16548	Q9BV73	CP250_HUMAN	503.6017247	3	0.858411431		
SWCQELEK	11.50866	Q9BV73	CP250_HUMAN	540.245498	2	0.742526352		
SWCQELEK	11.50866	Q9BV73	CP250_HUMAN	360.499607	3	0.742526352		
TQAESALCQMQLETEK	35.76569	Q9BV73	CP250_HUMAN	933.9327835	2	0.648548305		
TQAESALCQMQLETEK	35.76569	Q9BV73	CP250_HUMAN	622.9577973	3	0.648548305		
TSPMEEQSLK	-7.969189	Q9BV73	CP250_HUMAN	575.276995	2	0.692179918		
TSPMEEQSLK	-7.969189	Q9BV73	CP250_HUMAN	383.8539383	3	0.692179918		
VALTHLTLDLEER	49.48982	Q9BV73	CP250_HUMAN	755.4177545	2	0.895946145		
VALTHLTLDLEER	49.48982	Q9BV73	CP250_HUMAN	503.947778	3	0.895946145		
VGETSLLLSQR	33.14521	Q9BV73	CP250_HUMAN	601.841151	2	0.843306839		
VGETSLLLSQR	33.14521	Q9BV73	CP250_HUMAN	401.5633757	3	0.843306839		
VGLNQQLLQLEEENQSVCSR	64.79206	Q9BV73	CP250_HUMAN	1172.582262	2	0.678674161		
VGLNQQLLQLEEENQSVCSR	64.79206	Q9BV73	CP250_HUMAN	782.0574497	3	0.678674161		
VNVELQLQGDSAQGQK	31.77693	Q9BV73	CP250_HUMAN	857.4424895	2	0.799926937		
VNVELQLQGDSAQGQK	31.77693	Q9BV73	CP250_HUMAN	571.964268	3	0.799926937		
VQALEEVLGDLR	84.54144	Q9BV73	CP250_HUMAN	671.3728155	2	0.844959259		
VQALEEVLGDLR	84.54144	Q9BV73	CP250_HUMAN	447.9178187	3	0.844959259		
VSLLETLLQTQK	88.16241	Q9BV73	CP250_HUMAN	686.9064915	2	0.840650797		
VSLLETLLQTQK	88.16241	Q9BV73	CP250_HUMAN	458.2736027	3	0.840650797		
VTELSALLTQSQK	53.5567	Q9BV73	CP250_HUMAN	709.3990305	2	0.811199129		
VTELSALLTQSQK	53.5567	Q9BV73	CP250_HUMAN	473.2686287	3	0.811199129		
WENVEEPNLDELLVR	93.69655	Q9BV73	CP250_HUMAN	927.965796	2	0.645949364		
WENVEEPNLDELLVR	93.69655	Q9BV73	CP250_HUMAN	618.9798057	3	0.645949364		
YQEDVQQLQQALAQR	58.13527	Q9BV73	CP250_HUMAN	909.46121	2	0.812978923		
YQEDVQQLQQALAQR	58.13527	Q9BV73	CP250_HUMAN	606.643415	3	0.812978923		
AANNIASLLTK	42.40893	Q9BV44	THUM3_HUMAN	558.3251365	2	0.818405151		
AANNIASLLTK	42.40893	Q9BV44	THUM3_HUMAN	372.5526993	3	0.818405151		
AVLLTQDTK	2.926201	Q9BV44	THUM3_HUMAN	494.7878565	2	0.850675642		
AVLLTQDTK	2.926201	Q9BV44	THUM3_HUMAN	330.1945127	3	0.850675642		
DFGGAVQDYFK	65.52937	Q9BV44	THUM3_HUMAN	623.791123	2	0.803782582	2	Yes
DFGGAVQDYFK	65.52937	Q9BV44	THUM3_HUMAN	416.1966903	3	0.803782582	2	
EFTSHALDSHILDYYENPAIK	61.3215	Q9BV44	THUM3_HUMAN	1232.095519	2	0.706775367		
EFTSHALDSHILDYYENPAIK	61.3215	Q9BV44	THUM3_HUMAN	821.7329543	3	0.706775367		
LPWSNPLK	41.35558	Q9BV44	THUM3_HUMAN	477.774551	2	0.783557892	2	Yes
LPWSNPLK	41.35558	Q9BV44	THUM3_HUMAN	318.852309	3	0.783557892	2	
NITHFGPTTLR	14.41295	Q9BV44	THUM3_HUMAN	628.841486	2	0.654955983		
NITHFGPTTLR	14.41295	Q9BV44	THUM3_HUMAN	419.563599	3	0.654955983		
STLAYGMLR	32.87875	Q9BV44	THUM3_HUMAN	506.268772	2	0.604802012		
STLAYGMLR	32.87875	Q9BV44	THUM3_HUMAN	337.8484563	3	0.604802012		
TGSVDIIVTDLPFGK	94.58441	Q9BV44	THUM3_HUMAN	781.427788	2	0.686323345		
TGSVDIIVTDLPFGK	94.58441	Q9BV44	THUM3_HUMAN	521.2878003	3	0.686323345		
TPQAFVHPSEQDGER	-8.158787	Q9BV44	THUM3_HUMAN	849.398082	2	0.654411733		
TPQAFVHPSEQDGER	-8.158787	Q9BV44	THUM3_HUMAN	566.6013297	3	0.654411733		
VDTVWVNVGGLR	66.42132	Q9BV44	THUM3_HUMAN	657.862418	2	0.725798726		
VDTVWVNVGGLR	66.42132	Q9BV44	THUM3_HUMAN	438.910887	3	0.725798726		
SMYLMIK	37.39078	Q9BUT1	BDH2_HUMAN	443.232812	2	0.605256855		
SMYLMIK	37.39078	Q9BUT1	BDH2_HUMAN	295.824483	3	0.605256855		
SVAADFIQQGIR	52.35846	Q9BUT1	BDH2_HUMAN	652.8520505	2	0.855980277		
SVAADFIQQGIR	52.35846	Q9BUT1	BDH2_HUMAN	435.570642	3	0.855980277		
VIATDINESK	-3.307484	Q9BUT1	BDH2_HUMAN	545.293502	2	0.80971694	2	Yes
VIATDINESK	-3.307484	Q9BUT1	BDH2_HUMAN	363.864943	3	0.80971694	2	
VIILTAAAQGIGQAAALAFAR	122.7593	Q9BUT1	BDH2_HUMAN	1013.594575	2	0.761740565		
VIILTAAAQGIGQAAALAFAR	122.7593	Q9BUT1	BDH2_HUMAN	676.065658	3	0.761740565		

AFLLDPDR	40.35398	Q9BTY7	HGH1 HUMAN	473.753816	2	0.624859452		
AFLLDPDR	40.35398	Q9BTY7	HGH1 HUMAN	316.171819	3	0.624859452		
ALCTPGYNAR	-2.90493	Q9BTY7	HGH1 HUMAN	561.7722105	2	0.628914177		
ALCTPGYNAR	-2.90493	Q9BTY7	HGH1 HUMAN	374.8507487	3	0.628914177		
ALLAGQAALLQALMELAPASAPAR	162.1724	Q9BTY7	HGH1 HUMAN	1174.162495	2	0.66833812		
ALLAGQAALLQALMELAPASAPAR	162.1724	Q9BTY7	HGH1 HUMAN	783.1109383	3	0.66833812		
ALVNLAADPGLHETLLAADPGLPAR	84.6113	Q9BTY7	HGH1 HUMAN	1248.185013	2	0.799976885	3	
ALVNLAADPGLHETLLAADPGLPAR	84.6113	Q9BTY7	HGH1 HUMAN	832.4592833	3	0.799976885	3	Yes
DQGAYLILR	51.01478	Q9BTY7	HGH1 HUMAN	524.7934685	2	0.801456094	2	Yes
DQGAYLILR	51.01478	Q9BTY7	HGH1 HUMAN	350.198254	3	0.801456094	2	
ELAPEPWVER	41.66414	Q9BTY7	HGH1 HUMAN	613.314769	2	0.772082031	2	Yes
ELAPEPWVER	41.66414	Q9BTY7	HGH1 HUMAN	409.2124543	3	0.772082031	2	
ELHSWEPEPDVR	20.52735	Q9BTY7	HGH1 HUMAN	747.3551535	2	0.73059541		
ELHSWEPEPDVR	20.52735	Q9BTY7	HGH1 HUMAN	498.5727107	3	0.73059541		
EPAPCAALMAALAAAEPADSGLER	117.1573	Q9BTY7	HGH1 HUMAN	1191.575592	2	0.682213306		
EPAPCAALMAALAAAEPADSGLER	117.1573	Q9BTY7	HGH1 HUMAN	794.7196697	3	0.682213306		
GMENLLEVQVPEDVEQQLQQLDCR	123.5706	Q9BTY7	HGH1 HUMAN	1435.687131	2	0.698451161		
GMENLLEVQVPEDVEQQLQQLDCR	123.5706	Q9BTY7	HGH1 HUMAN	957.4606953	3	0.698451161		
HVLALTGCGPGR	2.584255	Q9BTY7	HGH1 HUMAN	619.327689	2	0.694213092		
HVLALTGCGPGR	2.584255	Q9BTY7	HGH1 HUMAN	413.2210677	3	0.694213092		
LIQVLIGDEPER	57.77056	Q9BTY7	HGH1 HUMAN	691.3884655	2	0.840624869		
LIQVLIGDEPER	57.77056	Q9BTY7	HGH1 HUMAN	461.2615853	3	0.840624869		
LLPFLAPGAR	68.48018	Q9BTY7	HGH1 HUMAN	527.824576	2	0.773032784	2	Yes
LLPFLAPGAR	68.48018	Q9BTY7	HGH1 HUMAN	352.2189923	3	0.773032784	2	
LLPLTQYPDSSVR	53.29695	Q9BTY7	HGH1 HUMAN	744.907018	2	0.84803158	2	Yes
LLPLTQYPDSSVR	53.29695	Q9BTY7	HGH1 HUMAN	496.9406203	3	0.84803158	2	
LPVDLQYLPPDK	67.91215	Q9BTY7	HGH1 HUMAN	699.38793	2	0.759294808	2	Yes
LPVDLQYLPPDK	67.91215	Q9BTY7	HGH1 HUMAN	466.5945617	3	0.759294808	2	
FEEMFASR	26.41409	Q9BTL3	RAMAC HUMAN	508.7294835	2	0.660353482		
FEEMFASR	26.41409	Q9BTL3	RAMAC HUMAN	339.4889307	3	0.660353482		
RPPESPPIVEEWNSR	31.00716	Q9BTL3	RAMAC HUMAN	896.953023	2	0.771619081	3	
RPPESPPIVEEWNSR	31.00716	Q9BTL3	RAMAC HUMAN	598.3046237	3	0.771619081	3	Yes
SWGNNYPQHR	-21.97368	Q9BTL3	RAMAC HUMAN	629.7897795	2	0.825111508	2	Yes
SWGNNYPQHR	-21.97368	Q9BTL3	RAMAC HUMAN	420.1957947	3	0.825111508	2	
AAAAATVVPPMVGGPPFVGPVGFGPGDR	118.7608	Q9BTD8	RBM42 HUMAN	1296.176135	2	0.738390565		
AAAAATVVPPMVGGPPFVGPVGFGPGDR	118.7608	Q9BTD8	RBM42 HUMAN	864.4533647	3	0.738390565		
APGPPLGSMAALRPPLEEPAAPR	66.04762	Q9BTD8	RBM42 HUMAN	1148.118087	2	0.616840482		
APGPPLGSMAALRPPLEEPAAPR	66.04762	Q9BTD8	RBM42 HUMAN	765.7479997	3	0.616840482		
APILRPAFVPHVLQR	47.64067	Q9BTD8	RBM42 HUMAN	857.5179405	2	0.742527366		
APILRPAFVPHVLQR	47.64067	Q9BTD8	RBM42 HUMAN	572.0145687	3	0.742527366		
ELGLGLGLGLK	79.74913	Q9BTD8	RBM42 HUMAN	535.3349735	2	0.812770128	2	Yes
ELGLGLGLGLK	79.74913	Q9BTD8	RBM42 HUMAN	357.225924	3	0.812770128	2	
GLLPPLR	40.70145	Q9BTD8	RBM42 HUMAN	383.253255	2	0.662621379		
GLLPPLR	40.70145	Q9BTD8	RBM42 HUMAN	255.8381117	3	0.662621379		
IFCGDLGNEVNDDILAR	71.87481	Q9BTD8	RBM42 HUMAN	960.9601885	2	0.705108106		
IFCGDLGNEVNDDILAR	71.87481	Q9BTD8	RBM42 HUMAN	640.9760673	3	0.705108106		
EAALANQEVWEETQGMAPPSR	55.44629	Q9BT25	HAUS8 HUMAN	1157.542606	2	0.606690228		
EAALANQEVWEETQGMAPPSR	55.44629	Q9BT25	HAUS8 HUMAN	772.0310123	3	0.606690228		
GDLQSTLLEGHGTAPPDLDLSAINDK	76.40245	Q9BT25	HAUS8 HUMAN	1339.169953	2	0.749187171		
GDLQSTLLEGHGTAPPDLDLSAINDK	76.40245	Q9BT25	HAUS8 HUMAN	893.1159103	3	0.749187171		
KPESTSFSAPR	-25.21492	Q9BT25	HAUS8 HUMAN	603.8098505	2	0.697823107		
KPESTSFSAPR	-25.21492	Q9BT25	HAUS8 HUMAN	402.875842	3	0.697823107		
LLGELDVGDSEENVQVLDLLSELK	164.5517	Q9BT25	HAUS8 HUMAN	1314.187279	2	0.645385385		
LLGELDVGDSEENVQVLDLLSELK	164.5517	Q9BT25	HAUS8 HUMAN	876.4607943	3	0.645385385		
MENNLAEFER	26.50162	Q9BT25	HAUS8 HUMAN	626.785518	2	0.788136363		
MENNLAEFER	26.50162	Q9BT25	HAUS8 HUMAN	418.1929537	3	0.788136363		
SFAQVLELSAEASK	72.64743	Q9BT25	HAUS8 HUMAN	740.388662	2	0.826808035		
SFAQVLELSAEASK	72.64743	Q9BT25	HAUS8 HUMAN	493.928383	3	0.826808035		
SIHLEGDGQQLLDALQHELVTTQR	93.19843	Q9BT25	HAUS8 HUMAN	1351.19938	2	0.839220583	4	
SIHLEGDGQQLLDALQHELVTTQR	93.19843	Q9BT25	HAUS8 HUMAN	901.1355283	3	0.839220583	4	
TFATALDTTR	19.5681	Q9BT25	HAUS8 HUMAN	548.7858455	2	0.799078465		
TFATALDTTR	19.5681	Q9BT25	HAUS8 HUMAN	366.193172	3	0.799078465		
WYFNQDSACR	26.88937	Q9BT25	HAUS8 HUMAN	673.783306	2	0.712309837		
WYFNQDSACR	26.88937	Q9BT25	HAUS8 HUMAN	449.5248123	3	0.712309837		
ATANLPSYNMDR	20.05365	Q9BSL1	UBAC1 HUMAN	676.8173455	2	0.797390461	2	Yes
ATANLPSYNMDR	20.05365	Q9BSL1	UBAC1 HUMAN	451.5475053	3	0.797390461	2	
AVISLMEMGFDEK	80.77011	Q9BSL1	UBAC1 HUMAN	735.3549195	2	0.827231407		
AVISLMEMGFDEK	80.77011	Q9BSL1	UBAC1 HUMAN	490.5725547	3	0.827231407		
DELTEIFK	63.992	Q9BSL1	UBAC1 HUMAN	497.758764	2	0.72137928		
DELTEIFK	63.992	Q9BSL1	UBAC1 HUMAN	332.1751177	3	0.72137928		
GIDPDSPLFQAILDNPVVQLGLTNPK	160.2855	Q9BSL1	UBAC1 HUMAN	1381.242724	2	0.611733794		
GIDPDSPLFQAILDNPVVQLGLTNPK	160.2855	Q9BSL1	UBAC1 HUMAN	921.1644243	3	0.611733794		
ILVSLIEVAQK	72.55698	Q9BSL1	UBAC1 HUMAN	606.8822875	2	0.678611875		
ILVSLIEVAQK	72.55698	Q9BSL1	UBAC1 HUMAN	404.9241333	3	0.678611875		
LLALNPDAVELFK	99.62361	Q9BSL1	UBAC1 HUMAN	721.9168585	2	0.884804964	2	Yes
LLALNPDAVELFK	99.62361	Q9BSL1	UBAC1 HUMAN	481.6138473	3	0.884804964	2	
QLTEMGFPENR	32.91477	Q9BSL1	UBAC1 HUMAN	661.3144435	2	0.625119209		
QLTEMGFPENR	32.91477	Q9BSL1	UBAC1 HUMAN	441.2122373	3	0.625119209		
TILEENIQDQDVLLLIK	114.1195	Q9BSL1	UBAC1 HUMAN	999.062437	2	0.728763461		
TILEENIQDQDVLLLIK	114.1195	Q9BSL1	UBAC1 HUMAN	666.3775663	3	0.728763461		
AGWYDEEAR	7.92477	Q9BRZ2	TRI56 HUMAN	548.73889	2	0.832597375		
AGWYDEEAR	7.92477	Q9BRZ2	TRI56 HUMAN	366.1618683	3	0.832597375		
ALSLSQASHAVAALPSGDR	37.76772	Q9BRZ2	TRI56 HUMAN	925.9901375	2	0.792401552		
ALSLSQASHAVAALPSGDR	37.76772	Q9BRZ2	TRI56 HUMAN	617.6627	3	0.792401552		
EAEILSLEGAIAQR	74.13533	Q9BRZ2	TRI56 HUMAN	750.4073865	2	0.825860918		
EAEILSLEGAIAQR	74.13533	Q9BRZ2	TRI56 HUMAN	500.6075327	3	0.825860918		
EILVADEQNR	11.365	Q9BRZ2	TRI56 HUMAN	593.807308	2	0.803783953		
EILVADEQNR	11.365	Q9BRZ2	TRI56 HUMAN	396.2074803	3	0.803783953		
ETVPVPPEGVASFK	53.94677	Q9BRZ2	TRI56 HUMAN	728.8882985	2	0.783430099		
ETVPVPPEGVASFK	53.94677	Q9BRZ2	TRI56 HUMAN	486.261474	3	0.783430099		
FLCQPCSQLLCR	47.53198	Q9BRZ2	TRI56 HUMAN	791.3709135	2	0.717576683		
FLCQPCSQLLCR	47.53198	Q9BRZ2	TRI56 HUMAN	527.9165507	3	0.717576683		
GPGLHGCQPGSVSVDK	-10.76883	Q9BRZ2	TRI56 HUMAN	797.886296	2	0.775590003		
GPGLHGCQPGSVSVDK	-10.76883	Q9BRZ2	TRI56 HUMAN	532.260139	3	0.775590003		
GSLLGDFLTAYHGLEKPR	71.96276	Q9BRZ2	TRI56 HUMAN	987.526352	2	0.795603752	4	
GSLLGDFLTAYHGLEKPR	71.96276	Q9BRZ2	TRI56 HUMAN	658.686843	3	0.795603752	4	
GYIFLTLR	69.19086	Q9BRZ2	TRI56 HUMAN	491.7901975	2	0.75896132		
GYIFLTLR	69.19086	Q9BRZ2	TRI56 HUMAN	328.1960733	3	0.75896132		
ITGLCPFGPR	41.69801	Q9BRZ2	TRI56 HUMAN	559.295326	2	0.787359715		
ITGLCPFGPR	41.69801	Q9BRZ2	TRI56 HUMAN	373.1994923	3	0.787359715		
LDPHLDHPCLPLAEAVR	51.16472	Q9BRZ2	TRI56 HUMAN	977.004734	2	0.765929818		

LDPHLDHPCLPLAEAVR	51.16472	Q9BRZ2	TRI56_HUMAN	651.672431	3	0.765929818		
LSFEEQQPQK	2.936626	Q9BRZ2	TRI56_HUMAN	617.309684	2	0.817332625		
LSFEEQQPQK	2.936626	Q9BRZ2	TRI56_HUMAN	411.875731	3	0.817332625		
LYLINPNGEVQWR	67.71593	Q9BRZ2	TRI56_HUMAN	801.425909	2	0.721323609		
LYLINPNGEVQWR	67.71593	Q9BRZ2	TRI56_HUMAN	534.619881	3	0.721323609		
VAVSVAGHVEVYNMEGSLATR	47.46156	Q9BRZ2	TRI56_HUMAN	1095.055148	2	0.634053111		
VAVSVAGHVEVYNMEGSLATR	47.46156	Q9BRZ2	TRI56_HUMAN	730.3727067	3	0.634053111		
VGTQVEEAAEGVLR	41.80762	Q9BRZ2	TRI56_HUMAN	729.383912	2	0.830549598		
VGTQVEEAAEGVLR	41.80762	Q9BRZ2	TRI56_HUMAN	486.591883	3	0.830549598		
VVDLVGYR	29.75464	Q9BRZ2	TRI56_HUMAN	460.7641795	2	0.692747533		
VVDLVGYR	29.75464	Q9BRZ2	TRI56_HUMAN	307.5120613	3	0.692747533		
AALEYLEDIDLK	81.27098	Q9BRX8	PXL2A_HUMAN	696.867027	2	0.833873451		
AALEYLEDIDLK	81.27098	Q9BRX8	PXL2A_HUMAN	464.9139597	3	0.833873451		
EEAADLSSLK	13.30689	Q9BRX8	PXL2A_HUMAN	531.7698595	2	0.61607939		
EEAADLSSLK	13.30689	Q9BRX8	PXL2A_HUMAN	354.8491813	3	0.61607939		
GEIFLDEK	23.43402	Q9BRX8	PXL2A_HUMAN	475.7456565	2	0.810784101		
GEIFLDEK	23.43402	Q9BRX8	PXL2A_HUMAN	317.4997127	3	0.810784101		
SMLDQLGVPLYAVVK	113.9255	Q9BRX8	PXL2A_HUMAN	816.955654	2	0.715986192		
SMLDQLGVPLYAVVK	113.9255	Q9BRX8	PXL2A_HUMAN	544.9730443	3	0.715986192		
VNLLSVLEAAK	91.52084	Q9BRX8	PXL2A_HUMAN	578.850987	2	0.827921569		
VNLLSVLEAAK	91.52084	Q9BRX8	PXL2A_HUMAN	386.2365997	3	0.827921569		
GLNDFQCWEK	38.55003	Q9BRX5	PSF3_HUMAN	648.7880615	2	0.844931483		
GLNDFQCWEK	38.55003	Q9BRX5	PSF3_HUMAN	432.861316	3	0.844931483		
GQASQITASNLVQNYK	34.52258	Q9BRX5	PSF3_HUMAN	861.4450275	2	0.858415186		
GQASQITASNLVQNYK	34.52258	Q9BRX5	PSF3_HUMAN	574.6326267	3	0.858415186		
ILSVELPK	40.6177	Q9BRX5	PSF3_HUMAN	449.7845845	2	0.772365689	2	Yes
ILSVELPK	40.6177	Q9BRX5	PSF3_HUMAN	300.1923313	3	0.772365689	2	
IMDSSQNAYNEDTSALVAR	38.69845	Q9BRX5	PSF3_HUMAN	1042.981845	2	0.702463806		
IMDSSQNAYNEDTSALVAR	38.69845	Q9BRX5	PSF3_HUMAN	695.6571713	3	0.702463806		
LELPLWLAK	94.78421	Q9BRX5	PSF3_HUMAN	541.834609	2	0.801064491	2	Yes
LELPLWLAK	94.78421	Q9BRX5	PSF3_HUMAN	361.5590143	3	0.801064491	2	
LGAFFLER	55.1857	Q9BRX5	PSF3_HUMAN	476.7667265	2	0.810290933	2	Yes
LGAFFLER	55.1857	Q9BRX5	PSF3_HUMAN	318.180426	3	0.810290933	2	
SAGAETDNAVPQGSK	-22.71804	Q9BRX5	PSF3_HUMAN	716.339701	2	0.637609065		
SAGAETDNAVPQGSK	-22.71804	Q9BRX5	PSF3_HUMAN	477.8957423	3	0.637609065		
TVFSADPNVVDLHK	37.85857	Q9BRX5	PSF3_HUMAN	771.402104	2	0.755765557		
TVFSADPNVVDLHK	37.85857	Q9BRX5	PSF3_HUMAN	514.604011	3	0.755765557		
VESGALGPEENFLSLDDILMSHEK	106.8024	Q9BRX5	PSF3_HUMAN	1315.636899	2	0.822377563		
VESGALGPEENFLSLDDILMSHEK	106.8024	Q9BRX5	PSF3_HUMAN	877.4272077	3	0.822377563		
DAPVHGSPTGPGAWTASK	13.6643	Q9BRP1	PDD2L_HUMAN	868.4241	2	0.828226328		
DAPVHGSPTGPGAWTASK	13.6643	Q9BRP1	PDD2L_HUMAN	579.2853417	3	0.828226328		
DFVNLDHAHSLLR	42.80684	Q9BRP1	PDD2L_HUMAN	768.9000625	2	0.817971349		
DFVNLDHAHSLLR	42.80684	Q9BRP1	PDD2L_HUMAN	512.9359833	3	0.817971349		
IAACQEQILR	14.56519	Q9BRP1	PDD2L_HUMAN	601.322072	2	0.76143533		
IAACQEQILR	14.56519	Q9BRP1	PDD2L_HUMAN	401.217323	3	0.76143533		
LGGIPDALPTVAAPRPVCQR	58.54742	Q9BRP1	PDD2L_HUMAN	1044.573316	2	0.605162144		
LGGIPDALPTVAAPRPVCQR	58.54742	Q9BRP1	PDD2L_HUMAN	696.7181523	3	0.605162144		
SQCLQVPER	-0.201656	Q9BRP1	PDD2L_HUMAN	558.777497	2	0.635679841		
SQCLQVPER	-0.201656	Q9BRP1	PDD2L_HUMAN	372.854273	3	0.635679841		
ALQALQQEHK	-25.66899	Q9BRG1	VPS25_HUMAN	583.3203865	2	0.706260026		
ALQALQQEHK	-25.66899	Q9BRG1	VPS25_HUMAN	389.2161993	3	0.706260026		
FPPFFTLQPNVDTR	91.91891	Q9BRG1	VPS25_HUMAN	839.933572	2	0.706522703	3	
FPPFFTLQPNVDTR	91.91891	Q9BRG1	VPS25_HUMAN	560.2916563	3	0.706522703	3	Yes
GNLEWLDK	34.20321	Q9BRG1	VPS25_HUMAN	487.751273	2	0.622438431		
GNLEWLDK	34.20321	Q9BRG1	VPS25_HUMAN	325.503457	3	0.622438431		
LIYQWVSR	43.96173	Q9BRG1	VPS25_HUMAN	532.7985535	2	0.719138682		
LIYQWVSR	43.96173	Q9BRG1	VPS25_HUMAN	355.5349773	3	0.719138682		
SSFLIMWR	75.18964	Q9BRG1	VPS25_HUMAN	520.273861	2	0.730836511		
SSFLIMWR	75.18964	Q9BRG1	VPS25_HUMAN	347.1851823	3	0.730836511		
AGTDPSHMPTGPQAASCLDLNLVTR	64.3662	Q9BQG0	MBB1A_HUMAN	1305.126512	2	0.720307887	3	
AGTDPSHMPTGPQAASCLDLNLVTR	64.3662	Q9BQG0	MBB1A_HUMAN	870.420283	3	0.720307887	3	Yes
ALVDILSEVSK	83.81236	Q9BQG0	MBB1A_HUMAN	587.340452	2	0.759860396	2	Yes
ALVDILSEVSK	83.81236	Q9BQG0	MBB1A_HUMAN	391.896243	3	0.759860396	2	
AQDSSLHMPER	-14.00223	Q9BQG0	MBB1A_HUMAN	635.7964175	2	0.794320524	2	Yes
AQDSSLHMPER	-14.00223	Q9BQG0	MBB1A_HUMAN	424.20022	3	0.794320524	2	
AQHQQALSSLELLNVLFR	112.5067	Q9BQG0	MBB1A_HUMAN	1034.071462	2	0.784915209	3	
AQHQQALSSLELLNVLFR	112.5067	Q9BQG0	MBB1A_HUMAN	689.7169163	3	0.784915209	3	Yes
ATLQEILPEVLK	88.40842	Q9BQG0	MBB1A_HUMAN	677.4035845	2	0.872231603	2	Yes
ATLQEILPEVLK	88.40842	Q9BQG0	MBB1A_HUMAN	451.9383313	3	0.872231603	2	
DGDVDQGFR	-0.128033	Q9BQG0	MBB1A_HUMAN	504.7232445	2	0.615810037		
DGDVDQGFR	-0.128033	Q9BQG0	MBB1A_HUMAN	336.8181047	3	0.615810037		
EQLHLVMQGDVIR	37.60917	Q9BQG0	MBB1A_HUMAN	769.4119495	2	0.778524041	2	Yes
EQLHLVMQGDVIR	37.60917	Q9BQG0	MBB1A_HUMAN	513.2772413	3	0.778524041	2	
EQLMTVLQAGK	43.06419	Q9BQG0	MBB1A_HUMAN	609.3321055	2	0.75746423	2	Yes
EQLMTVLQAGK	43.06419	Q9BQG0	MBB1A_HUMAN	406.5573453	3	0.75746423	2	
FAPEMDDYVGTFLEGCQDDPER	100.9735	Q9BQG0	MBB1A_HUMAN	1296.039232	2	0.713999987	3	
FAPEMDDYVGTFLEGCQDDPER	100.9735	Q9BQG0	MBB1A_HUMAN	864.3620963	3	0.713999987	3	Yes
FLSPPALQGYVAWLR	118.6683	Q9BQG0	MBB1A_HUMAN	859.4754025	2	0.709952772		
FLSPPALQGYVAWLR	118.6683	Q9BQG0	MBB1A_HUMAN	573.3195433	3	0.709952772		
GNTAEGCVHETQEK	-44.56256	Q9BQG0	MBB1A_HUMAN	780.3419185	2	0.717716813		
GNTAEGCVHETQEK	-44.56256	Q9BQG0	MBB1A_HUMAN	520.5638873	3	0.717716813		
HPFSFPLENQAR	35.72892	Q9BQG0	MBB1A_HUMAN	721.862949	2	0.724686861	2	Yes
HPFSFPLENQAR	35.72892	Q9BQG0	MBB1A_HUMAN	481.5779077	3	0.724686861	2	
LHDLYWQAMK	30.03103	Q9BQG0	MBB1A_HUMAN	652.8269855	2	0.796650112	3	
LHDLYWQAMK	30.03103	Q9BQG0	MBB1A_HUMAN	435.553932	3	0.796650112	3	Yes
LITGLGVGR	28.93742	Q9BQG0	MBB1A_HUMAN	443.2800015	2	0.734265864	2	Yes
LITGLGVGR	28.93742	Q9BQG0	MBB1A_HUMAN	295.8559427	3	0.734265864	2	
LLGAALPLLTK	80.62857	Q9BQG0	MBB1A_HUMAN	555.3688165	2	0.784552515	2	Yes
LLGAALPLLTK	80.62857	Q9BQG0	MBB1A_HUMAN	370.5818193	3	0.784552515	2	
LLQALAQYQNHLQEQPR	46.35355	Q9BQG0	MBB1A_HUMAN	1025.545608	2	0.899368525	3	
LLQALAQYQNHLQEQPR	46.35355	Q9BQG0	MBB1A_HUMAN	684.0330137	3	0.899368525	3	Yes
LPAIALDLLR	100.1775	Q9BQG0	MBB1A_HUMAN	547.8507905	2	0.817859054	2	Yes
LPAIALDLLR	100.1775	Q9BQG0	MBB1A_HUMAN	365.569802	3	0.817859054	2	
LVGSVNLFSDENVPR	65.48662	Q9BQG0	MBB1A_HUMAN	823.431392	2	0.88801384	3	
LVGSVNLFSDENVPR	65.48662	Q9BQG0	MBB1A_HUMAN	549.290203	3	0.88801384	3	Yes
LVSIVDSLHLEMEEALTEQVAR	117.7897	Q9BQG0	MBB1A_HUMAN	1241.647071	2	0.743858993		
LVSIVDSLHLEMEEALTEQVAR	117.7897	Q9BQG0	MBB1A_HUMAN	828.1006553	3	0.743858993		
SCFEDPEWK	27.71513	Q9BQG0	MBB1A_HUMAN	599.2482375	2	0.684385002	2	Yes
SCFEDPEWK	27.71513	Q9BQG0	MBB1A_HUMAN	399.8347667	3	0.684385002	2	
SPSLLQSGAK	-0.088966	Q9BQG0	MBB1A_HUMAN	494.277655	2	0.731837809	2	Yes
SPSLLQSGAK	-0.088966	Q9BQG0	MBB1A_HUMAN	329.8543783	3	0.731837809	2	

SVFGHICSHLTPR	13.72345	Q9BQG0	MBB1A HUMAN	755.883359	2	0.793466568	4	
SVFGHICSHLTPR	13.72345	Q9BQG0	MBB1A HUMAN	504.258181	3	0.793466568	4	
VLDLVEVLVTK	105.203	Q9BQG0	MBB1A HUMAN	614.3821205	2	0.808005631	2	Yes
VLDLVEVLVTK	105.203	Q9BQG0	MBB1A HUMAN	409.924022	3	0.808005631	2	
VYSTALSSFLTK	62.36906	Q9BQG0	MBB1A HUMAN	658.859005	2	0.827614963	2	Yes
VYSTALSSFLTK	62.36906	Q9BQG0	MBB1A HUMAN	439.5752783	3	0.827614963	2	
YDLHQVK	-23.72818	Q9BQG0	MBB1A HUMAN	451.7407045	2	0.740029454		
YDLHQVK	-23.72818	Q9BQG0	MBB1A HUMAN	301.4964113	3	0.740029454		
FIADQLDHLNVTK	46.01171	Q9BQC6	RT63 HUMAN	757.404647	2	0.815898895		
FIADQLDHLNVTK	46.01171	Q9BQC6	RT63 HUMAN	505.272373	3	0.815898895		
AAGPLLTDECR	14.91179	Q9BQ69	MACD1 HUMAN	601.795887	2	0.792469561		
AAGPLLTDECR	14.91179	Q9BQ69	MACD1 HUMAN	401.5331997	3	0.792469561		
EWLEQHK	-19.34369	Q9BQ69	MACD1 HUMAN	485.2436155	2	0.680134833		
EWLEQHK	-19.34369	Q9BQ69	MACD1 HUMAN	323.8316853	3	0.680134833		
SCYLSSLDLLLEHR	85.29713	Q9BQ69	MACD1 HUMAN	853.4330735	2	0.709962785	3	
SCYLSSLDLLLEHR	85.29713	Q9BQ69	MACD1 HUMAN	569.291324	3	0.709962785	3	Yes
SSTCGPPAFLGVFGR	79.91013	Q9BQ69	MACD1 HUMAN	776.883025	2	0.805790246		
SSTCGPPAFLGVFGR	79.91013	Q9BQ69	MACD1 HUMAN	518.2579583	3	0.805790246		
TSAGVGAWGAAAVGR	33.47998	Q9BQ69	MACD1 HUMAN	665.8473	2	0.853135705		
TSAGVGAWGAAAVGR	33.47998	Q9BQ69	MACD1 HUMAN	444.2341417	3	0.853135705		
TWAPLAMAAK	47.68628	Q9BQ69	MACD1 HUMAN	530.2869695	2	0.701767683		
TWAPLAMAAK	47.68628	Q9BQ69	MACD1 HUMAN	353.860588	3	0.701767683		
VDLSTSTDWK	27.50352	Q9BQ69	MACD1 HUMAN	576.2831345	2	0.780531049	2	Yes
VDLSTSTDWK	27.50352	Q9BQ69	MACD1 HUMAN	384.524698	3	0.780531049	2	
YVIHTVGPIAYGEPSASQAAELR	50.82497	Q9BQ69	MACD1 HUMAN	1215.127155	2	0.824278653	3	
YVIHTVGPIAYGEPSASQAAELR	50.82497	Q9BQ69	MACD1 HUMAN	810.4207113	3	0.824278653	3	Yes
YWAEISDTIISGTFHQWR	94.01598	Q99720	SGMR1 HUMAN	1105.537448	2	0.681973696	3	
YWAEISDTIISGTFHQWR	94.01598	Q99720	SGMR1 HUMAN	737.360907	3	0.681973696	3	Yes
DVNSSSPVMLAFK	65.83154	Q99598	TSNAX HUMAN	697.8534005	2	0.757552922		
DVNSSSPVMLAFK	65.83154	Q99598	TSNAX HUMAN	465.571542	3	0.757552922		
HMLADVFSVK	41.8226	Q99598	TSNAX HUMAN	573.802983	2	0.720832229	2	Yes
HMLADVFSVK	41.8226	Q99598	TSNAX HUMAN	382.8712637	3	0.720832229	2	
IFQVAQELSGEDMHQFHR	47.02009	Q99598	TSNAX HUMAN	1086.518737	2	0.792783439	4	
IFQVAQELSGEDMHQFHR	47.02009	Q99598	TSNAX HUMAN	724.6817663	3	0.792783439	4	
ITSAPDMEDILTESEIK	80.61699	Q99598	TSNAX HUMAN	946.464438	2	0.808643758	3	
ITSAPDMEDILTESEIK	80.61699	Q99598	TSNAX HUMAN	631.3122337	3	0.808643758	3	Yes
SFQQELDAR	10.21429	Q99598	TSNAX HUMAN	547.267819	2	0.793883741	2	Yes
SFQQELDAR	10.21429	Q99598	TSNAX HUMAN	365.1811543	3	0.793883741	2	
SLISMDEINK	38.29839	Q99598	TSNAX HUMAN	575.295187	2	0.792559862	2	Yes
SLISMDEINK	38.29839	Q99598	TSNAX HUMAN	383.8660663	3	0.792559862	2	
VTPVDYLLGVADLTGELMR	151.6586	Q99598	TSNAX HUMAN	1031.548636	2	0.657230258		
VTPVDYLLGVADLTGELMR	151.6586	Q99598	TSNAX HUMAN	688.0350323	3	0.657230258		
AQDVLVQEMEVVK	56.64204	Q99459	CDC5L HUMAN	744.392891	2	0.796369612	2	Yes
AQDVLVQEMEVVK	56.64204	Q99459	CDC5L HUMAN	496.597869	3	0.796369612	2	
EIDDTYIEDAADVDAR	51.93314	Q99459	CDC5L HUMAN	905.903055	2	0.80269891	2	Yes
EIDDTYIEDAADVDAR	51.93314	Q99459	CDC5L HUMAN	604.2713117	3	0.80269891	2	
ESDLPSAILQTSGVSEFTK	84.0332	Q99459	CDC5L HUMAN	1005.007859	2	0.623746097	3	
ESDLPSAILQTSGVSEFTK	84.0332	Q99459	CDC5L HUMAN	670.3411807	3	0.623746097	3	Yes
GGLNTPLHESDFSGVTPQR	36.35146	Q99459	CDC5L HUMAN	1006.495785	2	0.749745905		
GGLNTPLHESDFSGVTPQR	36.35146	Q99459	CDC5L HUMAN	671.3331313	3	0.749745905		
GVDYNAEIPFEK	50.14784	Q99459	CDC5L HUMAN	691.335894	2	0.812424541	2	Yes
GVDYNAEIPFEK	50.14784	Q99459	CDC5L HUMAN	461.2265377	3	0.812424541	2	
ILLGGYQSR	21.40701	Q99459	CDC5L HUMAN	503.788186	2	0.755997241	2	Yes
ILLGGYQSR	21.40701	Q99459	CDC5L HUMAN	336.1947323	3	0.755997241	2	
ILQEAQNLMALTNVDTPLK	96.26877	Q99459	CDC5L HUMAN	1056.572647	2	0.832503557	3	
ILQEAQNLMALTNVDTPLK	96.26877	Q99459	CDC5L HUMAN	704.717706	3	0.832503557	3	Yes
KPALGFYDTSEENYQALDADFR	64.77833	Q99459	CDC5L HUMAN	1275.593341	2	0.667931318		
KPALGFYDTSEENYQALDADFR	64.77833	Q99459	CDC5L HUMAN	850.7315023	3	0.667931318		
LGLLGLPAPK	67.9765	Q99459	CDC5L HUMAN	489.821502	2	0.819200039	2	Yes
LGLLGLPAPK	67.9765	Q99459	CDC5L HUMAN	326.8836097	3	0.819200039	2	
LMPTQWR	20.46827	Q99459	CDC5L HUMAN	466.2451045	2	0.741176069		
LMPTQWR	20.46827	Q99459	CDC5L HUMAN	311.1660113	3	0.741176069		
LNINPEDGMADYSDPSYVK	60.35512	Q99459	CDC5L HUMAN	1064.481142	2	0.803058267		
LNINPEDGMADYSDPSYVK	60.35512	Q99459	CDC5L HUMAN	709.990036	3	0.803058267		
LVLPAPQISDAELQEVVK	88.41769	Q99459	CDC5L HUMAN	975.0518725	2	0.843527794	3	
LVLPAPQISDAELQEVVK	88.41769	Q99459	CDC5L HUMAN	650.3705233	3	0.843527794	3	Yes
NDFEIVLPENAEK	64.62953	Q99459	CDC5L HUMAN	759.378294	2	0.803250313	2	Yes
NDFEIVLPENAEK	64.62953	Q99459	CDC5L HUMAN	506.5881377	3	0.803250313	2	
QLNDLWDQIEQAHLELR	94.47072	Q99459	CDC5L HUMAN	1061.040359	2	0.678421199		
QLNDLWDQIEQAHLELR	94.47072	Q99459	CDC5L HUMAN	707.696181	3	0.678421199		
TAAQCLEHYEFLLDK	58.78849	Q99459	CDC5L HUMAN	919.4436395	2	0.740059257		
TAAQCLEHYEFLLDK	58.78849	Q99459	CDC5L HUMAN	613.298368	3	0.740059257		
TVGFGTNNSEHITYLEHNPYEK	30.62027	Q99459	CDC5L HUMAN	1275.598958	2	0.821811497	4	
TVGFGTNNSEHITYLEHNPYEK	30.62027	Q99459	CDC5L HUMAN	850.7352467	3	0.821811497	4	
WYEWLDPSIK	81.24371	Q99459	CDC5L HUMAN	668.83279	2	0.719266176	2	Yes
WYEWLDPSIK	81.24371	Q99459	CDC5L HUMAN	446.2244683	3	0.719266176	2	
YADLLLEK	35.8473	Q99459	CDC5L HUMAN	482.77167	2	0.731979668	2	Yes
YADLLLEK	35.8473	Q99459	CDC5L HUMAN	322.1837217	3	0.731979668	2	
AEDLNIAPR	12.91196	Q96SZ5	AEDO HUMAN	499.7674545	2	0.835861921		
AEDLNIAPR	12.91196	Q96SZ5	AEDO HUMAN	333.5142447	3	0.835861921		
AEYTEASGPCILTPHR	22.40313	Q96SZ5	AEDO HUMAN	901.4310635	2	0.751859248	3	
AEYTEASGPCILTPHR	22.40313	Q96SZ5	AEDO HUMAN	601.289984	3	0.751859248	3	Yes
ALPPEQQFEPPLQPR	54.92152	Q96SZ5	AEDO HUMAN	873.962861	2	0.846816838		
ALPPEQQFEPPLQPR	54.92152	Q96SZ5	AEDO HUMAN	582.977849	3	0.846816838		
DAASGPEAPMQPGFPENLSK	59.68852	Q96SZ5	AEDO HUMAN	1021.978579	2	0.865818381		
DAASGPEAPMQPGFPENLSK	59.68852	Q96SZ5	AEDO HUMAN	681.654994	3	0.865818381		
DNLHQIDAVEGPAAFLDILAPPYDPDDGR	147.8255	Q96SZ5	AEDO HUMAN	1567.757451	2	0.797526062		
DNLHQIDAVEGPAAFLDILAPPYDPDDGR	147.8255	Q96SZ5	AEDO HUMAN	1045.507576	3	0.797526062		
DNMASLIQR	34.71767	Q96SZ5	AEDO HUMAN	524.2667645	2	0.812378705		
DNMASLIQR	34.71767	Q96SZ5	AEDO HUMAN	349.847118	3	0.812378705		
EASSSACDLPR	-14.17247	Q96SZ5	AEDO HUMAN	596.7673255	2	0.636554658	2	Yes
EASSSACDLPR	-14.17247	Q96SZ5	AEDO HUMAN	398.1808253	3	0.636554658	2	
EVWLLETPQADDFWCEGEPYPGPK	119.3185	Q96SZ5	AEDO HUMAN	1432.150171	2	0.753004074		
EVWLLETPQADDFWCEGEPYPGPK	119.3185	Q96SZ5	AEDO HUMAN	955.1027223	3	0.753004074		
SGTSIPLHDHPGMHGMLK	12.36601	Q96SZ5	AEDO HUMAN	957.969837	2	0.615233421		
SGTSIPLHDHPGMHGMLK	12.36601	Q96SZ5	AEDO HUMAN	638.9824997	3	0.615233421		
ETTNSMVESIK	7.36652	Q96SU4	OSBL9 HUMAN	619.800834	2	0.725537181		
ETTNSMVESIK	7.36652	Q96SU4	OSBL9 HUMAN	413.5364977	3	0.725537181		
HCIVLLQIAK	34.66667	Q96SU4	OSBL9 HUMAN	597.8555505	2	0.742585897		
HCIVLLQIAK	34.66667	Q96SU4	OSBL9 HUMAN	398.9063087	3	0.742585897		
HTLQLQGLDSGFVPSVQDFDK	79.82556	Q96SU4	OSBL9 HUMAN	1166.084964	2	0.768456638	3	

HTLQLQGLDSGFVPSVQDFDK	79.82556	Q96SU4	OSBL9 HUMAN	777.7259177	3	0.768456638	3	Yes
LFHEDGECWVYDEPLLK	72.10566	Q96SU4	OSBL9 HUMAN	1075.499143	2	0.8120628	3	
LFHEDGECWVYDEPLLK	72.10566	Q96SU4	OSBL9 HUMAN	717.33537	3	0.8120628	3	Yes
LIDSSGSASVLTHSSSGNSLK	20.47204	Q96SU4	OSBL9 HUMAN	1024.019288	2	0.703672886		
LIDSSGSASVLTHSSSGNSLK	20.47204	Q96SU4	OSBL9 HUMAN	683.0154667	3	0.703672886		
LTEADAYLQILIEQLK	154.5728	Q96SU4	OSBL9 HUMAN	931.020037	2	0.675966382		
LTEADAYLQILIEQLK	154.5728	Q96SU4	OSBL9 HUMAN	621.0159663	3	0.675966382		
SVIMHLLSQVR	50.23191	Q96SU4	OSBL9 HUMAN	641.8691885	2	0.776263058	3	
SVIMHLLSQVR	50.23191	Q96SU4	OSBL9 HUMAN	428.248734	3	0.776263058	3	Yes
TGYSANIIFHTKPFYGGK	40.54918	Q96SU4	OSBL9 HUMAN	1001.015616	2	0.768974423	3	
TGYSANIIFHTKPFYGGK	40.54918	Q96SU4	OSBL9 HUMAN	667.6796857	3	0.768974423	3	Yes
VVLPTFILER	86.60326	Q96SU4	OSBL9 HUMAN	593.863898	2	0.826049924	2	Yes
VVLPTFILER	86.60326	Q96SU4	OSBL9 HUMAN	396.245207	3	0.826049924	2	
WIHALEETILR	52.87793	Q96SU4	OSBL9 HUMAN	690.885893	2	0.705706358	3	
WIHALEETILR	52.87793	Q96SU4	OSBL9 HUMAN	460.926537	3	0.705706358	3	Yes
YATGENTVFVDTK	21.78641	Q96SU4	OSBL9 HUMAN	722.851909	2	0.893494487	2	Yes
YATGENTVFVDTK	21.78641	Q96SU4	OSBL9 HUMAN	482.2372143	3	0.893494487	2	
EAAAAALPAAVPGPGR	40.78722	Q96SL1	DIRC2 HUMAN	709.8917075	2	0.629451215	2	Yes
EAAAAALPAAVPGPGR	40.78722	Q96SL1	DIRC2 HUMAN	473.59708	3	0.629451215	2	
DFSLLYEEAR	68.16711	Q96SI1	KCD15 HUMAN	621.8042295	2	0.75651747		
DFSLLYEEAR	68.16711	Q96SI1	KCD15 HUMAN	414.8720947	3	0.75651747		
LLLPDDFK	56.80212	Q96SI1	KCD15 HUMAN	480.774217	2	0.816567779	2	Yes
LLLPDDFK	56.80212	Q96SI1	KCD15 HUMAN	320.8520863	3	0.816567779	2	
LNSVQVLER	19.00171	Q96SI1	KCD15 HUMAN	529.304204	2	0.742783904	2	Yes
LNSVQVLER	19.00171	Q96SI1	KCD15 HUMAN	353.205411	3	0.742783904	2	
SPVSPLAAQGIPLPAQLTK	88.11496	Q96SI1	KCD15 HUMAN	944.5493005	2	0.818390787		
SPVSPLAAQGIPLPAQLTK	88.11496	Q96SI1	KCD15 HUMAN	630.0354753	3	0.818390787		
VTPDLGER	-8.236759	Q96SI1	KCD15 HUMAN	443.7356235	2	0.648796141		
VTPDLGER	-8.236759	Q96SI1	KCD15 HUMAN	296.1596907	3	0.648796141		
YVLSFLR	58.87294	Q96SI1	KCD15 HUMAN	449.263815	2	0.703387022	2	Yes
YVLSFLR	58.87294	Q96SI1	KCD15 HUMAN	299.8451517	3	0.703387022	2	
YYQLQPMVR	33.4164	Q96SI1	KCD15 HUMAN	599.3084245	2	0.711641967		
YYQLQPMVR	33.4164	Q96SI1	KCD15 HUMAN	399.8748913	3	0.711641967		
APDVAPEEVDESK	4.51495	Q96SB3	NEB2 HUMAN	693.3257275	2	0.831297815		
APDVAPEEVDESK	4.51495	Q96SB3	NEB2 HUMAN	462.5530933	3	0.831297815		
AQLEQSVEENK	-15.33497	Q96SB3	NEB2 HUMAN	637.81533	2	0.655901968		
AQLEQSVEENK	-15.33497	Q96SB3	NEB2 HUMAN	425.5461617	3	0.655901968		
ASSLNENVDHSALLK	15.09678	Q96SB3	NEB2 HUMAN	799.413199	2	0.780621886		
ASSLNENVDHSALLK	15.09678	Q96SB3	NEB2 HUMAN	533.2780743	3	0.780621886		
DSEGLGISIIGMGAGADMGLEK	116.0968	Q96SB3	NEB2 HUMAN	1061.014304	2	0.680075884		
DSEGLGISIIGMGAGADMGLEK	116.0968	Q96SB3	NEB2 HUMAN	707.678811	3	0.680075884		
ERPGEQSEVAQLIQQTLEQER	79.26154	Q96SB3	NEB2 HUMAN	1234.622974	2	0.789936423		
ERPGEQSEVAQLIQQTLEQER	79.26154	Q96SB3	NEB2 HUMAN	823.417924	3	0.789936423		
ETQAQYQALER	1.029217	Q96SB3	NEB2 HUMAN	668.8287685	2	0.764909565	2	Yes
ETQAQYQALER	1.029217	Q96SB3	NEB2 HUMAN	446.2217873	3	0.764909565	2	
FDSKPAPSAQPAPPPHPPSR	-1.869457	Q96SB3	NEB2 HUMAN	1041.032337	2	0.622329772	4	
FDSKPAPSAQPAPPPHPPSR	-1.869457	Q96SB3	NEB2 HUMAN	694.3574997	3	0.622329772	4	
HAVTEAEIQQLK	4.211704	Q96SB3	NEB2 HUMAN	683.8704405	2	0.82554251		
HAVTEAEIQQLK	4.211704	Q96SB3	NEB2 HUMAN	456.2495687	3	0.82554251		
ISELEGNLQTLR	40.46487	Q96SB3	NEB2 HUMAN	686.875722	2	0.884422898	2	Yes
ISELEGNLQTLR	40.46487	Q96SB3	NEB2 HUMAN	458.2530897	3	0.884422898	2	
LDADAVSPTVSQLSAVFEK	93.0088	Q96SB3	NEB2 HUMAN	989.012944	2	0.7873137		
LDADAVSPTVSQLSAVFEK	93.0088	Q96SB3	NEB2 HUMAN	659.6779043	3	0.7873137		
LEGYWGEAQSLCQAVDEHLR	76.52164	Q96SB3	NEB2 HUMAN	1181.050594	2	0.740132213	3	
LEGYWGEAQSLCQAVDEHLR	76.52164	Q96SB3	NEB2 HUMAN	787.7030043	3	0.740132213	3	Yes
NEDVDPMAASAEYELEK	52.38607	Q96SB3	NEB2 HUMAN	955.92039	2	0.795295954		
NEDVDPMAASAEYELEK	52.38607	Q96SB3	NEB2 HUMAN	637.6162017	3	0.795295954		
SAYEAGIQALKPPDAPGPDEAPK	44.12971	Q96SB3	NEB2 HUMAN	1161.584793	2	0.785646319		
SAYEAGIQALKPPDAPGPDEAPK	44.12971	Q96SB3	NEB2 HUMAN	774.7258033	3	0.785646319		
SMFLQMGTTAGPSGEAGGGAGLAEAPR	66.97521	Q96SB3	NEB2 HUMAN	1261.094681	2	0.821818352		
SMFLQMGTTAGPSGEAGGGAGLAEAPR	66.97521	Q96SB3	NEB2 HUMAN	841.065729	3	0.821818352		
VFQPPPPPPPAPSGDAPAEK	38.78252	Q96SB3	NEB2 HUMAN	998.512915	2	0.710794628		
VFQPPPPPPPAPSGDAPAEK	38.78252	Q96SB3	NEB2 HUMAN	666.0112183	3	0.710794628		
VLEESELAR	6.130116	Q96SB3	NEB2 HUMAN	523.2803945	2	0.63929683		
VLEESELAR	6.130116	Q96SB3	NEB2 HUMAN	349.189538	3	0.63929683		
ALTQPLGLLR	57.44182	Q96S97	MYADM HUMAN	541.3405905	2	0.752912521	2	Yes
ALTQPLGLLR	57.44182	Q96S97	MYADM HUMAN	361.2296687	3	0.752912521	2	
ARPGEITGYMATVPGLLK	67.27772	Q96S97	MYADM HUMAN	937.5143995	2	0.752325416		
ARPGEITGYMATVPGLLK	67.27772	Q96S97	MYADM HUMAN	625.3455413	3	0.752325416		
TTITTTTTSSSGLGSPMIVGSPR	52.59523	Q96S97	MYADM HUMAN	1126.576115	2	0.693441629	3	
TTITTTTTSSSGLGSPMIVGSPR	52.59523	Q96S97	MYADM HUMAN	751.386685	3	0.693441629	3	Yes
DEYLSLVAR	52.85503	Q96RN5	MED15 HUMAN	533.282933	2	0.685638726		
DEYLSLVAR	52.85503	Q96RN5	MED15 HUMAN	355.857897	3	0.685638726		
DMESHVFLK	22.43742	Q96RN5	MED15 HUMAN	553.2715155	2	0.692718387		
DMESHVFLK	22.43742	Q96RN5	MED15 HUMAN	369.1836187	3	0.692718387		
FPPTTAVSAIPSSSIPLGR	73.88982	Q96RN5	MED15 HUMAN	949.523282	2	0.613692522		
FPPTTAVSAIPSSSIPLGR	73.88982	Q96RN5	MED15 HUMAN	633.351463	3	0.613692522		
LDDKDLPSVPPLELSVPADYPAQSPLWIDR	123.9298	Q96RN5	MED15 HUMAN	1673.864458	2	0.754663348		
LDDKDLPSVPPLELSVPADYPAQSPLWIDR	123.9298	Q96RN5	MED15 HUMAN	1116.24558	3	0.754663348		
LLQLPDK	25.69644	Q96RN5	MED15 HUMAN	413.7558275	2	0.678199828	2	Yes
LLQLPDK	25.69644	Q96RN5	MED15 HUMAN	276.17316	3	0.678199828	2	
LVSQIEDAMR	32.82142	Q96RN5	MED15 HUMAN	581.3008045	2	0.847164154	2	Yes
LVSQIEDAMR	32.82142	Q96RN5	MED15 HUMAN	387.8698113	3	0.847164154	2	
MDVSGQETDWR	22.92722	Q96RN5	MED15 HUMAN	662.285883	2	0.663874865		
MDVSGQETDWR	22.92722	Q96RN5	MED15 HUMAN	441.8598637	3	0.663874865		
QSIPSVLQGEVAR	43.32506	Q96RN5	MED15 HUMAN	692.3837145	2	0.658503532	2	Yes
QSIPSVLQGEVAR	43.32506	Q96RN5	MED15 HUMAN	461.9250847	3	0.658503532	2	
QWQYDANPFLQSVHR	54.52957	Q96RN5	MED15 HUMAN	944.9586365	2	0.687380314		
QWQYDANPFLQSVHR	54.52957	Q96RN5	MED15 HUMAN	630.308366	3	0.687380314		
SLLDILTDPSK	87.6532	Q96RN5	MED15 HUMAN	601.3379095	2	0.814624608		
SLLDILTDPSK	87.6532	Q96RN5	MED15 HUMAN	401.2278813	3	0.814624608		
SPVFNHSLYR	6.629978	Q96RN5	MED15 HUMAN	610.3150985	2	0.68871516		
SPVFNHSLYR	6.629978	Q96RN5	MED15 HUMAN	407.212674	3	0.68871516		
AAAEALSFTK	18.31833	Q96QE3	ATAD5 HUMAN	504.772206	2	0.60323751		
AAAEALSFTK	18.31833	Q96QE3	ATAD5 HUMAN	336.8507457	3	0.60323751		
AAALDVYNAVSTSFQR	68.33449	Q96QE3	ATAD5 HUMAN	856.9342955	2	0.770340621		
AAALDVYNAVSTSFQR	68.33449	Q96QE3	ATAD5 HUMAN	571.625472	3	0.770340621		
AADPVPSFDESSQDTSEK	27.77086	Q96QE3	ATAD5 HUMAN	955.419073	2	0.687650681		
AADPVPSFDESSQDTSEK	27.77086	Q96QE3	ATAD5 HUMAN	637.2819903	3	0.687650681		
AESEASLLNVSTPK	37.13757	Q96QE3	ATAD5 HUMAN	723.378294	2	0.735844731		
AESEASLLNVSTPK	37.13757	Q96QE3	ATAD5 HUMAN	482.5881377	3	0.735844731		

ALETLNSCK	-8.507862	Q96QE3	ATAD5 HUMAN	518.261148	2	0.779066503		
ALETLNSCK	-8.507862	Q96QE3	ATAD5 HUMAN	345.8433737	3	0.779066503		
DCTTPLEMFSNVEFK	100.0099	Q96QE3	ATAD5 HUMAN	909.408412	2	0.817912221		
DCTTPLEMFSNVEFK	100.0099	Q96QE3	ATAD5 HUMAN	606.6082163	3	0.817912221		
DDGCCLWHLKPPSCPLLTK	56.0133	Q96QE3	ATAD5 HUMAN	1149.047959	2	0.725983918		
DDGCCLWHLKPPSCPLLTK	56.0133	Q96QE3	ATAD5 HUMAN	766.3679143	3	0.725983918		
DFLMSGLPDLLK	127.0009	Q96QE3	ATAD5 HUMAN	674.8632375	2	0.847035587		
DFLMSGLPDLLK	127.0009	Q96QE3	ATAD5 HUMAN	450.2447667	3	0.847035587		
DFVTLLTANTCDIR	95.9924	Q96QE3	ATAD5 HUMAN	819.9119795	2	0.797807574		
DFVTLLTANTCDIR	95.9924	Q96QE3	ATAD5 HUMAN	546.943928	3	0.797807574		
DNVTEAAQLNDSIITVSYEEFLK	133.6826	Q96QE3	ATAD5 HUMAN	1300.142868	2	0.657455087		
DNVTEAAQLNDSIITVSYEEFLK	133.6826	Q96QE3	ATAD5 HUMAN	867.0978533	3	0.657455087		
DPTNDLTFYVSQK	62.58369	Q96QE3	ATAD5 HUMAN	764.3704655	2	0.86501503		
DPTNDLTFYVSQK	62.58369	Q96QE3	ATAD5 HUMAN	509.916252	3	0.86501503		
DSGTEDMLWTEK	43.08929	Q96QE3	ATAD5 HUMAN	706.306481	2	0.891937196		
DSGTEDMLWTEK	43.08929	Q96QE3	ATAD5 HUMAN	471.206929	3	0.891937196		
DVVDLSESLPLAEELNLLK	147.815	Q96QE3	ATAD5 HUMAN	1049.070458	2	0.638185501		
DVVDLSESLPLAEELNLLK	147.815	Q96QE3	ATAD5 HUMAN	699.716247	3	0.638185501		
EDYSLNNDFVESSTSVLR	72.8252	Q96QE3	ATAD5 HUMAN	1037.982367	2	0.809887648		
EDYSLNNDFVESSTSVLR	72.8252	Q96QE3	ATAD5 HUMAN	692.324186	3	0.809887648		
FIQLLTEFQMR	92.82545	Q96QE3	ATAD5 HUMAN	713.3821295	2	0.742511749		
FIQLLTEFQMR	92.82545	Q96QE3	ATAD5 HUMAN	475.924028	3	0.742511749		
FLHYFEGIHLDIPK	63.06415	Q96QE3	ATAD5 HUMAN	864.959585	2	0.758422136		
FLHYFEGIHLDIPK	63.06415	Q96QE3	ATAD5 HUMAN	576.975665	3	0.758422136		
GIDSDDVQDNSQLK	5.052521	Q96QE3	ATAD5 HUMAN	767.3555475	2	0.705803514		
GIDSDDVQDNSQLK	5.052521	Q96QE3	ATAD5 HUMAN	511.9063067	3	0.705803514		
HEDFSGGIDFK	21.72412	Q96QE3	ATAD5 HUMAN	626.2862085	2	0.810129285		
HEDFSGGIDFK	21.72412	Q96QE3	ATAD5 HUMAN	417.8600807	3	0.810129285		
HSLYTAELITVPFDSESPIR	88.71823	Q96QE3	ATAD5 HUMAN	1138.084428	2	0.64079982		
HSLYTAELITVPFDSESPIR	88.71823	Q96QE3	ATAD5 HUMAN	759.0588933	3	0.64079982		
IMENSGIQMVSK	26.63373	Q96QE3	ATAD5 HUMAN	668.834154	2	0.774525642		
IMENSGIQMVSK	26.63373	Q96QE3	ATAD5 HUMAN	446.2253777	3	0.774525642		
ISSTPTTETIR	-4.780251	Q96QE3	ATAD5 HUMAN	603.3227915	2	0.665067971		
ISSTPTTETIR	-4.780251	Q96QE3	ATAD5 HUMAN	402.551136	3	0.665067971		
LCNTVLITGPTGVGK	46.08847	Q96QE3	ATAD5 HUMAN	765.4219835	2	0.802282929		
LCNTVLITGPTGVGK	46.08847	Q96QE3	ATAD5 HUMAN	510.617264	3	0.802282929		
LQCLNDVLGK	33.81008	Q96QE3	ATAD5 HUMAN	580.3111725	2	0.671862483		
LQCLNDVLGK	33.81008	Q96QE3	ATAD5 HUMAN	387.2100567	3	0.671862483		
LSETEDSVIIIDSSPTALK	68.16021	Q96QE3	ATAD5 HUMAN	1009.531166	2	0.776028395		
LSETEDSVIIIDSSPTALK	68.16021	Q96QE3	ATAD5 HUMAN	673.356719	3	0.776028395		
NFCGPSVTVDASAATK	31.18805	Q96QE3	ATAD5 HUMAN	812.8859615	2	0.859129906		
NFCGPSVTVDASAATK	31.18805	Q96QE3	ATAD5 HUMAN	542.259916	3	0.859129906		
NIFSPSEDLFSFLK	136.1147	Q96QE3	ATAD5 HUMAN	822.4199615	2	0.803955495		
NIFSPSEDLFSFLK	136.1147	Q96QE3	ATAD5 HUMAN	548.615916	3	0.803955495		
NLLLEEIR	52.51539	Q96QE3	ATAD5 HUMAN	500.2958475	2	0.779348969		
NLLLEEIR	52.51539	Q96QE3	ATAD5 HUMAN	333.8665067	3	0.779348969		
NNEEIGMLLENNK	50.97137	Q96QE3	ATAD5 HUMAN	759.367403	2	0.742266536		
NNEEIGMLLENNK	50.97137	Q96QE3	ATAD5 HUMAN	506.580877	3	0.742266536		
NNVYFSQSAANLDNAWK	57.19357	Q96QE3	ATAD5 HUMAN	971.458665	2	0.836357892		
NNVYFSQSAANLDNAWK	57.19357	Q96QE3	ATAD5 HUMAN	647.9750517	3	0.836357892		
NVQLVCSEHGLDNK	1.284122	Q96QE3	ATAD5 HUMAN	806.8915775	2	0.640781462		
NVQLVCSEHGLDNK	1.284122	Q96QE3	ATAD5 HUMAN	538.26366	3	0.640781462		
QASIIEYLPTLR	85.80304	Q96QE3	ATAD5 HUMAN	702.398829	2	0.644753098		
QASIIEYLPTLR	85.80304	Q96QE3	ATAD5 HUMAN	468.6018277	3	0.644753098		
SEATDGGFTSQIR	13.70393	Q96QE3	ATAD5 HUMAN	684.823687	2	0.788888514		
SEATDGGFTSQIR	13.70393	Q96QE3	ATAD5 HUMAN	456.8850663	3	0.788888514		
SGGGVLEERPLTLYR	34.90706	Q96QE3	ATAD5 HUMAN	823.947206	2	0.795590281		
SGGGVLEERPLTLYR	34.90706	Q96QE3	ATAD5 HUMAN	549.634079	3	0.795590281		
SGYISESENSEISQQVR	26.29649	Q96QE3	ATAD5 HUMAN	956.948327	2	0.772796869		
SGYISESENSEISQQVR	26.29649	Q96QE3	ATAD5 HUMAN	638.301493	3	0.772796869		
SLLYVGNR	21.09611	Q96QE3	ATAD5 HUMAN	461.2618035	2	0.684906244		
SLLYVGNR	21.09611	Q96QE3	ATAD5 HUMAN	307.8438107	3	0.684906244		
SQPNTMTSLQNSK	-6.369881	Q96QE3	ATAD5 HUMAN	718.3464715	2	0.639298141		
SQPNTMTSLQNSK	-6.369881	Q96QE3	ATAD5 HUMAN	479.2335893	3	0.639298141		
SSTLFNNESLVYEDIANDDLLK	101.8127	Q96QE3	ATAD5 HUMAN	1250.60866	2	0.678436697		
SSTLFNNESLVYEDIANDDLLK	101.8127	Q96QE3	ATAD5 HUMAN	834.075048	3	0.678436697		
TAAVYACAQELGFK	40.69048	Q96QE3	ATAD5 HUMAN	764.8774045	2	0.667843759		
TAAVYACAQELGFK	40.69048	Q96QE3	ATAD5 HUMAN	510.2542113	3	0.667843759		
TENEAPIEISSDDSK	21.50374	Q96QE3	ATAD5 HUMAN	817.8737695	2	0.757512212		
TENEAPIEISSDDSK	21.50374	Q96QE3	ATAD5 HUMAN	545.5851213	3	0.757512212		
TLANYFK	17.41531	Q96QE3	ATAD5 HUMAN	428.732348	2	0.615401626		
TLANYFK	17.41531	Q96QE3	ATAD5 HUMAN	286.157507	3	0.615401626		
TVTVLAQVHPIPPK	34.36637	Q96QE3	ATAD5 HUMAN	750.451401	2	0.814846575		
TVTVLAQVHPIPPK	34.36637	Q96QE3	ATAD5 HUMAN	500.6368757	3	0.814846575		
VAPLFLVR	56.70562	Q96QE3	ATAD5 HUMAN	457.795287	2	0.719912112		
VAPLFLVR	56.70562	Q96QE3	ATAD5 HUMAN	305.5327997	3	0.719912112		
VEEIPDSTMSICVPSETVDEIVK	96.0782	Q96QE3	ATAD5 HUMAN	1289.119694	2	0.665934622		
VEEIPDSTMSICVPSETVDEIVK	96.0782	Q96QE3	ATAD5 HUMAN	859.749071	3	0.665934622		
VFAPPKPSNILDYFR	73.62642	Q96QE3	ATAD5 HUMAN	882.4781415	2	0.777949333	3	
VFAPPKPSNILDYFR	73.62642	Q96QE3	ATAD5 HUMAN	588.6547027	3	0.777949333	3	Yes
VTEEIAIPLR	50.87709	Q96QE3	ATAD5 HUMAN	570.8353375	2	0.771153331		
VTEEIAIPLR	50.87709	Q96QE3	ATAD5 HUMAN	380.8928333	3	0.771153331		
VTSGLCDEFSLESNDGWTSQSSGELK	74.25563	Q96QE3	ATAD5 HUMAN	1417.127624	2	0.681562841		
VTSGLCDEFSLESNDGWTSQSSGELK	74.25563	Q96QE3	ATAD5 HUMAN	945.087691	3	0.681562841		
YFPLLLK	72.0505	Q96QE3	ATAD5 HUMAN	447.278934	2	0.740379632		
YFPLLLK	72.0505	Q96QE3	ATAD5 HUMAN	298.5218977	3	0.740379632		
YQPQTASELIGNELAIK	67.7468	Q96QE3	ATAD5 HUMAN	937.997093	2	0.829833627		
YQPQTASELIGNELAIK	67.7468	Q96QE3	ATAD5 HUMAN	625.667337	3	0.829833627		
GISPMNRPPLSDK	11.25871	Q96QD9	UIF HUMAN	706.372292	2	0.667732835		
GISPMNRPPLSDK	11.25871	Q96QD9	UIF HUMAN	471.250803	3	0.667732835		
GVPLQFDINSVGK	63.47616	Q96QD9	UIF HUMAN	687.375358	2	0.790697396		
GVPLQFDINSVGK	63.47616	Q96QD9	UIF HUMAN	458.5861803	3	0.790697396		
IDMSLDDIIK	77.76839	Q96QD9	UIF HUMAN	581.805388	2	0.809421003		
IDMSLDDIIK	77.76839	Q96QD9	UIF HUMAN	388.2062003	3	0.809421003		
LLQQSGAQQFR	5.181831	Q96QD9	UIF HUMAN	638.344393	2	0.823790908	2	Yes
LLQQSGAQQFR	5.181831	Q96QD9	UIF HUMAN	425.8988703	3	0.823790908	2	
LVGATATSSPPPK	-7.294273	Q96QD9	UIF HUMAN	613.343527	2	0.782358706		
LVGATATSSPPPK	-7.294273	Q96QD9	UIF HUMAN	409.2316263	3	0.782358706		
NIEQYFPVLK	69.37941	Q96QD9	UIF HUMAN	625.843158	2	0.835602641		
NIEQYFPVLK	69.37941	Q96QD9	UIF HUMAN	417.5647137	3	0.835602641		
VQAQLNTEQLLDDVVAK	77.04303	Q96QD9	UIF HUMAN	942.5078295	2	0.842252791		

VQAQLNTEQLLDDVVAK	77.04303	Q96QD9	UIF_HUMAN	628.6744947	3	0.842252791		
WGIQQNSGFGK	26.68017	Q96QD9	UIF_HUMAN	611.304736	2	0.799364805		
WGIQQNSGFGK	26.68017	Q96QD9	UIF_HUMAN	407.8724323	3	0.799364805		
AAAANAVGLFSR	38.83293	Q96PE7	MCEE_HUMAN	574.3151035	2	0.833385587		
AAAANAVGLFSR	38.83293	Q96PE7	MCEE_HUMAN	383.2126773	3	0.833385587		
ASSTSQPLDQVTGSVWNLGR	79.30641	Q96PE7	MCEE_HUMAN	1052.027449	2	0.668297708		
ASSTSQPLDQVTGSVWNLGR	79.30641	Q96PE7	MCEE_HUMAN	701.687574	3	0.668297708		
DCGGVLVELEQA	102.7375	Q96PE7	MCEE_HUMAN	645.3062845	2	0.635819256		
DCGGVLVELEQA	102.7375	Q96PE7	MCEE_HUMAN	430.5401313	3	0.635819256		
DSPIAGFLQK	65.81223	Q96PE7	MCEE_HUMAN	538.2933055	2	0.80581069		
DSPIAGFLQK	65.81223	Q96PE7	MCEE_HUMAN	359.1981453	3	0.80581069		
IGAHGKPVIFLHPK	-0.370399	Q96PE7	MCEE_HUMAN	757.4542775	2	0.830239058	4	
IGAHGKPVIFLHPK	-0.370399	Q96PE7	MCEE_HUMAN	505.30546	3	0.830239058	4	
LNHVAIAVPDLEK	38.22876	Q96PE7	MCEE_HUMAN	709.9042825	2	0.83348316	3	
LNHVAIAVPDLEK	38.22876	Q96PE7	MCEE_HUMAN	473.6054633	3	0.83348316	3	Yes
LQAPIPTVR	30.95107	Q96PE7	MCEE_HUMAN	497.8063835	2	0.714849055		
LQAPIPTVR	30.95107	Q96PE7	MCEE_HUMAN	332.206864	3	0.714849055		
ASPETTLSSGFFVAVIER	103.7339	Q96P11	NSUN5_HUMAN	955.997097	2	0.708847702		
ASPETTLSSGFFVAVIER	103.7339	Q96P11	NSUN5_HUMAN	637.6673397	3	0.708847702		
ASSLDDLR	1.251774	Q96P11	NSUN5_HUMAN	438.725255	2	0.607701004		
ASSLDDLR	1.251774	Q96P11	NSUN5_HUMAN	292.819445	3	0.607701004		
DALQQNPGAFR	19.45621	Q96P11	NSUN5_HUMAN	608.807643	2	0.87471652		
DALQQNPGAFR	19.45621	Q96P11	NSUN5_HUMAN	406.2077037	3	0.87471652		
GLVYSSNFQNVK	28.48636	Q96P11	NSUN5_HUMAN	678.351878	2	0.850156784		
GLVYSSNFQNVK	28.48636	Q96P11	NSUN5_HUMAN	452.570527	3	0.850156784		
HFLLDPLMPELLVFPAQTDLHEHPLYR	117.3466	Q96P11	NSUN5_HUMAN	1621.347528	2	0.692686081	4	
HFLLDPLMPELLVFPAQTDLHEHPLYR	117.3466	Q96P11	NSUN5_HUMAN	1081.234294	3	0.692686081	4	
IFAFDLDAK	68.13033	Q96P11	NSUN5_HUMAN	520.277124	2	0.663995802		
IFAFDLDAK	68.13033	Q96P11	NSUN5_HUMAN	347.1873577	3	0.663995802		
LASMATLLAR	49.38032	Q96P11	NSUN5_HUMAN	523.805526	2	0.666738689	2	Yes
LASMATLLAR	49.38032	Q96P11	NSUN5_HUMAN	349.5396257	3	0.666738689	2	
NEDLLEVGSRPGPASQLPR	41.53557	Q96P11	NSUN5_HUMAN	1018.032534	2	0.752004445	3	
NEDLLEVGSRPGPASQLPR	41.53557	Q96P11	NSUN5_HUMAN	679.0242973	3	0.752004445	3	Yes
VLVYELLLGK	95.45042	Q96P11	NSUN5_HUMAN	573.8608195	2	0.755310655	2	Yes
VLVYELLLGK	95.45042	Q96P11	NSUN5_HUMAN	382.9098213	3	0.755310655	2	
YSAVLDAVIASAGLLR	120.0062	Q96P11	NSUN5_HUMAN	809.9623245	2	0.795402765		
YSAVLDAVIASAGLLR	120.0062	Q96P11	NSUN5_HUMAN	540.3108247	3	0.795402765		
ATLVPQVQPSTSAWTTNFDK	68.67513	Q96NB3	ZN830_HUMAN	1096.055676	2	0.816486835		
ATLVPQVQPSTSAWTTNFDK	68.67513	Q96NB3	ZN830_HUMAN	731.0397253	3	0.816486835		
ENTAEALPEGFFDDPEVDAR	83.15704	Q96NB3	ZN830_HUMAN	1111.498387	2	0.713995397		
ENTAEALPEGFFDDPEVDAR	83.15704	Q96NB3	ZN830_HUMAN	741.334866	3	0.713995397		
EVTSSVLPNDFFSTNPPK	75.73199	Q96NB3	ZN830_HUMAN	989.992011	2	0.797299445		
EVTSSVLPNDFFSTNPPK	75.73199	Q96NB3	ZN830_HUMAN	660.3306157	3	0.797299445		
LGQLSCALCNTPVK	35.30022	Q96NB3	ZN830_HUMAN	780.897818	2	0.821041763		
LGQLSCALCNTPVK	35.30022	Q96NB3	ZN830_HUMAN	520.934487	3	0.821041763		
QIGEIDEQIECYR	44.61588	Q96NB3	ZN830_HUMAN	826.8834145	2	0.681777775	2	Yes
QIGEIDEQIECYR	44.61588	Q96NB3	ZN830_HUMAN	551.5915513	3	0.681777775	2	
SELLWQTHVLGK	43.58168	Q96NB3	ZN830_HUMAN	705.8911755	2	0.792032897		
SELLWQTHVLGK	43.58168	Q96NB3	ZN830_HUMAN	470.9300587	3	0.792032897		
VINQEELR	-12.83503	Q96NB3	ZN830_HUMAN	500.7752795	2	0.637114942		
VINQEELR	-12.83503	Q96NB3	ZN830_HUMAN	334.186128	3	0.637114942		
GISPIVFDR	46.03668	Q96MU7	YTDC1_HUMAN	502.2827405	2	0.745462477		
GISPIVFDR	46.03668	Q96MU7	YTDC1_HUMAN	335.191102	3	0.745462477		
GVWSTLPVNEK	45.64276	Q96MU7	YTDC1_HUMAN	615.330419	2	0.808554173	2	Yes
GVWSTLPVNEK	45.64276	Q96MU7	YTDC1_HUMAN	410.556221	3	0.808554173	2	
IDYPPEFHQRPGYLK	26.41618	Q96MU7	YTDC1_HUMAN	930.4761265	2	0.732846439		
IDYPPEFHQRPGYLK	26.41618	Q96MU7	YTDC1_HUMAN	620.6533593	3	0.732846439		
RRPEDYDIHNSR	-33.94283	Q96MU7	YTDC1_HUMAN	779.380021	2	0.778012216		
RRPEDYDIHNSR	-33.94283	Q96MU7	YTDC1_HUMAN	519.9226223	3	0.778012216		
SAHLTNPWNEHKPVK	-23.21114	Q96MU7	YTDC1_HUMAN	879.458276	2	0.789063215	3	
SAHLTNPWNEHKPVK	-23.21114	Q96MU7	YTDC1_HUMAN	586.641459	3	0.789063215	3	Yes
SEASDSGSESVSFTDGSVR	22.68219	Q96MU7	YTDC1_HUMAN	952.4117785	2	0.738306046		
SEASDSGSESVSFTDGSVR	22.68219	Q96MU7	YTDC1_HUMAN	635.2771273	3	0.738306046		
YVLQDAR	-8.177391	Q96MU7	YTDC1_HUMAN	432.7328795	2	0.834540009		
YVLQDAR	-8.177391	Q96MU7	YTDC1_HUMAN	288.824528	3	0.834540009		
AQLTEVQEAYETLLQK	81.5024	Q96KN7	RPGR1_HUMAN	932.4891015	2	0.88023901		
AQLTEVQEAYETLLQK	81.5024	Q96KN7	RPGR1_HUMAN	621.9953427	3	0.88023901		
AQVYLSTDVLGGR	50.90005	Q96KN7	RPGR1_HUMAN	689.870436	2	0.685207963		
AQVYLSTDVLGGR	50.90005	Q96KN7	RPGR1_HUMAN	460.2495657	3	0.685207963		
ASFPSQDQMASPEVPIEAGQYR	62.28328	Q96KN7	RPGR1_HUMAN	1204.563534	2	0.88765645		
ASFPSQDQMASPEVPIEAGQYR	62.28328	Q96KN7	RPGR1_HUMAN	803.3782977	3	0.88765645		
DIDAIPLVLPASK	99.5219	Q96KN7	RPGR1_HUMAN	676.395759	2	0.797971308		
DIDAIPLVLPASK	99.5219	Q96KN7	RPGR1_HUMAN	451.2664477	3	0.797971308		
DILEQELDIVSPEDLATPIGR	129.5439	Q96KN7	RPGR1_HUMAN	1162.105562	2	0.775623918		
DILEQELDIVSPEDLATPIGR	129.5439	Q96KN7	RPGR1_HUMAN	775.0729827	3	0.775623918		
DMLILQR	43.33632	Q96KN7	RPGR1_HUMAN	444.752762	2	0.749955475		
DMLILQR	43.33632	Q96KN7	RPGR1_HUMAN	296.837783	3	0.749955475		
EALSIHVFDDEDLEPGSYLGR	80.0249	Q96KN7	RPGR1_HUMAN	1181.564056	2	0.660329819		
EALSIHVFDDEDLEPGSYLGR	80.0249	Q96KN7	RPGR1_HUMAN	788.0453123	3	0.660329819		
ESSEQGSEVSEAQTTDSDDVIVPPMSQK	50.74396	Q96KN7	RPGR1_HUMAN	1490.664768	2	0.607369363		
ESSEQGSEVSEAQTTDSDDVIVPPMSQK	50.74396	Q96KN7	RPGR1_HUMAN	994.1124537	3	0.607369363		
FFTFSDHDTAIIPASNNPYFR	80.17526	Q96KN7	RPGR1_HUMAN	1230.585126	2	0.815192938		
FFTFSDHDTAIIPASNNPYFR	80.17526	Q96KN7	RPGR1_HUMAN	820.7260257	3	0.815192938		
FPVLVTSDLDHYLR	76.11883	Q96KN7	RPGR1_HUMAN	837.9466745	2	0.77184242		
FPVLVTSDLDHYLR	76.11883	Q96KN7	RPGR1_HUMAN	558.967058	3	0.77184242		
FPYIPPESFLKPEAQTK	70.17445	Q96KN7	RPGR1_HUMAN	996.528029	2	0.782843351		
FPYIPPESFLKPEAQTK	70.17445	Q96KN7	RPGR1_HUMAN	664.687961	3	0.782843351		
FSETNSFIGDGFK	49.95795	Q96KN7	RPGR1_HUMAN	724.838805	2	0.839664459		
FSETNSFIGDGFK	49.95795	Q96KN7	RPGR1_HUMAN	483.5618117	3	0.839664459		
FTVVSDPLDEEK	48.41315	Q96KN7	RPGR1_HUMAN	689.8410135	2	0.708118141		
FTVVSDPLDEEK	48.41315	Q96KN7	RPGR1_HUMAN	460.2299507	3	0.708118141		
FYDLPLSETETPVSLR	85.17232	Q96KN7	RPGR1_HUMAN	933.9783685	2	0.798022032		
FYDLPLSETETPVSLR	85.17232	Q96KN7	RPGR1_HUMAN	622.9881873	3	0.798022032		
GDFNLTDPAEKPNGSIQVQLDWK	73.10455	Q96KN7	RPGR1_HUMAN	1286.638092	2	0.649422169		
GDFNLTDPAEKPNGSIQVQLDWK	73.10455	Q96KN7	RPGR1_HUMAN	858.0946693	3	0.649422169		
LDIHQAMASEHSTLAAGWICFDR	75.71254	Q96KN7	RPGR1_HUMAN	1315.11849	2	0.789347589		
LDIHQAMASEHSTLAAGWICFDR	75.71254	Q96KN7	RPGR1_HUMAN	877.0816013	3	0.789347589		
LEVTNILQK	41.89407	Q96KN7	RPGR1_HUMAN	529.3167805	2	0.746757269		
LEVTNILQK	41.89407	Q96KN7	RPGR1_HUMAN	353.2137953	3	0.746757269		
LLNDNYDK	-7.543785	Q96KN7	RPGR1_HUMAN	497.746183	2	0.607476056		
LLNDNYDK	-7.543785	Q96KN7	RPGR1_HUMAN	332.1667303	3	0.607476056		

LSMHQRPQMHR	-38.05114	Q96KN7	RPGR1 HUMAN	710.8566215	2	0.634899974		
LSMHQRPQMHR	-38.05114	Q96KN7	RPGR1 HUMAN	474.240356	3	0.634899974		
LSQVLNELQVSHAETTLELEK	65.27945	Q96KN7	RPGR1 HUMAN	1191.132111	2	0.765570879		
LSQVLNELQVSHAETTLELEK	65.27945	Q96KN7	RPGR1 HUMAN	794.4240153	3	0.765570879		
LSYTAPPSFK	29.69954	Q96KN7	RPGR1 HUMAN	555.7956765	2	0.837613761		
LSYTAPPSFK	29.69954	Q96KN7	RPGR1 HUMAN	370.8663927	3	0.837613761		
NQGILSAAHEALLK	36.80973	Q96KN7	RPGR1 HUMAN	732.912639	2	0.777187586		
NQGILSAAHEALLK	36.80973	Q96KN7	RPGR1 HUMAN	488.9443677	3	0.777187586		
QSEPATHPAVLQENTQIEPSEPK	24.01573	Q96KN7	RPGR1 HUMAN	1265.625182	2	0.612480164		
QSEPATHPAVLQENTQIEPSEPK	24.01573	Q96KN7	RPGR1 HUMAN	844.0860627	3	0.612480164		
SESWEPQNELWIEITK	90.67619	Q96KN7	RPGR1 HUMAN	994.984186	2	0.780715466		
SESWEPQNELWIEITK	90.67619	Q96KN7	RPGR1 HUMAN	663.6587323	3	0.780715466		
SQLEDVSILQMTLK	94.95087	Q96KN7	RPGR1 HUMAN	802.93238	2	0.657649875		
SQLEDVSILQMTLK	94.95087	Q96KN7	RPGR1 HUMAN	535.624195	3	0.657649875		
VAEEAAPLSETAR	9.32206	Q96KN7	RPGR1 HUMAN	672.344255	2	0.806707263		
VAEEAAPLSETAR	9.32206	Q96KN7	RPGR1 HUMAN	448.565445	3	0.806707263		
VIDLDPQEQQGR	21.14936	Q96KN7	RPGR1 HUMAN	699.3551545	2	0.734212756		
VIDLDPQEQQGR	21.14936	Q96KN7	RPGR1 HUMAN	466.5727113	3	0.734212756		
VLLELSR	33.37215	Q96KN7	RPGR1 HUMAN	415.2612765	2	0.635823607		
VLLELSR	33.37215	Q96KN7	RPGR1 HUMAN	277.1767927	3	0.635823607		
VSLQAAAVLHAIYK	63.83817	Q96KN7	RPGR1 HUMAN	742.435746	2	0.825724065		
VSLQAAAVLHAIYK	63.83817	Q96KN7	RPGR1 HUMAN	495.2931057	3	0.825724065		
WLGTQPSPYAVYR	51.80972	Q96KN7	RPGR1 HUMAN	769.394074	2	0.753000259		
WLGTQPSPYAVYR	51.80972	Q96KN7	RPGR1 HUMAN	513.2653243	3	0.753000259		
APKPEDIDEEDDDVPDLVENFDEASK	75.30105	Q96K17	BT3L4 HUMAN	1466.149276	2	0.731761932	3	
APKPEDIDEEDDDVPDLVENFDEASK	75.30105	Q96K17	BT3L4 HUMAN	977.7687923	3	0.731761932	3	Yes
DDGTVIHFNNPK	15.49975	Q96K17	BT3L4 HUMAN	678.8313145	2	0.741434336	2	Yes
DDGTVIHFNNPK	15.49975	Q96K17	BT3L4 HUMAN	452.8901513	3	0.741434336	2	
LAEQFPR	3.533947	Q96K17	BT3L4 HUMAN	430.7354265	2	0.747389257		
LAEQFPR	3.533947	Q96K17	BT3L4 HUMAN	287.4928927	3	0.747389257		
LAVNNIAGIEEVNMIK	77.2605	Q96K17	BT3L4 HUMAN	864.472203	2	0.86408031	3	
LAVNNIAGIEEVNMIK	77.2605	Q96K17	BT3L4 HUMAN	576.6507437	3	0.86408031	3	Yes
AGDSAVLVLPPSPGPR	57.16582	Q96JY6	PDLI2 HUMAN	766.9257465	2	0.69982785		
AGDSAVLVLPPSPGPR	57.16582	Q96JY6	PDLI2 HUMAN	511.6197727	3	0.69982785		
AGSPFSPPPSSSSLTGEAAISR	54.17624	Q96JY6	PDLI2 HUMAN	1052.021831	2	0.660106063		
AGSPFSPPPSSSSLTGEAAISR	54.17624	Q96JY6	PDLI2 HUMAN	701.683829	3	0.660106063		
DFHTPIMVTK	31.62177	Q96JY6	PDLI2 HUMAN	594.808266	2	0.689978957		
DFHTPIMVTK	31.62177	Q96JY6	PDLI2 HUMAN	396.8747857	3	0.689978957		
GGTPAFLPSSLSPQSSLPASR	72.05825	Q96JY6	PDLI2 HUMAN	1029.037285	2	0.647350907		
GGTPAFLPSSLSPQSSLPASR	72.05825	Q96JY6	PDLI2 HUMAN	686.360798	3	0.647350907		
GHFWVGDELYCEK	41.86046	Q96JY6	PDLI2 HUMAN	820.3646605	2	0.663043916	2	Yes
GHFWVGDELYCEK	41.86046	Q96JY6	PDLI2 HUMAN	547.2457153	3	0.663043916	2	
LLQEALEAEER	29.12206	Q96JY6	PDLI2 HUMAN	650.841348	2	0.757375598	2	Yes
LLQEALEAEER	29.12206	Q96JY6	PDLI2 HUMAN	434.2301737	3	0.757375598	2	
SFQSLACSPGLPAADR	48.59045	Q96JY6	PDLI2 HUMAN	838.907228	2	0.808288634		
SFQSLACSPGLPAADR	48.59045	Q96JY6	PDLI2 HUMAN	559.607427	3	0.808288634		
SSYSSPTSLSPR	10.35297	Q96JY6	PDLI2 HUMAN	634.8100425	2	0.820421755		
SSYSSPTSLSPR	10.35297	Q96JY6	PDLI2 HUMAN	423.5426367	3	0.820421755		
TYTESQSSLR	-22.95704	Q96JY6	PDLI2 HUMAN	586.2836615	2	0.674484372		
TYTESQSSLR	-22.95704	Q96JY6	PDLI2 HUMAN	391.191716	3	0.674484372		
AASSFFSTQAHVACEHPTGWSSMEER	41.92342	Q96JI7	SPTCS HUMAN	1455.632689	2	0.791739523		
AASSFFSTQAHVACEHPTGWSSMEER	41.92342	Q96JI7	SPTCS HUMAN	970.7577343	3	0.791739523		
ALASGEASMEDLHPEIHALLQSAELLEEEAPDIPL	124.8415	Q96JI7	SPTCS HUMAN	1947.975675	2	0.735817432		
ALASGEASMEDLHPEIHALLQSAELLEEEAPDIPL	124.8415	Q96JI7	SPTCS HUMAN	1298.986392	3	0.735817432		
ALTLMLDAAESYAK	77.62241	Q96JI7	SPTCS HUMAN	748.887437	2	0.822878242		
ALTLMLDAAESYAK	77.62241	Q96JI7	SPTCS HUMAN	499.594233	3	0.822878242		
AQAFISTQGLKPDTVAELVAEEVTR	99.60309	Q96JI7	SPTCS HUMAN	1337.208882	2	0.69730103		
AQAFISTQGLKPDTVAELVAEEVTR	99.60309	Q96JI7	SPTCS HUMAN	891.8085297	3	0.69730103		
AQLSSLNETIK	20.30534	Q96JI7	SPTCS HUMAN	602.3331585	2	0.742120564		
AQLSSLNETIK	20.30534	Q96JI7	SPTCS HUMAN	401.8913807	3	0.742120564		
DFLVEILK	97.15707	Q96JI7	SPTCS HUMAN	488.789867	2	0.778501093		
DFLVEILK	97.15707	Q96JI7	SPTCS HUMAN	326.195853	3	0.778501093		
EHLFSDGPDVK	9.116287	Q96JI7	SPTCS HUMAN	622.3018585	2	0.811756909		
EHLFSDGPDVK	9.116287	Q96JI7	SPTCS HUMAN	415.2038473	3	0.811756909		
EITQEMQTLK	16.91959	Q96JI7	SPTCS HUMAN	610.8137455	2	0.800331593		
EITQEMQTLK	16.91959	Q96JI7	SPTCS HUMAN	407.5451053	3	0.800331593		
ELFIHTEELDEHLQK	37.31084	Q96JI7	SPTCS HUMAN	940.973622	2	0.854887426		
ELFIHTEELDEHLQK	37.31084	Q96JI7	SPTCS HUMAN	627.6516897	3	0.854887426		
ELGCSYTDVAAQDGEAMLR	53.69411	Q96JI7	SPTCS HUMAN	1043.464975	2	0.738729835		
ELGCSYTDVAAQDGEAMLR	53.69411	Q96JI7	SPTCS HUMAN	695.979258	3	0.738729835		
ELLTSSQGTGHK	-27.68143	Q96JI7	SPTCS HUMAN	629.3258655	2	0.744386911		
ELLTSSQGTGHK	-27.68143	Q96JI7	SPTCS HUMAN	419.8865187	3	0.744386911		
FVTVPSSNEVVTNLEVLTSK	96.05598	Q96JI7	SPTCS HUMAN	1082.081358	2	0.815169811		
FVTVPSSNEVVTNLEVLTSK	96.05598	Q96JI7	SPTCS HUMAN	721.7235133	3	0.815169811		
FYEIVNVLLK	97.14514	Q96JI7	SPTCS HUMAN	619.363726	2	0.739768803		
FYEIVNVLLK	97.14514	Q96JI7	SPTCS HUMAN	413.2450923	3	0.739768803		
GDFNYLEEFK	60.69591	Q96JI7	SPTCS HUMAN	631.290955	2	0.841020226		
GDFNYLEEFK	60.69591	Q96JI7	SPTCS HUMAN	421.1965783	3	0.841020226		
GVDEDDPVNSAYNMK	24.65632	Q96JI7	SPTCS HUMAN	827.3572285	2	0.739733994		
GVDEDDPVNSAYNMK	24.65632	Q96JI7	SPTCS HUMAN	551.9074273	3	0.739733994		
GVNILTSYINELR	99.64023	Q96JI7	SPTCS HUMAN	746.412467	2	0.767540991		
GVNILTSYINELR	99.64023	Q96JI7	SPTCS HUMAN	497.944253	3	0.767540991		
HIEQWSLK	6.012066	Q96JI7	SPTCS HUMAN	520.780365	2	0.607362688		
HIEQWSLK	6.012066	Q96JI7	SPTCS HUMAN	347.5228517	3	0.607362688		
HLLLTLAGHWLAQEDVVPLDK	94.3261	Q96JI7	SPTCS HUMAN	1184.65536	2	0.714980662		
HLLLTLAGHWLAQEDVVPLDK	94.3261	Q96JI7	SPTCS HUMAN	790.1061813	3	0.714980662		
IGGVIQDTLPVQNYK	56.5368	Q96JI7	SPTCS HUMAN	822.9519575	2	0.878627837		
IGGVIQDTLPVQNYK	56.5368	Q96JI7	SPTCS HUMAN	548.97058	3	0.878627837		
ILEDLPIQGPK	52.92044	Q96JI7	SPTCS HUMAN	611.85627	2	0.802308798		
ILEDLPIQGPK	52.92044	Q96JI7	SPTCS HUMAN	408.2401217	3	0.802308798		
ILSFHNNTSLLFINK	61.84056	Q96JI7	SPTCS HUMAN	880.988877	2	0.760733664		
ILSFHNNTSLLFINK	61.84056	Q96JI7	SPTCS HUMAN	587.6618597	3	0.760733664		
IMHISEQEEPIELK	33.98772	Q96JI7	SPTCS HUMAN	848.4352865	2	0.776840985		
IMHISEQEEPIELK	33.98772	Q96JI7	SPTCS HUMAN	565.959466	3	0.776840985		
IPEAQTFFR	46.98454	Q96JI7	SPTCS HUMAN	554.793473	2	0.734882891		
IPEAQTFFR	46.98454	Q96JI7	SPTCS HUMAN	370.198257	3	0.734882891		
LAALNTSK	-16.84894	Q96JI7	SPTCS HUMAN	409.243084	2	0.610726774		
LAALNTSK	-16.84894	Q96JI7	SPTCS HUMAN	273.1646643	3	0.610726774		
LAFENLPSVPTSK	60.43703	Q96JI7	SPTCS HUMAN	701.8830155	2	0.841241598		
LAFENLPSVPTSK	60.43703	Q96JI7	SPTCS HUMAN	468.257952	3	0.841241598		
LFGWQSANTLAIGDAWSHLPHFSSPDLVNK	106.577	Q96JI7	SPTCS HUMAN	1654.828549	2	0.80107969		

LFGWQSANTLAIGDAWSHLPHFSSPDLVNK	106.577	Q96JI7	SPTCS_HUMAN	1103.554974	3	0.80107969		
LIESQPWEDSLK	47.84351	Q96JI7	SPTCS_HUMAN	722.870105	2	0.749827206		
LIESQPWEDSLK	47.84351	Q96JI7	SPTCS_HUMAN	482.249345	3	0.749827206		
LLDDGCVHEASR	-15.94627	Q96JI7	SPTCS_HUMAN	686.320257	2	0.827684402		
LLDDGCVHEASR	-15.94627	Q96JI7	SPTCS_HUMAN	457.8827797	3	0.827684402		
LLQLFVER	59.07364	Q96JI7	SPTCS_HUMAN	509.3087585	2	0.81877476		
LLQLFVER	59.07364	Q96JI7	SPTCS_HUMAN	339.875114	3	0.81877476		
LLTGIGR	12.00385	Q96JI7	SPTCS_HUMAN	365.2350625	2	0.612274468		
LLTGIGR	12.00385	Q96JI7	SPTCS_HUMAN	243.8259833	3	0.612274468		
LMLQQCK	-8.165859	Q96JI7	SPTCS_HUMAN	460.738798	2	0.799976885		
LMLQQCK	-8.165859	Q96JI7	SPTCS_HUMAN	307.4951403	3	0.799976885		
LQTDGQFALAR	28.35464	Q96JI7	SPTCS_HUMAN	610.325669	2	0.821051955		
LQTDGQFALAR	28.35464	Q96JI7	SPTCS_HUMAN	407.219721	3	0.821051955		
LSFEEVIASAILNNK	129.4203	Q96JI7	SPTCS_HUMAN	824.451793	2	0.779517829		
LSFEEVIASAILNNK	129.4203	Q96JI7	SPTCS_HUMAN	549.9704703	3	0.779517829		
LSPENCPFLEK	33.04135	Q96JI7	SPTCS_HUMAN	667.327019	2	0.816533089		
LSPENCPFLEK	33.04135	Q96JI7	SPTCS_HUMAN	445.220621	3	0.816533089		
LTDAIDEYDVHENVPK	34.65959	Q96JI7	SPTCS_HUMAN	929.4474325	2	0.840399742		
LTDAIDEYDVHENVPK	34.65959	Q96JI7	SPTCS_HUMAN	619.9675633	3	0.840399742		
LYLGHFQENMQIQSFPR	57.83842	Q96JI7	SPTCS_HUMAN	1054.523287	2	0.788803041		
LYLGHFQENMQIQSFPR	57.83842	Q96JI7	SPTCS_HUMAN	703.351466	3	0.788803041		
MALTPYPK	19.17879	Q96JI7	SPTCS_HUMAN	460.749684	2	0.701348484		
MALTPYPK	19.17879	Q96JI7	SPTCS_HUMAN	307.5023977	3	0.701348484		
NEDAQYSFIR	24.39336	Q96JI7	SPTCS_HUMAN	621.7916535	2	0.772305608		
NEDAQYSFIR	24.39336	Q96JI7	SPTCS_HUMAN	414.8637107	3	0.772305608		
NVEELIPALDLLCSAIR	156.1865	Q96JI7	SPTCS_HUMAN	963.5224245	2	0.655012846		
NVEELIPALDLLCSAIR	156.1865	Q96JI7	SPTCS_HUMAN	642.6842247	3	0.655012846		
SLETLNTAATK	7.416702	Q96JI7	SPTCS_HUMAN	574.8120595	2	0.801412106		
SLETLNTAATK	7.416702	Q96JI7	SPTCS_HUMAN	383.5439813	3	0.801412106		
SLIQYFSPVIQDHLR	79.35832	Q96JI7	SPTCS_HUMAN	908.4917805	2	0.789019942		
SLIQYFSPVIQDHLR	79.35832	Q96JI7	SPTCS_HUMAN	605.9971287	3	0.789019942		
SSIFEEISK	37.52187	Q96JI7	SPTCS_HUMAN	520.269495	2	0.716008842		
SSIFEEISK	37.52187	Q96JI7	SPTCS_HUMAN	347.1822717	3	0.716008842		
SSVSDQFDHLSSHLYLR	45.94156	Q96JI7	SPTCS_HUMAN	995.985047	2	0.823107004		
SSVSDQFDHLSSHLYLR	45.94156	Q96JI7	SPTCS_HUMAN	664.325973	3	0.823107004		
SVLDSFLK	63.5202	Q96JI7	SPTCS_HUMAN	454.7585665	2	0.675881922		
SVLDSFLK	63.5202	Q96JI7	SPTCS_HUMAN	303.5083193	3	0.675881922		
TALLDYIK	50.78254	Q96JI7	SPTCS_HUMAN	468.774213	2	0.732535183		
TALLDYIK	50.78254	Q96JI7	SPTCS_HUMAN	312.8520837	3	0.732535183		
TIDFVHQVEK	17.558	Q96JI7	SPTCS_HUMAN	608.3225945	2	0.755452991		
TIDFVHQVEK	17.558	Q96JI7	SPTCS_HUMAN	405.884338	3	0.755452991		
TTTEELLVLLEEGTWNSIQQQEIK	150.1524	Q96JI7	SPTCS_HUMAN	1401.724562	2	0.631622314		
TTTEELLVLLEEGTWNSIQQQEIK	150.1524	Q96JI7	SPTCS_HUMAN	934.8189827	3	0.631622314		
VAELAELPVDNLVIK	91.17691	Q96JI7	SPTCS_HUMAN	811.972362	2	0.900386572		
VAELAELPVDNLVIK	91.17691	Q96JI7	SPTCS_HUMAN	541.6508497	3	0.900386572		
VANIILSYK	38.84224	Q96JI7	SPTCS_HUMAN	510.8085865	2	0.645380497		
VANIILSYK	38.84224	Q96JI7	SPTCS_HUMAN	340.8749993	3	0.645380497		
VLQAAHMLTDNHLAPSEEYGLVVR	56.67684	Q96JI7	SPTCS_HUMAN	1332.184682	2	0.82228297		
VLQAAHMLTDNHLAPSEEYGLVVR	56.67684	Q96JI7	SPTCS_HUMAN	888.459063	3	0.82228297		
YDCKDEFNK	-28.96185	Q96JI7	SPTCS_HUMAN	609.758965	2	0.671350896		
YDCKDEFNK	-28.96185	Q96JI7	SPTCS_HUMAN	406.8419183	3	0.671350896		
YFHFYNPDVALVLHCR	63.54942	Q96JI7	SPTCS_HUMAN	1026.001986	2	0.610046506		
YFHFYNPDVALVLHCR	63.54942	Q96JI7	SPTCS_HUMAN	684.3372653	3	0.610046506		
YLELNSLPSK	40.10621	Q96JI7	SPTCS_HUMAN	582.319515	2	0.835623145		
YLELNSLPSK	40.10621	Q96JI7	SPTCS_HUMAN	388.5489517	3	0.835623145		
YNEMTYIFDLLHK	103.5956	Q96JI7	SPTCS_HUMAN	843.9139815	2	0.730463505		
YNEMTYIFDLLHK	103.5956	Q96JI7	SPTCS_HUMAN	562.9452627	3	0.730463505		
ALDYEQMLLLMANVK	144.0244	Q96JG6	VPS50_HUMAN	876.4577035	2	0.602220058		
ALDYEQMLLLMANVK	144.0244	Q96JG6	VPS50_HUMAN	584.6410773	3	0.602220058		
ALMQLDFQQFLMK	116.908	Q96JG6	VPS50_HUMAN	806.9154795	2	0.694778979		
ALMQLDFQQFLMK	116.908	Q96JG6	VPS50_HUMAN	538.2795947	3	0.694778979		
ALWEVMLSYYR	109.5241	Q96JG6	VPS50_HUMAN	715.8610205	2	0.77023983		
ALWEVMLSYYR	109.5241	Q96JG6	VPS50_HUMAN	477.576622	3	0.77023983		
AYYLTENDMER	24.4352	Q96JG6	VPS50_HUMAN	702.809182	2	0.858679235		
AYYLTENDMER	24.4352	Q96JG6	VPS50_HUMAN	468.8753963	3	0.858679235		
DYVDEQTGDGPVK	10.0909	Q96JG6	VPS50_HUMAN	711.8233485	2	0.666419089	2	Yes
DYVDEQTGDGPVK	10.0909	Q96JG6	VPS50_HUMAN	474.8848407	3	0.666419089	2	
EGFTQASLGLLANQR	65.78842	Q96JG6	VPS50_HUMAN	802.9237355	2	0.804738104		
EGFTQASLGLLANQR	65.78842	Q96JG6	VPS50_HUMAN	535.618432	3	0.804738104		
EIMSQHNIYVDALLK	54.84823	Q96JG6	VPS50_HUMAN	887.464374	2	0.732229948		
EIMSQHNIYVDALLK	54.84823	Q96JG6	VPS50_HUMAN	591.9788577	3	0.732229948		
IPLPVSNILWEHCIR	85.89017	Q96JG6	VPS50_HUMAN	924.004005	2	0.754782856	3	
IPLPVSNILWEHCIR	85.89017	Q96JG6	VPS50_HUMAN	616.3386117	3	0.754782856	3	Yes
IWQDVQLK	31.61285	Q96JG6	VPS50_HUMAN	515.290566	2	0.742179096		
IWQDVQLK	31.61285	Q96JG6	VPS50_HUMAN	343.8629857	3	0.742179096		
KPFLQQFYSQTVSTASELR	65.5406	Q96JG6	VPS50_HUMAN	1115.579313	2	0.747960687	3	
KPFLQQFYSQTVSTASELR	65.5406	Q96JG6	VPS50_HUMAN	744.0554837	3	0.747960687	3	Yes
KPIYWIVAGK	31.6223	Q96JG6	VPS50_HUMAN	587.853329	2	0.714948297		
KPIYWIVAGK	31.6223	Q96JG6	VPS50_HUMAN	392.238161	3	0.714948297		
LLAAIDDIDRPK	32.62955	Q96JG6	VPS50_HUMAN	670.383183	2	0.75206995		
LLAAIDDIDRPK	32.62955	Q96JG6	VPS50_HUMAN	447.2580637	3	0.75206995		
LMQVGEEFCGSK	30.87996	Q96JG6	VPS50_HUMAN	692.815962	2	0.801864684		
LMQVGEEFCGSK	30.87996	Q96JG6	VPS50_HUMAN	462.2132497	3	0.801864684		
LPPVLNLQELEAYR	92.71265	Q96JG6	VPS50_HUMAN	827.9623245	2	0.860417306	2	Yes
LPPVLNLQELEAYR	92.71265	Q96JG6	VPS50_HUMAN	552.3108247	3	0.860417306	2	
LQDTLEQIEEQLDVALSK	125.7104	Q96JG6	VPS50_HUMAN	1036.542066	2	0.799043834		
LQDTLEQIEEQLDVALSK	125.7104	Q96JG6	VPS50_HUMAN	691.3639857	3	0.799043834		
LTDIRPIPDK	5.657257	Q96JG6	VPS50_HUMAN	584.340787	2	0.688394785		
LTDIRPIPDK	5.657257	Q96JG6	VPS50_HUMAN	389.8964663	3	0.688394785		
MFLENETWELCPVK	81.52859	Q96JG6	VPS50_HUMAN	898.423865	2	0.798480868		
MFLENETWELCPVK	81.52859	Q96JG6	VPS50_HUMAN	599.285185	3	0.798480868		
NDSLESTGLGLSSSR	32.66622	Q96JG6	VPS50_HUMAN	761.871364	2	0.80947125		
NDSLESTGLGLSSSR	32.66622	Q96JG6	VPS50_HUMAN	508.2501843	3	0.80947125		
NFDINHYTK	3.877735	Q96JG6	VPS50_HUMAN	576.278182	2	0.636851788		
NFDINHYTK	3.877735	Q96JG6	VPS50_HUMAN	384.5213963	3	0.636851788		
SAYQEYDSDSDVPEELK	35.25591	Q96JG6	VPS50_HUMAN	987.926722	2	0.824282885		
SAYQEYDSDSDVPEELK	35.25591	Q96JG6	VPS50_HUMAN	658.9537563	3	0.824282885		
SEVLQESIR	9.236015	Q96JG6	VPS50_HUMAN	530.785844	2	0.758898079		
SEVLQESIR	9.236015	Q96JG6	VPS50_HUMAN	354.193171	3	0.758898079		
SNFSILQLHEFK	54.64926	Q96JG6	VPS50_HUMAN	731.888632	2	0.810947776	3	
SNFSILQLHEFK	54.64926	Q96JG6	VPS50_HUMAN	488.2616963	3	0.810947776	3	Yes

SPQESLSDLGAIESLR	73.45283	Q96JG6	VPS50 HUMAN	851.4368715	2	0.772724867		
SPQESLSDLGAIESLR	73.45283	Q96JG6	VPS50 HUMAN	567.9605227	3	0.772724867		
TVTLFEQYCSGGNPFEIQANHK	65.92662	Q96JG6	VPS50 HUMAN	1270.597909	2	0.60126549		
TVTLFEQYCSGGNPFEIQANHK	65.92662	Q96JG6	VPS50 HUMAN	847.401214	3	0.60126549		
TYLLGTDLSIFK	90.21915	Q96JG6	VPS50 HUMAN	685.8824805	2	0.725108683		
TYLLGTDLSIFK	90.21915	Q96JG6	VPS50 HUMAN	457.5909287	3	0.725108683		
VADLILEK	30.63663	Q96JG6	VPS50 HUMAN	450.774217	2	0.761876404		
VADLILEK	30.63663	Q96JG6	VPS50 HUMAN	300.8520863	3	0.761876404		
VNAPILTNTTLNVIR	69.98744	Q96JG6	VPS50 HUMAN	819.981053	2	0.840464294		
VNAPILTNTTLNVIR	69.98744	Q96JG6	VPS50 HUMAN	546.989977	3	0.840464294		
DNTYLVELSSLLVR	137.4745	Q96JB5	CK5P3 HUMAN	811.443964	2	0.661497772		
DNTYLVELSSLLVR	137.4745	Q96JB5	CK5P3 HUMAN	541.2985843	3	0.661497772		
DWQEIIALYEK	107.2176	Q96JB5	CK5P3 HUMAN	704.361912	2	0.603123784		
DWQEIIALYEK	107.2176	Q96JB5	CK5P3 HUMAN	469.9105497	3	0.603123784		
GNSTVYEWR	12.79994	Q96JB5	CK5P3 HUMAN	556.262532	2	0.67522639		
GNSTVYEWR	12.79994	Q96JB5	CK5P3 HUMAN	371.1776297	3	0.67522639		
GPDALTLLEYTETR	88.83105	Q96JB5	CK5P3 HUMAN	789.904673	2	0.821778655	2	Yes
GPDALTLLEYTETR	88.83105	Q96JB5	CK5P3 HUMAN	526.939057	3	0.821778655	2	
LDLLLEK	48.82323	Q96JB5	CK5P3 HUMAN	422.263485	2	0.65826875		
LDLLLEK	48.82323	Q96JB5	CK5P3 HUMAN	281.8449317	3	0.65826875		
LLDWLVDR	74.97868	Q96JB5	CK5P3 HUMAN	515.2905655	2	0.73578608		
LLDWLVDR	74.97868	Q96JB5	CK5P3 HUMAN	343.8629853	3	0.73578608		
MEDHQHVPIDIQTSK	12.55556	Q96JB5	CK5P3 HUMAN	889.4310675	2	0.772400796		
MEDHQHVPIDIQTSK	12.55556	Q96JB5	CK5P3 HUMAN	593.2899867	3	0.772400796		
NVNYEIPSLK	42.28342	Q96JB5	CK5P3 HUMAN	588.8171395	2	0.755406678	2	Yes
NVNYEIPSLK	42.28342	Q96JB5	CK5P3 HUMAN	392.8807013	3	0.755406678	2	
VTEFLQQK	9.539978	Q96JB5	CK5P3 HUMAN	496.774749	2	0.748216093	2	Yes
VTEFLQQK	9.539978	Q96JB5	CK5P3 HUMAN	331.5191077	3	0.748216093	2	
WQSLVLTIR	63.54837	Q96JB5	CK5P3 HUMAN	558.332765	2	0.691442132		
WQSLVLTIR	63.54837	Q96JB5	CK5P3 HUMAN	372.557785	3	0.691442132		
DANVSGTLVSSSTLEK	36.32516	Q96IZ0	PAWR HUMAN	804.4103225	2	0.79805851		
DANVSGTLVSSSTLEK	36.32516	Q96IZ0	PAWR HUMAN	536.60949	3	0.79805851		
DLDDIEDENEQLK	38.27135	Q96IZ0	PAWR HUMAN	788.355213	2	0.811166883	2	Yes
DLDDIEDENEQLK	38.27135	Q96IZ0	PAWR HUMAN	525.9060837	3	0.811166883	2	
EDAITQQNTIQNEAVNLLDPGSSYLLQEPPR	117.4536	Q96IZ0	PAWR HUMAN	1727.3608	2	0.64758718		
EDAITQQNTIQNEAVNLLDPGSSYLLQEPPR	117.4536	Q96IZ0	PAWR HUMAN	1151.909808	3	0.64758718		
TSSGLGGSTTDFLEEWK	75.01505	Q96IZ0	PAWR HUMAN	907.9263375	2	0.831412911		
TSSGLGGSTTDFLEEWK	75.01505	Q96IZ0	PAWR HUMAN	605.6201667	3	0.831412911		
AVAIDLPGLGHSK	40.03728	Q96IU4	ABHEB HUMAN	639.3647935	2	0.831975877	3	
AVAIDLPGLGHSK	40.03728	Q96IU4	ABHEB HUMAN	426.5791373	3	0.831975877	3	Yes
EGTIQVQGQALFFR	67.94648	Q96IU4	ABHEB HUMAN	797.423372	2	0.761568606	2	Yes
EGTIQVQGQALFFR	67.94648	Q96IU4	ABHEB HUMAN	531.951523	3	0.761568606	2	
FSSETWQNLGTLHR	40.2932	Q96IU4	ABHEB HUMAN	838.4135345	2	0.887060881		
FSSETWQNLGTLHR	40.2932	Q96IU4	ABHEB HUMAN	559.278298	3	0.887060881		
FSVLLLHGIR	58.15935	Q96IU4	ABHEB HUMAN	577.856407	2	0.834472358	2	Yes
FSVLLLHGIR	58.15935	Q96IU4	ABHEB HUMAN	385.5735463	3	0.834472358	2	
TPALIVYGDQDPMGQTSFEHLK	67.42848	Q96IU4	ABHEB HUMAN	1224.099753	2	0.802199006	3	
TPALIVYGDQDPMGQTSFEHLK	67.42848	Q96IU4	ABHEB HUMAN	816.4024437	3	0.802199006	3	Yes
HLQLNETSTANHIHSR	-28.99802	Q96HV5	TM41A HUMAN	929.4699035	2	0.755880773		
HLQLNETSTANHIHSR	-28.99802	Q96HV5	TM41A HUMAN	619.982544	3	0.755880773		
SLWFPSDLAELR	99.74818	Q96HV5	TM41A HUMAN	717.3753575	2	0.844407558		
SLWFPSDLAELR	99.74818	Q96HV5	TM41A HUMAN	478.58618	3	0.844407558		
AAVMATLLFPGR	82.20313	Q96GX9	MTNB HUMAN	623.853008	2	0.762465775		
AAVMATLLFPGR	82.20313	Q96GX9	MTNB HUMAN	416.237947	3	0.762465775		
HGDEIYIAPSGVQK	15.72786	Q96GX9	MTNB HUMAN	757.38645	2	0.830471337	2	Yes
HGDEIYIAPSGVQK	15.72786	Q96GX9	MTNB HUMAN	505.2602417	3	0.830471337	2	
IQPEDMFVCDINEK	65.27937	Q96GX9	MTNB HUMAN	869.3953045	2	0.720301628		
IQPEDMFVCDINEK	65.27937	Q96GX9	MTNB HUMAN	579.9328113	3	0.720301628		
MAHAMNEYPDSCAVLVR	32.92819	Q96GX9	MTNB HUMAN	982.445333	2	0.799305081		
MAHAMNEYPDSCAVLVR	32.92819	Q96GX9	MTNB HUMAN	655.299497	3	0.799305081		
RHGVYVWGETWEK	23.26724	Q96GX9	MTNB HUMAN	823.907884	2	0.826854706		
RHGVYVWGETWEK	23.26724	Q96GX9	MTNB HUMAN	549.6078643	3	0.826854706		
SQCTPLFMNAYTMR	59.98046	Q96GX9	MTNB HUMAN	860.386756	2	0.779269457		
SQCTPLFMNAYTMR	59.98046	Q96GX9	MTNB HUMAN	573.9271123	3	0.779269457		
VGLDPSQLPVGENGIV	96.80457	Q96GX9	MTNB HUMAN	797.428319	2	0.730214655		
VGLDPSQLPVGENGIV	96.80457	Q96GX9	MTNB HUMAN	531.954821	3	0.730214655		
YDDMLVVPIIENTPEEK	96.93513	Q96GX9	MTNB HUMAN	1002.995901	2	0.7927351	3	
YDDMLVVPIIENTPEEK	96.93513	Q96GX9	MTNB HUMAN	668.9998757	3	0.7927351	3	Yes
YLIPELCK	46.94395	Q96GX9	MTNB HUMAN	518.281348	2	0.661290348		
YLIPELCK	46.94395	Q96GX9	MTNB HUMAN	345.8568403	3	0.661290348		
AQAEDYALVSAVATLPK	85.0347	Q96GW9	SYMM HUMAN	873.9678045	2	0.801646948		
AQAEDYALVSAVATLPK	85.0347	Q96GW9	SYMM HUMAN	582.9811447	3	0.801646948		
FSTGTDEHGLK	-26.25715	Q96GW9	SYMM HUMAN	596.286209	2	0.817214608	2	Yes
FSTGTDEHGLK	-26.25715	Q96GW9	SYMM HUMAN	397.860081	3	0.817214608	2	
GVYEGWYCASDECFLPEAK	78.50426	Q96GW9	SYMM HUMAN	1140.982996	2	0.708631933		
GVYEGWYCASDECFLPEAK	78.50426	Q96GW9	SYMM HUMAN	760.991272	3	0.708631933		
IQQAAATAGLAPTELCDR	42.70966	Q96GW9	SYMM HUMAN	943.4760075	2	0.831576467		
IQQAAATAGLAPTELCDR	42.70966	Q96GW9	SYMM HUMAN	629.3199467	3	0.831576467		
LGPETGLLFPR	64.80872	Q96GW9	SYMM HUMAN	600.34333	2	0.807180882		
LGPETGLLFPR	64.80872	Q96GW9	SYMM HUMAN	400.5648283	3	0.807180882		
LLNSELADALGGLLNR	114.3633	Q96GW9	SYMM HUMAN	834.968142	2	0.770338893		
LLNSELADALGGLLNR	114.3633	Q96GW9	SYMM HUMAN	556.9813697	3	0.770338893		
LSLLEDFGPR	71.08412	Q96GW9	SYMM HUMAN	573.811862	2	0.847373545		
LSLLEDFGPR	71.08412	Q96GW9	SYMM HUMAN	382.877183	3	0.847373545		
SLGELYFLPR	81.4967	Q96GW9	SYMM HUMAN	597.8300505	2	0.86644876	2	Yes
SLGELYFLPR	81.4967	Q96GW9	SYMM HUMAN	398.8893087	3	0.86644876	2	
SLGNVVDPR	9.223759	Q96GW9	SYMM HUMAN	478.762172	2	0.775001287		
SLGNVVDPR	9.223759	Q96GW9	SYMM HUMAN	319.510723	3	0.775001287		
SWWPATSHIIGK	48.63895	Q96GW9	SYMM HUMAN	691.8649605	2	0.781748652	3	
SWWPATSHIIGK	48.63895	Q96GW9	SYMM HUMAN	461.5792487	3	0.781748652	3	Yes
VAVQHFWGVLK	48.22239	Q96GW9	SYMM HUMAN	642.3671395	2	0.700229526	3	
VAVQHFWGVLK	48.22239	Q96GW9	SYMM HUMAN	428.5807013	3	0.700229526	3	Yes
VFGTLLQPVTPSLADK	84.66533	Q96GW9	SYMM HUMAN	843.4778125	2	0.841093659		
VFGTLLQPVTPSLADK	84.66533	Q96GW9	SYMM HUMAN	562.6544833	3	0.841093659		
VSEQFQQLFQEAGISCTDFIR	107.3118	Q96GW9	SYMM HUMAN	1252.100289	2	0.723867834		
VSEQFQQLFQEAGISCTDFIR	107.3118	Q96GW9	SYMM HUMAN	835.0694673	3	0.723867834		
VTQQPGPSGDSFPVSLESGHPVSWTK	57.74461	Q96GW9	SYMM HUMAN	1362.667381	2	0.802860618	3	
VTQQPGPSGDSFPVSLESGHPVSWTK	57.74461	Q96GW9	SYMM HUMAN	908.780862	3	0.802860618	3	Yes
YYSSGSLSAGDDACDVR	15.27814	Q96GW9	SYMM HUMAN	911.881595	2	0.686570048		
YYSSGSLSAGDDACDVR	15.27814	Q96GW9	SYMM HUMAN	608.257005	3	0.686570048		
QPFESYITAPPGTAAAPAKPAPPATPGAPTSPAEI	57.39274	Q96GE9	DMAC1 HUMAN	1876.458111	2	0.739817619	4	

QPFESYITAPPGTAAAPAKPAPPATPGAPTSPAEI	57.39274	Q96GE9	DMAC1_HUMAN	1251.308015	3	0.739817619	4	
ASATCSSATAAASSGLEEWTSR	42.94176	Q96G74	OTUD5_HUMAN	1100.992946	2	0.653394222		
ASATCSSATAAASSGLEEWTSR	42.94176	Q96G74	OTUD5_HUMAN	734.3312387	3	0.653394222		
ASPPPQGPLPGPPGALHR	33.04661	Q96G74	OTUD5_HUMAN	873.47647	2	0.774672568		
ASPPPQGPLPGPPGALHR	33.04661	Q96G74	OTUD5_HUMAN	582.6535883	3	0.774672568		
ATDWEATNEAIEEQVAR	47.71103	Q96G74	OTUD5_HUMAN	966.9508755	2	0.892533481		
ATDWEATNEAIEEQVAR	47.71103	Q96G74	OTUD5_HUMAN	644.9698587	3	0.892533481		
ATIGVGLGLPSFKPGFAEQSLMK	93.62218	Q96G74	OTUD5_HUMAN	1174.638321	2	0.820291698	3	
ATIGVGLGLPSFKPGFAEQSLMK	93.62218	Q96G74	OTUD5_HUMAN	783.4281557	3	0.820291698	3	Yes
ATSPLVSLYPALECR	79.9254	Q96G74	OTUD5_HUMAN	838.9379925	2	0.754296958		
ATSPLVSLYPALECR	79.9254	Q96G74	OTUD5_HUMAN	559.6279367	3	0.754296958		
AVADQVYGDQDMHEVVR	25.30827	Q96G74	OTUD5_HUMAN	966.4499845	2	0.838701367	3	
AVADQVYGDQDMHEVVR	25.30827	Q96G74	OTUD5_HUMAN	644.6359313	3	0.838701367	3	Yes
ESYLQWLR	66.4206	Q96G74	OTUD5_HUMAN	547.785643	2	0.821923673		
ESYLQWLR	66.4206	Q96G74	OTUD5_HUMAN	365.5263703	3	0.821923673		
GGGVGVGGGGTGVGGGDR	-10.15026	Q96G74	OTUD5_HUMAN	686.832379	2	0.657478631		
GGGVGVGGGGTGVGGGDR	-10.15026	Q96G74	OTUD5_HUMAN	458.2241943	3	0.657478631		
IEAMDPATVEQQEHWFEK	54.06776	Q96G74	OTUD5_HUMAN	1094.504961	2	0.776004314	3	
IEAMDPATVEQQEHWFEK	54.06776	Q96G74	OTUD5_HUMAN	730.0059157	3	0.776004314	3	Yes
NADYFSNYVTEDFTTYINR	112.1138	Q96G74	OTUD5_HUMAN	1167.022016	2	0.744005203		
NADYFSNYVTEDFTTYINR	112.1138	Q96G74	OTUD5_HUMAN	778.3506187	3	0.744005203		
QAPGVGAVGGGSPER	-1.814426	Q96G74	OTUD5_HUMAN	669.8422145	2	0.633703232		
QAPGVGAVGGGSPER	-1.814426	Q96G74	OTUD5_HUMAN	446.897418	3	0.633703232		
PELHAELGMKPPSPGTVLALAKPPSPCAPGTSSQ	65.32481	Q96G74	OTUD5_HUMAN	2455.198367	2	0.620394647		
PELHAELGMKPPSPGTVLALAKPPSPCAPGTSSQ	65.32481	Q96G74	OTUD5_HUMAN	1637.134853	3	0.620394647		
TSEESWIEQQMLEDK	59.89816	Q96G74	OTUD5_HUMAN	926.917655	2	0.864337385		
TSEESWIEQQMLEDK	59.89816	Q96G74	OTUD5_HUMAN	618.281045	3	0.864337385		
DKPVTGEQIEVFANK	30.12683	Q96FV9	THOC1_HUMAN	837.939051	2	0.832537413	2	Yes
DKPVTGEQIEVFANK	30.12683	Q96FV9	THOC1_HUMAN	558.9619757	3	0.832537413	2	
FWSLQDYFR	87.36162	Q96FV9	THOC1_HUMAN	631.3042	2	0.758455157	2	Yes
FWSLQDYFR	87.36162	Q96FV9	THOC1_HUMAN	421.2054083	3	0.758455157	2	
ILAPYLEMK	55.43709	Q96FV9	THOC1_HUMAN	539.304823	2	0.730739295	2	Yes
ILAPYLEMK	55.43709	Q96FV9	THOC1_HUMAN	359.8724903	3	0.730739295	2	
ILMGNEELTR	26.59458	Q96FV9	THOC1_HUMAN	588.3086295	2	0.781962514		
ILMGNEELTR	26.59458	Q96FV9	THOC1_HUMAN	392.5416947	3	0.781962514		
LMDLQLSDSNFR	61.24299	Q96FV9	THOC1_HUMAN	719.853932	2	0.863187194		
LMDLQLSDSNFR	61.24299	Q96FV9	THOC1_HUMAN	480.238563	3	0.863187194		
LWNLCPDNMEACK	52.83604	Q96FV9	THOC1_HUMAN	825.857835	2	0.800860703	2	Yes
LWNLCPDNMEACK	52.83604	Q96FV9	THOC1_HUMAN	550.9078317	3	0.800860703	2	
MVEHILNTEENWNSWK	50.95537	Q96FV9	THOC1_HUMAN	1015.476005	2	0.842054009	3	
MVEHILNTEENWNSWK	50.95537	Q96FV9	THOC1_HUMAN	677.319945	3	0.842054009	3	Yes
SLPEYLENMVIK	83.11627	Q96FV9	THOC1_HUMAN	718.379247	2	0.838065505		
SLPEYLENMVIK	83.11627	Q96FV9	THOC1_HUMAN	479.2554397	3	0.838065505		
SPHFFQPTNQQFK	20.06385	Q96FV9	THOC1_HUMAN	803.394614	2	0.805275798	3	
SPHFFQPTNQQFK	20.06385	Q96FV9	THOC1_HUMAN	535.932351	3	0.805275798	3	Yes
SSNYVLTDEQSLWIEDTTK	76.85146	Q96FV9	THOC1_HUMAN	1115.032058	2	0.634507716		
SSNYVLTDEQSLWIEDTTK	76.85146	Q96FV9	THOC1_HUMAN	743.6906467	3	0.634507716		
SVYQLLSENPPDGER	52.52383	Q96FV9	THOC1_HUMAN	852.415935	2	0.876545787	2	Yes
SVYQLLSENPPDGER	52.52383	Q96FV9	THOC1_HUMAN	568.6132317	3	0.876545787	2	
YSEEVLAVFK	61.76734	Q96FV9	THOC1_HUMAN	592.814066	2	0.738340318	2	Yes
YSEEVLAVFK	61.76734	Q96FV9	THOC1_HUMAN	395.545319	3	0.738340318	2	
EPVGEGEALLGMDLLR	100.7763	Q96FV2	SCRN2_HUMAN	849.9407365	2	0.788367689	2	Yes
EPVGEGEALLGMDLLR	100.7763	Q96FV2	SCRN2_HUMAN	566.9630993	3	0.788367689	2	
GHQAALGLMER	4.712421	Q96FV2	SCRN2_HUMAN	591.806589	2	0.761323214		
GHQAALGLMER	4.712421	Q96FV2	SCRN2_HUMAN	394.8736677	3	0.761323214		
LQCTYIEVEQVSK	37.02363	Q96FV2	SCRN2_HUMAN	798.901076	2	0.70529145	2	Yes
LQCTYIEVEQVSK	37.02363	Q96FV2	SCRN2_HUMAN	532.936659	3	0.70529145	2	
NISNQLSIGTDISAQHPELR	47.43546	Q96FV2	SCRN2_HUMAN	1097.067105	2	0.801687241	3	
NISNQLSIGTDISAQHPELR	47.43546	Q96FV2	SCRN2_HUMAN	731.7140113	3	0.801687241	3	Yes
TEAWVLETAGR	40.62479	Q96FV2	SCRN2_HUMAN	616.8176765	2	0.790077269		
TEAWVLETAGR	40.62479	Q96FV2	SCRN2_HUMAN	411.547726	3	0.790077269		
NADMSEDMQQDAVDCATQAMEK	46.87428	Q96FJ2	DYL2_HUMAN	1244.490739	2	0.793859065	3	
NADMSEDMQQDAVDCATQAMEK	46.87428	Q96FJ2	DYL2_HUMAN	829.9964343	3	0.793859065	3	Yes
AGPILELEQWIDK	104.2094	Q96EY8	MMAB_HUMAN	756.409398	2	0.822011471	2	Yes
AGPILELEQWIDK	104.2094	Q96EY8	MMAB_HUMAN	504.6088737	3	0.822011471	2	
GFSSTFTGER	7.331188	Q96EY8	MMAB_HUMAN	544.7545445	2	0.755397081		
GFSSTFTGER	7.331188	Q96EY8	MMAB_HUMAN	363.505638	3	0.755397081		
GHTFAEELQK	-11.08072	Q96EY8	MMAB_HUMAN	580.2912945	2	0.80880928		
GHTFAEELQK	-11.08072	Q96EY8	MMAB_HUMAN	387.1968047	3	0.80880928		
LSDYLFTLAR	75.76077	Q96EY8	MMAB_HUMAN	599.827508	2	0.787131429	2	Yes
LSDYLFTLAR	75.76077	Q96EY8	MMAB_HUMAN	400.220947	3	0.787131429	2	
VVPLVQMGETDANVAK	49.63752	Q96EY8	MMAB_HUMAN	835.9432795	2	0.848843932	3	
VVPLVQMGETDANVAK	49.63752	Q96EY8	MMAB_HUMAN	557.6314613	3	0.848843932	3	Yes
AALLEFEER	39.88951	Q96EV2	RBM33_HUMAN	539.2829375	2	0.824118733		
AALLEFEER	39.88951	Q96EV2	RBM33_HUMAN	359.8579	3	0.824118733		
DPFFLGVSGEPR	74.8831	Q96EV2	RBM33_HUMAN	660.833326	2	0.769549489		
DPFFLGVSGEPR	74.8831	Q96EV2	RBM33_HUMAN	440.8914923	3	0.769549489		
ELPIAPSHVIEMSSSR	47.92941	Q96EV2	RBM33_HUMAN	876.9516345	2	0.746720672		
ELPIAPSHVIEMSSSR	47.92941	Q96EV2	RBM33_HUMAN	584.9703647	3	0.746720672		
EPAHALAFQQK	-13.24679	Q96EV2	RBM33_HUMAN	620.3282115	2	0.690328538		
EPAHALAFQQK	-13.24679	Q96EV2	RBM33_HUMAN	413.8880827	3	0.690328538		
FPGPPEFPQHTPGPVPNSFSQPPR	58.22404	Q96EV2	RBM33_HUMAN	1307.646055	2	0.670879722		
FPGPPEFPQHTPGPVPNSFSQPPR	58.22404	Q96EV2	RBM33_HUMAN	872.099978	3	0.670879722		
FPSHLFLEQR	30.32332	Q96EV2	RBM33_HUMAN	637.3385785	2	0.77929306		
FPSHLFLEQR	30.32332	Q96EV2	RBM33_HUMAN	425.2283273	3	0.77929306		
GGLQQPPHLPAGPHAHSPVPPGIK	20.73063	Q96EV2	RBM33_HUMAN	1195.148568	2	0.775895655		
GGLQQPPHLPAGPHAHSPVPPGIK	20.73063	Q96EV2	RBM33_HUMAN	797.1016537	3	0.775895655		
LPLQDQWR	34.4307	Q96EV2	RBM33_HUMAN	528.285815	2	0.772344649		
LPLQDQWR	34.4307	Q96EV2	RBM33_HUMAN	352.526485	3	0.772344649		
LPQPPEVGPQPAR	21.06413	Q96EV2	RBM33_HUMAN	693.3809755	2	0.763672113		
LPQPPEVGPQPAR	21.06413	Q96EV2	RBM33_HUMAN	462.5899253	3	0.763672113		
LSGGGGESDGFFHPEGQPQR	22.34713	Q96EV2	RBM33_HUMAN	1029.967392	2	0.863740861		
LSGGGGESDGFFHPEGQPQR	22.34713	Q96EV2	RBM33_HUMAN	686.9808693	3	0.863740861		
MMMTPPPVTPQQPK	39.48864	Q96EV2	RBM33_HUMAN	791.8936905	2	0.845405817		
MMMTPPPVTPQQPK	39.48864	Q96EV2	RBM33_HUMAN	528.2650687	3	0.845405817		
NIPETLELSAEAK	46.17339	Q96EV2	RBM33_HUMAN	707.8753875	2	0.810645044		
NIPETLELSAEAK	46.17339	Q96EV2	RBM33_HUMAN	472.2528667	3	0.810645044		
NQDVSISNVQPK	6.565224	Q96EV2	RBM33_HUMAN	664.8444215	2	0.837824345	2	Yes
NQDVSISNVQPK	6.565224	Q96EV2	RBM33_HUMAN	443.565556	3	0.837824345	2	
RPMQQMQPTAPR	-21.29398	Q96EV2	RBM33_HUMAN	720.864113	2	0.790977955		
RPMQQMQPTAPR	-21.29398	Q96EV2	RBM33_HUMAN	480.912017	3	0.790977955		

LFQPQPLQPLLPVQHPHHPSPPQGMHMPPQLETP	65.55598	Q96EV2	RBM33 HUMAN	2047.554378	2	0.677451611		
LFQPQPLQPLLPVQHPHHPSPPQGMHMPPQLETP	65.55598	Q96EV2	RBM33 HUMAN	1365.372194	3	0.677451611		
SLLMSVGPIQSLQMLPQQR	106.746	Q96EV2	RBM33 HUMAN	1063.577209	2	0.735078573	3	
SLLMSVGPIQSLQMLPQQR	106.746	Q96EV2	RBM33 HUMAN	709.3874143	3	0.735078573	3	Yes
TSNFVPSSANMQYQGQQMK	32.06667	Q96EV2	RBM33 HUMAN	1073.488784	2	0.73401767		
TSNFVPSSANMQYQGQQMK	32.06667	Q96EV2	RBM33 HUMAN	715.9951307	3	0.73401767		
TVPQSQTQPLHK	-27.27648	Q96EV2	RBM33 HUMAN	682.370608	2	0.719661474		
TVPQSQTQPLHK	-27.27648	Q96EV2	RBM33 HUMAN	455.2496803	3	0.719661474		
VLPIKPADVEEPAVPQTPR	46.67433	Q96EV2	RBM33 HUMAN	1028.576047	2	0.771731734		
VLPIKPADVEEPAVPQTPR	46.67433	Q96EV2	RBM33 HUMAN	686.053306	3	0.771731734		
GAHLTALEMLTAFASHIR	93.16458	Q96EL3	RM53 HUMAN	970.015102	2	0.785622418	3	
GAHLTALEMLTAFASHIR	93.16458	Q96EL3	RM53 HUMAN	647.0126763	3	0.785622418	3	Yes
HDGSEPCVDVLFGDGHR	37.75501	Q96EL3	RM53 HUMAN	948.9188555	2	0.750581443	3	
HDGSEPCVDVLFGDGHR	37.75501	Q96EL3	RM53 HUMAN	632.948512	3	0.750581443	3	Yes
TFLQTVSSEK	17.67205	Q96EL3	RM53 HUMAN	570.3013275	2	0.799265325	2	Yes
TFLQTVSSEK	17.67205	Q96EL3	RM53 HUMAN	380.5368267	3	0.799265325	2	
VQFCPFEK	29.74979	Q96EL3	RM53 HUMAN	527.755502	2	0.645179331	2	Yes
VQFCPFEK	29.74979	Q96EL3	RM53 HUMAN	352.172943	3	0.645179331	2	
AAQDFCQLVAQK	36.81802	Q96E22	NGBR HUMAN	689.843369	2	0.851721525		
AAQDFCQLVAQK	36.81802	Q96E22	NGBR HUMAN	460.231521	3	0.851721525		
FGPVDSTLGFLPWHIR	101.4085	Q96E22	NGBR HUMAN	921.4890455	2	0.761446834		
FGPVDSTLGFLPWHIR	101.4085	Q96E22	NGBR HUMAN	614.661972	3	0.761446834		
FGTWNWIWR	94.34058	Q96E22	NGBR HUMAN	633.3149065	2	0.74729073		
FGTWNWIWR	94.34058	Q96E22	NGBR HUMAN	422.5458793	3	0.74729073		
LMDEILK	33.07781	Q96E22	NGBR HUMAN	431.2416955	2	0.676010191		
LMDEILK	33.07781	Q96E22	NGBR HUMAN	287.8304053	3	0.676010191		
TLTSWLR	40.47417	Q96E22	NGBR HUMAN	438.7510765	2	0.674209714		
TLTSWLR	40.47417	Q96E22	NGBR HUMAN	292.8366593	3	0.674209714		
YSPEFANSNDKDDQVLNCHLAVK	28.47282	Q96E22	NGBR HUMAN	1332.622111	2	0.806217492		
YSPEFANSNDKDDQVLNCHLAVK	28.47282	Q96E22	NGBR HUMAN	888.750682	3	0.806217492		
AEQVDPNFYSK	15.92721	Q96DB5	RMD1 HUMAN	649.3071365	2	0.769245446		
AEQVDPNFYSK	15.92721	Q96DB5	RMD1 HUMAN	433.207366	3	0.769245446		
GFEVMGNPGTFK	45.64943	Q96DB5	RMD1 HUMAN	642.30863	2	0.789842606		
GFEVMGNPGTFK	45.64943	Q96DB5	RMD1 HUMAN	428.541695	3	0.789842606		
LAAFWLMK	71.11597	Q96DB5	RMD1 HUMAN	490.275873	2	0.674628675	2	Yes
LAAFWLMK	71.11597	Q96DB5	RMD1 HUMAN	327.1865237	3	0.674628675	2	
LLVYEALEYAK	68.75311	Q96DB5	RMD1 HUMAN	656.363919	2	0.783853531	2	Yes
LLVYEALEYAK	68.75311	Q96DB5	RMD1 HUMAN	437.9118877	3	0.783853531	2	
LYQLLTQYK	44.95358	Q96DB5	RMD1 HUMAN	585.3324225	2	0.830017149	2	Yes
LYQLLTQYK	44.95358	Q96DB5	RMD1 HUMAN	390.5575567	3	0.830017149	2	
MLFATPPSSTYEK	41.04725	Q96DB5	RMD1 HUMAN	736.361055	2	0.836824656	2	Yes
MLFATPPSSTYEK	41.04725	Q96DB5	RMD1 HUMAN	491.2433117	3	0.836824656	2	
NLLLLGK	44.1672	Q96DB5	RMD1 HUMAN	385.7609125	2	0.741675615		
NLLLLGK	44.1672	Q96DB5	RMD1 HUMAN	257.5098833	3	0.741675615		
VEEILEQADYLYESGETEK	73.6465	Q96DB5	RMD1 HUMAN	1123.023894	2	0.832848728		
VEEILEQADYLYESGETEK	73.6465	Q96DB5	RMD1 HUMAN	749.0185377	3	0.832848728		
WYAICLSDVGDYEGIK	88.44235	Q96DB5	RMD1 HUMAN	944.9434675	2	0.639385998		
WYAICLSDVGDYEGIK	88.44235	Q96DB5	RMD1 HUMAN	630.2982533	3	0.639385998		
DTSLASFIPAVNDLTSDLFR	178.553	Q96CS2	HAUS1 HUMAN	1091.555507	2	0.744385898		
DTSLASFIPAVNDLTSDLFR	178.553	Q96CS2	HAUS1 HUMAN	728.039613	3	0.744385898		
DVYLVIEDLK	83.3333	Q96CS2	HAUS1 HUMAN	603.8349985	2	0.709390283		
DVYLVIEDLK	83.3333	Q96CS2	HAUS1 HUMAN	402.8926073	3	0.709390283		
ELDSIEAELTR	49.57298	Q96CS2	HAUS1 HUMAN	638.3255305	2	0.780796528		
ELDSIEAELTR	49.57298	Q96CS2	HAUS1 HUMAN	425.8862953	3	0.780796528		
ETQVAAWLK	38.8108	Q96CS2	HAUS1 HUMAN	523.2880235	2	0.773228407		
ETQVAAWLK	38.8108	Q96CS2	HAUS1 HUMAN	349.194624	3	0.773228407		
GMDASLSHQSLVALSEK	35.31882	Q96CS2	HAUS1 HUMAN	886.9465495	2	0.746421576		
GMDASLSHQSLVALSEK	35.31882	Q96CS2	HAUS1 HUMAN	591.6336413	3	0.746421576		
IFGDHPIPQYEVNPR	35.70895	Q96CS2	HAUS1 HUMAN	891.4526555	2	0.863829613		
IFGDHPIPQYEVNPR	35.70895	Q96CS2	HAUS1 HUMAN	594.637712	3	0.863829613		
NLTATLVLEK	46.75095	Q96CS2	HAUS1 HUMAN	551.329888	2	0.768557429		
NLTATLVLEK	46.75095	Q96CS2	HAUS1 HUMAN	367.8892003	3	0.768557429		
YLNALVDSAVALETK	73.35949	Q96CS2	HAUS1 HUMAN	803.938515	2	0.768332362		
YLNALVDSAVALETK	73.35949	Q96CS2	HAUS1 HUMAN	536.2949517	3	0.768332362		
AMALLTALLQGASPVER	116.8282	Q96BJ8	ELMO3 HUMAN	870.9880215	2	0.83552897		
AMALLTALLQGASPVER	116.8282	Q96BJ8	ELMO3 HUMAN	580.9946227	3	0.83552897		
DAIPQLIQLDQAKPLAAVLK	101.8082	Q96BJ8	ELMO3 HUMAN	1073.136272	2	0.829755425		
DAIPQLIQLDQAKPLAAVLK	101.8082	Q96BJ8	ELMO3 HUMAN	715.7601227	3	0.829755425		
EVCDAWSLTHSER	19.92556	Q96BJ8	ELMO3 HUMAN	795.354828	2	0.78356564		
EVCDAWSLTHSER	19.92556	Q96BJ8	ELMO3 HUMAN	530.5724937	3	0.78356564		
GEEEAYLNFIAPSK	67.68031	Q96BJ8	ELMO3 HUMAN	784.386115	2	0.864242971		
GEEEAYLNFIAPSK	67.68031	Q96BJ8	ELMO3 HUMAN	523.2600183	3	0.864242971		
HMLDYLWQR	48.98991	Q96BJ8	ELMO3 HUMAN	631.3115025	2	0.662170172		
HMLDYLWQR	48.98991	Q96BJ8	ELMO3 HUMAN	421.2102767	3	0.662170172		
LDLEQLLTMETK	111.2523	Q96BJ8	ELMO3 HUMAN	717.381992	2	0.872025192		
LDLEQLLTMETK	111.2523	Q96BJ8	ELMO3 HUMAN	478.590603	3	0.872025192		
LGFSNSNPAQDLER	32.44438	Q96BJ8	ELMO3 HUMAN	774.376617	2	0.808165133		
LGFSNSNPAQDLER	32.44438	Q96BJ8	ELMO3 HUMAN	516.5870197	3	0.808165133		
LHQEGTLAPPILELR	56.83253	Q96BJ8	ELMO3 HUMAN	843.9810535	2	0.854323149		
LHQEGTLAPPILELR	56.83253	Q96BJ8	ELMO3 HUMAN	562.9899773	3	0.854323149		
LKPELMGLIR	47.55309	Q96BJ8	ELMO3 HUMAN	585.3579255	2	0.779720783		
LKPELMGLIR	47.55309	Q96BJ8	ELMO3 HUMAN	390.5745587	3	0.779720783		
LLQYGDMEEGASPPTLESLPEQLPVADMR	112.3622	Q96BJ8	ELMO3 HUMAN	1593.770856	2	0.712656438		
LLQYGDMEEGASPPTLESLPEQLPVADMR	112.3622	Q96BJ8	ELMO3 HUMAN	1062.849845	3	0.712656438		
LLVHLQVMNQQLQTK	43.853	Q96BJ8	ELMO3 HUMAN	897.0092885	2	0.838227689		
LLVHLQVMNQQLQTK	43.853	Q96BJ8	ELMO3 HUMAN	598.342134	3	0.838227689		
LVPLASDMIFAR	83.28999	Q96BJ8	ELMO3 HUMAN	666.871397	2	0.845276237		
LVPLASDMIFAR	83.28999	Q96BJ8	ELMO3 HUMAN	444.916873	3	0.845276237		
QAAFEVEGESSGAGLSADR	34.00632	Q96BJ8	ELMO3 HUMAN	940.935225	2	0.614643574		
QAAFEVEGESSGAGLSADR	34.00632	Q96BJ8	ELMO3 HUMAN	627.6260917	3	0.614643574		
TLALKPTSLELFR	63.67135	Q96BJ8	ELMO3 HUMAN	744.943408	2	0.838551044		
TLALKPTSLELFR	63.67135	Q96BJ8	ELMO3 HUMAN	496.9648803	3	0.838551044		
TPLDPYSQEQR	13.29138	Q96BJ8	ELMO3 HUMAN	667.3233185	2	0.746215224		
TPLDPYSQEQR	13.29138	Q96BJ8	ELMO3 HUMAN	445.218154	3	0.746215224		
VNALTYGEVLR	44.8871	Q96BJ8	ELMO3 HUMAN	617.8436895	2	0.804576993		
VNALTYGEVLR	44.8871	Q96BJ8	ELMO3 HUMAN	412.2317347	3	0.804576993		
VPPGLLALDNMLYFSR	130.2489	Q96BJ8	ELMO3 HUMAN	903.4851095	2	0.686822951		
VPPGLLALDNMLYFSR	130.2489	Q96BJ8	ELMO3 HUMAN	602.659348	3	0.686822951		
YALQFADGHR	9.030514	Q96BJ8	ELMO3 HUMAN	589.2916245	2	0.838367581		
YALQFADGHR	9.030514	Q96BJ8	ELMO3 HUMAN	393.1970247	3	0.838367581		
ANSPSLFGTEGKPK	-0.124279	Q96B97	SH3K1 HUMAN	716.875722	2	0.770592451		

ANSPSLFGTEGKPK	-0.124279	Q96B97	SH3K1 HUMAN	478.2530897	3	0.770592451		
APEKPLHEVPSGNSLLSSETILR	46.12325	Q96B97	SH3K1 HUMAN	1237.666651	2	0.79842329		
APEKPLHEVPSGNSLLSSETILR	46.12325	Q96B97	SH3K1 HUMAN	825.4470423	3	0.79842329		
LPPKPGTMAAGGGGPAPLSSAAPSPLSSSLGTAG	56.38695	Q96B97	SH3K1 HUMAN	1705.878445	2	0.71995908		
LPPKPGTMAAGGGGPAPLSSAAPSPLSSSLGTAG	56.38695	Q96B97	SH3K1 HUMAN	1137.588238	3	0.71995908		
EDGGWWEGQINGR	48.79324	Q96B97	SH3K1 HUMAN	752.334753	2	0.605884552		
EDGGWWEGQINGR	48.79324	Q96B97	SH3K1 HUMAN	501.8924437	3	0.605884552		
EGDIVTLINK	46.65475	Q96B97	SH3K1 HUMAN	551.311695	2	0.665457249		
EGDIVTLINK	46.65475	Q96B97	SH3K1 HUMAN	367.8770717	3	0.665457249		
ELSGESDELGISQDEQLSK	42.49441	Q96B97	SH3K1 HUMAN	1032.484944	2	0.753344774		
ELSGESDELGISQDEQLSK	42.49441	Q96B97	SH3K1 HUMAN	688.6592377	3	0.753344774		
GLFPDNFVR	60.27563	Q96B97	SH3K1 HUMAN	532.780365	2	0.842836261		
GLFPDNFVR	60.27563	Q96B97	SH3K1 HUMAN	355.5228517	3	0.842836261		
GVFPDNFVK	41.6747	Q96B97	SH3K1 HUMAN	511.769466	2	0.720034003		
GVFPDNFVK	41.6747	Q96B97	SH3K1 HUMAN	341.5155857	3	0.720034003		
GVGFGDIFK	59.21771	Q96B97	SH3K1 HUMAN	470.2509095	2	0.827052772		
GVGFGDIFK	59.21771	Q96B97	SH3K1 HUMAN	313.836548	3	0.827052772		
IDLAGSSLSGILDKDLSDR	78.58221	Q96B97	SH3K1 HUMAN	988.0212995	2	0.781700373		
IDLAGSSLSGILDKDLSDR	78.58221	Q96B97	SH3K1 HUMAN	659.016808	3	0.781700373		
KPPPPSAPVIK	-6.521366	Q96B97	SH3K1 HUMAN	565.8507905	2	0.790107191		
KPPPPSAPVIK	-6.521366	Q96B97	SH3K1 HUMAN	377.569802	3	0.790107191		
LDLQKPSVPAIPPK	40.14464	Q96B97	SH3K1 HUMAN	751.951233	2	0.790904522		
LDLQKPSVPAIPPK	40.14464	Q96B97	SH3K1 HUMAN	501.6367637	3	0.790904522		
LLPPDFEK	32.71819	Q96B97	SH3K1 HUMAN	479.766392	2	0.701194227		
LLPPDFEK	32.71819	Q96B97	SH3K1 HUMAN	320.180203	3	0.701194227		
LQMEVNDIK	22.63058	Q96B97	SH3K1 HUMAN	545.284623	2	0.748712242		
LQMEVNDIK	22.63058	Q96B97	SH3K1 HUMAN	363.8590237	3	0.748712242		
MEPAASSQAAVEELR	27.29272	Q96B97	SH3K1 HUMAN	794.885961	2	0.815372109		
MEPAASSQAAVEELR	27.29272	Q96B97	SH3K1 HUMAN	530.2599157	3	0.815372109		
RPERPVGPLTHTR	-28.5859	Q96B97	SH3K1 HUMAN	758.4293225	2	0.669669151		
RPERPVGPLTHTR	-28.5859	Q96B97	SH3K1 HUMAN	505.95549	3	0.669669151		
RPPSQSLTSSSLSSPDIFDSPSPEEDK	56.13942	Q96B97	SH3K1 HUMAN	1445.68362	2	0.687005639		
RPPSQSLTSSSLSSPDIFDSPSPEEDK	56.13942	Q96B97	SH3K1 HUMAN	964.1250213	3	0.687005639		
SIEVENDFLPVEK	62.66363	Q96B97	SH3K1 HUMAN	759.8884945	2	0.862689853		
SIEVENDFLPVEK	62.66363	Q96B97	SH3K1 HUMAN	506.9282713	3	0.862689853		
SNDIDLEGFDSVVSSTEK	71.48654	Q96B97	SH3K1 HUMAN	971.4503725	2	0.813518524		
SNDIDLEGFDSVVSSTEK	71.48654	Q96B97	SH3K1 HUMAN	647.9695233	3	0.813518524		
TGMFPSNFIK	53.88718	Q96B97	SH3K1 HUMAN	571.2897085	2	0.766147792		
TGMFPSNFIK	53.88718	Q96B97	SH3K1 HUMAN	381.1957473	3	0.766147792		
TVTISQVSDNK	-2.160229	Q96B97	SH3K1 HUMAN	596.314966	2	0.670830846		
TVTISQVSDNK	-2.160229	Q96B97	SH3K1 HUMAN	397.8792523	3	0.670830846		
VIFPYEAQNDDELTIK	71.22643	Q96B97	SH3K1 HUMAN	947.975826	2	0.845497489		
VIFPYEAQNDDELTIK	71.22643	Q96B97	SH3K1 HUMAN	632.3198257	3	0.845497489		
ALGTAADLGTLAPR	48.95237	Q96B70	LENG9 HUMAN	663.8729835	2	0.82604301		
ALGTAADLGTLAPR	48.95237	Q96B70	LENG9 HUMAN	442.9179307	3	0.82604301		
ALLAPGLNAPPR	45.14964	Q96B70	LENG9 HUMAN	595.3567715	2	0.81760323		
ALLAPGLNAPPR	45.14964	Q96B70	LENG9 HUMAN	397.240456	3	0.81760323		
EAQALGVPGGSAETTEAEWGPAAWPEDK	80.82054	Q96B70	LENG9 HUMAN	1427.662493	2	0.727662921		
EAQALGVPGGSAETTEAEWGPAAWPEDK	80.82054	Q96B70	LENG9 HUMAN	952.110937	3	0.727662921		
EPELPQEAPATEPAPPPACR	37.11395	Q96B70	LENG9 HUMAN	1079.018236	2	0.8089692		
EPELPQEAPATEPAPPPACR	37.11395	Q96B70	LENG9 HUMAN	719.6814323	3	0.8089692		
PTILDAPNTEGAHGAEGAEWTLAGTGQEQAAF	64.84792	Q96B70	LENG9 HUMAN	1708.821849	2	0.845169902		
PTILDAPNTEGAHGAEGAEWTLAGTGQEQAAF	64.84792	Q96B70	LENG9 HUMAN	1139.550507	3	0.845169902		
LAGAGEEAAAIGALR	40.73013	Q96B70	LENG9 HUMAN	685.37589	2	0.838047087		
LAGAGEEAAAIGALR	40.73013	Q96B70	LENG9 HUMAN	457.2532017	3	0.838047087		
LAGVTEEALKPTAATR	18.97671	Q96B70	LENG9 HUMAN	814.454869	2	0.796883702		
LAGVTEEALKPTAATR	18.97671	Q96B70	LENG9 HUMAN	543.3058543	3	0.796883702		
LDPADFSVGYVDR	66.84184	Q96B70	LENG9 HUMAN	727.3520755	2	0.810184181	2	Yes
LDPADFSVGYVDR	66.84184	Q96B70	LENG9 HUMAN	485.2373253	3	0.810184181	2	
LEAEGLSTLQSPGQLHPHLTVAK	45.26881	Q96B70	LENG9 HUMAN	1213.656088	2	0.748275578		
LEAEGLSTLQSPGQLHPHLTVAK	45.26881	Q96B70	LENG9 HUMAN	809.4400003	3	0.748275578		
TDLVFGSGSAAGR	24.71506	Q96B70	LENG9 HUMAN	619.312758	2	0.805119872		
TDLVFGSGSAAGR	24.71506	Q96B70	LENG9 HUMAN	413.2111137	3	0.805119872		
TGGPFQPLAEIR	55.55198	Q96B70	LENG9 HUMAN	643.349144	2	0.844409704		
TGGPFQPLAEIR	55.55198	Q96B70	LENG9 HUMAN	429.235371	3	0.844409704		
DFGNYLFNFASAATK	117.8098	Q96A49	SYAP1 HUMAN	833.399557	2	0.821533263	2	Yes
DFGNYLFNFASAATK	117.8098	Q96A49	SYAP1 HUMAN	555.9356463	3	0.821533263	2	
ELQQELQEYEVVTESEK	61.20184	Q96A49	SYAP1 HUMAN	1041.000227	2	0.815118253		
ELQQELQEYEVVTESEK	61.20184	Q96A49	SYAP1 HUMAN	694.3360927	3	0.815118253		
EMEQLVLDK	35.54372	Q96A49	SYAP1 HUMAN	552.784456	2	0.6302948	2	Yes
EMEQLVLDK	35.54372	Q96A49	SYAP1 HUMAN	368.8589123	3	0.6302948	2	
EQDLPLAEAVRPK	30.83001	Q96A49	SYAP1 HUMAN	733.404647	2	0.765350997	3	
EQDLPLAEAVRPK	30.83001	Q96A49	SYAP1 HUMAN	489.272373	3	0.765350997	3	Yes
ITESVAETAQTIK	19.58979	Q96A49	SYAP1 HUMAN	695.8753885	2	0.804114699	2	Yes
ITESVAETAQTIK	19.58979	Q96A49	SYAP1 HUMAN	464.2528673	3	0.804114699	2	
QSAQLTALAAQQQAAGK	22.45177	Q96A49	SYAP1 HUMAN	842.9530255	2	0.771352053		
QSAQLTALAAQQQAAGK	22.45177	Q96A49	SYAP1 HUMAN	562.3046253	3	0.771352053		
TIIGDFQK	21.64793	Q96A49	SYAP1 HUMAN	461.256192	2	0.721546948		
TIIGDFQK	21.64793	Q96A49	SYAP1 HUMAN	307.8400697	3	0.721546948		
ADGIVPETWIDGPK	67.31661	Q96A35	RM24 HUMAN	749.3833805	2	0.856778383	2	Yes
ADGIVPETWIDGPK	67.31661	Q96A35	RM24 HUMAN	499.924862	3	0.856778383	2	
GTMIPSEAPLLHR	38.47233	Q96A35	RM24 HUMAN	711.3826605	2	0.864591658		
GTMIPSEAPLLHR	38.47233	Q96A35	RM24 HUMAN	474.5910487	3	0.864591658		
IIPKPEFPR	15.84686	Q96A35	RM24 HUMAN	548.829858	2	0.735309958	2	Yes
IIPKPEFPR	15.84686	Q96A35	RM24 HUMAN	366.2225137	3	0.735309958	2	
LSALLALASK	54.12454	Q96A35	RM24 HUMAN	493.816416	2	0.696604431		
LSALLALASK	54.12454	Q96A35	RM24 HUMAN	329.5468857	3	0.696604431		
NWVVVGGLNTHYR	40.1089	Q96A35	RM24 HUMAN	757.897319	2	0.668513834	3	
NWVVVGGLNTHYR	40.1089	Q96A35	RM24 HUMAN	505.600821	3	0.668513834	3	Yes
TLQEEVMEAMGIK	76.74897	Q96A35	RM24 HUMAN	739.865652	2	0.802013516	2	Yes
TLQEEVMEAMGIK	76.74897	Q96A35	RM24 HUMAN	493.5797097	3	0.802013516	2	
APDMDQLVLPSK	51.09893	Q969Z3	MARC2 HUMAN	657.3426695	2	0.749132872	2	Yes
APDMDQLVLPSK	51.09893	Q969Z3	MARC2 HUMAN	438.564388	3	0.749132872	2	
LSPLFGIYYSVEK	90.34984	Q969Z3	MARC2 HUMAN	758.4088575	2	0.604980886		
LSPLFGIYYSVEK	90.34984	Q969Z3	MARC2 HUMAN	505.9418467	3	0.604980886		
LVQFETNMK	18.94825	Q969Z3	MARC2 HUMAN	555.287166	2	0.7735641	2	Yes
LVQFETNMK	18.94825	Q969Z3	MARC2 HUMAN	370.5273857	3	0.7735641	2	
LWIYPVK	50.50341	Q969Z3	MARC2 HUMAN	459.7765585	2	0.792385757	2	Yes
LWIYPVK	50.50341	Q969Z3	MARC2 HUMAN	306.8536473	3	0.792385757	2	
WFTNFLK	68.17191	Q969Z3	MARC2 HUMAN	478.2559945	2	0.657530785	2	Yes
WFTNFLK	68.17191	Q969Z3	MARC2 HUMAN	319.1732713	3	0.657530785	2	

ALQVALGAHLR	24.18829	Q969Y2	GTPB3 HUMAN	574.849114	2	0.815899193		
ALQVALGAHLR	24.18829	Q969Y2	GTPB3 HUMAN	383.5686843	3	0.815899193		
DLALAAEALR	57.26181	Q969Y2	GTPB3 HUMAN	521.798755	2	0.814867735		
DLALAAEALR	57.26181	Q969Y2	GTPB3 HUMAN	348.2017783	3	0.814867735		
DVLETPVDLAGFPVLLSDTAGLR	154.904	Q969Y2	GTPB3 HUMAN	1199.647397	2	0.639953196		
DVLETPVDLAGFPVLLSDTAGLR	154.904	Q969Y2	GTPB3 HUMAN	800.100873	3	0.639953196		
EGVGPVEQEGVR	2.41869	Q969Y2	GTPB3 HUMAN	628.3180405	2	0.776551843		
EGVGPVEQEGVR	2.41869	Q969Y2	GTPB3 HUMAN	419.2146353	3	0.776551843		
GLWTLAAQAAR	58.65088	Q969Y2	GTPB3 HUMAN	579.325472	2	0.672962427		
GLWTLAAQAAR	58.65088	Q969Y2	GTPB3 HUMAN	386.552923	3	0.672962427		
HQHHLQGCLDALGHYK	-5.978828	Q969Y2	GTPB3 HUMAN	957.463564	2	0.716748774		
HQHHLQGCLDALGHYK	-5.978828	Q969Y2	GTPB3 HUMAN	638.6449843	3	0.716748774		
KPVSIVSPEPGTTR	1.7887	Q969Y2	GTPB3 HUMAN	734.412472	2	0.78222847		
KPVSIVSPEPGTTR	1.7887	Q969Y2	GTPB3 HUMAN	489.9442563	3	0.78222847		
LLLVLNK	42.88177	Q969Y2	GTPB3 HUMAN	406.7843875	2	0.740012646		
LLLVLNK	42.88177	Q969Y2	GTPB3 HUMAN	271.5255333	3	0.740012646		
LNLTEVEGLADLIHAETEAQR	110.5319	Q969Y2	GTPB3 HUMAN	1161.600978	2	0.774010301	3	
LNLTEVEGLADLIHAETEAQR	110.5319	Q969Y2	GTPB3 HUMAN	774.7365937	3	0.774010301	3	Yes
SGVHVVVTGPPNAGK	-3.164875	Q969Y2	GTPB3 HUMAN	709.891707	2	0.756929398		
SGVHVVVTGPPNAGK	-3.164875	Q969Y2	GTPB3 HUMAN	473.5970797	3	0.756929398		
SSLVNLLSR	55.34797	Q969Y2	GTPB3 HUMAN	494.7934715	2	0.673733234		
SSLVNLLSR	55.34797	Q969Y2	GTPB3 HUMAN	330.198256	3	0.673733234		
AAQLVDKDSTFLSTLEHHLSR	51.98038	Q969V3	NCLN HUMAN	1184.616962	2	0.821046293	4	
AAQLVDKDSTFLSTLEHHLSR	51.98038	Q969V3	NCLN HUMAN	790.080583	3	0.821046293	4	
AMAAVPQDVVR	23.44008	Q969V3	NCLN HUMAN	578.81134	2	0.711532533	2	Yes
AMAAVPQDVVR	23.44008	Q969V3	NCLN HUMAN	386.2101683	3	0.711532533	2	
AVSDWLIASVEGR	78.18756	Q969V3	NCLN HUMAN	701.8704395	2	0.800034642	2	Yes
AVSDWLIASVEGR	78.18756	Q969V3	NCLN HUMAN	468.249568	3	0.800034642	2	
DPEFVFYDQLK	82.6442	Q969V3	NCLN HUMAN	700.8408125	2	0.804157734	2	Yes
DPEFVFYDQLK	82.6442	Q969V3	NCLN HUMAN	467.56315	3	0.804157734	2	
EGTLQHAFLR	12.69829	Q969V3	NCLN HUMAN	586.315104	2	0.782161117	3	
EGTLQHAFLR	12.69829	Q969V3	NCLN HUMAN	391.2126777	3	0.782161117	3	Yes
ELETVAAHQFPEVR	33.65962	Q969V3	NCLN HUMAN	813.418286	2	0.780053377	3	
ELETVAAHQFPEVR	33.65962	Q969V3	NCLN HUMAN	542.614799	3	0.780053377	3	Yes
GSSLHLHVSKPPR	-27.243	Q969V3	NCLN HUMAN	707.8998655	2	0.751979232	4	
GSSLHLHVSKPPR	-27.243	Q969V3	NCLN HUMAN	472.2691853	3	0.751979232	4	
INLAEDVLAWEHER	73.25594	Q969V3	NCLN HUMAN	847.929017	2	0.897981286	3	
INLAEDVLAWEHER	73.25594	Q969V3	NCLN HUMAN	565.621953	3	0.897981286	3	Yes
LLDFSYEQYQK	51.2522	Q969V3	NCLN HUMAN	717.35154	2	0.851113319	2	Yes
LLDFSYEQYQK	51.2522	Q969V3	NCLN HUMAN	478.5703017	3	0.851113319	2	
LPAFTLSHLESHR	30.22014	Q969V3	NCLN HUMAN	754.404981	2	0.726848662	4	
LPAFTLSHLESHR	30.22014	Q969V3	NCLN HUMAN	503.2725957	3	0.726848662	4	
MLEEAGEVLENMLK	96.61768	Q969V3	NCLN HUMAN	803.3973155	2	0.602897704		
MLEEAGEVLENMLK	96.61768	Q969V3	NCLN HUMAN	535.934152	3	0.602897704		
MQQYDLQGQPYGTR	22.55815	Q969V3	NCLN HUMAN	842.8915705	2	0.828818083		
MQQYDLQGQPYGTR	22.55815	Q969V3	NCLN HUMAN	562.2636553	3	0.828818083		
THAAYNLLFFASGGGK	61.94098	Q969V3	NCLN HUMAN	827.423367	2	0.792011738	3	
THAAYNLLFFASGGGK	61.94098	Q969V3	NCLN HUMAN	551.9515197	3	0.792011738	3	Yes
VIYNLTEK	15.10054	Q969V3	NCLN HUMAN	490.2771195	2	0.640404463		
VIYNLTEK	15.10054	Q969V3	NCLN HUMAN	327.1873547	3	0.640404463		
EIQMAVLLK	60.99644	Q969U7	PSMG2 HUMAN	522.810277	2	0.609951973	2	Yes
EIQMAVLLK	60.99644	Q969U7	PSMG2 HUMAN	348.8761263	3	0.609951973	2	
SLNWEEMEK	31.6039	Q969U7	PSMG2 HUMAN	583.263887	2	0.76069653	2	Yes
SLNWEEMEK	31.6039	Q969U7	PSMG2 HUMAN	389.178533	3	0.76069653	2	
TLYDESCSK	-16.58399	Q969U7	PSMG2 HUMAN	551.740241	2	0.633821309	2	Yes
TLYDESCSK	-16.58399	Q969U7	PSMG2 HUMAN	368.162769	3	0.633821309	2	
VIVLSSSHSYQR	-3.359997	Q969U7	PSMG2 HUMAN	688.370602	2	0.747095883	3	
VIVLSSSHSYQR	-3.359997	Q969U7	PSMG2 HUMAN	459.2496763	3	0.747095883	3	Yes
YLLTPSMQK	27.63078	Q969U7	PSMG2 HUMAN	540.79208	2	0.742859125	2	Yes
YLLTPSMQK	27.63078	Q969U7	PSMG2 HUMAN	360.863995	3	0.742859125	2	
AALMDASQLEPGEK	33.26058	Q969F9	HPS3 HUMAN	730.359048	2	0.873951077		
AALMDASQLEPGEK	33.26058	Q969F9	HPS3 HUMAN	487.2419737	3	0.873951077		
AELLEAFK	43.48032	Q969F9	HPS3 HUMAN	460.758567	2	0.681760073		
AELLEAFK	43.48032	Q969F9	HPS3 HUMAN	307.5083197	3	0.681760073		
DALFVAAGCK	33.50604	Q969F9	HPS3 HUMAN	526.266234	2	0.646575391		
DALFVAAGCK	33.50604	Q969F9	HPS3 HUMAN	351.1800977	3	0.646575391		
EEDPYMDTTLK	22.65176	Q969F9	HPS3 HUMAN	671.2981205	2	0.732243955		
EEDPYMDTTLK	22.65176	Q969F9	HPS3 HUMAN	447.868022	3	0.732243955		
ETQPGLLVASVLGLQK	96.10942	Q969F9	HPS3 HUMAN	826.983262	2	0.735575914		
ETQPGLLVASVLGLQK	96.10942	Q969F9	HPS3 HUMAN	551.6581163	3	0.735575914		
FAPDISSYVLSDDIK	85.70206	Q969F9	HPS3 HUMAN	835.4201545	2	0.827896535		
FAPDISSYVLSDDIK	85.70206	Q969F9	HPS3 HUMAN	557.2827113	3	0.827896535		
GDLLVGCTNK	11.9178	Q969F9	HPS3 HUMAN	538.774423	2	0.727339625		
GDLLVGCTNK	11.9178	Q969F9	HPS3 HUMAN	359.5188903	3	0.727339625		
GLIFYINHSLYENLDEELNEELAAK	119.1861	Q969F9	HPS3 HUMAN	1469.230001	2	0.690829813		
GLIFYINHSLYENLDEELNEELAAK	119.1861	Q969F9	HPS3 HUMAN	979.8226087	3	0.690829813		
GQIVPTELALHLK	52.87357	Q969F9	HPS3 HUMAN	709.922476	2	0.764232397		
GQIVPTELALHLK	52.87357	Q969F9	HPS3 HUMAN	473.6175923	3	0.764232397		
IGIEEADSFFK	64.26917	Q969F9	HPS3 HUMAN	628.3144345	2	0.843465567		
IGIEEADSFFK	64.26917	Q969F9	HPS3 HUMAN	419.2122313	3	0.843465567		
LAYSEAGDYLVAIEEK	73.34097	Q969F9	HPS3 HUMAN	885.94399	2	0.729897559		
LAYSEAGDYLVAIEEK	73.34097	Q969F9	HPS3 HUMAN	590.9652683	3	0.729897559		
LDSQHSHLTLPYYK	19.07972	Q969F9	HPS3 HUMAN	851.433927	2	0.863745093		
LDSQHSHLTLPYYK	19.07972	Q969F9	HPS3 HUMAN	567.9585597	3	0.863745093		
LDTSGFSSILVTLTK	110.3402	Q969F9	HPS3 HUMAN	791.440895	2	0.749313653		
LDTSGFSSILVTLTK	110.3402	Q969F9	HPS3 HUMAN	527.963205	3	0.749313653		
LHSLQLLPIYQTGSLTSDGK	69.91577	Q969F9	HPS3 HUMAN	1086.089514	2	0.780360222		
LHSLQLLPIYQTGSLTSDGK	69.91577	Q969F9	HPS3 HUMAN	724.3956173	3	0.780360222		
LLPELCQR	27.82895	Q969F9	HPS3 HUMAN	514.782051	2	0.783467054		
LLPELCQR	27.82895	Q969F9	HPS3 HUMAN	343.5239757	3	0.783467054		
LTSQYIWR	31.25539	Q969F9	HPS3 HUMAN	533.788186	2	0.776785612	2	Yes
LTSQYIWR	31.25539	Q969F9	HPS3 HUMAN	356.1947323	3	0.776785612	2	
LVCGFILEPR	62.38532	Q969F9	HPS3 HUMAN	602.331908	2	0.729893088		
LVCGFILEPR	62.38532	Q969F9	HPS3 HUMAN	401.890547	3	0.729893088		
LVLFSLK	59.28163	Q969F9	HPS3 HUMAN	410.271113	2	0.678073108		
LVLFSLK	59.28163	Q969F9	HPS3 HUMAN	273.850017	3	0.678073108		
MGDLDMHR	-15.78454	Q969F9	HPS3 HUMAN	487.713311	2	0.819505513		
MGDLDMHR	-15.78454	Q969F9	HPS3 HUMAN	325.478149	3	0.819505513		
MIGHNVEGPFSK	5.40852	Q969F9	HPS3 HUMAN	658.3273535	2	0.696478426		
MIGHNVEGPFSK	5.40852	Q969F9	HPS3 HUMAN	439.220844	3	0.696478426		
MSGLSMAEVLAR	65.02616	Q969F9	HPS3 HUMAN	632.8235895	2	0.779050469		

MSGLSMAEVLAR	65.02616	Q969F9	HPS3 HUMAN	422.2183347	3	0.779050469		
NINPLTAMSYLR	75.67186	Q969F9	HPS3 HUMAN	696.869381	2	0.801504493		
NINPLTAMSYLR	75.67186	Q969F9	HPS3 HUMAN	464.915529	3	0.801504493		
SEQSGLSVTLESTGLADEK	51.0952	Q969F9	HPS3 HUMAN	975.979298	2	0.762120247		
SEQSGLSVTLESTGLADEK	51.0952	Q969F9	HPS3 HUMAN	650.988807	3	0.762120247		
SVELMSVYQYPEK	54.73406	Q969F9	HPS3 HUMAN	786.8848895	2	0.852278531		
SVELMSVYQYPEK	54.73406	Q969F9	HPS3 HUMAN	524.925868	3	0.852278531		
TDWTVEDGLQK	34.31771	Q969F9	HPS3 HUMAN	646.312424	2	0.854721606		
TDWTVEDGLQK	34.31771	Q969F9	HPS3 HUMAN	431.210891	3	0.854721606		
TQSCIHLLSEAHLLVR	48.28203	Q969F9	HPS3 HUMAN	939.0072765	2	0.730333328		
TQSCIHLLSEAHLLVR	48.28203	Q969F9	HPS3 HUMAN	626.3407927	3	0.730333328		
VEAFAVAGQELCQPR	49.25671	Q969F9	HPS3 HUMAN	837.9175965	2	0.836936176		
VEAFAVAGQELCQPR	49.25671	Q969F9	HPS3 HUMAN	558.9476727	3	0.836936176		
VVQMFYVAEPK	50.87124	Q969F9	HPS3 HUMAN	655.844844	2	0.689817667		
VVQMFYVAEPK	50.87124	Q969F9	HPS3 HUMAN	437.5658377	3	0.689817667		
YQIINEEFSLLDFER	111.0105	Q969F9	HPS3 HUMAN	958.4759925	2	0.728485882		
YQIINEEFSLLDFER	111.0105	Q969F9	HPS3 HUMAN	639.3199367	3	0.728485882		
YQLYLSSLK	46.55826	Q969F9	HPS3 HUMAN	557.811322	2	0.630870163		
YQLYLSSLK	46.55826	Q969F9	HPS3 HUMAN	372.2101563	3	0.630870163		
EEDIVEDGWTIVR	72.02896	Q969E8	TSR2 HUMAN	780.8812015	2	0.727971196		
EEDIVEDGWTIVR	72.02896	Q969E8	TSR2 HUMAN	520.9234093	3	0.727971196		
ENTPSEANLQEEEVR	11.92558	Q93062	RBPMS HUMAN	872.903393	2	0.696696162	2	Yes
ENTPSEANLQEEEVR	11.92558	Q93062	RBPMS HUMAN	582.271537	3	0.696696162	2	
FDPEIPQTLR	48.47952	Q93062	RBPMS HUMAN	608.3225945	2	0.846392035		
FDPEIPQTLR	48.47952	Q93062	RBPMS HUMAN	405.884338	3	0.846392035		
LVGTPNPSTPLPNTVPQFIAR	83.09695	Q93062	RBPMS HUMAN	1110.113328	2	0.796500564		
LVGTPNPSTPLPNTVPQFIAR	83.09695	Q93062	RBPMS HUMAN	740.4114933	3	0.796500564		
TLFVSGLPLDIKPR	76.68266	Q93062	RBPMS HUMAN	778.4645075	2	0.807251215	3	
TLFVSGLPLDIKPR	76.68266	Q93062	RBPMS HUMAN	519.31228	3	0.807251215	3	Yes
WLPPSEATSQGWK	40.71047	Q93062	RBPMS HUMAN	743.87044	2	0.652286708		
WLPPSEATSQGWK	40.71047	Q93062	RBPMS HUMAN	496.2495683	3	0.652286708		
ATNNNLER	-50	Q92995	UBP13 HUMAN	466.2337785	2	0.600590765		
ATNNNLER	-50	Q92995	UBP13 HUMAN	311.1584607	3	0.600590765		
AYVGNLPR	5.199303	Q92995	UBP13 HUMAN	445.2486965	2	0.756845951		
AYVGNLPR	5.199303	Q92995	UBP13 HUMAN	297.168406	3	0.756845951		
DELIAYELTR	62.98335	Q92995	UBP13 HUMAN	611.81988	2	0.790236712		
DELIAYELTR	62.98335	Q92995	UBP13 HUMAN	408.2158617	3	0.790236712		
DLGYMYFYR	73.06752	Q92995	UBP13 HUMAN	614.279328	2	0.777043641		
DLGYMYFYR	73.06752	Q92995	UBP13 HUMAN	409.8554937	3	0.777043641		
FASFPEYLVVQIK	95.89073	Q92995	UBP13 HUMAN	770.9246795	2	0.802699685		
FASFPEYLVVQIK	95.89073	Q92995	UBP13 HUMAN	514.285728	3	0.802699685		
FDVSIDMPDLLDINHLR	119.5324	Q92995	UBP13 HUMAN	1007.009681	2	0.735761046		
FDVSIDMPDLLDINHLR	119.5324	Q92995	UBP13 HUMAN	671.675729	3	0.735761046		
GALFGMPGGSGGR	37.85017	Q92995	UBP13 HUMAN	582.2854895	2	0.730090141		
GALFGMPGGSGGR	37.85017	Q92995	UBP13 HUMAN	388.526268	3	0.730090141		
GLQPGEEELPDISPPIVIPDDSK	109.1266	Q92995	UBP13 HUMAN	1223.123951	2	0.774232745		
GLQPGEEELPDISPPIVIPDDSK	109.1266	Q92995	UBP13 HUMAN	815.751909	3	0.774232745		
IFDYSPLDPTQDFNTQMTK	92.11588	Q92995	UBP13 HUMAN	1131.025722	2	0.774423897		
IFDYSPLDPTQDFNTQMTK	92.11588	Q92995	UBP13 HUMAN	754.3530897	3	0.774423897		
IFLDLDTDDDLNSDDYEYEDEAK	87.19917	Q92995	UBP13 HUMAN	1377.07778	2	0.727880359		
IFLDLDTDDDLNSDDYEYEDEAK	87.19917	Q92995	UBP13 HUMAN	918.3877947	3	0.727880359		
IGSENPSDVFR	27.38239	Q92995	UBP13 HUMAN	610.7994825	2	0.751341641	2	Yes
IGSENPSDVFR	27.38239	Q92995	UBP13 HUMAN	407.5355967	3	0.751341641	2	
LGHGLLSGQYSKPPVK	5.413746	Q92995	UBP13 HUMAN	840.9757665	2	0.847216487		
LGHGLLSGQYSKPPVK	5.413746	Q92995	UBP13 HUMAN	560.9864527	3	0.847216487		
LGTITPDGADVYSFQEEEPVLDPHLAK	83.73149	Q92995	UBP13 HUMAN	1471.227464	2	0.81384021	3	
LGTITPDGADVYSFQEEEPVLDPHLAK	83.73149	Q92995	UBP13 HUMAN	981.154251	3	0.81384021	3	Yes
LKPMYGPGYTGLK	20.20881	Q92995	UBP13 HUMAN	712.8844965	2	0.869672179		
LKPMYGPGYTGLK	20.20881	Q92995	UBP13 HUMAN	475.5922727	3	0.869672179		
MAAGDIGELLVPHMPTIR	90.82072	Q92995	UBP13 HUMAN	961.0058885	2	0.718151152	3	
MAAGDIGELLVPHMPTIR	90.82072	Q92995	UBP13 HUMAN	641.006534	3	0.718151152	3	Yes
TGQSVYMHLK	-8.624073	Q92995	UBP13 HUMAN	582.298061	2	0.656932771		
TGQSVYMHLK	-8.624073	Q92995	UBP13 HUMAN	388.534649	3	0.656932771		
VDYLMQLPVAMEAATNK	97.44136	Q92995	UBP13 HUMAN	947.476625	2	0.78480345		
VDYLMQLPVAMEAATNK	97.44136	Q92995	UBP13 HUMAN	631.987025	3	0.78480345		
VSEWEVIQESGTK	38.55363	Q92995	UBP13 HUMAN	746.3704695	2	0.871944129	2	Yes
VSEWEVIQESGTK	38.55363	Q92995	UBP13 HUMAN	497.9162547	3	0.871944129	2	
YANNLTQLDNGVR	27.47565	Q92995	UBP13 HUMAN	739.3738735	2	0.758054137		
YANNLTQLDNGVR	27.47565	Q92995	UBP13 HUMAN	493.2518573	3	0.758054137		
ALVELLR	47.04771	Q92974	ARHG2 HUMAN	407.2638195	2	0.673542082		
ALVELLR	47.04771	Q92974	ARHG2 HUMAN	271.8451547	3	0.673542082		
DFAADSWSLAVDSSFLQQHK	101.5441	Q92974	ARHG2 HUMAN	1126.535106	2	0.64166826	3	
DFAADSWSLAVDSSFLQQHK	101.5441	Q92974	ARHG2 HUMAN	751.3593457	3	0.64166826	3	Yes
DLLVGPGVELLLTPR	134.9005	Q92974	ARHG2 HUMAN	796.4750725	2	0.819201291		
DLLVGPGVELLLTPR	134.9005	Q92974	ARHG2 HUMAN	531.3193233	3	0.819201291		
EDFPLIETEDEAYLR	86.59512	Q92974	ARHG2 HUMAN	920.4365335	2	0.765966177		
EDFPLIETEDEAYLR	86.59512	Q92974	ARHG2 HUMAN	613.9602973	3	0.765966177		
ELLSNVDEGIYQLEK	70.91041	Q92974	ARHG2 HUMAN	875.4494435	2	0.829848349		
ELLSNVDEGIYQLEK	70.91041	Q92974	ARHG2 HUMAN	583.968904	3	0.829848349		
EPALPLEPDSGGNTSPGVTANGEAR	49.63593	Q92974	ARHG2 HUMAN	1218.586056	2	0.676445961		
EPALPLEPDSGGNTSPGVTANGEAR	49.63593	Q92974	ARHG2 HUMAN	812.7266457	3	0.676445961		
ERPSSAIYPSDSFR	14.81406	Q92974	ARHG2 HUMAN	806.3922625	2	0.790097594	2	Yes
ERPSSAIYPSDSFR	14.81406	Q92974	ARHG2 HUMAN	537.9307833	3	0.790097594	2	
FLSQLLER	49.27174	Q92974	ARHG2 HUMAN	503.2905655	2	0.816831052	2	Yes
FLSQLLER	49.27174	Q92974	ARHG2 HUMAN	335.8629853	3	0.816831052	2	
GMFLISAAPPEMYEVHTASR	73.56078	Q92974	ARHG2 HUMAN	1104.03537	2	0.786595583	3	
GMFLISAAPPEMYEVHTASR	73.56078	Q92974	ARHG2 HUMAN	736.3595213	3	0.786595583	3	Yes
HGVQECILLVTQR	35.68809	Q92974	ARHG2 HUMAN	776.9173995	2	0.749038935		
HGVQECILLVTQR	35.68809	Q92974	ARHG2 HUMAN	518.2808747	3	0.749038935		
ILQHSHGIEEER	-29.28031	Q92974	ARHG2 HUMAN	724.3685955	2	0.787794888		
ILQHSHGIEEER	-29.28031	Q92974	ARHG2 HUMAN	483.2483387	3	0.787794888		
ILSQSTDSLNMR	22.21442	Q92974	ARHG2 HUMAN	682.846107	2	0.772201419	2	Yes
ILSQSTDSLNMR	22.21442	Q92974	ARHG2 HUMAN	455.5666797	3	0.772201419	2	
LDLPVTTR	31.39281	Q92974	ARHG2 HUMAN	457.7694665	2	0.758057296	2	Yes
LDLPVTTR	31.39281	Q92974	ARHG2 HUMAN	305.515586	3	0.758057296	2	
LGDLLISQFSGPSAEQMCK	82.38179	Q92974	ARHG2 HUMAN	1041.006282	2	0.661753416		
LGDLLISQFSGPSAEQMCK	82.38179	Q92974	ARHG2 HUMAN	694.3401297	3	0.661753416		
MQDIPEETESR	3.285862	Q92974	ARHG2 HUMAN	667.798823	2	0.726773024		
MQDIPEETESR	3.285862	Q92974	ARHG2 HUMAN	445.535157	3	0.726773024		
NNTALQSVSLR	17.26662	Q92974	ARHG2 HUMAN	601.8285745	2	0.766232848		
NNTALQSVSLR	17.26662	Q92974	ARHG2 HUMAN	401.5549913	3	0.766232848		

QLAALGQTEPLPAEAPWAR	72.84126	Q92974	ARHG2_HUMAN	1010.037089	2	0.639065087		
QLAALGQTEPLPAEAPWAR	72.84126	Q92974	ARHG2_HUMAN	673.694001	3	0.639065087		
SVSTTNIAGHFNDESPLGLR	50.81265	Q92974	ARHG2_HUMAN	1058.027448	2	0.793468177		
SVSTTNIAGHFNDESPLGLR	50.81265	Q92974	ARHG2_HUMAN	705.6875737	3	0.793468177		
VGLFAEMTHFQAEEDGGSGMALPTLPR	105.0479	Q92974	ARHG2_HUMAN	1431.184027	2	0.74535352		
VGLFAEMTHFQAEEDGGSGMALPTLPR	105.0479	Q92974	ARHG2_HUMAN	954.4586263	3	0.74535352		
YIFPTLDKPSVVSLQNLIVR	105.5069	Q92974	ARHG2_HUMAN	1151.662648	2	0.62998724	3	
YIFPTLDKPSVVSLQNLIVR	105.5069	Q92974	ARHG2_HUMAN	768.1110403	3	0.62998724	3	Yes
NIEEHASSDVER	-24.1926	Q92930	RAB8B_HUMAN	693.3187675	2	0.727149308		
NIEEHASSDVER	-24.1926	Q92930	RAB8B_HUMAN	462.5484533	3	0.727149308		
SSANVEEAFFTLAR	80.96759	Q92930	RAB8B_HUMAN	771.383911	2	0.833583474	2	Yes
SSANVEEAFFTLAR	80.96759	Q92930	RAB8B_HUMAN	514.5918823	3	0.833583474	2	
ALELAHLK	2.742207	Q92887	MRP2_HUMAN	447.7745515	2	0.725887179		
ALELAHLK	2.742207	Q92887	MRP2_HUMAN	298.8523093	3	0.725887179		
ALTLSNLAR	30.66887	Q92887	MRP2_HUMAN	479.78819	2	0.830859661		
ALTLSNLAR	30.66887	Q92887	MRP2_HUMAN	320.194735	3	0.830859661		
AMQFSEASFTWEHDSEATVR	54.31479	Q92887	MRP2_HUMAN	1165.013681	2	0.789886713		
AMQFSEASFTWEHDSEATVR	54.31479	Q92887	MRP2_HUMAN	777.011729	3	0.789886713		
ATYQNLDIYLLDDPLSAVDAHVGK	107.2869	Q92887	MRP2_HUMAN	1316.169216	2	0.688367784		
ATYQNLDIYLLDDPLSAVDAHVGK	107.2869	Q92887	MRP2_HUMAN	877.7820857	3	0.688367784		
DNILFGTEFNEK	66.80801	Q92887	MRP2_HUMAN	713.84663	2	0.85427922		
DNILFGTEFNEK	66.80801	Q92887	MRP2_HUMAN	476.233695	3	0.85427922		
EYTVGETVNLMSVDAQK	62.35827	Q92887	MRP2_HUMAN	942.4569435	2	0.799895525		
EYTVGETVNLMSVDAQK	62.35827	Q92887	MRP2_HUMAN	628.6405707	3	0.799895525		
FAGDISTVDDTLPQSLR	67.9838	Q92887	MRP2_HUMAN	917.9632545	2	0.82639128		
FAGDISTVDDTLPQSLR	67.9838	Q92887	MRP2_HUMAN	612.3114447	3	0.82639128		
GINLSGGQK	-14.48722	Q92887	MRP2_HUMAN	437.2436155	2	0.622626603		
GINLSGGQK	-14.48722	Q92887	MRP2_HUMAN	291.8316853	3	0.622626603		
GITCDIGSMEK	19.04111	Q92887	MRP2_HUMAN	605.7763055	2	0.772159636		
GITCDIGSMEK	19.04111	Q92887	MRP2_HUMAN	404.186812	3	0.772159636		
GSYSALLAK	14.32131	Q92887	MRP2_HUMAN	455.256187	2	0.722891688		
GSYSALLAK	14.32131	Q92887	MRP2_HUMAN	303.8400663	3	0.722891688		
GTTAYVPQQSWIQNGTIK	52.35143	Q92887	MRP2_HUMAN	996.5134425	2	0.683848619		
GTTAYVPQQSWIQNGTIK	52.35143	Q92887	MRP2_HUMAN	664.6782367	3	0.683848619		
IFNSTDYPASQR	12.95997	Q92887	MRP2_HUMAN	699.8365925	2	0.794143081		
IFNSTDYPASQR	12.95997	Q92887	MRP2_HUMAN	466.89367	3	0.794143081		
IMNEILSGIK	51.92709	Q92887	MRP2_HUMAN	559.3184655	2	0.74271071		
IMNEILSGIK	51.92709	Q92887	MRP2_HUMAN	373.2149187	3	0.74271071		
IQFNNYQVR	22.32888	Q92887	MRP2_HUMAN	591.307274	2	0.693022966		
IQFNNYQVR	22.32888	Q92887	MRP2_HUMAN	394.540791	3	0.693022966		
LTIIPQDPILFSGSLR	110.2139	Q92887	MRP2_HUMAN	885.512186	2	0.649423361		
LTIIPQDPILFSGSLR	110.2139	Q92887	MRP2_HUMAN	590.677399	3	0.649423361		
LVNDIFTFVSPQLLK	125.3903	Q92887	MRP2_HUMAN	867.4960045	2	0.672692597		
LVNDIFTFVSPQLLK	125.3903	Q92887	MRP2_HUMAN	578.6666113	3	0.672692597		
MNLDPFNNYSDEEIWK	89.98692	Q92887	MRP2_HUMAN	1007.946742	2	0.827585161		
MNLDPFNNYSDEEIWK	89.98692	Q92887	MRP2_HUMAN	672.300436	3	0.827585161		
MTSEIETNIVAVER	46.77233	Q92887	MRP2_HUMAN	796.4039865	2	0.804861307		
MTSEIETNIVAVER	46.77233	Q92887	MRP2_HUMAN	531.2719327	3	0.804861307		
NQSQSQDALVLEDVEK	39.82303	Q92887	MRP2_HUMAN	901.9425185	2	0.783644795		
NQSQSQDALVLEDVEK	39.82303	Q92887	MRP2_HUMAN	601.630954	3	0.783644795		
RPLTLEDVWEVDEEMK	72.64201	Q92887	MRP2_HUMAN	994.985872	2	0.798218429		
RPLTLEDVWEVDEEMK	72.64201	Q92887	MRP2_HUMAN	663.6598563	3	0.798218429		
SFVASLQLGLSHEVTEAGGNLSIGQR	85.40559	Q92887	MRP2_HUMAN	1335.696472	2	0.68928957		
SFVASLQLGLSHEVTEAGGNLSIGQR	85.40559	Q92887	MRP2_HUMAN	890.8002563	3	0.68928957		
SPIYSHFSETVSGLPVIR	62.33959	Q92887	MRP2_HUMAN	995.026184	2	0.809869528		
SPIYSHFSETVSGLPVIR	62.33959	Q92887	MRP2_HUMAN	663.686731	3	0.809869528		
SSLISAMLGEMENVHGHITIK	75.48911	Q92887	MRP2_HUMAN	1134.080312	2	0.621913195		
SSLISAMLGEMENVHGHITIK	75.48911	Q92887	MRP2_HUMAN	756.389483	3	0.621913195		
SSLTNCLFR	36.64731	Q92887	MRP2_HUMAN	549.2745895	2	0.798353791		
SSLTNCLFR	36.64731	Q92887	MRP2_HUMAN	366.5190013	3	0.798353791		
TFYMVLLK	68.73	Q92887	MRP2_HUMAN	507.788809	2	0.614511728		
TFYMVLLK	68.73	Q92887	MRP2_HUMAN	338.8618143	3	0.614511728		
VENEAPWVTDK	22.9133	Q92887	MRP2_HUMAN	644.314966	2	0.762147784		
VENEAPWVTDK	22.9133	Q92887	MRP2_HUMAN	429.8792523	3	0.762147784		
YFAWEPSFR	62.74496	Q92887	MRP2_HUMAN	601.785643	2	0.785732448	2	Yes
YFAWEPSFR	62.74496	Q92887	MRP2_HUMAN	401.5263703	3	0.785732448	2	
YLGGDDLDTSAIR	35.98898	Q92887	MRP2_HUMAN	698.341708	2	0.856254578		
YLGGDDLDTSAIR	35.98898	Q92887	MRP2_HUMAN	465.8970803	3	0.856254578		
YRPELDLVLR	46.63689	Q92887	MRP2_HUMAN	637.3673315	2	0.671228945		
YRPELDLVLR	46.63689	Q92887	MRP2_HUMAN	425.247496	3	0.671228945		
AALEQLLK	39.24062	Q92797	SYMPK_HUMAN	443.2743845	2	0.711057186	2	Yes
AALEQLLK	39.24062	Q92797	SYMPK_HUMAN	295.852198	3	0.711057186	2	
AEVLSFILEDVR	105.1247	Q92797	SYMPK_HUMAN	695.8830155	2	0.653322637	2	Yes
AEVLSFILEDVR	105.1247	Q92797	SYMPK_HUMAN	464.257952	3	0.653322637	2	
AILTMTQLYK	51.47919	Q92797	SYMPK_HUMAN	591.3341125	2	0.751882315	2	Yes
AILTMTQLYK	51.47919	Q92797	SYMPK_HUMAN	394.5586833	3	0.751882315	2	
ATNLCFAER	12.64334	Q92797	SYMPK_HUMAN	541.25894	2	0.791712284		
ATNLCFAER	12.64334	Q92797	SYMPK_HUMAN	361.175235	3	0.791712284		
AVACSGAAQVR	-27.5781	Q92797	SYMPK_HUMAN	545.2776645	2	0.651433945		
AVACSGAAQVR	-27.5781	Q92797	SYMPK_HUMAN	363.8543847	3	0.651433945		
DHPYIQYNVLWEEGK	61.09386	Q92797	SYMPK_HUMAN	945.9552235	2	0.682440698	3	
DHPYIQYNVLWEEGK	61.09386	Q92797	SYMPK_HUMAN	630.9727573	3	0.682440698	3	Yes
ETAAGGLTLK	3.99612	Q92797	SYMPK_HUMAN	480.7722065	2	0.76524967	2	Yes
ETAAGGLTLK	3.99612	Q92797	SYMPK_HUMAN	320.850746	3	0.76524967	2	
FLIPVLNGLEK	90.99335	Q92797	SYMPK_HUMAN	621.877005	2	0.736382902		
FLIPVLNGLEK	90.99335	Q92797	SYMPK_HUMAN	414.9206117	3	0.736382902		
FVEGLIVTLSPR	81.4783	Q92797	SYMPK_HUMAN	665.890644	2	0.780786455		
FVEGLIVTLSPR	81.4783	Q92797	SYMPK_HUMAN	444.2630377	3	0.780786455		
FVIGFIEEACK	76.06559	Q92797	SYMPK_HUMAN	656.8344805	2	0.644738734		
FVIGFIEEACK	76.06559	Q92797	SYMPK_HUMAN	438.2255953	3	0.644738734		
ILASLVTQFNSGLK	79.57104	Q92797	SYMPK_HUMAN	745.93304	2	0.6999439		
ILASLVTQFNSGLK	79.57104	Q92797	SYMPK_HUMAN	497.624635	3	0.6999439		
LEPNLGEDDEDKDLEPGPSGTSK	26.32191	Q92797	SYMPK_HUMAN	1221.561912	2	0.747619271	3	
LEPNLGEDDEDKDLEPGPSGTSK	26.32191	Q92797	SYMPK_HUMAN	814.7105493	3	0.747619271	3	Yes
LGGFVMNILSR	82.78947	Q92797	SYMPK_HUMAN	603.837357	2	0.724383593		
LGGFVMNILSR	82.78947	Q92797	SYMPK_HUMAN	402.8941797	3	0.724383593		
LHLLSVLK	36.14148	Q92797	SYMPK_HUMAN	461.808394	2	0.722362638		
LHLLSVLK	36.14148	Q92797	SYMPK_HUMAN	308.2082043	3	0.722362638		
LIANLNMLLR	81.8546	Q92797	SYMPK_HUMAN	585.8555495	2	0.847606003	2	Yes
LIANLNMLLR	81.8546	Q92797	SYMPK_HUMAN	390.906308	3	0.847606003	2	
LIHELAAVYTEAIADIK	87.90063	Q92797	SYMPK_HUMAN	935.520204	2	0.706403494		

LIHELAAVYTEAIADIK	87.90063	Q92797	SYMPK HUMAN	624.0160777	3	0.706403494		
LLGTQHGEGNSALSPLNPGELLIALHNIDSVK	107.273	Q92797	SYMPK HUMAN	1654.386428	2	0.642761648	4	
LLGTQHGEGNSALSPLNPGELLIALHNIDSVK	107.273	Q92797	SYMPK HUMAN	1103.260227	3	0.642761648	4	
LLSGLQEKPDQK	-3.165474	Q92797	SYMPK HUMAN	678.3806405	2	0.815840602		
LLSGLQEKPDQK	-3.165474	Q92797	SYMPK HUMAN	452.589702	3	0.815840602		
LMATQMTAAGLGPGVEQTK	47.70644	Q92797	SYMPK HUMAN	952.484987	2	0.746276319		
LMATQMTAAGLGPGVEQTK	47.70644	Q92797	SYMPK HUMAN	635.325933	3	0.746276319		
LSAQGQAISVVGSLSSMSPLEEEAPQAK	94.37387	Q92797	SYMPK HUMAN	1407.71367	2	0.758685112	3	
LSAQGQAISVVGSLSSMSPLEEEAPQAK	94.37387	Q92797	SYMPK HUMAN	938.8117217	3	0.758685112	3	Yes
LSDVLKPLTDAQVEAMK	52.49071	Q92797	SYMPK HUMAN	929.503701	2	0.863378406		
LSDVLKPLTDAQVEAMK	52.49071	Q92797	SYMPK HUMAN	620.0050757	3	0.863378406		
RPEPIIPVTQPR	21.76857	Q92797	SYMPK HUMAN	701.9124425	2	0.80503875	3	
RPEPIIPVTQPR	21.76857	Q92797	SYMPK HUMAN	468.27757	3	0.80503875	3	Yes
SFTPHQQAHIPNSIMTILEASGK	65.41243	Q92797	SYMPK HUMAN	1254.139748	2	0.631205261		
SFTPHQQAHIPNSIMTILEASGK	65.41243	Q92797	SYMPK HUMAN	836.4291067	3	0.631205261		
SPQTLAPVGEDAMK	27.51395	Q92797	SYMPK HUMAN	722.361591	2	0.801920891	2	Yes
SPQTLAPVGEDAMK	27.51395	Q92797	SYMPK HUMAN	481.9103357	3	0.801920891	2	
SQALLFIK	46.65001	Q92797	SYMPK HUMAN	460.284752	2	0.77000922		
SQALLFIK	46.65001	Q92797	SYMPK HUMAN	307.192443	3	0.77000922		
SVASQFFTQEEGPGIDGMTTSER	70.78527	Q92797	SYMPK HUMAN	1237.561193	2	0.852043986	3	
SVASQFFTQEEGPGIDGMTTSER	70.78527	Q92797	SYMPK HUMAN	825.376737	3	0.852043986	3	Yes
TVIQSLTMYPR	51.0381	Q92797	SYMPK HUMAN	654.8532005	2	0.782353699	2	Yes
TVIQSLTMYPR	51.0381	Q92797	SYMPK HUMAN	436.904742	3	0.782353699	2	
TYLGMSTLR	30.81783	Q92797	SYMPK HUMAN	521.2740545	2	0.759715915		
TYLGMSTLR	30.81783	Q92797	SYMPK HUMAN	347.851978	3	0.759715915		
VALQWMVK	48.93381	Q92797	SYMPK HUMAN	487.77878	2	0.705262184		
VALQWMVK	48.93381	Q92797	SYMPK HUMAN	325.521795	3	0.705262184		
VVDLLNQAALITNDSK	75.02979	Q92797	SYMPK HUMAN	857.4732575	2	0.885596812		
VVDLLNQAALITNDSK	75.02979	Q92797	SYMPK HUMAN	571.98478	3	0.885596812		
VVLEAPLITESALEVVR	111.2532	Q92797	SYMPK HUMAN	919.535858	2	0.829825282		
VVLEAPLITESALEVVR	111.2532	Q92797	SYMPK HUMAN	613.359847	3	0.829825282		
AGEIITVLDDSDPNWWK	102.7339	Q92783	STAM1 HUMAN	979.978904	2	0.728914022	3	
AGEIITVLDDSDPNWWK	102.7339	Q92783	STAM1 HUMAN	653.655211	3	0.728914022	3	Yes
AIELSLK	27.31213	Q92783	STAM1 HUMAN	387.2425525	2	0.616679192		
AIELSLK	27.31213	Q92783	STAM1 HUMAN	258.4976433	3	0.616679192		
AIYDFEAAEDNELTFK	75.93875	Q92783	STAM1 HUMAN	938.4365335	2	0.922393799		
AIYDFEAAEDNELTFK	75.93875	Q92783	STAM1 HUMAN	625.9602973	3	0.922393799		
ALMVEWTDEFK	75.80554	Q92783	STAM1 HUMAN	684.829395	2	0.816041946	2	Yes
ALMVEWTDEFK	75.80554	Q92783	STAM1 HUMAN	456.8888717	3	0.816041946	2	
DFASEVSNVLNK	70.76745	Q92783	STAM1 HUMAN	661.833522	2	0.854873061	2	Yes
DFASEVSNVLNK	70.76745	Q92783	STAM1 HUMAN	441.5582897	3	0.854873061	2	
EQGVTFPAIGSQAAEQAK	47.99512	Q92783	STAM1 HUMAN	916.4634225	2	0.725993991		
EQGVTFPAIGSQAAEQAK	47.99512	Q92783	STAM1 HUMAN	611.3115567	3	0.725993991		
GETHQGIGLFPSNFVTADLTAEPEMIK	102.6898	Q92783	STAM1 HUMAN	1451.718757	2	0.847134471	3	
GETHQGIGLFPSNFVTADLTAEPEMIK	102.6898	Q92783	STAM1 HUMAN	968.148446	3	0.847134471	3	Yes
LMNEDPMYSMYAK	44.17641	Q92783	STAM1 HUMAN	796.843853	2	0.830156565	2	Yes
LMNEDPMYSMYAK	44.17641	Q92783	STAM1 HUMAN	531.565177	3	0.830156565	2	
NDPQLSLISAMIK	88.03745	Q92783	STAM1 HUMAN	715.3901505	2	0.783512294	2	Yes
NDPQLSLISAMIK	88.03745	Q92783	STAM1 HUMAN	477.2627087	3	0.783512294	2	
TVQFSDDVQVETIEPEPEPAFIDEDK	88.60542	Q92783	STAM1 HUMAN	1489.196031	2	0.749219179		
TVQFSDDVQVETIEPEPEPAFIDEDK	88.60542	Q92783	STAM1 HUMAN	993.1332953	3	0.749219179		
VMEALSLYTK	45.73248	Q92783	STAM1 HUMAN	577.8104695	2	0.780907989	2	Yes
VMEALSLYTK	45.73248	Q92783	STAM1 HUMAN	385.5429213	3	0.780907989	2	
DGIDDESYGQIFKPIISK	77.2233	Q92769	HDAC2 HUMAN	1013.01294	2	0.844162285	3	
DGIDDESYGQIFKPIISK	77.2233	Q92769	HDAC2 HUMAN	675.6779013	3	0.844162285	3	Yes
LGCFNLTVK	40.47798	Q92769	HDAC2 HUMAN	526.2844265	2	0.692995608	2	Yes
LGCFNLTVK	40.47798	Q92769	HDAC2 HUMAN	351.192226	3	0.692995608	2	
LHISPSNMTNQNTPEYMEK	27.16514	Q92769	HDAC2 HUMAN	1117.514998	2	0.838745832	3	
LHISPSNMTNQNTPEYMEK	27.16514	Q92769	HDAC2 HUMAN	745.34594	3	0.838745832	3	Yes
TFNLPLLMLGGGGYTIR	128.2805	Q92769	HDAC2 HUMAN	911.998385	2	0.729486346		
TFNLPLLMLGGGGYTIR	128.2805	Q92769	HDAC2 HUMAN	608.334865	3	0.729486346		
YYAVNFPMR	48.76391	Q92769	HDAC2 HUMAN	580.782042	2	0.661028564	2	Yes
YYAVNFPMR	48.76391	Q92769	HDAC2 HUMAN	387.5239697	3	0.661028564	2	
AAHVTLLTSLVEGEAVHLAR	75.599	Q92754	AP2C HUMAN	1044.08457	2	0.786080837		
AAHVTLLTSLVEGEAVHLAR	75.599	Q92754	AP2C HUMAN	696.3923217	3	0.786080837		
EFTELLSQDR	42.92796	Q92754	AP2C HUMAN	619.307141	2	0.730243385		
EFTELLSQDR	42.92796	Q92754	AP2C HUMAN	413.207369	3	0.730243385		
NMLLAAQQLCK	37.45628	Q92754	AP2C HUMAN	645.3394075	2	0.724270642		
NMLLAAQQLCK	37.45628	Q92754	AP2C HUMAN	430.5622133	3	0.724270642		
NPLNLPCQK	15.19829	Q92754	AP2C HUMAN	542.2849575	2	0.748356164		
NPLNLPCQK	15.19829	Q92754	AP2C HUMAN	361.8592467	3	0.748356164		
SYMNPGDQSPADSNK	-1.99369	Q92754	AP2C HUMAN	805.8417455	2	0.823101163		
SYMNPGDQSPADSNK	-1.99369	Q92754	AP2C HUMAN	537.563772	3	0.823101163		
FCTTGIDGAMTIWDFK	101.2231	Q92747	ARC1A HUMAN	931.926773	2	0.695103347		
FCTTGIDGAMTIWDFK	101.2231	Q92747	ARC1A HUMAN	621.620457	3	0.695103347		
LAWVSHDSTVSVADASK	28.17126	Q92747	ARC1A HUMAN	886.944864	2	0.853948474		
LAWVSHDSTVSVADASK	28.17126	Q92747	ARC1A HUMAN	591.6325177	3	0.853948474		
NTALETLHQNSITQVSIYEVDK	61.27964	Q92747	ARC1A HUMAN	1252.13792	2	0.755489886		
NTALETLHQNSITQVSIYEVDK	61.27964	Q92747	ARC1A HUMAN	835.094555	3	0.755489886		
PTLVILR	38.31428	Q92747	ARC1A HUMAN	406.2741875	2	0.706324399		
PTLVILR	38.31428	Q92747	ARC1A HUMAN	271.1854	3	0.706324399		
TLESSIQGLR	25.28564	Q92747	ARC1A HUMAN	552.306944	2	0.746738434	2	Yes
TLESSIQGLR	25.28564	Q92747	ARC1A HUMAN	368.540571	3	0.746738434	2	
TQIALSPNNHEVHIYK	12.87508	Q92747	ARC1A HUMAN	932.489769	2	0.757940114		
TQIALSPNNHEVHIYK	12.87508	Q92747	ARC1A HUMAN	621.9957877	3	0.757940114		
WSPLENK	4.302425	Q92747	ARC1A HUMAN	437.2274335	2	0.602445543		
WSPLENK	4.302425	Q92747	ARC1A HUMAN	291.8208973	3	0.602445543		
AQLGEDIR	-7.239586	Q92734	TFG HUMAN	451.241073	2	0.7990309	2	Yes
AQLGEDIR	-7.239586	Q92734	TFG HUMAN	301.1633237	3	0.7990309	2	
IPIHNEDITYDELVLMMQR	92.57951	Q92734	TFG HUMAN	1165.580141	2	0.695867062	3	
IPIHNEDITYDELVLMMQR	92.57951	Q92734	TFG HUMAN	777.389369	3	0.695867062	3	Yes
LLDSLEPPGEPGPSTNIPENDTVDGR	70.15657	Q92734	TFG HUMAN	1360.157042	2	0.726924539	3	
LLDSLEPPGEPGPSTNIPENDTVDGR	70.15657	Q92734	TFG HUMAN	907.107303	3	0.726924539	3	Yes
LLSNDEVTIK	22.28314	Q92734	TFG HUMAN	566.316977	2	0.712372184	2	Yes
LLSNDEVTIK	22.28314	Q92734	TFG HUMAN	377.880593	3	0.712372184	2	
NRPPFGQGYTQPGPGYR	13.29129	Q92734	TFG HUMAN	946.4640825	2	0.834679365		
NRPPFGQGYTQPGPGYR	13.29129	Q92734	TFG HUMAN	631.3119967	3	0.834679365		
AFNDPFIQK	36.35353	Q92598	HS105 HUMAN	540.280198	2	0.780917883	2	Yes
AFNDPFIQK	36.35353	Q92598	HS105 HUMAN	360.5227403	3	0.780917883	2	
AGGIETIANEFSDR	53.05324	Q92598	HS105 HUMAN	740.3578935	2	0.790049911	2	Yes
AGGIETIANEFSDR	53.05324	Q92598	HS105 HUMAN	493.9078707	3	0.790049911	2	

DLLNMYIETEGK	88.18634	Q92598	HS105 HUMAN	713.350687	2	0.776522815	2	Yes
DLLNMYIETEGK	88.18634	Q92598	HS105 HUMAN	475.9030663	3	0.776522815	2	
ENLSYDLVPLK	64.10673	Q92598	HS105 HUMAN	645.8511795	2	0.715007961	2	Yes
ENLSYDLVPLK	64.10673	Q92598	HS105 HUMAN	430.9033947	3	0.715007961	2	
FICEQDHQNFLR	21.23121	Q92598	HS105 HUMAN	803.875731	2	0.7881037	3	
FICEQDHQNFLR	21.23121	Q92598	HS105 HUMAN	536.2530957	3	0.7881037	3	Yes
FVVQNVSAQK	5.62365	Q92598	HS105 HUMAN	560.3120295	2	0.689028144	2	Yes
FVVQNVSAQK	5.62365	Q92598	HS105 HUMAN	373.8772947	3	0.689028144	2	
IEVPLYSLLEQTHLK	92.48022	Q92598	HS105 HUMAN	892.0041895	2	0.794198751	3	
IEVPLYSLLEQTHLK	92.48022	Q92598	HS105 HUMAN	595.0054013	3	0.794198751	3	Yes
LLTETEDWLYEEGEDQAK	73.13569	Q92598	HS105 HUMAN	1084.997684	2	0.81624651	3	
LLTETEDWLYEEGEDQAK	73.13569	Q92598	HS105 HUMAN	723.667731	3	0.81624651	3	Yes
LMNDMTAVALNYGIYK	81.11748	Q92598	HS105 HUMAN	908.952781	2	0.720669508	3	
LMNDMTAVALNYGIYK	81.11748	Q92598	HS105 HUMAN	606.3044623	3	0.720669508	3	Yes
LVEHFCAEFK	19.68824	Q92598	HS105 HUMAN	640.3111725	2	0.755325913	3	
LVEHFCAEFK	19.68824	Q92598	HS105 HUMAN	427.2100567	3	0.755325913	3	Yes
MFEELGQR	19.23652	Q92598	HS105 HUMAN	505.2427585	2	0.774241924	2	Yes
MFEELGQR	19.23652	Q92598	HS105 HUMAN	337.1644473	3	0.774241924	2	
NAVEEYVYEFR	55.53129	Q92598	HS105 HUMAN	709.833514	2	0.795012712	2	Yes
NAVEEYVYEFR	55.53129	Q92598	HS105 HUMAN	473.5582843	3	0.795012712	2	
QQDNSEAGTQPQVQTDAQQTSQSPPSPELTSEE	31.24014	Q92598	HS105 HUMAN	1999.410669	2	0.680283487		
QQDNSEAGTQPQVQTDAQQTSQSPPSPELTSEE	31.24014	Q92598	HS105 HUMAN	1333.276387	3	0.680283487		
SQFEELCAELLQK	75.21365	Q92598	HS105 HUMAN	797.893255	2	0.844769061	2	Yes
SQFEELCAELLQK	75.21365	Q92598	HS105 HUMAN	532.2647783	3	0.844769061	2	
SVLDAAQIVGLNCLR	92.40904	Q92598	HS105 HUMAN	814.9436135	2	0.737844467	3	
SVLDAAQIVGLNCLR	92.40904	Q92598	HS105 HUMAN	543.631684	3	0.737844467	3	Yes
SVNEVMEWMNNVMNAQAK	102.0955	Q92598	HS105 HUMAN	1047.974458	2	0.74237901	3	
SVNEVMEWMNNVMNAQAK	102.0955	Q92598	HS105 HUMAN	698.9855803	3	0.74237901	3	Yes
VEDVSAVEIVGGATR	47.40424	Q92598	HS105 HUMAN	751.397019	2	0.830213249	2	Yes
VEDVSAVEIVGGATR	47.40424	Q92598	HS105 HUMAN	501.2672877	3	0.830213249	2	
VLGTAFDPFLGGK	81.75504	Q92598	HS105 HUMAN	661.36172	2	0.841247737	2	Yes
VLGTAFDPFLGGK	81.75504	Q92598	HS105 HUMAN	441.243755	3	0.841247737	2	
VMYMGEEHLFSVEQITAMLLTK	129.4882	Q92598	HS105 HUMAN	1285.639342	2	0.639853895	3	
VMYMGEEHLFSVEQITAMLLTK	129.4882	Q92598	HS105 HUMAN	857.4288363	3	0.639853895	3	Yes
VNTHGIFTISTASMVEK	49.04124	Q92598	HS105 HUMAN	917.972568	2	0.79712224		
VNTHGIFTISTASMVEK	49.04124	Q92598	HS105 HUMAN	612.3176537	3	0.79712224		
ALQVMEK	-7.192593	Q92552	RT27 HUMAN	409.726213	2	0.708252788		
ALQVMEK	-7.192593	Q92552	RT27 HUMAN	273.4867503	3	0.708252788		
ALTSADGASEEQSQNDEDNQGSEK	-12.21804	Q92552	RT27 HUMAN	1255.524046	2	0.681164384	3	
ALTSADGASEEQSQNDEDNQGSEK	-12.21804	Q92552	RT27 HUMAN	837.3519723	3	0.681164384	3	Yes
ALYTLVNK	20.87527	Q92552	RT27 HUMAN	461.27438	2	0.71739459	2	Yes
ALYTLVNK	20.87527	Q92552	RT27 HUMAN	307.852195	3	0.71739459	2	
AVYHNMPLIWK	40.55535	Q92552	RT27 HUMAN	686.3662775	2	0.860738516		
AVYHNMPLIWK	40.55535	Q92552	RT27 HUMAN	457.91346	3	0.860738516		
EALDVLGAVLK	87.46121	Q92552	RT27 HUMAN	564.337713	2	0.751866937	2	Yes
EALDVLGAVLK	87.46121	Q92552	RT27 HUMAN	376.5610837	3	0.751866937	2	
EEIDHAEYYLYK	32.67834	Q92552	RT27 HUMAN	786.865007	2	0.784054279	2	Yes
EEIDHAEYYLYK	32.67834	Q92552	RT27 HUMAN	524.912613	3	0.784054279	2	
HSPNCWYLR	7.039822	Q92552	RT27 HUMAN	616.7856515	2	0.624810398		
HSPNCWYLR	7.039822	Q92552	RT27 HUMAN	411.526376	3	0.624810398		
IESEGLLSLTTQLVK	95.5533	Q92552	RT27 HUMAN	815.967277	2	0.740331948	3	
IESEGLLSLTTQLVK	95.5533	Q92552	RT27 HUMAN	544.3141263	3	0.740331948	3	Yes
LIDNISSR	4.264729	Q92552	RT27 HUMAN	459.256722	2	0.805694997	2	Yes
LIDNISSR	4.264729	Q92552	RT27 HUMAN	306.5070897	3	0.805694997	2	
LPQYLER	12.40954	Q92552	RT27 HUMAN	459.7563545	2	0.843499064	2	Yes
LPQYLER	12.40954	Q92552	RT27 HUMAN	306.840178	3	0.843499064	2	
LVEQLDIEETEQSK	37.14787	Q92552	RT27 HUMAN	830.917981	2	0.875724971	2	Yes
LVEQLDIEETEQSK	37.14787	Q92552	RT27 HUMAN	554.2812623	3	0.875724971	2	
NFGASLLLPGLK	87.45634	Q92552	RT27 HUMAN	615.3668045	2	0.776064277		
NFGASLLLPGLK	87.45634	Q92552	RT27 HUMAN	410.580478	3	0.776064277		
NWTIHTWIR	41.41766	Q92552	RT27 HUMAN	613.8256385	2	0.732807279	3	
NWTIHTWIR	41.41766	Q92552	RT27 HUMAN	409.553034	3	0.732807279	3	Yes
TDFSWEEER	27.69189	Q92552	RT27 HUMAN	599.754741	2	0.656376243		
TDFSWEEER	27.69189	Q92552	RT27 HUMAN	400.1724357	3	0.656376243		
VELQQGLR	4.474735	Q92552	RT27 HUMAN	471.7725405	2	0.758469641	2	Yes
VELQQGLR	4.474735	Q92552	RT27 HUMAN	314.8509687	3	0.758469641	2	
YLLSSAYVDSHK	20.85468	Q92552	RT27 HUMAN	691.8517065	2	0.756951451		
YLLSSAYVDSHK	20.85468	Q92552	RT27 HUMAN	461.5704127	3	0.756951451		
ADVLFIAPR	56.68609	Q92542	NICA HUMAN	501.2931085	2	0.834585786	2	Yes
ADVLFIAPR	56.68609	Q92542	NICA HUMAN	334.5313473	3	0.834585786	2	
ALADVATVLGR	55.75714	Q92542	NICA HUMAN	543.319855	2	0.795091689	2	Yes
ALADVATVLGR	55.75714	Q92542	NICA HUMAN	362.5491783	3	0.795091689	2	
ALYELAGGTNFSDTVQADPQTVTR	68.29398	Q92542	NICA HUMAN	1277.625178	2	0.780719399		
ALYELAGGTNFSDTVQADPQTVTR	68.29398	Q92542	NICA HUMAN	852.0860603	3	0.780719399		
ANNSWFQSILR	77.36189	Q92542	NICA HUMAN	668.3443915	2	0.811914265		
ANNSWFQSILR	77.36189	Q92542	NICA HUMAN	445.8988693	3	0.811914265		
APDVTTLPR	13.39299	Q92542	NICA HUMAN	485.2723735	2	0.80818224	2	Yes
APDVTTLPR	13.39299	Q92542	NICA HUMAN	323.8508573	3	0.80818224	2	
DLYEYSWVQGPLHSNETDR	65.68494	Q92542	NICA HUMAN	1155.027637	2	0.633754253		
DLYEYSWVQGPLHSNETDR	65.68494	Q92542	NICA HUMAN	770.354366	3	0.633754253		
LLNATHQIGCQSSISGDTGVIHVVEK	41.83454	Q92542	NICA HUMAN	1382.20889	2	0.698430955		
LLNATHQIGCQSSISGDTGVIHVVEK	41.83454	Q92542	NICA HUMAN	921.808535	3	0.698430955		
NISGVVLADHSGAFHNK	13.27156	Q92542	NICA HUMAN	883.4531905	2	0.815792859		
NISGVVLADHSGAFHNK	13.27156	Q92542	NICA HUMAN	589.3047353	3	0.815792859		
NQVEDLLATLEK	84.04663	Q92542	NICA HUMAN	686.8701055	2	0.868537784	2	Yes
NQVEDLLATLEK	84.04663	Q92542	NICA HUMAN	458.2493453	3	0.868537784	2	
RPNQSQPLPPSSLQR	0.178371	Q92542	NICA HUMAN	852.961183	2	0.671885669		
RPNQSQPLPPSSLQR	0.178371	Q92542	NICA HUMAN	568.9767303	3	0.671885669		
SGAGVPAVILR	42.89669	Q92542	NICA HUMAN	520.317115	2	0.714553237	2	Yes
SGAGVPAVILR	42.89669	Q92542	NICA HUMAN	347.2140183	3	0.714553237	2	
TSLELWMHTDPVSQK	50.90578	Q92542	NICA HUMAN	886.438361	2	0.812837183		
TSLELWMHTDPVSQK	50.90578	Q92542	NICA HUMAN	591.294849	3	0.812837183		
YYQSIYDTAENINVSYPEWLSPEEDLNFVTDTAK	130.4841	Q92542	NICA HUMAN	2007.926353	2	0.651556849		
YYQSIYDTAENINVSYPEWLSPEEDLNFVTDTAK	130.4841	Q92542	NICA HUMAN	1338.95351	3	0.651556849		
AALAVNICAAR	30.40373	Q92526	TCPW HUMAN	565.311507	2	0.747499943		
AALAVNICAAR	30.40373	Q92526	TCPW HUMAN	377.2102797	3	0.747499943		
ALEVLEEVK	41.6098	Q92526	TCPW HUMAN	515.2955135	2	0.813989937		
ALEVLEEVK	41.6098	Q92526	TCPW HUMAN	343.866284	3	0.813989937		
DGNVLLDEMQIQHPTASLIAK	82.67297	Q92526	TCPW HUMAN	1147.097017	2	0.867777765		
DGNVLLDEMQIQHPTASLIAK	82.67297	Q92526	TCPW HUMAN	765.0672863	3	0.867777765		
GIDPFSLDSLAK	82.45184	Q92526	TCPW HUMAN	631.835534	2	0.796682358	2	Yes

GIDPFSLDSLAK	82.45184	Q92526	TCPW HUMAN	421.559631	3	0.796682358	2	
LGVQAFADALLIIPK	132.3526	Q92526	TCPW HUMAN	784.9747085	2	0.660481751		
LGVQAFADALLIIPK	132.3526	Q92526	TCPW HUMAN	523.652414	3	0.660481751		
LIQGLVLDHGAR	27.48779	Q92526	TCPW HUMAN	646.3782355	2	0.829236627		
LIQGLVLDHGAR	27.48779	Q92526	TCPW HUMAN	431.2547653	3	0.829236627		
VATAQDDVTGDGTTSNVLIIGELLK	119.3854	Q92526	TCPW HUMAN	1265.666516	2	0.794122934		
VATAQDDVTGDGTTSNVLIIGELLK	119.3854	Q92526	TCPW HUMAN	844.1136187	3	0.794122934		
VLAQNAGYDPQETLVK	37.09925	Q92526	TCPW HUMAN	873.457604	2	0.879021406		
VLAQNAGYDPQETLVK	37.09925	Q92526	TCPW HUMAN	582.641011	3	0.879021406		
ALVQNDTLLQVK	45.0024	Q92522	H1X HUMAN	671.3910085	2	0.854566097	2	Yes
ALVQNDTLLQVK	45.0024	Q92522	H1X HUMAN	447.9299473	3	0.854566097	2	
GAPAAATAPAPTAHK	-22.86025	Q92522	H1X HUMAN	666.357501	2	0.785446644	3	
GAPAAATAPAPTAHK	-22.86025	Q92522	H1X HUMAN	444.5742757	3	0.785446644	3	Yes
YSQLVVETIR	34.39473	Q92522	H1X HUMAN	604.33824	2	0.825718105	2	Yes
YSQLVVETIR	34.39473	Q92522	H1X HUMAN	403.2281017	3	0.825718105	2	
ALAFNGVMFGDRPLK	59.84385	Q8WXA9	SREK1 HUMAN	818.437967	2	0.605576754		
ALAFNGVMFGDRPLK	59.84385	Q8WXA9	SREK1 HUMAN	545.961253	3	0.605576754		
EAQSFISAAIEPESGK	51.21045	Q8WXA9	SREK1 HUMAN	832.4128655	2	0.767543614	3	
EAQSFISAAIEPESGK	51.21045	Q8WXA9	SREK1 HUMAN	555.277852	3	0.767543614	3	Yes
FAFVEFADQNSVPR	70.60456	Q8WXA9	SREK1 HUMAN	813.8997285	2	0.823385715	2	Yes
FAFVEFADQNSVPR	70.60456	Q8WXA9	SREK1 HUMAN	542.9357607	3	0.823385715	2	
TVYVGNLNSQTTADQLLEFFK	125.5509	Q8WXA9	SREK1 HUMAN	1245.132107	2	0.618359745		
TVYVGNLNSQTTADQLLEFFK	125.5509	Q8WXA9	SREK1 HUMAN	830.424013	3	0.618359745		
AWDAHVTAVCSQDASELVR	41.01535	Q8WWV3	RT4I1 HUMAN	1058.000377	2	0.747095644	3	
AWDAHVTAVCSQDASELVR	41.01535	Q8WWV3	RT4I1 HUMAN	705.6695263	3	0.747095644	3	Yes
DVSGVVMECGLDVK	59.86955	Q8WWV3	RT4I1 HUMAN	754.3607335	2	0.702128708		
DVSGVVMECGLDVK	59.86955	Q8WWV3	RT4I1 HUMAN	503.2430973	3	0.702128708		
FTQNMMMPIIHYPNEVIVK	80.9705	Q8WWV3	RT4I1 HUMAN	1153.081266	2	0.606434405		
FTQNMMMPIIHYPNEVIVK	80.9705	Q8WWV3	RT4I1 HUMAN	769.0567853	3	0.606434405		
GEEFPLTLGR	56.00307	Q8WWV3	RT4I1 HUMAN	559.7962125	2	0.812205434		
GEEFPLTLGR	56.00307	Q8WWV3	RT4I1 HUMAN	373.5334167	3	0.812205434		
IRPVIEQTFPFSK	50.68521	Q8WWV3	RT4I1 HUMAN	781.4410325	2	0.697552919		
IRPVIEQTFPFSK	50.68521	Q8WWV3	RT4I1 HUMAN	521.29663	3	0.697552919		
LGADDVIDYK	32.50262	Q8WWV3	RT4I1 HUMAN	554.7802235	2	0.790045381	2	Yes
LGADDVIDYK	32.50262	Q8WWV3	RT4I1 HUMAN	370.189424	3	0.790045381	2	
LGIADGMLQTGVTVGSK	65.40292	Q8WWV3	RT4I1 HUMAN	823.94328	2	0.884169996		
LGIADGMLQTGVTVGSK	65.40292	Q8WWV3	RT4I1 HUMAN	549.6314617	3	0.884169996		
SGSVEEQLK	-20.31606	Q8WWV3	RT4I1 HUMAN	488.75147	2	0.60404408		
SGSVEEQLK	-20.31606	Q8WWV3	RT4I1 HUMAN	326.170255	3	0.60404408		
SGYGATALNMK	12.79415	Q8WWV3	RT4I1 HUMAN	556.7744185	2	0.819281697		
SGYGATALNMK	12.79415	Q8WWV3	RT4I1 HUMAN	371.5188873	3	0.819281697		
STVMPAWVIDK	61.7625	Q8WWV3	RT4I1 HUMAN	623.829198	2	0.828097582		
STVMPAWVIDK	61.7625	Q8WWV3	RT4I1 HUMAN	416.2220737	3	0.828097582		
VHAASVNPIDVNMR	24.99015	Q8WWV3	RT4I1 HUMAN	761.893923	2	0.820974767	2	Yes
VHAASVNPIDVNMR	24.99015	Q8WWV3	RT4I1 HUMAN	508.2652237	3	0.820974767	2	
YFKPGDEVWAAVPPWK	72.39632	Q8WWV3	RT4I1 HUMAN	945.4834245	2	0.849175274		
YFKPGDEVWAAVPPWK	72.39632	Q8WWV3	RT4I1 HUMAN	630.6582247	3	0.849175274		
AEGLQVGQDAR	-5.545418	Q8WWM7	ATX2L HUMAN	572.291826	2	0.825017452	2	Yes
AEGLQVGQDAR	-5.545418	Q8WWM7	ATX2L HUMAN	381.8638257	3	0.825017452	2	
EFNPTKPLLSVNK	29.09599	Q8WWM7	ATX2L HUMAN	743.9173895	2	0.717779458	2	Yes
EFNPTKPLLSVNK	29.09599	Q8WWM7	ATX2L HUMAN	496.280868	3	0.717779458	2	
EFSLAGGIWHGR	48.53685	Q8WWM7	ATX2L HUMAN	665.33911	2	0.728924394		
EFSLAGGIWHGR	48.53685	Q8WWM7	ATX2L HUMAN	443.8953483	3	0.728924394		
FELAVDAVHR	28.58564	Q8WWM7	ATX2L HUMAN	578.809654	2	0.712676167	3	
FELAVDAVHR	28.58564	Q8WWM7	ATX2L HUMAN	386.2090443	3	0.712676167	3	Yes
FQLEELR	36.56446	Q8WWM7	ATX2L HUMAN	467.753816	2	0.662187397	2	Yes
FQLEELR	36.56446	Q8WWM7	ATX2L HUMAN	312.171819	3	0.662187397	2	
GAEGILAPQPPPPQQHQERPGAAAIGSAR	27.76301	Q8WWM7	ATX2L HUMAN	1451.258096	2	0.734608531	4	
GAEGILAPQPPPPQQHQERPGAAAIGSAR	27.76301	Q8WWM7	ATX2L HUMAN	967.8413387	3	0.734608531	4	
GEDKDEGPVAEQVK	-21.51718	Q8WWM7	ATX2L HUMAN	750.863009	2	0.835070848	2	Yes
GEDKDEGPVAEQVK	-21.51718	Q8WWM7	ATX2L HUMAN	500.911281	3	0.835070848	2	
GPPQSPVFEGVYNNSR	39.68027	Q8WWM7	ATX2L HUMAN	874.4240945	2	0.742250085		
GPPQSPVFEGVYNNSR	39.68027	Q8WWM7	ATX2L HUMAN	583.285338	3	0.742250085		
ISLAPTDVK	19.50154	Q8WWM7	ATX2L HUMAN	472.277124	2	0.628733337		
ISLAPTDVK	19.50154	Q8WWM7	ATX2L HUMAN	315.1873577	3	0.628733337		
LQPSSSPENSLDPFPPR	51.00014	Q8WWM7	ATX2L HUMAN	934.4634205	2	0.78268224		
LQPSSSPENSLDPFPPR	51.00014	Q8WWM7	ATX2L HUMAN	623.3115553	3	0.78268224		
MLHFLTAVVGSTCDVK	59.48408	Q8WWM7	ATX2L HUMAN	889.4529575	2	0.6767537	3	
MLHFLTAVVGSTCDVK	59.48408	Q8WWM7	ATX2L HUMAN	593.30458	3	0.6767537	3	Yes
NVDFNYATK	13.47584	Q8WWM7	ATX2L HUMAN	536.259458	2	0.795560539	2	Yes
NVDFNYATK	13.47584	Q8WWM7	ATX2L HUMAN	357.842247	3	0.795560539	2	
TLEPQELAR	13.33249	Q8WWM7	ATX2L HUMAN	528.7883875	2	0.780655861	2	Yes
TLEPQELAR	13.33249	Q8WWM7	ATX2L HUMAN	352.8615333	3	0.780655861	2	
TTYDSSLSSYTVPLEK	54.40223	Q8WWM7	ATX2L HUMAN	895.9389045	2	0.819952965		
TTYDSSLSSYTVPLEK	54.40223	Q8WWM7	ATX2L HUMAN	597.6285447	3	0.819952965		
VPGLQNEQK	-15.51881	Q8WWM7	ATX2L HUMAN	506.77528	2	0.781877518	2	Yes
VPGLQNEQK	-15.51881	Q8WWM7	ATX2L HUMAN	338.1861283	3	0.781877518	2	
DAFVILVENALR	125.243	Q8WWI5	CTL1 HUMAN	680.3857255	2	0.709456682		
DAFVILVENALR	125.243	Q8WWI5	CTL1 HUMAN	453.9264253	3	0.709456682		
ETVTPEQLQIAEDNLR	54.75748	Q8WWI5	CTL1 HUMAN	928.4739865	2	0.878171623		
ETVTPEQLQIAEDNLR	54.75748	Q8WWI5	CTL1 HUMAN	619.3185993	3	0.878171623		
FAEALITFVSDNSVLHR	85.73071	Q8WWI5	CTL1 HUMAN	960.005256	2	0.831217408	3	
FAEALITFVSDNSVLHR	85.73071	Q8WWI5	CTL1 HUMAN	640.3394457	3	0.831217408	3	Yes
LEAIPNSGMDHTQR	5.92783	Q8WWI5	CTL1 HUMAN	784.8784705	2	0.739370108	3	
LEAIPNSGMDHTQR	5.92783	Q8WWI5	CTL1 HUMAN	523.5882553	3	0.739370108	3	Yes
LPVPASAPIPFFHR	73.89722	Q8WWI5	CTL1 HUMAN	774.93846	2	0.803322077		
LPVPASAPIPFFHR	73.89722	Q8WWI5	CTL1 HUMAN	516.9615817	3	0.803322077		
LVSGYDSYGNICGQK	27.18303	Q8WWI5	CTL1 HUMAN	830.8859525	2	0.84101975		
LVSGYDSYGNICGQK	27.18303	Q8WWI5	CTL1 HUMAN	554.25991	3	0.84101975		
MILMYIHSQLK	46.69548	Q8WWI5	CTL1 HUMAN	688.875621	2	0.755086601		
MILMYIHSQLK	46.69548	Q8WWI5	CTL1 HUMAN	459.5863557	3	0.755086601		
NLPFTPILASVNR	86.63448	Q8WWI5	CTL1 HUMAN	721.4122745	2	0.831980646	2	Yes
NLPFTPILASVNR	86.63448	Q8WWI5	CTL1 HUMAN	481.277458	3	0.831980646	2	
SVALCVAACPR	21.54559	Q8WWI5	CTL1 HUMAN	602.302825	2	0.600598335		
SVALCVAACPR	21.54559	Q8WWI5	CTL1 HUMAN	401.8711583	3	0.600598335		
VALTIALFHVAGK	67.35811	Q8WWI5	CTL1 HUMAN	670.4090045	2	0.724094033		
VALTIALFHVAGK	67.35811	Q8WWI5	CTL1 HUMAN	447.275278	3	0.724094033		
VLMEFVENSR	48.26291	Q8WWI5	CTL1 HUMAN	612.308629	2	0.837533712		
VLMEFVENSR	48.26291	Q8WWI5	CTL1 HUMAN	408.5416943	3	0.837533712		
YVFFLDPCNLDLINR	121.1029	Q8WWI5	CTL1 HUMAN	949.9776485	2	0.611730516		
YVFFLDPCNLDLINR	121.1029	Q8WWI5	CTL1 HUMAN	633.654374	3	0.611730516		

AVTHLNNVK	-33.45615	Q8WVV9	HNRLL HUMAN	498.2858145	2	0.705866814		
AVTHLNNVK	-33.45615	Q8WVV9	HNRLL HUMAN	332.5264847	3	0.705866814		
GLCESVVEADLVEALEK	108.3458	Q8WVV9	HNRLL HUMAN	930.967148	2	0.779613197		
GLCESVVEADLVEALEK	108.3458	Q8WVV9	HNRLL HUMAN	620.980707	3	0.779613197		
HDGYGSHGPLLPLPSR	27.65488	Q8WVV9	HNRLL HUMAN	851.9371725	2	0.636714935	4	
HDGYGSHGPLLPLPSR	27.65488	Q8WVV9	HNRLL HUMAN	568.2940567	3	0.636714935	4	
LCNDHEVLTFIK	38.94032	Q8WVV9	HNRLL HUMAN	744.8799505	2	0.718783915	3	
LCNDHEVLTFIK	38.94032	Q8WVV9	HNRLL HUMAN	496.9225753	3	0.718783915	3	Yes
NDNDSWDYTKPYLGR	35.43366	Q8WVV9	HNRLL HUMAN	922.416462	2	0.815297961	3	
NDNDSWDYTKPYLGR	35.43366	Q8WVV9	HNRLL HUMAN	615.2802497	3	0.815297961	3	Yes
NIIQPPSCVLHYYNVPLCVTEETFTK	94.42871	Q8WVV9	HNRLL HUMAN	1561.770458	2	0.605491996	3	
NIIQPPSCVLHYYNVPLCVTEETFTK	94.42871	Q8WVV9	HNRLL HUMAN	1041.516247	3	0.605491996	3	Yes
QALVEFENIDSAK	48.86432	Q8WVV9	HNRLL HUMAN	732.373012	2	0.751506329	2	Yes
QALVEFENIDSAK	48.86432	Q8WVV9	HNRLL HUMAN	488.5846163	3	0.751506329	2	
TDAVEALTALNHYQIR	66.5705	Q8WVV9	HNRLL HUMAN	907.9739525	2	0.642752826		
TDAVEALTALNHYQIR	66.5705	Q8WVV9	HNRLL HUMAN	605.65191	3	0.642752826		
TEEGEIDYSAEEGENR	13.2021	Q8WVV9	HNRLL HUMAN	914.3799435	2	0.606001198	2	Yes
TEEGEIDYSAEEGENR	13.2021	Q8WVV9	HNRLL HUMAN	609.9225707	3	0.606001198	2	
TIPGTALVEMGDEYAVER	72.39954	Q8WVV9	HNRLL HUMAN	975.9780435	2	0.79080081		
TIPGTALVEMGDEYAVER	72.39954	Q8WVV9	HNRLL HUMAN	650.9879707	3	0.79080081		
VSVSPVVHVR	7.124752	Q8WVV9	HNRLL HUMAN	539.822564	2	0.754047275	2	Yes
VSVSPVVHVR	7.124752	Q8WVV9	HNRLL HUMAN	360.217651	3	0.754047275	2	
DLQEAFQR	24.80634	Q8WUX9	CHMP7 HUMAN	503.751805	2	0.723487377		
DLQEAFQR	24.80634	Q8WUX9	CHMP7 HUMAN	336.1704783	3	0.723487377		
EAEAPAGGDPAGLLPPEWEEDEER	78.45898	Q8WUX9	CHMP7 HUMAN	1282.575354	2	0.637875676	3	
EAEAPAGGDPAGLLPPEWEEDEER	78.45898	Q8WUX9	CHMP7 HUMAN	855.3861777	3	0.637875676	3	Yes
ELDILLQDTTK	66.36575	Q8WUX9	CHMP7 HUMAN	644.8539245	2	0.787163973		
ELDILLQDTTK	66.36575	Q8WUX9	CHMP7 HUMAN	430.238558	3	0.787163973		
EPLDLPDNPR	32.31678	Q8WUX9	CHMP7 HUMAN	583.296576	2	0.794553101		
EPLDLPDNPR	32.31678	Q8WUX9	CHMP7 HUMAN	389.2003257	3	0.794553101		
HFTNSVPNPR	-21.61787	Q8WUX9	CHMP7 HUMAN	584.7970775	2	0.659921408		
HFTNSVPNPR	-21.61787	Q8WUX9	CHMP7 HUMAN	390.20066	3	0.659921408		
ISDAELEAELEK	39.20347	Q8WUX9	CHMP7 HUMAN	673.8384705	2	0.82568872		
ISDAELEAELEK	39.20347	Q8WUX9	CHMP7 HUMAN	449.5615887	3	0.82568872		
LDTVQGILDR	47.02414	Q8WUX9	CHMP7 HUMAN	565.3147695	2	0.847407818		
LDTVQGILDR	47.02414	Q8WUX9	CHMP7 HUMAN	377.2124547	3	0.847407818		
LSLSEGGLVPSSK	40.42839	Q8WUX9	CHMP7 HUMAN	637.3540905	2	0.821351886		
LSLSEGGLVPSSK	40.42839	Q8WUX9	CHMP7 HUMAN	425.2386687	3	0.821351886		
LYQNSPLSSHPVVALSELSTLCANSCPDER	83.60446	Q8WUX9	CHMP7 HUMAN	1672.798466	2	0.768637776		
LYQNSPLSSHPVVALSELSTLCANSCPDER	83.60446	Q8WUX9	CHMP7 HUMAN	1115.534919	3	0.768637776		
MGFWAPLVLSHSR	74.2332	Q8WUX9	CHMP7 HUMAN	750.893195	2	0.777469099		
MGFWAPLVLSHSR	74.2332	Q8WUX9	CHMP7 HUMAN	500.931405	3	0.777469099		
MSFLFSAFK	85.61165	Q8WUX9	CHMP7 HUMAN	539.2760695	2	0.638261855		
MSFLFSAFK	85.61165	Q8WUX9	CHMP7 HUMAN	359.8533213	3	0.638261855		
TFYLVLLQLQK	103.4777	Q8WUX9	CHMP7 HUMAN	683.4112085	2	0.775844991		
TFYLVLLQLQK	103.4777	Q8WUX9	CHMP7 HUMAN	455.943414	3	0.775844991		
VPAEEVLVAVELLK	128.1697	Q8WUX9	CHMP7 HUMAN	754.9508985	2	0.769028604		
VPAEEVLVAVELLK	128.1697	Q8WUX9	CHMP7 HUMAN	503.6365407	3	0.769028604		
VSPVNDVDVGVYQLMQSEQLLSR	121.2555	Q8WUX9	CHMP7 HUMAN	1288.655423	2	0.624576509		
VSPVNDVDVGVYQLMQSEQLLSR	121.2555	Q8WUX9	CHMP7 HUMAN	859.4395567	3	0.624576509		
VTVLEQNGEK	-9.343212	Q8WUX9	CHMP7 HUMAN	558.798952	2	0.608377397		
VTVLEQNGEK	-9.343212	Q8WUX9	CHMP7 HUMAN	372.8685763	3	0.608377397		
WTLSNMLGDNK	58.80303	Q8WUX9	CHMP7 HUMAN	639.811536	2	0.77776289		
WTLSNMLGDNK	58.80303	Q8WUX9	CHMP7 HUMAN	426.8769657	3	0.77776289		
APESVSTIITAESIFYK	104.5274	Q8WUU5	GATD1 HUMAN	928.488569	2	0.763041079		
APESVSTIITAESIFYK	104.5274	Q8WUU5	GATD1 HUMAN	619.328321	3	0.763041079		
DQFDPASYIIGPEEDLPR	101.1699	Q8WUU5	GATD1 HUMAN	1031.492372	2	0.712760568		
DQFDPASYIIGPEEDLPR	101.1699	Q8WUU5	GATD1 HUMAN	687.9975227	3	0.712760568		
GFIQDQYCEK	14.16986	Q8WUU5	GATD1 HUMAN	644.2878905	2	0.809459686		
GFIQDQYCEK	14.16986	Q8WUU5	GATD1 HUMAN	429.861202	3	0.809459686		
GYIWTHVGPTPAITIK	54.57506	Q8WUU5	GATD1 HUMAN	877.485968	2	0.784372866		
GYIWTHVGPTPAITIK	54.57506	Q8WUU5	GATD1 HUMAN	585.326587	3	0.784372866		
MEYLEFVCHAPSEYFK	77.26824	Q8WUU5	GATD1 HUMAN	1025.458428	2	0.604099452		
MEYLEFVCHAPSEYFK	77.26824	Q8WUU5	GATD1 HUMAN	683.9748933	3	0.604099452		
SAALTWLIPTLSSPR	109.0369	Q8WUU5	GATD1 HUMAN	806.9570465	2	0.740466297		
SAALTWLIPTLSSPR	109.0369	Q8WUU5	GATD1 HUMAN	538.307306	3	0.740466297		
SSPFPTVPTRPEK	17.19278	Q8WUU5	GATD1 HUMAN	721.88609	2	0.743404448		
SSPFPTVPTRPEK	17.19278	Q8WUU5	GATD1 HUMAN	481.593335	3	0.743404448		
AAAAALSQQQSLQER	9.860516	Q8WUQ7	CATIN HUMAN	786.4109925	2	0.822085142		
AAAAALSQQQSLQER	9.860516	Q8WUQ7	CATIN HUMAN	524.6099367	3	0.822085142		
AGGPNLDMGYWESLLQQLR	151.702	Q8WUQ7	CATIN HUMAN	1074.531309	2	0.776881933		
AGGPNLDMGYWESLLQQLR	151.702	Q8WUQ7	CATIN HUMAN	716.6901473	3	0.776881933		
DMTTITEDEISK	33.82594	Q8WUQ7	CATIN HUMAN	691.821964	2	0.775738358		
DMTTITEDEISK	33.82594	Q8WUQ7	CATIN HUMAN	461.5505843	3	0.775738358		
EGMGQDEAQFSVEMPLTGK	75.06614	Q8WUQ7	CATIN HUMAN	1027.464448	2	0.746652365		
EGMGQDEAQFSVEMPLTGK	75.06614	Q8WUQ7	CATIN HUMAN	685.31224	3	0.746652365		
EQELEMLQR	26.40674	Q8WUQ7	CATIN HUMAN	588.290437	2	0.776770055		
EQELEMLQR	26.40674	Q8WUQ7	CATIN HUMAN	392.5295663	3	0.776770055		
FHAGPPYEDIAFK	37.1042	Q8WUQ7	CATIN HUMAN	746.367529	2	0.828712583	3	
FHAGPPYEDIAFK	37.1042	Q8WUQ7	CATIN HUMAN	497.9142943	3	0.828712583	3	Yes
FNIFYPDLIDK	95.45963	Q8WUQ7	CATIN HUMAN	692.8615475	2	0.763402581		
FNIFYPDLIDK	95.45963	Q8WUQ7	CATIN HUMAN	462.24364	3	0.763402581		
LLTAHELPLDAHVLEPDEDLQR	61.40983	Q8WUQ7	CATIN HUMAN	1262.656285	2	0.788411736	4	
LLTAHELPLDAHVLEPDEDLQR	61.40983	Q8WUQ7	CATIN HUMAN	842.106798	3	0.788411736	4	
MGWGEEYMGYTNTDNPFGDNNLLGTFIWNK	143.5664	Q8WUQ7	CATIN HUMAN	1742.766631	2	0.777433336		
MGWGEEYMGYTNTDNPFGDNNLLGTFIWNK	143.5664	Q8WUQ7	CATIN HUMAN	1162.180362	3	0.777433336		
STPEYFLEACADNK	42.62789	Q8WUQ7	CATIN HUMAN	822.86469	2	0.800735295		
STPEYFLEACADNK	42.62789	Q8WUQ7	CATIN HUMAN	548.9124017	3	0.800735295		
TWEEQEDNFHLQQAK	26.05005	Q8WUQ7	CATIN HUMAN	951.9350285	2	0.880419135		
TWEEQEDNFHLQQAK	26.05005	Q8WUQ7	CATIN HUMAN	634.959294	3	0.880419135		
TYNQLQVIFQGIEGK	97.7829	Q8WUQ7	CATIN HUMAN	869.4626895	2	0.777153909		
TYNQLQVIFQGIEGK	97.7829	Q8WUQ7	CATIN HUMAN	579.9777347	3	0.777153909		
VHTGFEWNK	0.476086	Q8WUQ7	CATIN HUMAN	559.275447	2	0.612343371		
VHTGFEWNK	0.476086	Q8WUQ7	CATIN HUMAN	373.1862397	3	0.612343371		
YNQTHYDFDNPPPK	7.326038	Q8WUQ7	CATIN HUMAN	868.3897165	2	0.723367929		
YNQTHYDFDNPPPK	7.326038	Q8WUQ7	CATIN HUMAN	579.2624193	3	0.723367929		
AFTLVSAVER	44.37701	Q8WUH2	TGFA1 HUMAN	546.80658	2	0.755964458		
AFTLVSAVER	44.37701	Q8WUH2	TGFA1 HUMAN	364.8736617	3	0.755964458		
EGHILQDFEGR	17.35605	Q8WUH2	TGFA1 HUMAN	650.8182075	2	0.765671432		
EGHILQDFEGR	17.35605	Q8WUH2	TGFA1 HUMAN	434.2147467	3	0.765671432		
FLMSYLNEVR	68.53154	Q8WUH2	TGFA1 HUMAN	636.3268175	2	0.798662782		

FLMSYLNEVR	68.53154	Q8WUH2	TGFA1 HUMAN	424.55382	3	0.798662782		
GVYILVPLPLEK	104.1986	Q8WUH2	TGFA1 HUMAN	670.9135835	2	0.706596792		
GVYILVPLPLEK	104.1986	Q8WUH2	TGFA1 HUMAN	447.611664	3	0.706596792		
LQGAGLPMESAILHGK	41.00488	Q8WUH2	TGFA1 HUMAN	811.440707	2	0.855171561		
LQGAGLPMESAILHGK	41.00488	Q8WUH2	TGFA1 HUMAN	541.296413	3	0.855171561		
NSFNPDDIINCLK	79.94061	Q8WUH2	TGFA1 HUMAN	775.369946	2	0.776523471		
NSFNPDDIINCLK	79.94061	Q8WUH2	TGFA1 HUMAN	517.249239	3	0.776523471		
SEEVGVQVFTK	34.42467	Q8WUH2	TGFA1 HUMAN	611.8198845	2	0.724475861		
SEEVGVQVFTK	34.42467	Q8WUH2	TGFA1 HUMAN	408.2158647	3	0.724475861		
SENLIYTYDK	26.39693	Q8WUH2	TGFA1 HUMAN	623.304058	2	0.801629901		
SENLIYTYDK	26.39693	Q8WUH2	TGFA1 HUMAN	415.8719803	3	0.801629901		
SHPPLHEYADLNQLTQGDQEK	25.59075	Q8WUH2	TGFA1 HUMAN	1210.57803	2	0.787606835		
SHPPLHEYADLNQLTQGDQEK	25.59075	Q8WUH2	TGFA1 HUMAN	807.3879617	3	0.787606835		
TIQMFLVYEDR	72.50464	Q8WUH2	TGFA1 HUMAN	707.85594	2	0.707740724		
TIQMFLVYEDR	72.50464	Q8WUH2	TGFA1 HUMAN	472.2399017	3	0.707740724		
TMQVALGLAR	39.3986	Q8WUH2	TGFA1 HUMAN	530.303151	2	0.735538185		
TMQVALGLAR	39.3986	Q8WUH2	TGFA1 HUMAN	353.8713757	3	0.735538185		
YLEHLVIDK	16.74878	Q8WUH2	TGFA1 HUMAN	565.316776	2	0.813046932		
YLEHLVIDK	16.74878	Q8WUH2	TGFA1 HUMAN	377.2137923	3	0.813046932		
ALAQVFCEESGLSPLLQK	88.05501	Q8WU76	SCFD2 HUMAN	995.5198825	2	0.611969113		
ALAQVFCEESGLSPLLQK	88.05501	Q8WU76	SCFD2 HUMAN	664.0158633	3	0.611969113		
ALWEALLNTK	81.04074	Q8WU76	SCFD2 HUMAN	579.830055	2	0.779435217	2	Yes
ALWEALLNTK	81.04074	Q8WU76	SCFD2 HUMAN	386.8893117	3	0.779435217	2	
ASVVFVDR	16.17923	Q8WU76	SCFD2 HUMAN	446.7485335	2	0.607577085		
ASVVFVDR	16.17923	Q8WU76	SCFD2 HUMAN	298.1682973	3	0.607577085		
DLVASLKPGTQVIVLSTR	74.8298	Q8WU76	SCFD2 HUMAN	949.060032	2	0.782263458		
DLVASLKPGTQVIVLSTR	74.8298	Q8WU76	SCFD2 HUMAN	633.0426297	3	0.782263458		
EFEPDAIGGGAK	20.31737	Q8WU76	SCFD2 HUMAN	595.7885845	2	0.759932637		
EFEPDAIGGGAK	20.31737	Q8WU76	SCFD2 HUMAN	397.5283313	3	0.759932637		
IAVDELFTSLR	85.72531	Q8WU76	SCFD2 HUMAN	632.3513515	2	0.758163571		
IAVDELFTSLR	85.72531	Q8WU76	SCFD2 HUMAN	421.9035093	3	0.758163571		
ITDWDSSINLTFHK	56.70634	Q8WU76	SCFD2 HUMAN	838.918118	2	0.760941088		
ITDWDSSINLTFHK	56.70634	Q8WU76	SCFD2 HUMAN	559.614687	3	0.760941088		
LLEAVGGPDCHLR	24.93985	Q8WU76	SCFD2 HUMAN	718.8699175	2	0.823540866		
LLEAVGGPDCHLR	24.93985	Q8WU76	SCFD2 HUMAN	479.5825533	3	0.823540866		
LLKPLNIPELLFATDR	106	Q8WU76	SCFD2 HUMAN	927.0489355	2	0.86339587		
LLKPLNIPELLFATDR	106	Q8WU76	SCFD2 HUMAN	618.3685653	3	0.86339587		
SVYVPGNHTHQASYKPLLK	3.582691	Q8WU76	SCFD2 HUMAN	1070.071454	2	0.616812348		
SVYVPGNHTHQASYKPLLK	3.582691	Q8WU76	SCFD2 HUMAN	713.7169107	3	0.616812348		
TLDLTGAVGHHGDNLVEK	27.00332	Q8WU76	SCFD2 HUMAN	938.482146	2	0.789322615		
TLDLTGAVGHHGDNLVEK	27.00332	Q8WU76	SCFD2 HUMAN	625.9907057	3	0.789322615		
VTPGQLMSYIQLFK	120.2295	Q8WU76	SCFD2 HUMAN	812.942547	2	0.794618487		
VTPGQLMSYIQLFK	120.2295	Q8WU76	SCFD2 HUMAN	542.2976397	3	0.794618487		
AELQSIEEGVQGQQDALNSAK	48.84926	Q8WTW3	COG1 HUMAN	1108.046036	2	0.819284856		
AELQSIEEGVQGQQDALNSAK	48.84926	Q8WTW3	COG1 HUMAN	739.0332987	3	0.819284856		
ALQLLYDLR	69.61958	Q8WTW3	COG1 HUMAN	552.8247685	2	0.711183608	2	Yes
ALQLLYDLR	69.61958	Q8WTW3	COG1 HUMAN	368.8857873	3	0.711183608	2	
AQAISPCVQNFCSALDSK	55.11642	Q8WTW3	COG1 HUMAN	998.467324	2	0.682703018		
AQAISPCVQNFCSALDSK	55.11642	Q8WTW3	COG1 HUMAN	665.9808243	3	0.682703018		
AQICSLVELLATTLK	141.1068	Q8WTW3	COG1 HUMAN	830.471673	2	0.716417253		
AQICSLVELLATTLK	141.1068	Q8WTW3	COG1 HUMAN	553.9837237	3	0.716417253		
DAMWELLTNESTNHSWDVLCR	105.4637	Q8WTW3	COG1 HUMAN	1289.079029	2	0.737953603		
DAMWELLTNESTNHSWDVLCR	105.4637	Q8WTW3	COG1 HUMAN	859.721961	3	0.737953603		
DLIEAADTIGQMR	78.379	Q8WTW3	COG1 HUMAN	716.8592155	2	0.86980623		
DLIEAADTIGQMR	78.379	Q8WTW3	COG1 HUMAN	478.2420853	3	0.86980623		
DPAALFETHGAEEIR	45.74892	Q8WTW3	COG1 HUMAN	828.4053755	2	0.814245343		
DPAALFETHGAEEIR	45.74892	Q8WTW3	COG1 HUMAN	552.606192	3	0.814245343		
EGFDSISSSSK	7.719456	Q8WTW3	COG1 HUMAN	572.262398	2	0.645124078		
EGFDSISSSSK	7.719456	Q8WTW3	COG1 HUMAN	381.844207	3	0.645124078		
ELLVSALQELESSTSNSPSNK	97.6134	Q8WTW3	COG1 HUMAN	1117.063892	2	0.665726781		
ELLVSALQELESSTSNSPSNK	97.6134	Q8WTW3	COG1 HUMAN	745.045203	3	0.665726781		
FGLLPLSMTSTR	87.03757	Q8WTW3	COG1 HUMAN	661.8610295	2	0.791931272		
FGLLPLSMTSTR	87.03757	Q8WTW3	COG1 HUMAN	441.576628	3	0.791931272		
FYSMAAQIK	26.13567	Q8WTW3	COG1 HUMAN	529.7711475	2	0.746121407		
FYSMAAQIK	26.13567	Q8WTW3	COG1 HUMAN	353.5167067	3	0.746121407		
GQFASSGLSMK	14.1277	Q8WTW3	COG1 HUMAN	556.7744225	2	0.791700482		
GQFASSGLSMK	14.1277	Q8WTW3	COG1 HUMAN	371.51889	3	0.791700482		
GTGVLQEEMK	8.220543	Q8WTW3	COG1 HUMAN	546.274256	2	0.8058604		
GTGVLQEEMK	8.220543	Q8WTW3	COG1 HUMAN	364.518779	3	0.8058604		
HLPASIVEFQPTLR	57.39212	Q8WTW3	COG1 HUMAN	804.449389	2	0.826260209		
HLPASIVEFQPTLR	57.39212	Q8WTW3	COG1 HUMAN	536.6355343	3	0.826260209		
LCQSLGELCPHLK	32.42326	Q8WTW3	COG1 HUMAN	777.8925355	2	0.622825027		
LCQSLGELCPHLK	32.42326	Q8WTW3	COG1 HUMAN	518.9309653	3	0.622825027		
LDDLLAYLPSDDSSLPK	109.1405	Q8WTW3	COG1 HUMAN	931.475658	2	0.831103325		
LDDLLAYLPSDDSSLPK	109.1405	Q8WTW3	COG1 HUMAN	621.3197137	3	0.831103325		
LGWLSSMTK	48.74129	Q8WTW3	COG1 HUMAN	511.7711515	2	0.693164825		
LGWLSSMTK	48.74129	Q8WTW3	COG1 HUMAN	341.5167093	3	0.693164825		
LLLEIPEK	51.53828	Q8WTW3	COG1 HUMAN	477.797692	2	0.809762061		
LLLEIPEK	51.53828	Q8WTW3	COG1 HUMAN	318.8677363	3	0.809762061		
NGITNLLMYVK	85.39267	Q8WTW3	COG1 HUMAN	633.350293	2	0.801331341		
NGITNLLMYVK	85.39267	Q8WTW3	COG1 HUMAN	422.5694703	3	0.801331341		
SCMVQVVAAYEK	40.90191	Q8WTW3	COG1 HUMAN	692.8341505	2	0.794759691		
SCMVQVVAAYEK	40.90191	Q8WTW3	COG1 HUMAN	462.2253753	3	0.794759691		
SSTFNSQEPHNILPLASSQIR	52.07931	Q8WTW3	COG1 HUMAN	1163.593486	2	0.779167414		
SSTFNSQEPHNILPLASSQIR	52.07931	Q8WTW3	COG1 HUMAN	776.0649323	3	0.779167414		
STAGDPTVPGSLFR	52.88849	Q8WTW3	COG1 HUMAN	702.8600725	2	0.763483405		
STAGDPTVPGSLFR	52.88849	Q8WTW3	COG1 HUMAN	468.9093233	3	0.763483405		
TLAHPISQEYLK	13.38243	Q8WTW3	COG1 HUMAN	700.383179	2	0.79086417		
TLAHPISQEYLK	13.38243	Q8WTW3	COG1 HUMAN	467.258061	3	0.79086417		
TSVLFGLVTGTENQLAPR	90.71117	Q8WTW3	COG1 HUMAN	952.0183645	2	0.767425776		
TSVLFGLVTGTENQLAPR	90.71117	Q8WTW3	COG1 HUMAN	635.0148513	3	0.767425776		
VTLQEMLK	37.91367	Q8WTW3	COG1 HUMAN	481.2735275	2	0.803204477		
VTLQEMLK	37.91367	Q8WTW3	COG1 HUMAN	321.18496	3	0.803204477		
YADAGTVQEMLR	36.69839	Q8WTW3	COG1 HUMAN	677.327547	2	0.840350807		
YADAGTVQEMLR	36.69839	Q8WTW3	COG1 HUMAN	451.8876397	3	0.840350807		
YLNIVLTAK	48.61208	Q8WTW3	COG1 HUMAN	517.816412	2	0.753691792		
YLNIVLTAK	48.61208	Q8WTW3	COG1 HUMAN	345.546883	3	0.753691792		
DSLGTAGSAHLIIK	30.73769	Q8TDH9	BL1S5 HUMAN	691.88609	2	0.844180048		
DSLGTAGSAHLIIK	30.73769	Q8TDH9	BL1S5 HUMAN	461.593335	3	0.844180048		
LLDHRPVIQGETR	-9.927528	Q8TDH9	BL1S5 HUMAN	767.428988	2	0.830681086		
LLDHRPVIQGETR	-9.927528	Q8TDH9	BL1S5 HUMAN	511.955267	3	0.830681086		

NMIHETNEHTLPK	-22.77027	Q8TDH9	BL1S5_HUMAN	782.3833885	2	0.660406113	3	
NMIHETNEHTLPK	-22.77027	Q8TDH9	BL1S5_HUMAN	521.9248673	3	0.660406113	3	Yes
AYITTLCPSIR	45.31904	Q8TDB6	DTX3L_HUMAN	647.8453755	2	0.799087107		
AYITTLCPSIR	45.31904	Q8TDB6	DTX3L_HUMAN	432.2328587	3	0.799087107		
DSLPGYESFGTIVITYSMK	121.1168	Q8TDB6	DTX3L_HUMAN	1054.516997	2	0.747407496		
DSLPGYESFGTIVITYSMK	121.1168	Q8TDB6	DTX3L_HUMAN	703.3472727	3	0.747407496		
ESFASEFQK	14.72033	Q8TDB6	DTX3L_HUMAN	536.75147	2	0.753760219		
ESFASEFQK	14.72033	Q8TDB6	DTX3L_HUMAN	358.170255	3	0.753760219		
FGGPEMYGYPDPSYLK	68.46767	Q8TDB6	DTX3L_HUMAN	910.9141745	2	0.86355269		
FGGPEMYGYPDPSYLK	68.46767	Q8TDB6	DTX3L_HUMAN	607.612058	3	0.86355269		
GGELTLLGTQDDISAAK	58.19685	Q8TDB6	DTX3L_HUMAN	844.9392485	2	0.897162795	2	Yes
GGELTLLGTQDDISAAK	58.19685	Q8TDB6	DTX3L_HUMAN	563.628774	3	0.897162795	2	
GGGMSSLAGK	-17.71931	Q8TDB6	DTX3L_HUMAN	432.7163765	2	0.654650688		
GGGMSSLAGK	-17.71931	Q8TDB6	DTX3L_HUMAN	288.813526	3	0.654650688		
GNQPEGSMVFTVSR	35.24096	Q8TDB6	DTX3L_HUMAN	754.862289	2	0.784666061		
GNQPEGSMVFTVSR	35.24096	Q8TDB6	DTX3L_HUMAN	503.5774677	3	0.784666061		
GSVSSEASELDK	-12.36671	Q8TDB6	DTX3L_HUMAN	604.7862375	2	0.695788562		
GSVSSEASELDK	-12.36671	Q8TDB6	DTX3L_HUMAN	403.5267667	3	0.695788562		
IHQFLSEQFLESEQK	46.35048	Q8TDB6	DTX3L_HUMAN	931.9683395	2	0.808613956	3	
IHQFLSEQFLESEQK	46.35048	Q8TDB6	DTX3L_HUMAN	621.648168	3	0.808613956	3	Yes
LFAANYMMNVIEVDSAHYK	80.26828	Q8TDB6	DTX3L_HUMAN	1108.529915	2	0.675629973		
LFAANYMMNVIEVDSAHYK	80.26828	Q8TDB6	DTX3L_HUMAN	739.355885	3	0.675629973		
LIFTVGYSR	46.28277	Q8TDB6	DTX3L_HUMAN	528.2983865	2	0.689984679		
LIFTVGYSR	46.28277	Q8TDB6	DTX3L_HUMAN	352.534866	3	0.689984679		
LLETELLQEISEIEK	133.1846	Q8TDB6	DTX3L_HUMAN	893.988406	2	0.852124929		
LLETELLQEISEIEK	133.1846	Q8TDB6	DTX3L_HUMAN	596.3282123	3	0.852124929		
SGDLEAAR	-29.94546	Q8TDB6	DTX3L_HUMAN	409.704323	2	0.625306845		
SGDLEAAR	-29.94546	Q8TDB6	DTX3L_HUMAN	273.472157	3	0.625306845		
SNYFEVPLPYFEYFK	135.1965	Q8TDB6	DTX3L_HUMAN	971.9672635	2	0.611742079		
SNYFEVPLPYFEYFK	135.1965	Q8TDB6	DTX3L_HUMAN	648.3141173	3	0.611742079		
SSGGGECTVSTQEHEAPGTFR	-5.356125	Q8TDB6	DTX3L_HUMAN	1097.477464	2	0.789977312		
SSGGGECTVSTQEHEAPGTFR	-5.356125	Q8TDB6	DTX3L_HUMAN	731.987584	3	0.789977312		
VCGDFQDIER	17.12786	Q8TDB6	DTX3L_HUMAN	619.777694	2	0.757151842		
VCGDFQDIER	17.12786	Q8TDB6	DTX3L_HUMAN	413.521071	3	0.757151842		
VLGVSDVITWNDIHHK	51.49085	Q8TDB6	DTX3L_HUMAN	916.9868665	2	0.774675071		
VLGVSDVITWNDIHHK	51.49085	Q8TDB6	DTX3L_HUMAN	611.6605193	3	0.774675071		
AGGGAAEQEELHYIPIR	37.68797	Q8TD19	NEK9_HUMAN	905.9583025	2	0.805090189	3	
AGGGAAEQEELHYIPIR	37.68797	Q8TD19	NEK9_HUMAN	604.3081433	3	0.805090189	3	Yes
DTLPYEELQGLK	62.17516	Q8TD19	NEK9_HUMAN	703.364652	2	0.825455666		
DTLPYEELQGLK	62.17516	Q8TD19	NEK9_HUMAN	469.2457097	3	0.825455666		
ELYTWVNMQGGTK	56.7998	Q8TD19	NEK9_HUMAN	763.869579	2	0.716299355		
ELYTWVNMQGGTK	56.7998	Q8TD19	NEK9_HUMAN	509.5823277	3	0.716299355		
GAFGEATLYR	25.27287	Q8TD19	NEK9_HUMAN	542.775276	2	0.825519025	2	Yes
GAFGEATLYR	25.27287	Q8TD19	NEK9_HUMAN	362.1861257	3	0.825519025	2	
GWHTILIVEK	36.64682	Q8TD19	NEK9_HUMAN	598.3458725	2	0.801034808		
GWHTILIVEK	36.64682	Q8TD19	NEK9_HUMAN	399.23319	3	0.801034808		
LGDYGLAK	5.781025	Q8TD19	NEK9_HUMAN	418.7298055	2	0.717669547		
LGDYGLAK	5.781025	Q8TD19	NEK9_HUMAN	279.4891453	3	0.717669547		
LGINLLGGPLGGK	79.6588	Q8TD19	NEK9_HUMAN	604.8722545	2	0.913645267	2	Yes
LGINLLGGPLGGK	79.6588	Q8TD19	NEK9_HUMAN	403.5841113	3	0.913645267	2	
LGLDSEEDYYTPQK	35.73286	Q8TD19	NEK9_HUMAN	829.3837655	2	0.893769324		
LGLDSEEDYYTPQK	35.73286	Q8TD19	NEK9_HUMAN	553.258452	3	0.893769324		
LGLNQCMSGIINHEAYHEVPYTTSFTLAK	75.09181	Q8TD19	NEK9_HUMAN	1647.800278	2	0.75838089		
LGLNQCMSGIINHEAYHEVPYTTSFTLAK	75.09181	Q8TD19	NEK9_HUMAN	1098.86946	3	0.75838089		
LHGQLGHGDK	-50	Q8TD19	NEK9_HUMAN	531.2785215	2	0.618117929		
LHGQLGHGDK	-50	Q8TD19	NEK9_HUMAN	354.5216227	3	0.618117929		
LLTFGCNK	19.64179	Q8TD19	NEK9_HUMAN	476.7502195	2	0.650135815		
LLTFGCNK	19.64179	Q8TD19	NEK9_HUMAN	318.1694213	3	0.650135815		
LQQENLQIFTQLQK	66.09843	Q8TD19	NEK9_HUMAN	865.9759685	2	0.882673502		
LQQENLQIFTQLQK	66.09843	Q8TD19	NEK9_HUMAN	577.653254	3	0.882673502		
SSTVTEAPIAVVTSR	45.60944	Q8TD19	NEK9_HUMAN	759.412669	2	0.84398824		
SSTVTEAPIAVVTSR	45.60944	Q8TD19	NEK9_HUMAN	506.6110543	3	0.84398824		
TFDATNPLNLCVK	63.10876	Q8TD19	NEK9_HUMAN	746.877408	2	0.833838105		
TFDATNPLNLCVK	63.10876	Q8TD19	NEK9_HUMAN	498.2542137	3	0.833838105		
TLNIFLTK	54.59645	Q8TD19	NEK9_HUMAN	475.2900345	2	0.664998114		
TLNIFLTK	54.59645	Q8TD19	NEK9_HUMAN	317.1959647	3	0.664998114		
TSEVYVWGGGK	27.73227	Q8TD19	NEK9_HUMAN	591.7936655	2	0.788893521	2	Yes
TSEVYVWGGGK	27.73227	Q8TD19	NEK9_HUMAN	394.865052	3	0.788893521	2	
VASEAPLEHKPQVEASSPR	-7.399143	Q8TD19	NEK9_HUMAN	1016.527084	2	0.715627551	3	
VASEAPLEHKPQVEASSPR	-7.399143	Q8TD19	NEK9_HUMAN	678.0206643	3	0.715627551	3	Yes
VLACGLNEFNK	28.97104	Q8TD19	NEK9_HUMAN	632.821904	2	0.690018117		
VLACGLNEFNK	28.97104	Q8TD19	NEK9_HUMAN	422.217211	3	0.690018117		
VTLLNAPTK	17.92525	Q8TD19	NEK9_HUMAN	478.7929415	2	0.730812669		
VTLLNAPTK	17.92525	Q8TD19	NEK9_HUMAN	319.531236	3	0.730812669		
AVVVPWGSCHFLEK	55.46404	Q8TCT8	SPP2A_HUMAN	814.9168675	2	0.709384918	3	
AVVVPWGSCHFLEK	55.46404	Q8TCT8	SPP2A_HUMAN	543.6138533	3	0.709384918	3	Yes
DMNQTLGDNITVK	39.54295	Q8TCT8	SPP2A_HUMAN	724.8566725	2	0.808151186		
DMNQTLGDNITVK	39.54295	Q8TCT8	SPP2A_HUMAN	483.5737233	3	0.808151186		
GNSYQMMDHLDCATNEENPVISGEQIVQQ	88.07626	Q8TCT8	SPP2A_HUMAN	1654.219193	2	0.626644135	3	
GNSYQMMDHLDCATNEENPVISGEQIVQQ	88.07626	Q8TCT8	SPP2A_HUMAN	1103.148737	3	0.626644135	3	Yes
ILIAFISYK	76.92965	Q8TCT8	SPP2A_HUMAN	534.329155	2	0.759624422		
ILIAFISYK	76.92965	Q8TCT8	SPP2A_HUMAN	356.5553783	3	0.759624422		
IPYGQCTIACR	16.09632	Q8TCT8	SPP2A_HUMAN	669.8188355	2	0.70508039		
IPYGQCTIACR	16.09632	Q8TCT8	SPP2A_HUMAN	446.881832	3	0.70508039		
LAEENPDLQEAYIAK	38.57975	Q8TB36	GDAP1_HUMAN	852.4285115	2	0.868307292	2	Yes
LAEENPDLQEAYIAK	38.57975	Q8TB36	GDAP1_HUMAN	568.621616	3	0.868307292	2	
LILYHWTHSFSSQK	32.88636	Q8TB36	GDAP1_HUMAN	873.952291	2	0.775585592		
LILYHWTHSFSSQK	32.88636	Q8TB36	GDAP1_HUMAN	582.9708023	3	0.775585592		
RPNLETYYER	2.11134	Q8TB36	GDAP1_HUMAN	670.8338485	2	0.806103051	2	Yes
RPNLETYYER	2.11134	Q8TB36	GDAP1_HUMAN	447.5585073	3	0.806103051	2	
VLDQVETELQR	33.4929	Q8TB36	GDAP1_HUMAN	665.354623	2	0.806625903	2	Yes
VLDQVETELQR	33.4929	Q8TB36	GDAP1_HUMAN	443.9056903	3	0.806625903	2	
VLGHVNNILISAVLPTAFR	99.97485	Q8TB36	GDAP1_HUMAN	1017.597116	2	0.721453369		
VLGHVNNILISAVLPTAFR	99.97485	Q8TB36	GDAP1_HUMAN	678.7340187	3	0.721453369		
ADGTSLDFSTYR	39.09572	Q8NG31	KNL1_HUMAN	666.807501	2	0.845045686		
ADGTSLDFSTYR	39.09572	Q8NG31	KNL1_HUMAN	444.8742757	3	0.845045686		
AFGIYLNK	37.71867	Q8NG31	KNL1_HUMAN	463.2612725	2	0.767900348		
AFGIYLNK	37.71867	Q8NG31	KNL1_HUMAN	309.17679	3	0.767900348		
ALIETYQK	5.216862	Q8NG31	KNL1_HUMAN	483.269295	2	0.726360261		
ALIETYQK	5.216862	Q8NG31	KNL1_HUMAN	322.5154717	3	0.726360261		
AQVESCQLNNR	-24.84446	Q8NG31	KNL1_HUMAN	659.812599	2	0.749013186		

AQVESCQLNNR	-24.84446	Q8NG31	KNL1_HUMAN	440.2110077	3	0.749013186
DCHLVPLAGTSETILYTCR	61.81419	Q8NG31	KNL1_HUMAN	1103.535735	2	0.707295597
DCHLVPLAGTSETILYTCR	61.81419	Q8NG31	KNL1_HUMAN	736.0264313	3	0.707295597
DKDWVLK	3.660316	Q8NG31	KNL1_HUMAN	452.250909	2	0.780552089
DKDWVLK	3.660316	Q8NG31	KNL1_HUMAN	301.8365477	3	0.780552089
DNSCVQEIAEK	4.989807	Q8NG31	KNL1_HUMAN	646.7935405	2	0.831895709
DNSCVQEIAEK	4.989807	Q8NG31	KNL1_HUMAN	431.5316353	3	0.831895709
DVQSPGFLNEPLSSK	57.66637	Q8NG31	KNL1_HUMAN	809.4101255	2	0.892409682
DVQSPGFLNEPLSSK	57.66637	Q8NG31	KNL1_HUMAN	539.942692	3	0.892409682
EFSIIEHTR	14.42129	Q8NG31	KNL1_HUMAN	566.2938365	2	0.722162127
EFSIIEHTR	14.42129	Q8NG31	KNL1_HUMAN	377.865166	3	0.722162127
EMMLQNLMTTSEDGK	67.1072	Q8NG31	KNL1_HUMAN	864.3866225	2	0.764922142
EMMLQNLMTTSEDGK	67.1072	Q8NG31	KNL1_HUMAN	576.59369	3	0.764922142
ENFEIPIYSK	55.29013	Q8NG31	KNL1_HUMAN	620.3169725	2	0.648574471
ENFEIPIYSK	55.29013	Q8NG31	KNL1_HUMAN	413.88059	3	0.648574471
ENIQTTNYNTALDFHSNSDVTK	37.84092	Q8NG31	KNL1_HUMAN	1256.583509	2	0.674492478
ENIQTTNYNTALDFHSNSDVTK	37.84092	Q8NG31	KNL1_HUMAN	838.0582807	3	0.674492478
EQTLAQIDFMQK	52.25624	Q8NG31	KNL1_HUMAN	726.364134	2	0.810634017
EQTLAQIDFMQK	52.25624	Q8NG31	KNL1_HUMAN	484.5786977	3	0.810634017
FSCLSLPETETGENLLLIQNK	98.79884	Q8NG31	KNL1_HUMAN	1203.615239	2	0.764744222
FSCLSLPETETGENLLLIQNK	98.79884	Q8NG31	KNL1_HUMAN	802.746101	3	0.764744222
GLLDNPISEK	34.30169	Q8NG31	KNL1_HUMAN	543.2960445	2	0.863728046
GLLDNPISEK	34.30169	Q8NG31	KNL1_HUMAN	362.5333047	3	0.863728046
GPVEVADNMELSK	32.73856	Q8NG31	KNL1_HUMAN	694.8404905	2	0.856775761
GPVEVADNMELSK	32.73856	Q8NG31	KNL1_HUMAN	463.5629353	3	0.856775761
GQLDCVITLHK	25.96803	Q8NG31	KNL1_HUMAN	642.3430045	2	0.752750814
GQLDCVITLHK	25.96803	Q8NG31	KNL1_HUMAN	428.5646113	3	0.752750814
IDFNDFIK	71.49322	Q8NG31	KNL1_HUMAN	506.2614735	2	0.810398042
IDFNDFIK	71.49322	Q8NG31	KNL1_HUMAN	337.8435907	3	0.810398042
IDNCLTEMETETK	28.19786	Q8NG31	KNL1_HUMAN	792.350563	2	0.76870209
IDNCLTEMETETK	28.19786	Q8NG31	KNL1_HUMAN	528.5696503	3	0.76870209
IDTTSFLANLK	71.94273	Q8NG31	KNL1_HUMAN	611.838077	2	0.681273282
IDTTSFLANLK	71.94273	Q8NG31	KNL1_HUMAN	408.227993	3	0.681273282
ILAMTPESIYSNPSIQGCK	63.19614	Q8NG31	KNL1_HUMAN	1055.021928	2	0.710744321
ILAMTPESIYSNPSIQGCK	63.19614	Q8NG31	KNL1_HUMAN	703.6838933	3	0.710744321
ILEENPK	-22.79507	Q8NG31	KNL1_HUMAN	421.7350915	2	0.634088457
ILEENPK	-22.79507	Q8NG31	KNL1_HUMAN	281.4926693	3	0.634088457
ILNSEEWFAAACK	57.28165	Q8NG31	KNL1_HUMAN	769.8695825	2	0.820878625
ILNSEEWFAAACK	57.28165	Q8NG31	KNL1_HUMAN	513.58233	3	0.820878625
IQQSLSNPLSISLTDR	69.66753	Q8NG31	KNL1_HUMAN	886.4816135	2	0.80369091
IQQSLSNPLSISLTDR	69.66753	Q8NG31	KNL1_HUMAN	591.323684	3	0.80369091
IVDVNFQSLLDEDQAPPSSLLVHK	105.5523	Q8NG31	KNL1_HUMAN	1332.698149	2	0.697248399
IVDVNFQSLLDEDQAPPSSLLVHK	105.5523	Q8NG31	KNL1_HUMAN	888.801374	3	0.697248399
IVLHTEQK	-28.81807	Q8NG31	KNL1_HUMAN	484.282741	2	0.70467782
IVLHTEQK	-28.81807	Q8NG31	KNL1_HUMAN	323.1911023	3	0.70467782
LEDNYCEITGMNTLLSAPIHTQMQQK	82.40192	Q8NG31	KNL1_HUMAN	1518.217737	2	0.689945042
LEDNYCEITGMNTLLSAPIHTQMQQK	82.40192	Q8NG31	KNL1_HUMAN	1012.481099	3	0.689945042
LIFQYVEEK	47.65512	Q8NG31	KNL1_HUMAN	584.816609	2	0.822788894
LIFQYVEEK	47.65512	Q8NG31	KNL1_HUMAN	390.213681	3	0.822788894
LLFSSSAAFAK	47.48605	Q8NG31	KNL1_HUMAN	571.31678	2	0.656503081
LLFSSSAAFAK	47.48605	Q8NG31	KNL1_HUMAN	381.213795	3	0.656503081
LLGEEIEYLK	58.29012	Q8NG31	KNL1_HUMAN	603.8349985	2	0.833793879
LLGEEIEYLK	58.29012	Q8NG31	KNL1_HUMAN	402.8926073	3	0.833793879
LLVDINK	24.06047	Q8NG31	KNL1_HUMAN	407.755827	2	0.643975854
LLVDINK	24.06047	Q8NG31	KNL1_HUMAN	272.1731597	3	0.643975854
LNLSPSQYINEENLPVYPDEINSSDSINIETEEK	104.9709	Q8NG31	KNL1_HUMAN	1947.426724	2	0.669251025
LNLSPSQYINEENLPVYPDEINSSDSINIETEEK	104.9709	Q8NG31	KNL1_HUMAN	1298.620424	3	0.669251025
LVANDSQLTPLEEWSNNR	67.16209	Q8NG31	KNL1_HUMAN	1043.514173	2	0.806359291
LVANDSQLTPLEEWSNNR	67.16209	Q8NG31	KNL1_HUMAN	696.012057	3	0.806359291
MYCNPDAMSSLTEK	36.4662	Q8NG31	KNL1_HUMAN	823.8471285	2	0.713807106
MYCNPDAMSSLTEK	36.4662	Q8NG31	KNL1_HUMAN	549.5673607	3	0.713807106
NLGVSFPK	30.4075	Q8NG31	KNL1_HUMAN	431.2456265	2	0.744232535
NLGVSFPK	30.4075	Q8NG31	KNL1_HUMAN	287.833026	3	0.744232535
NLLANQTLVYSQDLGEMTK	80.29797	Q8NG31	KNL1_HUMAN	1069.544082	2	0.797109962
NLLANQTLVYSQDLGEMTK	80.29797	Q8NG31	KNL1_HUMAN	713.3653293	3	0.797109962
NLLELEVQK	47.69299	Q8NG31	KNL1_HUMAN	543.3142375	2	0.814734638
NLLELEVQK	47.69299	Q8NG31	KNL1_HUMAN	362.5454333	3	0.814734638
NNPVEEWDSEMR	36.19875	Q8NG31	KNL1_HUMAN	753.320453	2	0.703931451
NNPVEEWDSEMR	36.19875	Q8NG31	KNL1_HUMAN	502.549577	3	0.703931451
NSLTDTWNK	12.62088	Q8NG31	KNL1_HUMAN	539.762369	2	0.798499763
NSLTDTWNK	12.62088	Q8NG31	KNL1_HUMAN	360.177521	3	0.798499763
SHIMGAETHIVSQTCNQDAR	-11.71531	Q8NG31	KNL1_HUMAN	1128.018776	2	0.745610178
SHIMGAETHIVSQTCNQDAR	-11.71531	Q8NG31	KNL1_HUMAN	752.3484587	3	0.745610178
SHTVAIDNQIFK	20.10493	Q8NG31	KNL1_HUMAN	686.8651575	2	0.841978967
SHTVAIDNQIFK	20.10493	Q8NG31	KNL1_HUMAN	458.2460467	3	0.841978967
SHTVVIGFGPSELQELGK	60.5669	Q8NG31	KNL1_HUMAN	949.5050895	2	0.767360568
SHTVVIGFGPSELQELGK	60.5669	Q8NG31	KNL1_HUMAN	633.3393347	3	0.767360568
SLNIIENSSAPICENKPK	34.16888	Q8NG31	KNL1_HUMAN	1007.51787	2	0.712253749
SLNIIENSSAPICENKPK	34.16888	Q8NG31	KNL1_HUMAN	672.0145213	3	0.712253749
SLSNPTPDYCHDK	-10.60698	Q8NG31	KNL1_HUMAN	767.3360995	2	0.754187107
SLSNPTPDYCHDK	-10.60698	Q8NG31	KNL1_HUMAN	511.8933413	3	0.754187107
SPLQDLR	13.59504	Q8NG31	KNL1_HUMAN	414.7328835	2	0.767400682
SPLQDLR	13.59504	Q8NG31	KNL1_HUMAN	276.8245307	3	0.767400682
SSQMESQFLR	20.02175	Q8NG31	KNL1_HUMAN	606.788061	2	0.834882796
SSQMESQFLR	20.02175	Q8NG31	KNL1_HUMAN	404.8613157	3	0.834882796
SVLGQNSK	-28.72742	Q8NG31	KNL1_HUMAN	416.7303405	2	0.685997307
SVLGQNSK	-28.72742	Q8NG31	KNL1_HUMAN	278.1561687	3	0.685997307
SYTIEINHRPLLEK	24.05256	Q8NG31	KNL1_HUMAN	856.9706805	2	0.804102659
SYTIEINHRPLLEK	24.05256	Q8NG31	KNL1_HUMAN	571.6497287	3	0.804102659
TDRPNFELSQR	4.158314	Q8NG31	KNL1_HUMAN	681.8422135	2	0.759497523
TDRPNFELSQR	4.158314	Q8NG31	KNL1_HUMAN	454.8974173	3	0.759497523
TGEFLAFQTVHLPPLPEQLLELGNK	134.3644	Q8NG31	KNL1_HUMAN	1396.255635	2	0.78644681
TGEFLAFQTVHLPPLPEQLLELGNK	134.3644	Q8NG31	KNL1_HUMAN	931.1730317	3	0.78644681
TILYSCGQDDMEITR	42.32394	Q8NG31	KNL1_HUMAN	901.4089395	2	0.838080525
TILYSCGQDDMEITR	42.32394	Q8NG31	KNL1_HUMAN	601.2752347	3	0.838080525
TIYSGEENMDITK	21.65119	Q8NG31	KNL1_HUMAN	750.8485085	2	0.75610292
TIYSGEENMDITK	21.65119	Q8NG31	KNL1_HUMAN	500.901614	3	0.75610292
TLLPNEIAIRPMDK	51.37717	Q8NG31	KNL1_HUMAN	805.950907	2	0.767831683
TLLPNEIAIRPMDK	51.37717	Q8NG31	KNL1_HUMAN	537.6365463	3	0.767831683
TNLEHTTGQLTTMNR	1.619457	Q8NG31	KNL1_HUMAN	858.9208675	2	0.815589905
TNLEHTTGQLTTMNR	1.619457	Q8NG31	KNL1_HUMAN	572.9498533	3	0.815589905

TPSSCSSSLDSIK	0.12542	Q8NG31	KNL1_HUMAN	684.8197545	2	0.645443618		
TPSSCSSSLDSIK	0.12542	Q8NG31	KNL1_HUMAN	456.8824447	3	0.645443618		
TVFYSSCNDAMEMTK	39.07526	Q8NG31	KNL1_HUMAN	892.370968	2	0.809690833		
TVFYSSCNDAMEMTK	39.07526	Q8NG31	KNL1_HUMAN	595.2499203	3	0.809690833		
TVLFTDNYSDLEVTDSHTVFIDCQATEK	80.07787	Q8NG31	KNL1_HUMAN	1624.751175	2	0.736699998		
TVLFTDNYSDLEVTDSHTVFIDCQATEK	80.07787	Q8NG31	KNL1_HUMAN	1083.503391	3	0.736699998		
TVVFVDNHVELEMTESHTVFIDYQEK	77.82016	Q8NG31	KNL1_HUMAN	1555.245331	2	0.641706526		
TVVFVDNHVELEMTESHTVFIDYQEK	77.82016	Q8NG31	KNL1_HUMAN	1037.166162	3	0.641706526		
TWVQEEEDIHK	5.305046	Q8NG31	KNL1_HUMAN	707.33643	2	0.630934298		
TWVQEEEDIHK	5.305046	Q8NG31	KNL1_HUMAN	471.8935617	3	0.630934298		
VPLENNYLK	22.43376	Q8NG31	KNL1_HUMAN	545.3011255	2	0.783592641		
VPLENNYLK	22.43376	Q8NG31	KNL1_HUMAN	363.8700253	3	0.783592641		
VVDQACTLEK	-11.97289	Q8NG31	KNL1_HUMAN	581.792813	2	0.610546172		
VVDQACTLEK	-11.97289	Q8NG31	KNL1_HUMAN	388.197817	3	0.610546172		
ASGGSLQGHDAVLR	-17.77166	Q8NEJ9	NGDN_HUMAN	684.3554885	2	0.819005668		
ASGGSLQGHDAVLR	-17.77166	Q8NEJ9	NGDN_HUMAN	456.572934	3	0.819005668		
EQYSDAPEEIR	5.379879	Q8NEJ9	NGDN_HUMAN	668.804958	2	0.705243468	2	Yes
EQYSDAPEEIR	5.379879	Q8NEJ9	NGDN_HUMAN	446.2059137	3	0.705243468	2	
INYEESMMVR	30.28697	Q8NEJ9	NGDN_HUMAN	636.2921175	2	0.725163043		
INYEESMMVR	30.28697	Q8NEJ9	NGDN_HUMAN	424.5306867	3	0.725163043		
LRPLDQK	-29.69913	Q8NEJ9	NGDN_HUMAN	435.264351	2	0.614076853		
LRPLDQK	-29.69913	Q8NEJ9	NGDN_HUMAN	290.5121757	3	0.614076853		
NLQEQVMAVTAQVK	60.04367	Q8NEJ9	NGDN_HUMAN	779.917065	2	0.778973758		
NLQEQVMAVTAQVK	60.04367	Q8NEJ9	NGDN_HUMAN	520.2806517	3	0.778973758		
TAVTGSLSENDPLR	27.80373	Q8NEJ9	NGDN_HUMAN	730.3735435	2	0.601507246	2	Yes
TAVTGSLSENDPLR	27.80373	Q8NEJ9	NGDN_HUMAN	487.2516373	3	0.601507246	2	
AAAAAASAGSSASSGNQPPQELGLGELLEEFSR	129.3083	Q8NCE2	MTMRE_HUMAN	1587.271458	2	0.735088348		
AAAAAASAGSSASSGNQPPQELGLGELLEEFSR	129.3083	Q8NCE2	MTMRE_HUMAN	1058.516913	3	0.735088348		
AVAPSPSGAIGGLLEQFAR	95.70923	Q8NCE2	MTMRE_HUMAN	920.9999745	2	0.84221077		
AVAPSPSGAIGGLLEQFAR	95.70923	Q8NCE2	MTMRE_HUMAN	614.3359247	3	0.84221077		
AVLHTDSSLPFSFPDELPNSCLLAALSDR	127.8453	Q8NCE2	MTMRE_HUMAN	1586.785158	2	0.628778577		
AVLHTDSSLPFSFPDELPNSCLLAALSDR	127.8453	Q8NCE2	MTMRE_HUMAN	1058.192713	3	0.628778577		
DGGFTLEDICMLR	97.36914	Q8NCE2	MTMRE_HUMAN	763.853076	2	0.757071495		
DGGFTLEDICMLR	97.36914	Q8NCE2	MTMRE_HUMAN	509.5713257	3	0.757071495		
DTFESTVQVSK	20.49458	Q8NCE2	MTMRE_HUMAN	620.806974	2	0.699057639		
DTFESTVQVSK	20.49458	Q8NCE2	MTMRE_HUMAN	414.2072577	3	0.699057639		
DYMAEGLIFNWK	109.6129	Q8NCE2	MTMRE_HUMAN	743.8559395	2	0.751970291		
DYMAEGLIFNWK	109.6129	Q8NCE2	MTMRE_HUMAN	496.2399013	3	0.751970291		
FGMNVTSSEK	6.465828	Q8NCE2	MTMRE_HUMAN	550.258605	2	0.687570572		
FGMNVTSSEK	6.465828	Q8NCE2	MTMRE_HUMAN	367.1750117	3	0.687570572		
FVCPVILFK	78.93167	Q8NCE2	MTMRE_HUMAN	561.8231875	2	0.640042722		
FVCPVILFK	78.93167	Q8NCE2	MTMRE_HUMAN	374.8847333	3	0.640042722		
HITSEEFSALK	11.27523	Q8NCE2	MTMRE_HUMAN	631.3253335	2	0.721790791		
HITSEEFSALK	11.27523	Q8NCE2	MTMRE_HUMAN	421.2194973	3	0.721790791		
HIVFLEYESSEK	32.3688	Q8NCE2	MTMRE_HUMAN	740.8701005	2	0.806989789		
HIVFLEYESSEK	32.3688	Q8NCE2	MTMRE_HUMAN	494.249342	3	0.806989789		
LPSQQGLAEAR	-1.894905	Q8NCE2	MTMRE_HUMAN	585.3178435	2	0.780098319		
LPSQQGLAEAR	-1.894905	Q8NCE2	MTMRE_HUMAN	390.5478373	3	0.780098319		
LQDLIHR	-3.537407	Q8NCE2	MTMRE_HUMAN	447.7619755	2	0.707855344		
LQDLIHR	-3.537407	Q8NCE2	MTMRE_HUMAN	298.8439253	3	0.707855344		
MGSSPLEVPKPR	13.46424	Q8NCE2	MTMRE_HUMAN	649.3508285	2	0.733297467		
MGSSPLEVPKPR	13.46424	Q8NCE2	MTMRE_HUMAN	433.236494	3	0.733297467		
SAFLAAYSSTVGLR	62.99802	Q8NCE2	MTMRE_HUMAN	721.8860855	2	0.785900176		
SAFLAAYSSTVGLR	62.99802	Q8NCE2	MTMRE_HUMAN	481.593332	3	0.785900176		
SATLAGWGELYGR	62.65214	Q8NCE2	MTMRE_HUMAN	690.8495035	2	0.721808791		
SATLAGWGELYGR	62.65214	Q8NCE2	MTMRE_HUMAN	460.9022773	3	0.721808791		
SGYNYFFSGGADDAWADVEDVTEEDCALR	115.4219	Q8NCE2	MTMRE_HUMAN	1630.175826	2	0.697262287		
SGYNYFFSGGADDAWADVEDVTEEDCALR	115.4219	Q8NCE2	MTMRE_HUMAN	1087.119826	3	0.697262287		
YICDLMVENK	35.39922	Q8NCE2	MTMRE_HUMAN	642.8023185	2	0.698373735		
YICDLMVENK	35.39922	Q8NCE2	MTMRE_HUMAN	428.8708207	3	0.698373735		
IINLSSLAHVAGHIDFDDLNWQTR	98.71486	Q8NBN7	RDH13_HUMAN	1368.199182	2	0.61467731		
IINLSSLAHVAGHIDFDDLNWQTR	98.71486	Q8NBN7	RDH13_HUMAN	912.4687293	3	0.61467731		
LAIVLFTK	63.39133	Q8NBN7	RDH13_HUMAN	452.7974955	2	0.67850095	2	Yes
LAIVLFTK	63.39133	Q8NBN7	RDH13_HUMAN	302.2009387	3	0.67850095	2	
LQGSGVTVNALHPGVAR	23.971	Q8NBN7	RDH13_HUMAN	838.466102	2	0.786137581		
LQGSGVTVNALHPGVAR	23.971	Q8NBN7	RDH13_HUMAN	559.313343	3	0.786137581		
LVGLEAPSVR	35.73686	Q8NBN7	RDH13_HUMAN	520.8091225	2	0.731141686		
LVGLEAPSVR	35.73686	Q8NBN7	RDH13_HUMAN	347.5420233	3	0.731141686		
SPELAAQPSTYLAVAEELADVSGK	127.2251	Q8NBN7	RDH13_HUMAN	1223.621572	2	0.794171453		
SPELAAQPSTYLAVAEELADVSGK	127.2251	Q8NBN7	RDH13_HUMAN	816.083656	3	0.794171453		
TVIVTGANTGIGK	18.15788	Q8NBN7	RDH13_HUMAN	615.856802	2	0.812367558	2	Yes
TVIVTGANTGIGK	18.15788	Q8NBN7	RDH13_HUMAN	410.907143	3	0.812367558	2	
VDILINNAGVMR	56.0126	Q8NBN7	RDH13_HUMAN	657.864103	2	0.604782403		
VDILINNAGVMR	56.0126	Q8NBN7	RDH13_HUMAN	438.9120103	3	0.604782403		
ALANESGLNFLAIK	74.35773	Q8NB90	AFG2H_HUMAN	730.9095645	2	0.844659865	2	Yes
ALANESGLNFLAIK	74.35773	Q8NB90	AFG2H_HUMAN	487.6089847	3	0.844659865	2	
APSAGSDFAATSGTLTVTNLLEK	85.22441	Q8NB90	AFG2H_HUMAN	1126.076805	2	0.754686892		
APSAGSDFAATSGTLTVTNLLEK	85.22441	Q8NB90	AFG2H_HUMAN	751.0538113	3	0.754686892		
ASDVLLDVTQSPGDGSGLMLEEVTGLK	122.4829	Q8NB90	AFG2H_HUMAN	1366.189497	2	0.815492153		
ASDVLLDVTQSPGDGSGLMLEEVTGLK	122.4829	Q8NB90	AFG2H_HUMAN	911.1289397	3	0.815492153		
AVANEVGAYVSVINGPEIISK	86.57104	Q8NB90	AFG2H_HUMAN	1065.576238	2	0.663808763		
AVANEVGAYVSVINGPEIISK	86.57104	Q8NB90	AFG2H_HUMAN	710.7201003	3	0.663808763		
DFLQAMNDIRPSAMR	69.96184	Q8NB90	AFG2H_HUMAN	882.93018	2	0.801645458	3	
DFLQAMNDIRPSAMR	69.96184	Q8NB90	AFG2H_HUMAN	588.9560617	3	0.801645458	3	Yes
DVTILAATNRPDR	21.69769	Q8NB90	AFG2H_HUMAN	721.392071	2	0.769430876	3	
DVTILAATNRPDR	21.69769	Q8NB90	AFG2H_HUMAN	481.263989	3	0.769430876	3	Yes
EAALLALEEDIQANLIMK	132.2311	Q8NB90	AFG2H_HUMAN	993.035365	2	0.722538173		
EAALLALEEDIQANLIMK	132.2311	Q8NB90	AFG2H_HUMAN	662.3595183	3	0.722538173		
EGNEQLTEEER	-23.35842	Q8NB90	AFG2H_HUMAN	667.2975015	2	0.681126773		
EGNEQLTEEER	-23.35842	Q8NB90	AFG2H_HUMAN	445.2009427	3	0.681126773		
EIIELPLK	59.65451	Q8NB90	AFG2H_HUMAN	477.797692	2	0.764678776		
EIIELPLK	59.65451	Q8NB90	AFG2H_HUMAN	318.8677363	3	0.764678776		
FYEDYQEK	-0.870113	Q8NB90	AFG2H_HUMAN	561.2434695	2	0.770385563		
FYEDYQEK	-0.870113	Q8NB90	AFG2H_HUMAN	374.4982547	3	0.770385563		
GADGMILGGPQSDSDTDAQR	33.15321	Q8NB90	AFG2H_HUMAN	995.942725	2	0.813254058		
GADGMILGGPQSDSDTDAQR	33.15321	Q8NB90	AFG2H_HUMAN	664.2977583	3	0.813254058		
GPELMNK	-18.78285	Q8NB90	AFG2H_HUMAN	394.7027375	2	0.61363703		
GPELMNK	-18.78285	Q8NB90	AFG2H_HUMAN	263.4711	3	0.61363703		
GSSLGAGNVADR	-15.4138	Q8NB90	AFG2H_HUMAN	552.276175	2	0.825123549		
GSSLGAGNVADR	-15.4138	Q8NB90	AFG2H_HUMAN	368.5200583	3	0.825123549		
GVLLYGPPGCSK	35.52659	Q8NB90	AFG2H_HUMAN	624.3268185	2	0.76069355	2	Yes

GVLLYGPPGCSK	35.52659	Q8NB90	AFG2H_HUMAN	416.5538207	3	0.76069355	2	
HFTQALSTVTPR	14.90846	Q8NB90	AFG2H_HUMAN	679.3653255	2	0.834307075		
HFTQALSTVTPR	14.90846	Q8NB90	AFG2H_HUMAN	453.2461587	3	0.834307075		
IIYVPLPDAATR	68.9771	Q8NB90	AFG2H_HUMAN	664.882815	2	0.787354946	2	Yes
IIYVPLPDAATR	68.9771	Q8NB90	AFG2H_HUMAN	443.5911517	3	0.787354946	2	
LEQAVEWPLK	52.04515	Q8NB90	AFG2H_HUMAN	606.8353375	2	0.768095493		
LEQAVEWPLK	52.04515	Q8NB90	AFG2H_HUMAN	404.8928333	3	0.768095493		
STPYKPIDDR	-17.79107	Q8NB90	AFG2H_HUMAN	596.304397	2	0.611572504		
STPYKPIDDR	-17.79107	Q8NB90	AFG2H_HUMAN	397.8722063	3	0.611572504		
SYGIPAPR	11.96386	Q8NB90	AFG2H_HUMAN	430.735422	2	0.662241399		
SYGIPAPR	11.96386	Q8NB90	AFG2H_HUMAN	287.4928897	3	0.662241399		
TFQNSLIHLGLNTMK	60.57117	Q8NB90	AFG2H_HUMAN	858.9592635	2	0.851416767		
TFQNSLIHLGLNTMK	60.57117	Q8NB90	AFG2H_HUMAN	572.9754507	3	0.851416767		
VGLSEMAQK	4.812302	Q8NB90	AFG2H_HUMAN	481.752959	2	0.779190779		
VGLSEMAQK	4.812302	Q8NB90	AFG2H_HUMAN	321.504581	3	0.779190779		
VLAQLLTEMDGIEQLK	107.433	Q8NB90	AFG2H_HUMAN	900.9929695	2	0.891107202		
VLAQLLTEMDGIEQLK	107.433	Q8NB90	AFG2H_HUMAN	600.9979213	3	0.891107202		
VLCNEAGLCALR	33.24211	Q8NB90	AFG2H_HUMAN	688.345221	2	0.853214145		
VLCNEAGLCALR	33.24211	Q8NB90	AFG2H_HUMAN	459.2327557	3	0.853214145		
VTYDMIGGLSSQLK	65.78131	Q8NB90	AFG2H_HUMAN	756.3928865	2	0.881891906		
VTYDMIGGLSSQLK	65.78131	Q8NB90	AFG2H_HUMAN	504.597866	3	0.881891906		
AQDTQPSDATSAPGAEGLEPPAAR	30.93633	Q8N884	CGAS_HUMAN	1169.05185	2	0.801024675		
AQDTQPSDATSAPGAEGLEPPAAR	30.93633	Q8N884	CGAS_HUMAN	779.7038417	3	0.801024675		
DDISTAAGMVK	27.85818	Q8N884	CGAS_HUMAN	554.271713	2	0.824111462		
DDISTAAGMVK	27.85818	Q8N884	CGAS_HUMAN	369.850417	3	0.824111462		
ENPLSQFLEGEILSASK	125.4536	Q8N884	CGAS_HUMAN	931.481279	2	0.662777662		
ENPLSQFLEGEILSASK	125.4536	Q8N884	CGAS_HUMAN	621.323461	3	0.662777662		
GAPMDPTESPAAPEAALPK	46.14046	Q8N884	CGAS_HUMAN	925.4542085	2	0.77046299		
GAPMDPTESPAAPEAALPK	46.14046	Q8N884	CGAS_HUMAN	617.305414	3	0.77046299		
GGSPAVTLLISEK	55.60419	Q8N884	CGAS_HUMAN	636.364459	2	0.697061837	2	Yes
GGSPAVTLLISEK	55.60419	Q8N884	CGAS_HUMAN	424.5789143	3	0.697061837	2	
GVGLLNTGSYYEHVK	34.68479	Q8N884	CGAS_HUMAN	818.9206525	2	0.890970945		
GVGLLNTGSYYEHVK	34.68479	Q8N884	CGAS_HUMAN	546.2830433	3	0.890970945		
GVVDHLLLR	35.48738	Q8N884	CGAS_HUMAN	511.3118325	2	0.711946487		
GVVDHLLLR	35.48738	Q8N884	CGAS_HUMAN	341.2104967	3	0.711946487		
IQLEEYSNTR	13.36424	Q8N884	CGAS_HUMAN	626.812586	2	0.76291573		
IQLEEYSNTR	13.36424	Q8N884	CGAS_HUMAN	418.210999	3	0.76291573		
ISAPNEFDVMFK	71.80917	Q8N884	CGAS_HUMAN	699.342669	2	0.87734586	2	Yes
ISAPNEFDVMFK	71.80917	Q8N884	CGAS_HUMAN	466.5643877	3	0.87734586	2	
LKPFYLVPK	33.13502	Q8N884	CGAS_HUMAN	552.8449725	2	0.733190417		
LKPFYLVPK	33.13502	Q8N884	CGAS_HUMAN	368.8992567	3	0.733190417		
LSFSHIEK	-6.889748	Q8N884	CGAS_HUMAN	480.7616405	2	0.769867539		
LSFSHIEK	-6.889748	Q8N884	CGAS_HUMAN	320.843702	3	0.769867539		
SSWPASTQEGLR	18.72732	Q8N884	CGAS_HUMAN	659.8234895	2	0.758610904	2	Yes
SSWPASTQEGLR	18.72732	Q8N884	CGAS_HUMAN	440.218268	3	0.758610904	2	
TAFFHVCTQNPQDSQWDR	41.27414	Q8N884	CGAS_HUMAN	1118.995627	2	0.795968175	3	
TAFFHVCTQNPQDSQWDR	41.27414	Q8N884	CGAS_HUMAN	746.333026	3	0.795968175	3	Yes
YLLEQLK	36.41196	Q8N884	CGAS_HUMAN	453.7689305	2	0.800416231		
YLLEQLK	36.41196	Q8N884	CGAS_HUMAN	302.848562	3	0.800416231		
ALLLQAK	13.99158	Q8N584	TT39C_HUMAN	378.753088	2	0.649867117		
ALLLQAK	13.99158	Q8N584	TT39C_HUMAN	252.8380003	3	0.649867117		
ALPNCSFPNLQR	41.62438	Q8N584	TT39C_HUMAN	708.8568095	2	0.7511127		
ALPNCSFPNLQR	41.62438	Q8N584	TT39C_HUMAN	472.907148	3	0.7511127		
EDFSGYDFENR	36.86576	Q8N584	TT39C_HUMAN	689.7814825	2	0.836847782	2	Yes
EDFSGYDFENR	36.86576	Q8N584	TT39C_HUMAN	460.1902633	3	0.836847782	2	
IINLLGFPGDR	80.62222	Q8N584	TT39C_HUMAN	607.848779	2	0.826344073	2	Yes
IINLLGFPGDR	80.62222	Q8N584	TT39C_HUMAN	405.568461	3	0.826344073	2	
LCESEEAGVIETIK	38.7831	Q8N584	TT39C_HUMAN	789.390545	2	0.798663616		
LCESEEAGVIETIK	38.7831	Q8N584	TT39C_HUMAN	526.596305	3	0.798663616		
LQGLSSLMYASESK	54.59583	Q8N584	TT39C_HUMAN	757.382518	2	0.765629292		
LQGLSSLMYASESK	54.59583	Q8N584	TT39C_HUMAN	505.2576203	3	0.765629292		
LTEESLTSDAANDNHIVAEGVSEESLNR	49.44271	Q8N584	TT39C_HUMAN	1500.705615	2	0.707619607	3	
LTEESLTSDAANDNHIVAEGVSEESLNR	49.44271	Q8N584	TT39C_HUMAN	1000.806352	3	0.707619607	3	Yes
MQLACDDLK	13.30239	Q8N584	TT39C_HUMAN	547.255009	2	0.758640409		
MQLACDDLK	13.30239	Q8N584	TT39C_HUMAN	365.1726143	3	0.758640409		
MSQACHEVDDSSVVGLK	13.25016	Q8N584	TT39C_HUMAN	931.4251245	2	0.65248853		
MSQACHEVDDSSVVGLK	13.25016	Q8N584	TT39C_HUMAN	621.2860247	3	0.65248853		
YLLLGAIHK	33.2063	Q8N584	TT39C_HUMAN	514.319122	2	0.753739715	2	Yes
YLLLGAIHK	33.2063	Q8N584	TT39C_HUMAN	343.2153563	3	0.753739715	2	
DSDGQVFGALASEPLK	71.6172	Q8N573	OXR1_HUMAN	817.4075835	2	0.864067674		
DSDGQVFGALASEPLK	71.6172	Q8N573	OXR1_HUMAN	545.2743307	3	0.864067674		
DSLNSIALK	33.66219	Q8N573	OXR1_HUMAN	480.772205	2	0.723640025		
DSLNSIALK	33.66219	Q8N573	OXR1_HUMAN	320.850745	3	0.723640025		
EDQIADNFQGISGPK	41.71202	Q8N573	OXR1_HUMAN	809.889558	2	0.791340232		
EDQIADNFQGISGPK	41.71202	Q8N573	OXR1_HUMAN	540.2623137	3	0.791340232		
ESLPIDIDQLSGR	71.00637	Q8N573	OXR1_HUMAN	721.8784615	2	0.758438826		
ESLPIDIDQLSGR	71.00637	Q8N573	OXR1_HUMAN	481.5882493	3	0.758438826		
EWEVVSVAEYHR	39.31416	Q8N573	OXR1_HUMAN	752.365517	2	0.697454989		
EWEVVSVAEYHR	39.31416	Q8N573	OXR1_HUMAN	501.912953	3	0.697454989		
FDTTPNELVQLNK	54.02218	Q8N573	OXR1_HUMAN	759.894112	2	0.869590044		
FDTTPNELVQLNK	54.02218	Q8N573	OXR1_HUMAN	506.9320163	3	0.869590044		
FYTIDTGQK	13.73384	Q8N573	OXR1_HUMAN	536.7696595	2	0.849023879		
FYTIDTGQK	13.73384	Q8N573	OXR1_HUMAN	358.1823813	3	0.849023879		
GTIEYTVESR	10.2154	Q8N573	OXR1_HUMAN	577.78858	2	0.747383654	2	Yes
GTIEYTVESR	10.2154	Q8N573	OXR1_HUMAN	385.5283283	3	0.747383654	2	
HEYWFAVPQER	40.15335	Q8N573	OXR1_HUMAN	731.3496705	2	0.791914821		
HEYWFAVPQER	40.15335	Q8N573	OXR1_HUMAN	487.9023887	3	0.791914821		
IDALNTEELR	29.79533	Q8N573	OXR1_HUMAN	587.3096835	2	0.803800106		
IDALNTEELR	29.79533	Q8N573	OXR1_HUMAN	391.8757307	3	0.803800106		
IEESETIEDSSNQAAAR	5.919868	Q8N573	OXR1_HUMAN	925.4246895	2	0.671916068		
IEESETIEDSSNQAAAR	5.919868	Q8N573	OXR1_HUMAN	617.2857347	3	0.671916068		
ITSADGHIESSALLK	23.41896	Q8N573	OXR1_HUMAN	771.4126685	2	0.612375021		
ITSADGHIESSALLK	23.41896	Q8N573	OXR1_HUMAN	514.611054	3	0.612375021		
SQSVDINAPGFNPLAGAGK	65.57134	Q8N573	OXR1_HUMAN	921.9714135	2	0.784040689		
SQSVDINAPGFNPLAGAGK	65.57134	Q8N573	OXR1_HUMAN	614.9835507	3	0.784040689		
STEESLSEDVFTESELSPIR	86.21967	Q8N573	OXR1_HUMAN	1128.032258	2	0.632555604		
STEESLSEDVFTESELSPIR	86.21967	Q8N573	OXR1_HUMAN	752.357447	3	0.632555604		
TDLNNLEMAIK	51.86594	Q8N573	OXR1_HUMAN	631.327019	2	0.836386681		
TDLNNLEMAIK	51.86594	Q8N573	OXR1_HUMAN	421.220621	3	0.836386681		
TFVSQASATMQQYAQR	47.50667	Q8N573	OXR1_HUMAN	908.9365135	2	0.790201247		
TFVSQASATMQQYAQR	47.50667	Q8N573	OXR1_HUMAN	606.2936173	3	0.790201247		

TIGYPWTLVYGTGK	82.98259	Q8N573	OXR1_HUMAN	778.411933	2	0.7606287		
TIGYPWTLVYGTGK	82.98259	Q8N573	OXR1_HUMAN	519.2772303	3	0.7606287		
TMTGLDTPVLMVIK	93.68332	Q8N573	OXR1_HUMAN	759.917688	2	0.709915161		
TMTGLDTPVLMVIK	93.68332	Q8N573	OXR1_HUMAN	506.9477337	3	0.709915161		
TTNPDVHPTEATPSSTFTGIRPAR	21.65087	Q8N573	OXR1_HUMAN	1277.138791	2	0.69618547		
TTNPDVHPTEATPSSTFTGIRPAR	21.65087	Q8N573	OXR1_HUMAN	851.7618023	3	0.69618547		
VVSSTSEEEEAFTEK	13.24337	Q8N573	OXR1_HUMAN	836.3839705	2	0.721794903	2	Yes
VVSSTSEEEEAFTEK	13.24337	Q8N573	OXR1_HUMAN	557.9252553	3	0.721794903	2	
WTGDNMFFIK	73.50552	Q8N573	OXR1_HUMAN	629.8004405	2	0.790381193		
WTGDNMFFIK	73.50552	Q8N573	OXR1_HUMAN	420.202902	3	0.790381193		
ELMNLDFHEK	33.80917	Q8N543	OGFD1_HUMAN	638.3060865	2	0.838734567		
ELMNLDFHEK	33.80917	Q8N543	OGFD1_HUMAN	425.8733327	3	0.838734567		
EVMAEFSDAVTEETLK	68.20007	Q8N543	OGFD1_HUMAN	899.924949	2	0.675854266	2	Yes
EVMAEFSDAVTEETLK	68.20007	Q8N543	OGFD1_HUMAN	600.2859077	3	0.675854266	2	
GEDEELLTVNPESNSLALVYR	93.53844	Q8N543	OGFD1_HUMAN	1174.584988	2	0.755055666		
GEDEELLTVNPESNSLALVYR	93.53844	Q8N543	OGFD1_HUMAN	783.3926003	3	0.755055666		
IDLESTIDMSCAK	56.80425	Q8N543	OGFD1_HUMAN	741.844916	2	0.837383032		
IDLESTIDMSCAK	56.80425	Q8N543	OGFD1_HUMAN	494.899219	3	0.837383032		
ILFEDFR	56.10298	Q8N543	OGFD1_HUMAN	470.250909	2	0.604140401		
ILFEDFR	56.10298	Q8N543	OGFD1_HUMAN	313.8365477	3	0.604140401		
LHFLAPSEEDEMNDK	29.40708	Q8N543	OGFD1_HUMAN	887.9018075	2	0.709195435		
LHFLAPSEEDEMNDK	29.40708	Q8N543	OGFD1_HUMAN	592.27048	3	0.709195435		
LSISGWFHGPSLTRPPNYFEPPIPR	95.77252	Q8N543	OGFD1_HUMAN	1433.245931	2	0.602883935		
LSISGWFHGPSLTRPPNYFEPPIPR	95.77252	Q8N543	OGFD1_HUMAN	955.8332287	3	0.602883935		
LVFFEVSPVSFHQVSEVLSEEK	108.045	Q8N543	OGFD1_HUMAN	1268.652678	2	0.717167497		
LVFFEVSPVSFHQVSEVLSEEK	108.045	Q8N543	OGFD1_HUMAN	846.1043937	3	0.717167497		
SEALFLLLSNFTGLK	148.3729	Q8N543	OGFD1_HUMAN	826.9670795	2	0.737414539	3	
SEALFLLLSNFTGLK	148.3729	Q8N543	OGFD1_HUMAN	551.647328	3	0.737414539	3	Yes
SMGGTLDLYSIDEHFQPK	66.9351	Q8N543	OGFD1_HUMAN	1019.483493	2	0.793924809	3	
SMGGTLDLYSIDEHFQPK	66.9351	Q8N543	OGFD1_HUMAN	679.9916033	3	0.793924809	3	Yes
TGHYTLIHDHSK	-37.27956	Q8N543	OGFD1_HUMAN	704.8525775	2	0.717399299		
TGHYTLIHDHSK	-37.27956	Q8N543	OGFD1_HUMAN	470.23766	3	0.717399299		
YEFTDALLCHDDELEGR	62.09342	Q8N543	OGFD1_HUMAN	1041.957839	2	0.64305836	3	
YEFTDALLCHDDELEGR	62.09342	Q8N543	OGFD1_HUMAN	694.974501	3	0.64305836	3	Yes
DCPVPLPGDGDLLVR	83.30455	Q8N4Q0	PTGR3_HUMAN	811.914522	2	0.693633556	2	Yes
DCPVPLPGDGDLLVR	83.30455	Q8N4Q0	PTGR3_HUMAN	541.6122897	3	0.693633556	2	
FTGLESIFR	67.19308	Q8N4Q0	PTGR3_HUMAN	535.288023	2	0.707739294		
FTGLESIFR	67.19308	Q8N4Q0	PTGR3_HUMAN	357.1946237	3	0.707739294		
FVGVNASDINYSAGR	38.86936	Q8N4Q0	PTGR3_HUMAN	785.3869805	2	0.860389948		
FVGVNASDINYSAGR	38.86936	Q8N4Q0	PTGR3_HUMAN	523.927262	3	0.860389948		
HFLDFQGSAIPQAMQK	58.84347	Q8N4Q0	PTGR3_HUMAN	909.454346	2	0.881718338		
HFLDFQGSAIPQAMQK	58.84347	Q8N4Q0	PTGR3_HUMAN	606.638839	3	0.881718338		
SASVQGFFLNHYLSK	56.64973	Q8N4Q0	PTGR3_HUMAN	849.4364735	2	0.715054929		
SASVQGFFLNHYLSK	56.64973	Q8N4Q0	PTGR3_HUMAN	566.626924	3	0.715054929		
SLGCDRPINYK	-13.12847	Q8N4Q0	PTGR3_HUMAN	661.830256	2	0.771522582		
SLGCDRPINYK	-13.12847	Q8N4Q0	PTGR3_HUMAN	441.5561123	3	0.771522582		
VLVTAAAGGTGQFAMQLSK	64.30022	Q8N4Q0	PTGR3_HUMAN	925.4962115	2	0.649336994		
VLVTAAAGGTGQFAMQLSK	64.30022	Q8N4Q0	PTGR3_HUMAN	617.333416	3	0.649336994		
LDPEEMK	-4.15678	Q8N4H5	TOM5_HUMAN	431.20531	2	0.623915911	2	Yes
LDPEEMK	-4.15678	Q8N4H5	TOM5_HUMAN	287.8061483	3	0.623915911	2	
ALVFDSTFDQYR	66.08264	Q8N442	GUF1_HUMAN	731.3546185	2	0.86474824		
ALVFDSTFDQYR	66.08264	Q8N442	GUF1_HUMAN	487.9056873	3	0.86474824		
AQTASLFYNCEGK	24.07861	Q8N442	GUF1_HUMAN	744.843561	2	0.725338817		
AQTASLFYNCEGK	24.07861	Q8N442	GUF1_HUMAN	496.8983157	3	0.725338817		
ATGAALLVAPGPR	37.17041	Q8N442	GUF1_HUMAN	597.3542295	2	0.825842798		
ATGAALLVAPGPR	37.17041	Q8N442	GUF1_HUMAN	398.5720947	3	0.825842798		
DSSLALGAGWR	51.09844	Q8N442	GUF1_HUMAN	566.791461	2	0.686335206		
DSSLALGAGWR	51.09844	Q8N442	GUF1_HUMAN	378.1969157	3	0.686335206		
DVTEAQIGDTLCLHK	41.10904	Q8N442	GUF1_HUMAN	850.420169	2	0.871127784		
DVTEAQIGDTLCLHK	41.10904	Q8N442	GUF1_HUMAN	567.282721	3	0.871127784		
EITIINPAQFPDK	70.47727	Q8N442	GUF1_HUMAN	743.401573	2	0.708387673		
EITIINPAQFPDK	70.47727	Q8N442	GUF1_HUMAN	495.9369903	3	0.708387673		
GVIANVALFDGVVSK	87.7692	Q8N442	GUF1_HUMAN	744.925215	2	0.755172968		
GVIANVALFDGVVSK	87.7692	Q8N442	GUF1_HUMAN	496.9527517	3	0.755172968		
IGNVEVPK	2.092369	Q8N442	GUF1_HUMAN	428.250909	2	0.64724797		
IGNVEVPK	2.092369	Q8N442	GUF1_HUMAN	285.8365477	3	0.64724797		
IMMLCEAR	25.28163	Q8N442	GUF1_HUMAN	512.24339	2	0.642816782		
IMMLCEAR	25.28163	Q8N442	GUF1_HUMAN	341.831535	3	0.642816782		
LEQEYNASVILTTPTVPYK	75.30117	Q8N442	GUF1_HUMAN	1083.570618	2	0.709796548		
LEQEYNASVILTTPTVPYK	75.30117	Q8N442	GUF1_HUMAN	722.7163537	3	0.709796548		
LGFLGLLHMEVFNQR	111.2191	Q8N442	GUF1_HUMAN	887.4776235	2	0.755574882		
LGFLGLLHMEVFNQR	111.2191	Q8N442	GUF1_HUMAN	591.9876907	3	0.755574882		
LGTNVESVLQAIIER	122.3571	Q8N442	GUF1_HUMAN	821.462693	2	0.822018862		
LGTNVESVLQAIIER	122.3571	Q8N442	GUF1_HUMAN	547.977737	3	0.822018862		
LLELTGTIDK	46.91297	Q8N442	GUF1_HUMAN	551.821896	2	0.824589729		
LLELTGTIDK	46.91297	Q8N442	GUF1_HUMAN	368.2172057	3	0.824589729		
LTLNDSSVTVHR	12.30984	Q8N442	GUF1_HUMAN	671.360239	2	0.802228093		
LTLNDSSVTVHR	12.30984	Q8N442	GUF1_HUMAN	447.9094343	3	0.802228093		
LYAGQVGYLIAGMK	73.85231	Q8N442	GUF1_HUMAN	742.4030535	2	0.780730665		
LYAGQVGYLIAGMK	73.85231	Q8N442	GUF1_HUMAN	495.2713107	3	0.780730665		
LYSSAEFK	1.15913	Q8N442	GUF1_HUMAN	472.7403695	2	0.69460994		
LYSSAEFK	1.15913	Q8N442	GUF1_HUMAN	315.496188	3	0.69460994		
NFSIVAHVDHGK	-4.05814	Q8N442	GUF1_HUMAN	662.344392	2	0.654257298		
NFSIVAHVDHGK	-4.05814	Q8N442	GUF1_HUMAN	441.8988697	3	0.654257298		
NMIFIDQNR	34.46493	Q8N442	GUF1_HUMAN	575.787864	2	0.813034177		
NMIFIDQNR	34.46493	Q8N442	GUF1_HUMAN	384.1945177	3	0.813034177		
SAKPMVFAGMYPLDQSEYNNLK	64.06167	Q8N442	GUF1_HUMAN	1252.103976	2	0.615457416		
SAKPMVFAGMYPLDQSEYNNLK	64.06167	Q8N442	GUF1_HUMAN	835.0719253	3	0.615457416		
SAPTLGAAPESWATDR	42.97531	Q8N442	GUF1_HUMAN	815.3975505	2	0.854459882		
SAPTLGAAPESWATDR	42.97531	Q8N442	GUF1_HUMAN	543.9343087	3	0.854459882		
SLSSGYASFDYEDAGYQTAELVK	71.84668	Q8N442	GUF1_HUMAN	1251.071903	2	0.723060548		
SLSSGYASFDYEDAGYQTAELVK	71.84668	Q8N442	GUF1_HUMAN	834.3838767	3	0.723060548		
TYEVNEVGVLNPNEQPTHK	32.79211	Q8N442	GUF1_HUMAN	1084.535102	2	0.782009721		
TYEVNEVGVLNPNEQPTHK	32.79211	Q8N442	GUF1_HUMAN	723.359343	3	0.782009721		
VFDIPSDECIK	46.51425	Q8N442	GUF1_HUMAN	661.819027	2	0.82453835		
VFDIPSDECIK	46.51425	Q8N442	GUF1_HUMAN	441.5486263	3	0.82453835		
VTEYLEPVVLGTIITPDEYTGK	116.1418	Q8N442	GUF1_HUMAN	1219.141605	2	0.6492607		
VTEYLEPVVLGTIITPDEYTGK	116.1418	Q8N442	GUF1_HUMAN	813.0970117	3	0.6492607		
ALITLGNNAAFSVNQAIIR	87.84532	Q8N2F6	ARM10_HUMAN	993.56073	2	0.612712085		
ALITLGNNAAFSVNQAIIR	87.84532	Q8N2F6	ARM10_HUMAN	662.7097617	3	0.612712085		
ALNALNNLSVNVENQIK	68.00089	Q8N2F6	ARM10_HUMAN	927.508162	2	0.873922765		

ALNALNNLSVNVENQIK	68.00089	Q8N2F6	ARM10_HUMAN	618.6747163	3	0.873922765		
AQVDSSFLSLYDSHVAK	55.0546	Q8N2F6	ARM10_HUMAN	933.965792	2	0.756135821	3	
AQVDSSFLSLYDSHVAK	55.0546	Q8N2F6	ARM10_HUMAN	622.979803	3	0.756135821	3	Yes
ELGGIPIVANK	45.25558	Q8N2F6	ARM10_HUMAN	555.830055	2	0.828025103	2	Yes
ELGGIPIVANK	45.25558	Q8N2F6	ARM10_HUMAN	370.8893117	3	0.828025103	2	
LLLNLSENPAMTEGLLR	101.0689	Q8N2F6	ARM10_HUMAN	942.517142	2	0.754013956		
LLLNLSENPAMTEGLLR	101.0689	Q8N2F6	ARM10_HUMAN	628.680703	3	0.754013956		
LLYLLESTEDPVIIER	103.5448	Q8N2F6	ARM10_HUMAN	952.0253185	2	0.836404383		
LLYLLESTEDPVIIER	103.5448	Q8N2F6	ARM10_HUMAN	635.0194873	3	0.836404383		
SAGALEEGTSEGQLCGR	12.42911	Q8N2F6	ARM10_HUMAN	861.3921395	2	0.646146894		
SAGALEEGTSEGQLCGR	12.42911	Q8N2F6	ARM10_HUMAN	574.597368	3	0.646146894		
SARPQTGGTWESQWSK	8.377659	Q8N2F6	ARM10_HUMAN	903.432456	2	0.634279251		
SARPQTGGTWESQWSK	8.377659	Q8N2F6	ARM10_HUMAN	602.6242457	3	0.634279251		
TSQPEDLTDGSYDDVLNAEQLQK	68.50215	Q8N2F6	ARM10_HUMAN	1283.59374	2	0.786522985		
TSQPEDLTDGSYDDVLNAEQLQK	68.50215	Q8N2F6	ARM10_HUMAN	856.0651013	3	0.786522985		
VLTLFQNIK	59.18393	Q8N2F6	ARM10_HUMAN	538.329691	2	0.86746347		
VLTLFQNIK	59.18393	Q8N2F6	ARM10_HUMAN	359.2224023	3	0.86746347		
ASALGLGDGEEEAPPSR	24.80397	Q8N1G0	ZN687_HUMAN	828.397747	2	0.815634727	2	Yes
ASALGLGDGEEEAPPSR	24.80397	Q8N1G0	ZN687_HUMAN	552.6011063	3	0.815634727	2	
ATDIPASASPPPVAGVPFFK	85.98521	Q8N1G0	ZN687_HUMAN	985.025658	2	0.683297694		
ATDIPASASPPPVAGVPFFK	85.98521	Q8N1G0	ZN687_HUMAN	657.0197137	3	0.683297694		
AVVLPGGTATSPK	17.49571	Q8N1G0	ZN687_HUMAN	599.34607	2	0.85223341		
AVVLPGGTATSPK	17.49571	Q8N1G0	ZN687_HUMAN	399.8999883	3	0.85223341		
GAGGALLTPK	4.854149	Q8N1G0	ZN687_HUMAN	442.7641845	2	0.798452854		
GAGGALLTPK	4.854149	Q8N1G0	ZN687_HUMAN	295.5120647	3	0.798452854		
GAITSSAITTVAAEAPVLPLSTEPPAAPATSAYTC	115.8666	Q8N1G0	ZN687_HUMAN	1938.472766	2	0.612535		
GAITSSAITTVAAEAPVLPLSTEPPAAPATSAYTC	115.8666	Q8N1G0	ZN687_HUMAN	1292.651119	3	0.612535		
GLVMQCSHLVMR	28.02171	Q8N1G0	ZN687_HUMAN	715.8574415	2	0.810185313		
GLVMQCSHLVMR	28.02171	Q8N1G0	ZN687_HUMAN	477.574236	3	0.810185313		
HGLQLGAQSPGR	-16.38672	Q8N1G0	ZN687_HUMAN	610.82891	2	0.794461608		
HGLQLGAQSPGR	-16.38672	Q8N1G0	ZN687_HUMAN	407.555215	3	0.794461608		
LSPATPTSEGPK	-12.60393	Q8N1G0	ZN687_HUMAN	592.8120595	2	0.802176476		
LSPATPTSEGPK	-12.60393	Q8N1G0	ZN687_HUMAN	395.5439813	3	0.802176476		
NLLPAYRPNLSPPAEAGLALPPTGYR	84.13797	Q8N1G0	ZN687_HUMAN	1374.74576	2	0.702301562		
NLLPAYRPNLSPPAEAGLALPPTGYR	84.13797	Q8N1G0	ZN687_HUMAN	916.8331147	3	0.702301562		
NRPPHVCPECGGNFLQANFQTHLR	33.87888	Q8N1G0	ZN687_HUMAN	1425.177736	2	0.669919074		
NRPPHVCPECGGNFLQANFQTHLR	33.87888	Q8N1G0	ZN687_HUMAN	950.4544323	3	0.669919074		
NTVCPEQSEALAGGSAGDGAQAAGVTK	26.19752	Q8N1G0	ZN687_HUMAN	1273.593557	2	0.68396455		
NTVCPEQSEALAGGSAGDGAQAAGVTK	26.19752	Q8N1G0	ZN687_HUMAN	849.3983127	3	0.68396455		
NVLGLVPQALPK	72.10343	Q8N1G0	ZN687_HUMAN	624.8879045	2	0.84178412		
NVLGLVPQALPK	72.10343	Q8N1G0	ZN687_HUMAN	416.927878	3	0.84178412		
VALDQMVGQPDITPLLPVAVPPVSGPLALPALG	160.93	Q8N1G0	ZN687_HUMAN	1735.988945	2	0.75635159		
VALDQMVGQPDITPLLPVAVPPVSGPLALPALG	160.93	Q8N1G0	ZN687_HUMAN	1157.661905	3	0.75635159		
SDPDGGDSPLPASGGPLTCK	40.02583	Q8N1G0	ZN687_HUMAN	964.4392865	2	0.74238193		
SDPDGGDSPLPASGGPLTCK	40.02583	Q8N1G0	ZN687_HUMAN	643.295466	3	0.74238193		
SGPSAHAHLYSQHPSFQTQQAK	-16.46044	Q8N1G0	ZN687_HUMAN	1204.081075	2	0.684531808		
SGPSAHAHLYSQHPSFQTQQAK	-16.46044	Q8N1G0	ZN687_HUMAN	803.056658	3	0.684531808		
SSSSTEQSLMMGLR	41.21387	Q8N1G0	ZN687_HUMAN	757.353439	2	0.63531512		
SSSSTEQSLMMGLR	41.21387	Q8N1G0	ZN687_HUMAN	505.2382343	3	0.63531512		
TATGPSTGGGTVISR	-5.872047	Q8N1G0	ZN687_HUMAN	681.355155	2	0.859633446		
TATGPSTGGGTVISR	-5.872047	Q8N1G0	ZN687_HUMAN	454.5727117	3	0.859633446		
TMLEHLK	-12.47284	Q8N1G0	ZN687_HUMAN	436.2394875	2	0.62092638		
TMLEHLK	-12.47284	Q8N1G0	ZN687_HUMAN	291.1622667	3	0.62092638		
TPLDLFAHFGPEPGDHSDPLPPSAPSPTR	87.94766	Q8N1G0	ZN687_HUMAN	1526.744151	2	0.668492794		
TPLDLFAHFGPEPGDHSDPLPPSAPSPTR	87.94766	Q8N1G0	ZN687_HUMAN	1018.165376	3	0.668492794		
TQSSLVEAFNK	27.58054	Q8N1G0	ZN687_HUMAN	612.3175085	2	0.777180851		
TQSSLVEAFNK	27.58054	Q8N1G0	ZN687_HUMAN	408.547614	3	0.777180851		
TVTQVPSDPDPPAPLAEGAFLAEASLLK	138.6621	Q8N1G0	ZN687_HUMAN	1417.745297	2	0.769001424		
TVTQVPSDPDPPAPLAEGAFLAEASLLK	138.6621	Q8N1G0	ZN687_HUMAN	945.499473	3	0.769001424		
VVSVQLGDGTR	15.6058	Q8N1G0	ZN687_HUMAN	565.812394	2	0.825927913		
VVSVQLGDGTR	15.6058	Q8N1G0	ZN687_HUMAN	377.5442043	3	0.825927913		
AATAQYLSPR	1.997948	Q8N1F8	S11IP_HUMAN	539.2885505	2	0.770654261		
AATAQYLSPR	1.997948	Q8N1F8	S11IP_HUMAN	359.861642	3	0.770654261		
ASISEPSDTDPEPR	3.755039	Q8N1F8	S11IP_HUMAN	750.8448155	2	0.691716373	2	Yes
ASISEPSDTDPEPR	3.755039	Q8N1F8	S11IP_HUMAN	500.899152	3	0.691716373	2	
DAATGFLLDGK	56.33971	Q8N1F8	S11IP_HUMAN	554.2882205	2	0.795252442	2	Yes
DAATGFLLDGK	56.33971	Q8N1F8	S11IP_HUMAN	369.861422	3	0.795252442	2	
DHGSWSLSPPPER	24.46519	Q8N1F8	S11IP_HUMAN	732.8474955	2	0.777713716		
DHGSWSLSPPPER	24.46519	Q8N1F8	S11IP_HUMAN	488.9009387	3	0.777713716		
EPWEELFSIGLR	121.068	Q8N1F8	S11IP_HUMAN	738.38064	2	0.663124502		
EPWEELFSIGLR	121.068	Q8N1F8	S11IP_HUMAN	492.5897017	3	0.663124502		
EQQPLSSLSSVLLYR	87.22517	Q8N1F8	S11IP_HUMAN	860.4679705	2	0.722818077		
EQQPLSSLSSVLLYR	87.22517	Q8N1F8	S11IP_HUMAN	573.9812553	3	0.722818077		
HLDLAYNLLEGHR	46.66282	Q8N1F8	S11IP_HUMAN	775.9078835	2	0.792911947		
HLDLAYNLLEGHR	46.66282	Q8N1F8	S11IP_HUMAN	517.607864	3	0.792911947		
LELQSLEAAEIEPEAQAQR	72.19193	Q8N1F8	S11IP_HUMAN	1063.042765	2	0.863537312		
LELQSLEAAEIEPEAQAQR	72.19193	Q8N1F8	S11IP_HUMAN	709.031118	3	0.863537312		
LESFWALR	63.75219	Q8N1F8	S11IP_HUMAN	511.277458	2	0.687403798		
LESFWALR	63.75219	Q8N1F8	S11IP_HUMAN	341.1875803	3	0.687403798		
LLFYDEVSR	48.94048	Q8N1F8	S11IP_HUMAN	571.298583	2	0.746528685		
LLFYDEVSR	48.94048	Q8N1F8	S11IP_HUMAN	381.2016637	3	0.746528685		
LVHVAGPGPTGPIK	12.05562	Q8N1F8	S11IP_HUMAN	671.8962615	2	0.734519362		
LVHVAGPGPTGPIK	12.05562	Q8N1F8	S11IP_HUMAN	448.2667827	3	0.734519362		
LYLEGNPLWFHPEHR	58.36534	Q8N1F8	S11IP_HUMAN	954.481747	2	0.855984032		
LYLEGNPLWFHPEHR	58.36534	Q8N1F8	S11IP_HUMAN	636.6571063	3	0.855984032		
MGLDSEEGWRPLFQK	56.82504	Q8N1F8	S11IP_HUMAN	896.938528	2	0.696824491		
MGLDSEEGWRPLFQK	56.82504	Q8N1F8	S11IP_HUMAN	598.2949603	3	0.696824491		
MGPSGAALGVLILR	85.74405	Q8N1F8	S11IP_HUMAN	677.8979465	2	0.737517118		
MGPSGAALGVLILR	85.74405	Q8N1F8	S11IP_HUMAN	452.267906	3	0.737517118		
TLNPSPAGWFVQQHPELELMSSFR	107.4784	Q8N1F8	S11IP_HUMAN	1386.184686	2	0.742112041		
TLNPSPAGWFVQQHPELELMSSFR	107.4784	Q8N1F8	S11IP_HUMAN	924.4590657	3	0.742112041		
TVIQEALALDR	55.91643	Q8N1F8	S11IP_HUMAN	614.8489765	2	0.747870922		
TVIQEALALDR	55.91643	Q8N1F8	S11IP_HUMAN	410.2352593	3	0.747870922		
VTSAHLFEVELQAAR	48.70496	Q8N1F8	S11IP_HUMAN	835.9472105	2	0.855370164		
VTSAHLFEVELQAAR	48.70496	Q8N1F8	S11IP_HUMAN	557.634082	3	0.855370164		
VVCQEQLTALLAWIR	129.4083	Q8N1F8	S11IP_HUMAN	900.496014	2	0.631313205		
VVCQEQLTALLAWIR	129.4083	Q8N1F8	S11IP_HUMAN	600.6666177	3	0.631313205		
YLVLEPDAHAAVQELLAVLTPVTNVAR	151.2737	Q8N1F8	S11IP_HUMAN	1451.806019	2	0.799565196		
YLVLEPDAHAAVQELLAVLTPVTNVAR	151.2737	Q8N1F8	S11IP_HUMAN	968.206621	3	0.799565196		
AAEAAPPTQEAQGETEPTEQAPDALEQAADTSR	48.6584	Q8N163	CCAR2_HUMAN	1690.772218	2	0.810910702	3	
AAEAAPPTQEAQGETEPTEQAPDALEQAADTSR	48.6584	Q8N163	CCAR2_HUMAN	1127.51742	3	0.810910702	3	Yes

AAYNPGQAVPWNAVK	44.92633	Q8N163	CCAR2 HUMAN	793.4102595	2	0.868116975	2	Yes
AAYNPGQAVPWNAVK	44.92633	Q8N163	CCAR2 HUMAN	529.2761147	3	0.868116975	2	
EAAPDAGAEPITADSDPAYSSK	29.38389	Q8N163	CCAR2 HUMAN	1081.99039	2	0.745419562	3	
EAAPDAGAEPITADSDPAYSSK	29.38389	Q8N163	CCAR2 HUMAN	721.6628683	3	0.745419562	3	Yes
FAEFQYLQPGPPR	58.08468	Q8N163	CCAR2 HUMAN	775.3940785	2	0.781481564	2	Yes
FAEFQYLQPGPPR	58.08468	Q8N163	CCAR2 HUMAN	517.2653273	3	0.781481564	2	
ILLTLGIR	64.22524	Q8N163	CCAR2 HUMAN	449.8083945	2	0.645761847	2	Yes
ILLTLGIR	64.22524	Q8N163	CCAR2 HUMAN	300.2082047	3	0.645761847	2	
IPPLFPQKPLSLFQTSHTLHLSHLNR	68.04492	Q8N163	CCAR2 HUMAN	1511.343441	2	0.737584651	6	
IPPLFPQKPLSLFQTSHTLHLSHLNR	68.04492	Q8N163	CCAR2 HUMAN	1007.898236	3	0.737584651	6	
LTPLQLEIQR	54.57441	Q8N163	CCAR2 HUMAN	605.861887	2	0.781800389	2	Yes
LTPLQLEIQR	54.57441	Q8N163	CCAR2 HUMAN	404.2438663	3	0.781800389	2	
MLLSLPEK	44.77116	Q8N163	CCAR2 HUMAN	465.77062	2	0.779454172	2	Yes
MLLSLPEK	44.77116	Q8N163	CCAR2 HUMAN	310.8496883	3	0.779454172	2	
NFSGTASTSLLGPPPGLLTPPVATELSQNAR	113.7871	Q8N163	CCAR2 HUMAN	1547.314941	2	0.646570981		
NFSGTASTSLLGPPPGLLTPPVATELSQNAR	113.7871	Q8N163	CCAR2 HUMAN	1031.879235	3	0.646570981		
SPAPPLLHVAALGQK	44.70982	Q8N163	CCAR2 HUMAN	749.9412	2	0.846772671		
SPAPPLLHVAALGQK	44.70982	Q8N163	CCAR2 HUMAN	500.2967417	3	0.846772671		
STKPGAAPTEHK	-50	Q8N163	CCAR2 HUMAN	612.323126	2	0.806815863	3	
STKPGAAPTEHK	-50	Q8N163	CCAR2 HUMAN	408.551359	3	0.806815863	3	Yes
SVASNQSEMEFSSLQDMPK	56.14221	Q8N163	CCAR2 HUMAN	1057.972635	2	0.781700671	3	
SVASNQSEMEFSSLQDMPK	56.14221	Q8N163	CCAR2 HUMAN	705.6510317	3	0.781700671	3	Yes
TLAAEMQELR	32.39004	Q8N163	CCAR2 HUMAN	581.300805	2	0.812658191		
TLAAEMQELR	32.39004	Q8N163	CCAR2 HUMAN	387.8698117	3	0.812658191		
VHLTPYTVDSPICDFLELQR	100.6331	Q8N163	CCAR2 HUMAN	1202.104838	2	0.697149158	3	
VHLTPYTVDSPICDFLELQR	100.6331	Q8N163	CCAR2 HUMAN	801.739167	3	0.697149158	3	Yes
VLLLSSPGLEELYR	91.32695	Q8N163	CCAR2 HUMAN	794.951425	2	0.797882199	3	
VLLLSSPGLEELYR	91.32695	Q8N163	CCAR2 HUMAN	530.3035583	3	0.797882199	3	Yes
VQTLSNQPLLK	22.64336	Q8N163	CCAR2 HUMAN	620.867169	2	0.81119895	2	Yes
VQTLSNQPLLK	22.64336	Q8N163	CCAR2 HUMAN	414.2473877	3	0.81119895	2	
VVTQNICQYR	3.449989	Q8N163	CCAR2 HUMAN	640.8249745	2	0.788519979	2	Yes
VVTQNICQYR	3.449989	Q8N163	CCAR2 HUMAN	427.5525913	3	0.788519979	2	
AHLVFDFHQAVDGLQEVQR	65.65916	Q8N142	PURA1 HUMAN	1105.061627	2	0.772571027		
AHLVFDFHQAVDGLQEVQR	65.65916	Q8N142	PURA1 HUMAN	737.0436927	3	0.772571027		
ASNDRPPGAGGVK	-35.19198	Q8N142	PURA1 HUMAN	613.3183745	2	0.742978573		
ASNDRPPGAGGVK	-35.19198	Q8N142	PURA1 HUMAN	409.214858	3	0.742978573		
DGVYFMYEALHGPPK	65.53347	Q8N142	PURA1 HUMAN	862.4116065	2	0.777472854		
DGVYFMYEALHGPPK	65.53347	Q8N142	PURA1 HUMAN	575.2770127	3	0.777472854		
EYDFHLLPSGIINTK	73.30689	Q8N142	PURA1 HUMAN	873.957239	2	0.863774478		
EYDFHLLPSGIINTK	73.30689	Q8N142	PURA1 HUMAN	582.974101	3	0.863774478		
FVENHVGVAVK	0.065487	Q8N142	PURA1 HUMAN	599.833129	2	0.781047225		
FVENHVGVAVK	0.065487	Q8N142	PURA1 HUMAN	400.2246943	3	0.781047225		
IPYFPANQEMLQK	55.80251	Q8N142	PURA1 HUMAN	789.9034215	2	0.816569686		
IPYFPANQEMLQK	55.80251	Q8N142	PURA1 HUMAN	526.9382227	3	0.816569686		
LDILDVLGEVK	130.3629	Q8N142	PURA1 HUMAN	607.3561025	2	0.826548815		
LDILDVLGEVK	130.3629	Q8N142	PURA1 HUMAN	405.24001	3	0.826548815		
VEVEYETLPGWK	60.02297	Q8N142	PURA1 HUMAN	725.3671945	2	0.795620978		
VEVEYETLPGWK	60.02297	Q8N142	PURA1 HUMAN	483.9140713	3	0.795620978		
VGIGAFPTEQINEIGGLLQTR	112.3831	Q8N142	PURA1 HUMAN	1107.100418	2	0.83960098		
VGIGAFPTEQINEIGGLLQTR	112.3831	Q8N142	PURA1 HUMAN	738.4028867	3	0.83960098		
VVDLLATDADIISR	77.27935	Q8N142	PURA1 HUMAN	750.917587	2	0.81986928		
VVDLLATDADIISR	77.27935	Q8N142	PURA1 HUMAN	500.9476663	3	0.81986928		
WEDLPPQAQNYIR	51.46997	Q8N142	PURA1 HUMAN	815.405174	2	0.771763146		
WEDLPPQAQNYIR	51.46997	Q8N142	PURA1 HUMAN	543.939391	3	0.771763146		
AWDLWDPETLPAEYCLPSAR	122.5482	Q8N0W3	FCSK HUMAN	1195.560263	2	0.734895527		
AWDLWDPETLPAEYCLPSAR	122.5482	Q8N0W3	FCSK HUMAN	797.376117	3	0.734895527		
DFPFDDCGR	45.9212	Q8N0W3	FCSK HUMAN	564.72493	2	0.6090343		
DFPFDDCGR	45.9212	Q8N0W3	FCSK HUMAN	376.8192283	3	0.6090343		
DLVLQGHHTR	-23.29375	Q8N0W3	FCSK HUMAN	588.318178	2	0.728771925		
DLVLQGHHTR	-23.29375	Q8N0W3	FCSK HUMAN	392.5480603	3	0.728771925		
DSVQVFQR	15.39671	Q8N0W3	FCSK HUMAN	489.7543475	2	0.788321257		
DSVQVFQR	15.39671	Q8N0W3	FCSK HUMAN	326.83884	3	0.788321257		
DYCQPHAPGALLK	26.90131	Q8N0W3	FCSK HUMAN	735.364464	2	0.726963282		
DYCQPHAPGALLK	26.90131	Q8N0W3	FCSK HUMAN	490.5789177	3	0.726963282		
EDFLVGRPPELGQGDADVAGYLQSAR	84.62495	Q8N0W3	FCSK HUMAN	1380.683558	2	0.75554496		
EDFLVGRPPELGQGDADVAGYLQSAR	84.62495	Q8N0W3	FCSK HUMAN	920.791647	3	0.75554496		
EGCPGPLLATLDQVAAGAGDPGVAAR	104.6651	Q8N0W3	FCSK HUMAN	1232.119014	2	0.757655799		
EGCPGPLLATLDQVAAGAGDPGVAAR	104.6651	Q8N0W3	FCSK HUMAN	821.7486173	3	0.757655799		
ELGPQDLLWMLDHQEDGGEALR	107.3809	Q8N0W3	FCSK HUMAN	1261.603195	2	0.683546066		
ELGPQDLLWMLDHQEDGGEALR	107.3809	Q8N0W3	FCSK HUMAN	841.4047383	3	0.683546066		
EQIPAGTLLLAVEDPEK	88.12569	Q8N0W3	FCSK HUMAN	911.9940235	2	0.725008667		
EQIPAGTLLLAVEDPEK	88.12569	Q8N0W3	FCSK HUMAN	608.3319573	3	0.725008667		
HYEGAGQILIR	14.18941	Q8N0W3	FCSK HUMAN	628.8414815	2	0.764493465		
HYEGAGQILIR	14.18941	Q8N0W3	FCSK HUMAN	419.563596	3	0.764493465		
IPEPELWLAVGPR	93.13889	Q8N0W3	FCSK HUMAN	738.9146505	2	0.858040214	2	Yes
IPEPELWLAVGPR	93.13889	Q8N0W3	FCSK HUMAN	492.9457087	3	0.858040214	2	
LFPVLHPSR	23.81507	Q8N0W3	FCSK HUMAN	533.314375	2	0.731664896		
LFPVLHPSR	23.81507	Q8N0W3	FCSK HUMAN	355.8788583	3	0.731664896		
LHGSPGHAFTLVGR	7.024754	Q8N0W3	FCSK HUMAN	724.8920415	2	0.738782346		
LHGSPGHAFTLVGR	7.024754	Q8N0W3	FCSK HUMAN	483.5973027	3	0.738782346		
LMAPGCEPLTVR	38.0664	Q8N0W3	FCSK HUMAN	672.34469	2	0.829166174		
LMAPGCEPLTVR	38.0664	Q8N0W3	FCSK HUMAN	448.565735	3	0.829166174		
LNDHLLLVYTGK	42.49092	Q8N0W3	FCSK HUMAN	693.3935465	2	0.813453674	3	
LNDHLLLVYTGK	42.49092	Q8N0W3	FCSK HUMAN	462.598306	3	0.813453674	3	Yes
LPAVVQNAHSLVR	22.73734	Q8N0W3	FCSK HUMAN	702.4100665	2	0.743398547	3	
LPAVVQNAHSLVR	22.73734	Q8N0W3	FCSK HUMAN	468.6093193	3	0.743398547	3	Yes
LSWEQLQPCLDR	67.2792	Q8N0W3	FCSK HUMAN	772.880482	2	0.74268645		
LSWEQLQPCLDR	67.2792	Q8N0W3	FCSK HUMAN	515.5895963	3	0.74268645		
NLLQDVLR	68.26833	Q8N0W3	FCSK HUMAN	485.78819	2	0.81233263	2	Yes
NLLQDVLR	68.26833	Q8N0W3	FCSK HUMAN	324.194735	3	0.81233263	2	
PFSYLECGDLAAGVEALAQER	102.0185	Q8N0W3	FCSK HUMAN	1148.549896	2	0.714922845		
PFSYLECGDLAAGVEALAQER	102.0185	Q8N0W3	FCSK HUMAN	766.035872	3	0.714922845		
SGPAANPEWMR	19.26662	Q8N0W3	FCSK HUMAN	608.282946	2	0.810076654		
SGPAANPEWMR	19.26662	Q8N0W3	FCSK HUMAN	405.8579057	3	0.810076654		
TEGLGNYSIHLVEVDTQGLSLK	75.90929	Q8N0W3	FCSK HUMAN	1187.118999	2	0.633085728		
TEGLGNYSIHLVEVDTQGLSLK	75.90929	Q8N0W3	FCSK HUMAN	791.7486077	3	0.633085728		
FGGGFELHTWSELPHGSGLGTSSILAGTALAALQ	112.5456	Q8N0W3	FCSK HUMAN	1820.4319	2	0.656737685		
FGGGFELHTWSELPHGSGLGTSSILAGTALAALQ	112.5456	Q8N0W3	FCSK HUMAN	1213.957208	3	0.656737685		
VEVEEVTVPEGFVQK	60.34541	Q8N0W3	FCSK HUMAN	844.9412595	2	0.813151598		
VEVEEVTVPEGFVQK	60.34541	Q8N0W3	FCSK HUMAN	563.6301147	3	0.813151598		
VGSGGATLNALLVAAEHLSAR	92.09764	Q8N0W3	FCSK HUMAN	1004.05327	2	0.770874321	3	

VGSGGATLNALLVAAEHLSAR	92.09764	Q8N0W3	FCSK HUMAN	669.704788	3	0.770874321	3	Yes
DAALFPGCER	33.38219	Q8N0V3	RBFA HUMAN	568.2642225	2	0.72812736		
DAALFPGCER	33.38219	Q8N0V3	RBFA HUMAN	379.1787567	3	0.72812736		
DNFVQNDFR	29.50861	Q8N0V3	RBFA HUMAN	577.765443	2	0.786994457		
DNFVQNDFR	29.50861	Q8N0V3	RBFA HUMAN	385.5129037	3	0.786994457		
DPDAPQPCGTTEPTTSSSLCGIDHEALNK	39.91134	Q8N0V3	RBFA HUMAN	1549.688056	2	0.773880541		
DPDAPQPCGTTEPTTSSSLCGIDHEALNK	39.91134	Q8N0V3	RBFA HUMAN	1033.461312	3	0.773880541		
GLGGLVWQGQVAELTTQMK	94.96274	Q8N0V3	RBFA HUMAN	1008.533326	2	0.835255504		
GLGGLVWQGQVAELTTQMK	94.96274	Q8N0V3	RBFA HUMAN	672.691492	3	0.835255504		
GNAALAELDQLLAVADFGPR	146.5044	Q8N0V3	RBFA HUMAN	1021.039828	2	0.723196983		
GNAALAELDQLLAVADFGPR	146.5044	Q8N0V3	RBFA HUMAN	681.0291603	3	0.723196983		
HLLMSQQTLR	3.466095	Q8N0V3	RBFA HUMAN	613.837889	2	0.780670941		
HLLMSQQTLR	3.466095	Q8N0V3	RBFA HUMAN	409.561201	3	0.780670941		
NVPPIVFVQDK	53.65775	Q8N0V3	RBFA HUMAN	628.356437	2	0.810525477		
NVPPIVFVQDK	53.65775	Q8N0V3	RBFA HUMAN	419.240233	3	0.810525477		
TTLSAEQNAHMEAVLQR	28.48001	Q8N0V3	RBFA HUMAN	949.973631	2	0.753748834		
TTLSAEQNAHMEAVLQR	28.48001	Q8N0V3	RBFA HUMAN	633.6516957	3	0.753748834		
VSLTPDFSACR	38.07125	Q8N0V3	RBFA HUMAN	626.8037115	2	0.742523432		
VSLTPDFSACR	38.07125	Q8N0V3	RBFA HUMAN	418.2050827	3	0.742523432		
VWYESPSLGSHSTYKPSK	16.82683	Q8N0V3	RBFA HUMAN	1027.005444	2	0.753352165		
VWYESPSLGSHSTYKPSK	16.82683	Q8N0V3	RBFA HUMAN	685.0062377	3	0.753352165		
ADDNIFETVK	37.67522	Q8IZU2	WDR17 HUMAN	576.2831345	2	0.849068522		
ADDNIFETVK	37.67522	Q8IZU2	WDR17 HUMAN	384.524698	3	0.849068522		
ADQLIQEDKDDVIPYCIAIGDVK	79.92432	Q8IZU2	WDR17 HUMAN	1309.655089	2	0.61756134		
ADQLIQEDKDDVIPYCIAIGDVK	79.92432	Q8IZU2	WDR17 HUMAN	873.4393343	3	0.61756134		
ALPIGISFVK	75.8088	Q8IZU2	WDR17 HUMAN	522.826784	2	0.779130816		
ALPIGISFVK	75.8088	Q8IZU2	WDR17 HUMAN	348.887131	3	0.779130816		
ALSIAPGVSVK	34.03928	Q8IZU2	WDR17 HUMAN	521.319323	2	0.848676205		
ALSIAPGVSVK	34.03928	Q8IZU2	WDR17 HUMAN	347.882157	3	0.848676205		
DLGHVETIFDCK	46.46429	Q8IZU2	WDR17 HUMAN	717.3406585	2	0.82543999		
DLGHVETIFDCK	46.46429	Q8IZU2	WDR17 HUMAN	478.5630473	3	0.82543999		
DMIATGCEDTNVR	17.24966	Q8IZU2	WDR17 HUMAN	741.32214	2	0.784015656		
DMIATGCEDTNVR	17.24966	Q8IZU2	WDR17 HUMAN	494.5507017	3	0.784015656		
EALLVAQAACEGNMQPLHVSVPK	64.03651	Q8IZU2	WDR17 HUMAN	1231.630702	2	0.627559245		
EALLVAQAACEGNMQPLHVSVPK	64.03651	Q8IZU2	WDR17 HUMAN	821.423076	3	0.627559245		
EDFNELLHK	27.96864	Q8IZU2	WDR17 HUMAN	572.785844	2	0.788887978		
EDFNELLHK	27.96864	Q8IZU2	WDR17 HUMAN	382.193171	3	0.788887978		
ELAEWYFQDGR	62.31677	Q8IZU2	WDR17 HUMAN	707.325861	2	0.809671342		
ELAEWYFQDGR	62.31677	Q8IZU2	WDR17 HUMAN	471.8865157	3	0.809671342		
FGGGIGVPAK	17.05621	Q8IZU2	WDR17 HUMAN	451.758902	2	0.793048739		
FGGGIGVPAK	17.05621	Q8IZU2	WDR17 HUMAN	301.508543	3	0.793048739		
FKPDDPNLLATASFDGTIK	69.17894	Q8IZU2	WDR17 HUMAN	1025.528762	2	0.827943981		
FKPDDPNLLATASFDGTIK	69.17894	Q8IZU2	WDR17 HUMAN	684.0217827	3	0.827943981		
GASYSDDIYK	3.855152	Q8IZU2	WDR17 HUMAN	559.754201	2	0.757267475		
GASYSDDIYK	3.855152	Q8IZU2	WDR17 HUMAN	373.505409	3	0.757267475		
GIIGPDYVTGSNLPSHSDIHISCLTGLK	69.74144	Q8IZU2	WDR17 HUMAN	1476.25075	2	0.798523128		
GIIGPDYVTGSNLPSHSDIHISCLTGLK	69.74144	Q8IZU2	WDR17 HUMAN	984.5031083	3	0.798523128		
GPLFIWTISGPDSGVIVHK	94.32904	Q8IZU2	WDR17 HUMAN	1012.05475	2	0.743989706		
GPLFIWTISGPDSGVIVHK	94.32904	Q8IZU2	WDR17 HUMAN	675.039108	3	0.743989706		
GQDDSLLPQNYCK	27.86185	Q8IZU2	WDR17 HUMAN	769.35175	2	0.848087549		
GQDDSLLPQNYCK	27.86185	Q8IZU2	WDR17 HUMAN	513.2371083	3	0.848087549		
IATCSSDGFCIIR	46.3978	Q8IZU2	WDR17 HUMAN	750.353243	2	0.786702871		
IATCSSDGFCIIR	46.3978	Q8IZU2	WDR17 HUMAN	500.571437	3	0.786702871		
IEYLSEELDAWR	77.81236	Q8IZU2	WDR17 HUMAN	762.3730075	2	0.804738402		
IEYLSEELDAWR	77.81236	Q8IZU2	WDR17 HUMAN	508.5846133	3	0.804738402		
IQGPVFFLEDGK	72.02778	Q8IZU2	WDR17 HUMAN	675.359177	2	0.837304235		
IQGPVFFLEDGK	72.02778	Q8IZU2	WDR17 HUMAN	450.575393	3	0.837304235		
LCAFYPGCTEEINDLHDK	46.07915	Q8IZU2	WDR17 HUMAN	1091.483167	2	0.759052157		
LCAFYPGCTEEINDLHDK	46.07915	Q8IZU2	WDR17 HUMAN	727.9913863	3	0.759052157		
LLLHTCTEAR	-10.41622	Q8IZU2	WDR17 HUMAN	607.322072	2	0.691280961		
LLLHTCTEAR	-10.41622	Q8IZU2	WDR17 HUMAN	405.217323	3	0.691280961		
LPTVEECMQLAETAR	58.34304	Q8IZU2	WDR17 HUMAN	874.4218545	2	0.779533625		
LPTVEECMQLAETAR	58.34304	Q8IZU2	WDR17 HUMAN	583.2838447	3	0.779533625		
LVHFFMSR	23.03214	Q8IZU2	WDR17 HUMAN	518.7740285	2	0.637614787		
LVHFFMSR	23.03214	Q8IZU2	WDR17 HUMAN	346.185294	3	0.637614787		
MIYATLLK	46.98317	Q8IZU2	WDR17 HUMAN	476.780984	2	0.765249729		
MIYATLLK	46.98317	Q8IZU2	WDR17 HUMAN	318.189931	3	0.765249729		
NGAFIWNVQK	47.60103	Q8IZU2	WDR17 HUMAN	588.8121965	2	0.763944507		
NGAFIWNVQK	47.60103	Q8IZU2	WDR17 HUMAN	392.877406	3	0.763944507		
NLAADLLLMIPDNELHLIK	137.0499	Q8IZU2	WDR17 HUMAN	1073.601206	2	0.751076698		
NLAADLLLMIPDNELHLIK	137.0499	Q8IZU2	WDR17 HUMAN	716.070079	3	0.751076698		
SAISLNDALMWAK	80.32719	Q8IZU2	WDR17 HUMAN	710.369218	2	0.782895505		
SAISLNDALMWAK	80.32719	Q8IZU2	WDR17 HUMAN	473.9154203	3	0.782895505		
SLEDSPYTPPSDSQR	17.54566	Q8IZU2	WDR17 HUMAN	839.881925	2	0.867353559		
SLEDSPYTPPSDSQR	17.54566	Q8IZU2	WDR17 HUMAN	560.257225	3	0.867353559		
TGFHCLHVLNSPPR	15.98072	Q8IZU2	WDR17 HUMAN	817.9151905	2	0.807925045		
TGFHCLHVLNSPPR	15.98072	Q8IZU2	WDR17 HUMAN	545.6127353	3	0.807925045		
TTPIDNLK	5.1273	Q8IZU2	WDR17 HUMAN	451.253649	2	0.749441683		
TTPIDNLK	5.1273	Q8IZU2	WDR17 HUMAN	301.1717077	3	0.749441683		
VNPFSPLGTGIR	59.93536	Q8IZU2	WDR17 HUMAN	629.351686	2	0.798369646		
VNPFSPLGTGIR	59.93536	Q8IZU2	WDR17 HUMAN	419.9037323	3	0.798369646		
VVFGHIDGSLSIFHPGNK	53.33822	Q8IZU2	WDR17 HUMAN	962.507966	2	0.772275329		
VVFGHIDGSLSIFHPGNK	53.33822	Q8IZU2	WDR17 HUMAN	642.007919	3	0.772275329		
VYYVATSSDQPLK	27.48863	Q8IZU2	WDR17 HUMAN	735.877922	2	0.826873422		
VYYVATSSDQPLK	27.48863	Q8IZU2	WDR17 HUMAN	490.921223	3	0.826873422		
YCELMVELGEWDK	86.39403	Q8IZU2	WDR17 HUMAN	836.3738365	2	0.612196028		
YCELMVELGEWDK	86.39403	Q8IZU2	WDR17 HUMAN	557.9184993	3	0.612196028		
YYLLSQEPEK	26.68143	Q8IZU2	WDR17 HUMAN	635.322251	2	0.863127232		
YYLLSQEPEK	26.68143	Q8IZU2	WDR17 HUMAN	423.884109	3	0.863127232		
DDVSPVMQFSSK	43.22667	Q8IZE3	PACE1 HUMAN	670.3141085	2	0.731741846		
DDVSPVMQFSSK	43.22667	Q8IZE3	PACE1 HUMAN	447.212014	3	0.731741846		
DHAQGETPCLLSPALFQSR	67.68159	Q8IZE3	PACE1 HUMAN	1064.01857	2	0.662042558		
DHAQGETPCLLSPALFQSR	67.68159	Q8IZE3	PACE1 HUMAN	709.681655	3	0.662042558		
DYYNTLLQTGDPFSQPIK	95.85497	Q8IZE3	PACE1 HUMAN	1050.518386	2	0.834666848		
DYYNTLLQTGDPFSQPIK	95.85497	Q8IZE3	PACE1 HUMAN	700.681532	3	0.834666848		
EPPFTLPSGLAVYPAVLQDGK	112.455	Q8IZE3	PACE1 HUMAN	1100.088983	2	0.699673772		
EPPFTLPSGLAVYPAVLQDGK	112.455	Q8IZE3	PACE1 HUMAN	733.7285967	3	0.699673772		
FAAAEITEGEAEGWEEEGELNWEDNNW	129.7807	Q8IZE3	PACE1 HUMAN	1563.150142	2	0.725663602		
FAAAEITEGEAEGWEEEGELNWEDNNW	129.7807	Q8IZE3	PACE1 HUMAN	1042.436036	3	0.725663602		
FLSCTVEADGIHLVTER	53.80997	Q8IZE3	PACE1 HUMAN	973.986207	2	0.747406065		
FLSCTVEADGIHLVTER	53.80997	Q8IZE3	PACE1 HUMAN	649.6600797	3	0.747406065		

GHLTHNNVCLSSVFVSEDGHWK	36.74661	Q8IZE3	PACE1 HUMAN	1262.095871	2	0.623455524		
GHLTHNNVCLSSVFVSEDGHWK	36.74661	Q8IZE3	PACE1 HUMAN	841.7331887	3	0.623455524		
LGGMETVCK	-6.807365	Q8IZE3	PACE1 HUMAN	497.738995	2	0.659173489		
LGGMETVCK	-6.807365	Q8IZE3	PACE1 HUMAN	332.1619383	3	0.659173489		
MVLLSHIEAYVEHFTQEQLK	95.27298	Q8IZE3	PACE1 HUMAN	1208.123031	2	0.842737615		
MVLLSHIEAYVEHFTQEQLK	95.27298	Q8IZE3	PACE1 HUMAN	805.7512953	3	0.842737615		
NDFLEVVNFLK	116.7856	Q8IZE3	PACE1 HUMAN	669.359176	2	0.768187404		
NDFLEVVNFLK	116.7856	Q8IZE3	PACE1 HUMAN	446.5753923	3	0.768187404		
SEEWPDWSEPEEPENQTVNIQIWPR	110.6195	Q8IZE3	PACE1 HUMAN	1548.205055	2	0.792356372		
SEEWPDWSEPEEPENQTVNIQIWPR	110.6195	Q8IZE3	PACE1 HUMAN	1032.472645	3	0.792356372		
SFLPYLLGPK	84.87346	Q8IZE3	PACE1 HUMAN	567.832062	2	0.777113259		
SFLPYLLGPK	84.87346	Q8IZE3	PACE1 HUMAN	378.8906497	3	0.777113259		
SQCTTLDVEESSWDDCEPSSLDTK	57.49152	Q8IZE3	PACE1 HUMAN	1395.074201	2	0.700511932		
SQCTTLDVEESSWDDCEPSSLDTK	57.49152	Q8IZE3	PACE1 HUMAN	930.3854087	3	0.700511932		
VILPQVLLGLR	105.0337	Q8IZE3	PACE1 HUMAN	610.90864	2	0.739259183		
VILPQVLLGLR	105.0337	Q8IZE3	PACE1 HUMAN	407.6083683	3	0.739259183		
VIPVLLQLFEVHEEHVR	89.74872	Q8IZE3	PACE1 HUMAN	1029.081299	2	0.713936746		
VIPVLLQLFEVHEEHVR	89.74872	Q8IZE3	PACE1 HUMAN	686.3901407	3	0.713936746		
VNPGGGITATKPVTSGEQKPIPALLSLTEESMPWI	86.87477	Q8IZE3	PACE1 HUMAN	1817.464019	2	0.738395214		
VNPGGGITATKPVTSGEQKPIPALLSLTEESMPWI	86.87477	Q8IZE3	PACE1 HUMAN	1211.978621	3	0.738395214		
VPSELGLGEEFTIQVK	79.98206	Q8IZE3	PACE1 HUMAN	873.470184	2	0.848265886		
VPSELGLGEEFTIQVK	79.98206	Q8IZE3	PACE1 HUMAN	582.6493977	3	0.848265886		
VSCLSEELIASR	37.97453	Q8IZE3	PACE1 HUMAN	682.3484825	2	0.73303473		
VSCLSEELIASR	37.97453	Q8IZE3	PACE1 HUMAN	455.23493	3	0.73303473		
VSQATPEFLR	27.88428	Q8IZE3	PACE1 HUMAN	574.309487	2	0.776589155		
VSQATPEFLR	27.88428	Q8IZE3	PACE1 HUMAN	383.208933	3	0.776589155		
AQPLAYYEAAVR	42.99202	Q8IZ73	RUSD2 HUMAN	676.3544175	2	0.761413455		
AQPLAYYEAAVR	42.99202	Q8IZ73	RUSD2 HUMAN	451.2388867	3	0.761413455		
AVETDVMNQETDPLCAECR	46.65575	Q8IZ73	RUSD2 HUMAN	1119.477761	2	0.755801439		
AVETDVMNQETDPLCAECR	46.65575	Q8IZ73	RUSD2 HUMAN	746.6544487	3	0.755801439		
DDLEELAAAAQK	60.76868	Q8IZ73	RUSD2 HUMAN	637.317706	2	0.825986505		
DDLEELAAAAQK	60.76868	Q8IZ73	RUSD2 HUMAN	425.2144123	3	0.825986505		
EPILVVSYK	40.43118	Q8IZ73	RUSD2 HUMAN	524.3084195	2	0.635064006		
EPILVVSYK	40.43118	Q8IZ73	RUSD2 HUMAN	349.874888	3	0.635064006		
EVEPAPVGGEHPSAAAPGPGK	3.582794	Q8IZ73	RUSD2 HUMAN	977.4874285	2	0.723733127		
EVEPAPVGGEHPSAAAPGPGK	3.582794	Q8IZ73	RUSD2 HUMAN	651.9942273	3	0.723733127		
GKPCETVFQR	-16.91648	Q8IZ73	RUSD2 HUMAN	611.306422	2	0.689948499		
GKPCETVFQR	-16.91648	Q8IZ73	RUSD2 HUMAN	407.8735563	3	0.689948499		
GPGFEYFSPMPAWAQDDWQK	107.924	Q8IZ73	RUSD2 HUMAN	1179.020774	2	0.68956542		
GPGFEYFSPMPAWAQDDWQK	107.924	Q8IZ73	RUSD2 HUMAN	786.349791	3	0.68956542		
GPMAETVSTQVGTEGGLR	35.70005	Q8IZ73	RUSD2 HUMAN	895.441633	2	0.732137322		
GPMAETVSTQVGTEGGLR	35.70005	Q8IZ73	RUSD2 HUMAN	597.2970303	3	0.732137322		
HNTVIFILGK	42.98666	Q8IZ73	RUSD2 HUMAN	571.34059	2	0.624175668		
HNTVIFILGK	42.98666	Q8IZ73	RUSD2 HUMAN	381.2296683	3	0.624175668		
LLAENEDVVVVDKPSSIPVHPCGR	47.11305	Q8IZ73	RUSD2 HUMAN	1315.684519	2	0.729496121		
LLAENEDVVVVDKPSSIPVHPCGR	47.11305	Q8IZ73	RUSD2 HUMAN	877.458954	3	0.729496121		
LQLNEKPVQDLNIVLK	58.39246	Q8IZ73	RUSD2 HUMAN	932.5492995	2	0.804648697		
LQLNEKPVQDLNIVLK	58.39246	Q8IZ73	RUSD2 HUMAN	622.0354747	3	0.804648697		
LTSGVLMFAK	47.92359	Q8IZ73	RUSD2 HUMAN	533.802452	2	0.746788502		
LTSGVLMFAK	47.92359	Q8IZ73	RUSD2 HUMAN	356.204243	3	0.746788502		
MEEVAEAAPQELDTIALASEK	74.34061	Q8IZ73	RUSD2 HUMAN	1123.049398	2	0.816058099		
MEEVAEAAPQELDTIALASEK	74.34061	Q8IZ73	RUSD2 HUMAN	749.0355403	3	0.816058099		
TGVSFGDEHFAETSYYFEGGLR	75.70815	Q8IZ73	RUSD2 HUMAN	1235.053852	2	0.803486943		
TGVSFGDEHFAETSYYFEGGLR	75.70815	Q8IZ73	RUSD2 HUMAN	823.7051763	3	0.803486943		
VEGEFPTEEVTCK	28.62694	Q8IZ73	RUSD2 HUMAN	762.8485135	2	0.727864206	2	Yes
VEGEFPTEEVTCK	28.62694	Q8IZ73	RUSD2 HUMAN	508.9016173	3	0.727864206	2	
VRPYYFDFR	38.45306	Q8IZ73	RUSD2 HUMAN	631.820013	2	0.713017046		
VRPYYFDFR	38.45306	Q8IZ73	RUSD2 HUMAN	421.5492837	3	0.713017046		
WVGHSLLHVFSTEFR	57.25558	Q8IZ73	RUSD2 HUMAN	907.9710195	2	0.811601996		
WVGHSLLHVFSTEFR	57.25558	Q8IZ73	RUSD2 HUMAN	605.6499547	3	0.811601996		
AKPEPDILEEEK	7.004974	Q8IY95	TM192 HUMAN	699.362113	2	0.765640974		
AKPEPDILEEEK	7.004974	Q8IY95	TM192 HUMAN	466.5773503	3	0.765640974		
IYAYPSNITSETGFR	47.88827	Q8IY95	TM192 HUMAN	859.923392	2	0.793107748		
IYAYPSNITSETGFR	47.88827	Q8IY95	TM192 HUMAN	573.618203	3	0.793107748		
LLALTSSDLGCQPSR	44.79794	Q8IY95	TM192 HUMAN	809.4174285	2	0.801014364		
LLALTSSDLGCQPSR	44.79794	Q8IY95	TM192 HUMAN	539.9475607	3	0.801014364		
TISSLEEIVEK	53.35779	Q8IY95	TM192 HUMAN	624.340649	2	0.774419188		
TISSLEEIVEK	53.35779	Q8IY95	TM192 HUMAN	416.563041	3	0.774419188		
AMFMSGLSESK	35.85381	Q8IY47	KBTB2 HUMAN	594.2759405	2	0.724585235		
AMFMSGLSESK	35.85381	Q8IY47	KBTB2 HUMAN	396.519902	3	0.724585235		
AVVISNSLCVFMR	80.89673	Q8IY47	KBTB2 HUMAN	748.3921705	2	0.666972339		
AVVISNSLCVFMR	80.89673	Q8IY47	KBTB2 HUMAN	499.2640553	3	0.666972339		
AWFQGLPPNDK	48.88714	Q8IY47	KBTB2 HUMAN	636.8227615	2	0.650413692		
AWFQGLPPNDK	48.88714	Q8IY47	KBTB2 HUMAN	424.8844493	3	0.650413692		
EAAMLWLEYNTESR	78.22089	Q8IY47	KBTB2 HUMAN	856.9016065	2	0.678007245		
EAAMLWLEYNTESR	78.22089	Q8IY47	KBTB2 HUMAN	571.6036793	3	0.678007245		
IDALSEVTQR	30.21608	Q8IY47	KBTB2 HUMAN	566.3044015	2	0.835134983		
IDALSEVTQR	30.21608	Q8IY47	KBTB2 HUMAN	377.8722093	3	0.835134983		
IFYIGGLHIATNSGIR	61.65987	Q8IY47	KBTB2 HUMAN	866.481216	2	0.854167461		
IFYIGGLHIATNSGIR	61.65987	Q8IY47	KBTB2 HUMAN	577.9900857	3	0.854167461		
LCSPPADLHK	-9.745483	Q8IY47	KBTB2 HUMAN	569.29024	2	0.685519457		
LCSPPADLHK	-9.745483	Q8IY47	KBTB2 HUMAN	379.8627683	3	0.685519457		
LLSFADLFSCEELK	118.3486	Q8IY47	KBTB2 HUMAN	836.419105	2	0.637696385		
LLSFADLFSCEELK	118.3486	Q8IY47	KBTB2 HUMAN	557.9486783	3	0.637696385		
LPSGTVDGSSVTVEIYDVNK	62.30006	Q8IY47	KBTB2 HUMAN	1040.526411	2	0.77336067		
LPSGTVDGSSVTVEIYDVNK	62.30006	Q8IY47	KBTB2 HUMAN	694.0202157	3	0.77336067		
LYPSCLEESPWK	52.19888	Q8IY47	KBTB2 HUMAN	754.858679	2	0.82612741		
LYPSCLEESPWK	52.19888	Q8IY47	KBTB2 HUMAN	503.575061	3	0.82612741		
MVLATCSSYFR	41.31199	Q8IY47	KBTB2 HUMAN	667.8157605	2	0.734072387		
MVLATCSSYFR	41.31199	Q8IY47	KBTB2 HUMAN	445.5464487	3	0.734072387		
SDSWVEMAMR	55.35482	Q8IY47	KBTB2 HUMAN	606.2633645	2	0.657241106		
SDSWVEMAMR	55.35482	Q8IY47	KBTB2 HUMAN	404.511518	3	0.657241106		
SFASAAAFGDK	20.90244	Q8IY47	KBTB2 HUMAN	536.2594625	2	0.693986535		
SFASAAAFGDK	20.90244	Q8IY47	KBTB2 HUMAN	357.84225	3	0.693986535		
SQYLSSVLSQIR	65.04008	Q8IY47	KBTB2 HUMAN	690.87826	2	0.875304282		
SQYLSSVLSQIR	65.04008	Q8IY47	KBTB2 HUMAN	460.9214483	3	0.875304282		
SVVVQGLYK	20.54551	Q8IY47	KBTB2 HUMAN	496.792937	2	0.748963773		
SVVVQGLYK	20.54551	Q8IY47	KBTB2 HUMAN	331.531233	3	0.748963773		
VGTVVTPDNDIYIAGGQVPLK	72.70358	Q8IY47	KBTB2 HUMAN	1078.584065	2	0.837928414		
VGTVVTPDNDIYIAGGQVPLK	72.70358	Q8IY47	KBTB2 HUMAN	719.3919847	3	0.837928414		
YVTYQYDLELDR	53.54224	Q8IY47	KBTB2 HUMAN	789.378282	2	0.780799389		

YVTYQYDLELDR	53.54224	Q8IY47	KBTB2_HUMAN	526.5881297	3	0.780799389		
AVASSCSYLHEASLK	9.774029	Q8IY45	AMN1_HUMAN	811.896324	2	0.857687652		
AVASSCSYLHEASLK	9.774029	Q8IY45	AMN1_HUMAN	541.6001577	3	0.857687652		
EVLEQLVGPNK	43.94576	Q8IY45	AMN1_HUMAN	613.3435265	2	0.828495622		
EVLEQLVGPNK	43.94576	Q8IY45	AMN1_HUMAN	409.231626	3	0.828495622		
ILLFHGCPLITDHSR	39.59175	Q8IY45	AMN1_HUMAN	889.9727055	2	0.774233103		
ILLFHGCPLITDHSR	39.59175	Q8IY45	AMN1_HUMAN	593.6510787	3	0.774233103		
IMSMQGQITDSNISEILHPEVQTLDLR	100.3589	Q8IY45	AMN1_HUMAN	1534.773737	2	0.776318848		
IMSMQGQITDSNISEILHPEVQTLDLR	100.3589	Q8IY45	AMN1_HUMAN	1023.518433	3	0.776318848		
YLTDIKPLPPNIK	45.61337	Q8IY45	AMN1_HUMAN	756.445779	2	0.816050947		
YLTDIKPLPPNIK	45.61337	Q8IY45	AMN1_HUMAN	504.6331277	3	0.816050947		
ADVLAFPSSGFTDLAEIVSR	137.9945	Q8IY17	PLPL6_HUMAN	1048.039493	2	0.731442332		
ADVLAFPSSGFTDLAEIVSR	137.9945	Q8IY17	PLPL6_HUMAN	699.028937	3	0.731442332		
AQPSVVLSAAHTVAAR	23.01745	Q8IY17	PLPL6_HUMAN	789.4420955	2	0.776342869	3	
AQPSVVLSAAHTVAAR	23.01745	Q8IY17	PLPL6_HUMAN	526.630672	3	0.776342869	3	Yes
AVFGGWSR	22.68787	Q8IY17	PLPL6_HUMAN	440.2277685	2	0.76771152		
AVFGGWSR	22.68787	Q8IY17	PLPL6_HUMAN	293.8211207	3	0.76771152		
DGFQDVLAPGEGSAGR	60.31973	Q8IY17	PLPL6_HUMAN	788.3740755	2	0.889179111		
DGFQDVLAPGEGSAGR	60.31973	Q8IY17	PLPL6_HUMAN	525.9186587	3	0.889179111		
DGHLLMDGGYINNLPADIAR	83.18697	Q8IY17	PLPL6_HUMAN	1078.034215	2	0.807697833		
DGHLLMDGGYINNLPADIAR	83.18697	Q8IY17	PLPL6_HUMAN	719.0254183	3	0.807697833		
EVVPGDSVNSLLSILDVITGHQHPQR	116.6449	Q8IY17	PLPL6_HUMAN	1405.743954	2	0.670487046	4	
EVVPGDSVNSLLSILDVITGHQHPQR	116.6449	Q8IY17	PLPL6_HUMAN	937.4985773	3	0.670487046	4	
FDQIYDVGYQYGK	49.15626	Q8IY17	PLPL6_HUMAN	798.373	2	0.84776473		
FDQIYDVGYQYGK	49.15626	Q8IY17	PLPL6_HUMAN	532.5846083	3	0.84776473		
GDLIGVVEALTR	98.65799	Q8IY17	PLPL6_HUMAN	621.8568015	2	0.732034206	2	Yes
GDLIGVVEALTR	98.65799	Q8IY17	PLPL6_HUMAN	414.9071427	3	0.732034206	2	
IEDPSLLNSR	27.54747	Q8IY17	PLPL6_HUMAN	572.3044005	2	0.763044715		
IEDPSLLNSR	27.54747	Q8IY17	PLPL6_HUMAN	381.8722087	3	0.763044715		
IEPPTSYVSDGCADGEESDCLTEYEEDAGPDCSR	65.30175	Q8IY17	PLPL6_HUMAN	1905.746555	2	0.729015529		
IEPPTSYVSDGCADGEESDCLTEYEEDAGPDCSR	65.30175	Q8IY17	PLPL6_HUMAN	1270.833645	3	0.729015529		
ISVSLQEEASGGSLAAPAR	48.2142	Q8IY17	PLPL6_HUMAN	921.981978	2	0.656236708		
ISVSLQEEASGGSLAAPAR	48.2142	Q8IY17	PLPL6_HUMAN	614.9905937	3	0.656236708		
IVLYQTDASLTPWTVR	85.8251	Q8IY17	PLPL6_HUMAN	932.0047215	2	0.83906877		
IVLYQTDASLTPWTVR	85.8251	Q8IY17	PLPL6_HUMAN	621.6724227	3	0.83906877		
LFPSPGLPTR	42.37031	Q8IY17	PLPL6_HUMAN	542.8116655	2	0.674984932	2	Yes
LFPSPGLPTR	42.37031	Q8IY17	PLPL6_HUMAN	362.2103853	3	0.674984932	2	
LGASALDSIQEFR	62.67054	Q8IY17	PLPL6_HUMAN	703.867897	2	0.857736051		
LGASALDSIQEFR	62.67054	Q8IY17	PLPL6_HUMAN	469.5812063	3	0.857736051		
LIHLLSQK	4.530174	Q8IY17	PLPL6_HUMAN	476.303476	2	0.756778955		
LIHLLSQK	4.530174	Q8IY17	PLPL6_HUMAN	317.8715923	3	0.756778955		
LPEGTLGHIK	1.171719	Q8IY17	PLPL6_HUMAN	532.809123	2	0.810883284		
LPEGTLGHIK	1.171719	Q8IY17	PLPL6_HUMAN	355.5420237	3	0.810883284		
LPVEAFSAVFTK	73.75645	Q8IY17	PLPL6_HUMAN	654.864095	2	0.747619271	2	Yes
LPVEAFSAVFTK	73.75645	Q8IY17	PLPL6_HUMAN	436.912005	3	0.747619271	2	
LSGWLAQQEDAHR	15.60244	Q8IY17	PLPL6_HUMAN	755.8740455	2	0.838050008		
LSGWLAQQEDAHR	15.60244	Q8IY17	PLPL6_HUMAN	504.251972	3	0.838050008		
SDFDMAYER	22.28742	Q8IY17	PLPL6_HUMAN	567.232583	2	0.76838398		
SDFDMAYER	22.28742	Q8IY17	PLPL6_HUMAN	378.490997	3	0.76838398		
SDFYEIMR	46.72779	Q8IY17	PLPL6_HUMAN	530.7425865	2	0.829226434		
SDFYEIMR	46.72779	Q8IY17	PLPL6_HUMAN	354.1643327	3	0.829226434		
SSSYCEYLRPPIDCFK	53.00986	Q8IY17	PLPL6_HUMAN	1011.458959	2	0.729605913	3	
SSSYCEYLRPPIDCFK	53.00986	Q8IY17	PLPL6_HUMAN	674.641914	3	0.729605913	3	Yes
VLGHFEKPLFLELCR	60.44452	Q8IY17	PLPL6_HUMAN	929.506381	2	0.748154521		
VLGHFEKPLFLELCR	60.44452	Q8IY17	PLPL6_HUMAN	620.0068623	3	0.748154521		
VLLHHAK	-50	Q8IY17	PLPL6_HUMAN	409.256329	2	0.615763068	2	Yes
VLLHHAK	-50	Q8IY17	PLPL6_HUMAN	273.1734943	3	0.615763068	2	
VLTGNTIALVLGGGGAR	71.44544	Q8IY17	PLPL6_HUMAN	784.9601215	2	0.814702213	3	
VLTGNTIALVLGGGGAR	71.44544	Q8IY17	PLPL6_HUMAN	523.6426893	3	0.814702213	3	Yes
VPDMAEIQSR	14.70858	Q8IY17	PLPL6_HUMAN	573.2851545	2	0.678706646	2	Yes
VPDMAEIQSR	14.70858	Q8IY17	PLPL6_HUMAN	382.5260447	3	0.678706646	2	
VSQSTSSLVDTSVSATSRPR	14.58529	Q8IY17	PLPL6_HUMAN	1033.030188	2	0.69528228		
VSQSTSSLVDTSVSATSRPR	14.58529	Q8IY17	PLPL6_HUMAN	689.0227333	3	0.69528228		
VTFLALHNYLGLTNELFSHEIQPLR	116.2255	Q8IY17	PLPL6_HUMAN	1463.285253	2	0.770154357		
VTFLALHNYLGLTNELFSHEIQPLR	116.2255	Q8IY17	PLPL6_HUMAN	975.8594437	3	0.770154357		
AMQDMQLLWEK	66.80302	Q8IWZ8	SUGP1_HUMAN	696.8366975	2	0.805386186		
AMQDMQLLWEK	66.80302	Q8IWZ8	SUGP1_HUMAN	464.89374	3	0.805386186		
ASSTGSFTAPDPGLK	28.34991	Q8IWZ8	SUGP1_HUMAN	718.3573625	2	0.782416642		
ASSTGSFTAPDPGLK	28.34991	Q8IWZ8	SUGP1_HUMAN	479.24085	3	0.782416642		
DNPAFAFLHDK	47.4259	Q8IWZ8	SUGP1_HUMAN	637.8123935	2	0.849776864		
DNPAFAFLHDK	47.4259	Q8IWZ8	SUGP1_HUMAN	425.544204	3	0.849776864		
DVDASPSPLSVQDLK	56.61752	Q8IWZ8	SUGP1_HUMAN	785.9021335	2	0.777265966		
DVDASPSPLSVQDLK	56.61752	Q8IWZ8	SUGP1_HUMAN	524.2706973	3	0.777265966		
EGEGLGSEGQGIK	-3.830673	Q8IWZ8	SUGP1_HUMAN	630.8075055	2	0.835530639		
EGEGLGSEGQGIK	-3.830673	Q8IWZ8	SUGP1_HUMAN	420.8742787	3	0.835530639		
ENQAFSFLYEPNSQGYK	71.16784	Q8IWZ8	SUGP1_HUMAN	1011.466152	2	0.747874558		
ENQAFSFLYEPNSQGYK	71.16784	Q8IWZ8	SUGP1_HUMAN	674.6467097	3	0.747874558		
EQQEMQQMYDMIMQHK	54.59342	Q8IWZ8	SUGP1_HUMAN	1049.447217	2	0.782980382		
EQQEMQQMYDMIMQHK	54.59342	Q8IWZ8	SUGP1_HUMAN	699.9674193	3	0.782980382		
FANDGSFLQQFLK	93.68925	Q8IWZ8	SUGP1_HUMAN	757.88609	2	0.857708454	2	Yes
FANDGSFLQQFLK	93.68925	Q8IWZ8	SUGP1_HUMAN	505.593335	3	0.857708454	2	
FIADGGPEVETIALQNNR	61.08939	Q8IWZ8	SUGP1_HUMAN	972.495253	2	0.832723141		
FIADGGPEVETIALQNNR	61.08939	Q8IWZ8	SUGP1_HUMAN	648.6661103	3	0.832723141		
FRPNPLNNPR	-4.36668	Q8IWZ8	SUGP1_HUMAN	612.833994	2	0.687509239		
FRPNPLNNPR	-4.36668	Q8IWZ8	SUGP1_HUMAN	408.8919377	3	0.687509239		
FVAEGGPELEK	16.44215	Q8IWZ8	SUGP1_HUMAN	588.3013275	2	0.766985416		
FVAEGGPELEK	16.44215	Q8IWZ8	SUGP1_HUMAN	392.5368267	3	0.766985416		
GKPVGLVGVTELSDAQK	35.28303	Q8IWZ8	SUGP1_HUMAN	849.475801	2	0.850014865	3	
GKPVGLVGVTELSDAQK	35.28303	Q8IWZ8	SUGP1_HUMAN	566.6531423	3	0.850014865	3	Yes
GTTTVDGAGFGIDRPAELSK	40.2027	Q8IWZ8	SUGP1_HUMAN	996.5058185	2	0.727805138		
GTTTVDGAGFGIDRPAELSK	40.2027	Q8IWZ8	SUGP1_HUMAN	664.673154	3	0.727805138		
HFIGDFLPPDELEK	70.239	Q8IWZ8	SUGP1_HUMAN	828.917587	2	0.864530921		
HFIGDFLPPDELEK	70.239	Q8IWZ8	SUGP1_HUMAN	552.9476663	3	0.864530921		
LTVENIGYQMLMK	73.79871	Q8IWZ8	SUGP1_HUMAN	770.3996575	2	0.800084591	2	Yes
LTVENIGYQMLMK	73.79871	Q8IWZ8	SUGP1_HUMAN	513.9357133	3	0.800084591	2	
MNMNILHQEELIAQK	48.12631	Q8IWZ8	SUGP1_HUMAN	906.461313	2	0.79108727		
MNMNILHQEELIAQK	48.12631	Q8IWZ8	SUGP1_HUMAN	604.6434837	3	0.79108727		
TGLGLASLPGPVK	59.58209	Q8IWZ8	SUGP1_HUMAN	605.3642625	2	0.873060346		
TGLGLASLPGPVK	59.58209	Q8IWZ8	SUGP1_HUMAN	403.9121167	3	0.873060346		
VELPPAELVQR	50.79092	Q8IWZ8	SUGP1_HUMAN	625.859344	2	0.77727294		
VELPPAELVQR	50.79092	Q8IWZ8	SUGP1_HUMAN	417.5755043	3	0.77727294		

AEQATVTSEFESYK	29.4307	Q8IWJ2	GCC2_HUMAN	795.3706625	2	0.805451035		
AEQATVTSEFESYK	29.4307	Q8IWJ2	GCC2_HUMAN	530.58305	3	0.805451035		
AETEQQCLSLK	4.9599	Q8IWJ2	GCC2_HUMAN	653.819559	2	0.764523566		
AETEQQCLSLK	4.9599	Q8IWJ2	GCC2_HUMAN	436.2156477	3	0.764523566		
DALLETVNR	30.35559	Q8IWJ2	GCC2_HUMAN	515.780562	2	0.811278045		
DALLETVNR	30.35559	Q8IWJ2	GCC2_HUMAN	344.1896497	3	0.811278045		
DAQQTTLMNMEIADYER	74.26915	Q8IWJ2	GCC2_HUMAN	1014.954243	2	0.787294447		
DAQQTTLMNMEIADYER	74.26915	Q8IWJ2	GCC2_HUMAN	676.9721033	3	0.787294447		
DEVTYMNNLK	28.11584	Q8IWJ2	GCC2_HUMAN	613.790265	2	0.721555531		
DEVTYMNNLK	28.11584	Q8IWJ2	GCC2_HUMAN	409.5294517	3	0.721555531		
DLEVFLSQK	64.49962	Q8IWJ2	GCC2_HUMAN	539.793138	2	0.793582082		
DLEVFLSQK	64.49962	Q8IWJ2	GCC2_HUMAN	360.1980337	3	0.793582082		
DLIQGAESYK	29.68967	Q8IWJ2	GCC2_HUMAN	562.285673	2	0.833554864		
DLIQGAESYK	29.68967	Q8IWJ2	GCC2_HUMAN	375.193057	3	0.833554864		
DVVNVLQAVGESLAK	121.0099	Q8IWJ2	GCC2_HUMAN	771.4308615	2	0.849372625		
DVVNVLQAVGESLAK	121.0099	Q8IWJ2	GCC2_HUMAN	514.6231827	3	0.849372625		
EALQSDLLEMK	49.70664	Q8IWJ2	GCC2_HUMAN	638.826852	2	0.792864323		
EALQSDLLEMK	49.70664	Q8IWJ2	GCC2_HUMAN	426.2205097	3	0.792864323		
EEGEGMETTDTESVSSASTYTQSLEQLLNSPETK	113.5798	Q8IWJ2	GCC2_HUMAN	1839.820907	2	0.654617488		
EEGEGMETTDTESVSSASTYTQSLEQLLNSPETK	113.5798	Q8IWJ2	GCC2_HUMAN	1226.883213	3	0.654617488		
EHATTVNELEELQVQLQK	52.28398	Q8IWJ2	GCC2_HUMAN	1055.045308	2	0.811738729		
EHATTVNELEELQVQLQK	52.28398	Q8IWJ2	GCC2_HUMAN	703.69948	3	0.811738729		
EHLEMLIDQLK	58.92863	Q8IWJ2	GCC2_HUMAN	684.863769	2	0.806476355		
EHLEMLIDQLK	58.92863	Q8IWJ2	GCC2_HUMAN	456.9117877	3	0.806476355		
EIMEILQTELGESAGK	91.32555	Q8IWJ2	GCC2_HUMAN	874.443309	2	0.758901477		
EIMEILQTELGESAGK	91.32555	Q8IWJ2	GCC2_HUMAN	583.2981477	3	0.758901477		
EISELNETFLSDSEK	59.19757	Q8IWJ2	GCC2_HUMAN	870.9128945	2	0.836349189		
EISELNETFLSDSEK	59.19757	Q8IWJ2	GCC2_HUMAN	580.944538	3	0.836349189		
ELEQSHINYVK	-2.239311	Q8IWJ2	GCC2_HUMAN	680.3493355	2	0.719369352		
ELEQSHINYVK	-2.239311	Q8IWJ2	GCC2_HUMAN	453.9021653	3	0.719369352		
ETEATNAILMEQIK	48.80967	Q8IWJ2	GCC2_HUMAN	795.9063625	2	0.821224749		
ETEATNAILMEQIK	48.80967	Q8IWJ2	GCC2_HUMAN	530.9401833	3	0.821224749		
ETVTQLQNIIEANSQHYQK	55.835	Q8IWJ2	GCC2_HUMAN	1122.566934	2	0.623462021	3	
ETVTQLQNIIEANSQHYQK	55.835	Q8IWJ2	GCC2_HUMAN	748.7138977	3	0.623462021	3	Yes
GELEASQQQVEVYK	19.64983	Q8IWJ2	GCC2_HUMAN	804.3997545	2	0.827217877		
GELEASQQQVEVYK	19.64983	Q8IWJ2	GCC2_HUMAN	536.6024447	3	0.827217877		
IEDLEQEIK	23.92204	Q8IWJ2	GCC2_HUMAN	558.793335	2	0.749459028		
IEDLEQEIK	23.92204	Q8IWJ2	GCC2_HUMAN	372.8648317	3	0.749459028		
IQLAEITSEK	26.66241	Q8IWJ2	GCC2_HUMAN	566.3169775	2	0.757344782		
IQLAEITSEK	26.66241	Q8IWJ2	GCC2_HUMAN	377.8805933	3	0.757344782		
IRPGFEEQILYLQK	56.69236	Q8IWJ2	GCC2_HUMAN	867.4834245	2	0.629723072		
IRPGFEEQILYLQK	56.69236	Q8IWJ2	GCC2_HUMAN	578.6582247	3	0.629723072		
ISQEFESMK	7.749348	Q8IWJ2	GCC2_HUMAN	549.7609805	2	0.773959816	2	Yes
ISQEFESMK	7.749348	Q8IWJ2	GCC2_HUMAN	366.843262	3	0.773959816	2	
LAAVAQGEEENASR	-8.624413	Q8IWJ2	GCC2_HUMAN	722.855518	2	0.713163435	2	Yes
LAAVAQGEEENASR	-8.624413	Q8IWJ2	GCC2_HUMAN	482.2396203	3	0.713163435	2	
LCSIQSENMMMK	29.75119	Q8IWJ2	GCC2_HUMAN	736.324782	2	0.689836681		
LCSIQSENMMMK	29.75119	Q8IWJ2	GCC2_HUMAN	491.2191297	3	0.689836681		
LDALLLEK	45.88979	Q8IWJ2	GCC2_HUMAN	457.782042	2	0.720228076	2	Yes
LDALLLEK	45.88979	Q8IWJ2	GCC2_HUMAN	305.5239697	3	0.720228076	2	
LENQNLLIQVEEVSQTCSK	70.06004	Q8IWJ2	GCC2_HUMAN	1116.563007	2	0.60217309		
LENQNLLIQVEEVSQTCSK	70.06004	Q8IWJ2	GCC2_HUMAN	744.7112793	3	0.60217309		
LEPPLWHAEFTK	45.08398	Q8IWJ2	GCC2_HUMAN	734.385726	2	0.827408791		
LEPPLWHAEFTK	45.08398	Q8IWJ2	GCC2_HUMAN	489.9264257	3	0.827408791		
LILELGK	42.4082	Q8IWJ2	GCC2_HUMAN	393.2607455	2	0.700841188		
LILELGK	42.4082	Q8IWJ2	GCC2_HUMAN	262.509772	3	0.700841188		
LLENEQVQK	-13.29959	Q8IWJ2	GCC2_HUMAN	550.8014945	2	0.794800937	2	Yes
LLENEQVQK	-13.29959	Q8IWJ2	GCC2_HUMAN	367.536938	3	0.794800937	2	
LLEVQILEVQR	73.69468	Q8IWJ2	GCC2_HUMAN	670.401376	2	0.814074516		
LLEVQILEVQR	73.69468	Q8IWJ2	GCC2_HUMAN	447.2701923	3	0.814074516		
LLSQQELVPELENTIK	76.41776	Q8IWJ2	GCC2_HUMAN	927.5151225	2	0.861484766		
LLSQQELVPELENTIK	76.41776	Q8IWJ2	GCC2_HUMAN	618.6793567	3	0.861484766		
LQDSQNNLQINVSELQTLQSEHDTLLER	84.6226	Q8IWJ2	GCC2_HUMAN	1633.318939	2	0.746475458		
LQDSQNNLQINVSELQTLQSEHDTLLER	84.6226	Q8IWJ2	GCC2_HUMAN	1089.215234	3	0.746475458		
LQLMVEEQDNLNK	40.3307	Q8IWJ2	GCC2_HUMAN	787.398704	2	0.765685558		
LQLMVEEQDNLNK	40.3307	Q8IWJ2	GCC2_HUMAN	525.268411	3	0.765685558		
LTLMFEIQGLK	95.49765	Q8IWJ2	GCC2_HUMAN	646.8683235	2	0.711245		
LTLMFEIQGLK	95.49765	Q8IWJ2	GCC2_HUMAN	431.5814907	3	0.711245		
MEAQLFQLK	51.58591	Q8IWJ2	GCC2_HUMAN	554.297534	2	0.799420714		
MEAQLFQLK	51.58591	Q8IWJ2	GCC2_HUMAN	369.867631	3	0.799420714		
MEDLVQDGVASPATPGTGK	43.42218	Q8IWJ2	GCC2_HUMAN	936.954573	2	0.693984687		
MEDLVQDGVASPATPGTGK	43.42218	Q8IWJ2	GCC2_HUMAN	624.9723237	3	0.693984687		
NGVYLLSLSQR	54.02618	Q8IWJ2	GCC2_HUMAN	625.3491385	2	0.654331505		
NGVYLLSLSQR	54.02618	Q8IWJ2	GCC2_HUMAN	417.2353673	3	0.654331505		
NINSLQEELLQLK	81.12251	Q8IWJ2	GCC2_HUMAN	771.430861	2	0.796415985		
NINSLQEELLQLK	81.12251	Q8IWJ2	GCC2_HUMAN	514.6231823	3	0.796415985		
NLLLEYEK	36.72324	Q8IWJ2	GCC2_HUMAN	511.2824015	2	0.835607886		
NLLLEYEK	36.72324	Q8IWJ2	GCC2_HUMAN	341.190876	3	0.835607886		
NSTLQCETINSDNEDLLAR	43.52177	Q8IWJ2	GCC2_HUMAN	1097.008595	2	0.649886727		
NSTLQCETINSDNEDLLAR	43.52177	Q8IWJ2	GCC2_HUMAN	731.675005	3	0.649886727		
NTDLPLLDMHTVTR	55.56774	Q8IWJ2	GCC2_HUMAN	813.4199715	2	0.807841718		
NTDLPLLDMHTVTR	55.56774	Q8IWJ2	GCC2_HUMAN	542.6159227	3	0.807841718		
SEHTQTVSQLTSQNEVLR	15.33661	Q8IWJ2	GCC2_HUMAN	1029.017081	2	0.736583531		
SEHTQTVSQLTSQNEVLR	15.33661	Q8IWJ2	GCC2_HUMAN	686.347329	3	0.736583531		
SKPVTEGTGDIIK	-8.194538	Q8IWJ2	GCC2_HUMAN	672.8726485	2	0.803612292	2	Yes
SKPVTEGTGDIIK	-8.194538	Q8IWJ2	GCC2_HUMAN	448.9177073	3	0.803612292	2	
SLYEENNK	-25.81817	Q8IWJ2	GCC2_HUMAN	498.735815	2	0.691339254		
SLYEENNK	-25.81817	Q8IWJ2	GCC2_HUMAN	332.826485	3	0.691339254		
SSGWASYLHSWSGLR	64.18333	Q8IWJ2	GCC2_HUMAN	847.408247	2	0.766287208	3	
SSGWASYLHSWSGLR	64.18333	Q8IWJ2	GCC2_HUMAN	565.274773	3	0.766287208	3	Yes
TLSAYQQR	-20.67972	Q8IWJ2	GCC2_HUMAN	483.7543435	2	0.668880582		
TLSAYQQR	-20.67972	Q8IWJ2	GCC2_HUMAN	322.8388373	3	0.668880582		
TMQELELVK	34.77563	Q8IWJ2	GCC2_HUMAN	545.794824	2	0.733602881		
TMQELELVK	34.77563	Q8IWJ2	GCC2_HUMAN	364.1991577	3	0.733602881		
TQLYGFLK	44.31159	Q8IWJ2	GCC2_HUMAN	485.2743805	2	0.784313202	2	Yes
TQLYGFLK	44.31159	Q8IWJ2	GCC2_HUMAN	323.8521953	3	0.784313202	2	
TVETLQQQLSK	13.5726	Q8IWJ2	GCC2_HUMAN	637.8517165	2	0.683677733		
TVETLQQQLSK	13.5726	Q8IWJ2	GCC2_HUMAN	425.5704193	3	0.683677733		
VEQTIQYNSELEQK	21.175	Q8IWJ2	GCC2_HUMAN	854.9235935	2	0.759370863		
VEQTIQYNSELEQK	21.175	Q8IWJ2	GCC2_HUMAN	570.285004	3	0.759370863		
VNELTGGLEETLK	47.87071	Q8IWJ2	GCC2_HUMAN	701.875388	2	0.863615453		

VNELTGGLEETLK	47.87071	Q8IWJ2	GCC2_HUMAN	468.252867	3	0.863615453		
YECELENLR	18.53683	Q8IWJ2	GCC2_HUMAN	613.2800645	2	0.70936358		
YECELENLR	18.53683	Q8IWJ2	GCC2_HUMAN	409.189318	3	0.70936358		
GEAGSDVSLVDLGFQTDFR	94.4485	Q8IWA5	CTL2_HUMAN	1006.982176	2	0.669400096		
GEAGSDVSLVDLGFQTDFR	94.4485	Q8IWA5	CTL2_HUMAN	671.657392	3	0.669400096		
GVAEVLQDGDCPAVLIPSKPLAR	70.27802	Q8IWA5	CTL2_HUMAN	1203.147042	2	0.799228787		
GVAEVLQDGDCPAVLIPSKPLAR	70.27802	Q8IWA5	CTL2_HUMAN	802.4339693	3	0.799228787		
GVLMVGNETTYEDGHGSR	20.33291	Q8IWA5	CTL2_HUMAN	961.4396165	2	0.817418337		
GVLMVGNETTYEDGHGSR	20.33291	Q8IWA5	CTL2_HUMAN	641.295686	3	0.817418337		
IFDDSPCPFTAK	44.00233	Q8IWA5	CTL2_HUMAN	699.324477	2	0.677921593		
IFDDSPCPFTAK	44.00233	Q8IWA5	CTL2_HUMAN	466.5522597	3	0.677921593		
KPDDLPAFPLFSAFGR	103.2817	Q8IWA5	CTL2_HUMAN	889.4677785	2	0.7351408	3	
KPDDLPAFPLFSAFGR	103.2817	Q8IWA5	CTL2_HUMAN	593.3144607	3	0.7351408	3	Yes
NDGSAERPYFMSSTLK	23.77557	Q8IWA5	CTL2_HUMAN	901.92307	2	0.716914773		
NDGSAERPYFMSSTLK	23.77557	Q8IWA5	CTL2_HUMAN	601.6179883	3	0.716914773		
TCNPETFPSSNESR	1.269657	Q8IWA5	CTL2_HUMAN	813.3471995	2	0.64855516		
TCNPETFPSSNESR	1.269657	Q8IWA5	CTL2_HUMAN	542.567408	3	0.64855516		
VILEYLDQR	52.86064	Q8IWA5	CTL2_HUMAN	574.819683	2	0.81632483		
VILEYLDQR	52.86064	Q8IWA5	CTL2_HUMAN	383.5490637	3	0.81632483		
VTDFLFLLGK	107.8179	Q8IWA5	CTL2_HUMAN	576.837349	2	0.627720177		
VTDFLFLLGK	107.8179	Q8IWA5	CTL2_HUMAN	384.8941743	3	0.627720177		
YLTYLNAR	22.87324	Q8IWA5	CTL2_HUMAN	507.274907	2	0.753401637		
YLTYLNAR	22.87324	Q8IWA5	CTL2_HUMAN	338.519213	3	0.753401637		
AEAMQEASEAVPSGMLSVLGQPQSK	88.44507	Q8IVS2	FABD_HUMAN	1273.117821	2	0.777771175		
AEAMQEASEAVPSGMLSVLGQPQSK	88.44507	Q8IVS2	FABD_HUMAN	849.0811553	3	0.777771175		
DATGAEEEAPWAATER	36.57019	Q8IVS2	FABD_HUMAN	852.379555	2	0.896634161		
DATGAEEEAPWAATER	36.57019	Q8IVS2	FABD_HUMAN	568.5889783	3	0.896634161		
FNFACLEAR	47.3772	Q8IVS2	FABD_HUMAN	564.2693075	2	0.732645094		
FNFACLEAR	47.3772	Q8IVS2	FABD_HUMAN	376.51548	3	0.732645094		
GASSFPVPPPGAQGVAELLR	94.3994	Q8IVS2	FABD_HUMAN	975.5263565	2	0.609627485		
GASSFPVPPPGAQGVAELLR	94.3994	Q8IVS2	FABD_HUMAN	650.686846	3	0.609627485		
GFPQTFEVGPGR	41.92631	Q8IVS2	FABD_HUMAN	646.325669	2	0.797228277	2	Yes
GFPQTFEVGPGR	41.92631	Q8IVS2	FABD_HUMAN	431.219721	3	0.797228277	2	
GLLNYPR	20.61227	Q8IVS2	FABD_HUMAN	416.7379645	2	0.760979414		
GLLNYPR	20.61227	Q8IVS2	FABD_HUMAN	278.1612513	3	0.760979414		
KPLVSVYSNVHAHR	-21.57502	Q8IVS2	FABD_HUMAN	803.9448	2	0.774726033	3	
KPLVSVYSNVHAHR	-21.57502	Q8IVS2	FABD_HUMAN	536.2991417	3	0.774726033	3	Yes
LLAQQLVSPVK	44.17033	Q8IVS2	FABD_HUMAN	598.37463	2	0.768571317		
LLAQQLVSPVK	44.17033	Q8IVS2	FABD_HUMAN	399.2523617	3	0.768571317		
LMEPAVEPLTQALK	69.71085	Q8IVS2	FABD_HUMAN	770.426734	2	0.907576025	2	Yes
LMEPAVEPLTQALK	69.71085	Q8IVS2	FABD_HUMAN	513.9537643	3	0.907576025	2	
MLPVSGAFHTR	17.16711	Q8IVS2	FABD_HUMAN	608.319332	2	0.777603686		
MLPVSGAFHTR	17.16711	Q8IVS2	FABD_HUMAN	405.882163	3	0.777603686		
SLGIENPVCEVSNYLFPDCR	109.8651	Q8IVS2	FABD_HUMAN	1185.049205	2	0.756992638	3	
SLGIENPVCEVSNYLFPDCR	109.8651	Q8IVS2	FABD_HUMAN	790.3687447	3	0.756992638	3	Yes
TVHCQPAIFVASLAAVEK	64.32402	Q8IVS2	FABD_HUMAN	971.0173105	2	0.637059152		
TVHCQPAIFVASLAAVEK	64.32402	Q8IVS2	FABD_HUMAN	647.6808153	3	0.637059152		
VLGYDLLELSLHGPQETLDR	99.75955	Q8IVS2	FABD_HUMAN	1134.597703	2	0.622282326	3	
VLGYDLLELSLHGPQETLDR	99.75955	Q8IVS2	FABD_HUMAN	756.73441	3	0.622282326	3	Yes
WEQTMHAIYER	14.16632	Q8IVS2	FABD_HUMAN	732.3409885	2	0.700988352	3	
WEQTMHAIYER	14.16632	Q8IVS2	FABD_HUMAN	488.5632673	3	0.700988352	3	Yes
EEADNPNTGIGAFR	27.01501	Q8IVB5	LIX1L_HUMAN	745.847693	2	0.81449759	2	Yes
EEADNPNTGIGAFR	27.01501	Q8IVB5	LIX1L_HUMAN	497.567737	3	0.81449759	2	
EQSVPGALSR	3.467606	Q8IVB5	LIX1L_HUMAN	522.2781865	2	0.700262487		
EQSVPGALSR	3.467606	Q8IVB5	LIX1L_HUMAN	348.5213993	3	0.700262487		
FMLESNK	6.758015	Q8IVB5	LIX1L_HUMAN	434.7158445	2	0.713396907		
FMLESNK	6.758015	Q8IVB5	LIX1L_HUMAN	290.1465047	3	0.713396907		
HQMALDWVSR	29.01423	Q8IVB5	LIX1L_HUMAN	621.8065885	2	0.776140749		
HQMALDWVSR	29.01423	Q8IVB5	LIX1L_HUMAN	414.8736673	3	0.776140749		
IALMNSVFNEHPSR	38.19125	Q8IVB5	LIX1L_HUMAN	807.90703	2	0.791059196		
IALMNSVFNEHPSR	38.19125	Q8IVB5	LIX1L_HUMAN	538.9406283	3	0.791059196		
LQPGVGTSGR	-22.8052	Q8IVB5	LIX1L_HUMAN	486.2676225	2	0.645325541		
LQPGVGTSGR	-22.8052	Q8IVB5	LIX1L_HUMAN	324.5143567	3	0.645325541		
VNVVEALQEFWQMK	129.5154	Q8IVB5	LIX1L_HUMAN	860.9405395	2	0.771900833		
VNVVEALQEFWQMK	129.5154	Q8IVB5	LIX1L_HUMAN	574.2963013	3	0.771900833		
AFLLSLAALR	95.05473	Q8IV08	PLD3_HUMAN	537.837683	2	0.683504522	2	Yes
AFLLSLAALR	95.05473	Q8IV08	PLD3_HUMAN	358.894397	3	0.683504522	2	
ALLNVVDNAR	32.0229	Q8IV08	PLD3_HUMAN	542.8096535	2	0.849896789		
ALLNVVDNAR	32.0229	Q8IV08	PLD3_HUMAN	362.209044	3	0.849896789		
FWPAIDDGLR	64.2266	Q8IV08	PLD3_HUMAN	595.3042045	2	0.785078406	2	Yes
FWPAIDDGLR	64.2266	Q8IV08	PLD3_HUMAN	397.2054113	3	0.785078406	2	
IAVSKPSGPQPQADLQALLQSGAQVR	69.0712	Q8IV08	PLD3_HUMAN	1330.230483	2	0.744472682	3	
IAVSKPSGPQPQADLQALLQSGAQVR	69.0712	Q8IV08	PLD3_HUMAN	887.1562637	3	0.744472682	3	Yes
IFEAYWFLGQAGSSIPSTWPR	136.7602	Q8IV08	PLD3_HUMAN	1207.102955	2	0.755763769		
IFEAYWFLGQAGSSIPSTWPR	136.7602	Q8IV08	PLD3_HUMAN	805.071245	3	0.755763769		
LFVVPADEAQAR	38.12845	Q8IV08	PLD3_HUMAN	658.354426	2	0.804095805	2	Yes
LFVVPADEAQAR	38.12845	Q8IV08	PLD3_HUMAN	439.2388923	3	0.804095805	2	
LLISCWGHSEPSMR	39.26112	Q8IV08	PLD3_HUMAN	836.900891	2	0.779493451		
LLISCWGHSEPSMR	39.26112	Q8IV08	PLD3_HUMAN	558.269869	3	0.779493451		
LMYQELK	11.18449	Q8IV08	PLD3_HUMAN	462.747141	2	0.730661929		
LMYQELK	11.18449	Q8IV08	PLD3_HUMAN	308.8340357	3	0.730661929		
SQLEAIFLR	65.76369	Q8IV08	PLD3_HUMAN	538.8091225	2	0.772423029	2	Yes
SQLEAIFLR	65.76369	Q8IV08	PLD3_HUMAN	359.5420233	3	0.772423029	2	
VPAEEPANELPMNEIEAWK	76.18426	Q8IV08	PLD3_HUMAN	1084.022986	2	0.827041209		
VPAEEPANELPMNEIEAWK	76.18426	Q8IV08	PLD3_HUMAN	723.017932	3	0.827041209		
ANLIVLGAVPR	63.45486	Q8IUR7	ARMC8_HUMAN	561.8538645	2	0.740114689		
ANLIVLGAVPR	63.45486	Q8IUR7	ARMC8_HUMAN	374.9051847	3	0.740114689		
DKPIEMQLTSAK	16.84715	Q8IUR7	ARMC8_HUMAN	680.8612265	2	0.7568717		
DKPIEMQLTSAK	16.84715	Q8IUR7	ARMC8_HUMAN	454.243426	3	0.7568717		
DLIMTNDDILQK	66.89787	Q8IUR7	ARMC8_HUMAN	709.863966	2	0.886939704		
DLIMTNDDILQK	66.89787	Q8IUR7	ARMC8_HUMAN	473.5785857	3	0.886939704		
DMGIVDILHK	63.05113	Q8IUR7	ARMC8_HUMAN	570.8082655	2	0.819412053		
DMGIVDILHK	63.05113	Q8IUR7	ARMC8_HUMAN	380.8747853	3	0.819412053		
EPILESGAVELLCGLTQSENPALR	131.1152	Q8IUR7	ARMC8_HUMAN	1298.668535	2	0.651601195		
EPILESGAVELLCGLTQSENPALR	131.1152	Q8IUR7	ARMC8_HUMAN	866.1149647	3	0.651601195		
IIETENMMDR	19.78521	Q8IUR7	ARMC8_HUMAN	626.2895795	2	0.781045318		
IIETENMMDR	19.78521	Q8IUR7	ARMC8_HUMAN	417.862328	3	0.781045318		
IVTGLSESSVK	8.004189	Q8IUR7	ARMC8_HUMAN	560.316977	2	0.620976567		
IVTGLSESSVK	8.004189	Q8IUR7	ARMC8_HUMAN	373.880593	3	0.620976567		
LFDPDPQK	13.67897	Q8IUR7	ARMC8_HUMAN	480.245824	2	0.732159317		
LFDPDPQK	13.67897	Q8IUR7	ARMC8_HUMAN	320.4998243	3	0.732159317		

LLSDSDLNVLMK	68.03265	Q8IUR7	ARMC8 HUMAN	674.363601	2	0.868006587		
LLSDSDLNVLMK	68.03265	Q8IUR7	ARMC8 HUMAN	449.9116757	3	0.868006587		
LLYLLQQETSSTELK	68.76738	Q8IUR7	ARMC8 HUMAN	883.483287	2	0.831394672		
LLYLLQQETSSTELK	68.76738	Q8IUR7	ARMC8 HUMAN	589.3247997	3	0.831394672		
LSQSPDSNLCDK	-6.773239	Q8IUR7	ARMC8 HUMAN	682.312097	2	0.85618782		
LSQSPDSNLCDK	-6.773239	Q8IUR7	ARMC8 HUMAN	455.210673	3	0.85618782		
LYASLGANDEDIR	30.88491	Q8IUR7	ARMC8 HUMAN	718.854982	2	0.805692315		
LYASLGANDEDIR	30.88491	Q8IUR7	ARMC8 HUMAN	479.5725963	3	0.805692315		
MSVLSEVTASSR	41.89294	Q8IUR7	ARMC8 HUMAN	633.8221005	2	0.619973898		
MSVLSEVTASSR	41.89294	Q8IUR7	ARMC8 HUMAN	422.8840087	3	0.619973898		
SLSTEQLFR	38.20824	Q8IUR7	ARMC8 HUMAN	540.788387	2	0.750963271		
SLSTEQLFR	38.20824	Q8IUR7	ARMC8 HUMAN	360.861533	3	0.750963271		
TDDNCIVLK	12.14508	Q8IUR7	ARMC8 HUMAN	539.2664305	2	0.727943897		
TDDNCIVLK	12.14508	Q8IUR7	ARMC8 HUMAN	359.8468953	3	0.727943897		
TIFTSPVTPEELLYTDATVIPHLMALLSR	162.3758	Q8IUR7	ARMC8 HUMAN	1614.865417	2	0.606450379		
TIFTSPVTPEELLYTDATVIPHLMALLSR	162.3758	Q8IUR7	ARMC8 HUMAN	1076.912886	3	0.606450379		
TSFQDHAVWK	7.455612	Q8IUR7	ARMC8 HUMAN	609.7992865	2	0.749134541		
TSFQDHAVWK	7.455612	Q8IUR7	ARMC8 HUMAN	406.8687993	3	0.749134541		
VEGAETLAYLIEPDVELQR	107.6212	Q8IUR7	ARMC8 HUMAN	1073.057879	2	0.679899156	3	
VEGAETLAYLIEPDVELQR	107.6212	Q8IUR7	ARMC8 HUMAN	715.707861	3	0.679899156	3	Yes
VLQGVIDMK	32.01699	Q8IUR7	ARMC8 HUMAN	501.786802	2	0.779848814		
VLQGVIDMK	32.01699	Q8IUR7	ARMC8 HUMAN	334.8604763	3	0.779848814		
YPSSVSAITDIK	36.82915	Q8IUR7	ARMC8 HUMAN	640.840812	2	0.663584352		
YPSSVSAITDIK	36.82915	Q8IUR7	ARMC8 HUMAN	427.5631497	3	0.663584352		
AALNQQILK	7.936676	Q8IUC4	RHPN2 HUMAN	499.8038405	2	0.771857619		
AALNQQILK	7.936676	Q8IUC4	RHPN2 HUMAN	333.538502	3	0.771857619		
DFILEHYSEDGYLYEDEIADLMDLR	142.4344	Q8IUC4	RHPN2 HUMAN	1532.692587	2	0.622139096		
DFILEHYSEDGYLYEDEIADLMDLR	142.4344	Q8IUC4	RHPN2 HUMAN	1022.131	3	0.622139096		
DTFTHTPSYDMSPAMLSVLVK	93.48436	Q8IUC4	RHPN2 HUMAN	1170.5667	2	0.627697766		
DTFTHTPSYDMSPAMLSVLVK	93.48436	Q8IUC4	RHPN2 HUMAN	780.7137413	3	0.627697766		
LELSFVNSDLQMLK	100.0589	Q8IUC4	RHPN2 HUMAN	818.934922	2	0.742217064		
LELSFVNSDLQMLK	100.0589	Q8IUC4	RHPN2 HUMAN	546.2925563	3	0.742217064		
LGPLSVFSANK	57.21593	Q8IUC4	RHPN2 HUMAN	566.8222295	2	0.687865019		
LGPLSVFSANK	57.21593	Q8IUC4	RHPN2 HUMAN	378.217428	3	0.687865019		
LTVTDFFQK	67.48343	Q8IUC4	RHPN2 HUMAN	549.7956815	2	0.710020185		
LTVTDFFQK	67.48343	Q8IUC4	RHPN2 HUMAN	366.866396	3	0.710020185		
LTYAQHQEEDDLLNLIDAPSVVAK	93.77763	Q8IUC4	RHPN2 HUMAN	1341.685235	2	0.803432226		
LTYAQHQEEDDLLNLIDAPSVVAK	93.77763	Q8IUC4	RHPN2 HUMAN	894.7927647	3	0.803432226		
MMLAQAQESVFEK	47.10619	Q8IUC4	RHPN2 HUMAN	756.365819	2	0.861115992		
MMLAQAQESVFEK	47.10619	Q8IUC4	RHPN2 HUMAN	504.579821	3	0.861115992		
QTQAGLESAIDAFQR	63.90412	Q8IUC4	RHPN2 HUMAN	817.9108255	2	0.652979791		
QTQAGLESAIDAFQR	63.90412	Q8IUC4	RHPN2 HUMAN	545.6098253	3	0.652979791		
TEQEVDIILPQFSK	81.50511	Q8IUC4	RHPN2 HUMAN	823.935977	2	0.614991665		
TEQEVDIILPQFSK	81.50511	Q8IUC4	RHPN2 HUMAN	549.626593	3	0.614991665		
TGAENLLK	0.486626	Q8IUC4	RHPN2 HUMAN	423.2405415	2	0.724888861		
TGAENLLK	0.486626	Q8IUC4	RHPN2 HUMAN	282.4963027	3	0.724888861		
VGEVYQQLHAAMSQAPVK	37.12846	Q8IUC4	RHPN2 HUMAN	978.504563	2	0.854756355		
VGEVYQQLHAAMSQAPVK	37.12846	Q8IUC4	RHPN2 HUMAN	652.672317	3	0.854756355		
WLTLSEVMK	67.16025	Q8IUC4	RHPN2 HUMAN	553.799909	2	0.768881023		
WLTLSEVMK	67.16025	Q8IUC4	RHPN2 HUMAN	369.535881	3	0.768881023		
IAAWPVSEFFLIR	133.4259	Q86YN1	DOPP1 HUMAN	774.932843	2	0.647618413		
IAAWPVSEFFLIR	133.4259	Q86YN1	DOPP1 HUMAN	516.957837	3	0.647618413		
AYSTTSIASVAGLTAAAYR	73.33018	Q86Y39	NDUAB HUMAN	937.4868885	2	0.736921608		
AYSTTSIASVAGLTAAAYR	73.33018	Q86Y39	NDUAB HUMAN	625.3272007	3	0.736921608		
EKPDDPLNYFLGGCAGGLTLGAR	93.12282	Q86Y39	NDUAB HUMAN	1211.097545	2	0.629598737	3	
EKPDDPLNYFLGGCAGGLTLGAR	93.12282	Q86Y39	NDUAB HUMAN	807.7343047	3	0.629598737	3	Yes
LEGWEVFAKPK	41.34506	Q86Y39	NDUAB HUMAN	652.356437	2	0.745271146		
LEGWEVFAKPK	41.34506	Q86Y39	NDUAB HUMAN	435.240233	3	0.745271146		
VTLNPPGTFLEGVAK	73.61757	Q86Y39	NDUAB HUMAN	771.930498	2	0.875658512		
VTLNPPGTFLEGVAK	73.61757	Q86Y39	NDUAB HUMAN	514.9562737	3	0.875658512		
ALQEGAEIVVCTPGR	40.66385	Q86XP3	DDX42 HUMAN	800.412147	2	0.74875766		
ALQEGAEIVVCTPGR	40.66385	Q86XP3	DDX42 HUMAN	533.9440397	3	0.74875766		
ANAEELANNLK	15.34856	Q86XP3	DDX42 HUMAN	593.8073075	2	0.773151398		
ANAEELANNLK	15.34856	Q86XP3	DDX42 HUMAN	396.20748	3	0.773151398		
DDIEEEDDQEAYFR	39.25382	Q86XP3	DDX42 HUMAN	887.35848	2	0.782738566	2	Yes
DDIEEEDDQEAYFR	39.25382	Q86XP3	DDX42 HUMAN	591.9082617	3	0.782738566	2	
DILIDPIR	66.84903	Q86XP3	DDX42 HUMAN	477.785116	2	0.712801695	2	Yes
DILIDPIR	66.84903	Q86XP3	DDX42 HUMAN	318.8593523	3	0.712801695	2	
DIPVLVATDVAAR	76.22813	Q86XP3	DDX42 HUMAN	670.3831835	2	0.692379296	2	Yes
DIPVLVATDVAAR	76.22813	Q86XP3	DDX42 HUMAN	447.258064	3	0.692379296	2	
DSNFAGDLVR	38.73537	Q86XP3	DDX42 HUMAN	547.2678185	2	0.841321051	2	Yes
DSNFAGDLVR	38.73537	Q86XP3	DDX42 HUMAN	365.181154	3	0.841321051	2	
ELEPGDGPIAVIVCPTR	78.79729	Q86XP3	DDX42 HUMAN	911.9725685	2	0.675886929		
ELEPGDGPIAVIVCPTR	78.79729	Q86XP3	DDX42 HUMAN	608.317654	3	0.675886929		
ELLDLAMQNAWFR	115.4477	Q86XP3	DDX42 HUMAN	803.9064995	2	0.789774716		
ELLDLAMQNAWFR	115.4477	Q86XP3	DDX42 HUMAN	536.273608	3	0.789774716		
GFGFGGFAISAGK	72.65084	Q86XP3	DDX42 HUMAN	608.31203	2	0.809743226		
GFGFGGFAISAGK	72.65084	Q86XP3	DDX42 HUMAN	405.877295	3	0.809743226		
IPGFGNTGNISGAPVTYPSAGAQGVNNTASGNNS	63.44096	Q86XP3	DDX42 HUMAN	1702.814883	2	0.66100955		
IPGFGNTGNISGAPVTYPSAGAQGVNNTASGNNS	63.44096	Q86XP3	DDX42 HUMAN	1135.545863	3	0.66100955		
GLDIPSIK	39.7592	Q86XP3	DDX42 HUMAN	421.7532845	2	0.728427112	2	Yes
GLDIPSIK	39.7592	Q86XP3	DDX42 HUMAN	281.504798	3	0.728427112	2	
GNNNVMSNYEAYKPSTGAMGDR	21.79818	Q86XP3	DDX42 HUMAN	1188.521338	2	0.746080995	3	
GNNNVMSNYEAYKPSTGAMGDR	21.79818	Q86XP3	DDX42 HUMAN	792.6835003	3	0.746080995	3	Yes
GVAYTLLTPK	41.54295	Q86XP3	DDX42 HUMAN	531.81387	2	0.846486866	2	Yes
GVAYTLLTPK	41.54295	Q86XP3	DDX42 HUMAN	354.8785217	3	0.846486866	2	
IIDPLPPIDHSEIDYPPFEK	86.70561	Q86XP3	DDX42 HUMAN	1168.097004	2	0.787074268	3	
IIDPLPPIDHSEIDYPPFEK	86.70561	Q86XP3	DDX42 HUMAN	779.0672773	3	0.787074268	3	Yes
LNIGGGGLGYR	31.21481	Q86XP3	DDX42 HUMAN	538.7965425	2	0.789079309		
LNIGGGGLGYR	31.21481	Q86XP3	DDX42 HUMAN	359.5336367	3	0.789079309		
LPQQSHSAFGATSSSSGFGK	6.577301	Q86XP3	DDX42 HUMAN	990.9746845	2	0.685394883		
LPQQSHSAFGATSSSSGFGK	6.577301	Q86XP3	DDX42 HUMAN	660.9857313	3	0.685394883		
MFDMGFEYQVR	72.35248	Q86XP3	DDX42 HUMAN	711.813218	2	0.729179502	2	Yes
MFDMGFEYQVR	72.35248	Q86XP3	DDX42 HUMAN	474.878087	3	0.729179502	2	
NFYNEHEEITNLTPQQLIDLR	84.99797	Q86XP3	DDX42 HUMAN	1294.143537	2	0.759902775	3	
NFYNEHEEITNLTPQQLIDLR	84.99797	Q86XP3	DDX42 HUMAN	863.0982993	3	0.759902775	3	Yes
SAPPQLPSFYK	43.89813	Q86XP3	DDX42 HUMAN	617.827508	2	0.802478313		
SAPPQLPSFYK	43.89813	Q86XP3	DDX42 HUMAN	412.220947	3	0.802478313		
SHFVAASLSNQK	-13.88999	Q86XP3	DDX42 HUMAN	644.8363995	2	0.743456423		
SHFVAASLSNQK	-13.88999	Q86XP3	DDX42 HUMAN	430.2268747	3	0.743456423		
SVAVYGGGSMWEQAK	40.18243	Q86XP3	DDX42 HUMAN	785.3724855	2	0.758753181		

SVAVYGGGSMWEQAK	40.18243	Q86XP3	DDX42 HUMAN	523.9175987	3	0.758753181		
TAAFIWPMLIHIMDQK	124.6806	Q86XP3	DDX42 HUMAN	958.0026175	2	0.737199664		
TAAFIWPMLIHIMDQK	124.6806	Q86XP3	DDX42 HUMAN	639.0043533	3	0.737199664		
TADGFAVPEPPK	33.68379	Q86XP3	DDX42 HUMAN	614.8146025	2	0.866073787		
TADGFAVPEPPK	33.68379	Q86XP3	DDX42 HUMAN	410.2123433	3	0.866073787		
TVINYDVAR	14.18366	Q86XP3	DDX42 HUMAN	525.7831005	2	0.636930048	2	Yes
TVINYDVAR	14.18366	Q86XP3	DDX42 HUMAN	350.8580087	3	0.636930048	2	
VSYLVFDEADR	53.84748	Q86XP3	DDX42 HUMAN	657.3227865	2	0.787689865	2	Yes
VSYLVFDEADR	53.84748	Q86XP3	DDX42 HUMAN	438.5511327	3	0.787689865	2	
VVQGDIGEANEDVTQIVEILHSGPSK	96.64197	Q86XP3	DDX42 HUMAN	1367.698878	2	0.770025671	3	
VVQGDIGEANEDVTQIVEILHSGPSK	96.64197	Q86XP3	DDX42 HUMAN	912.1351933	3	0.770025671	3	Yes
YMAENPTAGVVQEEEEDNLEYDSDGNPIAPTK	66.60957	Q86XP3	DDX42 HUMAN	1763.278477	2	0.71272701		
YMAENPTAGVVQEEEEDNLEYDSDGNPIAPTK	66.60957	Q86XP3	DDX42 HUMAN	1175.854926	3	0.71272701		
IVDFYPEDNNSLQTFPIPAAEVDR	109.121	Q86WQ0	NR2CA HUMAN	1375.669584	2	0.732842147	3	
IVDFYPEDNNSLQTFPIPAAEVDR	109.121	Q86WQ0	NR2CA HUMAN	917.4489977	3	0.732842147	3	Yes
VSQLQIQFQGGFSSR	58.20533	Q86WQ0	NR2CA HUMAN	841.43701	2	0.685607612		
VSQLQIQFQGGFSSR	58.20533	Q86WQ0	NR2CA HUMAN	561.2939483	3	0.685607612		
VTFEDATDFFGR	80.47986	Q86WQ0	NR2CA HUMAN	702.8256985	2	0.862579465	2	Yes
VTFEDATDFFGR	80.47986	Q86WQ0	NR2CA HUMAN	468.8864073	3	0.862579465	2	
VVIYHLR	3.565723	Q86WQ0	NR2CA HUMAN	450.277257	2	0.744135976		
VVIYHLR	3.565723	Q86WQ0	NR2CA HUMAN	300.5207797	3	0.744135976		
AAQQQQPSASPR	-38.16792	Q86U86	PB1 HUMAN	634.821282	2	0.803740859		
AAQQQQPSASPR	-38.16792	Q86U86	PB1 HUMAN	423.5501297	3	0.803740859		
AEDNFNLEK	9.007095	Q86U86	PB1 HUMAN	540.2543765	2	0.805422664		
AEDNFNLEK	9.007095	Q86U86	PB1 HUMAN	360.505526	3	0.805422664		
AQHPDYSFGELSR	22.71129	Q86U86	PB1 HUMAN	753.852774	2	0.838425457		
AQHPDYSFGELSR	22.71129	Q86U86	PB1 HUMAN	502.9044577	3	0.838425457		
DALVLHK	-8.631809	Q86U86	PB1 HUMAN	398.2403445	2	0.680358291		
DALVLHK	-8.631809	Q86U86	PB1 HUMAN	265.8295047	3	0.680358291		
DEDVFVCESR	27.86921	Q86U86	PB1 HUMAN	628.2671585	2	0.740338266		
DEDVFVCESR	27.86921	Q86U86	PB1 HUMAN	419.180714	3	0.740338266		
DGAAYFYGPIFIHPEETEHEPTK	63.47227	Q86U86	PB1 HUMAN	1324.61936	2	0.85147351	4	
DGAAYFYGPIFIHPEETEHEPTK	63.47227	Q86U86	PB1 HUMAN	883.4155147	3	0.85147351	4	
GAHTTMADALWR	25.3887	Q86U86	PB1 HUMAN	665.3226035	2	0.625346422		
GAHTTMADALWR	25.3887	Q86U86	PB1 HUMAN	443.884344	3	0.625346422		
IILEPMDLK	59.11427	Q86U86	PB1 HUMAN	536.3101095	2	0.8354491		
IILEPMDLK	59.11427	Q86U86	PB1 HUMAN	357.8760147	3	0.8354491		
ILFNVVLEAR	77.70526	Q86U86	PB1 HUMAN	587.353697	2	0.614016473		
ILFNVVLEAR	77.70526	Q86U86	PB1 HUMAN	391.905073	3	0.614016473		
LCDLFMVKPSK	37.46882	Q86U86	PB1 HUMAN	669.351983	2	0.773412049		
LCDLFMVKPSK	37.46882	Q86U86	PB1 HUMAN	446.570597	3	0.773412049		
LDLFQEHMFEVLER	98.79677	Q86U86	PB1 HUMAN	903.4487285	2	0.825920999		
LDLFQEHMFEVLER	98.79677	Q86U86	PB1 HUMAN	602.635094	3	0.825920999		
LISELFQK	41.49779	Q86U86	PB1 HUMAN	489.2874915	2	0.798315704		
LISELFQK	41.49779	Q86U86	PB1 HUMAN	326.5276027	3	0.798315704		
LLCELFIR	66.95277	Q86U86	PB1 HUMAN	532.302619	2	0.616024375		
LLCELFIR	66.95277	Q86U86	PB1 HUMAN	355.2043543	3	0.616024375		
LLHSEAYLK	-3.722157	Q86U86	PB1 HUMAN	537.3036685	2	0.603815079		
LLHSEAYLK	-3.722157	Q86U86	PB1 HUMAN	358.5383873	3	0.603815079		
LNEVYEAVK	9.355099	Q86U86	PB1 HUMAN	532.7853085	2	0.750767708		
LNEVYEAVK	9.355099	Q86U86	PB1 HUMAN	355.5261473	3	0.750767708		
LPSHWLK	1.834267	Q86U86	PB1 HUMAN	440.7561615	2	0.748298526		
LPSHWLK	1.834267	Q86U86	PB1 HUMAN	294.1733827	3	0.748298526		
LSAITMALQYGSESEEDAALAAAR	93.24905	Q86U86	PB1 HUMAN	1234.602856	2	0.624505579		
LSAITMALQYGSESEEDAALAAAR	93.24905	Q86U86	PB1 HUMAN	823.4045123	3	0.624505579		
LWTMPISSVR	65.34454	Q86U86	PB1 HUMAN	595.3240825	2	0.819073439		
LWTMPISSVR	65.34454	Q86U86	PB1 HUMAN	397.2186633	3	0.819073439		
MPISLQQIR	42.72487	Q86U86	PB1 HUMAN	543.3109755	2	0.847323477		
MPISLQQIR	42.72487	Q86U86	PB1 HUMAN	362.5432587	3	0.847323477		
NNQGQLIAEPFYHLPSK	53.61507	Q86U86	PB1 HUMAN	978.5028765	2	0.88501668		
NNQGQLIAEPFYHLPSK	53.61507	Q86U86	PB1 HUMAN	652.6711927	3	0.88501668		
NQEYETLDHLECDLNLMFENAK	96.01727	Q86U86	PB1 HUMAN	1363.607812	2	0.629895687		
NQEYETLDHLECDLNLMFENAK	96.01727	Q86U86	PB1 HUMAN	909.4078163	3	0.629895687		
NQPDYYEVVSQPIDLMK	84.19809	Q86U86	PB1 HUMAN	1019.993689	2	0.811682701		
NQPDYYEVVSQPIDLMK	84.19809	Q86U86	PB1 HUMAN	680.331734	3	0.811682701		
SEDSSGAAGLSGLHR	-3.588371	Q86U86	PB1 HUMAN	722.345317	2	0.748748243	3	
SEDSSGAAGLSGLHR	-3.588371	Q86U86	PB1 HUMAN	481.8994863	3	0.748748243	3	Yes
SELPDYYLTIK	67.71147	Q86U86	PB1 HUMAN	671.3510085	2	0.854029298		
SELPDYYLTIK	67.71147	Q86U86	PB1 HUMAN	447.9032807	3	0.854029298		
SYYKPDSPEYK	-15.71892	Q86U86	PB1 HUMAN	688.8226105	2	0.73187089		
SYYKPDSPEYK	-15.71892	Q86U86	PB1 HUMAN	459.5510153	3	0.73187089		
TDSEIYEDAVELQQFFIK	117.4126	Q86U86	PB1 HUMAN	1088.028787	2	0.720204771	3	
TDSEIYEDAVELQQFFIK	117.4126	Q86U86	PB1 HUMAN	725.6884663	3	0.720204771	3	Yes
VGDCVFIK	21.92554	Q86U86	PB1 HUMAN	469.24477	2	0.630040526		
VGDCVFIK	21.92554	Q86U86	PB1 HUMAN	313.1657883	3	0.630040526		
VQYPDYYAIIK	60.06253	Q86U86	PB1 HUMAN	686.8615395	2	0.852727771		
VQYPDYYAIIK	60.06253	Q86U86	PB1 HUMAN	458.2436347	3	0.852727771		
VVDDEIYYFR	54.75745	Q86U86	PB1 HUMAN	659.8198755	2	0.865687191	2	Yes
VVDDEIYYFR	54.75745	Q86U86	PB1 HUMAN	440.2158587	3	0.865687191	2	
YAGEEGMIEDMK	29.09479	Q86U86	PB1 HUMAN	686.792148	2	0.722365797		
YAGEEGMIEDMK	29.09479	Q86U86	PB1 HUMAN	458.1973737	3	0.722365797		
YIEGLSAESNSISK	28.00156	Q86U86	PB1 HUMAN	749.3757465	2	0.783798933	2	Yes
YIEGLSAESNSISK	28.00156	Q86U86	PB1 HUMAN	499.9197727	3	0.783798933	2	
YPDYYQQIK	20.27159	Q86U86	PB1 HUMAN	609.2960325	2	0.777126431		
YPDYYQQIK	20.27159	Q86U86	PB1 HUMAN	406.5332967	3	0.777126431		
AEQDSTTVAAFASSLVSGLNSSASEPAK	106.2909	Q86U44	MTA70 HUMAN	1363.162324	2	0.648709536		
AEQDSTTVAAFASSLVSGLNSSASEPAK	106.2909	Q86U44	MTA70 HUMAN	909.1108243	3	0.648709536		
AQVQEFCDYGTK	15.05801	Q86U44	MTA70 HUMAN	723.322462	2	0.751030743		
AQVQEFCDYGTK	15.05801	Q86U44	MTA70 HUMAN	482.5509163	3	0.751030743		
DHTPSQELALTQSVGGDSSADR	33.25798	Q86U44	MTA70 HUMAN	1136.028374	2	0.773485661	3	
DHTPSQELALTQSVGGDSSADR	33.25798	Q86U44	MTA70 HUMAN	757.688191	3	0.773485661	3	Yes
ECLNLWGYER	54.03751	Q86U44	MTA70 HUMAN	670.3091565	2	0.779708862		
ECLNLWGYER	54.03751	Q86U44	MTA70 HUMAN	447.2087127	3	0.779708862		
FAAQELIEVK	48.40253	Q86U44	MTA70 HUMAN	574.322063	2	0.772201359		
FAAQELIEVK	48.40253	Q86U44	MTA70 HUMAN	383.217317	3	0.772201359		
GLLQDDAHPTLVTYADHSK	34.92542	Q86U44	MTA70 HUMAN	1041.019089	2	0.828980446	4	
GLLQDDAHPTLVTYADHSK	34.92542	Q86U44	MTA70 HUMAN	694.3486673	3	0.828980446	4	
GNPQGFNQGLDCDVIVAEVR	69.96017	Q86U44	MTA70 HUMAN	1094.526759	2	0.623272717		
GNPQGFNQGLDCDVIVAEVR	69.96017	Q86U44	MTA70 HUMAN	730.0204477	3	0.623272717		
GPGEVAGTVTGQK	-13.88231	Q86U44	MTA70 HUMAN	600.8151345	2	0.708486915		
GPGEVAGTVTGQK	-13.88231	Q86U44	MTA70 HUMAN	400.8793647	3	0.708486915		

LSAMMGAVAEK	24.7849	Q86U44	MTA70_HUMAN	554.2810265	2	0.816323221	2	Yes
LSAMMGAVAEK	24.7849	Q86U44	MTA70_HUMAN	369.856626	3	0.816323221	2	
NPEAALSPTFR	30.55737	Q86U44	MTA70_HUMAN	601.8123935	2	0.810452342	2	Yes
NPEAALSPTFR	30.55737	Q86U44	MTA70_HUMAN	401.544204	3	0.810452342	2	
SDSPVPTAPTSGGPKPSTASAVPELATDPELEK	54.39958	Q86U44	MTA70_HUMAN	1617.307175	2	0.665222943		
SDSPVPTAPTSGGPKPSTASAVPELATDPELEK	54.39958	Q86U44	MTA70_HUMAN	1078.540725	3	0.665222943		
STSHKPDEIYGMIER	18.32076	Q86U44	MTA70_HUMAN	881.925613	2	0.836937726		
STSHKPDEIYGMIER	18.32076	Q86U44	MTA70_HUMAN	588.2863503	3	0.836937726		
VSQEILELLNTTTAK	89.23708	Q86U44	MTA70_HUMAN	830.462359	2	0.713621318		
VSQEILELLNTTTAK	89.23708	Q86U44	MTA70_HUMAN	553.9775143	3	0.713621318		
DIAVFITDK	62.96139	Q86U38	NOP9_HUMAN	511.2824065	2	0.658210099		
DIAVFITDK	62.96139	Q86U38	NOP9_HUMAN	341.1908793	3	0.658210099		
DLMVHNIMK	27.80006	Q86U38	NOP9_HUMAN	550.783736	2	0.773423433		
DLMVHNIMK	27.80006	Q86U38	NOP9_HUMAN	367.525099	3	0.773423433		
EAWEQQQGAVAK	-4.779823	Q86U38	NOP9_HUMAN	672.8313155	2	0.716361344		
EAWEQQQGAVAK	-4.779823	Q86U38	NOP9_HUMAN	448.890152	3	0.716361344		
EIAAELGEQNQELIR	49.54859	Q86U38	NOP9_HUMAN	856.9448645	2	0.82819581	2	Yes
EIAAELGEQNQELIR	49.54859	Q86U38	NOP9_HUMAN	571.632518	3	0.82819581	2	
EVETQALALSTNR	30.71246	Q86U38	NOP9_HUMAN	716.3760865	2	0.751103282		
EVETQALALSTNR	30.71246	Q86U38	NOP9_HUMAN	477.9199993	3	0.751103282		
GSSVDGSPLLLFLR	113.0116	Q86U38	NOP9_HUMAN	730.9095645	2	0.672874689	2	Yes
GSSVDGSPLLLFLR	113.0116	Q86U38	NOP9_HUMAN	487.6089847	3	0.672874689	2	
LQDLSSSFLK	47.16808	Q86U38	NOP9_HUMAN	569.3116945	2	0.808392584	2	Yes
LQDLSSSFLK	47.16808	Q86U38	NOP9_HUMAN	379.8770713	3	0.808392584	2	
LQSLFEEHLQGQLQTLAAHPIANFPLQR	94.26305	Q86U38	NOP9_HUMAN	1600.354735	2	0.717713833	4	
LQSLFEEHLQGQLQTLAAHPIANFPLQR	94.26305	Q86U38	NOP9_HUMAN	1067.239098	3	0.717713833	4	
NVALTTFLK	53.47602	Q86U38	NOP9_HUMAN	503.8007665	2	0.778919816	2	Yes
NVALTTFLK	53.47602	Q86U38	NOP9_HUMAN	336.2031193	3	0.778919816	2	
SEPAPDSHPHLSPEALGYFR	43.47308	Q86U38	NOP9_HUMAN	1104.029987	2	0.686578512		
SEPAPDSHPHLSPEALGYFR	43.47308	Q86U38	NOP9_HUMAN	736.3559327	3	0.686578512		
TGSEMLQELLGFSPLKPLCR	117.6884	Q86U38	NOP9_HUMAN	1138.593056	2	0.740286469		
TGSEMLQELLGFSPLKPLCR	117.6884	Q86U38	NOP9_HUMAN	759.397979	3	0.740286469		
TLLQVLGGTILESER	104.6986	Q86U38	NOP9_HUMAN	814.965069	2	0.825535655	3	
TLLQVLGGTILESER	104.6986	Q86U38	NOP9_HUMAN	543.6459877	3	0.825535655	3	Yes
TPAQECKPADFEVPETFLNR	60.92789	Q86U38	NOP9_HUMAN	1175.063175	2	0.642283976	3	
TPAQECKPADFEVPETFLNR	60.92789	Q86U38	NOP9_HUMAN	783.7113913	3	0.642283976	3	Yes
VLDAIWSGAALR	72.2516	Q86U38	NOP9_HUMAN	636.359511	2	0.832884073		
VLDAIWSGAALR	72.2516	Q86U38	NOP9_HUMAN	424.5756157	3	0.832884073		
VLQLLLEAFHCAEPSSR	83.31557	Q86U38	NOP9_HUMAN	985.5123915	2	0.633156836		
VLQLLLEAFHCAEPSSR	83.31557	Q86U38	NOP9_HUMAN	657.3442027	3	0.633156836		
FDVSGLTTEQMLR	76.39661	Q86TP1	PRUN1_HUMAN	748.8748655	2	0.868036509		
FDVSGLTTEQMLR	76.39661	Q86TP1	PRUN1_HUMAN	499.585852	3	0.868036509		
GDIVFFLQK	73.92943	Q86TP1	PRUN1_HUMAN	533.8007665	2	0.748513222		
GDIVFFLQK	73.92943	Q86TP1	PRUN1_HUMAN	356.2031193	3	0.748513222		
ILQGAPEILDR	43.1795	Q86TP1	PRUN1_HUMAN	612.851519	2	0.865589619	2	Yes
ILQGAPEILDR	43.1795	Q86TP1	PRUN1_HUMAN	408.903621	3	0.865589619	2	
LEALFPDLPK	74.91305	Q86TP1	PRUN1_HUMAN	571.826981	2	0.876751363	2	Yes
LEALFPDLPK	74.91305	Q86TP1	PRUN1_HUMAN	381.553929	3	0.876751363	2	
LLPLLQEALSAYFDSMK	162.9716	Q86TP1	PRUN1_HUMAN	970.0164395	2	0.735131145		
LLPLLQEALSAYFDSMK	162.9716	Q86TP1	PRUN1_HUMAN	647.013568	3	0.735131145		
LSAEAVFEK	24.4731	Q86TP1	PRUN1_HUMAN	497.266756	2	0.647489905	2	Yes
LSAEAVFEK	24.4731	Q86TP1	PRUN1_HUMAN	331.8471123	3	0.647489905	2	
MEDYLQGCR	12.15955	Q86TP1	PRUN1_HUMAN	586.247711	2	0.650794446		
MEDYLQGCR	12.15955	Q86TP1	PRUN1_HUMAN	391.167749	3	0.650794446		
NDIFDSLQK	44.89726	Q86TP1	PRUN1_HUMAN	540.2725695	2	0.712175608		
NDIFDSLQK	44.89726	Q86TP1	PRUN1_HUMAN	360.5176547	3	0.712175608		
SDTALEEAVAEVLDHRPIEPK	83.10783	Q86TP1	PRUN1_HUMAN	1160.095528	2	0.727164805		
SDTALEEAVAEVLDHRPIEPK	83.10783	Q86TP1	PRUN1_HUMAN	773.7329603	3	0.727164805		
TTEAEEVFVPVLNIK	90.46344	Q86TP1	PRUN1_HUMAN	844.959452	2	0.778782248		
TTEAEEVFVPVLNIK	90.46344	Q86TP1	PRUN1_HUMAN	563.642243	3	0.778782248		
VHIPESILIFR	68.90344	Q86TP1	PRUN1_HUMAN	662.3933535	2	0.806310773		
VHIPESILIFR	68.90344	Q86TP1	PRUN1_HUMAN	441.9315107	3	0.806310773		
AAEMEHLLER	14.72761	Q86TM6	SYVN1_HUMAN	599.798429	2	0.816054463		
AAEMEHLLER	14.72761	Q86TM6	SYVN1_HUMAN	400.201561	3	0.816054463		
HQFYPTVVYLTK	42.88947	Q86TM6	SYVN1_HUMAN	748.401368	2	0.810899019		
HQFYPTVVYLTK	42.88947	Q86TM6	SYVN1_HUMAN	499.270187	3	0.810899019		
LPCNHIFHTSCLR	11.72549	Q86TM6	SYVN1_HUMAN	827.901226	2	0.612870574		
LPCNHIFHTSCLR	11.72549	Q86TM6	SYVN1_HUMAN	552.2700923	3	0.612870574		
VFFGQLR	38.62211	Q86TM6	SYVN1_HUMAN	433.748337	2	0.782961369		
VFFGQLR	38.62211	Q86TM6	SYVN1_HUMAN	289.5014997	3	0.782961369		
YVLHSVDLQSENPWDNK	46.5232	Q86TM6	SYVN1_HUMAN	1022.492705	2	0.85559243		
YVLHSVDLQSENPWDNK	46.5232	Q86TM6	SYVN1_HUMAN	681.997745	3	0.85559243		
GNTLLLQHLK	15.60955	Q7Z7E8	UB2Q1_HUMAN	568.843497	2	0.857199311		
GNTLLLQHLK	15.60955	Q7Z7E8	UB2Q1_HUMAN	379.5649397	3	0.857199311		
LLESIFHR	32.98238	Q7Z7E8	UB2Q1_HUMAN	507.7907325	2	0.681934714		
LLESIFHR	32.98238	Q7Z7E8	UB2Q1_HUMAN	338.8630967	3	0.681934714		
VDQDSALHNDLQILK	42.04301	Q7Z7E8	UB2Q1_HUMAN	854.947407	2	0.878355682		
VDQDSALHNDLQILK	42.04301	Q7Z7E8	UB2Q1_HUMAN	570.3008797	3	0.878355682		
GDGPFLDSLAK	53.57094	Q7Z6K5	ARPIN_HUMAN	560.28822	2	0.822106838		
GDGPFLDSLAK	53.57094	Q7Z6K5	ARPIN_HUMAN	373.8614217	3	0.822106838		
GNEIEPNFSATR	20.90981	Q7Z6K5	ARPIN_HUMAN	667.8209465	2	0.77704066	2	Yes
GNEIEPNFSATR	20.90981	Q7Z6K5	ARPIN_HUMAN	445.549906	3	0.77704066	2	
LPGAWDPAAHQGGNGVLLEGELIDVSR	96.10327	Q7Z6K5	ARPIN_HUMAN	1386.209747	2	0.656060338	3	
LPGAWDPAAHQGGNGVLLEGELIDVSR	96.10327	Q7Z6K5	ARPIN_HUMAN	924.475773	3	0.656060338	3	Yes
TGASWTDNIMAQK	35.57762	Q7Z6K5	ARPIN_HUMAN	711.838283	2	0.856777072	2	Yes
TGASWTDNIMAQK	35.57762	Q7Z6K5	ARPIN_HUMAN	474.894797	3	0.856777072	2	
VNTGFLMSSYK	37.06423	Q7Z6K5	ARPIN_HUMAN	623.8110005	2	0.725768864		
VNTGFLMSSYK	37.06423	Q7Z6K5	ARPIN_HUMAN	416.209942	3	0.725768864		
AVIQVSQIVAR	41.41239	Q7Z4W1	DCXR_HUMAN	592.362054	2	0.823317409	2	Yes
AVIQVSQIVAR	41.41239	Q7Z4W1	DCXR_HUMAN	395.2439777	3	0.823317409	2	
GALDMLTK	32.07333	Q7Z4W1	DCXR_HUMAN	424.7314955	2	0.745597899	2	Yes
GALDMLTK	32.07333	Q7Z4W1	DCXR_HUMAN	283.490272	3	0.745597899	2	
GTVQALHATGAR	-26.13043	Q7Z4W1	DCXR_HUMAN	591.323461	2	0.665362179	3	
GTVQALHATGAR	-26.13043	Q7Z4W1	DCXR_HUMAN	394.5515823	3	0.665362179	3	Yes
GVPGAIVNVSSQCSQR	26.44699	Q7Z4W1	DCXR_HUMAN	829.918127	2	0.710037768		
GVPGAIVNVSSQCSQR	26.44699	Q7Z4W1	DCXR_HUMAN	553.614693	3	0.710037768		
SFEVNLR	23.47504	Q7Z4W1	DCXR_HUMAN	432.732883	2	0.642079532		
SFEVNLR	23.47504	Q7Z4W1	DCXR_HUMAN	288.8245303	3	0.642079532		
SGMTTGSTLPVEGGFWAC	99.40384	Q7Z4W1	DCXR_HUMAN	929.411487	2	0.715813935		
SGMTTGSTLPVEGGFWAC	99.40384	Q7Z4W1	DCXR_HUMAN	619.9435997	3	0.715813935		
TQADLDSLVR	32.04674	Q7Z4W1	DCXR_HUMAN	559.2965765	2	0.730990052	2	Yes

TQADLDSLVR	32.04674	Q7Z4W1	DCXR HUMAN	373.200326	3	0.730990052	2	
VMALELGPHK	16.9459	Q7Z4W1	DCXR HUMAN	547.805526	2	0.836805761	3	
VMALELGPHK	16.9459	Q7Z4W1	DCXR HUMAN	365.5396257	3	0.836805761	3	Yes
AFPLQCIHIAEGHTK	29.87283	Q7Z4S6	KI21A HUMAN	861.4437815	2	0.771481872		
AFPLQCIHIAEGHTK	29.87283	Q7Z4S6	KI21A HUMAN	574.631796	3	0.771481872		
AGEGNEEISNMIHSYIK	53.3941	Q7Z4S6	KI21A HUMAN	946.446909	2	0.780139983		
AGEGNEEISNMIHSYIK	53.3941	Q7Z4S6	KI21A HUMAN	631.3005477	3	0.780139983		
AMQETVDALR	14.21275	Q7Z4S6	KI21A HUMAN	567.285155	2	0.672002017		
AMQETVDALR	14.21275	Q7Z4S6	KI21A HUMAN	378.526045	3	0.672002017		
ANYQADLANITCEIAIK	77.75269	Q7Z4S6	KI21A HUMAN	954.480753	2	0.646271527		
ANYQADLANITCEIAIK	77.75269	Q7Z4S6	KI21A HUMAN	636.6564437	3	0.646271527		
APYFSGSSTFSPTILSSDK	69.78314	Q7Z4S6	KI21A HUMAN	996.4840145	2	0.685412228		
APYFSGSSTFSPTILSSDK	69.78314	Q7Z4S6	KI21A HUMAN	664.658618	3	0.685412228		
AVLCVDSTDDLLFTGSK	80.78159	Q7Z4S6	KI21A HUMAN	920.954041	2	0.729206264		
AVLCVDSTDDLLFTGSK	80.78159	Q7Z4S6	KI21A HUMAN	614.3053023	3	0.729206264		
DLLQQVPNAHK	19.39288	Q7Z4S6	KI21A HUMAN	631.846768	2	0.807568312		
DLLQQVPNAHK	19.39288	Q7Z4S6	KI21A HUMAN	421.5671203	3	0.807568312		
DQVLQNLGSVESYSEEK	59.72536	Q7Z4S6	KI21A HUMAN	962.9609035	2	0.866513371		
DQVLQNLGSVESYSEEK	59.72536	Q7Z4S6	KI21A HUMAN	642.3098773	3	0.866513371		
GIINPFPASK	41.07032	Q7Z4S6	KI21A HUMAN	522.2983905	2	0.777256012		
GIINPFPASK	41.07032	Q7Z4S6	KI21A HUMAN	348.5348687	3	0.777256012		
IEGCHICTSVTPGEPQVFLGK	51.30017	Q7Z4S6	KI21A HUMAN	1165.069946	2	0.603605211		
IEGCHICTSVTPGEPQVFLGK	51.30017	Q7Z4S6	KI21A HUMAN	777.049239	3	0.603605211		
IHEDSTGGIYTVGVTTR	23.44411	Q7Z4S6	KI21A HUMAN	903.455593	2	0.777248383		
IHEDSTGGIYTVGVTTR	23.44411	Q7Z4S6	KI21A HUMAN	602.6396703	3	0.777248383		
IIDEEGVESINDMFHENAMLQTENNNLR	89.9425	Q7Z4S6	KI21A HUMAN	1638.251353	2	0.713104308		
IIDEEGVESINDMFHENAMLQTENNNLR	89.9425	Q7Z4S6	KI21A HUMAN	1092.50351	3	0.713104308		
IISESAQMNEFETLTAK	59.29803	Q7Z4S6	KI21A HUMAN	956.4725975	2	0.828097284		
IISESAQMNEFETLTAK	59.29803	Q7Z4S6	KI21A HUMAN	637.98434	3	0.828097284		
ITQLVSDQANHVLAR	22.05465	Q7Z4S6	KI21A HUMAN	832.9581095	2	0.799393773	3	
ITQLVSDQANHVLAR	22.05465	Q7Z4S6	KI21A HUMAN	555.641348	3	0.799393773	3	Yes
LIDELENSQK	17.07967	Q7Z4S6	KI21A HUMAN	594.809516	2	0.765454233		
LIDELENSQK	17.07967	Q7Z4S6	KI21A HUMAN	396.875619	3	0.765454233		
LLESEAVNENLR	28.69191	Q7Z4S6	KI21A HUMAN	693.8653535	2	0.807712615	2	Yes
LLESEAVNENLR	28.69191	Q7Z4S6	KI21A HUMAN	462.912844	3	0.807712615	2	
LMMLQHK	-6.597397	Q7Z4S6	KI21A HUMAN	450.743883	2	0.744807601		
LMMLQHK	-6.597397	Q7Z4S6	KI21A HUMAN	300.8318637	3	0.744807601		
LQQDVMEMK	12.6839	Q7Z4S6	KI21A HUMAN	561.270659	2	0.800580978		
LQQDVMEMK	12.6839	Q7Z4S6	KI21A HUMAN	374.516381	3	0.800580978		
LTVSQGNTSVQQDK	-7.380772	Q7Z4S6	KI21A HUMAN	752.884276	2	0.730363011		
LTVSQGNTSVQQDK	-7.380772	Q7Z4S6	KI21A HUMAN	502.2587923	3	0.730363011		
VTEGALGTVSPTHNFEPPHYDGIEALTIQGDNLF[illegible]	107.0168	Q7Z4S6	KI21A HUMAN	2210.545513	2	0.730844378		
VTEGALGTVSPTHNFEPPHYDGIEALTIQGDNLF[illegible]	107.0168	Q7Z4S6	KI21A HUMAN	1474.03295	3	0.730844378		
MTISNMEADMNR	29.3025	Q7Z4S6	KI21A HUMAN	706.802528	2	0.820443034	2	Yes
MTISNMEADMNR	29.3025	Q7Z4S6	KI21A HUMAN	471.537627	3	0.820443034	2	
NELNVFNR	20.67532	Q7Z4S6	KI21A HUMAN	503.259796	2	0.630693257		
NELNVFNR	20.67532	Q7Z4S6	KI21A HUMAN	335.8424723	3	0.630693257		
NLQDGQISDTGDLGEDIASN	66.10913	Q7Z4S6	KI21A HUMAN	1031.464544	2	0.812985659		
NLQDGQISDTGDLGEDIASN	66.10913	Q7Z4S6	KI21A HUMAN	687.9789707	3	0.812985659		
TLTSSGQVTLGDACSASTSR	22.05763	Q7Z4S6	KI21A HUMAN	999.974025	2	0.702814698		
TLTSSGQVTLGDACSASTSR	22.05763	Q7Z4S6	KI21A HUMAN	666.9852917	3	0.702814698		
TVAIPSGENQINQIALNPTGTFLYAASGNAVR	108.0962	Q7Z4S6	KI21A HUMAN	1644.355124	2	0.668780744		
TVAIPSGENQINQIALNPTGTFLYAASGNAVR	108.0962	Q7Z4S6	KI21A HUMAN	1096.572691	3	0.668780744		
VCPQIDADNATDNK	-0.76627	Q7Z4S6	KI21A HUMAN	780.8521185	2	0.679773271	2	Yes
VCPQIDADNATDNK	-0.76627	Q7Z4S6	KI21A HUMAN	520.9040207	3	0.679773271	2	
VQALPTPATNGNR	7.698799	Q7Z4S6	KI21A HUMAN	669.860407	2	0.675415635		
VQALPTPATNGNR	7.698799	Q7Z4S6	KI21A HUMAN	446.9095463	3	0.675415635		
VWNLVTGQEIMSLGGHPNNVVSVK	83.52124	Q7Z4S6	KI21A HUMAN	1289.676497	2	0.798751473		
VWNLVTGQEIMSLGGHPNNVVSVK	83.52124	Q7Z4S6	KI21A HUMAN	860.1202727	3	0.798751473		
VWNMDTFMPVGEMK	91.26633	Q7Z4S6	KI21A HUMAN	842.880779	2	0.80111593		
VWNMDTFMPVGEMK	91.26633	Q7Z4S6	KI21A HUMAN	562.256461	3	0.80111593		
YLLDHFLSMGINK	78.89337	Q7Z4S6	KI21A HUMAN	775.9059635	2	0.784869909		
YLLDHFLSMGINK	78.89337	Q7Z4S6	KI21A HUMAN	517.606584	3	0.784869909		
AVLSDSPQLSEGK	15.87626	Q7Z4H7	HAUS6 HUMAN	665.84663	2	0.822126031		
AVLSDSPQLSEGK	15.87626	Q7Z4H7	HAUS6 HUMAN	444.233695	3	0.822126031		
DNDFGILHETLPEEVGHLSFNSSSSSEANFK	81.49313	Q7Z4H7	HAUS6 HUMAN	1704.287303	2	0.749415159		
DNDFGILHETLPEEVGHLSFNSSSSSEANFK	81.49313	Q7Z4H7	HAUS6 HUMAN	1136.527477	3	0.749415159		
EFLGLSPFSLIK	120.0881	Q7Z4H7	HAUS6 HUMAN	675.8875695	2	0.727263033		
EFLGLSPFSLIK	120.0881	Q7Z4H7	HAUS6 HUMAN	450.9276547	3	0.727263033		
EFSMADFLLETTVSDFGQSHLTEEK	126.7082	Q7Z4H7	HAUS6 HUMAN	1431.163479	2	0.636853218		
EFSMADFLLETTVSDFGQSHLTEEK	126.7082	Q7Z4H7	HAUS6 HUMAN	954.4449277	3	0.636853218		
EPIQMDAEHR	-13.80683	Q7Z4H7	HAUS6 HUMAN	613.285686	2	0.693388939		
EPIQMDAEHR	-13.80683	Q7Z4H7	HAUS6 HUMAN	409.1930657	3	0.693388939		
EVLPESLPVLHNQR	44.24641	Q7Z4H7	HAUS6 HUMAN	815.9497525	2	0.817098022		
EVLPESLPVLHNQR	44.24641	Q7Z4H7	HAUS6 HUMAN	544.3024433	3	0.817098022		
FCWPPFDQK	65.99462	Q7Z4H7	HAUS6 HUMAN	612.7795085	2	0.707805514		
FCWPPFDQK	65.99462	Q7Z4H7	HAUS6 HUMAN	408.855614	3	0.707805514		
FIHLMYHFAR	30.07766	Q7Z4H7	HAUS6 HUMAN	667.8455125	2	0.690792024		
FIHLMYHFAR	30.07766	Q7Z4H7	HAUS6 HUMAN	445.5662833	3	0.690792024		
FLQILQR	41.72016	Q7Z4H7	HAUS6 HUMAN	459.282544	2	0.733050466		
FLQILQR	41.72016	Q7Z4H7	HAUS6 HUMAN	306.5243043	3	0.733050466		
FSPVEQR	-14.19169	Q7Z4H7	HAUS6 HUMAN	431.7250585	2	0.743140936	2	Yes
FSPVEQR	-14.19169	Q7Z4H7	HAUS6 HUMAN	288.1526473	3	0.743140936	2	
KPELSPTPQNVQTDDTLNFLDTCDLHTEHIKPSL	52.90263	Q7Z4H7	HAUS6 HUMAN	2099.037662	2	0.719556868		
KPELSPTPQNVQTDDTLNFLDTCDLHTEHIKPSL	52.90263	Q7Z4H7	HAUS6 HUMAN	1399.694383	3	0.719556868		
HVLTSHIDEPPTQNQSDLLNK	19.0701	Q7Z4H7	HAUS6 HUMAN	1193.604052	2	0.773049951		
HVLTSHIDEPPTQNQSDLLNK	19.0701	Q7Z4H7	HAUS6 HUMAN	796.071976	3	0.773049951		
IDVMGGSEEEFMK	49.60364	Q7Z4H7	HAUS6 HUMAN	736.326359	2	0.80477798		
IDVMGGSEEEFMK	49.60364	Q7Z4H7	HAUS6 HUMAN	491.220181	3	0.80477798		
ILDHLEVSCNKPSTNK	-3.525852	Q7Z4H7	HAUS6 HUMAN	927.9730985	2	0.661368966		
ILDHLEVSCNKPSTNK	-3.525852	Q7Z4H7	HAUS6 HUMAN	618.984674	3	0.661368966		
IPEFEVENSPLSDVAK	66.6683	Q7Z4H7	HAUS6 HUMAN	887.4494475	2	0.811513484		
IPEFEVENSPLSDVAK	66.6683	Q7Z4H7	HAUS6 HUMAN	591.9689067	3	0.811513484		
ISGECGSSFPQVVGSLFLSPGGPK	111.617	Q7Z4H7	HAUS6 HUMAN	1204.102299	2	0.654534221		
ISGECGSSFPQVVGSLFLSPGGPK	111.617	Q7Z4H7	HAUS6 HUMAN	803.0708077	3	0.654534221		
IVSHTHLGVNMFDK	16.33936	Q7Z4H7	HAUS6 HUMAN	799.411949	2	0.752979219		
IVSHTHLGVNMFDK	16.33936	Q7Z4H7	HAUS6 HUMAN	533.277241	3	0.752979219		
LEELIDSLGSNPFLTR	100.3946	Q7Z4H7	HAUS6 HUMAN	902.4785395	2	0.80360049		
LEELIDSLGSNPFLTR	100.3946	Q7Z4H7	HAUS6 HUMAN	601.9883013	3	0.80360049		
LNLLTVIQLLNEVLK	191.9868	Q7Z4H7	HAUS6 HUMAN	862.040579	2	0.731296539		
LNLLTVIQLLNEVLK	191.9868	Q7Z4H7	HAUS6 HUMAN	575.029661	3	0.731296539		

LTVDLHYLEK	39.62968	Q7Z4H7	HAUS6_HUMAN	615.8406155	2	0.832407713		
LTVDLHYLEK	39.62968	Q7Z4H7	HAUS6_HUMAN	410.896352	3	0.832407713		
MEPYDDHSNMEEK	-16.04019	Q7Z4H7	HAUS6_HUMAN	812.816882	2	0.605275571		
MEPYDDHSNMEEK	-16.04019	Q7Z4H7	HAUS6_HUMAN	542.213863	3	0.605275571		
METFSPAVGNR	18.13977	Q7Z4H7	HAUS6_HUMAN	604.790604	2	0.766928017		
METFSPAVGNR	18.13977	Q7Z4H7	HAUS6_HUMAN	403.5296777	3	0.766928017		
NSSHHFVETFNIKPQDLHK	8.064259	Q7Z4H7	HAUS6_HUMAN	1139.572357	2	0.689157844		
NSSHHFVETFNIKPQDLHK	8.064259	Q7Z4H7	HAUS6_HUMAN	760.0508463	3	0.689157844		
NTESSAFGGSLPAK	18.91042	Q7Z4H7	HAUS6_HUMAN	683.3364295	2	0.732572854		
NTESSAFGGSLPAK	18.91042	Q7Z4H7	HAUS6_HUMAN	455.8935613	3	0.732572854		
QTTPESDFNLQALR	52.06549	Q7Z4H7	HAUS6_HUMAN	810.4053755	2	0.762887478		
QTTPESDFNLQALR	52.06549	Q7Z4H7	HAUS6_HUMAN	540.606192	3	0.762887478		
SECIGLENQIK	24.57548	Q7Z4H7	HAUS6_HUMAN	645.822101	2	0.79147625		
SECIGLENQIK	24.57548	Q7Z4H7	HAUS6_HUMAN	430.884009	3	0.79147625		
SILCQYPASLPDAHK	38.90128	Q7Z4H7	HAUS6_HUMAN	850.427792	2	0.885434031		
SILCQYPASLPDAHK	38.90128	Q7Z4H7	HAUS6_HUMAN	567.287803	3	0.885434031		
SLWASVNETLMFLEK	124.2875	Q7Z4H7	HAUS6_HUMAN	884.453479	2	0.719880402		
SLWASVNETLMFLEK	124.2875	Q7Z4H7	HAUS6_HUMAN	589.9715943	3	0.719880402		
TMLWNSFQISSGISSK	83.35004	Q7Z4H7	HAUS6_HUMAN	893.446185	2	0.61137259		
TMLWNSFQISSGISSK	83.35004	Q7Z4H7	HAUS6_HUMAN	595.9667317	3	0.61137259		
TPENLITEIR	47.96751	Q7Z4H7	HAUS6_HUMAN	593.3278765	2	0.796114922		
TPENLITEIR	47.96751	Q7Z4H7	HAUS6_HUMAN	395.8878593	3	0.796114922		
VISDCECVPQK	-1.495888	Q7Z4H7	HAUS6_HUMAN	667.808137	2	0.729402184		
VISDCECVPQK	-1.495888	Q7Z4H7	HAUS6_HUMAN	445.5413663	3	0.729402184		
YQENAQLSVK	-4.74313	Q7Z4H7	HAUS6_HUMAN	590.304397	2	0.790245652		
YQENAQLSVK	-4.74313	Q7Z4H7	HAUS6_HUMAN	393.8722063	3	0.790245652		
ELYELWEEYETQSSAEAK	81.64458	Q7Z4H3	HDDC2_HUMAN	1102.997679	2	0.799130261		
ELYELWEEYETQSSAEAK	81.64458	Q7Z4H3	HDDC2_HUMAN	735.6677277	3	0.799130261		
FNHPEIVQLVSELEAER	93.22575	Q7Z4H3	HDDC2_HUMAN	1005.518728	2	0.806171179	3	
FNHPEIVQLVSELEAER	93.22575	Q7Z4H3	HDDC2_HUMAN	670.68176	3	0.806171179	3	Yes
LALVHDMAECIVGDIAPADNIPK	91.83669	Q7Z4H3	HDDC2_HUMAN	1231.625085	2	0.772345901		
LALVHDMAECIVGDIAPADNIPK	91.83669	Q7Z4H3	HDDC2_HUMAN	821.4193313	3	0.772345901		
LQDFYDSTAGK	17.04034	Q7Z4H3	HDDC2_HUMAN	622.7938625	2	0.838842154		
LQDFYDSTAGK	17.04034	Q7Z4H3	HDDC2_HUMAN	415.53185	3	0.838842154		
NVQRPESVSDHMYR	-21.57324	Q7Z4H3	HDDC2_HUMAN	859.4079215	2	0.724394143	3	
NVQRPESVSDHMYR	-21.57324	Q7Z4H3	HDDC2_HUMAN	573.274556	3	0.724394143	3	Yes
QITQLLPEDLR	61.10609	Q7Z4H3	HDDC2_HUMAN	663.3753585	2	0.714309454	2	Yes
QITQLLPEDLR	61.10609	Q7Z4H3	HDDC2_HUMAN	442.5861807	3	0.714309454	2	
SLLQFLR	71.64271	Q7Z4H3	HDDC2_HUMAN	438.769269	2	0.804015756		
SLLQFLR	71.64271	Q7Z4H3	HDDC2_HUMAN	292.8487877	3	0.804015756		
ILGSASPEEEQEKPILDRPTR	20.64796	Q7Z422	SZRD1_HUMAN	1183.122077	2	0.743464351	4	
ILGSASPEEEQEKPILDRPTR	20.64796	Q7Z422	SZRD1_HUMAN	789.083993	3	0.743464351	4	
MEDEEVAESWEEAADSGEIDR	73.8074	Q7Z422	SZRD1_HUMAN	1198.987722	2	0.705154777		
MEDEEVAESWEEAADSGEIDR	73.8074	Q7Z422	SZRD1_HUMAN	799.6610897	3	0.705154777		
VPIVIQDDSLPAGPPPQIR	72.71767	Q7Z422	SZRD1_HUMAN	1006.562939	2	0.749619544		
VPIVIQDDSLPAGPPPQIR	72.71767	Q7Z422	SZRD1_HUMAN	671.377901	3	0.749619544		
AYANHMINNHVPR	-20.71339	Q7Z3K3	POGZ_HUMAN	768.878603	2	0.722190261	3	
AYANHMINNHVPR	-20.71339	Q7Z3K3	POGZ_HUMAN	512.921677	3	0.722190261	3	Yes
DVAENAGLFIDFVQR	112.8307	Q7Z3K3	POGZ_HUMAN	847.431393	2	0.771778047		
DVAENAGLFIDFVQR	112.8307	Q7Z3K3	POGZ_HUMAN	565.2902037	3	0.771778047		
EQQLPVNEETLFQK	53.25787	Q7Z3K3	POGZ_HUMAN	851.9365085	2	0.844749928		
EQQLPVNEETLFQK	53.25787	Q7Z3K3	POGZ_HUMAN	568.293614	3	0.844749928		
ESGYSDDEIMELWSTR	89.34088	Q7Z3K3	POGZ_HUMAN	959.412732	2	0.815269589		
ESGYSDDEIMELWSTR	89.34088	Q7Z3K3	POGZ_HUMAN	639.9444297	3	0.815269589		
GLTWIAHSR	12.61948	Q7Z3K3	POGZ_HUMAN	520.785982	2	0.647399306		
GLTWIAHSR	12.61948	Q7Z3K3	POGZ_HUMAN	347.5265963	3	0.647399306		
GMLVMDCHR	0.374569	Q7Z3K3	POGZ_HUMAN	559.749371	2	0.677468956		
GMLVMDCHR	0.374569	Q7Z3K3	POGZ_HUMAN	373.502189	3	0.677468956		
GQMDQPANMPDSILLEAK	70.81275	Q7Z3K3	POGZ_HUMAN	979.472075	2	0.773099363		
GQMDQPANMPDSILLEAK	70.81275	Q7Z3K3	POGZ_HUMAN	653.317325	3	0.773099363		
HLLCPYCLK	20.86362	Q7Z3K3	POGZ_HUMAN	602.304832	2	0.706106424		
HLLCPYCLK	20.86362	Q7Z3K3	POGZ_HUMAN	401.8724963	3	0.706106424		
HLVFNPSHR	-24.39019	Q7Z3K3	POGZ_HUMAN	553.7968805	2	0.625540018		
HLVFNPSHR	-24.39019	Q7Z3K3	POGZ_HUMAN	369.533862	3	0.625540018		
ICEWAFESEPLFLQHMK	87.73985	Q7Z3K3	POGZ_HUMAN	1083.01391	2	0.610084116	3	
ICEWAFESEPLFLQHMK	87.73985	Q7Z3K3	POGZ_HUMAN	722.345215	3	0.610084116	3	Yes
ISDVIEDSVVEDYNSVDK	70.46789	Q7Z3K3	POGZ_HUMAN	1013.479126	2	0.72832638	3	
ISDVIEDSVVEDYNSVDK	70.46789	Q7Z3K3	POGZ_HUMAN	675.988692	3	0.72832638	3	Yes
LACTSCTFVTSVGDAMAK	51.07053	Q7Z3K3	POGZ_HUMAN	959.9395545	2	0.715447903		
LACTSCTFVTSVGDAMAK	51.07053	Q7Z3K3	POGZ_HUMAN	640.2956447	3	0.715447903		
LAEWVLTQR	47.15295	Q7Z3K3	POGZ_HUMAN	558.3145725	2	0.795217156	2	Yes
LAEWVLTQR	47.15295	Q7Z3K3	POGZ_HUMAN	372.5456567	3	0.795217156	2	
LAPSFPSPPAVSIASFVTVK	118.8166	Q7Z3K3	POGZ_HUMAN	1008.064783	2	0.646024525		
LAPSFPSPPAVSIASFVTVK	118.8166	Q7Z3K3	POGZ_HUMAN	672.37913	3	0.646024525		
LIMLVDDFYYGR	96.1722	Q7Z3K3	POGZ_HUMAN	752.8794105	2	0.794006288		
LIMLVDDFYYGR	96.1722	Q7Z3K3	POGZ_HUMAN	502.2555487	3	0.794006288		
NADMQEELIASLEEQLK	120.4055	Q7Z3K3	POGZ_HUMAN	980.9807865	2	0.757111251		
NADMQEELIASLEEQLK	120.4055	Q7Z3K3	POGZ_HUMAN	654.3231327	3	0.757111251		
NMYPPPSFPTNK	36.1908	Q7Z3K3	POGZ_HUMAN	696.835007	2	0.621790171		
NMYPPPSFPTNK	36.1908	Q7Z3K3	POGZ_HUMAN	464.892613	3	0.621790171		
SFLVASVLPGPDGNINSPTR	86.46036	Q7Z3K3	POGZ_HUMAN	1021.039827	2	0.801496863		
SFLVASVLPGPDGNINSPTR	86.46036	Q7Z3K3	POGZ_HUMAN	681.0291597	3	0.801496863		
SLDSEPSVPSAAKPPSPEK	17.88206	Q7Z3K3	POGZ_HUMAN	961.989468	2	0.725022614		
SLDSEPSVPSAAKPPSPEK	17.88206	Q7Z3K3	POGZ_HUMAN	641.6622537	3	0.725022614		
SSLYSEVDVHFR	37.36575	Q7Z3K3	POGZ_HUMAN	719.852242	2	0.803387284		
SSLYSEVDVHFR	37.36575	Q7Z3K3	POGZ_HUMAN	480.2374363	3	0.803387284		
STPSTSTTPTATQPTSLGQLAVQSPGQSNQTTNPK	49.18372	Q7Z3K3	POGZ_HUMAN	1757.36936	2	0.72980094		
STPSTSTTPTATQPTSLGQLAVQSPGQSNQTTNPK	49.18372	Q7Z3K3	POGZ_HUMAN	1171.915515	3	0.72980094		
STVPQSQSQQTK	-35.70448	Q7Z3K3	POGZ_HUMAN	659.834055	2	0.620273888		
STVPQSQSQQTK	-35.70448	Q7Z3K3	POGZ_HUMAN	440.2253117	3	0.620273888		
TAPVASTPSSTPIPALSPPTK	53.45779	Q7Z3K3	POGZ_HUMAN	1010.552237	2	0.780431151		
TAPVASTPSSTPIPALSPPTK	53.45779	Q7Z3K3	POGZ_HUMAN	674.037433	3	0.780431151		
THLSEEVLAMLSASSTLPAVVPAGCSSK	113.8002	Q7Z3K3	POGZ_HUMAN	1421.720441	2	0.63034296		
THLSEEVLAMLSASSTLPAVVPAGCSSK	113.8002	Q7Z3K3	POGZ_HUMAN	948.149569	3	0.63034296		
VSQQPVSAPVPIAAHASVAGHLSTSTTVSSSGAQN	49.98884	Q7Z3K3	POGZ_HUMAN	2176.086592	2	0.603679061		
VSQQPVSAPVPIAAHASVAGHLSTSTTVSSSGAQN	49.98884	Q7Z3K3	POGZ_HUMAN	1451.060336	3	0.603679061		
VAQLTNFPK	19.38088	Q7Z3K3	POGZ_HUMAN	509.290566	2	0.800023973		
VAQLTNFPK	19.38088	Q7Z3K3	POGZ_HUMAN	339.8629857	3	0.800023973		
VPEPNENVGDAVQTK	14.64392	Q7Z3K3	POGZ_HUMAN	798.897383	2	0.761776567	2	Yes
VPEPNENVGDAVQTK	14.64392	Q7Z3K3	POGZ_HUMAN	532.934197	3	0.761776567	2	
VTSSIPVFDLQDGGR	68.90276	Q7Z3K3	POGZ_HUMAN	795.9102935	2	0.779457867		

VTSSIPVFDLQDGGR	68.90276	Q7Z3K3	POGZ HUMAN	530.942804	3	0.779457867		
YLSFEAEEK	20.52099	Q7Z3K3	POGZ HUMAN	558.2669485	2	0.735762		
YLSFEAEEK	20.52099	Q7Z3K3	POGZ HUMAN	372.5139073	3	0.735762		
ASEAGEVPFNHEILR	34.81962	Q7L5N1	CSN6 HUMAN	834.921192	2	0.87550354		
ASEAGEVPFNHEILR	34.81962	Q7L5N1	CSN6 HUMAN	556.9500697	3	0.87550354		
ELEFLGWYTTGGPPDPSDIHVHK	79.81728	Q7L5N1	CSN6 HUMAN	1298.129899	2	0.690896988	4	
ELEFLGWYTTGGPPDPSDIHVHK	79.81728	Q7L5N1	CSN6 HUMAN	865.755874	3	0.690896988	4	
FNVLYDR	27.73717	Q7L5N1	CSN6 HUMAN	463.740704	2	0.654751599	2	Yes
FNVLYDR	27.73717	Q7L5N1	CSN6 HUMAN	309.496411	3	0.654751599	2	
LILEYVK	40.44844	Q7L5N1	CSN6 HUMAN	439.2738485	2	0.688852251	2	Yes
LILEYVK	40.44844	Q7L5N1	CSN6 HUMAN	293.185174	3	0.688852251	2	
MTATGSGENSTVAEHLIAQHSAIK	24.67577	Q7L5N1	CSN6 HUMAN	1227.108645	2	0.638885975	4	
MTATGSGENSTVAEHLIAQHSAIK	24.67577	Q7L5N1	CSN6 HUMAN	818.4083713	3	0.638885975	4	
NIEVMNSFELLSHTVEEK	82.67398	Q7L5N1	CSN6 HUMAN	1060.022985	2	0.627447605	3	
NIEVMNSFELLSHTVEEK	82.67398	Q7L5N1	CSN6 HUMAN	707.0179317	3	0.627447605	3	Yes
TCNTMNQFVNK	3.70314	Q7L5N1	CSN6 HUMAN	678.8059285	2	0.720497191		
TCNTMNQFVNK	3.70314	Q7L5N1	CSN6 HUMAN	452.8732273	3	0.720497191		
AANPEMPLSFTISR	70.03127	Q7L5L3	GDPD3 HUMAN	767.390682	2	0.665022612		
AANPEMPLSFTISR	70.03127	Q7L5L3	GDPD3 HUMAN	511.9297297	3	0.665022612		
DVGSLDFEDLPLYK	105.267	Q7L5L3	GDPD3 HUMAN	805.901598	2	0.888693273		
DVGSLDFEDLPLYK	105.267	Q7L5L3	GDPD3 HUMAN	537.6036737	3	0.888693273		
GGSGELLENTMEAMENSMAQR	76.3078	Q7L5L3	GDPD3 HUMAN	1127.991034	2	0.730068862		
GGSGELLENTMEAMENSMAQR	76.3078	Q7L5L3	GDPD3 HUMAN	752.3299643	3	0.730068862		
LEDLFQR	31.74257	Q7L5L3	GDPD3 HUMAN	460.745991	2	0.78676784		
LEDLFQR	31.74257	Q7L5L3	GDPD3 HUMAN	307.4999357	3	0.78676784		
NEITIWASEK	37.75241	Q7L5L3	GDPD3 HUMAN	595.8067765	2	0.641235113		
NEITIWASEK	37.75241	Q7L5L3	GDPD3 HUMAN	397.5404593	3	0.641235113		
SDLLELDCQLTR	65.55286	Q7L5L3	GDPD3 HUMAN	731.8644975	2	0.836669207		
SDLLELDCQLTR	65.55286	Q7L5L3	GDPD3 HUMAN	488.2456067	3	0.836669207		
VVVVSHDENLCR	-6.711788	Q7L5L3	GDPD3 HUMAN	713.859549	2	0.807339668		
VVVVSHDENLCR	-6.711788	Q7L5L3	GDPD3 HUMAN	476.2423077	3	0.807339668		
ADLSGSVLDCANLQGVK	58.65688	Q7L273	KCTD9 HUMAN	873.938725	2	0.799372911		
ADLSGSVLDCANLQGVK	58.65688	Q7L273	KCTD9 HUMAN	582.9617583	3	0.799372911		
EPDSMLAHMFK	41.47417	Q7L273	KCTD9 HUMAN	653.30249	2	0.744660079		
EPDSMLAHMFK	41.47417	Q7L273	KCTD9 HUMAN	435.870935	3	0.744660079		
FFGIDSLIEHLEVAIK	140.2096	Q7L273	KCTD9 HUMAN	916.0041935	2	0.822879434		
FFGIDSLIEHLEVAIK	140.2096	Q7L273	KCTD9 HUMAN	611.005404	3	0.822879434		
FLLATPTK	32.39787	Q7L273	KCTD9 HUMAN	445.771478	2	0.739061177		
FLLATPTK	32.39787	Q7L273	KCTD9 HUMAN	297.516927	3	0.739061177		
GGLIDDIALIR	86.13333	Q7L273	KCTD9 HUMAN	578.340787	2	0.775335968		
GGLIDDIALIR	86.13333	Q7L273	KCTD9 HUMAN	385.8964663	3	0.775335968		
GVDMEGSQMTGINLR	49.20656	Q7L273	KCTD9 HUMAN	804.379989	2	0.837338626		
GVDMEGSQMTGINLR	49.20656	Q7L273	KCTD9 HUMAN	536.5892677	3	0.837338626		
HGQLIVNDGINLLGVLEEAR	104.1758	Q7L273	KCTD9 HUMAN	1080.592759	2	0.74348253		
HGQLIVNDGINLLGVLEEAR	104.1758	Q7L273	KCTD9 HUMAN	720.731114	3	0.74348253		
LCNFEDPSGLK	31.70467	Q7L273	KCTD9 HUMAN	640.303544	2	0.767665148		
LCNFEDPSGLK	31.70467	Q7L273	KCTD9 HUMAN	427.204971	3	0.767665148		
MLCSNAEGASLK	6.108822	Q7L273	KCTD9 HUMAN	640.802854	2	0.755407095		
MLCSNAEGASLK	6.108822	Q7L273	KCTD9 HUMAN	427.5378443	3	0.755407095		
NSQPPEDHSPISR	-21.2326	Q7L273	KCTD9 HUMAN	732.3478595	2	0.676308334		
NSQPPEDHSPISR	-21.2326	Q7L273	KCTD9 HUMAN	488.567848	3	0.676308334		
SPEYFEPILNYLR	103.3271	Q7L273	KCTD9 HUMAN	820.9201205	2	0.798672199		
SPEYFEPILNYLR	103.3271	Q7L273	KCTD9 HUMAN	547.616022	3	0.798672199		
VVAVYGTLSDLLSVASSK	112.1119	Q7L273	KCTD9 HUMAN	905.004386	2	0.647229791		
VVAVYGTLSDLLSVASSK	112.1119	Q7L273	KCTD9 HUMAN	603.672199	3	0.647229791		
AEVLSEEPILK	40.64005	Q7L1Q6	BZW1 HUMAN	614.3457345	2	0.837719679	2	Yes
AEVLSEEPILK	40.64005	Q7L1Q6	BZW1 HUMAN	409.8997647	3	0.837719679	2	
DIILYVK	57.34129	Q7L1Q6	BZW1 HUMAN	432.2660235	2	0.667356372	2	Yes
DIILYVK	57.34129	Q7L1Q6	BZW1 HUMAN	288.5132907	3	0.667356372	2	
DINAVAASLR	37.68116	Q7L1Q6	BZW1 HUMAN	515.288554	2	0.633242846	2	Yes
DINAVAASLR	37.68116	Q7L1Q6	BZW1 HUMAN	343.8616443	3	0.633242846	2	
FDPTQFQDCIIQGLTETGTDLEAVAK	139.1134	Q7L1Q6	BZW1 HUMAN	1449.197855	2	0.637182117		
FDPTQFQDCIIQGLTETGTDLEAVAK	139.1134	Q7L1Q6	BZW1 HUMAN	966.467845	3	0.637182117		
IQEYCYDNIHFMK	39.60641	Q7L1Q6	BZW1 HUMAN	880.8927235	2	0.80314678	3	
IQEYCYDNIHFMK	39.60641	Q7L1Q6	BZW1 HUMAN	587.5977573	3	0.80314678	3	Yes
LMELFPANK	51.149	Q7L1Q6	BZW1 HUMAN	531.7868015	2	0.813261509	2	Yes
LMELFPANK	51.149	Q7L1Q6	BZW1 HUMAN	354.860476	3	0.813261509	2	
SVFLEQMK	30.18008	Q7L1Q6	BZW1 HUMAN	491.257877	2	0.84980303		
SVFLEQMK	30.18008	Q7L1Q6	BZW1 HUMAN	327.841193	3	0.84980303		
DLLHPSLEEEK	27.55198	Q71UM5	RS27L HUMAN	655.335898	2	0.753773212	2	Yes
DLLHPSLEEEK	27.55198	Q71UM5	RS27L HUMAN	437.2265403	3	0.753773212	2	
AAESTEEVSSLRPPR	5.458916	Q709C8	VP13C HUMAN	814.9161065	2	0.757813632	3	
AAESTEEVSSLRPPR	5.458916	Q709C8	VP13C HUMAN	543.613346	3	0.757813632	3	Yes
AAEVPNEELINLLLK	110.8861	Q709C8	VP13C HUMAN	833.4752685	2	0.868900299		
AAEVPNEELINLLLK	110.8861	Q709C8	VP13C HUMAN	555.9861207	3	0.868900299		
AVPQSPENVAK	-14.61734	Q709C8	VP13C HUMAN	570.306944	2	0.714242756		
AVPQSPENVAK	-14.61734	Q709C8	VP13C HUMAN	380.540571	3	0.714242756		
AVSILGDEVFR	66.21996	Q709C8	VP13C HUMAN	603.330419	2	0.813238323		
AVSILGDEVFR	66.21996	Q709C8	VP13C HUMAN	402.556221	3	0.813238323		
AVSIMGNEVFR	47.78861	Q709C8	VP13C HUMAN	611.8166215	2	0.784946561		
AVSIMGNEVFR	47.78861	Q709C8	VP13C HUMAN	408.2136893	3	0.784946561		
DFLSGIQIEFK	104.2345	Q709C8	VP13C HUMAN	648.845902	2	0.697037339		
DFLSGIQIEFK	104.2345	Q709C8	VP13C HUMAN	432.8998763	3	0.697037339		
DIILFSFK	96.7813	Q709C8	VP13C HUMAN	491.7845845	2	0.677266836	2	Yes
DIILFSFK	96.7813	Q709C8	VP13C HUMAN	328.1923313	3	0.677266836	2	
DVLYLMNSIPLPQK	108.3192	Q709C8	VP13C HUMAN	815.9478285	2	0.605629861		
DVLYLMNSIPLPQK	108.3192	Q709C8	VP13C HUMAN	544.3011607	3	0.605629861		
EMENLWGIK	58.38157	Q709C8	VP13C HUMAN	560.2793405	2	0.718961239		
EMENLWGIK	58.38157	Q709C8	VP13C HUMAN	373.855502	3	0.718961239		
ENALSELDVPFK	72.32926	Q709C8	VP13C HUMAN	681.351548	2	0.829910338	2	Yes
ENALSELDVPFK	72.32926	Q709C8	VP13C HUMAN	454.570307	3	0.829910338	2	
EPLEISISQDVHDSK	35.52189	Q709C8	VP13C HUMAN	848.923597	2	0.751692116		
EPLEISISQDVHDSK	35.52189	Q709C8	VP13C HUMAN	566.2850063	3	0.751692116		
ESELKPLVGESR	4.09972	Q709C8	VP13C HUMAN	672.362447	2	0.78340441		
ESELKPLVGESR	4.09972	Q709C8	VP13C HUMAN	448.577573	3	0.78340441		
EVILEFTK	45.42974	Q709C8	VP13C HUMAN	489.7794995	2	0.735831857		
EVILEFTK	45.42974	Q709C8	VP13C HUMAN	326.855608	3	0.735831857		
FETNPEDSPADQTLIVQSQPVEVIYDAK	97.07039	Q709C8	VP13C HUMAN	1567.264775	2	0.860344887		
FETNPEDSPADQTLIVQSQPVEVIYDAK	97.07039	Q709C8	VP13C HUMAN	1045.179125	3	0.860344887		
FLGDYVENLNK	51.53239	Q709C8	VP13C HUMAN	656.333154	2	0.83659786	2	Yes
FLGDYVENLNK	51.53239	Q709C8	VP13C HUMAN	437.8913777	3	0.83659786	2	

FLMSLLNFLNNFQTAK	155.9818	Q709C8	VP13C HUMAN	951.0036705	2	0.60441792		
FLMSLLNFLNNFQTAK	155.9818	Q709C8	VP13C HUMAN	634.3383887	3	0.60441792		
FQHPSTMHILQPMDIHVELAK	53.65594	Q709C8	VP13C HUMAN	1236.630505	2	0.716293573		
FQHPSTMHILQPMDIHVELAK	53.65594	Q709C8	VP13C HUMAN	824.756278	3	0.716293573		
FQLTLYPDATEGEAYADMSK	79.99855	Q709C8	VP13C HUMAN	1125.517726	2	0.636654198		
FQLTLYPDATEGEAYADMSK	79.99855	Q709C8	VP13C HUMAN	750.681092	3	0.636654198		
FSLVPMEHYSLPPVIDK	82.36621	Q709C8	VP13C HUMAN	986.5166065	2	0.686506569		
FSLVPMEHYSLPPVIDK	82.36621	Q709C8	VP13C HUMAN	658.0136793	3	0.686506569		
GEPLHIINSSNVTDEPLLK	55.10931	Q709C8	VP13C HUMAN	1038.552767	2	0.820564866	3	
GEPLHIINSSNVTDEPLLK	55.10931	Q709C8	VP13C HUMAN	692.704453	3	0.820564866	3	Yes
GFSEGTASTFDYSLK	49.86607	Q709C8	VP13C HUMAN	805.373205	2	0.873392463		
GFSEGTASTFDYSLK	49.86607	Q709C8	VP13C HUMAN	537.2514117	3	0.873392463		
GGWFSGLWGK	81.13518	Q709C8	VP13C HUMAN	547.775083	2	0.851079285	2	Yes
GGWFSGLWGK	81.13518	Q709C8	VP13C HUMAN	365.5193303	3	0.851079285	2	
GHLDSMNVSLNQEDLNLLFR	89.45308	Q709C8	VP13C HUMAN	1158.076615	2	0.799176574		
GHLDSMNVSLNQEDLNLLFR	89.45308	Q709C8	VP13C HUMAN	772.387018	3	0.799176574		
GLAAITMDK	20.69534	Q709C8	VP13C HUMAN	460.2500525	2	0.758012593		
GLAAITMDK	20.69534	Q709C8	VP13C HUMAN	307.16931	3	0.758012593		
GLDLEQITSATLMK	83.90231	Q709C8	VP13C HUMAN	760.4059985	2	0.858981848		
GLDLEQITSATLMK	83.90231	Q709C8	VP13C HUMAN	507.273274	3	0.858981848		
GRPLHELNVLQLGMEAK	46.29292	Q709C8	VP13C HUMAN	953.0229265	2	0.766160369	3	
GRPLHELNVLQLGMEAK	46.29292	Q709C8	VP13C HUMAN	635.6845593	3	0.766160369	3	Yes
GTHSGEFIYGLENFVYK	80.05867	Q709C8	VP13C HUMAN	980.976156	2	0.751301765		
GTHSGEFIYGLENFVYK	80.05867	Q709C8	VP13C HUMAN	654.3200457	3	0.751301765		
GVVGGVTGIITKPVEGAK	38.2106	Q709C8	VP13C HUMAN	841.496537	2	0.77333957	3	
GVVGGVTGIITKPVEGAK	38.2106	Q709C8	VP13C HUMAN	561.333633	3	0.77333957	3	Yes
IGIARPEEEFHVPLDSYR	50.23286	Q709C8	VP13C HUMAN	1064.545273	2	0.669693172	4	
IGIARPEEEFHVPLDSYR	50.23286	Q709C8	VP13C HUMAN	710.03279	3	0.669693172	4	
IHGMDASISVKPK	-6.198437	Q709C8	VP13C HUMAN	691.8772105	2	0.804525733		
IHGMDASISVKPK	-6.198437	Q709C8	VP13C HUMAN	461.5874153	3	0.804525733		
ILLENLGEASSQPSPTQSVQETVR	64.23251	Q709C8	VP13C HUMAN	1292.167213	2	0.788286805	3	
ILLENLGEASSQPSPTQSVQETVR	64.23251	Q709C8	VP13C HUMAN	861.7807503	3	0.788286805	3	Yes
ILTENLCEGTEDLDK	40.06814	Q709C8	VP13C HUMAN	875.4147485	2	0.824269891	2	Yes
ILTENLCEGTEDLDK	40.06814	Q709C8	VP13C HUMAN	583.945774	3	0.824269891	2	
INLKPSYLVVPQTGFHHEK	31.79383	Q709C8	VP13C HUMAN	1104.102758	2	0.813509762		
INLKPSYLVVPQTGFHHEK	31.79383	Q709C8	VP13C HUMAN	736.404447	3	0.813509762		
IQGLDSSLSLQSR	40.95978	Q709C8	VP13C HUMAN	702.3786285	2	0.83235544		
IQGLDSSLSLQSR	40.95978	Q709C8	VP13C HUMAN	468.5883607	3	0.83235544		
ISGEIMELVLVK	90.8374	Q709C8	VP13C HUMAN	665.8867125	2	0.837560594		
ISGEIMELVLVK	90.8374	Q709C8	VP13C HUMAN	444.2604167	3	0.837560594		
ISLDYHEIEGSK	20.15671	Q709C8	VP13C HUMAN	695.8466255	2	0.796551526		
ISLDYHEIEGSK	20.15671	Q709C8	VP13C HUMAN	464.233692	3	0.796551526		
ISMQCFDFTDSK	57.99451	Q709C8	VP13C HUMAN	739.8187015	2	0.804192185		
ISMQCFDFTDSK	57.99451	Q709C8	VP13C HUMAN	493.5484093	3	0.804192185		
ITAELNAFNVFVCDQK	87.65048	Q709C8	VP13C HUMAN	934.964743	2	0.614851892		
ITAELNAFNVFVCDQK	87.65048	Q709C8	VP13C HUMAN	623.6457703	3	0.614851892		
IVTLTPFCTIANK	67.34691	Q709C8	VP13C HUMAN	739.4083445	2	0.660898685		
IVTLTPFCTIANK	67.34691	Q709C8	VP13C HUMAN	493.274838	3	0.660898685		
KPLHLISSSDKPGLDLLK	38.22057	Q709C8	VP13C HUMAN	981.075681	2	0.830809772		
KPLHLISSSDKPGLDLLK	38.22057	Q709C8	VP13C HUMAN	654.3863957	3	0.830809772		
LAYYEIR	19.50096	Q709C8	VP13C HUMAN	464.2509005	2	0.788228571		
LAYYEIR	19.50096	Q709C8	VP13C HUMAN	309.836542	3	0.788228571		
LDNNFEVNFDKDPMEMR	60.97035	Q709C8	VP13C HUMAN	1057.470063	2	0.868406951	3	
LDNNFEVNFDKDPMEMR	60.97035	Q709C8	VP13C HUMAN	705.3159833	3	0.868406951	3	Yes
LEHWYITGLR	38.84983	Q709C8	VP13C HUMAN	644.3463995	2	0.734223366		
LEHWYITGLR	38.84983	Q709C8	VP13C HUMAN	429.900208	3	0.734223366		
LENIIVTDVDPK	51.54042	Q709C8	VP13C HUMAN	678.3750235	2	0.772958994		
LENIIVTDVDPK	51.54042	Q709C8	VP13C HUMAN	452.5859573	3	0.772958994		
LEQMMEASVR	21.61987	Q709C8	VP13C HUMAN	597.28684	2	0.710638881		
LEQMMEASVR	21.61987	Q709C8	VP13C HUMAN	398.5271683	3	0.710638881		
LFAWADPTGTR	56.48274	Q709C8	VP13C HUMAN	617.814937	2	0.802523255		
LFAWADPTGTR	56.48274	Q709C8	VP13C HUMAN	412.2125663	3	0.802523255		
LFTAIGYSESTHNLTLPK	56.24364	Q709C8	VP13C HUMAN	996.5260175	2	0.782429814	3	
LFTAIGYSESTHNLTLPK	56.24364	Q709C8	VP13C HUMAN	664.68662	3	0.782429814	3	Yes
LGIWGGNVALDNLQIK	92.58644	Q709C8	VP13C HUMAN	855.981053	2	0.857195258		
LGIWGGNVALDNLQIK	92.58644	Q709C8	VP13C HUMAN	570.989977	3	0.857195258		
LKPMQVALSEDDLTVLMK	76.52164	Q709C8	VP13C HUMAN	1016.047419	2	0.640002668		
LKPMQVALSEDDLTVLMK	76.52164	Q709C8	VP13C HUMAN	677.7008873	3	0.640002668		
LLRPQLPS	31.74353	Q709C8	VP13C HUMAN	462.287826	2	0.634026587		
LLRPQLPS	31.74353	Q709C8	VP13C HUMAN	308.5278257	3	0.634026587		
LNAFCVIVCNEK	55.95688	Q709C8	VP13C HUMAN	733.8607035	2	0.646932781		
LNAFCVIVCNEK	55.95688	Q709C8	VP13C HUMAN	489.5764107	3	0.646932781		
LTWTYAANVGEHDLLK	59.80539	Q709C8	VP13C HUMAN	915.973421	2	0.844830036		
LTWTYAANVGEHDLLK	59.80539	Q709C8	VP13C HUMAN	610.984889	3	0.844830036		
LYMNPYAESELK	45.68532	Q709C8	VP13C HUMAN	729.353225	2	0.821392894	2	Yes
LYMNPYAESELK	45.68532	Q709C8	VP13C HUMAN	486.571425	3	0.821392894	2	
MNIELTQLK	44.76671	Q709C8	VP13C HUMAN	545.302816	2	0.826202631		
MNIELTQLK	44.76671	Q709C8	VP13C HUMAN	363.8711523	3	0.826202631		
NEILTSGNIPPNYQYIFQPISASAK	105.8148	Q709C8	VP13C HUMAN	1383.211415	2	0.65071857		
NEILTSGNIPPNYQYIFQPISASAK	105.8148	Q709C8	VP13C HUMAN	922.4768847	3	0.65071857		
NLAASWYHK	5.4702	Q709C8	VP13C HUMAN	545.277985	2	0.777556121		
NLAASWYHK	5.4702	Q709C8	VP13C HUMAN	363.8545983	3	0.777556121		
NLLPYSLR	46.85648	Q709C8	VP13C HUMAN	488.2852785	2	0.736007869		
NLLPYSLR	46.85648	Q709C8	VP13C HUMAN	325.8594607	3	0.736007869		
NTLTTGVEEIR	22.35739	Q709C8	VP13C HUMAN	616.828241	2	0.835649729	2	Yes
NTLTTGVEEIR	22.35739	Q709C8	VP13C HUMAN	411.554769	3	0.835649729	2	
NVQLLFAR	43.9996	Q709C8	VP13C HUMAN	480.7854505	2	0.726952314		
NVQLLFAR	43.9996	Q709C8	VP13C HUMAN	320.8595753	3	0.726952314		
SCLNVFNNLAK	45.07053	Q709C8	VP13C HUMAN	640.327353	2	0.819402993		
SCLNVFNNLAK	45.07053	Q709C8	VP13C HUMAN	427.2208437	3	0.819402993		
SIALDSEPKPFIDVSVITR	75.01838	Q709C8	VP13C HUMAN	1044.073336	2	0.610442281		
SIALDSEPKPFIDVSVITR	75.01838	Q709C8	VP13C HUMAN	696.384832	3	0.610442281		
SIGATLTDVDDLIFK	104.8999	Q709C8	VP13C HUMAN	804.4305275	2	0.818497181	2	Yes
SIGATLTDVDDLIFK	104.8999	Q709C8	VP13C HUMAN	536.62296	3	0.818497181	2	
SINDYNTWFLGVDTATEITESFK	133.8774	Q709C8	VP13C HUMAN	1326.129761	2	0.675455689		
SINDYNTWFLGVDTATEITESFK	133.8774	Q709C8	VP13C HUMAN	884.4224487	3	0.675455689		
SLFGHTVGGAAGVVSR	30.66042	Q709C8	VP13C HUMAN	757.9078885	2	0.830069482		
SLFGHTVGGAAGVVSR	30.66042	Q709C8	VP13C HUMAN	505.6078673	3	0.830069482		
SSLELEVGEIASDGSMPTNK	67.40743	Q709C8	VP13C HUMAN	1032.492458	2	0.805849671		
SSLELEVGEIASDGSMPTNK	67.40743	Q709C8	VP13C HUMAN	688.6654467	3	0.805849671		
TATGLTHIIETR	21.30254	Q709C8	VP13C HUMAN	656.8651585	2	0.849785686		

TATGLTHIIETR	21.30254	Q709C8	VP13C HUMAN	438.2460473	3	0.849785686		
TFDLTVVSYLK	91.81047	Q709C8	VP13C HUMAN	643.3560985	2	0.784483492		
TFDLTVVSYLK	91.81047	Q709C8	VP13C HUMAN	429.2400073	3	0.784483492		
TTNSSLEEIMDK	33.08252	Q709C8	VP13C HUMAN	684.3221305	2	0.697821498		
TTNSSLEEIMDK	33.08252	Q709C8	VP13C HUMAN	456.5506953	3	0.697821498		
TVIQPGIYHPDIQLLHPINLEFLVNR	110.0249	Q709C8	VP13C HUMAN	1520.343103	2	0.784511089		
TVIQPGIYHPDIQLLHPINLEFLVNR	110.0249	Q709C8	VP13C HUMAN	1013.89801	3	0.784511089		
TVNAVVEFFQSNK	66.03644	Q709C8	VP13C HUMAN	741.883547	2	0.766496658		
TVNAVVEFFQSNK	66.03644	Q709C8	VP13C HUMAN	494.924973	3	0.766496658		
TVPLLLAESK	48.3055	Q709C8	VP13C HUMAN	535.826981	2	0.806846976		
TVPLLLAESK	48.3055	Q709C8	VP13C HUMAN	357.553929	3	0.806846976		
VLACPFLR	41.70331	Q709C8	VP13C HUMAN	488.2764045	2	0.768932283		
VLACPFLR	41.70331	Q709C8	VP13C HUMAN	325.8535447	3	0.768932283		
VLLFTDDVALVSK	87.50336	Q709C8	VP13C HUMAN	710.4088665	2	0.823555231		
VLLFTDDVALVSK	87.50336	Q709C8	VP13C HUMAN	473.9418527	3	0.823555231		
VSGGLPLMHVR	22.54005	Q709C8	VP13C HUMAN	583.3296995	2	0.821764767		
VSGGLPLMHVR	22.54005	Q709C8	VP13C HUMAN	389.222408	3	0.821764767		
VVGCEGSSKPFFYNR	18.38632	Q709C8	VP13C HUMAN	873.917591	2	0.745089948		
VVGCEGSSKPFFYNR	18.38632	Q709C8	VP13C HUMAN	582.947669	3	0.745089948		
WNYIASSECLPFWPESLSGK	120.3041	Q709C8	VP13C HUMAN	1186.057355	2	0.655312419		
WNYIASSECLPFWPESLSGK	120.3041	Q709C8	VP13C HUMAN	791.040845	3	0.655312419		
YAIDSVLEVHIR	55.24133	Q709C8	VP13C HUMAN	707.888628	2	0.770964444		
YAIDSVLEVHIR	55.24133	Q709C8	VP13C HUMAN	472.2616937	3	0.770964444		
YFMVLIQEMALK	111.9192	Q709C8	VP13C HUMAN	743.3963865	2	0.732965231		
YFMVLIQEMALK	111.9192	Q709C8	VP13C HUMAN	495.9335327	3	0.732965231		
YLLEGTAETHELAEGSTADVLHSR	47.58068	Q709C8	VP13C HUMAN	1300.135909	2	0.858658314		
YLLEGTAETHELAEGSTADVLHSR	47.58068	Q709C8	VP13C HUMAN	867.0932143	3	0.858658314		
YTQMWSWSNIK	57.71704	Q709C8	VP13C HUMAN	722.3404565	2	0.770106614		
YTQMWSWSNIK	57.71704	Q709C8	VP13C HUMAN	481.896246	3	0.770106614		
ALELNPK	-1.247868	Q6ZXV5	TMTC3 HUMAN	392.732352	2	0.73024404		
ALELNPK	-1.247868	Q6ZXV5	TMTC3 HUMAN	262.1575097	3	0.73024404		
ALPILEELLR	109.7408	Q6ZXV5	TMTC3 HUMAN	583.861355	2	0.836029172	2	Yes
ALPILEELLR	109.7408	Q6ZXV5	TMTC3 HUMAN	389.576845	3	0.836029172	2	
FDNPAAVSPTPTR	21.59035	Q6ZXV5	TMTC3 HUMAN	686.846965	2	0.740398228		
FDNPAAVSPTPTR	21.59035	Q6ZXV5	TMTC3 HUMAN	458.2339183	3	0.740398228		
HNLCVVYFEEK	27.56628	Q6ZXV5	TMTC3 HUMAN	719.345739	2	0.62379545		
HNLCVVYFEEK	27.56628	Q6ZXV5	TMTC3 HUMAN	479.8997677	3	0.62379545		
ILEMDPSNVQGK	29.96522	Q6ZXV5	TMTC3 HUMAN	665.837751	2	0.761788487	2	Yes
ILEMDPSNVQGK	29.96522	Q6ZXV5	TMTC3 HUMAN	444.2277757	3	0.761788487	2	
ISSSSFIEPIFPTSK	82.7256	Q6ZXV5	TMTC3 HUMAN	820.433069	2	0.798246324		
ISSSSFIEPIFPTSK	82.7256	Q6ZXV5	TMTC3 HUMAN	547.291321	3	0.798246324		
LEEADQLYR	12.01811	Q6ZXV5	TMTC3 HUMAN	568.7832975	2	0.691065073		
LEEADQLYR	12.01811	Q6ZXV5	TMTC3 HUMAN	379.5248067	3	0.691065073		
LWNNVGHALENEK	17.8491	Q6ZXV5	TMTC3 HUMAN	762.384245	2	0.842253983	3	
LWNNVGHALENEK	17.8491	Q6ZXV5	TMTC3 HUMAN	508.592105	3	0.842253983	3	Yes
SALFNLALLYSQTAK	102.9478	Q6ZXV5	TMTC3 HUMAN	820.456875	2	0.783563793		
SALFNLALLYSQTAK	102.9478	Q6ZXV5	TMTC3 HUMAN	547.3071917	3	0.783563793		
SLMPQIIPGK	53.26723	Q6ZXV5	TMTC3 HUMAN	542.3157265	2	0.830255449	2	Yes
SLMPQIIPGK	53.26723	Q6ZXV5	TMTC3 HUMAN	361.8797593	3	0.830255449	2	
TLFQNDFWGTPMSEER	83.81239	Q6ZXV5	TMTC3 HUMAN	979.441632	2	0.809364319		
TLFQNDFWGTPMSEER	83.81239	Q6ZXV5	TMTC3 HUMAN	653.2970297	3	0.809364319		
VQVIQSQLPVFTR	66.99145	Q6ZXV5	TMTC3 HUMAN	757.9386575	2	0.871311784		
VQVIQSQLPVFTR	66.99145	Q6ZXV5	TMTC3 HUMAN	505.62838	3	0.871311784		
YFLQATHVQPDDIGAHMNVGR	40.78593	Q6ZXV5	TMTC3 HUMAN	1185.076947	2	0.748959005		
YFLQATHVQPDDIGAHMNVGR	40.78593	Q6ZXV5	TMTC3 HUMAN	790.3872393	3	0.748959005		
ALLCFLDQLEDEDVQTR	111.6	Q6ZS17	RIPR1 HUMAN	1032.999512	2	0.687141836		
ALLCFLDQLEDEDVQTR	111.6	Q6ZS17	RIPR1 HUMAN	689.0022827	3	0.687141836		
ALSSLLVHGNNK	8.369213	Q6ZS17	RIPR1 HUMAN	626.854592	2	0.672689855		
ALSSLLVHGNNK	8.369213	Q6ZS17	RIPR1 HUMAN	418.239003	3	0.672689855		
APEGIEPLVYLCQTDTEAVR	85.54938	Q6ZS17	RIPR1 HUMAN	1131.060097	2	0.706461787		
APEGIEPLVYLCQTDTEAVR	85.54938	Q6ZS17	RIPR1 HUMAN	754.376006	3	0.706461787		
DGAYNMVR	0.459393	Q6ZS17	RIPR1 HUMAN	463.2139965	2	0.662830591		
DGAYNMVR	0.459393	Q6ZS17	RIPR1 HUMAN	309.1452727	3	0.662830591		
DLFAALPQVVAVDINDLGTIK	161.2366	Q6ZS17	RIPR1 HUMAN	1106.615369	2	0.644866228		
DLFAALPQVVAVDINDLGTIK	161.2366	Q6ZS17	RIPR1 HUMAN	738.0795207	3	0.644866228		
EQAFYNMLR	43.46205	Q6ZS17	RIPR1 HUMAN	586.2824105	2	0.788640499		
EQAFYNMLR	43.46205	Q6ZS17	RIPR1 HUMAN	391.190882	3	0.788640499		
FLEDALGQK	32.22632	Q6ZS17	RIPR1 HUMAN	510.772206	2	0.73135525		
FLEDALGQK	32.22632	Q6ZS17	RIPR1 HUMAN	340.8507457	3	0.73135525		
FSTYSQSPPDTPSLR	33.76134	Q6ZS17	RIPR1 HUMAN	841.9052035	2	0.844089091		
FSTYSQSPPDTPSLR	33.76134	Q6ZS17	RIPR1 HUMAN	561.6060773	3	0.844089091		
GLANHVVVGSVSCETK	9.200119	Q6ZS17	RIPR1 HUMAN	828.922878	2	0.821783185		
GLANHVVVGSVSCETK	9.200119	Q6ZS17	RIPR1 HUMAN	552.9511937	3	0.821783185		
GLTAYLEVHQQEQEK	25.55774	Q6ZS17	RIPR1 HUMAN	886.944861	2	0.792093873		
GLTAYLEVHQQEQEK	25.55774	Q6ZS17	RIPR1 HUMAN	591.6325157	3	0.792093873		
GQFPELQGLEQEVTR	70.43431	Q6ZS17	RIPR1 HUMAN	865.939583	2	0.908899903		
GQFPELQGLEQEVTR	70.43431	Q6ZS17	RIPR1 HUMAN	577.628997	3	0.908899903		
IDELYEAYCVQR	42.28052	Q6ZS17	RIPR1 HUMAN	779.864489	2	0.792018235		
IDELYEAYCVQR	42.28052	Q6ZS17	RIPR1 HUMAN	520.245601	3	0.792018235		
IFGPGSMASTAF	82.08427	Q6ZS17	RIPR1 HUMAN	593.2846235	2	0.69293654		
IFGPGSMASTAF	82.08427	Q6ZS17	RIPR1 HUMAN	395.859024	3	0.69293654		
LDLVYTALK	60.04755	Q6ZS17	RIPR1 HUMAN	518.30842	2	0.779359221		
LDLVYTALK	60.04755	Q6ZS17	RIPR1 HUMAN	345.8748883	3	0.779359221		
LEESLDALPR	39.40091	Q6ZS17	RIPR1 HUMAN	571.8067765	2	0.752925754		
LEESLDALPR	39.40091	Q6ZS17	RIPR1 HUMAN	381.5404593	3	0.752925754		
LESLLMQR	31.41882	Q6ZS17	RIPR1 HUMAN	495.276601	2	0.761799872		
LESLLMQR	31.41882	Q6ZS17	RIPR1 HUMAN	330.5203423	3	0.761799872		
LGFLYDLDK	67.33358	Q6ZS17	RIPR1 HUMAN	542.290227	2	0.806505799	2	Yes
LGFLYDLDK	67.33358	Q6ZS17	RIPR1 HUMAN	361.8627597	3	0.806505799	2	
LGTFGPLR	29.949	Q6ZS17	RIPR1 HUMAN	430.7536195	2	0.737764418		
LGTFGPLR	29.949	Q6ZS17	RIPR1 HUMAN	287.5050213	3	0.737764418		
LSCFLCPVER	42.19357	Q6ZS17	RIPR1 HUMAN	640.8104825	2	0.604995728		
LSCFLCPVER	42.19357	Q6ZS17	RIPR1 HUMAN	427.54293	3	0.604995728		
LSLEVTWSPFDKDDQPSAASSVNK	72.03738	Q6ZS17	RIPR1 HUMAN	1311.140663	2	0.788195968		
LSLEVTWSPFDKDDQPSAASSVNK	72.03738	Q6ZS17	RIPR1 HUMAN	874.429717	3	0.788195968		
NLNSDDQAVVLK	19.08511	Q6ZS17	RIPR1 HUMAN	658.346797	2	0.676313996		
NLNSDDQAVVLK	19.08511	Q6ZS17	RIPR1 HUMAN	439.2338063	3	0.676313996		
SFPVFSPPGPPR	62.48553	Q6ZS17	RIPR1 HUMAN	642.840954	2	0.642120779		
SFPVFSPPGPPR	62.48553	Q6ZS17	RIPR1 HUMAN	428.8965777	3	0.642120779		
SLEEALGALMAALDDYR	160.5551	Q6ZS17	RIPR1 HUMAN	919.4542035	2	0.786360264		
SLEEALGALMAALDDYR	160.5551	Q6ZS17	RIPR1 HUMAN	613.3054107	3	0.786360264		

SLSLGPTFR	37.93159	Q6ZS17	RIPR1 HUMAN	489.2749155	2	0.761792004	2	Yes
SLSLGPTFR	37.93159	Q6ZS17	RIPR1 HUMAN	326.5192187	3	0.761792004	2	
SQSFAGVLGSHER	9.061726	Q6ZS17	RIPR1 HUMAN	687.8422135	2	0.760065556		
SQSFAGVLGSHER	9.061726	Q6ZS17	RIPR1 HUMAN	458.8974173	3	0.760065556		
VAGCLALGCIK	36.09206	Q6ZS17	RIPR1 HUMAN	581.310119	2	0.697042406		
VAGCLALGCIK	36.09206	Q6ZS17	RIPR1 HUMAN	387.876021	3	0.697042406		
VLEAVCEFSR	37.19269	Q6ZS17	RIPR1 HUMAN	605.3008045	2	0.713146448		
VLEAVCEFSR	37.19269	Q6ZS17	RIPR1 HUMAN	403.8698113	3	0.713146448		
VLLTFCNQYGAR	49.88756	Q6ZS17	RIPR1 HUMAN	721.3670065	2	0.803943336		
VLLTFCNQYGAR	49.88756	Q6ZS17	RIPR1 HUMAN	481.2472793	3	0.803943336		
VMAAVSTQLR	12.39986	Q6ZS17	RIPR1 HUMAN	538.300608	2	0.700575233		
VMAAVSTQLR	12.39986	Q6ZS17	RIPR1 HUMAN	359.2030137	3	0.700575233		
ACQVGSEEEPSSQLTSVFR	52.92871	Q6ZRQ5	MMS22 HUMAN	1055.989675	2	0.796929479		
ACQVGSEEEPSSQLTSVFR	52.92871	Q6ZRQ5	MMS22 HUMAN	704.329058	3	0.796929479		
AQVEYLSISEDPK	37.41067	Q6ZRQ5	MMS22 HUMAN	739.8728405	2	0.758307278		
AQVEYLSISEDPK	37.41067	Q6ZRQ5	MMS22 HUMAN	493.584502	3	0.758307278		
CLVLVSEPQVK	41.09566	Q6ZRQ5	MMS22 HUMAN	636.35558	2	0.606109619		
CLVLVSEPQVK	41.09566	Q6ZRQ5	MMS22 HUMAN	424.572995	3	0.606109619		
DNVDLFVQSSLSAK	68.50484	Q6ZRQ5	MMS22 HUMAN	761.8915685	2	0.793949842		
DNVDLFVQSSLSAK	68.50484	Q6ZRQ5	MMS22 HUMAN	508.263654	3	0.793949842		
ELFHLFR	43.47038	Q6ZRQ5	MMS22 HUMAN	481.2668935	2	0.616024256		
ELFHLFR	43.47038	Q6ZRQ5	MMS22 HUMAN	321.1805373	3	0.616024256		
ELPAPMLSAIQK	64.62543	Q6ZRQ5	MMS22 HUMAN	649.363405	2	0.753120422		
ELPAPMLSAIQK	64.62543	Q6ZRQ5	MMS22 HUMAN	433.2448783	3	0.753120422		
FFEAVGVTYGNVQTLSDK	74.00303	Q6ZRQ5	MMS22 HUMAN	987.9945505	2	0.6819309		
FFEAVGVTYGNVQTLSDK	74.00303	Q6ZRQ5	MMS22 HUMAN	658.9989753	3	0.6819309		
FLPASPYVSDLGQHPVLLALR	94.39554	Q6ZRQ5	MMS22 HUMAN	1147.139341	2	0.811397672		
FLPASPYVSDLGQHPVLLALR	94.39554	Q6ZRQ5	MMS22 HUMAN	765.095502	3	0.811397672		
HFSGESYLCSGALK	20.20184	Q6ZRQ5	MMS22 HUMAN	778.36466	2	0.774508119		
HFSGESYLCSGALK	20.20184	Q6ZRQ5	MMS22 HUMAN	519.245715	3	0.774508119		
LATENLQYMVK	38.66034	Q6ZRQ5	MMS22 HUMAN	655.345208	2	0.805428743		
LATENLQYMVK	38.66034	Q6ZRQ5	MMS22 HUMAN	437.232747	3	0.805428743		
LLFNLSEVK	64.93566	Q6ZRQ5	MMS22 HUMAN	531.813873	2	0.780207932		
LLFNLSEVK	64.93566	Q6ZRQ5	MMS22 HUMAN	354.8785237	3	0.780207932		
LLNDGFSMLLR	91.69392	Q6ZRQ5	MMS22 HUMAN	639.8479215	2	0.768660486		
LLNDGFSMLLR	91.69392	Q6ZRQ5	MMS22 HUMAN	426.9012227	3	0.768660486		
LLSHLGQMGQDEMQR	19.38143	Q6ZRQ5	MMS22 HUMAN	871.919813	2	0.869967401		
LLSHLGQMGQDEMQR	19.38143	Q6ZRQ5	MMS22 HUMAN	581.615817	3	0.869967401		
NLDIGVLAEK	48.97102	Q6ZRQ5	MMS22 HUMAN	536.3064125	2	0.83015722		
NLDIGVLAEK	48.97102	Q6ZRQ5	MMS22 HUMAN	357.87355	3	0.83015722		
NLNSSFSISWLPFK	108.2015	Q6ZRQ5	MMS22 HUMAN	820.4281205	2	0.725671232		
NLNSSFSISWLPFK	108.2015	Q6ZRQ5	MMS22 HUMAN	547.288022	3	0.725671232		
NLSGPDDLLIDK	53.59726	Q6ZRQ5	MMS22 HUMAN	650.343723	2	0.840877175		
NLSGPDDLLIDK	53.59726	Q6ZRQ5	MMS22 HUMAN	433.8984237	3	0.840877175		
NTATIPPISSLK	42.1974	Q6ZRQ5	MMS22 HUMAN	621.3591765	2	0.778206348		
NTATIPPISSLK	42.1974	Q6ZRQ5	MMS22 HUMAN	414.5753927	3	0.778206348		
SISVQGVILEEQLR	70.59455	Q6ZRQ5	MMS22 HUMAN	785.944136	2	0.715207934		
SISVQGVILEEQLR	70.59455	Q6ZRQ5	MMS22 HUMAN	524.298699	3	0.715207934		
SLEYLGEVLK	64.66586	Q6ZRQ5	MMS22 HUMAN	575.821891	2	0.855387688		
SLEYLGEVLK	64.66586	Q6ZRQ5	MMS22 HUMAN	384.2172023	3	0.855387688		
SMHQQLCQELQR	-8.141129	Q6ZRQ5	MMS22 HUMAN	779.3672165	2	0.826070845		
SMHQQLCQELQR	-8.141129	Q6ZRQ5	MMS22 HUMAN	519.914086	3	0.826070845		
SPLSMLEMVK	80.4008	Q6ZRQ5	MMS22 HUMAN	567.7990515	2	0.786994219		
SPLSMLEMVK	80.4008	Q6ZRQ5	MMS22 HUMAN	378.8686427	3	0.786994219		
SSMPVIQSR	7.784569	Q6ZRQ5	MMS22 HUMAN	502.7638575	2	0.624313593		
SSMPVIQSR	7.784569	Q6ZRQ5	MMS22 HUMAN	335.5118467	3	0.624313593		
SWAQIFATSK	52.75385	Q6ZRQ5	MMS22 HUMAN	569.798755	2	0.792319775		
SWAQIFATSK	52.75385	Q6ZRQ5	MMS22 HUMAN	380.2017783	3	0.792319775		
TVLSFLQAVLAR	123.2674	Q6ZRQ5	MMS22 HUMAN	659.3986365	2	0.760207474		
TVLSFLQAVLAR	123.2674	Q6ZRQ5	MMS22 HUMAN	439.9350327	3	0.760207474		
VESNWNFVEELLK	105.4431	Q6ZRQ5	MMS22 HUMAN	803.909761	2	0.723585606		
VESNWNFVEELLK	105.4431	Q6ZRQ5	MMS22 HUMAN	536.2757823	3	0.723585606		
ACIPYLK	24.71091	Q6YN16	HSDL2 HUMAN	432.7365765	2	0.685682178	2	Yes
ACIPYLK	24.71091	Q6YN16	HSDL2 HUMAN	288.8269927	3	0.685682178	2	
ALPCIVDVR	43.53547	Q6YN16	HSDL2 HUMAN	521.789876	2	0.690995693	2	Yes
ALPCIVDVR	43.53547	Q6YN16	HSDL2 HUMAN	348.195859	3	0.690995693	2	
DEQQISAAVEK	6.977577	Q6YN16	HSDL2 HUMAN	609.3045985	2	0.713031113	2	Yes
DEQQISAAVEK	6.977577	Q6YN16	HSDL2 HUMAN	406.5390073	3	0.713031113	2	
DGANIVIAAK	24.49332	Q6YN16	HSDL2 HUMAN	486.280198	2	0.732396066	2	Yes
DGANIVIAAK	24.49332	Q6YN16	HSDL2 HUMAN	324.5227403	3	0.732396066	2	
GEIAVNALWPK	61.37198	Q6YN16	HSDL2 HUMAN	599.3355045	2	0.837886095	2	Yes
GEIAVNALWPK	61.37198	Q6YN16	HSDL2 HUMAN	399.8929447	3	0.837886095	2	
GGNVGYGEPSDQADVVMSMTTDDFVK	85.67311	Q6YN16	HSDL2 HUMAN	1360.097278	2	0.667018712	3	
GGNVGYGEPSDQADVVMSMTTDDFVK	85.67311	Q6YN16	HSDL2 HUMAN	907.0674603	3	0.667018712	3	Yes
LAGCTVFITGASR	38.58919	Q6YN16	HSDL2 HUMAN	676.8537365	2	0.771170735	2	Yes
LAGCTVFITGASR	38.58919	Q6YN16	HSDL2 HUMAN	451.571766	3	0.771170735	2	
LDLMMNVNTR	48.33724	Q6YN16	HSDL2 HUMAN	603.802657	2	0.826076388		
LDLMMNVNTR	48.33724	Q6YN16	HSDL2 HUMAN	402.8710463	3	0.826076388		
LKPTMAFMSGK	18.8808	Q6YN16	HSDL2 HUMAN	605.820319	2	0.780888736	2	Yes
LKPTMAFMSGK	18.8808	Q6YN16	HSDL2 HUMAN	404.2161543	3	0.780888736	2	
LLGTIYTAAEEIEAVGGK	97.59932	Q6YN16	HSDL2 HUMAN	917.9940195	2	0.794972181	2	Yes
LLGTIYTAAEEIEAVGGK	97.59932	Q6YN16	HSDL2 HUMAN	612.3319547	3	0.794972181	2	
LMNQMNAR	-17.81923	Q6YN16	HSDL2 HUMAN	489.236953	2	0.664484382		
LMNQMNAR	-17.81923	Q6YN16	HSDL2 HUMAN	326.4939103	3	0.664484382		
SFTGNFVIDENILK	80.63943	Q6YN16	HSDL2 HUMAN	798.9175865	2	0.856894433	2	Yes
SFTGNFVIDENILK	80.63943	Q6YN16	HSDL2 HUMAN	532.947666	3	0.856894433	2	
TAIHTAAMDMLGGPGIESQCRK	39.85782	Q6YN16	HSDL2 HUMAN	1172.564505	2	0.756070912		
TAIHTAAMDMLGGPGIESQCRK	39.85782	Q6YN16	HSDL2 HUMAN	782.0456117	3	0.756070912		
VAHILNISPPLNLNPVWFK	103.7353	Q6YN16	HSDL2 HUMAN	1086.62059	2	0.696432769	3	
VAHILNISPPLNLNPVWFK	103.7353	Q6YN16	HSDL2 HUMAN	724.7496683	3	0.696432769	3	Yes
AETLEDLK	6.083817	Q6XZF7	DNMBP HUMAN	459.743114	2	0.676886439		
AETLEDLK	6.083817	Q6XZF7	DNMBP HUMAN	306.831351	3	0.676886439		
ALMGLSAQLDEELDFR	95.44153	Q6XZF7	DNMBP HUMAN	904.4489255	2	0.765597403		
ALMGLSAQLDEELDFR	95.44153	Q6XZF7	DNMBP HUMAN	603.301892	3	0.765597403		
AQEELNLMLEEK	50.3819	Q6XZF7	DNMBP HUMAN	723.861423	2	0.791353464		
AQEELNLMLEEK	50.3819	Q6XZF7	DNMBP HUMAN	482.9102237	3	0.791353464		
DLDMYSR	14.19092	Q6XZF7	DNMBP HUMAN	450.2005545	2	0.636687219		
DLDMYSR	14.19092	Q6XZF7	DNMBP HUMAN	300.4696447	3	0.636687219		
DLEMCIER	34.74456	Q6XZF7	DNMBP HUMAN	533.2393585	2	0.631327569		
DLEMCIER	34.74456	Q6XZF7	DNMBP HUMAN	355.8288473	3	0.631327569		
DLSLYLQHIR	52.57337	Q6XZF7	DNMBP HUMAN	629.3516815	2	0.838195443		

DLSLYLQHIR	52.57337	Q6XZF7	DNMBP_HUMAN	419.9037293	3	0.838195443		
DYASLPPK	13.13982	Q6XZF7	DNMBP_HUMAN	445.7350875	2	0.764573157		
DYASLPPK	13.13982	Q6XZF7	DNMBP_HUMAN	297.4926667	3	0.764573157		
EGNLIAIFHEEHSR	34.27082	Q6XZF7	DNMBP_HUMAN	826.413534	2	0.754416585		
EGNLIAIFHEEHSR	34.27082	Q6XZF7	DNMBP_HUMAN	551.2782977	3	0.754416585		
EINVNINEYK	32.46258	Q6XZF7	DNMBP_HUMAN	618.3175035	2	0.609914362		
EINVNINEYK	32.46258	Q6XZF7	DNMBP_HUMAN	412.5476107	3	0.609914362		
FCESNIESLNMELQQLR	83.41296	Q6XZF7	DNMBP_HUMAN	1055.998988	2	0.815714002		
FCESNIESLNMELQQLR	83.41296	Q6XZF7	DNMBP_HUMAN	704.3352667	3	0.815714002		
FQALEPNELDFEVGDK	77.04444	Q6XZF7	DNMBP_HUMAN	925.94453	2	0.856659055		
FQALEPNELDFEVGDK	77.04444	Q6XZF7	DNMBP_HUMAN	617.632295	3	0.856659055		
GFVYSSFLKPYNPR	56.07291	Q6XZF7	DNMBP_HUMAN	837.936105	2	0.774374962		
GFVYSSFLKPYNPR	56.07291	Q6XZF7	DNMBP_HUMAN	558.9600117	3	0.774374962		
GIFPEGFVELLGPLR	148.12	Q6XZF7	DNMBP_HUMAN	822.461965	2	0.6565817		
GIFPEGFVELLGPLR	148.12	Q6XZF7	DNMBP_HUMAN	548.6439183	3	0.6565817		
GYVPSNYIR	19.1032	Q6XZF7	DNMBP_HUMAN	534.7778135	2	0.730714202		
GYVPSNYIR	19.1032	Q6XZF7	DNMBP_HUMAN	356.854484	3	0.730714202		
HHTSSVYSISER	-24.78843	Q6XZF7	DNMBP_HUMAN	701.839666	2	0.682599723		
HHTSSVYSISER	-24.78843	Q6XZF7	DNMBP_HUMAN	468.2290523	3	0.682599723		
HLQDSLADLK	15.65382	Q6XZF7	DNMBP_HUMAN	570.306944	2	0.785393357		
HLQDSLADLK	15.65382	Q6XZF7	DNMBP_HUMAN	380.540571	3	0.785393357		
HLTGFAPQIK	10.69417	Q6XZF7	DNMBP_HUMAN	556.3171155	2	0.725556612		
HLTGFAPQIK	10.69417	Q6XZF7	DNMBP_HUMAN	371.2140187	3	0.725556612		
HPEIVGYSVPGR	18.04273	Q6XZF7	DNMBP_HUMAN	655.8467635	2	0.602091014		
HPEIVGYSVPGR	18.04273	Q6XZF7	DNMBP_HUMAN	437.5671173	3	0.602091014		
ILATLEDGWLEGSLK	92.87079	Q6XZF7	DNMBP_HUMAN	822.9463445	2	0.809955716		
ILATLEDGWLEGSLK	92.87079	Q6XZF7	DNMBP_HUMAN	548.966838	3	0.809955716		
IYCQNHDEAIALLEIYEK	74.10774	Q6XZF7	DNMBP_HUMAN	1111.544077	2	0.719239891		
IYCQNHDEAIALLEIYEK	74.10774	Q6XZF7	DNMBP_HUMAN	741.3653263	3	0.719239891		
KPLLGLPSYMLQSEELR	74.32823	Q6XZF7	DNMBP_HUMAN	987.540613	2	0.823312879		
KPLLGLPSYMLQSEELR	74.32823	Q6XZF7	DNMBP_HUMAN	658.6963503	3	0.823312879		
LITEQELPER	20.64993	Q6XZF7	DNMBP_HUMAN	614.333159	2	0.849557877		
LITEQELPER	20.64993	Q6XZF7	DNMBP_HUMAN	409.891381	3	0.849557877		
LLDFYNCTER	41.97313	Q6XZF7	DNMBP_HUMAN	665.8089895	2	0.753973901		
LLDFYNCTER	41.97313	Q6XZF7	DNMBP_HUMAN	444.2086013	3	0.753973901		
LNIHSIIK	14.38623	Q6XZF7	DNMBP_HUMAN	469.2956505	2	0.600954652		
LNIHSIIK	14.38623	Q6XZF7	DNMBP_HUMAN	313.1997087	3	0.600954652		
LTQQLIEFEK	41.40891	Q6XZF7	DNMBP_HUMAN	624.8459025	2	0.846246243		
LTQQLIEFEK	41.40891	Q6XZF7	DNMBP_HUMAN	416.8998767	3	0.846246243		
LVISPLNQLLSMFTGPHK	138.004	Q6XZF7	DNMBP_HUMAN	998.0589775	2	0.769953609		
LVISPLNQLLSMFTGPHK	138.004	Q6XZF7	DNMBP_HUMAN	665.7085933	3	0.769953609		
NNYEALNAQLLDELPK	86.36546	Q6XZF7	DNMBP_HUMAN	922.973617	2	0.816746473		
NNYEALNAQLLDELPK	86.36546	Q6XZF7	DNMBP_HUMAN	615.6516863	3	0.816746473		
NPNELSVSANQK	-3.403332	Q6XZF7	DNMBP_HUMAN	650.828771	2	0.758151054	2	Yes
NPNELSVSANQK	-3.403332	Q6XZF7	DNMBP_HUMAN	434.221789	3	0.758151054	2	
NSYQDEDTAGGPPR	-17.54243	Q6XZF7	DNMBP_HUMAN	753.8269535	2	0.709373534	2	Yes
NSYQDEDTAGGPPR	-17.54243	Q6XZF7	DNMBP_HUMAN	502.887244	3	0.709373534	2	
SLAGPGTEPDK	-12.61248	Q6XZF7	DNMBP_HUMAN	536.2700275	2	0.742018819	2	Yes
SLAGPGTEPDK	-12.61248	Q6XZF7	DNMBP_HUMAN	357.8492933	3	0.742018819	2	
SLDQTSPCPLVLVR	64.86738	Q6XZF7	DNMBP_HUMAN	792.9248895	2	0.785434663		
SLDQTSPCPLVLVR	64.86738	Q6XZF7	DNMBP_HUMAN	528.9525347	3	0.785434663		
SQYYSTVGGSHPHSEQYPDLLPLEAR	56.20568	Q6XZF7	DNMBP_HUMAN	1466.199563	2	0.708935022		
SQYYSTVGGSHPHSEQYPDLLPLEAR	56.20568	Q6XZF7	DNMBP_HUMAN	977.802317	3	0.708935022		
TLEELQSAR	5.950867	Q6XZF7	DNMBP_HUMAN	523.7780195	2	0.743895829		
TLEELQSAR	5.950867	Q6XZF7	DNMBP_HUMAN	349.521288	3	0.743895829		
TLQKPVLPLYR	30.31652	Q6XZF7	DNMBP_HUMAN	664.409	2	0.811673522		
TLQKPVLPLYR	30.31652	Q6XZF7	DNMBP_HUMAN	443.275275	3	0.811673522		
VEETMALPQEGSLAR	41.56916	Q6XZF7	DNMBP_HUMAN	815.9094365	2	0.783236682		
VEETMALPQEGSLAR	41.56916	Q6XZF7	DNMBP_HUMAN	544.275566	3	0.783236682		
VIEELLQTER	42.45792	Q6XZF7	DNMBP_HUMAN	615.340984	2	0.808094561	2	Yes
VIEELLQTER	42.45792	Q6XZF7	DNMBP_HUMAN	410.5632643	3	0.808094561	2	
VLQQLQVFTFFPESLPATK	132.6184	Q6XZF7	DNMBP_HUMAN	1097.101898	2	0.681231976		
VLQQLQVFTFFPESLPATK	132.6184	Q6XZF7	DNMBP_HUMAN	731.7372067	3	0.681231976		
VPLTNAVLAVK	41.73554	Q6XZF7	DNMBP_HUMAN	562.856073	2	0.783695102		
VPLTNAVLAVK	41.73554	Q6XZF7	DNMBP_HUMAN	375.5733237	3	0.783695102		
YISDQLFTNFK	67.13755	Q6XZF7	DNMBP_HUMAN	688.3488045	2	0.812466562		
YISDQLFTNFK	67.13755	Q6XZF7	DNMBP_HUMAN	459.2351447	3	0.812466562		
AEEQQLPPPLSPPSPSTPNHR	36.25718	Q6WCQ1	MPRIP_HUMAN	1140.07493	2	0.6223104		
AEEQQLPPPLSPPSPSTPNHR	36.25718	Q6WCQ1	MPRIP_HUMAN	760.385895	3	0.6223104		
AEHMETNAVGPSPSSDTR	-16.33384	Q6WCQ1	MPRIP_HUMAN	943.4214275	2	0.662197769		
AEHMETNAVGPSPSSDTR	-16.33384	Q6WCQ1	MPRIP_HUMAN	629.28356	3	0.662197769		
DAYELEVLLR	92.80894	Q6WCQ1	MPRIP_HUMAN	610.8302475	2	0.788514912		
DAYELEVLLR	92.80894	Q6WCQ1	MPRIP_HUMAN	407.5561067	3	0.788514912		
DELQTALR	19.29601	Q6WCQ1	MPRIP_HUMAN	473.2541805	2	0.600197852		
DELQTALR	19.29601	Q6WCQ1	MPRIP_HUMAN	315.8387287	3	0.600197852		
DFTNEAPPAPLPDASASPLSPHR	59.91814	Q6WCQ1	MPRIP_HUMAN	1194.085495	2	0.699220061		
DFTNEAPPAPLPDASASPLSPHR	59.91814	Q6WCQ1	MPRIP_HUMAN	796.392938	3	0.699220061		
DIYTELSIAK	56.58301	Q6WCQ1	MPRIP_HUMAN	576.8115235	2	0.746803761		
DIYTELSIAK	56.58301	Q6WCQ1	MPRIP_HUMAN	384.8769573	3	0.746803761		
DQPDGSSLSPAQSPSQSQPPAASSLR	35.8802	Q6WCQ1	MPRIP_HUMAN	1298.118251	2	0.650610924		
DQPDGSSLSPAQSPSQSQPPAASSLR	35.8802	Q6WCQ1	MPRIP_HUMAN	865.748109	3	0.650610924		
EASDLLEQNR	13.02165	Q6WCQ1	MPRIP_HUMAN	587.789115	2	0.784142852	2	Yes
EASDLLEQNR	13.02165	Q6WCQ1	MPRIP_HUMAN	392.1953517	3	0.784142852	2	
EGEFTLSAMTSGIR	61.41453	Q6WCQ1	MPRIP_HUMAN	749.8644975	2	0.806900978		
EGEFTLSAMTSGIR	61.41453	Q6WCQ1	MPRIP_HUMAN	500.2456067	3	0.806900978		
EGYVLQATCER	14.56277	Q6WCQ1	MPRIP_HUMAN	663.311897	2	0.781648576		
EGYVLQATCER	14.56277	Q6WCQ1	MPRIP_HUMAN	442.543873	3	0.781648576		
ELEVLSEQYSQK	33.01665	Q6WCQ1	MPRIP_HUMAN	726.8650155	2	0.85542047		
ELEVLSEQYSQK	33.01665	Q6WCQ1	MPRIP_HUMAN	484.9126187	3	0.85542047		
FFILYEHGLLR	68.03765	Q6WCQ1	MPRIP_HUMAN	704.3933495	2	0.784554243		
FFILYEHGLLR	68.03765	Q6WCQ1	MPRIP_HUMAN	469.931508	3	0.784554243		
FGMLDATDGPGTEDAALR	61.61931	Q6WCQ1	MPRIP_HUMAN	918.9258155	2	0.834851027		
FGMLDATDGPGTEDAALR	61.61931	Q6WCQ1	MPRIP_HUMAN	612.953152	3	0.834851027		
FSLCILTPEK	66.98653	Q6WCQ1	MPRIP_HUMAN	604.3237485	2	0.607777834		
FSLCILTPEK	66.98653	Q6WCQ1	MPRIP_HUMAN	403.2184407	3	0.607777834		
GFAAMEETHQK	-20.20241	Q6WCQ1	MPRIP_HUMAN	624.788062	2	0.730240881		
GFAAMEETHQK	-20.20241	Q6WCQ1	MPRIP_HUMAN	416.8613163	3	0.730240881		
HVHPTTAPDVTSSLPEEK	7.50087	Q6WCQ1	MPRIP_HUMAN	972.987261	2	0.784876704		
HVHPTTAPDVTSSLPEEK	7.50087	Q6WCQ1	MPRIP_HUMAN	648.9941157	3	0.784876704		
HWFVLADQSLR	51.20263	Q6WCQ1	MPRIP_HUMAN	686.362585	2	0.757843018		
HWFVLADQSLR	51.20263	Q6WCQ1	MPRIP_HUMAN	457.9109983	3	0.757843018		

LLAEETAATISAIEAMK	89.37276	Q6WCQ1	MPRIP HUMAN	881.469327	2	0.869079709		
LLAEETAATISAIEAMK	89.37276	Q6WCQ1	MPRIP HUMAN	587.9821597	3	0.869079709		
LLQDQLR	5.549461	Q6WCQ1	MPRIP HUMAN	443.2618085	2	0.772336245		
LLQDQLR	5.549461	Q6WCQ1	MPRIP HUMAN	295.843814	3	0.772336245		
LSTHELTSLLEK	37.34367	Q6WCQ1	MPRIP HUMAN	685.880473	2	0.879178822		
LSTHELTSLLEK	37.34367	Q6WCQ1	MPRIP HUMAN	457.5895903	3	0.879178822		
QYLEELQSVQR	35.61333	Q6WCQ1	MPRIP HUMAN	696.860068	2	0.789080858		
QYLEELQSVQR	35.61333	Q6WCQ1	MPRIP HUMAN	464.9093203	3	0.789080858		
SNPDFLK	6.955635	Q6WCQ1	MPRIP HUMAN	410.714159	2	0.716566265		
SNPDFLK	6.955635	Q6WCQ1	MPRIP HUMAN	274.145381	3	0.716566265		
SPDSATVSGYDIMK	36.37869	Q6WCQ1	MPRIP HUMAN	735.843226	2	0.830678463	2	Yes
SPDSATVSGYDIMK	36.37869	Q6WCQ1	MPRIP HUMAN	490.8980923	3	0.830678463	2	
SPGLPMSDLK	31.90209	Q6WCQ1	MPRIP HUMAN	522.773891	2	0.832588911		
SPGLPMSDLK	31.90209	Q6WCQ1	MPRIP HUMAN	348.851869	3	0.832588911		
SQISSVNSDVEALR	27.713	Q6WCQ1	MPRIP HUMAN	752.8842745	2	0.747074783	2	Yes
SQISSVNSDVEALR	27.713	Q6WCQ1	MPRIP HUMAN	502.2587913	3	0.747074783	2	
STEPSVTPDLLNFK	71.5353	Q6WCQ1	MPRIP HUMAN	774.4017695	2	0.838368475	2	Yes
STEPSVTPDLLNFK	71.5353	Q6WCQ1	MPRIP HUMAN	516.603788	3	0.838368475	2	
STLWQEEMR	32.2805	Q6WCQ1	MPRIP HUMAN	590.2773295	2	0.855012178		
STLWQEEMR	32.2805	Q6WCQ1	MPRIP HUMAN	393.8541613	3	0.855012178		
TFDWAEFRPIQQALAQER	86.97226	Q6WCQ1	MPRIP HUMAN	1103.556177	2	0.769316792	3	
TFDWAEFRPIQQALAQER	86.97226	Q6WCQ1	MPRIP HUMAN	736.0400597	3	0.769316792	3	Yes
TLLTGDGGGEATGSPLAQGK	35.39106	Q6WCQ1	MPRIP HUMAN	915.4661625	2	0.740828931		
TLLTGDGGGEATGSPLAQGK	35.39106	Q6WCQ1	MPRIP HUMAN	610.6467167	3	0.740828931		
VAVTSSSSSSSSSSIPSAEK	-0.554539	Q6WCQ1	MPRIP HUMAN	986.471835	2	0.623542309		
VAVTSSSSSSSSSSIPSAEK	-0.554539	Q6WCQ1	MPRIP HUMAN	657.9838317	3	0.623542309		
VESGYFSLEK	32.10116	Q6WCQ1	MPRIP HUMAN	579.788048	2	0.772032261		
VESGYFSLEK	32.10116	Q6WCQ1	MPRIP HUMAN	386.861307	3	0.772032261		
VGGVGPADTHEPLRPEAEPGELER	29.06071	Q6WCQ1	MPRIP HUMAN	1256.625516	2	0.857606053	4	
VGGVGPADTHEPLRPEAEPGELER	29.06071	Q6WCQ1	MPRIP HUMAN	838.0862857	3	0.857606053	4	
YALDEMPTTLPQGTINMNQCTDVVDGEGR	84.22488	Q6WCQ1	MPRIP HUMAN	1613.22903	2	0.711354554		
YALDEMPTTLPQGTINMNQCTDVVDGEGR	84.22488	Q6WCQ1	MPRIP HUMAN	1075.821962	3	0.711354554		
DPTLATPPAGQTLAVPSLPR	82.2993	Q6UXD5	SE6L2 HUMAN	1001.552572	2	0.728286386		
DPTLATPPAGQTLAVPSLPR	82.2993	Q6UXD5	SE6L2 HUMAN	668.0376563	3	0.728286386		
EGDMLTLFDGDGPSAR	78.65515	Q6UXD5	SE6L2 HUMAN	840.880876	2	0.832273841		
EGDMLTLFDGDGPSAR	78.65515	Q6UXD5	SE6L2 HUMAN	560.9231923	3	0.832273841		
ILLQVEILNVR	86.32643	Q6UXD5	SE6L2 HUMAN	655.414286	2	0.632444084		
ILLQVEILNVR	86.32643	Q6UXD5	SE6L2 HUMAN	437.278799	3	0.632444084		
IVSPEPGGAVGPNLTCR	36.38841	Q6UXD5	SE6L2 HUMAN	862.443978	2	0.800072134		
IVSPEPGGAVGPNLTCR	36.38841	Q6UXD5	SE6L2 HUMAN	575.2985937	3	0.800072134		
LLANSSMLGEGQVLR	55.47198	Q6UXD5	SE6L2 HUMAN	794.4303385	2	0.796277761		
LLANSSMLGEGQVLR	55.47198	Q6UXD5	SE6L2 HUMAN	529.9561673	3	0.796277761		
LLLHFQSPR	22.78682	Q6UXD5	SE6L2 HUMAN	555.825107	2	0.823862195		
LLLHFQSPR	22.78682	Q6UXD5	SE6L2 HUMAN	370.886013	3	0.823862195		
NDTCPELPPPEWGWR	71.68014	Q6UXD5	SE6L2 HUMAN	927.41796	2	0.749959886		
NDTCPELPPPEWGWR	71.68014	Q6UXD5	SE6L2 HUMAN	618.6145817	3	0.749959886		
SGGSPLSPVIYDSDMDDVPER	68.9286	Q6UXD5	SE6L2 HUMAN	1118.507892	2	0.684760153		
SGGSPLSPVIYDSDMDDVPER	68.9286	Q6UXD5	SE6L2 HUMAN	746.0078697	3	0.684760153		
SLFGFSGSHSYSPITVESDFSNPLYEAGDTR	98.86967	Q6UXD5	SE6L2 HUMAN	1684.273657	2	0.616946459	3	
SLFGFSGSHSYSPITVESDFSNPLYEAGDTR	98.86967	Q6UXD5	SE6L2 HUMAN	1123.185046	3	0.616946459	3	Yes
TASDAGFPVGSHVQYR	17.36105	Q6UXD5	SE6L2 HUMAN	846.410988	2	0.769692481	3	
TASDAGFPVGSHVQYR	17.36105	Q6UXD5	SE6L2 HUMAN	564.6099337	3	0.769692481	3	Yes
AAATYEQMK	-20.41254	Q6UWP2	DHR11 HUMAN	506.7425875	2	0.631689191		
AAATYEQMK	-20.41254	Q6UWP2	DHR11 HUMAN	338.1643333	3	0.631689191		
ALVQQGLK	-7.539291	Q6UWP2	DHR11 HUMAN	428.766727	2	0.745389223	2	Yes
ALVQQGLK	-7.539291	Q6UWP2	DHR11 HUMAN	286.1804263	3	0.745389223	2	
ATCISPGVVETQFAFK	68.39604	Q6UWP2	DHR11 HUMAN	877.94328	2	0.807366967		
ATCISPGVVETQFAFK	68.39604	Q6UWP2	DHR11 HUMAN	585.6314617	3	0.807366967		
LALVTGASGGIGAAVAR	51.88539	Q6UWP2	DHR11 HUMAN	742.4337395	2	0.858066142		
LALVTGASGGIGAAVAR	51.88539	Q6UWP2	DHR11 HUMAN	495.291768	3	0.858066142		
NVDDGHIININSMSGHR	20.92226	Q6UWP2	DHR11 HUMAN	939.947946	2	0.812646031		
NVDDGHIININSMSGHR	20.92226	Q6UWP2	DHR11 HUMAN	626.9679057	3	0.812646031		
SAGYPGTLIPYR	46.13412	Q6UWP2	DHR11 HUMAN	647.8436855	2	0.602784157		
SAGYPGTLIPYR	46.13412	Q6UWP2	DHR11 HUMAN	432.231732	3	0.602784157		
TVGNIEELAAECK	35.64397	Q6UWP2	DHR11 HUMAN	717.351223	2	0.865611553		
TVGNIEELAAECK	35.64397	Q6UWP2	DHR11 HUMAN	478.5700903	3	0.865611553		
VLPLSVTHFYSATK	52.45779	Q6UWP2	DHR11 HUMAN	781.933036	2	0.83474952	3	
VLPLSVTHFYSATK	52.45779	Q6UWP2	DHR11 HUMAN	521.6246323	3	0.83474952	3	Yes
YAVTALTEGLR	44.30972	Q6UWP2	DHR11 HUMAN	597.3304155	2	0.812134147		
YAVTALTEGLR	44.30972	Q6UWP2	DHR11 HUMAN	398.5562187	3	0.812134147		
ALLHVWAGGPWPQGATLR	70.86737	Q6UVK1	CSPG4 HUMAN	965.5264945	2	0.724237025		
ALLHVWAGGPWPQGATLR	70.86737	Q6UVK1	CSPG4 HUMAN	644.0202713	3	0.724237025		
APLEVPQALGR	41.19085	Q6UVK1	CSPG4 HUMAN	575.8331295	2	0.841769218	2	Yes
APLEVPQALGR	41.19085	Q6UVK1	CSPG4 HUMAN	384.2246947	3	0.841769218	2	
AQLSVVDPDSAPGEIEYEVQR	69.88611	Q6UVK1	CSPG4 HUMAN	1151.564057	2	0.773802161		
AQLSVVDPDSAPGEIEYEVQR	69.88611	Q6UVK1	CSPG4 HUMAN	768.0453127	3	0.773802161		
ASSSAGTDPQLLLYR	47.70045	Q6UVK1	CSPG4 HUMAN	789.910289	2	0.821091175		
ASSSAGTDPQLLLYR	47.70045	Q6UVK1	CSPG4 HUMAN	526.942801	3	0.821091175		
ATVWMLR	38.3204	Q6UVK1	CSPG4 HUMAN	438.7421975	2	0.643934309		
ATVWMLR	38.3204	Q6UVK1	CSPG4 HUMAN	292.83074	3	0.643934309		
DQLEAAQEAVPPADIVFSVK	98.33195	Q6UVK1	CSPG4 HUMAN	1064.052601	2	0.705471635		
DQLEAAQEAVPPADIVFSVK	98.33195	Q6UVK1	CSPG4 HUMAN	709.7043423	3	0.705471635		
DQPGEPATEFSCR	18.18446	Q6UVK1	CSPG4 HUMAN	747.3204545	2	0.653606713		
DQPGEPATEFSCR	18.18446	Q6UVK1	CSPG4 HUMAN	498.549578	3	0.653606713		
DVNERPPQPQASVPLR	19.01095	Q6UVK1	CSPG4 HUMAN	901.979573	2	0.788794696		
DVNERPPQPQASVPLR	19.01095	Q6UVK1	CSPG4 HUMAN	601.655657	3	0.788794696		
ELEAGSLVYVHR	31.89271	Q6UVK1	CSPG4 HUMAN	686.865153	2	0.698323786		
ELEAGSLVYVHR	31.89271	Q6UVK1	CSPG4 HUMAN	458.2460437	3	0.698323786		
GALADEPPSLDPVQSFSQEAVDTGR	85.53279	Q6UVK1	CSPG4 HUMAN	1293.620096	2	0.797328711	3	
GALADEPPSLDPVQSFSQEAVDTGR	85.53279	Q6UVK1	CSPG4 HUMAN	862.749339	3	0.797328711	3	Yes
GGPAQDLTFR	20.54929	Q6UVK1	CSPG4 HUMAN	531.272905	2	0.789245307		
GGPAQDLTFR	20.54929	Q6UVK1	CSPG4 HUMAN	354.5178783	3	0.789245307		
GLWVPEGQR	28.25915	Q6UVK1	CSPG4 HUMAN	521.27799	2	0.851233959		
GLWVPEGQR	28.25915	Q6UVK1	CSPG4 HUMAN	347.8546017	3	0.851233959		
GNLQLQGTR	-12.21442	Q6UVK1	CSPG4 HUMAN	493.773072	2	0.644733012		
GNLQLQGTR	-12.21442	Q6UVK1	CSPG4 HUMAN	329.5179897	3	0.644733012		
GQGTVLLHNSVPVADGQPHEVSVHINAHR	24.84319	Q6UVK1	CSPG4 HUMAN	1534.792833	2	0.691567719		
GQGTVLLHNSVPVADGQPHEVSVHINAHR	24.84319	Q6UVK1	CSPG4 HUMAN	1023.531163	3	0.691567719		
[illegible]EEPLHAGQPHFLQSQLAAGQLVYAHGGGGTQQ	68.38896	Q6UVK1	CSPG4 HUMAN	2342.166177	2	0.630798399		
[illegible]EEPLHAGQPHFLQSQLAAGQLVYAHGGGGTQQ	68.38896	Q6UVK1	CSPG4 HUMAN	1561.78006	3	0.630798399		
GSLLLGGLDAEASR	61.02208	Q6UVK1	CSPG4 HUMAN	679.867897	2	0.804387987		

GSLLLGGLDAEASR	61.02208	Q6UVK1	CSPG4_HUMAN	453.5812063	3	0.804387987		
GSQTLTVCPGSVQPLSSQTLR	53.62863	Q6UVK1	CSPG4_HUMAN	1108.571167	2	0.70198065		
GSQTLTVCPGSVQPLSSQTLR	53.62863	Q6UVK1	CSPG4_HUMAN	739.3833863	3	0.70198065		
GVLSYLEPR	45.84481	Q6UVK1	CSPG4_HUMAN	517.2880185	2	0.796509862	2	Yes
GVLSYLEPR	45.84481	Q6UVK1	CSPG4_HUMAN	345.1946207	3	0.796509862	2	
GWIQLQVSDGQHQATALLEVQASEPYLR	101.541	Q6UVK1	CSPG4_HUMAN	1569.304904	2	0.682694018		
GWIQLQVSDGQHQATALLEVQASEPYLR	101.541	Q6UVK1	CSPG4_HUMAN	1046.539211	3	0.682694018		
HDVQVLTAKPR	-22.39191	Q6UVK1	CSPG4_HUMAN	632.3625855	2	0.730757952		
HDVQVLTAKPR	-22.39191	Q6UVK1	CSPG4_HUMAN	421.9109987	3	0.730757952		
HGELELDIPGAQAR	35.86013	Q6UVK1	CSPG4_HUMAN	753.3895285	2	0.7981323		
HGELELDIPGAQAR	35.86013	Q6UVK1	CSPG4_HUMAN	502.5956273	3	0.7981323		
HVQPTLDLMEAELR	56.70177	Q6UVK1	CSPG4_HUMAN	826.4277965	2	0.759452581	3	
HVQPTLDLMEAELR	56.70177	Q6UVK1	CSPG4_HUMAN	551.287806	3	0.759452581	3	Yes
ITVAALDASNLLASVPSPQR	103.8156	Q6UVK1	CSPG4_HUMAN	1012.063303	2	0.836534262		
ITVAALDASNLLASVPSPQR	103.8156	Q6UVK1	CSPG4_HUMAN	675.04481	3	0.836534262		
IYVFQGEAAEIR	49.27419	Q6UVK1	CSPG4_HUMAN	698.367529	2	0.833817422	2	Yes
IYVFQGEAAEIR	49.27419	Q6UVK1	CSPG4_HUMAN	465.9142943	3	0.833817422	2	
LDPTVLDAGELANR	62.53927	Q6UVK1	CSPG4_HUMAN	742.3917365	2	0.839822114		
LDPTVLDAGELANR	62.53927	Q6UVK1	CSPG4_HUMAN	495.263766	3	0.839822114		
LEISVDQYPTHTSNR	22.36321	Q6UVK1	CSPG4_HUMAN	880.4346595	2	0.786577046		
LEISVDQYPTHTSNR	22.36321	Q6UVK1	CSPG4_HUMAN	587.2923813	3	0.786577046		
HTQNTQQETLTTAHLEATLEEAGPSPPTFHYEVV	76.16999	Q6UVK1	CSPG4_HUMAN	2341.162631	2	0.609356523		
HTQNTQQETLTTAHLEATLEEAGPSPPTFHYEVV	76.16999	Q6UVK1	CSPG4_HUMAN	1561.111029	3	0.609356523		
LGLEVGRPEGR	7.426025	Q6UVK1	CSPG4_HUMAN	591.8336605	2	0.644237101		
LGLEVGRPEGR	7.426025	Q6UVK1	CSPG4_HUMAN	394.8917153	3	0.644237101		
LLTTDDVAFSDADSGFADAQLVLTR	112.68	Q6UVK1	CSPG4_HUMAN	1321.153772	2	0.617295206		
LLTTDDVAFSDADSGFADAQLVLTR	112.68	Q6UVK1	CSPG4_HUMAN	881.105123	3	0.617295206		
LSDGEHTSPGHFFR	-0.784939	Q6UVK1	CSPG4_HUMAN	793.8715025	2	0.78117466	4	
LSDGEHTSPGHFFR	-0.784939	Q6UVK1	CSPG4_HUMAN	529.58361	3	0.78117466	4	
LSDGQGFTQDDIQAGR	26.06074	Q6UVK1	CSPG4_HUMAN	854.400822	2	0.90715903	2	Yes
LSDGQGFTQDDIQAGR	26.06074	Q6UVK1	CSPG4_HUMAN	569.9364897	3	0.90715903	2	
LVLGQEELR	27.27589	Q6UVK1	CSPG4_HUMAN	528.80658	2	0.831595778		
LVLGQEELR	27.27589	Q6UVK1	CSPG4_HUMAN	352.8736617	3	0.831595778		
LVYQHDDSETTEDDIPFVATR	52.92373	Q6UVK1	CSPG4_HUMAN	1226.069704	2	0.743774831	3	
LVYQHDDSETTEDDIPFVATR	52.92373	Q6UVK1	CSPG4_HUMAN	817.715744	3	0.743774831	3	Yes
MFTLLDVVNR	89.32076	Q6UVK1	CSPG4_HUMAN	604.329365	2	0.701368093		
MFTLLDVVNR	89.32076	Q6UVK1	CSPG4_HUMAN	403.222185	3	0.701368093		
QGAGGVEGAEWWATQAFHQR	58.73907	Q6UVK1	CSPG4_HUMAN	1093.512302	2	0.719942033		
QGAGGVEGAEWWATQAFHQR	58.73907	Q6UVK1	CSPG4_HUMAN	729.3441427	3	0.719942033		
SEHDVLFQVTQFPSR	56.03445	Q6UVK1	CSPG4_HUMAN	895.4475745	2	0.743014574		
SEHDVLFQVTQFPSR	56.03445	Q6UVK1	CSPG4_HUMAN	597.3009913	3	0.743014574		
SFTQAQLDGGLVLFSHR	68.35928	Q6UVK1	CSPG4_HUMAN	938.489774	2	0.759828269		
SFTQAQLDGGLVLFSHR	68.35928	Q6UVK1	CSPG4_HUMAN	625.995791	3	0.759828269		
SLNSASYLYEVMERPR	56.3133	Q6UVK1	CSPG4_HUMAN	957.9730895	2	0.758525372		
SLNSASYLYEVMERPR	56.3133	Q6UVK1	CSPG4_HUMAN	638.984668	3	0.758525372		
SPPSAGYLVMVSR	48.38918	Q6UVK1	CSPG4_HUMAN	682.3561065	2	0.667497277		
SPPSAGYLVMVSR	48.38918	Q6UVK1	CSPG4_HUMAN	455.2400127	3	0.667497277		
SQVLFSVTR	34.63631	Q6UVK1	CSPG4_HUMAN	518.7934725	2	0.726280451		
SQVLFSVTR	34.63631	Q6UVK1	CSPG4_HUMAN	346.1982567	3	0.726280451		
SSLSQQQLR	-19.52322	Q6UVK1	CSPG4_HUMAN	523.783636	2	0.740260959		
SSLSQQQLR	-19.52322	Q6UVK1	CSPG4_HUMAN	349.5250323	3	0.740260959		
TEAGKPESSTPTGEPGPMASSPEPAVAK	13.81033	Q6UVK1	CSPG4_HUMAN	1355.647997	2	0.76618576		
TEAGKPESSTPTGEPGPMASSPEPAVAK	13.81033	Q6UVK1	CSPG4_HUMAN	904.1012727	3	0.76618576		
TEPGGSQLVEQFTQQDLEDGR	67.95983	Q6UVK1	CSPG4_HUMAN	1167.546401	2	0.732207358		
TEPGGSQLVEQFTQQDLEDGR	67.95983	Q6UVK1	CSPG4_HUMAN	778.7002087	3	0.732207358		
TTMVTSFTNEDLLR	63.83426	Q6UVK1	CSPG4_HUMAN	814.403987	2	0.836391509		
TTMVTSFTNEDLLR	63.83426	Q6UVK1	CSPG4_HUMAN	543.271933	3	0.836391509		
VAIQPVNDHAPVQTISR	21.63274	Q6UVK1	CSPG4_HUMAN	923.0030485	2	0.869343042		
VAIQPVNDHAPVQTISR	21.63274	Q6UVK1	CSPG4_HUMAN	615.6713073	3	0.869343042		
VLFVHSGADR	-2.823132	Q6UVK1	CSPG4_HUMAN	550.7965465	2	0.789175808		
VLFVHSGADR	-2.823132	Q6UVK1	CSPG4_HUMAN	367.5336393	3	0.789175808		
VSDGLQASPPATLK	24.49706	Q6UVK1	CSPG4_HUMAN	692.378098	2	0.862971783	2	Yes
VSDGLQASPPATLK	24.49706	Q6UVK1	CSPG4_HUMAN	461.9213403	3	0.862971783	2	
VTGALQFGELQK	41.97138	Q6UVK1	CSPG4_HUMAN	645.856802	2	0.819951057	2	Yes
VTGALQFGELQK	41.97138	Q6UVK1	CSPG4_HUMAN	430.907143	3	0.819951057	2	
DGSGEVGTTLQGHTR	-12.87859	Q6PJI9	WDR59_HUMAN	757.8638755	2	0.832818687		
DGSGEVGTTLQGHTR	-12.87859	Q6PJI9	WDR59_HUMAN	505.5785253	3	0.832818687		
DSQATAMSVDCLGQHAVLSGR	43.30848	Q6PJI9	WDR59_HUMAN	1102.015702	2	0.795971453		
DSQATAMSVDCLGQHAVLSGR	43.30848	Q6PJI9	WDR59_HUMAN	735.013076	3	0.795971453		
DYQLVTWSR	53.46141	Q6PJI9	WDR59_HUMAN	584.2938325	2	0.751878977		
DYQLVTWSR	53.46141	Q6PJI9	WDR59_HUMAN	389.8651633	3	0.751878977		
EAEHLSSPWGESSPEELR	37.47251	Q6PJI9	WDR59_HUMAN	1020.469431	2	0.772786379		
EAEHLSSPWGESSPEELR	37.47251	Q6PJI9	WDR59_HUMAN	680.6488957	3	0.772786379		
EQVSISSFYYK	48.15882	Q6PJI9	WDR59_HUMAN	675.8329825	2	0.733865738		
EQVSISSFYYK	48.15882	Q6PJI9	WDR59_HUMAN	450.8912633	3	0.733865738		
FGSLTYSDPR	20.61243	Q6PJI9	WDR59_HUMAN	571.778015	2	0.753936768		
FGSLTYSDPR	20.61243	Q6PJI9	WDR59_HUMAN	381.521285	3	0.753936768		
FLYIVNLDAPFEGHR	86.04358	Q6PJI9	WDR59_HUMAN	895.9653985	2	0.72034061		
FLYIVNLDAPFEGHR	86.04358	Q6PJI9	WDR59_HUMAN	597.6462073	3	0.72034061		
FPAQYPNNAAPSFQFINPTTITSTMK	104.9738	Q6PJI9	WDR59_HUMAN	1443.71073	2	0.647485316		
FPAQYPNNAAPSFQFINPTTITSTMK	104.9738	Q6PJI9	WDR59_HUMAN	962.8097617	3	0.647485316		
FVSCPPDPHK	-18.79222	Q6PJI9	WDR59_HUMAN	592.282415	2	0.634146154		
FVSCPPDPHK	-18.79222	Q6PJI9	WDR59_HUMAN	395.190885	3	0.634146154		
GFTFQCAICHVAVR	48.05749	Q6PJI9	WDR59_HUMAN	833.4036025	2	0.602560818		
GFTFQCAICHVAVR	48.05749	Q6PJI9	WDR59_HUMAN	555.9383433	3	0.602560818		
IHGLDWHPDSEHILATSSQDNSVK	34.12607	Q6PJI9	WDR59_HUMAN	1343.646979	2	0.791144252		
IHGLDWHPDSEHILATSSQDNSVK	34.12607	Q6PJI9	WDR59_HUMAN	896.100594	3	0.791144252		
KPSTAVEYLAAHLSK	25.40914	Q6PJI9	WDR59_HUMAN	807.9466745	2	0.814567208		
KPSTAVEYLAAHLSK	25.40914	Q6PJI9	WDR59_HUMAN	538.967058	3	0.814567208		
KPTVALSAVAGASQVK	17.31521	Q6PJI9	WDR59_HUMAN	763.949222	2	0.841198862		
KPTVALSAVAGASQVK	17.31521	Q6PJI9	WDR59_HUMAN	509.635423	3	0.841198862		
LLDPANTQQFDDFK	55.26323	Q6PJI9	WDR59_HUMAN	826.4023015	2	0.862386286		
LLDPANTQQFDDFK	55.26323	Q6PJI9	WDR59_HUMAN	551.2708093	3	0.862386286		
NAASALLVGR	21.34664	Q6PJI9	WDR59_HUMAN	486.2858145	2	0.72885716		
NAASALLVGR	21.34664	Q6PJI9	WDR59_HUMAN	324.5264847	3	0.72885716		
NVNVEMDAADR	8.124794	Q6PJI9	WDR59_HUMAN	617.2806	2	0.719676673		
NVNVEMDAADR	8.124794	Q6PJI9	WDR59_HUMAN	411.8563417	3	0.719676673		
SDPDLETPWAR	45.01344	Q6PJI9	WDR59_HUMAN	643.8047655	2	0.828823209		
SDPDLETPWAR	45.01344	Q6PJI9	WDR59_HUMAN	429.5391187	3	0.828823209		
SLGELYILNVNDIQETCQK	91.22252	Q6PJI9	WDR59_HUMAN	1119.060096	2	0.686653018		
SLGELYILNVNDIQETCQK	91.22252	Q6PJI9	WDR59_HUMAN	746.3760053	3	0.686653018		

SLSALSAYHTGLIAPMK	58.71542	Q6PJI9	WDR59 HUMAN	880.474742	2	0.813361824		
SLSALSAYHTGLIAPMK	58.71542	Q6PJI9	WDR59 HUMAN	587.319103	3	0.813361824		
TEAPGNLR	-26.11866	Q6PJI9	WDR59 HUMAN	429.2279655	2	0.763880014		
TEAPGNLR	-26.11866	Q6PJI9	WDR59 HUMAN	286.4879187	3	0.763880014		
VIIQDIACLLPVHK	88.53954	Q6PJI9	WDR59 HUMAN	809.971643	2	0.669831634		
VIIQDIACLLPVHK	88.53954	Q6PJI9	WDR59 HUMAN	540.317037	3	0.669831634		
VTTAYGSYQDANIPFPR	59.01363	Q6PJI9	WDR59 HUMAN	950.465956	2	0.842081726		
VTTAYGSYQDANIPFPR	59.01363	Q6PJI9	WDR59 HUMAN	633.9799123	3	0.842081726		
WSSENVVVEFR	51.4444	Q6PJI9	WDR59 HUMAN	676.336232	2	0.656379282		
WSSENVVVEFR	51.4444	Q6PJI9	WDR59 HUMAN	451.226763	3	0.656379282		
ALLDAER	0.700516	Q6PJ69	TRI65 HUMAN	394.219609	2	0.712914646		
ALLDAER	0.700516	Q6PJ69	TRI65 HUMAN	263.1490143	3	0.712914646		
ASDHSVTLGVSYPQLPR	48.35883	Q6PJ69	TRI65 HUMAN	913.973952	2	0.804802895		
ASDHSVTLGVSYPQLPR	48.35883	Q6PJ69	TRI65 HUMAN	609.6519097	3	0.804802895		
ASLEVTQQQATQAEGQLLELR	69.69208	Q6PJ69	TRI65 HUMAN	1157.106429	2	0.822000921		
ASLEVTQQQATQAEGQLLELR	69.69208	Q6PJ69	TRI65 HUMAN	771.7402273	3	0.822000921		
EPFPDGAELR	31.85278	Q6PJ69	TRI65 HUMAN	565.7780195	2	0.792413414	2	Yes
EPFPDGAELR	31.85278	Q6PJ69	TRI65 HUMAN	377.521288	3	0.792413414	2	
FSSLLQALEIQHTTALR	79.60324	Q6PJ69	TRI65 HUMAN	964.5341815	2	0.799061477		
FSSLLQALEIQHTTALR	79.60324	Q6PJ69	TRI65 HUMAN	643.3587293	3	0.799061477		
NVALSGVLEVVR	71.65022	Q6PJ69	TRI65 HUMAN	628.372618	2	0.747703552	2	Yes
NVALSGVLEVVR	71.65022	Q6PJ69	TRI65 HUMAN	419.2510203	3	0.747703552	2	
TLTLCHQPGAVFPLGPQEEVLS	92.47258	Q6PJ69	TRI65 HUMAN	1197.112668	2	0.779458404		
TLTLCHQPGAVFPLGPQEEVLS	92.47258	Q6PJ69	TRI65 HUMAN	798.4110537	3	0.779458404		
DLELYENWNK	55.87622	Q6PIW4	FIGL1 HUMAN	662.314961	2	0.888565302		
DLELYENWNK	55.87622	Q6PIW4	FIGL1 HUMAN	441.879249	3	0.888565302		
DSLLEPALASVVIHK	82.86369	Q6PIW4	FIGL1 HUMAN	796.456879	2	0.834861755		
DSLLEPALASVVIHK	82.86369	Q6PIW4	FIGL1 HUMAN	531.3071943	3	0.834861755		
EQLWVDQQK	16.22889	Q6PIW4	FIGL1 HUMAN	587.2991195	2	0.761830449		
EQLWVDQQK	16.22889	Q6PIW4	FIGL1 HUMAN	391.868688	3	0.761830449		
FHVTPLFGNVK	39.84117	Q6PIW4	FIGL1 HUMAN	629.851322	2	0.843138456		
FHVTPLFGNVK	39.84117	Q6PIW4	FIGL1 HUMAN	420.236823	3	0.843138456		
ILVVGATNRPQEIDEAAR	29.4886	Q6PIW4	FIGL1 HUMAN	976.53217	2	0.718925834		
ILVVGATNRPQEIDEAAR	29.4886	Q6PIW4	FIGL1 HUMAN	651.3573883	3	0.718925834		
IQYAWANSEISQVCATK	54.2341	Q6PIW4	FIGL1 HUMAN	984.9783775	2	0.663163424		
IQYAWANSEISQVCATK	54.2341	Q6PIW4	FIGL1 HUMAN	656.9881933	3	0.663163424		
LLQNAQPPMVTNTAR	27.96175	Q6PIW4	FIGL1 HUMAN	827.4412385	2	0.823305011		
LLQNAQPPMVTNTAR	27.96175	Q6PIW4	FIGL1 HUMAN	551.963434	3	0.823305011		
LYIPLPEASAR	61.85602	Q6PIW4	FIGL1 HUMAN	615.3486075	2	0.814431667		
LYIPLPEASAR	61.85602	Q6PIW4	FIGL1 HUMAN	410.5683467	3	0.814431667		
SFYGSGTIDALSNPILNK	80.10677	Q6PIW4	FIGL1 HUMAN	948.989267	2	0.801747084		
SFYGSGTIDALSNPILNK	80.10677	Q6PIW4	FIGL1 HUMAN	632.995453	3	0.801747084		
SVHLSEWQK	-0.011929	Q6PIW4	FIGL1 HUMAN	557.288554	2	0.827111065		
SVHLSEWQK	-0.011929	Q6PIW4	FIGL1 HUMAN	371.8616443	3	0.827111065		
TCPTFSAPVGESATAK	22.74412	Q6PIW4	FIGL1 HUMAN	812.3883375	2	0.756632686		
TCPTFSAPVGESATAK	22.74412	Q6PIW4	FIGL1 HUMAN	541.9281667	3	0.756632686		
TEFLVQLDGATTSSEDR	62.1541	Q6PIW4	FIGL1 HUMAN	934.9478015	2	0.724521697		
TEFLVQLDGATTSSEDR	62.1541	Q6PIW4	FIGL1 HUMAN	623.634476	3	0.724521697		
WQSGLSINNVFK	60.62647	Q6PIW4	FIGL1 HUMAN	696.8676995	2	0.777569473		
WQSGLSINNVFK	60.62647	Q6PIW4	FIGL1 HUMAN	464.914408	3	0.777569473		
YSAIIDSDNVESGLNNYAENILTLAGSQQTDSDK	117.3876	Q6PIW4	FIGL1 HUMAN	1823.356103	2	0.711786747		
YSAIIDSDNVESGLNNYAENILTLAGSQQTDSDK	117.3876	Q6PIW4	FIGL1 HUMAN	1215.906677	3	0.711786747		
AAELEPDQLLAWQGLANLYEK	127.0096	Q6PGP7	TTC37 HUMAN	1186.61081	2	0.810142875		
AAELEPDQLLAWQGLANLYEK	127.0096	Q6PGP7	TTC37 HUMAN	791.409815	3	0.810142875		
AALNELLK	33.59864	Q6PGP7	TTC37 HUMAN	436.266559	2	0.810968637	2	Yes
AALNELLK	33.59864	Q6PGP7	TTC37 HUMAN	291.1803143	3	0.810968637	2	
AALVDYLDGK	48.5096	Q6PGP7	TTC37 HUMAN	532.785309	2	0.862909198		
AALVDYLDGK	48.5096	Q6PGP7	TTC37 HUMAN	355.5261477	3	0.862909198		
AGQHSQAVADLQAALR	29.53718	Q6PGP7	TTC37 HUMAN	818.4322595	2	0.757610321	3	
AGQHSQAVADLQAALR	29.53718	Q6PGP7	TTC37 HUMAN	545.957448	3	0.757610321	3	Yes
AHILTALAITEYK	49.33138	Q6PGP7	TTC37 HUMAN	722.4144795	2	0.800253034		
AHILTALAITEYK	49.33138	Q6PGP7	TTC37 HUMAN	481.9455947	3	0.800253034		
AILLLQTAEDQDTYNVAIR	81.09477	Q6PGP7	TTC37 HUMAN	1074.071321	2	0.802878618		
AILLLQTAEDQDTYNVAIR	81.09477	Q6PGP7	TTC37 HUMAN	716.383489	3	0.802878618		
ALELNQR	-9.064819	Q6PGP7	TTC37 HUMAN	422.238333	2	0.782117248	2	Yes
ALELNQR	-9.064819	Q6PGP7	TTC37 HUMAN	281.8281637	3	0.782117248	2	
ALEYFTCALQHR	38.07092	Q6PGP7	TTC37 HUMAN	754.8699135	2	0.773797452	3	
ALEYFTCALQHR	38.07092	Q6PGP7	TTC37 HUMAN	503.5825507	3	0.773797452	3	Yes
ALSIVESEQDK	13.92884	Q6PGP7	TTC37 HUMAN	609.8147985	2	0.792269588	2	Yes
ALSIVESEQDK	13.92884	Q6PGP7	TTC37 HUMAN	406.8791407	3	0.792269588	2	
ALTHFLK	-3.657574	Q6PGP7	TTC37 HUMAN	415.2507125	2	0.615216196	2	Yes
ALTHFLK	-3.657574	Q6PGP7	TTC37 HUMAN	277.16975	3	0.615216196	2	
ASELNPESIYSVFK	80.88426	Q6PGP7	TTC37 HUMAN	792.4017645	2	0.899847031		
ASELNPESIYSVFK	80.88426	Q6PGP7	TTC37 HUMAN	528.6037847	3	0.899847031		
AVHSNPGDPALWSLLSR	73.66672	Q6PGP7	TTC37 HUMAN	910.4766655	2	0.742519081	3	
AVHSNPGDPALWSLLSR	73.66672	Q6PGP7	TTC37 HUMAN	607.3203853	3	0.742519081	3	Yes
DDLPGVYQK	17.95302	Q6PGP7	TTC37 HUMAN	517.761834	2	0.768133879		
DDLPGVYQK	17.95302	Q6PGP7	TTC37 HUMAN	345.5104977	3	0.768133879		
DFNCWESLGEAYLSR	100.2266	Q6PGP7	TTC37 HUMAN	923.90742	2	0.762491941		
DFNCWESLGEAYLSR	100.2266	Q6PGP7	TTC37 HUMAN	616.2742217	3	0.762491941		
EAVAQYQMIIK	42.71796	Q6PGP7	TTC37 HUMAN	647.347751	2	0.767297626		
EAVAQYQMIIK	42.71796	Q6PGP7	TTC37 HUMAN	431.901109	3	0.767297626		
EAVLSCSQALK	12.33688	Q6PGP7	TTC37 HUMAN	603.313912	2	0.731958568		
EAVLSCSQALK	12.33688	Q6PGP7	TTC37 HUMAN	402.5452163	3	0.731958568		
FFENYNQSLEK	34.44466	Q6PGP7	TTC37 HUMAN	709.833518	2	0.861559987	2	Yes
FFENYNQSLEK	34.44466	Q6PGP7	TTC37 HUMAN	473.558287	3	0.861559987	2	
GGVVAGNVAHILDSNHGK	19.298	Q6PGP7	TTC37 HUMAN	872.9586405	2	0.768487334		
GGVVAGNVAHILDSNHGK	19.298	Q6PGP7	TTC37 HUMAN	582.3083687	3	0.768487334		
GLGECHLMMAK	8.591019	Q6PGP7	TTC37 HUMAN	623.791236	2	0.641681194		
GLGECHLMMAK	8.591019	Q6PGP7	TTC37 HUMAN	416.1967657	3	0.641681194		
IVDNLGASGNSLYQR	35.11743	Q6PGP7	TTC37 HUMAN	803.9133625	2	0.876134157	2	Yes
IVDNLGASGNSLYQR	35.11743	Q6PGP7	TTC37 HUMAN	536.2781833	3	0.876134157	2	
LAGDACTCLYAVAPSK	40.63261	Q6PGP7	TTC37 HUMAN	848.905836	2	0.69657433	2	Yes
LAGDACTCLYAVAPSK	40.63261	Q6PGP7	TTC37 HUMAN	566.2731657	3	0.69657433	2	
LALVNNTQPK	5.093254	Q6PGP7	TTC37 HUMAN	549.3198545	2	0.795562625		
LALVNNTQPK	5.093254	Q6PGP7	TTC37 HUMAN	366.549178	3	0.795562625		
LLCSTGEYDK	-0.25536	Q6PGP7	TTC37 HUMAN	593.276991	2	0.83518374	2	Yes
LLCSTGEYDK	-0.25536	Q6PGP7	TTC37 HUMAN	395.8539357	3	0.83518374	2	
LLDLYESVDK	51.4287	Q6PGP7	TTC37 HUMAN	597.8168055	2	0.807080865		
LLDLYESVDK	51.4287	Q6PGP7	TTC37 HUMAN	398.8804787	3	0.807080865		
LMSTSNTWCDLGINYYR	73.08581	Q6PGP7	TTC37 HUMAN	1047.47514	2	0.70789355		

LMSTSNTWCDLGINYYR	73.08581	Q6PGP7	TTC37 HUMAN	698.6527017	3	0.70789355		
LSDYDSSEEAIR	12.11755	Q6PGP7	TTC37 HUMAN	692.815522	2	0.870295644		
LSDYDSSEEAIR	12.11755	Q6PGP7	TTC37 HUMAN	462.2129563	3	0.870295644		
LVDLYYQEK	31.20568	Q6PGP7	TTC37 HUMAN	585.806237	2	0.801803648		
LVDLYYQEK	31.20568	Q6PGP7	TTC37 HUMAN	390.873433	3	0.801803648		
SGPGLIGLGIK	55.65492	Q6PGP7	TTC37 HUMAN	506.314041	2	0.845923424	2	Yes
SGPGLIGLGIK	55.65492	Q6PGP7	TTC37 HUMAN	337.8786357	3	0.845923424	2	
SNPDQPAVILLLR	87.40611	Q6PGP7	TTC37 HUMAN	718.417557	2	0.791499615	2	Yes
SNPDQPAVILLLR	87.40611	Q6PGP7	TTC37 HUMAN	479.2809797	3	0.791499615	2	
TLDQISDADNIPGLLVLK	109.2727	Q6PGP7	TTC37 HUMAN	963.0336795	2	0.826860547		
TLDQISDADNIPGLLVLK	109.2727	Q6PGP7	TTC37 HUMAN	642.3583947	3	0.826860547		
VAAIQQILGK	43.53587	Q6PGP7	TTC37 HUMAN	520.827316	2	0.780883193	2	Yes
VAAIQQILGK	43.53587	Q6PGP7	TTC37 HUMAN	347.5541523	3	0.780883193	2	
VCMANISNDHWPSLVQEATTEALK	84.73938	Q6PGP7	TTC37 HUMAN	1357.649819	2	0.628346026		
VCMANISNDHWPSLVQEATTEALK	84.73938	Q6PGP7	TTC37 HUMAN	905.435821	3	0.628346026		
VFCYLGHYYR	29.39419	Q6PGP7	TTC37 HUMAN	689.3246015	2	0.77864188	3	
VFCYLGHYYR	29.39419	Q6PGP7	TTC37 HUMAN	459.885676	3	0.77864188	3	Yes
VNVHVLGVLLGQK	56.67636	Q6PGP7	TTC37 HUMAN	688.4251855	2	0.771460116	3	
VNVHVLGVLLGQK	56.67636	Q6PGP7	TTC37 HUMAN	459.2860653	3	0.771460116	3	Yes
VVYQPGYPK	7.194221	Q6PGP7	TTC37 HUMAN	525.785108	2	0.726930797		
VVYQPGYPK	7.194221	Q6PGP7	TTC37 HUMAN	350.859347	3	0.726930797		
WSLSQAVTGLIDTGR	100.6354	Q6PGP7	TTC37 HUMAN	802.426111	2	0.817180336	2	Yes
WSLSQAVTGLIDTGR	100.6354	Q6PGP7	TTC37 HUMAN	535.2866823	3	0.817180336	2	
GSAALGGVLALAER	67.69074	Q6P582	MZT2A HUMAN	642.8677005	2	0.825978458		
GSAALGGVLALAER	67.69074	Q6P582	MZT2A HUMAN	428.9144087	3	0.825978458		
VLSTEEMELYELAQAAGGGIDPDVFK	138.5879	Q6P582	MZT2A HUMAN	1391.678761	2	0.661824167		
VLSTEEMELYELAQAAGGGIDPDVFK	138.5879	Q6P582	MZT2A HUMAN	928.1217823	3	0.661824167		
ACSGDGGVSYTQGQSPEPR	-4.992241	Q6P1X6	CH082 HUMAN	976.924331	2	0.730398417		
ACSGDGGVSYTQGQSPEPR	-4.992241	Q6P1X6	CH082 HUMAN	651.618829	3	0.730398417		
EYFYYVDHQGQLFLDDSK	70.59071	Q6P1X6	CH082 HUMAN	1134.018745	2	0.732246757	3	
EYFYYVDHQGQLFLDDSK	70.59071	Q6P1X6	CH082 HUMAN	756.3484383	3	0.732246757	3	Yes
YEAAFPFLSPCGR	76.02251	Q6P1X6	CH082 HUMAN	757.859014	2	0.740797043		
YEAAFPFLSPCGR	76.02251	Q6P1X6	CH082 HUMAN	505.5752843	3	0.740797043		
DVVAQQESILALEK	69.65608	Q6P1X5	TAF2 HUMAN	771.9228695	2	0.82058847		
DVVAQQESILALEK	69.65608	Q6P1X5	TAF2 HUMAN	514.951188	3	0.82058847		
EQAPLEMSMHPAASAPLSVFTK	75.25058	Q6P1X5	TAF2 HUMAN	1171.580146	2	0.744854391		
EQAPLEMSMHPAASAPLSVFTK	75.25058	Q6P1X5	TAF2 HUMAN	781.389372	3	0.744854391		
EVLTFILDLIK	144.9065	Q6P1X5	TAF2 HUMAN	652.3977705	2	0.663640618		
EVLTFILDLIK	144.9065	Q6P1X5	TAF2 HUMAN	435.2677887	3	0.663640618		
FSDNYYR	-5.40152	Q6P1X5	TAF2 HUMAN	482.712139	2	0.711984932		
FSDNYYR	-5.40152	Q6P1X5	TAF2 HUMAN	322.1440343	3	0.711984932		
FYGSFAFNR	43.36721	Q6P1X5	TAF2 HUMAN	554.7647105	2	0.795336962		
FYGSFAFNR	43.36721	Q6P1X5	TAF2 HUMAN	370.179082	3	0.795336962		
GGLHFVVPSVEGSMAER	52.66125	Q6P1X5	TAF2 HUMAN	886.4439775	2	0.820993185		
GGLHFVVPSVEGSMAER	52.66125	Q6P1X5	TAF2 HUMAN	591.2985933	3	0.820993185		
GISGYIYGLWMK	92.02092	Q6P1X5	TAF2 HUMAN	694.358114	2	0.745856404		
GISGYIYGLWMK	92.02092	Q6P1X5	TAF2 HUMAN	463.241351	3	0.745856404		
IAALEAVVDYTK	61.24005	Q6P1X5	TAF2 HUMAN	646.8590055	2	0.82362771		
IAALEAVVDYTK	61.24005	Q6P1X5	TAF2 HUMAN	431.5752787	3	0.82362771		
IANSMVSTWTGPPAMK	51.34443	Q6P1X5	TAF2 HUMAN	845.91875	2	0.732592702		
IANSMVSTWTGPPAMK	51.34443	Q6P1X5	TAF2 HUMAN	564.281775	3	0.732592702		
IDPDMSVLR	39.4667	Q6P1X5	TAF2 HUMAN	523.2715155	2	0.713907599		
IDPDMSVLR	39.4667	Q6P1X5	TAF2 HUMAN	349.1836187	3	0.713907599		
IHINFSLDQPK	36.60677	Q6P1X5	TAF2 HUMAN	656.356968	2	0.69603771		
IHINFSLDQPK	36.60677	Q6P1X5	TAF2 HUMAN	437.9072537	3	0.69603771		
INDLEAAFIYNDPTLEVCHSESK	73.55366	Q6P1X5	TAF2 HUMAN	1333.126695	2	0.796848536		
INDLEAAFIYNDPTLEVCHSESK	73.55366	Q6P1X5	TAF2 HUMAN	889.0870713	3	0.796848536		
IVAYELK	12.76227	Q6P1X5	TAF2 HUMAN	418.2503735	2	0.763915181		
IVAYELK	12.76227	Q6P1X5	TAF2 HUMAN	279.169524	3	0.763915181		
LALTDILEQEQCFYR	102.9143	Q6P1X5	TAF2 HUMAN	949.9700215	2	0.64177537		
LALTDILEQEQCFYR	102.9143	Q6P1X5	TAF2 HUMAN	633.6492893	3	0.64177537		
LILEEITR	46.81393	Q6P1X5	TAF2 HUMAN	493.7982235	2	0.849334955		
LILEEITR	46.81393	Q6P1X5	TAF2 HUMAN	329.5347573	3	0.849334955		
LLSLASTASSQK	17.56458	Q6P1X5	TAF2 HUMAN	603.3409835	2	0.790446401		
LLSLASTASSQK	17.56458	Q6P1X5	TAF2 HUMAN	402.563264	3	0.790446401		
MSWSDEWVLK	73.65839	Q6P1X5	TAF2 HUMAN	640.8031795	2	0.790986657		
MSWSDEWVLK	73.65839	Q6P1X5	TAF2 HUMAN	427.5380613	3	0.790986657		
NMESPLCNEALVDQLWK	93.6698	Q6P1X5	TAF2 HUMAN	1023.985349	2	0.785383105		
NMESPLCNEALVDQLWK	93.6698	Q6P1X5	TAF2 HUMAN	682.992841	3	0.785383105		
NVLELEIK	46.48368	Q6P1X5	TAF2 HUMAN	479.2849485	2	0.740392745		
NVLELEIK	46.48368	Q6P1X5	TAF2 HUMAN	319.8592407	3	0.740392745		
SALIPQHSAGCDSTPTTKPQWSLELAR	47.92773	Q6P1X5	TAF2 HUMAN	1476.238179	2	0.65947783		
SALIPQHSAGCDSTPTTKPQWSLELAR	47.92773	Q6P1X5	TAF2 HUMAN	984.4947277	3	0.65947783		
SYAEYGHFVDIR	35.68782	Q6P1X5	TAF2 HUMAN	728.846956	2	0.812977076		
SYAEYGHFVDIR	35.68782	Q6P1X5	TAF2 HUMAN	486.2339123	3	0.812977076		
TFGVNEYR	10.73438	Q6P1X5	TAF2 HUMAN	493.2410685	2	0.671762824		
TFGVNEYR	10.73438	Q6P1X5	TAF2 HUMAN	329.1633207	3	0.671762824		
TGGVLLHPIFGGGK	42.42101	Q6P1X5	TAF2 HUMAN	676.8884365	2	0.807139635		
TGGVLLHPIFGGGK	42.42101	Q6P1X5	TAF2 HUMAN	451.5948993	3	0.807139635		
TLDNLNPDVR	23.19836	Q6P1X5	TAF2 HUMAN	578.8020255	2	0.814689398		
TLDNLNPDVR	23.19836	Q6P1X5	TAF2 HUMAN	386.2039587	3	0.814689398		
TMPVAMALLR	73.44868	Q6P1X5	TAF2 HUMAN	551.8097545	2	0.802696347		
TMPVAMALLR	73.44868	Q6P1X5	TAF2 HUMAN	368.2091113	3	0.802696347		
TNNFMSFQSYFLQK	87.89694	Q6P1X5	TAF2 HUMAN	877.914517	2	0.766614914		
TNNFMSFQSYFLQK	87.89694	Q6P1X5	TAF2 HUMAN	585.6122863	3	0.766614914		
VTVQELDGSFNHTLQIEENSLK	65.05	Q6P1X5	TAF2 HUMAN	1251.130099	2	0.723599851	3	
VTVQELDGSFNHTLQIEENSLK	65.05	Q6P1X5	TAF2 HUMAN	834.4226743	3	0.723599851	3	Yes
AMLLTSYLPPPLLR	110.7188	Q6P1Q0	LTMD1 HUMAN	792.963282	2	0.793516457		
AMLLTSYLPPPLLR	110.7188	Q6P1Q0	LTMD1 HUMAN	528.9781297	3	0.793516457		
EAELSLLLHNVVLLSTNYLGTR	130.867	Q6P1Q0	LTMD1 HUMAN	1228.181933	2	0.649249971		
EAELSLLLHNVVLLSTNYLGTR	130.867	Q6P1Q0	LTMD1 HUMAN	819.123897	3	0.649249971		
GLQMLWADAK	54.47029	Q6P1Q0	LTMD1 HUMAN	566.7951585	2	0.774950266		
GLQMLWADAK	54.47029	Q6P1Q0	LTMD1 HUMAN	378.1993807	3	0.774950266		
GTHPAIHDILALR	33.17123	Q6P1Q0	LTMD1 HUMAN	707.402242	2	0.724655807		
GTHPAIHDILALR	33.17123	Q6P1Q0	LTMD1 HUMAN	471.9374363	3	0.724655807		
LGIGQLTAQEVK	45.44783	Q6P1Q0	LTMD1 HUMAN	628.864627	2	0.889381409	2	Yes
LGIGQLTAQEVK	45.44783	Q6P1Q0	LTMD1 HUMAN	419.5790263	3	0.889381409	2	
NLMSYVVTK	41.60362	Q6P1Q0	LTMD1 HUMAN	527.7842545	2	0.743896484	2	Yes
NLMSYVVTK	41.60362	Q6P1Q0	LTMD1 HUMAN	352.1921113	3	0.743896484	2	
QQTDFLDIYHAFR	70.29604	Q6P1Q0	LTMD1 HUMAN	827.4051745	2	0.743661702	3	
QQTDFLDIYHAFR	70.29604	Q6P1Q0	LTMD1 HUMAN	551.9393913	3	0.743661702	3	Yes

SAVWGSAVTPGHFVTR	40.34	Q6P1Q0	LTMD1 HUMAN	836.4342705	2	0.809253097		
SAVWGSAVTPGHFVTR	40.34	Q6P1Q0	LTMD1 HUMAN	557.9587887	3	0.809253097		
SGLAWGAPR	19.71753	Q6P1Q0	LTMD1 HUMAN	457.7463255	2	0.71484381		
SGLAWGAPR	19.71753	Q6P1Q0	LTMD1 HUMAN	305.5001587	3	0.71484381		
THTTVIHQLDK	-28.80138	Q6P1Q0	LTMD1 HUMAN	646.852051	2	0.767582655		
THTTVIHQLDK	-28.80138	Q6P1Q0	LTMD1 HUMAN	431.5706423	3	0.767582655		
VIPLISDAGLR	62.63392	Q6P1Q0	LTMD1 HUMAN	577.3511545	2	0.803287745	2	Yes
VIPLISDAGLR	62.63392	Q6P1Q0	LTMD1 HUMAN	385.2367113	3	0.803287745	2	
AQLEQGGVGIR	6.031139	Q6P1N0	C2D1A HUMAN	564.3125615	2	0.734598458	2	Yes
AQLEQGGVGIR	6.031139	Q6P1N0	C2D1A HUMAN	376.544316	3	0.734598458	2	
AQQQLAFLEGR	37.36835	Q6P1N0	C2D1A HUMAN	630.8389435	2	0.798017263		
AQQQLAFLEGR	37.36835	Q6P1N0	C2D1A HUMAN	420.8952373	3	0.798017263		
ASETPPPVAQPKPEAPHPGLETTLQER	32.26967	Q6P1N0	C2D1A HUMAN	1439.241245	2	0.82764256		
ASETPPPVAQPKPEAPHPGLETTLQER	32.26967	Q6P1N0	C2D1A HUMAN	959.830105	3	0.82764256		
DVAELPVPPGFPPIQGLEATKPTQQSLVGVLETA	147.2991	Q6P1N0	C2D1A HUMAN	1964.561192	2	0.748326242		
DVAELPVPPGFPPIQGLEATKPTQQSLVGVLETA	147.2991	Q6P1N0	C2D1A HUMAN	1310.043403	3	0.748326242		
DDFALVQRPGPGLSQEAAR	47.84721	Q6P1N0	C2D1A HUMAN	1014.019427	2	0.65368551		
DDFALVQRPGPGLSQEAAR	47.84721	Q6P1N0	C2D1A HUMAN	676.348893	3	0.65368551		
EPLTAQQLETTER	22.40813	Q6P1N0	C2D1A HUMAN	808.9104915	2	0.807285249		
EPLTAQQLETTER	22.40813	Q6P1N0	C2D1A HUMAN	539.6096027	3	0.807285249		
FDFPYPNVEEAQK	64.87036	Q6P1N0	C2D1A HUMAN	792.373008	2	0.828622699		
FDFPYPNVEEAQK	64.87036	Q6P1N0	C2D1A HUMAN	528.5846137	3	0.828622699		
GINLPTPPGLSPGDLDVFVR	121.5394	Q6P1N0	C2D1A HUMAN	1032.560396	2	0.734451175		
GINLPTPPGLSPGDLDVFVR	121.5394	Q6P1N0	C2D1A HUMAN	688.709539	3	0.734451175		
GLEPMLEASR	32.49892	Q6P1N0	C2D1A HUMAN	551.7822475	2	0.791132629		
GLEPMLEASR	32.49892	Q6P1N0	C2D1A HUMAN	368.1907733	3	0.791132629		
GNAIDEADIPPPVAIGK	61.06078	Q6P1N0	C2D1A HUMAN	838.946876	2	0.626113892		
GNAIDEADIPPPVAIGK	61.06078	Q6P1N0	C2D1A HUMAN	559.633859	3	0.626113892		
GPASTPTYSPAPTQPAPR	13.6759	Q6P1N0	C2D1A HUMAN	898.4528535	2	0.778462529		
GPASTPTYSPAPTQPAPR	13.6759	Q6P1N0	C2D1A HUMAN	599.3045107	3	0.778462529		
GPLPMEAIEK	36.5508	Q6P1N0	C2D1A HUMAN	542.7895415	2	0.651548803		
GPLPMEAIEK	36.5508	Q6P1N0	C2D1A HUMAN	362.195636	3	0.651548803		
LALYQTAIESAR	42.51257	Q6P1N0	C2D1A HUMAN	668.367529	2	0.794982016	2	Yes
LALYQTAIESAR	42.51257	Q6P1N0	C2D1A HUMAN	445.9142943	3	0.794982016	2	
LANQDEGPEDEEDEVPK	9.770424	Q6P1N0	C2D1A HUMAN	957.4165305	2	0.74411279	2	Yes
LANQDEGPEDEEDEVPK	9.770424	Q6P1N0	C2D1A HUMAN	638.6136287	3	0.74411279	2	
LDALEIACEVR	58.61221	Q6P1N0	C2D1A HUMAN	644.832469	2	0.707304657		
LDALEIACEVR	58.61221	Q6P1N0	C2D1A HUMAN	430.2242543	3	0.707304657		
NLVESELQR	14.93959	Q6P1N0	C2D1A HUMAN	544.2912935	2	0.744949877		
NLVESELQR	14.93959	Q6P1N0	C2D1A HUMAN	363.196804	3	0.744949877		
RPVPPEVAQQYQDIMQR	46.89119	Q6P1N0	C2D1A HUMAN	1028.026194	2	0.779049337		
RPVPPEVAQQYQDIMQR	46.89119	Q6P1N0	C2D1A HUMAN	685.6867377	3	0.779049337		
SARPLHSLSVLAFDQER	37.26626	Q6P1N0	C2D1A HUMAN	963.5137795	2	0.756672561		
SARPLHSLSVLAFDQER	37.26626	Q6P1N0	C2D1A HUMAN	642.6784613	3	0.756672561		
SFDAVLEALSR	85.08499	Q6P1N0	C2D1A HUMAN	604.320051	2	0.766402066		
SFDAVLEALSR	85.08499	Q6P1N0	C2D1A HUMAN	403.2159757	3	0.766402066		
TLENLLASIR	74.88184	Q6P1N0	C2D1A HUMAN	565.3329615	2	0.798303545		
TLENLLASIR	74.88184	Q6P1N0	C2D1A HUMAN	377.2245827	3	0.798303545		
TLLEALEQR	47.49347	Q6P1N0	C2D1A HUMAN	536.8040375	2	0.794040084	2	Yes
TLLEALEQR	47.49347	Q6P1N0	C2D1A HUMAN	358.2053	3	0.794040084	2	
LEGPSATAPASSPGLAKPQMPPGPCSPGPLAQLC	71.43787	Q6P1N0	C2D1A HUMAN	1876.95911	2	0.690629303		
LEGPSATAPASSPGLAKPQMPPGPCSPGPLAQLC	71.43787	Q6P1N0	C2D1A HUMAN	1251.642015	3	0.690629303		
WLVIDPVPAAVPTQVAGPK	96.23718	Q6P1N0	C2D1A HUMAN	979.5596685	2	0.711222112		
WLVIDPVPAAVPTQVAGPK	96.23718	Q6P1N0	C2D1A HUMAN	653.3757207	3	0.711222112		
ALDVADLQK	21.31567	Q6NUQ4	TM214 HUMAN	486.772206	2	0.829549193	2	Yes
ALDVADLQK	21.31567	Q6NUQ4	TM214 HUMAN	324.8507457	3	0.829549193	2	
ATPSCPPEMK	-13.14977	Q6NUQ4	TM214 HUMAN	559.255009	2	0.708551347	2	Yes
ATPSCPPEMK	-13.14977	Q6NUQ4	TM214 HUMAN	373.1726143	3	0.708551347	2	
DLASYLNYK	56.78429	Q6NUQ4	TM214 HUMAN	543.777479	2	0.776394248	2	Yes
DLASYLNYK	56.78429	Q6NUQ4	TM214 HUMAN	362.854261	3	0.776394248	2	
GFENIMK	15.6132	Q6NUQ4	TM214 HUMAN	419.7105625	2	0.762122095		
GFENIMK	15.6132	Q6NUQ4	TM214 HUMAN	280.1429833	3	0.762122095		
GFGMIGPK	24.55966	Q6NUQ4	TM214 HUMAN	403.7156485	2	0.824465036	2	Yes
GFGMIGPK	24.55966	Q6NUQ4	TM214 HUMAN	269.4797073	3	0.824465036	2	
GLLQQVQGPR	22.59123	Q6NUQ4	TM214 HUMAN	548.317647	2	0.80455631	2	Yes
GLLQQVQGPR	22.59123	Q6NUQ4	TM214 HUMAN	365.8810397	3	0.80455631	2	
HLSQSSLLLEHLLSSWEQIPK	102.8324	Q6NUQ4	TM214 HUMAN	1223.161005	2	0.753201127		
HLSQSSLLLEHLLSSWEQIPK	102.8324	Q6NUQ4	TM214 HUMAN	815.7766113	3	0.753201127		
ICIQAILQDKPK	43.53178	Q6NUQ4	TM214 HUMAN	713.9085115	2	0.765350461	3	
ICIQAILQDKPK	43.53178	Q6NUQ4	TM214 HUMAN	476.2749493	3	0.765350461	3	Yes
LLLMHPNLTK	29.07317	Q6NUQ4	TM214 HUMAN	590.3501005	2	0.761910439	2	Yes
LLLMHPNLTK	29.07317	Q6NUQ4	TM214 HUMAN	393.9026753	3	0.761910439	2	
LQAPLSEPTLSQHTHDYPYSLVSR	50.94775	Q6NUQ4	TM214 HUMAN	1370.191014	2	0.728829205	4	
LQAPLSEPTLSQHTHDYPYSLVSR	50.94775	Q6NUQ4	TM214 HUMAN	913.7966177	3	0.728829205	4	
LQIQLPDSVNQLLR	92.57155	Q6NUQ4	TM214 HUMAN	818.973228	2	0.821861625		
LQIQLPDSVNQLLR	92.57155	Q6NUQ4	TM214 HUMAN	546.3180937	3	0.821861625		
SLQETIQSLK	32.04958	Q6NUQ4	TM214 HUMAN	573.822427	2	0.751875997	2	Yes
SLQETIQSLK	32.04958	Q6NUQ4	TM214 HUMAN	382.8842263	3	0.751875997	2	
SLSPFAITYLDR	88.53152	Q6NUQ4	TM214 HUMAN	691.869904	2	0.739073813	2	Yes
SLSPFAITYLDR	88.53152	Q6NUQ4	TM214 HUMAN	461.5825443	3	0.739073813	2	
SQSVFSGNPSIWLK	65.81949	Q6NUQ4	TM214 HUMAN	775.404646	2	0.76154238		
SQSVFSGNPSIWLK	65.81949	Q6NUQ4	TM214 HUMAN	517.2723723	3	0.76154238		
SSGFLPASQQACAK	13.61283	Q6NUQ4	TM214 HUMAN	726.3515575	2	0.744556487		
SSGFLPASQQACAK	13.61283	Q6NUQ4	TM214 HUMAN	484.5703133	3	0.744556487		
YDLTPAIQTTSTLYER	65.7774	Q6NUQ4	TM214 HUMAN	936.473447	2	0.773340225		
YDLTPAIQTTSTLYER	65.7774	Q6NUQ4	TM214 HUMAN	624.651573	3	0.773340225		
AGGGGGLGAGSPALSGGQGR	7.468624	Q6IQ22	RAB12 HUMAN	792.398417	2	0.659625292	2	Yes
AGGGGGLGAGSPALSGGQGR	7.468624	Q6IQ22	RAB12 HUMAN	528.601553	3	0.659625292	2	
DNFNVDEIFLK	93.48581	Q6IQ22	RAB12 HUMAN	677.3384405	2	0.848358035	2	Yes
DNFNVDEIFLK	93.48581	Q6IQ22	RAB12 HUMAN	451.894902	3	0.848358035	2	
ETFDDLPK	22.66081	Q6IQ22	RAB12 HUMAN	482.735289	2	0.601921916		
ETFDDLPK	22.66081	Q6IQ22	RAB12 HUMAN	322.1594677	3	0.601921916		
FAQQITGMR	10.28293	Q6IQ22	RAB12 HUMAN	526.271851	2	0.783428192	2	Yes
FAQQITGMR	10.28293	Q6IQ22	RAB12 HUMAN	351.1838423	3	0.783428192	2	
FNSITSAYYR	24.84116	Q6IQ22	RAB12 HUMAN	611.29911	2	0.724662066		
FNSITSAYYR	24.84116	Q6IQ22	RAB12 HUMAN	407.8686817	3	0.724662066		
GIILVYDITK	70.41976	Q6IQ22	RAB12 HUMAN	567.842627	2	0.771373034		
GIILVYDITK	70.41976	Q6IQ22	RAB12 HUMAN	378.897693	3	0.771373034		
LQVIIIGSR	47.98277	Q6IQ22	RAB12 HUMAN	499.822033	2	0.631322086	2	Yes
LQVIIIGSR	47.98277	Q6IQ22	RAB12 HUMAN	333.5506303	3	0.631322086	2	
YASEDAELLLVGNK	58.57398	Q6IQ22	RAB12 HUMAN	761.39394	2	0.861065805		

YASEDAELLLVGNK	58.57398	Q6IQ22	RAB12 HUMAN	507.9319017	3	0.861065805		
AVHLQGHEGPVYAVHAVYQR	12.45056	Q6IA86	ELP2 HUMAN	1116.077603	2	0.807623386	4	
AVHLQGHEGPVYAVHAVYQR	12.45056	Q6IA86	ELP2 HUMAN	744.3876767	3	0.807623386	4	
DGVLQQPVR	12.87701	Q6IA86	ELP2 HUMAN	506.2832725	2	0.787788391	2	Yes
DGVLQQPVR	12.87701	Q6IA86	ELP2 HUMAN	337.8581233	3	0.787788391	2	
DLFLASCSQDCLIR	77.11227	Q6IA86	ELP2 HUMAN	849.403464	2	0.725876212		
DLFLASCSQDCLIR	77.11227	Q6IA86	ELP2 HUMAN	566.6049177	3	0.725876212		
EAEGAEWLHFASCGEDHTVK	40.43394	Q6IA86	ELP2 HUMAN	1137.000574	2	0.74263823	4	
EAEGAEWLHFASCGEDHTVK	40.43394	Q6IA86	ELP2 HUMAN	758.3363243	3	0.74263823	4	
ENTFTIENESVK	22.80737	Q6IA86	ELP2 HUMAN	705.8415445	2	0.690316558	2	Yes
ENTFTIENESVK	22.80737	Q6IA86	ELP2 HUMAN	470.8969713	3	0.690316558	2	
FQFVSGADEK	18.40902	Q6IA86	ELP2 HUMAN	564.27257	2	0.751568496	2	Yes
FQFVSGADEK	18.40902	Q6IA86	ELP2 HUMAN	376.517655	3	0.751568496	2	
GVLNWSSGPR	29.65101	Q6IA86	ELP2 HUMAN	536.780896	2	0.77808392	2	Yes
GVLNWSSGPR	29.65101	Q6IA86	ELP2 HUMAN	358.1898723	3	0.77808392	2	
IHIFAQQNDQFQK	18.08266	Q6IA86	ELP2 HUMAN	808.913171	2	0.844471455		
IHIFAQQNDQFQK	18.08266	Q6IA86	ELP2 HUMAN	539.611389	3	0.844471455		
IIWSCDWSPDSK	55.36292	Q6IA86	ELP2 HUMAN	747.3406575	2	0.750840664	2	Yes
IIWSCDWSPDSK	55.36292	Q6IA86	ELP2 HUMAN	498.5630467	3	0.750840664	2	
QDGSPSTELVSGGSDNQVIHWEIEDNQLLK	86.30478	Q6IA86	ELP2 HUMAN	1648.289847	2	0.623236835		
QDGSPSTELVSGGSDNQVIHWEIEDNQLLK	86.30478	Q6IA86	ELP2 HUMAN	1099.19584	3	0.623236835		
STSLETQDDDNIR	0.447662	Q6IA86	ELP2 HUMAN	747.3398975	2	0.815552592	2	Yes
STSLETQDDDNIR	0.447662	Q6IA86	ELP2 HUMAN	498.56254	3	0.815552592	2	
TMILWAPDEESGVWLEQVR	116.1632	Q6IA86	ELP2 HUMAN	1130.059903	2	0.776512325		
TMILWAPDEESGVWLEQVR	116.1632	Q6IA86	ELP2 HUMAN	753.7092103	3	0.776512325		
VLSLCGHEDWIR	40.49668	Q6IA86	ELP2 HUMAN	742.869917	2	0.769512951	3	
VLSLCGHEDWIR	40.49668	Q6IA86	ELP2 HUMAN	495.582553	3	0.769512951	3	Yes
VVVTNLNGHTAR	-23.72125	Q6IA86	ELP2 HUMAN	640.857667	2	0.651783049		
VVVTNLNGHTAR	-23.72125	Q6IA86	ELP2 HUMAN	427.5743863	3	0.651783049		
YFFTGSR	15.73468	Q6IA86	ELP2 HUMAN	439.2143225	2	0.62486428		
YFFTGSR	15.73468	Q6IA86	ELP2 HUMAN	293.14549	3	0.62486428		
YVVAVGLECGK	33.55986	Q6IA86	ELP2 HUMAN	597.813544	2	0.768595219		
YVVAVGLECGK	33.55986	Q6IA86	ELP2 HUMAN	398.8783043	3	0.768595219		
ACAVETSVDLSEISSK	42.53625	Q69YH5	CDCA2 HUMAN	848.4094655	2	0.761785686		
ACAVETSVDLSEISSK	42.53625	Q69YH5	CDCA2 HUMAN	565.942252	3	0.761785686		
AGTDSPVSCASVTEER	9.806801	Q69YH5	CDCA2 HUMAN	833.373415	2	0.738279343		
AGTDSPVSCASVTEER	9.806801	Q69YH5	CDCA2 HUMAN	555.9182183	3	0.738279343		
DCHCLGDVLIENTK	38.00352	Q69YH5	CDCA2 HUMAN	837.3852715	2	0.671737492		
DCHCLGDVLIENTK	38.00352	Q69YH5	CDCA2 HUMAN	558.5927893	3	0.671737492		
DFSGLSSLLLEQSPVPEPLPQPDFDDK	155.5625	Q69YH5	CDCA2 HUMAN	1485.735125	2	0.626690269		
DFSGLSSLLLEQSPVPEPLPQPDFDDK	155.5625	Q69YH5	CDCA2 HUMAN	990.826025	3	0.626690269		
DLENEGLVWISLPLPSTSQK	142.5527	Q69YH5	CDCA2 HUMAN	1113.586807	2	0.677668869		
DLENEGLVWISLPLPSTSQK	142.5527	Q69YH5	CDCA2 HUMAN	742.7271463	3	0.677668869		
DLSDAIEQTFQR	79.11351	Q69YH5	CDCA2 HUMAN	711.847162	2	0.828593135		
DLSDAIEQTFQR	79.11351	Q69YH5	CDCA2 HUMAN	474.9007163	3	0.828593135		
EAEDEENFEAPAFLNMR	81.35861	Q69YH5	CDCA2 HUMAN	1006.439286	2	0.735576928		
EAEDEENFEAPAFLNMR	81.35861	Q69YH5	CDCA2 HUMAN	671.2954653	3	0.735576928		
EGLSACQQSGFPAVLSSK	53.48636	Q69YH5	CDCA2 HUMAN	933.457282	2	0.617665708		
EGLSACQQSGFPAVLSSK	53.48636	Q69YH5	CDCA2 HUMAN	622.6407963	3	0.617665708		
ESAMNNAGNASFILGTGK	51.22212	Q69YH5	CDCA2 HUMAN	891.428524	2	0.800296843		
ESAMNNAGNASFILGTGK	51.22212	Q69YH5	CDCA2 HUMAN	594.6216243	3	0.800296843		
GESSLTALER	12.70943	Q69YH5	CDCA2 HUMAN	531.7754765	2	0.686818719		
GESSLTALER	12.70943	Q69YH5	CDCA2 HUMAN	354.852926	3	0.686818719		
GSSDAVSPDTFTAEVSSDAVPDVR	69.57758	Q69YH5	CDCA2 HUMAN	1205.056797	2	0.73643887		
GSSDAVSPDTFTAEVSSDAVPDVR	69.57758	Q69YH5	CDCA2 HUMAN	803.7071393	3	0.73643887		
HAELPPNPCTPDTFK	24.46771	Q69YH5	CDCA2 HUMAN	862.409604	2	0.733394623		
HAELPPNPCTPDTFK	24.46771	Q69YH5	CDCA2 HUMAN	575.2756777	3	0.733394623		
ISAFQSAFHSIK	37.838	Q69YH5	CDCA2 HUMAN	668.356968	2	0.804999053		
ISAFQSAFHSIK	37.838	Q69YH5	CDCA2 HUMAN	445.9072537	3	0.804999053		
KPLLSPIPELPEVPEMTPSIPSIR	114.6329	Q69YH5	CDCA2 HUMAN	1320.738231	2	0.825004458	3	
KPLLSPIPELPEVPEMTPSIPSIR	114.6329	Q69YH5	CDCA2 HUMAN	880.8280957	3	0.825004458	3	Yes
KPMESSSVVSCR	-26.71034	Q69YH5	CDCA2 HUMAN	683.82686	2	0.749668896		
KPMESSSVVSCR	-26.71034	Q69YH5	CDCA2 HUMAN	456.220515	3	0.749668896		
LGSTQSGFLVEESLPLSELTETSNALK	123.3804	Q69YH5	CDCA2 HUMAN	1425.735125	2	0.724501371		
LGSTQSGFLVEESLPLSELTETSNALK	123.3804	Q69YH5	CDCA2 HUMAN	950.826025	3	0.724501371		
SFCISTLANTK	36.67496	Q69YH5	CDCA2 HUMAN	621.313912	2	0.604846656		
SFCISTLANTK	36.67496	Q69YH5	CDCA2 HUMAN	414.5452163	3	0.604846656		
SSSSLGNATSDEDPNTNIMNINENK	46.29458	Q69YH5	CDCA2 HUMAN	1326.588665	2	0.674143136		
SSSSLGNATSDEDPNTNIMNINENK	46.29458	Q69YH5	CDCA2 HUMAN	884.7283847	3	0.674143136		
TICTFDSSGFESMSPIK	72.10176	Q69YH5	CDCA2 HUMAN	953.932251	2	0.714846909		
TICTFDSSGFESMSPIK	72.10176	Q69YH5	CDCA2 HUMAN	636.2907757	3	0.714846909		
VIGLQIFNIDTDR	95.47214	Q69YH5	CDCA2 HUMAN	752.412472	2	0.794566154		
VIGLQIFNIDTDR	95.47214	Q69YH5	CDCA2 HUMAN	501.9442563	3	0.794566154		
VTFGEDLSPEVFDESLPANTPLR	108.9985	Q69YH5	CDCA2 HUMAN	1267.127025	2	0.721858263		
VTFGEDLSPEVFDESLPANTPLR	108.9985	Q69YH5	CDCA2 HUMAN	845.0872917	3	0.721858263		
YDVSEFCSYIK	56.90401	Q69YH5	CDCA2 HUMAN	705.8164755	2	0.702101171		
YDVSEFCSYIK	56.90401	Q69YH5	CDCA2 HUMAN	470.8802587	3	0.702101171		
DIVENLETQSK	37.03938	Q68CZ6	HAUS3 HUMAN	638.3255305	2	0.751327753		
DIVENLETQSK	37.03938	Q68CZ6	HAUS3 HUMAN	425.8862953	3	0.751327753		
ELEAFSILQK	66.853	Q68CZ6	HAUS3 HUMAN	589.327345	2	0.760443509		
ELEAFSILQK	66.853	Q68CZ6	HAUS3 HUMAN	393.2208383	3	0.760443509		
ELFLTHGNLEEVAEK	43.61778	Q68CZ6	HAUS3 HUMAN	864.944333	2	0.726821482		
ELFLTHGNLEEVAEK	43.61778	Q68CZ6	HAUS3 HUMAN	576.965497	3	0.726821482		
ENAQLLNMPVVK	56.27122	Q68CZ6	HAUS3 HUMAN	678.371761	2	0.815837383		
ENAQLLNMPVVK	56.27122	Q68CZ6	HAUS3 HUMAN	452.5837823	3	0.815837383		
GDFDLQIAK	39.29706	Q68CZ6	HAUS3 HUMAN	503.764381	2	0.769398212		
GDFDLQIAK	39.29706	Q68CZ6	HAUS3 HUMAN	336.1788623	3	0.769398212		
HSNLGQGTNPLVFLSQFSLEK	101.5887	Q68CZ6	HAUS3 HUMAN	1158.603323	2	0.78308475		
HSNLGQGTNPLVFLSQFSLEK	101.5887	Q68CZ6	HAUS3 HUMAN	772.738157	3	0.78308475		
ISSLTSEIMK	34.80324	Q68CZ6	HAUS3 HUMAN	554.8001055	2	0.834890366		
ISSLTSEIMK	34.80324	Q68CZ6	HAUS3 HUMAN	370.2026787	3	0.834890366		
LNHLLTDILADVK	72.1272	Q68CZ6	HAUS3 HUMAN	732.925215	2	0.837740064	3	
LNHLLTDILADVK	72.1272	Q68CZ6	HAUS3 HUMAN	488.9527517	3	0.837740064	3	Yes
LYQVLEGENK	18.4992	Q68CZ6	HAUS3 HUMAN	596.8145975	2	0.820312858		
LYQVLEGENK	18.4992	Q68CZ6	HAUS3 HUMAN	398.21234	3	0.820312858		
QELVLNQLIK	57.91359	Q68CZ6	HAUS3 HUMAN	599.364262	2	0.760896623		
QELVLNQLIK	57.91359	Q68CZ6	HAUS3 HUMAN	399.9121163	3	0.760896623		
QSQGILNAMITK	41.10963	Q68CZ6	HAUS3 HUMAN	652.3561115	2	0.70507884		
QSQGILNAMITK	41.10963	Q68CZ6	HAUS3 HUMAN	435.240016	3	0.70507884		
SGKPILEGAALDEALK	45.08625	Q68CZ6	HAUS3 HUMAN	806.451794	2	0.842420578		
SGKPILEGAALDEALK	45.08625	Q68CZ6	HAUS3 HUMAN	537.970471	3	0.842420578		

WAEESLHSLTSK	17.18971	Q68CZ6	HAUS3 HUMAN	694.346797	2	0.783373296		
WAEESLHSLTSK	17.18971	Q68CZ6	HAUS3 HUMAN	463.2338063	3	0.783373296		
WFCGNVNEQNVLSER	50.20625	Q68CZ6	HAUS3 HUMAN	926.4263155	2	0.755723119		
WFCGNVNEQNVLSER	50.20625	Q68CZ6	HAUS3 HUMAN	617.9534853	3	0.755723119		
YLSQEEQSTAALTLYTK	50.78387	Q68CZ6	HAUS3 HUMAN	973.4918365	2	0.79849273		
YLSQEEQSTAALTLYTK	50.78387	Q68CZ6	HAUS3 HUMAN	649.3304993	3	0.79849273		
AGYSPIMLTALATLK	112.1616	Q63ZY3	KANK2 HUMAN	775.4370975	2	0.661406815		
AGYSPIMLTALATLK	112.1616	Q63ZY3	KANK2 HUMAN	517.2940067	3	0.661406815		
ALAMPGRPESPPVFR	41.6762	Q63ZY3	KANK2 HUMAN	812.9355915	2	0.761235654		
ALAMPGRPESPPVFR	41.6762	Q63ZY3	KANK2 HUMAN	542.2930027	3	0.761235654		
ALQELQAAQAR	9.164196	Q63ZY3	KANK2 HUMAN	599.8311185	2	0.773048341	2	Yes
ALQELQAAQAR	9.164196	Q63ZY3	KANK2 HUMAN	400.223354	3	0.773048341	2	
AQSLEPYGTGLR	24.20865	Q63ZY3	KANK2 HUMAN	646.336229	2	0.836043		
AQSLEPYGTGLR	24.20865	Q63ZY3	KANK2 HUMAN	431.226761	3	0.836043		
DLGMPDGEAALAAK	52.56429	Q63ZY3	KANK2 HUMAN	679.835209	2	0.8196944		
DLGMPDGEAALAAK	52.56429	Q63ZY3	KANK2 HUMAN	453.5594143	3	0.8196944		
DPDPPYSVETPYGYR	47.82644	Q63ZY3	KANK2 HUMAN	878.397203	2	0.746617198		
DPDPPYSVETPYGYR	47.82644	Q63ZY3	KANK2 HUMAN	585.934077	3	0.746617198		
ESQHIPTAEGASGSNTEEEIR	1.440037	Q63ZY3	KANK2 HUMAN	1121.515099	2	0.79821229		
ESQHIPTAEGASGSNTEEEIR	1.440037	Q63ZY3	KANK2 HUMAN	748.0126743	3	0.79821229		
EVEVVASTAAGAPAQR	18.68443	Q63ZY3	KANK2 HUMAN	778.4079185	2	0.695114434		
EVEVVASTAAGAPAQR	18.68443	Q63ZY3	KANK2 HUMAN	519.274554	3	0.695114434		
GFYPQYGALETR	41.68082	Q63ZY3	KANK2 HUMAN	701.34405	2	0.799754858		
GFYPQYGALETR	41.68082	Q63ZY3	KANK2 HUMAN	467.8986417	3	0.799754858		
LDLDFLK	70.67242	Q63ZY3	KANK2 HUMAN	432.247835	2	0.807036996	2	Yes
LDLDFLK	70.67242	Q63ZY3	KANK2 HUMAN	288.501165	3	0.807036996	2	
LIPVLQVK	46.95482	Q63ZY3	KANK2 HUMAN	455.31077	2	0.74464488		
LIPVLQVK	46.95482	Q63ZY3	KANK2 HUMAN	303.876455	3	0.74464488		
LSVLQEEK	5.409603	Q63ZY3	KANK2 HUMAN	473.266756	2	0.805099726		
LSVLQEEK	5.409603	Q63ZY3	KANK2 HUMAN	315.8471123	3	0.805099726		
SCDGAAGLPEVPAESSSSPPGSEVASLTQPEK	66.80529	Q63ZY3	KANK2 HUMAN	1571.23061	2	0.750725389	3	
SCDGAAGLPEVPAESSSSPPGSEVASLTQPEK	66.80529	Q63ZY3	KANK2 HUMAN	1047.823015	3	0.750725389	3	Yes
SELCLDLPDPPEDPVALETR	103.7442	Q63ZY3	KANK2 HUMAN	1133.549565	2	0.753781378		
SELCLDLPDPPEDPVALETR	103.7442	Q63ZY3	KANK2 HUMAN	756.0356517	3	0.753781378		
SQEVVETMCPVPAAATSNVHMVK	48.31253	Q63ZY3	KANK2 HUMAN	1243.098377	2	0.641391933		
SQEVVETMCPVPAAATSNVHMVK	48.31253	Q63ZY3	KANK2 HUMAN	829.068193	3	0.641391933		
TQDDIETVLQLFR	132.9225	Q63ZY3	KANK2 HUMAN	789.4126695	2	0.773729563		
TQDDIETVLQLFR	132.9225	Q63ZY3	KANK2 HUMAN	526.6110547	3	0.773729563		
VAVLETQLK	22.69067	Q63ZY3	KANK2 HUMAN	500.806049	2	0.811758518	2	Yes
VAVLETQLK	22.69067	Q63ZY3	KANK2 HUMAN	334.206641	3	0.811758518	2	
VAYTTVLQEWLR	91.69093	Q63ZY3	KANK2 HUMAN	739.904279	2	0.74334389		
VAYTTVLQEWLR	91.69093	Q63ZY3	KANK2 HUMAN	493.605461	3	0.74334389		
VPSVAEAPQLRPAGTAAAK	18.38851	Q63ZY3	KANK2 HUMAN	917.5132495	2	0.700179398		
VPSVAEAPQLRPAGTAAAK	18.38851	Q63ZY3	KANK2 HUMAN	612.0114413	3	0.700179398		
YLDNPNALTER	23.07136	Q63ZY3	KANK2 HUMAN	653.3258605	2	0.883873582	2	Yes
YLDNPNALTER	23.07136	Q63ZY3	KANK2 HUMAN	435.8865153	3	0.883873582	2	
AGEQLAPFLPQLVPR	100.9084	Q5VYK3	ECM29 HUMAN	818.4650395	2	0.863079548		
AGEQLAPFLPQLVPR	100.9084	Q5VYK3	ECM29 HUMAN	545.9793013	3	0.863079548		
AIQTLGYFPVGDGDFPHQK	64.55278	Q5VYK3	ECM29 HUMAN	1045.521268	2	0.815879703	3	
AIQTLGYFPVGDGDFPHQK	64.55278	Q5VYK3	ECM29 HUMAN	697.35012	3	0.815879703	3	Yes
ALSINTLVK	39.63074	Q5VYK3	ECM29 HUMAN	479.800766	2	0.773624957	2	Yes
ALSINTLVK	39.63074	Q5VYK3	ECM29 HUMAN	320.203119	3	0.773624957	2	
DAWQMTEEEYTPPAGAK	51.46868	Q5VYK3	ECM29 HUMAN	962.425644	2	0.848101556		
DAWQMTEEEYTPPAGAK	51.46868	Q5VYK3	ECM29 HUMAN	641.9530377	3	0.848101556		
DIALVQQLFEALCK	154.6438	Q5VYK3	ECM29 HUMAN	824.4429155	2	0.720703602		
DIALVQQLFEALCK	154.6438	Q5VYK3	ECM29 HUMAN	549.964552	3	0.720703602		
DNHSPEIQHGSLLALGFTVGR	63.14971	Q5VYK3	ECM29 HUMAN	1124.57764	2	0.707909226		
DNHSPEIQHGSLLALGFTVGR	63.14971	Q5VYK3	ECM29 HUMAN	750.0543683	3	0.707909226		
EGAVLKPELK	2.831413	Q5VYK3	ECM29 HUMAN	542.3246055	2	0.613013089	2	Yes
EGAVLKPELK	2.831413	Q5VYK3	ECM29 HUMAN	361.8856787	3	0.613013089	2	
ELCSLASDLSQPDLVYK	75.77087	Q5VYK3	ECM29 HUMAN	969.480418	2	0.792013407		
ELCSLASDLSQPDLVYK	75.77087	Q5VYK3	ECM29 HUMAN	646.6562203	3	0.792013407		
ESSCLALNDLLR	69.78352	Q5VYK3	ECM29 HUMAN	695.853932	2	0.746427536	2	Yes
ESSCLALNDLLR	69.78352	Q5VYK3	ECM29 HUMAN	464.238563	3	0.746427536	2	
ESTSEQMPSFPEMVYYIQEK	99.02823	Q5VYK3	ECM29 HUMAN	1212.04325	2	0.77696234		
ESTSEQMPSFPEMVYYIQEK	99.02823	Q5VYK3	ECM29 HUMAN	808.3647747	3	0.77696234		
EVLPLAFLGMHEIADEEK	98.60968	Q5VYK3	ECM29 HUMAN	1021.019715	2	0.813847303	3	
EVLPLAFLGMHEIADEEK	98.60968	Q5VYK3	ECM29 HUMAN	681.0157517	3	0.813847303	3	Yes
FASTVFPSDHIPSR	33.34622	Q5VYK3	ECM29 HUMAN	780.894446	2	0.830285549		
FASTVFPSDHIPSR	33.34622	Q5VYK3	ECM29 HUMAN	520.932239	3	0.830285549		
FLPPVLLK	66.97907	Q5VYK3	ECM29 HUMAN	463.807863	2	0.790745139	2	Yes
FLPPVLLK	66.97907	Q5VYK3	ECM29 HUMAN	309.5411837	3	0.790745139	2	
FQEFSNIVIPLIK	106.2305	Q5VYK3	ECM29 HUMAN	774.445783	2	0.820947051		
FQEFSNIVIPLIK	106.2305	Q5VYK3	ECM29 HUMAN	516.6331303	3	0.820947051		
GAAFGFNVIATR	60.05044	Q5VYK3	ECM29 HUMAN	612.330754	2	0.792945325	2	Yes
GAAFGFNVIATR	60.05044	Q5VYK3	ECM29 HUMAN	408.5564443	3	0.792945325	2	
GGCASVIVSLTTQCPQDLTPYSGK	80.14655	Q5VYK3	ECM29 HUMAN	1270.112535	2	0.766056597		
GGCASVIVSLTTQCPQDLTPYSGK	80.14655	Q5VYK3	ECM29 HUMAN	847.0776317	3	0.766056597		
GMMSTVTEVR	18.92233	Q5VYK3	ECM29 HUMAN	555.7682835	2	0.766326368	2	Yes
GMMSTVTEVR	18.92233	Q5VYK3	ECM29 HUMAN	370.8481307	3	0.766326368	2	
GRPLDDIIDK	14.97647	Q5VYK3	ECM29 HUMAN	571.314769	2	0.629258811		
GRPLDDIIDK	14.97647	Q5VYK3	ECM29 HUMAN	381.2124543	3	0.629258811		
HEVSGETVVFQGGALGK	27.90256	Q5VYK3	ECM29 HUMAN	857.9421255	2	0.835955739		
HEVSGETVVFQGGALGK	27.90256	Q5VYK3	ECM29 HUMAN	572.2973587	3	0.835955739		
HSVATAADLELK	11.17977	Q5VYK3	ECM29 HUMAN	627.8386085	2	0.763418019	2	Yes
HSVATAADLELK	11.17977	Q5VYK3	ECM29 HUMAN	418.895014	3	0.763418019	2	
IKPLGPMLLNGLTK	57.07216	Q5VYK3	ECM29 HUMAN	747.958004	2	0.781927705		
IKPLGPMLLNGLTK	57.07216	Q5VYK3	ECM29 HUMAN	498.974611	3	0.781927705		
IVAISCAADILK	64.79647	Q5VYK3	ECM29 HUMAN	637.363405	2	0.805437744	2	Yes
IVAISCAADILK	64.79647	Q5VYK3	ECM29 HUMAN	425.2448783	3	0.805437744	2	
LAIQEALSMMVGAYSTLEGAQR	147.6307	Q5VYK3	ECM29 HUMAN	1170.090874	2	0.681939125		
LAIQEALSMMVGAYSTLEGAQR	147.6307	Q5VYK3	ECM29 HUMAN	780.396524	3	0.681939125		
LGHAETDEQLQNIISK	28.58553	Q5VYK3	ECM29 HUMAN	898.4634215	2	0.76819557		
LGHAETDEQLQNIISK	28.58553	Q5VYK3	ECM29 HUMAN	599.311556	3	0.76819557		
LHLVESLLSR	42.48229	Q5VYK3	ECM29 HUMAN	583.8487785	2	0.74967587	2	Yes
LHLVESLLSR	42.48229	Q5VYK3	ECM29 HUMAN	389.5684607	3	0.74967587	2	
LLLQGLMDSVEAK	86.65588	Q5VYK3	ECM29 HUMAN	708.8925265	2	0.842615664	2	Yes
LLLQGLMDSVEAK	86.65588	Q5VYK3	ECM29 HUMAN	472.9309593	3	0.842615664	2	
LLSMAYSAVGK	36.43988	Q5VYK3	ECM29 HUMAN	570.3106365	2	0.74363482	2	Yes
LLSMAYSAVGK	36.43988	Q5VYK3	ECM29 HUMAN	380.5430327	3	0.74363482	2	
LMSALLSGLTDR	73.44689	Q5VYK3	ECM29 HUMAN	638.8506615	2	0.836227179	2	Yes

LMSALLSGLTDR	73.44689	Q5VYK3	ECM29 HUMAN	426.2363827	3	0.836227179	2	
LPEIWETLFR	118.6663	Q5VYK3	ECM29 HUMAN	652.356437	2	0.798201442		
LPEIWETLFR	118.6663	Q5VYK3	ECM29 HUMAN	435.240233	3	0.798201442		
LYLQELITITQK	89.9332	Q5VYK3	ECM29 HUMAN	731.9299625	2	0.745102882	2	Yes
LYLQELITITQK	89.9332	Q5VYK3	ECM29 HUMAN	488.28925	3	0.745102882	2	
NLTSNMWR	20.03204	Q5VYK3	ECM29 HUMAN	511.2483745	2	0.739372551		
NLTSNMWR	20.03204	Q5VYK3	ECM29 HUMAN	341.1681913	3	0.739372551		
QSLIDWNNPAIINK	75.08528	Q5VYK3	ECM29 HUMAN	813.4364775	2	0.72713542		
QSLIDWNNPAIINK	75.08528	Q5VYK3	ECM29 HUMAN	542.6269267	3	0.72713542		
SAGAMLKPHAPK	-25.92698	Q5VYK3	ECM29 HUMAN	604.334982	2	0.799951673		
SAGAMLKPHAPK	-25.92698	Q5VYK3	ECM29 HUMAN	403.2259297	3	0.799951673		
SASPFNLAEKPK	13.16043	Q5VYK3	ECM29 HUMAN	644.8489755	2	0.802131414		
SASPFNLAEKPK	13.16043	Q5VYK3	ECM29 HUMAN	430.2352587	3	0.802131414		
SMIEQLIK	47.64589	Q5VYK3	ECM29 HUMAN	481.273527	2	0.741174042		
SMIEQLIK	47.64589	Q5VYK3	ECM29 HUMAN	321.1849597	3	0.741174042		
SVPNQPSTNEILQAVLK	78.63927	Q5VYK3	ECM29 HUMAN	919.505089	2	0.87227422	2	Yes
SVPNQPSTNEILQAVLK	78.63927	Q5VYK3	ECM29 HUMAN	613.3393343	3	0.87227422	2	
TEALSVIELLLK	131.9681	Q5VYK3	ECM29 HUMAN	664.9059595	2	0.787714064	2	Yes
TEALSVIELLLK	131.9681	Q5VYK3	ECM29 HUMAN	443.6065813	3	0.787714064	2	
TLMEALVASYLIKPEVQVR	112.2685	Q5VYK3	ECM29 HUMAN	1080.609028	2	0.661939979	3	
TLMEALVASYLIKPEVQVR	112.2685	Q5VYK3	ECM29 HUMAN	720.74196	3	0.661939979	3	Yes
TLMSSGQMAPSSSNK	1.382835	Q5VYK3	ECM29 HUMAN	763.353439	2	0.786153913	2	Yes
TLMSSGQMAPSSSNK	1.382835	Q5VYK3	ECM29 HUMAN	509.2382343	3	0.786153913	2	
TPDGQGLSTYK	-1.057686	Q5VYK3	ECM29 HUMAN	583.7885805	2	0.796970129	2	Yes
TPDGQGLSTYK	-1.057686	Q5VYK3	ECM29 HUMAN	389.5283287	3	0.796970129	2	
TSCALTIHAIGR	13.90913	Q5VYK3	ECM29 HUMAN	650.3460785	2	0.650265038		
TSCALTIHAIGR	13.90913	Q5VYK3	ECM29 HUMAN	433.899994	3	0.650265038		
VIGDNPWTPEQLEQCK	56.28213	Q5VYK3	ECM29 HUMAN	957.4572825	2	0.906639695		
VIGDNPWTPEQLEQCK	56.28213	Q5VYK3	ECM29 HUMAN	638.6407967	3	0.906639695		
VLLIESLATMEPDSRPELQEK	80.73492	Q5VYK3	ECM29 HUMAN	1199.630889	2	0.641805708	3	
VLLIESLATMEPDSRPELQEK	80.73492	Q5VYK3	ECM29 HUMAN	800.0898677	3	0.641805708	3	Yes
VMELLVHLNK	40.7221	Q5VYK3	ECM29 HUMAN	598.3475575	2	0.783948004		
VMELLVHLNK	40.7221	Q5VYK3	ECM29 HUMAN	399.2343133	3	0.783948004		
VYLGDIPLK	50.79038	Q5VYK3	ECM29 HUMAN	509.3031375	2	0.839886546		
VYLGDIPLK	50.79038	Q5VYK3	ECM29 HUMAN	339.8713667	3	0.839886546		
YLLLLAAGDPR	69.11876	Q5VYK3	ECM29 HUMAN	601.3511505	2	0.818747997	2	Yes
YLLLLAAGDPR	69.11876	Q5VYK3	ECM29 HUMAN	401.2367087	3	0.818747997	2	
YQFDPNLGIR	51.32363	Q5VYK3	ECM29 HUMAN	611.814932	2	0.744928777	2	Yes
YQFDPNLGIR	51.32363	Q5VYK3	ECM29 HUMAN	408.212563	3	0.744928777	2	
AGITAAINLAK	38.70748	Q5VW38	GP107 HUMAN	521.816948	2	0.752010524		
AGITAAINLAK	38.70748	Q5VW38	GP107 HUMAN	348.213907	3	0.752010524		
GDSMGPLQQR	-0.553883	Q5VW38	GP107 HUMAN	544.761847	2	0.830809832		
GDSMGPLQQR	-0.553883	Q5VW38	GP107 HUMAN	363.5105063	3	0.830809832		
IESHHFAQADLELLASSCPPASVSQR	52.40468	Q5VW38	GP107 HUMAN	1425.696146	2	0.759942472		
IESHHFAQADLELLASSCPPASVSQR	52.40468	Q5VW38	GP107 HUMAN	950.800039	3	0.759942472		
IHWLMAALPFTK	80.26479	Q5VW38	GP107 HUMAN	714.3975825	2	0.73997122		
IHWLMAALPFTK	80.26479	Q5VW38	GP107 HUMAN	476.6009967	3	0.73997122		
NPDSYLSAGEIPLPK	68.27394	Q5VW38	GP107 HUMAN	800.9150395	2	0.777265191		
NPDSYLSAGEIPLPK	68.27394	Q5VW38	GP107 HUMAN	534.2793013	3	0.777265191		
VLGQSQEPNVNPASAGNQTQK	8.911736	Q5VW38	GP107 HUMAN	1084.041088	2	0.802803516		
VLGQSQEPNVNPASAGNQTQK	8.911736	Q5VW38	GP107 HUMAN	723.03	3	0.802803516		
ALCELESGIPAAESQIVYAERPLTDNHR	64.15602	Q5TDH0	DDI2 HUMAN	1570.278028	2	0.656754315	4	
ALCELESGIPAAESQIVYAERPLTDNHR	64.15602	Q5TDH0	DDI2 HUMAN	1047.18796	3	0.656754315	4	
DGDVVILR	34.1712	Q5TDH0	DDI2 HUMAN	443.753816	2	0.707598269		
DGDVVILR	34.1712	Q5TDH0	DDI2 HUMAN	296.171819	3	0.707598269		
DMLLANPHELSLLK	73.21266	Q5TDH0	DDI2 HUMAN	797.437632	2	0.784905732	3	
DMLLANPHELSLLK	73.21266	Q5TDH0	DDI2 HUMAN	531.9610297	3	0.784905732	3	Yes
EDVRPEEIADQELAEALQK	67.9785	Q5TDH0	DDI2 HUMAN	1092.045504	2	0.787301719		
EDVRPEEIADQELAEALQK	67.9785	Q5TDH0	DDI2 HUMAN	728.3662777	3	0.787301719		
IDFSSIAVPGTSSPR	66.69134	Q5TDH0	DDI2 HUMAN	767.399561	2	0.826894701		
IDFSSIAVPGTSSPR	66.69134	Q5TDH0	DDI2 HUMAN	511.935649	3	0.826894701		
LFSADPFDLEAQAK	78.98682	Q5TDH0	DDI2 HUMAN	776.3886625	2	0.84390831	2	Yes
LFSADPFDLEAQAK	78.98682	Q5TDH0	DDI2 HUMAN	517.9283833	3	0.84390831	2	
NPPLAEALLSGDLEK	80.52606	Q5TDH0	DDI2 HUMAN	783.922869	2	0.85489732		
NPPLAEALLSGDLEK	80.52606	Q5TDH0	DDI2 HUMAN	522.9511877	3	0.85489732		
NVLVIGTTGSQTTFLPEGELPECAR	93.4012	Q5TDH0	DDI2 HUMAN	1345.179268	2	0.836626589		
NVLVIGTTGSQTTFLPEGELPECAR	93.4012	Q5TDH0	DDI2 HUMAN	897.12212	3	0.836626589		
VLVEQQQDR	-23.15276	Q5TDH0	DDI2 HUMAN	557.796744	2	0.753727973	2	Yes
VLVEQQQDR	-23.15276	Q5TDH0	DDI2 HUMAN	372.2004377	3	0.753727973	2	
DADKDWPR	-17.51726	Q5T200	ZC3HD HUMAN	501.7361545	2	0.603884518		
DADKDWPR	-17.51726	Q5T200	ZC3HD HUMAN	334.8267113	3	0.603884518		
DADNLFEHELGALNMAALLR	136.1316	Q5T200	ZC3HD HUMAN	1107.055152	2	0.670771718		
DADNLFEHELGALNMAALLR	136.1316	Q5T200	ZC3HD HUMAN	738.3727093	3	0.670771718		
EQTEILESSR	5.452187	Q5T200	ZC3HD HUMAN	596.296773	2	0.635690808		
EQTEILESSR	5.452187	Q5T200	ZC3HD HUMAN	397.8671237	3	0.635690808		
FTPGAVMLR	38.59612	Q5T200	ZC3HD HUMAN	496.273862	2	0.789401412	2	Yes
FTPGAVMLR	38.59612	Q5T200	ZC3HD HUMAN	331.185183	3	0.789401412	2	
GDSDISDEEAAQQSK	-10.12006	Q5T200	ZC3HD HUMAN	790.3400945	2	0.748834908		
GDSDISDEEAAQQSK	-10.12006	Q5T200	ZC3HD HUMAN	527.229338	3	0.748834908		
GNIETTSEDGQVFSPK	27.17664	Q5T200	ZC3HD HUMAN	854.905405	2	0.831392527		
GNIETTSEDGQVFSPK	27.17664	Q5T200	ZC3HD HUMAN	570.2728783	3	0.831392527		
LAGSELFAK	24.58933	Q5T200	ZC3HD HUMAN	468.2640165	2	0.757717907	2	Yes
LAGSELFAK	24.58933	Q5T200	ZC3HD HUMAN	312.5119527	3	0.757717907	2	
LDDAHSLGSGAGEGYEPISDDELDEILAGDAEK	94.67699	Q5T200	ZC3HD HUMAN	1694.763521	2	0.823108315		
LDDAHSLGSGAGEGYEPISDDELDEILAGDAEK	94.67699	Q5T200	ZC3HD HUMAN	1130.178289	3	0.823108315		
LGPSTGSTAETQCR	-24.81062	Q5T200	ZC3HD HUMAN	732.8415545	2	0.748722434	2	Yes
LGPSTGSTAETQCR	-24.81062	Q5T200	ZC3HD HUMAN	488.896978	3	0.748722434	2	
MDKDLGSVQGFEETNK	21.68442	Q5T200	ZC3HD HUMAN	899.420365	2	0.837331414		
MDKDLGSVQGFEETNK	21.68442	Q5T200	ZC3HD HUMAN	599.9495183	3	0.837331414		
QAYTSAPMVDNELLR	52.92793	Q5T200	ZC3HD HUMAN	854.4227065	2	0.715885162		
QAYTSAPMVDNELLR	52.92793	Q5T200	ZC3HD HUMAN	569.9510793	3	0.715885162		
TFESSQIESVK	14.0351	Q5T200	ZC3HD HUMAN	627.8147985	2	0.760791242		
TFESSQIESVK	14.0351	Q5T200	ZC3HD HUMAN	418.8791407	3	0.760791242		
TGNCLYGNTCR	-16.42426	Q5T200	ZC3HD HUMAN	658.280074	2	0.770499647		
TGNCLYGNTCR	-16.42426	Q5T200	ZC3HD HUMAN	439.1893243	3	0.770499647		
TPSPPPPIPEDIALGK	65.80408	Q5T200	ZC3HD HUMAN	814.9488875	2	0.763184786		
TPSPPPPIPEDIALGK	65.80408	Q5T200	ZC3HD HUMAN	543.6352	3	0.763184786		
TSAVSSPLLDQQR	20.81862	Q5T200	ZC3HD HUMAN	701.370804	2	0.715346992		
TSAVSSPLLDQQR	20.81862	Q5T200	ZC3HD HUMAN	467.9164777	3	0.715346992		
VETPHVTIEDAQHR	-7.67432	Q5T200	ZC3HD HUMAN	816.4109925	2	0.614437938		
VETPHVTIEDAQHR	-7.67432	Q5T200	ZC3HD HUMAN	544.6099367	3	0.614437938		

ANALYTHSR	-28.00456	Q5T0W9	FA83B HUMAN	516.765242	2	0.639344215		
ANALYTHSR	-28.00456	Q5T0W9	FA83B HUMAN	344.846103	3	0.639344215		
EDVTVSPSQEINAPPDENK	31.75131	Q5T0W9	FA83B HUMAN	1034.987654	2	0.72400856		
EDVTVSPSQEINAPPDENK	31.75131	Q5T0W9	FA83B HUMAN	690.327711	3	0.72400856		
EVPDTPTNVQHLTDKPLPESIPK	40.53567	Q5T0W9	FA83B HUMAN	1278.171768	2	0.808729708		
EVPDTPTNVQHLTDKPLPESIPK	40.53567	Q5T0W9	FA83B HUMAN	852.4504533	3	0.808729708		
FEGYDNPENLK	22.26382	Q5T0W9	FA83B HUMAN	663.3045935	2	0.773501575		
FEGYDNPENLK	22.26382	Q5T0W9	FA83B HUMAN	442.539004	3	0.773501575		
FNTEQIQYR	13.33348	Q5T0W9	FA83B HUMAN	599.7967395	2	0.691889107		
FNTEQIQYR	13.33348	Q5T0W9	FA83B HUMAN	400.2004347	3	0.691889107		
GIYTLNEHDK	-11.89629	Q5T0W9	FA83B HUMAN	595.296572	2	0.811978221		
GIYTLNEHDK	-11.89629	Q5T0W9	FA83B HUMAN	397.200323	3	0.811978221		
HYVYSTLTR	-6.160252	Q5T0W9	FA83B HUMAN	570.296371	2	0.826053023		
HYVYSTLTR	-6.160252	Q5T0W9	FA83B HUMAN	380.5335223	3	0.826053023		
KPSDSLSVASSSR	-26.05404	Q5T0W9	FA83B HUMAN	660.841878	2	0.628631651		
KPSDSLSVASSSR	-26.05404	Q5T0W9	FA83B HUMAN	440.8971937	3	0.628631651		
LPLQSEAPK	0.73484	Q5T0W9	FA83B HUMAN	491.7825735	2	0.77789849		
LPLQSEAPK	0.73484	Q5T0W9	FA83B HUMAN	328.1909907	3	0.77789849		
METSSMLSSLNDECK	44.34813	Q5T0W9	FA83B HUMAN	866.365886	2	0.642805636		
METSSMLSSLNDECK	44.34813	Q5T0W9	FA83B HUMAN	577.913199	3	0.642805636		
SATMGNSYGR	-26.09496	Q5T0W9	FA83B HUMAN	522.2329175	2	0.687507272		
SATMGNSYGR	-26.09496	Q5T0W9	FA83B HUMAN	348.49122	3	0.687507272		
SCVPSSFAQEESAR	15.70406	Q5T0W9	FA83B HUMAN	777.8468355	2	0.811326325		
SCVPSSFAQEESAR	15.70406	Q5T0W9	FA83B HUMAN	518.9004987	3	0.811326325		
SFPLFDNSK	48.92894	Q5T0W9	FA83B HUMAN	527.76438	2	0.803110421		
SFPLFDNSK	48.92894	Q5T0W9	FA83B HUMAN	352.1788617	3	0.803110421		
SLLSLTPDK	38.96159	Q5T0W9	FA83B HUMAN	487.282406	2	0.773290038		
SLLSLTPDK	38.96159	Q5T0W9	FA83B HUMAN	325.190879	3	0.773290038		
SMHNVTHNLEEDEEEVTK	-5.60619	Q5T0W9	FA83B HUMAN	1070.976764	2	0.683058977		
SMHNVTHNLEEDEEEVTK	-5.60619	Q5T0W9	FA83B HUMAN	714.3204507	3	0.683058977		
SSLVFKPTLPEQK	33.0471	Q5T0W9	FA83B HUMAN	737.4197655	2	0.783388555		
SSLVFKPTLPEQK	33.0471	Q5T0W9	FA83B HUMAN	491.9491187	3	0.783388555		
SSPLLNYNTGVYR	41.80637	Q5T0W9	FA83B HUMAN	742.3811625	2	0.795476437		
SSPLLNYNTGVYR	41.80637	Q5T0W9	FA83B HUMAN	495.2567167	3	0.795476437		
SVSIAALLDVNK	78.38666	Q5T0W9	FA83B HUMAN	615.359176	2	0.777422309		
SVSIAALLDVNK	78.38666	Q5T0W9	FA83B HUMAN	410.5753923	3	0.777422309		
VSDFLAEEEINYILK	104.4069	Q5T0W9	FA83B HUMAN	891.9621865	2	0.866061628		
VSDFLAEEEINYILK	104.4069	Q5T0W9	FA83B HUMAN	594.9773993	3	0.866061628		
DVYAQFLEMTDDK	90.8669	Q5MNZ6	WIPI3 HUMAN	787.856334	2	0.718175292		
DVYAQFLEMTDDK	90.8669	Q5MNZ6	WIPI3 HUMAN	525.5734977	3	0.718175292		
IFDTSSGHLIQELR	46.24984	Q5MNZ6	WIPI3 HUMAN	808.4261105	2	0.835180402	3	
IFDTSSGHLIQELR	46.24984	Q5MNZ6	WIPI3 HUMAN	539.286682	3	0.835180402	3	Yes
IVVVLDSMIK	71.62921	Q5MNZ6	WIPI3 HUMAN	558.839034	2	0.764990091		
IVVVLDSMIK	71.62921	Q5MNZ6	WIPI3 HUMAN	372.8952977	3	0.764990091		
TVIEIEFSTEVK	66.12959	Q5MNZ6	WIPI3 HUMAN	697.8748565	2	0.700468421		
TVIEIEFSTEVK	66.12959	Q5MNZ6	WIPI3 HUMAN	465.585846	3	0.700468421		
VFTFTHNPHQLHVFETCYNPK	36.96953	Q5MNZ6	WIPI3 HUMAN	1308.626804	2	0.656985581		
VFTFTHNPHQLHVFETCYNPK	36.96953	Q5MNZ6	WIPI3 HUMAN	872.7538107	3	0.656985581		
VMIWDDLK	58.94966	Q5MNZ6	WIPI3 HUMAN	510.265702	2	0.68715179	2	Yes
VMIWDDLK	58.94966	Q5MNZ6	WIPI3 HUMAN	340.5130763	3	0.68715179	2	
AFMDIMSAQASSGSTSVLR	73.26109	Q5JTH9	RRP12 HUMAN	979.969699	2	0.832761288		
AFMDIMSAQASSGSTSVLR	73.26109	Q5JTH9	RRP12 HUMAN	653.6490743	3	0.832761288		
AMDLAQAGSTVESK	13.77399	Q5JTH9	RRP12 HUMAN	704.343398	2	0.851195931	2	Yes
AMDLAQAGSTVESK	13.77399	Q5JTH9	RRP12 HUMAN	469.898207	3	0.851195931	2	
DLLPCFPEGLVK	100.183	Q5JTH9	RRP12 HUMAN	694.3686875	2	0.719587386		
DLLPCFPEGLVK	100.183	Q5JTH9	RRP12 HUMAN	463.2484	3	0.719587386		
EATTTLHMLTLLK	63.73251	Q5JTH9	RRP12 HUMAN	736.413627	2	0.757931709		
EATTTLHMLTLLK	63.73251	Q5JTH9	RRP12 HUMAN	491.2783597	3	0.757931709		
EGGGDEPLNFLDPK	65.22264	Q5JTH9	RRP12 HUMAN	744.3548195	2	0.763391852	2	Yes
EGGGDEPLNFLDPK	65.22264	Q5JTH9	RRP12 HUMAN	496.572488	3	0.763391852	2	
FCIQEIEK	32.71946	Q5JTH9	RRP12 HUMAN	533.7660665	2	0.737617075	2	Yes
FCIQEIEK	32.71946	Q5JTH9	RRP12 HUMAN	356.179986	3	0.737617075	2	
GEDEEMADPMEDVIIR	70.58058	Q5JTH9	RRP12 HUMAN	924.9036905	2	0.834423423		
GEDEEMADPMEDVIIR	70.58058	Q5JTH9	RRP12 HUMAN	616.938402	3	0.834423423		
GSEFMFEK	24.04813	Q5JTH9	RRP12 HUMAN	487.7185845	2	0.781080902	2	Yes
GSEFMFEK	24.04813	Q5JTH9	RRP12 HUMAN	325.4816647	3	0.781080902	2	
LGFFTTYFLPLANTLK	147.6335	Q5JTH9	RRP12 HUMAN	923.511651	2	0.649361134		
LGFFTTYFLPLANTLK	147.6335	Q5JTH9	RRP12 HUMAN	616.0103757	3	0.649361134		
LHNELQSGSLR	-21.29086	Q5JTH9	RRP12 HUMAN	627.334024	2	0.812609196		
LHNELQSGSLR	-21.29086	Q5JTH9	RRP12 HUMAN	418.5586243	3	0.812609196		
LLPEEYHR	-7.969238	Q5JTH9	RRP12 HUMAN	528.777818	2	0.826812029		
LLPEEYHR	-7.969238	Q5JTH9	RRP12 HUMAN	352.854487	3	0.826812029		
LQWDLGLGHLPR	64.70151	Q5JTH9	RRP12 HUMAN	702.89151	2	0.80700767	3	
LQWDLGLGHLPR	64.70151	Q5JTH9	RRP12 HUMAN	468.9302817	3	0.80700767	3	Yes
LYSTIRPYLESK	26.40164	Q5JTH9	RRP12 HUMAN	735.4041065	2	0.77572453	3	
LYSTIRPYLESK	26.40164	Q5JTH9	RRP12 HUMAN	490.605346	3	0.77572453	3	Yes
SEAPETPMEEEAELVLTEK	77.76144	Q5JTH9	RRP12 HUMAN	1066.501749	2	0.80790931	3	
SEAPETPMEEEAELVLTEK	77.76144	Q5JTH9	RRP12 HUMAN	711.3371077	3	0.80790931	3	Yes
SSGTFLSGLSDCTNVTFSK	69.78755	Q5JTH9	RRP12 HUMAN	1004.470586	2	0.728210449		
SSGTFLSGLSDCTNVTFSK	69.78755	Q5JTH9	RRP12 HUMAN	669.982999	3	0.728210449		
SWLLPVIR	79.58034	Q5JTH9	RRP12 HUMAN	492.3060185	2	0.759881914	2	Yes
SWLLPVIR	79.58034	Q5JTH9	RRP12 HUMAN	328.539954	3	0.759881914	2	
TLGMAISERPDLR	30.38447	Q5JTH9	RRP12 HUMAN	729.8908495	2	0.801873088		
TLGMAISERPDLR	30.38447	Q5JTH9	RRP12 HUMAN	486.9298413	3	0.801873088		
TYLTITDTQLVNSLLEK	108.3965	Q5JTH9	RRP12 HUMAN	976.5335085	2	0.738680243	3	
TYLTITDTQLVNSLLEK	108.3965	Q5JTH9	RRP12 HUMAN	651.3582807	3	0.738680243	3	Yes
VAVTVMDVAHLAK	46.11449	Q5JTH9	RRP12 HUMAN	677.3821295	2	0.796454549	3	
VAVTVMDVAHLAK	46.11449	Q5JTH9	RRP12 HUMAN	451.924028	3	0.796454549	3	Yes
VLDPASSDFTR	28.06014	Q5JTH9	RRP12 HUMAN	604.3018585	2	0.739353359	2	Yes
VLDPASSDFTR	28.06014	Q5JTH9	RRP12 HUMAN	403.2038473	3	0.739353359	2	
VLEEVCASPQGPGALFVQSHLEDLK	77.50562	Q5JTH9	RRP12 HUMAN	1362.189635	2	0.713438392	3	
VLEEVCASPQGPGALFVQSHLEDLK	77.50562	Q5JTH9	RRP12 HUMAN	908.4623647	3	0.713438392	3	Yes
VTVCQALR	2.580936	Q5JTH9	RRP12 HUMAN	473.761119	2	0.699268043		
VTVCQALR	2.580936	Q5JTH9	RRP12 HUMAN	316.1766877	3	0.699268043		
DPLLQEPPAWFK	86.56024	Q5BJF2	SGMR2 HUMAN	720.8802765	2	0.708088636		
DPLLQEPPAWFK	86.56024	Q5BJF2	SGMR2 HUMAN	480.9227927	3	0.708088636		
ELYPVEFR	45.703	Q5BJF2	SGMR2 HUMAN	526.774744	2	0.762693822	2	Yes
ELYPVEFR	45.703	Q5BJF2	SGMR2 HUMAN	351.5191043	3	0.762693822	2	
GQRPETLHER	-50	Q5BJF2	SGMR2 HUMAN	611.818542	2	0.653540015	2	Yes
GQRPETLHER	-50	Q5BJF2	SGMR2 HUMAN	408.2149697	3	0.653540015	2	
FHFFEDQLR	39.09705	Q56VL3	OCAD2 HUMAN	619.801829	2	0.79767096		

FHFFEDQLR	39.09705	Q56VL3	OCAD2 HUMAN	413.537161	3	0.79767096		
GAGFGPQHNR	-31.82833	Q56VL3	OCAD2 HUMAN	520.7552135	2	0.626876414		
GAGFGPQHNR	-31.82833	Q56VL3	OCAD2 HUMAN	347.506084	3	0.626876414		
GNQDKDAHFPPPSK	-22.22969	Q56VL3	OCAD2 HUMAN	769.373878	2	0.732242942		
GNQDKDAHFPPPSK	-22.22969	Q56VL3	OCAD2 HUMAN	513.2518603	3	0.732242942		
VALAGLLGFGLGK	105.8722	Q56VL3	OCAD2 HUMAN	608.377173	2	0.828963995		
VALAGLLGFGLGK	105.8722	Q56VL3	OCAD2 HUMAN	405.9207237	3	0.828963995		
VSYIGVCQSK	8.583755	Q56VL3	OCAD2 HUMAN	570.7900685	2	0.783586383		
VSYIGVCQSK	8.583755	Q56VL3	OCAD2 HUMAN	380.862654	3	0.783586383		
AAAHQLCLK	-25.89372	Q53GG5	PDLI3 HUMAN	506.2743935	2	0.602444291		
AAAHQLCLK	-25.89372	Q53GG5	PDLI3 HUMAN	337.852204	3	0.602444291		
GETHLWSPQVSEDGK	17.67634	Q53GG5	PDLI3 HUMAN	835.3950075	2	0.814777374		
GETHLWSPQVSEDGK	17.67634	Q53GG5	PDLI3 HUMAN	557.2659467	3	0.814777374		
INLESEPQDGNYFEHK	29.92931	Q53GG5	PDLI3 HUMAN	960.442681	2	0.884471893		
INLESEPQDGNYFEHK	29.92931	Q53GG5	PDLI3 HUMAN	640.6310623	3	0.884471893		
LAPNIPLEMELPGVK	99.23608	Q53GG5	PDLI3 HUMAN	810.955658	2	0.863652766		
LAPNIPLEMELPGVK	99.23608	Q53GG5	PDLI3 HUMAN	540.973047	3	0.863652766		
LSGGIDFNQPLVITR	74.95742	Q53GG5	PDLI3 HUMAN	815.4521285	2	0.842930675		
LSGGIDFNQPLVITR	74.95742	Q53GG5	PDLI3 HUMAN	543.970694	3	0.842930675		
SSGCSTPSGIDCGSGR	-22.35227	Q53GG5	PDLI3 HUMAN	792.823035	2	0.750734627		
SSGCSTPSGIDCGSGR	-22.35227	Q53GG5	PDLI3 HUMAN	528.8846317	3	0.750734627		
STPSSVSTVSTICPGDLK	38.76468	Q53GG5	PDLI3 HUMAN	918.4569475	2	0.797655344		
STPSSVSTVSTICPGDLK	38.76468	Q53GG5	PDLI3 HUMAN	612.6405733	3	0.797655344		
TKPPEGYDTVTLYPK	27.30243	Q53GG5	PDLI3 HUMAN	854.943794	2	0.86456418		
TKPPEGYDTVTLYPK	27.30243	Q53GG5	PDLI3 HUMAN	570.298471	3	0.86456418		
LAQNPLFWK	56.52843	Q53FT3	HIKES HUMAN	558.814208	2	0.816554487		
LAQNPLFWK	56.52843	Q53FT3	HIKES HUMAN	372.878747	3	0.816554487		
LVQTAAQQVAEDK	4.666531	Q53FT3	HIKES HUMAN	700.8731805	2	0.782256484	2	Yes
LVQTAAQQVAEDK	4.666531	Q53FT3	HIKES HUMAN	467.5847287	3	0.782256484	2	
SGEGSQHPFGAMNIVR	25.88277	Q53FT3	HIKES HUMAN	843.9050195	2	0.729488194	3	
SGEGSQHPFGAMNIVR	25.88277	Q53FT3	HIKES HUMAN	562.939288	3	0.729488194	3	Yes
WYENFQR	16.11954	Q53FT3	HIKES HUMAN	521.7412355	2	0.795868337		
WYENFQR	16.11954	Q53FT3	HIKES HUMAN	348.163432	3	0.795868337		
EVAKPSPGEGEVLLK	12.87732	Q53FA7	QORX HUMAN	776.933237	2	0.731646299		
EVAKPSPGEGEVLLK	12.87732	Q53FA7	QORX HUMAN	518.291433	3	0.731646299		
GAGVNLILDCIGGSYWEK	109.84	Q53FA7	QORX HUMAN	976.4832955	2	0.740558743		
GAGVNLILDCIGGSYWEK	109.84	Q53FA7	QORX HUMAN	651.3248053	3	0.740558743		
GSLITSLLR	65.09616	Q53FA7	QORX HUMAN	480.2983905	2	0.661883473		
GSLITSLLR	65.09616	Q53FA7	QORX HUMAN	320.5348687	3	0.661883473		
IVLELPQ	80.93716	Q53FA7	QORX HUMAN	406.250378	2	0.649914563		
IVLELPQ	80.93716	Q53FA7	QORX HUMAN	271.169527	3	0.649914563		
IYPVTEIQEAHK	11.72234	Q53FA7	QORX HUMAN	714.3806365	2	0.858002543		
IYPVTEIQEAHK	11.72234	Q53FA7	QORX HUMAN	476.5896993	3	0.858002543		
LGAAAGFNYK	14.70224	Q53FA7	QORX HUMAN	506.2670865	2	0.821711481		
LGAAAGFNYK	14.70224	Q53FA7	QORX HUMAN	337.8473327	3	0.821711481		
LLPVLDR	36.53947	Q53FA7	QORX HUMAN	413.2638195	2	0.665796936		
LLPVLDR	36.53947	Q53FA7	QORX HUMAN	275.8451547	3	0.665796936		
MAGAIPLVTAGSQK	42.01813	Q53FA7	QORX HUMAN	672.371762	2	0.736797214		
MAGAIPLVTAGSQK	42.01813	Q53FA7	QORX HUMAN	448.583783	3	0.736797214		
MLAVHFDKPGGPENLYVK	41.754	Q53FA7	QORX HUMAN	1008.025131	2	0.63770628	3	
MLAVHFDKPGGPENLYVK	41.754	Q53FA7	QORX HUMAN	672.3526953	3	0.63770628	3	Yes
MLLSQNESQK	-8.853088	Q32NC0	CR021 HUMAN	589.2982615	2	0.798117399		
MLLSQNESQK	-8.853088	Q32NC0	CR021 HUMAN	393.2014493	3	0.798117399		
NFLSSLK	31.23122	Q32NC0	CR021 HUMAN	404.7323515	2	0.767701507		
NFLSSLK	31.23122	Q32NC0	CR021 HUMAN	270.1575093	3	0.767701507		
SVLLITCK	27.02077	Q32NC0	CR021 HUMAN	467.27607	2	0.739554048		
SVLLITCK	27.02077	Q32NC0	CR021 HUMAN	311.8533217	3	0.739554048		
YLLWAYTSSHDDK	39.60778	Q32NC0	CR021 HUMAN	799.878453	2	0.642178893		
YLLWAYTSSHDDK	39.60778	Q32NC0	CR021 HUMAN	533.5882437	3	0.642178893		
DSKPHFTTVFQNSVYK	28.82386	Q2PZI1	D19L1 HUMAN	949.4763275	2	0.852514088		
DSKPHFTTVFQNSVYK	28.82386	Q2PZI1	D19L1 HUMAN	633.32016	3	0.852514088		
FNLYPEVILASWYR	133.9796	Q2PZI1	D19L1 HUMAN	885.9648625	2	0.721631825		
FNLYPEVILASWYR	133.9796	Q2PZI1	D19L1 HUMAN	590.9791833	3	0.721631825		
IFGIADDAHIGNLLTSK	75.3593	Q2PZI1	D19L1 HUMAN	892.98125	2	0.892944515		
IFGIADDAHIGNLLTSK	75.3593	Q2PZI1	D19L1 HUMAN	595.656775	3	0.892944515		
IISDMWGVLAK	76.92237	Q2PZI1	D19L1 HUMAN	616.8395655	2	0.683589935		
IISDMWGVLAK	76.92237	Q2PZI1	D19L1 HUMAN	411.5623187	3	0.683589935		
IMDLIGIQTK	58.8506	Q2PZI1	D19L1 HUMAN	566.3262915	2	0.849098086		
IMDLIGIQTK	58.8506	Q2PZI1	D19L1 HUMAN	377.8868027	3	0.849098086		
LSALRPIVNHPHYEDAGLR	19.39335	Q2PZI1	D19L1 HUMAN	1079.579981	2	0.695065677	4	
LSALRPIVNHPHYEDAGLR	19.39335	Q2PZI1	D19L1 HUMAN	720.055929	3	0.695065677	4	
LTEYPLVINTLK	70.99368	Q2PZI1	D19L1 HUMAN	702.411405	2	0.845781386	2	Yes
LTEYPLVINTLK	70.99368	Q2PZI1	D19L1 HUMAN	468.6102117	3	0.845781386	2	
SKPGCSMPEIWDVEDPANAGK	52.43292	Q2PZI1	D19L1 HUMAN	1144.520285	2	0.731907904		
SKPGCSMPEIWDVEDPANAGK	52.43292	Q2PZI1	D19L1 HUMAN	763.3494647	3	0.731907904		
TPLCNLLVK	50.02737	Q2PZI1	D19L1 HUMAN	529.3079015	2	0.742116094	2	Yes
TPLCNLLVK	50.02737	Q2PZI1	D19L1 HUMAN	353.207876	3	0.742116094	2	
YSTKPDAVFAGAMPTMASVK	55.45182	Q2PZI1	D19L1 HUMAN	1036.513739	2	0.778729439		
YSTKPDAVFAGAMPTMASVK	55.45182	Q2PZI1	D19L1 HUMAN	691.345101	3	0.778729439		
ENTFECQNPR	-18.00586	Q17RY6	LY6K HUMAN	647.778225	2	0.650916755	2	Yes
ENTFECQNPR	-18.00586	Q17RY6	LY6K HUMAN	432.1880917	3	0.650916755	2	
VWTDANLTAR	25.93064	Q17RY6	LY6K HUMAN	573.7992865	2	0.652697027		
VWTDANLTAR	25.93064	Q17RY6	LY6K HUMAN	382.8687993	3	0.652697027		
YCNLEGPPINSSVFK	54.18008	Q17RY6	LY6K HUMAN	862.919799	2	0.755165696		
YCNLEGPPINSSVFK	54.18008	Q17RY6	LY6K HUMAN	575.6158077	3	0.755165696		
ADNIFPFPDRPR	46.10257	Q17RY0	CPEB4 HUMAN	722.870774	2	0.834662497	3	
ADNIFPFPDRPR	46.10257	Q17RY0	CPEB4 HUMAN	482.249791	3	0.834662497	3	Yes
FGPLIVDWPHK	62.79926	Q17RY0	CPEB4 HUMAN	654.859147	2	0.821147799		
FGPLIVDWPHK	62.79926	Q17RY0	CPEB4 HUMAN	436.9087063	3	0.821147799		
FVQLQHGEIDK	1.997463	Q17RY0	CPEB4 HUMAN	657.346601	2	0.825292289		
FVQLQHGEIDK	1.997463	Q17RY0	CPEB4 HUMAN	438.567009	3	0.825292289		
GLNGGITPLNSISPLK	70.41323	Q17RY0	CPEB4 HUMAN	790.9545035	2	0.678422511		
GLNGGITPLNSISPLK	70.41323	Q17RY0	CPEB4 HUMAN	527.638944	3	0.678422511		
GQSSLFPMEDGFLDDGR	85.55568	Q17RY0	CPEB4 HUMAN	935.9179895	2	0.732998848		
GQSSLFPMEDGFLDDGR	85.55568	Q17RY0	CPEB4 HUMAN	624.281268	3	0.732998848		
LNYSYPGSDSSLLINAR	62.46357	Q17RY0	CPEB4 HUMAN	935.4712365	2	0.76375258		
LNYSYPGSDSSLLINAR	62.46357	Q17RY0	CPEB4 HUMAN	623.9834327	3	0.76375258		
NFASNHIQLQK	-4.068256	Q17RY0	CPEB4 HUMAN	650.344392	2	0.637349725		
NFASNHIQLQK	-4.068256	Q17RY0	CPEB4 HUMAN	433.8988697	3	0.637349725		
SWMEDSLNR	30.7534	Q17RY0	CPEB4 HUMAN	569.2538535	2	0.723268747		
SWMEDSLNR	30.7534	Q17RY0	CPEB4 HUMAN	379.8385107	3	0.723268747		

TFDMHSLESSLIDIMR	105.2666	Q17RY0	CPEB4_HUMAN	947.95606	2	0.762991905		
TFDMHSLESSLIDIMR	105.2666	Q17RY0	CPEB4_HUMAN	632.3066483	3	0.762991905		
VEVKPYVLDDQLCDECQGAR	40.95258	Q17RY0	CPEB4_HUMAN	1197.557395	2	0.640248179		
VEVKPYVLDDQLCDECQGAR	40.95258	Q17RY0	CPEB4_HUMAN	798.7075383	3	0.640248179		
ALEGTLSELAAETDLPVVFVK	124.284	Q16881	TRXR1_HUMAN	1101.599384	2	0.761859715		
ALEGTLSELAAETDLPVVFVK	124.284	Q16881	TRXR1_HUMAN	734.735531	3	0.761859715		
AVAAAAPTELQTK	7.725166	Q16881	TRXR1_HUMAN	635.8542595	2	0.848153353		
AVAAAAPTELQTK	7.725166	Q16881	TRXR1_HUMAN	424.2387813	3	0.848153353		
FLIATGERPR	8.646347	Q16881	TRXR1_HUMAN	580.3332965	2	0.780914903	3	
FLIATGERPR	8.646347	Q16881	TRXR1_HUMAN	387.224806	3	0.780914903	3	Yes
GFDQDMANK	-11.10138	Q16881	TRXR1_HUMAN	513.222023	2	0.756016135	2	Yes
GFDQDMANK	-11.10138	Q16881	TRXR1_HUMAN	342.483957	3	0.756016135	2	
IGEHMEEHGIK	-30.38715	Q16881	TRXR1_HUMAN	640.309161	2	0.800023735	3	
IGEHMEEHGIK	-30.38715	Q16881	TRXR1_HUMAN	427.2087157	3	0.800023735	3	Yes
IGLETVGVK	27.68463	Q16881	TRXR1_HUMAN	458.279667	2	0.79184401	2	Yes
IGLETVGVK	27.68463	Q16881	TRXR1_HUMAN	305.8557197	3	0.79184401	2	
IPVTDEEQTNVPYIYAIGDILEDK	133.4657	Q16881	TRXR1_HUMAN	1368.187272	2	0.773392558		
IPVTDEEQTNVPYIYAIGDILEDK	133.4657	Q16881	TRXR1_HUMAN	912.4607893	3	0.773392558		
LMHQAALLGQALQDSR	35.34029	Q16881	TRXR1_HUMAN	876.465245	2	0.811245441	3	
LMHQAALLGQALQDSR	35.34029	Q16881	TRXR1_HUMAN	584.646105	3	0.811245441	3	Yes
MIEAVQNHIGSLNWGYR	56.91123	Q16881	TRXR1_HUMAN	994.4945285	2	0.749730349		
MIEAVQNHIGSLNWGYR	56.91123	Q16881	TRXR1_HUMAN	663.332294	3	0.749730349		
TLPENPAGFTSTATADSR	36.72118	Q16881	TRXR1_HUMAN	918.4426865	2	0.823224008		
TLPENPAGFTSTATADSR	36.72118	Q16881	TRXR1_HUMAN	612.631066	3	0.823224008		
VELTPVAIQAGR	42.02681	Q16881	TRXR1_HUMAN	627.364794	2	0.742684722	2	Yes
VELTPVAIQAGR	42.02681	Q16881	TRXR1_HUMAN	418.5791377	3	0.742684722	2	
VMVLDFVTPTPLGTR	105.1276	Q16881	TRXR1_HUMAN	823.4532835	2	0.610852897	2	Yes
VMVLDFVTPTPLGTR	105.1276	Q16881	TRXR1_HUMAN	549.3047973	3	0.610852897	2	
VVGFHVLGPNAGEVTQGFAAALK	81.67351	Q16881	TRXR1_HUMAN	1141.618778	2	0.644826412	3	
VVGFHVLGPNAGEVTQGFAAALK	81.67351	Q16881	TRXR1_HUMAN	761.4151267	3	0.644826412	3	Yes
VVYENAYGQFIGPHR	35.88179	Q16881	TRXR1_HUMAN	875.439544	2	0.806864262		
VVYENAYGQFIGPHR	35.88179	Q16881	TRXR1_HUMAN	583.9623043	3	0.806864262		
AAEQLPYLK	29.29018	Q16877	F264_HUMAN	516.7903945	2	0.742877185		
AAEQLPYLK	29.29018	Q16877	F264_HUMAN	344.8628713	3	0.742877185		
ATIFNFGEQNGYK	51.60429	Q16877	F264_HUMAN	744.860068	2	0.716672301		
ATIFNFGEQNGYK	51.60429	Q16877	F264_HUMAN	496.9093203	3	0.716672301		
DRPQNVDISRPPEEALVTVPAHQ	46.96095	Q16877	F264_HUMAN	1284.662433	2	0.667332649		
DRPQNVDISRPPEEALVTVPAHQ	46.96095	Q16877	F264_HUMAN	856.7775633	3	0.667332649		
DSDEATEDFMR	25.66851	Q16877	F264_HUMAN	658.2595305	2	0.792785704		
DSDEATEDFMR	25.66851	Q16877	F264_HUMAN	439.1756287	3	0.792785704		
ELTQNPLK	-0.708157	Q16877	F264_HUMAN	471.7669235	2	0.805652857		
ELTQNPLK	-0.708157	Q16877	F264_HUMAN	314.847224	3	0.805652857		
FLSEEGGHVAVFDATNTTR	41.03693	Q16877	F264_HUMAN	1025.995618	2	0.755361557		
FLSEEGGHVAVFDATNTTR	41.03693	Q16877	F264_HUMAN	684.33302	3	0.755361557		
IMDVGQSYVVNR	33.67957	Q16877	F264_HUMAN	690.8511885	2	0.852546573		
IMDVGQSYVVNR	33.67957	Q16877	F264_HUMAN	460.9034007	3	0.852546573		
IVYYLMNIHVTPR	56.83482	Q16877	F264_HUMAN	809.9428765	2	0.695207536		
IVYYLMNIHVTPR	56.83482	Q16877	F264_HUMAN	540.2978593	3	0.695207536		
SFEFFLPDNEEGLK	95.8096	Q16877	F264_HUMAN	836.3992265	2	0.879948914		
SFEFFLPDNEEGLK	95.8096	Q16877	F264_HUMAN	557.935426	3	0.879948914		
SLAQFISDQNIK	52.38187	Q16877	F264_HUMAN	682.36499	2	0.823011279		
SLAQFISDQNIK	52.38187	Q16877	F264_HUMAN	455.245935	3	0.823011279		
VESIFLNVAAVNTHR	57.36436	Q16877	F264_HUMAN	835.455202	2	0.633824527		
VESIFLNVAAVNTHR	57.36436	Q16877	F264_HUMAN	557.3060763	3	0.633824527		
VWTSQMK	-8.737511	Q16877	F264_HUMAN	440.2238375	2	0.655087829		
VWTSQMK	-8.737511	Q16877	F264_HUMAN	293.8185	3	0.655087829		
YLNWIGVPTR	65.44158	Q16877	F264_HUMAN	609.8356675	2	0.774411738		
YLNWIGVPTR	65.44158	Q16877	F264_HUMAN	406.8930533	3	0.774411738		
ASAETVDPASLWEY	102.2009	Q16658	FSCN1_HUMAN	769.854648	2	0.691448569	2	Yes
ASAETVDPASLWEY	102.2009	Q16658	FSCN1_HUMAN	513.5723737	3	0.691448569	2	
DVPWGVDSLITLAFQDQR	161.2795	Q16658	FSCN1_HUMAN	1030.526554	2	0.661754191		
DVPWGVDSLITLAFQDQR	161.2795	Q16658	FSCN1_HUMAN	687.353644	3	0.661754191		
FLIVAHDDGR	12.12541	Q16658	FSCN1_HUMAN	571.801829	2	0.74863857	3	
FLIVAHDDGR	12.12541	Q16658	FSCN1_HUMAN	381.537161	3	0.74863857	3	Yes
GEHGFIGCRK	-31.3365	Q16658	FSCN1_HUMAN	580.7856565	2	0.601987958		
GEHGFIGCRK	-31.3365	Q16658	FSCN1_HUMAN	387.5263793	3	0.601987958		
LINRPIIVFR	43.17773	Q16658	FSCN1_HUMAN	620.898606	2	0.706277907	3	
LINRPIIVFR	43.17773	Q16658	FSCN1_HUMAN	414.2683457	3	0.706277907	3	Yes
LSCFAQTVSPAEK	21.10289	Q16658	FSCN1_HUMAN	719.3563085	2	0.834035039	2	Yes
LSCFAQTVSPAEK	21.10289	Q16658	FSCN1_HUMAN	479.906814	3	0.834035039	2	
LVARPEPATGYTLEFR	39.9524	Q16658	FSCN1_HUMAN	910.4892385	2	0.746986985	3	
LVARPEPATGYTLEFR	39.9524	Q16658	FSCN1_HUMAN	607.3287673	3	0.746986985	3	Yes
NASCYFDIEWR	67.50948	Q16658	FSCN1_HUMAN	730.8173455	2	0.713461697	2	Yes
NASCYFDIEWR	67.50948	Q16658	FSCN1_HUMAN	487.5475053	3	0.713461697	2	
SSYDVFQLEFNDGAYNIK	91.91936	Q16658	FSCN1_HUMAN	1055.492367	2	0.671554208	3	
SSYDVFQLEFNDGAYNIK	91.91936	Q16658	FSCN1_HUMAN	703.9975193	3	0.671554208	3	Yes
WSLQSEAHR	-12.99445	Q16658	FSCN1_HUMAN	557.275978	2	0.692922115	2	Yes
WSLQSEAHR	-12.99445	Q16658	FSCN1_HUMAN	371.8532603	3	0.692922115	2	
YFGGTEDR	-16.57505	Q16658	FSCN1_HUMAN	472.7096015	2	0.635671377	2	Yes
YFGGTEDR	-16.57505	Q16658	FSCN1_HUMAN	315.475676	3	0.635671377	2	
YLAADKDGNVTCER	-18.89311	Q16658	FSCN1_HUMAN	806.3757565	2	0.715783477	2	Yes
YLAADKDGNVTCER	-18.89311	Q16658	FSCN1_HUMAN	537.9197793	3	0.715783477	2	
YLAPSGPSGTLK	14.9657	Q16658	FSCN1_HUMAN	595.8249655	2	0.814396143	2	Yes
YLAPSGPSGTLK	14.9657	Q16658	FSCN1_HUMAN	397.5525853	3	0.814396143	2	
YLTAEAFGFK	54.98943	Q16658	FSCN1_HUMAN	573.795677	2	0.808411717	2	Yes
YLTAEAFGFK	54.98943	Q16658	FSCN1_HUMAN	382.866393	3	0.808411717	2	
YWTLTATGGVQSTASSK	43.03445	Q16658	FSCN1_HUMAN	879.4394115	2	0.821883321		
YWTLTATGGVQSTASSK	43.03445	Q16658	FSCN1_HUMAN	586.6288827	3	0.821883321		
AFIPEMLK	56.50883	Q16656	NRF1_HUMAN	474.765338	2	0.866339326		
AFIPEMLK	56.50883	Q16656	NRF1_HUMAN	316.846167	3	0.866339326		
ATLDEYTTR	3.949642	Q16656	NRF1_HUMAN	535.2621985	2	0.831562459		
ATLDEYTTR	3.949642	Q16656	NRF1_HUMAN	357.1774073	3	0.831562459		
ESCKPIWWPEDIPWANVR	91.71545	Q16656	NRF1_HUMAN	1142.054955	2	0.676003516	3	
ESCKPIWWPEDIPWANVR	91.71545	Q16656	NRF1_HUMAN	761.7059113	3	0.676003516	3	Yes
ISDSAVTMDGQAVEVVTLEQ	101.3243	Q16656	NRF1_HUMAN	1046.509909	2	0.648878932		
ISDSAVTMDGQAVEVVTLEQ	101.3243	Q16656	NRF1_HUMAN	698.0092143	3	0.648878932		
ILEDLESALAEHAPAPQEVNSELPPLTIDGIPVSV	153.1701	Q16656	NRF1_HUMAN	2063.05158	2	0.798131406		
ILEDLESALAEHAPAPQEVNSELPPLTIDGIPVSV	153.1701	Q16656	NRF1_HUMAN	1375.703661	3	0.798131406		
VFGAAPLENVVR	53.74574	Q16656	NRF1_HUMAN	636.359511	2	0.817246318	2	Yes
VFGAAPLENVVR	53.74574	Q16656	NRF1_HUMAN	424.5756157	3	0.817246318	2	
VGQQAIVLCISPSKPNPVFK	59.32336	Q16656	NRF1_HUMAN	1091.606824	2	0.681345403	3	

VGQQAIVLCISPSKPNPVFK	59.32336	Q16656	NRF1_HUMAN	728.073824	3	0.681345403	3	Yes
EISDAQWEDVVQK	46.23333	Q16626	MEA1_HUMAN	773.8733765	2	0.778300285	2	Yes
EISDAQWEDVVQK	46.23333	Q16626	MEA1_HUMAN	516.251526	3	0.778300285	2	
MATVVLGGDTMGPER	41.16825	Q16626	MEA1_HUMAN	767.374176	2	0.845251083		
MATVVLGGDTMGPER	41.16825	Q16626	MEA1_HUMAN	511.9187257	3	0.845251083		
TMAGVSLPAPGVPAWAR	81.73083	Q16626	MEA1_HUMAN	840.9486995	2	0.62255162	2	Yes
TMAGVSLPAPGVPAWAR	81.73083	Q16626	MEA1_HUMAN	560.968408	3	0.62255162	2	
AEAENTSEVSTVLK	18.08328	Q16512	PKN1_HUMAN	739.373209	2	0.676663637		
AEAENTSEVSTVLK	18.08328	Q16512	PKN1_HUMAN	493.2514143	3	0.676663637		
ALQAGQLENQAAPDDTQGSPDLGAVELR	65.93883	Q16512	PKN1_HUMAN	1432.705224	2	0.817255139		
ALQAGQLENQAAPDDTQGSPDLGAVELR	65.93883	Q16512	PKN1_HUMAN	955.4727573	3	0.817255139		
[illegible]PPSSPSSLSSPIQESTAPELPSETQETPGPALCSPL	93.05234	Q16512	PKN1_HUMAN	1973.947312	2	0.7073614		
[illegible]PPSSPSSLSSPIQESTAPELPSETQETPGPALCSPL	93.05234	Q16512	PKN1_HUMAN	1316.300816	3	0.7073614		
EELAAASSAAFSTR	29.80411	Q16512	PKN1_HUMAN	705.8471615	2	0.72638309		
EELAAASSAAFSTR	29.80411	Q16512	PKN1_HUMAN	470.900716	3	0.72638309		
FLSAEAIGIMR	65.41202	Q16512	PKN1_HUMAN	604.329365	2	0.850012302		
FLSAEAIGIMR	65.41202	Q16512	PKN1_HUMAN	403.222185	3	0.850012302		
HEVQLDMEPQGCLVAEVTFR	76.38266	Q16512	PKN1_HUMAN	1179.565028	2	0.609937489		
HEVQLDMEPQGCLVAEVTFR	76.38266	Q16512	PKN1_HUMAN	786.7126267	3	0.609937489		
LAGPFPATHYSTLCKPAPLTGTLEVR	59.57188	Q16512	PKN1_HUMAN	1399.239459	2	0.781805992		
LAGPFPATHYSTLCKPAPLTGTLEVR	59.57188	Q16512	PKN1_HUMAN	933.1622473	3	0.781805992		
[illegible]QLQELHAHVVLPDPAATHDGPQSPGAGGPTCS[illegible]	69.01485	Q16512	PKN1_HUMAN	2303.646964	2	0.664848804		
[illegible]QLQELHAHVVLPDPAATHDGPQSPGAGGPTCS[illegible]	69.01485	Q16512	PKN1_HUMAN	1536.100584	3	0.664848804		
LDNLLLDTEGYVK	72.40147	Q16512	PKN1_HUMAN	746.8988585	2	0.880185425		
LDNLLLDTEGYVK	72.40147	Q16512	PKN1_HUMAN	498.268514	3	0.880185425		
LDNTVVGQTSWK	28.173	Q16512	PKN1_HUMAN	674.3493405	2	0.805542767		
LDNTVVGQTSWK	28.173	Q16512	PKN1_HUMAN	449.9021687	3	0.805542767		
LEDFLDNER	33.83891	Q16512	PKN1_HUMAN	575.7729335	2	0.744730294	2	Yes
LEDFLDNER	33.83891	Q16512	PKN1_HUMAN	384.184564	3	0.744730294	2	
LGELPADHPK	-13.83566	Q16512	PKN1_HUMAN	538.79093	2	0.614962518		
LGELPADHPK	-13.83566	Q16512	PKN1_HUMAN	359.529895	3	0.614962518		
LIPNATGTGTFSPGASPGSEAR	40.65915	Q16512	PKN1_HUMAN	1044.522	2	0.76348263		
LIPNATGTGTFSPGASPGSEAR	40.65915	Q16512	PKN1_HUMAN	696.6839413	3	0.76348263		
LLLTAQQMLQDSK	58.80127	Q16512	PKN1_HUMAN	744.9087085	2	0.824492455		
LLLTAQQMLQDSK	58.80127	Q16512	PKN1_HUMAN	496.9417473	3	0.824492455		
LNLGTDSDSSPQK	0.69326	Q16512	PKN1_HUMAN	681.331344	2	0.74748528		
LNLGTDSDSSPQK	0.69326	Q16512	PKN1_HUMAN	454.5568377	3	0.74748528		
PCGPNAWDQSFTLELER	82.54408	Q16512	PKN1_HUMAN	1010.465639	2	0.744322419		
PCGPNAWDQSFTLELER	82.54408	Q16512	PKN1_HUMAN	673.9797007	3	0.744322419		
SLGPVELLLR	86.6544	Q16512	PKN1_HUMAN	548.8404225	2	0.748967469	2	Yes
SLGPVELLLR	86.6544	Q16512	PKN1_HUMAN	366.2295567	3	0.748967469	2	
SPLTLEDFK	44.31118	Q16512	PKN1_HUMAN	525.2798635	2	0.777472675		
SPLTLEDFK	44.31118	Q16512	PKN1_HUMAN	350.5225173	3	0.777472675		
TDVSNFDEEFTGEAPTLSPPR	72.20645	Q16512	PKN1_HUMAN	1155.032594	2	0.886049151		
TDVSNFDEEFTGEAPTLSPPR	72.20645	Q16512	PKN1_HUMAN	770.3576707	3	0.886049151		
TLGWEALLAR	80.36897	Q16512	PKN1_HUMAN	565.3223975	2	0.665027916		
TLGWEALLAR	80.36897	Q16512	PKN1_HUMAN	377.21754	3	0.665027916		
TSTFCGTPEFLAPEVLTDTSYTR	98.26846	Q16512	PKN1_HUMAN	1297.110515	2	0.825092435		
TSTFCGTPEFLAPEVLTDTSYTR	98.26846	Q16512	PKN1_HUMAN	865.076285	3	0.825092435		
VLLSEFRPSGELFAIK	75.91979	Q16512	PKN1_HUMAN	903.5121855	2	0.746238828		
VLLSEFRPSGELFAIK	75.91979	Q16512	PKN1_HUMAN	602.6773987	3	0.746238828		
DKPETWENQWK	14.08431	Q15800	MSMO1_HUMAN	730.844422	2	0.688158453		
DKPETWENQWK	14.08431	Q15800	MSMO1_HUMAN	487.5655563	3	0.688158453		
IFGTDSQYNAYNEK	23.10493	Q15800	MSMO1_HUMAN	825.3762745	2	0.840395331	2	Yes
IFGTDSQYNAYNEK	23.10493	Q15800	MSMO1_HUMAN	550.5867913	3	0.840395331	2	
NAWNYMLNNYTK	58.3652	Q15800	MSMO1_HUMAN	766.3540905	2	0.659142792	2	Yes
NAWNYMLNNYTK	58.3652	Q15800	MSMO1_HUMAN	511.2386687	3	0.659142792	2	
ALQAALAESGGSPDVLQMLK	91.76231	Q15628	TRADD_HUMAN	1000.030615	2	0.780297041		
ALQAALAESGGSPDVLQMLK	91.76231	Q15628	TRADD_HUMAN	667.023018	3	0.780297041		
DEELAELEDALR	88.70901	Q15628	TRADD_HUMAN	701.839002	2	0.852209806		
DEELAELEDALR	88.70901	Q15628	TRADD_HUMAN	468.2286097	3	0.852209806		
DPALDSLAYEYER	62.94396	Q15628	TRADD_HUMAN	771.360093	2	0.842072845		
DPALDSLAYEYER	62.94396	Q15628	TRADD_HUMAN	514.5760037	3	0.842072845		
EGLYEQAFQLLR	93.46953	Q15628	TRADD_HUMAN	733.886086	2	0.830136418		
EGLYEQAFQLLR	93.46953	Q15628	TRADD_HUMAN	489.5933323	3	0.830136418		
LDALLADEER	31.96922	Q15628	TRADD_HUMAN	572.796409	2	0.715090573	2	Yes
LDALLADEER	31.96922	Q15628	TRADD_HUMAN	382.2002143	3	0.715090573	2	
SDPQLIVQLR	47.39808	Q15628	TRADD_HUMAN	584.8384115	2	0.800594509		
SDPQLIVQLR	47.39808	Q15628	TRADD_HUMAN	390.228216	3	0.800594509		
SLAAALAQHSVPLQLELR	76.05877	Q15628	TRADD_HUMAN	959.0499985	2	0.618937314		
SLAAALAQHSVPLQLELR	76.05877	Q15628	TRADD_HUMAN	639.7026073	3	0.618937314		
VVLSDAYAHPQQK	-0.30896	Q15628	TRADD_HUMAN	728.3837105	2	0.812275171	3	
VVLSDAYAHPQQK	-0.30896	Q15628	TRADD_HUMAN	485.925082	3	0.812275171	3	Yes
MLDDNNHLIQCIMDSQNK	61.25548	Q15532	SSXT_HUMAN	1094.99338	2	0.677533209	3	
MLDDNNHLIQCIMDSQNK	61.25548	Q15532	SSXT_HUMAN	730.331528	3	0.677533209	3	Yes
ALCKYEECQK	-28.7043	Q15527	SURF2_HUMAN	664.8028505	2	0.782755673		
ALCKYEECQK	-28.7043	Q15527	SURF2_HUMAN	443.537842	3	0.782755673		
ASPAFDYAEFEPHIVPSTK	62.3726	Q15527	SURF2_HUMAN	1053.513107	2	0.765409052		
ASPAFDYAEFEPHIVPSTK	62.3726	Q15527	SURF2_HUMAN	702.678013	3	0.765409052		
DLGSTEDGDGTDDFLTDK	50.67549	Q15527	SURF2_HUMAN	950.900714	2	0.701597691		
DLGSTEDGDGTDDFLTDK	50.67549	Q15527	SURF2_HUMAN	634.269751	3	0.701597691		
EAFWEPTSSDEGGAASDDSMTDLYPPELFTR	118.6495	Q15527	SURF2_HUMAN	1711.238628	2	0.684792101		
EAFWEPTSSDEGGAASDDSMTDLYPPELFTR	118.6495	Q15527	SURF2_HUMAN	1141.161693	3	0.684792101		
LPELQVYTR	41.85219	Q15527	SURF2_HUMAN	559.814401	2	0.800045133	2	Yes
LPELQVYTR	41.85219	Q15527	SURF2_HUMAN	373.5455423	3	0.800045133	2	
ALYLSDNDFEILPPDIGK	110.4213	Q15404	RSU1_HUMAN	1010.517858	2	0.812819242	3	
ALYLSDNDFEILPPDIGK	110.4213	Q15404	RSU1_HUMAN	674.0145133	3	0.812819242	3	Yes
DNDLISLPK	49.65842	Q15404	RSU1_HUMAN	507.7774875	2	0.836413264	2	Yes
DNDLISLPK	49.65842	Q15404	RSU1_HUMAN	338.8542667	3	0.836413264	2	
EIGELTQLK	32.34277	Q15404	RSU1_HUMAN	515.7931385	2	0.809419274	2	Yes
EIGELTQLK	32.34277	Q15404	RSU1_HUMAN	344.198034	3	0.809419274	2	
HMQANPEPPK	-30.37744	Q15404	RSU1_HUMAN	574.7800395	2	0.695231736		
HMQANPEPPK	-30.37744	Q15404	RSU1_HUMAN	383.5226347	3	0.695231736		
LTMVPPNIAELK	67.62247	Q15404	RSU1_HUMAN	663.379055	2	0.806825697		
LTMVPPNIAELK	67.62247	Q15404	RSU1_HUMAN	442.588645	3	0.806825697		
LTVLPPELGNLDLTGQK	96.01543	Q15404	RSU1_HUMAN	904.5123835	2	0.886965752	3	
LTVLPPELGNLDLTGQK	96.01543	Q15404	RSU1_HUMAN	603.3441973	3	0.886965752	3	Yes
NLEVLNFFNNQIEELPTQISSLQK	136.6798	Q15404	RSU1_HUMAN	1409.735262	2	0.761810839	3	
NLEVLNFFNNQIEELPTQISSLQK	136.6798	Q15404	RSU1_HUMAN	940.1594497	3	0.761810839	3	Yes
AETLVTLDFTSR	62.92467	Q15334	L2GL1_HUMAN	676.8569985	2	0.77542007		
AETLVTLDFTSR	62.92467	Q15334	L2GL1_HUMAN	451.5739407	3	0.77542007		

ALGPVESLQGHLR	31.03688	Q15334	L2GL1 HUMAN	688.8864245	2	0.817090869		
ALGPVESLQGHLR	31.03688	Q15334	L2GL1 HUMAN	459.593558	3	0.817090869		
APVVAIAVLDGR	66.98761	Q15334	L2GL1 HUMAN	590.8566045	2	0.715643764	2	Yes
APVVAIAVLDGR	66.98761	Q15334	L2GL1 HUMAN	394.2403447	3	0.715643764	2	
DAATVTQMHFLTGQGR	44.03671	Q15334	L2GL1 HUMAN	866.9259535	2	0.816811562		
DAATVTQMHFLTGQGR	44.03671	Q15334	L2GL1 HUMAN	578.2865773	3	0.816811562		
DFLGSSEESEK	15.97427	Q15334	L2GL1 HUMAN	614.272963	2	0.767846763		
DFLGSSEESEK	15.97427	Q15334	L2GL1 HUMAN	409.8512503	3	0.767846763		
GAHHGPTMWAGTNSGSVFAYALEVPAAAVGGE	94.90822	Q15334	L2GL1 HUMAN	1678.29421	2	0.674018621		
GAHHGPTMWAGTNSGSVFAYALEVPAAAVGGE	94.90822	Q15334	L2GL1 HUMAN	1119.198748	3	0.674018621		
DLAQAPDMQGGHAVLIASEEQFK	58.98391	Q15334	L2GL1 HUMAN	1228.100289	2	0.820129633	3	
DLAQAPDMQGGHAVLIASEEQFK	58.98391	Q15334	L2GL1 HUMAN	819.0694673	3	0.820129633	3	Yes
EDISGIASCVFTR	72.33475	Q15334	L2GL1 HUMAN	727.85139	2	0.648989618		
EDISGIASCVFTR	72.33475	Q15334	L2GL1 HUMAN	485.5702017	3	0.648989618		
FWDASGVALRPLYK	59.76289	Q15334	L2GL1 HUMAN	811.9386525	2	0.680641532		
FWDASGVALRPLYK	59.76289	Q15334	L2GL1 HUMAN	541.6283767	3	0.680641532		
GLLLTGHEDGTVR	14.42763	Q15334	L2GL1 HUMAN	684.368065	2	0.792629719		
GLLLTGHEDGTVR	14.42763	Q15334	L2GL1 HUMAN	456.5813183	3	0.792629719		
GRPLPEPYEASR	-3.46114	Q15334	L2GL1 HUMAN	686.3549525	2	0.736920714		
GRPLPEPYEASR	-3.46114	Q15334	L2GL1 HUMAN	457.90591	3	0.736920714		
HGQGFYLISPSEFER	58.17532	Q15334	L2GL1 HUMAN	883.929013	2	0.838991344		
HGQGFYLISPSEFER	58.17532	Q15334	L2GL1 HUMAN	589.6219503	3	0.838991344		
IYGAPGVEFTGLHR	43.46298	Q15334	L2GL1 HUMAN	758.899528	2	0.837545574	3	
IYGAPGVEFTGLHR	43.46298	Q15334	L2GL1 HUMAN	506.2689603	3	0.837545574	3	Yes
LSTAGLFQTDCEHADSLAQAAEDDWPPFR	100.6407	Q15334	L2GL1 HUMAN	1624.733655	2	0.787177444		
LSTAGLFQTDCEHADSLAQAAEDDWPPFR	100.6407	Q15334	L2GL1 HUMAN	1083.491712	3	0.787177444		
NITEPLCSLDINWPR	90.52782	Q15334	L2GL1 HUMAN	914.4570845	2	0.840724349		
NITEPLCSLDINWPR	90.52782	Q15334	L2GL1 HUMAN	609.973998	3	0.840724349		
NLAEDEAHACAILIK	34.95452	Q15334	L2GL1 HUMAN	834.4252535	2	0.72419709	3	
NLAEDEAHACAILIK	34.95452	Q15334	L2GL1 HUMAN	556.619444	3	0.72419709	3	Yes
NLAQEPSQR	-26.53721	Q15334	L2GL1 HUMAN	521.767986	2	0.613410175		
NLAQEPSQR	-26.53721	Q15334	L2GL1 HUMAN	348.1812657	3	0.613410175		
RPEQAVEAVLGK	14.22382	Q15334	L2GL1 HUMAN	648.8677005	2	0.719264388	2	Yes
RPEQAVEAVLGK	14.22382	Q15334	L2GL1 HUMAN	432.9144087	3	0.719264388	2	
TGPLPWPAGFQPR	74.23348	Q15334	L2GL1 HUMAN	712.378236	2	0.796841264		
TGPLPWPAGFQPR	74.23348	Q15334	L2GL1 HUMAN	475.2547657	3	0.796841264		
TVEHGFPNQPSALAFDPELR	62.65727	Q15334	L2GL1 HUMAN	1113.053467	2	0.824093401	3	
TVEHGFPNQPSALAFDPELR	62.65727	Q15334	L2GL1 HUMAN	742.371586	3	0.824093401	3	Yes
VGCFDPYSDDPR	27.7303	Q15334	L2GL1 HUMAN	714.298986	2	0.716391146		
VGCFDPYSDDPR	27.7303	Q15334	L2GL1 HUMAN	476.5352657	3	0.716391146		
ELDENVEYEER	11.29166	Q15291	RBBP5 HUMAN	712.8129795	2	0.755448401	2	Yes
ELDENVEYEER	11.29166	Q15291	RBBP5 HUMAN	475.5445947	3	0.755448401	2	
GLPLEGSAK	3.591694	Q15291	RBBP5 HUMAN	436.2483665	2	0.727491438		
GLPLEGSAK	3.591694	Q15291	RBBP5 HUMAN	291.168186	3	0.727491438		
GSCFLINTADR	32.03423	Q15291	RBBP5 HUMAN	627.301336	2	0.792016089		
GSCFLINTADR	32.03423	Q15291	RBBP5 HUMAN	418.5368323	3	0.792016089		
HVVLPVDDDSDLNVVASFDR	76.35538	Q15291	RBBP5 HUMAN	1106.548213	2	0.796438038		
HVVLPVDDDSDLNVVASFDR	76.35538	Q15291	RBBP5 HUMAN	738.0347503	3	0.796438038		
IVIWDFLTR	106.9018	Q15291	RBBP5 HUMAN	581.8351405	2	0.699149489	2	Yes
IVIWDFLTR	106.9018	Q15291	RBBP5 HUMAN	388.2260353	3	0.699149489	2	
LQDLVNR	-0.931843	Q15291	RBBP5 HUMAN	429.246158	2	0.751042485		
LQDLVNR	-0.931843	Q15291	RBBP5 HUMAN	286.500047	3	0.751042485		
LVSASTDNIVSQWDVLSGDCDQR	84.28268	Q15291	RBBP5 HUMAN	1283.098473	2	0.792817712	3	
LVSASTDNIVSQWDVLSGDCDQR	84.28268	Q15291	RBBP5 HUMAN	855.7349237	3	0.792817712	3	Yes
SAPVMLTLSDSK	42.64629	Q15291	RBBP5 HUMAN	624.8293945	2	0.720405221	2	Yes
SAPVMLTLSDSK	42.64629	Q15291	RBBP5 HUMAN	416.8888713	3	0.720405221	2	
TDSQDLVASFR	39.71049	Q15291	RBBP5 HUMAN	619.8047655	2	0.721446335		
TDSQDLVASFR	39.71049	Q15291	RBBP5 HUMAN	413.5391187	3	0.721446335		
TTNIELQGVPNDEVHPLLGVK	64.91315	Q15291	RBBP5 HUMAN	1137.110982	2	0.773163199	3	
TTNIELQGVPNDEVHPLLGVK	64.91315	Q15291	RBBP5 HUMAN	758.4099293	3	0.773163199	3	Yes
VTTGTSNTTAIK	-24.59307	Q15291	RBBP5 HUMAN	597.322792	2	0.657022476		
VTTGTSNTTAIK	-24.59307	Q15291	RBBP5 HUMAN	398.5511363	3	0.657022476		
WGTLLAVGCNDGR	52.43399	Q15291	RBBP5 HUMAN	709.8464425	2	0.76641643	2	Yes
WGTLLAVGCNDGR	52.43399	Q15291	RBBP5 HUMAN	473.5669033	3	0.76641643	2	
AAPFSLEYR	39.01399	Q15181	IPYR HUMAN	527.2723685	2	0.82498914	2	Yes
AAPFSLEYR	39.01399	Q15181	IPYR HUMAN	351.850854	3	0.82498914	2	
DKDFAIDIIK	47.51456	Q15181	IPYR HUMAN	589.327345	2	0.851270378	2	Yes
DKDFAIDIIK	47.51456	Q15181	IPYR HUMAN	393.2208383	3	0.851270378	2	
GISCMNTTLSESPFK	49.41762	Q15181	IPYR HUMAN	836.390022	2	0.720572472	2	Yes
GISCMNTTLSESPFK	49.41762	Q15181	IPYR HUMAN	557.9292897	3	0.720572472	2	
GQYISPFHDIPIYADKDVFHMVVEVPR	97.21132	Q15181	IPYR HUMAN	1586.800406	2	0.675619662	4	
GQYISPFHDIPIYADKDVFHMVVEVPR	97.21132	Q15181	IPYR HUMAN	1058.202879	3	0.675619662	4	
LKPGYLEATVDWFR	74.43607	Q15181	IPYR HUMAN	847.9492175	2	0.835193396		
LKPGYLEATVDWFR	74.43607	Q15181	IPYR HUMAN	565.63542	3	0.835193396		
VIAINVDDPDAANYNDINDVK	65.3496	Q15181	IPYR HUMAN	1144.556231	2	0.817131877	3	
VIAINVDDPDAANYNDINDVK	65.3496	Q15181	IPYR HUMAN	763.3734287	3	0.817131877	3	Yes
VLGILAMIDEGETDWK	126.5982	Q15181	IPYR HUMAN	895.4562195	2	0.803121448	3	Yes
VLGILAMIDEGETDWK	126.5982	Q15181	IPYR HUMAN	597.3067547	3	0.803121448	3	Yes
VPDGKPENEFAFNAEFK	47.99135	Q15181	IPYR HUMAN	969.9657965	2	0.799581528		
VPDGKPENEFAFNAEFK	47.99135	Q15181	IPYR HUMAN	646.979806	3	0.799581528		
YVANLFPYK	51.21655	Q15181	IPYR HUMAN	557.8007575	2	0.792594075		
YVANLFPYK	51.21655	Q15181	IPYR HUMAN	372.2031133	3	0.792594075		
AALAHSEEVTASQVAATK	1.66996	Q15149	PLEC HUMAN	892.463422	2	0.808204114	3	
AALAHSEEVTASQVAATK	1.66996	Q15149	PLEC HUMAN	595.3115563	3	0.808204114	3	Yes
AEAELLQQQK	1.173294	Q15149	PLEC HUMAN	579.312227	2	0.812918901		
AEAELLQQQK	1.173294	Q15149	PLEC HUMAN	386.544093	3	0.812918901		
AELELELGR	43.0653	Q15149	PLEC HUMAN	515.2829375	2	0.794783533		
AELELELGR	43.0653	Q15149	PLEC HUMAN	343.8579	3	0.794783533		
AEMEVLLASK	43.4632	Q15149	PLEC HUMAN	545.7948235	2	0.751607001	2	Yes
AEMEVLLASK	43.4632	Q15149	PLEC HUMAN	364.1991573	3	0.751607001	2	
AEVVETTQVYTEEETR	25.61976	Q15149	PLEC HUMAN	942.4476305	2	0.693812907	2	Yes
AEVVETTQVYTEEETR	25.61976	Q15149	PLEC HUMAN	628.634362	3	0.693812907	2	
AFCGFEDPR	23.14899	Q15149	PLEC HUMAN	549.7378405	2	0.80930531	2	Yes
AFCGFEDPR	23.14899	Q15149	PLEC HUMAN	366.8278353	3	0.80930531	2	
AGLVGPEFHEK	5.149662	Q15149	PLEC HUMAN	592.309487	2	0.888660848	2	Yes
AGLVGPEFHEK	5.149662	Q15149	PLEC HUMAN	395.208933	3	0.888660848	2	
AGVAAPATQVAQVTLQSVQR	59.53548	Q15149	PLEC HUMAN	998.053271	2	0.764229655	3	
AGVAAPATQVAQVTLQSVQR	59.53548	Q15149	PLEC HUMAN	665.704789	3	0.764229655	3	Yes
AGVVGPELHEQLLSAEK	39.36015	Q15149	PLEC HUMAN	888.9787075	2	0.922146082	3	
AGVVGPELHEQLLSAEK	39.36015	Q15149	PLEC HUMAN	592.9884133	3	0.922146082	3	Yes
AIYEVLFR	64.03775	Q15149	PLEC HUMAN	505.7876545	2	0.776292264	2	Yes

AIYEVLFR	64.03775	Q15149	PLEC HUMAN	337.5277113	3	0.776292264	2	
ALQALEELR	38.4229	Q15149	PLEC HUMAN	521.798755	2	0.77985394		
ALQALEELR	38.4229	Q15149	PLEC HUMAN	348.2017783	3	0.77985394		
APVPASELLASGVLSR	78.11517	Q15149	PLEC HUMAN	783.9466785	2	0.871645927	3	
APVPASELLASGVLSR	78.11517	Q15149	PLEC HUMAN	522.9670607	3	0.871645927	3	Yes
AQAEAQQPTFDALR	31.10101	Q15149	PLEC HUMAN	773.3869865	2	0.833987951	2	Yes
AQAEAQQPTFDALR	31.10101	Q15149	PLEC HUMAN	515.927266	3	0.833987951	2	
AQLEPVASPAK	4.063995	Q15149	PLEC HUMAN	555.8118625	2	0.693509936	2	Yes
AQLEPVASPAK	4.063995	Q15149	PLEC HUMAN	370.8771833	3	0.693509936	2	
AQLMADFQAGR	31.40871	Q15149	PLEC HUMAN	604.298597	2	0.810850739	2	Yes
AQLMADFQAGR	31.40871	Q15149	PLEC HUMAN	403.201673	3	0.810850739	2	
AQVEQELTTLR	28.22871	Q15149	PLEC HUMAN	644.349341	2	0.778137565	2	Yes
AQVEQELTTLR	28.22871	Q15149	PLEC HUMAN	429.902169	3	0.778137565	2	
AYSDPSTGEPATYGELQQR	29.56798	Q15149	PLEC HUMAN	1035.474707	2	0.815067053	3	
AYSDPSTGEPATYGELQQR	29.56798	Q15149	PLEC HUMAN	690.6524127	3	0.815067053	3	Yes
DALDGPAAEAEPEHSFDGLR	48.16313	Q15149	PLEC HUMAN	1048.980164	2	0.808777094	3	
DALDGPAAEAEPEHSFDGLR	48.16313	Q15149	PLEC HUMAN	699.656051	3	0.808777094	3	Yes
DDGTGQLLLPLSDAR	82.77542	Q15149	PLEC HUMAN	785.907751	2	0.780814528		
DDGTGQLLLPLSDAR	82.77542	Q15149	PLEC HUMAN	524.2744423	3	0.780814528		
DGHNLISLLEVLSGDSLPR	138.8126	Q15149	PLEC HUMAN	1018.045109	2	0.805459499		
DGHNLISLLEVLSGDSLPR	138.8126	Q15149	PLEC HUMAN	679.032681	3	0.805459499		
DLLPSDMAVALLEAQAGTGHIIDPATSAR	136.7028	Q15149	PLEC HUMAN	1467.256038	2	0.655766845	4	
DLLPSDMAVALLEAQAGTGHIIDPATSAR	136.7028	Q15149	PLEC HUMAN	978.5066333	3	0.655766845	4	
DLSELGSVR	32.35667	Q15149	PLEC HUMAN	488.259462	2	0.643602908		
DLSELGSVR	32.35667	Q15149	PLEC HUMAN	325.8422497	3	0.643602908		
DPYSGSTISLFQAMQK	86.90631	Q15149	PLEC HUMAN	886.9303645	2	0.788198471		
DPYSGSTISLFQAMQK	86.90631	Q15149	PLEC HUMAN	591.6228513	3	0.788198471		
DPYTEQTISLFQAMK	91.98172	Q15149	PLEC HUMAN	886.4327405	2	0.815172434	2	Yes
DPYTEQTISLFQAMK	91.98172	Q15149	PLEC HUMAN	591.291102	3	0.815172434	2	
DPYTGQQISLFQAMQK	80.1507	Q15149	PLEC HUMAN	927.9569145	2	0.734378815	3	
DPYTGQQISLFQAMQK	80.1507	Q15149	PLEC HUMAN	618.9738847	3	0.734378815	3	Yes
DPYTGQSVSLFQALK	84.9832	Q15149	PLEC HUMAN	827.428315	2	0.820257187	2	Yes
DPYTGQSVSLFQALK	84.9832	Q15149	PLEC HUMAN	551.9548183	3	0.820257187	2	
DSQDAGGFGPEDR	5.138458	Q15149	PLEC HUMAN	675.782019	2	0.783518732	2	Yes
DSQDAGGFGPEDR	5.138458	Q15149	PLEC HUMAN	450.8572877	3	0.783518732	2	
DYELQLVTYK	62.73724	Q15149	PLEC HUMAN	636.3300765	2	0.811425567	2	Yes
DYELQLVTYK	62.73724	Q15149	PLEC HUMAN	424.5559927	3	0.811425567	2	
EAIAELER	9.539585	Q15149	PLEC HUMAN	465.7487305	2	0.746421576		
EAIAELER	9.539585	Q15149	PLEC HUMAN	310.8350953	3	0.746421576		
EALEAESAWCYLYGTGSVAGVYLPGSR	126.7056	Q15149	PLEC HUMAN	1453.687443	2	0.609767735		
EALEAESAWCYLYGTGSVAGVYLPGSR	126.7056	Q15149	PLEC HUMAN	969.4609037	3	0.609767735		
EAQAVPATLPELEATK	53.12871	Q15149	PLEC HUMAN	834.4467095	2	0.832178235		
EAQAVPATLPELEATK	53.12871	Q15149	PLEC HUMAN	556.633748	3	0.832178235		
EGLTSIEEVTK	35.15405	Q15149	PLEC HUMAN	603.3171745	2	0.814067364	2	Yes
EGLTSIEEVTK	35.15405	Q15149	PLEC HUMAN	402.5473913	3	0.814067364	2	
EGVVGPELHHK	-28.42582	Q15149	PLEC HUMAN	601.320386	2	0.78816539	2	Yes
EGVVGPELHHK	-28.42582	Q15149	PLEC HUMAN	401.216199	3	0.78816539	2	
ELEEVSPETPVVPATTQR	49.02307	Q15149	PLEC HUMAN	991.5080265	2	0.815629959		
ELEEVSPETPVVPATTQR	49.02307	Q15149	PLEC HUMAN	661.3412927	3	0.815629959		
ELIPTEEALR	39.39141	Q15149	PLEC HUMAN	585.822427	2	0.618172705	2	Yes
ELIPTEEALR	39.39141	Q15149	PLEC HUMAN	390.8842263	3	0.618172705	2	
ELMWLNEK	49.9369	Q15149	PLEC HUMAN	531.7686085	2	0.637041986		
ELMWLNEK	49.9369	Q15149	PLEC HUMAN	354.8483473	3	0.637041986		
ELYQQLQR	6.729431	Q15149	PLEC HUMAN	539.2885505	2	0.752949595	2	Yes
ELYQQLQR	6.729431	Q15149	PLEC HUMAN	359.861642	3	0.752949595	2	
EMSVYEAYR	20.45044	Q15149	PLEC HUMAN	574.2585965	2	0.816283941	2	Yes
EMSVYEAYR	20.45044	Q15149	PLEC HUMAN	383.175006	3	0.816283941	2	
ENAAYFQFFSDVR	87.12001	Q15149	PLEC HUMAN	797.3707995	2	0.738133669	2	Yes
ENAAYFQFFSDVR	87.12001	Q15149	PLEC HUMAN	531.9164747	3	0.738133669	2	
EQELQQTLQQEQSVLDQLR	91.37123	Q15149	PLEC HUMAN	1157.088236	2	0.767657638	3	
EQELQQTLQQEQSVLDQLR	91.37123	Q15149	PLEC HUMAN	771.7280987	3	0.767657638	3	Yes
EQLQLLEEQHR	16.64899	Q15149	PLEC HUMAN	711.8709715	2	0.819112837	3	
EQLQLLEEQHR	16.64899	Q15149	PLEC HUMAN	474.9165893	3	0.819112837	3	Yes
EQMAQQLAEETQGFQR	50.04446	Q15149	PLEC HUMAN	947.4421645	2	0.689320087		
EQMAQQLAEETQGFQR	50.04446	Q15149	PLEC HUMAN	631.9640513	3	0.689320087		
ESADPLGAWLQDAR	87.91528	Q15149	PLEC HUMAN	764.873711	2	0.78451252	2	Yes
ESADPLGAWLQDAR	87.91528	Q15149	PLEC HUMAN	510.251749	3	0.78451252	2	
FLEGTSCIAGVFVDATK	87.46884	Q15149	PLEC HUMAN	907.9538445	2	0.645892441	3	
FLEGTSCIAGVFVDATK	87.46884	Q15149	PLEC HUMAN	605.6385047	3	0.645892441	3	Yes
FLEVQYLTGGLIEPDTPGR	105.2067	Q15149	PLEC HUMAN	1053.049858	2	0.783208728	3	
FLEVQYLTGGLIEPDTPGR	105.2067	Q15149	PLEC HUMAN	702.36918	3	0.783208728	3	Yes
GANVIAGVWLEEAGQK	74.37157	Q15149	PLEC HUMAN	821.433936	2	0.825860083		
GANVIAGVWLEEAGQK	74.37157	Q15149	PLEC HUMAN	547.9585657	3	0.825860083		
GDECQLVGPAQPSHWK	22.12834	Q15149	PLEC HUMAN	904.92341	2	0.754281521	3	
GDECQLVGPAQPSHWK	22.12834	Q15149	PLEC HUMAN	603.618215	3	0.754281521	3	Yes
GFFDPNTEENLTYLQLMER	119.2513	Q15149	PLEC HUMAN	1159.044446	2	0.762949824	3	
GFFDPNTEENLTYLQLMER	119.2513	Q15149	PLEC HUMAN	773.0322387	3	0.762949824	3	Yes
GFFDPNTHENLTYR	40.38208	Q15149	PLEC HUMAN	855.897713	2	0.837671399	3	
GFFDPNTHENLTYR	40.38208	Q15149	PLEC HUMAN	570.934417	3	0.837671399	3	Yes
GGAEGELQALR	16.51053	Q15149	PLEC HUMAN	550.788919	2	0.75635016	2	Yes
GGAEGELQALR	16.51053	Q15149	PLEC HUMAN	367.5285543	3	0.75635016	2	
GGELVYTDSEAR	7.530426	Q15149	PLEC HUMAN	648.807501	2	0.81611836	2	Yes
GGELVYTDSEAR	7.530426	Q15149	PLEC HUMAN	432.8742757	3	0.81611836	2	
GIYQSLEGAVQAGQLK	61.33317	Q15149	PLEC HUMAN	831.4470395	2	0.865474224	3	
GIYQSLEGAVQAGQLK	61.33317	Q15149	PLEC HUMAN	554.633968	3	0.865474224	3	Yes
GLFDEEMNEILTDPSDDTK	104.4731	Q15149	PLEC HUMAN	1084.981181	2	0.82207936	3	
GLFDEEMNEILTDPSDDTK	104.4731	Q15149	PLEC HUMAN	723.6567287	3	0.82207936	3	Yes
GLHQSIEEFR	2.423477	Q15149	PLEC HUMAN	608.310018	2	0.721627176		
GLHQSIEEFR	2.423477	Q15149	PLEC HUMAN	405.8759537	3	0.721627176		
GLIDHQTYLELSEQECEWEEITISSSDGVVK	96.83588	Q15149	PLEC HUMAN	1797.843791	2	0.621379852		
GLIDHQTYLELSEQECEWEEITISSSDGVVK	96.83588	Q15149	PLEC HUMAN	1198.898469	3	0.621379852		
GLLSAEVAR	21.73054	Q15149	PLEC HUMAN	458.2670905	2	0.616513312	2	Yes
GLLSAEVAR	21.73054	Q15149	PLEC HUMAN	305.8473353	3	0.616513312	2	
GLVEDTLR	18.28008	Q15149	PLEC HUMAN	451.7512735	2	0.755239129	2	Yes
GLVEDTLR	18.28008	Q15149	PLEC HUMAN	301.5034573	3	0.755239129	2	
GLVGPELHDR	-0.780533	Q15149	PLEC HUMAN	546.794004	2	0.861412227		
GLVGPELHDR	-0.780533	Q15149	PLEC HUMAN	364.8652777	3	0.861412227		
GTQGAEEVLR	-2.921825	Q15149	PLEC HUMAN	530.2756445	2	0.755676031	2	Yes
GTQGAEEVLR	-2.921825	Q15149	PLEC HUMAN	353.853038	3	0.755676031	2	
GWLYYEAGQR	36.80594	Q15149	PLEC HUMAN	621.799278	2	0.678850293	2	Yes
GWLYYEAGQR	36.80594	Q15149	PLEC HUMAN	414.8687937	3	0.678850293	2	

GYFSEEMNR	12.62786	Q15149	PLEC_HUMAN	566.740575	2	0.725113273	2	Yes
GYFSEEMNR	12.62786	Q15149	PLEC_HUMAN	378.1629917	3	0.725113273	2	
HISDLYEDLR	28.93624	Q15149	PLEC_HUMAN	630.8151285	2	0.784194708	2	Yes
HISDLYEDLR	28.93624	Q15149	PLEC_HUMAN	420.8793607	3	0.784194708	2	
HKPLLIDMNK	2.091766	Q15149	PLEC_HUMAN	604.845182	2	0.78406918	3	
HKPLLIDMNK	2.091766	Q15149	PLEC_HUMAN	403.566063	3	0.78406918	3	Yes
IIITVVEEQEQK	39.91396	Q15149	PLEC_HUMAN	714.901406	2	0.720273435		
IIITVVEEQEQK	39.91396	Q15149	PLEC_HUMAN	476.936879	3	0.720273435		
IISLETYNLLR	80.02846	Q15149	PLEC_HUMAN	667.8880965	2	0.807108581	2	Yes
IISLETYNLLR	80.02846	Q15149	PLEC_HUMAN	445.5946727	3	0.807108581	2	
ILITIVEEVETLR	109.4746	Q15149	PLEC_HUMAN	764.4538055	2	0.792528093	3	
ILITIVEEVETLR	109.4746	Q15149	PLEC_HUMAN	509.971812	3	0.792528093	3	Yes
LAAEQELIR	20.75465	Q15149	PLEC_HUMAN	521.798755	2	0.731216729	2	Yes
LAAEQELIR	20.75465	Q15149	PLEC_HUMAN	348.2017783	3	0.731216729	2	
LAAIGEATR	-2.643394	Q15149	PLEC_HUMAN	451.259266	2	0.692516565	2	Yes
LAAIGEATR	-2.643394	Q15149	PLEC_HUMAN	301.1754523	3	0.692516565	2	
LAEDEAFQR	0.434555	Q15149	PLEC_HUMAN	539.7623695	2	0.681464314	2	Yes
LAEDEAFQR	0.434555	Q15149	PLEC_HUMAN	360.1775213	3	0.681464314	2	
LAEVEAALEK	22.77354	Q15149	PLEC_HUMAN	536.7984205	2	0.723375797	2	Yes
LAEVEAALEK	22.77354	Q15149	PLEC_HUMAN	358.2015553	3	0.723375797	2	
LAQGHTTVDELAR	-8.340302	Q15149	PLEC_HUMAN	705.870972	2	0.724725664	2	Yes
LAQGHTTVDELAR	-8.340302	Q15149	PLEC_HUMAN	470.9165897	3	0.724725664	2	
LDLQYAK	12.96272	Q15149	PLEC_HUMAN	425.7376305	2	0.770148516	2	Yes
LDLQYAK	12.96272	Q15149	PLEC_HUMAN	284.1610287	3	0.770148516	2	
LEAQHQALVTLWHQLHVDMK	55.63319	Q15149	PLEC_HUMAN	1199.128987	2	0.749640226	4	
LEAQHQALVTLWHQLHVDMK	55.63319	Q15149	PLEC_HUMAN	799.755266	3	0.749640226	4	
LEDLLQDAQDEK	40.55113	Q15149	PLEC_HUMAN	708.8468275	2	0.822469592	2	Yes
LEDLLQDAQDEK	40.55113	Q15149	PLEC_HUMAN	472.9004933	3	0.822469592	2	
LEQLFQDEVAK	41.03405	Q15149	PLEC_HUMAN	660.3462665	2	0.841647983	2	Yes
LEQLFQDEVAK	41.03405	Q15149	PLEC_HUMAN	440.566786	3	0.841647983	2	
LGFHLPLEVAYQR	66.9245	Q15149	PLEC_HUMAN	771.9255455	2	0.841200471	2	Yes
LGFHLPLEVAYQR	66.9245	Q15149	PLEC_HUMAN	514.952972	3	0.841200471	2	
LISLFQAMK	70.2678	Q15149	PLEC_HUMAN	525.8049945	2	0.842398167	2	Yes
LISLFQAMK	70.2678	Q15149	PLEC_HUMAN	350.8726047	3	0.842398167	2	
LLDAQLATGGIVDPR	63.07604	Q15149	PLEC_HUMAN	769.9310295	2	0.861518025		
LLDAQLATGGIVDPR	63.07604	Q15149	PLEC_HUMAN	513.6232947	3	0.861518025		
LLDAQLSTGGIVDPSK	56.55577	Q15149	PLEC_HUMAN	807.4414265	2	0.847242713		
LLDAQLSTGGIVDPSK	56.55577	Q15149	PLEC_HUMAN	538.630226	3	0.847242713		
LLDPEDVDVPQPDEK	52.33714	Q15149	PLEC_HUMAN	854.917981	2	0.847038627	3	
LLDPEDVDVPQPDEK	52.33714	Q15149	PLEC_HUMAN	570.2812623	3	0.847038627	3	Yes
LLEAAAQSTK	-4.789211	Q15149	PLEC_HUMAN	516.290763	2	0.784965992	2	Yes
LLEAAAQSTK	-4.789211	Q15149	PLEC_HUMAN	344.5297837	3	0.784965992	2	
LLEAQACTGGIIDPSTGER	49.94079	Q15149	PLEC_HUMAN	994.491854	2	0.71072942	3	
LLEAQACTGGIIDPSTGER	49.94079	Q15149	PLEC_HUMAN	663.330511	3	0.71072942	3	Yes
LLEAQIATGGIIDPEESHR	56.56221	Q15149	PLEC_HUMAN	1025.034743	2	0.752387762	3	
LLEAQIATGGIIDPEESHR	56.56221	Q15149	PLEC_HUMAN	683.6924367	3	0.752387762	3	Yes
LLFNDVQTLK	56.12829	Q15149	PLEC_HUMAN	595.8431625	2	0.828496575		
LLFNDVQTLK	56.12829	Q15149	PLEC_HUMAN	397.5647167	3	0.828496575		
LLLEAQAATGFLLDPVK	111.8973	Q15149	PLEC_HUMAN	900.0198445	2	0.796790481	3	
LLLEAQAATGFLLDPVK	111.8973	Q15149	PLEC_HUMAN	600.3491713	3	0.796790481	3	Yes
LLLWSQR	42.49838	Q15149	PLEC_HUMAN	458.2747185	2	0.774297237		
LLLWSQR	42.49838	Q15149	PLEC_HUMAN	305.8524207	3	0.774297237		
LPLLAVCDYK	60.62039	Q15149	PLEC_HUMAN	596.326287	2	0.732799351		
LPLLAVCDYK	60.62039	Q15149	PLEC_HUMAN	397.8867997	3	0.732799351		
LQAEEVAQQK	-15.10317	Q15149	PLEC_HUMAN	572.304402	2	0.690474927	2	Yes
LQAEEVAQQK	-15.10317	Q15149	PLEC_HUMAN	381.8722097	3	0.690474927	2	
LQEAGILSAEELQR	50.24107	Q15149	PLEC_HUMAN	778.9181185	2	0.780333161	2	Yes
LQEAGILSAEELQR	50.24107	Q15149	PLEC_HUMAN	519.6146873	3	0.780333161	2	
LQLEACETR	4.321171	Q15149	PLEC_HUMAN	560.27733	2	0.759248137	2	Yes
LQLEACETR	4.321171	Q15149	PLEC_HUMAN	373.8541617	3	0.759248137	2	
LQLEATER	0.126053	Q15149	PLEC_HUMAN	480.2620055	2	0.668089986	2	Yes
LQLEATER	0.126053	Q15149	PLEC_HUMAN	320.510612	3	0.668089986	2	
LQLEETDHQK	-24.90871	Q15149	PLEC_HUMAN	620.812591	2	0.700128675	3	
LQLEETDHQK	-24.90871	Q15149	PLEC_HUMAN	414.2110023	3	0.700128675	3	Yes
LQNVQIALDYLR	79.80418	Q15149	PLEC_HUMAN	723.409728	2	0.770241559	2	Yes
LQNVQIALDYLR	79.80418	Q15149	PLEC_HUMAN	482.6090937	3	0.770241559	2	
LSIYNALK	36.44466	Q15149	PLEC_HUMAN	461.2743795	2	0.767345011	2	Yes
LSIYNALK	36.44466	Q15149	PLEC_HUMAN	307.8521947	3	0.767345011	2	
LSVAAQEAAR	-6.378117	Q15149	PLEC_HUMAN	508.2807295	2	0.773158789	2	Yes
LSVAAQEAAR	-6.378117	Q15149	PLEC_HUMAN	339.1897613	3	0.773158789	2	
LSVYAALQR	33.69296	Q15149	PLEC_HUMAN	510.796011	2	0.706783295	2	Yes
LSVYAALQR	33.69296	Q15149	PLEC_HUMAN	340.8666157	3	0.706783295	2	
LSVYQAMK	16.08047	Q15149	PLEC_HUMAN	470.2525905	2	0.787549019	2	Yes
LSVYQAMK	16.08047	Q15149	PLEC_HUMAN	313.8376687	3	0.787549019	2	
LSYTQLLR	41.5455	Q15149	PLEC_HUMAN	497.2905615	2	0.770026922	2	Yes
LSYTQLLR	41.5455	Q15149	PLEC_HUMAN	331.8629827	3	0.770026922	2	
LTAEDLFEAR	54.12645	Q15149	PLEC_HUMAN	582.798952	2	0.8203637	2	Yes
LTAEDLFEAR	54.12645	Q15149	PLEC_HUMAN	388.8685763	3	0.8203637	2	
LTVNEAVK	-3.878784	Q15149	PLEC_HUMAN	437.2561915	2	0.694727421	2	Yes
LTVNEAVK	-3.878784	Q15149	PLEC_HUMAN	291.8400693	3	0.694727421	2	
LVASMEEAR	-0.261387	Q15149	PLEC_HUMAN	503.2558655	2	0.704171479	2	Yes
LVASMEEAR	-0.261387	Q15149	PLEC_HUMAN	335.839852	3	0.704171479	2	
MGIVGPEFK	39.8335	Q15149	PLEC_HUMAN	489.26042	2	0.817737639	2	Yes
MGIVGPEFK	39.8335	Q15149	PLEC_HUMAN	326.509555	3	0.817737639	2	
NLLDEELQR	39.15592	Q15149	PLEC_HUMAN	565.296576	2	0.857702494	2	Yes
NLLDEELQR	39.15592	Q15149	PLEC_HUMAN	377.2003257	3	0.857702494	2	
NLVDNITGQR	24.75463	Q15149	PLEC_HUMAN	565.302193	2	0.844066739	2	Yes
NLVDNITGQR	24.75463	Q15149	PLEC_HUMAN	377.2040703	3	0.844066739	2	
QEELYSELQAR	28.76924	Q15149	PLEC_HUMAN	683.3364255	2	0.60281384	2	Yes
QEELYSELQAR	28.76924	Q15149	PLEC_HUMAN	455.8935587	3	0.60281384	2	
QYLHLPPEIVPASLQR	66.31934	Q15149	PLEC_HUMAN	931.0207055	2	0.801885724		
QYLHLPPEIVPASLQR	66.31934	Q15149	PLEC_HUMAN	621.016412	3	0.801885724		
RPELEDSTLR	-8.944317	Q15149	PLEC_HUMAN	608.3205825	2	0.629313529	2	Yes
RPELEDSTLR	-8.944317	Q15149	PLEC_HUMAN	405.8829967	3	0.629313529	2	
SALDQYR	-9.57626	Q15149	PLEC_HUMAN	426.7146865	2	0.730269432	2	Yes
SALDQYR	-9.57626	Q15149	PLEC_HUMAN	284.8123993	3	0.730269432	2	
SDEGQLSPATR	-13.78598	Q15149	PLEC_HUMAN	580.7812905	2	0.769592226	2	Yes
SDEGQLSPATR	-13.78598	Q15149	PLEC_HUMAN	387.5234687	3	0.769592226	2	
SEEMQTVQQEQLLQETQALQQSFLSEK	115.5755	Q15149	PLEC_HUMAN	1590.772446	2	0.691263139		
SEEMQTVQQEQLLQETQALQQSFLSEK	115.5755	Q15149	PLEC_HUMAN	1060.850906	3	0.691263139		
SELELTLGK	37.43087	Q15149	PLEC_HUMAN	495.2798635	2	0.651838541	2	Yes

SELELTLGK	37.43087	Q15149	PLEC_HUMAN	330.5225173	3	0.651838541	2	
SIITYVSSLYDAMPR	113.1576	Q15149	PLEC_HUMAN	858.4378205	2	0.726813912		
SIITYVSSLYDAMPR	113.1576	Q15149	PLEC_HUMAN	572.627822	3	0.726813912		
SIQEELQQLR	40.71775	Q15149	PLEC_HUMAN	622.336233	2	0.805928111		
SIQEELQQLR	40.71775	Q15149	PLEC_HUMAN	415.2267637	3	0.805928111		
SLESLHSFVAAATK	42.16465	Q15149	PLEC_HUMAN	730.891372	2	0.764240861	2	Yes
SLESLHSFVAAATK	42.16465	Q15149	PLEC_HUMAN	487.5968563	3	0.764240861	2	
SLLAWQSLR	59.25598	Q15149	PLEC_HUMAN	537.3092895	2	0.844464898		
SLLAWQSLR	59.25598	Q15149	PLEC_HUMAN	358.5421347	3	0.844464898		
SLQEEHVAVAQLR	16.29172	Q15149	PLEC_HUMAN	740.399896	2	0.777747869	3	
SLQEEHVAVAQLR	16.29172	Q15149	PLEC_HUMAN	493.9358723	3	0.777747869	3	Yes
SLVPAAELLESR	70.03131	Q15149	PLEC_HUMAN	642.862083	2	0.848119318	2	Yes
SLVPAAELLESR	70.03131	Q15149	PLEC_HUMAN	428.9106637	3	0.848119318	2	
SMVEEGTGLR	3.571266	Q15149	PLEC_HUMAN	539.764055	2	0.783545673	2	Yes
SMVEEGTGLR	3.571266	Q15149	PLEC_HUMAN	360.178645	3	0.783545673	2	
SQVEEELFSVR	47.11428	Q15149	PLEC_HUMAN	661.8335225	2	0.747169673	3	
SQVEEELFSVR	47.11428	Q15149	PLEC_HUMAN	441.55829	3	0.747169673	3	Yes
SQVMDEATALQLR	46.94559	Q15149	PLEC_HUMAN	731.37249	2	0.813666463	2	Yes
SQVMDEATALQLR	46.94559	Q15149	PLEC_HUMAN	487.9176017	3	0.813666463	2	
SSIAGLLLK	58.57608	Q15149	PLEC_HUMAN	451.290034	2	0.76260072	2	Yes
SSIAGLLLK	58.57608	Q15149	PLEC_HUMAN	301.1959643	3	0.76260072	2	
SSSVGSSSSYPISPAVSR	27.16103	Q15149	PLEC_HUMAN	877.931948	2	0.790776014	3	
SSSVGSSSSYPISPAVSR	27.16103	Q15149	PLEC_HUMAN	585.623907	3	0.790776014	3	Yes
THYSELTTLTSQYIK	40.06619	Q15149	PLEC_HUMAN	892.9574325	2	0.815984309		
THYSELTTLTSQYIK	40.06619	Q15149	PLEC_HUMAN	595.6408967	3	0.815984309		
TLLQGSGCLAGIYLEDTK	78.57428	Q15149	PLEC_HUMAN	969.9962365	2	0.6725173	3	
TLLQGSGCLAGIYLEDTK	78.57428	Q15149	PLEC_HUMAN	647.0000993	3	0.6725173	3	Yes
TQLASWSDPTEETGPVAGILDTETLEK	103.5676	Q15149	PLEC_HUMAN	1444.706566	2	0.81934917		
TQLASWSDPTEETGPVAGILDTETLEK	103.5676	Q15149	PLEC_HUMAN	963.4736523	3	0.81934917		
TVTIWEIINSEYFTAEQR	138.5388	Q15149	PLEC_HUMAN	1100.550221	2	0.644903183		
TVTIWEIINSEYFTAEQR	138.5388	Q15149	PLEC_HUMAN	734.036089	3	0.644903183		
TVTVWELISSEYFTAEQR	130.8632	Q15149	PLEC_HUMAN	1080.036947	2	0.610636294	3	
TVTVWELISSEYFTAEQR	130.8632	Q15149	PLEC_HUMAN	720.3605727	3	0.610636294	3	Yes
VDGAEWGVDLPSVEAQLGSHR	78.84174	Q15149	PLEC_HUMAN	1111.546006	2	0.762854695	3	
VDGAEWGVDLPSVEAQLGSHR	78.84174	Q15149	PLEC_HUMAN	741.366612	3	0.762854695	3	Yes
VLALPEPSPAAPTLR	63.85163	Q15149	PLEC_HUMAN	766.446315	2	0.880589724	3	
VLALPEPSPAAPTLR	63.85163	Q15149	PLEC_HUMAN	511.3001517	3	0.880589724	3	Yes
VPLDEALQR	23.2466	Q15149	PLEC_HUMAN	520.79093	2	0.680222094	2	Yes
VPLDEALQR	23.2466	Q15149	PLEC_HUMAN	347.529895	3	0.680222094	2	
VPPGYHPLDVEK	12.76667	Q15149	PLEC_HUMAN	675.856797	2	0.733391881	3	
VPPGYHPLDVEK	12.76667	Q15149	PLEC_HUMAN	450.9071397	3	0.733391881	3	Yes
VQSGSESVIQEYVDLR	63.7784	Q15149	PLEC_HUMAN	904.9554245	2	0.807866633	3	
VQSGSESVIQEYVDLR	63.7784	Q15149	PLEC_HUMAN	603.639558	3	0.807866633	3	Yes
VSIYEAMR	23.60874	Q15149	PLEC_HUMAN	484.747672	2	0.635514617		
VSIYEAMR	23.60874	Q15149	PLEC_HUMAN	323.5010563	3	0.635514617		
VTLVQTLEIQR	54.41348	Q15149	PLEC_HUMAN	650.3857265	2	0.776452065	2	Yes
VTLVQTLEIQR	54.41348	Q15149	PLEC_HUMAN	433.926426	3	0.776452065	2	
VVIVDPETGK	15.82909	Q15149	PLEC_HUMAN	528.8009635	2	0.621383429	2	Yes
VVIVDPETGK	15.82909	Q15149	PLEC_HUMAN	352.8699173	3	0.621383429	2	
WQAVLAQTDVR	35.87664	Q15149	PLEC_HUMAN	643.8467685	2	0.778132141	2	Yes
WQAVLAQTDVR	35.87664	Q15149	PLEC_HUMAN	429.5671207	3	0.778132141	2	
YASGSSASLGGPESAVA	37.22154	Q15149	PLEC_HUMAN	755.855179	2	0.687370658	2	Yes
YASGSSASLGGPESAVA	37.22154	Q15149	PLEC_HUMAN	504.2393943	3	0.687370658	2	
YLQDLLAWVEENQHR	96.39035	Q15149	PLEC_HUMAN	957.4794015	2	0.818459511	3	
YLQDLLAWVEENQHR	96.39035	Q15149	PLEC_HUMAN	638.6555427	3	0.818459511	3	Yes
AVHEQLAALSQAPVNKPK	6.530964	Q15059	BRD3_HUMAN	951.0343485	2	0.83865875		
AVHEQLAALSQAPVNKPK	6.530964	Q15059	BRD3_HUMAN	634.3588407	3	0.83865875		
DSNPDEIEIDFETLKPTTLR	81.75467	Q15059	BRD3_HUMAN	1167.079544	2	0.80705452	3	
DSNPDEIEIDFETLKPTTLR	81.75467	Q15059	BRD3_HUMAN	778.3889707	3	0.80705452	3	Yes
EYPDAQGFAADVR	37.83262	Q15059	BRD3_HUMAN	719.8340505	2	0.751781285	2	Yes
EYPDAQGFAADVR	37.83262	Q15059	BRD3_HUMAN	480.2253087	3	0.751781285	2	
HQFAWPFYQPVDAIK	81.38406	Q15059	BRD3_HUMAN	923.967942	2	0.84290123	3	
HQFAWPFYQPVDAIK	81.38406	Q15059	BRD3_HUMAN	616.3145697	3	0.84290123	3	Yes
LNLPDYHK	5.499279	Q15059	BRD3_HUMAN	500.267086	2	0.837204874		
LNLPDYHK	5.499279	Q15059	BRD3_HUMAN	333.8473323	3	0.837204874		
LQDVSGQLSSSK	0.520985	Q15059	BRD3_HUMAN	624.8256975	2	0.7701419	2	Yes
LQDVSGQLSSSK	0.520985	Q15059	BRD3_HUMAN	416.8864067	3	0.7701419	2	
SESPPPLSDPK	4.597885	Q15059	BRD3_HUMAN	577.290959	2	0.663662493		
SESPPPLSDPK	4.597885	Q15059	BRD3_HUMAN	385.196581	3	0.663662493		
TNQLQYMQNVVVK	47.24092	Q15059	BRD3_HUMAN	782.911778	2	0.744879961	2	Yes
TNQLQYMQNVVVK	47.24092	Q15059	BRD3_HUMAN	522.277127	3	0.744879961	2	
VAQMPQEEVELLPPAPK	66.81485	Q15059	BRD3_HUMAN	938.498419	2	0.839507878		
VAQMPQEEVELLPPAPK	66.81485	Q15059	BRD3_HUMAN	626.0015543	3	0.839507878		
WTTEQQQR	-25.05512	Q15041	AR6P1_HUMAN	538.760162	2	0.67145431		
WTTEQQQR	-25.05512	Q15041	AR6P1_HUMAN	359.509383	3	0.67145431		
AIYQVYNALQEK	42.99863	Q15018	ABRX2_HUMAN	720.380632	2	0.86006546		
AIYQVYNALQEK	42.99863	Q15018	ABRX2_HUMAN	480.5896963	3	0.86006546		
EHGTDFFDKDGVMK	14.01884	Q15018	ABRX2_HUMAN	813.3674045	2	0.7736817		
EHGTDFFDKDGVMK	14.01884	Q15018	ABRX2_HUMAN	542.580878	3	0.7736817		
ISLAIPNLGNTSQQEYK	67.39237	Q15018	ABRX2_HUMAN	938.494717	2	0.685393572		
ISLAIPNLGNTSQQEYK	67.39237	Q15018	ABRX2_HUMAN	625.9990863	3	0.685393572		
LFSFYDYASK	63.10353	Q15018	ABRX2_HUMAN	620.7984115	2	0.794077516	2	Yes
LFSFYDYASK	63.10353	Q15018	ABRX2_HUMAN	414.2015493	3	0.794077516	2	
STLGDAEASDPPPPYSDFHPNNQESTLSHSR	34.63539	Q15018	ABRX2_HUMAN	1677.251249	2	0.736960053	4	
STLGDAEASDPPPPYSDFHPNNQESTLSHSR	34.63539	Q15018	ABRX2_HUMAN	1118.503441	3	0.736960053	4	
SVFMPRPQAVGSSNYASTSAGLK	37.775	Q15018	ABRX2_HUMAN	1178.092261	2	0.67160809		
SVFMPRPQAVGSSNYASTSAGLK	37.775	Q15018	ABRX2_HUMAN	785.7307823	3	0.67160809		
VSSVPNTSQSYAK	-12.65065	Q15018	ABRX2_HUMAN	684.34425	2	0.682341039	2	Yes
VSSVPNTSQSYAK	-12.65065	Q15018	ABRX2_HUMAN	456.5654417	3	0.682341039	2	
YPGSGADLPPPQR	17.69807	Q15018	ABRX2_HUMAN	677.8416785	2	0.730167687		
YPGSGADLPPPQR	17.69807	Q15018	ABRX2_HUMAN	452.230394	3	0.730167687		
ALQIPEVYLR	69.53056	Q14C86	GAPD1_HUMAN	601.3511505	2	0.819133043		
ALQIPEVYLR	69.53056	Q14C86	GAPD1_HUMAN	401.2367087	3	0.819133043		
APYIAYLTR	44.28229	Q14C86	GAPD1_HUMAN	534.2983825	2	0.753009379	2	Yes
APYIAYLTR	44.28229	Q14C86	GAPD1_HUMAN	356.5348633	3	0.753009379	2	
AVETPPLSSVNLLEGLSR	100.5715	Q14C86	GAPD1_HUMAN	941.518196	2	0.773665786		
AVETPPLSSVNLLEGLSR	100.5715	Q14C86	GAPD1_HUMAN	628.014739	3	0.773665786		
DEALQNISADDLPDSASQAAHPQDSAFSYR	65.01591	Q14C86	GAPD1_HUMAN	1610.227243	2	0.617245734	3	
DEALQNISADDLPDSASQAAHPQDSAFSYR	65.01591	Q14C86	GAPD1_HUMAN	1073.82077	3	0.617245734	3	Yes
DISETVSETWSTDVLGSDFDPNIDEDR	123.3283	Q14C86	GAPD1_HUMAN	1521.670907	2	0.612269104		
DISETVSETWSTDVLGSDFDPNIDEDR	123.3283	Q14C86	GAPD1_HUMAN	1014.783213	3	0.612269104		

DKDDLGPDR	-29.52048	Q14C86	GAPD1_HUMAN	515.7441765	2	0.668252766		
DKDDLGPDR	-29.52048	Q14C86	GAPD1_HUMAN	344.1653927	3	0.668252766		
DMMGLTDDR	29.64949	Q14C86	GAPD1_HUMAN	527.221166	2	0.771703184		
DMMGLTDDR	29.64949	Q14C86	GAPD1_HUMAN	351.816719	3	0.771703184		
DQVLHEHIQR	-25.96403	Q14C86	GAPD1_HUMAN	637.834192	2	0.800429881	2	Yes
DQVLHEHIQR	-25.96403	Q14C86	GAPD1_HUMAN	425.5587363	3	0.800429881	2	
EAPWPSAQSEIR	33.33569	Q14C86	GAPD1_HUMAN	685.8391395	2	0.837433279		
EAPWPSAQSEIR	33.33569	Q14C86	GAPD1_HUMAN	457.5620347	3	0.837433279		
EFIQDFQK	31.22002	Q14C86	GAPD1_HUMAN	527.764381	2	0.662304461	2	Yes
EFIQDFQK	31.22002	Q14C86	GAPD1_HUMAN	352.1788623	3	0.662304461	2	
FSLCSDNLEGISEGPSNR	53.67365	Q14C86	GAPD1_HUMAN	991.450184	2	0.845843792		
FSLCSDNLEGISEGPSNR	53.67365	Q14C86	GAPD1_HUMAN	661.302731	3	0.845843792		
FSTLTDDPSPR	19.7666	Q14C86	GAPD1_HUMAN	618.299316	2	0.802879035	2	Yes
FSTLTDDPSPR	19.7666	Q14C86	GAPD1_HUMAN	412.5354857	3	0.802879035	2	
ILEDTQFVDGYK	44.60683	Q14C86	GAPD1_HUMAN	714.356827	2	0.836619139	2	Yes
ILEDTQFVDGYK	44.60683	Q14C86	GAPD1_HUMAN	476.5738263	3	0.836619139	2	
LAFYPNQDGDILR	61.4079	Q14C86	GAPD1_HUMAN	761.3889925	2	0.864204526	2	Yes
LAFYPNQDGDILR	61.4079	Q14C86	GAPD1_HUMAN	507.9286033	3	0.864204526	2	
LALCSADSVAFPVLTHSTR	65.56258	Q14C86	GAPD1_HUMAN	1023.028406	2	0.742746532		
LALCSADSVAFPVLTHSTR	65.56258	Q14C86	GAPD1_HUMAN	682.354879	3	0.742746532		
LDIHTLAHHLK	10.99495	Q14C86	GAPD1_HUMAN	649.372953	2	0.792668045	3	
LDIHTLAHHLK	10.99495	Q14C86	GAPD1_HUMAN	433.2512437	3	0.792668045	3	Yes
LFLTATLHEPIMQLLVEDEDHLETDPNK	110.8783	Q14C86	GAPD1_HUMAN	1631.321574	2	0.713745534		
LFLTATLHEPIMQLLVEDEDHLETDPNK	110.8783	Q14C86	GAPD1_HUMAN	1087.883658	3	0.713745534		
LFSEGLFSAK	54.0415	Q14C86	GAPD1_HUMAN	549.7956805	2	0.766000688	2	Yes
LFSEGLFSAK	54.0415	Q14C86	GAPD1_HUMAN	366.8663953	3	0.766000688	2	
LIASSLVAGEK	23.38637	Q14C86	GAPD1_HUMAN	544.3220625	2	0.67619729	2	Yes
LIASSLVAGEK	23.38637	Q14C86	GAPD1_HUMAN	363.2173167	3	0.67619729	2	
LIITSAEASPAECCQHAK	7.787598	Q14C86	GAPD1_HUMAN	993.4751495	2	0.838135123		
LIITSAEASPAECCQHAK	7.787598	Q14C86	GAPD1_HUMAN	662.652708	3	0.838135123		
LLASIAEDYR	38.78719	Q14C86	GAPD1_HUMAN	575.809315	2	0.734254479	2	Yes
LLASIAEDYR	38.78719	Q14C86	GAPD1_HUMAN	384.2088183	3	0.734254479	2	
LLQQLAMTGSEEGDPR	42.4502	Q14C86	GAPD1_HUMAN	872.9309005	2	0.761574805	3	
LLQQLAMTGSEEGDPR	42.4502	Q14C86	GAPD1_HUMAN	582.2898753	3	0.761574805	3	Yes
LPNFGSHVLTPAEMEAFK	65.85295	Q14C86	GAPD1_HUMAN	994.5014925	2	0.640684485		
LPNFGSHVLTPAEMEAFK	65.85295	Q14C86	GAPD1_HUMAN	663.3369367	3	0.640684485		
LQELESCSGLGSTSDDTDVR	34.46375	Q14C86	GAPD1_HUMAN	1084.984786	2	0.708748162	3	
LQELESCSGLGSTSDDTDVR	34.46375	Q14C86	GAPD1_HUMAN	723.6591323	3	0.708748162	3	Yes
LSAQAQVAEDILDK	52.44657	Q14C86	GAPD1_HUMAN	750.8993945	2	0.836746037	2	Yes
LSAQAQVAEDILDK	52.44657	Q14C86	GAPD1_HUMAN	500.935538	3	0.836746037	2	
MALDNLLANLPPAKPGK	73.08328	Q14C86	GAPD1_HUMAN	881.9983885	2	0.821901441	3	
MALDNLLANLPPAKPGK	73.08328	Q14C86	GAPD1_HUMAN	588.3348673	3	0.821901441	3	Yes
NLMAQLQETMR	60.62817	Q14C86	GAPD1_HUMAN	667.8319465	2	0.837150455	2	Yes
NLMAQLQETMR	60.62817	Q14C86	GAPD1_HUMAN	445.5572393	3	0.837150455	2	
QLGFQETAYGEFLSR	74.31226	Q14C86	GAPD1_HUMAN	873.4288465	2	0.705442965		
QLGFQETAYGEFLSR	74.31226	Q14C86	GAPD1_HUMAN	582.6218393	3	0.705442965		
TSPSDGAMANYESTGDNHDR	-3.404751	Q14C86	GAPD1_HUMAN	1062.930341	2	0.662154973		
TSPSDGAMANYESTGDNHDR	-3.404751	Q14C86	GAPD1_HUMAN	708.9561687	3	0.662154973		
VQEMVESNEAK	-18.62231	Q14C86	GAPD1_HUMAN	632.2984585	2	0.670896649		
VQEMVESNEAK	-18.62231	Q14C86	GAPD1_HUMAN	421.8682473	3	0.670896649		
VQIAEAINLQDK	42.13863	Q14C86	GAPD1_HUMAN	671.3728155	2	0.790725589		
VQIAEAINLQDK	42.13863	Q14C86	GAPD1_HUMAN	447.9178187	3	0.790725589		
YLIEFELK	68.7528	Q14C86	GAPD1_HUMAN	527.795145	2	0.663844347	2	Yes
YLIEFELK	68.7528	Q14C86	GAPD1_HUMAN	352.1993717	3	0.663844347	2	
AAVENLPTFLVELSR	109.7508	Q14974	IMB1_HUMAN	829.959786	2	0.778435349	3	
AAVENLPTFLVELSR	109.7508	Q14974	IMB1_HUMAN	553.6424657	3	0.778435349	3	Yes
ESCLEAYTGIVQGLK	76.20672	Q14974	IMB1_HUMAN	834.419633	2	0.826410532	3	
ESCLEAYTGIVQGLK	76.20672	Q14974	IMB1_HUMAN	556.615697	3	0.826410532	3	Yes
GDQENVHPDVMLVQPR	33.64147	Q14974	IMB1_HUMAN	917.4497915	2	0.697990417	3	
GDQENVHPDVMLVQPR	33.64147	Q14974	IMB1_HUMAN	611.969136	3	0.697990417	3	Yes
HFIMQVVCEATQCPDTR	54.49364	Q14974	IMB1_HUMAN	1046.474628	2	0.732151508	3	
HFIMQVVCEATQCPDTR	54.49364	Q14974	IMB1_HUMAN	697.9856933	3	0.732151508	3	Yes
LAATNALLNSLEFTK	81.30887	Q14974	IMB1_HUMAN	803.4465115	2	0.84130013	2	Yes
LAATNALLNSLEFTK	81.30887	Q14974	IMB1_HUMAN	535.9669493	3	0.84130013	2	
LLETTDRPDGHQNNLR	-18.50889	Q14974	IMB1_HUMAN	939.975019	2	0.698435187	3	
LLETTDRPDGHQNNLR	-18.50889	Q14974	IMB1_HUMAN	626.9859543	3	0.698435187	3	Yes
LQQVLQMESHIQSTSDR	28.95264	Q14974	IMB1_HUMAN	1000.49747	2	0.847187519	3	
LQQVLQMESHIQSTSDR	28.95264	Q14974	IMB1_HUMAN	667.334255	3	0.847187519	3	Yes
LVEARPMIHELLTEGR	42.48782	Q14974	IMB1_HUMAN	932.509652	2	0.679848194		
LVEARPMIHELLTEGR	42.48782	Q14974	IMB1_HUMAN	622.009043	3	0.679848194		
MELITILEK	72.7197	Q14974	IMB1_HUMAN	545.315392	2	0.768064737		
MELITILEK	72.7197	Q14974	IMB1_HUMAN	363.8795363	3	0.768064737		
SDYDMVDYLNELR	92.03554	Q14974	IMB1_HUMAN	816.864685	2	0.811429262	2	Yes
SDYDMVDYLNELR	92.03554	Q14974	IMB1_HUMAN	544.9123983	3	0.811429262	2	
SNEILTAIIQGMR	95.68665	Q14974	IMB1_HUMAN	723.393225	2	0.754688978	2	Yes
SNEILTAIIQGMR	95.68665	Q14974	IMB1_HUMAN	482.5980917	3	0.754688978	2	
SSAYESLMEIVK	66.54329	Q14974	IMB1_HUMAN	678.8399545	2	0.832997918	2	Yes
SSAYESLMEIVK	66.54329	Q14974	IMB1_HUMAN	452.8959113	3	0.832997918	2	
VAALQNLVK	28.91479	Q14974	IMB1_HUMAN	478.3009335	2	0.787569821	2	Yes
VAALQNLVK	28.91479	Q14974	IMB1_HUMAN	319.2032307	3	0.787569821	2	
VLANPGNSQVAR	-10.66841	Q14974	IMB1_HUMAN	613.336567	2	0.747842371	2	Yes
VLANPGNSQVAR	-10.66841	Q14974	IMB1_HUMAN	409.2269863	3	0.747842371	2	
VQHQDALQISDVVMASLLR	107.055	Q14974	IMB1_HUMAN	1062.068063	2	0.704266191	4	
VQHQDALQISDVVMASLLR	107.055	Q14974	IMB1_HUMAN	708.3813167	3	0.704266191	4	
YLEVVLNTLQQASQAQVDK	90.8111	Q14974	IMB1_HUMAN	1074.071321	2	0.650523663	3	
YLEVVLNTLQQASQAQVDK	90.8111	Q14974	IMB1_HUMAN	716.383489	3	0.650523663	3	Yes
YMEAFKPFLGIGLK	81.58061	Q14974	IMB1_HUMAN	807.4421825	2	0.780678928	2	Yes
YMEAFKPFLGIGLK	81.58061	Q14974	IMB1_HUMAN	538.63073	3	0.780678928	2	
DLSEPYSIYTYR	59.37065	Q147X3	NAA30_HUMAN	753.8597245	2	0.883920908	2	Yes
DLSEPYSIYTYR	59.37065	Q147X3	NAA30_HUMAN	502.9090913	3	0.883920908	2	
GYIAMLAVDSK	56.60794	Q147X3	NAA30_HUMAN	584.308094	2	0.78883338	2	Yes
GYIAMLAVDSK	56.60794	Q147X3	NAA30_HUMAN	389.874671	3	0.78883338	2	
LLSSSLTADCSLR	35.95297	Q147X3	NAA30_HUMAN	711.8670395	2	0.748575509		
LLSSSLTADCSLR	35.95297	Q147X3	NAA30_HUMAN	474.913968	3	0.748575509		
LYENLGFVR	47.05616	Q147X3	NAA30_HUMAN	555.801293	2	0.825476825		
LYENLGFVR	47.05616	Q147X3	NAA30_HUMAN	370.870137	3	0.825476825		
TAVPSPVEAAAASDPAAAR	38.68564	Q147X3	NAA30_HUMAN	876.4503145	2	0.704425871		
TAVPSPVEAAAASDPAAAR	38.68564	Q147X3	NAA30_HUMAN	584.6361513	3	0.704425871		
VLSVAEVAATTATPDGGPR	54.61583	Q147X3	NAA30_HUMAN	906.4790725	2	0.740011692		
VLSVAEVAATTATPDGGPR	54.61583	Q147X3	NAA30_HUMAN	604.6553233	3	0.740011692		
YESELQMPDIMR	64.10768	Q147X3	NAA30_HUMAN	756.3476215	2	0.831069231		

YESELQMPDIMR	64.10768	Q147X3	NAA30 HUMAN	504.5676893	3	0.831069231		
YYLNGVDALR	43.24317	Q147X3	NAA30 HUMAN	592.309478	2	0.708369911		
YYLNGVDALR	43.24317	Q147X3	NAA30 HUMAN	395.208927	3	0.708369911		
ALNQLSVPLGQLR	68.36433	Q14746	COG2 HUMAN	704.9177245	2	0.861923814		
ALNQLSVPLGQLR	68.36433	Q14746	COG2 HUMAN	470.2810913	3	0.861923814		
DDLELYYK	43.40725	Q14746	COG2 HUMAN	529.7562125	2	0.716152787		
DDLELYYK	43.40725	Q14746	COG2 HUMAN	353.50675	3	0.716152787		
DYADFVNLSTNLVGMDK	114.8328	Q14746	COG2 HUMAN	951.4516605	2	0.792178273		
DYADFVNLSTNLVGMDK	114.8328	Q14746	COG2 HUMAN	634.6370487	3	0.792178273		
EDFDVDHFVSDCRK	28.08381	Q14746	COG2 HUMAN	884.8839495	2	0.692383468		
EDFDVDHFVSDCRK	28.08381	Q14746	COG2 HUMAN	590.2585747	3	0.692383468		
EPSITQGNTEDQGSGPSETKPVVSISR	25.32383	Q14746	COG2 HUMAN	1400.683956	2	0.720685601	3	
EPSITQGNTEDQGSGPSETKPVVSISR	25.32383	Q14746	COG2 HUMAN	934.1252453	3	0.720685601	3	Yes
ETSALEASSPLLTGQILER	83.18893	Q14746	COG2 HUMAN	1008.036951	2	0.772381961		
ETSALEASSPLLTGQILER	83.18893	Q14746	COG2 HUMAN	672.3605753	3	0.772381961		
EVPTTASSYVDSALKPLFQLQSGHK	79.17345	Q14746	COG2 HUMAN	1352.203595	2	0.722566783		
EVPTTASSYVDSALKPLFQLQSGHK	79.17345	Q14746	COG2 HUMAN	901.8050047	3	0.722566783		
GPDTLCFDKDEFMK	38.34149	Q14746	COG2 HUMAN	851.876748	2	0.814271331		
GPDTLCFDKDEFMK	38.34149	Q14746	COG2 HUMAN	568.2537737	3	0.814271331		
IATEFNQLQFHAVQSK	41.95803	Q14746	COG2 HUMAN	930.9843245	2	0.798517168		
IATEFNQLQFHAVQSK	41.95803	Q14746	COG2 HUMAN	620.992158	3	0.798517168		
IIQDLSDSCFGFLK	83.77481	Q14746	COG2 HUMAN	821.9114475	2	0.728485346		
IIQDLSDSCFGFLK	83.77481	Q14746	COG2 HUMAN	548.2769067	3	0.728485346		
LGLQASDIK	17.00082	Q14746	COG2 HUMAN	472.7747485	2	0.79488188		
LGLQASDIK	17.00082	Q14746	COG2 HUMAN	315.5191073	3	0.79488188		
LPSLFNPGNPDAFHEK	54.08981	Q14746	COG2 HUMAN	891.944667	2	0.856683254	3	
LPSLFNPGNPDAFHEK	54.08981	Q14746	COG2 HUMAN	594.9657197	3	0.856683254	3	Yes
LQEQLPELLEIIKPK	87.01077	Q14746	COG2 HUMAN	896.035494	2	0.864420056	3	
LQEQLPELLEIIKPK	87.01077	Q14746	COG2 HUMAN	597.6929377	3	0.864420056	3	Yes
LQLALDVEYLGEQIQK	95.23599	Q14746	COG2 HUMAN	930.5098365	2	0.790272951		
LQLALDVEYLGEQIQK	95.23599	Q14746	COG2 HUMAN	620.6758327	3	0.790272951		
SFSALAELVAAAK	90.17233	Q14746	COG2 HUMAN	639.3591765	2	0.788124442		
SFSALAELVAAAK	90.17233	Q14746	COG2 HUMAN	426.5753927	3	0.788124442		
TQLVYVVADLDK	67.62922	Q14746	COG2 HUMAN	682.3775625	2	0.744842589		
TQLVYVVADLDK	67.62922	Q14746	COG2 HUMAN	455.2543167	3	0.744842589		
WNLPVYFQIR	96.03688	Q14746	COG2 HUMAN	668.364592	2	0.786349177		
WNLPVYFQIR	96.03688	Q14746	COG2 HUMAN	445.9123363	3	0.786349177		
YSVFVNELSLRPISNESPK	69.45296	Q14746	COG2 HUMAN	1090.073862	2	0.645145297		
YSVFVNELSLRPISNESPK	69.45296	Q14746	COG2 HUMAN	727.0518493	3	0.645145297		
YTISMDFVR	56.50413	Q14746	COG2 HUMAN	566.2793365	2	0.78795296		
YTISMDFVR	56.50413	Q14746	COG2 HUMAN	377.8554993	3	0.78795296		
YYETVSDVLNSVK	63.14695	Q14746	COG2 HUMAN	758.880661	2	0.840725362		
YYETVSDVLNSVK	63.14695	Q14746	COG2 HUMAN	506.2563823	3	0.840725362		
APSIALGFR	42.41754	Q14728	MFS10 HUMAN	466.272176	2	0.693841219	2	Yes
APSIALGFR	42.41754	Q14728	MFS10 HUMAN	311.184059	3	0.693841219	2	
DAADLLSPLALLR	143.9774	Q14728	MFS10 HUMAN	684.398833	2	0.778303385	2	Yes
DAADLLSPLALLR	143.9774	Q14728	MFS10 HUMAN	456.6018303	3	0.778303385	2	
FQFSSLQQGK	27.89658	Q14728	MFS10 HUMAN	585.301662	2	0.755818546		
FQFSSLQQGK	27.89658	Q14728	MFS10 HUMAN	390.5370497	3	0.755818546		
GNVSLSTAIVADLGSPLAR	100.2965	Q14728	MFS10 HUMAN	921.0105385	2	0.626786232		
GNVSLSTAIVADLGSPLAR	100.2965	Q14728	MFS10 HUMAN	614.3429673	3	0.626786232		
LSYPAQTLK	14.53011	Q14728	MFS10 HUMAN	510.7903945	2	0.61181432		
LSYPAQTLK	14.53011	Q14728	MFS10 HUMAN	340.8628713	3	0.61181432		
SFAAFLASR	53.86353	Q14728	MFS10 HUMAN	485.261808	2	0.722055793		
SFAAFLASR	53.86353	Q14728	MFS10 HUMAN	323.8438137	3	0.722055793		
AFFAGSQR	3.970867	Q14697	GANAB HUMAN	442.225226	2	0.603828609	2	Yes
AFFAGSQR	3.970867	Q14697	GANAB HUMAN	295.152759	3	0.603828609	2	
ALLDSLQLGPDSLTVHLIHEVTK	103.0224	Q14697	GANAB HUMAN	1250.195046	2	0.894791603	4	
ALLDSLQLGPDSLTVHLIHEVTK	103.0224	Q14697	GANAB HUMAN	833.7993053	3	0.894791603	4	
DAQHYGGWEHR	-25.2031	Q14697	GANAB HUMAN	678.2979695	2	0.753868401	3	
DAQHYGGWEHR	-25.2031	Q14697	GANAB HUMAN	452.534588	3	0.753868401	3	Yes
DENSVELTMAEGPYK	54.00921	Q14697	GANAB HUMAN	841.8830795	2	0.849718928	2	Yes
DENSVELTMAEGPYK	54.00921	Q14697	GANAB HUMAN	561.591328	3	0.849718928	2	
DEPGAWEETFK	44.4494	Q14697	GANAB HUMAN	654.791324	2	0.743719459	2	Yes
DEPGAWEETFK	44.4494	Q14697	GANAB HUMAN	436.863491	3	0.743719459	2	
DGSDYEGWCWPGSAGYPDFTNPTMR	111.9645	Q14697	GANAB HUMAN	1433.579583	2	0.756609082	3	
DGSDYEGWCWPGSAGYPDFTNPTMR	111.9645	Q14697	GANAB HUMAN	956.0556633	3	0.756609082	3	Yes
DVHNIYGLYVHMATADGLR	67.96057	Q14697	GANAB HUMAN	1073.031472	2	0.736326396	4	
DVHNIYGLYVHMATADGLR	67.96057	Q14697	GANAB HUMAN	715.690256	3	0.736326396	4	
EPWLLPSQHNDIIR	57.23116	Q14697	GANAB HUMAN	859.455202	2	0.789400578		
EPWLLPSQHNDIIR	57.23116	Q14697	GANAB HUMAN	573.3060763	3	0.789400578		
FGAVWTGDNTAEWDHLK	56.39708	Q14697	GANAB HUMAN	973.955764	2	0.877814412	3	
FGAVWTGDNTAEWDHLK	56.39708	Q14697	GANAB HUMAN	649.6397843	3	0.877814412	3	Yes
FSFSGNTLVSSSADPEGHFETPIWIER	92.34744	Q14697	GANAB HUMAN	1505.715058	2	0.686998665	3	
FSFSGNTLVSSSADPEGHFETPIWIER	92.34744	Q14697	GANAB HUMAN	1004.14598	3	0.686998665	3	Yes
GLLEFEHQR	14.05177	Q14697	GANAB HUMAN	564.794004	2	0.799047172	2	Yes
GLLEFEHQR	14.05177	Q14697	GANAB HUMAN	376.8652777	3	0.799047172	2	
IDELEPR	-1.303143	Q14697	GANAB HUMAN	436.2301735	2	0.644224405	2	Yes
IDELEPR	-1.303143	Q14697	GANAB HUMAN	291.1560573	3	0.644224405	2	
KPGINVASDWSIHLR	43.68218	Q14697	GANAB HUMAN	846.963194	2	0.814737618	3	
KPGINVASDWSIHLR	43.68218	Q14697	GANAB HUMAN	564.978071	3	0.814737618	3	Yes
LDLLEDR	30.29997	Q14697	GANAB HUMAN	437.2379985	2	0.686971784	2	Yes
LDLLEDR	30.29997	Q14697	GANAB HUMAN	291.8279407	3	0.686971784	2	
LSFQHDPETSVLVLR	52.14907	Q14697	GANAB HUMAN	870.9681425	2	0.832886934		
LSFQHDPETSVLVLR	52.14907	Q14697	GANAB HUMAN	580.98137	3	0.832886934		
MMDYLQGSGETPQTDVR	41.9642	Q14697	GANAB HUMAN	964.4304035	2	0.793131113		
MMDYLQGSGETPQTDVR	41.9642	Q14697	GANAB HUMAN	643.289544	3	0.793131113		
SGGMERPFVLAR	24.21402	Q14697	GANAB HUMAN	660.3486205	2	0.759019852	3	
SGGMERPFVLAR	24.21402	Q14697	GANAB HUMAN	440.5683553	3	0.759019852	3	Yes
SLLLSVNAR	36.69685	Q14697	GANAB HUMAN	486.7960145	2	0.685329318	2	Yes
SLLLSVNAR	36.69685	Q14697	GANAB HUMAN	324.866618	3	0.685329318	2	
VLLVLELQGLQK	93.56456	Q14697	GANAB HUMAN	676.9297695	2	0.775518298	2	Yes
VLLVLELQGLQK	93.56456	Q14697	GANAB HUMAN	451.6224547	3	0.775518298	2	
VPDVLVADPPIAR	61.92553	Q14697	GANAB HUMAN	681.393551	2	0.825142026	2	Yes
VPDVLVADPPIAR	61.92553	Q14697	GANAB HUMAN	454.598309	3	0.825142026	2	
VVIIGAGKPAAVVLQTK	43.32671	Q14697	GANAB HUMAN	832.52764	2	0.738397658	2	Yes
VVIIGAGKPAAVVLQTK	43.32671	Q14697	GANAB HUMAN	555.3543683	3	0.738397658	2	
WYQMGAYQPFFR	78.76035	Q14697	GANAB HUMAN	797.369545	2	0.764782488	2	Yes
WYQMGAYQPFFR	78.76035	Q14697	GANAB HUMAN	531.9156383	3	0.764782488	2	
YFTWDPSR	37.0714	Q14697	GANAB HUMAN	536.2488935	2	0.666979492	2	Yes
YFTWDPSR	37.0714	Q14697	GANAB HUMAN	357.835204	3	0.666979492	2	

AGLMLQEADK	15.06779	Q14651	PLSI_HUMAN	538.2767985	2	0.855995357		
AGLMLQEADK	15.06779	Q14651	PLSI_HUMAN	359.1871407	3	0.855995357		
ALENDPDCKHLIPMNPNDDSLFK	55.81131	Q14651	PLSI_HUMAN	1342.128719	2	0.720487177		
ALENDPDCKHLIPMNPNDDSLFK	55.81131	Q14651	PLSI_HUMAN	895.0884207	3	0.720487177		
EASLPLPGYK	42.43637	Q14651	PLSI_HUMAN	537.7956765	2	0.64027077		
EASLPLPGYK	42.43637	Q14651	PLSI_HUMAN	358.8663927	3	0.64027077		
EELEELQEAFNK	62.96346	Q14651	PLSI_HUMAN	739.854652	2	0.781267822	2	Yes
EELEELQEAFNK	62.96346	Q14651	PLSI_HUMAN	493.5723763	3	0.781267822	2	
EGITAIGGTSTISSEGTQHSYSEEEK	27.25265	Q14651	PLSI_HUMAN	1349.620486	2	0.809502423	3	
EGITAIGGTSTISSEGTQHSYSEEEK	27.25265	Q14651	PLSI_HUMAN	900.082932	3	0.809502423	3	Yes
GGEDGPAIAIDLSGINETNDLK	82.25166	Q14651	PLSI_HUMAN	1100.042961	2	0.804323852		
GGEDGPAIAIDLSGINETNDLK	82.25166	Q14651	PLSI_HUMAN	733.6979157	3	0.804323852		
IDIDNSGYVSDYELQDLFK	118.2695	Q14651	PLSI_HUMAN	1117.529146	2	0.761787951		
IDIDNSGYVSDYELQDLFK	118.2695	Q14651	PLSI_HUMAN	745.3553723	3	0.761787951		
ISFEEFVSLMQELK	152.8617	Q14651	PLSI_HUMAN	850.434755	2	0.66787529		
ISFEEFVSLMQELK	152.8617	Q14651	PLSI_HUMAN	567.292445	3	0.66787529		
IYALPDDLVEVKPK	60.08987	Q14651	PLSI_HUMAN	800.453801	2	0.865490794	3	
IYALPDDLVEVKPK	60.08987	Q14651	PLSI_HUMAN	533.971809	3	0.865490794	3	Yes
MINLSEPDTIDER	42.10323	Q14651	PLSI_HUMAN	766.8672365	2	0.844335079	2	Yes
MINLSEPDTIDER	42.10323	Q14651	PLSI_HUMAN	511.580766	3	0.844335079	2	
NEALIALLNEGEELEELMK	146.9549	Q14651	PLSI_HUMAN	1079.551576	2	0.723025978		
NEALIALLNEGEELEELMK	146.9549	Q14651	PLSI_HUMAN	720.036992	3	0.723025978		
SLADGILLCK	54.17847	Q14651	PLSI_HUMAN	545.302816	2	0.613761485		
SLADGILLCK	54.17847	Q14651	PLSI_HUMAN	363.8711523	3	0.613761485		
VGLFADIEISR	74.13888	Q14651	PLSI_HUMAN	610.338244	2	0.829008222		
VGLFADIEISR	74.13888	Q14651	PLSI_HUMAN	407.2281043	3	0.829008222		
VPVNWSHVNKPPYPALGGNMK	38.44176	Q14651	PLSI_HUMAN	1153.099692	2	0.622003078		
VPVNWSHVNKPPYPALGGNMK	38.44176	Q14651	PLSI_HUMAN	769.0690697	3	0.622003078		
YTLNVLSDLGEGEK	71.05912	Q14651	PLSI_HUMAN	769.3913975	2	0.857004046		
YTLNVLSDLGEGEK	71.05912	Q14651	PLSI_HUMAN	513.26354	3	0.857004046		
AATSPALFNR	14.94769	Q14204	DYHC1_HUMAN	524.283272	2	0.74640131	2	Yes
AATSPALFNR	14.94769	Q14204	DYHC1_HUMAN	349.858123	3	0.74640131	2	
AELGEYIR	19.60252	Q14204	DYHC1_HUMAN	475.751269	2	0.795911193	2	Yes
AELGEYIR	19.60252	Q14204	DYHC1_HUMAN	317.5034543	3	0.795911193	2	
ALEHAFQLEHIMDLTR	66.16921	Q14204	DYHC1_HUMAN	962.4914595	2	0.881033063	4	
ALEHAFQLEHIMDLTR	66.16921	Q14204	DYHC1_HUMAN	641.9969147	3	0.881033063	4	
ALGEYLER	19.84064	Q14204	DYHC1_HUMAN	475.751269	2	0.796728134	2	Yes
ALGEYLER	19.84064	Q14204	DYHC1_HUMAN	317.5034543	3	0.796728134	2	
AMSRPILYSNWLSK	52.10829	Q14204	DYHC1_HUMAN	833.443244	2	0.601840615		
AMSRPILYSNWLSK	52.10829	Q14204	DYHC1_HUMAN	555.964771	3	0.601840615		
ANEVEQMIR	18.05532	Q14204	DYHC1_HUMAN	545.272047	2	0.739772856	2	Yes
ANEVEQMIR	18.05532	Q14204	DYHC1_HUMAN	363.8506397	3	0.739772856	2	
ASLACGPMVK	10.25996	Q14204	DYHC1_HUMAN	517.262637	2	0.794294596		
ASLACGPMVK	10.25996	Q14204	DYHC1_HUMAN	345.1776997	3	0.794294596		
ATSIDPNTYITWIDK	82.5562	Q14204	DYHC1_HUMAN	869.4388795	2	0.742805004		
ATSIDPNTYITWIDK	82.5562	Q14204	DYHC1_HUMAN	579.9618613	3	0.742805004		
AVDDLNLHSYSNLPIWVNK	74.08006	Q14204	DYHC1_HUMAN	1099.566204	2	0.827686906	3	
AVDDLNLHSYSNLPIWVNK	74.08006	Q14204	DYHC1_HUMAN	733.3800777	3	0.827686906	3	Yes
AWTQVLLGQAEDK	58.92029	Q14204	DYHC1_HUMAN	729.883548	2	0.745819926		
AWTQVLLGQAEDK	58.92029	Q14204	DYHC1_HUMAN	486.9249737	3	0.745819926		
DFPLNDLLSATELDK	122.2478	Q14204	DYHC1_HUMAN	845.930891	2	0.846534073		
DFPLNDLLSATELDK	122.2478	Q14204	DYHC1_HUMAN	564.289869	3	0.846534073		
DHLYGTLDPNTR	13.93645	Q14204	DYHC1_HUMAN	701.3420425	2	0.821171284	3	
DHLYGTLDPNTR	13.93645	Q14204	DYHC1_HUMAN	467.8973033	3	0.821171284	3	Yes
DIQMPDGIR	37.06676	Q14204	DYHC1_HUMAN	522.7613155	2	0.706439316	2	Yes
DIQMPDGIR	37.06676	Q14204	DYHC1_HUMAN	348.8434853	3	0.706439316	2	
DLFQVAFNR	76.04966	Q14204	DYHC1_HUMAN	555.291097	2	0.778213084	2	Yes
DLFQVAFNR	76.04966	Q14204	DYHC1_HUMAN	370.5300063	3	0.778213084	2	
DLPPVSGSIIWAK	80.08511	Q14204	DYHC1_HUMAN	691.888101	2	0.815185547	2	Yes
DLPPVSGSIIWAK	80.08511	Q14204	DYHC1_HUMAN	461.5946757	3	0.815185547	2	
DLSSQLLK	38.76784	Q14204	DYHC1_HUMAN	452.2614735	2	0.663941324	2	Yes
DLSSQLLK	38.76784	Q14204	DYHC1_HUMAN	301.8435907	3	0.663941324	2	
DPTVEFPPDLCSR	64.64858	Q14204	DYHC1_HUMAN	766.8566725	2	0.712359369	2	Yes
DPTVEFPPDLCSR	64.64858	Q14204	DYHC1_HUMAN	511.5737233	3	0.712359369	2	
DSAIQQQVANLQMK	50.85986	Q14204	DYHC1_HUMAN	787.4043215	2	0.785880744	3	
DSAIQQQVANLQMK	50.85986	Q14204	DYHC1_HUMAN	525.272156	3	0.785880744	3	Yes
DVLLVAQGEMALEEFLK	151.0796	Q14204	DYHC1_HUMAN	953.0060765	2	0.692887008		
DVLLVAQGEMALEEFLK	151.0796	Q14204	DYHC1_HUMAN	635.673326	3	0.692887008		
DYIPVDQEELR	46.47704	Q14204	DYHC1_HUMAN	688.838801	2	0.839713931	2	Yes
DYIPVDQEELR	46.47704	Q14204	DYHC1_HUMAN	459.561809	3	0.839713931	2	
EALELTDTGLLSGSEER	65.37215	Q14204	DYHC1_HUMAN	910.4501765	2	0.82831198	3	
EALELTDTGLLSGSEER	65.37215	Q14204	DYHC1_HUMAN	607.302726	3	0.82831198	3	Yes
EDEGEEAASPMLQIQR	41.77577	Q14204	DYHC1_HUMAN	901.915447	2	0.639621437		
EDEGEEAASPMLQIQR	41.77577	Q14204	DYHC1_HUMAN	601.6129063	3	0.639621437		
EEYAVLISEAQAIK	77.19319	Q14204	DYHC1_HUMAN	782.4174155	2	0.77474457		
EEYAVLISEAQAIK	77.19319	Q14204	DYHC1_HUMAN	521.947552	3	0.77474457		
EFGPVVIDYGK	52.19479	Q14204	DYHC1_HUMAN	612.319516	2	0.758067608	2	Yes
EFGPVVIDYGK	52.19479	Q14204	DYHC1_HUMAN	408.5489523	3	0.758067608	2	
EGLMLDSHEELYK	32.43663	Q14204	DYHC1_HUMAN	782.3721505	2	0.84901762		
EGLMLDSHEELYK	32.43663	Q14204	DYHC1_HUMAN	521.9173753	3	0.84901762		
EGTEAWEAAMK	25.42661	Q14204	DYHC1_HUMAN	611.77462	2	0.711947024	2	Yes
EGTEAWEAAMK	25.42661	Q14204	DYHC1_HUMAN	408.1856883	3	0.711947024	2	
ENFIPTIVNFSAEEISDAIR	139.6172	Q14204	DYHC1_HUMAN	1133.074064	2	0.62261945	3	
ENFIPTIVNFSAEEISDAIR	139.6172	Q14204	DYHC1_HUMAN	755.7186507	3	0.62261945	3	Yes
EQPWVSVQPR	30.41912	Q14204	DYHC1_HUMAN	613.320386	2	0.83657825		
EQPWVSVQPR	30.41912	Q14204	DYHC1_HUMAN	409.216199	3	0.83657825		
ESPEVLLTLDILK	116.6874	Q14204	DYHC1_HUMAN	735.427256	2	0.725039959		
ESPEVLLTLDILK	116.6874	Q14204	DYHC1_HUMAN	490.620779	3	0.725039959		
EVWNTYELDLVNYQNK	82.43587	Q14204	DYHC1_HUMAN	1014.489627	2	0.785155416	3	
EVWNTYELDLVNYQNK	82.43587	Q14204	DYHC1_HUMAN	676.6623597	3	0.785155416	3	Yes
EWTDGLFTHVLR	70.38038	Q14204	DYHC1_HUMAN	737.3784325	2	0.79123795	3	
EWTDGLFTHVLR	70.38038	Q14204	DYHC1_HUMAN	491.9215633	3	0.79123795	3	Yes
EYQTQLIQR	12.26854	Q14204	DYHC1_HUMAN	589.81239	2	0.649310529		
EYQTQLIQR	12.26854	Q14204	DYHC1_HUMAN	393.5442017	3	0.649310529		
FGNPLLVQDVESYDPVLNPVLNR	126.1545	Q14204	DYHC1_HUMAN	1299.682297	2	0.624827385	3	
FGNPLLVQDVESYDPVLNPVLNR	126.1545	Q14204	DYHC1_HUMAN	866.7908063	3	0.624827385	3	Yes
FGQMLGSNMTEFHSQISK	46.17528	Q14204	DYHC1_HUMAN	1021.477692	2	0.754073322	3	
FGQMLGSNMTEFHSQISK	46.17528	Q14204	DYHC1_HUMAN	681.3210693	3	0.754073322	3	Yes
FHATVSFDTDTGLK	28.16813	Q14204	DYHC1_HUMAN	769.8784625	2	0.761157572		
FHATVSFDTDTGLK	28.16813	Q14204	DYHC1_HUMAN	513.58825	3	0.761157572		
FLSDPQVHTVLVER	39.81633	Q14204	DYHC1_HUMAN	820.4443035	2	0.748707414		

FLSDPQVHTVLVER	39.81633	Q14204	DYHC1 HUMAN	547.2988107	3	0.748707414		
FNYGFEYLGVQDK	69.81242	Q14204	DYHC1 HUMAN	790.3755465	2	0.8487764	2	Yes
FNYGFEYLGVQDK	69.81242	Q14204	DYHC1 HUMAN	527.2529727	3	0.8487764	2	
FQSISTEFLALMK	103.2712	Q14204	DYHC1 HUMAN	757.9003515	2	0.78049171	2	Yes
FQSISTEFLALMK	103.2712	Q14204	DYHC1 HUMAN	505.6028427	3	0.78049171	2	
FTQDTQPHYIYSPR	15.53241	Q14204	DYHC1 HUMAN	876.9211845	2	0.795760036		
FTQDTQPHYIYSPR	15.53241	Q14204	DYHC1 HUMAN	584.9500647	3	0.795760036		
FVAWMNGLSVYQIK	97.76935	Q14204	DYHC1 HUMAN	828.4348885	2	0.603776515		
FVAWMNGLSVYQIK	97.76935	Q14204	DYHC1 HUMAN	552.6258673	3	0.603776515		
GIFEALRPLETLPVEGLIR	118.9561	Q14204	DYHC1 HUMAN	1062.115339	2	0.652321339		
GIFEALRPLETLPVEGLIR	118.9561	Q14204	DYHC1 HUMAN	708.412834	3	0.652321339		
GMLHQDHITFAMLLAR	63.46001	Q14204	DYHC1 HUMAN	927.479841	2	0.774243534	4	
GMLHQDHITFAMLLAR	63.46001	Q14204	DYHC1 HUMAN	618.6558357	3	0.774243534	4	
GNEIVLSAGSTPR	22.5629	Q14204	DYHC1 HUMAN	650.8469645	2	0.794673085		
GNEIVLSAGSTPR	22.5629	Q14204	DYHC1 HUMAN	434.233918	3	0.794673085		
GTVGEPTYDAEFQHFLR	58.0766	Q14204	DYHC1 HUMAN	983.9688675	2	0.861278355	3	
GTVGEPTYDAEFQHFLR	58.0766	Q14204	DYHC1 HUMAN	656.3151867	3	0.861278355	3	Yes
GVWSELSK	25.13734	Q14204	DYHC1 HUMAN	453.240541	2	0.779048443	2	Yes
GVWSELSK	25.13734	Q14204	DYHC1 HUMAN	302.4963023	3	0.779048443	2	
GWDDLFNK	57.90119	Q14204	DYHC1 HUMAN	497.735623	2	0.747885585	2	Yes
GWDDLFNK	57.90119	Q14204	DYHC1 HUMAN	332.1596903	3	0.747885585	2	
GWENHVEGQK	-28.5639	Q14204	DYHC1 HUMAN	592.278718	2	0.756444156	3	
GWENHVEGQK	-28.5639	Q14204	DYHC1 HUMAN	395.1884203	3	0.756444156	3	Yes
HLLPVETQR	-1.879841	Q14204	DYHC1 HUMAN	546.812197	2	0.819027901	2	Yes
HLLPVETQR	-1.879841	Q14204	DYHC1 HUMAN	364.8774063	3	0.819027901	2	
HYLDFINHYANLFHEK	62.01892	Q14204	DYHC1 HUMAN	1031.003039	2	0.805409014	4	
HYLDFINHYANLFHEK	62.01892	Q14204	DYHC1 HUMAN	687.671301	3	0.805409014	4	
IAFIMDESNVLDSGFLER	112.8937	Q14204	DYHC1 HUMAN	1028.506971	2	0.801525712		
IAFIMDESNVLDSGFLER	112.8937	Q14204	DYHC1 HUMAN	686.0072557	3	0.801525712		
IFTIESTR	21.1315	Q14204	DYHC1 HUMAN	483.7669235	2	0.60236156	2	Yes
IFTIESTR	21.1315	Q14204	DYHC1 HUMAN	322.847224	3	0.60236156	2	
IFVFEPPPGVK	65.31596	Q14204	DYHC1 HUMAN	615.3506235	2	0.778235137		
IFVFEPPPGVK	65.31596	Q14204	DYHC1 HUMAN	410.5696907	3	0.778235137		
ILDDDTIITTLENLK	116.6075	Q14204	DYHC1 HUMAN	858.967474	2	0.807320178		
ILDDDTIITTLENLK	116.6075	Q14204	DYHC1 HUMAN	572.9809243	3	0.807320178		
IMFEVQDLK	53.20604	Q14204	DYHC1 HUMAN	561.7973665	2	0.78302449		
IMFEVQDLK	53.20604	Q14204	DYHC1 HUMAN	374.8675193	3	0.78302449		
INEWLTLVEK	73.22729	Q14204	DYHC1 HUMAN	622.8484445	2	0.823059082		
INEWLTLVEK	73.22729	Q14204	DYHC1 HUMAN	415.568238	3	0.823059082		
INMLVIELK	72.45689	Q14204	DYHC1 HUMAN	536.8259265	2	0.76256609	2	Yes
INMLVIELK	72.45689	Q14204	DYHC1 HUMAN	358.2198927	3	0.76256609	2	
IQFVGACNPPTDPGR	36.22598	Q14204	DYHC1 HUMAN	814.896664	2	0.759656906		
IQFVGACNPPTDPGR	36.22598	Q14204	DYHC1 HUMAN	543.6003843	3	0.759656906		
IQGLTVEQAEAVVR	51.79818	Q14204	DYHC1 HUMAN	756.9232045	2	0.825837195	3	
IQGLTVEQAEAVVR	51.79818	Q14204	DYHC1 HUMAN	504.9514113	3	0.825837195	3	Yes
ITNQVIYLNPPIEECR	64.13808	Q14204	DYHC1 HUMAN	980.004395	2	0.769438744	3	
ITNQVIYLNPPIEECR	64.13808	Q14204	DYHC1 HUMAN	653.672205	3	0.769438744	3	Yes
LAETVFNFQEK	49.41235	Q14204	DYHC1 HUMAN	663.340984	2	0.78355217	2	Yes
LAETVFNFQEK	49.41235	Q14204	DYHC1 HUMAN	442.5632643	3	0.78355217	2	
LALESICLLLGESTTDWK	125.9532	Q14204	DYHC1 HUMAN	1025.032823	2	0.690519333		
LALESICLLLGESTTDWK	125.9532	Q14204	DYHC1 HUMAN	683.6911567	3	0.690519333		
LCDEQLSSQSHYDFGLR	40.22572	Q14204	DYHC1 HUMAN	1027.965998	2	0.784332931	3	
LCDEQLSSQSHYDFGLR	40.22572	Q14204	DYHC1 HUMAN	685.646607	3	0.784332931	3	Yes
LDMEIER	16.54761	Q14204	DYHC1 HUMAN	453.224034	2	0.720649302	2	Yes
LDMEIER	16.54761	Q14204	DYHC1 HUMAN	302.4852977	3	0.720649302	2	
LEGVEGVAHIIDPK	48.53838	Q14204	DYHC1 HUMAN	738.9070225	2	0.804887056	3	
LEGVEGVAHIIDPK	48.53838	Q14204	DYHC1 HUMAN	492.9406233	3	0.804887056	3	Yes
LEHLITELVHQR	28.86732	Q14204	DYHC1 HUMAN	744.4206315	2	0.760076225	4	
LEHLITELVHQR	28.86732	Q14204	DYHC1 HUMAN	496.6163627	3	0.760076225	4	
LFLTMEINPK	66.52016	Q14204	DYHC1 HUMAN	603.334116	2	0.655911922		
LFLTMEINPK	66.52016	Q14204	DYHC1 HUMAN	402.5586857	3	0.655911922		
LGEDLNK	-20.22256	Q14204	DYHC1 HUMAN	394.7116165	2	0.665132284	2	Yes
LGEDLNK	-20.22256	Q14204	DYHC1 HUMAN	263.4770193	3	0.665132284	2	
LGGSPFGPAGTGK	18.35236	Q14204	DYHC1 HUMAN	573.3016625	2	0.779757738	2	Yes
LGGSPFGPAGTGK	18.35236	Q14204	DYHC1 HUMAN	382.53705	3	0.779757738	2	
LLAESVTEVEIFGK	79.1395	Q14204	DYHC1 HUMAN	767.922338	2	0.767812133	2	Yes
LLAESVTEVEIFGK	79.1395	Q14204	DYHC1 HUMAN	512.284167	3	0.767812133	2	
LLNTFLER	44.46826	Q14204	DYHC1 HUMAN	503.2905655	2	0.780075312	2	Yes
LLNTFLER	44.46826	Q14204	DYHC1 HUMAN	335.8629853	3	0.780075312	2	
LLTLPNGER	26.56378	Q14204	DYHC1 HUMAN	506.7934725	2	0.698289752	2	Yes
LLTLPNGER	26.56378	Q14204	DYHC1 HUMAN	338.1982567	3	0.698289752	2	
LMHVAYEEFEK	20.16564	Q14204	DYHC1 HUMAN	698.33484	2	0.745572031	3	
LMHVAYEEFEK	20.16564	Q14204	DYHC1 HUMAN	465.8925017	3	0.745572031	3	Yes
LNTQEIFDDWAR	74.65909	Q14204	DYHC1 HUMAN	754.362979	2	0.871725261	3	
LNTQEIFDDWAR	74.65909	Q14204	DYHC1 HUMAN	503.2445943	3	0.871725261	3	Yes
LQGEFQLR	21.585	Q14204	DYHC1 HUMAN	495.7725405	2	0.765802085	2	Yes
LQGEFQLR	21.585	Q14204	DYHC1 HUMAN	330.8509687	3	0.765802085	2	
LSCLPAFK	32.08472	Q14204	DYHC1 HUMAN	468.2551375	2	0.613473892	2	Yes
LSCLPAFK	32.08472	Q14204	DYHC1 HUMAN	312.5060333	3	0.613473892	2	
LSLPPNVR	26.11251	Q14204	DYHC1 HUMAN	448.2721755	2	0.727818549	2	Yes
LSLPPNVR	26.11251	Q14204	DYHC1 HUMAN	299.1840587	3	0.727818549	2	
LSLSNAISTALPLTQLR	107.1252	Q14204	DYHC1 HUMAN	899.5258245	2	0.788037837	3	
LSLSNAISTALPLTQLR	107.1252	Q14204	DYHC1 HUMAN	600.0198247	3	0.788037837	3	Yes
LVQTPLTDR	12.52404	Q14204	DYHC1 HUMAN	521.7987555	2	0.802434802	2	Yes
LVQTPLTDR	12.52404	Q14204	DYHC1 HUMAN	348.2017787	3	0.802434802	2	
LYQEMFAWK	55.987	Q14204	DYHC1 HUMAN	608.2975295	2	0.732670903		
LYQEMFAWK	55.987	Q14204	DYHC1 HUMAN	405.867628	3	0.732670903		
MDLEKPNYIVPDYMPVVYDK	82.93254	Q14204	DYHC1 HUMAN	1215.092541	2	0.793707967	3	
MDLEKPNYIVPDYMPVVYDK	82.93254	Q14204	DYHC1 HUMAN	810.3976357	3	0.793707967	3	Yes
MLSAVSQQVQCIQEALR	75.94354	Q14204	DYHC1 HUMAN	981.0013345	2	0.74526155	3	
MLSAVSQQVQCIQEALR	75.94354	Q14204	DYHC1 HUMAN	654.3368313	3	0.74526155	3	Yes
MNTLLANGEVPGLFEGDEYATLMTQCK	117.6669	Q14204	DYHC1 HUMAN	1501.701388	2	0.622061968		
MNTLLANGEVPGLFEGDEYATLMTQCK	117.6669	Q14204	DYHC1 HUMAN	1001.4702	3	0.622061968		
MQMLEDEDDLAYAETEK	53.64339	Q14204	DYHC1 HUMAN	1015.932641	2	0.829455018	3	
MQMLEDEDDLAYAETEK	53.64339	Q14204	DYHC1 HUMAN	677.624369	3	0.829455018	3	Yes
MVVLSLPR	48.67835	Q14204	DYHC1 HUMAN	457.7787795	2	0.636793613	2	Yes
MVVLSLPR	48.67835	Q14204	DYHC1 HUMAN	305.5217947	3	0.636793613	2	
NTISLLVAGLK	77.2589	Q14204	DYHC1 HUMAN	564.85353	2	0.81594646		
NTISLLVAGLK	77.2589	Q14204	DYHC1 HUMAN	376.9049617	3	0.81594646		
NVAQYNANHPDFPMQIEQLER	64.68559	Q14204	DYHC1 HUMAN	1257.5957	2	0.820385158	3	
NVAQYNANHPDFPMQIEQLER	64.68559	Q14204	DYHC1 HUMAN	838.7330747	3	0.820385158	3	Yes

NVHLAPGWLMQLEK	65.82701	Q14204	DYHC1 HUMAN	818.437967	2	0.674374282	3	
NVHLAPGWLMQLEK	65.82701	Q14204	DYHC1 HUMAN	545.961253	3	0.674374282	3	Yes
NYMSNPSYNYEIVNR	41.11416	Q14204	DYHC1 HUMAN	932.420685	2	0.64584291	2	Yes
NYMSNPSYNYEIVNR	41.11416	Q14204	DYHC1 HUMAN	621.9497317	3	0.64584291	2	
QALETVNDYNPLMK	49.59608	Q14204	DYHC1 HUMAN	818.406525	2	0.782257915	2	Yes
QALETVNDYNPLMK	49.59608	Q14204	DYHC1 HUMAN	545.9402917	3	0.782257915	2	
QNLFTTWSHHLQQANIQFR	58.38833	Q14204	DYHC1 HUMAN	1185.099075	2	0.615825176		
QNLFTTWSHHLQQANIQFR	58.38833	Q14204	DYHC1 HUMAN	790.4019913	3	0.615825176		
SACDTVDTWLDDTAK	54.86307	Q14204	DYHC1 HUMAN	849.3703415	2	0.798018277		
SACDTVDTWLDDTAK	54.86307	Q14204	DYHC1 HUMAN	566.582836	3	0.798018277		
SALEQMR	-11.58881	Q14204	DYHC1 HUMAN	417.711094	2	0.675011814	2	Yes
SALEQMR	-11.58881	Q14204	DYHC1 HUMAN	278.8100043	3	0.675011814	2	
SELEEQQMHLNVGLR	37.1357	Q14204	DYHC1 HUMAN	891.9443415	2	0.841745615	2	Yes
SELEEQQMHLNVGLR	37.1357	Q14204	DYHC1 HUMAN	594.9655027	3	0.841745615	2	
SFEWLSQMR	62.81209	Q14204	DYHC1 HUMAN	592.2824145	2	0.863016605	2	Yes
SFEWLSQMR	62.81209	Q14204	DYHC1 HUMAN	395.1908847	3	0.863016605	2	
SLETCMYDHK	-3.557766	Q14204	DYHC1 HUMAN	642.2739255	2	0.766755283		
SLETCMYDHK	-3.557766	Q14204	DYHC1 HUMAN	428.5185587	3	0.766755283		
SLLQALNEVK	58.82709	Q14204	DYHC1 HUMAN	557.827512	2	0.80422473	2	Yes
SLLQALNEVK	58.82709	Q14204	DYHC1 HUMAN	372.2209497	3	0.80422473	2	
SNLPDNLK	-0.054688	Q14204	DYHC1 HUMAN	450.7434475	2	0.625100374		
SNLPDNLK	-0.054688	Q14204	DYHC1 HUMAN	300.8315733	3	0.625100374		
SPLVMDVLNIQGVQR	91.78529	Q14204	DYHC1 HUMAN	834.9592635	2	0.793838501	2	Yes
SPLVMDVLNIQGVQR	91.78529	Q14204	DYHC1 HUMAN	556.9754507	3	0.793838501	2	
SSLQSQCLNEVLK	38.28062	Q14204	DYHC1 HUMAN	753.3855965	2	0.841816425	2	Yes
SSLQSQCLNEVLK	38.28062	Q14204	DYHC1 HUMAN	502.593006	3	0.841816425	2	
SVLVSAGNVK	1.916821	Q14204	DYHC1 HUMAN	487.2880225	2	0.676037848	2	Yes
SVLVSAGNVK	1.916821	Q14204	DYHC1 HUMAN	325.1946233	3	0.676037848	2	
TEYLSNADER	-7.635185	Q14204	DYHC1 HUMAN	599.2732935	2	0.795491099	2	Yes
TEYLSNADER	-7.635185	Q14204	DYHC1 HUMAN	399.8514707	3	0.795491099	2	
TFSEILNR	29.6535	Q14204	DYHC1 HUMAN	490.2645475	2	0.658934593	2	Yes
TFSEILNR	29.6535	Q14204	DYHC1 HUMAN	327.1789733	3	0.658934593	2	
TKPVTGNLRPEEALQALTIYEGK	65.96179	Q14204	DYHC1 HUMAN	1264.690124	2	0.690192282	3	
TKPVTGNLRPEEALQALTIYEGK	65.96179	Q14204	DYHC1 HUMAN	843.4626907	3	0.690192282	3	Yes
TLHTTASNWLHLIPQTLSHLK	69.83891	Q14204	DYHC1 HUMAN	1206.163883	2	0.733277082	4	
TLHTTASNWLHLIPQTLSHLK	69.83891	Q14204	DYHC1 HUMAN	804.445197	3	0.733277082	4	
TLINELVK	42.66248	Q14204	DYHC1 HUMAN	465.2874915	2	0.757052898	2	Yes
TLINELVK	42.66248	Q14204	DYHC1 HUMAN	310.5276027	3	0.757052898	2	
TLMAQSIYGGR	31.10479	Q14204	DYHC1 HUMAN	598.808793	2	0.756762266	2	Yes
TLMAQSIYGGR	31.10479	Q14204	DYHC1 HUMAN	399.5418037	3	0.756762266	2	
TPVIDADKPVSSQLR	19.56249	Q14204	DYHC1 HUMAN	813.447043	2	0.657993793	3	
TPVIDADKPVSSQLR	19.56249	Q14204	DYHC1 HUMAN	542.6339703	3	0.657993793	3	Yes
TPVSITEHPK	-23.51027	Q14204	DYHC1 HUMAN	554.8040375	2	0.703544557	2	Yes
TPVSITEHPK	-23.51027	Q14204	DYHC1 HUMAN	370.2053	3	0.703544557	2	
TSAPITCELLNK	37.0553	Q14204	DYHC1 HUMAN	673.8534015	2	0.783716798	2	Yes
TSAPITCELLNK	37.0553	Q14204	DYHC1 HUMAN	449.5715427	3	0.783716798	2	
TSFLDDAFR	58.30866	Q14204	DYHC1 HUMAN	536.2594625	2	0.812283158	2	Yes
TSFLDDAFR	58.30866	Q14204	DYHC1 HUMAN	357.84225	3	0.812283158	2	
TTDLLTDWEK	53.84381	Q14204	DYHC1 HUMAN	611.3040675	2	0.85429889	2	Yes
TTDLLTDWEK	53.84381	Q14204	DYHC1 HUMAN	407.8719867	3	0.85429889	2	
VAAPDVVVPTLDTVR	71.22681	Q14204	DYHC1 HUMAN	776.44123	2	0.844631076	2	Yes
VAAPDVVVPTLDTVR	71.22681	Q14204	DYHC1 HUMAN	517.9634283	3	0.844631076	2	
VAEVLFDAADANAIEEVNLAYENVK	115.1282	Q14204	DYHC1 HUMAN	1354.177242	2	0.724655986	4	
VAEVLFDAADANAIEEVNLAYENVK	115.1282	Q14204	DYHC1 HUMAN	903.1207693	3	0.724655986	4	
VDDLLIIEEK	57.36046	Q14204	DYHC1 HUMAN	593.83246	2	0.804990411	2	Yes
VDDLLIIEEK	57.36046	Q14204	DYHC1 HUMAN	396.2242483	3	0.804990411	2	
VDNEFDQR	-14.61151	Q14204	DYHC1 HUMAN	511.731069	2	0.69312191	2	Yes
VDNEFDQR	-14.61151	Q14204	DYHC1 HUMAN	341.4899877	3	0.69312191	2	
VEDPTFLNQLQSGVNR	65.93926	Q14204	DYHC1 HUMAN	908.9635885	2	0.843191981		
VEDPTFLNQLQSGVNR	65.93926	Q14204	DYHC1 HUMAN	606.3116673	3	0.843191981		
VEPAVIEAQNAVK	25.74974	Q14204	DYHC1 HUMAN	684.3806405	2	0.837722063	2	Yes
VEPAVIEAQNAVK	25.74974	Q14204	DYHC1 HUMAN	456.589702	3	0.837722063	2	
VFEEDALSWEDK	55.38136	Q14204	DYHC1 HUMAN	734.336095	2	0.823435009		
VFEEDALSWEDK	55.38136	Q14204	DYHC1 HUMAN	489.8933383	3	0.823435009		
VLLTTQGVDMISK	54.03974	Q14204	DYHC1 HUMAN	702.892527	2	0.863478541	2	Yes
VLLTTQGVDMISK	54.03974	Q14204	DYHC1 HUMAN	468.9309597	3	0.863478541	2	
VLQLYQITQINHGLMMVGPSGSGK	84.80444	Q14204	DYHC1 HUMAN	1286.175272	2	0.607991695	3	
VLQLYQITQINHGLMMVGPSGSGK	84.80444	Q14204	DYHC1 HUMAN	857.786123	3	0.607991695	3	Yes
VLRPQVTAVAQQNQGEVPEPQDMK	35.60966	Q14204	DYHC1 HUMAN	1331.685052	2	0.839578927	3	
VLRPQVTAVAQQNQGEVPEPQDMK	35.60966	Q14204	DYHC1 HUMAN	888.125976	3	0.839578927	3	Yes
VLTLSEDSPYETLHSFISNAVAPFFK	140.3878	Q14204	DYHC1 HUMAN	1456.740009	2	0.740928173		
VLTLSEDSPYETLHSFISNAVAPFFK	140.3878	Q14204	DYHC1 HUMAN	971.4959477	3	0.740928173		
VMSQEIQEQLHK	5.817623	Q14204	DYHC1 HUMAN	735.3750325	2	0.70470649	3	
VMSQEIQEQLHK	5.817623	Q14204	DYHC1 HUMAN	490.5859633	3	0.70470649	3	Yes
VNFLPEIITLSK	98.29497	Q14204	DYHC1 HUMAN	687.4061265	2	0.861522019	2	Yes
VNFLPEIITLSK	98.29497	Q14204	DYHC1 HUMAN	458.6066927	3	0.861522019	2	
VPQIEVETHK	-6.647289	Q14204	DYHC1 HUMAN	590.3225945	2	0.730380416	3	
VPQIEVETHK	-6.647289	Q14204	DYHC1 HUMAN	393.884338	3	0.730380416	3	Yes
VQVALEELQDLK	66.18037	Q14204	DYHC1 HUMAN	692.8882985	2	0.847001791	2	Yes
VQVALEELQDLK	66.18037	Q14204	DYHC1 HUMAN	462.261474	3	0.847001791	2	
VSPDMAIFITMNPGYAGR	103.4499	Q14204	DYHC1 HUMAN	970.4744165	2	0.709392011	3	
VSPDMAIFITMNPGYAGR	103.4499	Q14204	DYHC1 HUMAN	647.318886	3	0.709392011	3	Yes
VTFVNFTVTR	55.92899	Q14204	DYHC1 HUMAN	592.32768	2	0.737240911	2	Yes
VTFVNFTVTR	55.92899	Q14204	DYHC1 HUMAN	395.2210617	3	0.737240911	2	
VWEQIDQMK	30.16319	Q14204	DYHC1 HUMAN	588.790073	2	0.769679606	2	Yes
VWEQIDQMK	30.16319	Q14204	DYHC1 HUMAN	392.862657	3	0.769679606	2	
WAIAQLNYADMLK	90.08197	Q14204	DYHC1 HUMAN	768.898139	2	0.821886301		
WAIAQLNYADMLK	90.08197	Q14204	DYHC1 HUMAN	512.934701	3	0.821886301		
WTDENIDTVALK	48.58138	Q14204	DYHC1 HUMAN	702.8544555	2	0.730789304	2	Yes
WTDENIDTVALK	48.58138	Q14204	DYHC1 HUMAN	468.9055787	3	0.730789304	2	
WVYLEGIFTGSADIK	104.3585	Q14204	DYHC1 HUMAN	849.941058	2	0.715345979		
WVYLEGIFTGSADIK	104.3585	Q14204	DYHC1 HUMAN	566.9633137	3	0.715345979		
YATLATVSR	4.966217	Q14204	DYHC1 HUMAN	491.272369	2	0.600307822	2	Yes
YATLATVSR	4.966217	Q14204	DYHC1 HUMAN	327.8508543	3	0.600307822	2	
YPLIIDPSGQATEFIMNEYK	113.8141	Q14204	DYHC1 HUMAN	1165.07521	2	0.639592767		
YPLIIDPSGQATEFIMNEYK	113.8141	Q14204	DYHC1 HUMAN	777.0527483	3	0.639592767		
YQVGVHYELTEEEK	23.0755	Q14204	DYHC1 HUMAN	862.4128575	2	0.804237664		
YQVGVHYELTEEEK	23.0755	Q14204	DYHC1 HUMAN	575.2778467	3	0.804237664		
AISTPETPLTK	16.46645	Q14181	DPOA2 HUMAN	579.324803	2	0.79368782	2	Yes
AISTPETPLTK	16.46645	Q14181	DPOA2 HUMAN	386.552477	3	0.79368782	2	
DVHHEPVYPQPPFSYSDLSR	51.2095	Q14181	DPOA2 HUMAN	1185.561647	2	0.788816452	3	

DVHHEPVYPQPPFSYSDLSR	51.2095	Q14181	DPOA2 HUMAN	790.7103727	3	0.788816452	3	Yes
EHSSGAQIPVDLSELK	46.4998	Q14181	DPOA2 HUMAN	855.4394145	2	0.721558034	3	
EHSSGAQIPVDLSELK	46.4998	Q14181	DPOA2 HUMAN	570.6288847	3	0.721558034	3	Yes
GQVGGTFAR	-20.01995	Q14181	DPOA2 HUMAN	446.7359585	2	0.689921916	2	Yes
GQVGGTFAR	-20.01995	Q14181	DPOA2 HUMAN	298.159914	3	0.689921916	2	
HEQVENCLLTSPFEDIFK	95.71884	Q14181	DPOA2 HUMAN	1103.528436	2	0.733562469	3	
HEQVENCLLTSPFEDIFK	95.71884	Q14181	DPOA2 HUMAN	736.0215653	3	0.733562469	3	Yes
IEELGSELK	17.66039	Q14181	DPOA2 HUMAN	509.2773205	2	0.739458203		
IEELGSELK	17.66039	Q14181	DPOA2 HUMAN	339.8541553	3	0.739458203		
SPHQLLSPSSFSPSATPSQK	31.3736	Q14181	DPOA2 HUMAN	1042.026917	2	0.708524168		
SPHQLLSPSSFSPSATPSQK	31.3736	Q14181	DPOA2 HUMAN	695.0205527	3	0.708524168		
SSGSHLVFVPSLR	40.88052	Q14181	DPOA2 HUMAN	693.380974	2	0.820540845	3	
SSGSHLVFVPSLR	40.88052	Q14181	DPOA2 HUMAN	462.5899243	3	0.820540845	3	Yes
SVILEGDR	5.153233	Q14181	DPOA2 HUMAN	444.743448	2	0.645681977		
SVILEGDR	5.153233	Q14181	DPOA2 HUMAN	296.8315737	3	0.645681977		
VGLTSEILNSFEHEFLSK	122.5075	Q14181	DPOA2 HUMAN	1025.528761	2	0.875367403	3	
VGLTSEILNSFEHEFLSK	122.5075	Q14181	DPOA2 HUMAN	684.021782	3	0.875367403	3	Yes
VLGCPEALTGSYK	30.66223	Q14181	DPOA2 HUMAN	697.8533975	2	0.85403657	2	Yes
VLGCPEALTGSYK	30.66223	Q14181	DPOA2 HUMAN	465.57154	3	0.85403657	2	
EQYQQQQQWGSR	-9.557449	Q14103	HNRPD HUMAN	783.358756	2	0.763663888	2	Yes
EQYQQQQQWGSR	-9.557449	Q14103	HNRPD HUMAN	522.5751123	3	0.763663888	2	
EYFGGFGEVESIELPMDNK	99.81783	Q14103	HNRPD HUMAN	1080.99389	2	0.822208643	3	
EYFGGFGEVESIELPMDNK	99.81783	Q14103	HNRPD HUMAN	720.9985347	3	0.822208643	3	Yes
FGEVVDCTLK	30.53241	Q14103	HNRPD HUMAN	584.289906	2	0.716195107	2	Yes
FGEVVDCTLK	30.53241	Q14103	HNRPD HUMAN	389.8625457	3	0.716195107	2	
IFVGGLSPDTPEEK	45.26139	Q14103	HNRPD HUMAN	744.883213	2	0.846230745	2	Yes
IFVGGLSPDTPEEK	45.26139	Q14103	HNRPD HUMAN	496.9247503	3	0.846230745	2	
MFIGGLSWDTTK	74.60756	Q14103	HNRPD HUMAN	678.3373875	2	0.756611466	2	Yes
MFIGGLSWDTTK	74.60756	Q14103	HNRPD HUMAN	452.5608667	3	0.756611466	2	
ALFKPPEDSQDDESDSDAEEEQTTK	19.99274	Q13769	THOC5 HUMAN	1406.102327	2	0.622746348		
ALFKPPEDSQDDESDSDAEEEQTTK	19.99274	Q13769	THOC5 HUMAN	937.7374927	3	0.622746348		
DVAIEIEER	30.9557	Q13769	THOC5 HUMAN	537.277852	2	0.770307183		
DVAIEIEER	30.9557	Q13769	THOC5 HUMAN	358.5211763	3	0.770307183		
ELCGPWPSHQLLTNQLQR	57.88002	Q13769	THOC5 HUMAN	1089.050205	2	0.739456773	3	
ELCGPWPSHQLLTNQLQR	57.88002	Q13769	THOC5 HUMAN	726.3694113	3	0.739456773	3	Yes
EQPQQTVIADHSLSASHMETTMK	19.86979	Q13769	THOC5 HUMAN	1285.105245	2	0.725492597	4	
EQPQQTVIADHSLSASHMETTMK	19.86979	Q13769	THOC5 HUMAN	857.0727717	3	0.725492597	4	
EYLSSLQPR	21.43553	Q13769	THOC5 HUMAN	546.7883825	2	0.762741566		
EYLSSLQPR	21.43553	Q13769	THOC5 HUMAN	364.86153	3	0.762741566		
HEEIDLVSLEEFYK	80.01149	Q13769	THOC5 HUMAN	875.9308865	2	0.69103682	3	
HEEIDLVSLEEFYK	80.01149	Q13769	THOC5 HUMAN	584.289866	3	0.69103682	3	Yes
HPLSVMLDLK	43.97201	Q13769	THOC5 HUMAN	576.826458	2	0.612473011	2	Yes
HPLSVMLDLK	43.97201	Q13769	THOC5 HUMAN	384.8869137	3	0.612473011	2	
LDWELEQR	40.16335	Q13769	THOC5 HUMAN	544.772737	2	0.840943694	2	Yes
LDWELEQR	40.16335	Q13769	THOC5 HUMAN	363.5177663	3	0.840943694	2	
LGGLHFPK	13.07479	Q13769	THOC5 HUMAN	434.756162	2	0.800878167		
LGGLHFPK	13.07479	Q13769	THOC5 HUMAN	290.173383	3	0.800878167		
LMAEIQDLK	34.17845	Q13769	THOC5 HUMAN	530.7895415	2	0.759519517		
LMAEIQDLK	34.17845	Q13769	THOC5 HUMAN	354.195636	3	0.759519517		
RRPTLGVQLDDK	-1.819698	Q13769	THOC5 HUMAN	699.3971565	2	0.756758392		
RRPTLGVQLDDK	-1.819698	Q13769	THOC5 HUMAN	466.6007127	3	0.756758392		
TLSVAIEGSVDEAK	36.45473	Q13769	THOC5 HUMAN	709.872845	2	0.776039124		
TLSVAIEGSVDEAK	36.45473	Q13769	THOC5 HUMAN	473.584505	3	0.776039124		
TPNPANQYQFDK	11.27023	Q13769	THOC5 HUMAN	711.836593	2	0.674014866	2	Yes
TPNPANQYQFDK	11.27023	Q13769	THOC5 HUMAN	474.8936703	3	0.674014866	2	
VDAYHLQLQNLLYEVMHLQK	108.3533	Q13769	THOC5 HUMAN	1228.144293	2	0.83643645		
VDAYHLQLQNLLYEVMHLQK	108.3533	Q13769	THOC5 HUMAN	819.0988037	3	0.83643645		
WVTVAHEDYMELHFTK	50.36521	Q13769	THOC5 HUMAN	1003.478014	2	0.834985614		
WVTVAHEDYMELHFTK	50.36521	Q13769	THOC5 HUMAN	669.321284	3	0.834985614		
YNHPQGFFSHR	-19.32023	Q13769	THOC5 HUMAN	695.3265295	2	0.626978159	3	
YNHPQGFFSHR	-19.32023	Q13769	THOC5 HUMAN	463.8869613	3	0.626978159	3	Yes
YYSEEAEVDLR	24.30683	Q13769	THOC5 HUMAN	687.315154	2	0.773629189	2	Yes
YYSEEAEVDLR	24.30683	Q13769	THOC5 HUMAN	458.5460443	3	0.773629189	2	
DIMVHFK	19.82002	Q13619	CUL4A HUMAN	445.234205	2	0.66101855		
DIMVHFK	19.82002	Q13619	CUL4A HUMAN	297.158745	3	0.66101855		
DKDMVQDLLDFK	77.00755	Q13619	CUL4A HUMAN	733.863966	2	0.848155379	3	
DKDMVQDLLDFK	77.00755	Q13619	CUL4A HUMAN	489.5785857	3	0.848155379	3	Yes
DKDNPNQYHYVA	-5.349628	Q13619	CUL4A HUMAN	732.33167	2	0.742949128	2	Yes
DKDNPNQYHYVA	-5.349628	Q13619	CUL4A HUMAN	488.557055	3	0.742949128	2	
ESFETFINK	38.78583	Q13619	CUL4A HUMAN	557.774945	2	0.732615411	2	Yes
ESFETFINK	38.78583	Q13619	CUL4A HUMAN	372.185905	3	0.732615411	2	
ETVEEQVSTTER	-12.00505	Q13619	CUL4A HUMAN	704.3340845	2	0.745939493	2	Yes
ETVEEQVSTTER	-12.00505	Q13619	CUL4A HUMAN	469.891998	3	0.745939493	2	
EVPEYLNHVSK	6.768585	Q13619	CUL4A HUMAN	657.8386035	2	0.768104434	2	Yes
EVPEYLNHVSK	6.768585	Q13619	CUL4A HUMAN	438.8950107	3	0.768104434	2	
FLEETNCLYAAEGQR	45.71652	Q13619	CUL4A HUMAN	900.915246	2	0.798564076	2	Yes
FLEETNCLYAAEGQR	45.71652	Q13619	CUL4A HUMAN	600.9461057	3	0.798564076	2	
FPVKPGDLK	5.229961	Q13619	CUL4A HUMAN	500.795484	2	0.6606251	2	Yes
FPVKPGDLK	5.229961	Q13619	CUL4A HUMAN	334.1995977	3	0.6606251	2	
GGQQALLQHWSEYIK	58.42304	Q13619	CUL4A HUMAN	879.452656	2	0.813401759	3	
GGQQALLQHWSEYIK	58.42304	Q13619	CUL4A HUMAN	586.6377123	3	0.813401759	3	Yes
GLDHLLDENR	14.60175	Q13619	CUL4A HUMAN	591.29965	2	0.801763952	3	
GLDHLLDENR	14.60175	Q13619	CUL4A HUMAN	394.5357083	3	0.801763952	3	Yes
INTCWQDHCR	-14.47877	Q13619	CUL4A HUMAN	695.29352	2	0.687563419		
INTCWQDHCR	-14.47877	Q13619	CUL4A HUMAN	463.864955	3	0.687563419		
LPDNYTQDTWR	28.27375	Q13619	CUL4A HUMAN	704.828768	2	0.803419113	2	Yes
LPDNYTQDTWR	28.27375	Q13619	CUL4A HUMAN	470.221787	3	0.803419113	2	
LQWQTTLGHAVLK	44.25261	Q13619	CUL4A HUMAN	747.9255505	2	0.822924852	3	
LQWQTTLGHAVLK	44.25261	Q13619	CUL4A HUMAN	498.9529753	3	0.822924852	3	Yes
MATGIEDSELR	17.37412	Q13619	CUL4A HUMAN	611.2931765	2	0.804661989		
MATGIEDSELR	17.37412	Q13619	CUL4A HUMAN	407.864726	3	0.804661989		
SLLGMLSDLQVYK	103.833	Q13619	CUL4A HUMAN	733.900347	2	0.767390311	2	Yes
SLLGMLSDLQVYK	103.833	Q13619	CUL4A HUMAN	489.6028397	3	0.767390311	2	
TFGTAIVINPEK	49.43834	Q13619	CUL4A HUMAN	645.359177	2	0.786564112		
TFGTAIVINPEK	49.43834	Q13619	CUL4A HUMAN	430.575393	3	0.786564112		
TLGHNLLVSELYNQLK	70.75513	Q13619	CUL4A HUMAN	921.51017	2	0.76835829	3	
TLGHNLLVSELYNQLK	70.75513	Q13619	CUL4A HUMAN	614.676055	3	0.76835829	3	Yes
VDHVIEVCFQK	27.58101	Q13619	CUL4A HUMAN	687.3482865	2	0.798201084		
VDHVIEVCFQK	27.58101	Q13619	CUL4A HUMAN	458.5681327	3	0.798201084		
VITYLDHSTQKPLIACVEK	35.47344	Q13619	CUL4A HUMAN	1108.093742	2	0.834872365	4	
VITYLDHSTQKPLIACVEK	35.47344	Q13619	CUL4A HUMAN	739.065103	3	0.834872365	4	

VPDLAQMYQLFSR	110.0066	Q13619	CUL4A_HUMAN	784.401046	2	0.684153676	2	Yes
VPDLAQMYQLFSR	110.0066	Q13619	CUL4A_HUMAN	523.2699723	3	0.684153676	2	
YNLEELYQAVENLCSHK	102.9084	Q13619	CUL4A_HUMAN	1055.499669	2	0.8487432	3	
YNLEELYQAVENLCSHK	102.9084	Q13619	CUL4A_HUMAN	704.0023877	3	0.8487432	3	Yes
DNTTLLTQVQTTMR	61.0256	Q13561	DCTN2_HUMAN	811.414887	2	0.800558805		
DNTTLLTQVQTTMR	61.0256	Q13561	DCTN2_HUMAN	541.2791997	3	0.800558805		
ENLATVEGNFASIDER	54.39765	Q13561	DCTN2_HUMAN	882.9241285	2	0.860572338		
ENLATVEGNFASIDER	54.39765	Q13561	DCTN2_HUMAN	588.9520273	3	0.860572338		
LLGPDAAINLTDPDGALAK	84.97116	Q13561	DCTN2_HUMAN	933.0049225	2	0.828725278	3	
LLGPDAAINLTDPDGALAK	84.97116	Q13561	DCTN2_HUMAN	622.3392233	3	0.828725278	3	Yes
LLHEVQELTTEVEK	39.22878	Q13561	DCTN2_HUMAN	834.446709	2	0.832033515	2	Yes
LLHEVQELTTEVEK	39.22878	Q13561	DCTN2_HUMAN	556.6337477	3	0.832033515	2	
LLLQLEATK	48.75311	Q13561	DCTN2_HUMAN	514.821699	2	0.772977889	2	Yes
LLLQLEATK	48.75311	Q13561	DCTN2_HUMAN	343.5504077	3	0.772977889	2	
VYETSDLPEDDQAEFDAEELTSTSVEHIIVNPNA	99.06183	Q13561	DCTN2_HUMAN	2355.55484	2	0.722670257	4	
VYETSDLPEDDQAEFDAEELTSTSVEHIIVNPNA	99.06183	Q13561	DCTN2_HUMAN	1570.705835	3	0.722670257	4	
TGYESGEYEMLGEGLGVK	67.09792	Q13561	DCTN2_HUMAN	959.941122	2	0.84493053		
TGYESGEYEMLGEGLGVK	67.09792	Q13561	DCTN2_HUMAN	640.2966897	3	0.84493053		
TTGTPPDSSLVTYELHSRPEQDK	30.39389	Q13561	DCTN2_HUMAN	1279.622635	2	0.74620986	4	
TTGTPPDSSLVTYELHSRPEQDK	30.39389	Q13561	DCTN2_HUMAN	853.417698	3	0.74620986	4	
VHQLYETIQR	-3.84819	Q13561	DCTN2_HUMAN	643.846764	2	0.806916058	2	Yes
VHQLYETIQR	-3.84819	Q13561	DCTN2_HUMAN	429.5671177	3	0.806916058	2	
VSALDLAVLDQVEAR	95.58737	Q13561	DCTN2_HUMAN	799.9415935	2	0.867871702	3	
VSALDLAVLDQVEAR	95.58737	Q13561	DCTN2_HUMAN	533.6303373	3	0.867871702	3	Yes
WSPIASTLPELVQR	87.0208	Q13561	DCTN2_HUMAN	798.9413965	2	0.796416283	2	Yes
WSPIASTLPELVQR	87.0208	Q13561	DCTN2_HUMAN	532.9635393	3	0.796416283	2	
YADLPGIAR	29.88615	Q13561	DCTN2_HUMAN	488.2670865	2	0.786959291	2	Yes
YADLPGIAR	29.88615	Q13561	DCTN2_HUMAN	325.8473327	3	0.786959291	2	
AELEADLK	5.552708	Q13472	TOP3A_HUMAN	444.7378315	2	0.699036956		
AELEADLK	5.552708	Q13472	TOP3A_HUMAN	296.8278293	3	0.699036956		
AIQAFVPEIFHR	66.07339	Q13472	TOP3A_HUMAN	714.3938855	2	0.810353696		
AIQAFVPEIFHR	66.07339	Q13472	TOP3A_HUMAN	476.598532	3	0.810353696		
ALAQTLPPPTAAGESNSVTCNCGQEAVLLTVR	79.10132	Q13472	TOP3A_HUMAN	1663.32994	2	0.622482896		
ALAQTLPPPTAAGESNSVTCNCGQEAVLLTVR	79.10132	Q13472	TOP3A_HUMAN	1109.222568	3	0.622482896		
AVKPNLQVLR	9.162922	Q13472	TOP3A_HUMAN	569.359314	2	0.781403422	3	
AVKPNLQVLR	9.162922	Q13472	TOP3A_HUMAN	379.9088177	3	0.781403422	3	Yes
DGIVEFNWK	65.55638	Q13472	TOP3A_HUMAN	554.277655	2	0.771297097		
DGIVEFNWK	65.55638	Q13472	TOP3A_HUMAN	369.8543783	3	0.771297097		
DLNLTVLVEQQTPDPR	90.23586	Q13472	TOP3A_HUMAN	919.486897	2	0.69023174		
DLNLTVLVEQQTPDPR	90.23586	Q13472	TOP3A_HUMAN	613.3272063	3	0.69023174		
DSSVCPVCQPHPVYR	12.93844	Q13472	TOP3A_HUMAN	900.9119835	2	0.668464839		
DSSVCPVCQPHPVYR	12.93844	Q13472	TOP3A_HUMAN	600.9439307	3	0.668464839		
EQQCGFFQWVDENTAPGTSGAPSWTGDR	98.07758	Q13472	TOP3A_HUMAN	1564.676141	2	0.615480542		
EQQCGFFQWVDENTAPGTSGAPSWTGDR	98.07758	Q13472	TOP3A_HUMAN	1043.453369	3	0.615480542		
FLPGHLGMGLVEGYDSMGYEMSKPDLR	78.55418	Q13472	TOP3A_HUMAN	1500.209178	2	0.714130282		
FLPGHLGMGLVEGYDSMGYEMSKPDLR	78.55418	Q13472	TOP3A_HUMAN	1000.475394	3	0.714130282		
FSEITPHAVR	-0.18932	Q13472	TOP3A_HUMAN	578.809654	2	0.6837973		
FSEITPHAVR	-0.18932	Q13472	TOP3A_HUMAN	386.2090443	3	0.6837973		
FVAHGLMILAR	43.56779	Q13472	TOP3A_HUMAN	614.3557175	2	0.783491492		
FVAHGLMILAR	43.56779	Q13472	TOP3A_HUMAN	409.90642	3	0.783491492		
GIADLLSNGR	51.5304	Q13472	TOP3A_HUMAN	508.280729	2	0.634827614		
GIADLLSNGR	51.5304	Q13472	TOP3A_HUMAN	339.189761	3	0.634827614		
HFLACCSQDAQGQETTVEIDIAQER	48.04298	Q13472	TOP3A_HUMAN	1453.656363	2	0.606137455		
HFLACCSQDAQGQETTVEIDIAQER	48.04298	Q13472	TOP3A_HUMAN	969.4401833	3	0.606137455		
HGIGTDATHAEHIETIK	-20.02873	Q13472	TOP3A_HUMAN	915.461214	2	0.849291027		
HGIGTDATHAEHIETIK	-20.02873	Q13472	TOP3A_HUMAN	610.6434177	3	0.849291027		
ILPVYEQGSHFQPSTVEMVDGETSPPK	60.50873	Q13472	TOP3A_HUMAN	1486.721492	2	0.725824893		
ILPVYEQGSHFQPSTVEMVDGETSPPK	60.50873	Q13472	TOP3A_HUMAN	991.4836027	3	0.725824893		
LLTEADLIALMEK	104.2707	Q13472	TOP3A_HUMAN	730.4080095	2	0.832992136		
LLTEADLIALMEK	104.2707	Q13472	TOP3A_HUMAN	487.2746147	3	0.832992136		
MYVGLTPDK	25.38226	Q13472	TOP3A_HUMAN	512.2631555	2	0.809702873		
MYVGLTPDK	25.38226	Q13472	TOP3A_HUMAN	341.844712	3	0.809702873		
NYLDVYPYDHWSDK	54.64206	Q13472	TOP3A_HUMAN	907.9051945	2	0.83611834		
NYLDVYPYDHWSDK	54.64206	Q13472	TOP3A_HUMAN	605.6060713	3	0.83611834		
SAVWLPDSVLEASR	80.50873	Q13472	TOP3A_HUMAN	765.4021035	2	0.809823573		
SAVWLPDSVLEASR	80.50873	Q13472	TOP3A_HUMAN	510.6040107	3	0.809823573		
TETNIFPR	15.32761	Q13472	TOP3A_HUMAN	489.256723	2	0.728787482		
TETNIFPR	15.32761	Q13472	TOP3A_HUMAN	326.5070903	3	0.728787482		
WGAFAQSILER	69.42785	Q13472	TOP3A_HUMAN	639.336036	2	0.781566441		
WGAFAQSILER	69.42785	Q13472	TOP3A_HUMAN	426.5599657	3	0.781566441		
WQSCNPLVLFEAEIEK	107.4543	Q13472	TOP3A_HUMAN	981.985675	2	0.606066644		
WQSCNPLVLFEAEIEK	107.4543	Q13472	TOP3A_HUMAN	654.9930583	3	0.606066644		
WRPQALDTVELEK	36.55512	Q13472	TOP3A_HUMAN	792.923204	2	0.827964723		
WRPQALDTVELEK	36.55512	Q13472	TOP3A_HUMAN	528.951411	3	0.827964723		
YTNNLQGDEQR	-25.49891	Q13472	TOP3A_HUMAN	669.3081995	2	0.825588524		
YTNNLQGDEQR	-25.49891	Q13472	TOP3A_HUMAN	446.541408	3	0.825588524		
AAALEQFK	5.686993	Q13423	NNTM_HUMAN	439.2430845	2	0.800768733		
AAALEQFK	5.686993	Q13423	NNTM_HUMAN	293.1646647	3	0.800768733		
AISPDKDNFYFDVK	44.34457	Q13423	NNTM_HUMAN	829.9072145	2	0.843269825		
AISPDKDNFYFDVK	44.34457	Q13423	NNTM_HUMAN	553.607418	3	0.843269825		
APMVNPTLGVHEADLLK	56.8857	Q13423	NNTM_HUMAN	902.9854785	2	0.830160499	3	
APMVNPTLGVHEADLLK	56.8857	Q13423	NNTM_HUMAN	602.3262607	3	0.830160499	3	Yes
APVKPGIPYK	-2.376133	Q13423	NNTM_HUMAN	535.3244045	2	0.701561093		
APVKPGIPYK	-2.376133	Q13423	NNTM_HUMAN	357.218878	3	0.701561093		
APVLFNK	12.62107	Q13423	NNTM_HUMAN	394.7374375	2	0.635709047		
APVLFNK	12.62107	Q13423	NNTM_HUMAN	263.4942333	3	0.635709047		
AQYPIADLVK	47.4013	Q13423	NNTM_HUMAN	559.3167765	2	0.819818079	2	Yes
AQYPIADLVK	47.4013	Q13423	NNTM_HUMAN	373.2137927	3	0.819818079	2	
AVVLAANHFGR	7.664673	Q13423	NNTM_HUMAN	577.8256385	2	0.852212429		
AVVLAANHFGR	7.664673	Q13423	NNTM_HUMAN	385.553034	3	0.852212429		
DDFDFGTMGHVIR	58.97361	Q13423	NNTM_HUMAN	755.3437325	2	0.759075642	3	
DDFDFGTMGHVIR	58.97361	Q13423	NNTM_HUMAN	503.89843	3	0.759075642	3	Yes
EVLASDLVVK	39.48311	Q13423	NNTM_HUMAN	536.816613	2	0.772871017		
EVLASDLVVK	39.48311	Q13423	NNTM_HUMAN	358.2136837	3	0.772871017		
FFTGQITAAGK	28.97917	Q13423	NNTM_HUMAN	570.806581	2	0.810701489		
FFTGQITAAGK	28.97917	Q13423	NNTM_HUMAN	380.8736623	3	0.810701489		
GITHIGYTDLPSR	22.5134	Q13423	NNTM_HUMAN	715.3758855	2	0.728292108	3	
GITHIGYTDLPSR	22.5134	Q13423	NNTM_HUMAN	477.2531987	3	0.728292108	3	Yes
ILIVGGGVAGLASAGAAK	67.40706	Q13423	NNTM_HUMAN	762.95959	2	0.792511642		
ILIVGGGVAGLASAGAAK	67.40706	Q13423	NNTM_HUMAN	508.9756683	3	0.792511642		
LFAQQCK	-21.67484	Q13423	NNTM_HUMAN	447.7292875	2	0.679921448		

LFAQQCK	-21.67484	Q13423	NNTM HUMAN	298.8221333	3	0.679921448		
MATQASTLYSNNITK	18.71416	Q13423	NNTM HUMAN	821.909432	2	0.811468065	2	Yes
MATQASTLYSNNITK	18.71416	Q13423	NNTM HUMAN	548.275563	3	0.811468065	2	
SAPLLLPGR	38.14706	Q13423	NNTM HUMAN	462.287826	2	0.688251078	2	Yes
SAPLLLPGR	38.14706	Q13423	NNTM HUMAN	308.5278257	3	0.688251078	2	
SLGAEPLEVDLK	50.86072	Q13423	NNTM HUMAN	635.8486415	2	0.854704797		
SLGAEPLEVDLK	50.86072	Q13423	NNTM HUMAN	424.235036	3	0.854704797		
TFYTHQELWCK	21.67212	Q13423	NNTM HUMAN	756.8511895	2	0.81881845		
TFYTHQELWCK	21.67212	Q13423	NNTM HUMAN	504.9034013	3	0.81881845		
TTVLAMDQVPR	37.22011	Q13423	NNTM HUMAN	615.82973	2	0.793578148		
TTVLAMDQVPR	37.22011	Q13423	NNTM HUMAN	410.889095	3	0.793578148		
TVVTGCSCPLLSNLGSCK	51.46742	Q13423	NNTM HUMAN	976.966103	2	0.639582694		
TVVTGCSCPLLSNLGSCK	51.46742	Q13423	NNTM HUMAN	651.646677	3	0.639582694		
VAGAQIQGAK	-26.2228	Q13423	NNTM HUMAN	471.772541	2	0.606436491		
VAGAQIQGAK	-26.2228	Q13423	NNTM HUMAN	314.850969	3	0.606436491		
VALSPAGVQNLVK	49.25748	Q13423	NNTM HUMAN	648.3882685	2	0.809657335	2	Yes
VALSPAGVQNLVK	49.25748	Q13423	NNTM HUMAN	432.5947873	3	0.809657335	2	
VIFPAPTPK	33.86147	Q13423	NNTM HUMAN	485.2925775	2	0.684904873		
VIFPAPTPK	33.86147	Q13423	NNTM HUMAN	323.8643267	3	0.684904873		
VTIAQGYDALSSMANIAGYK	95.8219	Q13423	NNTM HUMAN	1037.020238	2	0.732680202	3	
VTIAQGYDALSSMANIAGYK	95.8219	Q13423	NNTM HUMAN	691.682767	3	0.732680202	3	Yes
AALQGGGPPYGPPSTWSVSTMDALR	81.14047	Q13421	MSLN HUMAN	1258.616102	2	0.730514228		
AALQGGGPPYGPPSTWSVSTMDALR	81.14047	Q13421	MSLN HUMAN	839.4133427	3	0.730514228		
ALGGLACDLPGR	38.66799	Q13421	MSLN HUMAN	600.314247	2	0.755418479		
ALGGLACDLPGR	38.66799	Q13421	MSLN HUMAN	400.5454397	3	0.755418479		
ALLEVNK	4.644299	Q13421	MSLN HUMAN	393.740177	2	0.707128525		
ALLEVNK	4.644299	Q13421	MSLN HUMAN	262.829393	3	0.707128525		
ALSQQNVSMDLATFMK	76.26684	Q13421	MSLN HUMAN	892.4400465	2	0.807126403		
ALSQQNVSMDLATFMK	76.26684	Q13421	MSLN HUMAN	595.2959727	3	0.807126403		
ANVDLLPR	25.12029	Q13421	MSLN HUMAN	449.261808	2	0.759138763		
ANVDLLPR	25.12029	Q13421	MSLN HUMAN	299.8438137	3	0.759138763		
EIDESLIFYK	65.2032	Q13421	MSLN HUMAN	628.8246305	2	0.627307534	2	Yes
EIDESLIFYK	65.2032	Q13421	MSLN HUMAN	419.552362	3	0.627307534	2	
ELAVALAQK	21.43401	Q13421	MSLN HUMAN	471.7851165	2	0.627320051		
ELAVALAQK	21.43401	Q13421	MSLN HUMAN	314.8593527	3	0.627320051		
FVAESAEVLLPR	63.93542	Q13421	MSLN HUMAN	665.872451	2	0.82777226		
FVAESAEVLLPR	63.93542	Q13421	MSLN HUMAN	444.250909	3	0.82777226		
GLLPVLGQPIIR	88.40193	Q13421	MSLN HUMAN	638.411547	2	0.833887935	2	Yes
GLLPVLGQPIIR	88.40193	Q13421	MSLN HUMAN	425.9436397	3	0.833887935	2	
GSLLSEADVR	23.49751	Q13421	MSLN HUMAN	523.778019	2	0.662900507		
GSLLSEADVR	23.49751	Q13421	MSLN HUMAN	349.5212877	3	0.662900507		
IQSFLGGAPTEDLK	50.37067	Q13421	MSLN HUMAN	738.3912055	2	0.863648772		
IQSFLGGAPTEDLK	50.37067	Q13421	MSLN HUMAN	492.5967453	3	0.863648772		
LAFQNMNGSEYFVK	54.33771	Q13421	MSLN HUMAN	824.39596	2	0.739664674		
LAFQNMNGSEYFVK	54.33771	Q13421	MSLN HUMAN	549.9332483	3	0.739664674		
LDELYPQGYPESVIQHLGYLFLK	137.9884	Q13421	MSLN HUMAN	1361.710516	2	0.614088774		
LDELYPQGYPESVIQHLGYLFLK	137.9884	Q13421	MSLN HUMAN	908.142952	3	0.614088774		
LLGPHVEGLK	12.25904	Q13421	MSLN HUMAN	531.8194905	2	0.65960139	3	
LLGPHVEGLK	12.25904	Q13421	MSLN HUMAN	354.8822687	3	0.65960139	3	Yes
LVSCPGPLDQDQQEAAR	23.37191	Q13421	MSLN HUMAN	942.449989	2	0.762572467		
LVSCPGPLDQDQQEAAR	23.37191	Q13421	MSLN HUMAN	628.6359343	3	0.762572467		
RPLPQVATLIDR	36.53722	Q13421	MSLN HUMAN	689.9124425	2	0.773255348		
RPLPQVATLIDR	36.53722	Q13421	MSLN HUMAN	460.27757	3	0.773255348		
SIPQGIVAAWR	60.7543	Q13421	MSLN HUMAN	599.3411215	2	0.747163951		
SIPQGIVAAWR	60.7543	Q13421	MSLN HUMAN	399.8966893	3	0.747163951		
TDAVLPLTVAEVQK	65.64465	Q13421	MSLN HUMAN	742.422506	2	0.894550025	2	Yes
TDAVLPLTVAEVQK	65.64465	Q13421	MSLN HUMAN	495.284279	3	0.894550025	2	
TLAGETGQEAAPLDGVLANPPNISSLSPR	97.07701	Q13421	MSLN HUMAN	1438.243984	2	0.801192641		
TLAGETGQEAAPLDGVLANPPNISSLSPR	97.07701	Q13421	MSLN HUMAN	959.165264	3	0.801192641		
VNAIPFTYEQLDVLK	95.19231	Q13421	MSLN HUMAN	875.475265	2	0.760345936		
VNAIPFTYEQLDVLK	95.19231	Q13421	MSLN HUMAN	583.9861183	3	0.760345936		
WNVTSLETLK	56.27923	Q13421	MSLN HUMAN	595.8249695	2	0.775053263		
WNVTSLETLK	56.27923	Q13421	MSLN HUMAN	397.552588	3	0.775053263		
DPPPNLPYFVR	69.76235	Q13405	RM49 HUMAN	657.846232	2	0.669603348		
DPPPNLPYFVR	69.76235	Q13405	RM49 HUMAN	438.9000963	3	0.669603348		
DVEDFLSPLLGK	128.536	Q13405	RM49 HUMAN	666.8564665	2	0.793380141	2	Yes
DVEDFLSPLLGK	128.536	Q13405	RM49 HUMAN	444.9069193	3	0.793380141	2	
FVESVDEYQFVER	55.62202	Q13405	RM49 HUMAN	823.889022	2	0.819219351	2	Yes
FVESVDEYQFVER	55.62202	Q13405	RM49 HUMAN	549.5952897	3	0.819219351	2	
GYFDQELK	23.68307	Q13405	RM49 HUMAN	500.243277	2	0.812584996	2	Yes
GYFDQELK	23.68307	Q13405	RM49 HUMAN	333.8314597	3	0.812584996	2	
HEHYPTPSGWQPPR	6.672882	Q13405	RM49 HUMAN	844.9005905	2	0.739367306		
HEHYPTPSGWQPPR	6.672882	Q13405	RM49 HUMAN	563.603002	3	0.739367306		
LLSQTQGPPDYPR	22.91261	Q13405	RM49 HUMAN	736.381168	2	0.843555212		
LLSQTQGPPDYPR	22.91261	Q13405	RM49 HUMAN	491.2567203	3	0.843555212		
TPVTQVNEVTGTLR	34.31395	Q13405	RM49 HUMAN	757.912837	2	0.808698535	2	Yes
TPVTQVNEVTGTLR	34.31395	Q13405	RM49 HUMAN	505.6111663	3	0.808698535	2	
VEGDIWALQK	46.06496	Q13405	RM49 HUMAN	579.8118625	2	0.623646259		
VEGDIWALQK	46.06496	Q13405	RM49 HUMAN	386.8771833	3	0.623646259		
AEVVLGNIIK	50.22852	Q13303	KCAB2 HUMAN	528.327148	2	0.797658384		
AEVVLGNIIK	50.22852	Q13303	KCAB2 HUMAN	352.5540403	3	0.797658384		
LSSSIIHEIDSILGNKPYSK	73.82919	Q13303	KCAB2 HUMAN	1101.094794	2	0.758962274		
LSSSIIHEIDSILGNKPYSK	73.82919	Q13303	KCAB2 HUMAN	734.3991377	3	0.758962274		
VEVQLPELFHK	56.72247	Q13303	KCAB2 HUMAN	669.874994	2	0.785308719		
VEVQLPELFHK	56.72247	Q13303	KCAB2 HUMAN	446.919271	3	0.785308719		
WSSMEIMEAYSVAR	74.68972	Q13303	KCAB2 HUMAN	830.3794525	2	0.778964043		
WSSMEIMEAYSVAR	74.68972	Q13303	KCAB2 HUMAN	553.9222433	3	0.778964043		
YDSGIPPYSR	16.18253	Q13303	KCAB2 HUMAN	577.7780105	2	0.714731157		
YDSGIPPYSR	16.18253	Q13303	KCAB2 HUMAN	385.521282	3	0.714731157		
NGYGFVEFEDSR	48.92593	Q13247	SRSF6 HUMAN	710.31295	2	0.779626787	2	Yes
NGYGFVEFEDSR	48.92593	Q13247	SRSF6 HUMAN	473.8779083	3	0.779626787	2	
TNEGVIEFR	21.79175	Q13247	SRSF6 HUMAN	532.772737	2	0.668357491	2	Yes
TNEGVIEFR	21.79175	Q13247	SRSF6 HUMAN	355.5177663	3	0.668357491	2	
AQSLAEQTSDTAGLESSTR	19.90045	Q12986	NFX1 HUMAN	976.464347	2	0.726783276		
AQSLAEQTSDTAGLESSTR	19.90045	Q12986	NFX1 HUMAN	651.312173	3	0.726783276		
ATQFVYSYGR	21.76184	Q12986	NFX1 HUMAN	596.2938285	2	0.764962733		
ATQFVYSYGR	21.76184	Q12986	NFX1 HUMAN	397.8651607	3	0.764962733		
DVLCGTDVGK	13.01906	Q12986	NFX1 HUMAN	532.258606	2	0.733758628		
DVLCGTDVGK	13.01906	Q12986	NFX1 HUMAN	355.1750123	3	0.733758628		
FNTDAAEFIPQEK	49.49178	Q12986	NFX1 HUMAN	755.3651875	2	0.793035626		
FNTDAAEFIPQEK	49.49178	Q12986	NFX1 HUMAN	503.9127333	3	0.793035626		

GECLVDEPCK	2.350227	Q12986	NFX1 HUMAN	603.7606555	2	0.647889018		
GECLVDEPCK	2.350227	Q12986	NFX1 HUMAN	402.8430453	3	0.647889018		
GVLDGYGAR	6.115025	Q12986	NFX1 HUMAN	454.2357865	2	0.720661283		
GVLDGYGAR	6.115025	Q12986	NFX1 HUMAN	303.1597993	3	0.720661283		
IIHDLAQVYGLESVSYDSEPK	67.48502	Q12986	NFX1 HUMAN	1182.092445	2	0.748784184		
IIHDLAQVYGLESVSYDSEPK	67.48502	Q12986	NFX1 HUMAN	788.3975717	3	0.748784184		
ITDMQLGGSVEISK	40.60976	Q12986	NFX1 HUMAN	739.382523	2	0.839886904		
ITDMQLGGSVEISK	40.60976	Q12986	NFX1 HUMAN	493.2576237	3	0.839886904		
LAEAFHISEDSDPFNIR	62.81416	Q12986	NFX1 HUMAN	980.9741525	2	0.669203818		
LAEAFHISEDSDPFNIR	62.81416	Q12986	NFX1 HUMAN	654.31871	3	0.669203818		
NVETHTGSLIEQLTTEK	45.75304	Q12986	NFX1 HUMAN	950.487094	2	0.76578021		
NVETHTGSLIEQLTTEK	45.75304	Q12986	NFX1 HUMAN	633.9940043	3	0.76578021		
SDGFGDFSCLK	47.6982	Q12986	NFX1 HUMAN	616.7667945	2	0.856494248	2	Yes
SDGFGDFSCLK	47.6982	Q12986	NFX1 HUMAN	411.5138047	3	0.856494248	2	
SEDATFMCDK	3.580467	Q12986	NFX1 HUMAN	602.237013	2	0.727064848		
SEDATFMCDK	3.580467	Q12986	NFX1 HUMAN	401.8272837	3	0.727064848		
SHGLQNQPWQK	-21.59246	Q12986	NFX1 HUMAN	661.834192	2	0.817642093		
SHGLQNQPWQK	-21.59246	Q12986	NFX1 HUMAN	441.5587363	3	0.817642093		
STCDSENLAVINK	17.59328	Q12986	NFX1 HUMAN	725.846304	2	0.673929274		
STCDSENLAVINK	17.59328	Q12986	NFX1 HUMAN	484.2334777	3	0.673929274		
SVCPPTTLTGVLER	55.59042	Q12986	NFX1 HUMAN	765.4037905	2	0.815298259		
SVCPPTTLTGVLER	55.59042	Q12986	NFX1 HUMAN	510.6051353	3	0.815298259		
ILLDAQHESGR	-8.371716	Q12983	BNIP3 HUMAN	619.828575	2	0.68557477		
ILLDAQHESGR	-8.371716	Q12983	BNIP3 HUMAN	413.5549917	3	0.68557477		
DNFHGLAIFLDTYPNDETTER	92.19113	Q12907	LMAN2 HUMAN	1234.572413	2	0.755075574	3	
DNFHGLAIFLDTYPNDETTER	92.19113	Q12907	LMAN2 HUMAN	823.384217	3	0.755075574	3	Yes
DNVDDPTGNFR	14.85484	Q12907	LMAN2 HUMAN	625.276372	2	0.696677506	2	Yes
DNVDDPTGNFR	14.85484	Q12907	LMAN2 HUMAN	417.1868563	3	0.696677506	2	
DWEMHVHFK	22.22335	Q12907	LMAN2 HUMAN	614.782582	2	0.717342436	3	
DWEMHVHFK	22.22335	Q12907	LMAN2 HUMAN	410.1909963	3	0.717342436	3	Yes
EGSIWNHQPCFLK	35.95141	Q12907	LMAN2 HUMAN	808.388474	2	0.645530045	2	Yes
EGSIWNHQPCFLK	35.95141	Q12907	LMAN2 HUMAN	539.261591	3	0.645530045	2	
IEPSVNFLK	46.03513	Q12907	LMAN2 HUMAN	523.798223	2	0.720784307	2	Yes
IEPSVNFLK	46.03513	Q12907	LMAN2 HUMAN	349.534757	3	0.720784307	2	
LFQLMVEHTPDEESIDWTK	76.00558	Q12907	LMAN2 HUMAN	1159.554651	2	0.828117847	3	
LFQLMVEHTPDEESIDWTK	76.00558	Q12907	LMAN2 HUMAN	773.3723753	3	0.828117847	3	Yes
LPTGYYFGASAGTGDLSDNHDIISMK	73.66543	Q12907	LMAN2 HUMAN	1365.639966	2	0.619461536	3	
LPTGYYFGASAGTGDLSDNHDIISMK	73.66543	Q12907	LMAN2 HUMAN	910.7625853	3	0.619461536	3	Yes
LTVMTDLEDK	37.47005	Q12907	LMAN2 HUMAN	582.795021	2	0.799366891	2	Yes
LTVMTDLEDK	37.47005	Q12907	LMAN2 HUMAN	388.8659557	3	0.799366891	2	
LVPGPVFGSK	32.06508	Q12907	LMAN2 HUMAN	500.795484	2	0.778994799	2	Yes
LVPGPVFGSK	32.06508	Q12907	LMAN2 HUMAN	334.1995977	3	0.778994799	2	
NLHGDGIALWYTR	44.55814	Q12907	LMAN2 HUMAN	758.389327	2	0.787612319	2	Yes
NLHGDGIALWYTR	44.55814	Q12907	LMAN2 HUMAN	505.9288263	3	0.787612319	2	
WTELAGCTADFR	44.62075	Q12907	LMAN2 HUMAN	713.825176	2	0.682785153	2	Yes
WTELAGCTADFR	44.62075	Q12907	LMAN2 HUMAN	476.2193923	3	0.682785153	2	
AQDPSEVLTMLTNETGFEISSSDATVK	124.8256	Q12905	ILF2 HUMAN	1435.684776	2	0.733014822	4	
AQDPSEVLTMLTNETGFEISSSDATVK	124.8256	Q12905	ILF2 HUMAN	957.4591253	3	0.733014822	4	
AYEKPPEK	-37.93341	Q12905	ILF2 HUMAN	481.2536445	2	0.682943285		
AYEKPPEK	-37.93341	Q12905	ILF2 HUMAN	321.1717047	3	0.682943285		
GTMTTGHNVADLVVILK	66.24673	Q12905	ILF2 HUMAN	884.985479	2	0.778889954	3	
GTMTTGHNVADLVVILK	66.24673	Q12905	ILF2 HUMAN	590.326261	3	0.778889954	3	Yes
ILGQEGDASYLASEISTWDGVIVTPSEK	120.8809	Q12905	ILF2 HUMAN	1483.238028	2	0.613357902	4	
ILGQEGDASYLASEISTWDGVIVTPSEK	120.8809	Q12905	ILF2 HUMAN	989.1612937	3	0.613357902	4	
ILITTVPPNLR	56.30669	Q12905	ILF2 HUMAN	618.8879045	2	0.782571018	2	Yes
ILITTVPPNLR	56.30669	Q12905	ILF2 HUMAN	412.927878	3	0.782571018	2	
ILPTLEAVAALGNK	84.07607	Q12905	ILF2 HUMAN	705.4223085	2	0.806336284	3	
ILPTLEAVAALGNK	84.07607	Q12905	ILF2 HUMAN	470.6174807	3	0.806336284	3	Yes
LDPELHLDIK	43.7334	Q12905	ILF2 HUMAN	596.8327945	2	0.8228302	2	Yes
LDPELHLDIK	43.7334	Q12905	ILF2 HUMAN	398.2244713	3	0.8228302	2	
NQDLAPNSAEQASILSLVTK	89.65648	Q12905	ILF2 HUMAN	1050.053132	2	0.703864157	3	
NQDLAPNSAEQASILSLVTK	89.65648	Q12905	ILF2 HUMAN	700.3713627	3	0.703864157	3	Yes
QPLALNVAYR	36.35287	Q12905	ILF2 HUMAN	572.8278425	2	0.780437231	2	Yes
QPLALNVAYR	36.35287	Q12905	ILF2 HUMAN	382.22117	3	0.780437231	2	
VKPAPDETSFSEALLK	43.43059	Q12905	ILF2 HUMAN	866.4623585	2	0.813146293	2	Yes
VKPAPDETSFSEALLK	43.43059	Q12905	ILF2 HUMAN	577.977514	3	0.813146293	2	
VLQSALAAIR	35.54192	Q12905	ILF2 HUMAN	521.32494	2	0.804895759	2	Yes
VLQSALAAIR	35.54192	Q12905	ILF2 HUMAN	347.8859017	3	0.804895759	2	
WFEENASQSTVK	18.74146	Q12905	ILF2 HUMAN	713.3364295	2	0.647431076	2	Yes
WFEENASQSTVK	18.74146	Q12905	ILF2 HUMAN	475.8935613	3	0.647431076	2	
DTVENAIQITSGK	36.89415	Q12884	SEPR HUMAN	688.3573625	2	0.705944896		
DTVENAIQITSGK	36.89415	Q12884	SEPR HUMAN	459.24085	3	0.705944896		
EDWQTWDCPK	34.54877	Q12884	SEPR HUMAN	682.7829765	2	0.783507526		
EDWQTWDCPK	34.54877	Q12884	SEPR HUMAN	455.5245927	3	0.783507526		
EGMVIALVDGR	62.63351	Q12884	SEPR HUMAN	580.3111725	2	0.649313271		
EGMVIALVDGR	62.63351	Q12884	SEPR HUMAN	387.2100567	3	0.649313271		
FIEMGFIDEK	70.8205	Q12884	SEPR HUMAN	614.800106	2	0.838332415		
FIEMGFIDEK	70.8205	Q12884	SEPR HUMAN	410.202679	3	0.838332415		
IFNGIPDWVYEEEMLATK	120.1619	Q12884	SEPR HUMAN	1078.024993	2	0.619446337		
IFNGIPDWVYEEEMLATK	120.1619	Q12884	SEPR HUMAN	719.0192703	3	0.619446337		
ISIGSYPPSK	18.50002	Q12884	SEPR HUMAN	524.787851	2	0.62502718		
ISIGSYPPSK	18.50002	Q12884	SEPR HUMAN	350.194509	3	0.62502718		
LAYVYQNNIYLK	49.16947	Q12884	SEPR HUMAN	751.406645	2	0.753635466		
LAYVYQNNIYLK	49.16947	Q12884	SEPR HUMAN	501.273705	3	0.753635466		
LEVDEITLWYK	87.69789	Q12884	SEPR HUMAN	704.8721125	2	0.734703362		
LEVDEITLWYK	87.69789	Q12884	SEPR HUMAN	470.2506833	3	0.734703362		
LGVYEVEDQITAVR	64.11987	Q12884	SEPR HUMAN	796.42049	2	0.851669908		
LGVYEVEDQITAVR	64.11987	Q12884	SEPR HUMAN	531.282935	3	0.851669908		
MILPPQFDR	48.50059	Q12884	SEPR HUMAN	558.797701	2	0.821192861		
MILPPQFDR	48.50059	Q12884	SEPR HUMAN	372.8677423	3	0.821192861		
NVDYLLIHGTADDNVHFQNSAQIAK	49.71747	Q12884	SEPR HUMAN	1392.19155	2	0.816941381		
NVDYLLIHGTADDNVHFQNSAQIAK	49.71747	Q12884	SEPR HUMAN	928.4636413	3	0.816941381		
SVNASNYGLSPDR	11.9096	Q12884	SEPR HUMAN	690.3316735	2	0.611912549		
SVNASNYGLSPDR	11.9096	Q12884	SEPR HUMAN	460.5570573	3	0.611912549		
TINIPYPK	32.22239	Q12884	SEPR HUMAN	473.27438	2	0.704996169		
TINIPYPK	32.22239	Q12884	SEPR HUMAN	315.852195	3	0.704996169		
VCLQWLK	47.567	Q12884	SEPR HUMAN	473.76313	2	0.755696237		
VCLQWLK	47.567	Q12884	SEPR HUMAN	316.1780283	3	0.755696237		
VTQDSLFYSSNEFEEYPGR	66.58702	Q12884	SEPR HUMAN	1134.508745	2	0.804195404		
VTQDSLFYSSNEFEEYPGR	66.58702	Q12884	SEPR HUMAN	756.675105	3	0.804195404		
AALEALLPYTER	69.94402	Q12788	TBL3 HUMAN	673.8699045	2	0.795068026	2	Yes

AALEALLPYTER	69.94402	Q12788	TBL3_HUMAN	449.5825447	3	0.795068026	2	
ALLLAQWAWQEGSVTR	95.76208	Q12788	TBL3_HUMAN	914.98941	2	0.744624972		
ALLLAQWAWQEGSVTR	95.76208	Q12788	TBL3_HUMAN	610.3288817	3	0.744624972		
DVTEAEQAEEQAR	2.757202	Q12788	TBL3_HUMAN	738.334616	2	0.683423221		
DVTEAEQAEEQAR	2.757202	Q12788	TBL3_HUMAN	492.559019	3	0.683423221		
EAPEELLAYEGVR	60.31719	Q12788	TBL3_HUMAN	738.373008	2	0.847315073	2	Yes
EAPEELLAYEGVR	60.31719	Q12788	TBL3_HUMAN	492.5846137	3	0.847315073	2	
ESFLVTGSQDCTVK	35.05863	Q12788	TBL3_HUMAN	785.8750625	2	0.623958528	2	Yes
ESFLVTGSQDCTVK	35.05863	Q12788	TBL3_HUMAN	524.25265	3	0.623958528	2	
FLGPEDSHVVVASNSPCLK	41.56951	Q12788	TBL3_HUMAN	1028.512588	2	0.806840897	3	
FLGPEDSHVVVASNSPCLK	41.56951	Q12788	TBL3_HUMAN	686.0110003	3	0.806840897	3	Yes
GSPGVVHLVAFHPDPTR	32.29832	Q12788	TBL3_HUMAN	893.473927	2	0.781334996	4	
GSPGVVHLVAFHPDPTR	32.29832	Q12788	TBL3_HUMAN	595.9852263	3	0.781334996	4	
GTQLLSSGSDGLVK	32.89311	Q12788	TBL3_HUMAN	681.36773	2	0.849896848	2	Yes
GTQLLSSGSDGLVK	32.89311	Q12788	TBL3_HUMAN	454.581095	3	0.849896848	2	
LDDHALTGASDSR	-10.99808	Q12788	TBL3_HUMAN	679.321311	2	0.855326056	3	
LDDHALTGASDSR	-10.99808	Q12788	TBL3_HUMAN	453.2168157	3	0.855326056	3	Yes
LLLFSSATDAAIR	69.38877	Q12788	TBL3_HUMAN	689.391008	2	0.828984976	2	Yes
LLLFSSATDAAIR	69.38877	Q12788	TBL3_HUMAN	459.929947	3	0.828984976	2	
LPVPAAAPTPWETHK	40.94707	Q12788	TBL3_HUMAN	807.936115	2	0.851220846	3	
LPVPAAAPTPWETHK	40.94707	Q12788	TBL3_HUMAN	538.9600183	3	0.851220846	3	Yes
LWALPQCQLLGVFSGHR	92.48199	Q12788	TBL3_HUMAN	991.525637	2	0.632824838	3	
LWALPQCQLLGVFSGHR	92.48199	Q12788	TBL3_HUMAN	661.353033	3	0.632824838	3	Yes
LWALQDFSCLK	87.23175	Q12788	TBL3_HUMAN	690.8532045	2	0.859053671	2	Yes
LWALQDFSCLK	87.23175	Q12788	TBL3_HUMAN	460.9047447	3	0.859053671	2	
NTAPDNGPILLQAQTTQR	39.88076	Q12788	TBL3_HUMAN	969.506153	2	0.778381944		
NTAPDNGPILLQAQTTQR	39.88076	Q12788	TBL3_HUMAN	646.673377	3	0.778381944		
SLEQEDQEDITAFDLSPDNEVLVTASR	102.4867	Q12788	TBL3_HUMAN	1511.212743	2	0.793349564	3	
SLEQEDQEDITAFDLSPDNEVLVTASR	102.4867	Q12788	TBL3_HUMAN	1007.811103	3	0.793349564	3	Yes
SPGLYFLTAGDQGTLR	73.50938	Q12788	TBL3_HUMAN	848.4392145	2	0.785735726	2	Yes
SPGLYFLTAGDQGTLR	73.50938	Q12788	TBL3_HUMAN	565.9620847	3	0.785735726	2	
TFEGHDASVLK	-5.15625	Q12788	TBL3_HUMAN	602.3044015	2	0.769988358	2	Yes
TFEGHDASVLK	-5.15625	Q12788	TBL3_HUMAN	401.8722093	3	0.769988358	2	
TVPVFESVEAAVLLPEEPVSQLGVK	139.1352	Q12788	TBL3_HUMAN	1319.223469	2	0.773667991		
TVPVFESVEAAVLLPEEPVSQLGVK	139.1352	Q12788	TBL3_HUMAN	879.818254	3	0.773667991		
VNILEVASGAVLR	85.60519	Q12788	TBL3_HUMAN	670.899	2	0.827442527	2	Yes
VNILEVASGAVLR	85.60519	Q12788	TBL3_HUMAN	447.6019417	3	0.827442527	2	
VWSLQDR	17.3499	Q12788	TBL3_HUMAN	452.238333	2	0.621774316		
VWSLQDR	17.3499	Q12788	TBL3_HUMAN	301.8281637	3	0.621774316		
AQSMETLPPGK	2.346378	Q10471	GALT2_HUMAN	579.7953555	2	0.608035445		
AQSMETLPPGK	2.346378	Q10471	GALT2_HUMAN	386.8661787	3	0.608035445		
EDWNEIDPIK	50.23217	Q10471	GALT2_HUMAN	629.801691	2	0.740628719		
EDWNEIDPIK	50.23217	Q10471	GALT2_HUMAN	420.2037357	3	0.740628719		
EIILVDDYSNDPEDGALLGK	85.23824	Q10471	GALT2_HUMAN	1088.536976	2	0.696601808	3	
EIILVDDYSNDPEDGALLGK	85.23824	Q10471	GALT2_HUMAN	726.0272587	3	0.696601808	3	Yes
FYFEELGK	51.52192	Q10471	GALT2_HUMAN	516.75602	2	0.733108759	2	Yes
FYFEELGK	51.52192	Q10471	GALT2_HUMAN	344.839955	3	0.733108759	2	
GGFDWNLVFK	91.78555	Q10471	GALT2_HUMAN	591.8012975	2	0.757544637	2	Yes
GGFDWNLVFK	91.78555	Q10471	GALT2_HUMAN	394.87014	3	0.757544637	2	
HMDLCLTVVDR	41.35477	Q10471	GALT2_HUMAN	679.8319465	2	0.663074195		
HMDLCLTVVDR	41.35477	Q10471	GALT2_HUMAN	453.5572393	3	0.663074195		
HVGSNLCLDSR	-10.88714	Q10471	GALT2_HUMAN	629.3044095	2	0.818185329	2	Yes
HVGSNLCLDSR	-10.88714	Q10471	GALT2_HUMAN	419.8722147	3	0.818185329	2	
NFYYAAVPSAR	33.80223	Q10471	GALT2_HUMAN	629.8149275	2	0.772176385		
NFYYAAVPSAR	33.80223	Q10471	GALT2_HUMAN	420.21256	3	0.772176385		
NVPYGNIQSR	0.071812	Q10471	GALT2_HUMAN	574.296906	2	0.779071212		
NVPYGNIQSR	0.071812	Q10471	GALT2_HUMAN	383.2005457	3	0.779071212		
SGGLSVEVCGPALSQQWK	58.59438	Q10471	GALT2_HUMAN	951.9730995	2	0.676926792		
SGGLSVEVCGPALSQQWK	58.59438	Q10471	GALT2_HUMAN	634.9846747	3	0.676926792		
TPMIAGGLFVMDK	75.67249	Q10471	GALT2_HUMAN	690.357266	2	0.629819155	2	Yes
TPMIAGGLFVMDK	75.67249	Q10471	GALT2_HUMAN	460.574119	3	0.629819155	2	
VDLPATSVVITFHNEAR	62.08793	Q10471	GALT2_HUMAN	934.9974315	2	0.790855885		
VDLPATSVVITFHNEAR	62.08793	Q10471	GALT2_HUMAN	623.6675627	3	0.790855885		
VLTFLDSHCECNEHWLEPLLER	81.73778	Q10471	GALT2_HUMAN	1399.157811	2	0.738478899	4	
VLTFLDSHCECNEHWLEPLLER	81.73778	Q10471	GALT2_HUMAN	933.1078157	3	0.738478899	4	
WDYMTPEQR	24.73921	Q10471	GALT2_HUMAN	613.2695005	2	0.744024575	2	Yes
WDYMTPEQR	24.73921	Q10471	GALT2_HUMAN	409.1822753	3	0.744024575	2	
WPDFNQEAYVGGTMVR	73.01759	Q10471	GALT2_HUMAN	935.433606	2	0.767587185	2	Yes
WPDFNQEAYVGGTMVR	73.01759	Q10471	GALT2_HUMAN	623.9583457	3	0.767587185	2	
WYLENVYPELR	79.51321	Q10471	GALT2_HUMAN	741.375349	2	0.794387341	2	Yes
WYLENVYPELR	79.51321	Q10471	GALT2_HUMAN	494.5861743	3	0.794387341	2	
DIYTFDGALNK	54.25601	Q08722	CD47_HUMAN	628.812055	2	0.80067569		
DIYTFDGALNK	54.25601	Q08722	CD47_HUMAN	419.5439783	3	0.80067569		
STVPTDFSSAK	8.813107	Q08722	CD47_HUMAN	570.2831345	2	0.62364769	2	Yes
STVPTDFSSAK	8.813107	Q08722	CD47_HUMAN	380.524698	3	0.62364769	2	
AAELWGEQAEAR	21.56377	Q08379	GOGA2_HUMAN	665.82349	2	0.877516747		
AAELWGEQAEAR	21.56377	Q08379	GOGA2_HUMAN	444.2182683	3	0.877516747		
AELQTALAHTQHAAR	-15.23362	Q08379	GOGA2_HUMAN	809.4269775	2	0.763120651	3	
AELQTALAHTQHAAR	-15.23362	Q08379	GOGA2_HUMAN	539.9539267	3	0.763120651	3	Yes
AGMQLNLEELQK	51.2217	Q08379	GOGA2_HUMAN	687.358851	2	0.872714043	2	Yes
AGMQLNLEELQK	51.2217	Q08379	GOGA2_HUMAN	458.5751757	3	0.872714043	2	
AQLEAHLGQVMESVR	39.40128	Q08379	GOGA2_HUMAN	834.4308705	2	0.825285077		
AQLEAHLGQVMESVR	39.40128	Q08379	GOGA2_HUMAN	556.6231887	3	0.825285077		
CEAPDANQQLQQAMEER	36.04537	Q08379	GOGA2_HUMAN	1009.439296	2	0.640303612		
CEAPDANQQLQQAMEER	36.04537	Q08379	GOGA2_HUMAN	673.295472	3	0.640303612		
PSDDTVLPGGVPSPGASLTSMAASQNHDADNVP	99.74284	Q08379	GOGA2_HUMAN	2375.098713	2	0.806978106		
PSDDTVLPGGVPSPGASLTSMAASQNHDADNVP	99.74284	Q08379	GOGA2_HUMAN	1583.735083	3	0.806978106		
DNPTAQQIMQLLR	103.1656	Q08379	GOGA2_HUMAN	764.401582	2	0.821196198	3	
DNPTAQQIMQLLR	103.1656	Q08379	GOGA2_HUMAN	509.9369963	3	0.821196198	3	Yes
DQYLGHLQQYVAAYQQLTSEK	88.44591	Q08379	GOGA2_HUMAN	1242.114241	2	0.774889827	3	
DQYLGHLQQYVAAYQQLTSEK	88.44591	Q08379	GOGA2_HUMAN	828.412102	3	0.774889827	3	Yes
ELEGLAGQLQAQVQDNEGLSR	68.7757	Q08379	GOGA2_HUMAN	1128.067303	2	0.66676265	3	
ELEGLAGQLQAQVQDNEGLSR	68.7757	Q08379	GOGA2_HUMAN	752.38081	3	0.66676265	3	Yes
EQLAELQSGFVK	44.41238	Q08379	GOGA2_HUMAN	674.859541	2	0.807827413		
EQLAELQSGFVK	44.41238	Q08379	GOGA2_HUMAN	450.2423023	3	0.807827413		
EVLHNQLLLQTQLVDQLQQQEAQGK	75.54631	Q08379	GOGA2_HUMAN	1451.27562	2	0.752528787	3	
EVLHNQLLLQTQLVDQLQQQEAQGK	75.54631	Q08379	GOGA2_HUMAN	967.8530213	3	0.752528787	3	Yes
PADEPTSGAPAPQELGAANQQGDLCEVSLAGSVI	90.65402	Q08379	GOGA2_HUMAN	2395.634113	2	0.814773738		
PADEPTSGAPAPQELGAANQQGDLCEVSLAGSVI	90.65402	Q08379	GOGA2_HUMAN	1597.42535	3	0.814773738		
FMELMQEK	30.04846	Q08379	GOGA2_HUMAN	528.249195	2	0.806482196	2	Yes
FMELMQEK	30.04846	Q08379	GOGA2_HUMAN	352.5020717	3	0.806482196	2	

LEAATQQNQQLR	-13.00313	Q08379	GOGA2 HUMAN	700.3685965	2	0.79701817		
LEAATQQNQQLR	-13.00313	Q08379	GOGA2 HUMAN	467.2483393	3	0.79701817		
LGELQEK	-19.0743	Q08379	GOGA2 HUMAN	408.727267	2	0.680246651		
LGELQEK	-19.0743	Q08379	GOGA2 HUMAN	272.8207863	3	0.680246651		
LLELQELVLR	87.31902	Q08379	GOGA2 HUMAN	613.379912	2	0.824425817	2	Yes
LLELQELVLR	87.31902	Q08379	GOGA2 HUMAN	409.255883	3	0.824425817	2	
LTNENMEITSALQSEQHVK	36.74564	Q08379	GOGA2 HUMAN	1086.534249	2	0.76436162	3	
LTNENMEITSALQSEQHVK	36.74564	Q08379	GOGA2 HUMAN	724.6921077	3	0.76436162	3	Yes
MQQMSEQVHTLR	-7.398735	Q08379	GOGA2 HUMAN	744.35886	2	0.76872319	3	
MQQMSEQVHTLR	-7.398735	Q08379	GOGA2 HUMAN	496.5751817	3	0.76872319	3	Yes
NQMAEPPPPEPPAGPSEVEQQLQAEAEHLR	72.48881	Q08379	GOGA2 HUMAN	1638.783679	2	0.719749629	3	
NQMAEPPPPEPPAGPSEVEQQLQAEAEHLR	72.48881	Q08379	GOGA2 HUMAN	1092.858394	3	0.719749629	3	Yes
SQEAQSLQQQR	-26.21084	Q08379	GOGA2 HUMAN	651.8240215	2	0.624537766		
SQEAQSLQQQR	-26.21084	Q08379	GOGA2 HUMAN	434.8852893	3	0.624537766		
VQELETSLAELR	52.72174	Q08379	GOGA2 HUMAN	694.375555	2	0.812970817		
VQELETSLAELR	52.72174	Q08379	GOGA2 HUMAN	463.2529783	3	0.812970817		
YQQLAVALDSSYVTNK	57.1317	Q08379	GOGA2 HUMAN	900.4628815	2	0.704811335		
YQQLAVALDSSYVTNK	57.1317	Q08379	GOGA2 HUMAN	600.6445293	3	0.704811335		
ADGATSDDLDLHDDR	3.607307	Q07157	ZO1 HUMAN	808.3457115	2	0.809816957	3	
ADGATSDDLDLHDDR	3.607307	Q07157	ZO1 HUMAN	539.2330827	3	0.809816957	3	Yes
AEASSPVPYLSPETNPASSTSAVNHNVNLTNVR	58.77838	Q07157	ZO1 HUMAN	1712.34294	2	0.612246692	3	
AEASSPVPYLSPETNPASSTSAVNHNVNLTNVR	58.77838	Q07157	ZO1 HUMAN	1141.897902	3	0.612246692	3	Yes
AEQLASVQYTLPK	41.91815	Q07157	ZO1 HUMAN	724.393744	2	0.843435109		
AEQLASVQYTLPK	41.91815	Q07157	ZO1 HUMAN	483.2651043	3	0.843435109		
APGFGFGIAISGGR	75.1204	Q07157	ZO1 HUMAN	653.849311	2	0.763264239		
APGFGFGIAISGGR	75.1204	Q07157	ZO1 HUMAN	436.2354823	3	0.763264239		
ATLLNVPDLSDSIHSANASER	60.62541	Q07157	ZO1 HUMAN	1105.556569	2	0.741519988		
ATLLNVPDLSDSIHSANASER	60.62541	Q07157	ZO1 HUMAN	737.3736543	3	0.741519988		
DDISEIQSLASDHSGR	47.60184	Q07157	ZO1 HUMAN	865.40356	2	0.776932716		
DDISEIQSLASDHSGR	47.60184	Q07157	ZO1 HUMAN	577.2716483	3	0.776932716		
DGNIQEGDVVLK	29.23469	Q07157	ZO1 HUMAN	643.833523	2	0.768259048		
DGNIQEGDVVLK	29.23469	Q07157	ZO1 HUMAN	429.5582903	3	0.768259048		
DLEQPTYR	5.30479	Q07157	ZO1 HUMAN	511.2516335	2	0.744954467		
DLEQPTYR	5.30479	Q07157	ZO1 HUMAN	341.170364	3	0.744954467		
DNPHFQSGETSIVISDVLK	69.05596	Q07157	ZO1 HUMAN	1043.52675	2	0.829296529		
DNPHFQSGETSIVISDVLK	69.05596	Q07157	ZO1 HUMAN	696.0204413	3	0.829296529		
DNSILPPLDK	49.48026	Q07157	ZO1 HUMAN	556.3038695	2	0.751889348	2	Yes
DNSILPPLDK	49.48026	Q07157	ZO1 HUMAN	371.205188	3	0.751889348	2	
DQEPSLSSHVDPTK	0.69046	Q07157	ZO1 HUMAN	770.368458	2	0.663450658		
DQEPSLSSHVDPTK	0.69046	Q07157	ZO1 HUMAN	513.9149137	3	0.663450658		
EAGFLRPVTIFGPIADVAR	97.45525	Q07157	ZO1 HUMAN	1015.065649	2	0.619907081	3	
EAGFLRPVTIFGPIADVAR	97.45525	Q07157	ZO1 HUMAN	677.0463743	3	0.619907081	3	Yes
EAIQQQQNQLVWVSEGK	47.0953	Q07157	ZO1 HUMAN	993.008528	2	0.752300978	3	
EAIQQQQNQLVWVSEGK	47.0953	Q07157	ZO1 HUMAN	662.341627	3	0.752300978	3	Yes
EDLSAQPVQTK	-4.121052	Q07157	ZO1 HUMAN	608.3149665	2	0.747730196	2	Yes
EDLSAQPVQTK	-4.121052	Q07157	ZO1 HUMAN	405.8792527	3	0.747730196	2	
EDTAQAAFYPQK	18.85363	Q07157	ZO1 HUMAN	684.8256945	2	0.653173864		
EDTAQAAFYPQK	18.85363	Q07157	ZO1 HUMAN	456.8864047	3	0.653173864		
EEPDIYQIAK	32.27885	Q07157	ZO1 HUMAN	603.3066055	2	0.802048147		
EEPDIYQIAK	32.27885	Q07157	ZO1 HUMAN	402.5403453	3	0.802048147		
EGLEEGDQILR	33.50414	Q07157	ZO1 HUMAN	629.817873	2	0.816495657		
EGLEEGDQILR	33.50414	Q07157	ZO1 HUMAN	420.2145237	3	0.816495657		
EISQDSLAAR	-4.449047	Q07157	ZO1 HUMAN	545.280926	2	0.756269813		
EISQDSLAAR	-4.449047	Q07157	ZO1 HUMAN	363.856559	3	0.756269813		
FEEPAPLSYDSRPR	25.90118	Q07157	ZO1 HUMAN	832.407913	2	0.696629286	3	
FEEPAPLSYDSRPR	25.90118	Q07157	ZO1 HUMAN	555.2745503	3	0.696629286	3	Yes
FLKPVELR	17.27621	Q07157	ZO1 HUMAN	501.311301	2	0.690596581		
FLKPVELR	17.27621	Q07157	ZO1 HUMAN	334.5434757	3	0.690596581		
FNHNLLPSETAHKPDLSSK	4.475033	Q07157	ZO1 HUMAN	1068.048183	2	0.713983059		
FNHNLLPSETAHKPDLSSK	4.475033	Q07157	ZO1 HUMAN	712.3680637	3	0.713983059		
GEEVTILAQK	21.37068	Q07157	ZO1 HUMAN	544.3038705	2	0.708823085		
GEEVTILAQK	21.37068	Q07157	ZO1 HUMAN	363.2051887	3	0.708823085		
GGPAEGQLQENDR	-17.49728	Q07157	ZO1 HUMAN	685.818936	2	0.824614763	2	Yes
GGPAEGQLQENDR	-17.49728	Q07157	ZO1 HUMAN	457.5485657	3	0.824614763	2	
HALLDVTPNAVDR	27.48994	Q07157	ZO1 HUMAN	710.881339	2	0.809323311		
HALLDVTPNAVDR	27.48994	Q07157	ZO1 HUMAN	474.2568343	3	0.809323311		
HEEQPAPGYDTHGR	-32.00414	Q07157	ZO1 HUMAN	797.356213	2	0.641442299		
HEEQPAPGYDTHGR	-32.00414	Q07157	ZO1 HUMAN	531.9067503	3	0.641442299		
INGTVTENMSLTDAK	27.80757	Q07157	ZO1 HUMAN	797.393619	2	0.72764349		
INGTVTENMSLTDAK	27.80757	Q07157	ZO1 HUMAN	531.9316877	3	0.72764349		
IPEPQKPQLKPPEDIVR	28.29492	Q07157	ZO1 HUMAN	992.5654815	2	0.80579567		
IPEPQKPQLKPPEDIVR	28.29492	Q07157	ZO1 HUMAN	662.0462627	3	0.80579567		
ISKPGAVSTPVK	-22.50387	Q07157	ZO1 HUMAN	592.356437	2	0.676232934	3	
ISKPGAVSTPVK	-22.50387	Q07157	ZO1 HUMAN	395.240233	3	0.676232934	3	Yes
IVESDVGDSFYIR	54.54585	Q07157	ZO1 HUMAN	750.3730075	2	0.810324192	2	Yes
IVESDVGDSFYIR	54.54585	Q07157	ZO1 HUMAN	500.5846133	3	0.810324192	2	
LAGGNDVGIFVAGVLEDSPAAK	105.5684	Q07157	ZO1 HUMAN	1050.552768	2	0.688146114	3	
LAGGNDVGIFVAGVLEDSPAAK	105.5684	Q07157	ZO1 HUMAN	700.7044537	3	0.688146114	3	Yes
LEEPTPAPSTSYSPQADSLR	40.8779	Q07157	ZO1 HUMAN	1073.519118	2	0.821712255		
LEEPTPAPSTSYSPQADSLR	40.8779	Q07157	ZO1 HUMAN	716.0153533	3	0.821712255		
LGSWLAIR	51.9391	Q07157	ZO1 HUMAN	458.2747185	2	0.751599312	2	Yes
LGSWLAIR	51.9391	Q07157	ZO1 HUMAN	305.8524207	3	0.751599312	2	
LSYLSAPGSEYSMYSTDSR	55.64384	Q07157	ZO1 HUMAN	1057.473312	2	0.724118412		
LSYLSAPGSEYSMYSTDSR	55.64384	Q07157	ZO1 HUMAN	705.3181497	3	0.724118412		
FENKPPAHIAASHLSEPAKPAHSQNQSNFSSYSSI	10.92822	Q07157	ZO1 HUMAN	1940.936059	2	0.696746945	5	
FENKPPAHIAASHLSEPAKPAHSQNQSNFSSYSSI	10.92822	Q07157	ZO1 HUMAN	1294.293314	3	0.696746945	5	
SHSLAQPPEFDSGVETFSIHAEKPK	35.33398	Q07157	ZO1 HUMAN	1369.675205	2	0.791986465	3	
SHSLAQPPEFDSGVETFSIHAEKPK	35.33398	Q07157	ZO1 HUMAN	913.452745	3	0.791986465	3	Yes
SNHYDPEEDEEYYR	5.106972	Q07157	ZO1 HUMAN	923.3640875	2	0.748391628	3	
SNHYDPEEDEEYYR	5.106972	Q07157	ZO1 HUMAN	615.912	3	0.748391628	3	Yes
STAMEETAIWEQHTVTLHR	38.49612	Q07157	ZO1 HUMAN	1120.54241	2	0.86616534	4	
STAMEETAIWEQHTVTLHR	38.49612	Q07157	ZO1 HUMAN	747.3642147	3	0.86616534	4	
SYEQVPPQGFTSR	25.25413	Q07157	ZO1 HUMAN	748.362975	2	0.829014301		
SYEQVPPQGFTSR	25.25413	Q07157	ZO1 HUMAN	499.2445917	3	0.829014301		
TPSTEAAHIMLR	13.09798	Q07157	ZO1 HUMAN	663.845911	2	0.725882649		
TPSTEAAHIMLR	13.09798	Q07157	ZO1 HUMAN	442.8998823	3	0.725882649		
VNNVDFTNIIR	57.17362	Q07157	ZO1 HUMAN	652.8520495	2	0.865206122	2	Yes
VNNVDFTNIIR	57.17362	Q07157	ZO1 HUMAN	435.5706413	3	0.865206122	2	
VPMYEEQWSYYDDK	52.22652	Q07157	ZO1 HUMAN	926.890896	2	0.783027291		
VPMYEEQWSYYDDK	52.22652	Q07157	ZO1 HUMAN	618.2632057	3	0.783027291		
YESSSYTDQFSR	13.0975	Q07157	ZO1 HUMAN	735.3131425	2	0.798701644	2	Yes

YESSSYTDQFSR	13.0975	Q07157	ZO1_HUMAN	490.5447033	3	0.798701644	2	
YQINNISTVPK	27.12043	Q07157	ZO1_HUMAN	638.8489715	2	0.824292362	2	Yes
YQINNISTVPK	27.12043	Q07157	ZO1_HUMAN	426.235256	3	0.824292362	2	
ATAVMPDGQFK	18.0719	Q06830	PRDX1_HUMAN	582.7900735	2	0.778160334	2	Yes
ATAVMPDGQFK	18.0719	Q06830	PRDX1_HUMAN	388.8626573	3	0.778160334	2	
DISLSDYK	29.50476	Q06830	PRDX1_HUMAN	470.735284	2	0.775766969	2	Yes
DISLSDYK	29.50476	Q06830	PRDX1_HUMAN	314.1594643	3	0.775766969	2	
LVQAFQFTDK	41.37892	Q06830	PRDX1_HUMAN	598.819688	2	0.757398248	2	Yes
LVQAFQFTDK	41.37892	Q06830	PRDX1_HUMAN	399.549067	3	0.757398248	2	
PGSDTIKPDVQK	-25.85869	Q06830	PRDX1_HUMAN	642.843891	2	0.818786442		
PGSDTIKPDVQK	-25.85869	Q06830	PRDX1_HUMAN	428.8985357	3	0.818786442		
QGGLGPMNIPLVSDPK	71.62249	Q06830	PRDX1_HUMAN	811.9327145	2	0.600788474		
QGGLGPMNIPLVSDPK	71.62249	Q06830	PRDX1_HUMAN	541.624418	3	0.600788474		
TIAQDYGVLK	28.53538	Q06830	PRDX1_HUMAN	554.306409	2	0.777716696	2	Yes
TIAQDYGVLK	28.53538	Q06830	PRDX1_HUMAN	369.8735477	3	0.777716696	2	
ADLEMQIENLK	50.04572	Q04695	K1C17_HUMAN	652.3323015	2	0.817333937	2	Yes
ADLEMQIENLK	50.04572	Q04695	K1C17_HUMAN	435.2241427	3	0.817333937	2	
ALEEANTELEVK	23.09835	Q04695	K1C17_HUMAN	673.346463	2	0.806277096	2	Yes
ALEEANTELEVK	23.09835	Q04695	K1C17_HUMAN	449.2335837	3	0.806277096	2	
ASLEGNLAETENR	13.47358	Q04695	K1C17_HUMAN	702.342243	2	0.872435927	2	Yes
ASLEGNLAETENR	13.47358	Q04695	K1C17_HUMAN	468.5641037	3	0.872435927	2	
DAEDWFFSK	77.22592	Q04695	K1C17_HUMAN	572.75147	2	0.697920084	2	Yes
DAEDWFFSK	77.22592	Q04695	K1C17_HUMAN	382.170255	3	0.697920084	2	
DYSQYYR	0.241081	Q04695	K1C17_HUMAN	497.7174175	2	0.699367285	2	Yes
DYSQYYR	0.241081	Q04695	K1C17_HUMAN	332.1475533	3	0.699367285	2	
GQVGGEINVEMDAAPGVDLSR	64.02264	Q04695	K1C17_HUMAN	1057.513317	2	0.782137454		
GQVGGEINVEMDAAPGVDLSR	64.02264	Q04695	K1C17_HUMAN	705.3448197	3	0.782137454		
GSSGLGGGSSR	-50	Q04695	K1C17_HUMAN	461.223411	2	0.649920881	2	Yes
GSSGLGGGSSR	-50	Q04695	K1C17_HUMAN	307.8182157	3	0.649920881	2	
ILTATVDNANILLQIDNAR	91.14633	Q04695	K1C17_HUMAN	1034.574035	2	0.766398787	3	
ILTATVDNANILLQIDNAR	91.14633	Q04695	K1C17_HUMAN	690.0519647	3	0.766398787	3	Yes
LLEGEDAHLTQYK	16.19505	Q04695	K1C17_HUMAN	758.886283	2	0.886599183		
LLEGEDAHLTQYK	16.19505	Q04695	K1C17_HUMAN	506.2601303	3	0.886599183		
LSGGLGAGSCR	-18.1794	Q04695	K1C17_HUMAN	517.7565645	2	0.681147099	2	Yes
LSGGLGAGSCR	-18.1794	Q04695	K1C17_HUMAN	345.5069847	3	0.681147099	2	
LSVEADINGLR	47.29994	Q04695	K1C17_HUMAN	593.8255005	2	0.740645647		
LSVEADINGLR	47.29994	Q04695	K1C17_HUMAN	396.2196087	3	0.740645647		
TIVEEVQDGK	6.530891	Q04695	K1C17_HUMAN	559.29096	2	0.74253267	2	Yes
TIVEEVQDGK	6.530891	Q04695	K1C17_HUMAN	373.1965817	3	0.74253267	2	
TMQALEIELQSQLSMK	85.06081	Q04695	K1C17_HUMAN	925.4740865	2	0.716385603		
TMQALEIELQSQLSMK	85.06081	Q04695	K1C17_HUMAN	617.318666	3	0.716385603		
APAAHPEGQLK	-31.28705	Q02818	NUCB1_HUMAN	559.8018295	2	0.65521574		
APAAHPEGQLK	-31.28705	Q02818	NUCB1_HUMAN	373.5371613	3	0.65521574		
AVLAVPLER	42.46606	Q02818	NUCB1_HUMAN	484.3009335	2	0.696007729		
AVLAVPLER	42.46606	Q02818	NUCB1_HUMAN	323.2032307	3	0.696007729		
DLAQYDAAHHEEFK	6.948586	Q02818	NUCB1_HUMAN	837.381896	2	0.830717683		
DLAQYDAAHHEEFK	6.948586	Q02818	NUCB1_HUMAN	558.590539	3	0.830717683		
DLELLIQTATR	85.74358	Q02818	NUCB1_HUMAN	636.862084	2	0.777347565		
DLELLIQTATR	85.74358	Q02818	NUCB1_HUMAN	424.9106643	3	0.777347565		
EETPATESPDTGLYYHR	16.51529	Q02818	NUCB1_HUMAN	983.4454175	2	0.689444721		
EETPATESPDTGLYYHR	16.51529	Q02818	NUCB1_HUMAN	655.96622	3	0.689444721		
EFGDTGEGWETVEMHPAYTEEELR	64.3662	Q02818	NUCB1_HUMAN	1406.606325	2	0.815141737		
EFGDTGEGWETVEMHPAYTEEELR	64.3662	Q02818	NUCB1_HUMAN	938.0734913	3	0.815141737		
ELQQAVLHMEQR	20.54989	Q02818	NUCB1_HUMAN	741.3806495	2	0.830139279	3	
ELQQAVLHMEQR	20.54989	Q02818	NUCB1_HUMAN	494.589708	3	0.830139279	3	Yes
EVWEELDGLDPNR	72.09244	Q02818	NUCB1_HUMAN	786.3710005	2	0.777239919	2	Yes
EVWEELDGLDPNR	72.09244	Q02818	NUCB1_HUMAN	524.5832753	3	0.777239919	2	
LPEVEVPQHL	49.53278	Q02818	NUCB1_HUMAN	580.8196875	2	0.712386429		
LPEVEVPQHL	49.53278	Q02818	NUCB1_HUMAN	387.5490667	3	0.712386429		
LSQETEALGR	-5.127125	Q02818	NUCB1_HUMAN	552.2887515	2	0.760222256	2	Yes
LSQETEALGR	-5.127125	Q02818	NUCB1_HUMAN	368.5284427	3	0.760222256	2	
LVTLEEFLASTQR	97.34357	Q02818	NUCB1_HUMAN	753.912305	2	0.90596813	3	
LVTLEEFLASTQR	97.34357	Q02818	NUCB1_HUMAN	502.944145	3	0.90596813	3	Yes
MDAEQDPNVQVDHLNLLK	55.17487	Q02818	NUCB1_HUMAN	1040.012953	2	0.774724603	3	
MDAEQDPNVQVDHLNLLK	55.17487	Q02818	NUCB1_HUMAN	693.67791	3	0.774724603	3	Yes
QFEHLDPQNQHTFEAR	12.14138	Q02818	NUCB1_HUMAN	998.9671945	2	0.640696526		
QFEHLDPQNQHTFEAR	12.14138	Q02818	NUCB1_HUMAN	666.3140713	3	0.640696526		
TFFILHDINSDGVLDEQELEALFTK	147.6838	Q02818	NUCB1_HUMAN	1447.727104	2	0.72140497		
TFFILHDINSDGVLDEQELEALFTK	147.6838	Q02818	NUCB1_HUMAN	965.487344	3	0.72140497		
VNVPGSQAQLK	3.073502	Q02818	NUCB1_HUMAN	570.8227615	2	0.618714392	2	Yes
VNVPGSQAQLK	3.073502	Q02818	NUCB1_HUMAN	380.8844493	3	0.618714392	2	
YLESLGEEQR	15.85374	Q02818	NUCB1_HUMAN	612.2993115	2	0.789694607	2	Yes
YLESLGEEQR	15.85374	Q02818	NUCB1_HUMAN	408.5354827	3	0.789694607	2	
YLQEVIDVLETDGHFR	89.44571	Q02818	NUCB1_HUMAN	967.4868925	2	0.778694212	3	
YLQEVIDVLETDGHFR	89.44571	Q02818	NUCB1_HUMAN	645.3272033	3	0.778694212	3	Yes
DLSTQTIR	5.31237	Q02127	PYRD_HUMAN	467.2541805	2	0.643317401		
DLSTQTIR	5.31237	Q02127	PYRD_HUMAN	311.8387287	3	0.643317401		
EQGFGGVTDAIGADHR	27.11909	Q02127	PYRD_HUMAN	815.384975	2	0.760226548	3	
EQGFGGVTDAIGADHR	27.11909	Q02127	PYRD_HUMAN	543.925925	3	0.760226548	3	Yes
FQDSDMLEVR	36.70277	Q02127	PYRD_HUMAN	620.287894	2	0.771461308	2	Yes
FQDSDMLEVR	36.70277	Q02127	PYRD_HUMAN	413.8612043	3	0.771461308	2	
FTSLGLLPR	58.69143	Q02127	PYRD_HUMAN	502.3009335	2	0.738441408		
FTSLGLLPR	58.69143	Q02127	PYRD_HUMAN	335.2032307	3	0.738441408		
FYAEHLMPTLQGLLDPESAHR	76.14331	Q02127	PYRD_HUMAN	1213.10263	2	0.62968123	4	
FYAEHLMPTLQGLLDPESAHR	76.14331	Q02127	PYRD_HUMAN	809.071028	3	0.62968123	4	
IAPDLTSQDK	0.704258	Q02127	PYRD_HUMAN	544.2856775	2	0.679552197		
IAPDLTSQDK	0.704258	Q02127	PYRD_HUMAN	363.19306	3	0.679552197		
LPEDQAVINR	12.57759	Q02127	PYRD_HUMAN	577.8123935	2	0.733977139	2	Yes
LPEDQAVINR	12.57759	Q02127	PYRD_HUMAN	385.544204	3	0.733977139	2	
LTEDGLPLGVNLGK	68.18612	Q02127	PYRD_HUMAN	713.401573	2	0.809904933	2	Yes
LTEDGLPLGVNLGK	68.18612	Q02127	PYRD_HUMAN	475.9369903	3	0.809904933	2	
NPVGIAAGFDK	31.47589	Q02127	PYRD_HUMAN	544.79093	2	0.682678998		
NPVGIAAGFDK	31.47589	Q02127	PYRD_HUMAN	363.529895	3	0.682678998		
VPIIGVGGVSSGQDALEK	59.23971	Q02127	PYRD_HUMAN	863.473258	2	0.746072054	2	Yes
VPIIGVGGVSSGQDALEK	59.23971	Q02127	PYRD_HUMAN	575.9847803	3	0.746072054	2	
YGFNSHGLSVVEHR	9.78688	Q02127	PYRD_HUMAN	801.39514	2	0.84537816		
YGFNSHGLSVVEHR	9.78688	Q02127	PYRD_HUMAN	534.5993683	3	0.84537816		
AFVDFNEGEIK	47.65112	Q01968	OCRL_HUMAN	634.812059	2	0.8819381		
AFVDFNEGEIK	47.65112	Q01968	OCRL_HUMAN	423.543981	3	0.8819381		
AQSQLLVPEQK	14.66074	Q01968	OCRL_HUMAN	620.8489765	2	0.718699396		
AQSQLLVPEQK	14.66074	Q01968	OCRL_HUMAN	414.2352593	3	0.718699396		

DIATETVGTGIMGK	50.34399	Q01968	OCRL_HUMAN	696.856142	2	0.859529257	2	Yes
DIATETVGTGIMGK	50.34399	Q01968	OCRL_HUMAN	464.906703	3	0.859529257	2	
DKPSVFSGLLGFEDNFSSMNLDK	116.9328	Q01968	OCRL_HUMAN	1274.107777	2	0.610266507		
DKPSVFSGLLGFEDNFSSMNLDK	116.9328	Q01968	OCRL_HUMAN	849.7411263	3	0.610266507		
DSVTILNSGEDK	27.6274	Q01968	OCRL_HUMAN	639.3151625	2	0.767558396		
DSVTILNSGEDK	27.6274	Q01968	OCRL_HUMAN	426.54605	3	0.767558396		
EIWLLVDHLFK	106.5688	Q01968	OCRL_HUMAN	706.9010115	2	0.768276989		
EIWLLVDHLFK	106.5688	Q01968	OCRL_HUMAN	471.6032827	3	0.768276989		
FDQLNIQR	19.17955	Q01968	OCRL_HUMAN	517.275447	2	0.827606082		
FDQLNIQR	19.17955	Q01968	OCRL_HUMAN	345.1862397	3	0.827606082		
FEIPDEEHCLK	29.04762	Q01968	OCRL_HUMAN	708.8273835	2	0.619238973		
FEIPDEEHCLK	29.04762	Q01968	OCRL_HUMAN	472.8875307	3	0.619238973		
FLSAVLAAQK	40.57062	Q01968	OCRL_HUMAN	524.314041	2	0.724953353	2	Yes
FLSAVLAAQK	40.57062	Q01968	OCRL_HUMAN	349.8786357	3	0.724953353	2	
GTNVNQLNYR	-1.618046	Q01968	OCRL_HUMAN	589.799813	2	0.728064895	2	Yes
GTNVNQLNYR	-1.618046	Q01968	OCRL_HUMAN	393.535817	3	0.728064895	2	
LFVPNTQSGQR	13.08112	Q01968	OCRL_HUMAN	623.831118	2	0.694268584	2	Yes
LFVPNTQSGQR	13.08112	Q01968	OCRL_HUMAN	416.2233537	3	0.694268584	2	
LIDLEEDSFLEK	73.19968	Q01968	OCRL_HUMAN	725.8697705	2	0.872116923		
LIDLEEDSFLEK	73.19968	Q01968	OCRL_HUMAN	484.249122	3	0.872116923		
LNDSQYCKPWLR	25.51839	Q01968	OCRL_HUMAN	790.3884695	2	0.708589554		
LNDSQYCKPWLR	25.51839	Q01968	OCRL_HUMAN	527.261588	3	0.708589554		
MENDFLPSLELSR	85.67393	Q01968	OCRL_HUMAN	775.8801465	2	0.803592145	2	Yes
MENDFLPSLELSR	85.67393	Q01968	OCRL_HUMAN	517.5893727	3	0.803592145	2	
MEPPLPVGAQPLATVEGMEMK	96.05072	Q01968	OCRL_HUMAN	1113.054919	2	0.708993495		
MEPPLPVGAQPLATVEGMEMK	96.05072	Q01968	OCRL_HUMAN	742.372554	3	0.708993495		
SLLQMVPLDEGASERPLQVPK	80.2979	Q01968	OCRL_HUMAN	1154.123035	2	0.693354309	3	
SLLQMVPLDEGASERPLQVPK	80.2979	Q01968	OCRL_HUMAN	769.751298	3	0.693354309	3	Yes
TSDHKPVSALFHIGVK	27.77082	Q01968	OCRL_HUMAN	868.4786775	2	0.760706365		
TSDHKPVSALFHIGVK	27.77082	Q01968	OCRL_HUMAN	579.3217267	3	0.760706365		
DAEDAMDAMDGAVLDGR	79.28984	Q01130	SRSF2_HUMAN	876.364733	2	0.778689444	2	Yes
DAEDAMDAMDGAVLDGR	79.28984	Q01130	SRSF2_HUMAN	584.579097	3	0.778689444	2	
AELFTQSCADLDK	34.3084	Q01082	SPTB2_HUMAN	749.3486805	2	0.819384992		
AELFTQSCADLDK	34.3084	Q01082	SPTB2_HUMAN	499.9017287	3	0.819384992		
AFEDEMSGR	2.778141	Q01082	SPTB2_HUMAN	521.21948	2	0.688410759	2	Yes
AFEDEMSGR	2.778141	Q01082	SPTB2_HUMAN	347.815595	3	0.688410759	2	
AQTLPTSVVTITSESSPGK	48.35177	Q01082	SPTB2_HUMAN	952.0051195	2	0.651573241	3	
AQTLPTSVVTITSESSPGK	48.35177	Q01082	SPTB2_HUMAN	635.0060213	3	0.651573241	3	Yes
DALLSALSIQNYHLECNETK	78.08635	Q01082	SPTB2_HUMAN	1160.068452	2	0.660659432	3	
DALLSALSIQNYHLECNETK	78.08635	Q01082	SPTB2_HUMAN	773.7149093	3	0.660659432	3	Yes
DASVAEAWLLGQEPYLSSR	113.1733	Q01082	SPTB2_HUMAN	1046.521463	2	0.81123364	3	
DASVAEAWLLGQEPYLSSR	113.1733	Q01082	SPTB2_HUMAN	698.016917	3	0.81123364	3	Yes
DEQSAVSMLK	25.57032	Q01082	SPTB2_HUMAN	554.2717125	2	0.628818631		
DEQSAVSMLK	25.57032	Q01082	SPTB2_HUMAN	369.8504167	3	0.628818631		
DGLNEAWADLLELIDTR	181.2006	Q01082	SPTB2_HUMAN	972.489636	2	0.830325603		
DGLNEAWADLLELIDTR	181.2006	Q01082	SPTB2_HUMAN	648.6623657	3	0.830325603		
DGMAFNALIHK	42.85432	Q01082	SPTB2_HUMAN	608.8113395	2	0.758060038	2	Yes
DGMAFNALIHK	42.85432	Q01082	SPTB2_HUMAN	406.210168	3	0.758060038	2	
DLDDFQSWLSR	100.7609	Q01082	SPTB2_HUMAN	691.323322	2	0.751335621	2	Yes
DLDDFQSWLSR	100.7609	Q01082	SPTB2_HUMAN	461.2181563	3	0.751335621	2	
DLMLWMEDVIR	132.2443	Q01082	SPTB2_HUMAN	710.852347	2	0.76729691	2	Yes
DLMLWMEDVIR	132.2443	Q01082	SPTB2_HUMAN	474.2375063	3	0.76729691	2	
DMGEMVTQGQTDAQYMFLR	87.47093	Q01082	SPTB2_HUMAN	1110.990303	2	0.769197166	3	
DMGEMVTQGQTDAQYMFLR	87.47093	Q01082	SPTB2_HUMAN	740.9961437	3	0.769197166	3	Yes
DQNTVETLQR	-2.355854	Q01082	SPTB2_HUMAN	602.3023905	2	0.666745961		
DQNTVETLQR	-2.355854	Q01082	SPTB2_HUMAN	401.8708687	3	0.666745961		
DVAEEIANYRPTLDTLHEQASALPQEHAESPDVR	63.91873	Q01082	SPTB2_HUMAN	1901.419909	2	0.638636053	4	
DVAEEIANYRPTLDTLHEQASALPQEHAESPDVR	63.91873	Q01082	SPTB2_HUMAN	1267.949214	3	0.638636053	4	
DVEDEILWVGER	86.77251	Q01082	SPTB2_HUMAN	730.357362	2	0.82775861	3	
DVEDEILWVGER	86.77251	Q01082	SPTB2_HUMAN	487.2408497	3	0.82775861	3	Yes
DVSSVELLMNNHQGIK	53.49419	Q01082	SPTB2_HUMAN	892.4545415	2	0.76326561	3	
DVSSVELLMNNHQGIK	53.49419	Q01082	SPTB2_HUMAN	595.305636	3	0.76326561	3	Yes
EAVCEVALDYK	31.64947	Q01082	SPTB2_HUMAN	648.811198	2	0.775731087	2	Yes
EAVCEVALDYK	31.64947	Q01082	SPTB2_HUMAN	432.8767403	3	0.775731087	2	
EGEDMIAEEHFGSEK	21.79193	Q01082	SPTB2_HUMAN	854.3625155	2	0.845982909	3	
EGEDMIAEEHFGSEK	21.79193	Q01082	SPTB2_HUMAN	569.910952	3	0.845982909	3	Yes
EGMQLISEKPETEAVVK	34.4358	Q01082	SPTB2_HUMAN	944.4907905	2	0.772707522	2	Yes
EGMQLISEKPETEAVVK	34.4358	Q01082	SPTB2_HUMAN	629.9964687	3	0.772707522	2	
EIEELQSQAQALSQEGK	39.55436	Q01082	SPTB2_HUMAN	944.468901	2	0.726458132		
EIEELQSQAQALSQEGK	39.55436	Q01082	SPTB2_HUMAN	629.9818757	3	0.726458132		
EIGQSVDEVEK	1.719982	Q01082	SPTB2_HUMAN	616.804431	2	0.786846995	2	Yes
EIGQSVDEVEK	1.719982	Q01082	SPTB2_HUMAN	411.5388957	3	0.786846995	2	
ELEAENYHDIK	-2.961102	Q01082	SPTB2_HUMAN	680.8231505	2	0.693843663		
ELEAENYHDIK	-2.961102	Q01082	SPTB2_HUMAN	454.218042	3	0.693843663		
EQWANLEQLSAIR	69.24048	Q01082	SPTB2_HUMAN	779.405178	2	0.864045143	2	Yes
EQWANLEQLSAIR	69.24048	Q01082	SPTB2_HUMAN	519.9393937	3	0.864045143	2	
EQWLNNMQIPEK	52.5728	Q01082	SPTB2_HUMAN	765.375032	2	0.818138182	2	Yes
EQWLNNMQIPEK	52.5728	Q01082	SPTB2_HUMAN	510.585963	3	0.818138182	2	
ETASELLMR	27.28191	Q01082	SPTB2_HUMAN	525.268973	2	0.648698866	2	Yes
ETASELLMR	27.28191	Q01082	SPTB2_HUMAN	350.515257	3	0.648698866	2	
EVDDLEQWIAER	78.81927	Q01082	SPTB2_HUMAN	751.860269	2	0.855985641	2	Yes
EVDDLEQWIAER	78.81927	Q01082	SPTB2_HUMAN	501.576121	3	0.855985641	2	
EVVAGSHELGQDYEHVTMLQER	35.21519	Q01082	SPTB2_HUMAN	1264.098273	2	0.809977233	4	
EVVAGSHELGQDYEHVTMLQER	35.21519	Q01082	SPTB2_HUMAN	843.0681233	3	0.809977233	4	
FANSLVGVQQQLQAFNTYR	84.41064	Q01082	SPTB2_HUMAN	1092.563998	2	0.682214677	3	
FANSLVGVQQQLQAFNTYR	84.41064	Q01082	SPTB2_HUMAN	728.71194	3	0.682214677	3	Yes
FATDGEGYKPCDPQVIR	27.59361	Q01082	SPTB2_HUMAN	976.962728	2	0.717101753	2	Yes
FATDGEGYKPCDPQVIR	27.59361	Q01082	SPTB2_HUMAN	651.644427	3	0.717101753	2	
FESLEPEMNNQASR	25.1681	Q01082	SPTB2_HUMAN	826.373217	2	0.840103269	2	Yes
FESLEPEMNNQASR	25.1681	Q01082	SPTB2_HUMAN	551.2514197	3	0.840103269	2	
FFWEMAEEEGWIR	109.0535	Q01082	SPTB2_HUMAN	865.3881395	2	0.789883673		
FFWEMAEEEGWIR	109.0535	Q01082	SPTB2_HUMAN	577.261368	3	0.789883673		
FMELLEPLNER	79.02513	Q01082	SPTB2_HUMAN	695.8559435	2	0.864439189	2	Yes
FMELLEPLNER	79.02513	Q01082	SPTB2_HUMAN	464.239904	3	0.864439189	2	
HEAIETDIAAYEER	24.87614	Q01082	SPTB2_HUMAN	823.887011	2	0.781725228	3	
HEAIETDIAAYEER	24.87614	Q01082	SPTB2_HUMAN	549.593949	3	0.781725228	3	Yes
HLLGVEDLLQK	54.3349	Q01082	SPTB2_HUMAN	632.867169	2	0.84864527	2	Yes
HLLGVEDLLQK	54.3349	Q01082	SPTB2_HUMAN	422.2473877	3	0.84864527	2	
HQAFMAELASNK	13.18328	Q01082	SPTB2_HUMAN	673.8302605	2	0.726991117	2	Yes
HQAFMAELASNK	13.18328	Q01082	SPTB2_HUMAN	449.5561153	3	0.726991117	2	
HQILEQAVEDYAETVHQLSK	81.16681	Q01082	SPTB2_HUMAN	1169.587867	2	0.785331607	3	

HQILEQAVEDYAETVHQLSK	81.16681	Q01082	SPTB2 HUMAN	780.061186	3	0.785331607	3	Yes
HTLVEADIGIQAER	31.24438	Q01082	SPTB2 HUMAN	776.410461	2	0.830015779	2	Yes
HTLVEADIGIQAER	31.24438	Q01082	SPTB2 HUMAN	517.9429157	3	0.830015779	2	
IDDIFER	30.11745	Q01082	SPTB2 HUMAN	454.2301735	2	0.616241574	2	Yes
IDDIFER	30.11745	Q01082	SPTB2 HUMAN	303.1560573	3	0.616241574	2	
ITDLYTDLR	41.3652	Q01082	SPTB2 HUMAN	555.296041	2	0.783029199	2	Yes
ITDLYTDLR	41.3652	Q01082	SPTB2 HUMAN	370.5333023	3	0.783029199	2	
IVSSSDVGHDEYSTQSLVK	22.48032	Q01082	SPTB2 HUMAN	1026.00056	2	0.788055241	3	
IVSSSDVGHDEYSTQSLVK	22.48032	Q01082	SPTB2 HUMAN	684.336315	3	0.788055241	3	Yes
LAEISDVWEEMK	74.78682	Q01082	SPTB2 HUMAN	725.350691	2	0.828987062	2	Yes
LAEISDVWEEMK	74.78682	Q01082	SPTB2 HUMAN	483.903069	3	0.828987062	2	
LEDLEVIQHR	19.60546	Q01082	SPTB2 HUMAN	626.3387755	2	0.820448756	2	Yes
LEDLEVIQHR	19.60546	Q01082	SPTB2 HUMAN	417.8951253	3	0.820448756	2	
LEMNLGLQK	35.66513	Q01082	SPTB2 HUMAN	523.2897085	2	0.767302394	2	Yes
LEMNLGLQK	35.66513	Q01082	SPTB2 HUMAN	349.1957473	3	0.767302394	2	
LESEHPDQAQAILSR	15.11815	Q01082	SPTB2 HUMAN	847.4293815	2	0.722542703	2	Yes
LESEHPDQAQAILSR	15.11815	Q01082	SPTB2 HUMAN	565.2888627	3	0.722542703	2	
LILEVHQFSR	33.24282	Q01082	SPTB2 HUMAN	621.3542285	2	0.801902354	3	
LILEVHQFSR	33.24282	Q01082	SPTB2 HUMAN	414.572094	3	0.801902354	3	Yes
LLDPEDISVDHPDEK	38.10355	Q01082	SPTB2 HUMAN	861.415605	2	0.841107488	3	
LLDPEDISVDHPDEK	38.10355	Q01082	SPTB2 HUMAN	574.6130117	3	0.841107488	3	Yes
LLEVLSGER	37.33406	Q01082	SPTB2 HUMAN	508.293305	2	0.782376945	2	Yes
LLEVLSGER	37.33406	Q01082	SPTB2 HUMAN	339.198145	3	0.782376945	2	
LNDGNEYLFQAK	34.29536	Q01082	SPTB2 HUMAN	706.346793	2	0.888366282	2	Yes
LNDGNEYLFQAK	34.29536	Q01082	SPTB2 HUMAN	471.2338037	3	0.888366282	2	
LQALDTGWNELHK	38.38439	Q01082	SPTB2 HUMAN	762.8944465	2	0.887648642		
LQALDTGWNELHK	38.38439	Q01082	SPTB2 HUMAN	508.9322393	3	0.887648642		
LSGIEER	-18.07326	Q01082	SPTB2 HUMAN	402.217066	2	0.679379642	2	Yes
LSGIEER	-18.07326	Q01082	SPTB2 HUMAN	268.4806523	3	0.679379642	2	
LTTLELLEVR	73.37354	Q01082	SPTB2 HUMAN	593.85627	2	0.845218182	2	Yes
LTTLELLEVR	73.37354	Q01082	SPTB2 HUMAN	396.2401217	3	0.845218182	2	
LVSDGNINSDR	-15.62778	Q01082	SPTB2 HUMAN	595.294564	2	0.862954378	2	Yes
LVSDGNINSDR	-15.62778	Q01082	SPTB2 HUMAN	397.1989843	3	0.862954378	2	
LVSQDNFGFDLPAVEAATK	95.2818	Q01082	SPTB2 HUMAN	1011.513112	2	0.791366994	3	
LVSQDNFGFDLPAVEAATK	95.2818	Q01082	SPTB2 HUMAN	674.678016	3	0.791366994	3	Yes
LWEYLLELLR	149.3648	Q01082	SPTB2 HUMAN	674.3877325	2	0.758569837	2	Yes
LWEYLLELLR	149.3648	Q01082	SPTB2 HUMAN	449.9277633	3	0.758569837	2	
MFSEADACELWIDEK	72.5935	Q01082	SPTB2 HUMAN	922.398044	2	0.692526221		
MFSEADACELWIDEK	72.5935	Q01082	SPTB2 HUMAN	615.267971	3	0.692526221		
MHTTFEHDIQALGTQVR	31.3672	Q01082	SPTB2 HUMAN	992.489449	2	0.804772377	4	
MHTTFEHDIQALGTQVR	31.3672	Q01082	SPTB2 HUMAN	661.9955743	3	0.804772377	4	
MLTAQDMSYDEAR	25.0451	Q01082	SPTB2 HUMAN	765.832336	2	0.777696609	2	Yes
MLTAQDMSYDEAR	25.0451	Q01082	SPTB2 HUMAN	510.8908323	3	0.777696609	2	
MWEVLESTTQTK	47.42798	Q01082	SPTB2 HUMAN	726.8561415	2	0.849972367	2	Yes
MWEVLESTTQTK	47.42798	Q01082	SPTB2 HUMAN	484.9067027	3	0.849972367	2	
NDSFTTCIELGK	43.06824	Q01082	SPTB2 HUMAN	692.824841	2	0.703753591	2	Yes
NDSFTTCIELGK	43.06824	Q01082	SPTB2 HUMAN	462.219169	3	0.703753591	2	
NEIDNYEEDYQK	12.90908	Q01082	SPTB2 HUMAN	780.3289895	2	0.643368363	2	Yes
NEIDNYEEDYQK	12.90908	Q01082	SPTB2 HUMAN	520.555268	3	0.643368363	2	
QALQDTLALYK	46.33131	Q01082	SPTB2 HUMAN	632.351348	2	0.638849258	2	Yes
QALQDTLALYK	46.33131	Q01082	SPTB2 HUMAN	421.903507	3	0.638849258	2	
QNLLSQSHAYQQFLR	46.3147	Q01082	SPTB2 HUMAN	916.9742865	2	0.709523678	3	
QNLLSQSHAYQQFLR	46.3147	Q01082	SPTB2 HUMAN	611.6521327	3	0.709523678	3	Yes
SAATWDER	-16.75508	Q01082	SPTB2 HUMAN	468.215055	2	0.613347292	2	Yes
SAATWDER	-16.75508	Q01082	SPTB2 HUMAN	312.4793117	3	0.613347292	2	
SLLDACESR	9.955544	Q01082	SPTB2 HUMAN	525.7484045	2	0.664771438	2	Yes
SLLDACESR	9.955544	Q01082	SPTB2 HUMAN	350.834878	3	0.664771438	2	
SNAHYNLQNAFNLAEQHLGLTK	65.75072	Q01082	SPTB2 HUMAN	1242.125481	2	0.726415813	4	
SNAHYNLQNAFNLAEQHLGLTK	65.75072	Q01082	SPTB2 HUMAN	828.4195957	3	0.726415813	4	
SQNIVTDSSSLSAEAIR	42.05544	Q01082	SPTB2 HUMAN	889.45051	2	0.856509268	3	
SQNIVTDSSSLSAEAIR	42.05544	Q01082	SPTB2 HUMAN	593.3029483	3	0.856509268	3	Yes
SWHNVYCVINNQEMGFYK	59.31213	Q01082	SPTB2 HUMAN	1145.014964	2	0.645950437	3	
SWHNVYCVINNQEMGFYK	59.31213	Q01082	SPTB2 HUMAN	763.6792507	3	0.645950437	3	Yes
TAASGIPYHSEVPVSLK	35.35142	Q01082	SPTB2 HUMAN	878.467971	2	0.755052269	3	
TAASGIPYHSEVPVSLK	35.35142	Q01082	SPTB2 HUMAN	585.9812557	3	0.755052269	3	Yes
TAGYPNVNIHNFTTSWR	52.00199	Q01082	SPTB2 HUMAN	989.4824755	2	0.676295459		
TAGYPNVNIHNFTTSWR	52.00199	Q01082	SPTB2 HUMAN	659.9909253	3	0.676295459		
TALPAQSAATLPAR	26.48252	Q01082	SPTB2 HUMAN	684.386258	2	0.761288941	2	Yes
TALPAQSAATLPAR	26.48252	Q01082	SPTB2 HUMAN	456.593447	3	0.761288941	2	
TQETPSAQMEGFLNR	43.88152	Q01082	SPTB2 HUMAN	854.902143	2	0.832341909	3	
TQETPSAQMEGFLNR	43.88152	Q01082	SPTB2 HUMAN	570.2707037	3	0.832341909	3	Yes
TQILAASYELHK	20.46085	Q01082	SPTB2 HUMAN	687.375354	2	0.890476525	3	
TQILAASYELHK	20.46085	Q01082	SPTB2 HUMAN	458.5861777	3	0.890476525	3	Yes
TQTAIASEDMPNTLTEAEK	41.4483	Q01082	SPTB2 HUMAN	1025.486434	2	0.797148585		
TQTAIASEDMPNTLTEAEK	41.4483	Q01082	SPTB2 HUMAN	683.9935643	3	0.797148585		
VAVVNQIAR	4.419128	Q01082	SPTB2 HUMAN	485.2961825	2	0.742710233	2	Yes
VAVVNQIAR	4.419128	Q01082	SPTB2 HUMAN	323.86673	3	0.742710233	2	
VDTVNHLADELINSGHSDAATIAEWK	75.91957	Q01082	SPTB2 HUMAN	1403.686301	2	0.769661546	3	
VDTVNHLADELINSGHSDAATIAEWK	75.91957	Q01082	SPTB2 HUMAN	936.126809	3	0.769661546	3	Yes
VIESTQDLGNDLAGVMALQR	91.32181	Q01082	SPTB2 HUMAN	1065.54716	2	0.788394928	3	
VIESTQDLGNDLAGVMALQR	91.32181	Q01082	SPTB2 HUMAN	710.700715	3	0.788394928	3	Yes
VLDNAIETEK	6.624043	Q01082	SPTB2 HUMAN	566.2987845	2	0.829248846	2	Yes
VLDNAIETEK	6.624043	Q01082	SPTB2 HUMAN	377.8684647	3	0.829248846	2	
VLVLSQDYGK	27.33483	Q01082	SPTB2 HUMAN	561.3142335	2	0.840219021	2	Yes
VLVLSQDYGK	27.33483	Q01082	SPTB2 HUMAN	374.5454307	3	0.840219021	2	
VSEEAESQQQWDTSK	0.463505	Q01082	SPTB2 HUMAN	876.390119	2	0.773060203	2	Yes
VSEEAESQQQWDTSK	0.463505	Q01082	SPTB2 HUMAN	584.596021	3	0.773060203	2	
WDVDDWDNENSSAR	43.90791	Q01082	SPTB2 HUMAN	854.8458775	2	0.727525115	2	Yes
WDVDDWDNENSSAR	43.90791	Q01082	SPTB2 HUMAN	570.2331933	3	0.727525115	2	
WLHGLESQIQSDDYGK	35.54193	Q01082	SPTB2 HUMAN	938.447767	2	0.801666498		
WLHGLESQIQSDDYGK	35.54193	Q01082	SPTB2 HUMAN	625.9677863	3	0.801666498		
DIDIHEVR	4.059757	Q00839	HNRPU HUMAN	498.7596295	2	0.770239055	2	Yes
DIDIHEVR	4.059757	Q00839	HNRPU HUMAN	332.8423613	3	0.770239055	2	
EKPYFPIPEEYTFIQNVPLEDR	101.8831	Q00839	HNRPU HUMAN	1362.681959	2	0.801896811	3	
EKPYFPIPEEYTFIQNVPLEDR	101.8831	Q00839	HNRPU HUMAN	908.790581	3	0.801896811	3	Yes
GGGGGGSGGIGYPYPR	23.71689	Q00839	HNRPU HUMAN	704.834381	2	0.611320019		
GGGGGGSGGIGYPYPR	23.71689	Q00839	HNRPU HUMAN	470.225529	3	0.611320019		
GNYNQNFR	-21.75525	Q00839	HNRPU HUMAN	506.7339415	2	0.750189185		
GNYNQNFR	-21.75525	Q00839	HNRPU HUMAN	338.1585693	3	0.750189185		
GYFEYIEENK	39.55972	Q00839	HNRPU HUMAN	646.296233	2	0.780416667	2	Yes
GYFEYIEENK	39.55972	Q00839	HNRPU HUMAN	431.200097	3	0.780416667	2	

LNTLLQR	10.48223	Q00839	HNRPU_HUMAN	429.264351	2	0.789446354	2	Yes
LNTLLQR	10.48223	Q00839	HNRPU_HUMAN	286.5121757	3	0.789446354	2	
NFILDQTNVSAAAQR	47.56354	Q00839	HNRPU_HUMAN	824.426642	2	0.865734339	3	
NFILDQTNVSAAAQR	47.56354	Q00839	HNRPU_HUMAN	549.953703	3	0.865734339	3	Yes
SSGPTSLFAVTVAPPGAR	72.53011	Q00839	HNRPU_HUMAN	857.960318	2	0.756325126	2	Yes
SSGPTSLFAVTVAPPGAR	72.53011	Q00839	HNRPU_HUMAN	572.309487	3	0.756325126	2	
YNILGTNTIMDK	49.66526	Q00839	HNRPU_HUMAN	691.853397	2	0.85527432	2	Yes
YNILGTNTIMDK	49.66526	Q00839	HNRPU_HUMAN	461.5715397	3	0.85527432	2	
AWTVEQLR	27.87881	Q00688	FKBP3_HUMAN	501.7725405	2	0.779686928		
AWTVEQLR	27.87881	Q00688	FKBP3_HUMAN	334.8509687	3	0.779686928		
DHLVTAYNHLFETK	35.0481	Q00688	FKBP3_HUMAN	844.4261065	2	0.880387723	3	
DHLVTAYNHLFETK	35.0481	Q00688	FKBP3_HUMAN	563.2866793	3	0.880387723	3	Yes
FLQEHGSDSFLAEHK	12.52456	Q00688	FKBP3_HUMAN	872.9186495	2	0.853012145	3	
FLQEHGSDSFLAEHK	12.52456	Q00688	FKBP3_HUMAN	582.281708	3	0.853012145	3	Yes
GWDEALLTMSK	67.80519	Q00688	FKBP3_HUMAN	625.8084625	2	0.817559063	2	Yes
GWDEALLTMSK	67.80519	Q00688	FKBP3_HUMAN	417.5415833	3	0.817559063	2	
LEIEPEWAYGK	57.44881	Q00688	FKBP3_HUMAN	667.83553	2	0.852824926	2	Yes
LEIEPEWAYGK	57.44881	Q00688	FKBP3_HUMAN	445.5596283	3	0.852824926	2	
SEETLDEGPPK	-4.851044	Q00688	FKBP3_HUMAN	601.2833315	2	0.759319186	2	Yes
SEETLDEGPPK	-4.851044	Q00688	FKBP3_HUMAN	401.191496	3	0.759319186	2	
ASWESLDEEWR	58.56689	Q00587	BORG5_HUMAN	704.312954	2	0.775069594	2	Yes
ASWESLDEEWR	58.56689	Q00587	BORG5_HUMAN	469.877911	3	0.775069594	2	
DGSFPSEPGLR	31.52771	Q00587	BORG5_HUMAN	581.280926	2	0.716188729		
DGSFPSEPGLR	31.52771	Q00587	BORG5_HUMAN	387.856559	3	0.716188729		
GGDVFGDTSFLSNHGGSSGSTHR	28.50107	Q00587	BORG5_HUMAN	1140.007776	2	0.831887603		
GGDVFGDTSFLSNHGGSSGSTHR	28.50107	Q00587	BORG5_HUMAN	760.3411253	3	0.831887603		
HWGAGWDGGHHYPEMDAR	14.56689	Q00587	BORG5_HUMAN	1039.93791	2	0.713553905		
HWGAGWDGGHHYPEMDAR	14.56689	Q00587	BORG5_HUMAN	693.6278817	3	0.713553905		
LSPVGWVSSSQGK	38.05637	Q00587	BORG5_HUMAN	666.3518825	2	0.768178761		
LSPVGWVSSSQGK	38.05637	Q00587	BORG5_HUMAN	444.57053	3	0.768178761		
LTADMISHPLGDFR	50.93884	Q00587	BORG5_HUMAN	786.896132	2	0.838358045	3	
LTADMISHPLGDFR	50.93884	Q00587	BORG5_HUMAN	524.933363	3	0.838358045	3	Yes
MASPPAPSPAPPAISPIIK	65.80806	Q00587	BORG5_HUMAN	921.5138715	2	0.651236475		
MASPPAPSPAPPAISPIIK	65.80806	Q00587	BORG5_HUMAN	614.6785227	3	0.651236475		
NAISLPQLNQAAYDSLVVGK	95.35043	Q00587	BORG5_HUMAN	1051.068581	2	0.638441503	3	
NAISLPQLNQAAYDSLVVGK	95.35043	Q00587	BORG5_HUMAN	701.0483287	3	0.638441503	3	Yes
TPVPSTVQANTFEFADAEEDDEVK	67.5426	Q00587	BORG5_HUMAN	1320.103945	2	0.744112372		
TPVPSTVQANTFEFADAEEDDEVK	67.5426	Q00587	BORG5_HUMAN	880.405238	3	0.744112372		
ETFGEMSDGDVQEQLR	42.19865	P84157	MXRA7_HUMAN	920.9050795	2	0.831371903		
ETFGEMSDGDVQEQLR	42.19865	P84157	MXRA7_HUMAN	614.2726613	3	0.831371903		
GPSSEGPEEEDGEGFSFK	35.24049	P84157	MXRA7_HUMAN	942.892691	2	0.772179961	2	Yes
GPSSEGPEEEDGEGFSFK	35.24049	P84157	MXRA7_HUMAN	628.931069	3	0.772179961	2	
LGLQHLR	-9.445061	P84085	ARF5_HUMAN	418.759236	2	0.773560882	2	Yes
LGLQHLR	-9.445061	P84085	ARF5_HUMAN	279.5087657	3	0.773560882	2	
MLQEDELR	5.264801	P84085	ARF5_HUMAN	517.253323	2	0.739673734	2	Yes
MLQEDELR	5.264801	P84085	ARF5_HUMAN	345.1714903	3	0.739673734	2	
NICFTVWDVGGQDK	75.80289	P84085	ARF5_HUMAN	819.883222	2	0.740852892		
NICFTVWDVGGQDK	75.80289	P84085	ARF5_HUMAN	546.9247563	3	0.740852892		
VQESADELQK	-22.44673	P84085	ARF5_HUMAN	573.7860415	2	0.780642092	2	Yes
VQESADELQK	-22.44673	P84085	ARF5_HUMAN	382.8599693	3	0.780642092	2	
AAEAAAAPAESAAPAAGEEPSK	10.17715	P80723	BASP1_HUMAN	983.971808	2	0.673553109		
AAEAAAAPAESAAPAAGEEPSK	10.17715	P80723	BASP1_HUMAN	656.317147	3	0.673553109		
APEQEQAAPGPAAGGEAPK	-8.811485	P80723	BASP1_HUMAN	888.4321225	2	0.79315716	3	
APEQEQAAPGPAAGGEAPK	-8.811485	P80723	BASP1_HUMAN	592.6240233	3	0.79315716	3	Yes
AQGPAASAEEPKPVEAPAANSDQTVTVK	18.60426	P80723	BASP1_HUMAN	1382.19396	2	0.668335617	3	
AQGPAASAEEPKPVEAPAANSDQTVTVK	18.60426	P80723	BASP1_HUMAN	921.7985817	3	0.668335617	3	Yes
ESEPQAAAEPAEAK	-20.3571	P80723	BASP1_HUMAN	714.336627	2	0.812420666	2	Yes
ESEPQAAAEPAEAK	-20.3571	P80723	BASP1_HUMAN	476.5603597	3	0.812420666	2	
ETPAATEAPSSTPK	-17.99362	P80723	BASP1_HUMAN	693.8415455	2	0.741351604	2	Yes
ETPAATEAPSSTPK	-17.99362	P80723	BASP1_HUMAN	462.896972	3	0.741351604	2	
EIGDVENWAR	33.84227	P78537	BL1S1_HUMAN	594.7863755	2	0.620196521	2	Yes
EIGDVENWAR	33.84227	P78537	BL1S1_HUMAN	396.860192	3	0.620196521	2	
GPGVPSPQPDVTMLSR	53.21481	P78537	BL1S1_HUMAN	819.4199715	2	0.78733021		
GPGVPSPQPDVTMLSR	53.21481	P78537	BL1S1_HUMAN	546.6159227	3	0.78733021		
SIELDMR	20.31402	P78537	BL1S1_HUMAN	432.2187515	2	0.736547589		
SIELDMR	20.31402	P78537	BL1S1_HUMAN	288.481776	3	0.736547589		
TIATALEYVYK	60.14793	P78537	BL1S1_HUMAN	636.3482695	2	0.832230449		
TIATALEYVYK	60.14793	P78537	BL1S1_HUMAN	424.5681213	3	0.832230449		
TLQVQAAQFAK	23.59346	P78537	BL1S1_HUMAN	602.8384125	2	0.760889649	2	Yes
TLQVQAAQFAK	23.59346	P78537	BL1S1_HUMAN	402.2282167	3	0.760889649	2	
GSAPPGPVPEGSIR	15.83169	P78417	GSTO1_HUMAN	660.8495075	2	0.731597185	2	Yes
GSAPPGPVPEGSIR	15.83169	P78417	GSTO1_HUMAN	440.90228	3	0.731597185	2	
LLPDDPYEK	19.25006	P78417	GSTO1_HUMAN	545.2773165	2	0.794667602	2	Yes
LLPDDPYEK	19.25006	P78417	GSTO1_HUMAN	363.8541527	3	0.794667602	2	
LNECVDHTPK	-27.79697	P78417	GSTO1_HUMAN	606.7880615	2	0.686882377	3	
LNECVDHTPK	-27.79697	P78417	GSTO1_HUMAN	404.861316	3	0.686882377	3	Yes
NKPEWFFK	26.9498	P78417	GSTO1_HUMAN	548.285283	2	0.703282833	2	Yes
NKPEWFFK	26.9498	P78417	GSTO1_HUMAN	365.8594637	3	0.703282833	2	
VPSLVGSFIR	62.96925	P78417	GSTO1_HUMAN	537.81949	2	0.810770631	2	Yes
VPSLVGSFIR	62.96925	P78417	GSTO1_HUMAN	358.8822683	3	0.810770631	2	
EQMELHHQNLNLQETLK	26.4584	P78380	OLR1_HUMAN	1053.026395	2	0.746195078		
EQMELHHQNLNLQETLK	26.4584	P78380	OLR1_HUMAN	702.353538	3	0.746195078		
GAVSQTYPSGTCAYIQR	25.33859	P78380	OLR1_HUMAN	929.9417915	2	0.773151577		
GAVSQTYPSGTCAYIQR	25.33859	P78380	OLR1_HUMAN	620.297136	3	0.773151577		
AAHSEGNTTAGLDMR	-20.37479	P78371	TCPB_HUMAN	765.852453	2	0.744087338	2	Yes
AAHSEGNTTAGLDMR	-20.37479	P78371	TCPB_HUMAN	510.9042437	3	0.744087338	2	
DASLMVTNDGATILK	61.54175	P78371	TCPB_HUMAN	774.90108	2	0.823661208	2	Yes
DASLMVTNDGATILK	61.54175	P78371	TCPB_HUMAN	516.9366617	3	0.823661208	2	
EALLSSAVDHGSDEVK	18.30718	P78371	TCPB_HUMAN	828.907947	2	0.729450881	3	
EALLSSAVDHGSDEVK	18.30718	P78371	TCPB_HUMAN	552.9412397	3	0.729450881	3	Yes
EGTIGDMAILGITESFQVK	117.817	P78371	TCPB_HUMAN	1005.017173	2	0.724155843	3	
EGTIGDMAILGITESFQVK	117.817	P78371	TCPB_HUMAN	670.3473903	3	0.724155843	3	Yes
GATQQILDEAER	23.80698	P78371	TCPB_HUMAN	665.834055	2	0.852703929	2	Yes
GATQQILDEAER	23.80698	P78371	TCPB_HUMAN	444.2253117	3	0.852703929	2	
GSGNLEAIHIIK	26.91788	P78371	TCPB_HUMAN	626.356968	2	0.838713109	2	Yes
GSGNLEAIHIIK	26.91788	P78371	TCPB_HUMAN	417.9072537	3	0.838713109	2	
IHPQTIIAGWR	33.27761	P78371	TCPB_HUMAN	646.367671	2	0.603693187	2	Yes
IHPQTIIAGWR	33.27761	P78371	TCPB_HUMAN	431.2477223	3	0.603693187	2	
ILIANTGMDTDK	24.61386	P78371	TCPB_HUMAN	646.332302	2	0.784874082	2	Yes
ILIANTGMDTDK	24.61386	P78371	TCPB_HUMAN	431.224143	3	0.784874082	2	
LALVTGGEIASTFDHPELVK	70.47255	P78371	TCPB_HUMAN	1049.065512	2	0.88036263	3	

LALVTGGEIASTFDHPELVK	70.47255	P78371	TCPB HUMAN	699.7129493	3	0.88036263	3	Yes
LGGSLADSYLDEGFLLDK	102.8985	P78371	TCPB HUMAN	956.981108	2	0.804841995	3	
LGGSLADSYLDEGFLLDK	102.8985	P78371	TCPB HUMAN	638.323347	3	0.804841995	3	Yes
LIEEVMIGEDK	47.17355	P78371	TCPB HUMAN	638.3292275	2	0.803508043	2	Yes
LIEEVMIGEDK	47.17355	P78371	TCPB HUMAN	425.88876	3	0.803508043	2	
LTSFIGAIAIGDLVK	122.8848	P78371	TCPB HUMAN	759.451066	2	0.759040892	3	
LTSFIGAIAIGDLVK	122.8848	P78371	TCPB HUMAN	506.6366523	3	0.759040892	3	Yes
MLPTIIADNAGYDSADLVAQLR	107.6553	P78371	TCPB HUMAN	1174.102295	2	0.62849915	3	
MLPTIIADNAGYDSADLVAQLR	107.6553	P78371	TCPB HUMAN	783.070805	3	0.62849915	3	Yes
QDLMNIAGTTLSSK	52.43528	P78371	TCPB HUMAN	739.8801475	2	0.796580434	3	
QDLMNIAGTTLSSK	52.43528	P78371	TCPB HUMAN	493.5893733	3	0.796580434	3	Yes
QVLLSAAEAAEVILR	94.94539	P78371	TCPB HUMAN	791.962329	2	0.704958558	2	Yes
QVLLSAAEAAEVILR	94.94539	P78371	TCPB HUMAN	528.3108277	3	0.704958558	2	
SLHDALCVLAQTVK	59.95033	P78371	TCPB HUMAN	777.9196075	2	0.743365407		
SLHDALCVLAQTVK	59.95033	P78371	TCPB HUMAN	518.9490133	3	0.743365407		
TVYGGGCSEMLMAHAVTQLANR	64.75644	P78371	TCPB HUMAN	1183.556676	2	0.746385932	3	
TVYGGGCSEMLMAHAVTQLANR	64.75644	P78371	TCPB HUMAN	789.3737253	3	0.746385932	3	Yes
VAEIEHAEK	-27.54433	P78371	TCPB HUMAN	513.2672875	2	0.610456467		
VAEIEHAEK	-27.54433	P78371	TCPB HUMAN	342.5141333	3	0.610456467		
VLVDMSR	7.177055	P78371	TCPB HUMAN	410.223837	2	0.65216738	2	Yes
VLVDMSR	7.177055	P78371	TCPB HUMAN	273.8184997	3	0.65216738	2	
VQDDEVGDGTTSVTVLAAELLR	126.1423	P78371	TCPB HUMAN	1144.584994	2	0.786425352	3	
VQDDEVGDGTTSVTVLAAELLR	126.1423	P78371	TCPB HUMAN	763.3926043	3	0.786425352	3	Yes
AAEMLSQLDLFSR	85.65843	P78318	IGBP1 HUMAN	740.8774075	2	0.807424903	3	
AAEMLSQLDLFSR	85.65843	P78318	IGBP1 HUMAN	494.2542133	3	0.807424903	3	Yes
EYYLLHLQR	32.545	P78318	IGBP1 HUMAN	617.8331205	2	0.739714384	3	
EYYLLHLQR	32.545	P78318	IGBP1 HUMAN	412.2246887	3	0.739714384	3	Yes
GLDLLEK	32.14311	P78318	IGBP1 HUMAN	394.232185	2	0.636922896		
GLDLLEK	32.14311	P78318	IGBP1 HUMAN	263.1573983	3	0.636922896		
LPELFETGR	47.1976	P78318	IGBP1 HUMAN	531.2854805	2	0.641341448	2	Yes
LPELFETGR	47.1976	P78318	IGBP1 HUMAN	354.526262	3	0.641341448	2	
NEDLEEIASTDLK	48.61256	P78318	IGBP1 HUMAN	738.8573915	2	0.837928474	2	Yes
NEDLEEIASTDLK	48.61256	P78318	IGBP1 HUMAN	492.907536	3	0.837928474	2	
QLLDEVEVATEPAGSR	60.11293	P78318	IGBP1 HUMAN	857.4368725	2	0.718781471		
QLLDEVEVATEPAGSR	60.11293	P78318	IGBP1 HUMAN	571.9605233	3	0.718781471		
VFGAGYPSLPTMTVSDWYEQHR	87.44107	P78318	IGBP1 HUMAN	1271.09754	2	0.753104091		
VFGAGYPSLPTMTVSDWYEQHR	87.44107	P78318	IGBP1 HUMAN	847.7343017	3	0.753104091		
YGALPDQGIAK	18.42028	P78318	IGBP1 HUMAN	566.8040335	2	0.797118723		
YGALPDQGIAK	18.42028	P78318	IGBP1 HUMAN	378.2052973	3	0.797118723		
YLLVPAFQGALTMK	101.1638	P78318	IGBP1 HUMAN	776.434358	2	0.772255421	2	Yes
YLLVPAFQGALTMK	101.1638	P78318	IGBP1 HUMAN	517.958847	3	0.772255421	2	
FLASVSTVLTSK	56.59771	P69905	HBA HUMAN	626.861552	2	0.771673143		
FLASVSTVLTSK	56.59771	P69905	HBA HUMAN	418.243643	3	0.771673143		
MFLSFPTTK	61.4627	P69905	HBA HUMAN	536.2813525	2	0.753426671		
MFLSFPTTK	61.4627	P69905	HBA HUMAN	357.8568433	3	0.753426671		
TYFPHFDLSHGSAQVK	33.44427	P69905	HBA HUMAN	917.450113	2	0.814358652		
TYFPHFDLSHGSAQVK	33.44427	P69905	HBA HUMAN	611.9693503	3	0.814358652		
VADALTNAVAHVDDMPNALSALSDLHAHK	81.15848	P69905	HBA HUMAN	1498.74891	2	0.766811013		
VADALTNAVAHVDDMPNALSALSDLHAHK	81.15848	P69905	HBA HUMAN	999.5018817	3	0.766811013		
VGAHAGEYGAEALER	1.699154	P69905	HBA HUMAN	765.3713315	2	0.800367773		
VGAHAGEYGAEALER	1.699154	P69905	HBA HUMAN	510.583496	3	0.800367773		
INVYYNEATGGK	8.642017	P68371	TBB4B HUMAN	664.828232	2	0.805634141	2	Yes
INVYYNEATGGK	8.642017	P68371	TBB4B HUMAN	443.554763	3	0.805634141	2	
AADPPAENSSAPEAEQGGAE	7.845688	P67809	YBOX1 HUMAN	949.4064975	2	0.723988175	2	Yes
AADPPAENSSAPEAEQGGAE	7.845688	P67809	YBOX1 HUMAN	633.2736067	3	0.723988175	2	
GAEAANVTGPGGVPVQGSK	14.84335	P67809	YBOX1 HUMAN	848.4372075	2	0.743331432	2	Yes
GAEAANVTGPGGVPVQGSK	14.84335	P67809	YBOX1 HUMAN	565.9607467	3	0.743331432	2	
ELDQWIEQLNECK	75.91021	P67775	PP2AA HUMAN	852.8990685	2	0.730489492	3	
ELDQWIEQLNECK	75.91021	P67775	PP2AA HUMAN	568.9353207	3	0.730489492	3	Yes
DHASIQMNVAEVDK	22.54702	P63220	RS21 HUMAN	778.8728535	2	0.808318436	2	Yes
DHASIQMNVAEVDK	22.54702	P63220	RS21 HUMAN	519.5845107	3	0.808318436	2	
MGESDDSILR	14.63738	P63220	RS21 HUMAN	561.758969	2	0.606169164	2	Yes
MGESDDSILR	14.63738	P63220	RS21 HUMAN	374.841921	3	0.606169164	2	
MQNDAGEFVDLYVPR	75.20144	P63220	RS21 HUMAN	877.4148815	2	0.747499824		
MQNDAGEFVDLYVPR	75.20144	P63220	RS21 HUMAN	585.279196	3	0.747499824		
TYAICGAIR	18.26191	P63220	RS21 HUMAN	512.766397	2	0.698225796	2	Yes
TYAICGAIR	18.26191	P63220	RS21 HUMAN	342.1802063	3	0.698225796	2	
GGIIEPSLR	30.17297	P62987	RL40 HUMAN	471.2749155	2	0.770080507		
GGIIEPSLR	30.17297	P62987	RL40 HUMAN	314.5192187	3	0.770080507		
ECPSDECGAGVFMASHFDR	42.84293	P62979	RS27A HUMAN	1086.433156	2	0.699238181	3	
ECPSDECGAGVFMASHFDR	42.84293	P62979	RS27A HUMAN	724.624712	3	0.699238181	3	Yes
EGMNIVEAMER	44.39576	P62937	PPIA HUMAN	639.795029	2	0.802343726	2	Yes
EGMNIVEAMER	44.39576	P62937	PPIA HUMAN	426.865961	3	0.802343726	2	
FEDENFILK	50.46339	P62937	PPIA HUMAN	577.790595	2	0.604852021	2	Yes
FEDENFILK	50.46339	P62937	PPIA HUMAN	385.5296717	3	0.604852021	2	
VSFELFADK	57.84905	P62937	PPIA HUMAN	528.274581	2	0.700792432	2	Yes
VSFELFADK	57.84905	P62937	PPIA HUMAN	352.5189957	3	0.700792432	2	
FLVHNVK	-24.32067	P62910	RL32 HUMAN	428.7561615	2	0.616928399	2	Yes
FLVHNVK	-24.32067	P62910	RL32 HUMAN	286.1733827	3	0.616928399	2	
GQILMPNIGYGSNK	51.07578	P62910	RL32 HUMAN	746.3853955	2	0.745146215	2	Yes
GQILMPNIGYGSNK	51.07578	P62910	RL32 HUMAN	497.9262053	3	0.745146215	2	
SYCAEIAHNVSSK	-11.55039	P62910	RL32 HUMAN	733.3411845	2	0.679834545	2	Yes
SYCAEIAHNVSSK	-11.55039	P62910	RL32 HUMAN	489.2300647	3	0.679834545	2	
FVNVVPTFGK	52.45173	P62861	RS30 HUMAN	554.314041	2	0.757716179	2	Yes
FVNVVPTFGK	52.45173	P62861	RS30 HUMAN	369.8786357	3	0.757716179	2	
TTGFGMIYDSLDYAK	79.08322	P62847	RS24 HUMAN	841.3930795	2	0.816960216	3	
TTGFGMIYDSLDYAK	79.08322	P62847	RS24 HUMAN	561.2646613	3	0.816960216	3	Yes
DAVTYTEHAK	-26.42645	P62805	H4 HUMAN	567.775473	2	0.752340794	2	Yes
DAVTYTEHAK	-26.42645	P62805	H4 HUMAN	378.8529237	3	0.752340794	2	
DNIQGITKPAIR	15.95101	P62805	H4 HUMAN	663.380975	2	0.804561436	3	
DNIQGITKPAIR	15.95101	P62805	H4 HUMAN	442.589925	3	0.804561436	3	Yes
ISGLIYEETR	31.57546	P62805	H4 HUMAN	590.8145975	2	0.824155211	2	Yes
ISGLIYEETR	31.57546	P62805	H4 HUMAN	394.21234	3	0.824155211	2	
TVTAMDVVYALK	70.29927	P62805	H4 HUMAN	655.855409	2	0.723278105	2	Yes
TVTAMDVVYALK	70.29927	P62805	H4 HUMAN	437.572881	3	0.723278105	2	
VFLENVIR	49.08236	P62805	H4 HUMAN	495.293108	2	0.771375597	2	Yes
VFLENVIR	49.08236	P62805	H4 HUMAN	330.531347	3	0.771375597	2	
FLILPDMLK	94.9111	P62318	SMD3 HUMAN	545.32302	2	0.809351683	2	Yes
FLILPDMLK	94.9111	P62318	SMD3 HUMAN	363.8846217	3	0.809351683	2	
LIEAEDNMNCQMSNITVTYR	57.98883	P62318	SMD3 HUMAN	1201.54343	2	0.67652297	3	
LIEAEDNMNCQMSNITVTYR	57.98883	P62318	SMD3 HUMAN	801.3648947	3	0.67652297	3	Yes

VAQLEQVYIR	35.46495	P62318	SMD3_HUMAN	609.8462325	2	0.79541564	2	Yes
VAQLEQVYIR	35.46495	P62318	SMD3_HUMAN	406.9000967	3	0.79541564	2	
VLHEAEGHIVTCETNTGEVYR	6.936039	P62318	SMD3_HUMAN	1207.574434	2	0.778580844	3	
VLHEAEGHIVTCETNTGEVYR	6.936039	P62318	SMD3_HUMAN	805.385564	3	0.778580844	3	Yes
EEEEFNTGPLSVLTQSVK	87.31953	P62316	SMD2_HUMAN	1004.000034	2	0.727903068	3	
EEEEFNTGPLSVLTQSVK	87.31953	P62316	SMD2_HUMAN	669.6692973	3	0.727903068	3	Yes
EMWTEVPK	29.84536	P62316	SMD2_HUMAN	510.2475095	2	0.671382606		
EMWTEVPK	29.84536	P62316	SMD2_HUMAN	340.500948	3	0.671382606		
NNTQVLINCR	4.094009	P62316	SMD2_HUMAN	616.3147775	2	0.761575639	2	Yes
NNTQVLINCR	4.094009	P62316	SMD2_HUMAN	411.21246	3	0.761575639	2	
SEMTPEELQK	4.509537	P62316	SMD2_HUMAN	596.2822775	2	0.809141874	2	Yes
SEMTPEELQK	4.509537	P62316	SMD2_HUMAN	397.85746	3	0.809141874	2	
DVQIGDIVTVGECRPLSK	63.55891	P62280	RS11_HUMAN	993.520414	2	0.660500824	3	
DVQIGDIVTVGECRPLSK	63.55891	P62280	RS11_HUMAN	662.6828843	3	0.660500824	3	Yes
DYLHYIR	19.6754	P62280	RS11_HUMAN	490.2539745	2	0.844481587		
DYLHYIR	19.6754	P62280	RS11_HUMAN	327.1719247	3	0.844481587		
EAIEGTYIDK	11.64574	P62280	RS11_HUMAN	569.785506	2	0.794220507	2	Yes
EAIEGTYIDK	11.64574	P62280	RS11_HUMAN	380.1929457	3	0.794220507	2	
VLLGETGK	-3.263653	P62280	RS11_HUMAN	408.74546	2	0.767480075	2	Yes
VLLGETGK	-3.263653	P62280	RS11_HUMAN	272.832915	3	0.767480075	2	
ALVAYYQK	8.612968	P62249	RS16_HUMAN	478.266551	2	0.820589483	2	Yes
ALVAYYQK	8.612968	P62249	RS16_HUMAN	319.180309	3	0.820589483	2	
DILIQYDR	40.4668	P62249	RS16_HUMAN	518.277651	2	0.776140153	2	Yes
DILIQYDR	40.4668	P62249	RS16_HUMAN	345.8543757	3	0.776140153	2	
GGGHVAQIYAIR	6.496441	P62249	RS16_HUMAN	621.341649	2	0.859722018	2	Yes
GGGHVAQIYAIR	6.496441	P62249	RS16_HUMAN	414.5637077	3	0.859722018	2	
GPLQSVQVFGR	44.32964	P62249	RS16_HUMAN	594.330754	2	0.791342854	2	Yes
GPLQSVQVFGR	44.32964	P62249	RS16_HUMAN	396.5564443	3	0.791342854	2	
LLEPVLLLGK	89.09196	P62249	RS16_HUMAN	547.8633665	2	0.80871731	2	Yes
LLEPVLLLGK	89.09196	P62249	RS16_HUMAN	365.578186	3	0.80871731	2	
TLLVADPR	20.17693	P62249	RS16_HUMAN	442.764184	2	0.728368044	2	Yes
TLLVADPR	20.17693	P62249	RS16_HUMAN	295.5120643	3	0.728368044	2	
FLTVMMK	39.34857	P62244	RS15A_HUMAN	435.2353595	2	0.619708121	2	Yes
FLTVMMK	39.34857	P62244	RS15A_HUMAN	290.492848	3	0.619708121	2	
HGYIGEFEIIDDHR	40.76973	P62244	RS15A_HUMAN	850.905538	2	0.786103725	3	
HGYIGEFEIIDDHR	40.76973	P62244	RS15A_HUMAN	567.6063003	3	0.786103725	3	Yes
MNVLADALK	48.11214	P62244	RS15A_HUMAN	487.7711515	2	0.79048723	2	Yes
MNVLADALK	48.11214	P62244	RS15A_HUMAN	325.5167093	3	0.79048723	2	
LTELSMQDEELMK	50.19908	P62072	TIM10_HUMAN	783.8736735	2	0.851507306	2	Yes
LTELSMQDEELMK	50.19908	P62072	TIM10_HUMAN	522.9183907	3	0.851507306	2	
YLDIHER	-16.88102	P62072	TIM10_HUMAN	473.243611	2	0.852296591	2	Yes
YLDIHER	-16.88102	P62072	TIM10_HUMAN	315.8316823	3	0.852296591	2	
AYSEAHEISK	-27.98074	P61981	1433G_HUMAN	567.775472	2	0.644072354	3	
AYSEAHEISK	-27.98074	P61981	1433G_HUMAN	378.852923	3	0.644072354	3	Yes
NVTELNEPLSNEER	23.98	P61981	1433G_HUMAN	822.397746	2	0.89221704	2	Yes
NVTELNEPLSNEER	23.98	P61981	1433G_HUMAN	548.6011057	3	0.89221704	2	
TAFDDAIAELDTLNEDSYK	104.2761	P61981	1433G_HUMAN	1065.989859	2	0.77464211	3	
TAFDDAIAELDTLNEDSYK	104.2761	P61981	1433G_HUMAN	710.9958473	3	0.77464211	3	Yes
YLAEVATGEK	5.66016	P61981	1433G_HUMAN	540.7827665	2	0.796582222	2	Yes
YLAEVATGEK	5.66016	P61981	1433G_HUMAN	360.857786	3	0.796582222	2	
AQPYDPNFYDETYDYGGFTMMFDDR	120.2235	P61978	HNRPK_HUMAN	1529.61328	2	0.694010437	3	
AQPYDPNFYDETYDYGGFTMMFDDR	120.2235	P61978	HNRPK_HUMAN	1020.078128	3	0.694010437	3	Yes
DYDDMSPR	2.40897	P61978	HNRPK_HUMAN	499.698376	2	0.747232854	2	Yes
DYDDMSPR	2.40897	P61978	HNRPK_HUMAN	333.4681923	3	0.747232854	2	
GGDLMAYDR	12.68789	P61978	HNRPK_HUMAN	499.2245615	2	0.755221665	2	Yes
GGDLMAYDR	12.68789	P61978	HNRPK_HUMAN	333.152316	3	0.755221665	2	
GSDFDCELR	12.31253	P61978	HNRPK_HUMAN	549.730212	2	0.743704319	2	Yes
GSDFDCELR	12.31253	P61978	HNRPK_HUMAN	366.8227497	3	0.743704319	2	
GSYGDLGGPIITTQVTIPK	80.16386	P61978	HNRPK_HUMAN	959.020569	2	0.849741876	3	
GSYGDLGGPIITTQVTIPK	80.16386	P61978	HNRPK_HUMAN	639.6829877	3	0.849741876	3	Yes
IDEPLEGSEDR	7.218559	P61978	HNRPK_HUMAN	630.2916875	2	0.742862582	2	Yes
IDEPLEGSEDR	7.218559	P61978	HNRPK_HUMAN	420.5304	3	0.742862582	2	
IILDLISESPIK	92.45932	P61978	HNRPK_HUMAN	670.905959	2	0.826159	2	Yes
IILDLISESPIK	92.45932	P61978	HNRPK_HUMAN	447.606581	3	0.826159	2	
IPTLEEGLQLPSPTATSQLPLESDAVECLNYQHYI	113.4474	P61978	HNRPK_HUMAN	2028.020075	2	0.634408236	5	
IPTLEEGLQLPSPTATSQLPLESDAVECLNYQHYI	113.4474	P61978	HNRPK_HUMAN	1352.349325	3	0.634408236	5	
IITITGTQDQIQNAQYLLQNSVK	89.95166	P61978	HNRPK_HUMAN	1295.198313	2	0.71745199	3	
IITITGTQDQIQNAQYLLQNSVK	89.95166	P61978	HNRPK_HUMAN	863.8014837	3	0.71745199	3	Yes
ILSISADIETIGEILK	124.4828	P61978	HNRPK_HUMAN	857.996034	2	0.742307007	3	
ILSISADIETIGEILK	124.4828	P61978	HNRPK_HUMAN	572.3332977	3	0.742307007	3	Yes
LFQECCPHSTDR	-21.18339	P61978	HNRPK_HUMAN	775.3302995	2	0.732026517	3	
LFQECCPHSTDR	-21.18339	P61978	HNRPK_HUMAN	517.222808	3	0.732026517	3	Yes
LLIHQSLAGGIIGVK	52.52035	P61978	HNRPK_HUMAN	759.9725	2	0.805845439	3	
LLIHQSLAGGIIGVK	52.52035	P61978	HNRPK_HUMAN	506.984275	3	0.805845439	3	Yes
NTDEMVELR	14.51973	P61978	HNRPK_HUMAN	553.761512	2	0.711389899	2	Yes
NTDEMVELR	14.51973	P61978	HNRPK_HUMAN	369.510283	3	0.711389899	2	
RPAEDMEEEQAFK	-1.143776	P61978	HNRPK_HUMAN	790.3570365	2	0.662894726	3	
RPAEDMEEEQAFK	-1.143776	P61978	HNRPK_HUMAN	527.2406327	3	0.662894726	3	Yes
TDYNASVSVPDSSGPER	21.33302	P61978	HNRPK_HUMAN	890.9033885	2	0.865503192	2	Yes
TDYNASVSVPDSSGPER	21.33302	P61978	HNRPK_HUMAN	594.271534	3	0.865503192	2	
VVLIGGKPDR	-3.22234	P61978	HNRPK_HUMAN	527.32494	2	0.860850573	3	
VVLIGGKPDR	-3.22234	P61978	HNRPK_HUMAN	351.8859017	3	0.860850573	3	Yes
DMFASVGADGSVR	43.76618	P61962	DCAF7_HUMAN	656.3040755	2	0.805204511	2	Yes
DMFASVGADGSVR	43.76618	P61962	DCAF7_HUMAN	437.871992	3	0.805204511	2	
GVYPDLLATSGDYLR	77.97632	P61962	DCAF7_HUMAN	820.4204855	2	0.897301912	2	Yes
GVYPDLLATSGDYLR	77.97632	P61962	DCAF7_HUMAN	547.282932	3	0.897301912	2	
HLEHSTIIYEDPQHHPLLR	10.96251	P61962	DCAF7_HUMAN	1168.101278	2	0.824271202		
HLEHSTIIYEDPQHHPLLR	10.96251	P61962	DCAF7_HUMAN	779.070127	3	0.824271202		
LALGSFVEEYNNK	53.97322	P61962	DCAF7_HUMAN	742.37555	2	0.836942136		
LALGSFVEEYNNK	53.97322	P61962	DCAF7_HUMAN	495.252975	3	0.836942136		
LECLLNNNK	10.89274	P61962	DCAF7_HUMAN	559.2876965	2	0.67453289	2	Yes
LECLLNNNK	10.89274	P61962	DCAF7_HUMAN	373.194406	3	0.67453289	2	
LMWIPDTK	51.95117	P61962	DCAF7_HUMAN	502.268245	2	0.772035241	2	Yes
LMWIPDTK	51.95117	P61962	DCAF7_HUMAN	335.1814383	3	0.772035241	2	
VQLVGLDEESSEFICR	74.2374	P61962	DCAF7_HUMAN	940.9571145	2	0.800049126		
VQLVGLDEESSEFICR	74.2374	P61962	DCAF7_HUMAN	627.6406847	3	0.800049126		
AAHVFFTDSCPDALFNELVK	89.42876	P61764	STXB1_HUMAN	1141.052078	2	0.735553026	3	
AAHVFFTDSCPDALFNELVK	89.42876	P61764	STXB1_HUMAN	761.037327	3	0.735553026	3	Yes
AIVPILLDANVSTYDK	92.82713	P61764	STXB1_HUMAN	866.480547	2	0.850895584	3	
AIVPILLDANVSTYDK	92.82713	P61764	STXB1_HUMAN	577.9896397	3	0.850895584	3	Yes
DIMEDTIEDK	35.68474	P61764	STXB1_HUMAN	604.7717425	2	0.809238791	2	Yes

DIMEDTIEDK	35.68474	P61764	STXB1 HUMAN	403.5171033	3	0.809238791	2	
DLSQMLK	34.74946	P61764	STXB1 HUMAN	417.72367	2	0.827778459		
DLSQMLK	34.74946	P61764	STXB1 HUMAN	278.8183883	3	0.827778459		
DNALLAQLIQDK	86.45926	P61764	STXB1 HUMAN	671.3728155	2	0.803012908		
DNALLAQLIQDK	86.45926	P61764	STXB1 HUMAN	447.9178187	3	0.803012908		
EPLPSLEAVYLITPSEK	106.4751	P61764	STXB1 HUMAN	943.512044	2	0.699887991	2	Yes
EPLPSLEAVYLITPSEK	106.4751	P61764	STXB1 HUMAN	629.343971	3	0.699887991	2	
HYPYISTR	-9.848656	P61764	STXB1 HUMAN	518.7647065	2	0.669709802	2	Yes
HYPYISTR	-9.848656	P61764	STXB1 HUMAN	346.1790793	3	0.669709802	2	
ISEQTYQLSR	4.762077	P61764	STXB1 HUMAN	612.815129	2	0.768440545		
ISEQTYQLSR	4.762077	P61764	STXB1 HUMAN	408.879361	3	0.768440545		
LAEQIATLCATLK	57.54974	P61764	STXB1 HUMAN	716.397977	2	0.780270875		
LAEQIATLCATLK	57.54974	P61764	STXB1 HUMAN	477.934593	3	0.780270875		
LIQHAQIPPEDSEIITNMAHLGVPIVTDSTLR	98.10216	P61764	STXB1 HUMAN	1754.927604	2	0.690022171	4	
LIQHAQIPPEDSEIITNMAHLGVPIVTDSTLR	98.10216	P61764	STXB1 HUMAN	1170.287677	3	0.690022171	4	
MTDIMTEGITIVEDINK	98.37289	P61764	STXB1 HUMAN	961.976659	2	0.755381107		
MTDIMTEGITIVEDINK	98.37289	P61764	STXB1 HUMAN	641.6537143	3	0.755381107		
SQLLILDR	42.24141	P61764	STXB1 HUMAN	479.2905655	2	0.786557972	2	Yes
SQLLILDR	42.24141	P61764	STXB1 HUMAN	319.8629853	3	0.786557972	2	
SSASFSTTAVSAR	6.634975	P61764	STXB1 HUMAN	636.315497	2	0.813495159	2	Yes
SSASFSTTAVSAR	6.634975	P61764	STXB1 HUMAN	424.546273	3	0.813495159	2	
SVHSLISDFK	20.28265	P61764	STXB1 HUMAN	566.8040365	2	0.72820574		
SVHSLISDFK	20.28265	P61764	STXB1 HUMAN	378.2052993	3	0.72820574		
VEQDLAMGTDAEGEK	13.96988	P61764	STXB1 HUMAN	796.8596095	2	0.803058922		
VEQDLAMGTDAEGEK	13.96988	P61764	STXB1 HUMAN	531.5756813	3	0.803058922		
VLVVDQLSMR	51.16582	P61764	STXB1 HUMAN	580.329365	2	0.735184491	2	Yes
VLVVDQLSMR	51.16582	P61764	STXB1 HUMAN	387.222185	3	0.735184491	2	
WEVLIGSTHILTPQK	61.55901	P61764	STXB1 HUMAN	861.483429	2	0.821600914	3	
WEVLIGSTHILTPQK	61.55901	P61764	STXB1 HUMAN	574.6582277	3	0.821600914	3	Yes
YETSGIGEAR	-12.87125	P61764	STXB1 HUMAN	541.7598225	2	0.648512006	2	Yes
YETSGIGEAR	-12.87125	P61764	STXB1 HUMAN	361.5091567	3	0.648512006	2	
YSTHLHLAEDCMK	-1.746105	P61764	STXB1 HUMAN	802.8639705	2	0.798248947	4	
YSTHLHLAEDCMK	-1.746105	P61764	STXB1 HUMAN	535.5785887	3	0.798248947	4	
ETSMVHELNR	-18.34282	P61619	S61A1 HUMAN	608.2935105	2	0.803577065		
ETSMVHELNR	-18.34282	P61619	S61A1 HUMAN	405.8649487	3	0.803577065		
GQYNTYPIK	1.428108	P61619	S61A1 HUMAN	542.277647	2	0.734050989	2	Yes
GQYNTYPIK	1.428108	P61619	S61A1 HUMAN	361.854373	3	0.734050989	2	
FLPLFDR	63.73831	P61604	CH10 HUMAN	454.2559945	2	0.76901859	2	Yes
FLPLFDR	63.73831	P61604	CH10 HUMAN	303.1732713	3	0.76901859	2	
GGEIQPVSVK	3.297745	P61604	CH10 HUMAN	507.2854805	2	0.690318942	2	Yes
GGEIQPVSVK	3.297745	P61604	CH10 HUMAN	338.526262	3	0.690318942	2	
GGIMLPEK	14.67692	P61604	CH10 HUMAN	422.734038	2	0.799198329	2	Yes
GGIMLPEK	14.67692	P61604	CH10 HUMAN	282.1586337	3	0.799198329	2	
VLLPEYGGTK	29.5063	P61604	CH10 HUMAN	538.803502	2	0.819718063	2	Yes
VLLPEYGGTK	29.5063	P61604	CH10 HUMAN	359.5382763	3	0.819718063	2	
VLQATVVAVGSGSK	22.617	P61604	CH10 HUMAN	658.3831835	2	0.831392884	2	Yes
VLQATVVAVGSGSK	22.617	P61604	CH10 HUMAN	439.258064	3	0.831392884	2	
VVLDDKDYFLFR	65.00797	P61604	CH10 HUMAN	765.404111	2	0.896983087	2	Yes
VVLDDKDYFLFR	65.00797	P61604	CH10 HUMAN	510.605349	3	0.896983087	2	
AFIITINSFGTELSK	94.42245	P61421	VA0D1 HUMAN	820.948887	2	0.813413978		
AFIITINSFGTELSK	94.42245	P61421	VA0D1 HUMAN	547.6351997	3	0.813413978		
AGVLSQADYLNLVQCETLEDLK	114.2981	P61421	VA0D1 HUMAN	1240.123425	2	0.652771294		
AGVLSQADYLNLVQCETLEDLK	114.2981	P61421	VA0D1 HUMAN	827.0848913	3	0.652771294		
AYLESFYK	32.68649	P61421	VA0D1 HUMAN	510.7560155	2	0.601374924		
AYLESFYK	32.68649	P61421	VA0D1 HUMAN	340.839952	3	0.601374924		
FFEHEVK	-18.97584	P61421	VA0D1 HUMAN	468.235259	2	0.710097671	2	Yes
FFEHEVK	-18.97584	P61421	VA0D1 HUMAN	312.492781	3	0.710097671	2	
LHLQSTDYGNFLANEASPLTVSVIDDR	92.71751	P61421	VA0D1 HUMAN	1488.241436	2	0.80280757	3	
LHLQSTDYGNFLANEASPLTVSVIDDR	92.71751	P61421	VA0D1 HUMAN	992.496899	3	0.80280757	3	Yes
LLFEGAGSNPGDK	28.74165	P61421	VA0D1 HUMAN	652.8282405	2	0.745278478	2	Yes
LLFEGAGSNPGDK	28.74165	P61421	VA0D1 HUMAN	435.5547687	3	0.745278478	2	
LYPEGLAQLAR	51.46165	P61421	VA0D1 HUMAN	615.8462325	2	0.808538854	2	Yes
LYPEGLAQLAR	51.46165	P61421	VA0D1 HUMAN	410.9000967	3	0.808538854	2	
NIVWIAECIAQR	75.50322	P61421	VA0D1 HUMAN	736.88811	2	0.6639902	3	
NIVWIAECIAQR	75.50322	P61421	VA0D1 HUMAN	491.5946817	3	0.6639902	3	Yes
NVADYYPEYK	24.38059	P61421	VA0D1 HUMAN	631.2909465	2	0.817671716	2	Yes
NVADYYPEYK	24.38059	P61421	VA0D1 HUMAN	421.1965727	3	0.817671716	2	
SIAELVPK	29.79648	P61421	VA0D1 HUMAN	428.7611095	2	0.673116744		
SIAELVPK	29.79648	P61421	VA0D1 HUMAN	286.1766813	3	0.673116744		
DKDNFWMTR	22.02659	P61160	ARP2 HUMAN	606.7774965	2	0.860761285	2	Yes
DKDNFWMTR	22.02659	P61160	ARP2 HUMAN	404.8542727	3	0.860761285	2	
DLMVGDEASELR	47.86134	P61160	ARP2 HUMAN	667.8170155	2	0.796776414	2	Yes
DLMVGDEASELR	47.86134	P61160	ARP2 HUMAN	445.5472853	3	0.796776414	2	
GYAFNHSADFETVR	21.28088	P61160	ARP2 HUMAN	807.371331	2	0.793062031	2	Yes
GYAFNHSADFETVR	21.28088	P61160	ARP2 HUMAN	538.5834957	3	0.793062031	2	
HIVLSGGSTMYPGLPSR	43.91408	P61160	ARP2 HUMAN	886.462166	2	0.742732525	3	
HIVLSGGSTMYPGLPSR	43.91408	P61160	ARP2 HUMAN	591.310719	3	0.742732525	3	Yes
HLWDYTFGPEK	48.12243	P61160	ARP2 HUMAN	696.833322	2	0.836645305	2	Yes
HLWDYTFGPEK	48.12243	P61160	ARP2 HUMAN	464.8914897	3	0.836645305	2	
ILLTEPPMNPTK	46.8833	P61160	ARP2 HUMAN	677.3765125	2	0.786336184	2	Yes
ILLTEPPMNPTK	46.8833	P61160	ARP2 HUMAN	451.9202833	3	0.786336184	2	
LALETTVLVESYTLPDGR	95.4644	P61160	ARP2 HUMAN	989.031133	2	0.758355141	3	
LALETTVLVESYTLPDGR	95.4644	P61160	ARP2 HUMAN	659.6900303	3	0.758355141	3	Yes
LCYVGYNIEQEQK	32.3659	P61160	ARP2 HUMAN	822.390871	2	0.721419871		
LCYVGYNIEQEQK	32.3659	P61160	ARP2 HUMAN	548.5965223	3	0.721419871		
SMLEVNYPMENGIVR	67.53616	P61160	ARP2 HUMAN	876.426934	2	0.741315484		
SMLEVNYPMENGIVR	67.53616	P61160	ARP2 HUMAN	584.6205643	3	0.741315484		
DMYEQFQNIMK	68.55354	P61011	SRP54 HUMAN	723.8237825	2	0.77585876	2	Yes
DMYEQFQNIMK	68.55354	P61011	SRP54 HUMAN	482.88513	3	0.77585876	2	
DVQELLTQYTK	71.25954	P61011	SRP54 HUMAN	669.351545	2	0.838057518	2	Yes
DVQELLTQYTK	71.25954	P61011	SRP54 HUMAN	446.570305	3	0.838057518	2	
EVCTALLEADVNIK	60.56862	P61011	SRP54 HUMAN	787.908905	2	0.711466491		
EVCTALLEADVNIK	60.56862	P61011	SRP54 HUMAN	525.608545	3	0.711466491		
GGGALSAVAATK	4.870354	P61011	SRP54 HUMAN	501.7831055	2	0.809664369	2	Yes
GGGALSAVAATK	4.870354	P61011	SRP54 HUMAN	334.858012	3	0.809664369	2	
LAYYYQR	3.51479	P61011	SRP54 HUMAN	488.748521	2	0.780228853		
LAYYYQR	3.51479	P61011	SRP54 HUMAN	326.168289	3	0.780228853		
LDDNEALIEK	18.55634	P61011	SRP54 HUMAN	580.2962415	2	0.840691745		
LDDNEALIEK	18.55634	P61011	SRP54 HUMAN	387.2001027	3	0.840691745		
LLGMGDIEGLIDK	98.47617	P61011	SRP54 HUMAN	687.371427	2	0.875890195	2	Yes
LLGMGDIEGLIDK	98.47617	P61011	SRP54 HUMAN	458.5835597	3	0.875890195	2	

LMTIMDSMNDQELDSTDGAK	65.52546	P61011	SRP54 HUMAN	1107.982344	2	0.695553005		
LMTIMDSMNDQELDSTDGAK	65.52546	P61011	SRP54 HUMAN	738.9908373	3	0.695553005		
SAIDLEEMASGLNK	69.12026	P61011	SRP54 HUMAN	739.3643295	2	0.868591368		
SAIDLEEMASGLNK	69.12026	P61011	SRP54 HUMAN	493.2454947	3	0.868591368		
SLSNATIINEEVLNAMLK	102.0768	P61011	SRP54 HUMAN	980.5251635	2	0.708524704	3	
SLSNATIINEEVLNAMLK	102.0768	P61011	SRP54 HUMAN	654.019384	3	0.708524704	3	Yes
SPIIFIGTGEHIDDFEPFK	93.41452	P61011	SRP54 HUMAN	1081.544412	2	0.696895719	3	
SPIIFIGTGEHIDDFEPFK	93.41452	P61011	SRP54 HUMAN	721.3655493	3	0.696895719	3	Yes
VDVASVIVTK	39.75853	P61011	SRP54 HUMAN	515.811331	2	0.646310985		
VDVASVIVTK	39.75853	P61011	SRP54 HUMAN	344.2101623	3	0.646310985		
DCGATWVVLGHSER	37.84312	P60174	TPIS HUMAN	793.8731885	2	0.696888328	3	
DCGATWVVLGHSER	37.84312	P60174	TPIS HUMAN	529.584734	3	0.696888328	3	Yes
IAVAAQNCYK	-7.293015	P60174	TPIS HUMAN	569.290236	2	0.655707717	2	Yes
IAVAAQNCYK	-7.293015	P60174	TPIS HUMAN	379.8627657	3	0.655707717	2	
IIYGGSVTGATCK	13.87489	P60174	TPIS HUMAN	663.8402905	2	0.742629647	2	Yes
IIYGGSVTGATCK	13.87489	P60174	TPIS HUMAN	442.8961353	3	0.742629647	2	
QSLGELIGTLNAAK	73.78472	P60174	TPIS HUMAN	707.8991975	2	0.731733024	2	Yes
QSLGELIGTLNAAK	73.78472	P60174	TPIS HUMAN	472.26874	3	0.731733024	2	
RHVFGESDELIGQK	5.741459	P60174	TPIS HUMAN	807.91591	2	0.82019484	4	
RHVFGESDELIGQK	5.741459	P60174	TPIS HUMAN	538.9465483	3	0.82019484	4	
SNVSDAVAQSTR	-12.22345	P60174	TPIS HUMAN	617.8052965	2	0.763942599	2	Yes
SNVSDAVAQSTR	-12.22345	P60174	TPIS HUMAN	412.2061393	3	0.763942599	2	
TATPQQAQEVHEK	-32.23933	P60174	TPIS HUMAN	733.865887	2	0.708052158	3	
TATPQQAQEVHEK	-32.23933	P60174	TPIS HUMAN	489.5798663	3	0.708052158	3	Yes
VAHALAEGLGVIACIGEK	63.98003	P60174	TPIS HUMAN	904.4909285	2	0.772728264	3	
VAHALAEGLGVIACIGEK	63.98003	P60174	TPIS HUMAN	603.329894	3	0.772728264	3	Yes
VTNGAFTGEISPGMIK	50.84937	P60174	TPIS HUMAN	811.4168975	2	0.749790192	2	Yes
VTNGAFTGEISPGMIK	50.84937	P60174	TPIS HUMAN	541.28054	3	0.749790192	2	
VVLAYEPVWAIGTGK	84.13374	P60174	TPIS HUMAN	801.9486865	2	0.807594478	2	Yes
VVLAYEPVWAIGTGK	84.13374	P60174	TPIS HUMAN	534.9683993	3	0.807594478	2	
AIYAALSGNLK	36.51286	P57740	NU107 HUMAN	560.8222255	2	0.761926413	2	Yes
AIYAALSGNLK	36.51286	P57740	NU107 HUMAN	374.2174253	3	0.761926413	2	
AYLEAHETFNEWFK	59.14044	P57740	NU107 HUMAN	892.918114	2	0.831304312		
AYLEAHETFNEWFK	59.14044	P57740	NU107 HUMAN	595.6146843	3	0.831304312		
DEIGEFSDNIEFYAK	83.17511	P57740	NU107 HUMAN	888.902326	2	0.786276102		
DEIGEFSDNIEFYAK	83.17511	P57740	NU107 HUMAN	592.9374923	3	0.786276102		
EADLDVATITK	34.50698	P57740	NU107 HUMAN	588.3118925	2	0.786547661		
EADLDVATITK	34.50698	P57740	NU107 HUMAN	392.54387	3	0.786547661		
EYLGANWTLEK	50.24612	P57740	NU107 HUMAN	662.3331545	2	0.802914739	2	Yes
EYLGANWTLEK	50.24612	P57740	NU107 HUMAN	441.891378	3	0.802914739	2	
FMTHLILFFR	77.69286	P57740	NU107 HUMAN	662.865918	2	0.646247625		
FMTHLILFFR	77.69286	P57740	NU107 HUMAN	442.2465537	3	0.646247625		
GHLDALTADVK	6.039001	P57740	NU107 HUMAN	570.3069445	2	0.735511303	2	Yes
GHLDALTADVK	6.039001	P57740	NU107 HUMAN	380.5405713	3	0.735511303	2	
IDVIDWLVFDPAQR	151.3647	P57740	NU107 HUMAN	843.946679	2	0.634820998	3	
IDVIDWLVFDPAQR	151.3647	P57740	NU107 HUMAN	562.967061	3	0.634820998	3	Yes
IPQDSIAEIYNQCEEQGMESPLPAEDDNAIR	83.13362	P57740	NU107 HUMAN	1766.796318	2	0.783388615	3	
IPQDSIAEIYNQCEEQGMESPLPAEDDNAIR	83.13362	P57740	NU107 HUMAN	1178.200154	3	0.783388615	3	Yes
IQSALEEESVFAVTAVNASEK	81.10747	P57740	NU107 HUMAN	1111.563529	2	0.686844289		
IQSALEEESVFAVTAVNASEK	81.10747	P57740	NU107 HUMAN	741.3782943	3	0.686844289		
LYHDPNVNGGTELEPVEGNPYR	35.61839	P57740	NU107 HUMAN	1235.58585	2	0.604839027	3	
LYHDPNVNGGTELEPVEGNPYR	35.61839	P57740	NU107 HUMAN	824.0598417	3	0.604839027	3	Yes
MAEDELFNR	25.68188	P57740	NU107 HUMAN	562.7562295	2	0.739177644	2	Yes
MAEDELFNR	25.68188	P57740	NU107 HUMAN	375.5067613	3	0.739177644	2	
NNLPGHLLR	4.237038	P57740	NU107 HUMAN	517.299256	2	0.659819365	2	Yes
NNLPGHLLR	4.237038	P57740	NU107 HUMAN	345.2021123	3	0.659819365	2	
SGFGEISSPVIR	45.853	P57740	NU107 HUMAN	624.8333255	2	0.880163193	2	Yes
SGFGEISSPVIR	45.853	P57740	NU107 HUMAN	416.891492	3	0.880163193	2	
SGLFTNTEPHSITEDVTISAVMLR	94.01181	P57740	NU107 HUMAN	1309.66071	2	0.721502602		
SGLFTNTEPHSITEDVTISAVMLR	94.01181	P57740	NU107 HUMAN	873.4430813	3	0.721502602		
SVYWENTLHTLK	40.02707	P57740	NU107 HUMAN	745.8860855	2	0.827461362	3	
SVYWENTLHTLK	40.02707	P57740	NU107 HUMAN	497.593332	3	0.827461362	3	Yes
TVVEALFQR	50.35355	P57740	NU107 HUMAN	531.801298	2	0.711984456	2	Yes
TVVEALFQR	50.35355	P57740	NU107 HUMAN	354.8701403	3	0.711984456	2	
VFEELQATDK	19.45381	P57740	NU107 HUMAN	590.298785	2	0.74424541	2	Yes
VFEELQATDK	19.45381	P57740	NU107 HUMAN	393.868465	3	0.74424541	2	
VLEENQEHYHIVQK	-13.25742	P57740	NU107 HUMAN	883.44757	2	0.690235436	3	
VLEENQEHYHIVQK	-13.25742	P57740	NU107 HUMAN	589.3009883	3	0.690235436	3	Yes
VLLQASQDENFGNTTPR	35.71809	P57740	NU107 HUMAN	945.471778	2	0.868777215		
VLLQASQDENFGNTTPR	35.71809	P57740	NU107 HUMAN	630.6504603	3	0.868777215		
VMVDSLVEQEIQTSVATLDETEELPR	137.9687	P57740	NU107 HUMAN	1466.229351	2	0.687813461		
VMVDSLVEQEIQTSVATLDETEELPR	137.9687	P57740	NU107 HUMAN	977.8221753	3	0.687813461		
YEMDFGIWK	76.05882	P57740	NU107 HUMAN	594.773887	2	0.674214184		
YEMDFGIWK	76.05882	P57740	NU107 HUMAN	396.8518663	3	0.674214184		
YLFTLIR	66.49239	P57740	NU107 HUMAN	463.2794655	2	0.617937326	2	Yes
YLFTLIR	66.49239	P57740	NU107 HUMAN	309.1889187	3	0.617937326	2	
APSSAQYLEEK	-1.26326	P57737	CORO7 HUMAN	611.801687	2	0.822897434		
APSSAQYLEEK	-1.26326	P57737	CORO7 HUMAN	408.203733	3	0.822897434		
VEFHEDLFPDTAGCVPATDPHSWWAGDNQQVQ	77.43111	P57737	CORO7 HUMAN	1926.871546	2	0.746769905		
VEFHEDLFPDTAGCVPATDPHSWWAGDNQQVQ	77.43111	P57737	CORO7 HUMAN	1284.916972	3	0.746769905		
CLLVSGFDSQSER	44.18696	P57737	CORO7 HUMAN	749.3542965	2	0.65581882		
CLLVSGFDSQSER	44.18696	P57737	CORO7 HUMAN	499.9054727	3	0.65581882		
DGALVGTACKDK	-22.57285	P57737	CORO7 HUMAN	617.8089945	2	0.728587389		
DGALVGTACKDK	-22.57285	P57737	CORO7 HUMAN	412.2086047	3	0.728587389		
FHPLAANVLASSSYDLTVR	65.29727	P57737	CORO7 HUMAN	1031.042366	2	0.721490443		
FHPLAANVLASSSYDLTVR	65.29727	P57737	CORO7 HUMAN	687.6975187	3	0.721490443		
GLNLTTPGESDGFCANK	45.09697	P57737	CORO7 HUMAN	890.9127075	2	0.803823352		
GLNLTTPGESDGFCANK	45.09697	P57737	CORO7 HUMAN	594.2777467	3	0.803823352		
GLVLLPK	41.22051	P57737	CORO7 HUMAN	370.258006	2	0.722358704		
GLVLLPK	41.22051	P57737	CORO7 HUMAN	247.1746123	3	0.722358704		
IWDLQAGADR	33.95038	P57737	CORO7 HUMAN	572.7914615	2	0.862125158		
IWDLQAGADR	33.95038	P57737	CORO7 HUMAN	382.196916	3	0.862125158		
LAWMGTWEHLVSTGFNQMR	106.8797	P57737	CORO7 HUMAN	1132.541158	2	0.759314239		
LAWMGTWEHLVSTGFNQMR	106.8797	P57737	CORO7 HUMAN	755.3633803	3	0.759314239		
LQGHQDQIFSLAWSPDGQQLATVCKDGR	72.07849	P57737	CORO7 HUMAN	1578.270543	2	0.712981701		
LQGHQDQIFSLAWSPDGQQLATVCKDGR	72.07849	P57737	CORO7 HUMAN	1052.516303	3	0.712981701		
SGPEPLQEGPGPK	0.970016	P57737	CORO7 HUMAN	646.828241	2	0.77419579		
SGPEPLQEGPGPK	0.970016	P57737	CORO7 HUMAN	431.554769	3	0.77419579		
SLQSLLGPSSK	37.67152	P57737	CORO7 HUMAN	558.817144	2	0.811850905	2	Yes
SLQSLLGPSSK	37.67152	P57737	CORO7 HUMAN	372.8807043	3	0.811850905	2	
VLQLSDTAIVPIGYHVPR	71.35921	P57737	CORO7 HUMAN	989.560195	2	0.841296196	3	

VLQLSDTAIVPIGYHVPR	71.35921	P57737	CORO7_HUMAN	660.0427383	3	0.841296196	3	Yes
VPAEGLEEVLTTPETVLTGHTEK	76.89283	P57737	CORO7_HUMAN	1225.637227	2	0.766622722	3	
VPAEGLEEVLTTPETVLTGHTEK	76.89283	P57737	CORO7_HUMAN	817.4274263	3	0.766622722	3	Yes
DALDQLR	19.61835	P57076	CF298_HUMAN	415.722516	2	0.626077414		
DALDQLR	19.61835	P57076	CF298_HUMAN	277.4842857	3	0.626077414		
EAEAQLWWAAK	54.68061	P57076	CF298_HUMAN	651.828044	2	0.802550018		
EAEAQLWWAAK	54.68061	P57076	CF298_HUMAN	434.887971	3	0.802550018		
EDLSGTQAGLNVIK	40.42943	P57076	CF298_HUMAN	722.886287	2	0.772006214	2	Yes
EDLSGTQAGLNVIK	40.42943	P57076	CF298_HUMAN	482.260133	3	0.772006214	2	
GDESQFLLQAPGSTELEELTVQVAR	121.4276	P57076	CF298_HUMAN	1359.185604	2	0.728402138		
GDESQFLLQAPGSTELEELTVQVAR	121.4276	P57076	CF298_HUMAN	906.4596773	3	0.728402138		
LCSEMEELAEHGIFLPPNMQGLTDDQIEELK	117.0307	P57076	CF298_HUMAN	1793.841688	2	0.678570032		
LCSEMEELAEHGIFLPPNMQGLTDDQIEELK	117.0307	P57076	CF298_HUMAN	1196.2304	3	0.678570032		
LEENDDDAYLNSPWADNTALK	68.69006	P57076	CF298_HUMAN	1197.540778	2	0.847519815	3	
LEENDDDAYLNSPWADNTALK	68.69006	P57076	CF298_HUMAN	798.69646	3	0.847519815	3	Yes
ALFFGLFSR	99.93468	P56937	DHB7_HUMAN	529.295651	2	0.764631271		
ALFFGLFSR	99.93468	P56937	DHB7_HUMAN	353.199709	3	0.764631271		
SNFSLEDFQHSK	28.17663	P56937	DHB7_HUMAN	719.8340535	2	0.80538106		
SNFSLEDFQHSK	28.17663	P56937	DHB7_HUMAN	480.2253107	3	0.80538106		
VIHMFSTAEGLLTQGDK	50.46717	P56937	DHB7_HUMAN	923.9725685	2	0.735463858		
VIHMFSTAEGLLTQGDK	50.46717	P56937	DHB7_HUMAN	616.317654	3	0.735463858		
VVLITGASSGIGLALCK	78.94025	P56937	DHB7_HUMAN	829.979665	2	0.768596649		
VVLITGASSGIGLALCK	78.94025	P56937	DHB7_HUMAN	553.6557183	3	0.768596649		
YATDLLSVALNR	72.51012	P56937	DHB7_HUMAN	668.3675285	2	0.848997116	2	Yes
YATDLLSVALNR	72.51012	P56937	DHB7_HUMAN	445.914294	3	0.848997116	2	
YLSATTGFGR	17.17938	P56937	DHB7_HUMAN	536.775276	2	0.782053292	2	Yes
YLSATTGFGR	17.17938	P56937	DHB7_HUMAN	358.1861257	3	0.782053292	2	
EVPNTVHQFQLDITVK	54.96489	P56556	NDUA6_HUMAN	934.4998075	2	0.781132221	2	Yes
EVPNTVHQFQLDITVK	54.96489	P56556	NDUA6_HUMAN	623.3358133	3	0.781132221	2	
VVDLLVIK	58.17064	P56556	NDUA6_HUMAN	449.8027775	2	0.690472782	2	Yes
VVDLLVIK	58.17064	P56556	NDUA6_HUMAN	300.20446	3	0.690472782	2	
GVGGLMNSFPPPQGHAQNPLQVGAELQSR	73.97315	P56270	MAZ_HUMAN	1493.751788	2	0.70835638		
GVGGLMNSFPPPQGHAQNPLQVGAELQSR	73.97315	P56270	MAZ_HUMAN	996.170467	3	0.70835638		
MLSSAYISDHMK	15.12213	P56270	MAZ_HUMAN	691.8263245	2	0.747458875		
MLSSAYISDHMK	15.12213	P56270	MAZ_HUMAN	461.5534913	3	0.747458875		
AAGGFTHDELLK	13.04554	P56182	RRP1_HUMAN	629.8255015	2	0.793310463	3	
AAGGFTHDELLK	13.04554	P56182	RRP1_HUMAN	420.2196093	3	0.793310463	3	Yes
DSGGPVLQFDYEAVANR	73.14193	P56182	RRP1_HUMAN	919.439942	2	0.818913221	3	
DSGGPVLQFDYEAVANR	73.14193	P56182	RRP1_HUMAN	613.295903	3	0.818913221	3	Yes
DSLVLNNITR	43.30087	P56182	RRP1_HUMAN	572.820218	2	0.819786966	2	Yes
DSLVLNNITR	43.30087	P56182	RRP1_HUMAN	382.216087	3	0.819786966	2	
LQDLAGGIFPEDEIPEK	79.44824	P56182	RRP1_HUMAN	935.9758305	2	0.804114044	3	
LQDLAGGIFPEDEIPEK	79.44824	P56182	RRP1_HUMAN	624.3198287	3	0.804114044	3	Yes
MQGWEER	-5.204964	P56182	RRP1_HUMAN	468.206176	2	0.618551791	2	Yes
MQGWEER	-5.204964	P56182	RRP1_HUMAN	312.4733923	3	0.618551791	2	
MVLNESLK	16.85902	P56182	RRP1_HUMAN	467.2578765	2	0.768805444	2	Yes
MVLNESLK	16.85902	P56182	RRP1_HUMAN	311.8411927	3	0.768805444	2	
SEKPPAGSICR	-27.55412	P56182	RRP1_HUMAN	601.3038785	2	0.656043947		
SEKPPAGSICR	-27.55412	P56182	RRP1_HUMAN	401.205194	3	0.656043947		
SHFIEIFLEELTK	107.6894	P56182	RRP1_HUMAN	803.43033	2	0.726769686	3	
SHFIEIFLEELTK	107.6894	P56182	RRP1_HUMAN	535.9561617	3	0.726769686	3	Yes
VGAEELTADQNLK	21.94711	P56182	RRP1_HUMAN	694.3573625	2	0.878834307	2	Yes
VGAEELTADQNLK	21.94711	P56182	RRP1_HUMAN	463.24085	3	0.878834307	2	
VQLPPEIQLAQR	54.74184	P56182	RRP1_HUMAN	696.4044505	2	0.878853798		
VQLPPEIQLAQR	54.74184	P56182	RRP1_HUMAN	464.6055753	3	0.878853798		
DLATALEQLLQAYPR	175.4748	P55957	BID_HUMAN	851.4626895	2	0.828607261		
DLATALEQLLQAYPR	175.4748	P55957	BID_HUMAN	567.9777347	3	0.828607261		
DVFHTTVNFINQNLR	71.69318	P55957	BID_HUMAN	909.468841	2	0.837490439	3	
DVFHTTVNFINQNLR	71.69318	P55957	BID_HUMAN	606.6485023	3	0.837490439	3	Yes
ELDALGHELPVLAPQWEGYDELQTDGNR	102.4417	P55957	BID_HUMAN	1583.260358	2	0.687729478	3	
ELDALGHELPVLAPQWEGYDELQTDGNR	102.4417	P55957	BID_HUMAN	1055.842847	3	0.687729478	3	Yes
HLAQVGDSMDR	-18.13775	P55957	BID_HUMAN	614.7911355	2	0.766516387	2	Yes
HLAQVGDSMDR	-18.13775	P55957	BID_HUMAN	410.1966987	3	0.766516387	2	
IEADSESQEDIIR	21.08763	P55957	BID_HUMAN	752.8604655	2	0.713863671		
IEADSESQEDIIR	21.08763	P55957	BID_HUMAN	502.2429187	3	0.713863671		
TMLVLALLLAK	125.3549	P55957	BID_HUMAN	593.386152	2	0.601788819		
TMLVLALLLAK	125.3549	P55957	BID_HUMAN	395.9267097	3	0.601788819		
DFSWSPGGNIIAFWVPEDKDIPAR	129.9472	P55884	EIF3B_HUMAN	1359.172101	2	0.609280229		
DFSWSPGGNIIAFWVPEDKDIPAR	129.9472	P55884	EIF3B_HUMAN	906.4506757	3	0.609280229		
DQYSVIFESGDR	53.19341	P55884	EIF3B_HUMAN	708.3260575	2	0.770198107	2	Yes
DQYSVIFESGDR	53.19341	P55884	EIF3B_HUMAN	472.5533133	3	0.770198107	2	
DRPQEADGIDSVIVVDNVPQVGPDR	70.75166	P55884	EIF3B_HUMAN	1345.673194	2	0.816218436	3	
DRPQEADGIDSVIVVDNVPQVGPDR	70.75166	P55884	EIF3B_HUMAN	897.4514043	3	0.816218436	3	Yes
FAVLHGEAPR	-1.160973	P55884	EIF3B_HUMAN	548.7990895	2	0.830086589	2	Yes
FAVLHGEAPR	-1.160973	P55884	EIF3B_HUMAN	366.2020013	3	0.830086589	2	
FSHQGVQLIDFSPCER	47.89937	P55884	EIF3B_HUMAN	960.4576165	2	0.752785802	3	
FSHQGVQLIDFSPCER	47.89937	P55884	EIF3B_HUMAN	640.6410193	3	0.752785802	3	Yes
GFHCESSAHWPIFK	27.86292	P55884	EIF3B_HUMAN	851.8939235	2	0.621449053	4	
GFHCESSAHWPIFK	27.86292	P55884	EIF3B_HUMAN	568.265224	3	0.621449053	4	
GIALWGGEK	34.49918	P55884	EIF3B_HUMAN	465.756359	2	0.807559967	2	Yes
GIALWGGEK	34.49918	P55884	EIF3B_HUMAN	310.840181	3	0.807559967	2	
GTQGVVTNFEIFR	65.96956	P55884	EIF3B_HUMAN	734.383715	2	0.816186547		
GTQGVVTNFEIFR	65.96956	P55884	EIF3B_HUMAN	489.925085	3	0.816186547		
GTYLATFHQR	2.132957	P55884	EIF3B_HUMAN	597.307275	2	0.789192796	3	
GTYLATFHQR	2.132957	P55884	EIF3B_HUMAN	398.5407917	3	0.789192796	3	Yes
GYIFLEYASPAHAVDAVK	68.02218	P55884	EIF3B_HUMAN	976.002174	2	0.805303454	3	
GYIFLEYASPAHAVDAVK	68.02218	P55884	EIF3B_HUMAN	651.0040577	3	0.805303454	3	Yes
ISVSFYHVK	18.3923	P55884	EIF3B_HUMAN	540.298386	2	0.706910372	3	
ISVSFYHVK	18.3923	P55884	EIF3B_HUMAN	360.5348657	3	0.706910372	3	Yes
ITNDFYPEEDGK	23.61835	P55884	EIF3B_HUMAN	714.320441	2	0.830099702	2	Yes
ITNDFYPEEDGK	23.61835	P55884	EIF3B_HUMAN	476.549569	3	0.830099702	2	
MAQELYMEQK	13.90842	P55884	EIF3B_HUMAN	635.794494	2	0.733911276	2	Yes
MAQELYMEQK	13.90842	P55884	EIF3B_HUMAN	424.1989377	3	0.733911276	2	
MQDAENVAVPEAAEER	28.31019	P55884	EIF3B_HUMAN	879.9023395	2	0.788317382	3	
MQDAENVAVPEAAEER	28.31019	P55884	EIF3B_HUMAN	586.9375013	3	0.788317382	3	Yes
MTLDTLSIYETPSMGLLDK	111.7484	P55884	EIF3B_HUMAN	1064.531794	2	0.699674368		
MTLDTLSIYETPSMGLLDK	111.7484	P55884	EIF3B_HUMAN	710.0238043	3	0.699674368		
NLFNVVDCK	43.45039	P55884	EIF3B_HUMAN	554.776965	2	0.76645124	2	Yes
NLFNVVDCK	43.45039	P55884	EIF3B_HUMAN	370.1872517	3	0.76645124	2	
AEAASGPSESPSPPAAEELPGSHAEPPVPAQGEAI	50.99673	P55884	EIF3B_HUMAN	2229.534389	2	0.692106664	4	
AEAASGPSESPSPPAAEELPGSHAEPPVPAQGEAI	50.99673	P55884	EIF3B_HUMAN	1486.692201	3	0.692106664	4	

TSIFWNDVK	47.02747	P55884	EIF3B_HUMAN	555.28548	2	0.759983063	2	Yes
TSIFWNDVK	47.02747	P55884	EIF3B_HUMAN	370.5262617	3	0.759983063	2	
VDNAYWLWTFQGR	96.78938	P55884	EIF3B_HUMAN	828.4024345	2	0.751473784	3	
VDNAYWLWTFQGR	96.78938	P55884	EIF3B_HUMAN	552.6042313	3	0.751473784	3	Yes
VNLFTDFDK	58.60252	P55884	EIF3B_HUMAN	549.777488	2	0.803367674	2	Yes
VNLFTDFDK	58.60252	P55884	EIF3B_HUMAN	366.854267	3	0.803367674	2	
VTLMQLPTR	44.38602	P55884	EIF3B_HUMAN	529.8055265	2	0.815362692	2	Yes
VTLMQLPTR	44.38602	P55884	EIF3B_HUMAN	353.539626	3	0.815362692	2	
YMTISDEWDIPEK	67.34376	P55884	EIF3B_HUMAN	813.8719835	2	0.827093363	2	Yes
YMTISDEWDIPEK	67.34376	P55884	EIF3B_HUMAN	542.917264	3	0.827093363	2	
YVVTSVSWWSHK	42.47933	P55884	EIF3B_HUMAN	739.8755205	2	0.766429603		
YVVTSVSWWSHK	42.47933	P55884	EIF3B_HUMAN	493.5862887	3	0.766429603		
YWLEEAECR	32.44949	P55884	EIF3B_HUMAN	628.2747825	2	0.726613641	2	Yes
YWLEEAECR	32.44949	P55884	EIF3B_HUMAN	419.1857967	3	0.726613641	2	
AADEEAFEDNSEEYIR	36.23219	P55060	XPO2_HUMAN	944.398136	2	0.849760175	2	Yes
AADEEAFEDNSEEYIR	36.23219	P55060	XPO2_HUMAN	629.934699	3	0.849760175	2	
ALTLPGSSENEYIMK	54.04642	P55060	XPO2_HUMAN	826.9141825	2	0.77660042	2	Yes
ALTLPGSSENEYIMK	54.04642	P55060	XPO2_HUMAN	551.6120633	3	0.77660042	2	
ANDHQGFYLLNSIIEHMPPESVDQYR	99.12296	P55060	XPO2_HUMAN	1537.227802	2	0.751933575	4	
ANDHQGFYLLNSIIEHMPPESVDQYR	99.12296	P55060	XPO2_HUMAN	1025.154476	3	0.751933575	4	
ANIVHLMLSSPEQIQK	54.50389	P55060	XPO2_HUMAN	904.4909275	2	0.792654097	3	
ANIVHLMLSSPEQIQK	54.50389	P55060	XPO2_HUMAN	603.3298933	3	0.792654097	3	Yes
ATIELCSTHANDASALR	14.84447	P55060	XPO2_HUMAN	915.444706	2	0.732446194	2	Yes
ATIELCSTHANDASALR	14.84447	P55060	XPO2_HUMAN	610.6324123	3	0.732446194	2	
DAAIYLVTSLASK	89.30716	P55060	XPO2_HUMAN	676.377562	2	0.707887113		
DAAIYLVTSLASK	89.30716	P55060	XPO2_HUMAN	451.2543163	3	0.707887113		
EHDPVGQMVNNPK	-6.24852	P55060	XPO2_HUMAN	732.8491815	2	0.78493011	3	
EHDPVGQMVNNPK	-6.24852	P55060	XPO2_HUMAN	488.9020627	3	0.78493011	3	Yes
FQSGDFHVINGVLR	51.07505	P55060	XPO2_HUMAN	794.915713	2	0.768169165	2	Yes
FQSGDFHVINGVLR	51.07505	P55060	XPO2_HUMAN	530.2797503	3	0.768169165	2	
HGITQANELVNLTEFFVNHILPDLK	132.5165	P55060	XPO2_HUMAN	1431.761615	2	0.660065889	4	
HGITQANELVNLTEFFVNHILPDLK	132.5165	P55060	XPO2_HUMAN	954.843685	3	0.660065889	4	
IHLAQSLHK	-24.77453	P55060	XPO2_HUMAN	523.809457	2	0.701914012	2	Yes
IHLAQSLHK	-24.77453	P55060	XPO2_HUMAN	349.5422463	3	0.701914012	2	
IIIPEIQK	41.91656	P55060	XPO2_HUMAN	477.3056845	2	0.797024488	2	Yes
IIIPEIQK	41.91656	P55060	XPO2_HUMAN	318.5397313	3	0.797024488	2	
IPGLLGVFQK	77.92301	P55060	XPO2_HUMAN	536.332234	2	0.798977852	2	Yes
IPGLLGVFQK	77.92301	P55060	XPO2_HUMAN	357.8907643	3	0.798977852	2	
LLQAFLER	48.74213	P55060	XPO2_HUMAN	495.2931085	2	0.809452057	2	Yes
LLQAFLER	48.74213	P55060	XPO2_HUMAN	330.5313473	3	0.809452057	2	
LLQTDDEEEAGLLELLK	110.9749	P55060	XPO2_HUMAN	965.0073275	2	0.794033587	3	
LLQTDDEEEAGLLELLK	110.9749	P55060	XPO2_HUMAN	643.67416	3	0.794033587	3	Yes
LLTECPPMMDTEYTK	48.3692	P55060	XPO2_HUMAN	914.9124695	2	0.848441958		
LLTECPPMMDTEYTK	48.3692	P55060	XPO2_HUMAN	610.277588	3	0.848441958		
LVLDAFALPLTNLFK	155.7672	P55060	XPO2_HUMAN	837.9956405	2	0.782905459	3	
LVLDAFALPLTNLFK	155.7672	P55060	XPO2_HUMAN	558.999702	3	0.782905459	3	Yes
MELSDANLQTLTEYLK	102.312	P55060	XPO2_HUMAN	934.9696865	2	0.903600991		
MELSDANLQTLTEYLK	102.312	P55060	XPO2_HUMAN	623.649066	3	0.903600991		
NLFEDQNTLTSICEK	58.24988	P55060	XPO2_HUMAN	906.42819	2	0.868779838		
NLFEDQNTLTSICEK	58.24988	P55060	XPO2_HUMAN	604.6214017	3	0.868779838		
SANVNEFPVLK	41.61839	P55060	XPO2_HUMAN	609.3304185	2	0.841705561	2	Yes
SANVNEFPVLK	41.61839	P55060	XPO2_HUMAN	406.5562207	3	0.841705561	2	
SNELWTEIK	39.03633	P55060	XPO2_HUMAN	560.2882195	2	0.645933449	2	Yes
SNELWTEIK	39.03633	P55060	XPO2_HUMAN	373.8614213	3	0.645933449	2	
SQICDNAALYAQK	16.49426	P55060	XPO2_HUMAN	741.3568355	2	0.815219104	2	Yes
SQICDNAALYAQK	16.49426	P55060	XPO2_HUMAN	494.573832	3	0.815219104	2	
VIVPNMEFR	49.804	P55060	XPO2_HUMAN	552.7977005	2	0.675490141	2	Yes
VIVPNMEFR	49.804	P55060	XPO2_HUMAN	368.867742	3	0.675490141	2	
WPDLLTEMVNR	98.05264	P55060	XPO2_HUMAN	687.348286	2	0.776114345	2	Yes
WPDLLTEMVNR	98.05264	P55060	XPO2_HUMAN	458.5681323	3	0.776114345	2	
YDEEFQR	-10.97978	P55060	XPO2_HUMAN	493.7148835	2	0.689118445	2	Yes
YDEEFQR	-10.97978	P55060	XPO2_HUMAN	329.4791973	3	0.689118445	2	
YGALALQEIFDGIQPK	115.797	P55060	XPO2_HUMAN	881.97289	2	0.841329873		
YGALALQEIFDGIQPK	115.797	P55060	XPO2_HUMAN	588.3178683	3	0.841329873		
AQLLEPSK	-0.073589	P55039	DRG2_HUMAN	443.2561915	2	0.749879956	2	Yes
AQLLEPSK	-0.073589	P55039	DRG2_HUMAN	295.8400693	3	0.749879956	2	
ATEYHLGLLK	25.23691	P55039	DRG2_HUMAN	572.822226	2	0.763363242		
ATEYHLGLLK	25.23691	P55039	DRG2_HUMAN	382.2174257	3	0.763363242		
EDCSPDEFIDVIVGNR	101.3495	P55039	DRG2_HUMAN	932.9232715	2	0.836693347		
EDCSPDEFIDVIVGNR	101.3495	P55039	DRG2_HUMAN	622.2847893	3	0.836693347		
GANIQLLDLPGIIEGAAQGK	119.8746	P55039	DRG2_HUMAN	989.5525715	2	0.834384263	3	
GANIQLLDLPGIIEGAAQGK	119.8746	P55039	DRG2_HUMAN	660.037656	3	0.834384263	3	Yes
GGGISFNSTVTLTQCSEK	46.04238	P55039	DRG2_HUMAN	943.452197	2	0.647254825		
GGGISFNSTVTLTQCSEK	46.04238	P55039	DRG2_HUMAN	629.304073	3	0.647254825		
GQRPDFTDAIILR	50.24715	P55039	DRG2_HUMAN	751.410264	2	0.820070446		
GQRPDFTDAIILR	50.24715	P55039	DRG2_HUMAN	501.2761177	3	0.820070446		
HKPNIYFKPK	-28.20424	P55039	DRG2_HUMAN	636.3671345	2	0.805641294		
HKPNIYFKPK	-28.20424	P55039	DRG2_HUMAN	424.580698	3	0.805641294		
IDQISMEEVDR	29.5382	P55039	DRG2_HUMAN	667.8170155	2	0.765703857	2	Yes
IDQISMEEVDR	29.5382	P55039	DRG2_HUMAN	445.5472853	3	0.765703857	2	
IFNAEVLFR	69.7968	P55039	DRG2_HUMAN	554.811665	2	0.666193724	2	Yes
IFNAEVLFR	69.7968	P55039	DRG2_HUMAN	370.210385	3	0.666193724	2	
KPNSVVISCGMK	-10.70195	P55039	DRG2_HUMAN	660.344689	2	0.709098935	2	Yes
KPNSVVISCGMK	-10.70195	P55039	DRG2_HUMAN	440.5657343	3	0.709098935	2	
LVQLILHEYK	33.69908	P55039	DRG2_HUMAN	628.3746255	2	0.813530803		
LVQLILHEYK	33.69908	P55039	DRG2_HUMAN	419.2523587	3	0.813530803		
VALIGFPSVGK	67.75036	P55039	DRG2_HUMAN	544.329691	2	0.811809301		
VALIGFPSVGK	67.75036	P55039	DRG2_HUMAN	363.2224023	3	0.811809301		
VGLTHTMEHEDVIQIVK	38.28609	P55039	DRG2_HUMAN	975.012225	2	0.796500802	3	
VGLTHTMEHEDVIQIVK	38.28609	P55039	DRG2_HUMAN	650.3440917	3	0.796500802	3	Yes
YALVWGTSTK	38.01875	P55039	DRG2_HUMAN	563.3011265	2	0.815223813		
YALVWGTSTK	38.01875	P55039	DRG2_HUMAN	375.870026	3	0.815223813		
AMVASGSELGK	-3.844795	P54819	KAD2_HUMAN	525.268973	2	0.668351054	2	Yes
AMVASGSELGK	-3.844795	P54819	KAD2_HUMAN	350.515257	3	0.668351054	2	
AVLLGPPGAGK	23.17274	P54819	KAD2_HUMAN	490.300934	2	0.857319593	2	Yes
AVLLGPPGAGK	23.17274	P54819	KAD2_HUMAN	327.203231	3	0.857319593	2	
DDITGEPLIR	40.2214	P54819	KAD2_HUMAN	564.798952	2	0.802716851	2	Yes
DDITGEPLIR	40.2214	P54819	KAD2_HUMAN	376.8685763	3	0.802716851	2	
LQAYHTQTTPLIEYYR	38.39822	P54819	KAD2_HUMAN	999.010527	2	0.884644389	3	
LQAYHTQTTPLIEYYR	38.39822	P54819	KAD2_HUMAN	666.3429597	3	0.884644389	3	Yes
LVSDEMVVELIEK	83.22696	P54819	KAD2_HUMAN	752.4029235	2	0.836236358	2	Yes

LVSDEMVVELIEK	83.22696	P54819	KAD2 HUMAN	501.9378907	3	0.836236358	2	
NGFLLDGFPR	74.88868	P54819	KAD2 HUMAN	568.298922	2	0.751894474	2	Yes
NGFLLDGFPR	74.88868	P54819	KAD2 HUMAN	379.2018897	3	0.751894474	2	
SYHEEFNPPK	-10.67899	P54819	KAD2 HUMAN	624.2887465	2	0.688416123		
SYHEEFNPPK	-10.67899	P54819	KAD2 HUMAN	416.5284393	3	0.688416123		
DDNMFQIGK	42.1794	P53999	TCP4 HUMAN	534.245498	2	0.745640635	2	Yes
DDNMFQIGK	42.1794	P53999	TCP4 HUMAN	356.499607	3	0.745640635	2	
EQISDIDDAVR	24.9323	P53999	TCP4 HUMAN	630.807505	2	0.682565987	2	Yes
EQISDIDDAVR	24.9323	P53999	TCP4 HUMAN	420.8742783	3	0.682565987	2	
EYWMDPEGEMKPGR	33.84878	P53999	TCP4 HUMAN	862.874535	2	0.754988909	3	
EYWMDPEGEMKPGR	33.84878	P53999	TCP4 HUMAN	575.5856317	3	0.754988909	3	Yes
GISLNPEQWSQLK	60.63383	P53999	TCP4 HUMAN	750.3968215	2	0.818848491	2	Yes
GISLNPEQWSQLK	60.63383	P53999	TCP4 HUMAN	500.6004893	3	0.818848491	2	
ASNLENSTYDLYTIPK	57.47325	P53621	COPA HUMAN	914.952346	2	0.812491894	3	
ASNLENSTYDLYTIPK	57.47325	P53621	COPA HUMAN	610.3041723	3	0.812491894	3	Yes
AWEVDTCR	5.182159	P53621	COPA HUMAN	518.7300155	2	0.725952506	2	Yes
AWEVDTCR	5.182159	P53621	COPA HUMAN	346.155952	3	0.725952506	2	
DADSITLFDVQQK	68.85867	P53621	COPA HUMAN	740.37047	2	0.815659285		
DADSITLFDVQQK	68.85867	P53621	COPA HUMAN	493.916255	3	0.815659285		
DMSGHYQNALYLGDVSER	44.74877	P53621	COPA HUMAN	1027.965994	2	0.776405513		
DMSGHYQNALYLGDVSER	44.74877	P53621	COPA HUMAN	685.646604	3	0.776405513		
DVAVMQLR	34.30379	P53621	COPA HUMAN	466.255669	2	0.736419976	2	Yes
DVAVMQLR	34.30379	P53621	COPA HUMAN	311.1730543	3	0.736419976	2	
ETIPDIDPNAK	30.27518	P53621	COPA HUMAN	606.8095165	2	0.672962189		
ETIPDIDPNAK	30.27518	P53621	COPA HUMAN	404.8756193	3	0.672962189		
EYIVGLSVETER	51.28608	P53621	COPA HUMAN	697.862276	2	0.838185191		
EYIVGLSVETER	51.28608	P53621	COPA HUMAN	465.577459	3	0.838185191		
GFFEGTIASK	34.68614	P53621	COPA HUMAN	528.772206	2	0.815961242	2	Yes
GFFEGTIASK	34.68614	P53621	COPA HUMAN	352.8507457	3	0.815961242	2	
GHYNNVSCAVFHPR	-2.817963	P53621	COPA HUMAN	829.3867925	2	0.765670419		
GHYNNVSCAVFHPR	-2.817963	P53621	COPA HUMAN	553.26047	3	0.765670419		
GITGVDLFGTTDAVVK	77.16082	P53621	COPA HUMAN	796.9306955	2	0.872381091	2	Yes
GITGVDLFGTTDAVVK	77.16082	P53621	COPA HUMAN	531.623072	3	0.872381091	2	
GNNVYCLDR	1.643635	P53621	COPA HUMAN	555.754017	2	0.75677824		
GNNVYCLDR	1.643635	P53621	COPA HUMAN	370.8386197	3	0.75677824		
GTSPTQIWCNNSQLPVDHILAGSFETAMR	98.62523	P53621	COPA HUMAN	1615.77206	2	0.737420857	3	
GTSPTQIWCNNSQLPVDHILAGSFETAMR	98.62523	P53621	COPA HUMAN	1077.517315	3	0.737420857	3	Yes
GVNWAAFHPTMPLIVSGADDR	84.99303	P53621	COPA HUMAN	1127.557862	2	0.703341544	3	
GVNWAAFHPTMPLIVSGADDR	84.99303	P53621	COPA HUMAN	752.041183	3	0.703341544	3	Yes
GYPEVALHFVK	42.30247	P53621	COPA HUMAN	630.3433255	2	0.690309227	3	
GYPEVALHFVK	42.30247	P53621	COPA HUMAN	420.5648253	3	0.690309227	3	Yes
LDALCNIHENIR	26.51328	P53621	COPA HUMAN	734.3728235	2	0.726153553	3	
LDALCNIHENIR	26.51328	P53621	COPA HUMAN	489.917824	3	0.726153553	3	Yes
LGEVALLQGNHQIVEMCYQR	61.84805	P53621	COPA HUMAN	1179.588833	2	0.818731189		
LGEVALLQGNHQIVEMCYQR	61.84805	P53621	COPA HUMAN	786.7284967	3	0.818731189		
LLELGPKPEVAQQTR	25.92763	P53621	COPA HUMAN	839.978511	2	0.835916162	2	Yes
LLELGPKPEVAQQTR	25.92763	P53621	COPA HUMAN	560.3216157	3	0.835916162	2	
LLHDQVGVIQFGPYK	50.52145	P53621	COPA HUMAN	857.4703175	2	0.790824473	2	Yes
LLHDQVGVIQFGPYK	50.52145	P53621	COPA HUMAN	571.98282	3	0.790824473	2	
LLQPPAPIMPLDTNWPLLTVSK	141.5573	P53621	COPA HUMAN	1222.685271	2	0.738682866	3	
LLQPPAPIMPLDTNWPLLTVSK	141.5573	P53621	COPA HUMAN	815.4594557	3	0.738682866	3	Yes
LQLCYQLTTVGK	50.03442	P53621	COPA HUMAN	712.3848655	2	0.736293316	2	Yes
LQLCYQLTTVGK	50.03442	P53621	COPA HUMAN	475.2591853	3	0.736293316	2	
LSFLYLITGNLEK	115.7489	P53621	COPA HUMAN	755.9299615	2	0.760119319		
LSFLYLITGNLEK	115.7489	P53621	COPA HUMAN	504.2892493	3	0.760119319		
LVGQSIIAYLQK	73.31879	P53621	COPA HUMAN	666.898465	2	0.83076483	2	Yes
LVGQSIIAYLQK	73.31879	P53621	COPA HUMAN	444.9349183	3	0.83076483	2	
QEIAEAQQLITICR	57.32649	P53621	COPA HUMAN	836.938529	2	0.70528698		
QEIAEAQQLITICR	57.32649	P53621	COPA HUMAN	558.294961	3	0.70528698		
QLFLQTYAR	40.8222	P53621	COPA HUMAN	570.3145685	2	0.663750708		
QLFLQTYAR	40.8222	P53621	COPA HUMAN	380.545654	3	0.663750708		
SGAWDESGVFIYTTSNHIK	58.30493	P53621	COPA HUMAN	1056.505813	2	0.761065781	3	
SGAWDESGVFIYTTSNHIK	58.30493	P53621	COPA HUMAN	704.6731503	3	0.761065781	3	Yes
SLAYLTAATHGLDEEAESLK	64.36715	P53621	COPA HUMAN	1060.031861	2	0.716902852	3	
SLAYLTAATHGLDEEAESLK	64.36715	P53621	COPA HUMAN	707.023849	3	0.716902852	3	Yes
SSGLTAVWVAR	44.11806	P53621	COPA HUMAN	573.817479	2	0.818984032	2	Yes
SSGLTAVWVAR	44.11806	P53621	COPA HUMAN	382.8809277	3	0.818984032	2	
TALNLFFK	65.68163	P53621	COPA HUMAN	477.276927	2	0.817913234	2	Yes
TALNLFFK	65.68163	P53621	COPA HUMAN	318.5205597	3	0.817913234	2	
TLDLPIYVTR	69.94936	P53621	COPA HUMAN	595.8431585	2	0.836154461	2	Yes
TLDLPIYVTR	69.94936	P53621	COPA HUMAN	397.564714	3	0.836154461	2	
TTFFHHEYPWILSASDDQTIR	65.85732	P53621	COPA HUMAN	1282.614416	2	0.837187469	4	
TTFFHHEYPWILSASDDQTIR	65.85732	P53621	COPA HUMAN	855.4122187	3	0.837187469	4	
TTYQALPCLPSMYGYPNR	78.0229	P53621	COPA HUMAN	1066.501155	2	0.630760431		
TTYQALPCLPSMYGYPNR	78.0229	P53621	COPA HUMAN	711.3367113	3	0.630760431		
VLTIDPTEFK	52.60645	P53621	COPA HUMAN	581.821896	2	0.790117145	2	Yes
VLTIDPTEFK	52.60645	P53621	COPA HUMAN	388.2172057	3	0.790117145	2	
VQVPNCDEIFYAGTGNLLLR	101.1099	P53621	COPA HUMAN	1140.078623	2	0.786173105		
VQVPNCDEIFYAGTGNLLLR	101.1099	P53621	COPA HUMAN	760.388357	3	0.786173105		
VWDISGLR	48.43194	P53621	COPA HUMAN	473.261808	2	0.743301928	2	Yes
VWDISGLR	48.43194	P53621	COPA HUMAN	315.8438137	3	0.743301928	2	
VWNWQSR	21.33144	P53621	COPA HUMAN	488.2439495	2	0.636196434	2	Yes
VWNWQSR	21.33144	P53621	COPA HUMAN	325.831908	3	0.636196434	2	
YAVTTGDHGIIR	5.073692	P53621	COPA HUMAN	651.8442215	2	0.807757854	2	Yes
YAVTTGDHGIIR	5.073692	P53621	COPA HUMAN	434.898756	3	0.807757854	2	
YDEVLHMVR	20.47536	P53621	COPA HUMAN	581.2902355	2	0.765463948		
YDEVLHMVR	20.47536	P53621	COPA HUMAN	387.8627653	3	0.765463948		
YVIWSADMSHVALLAK	77.52116	P53621	COPA HUMAN	902.4772845	2	0.745208025	3	
YVIWSADMSHVALLAK	77.52116	P53621	COPA HUMAN	601.9874647	3	0.745208025	3	Yes
AFDSGIIPMEFVNK	89.56622	P53396	ACLY HUMAN	784.395433	2	0.792259514		
AFDSGIIPMEFVNK	89.56622	P53396	ACLY HUMAN	523.2662303	3	0.792259514		
AIVWGMQTR	36.53511	P53396	ACLY HUMAN	531.2822185	2	0.746329367	2	Yes
AIVWGMQTR	36.53511	P53396	ACLY HUMAN	354.5240873	3	0.746329367	2	
AKPAMPQDSVPSPR	-2.273708	P53396	ACLY HUMAN	740.8830245	2	0.731015682	2	Yes
AKPAMPQDSVPSPR	-2.273708	P53396	ACLY HUMAN	494.257958	3	0.731015682	2	
AVQGMLDFDYVCSR	70.27719	P53396	ACLY HUMAN	830.877078	2	0.620312333	2	Yes
AVQGMLDFDYVCSR	70.27719	P53396	ACLY HUMAN	554.2539937	3	0.620312333	2	
DEPSVAAMVYPFTGDHK	69.14401	P53396	ACLY HUMAN	932.4332715	2	0.748795152	3	
DEPSVAAMVYPFTGDHK	69.14401	P53396	ACLY HUMAN	621.9581227	3	0.748795152	3	Yes
DGVYVLDLAAK	68.74319	P53396	ACLY HUMAN	582.319516	2	0.812277973		
DGVYVLDLAAK	68.74319	P53396	ACLY HUMAN	388.5489523	3	0.812277973		

DLVSSLTSGLLTIGDR	142.2962	P53396	ACLY HUMAN	823.952158	2	0.690579593	3	
DLVSSLTSGLLTIGDR	142.2962	P53396	ACLY HUMAN	549.6373803	3	0.690579593	3	Yes
EAYPEEAYIADLDAK	59.70237	P53396	ACLY HUMAN	849.3994155	2	0.852737248	2	Yes
EAYPEEAYIADLDAK	59.70237	P53396	ACLY HUMAN	566.6022187	3	0.852737248	2	
EGDYVLFHHEGGVDVGDVDAK	39.27678	P53396	ACLY HUMAN	1129.522192	2	0.70173949	4	
EGDYVLFHHEGGVDVGDVDAK	39.27678	P53396	ACLY HUMAN	753.3507363	3	0.70173949	4	
FGGALDAAAK	8.290054	P53396	ACLY HUMAN	460.7459915	2	0.760013998		
FGGALDAAAK	8.290054	P53396	ACLY HUMAN	307.499936	3	0.760013998		
FICTTSAIQNR	12.01345	P53396	ACLY HUMAN	655.830261	2	0.795939207		
FICTTSAIQNR	12.01345	P53396	ACLY HUMAN	437.5561157	3	0.795939207		
GGPNYQEGLR	-5.882336	P53396	ACLY HUMAN	545.767982	2	0.765880704	2	Yes
GGPNYQEGLR	-5.882336	P53396	ACLY HUMAN	364.181263	3	0.765880704	2	
GQELIYAGMPITEVFK	102.7693	P53396	ACLY HUMAN	898.469126	2	0.89381969		
GQELIYAGMPITEVFK	102.7693	P53396	ACLY HUMAN	599.315359	3	0.89381969		
GVTIIGPATVGGIKPGCFK	56.9845	P53396	ACLY HUMAN	936.524772	2	0.815413594	3	
GVTIIGPATVGGIKPGCFK	56.9845	P53396	ACLY HUMAN	624.6857897	3	0.815413594	3	Yes
HLLVHAPEDK	-25.61667	P53396	ACLY HUMAN	579.817479	2	0.775974989	2	Yes
HLLVHAPEDK	-25.61667	P53396	ACLY HUMAN	386.8809277	3	0.775974989	2	
IGNTGGMLDNILASK	62.11951	P53396	ACLY HUMAN	752.3959645	2	0.823793888	2	Yes
IGNTGGMLDNILASK	62.11951	P53396	ACLY HUMAN	501.9332513	3	0.823793888	2	
ILIIGGSIANFTNVAATFK	121.0137	P53396	ACLY HUMAN	975.557125	2	0.61339277		
ILIIGGSIANFTNVAATFK	121.0137	P53396	ACLY HUMAN	650.7073583	3	0.61339277		
KPASFMTSICDER	17.30928	P53396	ACLY HUMAN	771.358525	2	0.715970039	2	Yes
KPASFMTSICDER	17.30928	P53396	ACLY HUMAN	514.5749583	3	0.715970039	2	
LGLVGVNLTLDGVK	86.56984	P53396	ACLY HUMAN	699.4223085	2	0.805363238	2	Yes
LGLVGVNLTLDGVK	86.56984	P53396	ACLY HUMAN	466.6174807	3	0.805363238	2	
LIMGIGHR	3.360237	P53396	ACLY HUMAN	448.7609215	2	0.72315073	2	Yes
LIMGIGHR	3.360237	P53396	ACLY HUMAN	299.5098893	3	0.72315073	2	
LLQDHPWLLSQNLVVKPDQLIK	82.36571	P53396	ACLY HUMAN	1299.244873	2	0.8171525	3	
LLQDHPWLLSQNLVVKPDQLIK	82.36571	P53396	ACLY HUMAN	866.49919	3	0.8171525	3	Yes
LLVGVDEK	15.42131	P53396	ACLY HUMAN	436.758567	2	0.661026955		
LLVGVDEK	15.42131	P53396	ACLY HUMAN	291.5083197	3	0.661026955		
LTLLNPK	20.98214	P53396	ACLY HUMAN	399.75837	2	0.716180563	2	Yes
LTLLNPK	20.98214	P53396	ACLY HUMAN	266.8415217	3	0.716180563	2	
LYRPGSVAYVSR	6.083698	P53396	ACLY HUMAN	684.3756835	2	0.609025896	3	
LYRPGSVAYVSR	6.083698	P53396	ACLY HUMAN	456.5863973	3	0.609025896	3	Yes
MIVVLGEIGGTEEYK	73.9495	P53396	ACLY HUMAN	819.4269265	2	0.790176749		
MIVVLGEIGGTEEYK	73.9495	P53396	ACLY HUMAN	546.6205593	3	0.790176749		
QHFPATPLLDYALEVEK	78.06909	P53396	ACLY HUMAN	986.015286	2	0.648740709	3	
QHFPATPLLDYALEVEK	78.06909	P53396	ACLY HUMAN	657.6794657	3	0.648740709	3	Yes
SAYDSTMETMNYAQIR	43.57004	P53396	ACLY HUMAN	940.9118415	2	0.861425579	2	Yes
SAYDSTMETMNYAQIR	43.57004	P53396	ACLY HUMAN	627.6105027	3	0.861425579	2	
SGGMSNELNNIISR	42.49583	P53396	ACLY HUMAN	746.3651945	2	0.802893043	2	Yes
SGGMSNELNNIISR	42.49583	P53396	ACLY HUMAN	497.912738	3	0.802893043	2	
SMGFIGHYLDQK	39.02647	P53396	ACLY HUMAN	698.340457	2	0.818511665	3	
SMGFIGHYLDQK	39.02647	P53396	ACLY HUMAN	465.8962463	3	0.818511665	3	Yes
TIAIIAEGIPEALTR	89.51794	P53396	ACLY HUMAN	784.45688	2	0.832158089	3	
TIAIIAEGIPEALTR	89.51794	P53396	ACLY HUMAN	523.307195	3	0.832158089	3	Yes
TILSLMTR	51.86865	P53396	ACLY HUMAN	467.7736945	2	0.686628759	2	Yes
TILSLMTR	51.86865	P53396	ACLY HUMAN	312.1850713	3	0.686628759	2	
TTDGVYEGVAIGGDR	30.66789	P53396	ACLY HUMAN	755.3631725	2	0.825149655	2	Yes
TTDGVYEGVAIGGDR	30.66789	P53396	ACLY HUMAN	503.91139	3	0.825149655	2	
VDATADYICK	13.72322	P53396	ACLY HUMAN	578.271709	2	0.788368702	2	Yes
VDATADYICK	13.72322	P53396	ACLY HUMAN	385.8504143	3	0.788368702	2	
VTPDTDWAR	12.80758	P53396	ACLY HUMAN	530.7570875	2	0.787065268	2	Yes
VTPDTDWAR	12.80758	P53396	ACLY HUMAN	354.174	3	0.787065268	2	
WGDIEFPPPFGR	91.19626	P53396	ACLY HUMAN	709.3491435	2	0.768251479	2	Yes
WGDIEFPPPFGR	91.19626	P53396	ACLY HUMAN	473.2353707	3	0.768251479	2	
YPGSTFMDHVLR	37.80229	P53396	ACLY HUMAN	711.8459065	2	0.806337714	3	
YPGSTFMDHVLR	37.80229	P53396	ACLY HUMAN	474.8998793	3	0.806337714	3	Yes
DVDWGEVDYLIVDTPPGTSDEHLSVVR	114.0816	P53384	NUBP1 HUMAN	1507.225452	2	0.673932254	3	
DVDWGEVDYLIVDTPPGTSDEHLSVVR	114.0816	P53384	NUBP1 HUMAN	1005.15291	3	0.673932254	3	Yes
GQSFFIDAPDSPATLAYR	81.46872	P53384	NUBP1 HUMAN	978.4790675	2	0.712851465	3	
GQSFFIDAPDSPATLAYR	81.46872	P53384	NUBP1 HUMAN	652.65532	3	0.712851465	3	Yes
IQEFCNLHQSK	-9.09568	P53384	NUBP1 HUMAN	702.3409925	2	0.687220812		
IQEFCNLHQSK	-9.09568	P53384	NUBP1 HUMAN	468.56327	3	0.687220812		
LCASGAGATPDTAIEEIK	40.31418	P53384	NUBP1 HUMAN	902.443841	2	0.643950105		
LCASGAGATPDTAIEEIK	40.31418	P53384	NUBP1 HUMAN	601.965169	3	0.643950105		
MEEVPHDCPGADSAQAGR	-4.90255	P53384	NUBP1 HUMAN	963.907631	2	0.814456761	3	
MEEVPHDCPGADSAQAGR	-4.90255	P53384	NUBP1 HUMAN	642.941029	3	0.814456761	3	Yes
YLATAHIDGAVIITTPQEVSLQDVR	78.8826	P53384	NUBP1 HUMAN	1355.724695	2	0.658302963		
YLATAHIDGAVIITTPQEVSLQDVR	78.8826	P53384	NUBP1 HUMAN	904.152405	3	0.658302963		
AAQLCGAGMAAVVDR	36.73858	P52789	HXK2 HUMAN	745.3666855	2	0.776100099		
AAQLCGAGMAAVVDR	36.73858	P52789	HXK2 HUMAN	497.2470653	3	0.776100099		
AILQHLGLESTCDDSIIVK	60.35885	P52789	HXK2 HUMAN	1056.554454	2	0.783536792		
AILQHLGLESTCDDSIIVK	60.35885	P52789	HXK2 HUMAN	704.7055773	3	0.783536792		
ASGCEGEDVVTLLK	52.6182	P52789	HXK2 HUMAN	739.3643305	2	0.709909499		
ASGCEGEDVVTLLK	52.6182	P52789	HXK2 HUMAN	493.2454953	3	0.709909499		
FLSQIESDCLALLQVR	97.74971	P52789	HXK2 HUMAN	946.5014925	2	0.718319297	3	
FLSQIESDCLALLQVR	97.74971	P52789	HXK2 HUMAN	631.3369367	3	0.718319297	3	Yes
GAALITAVACR	30.55802	P52789	HXK2 HUMAN	551.806058	2	0.729946136	2	Yes
GAALITAVACR	30.55802	P52789	HXK2 HUMAN	368.206647	3	0.729946136	2	
GSGTQLFDHIAECLANFMDK	110.6256	P52789	HXK2 HUMAN	1127.517546	2	0.696298301		
GSGTQLFDHIAECLANFMDK	110.6256	P52789	HXK2 HUMAN	752.0143053	3	0.696298301		
LDESFLVSWTK	86.40909	P52789	HXK2 HUMAN	662.843359	2	0.792918324	2	Yes
LDESFLVSWTK	86.40909	P52789	HXK2 HUMAN	442.2315143	3	0.792918324	2	
LGLDPTQEDCVATHR	18.13702	P52789	HXK2 HUMAN	856.407593	2	0.834763288	3	
LGLDPTQEDCVATHR	18.13702	P52789	HXK2 HUMAN	571.274337	3	0.834763288	3	Yes
LSDETLLEISK	52.63583	P52789	HXK2 HUMAN	624.340649	2	0.860798299	2	Yes
LSDETLLEISK	52.63583	P52789	HXK2 HUMAN	416.563041	3	0.860798299	2	
LSPELLNTGR	30.75564	P52789	HXK2 HUMAN	550.3094865	2	0.818938911	2	Yes
LSPELLNTGR	30.75564	P52789	HXK2 HUMAN	367.2089327	3	0.818938911	2	
MISGMYMGELVR	63.08675	P52789	HXK2 HUMAN	693.833096	2	0.661439776	2	Yes
MISGMYMGELVR	63.08675	P52789	HXK2 HUMAN	462.891339	3	0.661439776	2	
MLPTYVCATPDGTEK	37.60861	P52789	HXK2 HUMAN	841.8923945	2	0.704578459		
MLPTYVCATPDGTEK	37.60861	P52789	HXK2 HUMAN	561.597538	3	0.704578459		
NVELVEGEEGR	8.003937	P52789	HXK2 HUMAN	615.8022225	2	0.678014398	2	Yes
NVELVEGEEGR	8.003937	P52789	HXK2 HUMAN	410.8707567	3	0.678014398	2	
SASLCAATLAAVLQR	75.80471	P52789	HXK2 HUMAN	766.417232	2	0.773185611	3	
SASLCAATLAAVLQR	75.80471	P52789	HXK2 HUMAN	511.280763	3	0.773185611	3	Yes
STIGVDGSVYK	14.05943	P52789	HXK2 HUMAN	563.293498	2	0.751038074	2	Yes

STIGVDGSVYK	14.05943	P52789	HXK2 HUMAN	375.8649403	3	0.751038074	2	
STPDGTEHGEFLALDLGGTNFR	76.6191	P52789	HXK2 HUMAN	1167.554028	2	0.737343073		
STPDGTEHGEFLALDLGGTNFR	76.6191	P52789	HXK2 HUMAN	778.7052937	3	0.737343073		
TEFDQEIDMGSLNPGK	59.17876	P52789	HXK2 HUMAN	890.9070905	2	0.799995244		
TEFDQEIDMGSLNPGK	59.17876	P52789	HXK2 HUMAN	594.274002	3	0.799995244		
TEFDVAVDELSLNPGK	82.51367	P52789	HXK2 HUMAN	867.433798	2	0.746357322		
TEFDVAVDELSLNPGK	82.51367	P52789	HXK2 HUMAN	578.6251403	3	0.746357322		
TLEHLQLSHDQLLEVK	37.85808	P52789	HXK2 HUMAN	952.018364	2	0.823563814		
TLEHLQLSHDQLLEVK	37.85808	P52789	HXK2 HUMAN	635.014851	3	0.823563814		
VDQYLYHMR	14.48006	P52789	HXK2 HUMAN	612.795681	2	0.763258278	3	
VDQYLYHMR	14.48006	P52789	HXK2 HUMAN	408.8663957	3	0.763258278	3	Yes
VEMENQIYAIPEDIMR	86.35643	P52789	HXK2 HUMAN	975.9691635	2	0.833831549	3	
VEMENQIYAIPEDIMR	86.35643	P52789	HXK2 HUMAN	650.9820507	3	0.833831549	3	Yes
VTVGVDGTLYK	32.56071	P52789	HXK2 HUMAN	576.3195165	2	0.813155055	2	Yes
VTVGVDGTLYK	32.56071	P52789	HXK2 HUMAN	384.5489527	3	0.813155055	2	
AAGCDFTNVVK	21.03508	P52758	RIDA HUMAN	591.285155	2	0.81392926	2	Yes
AAGCDFTNVVK	21.03508	P52758	RIDA HUMAN	394.526045	3	0.81392926	2	
AAYQVAALPK	20.50066	P52758	RIDA HUMAN	516.298387	2	0.779733419	2	Yes
AAYQVAALPK	20.50066	P52758	RIDA HUMAN	344.5348663	3	0.779733419	2	
APGAIGPYSQAVLVDR	50.98875	P52758	RIDA HUMAN	807.4364745	2	0.8605178	2	Yes
APGAIGPYSQAVLVDR	50.98875	P52758	RIDA HUMAN	538.6269247	3	0.8605178	2	
TTVLLADINDFNTVNEIYK	102.8151	P52758	RIDA HUMAN	1092.065704	2	0.812648177	3	
TTVLLADINDFNTVNEIYK	102.8151	P52758	RIDA HUMAN	728.379744	3	0.812648177	3	Yes
AGFDSDYDQALADIR	65.10346	P52701	MSH6 HUMAN	828.879186	2	0.880431294	2	Yes
AGFDSDYDQALADIR	65.10346	P52701	MSH6 HUMAN	552.9220657	3	0.880431294	2	
AIMYEETTYSK	13.18961	P52701	MSH6 HUMAN	668.3110265	2	0.801614046	2	Yes
AIMYEETTYSK	13.18961	P52701	MSH6 HUMAN	445.876626	3	0.801614046	2	
AYGVCFVDTSLGK	57.16817	P52701	MSH6 HUMAN	708.8455725	2	0.734020591	2	Yes
AYGVCFVDTSLGK	57.16817	P52701	MSH6 HUMAN	472.8996567	3	0.734020591	2	
EEGSSDEISSGVGDSESEGLNSPVK	33.22768	P52701	MSH6 HUMAN	1248.049364	2	0.621922731		
EEGSSDEISSGVGDSESEGLNSPVK	33.22768	P52701	MSH6 HUMAN	832.368851	3	0.621922731		
ENEQSLLEYLEK	72.09358	P52701	MSH6 HUMAN	747.8702975	2	0.779965281	2	Yes
ENEQSLLEYLEK	72.09358	P52701	MSH6 HUMAN	498.91614	3	0.779965281	2	
FFIGQFSDDR	60.1163	P52701	MSH6 HUMAN	616.291294	2	0.791053653	2	Yes
FFIGQFSDDR	60.1163	P52701	MSH6 HUMAN	411.1968043	3	0.791053653	2	
FPDLTVELNR	64.27765	P52701	MSH6 HUMAN	602.322594	2	0.808636606	2	Yes
FPDLTVELNR	64.27765	P52701	MSH6 HUMAN	401.8843377	3	0.808636606	2	
GGHFYSAKPEILR	5.721832	P52701	MSH6 HUMAN	737.894245	2	0.701571703	4	
GGHFYSAKPEILR	5.721832	P52701	MSH6 HUMAN	492.2654383	3	0.701571703	4	
GMTSESDSIGLTPGEK	23.68439	P52701	MSH6 HUMAN	804.875259	2	0.833396256	2	Yes
GMTSESDSIGLTPGEK	23.68439	P52701	MSH6 HUMAN	536.9194477	3	0.833396256	2	
GNWAHSGFPEIAFGR	54.54385	P52701	MSH6 HUMAN	823.3976875	2	0.839729786	3	
GNWAHSGFPEIAFGR	54.54385	P52701	MSH6 HUMAN	549.2677333	3	0.839729786	3	Yes
GTATFDGTAIANAVVK	50.47159	P52701	MSH6 HUMAN	768.4073875	2	0.875148952	2	Yes
GTATFDGTAIANAVVK	50.47159	P52701	MSH6 HUMAN	512.6075333	3	0.875148952	2	
GTQTYSVLEGDPSENYSK	35.78251	P52701	MSH6 HUMAN	987.950532	2	0.736739993		
GTQTYSVLEGDPSENYSK	35.78251	P52701	MSH6 HUMAN	658.9696297	3	0.736739993		
IIDFLSALEGFK	140.6219	P52701	MSH6 HUMAN	676.877202	2	0.795865476	2	Yes
IIDFLSALEGFK	140.6219	P52701	MSH6 HUMAN	451.5874097	3	0.795865476	2	
IIGIMEEVADGFK	92.0979	P52701	MSH6 HUMAN	711.371427	2	0.793204904	2	Yes
IIGIMEEVADGFK	92.0979	P52701	MSH6 HUMAN	474.5835597	3	0.793204904	2	
ISEVVELLK	56.53854	P52701	MSH6 HUMAN	515.313706	2	0.772615373	2	Yes
ISEVVELLK	56.53854	P52701	MSH6 HUMAN	343.8784123	3	0.772615373	2	
LANLINAEER	22.95467	P52701	MSH6 HUMAN	571.812393	2	0.761164129	2	Yes
LANLINAEER	22.95467	P52701	MSH6 HUMAN	381.5442037	3	0.761164129	2	
LANLPEEVIQK	39.89668	P52701	MSH6 HUMAN	627.3591765	2	0.861507654	2	Yes
LANLPEEVIQK	39.89668	P52701	MSH6 HUMAN	418.5753927	3	0.861507654	2	
LDAIEDLMVVPDK	92.95595	P52701	MSH6 HUMAN	729.3819915	2	0.875255346	2	Yes
LDAIEDLMVVPDK	92.95595	P52701	MSH6 HUMAN	486.5906027	3	0.875255346	2	
LFYNFDK	34.00109	P52701	MSH6 HUMAN	473.73763	2	0.613189876	2	Yes
LFYNFDK	34.00109	P52701	MSH6 HUMAN	316.1610283	3	0.613189876	2	
LSDGIGVMLPQVLK	93.43021	P52701	MSH6 HUMAN	735.423994	2	0.74326396	2	Yes
LSDGIGVMLPQVLK	93.43021	P52701	MSH6 HUMAN	490.6186043	3	0.74326396	2	
NLPEEYELK	23.81358	P52701	MSH6 HUMAN	567.788048	2	0.848475933	2	Yes
NLPEEYELK	23.81358	P52701	MSH6 HUMAN	378.861307	3	0.848475933	2	
SVAPAAPTSCDFSPGDLVWAK	81.88124	P52701	MSH6 HUMAN	1088.523154	2	0.727410316	3	
SVAPAAPTSCDFSPGDLVWAK	81.88124	P52701	MSH6 HUMAN	726.018044	3	0.727410316	3	Yes
TLLEEEYFR	50.56032	P52701	MSH6 HUMAN	600.301323	2	0.815668225	2	Yes
TLLEEEYFR	50.56032	P52701	MSH6 HUMAN	400.5368237	3	0.815668225	2	
TLVAHYPPVQVLFEK	64.12589	P52701	MSH6 HUMAN	870.988343	2	0.773098469		
TLVAHYPPVQVLFEK	64.12589	P52701	MSH6 HUMAN	580.994837	3	0.773098469		
VEQTETPEMMEAR	13.00101	P52701	MSH6 HUMAN	775.845448	2	0.840362787		
VEQTETPEMMEAR	13.00101	P52701	MSH6 HUMAN	517.5662403	3	0.840362787		
VHVQFFDDSPTR	27.3265	P52701	MSH6 HUMAN	724.3524145	2	0.76532954	3	
VHVQFFDDSPTR	27.3265	P52701	MSH6 HUMAN	483.2375513	3	0.76532954	3	Yes
VISDSESDIGGSDVEFKPDTK	33.47649	P52701	MSH6 HUMAN	1113.026976	2	0.748034596	3	
VISDSESDIGGSDVEFKPDTK	33.47649	P52701	MSH6 HUMAN	742.3539257	3	0.748034596	3	Yes
YQLEIPENFTTR	62.18228	P52701	MSH6 HUMAN	755.8810005	2	0.849707603	2	Yes
YQLEIPENFTTR	62.18228	P52701	MSH6 HUMAN	504.2566087	3	0.849707603	2	
ACISIGNQNFEVK	39.3316	P52564	MP2K6 HUMAN	740.367207	2	0.815597773		
ACISIGNQNFEVK	39.3316	P52564	MP2K6 HUMAN	493.9140797	3	0.815597773		
ADDLEPIMELGR	69.89508	P52564	MP2K6 HUMAN	679.8352085	2	0.816631377	2	Yes
ADDLEPIMELGR	69.89508	P52564	MP2K6 HUMAN	453.559414	3	0.816631377	2	
DVKPSNVLINALGQVK	70.15924	P52564	MP2K6 HUMAN	847.99416	2	0.845893621	3	
DVKPSNVLINALGQVK	70.15924	P52564	MP2K6 HUMAN	565.6653817	3	0.845893621	3	Yes
EAFEQPQTSSTPPR	4.134136	P52564	MP2K6 HUMAN	787.876451	2	0.759856999	2	Yes
EAFEQPQTSSTPPR	4.134136	P52564	MP2K6 HUMAN	525.586909	3	0.759856999	2	
ERPTYPELMQHPFFTLHESK	44.16535	P52564	MP2K6 HUMAN	1244.110455	2	0.753194809	4	
ERPTYPELMQHPFFTLHESK	44.16535	P52564	MP2K6 HUMAN	829.7429113	3	0.753194809	4	
FPYDSWGTPFQQLK	85.11755	P52564	MP2K6 HUMAN	857.4177505	2	0.781665802		
FPYDSWGTPFQQLK	85.11755	P52564	MP2K6 HUMAN	571.9477753	3	0.781665802		
FSAEFVDFTSQCLK	77.75233	P52564	MP2K6 HUMAN	839.893255	2	0.788128614		
FSAEFVDFTSQCLK	77.75233	P52564	MP2K6 HUMAN	560.2647783	3	0.788128614		
GQTIPEDILGK	44.45207	P52564	MP2K6 HUMAN	585.8224275	2	0.830715299	2	Yes
GQTIPEDILGK	44.45207	P52564	MP2K6 HUMAN	390.8842267	3	0.830715299	2	
HVPSGQIMAVK	-6.873314	P52564	MP2K6 HUMAN	583.8217075	2	0.758706093		
HVPSGQIMAVK	-6.873314	P52564	MP2K6 HUMAN	389.5504133	3	0.758706093		
LLMDLDISMR	82.25111	P52564	MP2K6 HUMAN	603.815233	2	0.652168691		
LLMDLDISMR	82.25111	P52564	MP2K6 HUMAN	402.8794303	3	0.652168691		
ADILEDKDGK	-20.38612	P52272	HNRPM HUMAN	552.2831345	2	0.801562071	2	Yes
ADILEDKDGK	-20.38612	P52272	HNRPM HUMAN	368.524698	3	0.801562071	2	

AFITNIPFDVK	79.50117	P52272	HNRPM HUMAN	632.8509875	2	0.854815364	2	Yes
AFITNIPFDVK	79.50117	P52272	HNRPM HUMAN	422.2366	3	0.854815364	2	
EVFSMAGVVVR	58.73282	P52272	HNRPM HUMAN	597.32154	2	0.690722764	2	Yes
EVFSMAGVVVR	58.73282	P52272	HNRPM HUMAN	398.5503017	3	0.690722764	2	
FGSGMNMGR	1.455391	P52272	HNRPM HUMAN	478.7080285	2	0.618254185	2	Yes
FGSGMNMGR	1.455391	P52272	HNRPM HUMAN	319.4746273	3	0.618254185	2	
FNECGHVLYADIK	25.00174	P52272	HNRPM HUMAN	783.375028	2	0.724099636	3	
FNECGHVLYADIK	25.00174	P52272	HNRPM HUMAN	522.5859603	3	0.724099636	3	Yes
[illegible]FFPPERPQQLPHGLGGIGMGLGPGGQPIDANHL[illegible]	71.90259	P52272	HNRPM HUMAN	1909.963376	2	0.703574181	4	
[illegible]FFPPERPQQLPHGLGGIGMGLGPGGQPIDANHL[illegible]	71.90259	P52272	HNRPM HUMAN	1273.644859	3	0.703574181	4	
GIGMGNIGPAGMGMEGIGFGINK	97.0499	P52272	HNRPM HUMAN	1089.529402	2	0.816257954	3	
GIGMGNIGPAGMGMEGIGFGINK	97.0499	P52272	HNRPM HUMAN	726.6888763	3	0.816257954	3	Yes
GNFGGSFAGSFGGAGGHAPGVAR	35.80299	P52272	HNRPM HUMAN	1017.980637	2	0.749588788	3	
GNFGGSFAGSFGGAGGHAPGVAR	35.80299	P52272	HNRPM HUMAN	678.9896993	3	0.749588788	3	Yes
INEILSNALK	39.09532	P52272	HNRPM HUMAN	557.8275115	2	0.710564315		
INEILSNALK	39.09532	P52272	HNRPM HUMAN	372.2209493	3	0.710564315		
LGGAGMER	-26.69829	P52272	HNRPM HUMAN	395.697987	2	0.678928375	2	Yes
LGGAGMER	-26.69829	P52272	HNRPM HUMAN	264.1345997	3	0.678928375	2	
LGSTVFVANLDYK	62.0945	P52272	HNRPM HUMAN	713.8830115	2	0.793584406	2	Yes
LGSTVFVANLDYK	62.0945	P52272	HNRPM HUMAN	476.2579493	3	0.793584406	2	
MGAGMGFGLER	41.90176	P52272	HNRPM HUMAN	563.2631685	2	0.801821589	2	Yes
MGAGMGFGLER	41.90176	P52272	HNRPM HUMAN	375.8447207	3	0.801821589	2	
MGANNLER	-22.92445	P52272	HNRPM HUMAN	452.71945	2	0.690706372		
MGANNLER	-22.92445	P52272	HNRPM HUMAN	302.1489083	3	0.690706372		
MGGMEGPFGGGMENMGR	53.50647	P52272	HNRPM HUMAN	857.8444025	2	0.818417907	2	Yes
MGGMEGPFGGGMENMGR	53.50647	P52272	HNRPM HUMAN	572.23221	3	0.818417907	2	
MGLAMGGGGGASFDR	35.55199	P52272	HNRPM HUMAN	692.3113785	2	0.781559289	2	Yes
MGLAMGGGGGASFDR	35.55199	P52272	HNRPM HUMAN	461.8768607	3	0.781559289	2	
MGLSMER	6.038498	P52272	HNRPM HUMAN	412.1942225	2	0.655175865	2	Yes
MGLSMER	6.038498	P52272	HNRPM HUMAN	275.13209	3	0.655175865	2	
MGPAMGPALGAGIER	42.59888	P52272	HNRPM HUMAN	714.3608715	2	0.842429638	2	Yes
MGPAMGPALGAGIER	42.59888	P52272	HNRPM HUMAN	476.5765227	3	0.842429638	2	
MGPLGLDHMASSIER	51.24601	P52272	HNRPM HUMAN	807.392899	2	0.887948334	3	
MGPLGLDHMASSIER	51.24601	P52272	HNRPM HUMAN	538.5978743	3	0.887948334	3	Yes
MVPAGMGAGLER	26.8386	P52272	HNRPM HUMAN	594.7973755	2	0.783008456	2	Yes
MVPAGMGAGLER	26.8386	P52272	HNRPM HUMAN	396.8675253	3	0.783008456	2	
NLPFDFTWK	87.30171	P52272	HNRPM HUMAN	584.295848	2	0.80762291	2	Yes
NLPFDFTWK	87.30171	P52272	HNRPM HUMAN	389.866507	3	0.80762291	2	
AFNFLNEIK	66.85172	P51809	VAMP7 HUMAN	548.2958475	2	0.841625571	2	Yes
AFNFLNEIK	66.85172	P51809	VAMP7 HUMAN	365.8665067	3	0.841625571	2	
HAWCGGNFLEVTEQILAK	87.20737	P51809	VAMP7 HUMAN	1037.015299	2	0.700232029	3	
HAWCGGNFLEVTEQILAK	87.20737	P51809	VAMP7 HUMAN	691.6794743	3	0.700232029	3	Yes
LTYSHGNYLFHYICQDR	42.63717	P51809	VAMP7 HUMAN	1094.007992	2	0.819839954	3	
LTYSHGNYLFHYICQDR	42.63717	P51809	VAMP7 HUMAN	729.674603	3	0.819839954	3	Yes
NIDLVAQR	14.90591	P51809	VAMP7 HUMAN	464.764715	2	0.687618315	2	Yes
NIDLVAQR	14.90591	P51809	VAMP7 HUMAN	310.179085	3	0.687618315	2	
TENLVDSSVTFK	36.87969	P51809	VAMP7 HUMAN	670.3411805	2	0.83534807	2	Yes
TENLVDSSVTFK	36.87969	P51809	VAMP7 HUMAN	447.230062	3	0.83534807	2	
VMETQAQVDELK	17.17542	P51809	VAMP7 HUMAN	695.8483165	2	0.833930612	2	Yes
VMETQAQVDELK	17.17542	P51809	VAMP7 HUMAN	464.2348193	3	0.833930612	2	
DILTAIAADLCK	113.8043	P51648	AL3A2 HUMAN	652.350495	2	0.735355139		
DILTAIAADLCK	113.8043	P51648	AL3A2 HUMAN	435.2362717	3	0.735355139		
EFYGENIK	9.997749	P51648	AL3A2 HUMAN	500.2432765	2	0.755317688	2	Yes
EFYGENIK	9.997749	P51648	AL3A2 HUMAN	333.8314593	3	0.755317688	2	
EKPLALYVFSHNHK	14.97298	P51648	AL3A2 HUMAN	841.9548335	2	0.722091973	3	
EKPLALYVFSHNHK	14.97298	P51648	AL3A2 HUMAN	561.639164	3	0.722091973	3	Yes
FDHIFYTGNTAVGK	31.04494	P51648	AL3A2 HUMAN	785.388993	2	0.841425002	2	Yes
FDHIFYTGNTAVGK	31.04494	P51648	AL3A2 HUMAN	523.9286037	3	0.841425002	2	
IAFGGETDEATR	12.78542	P51648	AL3A2 HUMAN	633.8022235	2	0.811614513	2	Yes
IAFGGETDEATR	12.78542	P51648	AL3A2 HUMAN	422.8707573	3	0.811614513	2	
ILSLLEGQK	43.37402	P51648	AL3A2 HUMAN	500.8060485	2	0.786518574	2	Yes
ILSLLEGQK	43.37402	P51648	AL3A2 HUMAN	334.2066407	3	0.786518574	2	
LQQLEALR	14.85968	P51648	AL3A2 HUMAN	485.7881905	2	0.745764017	2	Yes
LQQLEALR	14.85968	P51648	AL3A2 HUMAN	324.1947353	3	0.745764017	2	
NVDEAINFINER	55.31206	P51648	AL3A2 HUMAN	717.355153	2	0.79879576		
NVDEAINFINER	55.31206	P51648	AL3A2 HUMAN	478.5727103	3	0.79879576		
VMQEEIFGPILPIVPVK	128.4627	P51648	AL3A2 HUMAN	955.0475475	2	0.85591805		
VMQEEIFGPILPIVPVK	128.4627	P51648	AL3A2 HUMAN	637.0343067	3	0.85591805		
YIAPTVLTDVDPK	55.78238	P51648	AL3A2 HUMAN	716.39067	2	0.850307345		
YIAPTVLTDVDPK	55.78238	P51648	AL3A2 HUMAN	477.9297217	3	0.850307345		
APVTVTSLPAGVR	39.85941	P51610	HCFC1 HUMAN	634.372619	2	0.781533778	2	Yes
APVTVTSLPAGVR	39.85941	P51610	HCFC1 HUMAN	423.251021	3	0.781533778	2	
AWNNQVCCKDLWYLETEKPPPPAR	53.4846	P51610	HCFC1 HUMAN	1486.713281	2	0.694775522		
AWNNQVCCKDLWYLETEKPPPPAR	53.4846	P51610	HCFC1 HUMAN	991.478129	3	0.694775522		
ENQWFDVGVIK	74.25003	P51610	HCFC1 HUMAN	667.841151	2	0.780119777	2	Yes
ENQWFDVGVIK	74.25003	P51610	HCFC1 HUMAN	445.5633757	3	0.780119777	2	
GAPGQPGTILR	12.04782	P51610	HCFC1 HUMAN	533.8043725	2	0.788788557	2	Yes
GAPGQPGTILR	12.04782	P51610	HCFC1 HUMAN	356.2055233	3	0.788788557	2	
GPFSEISAFK	50.03933	P51610	HCFC1 HUMAN	541.7800305	2	0.808453977	2	Yes
GPFSEISAFK	50.03933	P51610	HCFC1 HUMAN	361.5226287	3	0.808453977	2	
GPLPAGTILK	34.24776	P51610	HCFC1 HUMAN	483.8033095	2	0.777503848	2	Yes
GPLPAGTILK	34.24776	P51610	HCFC1 HUMAN	322.8714813	3	0.777503848	2	
[illegible]GTNVMVTHYFLPPDDAVPSDDDLGTVPDYNQL[illegible]	91.75847	P51610	HCFC1 HUMAN	1817.356865	2	0.645356715		
[illegible]GTNVMVTHYFLPPDDAVPSDDDLGTVPDYNQL[illegible]	91.75847	P51610	HCFC1 HUMAN	1211.907185	3	0.645356715		
IATGHGQQGVTQVVLK	8.667164	P51610	HCFC1 HUMAN	818.463029	2	0.80154264	2	Yes
IATGHGQQGVTQVVLK	8.667164	P51610	HCFC1 HUMAN	545.977961	3	0.80154264	2	
IPPSSAPTVLSVPAGTTIVK	70.50319	P51610	HCFC1 HUMAN	968.0622405	2	0.823019385	3	
IPPSSAPTVLSVPAGTTIVK	70.50319	P51610	HCFC1 HUMAN	645.7107687	3	0.823019385	3	Yes
ISVATGALEAAQGSK	31.48453	P51610	HCFC1 HUMAN	701.881005	2	0.738506496	2	Yes
ISVATGALEAAQGSK	31.48453	P51610	HCFC1 HUMAN	468.2566117	3	0.738506496	2	
LLVFGGMVEYGK	77.08163	P51610	HCFC1 HUMAN	656.852669	2	0.79963702		
LLVFGGMVEYGK	77.08163	P51610	HCFC1 HUMAN	438.237721	3	0.79963702		
LVIYGGMSGCR	30.38046	P51610	HCFC1 HUMAN	606.797371	2	0.748770595		
LVIYGGMSGCR	30.38046	P51610	HCFC1 HUMAN	404.8675223	3	0.748770595		
LVTPVTVSAVKPAVTTLVVK	66.12371	P51610	HCFC1 HUMAN	1011.632834	2	0.772673666		
LVTPVTVSAVKPAVTTLVVK	66.12371	P51610	HCFC1 HUMAN	674.7578307	3	0.772673666		
DGKPTTIITTTQASGAGTKPTILGISSVSPSTTKPG	58.45006	P51610	HCFC1 HUMAN	2300.271321	2	0.629719913		
DGKPTTIITTTQASGAGTKPTILGISSVSPSTTKPG	58.45006	P51610	HCFC1 HUMAN	1533.850155	3	0.629719913		
SGTVTVAQQAQVVTTVVGGVTK	81.38208	P51610	HCFC1 HUMAN	1065.592427	2	0.695695996		
SGTVTVAQQAQVVTTVVGGVTK	81.38208	P51610	HCFC1 HUMAN	710.7308927	3	0.695695996		
SPAFVQLAPLSSK	60.95094	P51610	HCFC1 HUMAN	672.880276	2	0.823923349		

SPAFVQLAPLSSK	60.95094	P51610	HCFC1 HUMAN	448.9227923	3	0.823923349		
SPDGAHLTWEPPSVTSGK	37.86879	P51610	HCFC1 HUMAN	933.4555965	2	0.827419519		
SPDGAHLTWEPPSVTSGK	37.86879	P51610	HCFC1 HUMAN	622.6396727	3	0.827419519		
SPISVPGGSALISNLGK	69.37057	P51610	HCFC1 HUMAN	798.9519605	2	0.768983662		
SPISVPGGSALISNLGK	69.37057	P51610	HCFC1 HUMAN	532.970582	3	0.768983662		
SSVGAGEPR	-48.19242	P51610	HCFC1 HUMAN	430.2175975	2	0.733573139		
SSVGAGEPR	-48.19242	P51610	HCFC1 HUMAN	287.1476733	3	0.733573139		
TAAAQVGTSVSSATNTSTRPIITVHK	19.36752	P51610	HCFC1 HUMAN	1299.696473	2	0.66868645		
TAAAQVGTSVSSATNTSTRPIITVHK	19.36752	P51610	HCFC1 HUMAN	866.800257	3	0.66868645		
TCLPGFPGAPCAIK	58.42616	P51610	HCFC1 HUMAN	744.8710725	2	0.843147695		
TCLPGFPGAPCAIK	58.42616	P51610	HCFC1 HUMAN	496.9166567	3	0.843147695		
TIPMSAIITQAGATGVTSSPGIK	77.67496	P51610	HCFC1 HUMAN	1101.096487	2	0.746358454		
TIPMSAIITQAGATGVTSSPGIK	77.67496	P51610	HCFC1 HUMAN	734.400266	3	0.746358454		
TMAVTPGTTTLPATVK	39.06763	P51610	HCFC1 HUMAN	794.9349245	2	0.834793806		
TMAVTPGTTTLPATVK	39.06763	P51610	HCFC1 HUMAN	530.292558	3	0.834793806		
TVESGLEVAAAPSVTPQAGTALLAPFPTQR	105.6431	P51610	HCFC1 HUMAN	1490.293478	2	0.793352604		
TVESGLEVAAAPSVTPQAGTALLAPFPTQR	105.6431	P51610	HCFC1 HUMAN	993.864927	3	0.793352604		
VASSPVMVSNPATR	13.472	P51610	HCFC1 HUMAN	708.3697495	2	0.792822301	2	Yes
VASSPVMVSNPATR	13.472	P51610	HCFC1 HUMAN	472.5824413	3	0.792822301	2	
VMTSGTGAPAK	-28.93322	P51610	HCFC1 HUMAN	510.2636915	2	0.650536299		
VMTSGTGAPAK	-28.93322	P51610	HCFC1 HUMAN	340.511736	3	0.650536299		
VTGPQATTGTPLVTMR	38.01437	P51610	HCFC1 HUMAN	815.435623	2	0.857165217		
VTGPQATTGTPLVTMR	38.01437	P51610	HCFC1 HUMAN	543.9596903	3	0.857165217		
YDIPATAATATSPTPNPVPSVPANPPK	66.65201	P51610	HCFC1 HUMAN	1337.690321	2	0.640057504		
YDIPATAATATSPTPNPVPSVPANPPK	66.65201	P51610	HCFC1 HUMAN	892.1294887	3	0.640057504		
YSNDLYELQASR	33.75822	P51610	HCFC1 HUMAN	729.8471525	2	0.909969151	2	Yes
YSNDLYELQASR	33.75822	P51610	HCFC1 HUMAN	486.90071	3	0.909969151	2	
DFMGCQEHVEPGPR	15.8924	P51511	MMP15 HUMAN	829.8566815	2	0.727269411		
DFMGCQEHVEPGPR	15.8924	P51511	MMP15 HUMAN	553.5737293	3	0.727269411		
EANLEPGYPQPLTSYGLGIPYDR	93.59627	P51511	MMP15 HUMAN	1275.629723	2	0.746652603		
EANLEPGYPQPLTSYGLGIPYDR	93.59627	P51511	MMP15 HUMAN	850.7557567	3	0.746652603		
FYGIPVTGVLDEETK	82.97743	P51511	MMP15 HUMAN	834.4305235	2	0.79106307	2	Yes
FYGIPVTGVLDEETK	82.97743	P51511	MMP15 HUMAN	556.6229573	3	0.79106307	2	
GAFLSNDAAYTYFYK	69.06291	P51511	MMP15 HUMAN	865.9072065	2	0.888815641		
GAFLSNDAAYTYFYK	69.06291	P51511	MMP15 HUMAN	577.6074127	3	0.888815641		
GDPGYPKPISVWQGIPASPK	61.24876	P51511	MMP15 HUMAN	1047.55511	2	0.624847293		
GDPGYPKPISVWQGIPASPK	61.24876	P51511	MMP15 HUMAN	698.7060147	3	0.624847293		
GIQQLYGTPDGQPQPTQPLPTVTPR	66.76653	P51511	MMP15 HUMAN	1345.201389	2	0.870381951		
GIQQLYGTPDGQPQPTQPLPTVTPR	66.76653	P51511	MMP15 HUMAN	897.1368673	3	0.870381951		
GLPGDISAAYER	32.88068	P51511	MMP15 HUMAN	624.815129	2	0.814883471	2	Yes
GLPGDISAAYER	32.88068	P51511	MMP15 HUMAN	416.879361	3	0.814883471	2	
IDTAIWWEPTGHTFFFQEDR	110.8486	P51511	MMP15 HUMAN	1248.585132	2	0.617651403		
IDTAIWWEPTGHTFFFQEDR	110.8486	P51511	MMP15 HUMAN	832.7260293	3	0.617651403		
LGWYHSMEAVR	25.79794	P51511	MMP15 HUMAN	674.8275165	2	0.791781604		
LGWYHSMEAVR	25.79794	P51511	MMP15 HUMAN	450.2209527	3	0.791781604		
LYGYLPQPSR	32.29057	P51511	MMP15 HUMAN	597.319846	2	0.683235168	2	Yes
LYGYLPQPSR	32.29057	P51511	MMP15 HUMAN	398.5491723	3	0.683235168	2	
SAQILASALAEMQR	66.77139	P51511	MMP15 HUMAN	744.896132	2	0.835285723		
SAQILASALAEMQR	66.77139	P51511	MMP15 HUMAN	496.933363	3	0.835285723		
VLDNYPMPIGHFWR	75.14417	P51511	MMP15 HUMAN	872.935587	2	0.813894272	3	
VLDNYPMPIGHFWR	75.14417	P51511	MMP15 HUMAN	582.2929997	3	0.813894272	3	Yes
VVVQMEEVAR	22.6815	P51511	MMP15 HUMAN	580.3111725	2	0.796177626		
VVVQMEEVAR	22.6815	P51511	MMP15 HUMAN	387.2100567	3	0.796177626		
VWEQATPLVFQEVPYEDIR	101.1205	P51511	MMP15 HUMAN	1160.086971	2	0.721637547	3	
VWEQATPLVFQEVPYEDIR	101.1205	P51511	MMP15 HUMAN	773.7272557	3	0.721637547	3	Yes
AANVLITR	6.054897	P50750	CDK9 HUMAN	429.264351	2	0.757995546		
AANVLITR	6.054897	P50750	CDK9 HUMAN	286.5121757	3	0.757995546		
DPYALDLIDK	72.37526	P50750	CDK9 HUMAN	581.8036985	2	0.749220014		
DPYALDLIDK	72.37526	P50750	CDK9 HUMAN	388.205074	3	0.749220014		
GMLSTHLTSMFEYLAPPR	92.52409	P50750	CDK9 HUMAN	1026.008624	2	0.764794469		
GMLSTHLTSMFEYLAPPR	92.52409	P50750	CDK9 HUMAN	684.3416907	3	0.764794469		
HENVVNLIEICR	44.32218	P50750	CDK9 HUMAN	748.3884735	2	0.69049418		
HENVVNLIEICR	44.32218	P50750	CDK9 HUMAN	499.2615907	3	0.69049418		
IDSDDALNHDFFWSDPMPSDLK	95.1104	P50750	CDK9 HUMAN	1283.06611	2	0.731654525	3	
IDSDDALNHDFFWSDPMPSDLK	95.1104	P50750	CDK9 HUMAN	855.713348	3	0.731654525	3	Yes
IGQGTFGEVFK	43.65757	P50750	CDK9 HUMAN	591.811863	2	0.890933752		
IGQGTFGEVFK	43.65757	P50750	CDK9 HUMAN	394.8771837	3	0.890933752		
LLVLDPAQR	41.94851	P50750	CDK9 HUMAN	512.8116655	2	0.772555888	2	Yes
LLVLDPAQR	41.94851	P50750	CDK9 HUMAN	342.2103853	3	0.772555888	2	
NPATTNQTEFER	-9.038235	P50750	CDK9 HUMAN	704.3291365	2	0.790869296	2	Yes
NPATTNQTEFER	-9.038235	P50750	CDK9 HUMAN	469.8886993	3	0.790869296	2	
VLMENEK	-22.9684	P50750	CDK9 HUMAN	431.721127	2	0.627188087		
VLMENEK	-22.9684	P50750	CDK9 HUMAN	288.1500263	3	0.627188087		
VVTLWYRPPELLLGER	89.77136	P50750	CDK9 HUMAN	971.0520055	2	0.765341401		
VVTLWYRPPELLLGER	89.77136	P50750	CDK9 HUMAN	647.7039453	3	0.765341401		
ALEMVPLLTSTK	75.54455	P50748	KNTC1 HUMAN	651.871063	2	0.812878132	2	Yes
ALEMVPLLTSTK	75.54455	P50748	KNTC1 HUMAN	434.9166503	3	0.812878132	2	
AQWITYETTQEMLNYAK	80.63754	P50748	KNTC1 HUMAN	1045.499139	2	0.789153576		
AQWITYETTQEMLNYAK	80.63754	P50748	KNTC1 HUMAN	697.3353677	3	0.789153576		
AWQNYDK	-17.44597	P50748	KNTC1 HUMAN	462.7146865	2	0.692761958		
AWQNYDK	-17.44597	P50748	KNTC1 HUMAN	308.8123993	3	0.692761958		
DIFLQLK	65.15441	P50748	KNTC1 HUMAN	438.7636525	2	0.800083876		
DIFLQLK	65.15441	P50748	KNTC1 HUMAN	292.8450433	3	0.800083876		
DLVISLSGILHK	79.65581	P50748	KNTC1 HUMAN	647.8906435	2	0.744078338		
DLVISLSGILHK	79.65581	P50748	KNTC1 HUMAN	432.2630373	3	0.744078338		
DVFENLWK	80.08437	P50748	KNTC1 HUMAN	525.766923	2	0.643267989		
DVFENLWK	80.08437	P50748	KNTC1 HUMAN	350.8472237	3	0.643267989		
DYQNTEEVCQLR	20.12581	P50748	KNTC1 HUMAN	777.846832	2	0.788434744	2	Yes
DYQNTEEVCQLR	20.12581	P50748	KNTC1 HUMAN	518.9004963	3	0.788434744	2	
EIAEVNEINLEK	38.9453	P50748	KNTC1 HUMAN	700.867562	2	0.826812625		
EIAEVNEINLEK	38.9453	P50748	KNTC1 HUMAN	467.580983	3	0.826812625		
ELSTDAQWGIR	38.88367	P50748	KNTC1 HUMAN	638.320583	2	0.774082541		
ELSTDAQWGIR	38.88367	P50748	KNTC1 HUMAN	425.882997	3	0.774082541		
FLLVGER	33.1914	P50748	KNTC1 HUMAN	417.2481695	2	0.744945526	2	Yes
FLLVGER	33.1914	P50748	KNTC1 HUMAN	278.501388	3	0.744945526	2	
FSGSSWIEFLNNEDDLK	105.99	P50748	KNTC1 HUMAN	1000.965993	2	0.767513275		
FSGSSWIEFLNNEDDLK	105.99	P50748	KNTC1 HUMAN	667.6466033	3	0.767513275		
FSLDTLYVSTAK	66.07573	P50748	KNTC1 HUMAN	672.8564625	2	0.758101642		
FSLDTLYVSTAK	66.07573	P50748	KNTC1 HUMAN	448.9069167	3	0.758101642		
HKPGSTPEPIAAEVR	-19.50617	P50748	KNTC1 HUMAN	794.926278	2	0.780587494		
HKPGSTPEPIAAEVR	-19.50617	P50748	KNTC1 HUMAN	530.2867937	3	0.780587494		

IQNSSGTDYPDIHAAAK	6.867565	P50748	KNTC1 HUMAN	894.432117	2	0.688670039		
IQNSSGTDYPDIHAAAK	6.867565	P50748	KNTC1 HUMAN	596.6240197	3	0.688670039		
IQQAIENVDFSTAK	37.98231	P50748	KNTC1 HUMAN	782.404844	2	0.804833889	2	Yes
IQQAIENVDFSTAK	37.98231	P50748	KNTC1 HUMAN	521.939171	3	0.804833889	2	
ITNININQALSILK	93.47763	P50748	KNTC1 HUMAN	777.964871	2	0.815155566		
ITNININQALSILK	93.47763	P50748	KNTC1 HUMAN	518.979189	3	0.815155566		
LALQEEPDHSK	-21.4659	P50748	KNTC1 HUMAN	633.8204155	2	0.796544313		
LALQEEPDHSK	-21.4659	P50748	KNTC1 HUMAN	422.8828853	3	0.796544313		
LALSDFEK	31.688	P50748	KNTC1 HUMAN	461.748199	2	0.695740819	2	Yes
LALSDFEK	31.688	P50748	KNTC1 HUMAN	308.1680743	3	0.695740819	2	
LALSSVDASEQTEWQQLVDDAK	89.54759	P50748	KNTC1 HUMAN	1217.093183	2	0.812558293	3	
LALSSVDASEQTEWQQLVDDAK	89.54759	P50748	KNTC1 HUMAN	811.7313967	3	0.812558293	3	Yes
LDPYDYEMIEVVLK	126.2223	P50748	KNTC1 HUMAN	863.9345795	2	0.601236045		
LDPYDYEMIEVVLK	126.2223	P50748	KNTC1 HUMAN	576.292328	3	0.601236045		
LFGETTLVK	36.23257	P50748	KNTC1 HUMAN	504.2927745	2	0.735164225	2	Yes
LFGETTLVK	36.23257	P50748	KNTC1 HUMAN	336.5311247	3	0.735164225	2	
LGISFQPVFR	75.21219	P50748	KNTC1 HUMAN	582.332765	2	0.836527288		
LGISFQPVFR	75.21219	P50748	KNTC1 HUMAN	388.557785	3	0.836527288		
LIALTASANK	16.19253	P50748	KNTC1 HUMAN	501.303673	2	0.607290745		
LIALTASANK	16.19253	P50748	KNTC1 HUMAN	334.5383903	3	0.607290745		
LLFLTCQK	44.6172	P50748	KNTC1 HUMAN	511.789345	2	0.632687688		
LLFLTCQK	44.6172	P50748	KNTC1 HUMAN	341.5288383	3	0.632687688		
LLGFNMIPYLR	105.7923	P50748	KNTC1 HUMAN	668.876478	2	0.717613995		
LLGFNMIPYLR	105.7923	P50748	KNTC1 HUMAN	446.2535937	3	0.717613995		
LNTEEYLR	13.82307	P50748	KNTC1 HUMAN	519.267283	2	0.813350797		
LNTEEYLR	13.82307	P50748	KNTC1 HUMAN	346.5141303	3	0.813350797		
LSEDSVSVLVLR	64.22346	P50748	KNTC1 HUMAN	658.87519	2	0.78590554		
LSEDSVSVLVLR	64.22346	P50748	KNTC1 HUMAN	439.5860683	3	0.78590554		
LTTFYGAFGPEK	48.57546	P50748	KNTC1 HUMAN	665.8380735	2	0.872306228	2	Yes
LTTFYGAFGPEK	48.57546	P50748	KNTC1 HUMAN	444.2279907	3	0.872306228	2	
LVTELCLEYK	46.84087	P50748	KNTC1 HUMAN	634.334309	2	0.689810753		
LVTELCLEYK	46.84087	P50748	KNTC1 HUMAN	423.225481	3	0.689810753		
NDVIPFVR	47.33485	P50748	KNTC1 HUMAN	480.269633	2	0.612919986		
NDVIPFVR	47.33485	P50748	KNTC1 HUMAN	320.515697	3	0.612919986		
NFLGSCDPQVILK	60.99686	P50748	KNTC1 HUMAN	745.8877755	2	0.864866972		
NFLGSCDPQVILK	60.99686	P50748	KNTC1 HUMAN	497.5944587	3	0.864866972		
NLIDAEIIK	51.7327	P50748	KNTC1 HUMAN	514.8035055	2	0.800463736		
NLIDAEIIK	51.7327	P50748	KNTC1 HUMAN	343.5382787	3	0.800463736		
QELEAELTLR	40.82567	P50748	KNTC1 HUMAN	601.325334	2	0.631394267		
QELEAELTLR	40.82567	P50748	KNTC1 HUMAN	401.2194977	3	0.631394267		
QTLLTNAFVQK	43.69016	P50748	KNTC1 HUMAN	631.8593445	2	0.692711949		
QTLLTNAFVQK	43.69016	P50748	KNTC1 HUMAN	421.5755047	3	0.692711949		
SGNLHLIHVTSK	-9.03405	P50748	KNTC1 HUMAN	653.367867	2	0.827623844		
SGNLHLIHVTSK	-9.03405	P50748	KNTC1 HUMAN	435.9145197	3	0.827623844		
SGTEAVLIAHK	-6.203289	P50748	KNTC1 HUMAN	563.317312	2	0.776161313		
SGTEAVLIAHK	-6.203289	P50748	KNTC1 HUMAN	375.8808163	3	0.776161313		
SLILNNIINK	53.4492	P50748	KNTC1 HUMAN	571.3511535	2	0.790981054	2	Yes
SLILNNIINK	53.4492	P50748	KNTC1 HUMAN	381.2367107	3	0.790981054	2	
STSLFETAWEAK	56.27939	P50748	KNTC1 HUMAN	685.3358985	2	0.858587384		
STSLFETAWEAK	56.27939	P50748	KNTC1 HUMAN	457.2265407	3	0.858587384		
TALIYSDGLK	31.22645	P50748	KNTC1 HUMAN	540.800959	2	0.775347531		
TALIYSDGLK	31.22645	P50748	KNTC1 HUMAN	360.8699143	3	0.775347531		
TDELGLASSWHWISLK	87.69046	P50748	KNTC1 HUMAN	921.9734245	2	0.706843257	3	
TDELGLASSWHWISLK	87.69046	P50748	KNTC1 HUMAN	614.9848913	3	0.706843257	3	Yes
TVPEGQIILAK	34.35657	P50748	KNTC1 HUMAN	584.850988	2	0.726296902	2	Yes
TVPEGQIILAK	34.35657	P50748	KNTC1 HUMAN	390.2366003	3	0.726296902	2	
TYQNLVIEK	19.39548	P50748	KNTC1 HUMAN	554.3064085	2	0.779682398		
TYQNLVIEK	19.39548	P50748	KNTC1 HUMAN	369.8735473	3	0.779682398		
VAQAFMLSDDEIYSLR	80.75423	P50748	KNTC1 HUMAN	929.456746	2	0.798164248		
VAQAFMLSDDEIYSLR	80.75423	P50748	KNTC1 HUMAN	619.9737723	3	0.798164248		
VIGKPAHLIVSLYEHPSINQR	42.36702	P50748	KNTC1 HUMAN	1186.166421	2	0.684920311		
VIGKPAHLIVSLYEHPSINQR	42.36702	P50748	KNTC1 HUMAN	791.1135553	3	0.684920311		
VLAPELIPSILEK	98.81503	P50748	KNTC1 HUMAN	711.434884	2	0.812122583	2	Yes
VLAPELIPSILEK	98.81503	P50748	KNTC1 HUMAN	474.6258643	3	0.812122583	2	
WLCPSTKPGEKPSELFELQEDEALR	64.12718	P50748	KNTC1 HUMAN	1480.229488	2	0.7115978		
WLCPSTKPGEKPSELFELQEDEALR	64.12718	P50748	KNTC1 HUMAN	987.1556	3	0.7115978		
WLQNIPSQDEK	29.82092	P50748	KNTC1 HUMAN	679.341515	2	0.749421597		
WLQNIPSQDEK	29.82092	P50748	KNTC1 HUMAN	453.230285	3	0.749421597		
DLGTESQIFISR	58.758	P50395	GDIB HUMAN	683.3546225	2	0.87763834	2	Yes
DLGTESQIFISR	58.758	P50395	GDIB HUMAN	455.90569	3	0.87763834	2	
EIRPALELLEPIEQK	69.63787	P50395	GDIB HUMAN	889.5071005	2	0.63120544	3	
EIRPALELLEPIEQK	69.63787	P50395	GDIB HUMAN	593.3406753	3	0.63120544	3	Yes
FLVYVANFDEK	67.3605	P50395	GDIB HUMAN	672.8458975	2	0.718565822	2	Yes
FLVYVANFDEK	67.3605	P50395	GDIB HUMAN	448.8998733	3	0.718565822	2	
FVSISDLLVPK	93.96185	P50395	GDIB HUMAN	609.3611875	2	0.801981688	2	Yes
FVSISDLLVPK	93.96185	P50395	GDIB HUMAN	406.5767333	3	0.801981688	2	
MTGSEFDFEEMK	51.96924	P50395	GDIB HUMAN	725.7974345	2	0.857193649	2	Yes
MTGSEFDFEEMK	51.96924	P50395	GDIB HUMAN	484.200898	3	0.857193649	2	
NPYYGGESASITPLEDLYK	79.03722	P50395	GDIB HUMAN	1059.007846	2	0.737279594	3	
NPYYGGESASITPLEDLYK	79.03722	P50395	GDIB HUMAN	706.341172	3	0.737279594	3	Yes
TDDYLDQPCYETINR	41.01892	P50395	GDIB HUMAN	951.912896	2	0.822779655	3	
TDDYLDQPCYETINR	41.01892	P50395	GDIB HUMAN	634.944539	3	0.822779655	3	Yes
TFEGIDPK	10.07382	P50395	GDIB HUMAN	453.7325495	2	0.70701766	2	Yes
TFEGIDPK	10.07382	P50395	GDIB HUMAN	302.824308	3	0.70701766	2	
TYDATTHFETTCDDIK	14.97665	P50395	GDIB HUMAN	959.412734	2	0.874049485		
TYDATTHFETTCDDIK	14.97665	P50395	GDIB HUMAN	639.944431	3	0.874049485		
VICILSHPIK	25.74075	P50395	GDIB HUMAN	590.3501005	2	0.711605728	3	
VICILSHPIK	25.74075	P50395	GDIB HUMAN	393.9026753	3	0.711605728	3	Yes
VPSTEAEALASSLMGLFEK	126.7785	P50395	GDIB HUMAN	990.5038975	2	0.764208794	3	
VPSTEAEALASSLMGLFEK	126.7785	P50395	GDIB HUMAN	660.6718733	3	0.764208794	3	Yes
VTEGSFVYK	7.60038	P50395	GDIB HUMAN	515.266752	2	0.723742306	2	Yes
VTEGSFVYK	7.60038	P50395	GDIB HUMAN	343.8471097	3	0.723742306	2	
YIAIVSTTVETK	36.44397	P50395	GDIB HUMAN	662.872113	2	0.755730093	2	Yes
YIAIVSTTVETK	36.44397	P50395	GDIB HUMAN	442.2506837	3	0.755730093	2	
DIKPQNLLLDPDTAVLK	78.46362	P49841	GSK3B HUMAN	947.038765	2	0.838328481		
DIKPQNLLLDPDTAVLK	78.46362	P49841	GSK3B HUMAN	631.6951183	3	0.838328481		
IQAAASTPTNATAASDANTGDR	1.340813	P49841	GSK3B HUMAN	1052.499253	2	0.72593677		
IQAAASTPTNATAASDANTGDR	1.340813	P49841	GSK3B HUMAN	702.0021103	3	0.72593677		
LCDSGELVAIK	32.49565	P49841	GSK3B HUMAN	602.8162875	2	0.720347166	2	Yes
LCDSGELVAIK	32.49565	P49841	GSK3B HUMAN	402.2134667	3	0.720347166	2	
LLEYTPTAR	18.57611	P49841	GSK3B HUMAN	532.2933015	2	0.69716686	2	Yes

LLEYTPTAR	18.57611	P49841	GSK3B_HUMAN	355.1981427	3	0.69716686	2	
LTPLEACAHSFFDELR	76.64034	P49841	GSK3B_HUMAN	953.4623675	2	0.746244013		
LTPLEACAHSFFDELR	76.64034	P49841	GSK3B_HUMAN	635.97752	3	0.746244013		
LYMYQLFR	69.95413	P49841	GSK3B_HUMAN	567.2947855	2	0.69295156		
LYMYQLFR	69.95413	P49841	GSK3B_HUMAN	378.5324653	3	0.69295156		
SLAYIHSFGICHR	25.12958	P49841	GSK3B_HUMAN	780.8911795	2	0.68860364	3	
SLAYIHSFGICHR	25.12958	P49841	GSK3B_HUMAN	520.9300613	3	0.68860364	3	Yes
TTSFAESCKPVQQPSAFGSMK	30.92767	P49841	GSK3B_HUMAN	1144.538478	2	0.70866257		
TTSFAESCKPVQQPSAFGSMK	30.92767	P49841	GSK3B_HUMAN	763.3615937	3	0.70866257		
VIGNGSFGVVYQAK	39.60768	P49841	GSK3B_HUMAN	719.8886285	2	0.757340789		
VIGNGSFGVVYQAK	39.60768	P49841	GSK3B_HUMAN	480.261694	3	0.757340789		
VTTVVATPGQGPDRPQEVSYTDTK	23.46607	P49841	GSK3B_HUMAN	1273.640829	2	0.641858518	3	
VTTVVATPGQGPDRPQEVSYTDTK	23.46607	P49841	GSK3B_HUMAN	849.4298273	3	0.641858518	3	Yes
AAVQELSSSILAGEDPEER	61.93228	P49768	PSN1_HUMAN	1000.99274	2	0.638263702	3	
AAVQELSSSILAGEDPEER	61.93228	P49768	PSN1_HUMAN	667.6644347	3	0.638263702	3	Yes
DGQLIYTPFTEDTETVGQR	75.3916	P49768	PSN1_HUMAN	1085.519119	2	0.668960214		
DGQLIYTPFTEDTETVGQR	75.3916	P49768	PSN1_HUMAN	724.0153543	3	0.668960214		
ESQDTVAENDDGGFSEEWEAQR	42.43114	P49768	PSN1_HUMAN	1250.013118	2	0.791909337		
ESQDTVAENDDGGFSEEWEAQR	42.43114	P49768	PSN1_HUMAN	833.67802	3	0.791909337		
AAQSNENLSDSQQEPPK	-14.65197	P49750	YLPM1_HUMAN	921.9273995	2	0.777418137		
AAQSNENLSDSQQEPPK	-14.65197	P49750	YLPM1_HUMAN	614.954208	3	0.777418137		
AIGFVVGQTDWEK	62.78336	P49750	YLPM1_HUMAN	725.372816	2	0.854565084		
AIGFVVGQTDWEK	62.78336	P49750	YLPM1_HUMAN	483.917819	3	0.854565084		
AQAVTQPVPLANKPVPAQSTFPSK	39.35329	P49750	YLPM1_HUMAN	1238.682106	2	0.700413644		
AQAVTQPVPLANKPVPAQSTFPSK	39.35329	P49750	YLPM1_HUMAN	826.1240123	3	0.700413644		
ATQSYLQEK	-16.5508	P49750	YLPM1_HUMAN	534.272566	2	0.639429152	2	Yes
ATQSYLQEK	-16.5508	P49750	YLPM1_HUMAN	356.5176523	3	0.639429152	2	
DAEEEESELGYIPK	47.35937	P49750	YLPM1_HUMAN	804.867952	2	0.66795814		
DAEEEESELGYIPK	47.35937	P49750	YLPM1_HUMAN	536.9145763	3	0.66795814		
DFGSEPQMADHLPPQESR	37.43903	P49750	YLPM1_HUMAN	1020.958178	2	0.684473097		
DFGSEPQMADHLPPQESR	37.43903	P49750	YLPM1_HUMAN	680.9747267	3	0.684473097		
DQLQEYEK	-3.557129	P49750	YLPM1_HUMAN	526.7489235	2	0.74775058		
DQLQEYEK	-3.557129	P49750	YLPM1_HUMAN	351.5018907	3	0.74775058		
DYGRPLDEQESQFR	16.49431	P49750	YLPM1_HUMAN	870.4033595	2	0.743825197		
DYGRPLDEQESQFR	16.49431	P49750	YLPM1_HUMAN	580.604848	3	0.743825197		
DYQDDTLELYNR	47.38536	P49750	YLPM1_HUMAN	772.84735	2	0.805440903		
DYQDDTLELYNR	47.38536	P49750	YLPM1_HUMAN	515.5675083	3	0.805440903		
EFQLWEEQLHSYPHK	44.83331	P49750	YLPM1_HUMAN	985.973952	2	0.745879054		
EFQLWEEQLHSYPHK	44.83331	P49750	YLPM1_HUMAN	657.6519097	3	0.745879054		
EPLADTSSNQQK	-27.29048	P49750	YLPM1_HUMAN	659.318237	2	0.671178579		
EPLADTSSNQQK	-27.29048	P49750	YLPM1_HUMAN	439.881433	3	0.671178579		
EQHLAQLQQLQQMHQK	9.934448	P49750	YLPM1_HUMAN	994.510716	2	0.812425971		
EQHLAQLQQLQQMHQK	9.934448	P49750	YLPM1_HUMAN	663.3430857	3	0.812425971		
GFEVYLAEMSADNQTCGK	67.05392	P49750	YLPM1_HUMAN	1010.443511	2	0.666568398		
GFEVYLAEMSADNQTCGK	67.05392	P49750	YLPM1_HUMAN	673.9649487	3	0.666568398		
GPASQFYITPSTSLSPR	55.03625	P49750	YLPM1_HUMAN	904.963053	2	0.776859581		
GPASQFYITPSTSLSPR	55.03625	P49750	YLPM1_HUMAN	603.6446437	3	0.776859581		
GPKPAFGQQHQQQPK	-31.8035	P49750	YLPM1_HUMAN	838.4373455	2	0.619524717		
GPKPAFGQQHQQQPK	-31.8035	P49750	YLPM1_HUMAN	559.294172	3	0.619524717		
GVIDYDR	0.64978	P49750	YLPM1_HUMAN	419.209237	2	0.698987365		
GVIDYDR	0.64978	P49750	YLPM1_HUMAN	279.8087663	3	0.698987365		
HFDQFWSAAK	28.65766	P49750	YLPM1_HUMAN	618.794004	2	0.701981127		
HFDQFWSAAK	28.65766	P49750	YLPM1_HUMAN	412.8652777	3	0.701981127		
HYEMQQQQFQHLYQEWER	43.70885	P49750	YLPM1_HUMAN	1254.561657	2	0.786355853	4	
HYEMQQQQFQHLYQEWER	43.70885	P49750	YLPM1_HUMAN	836.7103793	3	0.786355853	4	
ISRPMDMYDR	4.245323	P49750	YLPM1_HUMAN	642.2977345	2	0.666487098		
ISRPMDMYDR	4.245323	P49750	YLPM1_HUMAN	428.5344313	3	0.666487098		
ITLRPDPLPER	23.09972	P49750	YLPM1_HUMAN	653.878068	2	0.746825814	3	
ITLRPDPLPER	23.09972	P49750	YLPM1_HUMAN	436.2546537	3	0.746825814	3	Yes
MADHWETAPR	-4.437191	P49750	YLPM1_HUMAN	607.2751215	2	0.64673233	3	
MADHWETAPR	-4.437191	P49750	YLPM1_HUMAN	405.1860227	3	0.64673233	3	Yes
MEDYLQLPDDYDTR	67.23325	P49750	YLPM1_HUMAN	887.3859825	2	0.819304287		
MEDYLQLPDDYDTR	67.23325	P49750	YLPM1_HUMAN	591.9265967	3	0.819304287		
MPLPAPSLSHQPPPAPR	35.83136	P49750	YLPM1_HUMAN	896.9805305	2	0.69085449		
MPLPAPSLSHQPPPAPR	35.83136	P49750	YLPM1_HUMAN	598.322962	3	0.69085449		
MQSAAFSIAADVK	47.64082	P49750	YLPM1_HUMAN	669.840294	2	0.769227386		
MQSAAFSIAADVK	47.64082	P49750	YLPM1_HUMAN	446.8961377	3	0.769227386		
NVDDILKPPGR	15.78333	P49750	YLPM1_HUMAN	612.341318	2	0.773111284		
NVDDILKPPGR	15.78333	P49750	YLPM1_HUMAN	408.563487	3	0.773111284		
SALPYSSFSSDQGLGESSAAPSQPITAVK	68.29929	P49750	YLPM1_HUMAN	1441.706895	2	0.735766232	3	
SALPYSSFSSDQGLGESSAAPSQPITAVK	68.29929	P49750	YLPM1_HUMAN	961.4738713	3	0.735766232	3	Yes
SDRPVYEGPSMFGGER	25.01007	P49750	YLPM1_HUMAN	892.4075875	2	0.813928723		
SDRPVYEGPSMFGGER	25.01007	P49750	YLPM1_HUMAN	595.2743333	3	0.813928723		
SEVSEGPVEPSNWDQNVQSMETQIDK	62.37795	P49750	YLPM1_HUMAN	1467.159456	2	0.876387715		
SEVSEGPVEPSNWDQNVQSMETQIDK	62.37795	P49750	YLPM1_HUMAN	978.4422453	3	0.876387715		
SGGLLPDPPR	27.35785	P49750	YLPM1_HUMAN	504.7778225	2	0.769404888		
SGGLLPDPPR	27.35785	P49750	YLPM1_HUMAN	336.85449	3	0.769404888		
SLDNEWDR	8.631794	P49750	YLPM1_HUMAN	517.7310685	2	0.771471381		
SLDNEWDR	8.631794	P49750	YLPM1_HUMAN	345.4899873	3	0.771471381		
SLLQDAAIEEVEMEDFDANIEEQK	115.1898	P49750	YLPM1_HUMAN	1383.637294	2	0.808021188		
SLLQDAAIEEVEMEDFDANIEEQK	115.1898	P49750	YLPM1_HUMAN	922.760804	3	0.808021188		
SQLLAPPPPSAPPGNK	31.93835	P49750	YLPM1_HUMAN	785.9335715	2	0.826426744		
SQLLAPPPPSAPPGNK	31.93835	P49750	YLPM1_HUMAN	524.291656	3	0.826426744		
SSYLESPR	-6.887657	P49750	YLPM1_HUMAN	469.7330755	2	0.811174452		
SSYLESPR	-6.887657	P49750	YLPM1_HUMAN	313.4913253	3	0.811174452		
STEQQQAAPEPDPSTMTPQEQQQYWYR	51.33595	P49750	YLPM1_HUMAN	1612.715455	2	0.838889003		
STEQQQAAPEPDPSTMTPQEQQQYWYR	51.33595	P49750	YLPM1_HUMAN	1075.479578	3	0.838889003		
TTVQQEPLESGAK	-6.667267	P49750	YLPM1_HUMAN	694.357363	2	0.669730067	2	Yes
TTVQQEPLESGAK	-6.667267	P49750	YLPM1_HUMAN	463.2408503	3	0.669730067	2	
VEQIPYGER	6.005051	P49750	YLPM1_HUMAN	545.780558	2	0.611763477		
VEQIPYGER	6.005051	P49750	YLPM1_HUMAN	364.189647	3	0.611763477		
VGFQYQGIMQK	38.29474	P49750	YLPM1_HUMAN	649.8322685	2	0.818405926		
VGFQYQGIMQK	38.29474	P49750	YLPM1_HUMAN	433.557454	3	0.818405926		
VLSLDDYFITEVEK	99.16403	P49750	YLPM1_HUMAN	835.9303555	2	0.786114872		
VLSLDDYFITEVEK	99.16403	P49750	YLPM1_HUMAN	557.6228453	3	0.786114872		
VMEYEYEAEMEETYR	51.56818	P49750	YLPM1_HUMAN	986.4035105	2	0.850659728		
VMEYEYEAEMEETYR	51.56818	P49750	YLPM1_HUMAN	657.938282	3	0.850659728		
WDEDSFYGLWDTNDEQGLNSEFK	112.9518	P49750	YLPM1_HUMAN	1398.091363	2	0.711091995		
WDEDSFYGLWDTNDEQGLNSEFK	112.9518	P49750	YLPM1_HUMAN	932.3968503	3	0.711091995		
FILNLPTFSVR	102.5085	P49721	PSB2_HUMAN	653.880079	2	0.785977423	2	Yes
FILNLPTFSVR	102.5085	P49721	PSB2_HUMAN	436.2559943	3	0.785977423	2	

NGIHDLDNISFPK	44.3062	P49721	PSB2_HUMAN	735.373346	2	0.791236579	2	Yes
NGIHDLDNISFPK	44.3062	P49721	PSB2_HUMAN	490.584839	3	0.791236579	2	
NGYELSPTAAANFTR	45.98387	P49721	PSB2_HUMAN	806.3922635	2	0.631481469		
NGYELSPTAAANFTR	45.98387	P49721	PSB2_HUMAN	537.930784	3	0.631481469		
NLADCLR	11.70372	P49721	PSB2_HUMAN	431.2165435	2	0.665142298	2	Yes
NLADCLR	11.70372	P49721	PSB2_HUMAN	287.8136373	3	0.665142298	2	
VAASNIVQMK	13.11026	P49721	PSB2_HUMAN	530.795158	2	0.772960365	2	Yes
VAASNIVQMK	13.11026	P49721	PSB2_HUMAN	354.1993803	3	0.772960365	2	
YYTPTISR	10.6915	P49721	PSB2_HUMAN	500.75909	2	0.663456023	2	Yes
YYTPTISR	10.6915	P49721	PSB2_HUMAN	334.175335	3	0.663456023	2	
AEEIANEMIEAAK	40.20998	P49588	SYAC_HUMAN	709.845773	2	0.775470316	2	Yes
AEEIANEMIEAAK	40.20998	P49588	SYAC_HUMAN	473.566457	3	0.775470316	2	
ASEWVQQVSGLMDGK	70.07489	P49588	SYAC_HUMAN	817.896329	2	0.879701853	2	Yes
ASEWVQQVSGLMDGK	70.07489	P49588	SYAC_HUMAN	545.600161	3	0.879701853	2	
AVFDETYPDPVR	45.46853	P49588	SYAC_HUMAN	704.841344	2	0.834445834	2	Yes
AVFDETYPDPVR	45.46853	P49588	SYAC_HUMAN	470.230171	3	0.834445834	2	
AVYTQDCPLAAAK	15.80135	P49588	SYAC_HUMAN	704.3510225	2	0.854614615	2	Yes
AVYTQDCPLAAAK	15.80135	P49588	SYAC_HUMAN	469.90329	3	0.854614615	2	
DIINEEEVQFLK	73.40961	P49588	SYAC_HUMAN	738.8832125	2	0.740127861	2	Yes
DIINEEEVQFLK	73.40961	P49588	SYAC_HUMAN	492.92475	3	0.740127861	2	
DNFWEMGDTGPCGPCSEIHYDR	64.56892	P49588	SYAC_HUMAN	1322.020851	2	0.75922811	3	
DNFWEMGDTGPCGPCSEIHYDR	64.56892	P49588	SYAC_HUMAN	881.6831753	3	0.75922811	3	Yes
EADGILKPLPK	20.07874	P49588	SYAC_HUMAN	590.8509875	2	0.75650239	2	Yes
EADGILKPLPK	20.07874	P49588	SYAC_HUMAN	394.2366	3	0.75650239	2	
EIADLGEALATAVIPQWQK	127.6071	P49588	SYAC_HUMAN	1027.052405	2	0.794047475	3	
EIADLGEALATAVIPQWQK	127.6071	P49588	SYAC_HUMAN	685.0375447	3	0.794047475	3	Yes
GGYVLHIGTIYGDLK	56.41522	P49588	SYAC_HUMAN	803.435939	2	0.846411824	3	
GGYVLHIGTIYGDLK	56.41522	P49588	SYAC_HUMAN	535.959901	3	0.846411824	3	Yes
GLEVTDDSPK	-0.407177	P49588	SYAC_HUMAN	530.762035	2	0.760662973	2	Yes
GLEVTDDSPK	-0.407177	P49588	SYAC_HUMAN	354.1772983	3	0.760662973	2	
GLVVDMDGFEEER	59.54375	P49588	SYAC_HUMAN	748.340855	2	0.844999671	2	Yes
GLVVDMDGFEEER	59.54375	P49588	SYAC_HUMAN	499.229845	3	0.844999671	2	
ITCLCQVPQNAANR	17.04887	P49588	SYAC_HUMAN	822.9014235	2	0.749706805		
ITCLCQVPQNAANR	17.04887	P49588	SYAC_HUMAN	548.9368907	3	0.749706805		
IVAVTGAEAQK	-6.312031	P49588	SYAC_HUMAN	543.811863	2	0.786970377	2	Yes
IVAVTGAEAQK	-6.312031	P49588	SYAC_HUMAN	362.8771837	3	0.786970377	2	
LVSVLQNK	8.65456	P49588	SYAC_HUMAN	450.7798335	2	0.801260591	2	Yes
LVSVLQNK	8.65456	P49588	SYAC_HUMAN	300.8558307	3	0.801260591	2	
LYVTYFGGDEAAGLEADLECK	87.01656	P49588	SYAC_HUMAN	1161.036283	2	0.647476733		
LYVTYFGGDEAAGLEADLECK	87.01656	P49588	SYAC_HUMAN	774.36013	3	0.647476733		
MALELLTQEFGIPIER	122.2705	P49588	SYAC_HUMAN	930.5009615	2	0.803375065	3	
MALELLTQEFGIPIER	122.2705	P49588	SYAC_HUMAN	620.669916	3	0.803375065	3	Yes
MFVEEVSTGQECGVVLDK	60.72511	P49588	SYAC_HUMAN	1013.977191	2	0.694800019	3	
MFVEEVSTGQECGVVLDK	60.72511	P49588	SYAC_HUMAN	676.3207353	3	0.694800019	3	Yes
MHSPQTSAMLFTVDNEAGK	40.40278	P49588	SYAC_HUMAN	1032.480432	2	0.735491455	3	
MHSPQTSAMLFTVDNEAGK	40.40278	P49588	SYAC_HUMAN	688.6562293	3	0.735491455	3	Yes
NSSHAGAFVIVTEEAIAK	44.92861	P49588	SYAC_HUMAN	922.481614	2	0.775187373	2	Yes
NSSHAGAFVIVTEEAIAK	44.92861	P49588	SYAC_HUMAN	615.3236843	3	0.775187373	2	
QIWQNLGLDDTK	56.06342	P49588	SYAC_HUMAN	715.8678975	2	0.746074319	2	Yes
QIWQNLGLDDTK	56.06342	P49588	SYAC_HUMAN	477.5812067	3	0.746074319	2	
SIDTGMGLER	17.56541	P49588	SYAC_HUMAN	539.764055	2	0.771775484	2	Yes
SIDTGMGLER	17.56541	P49588	SYAC_HUMAN	360.178645	3	0.771775484	2	
SVLGEADQK	-16.22094	P49588	SYAC_HUMAN	473.746188	2	0.779441833	2	Yes
SVLGEADQK	-16.22094	P49588	SYAC_HUMAN	316.1667337	3	0.779441833	2	
TITVALADGGRPDNTGR	16.91705	P49588	SYAC_HUMAN	857.4481065	2	0.696777344	3	
TITVALADGGRPDNTGR	16.91705	P49588	SYAC_HUMAN	571.9680127	3	0.696777344	3	Yes
VGAEDADGIDMAYR	33.78326	P49588	SYAC_HUMAN	741.8306505	2	0.842177689	2	Yes
VGAEDADGIDMAYR	33.78326	P49588	SYAC_HUMAN	494.8897087	3	0.842177689	2	
VGDQVWLFIDEPR	91.73003	P49588	SYAC_HUMAN	787.404647	2	0.794885337	3	
VGDQVWLFIDEPR	91.73003	P49588	SYAC_HUMAN	525.272373	3	0.794885337	3	Yes
AAVEGTVEAGATVESTAC	35.94945	P49321	NASP_HUMAN	861.896724	2	0.734417379		
AAVEGTVEAGATVESTAC	35.94945	P49321	NASP_HUMAN	574.9337577	3	0.734417379		
ATLVESSTSGFTPGGGGSSVSMIASR	62.49714	P49321	NASP_HUMAN	1222.09266	2	0.736911058		
ATLVESSTSGFTPGGGGSSVSMIASR	62.49714	P49321	NASP_HUMAN	815.0643813	3	0.736911058		
DGAVNGPSVVGDQTPIEPQTSIER	55.08754	P49321	NASP_HUMAN	1233.609532	2	0.75691247	3	
DGAVNGPSVVGDQTPIEPQTSIER	55.08754	P49321	NASP_HUMAN	822.742296	3	0.75691247	3	Yes
EAQLYAAQAHLK	0.461052	P49321	NASP_HUMAN	671.8598715	2	0.799685001		
EAQLYAAQAHLK	0.461052	P49321	NASP_HUMAN	448.2425227	3	0.799685001		
EQVYDAMGEK	4.743694	P49321	NASP_HUMAN	585.261341	2	0.758204639	2	Yes
EQVYDAMGEK	4.743694	P49321	NASP_HUMAN	390.510169	3	0.758204639	2	
EVSEEQPVVTLEK	29.20409	P49321	NASP_HUMAN	743.8859525	2	0.795480609	2	Yes
EVSEEQPVVTLEK	29.20409	P49321	NASP_HUMAN	496.25991	3	0.795480609	2	
GGAAPEGPNEAEVTSGKPEQEVPDAEEEK	18.2102	P49321	NASP_HUMAN	1476.173618	2	0.688890994	3	
GGAAPEGPNEAEVTSGKPEQEVPDAEEEK	18.2102	P49321	NASP_HUMAN	984.451687	3	0.688890994	3	Yes
HLVMGDIPAAVNAFQEAASLLGK	125.1824	P49321	NASP_HUMAN	1176.623202	2	0.691854954	3	
HLVMGDIPAAVNAFQEAASLLGK	125.1824	P49321	NASP_HUMAN	784.7514097	3	0.691854954	3	Yes
KPTDGASSSNCVTDISHLVR	22.15453	P49321	NASP_HUMAN	1072.524216	2	0.765657604	3	
KPTDGASSSNCVTDISHLVR	22.15453	P49321	NASP_HUMAN	715.3520853	3	0.765657604	3	Yes
LLAETHYQLGLAYGYNSQYDEAVAQFSK	80.37979	P49321	NASP_HUMAN	1590.270182	2	0.635262132	3	
LLAETHYQLGLAYGYNSQYDEAVAQFSK	80.37979	P49321	NASP_HUMAN	1060.516063	3	0.635262132	3	Yes
LSVEESEAAGDGVDTK	12.91661	P49321	NASP_HUMAN	803.876313	2	0.852491617	2	Yes
LSVEESEAAGDGVDTK	12.91661	P49321	NASP_HUMAN	536.2534837	3	0.852491617	2	
MAVLNEQVK	9.51823	P49321	NASP_HUMAN	516.2818835	2	0.694250047	2	Yes
MAVLNEQVK	9.51823	P49321	NASP_HUMAN	344.523864	3	0.694250047	2	
SGNVAELALK	23.54135	P49321	NASP_HUMAN	501.28548	2	0.791445613	2	Yes
SGNVAELALK	23.54135	P49321	NASP_HUMAN	334.5262617	3	0.791445613	2	
TEEMPNDSVLENK	19.43036	P49321	NASP_HUMAN	753.343594	2	0.716124296		
TEEMPNDSVLENK	19.43036	P49321	NASP_HUMAN	502.5650043	3	0.716124296		
VDLTLDWLTETSEEAK	101.0048	P49321	NASP_HUMAN	925.4574705	2	0.761498809		
VDLTLDWLTETSEEAK	101.0048	P49321	NASP_HUMAN	617.3075887	3	0.761498809		
YGETANECGEAFFFYGK	70.24104	P49321	NASP_HUMAN	995.420357	2	0.791011333	2	Yes
YGETANECGEAFFFYGK	70.24104	P49321	NASP_HUMAN	663.949513	3	0.791011333	2	
AAFENWEVEVTFR	84.85129	P49257	LMAN1_HUMAN	799.386454	2	0.821323156		
AAFENWEVEVTFR	84.85129	P49257	LMAN1_HUMAN	533.2602443	3	0.821323156		
DIDNLVQR	25.50906	P49257	LMAN1_HUMAN	486.7596295	2	0.763694704	2	Yes
DIDNLVQR	25.50906	P49257	LMAN1_HUMAN	324.8423613	3	0.763694704	2	
GAGMPGQHGQITQQELDTVVK	29.83826	P49257	LMAN1_HUMAN	1097.550236	2	0.820105612	3	
GAGMPGQHGQITQQELDTVVK	29.83826	P49257	LMAN1_HUMAN	732.0360987	3	0.820105612	3	Yes
GDGVGGDPAVALPHR	17.41521	P49257	LMAN1_HUMAN	709.363314	2	0.619936824		
GDGVGGDPAVALPHR	17.41521	P49257	LMAN1_HUMAN	473.2448177	3	0.619936824		
GHPDLQGQPAEEIFESVGDR	63.52486	P49257	LMAN1_HUMAN	1091.014539	2	0.763283789	3	

GHPDLQGQPAEEIFESVGDR	63.52486	P49257	LMAN1_HUMAN	727.6789673	3	0.763283789	3	Yes
GPHLVQSDGTVPFWAHAGNAIPSSDQIR	57.77867	P49257	LMAN1_HUMAN	1479.236828	2	0.668447018	3	
GPHLVQSDGTVPFWAHAGNAIPSSDQIR	57.77867	P49257	LMAN1_HUMAN	986.4938267	3	0.668447018	3	Yes
LVSGMQHPGSAGGVYETTQHFIDIK	48.05038	P49257	LMAN1_HUMAN	1336.661041	2	0.786738634	4	
LVSGMQHPGSAGGVYETTQHFIDIK	48.05038	P49257	LMAN1_HUMAN	891.443302	3	0.786738634	4	
YQEEFEHFQQELDK	38.25538	P49257	LMAN1_HUMAN	935.4186755	2	0.820056677		
YQEEFEHFQQELDK	38.25538	P49257	LMAN1_HUMAN	623.948392	3	0.820056677		
YVSSLTEEISK	27.47924	P49257	LMAN1_HUMAN	628.3249945	2	0.695746779	2	Yes
YVSSLTEEISK	27.47924	P49257	LMAN1_HUMAN	419.2192713	3	0.695746779	2	
AEASAASEEEAGPQATEPSTPSGPESGPTPASAEQ	37.98353	P49006	MRP_HUMAN	1813.2808	2	0.629270434	3	
AEASAASEEEAGPQATEPSTPSGPESGPTPASAEQ	37.98353	P49006	MRP_HUMAN	1209.189808	3	0.629270434	3	Yes
GDVTAEEAAGASPAK	-2.011616	P49006	MRP_HUMAN	687.331345	2	0.75441587	2	Yes
GDVTAEEAAGASPAK	-2.011616	P49006	MRP_HUMAN	458.5568383	3	0.75441587	2	
EIEQEAAVELSQLR	62.89928	P47985	UCRI_HUMAN	807.920858	2	0.844332337	2	Yes
EIEQEAAVELSQLR	62.89928	P47985	UCRI_HUMAN	538.949847	3	0.844332337	2	
AQAAAPASVPAQAPK	-0.56728	P47914	RL29_HUMAN	689.378433	2	0.800784051	2	Yes
AQAAAPASVPAQAPK	-0.56728	P47914	RL29_HUMAN	459.9215637	3	0.800784051	2	
DIICQIAYAR	57.64346	P46777	RL5_HUMAN	611.816618	2	0.791116595	2	Yes
DIICQIAYAR	57.64346	P46777	RL5_HUMAN	408.213687	3	0.791116595	2	
GAVDGGLSIPHSTK	9.692253	P46777	RL5_HUMAN	669.85479	2	0.814282596	3	
GAVDGGLSIPHSTK	9.692253	P46777	RL5_HUMAN	446.9058017	3	0.814282596	3	Yes
HIMGQNVADYMR	18.95478	P46777	RL5_HUMAN	717.835016	2	0.778046131	2	Yes
HIMGQNVADYMR	18.95478	P46777	RL5_HUMAN	478.892619	3	0.778046131	2	
IEGDMIVCAAYAHELPK	53.98376	P46777	RL5_HUMAN	958.9664245	2	0.706006885	3	
IEGDMIVCAAYAHELPK	53.98376	P46777	RL5_HUMAN	639.6468913	3	0.706006885	3	Yes
NSVTPDMMEEMYK	47.6757	P46777	RL5_HUMAN	787.830947	2	0.852159858	2	Yes
NSVTPDMMEEMYK	47.6757	P46777	RL5_HUMAN	525.556573	3	0.852159858	2	
VGLTNYAAAYCTGLLLAR	89.72658	P46777	RL5_HUMAN	964.009477	2	0.714992046	3	
VGLTNYAAAYCTGLLLAR	89.72658	P46777	RL5_HUMAN	643.0089263	3	0.714992046	3	Yes
YLMEEDEDAYK	21.56796	P46777	RL5_HUMAN	703.295573	2	0.730006158	2	Yes
YLMEEDEDAYK	21.56796	P46777	RL5_HUMAN	469.199657	3	0.730006158	2	
ALFDFNGNDEEDLPFK	91.65511	P46108	CRK_HUMAN	935.9288795	2	0.72729075		
ALFDFNGNDEEDLPFK	91.65511	P46108	CRK_HUMAN	624.288528	3	0.72729075		
DKPEEQWWNAEDSEGK	29.05555	P46108	CRK_HUMAN	974.42195	2	0.646266878		
DKPEEQWWNAEDSEGK	29.05555	P46108	CRK_HUMAN	649.950575	3	0.646266878		
DSSTSPGDYVLSVSENSR	50.2769	P46108	CRK_HUMAN	950.4325095	2	0.760192752		
DSSTSPGDYVLSVSENSR	50.2769	P46108	CRK_HUMAN	633.9576147	3	0.760192752		
GHFPFTHVR	-10.05992	P46108	CRK_HUMAN	549.2861495	2	0.707811117		
GHFPFTHVR	-10.05992	P46108	CRK_HUMAN	366.526708	3	0.707811117		
GMIPVPYVEK	50.93238	P46108	CRK_HUMAN	566.80773	2	0.74500823		
GMIPVPYVEK	50.93238	P46108	CRK_HUMAN	378.2077617	3	0.74500823		
IGDQEFDSLPALLEFYK	124.0911	P46108	CRK_HUMAN	992.999301	2	0.747348487		
IGDQEFDSLPALLEFYK	124.0911	P46108	CRK_HUMAN	662.3354757	3	0.747348487		
IHYLDTTTLIEPVSR	52.40678	P46108	CRK_HUMAN	879.4757965	2	0.835397899	3	
IHYLDTTTLIEPVSR	52.40678	P46108	CRK_HUMAN	586.6531393	3	0.835397899	3	Yes
LLDQQNPDEDFS	52.12517	P46108	CRK_HUMAN	710.815527	2	0.683185756		
LLDQQNPDEDFS	52.12517	P46108	CRK_HUMAN	474.2129597	3	0.683185756		
SSWYWGR	32.3821	P46108	CRK_HUMAN	471.217396	2	0.798565149	2	Yes
SSWYWGR	32.3821	P46108	CRK_HUMAN	314.4808723	3	0.798565149	2	
TALALEVGELVK	72.1828	P46108	CRK_HUMAN	621.8693775	2	0.851810992	2	Yes
TALALEVGELVK	72.1828	P46108	CRK_HUMAN	414.9155267	3	0.851810992	2	
AAWAEYEAEK	13.13028	P46100	ATRX_HUMAN	584.270023	2	0.861617565		
AAWAEYEAEK	13.13028	P46100	ATRX_HUMAN	389.8492903	3	0.861617565		
AGSLGINLVAANR	47.66403	P46100	ATRX_HUMAN	628.360042	2	0.860818267		
AGSLGINLVAANR	47.66403	P46100	ATRX_HUMAN	419.2426363	3	0.860818267		
AHILYEMLAGCVQR	52.9124	P46100	ATRX_HUMAN	830.9190805	2	0.766767204		
AHILYEMLAGCVQR	52.9124	P46100	ATRX_HUMAN	554.2819953	3	0.766767204		
AHLALEEDLNSEFR	44.04021	P46100	ATRX_HUMAN	822.4053745	2	0.775859535		
AHLALEEDLNSEFR	44.04021	P46100	ATRX_HUMAN	548.6061913	3	0.775859535		
ALVDPGPDFVVCDEGHILK	68.79196	P46100	ATRX_HUMAN	1041.02279	2	0.793180525	3	
ALVDPGPDFVVCDEGHILK	68.79196	P46100	ATRX_HUMAN	694.3511347	3	0.793180525	3	Yes
ATSSSNPSSPAPDWYK	23.3456	P46100	ATRX_HUMAN	847.88701	2	0.76033026		
ATSSSNPSSPAPDWYK	23.3456	P46100	ATRX_HUMAN	565.5939483	3	0.76033026		
DFVTDADAEVLEHSGK	50.28352	P46100	ATRX_HUMAN	866.905405	2	0.849390507	3	
DFVTDADAEVLEHSGK	50.28352	P46100	ATRX_HUMAN	578.2728783	3	0.849390507	3	Yes
DSDGMDEQCR	-34.0363	P46100	ATRX_HUMAN	606.716976	2	0.761554718		
DSDGMDEQCR	-34.0363	P46100	ATRX_HUMAN	404.8139257	3	0.761554718		
DTILAELLQIHK	98.26369	P46100	ATRX_HUMAN	697.4066585	2	0.843246102	3	
DTILAELLQIHK	98.26369	P46100	ATRX_HUMAN	465.273714	3	0.843246102	3	Yes
EAIYNDVLTK	27.50349	P46100	ATRX_HUMAN	583.309148	2	0.664415061		
EAIYNDVLTK	27.50349	P46100	ATRX_HUMAN	389.208707	3	0.664415061		
ENMNLSEAQVQALALSR	66.72372	P46100	ATRX_HUMAN	937.4760055	2	0.732545316		
ENMNLSEAQVQALALSR	66.72372	P46100	ATRX_HUMAN	625.3199453	3	0.732545316		
EQSFTSLEVR	29.72566	P46100	ATRX_HUMAN	598.3018585	2	0.834684193		
EQSFTSLEVR	29.72566	P46100	ATRX_HUMAN	399.2038473	3	0.834684193		
ETFSSAEGTVDKDTTIMELR	48.43365	P46100	ATRX_HUMAN	1115.531373	2	0.860850871	3	
ETFSSAEGTVDKDTTIMELR	48.43365	P46100	ATRX_HUMAN	744.0235233	3	0.860850871	3	Yes
EVIEIEDASPTK	29.92015	P46100	ATRX_HUMAN	665.8410135	2	0.826039255		
EVIEIEDASPTK	29.92015	P46100	ATRX_HUMAN	444.2299507	3	0.826039255		
FGQTKPVYVYR	2.156036	P46100	ATRX_HUMAN	679.367328	2	0.799587727		
FGQTKPVYVYR	2.156036	P46100	ATRX_HUMAN	453.2474937	3	0.799587727		
FLAQGTMEDK	8.375435	P46100	ATRX_HUMAN	570.274256	2	0.791824698	2	Yes
FLAQGTMEDK	8.375435	P46100	ATRX_HUMAN	380.518779	3	0.791824698	2	
GADCQEVPQDKDGYK	-24.59844	P46100	ATRX_HUMAN	855.375954	2	0.791131854		
GADCQEVPQDKDGYK	-24.59844	P46100	ATRX_HUMAN	570.5865777	3	0.791131854		
GGGEGNVDETGNNPSVSLK	13.70025	P46100	ATRX_HUMAN	915.9273995	2	0.662193656	2	Yes
GGGEGNVDETGNNPSVSLK	13.70025	P46100	ATRX_HUMAN	610.954208	3	0.662193656	2	
GMYQPVAGGMQPPPLQR	47.25328	P46100	ATRX_HUMAN	913.956195	2	0.78138566		
GMYQPVAGGMQPPPLQR	47.25328	P46100	ATRX_HUMAN	609.6400717	3	0.78138566		
GTVIVQPEPVLNEDKDDFK	46.6457	P46100	ATRX_HUMAN	1072.050058	2	0.778980017		
GTVIVQPEPVLNEDKDDFK	46.6457	P46100	ATRX_HUMAN	715.0359803	3	0.778980017		
GYFDEDSMDEFIASDSDETSMSLSSDDYTK	103.7833	P46100	ATRX_HUMAN	1694.164128	2	0.633339763		
GYFDEDSMDEFIASDSDETSMSLSSDDYTK	103.7833	P46100	ATRX_HUMAN	1129.778694	3	0.633339763		
IKPVTENLVLSSHTGFCQSSGDEALSK	37.64421	P46100	ATRX_HUMAN	1452.724569	2	0.629868984		
IKPVTENLVLSSHTGFCQSSGDEALSK	37.64421	P46100	ATRX_HUMAN	968.8189877	3	0.629868984		
IQPLEDIISAVWK	129.1799	P46100	ATRX_HUMAN	756.4275905	2	0.81156987		
IQPLEDIISAVWK	129.1799	P46100	ATRX_HUMAN	504.621002	3	0.81156987		
LDDSCSGSVTYSYSALIVPK	71.51192	P46100	ATRX_HUMAN	1081.520267	2	0.71786958		
LDDSCSGSVTYSYSALIVPK	71.51192	P46100	ATRX_HUMAN	721.349453	3	0.71786958		
LDGSTTAQSR	-34.11767	P46100	ATRX_HUMAN	518.2574515	2	0.615906894		
LDGSTTAQSR	-34.11767	P46100	ATRX_HUMAN	345.8409093	3	0.615906894		

LFQDFQMLSR	70.86375	P46100	ATRX_HUMAN	642.824447	2	0.779432774		
LFQDFQMLSR	70.86375	P46100	ATRX_HUMAN	428.885573	3	0.779432774		
LIETTANMNSSYVK	24.84914	P46100	ATRX_HUMAN	785.89325	2	0.734960318	2	Yes
LIETTANMNSSYVK	24.84914	P46100	ATRX_HUMAN	524.264775	3	0.734960318	2	
LNTLVQK	-12.8055	P46100	ATRX_HUMAN	408.253452	2	0.632634401		
LNTLVQK	-12.8055	P46100	ATRX_HUMAN	272.5049097	3	0.632634401		
LTPVSLSNSPIK	38.26159	P46100	ATRX_HUMAN	628.367001	2	0.81251967	2	Yes
LTPVSLSNSPIK	38.26159	P46100	ATRX_HUMAN	419.2472757	3	0.81251967	2	
LVLDEDEETK	9.927021	P46100	ATRX_HUMAN	595.793532	2	0.779556751		
LVLDEDEETK	9.927021	P46100	ATRX_HUMAN	397.5316297	3	0.779556751		
LYQYYLDHLTGVGNNSEGGR	48.75598	P46100	ATRX_HUMAN	1128.538168	2	0.641040683		
LYQYYLDHLTGVGNNSEGGR	48.75598	P46100	ATRX_HUMAN	752.69472	3	0.641040683		
MEQQYESSSDGTEK	-24.19253	P46100	ATRX_HUMAN	809.831044	2	0.8111642		
MEQQYESSSDGTEK	-24.19253	P46100	ATRX_HUMAN	540.2233043	3	0.8111642		
MVLLFEILR	111.7581	P46100	ATRX_HUMAN	567.341744	2	0.709182739		
MVLLFEILR	111.7581	P46100	ATRX_HUMAN	378.563771	3	0.709182739		
VQDGLSDIAEK	18.06854	P46100	ATRX_HUMAN	587.8016915	2	0.822265983		
VQDGLSDIAEK	18.06854	P46100	ATRX_HUMAN	392.203736	3	0.822265983		
WAEEFNDETNVR	29.26714	P46100	ATRX_HUMAN	755.334418	2	0.745489359	2	Yes
WAEEFNDETNVR	29.26714	P46100	ATRX_HUMAN	503.8922203	3	0.745489359	2	
VESDDEKPLDDETVNEDASNENSENDITMQSLP	53.34904	P46100	ATRX_HUMAN	1985.86129	2	0.603444695		
VESDDEKPLDDETVNEDASNENSENDITMQSLP	53.34904	P46100	ATRX_HUMAN	1324.243468	3	0.603444695		
AANMLQQSGSK	-21.88347	P46063	RECQ1_HUMAN	567.782779	2	0.759828627		
AANMLQQSGSK	-21.88347	P46063	RECQ1_HUMAN	378.8577943	3	0.759828627		
ANLLNNEAHAITMQVTK	41.01839	P46063	RECQ1_HUMAN	934.4889165	2	0.869758248	2	Yes
ANLLNNEAHAITMQVTK	41.01839	P46063	RECQ1_HUMAN	623.3285527	3	0.869758248	2	
DILQNVFK	74.23501	P46063	RECQ1_HUMAN	488.777291	2	0.791163445	2	Yes
DILQNVFK	74.23501	P46063	RECQ1_HUMAN	326.187469	3	0.791163445	2	
DSEQVTVSLQNLGIHAGAYHANLEPEDK	63.78055	P46063	RECQ1_HUMAN	1518.239425	2	0.679171503		
DSEQVTVSLQNLGIHAGAYHANLEPEDK	63.78055	P46063	RECQ1_HUMAN	1012.495558	3	0.679171503		
EDYSFTAYATISYLK	94.19196	P46063	RECQ1_HUMAN	886.425429	2	0.620113134		
EDYSFTAYATISYLK	94.19196	P46063	RECQ1_HUMAN	591.2862277	3	0.620113134		
EVFLVMPTGGGK	57.54719	P46063	RECQ1_HUMAN	617.8291985	2	0.755870998	2	Yes
EVFLVMPTGGGK	57.54719	P46063	RECQ1_HUMAN	412.222074	3	0.755870998	2	
FRPLQLETINVTMAGK	59.64637	P46063	RECQ1_HUMAN	909.501296	2	0.823028088	3	
FRPLQLETINVTMAGK	59.64637	P46063	RECQ1_HUMAN	606.670139	3	0.823028088	3	Yes
GQSGIIYCFSQK	37.22713	P46063	RECQ1_HUMAN	694.3379145	2	0.67953223		
GQSGIIYCFSQK	37.22713	P46063	RECQ1_HUMAN	463.2278847	3	0.67953223		
IIAHFLIQQYLK	65.11604	P46063	RECQ1_HUMAN	743.943207	2	0.80412674	3	
IIAHFLIQQYLK	65.11604	P46063	RECQ1_HUMAN	496.2980797	3	0.80412674	3	Yes
ISSMVVMENVGQQK	39.44	P46063	RECQ1_HUMAN	775.389825	2	0.788425565	3	
ISSMVVMENVGQQK	39.44	P46063	RECQ1_HUMAN	517.2624917	3	0.788425565	3	Yes
LIDSWMGK	36.82971	P46063	RECQ1_HUMAN	475.2447695	2	0.749040484		
LIDSWMGK	36.82971	P46063	RECQ1_HUMAN	317.165788	3	0.749040484		
LIYVTPEK	17.04199	P46063	RECQ1_HUMAN	481.782038	2	0.685492635	2	Yes
LIYVTPEK	17.04199	P46063	RECQ1_HUMAN	321.523967	3	0.685492635	2	
SMENYYQESGR	0.58865	P46063	RECQ1_HUMAN	682.283331	2	0.766894996	2	Yes
SMENYYQESGR	0.58865	P46063	RECQ1_HUMAN	455.1914957	3	0.766894996	2	
VAGVVAPTLPR	34.30701	P46063	RECQ1_HUMAN	540.3327655	2	0.767031968	2	Yes
VAGVVAPTLPR	34.30701	P46063	RECQ1_HUMAN	360.5577853	3	0.767031968	2	
VLMAQHFDEVWNSEACNK	48.04266	P46063	RECQ1_HUMAN	1089.49133	2	0.85936451	3	
VLMAQHFDEVWNSEACNK	48.04266	P46063	RECQ1_HUMAN	726.663495	3	0.85936451	3	Yes
ASSTCPLTFENVK	31.25969	P45954	ACDSB_HUMAN	727.3537655	2	0.835135043	2	Yes
ASSTCPLTFENVK	31.25969	P45954	ACDSB_HUMAN	485.238452	3	0.835135043	2	
ATYLPQLTTEK	35.38111	P45954	ACDSB_HUMAN	632.843356	2	0.857242286	2	Yes
ATYLPQLTTEK	35.38111	P45954	ACDSB_HUMAN	422.2315123	3	0.857242286	2	
DTPGLHIGKPENK	-12.98096	P45954	ACDSB_HUMAN	703.3758895	2	0.638251305	3	
DTPGLHIGKPENK	-12.98096	P45954	ACDSB_HUMAN	469.2532013	3	0.638251305	3	Yes
FAQEQIAPLVSTMDENSK	63.93945	P45954	ACDSB_HUMAN	1004.488779	2	0.832668424		
FAQEQIAPLVSTMDENSK	63.93945	P45954	ACDSB_HUMAN	669.9951277	3	0.832668424		
GITSFLVDR	51.72868	P45954	ACDSB_HUMAN	504.280198	2	0.747443557		
GITSFLVDR	51.72868	P45954	ACDSB_HUMAN	336.5227403	3	0.747443557		
IGTIYEGASNIQLNTIAK	60.08117	P45954	ACDSB_HUMAN	953.5181925	2	0.881755114		
IGTIYEGASNIQLNTIAK	60.08117	P45954	ACDSB_HUMAN	636.0147367	3	0.881755114		
LFDFQGLQHQVAHVATQLEAAR	82.73323	P45954	ACDSB_HUMAN	1240.146223	2	0.810804129		
LFDFQGLQHQVAHVATQLEAAR	82.73323	P45954	ACDSB_HUMAN	827.10009	3	0.810804129		
LLEAGKPFIK	19.35366	P45954	ACDSB_HUMAN	558.345341	2	0.819476962	3	
LLEAGKPFIK	19.35366	P45954	ACDSB_HUMAN	372.566169	3	0.819476962	3	Yes
LLTYNAAR	0.318802	P45954	ACDSB_HUMAN	461.261804	2	0.621070266		
LLTYNAAR	0.318802	P45954	ACDSB_HUMAN	307.843811	3	0.621070266		
VPEANILGQIGHGYK	41.94198	P45954	ACDSB_HUMAN	798.431192	2	0.873484254		
VPEANILGQIGHGYK	41.94198	P45954	ACDSB_HUMAN	532.623403	3	0.873484254		
YAIGSLNEGR	15.26899	P45954	ACDSB_HUMAN	540.278182	2	0.777140021	2	Yes
YAIGSLNEGR	15.26899	P45954	ACDSB_HUMAN	360.5213963	3	0.777140021	2	
YYASEIAGQTTSK	5.040298	P45954	ACDSB_HUMAN	709.8440795	2	0.875551999	2	Yes
YYASEIAGQTTSK	5.040298	P45954	ACDSB_HUMAN	473.565328	3	0.875551999	2	
DFMIQGGDITTGDGTGGVSIYGETFPDENFK	109.6133	P45877	PPIC_HUMAN	1634.735525	2	0.701622605		
DFMIQGGDITTGDGTGGVSIYGETFPDENFK	109.6133	P45877	PPIC_HUMAN	1090.159625	3	0.701622605		
IVIGLFGK	61.17971	P45877	PPIC_HUMAN	423.776563	2	0.717350185	2	Yes
IVIGLFGK	61.17971	P45877	PPIC_HUMAN	282.8536503	3	0.717350185	2	
TVENFVALATGEK	58.67036	P45877	PPIC_HUMAN	689.8648235	2	0.83108902		
TVENFVALATGEK	58.67036	P45877	PPIC_HUMAN	460.245824	3	0.83108902		
VIDGMTVVHSIELQATDGHDRPLTNCSIINSGK	58.28612	P45877	PPIC_HUMAN	1789.391059	2	0.677737951		
VIDGMTVVHSIELQATDGHDRPLTNCSIINSGK	58.28612	P45877	PPIC_HUMAN	1193.263314	3	0.677737951		
ADTLDPALLRPGR	34.79872	P43686	PRS6B_HUMAN	697.891707	2	0.790742159	3	
ADTLDPALLRPGR	34.79872	P43686	PRS6B_HUMAN	465.5970797	3	0.790742159	3	Yes
EAVELPLTHFELYK	72.56999	P43686	PRS6B_HUMAN	844.948883	2	0.769112706	3	
EAVELPLTHFELYK	72.56999	P43686	PRS6B_HUMAN	563.635197	3	0.769112706	3	Yes
EFLHAQEEVK	-0.960323	P43686	PRS6B_HUMAN	615.3122265	2	0.740613937	2	Yes
EFLHAQEEVK	-0.960323	P43686	PRS6B_HUMAN	410.5440927	3	0.740613937	2	
ELLKPNASVALHK	7.814098	P43686	PRS6B_HUMAN	710.4200995	2	0.735368311	3	
ELLKPNASVALHK	7.814098	P43686	PRS6B_HUMAN	473.9493413	3	0.735368311	3	Yes
ENAPAIIFIDEIDAIATK	139.1182	P43686	PRS6B_HUMAN	972.520405	2	0.631122708	3	
ENAPAIIFIDEIDAIATK	139.1182	P43686	PRS6B_HUMAN	648.6828783	3	0.631122708	3	Yes
GVLMYGPPGCGK	29.32289	P43686	PRS6B_HUMAN	618.299747	2	0.729296327	2	Yes
GVLMYGPPGCGK	29.32289	P43686	PRS6B_HUMAN	412.535773	3	0.729296327	2	
ILLELLNQMDGFDQNVNVK	117.8225	P43686	PRS6B_HUMAN	1102.075553	2	0.73704952		
ILLELLNQMDGFDQNVNVK	117.8225	P43686	PRS6B_HUMAN	735.0529767	3	0.73704952		
ISGADINSICQESGMLAVR	64.53586	P43686	PRS6B_HUMAN	1010.993706	2	0.795441508	3	
ISGADINSICQESGMLAVR	64.53586	P43686	PRS6B_HUMAN	674.3317453	3	0.795441508	3	Yes
LIFSTITSK	40.62464	P43686	PRS6B_HUMAN	505.300599	2	0.639507949	2	Yes

LIFSTITSK	40.62464	P43686	PRS6B HUMAN	337.2030077	3	0.639507949	2	
LQQELEFLEVQEEYIK	92.18709	P43686	PRS6B HUMAN	1019.523141	2	0.772002578		
LQQELEFLEVQEEYIK	92.18709	P43686	PRS6B HUMAN	680.0180353	3	0.772002578		
MEEIGILVEK	56.63541	P43686	PRS6B HUMAN	580.815756	2	0.697111845		
MEEIGILVEK	56.63541	P43686	PRS6B HUMAN	387.5464457	3	0.697111845		
MNLSEEVDLEDYVARPDK	62.37838	P43686	PRS6B HUMAN	1062.002246	2	0.695375562	3	
MNLSEEVDLEDYVARPDK	62.37838	P43686	PRS6B HUMAN	708.3374387	3	0.695375562	3	Yes
DDSFFGETSHNYHK	8.92453	P43243	MATR3 HUMAN	842.3558775	2	0.683340311	3	
DDSFFGETSHNYHK	8.92453	P43243	MATR3 HUMAN	561.9065267	3	0.683340311	3	Yes
DDYTIPDEYR	35.42743	P43243	MATR3 HUMAN	643.7809475	2	0.612681329		
DDYTIPDEYR	35.42743	P43243	MATR3 HUMAN	429.52324	3	0.612681329		
DLSAAGIGLLAAATQSLSMPASLGR	164.959	P43243	MATR3 HUMAN	1186.136674	2	0.708977938	3	
DLSAAGIGLLAAATQSLSMPASLGR	164.959	P43243	MATR3 HUMAN	791.093724	3	0.708977938	3	Yes
EDAMAMVDHCLK	28.94317	P43243	MATR3 HUMAN	710.307447	2	0.688406825	3	
EDAMAMVDHCLK	28.94317	P43243	MATR3 HUMAN	473.8742397	3	0.688406825	3	Yes
GAPPSSNIEDFHGLLPK	53.49295	P43243	MATR3 HUMAN	889.9577745	2	0.77119422	3	
GAPPSSNIEDFHGLLPK	53.49295	P43243	MATR3 HUMAN	593.6411247	3	0.77119422	3	Yes
GDADQASNILASFGLSAR	103.2995	P43243	MATR3 HUMAN	896.9453955	2	0.765230834	3	
GDADQASNILASFGLSAR	103.2995	P43243	MATR3 HUMAN	598.2995387	3	0.765230834	3	Yes
GNLGAGNGNLQGPR	-8.291199	P43243	MATR3 HUMAN	662.8400055	2	0.750432849	2	Yes
GNLGAGNGNLQGPR	-8.291199	P43243	MATR3 HUMAN	442.2292787	3	0.750432849	2	
GPGPLQER	-22.57842	P43243	MATR3 HUMAN	427.2305085	2	0.74095124	2	Yes
GPGPLQER	-22.57842	P43243	MATR3 HUMAN	285.1562807	3	0.74095124	2	
GPLPLSSQHR	-11.55293	P43243	MATR3 HUMAN	546.301996	2	0.647597492	2	Yes
GPLPLSSQHR	-11.55293	P43243	MATR3 HUMAN	364.5372723	3	0.647597492	2	
GPSLNPVLDYDHGSR	31.84431	P43243	MATR3 HUMAN	813.8977125	2	0.773834944	3	
GPSLNPVLDYDHGSR	31.84431	P43243	MATR3 HUMAN	542.9344167	3	0.773834944	3	Yes
IGPYQPNVPVGIDYVIPK	91.62764	P43243	MATR3 HUMAN	985.043842	2	0.769255042	3	
IGPYQPNVPVGIDYVIPK	91.62764	P43243	MATR3 HUMAN	657.0318363	3	0.769255042	3	Yes
INEAFIEMATTEDAQAAVDYYTTTPALVFGKPVF	126.1818	P43243	MATR3 HUMAN	1866.927459	2	0.616412699		
INEAFIEMATTEDAQAAVDYYTTTPALVFGKPVF	126.1818	P43243	MATR3 HUMAN	1244.954247	3	0.616412699		
ITPENLPQILLQLK	114.6407	P43243	MATR3 HUMAN	810.4907225	2	0.828389168	3	
ITPENLPQILLQLK	114.6407	P43243	MATR3 HUMAN	540.66309	3	0.828389168	3	Yes
LCSLFYTNEEVAK	47.35005	P43243	MATR3 HUMAN	787.3825185	2	0.700890243	2	Yes
LCSLFYTNEEVAK	47.35005	P43243	MATR3 HUMAN	525.2576207	3	0.700890243	2	
SQAFIEMETR	29.33346	P43243	MATR3 HUMAN	606.290437	2	0.76300323	2	Yes
SQAFIEMETR	29.33346	P43243	MATR3 HUMAN	404.5295663	3	0.76300323	2	
VIHLSNLPHSGYSDSAVLK	32.13586	P43243	MATR3 HUMAN	1019.042365	2	0.773637533		
VIHLSNLPHSGYSDSAVLK	32.13586	P43243	MATR3 HUMAN	679.6975183	3	0.773637533		
VVHIMDFQR	19.90028	P43243	MATR3 HUMAN	572.800775	2	0.814302742	3	
VVHIMDFQR	19.90028	P43243	MATR3 HUMAN	382.203125	3	0.814302742	3	Yes
YQLLQLVEPFGVISNHLILNK	129.6756	P43243	MATR3 HUMAN	1219.69448	2	0.767920434	3	
YQLLQLVEPFGVISNHLILNK	129.6756	P43243	MATR3 HUMAN	813.4655947	3	0.767920434	3	Yes
DVEMGNSVIEENEMK	42.97306	P43003	EAA1 HUMAN	862.3798505	2	0.771474242	2	Yes
DVEMGNSVIEENEMK	42.97306	P43003	EAA1 HUMAN	575.255842	3	0.771474242	2	
EFFDSLNEAIMR	90.46686	P43003	EAA1 HUMAN	736.3484825	2	0.770861387		
EFFDSLNEAIMR	90.46686	P43003	EAA1 HUMAN	491.23493	3	0.770861387		
TTTNVLGDSLGAGIVEHLSR	67.26403	P43003	EAA1 HUMAN	1020.540192	2	0.868165135	3	
TTTNVLGDSLGAGIVEHLSR	67.26403	P43003	EAA1 HUMAN	680.6960697	3	0.868165135	3	Yes
VPIQANETLVGAVINNVSEAMETLTR	150.22	P43003	EAA1 HUMAN	1385.226748	2	0.714370072		
VPIQANETLVGAVINNVSEAMETLTR	150.22	P43003	EAA1 HUMAN	923.8204403	3	0.714370072		
VTAADAFLDLIR	101.8481	P43003	EAA1 HUMAN	652.8646265	2	0.866152585		
VTAADAFLDLIR	101.8481	P43003	EAA1 HUMAN	435.579026	3	0.866152585		
AAAVLGMDK	5.511314	P42858	HD HUMAN	438.236945	2	0.788715363		
AAAVLGMDK	5.511314	P42858	HD HUMAN	292.493905	3	0.788715363		
AALPSLTNPPSLSPIR	74.01199	P42858	HD HUMAN	817.467778	2	0.868432581		
AALPSLTNPPSLSPIR	74.01199	P42858	HD HUMAN	545.3144603	3	0.868432581		
AFQSVLEVVAAPGSPYHR	66.44599	P42858	HD HUMAN	964.5054195	2	0.779642045		
AFQSVLEVVAAPGSPYHR	66.44599	P42858	HD HUMAN	643.3395547	3	0.779642045		
ALALSCVGAAVALHPESFFSK	86.67818	P42858	HD HUMAN	1088.067531	2	0.750742078		
ALALSCVGAAVALHPESFFSK	86.67818	P42858	HD HUMAN	725.7142957	3	0.750742078		
ALAQYLVVVSK	59.94318	P42858	HD HUMAN	595.861351	2	0.78334564		
ALAQYLVVVSK	59.94318	P42858	HD HUMAN	397.5768423	3	0.78334564		
ALMDSNLPR	20.96033	P42858	HD HUMAN	508.7638575	2	0.846568167		
ALMDSNLPR	20.96033	P42858	HD HUMAN	339.5118467	3	0.846568167		
ALVPMVEQLFSHLLK	132.5151	P42858	HD HUMAN	862.992575	2	0.789785087		
ALVPMVEQLFSHLLK	132.5151	P42858	HD HUMAN	575.664325	3	0.789785087		
AVTHAIPALQPIVHDLFVLR	93.58333	P42858	HD HUMAN	1105.644598	2	0.60058248	4	
AVTHAIPALQPIVHDLFVLR	93.58333	P42858	HD HUMAN	737.4323403	3	0.60058248	4	
DEISGELAASSGVSTPGSAGHDIITEQPR	55.7816	P42858	HD HUMAN	1441.194688	2	0.748007894		
DEISGELAASSGVSTPGSAGHDIITEQPR	55.7816	P42858	HD HUMAN	961.1324	3	0.748007894		
DGDSTSTLEEHSEGK	-20.65523	P42858	HD HUMAN	796.3400945	2	0.627430558		
DGDSTSTLEEHSEGK	-20.65523	P42858	HD HUMAN	531.229338	3	0.627430558		
ENIATHHLYQAWDPVPSLSPATTGALISHEK	66.76583	P42858	HD HUMAN	1692.355124	2	0.600639224		
ENIATHHLYQAWDPVPSLSPATTGALISHEK	66.76583	P42858	HD HUMAN	1128.572691	3	0.600639224		
FHVGDWMGTIR	56.16834	P42858	HD HUMAN	659.822239	2	0.826111734		
FHVGDWMGTIR	56.16834	P42858	HD HUMAN	440.2174343	3	0.826111734		
GDIGQSTDDDSAPLVHCVR	23.04846	P42858	HD HUMAN	1021.466367	2	0.775847673		
GDIGQSTDDDSAPLVHCVR	23.04846	P42858	HD HUMAN	681.3135197	3	0.775847673		
GIVEQEIQAMVSK	67.16266	P42858	HD HUMAN	716.3797835	2	0.794598222		
GIVEQEIQAMVSK	67.16266	P42858	HD HUMAN	477.922464	3	0.794598222		
GYNLLPSITDVTMENNLSR	96.19748	P42858	HD HUMAN	1069.03388	2	0.680766463		
GYNLLPSITDVTMENNLSR	96.19748	P42858	HD HUMAN	713.025195	3	0.680766463		
IIQLCDGIMASGR	54.68703	P42858	HD HUMAN	717.3661535	2	0.745543718		
IIQLCDGIMASGR	54.68703	P42858	HD HUMAN	478.580044	3	0.745543718		
ILPQFLDDFFPPQDIMNK	149.9955	P42858	HD HUMAN	1089.551183	2	0.768766165		
ILPQFLDDFFPPQDIMNK	149.9955	P42858	HD HUMAN	726.7033967	3	0.768766165		
IMASFGNFANDNEIK	55.19914	P42858	HD HUMAN	835.896328	2	0.790823817		
IMASFGNFANDNEIK	55.19914	P42858	HD HUMAN	557.6001603	3	0.790823817		
INTLGWTSR	27.82257	P42858	HD HUMAN	524.283272	2	0.780250669		
INTLGWTSR	27.82257	P42858	HD HUMAN	349.858123	3	0.780250669		
IQEYLQSSGLAQR	29.18857	P42858	HD HUMAN	746.8918995	2	0.790893912		
IQEYLQSSGLAQR	29.18857	P42858	HD HUMAN	498.2638747	3	0.790893912		
LDAESLVK	17.5567	P42858	HD HUMAN	437.748199	2	0.641333342		
LDAESLVK	17.5567	P42858	HD HUMAN	292.1680743	3	0.641333342		
LFEPLVIK	64.70583	P42858	HD HUMAN	479.8027775	2	0.820491731	2	Yes
LFEPLVIK	64.70583	P42858	HD HUMAN	320.20446	3	0.820491731	2	
LGQVSIHSVWLGNSITPLR	74.51176	P42858	HD HUMAN	1039.08183	2	0.725070238		
LGQVSIHSVWLGNSITPLR	74.51176	P42858	HD HUMAN	693.0571613	3	0.725070238		
LGWSPKPGGDFGTAFPEIPVEFLQEK	122.3393	P42858	HD HUMAN	1423.724168	2	0.721164525		
LGWSPKPGGDFGTAFPEIPVEFLQEK	122.3393	P42858	HD HUMAN	949.4853867	3	0.721164525		

LLLQINPER	45.62943	P42858	HD_HUMAN	548.330222	2	0.778636217		
LLLQINPER	45.62943	P42858	HD_HUMAN	365.889423	3	0.778636217		
LLLSEQLSR	34.49435	P42858	HD_HUMAN	529.8144045	2	0.723427951		
LLLSEQLSR	34.49435	P42858	HD_HUMAN	353.5455447	3	0.723427951		
LLMHETQPPSHFSVSTITR	31.63165	P42858	HD_HUMAN	1091.060238	2	0.811529994		
LLMHETQPPSHFSVSTITR	31.63165	P42858	HD_HUMAN	727.7094333	3	0.811529994		
LLSASFLLTGGK	71.23564	P42858	HD_HUMAN	603.8588125	2	0.753177106		
LLSASFLLTGGK	71.23564	P42858	HD_HUMAN	402.9084833	3	0.753177106		
LLSPQMSGEEEDSDLAAK	37.73892	P42858	HD_HUMAN	960.449319	2	0.730880022		
LLSPQMSGEEEDSDLAAK	37.73892	P42858	HD_HUMAN	640.6354877	3	0.730880022		
LNDLFGDAALYQSLPTLAR	116.1418	P42858	HD_HUMAN	1039.550024	2	0.739911914		
LNDLFGDAALYQSLPTLAR	116.1418	P42858	HD_HUMAN	693.369291	3	0.739911914		
LPLVNSYTR	30.26383	P42858	HD_HUMAN	531.801293	2	0.752784789		
LPLVNSYTR	30.26383	P42858	HD_HUMAN	354.870137	3	0.752784789		
LPSHLHLPPEK	2.141933	P42858	HD_HUMAN	634.3620535	2	0.839352846		
LPSHLHLPPEK	2.141933	P42858	HD_HUMAN	423.2439773	3	0.839352846		
LQLELYK	36.49065	P42858	HD_HUMAN	453.7689305	2	0.709973633		
LQLELYK	36.49065	P42858	HD_HUMAN	302.848562	3	0.709973633		
QDSLSPSPPVSSHPLDGDGHVSLETVSPDKDWYV	72.35076	P42858	HD_HUMAN	2279.116301	2	0.612191916		
QDSLSPSPPVSSHPLDGDGHVSLETVSPDKDWYV	72.35076	P42858	HD_HUMAN	1519.746809	3	0.612191916		
LVSFLEAK	36.65893	P42858	HD_HUMAN	453.7689345	2	0.673425138		
LVSFLEAK	36.65893	P42858	HD_HUMAN	302.8485647	3	0.673425138		
LYSLLDR	35.56327	P42858	HD_HUMAN	440.2509045	2	0.675549328		
LYSLLDR	35.56327	P42858	HD_HUMAN	293.8365447	3	0.675549328		
MVADECLNK	-7.00988	P42858	HD_HUMAN	540.2471835	2	0.72063905		
MVADECLNK	-7.00988	P42858	HD_HUMAN	360.5007307	3	0.72063905		
NLPEETFSR	15.80131	P42858	HD_HUMAN	546.770194	2	0.642340779		
NLPEETFSR	15.80131	P42858	HD_HUMAN	364.8494043	3	0.642340779		
NMVQAEQENDTSGWFDVLQK	87.79855	P42858	HD_HUMAN	1170.034614	2	0.71131748		
NMVQAEQENDTSGWFDVLQK	87.79855	P42858	HD_HUMAN	780.3590173	3	0.71131748		
NQFELMYVTLTELR	113.0433	P42858	HD_HUMAN	878.9511	2	0.696735203		
NQFELMYVTLTELR	113.0433	P42858	HD_HUMAN	586.3033417	3	0.696735203		
NSAASGLFIQAIQSR	66.32114	P42858	HD_HUMAN	781.9184525	2	0.7922405		
NSAASGLFIQAIQSR	66.32114	P42858	HD_HUMAN	521.61491	3	0.7922405		
NSGVPAFLTPLLR	93.64674	P42858	HD_HUMAN	692.901543	2	0.687555015		
NSGVPAFLTPLLR	93.64674	P42858	HD_HUMAN	462.2703037	3	0.687555015		
NSSMALQQAHLLK	18.82298	P42858	HD_HUMAN	720.885567	2	0.785315573		
NSSMALQQAHLLK	18.82298	P42858	HD_HUMAN	480.9263197	3	0.785315573		
NSSYWLVR	31.89469	P42858	HD_HUMAN	512.76471	2	0.682987094		
NSSYWLVR	31.89469	P42858	HD_HUMAN	342.1790817	3	0.682987094		
RPEESVQETLAAAVPK	36.94266	P42858	HD_HUMAN	862.9630575	2	0.832640648		
RPEESVQETLAAAVPK	36.94266	P42858	HD_HUMAN	575.6446467	3	0.832640648		
SALFEAAR	13.26715	P42858	HD_HUMAN	432.7328835	2	0.697450876		
SALFEAAR	13.26715	P42858	HD_HUMAN	288.8245307	3	0.697450876		
SDGCGGSFYTLDSLNLR	75.07935	P42858	HD_HUMAN	931.4234345	2	0.738454819		
SDGCGGSFYTLDSLNLR	75.07935	P42858	HD_HUMAN	621.284898	3	0.738454819		
SDSALLEGAELVNR	56.28638	P42858	HD_HUMAN	737.381368	2	0.848651409	2	Yes
SDSALLEGAELVNR	56.28638	P42858	HD_HUMAN	491.9235203	3	0.848651409	2	
SDVSSSALTASVK	14.39536	P42858	HD_HUMAN	626.32553	2	0.752949536		
SDVSSSALTASVK	14.39536	P42858	HD_HUMAN	417.886295	3	0.752949536		
SGSIVELIAGGGSSCSPVLSR	85.89102	P42858	HD_HUMAN	1017.020777	2	0.658596039		
SGSIVELIAGGGSSCSPVLSR	85.89102	P42858	HD_HUMAN	678.349793	3	0.658596039		
SLLVVSDLFTER	96.13483	P42858	HD_HUMAN	689.8830155	2	0.841243744		
SLLVVSDLFTER	96.13483	P42858	HD_HUMAN	460.257952	3	0.841243744		
SSSLGSFYHLPSYLK	62.17721	P42858	HD_HUMAN	843.430852	2	0.868524551	3	
SSSLGSFYHLPSYLK	62.17721	P42858	HD_HUMAN	562.6231763	3	0.868524551	3	Yes
SSWASEEEANPAATK	6.167507	P42858	HD_HUMAN	789.35809	2	0.730807364	2	Yes
SSWASEEEANPAATK	6.167507	P42858	HD_HUMAN	526.5746683	3	0.730807364	2	
TELLETLAEIDFR	107.319	P42858	HD_HUMAN	775.409595	2	0.781908512		
TELLETLAEIDFR	107.319	P42858	HD_HUMAN	517.2756717	3	0.781908512		
TLFGTNLASQFDGLSSNPSK	85.38458	P42858	HD_HUMAN	1042.518925	2	0.736929178		
TLFGTNLASQFDGLSSNPSK	85.38458	P42858	HD_HUMAN	695.348558	3	0.736929178		
TLTGNTFSLADCIPLLR	106.8535	P42858	HD_HUMAN	946.501493	2	0.754682302		
TLTGNTFSLADCIPLLR	106.8535	P42858	HD_HUMAN	631.336937	3	0.754682302		
TSDPNPAAPDSESVIVAMER	59.62101	P42858	HD_HUMAN	1043.49205	2	0.798087895		
TSDPNPAAPDSESVIVAMER	59.62101	P42858	HD_HUMAN	695.997308	3	0.798087895		
VEMLLAANLQSSMAQLPMEELNR	119.444	P42858	HD_HUMAN	1294.648233	2	0.702706456		
VEMLLAANLQSSMAQLPMEELNR	119.444	P42858	HD_HUMAN	863.4347637	3	0.702706456		
VFQTLHSTGQSSMVR	7.327927	P42858	HD_HUMAN	839.4230455	2	0.872948945		
VFQTLHSTGQSSMVR	7.327927	P42858	HD_HUMAN	559.9513053	3	0.872948945		
VHPSEDEILAQYLVPATCK	65.21049	P42858	HD_HUMAN	1085.546625	2	0.806802511		
VHPSEDEILAQYLVPATCK	65.21049	P42858	HD_HUMAN	724.0336913	3	0.806802511		
VIAAVSHELITSTTR	30.54372	P42858	HD_HUMAN	799.449586	2	0.811108947		
VIAAVSHELITSTTR	30.54372	P42858	HD_HUMAN	533.3023323	3	0.811108947		
VINICAHVLDDVAPGPAIK	65.96571	P42858	HD_HUMAN	1001.543692	2	0.714107215		
VINICAHVLDDVAPGPAIK	65.96571	P42858	HD_HUMAN	668.0317363	3	0.714107215		
VLISQSTEDIVLSR	55.32774	P42858	HD_HUMAN	780.4361435	2	0.885645688		
VLISQSTEDIVLSR	55.32774	P42858	HD_HUMAN	520.626704	3	0.885645688		
VLLGEEEALEDDSESR	41.75498	P42858	HD_HUMAN	895.9187085	2	0.752035081		
VLLGEEEALEDDSESR	41.75498	P42858	HD_HUMAN	597.6150807	3	0.752035081		
VLNNVVIHLLGDEDPR	66.15186	P42858	HD_HUMAN	901.9921485	2	0.764469862	3	
VLNNVVIHLLGDEDPR	66.15186	P42858	HD_HUMAN	601.6640407	3	0.764469862	3	Yes
VPLDTTEYPEEQYVSDILNYIDHGDPQVR	118.7688	P42858	HD_HUMAN	1703.310236	2	0.784995317		
VPLDTTEYPEEQYVSDILNYIDHGDPQVR	118.7688	P42858	HD_HUMAN	1135.876099	3	0.784995317		
VSGTVQQLPAVHHVFQPELPAEPAAYWSK	72.98717	P42858	HD_HUMAN	1593.822732	2	0.812986434		
VSGTVQQLPAVHHVFQPELPAEPAAYWSK	72.98717	P42858	HD_HUMAN	1062.88443	3	0.812986434		
VSVLFDR	30.69141	P42858	HD_HUMAN	418.2378015	2	0.634188235		
VSVLFDR	30.69141	P42858	HD_HUMAN	279.1611427	3	0.634188235		
VTLDLQNSTEK	17.13102	P42858	HD_HUMAN	624.3280735	2	0.85012567		
VTLDLQNSTEK	17.13102	P42858	HD_HUMAN	416.5546573	3	0.85012567		
WILPSSSAR	32.42793	P42858	HD_HUMAN	508.7803645	2	0.623737991		
WILPSSSAR	32.42793	P42858	HD_HUMAN	339.5228513	3	0.623737991		
WWAEVQQTPK	33.42467	P42858	HD_HUMAN	636.822762	2	0.770635724		
WWAEVQQTPK	33.42467	P42858	HD_HUMAN	424.8844497	3	0.770635724		
YLVPLLQQQVK	63.53535	P42858	HD_HUMAN	664.901008	2	0.801833391		
YLVPLLQQQVK	63.53535	P42858	HD_HUMAN	443.6032803	3	0.801833391		
ATSYTLVESFSGK	43.63615	P42702	LIFR_HUMAN	695.3490015	2	0.762549996		
ATSYTLVESFSGK	43.63615	P42702	LIFR_HUMAN	463.9019427	3	0.762549996		
AYTDGGVGPEK	-17.89304	P42702	LIFR_HUMAN	547.2621985	2	0.772087932		
AYTDGGVGPEK	-17.89304	P42702	LIFR_HUMAN	365.1774073	3	0.772087932		
ETFYPDIPNPENCK	53.94986	P42702	LIFR_HUMAN	862.3857895	2	0.671762526		

ETFYPDIPNPENCK	53.94986	P42702	LIFR HUMAN	575.2598013	3	0.671762526		
GVENSSYLVALDK	43.93057	P42702	LIFR HUMAN	697.8622755	2	0.811704814		
GVENSSYLVALDK	43.93057	P42702	LIFR HUMAN	465.5774587	3	0.811704814		
IASMEIPNDDLK	40.7806	P42702	LIFR HUMAN	673.3375835	2	0.742691934		
IASMEIPNDDLK	40.7806	P42702	LIFR HUMAN	449.227664	3	0.742691934		
IEDTEIISPVAERPEDR	36.97682	P42702	LIFR HUMAN	984.997825	2	0.616856039	3	
IEDTEIISPVAERPEDR	36.97682	P42702	LIFR HUMAN	657.0011583	3	0.616856039	3	Yes
IEQVVGMGK	6.819355	P42702	LIFR HUMAN	480.763327	2	0.68734324		
IEQVVGMGK	6.819355	P42702	LIFR HUMAN	320.8448263	3	0.68734324		
INFLCEIEIK	74.68183	P42702	LIFR HUMAN	639.842305	2	0.779783666		
INFLCEIEIK	74.68183	P42702	LIFR HUMAN	426.8974783	3	0.779783666		
IPALSHGDYEITINSLHDFGSSTSK	61.70989	P42702	LIFR HUMAN	1345.159383	2	0.752789915	4	
IPALSHGDYEITINSLHDFGSSTSK	61.70989	P42702	LIFR HUMAN	897.1088637	3	0.752789915	4	
LNPYTLYTFR	58.54878	P42702	LIFR HUMAN	644.3407785	2	0.792802334		
LNPYTLYTFR	58.54878	P42702	LIFR HUMAN	429.8964607	3	0.792802334		
LSWHLPGNFAK	44.05505	P42702	LIFR HUMAN	635.341121	2	0.889284849		
LSWHLPGNFAK	44.05505	P42702	LIFR HUMAN	423.896689	3	0.889284849		
NDYIISVVAK	49.60915	P42702	LIFR HUMAN	561.314233	2	0.663654149		
NDYIISVVAK	49.60915	P42702	LIFR HUMAN	374.5454303	3	0.663654149		
NISWIPDSQTK	39.81203	P42702	LIFR HUMAN	644.830783	2	0.83863759		
NISWIPDSQTK	39.81203	P42702	LIFR HUMAN	430.2231303	3	0.83863759		
NLIIYWK	58.56892	P42702	LIFR HUMAN	475.279465	2	0.75653255		
NLIIYWK	58.56892	P42702	LIFR HUMAN	317.1889183	3	0.75653255		
PLPINEANGK	2.077969	P42702	LIFR HUMAN	526.7909295	2	0.672572076		
PLPINEANGK	2.077969	P42702	LIFR HUMAN	351.5298947	3	0.672572076		
SEPCLMDWR	46.33041	P42702	LIFR HUMAN	597.2580825	2	0.751677155		
SEPCLMDWR	46.33041	P42702	LIFR HUMAN	398.5079967	3	0.751677155		
SQSTILVNITEK	41.99149	P42702	LIFR HUMAN	666.872648	2	0.700722337		
SQSTILVNITEK	41.99149	P42702	LIFR HUMAN	444.917707	3	0.700722337		
TLEMNPCTPNNVEVLETR	57.66542	P42702	LIFR HUMAN	1059.004271	2	0.744094551		
TLEMNPCTPNNVEVLETR	57.66542	P42702	LIFR HUMAN	706.3387887	3	0.744094551		
TSYHLVLR	2.987087	P42702	LIFR HUMAN	494.7829035	2	0.717769206		
TSYHLVLR	2.987087	P42702	LIFR HUMAN	330.1912107	3	0.717769206		
VLSALIGHTNCPLIHLDGENVAIK	67.51207	P42702	LIFR HUMAN	1292.699972	2	0.782260418		
VLSALIGHTNCPLIHLDGENVAIK	67.51207	P42702	LIFR HUMAN	862.135923	3	0.782260418		
VYPHTPTSFK	-4.288143	P42702	LIFR HUMAN	588.806576	2	0.700621068		
VYPHTPTSFK	-4.288143	P42702	LIFR HUMAN	392.873659	3	0.700621068		
WEDIPVEELR	58.2093	P42702	LIFR HUMAN	643.3253335	2	0.780154407		
WEDIPVEELR	58.2093	P42702	LIFR HUMAN	429.2194973	3	0.780154407		
YNFFLYGCR	60.77869	P42702	LIFR HUMAN	620.284949	2	0.613950372		
YNFFLYGCR	60.77869	P42702	LIFR HUMAN	413.859241	3	0.613950372		
AALGAVPISGK	21.20753	P42684	ABL2 HUMAN	492.298391	2	0.815117955		
AALGAVPISGK	21.20753	P42684	ABL2 HUMAN	328.534869	3	0.815117955		
AAPVLPTTHNHK	-28.3946	P42684	ABL2 HUMAN	643.3547605	2	0.649311066		
AAPVLPTTHNHK	-28.3946	P42684	ABL2 HUMAN	429.2391153	3	0.649311066		
AASSSSVVPYLPR	40.87169	P42684	ABL2 HUMAN	667.359703	2	0.679567814		
AASSSSVVPYLPR	40.87169	P42684	ABL2 HUMAN	445.2424103	3	0.679567814		
AGRPVMPPPQVPLPTSSISPAK	53.68784	P42684	ABL2 HUMAN	1114.117556	2	0.613659322		
AGRPVMPPPQVPLPTSSISPAK	53.68784	P42684	ABL2 HUMAN	743.0809787	3	0.613659322		
DPPGVGVAGVAAAPK	36.65816	P42684	ABL2 HUMAN	653.3622515	2	0.773014367		
DPPGVGVAGVAAAPK	36.65816	P42684	ABL2 HUMAN	435.910776	3	0.773014367		
ENIEGAQDATENSASSLAPGFIR	64.70585	P42684	ABL2 HUMAN	1189.067499	2	0.65867424		
ENIEGAQDATENSASSLAPGFIR	64.70585	P42684	ABL2 HUMAN	793.0476073	3	0.65867424		
ESESSPGQLSISLR	45.36269	P42684	ABL2 HUMAN	745.378825	2	0.839254558		
ESESSPGQLSISLR	45.36269	P42684	ABL2 HUMAN	497.2551583	3	0.839254558		
FSTLAELVHHHSTVADGLVTTLHYPAPK	69.44798	P42684	ABL2 HUMAN	1521.29635	2	0.68856287		
FSTLAELVHHHSTVADGLVTTLHYPAPK	69.44798	P42684	ABL2 HUMAN	1014.533508	3	0.68856287		
GGFFSSFMK	62.23726	P42684	ABL2 HUMAN	504.2369445	2	0.76733917		
GGFFSSFMK	62.23726	P42684	ABL2 HUMAN	336.4939047	3	0.76733917		
HTPADVQLIGTDSQGNK	14.00096	P42684	ABL2 HUMAN	890.9453965	2	0.826344848		
HTPADVQLIGTDSQGNK	14.00096	P42684	ABL2 HUMAN	594.2995393	3	0.826344848		
LGGGQYGEVYVGVWK	63.82854	P42684	ABL2 HUMAN	806.412464	2	0.783958912		
LGGGQYGEVYVGVWK	63.82854	P42684	ABL2 HUMAN	537.944251	3	0.783958912		
LGMAGVPEDGEQPGWPSPAK	56.72388	P42684	ABL2 HUMAN	1011.983665	2	0.722848952		
LGMAGVPEDGEQPGWPSPAK	56.72388	P42684	ABL2 HUMAN	674.991718	3	0.722848952		
LLQHPSICSDPTEEPTALTAGQSTSETQEGGK	36.10811	P42684	ABL2 HUMAN	1685.291732	2	0.747076035		
LLQHPSICSDPTEEPTALTAGQSTSETQEGGK	36.10811	P42684	ABL2 HUMAN	1123.863763	3	0.747076035		
LLSEHQVTSSGDKDRPR	-32.02122	P42684	ABL2 HUMAN	962.995951	2	0.785687923		
LLSEHQVTSSGDKDRPR	-32.02122	P42684	ABL2 HUMAN	642.3332423	3	0.785687923		
LPILPSK	29.84274	P42684	ABL2 HUMAN	384.255463	2	0.603538334		
LPILPSK	29.84274	P42684	ABL2 HUMAN	256.5062503	3	0.603538334		
MEQPEGCPPK	-26.15942	P42684	ABL2 HUMAN	586.757916	2	0.702480972		
MEQPEGCPPK	-26.15942	P42684	ABL2 HUMAN	391.5078857	3	0.702480972		
SPSSLLEDAK	16.34566	P42684	ABL2 HUMAN	523.772402	2	0.638096809		
SPSSLLEDAK	16.34566	P42684	ABL2 HUMAN	349.517543	3	0.638096809		
TPSGDLAITEK	6.791759	P42684	ABL2 HUMAN	566.298785	2	0.71179378		
TPSGDLAITEK	6.791759	P42684	ABL2 HUMAN	377.868465	3	0.71179378		
TTETGFNIFTQHDHFASCVEDGFEGDK	64.19072	P42684	ABL2 HUMAN	1545.165074	2	0.761852145		
TTETGFNIFTQHDHFASCVEDGFEGDK	64.19072	P42684	ABL2 HUMAN	1030.445991	3	0.761852145		
VGEAPGLQQPQPR	6.743992	P42684	ABL2 HUMAN	688.8682325	2	0.839205623		
VGEAPGLQQPQPR	6.743992	P42684	ABL2 HUMAN	459.58143	3	0.839205623		
VLGYNQNGEWSEVR	35.38528	P42684	ABL2 HUMAN	825.8977125	2	0.687791944		
VLGYNQNGEWSEVR	35.38528	P42684	ABL2 HUMAN	550.9344167	3	0.687791944		
VPVLISPTLK	54.79179	P42684	ABL2 HUMAN	533.8477165	2	0.720941961	2	Yes
VPVLISPTLK	54.79179	P42684	ABL2 HUMAN	356.2344193	3	0.720941961	2	
WTAPESLAYNTFSIK	76.50197	P42684	ABL2 HUMAN	864.4361395	2	0.817888379		
WTAPESLAYNTFSIK	76.50197	P42684	ABL2 HUMAN	576.6267013	3	0.817888379		
DLDVAILVGSMPR	109.5532	P40925	MDHC HUMAN	693.3770435	2	0.716658115		
DLDVAILVGSMPR	109.5532	P40925	MDHC HUMAN	462.587304	3	0.716658115		
EVGVYEALK	28.82322	P40925	MDHC HUMAN	504.274577	2	0.649794102	2	Yes
EVGVYEALK	28.82322	P40925	MDHC HUMAN	336.518993	3	0.649794102	2	
FVEGLPINDFSR	67.75051	P40925	MDHC HUMAN	697.3597075	2	0.826903522	2	Yes
FVEGLPINDFSR	67.75051	P40925	MDHC HUMAN	465.2424133	3	0.826903522	2	
GEFVTTVQQR	8.49855	P40925	MDHC HUMAN	582.8045695	2	0.800335586	2	Yes
GEFVTTVQQR	8.49855	P40925	MDHC HUMAN	388.8723213	3	0.800335586	2	
LGVTANDVK	-3.610271	P40925	MDHC HUMAN	458.7590985	2	0.73446095	2	Yes
LGVTANDVK	-3.610271	P40925	MDHC HUMAN	306.1753407	3	0.73446095	2	
NVIIWGNHSSTQYPDVNHAK	24.16879	P40925	MDHC HUMAN	1140.561985	2	0.784568191	3	
NVIIWGNHSSTQYPDVNHAK	24.16879	P40925	MDHC HUMAN	760.7105983	3	0.784568191	3	Yes
VIVVGNPANTNCLTASK	35.69318	P40925	MDHC HUMAN	879.46491	2	0.720009506	3	
VIVVGNPANTNCLTASK	35.69318	P40925	MDHC HUMAN	586.6458817	3	0.720009506	3	Yes

ALQFLEEVK	51.42191	P40227	TCPZ HUMAN	538.803506	2	0.812105298	2	Yes
ALQFLEEVK	51.42191	P40227	TCPZ HUMAN	359.538279	3	0.812105298	2	
AQAALAVNISAAR	34.19493	P40227	TCPZ HUMAN	628.3600425	2	0.73898077	2	Yes
AQAALAVNISAAR	34.19493	P40227	TCPZ HUMAN	419.2426367	3	0.73898077	2	
AQLGVQAFADALLIIPK	135.1754	P40227	TCPZ HUMAN	884.5225545	2	0.610518456		
AQLGVQAFADALLIIPK	135.1754	P40227	TCPZ HUMAN	590.0176447	3	0.610518456		
DGNVLLHEMQIQHPTASLIAK	56.08802	P40227	TCPZ HUMAN	1158.113002	2	0.855944335	3	
DGNVLLHEMQIQHPTASLIAK	56.08802	P40227	TCPZ HUMAN	772.411276	3	0.855944335	3	Yes
GIDPFSLDALSK	84.75192	P40227	TCPZ HUMAN	631.835534	2	0.796854317	2	Yes
GIDPFSLDALSK	84.75192	P40227	TCPZ HUMAN	421.559631	3	0.796854317	2	
GLVLDHGAR	-20.52377	P40227	TCPZ HUMAN	469.2648825	2	0.691382527	2	Yes
GLVLDHGAR	-20.52377	P40227	TCPZ HUMAN	313.1791967	3	0.691382527	2	
NAIDDGCVVPGAGAVEVAMAEALIK	137.597	P40227	TCPZ HUMAN	1235.62	2	0.703739226		
NAIDDGCVVPGAGAVEVAMAEALIK	137.597	P40227	TCPZ HUMAN	824.082608	3	0.703739226		
VATAQDDITGDGTTSNVLIIGELLK	124.3944	P40227	TCPZ HUMAN	1272.674341	2	0.843271017	3	
VATAQDDITGDGTTSNVLIIGELLK	124.3944	P40227	TCPZ HUMAN	848.785502	3	0.843271017	3	Yes
VEDAYILTCNVSLEYEK	67.48104	P40227	TCPZ HUMAN	1023.490979	2	0.731562257		
VEDAYILTCNVSLEYEK	67.48104	P40227	TCPZ HUMAN	682.6632607	3	0.731562257		
VLAQNSGFDLQETLVK	67.19672	P40227	TCPZ HUMAN	881.473258	2	0.921638191	3	
VLAQNSGFDLQETLVK	67.19672	P40227	TCPZ HUMAN	587.9847803	3	0.921638191	3	Yes
EAHQLFLEPEVLDPESVELK	84.86255	P39748	FEN1 HUMAN	1161.597372	2	0.62119031	3	
EAHQLFLEPEVLDPESVELK	84.86255	P39748	FEN1 HUMAN	774.7341897	3	0.62119031	3	Yes
LIADVAPSAIR	35.57188	P39748	FEN1 HUMAN	563.3355045	2	0.827224135	2	Yes
LIADVAPSAIR	35.57188	P39748	FEN1 HUMAN	375.8929447	3	0.827224135	2	
LPIQEFHLSR	34.05804	P39748	FEN1 HUMAN	620.3464035	2	0.709675789	3	
LPIQEFHLSR	34.05804	P39748	FEN1 HUMAN	413.9002107	3	0.709675789	3	Yes
QLQQAQAAGAEQEVEK	-0.621872	P39748	FEN1 HUMAN	864.4321225	2	0.643569529	2	Yes
QLQQAQAAGAEQEVEK	-0.621872	P39748	FEN1 HUMAN	576.6240233	3	0.643569529	2	
WSEPNEEELIK	35.62635	P39748	FEN1 HUMAN	687.333355	2	0.76988095	2	Yes
WSEPNEEELIK	35.62635	P39748	FEN1 HUMAN	458.5581783	3	0.76988095	2	
YPVPENWLHK	34.35778	P39748	FEN1 HUMAN	641.8331245	2	0.790919423	3	
YPVPENWLHK	34.35778	P39748	FEN1 HUMAN	428.2246913	3	0.790919423	3	Yes
ALAAFLK	33.73175	P39019	RS19 HUMAN	367.234531	2	0.654371023	2	Yes
ALAAFLK	33.73175	P39019	RS19 HUMAN	245.1589623	3	0.654371023	2	
DVNQQEFVR	9.447075	P39019	RS19 HUMAN	567.7810935	2	0.729920328	2	Yes
DVNQQEFVR	9.447075	P39019	RS19 HUMAN	378.8566707	3	0.729920328	2	
ELAPYDENWFYTR	73.33891	P39019	RS19 HUMAN	852.389185	2	0.820724607	2	Yes
ELAPYDENWFYTR	73.33891	P39019	RS19 HUMAN	568.5953983	3	0.820724607	2	
VLQALEGLK	38.84113	P39019	RS19 HUMAN	485.8007665	2	0.842284858	2	Yes
VLQALEGLK	38.84113	P39019	RS19 HUMAN	324.2031193	3	0.842284858	2	
ALDEYYDK	2.909691	P38606	VATA HUMAN	508.7327375	2	0.801618814	2	Yes
ALDEYYDK	2.909691	P38606	VATA HUMAN	339.4911	3	0.801618814	2	
DDFLQQNGYTPYDR	52.24078	P38606	VATA HUMAN	866.3846315	2	0.749326468		
DDFLQQNGYTPYDR	52.24078	P38606	VATA HUMAN	577.925696	3	0.749326468		
DFPELTMEVDGK	70.91026	P38606	VATA HUMAN	690.821767	2	0.80233562		
DFPELTMEVDGK	70.91026	P38606	VATA HUMAN	460.8837863	3	0.80233562		
EASIYTGITLSEYFR	95.73944	P38606	VATA HUMAN	875.438875	2	0.820930481		
EASIYTGITLSEYFR	95.73944	P38606	VATA HUMAN	583.9618583	3	0.820930481		
EILQEEEDLAEIVQLVGK	137.3104	P38606	VATA HUMAN	1028.046984	2	0.766257226		
EILQEEEDLAEIVQLVGK	137.3104	P38606	VATA HUMAN	685.7005977	3	0.766257226		
FTMVQVWPVR	75.62029	P38606	VATA HUMAN	631.8399005	2	0.702114284	2	Yes
FTMVQVWPVR	75.62029	P38606	VATA HUMAN	421.562542	3	0.702114284	2	
HFPSVNWLISYSK	69.25269	P38606	VATA HUMAN	789.409727	2	0.709413767		
HFPSVNWLISYSK	69.25269	P38606	VATA HUMAN	526.609093	3	0.709413767		
LAEMPADSGYPAYLGAR	59.42595	P38606	VATA HUMAN	891.4305275	2	0.851003766	3	
LAEMPADSGYPAYLGAR	59.42595	P38606	VATA HUMAN	594.62296	3	0.851003766	3	Yes
LASFYER	5.383564	P38606	VATA HUMAN	443.2274295	2	0.628789961		
LASFYER	5.383564	P38606	VATA HUMAN	295.8208947	3	0.628789961		
LPANHPLLTGQR	0.024467	P38606	VATA HUMAN	658.87586	2	0.788668036	3	
LPANHPLLTGQR	0.024467	P38606	VATA HUMAN	439.586515	3	0.788668036	3	Yes
SDYAQLLEDMQNAFR	120.2211	P38606	VATA HUMAN	900.9152455	2	0.814826548	3	
SDYAQLLEDMQNAFR	120.2211	P38606	VATA HUMAN	600.9461053	3	0.814826548	3	Yes
TALVANTSNMPVAAR	29.52399	P38606	VATA HUMAN	758.4015815	2	0.796239316	2	Yes
TALVANTSNMPVAAR	29.52399	P38606	VATA HUMAN	505.936996	3	0.796239316	2	
VGHSELVGEIIR	28.03143	P38606	VATA HUMAN	654.8677	2	0.832461238	2	Yes
VGHSELVGEIIR	28.03143	P38606	VATA HUMAN	436.9144083	3	0.832461238	2	
VGSHITGGDIYGIVSENSLIK	66.58475	P38606	VATA HUMAN	1080.07132	2	0.735845447		
VGSHITGGDIYGIVSENSLIK	66.58475	P38606	VATA HUMAN	720.3834883	3	0.735845447		
VLDALFPCVQGGTTAIPGAFGCGK	108.9564	P38606	VATA HUMAN	1218.606697	2	0.701171279		
VLDALFPCVQGGTTAIPGAFGCGK	108.9564	P38606	VATA HUMAN	812.740406	3	0.701171279		
YSNSDVIIYVGCGER	49.61628	P38606	VATA HUMAN	866.404509	2	0.624686897		
YSNSDVIIYVGCGER	49.61628	P38606	VATA HUMAN	577.9389477	3	0.624686897		
VEQATKPSFESGR	-22.84923	P38159	RBMX HUMAN	718.362979	2	0.671391308	3	
VEQATKPSFESGR	-22.84923	P38159	RBMX HUMAN	479.2445943	3	0.671391308	3	Yes
EIDGGLETLR	30.75113	P38117	ETFB HUMAN	551.791127	2	0.751408637	2	Yes
EIDGGLETLR	30.75113	P38117	ETFB HUMAN	368.196693	3	0.751408637	2	
EVIAVSCGPAQCQETIR	29.06155	P38117	ETFB HUMAN	959.4620425	2	0.728409767		
EVIAVSCGPAQCQETIR	29.06155	P38117	ETFB HUMAN	639.9773033	3	0.728409767		
GIHVEVPPAEAER	13.25185	P38117	ETFB HUMAN	702.3680645	2	0.835191011	2	Yes
GIHVEVPPAEAER	13.25185	P38117	ETFB HUMAN	468.581318	3	0.835191011	2	
HSMNPFCEIAVEEAVR	57.96602	P38117	ETFB HUMAN	944.938202	2	0.799901366	3	
HSMNPFCEIAVEEAVR	57.96602	P38117	ETFB HUMAN	630.294743	3	0.799901366	3	Yes
IEVIKPGDLGVDLTSK	53.87259	P38117	ETFB HUMAN	842.4805515	2	0.797281146		
IEVIKPGDLGVDLTSK	53.87259	P38117	ETFB HUMAN	561.9896427	3	0.797281146		
LGPLQVAR	17.02267	P38117	ETFB HUMAN	427.266894	2	0.797053695	2	Yes
LGPLQVAR	17.02267	P38117	ETFB HUMAN	285.1805377	3	0.797053695	2	
LPAVVTADLR	41.98364	P38117	ETFB HUMAN	527.816948	2	0.762563586	2	Yes
LPAVVTADLR	41.98364	P38117	ETFB HUMAN	352.213907	3	0.762563586	2	
LSVISVEDPPQR	41.02915	P38117	ETFB HUMAN	670.36499	2	0.850785255	2	Yes
LSVISVEDPPQR	41.02915	P38117	ETFB HUMAN	447.245935	3	0.850785255	2	
VDLVLLGK	56.83591	P38117	ETFB HUMAN	428.7793025	2	0.679538906	2	Yes
VDLVLLGK	56.83591	P38117	ETFB HUMAN	286.18881	3	0.679538906	2	
YATLPNIMK	41.98866	P38117	ETFB HUMAN	525.7867975	2	0.809611022		
YATLPNIMK	41.98866	P38117	ETFB HUMAN	350.8604733	3	0.809611022		
DDGLFSGDPNWFPK	99.96834	P37802	TAGL2 HUMAN	797.8628115	2	0.874990463	2	Yes
DDGLFSGDPNWFPK	99.96834	P37802	TAGL2 HUMAN	532.2444827	3	0.874990463	2	
DGTVLCELINALYPEGQAPVK	151.8938	P37802	TAGL2 HUMAN	1144.086114	2	0.743412733	3	
DGTVLCELINALYPEGQAPVK	151.8938	P37802	TAGL2 HUMAN	763.0600177	3	0.743412733	3	Yes
ENFQNWLK	42.27927	P37802	TAGL2 HUMAN	539.769997	2	0.781202793	2	Yes
ENFQNWLK	42.27927	P37802	TAGL2 HUMAN	360.1826063	3	0.781202793	2	
GPAYGLSR	-11.68863	P37802	TAGL2 HUMAN	410.719772	2	0.656606972	2	Yes

GPAYGLSR	-11.68863	P37802	TAGL2_HUMAN	274.149123	3	0.656606972	2	
NFSDNQLQEGK	-0.51828	P37802	TAGL2_HUMAN	640.299847	2	0.80305016	2	Yes
NFSDNQLQEGK	-0.51828	P37802	TAGL2_HUMAN	427.2025063	3	0.80305016	2	
NVIGLQMGTNR	28.16827	P37802	TAGL2_HUMAN	601.819696	2	0.816002429	2	Yes
NVIGLQMGTNR	28.16827	P37802	TAGL2_HUMAN	401.5490723	3	0.816002429	2	
TLMNLGGLAVAR	65.78429	P37802	TAGL2_HUMAN	608.3480895	2	0.815506101	2	Yes
TLMNLGGLAVAR	65.78429	P37802	TAGL2_HUMAN	405.9013347	3	0.815506101	2	
YGINTTDIFQTVDLWEGK	123.3853	P37802	TAGL2_HUMAN	1050.51839	2	0.780201614	3	
YGINTTDIFQTVDLWEGK	123.3853	P37802	TAGL2_HUMAN	700.681535	3	0.780201614	3	Yes
LNIDSIIQR	52.97425	P36873	PP1G_HUMAN	536.312029	2	0.77833271	2	Yes
LNIDSIIQR	52.97425	P36873	PP1G_HUMAN	357.8772943	3	0.77833271	2	
NVQLQENEIR	5.142982	P36873	PP1G_HUMAN	621.826032	2	0.819374263	2	Yes
NVQLQENEIR	5.142982	P36873	PP1G_HUMAN	414.8866297	3	0.819374263	2	
PTDVPDQGLLCDLLWSDPDKDVLGWGENDR	132.2169	P36873	PP1G_HUMAN	1713.301901	2	0.680066764		
PTDVPDQGLLCDLLWSDPDKDVLGWGENDR	132.2169	P36873	PP1G_HUMAN	1142.537209	3	0.680066764		
ELQSLLVEER	44.32616	P36404	ARL2_HUMAN	608.3331585	2	0.69970721	2	Yes
ELQSLLVEER	44.32616	P36404	ARL2_HUMAN	405.8913807	3	0.69970721	2	
EVLELDSIR	44.14501	P36404	ARL2_HUMAN	537.2960445	2	0.842944086		
EVLELDSIR	44.14501	P36404	ARL2_HUMAN	358.5333047	3	0.842944086		
LAGATLLIFANK	76.84142	P36404	ARL2_HUMAN	616.37463	2	0.714630365	2	Yes
LAGATLLIFANK	76.84142	P36404	ARL2_HUMAN	411.2523617	3	0.714630365	2	
LLMLGLDNAGK	59.79444	P36404	ARL2_HUMAN	572.8239155	2	0.842311025	2	Yes
LLMLGLDNAGK	59.79444	P36404	ARL2_HUMAN	382.218552	3	0.842311025	2	
LNIWDVGGQK	51.313	P36404	ARL2_HUMAN	565.3042045	2	0.821824312		
LNIWDVGGQK	51.313	P36404	ARL2_HUMAN	377.2054113	3	0.821824312		
ILNFLMHPKPSGKPLPK	28.35065	P35659	DEK_HUMAN	959.061323	2	0.641536176		
ILNFLMHPKPSGKPLPK	28.35065	P35659	DEK_HUMAN	639.710157	3	0.641536176		
LLASANLEEVTMK	50.25179	P35659	DEK_HUMAN	709.8821585	2	0.809877396	2	Yes
LLASANLEEVTMK	50.25179	P35659	DEK_HUMAN	473.590714	3	0.809877396	2	
LLYNRPGTVSSLK	13.26023	P35659	DEK_HUMAN	724.4175525	2	0.749128222	3	
LLYNRPGTVSSLK	13.26023	P35659	DEK_HUMAN	483.2809767	3	0.749128222	3	Yes
LTMQVSSLQR	22.95978	P35659	DEK_HUMAN	581.816622	2	0.793102324	2	Yes
LTMQVSSLQR	22.95978	P35659	DEK_HUMAN	388.2136897	3	0.793102324	2	
NVGQFSGFPFEK	62.08956	P35659	DEK_HUMAN	678.833326	2	0.804519713	2	Yes
NVGQFSGFPFEK	62.08956	P35659	DEK_HUMAN	452.8914923	3	0.804519713	2	
SICEVLDLER	58.40865	P35659	DEK_HUMAN	617.311369	2	0.771408379	2	Yes
SICEVLDLER	58.40865	P35659	DEK_HUMAN	411.8768543	3	0.771408379	2	
VYENYPTYDLTER	39.484	P35659	DEK_HUMAN	831.886471	2	0.846776843	2	Yes
VYENYPTYDLTER	39.484	P35659	DEK_HUMAN	554.9269223	3	0.846776843	2	
GGFPSGGGGGGGGQQR	-25.72544	P35637	FUS_HUMAN	638.295432	2	0.669206798		
GGFPSGGGGGGGGQQR	-25.72544	P35637	FUS_HUMAN	425.8662297	3	0.669206798		
SSGGYEPR	-28.82903	P35637	FUS_HUMAN	426.6964935	2	0.689509034		
SSGGYEPR	-28.82903	P35637	FUS_HUMAN	284.8002707	3	0.689509034		
TGQPMINLYTDR	40.9941	P35637	FUS_HUMAN	704.8486465	2	0.810406268		
TGQPMINLYTDR	40.9941	P35637	FUS_HUMAN	470.2350393	3	0.810406268		
GSDQAIITLR	30.59912	P35613	BASI_HUMAN	537.301662	2	0.799928188	2	Yes
GSDQAIITLR	30.59912	P35613	BASI_HUMAN	358.5370497	3	0.799928188	2	
KPEDVLDDDDAGSAPLK	22.22891	P35613	BASI_HUMAN	892.9316195	2	0.724097848	3	
KPEDVLDDDDAGSAPLK	22.22891	P35613	BASI_HUMAN	595.623688	3	0.724097848	3	Yes
SELHIENLNMEADPGQYR	46.3951	P35613	BASI_HUMAN	1058.49238	2	0.73236835	3	
SELHIENLNMEADPGQYR	46.3951	P35613	BASI_HUMAN	705.997528	3	0.73236835	3	Yes
SESVPPVTDWAWYK	73.68922	P35613	BASI_HUMAN	832.9019325	2	0.726562917	2	Yes
SESVPPVTDWAWYK	73.68922	P35613	BASI_HUMAN	555.6038967	3	0.726562917	2	
SSEHINEGETAMLVCK	15.25613	P35613	BASI_HUMAN	902.9143925	2	0.811509728	3	
SSEHINEGETAMLVCK	15.25613	P35613	BASI_HUMAN	602.27887	3	0.811509728	3	Yes
AAVVTSPPPTTAPHK	-8.223427	P35611	ADDA_HUMAN	737.4071905	2	0.796667337		
AAVVTSPPPTTAPHK	-8.223427	P35611	ADDA_HUMAN	491.9407353	3	0.796667337		
EYQPHVIVSTTGPNPFTTLTDR	65.08322	P35611	ADDA_HUMAN	1237.122074	2	0.702474475	3	
EYQPHVIVSTTGPNPFTTLTDR	65.08322	P35611	ADDA_HUMAN	825.083991	3	0.702474475	3	Yes
GSTNLGVNQAGFTLHSAIYAARPDVK	48.08674	P35611	ADDA_HUMAN	1344.199178	2	0.862298965		
GSTNLGVNQAGFTLHSAIYAARPDVK	48.08674	P35611	ADDA_HUMAN	896.468727	3	0.862298965		
INLQGDIVDR	31.38631	P35611	ADDA_HUMAN	571.8123935	2	0.826385796	2	Yes
INLQGDIVDR	31.38631	P35611	ADDA_HUMAN	381.544204	3	0.826385796	2	
LADLFGWSQLIYNHITTR	121.3428	P35611	ADDA_HUMAN	1074.566009	2	0.718378842		
LADLFGWSQLIYNHITTR	121.3428	P35611	ADDA_HUMAN	716.7132807	3	0.718378842		
SLVQGELVTASK	27.82793	P35611	ADDA_HUMAN	616.348809	2	0.7920627	2	Yes
SLVQGELVTASK	27.82793	P35611	ADDA_HUMAN	411.2351477	3	0.7920627	2	
SPGSPVGEGTGSPPK	-10.52838	P35611	ADDA_HUMAN	677.33643	2	0.799921632	2	Yes
SPGSPVGEGTGSPPK	-10.52838	P35611	ADDA_HUMAN	451.8935617	3	0.799921632	2	
TLASAGGPDNLVLLNPEK	67.10553	P35611	ADDA_HUMAN	904.9918145	2	0.861065626		
TLASAGGPDNLVLLNPEK	67.10553	P35611	ADDA_HUMAN	603.663818	3	0.861065626		
TSTSAVPNLFVPLNTNPK	78.27025	P35611	ADDA_HUMAN	950.512914	2	0.662881076		
TSTSAVPNLFVPLNTNPK	78.27025	P35611	ADDA_HUMAN	634.0112177	3	0.662881076		
VDENNPEYLR	11.75656	P35611	ADDA_HUMAN	624.7969355	2	0.847739279	2	Yes
VDENNPEYLR	11.75656	P35611	ADDA_HUMAN	416.867232	3	0.847739279	2	
WQIGEQEFEALMR	88.50406	P35611	ADDA_HUMAN	818.8935895	2	0.74023658		
WQIGEQEFEALMR	88.50406	P35611	ADDA_HUMAN	546.2650013	3	0.74023658		
YSDVEVPASVTGYSFASDGDSGTCSPLR	76.95111	P35611	ADDA_HUMAN	1462.648716	2	0.631637752		
YSDVEVPASVTGYSFASDGDSGTCSPLR	76.95111	P35611	ADDA_HUMAN	975.4350853	3	0.631637752		
AITFLQSATR	37.93302	P35249	RFC4_HUMAN	554.31203	2	0.810701013	2	Yes
AITFLQSATR	37.93302	P35249	RFC4_HUMAN	369.877295	3	0.810701013	2	
DLIDEGHAATQLVNQLHDVVVENNLSDK	100.3419	P35249	RFC4_HUMAN	1543.773636	2	0.775402486		
DLIDEGHAATQLVNQLHDVVVENNLSDK	100.3419	P35249	RFC4_HUMAN	1029.518366	3	0.775402486		
ELFGPELFR	75.01009	P35249	RFC4_HUMAN	554.295848	2	0.801248074	2	Yes
ELFGPELFR	75.01009	P35249	RFC4_HUMAN	369.866507	3	0.801248074	2	
GTSISTKPPLTK	-13.45382	P35249	RFC4_HUMAN	615.359177	2	0.755185127	2	Yes
GTSISTKPPLTK	-13.45382	P35249	RFC4_HUMAN	410.575393	3	0.755185127	2	
IDGVFAACQSGSFDK	40.93426	P35249	RFC4_HUMAN	801.3674045	2	0.670725346	2	Yes
IDGVFAACQSGSFDK	40.93426	P35249	RFC4_HUMAN	534.580878	3	0.670725346	2	
IIEPLTSR	17.46129	P35249	RFC4_HUMAN	464.777291	2	0.764986396	2	Yes
IIEPLTSR	17.46129	P35249	RFC4_HUMAN	310.187469	3	0.764986396	2	
ISDEGIAYLVK	54.23486	P35249	RFC4_HUMAN	604.332623	2	0.836144507	2	Yes
ISDEGIAYLVK	54.23486	P35249	RFC4_HUMAN	403.224357	3	0.836144507	2	
IVILDEADSMTSAAQAALR	86.8576	P35249	RFC4_HUMAN	988.0124215	2	0.779301167	3	
IVILDEADSMTSAAQAALR	86.8576	P35249	RFC4_HUMAN	659.0108893	3	0.779301167	3	Yes
NFAQLTVSGSR	23.57742	P35249	RFC4_HUMAN	590.310018	2	0.760875344	2	Yes
NFAQLTVSGSR	23.57742	P35249	RFC4_HUMAN	393.8759537	3	0.760875344	2	
SLEGADLPNLLFYGPPGTGK	110.7364	P35249	RFC4_HUMAN	1023.5313	2	0.768901765	3	
SLEGADLPNLLFYGPPGTGK	110.7364	P35249	RFC4_HUMAN	682.6901417	3	0.768901765	3	Yes
VITDIAGVIPAEK	62.00974	P35249	RFC4_HUMAN	663.3879345	2	0.871251881	2	Yes
VITDIAGVIPAEK	62.00974	P35249	RFC4_HUMAN	442.5945647	3	0.871251881	2	

VLELNASDER	14.9566	P35249	RFC4 HUMAN	573.294033	2	0.826131105	2	Yes
VLELNASDER	14.9566	P35249	RFC4 HUMAN	382.5319637	3	0.826131105	2	
AAELIANSLATAGDGLIELR	100.6523	P35232	PHB HUMAN	999.5474855	2	0.806728303	3	
AAELIANSLATAGDGLIELR	100.6523	P35232	PHB HUMAN	666.700932	3	0.806728303	3	Yes
DLQNVNITLR	45.81733	P35232	PHB HUMAN	593.333493	2	0.790069401	2	Yes
DLQNVNITLR	45.81733	P35232	PHB HUMAN	395.8916037	3	0.790069401	2	
FDAGELITQR	43.23525	P35232	PHB HUMAN	575.2991195	2	0.834507287	2	Yes
FDAGELITQR	43.23525	P35232	PHB HUMAN	383.868688	3	0.834507287	2	
IFTSIGEDYDER	36.40862	P35232	PHB HUMAN	722.8337155	2	0.791299224	2	Yes
IFTSIGEDYDER	36.40862	P35232	PHB HUMAN	482.2250853	3	0.791299224	2	
ILFRPVASQLPR	39.58369	P35232	PHB HUMAN	698.9253525	2	0.791340113	2	Yes
ILFRPVASQLPR	39.58369	P35232	PHB HUMAN	466.2861767	3	0.791340113	2	
LEAAEDIAYQLSR	55.50978	P35232	PHB HUMAN	739.8784575	2	0.79581821	2	Yes
LEAAEDIAYQLSR	55.50978	P35232	PHB HUMAN	493.5882467	3	0.79581821	2	
NITYLPAGQSVLLQLPQ	125.6758	P35232	PHB HUMAN	928.0203715	2	0.720674753		
NITYLPAGQSVLLQLPQ	125.6758	P35232	PHB HUMAN	619.0161893	3	0.720674753		
VLPSITTEILK	70.18142	P35232	PHB HUMAN	607.3742955	2	0.834428966	2	Yes
VLPSITTEILK	70.18142	P35232	PHB HUMAN	405.2521387	3	0.834428966	2	
LGVQQSLGWVHYMIHEPEPHILLFR	94.36066	P33552	CKS2 HUMAN	1500.289816	2	0.724418521		
LGVQQSLGWVHYMIHEPEPHILLFR	94.36066	P33552	CKS2 HUMAN	1000.529152	3	0.724418521		
YFDEHYEYR	5.424236	P33552	CKS2 HUMAN	661.2783705	2	0.786310852	3	
YFDEHYEYR	5.424236	P33552	CKS2 HUMAN	441.1881887	3	0.786310852	3	Yes
AAGYDLYSAYDYTIPPMEK	84.02727	P33316	DUT HUMAN	1084.498796	2	0.749329805		
AAGYDLYSAYDYTIPPMEK	84.02727	P33316	DUT HUMAN	723.3351387	3	0.749329805		
AEAAVLSGPGPPLGR	41.86907	P33316	DUT HUMAN	696.3862575	2	0.753199279		
AEAAVLSGPGPPLGR	41.86907	P33316	DUT HUMAN	464.5934467	3	0.753199279		
AGGSPAPGPETPAISPSK	15.9567	P33316	DUT HUMAN	810.915576	2	0.669657946	2	Yes
AGGSPAPGPETPAISPSK	15.9567	P33316	DUT HUMAN	540.9463257	3	0.669657946	2	
ARPAEVGGMQLR	1.95961	P33316	DUT HUMAN	642.8462455	2	0.678718805	3	
ARPAEVGGMQLR	1.95961	P33316	DUT HUMAN	428.9001053	3	0.678718805	3	Yes
GNVGVVLFNFGK	74.52748	P33316	DUT HUMAN	625.848779	2	0.773450792	2	Yes
GNVGVVLFNFGK	74.52748	P33316	DUT HUMAN	417.568461	3	0.773450792	2	
GSGGFGSTGK	-34.58472	P33316	DUT HUMAN	427.7043235	2	0.752936959	2	Yes
GSGGFGSTGK	-34.58472	P33316	DUT HUMAN	285.4721573	3	0.752936959	2	
HFIDVGAGVIDEDYR	53.83494	P33316	DUT HUMAN	853.413196	2	0.824210107	3	
HFIDVGAGVIDEDYR	53.83494	P33316	DUT HUMAN	569.2780723	3	0.824210107	3	Yes
IAQLICER	12.93819	P33316	DUT HUMAN	501.774226	2	0.790088952	2	Yes
IAQLICER	12.93819	P33316	DUT HUMAN	334.8520923	3	0.790088952	2	
IFYPEIEEVQALDDTER	87.26904	P33316	DUT HUMAN	1034.00003	2	0.816927195	3	
IFYPEIEEVQALDDTER	87.26904	P33316	DUT HUMAN	689.6692947	3	0.816927195	3	Yes
TDIQIALPSGCYGR	57.71639	P33316	DUT HUMAN	775.8857605	2	0.777321577	2	Yes
TDIQIALPSGCYGR	57.71639	P33316	DUT HUMAN	517.5931153	3	0.777321577	2	
DPNSQVGACIVNSENK	23.24648	P32321	DCTD HUMAN	866.402506	2	0.659577072		
DPNSQVGACIVNSENK	23.24648	P32321	DCTD HUMAN	577.9376123	3	0.659577072		
EVIFMSDK	26.25731	P32321	DCTD HUMAN	484.7420595	2	0.679919779		
EVIFMSDK	26.25731	P32321	DCTD HUMAN	323.4973147	3	0.679919779		
LLFNMAGVTFR	88.10205	P32321	DCTD HUMAN	634.8451825	2	0.783779204	2	Yes
LLFNMAGVTFR	88.10205	P32321	DCTD HUMAN	423.5660633	3	0.783779204	2	
YPYVCHAELNAIMNK	37.01732	P32321	DCTD HUMAN	911.9349225	2	0.73513329		
YPYVCHAELNAIMNK	37.01732	P32321	DCTD HUMAN	608.2925567	3	0.73513329		
DGYNYTLSK	12.73878	P31949	S10AB HUMAN	530.7514615	2	0.690800667	2	Yes
DGYNYTLSK	12.73878	P31949	S10AB HUMAN	354.1702493	3	0.690800667	2	
TEFLSFMNTELAAFTK	120.4516	P31949	S10AB HUMAN	925.4562195	2	0.785956144	2	Yes
TEFLSFMNTELAAFTK	120.4516	P31949	S10AB HUMAN	617.3067547	3	0.785956144	2	
AVTEQGHELSNEER	-26.09404	P31946	1433B HUMAN	799.874439	2	0.79179889	2	Yes
AVTEQGHELSNEER	-26.09404	P31946	1433B HUMAN	533.5855677	3	0.79179889	2	
TAFDEAIAELDTLNEESYK	111.4304	P31946	1433B HUMAN	1080.005509	2	0.763056993	3	
TAFDEAIAELDTLNEESYK	111.4304	P31946	1433B HUMAN	720.339614	3	0.763056993	3	Yes
YLIPNATQPESK	27.40434	P31946	1433B HUMAN	680.8595365	2	0.736509979	2	Yes
YLIPNATQPESK	27.40434	P31946	1433B HUMAN	454.2422993	3	0.736509979	2	
YLSEVASGDNK	-5.046291	P31946	1433B HUMAN	591.7860365	2	0.745172858	2	Yes
YLSEVASGDNK	-5.046291	P31946	1433B HUMAN	394.859966	3	0.745172858	2	
AADTIGYPVMIR	53.49166	P31327	CPSM HUMAN	653.8453755	2	0.812980533	2	Yes
AADTIGYPVMIR	53.49166	P31327	CPSM HUMAN	436.2328587	3	0.812980533	2	
AFAISGPFNVQFLVK	110.549	P31327	CPSM HUMAN	819.4566825	2	0.732668281	2	Yes
AFAISGPFNVQFLVK	110.549	P31327	CPSM HUMAN	546.6403967	3	0.732668281	2	
AFAMTNQILVEK	48.54245	P31327	CPSM HUMAN	682.866312	2	0.798920989	2	Yes
AFAMTNQILVEK	48.54245	P31327	CPSM HUMAN	455.5801497	3	0.798920989	2	
AIDDNMSLDEIEK	42.82765	P31327	CPSM HUMAN	746.8459695	2	0.894123673	2	Yes
AIDDNMSLDEIEK	42.82765	P31327	CPSM HUMAN	498.2332547	3	0.894123673	2	
APMFSWPR	52.56927	P31327	CPSM HUMAN	496.245104	2	0.769570112	2	Yes
APMFSWPR	52.56927	P31327	CPSM HUMAN	331.166011	3	0.769570112	2	
AQTAHIVLEDGTK	-3.312157	P31327	CPSM HUMAN	691.867898	2	0.779044092	2	Yes
AQTAHIVLEDGTK	-3.312157	P31327	CPSM HUMAN	461.581207	3	0.779044092	2	
ATGYPLAFIAAK	63.85324	P31327	CPSM HUMAN	611.8457015	2	0.784308314	2	Yes
ATGYPLAFIAAK	63.85324	P31327	CPSM HUMAN	408.233076	3	0.784308314	2	
ATTITSVLPKPALVASR	50.42165	P31327	CPSM HUMAN	863.017636	2	0.705622077	3	
ATTITSVLPKPALVASR	50.42165	P31327	CPSM HUMAN	575.6810323	3	0.705622077	3	Yes
AVNTLNEALEFAK	61.00993	P31327	CPSM HUMAN	710.3780975	2	0.84047246	2	Yes
AVNTLNEALEFAK	61.00993	P31327	CPSM HUMAN	473.92134	3	0.84047246	2	
DGSIDLVINLPNNNTK	84.80043	P31327	CPSM HUMAN	863.9526885	2	0.699506402		
DGSIDLVINLPNNNTK	84.80043	P31327	CPSM HUMAN	576.3044007	3	0.699506402		
DILNMEK	22.75304	P31327	CPSM HUMAN	431.721127	2	0.695545375	2	Yes
DILNMEK	22.75304	P31327	CPSM HUMAN	288.1500263	3	0.695545375	2	
DYNHWLATK	13.26661	P31327	CPSM HUMAN	574.280725	2	0.694595397	2	Yes
DYNHWLATK	13.26661	P31327	CPSM HUMAN	383.1897583	3	0.694595397	2	
EPLFGISTGNLITGLAAGAK	112.0827	P31327	CPSM HUMAN	965.53639	2	0.733957469	3	
EPLFGISTGNLITGLAAGAK	112.0827	P31327	CPSM HUMAN	644.0268683	3	0.733957469	3	Yes
ETLMDLSTK	32.02293	P31327	CPSM HUMAN	519.2633565	2	0.617773712	2	Yes
ETLMDLSTK	32.02293	P31327	CPSM HUMAN	346.5115127	3	0.617773712	2	
EWPSNLDLR	45.54797	P31327	CPSM HUMAN	565.286011	2	0.81804812	2	Yes
EWPSNLDLR	45.54797	P31327	CPSM HUMAN	377.1932823	3	0.81804812	2	
FLGVAEQLHNEGFK	38.50061	P31327	CPSM HUMAN	794.9100965	2	0.820049405	2	Yes
FLGVAEQLHNEGFK	38.50061	P31327	CPSM HUMAN	530.276006	3	0.820049405	2	
GAEVHLVPWNHDFTK	33.68636	P31327	CPSM HUMAN	875.4395525	2	0.872148752	4	
GAEVHLVPWNHDFTK	33.68636	P31327	CPSM HUMAN	583.96231	3	0.872148752	4	
GILIGIQQSFRPR	51.2687	P31327	CPSM HUMAN	742.9389915	2	0.673751831	3	
GILIGIQQSFRPR	51.2687	P31327	CPSM HUMAN	495.6286027	3	0.673751831	3	Yes
GLNSESMTEETLK	15.24609	P31327	CPSM HUMAN	719.8406875	2	0.751508355	2	Yes
GLNSESMTEETLK	15.24609	P31327	CPSM HUMAN	480.2297333	3	0.751508355	2	
GNDVLVIECNLR	52.94745	P31327	CPSM HUMAN	701.3619245	2	0.743325233	2	Yes

GNDVLVIECNLR	52.94745	P31327	CPSM HUMAN	467.910558	3	0.743325233	2	
GQILTMANPIIGNGGAPDTTALDELGLSK	113.2012	P31327	CPSM HUMAN	1434.245139	2	0.677564919	3	
GQILTMANPIIGNGGAPDTTALDELGLSK	113.2012	P31327	CPSM HUMAN	956.4993673	3	0.677564919	3	Yes
GQNQPVLNITNK	18.08495	P31327	CPSM HUMAN	663.362782	2	0.815827787	2	Yes
GQNQPVLNITNK	18.08495	P31327	CPSM HUMAN	442.5777963	3	0.815827787	2	
GYSFGHPSSVAGEVVFNTGLGGYPEAITDPAYK	89.69807	P31327	CPSM HUMAN	1694.312582	2	0.605295897	3	
GYSFGHPSSVAGEVVFNTGLGGYPEAITDPAYK	89.69807	P31327	CPSM HUMAN	1129.877663	3	0.605295897	3	Yes
HLPTLDHPIIPADYVAIK	63.9117	P31327	CPSM HUMAN	1007.062571	2	0.822452188	3	
HLPTLDHPIIPADYVAIK	63.9117	P31327	CPSM HUMAN	671.7109887	3	0.822452188	3	Yes
IALGIPLPEIK	92.33151	P31327	CPSM HUMAN	582.3740985	2	0.841398597	2	Yes
IALGIPLPEIK	92.33151	P31327	CPSM HUMAN	388.5853407	3	0.841398597	2	
IAPSFAVESIEDALK	92.91351	P31327	CPSM HUMAN	795.4252445	2	0.814167857	3	
IAPSFAVESIEDALK	92.91351	P31327	CPSM HUMAN	530.619438	3	0.814167857	3	Yes
IEFEGQPVDFVDPNK	69.13755	P31327	CPSM HUMAN	867.4232335	2	0.843814254	3	
IEFEGQPVDFVDPNK	69.13755	P31327	CPSM HUMAN	578.6180973	3	0.843814254	3	Yes
IMGTSPLQIDR	37.12923	P31327	CPSM HUMAN	615.8297295	2	0.82455337	2	Yes
IMGTSPLQIDR	37.12923	P31327	CPSM HUMAN	410.8890947	3	0.82455337	2	
ATEATSDWLNANNVPATPVAWPSQEGQNPSLS	116.6371	P31327	CPSM HUMAN	1984.974855	2	0.642731726	4	
ATEATSDWLNANNVPATPVAWPSQEGQNPSLS	116.6371	P31327	CPSM HUMAN	1323.652512	3	0.642731726	4	
LYFEELSLER	67.70554	P31327	CPSM HUMAN	649.8355295	2	0.811046422	2	Yes
LYFEELSLER	67.70554	P31327	CPSM HUMAN	433.559628	3	0.811046422	2	
MEYDGILIAGGPGNPALAEPLIQNVR	117.248	P31327	CPSM HUMAN	1354.70799	2	0.76355207		
MEYDGILIAGGPGNPALAEPLIQNVR	117.248	P31327	CPSM HUMAN	903.4746017	3	0.76355207		
SAYALGGLGSGICPNR	46.41872	P31327	CPSM HUMAN	796.896659	2	0.835450113	2	Yes
SAYALGGLGSGICPNR	46.41872	P31327	CPSM HUMAN	531.600381	3	0.835450113	2	
SIFSAVLDELK	114.448	P31327	CPSM HUMAN	611.340452	2	0.77011317	2	Yes
SIFSAVLDELK	114.448	P31327	CPSM HUMAN	407.896243	3	0.77011317	2	
SLGQWLQEEK	43.43196	P31327	CPSM HUMAN	609.3122265	2	0.857541323	2	Yes
SLGQWLQEEK	43.43196	P31327	CPSM HUMAN	406.5440927	3	0.857541323	2	
SVGEVMAIGR	25.78175	P31327	CPSM HUMAN	509.771683	2	0.632351637	2	Yes
SVGEVMAIGR	25.78175	P31327	CPSM HUMAN	340.1837303	3	0.632351637	2	
TAVDSGIPLLTNFQVTK	90.30721	P31327	CPSM HUMAN	902.4967335	2	0.743848443	3	
TAVDSGIPLLTNFQVTK	90.30721	P31327	CPSM HUMAN	602.0004307	3	0.743848443	3	Yes
TFEESFQK	2.780144	P31327	CPSM HUMAN	508.2407385	2	0.7382195	2	Yes
TFEESFQK	2.780144	P31327	CPSM HUMAN	339.1631007	3	0.7382195	2	
TGFGFTNVTAHQK	15.26267	P31327	CPSM HUMAN	704.354958	2	0.83336401		
TGFGFTNVTAHQK	15.26267	P31327	CPSM HUMAN	469.9059137	3	0.83336401		
TLGVDFIDVATK	78.38405	P31327	CPSM HUMAN	639.851185	2	0.810617507	2	Yes
TLGVDFIDVATK	78.38405	P31327	CPSM HUMAN	426.9033983	3	0.810617507	2	
TSACFEPSLDYMVTK	65.29431	P31327	CPSM HUMAN	874.897676	2	0.850611448	2	Yes
TSACFEPSLDYMVTK	65.29431	P31327	CPSM HUMAN	583.601059	3	0.850611448	2	
TVLMNPNIASVQTNEVGLK	66.69854	P31327	CPSM HUMAN	1014.543889	2	0.811693907	3	
TVLMNPNIASVQTNEVGLK	66.69854	P31327	CPSM HUMAN	676.698534	3	0.811693907	3	Yes
VISHAISEHVEDAGVHSGDATLMLPTQTISQGAIEI	56.2557	P31327	CPSM HUMAN	1871.441804	2	0.639797986	5	
VISHAISEHVEDAGVHSGDATLMLPTQTISQGAIEI	56.2557	P31327	CPSM HUMAN	1247.963811	3	0.639797986	5	
VLGTSVESIMATEDR	58.45895	P31327	CPSM HUMAN	804.401444	2	0.826182365	2	Yes
VLGTSVESIMATEDR	58.45895	P31327	CPSM HUMAN	536.603571	3	0.826182365	2	
VLILGSGGLSIGQAGEFDYSGSQAVK	96.07103	P31327	CPSM HUMAN	1277.163938	2	0.680925369		
VLILGSGGLSIGQAGEFDYSGSQAVK	96.07103	P31327	CPSM HUMAN	851.778567	3	0.680925369		
VMIGENVDEK	5.628777	P31327	CPSM HUMAN	567.2795375	2	0.767688811	2	Yes
VMIGENVDEK	5.628777	P31327	CPSM HUMAN	378.5223	3	0.767688811	2	
VPAIYGVDTR	28.83699	P31327	CPSM HUMAN	545.798751	2	0.783439815	2	Yes
VPAIYGVDTR	28.83699	P31327	CPSM HUMAN	364.2017757	3	0.783439815	2	
VSGLLVLDYSK	62.52414	P31327	CPSM HUMAN	597.3429905	2	0.756232977	2	Yes
VSGLLVLDYSK	62.52414	P31327	CPSM HUMAN	398.564602	3	0.756232977	2	
VSQEHPVVLTK	-15.28352	P31327	CPSM HUMAN	618.851519	2	0.763576925	3	
VSQEHPVVLTK	-15.28352	P31327	CPSM HUMAN	412.903621	3	0.763576925	3	Yes
VVAVDCGIK	2.837776	P31327	CPSM HUMAN	480.763327	2	0.635628521	2	Yes
VVAVDCGIK	2.837776	P31327	CPSM HUMAN	320.8448263	3	0.635628521	2	
AAQTAEDAMQIMEQMTK	80.94995	P30622	CLIP1 HUMAN	948.929187	2	0.874713361	3	
AAQTAEDAMQIMEQMTK	80.94995	P30622	CLIP1 HUMAN	632.9553997	3	0.874713361	3	Yes
AEDQHLVEMEDTLNK	29.09462	P30622	CLIP1 HUMAN	886.41254	2	0.849194646	2	Yes
AEDQHLVEMEDTLNK	29.09462	P30622	CLIP1 HUMAN	591.277635	3	0.849194646	2	
ANENASFLQK	0.173134	P30622	CLIP1 HUMAN	561.2834685	2	0.818266511	2	Yes
ANENASFLQK	0.173134	P30622	CLIP1 HUMAN	374.5249207	3	0.818266511	2	
AQESQIDFLNSVIVDLQR	128.0081	P30622	CLIP1 HUMAN	1038.042567	2	0.784609258		
AQESQIDFLNSVIVDLQR	128.0081	P30622	CLIP1 HUMAN	692.3643197	3	0.784609258		
ASSTPSSETQEEFVDDFR	45.41251	P30622	CLIP1 HUMAN	1016.44308	2	0.780560136	3	
ASSTPSSETQEEFVDDFR	45.41251	P30622	CLIP1 HUMAN	677.9646613	3	0.780560136	3	Yes
ATSHVGEIEQELALAR	40.98193	P30622	CLIP1 HUMAN	862.452857	2	0.888153911	3	
ATSHVGEIEQELALAR	40.98193	P30622	CLIP1 HUMAN	575.304513	3	0.888153911	3	Yes
ATSPLCTSTASMVSSSPSTPSNIPQKPSQPAAK	38.40835	P30622	CLIP1 HUMAN	1658.313954	2	0.748081148		
ATSPLCTSTASMVSSSPSTPSNIPQKPSQPAAK	38.40835	P30622	CLIP1 HUMAN	1105.878578	3	0.748081148		
DGHDQHVLELEAK	1.480156	P30622	CLIP1 HUMAN	745.865886	2	0.767313957	3	
DGHDQHVLELEAK	1.480156	P30622	CLIP1 HUMAN	497.5798657	3	0.767313957	3	Yes
EENSGLLQELEELR	94.50085	P30622	CLIP1 HUMAN	829.915772	2	0.667185068	2	Yes
EENSGLLQELEELR	94.50085	P30622	CLIP1 HUMAN	553.613123	3	0.667185068	2	
EEQFNMLSSDLEK	53.18234	P30622	CLIP1 HUMAN	785.359244	2	0.819381118		
EEQFNMLSSDLEK	53.18234	P30622	CLIP1 HUMAN	523.908771	3	0.819381118		
ELEQSLLFEK	53.19452	P30622	CLIP1 HUMAN	618.3300845	2	0.730290413		
ELEQSLLFEK	53.19452	P30622	CLIP1 HUMAN	412.555998	3	0.730290413		
ENLADMEAK	1.513309	P30622	CLIP1 HUMAN	510.7375055	2	0.670977414	2	Yes
ENLADMEAK	1.513309	P30622	CLIP1 HUMAN	340.827612	3	0.670977414	2	
EPSATPPISNLTK	31.01714	P30622	CLIP1 HUMAN	677.864823	2	0.77987349		
EPSATPPISNLTK	31.01714	P30622	CLIP1 HUMAN	452.2458237	3	0.77987349		
FAEASEEAVSVQR	13.56183	P30622	CLIP1 HUMAN	711.8471615	2	0.776124954	2	Yes
FAEASEEAVSVQR	13.56183	P30622	CLIP1 HUMAN	474.900716	3	0.776124954	2	
FLGETDFAK	33.95163	P30622	CLIP1 HUMAN	514.2589315	2	0.723548889		
FLGETDFAK	33.95163	P30622	CLIP1 HUMAN	343.1752293	3	0.723548889		
GLGTETAEFAELK	48.50237	P30622	CLIP1 HUMAN	683.3490065	2	0.883467913	2	Yes
GLGTETAEFAELK	48.50237	P30622	CLIP1 HUMAN	455.901946	3	0.883467913	2	
HEEILQNLQK	6.940907	P30622	CLIP1 HUMAN	626.3387755	2	0.792109728		
HEEILQNLQK	6.940907	P30622	CLIP1 HUMAN	417.8951253	3	0.792109728		
ILKPGSTALK	-14.6279	P30622	CLIP1 HUMAN	514.329691	2	0.681166589	2	Yes
ILKPGSTALK	-14.6279	P30622	CLIP1 HUMAN	343.2224023	3	0.681166589	2	
LDYQHEIENLQNQQDSER	37.70593	P30622	CLIP1 HUMAN	1130.017805	2	0.75739187	3	
LDYQHEIENLQNQQDSER	37.70593	P30622	CLIP1 HUMAN	753.6811447	3	0.75739187	3	Yes
LENDIAEIMK	50.34956	P30622	CLIP1 HUMAN	588.3030125	2	0.740721822		
LENDIAEIMK	50.34956	P30622	CLIP1 HUMAN	392.53795	3	0.740721822		
LETAIASHQQAMEELK	20.1649	P30622	CLIP1 HUMAN	899.9543755	2	0.783929884		
LETAIASHQQAMEELK	20.1649	P30622	CLIP1 HUMAN	600.3055253	3	0.783929884		

LLDLDALR	55.39825	P30622	CLIP1 HUMAN	464.777291	2	0.798874974	2	Yes
LLDLDALR	55.39825	P30622	CLIP1 HUMAN	310.187469	3	0.798874974	2	
LQNELDTLK	18.92168	P30622	CLIP1 HUMAN	537.296045	2	0.757849813		
LQNELDTLK	18.92168	P30622	CLIP1 HUMAN	358.533305	3	0.757849813		
LTNLQENLSEVSQVK	44.66544	P30622	CLIP1 HUMAN	851.4550645	2	0.830799162		
LTNLQENLSEVSQVK	44.66544	P30622	CLIP1 HUMAN	567.9726513	3	0.830799162		
NLELQLK	28.87351	P30622	CLIP1 HUMAN	429.258734	2	0.708249569	2	Yes
NLELQLK	28.87351	P30622	CLIP1 HUMAN	286.508431	3	0.708249569	2	
SISITSALLTEK	63.0842	P30622	CLIP1 HUMAN	631.8642915	2	0.725716829		
SISITSALLTEK	63.0842	P30622	CLIP1 HUMAN	421.5788027	3	0.725716829		
SLHSVVQTLESDK	17.11211	P30622	CLIP1 HUMAN	721.8784615	2	0.677647293	2	Yes
SLHSVVQTLESDK	17.11211	P30622	CLIP1 HUMAN	481.5882493	3	0.677647293	2	
SQQLSALQEENVK	13.40496	P30622	CLIP1 HUMAN	737.3813685	2	0.770833015	2	Yes
SQQLSALQEENVK	13.40496	P30622	CLIP1 HUMAN	491.9235207	3	0.770833015	2	
SVLNNQLLEMK	51.17578	P30622	CLIP1 HUMAN	644.850661	2	0.897995532	2	Yes
SVLNNQLLEMK	51.17578	P30622	CLIP1 HUMAN	430.2363823	3	0.897995532	2	
TASESISNLSEAGSIK	28.34656	P30622	CLIP1 HUMAN	797.402497	2	0.722432375		
TASESISNLSEAGSIK	28.34656	P30622	CLIP1 HUMAN	531.9376063	3	0.722432375		
TETLASLEDTK	15.77238	P30622	CLIP1 HUMAN	604.306807	2	0.71369648		
TETLASLEDTK	15.77238	P30622	CLIP1 HUMAN	403.2071463	3	0.71369648		
TGLLTETSSR	3.729057	P30622	CLIP1 HUMAN	532.783302	2	0.768428147	2	Yes
TGLLTETSSR	3.729057	P30622	CLIP1 HUMAN	355.5248097	3	0.768428147	2	
VELLNQLEEEK	45.43739	P30622	CLIP1 HUMAN	672.3568305	2	0.797103286		
VELLNQLEEEK	45.43739	P30622	CLIP1 HUMAN	448.5738287	3	0.797103286		
VIDNFTSQLK	32.17551	P30622	CLIP1 HUMAN	582.8171445	2	0.843447685	2	Yes
VIDNFTSQLK	32.17551	P30622	CLIP1 HUMAN	388.8807047	3	0.843447685	2	
YFQCEPLK	11.60576	P30622	CLIP1 HUMAN	542.76078	2	0.683887303		
YFQCEPLK	11.60576	P30622	CLIP1 HUMAN	362.1764617	3	0.683887303		
YGLFAPVHK	18.10879	P30622	CLIP1 HUMAN	516.287822	2	0.813565373		
YGLFAPVHK	18.10879	P30622	CLIP1 HUMAN	344.527823	3	0.813565373		
AFEYNMQIFNELDQAGSTLAR	108.1959	P30519	HMOX2 HUMAN	1209.573902	2	0.787508905		
AFEYNMQIFNELDQAGSTLAR	108.1959	P30519	HMOX2 HUMAN	806.7185427	3	0.787508905		
DHPAFAPLYFPMELHR	74.8625	P30519	HMOX2 HUMAN	970.9779835	2	0.737325132	4	
DHPAFAPLYFPMELHR	74.8625	P30519	HMOX2 HUMAN	647.6545973	3	0.737325132	4	
DMEYFFGENWEEQVQCPK	95.83852	P30519	HMOX2 HUMAN	1168.485907	2	0.787029624		
DMEYFFGENWEEQVQCPK	95.83852	P30519	HMOX2 HUMAN	779.326546	3	0.787029624		
ETLEDGFPVHDGK	21.1923	P30519	HMOX2 HUMAN	722.3417125	2	0.745145023	3	
ETLEDGFPVHDGK	21.1923	P30519	HMOX2 HUMAN	481.8970833	3	0.745145023	3	Yes
GALEGSSCPFR	13.3543	P30519	HMOX2 HUMAN	590.774954	2	0.718078077		
GALEGSSCPFR	13.3543	P30519	HMOX2 HUMAN	394.185911	3	0.718078077		
IHYIGQNEPELLVAHAYTR	45.03333	P30519	HMOX2 HUMAN	1112.579643	2	0.778196156	3	
IHYIGQNEPELLVAHAYTR	45.03333	P30519	HMOX2 HUMAN	742.0557037	3	0.778196156	3	Yes
LPSTGEGTQFYLFENVDNAQQFK	89.95657	P30519	HMOX2 HUMAN	1317.130096	2	0.705252647	3	
LPSTGEGTQFYLFENVDNAQQFK	89.95657	P30519	HMOX2 HUMAN	878.422672	3	0.705252647	3	Yes
MADLSELLK	61.22178	P30519	HMOX2 HUMAN	510.2762665	2	0.712616861		
MADLSELLK	61.22178	P30519	HMOX2 HUMAN	340.5201193	3	0.712616861		
MNALDLNMK	37.63981	P30519	HMOX2 HUMAN	525.2600935	2	0.836301148	2	Yes
MNALDLNMK	37.63981	P30519	HMOX2 HUMAN	350.5093373	3	0.836301148	2	
YMGDLSGGQVLK	33.78867	P30519	HMOX2 HUMAN	634.321733	2	0.811552405	2	Yes
YMGDLSGGQVLK	33.78867	P30519	HMOX2 HUMAN	423.217097	3	0.811552405	2	
EAATNNLMK	-21.57163	P30154	2AAB HUMAN	496.2480405	2	0.743002594	2	Yes
EAATNNLMK	-21.57163	P30154	2AAB HUMAN	331.1679687	3	0.743002594	2	
ELGENLPIEDR	36.88073	P30154	2AAB HUMAN	642.8256975	2	0.726494014	2	Yes
ELGENLPIEDR	36.88073	P30154	2AAB HUMAN	428.8864067	3	0.726494014	2	
ELVSDTNQHVK	-27.77763	P30154	2AAB HUMAN	635.325865	2	0.68915844	2	Yes
ELVSDTNQHVK	-27.77763	P30154	2AAB HUMAN	423.8865183	3	0.68915844	2	
FGTEWAQNTIVPK	50.07477	P30154	2AAB HUMAN	745.8860905	2	0.900250852	2	Yes
FGTEWAQNTIVPK	50.07477	P30154	2AAB HUMAN	497.5933353	3	0.900250852	2	
IGPILDTNALQGEVKPVLQK	67.00552	P30154	2AAB HUMAN	1067.118079	2	0.840799332		
IGPILDTNALQGEVKPVLQK	67.00552	P30154	2AAB HUMAN	711.747994	3	0.840799332		
ITLNDLIPAFQNLLK	151.1909	P30154	2AAB HUMAN	857.001454	2	0.827778041		
ITLNDLIPAFQNLLK	151.1909	P30154	2AAB HUMAN	571.6702443	3	0.827778041		
LASGDWFTSR	38.52235	P30154	2AAB HUMAN	570.2781865	2	0.784617901		
LASGDWFTSR	38.52235	P30154	2AAB HUMAN	380.5213993	3	0.784617901		
SALASVIMGLSTILGK	133.8432	P30154	2AAB HUMAN	780.955658	2	0.715787113		
SALASVIMGLSTILGK	133.8432	P30154	2AAB HUMAN	520.973047	3	0.715787113		
SEIVPLFTSLASDEQDSVR	101.2969	P30154	2AAB HUMAN	1047.02404	2	0.746038854		
SEIVPLFTSLASDEQDSVR	101.2969	P30154	2AAB HUMAN	698.351968	3	0.746038854		
SLCSDDTPMVR	14.20267	P30154	2AAB HUMAN	640.7846615	2	0.756331205	2	Yes
SLCSDDTPMVR	14.20267	P30154	2AAB HUMAN	427.525716	3	0.756331205	2	
VLELDSVK	23.283	P30154	2AAB HUMAN	451.763849	2	0.717880964		
VLELDSVK	23.283	P30154	2AAB HUMAN	301.511841	3	0.717880964		
VLVMANDPNYLHR	31.12418	P30154	2AAB HUMAN	771.398837	2	0.799321055		
VLVMANDPNYLHR	31.12418	P30154	2AAB HUMAN	514.601833	3	0.799321055		
AFQYVETHGEVCPANWTPDSPTIKPSPAASK	49.54244	P30048	PRDX3 HUMAN	1693.312068	2	0.643515468	4	
AFQYVETHGEVCPANWTPDSPTIKPSPAASK	49.54244	P30048	PRDX3 HUMAN	1129.210654	3	0.643515468	4	
ANEFHDVNCEVVAVSVDSHFSHLAWINTPR	75.92912	P30048	PRDX3 HUMAN	1725.818386	2	0.684264958	4	
ANEFHDVNCEVVAVSVDSHFSHLAWINTPR	75.92912	P30048	PRDX3 HUMAN	1150.881532	3	0.684264958	4	
DLSLDDFK	53.12335	P30048	PRDX3 HUMAN	476.7352885	2	0.750797689	2	Yes
DLSLDDFK	53.12335	P30048	PRDX3 HUMAN	318.1594673	3	0.750797689	2	
DYGVLLEGSGLALR	91.68558	P30048	PRDX3 HUMAN	731.899193	2	0.814754307	2	Yes
DYGVLLEGSGLALR	91.68558	P30048	PRDX3 HUMAN	488.268737	3	0.814754307	2	
GLFIIDPNGVIK	89.7616	P30048	PRDX3 HUMAN	643.379912	2	0.628006697	2	Yes
GLFIIDPNGVIK	89.7616	P30048	PRDX3 HUMAN	429.255883	3	0.628006697	2	
HLSVNDLPVGR	20.51985	P30048	PRDX3 HUMAN	603.83366	2	0.769415975	2	Yes
HLSVNDLPVGR	20.51985	P30048	PRDX3 HUMAN	402.891715	3	0.769415975	2	
LFSTSSSCHAPAVTQHAPYFK	23.08885	P30048	PRDX3 HUMAN	1168.560598	2	0.823629439		
LFSTSSSCHAPAVTQHAPYFK	23.08885	P30048	PRDX3 HUMAN	779.37634	3	0.823629439		
TSLTNLLCSGSSQAK	46.31272	P30048	PRDX3 HUMAN	783.893786	2	0.835074663		
TSLTNLLCSGSSQAK	46.31272	P30048	PRDX3 HUMAN	522.931799	3	0.835074663		
ALNVEPDGTGLTCSLAPNIISQL	150.9214	P30044	PRDX5 HUMAN	1192.112864	2	0.752260327	3	
ALNVEPDGTGLTCSLAPNIISQL	150.9214	P30044	PRDX5 HUMAN	795.077851	3	0.752260327	3	Yes
ETDLLLDDSLVSIFGNR	139.6026	P30044	PRDX5 HUMAN	953.992011	2	0.69447428	3	
ETDLLLDDSLVSIFGNR	139.6026	P30044	PRDX5 HUMAN	636.3306157	3	0.69447428	3	Yes
FSMVVQDGIVK	49.52103	P30044	PRDX5 HUMAN	611.829198	2	0.757140577	2	Yes
FSMVVQDGIVK	49.52103	P30044	PRDX5 HUMAN	408.2220737	3	0.757140577	2	
GVLFGVPGAFTPGCSK	77.18358	P30044	PRDX5 HUMAN	797.408876	2	0.789111793	2	Yes
GVLFGVPGAFTPGCSK	77.18358	P30044	PRDX5 HUMAN	531.941859	3	0.789111793	2	
LLADPTGAFGK	37.8754	P30044	PRDX5 HUMAN	545.301131	2	0.747311711	2	Yes
LLADPTGAFGK	37.8754	P30044	PRDX5 HUMAN	363.870029	3	0.747311711	2	
SAGYILVGGAGGQSAAAAAR	41.69032	P30044	PRDX5 HUMAN	874.45847	2	0.781748831		

SAGYILVGGAGGQSAAAAAR	41.69032	P30044	PRDX5 HUMAN	583.308255	3	0.781748831		
THLPGFVEQAEALK	42.64497	P30044	PRDX5 HUMAN	770.4124725	2	0.876034796	2	Yes
THLPGFVEQAEALK	42.64497	P30044	PRDX5 HUMAN	513.9442567	3	0.876034796	2	
VGDAIPAVEVFEGEPGNK	72.71941	P30044	PRDX5 HUMAN	914.4603475	2	0.820335448	3	
VGDAIPAVEVFEGEPGNK	72.71941	P30044	PRDX5 HUMAN	609.9761733	3	0.820335448	3	Yes
VNLAELFK	69.9832	P30044	PRDX5 HUMAN	467.274384	2	0.812558949	2	Yes
VNLAELFK	69.9832	P30044	PRDX5 HUMAN	311.8521977	3	0.812558949	2	
YSEGEWASGGVR	12.16927	P30044	PRDX5 HUMAN	649.2945605	2	0.680722475		
YSEGEWASGGVR	12.16927	P30044	PRDX5 HUMAN	433.198982	3	0.680722475		
DFTPVCTTELGR	50.28436	P30041	PRDX6 HUMAN	698.332834	2	0.7433483	2	Yes
DFTPVCTTELGR	50.28436	P30041	PRDX6 HUMAN	465.8911643	3	0.7433483	2	
DGDSVMVLPTIPEEEAK	83.56566	P30041	PRDX6 HUMAN	915.4460485	2	0.800402522	3	
DGDSVMVLPTIPEEEAK	83.56566	P30041	PRDX6 HUMAN	610.6333073	3	0.800402522	3	Yes
DINAYNCEEPTEK	11.99167	P30041	PRDX6 HUMAN	791.838672	2	0.720268965	2	Yes
DINAYNCEEPTEK	11.99167	P30041	PRDX6 HUMAN	528.2283897	3	0.720268965	2	
LIALSIDSVEDHLAWSK	83.55875	P30041	PRDX6 HUMAN	949.007464	2	0.752232432	3	
LIALSIDSVEDHLAWSK	83.55875	P30041	PRDX6 HUMAN	633.0075843	3	0.752232432	3	Yes
LPFPIIDDR	70.6536	P30041	PRDX6 HUMAN	543.303673	2	0.78483212	2	Yes
LPFPIIDDR	70.6536	P30041	PRDX6 HUMAN	362.5383903	3	0.78483212	2	
LSILYPATTGR	44.71997	P30041	PRDX6 HUMAN	596.340783	2	0.832563758	2	Yes
LSILYPATTGR	44.71997	P30041	PRDX6 HUMAN	397.8964637	3	0.832563758	2	
VVFVFGPDK	50.18234	P30041	PRDX6 HUMAN	504.2822095	2	0.753899992	2	Yes
VVFVFGPDK	50.18234	P30041	PRDX6 HUMAN	336.5240813	3	0.753899992	2	
VVISLQLTAEK	52.75014	P30041	PRDX6 HUMAN	600.864095	2	0.814753115	2	Yes
VVISLQLTAEK	52.75014	P30041	PRDX6 HUMAN	400.912005	3	0.814753115	2	
AVELAANTK	-17.68027	P29401	TKT HUMAN	458.7590985	2	0.675738335	2	Yes
AVELAANTK	-17.68027	P29401	TKT HUMAN	306.1753407	3	0.675738335	2	
DDQVTVIGAGVTLHEALAAAELLK	138.868	P29401	TKT HUMAN	1217.663579	2	0.706629217		
DDQVTVIGAGVTLHEALAAAELLK	138.868	P29401	TKT HUMAN	812.111661	3	0.706629217		
IIALDGDTK	12.98574	P29401	TKT HUMAN	473.2667565	2	0.710436583	2	Yes
IIALDGDTK	12.98574	P29401	TKT HUMAN	315.8471127	3	0.710436583	2	
ILATPPQEDAPSVDIANIR	64.31986	P29401	TKT HUMAN	1010.539661	2	0.771794498	3	
ILATPPQEDAPSVDIANIR	64.31986	P29401	TKT HUMAN	674.0290487	3	0.771794498	3	Yes
LTVEDHYYEGGIGEAVSSAVVGEPGITVTHLAVN	94.33009	P29401	TKT HUMAN	1876.960678	2	0.686283767	5	
LTVEDHYYEGGIGEAVSSAVVGEPGITVTHLAVN	94.33009	P29401	TKT HUMAN	1251.64306	3	0.686283767	5	
ISSDLDGHPVPK	-9.085918	P29401	TKT HUMAN	632.830783	2	0.705238998	3	
ISSDLDGHPVPK	-9.085918	P29401	TKT HUMAN	422.2231303	3	0.705238998	3	Yes
LDNLVAILDINR	98.59485	P29401	TKT HUMAN	684.896457	2	0.816006541	2	Yes
LDNLVAILDINR	98.59485	P29401	TKT HUMAN	456.9335797	3	0.816006541	2	
LGQSDPAPLQHQMDIYQK	32.52698	P29401	TKT HUMAN	1035.01021	2	0.812808871	3	
LGQSDPAPLQHQMDIYQK	32.52698	P29401	TKT HUMAN	690.342748	3	0.812808871	3	Yes
NMAEQIIQEIYSQIQSK	131.3064	P29401	TKT HUMAN	1012.012417	2	0.674727023	3	
NMAEQIIQEIYSQIQSK	131.3064	P29401	TKT HUMAN	675.0108863	3	0.674727023	3	Yes
NSTFSEIFK	43.3986	P29401	TKT HUMAN	536.7696625	2	0.721201479	2	Yes
NSTFSEIFK	43.3986	P29401	TKT HUMAN	358.1823833	3	0.721201479	2	
SVPTSTVFYPSDGVATEK	47.09126	P29401	TKT HUMAN	942.9654585	2	0.804343402	3	
SVPTSTVFYPSDGVATEK	47.09126	P29401	TKT HUMAN	628.9795807	3	0.804343402	3	Yes
TSRPENAIIYNNNEDFQVGQAK	32.60497	P29401	TKT HUMAN	1254.609861	2	0.707101166	3	
TSRPENAIIYNNNEDFQVGQAK	32.60497	P29401	TKT HUMAN	836.7425153	3	0.707101166	3	Yes
VLDPFTIKPLDR	61.43948	P29401	TKT HUMAN	707.409201	2	0.779803514	3	
VLDPFTIKPLDR	61.43948	P29401	TKT HUMAN	471.9420757	3	0.779803514	3	Yes
FQIATVTEK	23.17265	P28070	PSB4 HUMAN	518.7878565	2	0.651558816	2	Yes
FQIATVTEK	23.17265	P28070	PSB4 HUMAN	346.1945127	3	0.651558816	2	
IPSTPDSFMDPASALYR	81.98755	P28070	PSB4 HUMAN	934.448921	2	0.670050502		
IPSTPDSFMDPASALYR	81.98755	P28070	PSB4 HUMAN	623.301889	3	0.670050502		
QVLGQMVIDEELLGDGHSYSPR	75.0721	P28070	PSB4 HUMAN	1222.100284	2	0.685326636	3	
QVLGQMVIDEELLGDGHSYSPR	75.0721	P28070	PSB4 HUMAN	815.069464	3	0.685326636	3	Yes
SGLWAGGPAPGQFYR	58.11746	P28070	PSB4 HUMAN	782.3893275	2	0.851626635		
SGLWAGGPAPGQFYR	58.11746	P28070	PSB4 HUMAN	521.9288267	3	0.851626635		
TQNPMVTGTSVLGVK	43.29046	P28070	PSB4 HUMAN	766.4116155	2	0.851636887	2	Yes
TQNPMVTGTSVLGVK	43.29046	P28070	PSB4 HUMAN	511.2770187	3	0.851636887	2	
VNNSTMLGASGDYADFQYLK	72.44733	P28070	PSB4 HUMAN	1097.510234	2	0.744956493	3	
VNNSTMLGASGDYADFQYLK	72.44733	P28070	PSB4 HUMAN	732.009431	3	0.744956493	3	Yes
APVPTGEVYFADSFDR	69.41748	P27824	CALX HUMAN	885.920854	2	0.817604661	3	
APVPTGEVYFADSFDR	69.41748	P27824	CALX HUMAN	590.9498443	3	0.817604661	3	Yes
GLVLMSR	22.92014	P27824	CALX HUMAN	388.2289225	2	0.725752831		
GLVLMSR	22.92014	P27824	CALX HUMAN	259.1552233	3	0.725752831		
GTLSGWILSK	53.24676	P27824	CALX HUMAN	531.303673	2	0.722663701	2	Yes
GTLSGWILSK	53.24676	P27824	CALX HUMAN	354.5383903	3	0.722663701	2	
IPDPEAVKPDDWDEDAPAK	39.60244	P27824	CALX HUMAN	1054.495116	2	0.717928648		
IPDPEAVKPDDWDEDAPAK	39.60244	P27824	CALX HUMAN	703.3326853	3	0.717928648		
IPNPDFFEDLEPFR	114.2857	P27824	CALX HUMAN	868.4204935	2	0.715076029	2	Yes
IPNPDFFEDLEPFR	114.2857	P27824	CALX HUMAN	579.2829373	3	0.715076029	2	
IVDDWANDGWGLK	67.2606	P27824	CALX HUMAN	744.860072	2	0.825623155	2	Yes
IVDDWANDGWGLK	67.2606	P27824	CALX HUMAN	496.909323	3	0.825623155	2	
PPMIDNPSYQGIWK	63.91122	P27824	CALX HUMAN	823.406328	2	0.878774285		
PPMIDNPSYQGIWK	63.91122	P27824	CALX HUMAN	549.2734937	3	0.878774285		
TGIYEEK	-24.71299	P27824	CALX HUMAN	420.2114455	2	0.607797265	2	Yes
TGIYEEK	-24.71299	P27824	CALX HUMAN	280.4769053	3	0.607797265	2	
TPELNLDQFHDK	31.08899	P27824	CALX HUMAN	728.8575295	2	0.79569602	3	
TPELNLDQFHDK	31.08899	P27824	CALX HUMAN	486.2409613	3	0.79569602	3	Yes
TPYTIMFGPDK	54.31815	P27824	CALX HUMAN	635.313377	2	0.7328462	2	Yes
TPYTIMFGPDK	54.31815	P27824	CALX HUMAN	423.878193	3	0.7328462	2	
DAEKPVLFFER	48.11201	P27707	DCK HUMAN	675.856801	2	0.8439008		
DAEKPVLFFER	48.11201	P27707	DCK HUMAN	450.9071423	3	0.8439008		
HESWLLHR	-4.403755	P27707	DCK HUMAN	539.283606	2	0.707248986	3	
HESWLLHR	-4.403755	P27707	DCK HUMAN	359.8583457	3	0.707248986	3	Yes
ISIEGNIAAGK	22.32219	P27707	DCK HUMAN	536.804037	2	0.706623197		
ISIEGNIAAGK	22.32219	P27707	DCK HUMAN	358.2052997	3	0.706623197		
NEEQGIPLEYLEK	59.6683	P27707	DCK HUMAN	781.3913975	2	0.81748426	2	Yes
NEEQGIPLEYLEK	59.6683	P27707	DCK HUMAN	521.26354	3	0.81748426	2	
NGGNVLQMMYEKPER	34.62526	P27707	DCK HUMAN	883.4221835	2	0.749558806		
NGGNVLQMMYEKPER	34.62526	P27707	DCK HUMAN	589.284064	3	0.749558806		
QLCEDWEVVPEPVAR	65.53843	P27707	DCK HUMAN	913.941268	2	0.611737132		
QLCEDWEVVPEPVAR	65.53843	P27707	DCK HUMAN	609.6301203	3	0.611737132		
STFVNILK	45.65401	P27707	DCK HUMAN	461.274384	2	0.60480231		
STFVNILK	45.65401	P27707	DCK HUMAN	307.8521977	3	0.60480231		
WCNVQSTQDEFEELTMSQK	68.42225	P27707	DCK HUMAN	1180.512657	2	0.621163964		
WCNVQSTQDEFEELTMSQK	68.42225	P27707	DCK HUMAN	787.3443797	3	0.621163964		
FNADEFEDMVAEK	63.11171	P27635	RL10 HUMAN	772.8328625	2	0.82725054	2	Yes
FNADEFEDMVAEK	63.11171	P27635	RL10 HUMAN	515.55785	3	0.82725054	2	

LIPDGCGVK	1.744072	P27635	RL10_HUMAN	479.755502	2	0.73119545	2	Yes
LIPDGCGVK	1.744072	P27635	RL10_HUMAN	320.172943	3	0.73119545	2	
VDEFPLCGHMVSDEYEQLSSEALEAAR	93.05029	P27635	RL10_HUMAN	1541.692605	2	0.683655977	3	
VDEFPLCGHMVSDEYEQLSSEALEAAR	93.05029	P27635	RL10_HUMAN	1028.131011	3	0.683655977	3	Yes
AWPPAGVSLPDDDPEAGQGSK	59.68921	P27540	ARNT_HUMAN	1047.492908	2	0.794001043		
AWPPAGVSLPDDDPEAGQGSK	59.68921	P27540	ARNT_HUMAN	698.6645467	3	0.794001043		
DGLASYNHSQVVQPVTTTGPEHSKPLEK	21.71252	P27540	ARNT_HUMAN	1510.260161	2	0.775885701		
DGLASYNHSQVVQPVTTTGPEHSKPLEK	21.71252	P27540	ARNT_HUMAN	1007.176049	3	0.775885701		
DSFQQVVK	13.19926	P27540	ARNT_HUMAN	475.7512735	2	0.732978404		
DSFQQVVK	13.19926	P27540	ARNT_HUMAN	317.5034573	3	0.732978404		
EQLSTSENALTGR	8.247375	P27540	ARNT_HUMAN	703.3500685	2	0.756761312	2	Yes
EQLSTSENALTGR	8.247375	P27540	ARNT_HUMAN	469.2359873	3	0.756761312	2	
FSEIYHNINADQSK	11.63317	P27540	ARNT_HUMAN	833.397545	2	0.675004363		
FSEIYHNINADQSK	11.63317	P27540	ARNT_HUMAN	555.934305	3	0.675004363		
GISSSTVPATQQLFSQGNTFPPTPRPAENFR	71.64082	P27540	ARNT_HUMAN	1666.837103	2	0.740773737		
GISSSTVPATQQLFSQGNTFPPTPRPAENFR	71.64082	P27540	ARNT_HUMAN	1111.560677	3	0.740773737		
GQVLSVMFR	51.16808	P27540	ARNT_HUMAN	518.7845935	2	0.764151752		
GQVLSVMFR	51.16808	P27540	ARNT_HUMAN	346.1923373	3	0.764151752		
GSNFAPETGQTAGQFQTR	22.46338	P27540	ARNT_HUMAN	948.9459285	2	0.84001106		
GSNFAPETGQTAGQFQTR	22.46338	P27540	ARNT_HUMAN	632.9665607	3	0.84001106		
GTGNTSTDGSYKPSFLTDQELK	35.60774	P27540	ARNT_HUMAN	1173.558972	2	0.715160906	3	
GTGNTSTDGSYKPSFLTDQELK	35.60774	P27540	ARNT_HUMAN	782.7085893	3	0.715160906	3	Yes
HNIEGIFTFVDHR	57.35181	P27540	ARNT_HUMAN	792.900063	2	0.839054465		
HNIEGIFTFVDHR	57.35181	P27540	ARNT_HUMAN	528.9359837	3	0.839054465		
LQVTSSPNCTDMSNVCQPTEFISR	64.26902	P27540	ARNT_HUMAN	1386.125851	2	0.681035817		
LQVTSSPNCTDMSNVCQPTEFISR	64.26902	P27540	ARNT_HUMAN	924.4198423	3	0.681035817		
NIVEFCHPEDQQLLR	46.55112	P27540	ARNT_HUMAN	949.4654415	2	0.79122746		
NIVEFCHPEDQQLLR	46.55112	P27540	ARNT_HUMAN	633.3129027	3	0.79122746		
NQEWLWMR	58.00349	P27540	ARNT_HUMAN	581.7772995	2	0.741883099		
NQEWLWMR	58.00349	P27540	ARNT_HUMAN	388.1874747	3	0.741883099		
QQQQQTELDMVPGR	26.96864	P27540	ARNT_HUMAN	829.402311	2	0.612241149		
QQQQQTELDMVPGR	26.96864	P27540	ARNT_HUMAN	553.2708157	3	0.612241149		
SDGLFAQDR	12.89748	P27540	ARNT_HUMAN	504.741437	2	0.717245936		
SDGLFAQDR	12.89748	P27540	ARNT_HUMAN	336.830233	3	0.717245936		
SGFSAQQVATQATAK	15.15668	P27540	ARNT_HUMAN	747.881537	2	0.793016076		
SGFSAQQVATQATAK	15.15668	P27540	ARNT_HUMAN	498.923633	3	0.793016076		
TAEGVGVWPQWQGQQPHHR	30.13037	P27540	ARNT_HUMAN	1099.536111	2	0.783241749		
TAEGVGVWPQWQGQQPHHR	30.13037	P27540	ARNT_HUMAN	733.3600157	3	0.783241749		
QFGVGSFQTPSSFSSMSLPGAPTASPGAAAYPSLT	106.9161	P27540	ARNT_HUMAN	1931.423606	2	0.726774633		
QFGVGSFQTPSSFSSMSLPGAPTASPGAAAYPSLT	106.9161	P27540	ARNT_HUMAN	1287.951679	3	0.726774633		
ADMETLQR	-7.513237	P26639	SYTC_HUMAN	482.232391	2	0.726481915	2	Yes
ADMETLQR	-7.513237	P26639	SYTC_HUMAN	321.8242023	3	0.726481915	2	
AELNPWPEYIYTR	78.70187	P26639	SYTC_HUMAN	826.4099205	2	0.807783723		
AELNPWPEYIYTR	78.70187	P26639	SYTC_HUMAN	551.2758887	3	0.807783723		
DQELYFFHELSPGSCFFLPK	124.4208	P26639	SYTC_HUMAN	1231.080831	2	0.634810209	3	
DQELYFFHELSPGSCFFLPK	124.4208	P26639	SYTC_HUMAN	821.0564957	3	0.634810209	3	Yes
FEDEEAQAVYWHSSAHIMGEAMER	47.99377	P26639	SYTC_HUMAN	1412.111054	2	0.747915924	4	
FEDEEAQAVYWHSSAHIMGEAMER	47.99377	P26639	SYTC_HUMAN	941.7433107	3	0.747915924	4	
FLGDIEVWDQAEK	75.24211	P26639	SYTC_HUMAN	775.3808375	2	0.82481432	2	Yes
FLGDIEVWDQAEK	75.24211	P26639	SYTC_HUMAN	517.2565	3	0.82481432	2	
FMADIDLDPGCTLNK	67.15253	P26639	SYTC_HUMAN	855.3978475	2	0.688288569		
FMADIDLDPGCTLNK	67.15253	P26639	SYTC_HUMAN	570.6011733	3	0.688288569		
FQQDDAHIFCAMEQIEDEIK	95.9418	P26639	SYTC_HUMAN	1234.049407	2	0.678202927	3	
FQQDDAHIFCAMEQIEDEIK	95.9418	P26639	SYTC_HUMAN	823.0355463	3	0.678202927	3	Yes
GAYIYNALIEFIR	122.7689	P26639	SYTC_HUMAN	771.919924	2	0.71068722	3	
GAYIYNALIEFIR	122.7689	P26639	SYTC_HUMAN	514.9492243	3	0.71068722	3	Yes
GCLDFLR	40.16719	P26639	SYTC_HUMAN	440.721462	2	0.641094506		
GCLDFLR	40.16719	P26639	SYTC_HUMAN	294.1502497	3	0.641094506		
GFQEVVTPNIFNSR	70.5184	P26639	SYTC_HUMAN	804.413003	2	0.654613078	3	
GFQEVVTPNIFNSR	70.5184	P26639	SYTC_HUMAN	536.611277	3	0.654613078	3	Yes
IYGISFPDPK	57.07684	P26639	SYTC_HUMAN	568.8035015	2	0.867462993	2	Yes
IYGISFPDPK	57.07684	P26639	SYTC_HUMAN	379.538276	3	0.867462993	2	
LADFGVLHR	24.91247	P26639	SYTC_HUMAN	514.2883575	2	0.807648242	2	Yes
LADFGVLHR	24.91247	P26639	SYTC_HUMAN	343.1948467	3	0.807648242	2	
LEMYNILK	52.79916	P26639	SYTC_HUMAN	512.2813475	2	0.784149945	2	Yes
LEMYNILK	52.79916	P26639	SYTC_HUMAN	341.85684	3	0.784149945	2	
LNLSTRPEK	-24.66364	P26639	SYTC_HUMAN	529.304204	2	0.759261727	2	Yes
LNLSTRPEK	-24.66364	P26639	SYTC_HUMAN	353.205411	3	0.759261727	2	
MIAILTENYGGK	49.84609	P26639	SYTC_HUMAN	655.345208	2	0.780105472	2	Yes
MIAILTENYGGK	49.84609	P26639	SYTC_HUMAN	437.232747	3	0.780105472	2	
NELSGALTGLTR	50.06664	P26639	SYTC_HUMAN	616.336233	2	0.827863276		
NELSGALTGLTR	50.06664	P26639	SYTC_HUMAN	411.2267637	3	0.827863276		
TTPYQIACGISQGLADNTVIAK	81.26305	P26639	SYTC_HUMAN	1161.094471	2	0.643688798	3	
TTPYQIACGISQGLADNTVIAK	81.26305	P26639	SYTC_HUMAN	774.3989223	3	0.643688798	3	Yes
WELNSGDGAFYGPK	51.19377	P26639	SYTC_HUMAN	770.857525	2	0.783749878	2	Yes
WELNSGDGAFYGPK	51.19377	P26639	SYTC_HUMAN	514.2409583	3	0.783749878	2	
DYGNSPLHR	-21.62014	P26599	PTBP1_HUMAN	529.754874	2	0.712540567	2	Yes
DYGNSPLHR	-21.62014	P26599	PTBP1_HUMAN	353.5058577	3	0.712540567	2	
IAIPGLAGAGNSVLLVSNLNPER	109.797	P26599	PTBP1_HUMAN	1138.142615	2	0.733430862	3	
IAIPGLAGAGNSVLLVSNLNPER	109.797	P26599	PTBP1_HUMAN	759.097685	3	0.733430862	3	Yes
LSLDGQNIYNACCTLR	54.34611	P26599	PTBP1_HUMAN	949.44893	2	0.73033756	2	Yes
LSLDGQNIYNACCTLR	54.34611	P26599	PTBP1_HUMAN	633.301895	3	0.73033756	2	
NFQNIFPPSATLHLSNIPPSVSEEDLK	92.12396	P26599	PTBP1_HUMAN	1497.764551	2	0.763269782	3	
NFQNIFPPSATLHLSNIPPSVSEEDLK	92.12396	P26599	PTBP1_HUMAN	998.845642	3	0.763269782	3	Yes
NNQFQALLQYADPVSAQHAK	70.31664	P26599	PTBP1_HUMAN	1122.064362	2	0.768502355	3	
NNQFQALLQYADPVSAQHAK	70.31664	P26599	PTBP1_HUMAN	748.3788493	3	0.768502355	3	Yes
VLFSSNGGVVK	22.82175	P26599	PTBP1_HUMAN	553.8144045	2	0.616866231	2	Yes
VLFSSNGGVVK	22.82175	P26599	PTBP1_HUMAN	369.5455447	3	0.616866231	2	
ALDVMVSTFHK	32.41552	P26447	S10A4_HUMAN	624.3268225	2	0.829923034	3	
ALDVMVSTFHK	32.41552	P26447	S10A4_HUMAN	416.5538233	3	0.829923034	3	Yes
LMSNLDSNR	-3.929272	P26447	S10A4_HUMAN	525.256396	2	0.770633996	2	Yes
LMSNLDSNR	-3.929272	P26447	S10A4_HUMAN	350.5068723	3	0.770633996	2	
ELLTSFGPLK	62.37876	P26368	U2AF2_HUMAN	552.819156	2	0.829052269	2	Yes
ELLTSFGPLK	62.37876	P26368	U2AF2_HUMAN	368.8820457	3	0.829052269	2	
IFVEFTSVFDCQK	91.20737	P26368	U2AF2_HUMAN	810.392891	2	0.681906939		
IFVEFTSVFDCQK	91.20737	P26368	U2AF2_HUMAN	540.597869	3	0.681906939		
LFIGGLPNYLNDDQVK	89.09721	P26368	U2AF2_HUMAN	903.475796	2	0.832028031	3	
LFIGGLPNYLNDDQVK	89.09721	P26368	U2AF2_HUMAN	602.653139	3	0.832028031	3	Yes
LGGLTQAPGNPVLAVQINQDK	69.9017	P26368	U2AF2_HUMAN	1067.08731	2	0.765357554	3	
LGGLTQAPGNPVLAVQINQDK	69.9017	P26368	U2AF2_HUMAN	711.7274817	3	0.765357554	3	Yes
NFAFLEFR	75.6396	P26368	U2AF2_HUMAN	522.269633	2	0.773386836	2	Yes

NFAFLEFR	75.6396	P26368	U2AF2 HUMAN	348.515697	3	0.773386836	2	
SVDETTQAMAFDGIIFQGQSLK	108.8436	P26368	U2AF2 HUMAN	1193.58394	2	0.740296602	3	
SVDETTQAMAFDGIIFQGQSLK	108.8436	P26368	U2AF2 HUMAN	796.0585683	3	0.740296602	3	Yes
YWDVPPPGFEHITPMQYK	72.2079	P26368	U2AF2 HUMAN	1103.027867	2	0.797391891	3	
YWDVPPPGFEHITPMQYK	72.2079	P26368	U2AF2 HUMAN	735.6878527	3	0.797391891	3	Yes
AIILAAAPGEK	30.03691	P26358	DNMT1 HUMAN	527.3193235	2	0.755121231	2	Yes
AIILAAAPGEK	30.03691	P26358	DNMT1 HUMAN	351.8821573	3	0.755121231	2	
APSENWAMEGGMDPESLLEGDDGK	91.11469	P26358	DNMT1 HUMAN	1268.036693	2	0.721708238		
APSENWAMEGGMDPESLLEGDDGK	91.11469	P26358	DNMT1 HUMAN	845.6937367	3	0.721708238		
DHICKDMSALVAAR	16.66331	P26358	DNMT1 HUMAN	793.8930665	2	0.710895956		
DHICKDMSALVAAR	16.66331	P26358	DNMT1 HUMAN	529.597986	3	0.710895956		
DLPNIEVR	38.39708	P26358	DNMT1 HUMAN	478.2645475	2	0.703524768	2	Yes
DLPNIEVR	38.39708	P26358	DNMT1 HUMAN	319.1789733	3	0.703524768	2	
DTMSDLPEVR	37.5755	P26358	DNMT1 HUMAN	581.7746195	2	0.675250173	2	Yes
DTMSDLPEVR	37.5755	P26358	DNMT1 HUMAN	388.185688	3	0.675250173	2	
EADDDEEVDDNIPEMPSPK	47.32935	P26358	DNMT1 HUMAN	1072.944795	2	0.753950179		
EADDDEEVDDNIPEMPSPK	47.32935	P26358	DNMT1 HUMAN	715.6324713	3	0.753950179		
FFLLENVR	63.86393	P26358	DNMT1 HUMAN	519.293108	2	0.772819161		
FFLLENVR	63.86393	P26358	DNMT1 HUMAN	346.531347	3	0.772819161		
FYFLEAYNAK	59.29463	P26358	DNMT1 HUMAN	633.314229	2	0.799546361	2	Yes
FYFLEAYNAK	59.29463	P26358	DNMT1 HUMAN	422.5454277	3	0.799546361	2	
GAQYQPILR	16.57978	P26358	DNMT1 HUMAN	523.293636	2	0.64151293		
GAQYQPILR	16.57978	P26358	DNMT1 HUMAN	349.1983657	3	0.64151293		
GDVEMLCGGPPCQGFSGMNR	56.57039	P26358	DNMT1 HUMAN	1084.953202	2	0.71162653		
GDVEMLCGGPPCQGFSGMNR	56.57039	P26358	DNMT1 HUMAN	723.638076	3	0.71162653		
GSNLDAPEPYR	7.757305	P26358	DNMT1 HUMAN	609.7916535	2	0.840948224	2	Yes
GSNLDAPEPYR	7.757305	P26358	DNMT1 HUMAN	406.8637107	3	0.840948224	2	
IETTVPPSGLNLNR	43.21852	P26358	DNMT1 HUMAN	755.9153785	2	0.792879224		
IETTVPPSGLNLNR	43.21852	P26358	DNMT1 HUMAN	504.2795273	3	0.792879224		
ISWVGEAVK	32.44746	P26358	DNMT1 HUMAN	494.777291	2	0.777038097	2	Yes
ISWVGEAVK	32.44746	P26358	DNMT1 HUMAN	330.187469	3	0.777038097	2	
IVVEFLQSNSDSTYEDLINK	94.76653	P26358	DNMT1 HUMAN	1157.576632	2	0.785636902		
IVVEFLQSNSDSTYEDLINK	94.76653	P26358	DNMT1 HUMAN	772.053696	3	0.785636902		
LAGVTLGQR	10.35929	P26358	DNMT1 HUMAN	457.7750835	2	0.803782761	2	Yes
LAGVTLGQR	10.35929	P26358	DNMT1 HUMAN	305.5193307	3	0.803782761	2	
LEWDGFFSTTVTNPEPMGK	100.0007	P26358	DNMT1 HUMAN	1078.50443	2	0.638716161		
LEWDGFFSTTVTNPEPMGK	100.0007	P26358	DNMT1 HUMAN	719.3388947	3	0.638716161		
LFGNILDK	44.43217	P26358	DNMT1 HUMAN	460.266559	2	0.825846791	2	Yes
LFGNILDK	44.43217	P26358	DNMT1 HUMAN	307.1803143	3	0.825846791	2	
LNLLHEFLQTEIK	84.22247	P26358	DNMT1 HUMAN	799.451597	2	0.861244321	3	
LNLLHEFLQTEIK	84.22247	P26358	DNMT1 HUMAN	533.303673	3	0.861244321	3	Yes
LNNPGSTVFTEDCNILLK	72.35385	P26358	DNMT1 HUMAN	1018.012421	2	0.796043873	3	
LNNPGSTVFTEDCNILLK	72.35385	P26358	DNMT1 HUMAN	679.010889	3	0.796043873	3	Yes
LPLFPEPLHVFAPR	85.66809	P26358	DNMT1 HUMAN	816.9672175	2	0.845855176	3	
LPLFPEPLHVFAPR	85.66809	P26358	DNMT1 HUMAN	544.9807533	3	0.845855176	3	Yes
LSDGTMAR	-25.13585	P26358	DNMT1 HUMAN	425.7085515	2	0.776628017		
LSDGTMAR	-25.13585	P26358	DNMT1 HUMAN	284.1416427	3	0.776628017		
LSIFDANESGFESYEALPQHK	72.48412	P26358	DNMT1 HUMAN	1191.566599	2	0.872413099	3	
LSIFDANESGFESYEALPQHK	72.48412	P26358	DNMT1 HUMAN	794.713674	3	0.872413099	3	Yes
NLGPINEWWITGFDGGEK	116.52	P26358	DNMT1 HUMAN	1016.992346	2	0.814144015		
NLGPINEWWITGFDGGEK	116.52	P26358	DNMT1 HUMAN	678.330839	3	0.814144015		
NQLCDLETK	5.06279	P26358	DNMT1 HUMAN	560.7693375	2	0.79784143		
NQLCDLETK	5.06279	P26358	DNMT1 HUMAN	374.1821667	3	0.79784143		
SQACEPSEPEIEIK	26.44022	P26358	DNMT1 HUMAN	808.8778015	2	0.764376998	2	Yes
SQACEPSEPEIEIK	26.44022	P26358	DNMT1 HUMAN	539.5878093	3	0.764376998	2	
SQGFPDTYR	8.675385	P26358	DNMT1 HUMAN	535.749258	2	0.756641269		
SQGFPDTYR	8.675385	P26358	DNMT1 HUMAN	357.5021137	3	0.756641269		
STPASYHADINLLYWSDEEAVVDFK	104.2204	P26358	DNMT1 HUMAN	1435.680144	2	0.753155231		
STPASYHADINLLYWSDEEAVVDFK	104.2204	P26358	DNMT1 HUMAN	957.4560377	3	0.753155231		
TYFYQLWYDQDYAR	87.63168	P26358	DNMT1 HUMAN	966.434116	2	0.780822814		
TYFYQLWYDQDYAR	87.63168	P26358	DNMT1 HUMAN	644.6253523	3	0.780822814		
VGDGVYLPPEAFTFNIK	100.2898	P26358	DNMT1 HUMAN	933.985997	2	0.809632659		
VGDGVYLPPEAFTFNIK	100.2898	P26358	DNMT1 HUMAN	622.993273	3	0.809632659		
VGMADANSPPKPLSKPR	-4.777485	P26358	DNMT1 HUMAN	882.9754445	2	0.802021384		
VGMADANSPPKPLSKPR	-4.777485	P26358	DNMT1 HUMAN	588.986238	3	0.802021384		
VLEQLEDLDSR	45.43773	P26358	DNMT1 HUMAN	658.838805	2	0.817237496	2	Yes
VLEQLEDLDSR	45.43773	P26358	DNMT1 HUMAN	439.5618117	3	0.817237496	2	
VPTLAVPAISLPDDVR	97.35388	P26358	DNMT1 HUMAN	831.9754365	2	0.639729798	3	
VPTLAVPAISLPDDVR	97.35388	P26358	DNMT1 HUMAN	554.9862327	3	0.639729798	3	Yes
YGQHPPDAVDEPQMLTNEK	27.34789	P26358	DNMT1 HUMAN	1085.000039	2	0.870945096	3	
YGQHPPDAVDEPQMLTNEK	27.34789	P26358	DNMT1 HUMAN	723.6693007	3	0.870945096	3	Yes
ALELEQER	1.352543	P26038	MOES HUMAN	494.2594625	2	0.817035675	2	Yes
ALELEQER	1.352543	P26038	MOES HUMAN	329.84225	3	0.817035675	2	
ALTSELANAR	6.143677	P26038	MOES HUMAN	523.2860115	2	0.781736135	2	Yes
ALTSELANAR	6.143677	P26038	MOES HUMAN	349.1932827	3	0.781736135	2	
AMLENEK	-24.68851	P26038	MOES HUMAN	417.705477	2	0.626455188		
AMLENEK	-24.68851	P26038	MOES HUMAN	278.8062597	3	0.626455188		
AQMVQEDLEK	1.817394	P26038	MOES HUMAN	595.79027	2	0.760332882	2	Yes
AQMVQEDLEK	1.817394	P26038	MOES HUMAN	397.529455	3	0.760332882	2	
EDAVLEYLK	53.49382	P26038	MOES HUMAN	540.2851415	2	0.778881609	2	Yes
EDAVLEYLK	53.49382	P26038	MOES HUMAN	360.526036	3	0.778881609	2	
EGILNDDIYCPPETAVLLASYAVQSK	131.404	P26038	MOES HUMAN	1433.713131	2	0.736327648		
EGILNDDIYCPPETAVLLASYAVQSK	131.404	P26038	MOES HUMAN	956.1446953	3	0.736327648		
EVWFFGLQYQDTK	94.67184	P26038	MOES HUMAN	830.904476	2	0.726715803	3	
EVWFFGLQYQDTK	94.67184	P26038	MOES HUMAN	554.272259	3	0.726715803	3	Yes
FYPEDVSEELIQDITQR	115.4221	P26038	MOES HUMAN	1041.505479	2	0.811520219	3	
FYPEDVSEELIQDITQR	115.4221	P26038	MOES HUMAN	694.6729277	3	0.811520219	3	Yes
GFSTWLK	39.64767	P26038	MOES HUMAN	419.72707	2	0.783878744		
GFSTWLK	39.64767	P26038	MOES HUMAN	280.1539883	3	0.783878744		
IAQDLEMYGVNYFSIK	95.49295	P26038	MOES HUMAN	945.969485	2	0.78054148	3	
IAQDLEMYGVNYFSIK	95.49295	P26038	MOES HUMAN	630.982265	3	0.78054148	3	Yes
IQVWHEEHR	-26.05934	P26038	MOES HUMAN	617.3103525	2	0.802588701		
IQVWHEEHR	-26.05934	P26038	MOES HUMAN	411.8761767	3	0.802588701		
ISQLEMAR	2.750988	P26038	MOES HUMAN	474.253126	2	0.791667521	2	Yes
ISQLEMAR	2.750988	P26038	MOES HUMAN	316.5046923	3	0.791667521	2	
TQEQLALEMAELTAR	77.46362	P26038	MOES HUMAN	852.435819	2	0.830799341	2	Yes
TQEQLALEMAELTAR	77.46362	P26038	MOES HUMAN	568.6264877	3	0.830799341	2	
ELPGFLQSGK	42.76729	P25815	S100P HUMAN	538.2933055	2	0.809416831	2	Yes
ELPGFLQSGK	42.76729	P25815	S100P HUMAN	359.1981453	3	0.809416831	2	
YSGSEGSTQTLTK	-23.71296	P25815	S100P HUMAN	679.825891	2	0.620237827	2	Yes
YSGSEGSTQTLTK	-23.71296	P25815	S100P HUMAN	453.5532023	3	0.620237827	2	

AVDSLVPIGR	41.05027	P25705	ATPA HUMAN	513.8012975	2	0.750847995	2	Yes
AVDSLVPIGR	41.05027	P25705	ATPA HUMAN	342.87014	3	0.750847995	2	
EAYPGDVFYLHSR	41.58678	P25705	ATPA HUMAN	777.373338	2	0.787360191	3	
EAYPGDVFYLHSR	41.58678	P25705	ATPA HUMAN	518.5848337	3	0.787360191	3	Yes
EVAAFAQFGSDLDAATQQLLSR	112.8344	P25705	ATPA HUMAN	1169.587871	2	0.694374621	3	
EVAAFAQFGSDLDAATQQLLSR	112.8344	P25705	ATPA HUMAN	780.061189	3	0.694374621	3	Yes
FENAFLSHVVSQHQALLGTIR	67.56543	P25705	ATPA HUMAN	1184.132583	2	0.708597779	3	
FENAFLSHVVSQHQALLGTIR	67.56543	P25705	ATPA HUMAN	789.7576633	3	0.708597779	3	Yes
GIRPAINVGLSVSR	35.6446	P25705	ATPA HUMAN	719.928623	2	0.621776581	2	Yes
GIRPAINVGLSVSR	35.6446	P25705	ATPA HUMAN	480.288357	3	0.621776581	2	
GMSLNLEPDNVGVVVFGNDK	91.21059	P25705	ATPA HUMAN	1052.523153	2	0.722641528	3	
GMSLNLEPDNVGVVVFGNDK	91.21059	P25705	ATPA HUMAN	702.0180433	3	0.722641528	3	Yes
HALIIYDDLSK	34.50319	P25705	ATPA HUMAN	644.351347	2	0.81409353	2	Yes
HALIIYDDLSK	34.50319	P25705	ATPA HUMAN	429.9035063	3	0.81409353	2	
ILGADTSVDLEETGR	41.90472	P25705	ATPA HUMAN	788.397216	2	0.805320919	2	Yes
ILGADTSVDLEETGR	41.90472	P25705	ATPA HUMAN	525.9340857	3	0.805320919	2	
LELAQYR	13.92721	P25705	ATPA HUMAN	446.7485295	2	0.662174642	2	Yes
LELAQYR	13.92721	P25705	ATPA HUMAN	298.1682947	3	0.662174642	2	
NALGSSFIAAR	35.1492	P25705	ATPA HUMAN	553.8018285	2	0.685457587		
NALGSSFIAAR	35.1492	P25705	ATPA HUMAN	369.5371607	3	0.685457587		
NVQAEEMVEFSSGLK	65.19656	P25705	ATPA HUMAN	834.4014435	2	0.837810159	3	
NVQAEEMVEFSSGLK	65.19656	P25705	ATPA HUMAN	556.6035707	3	0.837810159	3	Yes
TGAIVDVPVGEELLGR	83.83258	P25705	ATPA HUMAN	812.949419	2	0.858171046	2	Yes
TGAIVDVPVGEELLGR	83.83258	P25705	ATPA HUMAN	542.302221	3	0.858171046	2	
TGTAEMSSILEER	41.04389	P25705	ATPA HUMAN	712.340855	2	0.798409343	2	Yes
TGTAEMSSILEER	41.04389	P25705	ATPA HUMAN	475.229845	3	0.798409343	2	
TSIAIDTIINQK	48.66494	P25705	ATPA HUMAN	658.875191	2	0.767754853	2	Yes
TSIAIDTIINQK	48.66494	P25705	ATPA HUMAN	439.586069	3	0.767754853	2	
VLSIGDGIAR	33.64732	P25705	ATPA HUMAN	500.7934725	2	0.859860599	2	Yes
VLSIGDGIAR	33.64732	P25705	ATPA HUMAN	334.1982567	3	0.859860599	2	
VVDALGNAIDGK	30.64028	P25705	ATPA HUMAN	586.3200515	2	0.796033323	2	Yes
VVDALGNAIDGK	30.64028	P25705	ATPA HUMAN	391.215976	3	0.796033323	2	
ANLCTLAEK	4.35331	P25445	TNR6 HUMAN	510.263691	2	0.703811407		
ANLCTLAEK	4.35331	P25445	TNR6 HUMAN	340.5117357	3	0.703811407		
NDNVQDTAEQK	-33.64417	P25445	TNR6 HUMAN	631.286937	2	0.633736432		
NDNVQDTAEQK	-33.64417	P25445	TNR6 HUMAN	421.1938997	3	0.633736432		
YITTIAGVMTLSQVK	83.05066	P25445	TNR6 HUMAN	812.953112	2	0.756172121		
YITTIAGVMTLSQVK	83.05066	P25445	TNR6 HUMAN	542.304683	3	0.756172121		
AEIQELAMVPR	51.4725	P24928	RPB1 HUMAN	628.8375545	2	0.82925725		
AEIQELAMVPR	51.4725	P24928	RPB1 HUMAN	419.560978	3	0.82925725		
AHNNELEPTPGNTLR	-1.659477	P24928	RPB1 HUMAN	831.9138985	2	0.779583216		
AHNNELEPTPGNTLR	-1.659477	P24928	RPB1 HUMAN	554.9452073	3	0.779583216		
DVLSNAHIQNELER	34.59608	P24928	RPB1 HUMAN	819.4162735	2	0.813330889	3	
DVLSNAHIQNELER	34.59608	P24928	RPB1 HUMAN	546.6134573	3	0.813330889	3	Yes
FDYTNER	-9.159206	P24928	RPB1 HUMAN	472.709601	2	0.608473122		
FDYTNER	-9.159206	P24928	RPB1 HUMAN	315.4756757	3	0.608473122		
FGVEQPEGDEDLTK	28.40449	P24928	RPB1 HUMAN	782.362842	2	0.850077629	2	Yes
FGVEQPEGDEDLTK	28.40449	P24928	RPB1 HUMAN	521.9111697	3	0.850077629	2	
FHPKPSDLHLQTGYK	1.821838	P24928	RPB1 HUMAN	884.4630235	2	0.800770998		
FHPKPSDLHLQTGYK	1.821838	P24928	RPB1 HUMAN	589.9779573	3	0.800770998		
HLALLCDTMTCR	32.67167	P24928	RPB1 HUMAN	745.849814	2	0.743006527		
HLALLCDTMTCR	32.67167	P24928	RPB1 HUMAN	497.569151	3	0.743006527		
HMCDGDIVIFNR	34.23924	P24928	RPB1 HUMAN	738.8403025	2	0.611241281		
HMCDGDIVIFNR	34.23924	P24928	RPB1 HUMAN	492.8961433	3	0.611241281		
ILPWSTFR	63.54272	P24928	RPB1 HUMAN	510.287826	2	0.664943337	2	Yes
ILPWSTFR	63.54272	P24928	RPB1 HUMAN	340.5278257	3	0.664943337	2	
INISQVIAVVGQQNVEGK	86.40056	P24928	RPB1 HUMAN	948.5316385	2	0.750243664	3	
INISQVIAVVGQQNVEGK	86.40056	P24928	RPB1 HUMAN	632.6903673	3	0.750243664	3	Yes
ISDEECFVLGMEPR	67.51392	P24928	RPB1 HUMAN	841.382197	2	0.844686866		
ISDEECFVLGMEPR	67.51392	P24928	RPB1 HUMAN	561.2574063	3	0.844686866		
ISPWLLR	52.25684	P24928	RPB1 HUMAN	442.7718115	2	0.682987928		
ISPWLLR	52.25684	P24928	RPB1 HUMAN	295.5171493	3	0.682987928		
LGGLMDPR	21.09956	P24928	RPB1 HUMAN	429.729287	2	0.80037874		
LGGLMDPR	21.09956	P24928	RPB1 HUMAN	286.822133	3	0.80037874		
LLQFHVATMVDNELPGLPR	82.18384	P24928	RPB1 HUMAN	1075.575524	2	0.789634943	3	
LLQFHVATMVDNELPGLPR	82.18384	P24928	RPB1 HUMAN	717.3862907	3	0.789634943	3	Yes
LLVDSNNPK	-3.277145	P24928	RPB1 HUMAN	500.2776545	2	0.810171247	2	Yes
LLVDSNNPK	-3.277145	P24928	RPB1 HUMAN	333.854378	3	0.810171247	2	
LNLSVTTPYNADFDGDEMNLHLPQSLETR	94.75224	P24928	RPB1 HUMAN	1645.785882	2	0.640620708		
LNLSVTTPYNADFDGDEMNLHLPQSLETR	94.75224	P24928	RPB1 HUMAN	1097.52653	3	0.640620708		
LPSDLHPIK	5.693752	P24928	RPB1 HUMAN	510.2983905	2	0.630163074		
LPSDLHPIK	5.693752	P24928	RPB1 HUMAN	340.5348687	3	0.630163074		
LSGEAFDWLLGEIESK	135.0547	P24928	RPB1 HUMAN	897.4519905	2	0.761624336		
LSGEAFDWLLGEIESK	135.0547	P24928	RPB1 HUMAN	598.6372687	3	0.761624336		
LTHVYDLCK	0.627113	P24928	RPB1 HUMAN	574.7926115	2	0.699997067		
LTHVYDLCK	0.627113	P24928	RPB1 HUMAN	383.531016	3	0.699997067		
LTMEQIAEK	16.51389	P24928	RPB1 HUMAN	531.779174	2	0.807789743		
LTMEQIAEK	16.51389	P24928	RPB1 HUMAN	354.855391	3	0.807789743		
NSINQVVQLR	28.54543	P24928	RPB1 HUMAN	585.83366	2	0.801477075	2	Yes
NSINQVVQLR	28.54543	P24928	RPB1 HUMAN	390.891715	3	0.801477075	2	
SGLELYAEWK	57.4882	P24928	RPB1 HUMAN	598.3038655	2	0.781566739		
SGLELYAEWK	57.4882	P24928	RPB1 HUMAN	399.2051853	3	0.781566739		
SLSEYNNFK	11.22391	P24928	RPB1 HUMAN	551.2647395	2	0.784729004	2	Yes
SLSEYNNFK	11.22391	P24928	RPB1 HUMAN	367.845768	3	0.784729004	2	
TVITPDPNLSIDQVGVPR	73.49052	P24928	RPB1 HUMAN	961.0236465	2	0.747471631		
TVITPDPNLSIDQVGVPR	73.49052	P24928	RPB1 HUMAN	641.0183727	3	0.747471631		
TYQDIQNTIK	12.97832	P24928	RPB1 HUMAN	612.317505	2	0.783876717		
TYQDIQNTIK	12.97832	P24928	RPB1 HUMAN	408.5476117	3	0.783876717		
VIFPTGDSK	18.94715	P24928	RPB1 HUMAN	482.261474	2	0.738765359	2	Yes
VIFPTGDSK	18.94715	P24928	RPB1 HUMAN	321.843591	3	0.738765359	2	
VQFGVLSPDELK	61.90679	P24928	RPB1 HUMAN	666.364459	2	0.820391715		
VQFGVLSPDELK	61.90679	P24928	RPB1 HUMAN	444.5789143	3	0.820391715		
VVLPCNLLR	53.98257	P24928	RPB1 HUMAN	542.321343	2	0.841126323	2	Yes
VVLPCNLLR	53.98257	P24928	RPB1 HUMAN	361.8835037	3	0.841126323	2	
VYMHLPQTDNK	3.678883	P24928	RPB1 HUMAN	673.332632	2	0.831080437		
VYMHLPQTDNK	3.678883	P24928	RPB1 HUMAN	449.224363	3	0.831080437		
YGEDGLAGESVEFQNLATLKPSNK	65.30914	P24928	RPB1 HUMAN	1284.135377	2	0.718208849	3	
YGEDGLAGESVEFQNLATLKPSNK	65.30914	P24928	RPB1 HUMAN	856.426193	3	0.718208849	3	Yes
YSPTSPTYSPTSPVYTPTSPK	43.88979	P24928	RPB1 HUMAN	1129.547336	2	0.853878379		
YSPTSPTYSPTSPVYTPTSPK	43.88979	P24928	RPB1 HUMAN	753.3674987	3	0.853878379		
YSPTSPTYSPTTPK	13.9315	P24928	RPB1 HUMAN	763.872837	2	0.852863312		

YSPTSPTYSPTTPK	13.9315	P24928	RPB1 HUMAN	509.5844997	3	0.852863312		
YTPQSPTYTPSSPSYSPSSPSYSPASPK	40.9651	P24928	RPB1 HUMAN	1474.67779	2	0.676524222		
YTPQSPTYTPSSPSYSPSSPSYSPASPK	40.9651	P24928	RPB1 HUMAN	983.454468	3	0.676524222		
YTPTSPSYSPSSPEYTPTSPK	31.8924	P24928	RPB1 HUMAN	1137.5266	2	0.80103159	3	
YTPTSPSYSPSSPEYTPTSPK	31.8924	P24928	RPB1 HUMAN	758.687008	3	0.80103159	3	Yes
IELLGSYDPQK	43.22527	P24666	PPAC HUMAN	631.83553	2	0.843910992	2	Yes
IELLGSYDPQK	43.22527	P24666	PPAC HUMAN	421.5596283	3	0.843910992	2	
LVTDQNISENWR	31.33517	P24666	PPAC HUMAN	737.868428	2	0.83753258	2	Yes
LVTDQNISENWR	31.33517	P24666	PPAC HUMAN	492.248227	3	0.83753258	2	
VDSAATSGYEIGNPPDYR	36.72252	P24666	PPAC HUMAN	956.4401345	2	0.842646301	3	
VDSAATSGYEIGNPPDYR	36.72252	P24666	PPAC HUMAN	637.962698	3	0.842646301	3	Yes
AFHSSLHESIQKPYNQK	-20.57958	P23634	AT2B4 HUMAN	1007.511233	2	0.628940642		
AFHSSLHESIQKPYNQK	-20.57958	P23634	AT2B4 HUMAN	672.0100967	3	0.628940642		
DAEGLDEIDHAEMELR	58.52134	P23634	AT2B4 HUMAN	921.912904	2	0.87967205		
DAEGLDEIDHAEMELR	58.52134	P23634	AT2B4 HUMAN	614.9445443	3	0.87967205		
DALTQINVHYGGVQNLCSR	49.97046	P23634	AT2B4 HUMAN	1073.029465	2	0.700023055	3	
DALTQINVHYGGVQNLCSR	49.97046	P23634	AT2B4 HUMAN	715.688918	3	0.700023055	3	Yes
EGDFGCTVMELR	52.04434	P23634	AT2B4 HUMAN	707.311044	2	0.792731524	2	Yes
EGDFGCTVMELR	52.04434	P23634	AT2B4 HUMAN	471.8766377	3	0.792731524	2	
GIIDSTVGEHR	1.641823	P23634	AT2B4 HUMAN	592.3074755	2	0.797560155		
GIIDSTVGEHR	1.641823	P23634	AT2B4 HUMAN	395.207592	3	0.797560155		
IQTQIDVINTFQTGASFK	80.62259	P23634	AT2B4 HUMAN	1006.02893	2	0.718556941		
IQTQIDVINTFQTGASFK	80.62259	P23634	AT2B4 HUMAN	671.0218947	3	0.718556941		
MTVVQAYIGGIHYR	53.19733	P23634	AT2B4 HUMAN	804.422309	2	0.672582984		
MTVVQAYIGGIHYR	53.19733	P23634	AT2B4 HUMAN	536.617481	3	0.672582984		
SIHSFMTHPEFAIEEELPR	61.62486	P23634	AT2B4 HUMAN	1135.549702	2	0.68490392		
SIHSFMTHPEFAIEEELPR	61.62486	P23634	AT2B4 HUMAN	757.3690763	3	0.68490392		
SLDKDPMLLSGTHVMEGSGR	36.67439	P23634	AT2B4 HUMAN	1065.520088	2	0.809899569		
SLDKDPMLLSGTHVMEGSGR	36.67439	P23634	AT2B4 HUMAN	710.6826667	3	0.809899569		
TPLLDEEEEENPDK	25.47061	P23634	AT2B4 HUMAN	829.3761455	2	0.606661975	2	Yes
TPLLDEEEEENPDK	25.47061	P23634	AT2B4 HUMAN	553.253372	3	0.606661975	2	
TQDGVALEIQPLNSQEGIDNEEK	60.85448	P23634	AT2B4 HUMAN	1264.112104	2	0.827115893		
TQDGVALEIQPLNSQEGIDNEEK	60.85448	P23634	AT2B4 HUMAN	843.077344	3	0.827115893		
TSPVEGLSGNPADLEK	37.98108	P23634	AT2B4 HUMAN	807.4050405	2	0.819365025	2	Yes
TSPVEGLSGNPADLEK	37.98108	P23634	AT2B4 HUMAN	538.6059687	3	0.819365025	2	
TVIEPMACDGLR	35.60503	P23634	AT2B4 HUMAN	681.3317795	2	0.693488657		
TVIEPMACDGLR	35.60503	P23634	AT2B4 HUMAN	454.557128	3	0.693488657		
AASIFGGAKPVDTAAR	16.85793	P23588	IF4B HUMAN	766.4155465	2	0.736562371		
AASIFGGAKPVDTAAR	16.85793	P23588	IF4B HUMAN	511.2796393	3	0.736562371		
ARPATDSFDDYPPR	10.96766	P23588	IF4B HUMAN	804.3766135	2	0.657136977	3	
ARPATDSFDDYPPR	10.96766	P23588	IF4B HUMAN	536.5870173	3	0.657136977	3	Yes
SILPTAPR	14.62068	P23588	IF4B HUMAN	427.7589015	2	0.623545706	2	Yes
SILPTAPR	14.62068	P23588	IF4B HUMAN	285.5085427	3	0.623545706	2	
SPPYTAFLGNLPYDVTEESIK	105.5722	P23588	IF4B HUMAN	1171.084089	2	0.683692813	3	
SPPYTAFLGNLPYDVTEESIK	105.5722	P23588	IF4B HUMAN	781.0586677	3	0.683692813	3	Yes
VAPAQPSEEGPGR	-22.98108	P23588	IF4B HUMAN	647.82349	2	0.687863588	2	Yes
VAPAQPSEEGPGR	-22.98108	P23588	IF4B HUMAN	432.2182683	3	0.687863588	2	
YAALSVDGEDENEGEDYAE	53.50793	P23588	IF4B HUMAN	1038.414176	2	0.616419911		
YAALSVDGEDENEGEDYAE	53.50793	P23588	IF4B HUMAN	692.612059	3	0.616419911		
AGIPVYAWK	44.30012	P23526	SAHH HUMAN	502.7823725	2	0.696017921	2	Yes
AGIPVYAWK	44.30012	P23526	SAHH HUMAN	335.52419	3	0.696017921	2	
ALDIAENEMPGLMR	73.80848	P23526	SAHH HUMAN	780.382	2	0.811029732	2	Yes
ALDIAENEMPGLMR	73.80848	P23526	SAHH HUMAN	520.5906083	3	0.811029732	2	
DGPLNMILDDGGDLTNLIHTK	129.3398	P23526	SAHH HUMAN	1126.56555	2	0.730061769	3	
DGPLNMILDDGGDLTNLIHTK	129.3398	P23526	SAHH HUMAN	751.3796413	3	0.730061769	3	Yes
FDNLYGCR	10.95227	P23526	SAHH HUMAN	522.7325535	2	0.782598853	2	Yes
FDNLYGCR	10.95227	P23526	SAHH HUMAN	348.8243107	3	0.782598853	2	
GISEETTTGVHNLYK	10.11566	P23526	SAHH HUMAN	824.913029	2	0.862179756	2	Yes
GISEETTTGVHNLYK	10.11566	P23526	SAHH HUMAN	550.277961	3	0.862179756	2	
IILLAEGR	34.97099	P23526	SAHH HUMAN	442.7823765	2	0.68821156	2	Yes
IILLAEGR	34.97099	P23526	SAHH HUMAN	295.5241927	3	0.68821156	2	
LDEAVAEAHLGK	7.07888	P23526	SAHH HUMAN	626.8307835	2	0.653546512	3	
LDEAVAEAHLGK	7.07888	P23526	SAHH HUMAN	418.2231307	3	0.653546512	3	Yes
VADIGLAAWGR	59.13634	P23526	SAHH HUMAN	564.812197	2	0.818363667	2	Yes
VADIGLAAWGR	59.13634	P23526	SAHH HUMAN	376.8774063	3	0.818363667	2	
VAVVAGYGDVGK	18.42887	P23526	SAHH HUMAN	567.8118585	2	0.761518955	2	Yes
VAVVAGYGDVGK	18.42887	P23526	SAHH HUMAN	378.8771807	3	0.761518955	2	
VPAINVNDSVTK	23.88462	P23526	SAHH HUMAN	628.846433	2	0.758900523	2	Yes
VPAINVNDSVTK	23.88462	P23526	SAHH HUMAN	419.566897	3	0.758900523	2	
YPQLLPGIR	54.70252	P23526	SAHH HUMAN	528.814204	2	0.767870963	2	Yes
YPQLLPGIR	54.70252	P23526	SAHH HUMAN	352.8787443	3	0.767870963	2	
YPVGVHFLPK	34.02301	P23526	SAHH HUMAN	578.829854	2	0.703578949	2	Yes
YPVGVHFLPK	34.02301	P23526	SAHH HUMAN	386.222511	3	0.703578949	2	
AELDDTPMR	3.86396	P23246	SFPQ HUMAN	524.2429555	2	0.73444438	2	Yes
AELDDTPMR	3.86396	P23246	SFPQ HUMAN	349.8312453	3	0.73444438	2	
FAQHGTFEYEYSQR	11.24165	P23246	SFPQ HUMAN	881.8951665	2	0.826282024	3	
FAQHGTFEYEYSQR	11.24165	P23246	SFPQ HUMAN	588.2660527	3	0.826282024	3	Yes
FGQGGAGPVGGQGPR	-3.141766	P23246	SFPQ HUMAN	671.3371	2	0.813242555	2	Yes
FGQGGAGPVGGQGPR	-3.141766	P23246	SFPQ HUMAN	447.8940083	3	0.813242555	2	
GIVEFASKPAAR	9.28968	P23246	SFPQ HUMAN	623.351686	2	0.791781723	3	
GIVEFASKPAAR	9.28968	P23246	SFPQ HUMAN	415.9037323	3	0.791781723	3	Yes
GMGPGTPAGYGR	0.902046	P23246	SFPQ HUMAN	560.764386	2	0.718506098	2	Yes
GMGPGTPAGYGR	0.902046	P23246	SFPQ HUMAN	374.1788657	3	0.718506098	2	
LESEMEDAYHEHQANLLR	25.61148	P23246	SFPQ HUMAN	1093.003112	2	0.769705176	3	
LESEMEDAYHEHQANLLR	25.61148	P23246	SFPQ HUMAN	729.0046827	3	0.769705176	3	Yes
LFVGNLPADITEDEFK	89.01917	P23246	SFPQ HUMAN	904.459816	2	0.802326918	3	
LFVGNLPADITEDEFK	89.01917	P23246	SFPQ HUMAN	603.3091523	3	0.802326918	3	Yes
MGGGGAMNMGDPYGSGGQK	17.95263	P23246	SFPQ HUMAN	886.3640095	2	0.637271821	2	Yes
MGGGGAMNMGDPYGSGGQK	17.95263	P23246	SFPQ HUMAN	591.2452813	3	0.637271821	2	
SPPPGMGLNQNR	4.276485	P23246	SFPQ HUMAN	634.3147775	2	0.691550493	2	Yes
SPPPGMGLNQNR	4.276485	P23246	SFPQ HUMAN	423.21246	3	0.691550493	2	
YGEPGEVFINK	33.22499	P23246	SFPQ HUMAN	626.8145975	2	0.782400548		
YGEPGEVFINK	33.22499	P23246	SFPQ HUMAN	418.21234	3	0.782400548		
DYFEEYGK	29.69984	P22626	ROA2 HUMAN	525.7249125	2	0.7862674	2	Yes
DYFEEYGK	29.69984	P22626	ROA2 HUMAN	350.8192167	3	0.7862674	2	
EESGKPGAHVTVK	-34.23161	P22626	ROA2 HUMAN	669.85479	2	0.647873759	3	
EESGKPGAHVTVK	-34.23161	P22626	ROA2 HUMAN	446.9058017	3	0.647873759	3	Yes
GFGFVTFDDHDPVDK	59.8037	P22626	ROA2 HUMAN	848.3866515	2	0.840932071	3	
GFGFVTFDDHDPVDK	59.8037	P22626	ROA2 HUMAN	565.9270427	3	0.840932071	3	Yes
GGGGNFGPGPGSNFR	16.1304	P22626	ROA2 HUMAN	689.318906	2	0.763441801	2	Yes
GGGGNFGPGPGSNFR	16.1304	P22626	ROA2 HUMAN	459.881879	3	0.763441801	2	

GGNFGFGDSR	12.96337	P22626	ROA2 HUMAN	507.225954	2	0.813772857	2	Yes
GGNFGFGDSR	12.96337	P22626	ROA2 HUMAN	338.4865777	3	0.813772857	2	
IDTIEIITDR	52.50594	P22626	ROA2 HUMAN	594.8277095	2	0.785654783	2	Yes
IDTIEIITDR	52.50594	P22626	ROA2 HUMAN	396.887748	3	0.785654783	2	
LFIGGLSFETTEESLR	92.65704	P22626	ROA2 HUMAN	899.9652655	2	0.744783044	2	Yes
LFIGGLSFETTEESLR	92.65704	P22626	ROA2 HUMAN	600.3127853	3	0.744783044	2	
NYYEQWGK	9.624119	P22626	ROA2 HUMAN	544.2463465	2	0.78286016	2	Yes
NYYEQWGK	9.624119	P22626	ROA2 HUMAN	363.1668393	3	0.78286016	2	
TLETVPLER	27.87968	P22626	ROA2 HUMAN	529.298588	2	0.789944768	2	Yes
TLETVPLER	27.87968	P22626	ROA2 HUMAN	353.201667	3	0.789944768	2	
ACGNFGIPCELR	47.53613	P22234	PUR6 HUMAN	697.321746	2	0.658271372	2	Yes
ACGNFGIPCELR	47.53613	P22234	PUR6 HUMAN	465.2171057	3	0.658271372	2	
AEYEGDGIPTVFVAVAGR	88.17508	P22234	PUR6 HUMAN	925.968336	2	0.697487831	2	Yes
AEYEGDGIPTVFVAVAGR	88.17508	P22234	PUR6 HUMAN	617.6481657	3	0.697487831	2	
ASILNTWISLK	90.50209	P22234	PUR6 HUMAN	623.3642615	2	0.833675027	2	Yes
ASILNTWISLK	90.50209	P22234	PUR6 HUMAN	415.912116	3	0.833675027	2	
DDANNDPQWSEEQLIAAK	56.39324	P22234	PUR6 HUMAN	1022.466889	2	0.83707428	3	
DDANNDPQWSEEQLIAAK	56.39324	P22234	PUR6 HUMAN	681.9805343	3	0.83707428	3	Yes
EIVLADVIDNDSWR	86.74792	P22234	PUR6 HUMAN	822.915575	2	0.757594347	2	Yes
EIVLADVIDNDSWR	86.74792	P22234	PUR6 HUMAN	548.946325	3	0.757594347	2	
EVTPEGLQMVK	34.7445	P22234	PUR6 HUMAN	615.824113	2	0.850822031	2	Yes
EVTPEGLQMVK	34.7445	P22234	PUR6 HUMAN	410.8853503	3	0.850822031	2	
EVYELLDSPGK	47.9298	P22234	PUR6 HUMAN	625.3197125	2	0.848699868	2	Yes
EVYELLDSPGK	47.9298	P22234	PUR6 HUMAN	417.21575	3	0.848699868	2	
IEFGVDVTTK	40.80908	P22234	PUR6 HUMAN	554.798421	2	0.706267178	2	Yes
IEFGVDVTTK	40.80908	P22234	PUR6 HUMAN	370.2015557	3	0.706267178	2	
ITSCIFQLLQEAGIK	120.8567	P22234	PUR6 HUMAN	860.9692975	2	0.674688458	3	
ITSCIFQLLQEAGIK	120.8567	P22234	PUR6 HUMAN	574.3154733	3	0.674688458	3	Yes
NFEWVAER	34.03243	P22234	PUR6 HUMAN	525.754347	2	0.735507607	2	Yes
NFEWVAER	34.03243	P22234	PUR6 HUMAN	350.8388397	3	0.735507607	2	
SWLPQNCTLVDMK	66.75658	P22234	PUR6 HUMAN	796.384543	2	0.803363919		
SWLPQNCTLVDMK	66.75658	P22234	PUR6 HUMAN	531.2589703	3	0.803363919		
VVVLMGSTSDLGHCEK	28.37211	P22234	PUR6 HUMAN	866.4243965	2	0.791309118	3	
VVVLMGSTSDLGHCEK	28.37211	P22234	PUR6 HUMAN	577.952206	3	0.791309118	3	Yes
FSWFAGEK	42.73578	P21266	GSTM3 HUMAN	486.235259	2	0.665388584	2	Yes
FSWFAGEK	42.73578	P21266	GSTM3 HUMAN	324.492781	3	0.665388584	2	
IAAYLQSDQFCK	34.30801	P21266	GSTM3 HUMAN	722.351022	2	0.754775405	2	Yes
IAAYLQSDQFCK	34.30801	P21266	GSTM3 HUMAN	481.9032897	3	0.754775405	2	
LDLDFPNLPYLLDGK	141.0258	P21266	GSTM3 HUMAN	866.96199	2	0.796174705		
LDLDFPNLPYLLDGK	141.0258	P21266	GSTM3 HUMAN	578.3106017	3	0.796174705		
LKPQYLEELPGQLK	46.48866	P21266	GSTM3 HUMAN	828.4725255	2	0.881274223	3	
LKPQYLEELPGQLK	46.48866	P21266	GSTM3 HUMAN	552.6509587	3	0.881274223	3	Yes
LLLEFTDTSYEEK	68.35236	P21266	GSTM3 HUMAN	794.393606	2	0.890347838		
LLLEFTDTSYEEK	68.35236	P21266	GSTM3 HUMAN	529.931679	3	0.890347838		
LTFVDFLTYDILDQNR	152.5318	P21266	GSTM3 HUMAN	987.004918	2	0.695771158	3	
LTFVDFLTYDILDQNR	152.5318	P21266	GSTM3 HUMAN	658.3392203	3	0.695771158	3	Yes
SQWLDVK	25.23173	P21266	GSTM3 HUMAN	438.235259	2	0.670704186	2	Yes
SQWLDVK	25.23173	P21266	GSTM3 HUMAN	292.492781	3	0.670704186	2	
VDIIENQVMDFR	73.1796	P21266	GSTM3 HUMAN	739.8695825	2	0.8037678	2	Yes
VDIIENQVMDFR	73.1796	P21266	GSTM3 HUMAN	493.58233	3	0.8037678	2	
DENQSINHQMAQEDAQR	-15.79695	P20073	ANXA7 HUMAN	1007.438141	2	0.751388431	2	Yes
DENQSINHQMAQEDAQR	-15.79695	P20073	ANXA7 HUMAN	671.9613687	3	0.751388431	2	
EFSGYVESGLK	32.42965	P20073	ANXA7 HUMAN	608.29878	2	0.791213274	2	Yes
EFSGYVESGLK	32.42965	P20073	ANXA7 HUMAN	405.8684617	3	0.791213274	2	
GAGTDDSTLVR	-6.68961	P20073	ANXA7 HUMAN	546.270559	2	0.73921442	2	Yes
GAGTDDSTLVR	-6.68961	P20073	ANXA7 HUMAN	364.5163143	3	0.73921442	2	
GFGTDEQAIVDVVANR	68.62261	P20073	ANXA7 HUMAN	845.9239325	2	0.890075147	3	
GFGTDEQAIVDVVANR	68.62261	P20073	ANXA7 HUMAN	564.28523	3	0.890075147	3	Yes
QMFAQMYQK	21.37295	P20073	ANXA7 HUMAN	587.773365	2	0.620434165	2	Yes
QMFAQMYQK	21.37295	P20073	ANXA7 HUMAN	392.1848517	3	0.620434165	2	
SEIDLVQIK	45.49976	P20073	ANXA7 HUMAN	522.800963	2	0.697583139	2	Yes
SEIDLVQIK	45.49976	P20073	ANXA7 HUMAN	348.869917	3	0.697583139	2	
TILQCALNRPAFFAER	64.94884	P20073	ANXA7 HUMAN	954.0019945	2	0.632291973		
TILQCALNRPAFFAER	64.94884	P20073	ANXA7 HUMAN	636.3372713	3	0.632291973		
TLGTMIAGDTSGDYR	36.51997	P20073	ANXA7 HUMAN	779.364858	2	0.860461712	2	Yes
TLGTMIAGDTSGDYR	36.51997	P20073	ANXA7 HUMAN	519.9125137	3	0.860461712	2	
VLIEILCTR	66.04339	P20073	ANXA7 HUMAN	558.8264585	2	0.648626924		
VLIEILCTR	66.04339	P20073	ANXA7 HUMAN	372.886914	3	0.648626924		
ALPLALVLHELGAGR	98.40775	P19971	TYPH HUMAN	765.4622995	2	0.801802278	3	
ALPLALVLHELGAGR	98.40775	P19971	TYPH HUMAN	510.6441413	3	0.801802278	3	Yes
ALQEALVLSDR	42.39615	P19971	TYPH HUMAN	607.841151	2	0.841044188		
ALQEALVLSDR	42.39615	P19971	TYPH HUMAN	405.5633757	3	0.841044188		
APFAAPSPFAELVLPPQQ	141.618	P19971	TYPH HUMAN	940.501819	2	0.680216432		
APFAAPSPFAELVLPPQQ	141.618	P19971	TYPH HUMAN	627.3371543	3	0.680216432		
DGPALSGPQSR	-0.360767	P19971	TYPH HUMAN	542.7732685	2	0.777075648	2	Yes
DGPALSGPQSR	-0.360767	P19971	TYPH HUMAN	362.1847873	3	0.777075648	2	
DVTATVDSLPLITASILSK	149.9167	P19971	TYPH HUMAN	972.5491625	2	0.740548074		
DVTATVDSLPLITASILSK	149.9167	P19971	TYPH HUMAN	648.70205	3	0.740548074		
EQEELLAPADGTVELVR	67.43791	P19971	TYPH HUMAN	934.984187	2	0.651977599		
EQEELLAPADGTVELVR	67.43791	P19971	TYPH HUMAN	623.658733	3	0.651977599		
FGGAAVFPNQEQAR	31.11321	P19971	TYPH HUMAN	746.371139	2	0.878775954		
FGGAAVFPNQEQAR	31.11321	P19971	TYPH HUMAN	497.916701	3	0.878775954		
GMDLEETSVLTQALAQSGQQLEWPEAWR	145.439	P19971	TYPH HUMAN	1587.264591	2	0.784210443		
GMDLEETSVLTQALAQSGQQLEWPEAWR	145.439	P19971	TYPH HUMAN	1058.512336	3	0.784210443		
LGVGAELLVDVGQR	79.03934	P19971	TYPH HUMAN	713.40719	2	0.792134166		
LGVGAELLVDVGQR	79.03934	P19971	TYPH HUMAN	475.940735	3	0.792134166		
LVEGLSALVVDVK	84.84583	P19971	TYPH HUMAN	671.403584	2	0.785551727		
LVEGLSALVVDVK	84.84583	P19971	TYPH HUMAN	447.938331	3	0.785551727		
MLAAQGVDPGLAR	31.73443	P19971	TYPH HUMAN	649.848454	2	0.840020418	2	Yes
MLAAQGVDPGLAR	31.73443	P19971	TYPH HUMAN	433.5682443	3	0.840020418	2	
TLVGVGASLGLR	53.63078	P19971	TYPH HUMAN	571.8487795	2	0.798438311		
TLVGVGASLGLR	53.63078	P19971	TYPH HUMAN	381.5684613	3	0.798438311		
VAAALDDGSALGR	20.37743	P19971	TYPH HUMAN	608.320583	2	0.875054359	2	Yes
VAAALDDGSALGR	20.37743	P19971	TYPH HUMAN	405.882997	3	0.875054359	2	
VAAALTAMDKPLGR	26.17966	P19971	TYPH HUMAN	707.398311	2	0.835223973	3	
VAAALTAMDKPLGR	26.17966	P19971	TYPH HUMAN	471.9348157	3	0.835223973	3	Yes
ALELTGLK	31.26167	P19338	NUCL HUMAN	422.76111	2	0.805947185	2	Yes
ALELTGLK	31.26167	P19338	NUCL HUMAN	282.1766817	3	0.805947185	2	
EAMEDGEIDGNK	-8.64558	P19338	NUCL HUMAN	654.2751805	2	0.779928863	2	Yes
EAMEDGEIDGNK	-8.64558	P19338	NUCL HUMAN	436.5193953	3	0.779928863	2	
EVFEDAAEIR	32.50536	P19338	NUCL HUMAN	589.788584	2	0.776084423	2	Yes

EVFEDAAEIR	32.50536	P19338	NUCL HUMAN	393.528331	3	0.776084423	2	
FGYVDFESAEDLEK	69.56702	P19338	NUCL HUMAN	824.8730375	2	0.821330905	2	Yes
FGYVDFESAEDLEK	69.56702	P19338	NUCL HUMAN	550.2513	3	0.821330905	2	
GFGFVDFNSEEDAK	64.78033	P19338	NUCL HUMAN	781.3444515	2	0.86156261	2	Yes
GFGFVDFNSEEDAK	64.78033	P19338	NUCL HUMAN	521.2322427	3	0.86156261	2	
GIAYIEFK	44.2711	P19338	NUCL HUMAN	470.7611055	2	0.71561271	2	Yes
GIAYIEFK	44.2711	P19338	NUCL HUMAN	314.1766787	3	0.71561271	2	
GLSEDTTEETLK	6.477409	P19338	NUCL HUMAN	661.8202785	2	0.818032146	2	Yes
GLSEDTTEETLK	6.477409	P19338	NUCL HUMAN	441.5494607	3	0.818032146	2	
GYAFIEFASFEDAK	91.28304	P19338	NUCL HUMAN	797.8753835	2	0.793235064	2	Yes
GYAFIEFASFEDAK	91.28304	P19338	NUCL HUMAN	532.252864	3	0.793235064	2	
NDLAVVDVR	28.08115	P19338	NUCL HUMAN	500.7752795	2	0.764240503	2	Yes
NDLAVVDVR	28.08115	P19338	NUCL HUMAN	334.186128	3	0.764240503	2	
SISLYYTGEK	24.56956	P19338	NUCL HUMAN	580.795869	2	0.748617828	2	Yes
SISLYYTGEK	24.56956	P19338	NUCL HUMAN	387.5331877	3	0.748617828	2	
TLVLSNLSYSATEETLQEVFEK	123.5815	P19338	NUCL HUMAN	1251.137054	2	0.689516425	3	
TLVLSNLSYSATEETLQEVFEK	123.5815	P19338	NUCL HUMAN	834.427311	3	0.689516425	3	Yes
VEGTEPTTAFNLFVGNLNFNK	113.6765	P19338	NUCL HUMAN	1156.582057	2	0.712517858	3	
VEGTEPTTAFNLFVGNLNFNK	113.6765	P19338	NUCL HUMAN	771.390646	3	0.712517858	3	Yes
VTLDWAKPK	14.72201	P19338	NUCL HUMAN	529.306216	2	0.691475451	3	
VTLDWAKPK	14.72201	P19338	NUCL HUMAN	353.2067523	3	0.691475451	3	Yes
GLIMYSWDCPMEQCK	71.90249	P19256	LFA3 HUMAN	959.4042885	2	0.82665652		
GLIMYSWDCPMEQCK	71.90249	P19256	LFA3 HUMAN	639.9388007	3	0.82665652		
MENDLPQK	-15.96227	P19256	LFA3 HUMAN	487.734766	2	0.656680882		
MENDLPQK	-15.96227	P19256	LFA3 HUMAN	325.4924523	3	0.656680882		
VAELENSEFR	15.81056	P19256	LFA3 HUMAN	597.294033	2	0.762902737	2	Yes
VAELENSEFR	15.81056	P19256	LFA3 HUMAN	398.5319637	3	0.762902737	2	
APQPGSAVQGAHISHIAQQVSSLSESEESQDSSDS	50.78422	P18846	ATF1 HUMAN	2379.098809	2	0.6365906		
APQPGSAVQGAHISHIAQQVSSLSESEESQDSSDS	50.78422	P18846	ATF1 HUMAN	1586.401814	3	0.6365906		
TTPSATSLPQTVVMTSPVTLTSQTTK	76.5965	P18846	ATF1 HUMAN	1339.202409	2	0.692923129		
TTPSATSLPQTVVMTSPVTLTSQTTK	76.5965	P18846	ATF1 HUMAN	893.1375477	3	0.692923129		
DNNGVIGLLEPMK	86.55853	P18754	RCC1 HUMAN	700.3666755	2	0.659727693	2	Yes
DNNGVIGLLEPMK	86.55853	P18754	RCC1 HUMAN	467.2470587	3	0.659727693	2	
DTSVEGSEMVPGK	15.37804	P18754	RCC1 HUMAN	668.3090235	2	0.764224827	2	Yes
DTSVEGSEMVPGK	15.37804	P18754	RCC1 HUMAN	445.8752907	3	0.764224827	2	
LGLGEGAEEK	-1.778301	P18754	RCC1 HUMAN	501.7592955	2	0.829652786	2	Yes
LGLGEGAEEK	-1.778301	P18754	RCC1 HUMAN	334.8421387	3	0.829652786	2	
LPAVSSVACGASVGYAVTK	51.69016	P18754	RCC1 HUMAN	918.980389	2	0.700702965	3	
LPAVSSVACGASVGYAVTK	51.69016	P18754	RCC1 HUMAN	612.9895343	3	0.700702965	3	Yes
SGQVYSFGCNDEGALGR	34.88512	P18754	RCC1 HUMAN	908.900127	2	0.782445073		
SGQVYSFGCNDEGALGR	34.88512	P18754	RCC1 HUMAN	606.2693597	3	0.782445073		
SHSTEPGLVLTLGQGDVGQLGLGENVMER	93.76416	P18754	RCC1 HUMAN	1497.254026	2	0.705553949	3	
SHSTEPGLVLTLGQGDVGQLGLGENVMER	93.76416	P18754	RCC1 HUMAN	998.5052923	3	0.705553949	3	Yes
SMVPVQVQLDVPVVK	84.15662	P18754	RCC1 HUMAN	819.468933	2	0.705603182		
SMVPVQVQLDVPVVK	84.15662	P18754	RCC1 HUMAN	546.6485637	3	0.705603182		
VFAWGMGTNYQLGTGQDEDAWSPVEMMGK	122.8392	P18754	RCC1 HUMAN	1603.207369	2	0.752895474		
VFAWGMGTNYQLGTGQDEDAWSPVEMMGK	122.8392	P18754	RCC1 HUMAN	1069.140854	3	0.752895474		
VPELFANR	25.14066	P18754	RCC1 HUMAN	473.261808	2	0.767097116	2	Yes
VPELFANR	25.14066	P18754	RCC1 HUMAN	315.8438137	3	0.767097116	2	
VVLSVSSGGQHTVLLVK	39.63258	P18754	RCC1 HUMAN	862.0098105	2	0.857920051	3	
VVLSVSSGGQHTVLLVK	39.63258	P18754	RCC1 HUMAN	575.0091487	3	0.857920051	3	Yes
VVQVSAGDSHTAALTDDGR	7.188278	P18754	RCC1 HUMAN	949.9643175	2	0.814511538	3	
VVQVSAGDSHTAALTDDGR	7.188278	P18754	RCC1 HUMAN	633.6454867	3	0.814511538	3	Yes
DQPQVPCVFR	44.37226	P18440	ARY1 HUMAN	623.306422	2	0.698055208		
DQPQVPCVFR	44.37226	P18440	ARY1 HUMAN	415.8735563	3	0.698055208		
EQYIPNEEFLHSDLLEDSK	71.85862	P18440	ARY1 HUMAN	1153.545332	2	0.711001396		
EQYIPNEEFLHSDLLEDSK	71.85862	P18440	ARY1 HUMAN	769.3661627	3	0.711001396		
IYSFTLKPR	22.89812	P18440	ARY1 HUMAN	562.827311	2	0.757023335		
IYSFTLKPR	22.89812	P18440	ARY1 HUMAN	375.554149	3	0.757023335		
LDLETLTDILQHQIR	114.63	P18440	ARY1 HUMAN	904.4998075	2	0.878362775		
LDLETLTDILQHQIR	114.63	P18440	ARY1 HUMAN	603.3358133	3	0.878362775		
LTEENGFWYLDQIR	86.55634	P18440	ARY1 HUMAN	892.436671	2	0.688270807		
LTEENGFWYLDQIR	86.55634	P18440	ARY1 HUMAN	595.2937223	3	0.688270807		
NIFNISLQR	57.35007	P18440	ARY1 HUMAN	552.812196	2	0.772917807		
NIFNISLQR	57.35007	P18440	ARY1 HUMAN	368.8774057	3	0.772917807		
SYQMWQPLELISGK	100.7696	P18440	ARY1 HUMAN	840.4272605	2	0.753280818		
SYQMWQPLELISGK	100.7696	P18440	ARY1 HUMAN	560.620782	3	0.753280818		
TIEDFESMNTYLQTSPSSVFTSK	100.3952	P18440	ARY1 HUMAN	1306.607804	2	0.728990078		
TIEDFESMNTYLQTSPSSVFTSK	100.3952	P18440	ARY1 HUMAN	871.4078107	3	0.728990078		
YSTGMIHLLLQVTIDGR	102.3185	P18440	ARY1 HUMAN	959.017306	2	0.673759878		
YSTGMIHLLLQVTIDGR	102.3185	P18440	ARY1 HUMAN	639.6808123	3	0.673759878		
DETEFYLGK	39.46938	P18077	RL35A HUMAN	551.259124	2	0.702650487	2	Yes
DETEFYLGK	39.46938	P18077	RL35A HUMAN	367.8420243	3	0.702650487	2	
DVSPYDHSR	-25.16103	P17706	PTN2 HUMAN	538.244339	2	0.666037917		
DVSPYDHSR	-25.16103	P17706	PTN2 HUMAN	359.165501	3	0.666037917		
EDLSPAFDHSPNK	8.876812	P17706	PTN2 HUMAN	728.839336	2	0.639913797		
EDLSPAFDHSPNK	8.876812	P17706	PTN2 HUMAN	486.2288323	3	0.639913797		
EFEELDTQR	12.90471	P17706	PTN2 HUMAN	583.7703915	2	0.670644462		
EFEELDTQR	12.90471	P17706	PTN2 HUMAN	389.5162027	3	0.670644462		
FSYMAIIEGAK	70.54729	P17706	PTN2 HUMAN	615.315919	2	0.697313428		
FSYMAIIEGAK	70.54729	P17706	PTN2 HUMAN	410.5465543	3	0.697313428		
IGLEEEK	-9.948261	P17706	PTN2 HUMAN	409.2192745	2	0.753419757		
IGLEEEK	-9.948261	P17706	PTN2 HUMAN	273.1487913	3	0.753419757		
LQNAENDYINASLVDIEEAQR	74.00713	P17706	PTN2 HUMAN	1203.083144	2	0.717634439	3	
LQNAENDYINASLVDIEEAQR	74.00713	P17706	PTN2 HUMAN	802.391371	3	0.717634439	3	Yes
MGLIQTPDQLR	46.59396	P17706	PTN2 HUMAN	636.3430045	2	0.826760769		
MGLIQTPDQLR	46.59396	P17706	PTN2 HUMAN	424.5646113	3	0.826760769		
MQDTMEENSESALR	8.954697	P17706	PTN2 HUMAN	820.848718	2	0.739592791	2	Yes
MQDTMEENSESALR	8.954697	P17706	PTN2 HUMAN	547.5684203	3	0.739592791	2	
SYYTVHLLQLENINSGETR	63.78174	P17706	PTN2 HUMAN	1119.064022	2	0.645799696		
SYYTVHLLQLENINSGETR	63.78174	P17706	PTN2 HUMAN	746.378623	3	0.645799696		
AAAGPLDMSLPSTPDIK	73.88452	P17544	ATF7 HUMAN	842.435287	2	0.866816103		
AAAGPLDMSLPSTPDIK	73.88452	P17544	ATF7 HUMAN	561.9594663	3	0.866816103		
AEELTSQNIQLSNEVTLLR	76.09818	P17544	ATF7 HUMAN	1079.571689	2	0.756393373		
AEELTSQNIQLSNEVTLLR	76.09818	P17544	ATF7 HUMAN	720.0504007	3	0.756393373		
NCEEVGLFNELASSFEHEFK	110.3105	P17544	ATF7 HUMAN	1193.536988	2	0.607483089		
NCEEVGLFNELASSFEHEFK	110.3105	P17544	ATF7 HUMAN	796.027267	3	0.607483089		
SAAEAVATSVLTQMASQR	78.53059	P17544	ATF7 HUMAN	910.962732	2	0.778152466		
SAAEAVATSVLTQMASQR	78.53059	P17544	ATF7 HUMAN	607.6444297	3	0.778152466		
TELSMPIQSHVIMTPQSQSAGR	49.49847	P17544	ATF7 HUMAN	1199.596859	2	0.666028559	3	
TELSMPIQSHVIMTPQSQSAGR	49.49847	P17544	ATF7 HUMAN	800.0671807	3	0.666028559	3	Yes

EPESILQVLSQMEK	116.4649	P17174	AATC HUMAN	815.922012	2	0.872131705	2	Yes
EPESILQVLSQMEK	116.4649	P17174	AATC HUMAN	544.2839497	3	0.872131705	2	
HIYLLPSGR	17.98296	P17174	AATC HUMAN	528.304003	2	0.605371952		
HIYLLPSGR	17.98296	P17174	AATC HUMAN	352.5386103	3	0.605371952		
IANDNSLNHEYLPILGLAEFR	101.4126	P17174	AATC HUMAN	1200.121875	2	0.728753984	3	
IANDNSLNHEYLPILGLAEFR	101.4126	P17174	AATC HUMAN	800.4171917	3	0.728753984	3	Yes
ITWSNPPAQGAR	17.90433	P17174	AATC HUMAN	649.3365675	2	0.826249361	2	Yes
ITWSNPPAQGAR	17.90433	P17174	AATC HUMAN	433.2269867	3	0.826249361	2	
IVASTLSNPELFEEWTGNVK	93.10056	P17174	AATC HUMAN	1117.571157	2	0.76831919	3	
IVASTLSNPELFEEWTGNVK	93.10056	P17174	AATC HUMAN	745.3833797	3	0.76831919	3	Yes
LALGDDSPALK	29.76248	P17174	AATC HUMAN	550.30387	2	0.853897929	2	Yes
LALGDDSPALK	29.76248	P17174	AATC HUMAN	367.2051883	3	0.853897929	2	
NFGLYNER	16.86757	P17174	AATC HUMAN	506.7465175	2	0.805410385	2	Yes
NFGLYNER	16.86757	P17174	AATC HUMAN	338.1669533	3	0.805410385	2	
NLDYVATSIHEAVTK	38.83546	P17174	AATC HUMAN	830.9312215	2	0.884241462	3	
NLDYVATSIHEAVTK	38.83546	P17174	AATC HUMAN	554.2900893	3	0.884241462	3	Yes
NTPVYVSSPTWENHNAVFSAAGFK	63.11164	P17174	AATC HUMAN	1312.132972	2	0.737238646	3	
NTPVYVSSPTWENHNAVFSAAGFK	63.11164	P17174	AATC HUMAN	875.091256	3	0.737238646	3	Yes
TDDCHPWVLPVVK	58.4735	P17174	AATC HUMAN	783.3932255	2	0.827618003	3	
TDDCHPWVLPVVK	58.4735	P17174	AATC HUMAN	522.598092	3	0.827618003	3	Yes
VGGVQSLGGTGALR	23.23134	P17174	AATC HUMAN	636.3575005	2	0.798852742	2	Yes
VGGVQSLGGTGALR	23.23134	P17174	AATC HUMAN	424.5742753	3	0.798852742	2	
VNLGVGAYR	15.49693	P17174	AATC HUMAN	474.7672535	2	0.606573105	2	Yes
VNLGVGAYR	15.49693	P17174	AATC HUMAN	316.847444	3	0.606573105	2	
AHEHIFGMVLMNDWSAR	66.18307	P16930	FAAA HUMAN	1007.475286	2	0.674912632		
AHEHIFGMVLMNDWSAR	66.18307	P16930	FAAA HUMAN	671.9861323	3	0.674912632		
ASSVVVSGTPIR	17.22488	P16930	FAAA HUMAN	586.8358685	2	0.764223278	2	Yes
ASSVVVSGTPIR	17.22488	P16930	FAAA HUMAN	391.559854	3	0.764223278	2	
FLLDGDEVIITGYCQGDGYR	95.79022	P16930	FAAA HUMAN	1146.036617	2	0.634819806		
FLLDGDEVIITGYCQGDGYR	95.79022	P16930	FAAA HUMAN	764.360353	3	0.634819806		
GEGMSQAATICK	-6.463402	P16930	FAAA HUMAN	626.787205	2	0.665717244	2	Yes
GEGMSQAATICK	-6.463402	P16930	FAAA HUMAN	418.1940783	3	0.665717244	2	
GTKPIDLGNGQTR	-17.13587	P16930	FAAA HUMAN	678.8656895	2	0.704990268		
GTKPIDLGNGQTR	-17.13587	P16930	FAAA HUMAN	452.913068	3	0.704990268		
HLFTGPVLSK	22.75699	P16930	FAAA HUMAN	549.8194905	2	0.781589687		
HLFTGPVLSK	22.75699	P16930	FAAA HUMAN	366.8822687	3	0.781589687		
HQDVFNQPTLNSFMGLGQAAWK	92.16687	P16930	FAAA HUMAN	1245.105708	2	0.823487639		
HQDVFNQPTLNSFMGLGQAAWK	92.16687	P16930	FAAA HUMAN	830.4064137	3	0.823487639		
IGFGQCAGK	-5.975769	P16930	FAAA HUMAN	469.2321945	2	0.70765847		
IGFGQCAGK	-5.975769	P16930	FAAA HUMAN	313.1574047	3	0.70765847		
LGEPIPISK	27.7478	P16930	FAAA HUMAN	477.2874915	2	0.708193481	2	Yes
LGEPIPISK	27.7478	P16930	FAAA HUMAN	318.5276027	3	0.708193481	2	
VFLQNLLSVSQAR	81.49113	P16930	FAAA HUMAN	737.9230065	2	0.868168354	3	
VFLQNLLSVSQAR	81.49113	P16930	FAAA HUMAN	492.2846127	3	0.868168354	3	Yes
VLPALLPS	87.96375	P16930	FAAA HUMAN	405.2607455	2	0.675948918		
VLPALLPS	87.96375	P16930	FAAA HUMAN	270.509772	3	0.675948918		
WEYVPLGPFLGK	104.6696	P16930	FAAA HUMAN	703.379908	2	0.784330249	2	Yes
WEYVPLGPFLGK	104.6696	P16930	FAAA HUMAN	469.2558803	3	0.784330249	2	
EWLLAHEGHR	-4.133846	P16455	MGMT HUMAN	624.3181775	2	0.697593808		
EWLLAHEGHR	-4.133846	P16455	MGMT HUMAN	416.54806	3	0.697593808		
FGEVISYQQLAALAGNPK	85.2312	P16455	MGMT HUMAN	953.507628	2	0.840139687		
FGEVISYQQLAALAGNPK	85.2312	P16455	MGMT HUMAN	636.0076937	3	0.840139687		
GNPVPILIPCHR	42.40385	P16455	MGMT HUMAN	686.880088	2	0.667658985		
GNPVPILIPCHR	42.40385	P16455	MGMT HUMAN	458.2560003	3	0.667658985		
LELSGCEQGLHEIK	23.82313	P16455	MGMT HUMAN	806.904154	2	0.776500881		
LELSGCEQGLHEIK	23.82313	P16455	MGMT HUMAN	538.2720443	3	0.776500881		
LGKPGLGGSSGLAGAWLK	46.60488	P16455	MGMT HUMAN	834.975771	2	0.705508947		
LGKPGLGGSSGLAGAWLK	46.60488	P16455	MGMT HUMAN	556.9864557	3	0.705508947		
VVCSSGAVGNYSGGLAVK	26.91995	P16455	MGMT HUMAN	862.935981	2	0.655271292		
VVCSSGAVGNYSGGLAVK	26.91995	P16455	MGMT HUMAN	575.6265957	3	0.655271292		
AKPEGALQNNDGLYDPDCDESGLFK	54.71185	P16422	EPCAM HUMAN	1377.122141	2	0.727037728		
AKPEGALQNNDGLYDPDCDESGLFK	54.71185	P16422	EPCAM HUMAN	918.417369	3	0.727037728		
APEFSMQGLK	35.77844	P16422	EPCAM HUMAN	554.279341	2	0.770282567		
APEFSMQGLK	35.77844	P16422	EPCAM HUMAN	369.8555023	3	0.770282567		
TDKDTEITCSER	-27.56634	P16422	EPCAM HUMAN	727.8255695	2	0.717084587		
TDKDTEITCSER	-27.56634	P16422	EPCAM HUMAN	485.552988	3	0.717084587		
TQNDVDIADVAYYFEK	91.6571	P16422	EPCAM HUMAN	945.941979	2	0.787173808		
TQNDVDIADVAYYFEK	91.6571	P16422	EPCAM HUMAN	630.9639277	3	0.787173808		
TYWIIIELK	93.75095	P16422	EPCAM HUMAN	589.8451695	2	0.601833344		
TYWIIIELK	93.75095	P16422	EPCAM HUMAN	393.5660547	3	0.601833344		
DDSSLPEYSAFNTSVHAAIR	58.02235	P15848	ARSB HUMAN	1090.516909	2	0.751826584		
DDSSLPEYSAFNTSVHAAIR	58.02235	P15848	ARSB HUMAN	727.347214	3	0.751826584		
ELIHISDWLPTLVK	100.4417	P15848	ARSB HUMAN	832.475072	2	0.810034096		
ELIHISDWLPTLVK	100.4417	P15848	ARSB HUMAN	555.319323	3	0.810034096		
GVGFVASPLLK	61.82175	P15848	ARSB HUMAN	544.329691	2	0.798642755		
GVGFVASPLLK	61.82175	P15848	ARSB HUMAN	363.2224023	3	0.798642755		
HHYAGMVSLMDEAVGNVTAALK	88.7856	P15848	ARSB HUMAN	1157.570108	2	0.631202519		
HHYAGMVSLMDEAVGNVTAALK	88.7856	P15848	ARSB HUMAN	772.049347	3	0.631202519		
HSVPVYFPAQDPR	30.07181	P15848	ARSB HUMAN	756.8838775	2	0.644092679		
HSVPVYFPAQDPR	30.07181	P15848	ARSB HUMAN	504.9251933	3	0.644092679		
LLPQLLK	46.64366	P15848	ARSB HUMAN	412.784388	2	0.762219191		
LLPQLLK	46.64366	P15848	ARSB HUMAN	275.5255337	3	0.762219191		
NMYSTNIFTK	32.86584	P15848	ARSB HUMAN	609.7953505	2	0.806087136		
NMYSTNIFTK	32.86584	P15848	ARSB HUMAN	406.8661753	3	0.806087136		
TGLQHQIIWPCQPSCVPLDEK	57.54801	P15848	ARSB HUMAN	1253.615052	2	0.6535061		
TGLQHQIIWPCQPSCVPLDEK	57.54801	P15848	ARSB HUMAN	836.0793097	3	0.6535061		
TLWLFDIDR	100.0171	P15848	ARSB HUMAN	589.814405	2	0.717123151		
TLWLFDIDR	100.0171	P15848	ARSB HUMAN	393.545545	3	0.717123151		
TPHLDALAAGGVLLDNYYTQPLCTPSR	92.76695	P15848	ARSB HUMAN	1472.23764	2	0.663535416		
TPHLDALAAGGVLLDNYYTQPLCTPSR	92.76695	P15848	ARSB HUMAN	981.8277013	3	0.663535416		
WSLWEGGVR	53.88775	P15848	ARSB HUMAN	545.2779895	2	0.728658438		
WSLWEGGVR	53.88775	P15848	ARSB HUMAN	363.8546013	3	0.728658438		
ALIVLAHSER	8.223129	P15559	NQO1 HUMAN	554.8278465	2	0.838588834	3	
ALIVLAHSER	8.223129	P15559	NQO1 HUMAN	370.2211727	3	0.838588834	3	Yes
DPANFQYPAESVLAYK	74.07718	P15559	NQO1 HUMAN	906.9443245	2	0.768872619		
DPANFQYPAESVLAYK	74.07718	P15559	NQO1 HUMAN	604.9654913	3	0.768872619		
EGHLSPDIVAEQK	6.99041	P15559	NQO1 HUMAN	711.8653545	2	0.661278129	3	
EGHLSPDIVAEQK	6.99041	P15559	NQO1 HUMAN	474.9128447	3	0.661278129	3	Yes
TSFNYAMK	8.511173	P15559	NQO1 HUMAN	481.2265725	2	0.665756345	2	Yes
TSFNYAMK	8.511173	P15559	NQO1 HUMAN	321.1536567	3	0.665756345	2	
VFIGEFAYTYAAMYDK	99.4621	P15559	NQO1 HUMAN	944.945475	2	0.73599261		

VFIGEFAYTYAAMYDK	99.4621	P15559	NQO1 HUMAN	630.2995917	3	0.73599261		
DRPFFAGLVK	46.05531	P15531	NDKA HUMAN	575.32494	2	0.756507158	3	
DRPFFAGLVK	46.05531	P15531	NDKA HUMAN	383.8859017	3	0.756507158	3	Yes
FMQASEDLLK	37.89802	P15531	NDKA HUMAN	591.2977305	2	0.808172405	2	Yes
FMQASEDLLK	37.89802	P15531	NDKA HUMAN	394.5344287	3	0.808172405	2	
YMHSGPVVAMVWEGLNVVK	94.6888	P15531	NDKA HUMAN	1058.540091	2	0.702095926	3	
YMHSGPVVAMVWEGLNVVK	94.6888	P15531	NDKA HUMAN	706.0293353	3	0.702095926	3	Yes
EDCEQWWEDCR	36.6468	P15328	FOLR1 HUMAN	806.7937465	2	0.631882548	2	Yes
EDCEQWWEDCR	36.6468	P15328	FOLR1 HUMAN	538.1984393	3	0.631882548	2	
GWNWTSGFNK	44.20181	P15328	FOLR1 HUMAN	598.7783535	2	0.639930665		
GWNWTSGFNK	44.20181	P15328	FOLR1 HUMAN	399.5215107	3	0.639930665		
VLNVPLCK	27.13322	P15328	FOLR1 HUMAN	471.776237	2	0.616034985	2	Yes
VLNVPLCK	27.13322	P15328	FOLR1 HUMAN	314.853433	3	0.616034985	2	
ALQLEEER	-0.651733	P15311	EZRI HUMAN	494.2594625	2	0.785384715		
ALQLEEER	-0.651733	P15311	EZRI HUMAN	329.84225	3	0.785384715		
DNAMLEYLK	56.32492	P15311	EZRI HUMAN	548.771344	2	0.804412544	2	Yes
DNAMLEYLK	56.32492	P15311	EZRI HUMAN	366.1835043	3	0.804412544	2	
EVWYFGLHYVDNK	65.06567	P15311	EZRI HUMAN	835.404638	2	0.769107223	3	
EVWYFGLHYVDNK	65.06567	P15311	EZRI HUMAN	557.272367	3	0.769107223	3	Yes
FYPEDVAEELIQDITQK	140.0631	P15311	EZRI HUMAN	1019.504948	2	0.812533319	3	
FYPEDVAEELIQDITQK	140.0631	P15311	EZRI HUMAN	680.005907	3	0.812533319	3	Yes
GFPTWLK	52.94671	P15311	EZRI HUMAN	424.737438	2	0.809835732	2	Yes
GFPTWLK	52.94671	P15311	EZRI HUMAN	283.4942337	3	0.809835732	2	
GTDLWLGVDALGLNIYEK	128.1177	P15311	EZRI HUMAN	989.020568	2	0.650487363		
GTDLWLGVDALGLNIYEK	128.1177	P15311	EZRI HUMAN	659.682987	3	0.650487363		
IALLEEAR	26.40166	P15311	EZRI HUMAN	457.769466	2	0.771959662		
IALLEEAR	26.40166	P15311	EZRI HUMAN	305.5155857	3	0.771959662		
IAQDLEMYGINYFEIK	104.0972	P15311	EZRI HUMAN	973.9825925	2	0.802850842	3	
IAQDLEMYGINYFEIK	104.0972	P15311	EZRI HUMAN	649.65767	3	0.802850842	3	Yes
ILQLCMGNHELYMR	47.74475	P15311	EZRI HUMAN	889.4314975	2	0.705573499	3	
ILQLCMGNHELYMR	47.74475	P15311	EZRI HUMAN	593.2902733	3	0.705573499	3	Yes
IQVWHAEHR	-25.54638	P15311	EZRI HUMAN	588.307613	2	0.664159834	3	
IQVWHAEHR	-25.54638	P15311	EZRI HUMAN	392.541017	3	0.664159834	3	Yes
LQDYEEK	-25.42891	P15311	EZRI HUMAN	462.7196345	2	0.765949667		
LQDYEEK	-25.42891	P15311	EZRI HUMAN	308.815698	3	0.765949667		
QLLTLSSELSQAR	54.58743	P15311	EZRI HUMAN	723.402104	2	0.749694109	3	
QLLTLSSELSQAR	54.58743	P15311	EZRI HUMAN	482.604011	3	0.749694109	3	Yes
SQEQLAAELAEYTAK	58.09705	P15311	EZRI HUMAN	826.412862	2	0.905525923	2	Yes
SQEQLAAELAEYTAK	58.09705	P15311	EZRI HUMAN	551.2778497	3	0.905525923	2	
AITHLNNNFMFGQK	31.35694	P14866	HNRPL HUMAN	817.9095735	2	0.858773172	2	Yes
AITHLNNNFMFGQK	31.35694	P14866	HNRPL HUMAN	545.6089907	3	0.858773172	2	
LCFSTAQHAS	3.405445	P14866	HNRPL HUMAN	561.2563975	2	0.609978437	2	Yes
LCFSTAQHAS	3.405445	P14866	HNRPL HUMAN	374.5068733	3	0.609978437	2	
NDQDTWDYTNPNLSGQGDPGSNPNK	42.28114	P14866	HNRPL HUMAN	1367.584768	2	0.705282688	3	
NDQDTWDYTNPNLSGQGDPGSNPNK	42.28114	P14866	HNRPL HUMAN	912.0591203	3	0.705282688	3	Yes
SDALETLGFLNHYQMK	71.52135	P14866	HNRPL HUMAN	933.9569135	2	0.662317634	3	
SDALETLGFLNHYQMK	71.52135	P14866	HNRPL HUMAN	622.973884	3	0.662317634	3	Yes
SKPGAAMVEMADGYAVDR	35.43111	P14866	HNRPL HUMAN	934.438031	2	0.765675306		
SKPGAAMVEMADGYAVDR	35.43111	P14866	HNRPL HUMAN	623.294629	3	0.765675306		
SSSGLLEWESK	39.59901	P14866	HNRPL HUMAN	611.8016905	2	0.748799562	2	Yes
SSSGLLEWESK	39.59901	P14866	HNRPL HUMAN	408.2037353	3	0.748799562	2	
TPASPVVHIR	-1.930237	P14866	HNRPL HUMAN	538.8147395	2	0.678518355	2	Yes
TPASPVVHIR	-1.930237	P14866	HNRPL HUMAN	359.545768	3	0.678518355	2	
GPQYGHPPPPPPPPEYGPHADSPVLMVYGLDQS	71.84584	P14866	HNRPL HUMAN	1892.909394	2	0.600872278	4	
GPQYGHPPPPPPPPEYGPHADSPVLMVYGLDQS	71.84584	P14866	HNRPL HUMAN	1262.275538	3	0.600872278	4	
AGKPVICATQMLESMIK	72.45424	P14618	KPYM HUMAN	938.98885	2	0.679130316	3	
AGKPVICATQMLESMIK	72.45424	P14618	KPYM HUMAN	626.3285083	3	0.679130316	3	Yes
EAEAAIYHLQLFEELR	80.64737	P14618	KPYM HUMAN	966.49726	2	0.734344006	3	
EAEAAIYHLQLFEELR	80.64737	P14618	KPYM HUMAN	644.6674483	3	0.734344006	3	Yes
FDEILEASDGIMVAR	82.19858	P14618	KPYM HUMAN	833.4118115	2	0.796442389	2	Yes
FDEILEASDGIMVAR	82.19858	P14618	KPYM HUMAN	555.943816	3	0.796442389	2	
FGVEQDVDMVFASFIR	143.5969	P14618	KPYM HUMAN	930.454011	2	0.68107909	3	
FGVEQDVDMVFASFIR	143.5969	P14618	KPYM HUMAN	620.6386157	3	0.68107909	3	Yes
GADFLVTEVENGGSLGSK	64.95232	P14618	KPYM HUMAN	890.4421545	2	0.641197681	3	
GADFLVTEVENGGSLGSK	64.95232	P14618	KPYM HUMAN	593.9640447	3	0.641197681	3	Yes
GDYPLEAVR	25.37395	P14618	KPYM HUMAN	510.262001	2	0.792404771	2	Yes
GDYPLEAVR	25.37395	P14618	KPYM HUMAN	340.510609	3	0.792404771	2	
GIFPVLCKDPVQEAWAEDVDLR	97.62167	P14618	KPYM HUMAN	1279.141956	2	0.734769464	3	
GIFPVLCKDPVQEAWAEDVDLR	97.62167	P14618	KPYM HUMAN	853.0972457	3	0.734769464	3	Yes
GSGTAEVELK	-6.693859	P14618	KPYM HUMAN	495.7592955	2	0.747893572	2	Yes
GSGTAEVELK	-6.693859	P14618	KPYM HUMAN	330.8421387	3	0.747893572	2	
GVNLPGAAVDLPAVSEK	70.81245	P14618	KPYM HUMAN	818.9494185	2	0.844434977	2	Yes
GVNLPGAAVDLPAVSEK	70.81245	P14618	KPYM HUMAN	546.3022207	3	0.844434977	2	
ITLDNAYMEK	24.46967	P14618	KPYM HUMAN	599.2951835	2	0.809906542	2	Yes
ITLDNAYMEK	24.46967	P14618	KPYM HUMAN	399.866064	3	0.809906542	2	
IYVDDGLISLQVK	76.85194	P14618	KPYM HUMAN	731.911769	2	0.85082227		
IYVDDGLISLQVK	76.85194	P14618	KPYM HUMAN	488.277121	3	0.85082227		
LAPITSDPTEATAVGAVEASFK	78.94711	P14618	KPYM HUMAN	1088.063166	2	0.744380474	3	
LAPITSDPTEATAVGAVEASFK	78.94711	P14618	KPYM HUMAN	725.7113857	3	0.744380474	3	Yes
LDIDSPPITAR	41.93307	P14618	KPYM HUMAN	599.3278765	2	0.795304775	2	Yes
LDIDSPPITAR	41.93307	P14618	KPYM HUMAN	399.8878593	3	0.795304775	2	
LNFSHGTHEYHAETIK	-18.5122	P14618	KPYM HUMAN	942.4559265	2	0.815846443	3	
LNFSHGTHEYHAETIK	-18.5122	P14618	KPYM HUMAN	628.6398927	3	0.815846443	3	Yes
MQHLIAR	-25.46981	P14618	KPYM HUMAN	434.7452715	2	0.642911315	2	Yes
MQHLIAR	-25.46981	P14618	KPYM HUMAN	290.1661227	3	0.642911315	2	
NTGIICTIGPASR	35.72297	P14618	KPYM HUMAN	680.356643	2	0.786904097	2	Yes
NTGIICTIGPASR	35.72297	P14618	KPYM HUMAN	453.907037	3	0.786904097	2	
TATESFASDPILYRPVAVALDTK	78.5748	P14618	KPYM HUMAN	1233.1503	2	0.623684466	3	
TATESFASDPILYRPVAVALDTK	78.5748	P14618	KPYM HUMAN	822.4361413	3	0.623684466	3	Yes
VNFAMNVGK	24.48361	P14618	KPYM HUMAN	490.2556685	2	0.776169419	2	Yes
VNFAMNVGK	24.48361	P14618	KPYM HUMAN	327.173054	3	0.776169419	2	
AELLSLTQMER	55.01675	P14373	TRI27 HUMAN	645.840294	2	0.865272701		
AELLSLTQMER	55.01675	P14373	TRI27 HUMAN	430.8961377	3	0.865272701		
ELLQDIGDTLSR	72.79515	P14373	TRI27 HUMAN	680.359905	2	0.820451856		
ELLQDIGDTLSR	72.79515	P14373	TRI27 HUMAN	453.9092117	3	0.820451856		
EQIQNQLDHLK	6.212734	P14373	TRI27 HUMAN	683.3602395	2	0.830156982		
EQIQNQLDHLK	6.212734	P14373	TRI27 HUMAN	455.9094347	3	0.830156982		
EYWALTSPMTALPLR	103.2245	P14373	TRI27 HUMAN	874.9561855	2	0.737643063		
EYWALTSPMTALPLR	103.2245	P14373	TRI27 HUMAN	583.6400653	3	0.737643063		
GHSVLPLEEAVEGFK	58.08804	P14373	TRI27 HUMAN	806.4230365	2	0.750486374	3	
GHSVLPLEEAVEGFK	58.08804	P14373	TRI27 HUMAN	537.9512993	3	0.750486374	3	Yes

HLANVTQLVK	10.91533	P14373	TRI27 HUMAN	561.835672	2	0.681625724	2	Yes
HLANVTQLVK	10.91533	P14373	TRI27 HUMAN	374.8930563	3	0.681625724	2	
IPEPWITPPDLQEK	79.79367	P14373	TRI27 HUMAN	831.9410625	2	0.893051326		
IPEPWITPPDLQEK	79.79367	P14373	TRI27 HUMAN	554.9633167	3	0.893051326		
TERPSGPGGEMGVCEK	-23.84919	P14373	TRI27 HUMAN	845.8803535	2	0.684094548		
TERPSGPGGEMGVCEK	-23.84919	P14373	TRI27 HUMAN	564.2561773	3	0.684094548		
YSYLQQDLPDNPER	40.00224	P14373	TRI27 HUMAN	869.408106	2	0.903418541		
YSYLQQDLPDNPER	40.00224	P14373	TRI27 HUMAN	579.9413457	3	0.903418541		
AQQEQELAADAFK	27.36054	P14314	GLU2B HUMAN	724.8549875	2	0.845526457	2	Yes
AQQEQELAADAFK	27.36054	P14314	GLU2B HUMAN	483.5726	3	0.845526457	2	
ESLQQMAEVTR	30.93304	P14314	GLU2B HUMAN	646.319726	2	0.848391533	2	Yes
ESLQQMAEVTR	30.93304	P14314	GLU2B HUMAN	431.215759	3	0.848391533	2	
ETMVTSTTEPSR	-7.809513	P14314	GLU2B HUMAN	669.8144735	2	0.65972966	2	Yes
ETMVTSTTEPSR	-7.809513	P14314	GLU2B HUMAN	446.878924	3	0.65972966	2	
LGGSPTSLGTWGSWIGPDHDK	68.15649	P14314	GLU2B HUMAN	1084.524542	2	0.735640407	3	
LGGSPTSLGTWGSWIGPDHDK	68.15649	P14314	GLU2B HUMAN	723.352303	3	0.735640407	3	Yes
LIELQAGK	13.07903	P14314	GLU2B HUMAN	436.2665595	2	0.734426737	2	Yes
LIELQAGK	13.07903	P14314	GLU2B HUMAN	291.1803147	3	0.734426737	2	
LWEEQLAAAK	30.85705	P14314	GLU2B HUMAN	579.8118625	2	0.817444384	2	Yes
LWEEQLAAAK	30.85705	P14314	GLU2B HUMAN	386.8771833	3	0.817444384	2	
MPPYDEQTQAFIDAAQEAR	63.00714	P14314	GLU2B HUMAN	1091.000039	2	0.717448473	3	
MPPYDEQTQAFIDAAQEAR	63.00714	P14314	GLU2B HUMAN	727.669301	3	0.717448473	3	Yes
SEALPTDLPAPSAPDLTEPK	75.91972	P14314	GLU2B HUMAN	1025.023509	2	0.818948388	2	Yes
SEALPTDLPAPSAPDLTEPK	75.91972	P14314	GLU2B HUMAN	683.6849477	3	0.818948388	2	
SLEDQVEMLR	45.5861	P14314	GLU2B HUMAN	610.303544	2	0.795887113	2	Yes
SLEDQVEMLR	45.5861	P14314	GLU2B HUMAN	407.204971	3	0.795887113	2	
YEQGTGCWQGPNR	1.582706	P14314	GLU2B HUMAN	776.8340595	2	0.746667266	2	Yes
YEQGTGCWQGPNR	1.582706	P14314	GLU2B HUMAN	518.2253147	3	0.746667266	2	
LLCGLLAER	48.04722	P14174	MIF HUMAN	522.797701	2	0.741070509	2	Yes
LLCGLLAER	48.04722	P14174	MIF HUMAN	348.8677423	3	0.741070509	2	
VYINYYDMNAANVGWNNSTFA	109.269	P14174	MIF HUMAN	1214.039685	2	0.604837477	3	
VYINYYDMNAANVGWNNSTFA	109.269	P14174	MIF HUMAN	809.6957317	3	0.604837477	3	Yes
AVENINNTLGPALLQK	56.17719	P13929	ENOB HUMAN	847.9759675	2	0.884412885	3	
AVENINNTLGPALLQK	56.17719	P13929	ENOB HUMAN	565.6532533	3	0.884412885	3	Yes
DATNVGDEGGFAPNILENNEALELLK	124.9303	P13929	ENOB HUMAN	1372.175235	2	0.834872723		
DATNVGDEGGFAPNILENNEALELLK	124.9303	P13929	ENOB HUMAN	915.1194313	3	0.834872723		
FMIELDGTENK	47.26331	P13929	ENOB HUMAN	648.811202	2	0.835074067	2	Yes
FMIELDGTENK	47.26331	P13929	ENOB HUMAN	432.876743	3	0.835074067	2	
GNPTVEVDLHTAK	10.78226	P13929	ENOB HUMAN	690.8600725	2	0.74815309	3	
GNPTVEVDLHTAK	10.78226	P13929	ENOB HUMAN	460.9093233	3	0.74815309	3	Yes
IGAEVYHHLK	-19.61947	P13929	ENOB HUMAN	583.8200175	2	0.732637107	2	Yes
IGAEVYHHLK	-19.61947	P13929	ENOB HUMAN	389.5492867	3	0.732637107	2	
LAMQEFMILPVGASSFK	117.0322	P13929	ENOB HUMAN	934.9866325	2	0.812426686		
LAMQEFMILPVGASSFK	117.0322	P13929	ENOB HUMAN	623.6603633	3	0.812426686		
TAIQAAGYPDK	-0.167496	P13929	ENOB HUMAN	567.793666	2	0.630621016	2	Yes
TAIQAAGYPDK	-0.167496	P13929	ENOB HUMAN	378.8650523	3	0.630621016	2	
VNQIGSVTESIQACK	28.89082	P13929	ENOB HUMAN	817.414886	2	0.735985041	2	Yes
VNQIGSVTESIQACK	28.89082	P13929	ENOB HUMAN	545.279199	3	0.735985041	2	
VVIGMDVAASEFYR	72.50993	P13929	ENOB HUMAN	778.8930535	2	0.779388666		
VVIGMDVAASEFYR	72.50993	P13929	ENOB HUMAN	519.5979773	3	0.779388666		
DSPCQLEALK	25.78413	P13489	RINI HUMAN	580.7849875	2	0.758947194		
DSPCQLEALK	25.78413	P13489	RINI HUMAN	387.5259333	3	0.758947194		
ELCQGLGQPGSVLR	41.01073	P13489	RINI HUMAN	757.393757	2	0.790543199	2	Yes
ELCQGLGQPGSVLR	41.01073	P13489	RINI HUMAN	505.265113	3	0.790543199	2	
ELSLAGNELGDEGAR	39.09532	P13489	RINI HUMAN	765.8739075	2	0.873939633	2	Yes
ELSLAGNELGDEGAR	39.09532	P13489	RINI HUMAN	510.9185467	3	0.873939633	2	
ELTVSNNDINEAGVR	24.41898	P13489	RINI HUMAN	815.9057385	2	0.785413265	2	Yes
ELTVSNNDINEAGVR	24.41898	P13489	RINI HUMAN	544.2731007	3	0.785413265	2	
FLLELQISNNR	73.55366	P13489	RINI HUMAN	673.8755245	2	0.714006662		
FLLELQISNNR	73.55366	P13489	RINI HUMAN	449.5862913	3	0.714006662		
LDDCGLTEAR	4.510498	P13489	RINI HUMAN	575.2644195	2	0.7682271	2	Yes
LDDCGLTEAR	4.510498	P13489	RINI HUMAN	383.8455547	3	0.7682271	2	
LGDVGMAELCPGLLHPSSR	63.29791	P13489	RINI HUMAN	1005.001334	2	0.775436401	3	
LGDVGMAELCPGLLHPSSR	63.29791	P13489	RINI HUMAN	670.336831	3	0.775436401	3	Yes
SNELGDVGVHCVLQGLQTPSCK	59.2088	P13489	RINI HUMAN	1199.578666	2	0.676991224	3	
SNELGDVGVHCVLQGLQTPSCK	59.2088	P13489	RINI HUMAN	800.0550523	3	0.676991224	3	Yes
VNPALAELNLR	49.93388	P13489	RINI HUMAN	605.3516855	2	0.873543918	2	Yes
VNPALAELNLR	49.93388	P13489	RINI HUMAN	403.903732	3	0.873543918	2	
ANPQVGVAFPHIK	34.03925	P13010	XRCC5 HUMAN	689.3860605	2	0.828294814	3	
ANPQVGVAFPHIK	34.03925	P13010	XRCC5 HUMAN	459.9266487	3	0.828294814	3	Yes
DQVTAQEIFQDNHEDGPTAK	34.77837	P13010	XRCC5 HUMAN	1122.014736	2	0.688171446	3	
DQVTAQEIFQDNHEDGPTAK	34.77837	P13010	XRCC5 HUMAN	748.3457657	3	0.688171446	3	Yes
EPLPPIQQHIWNMLNPPAEVTTK	94.36192	P13010	XRCC5 HUMAN	1327.194523	2	0.810167968	3	
EPLPPIQQHIWNMLNPPAEVTTK	94.36192	P13010	XRCC5 HUMAN	885.1322903	3	0.810167968	3	Yes
ETVYCLNDDDETEVLK	50.28268	P13010	XRCC5 HUMAN	971.933498	2	0.602074921		
ETVYCLNDDDETEVLK	50.28268	P13010	XRCC5 HUMAN	648.291607	3	0.602074921		
FFMGNQVLK	42.77238	P13010	XRCC5 HUMAN	542.286969	2	0.859838367	2	Yes
FFMGNQVLK	42.77238	P13010	XRCC5 HUMAN	361.8605877	3	0.859838367	2	
HIEIFTDLSSR	44.25285	P13010	XRCC5 HUMAN	659.3440575	2	0.756121755	2	Yes
HIEIFTDLSSR	44.25285	P13010	XRCC5 HUMAN	439.8986467	3	0.756121755	2	
HLMLPDFDLLEDIESK	114.5488	P13010	XRCC5 HUMAN	957.980058	2	0.677842677	3	
HLMLPDFDLLEDIESK	114.5488	P13010	XRCC5 HUMAN	638.9893137	3	0.677842677	3	Yes
LFQCLLHR	25.0053	P13010	XRCC5 HUMAN	543.7980355	2	0.677411556	3	
LFQCLLHR	25.0053	P13010	XRCC5 HUMAN	362.8679653	3	0.677411556	3	Yes
LGGHGPSFPLK	11.65707	P13010	XRCC5 HUMAN	555.30929	2	0.848479927	2	Yes
LGGHGPSFPLK	11.65707	P13010	XRCC5 HUMAN	370.542135	3	0.848479927	2	
LTIGSNLSIR	40.19536	P13010	XRCC5 HUMAN	537.319854	2	0.728479505	2	Yes
LTIGSNLSIR	40.19536	P13010	XRCC5 HUMAN	358.5491777	3	0.728479505	2	
SQLDIIIHSLK	58.89714	P13010	XRCC5 HUMAN	633.8749935	2	0.782736778	3	
SQLDIIIHSLK	58.89714	P13010	XRCC5 HUMAN	422.9192707	3	0.782736778	3	Yes
TDTLEDLFPTTK	75.58978	P13010	XRCC5 HUMAN	690.8488395	2	0.857096016	2	Yes
TDTLEDLFPTTK	75.58978	P13010	XRCC5 HUMAN	460.9018347	3	0.857096016	2	
TLFPLIEAK	72.7345	P13010	XRCC5 HUMAN	516.310967	2	0.68331337	2	Yes
TLFPLIEAK	72.7345	P13010	XRCC5 HUMAN	344.543253	3	0.68331337	2	
VITMFVQR	35.00637	P13010	XRCC5 HUMAN	497.281687	2	0.696104765	2	Yes
VITMFVQR	35.00637	P13010	XRCC5 HUMAN	331.8570663	3	0.696104765	2	
YAPTEAQLNAVDALIDSMSLAK	120.4442	P13010	XRCC5 HUMAN	1161.088853	2	0.751354456	3	
YAPTEAQLNAVDALIDSMSLAK	120.4442	P13010	XRCC5 HUMAN	774.395177	3	0.751354456	3	Yes
YGSDIVPFSK	39.93248	P13010	XRCC5 HUMAN	556.7853085	2	0.728216887	2	Yes
YGSDIVPFSK	39.93248	P13010	XRCC5 HUMAN	371.5261473	3	0.728216887	2	
ACLISLGYDIGNDPQGEAEFAR	87.96594	P12814	ACTN1 HUMAN	1198.563534	2	0.733830869	3	

ACLISLGYDIGNDPQGEAEFAR	87.96594	P12814	ACTN1 HUMAN	799.3782977	3	0.733830869	3	Yes
DDPLTNLNTAFDVAEK	87.70764	P12814	ACTN1 HUMAN	881.92888	2	0.83611083		
DDPLTNLNTAFDVAEK	87.70764	P12814	ACTN1 HUMAN	588.2885283	3	0.83611083		
DGLGFCALIHR	57.98376	P12814	ACTN1 HUMAN	629.822239	2	0.709484696	3	
DGLGFCALIHR	57.98376	P12814	ACTN1 HUMAN	420.2174343	3	0.709484696	3	Yes
DHSGTLGPEEFK	9.598667	P12814	ACTN1 HUMAN	658.810048	2	0.838975668	2	Yes
DHSGTLGPEEFK	9.598667	P12814	ACTN1 HUMAN	439.5426403	3	0.838975668	2	
DYETATLSEIK	39.24749	P12814	ACTN1 HUMAN	635.3146275	2	0.836488485	2	Yes
DYETATLSEIK	39.24749	P12814	ACTN1 HUMAN	423.8790267	3	0.836488485	2	
ETADTDTADQVMASFK	52.06093	P12814	ACTN1 HUMAN	865.383449	2	0.796266258	2	Yes
ETADTDTADQVMASFK	52.06093	P12814	ACTN1 HUMAN	577.258241	3	0.796266258	2	
GISQEQMNEFR	14.78426	P12814	ACTN1 HUMAN	669.809525	2	0.899764061	2	Yes
GISQEQMNEFR	14.78426	P12814	ACTN1 HUMAN	446.875625	3	0.899764061	2	
ICDQWDNLGALTQK	60.70512	P12814	ACTN1 HUMAN	831.401779	2	0.791930199		
ICDQWDNLGALTQK	60.70512	P12814	ACTN1 HUMAN	554.6037943	3	0.791930199		
IDQLEGDHQLIQEALIFDNK	87.36646	P12814	ACTN1 HUMAN	1170.098071	2	0.849175692	3	
IDQLEGDHQLIQEALIFDNK	87.36646	P12814	ACTN1 HUMAN	780.4013223	3	0.849175692	3	Yes
ISIEMHGTLEDQLSHLR	50.31978	P12814	ACTN1 HUMAN	990.0049305	2	0.811029077	3	
ISIEMHGTLEDQLSHLR	50.31978	P12814	ACTN1 HUMAN	660.3392287	3	0.811029077	3	Yes
LAILGIHNEVSK	29.53559	P12814	ACTN1 HUMAN	647.380443	2	0.868029237	3	
LAILGIHNEVSK	29.53559	P12814	ACTN1 HUMAN	431.9229037	3	0.868029237	3	Yes
LSNRPAFMPSEGR	2.65934	P12814	ACTN1 HUMAN	731.367541	2	0.725880384	2	Yes
LSNRPAFMPSEGR	2.65934	P12814	ACTN1 HUMAN	487.9143023	3	0.725880384	2	
MLDAEDIVGTARPDEK	41.20673	P12814	ACTN1 HUMAN	880.430733	2	0.863448918	3	
MLDAEDIVGTARPDEK	41.20673	P12814	ACTN1 HUMAN	587.2897637	3	0.863448918	3	Yes
MVSDINNAWGCLEQVEK	70.33443	P12814	ACTN1 HUMAN	996.961874	2	0.747350693	3	
MVSDINNAWGCLEQVEK	70.33443	P12814	ACTN1 HUMAN	664.977191	3	0.747350693	3	Yes
NVNIQNFHISWK	48.71704	P12814	ACTN1 HUMAN	750.391873	2	0.767817676	2	Yes
NVNIQNFHISWK	48.71704	P12814	ACTN1 HUMAN	500.5971903	3	0.767817676	2	
NYITMDELR	37.73477	P12814	ACTN1 HUMAN	577.7797005	2	0.793298423	2	Yes
NYITMDELR	37.73477	P12814	ACTN1 HUMAN	385.5224087	3	0.793298423	2	
QFGAQANVIGPWIQTK	71.37165	P12814	ACTN1 HUMAN	879.4708535	2	0.657063782		
QFGAQANVIGPWIQTK	71.37165	P12814	ACTN1 HUMAN	586.649844	3	0.657063782		
VEQIAAIAQELNELDYYDSPSVNAR	116.2002	P12814	ACTN1 HUMAN	1404.688501	2	0.800530851	4	
VEQIAAIAQELNELDYYDSPSVNAR	116.2002	P12814	ACTN1 HUMAN	936.794942	3	0.800530851	4	
VLAVNQENEQLMEDYEK	44.32195	P12814	ACTN1 HUMAN	1026.483689	2	0.853909731	3	
VLAVNQENEQLMEDYEK	44.32195	P12814	ACTN1 HUMAN	684.658401	3	0.853909731	3	Yes
DFLAGGIAAAISK	86.30862	P12236	ADT3 HUMAN	617.3460695	2	0.831903934	2	Yes
DFLAGGIAAAISK	86.30862	P12236	ADT3 HUMAN	411.899988	3	0.831903934	2	
AAYYPPSQIAQLRPSPR	36.56727	P11940	PABP1 HUMAN	958.0134075	2	0.68318063		
AAYYPPSQIAQLRPSPR	36.56727	P11940	PABP1 HUMAN	639.0115467	3	0.68318063		
AVPNPVINPYQPAPPSGYFMAAIPQTQNR	103.6463	P11940	PABP1 HUMAN	1569.795656	2	0.65054512		
AVPNPVINPYQPAPPSGYFMAAIPQTQNR	103.6463	P11940	PABP1 HUMAN	1046.866379	3	0.65054512		
EFSPFGTITSAK	56.25534	P11940	PABP1 HUMAN	642.8277095	2	0.788303077	2	Yes
EFSPFGTITSAK	56.25534	P11940	PABP1 HUMAN	428.887748	3	0.788303077	2	
GYGFVHFETQEAAER	32.48169	P11940	PABP1 HUMAN	870.902996	2	0.898307979	2	Yes
GYGFVHFETQEAAER	32.48169	P11940	PABP1 HUMAN	580.937939	3	0.898307979	2	
HLNAQPQVTMQQPAVHVQGQEPLTASMLASAPI	58.2511	P11940	PABP1 HUMAN	2229.108629	2	0.703772068	4	
HLNAQPQVTMQQPAVHVQGQEPLTASMLASAPI	58.2511	P11940	PABP1 HUMAN	1486.408361	3	0.703772068	4	
PASSQVPR	-27.90002	P11940	PABP1 HUMAN	421.230508	2	0.613185167		
PASSQVPR	-27.90002	P11940	PABP1 HUMAN	281.1562803	3	0.613185167		
AAQAPSSFQLLYDLK	81.62953	P11586	C1TC HUMAN	826.4386825	2	0.781799793	3	
AAQAPSSFQLLYDLK	81.62953	P11586	C1TC HUMAN	551.2950633	3	0.781799793	3	Yes
AYIQENLELVEK	47.77184	P11586	C1TC HUMAN	724.885751	2	0.835540175	2	Yes
AYIQENLELVEK	47.77184	P11586	C1TC HUMAN	483.593109	3	0.835540175	2	
DDSNLYINVK	36.24037	P11586	C1TC HUMAN	590.796404	2	0.793407738	2	Yes
DDSNLYINVK	36.24037	P11586	C1TC HUMAN	394.200211	3	0.793407738	2	
DVDGLTSINAGR	34.23125	P11586	C1TC HUMAN	609.310215	2	0.792501509	2	Yes
DVDGLTSINAGR	34.23125	P11586	C1TC HUMAN	406.5427517	3	0.792501509	2	
EIGLLSEEVELYGETK	86.08932	P11586	C1TC HUMAN	904.962384	2	0.780439079	2	Yes
EIGLLSEEVELYGETK	86.08932	P11586	C1TC HUMAN	603.6441977	3	0.780439079	2	
FSDIQIR	19.02338	P11586	C1TC HUMAN	439.7407085	2	0.711368084	2	Yes
FSDIQIR	19.02338	P11586	C1TC HUMAN	293.496414	3	0.711368084	2	
GALALAQAVQR	32.94287	P11586	C1TC HUMAN	549.325472	2	0.797535837	2	Yes
GALALAQAVQR	32.94287	P11586	C1TC HUMAN	366.552923	3	0.797535837	2	
GDILVVATGQPEMVK	59.94871	P11586	C1TC HUMAN	778.921816	2	0.841936827		
GDILVVATGQPEMVK	59.94871	P11586	C1TC HUMAN	519.6171523	3	0.841936827		
GDLNDCFIPCTPK	50.64142	P11586	C1TC HUMAN	768.845251	2	0.810899794		
GDLNDCFIPCTPK	50.64142	P11586	C1TC HUMAN	512.8994423	3	0.810899794		
GVPTGFILPIR	81.8394	P11586	C1TC HUMAN	585.3562405	2	0.733844399	2	Yes
GVPTGFILPIR	81.8394	P11586	C1TC HUMAN	390.5734353	3	0.733844399	2	
IFHELTQTDK	-7.839054	P11586	C1TC HUMAN	616.320052	2	0.779684901	2	Yes
IFHELTQTDK	-7.839054	P11586	C1TC HUMAN	411.2159763	3	0.779684901	2	
ITIGQAPTEK	2.32312	P11586	C1TC HUMAN	529.2985885	2	0.722681224	2	Yes
ITIGQAPTEK	2.32312	P11586	C1TC HUMAN	353.2016673	3	0.722681224	2	
IVGAPMHDLLLWNNATVTTCHSK	61.70516	P11586	C1TC HUMAN	1289.649426	2	0.764522135	3	
IVGAPMHDLLLWNNATVTTCHSK	61.70516	P11586	C1TC HUMAN	860.1022253	3	0.764522135	3	Yes
IYGADDIELLPEAQHK	53.7367	P11586	C1TC HUMAN	906.462886	2	0.858627796	3	
IYGADDIELLPEAQHK	53.7367	P11586	C1TC HUMAN	604.6445323	3	0.858627796	3	Yes
LAILQVGNR	33.79903	P11586	C1TC HUMAN	492.3040075	2	0.729706883	2	Yes
LAILQVGNR	33.79903	P11586	C1TC HUMAN	328.5386133	3	0.729706883	2	
LDIDPETITWQR	68.25211	P11586	C1TC HUMAN	743.881005	2	0.868245959	2	Yes
LDIDPETITWQR	68.25211	P11586	C1TC HUMAN	496.2566117	3	0.868245959	2	
LVGPEGFVVTEAGFGADIGMEK	103.9834	P11586	C1TC HUMAN	1112.054287	2	0.785152435	3	
LVGPEGFVVTEAGFGADIGMEK	103.9834	P11586	C1TC HUMAN	741.7054663	3	0.785152435	3	Yes
MFGIPVVVAVNAFK	124.7833	P11586	C1TC HUMAN	746.423797	2	0.697876692	2	Yes
MFGIPVVVAVNAFK	124.7833	P11586	C1TC HUMAN	497.9518063	3	0.697876692	2	
MHGGGPTVTAGLPLPK	35.70094	P11586	C1TC HUMAN	766.9168685	2	0.77126193	3	
MHGGGPTVTAGLPLPK	35.70094	P11586	C1TC HUMAN	511.613854	3	0.77126193	3	Yes
QGFGNLPICMAK	58.33933	P11586	C1TC HUMAN	668.3315825	2	0.62257117	2	Yes
QGFGNLPICMAK	58.33933	P11586	C1TC HUMAN	445.89033	3	0.62257117	2	
TDPTTLTDEEINR	23.93061	P11586	C1TC HUMAN	752.8604665	2	0.852999806	2	Yes
TDPTTLTDEEINR	23.93061	P11586	C1TC HUMAN	502.2429193	3	0.852999806	2	
TDTESELDLISR	48.43575	P11586	C1TC HUMAN	689.839002	2	0.869541407	2	Yes
TDTESELDLISR	48.43575	P11586	C1TC HUMAN	460.2286097	3	0.869541407	2	
TPVPSDIDISR	32.48268	P11586	C1TC HUMAN	600.3175085	2	0.680027544		
TPVPSDIDISR	32.48268	P11586	C1TC HUMAN	400.547614	3	0.680027544		
VLLSALER	34.30193	P11586	C1TC HUMAN	450.7798335	2	0.615884483	2	Yes
VLLSALER	34.30193	P11586	C1TC HUMAN	300.8558307	3	0.615884483	2	
VVGDVAYDEAK	2.697678	P11586	C1TC HUMAN	583.2909555	2	0.768449664	2	Yes
VVGDVAYDEAK	2.697678	P11586	C1TC HUMAN	389.1965787	3	0.768449664	2	

WMIQYNNLNLK	64.19611	P11586	C1TC HUMAN	718.8719235	2	0.655542731		
WMIQYNNLNLK	64.19611	P11586	C1TC HUMAN	479.5838907	3	0.655542731		
YYVVTGITPTPLGEGK	64.17415	P11586	C1TC HUMAN	815.956709	2	0.845344007	2	Yes
YYVVTGITPTPLGEGK	64.17415	P11586	C1TC HUMAN	544.307081	3	0.845344007	2	
AAIGCGIVESILNWVK	136.4085	P11388	TOP2A HUMAN	865.469464	2	0.747447729		
AAIGCGIVESILNWVK	136.4085	P11388	TOP2A HUMAN	577.3155843	3	0.747447729		
AYDIAGSTK	-12.24951	P11388	TOP2A HUMAN	463.235452	2	0.72989428	2	Yes
AYDIAGSTK	-12.24951	P11388	TOP2A HUMAN	309.1595763	3	0.72989428	2	
DPALNSGVSQKPDPAK	-4.656124	P11388	TOP2A HUMAN	812.421025	2	0.681212604	3	
DPALNSGVSQKPDPAK	-4.656124	P11388	TOP2A HUMAN	541.9499583	3	0.681212604	3	Yes
EDLATFIEELEAVEAK	158.5627	P11388	TOP2A HUMAN	903.9545635	2	0.724359334		
EDLATFIEELEAVEAK	158.5627	P11388	TOP2A HUMAN	602.9723173	3	0.724359334		
EWLLGMLGAESAK	94.3923	P11388	TOP2A HUMAN	702.863769	2	0.816938758	2	Yes
EWLLGMLGAESAK	94.3923	P11388	TOP2A HUMAN	468.9117877	3	0.816938758	2	
FLEEFITPIVK	97.97601	P11388	TOP2A HUMAN	668.3821205	2	0.830149293	2	Yes
FLEEFITPIVK	97.97601	P11388	TOP2A HUMAN	445.924022	3	0.830149293	2	
GESDDFHMDFDSAVAPR	50.52346	P11388	TOP2A HUMAN	948.397421	2	0.802415013	3	
GESDDFHMDFDSAVAPR	50.52346	P11388	TOP2A HUMAN	632.600889	3	0.802415013	3	Yes
GFQQISFVNSIATSK	66.0155	P11388	TOP2A HUMAN	813.928486	2	0.768842638	2	Yes
GFQQISFVNSIATSK	66.0155	P11388	TOP2A HUMAN	542.9549323	3	0.768842638	2	
GSVPLSSSPPATHFPDETEITNPVPK	60.19771	P11388	TOP2A HUMAN	1352.677414	2	0.781470656	3	
GSVPLSSSPPATHFPDETEITNPVPK	60.19771	P11388	TOP2A HUMAN	902.120884	3	0.781470656	3	Yes
HVDYVADQIVTK	20.65558	P11388	TOP2A HUMAN	694.3649865	2	0.806632757		
HVDYVADQIVTK	20.65558	P11388	TOP2A HUMAN	463.2459327	3	0.806632757		
LLGLPEDYLYGQTTTYLTYNDFINK	133.0922	P11388	TOP2A HUMAN	1478.237288	2	0.76059258		
LLGLPEDYLYGQTTTYLTYNDFINK	133.0922	P11388	TOP2A HUMAN	985.8274667	3	0.76059258		
LMDGEEPLPMLPSYK	81.30327	P11388	TOP2A HUMAN	860.4207865	2	0.863403797	2	Yes
LMDGEEPLPMLPSYK	81.30327	P11388	TOP2A HUMAN	573.9497993	3	0.863403797	2	
LQTSLTCNSMVLFDHVGCLK	72.91116	P11388	TOP2A HUMAN	1162.066349	2	0.708826005	3	
LQTSLTCNSMVLFDHVGCLK	72.91116	P11388	TOP2A HUMAN	775.0468407	3	0.708826005	3	Yes
MEVSPLQPVNENMQVNK	50.33366	P11388	TOP2A HUMAN	978.9800665	2	0.80466032	3	
MEVSPLQPVNENMQVNK	50.33366	P11388	TOP2A HUMAN	652.9893193	3	0.80466032	3	Yes
MQSLDKDIVALMVR	68.83292	P11388	TOP2A HUMAN	809.9369425	2	0.72420162	3	
MQSLDKDIVALMVR	68.83292	P11388	TOP2A HUMAN	540.2939033	3	0.72420162	3	Yes
NSTECTLILTEGDSAK	34.38005	P11388	TOP2A HUMAN	869.912373	2	0.6506055		
NSTECTLILTEGDSAK	34.38005	P11388	TOP2A HUMAN	580.2775237	3	0.6506055		
SDSVTDSGPTFNYLLDMPLWYLTK	159.9929	P11388	TOP2A HUMAN	1382.165285	2	0.679752767		
SDSVTDSGPTFNYLLDMPLWYLTK	159.9929	P11388	TOP2A HUMAN	921.7794647	3	0.679752767		
SFGSTCQLSEK	0.296814	P11388	TOP2A HUMAN	622.2853515	2	0.78475076		
SFGSTCQLSEK	0.296814	P11388	TOP2A HUMAN	415.1928427	3	0.78475076		
SIPSMVDGLKPGQR	31.69471	P11388	TOP2A HUMAN	742.8986745	2	0.781771839	3	
SIPSMVDGLKPGQR	31.69471	P11388	TOP2A HUMAN	495.6017247	3	0.781771839	3	Yes
SSDESNFDVPPR	18.89822	P11388	TOP2A HUMAN	675.302586	2	0.613032877		
SSDESNFDVPPR	18.89822	P11388	TOP2A HUMAN	450.5376657	3	0.613032877		
SVVSDLEADDVK	30.21338	P11388	TOP2A HUMAN	638.817538	2	0.787831962	2	Yes
SVVSDLEADDVK	30.21338	P11388	TOP2A HUMAN	426.2143003	3	0.787831962	2	
SYVDMYLK	40.42436	P11388	TOP2A HUMAN	509.749876	2	0.774448454	2	Yes
SYVDMYLK	40.42436	P11388	TOP2A HUMAN	340.1691923	3	0.774448454	2	
TLAVSGLGVVGR	47.43346	P11388	TOP2A HUMAN	564.8409545	2	0.793735325		
TLAVSGLGVVGR	47.43346	P11388	TOP2A HUMAN	376.896578	3	0.793735325		
TPPLITDYR	30.67571	P11388	TOP2A HUMAN	538.2933015	2	0.699394584	2	Yes
TPPLITDYR	30.67571	P11388	TOP2A HUMAN	359.1981427	3	0.699394584	2	
TQMAEVLPSPR	33.03406	P11388	TOP2A HUMAN	614.8219045	2	0.685180724		
TQMAEVLPSPR	33.03406	P11388	TOP2A HUMAN	410.2172113	3	0.685180724		
YDTVLDILR	78.74768	P11388	TOP2A HUMAN	554.3064085	2	0.761934996	2	Yes
YDTVLDILR	78.74768	P11388	TOP2A HUMAN	369.8735473	3	0.761934996	2	
YIFTMLSSLAR	96.38026	P11388	TOP2A HUMAN	651.350293	2	0.707479835	2	Yes
YIFTMLSSLAR	96.38026	P11388	TOP2A HUMAN	434.5694703	3	0.707479835	2	
YSGPEDDAAISLAFSK	65.30094	P11388	TOP2A HUMAN	835.8995865	2	0.79771769	2	Yes
YSGPEDDAAISLAFSK	65.30094	P11388	TOP2A HUMAN	557.6023327	3	0.79771769	2	
AEEVATFFAK	43.19925	P11387	TOP1 HUMAN	556.7853135	2	0.682252824	2	Yes
AEEVATFFAK	43.19925	P11387	TOP1 HUMAN	371.5261507	3	0.682252824	2	
AVAILCNHQR	-13.05191	P11387	TOP1 HUMAN	591.314581	2	0.7821666	3	
AVAILCNHQR	-13.05191	P11387	TOP1 HUMAN	394.5456623	3	0.7821666	3	Yes
ELTAPDENIPAK	19.81629	P11387	TOP1 HUMAN	649.3358985	2	0.853265166		
ELTAPDENIPAK	19.81629	P11387	TOP1 HUMAN	433.2265407	3	0.853265166		
EYGFCIMDNHK	28.09346	P11387	TOP1 HUMAN	707.3004745	2	0.698593497	2	Yes
EYGFCIMDNHK	28.09346	P11387	TOP1 HUMAN	471.8695913	3	0.698593497	2	
GPVFAPPYEPLPENVK	65.95399	P11387	TOP1 HUMAN	877.4621575	2	0.873073459		
GPVFAPPYEPLPENVK	65.95399	P11387	TOP1 HUMAN	585.3107133	3	0.873073459		
HLQDLMEGLTAK	42.18859	P11387	TOP1 HUMAN	678.353569	2	0.870405078	2	Yes
HLQDLMEGLTAK	42.18859	P11387	TOP1 HUMAN	452.5716543	3	0.870405078	2	
IMPEDIIINCSK	58.73589	P11387	TOP1 HUMAN	716.8629115	2	0.62427026	2	Yes
IMPEDIIINCSK	58.73589	P11387	TOP1 HUMAN	478.2445493	3	0.62427026	2	
LNTGILNK	5.233074	P11387	TOP1 HUMAN	436.7641835	2	0.714134872	2	Yes
LNTGILNK	5.233074	P11387	TOP1 HUMAN	291.512064	3	0.714134872	2	
SMMNLQTK	2.181602	P11387	TOP1 HUMAN	476.733712	2	0.782022119	2	Yes
SMMNLQTK	2.181602	P11387	TOP1 HUMAN	318.1584163	3	0.782022119	2	
TYNASITLQQQLK	36.56064	P11387	TOP1 HUMAN	754.4099255	2	0.725229144		
TYNASITLQQQLK	36.56064	P11387	TOP1 HUMAN	503.275892	3	0.725229144		
VEHINLHPELDGQEYVVEFDFLGK	91.78793	P11387	TOP1 HUMAN	1414.201052	2	0.682310879	3	
VEHINLHPELDGQEYVVEFDFLGK	91.78793	P11387	TOP1 HUMAN	943.1366427	3	0.682310879	3	Yes
VTWLVSWTENIQGSIK	103.4333	P11387	TOP1 HUMAN	930.9969	2	0.710918725		
VTWLVSWTENIQGSIK	103.4333	P11387	TOP1 HUMAN	621.0005417	3	0.710918725		
AAVEEGIVLGGGCALLR	72.39213	P10809	CH60 HUMAN	842.9567215	2	0.640659869	3	
AAVEEGIVLGGGCALLR	72.39213	P10809	CH60 HUMAN	562.3070893	3	0.640659869	3	Yes
APGFGDNR	-18.99878	P10809	CH60 HUMAN	417.199208	2	0.603517175	2	Yes
APGFGDNR	-18.99878	P10809	CH60 HUMAN	278.468747	3	0.603517175	2	
DMAIATGGAVFGEEGLTLNLEDVQPHDLGK	111.6969	P10809	CH60 HUMAN	1549.261517	2	0.697573543	4	
DMAIATGGAVFGEEGLTLNLEDVQPHDLGK	111.6969	P10809	CH60 HUMAN	1033.176953	3	0.697573543	4	
EIGNIISDAMK	51.95425	P10809	CH60 HUMAN	595.808462	2	0.779970765	2	Yes
EIGNIISDAMK	51.95425	P10809	CH60 HUMAN	397.541583	3	0.779970765	2	
GVMLAVDAVIAELK	112.2984	P10809	CH60 HUMAN	714.9107195	2	0.757245779	3	
GVMLAVDAVIAELK	112.2984	P10809	CH60 HUMAN	476.943088	3	0.757245779	3	Yes
GYISPYFINTSK	52.88911	P10809	CH60 HUMAN	695.356625	2	0.775307059	2	Yes
GYISPYFINTSK	52.88911	P10809	CH60 HUMAN	463.907025	3	0.775307059	2	
IMQSSSEVGYDAMAGDFVNMVEK	95.20261	P10809	CH60 HUMAN	1254.558745	2	0.660708904	3	
IMQSSSEVGYDAMAGDFVNMVEK	95.20261	P10809	CH60 HUMAN	836.7084383	3	0.660708904	3	Yes
IQEIIEQLDVTTSEYEK	75.65596	P10809	CH60 HUMAN	1019.515513	2	0.806986451	3	
IQEIIEQLDVTTSEYEK	75.65596	P10809	CH60 HUMAN	680.01295	3	0.806986451	3	Yes
ISSIQSIVPALEIANAHR	76.9584	P10809	CH60 HUMAN	960.03963	2	0.719776511	3	

ISSIQSIVPALEIANAHR	76.9584	P10809	CH60 HUMAN	640.3623617	3	0.719776511	3	Yes
LSDGVAVLK	20.3592	P10809	CH60 HUMAN	451.2718415	2	0.800335884	2	Yes
LSDGVAVLK	20.3592	P10809	CH60 HUMAN	301.183836	3	0.800335884	2	
LVQDVANNTNEEAGDGTTTATVLAR	40.51093	P10809	CH60 HUMAN	1280.628453	2	0.709515035	3	
LVQDVANNTNEEAGDGTTTATVLAR	40.51093	P10809	CH60 HUMAN	854.0882437	3	0.709515035	3	Yes
NAGVEGSLIVEK	25.81135	P10809	CH60 HUMAN	608.3331585	2	0.799240649	2	Yes
NAGVEGSLIVEK	25.81135	P10809	CH60 HUMAN	405.8913807	3	0.799240649	2	
TLNDELEIIEGMK	80.78877	P10809	CH60 HUMAN	752.8823555	2	0.85857594	2	Yes
TLNDELEIIEGMK	80.78877	P10809	CH60 HUMAN	502.257512	3	0.85857594	2	
TVIIEQSWGSPK	40.31388	P10809	CH60 HUMAN	672.8620835	2	0.789186001	2	Yes
TVIIEQSWGSPK	40.31388	P10809	CH60 HUMAN	448.910664	3	0.789186001	2	
VGGTSDVEVNEK	-19.86565	P10809	CH60 HUMAN	617.3020555	2	0.655398369	2	Yes
VGGTSDVEVNEK	-19.86565	P10809	CH60 HUMAN	411.8706453	3	0.655398369	2	
VTDALNATR	-11.48738	P10809	CH60 HUMAN	480.75963	2	0.740640998	2	Yes
VTDALNATR	-11.48738	P10809	CH60 HUMAN	320.8423617	3	0.740640998	2	
AFSGYLGTDQSK	18.87154	P10768	ESTD HUMAN	637.307137	2	0.822127342	2	Yes
AFSGYLGTDQSK	18.87154	P10768	ESTD HUMAN	425.2073663	3	0.822127342	2	
AYDATHLVK	-10.50758	P10768	ESTD HUMAN	509.272369	2	0.612420678	2	Yes
AYDATHLVK	-10.50758	P10768	ESTD HUMAN	339.8508543	3	0.612420678	2	
DDQFLLDGQLLPDNFIAACTEK	142.8979	P10768	ESTD HUMAN	1262.107779	2	0.66159302		
DDQFLLDGQLLPDNFIAACTEK	142.8979	P10768	ESTD HUMAN	841.7411277	3	0.66159302		
GEDESWDFGTGAGFYVDATEDPWK	114.0265	P10768	ESTD HUMAN	1340.062076	2	0.65340507		
GEDESWDFGTGAGFYVDATEDPWK	114.0265	P10768	ESTD HUMAN	893.7106587	3	0.65340507		
MYSYVTEELPQLINANFPVDPQR	120.1322	P10768	ESTD HUMAN	1362.671069	2	0.701609194		
MYSYVTEELPQLINANFPVDPQR	120.1322	P10768	ESTD HUMAN	908.7833207	3	0.701609194		
SGYHQSASEHGLVVIAPDTSPR	18.17898	P10768	ESTD HUMAN	1154.570008	2	0.680572808	4	
SGYHQSASEHGLVVIAPDTSPR	18.17898	P10768	ESTD HUMAN	770.04928	3	0.680572808	4	
SYPGSQLDILIDQGK	66.91618	P10768	ESTD HUMAN	817.425772	2	0.740175903		
SYPGSQLDILIDQGK	66.91618	P10768	ESTD HUMAN	545.2864563	3	0.740175903		
VFEHDSVELNCK	4.852676	P10768	ESTD HUMAN	738.8435645	2	0.730224133	3	
VFEHDSVELNCK	4.852676	P10768	ESTD HUMAN	492.898318	3	0.730224133	3	Yes
APWVEQEGPEYWDR	53.51176	P10321	HLAC HUMAN	881.397546	2	0.875633001		
APWVEQEGPEYWDR	53.51176	P10321	HLAC HUMAN	587.9343057	3	0.875633001		
AYLEGTCVEWLR	64.34608	P10321	HLAC HUMAN	748.8642965	2	0.787723958	2	Yes
AYLEGTCVEWLR	64.34608	P10321	HLAC HUMAN	499.578806	3	0.787723958	2	
MSGCDLGPDGR	0.185936	P10321	HLAC HUMAN	582.742797	2	0.717813969		
MSGCDLGPDGR	0.185936	P10321	HLAC HUMAN	388.8311397	3	0.717813969		
THVTHHPLSDHEATLR	-31.39191	P10321	HLAC HUMAN	925.9669975	2	0.614944816		
THVTHHPLSDHEATLR	-31.39191	P10321	HLAC HUMAN	617.6472733	3	0.614944816		
WAAVVVPSGQEQR	33.46513	P10321	HLAC HUMAN	713.876057	2	0.835873604		
WAAVVVPSGQEQR	33.46513	P10321	HLAC HUMAN	476.253313	3	0.835873604		
GYEFLGVAFR	77.52872	P0C2W1	FBSP1 HUMAN	579.8012935	2	0.749134302		
GYEFLGVAFR	77.52872	P0C2W1	FBSP1 HUMAN	386.8701373	3	0.749134302		
TDILCNLPSYK	52.83916	P0C2W1	FBSP1 HUMAN	662.33484	2	0.869445324		
TDILCNLPSYK	52.83916	P0C2W1	FBSP1 HUMAN	441.8925017	3	0.869445324		
AYADFYR	13.31638	P09669	COX6C HUMAN	453.2117755	2	0.652293146	2	Yes
AYADFYR	13.31638	P09669	COX6C HUMAN	302.4771253	3	0.652293146	2	
GGGFGGNDNFGR	4.597301	P09651	ROA1 HUMAN	577.7528675	2	0.76867795	2	Yes
GGGFGGNDNFGR	4.597301	P09651	ROA1 HUMAN	385.50452	3	0.76867795	2	
SSGPYGGGGQYFAKPR	1.867001	P09651	ROA1 HUMAN	814.8949695	2	0.719521701	3	
SSGPYGGGGQYFAKPR	1.867001	P09651	ROA1 HUMAN	543.5992547	3	0.719521701	3	Yes
ADHQPLTEASYVNLPTIALCNTDSPLR	89.38031	P08865	RSSA HUMAN	1498.74329	2	0.7935763	3	
ADHQPLTEASYVNLPTIALCNTDSPLR	89.38031	P08865	RSSA HUMAN	999.4981347	3	0.7935763	3	Yes
AIVAIENPADVSVISSR	69.92838	P08865	RSSA HUMAN	870.9787065	2	0.703807533	3	
AIVAIENPADVSVISSR	69.92838	P08865	RSSA HUMAN	580.9884127	3	0.703807533	3	Yes
EHPWEVMPDLYFYR	82.6673	P08865	RSSA HUMAN	941.4356125	2	0.664307058	3	
EHPWEVMPDLYFYR	82.6673	P08865	RSSA HUMAN	627.9596833	3	0.664307058	3	Yes
FAAATGATPIAGR	17.78372	P08865	RSSA HUMAN	602.328212	2	0.70069617	2	Yes
FAAATGATPIAGR	17.78372	P08865	RSSA HUMAN	401.888083	3	0.70069617	2	
FLAAGTHLGGTNLDFQMEQYIYK	80.72107	P08865	RSSA HUMAN	1309.141948	2	0.616930842	3	
FLAAGTHLGGTNLDFQMEQYIYK	80.72107	P08865	RSSA HUMAN	873.0972403	3	0.616930842	3	Yes
FTPGTFTNQIQAAFR	78.79257	P08865	RSSA HUMAN	849.934104	2	0.849448442	3	
FTPGTFTNQIQAAFR	78.79257	P08865	RSSA HUMAN	566.9586777	3	0.849448442	3	Yes
LLVVTDPR	24.60429	P08865	RSSA HUMAN	456.779834	2	0.618509769	2	Yes
LLVVTDPR	24.60429	P08865	RSSA HUMAN	304.855831	3	0.618509769	2	
SDGIYIINLK	60.99455	P08865	RSSA HUMAN	568.322058	2	0.733641684	2	Yes
SDGIYIINLK	60.99455	P08865	RSSA HUMAN	379.2173137	3	0.733641684	2	
YVDIAIPCNNK	37.60023	P08865	RSSA HUMAN	653.827182	2	0.755975664	2	Yes
YVDIAIPCNNK	37.60023	P08865	RSSA HUMAN	436.2207297	3	0.755975664	2	
EILAELTGR	42.80586	P08559	ODPA HUMAN	501.2854805	2	0.701944709	2	Yes
EILAELTGR	42.80586	P08559	ODPA HUMAN	334.526262	3	0.701944709	2	
LEEGPPVTTVLTR	44.41142	P08559	ODPA HUMAN	706.3937485	2	0.807588696	2	Yes
LEEGPPVTTVLTR	44.41142	P08559	ODPA HUMAN	471.2651073	3	0.807588696	2	
MVNSNLASVEELK	39.87165	P08559	ODPA HUMAN	717.3694145	2	0.777920485	2	Yes
MVNSNLASVEELK	39.87165	P08559	ODPA HUMAN	478.582218	3	0.777920485	2	
NFANDATFEIK	39.75746	P08559	ODPA HUMAN	635.3096835	2	0.809062779		
NFANDATFEIK	39.75746	P08559	ODPA HUMAN	423.8757307	3	0.809062779		
SDPIMLLK	52.48924	P08559	ODPA HUMAN	458.762795	2	0.645743251	2	Yes
SDPIMLLK	52.48924	P08559	ODPA HUMAN	306.177805	3	0.645743251	2	
VDGMDILCVR	57.87928	P08559	ODPA HUMAN	589.289383	2	0.757010579	2	Yes
VDGMDILCVR	57.87928	P08559	ODPA HUMAN	393.1955303	3	0.757010579	2	
DLPTVLPPGFTIGAICK	120.3459	P08397	HEM3 HUMAN	899.992773	2	0.746016622		
DLPTVLPPGFTIGAICK	120.3459	P08397	HEM3 HUMAN	600.3311237	3	0.746016622		
DQDILDLVGVLHDPETLLR	147.5871	P08397	HEM3 HUMAN	1081.07915	2	0.637309849		
DQDILDLVGVLHDPETLLR	147.5871	P08397	HEM3 HUMAN	721.055375	3	0.637309849		
GPQLAAQNLGISLANLLLSK	129.4235	P08397	HEM3 HUMAN	1011.091863	2	0.710958779	3	
GPQLAAQNLGISLANLLLSK	129.4235	P08397	HEM3 HUMAN	674.3971837	3	0.710958779	3	Yes
HLEGGCSVPVAVHTAMK	7.698429	P08397	HEM3 HUMAN	896.945831	2	0.808989525		
HLEGGCSVPVAVHTAMK	7.698429	P08397	HEM3 HUMAN	598.299829	3	0.808989525		
NEVDLVVHSLK	32.61372	P08397	HEM3 HUMAN	626.8489755	2	0.73859942		
NEVDLVVHSLK	32.61372	P08397	HEM3 HUMAN	418.2352587	3	0.73859942		
SVVGTSSLR	-8.072792	P08397	HEM3 HUMAN	453.2567225	2	0.627653599	2	Yes
SVVGTSSLR	-8.072792	P08397	HEM3 HUMAN	302.50709	3	0.627653599	2	
TLETLPEK	6.283443	P08397	HEM3 HUMAN	465.761307	2	0.662628889		
TLETLPEK	6.283443	P08397	HEM3 HUMAN	310.8434797	3	0.662628889		
VGQILHPEECMYAVGQGALGVEVR	68.35738	P08397	HEM3 HUMAN	1306.652162	2	0.823005319		
VGQILHPEECMYAVGQGALGVEVR	68.35738	P08397	HEM3 HUMAN	871.4373827	3	0.823005319		
ALLFIPR	59.27701	P08238	HS90B HUMAN	415.268905	2	0.729117751	2	Yes
ALLFIPR	59.27701	P08238	HS90B HUMAN	277.1818783	3	0.729117751	2	
HLEINPDHPIVETLR	35.14265	P08238	HS90B HUMAN	891.9790415	2	0.841995478	3	
HLEINPDHPIVETLR	35.14265	P08238	HS90B HUMAN	594.988636	3	0.841995478	3	Yes

LGLGIDEDEVAAEEPNAAVPDEIPPLEGDEDASR	102.0397	P08238	HS90B HUMAN	1766.826657	2	0.759397626	3	
LGLGIDEDEVAAEEPNAAVPDEIPPLEGDEDASR	102.0397	P08238	HS90B HUMAN	1178.220379	3	0.759397626	3	Yes
NPDDITQEEYGEFYK	49.63721	P08238	HS90B HUMAN	924.4026865	2	0.810590386	2	Yes
NPDDITQEEYGEFYK	49.63721	P08238	HS90B HUMAN	616.6043993	3	0.810590386	2	
YHTSQSGDEMTSLSEYVSR	32.99226	P08238	HS90B HUMAN	1088.976755	2	0.730414093	3	
YHTSQSGDEMTSLSEYVSR	32.99226	P08238	HS90B HUMAN	726.320445	3	0.730414093	3	Yes
ADLLLSTQPGR	33.2951	P08195	4F2 HUMAN	585.828044	2	0.814855516	2	Yes
ADLLLSTQPGR	33.2951	P08195	4F2 HUMAN	390.887971	3	0.814855516	2	
DASSFLAEWQNITK	108.3055	P08195	4F2 HUMAN	805.3970185	2	0.810197711		
DASSFLAEWQNITK	108.3055	P08195	4F2 HUMAN	537.2672873	3	0.810197711		
DDVAQTDLLQIDPNFGSK	87.73436	P08195	4F2 HUMAN	988.484551	2	0.755187094	3	
DDVAQTDLLQIDPNFGSK	87.73436	P08195	4F2 HUMAN	659.3256423	3	0.755187094	3	Yes
DLLLTSSYLSDSGSTGEHTK	55.96977	P08195	4F2 HUMAN	1056.011125	2	0.779433846	3	
DLLLTSSYLSDSGSTGEHTK	55.96977	P08195	4F2 HUMAN	704.3433583	3	0.779433846	3	Yes
EDFDSLLQSAK	61.70457	P08195	4F2 HUMAN	626.8069735	2	0.792586565	2	Yes
EDFDSLLQSAK	61.70457	P08195	4F2 HUMAN	418.2072573	3	0.792586565	2	
EEGSPLELER	24.98359	P08195	4F2 HUMAN	579.786041	2	0.659790337	2	Yes
EEGSPLELER	24.98359	P08195	4F2 HUMAN	386.859969	3	0.659790337	2	
EVELNELEPEK	32.71091	P08195	4F2 HUMAN	664.833188	2	0.838514268	2	Yes
EVELNELEPEK	32.71091	P08195	4F2 HUMAN	443.558067	3	0.838514268	2	
GENSWFSTQVDTVATK	50.95303	P08195	4F2 HUMAN	885.4212225	2	0.803876042	2	Yes
GENSWFSTQVDTVATK	50.95303	P08195	4F2 HUMAN	590.6167567	3	0.803876042	2	
GLVLGPIHK	11.14585	P08195	4F2 HUMAN	467.298194	2	0.804713488	2	Yes
GLVLGPIHK	11.14585	P08195	4F2 HUMAN	311.868071	3	0.804713488	2	
GQSEDPGSLLSLFR	102.5223	P08195	4F2 HUMAN	753.383911	2	0.777688086	3	
GQSEDPGSLLSLFR	102.5223	P08195	4F2 HUMAN	502.5918823	3	0.777688086	3	Yes
IGDLQAFQGHGAGNLAGLK	46.80568	P08195	4F2 HUMAN	933.995224	2	0.800316274		
IGDLQAFQGHGAGNLAGLK	46.80568	P08195	4F2 HUMAN	622.9994243	3	0.800316274		
LDYLSSLK	40.00719	P08195	4F2 HUMAN	469.7638445	2	0.828858852	2	Yes
LDYLSSLK	40.00719	P08195	4F2 HUMAN	313.511838	3	0.828858852	2	
LEPHEGLLLR	24.98106	P08195	4F2 HUMAN	588.840954	2	0.71411258		
LEPHEGLLLR	24.98106	P08195	4F2 HUMAN	392.8965777	3	0.71411258		
LLIAGTNSSDLQQILSLLESNK	153.3673	P08195	4F2 HUMAN	1179.150303	2	0.692844272	3	
LLIAGTNSSDLQQILSLLESNK	153.3673	P08195	4F2 HUMAN	786.4361433	3	0.692844272	3	Yes
MELQPPEASIAVVSIPR	87.35921	P08195	4F2 HUMAN	918.9985855	2	0.650808871	3	
MELQPPEASIAVVSIPR	87.35921	P08195	4F2 HUMAN	613.0016653	3	0.650808871	3	Yes
NAEVTGTMSQDTEVDMK	24.58983	P08195	4F2 HUMAN	928.406598	2	0.796847045	2	Yes
NAEVTGTMSQDTEVDMK	24.58983	P08195	4F2 HUMAN	619.2736737	3	0.796847045	2	
SLLHGDFHAFSAGPGLFSYIR	82.70102	P08195	4F2 HUMAN	1146.58219	2	0.693347037	3	
SLLHGDFHAFSAGPGLFSYIR	82.70102	P08195	4F2 HUMAN	764.724068	3	0.693347037	3	Yes
SLVTQYLNATGNR	48.03305	P08195	4F2 HUMAN	718.878792	2	0.810308874	2	Yes
SLVTQYLNATGNR	48.03305	P08195	4F2 HUMAN	479.5884697	3	0.810308874	2	
VILDLTPNYR	55.5181	P08195	4F2 HUMAN	602.3407825	2	0.775500953	2	Yes
VILDLTPNYR	55.5181	P08195	4F2 HUMAN	401.8964633	3	0.775500953	2	
AANVLVSESLMCK	46.26758	P07948	LYN HUMAN	711.360536	2	0.82848835		
AANVLVSESLMCK	46.26758	P07948	LYN HUMAN	474.576299	3	0.82848835		
DSLSDDGVDLK	29.98714	P07948	LYN HUMAN	582.275506	2	0.786273479		
DSLSDDGVDLK	29.98714	P07948	LYN HUMAN	388.5196123	3	0.786273479		
EEPIYIITEYMAK	93.27124	P07948	LYN HUMAN	800.4029155	2	0.762753606	2	Yes
EEPIYIITEYMAK	93.27124	P07948	LYN HUMAN	533.9378853	3	0.762753606	2	
EGFIPSNYVAK	39.53115	P07948	LYN HUMAN	612.81714	2	0.781748831	2	Yes
EGFIPSNYVAK	39.53115	P07948	LYN HUMAN	408.8807017	3	0.781748831	2	
ITFPCISDMIK	85.37791	P07948	LYN HUMAN	662.836166	2	0.70817554	2	Yes
ITFPCISDMIK	85.37791	P07948	LYN HUMAN	442.226719	3	0.70817554	2	
LGAGQFGEVWMGYYNNSTK	80.86899	P07948	LYN HUMAN	1061.489105	2	0.700361967		
LGAGQFGEVWMGYYNNSTK	80.86899	P07948	LYN HUMAN	707.995345	3	0.700361967		
LIDFSAQIAEGMAYIER	125.524	P07948	LYN HUMAN	963.985671	2	0.819149733		
LIDFSAQIAEGMAYIER	125.524	P07948	LYN HUMAN	642.9930557	3	0.819149733		
LNTLETEEWFFK	85.72681	P07948	LYN HUMAN	778.8857555	2	0.868990302		
LNTLETEEWFFK	85.72681	P07948	LYN HUMAN	519.593112	3	0.868990302		
QLLAPGNSAGAFLIR	69.92974	P07948	LYN HUMAN	764.4362815	2	0.610313654		
QLLAPGNSAGAFLIR	69.92974	P07948	LYN HUMAN	509.9601293	3	0.610313654		
TLKPGTMSVQAFLEEANLMK	91.25647	P07948	LYN HUMAN	1104.574332	2	0.687752903		
TLKPGTMSVQAFLEEANLMK	91.25647	P07948	LYN HUMAN	736.7188297	3	0.687752903		
TNADVMTALSQGYR	50.04201	P07948	LYN HUMAN	763.8675675	2	0.716337442		
TNADVMTALSQGYR	50.04201	P07948	LYN HUMAN	509.5809867	3	0.716337442		
VENCPDELYDIMK	59.1927	P07948	LYN HUMAN	813.3634685	2	0.891062737	2	Yes
VENCPDELYDIMK	59.1927	P07948	LYN HUMAN	542.578254	3	0.891062737	2	
VLEEHGEWWK	24.9774	P07948	LYN HUMAN	656.8202185	2	0.856220484	3	
VLEEHGEWWK	24.9774	P07948	LYN HUMAN	438.2160873	3	0.856220484	3	Yes
WTAPEAINFGCFTIK	94.93239	P07948	LYN HUMAN	877.932715	2	0.700303018		
WTAPEAINFGCFTIK	94.93239	P07948	LYN HUMAN	585.6244183	3	0.700303018		
GDDQLELIK	33.17355	P07910	HNRPC HUMAN	515.7749455	2	0.636094749		
GDDQLELIK	33.17355	P07910	HNRPC HUMAN	344.1859053	3	0.636094749		
GFAFVQYVNER	57.15112	P07910	HNRPC HUMAN	665.333489	2	0.797237992	2	Yes
GFAFVQYVNER	57.15112	P07910	HNRPC HUMAN	443.891601	3	0.797237992	2	
MIAGQVLDINLAAEPK	78.44815	P07910	HNRPC HUMAN	841.961472	2	0.822185636	2	Yes
MIAGQVLDINLAAEPK	78.44815	P07910	HNRPC HUMAN	561.6435897	3	0.822185636	2	
EQWPQCPTIK	25.09866	P07858	CATB HUMAN	643.81408	2	0.78482306	2	Yes
EQWPQCPTIK	25.09866	P07858	CATB HUMAN	429.5453283	3	0.78482306	2	
GQDHCGIESEVVAGIPR	40.38219	P07858	CATB HUMAN	912.439424	2	0.747249305	3	
GQDHCGIESEVVAGIPR	40.38219	P07858	CATB HUMAN	608.628891	3	0.747249305	3	Yes
ICEPGYSPTYK	9.187435	P07858	CATB HUMAN	657.8059115	2	0.713066995	2	Yes
ICEPGYSPTYK	9.187435	P07858	CATB HUMAN	438.873216	3	0.713066995	2	
LCGTFLGGPKPPQR	22.19392	P07858	CATB HUMAN	764.4092105	2	0.855617523		
LCGTFLGGPKPPQR	22.19392	P07858	CATB HUMAN	509.942082	3	0.855617523		
NTTWQAGHNFYNVDMSYLK	61.43451	P07858	CATB HUMAN	1145.023843	2	0.694408059		
NTTWQAGHNFYNVDMSYLK	61.43451	P07858	CATB HUMAN	763.6851703	3	0.694408059		
SGVYQHVTGEMMGGHAIR	6.642921	P07858	CATB HUMAN	965.45709	2	0.858323812	3	
SGVYQHVTGEMMGGHAIR	6.642921	P07858	CATB HUMAN	643.9740017	3	0.858323812	3	Yes
SRPSFHPLSDELVNYVNK	49.63807	P07858	CATB HUMAN	1051.537447	2	0.740328848		
SRPSFHPLSDELVNYVNK	49.63807	P07858	CATB HUMAN	701.360906	3	0.740328848		
VMFTEDLK	29.45106	P07858	CATB HUMAN	491.749885	2	0.775689125		
VMFTEDLK	29.45106	P07858	CATB HUMAN	328.1691983	3	0.775689125		
IDYIAGLDSR	40.06899	P07741	APT HUMAN	561.793665	2	0.796797395	2	Yes
IDYIAGLDSR	40.06899	P07741	APT HUMAN	374.8650517	3	0.796797395	2	
LPGPTLWASYSLEYGK	87.58591	P07741	APT HUMAN	891.4596105	2	0.837212086	2	Yes
LPGPTLWASYSLEYGK	87.58591	P07741	APT HUMAN	594.6423487	3	0.837212086	2	
SFPDFPTPGVVFR	91.4447	P07741	APT HUMAN	733.377901	2	0.74283731	2	Yes
SFPDFPTPGVVFR	91.4447	P07741	APT HUMAN	489.2545423	3	0.74283731	2	
DSLLQDGEFSMDLR	85.65889	P07737	PROF1 HUMAN	813.3779685	2	0.890587211	2	Yes

DSLLQDGEFSMDLR	85.65889	P07737	PROF1 HUMAN	542.5879207	3	0.890587211	2	
DSPSVWAAVPGK	48.83871	P07737	PROF1 HUMAN	607.314769	2	0.803867817	2	Yes
DSPSVWAAVPGK	48.83871	P07737	PROF1 HUMAN	405.2124543	3	0.803867817	2	
SSFYVNGLTLGGQK	54.78084	P07737	PROF1 HUMAN	735.883543	2	0.740254939	2	Yes
SSFYVNGLTLGGQK	54.78084	P07737	PROF1 HUMAN	490.9249703	3	0.740254939	2	
STGGAPTFNVTVTK	31.04638	P07737	PROF1 HUMAN	690.3624485	2	0.831812561	2	Yes
STGGAPTFNVTVTK	31.04638	P07737	PROF1 HUMAN	460.577574	3	0.831812561	2	
TFVNITPAEVGVLVGK	90.05203	P07737	PROF1 HUMAN	822.47253	2	0.772576034	2	Yes
TFVNITPAEVGVLVGK	90.05203	P07737	PROF1 HUMAN	548.6509617	3	0.772576034	2	
TLVLLMGK	52.08149	P07737	PROF1 HUMAN	437.775706	2	0.757099152	2	Yes
TLVLLMGK	52.08149	P07737	PROF1 HUMAN	292.1864123	3	0.757099152	2	
DGGFCEVCK	2.159054	P07602	SAP HUMAN	536.215884	2	0.741240084	2	Yes
DGGFCEVCK	2.159054	P07602	SAP HUMAN	357.8131977	3	0.741240084	2	
DNATEEEILVYLEK	96.05443	P07602	SAP HUMAN	833.4150695	2	0.777853251	2	Yes
DNATEEEILVYLEK	96.05443	P07602	SAP HUMAN	555.945988	3	0.777853251	2	
EIVDSYLPVILDIIK	156.035	P07602	SAP HUMAN	865.5034905	2	0.617390752		
EIVDSYLPVILDIIK	156.035	P07602	SAP HUMAN	577.3382687	3	0.617390752		
EMPMQTLVPAK	42.39752	P07602	SAP HUMAN	622.823059	2	0.771602213	2	Yes
EMPMQTLVPAK	42.39752	P07602	SAP HUMAN	415.5513143	3	0.771602213	2	
GCSFLPDPYQK	40.32401	P07602	SAP HUMAN	656.306083	2	0.693860888	2	Yes
GCSFLPDPYQK	40.32401	P07602	SAP HUMAN	437.8733303	3	0.693860888	2	
GSAVWCQNVK	4.838627	P07602	SAP HUMAN	574.7800395	2	0.72020185		
GSAVWCQNVK	4.838627	P07602	SAP HUMAN	383.5226347	3	0.72020185		
HCLQTVWNKPTVK	-1.832588	P07602	SAP HUMAN	805.927767	2	0.787445664	3	
HCLQTVWNKPTVK	-1.832588	P07602	SAP HUMAN	537.6211197	3	0.787445664	3	Yes
IGACPSAHKPLLGTEK	-14.15232	P07602	SAP HUMAN	839.951439	2	0.751660705		
IGACPSAHKPLLGTEK	-14.15232	P07602	SAP HUMAN	560.3035677	3	0.751660705		
LGPGMADICK	20.19521	P07602	SAP HUMAN	531.2600945	2	0.834996164	2	Yes
LGPGMADICK	20.19521	P07602	SAP HUMAN	354.509338	3	0.834996164	2	
LPALTVHVTQPK	24.86388	P07602	SAP HUMAN	652.390812	2	0.835241616		
LPALTVHVTQPK	24.86388	P07602	SAP HUMAN	435.2631497	3	0.835241616		
NVIPALELVEPIK	95.03503	P07602	SAP HUMAN	717.9325085	2	0.847024322	2	Yes
NVIPALELVEPIK	95.03503	P07602	SAP HUMAN	478.957614	3	0.847024322	2	
TNSTFVQALVEHVK	66.49789	P07602	SAP HUMAN	786.923204	2	0.823381245		
TNSTFVQALVEHVK	66.49789	P07602	SAP HUMAN	524.951411	3	0.823381245		
ADAPEEEDHVLVLR	28.05852	P07237	PDIA1 HUMAN	796.8999255	2	0.822888851		
ADAPEEEDHVLVLR	28.05852	P07237	PDIA1 HUMAN	531.6025587	3	0.822888851		
EADDIVNWLK	76.68092	P07237	PDIA1 HUMAN	601.8067765	2	0.814843833	2	Yes
EADDIVNWLK	76.68092	P07237	PDIA1 HUMAN	401.5404593	3	0.814843833	2	
ENLLDFIK	80.59482	P07237	PDIA1 HUMAN	496.2771235	2	0.661832988	2	Yes
ENLLDFIK	80.59482	P07237	PDIA1 HUMAN	331.1873573	3	0.661832988	2	
HNQLPLVIEFTEQTAPK	68.46318	P07237	PDIA1 HUMAN	983.0261895	2	0.680652797	3	
HNQLPLVIEFTEQTAPK	68.46318	P07237	PDIA1 HUMAN	655.6867347	3	0.680652797	3	Yes
IKPHLMSQELPEDWDK	35.53694	P07237	PDIA1 HUMAN	983.4911245	2	0.823733926	4	
IKPHLMSQELPEDWDK	35.53694	P07237	PDIA1 HUMAN	655.9966913	3	0.823733926	4	
ILEFFGLK	82.00824	P07237	PDIA1 HUMAN	483.7871275	2	0.731458843	2	Yes
ILEFFGLK	82.00824	P07237	PDIA1 HUMAN	322.8606933	3	0.731458843	2	
ILFIFIDSDHTDNQR	68.41287	P07237	PDIA1 HUMAN	917.4606815	2	0.739679158	3	
ILFIFIDSDHTDNQR	68.41287	P07237	PDIA1 HUMAN	611.976396	3	0.739679158	3	Yes
LITLEEEMTK	42.60542	P07237	PDIA1 HUMAN	603.818496	2	0.823664784	2	Yes
LITLEEEMTK	42.60542	P07237	PDIA1 HUMAN	402.8816057	3	0.823664784	2	
MDSTANEVEAVK	4.858093	P07237	PDIA1 HUMAN	647.303741	2	0.632305443	2	Yes
MDSTANEVEAVK	4.858093	P07237	PDIA1 HUMAN	431.871769	3	0.632305443	2	
NFEDVAFDEK	33.28993	P07237	PDIA1 HUMAN	607.2727665	2	0.849060416	2	Yes
NFEDVAFDEK	33.28993	P07237	PDIA1 HUMAN	405.1844527	3	0.849060416	2	
SNFAEALAAHK	9.791546	P07237	PDIA1 HUMAN	579.799286	2	0.795329273	3	
SNFAEALAAHK	9.791546	P07237	PDIA1 HUMAN	386.868799	3	0.795329273	3	Yes
TGPAATTLPDGAAAESLVESSEVAVIGFFK	151.0844	P07237	PDIA1 HUMAN	1468.250944	2	0.63915056		
TGPAATTLPDGAAAESLVESSEVAVIGFFK	151.0844	P07237	PDIA1 HUMAN	979.169904	3	0.63915056		
THILLFLPK	51.58119	P07237	PDIA1 HUMAN	541.3426015	2	0.760546029	2	Yes
THILLFLPK	51.58119	P07237	PDIA1 HUMAN	361.2310093	3	0.760546029	2	
VDATEESDLAQQYGVR	35.32011	P07237	PDIA1 HUMAN	890.9215825	2	0.746439338	2	Yes
VDATEESDLAQQYGVR	35.32011	P07237	PDIA1 HUMAN	594.2836633	3	0.746439338	2	
YKPESEELTAER	-13.11604	P07237	PDIA1 HUMAN	726.354815	2	0.799378157	2	Yes
YKPESEELTAER	-13.11604	P07237	PDIA1 HUMAN	484.572485	3	0.799378157	2	
YQLDKDGVVLFK	40.83326	P07237	PDIA1 HUMAN	712.8933795	2	0.825295806	2	Yes
YQLDKDGVVLFK	40.83326	P07237	PDIA1 HUMAN	475.5981947	3	0.825295806	2	
AADAEAEVASLNR	16.799	P06753	TPM3 HUMAN	658.826229	2	0.847021937	2	Yes
AADAEAEVASLNR	16.799	P06753	TPM3 HUMAN	439.5534277	3	0.847021937	2	
LVIIEGDLER	50.91866	P06753	TPM3 HUMAN	578.8327945	2	0.746821702	2	Yes
LVIIEGDLER	50.91866	P06753	TPM3 HUMAN	386.2244713	3	0.746821702	2	
MELQEIQLK	42.45444	P06753	TPM3 HUMAN	566.3080985	2	0.773135126	2	Yes
MELQEIQLK	42.45444	P06753	TPM3 HUMAN	377.874674	3	0.773135126	2	
TIDDLEDELYAQK	61.48567	P06753	TPM3 HUMAN	776.873038	2	0.848283887		
TIDDLEDELYAQK	61.48567	P06753	TPM3 HUMAN	518.2513003	3	0.848283887		
ADKDYHFK	-30.15972	P06748	NPM HUMAN	512.2488935	2	0.668327689	2	Yes
ADKDYHFK	-30.15972	P06748	NPM HUMAN	341.835204	3	0.668327689	2	
DELHIVEAEAMNYEGSPIK	69.22464	P06748	NPM HUMAN	1073.012614	2	0.805323839	3	
DELHIVEAEAMNYEGSPIK	69.22464	P06748	NPM HUMAN	715.677684	3	0.805323839	3	Yes
MTDQEAIQDLWQWR	95.57669	P06748	NPM HUMAN	910.4257855	2	0.853317261	2	Yes
MTDQEAIQDLWQWR	95.57669	P06748	NPM HUMAN	607.2864653	3	0.853317261	2	
DATNVGDEGGFAPNILENK	66.27479	P06733	ENOA HUMAN	980.966525	2	0.855866849	3	
DATNVGDEGGFAPNILENK	66.27479	P06733	ENOA HUMAN	654.313625	3	0.855866849	3	Yes
DYPVVSIEDPFDQDDWGAWQK	126.2548	P06733	ENOA HUMAN	1255.561514	2	0.755000234	3	
DYPVVSIEDPFDQDDWGAWQK	126.2548	P06733	ENOA HUMAN	837.376951	3	0.755000234	3	Yes
GNPTVEVDLFTSK	57.73852	P06733	ENOA HUMAN	703.8622805	2	0.691246033	2	Yes
GNPTVEVDLFTSK	57.73852	P06733	ENOA HUMAN	469.577462	3	0.691246033	2	
IGAEVYHNLK	-1.909683	P06733	ENOA HUMAN	572.312025	2	0.739076436	2	Yes
IGAEVYHNLK	-1.909683	P06733	ENOA HUMAN	381.8772917	3	0.739076436	2	
LAMQEFMILPVGAANFR	119.9737	P06733	ENOA HUMAN	954.497699	2	0.76640892	3	
LAMQEFMILPVGAANFR	119.9737	P06733	ENOA HUMAN	636.667741	3	0.76640892	3	Yes
LMIEMDGTENK	28.33332	P06733	ENOA HUMAN	640.7972375	2	0.775396287	2	Yes
LMIEMDGTENK	28.33332	P06733	ENOA HUMAN	427.5341	3	0.775396287	2	
VNQIGSVTESLQACK	32.33532	P06733	ENOA HUMAN	817.414886	2	0.709308088		
VNQIGSVTESLQACK	32.33532	P06733	ENOA HUMAN	545.279199	3	0.709308088		
VVIGMDVAASEFFR	92.72241	P06733	ENOA HUMAN	770.8956005	2	0.804796755	2	Yes
VVIGMDVAASEFFR	92.72241	P06733	ENOA HUMAN	514.266342	3	0.804796755	2	
YISPDQLADLYK	62.93488	P06733	ENOA HUMAN	713.36719	2	0.852310836	2	Yes
YISPDQLADLYK	62.93488	P06733	ENOA HUMAN	475.9140683	3	0.852310836	2	
ALVQQMEQLR	32.43973	P06727	APOA4 HUMAN	608.329897	2	0.832923472		
ALVQQMEQLR	32.43973	P06727	APOA4 HUMAN	405.8892063	3	0.832923472		

ALVQQMEQLR	32.43973	P06727	APOA4 HUMAN	608.329897	2	0.832923472		
ALVQQMEQLR	32.43973	P06727	APOA4 HUMAN	405.8892063	3	0.832923472		
IDQNVEELK	5.228924	P06727	APOA4 HUMAN	544.285677	2	0.773920715		
IDQNVEELK	5.228924	P06727	APOA4 HUMAN	363.1930597	3	0.773920715		
IDQNVEELK	5.228924	P06727	APOA4 HUMAN	544.285677	2	0.773920715		
IDQNVEELK	5.228924	P06727	APOA4 HUMAN	363.1930597	3	0.773920715		
IDQTVEELR	15.01054	P06727	APOA4 HUMAN	551.791127	2	0.695162654		
IDQTVEELR	15.01054	P06727	APOA4 HUMAN	368.196693	3	0.695162654		
IDQTVEELR	15.01054	P06727	APOA4 HUMAN	551.791127	2	0.695162654		
IDQTVEELR	15.01054	P06727	APOA4 HUMAN	368.196693	3	0.695162654		
ISASAEELR	-1.654205	P06727	APOA4 HUMAN	488.259462	2	0.649228215		
ISASAEELR	-1.654205	P06727	APOA4 HUMAN	325.8422497	3	0.649228215		
ISASAEELR	-1.654205	P06727	APOA4 HUMAN	488.259462	2	0.649228215		
ISASAEELR	-1.654205	P06727	APOA4 HUMAN	325.8422497	3	0.649228215		
LEPYADQLR	17.43387	P06727	APOA4 HUMAN	552.788383	2	0.774389744		
LEPYADQLR	17.43387	P06727	APOA4 HUMAN	368.8615303	3	0.774389744		
LEPYADQLR	17.43387	P06727	APOA4 HUMAN	552.788383	2	0.774389744		
LEPYADQLR	17.43387	P06727	APOA4 HUMAN	368.8615303	3	0.774389744		
LGEVNTYAGDLQK	25.42849	P06727	APOA4 HUMAN	704.359901	2	0.876231253		
LGEVNTYAGDLQK	25.42849	P06727	APOA4 HUMAN	469.909209	3	0.876231253		
LGEVNTYAGDLQK	25.42849	P06727	APOA4 HUMAN	704.359901	2	0.876231253		
LGEVNTYAGDLQK	25.42849	P06727	APOA4 HUMAN	469.909209	3	0.876231253		
LGPHAGDVEGHLSFLEK	37.31583	P06727	APOA4 HUMAN	903.4632245	2	0.788745403		
LGPHAGDVEGHLSFLEK	37.31583	P06727	APOA4 HUMAN	602.644758	3	0.788745403		
LGPHAGDVEGHLSFLEK	37.31583	P06727	APOA4 HUMAN	903.4632245	2	0.788745403		
LGPHAGDVEGHLSFLEK	37.31583	P06727	APOA4 HUMAN	602.644758	3	0.788745403		
LLPHANEVSQK	-22.07599	P06727	APOA4 HUMAN	618.341318	2	0.634088099		
LLPHANEVSQK	-22.07599	P06727	APOA4 HUMAN	412.563487	3	0.634088099		
LLPHANEVSQK	-22.07599	P06727	APOA4 HUMAN	618.341318	2	0.634088099		
LLPHANEVSQK	-22.07599	P06727	APOA4 HUMAN	412.563487	3	0.634088099		
LNHQLEGLTFQMK	40.04355	P06727	APOA4 HUMAN	779.9065	2	0.806743026		
LNHQLEGLTFQMK	40.04355	P06727	APOA4 HUMAN	520.2736083	3	0.806743026		
LNHQLEGLTFQMK	40.04355	P06727	APOA4 HUMAN	779.9065	2	0.806743026		
LNHQLEGLTFQMK	40.04355	P06727	APOA4 HUMAN	520.2736083	3	0.806743026		
LTPYADEFK	26.02764	P06727	APOA4 HUMAN	542.2720345	2	0.77775079		
LTPYADEFK	26.02764	P06727	APOA4 HUMAN	361.8506313	3	0.77775079		
LTPYADEFK	26.02764	P06727	APOA4 HUMAN	542.2720345	2	0.77775079		
LTPYADEFK	26.02764	P06727	APOA4 HUMAN	361.8506313	3	0.77775079		
LVPFATELHER	30.52245	P06727	APOA4 HUMAN	656.3569685	2	0.8129462		
LVPFATELHER	30.52245	P06727	APOA4 HUMAN	437.907254	3	0.8129462		
LVPFATELHER	30.52245	P06727	APOA4 HUMAN	656.3569685	2	0.8129462		
LVPFATELHER	30.52245	P06727	APOA4 HUMAN	437.907254	3	0.8129462		
SELTQQLNALFQDK	76.34454	P06727	APOA4 HUMAN	817.923401	2	0.855960429		
SELTQQLNALFQDK	76.34454	P06727	APOA4 HUMAN	545.618209	3	0.855960429		
SELTQQLNALFQDK	76.34454	P06727	APOA4 HUMAN	817.923401	2	0.855960429		
SELTQQLNALFQDK	76.34454	P06727	APOA4 HUMAN	545.618209	3	0.855960429		
SLAELGGHLDQQVEEFR	52.22967	P06727	APOA4 HUMAN	964.479603	2	0.793740153		
SLAELGGHLDQQVEEFR	52.22967	P06727	APOA4 HUMAN	643.3223437	3	0.793740153		
SLAELGGHLDQQVEEFR	52.22967	P06727	APOA4 HUMAN	964.479603	2	0.793740153		
SLAELGGHLDQQVEEFR	52.22967	P06727	APOA4 HUMAN	643.3223437	3	0.793740153		
SLAPYAQDTQEK	2.01437	P06727	APOA4 HUMAN	675.8309765	2	0.7291435		
SLAPYAQDTQEK	2.01437	P06727	APOA4 HUMAN	450.889926	3	0.7291435		
SLAPYAQDTQEK	2.01437	P06727	APOA4 HUMAN	675.8309765	2	0.7291435		
SLAPYAQDTQEK	2.01437	P06727	APOA4 HUMAN	450.889926	3	0.7291435		
TLSLPELEQQQEQQQEQQQEQVQMLAPLES	118.439	P06727	APOA4 HUMAN	1769.354864	2	0.684677243		
TLSLPELEQQQEQQQEQQQEQVQMLAPLES	118.439	P06727	APOA4 HUMAN	1179.905851	3	0.684677243		
TLSLPELEQQQEQQQEQQQEQVQMLAPLES	118.439	P06727	APOA4 HUMAN	1769.354864	2	0.684677243		
TLSLPELEQQQEQQQEQQQEQVQMLAPLES	118.439	P06727	APOA4 HUMAN	1179.905851	3	0.684677243		
VEPYGENFNK	2.974583	P06727	APOA4 HUMAN	598.783297	2	0.6181795		
VEPYGENFNK	2.974583	P06727	APOA4 HUMAN	399.5248063	3	0.6181795		
VEPYGENFNK	2.974583	P06727	APOA4 HUMAN	598.783297	2	0.6181795		
VEPYGENFNK	2.974583	P06727	APOA4 HUMAN	399.5248063	3	0.6181795		
AEAESMYQIK	13.34728	P05787	K2C8 HUMAN	585.2795335	2	0.814302564	2	Yes
AEAESMYQIK	13.34728	P05787	K2C8 HUMAN	390.5222973	3	0.814302564	2	
ASLEAAIADAEQR	41.27395	P05787	K2C8 HUMAN	672.8418795	2	0.845234931	3	
ASLEAAIADAEQR	41.27395	P05787	K2C8 HUMAN	448.8971947	3	0.845234931	3	Yes
ELQSQISDTSVVLSMDNSR	61.30469	P05787	K2C8 HUMAN	1055.010606	2	0.705768108	3	
ELQSQISDTSVVLSMDNSR	61.30469	P05787	K2C8 HUMAN	703.6763457	3	0.705768108	3	Yes
LEAELGNMQGLVEDFK	86.078	P05787	K2C8 HUMAN	896.943476	2	0.813484907	2	Yes
LEAELGNMQGLVEDFK	86.078	P05787	K2C8 HUMAN	598.298259	3	0.813484907	2	
LEGLTDEINFLR	87.43553	P05787	K2C8 HUMAN	710.3780975	2	0.857954264	2	Yes
LEGLTDEINFLR	87.43553	P05787	K2C8 HUMAN	473.92134	3	0.857954264	2	
LESGMQNMSIHTK	-1.64949	P05787	K2C8 HUMAN	738.3532425	2	0.71773386	3	
LESGMQNMSIHTK	-1.64949	P05787	K2C8 HUMAN	492.5714367	3	0.71773386	3	Yes
LQAEIEGLK	22.15864	P05787	K2C8 HUMAN	500.787856	2	0.797731161	2	Yes
LQAEIEGLK	22.15864	P05787	K2C8 HUMAN	334.1945123	3	0.797731161	2	
LSELEAALQR	35.34605	P05787	K2C8 HUMAN	565.314769	2	0.836390734	2	Yes
LSELEAALQR	35.34605	P05787	K2C8 HUMAN	377.2124543	3	0.836390734	2	
LVSESSDVLPK	19.39658	P05787	K2C8 HUMAN	587.322259	2	0.679458976	2	Yes
LVSESSDVLPK	19.39658	P05787	K2C8 HUMAN	391.8841143	3	0.679458976	2	
SLDMDSIIAEVK	78.40932	P05787	K2C8 HUMAN	660.839959	2	0.778228998	2	Yes
SLDMDSIIAEVK	78.40932	P05787	K2C8 HUMAN	440.8959143	3	0.778228998	2	
SNMDNMFESYINNLR	94.08121	P05787	K2C8 HUMAN	924.406729	2	0.845485687	3	
SNMDNMFESYINNLR	94.08121	P05787	K2C8 HUMAN	616.6070943	3	0.845485687	3	Yes
TEMENEFVLIK	60.45892	P05787	K2C8 HUMAN	676.842502	2	0.639327168	2	Yes
TEMENEFVLIK	60.45892	P05787	K2C8 HUMAN	451.5642763	3	0.639327168	2	
WSLLQQQK	28.32258	P05787	K2C8 HUMAN	515.7881905	2	0.792124867	2	Yes
WSLLQQQK	28.32258	P05787	K2C8 HUMAN	344.1947353	3	0.792124867	2	
YEELQSLAGK	18.25239	P05787	K2C8 HUMAN	569.293498	2	0.768844426	2	Yes
YEELQSLAGK	18.25239	P05787	K2C8 HUMAN	379.8649403	3	0.768844426	2	
AQIFANTVDNAR	18.73503	P05783	K1C18 HUMAN	660.339307	2	0.854479432	2	Yes
AQIFANTVDNAR	18.73503	P05783	K1C18 HUMAN	440.5621463	3	0.854479432	2	
AQYDELAR	-2.149113	P05783	K1C18 HUMAN	483.238526	2	0.841050982	2	Yes
AQYDELAR	-2.149113	P05783	K1C18 HUMAN	322.494959	3	0.841050982	2	
ASLENSLR	-4.146202	P05783	K1C18 HUMAN	445.241072	2	0.751710296	2	Yes
ASLENSLR	-4.146202	P05783	K1C18 HUMAN	297.163323	3	0.751710296	2	
DWSHYFK	19.2223	P05783	K1C18 HUMAN	491.725054	2	0.782645702	2	Yes
DWSHYFK	19.2223	P05783	K1C18 HUMAN	328.1526443	3	0.782645702	2	
GGMGSGGLATGIAGGLAGMGGIQNEK	76.22939	P05783	K1C18 HUMAN	1131.054828	2	0.720859528	3	
GGMGSGGLATGIAGGLAGMGGIQNEK	76.22939	P05783	K1C18 HUMAN	754.3724933	3	0.720859528	3	Yes
GLQAQIASSGLTVEVDAPK	63.88007	P05783	K1C18 HUMAN	942.5078295	2	0.768933117	3	

GLQAQIASSGLTVEVDAPK	63.88007	P05783	K1C18_HUMAN	628.6744947	3	0.768933117	3	Yes
LLEDGEDFNLGDALDSSNSMQTIQK	91.73982	P05783	K1C18_HUMAN	1370.635085	2	0.765231252	3	
LLEDGEDFNLGDALDSSNSMQTIQK	91.73982	P05783	K1C18_HUMAN	914.092665	3	0.765231252	3	Yes
LQLETEIEALK	60.74223	P05783	K1C18_HUMAN	643.864292	2	0.861041248	2	Yes
LQLETEIEALK	60.74223	P05783	K1C18_HUMAN	429.578803	3	0.861041248	2	
QAQEYEALLNIK	60.89687	P05783	K1C18_HUMAN	710.3780935	2	0.617017627	2	Yes
QAQEYEALLNIK	60.89687	P05783	K1C18_HUMAN	473.9213373	3	0.617017627	2	
STFSTNYR	-15.4192	P05783	K1C18_HUMAN	488.2307005	2	0.628558338	2	Yes
STFSTNYR	-15.4192	P05783	K1C18_HUMAN	325.8230753	3	0.628558338	2	
TVQSLEIDLDSMR	64.22776	P05783	K1C18_HUMAN	753.8776045	2	0.799164474	2	Yes
TVQSLEIDLDSMR	64.22776	P05783	K1C18_HUMAN	502.9210113	3	0.799164474	2	
YWSQQIEESTTVVTTQSAEVGAAETTLTELR	103.635	P05783	K1C18_HUMAN	1714.839368	2	0.734517932		
YWSQQIEESTTVVTTQSAEVGAAETTLTELR	103.635	P05783	K1C18_HUMAN	1143.562187	3	0.734517932		
AVVLMGK	2.108395	P05388	RLA0_HUMAN	359.2205665	2	0.61225003		
AVVLMGK	2.108395	P05388	RLA0_HUMAN	239.8163193	3	0.61225003		
GNVGFVFTK	35.99153	P05388	RLA0_HUMAN	484.764184	2	0.762713313	2	Yes
GNVGFVFTK	35.99153	P05388	RLA0_HUMAN	323.5120643	3	0.762713313	2	
VLALSVETDYTFPLAEK	94.93787	P05388	RLA0_HUMAN	948.5042195	2	0.841686487	3	
VLALSVETDYTFPLAEK	94.93787	P05388	RLA0_HUMAN	632.672088	3	0.841686487	3	Yes
ILDSVGIEADDDR	39.21593	P05387	RLA2_HUMAN	709.3444515	2	0.730770588	2	Yes
ILDSVGIEADDDR	39.21593	P05387	RLA2_HUMAN	473.2322427	3	0.730770588	2	
NIEDVIAQGIGK	62.53195	P05387	RLA2_HUMAN	628.8464335	2	0.848021686	2	Yes
NIEDVIAQGIGK	62.53195	P05387	RLA2_HUMAN	419.5668973	3	0.848021686	2	
YVASYLLAALGGNSSPSAK	92.20299	P05387	RLA2_HUMAN	934.9918055	2	0.640435934	3	
YVASYLLAALGGNSSPSAK	92.20299	P05387	RLA2_HUMAN	623.663812	3	0.640435934	3	Yes
IGVHAFQQR	-14.71329	P05161	ISG15_HUMAN	528.291432	2	0.799132705		
IGVHAFQQR	-14.71329	P05161	ISG15_HUMAN	352.5302297	3	0.799132705		
LAVHPSGVALQDR	10.60956	P05161	ISG15_HUMAN	681.8785995	2	0.831898689		
LAVHPSGVALQDR	10.60956	P05161	ISG15_HUMAN	454.9216747	3	0.831898689		
LTQTVAHLK	-23.80641	P05161	ISG15_HUMAN	505.803841	2	0.631632745		
LTQTVAHLK	-23.80641	P05161	ISG15_HUMAN	337.5385023	3	0.631632745		
VPLASQGLGPGSTVLLVVDK	86.63715	P05161	ISG15_HUMAN	975.56769	2	0.7137205	3	
VPLASQGLGPGSTVLLVVDK	86.63715	P05161	ISG15_HUMAN	650.7144017	3	0.7137205	3	Yes
ALTSEIALLQSR	58.43472	P04843	RPN1_HUMAN	651.375358	2	0.862691045	3	
ALTSEIALLQSR	58.43472	P04843	RPN1_HUMAN	434.5861803	3	0.862691045	3	Yes
APDELHYTYLDTFGRPVIVAYK	71.0914	P04843	RPN1_HUMAN	1284.660826	2	0.625326455	4	
APDELHYTYLDTFGRPVIVAYK	71.0914	P04843	RPN1_HUMAN	856.7764923	3	0.625326455	4	
ATSFLLALEPELEAR	102.2127	P04843	RPN1_HUMAN	830.451794	2	0.718181908	3	
ATSFLLALEPELEAR	102.2127	P04843	RPN1_HUMAN	553.970471	3	0.718181908	3	Yes
DISTLNSGK	-2.270653	P04843	RPN1_HUMAN	467.7461875	2	0.777479172	2	Yes
DISTLNSGK	-2.270653	P04843	RPN1_HUMAN	312.1667333	3	0.777479172	2	
DVPAYSQDTFK	24.87366	P04843	RPN1_HUMAN	635.8016875	2	0.780052066	2	Yes
DVPAYSQDTFK	24.87366	P04843	RPN1_HUMAN	424.2037333	3	0.780052066	2	
FPLFGGWK	71.23923	P04843	RPN1_HUMAN	476.2585375	2	0.720484436	2	Yes
FPLFGGWK	71.23923	P04843	RPN1_HUMAN	317.8416333	3	0.720484436	2	
FVDHVFDEQVIDSLTVK	81.923	P04843	RPN1_HUMAN	996.0102045	2	0.805391192	3	
FVDHVFDEQVIDSLTVK	81.923	P04843	RPN1_HUMAN	664.3427447	3	0.805391192	3	Yes
ISVIVETVYTHVLHPYPTQITQSEK	77.50037	P04843	RPN1_HUMAN	1441.769098	2	0.733739018	3	
ISVIVETVYTHVLHPYPTQITQSEK	77.50037	P04843	RPN1_HUMAN	961.5153403	3	0.733739018	3	Yes
LPVALDPGAK	31.01718	P04843	RPN1_HUMAN	490.7929415	2	0.758796334		
LPVALDPGAK	31.01718	P04843	RPN1_HUMAN	327.531236	3	0.758796334		
NIEIDSPYEISR	46.86949	P04843	RPN1_HUMAN	718.357357	2	0.824997485	2	Yes
NIEIDSPYEISR	46.86949	P04843	RPN1_HUMAN	479.2408463	3	0.824997485	2	
NLVEQHIQDIVVHYTFNK	67.94519	P04843	RPN1_HUMAN	1099.074198	2	0.756051421	3	
NLVEQHIQDIVVHYTFNK	67.94519	P04843	RPN1_HUMAN	733.0520733	3	0.756051421	3	Yes
SEDLLDYGPFR	68.28711	P04843	RPN1_HUMAN	656.3149615	2	0.837925017	2	Yes
SEDLLDYGPFR	68.28711	P04843	RPN1_HUMAN	437.8792493	3	0.837925017	2	
THYIVGYNLPSYEYLYNLGDQYALK	106.1874	P04843	RPN1_HUMAN	1499.237613	2	0.707564533	3	
THYIVGYNLPSYEYLYNLGDQYALK	106.1874	P04843	RPN1_HUMAN	999.8276833	3	0.707564533	3	Yes
TILPAAAQDVYYR	49.04073	P04843	RPN1_HUMAN	740.893907	2	0.844557166	2	Yes
TILPAAAQDVYYR	49.04073	P04843	RPN1_HUMAN	494.265213	3	0.844557166	2	
VACITEQVLTLVNK	79.8177	P04843	RPN1_HUMAN	794.4429155	2	0.661933064	3	
VACITEQVLTLVNK	79.8177	P04843	RPN1_HUMAN	529.964552	3	0.661933064	3	Yes
VHYENNSPFLTITSMTR	57.07503	P04843	RPN1_HUMAN	1005.491652	2	0.796758473	3	
VHYENNSPFLTITSMTR	57.07503	P04843	RPN1_HUMAN	670.6637093	3	0.796758473	3	Yes
VIEVSHWGNIAVEENVDLK	64.74695	P04843	RPN1_HUMAN	1076.058217	2	0.732585073		
VIEVSHWGNIAVEENVDLK	64.74695	P04843	RPN1_HUMAN	717.708086	3	0.732585073		
VTAEVVLAHLGGGSTSR	42.28079	P04843	RPN1_HUMAN	827.4501175	2	0.775820613		
VTAEVVLAHLGGGSTSR	42.28079	P04843	RPN1_HUMAN	551.9693533	3	0.775820613		
AQLGGPEAAK	-24.1167	P04792	HSPB1_HUMAN	471.2567235	2	0.641309798	2	Yes
AQLGGPEAAK	-24.1167	P04792	HSPB1_HUMAN	314.5070907	3	0.641309798	2	
DGVVEITGK	14.65854	P04792	HSPB1_HUMAN	459.2511065	2	0.661195755	2	Yes
DGVVEITGK	14.65854	P04792	HSPB1_HUMAN	306.503346	3	0.661195755	2	
LATQSNEITIPVTFESR	71.58147	P04792	HSPB1_HUMAN	953.500004	2	0.827628016	3	
LATQSNEITIPVTFESR	71.58147	P04792	HSPB1_HUMAN	636.002611	3	0.827628016	3	Yes
LFDQAFGLPR	71.28941	P04792	HSPB1_HUMAN	582.3145725	2	0.834599137	2	Yes
LFDQAFGLPR	71.28941	P04792	HSPB1_HUMAN	388.5456567	3	0.834599137	2	
VSLDVNHFAPDELTVK	57.26463	P04792	HSPB1_HUMAN	892.4654325	2	0.872214854	2	Yes
VSLDVNHFAPDELTVK	57.26463	P04792	HSPB1_HUMAN	595.3128967	3	0.872214854	2	
AGAHLQGGAK	-50	P04406	G3P_HUMAN	455.249233	2	0.608088374	2	Yes
AGAHLQGGAK	-50	P04406	G3P_HUMAN	303.8354303	3	0.608088374	2	
GALQNIIPASTGAAK	40.56278	P04406	G3P_HUMAN	706.399365	2	0.869713783	2	Yes
GALQNIIPASTGAAK	40.56278	P04406	G3P_HUMAN	471.2688517	3	0.869713783	2	
IISNASCTTNCLAPLAK	38.02053	P04406	G3P_HUMAN	917.464053	2	0.784035385	3	
IISNASCTTNCLAPLAK	38.02053	P04406	G3P_HUMAN	611.9786437	3	0.784035385	3	Yes
LISWYDNEFGYSNR	71.13669	P04406	G3P_HUMAN	882.4053655	2	0.869633794	2	Yes
LISWYDNEFGYSNR	71.13669	P04406	G3P_HUMAN	588.6061853	3	0.869633794	2	
LVINGNPITIFQER	82.47678	P04406	G3P_HUMAN	807.454671	2	0.75553149	2	Yes
LVINGNPITIFQER	82.47678	P04406	G3P_HUMAN	538.6390557	3	0.75553149	2	
VIHDNFGIVEGLMTTVHAITATQK	102.3628	P04406	G3P_HUMAN	1298.184156	2	0.773056328	4	
VIHDNFGIVEGLMTTVHAITATQK	102.3628	P04406	G3P_HUMAN	865.7920457	3	0.773056328	4	
VPTANVSVVDLTCR	51.30193	P04406	G3P_HUMAN	765.9014145	2	0.8059237	2	Yes
VPTANVSVVDLTCR	51.30193	P04406	G3P_HUMAN	510.9368847	3	0.8059237	2	
VVDLMAHMASK	22.00478	P04406	G3P_HUMAN	601.3075755	2	0.788415909	2	Yes
VVDLMAHMASK	22.00478	P04406	G3P_HUMAN	401.2076587	3	0.788415909	2	
WGDAGAEYVVESTGVFTTMEK	94.09442	P04406	G3P_HUMAN	1139.02318	2	0.779943228	3	
WGDAGAEYVVESTGVFTTMEK	94.09442	P04406	G3P_HUMAN	759.684728	3	0.779943228	3	Yes
SQVVAGTNYFIK	38.56919	P04080	CYTB_HUMAN	663.856797	2	0.817462683	2	Yes
SQVVAGTNYFIK	38.56919	P04080	CYTB_HUMAN	442.9071397	3	0.817462683	2	
VFQSLPHENKPLTLSNYQTNK	28.81261	P04080	CYTB_HUMAN	1229.640433	2	0.804294586	4	
VFQSLPHENKPLTLSNYQTNK	28.81261	P04080	CYTB_HUMAN	820.09623	3	0.804294586	4	

VHVGDEDFVHLR	24.57294	P04080	CYTB HUMAN	711.8604065	2	0.821447492	3	
VHVGDEDFVHLR	24.57294	P04080	CYTB HUMAN	474.909546	3	0.821447492	3	Yes
ADDGRPFPQVIK	22.33302	P04075	ALDOA HUMAN	671.8598755	2	0.808172226	3	
ADDGRPFPQVIK	22.33302	P04075	ALDOA HUMAN	448.2425253	3	0.808172226	3	Yes
ALANSLACQGK	-1.341991	P04075	ALDOA HUMAN	566.793147	2	0.760767519	2	Yes
ALANSLACQGK	-1.341991	P04075	ALDOA HUMAN	378.1980397	3	0.760767519	2	
ALSDHHIYLEGTLLKPNMVTPGHACTQK	36.98582	P04075	ALDOA HUMAN	1566.292428	2	0.682544827	5	
ALSDHHIYLEGTLLKPNMVTPGHACTQK	36.98582	P04075	ALDOA HUMAN	1044.530893	3	0.682544827	5	
FSHEEIAMATVTALR	50.69332	P04075	ALDOA HUMAN	838.4277965	2	0.823155165	3	
FSHEEIAMATVTALR	50.69332	P04075	ALDOA HUMAN	559.287806	3	0.823155165	3	Yes
GILAADESTGSIAK	25.3692	P04075	ALDOA HUMAN	666.8544555	2	0.805260539	2	Yes
GILAADESTGSIAK	25.3692	P04075	ALDOA HUMAN	444.9055787	3	0.805260539	2	
GVVPLAGTNGETTTQGLDGLSER	59.45242	P04075	ALDOA HUMAN	1136.574961	2	0.75028199	3	
GVVPLAGTNGETTTQGLDGLSER	59.45242	P04075	ALDOA HUMAN	758.0525823	3	0.75028199	3	Yes
IGEHTPSALAIMENANVLAR	72.61312	P04075	ALDOA HUMAN	1054.052412	2	0.866910636	3	
IGEHTPSALAIMENANVLAR	72.61312	P04075	ALDOA HUMAN	703.0375497	3	0.866910636	3	Yes
LQSIGTENTEENR	-10.44248	P04075	ALDOA HUMAN	745.8582575	2	0.777069688	2	Yes
LQSIGTENTEENR	-10.44248	P04075	ALDOA HUMAN	497.57478	3	0.777069688	2	
TVPPAVTGITFLSGGQSEEEASINLNAINK	107.0166	P04075	ALDOA HUMAN	1529.291131	2	0.711512387	4	
TVPPAVTGITFLSGGQSEEEASINLNAINK	107.0166	P04075	ALDOA HUMAN	1019.863362	3	0.711512387	4	
VNPCIGGVILFHETLYQK	75.48584	P04075	ALDOA HUMAN	1044.551513	2	0.719127119	3	
VNPCIGGVILFHETLYQK	75.48584	P04075	ALDOA HUMAN	696.703617	3	0.719127119	3	Yes
ADANTAAIQAILYNR	70.57243	P03915	NU5M HUMAN	802.923731	2	0.792971969		
ADANTAAIQAILYNR	70.57243	P03915	NU5M HUMAN	535.618429	3	0.792971969		
FPTLTNINENNPTLLNPIK	88.14008	P03915	NU5M HUMAN	1077.084235	2	0.76132834	3	
FPTLTNINENNPTLLNPIK	88.14008	P03915	NU5M HUMAN	718.392098	3	0.76132834	3	Yes
MILLTLTGQPR	66.20765	P03915	NU5M HUMAN	621.8661155	2	0.817648351		
MILLTLTGQPR	66.20765	P03915	NU5M HUMAN	414.913352	3	0.817648351		
TISQHQISTSIITSTQK	20.03426	P03915	NU5M HUMAN	937.005454	2	0.85556072	3	
TISQHQISTSIITSTQK	20.03426	P03915	NU5M HUMAN	625.0062443	3	0.85556072	3	Yes
ELGDHVTNLR	-0.739685	P02794	FRIH HUMAN	577.302193	2	0.668261707	3	
ELGDHVTNLR	-0.739685	P02794	FRIH HUMAN	385.2040703	3	0.668261707	3	Yes
IFLQDIK	38.41295	P02794	FRIH HUMAN	438.7636525	2	0.757460058	2	Yes
IFLQDIK	38.41295	P02794	FRIH HUMAN	292.8450433	3	0.757460058	2	
MGAPESGLAEYLFDK	83.85938	P02794	FRIH HUMAN	814.387801	2	0.793075621	2	Yes
MGAPESGLAEYLFDK	83.85938	P02794	FRIH HUMAN	543.2611423	3	0.793075621	2	
NDPHLCDFIETHYLNEQVK	55.1558	P02794	FRIH HUMAN	1186.552969	2	0.710580468	4	
NDPHLCDFIETHYLNEQVK	55.1558	P02794	FRIH HUMAN	791.3712543	3	0.710580468	4	
NVNQSLLELHK	18.3259	P02794	FRIH HUMAN	647.8598745	2	0.818531275	3	
NVNQSLLELHK	18.3259	P02794	FRIH HUMAN	432.2425247	3	0.818531275	3	Yes
YFLHQSHEER	-28.69016	P02794	FRIH HUMAN	673.31837	2	0.651168644	3	
YFLHQSHEER	-28.69016	P02794	FRIH HUMAN	449.214855	3	0.651168644	3	Yes
ASEAEDASLLSFMQGYMK	109.2932	P02656	APOC3 HUMAN	989.4508035	2	0.687343955		
ASEAEDASLLSFMQGYMK	109.2932	P02656	APOC3 HUMAN	659.9698107	3	0.687343955		
DALSSVQESQVAQQAR	30.29684	P02656	APOC3 HUMAN	858.929746	2	0.827313781		
DALSSVQESQVAQQAR	30.29684	P02656	APOC3 HUMAN	572.9557723	3	0.827313781		
FSEFWDLDPEVRPTSAVAA	103.1092	P02656	APOC3 HUMAN	1069.016018	2	0.674513102		
FSEFWDLDPEVRPTSAVAA	103.1092	P02656	APOC3 HUMAN	713.013287	3	0.674513102		
GWVTDGFSSLK	57.31206	P02656	APOC3 HUMAN	598.8014945	2	0.808169484		
GWVTDGFSSLK	57.31206	P02656	APOC3 HUMAN	399.536938	3	0.808169484		
HGATVLTALGGILK	67.20882	P02144	MYG HUMAN	675.909369	2	0.820262074		
HGATVLTALGGILK	67.20882	P02144	MYG HUMAN	450.9421877	3	0.820262074		
HPGDFGADAQGAMNK	-5.917854	P02144	MYG HUMAN	758.336439	2	0.760045588		
HPGDFGADAQGAMNK	-5.917854	P02144	MYG HUMAN	505.8935677	3	0.760045588		
VEADIPGHGQEVLIR	29.71694	P02144	MYG HUMAN	816.9393855	2	0.787609696		
VEADIPGHGQEVLIR	29.71694	P02144	MYG HUMAN	544.9621987	3	0.787609696		
FVYHLSDLCK	26.74086	P01591	IGJ HUMAN	641.318993	2	0.774474978		
FVYHLSDLCK	26.74086	P01591	IGJ HUMAN	427.881937	3	0.774474978		
IIVPLNNR	27.21965	P01591	IGJ HUMAN	469.793275	2	0.685689747		
IIVPLNNR	27.21965	P01591	IGJ HUMAN	313.5314583	3	0.685689747		
IVLVDNK	5.214127	P01591	IGJ HUMAN	400.748002	2	0.733171999		
IVLVDNK	5.214127	P01591	IGJ HUMAN	267.5012763	3	0.733171999		
MVETALTPDACYPD	60.6386	P01591	IGJ HUMAN	791.84237	2	0.71831584		
MVETALTPDACYPD	60.6386	P01591	IGJ HUMAN	528.230855	3	0.71831584		
ACANPAAGSVILLENLR	81.25775	P00558	PGK1 HUMAN	884.972902	2	0.611929595		
ACANPAAGSVILLENLR	81.25775	P00558	PGK1 HUMAN	590.3178763	3	0.611929595		
AHSSMVGVNLPQK	9.501839	P00558	PGK1 HUMAN	684.359185	2	0.738327205	2	Yes
AHSSMVGVNLPQK	9.501839	P00558	PGK1 HUMAN	456.5753983	3	0.738327205	2	
ALESPERPFLAILGGAK	74.9851	P00558	PGK1 HUMAN	885.0019855	2	0.667492628	2	Yes
ALESPERPFLAILGGAK	74.9851	P00558	PGK1 HUMAN	590.3372653	3	0.667492628	2	
DCVGPEVEK	-12.88567	P00558	PGK1 HUMAN	516.737506	2	0.639325917	2	Yes
DCVGPEVEK	-12.88567	P00558	PGK1 HUMAN	344.8276123	3	0.639325917	2	
IQLINNMLDK	53.25818	P00558	PGK1 HUMAN	601.334647	2	0.792678237	2	Yes
IQLINNMLDK	53.25818	P00558	PGK1 HUMAN	401.2257063	3	0.792678237	2	
ITLPVDFVTADK	78.936	P00558	PGK1 HUMAN	659.866835	2	0.848811448	2	Yes
ITLPVDFVTADK	78.936	P00558	PGK1 HUMAN	440.247165	3	0.848811448	2	
SVVLMSHLGRPDGVPMPDK	42.8671	P00558	PGK1 HUMAN	1018.027352	2	0.682786047	3	
SVVLMSHLGRPDGVPMPDK	42.8671	P00558	PGK1 HUMAN	679.0208427	3	0.682786047	3	Yes
TGQATVASGIPAGWMGLDCGPESSK	76.98347	P00558	PGK1 HUMAN	1239.075957	2	0.630634665	3	
TGQATVASGIPAGWMGLDCGPESSK	76.98347	P00558	PGK1 HUMAN	826.3865793	3	0.630634665	3	Yes
VLNNMEIGTSLFDEEGAK	73.35921	P00558	PGK1 HUMAN	983.975504	2	0.820089281	3	
VLNNMEIGTSLFDEEGAK	73.35921	P00558	PGK1 HUMAN	656.319611	3	0.820089281	3	Yes
YSLEPVAVELK	57.69006	P00558	PGK1 HUMAN	624.348273	2	0.798168778	2	Yes
YSLEPVAVELK	57.69006	P00558	PGK1 HUMAN	416.5681237	3	0.798168778	2	
ACGADSYEMEEDGVR	13.05274	P00533	EGFR HUMAN	844.8305215	2	0.726624608	2	Yes
ACGADSYEMEEDGVR	13.05274	P00533	EGFR HUMAN	563.5562893	3	0.726624608	2	
ALMDEEDMDDVVDADEYLIPQQGFFSSPSTSR	127.384	P00533	EGFR HUMAN	1804.290534	2	0.60502547		
ALMDEEDMDDVVDADEYLIPQQGFFSSPSTSR	127.384	P00533	EGFR HUMAN	1203.196298	3	0.60502547		
DEATCKDTCPPLMLYNPTTYQMDVNPEGK	76.87266	P00533	EGFR HUMAN	1694.746194	2	0.645727158		
DEATCKDTCPPLMLYNPTTYQMDVNPEGK	76.87266	P00533	EGFR HUMAN	1130.166738	3	0.645727158		
YQDPHSTAVGNPEYLNTVQPTCVNSTFDSPAHW	58.87326	P00533	EGFR HUMAN	2205.0018	2	0.615160048	4	
YQDPHSTAVGNPEYLNTVQPTCVNSTFDSPAHW	58.87326	P00533	EGFR HUMAN	1470.337141	3	0.615160048	4	
DSLSINATNIK	31.18591	P00533	EGFR HUMAN	588.317508	2	0.738349557		
DSLSINATNIK	31.18591	P00533	EGFR HUMAN	392.5476137	3	0.738349557		
EISDGDVIISGNK	31.41605	P00533	EGFR HUMAN	673.844087	2	0.78958571	2	Yes
EISDGDVIISGNK	31.41605	P00533	EGFR HUMAN	449.565333	3	0.78958571	2	
EITGFLLIQAWPENR	118.6042	P00533	EGFR HUMAN	893.9785105	2	0.785727739		
EITGFLLIQAWPENR	118.6042	P00533	EGFR HUMAN	596.3216153	3	0.785727739		
ELVEPLTPSGEAPNQALLR	68.86809	P00533	EGFR HUMAN	1017.547486	2	0.776645839		
ELVEPLTPSGEAPNQALLR	68.86809	P00533	EGFR HUMAN	678.700932	3	0.776645839		
FSNNPALCNVESIQWR	63.66692	P00533	EGFR HUMAN	967.963065	2	0.751725852	3	

FSNNPALCNVESIQWR	63.66692	P00533	EGFR HUMAN	645.6446517	3	0.751725852	3	Yes
GDSFTHTPPLDPQELDILK	72.82618	P00533	EGFR HUMAN	1062.036951	2	0.734418511		
GDSFTHTPPLDPQELDILK	72.82618	P00533	EGFR HUMAN	708.3605757	3	0.734418511		
GLWIPEGEK	39.39947	P00533	EGFR HUMAN	514.7747485	2	0.820319891	2	Yes
GLWIPEGEK	39.39947	P00533	EGFR HUMAN	343.5191073	3	0.820319891	2	
GMNYLEDR	9.067917	P00533	EGFR HUMAN	499.224561	2	0.732902646	2	Yes
GMNYLEDR	9.067917	P00533	EGFR HUMAN	333.1523157	3	0.732902646	2	
GNMYYENSYALAVLSNYDANK	91.33908	P00533	EGFR HUMAN	1200.544796	2	0.701860487		
GNMYYENSYALAVLSNYDANK	91.33908	P00533	EGFR HUMAN	800.6991387	3	0.701860487		
GSHQISLDNPDYQQDFFPK	56.57287	P00533	EGFR HUMAN	1118.519452	2	0.747230947	3	
GSHQISLDNPDYQQDFFPK	56.57287	P00533	EGFR HUMAN	746.0155763	3	0.747230947	3	Yes
GSTAENAEYLR	-4.72678	P00533	EGFR HUMAN	605.789111	2	0.805278599	2	Yes
GSTAENAEYLR	-4.72678	P00533	EGFR HUMAN	404.195349	3	0.805278599	2	
IPLENLQIIR	65.45131	P00533	EGFR HUMAN	604.872254	2	0.838564932	2	Yes
IPLENLQIIR	65.45131	P00533	EGFR HUMAN	403.584111	3	0.838564932	2	
LTQLGTFEDHFLSLQR	68.95669	P00533	EGFR HUMAN	952.997432	2	0.893937409		
LTQLGTFEDHFLSLQR	68.95669	P00533	EGFR HUMAN	635.667563	3	0.893937409		
MHLPSPTDSNFYR	32.56789	P00533	EGFR HUMAN	782.864827	2	0.863461375	3	
MHLPSPTDSNFYR	32.56789	P00533	EGFR HUMAN	522.2458263	3	0.863461375	3	Yes
NLCYANTINWK	41.17251	P00533	EGFR HUMAN	698.838081	2	0.727010787	2	Yes
NLCYANTINWK	41.17251	P00533	EGFR HUMAN	466.2279957	3	0.727010787	2	
NLQEILHGAVR	30.32581	P00533	EGFR HUMAN	625.35476	2	0.808926463	3	
NLQEILHGAVR	30.32581	P00533	EGFR HUMAN	417.239115	3	0.808926463	3	Yes
NYDLSFLK	52.64098	P00533	EGFR HUMAN	500.261469	2	0.601941407	2	Yes
NYDLSFLK	52.64098	P00533	EGFR HUMAN	333.8435877	3	0.601941407	2	
TDLHAFENLEIIR	65.20689	P00533	EGFR HUMAN	785.9153785	2	0.892940879	3	
TDLHAFENLEIIR	65.20689	P00533	EGFR HUMAN	524.2795273	3	0.892940879	3	Yes
VAPQSSEFIGA	43.08828	P00533	EGFR HUMAN	553.280395	2	0.649134576		
VAPQSSEFIGA	43.08828	P00533	EGFR HUMAN	369.1895383	3	0.649134576		
WMALESILHR	60.78718	P00533	EGFR HUMAN	628.3349815	2	0.804007888	3	
WMALESILHR	60.78718	P00533	EGFR HUMAN	419.2259293	3	0.804007888	3	Yes
YLVIQGDER	15.46825	P00533	EGFR HUMAN	546.788383	2	0.774051309	2	Yes
YLVIQGDER	15.46825	P00533	EGFR HUMAN	364.8615303	3	0.774051309	2	
YSFGATCVK	11.33798	P00533	EGFR HUMAN	516.74513	2	0.768482447	2	Yes
YSFGATCVK	11.33798	P00533	EGFR HUMAN	344.832695	3	0.768482447	2	
YSSDPTGALTEDSIDDTFLPVPEYINQSVPK	125.1945	P00533	EGFR HUMAN	1699.812281	2	0.61805582	3	
YSSDPTGALTEDSIDDTFLPVPEYINQSVPK	125.1945	P00533	EGFR HUMAN	1133.544129	3	0.61805582	3	Yes
ASAELALGENSEVLK	42.91487	P00505	AATM HUMAN	765.904676	2	0.836747944	2	Yes
ASAELALGENSEVLK	42.91487	P00505	AATM HUMAN	510.939059	3	0.836747944	2	
ASSWWTHVEMGPPDPILGVTEAFK	112.7784	P00505	AATM HUMAN	1328.149781	2	0.625700116		
ASSWWTHVEMGPPDPILGVTEAFK	112.7784	P00505	AATM HUMAN	885.769129	3	0.625700116		
DAGMQLQGYR	17.23036	P00505	AATM HUMAN	569.769668	2	0.767995894	2	Yes
DAGMQLQGYR	17.23036	P00505	AATM HUMAN	380.182387	3	0.767995894	2	
DVFLPKPTWGNHTPIFR	64.18662	P00505	AATM HUMAN	1013.039434	2	0.761897206		
DVFLPKPTWGNHTPIFR	64.18662	P00505	AATM HUMAN	675.6955643	3	0.761897206		
EYLPIGGLAEFCK	81.47823	P00505	AATM HUMAN	748.8768725	2	0.785716355	2	Yes
EYLPIGGLAEFCK	81.47823	P00505	AATM HUMAN	499.58719	3	0.785716355	2	
FVTVQTISGTGALR	47.74602	P00505	AATM HUMAN	725.4071905	2	0.832601488	2	Yes
FVTVQTISGTGALR	47.74602	P00505	AATM HUMAN	483.9407353	3	0.832601488	2	
IAAAILNTPDLR	55.01173	P00505	AATM HUMAN	634.3726185	2	0.849722981	2	Yes
IAAAILNTPDLR	55.01173	P00505	AATM HUMAN	423.2510207	3	0.849722981	2	
IGASFLQR	21.82628	P00505	AATM HUMAN	446.256526	2	0.793538511	2	Yes
IGASFLQR	21.82628	P00505	AATM HUMAN	297.8402923	3	0.793538511	2	
ISVAGVTSSNVGYLAHAIHQVTK	63.61349	P00505	AATM HUMAN	1176.637694	2	0.780975521	4	
ISVAGVTSSNVGYLAHAIHQVTK	63.61349	P00505	AATM HUMAN	784.7610707	3	0.780975521	4	
MNLGVGAYR	20.71503	P00505	AATM HUMAN	490.753289	2	0.719320536	2	Yes
MNLGVGAYR	20.71503	P00505	AATM HUMAN	327.504801	3	0.719320536	2	
TCGFDFTGAVEDISK	72.86453	P00505	AATM HUMAN	823.87252	2	0.840289116	3	
TCGFDFTGAVEDISK	72.86453	P00505	AATM HUMAN	549.5842883	3	0.840289116	3	Yes
TQLVSNLK	-2.180458	P00505	AATM HUMAN	451.769466	2	0.620204508	2	Yes
TQLVSNLK	-2.180458	P00505	AATM HUMAN	301.5155857	3	0.620204508	2	
VGAFTMVCKDADEAK	16.26566	P00505	AATM HUMAN	821.3847405	2	0.726706922		
VGAFTMVCKDADEAK	16.26566	P00505	AATM HUMAN	547.9257687	3	0.726706922		
VLPGIAAAFHPGLAAAASAR	71.21453	P00505	AATM HUMAN	931.026327	2	0.765410542		
VLPGIAAAFHPGLAAAASAR	71.21453	P00505	AATM HUMAN	621.0201597	3	0.765410542		
DHINLPGFSGQNPLR	55.44002	P00491	PNPH HUMAN	832.9293515	2	0.774587095	2	Yes
DHINLPGFSGQNPLR	55.44002	P00491	PNPH HUMAN	555.622176	3	0.774587095	2	
ELQEGTYVMVAGPSFETVAECR	78.19441	P00491	PNPH HUMAN	1237.072878	2	0.658035457	3	
ELQEGTYVMVAGPSFETVAECR	78.19441	P00491	PNPH HUMAN	825.0511937	3	0.658035457	3	Yes
FEVGDIMLIR	88.52144	P00491	PNPH HUMAN	596.8239155	2	0.841933012	2	Yes
FEVGDIMLIR	88.52144	P00491	PNPH HUMAN	398.218552	3	0.841933012	2	
FHMYEGYPLWK	50.76549	P00491	PNPH HUMAN	735.8479135	2	0.696148098	2	Yes
FHMYEGYPLWK	50.76549	P00491	PNPH HUMAN	490.9012173	3	0.696148098	2	
FPAMSDAYDR	25.42211	P00491	PNPH HUMAN	586.7562255	2	0.70705992	2	Yes
FPAMSDAYDR	25.42211	P00491	PNPH HUMAN	391.5067587	3	0.70705992	2	
LGADAVGMSTVPEVIVAR	73.7455	P00491	PNPH HUMAN	892.982936	2	0.86477232		
LGADAVGMSTVPEVIVAR	73.7455	P00491	PNPH HUMAN	595.657899	3	0.86477232		
LTQAQIFDYGEIPNFPR	92.90703	P00491	PNPH HUMAN	1005.010535	2	0.842785299	3	
LTQAQIFDYGEIPNFPR	92.90703	P00491	PNPH HUMAN	670.342965	3	0.842785299	3	Yes
NTAEWLLSHTK	23.18386	P00491	PNPH HUMAN	650.3387755	2	0.753525615	3	
NTAEWLLSHTK	23.18386	P00491	PNPH HUMAN	433.8951253	3	0.753525615	3	Yes
VFGFSLITNK	72.13862	P00491	PNPH HUMAN	563.319323	2	0.749308586	2	Yes
VFGFSLITNK	72.13862	P00491	PNPH HUMAN	375.882157	3	0.749308586	2	
VFHLLGVDTLVVTNAAGGLNPK	87.71082	P00491	PNPH HUMAN	1118.128978	2	0.667365491	3	
VFHLLGVDTLVVTNAAGGLNPK	87.71082	P00491	PNPH HUMAN	745.75526	3	0.667365491	3	Yes
VIMDYESLEK	38.13499	P00491	PNPH HUMAN	613.802841	2	0.84081012	2	Yes
VIMDYESLEK	38.13499	P00491	PNPH HUMAN	409.5378357	3	0.84081012	2	
ILYMTDEVNDPSLTIK	70.68282	P00403	COX2 HUMAN	926.4746045	2	0.77185303		
ILYMTDEVNDPSLTIK	70.68282	P00403	COX2 HUMAN	617.985678	3	0.77185303		
LLDVDNR	4.33139	P00403	COX2 HUMAN	422.7303405	2	0.746050417	2	Yes
LLDVDNR	4.33139	P00403	COX2 HUMAN	282.1561687	3	0.746050417	2	
VVLPIEAPIR	64.46657	P00403	COX2 HUMAN	553.8507905	2	0.88845396	2	Yes
VVLPIEAPIR	64.46657	P00403	COX2 HUMAN	369.569802	3	0.88845396	2	
DLADELALVDVIEDK	133.5257	P00338	LDHA HUMAN	829.430724	2	0.77914089	3	
DLADELALVDVIEDK	133.5257	P00338	LDHA HUMAN	553.2897577	3	0.77914089	3	Yes
DQLIYNLLK	79.06772	P00338	LDHA HUMAN	560.324601	2	0.739357948	2	Yes
DQLIYNLLK	79.06772	P00338	LDHA HUMAN	373.8856757	3	0.739357948	2	
FIIPNVVK	54.73213	P00338	LDHA HUMAN	465.2951195	2	0.755534172	2	Yes
FIIPNVVK	54.73213	P00338	LDHA HUMAN	310.532688	3	0.755534172	2	
GEMMDLQHGSLFLR	58.44989	P00338	LDHA HUMAN	817.395442	2	0.778468132	2	Yes
GEMMDLQHGSLFLR	58.44989	P00338	LDHA HUMAN	545.2662363	3	0.778468132	2	

SADTLWGIQK	42.36584	P00338	LDHA HUMAN	559.7962125	2	0.824763775	2	Yes
SADTLWGIQK	42.36584	P00338	LDHA HUMAN	373.5334167	3	0.824763775	2	
TLHPDLGTDKDK	-26.55528	P00338	LDHA HUMAN	670.346798	2	0.67763418	2	Yes
TLHPDLGTDKDK	-26.55528	P00338	LDHA HUMAN	447.233807	3	0.67763418	2	
VTLTSEEEAR	-11.37031	P00338	LDHA HUMAN	567.7860415	2	0.789091766	2	Yes
VTLTSEEEAR	-11.37031	P00338	LDHA HUMAN	378.8599693	3	0.789091766	2	
ASDQLQVGVEFEASTR	53.3755	O96008	TOM40 HUMAN	868.926672	2	0.813012004		
ASDQLQVGVEFEASTR	53.3755	O96008	TOM40 HUMAN	579.6203897	3	0.813012004		
ELFPIQMEGVK	70.12942	O96008	TOM40 HUMAN	645.8423055	2	0.728513896	2	Yes
ELFPIQMEGVK	70.12942	O96008	TOM40 HUMAN	430.8974787	3	0.728513896	2	
FVNWQVDGEYR	47.84018	O96008	TOM40 HUMAN	706.833853	2	0.781535149	2	Yes
FVNWQVDGEYR	47.84018	O96008	TOM40 HUMAN	471.5585103	3	0.781535149	2	
GLSNHFQVNHTVALSTIGESNYHFGVTYVGTK	57.57463	O96008	TOM40 HUMAN	1739.363476	2	0.680556417		
GLSNHFQVNHTVALSTIGESNYHFGVTYVGTK	57.57463	O96008	TOM40 HUMAN	1159.911592	3	0.680556417		
GSVDSNWIVGATLEK	64.30679	O96008	TOM40 HUMAN	788.4048435	2	0.886794508	2	Yes
GSVDSNWIVGATLEK	64.30679	O96008	TOM40 HUMAN	525.9391707	3	0.886794508	2	
MAIQTQQSK	-25.31464	O96008	TOM40 HUMAN	517.769141	2	0.623929203		
MAIQTQQSK	-25.31464	O96008	TOM40 HUMAN	345.515369	3	0.623929203		
MQDTSVSFGYQLDLPK	74.39954	O96008	TOM40 HUMAN	914.943472	2	0.738248944		
MQDTSVSFGYQLDLPK	74.39954	O96008	TOM40 HUMAN	610.2982563	3	0.738248944		
RPGEEGTVMSLAGK	4.982746	O96008	TOM40 HUMAN	716.3672075	2	0.765924633	3	
RPGEEGTVMSLAGK	4.982746	O96008	TOM40 HUMAN	477.91408	3	0.765924633	3	Yes
GLDTLVESIR	56.54039	O95999	BCL10 HUMAN	551.8093195	2	0.809971392	2	Yes
GLDTLVESIR	56.54039	O95999	BCL10 HUMAN	368.2088213	3	0.809971392	2	
LLDYLQENPK	38.59147	O95999	BCL10 HUMAN	616.8302475	2	0.83878541		
LLDYLQENPK	38.59147	O95999	BCL10 HUMAN	411.5561067	3	0.83878541		
MEPTAPSLTEEDLTEVK	60.28001	O95999	BCL10 HUMAN	945.4566135	2	0.822592199		
MEPTAPSLTEEDLTEVK	60.28001	O95999	BCL10 HUMAN	630.6403507	3	0.822592199		
DLPDGPDAPADR	16.90034	O95721	SNP29 HUMAN	619.786573	2	0.777430594	2	Yes
DLPDGPDAPADR	16.90034	O95721	SNP29 HUMAN	413.5269903	3	0.777430594	2	
GAGSAMSTDAYPK	-2.647995	O95721	SNP29 HUMAN	628.2853475	2	0.773877203	2	Yes
GAGSAMSTDAYPK	-2.647995	O95721	SNP29 HUMAN	419.19284	3	0.773877203	2	
IDSNLDELSMGLGR	71.48077	O95721	SNP29 HUMAN	760.3752285	2	0.864822626	2	Yes
IDSNLDELSMGLGR	71.48077	O95721	SNP29 HUMAN	507.2527607	3	0.864822626	2	
SKPVETPPEQNGTLTSQPNNR	-7.862263	O95721	SNP29 HUMAN	1147.572752	2	0.636803389		
SKPVETPPEQNGTLTSQPNNR	-7.862263	O95721	SNP29 HUMAN	765.3844427	3	0.636803389		
SLALMYESEK	29.98246	O95721	SNP29 HUMAN	585.7897335	2	0.797201276	2	Yes
SLALMYESEK	29.98246	O95721	SNP29 HUMAN	390.8624307	3	0.797201276	2	
SVFGGLVNYFK	87.31995	O95721	SNP29 HUMAN	615.8300505	2	0.833782554		
SVFGGLVNYFK	87.31995	O95721	SNP29 HUMAN	410.8893087	3	0.833782554		
VGVASSEELAR	1.200157	O95721	SNP29 HUMAN	559.296576	2	0.784359157		
VGVASSEELAR	1.200157	O95721	SNP29 HUMAN	373.2003257	3	0.784359157		
ECPFLHIDPESK	36.0778	O95639	CPSF4 HUMAN	736.348483	2	0.774503052	3	
ECPFLHIDPESK	36.0778	O95639	CPSF4 HUMAN	491.2349303	3	0.774503052	3	Yes
FELPMGTTEQPPLPQQTQPPAK	67.11295	O95639	CPSF4 HUMAN	1218.117951	2	0.8398121	3	
FELPMGTTEQPPLPQQTQPPAK	67.11295	O95639	CPSF4 HUMAN	812.4145757	3	0.8398121	3	Yes
GDQCEFLHEYDMTK	30.17529	O95639	CPSF4 HUMAN	886.8669075	2	0.715602756		
GDQCEFLHEYDMTK	30.17529	O95639	CPSF4 HUMAN	591.5805467	3	0.715602756		
GFCKHGPLCR	-30.59781	O95639	CPSF4 HUMAN	616.2953345	2	0.606398523		
GFCKHGPLCR	-30.59781	O95639	CPSF4 HUMAN	411.199498	3	0.606398523		
MQEIIASVDHIK	33.46562	O95639	CPSF4 HUMAN	692.3692185	2	0.809560597		
MQEIIASVDHIK	33.46562	O95639	CPSF4 HUMAN	461.9154207	3	0.809560597		
SGAAVCEFFLK	61.3831	O95639	CPSF4 HUMAN	614.805723	2	0.677012324		
SGAAVCEFFLK	61.3831	O95639	CPSF4 HUMAN	410.2064237	3	0.677012324		
ALTPLTSSYR	23.98126	O95486	SC24A HUMAN	554.804033	2	0.777779222		
ALTPLTSSYR	23.98126	O95486	SC24A HUMAN	370.205297	3	0.777779222		
ANFLQNMIEDR	68.65892	O95486	SC24A HUMAN	675.8277175	2	0.845320344		
ANFLQNMIEDR	68.65892	O95486	SC24A HUMAN	450.8877533	3	0.845320344		
ASSQPTVSGNTSLTTNHQYVSSGYPSLQNSFIK	58.87947	O95486	SC24A HUMAN	1750.850595	2	0.658477545		
ASSQPTVSGNTSLTTNHQYVSSGYPSLQNSFIK	58.87947	O95486	SC24A HUMAN	1167.569671	3	0.658477545		
DALVNAVIDSLSAYR	116.4998	O95486	SC24A HUMAN	803.9259385	2	0.812765598		
DALVNAVIDSLSAYR	116.4998	O95486	SC24A HUMAN	536.2865673	3	0.812765598		
DIHMTPSTDFYK	32.189	O95486	SC24A HUMAN	727.8352045	2	0.805078745		
DIHMTPSTDFYK	32.189	O95486	SC24A HUMAN	485.5594113	3	0.805078745		
DLVQLPVVTSSTIVR	98.1528	O95486	SC24A HUMAN	813.9754365	2	0.872119784		
DLVQLPVVTSSTIVR	98.1528	O95486	SC24A HUMAN	542.9862327	3	0.872119784		
GLSIHTFHGNFFVR	42.22283	O95486	SC24A HUMAN	816.426248	2	0.710844517	3	
GLSIHTFHGNFFVR	42.22283	O95486	SC24A HUMAN	544.620107	3	0.710844517	3	Yes
LNCNPELFR	30.04517	O95486	SC24A HUMAN	581.787864	2	0.611194432		
LNCNPELFR	30.04517	O95486	SC24A HUMAN	388.1945177	3	0.611194432		
LPLGLLLHPFK	76.58064	O95486	SC24A HUMAN	624.397908	2	0.80032599	3	
LPLGLLLHPFK	76.58064	O95486	SC24A HUMAN	416.6012137	3	0.80032599	3	Yes
NMLPSTPLKPPVPNLHEDIQK	50.77946	O95486	SC24A HUMAN	1184.638852	2	0.848877311		
NMLPSTPLKPPVPNLHEDIQK	50.77946	O95486	SC24A HUMAN	790.095176	3	0.848877311		
NQPLVYLMLTTHPSLYR	86.25443	O95486	SC24A HUMAN	1023.546226	2	0.728864491		
NQPLVYLMLTTHPSLYR	86.25443	O95486	SC24A HUMAN	682.7000923	3	0.728864491		
SGPSVPPLVNPPLPTTFQPGAPHGPPPAGGPPPVR	84.79327	O95486	SC24A HUMAN	1693.904593	2	0.729692817		
SGPSVPPLVNPPLPTTFQPGAPHGPPPAGGPPPVR	84.79327	O95486	SC24A HUMAN	1129.60567	3	0.729692817		
SMTASLSDAR	-1.006729	O95486	SC24A HUMAN	519.7484045	2	0.759074569	2	Yes
SMTASLSDAR	-1.006729	O95486	SC24A HUMAN	346.834878	3	0.759074569	2	
TIPQPPILQLSVEK	75.6861	O95486	SC24A HUMAN	781.961798	2	0.856378675		
TIPQPPILQLSVEK	75.6861	O95486	SC24A HUMAN	521.643807	3	0.856378675		
TLETQSALGPALQAAFK	72.71964	O95486	SC24A HUMAN	873.4758015	2	0.846218586		
TLETQSALGPALQAAFK	72.71964	O95486	SC24A HUMAN	582.6531427	3	0.846218586		
VSGQSNYGGSQGSGQTLNRPPVASNPVTPSLHSC	42.57092	O95486	SC24A HUMAN	2092.547569	2	0.617205083		
VSGQSNYGGSQGSGQTLNRPPVASNPVTPSLHSC	42.57092	O95486	SC24A HUMAN	1395.367654	3	0.617205083		
TLPQMFTK	27.05695	O95486	SC24A HUMAN	483.2604205	2	0.703672051		
TLPQMFTK	27.05695	O95486	SC24A HUMAN	322.5095553	3	0.703672051		
TYINPFVSFLDQR	107.3159	O95486	SC24A HUMAN	800.4124675	2	0.672292113		
TYINPFVSFLDQR	107.3159	O95486	SC24A HUMAN	533.9442533	3	0.672292113		
VDNLSDEGALNISDR	41.34415	O95486	SC24A HUMAN	809.389921	2	0.860750437		
VDNLSDEGALNISDR	41.34415	O95486	SC24A HUMAN	539.9292223	3	0.860750437		
VNDVPEEFLYNPLTR	86.02419	O95486	SC24A HUMAN	903.457603	2	0.838559508	3	
VNDVPEEFLYNPLTR	86.02419	O95486	SC24A HUMAN	602.6410103	3	0.838559508	3	Yes
VVNLLQER	20.17078	O95486	SC24A HUMAN	485.78819	2	0.793367684	2	Yes
VVNLLQER	20.17078	O95486	SC24A HUMAN	324.194735	3	0.793367684	2	
YSAGSVYYYPSYHHQHNPVQVQK	12.77549	O95486	SC24A HUMAN	1376.649304	2	0.607694626		
YSAGSVYYYPSYHHQHNPVQVQK	12.77549	O95486	SC24A HUMAN	918.102144	3	0.607694626		
ATFCLPILGVK	90.4375	O95478	NSA2 HUMAN	609.849934	2	0.74053973	2	Yes
ATFCLPILGVK	90.4375	O95478	NSA2 HUMAN	406.9025643	3	0.74053973	2	
NPSSPLYTTLGVITK	66.71827	O95478	NSA2 HUMAN	795.941058	2	0.716856539	2	Yes

NPSSPLYTTLGVITK	66.71827	O95478	NSA2 HUMAN	530.9633137	3	0.716856539	2	
TPQGAVPAYLLDR	52.4004	O95478	NSA2 HUMAN	700.880804	2	0.700565279	2	Yes
TPQGAVPAYLLDR	52.4004	O95478	NSA2 HUMAN	467.589811	3	0.700565279	2	
VCFVGDGFTR	37.63432	O95478	NSA2 HUMAN	579.2745905	2	0.752282321	2	Yes
VCFVGDGFTR	37.63432	O95478	NSA2 HUMAN	386.519002	3	0.752282321	2	
AGSVSLDSVLADVR	69.56522	O95466	FMNL1 HUMAN	694.873179	2	0.740872324		
AGSVSLDSVLADVR	69.56522	O95466	FMNL1 HUMAN	463.5847277	3	0.740872324		
APPAAPTRPSALELK	16.44427	O95466	FMNL1 HUMAN	759.9361145	2	0.654666126	3	
APPAAPTRPSALELK	16.44427	O95466	FMNL1 HUMAN	506.960018	3	0.654666126	3	Yes
DGAIEDIITVIK	110.7019	O95466	FMNL1 HUMAN	643.864292	2	0.836555004		
DGAIEDIITVIK	110.7019	O95466	FMNL1 HUMAN	429.578803	3	0.836555004		
EPLIYESDR	17.19895	O95466	FMNL1 HUMAN	561.2778475	2	0.621784925		
EPLIYESDR	17.19895	O95466	FMNL1 HUMAN	374.5211733	3	0.621784925		
FLPTEYER	18.78181	O95466	FMNL1 HUMAN	527.7643765	2	0.844764888		
FLPTEYER	18.78181	O95466	FMNL1 HUMAN	352.1788593	3	0.844764888		
FSESTAMGPSR	-2.87265	O95466	FMNL1 HUMAN	585.2669615	2	0.695912123		
FSESTAMGPSR	-2.87265	O95466	FMNL1 HUMAN	390.513916	3	0.695912123		
GGHDIILAAFDNFK	74.6856	O95466	FMNL1 HUMAN	759.3915395	2	0.792942524	3	
GGHDIILAAFDNFK	74.6856	O95466	FMNL1 HUMAN	506.596968	3	0.792942524	3	Yes
GLELTQR	1.508343	O95466	FMNL1 HUMAN	408.732884	2	0.647851765		
GLELTQR	1.508343	O95466	FMNL1 HUMAN	272.824531	3	0.647851765		
LLCEASLGEEMPL	92.68095	O95466	FMNL1 HUMAN	731.352377	2	0.735140085		
LLCEASLGEEMPL	92.68095	O95466	FMNL1 HUMAN	487.904193	3	0.735140085		
LQSLDALLEMK	79.1996	O95466	FMNL1 HUMAN	630.8475875	2	0.790460527	2	Yes
LQSLDALLEMK	79.1996	O95466	FMNL1 HUMAN	420.901	3	0.790460527	2	
NAVLEHMEELQEQVALLTER	98.08908	O95466	FMNL1 HUMAN	1176.597381	2	0.741152644		
NAVLEHMEELQEQVALLTER	98.08908	O95466	FMNL1 HUMAN	784.7341957	3	0.741152644		
NKPLEQSVEDLSK	9.41291	O95466	FMNL1 HUMAN	743.8915685	2	0.77260375		
NKPLEQSVEDLSK	9.41291	O95466	FMNL1 HUMAN	496.263654	3	0.77260375		
NPPAAYIQK	-5.539032	O95466	FMNL1 HUMAN	501.2749115	2	0.727463186		
NPPAAYIQK	-5.539032	O95466	FMNL1 HUMAN	334.519216	3	0.727463186		
RPQMDLISELK	40.81354	O95466	FMNL1 HUMAN	665.363936	2	0.688597858		
RPQMDLISELK	40.81354	O95466	FMNL1 HUMAN	443.911899	3	0.688597858		
SQGPSLDLSALK	43.91759	O95466	FMNL1 HUMAN	608.3331585	2	0.809325993	2	Yes
SQGPSLDLSALK	43.91759	O95466	FMNL1 HUMAN	405.8913807	3	0.809325993	2	
TAQEAFESVVEYFGENPK	94.51411	O95466	FMNL1 HUMAN	1022.979097	2	0.757806659		
TAQEAFESVVEYFGENPK	94.51411	O95466	FMNL1 HUMAN	682.3220063	3	0.757806659		
TNHIGWVQEFLNEENR	64.60774	O95466	FMNL1 HUMAN	993.477394	2	0.654517174	3	
TNHIGWVQEFLNEENR	64.60774	O95466	FMNL1 HUMAN	662.6542043	3	0.654517174	3	Yes
TTSPGLFFSLFSR	114.1375	O95466	FMNL1 HUMAN	730.383183	2	0.747973859		
TTSPGLFFSLFSR	114.1375	O95466	FMNL1 HUMAN	487.2580637	3	0.747973859		
VAADWMSNLGFK	71.26584	O95466	FMNL1 HUMAN	669.829729	2	0.807622313		
VAADWMSNLGFK	71.26584	O95466	FMNL1 HUMAN	446.8890943	3	0.807622313		
VLQELDMSDFEEQFK	86.41159	O95466	FMNL1 HUMAN	929.432941	2	0.881170332		
VLQELDMSDFEEQFK	86.41159	O95466	FMNL1 HUMAN	619.9579023	3	0.881170332		
VQLLSQYDNEK	22.21694	O95466	FMNL1 HUMAN	668.8413435	2	0.854845405		
VQLLSQYDNEK	22.21694	O95466	FMNL1 HUMAN	446.2301707	3	0.854845405		
YPQLTGFHSDLHFLDK	53.70183	O95466	FMNL1 HUMAN	959.4788705	2	0.822816133	4	
YPQLTGFHSDLHFLDK	53.70183	O95466	FMNL1 HUMAN	639.988522	3	0.822816133	4	
DHVLHVTFPK	13.39126	O95453	PARN HUMAN	596.827847	2	0.779935837		
DHVLHVTFPK	13.39126	O95453	PARN HUMAN	398.221173	3	0.779935837		
DIINNTSLAELEK	60.22968	O95453	PARN HUMAN	730.386119	2	0.838728905	2	Yes
DIINNTSLAELEK	60.22968	O95453	PARN HUMAN	487.260021	3	0.838728905	2	
EMTTCVFPR	31.51455	O95453	PARN HUMAN	570.7630015	2	0.676600218	2	Yes
EMTTCVFPR	31.51455	O95453	PARN HUMAN	380.8446093	3	0.676600218	2	
EQEELNDAVGFSR	33.08704	O95453	PARN HUMAN	747.3475255	2	0.755609632	2	Yes
EQEELNDAVGFSR	33.08704	O95453	PARN HUMAN	498.5676253	3	0.755609632	2	
FVCQSSSIDFLASQGFDFNK	94.41252	O95453	PARN HUMAN	1149.031342	2	0.612855673		
FVCQSSSIDFLASQGFDFNK	94.41252	O95453	PARN HUMAN	766.3568363	3	0.612855673		
GIHVETLETEK	0.038208	O95453	PARN HUMAN	628.3306165	2	0.822253346	3	
GIHVETLETEK	0.038208	O95453	PARN HUMAN	419.2230193	3	0.822253346	3	Yes
IEDLLQSEENK	26.43867	O95453	PARN HUMAN	659.3308125	2	0.753519177	2	Yes
IEDLLQSEENK	26.43867	O95453	PARN HUMAN	439.8898167	3	0.753519177	2	
IQTYAEYMGR	23.70083	O95453	PARN HUMAN	616.2929715	2	0.788275242		
IQTYAEYMGR	23.70083	O95453	PARN HUMAN	411.1979227	3	0.788275242		
LIEPFFNK	54.35979	O95453	PARN HUMAN	504.282209	2	0.63738656	2	Yes
LIEPFFNK	54.35979	O95453	PARN HUMAN	336.524081	3	0.63738656	2	
LIYQTLSWK	50.69048	O95453	PARN HUMAN	576.327144	2	0.751882911		
LIYQTLSWK	50.69048	O95453	PARN HUMAN	384.5540377	3	0.751882911		
LNPQCIPYTLQNHYYR	46.19767	O95453	PARN HUMAN	1040.507628	2	0.773386598		
LNPQCIPYTLQNHYYR	46.19767	O95453	PARN HUMAN	694.0076937	3	0.773386598		
NLDLEPCTGFQR	42.0426	O95453	PARN HUMAN	725.3437325	2	0.738241971	2	Yes
NLDLEPCTGFQR	42.0426	O95453	PARN HUMAN	483.89843	3	0.738241971	2	
NNSFTAPSTVGK	2.201485	O95453	PARN HUMAN	611.807308	2	0.619126439		
NNSFTAPSTVGK	2.201485	O95453	PARN HUMAN	408.2074803	3	0.619126439		
NSPATLFEVPDTW	127.522	O95453	PARN HUMAN	738.8544555	2	0.768957078		
NSPATLFEVPDTW	127.522	O95453	PARN HUMAN	492.9055787	3	0.768957078		
SFNFYVFPKPFNR	75.85354	O95453	PARN HUMAN	831.9255445	2	0.678050995		
SFNFYVFPKPFNR	75.85354	O95453	PARN HUMAN	554.9529713	3	0.678050995		
VMDIPYLNLEGPDLQPK	100.4168	O95453	PARN HUMAN	971.5036965	2	0.809515178	3	
VMDIPYLNLEGPDLQPK	100.4168	O95453	PARN HUMAN	648.0050727	3	0.809515178	3	Yes
ADALYELDENGNSR	32.38358	O95427	PIGN HUMAN	783.85571	2	0.729993165		
ADALYELDENGNSR	32.38358	O95427	PIGN HUMAN	522.906415	3	0.729993165		
AESMFTNAVQILEQFK	131.0859	O95427	PIGN HUMAN	928.4671185	2	0.757537484		
AESMFTNAVQILEQFK	131.0859	O95427	PIGN HUMAN	619.3140207	3	0.757537484		
ELIHLALK	27.69002	O95427	PIGN HUMAN	468.7980265	2	0.7110461		
ELIHLALK	27.69002	O95427	PIGN HUMAN	312.8679593	3	0.7110461		
ENPVEFDSLFNESK	80.74306	O95427	PIGN HUMAN	827.88394	2	0.760415673		
ENPVEFDSLFNESK	80.74306	O95427	PIGN HUMAN	552.2585683	3	0.760415673		
EVTLPFLFTPFK	128.485	O95427	PIGN HUMAN	719.9032205	2	0.696899951		
EVTLPFLFTPFK	128.485	O95427	PIGN HUMAN	480.271422	3	0.696899951		
GASGDHVYTYSYDAK	-5.644753	O95427	PIGN HUMAN	817.3606205	2	0.838803589		
GASGDHVYTYSYDAK	-5.644753	O95427	PIGN HUMAN	545.243022	3	0.838803589		
GLSYYHTYDR	-0.429409	O95427	PIGN HUMAN	637.794188	2	0.735003173		
GLSYYHTYDR	-0.429409	O95427	PIGN HUMAN	425.532067	3	0.735003173		
LDTWVFDNVK	65.09669	O95427	PIGN HUMAN	618.8171445	2	0.859056115		
LDTWVFDNVK	65.09669	O95427	PIGN HUMAN	412.8807047	3	0.859056115		
LVLFVADGLR	74.04866	O95427	PIGN HUMAN	551.8351405	2	0.757091939		
LVLFVADGLR	74.04866	O95427	PIGN HUMAN	368.2260353	3	0.757091939		
NIIMHEGSWGISHTR	17.16332	O95427	PIGN HUMAN	869.4286615	2	0.808270097		
NIIMHEGSWGISHTR	17.16332	O95427	PIGN HUMAN	579.9550493	3	0.808270097		

FIFTSDHGMTDWGSHGAGHPSETLTPLVTWGAC	86.9617	O95427	PIGN_HUMAN	1956.93669	2	0.677604616		
FIFTSDHGMTDWGSHGAGHPSETLTPLVTWGAC	86.9617	O95427	PIGN_HUMAN	1304.960401	3	0.677604616		
VSAQQFDDAFLK	50.33722	O95427	PIGN_HUMAN	684.843891	2	0.851749301		
VSAQQFDDAFLK	50.33722	O95427	PIGN_HUMAN	456.8985357	3	0.851749301		
DYTGCSTSESLSPVK	24.05484	O95297	MPZL1_HUMAN	815.8674295	2	0.740755737		
DYTGCSTSESLSPVK	24.05484	O95297	MPZL1_HUMAN	544.2475613	3	0.740755737		
ISWAGDLDK	30.84199	O95297	MPZL1_HUMAN	502.7565555	2	0.766676068	2	Yes
ISWAGDLDK	30.84199	O95297	MPZL1_HUMAN	335.5069787	3	0.766676068	2	
NPPDIVVQPGHIR	23.32202	O95297	MPZL1_HUMAN	721.399699	2	0.778465807	3	
NPPDIVVQPGHIR	23.32202	O95297	MPZL1_HUMAN	481.2690743	3	0.778465807	3	Yes
DILQDYTHEFHK	41.48189	O95249	GOSR1_HUMAN	773.3708	2	0.827499151	3	
DILQDYTHEFHK	41.48189	O95249	GOSR1_HUMAN	515.916475	3	0.827499151	3	Yes
FPAVNSLIQR	47.43902	O95249	GOSR1_HUMAN	572.8278465	2	0.847376347		
FPAVNSLIQR	47.43902	O95249	GOSR1_HUMAN	382.2211727	3	0.847376347		
LIEETISIAMATK	66.03907	O95249	GOSR1_HUMAN	710.3923595	2	0.782842457		
LIEETISIAMATK	66.03907	O95249	GOSR1_HUMAN	473.930848	3	0.782842457		
MAEYTNSAGVPSLNAALMHTLQR	76.32656	O95249	GOSR1_HUMAN	1238.110129	2	0.677088141	3	
MAEYTNSAGVPSLNAALMHTLQR	76.32656	O95249	GOSR1_HUMAN	825.742694	3	0.677088141	3	Yes
MFETMAIEIEQLLAR	136.9361	O95249	GOSR1_HUMAN	897.960615	2	0.622849941		
MFETMAIEIEQLLAR	136.9361	O95249	GOSR1_HUMAN	598.9763517	3	0.622849941		
AACIAEQYHTVLK	15.92171	O95235	KI20A_HUMAN	752.3853965	2	0.840900242		
AACIAEQYHTVLK	15.92171	O95235	KI20A_HUMAN	501.926206	3	0.840900242		
ADTGLDDDIENEADISMYGK	66.68134	O95235	KI20A_HUMAN	1086.468433	2	0.812250137		
ADTGLDDDIENEADISMYGK	66.68134	O95235	KI20A_HUMAN	724.6482303	3	0.812250137		
AELNSTTEELHK	-16.13541	O95235	KI20A_HUMAN	686.341712	2	0.78293252		
AELNSTTEELHK	-16.13541	O95235	KI20A_HUMAN	457.897083	3	0.78293252		
DEICNEMVEQMQQR	44.9441	O95235	KI20A_HUMAN	905.382403	2	0.782525182		
DEICNEMVEQMQQR	44.9441	O95235	KI20A_HUMAN	603.9242103	3	0.782525182		
DLNWIHVQDAEEAWK	73.23682	O95235	KI20A_HUMAN	927.4450315	2	0.81870234		
DLNWIHVQDAEEAWK	73.23682	O95235	KI20A_HUMAN	618.6326293	3	0.81870234		
EAGNINTSLHTLGR	15.63528	O95235	KI20A_HUMAN	741.8871525	2	0.622303426		
EAGNINTSLHTLGR	15.63528	O95235	KI20A_HUMAN	494.9273767	3	0.622303426		
EELLQVVEAMK	79.96579	O95235	KI20A_HUMAN	644.845045	2	0.808713913		
EELLQVVEAMK	79.96579	O95235	KI20A_HUMAN	430.2326383	3	0.808713913		
EHSLQVSPSLEK	1.20219	O95235	KI20A_HUMAN	677.354622	2	0.778558493		
EHSLQVSPSLEK	1.20219	O95235	KI20A_HUMAN	451.9056897	3	0.778558493		
ELLEEMYEEK	46.14579	O95235	KI20A_HUMAN	656.803038	2	0.810356736		
ELLEEMYEEK	46.14579	O95235	KI20A_HUMAN	438.2046337	3	0.810356736		
EQWCSEHLDTQK	-6.49976	O95235	KI20A_HUMAN	780.841554	2	0.767236888		
EQWCSEHLDTQK	-6.49976	O95235	KI20A_HUMAN	520.8969777	3	0.767236888		
ESLTSFYQEEIQER	58.21273	O95235	KI20A_HUMAN	879.9132255	2	0.85185796		
ESLTSFYQEEIQER	58.21273	O95235	KI20A_HUMAN	586.9447587	3	0.85185796		
FSAIASQLVHAPPMQLGFPSLHSFIK	104.7385	O95235	KI20A_HUMAN	1412.254915	2	0.657623053		
FSAIASQLVHAPPMQLGFPSLHSFIK	104.7385	O95235	KI20A_HUMAN	941.839218	3	0.657623053		
IEELEALLQEAR	81.5406	O95235	KI20A_HUMAN	707.38338	2	0.767772675		
IEELEALLQEAR	81.5406	O95235	KI20A_HUMAN	471.9248617	3	0.767772675		
IENVETLVLQAPK	61.12566	O95235	KI20A_HUMAN	727.417223	2	0.806297481		
IENVETLVLQAPK	61.12566	O95235	KI20A_HUMAN	485.280757	3	0.806297481		
ILHLQGEGDIVPK	27.71948	O95235	KI20A_HUMAN	709.904283	2	0.865563095		
ILHLQGEGDIVPK	27.71948	O95235	KI20A_HUMAN	473.6054637	3	0.865563095		
ISELSLCDLAGSER	57.94885	O95235	KI20A_HUMAN	775.380511	2	0.780359387		
ISELSLCDLAGSER	57.94885	O95235	KI20A_HUMAN	517.2562823	3	0.780359387		
LAASASTQQLQEVK	12.77361	O95235	KI20A_HUMAN	737.399562	2	0.799156606		
LAASASTQQLQEVK	12.77361	O95235	KI20A_HUMAN	491.9356497	3	0.799156606		
LGESLQSAER	-5.81842	O95235	KI20A_HUMAN	545.280926	2	0.787197888		
LGESLQSAER	-5.81842	O95235	KI20A_HUMAN	363.856559	3	0.787197888		
LGTNQENQQPNQQPPGK	-22.19372	O95235	KI20A_HUMAN	939.4592025	2	0.71218735		
LGTNQENQQPNQQPPGK	-22.19372	O95235	KI20A_HUMAN	626.6420767	3	0.71218735		
LQLEMHLR	24.07121	O95235	KI20A_HUMAN	520.290043	2	0.753462851		
LQLEMHLR	24.07121	O95235	KI20A_HUMAN	347.1959703	3	0.753462851		
LSLLNGGLQEEELSTSLK	80.00031	O95235	KI20A_HUMAN	966.0207685	2	0.62835288		
LSLLNGGLQEEELSTSLK	80.00031	O95235	KI20A_HUMAN	644.3497873	3	0.62835288		
MLEPPPSAKPFTIDVDK	49.54304	O95235	KI20A_HUMAN	942.992969	2	0.783156335		
MLEPPPSAKPFTIDVDK	49.54304	O95235	KI20A_HUMAN	628.997921	3	0.783156335		
NLLSDCSVVSTSLEDK	60.79843	O95235	KI20A_HUMAN	883.928022	2	0.763270795		
NLLSDCSVVSTSLEDK	60.79843	O95235	KI20A_HUMAN	589.6212897	3	0.763270795		
QALTTCDDILIK	45.04401	O95235	KI20A_HUMAN	695.8665095	2	0.605385244		
QALTTCDDILIK	45.04401	O95235	KI20A_HUMAN	464.246948	3	0.605385244		
SVYIESR	-9.782696	O95235	KI20A_HUMAN	427.2248865	2	0.654347599		
SVYIESR	-9.782696	O95235	KI20A_HUMAN	285.1525327	3	0.654347599		
VFQGFFTGR	48.40929	O95235	KI20A_HUMAN	529.7750835	2	0.718365431		
VFQGFFTGR	48.40929	O95235	KI20A_HUMAN	353.5193307	3	0.718365431		
VRPLLPSELER	27.94905	O95235	KI20A_HUMAN	654.8858925	2	0.805016041		
VRPLLPSELER	27.94905	O95235	KI20A_HUMAN	436.9265367	3	0.805016041		
WAQPDTAPLPVPANIR	68.75546	O95235	KI20A_HUMAN	873.470853	2	0.730901599		
WAQPDTAPLPVPANIR	68.75546	O95235	KI20A_HUMAN	582.6498437	3	0.730901599		
IFPGDTILETGEVIPPMK	97.40646	O95139	NDUB6_HUMAN	979.021727	2	0.825025976	3	
IFPGDTILETGEVIPPMK	97.40646	O95139	NDUB6_HUMAN	653.017093	3	0.825025976	3	Yes
YHVSEKPYGIVEK	-3.673233	O95139	NDUB6_HUMAN	774.9070135	2	0.776281416		
YHVSEKPYGIVEK	-3.673233	O95139	NDUB6_HUMAN	516.9406173	3	0.776281416		
LWSSLTLLGSYK	84.12968	O95059	RPP14_HUMAN	684.382647	2	0.845176101		
LWSSLTLLGSYK	84.12968	O95059	RPP14_HUMAN	456.5910397	3	0.845176101		
AAQTTIETAIHSLIETLK	103.1676	O94915	FRYL_HUMAN	970.53913	2	0.84263289		
AAQTTIETAIHSLIETLK	103.1676	O94915	FRYL_HUMAN	647.3620283	3	0.84263289		
AIANVFQNR	17.79094	O94915	FRYL_HUMAN	516.783439	2	0.680141568		
AIANVFQNR	17.79094	O94915	FRYL_HUMAN	344.8582343	3	0.680141568		
ALFALLEIPK	102.1801	O94915	FRYL_HUMAN	557.8477165	2	0.750583768		
ALFALLEIPK	102.1801	O94915	FRYL_HUMAN	372.2344193	3	0.750583768		
DCSNWINVVCR	57.84085	O94915	FRYL_HUMAN	711.816827	2	0.654389799		
DCSNWINVVCR	57.84085	O94915	FRYL_HUMAN	474.880493	3	0.654389799		
DETPTLEASLDNANSR	41.17887	O94915	FRYL_HUMAN	866.903393	2	0.703128994		
DETPTLEASLDNANSR	41.17887	O94915	FRYL_HUMAN	578.271537	3	0.703128994		
DGEPPMPTTGVILPSGNTLR	81.01604	O94915	FRYL_HUMAN	1026.525697	2	0.832234263		
DGEPPMPTTGVILPSGNTLR	81.01604	O94915	FRYL_HUMAN	684.686406	3	0.832234263		
EAAEQWLDDCK	31.69669	O94915	FRYL_HUMAN	682.793541	2	0.856197238		
EAAEQWLDDCK	31.69669	O94915	FRYL_HUMAN	455.5316357	3	0.856197238		
ELIEELHPIIK	53.44701	O94915	FRYL_HUMAN	667.3904765	2	0.805608273		
ELIEELHPIIK	53.44701	O94915	FRYL_HUMAN	445.262926	3	0.805608273		
EVTDVHPTLLDNAVK	38.94023	O94915	FRYL_HUMAN	825.939051	2	0.842427373		
EVTDVHPTLLDNAVK	38.94023	O94915	FRYL_HUMAN	550.9619757	3	0.842427373		
FGEITNEAVSFLGDSLQR	97.22768	O94915	FRYL_HUMAN	991.9950855	2	0.778289557		

FGEITNEAVSFLGDSLQR	97.22768	O94915	FRYL_HUMAN	661.6659987	3	0.778289557		
FGVLELQEHLDTYNVK	68.18013	O94915	FRYL_HUMAN	952.9918105	2	0.881019711		
FGVLELQEHLDTYNVK	68.18013	O94915	FRYL_HUMAN	635.6638153	3	0.881019711		
GDDELAIDVMDR	55.19159	O94915	FRYL_HUMAN	674.806648	2	0.812605858		
GDDELAIDVMDR	55.19159	O94915	FRYL_HUMAN	450.2070403	3	0.812605858		
GFTSASTQEMTVHLLSK	46.65385	O94915	FRYL_HUMAN	918.9622005	2	0.830617666		
GFTSASTQEMTVHLLSK	46.65385	O94915	FRYL_HUMAN	612.9774087	3	0.830617666		
GPLWNHEDVSAK	4.015411	O94915	FRYL_HUMAN	676.833857	2	0.633799434		
GPLWNHEDVSAK	4.015411	O94915	FRYL_HUMAN	451.558513	3	0.633799434		
HYDLLSALSQTSYHDPIMGNK	71.01962	O94915	FRYL_HUMAN	1195.57644	2	0.646574318		
HYDLLSALSQTSYHDPIMGNK	71.01962	O94915	FRYL_HUMAN	797.3869013	3	0.646574318		
IIGIPSPSSLFK	84.15189	O94915	FRYL_HUMAN	629.874462	2	0.769005656		
IIGIPSPSSLFK	84.15189	O94915	FRYL_HUMAN	420.2522497	3	0.769005656		
IPSVTSGTTSSSNTMVAPTDGNPDNKPIK	31.63492	O94915	FRYL_HUMAN	1458.716941	2	0.759709716		
IPSVTSGTTSSSNTMVAPTDGNPDNKPIK	31.63492	O94915	FRYL_HUMAN	972.8139023	3	0.759709716		
LFQTIQR	9.302055	O94915	FRYL_HUMAN	453.2643515	2	0.788043678		
LFQTIQR	9.302055	O94915	FRYL_HUMAN	302.512176	3	0.788043678		
LINQVNTIK	5.388214	O94915	FRYL_HUMAN	521.8169475	2	0.627680302		
LINQVNTIK	5.388214	O94915	FRYL_HUMAN	348.2139067	3	0.627680302		
LLIHLPLDK	46.12747	O94915	FRYL_HUMAN	531.3400585	2	0.775710881		
LLIHLPLDK	46.12747	O94915	FRYL_HUMAN	354.5626473	3	0.775710881		
LLWVYYVIR	84.70073	O94915	FRYL_HUMAN	531.3294895	2	0.600894332		
LLWVYYVIR	84.70073	O94915	FRYL_HUMAN	354.5556013	3	0.600894332		
LMELNLEIR	63.41353	O94915	FRYL_HUMAN	565.81609	2	0.739730656		
LMELNLEIR	63.41353	O94915	FRYL_HUMAN	377.5466683	3	0.739730656		
LMNVLSLCGPESGLPK	82.1873	O94915	FRYL_HUMAN	857.947507	2	0.772450924		
LMNVLSLCGPESGLPK	82.1873	O94915	FRYL_HUMAN	572.3009463	3	0.772450924		
LMSIVSALFPK	102.2752	O94915	FRYL_HUMAN	603.3523085	2	0.781985939		
LMSIVSALFPK	102.2752	O94915	FRYL_HUMAN	402.570814	3	0.781985939		
LPEDTTSVLK	23.43487	O94915	FRYL_HUMAN	551.803703	2	0.74379611		
LPEDTTSVLK	23.43487	O94915	FRYL_HUMAN	368.205077	3	0.74379611		
LQLLSPQVDINSPINAK	80.64273	O94915	FRYL_HUMAN	925.5232815	2	0.827427387		
LQLLSPQVDINSPINAK	80.64273	O94915	FRYL_HUMAN	617.3514627	3	0.827427387		
LTIHMDEELR	28.89678	O94915	FRYL_HUMAN	628.8193615	2	0.784966111		
LTIHMDEELR	28.89678	O94915	FRYL_HUMAN	419.5488493	3	0.784966111		
MLVQLINQWK	80.59628	O94915	FRYL_HUMAN	636.8608325	2	0.75279355		
MLVQLINQWK	80.59628	O94915	FRYL_HUMAN	424.90983	3	0.75279355		
NEAEVINMSEELAQLESILK	148.0656	O94915	FRYL_HUMAN	1130.573039	2	0.682436585		
NEAEVINMSEELAQLESILK	148.0656	O94915	FRYL_HUMAN	754.051301	3	0.682436585		
SAEQLTTFLK	47.26174	O94915	FRYL_HUMAN	569.3116955	2	0.824092746	2	Yes
SAEQLTTFLK	47.26174	O94915	FRYL_HUMAN	379.877072	3	0.824092746	2	
SASLVVPSDIPK	40.18883	O94915	FRYL_HUMAN	606.8459015	2	0.840404868		
SASLVVPSDIPK	40.18883	O94915	FRYL_HUMAN	404.899876	3	0.840404868		
SDSMPLYSNWR	45.02418	O94915	FRYL_HUMAN	678.306613	2	0.804599881		
SDSMPLYSNWR	45.02418	O94915	FRYL_HUMAN	452.5403503	3	0.804599881		
SIFPQQSLR	28.18887	O94915	FRYL_HUMAN	538.298922	2	0.745876908		
SIFPQQSLR	28.18887	O94915	FRYL_HUMAN	359.2018897	3	0.745876908		
SIYEVAMQLLQILEPK	168.0225	O94915	FRYL_HUMAN	938.0189825	2	0.725100875		
SIYEVAMQLLQILEPK	168.0225	O94915	FRYL_HUMAN	625.68193	3	0.725100875		
SLFAEFAVQAEK	73.28756	O94915	FRYL_HUMAN	670.348809	2	0.82755518		
SLFAEFAVQAEK	73.28756	O94915	FRYL_HUMAN	447.2351477	3	0.82755518		
SNTLDIMDGR	29.53211	O94915	FRYL_HUMAN	561.2669615	2	0.807523906		
SNTLDIMDGR	29.53211	O94915	FRYL_HUMAN	374.513916	3	0.807523906		
STSSTSSGSNSNALVPVSWK	47.22339	O94915	FRYL_HUMAN	998.4850805	2	0.759302855		
STSSTSSGSNSNALVPVSWK	47.22339	O94915	FRYL_HUMAN	665.992662	3	0.759302855		
TDLIELLAR	86.75948	O94915	FRYL_HUMAN	522.3089555	2	0.783089936	2	Yes
TDLIELLAR	86.75948	O94915	FRYL_HUMAN	348.541912	3	0.783089936	2	
TFTINPER	13.51138	O94915	FRYL_HUMAN	489.256723	2	0.613840938		
TFTINPER	13.51138	O94915	FRYL_HUMAN	326.5070903	3	0.613840938		
TLDFHFDISETPIIGNK	85.9666	O94915	FRYL_HUMAN	973.997097	2	0.856945753		
TLDFHFDISETPIIGNK	85.9666	O94915	FRYL_HUMAN	649.6673397	3	0.856945753		
TLFDWYR	71.08936	O94915	FRYL_HUMAN	500.7485295	2	0.688278735		
TLFDWYR	71.08936	O94915	FRYL_HUMAN	334.1682947	3	0.688278735		
TLMEFITSR	65.39052	O94915	FRYL_HUMAN	549.287166	2	0.721651018		
TLMEFITSR	65.39052	O94915	FRYL_HUMAN	366.5273857	3	0.721651018		
TLTDEEAK	-26.61802	O94915	FRYL_HUMAN	453.7249215	2	0.63072443		
TLTDEEAK	-26.61802	O94915	FRYL_HUMAN	302.8192227	3	0.63072443		
MLNSDSATDETIPDHPDLLLQSEDSTGSITTEEVL	105.9484	O94915	FRYL_HUMAN	2200.550859	2	0.710798144		
MLNSDSATDETIPDHPDLLLQSEDSTGSITTEEVL	105.9484	O94915	FRYL_HUMAN	1467.369847	3	0.710798144		
TVASVLLR	30.66648	O94915	FRYL_HUMAN	429.7745515	2	0.781760037		
TVASVLLR	30.66648	O94915	FRYL_HUMAN	286.8523093	3	0.781760037		
TYGGDTGSPEISFTK	33.11458	O94915	FRYL_HUMAN	780.3653805	2	0.819593191		
TYGGDTGSPEISFTK	33.11458	O94915	FRYL_HUMAN	520.5795287	3	0.819593191		
VFLVIADSLQQK	72.79623	O94915	FRYL_HUMAN	680.8959265	2	0.825699449		
VFLVIADSLQQK	72.79623	O94915	FRYL_HUMAN	454.2665593	3	0.825699449		
VIGMSVYYPQVR	54.83537	O94915	FRYL_HUMAN	706.3742955	2	0.725799441		
VIGMSVYYPQVR	54.83537	O94915	FRYL_HUMAN	471.2521387	3	0.725799441		
VNTTTSSDSYIGLWR	60.67022	O94915	FRYL_HUMAN	850.418478	2	0.649892509		
VNTTTSSDSYIGLWR	60.67022	O94915	FRYL_HUMAN	567.2815937	3	0.649892509		
WLDNILDSLDK	98.91545	O94915	FRYL_HUMAN	666.3462655	2	0.807559371		
WLDNILDSLDK	98.91545	O94915	FRYL_HUMAN	444.5667853	3	0.807559371		
WTNFPGLQQLFLK	113.4783	O94915	FRYL_HUMAN	796.4357505	2	0.710885942		
WTNFPGLQQLFLK	113.4783	O94915	FRYL_HUMAN	531.2931087	3	0.710885942		
YGDELAWSEVENVWTTLADGWPK	156.8999	O94915	FRYL_HUMAN	1333.624646	2	0.747664511		
YGDELAWSEVENVWTTLADGWPK	156.8999	O94915	FRYL_HUMAN	889.4190387	3	0.747664511		
AQLAVSLSVQQETSNLGPGSAPSK	60.61041	O94913	PCF11_HUMAN	1185.119535	2	0.767485142		
AQLAVSLSVQQETSNLGPGSAPSK	60.61041	O94913	PCF11_HUMAN	790.4156313	3	0.767485142		
AYPDNHLSQVDVNELFSK	60.02376	O94913	PCF11_HUMAN	1038.505813	2	0.847093701	3	
AYPDNHLSQVDVNELFSK	60.02376	O94913	PCF11_HUMAN	692.67315	3	0.847093701	3	Yes
DYQSSLEDLTFNSKPHINMLTILAEENLPFAK	121.4876	O94913	PCF11_HUMAN	1839.922178	2	0.621683896		
DYQSSLEDLTFNSKPHINMLTILAEENLPFAK	121.4876	O94913	PCF11_HUMAN	1226.950727	3	0.621683896		
EFLMNTLNQSDTK	45.9538	O94913	PCF11_HUMAN	770.8697795	2	0.730079293		
EFLMNTLNQSDTK	45.9538	O94913	PCF11_HUMAN	514.249128	3	0.730079293		
EIVSLIEAQTAK	51.40657	O94913	PCF11_HUMAN	651.3697415	2	0.69991082		
EIVSLIEAQTAK	51.40657	O94913	PCF11_HUMAN	434.582436	3	0.69991082		
FAGLDTNQR	3.394623	O94913	PCF11_HUMAN	511.2572545	2	0.795329392		
FAGLDTNQR	3.394623	O94913	PCF11_HUMAN	341.1741113	3	0.795329392		
FDGLHGQPGPR	-9.277863	O94913	PCF11_HUMAN	590.7970785	2	0.775912464		
FDGLHGQPGPR	-9.277863	O94913	PCF11_HUMAN	394.2006607	3	0.775912464		
FDGPHGQPGGGIR	-17.12349	O94913	PCF11_HUMAN	647.8185425	2	0.737194657		
FDGPHGQPGGGIR	-17.12349	O94913	PCF11_HUMAN	432.21497	3	0.737194657		

FDGPPGQQVQPR	10.46134	O94913	PCF11_HUMAN	663.3340255	2	0.766401649		
FDGPPGQQVQPR	10.46134	O94913	PCF11_HUMAN	442.5586253	3	0.766401649		
FDGQPGQPSLLPR	43.58162	O94913	PCF11_HUMAN	706.3706075	2	0.842247128		
FDGQPGQPSLLPR	43.58162	O94913	PCF11_HUMAN	471.24968	3	0.842247128		
FDGSPGQMGGGGPLR	31.48929	O94913	PCF11_HUMAN	716.836075	2	0.791665673		
FDGSPGQMGGGGPLR	31.48929	O94913	PCF11_HUMAN	478.2266583	3	0.791665673		
FDIPLGLQGTR	74.38823	O94913	PCF11_HUMAN	608.838412	2	0.842237592		
FDIPLGLQGTR	74.38823	O94913	PCF11_HUMAN	406.2282163	3	0.842237592		
FEGGHGPSGAAIR	-22.62408	O94913	PCF11_HUMAN	628.3130925	2	0.756922603		
FEGGHGPSGAAIR	-22.62408	O94913	PCF11_HUMAN	419.2113367	3	0.756922603		
FEGPGGQPVGGLR	24.57568	O94913	PCF11_HUMAN	635.8311185	2	0.770380378		
FEGPGGQPVGGLR	24.57568	O94913	PCF11_HUMAN	424.223354	3	0.770380378		
FEGPHGQPGVGIR	0.70792	O94913	PCF11_HUMAN	675.8498425	2	0.777505577		
FEGPHGQPGVGIR	0.70792	O94913	PCF11_HUMAN	450.9025033	3	0.777505577		
FEGPHGQPVGGLR	-0.419727	O94913	PCF11_HUMAN	675.8498425	2	0.71733892		
FEGPHGQPVGGLR	-0.419727	O94913	PCF11_HUMAN	450.9025033	3	0.71733892		
FEGPHGQSVAGLR	-0.709965	O94913	PCF11_HUMAN	677.8472995	2	0.772553444		
FEGPHGQSVAGLR	-0.709965	O94913	PCF11_HUMAN	452.2341413	3	0.772553444		
FEGPIGQAGGGGFR	34.04931	O94913	PCF11_HUMAN	675.3340255	2	0.803144157		
FEGPIGQAGGGGFR	34.04931	O94913	PCF11_HUMAN	450.5586253	3	0.803144157		
FEGPLLQQGVGMR	55.38435	O94913	PCF11_HUMAN	716.374836	2	0.834585905		
FEGPLLQQGVGMR	55.38435	O94913	PCF11_HUMAN	477.9191657	3	0.834585905		
FEGPLVQQGGGMR	31.38708	O94913	PCF11_HUMAN	688.343536	2	0.817684829		
FEGPLVQQGGGMR	31.38708	O94913	PCF11_HUMAN	459.2316323	3	0.817684829		
FEGPPGPVGTPLR	41.49217	O94913	PCF11_HUMAN	662.356969	2	0.760934234		
FEGPPGPVGTPLR	41.49217	O94913	PCF11_HUMAN	441.9072543	3	0.760934234		
FEGPQGQLGGGCPLR	32.46114	O94913	PCF11_HUMAN	786.883557	2	0.808832407		
FEGPQGQLGGGCPLR	32.46114	O94913	PCF11_HUMAN	524.9249797	3	0.808832407		
FEGPSVPGGGLR	31.07935	O94913	PCF11_HUMAN	586.8071115	2	0.799668193		
FEGPSVPGGGLR	31.07935	O94913	PCF11_HUMAN	391.5406827	3	0.799668193		
FEGQHNQLGGNLR	-10.2285	O94913	PCF11_HUMAN	735.3663875	2	0.803676486		
FEGQHNQLGGNLR	-10.2285	O94913	PCF11_HUMAN	490.5802	3	0.803676486		
FTTSQTDVYADHLDWHYR	42.49039	O94913	PCF11_HUMAN	1128.011791	2	0.815007746		
FTTSQTDVYADHLDWHYR	42.49039	O94913	PCF11_HUMAN	752.343802	3	0.815007746		
HEQIFDSPQGPNFNGPHGPGNQSFSNPLNR	48.68256	O94913	PCF11_HUMAN	1645.262093	2	0.663963199		
HEQIFDSPQGPNFNGPHGPGNQSFSNPLNR	48.68256	O94913	PCF11_HUMAN	1097.177337	3	0.663963199		
IDGPPTPASLR	22.77318	O94913	PCF11_HUMAN	562.309487	2	0.719301403	2	Yes
IDGPPTPASLR	22.77318	O94913	PCF11_HUMAN	375.208933	3	0.719301403	2	
IDTPPACTEESIATPSEIK	41.96825	O94913	PCF11_HUMAN	1029.999177	2	0.747165442		
IDTPPACTEESIATPSEIK	41.96825	O94913	PCF11_HUMAN	687.0020597	3	0.747165442		
IEGPLGQGGPR	8.184288	O94913	PCF11_HUMAN	540.7940045	2	0.838816047		
IEGPLGQGGPR	8.184288	O94913	PCF11_HUMAN	360.865278	3	0.838816047		
LASGEITQDDFLVVVHQIR	77.06456	O94913	PCF11_HUMAN	1070.574035	2	0.74274379	3	
LASGEITQDDFLVVVHQIR	77.06456	O94913	PCF11_HUMAN	714.051965	3	0.74274379	3	Yes
LELELEQAK	33.36246	O94913	PCF11_HUMAN	536.7984205	2	0.773808181		
LELELEQAK	33.36246	O94913	PCF11_HUMAN	358.2015553	3	0.773808181		
LHVSQIPPMAVK	26.98804	O94913	PCF11_HUMAN	660.3793895	2	0.74034965		
LHVSQIPPMAVK	26.98804	O94913	PCF11_HUMAN	440.588868	3	0.74034965		
LSVDANLQIPK	48.31272	O94913	PCF11_HUMAN	599.346069	2	0.840096951		
LSVDANLQIPK	48.31272	O94913	PCF11_HUMAN	399.8999877	3	0.840096951		
MIFEGPNK	12.82696	O94913	PCF11_HUMAN	468.2369445	2	0.695438623		
MIFEGPNK	12.82696	O94913	PCF11_HUMAN	312.4939047	3	0.695438623		
NLTQEQLIR	17.69022	O94913	PCF11_HUMAN	557.8149365	2	0.810591578		
NLTQEQLIR	17.69022	O94913	PCF11_HUMAN	372.212566	3	0.810591578		
SLQQVDEHSKPPHLR	-20.13175	O94913	PCF11_HUMAN	885.9664655	2	0.796744525		
SLQQVDEHSKPPHLR	-20.13175	O94913	PCF11_HUMAN	590.980252	3	0.796744525		
SPEEPSTPGTVVSSPSISTPPIVPDIQK	81.70819	O94913	PCF11_HUMAN	1423.737668	2	0.758818388		
SPEEPSTPGTVVSSPSISTPPIVPDIQK	81.70819	O94913	PCF11_HUMAN	949.494387	3	0.758818388		
SRPGPSLQIQDLK	22.01822	O94913	PCF11_HUMAN	719.904814	2	0.728670597	3	
SRPGPSLQIQDLK	22.01822	O94913	PCF11_HUMAN	480.2724843	3	0.728670597	3	Yes
STWDEIFPLK	85.81094	O94913	PCF11_HUMAN	618.31952	2	0.81686604		
STWDEIFPLK	85.81094	O94913	PCF11_HUMAN	412.548955	3	0.81686604		
TPVENPLNIMLNIVK	120.1283	O94913	PCF11_HUMAN	847.979664	2	0.753823817		
TPVENPLNIMLNIVK	120.1283	O94913	PCF11_HUMAN	565.6557177	3	0.753823817		
YEDSDKPFVDSPASR	8.482979	O94913	PCF11_HUMAN	856.8922925	2	0.771408141		
YEDSDKPFVDSPASR	8.482979	O94913	PCF11_HUMAN	571.59747	3	0.771408141		
DLQLQILR	65.41161	O94901	SUN1_HUMAN	499.8038405	2	0.751440942		
DLQLQILR	65.41161	O94901	SUN1_HUMAN	333.538502	3	0.751440942		
DVLTAHPAAPGPVSR	17.42523	O94901	SUN1_HUMAN	744.402439	2	0.710267782		
DVLTAHPAAPGPVSR	17.42523	O94901	SUN1_HUMAN	496.6042343	3	0.710267782		
ELTTLLQK	29.66821	O94901	SUN1_HUMAN	473.2849495	2	0.659602046	2	Yes
ELTTLLQK	29.66821	O94901	SUN1_HUMAN	315.8592413	3	0.659602046	2	
ETDFMAFHQEHEVR	13.74432	O94901	SUN1_HUMAN	888.394485	2	0.669140816		
ETDFMAFHQEHEVR	13.74432	O94901	SUN1_HUMAN	592.5989317	3	0.669140816		
GDLQTMLR	25.66933	O94901	SUN1_HUMAN	467.2453015	2	0.638204277	2	Yes
GDLQTMLR	25.66933	O94901	SUN1_HUMAN	311.8328093	3	0.638204277	2	
GSQGYLVVR	8.873844	O94901	SUN1_HUMAN	489.772536	2	0.62529701		
GSQGYLVVR	8.873844	O94901	SUN1_HUMAN	326.8509657	3	0.62529701		
IFSNWGHPEYTCLYR	47.11966	O94901	SUN1_HUMAN	971.9494185	2	0.813567281		
IFSNWGHPEYTCLYR	47.11966	O94901	SUN1_HUMAN	648.3022207	3	0.813567281		
LATTACTLGDGEAVGADSGTSSAVSLK	47.81343	O94901	SUN1_HUMAN	1270.11379	2	0.660507679		
LATTACTLGDGEAVGADSGTSSAVSLK	47.81343	O94901	SUN1_HUMAN	847.0784683	3	0.660507679		
LDPVFDSPR	31.03072	O94901	SUN1_HUMAN	523.26983	2	0.733139634		
LDPVFDSPR	31.03072	O94901	SUN1_HUMAN	349.182495	3	0.733139634		
LLFSEDQQGGSLEQLLQR	92.56433	O94901	SUN1_HUMAN	1031.034743	2	0.807685375	3	
LLFSEDQQGGSLEQLLQR	92.56433	O94901	SUN1_HUMAN	687.6924367	3	0.807685375	3	Yes
LSMMIHPAAFTLEHIPK	63.91344	O94901	SUN1_HUMAN	968.513349	2	0.816613436		
LSMMIHPAAFTLEHIPK	63.91344	O94901	SUN1_HUMAN	646.0115077	3	0.816613436		
MSHLEDILGK	28.54633	O94901	SUN1_HUMAN	571.7978975	2	0.748636484		
MSHLEDILGK	28.54633	O94901	SUN1_HUMAN	381.53454	3	0.748636484		
QLPTSEAVVSAVSEAGASGITEAQAR	69.09785	O94901	SUN1_HUMAN	1265.143739	2	0.612412035		
QLPTSEAVVSAVSEAGASGITEAQAR	69.09785	O94901	SUN1_HUMAN	843.7651007	3	0.612412035		
RPDDTAFQIVELR	54.03949	O94901	SUN1_HUMAN	780.4130035	2	0.760170519	2	Yes
RPDDTAFQIVELR	54.03949	O94901	SUN1_HUMAN	520.6112773	3	0.760170519	2	
RPPVLDESWIR	38.54543	O94901	SUN1_HUMAN	684.375692	2	0.86507535	3	
RPPVLDESWIR	38.54543	O94901	SUN1_HUMAN	456.586403	3	0.86507535	3	Yes
TAWSALWLAVVAPGK	124.2495	O94901	SUN1_HUMAN	785.4435755	2	0.744596064		
TAWSALWLAVVAPGK	124.2495	O94901	SUN1_HUMAN	523.964992	3	0.744596064		
TGMVDFALESGGGSILSTR	90.82265	O94901	SUN1_HUMAN	949.4703895	2	0.740922987		
TGMVDFALESGGGSILSTR	90.82265	O94901	SUN1_HUMAN	633.3162013	3	0.740922987		
TISAVGEQLLPTVEHLQLELDQLK	120.22	O94901	SUN1_HUMAN	1337.737275	2	0.715768218		

TISAVGEQLLPTVEHLQLELDQLK	120.22	O94901	SUN1 HUMAN	892.1607917	3	0.715768218		
TLSPTGNISSAPK	11.21578	O94901	SUN1 HUMAN	636.8438905	2	0.74926579		
TLSPTGNISSAPK	11.21578	O94901	SUN1 HUMAN	424.8985353	3	0.74926579		
VDDPQDVFKPTTSR	21.45232	O94901	SUN1 HUMAN	802.899926	2	0.75232935		
VDDPQDVFKPTTSR	21.45232	O94901	SUN1 HUMAN	535.602559	3	0.75232935		
VDQMEGGAAGPSASVR	3.56427	O94901	SUN1 HUMAN	766.3626535	2	0.789428949		
VDQMEGGAAGPSASVR	3.56427	O94901	SUN1 HUMAN	511.2443773	3	0.789428949		
VVIQPDIYPGNCWAFK	81.31281	O94901	SUN1 HUMAN	953.980192	2	0.759185374		
VVIQPDIYPGNCWAFK	81.31281	O94901	SUN1 HUMAN	636.3227363	3	0.759185374		
AQFAEATQR	-20.03746	O94874	UFL1 HUMAN	511.257255	2	0.621529579		
AQFAEATQR	-20.03746	O94874	UFL1 HUMAN	341.1741117	3	0.621529579		
AVFVPDIYSR	56.21471	O94874	UFL1 HUMAN	583.8144005	2	0.760804176	2	Yes
AVFVPDIYSR	56.21471	O94874	UFL1 HUMAN	389.545542	3	0.760804176	2	
DLQEEVSNLYNNIR	83.81343	O94874	UFL1 HUMAN	853.921384	2	0.824065089		
DLQEEVSNLYNNIR	83.81343	O94874	UFL1 HUMAN	569.6168643	3	0.824065089		
EYITPAQISK	21.1309	O94874	UFL1 HUMAN	575.311691	2	0.758681655	2	Yes
EYITPAQISK	21.1309	O94874	UFL1 HUMAN	383.877069	3	0.758681655	2	
FFADDTQAALTK	37.16868	O94874	UFL1 HUMAN	664.330617	2	0.816208363		
FFADDTQAALTK	37.16868	O94874	UFL1 HUMAN	443.2230197	3	0.816208363		
FINDCTELFR	50.25851	O94874	UFL1 HUMAN	657.8115365	2	0.79291898		
FINDCTELFR	50.25851	O94874	UFL1 HUMAN	438.876966	3	0.79291898		
GLFSAITRPTAVNSLISK	68.50653	O94874	UFL1 HUMAN	938.039099	2	0.708985448		
GLFSAITRPTAVNSLISK	68.50653	O94874	UFL1 HUMAN	625.695341	3	0.708985448		
GVIFTEAFVAR	66.98409	O94874	UFL1 HUMAN	605.335505	2	0.792460918	2	Yes
GVIFTEAFVAR	66.98409	O94874	UFL1 HUMAN	403.892945	3	0.792460918	2	
HIQDAPEEFISELAEYLIKPLNK	138.6187	O94874	UFL1 HUMAN	1349.210888	2	0.817937911		
HIQDAPEEFISELAEYLIKPLNK	138.6187	O94874	UFL1 HUMAN	899.8098667	3	0.817937911		
HVQLVLGQLIDENYLDR	82.39633	O94874	UFL1 HUMAN	1013.042366	2	0.778493464	3	
HVQLVLGQLIDENYLDR	82.39633	O94874	UFL1 HUMAN	675.697519	3	0.778493464	3	Yes
IISGHIDLDNR	10.20469	O94874	UFL1 HUMAN	626.8363995	2	0.786084592	2	Yes
IISGHIDLDNR	10.20469	O94874	UFL1 HUMAN	418.2268747	3	0.786084592	2	
IPEDQHALLVK	12.04938	O94874	UFL1 HUMAN	631.859344	2	0.772479892		
IPEDQHALLVK	12.04938	O94874	UFL1 HUMAN	421.5755043	3	0.772479892		
LAADFQR	-4.047249	O94874	UFL1 HUMAN	410.7197765	2	0.681425929		
LAADFQR	-4.047249	O94874	UFL1 HUMAN	274.149126	3	0.681425929		
LGIPDAVSYIK	67.04648	O94874	UFL1 HUMAN	588.3377085	2	0.83197844		
LGIPDAVSYIK	67.04648	O94874	UFL1 HUMAN	392.5610807	3	0.83197844		
LQESGQVTISELCK	33.39201	O94874	UFL1 HUMAN	796.403987	2	0.844175696		
LQESGQVTISELCK	33.39201	O94874	UFL1 HUMAN	531.271933	3	0.844175696		
NNPVHLITEEDLK	27.62791	O94874	UFL1 HUMAN	761.399561	2	0.765377402	3	
NNPVHLITEEDLK	27.62791	O94874	UFL1 HUMAN	507.935649	3	0.765377402	3	Yes
SVFMSSTTSASGTGR	9.350456	O94874	UFL1 HUMAN	738.343929	2	0.807370842	2	Yes
SVFMSSTTSASGTGR	9.350456	O94874	UFL1 HUMAN	492.5652277	3	0.807370842	2	
TGQGDYPLNNELDK	25.99918	O94874	UFL1 HUMAN	782.368454	2	0.767464876		
TGQGDYPLNNELDK	25.99918	O94874	UFL1 HUMAN	521.914911	3	0.767464876		
TQSTWVDSFFR	73.96978	O94874	UFL1 HUMAN	687.328408	2	0.790573597	2	Yes
TQSTWVDSFFR	73.96978	O94874	UFL1 HUMAN	458.5548803	3	0.790573597	2	
TTQLLFLK	51.53743	O94874	UFL1 HUMAN	482.29786	2	0.767349899	2	Yes
TTQLLFLK	51.53743	O94874	UFL1 HUMAN	321.8678483	3	0.767349899	2	
TYDLPGNFLTQALTQR	101.8885	O94874	UFL1 HUMAN	919.4763285	2	0.831780553		
TYDLPGNFLTQALTQR	101.8885	O94874	UFL1 HUMAN	613.3201607	3	0.831780553		
VNIVDLQQVINVDLIHIENR	129.0726	O94874	UFL1 HUMAN	1172.653347	2	0.703210413	3	
VNIVDLQQVINVDLIHIENR	129.0726	O94874	UFL1 HUMAN	782.1048397	3	0.703210413	3	Yes
EAAAALVEEETR	17.0353	O75934	SPF27 HUMAN	644.8231555	2	0.608186245	2	Yes
EAAAALVEEETR	17.0353	O75934	SPF27 HUMAN	430.2180453	3	0.608186245	2	
EMESNWVSLVSK	64.03909	O75934	SPF27 HUMAN	704.8430325	2	0.821378529	2	Yes
EMESNWVSLVSK	64.03909	O75934	SPF27 HUMAN	470.2312967	3	0.821378529	2	
HIQDLNWQR	8.629787	O75934	SPF27 HUMAN	605.3103525	2	0.852929831		
HIQDLNWQR	8.629787	O75934	SPF27 HUMAN	403.8761767	3	0.852929831		
IENLELMSQHGCNAWK	36.06307	O75934	SPF27 HUMAN	965.4514765	2	0.675183058		
IENLELMSQHGCNAWK	36.06307	O75934	SPF27 HUMAN	643.9702593	3	0.675183058		
NDITAWQECVNNSMAQLEHQAVR	78.21452	O75934	SPF27 HUMAN	1357.624667	2	0.782148242	3	
NDITAWQECVNNSMAQLEHQAVR	78.21452	O75934	SPF27 HUMAN	905.419053	3	0.782148242	3	Yes
NYLSYLTAPDYSAFETDIMR	115.7262	O75934	SPF27 HUMAN	1185.552095	2	0.696891606		
NYLSYLTAPDYSAFETDIMR	115.7262	O75934	SPF27 HUMAN	790.7040047	3	0.696891606		
TIVQLENEIYQIK	74.76711	O75934	SPF27 HUMAN	795.941058	2	0.876223087		
TIVQLENEIYQIK	74.76711	O75934	SPF27 HUMAN	530.9633137	3	0.876223087		
VYNENLVHMIEHAQK	36.1963	O75934	SPF27 HUMAN	912.9572475	2	0.788852453	3	
VYNENLVHMIEHAQK	36.1963	O75934	SPF27 HUMAN	608.9741067	3	0.788852453	3	Yes
YELPAPSSGQK	9.689117	O75934	SPF27 HUMAN	588.7989475	2	0.675150931	2	Yes
YELPAPSSGQK	9.689117	O75934	SPF27 HUMAN	392.8685733	3	0.675150931	2	
FEFQADAFAK	48.40814	O75844	FACE1 HUMAN	587.282938	2	0.705876708		
FEFQADAFAK	48.40814	O75844	FACE1 HUMAN	391.8579003	3	0.705876708		
HGFNQQTLGFFMK	54.18167	O75844	FACE1 HUMAN	777.8802855	2	0.779394031	3	
HGFNQQTLGFFMK	54.18167	O75844	FACE1 HUMAN	518.9227987	3	0.779394031	3	Yes
IVLFDTLLEEYSVLNK	139.5284	O75844	FACE1 HUMAN	948.5224115	2	0.676823616	3	
IVLFDTLLEEYSVLNK	139.5284	O75844	FACE1 HUMAN	632.684216	3	0.676823616	3	Yes
NEEVLAVLGHELGHWK	64.36889	O75844	FACE1 HUMAN	915.9790415	2	0.628175139	4	
NEEVLAVLGHELGHWK	64.36889	O75844	FACE1 HUMAN	610.988636	3	0.628175139	4	
SIDFPLTK	46.03741	O75844	FACE1 HUMAN	460.758567	2	0.740081668	2	Yes
SIDFPLTK	46.03741	O75844	FACE1 HUMAN	307.5083197	3	0.740081668	2	
SSHSNAYFYGFFK	48.96048	O75844	FACE1 HUMAN	777.8547805	2	0.837753534	3	
SSHSNAYFYGFFK	48.96048	O75844	FACE1 HUMAN	518.9057953	3	0.837753534	3	Yes
TTTHVPPELGQIMDSETFEK	62.01823	O75844	FACE1 HUMAN	1130.544284	2	0.802211285	3	
TTTHVPPELGQIMDSETFEK	62.01823	O75844	FACE1 HUMAN	754.0321307	3	0.802211285	3	Yes
AEEATEAQEVVEATPEGACTEPR	39.17338	O75683	SURF6 HUMAN	1237.553566	2	0.610580504		
AEEATEAQEVVEATPEGACTEPR	39.17338	O75683	SURF6 HUMAN	825.371652	3	0.610580504		
DAYLQSLAK	32.60512	O75683	SURF6 HUMAN	504.7722015	2	0.803245187		
DAYLQSLAK	32.60512	O75683	SURF6 HUMAN	336.8507427	3	0.803245187		
EEAAWASSSAGNPADGLATEPESVFALDVLR	131.6529	O75683	SURF6 HUMAN	1580.757652	2	0.677603543		
EEAAWASSSAGNPADGLATEPESVFALDVLR	131.6529	O75683	SURF6 HUMAN	1054.174376	3	0.677603543		
EPPGLIFNK	38.27195	O75683	SURF6 HUMAN	507.785116	2	0.600984275		
EPPGLIFNK	38.27195	O75683	SURF6 HUMAN	338.8593523	3	0.600984275		
ILPQDLER	19.9472	O75683	SURF6 HUMAN	492.280198	2	0.779678106	2	Yes
ILPQDLER	19.9472	O75683	SURF6 HUMAN	328.5227403	3	0.779678106	2	
AALETDENLLLCAPTGAGK	67.44757	O75643	U520 HUMAN	972.4913225	2	0.840220273	3	
AALETDENLLLCAPTGAGK	67.44757	O75643	U520 HUMAN	648.66349	3	0.840220273	3	Yes
ADEVLEILK	62.12646	O75643	U520 HUMAN	515.2955135	2	0.818062961	2	Yes
ADEVLEILK	62.12646	O75643	U520 HUMAN	343.866284	3	0.818062961	2	
ANSNLVLQADR	11.62881	O75643	U520 HUMAN	600.8207495	2	0.856151283	2	Yes
ANSNLVLQADR	11.62881	O75643	U520 HUMAN	400.883108	3	0.856151283	2	

DEPTGEVLSLVGK	71.38479	O75643	U520 HUMAN	672.356831	2	0.722153008	2	Yes
DEPTGEVLSLVGK	71.38479	O75643	U520 HUMAN	448.573829	3	0.722153008	2	
DIDAFWLQR	82.72675	O75643	U520 HUMAN	582.2963795	2	0.683922052	2	Yes
DIDAFWLQR	82.72675	O75643	U520 HUMAN	388.533528	3	0.683922052	2	
DILCGAADEVLAVLK	141.8489	O75643	U520 HUMAN	793.927098	2	0.816913068	3	
DILCGAADEVLAVLK	141.8489	O75643	U520 HUMAN	529.6206737	3	0.816913068	3	Yes
DLIPYLEK	61.74155	O75643	U520 HUMAN	495.779495	2	0.68245554	2	Yes
DLIPYLEK	61.74155	O75643	U520 HUMAN	330.855605	3	0.68245554	2	
DTLGLFLR	79.28726	O75643	U520 HUMAN	467.772009	2	0.750479281	2	Yes
DTLGLFLR	79.28726	O75643	U520 HUMAN	312.1839477	3	0.750479281	2	
EEASDDDMEGDEAVVR	14.81172	O75643	U520 HUMAN	883.8552515	2	0.673359454	2	Yes
EEASDDDMEGDEAVVR	14.81172	O75643	U520 HUMAN	589.572776	3	0.673359454	2	
EEEVTGPVIAPLFPQK	78.98779	O75643	U520 HUMAN	877.472727	2	0.852434456	3	
EEEVTGPVIAPLFPQK	78.98779	O75643	U520 HUMAN	585.3177597	3	0.852434456	3	Yes
EEGWWVVIGDAK	88.6935	O75643	U520 HUMAN	694.8464335	2	0.66918087	2	Yes
EEGWWVVIGDAK	88.6935	O75643	U520 HUMAN	463.5668973	3	0.66918087	2	
EIDLLLGQTDDTR	68.33288	O75643	U520 HUMAN	744.881202	2	0.798561096		
EIDLLLGQTDDTR	68.33288	O75643	U520 HUMAN	496.9234097	3	0.798561096		
FLYQLHETEK	13.08175	O75643	U520 HUMAN	654.3356975	2	0.773814082	3	
FLYQLHETEK	13.08175	O75643	U520 HUMAN	436.55974	3	0.773814082	3	Yes
FQIMNEIVYEK	64.78265	O75643	U520 HUMAN	707.358315	2	0.836890697	2	Yes
FQIMNEIVYEK	64.78265	O75643	U520 HUMAN	471.9081517	3	0.836890697	2	
FYDDAIVSQK	23.57089	O75643	U520 HUMAN	593.293498	2	0.728718162	2	Yes
FYDDAIVSQK	23.57089	O75643	U520 HUMAN	395.8649403	3	0.728718162	2	
GDPLLDQR	2.502548	O75643	U520 HUMAN	457.241073	2	0.791011035	2	Yes
GDPLLDQR	2.502548	O75643	U520 HUMAN	305.1633237	3	0.791011035	2	
GLIEIISNAAEYENIPIR	113.6811	O75643	U520 HUMAN	1008.044574	2	0.687421858	3	
GLIEIISNAAEYENIPIR	113.6811	O75643	U520 HUMAN	672.3656573	3	0.687421858	3	Yes
GNIIISTPEK	21.64388	O75643	U520 HUMAN	536.3064125	2	0.699398577	2	Yes
GNIIISTPEK	21.64388	O75643	U520 HUMAN	357.87355	3	0.699398577	2	
GPVLEALVAR	51.4211	O75643	U520 HUMAN	512.8116655	2	0.793797255	2	Yes
GPVLEALVAR	51.4211	O75643	U520 HUMAN	342.2103853	3	0.793797255	2	
GVESVFDIMEMEDEER	106.7075	O75643	U520 HUMAN	957.9089725	2	0.790600121	3	
GVESVFDIMEMEDEER	106.7075	O75643	U520 HUMAN	638.9419233	3	0.790600121	3	Yes
GWAQLTDK	14.18123	O75643	U520 HUMAN	459.7381665	2	0.773908973	2	Yes
GWAQLTDK	14.18123	O75643	U520 HUMAN	306.8280527	3	0.773908973	2	
GYEEVHVPALKPKPFGSEEQLLPVEK	56.51894	O75643	U520 HUMAN	1460.776927	2	0.611019731	4	
GYEEVHVPALKPKPFGSEEQLLPVEK	56.51894	O75643	U520 HUMAN	974.187226	3	0.611019731	4	
GYTLLSEGIDEMVGIIYKPK	105.2626	O75643	U520 HUMAN	1113.590496	2	0.64677906	3	
GYTLLSEGIDEMVGIIYKPK	105.2626	O75643	U520 HUMAN	742.7296057	3	0.64677906	3	Yes
HINMDGTINVDDFK	38.27195	O75643	U520 HUMAN	809.8806785	2	0.798789978	3	
HINMDGTINVDDFK	38.27195	O75643	U520 HUMAN	540.256394	3	0.798789978	3	Yes
HLSDHLSELVEQTLSDLEQSK	85.51993	O75643	U520 HUMAN	1204.601174	2	0.692887187	3	
HLSDHLSELVEQTLSDLEQSK	85.51993	O75643	U520 HUMAN	803.403391	3	0.692887187	3	Yes
IASHYYITNDTVQTYNQLLKPTLSEIELFR	103.8769	O75643	U520 HUMAN	1785.928113	2	0.694990993	5	
IASHYYITNDTVQTYNQLLKPTLSEIELFR	103.8769	O75643	U520 HUMAN	1190.954684	3	0.694990993	5	
INVLLQAFISQLK	132.5055	O75643	U520 HUMAN	743.9537755	2	0.784085274		
INVLLQAFISQLK	132.5055	O75643	U520 HUMAN	496.3051253	3	0.784085274		
IVALSSSLSNAK	23.45725	O75643	U520 HUMAN	595.3435255	2	0.786249936	2	Yes
IVALSSSLSNAK	23.45725	O75643	U520 HUMAN	397.2316253	3	0.786249936	2	
KPVIVFVPSR	27.37937	O75643	U520 HUMAN	571.3587825	2	0.630227029	2	Yes
KPVIVFVPSR	27.37937	O75643	U520 HUMAN	381.2417967	3	0.630227029	2	
LATYGITVAELTGDHQLCK	57.65741	O75643	U520 HUMAN	1045.533518	2	0.76018548	3	
LATYGITVAELTGDHQLCK	57.65741	O75643	U520 HUMAN	697.358287	3	0.76018548	3	Yes
LDLVHTAALMLDK	67.72993	O75643	U520 HUMAN	720.400519	2	0.835566938	2	Yes
LDLVHTAALMLDK	67.72993	O75643	U520 HUMAN	480.6029543	3	0.835566938	2	
LELSVHLQPITR	48.01176	O75643	U520 HUMAN	703.412275	2	0.791603923	3	
LELSVHLQPITR	48.01176	O75643	U520 HUMAN	469.2774583	3	0.791603923	3	Yes
LIGLSATLPNYEDVATFLR	130.9628	O75643	U520 HUMAN	1047.06805	2	0.77113384		
LIGLSATLPNYEDVATFLR	130.9628	O75643	U520 HUMAN	698.381308	3	0.77113384		
LIILDEIHLLHDDR	72.98763	O75643	U520 HUMAN	857.97851	2	0.808632493	4	
LIILDEIHLLHDDR	72.98763	O75643	U520 HUMAN	572.321615	3	0.808632493	4	
LLSMAKPVYHAITK	16.91801	O75643	U520 HUMAN	786.4530815	2	0.783130884		
LLSMAKPVYHAITK	16.91801	O75643	U520 HUMAN	524.637996	3	0.783130884		
LPDMLNAEIVLGNVQNAK	98.85611	O75643	U520 HUMAN	970.020049	2	0.759604216	3	
LPDMLNAEIVLGNVQNAK	98.85611	O75643	U520 HUMAN	647.0159743	3	0.759604216	3	Yes
LTAIDILTTCAADIQR	87.53793	O75643	U520 HUMAN	887.972569	2	0.802062869	3	
LTAIDILTTCAADIQR	87.53793	O75643	U520 HUMAN	592.3176543	3	0.802062869	3	Yes
LYDLNHNEIGELIR	60.8747	O75643	U520 HUMAN	849.9446625	2	0.864194274	2	Yes
LYDLNHNEIGELIR	60.8747	O75643	U520 HUMAN	566.9657167	3	0.864194274	2	
MDTDLETMDLDQGGEALAPR	70.43121	O75643	U520 HUMAN	1089.488651	2	0.792657554	3	
MDTDLETMDLDQGGEALAPR	70.43121	O75643	U520 HUMAN	726.661709	3	0.792657554	3	Yes
MLLQSSEGR	-2.417118	O75643	U520 HUMAN	510.761315	2	0.723956108	2	Yes
MLLQSSEGR	-2.417118	O75643	U520 HUMAN	340.843485	3	0.723956108	2	
MQLSAELQSDTEEILSK	71.00184	O75643	U520 HUMAN	961.475337	2	0.83390528		
MQLSAELQSDTEEILSK	71.00184	O75643	U520 HUMAN	641.3194997	3	0.83390528		
MTQNPNYYNLQGISHR	25.21439	O75643	U520 HUMAN	968.4606815	2	0.827042103	3	
MTQNPNYYNLQGISHR	25.21439	O75643	U520 HUMAN	645.976396	3	0.827042103	3	Yes
MWQSMCPLR	39.71661	O75643	U520 HUMAN	604.772846	2	0.687800229	2	Yes
MWQSMCPLR	39.71661	O75643	U520 HUMAN	403.517839	3	0.687800229	2	
NALLQLTDSQIADVAR	76.19703	O75643	U520 HUMAN	864.468507	2	0.874259412	3	
NALLQLTDSQIADVAR	76.19703	O75643	U520 HUMAN	576.6482797	3	0.874259412	3	Yes
NIEMTQEDVR	5.495296	O75643	U520 HUMAN	617.790801	2	0.776140094	2	Yes
NIEMTQEDVR	5.495296	O75643	U520 HUMAN	412.1964757	3	0.776140094	2	
NQVLVFVHSR	18.44741	O75643	U520 HUMAN	599.8387455	2	0.795906961	3	
NQVLVFVHSR	18.44741	O75643	U520 HUMAN	400.2284387	3	0.795906961	3	Yes
NSAFESLYQDK	31.62013	O75643	U520 HUMAN	651.3045935	2	0.773327827		
NSAFESLYQDK	31.62013	O75643	U520 HUMAN	434.539004	3	0.773327827		
SGGPVVVLVQLER	72.16635	O75643	U520 HUMAN	676.8990005	2	0.767234445	2	Yes
SGGPVVVLVQLER	72.16635	O75643	U520 HUMAN	451.601942	3	0.767234445	2	
SLQYEYK	-7.600342	O75643	U520 HUMAN	465.7325405	2	0.700269222		
SLQYEYK	-7.600342	O75643	U520 HUMAN	310.824302	3	0.700269222		
SLVQEMVGSFGK	74.28644	O75643	U520 HUMAN	641.329562	2	0.778223276		
SLVQEMVGSFGK	74.28644	O75643	U520 HUMAN	427.888983	3	0.778223276		
SPTLYGISHDDLK	23.7704	O75643	U520 HUMAN	723.3677255	2	0.81642127	3	
SPTLYGISHDDLK	23.7704	O75643	U520 HUMAN	482.581092	3	0.81642127	3	Yes
TGNFQVTELGR	28.51978	O75643	U520 HUMAN	611.315301	2	0.810124099	2	Yes
TGNFQVTELGR	28.51978	O75643	U520 HUMAN	407.8794757	3	0.810124099	2	
TLVEDLFADK	69.60025	O75643	U520 HUMAN	575.803703	2	0.845294237	2	Yes
TLVEDLFADK	69.60025	O75643	U520 HUMAN	384.205077	3	0.845294237	2	
TNLLLQAHLSR	26.30423	O75643	U520 HUMAN	633.37041	2	0.805086136	3	

TNLLLQAHLSR	26.30423	O75643	U520_HUMAN	422.5828817	3	0.805086136	3	Yes
TNVALMCMLR	55.91615	O75643	U520_HUMAN	604.8016035	2	0.646733224		
TNVALMCMLR	55.91615	O75643	U520_HUMAN	403.5370107	3	0.646733224		
VELTITPDFQWDEK	85.66599	O75643	U520_HUMAN	860.9256095	2	0.803570986		
VELTITPDFQWDEK	85.66599	O75643	U520_HUMAN	574.286348	3	0.803570986		
VFSLSSEFK	41.5721	O75643	U520_HUMAN	522.2745805	2	0.733745217	2	Yes
VFSLSSEFK	41.5721	O75643	U520_HUMAN	348.5189953	3	0.733745217	2	
VVLLTGETSTDLK	41.63831	O75643	U520_HUMAN	688.3881315	2	0.910667241	2	Yes
VVLLTGETSTDLK	41.63831	O75643	U520_HUMAN	459.2613627	3	0.910667241	2	
WLSCETQLPVSFR	71.75398	O75643	U520_HUMAN	811.903957	2	0.715757668	2	Yes
WLSCETQLPVSFR	71.75398	O75643	U520_HUMAN	541.6052463	3	0.715757668	2	
YAQAGFEGFK	22.1725	O75643	U520_HUMAN	559.2698265	2	0.757605791	2	Yes
YAQAGFEGFK	22.1725	O75643	U520_HUMAN	373.1824927	3	0.757605791	2	
YHVLVNLGK	16.71312	O75643	U520_HUMAN	521.8063785	2	0.732612848		
YHVLVNLGK	16.71312	O75643	U520_HUMAN	348.2068607	3	0.732612848		
YISSQIERPIR	5.869041	O75643	U520_HUMAN	681.38097	2	0.695756257		
YISSQIERPIR	5.869041	O75643	U520_HUMAN	454.5899217	3	0.695756257		
YPNIELSYEVVDKDSIR	58.1491	O75643	U520_HUMAN	1020.518384	2	0.717876554	3	
YPNIELSYEVVDKDSIR	58.1491	O75643	U520_HUMAN	680.681531	3	0.717876554	3	Yes
YPPPTELLDLQPLPVSALR	123.2326	O75643	U520_HUMAN	1060.094068	2	0.726222932	3	
YPPPTELLDLQPLPVSALR	123.2326	O75643	U520_HUMAN	707.06532	3	0.726222932	3	Yes
YVHLFPK	6.215714	O75643	U520_HUMAN	452.258533	2	0.684643328	2	Yes
YVHLFPK	6.215714	O75643	U520_HUMAN	301.8416303	3	0.684643328	2	
EYQTLEQCLQHIPVNEENR	51.00956	O75570	RF1M_HUMAN	1200.566608	2	0.656297743		
EYQTLEQCLQHIPVNEENR	51.00956	O75570	RF1M_HUMAN	800.7136803	3	0.656297743		
GLDQLIQR	29.47497	O75570	RF1M_HUMAN	471.7725405	2	0.756379128		
GLDQLIQR	29.47497	O75570	RF1M_HUMAN	314.8509687	3	0.756379128		
IHTGTMSVIVLPQPDEVDVK	65.52406	O75570	RF1M_HUMAN	1089.577929	2	0.686045229		
IHTGTMSVIVLPQPDEVDVK	65.52406	O75570	RF1M_HUMAN	726.7212277	3	0.686045229		
INMLYNELFQSLVPK	128.6287	O75570	RF1M_HUMAN	904.984942	2	0.822480738		
INMLYNELFQSLVPK	128.6287	O75570	RF1M_HUMAN	603.6592363	3	0.822480738		
LLQSADEEAIAELLDEHLK	95.26532	O75570	RF1M_HUMAN	1069.05534	2	0.855163574		
LLQSADEEAIAELLDEHLK	95.26532	O75570	RF1M_HUMAN	713.0395017	3	0.855163574		
LVHIPTGLVVECQQER	44.67225	O75570	RF1M_HUMAN	939.499285	2	0.808951497		
LVHIPTGLVVECQQER	44.67225	O75570	RF1M_HUMAN	626.6687983	3	0.808951497		
LYQQIIEK	12.11414	O75570	RF1M_HUMAN	517.7982195	2	0.773119926		
LYQQIIEK	12.11414	O75570	RF1M_HUMAN	345.5347547	3	0.773119926		
NDVILEVTAGR	44.76055	O75570	RF1M_HUMAN	593.825501	2	0.763837755		
NDVILEVTAGR	44.76055	O75570	RF1M_HUMAN	396.219609	3	0.763837755		
TTGGDICQQFTR	15.33227	O75570	RF1M_HUMAN	692.3202585	2	0.794707358		
TTGGDICQQFTR	15.33227	O75570	RF1M_HUMAN	461.8827807	3	0.794707358		
AYVVLGQFLVLK	102.1449	O75531	BAF_HUMAN	675.413751	2	0.605954468	3	
AYVVLGQFLVLK	102.1449	O75531	BAF_HUMAN	450.6117757	3	0.605954468	3	Yes
DFVAEPMGEKPVGSLAGIGEVLGK	91.20268	O75531	BAF_HUMAN	1200.62815	2	0.780001163	3	
DFVAEPMGEKPVGSLAGIGEVLGK	91.20268	O75531	BAF_HUMAN	800.7547083	3	0.780001163	3	Yes
DLQQYQSQAK	-13.25126	O75396	SC22B_HUMAN	604.7994795	2	0.808874846	2	Yes
DLQQYQSQAK	-13.25126	O75396	SC22B_HUMAN	403.5355947	3	0.808874846	2	
GEALSALDSK	7.771606	O75396	SC22B_HUMAN	495.759295	2	0.744201541	2	Yes
GEALSALDSK	7.771606	O75396	SC22B_HUMAN	330.8421383	3	0.744201541	2	
IMVANIEEVLQR	71.70692	O75396	SC22B_HUMAN	707.890318	2	0.839669764	2	Yes
IMVANIEEVLQR	71.70692	O75396	SC22B_HUMAN	472.2628203	3	0.839669764	2	
NLGSINTELQDVQR	44.5868	O75396	SC22B_HUMAN	793.9108245	2	0.787521124	2	Yes
NLGSINTELQDVQR	44.5868	O75396	SC22B_HUMAN	529.6098247	3	0.787521124	2	
TLAFAYLEDLHSEFDEQHGK	73.06184	O75396	SC22B_HUMAN	1175.553492	2	0.747992933		
TLAFAYLEDLHSEFDEQHGK	73.06184	O75396	SC22B_HUMAN	784.0382697	3	0.747992933		
VADGLPLAASMQEDEQSGR	51.03749	O75396	SC22B_HUMAN	987.4658355	2	0.764543831	3	
VADGLPLAASMQEDEQSGR	51.03749	O75396	SC22B_HUMAN	658.6464987	3	0.764543831	3	Yes
YLNMHSTYAK	-19.23765	O75396	SC22B_HUMAN	614.2955135	2	0.728240311		
YLNMHSTYAK	-19.23765	O75396	SC22B_HUMAN	409.866284	3	0.728240311		
ATTSTNWILESQNINELK	70.38232	O75381	PEX14_HUMAN	1031.52675	2	0.817089975	3	
ATTSTNWILESQNINELK	70.38232	O75381	PEX14_HUMAN	688.0204413	3	0.817089975	3	Yes
GGDGQINEQVEK	-21.39886	O75381	PEX14_HUMAN	637.30513	2	0.79354459		
GGDGQINEQVEK	-21.39886	O75381	PEX14_HUMAN	425.2060283	3	0.79354459		
[illegible]MAFQQSGTAADEPSSLGPATQVVPVQPPHLISQI	107.2637	O75381	PEX14_HUMAN	2538.730748	2	0.613712847		
[illegible]MAFQQSGTAADEPSSLGPATQVVPVQPPHLISQI	107.2637	O75381	PEX14_HUMAN	1692.823107	3	0.613712847		
IPSWQIPVK	56.17604	O75381	PEX14_HUMAN	534.3165835	2	0.639446437		
IPSWQIPVK	56.17604	O75381	PEX14_HUMAN	356.5469973	3	0.639446437		
IQELAHELAAAK	14.39052	O75381	PEX14_HUMAN	647.362251	2	0.797787547	3	
IQELAHELAAAK	14.39052	O75381	PEX14_HUMAN	431.9107757	3	0.797787547	3	Yes
YLLPLILGGR	97.28453	O75381	PEX14_HUMAN	557.853329	2	0.728444159		
YLLPLILGGR	97.28453	O75381	PEX14_HUMAN	372.238161	3	0.728444159		
AAGSGELGVTMK	11.69487	O75369	FLNB_HUMAN	560.7875305	2	0.777877033	2	Yes
AAGSGELGVTMK	11.69487	O75369	FLNB_HUMAN	374.1942953	3	0.777877033	2	
ADIEMPFDPSK	58.09164	O75369	FLNB_HUMAN	625.292645	2	0.791538835	2	Yes
ADIEMPFDPSK	58.09164	O75369	FLNB_HUMAN	417.197705	3	0.791538835	2	
AEISCIDNK	-8.818417	O75369	FLNB_HUMAN	525.25078	2	0.802055001	2	Yes
AEISCIDNK	-8.818417	O75369	FLNB_HUMAN	350.5031283	3	0.802055001	2	
AEITFDDHK	-4.165031	O75369	FLNB_HUMAN	538.25692	2	0.701427817	2	Yes
AEITFDDHK	-4.165031	O75369	FLNB_HUMAN	359.1738883	3	0.701427817	2	
AGLAPLEVR	30.03179	O75369	FLNB_HUMAN	463.2774585	2	0.7477054	2	Yes
AGLAPLEVR	30.03179	O75369	FLNB_HUMAN	309.1875807	3	0.7477054	2	
AGPGTLSVTIEGPSK	36.33746	O75369	FLNB_HUMAN	707.3833805	2	0.82771349	2	Yes
AGPGTLSVTIEGPSK	36.33746	O75369	FLNB_HUMAN	471.924862	3	0.82771349	2	
AHGPGLEGGLVGKPAEFTIDTK	40.74789	O75369	FLNB_HUMAN	1097.579318	2	0.740699291	3	
AHGPGLEGGLVGKPAEFTIDTK	40.74789	O75369	FLNB_HUMAN	732.055487	3	0.740699291	3	Yes
AHIANPSGASTECFVTDNADGTYQVEYTPFEK	63.89818	O75369	FLNB_HUMAN	1760.28644	2	0.715520263	3	
AHIANPSGASTECFVTDNADGTYQVEYTPFEK	63.89818	O75369	FLNB_HUMAN	1173.860235	3	0.715520263	3	Yes
ALGALVDSCAPGLCPDWESWDPQKPVDNAR	89.09354	O75369	FLNB_HUMAN	1662.7748	2	0.737719774	3	
ALGALVDSCAPGLCPDWESWDPQKPVDNAR	89.09354	O75369	FLNB_HUMAN	1108.852475	3	0.737719774	3	Yes
ANEPTHFTVDCTEAGEGDVSVGIK	40.53291	O75369	FLNB_HUMAN	1267.07975	2	0.757302642	3	
ANEPTHFTVDCTEAGEGDVSVGIK	40.53291	O75369	FLNB_HUMAN	845.055775	3	0.757302642	3	Yes
APLNVQFNSPLPGDAVK	66.77222	O75369	FLNB_HUMAN	883.9759675	2	0.891704679	3	
APLNVQFNSPLPGDAVK	66.77222	O75369	FLNB_HUMAN	589.6532533	3	0.891704679	3	Yes
APSVATVGSICDLNLK	60.87952	O75369	FLNB_HUMAN	822.935454	2	0.783651531	3	
APSVATVGSICDLNLK	60.87952	O75369	FLNB_HUMAN	548.9595777	3	0.783651531	3	Yes
AWGPGLHGGIVGR	28.17035	O75369	FLNB_HUMAN	638.8496455	2	0.853955626	2	Yes
AWGPGLHGGIVGR	28.17035	O75369	FLNB_HUMAN	426.2357053	3	0.853955626	2	
AYGPGLEK	-11.00579	O75369	FLNB_HUMAN	417.7219805	2	0.677040458	2	Yes
AYGPGLEK	-11.00579	O75369	FLNB_HUMAN	278.817262	3	0.677040458	2	
CLATGPGIASTVK	22.68864	O75369	FLNB_HUMAN	637.8428375	2	0.6943928	2	Yes
CLATGPGIASTVK	22.68864	O75369	FLNB_HUMAN	425.5645	3	0.6943928	2	

DAGEGLLAVQITDQEGKPK	51.48177	O75369	FLNB HUMAN	985.016019	2	0.759206653	3	
DAGEGLLAVQITDQEGKPK	51.48177	O75369	FLNB HUMAN	657.0132877	3	0.759206653	3	Yes
DAGYGGISLAVEGPSK	55.10976	O75369	FLNB HUMAN	760.88374	2	0.836794913	2	Yes
DAGYGGISLAVEGPSK	55.10976	O75369	FLNB HUMAN	507.5917683	3	0.836794913	2	
DEPCLLK	15.39601	O75369	FLNB HUMAN	437.7211275	2	0.642805338		
DEPCLLK	15.39601	O75369	FLNB HUMAN	292.1500267	3	0.642805338		
DFLDGVYAFEYYPSTPGR	120.7419	O75369	FLNB HUMAN	1048.984174	2	0.71674937	3	
DFLDGVYAFEYYPSTPGR	120.7419	O75369	FLNB HUMAN	699.6587243	3	0.71674937	3	Yes
DGSCSAEYIPFAPGDYDVNITYGGAHIPGSPFR	111.9353	O75369	FLNB HUMAN	1765.802056	2	0.748743474	3	
DGSCSAEYIPFAPGDYDVNITYGGAHIPGSPFR	111.9353	O75369	FLNB HUMAN	1177.537312	3	0.748743474	3	Yes
DGTCTVTYLPTLPGDYSILVK	121.552	O75369	FLNB HUMAN	1157.088319	2	0.656282783	3	
DGTCTVTYLPTLPGDYSILVK	121.552	O75369	FLNB HUMAN	771.728154	3	0.656282783	3	Yes
DGTYAVTYIPDK	43.9081	O75369	FLNB HUMAN	671.830441	2	0.714498281	2	Yes
DGTYAVTYIPDK	43.9081	O75369	FLNB HUMAN	448.2229023	3	0.714498281	2	
DLDIIDNYDYSHTVK	56.51324	O75369	FLNB HUMAN	905.928871	2	0.805567145	3	
DLDIIDNYDYSHTVK	56.51324	O75369	FLNB HUMAN	604.2885223	3	0.805567145	3	Yes
DQEFTVDTR	13.51694	O75369	FLNB HUMAN	555.7572845	2	0.75241673	2	Yes
DQEFTVDTR	13.51694	O75369	FLNB HUMAN	370.840798	3	0.75241673	2	
DSPYMAFIHPATGGYNPDLVR	81.51787	O75369	FLNB HUMAN	1161.055144	2	0.714529514		
DSPYMAFIHPATGGYNPDLVR	81.51787	O75369	FLNB HUMAN	774.372704	3	0.714529514		
EAFTNKPNVFTVVTR	39.80408	O75369	FLNB HUMAN	861.9628605	2	0.839033008	2	Yes
EAFTNKPNVFTVVTR	39.80408	O75369	FLNB HUMAN	574.9778487	3	0.839033008	2	
EATTDFTVDSRPLTQVGGDHIK	33.72504	O75369	FLNB HUMAN	1194.096061	2	0.789947629	3	
EATTDFTVDSRPLTQVGGDHIK	33.72504	O75369	FLNB HUMAN	796.399982	3	0.789947629	3	Yes
FADEHVPGSPFTVK	33.84625	O75369	FLNB HUMAN	765.8835475	2	0.803258955		
FADEHVPGSPFTVK	33.84625	O75369	FLNB HUMAN	510.9249733	3	0.803258955		
FNDEHIPESPYLVPVIAPSDDAR	76.77583	O75369	FLNB HUMAN	1291.132637	2	0.867096424	3	
FNDEHIPESPYLVPVIAPSDDAR	76.77583	O75369	FLNB HUMAN	861.091033	3	0.867096424	3	Yes
FVPQEMGVHTVSVK	26.98675	O75369	FLNB HUMAN	779.4088755	2	0.841395974		
FVPQEMGVHTVSVK	26.98675	O75369	FLNB HUMAN	519.9418587	3	0.841395974		
GAGIGGLGITVEGPSESK	52.21529	O75369	FLNB HUMAN	814.9286835	2	0.828995407	2	Yes
GAGIGGLGITVEGPSESK	52.21529	O75369	FLNB HUMAN	543.6217307	3	0.828995407	2	
GEAGVPAEFSIWTR	72.40703	O75369	FLNB HUMAN	760.381172	2	0.80153048	2	Yes
GEAGVPAEFSIWTR	72.40703	O75369	FLNB HUMAN	507.256723	3	0.80153048	2	
GIEPTGNMVK	5.868065	O75369	FLNB HUMAN	523.271516	2	0.673797011	2	Yes
GIEPTGNMVK	5.868065	O75369	FLNB HUMAN	349.183619	3	0.673797011	2	
GLHVVEVTYDDVPIPNSPFK	76.90754	O75369	FLNB HUMAN	1113.576239	2	0.816362917	3	
GLHVVEVTYDDVPIPNSPFK	76.90754	O75369	FLNB HUMAN	742.7201007	3	0.816362917	3	Yes
GLVEPVNVVDNGDGTHTVTYTPSQEGPYMVSVK	71.15481	O75369	FLNB HUMAN	1745.346301	2	0.614658833		
GLVEPVNVVDNGDGTHTVTYTPSQEGPYMVSVK	71.15481	O75369	FLNB HUMAN	1163.900142	3	0.614658833		
GQHVTGSPFQFTVGPLGEGGAHK	47.89411	O75369	FLNB HUMAN	1154.577642	2	0.738273025	4	
GQHVTGSPFQFTVGPLGEGGAHK	47.89411	O75369	FLNB HUMAN	770.0543693	3	0.738273025	4	
HTIAVVWGGVNIPHSPYR	50.99494	O75369	FLNB HUMAN	1002.034679	2	0.715094149	3	
HTIAVVWGGVNIPHSPYR	50.99494	O75369	FLNB HUMAN	668.3590607	3	0.715094149	3	Yes
HVGNQQYNVTYVVK	10.54977	O75369	FLNB HUMAN	824.9262695	2	0.809395254	2	Yes
HVGNQQYNVTYVVK	10.54977	O75369	FLNB HUMAN	550.286788	3	0.809395254	2	
IAGPGLGSGVR	12.90753	O75369	FLNB HUMAN	492.285815	2	0.700395763	2	Yes
IAGPGLGSGVR	12.90753	O75369	FLNB HUMAN	328.526485	3	0.700395763	2	
IEYNDQNDGSCDVK	-10.62885	O75369	FLNB HUMAN	828.8444855	2	0.771760285	2	Yes
IEYNDQNDGSCDVK	-10.62885	O75369	FLNB HUMAN	552.898932	3	0.771760285	2	
IFAQDGEGQR	-14.64151	O75369	FLNB HUMAN	560.773269	2	0.697246075	2	Yes
IFAQDGEGQR	-14.64151	O75369	FLNB HUMAN	374.1847877	3	0.697246075	2	
IFFAGDTIPK	51.81162	O75369	FLNB HUMAN	554.806049	2	0.869869113	2	Yes
IFFAGDTIPK	51.81162	O75369	FLNB HUMAN	370.206641	3	0.869869113	2	
IGNLQTDLSDGLR	49.53728	O75369	FLNB HUMAN	701.370804	2	0.818823278	2	Yes
IGNLQTDLSDGLR	49.53728	O75369	FLNB HUMAN	467.9164777	3	0.818823278	2	
IPEINSSDMSAHVTSPSGR	26.59369	O75369	FLNB HUMAN	992.9738265	2	0.708506525		
IPEINSSDMSAHVTSPSGR	26.59369	O75369	FLNB HUMAN	662.3184927	3	0.708506525		
IPYLPITNFNQNWQDGK	87.1591	O75369	FLNB HUMAN	1024.515984	2	0.85060823	3	
IPYLPITNFNQNWQDGK	87.1591	O75369	FLNB HUMAN	683.3465977	3	0.85060823	3	Yes
LDVTILSPSR	52.71249	O75369	FLNB HUMAN	550.819687	2	0.753076077	2	Yes
LDVTILSPSR	52.71249	O75369	FLNB HUMAN	367.5490663	3	0.753076077	2	
LKPGAPLKPK	-27.6831	O75369	FLNB HUMAN	524.848051	2	0.777250051	2	Yes
LKPGAPLKPK	-27.6831	O75369	FLNB HUMAN	350.2346423	3	0.777250051	2	
LPNNHIGISFIPR	47.72252	O75369	FLNB HUMAN	739.417891	2	0.83805275		
LPNNHIGISFIPR	47.72252	O75369	FLNB HUMAN	493.2812023	3	0.83805275		
LTVMSLQESGLK	48.97484	O75369	FLNB HUMAN	653.3583195	2	0.831425011	2	Yes
LTVMSLQESGLK	48.97484	O75369	FLNB HUMAN	435.9081547	3	0.831425011	2	
LVSPGSANETSSILVESVTR	67.93507	O75369	FLNB HUMAN	1023.539857	2	0.680442393	3	
LVSPGSANETSSILVESVTR	67.93507	O75369	FLNB HUMAN	682.695846	3	0.680442393	3	Yes
MDCQETPEGYK	-12.06428	O75369	FLNB HUMAN	679.274123	2	0.789198458	2	Yes
MDCQETPEGYK	-12.06428	O75369	FLNB HUMAN	453.185357	3	0.789198458	2	
MDGTYACSYTPVK	22.82537	O75369	FLNB HUMAN	746.8265185	2	0.777821124	2	Yes
MDGTYACSYTPVK	22.82537	O75369	FLNB HUMAN	498.2202873	3	0.777821124	2	
NTVELLVEDK	36.49838	O75369	FLNB HUMAN	580.3144345	2	0.679082692		
NTVELLVEDK	36.49838	O75369	FLNB HUMAN	387.2122313	3	0.679082692		
SGCIVNNLAEFTVDPK	71.15722	O75369	FLNB HUMAN	882.435818	2	0.734823525		
SGCIVNNLAEFTVDPK	71.15722	O75369	FLNB HUMAN	588.626487	3	0.734823525		
SPFEVQVGPEAGMQK	44.92677	O75369	FLNB HUMAN	802.3934225	2	0.824041605	2	Yes
SPFEVQVGPEAGMQK	44.92677	O75369	FLNB HUMAN	535.26489	3	0.824041605	2	
SPFTVGVAAPLDLSK	82.04901	O75369	FLNB HUMAN	751.417223	2	0.779442251	2	Yes
SPFTVGVAAPLDLSK	82.04901	O75369	FLNB HUMAN	501.280757	3	0.779442251	2	
SPFVVQVGEACNPNACR	42.11913	O75369	FLNB HUMAN	952.9412765	2	0.726762533	3	
SPFVVQVGEACNPNACR	42.11913	O75369	FLNB HUMAN	635.630126	3	0.726762533	3	Yes
SSFLVDCSK	13.50303	O75369	FLNB HUMAN	521.747873	2	0.611437261	2	Yes
SSFLVDCSK	13.50303	O75369	FLNB HUMAN	348.167857	3	0.611437261	2	
SSTETCYSAIPK	3.916985	O75369	FLNB HUMAN	672.311562	2	0.829028368	2	Yes
SSTETCYSAIPK	3.916985	O75369	FLNB HUMAN	448.5436497	3	0.829028368	2	
TATPEIVDNK	-4.652534	O75369	FLNB HUMAN	544.2856775	2	0.699802041	2	Yes
TATPEIVDNK	-4.652534	O75369	FLNB HUMAN	363.19306	3	0.699802041	2	
TFEMSDFIVDTR	77.70653	O75369	FLNB HUMAN	730.840491	2	0.835240006	2	Yes
TFEMSDFIVDTR	77.70653	O75369	FLNB HUMAN	487.5629357	3	0.835240006	2	
TGEEVGFVVDAK	34.95842	O75369	FLNB HUMAN	625.817342	2	0.835317314	2	Yes
TGEEVGFVVDAK	34.95842	O75369	FLNB HUMAN	417.547503	3	0.835317314	2	
TYSVEYLPK	31.10507	O75369	FLNB HUMAN	550.28768	2	0.76316607	2	Yes
TYSVEYLPK	31.10507	O75369	FLNB HUMAN	367.194395	3	0.76316607	2	
VDIQTEDLEDGTCK	28.4296	O75369	FLNB HUMAN	811.864892	2	0.808645368		
VDIQTEDLEDGTCK	28.4296	O75369	FLNB HUMAN	541.579203	3	0.808645368		
VFGPGIEGK	14.64826	O75369	FLNB HUMAN	452.2509095	2	0.769922435	2	Yes
VFGPGIEGK	14.64826	O75369	FLNB HUMAN	301.836548	3	0.769922435	2	
VHSPSGAVEECHVSELEPDK	6.796181	O75369	FLNB HUMAN	1103.508231	2	0.743250728	4	

VHSPSGAVEECHVSELEPDK	6.796181	O75369	FLNB HUMAN	736.0080957	3	0.743250728	4	
VLFASQEIPASPFR	75.26878	O75369	FLNB HUMAN	781.4228395	2	0.926880777	2	Yes
VLFASQEIPASPFR	75.26878	O75369	FLNB HUMAN	521.2845013	3	0.926880777	2	
VLPTYDASK	0.464321	O75369	FLNB HUMAN	497.266752	2	0.795837641		
VLPTYDASK	0.464321	O75369	FLNB HUMAN	331.8471097	3	0.795837641		
VLQSFTVDSSK	21.38763	O75369	FLNB HUMAN	605.819884	2	0.851889491		
VLQSFTVDSSK	21.38763	O75369	FLNB HUMAN	404.2158643	3	0.851889491		
VLSEDEEDVDFDIIHNANDTFTVK	80.30247	O75369	FLNB HUMAN	1383.1436	2	0.678185999	3	
VLSEDEEDVDFDIIHNANDTFTVK	80.30247	O75369	FLNB HUMAN	922.431675	3	0.678185999	3	Yes
VMYTPMAPGNYLISVK	78.88557	O75369	FLNB HUMAN	892.460242	2	0.795036077	3	
VMYTPMAPGNYLISVK	78.88557	O75369	FLNB HUMAN	595.3094363	3	0.795036077	3	Yes
VNIGQGSHPQK	-31.78474	O75369	FLNB HUMAN	582.8101855	2	0.74418509		
VNIGQGSHPQK	-31.78474	O75369	FLNB HUMAN	388.8760653	3	0.74418509		
VNQPASFAIR	21.30868	O75369	FLNB HUMAN	551.8043715	2	0.738766372	2	Yes
VNQPASFAIR	21.30868	O75369	FLNB HUMAN	368.2055227	3	0.738766372	2	
VQAQGPGLK	-25.37325	O75369	FLNB HUMAN	449.261809	2	0.70672971	2	Yes
VQAQGPGLK	-25.37325	O75369	FLNB HUMAN	299.8438143	3	0.70672971	2	
VSYFPTVPGVYIVSTK	93.03474	O75369	FLNB HUMAN	878.980179	2	0.791791201	3	
VSYFPTVPGVYIVSTK	93.03474	O75369	FLNB HUMAN	586.3227277	3	0.791791201	3	Yes
VTASGPGLSSYGVPASLPVDFAIDAR	106.4035	O75369	FLNB HUMAN	1274.158656	2	0.787092686	3	
VTASGPGLSSYGVPASLPVDFAIDAR	106.4035	O75369	FLNB HUMAN	849.7750453	3	0.787092686	3	Yes
VTEAEIVPMGK	29.50228	O75369	FLNB HUMAN	587.313381	2	0.704153836	2	Yes
VTEAEIVPMGK	29.50228	O75369	FLNB HUMAN	391.8781957	3	0.704153836	2	
VTVLFAGQHISK	23.17395	O75369	FLNB HUMAN	650.3751615	2	0.873451531	3	
VTVLFAGQHISK	23.17395	O75369	FLNB HUMAN	433.9193827	3	0.873451531	3	Yes
VVASGPGLEHGK	-27.20562	O75369	FLNB HUMAN	575.8149365	2	0.833516896	3	
VVASGPGLEHGK	-27.20562	O75369	FLNB HUMAN	384.212566	3	0.833516896	3	Yes
VVPCLVTPVTGR	48.71743	O75369	FLNB HUMAN	649.3690225	2	0.617010295	2	Yes
VVPCLVTPVTGR	48.71743	O75369	FLNB HUMAN	433.2486233	3	0.617010295	2	
YADEEIPR	-0.080544	O75369	FLNB HUMAN	496.7383585	2	0.785312057		
YADEEIPR	-0.080544	O75369	FLNB HUMAN	331.4948473	3	0.785312057		
YAPTEVGLHEMHIK	15.29621	O75369	FLNB HUMAN	812.911778	2	0.847094893	3	
YAPTEVGLHEMHIK	15.29621	O75369	FLNB HUMAN	542.277127	3	0.847094893	3	Yes
YGGELVPHFPAR	33.1151	O75369	FLNB HUMAN	671.8493065	2	0.787401319	3	
YGGELVPHFPAR	33.1151	O75369	FLNB HUMAN	448.2354793	3	0.787401319	3	Yes
YGGPNHIVGSPFK	18.25402	O75369	FLNB HUMAN	686.8545885	2	0.71948278		
YGGPNHIVGSPFK	18.25402	O75369	FLNB HUMAN	458.2390007	3	0.71948278		
YMIGVTYGGDDIPLSPYR	85.94522	O75369	FLNB HUMAN	1008.990945	2	0.779216468	3	
YMIGVTYGGDDIPLSPYR	85.94522	O75369	FLNB HUMAN	672.9965717	3	0.779216468	3	Yes
YSIAITWGGHHIPK	34.01486	O75369	FLNB HUMAN	790.42317	2	0.796570301	3	
YSIAITWGGHHIPK	34.01486	O75369	FLNB HUMAN	527.2847217	3	0.796570301	3	Yes
YTPTQQGNMQVLVTYGGDPIPK	71.15932	O75369	FLNB HUMAN	1204.102292	2	0.695031703	3	
YTPTQQGNMQVLVTYGGDPIPK	71.15932	O75369	FLNB HUMAN	803.070803	3	0.695031703	3	Yes
LEAAYLDLQR	45.71084	O75347	TBCA HUMAN	596.32259	2	0.835852742	2	Yes
LEAAYLDLQR	45.71084	O75347	TBCA HUMAN	397.884335	3	0.835852742	2	
AFTDVPSIQIYSSR	65.70468	O75122	CLAP2 HUMAN	792.407382	2	0.780468047		
AFTDVPSIQIYSSR	65.70468	O75122	CLAP2 HUMAN	528.6075297	3	0.780468047		
AGGDATDSSQTALDNK	-16.54473	O75122	CLAP2 HUMAN	775.85063	2	0.600278616		
AGGDATDSSQTALDNK	-16.54473	O75122	CLAP2 HUMAN	517.569695	3	0.600278616		
ASSLPGSLQR	7.34557	O75122	CLAP2 HUMAN	508.280729	2	0.683512211		
ASSLPGSLQR	7.34557	O75122	CLAP2 HUMAN	339.189761	3	0.683512211		
DYNPYNYSDSISPFNK	64.48347	O75122	CLAP2 HUMAN	962.4239475	2	0.740255296		
DYNPYNYSDSISPFNK	64.48347	O75122	CLAP2 HUMAN	641.9519067	3	0.740255296		
EACITVAHLSTVLGNK	46.70679	O75122	CLAP2 HUMAN	856.9541785	2	0.717083216		
EACITVAHLSTVLGNK	46.70679	O75122	CLAP2 HUMAN	571.6387273	3	0.717083216		
EAMFDDDADQFPDDLSLDHSDLVAELLK	141.2443	O75122	CLAP2 HUMAN	1582.716804	2	0.78240037		
EAMFDDDADQFPDDLSLDHSDLVAELLK	141.2443	O75122	CLAP2 HUMAN	1055.480477	3	0.78240037		
EGGAGAVDEDDFIK	30.87463	O75122	CLAP2 HUMAN	711.8233525	2	0.692400455		
EGGAGAVDEDDFIK	30.87463	O75122	CLAP2 HUMAN	474.8848433	3	0.692400455		
ESFPNDLQFNILMR	102.96	O75122	CLAP2 HUMAN	862.4277955	2	0.671510696		
ESFPNDLQFNILMR	102.96	O75122	CLAP2 HUMAN	575.2878053	3	0.671510696		
ETLNLLLPEIMPGLIQGYDNSESSVR	155.4729	O75122	CLAP2 HUMAN	1444.739683	2	0.652162194		
ETLNLLLPEIMPGLIQGYDNSESSVR	155.4729	O75122	CLAP2 HUMAN	963.4957303	3	0.652162194		
FDHGAEAIVPTLFNLVPNSAK	104.8191	O75122	CLAP2 HUMAN	1120.589685	2	0.751287103		
FDHGAEAIVPTLFNLVPNSAK	104.8191	O75122	CLAP2 HUMAN	747.3957313	3	0.751287103		
FTVDQTQTPSLK	27.2692	O75122	CLAP2 HUMAN	682.856999	2	0.820761919	2	Yes
FTVDQTQTPSLK	27.2692	O75122	CLAP2 HUMAN	455.573941	3	0.820761919	2	
GVTEAIQNFSFR	58.49829	O75122	CLAP2 HUMAN	684.8495075	2	0.74220556		
GVTEAIQNFSFR	58.49829	O75122	CLAP2 HUMAN	456.90228	3	0.74220556		
IALYELMK	57.13728	O75122	CLAP2 HUMAN	490.778441	2	0.744819641		
IALYELMK	57.13728	O75122	CLAP2 HUMAN	327.521569	3	0.744819641		
LGAGALNAGSYASLEDTSDK	50.02074	O75122	CLAP2 HUMAN	970.4663535	2	0.754225492		
LGAGALNAGSYASLEDTSDK	50.02074	O75122	CLAP2 HUMAN	647.3135107	3	0.754225492		
LIPLITSNCTSK	45.8373	O75122	CLAP2 HUMAN	673.871594	2	0.639636397		
LIPLITSNCTSK	45.8373	O75122	CLAP2 HUMAN	449.583671	3	0.639636397		
LSAPLAGMGNAK	25.97941	O75122	CLAP2 HUMAN	565.30589	2	0.684674919		
LSAPLAGMGNAK	25.97941	O75122	CLAP2 HUMAN	377.206535	3	0.684674919		
LTQEESFSVWDEHFK	50.40951	O75122	CLAP2 HUMAN	941.436872	2	0.826672554		
LTQEESFSVWDEHFK	50.40951	O75122	CLAP2 HUMAN	627.960523	3	0.826672554		
NHFPGEAETLYNSLEPSYQK	52.83697	O75122	CLAP2 HUMAN	1162.545662	2	0.730937958		
NHFPGEAETLYNSLEPSYQK	52.83697	O75122	CLAP2 HUMAN	775.366383	3	0.730937958		
NYAELTVMK	28.05439	O75122	CLAP2 HUMAN	534.773887	2	0.739394307		
NYAELTVMK	28.05439	O75122	CLAP2 HUMAN	356.8518663	3	0.739394307		
SAEEAASVLATSISPEQCIK	68.43088	O75122	CLAP2 HUMAN	1046.017901	2	0.731657445		
SAEEAASVLATSISPEQCIK	68.43088	O75122	CLAP2 HUMAN	697.6812087	3	0.731657445		
SDIDVNAAAGAK	3.175938	O75122	CLAP2 HUMAN	566.2862085	2	0.739650905		
SDIDVNAAAGAK	3.175938	O75122	CLAP2 HUMAN	377.8600807	3	0.739650905		
SFEFLDLLLQEWQTHSLER	156.7077	O75122	CLAP2 HUMAN	1196.103156	2	0.610617816		
SFEFLDLLLQEWQTHSLER	156.7077	O75122	CLAP2 HUMAN	797.7380457	3	0.610617816		
SLQTYLK	10.41244	O75122	CLAP2 HUMAN	426.7454555	2	0.645069361		
SLQTYLK	10.41244	O75122	CLAP2 HUMAN	284.832912	3	0.645069361		
SQEDMNEPLK	-4.637451	O75122	CLAP2 HUMAN	595.7720765	2	0.727173269		
SQEDMNEPLK	-4.637451	O75122	CLAP2 HUMAN	397.517326	3	0.727173269		
SSGSVASLPQSDR	2.373772	O75122	CLAP2 HUMAN	645.8184035	2	0.753031135	2	Yes
SSGSVASLPQSDR	2.373772	O75122	CLAP2 HUMAN	430.881544	3	0.753031135	2	
STGALYAPEVYGASGPGYGISQSSR	58.87196	O75122	CLAP2 HUMAN	1238.093504	2	0.819983125		
STGALYAPEVYGASGPGYGISQSSR	58.87196	O75122	CLAP2 HUMAN	825.731611	3	0.819983125		
VITWTTEPK	23.27676	O75122	CLAP2 HUMAN	537.7956815	2	0.712219596		
VITWTTEPK	23.27676	O75122	CLAP2 HUMAN	358.866396	3	0.712219596		
VLNTGSDVEEAVADALK	75.72772	O75122	CLAP2 HUMAN	865.94453	2	0.827240348	2	Yes
VLNTGSDVEEAVADALK	75.72772	O75122	CLAP2 HUMAN	577.632295	3	0.827240348	2	

VLTTTALSTVSSGVQR	38.33479	O75122	CLAP2 HUMAN	810.452326	2	0.783657134		
VLTTTALSTVSSGVQR	38.33479	O75122	CLAP2 HUMAN	540.6374923	3	0.783657134		
VMATSGCAAIR	-7.647652	O75122	CLAP2 HUMAN	568.7817255	2	0.652996898		
VMATSGCAAIR	-7.647652	O75122	CLAP2 HUMAN	379.5237587	3	0.652996898		
WSTANPSTVAGR	6.484295	O75122	CLAP2 HUMAN	623.812925	2	0.602852941		
WSTANPSTVAGR	6.484295	O75122	CLAP2 HUMAN	416.211225	3	0.602852941		
ASDPDEAGGPEGSEAVQSGTPEEPEPELEAEASK	43.98377	O60936	NOL3 HUMAN	1698.740247	2	0.691343963	3	
ASDPDEAGGPEGSEAVQSGTPEEPEPELEAEASK	43.98377	O60936	NOL3 HUMAN	1132.82944	3	0.691343963	3	Yes
GEAACQELLR	9.786541	O60936	NOL3 HUMAN	573.7827795	2	0.720083654	2	Yes
GEAACQELLR	9.786541	O60936	NOL3 HUMAN	382.8577947	3	0.720083654	2	
GVLTGPEYEALDALPDAER	81.46675	O60936	NOL3 HUMAN	1008.500197	2	0.847181082	3	
GVLTGPEYEALDALPDAER	81.46675	O60936	NOL3 HUMAN	672.6694063	3	0.847181082	3	Yes
LLLLVQGK	41.65546	O60936	NOL3 HUMAN	442.302945	2	0.707832992	2	Yes
LLLLVQGK	41.65546	O60936	NOL3 HUMAN	295.2045717	3	0.707832992	2	
SYDPPCPGHWTPEAPGSGTTCPGLPR	48.24362	O60936	NOL3 HUMAN	1397.62159	2	0.731293797	3	
SYDPPCPGHWTPEAPGSGTTCPGLPR	48.24362	O60936	NOL3 HUMAN	932.0836683	3	0.731293797	3	Yes
TAGAPDPAWDWQHVGPGYR	52.75929	O60936	NOL3 HUMAN	1040.985384	2	0.81258446		
TAGAPDPAWDWQHVGPGYR	52.75929	O60936	NOL3 HUMAN	694.3261973	3	0.81258446		
DTKPELEIDVK	27.60317	O60934	NBN HUMAN	643.846099	2	0.780827463		
DTKPELEIDVK	27.60317	O60934	NBN HUMAN	429.5666743	3	0.780827463		
EESLADDLFR	67.40031	O60934	NBN HUMAN	597.786041	2	0.633158147	2	Yes
EESLADDLFR	67.40031	O60934	NBN HUMAN	398.859969	3	0.633158147	2	
EQHLSENEPVDTNSDNNLFTDTDLK	49.18667	O60934	NBN HUMAN	1438.147402	2	0.675623357		
EQHLSENEPVDTNSDNNLFTDTDLK	49.18667	O60934	NBN HUMAN	959.1008763	3	0.675623357		
IETSCSLLEQTQPATPSLWK	74.17342	O60934	NBN HUMAN	1145.075751	2	0.772901833		
IETSCSLLEQTQPATPSLWK	74.17342	O60934	NBN HUMAN	763.7197753	3	0.772901833		
IPNYQLSPTK	19.47038	O60934	NBN HUMAN	580.819683	2	0.781436205	2	Yes
IPNYQLSPTK	19.47038	O60934	NBN HUMAN	387.5490637	3	0.781436205	2	
LLLTEFR	47.50732	O60934	NBN HUMAN	446.269102	2	0.705215514		
LLLTEFR	47.50732	O60934	NBN HUMAN	297.8486763	3	0.705215514		
LLPAAGPAGGEPYR	26.46813	O60934	NBN HUMAN	684.8676965	2	0.729786396	2	Yes
LLPAAGPAGGEPYR	26.46813	O60934	NBN HUMAN	456.914406	3	0.729786396	2	
LLTGVEYVVGR	52.05331	O60934	NBN HUMAN	603.348608	2	0.798430681	2	Yes
LLTGVEYVVGR	52.05331	O60934	NBN HUMAN	402.568347	3	0.798430681	2	
LMPSAPVNTTTYVADTESEQADTWDLSERPK	67.6488	O60934	NBN HUMAN	1726.812295	2	0.632938206	4	
LMPSAPVNTTTYVADTESEQADTWDLSERPK	67.6488	O60934	NBN HUMAN	1151.544138	3	0.632938206	4	
LPHIIGGSDLIAHHAR	19.63849	O60934	NBN HUMAN	853.9766365	2	0.793499768	4	
LPHIIGGSDLIAHHAR	19.63849	O60934	NBN HUMAN	569.6536993	3	0.793499768	4	
LQDDSEMLPK	14.18636	O60934	NBN HUMAN	588.28482	2	0.810711026		
LQDDSEMLPK	14.18636	O60934	NBN HUMAN	392.5258217	3	0.810711026		
LSSAVVFGGGEAR	26.52339	O60934	NBN HUMAN	625.3309505	2	0.646336913	2	Yes
LSSAVVFGGGEAR	26.52339	O60934	NBN HUMAN	417.223242	3	0.646336913	2	
MDIETNDTFSDEAVPESSK	48.70966	O60934	NBN HUMAN	1057.957705	2	0.793860912		
MDIETNDTFSDEAVPESSK	48.70966	O60934	NBN HUMAN	705.6410783	3	0.793860912		
MLSQDAPTVK	-0.244156	O60934	NBN HUMAN	545.2846235	2	0.751275539	2	Yes
MLSQDAPTVK	-0.244156	O60934	NBN HUMAN	363.859024	3	0.751275539	2	
NPSGINDDYGQLK	19.64276	O60934	NBN HUMAN	710.839332	2	0.83860743		
NPSGINDDYGQLK	19.64276	O60934	NBN HUMAN	474.2288297	3	0.83860743		
NTELEEWLR	58.70802	O60934	NBN HUMAN	595.296576	2	0.723431408	2	Yes
NTELEEWLR	58.70802	O60934	NBN HUMAN	397.2003257	3	0.723431408	2	
SGDGITFGVFGSK	58.32374	O60934	NBN HUMAN	636.317509	2	0.804159164		
SGDGITFGVFGSK	58.32374	O60934	NBN HUMAN	424.5476143	3	0.804159164		
TTTPGPSLSQGVSVDEK	24.98924	O60934	NBN HUMAN	851.9288805	2	0.807273448		
TTTPGPSLSQGVSVDEK	24.98924	O60934	NBN HUMAN	568.2885287	3	0.807273448		
YGTFVNEEK	-0.255783	O60934	NBN HUMAN	543.759291	2	0.756746054	2	Yes
YGTFVNEEK	-0.255783	O60934	NBN HUMAN	362.8421357	3	0.756746054	2	
AYLTEENGQIAVFDATNTTR	61.51085	O60825	F262 HUMAN	1107.537843	2	0.680229902		
AYLTEENGQIAVFDATNTTR	61.51085	O60825	F262 HUMAN	738.6945033	3	0.680229902		
DMILNFAEQNSFK	82.38103	O60825	F262 HUMAN	778.8748645	2	0.82506752		
DMILNFAEQNSFK	82.38103	O60825	F262 HUMAN	519.5858513	3	0.82506752		
FLEEQEITDLK	52.25749	O60825	F262 HUMAN	682.8513815	2	0.854372084		
FLEEQEITDLK	52.25749	O60825	F262 HUMAN	455.570196	3	0.854372084		
GADELPYLR	37.82588	O60825	F262 HUMAN	517.269826	2	0.71173209		
GADELPYLR	37.82588	O60825	F262 HUMAN	345.1824923	3	0.71173209		
HGESEFNLLGK	23.14776	O60825	F262 HUMAN	615.8098505	2	0.741396844	2	Yes
HGESEFNLLGK	23.14776	O60825	F262 HUMAN	410.875842	3	0.741396844	2	
NSFTPLSSSNTIR	31.20599	O60825	F262 HUMAN	712.362978	2	0.797352731		
NSFTPLSSSNTIR	31.20599	O60825	F262 HUMAN	475.2445937	3	0.797352731		
TIQTAESLGVPYEQWK	61.87206	O60825	F262 HUMAN	925.4707115	2	0.848869324		
TIQTAESLGVPYEQWK	61.87206	O60825	F262 HUMAN	617.316416	3	0.848869324		
VFNLGVYR	39.40757	O60825	F262 HUMAN	484.2721715	2	0.810319304	2	Yes
VFNLGVYR	39.40757	O60825	F262 HUMAN	323.184056	3	0.810319304	2	
VQDYIQSK	-13.13998	O60825	F262 HUMAN	490.7565515	2	0.720351577		
VQDYIQSK	-13.13998	O60825	F262 HUMAN	327.506976	3	0.720351577		
YPGGESYQDLVQR	32.48838	O60825	F262 HUMAN	756.360428	2	0.798890769	2	Yes
YPGGESYQDLVQR	32.48838	O60825	F262 HUMAN	504.576227	3	0.798890769	2	
EGNFDIVSGTR	29.28572	O60762	DPM1 HUMAN	597.791658	2	0.630741119	2	Yes
EGNFDIVSGTR	29.28572	O60762	DPM1 HUMAN	398.8637137	3	0.630741119	2	
FIPEFIR	55.23841	O60762	DPM1 HUMAN	461.2638195	2	0.735792279		
FIPEFIR	55.23841	O60762	DPM1 HUMAN	307.8451547	3	0.735792279		
GYVFQMEMIVR	84.21461	O60762	DPM1 HUMAN	686.8417785	2	0.710530102		
GYVFQMEMIVR	84.21461	O60762	DPM1 HUMAN	458.2304607	3	0.710530102		
HATGNYIIIMDADLSHHPK	32.01497	O60762	DPM1 HUMAN	1067.031476	2	0.668651044	4	
HATGNYIIIMDADLSHHPK	32.01497	O60762	DPM1 HUMAN	711.6902587	3	0.668651044	4	
LGGNEIVSFLK	70.71111	O60762	DPM1 HUMAN	588.835337	2	0.762973309	2	Yes
LGGNEIVSFLK	70.71111	O60762	DPM1 HUMAN	392.892833	3	0.762973309	2	
LGLGTAYIHGMK	29.65797	O60762	DPM1 HUMAN	630.842636	2	0.797412634	3	
LGLGTAYIHGMK	29.65797	O60762	DPM1 HUMAN	420.897699	3	0.797412634	3	Yes
YSVLLPTYNER	49.798	O60762	DPM1 HUMAN	677.8542495	2	0.883696675	2	Yes
YSVLLPTYNER	49.798	O60762	DPM1 HUMAN	452.2387747	3	0.883696675	2	
ADAIFQEGIQQK	33.0739	O60566	BUB1B HUMAN	674.349341	2	0.831954062	2	Yes
ADAIFQEGIQQK	33.0739	O60566	BUB1B HUMAN	449.902169	3	0.831954062	2	
AEIVHGDLSPR	-0.999237	O60566	BUB1B HUMAN	597.317843	2	0.742763817	3	
AEIVHGDLSPR	-0.999237	O60566	BUB1B HUMAN	398.547837	3	0.742763817	3	Yes
AFEYEIR	20.37074	O60566	BUB1B HUMAN	464.232712	2	0.811991572		
AFEYEIR	20.37074	O60566	BUB1B HUMAN	309.8244163	3	0.811991572		
DGELWNK	11.24323	O60566	BUB1B HUMAN	431.209241	2	0.717566192		
DGELWNK	11.24323	O60566	BUB1B HUMAN	287.808769	3	0.717566192		
EATHSSGFSGSSASVASTSSIK	-6.140575	O60566	BUB1B HUMAN	1042.990728	2	0.779094815	3	
EATHSSGFSGSSASVASTSSIK	-6.140575	O60566	BUB1B HUMAN	695.6630933	3	0.779094815	3	Yes
EEEEEVFESSVPQR	35.38794	O60566	BUB1B HUMAN	847.381762	2	0.694930792		

EEEEEVFESSVPQR	35.38794	O60566	BUB1B_HUMAN	565.2571163	3	0.694930792		
EGGALSEAMSLEGDEWELSK	86.28938	O60566	BUB1B_HUMAN	1069.48389	2	0.63807106		
EGGALSEAMSLEGDEWELSK	86.28938	O60566	BUB1B_HUMAN	713.3252017	3	0.63807106		
EIELGNEDYCIK	41.92358	O60566	BUB1B_HUMAN	741.843226	2	0.765305698	2	Yes
EIELGNEDYCIK	41.92358	O60566	BUB1B_HUMAN	494.8980923	3	0.765305698	2	
ENELQAGPWNTGR	27.7817	O60566	BUB1B_HUMAN	736.350403	2	0.64718473		
ENELQAGPWNTGR	27.7817	O60566	BUB1B_HUMAN	491.2362103	3	0.64718473		
ESNMSTLLER	28.67267	O60566	BUB1B_HUMAN	590.2878935	2	0.636783361		
ESNMSTLLER	28.67267	O60566	BUB1B_HUMAN	393.861204	3	0.636783361		
ETSLAENIWQEQPHSK	31.97556	O60566	BUB1B_HUMAN	948.958503	2	0.766897142		
ETSLAENIWQEQPHSK	31.97556	O60566	BUB1B_HUMAN	632.9749437	3	0.766897142		
EYLICEDYK	27.15878	O60566	BUB1B_HUMAN	616.779362	2	0.668824613		
EYLICEDYK	27.15878	O60566	BUB1B_HUMAN	411.522183	3	0.668824613		
FLNLWLK	74.66595	O60566	BUB1B_HUMAN	467.282012	2	0.745529652		
FLNLWLK	74.66595	O60566	BUB1B_HUMAN	311.857283	3	0.745529652		
FVSTPFHEIMSLK	62.17377	O60566	BUB1B_HUMAN	768.4005185	2	0.722838879		
FVSTPFHEIMSLK	62.17377	O60566	BUB1B_HUMAN	512.602954	3	0.722838879		
FYTGNDPLDVWDR	70.89553	O60566	BUB1B_HUMAN	799.368257	2	0.836104035		
FYTGNDPLDVWDR	70.89553	O60566	BUB1B_HUMAN	533.248113	3	0.836104035		
GLQNPFPQQMQNNSR	31.72614	O60566	BUB1B_HUMAN	879.921201	2	0.790716469		
GLQNPFPQQMQNNSR	31.72614	O60566	BUB1B_HUMAN	586.9500757	3	0.790716469		
GNTASLIAVPAVLPSFTPYVEETAR	134.6196	O60566	BUB1B_HUMAN	1302.189956	2	0.619213879		
GNTASLIAVPAVLPSFTPYVEETAR	134.6196	O60566	BUB1B_HUMAN	868.462579	3	0.619213879		
IEPSINHILSTR	32.32837	O60566	BUB1B_HUMAN	690.3862565	2	0.771540105		
IEPSINHILSTR	32.32837	O60566	BUB1B_HUMAN	460.593446	3	0.771540105		
IMSTLQGALAQESACNNTLQQQK	57.77698	O60566	BUB1B_HUMAN	1267.621063	2	0.645482481		
IMSTLQGALAQESACNNTLQQQK	57.77698	O60566	BUB1B_HUMAN	845.41665	3	0.645482481		
ITVFDENADEASTAELSKPTVQPWIAPPMPR	90.97977	O60566	BUB1B_HUMAN	1705.85103	2	0.721438825		
ITVFDENADEASTAELSKPTVQPWIAPPMPR	90.97977	O60566	BUB1B_HUMAN	1137.569962	3	0.721438825		
IVDFSYSVDLR	65.71059	O60566	BUB1B_HUMAN	657.340979	2	0.789696336		
IVDFSYSVDLR	65.71059	O60566	BUB1B_HUMAN	438.563261	3	0.789696336		
IYAGVGEFSFEEIR	78.69238	O60566	BUB1B_HUMAN	808.9019325	2	0.734650075		
IYAGVGEFSFEEIR	78.69238	O60566	BUB1B_HUMAN	539.6038967	3	0.734650075		
LFWVAPR	50.30695	O60566	BUB1B_HUMAN	444.7587045	2	0.740602255		
LFWVAPR	50.30695	O60566	BUB1B_HUMAN	296.8417447	3	0.740602255		
LLPEEDLDVK	38.04776	O60566	BUB1B_HUMAN	585.81681	2	0.783209324	2	Yes
LLPEEDLDVK	38.04776	O60566	BUB1B_HUMAN	390.8804817	3	0.783209324	2	
LSPIIEDSR	19.4329	O60566	BUB1B_HUMAN	515.282937	2	0.653382659		
LSPIIEDSR	19.4329	O60566	BUB1B_HUMAN	343.8578997	3	0.653382659		
LSQNISELK	6.281303	O60566	BUB1B_HUMAN	516.290762	2	0.763465643		
LSQNISELK	6.281303	O60566	BUB1B_HUMAN	344.529783	3	0.763465643		
TSEDQQTACGTIYSQTLSIK	45.35464	O60566	BUB1B_HUMAN	1116.028994	2	0.694822311		
TSEDQQTACGTIYSQTLSIK	45.35464	O60566	BUB1B_HUMAN	744.3552707	3	0.694822311		
TVQILEGQK	9.898579	O60566	BUB1B_HUMAN	508.293306	2	0.735769391		
TVQILEGQK	9.898579	O60566	BUB1B_HUMAN	339.1981457	3	0.735769391		
VQLDVFTLSGFR	96.09151	O60566	BUB1B_HUMAN	691.377901	2	0.711958468		
VQLDVFTLSGFR	96.09151	O60566	BUB1B_HUMAN	461.2545423	3	0.711958468		
YISWTEQNYPQGGK	31.99442	O60566	BUB1B_HUMAN	835.894635	2	0.782136738		
YISWTEQNYPQGGK	31.99442	O60566	BUB1B_HUMAN	557.5990317	3	0.782136738		
AAFAHFPELYDFALSNVAEVDTR	108.7315	O60306	AQR_HUMAN	1292.129898	2	0.814781249	3	
AAFAHFPELYDFALSNVAEVDTR	108.7315	O60306	AQR_HUMAN	861.7558737	3	0.814781249	3	Yes
ADVTINLNVR	38.07771	O60306	AQR_HUMAN	557.814936	2	0.842581749		
ADVTINLNVR	38.07771	O60306	AQR_HUMAN	372.2125657	3	0.842581749		
AGMQPGLTMVVGPPGTGK	58.17717	O60306	AQR_HUMAN	849.439851	2	0.755251884		
AGMQPGLTMVVGPPGTGK	58.17717	O60306	AQR_HUMAN	566.6291757	3	0.755251884		
ASLCNLYNWR	49.24255	O60306	AQR_HUMAN	648.811866	2	0.762441218	2	Yes
ASLCNLYNWR	49.24255	O60306	AQR_HUMAN	432.8771857	3	0.762441218	2	
ENVPAWEIFK	71.14925	O60306	AQR_HUMAN	616.819687	2	0.829094291	2	Yes
ENVPAWEIFK	71.14925	O60306	AQR_HUMAN	411.5490663	3	0.829094291	2	
FLSQLIQK	36.86738	O60306	AQR_HUMAN	488.795484	2	0.664558589	2	Yes
FLSQLIQK	36.86738	O60306	AQR_HUMAN	326.1995977	3	0.664558589	2	
FQGQQNDYILLSLVR	99.49486	O60306	AQR_HUMAN	897.481413	2	0.742741644		
FQGQQNDYILLSLVR	99.49486	O60306	AQR_HUMAN	598.6568837	3	0.742741644		
FYTGFEINDQTGNALTENEMTTIHYDR	72.82746	O60306	AQR_HUMAN	1590.714922	2	0.805663228		
FYTGFEINDQTGNALTENEMTTIHYDR	72.82746	O60306	AQR_HUMAN	1060.812556	3	0.805663228		
GCEIQGMLDDK	27.36595	O60306	AQR_HUMAN	633.2792125	2	0.753148496		
GCEIQGMLDDK	27.36595	O60306	AQR_HUMAN	422.5220833	3	0.753148496		
GPYPYNQPK	-14.54776	O60306	AQR_HUMAN	532.2645395	2	0.750528932	2	Yes
GPYPYNQPK	-14.54776	O60306	AQR_HUMAN	355.178968	3	0.750528932	2	
GSTLPDVTEVSTFFPFHEYFANAPQPIFK	137.2711	O60306	AQR_HUMAN	1643.808956	2	0.609638512		
GSTLPDVTEVSTFFPFHEYFANAPQPIFK	137.2711	O60306	AQR_HUMAN	1096.208579	3	0.609638512		
IFTQLEEFR	51.79603	O60306	AQR_HUMAN	591.8118625	2	0.805237293	2	Yes
IFTQLEEFR	51.79603	O60306	AQR_HUMAN	394.8771833	3	0.805237293	2	
IIWDENIVPTEYYSGEGCLALPK	106.5182	O60306	AQR_HUMAN	1334.15472	2	0.784599006		
IIWDENIVPTEYYSGEGCLALPK	106.5182	O60306	AQR_HUMAN	889.7724217	3	0.784599006		
IMALDIDER	39.26728	O60306	AQR_HUMAN	538.276798	2	0.758671463		
IMALDIDER	39.26728	O60306	AQR_HUMAN	359.1871403	3	0.758671463		
ISILTTYNGQK	30.71785	O60306	AQR_HUMAN	619.3435225	2	0.732743502		
ISILTTYNGQK	30.71785	O60306	AQR_HUMAN	413.2316233	3	0.732743502		
ISQIQQLNQMPLYPTEK	63.82465	O60306	AQR_HUMAN	1016.033153	2	0.852282405		
ISQIQQLNQMPLYPTEK	63.82465	O60306	AQR_HUMAN	677.691377	3	0.852282405		
KPDHFPFFFK	42.37353	O60306	AQR_HUMAN	655.34059	2	0.788493156	3	
KPDHFPFFFK	42.37353	O60306	AQR_HUMAN	437.2296683	3	0.788493156	3	Yes
LESTYEIR	4.913933	O60306	AQR_HUMAN	505.7618335	2	0.733275175	2	Yes
LESTYEIR	4.913933	O60306	AQR_HUMAN	337.5104973	3	0.733275175	2	
LNLQFLTLHDYLLR	98.6142	O60306	AQR_HUMAN	879.9992415	2	0.79765451	3	
LNLQFLTLHDYLLR	98.6142	O60306	AQR_HUMAN	587.0021027	3	0.79765451	3	Yes
MPNQIATLDFNDTFLSIEHLK	105.7822	O60306	AQR_HUMAN	1224.117949	2	0.793014348	3	
MPNQIATLDFNDTFLSIEHLK	105.7822	O60306	AQR_HUMAN	816.4145743	3	0.793014348	3	Yes
NTIQFTHTQIEAIR	31.49899	O60306	AQR_HUMAN	836.4448355	2	0.822088361		
NTIQFTHTQIEAIR	31.49899	O60306	AQR_HUMAN	557.965832	3	0.822088361		
RRPFIEQVGLVYVR	44.95181	O60306	AQR_HUMAN	866.5050255	2	0.66350764		
RRPFIEQVGLVYVR	44.95181	O60306	AQR_HUMAN	578.0059587	3	0.66350764		
SVPLSEPVTMDK	31.37244	O60306	AQR_HUMAN	651.834677	2	0.811111152	2	Yes
SVPLSEPVTMDK	31.37244	O60306	AQR_HUMAN	434.892393	3	0.811111152	2	
SYEEDMEIAEGCFR	52.9732	O60306	AQR_HUMAN	868.3510895	2	0.772940159	2	Yes
SYEEDMEIAEGCFR	52.9732	O60306	AQR_HUMAN	579.236668	3	0.772940159	2	
TDVAVQIISNIYHNFPEQR	101.4055	O60306	AQR_HUMAN	1122.574562	2	0.755895972	3	
TDVAVQIISNIYHNFPEQR	101.4055	O60306	AQR_HUMAN	748.7189827	3	0.755895972	3	Yes
TLIVEPHVIPNR	31.25598	O60306	AQR_HUMAN	694.4069925	2	0.755108237	3	
TLIVEPHVIPNR	31.25598	O60306	AQR_HUMAN	463.2739367	3	0.755108237	3	Yes

TLIVTHSNQALNQLFEK	51.97525	O60306	AQR HUMAN	978.5316385	2	0.845512688		
TLIVTHSNQALNQLFEK	51.97525	O60306	AQR HUMAN	652.6903673	3	0.845512688		
VGVPTVDLDAQGR	42.98669	O60306	AQR HUMAN	663.8547905	2	0.710102022	2	Yes
VGVPTVDLDAQGR	42.98669	O60306	AQR HUMAN	442.905802	3	0.710102022	2	
VIEDIYEK	14.63458	O60306	AQR HUMAN	504.7665845	2	0.819548726	2	Yes
VIEDIYEK	14.63458	O60306	AQR HUMAN	336.846998	3	0.819548726	2	
VTVEDPALQIPPFR	78.82707	O60306	AQR HUMAN	791.4359475	2	0.769925356		
VTVEDPALQIPPFR	78.82707	O60306	AQR HUMAN	527.9599067	3	0.769925356		
WIMIGDHHQLPPVIK	53.44346	O60306	AQR HUMAN	892.4879915	2	0.72302264	4	
WIMIGDHHQLPPVIK	53.44346	O60306	AQR HUMAN	595.327936	3	0.72302264	4	
YSNMEQSLFTR	38.8239	O60306	AQR HUMAN	688.319721	2	0.840614438		
YSNMEQSLFTR	38.8239	O60306	AQR HUMAN	459.2157557	3	0.840614438		
AAVWEVDFPDVAR	79.98474	O60294	TYW4 HUMAN	737.87044	2	0.853703499	2	Yes
AAVWEVDFPDVAR	79.98474	O60294	TYW4 HUMAN	492.2495683	3	0.853703499	2	
AFLEQIGAPQAALR	67.48679	O60294	TYW4 HUMAN	742.9151825	2	0.847416937		
AFLEQIGAPQAALR	67.48679	O60294	TYW4 HUMAN	495.61273	3	0.847416937		
AQILSLGAGFDSLYFR	112.3601	O60294	TYW4 HUMAN	879.4652315	2	0.705035985		
AQILSLGAGFDSLYFR	112.3601	O60294	TYW4 HUMAN	586.646096	3	0.705035985		
FPNALFVVYEQMR	93.3063	O60294	TYW4 HUMAN	807.4114135	2	0.732608199		
FPNALFVVYEQMR	93.3063	O60294	TYW4 HUMAN	538.6102173	3	0.732608199		
GDTLSHTLVFPSSEAFPR	62.84518	O60294	TYW4 HUMAN	980.992346	2	0.773593664		
GDTLSHTLVFPSSEAFPR	62.84518	O60294	TYW4 HUMAN	654.330839	3	0.773593664		
GYVQDPFAALLVPGAAR	109.7909	O60294	TYW4 HUMAN	872.9732245	2	0.743115306		
GYVQDPFAALLVPGAAR	109.7909	O60294	TYW4 HUMAN	582.3180913	3	0.743115306		
IGETPELCALTGPFER	74.73441	O60294	TYW4 HUMAN	895.443644	2	0.830958903		
IGETPELCALTGPFER	74.73441	O60294	TYW4 HUMAN	597.298371	3	0.830958903		
LSPVSPALGVLQLHFFK	100.0105	O60294	TYW4 HUMAN	927.038371	2	0.718668342		
LSPVSPALGVLQLHFFK	100.0105	O60294	TYW4 HUMAN	618.3615223	3	0.718668342		
PQDAFGQFMLQHFR	70.39828	O60294	TYW4 HUMAN	861.415024	2	0.796494842		
PQDAFGQFMLQHFR	70.39828	O60294	TYW4 HUMAN	574.6126243	3	0.796494842		
SVVEPVLSDWHFLHVGTMAWVR	101.9709	O60294	TYW4 HUMAN	1283.157743	2	0.691032887		
SVVEPVLSDWHFLHVGTMAWVR	101.9709	O60294	TYW4 HUMAN	855.774437	3	0.691032887		
VENIEPFDEFEEWHLK	82.21185	O60294	TYW4 HUMAN	1030.984186	2	0.800325155		
VENIEPFDEFEEWHLK	82.21185	O60294	TYW4 HUMAN	687.6587323	3	0.800325155		
VNPASPSGVFPASVVSSEGQVPNLK	78.19897	O60294	TYW4 HUMAN	1234.145552	2	0.742216825		
VNPASPSGVFPASVVSSEGQVPNLK	78.19897	O60294	TYW4 HUMAN	823.0996427	3	0.742216825		
AFDCPETEYPVK	26.59036	O43847	NRDC HUMAN	728.3272125	2	0.808055282	2	Yes
AFDCPETEYPVK	26.59036	O43847	NRDC HUMAN	485.8874167	3	0.808055282	2	
AFTTTLNLLPYHK	58.22453	O43847	NRDC HUMAN	759.919929	2	0.822523534	3	
AFTTTLNLLPYHK	58.22453	O43847	NRDC HUMAN	506.9492277	3	0.822523534	3	Yes
ANLVLLSGANEGK	39.1903	O43847	NRDC HUMAN	643.3597075	2	0.773727953	2	Yes
ANLVLLSGANEGK	39.1903	O43847	NRDC HUMAN	429.2424133	3	0.773727953	2	
ELAALWGIETR	74.661	O43847	NRDC HUMAN	629.843694	2	0.778317988		
ELAALWGIETR	74.661	O43847	NRDC HUMAN	420.2317377	3	0.778317988		
FFWGNAETLK	58.51016	O43847	NRDC HUMAN	606.80658	2	0.796666622		
FFWGNAETLK	58.51016	O43847	NRDC HUMAN	404.8736617	3	0.796666622		
FHLISPLIQK	46.53777	O43847	NRDC HUMAN	598.364065	2	0.825330138	2	Yes
FHLISPLIQK	46.53777	O43847	NRDC HUMAN	399.2453183	3	0.825330138	2	
GDANSEVTVYYQSGTR	21.77869	O43847	NRDC HUMAN	873.9006455	2	0.848892212	2	Yes
GDANSEVTVYYQSGTR	21.77869	O43847	NRDC HUMAN	582.936372	3	0.848892212	2	
GSILSFLR	69.64745	O43847	NRDC HUMAN	446.766726	2	0.758648813	2	Yes
GSILSFLR	69.64745	O43847	NRDC HUMAN	298.1804257	3	0.758648813	2	
GSLSNAGDPEIVK	13.42571	O43847	NRDC HUMAN	643.8335225	2	0.752019644	2	Yes
GSLSNAGDPEIVK	13.42571	O43847	NRDC HUMAN	429.55829	3	0.752019644	2	
IEEFLSSFEEK	63.46696	O43847	NRDC HUMAN	679.330281	2	0.768076777	2	Yes
IEEFLSSFEEK	63.46696	O43847	NRDC HUMAN	453.2227957	3	0.768076777	2	
IENLTEEAFNTQVTALIK	94.31258	O43847	NRDC HUMAN	1017.541869	2	0.743403077		
IENLTEEAFNTQVTALIK	94.31258	O43847	NRDC HUMAN	678.6971877	3	0.743403077		
IVNTPQGCLWYK	40.514	O43847	NRDC HUMAN	739.877207	2	0.826053262	2	Yes
IVNTPQGCLWYK	40.514	O43847	NRDC HUMAN	493.587413	3	0.826053262	2	
LLILEYAR	55.60956	O43847	NRDC HUMAN	495.8033045	2	0.817854822	2	Yes
LLILEYAR	55.60956	O43847	NRDC HUMAN	330.871478	3	0.817854822	2	
LVAGEHGLIIR	17.47887	O43847	NRDC HUMAN	589.3567715	2	0.78875494	3	
LVAGEHGLIIR	17.47887	O43847	NRDC HUMAN	393.240456	3	0.78875494	3	Yes
MLSVHVVGYGK	20.27224	O43847	NRDC HUMAN	595.3240785	2	0.754938006	3	
MLSVHVVGYGK	20.27224	O43847	NRDC HUMAN	397.2186607	3	0.754938006	3	Yes
NWNEVVTQQYLFDR	76.58151	O43847	NRDC HUMAN	906.439745	2	0.811861575	3	
NWNEVVTQQYLFDR	76.58151	O43847	NRDC HUMAN	604.629105	3	0.811861575	3	Yes
SDLVNWFK	70.84566	O43847	NRDC HUMAN	504.7616405	2	0.790564835	2	Yes
SDLVNWFK	70.84566	O43847	NRDC HUMAN	336.843702	3	0.790564835	2	
TVFQFDVQR	44.59607	O43847	NRDC HUMAN	570.29638	2	0.817751408	2	Yes
TVFQFDVQR	44.59607	O43847	NRDC HUMAN	380.5335283	3	0.817751408	2	
TYFNILIKPETLAK	67.06345	O43847	NRDC HUMAN	825.9774435	2	0.803168654		
TYFNILIKPETLAK	67.06345	O43847	NRDC HUMAN	550.9875707	3	0.803168654		
VKPLHYISWLVGHEGK	36.35323	O43847	NRDC HUMAN	932.0179655	2	0.806078911	4	
VKPLHYISWLVGHEGK	36.35323	O43847	NRDC HUMAN	621.681252	3	0.806078911	4	
WAQFFIHPLMIR	82.22253	O43847	NRDC HUMAN	779.921756	2	0.653198063	3	
WAQFFIHPLMIR	82.22253	O43847	NRDC HUMAN	520.283779	3	0.653198063	3	Yes
YIATDFTLK	40.09721	O43847	NRDC HUMAN	536.2902275	2	0.714923143	2	Yes
YIATDFTLK	40.09721	O43847	NRDC HUMAN	357.86276	3	0.714923143	2	
YPDENGFDAFLK	67.26642	O43847	NRDC HUMAN	708.328069	2	0.679331839		
YPDENGFDAFLK	67.26642	O43847	NRDC HUMAN	472.5546543	3	0.679331839		
YYSSHYMTLVVQSK	28.54542	O43847	NRDC HUMAN	853.416884	2	0.770142734		
YYSSHYMTLVVQSK	28.54542	O43847	NRDC HUMAN	569.280531	3	0.770142734		
ADEVEALTFPPSSGK	53.10044	O43815	STRN HUMAN	774.383577	2	0.808436036		
ADEVEALTFPPSSGK	53.10044	O43815	STRN HUMAN	516.5916597	3	0.808436036		
AELQAQIAFLQGER	66.49482	O43815	STRN HUMAN	787.420829	2	0.726166487		
AELQAQIAFLQGER	66.49482	O43815	STRN HUMAN	525.283161	3	0.726166487		
ALAFHPIEPVLITASEDHTLK	64.26852	O43815	STRN HUMAN	1151.626268	2	0.803493559	3	
ALAFHPIEPVLITASEDHTLK	64.26852	O43815	STRN HUMAN	768.0867867	3	0.803493559	3	Yes
ALLGFSSDVTDR	49.28963	O43815	STRN HUMAN	640.8282405	2	0.796430945		
ALLGFSSDVTDR	49.28963	O43815	STRN HUMAN	427.5547687	3	0.796430945		
AQYSLPGILHFLQHEWAR	98.32597	O43815	STRN HUMAN	1083.566343	2	0.703868508		
AQYSLPGILHFLQHEWAR	98.32597	O43815	STRN HUMAN	722.7135037	3	0.703868508		
CYIASAGADALAK	25.07015	O43815	STRN HUMAN	655.82464	2	0.612416923		
CYIASAGADALAK	25.07015	O43815	STRN HUMAN	437.5523683	3	0.612416923		
EDQCLMPEAWNVDQGVITK	81.05524	O43815	STRN HUMAN	1117.017379	2	0.760933936		
EDQCLMPEAWNVDQGVITK	81.05524	O43815	STRN HUMAN	745.014194	3	0.760933936		
FEESIHDVAFHPSK	19.00799	O43815	STRN HUMAN	821.8971855	2	0.631485283		
FEESIHDVAFHPSK	19.00799	O43815	STRN HUMAN	548.2673987	3	0.631485283		
FYDNNTGK	-25.97208	O43815	STRN HUMAN	479.717426	2	0.773373663		

FYDNNTGK	-25.97208	O43815	STRN HUMAN	320.147559	3	0.773373663		
GLGPLAEAAAAGDGAAAAGAAR	51.19879	O43815	STRN HUMAN	904.966664	2	0.817787468		
GLGPLAEAAAAGDGAAAAGAAR	51.19879	O43815	STRN HUMAN	603.647051	3	0.817787468		
GPLLGHTDAVWGLAYSAAHQR	53.27778	O43815	STRN HUMAN	1110.569615	2	0.706224203		
GPLLGHTDAVWGLAYSAAHQR	53.27778	O43815	STRN HUMAN	740.7156847	3	0.706224203		
GYTSIFNMETQQR	48.47951	O43815	STRN HUMAN	787.8675675	2	0.814810514		
GYTSIFNMETQQR	48.47951	O43815	STRN HUMAN	525.5809867	3	0.814810514		
ILTLESNVDTTANSSCQINR	43.24483	O43815	STRN HUMAN	1118.547888	2	0.707014322		
ILTLESNVDTTANSSCQINR	43.24483	O43815	STRN HUMAN	746.0345333	3	0.707014322		
LLSCSADGTLR	10.89795	O43815	STRN HUMAN	596.8037115	2	0.605352104		
LLSCSADGTLR	10.89795	O43815	STRN HUMAN	398.2050827	3	0.605352104		
LQDMLANLR	45.73109	O43815	STRN HUMAN	537.2927825	2	0.792978168	2	Yes
LQDMLANLR	45.73109	O43815	STRN HUMAN	358.53113	3	0.792978168	2	
LWNLESK	24.14653	O43815	STRN HUMAN	445.2430835	2	0.67132473	2	Yes
LWNLESK	24.14653	O43815	STRN HUMAN	297.164664	3	0.67132473	2	
LWNTTEVAPALSVFNDTK	91.07794	O43815	STRN HUMAN	1003.515654	2	0.737966478		
LWNTTEVAPALSVFNDTK	91.07794	O43815	STRN HUMAN	669.3463777	3	0.737966478		
QYLQEVGYTDTILDVK	76.32601	O43815	STRN HUMAN	942.9836475	2	0.679148078		
QYLQEVGYTDTILDVK	76.32601	O43815	STRN HUMAN	628.9917067	3	0.679148078		
SAGDGTDWEK	-14.84931	O43815	STRN HUMAN	533.2283595	2	0.719611168		
SAGDGTDWEK	-14.84931	O43815	STRN HUMAN	355.8215147	3	0.719611168		
SELTDSASVLDNFK	61.16428	O43815	STRN HUMAN	763.3732085	2	0.844837189		
SELTDSASVLDNFK	61.16428	O43815	STRN HUMAN	509.251414	3	0.844837189		
STSLDVEPIYTFR	65.39063	O43815	STRN HUMAN	764.388658	2	0.858061731	2	Yes
STSLDVEPIYTFR	65.39063	O43815	STRN HUMAN	509.9283803	3	0.858061731	2	
SVIDTSTIVR	26.6845	O43815	STRN HUMAN	545.8093195	2	0.704367995	2	Yes
SVIDTSTIVR	26.6845	O43815	STRN HUMAN	364.2088213	3	0.704367995	2	
TCIQEFTAHR	0.791134	O43815	STRN HUMAN	631.801504	2	0.744827092	3	
TCIQEFTAHR	0.791134	O43815	STRN HUMAN	421.5369443	3	0.744827092	3	Yes
VISHPTLPISITAHEDR	33.37775	O43815	STRN HUMAN	943.510706	2	0.755476117	4	
VISHPTLPISITAHEDR	33.37775	O43815	STRN HUMAN	629.343079	3	0.755476117	4	
TELNQGDMKPPSYDSDEGNETEVQPQQNSQLM'	59.89902	O43815	STRN HUMAN	2072.413302	2	0.741088748		
TELNQGDMKPPSYDSDEGNETEVQPQQNSQLM'	59.89902	O43815	STRN HUMAN	1381.94481	3	0.741088748		
AAHIFNEALVCHQIR	25.4798	O43795	MYO1B HUMAN	889.9601295	2	0.830815136	3	
AAHIFNEALVCHQIR	25.4798	O43795	MYO1B HUMAN	593.6426947	3	0.830815136	3	Yes
ALYPSSVGQPFQGAYLEINK	77.96447	O43795	MYO1B HUMAN	1091.563127	2	0.731441498		
ALYPSSVGQPFQGAYLEINK	77.96447	O43795	MYO1B HUMAN	728.044693	3	0.731441498		
DLSQAMWK	39.97507	O43795	MYO1B HUMAN	489.7398515	2	0.664485097	2	Yes
DLSQAMWK	39.97507	O43795	MYO1B HUMAN	326.829176	3	0.664485097	2	
DQDKDQCILITGESGAGK	21.48277	O43795	MYO1B HUMAN	967.9603865	2	0.741308212		
DQDKDQCILITGESGAGK	21.48277	O43795	MYO1B HUMAN	645.642866	3	0.741308212		
DQFTDQQK	-22.57591	O43795	MYO1B HUMAN	505.2334455	2	0.628580272		
DQFTDQQK	-22.57591	O43795	MYO1B HUMAN	337.1582387	3	0.628580272		
EICELTGIDQSVLER	69.35824	O43795	MYO1B HUMAN	881.438558	2	0.781304181		
EICELTGIDQSVLER	69.35824	O43795	MYO1B HUMAN	587.961647	3	0.781304181		
FLNDTSLPHSCFR	33.47865	O43795	MYO1B HUMAN	797.378106	2	0.742195785	3	
FLNDTSLPHSCFR	33.47865	O43795	MYO1B HUMAN	531.9213457	3	0.742195785	3	Yes
GDFLFSSDHLIEMATK	77.27744	O43795	MYO1B HUMAN	905.9381935	2	0.874573827	3	
GDFLFSSDHLIEMATK	77.27744	O43795	MYO1B HUMAN	604.2947373	3	0.874573827	3	Yes
GDPLGGVISNYLLEK	94.76826	O43795	MYO1B HUMAN	787.9254075	2	0.830270767	2	Yes
GDPLGGVISNYLLEK	94.76826	O43795	MYO1B HUMAN	525.6195467	3	0.830270767	2	
IFLLTNNNLLLADQK	94.14244	O43795	MYO1B HUMAN	865.4965355	2	0.861474991		
IFLLTNNNLLLADQK	94.14244	O43795	MYO1B HUMAN	577.333632	3	0.861474991		
IYEFTLQR	39.42433	O43795	MYO1B HUMAN	535.288019	2	0.784243286	2	Yes
IYEFTLQR	39.42433	O43795	MYO1B HUMAN	357.194621	3	0.784243286	2	
LEDLATLIQK	60.25207	O43795	MYO1B HUMAN	572.3351705	2	0.813407063	2	Yes
LEDLATLIQK	60.25207	O43795	MYO1B HUMAN	381.892722	3	0.813407063	2	
LFSWLVNR	71.68834	O43795	MYO1B HUMAN	517.793275	2	0.790417194		
LFSWLVNR	71.68834	O43795	MYO1B HUMAN	345.5314583	3	0.790417194		
LGNIEFKPESR	12.89359	O43795	MYO1B HUMAN	645.3466	2	0.803801358		
LGNIEFKPESR	12.89359	O43795	MYO1B HUMAN	430.5670083	3	0.803801358		
LNIEISDEFLVQFR	113.8332	O43795	MYO1B HUMAN	861.957243	2	0.622699678		
LNIEISDEFLVQFR	113.8332	O43795	MYO1B HUMAN	574.9741037	3	0.622699678		
LNQVCATHQHFESR	-24.56787	O43795	MYO1B HUMAN	863.908094	2	0.725778937		
LNQVCATHQHFESR	-24.56787	O43795	MYO1B HUMAN	576.274671	3	0.725778937		
NFYELSPHIFALSDEAYR	83.35504	O43795	MYO1B HUMAN	1086.524001	2	0.681386054		
NFYELSPHIFALSDEAYR	83.35504	O43795	MYO1B HUMAN	724.6852757	3	0.681386054		
NWPSRPYLFLDSTHK	44.30041	O43795	MYO1B HUMAN	930.9737545	2	0.661291122		
NWPSRPYLFLDSTHK	44.30041	O43795	MYO1B HUMAN	620.9851113	3	0.661291122		
SEVPLVDVTK	35.29249	O43795	MYO1B HUMAN	543.8062455	2	0.79216361	2	Yes
SEVPLVDVTK	35.29249	O43795	MYO1B HUMAN	362.8734387	3	0.79216361	2	
SLFPEGNPAK	20.66967	O43795	MYO1B HUMAN	530.277655	2	0.805516243	2	Yes
SLFPEGNPAK	20.66967	O43795	MYO1B HUMAN	353.8543783	3	0.805516243	2	
SLPIYSPEK	26.65769	O43795	MYO1B HUMAN	517.2824015	2	0.74104023		
SLPIYSPEK	26.65769	O43795	MYO1B HUMAN	345.190876	3	0.74104023		
SQIVIAAWYR	59.62491	O43795	MYO1B HUMAN	603.8356675	2	0.751275241		
SQIVIAAWYR	59.62491	O43795	MYO1B HUMAN	402.8930533	3	0.751275241		
SSALVIQSYIR	51.45764	O43795	MYO1B HUMAN	618.851514	2	0.823153257	2	Yes
SSALVIQSYIR	51.45764	O43795	MYO1B HUMAN	412.9036177	3	0.823153257	2	
VLYQVEGFVDK	51.33147	O43795	MYO1B HUMAN	648.845898	2	0.80014348		
VLYQVEGFVDK	51.33147	O43795	MYO1B HUMAN	432.8998737	3	0.80014348		
VSMSSQNDGFFAVHLK	48.67206	O43795	MYO1B HUMAN	883.9307025	2	0.838243604		
VSMSSQNDGFFAVHLK	48.67206	O43795	MYO1B HUMAN	589.6230767	3	0.838243604		
VSTTLNVAQAYYAR	40.73951	O43795	MYO1B HUMAN	778.9075455	2	0.898356736	2	Yes
VSTTLNVAQAYYAR	40.73951	O43795	MYO1B HUMAN	519.6076387	3	0.898356736	2	
YNYLSLDSAK	33.92934	O43795	MYO1B HUMAN	587.293493	2	0.739964187	2	Yes
YNYLSLDSAK	33.92934	O43795	MYO1B HUMAN	391.864937	3	0.739964187	2	
EIPIEGLEFMGHGK	63.80075	O43715	TRIA1 HUMAN	778.893058	2	0.699885607		
EIPIEGLEFMGHGK	63.80075	O43715	TRIA1 HUMAN	519.5979803	3	0.699885607		
GDSSGDPCTDLFK	31.22177	O43715	TRIA1 HUMAN	699.7962805	2	0.684144735	2	Yes
GDSSGDPCTDLFK	31.22177	O43715	TRIA1 HUMAN	466.8667953	3	0.684144735	2	
MNSVGEACTDMK	-3.975433	O43715	TRIA1 HUMAN	671.7759795	2	0.662395537		
MNSVGEACTDMK	-3.975433	O43715	TRIA1 HUMAN	448.1865947	3	0.662395537		
ALLTSGTSDPR	3.253139	O43464	HTRA2 HUMAN	559.2965765	2	0.81293714		
ALLTSGTSDPR	3.253139	O43464	HTRA2 HUMAN	373.200326	3	0.81293714		
AQLTAVTPDTR	10.33751	O43464	HTRA2 HUMAN	586.817677	2	0.743478417		
AQLTAVTPDTR	10.33751	O43464	HTRA2 HUMAN	391.5477263	3	0.743478417		
EPLPTLPLGR	57.62602	O43464	HTRA2 HUMAN	546.824773	2	0.767970145	2	Yes
EPLPTLPLGR	57.62602	O43464	HTRA2 HUMAN	364.8857903	3	0.767970145	2	
EPSFPDVQHGVLIHK	32.35099	O43464	HTRA2 HUMAN	851.949753	2	0.834882379		
EPSFPDVQHGVLIHK	32.35099	O43464	HTRA2 HUMAN	568.3024437	3	0.834882379		

LLSGDTYEAVVTAVDPVADIATLR	126.726	O43464	HTRA2_HUMAN	1245.160865	2	0.634751379		
LLSGDTYEAVVTAVDPVADIATLR	126.726	O43464	HTRA2_HUMAN	830.4431847	3	0.634751379		
LSVGVTEPR	9.593559	O43464	HTRA2_HUMAN	479.272373	2	0.778955877		
LSVGVTEPR	9.593559	O43464	HTRA2_HUMAN	319.850857	3	0.778955877		
SQYNFIADVVEK	63.64333	O43464	HTRA2_HUMAN	706.8569935	2	0.837408602	2	Yes
SQYNFIADVVEK	63.64333	O43464	HTRA2_HUMAN	471.5739373	3	0.837408602	2	
TQSQLAVQIR	13.86098	O43464	HTRA2_HUMAN	572.3282115	2	0.79194957	2	Yes
TQSQLAVQIR	13.86098	O43464	HTRA2_HUMAN	381.8880827	3	0.79194957	2	
VTYGTPSLWAR	46.69653	O43464	HTRA2_HUMAN	625.8305825	2	0.765323997		
VTYGTPSLWAR	46.69653	O43464	HTRA2_HUMAN	417.55633	3	0.765323997		
DLYEDELVPLFEK	113.984	O43390	HNRPR_HUMAN	805.4039735	2	0.831601858		
DLYEDELVPLFEK	113.984	O43390	HNRPR_HUMAN	537.271924	3	0.831601858		
DYAFVHFEDR	40.95207	O43390	HNRPR_HUMAN	649.7941965	2	0.810224056	3	
DYAFVHFEDR	40.95207	O43390	HNRPR_HUMAN	433.5320727	3	0.810224056	3	Yes
EEEEPMDTSSVTHTEHYK	-5.311668	O43390	HNRPR_HUMAN	1074.955493	2	0.747889996	3	
EEEEPMDTSSVTHTEHYK	-5.311668	O43390	HNRPR_HUMAN	716.972937	3	0.747889996	3	Yes
EFNEEGALSVLQQFK	96.46602	O43390	HNRPR_HUMAN	869.936508	2	0.824579	3	
EFNEEGALSVLQQFK	96.46602	O43390	HNRPR_HUMAN	580.2936137	3	0.824579	3	Yes
EIEGEEIEIVLAKPPDK	60.40498	O43390	HNRPR_HUMAN	955.0124125	2	0.740487754	3	
EIEGEEIEIVLAKPPDK	60.40498	O43390	HNRPR_HUMAN	637.0108833	3	0.740487754	3	Yes
GGYEDPYYGYDDGYAVR	45.83412	O43390	HNRPR_HUMAN	980.405748	2	0.826138258		
GGYEDPYYGYDDGYAVR	45.83412	O43390	HNRPR_HUMAN	653.9397737	3	0.826138258		
LCDSYEIRPGK	-0.62759	O43390	HNRPR_HUMAN	669.330089	2	0.786512613	2	Yes
LCDSYEIRPGK	-0.62759	O43390	HNRPR_HUMAN	446.556001	3	0.786512613	2	
LMMDPLSGQNR	37.35767	O43390	HNRPR_HUMAN	631.305564	2	0.718166351	2	Yes
LMMDPLSGQNR	37.35767	O43390	HNRPR_HUMAN	421.2063177	3	0.718166351	2	
NLATTVTEEILEK	71.61414	O43390	HNRPR_HUMAN	730.8963205	2	0.794221163	2	Yes
NLATTVTEEILEK	71.61414	O43390	HNRPR_HUMAN	487.6001553	3	0.794221163	2	
STAYEDYYYHPPPR	19.27337	O43390	HNRPR_HUMAN	879.8920835	2	0.779537976	3	
STAYEDYYYHPPPR	19.27337	O43390	HNRPR_HUMAN	586.930664	3	0.779537976	3	Yes
TLIEAGLPQK	30.85944	O43390	HNRPR_HUMAN	535.316781	2	0.778437674	2	Yes
TLIEAGLPQK	30.85944	O43390	HNRPR_HUMAN	357.2137957	3	0.778437674	2	
VTEGLVDVILYHQPDDK	67.02449	O43390	HNRPR_HUMAN	971.0023755	2	0.728989124	3	
VTEGLVDVILYHQPDDK	67.02449	O43390	HNRPR_HUMAN	647.6708587	3	0.728989124	3	Yes
YGGPPPDSVYSGVQPGIGTEVFVGK	81.61702	O43390	HNRPR_HUMAN	1254.126821	2	0.76129353	3	
YGGPPPDSVYSGVQPGIGTEVFVGK	81.61702	O43390	HNRPR_HUMAN	836.4204887	3	0.76129353	3	Yes
EESDSLGQDLR	11.97379	O43293	DAPK3_HUMAN	624.789312	2	0.66774869	2	Yes
EESDSLGQDLR	11.97379	O43293	DAPK3_HUMAN	416.8621497	3	0.66774869	2	
ESLTEDEATQFLK	54.00798	O43293	DAPK3_HUMAN	755.86776	2	0.803720593	2	Yes
ESLTEDEATQFLK	54.00798	O43293	DAPK3_HUMAN	504.2477817	3	0.803720593	2	
GALLGTSGLK	17.68749	O43293	DAPK3_HUMAN	458.7772915	2	0.790777743		
GALLGTSGLK	17.68749	O43293	DAPK3_HUMAN	306.1874693	3	0.790777743		
HPNIITLHDIFENK	46.97165	O43293	DAPK3_HUMAN	845.9497525	2	0.731621742		
HPNIITLHDIFENK	46.97165	O43293	DAPK3_HUMAN	564.3024433	3	0.731621742		
LCHEDVEALAAIYEEK	61.8497	O43293	DAPK3_HUMAN	945.451661	2	0.757286191		
LCHEDVEALAAIYEEK	61.8497	O43293	DAPK3_HUMAN	630.637049	3	0.757286191		
LIDFGIAHK	30.51155	O43293	DAPK3_HUMAN	507.2931085	2	0.778903067		
LIDFGIAHK	30.51155	O43293	DAPK3_HUMAN	338.5313473	3	0.778903067		
MTIAQSLEHSWIK	56.35612	O43293	DAPK3_HUMAN	772.40105	2	0.83518672		
MTIAQSLEHSWIK	56.35612	O43293	DAPK3_HUMAN	515.269975	3	0.83518672		
SHSSLPPNNSYADFER	16.58318	O43293	DAPK3_HUMAN	910.91409	2	0.74695915		
SHSSLPPNNSYADFER	16.58318	O43293	DAPK3_HUMAN	607.6120017	3	0.74695915		
VLEEAAAAEEGLR	29.37482	O43293	DAPK3_HUMAN	679.35208	2	0.825436473	2	Yes
VLEEAAAAEEGLR	29.37482	O43293	DAPK3_HUMAN	453.2373283	3	0.825436473	2	
ALFQNTER	-1.491352	O43264	ZW10_HUMAN	489.7543475	2	0.784962177		
ALFQNTER	-1.491352	O43264	ZW10_HUMAN	326.83884	3	0.784962177		
AMGTLLNTAISEVIGK	101.1015	O43264	ZW10_HUMAN	809.4481975	2	0.75201726	2	Yes
AMGTLLNTAISEVIGK	101.1015	O43264	ZW10_HUMAN	539.9680733	3	0.75201726	2	
DLHVSTGEFTDLK	38.2114	O43264	ZW10_HUMAN	731.3651875	2	0.859480023	3	
DLHVSTGEFTDLK	38.2114	O43264	ZW10_HUMAN	487.9127333	3	0.859480023	3	Yes
DSVVLSLLK	75.81315	O43264	ZW10_HUMAN	487.3005985	2	0.75339973	2	Yes
DSVVLSLLK	75.81315	O43264	ZW10_HUMAN	325.2030073	3	0.75339973	2	
DTSSLESYLQTELHLYTEQSHK	95.71194	O43264	ZW10_HUMAN	1305.122463	2	0.752078772		
DTSSLESYLQTELHLYTEQSHK	95.71194	O43264	ZW10_HUMAN	870.4175833	3	0.752078772		
ELMMMLQASLQEIGDR	108.7744	O43264	ZW10_HUMAN	932.952464	2	0.643931568		
ELMMMLQASLQEIGDR	108.7744	O43264	ZW10_HUMAN	622.304251	3	0.643931568		
FESIMTNLEYPSPSEVFTK	95.39061	O43264	ZW10_HUMAN	1110.033015	2	0.786337018		
FESIMTNLEYPSPSEVFTK	95.39061	O43264	ZW10_HUMAN	740.3579513	3	0.786337018		
GPLAAAFSSSEVK	40.17489	O43264	ZW10_HUMAN	632.3331585	2	0.770099998	2	Yes
GPLAAAFSSSEVK	40.17489	O43264	ZW10_HUMAN	421.8913807	3	0.770099998	2	
INVPELPTPDEDNK	53.92528	O43264	ZW10_HUMAN	790.8943085	2	0.824991345		
INVPELPTPDEDNK	53.92528	O43264	ZW10_HUMAN	527.598814	3	0.824991345		
ITALEDISTEDGDR	36.28646	O43264	ZW10_HUMAN	767.8657485	2	0.836229503	2	Yes
ITALEDISTEDGDR	36.28646	O43264	ZW10_HUMAN	512.2464407	3	0.836229503	2	
LGTECFLAQMR	54.64318	O43264	ZW10_HUMAN	663.321215	2	0.792321205		
LGTECFLAQMR	54.64318	O43264	ZW10_HUMAN	442.550085	3	0.792321205		
LQQYEEIIQSTEEFENALK	102.7406	O43264	ZW10_HUMAN	1156.568808	2	0.813224494	3	
LQQYEEIIQSTEEFENALK	102.7406	O43264	ZW10_HUMAN	771.3818133	3	0.813224494	3	Yes
LSEDIDLLK	47.56289	O43264	ZW10_HUMAN	523.2929705	2	0.768591166	2	Yes
LSEDIDLLK	47.56289	O43264	ZW10_HUMAN	349.197922	3	0.768591166	2	
LVLEVLQK	46.7652	O43264	ZW10_HUMAN	471.3056845	2	0.773389101	2	Yes
LVLEVLQK	46.7652	O43264	ZW10_HUMAN	314.5397313	3	0.773389101	2	
NIFHLFHDVVPTYHK	51.69926	O43264	ZW10_HUMAN	933.9866655	2	0.825893342		
NIFHLFHDVVPTYHK	51.69926	O43264	ZW10_HUMAN	622.9937187	3	0.825893342		
NLMTSEIHNTVK	6.381832	O43264	ZW10_HUMAN	693.856475	2	0.81336844	3	
NLMTSEIHNTVK	6.381832	O43264	ZW10_HUMAN	462.906925	3	0.81336844	3	Yes
SFGQMLLK	43.68997	O43264	ZW10_HUMAN	462.2551375	2	0.631134748		
SFGQMLLK	43.68997	O43264	ZW10_HUMAN	308.5060333	3	0.631134748		
SLSMELTIQK	44.33399	O43264	ZW10_HUMAN	575.3133805	2	0.692695439		
SLSMELTIQK	44.33399	O43264	ZW10_HUMAN	383.8781953	3	0.692695439		
TVMDEGPQVFAPLSEESK	67.51006	O43264	ZW10_HUMAN	982.470055	2	0.814224243		
TVMDEGPQVFAPLSEESK	67.51006	O43264	ZW10_HUMAN	655.3159783	3	0.814224243		
VSNTQYHEVMNLEPENTLDQHSFSLPTCR	61.25997	O43264	ZW10_HUMAN	1723.791173	2	0.793422341	4	
VSNTQYHEVMNLEPENTLDQHSFSLPTCR	61.25997	O43264	ZW10_HUMAN	1149.530057	3	0.793422341	4	
YQEEVPVYVPK	36.8325	O43264	ZW10_HUMAN	675.851176	2	0.81991148		
YQEEVPVYVPK	36.8325	O43264	ZW10_HUMAN	450.9033923	3	0.81991148		
YSEFLPSMQSAQGLITQVDK	95.34274	O43264	ZW10_HUMAN	1121.557189	2	0.76522994	3	
YSEFLPSMQSAQGLITQVDK	95.34274	O43264	ZW10_HUMAN	748.0407343	3	0.76522994	3	Yes
AILESLFSQYSGK	83.35233	O43149	ZZEF1_HUMAN	721.8804685	2	0.802876472		
AILESLFSQYSGK	83.35233	O43149	ZZEF1_HUMAN	481.5895873	3	0.802876472		
AIYELQMK	28.88303	O43149	ZZEF1_HUMAN	498.265698	2	0.770241022		

AIYELQMK	28.88303	O43149	ZZEF1 HUMAN	332.5130737	3	0.770241022		
ALTELFFVTENR	83.91736	O43149	ZZEF1 HUMAN	720.3806405	2	0.812708139		
ALTELFFVTENR	83.91736	O43149	ZZEF1 HUMAN	480.589702	3	0.812708139		
AQAQSILEVLK	65.17834	O43149	ZZEF1 HUMAN	600.3538945	2	0.828037024		
AQAQSILEVLK	65.17834	O43149	ZZEF1 HUMAN	400.5718713	3	0.828037024		
AQELGVLQDYLLALTTDDHLLR	135.3062	O43149	ZZEF1 HUMAN	1249.169024	2	0.613506198		
AQELGVLQDYLLALTTDDHLLR	135.3062	O43149	ZZEF1 HUMAN	833.115291	3	0.613506198		
ASTIFSTGTESAFQVTQIR	70.03009	O43149	ZZEF1 HUMAN	1022.521469	2	0.867005765		
ASTIFSTGTESAFQVTQIR	70.03009	O43149	ZZEF1 HUMAN	682.0169207	3	0.867005765		
ATFLQTDLLK	60.92356	O43149	ZZEF1 HUMAN	575.3298885	2	0.853378594	2	Yes
ATFLQTDLLK	60.92356	O43149	ZZEF1 HUMAN	383.8892007	3	0.853378594	2	
AVHEEIRPVDFK	0.791855	O43149	ZZEF1 HUMAN	720.386257	2	0.61462605		
AVHEEIRPVDFK	0.791855	O43149	ZZEF1 HUMAN	480.5934463	3	0.61462605		
AVTPSPEQVFAECSQK	36.37558	O43149	ZZEF1 HUMAN	889.425451	2	0.8355937		
AVTPSPEQVFAECSQK	36.37558	O43149	ZZEF1 HUMAN	593.2862423	3	0.8355937		
DFELPGDTLYYR	80.72379	O43149	ZZEF1 HUMAN	744.854447	2	0.800855875		
DFELPGDTLYYR	80.72379	O43149	ZZEF1 HUMAN	496.905573	3	0.800855875		
DFTNYFGHLEGCGADLHK	53.97453	O43149	ZZEF1 HUMAN	1040.963259	2	0.770760179		
DFTNYFGHLEGCGADLHK	53.97453	O43149	ZZEF1 HUMAN	694.3114477	3	0.770760179		
DGFLTETGK	18.02575	O43149	ZZEF1 HUMAN	484.240739	2	0.802608609		
DGFLTETGK	18.02575	O43149	ZZEF1 HUMAN	323.163101	3	0.802608609		
DLAVDLIEK	66.93172	O43149	ZZEF1 HUMAN	508.2876885	2	0.743809462		
DLAVDLIEK	66.93172	O43149	ZZEF1 HUMAN	339.1944007	3	0.743809462		
DVIATFTDHCIK	38.73485	O43149	ZZEF1 HUMAN	710.3510265	2	0.660209656		
DVIATFTDHCIK	38.73485	O43149	ZZEF1 HUMAN	473.9032927	3	0.660209656		
DYQLVQK	3.406914	O43149	ZZEF1 HUMAN	447.2405375	2	0.648221374		
DYQLVQK	3.406914	O43149	ZZEF1 HUMAN	298.4963	3	0.648221374		
EFSVLTELLK	100.1326	O43149	ZZEF1 HUMAN	589.8375455	2	0.777604878		
EFSVLTELLK	100.1326	O43149	ZZEF1 HUMAN	393.560972	3	0.777604878		
EGLDIHSSMILR	42.80492	O43149	ZZEF1 HUMAN	685.8590175	2	0.78990829		
EGLDIHSSMILR	42.80492	O43149	ZZEF1 HUMAN	457.5752867	3	0.78990829		
ELYTHLCDVVDK	32.12061	O43149	ZZEF1 HUMAN	746.3615865	2	0.733884096		
ELYTHLCDVVDK	32.12061	O43149	ZZEF1 HUMAN	497.9103327	3	0.733884096		
ESLDQLVQK	28.85641	O43149	ZZEF1 HUMAN	530.28822	2	0.730058253		
ESLDQLVQK	28.85641	O43149	ZZEF1 HUMAN	353.8614217	3	0.730058253		
FCAEEHFK	-19.42323	O43149	ZZEF1 HUMAN	534.2349335	2	0.697612286		
FCAEEHFK	-19.42323	O43149	ZZEF1 HUMAN	356.492564	3	0.697612286		
FDGDELTTDER	15.94787	O43149	ZZEF1 HUMAN	649.2813205	2	0.79560113		
FDGDELTTDER	15.94787	O43149	ZZEF1 HUMAN	433.1901553	3	0.79560113		
FDSQCNTEEGCDELAMSSSSDFQQDR	47.43531	O43149	ZZEF1 HUMAN	1522.085305	2	0.641216934		
FDSQCNTEEGCDELAMSSSSDFQQDR	47.43531	O43149	ZZEF1 HUMAN	1015.059478	3	0.641216934		
FLPTGISSK	20.07235	O43149	ZZEF1 HUMAN	475.2718415	2	0.772111475		
FLPTGISSK	20.07235	O43149	ZZEF1 HUMAN	317.183836	3	0.772111475		
FQTGFEILK	54.85983	O43149	ZZEF1 HUMAN	541.798224	2	0.773831964		
FQTGFEILK	54.85983	O43149	ZZEF1 HUMAN	361.5347577	3	0.773831964		
FTSDMSNTEWGYR	38.23182	O43149	ZZEF1 HUMAN	797.3360995	2	0.855567932		
FTSDMSNTEWGYR	38.23182	O43149	ZZEF1 HUMAN	531.8933413	3	0.855567932		
GAGCSSEQFEEAFAQFDAEGDGTVDAENMLEALI	127.7309	O43149	ZZEF1 HUMAN	1812.275421	2	0.630224705		
GAGCSSEQFEEAFAQFDAEGDGTVDAENMLEALI	127.7309	O43149	ZZEF1 HUMAN	1208.519555	3	0.630224705		
GEESVTLEQFR	33.13031	O43149	ZZEF1 HUMAN	647.817873	2	0.744098544		
GEESVTLEQFR	33.13031	O43149	ZZEF1 HUMAN	432.2145237	3	0.744098544		
GFSSLNDR	-4.887356	O43149	ZZEF1 HUMAN	448.217597	2	0.686795115		
GFSSLNDR	-4.887356	O43149	ZZEF1 HUMAN	299.147673	3	0.686795115		
HFLLDFAQSEPAQNFCGPYSELFK	114.0014	O43149	ZZEF1 HUMAN	1423.168698	2	0.682420492		
HFLLDFAQSEPAQNFCGPYSELFK	114.0014	O43149	ZZEF1 HUMAN	949.1150733	3	0.682420492		
HLSIAVAATDQSYMPQQVTVAVGR	64.15651	O43149	ZZEF1 HUMAN	1271.658301	2	0.60465914		
HLSIAVAATDQSYMPQQVTVAVGR	64.15651	O43149	ZZEF1 HUMAN	848.1081423	3	0.60465914		
HNLFAAGDSSIVPDGWK	58.89616	O43149	ZZEF1 HUMAN	907.447574	2	0.8570171		
HNLFAAGDSSIVPDGWK	58.89616	O43149	ZZEF1 HUMAN	605.300991	3	0.8570171		
HSSEATEVNPESLAK	-3.176613	O43149	ZZEF1 HUMAN	799.8870145	2	0.642796934		
HSSEATEVNPESLAK	-3.176613	O43149	ZZEF1 HUMAN	533.5939513	3	0.642796934		
IALSEEFAQVYSLADGIR	114.0921	O43149	ZZEF1 HUMAN	991.5156495	2	0.725947559		
IALSEEFAQVYSLADGIR	114.0921	O43149	ZZEF1 HUMAN	661.3463747	3	0.725947559		
ICFLMAHDALNAPLHILR	79.53539	O43149	ZZEF1 HUMAN	1053.061529	2	0.730530143		
ICFLMAHDALNAPLHILR	79.53539	O43149	ZZEF1 HUMAN	702.376961	3	0.730530143		
IWMLEMK	59.90424	O43149	ZZEF1 HUMAN	475.746091	2	0.60048461		
IWMLEMK	59.90424	O43149	ZZEF1 HUMAN	317.5000023	3	0.60048461		
LDPLEGLDEPTR	52.84877	O43149	ZZEF1 HUMAN	677.8466305	2	0.829350352		
LDPLEGLDEPTR	52.84877	O43149	ZZEF1 HUMAN	452.2336953	3	0.829350352		
LDVETWQQEQPVVLHTWTK	73.21964	O43149	ZZEF1 HUMAN	1169.097875	2	0.825110614		
LDVETWQQEQPVVLHTWTK	73.21964	O43149	ZZEF1 HUMAN	779.734525	3	0.825110614		
LETADETSHLQPLNK	11.04623	O43149	ZZEF1 HUMAN	848.43159	2	0.756931484		
LETADETSHLQPLNK	11.04623	O43149	ZZEF1 HUMAN	565.9570017	3	0.756931484		
LGVQGLTISGYLRPAR	63.51129	O43149	ZZEF1 HUMAN	850.994491	2	0.647930384		
LGVQGLTISGYLRPAR	63.51129	O43149	ZZEF1 HUMAN	567.6656023	3	0.647930384		
LISSTESELQQSYAK	25.65685	O43149	ZZEF1 HUMAN	842.4259685	2	0.886511207		
LISSTESELQQSYAK	25.65685	O43149	ZZEF1 HUMAN	561.953254	3	0.886511207		
LLPAEVDAAVIK	54.44788	O43149	ZZEF1 HUMAN	619.87192	2	0.857228279		
LLPAEVDAAVIK	54.44788	O43149	ZZEF1 HUMAN	413.5838883	3	0.857228279		
LPSSSGLPAADVSPATAEEPLSPSTPTR	63.56282	O43149	ZZEF1 HUMAN	1368.190885	2	0.817667961		
LPSSSGLPAADVSPATAEEPLSPSTPTR	63.56282	O43149	ZZEF1 HUMAN	912.4631983	3	0.817667961		
LQFLFHSDSSHNEWGYK	42.17024	O43149	ZZEF1 HUMAN	1047.98759	2	0.798082888		
LQFLFHSDSSHNEWGYK	42.17024	O43149	ZZEF1 HUMAN	698.994335	3	0.798082888		
MALVLSSPLWK	83.87874	O43149	ZZEF1 HUMAN	622.857758	2	0.822038829		
MALVLSSPLWK	83.87874	O43149	ZZEF1 HUMAN	415.574447	3	0.822038829		
MSQENISVHDSVISQWSEEDELADAK	62.20856	O43149	ZZEF1 HUMAN	1474.16728	2	0.78840965		
MSQENISVHDSVISQWSEEDELADAK	62.20856	O43149	ZZEF1 HUMAN	983.1141283	3	0.78840965		
NASDLQEVR	-7.821308	O43149	ZZEF1 HUMAN	516.2599935	2	0.692263007		
NASDLQEVR	-7.821308	O43149	ZZEF1 HUMAN	344.5092707	3	0.692263007		
NHLFTMMNVTEQEHK	21.71642	O43149	ZZEF1 HUMAN	929.93292	2	0.827341914		
NHLFTMMNVTEQEHK	21.71642	O43149	ZZEF1 HUMAN	620.2912217	3	0.827341914		
NSSGANLQGELSHIIR	44.02473	O43149	ZZEF1 HUMAN	848.4428225	2	0.751017094		
NSSGANLQGELSHIIR	44.02473	O43149	ZZEF1 HUMAN	565.96449	3	0.751017094		
SGPENLLVEPWTR	71.3	O43149	ZZEF1 HUMAN	749.3889965	2	0.866832852		
SGPENLLVEPWTR	71.3	O43149	ZZEF1 HUMAN	499.928606	3	0.866832852		
SLCTYFSDKDPGGLLLLPEK	81.12877	O43149	ZZEF1 HUMAN	1127.077757	2	0.719649494		
SLCTYFSDKDPGGLLLLPEK	81.12877	O43149	ZZEF1 HUMAN	751.721113	3	0.719649494		
SLMSLGNEAEEK	24.22006	O43149	ZZEF1 HUMAN	654.3115655	2	0.713869333		
SLMSLGNEAEEK	24.22006	O43149	ZZEF1 HUMAN	436.543652	3	0.713869333		
SQCMQLVGDCLMK	47.99698	O43149	ZZEF1 HUMAN	785.3487895	2	0.604156852		
SQCMQLVGDCLMK	47.99698	O43149	ZZEF1 HUMAN	523.9018013	3	0.604156852		

CFLGGVKPEGHGDDHEMVNMEFTCDHCQGLIIG	57.73705	O43149	ZZEF1_HUMAN	2008.884437	2	0.616550744		
CFLGGVKPEGHGDDHEMVNMEFTCDHCQGLIIG	57.73705	O43149	ZZEF1_HUMAN	1339.592233	3	0.616550744		
TDYFFLEVQK	76.07541	O43149	ZZEF1_HUMAN	645.3247985	2	0.781316578		
TDYFFLEVQK	76.07541	O43149	ZZEF1_HUMAN	430.552474	3	0.781316578		
TLQFIQQLAHDLVQQK	90.41016	O43149	ZZEF1_HUMAN	955.5289	2	0.851846278		
TLQFIQQLAHDLVQQK	90.41016	O43149	ZZEF1_HUMAN	637.3552083	3	0.851846278		
TSSVVEEHFQASVSPTEAAPPATGDQSPGLGTQPI	48.90696	O43149	ZZEF1_HUMAN	1754.347895	2	0.775814593		
TSSVVEEHFQASVSPTEAAPPATGDQSPGLGTQPI	48.90696	O43149	ZZEF1_HUMAN	1169.901205	3	0.775814593		
VDGDSVPMEILK	58.76069	O43149	ZZEF1_HUMAN	651.834677	2	0.755669057		
VDGDSVPMEILK	58.76069	O43149	ZZEF1_HUMAN	434.892393	3	0.755669057		
VGLDWACSMAEILR	115.4919	O43149	ZZEF1_HUMAN	810.8978175	2	0.616011798		
VGLDWACSMAEILR	115.4919	O43149	ZZEF1_HUMAN	540.9344867	3	0.616011798		
VHIPGAIYLSIK	55.49717	O43149	ZZEF1_HUMAN	655.895725	2	0.743012011		
VHIPGAIYLSIK	55.49717	O43149	ZZEF1_HUMAN	437.5997583	3	0.743012011		
VNEATAVLYAR	21.88011	O43149	ZZEF1_HUMAN	603.8280395	2	0.767357528		
VNEATAVLYAR	21.88011	O43149	ZZEF1_HUMAN	402.887968	3	0.767357528		
VVPHLPLAK	15.9988	O43149	ZZEF1_HUMAN	487.313844	2	0.729998589		
VVPHLPLAK	15.9988	O43149	ZZEF1_HUMAN	325.2118377	3	0.729998589		
YDYLEFTDAR	48.83527	O43149	ZZEF1_HUMAN	646.793858	2	0.807618976		
YDYLEFTDAR	48.83527	O43149	ZZEF1_HUMAN	431.531847	3	0.807618976		
YGKPLQLTLQACDVK	35.5146	O43149	ZZEF1_HUMAN	867.466918	2	0.693815172		
YGKPLQLTLQACDVK	35.5146	O43149	ZZEF1_HUMAN	578.6472203	3	0.693815172		
YSYGHLPTHSITAHPMVTIR	27.10999	O43149	ZZEF1_HUMAN	1141.081496	2	0.725560367		
YSYGHLPTHSITAHPMVTIR	27.10999	O43149	ZZEF1_HUMAN	761.056939	3	0.725560367		
YVGQFLASMR	46.74123	O43149	ZZEF1_HUMAN	586.3006035	2	0.729721606		
YVGQFLASMR	46.74123	O43149	ZZEF1_HUMAN	391.2030107	3	0.729721606		
EFGSLPTTPSEQR	31.43264	O15400	STX7_HUMAN	724.854987	2	0.776081562	2	Yes
EFGSLPTTPSEQR	31.43264	O15400	STX7_HUMAN	483.5725997	3	0.776081562	2	
ITQCSVEIQR	0.279087	O15400	STX7_HUMAN	617.3169865	2	0.790320158		
ITQCSVEIQR	0.279087	O15400	STX7_HUMAN	411.8805993	3	0.790320158		
LVAEFTTSLTNFQK	70.3176	O15400	STX7_HUMAN	799.9254125	2	0.831262589		
LVAEFTTSLTNFQK	70.3176	O15400	STX7_HUMAN	533.61955	3	0.831262589		
NLVSWESQTQPQVQVQDEEITEDDLR	77.00629	O15400	STX7_HUMAN	1543.731635	2	0.793251276	3	
NLVSWESQTQPQVQVQDEEITEDDLR	77.00629	O15400	STX7_HUMAN	1029.490365	3	0.793251276	3	Yes
QLEADIMDINEIFK	112.1132	O15400	STX7_HUMAN	839.922012	2	0.725771129		
QLEADIMDINEIFK	112.1132	O15400	STX7_HUMAN	560.2839497	3	0.725771129		
TLNQLGTPQDSPELR	34.90993	O15400	STX7_HUMAN	834.9317575	2	0.869002581		
TLNQLGTPQDSPELR	34.90993	O15400	STX7_HUMAN	556.9571133	3	0.869002581		
VSGSFPEDSSK	-9.420208	O15400	STX7_HUMAN	570.264941	2	0.707989097	2	Yes
VSGSFPEDSSK	-9.420208	O15400	STX7_HUMAN	380.512569	3	0.707989097	2	
EMDDFLQK	30.79907	O15305	PMM2_HUMAN	513.234599	2	0.700375319	2	Yes
EMDDFLQK	30.79907	O15305	PMM2_HUMAN	342.492341	3	0.700375319	2	
IEFYELDK	44.03044	O15305	PMM2_HUMAN	528.7665845	2	0.800390899	2	Yes
IEFYELDK	44.03044	O15305	PMM2_HUMAN	352.846998	3	0.800390899	2	
IGVVGGSDFEK	22.59411	O15305	PMM2_HUMAN	554.28822	2	0.762763441		
IGVVGGSDFEK	22.59411	O15305	PMM2_HUMAN	369.8614217	3	0.762763441		
NGMLNVSPIGR	39.00851	O15305	PMM2_HUMAN	579.3089635	2	0.636483014	2	Yes
NGMLNVSPIGR	39.00851	O15305	PMM2_HUMAN	386.5419173	3	0.636483014	2	
TIYFFGDK	48.36372	O15305	PMM2_HUMAN	495.750738	2	0.785508931	2	Yes
TIYFFGDK	48.36372	O15305	PMM2_HUMAN	330.8364337	3	0.785508931	2	
TMGYSVTAPEDTR	18.33121	O15305	PMM2_HUMAN	714.327744	2	0.696284175	2	Yes
TMGYSVTAPEDTR	18.33121	O15305	PMM2_HUMAN	476.5544377	3	0.696284175	2	
TMPGGNDHEIFTDPR	20.42946	O15305	PMM2_HUMAN	843.8812105	2	0.800038934		
TMPGGNDHEIFTDPR	20.42946	O15305	PMM2_HUMAN	562.9234153	3	0.800038934		
VQEQLGNDVVEK	1.5481	O15305	PMM2_HUMAN	679.35208	2	0.840521872	2	Yes
VQEQLGNDVVEK	1.5481	O15305	PMM2_HUMAN	453.2373283	3	0.840521872	2	
YDYVFPENGLVAYK	74.40536	O15305	PMM2_HUMAN	839.4121245	2	0.805380225		
YDYVFPENGLVAYK	74.40536	O15305	PMM2_HUMAN	559.9440247	3	0.805380225		
EAVCFIPGEGHTLQEHQIVLVEGGR	57.10847	O15235	RT12_HUMAN	1388.198326	2	0.63982743	4	
EAVCFIPGEGHTLQEHQIVLVEGGR	57.10847	O15235	RT12_HUMAN	925.8014923	3	0.63982743	4	
GVVLCTFTR	35.11005	O15235	RT12_HUMAN	526.7820515	2	0.655492783		
GVVLCTFTR	35.11005	O15235	RT12_HUMAN	351.523976	3	0.655492783		
EELALLDGSNVVFK	84.58708	O15212	PFD6_HUMAN	767.412137	2	0.83786869	2	Yes
EELALLDGSNVVFK	84.58708	O15212	PFD6_HUMAN	511.944033	3	0.83786869	2	
ETLAQLQQEFQR	56.49197	O15212	PFD6_HUMAN	745.8840795	2	0.833791852		
ETLAQLQQEFQR	56.49197	O15212	PFD6_HUMAN	497.5919947	3	0.833791852		
LDYITAEIK	38.49617	O15212	PFD6_HUMAN	533.2955095	2	0.758705139	2	Yes
LDYITAEIK	38.49617	O15212	PFD6_HUMAN	355.8662813	3	0.758705139	2	
LEAQLTENNIVK	28.91112	O15212	PFD6_HUMAN	686.3780975	2	0.839115322	2	Yes
LEAQLTENNIVK	28.91112	O15212	PFD6_HUMAN	457.92134	3	0.839115322	2	
LLGPVLVK	38.98093	O15212	PFD6_HUMAN	419.792213	2	0.733269691	2	Yes
LLGPVLVK	38.98093	O15212	PFD6_HUMAN	280.197417	3	0.733269691	2	
DEYDDLSDLNAVQMESVR	81.09772	O15173	PGRC2_HUMAN	1049.96586	2	0.754586816	3	
DEYDDLSDLNAVQMESVR	81.09772	O15173	PGRC2_HUMAN	700.3131817	3	0.754586816	3	Yes
DFSLEQLR	53.77104	O15173	PGRC2_HUMAN	504.262005	2	0.769051671	2	Yes
DFSLEQLR	53.77104	O15173	PGRC2_HUMAN	336.5106117	3	0.769051671	2	
EWEMQFK	37.04803	O15173	PGRC2_HUMAN	499.226577	2	0.645411372		
EWEMQFK	37.04803	O15173	PGRC2_HUMAN	333.1536597	3	0.645411372		
FYGPAGPYGIFAGR	71.02377	O15173	PGRC2_HUMAN	736.870235	2	0.792919993	2	Yes
FYGPAGPYGIFAGR	71.02377	O15173	PGRC2_HUMAN	491.582765	3	0.792919993	2	
GLATFCLDKDALR	43.92506	O15173	PGRC2_HUMAN	740.3854005	2	0.804710865		
GLATFCLDKDALR	43.92506	O15173	PGRC2_HUMAN	493.9262087	3	0.804710865		
GLGAGAGAGEESPATSLPR	28.21286	O15173	PGRC2_HUMAN	849.4268395	2	0.697924256	2	Yes
GLGAGAGAGEESPATSLPR	28.21286	O15173	PGRC2_HUMAN	566.6205013	3	0.697924256	2	
LLKPGEEPSEYTDEEDTK	12.29863	O15173	PGRC2_HUMAN	1040.484409	2	0.777931452	3	
LLKPGEEPSEYTDEEDTK	12.29863	O15173	PGRC2_HUMAN	693.9922143	3	0.777931452	3	Yes
DSSDPNELYVNHK	5.189671	O15160	RPAC1_HUMAN	759.3475205	2	0.76271379	3	
DSSDPNELYVNHK	5.189671	O15160	RPAC1_HUMAN	506.567622	3	0.76271379	3	Yes
ILLAEVPTMAVEK	71.26961	O15160	RPAC1_HUMAN	707.40527	2	0.801822543	2	Yes
ILLAEVPTMAVEK	71.26961	O15160	RPAC1_HUMAN	471.939455	3	0.801822543	2	
LGLIPIHADPR	37.34236	O15160	RPAC1_HUMAN	601.3567715	2	0.774379253		
LGLIPIHADPR	37.34236	O15160	RPAC1_HUMAN	401.240456	3	0.774379253		
LLPDITLLEPVEGEAAEELSR	138.7512	O15160	RPAC1_HUMAN	1147.61048	2	0.703644395		
LLPDITLLEPVEGEAAEELSR	138.7512	O15160	RPAC1_HUMAN	765.4095947	3	0.703644395		
NQGDEEGTEIDTLQFR	48.94148	O15160	RPAC1_HUMAN	926.421951	2	0.811682105	2	Yes
NQGDEEGTEIDTLQFR	48.94148	O15160	RPAC1_HUMAN	617.9505757	3	0.811682105	2	
NVHTTDFPGNYSGYDDAWDQDR	47.09189	O15160	RPAC1_HUMAN	1287.034179	2	0.711407244		
NVHTTDFPGNYSGYDDAWDQDR	47.09189	O15160	RPAC1_HUMAN	858.3587277	3	0.711407244		
VLVYNNTSIVQDEILAHR	57.4296	O15160	RPAC1_HUMAN	1042.560923	2	0.770242095	3	
VLVYNNTSIVQDEILAHR	57.4296	O15160	RPAC1_HUMAN	695.3765567	3	0.770242095	3	Yes
VVLGEFGVR	42.06909	O15160	RPAC1_HUMAN	488.2852835	2	0.806536674		

VVLGEFGVR	42.06909	O15160	RPAC1_HUMAN	325.859464	3	0.806536674		
AQQEFATGVMSNK	14.47099	O15126	SCAM1_HUMAN	705.838283	2	0.807204545	2	Yes
AQQEFATGVMSNK	14.47099	O15126	SCAM1_HUMAN	470.894797	3	0.807204545	2	
EHALAQAELLK	13.09216	O15126	SCAM1_HUMAN	611.843694	2	0.77127254	2	Yes
EHALAQAELLK	13.09216	O15126	SCAM1_HUMAN	408.2317377	3	0.77127254	2	
MPNVPNTQPAIMKPTEEHPAYTQIAK	41.1217	O15126	SCAM1_HUMAN	1453.73114	2	0.704511881	3	
MPNVPNTQPAIMKPTEEHPAYTQIAK	41.1217	O15126	SCAM1_HUMAN	969.490035	3	0.704511881	3	Yes
NVPPGLDEYNPFSDSR	63.64484	O15126	SCAM1_HUMAN	903.9188415	2	0.763726175		
NVPPGLDEYNPFSDSR	63.64484	O15126	SCAM1_HUMAN	602.9485027	3	0.763726175		
TVQTAAANAASTAASSAAQNAFK	36.10625	O15126	SCAM1_HUMAN	1076.535639	2	0.806853116	3	
TVQTAAANAASTAASSAAQNAFK	36.10625	O15126	SCAM1_HUMAN	718.0263673	3	0.806853116	3	Yes
AHYQSFPPPVADFIK	59.33075	O14787	TNPO2_HUMAN	858.9413925	2	0.788105905		
AHYQSFPPPVADFIK	59.33075	O14787	TNPO2_HUMAN	572.9635367	3	0.788105905		
ATIGILITTIASK	83.38902	O14787	TNPO2_HUMAN	651.4061275	2	0.621586859		
ATIGILITTIASK	83.38902	O14787	TNPO2_HUMAN	434.6066933	3	0.621586859		
DQVGEDNWQQFSEQFPPLLK	117.6284	O14787	TNPO2_HUMAN	1203.074596	2	0.810798645		
DQVGEDNWQQFSEQFPPLLK	117.6284	O14787	TNPO2_HUMAN	802.3856723	3	0.810798645		
EELLPHLLPLLK	90.01295	O14787	TNPO2_HUMAN	707.937594	2	0.728511095	2	Yes
EELLPHLLPLLK	90.01295	O14787	TNPO2_HUMAN	472.2943377	3	0.728511095	2	
GDVEEDEAVPDSEQDIKPR	15.42393	O14787	TNPO2_HUMAN	1064.488019	2	0.764645338	3	
GDVEEDEAVPDSEQDIKPR	15.42393	O14787	TNPO2_HUMAN	709.9946207	3	0.764645338	3	Yes
GLLFHPEWVVK	59.27153	O14787	TNPO2_HUMAN	662.874797	2	0.813104093		
GLLFHPEWVVK	59.27153	O14787	TNPO2_HUMAN	442.252473	3	0.813104093		
LIPHMHSIIQYMLQR	66.62865	O14787	TNPO2_HUMAN	940.505854	2	0.621868491		
LIPHMHSIIQYMLQR	66.62865	O14787	TNPO2_HUMAN	627.3398443	3	0.621868491		
LMPPLIQK	37.2504	O14787	TNPO2_HUMAN	470.2889805	2	0.731172144		
LMPPLIQK	37.2504	O14787	TNPO2_HUMAN	313.8619287	3	0.731172144		
LTSPSAIPAITIGR	62.92075	O14787	TNPO2_HUMAN	698.912108	2	0.835804641		
LTSPSAIPAITIGR	62.92075	O14787	TNPO2_HUMAN	466.277347	3	0.835804641		
YAHWVVSQPPDMHLKPLMTELLK	73.58408	O14787	TNPO2_HUMAN	1367.21694	2	0.63171792	4	
YAHWVVSQPPDMHLKPLMTELLK	73.58408	O14787	TNPO2_HUMAN	911.8139013	3	0.63171792	4	
DIKPENLLLDER	51.12729	O14757	CHK1_HUMAN	727.896654	2	0.838302076	2	Yes
DIKPENLLLDER	51.12729	O14757	CHK1_HUMAN	485.6003777	3	0.838302076	2	
FFHQLMAGVVYLHGIGITHR	67.64853	O14757	CHK1_HUMAN	1148.612771	2	0.660023212		
FFHQLMAGVVYLHGIGITHR	67.64853	O14757	CHK1_HUMAN	766.077789	3	0.660023212		
GDGLEFK	6.743401	O14757	CHK1_HUMAN	383.19306	2	0.695525825		
GDGLEFK	6.743401	O14757	CHK1_HUMAN	255.7979817	3	0.695525825		
HIQSNLDFSPVNSASSEENVK	33.53595	O14757	CHK1_HUMAN	1151.551483	2	0.689245582	3	
HIQSNLDFSPVNSASSEENVK	33.53595	O14757	CHK1_HUMAN	768.0369303	3	0.689245582	3	Yes
IDSAPLALLHK	41.65643	O14757	CHK1_HUMAN	589.3511545	2	0.724539876		
IDSAPLALLHK	41.65643	O14757	CHK1_HUMAN	393.2367113	3	0.724539876		
IEPDIGMPEPDAQR	44.60203	O14757	CHK1_HUMAN	784.3752295	2	0.855145156		
IEPDIGMPEPDAQR	44.60203	O14757	CHK1_HUMAN	523.2527613	3	0.855145156		
ISDFGLATVFR	79.43849	O14757	CHK1_HUMAN	613.332962	2	0.837160766	2	Yes
ISDFGLATVFR	79.43849	O14757	CHK1_HUMAN	409.224583	3	0.837160766	2	
LIDIVSSQK	26.94852	O14757	CHK1_HUMAN	501.7956805	2	0.71193862		
LIDIVSSQK	26.94852	O14757	CHK1_HUMAN	334.8663953	3	0.71193862		
SCMNQVTISTTDR	13.48494	O14757	CHK1_HUMAN	756.8432395	2	0.800792217		
SCMNQVTISTTDR	13.48494	O14757	CHK1_HUMAN	504.8981013	3	0.800792217		
TGLSLWDTSPSYIDK	74.08151	O14757	CHK1_HUMAN	841.9177795	2	0.861529231		
TGLSLWDTSPSYIDK	74.08151	O14757	CHK1_HUMAN	561.6144613	3	0.861529231		
VNLLEMDDK	36.96678	O14757	CHK1_HUMAN	538.7688055	2	0.799942255		
VNLLEMDDK	36.96678	O14757	CHK1_HUMAN	359.5151453	3	0.799942255		
VTSGGVSESPSGFSK	3.238464	O14757	CHK1_HUMAN	713.346994	2	0.804504395		
VTSGGVSESPSGFSK	3.238464	O14757	CHK1_HUMAN	475.9006043	3	0.804504395		
AMFIFLSNAGAER	77.18002	O14656	TOR1A_HUMAN	713.8615605	2	0.823135018		
AMFIFLSNAGAER	77.18002	O14656	TOR1A_HUMAN	476.2436487	3	0.823135018		
DIEHALSVSVFNNK	60.91029	O14656	TOR1A_HUMAN	786.90501	2	0.767166734		
DIEHALSVSVFNNK	60.91029	O14656	TOR1A_HUMAN	524.9392817	3	0.767166734		
DLDDNLFGQHLAK	49.2254	O14656	TOR1A_HUMAN	743.370804	2	0.911741793		
DLDDNLFGQHLAK	49.2254	O14656	TOR1A_HUMAN	495.9164777	3	0.911741793		
DQLQLWIR	71.47205	O14656	TOR1A_HUMAN	536.301465	2	0.650918782		
DQLQLWIR	71.47205	O14656	TOR1A_HUMAN	357.8702517	3	0.650918782		
GYEIDEDIVSR	34.15444	O14656	TOR1A_HUMAN	648.309876	2	0.737336814		
GYEIDEDIVSR	34.15444	O14656	TOR1A_HUMAN	432.5425257	3	0.737336814		
IIAENIYEGGLNSDYVHLFVATLHFPHASNITLYK	110.1142	O14656	TOR1A_HUMAN	1980.5207	2	0.621845901		
IIAENIYEGGLNSDYVHLFVATLHFPHASNITLYK	110.1142	O14656	TOR1A_HUMAN	1320.683075	3	0.621845901		
ITDVALDFWR	82.71649	O14656	TOR1A_HUMAN	618.325137	2	0.756234765		
ITDVALDFWR	82.71649	O14656	TOR1A_HUMAN	412.5526997	3	0.756234765		
KPLTLSLHGWTGTGK	26.52874	O14656	TOR1A_HUMAN	798.4493895	2	0.817305565		
KPLTLSLHGWTGTGK	26.52874	O14656	TOR1A_HUMAN	532.6355347	3	0.817305565		
LYCLFAECCGQK	44.52612	O14656	TOR1A_HUMAN	774.836368	2	0.674674511		
LYCLFAECCGQK	44.52612	O14656	TOR1A_HUMAN	516.8935203	3	0.674674511		
NSGFWHSSLIDR	34.88399	O14656	TOR1A_HUMAN	709.8447555	2	0.807192206		
NSGFWHSSLIDR	34.88399	O14656	TOR1A_HUMAN	473.5657787	3	0.807192206		
SIFIFDEMDK	81.51741	O14656	TOR1A_HUMAN	622.797563	2	0.766034007		
SIFIFDEMDK	81.51741	O14656	TOR1A_HUMAN	415.534317	3	0.766034007		
VAEEMTFFPK	50.6223	O14656	TOR1A_HUMAN	599.794824	2	0.834763765		
VAEEMTFFPK	50.6223	O14656	TOR1A_HUMAN	400.1991577	3	0.834763765		
ALDIDLDKPLADSEK	44.45566	O14617	AP3D1_HUMAN	821.930891	2	0.835897565		
ALDIDLDKPLADSEK	44.45566	O14617	AP3D1_HUMAN	548.289869	3	0.835897565		
ALDLLYGMVSK	80.6805	O14617	AP3D1_HUMAN	605.331569	2	0.752809048		
ALDLLYGMVSK	80.6805	O14617	AP3D1_HUMAN	403.890321	3	0.752809048		
ASCILQLVK	50.31319	O14617	AP3D1_HUMAN	516.3000765	2	0.778456807		
ASCILQLVK	50.31319	O14617	AP3D1_HUMAN	344.5359927	3	0.778456807		
AVLIMYK	27.11094	O14617	AP3D1_HUMAN	419.2493195	2	0.753428638	2	Yes
AVLIMYK	27.11094	O14617	AP3D1_HUMAN	279.835488	3	0.753428638	2	
DLANDIMTLMSHTKPYIR	97.07076	O14617	AP3D1_HUMAN	1060.037912	2	0.771502793	4	
DLANDIMTLMSHTKPYIR	97.07076	O14617	AP3D1_HUMAN	707.027883	3	0.771502793	4	
DLILQCLDDKDESIR	61.31636	O14617	AP3D1_HUMAN	916.9571145	2	0.833055973	3	
DLILQCLDDKDESIR	61.31636	O14617	AP3D1_HUMAN	611.6406847	3	0.833055973	3	Yes
DLSSPSQYDTGVALTGLSCFVTPDLAR	130.8263	O14617	AP3D1_HUMAN	1435.698017	2	0.603522062		
DLSSPSQYDTGVALTGLSCFVTPDLAR	130.8263	O14617	AP3D1_HUMAN	957.4679527	3	0.603522062		
DVPVAEEVSALFAGELNPVAPK	147.9045	O14617	AP3D1_HUMAN	1126.594633	2	0.739028573	3	
DVPVAEEVSALFAGELNPVAPK	147.9045	O14617	AP3D1_HUMAN	751.39903	3	0.739028573	3	Yes
EQAGEAEGAQAVTQLMVDR	55.44107	O14617	AP3D1_HUMAN	1001.979111	2	0.769070566		
EQAGEAEGAQAVTQLMVDR	55.44107	O14617	AP3D1_HUMAN	668.3220157	3	0.769070566		
FAVSQMSALLDSAHLLASSTQR	104.5798	O14617	AP3D1_HUMAN	1167.100091	2	0.745248854	3	
FAVSQMSALLDSAHLLASSTQR	104.5798	O14617	AP3D1_HUMAN	778.4026687	3	0.745248854	3	Yes
GMELSVLDSLNAR	76.79513	O14617	AP3D1_HUMAN	702.861757	2	0.721143723		
GMELSVLDSLNAR	76.79513	O14617	AP3D1_HUMAN	468.9104463	3	0.721143723		

GSLQEDSQVTVAIVLENR	76.85017	O14617	AP3D1 HUMAN	979.5136425	2	0.702285409		
GSLQEDSQVTVAIVLENR	76.85017	O14617	AP3D1 HUMAN	653.3450367	3	0.702285409		
HGHLIAAQMLDVAIR	43.97513	O14617	AP3D1 HUMAN	822.954316	2	0.732834816		
HGHLIAAQMLDVAIR	43.97513	O14617	AP3D1 HUMAN	548.9721523	3	0.732834816		
IGYLAASQSFHEGTDVIMLTTNQIR	80.75128	O14617	AP3D1 HUMAN	1383.20053	2	0.676024973		
IGYLAASQSFHEGTDVIMLTTNQIR	80.75128	O14617	AP3D1 HUMAN	922.469628	3	0.676024973		
ILIEDSDQNLK	29.32325	O14617	AP3D1 HUMAN	644.343723	2	0.763663709	2	Yes
ILIEDSDQNLK	29.32325	O14617	AP3D1 HUMAN	429.8984237	3	0.763663709	2	
LEDPDPGVQSAAVNVICELAR	103.8222	O14617	AP3D1 HUMAN	1127.063174	2	0.675991416		
LEDPDPGVQSAAVNVICELAR	103.8222	O14617	AP3D1 HUMAN	751.711391	3	0.675991416		
LFGALTPLEPR	69.21253	O14617	AP3D1 HUMAN	607.351155	2	0.907584786	2	Yes
LFGALTPLEPR	69.21253	O14617	AP3D1 HUMAN	405.2367117	3	0.907584786	2	
LLESGDLSMSSIK	46.73632	O14617	AP3D1 HUMAN	690.3585155	2	0.812753141	2	Yes
LLESGDLSMSSIK	46.73632	O14617	AP3D1 HUMAN	460.574952	3	0.812753141	2	
LMTSSTNNWVLIK	57.12745	O14617	AP3D1 HUMAN	753.903425	2	0.830751717	2	Yes
LMTSSTNNWVLIK	57.12745	O14617	AP3D1 HUMAN	502.938225	3	0.830751717	2	
LPQFVQSADLEVQER	56.18928	O14617	AP3D1 HUMAN	879.9552325	2	0.877358556	3	
LPQFVQSADLEVQER	56.18928	O14617	AP3D1 HUMAN	586.9727633	3	0.877358556	3	Yes
LYASILQQK	29.91901	O14617	AP3D1 HUMAN	532.311494	2	0.796642959	2	Yes
LYASILQQK	29.91901	O14617	AP3D1 HUMAN	355.210271	3	0.796642959	2	
MSFQNLLAK	56.70187	O14617	AP3D1 HUMAN	526.284426	2	0.786793888		
MSFQNLLAK	56.70187	O14617	AP3D1 HUMAN	351.1922257	3	0.786793888		
NLQDLVR	22.72632	O14617	AP3D1 HUMAN	429.246158	2	0.673359752		
NLQDLVR	22.72632	O14617	AP3D1 HUMAN	286.500047	3	0.673359752		
NYLSLAPLFFK	106.0714	O14617	AP3D1 HUMAN	656.8691755	2	0.71847403		
NYLSLAPLFFK	106.0714	O14617	AP3D1 HUMAN	438.2487253	3	0.71847403		
VDSCASMYSR	-13.25053	O14617	AP3D1 HUMAN	588.2451675	2	0.688944638	2	Yes
VDSCASMYSR	-13.25053	O14617	AP3D1 HUMAN	392.4993867	3	0.688944638	2	
VPGLPMSDQYVK	46.00183	O14617	AP3D1 HUMAN	667.345208	2	0.834319115		
VPGLPMSDQYVK	46.00183	O14617	AP3D1 HUMAN	445.232747	3	0.834319115		
VTTLPGHIQAVYVQNVVK	49.41413	O14617	AP3D1 HUMAN	983.5601955	2	0.782618761	3	
VTTLPGHIQAVYVQNVVK	49.41413	O14617	AP3D1 HUMAN	656.0427387	3	0.782618761	3	Yes
YISQCIDEIK	24.68612	O14617	AP3D1 HUMAN	634.8137405	2	0.728855073		
YISQCIDEIK	24.68612	O14617	AP3D1 HUMAN	423.545102	3	0.728855073		
YLGLLAMSK	56.63009	O14617	AP3D1 HUMAN	498.2838905	2	0.736943781	2	Yes
YLGLLAMSK	56.63009	O14617	AP3D1 HUMAN	332.525202	3	0.736943781	2	
YPESLRPAFPR	27.06428	O14617	AP3D1 HUMAN	666.857131	2	0.758789182		
YPESLRPAFPR	27.06428	O14617	AP3D1 HUMAN	444.9073623	3	0.758789182		
YQDTPGVEHIPVVQIDLSVPLK	91.31801	O14617	AP3D1 HUMAN	1224.16321	2	0.673534155	3	
YQDTPGVEHIPVVQIDLSVPLK	91.31801	O14617	AP3D1 HUMAN	816.4447483	3	0.673534155	3	Yes
FPLDSTGSELK	34.73145	O14562	UBFD1 HUMAN	597.3066095	2	0.809569478	2	Yes
FPLDSTGSELK	34.73145	O14562	UBFD1 HUMAN	398.540348	3	0.809569478	2	
GKPEDVMPSVK	-0.816544	O14562	UBFD1 HUMAN	593.811005	2	0.773505807		
GKPEDVMPSVK	-0.816544	O14562	UBFD1 HUMAN	396.209945	3	0.773505807		
GSLQPAPAQPPGDPAAQASVSNGEDAGGGAGR	30.47221	O14562	UBFD1 HUMAN	1444.182447	2	0.621929467	3	
GSLQPAPAQPPGDPAAQASVSNGEDAGGGAGR	30.47221	O14562	UBFD1 HUMAN	963.1242397	3	0.621929467	3	Yes
IHSITGLPPAMQK	19.4922	O14562	UBFD1 HUMAN	696.887579	2	0.760783374		
IHSITGLPPAMQK	19.4922	O14562	UBFD1 HUMAN	464.927661	3	0.760783374		
IMVVGSTINDVLAVNTPK	85.73262	O14562	UBFD1 HUMAN	936.019518	2	0.754094064	3	
IMVVGSTINDVLAVNTPK	85.73262	O14562	UBFD1 HUMAN	624.3489537	3	0.754094064	3	Yes
LEQDQLWIGTK	45.47515	O14562	UBFD1 HUMAN	665.854259	2	0.858615756		
LEQDQLWIGTK	45.47515	O14562	UBFD1 HUMAN	444.238781	3	0.858615756		
LPTVPLSGMYNK	51.45285	O14562	UBFD1 HUMAN	660.3555755	2	0.833949745	2	Yes
LPTVPLSGMYNK	51.45285	O14562	UBFD1 HUMAN	440.572992	3	0.833949745	2	
ATGRPECFLTIQEGLASK	49.86183	O00566	MPP10 HUMAN	989.507307	2	0.752962828		
ATGRPECFLTIQEGLASK	49.86183	O00566	MPP10 HUMAN	660.0074797	3	0.752962828		
KPWQLQGEVTAQK	11.09513	O00566	MPP10 HUMAN	756.91264	2	0.870164573	3	
KPWQLQGEVTAQK	11.09513	O00566	MPP10 HUMAN	504.9443683	3	0.870164573	3	Yes
LDALSNFHFIPKPPVPEIK	76.14379	O00566	MPP10 HUMAN	1081.604606	2	0.775329351	4	
LDALSNFHFIPKPPVPEIK	76.14379	O00566	MPP10 HUMAN	721.405679	3	0.775329351	4	
LSEMEAYLENIEK	69.33022	O00566	MPP10 HUMAN	784.879808	2	0.817018092		
LSEMEAYLENIEK	69.33022	O00566	MPP10 HUMAN	523.589147	3	0.817018092		
LSLAEIYEQEYIK	81.36049	O00566	MPP10 HUMAN	799.9197865	2	0.889618397		
LSLAEIYEQEYIK	81.36049	O00566	MPP10 HUMAN	533.6157993	3	0.889618397		
MAPVITEETTLQLEDIIK	102.6973	O00566	MPP10 HUMAN	1022.548306	2	0.756282151	3	
MAPVITEETTLQLEDIIK	102.6973	O00566	MPP10 HUMAN	682.0348123	3	0.756282151	3	Yes
RPENSLLEETLHFDHAVR	41.64643	O00566	MPP10 HUMAN	1082.051258	2	0.7875579	4	
RPENSLLEETLHFDHAVR	41.64643	O00566	MPP10 HUMAN	721.7034467	3	0.7875579	4	
SPVFSDEDSDLDFDISK	73.08333	O00566	MPP10 HUMAN	958.426366	2	0.688996077		
SPVFSDEDSDLDFDISK	73.08333	O00566	MPP10 HUMAN	639.2868523	3	0.688996077		
VLYDFNK	20.28148	O00566	MPP10 HUMAN	449.73763	2	0.760580242	2	Yes
VLYDFNK	20.28148	O00566	MPP10 HUMAN	300.1610283	3	0.760580242	2	
VTFALPDDAETEDTGVLNVK	81.61397	O00566	MPP10 HUMAN	1067.531699	2	0.792703748		
VTFALPDDAETEDTGVLNVK	81.61397	O00566	MPP10 HUMAN	712.0237407	3	0.792703748		
VVSNLPAITMEEVAPVSVSDAALLAPEEIK	134.8451	O00566	MPP10 HUMAN	1546.82596	2	0.643115938		
VVSNLPAITMEEVAPVSVSDAALLAPEEIK	134.8451	O00566	MPP10 HUMAN	1031.553248	3	0.643115938		
DSQVVQVVLDGLK	86.26971	O00505	IMA4 HUMAN	700.393748	2	0.733321011		
DSQVVQVVLDGLK	86.26971	O00505	IMA4 HUMAN	467.265107	3	0.733321011		
IEVLQQHENEDIYK	19.9417	O00505	IMA4 HUMAN	879.4394105	2	0.800674677	2	Yes
IEVLQQHENEDIYK	19.9417	O00505	IMA4 HUMAN	586.628882	3	0.800674677	2	
NVPQEESLEDSDVDADFK	44.07069	O00505	IMA4 HUMAN	1018.950737	2	0.759849608		
NVPQEESLEDSDVDADFK	44.07069	O00505	IMA4 HUMAN	679.636433	3	0.759849608		
NVTWVIVNLCR	77.83635	O00505	IMA4 HUMAN	687.3720955	2	0.620492756		
NVTWVIVNLCR	77.83635	O00505	IMA4 HUMAN	458.5840053	3	0.620492756		
SGILPILVK	66.81659	O00505	IMA4 HUMAN	470.316052	2	0.67814672	2	Yes
SGILPILVK	66.81659	O00505	IMA4 HUMAN	313.8799763	3	0.67814672	2	
DQTPDENDQVVVK	9.288864	O00425	IF2B3 HUMAN	743.855184	2	0.746005118	2	Yes
DQTPDENDQVVVK	9.288864	O00425	IF2B3 HUMAN	496.2393977	3	0.746005118	2	
FTEEIPLK	31.77435	O00425	IF2B3 HUMAN	488.7716745	2	0.708772182	2	Yes
FTEEIPLK	31.77435	O00425	IF2B3 HUMAN	326.1837247	3	0.708772182	2	
IAPAEAPDAK	-19.41247	O00425	IF2B3 HUMAN	491.764381	2	0.72353363	2	Yes
IAPAEAPDAK	-19.41247	O00425	IF2B3 HUMAN	328.1788623	3	0.72353363	2	
IELHGKPIEVEHSVPK	2.873699	O00425	IF2B3 HUMAN	906.504892	2	0.759756684	3	
IELHGKPIEVEHSVPK	2.873699	O00425	IF2B3 HUMAN	604.6725363	3	0.759756684	3	Yes
IPVSGPFLVK	53.61926	O00425	IF2B3 HUMAN	528.826784	2	0.748477101		
IPVSGPFLVK	53.61926	O00425	IF2B3 HUMAN	352.887131	3	0.748477101		
ITGHFYACQVAQR	5.743675	O00425	IF2B3 HUMAN	775.880813	2	0.836399198		
ITGHFYACQVAQR	5.743675	O00425	IF2B3 HUMAN	517.589817	3	0.836399198		
ITISPLQELTLYNPER	103.0123	O00425	IF2B3 HUMAN	944.0152855	2	0.870638728	3	
ITISPLQELTLYNPER	103.0123	O00425	IF2B3 HUMAN	629.6794653	3	0.870638728	3	Yes
LNGFQLENFTLK	78.09584	O00425	IF2B3 HUMAN	712.383183	2	0.650612473		

LNGFQLENFTLK	78.09584	O00425	IF2B3_HUMAN	475.2580637	3	0.650612473		
LYIGNLSENAAPSDLESIFK	108.7	O00425	IF2B3_HUMAN	1091.057878	2	0.820680261	3	
LYIGNLSENAAPSDLESIFK	108.7	O00425	IF2B3_HUMAN	727.70786	3	0.820680261	3	Yes
SITILSTPEGTSAACK	40.76854	O00425	IF2B3_HUMAN	818.4170945	2	0.821486235		
SITILSTPEGTSAACK	40.76854	O00425	IF2B3_HUMAN	545.947338	3	0.821486235		
TGYAFVDCPDESWALK	71.82286	O00425	IF2B3_HUMAN	929.919997	2	0.857038021		
TGYAFVDCPDESWALK	71.82286	O00425	IF2B3_HUMAN	620.2826063	3	0.857038021		
TVNELQNLSSAEVVVPR	64.57229	O00425	IF2B3_HUMAN	928.0001705	2	0.827672005	3	
TVNELQNLSSAEVVVPR	64.57229	O00425	IF2B3_HUMAN	619.002722	3	0.827672005	3	Yes
VAYIPDEMAAQQNPLQQPR	60.88879	O00425	IF2B3_HUMAN	1085.042041	2	0.91059953	3	
VAYIPDEMAAQQNPLQQPR	60.88879	O00425	IF2B3_HUMAN	723.6973023	3	0.91059953	3	Yes
ASCLVQTPPGQYSGNSFK	32.97385	O00311	CDC7_HUMAN	970.9627275	2	0.795186996		
ASCLVQTPPGQYSGNSFK	32.97385	O00311	CDC7_HUMAN	647.6444267	3	0.795186996		
ASDDLTALAQIMTIR	114.9228	O00311	CDC7_HUMAN	809.9276295	2	0.836591184		
ASDDLTALAQIMTIR	114.9228	O00311	CDC7_HUMAN	540.2876947	3	0.836591184		
DVKPSNFLYNR	25.4418	O00311	CDC7_HUMAN	676.852045	2	0.871255279		
DVKPSNFLYNR	25.4418	O00311	CDC7_HUMAN	451.5706383	3	0.871255279		
EYMLNLFK	70.89967	O00311	CDC7_HUMAN	529.2735225	2	0.714370131		
EYMLNLFK	70.89967	O00311	CDC7_HUMAN	353.1849567	3	0.714370131		
HLIPTSHPIR	-19.71446	O00311	CDC7_HUMAN	585.8412885	2	0.786295533		
HLIPTSHPIR	-19.71446	O00311	CDC7_HUMAN	390.8968007	3	0.786295533		
IAAELQCLTVAGGQDNVMGVK	71.36862	O00311	CDC7_HUMAN	1087.551389	2	0.671658635		
IAAELQCLTVAGGQDNVMGVK	71.36862	O00311	CDC7_HUMAN	725.370201	3	0.671658635		
IGEGTFSSVYLATAQLQVGPEEK	90.00809	O00311	CDC7_HUMAN	1212.618833	2	0.800255954		
IGEGTFSSVYLATAQLQVGPEEK	90.00809	O00311	CDC7_HUMAN	808.7484967	3	0.800255954		
IHQFGIVHR	-19.26523	O00311	CDC7_HUMAN	553.815074	2	0.609667182		
IHQFGIVHR	-19.26523	O00311	CDC7_HUMAN	369.545991	3	0.609667182		
IPLSGPVPK	22.13586	O00311	CDC7_HUMAN	454.284752	2	0.669386387		
IPLSGPVPK	22.13586	O00311	CDC7_HUMAN	303.192443	3	0.669386387		
ITAEEALLHPFFK	65.04992	O00311	CDC7_HUMAN	758.4144835	2	0.780988932		
ITAEEALLHPFFK	65.04992	O00311	CDC7_HUMAN	505.9455973	3	0.780988932		
LLDLNPASR	32.68091	O00311	CDC7_HUMAN	499.785647	2	0.805346608		
LLDLNPASR	32.68091	O00311	CDC7_HUMAN	333.526373	3	0.805346608		
LTSDIQGHASHQPAISEK	-19.4921	O00311	CDC7_HUMAN	959.9850525	2	0.784557998		
LTSDIQGHASHQPAISEK	-19.4921	O00311	CDC7_HUMAN	640.3259767	3	0.784557998		
LYEAVPQLSNVFK	74.78612	O00311	CDC7_HUMAN	754.411936	2	0.841804385		
LYEAVPQLSNVFK	74.78612	O00311	CDC7_HUMAN	503.2772323	3	0.841804385		
NFNIHSSISHESPAVK	-0.071854	O00311	CDC7_HUMAN	883.9451975	2	0.740998745		
NFNIHSSISHESPAVK	-0.071854	O00311	CDC7_HUMAN	589.63274	3	0.740998745		
YALVDFGLAQGTHDTK	54.40405	O00311	CDC7_HUMAN	868.436672	2	0.785589635		
YALVDFGLAQGTHDTK	54.40405	O00311	CDC7_HUMAN	579.293723	3	0.785589635		
AYVSTLMGVPGR	46.64738	O00303	EIF3F_HUMAN	625.832268	2	0.780347407	2	Yes
AYVSTLMGVPGR	46.64738	O00303	EIF3F_HUMAN	417.5574537	3	0.780347407	2	
EAPNPIHLTVDTSLQNGR	43.84657	O00303	EIF3F_HUMAN	981.506152	2	0.709132373	3	
EAPNPIHLTVDTSLQNGR	43.84657	O00303	EIF3F_HUMAN	654.6733763	3	0.709132373	3	Yes
FLMSLVNQVPK	73.02351	O00303	EIF3F_HUMAN	638.360665	2	0.852987766	2	Yes
FLMSLVNQVPK	73.02351	O00303	EIF3F_HUMAN	425.9097183	3	0.852987766	2	
IGVDLIMK	50.41669	O00303	EIF3F_HUMAN	444.765338	2	0.647426128		
IGVDLIMK	50.41669	O00303	EIF3F_HUMAN	296.846167	3	0.647426128		
IQDALSTVLQYAEDVLSGK	158.5221	O00303	EIF3F_HUMAN	1025.539322	2	0.60545671	3	
IQDALSTVLQYAEDVLSGK	158.5221	O00303	EIF3F_HUMAN	684.028823	3	0.60545671	3	Yes
LHPVILASIVDSYER	70.40039	O00303	EIF3F_HUMAN	856.473056	2	0.801579714	3	
LHPVILASIVDSYER	70.40039	O00303	EIF3F_HUMAN	571.317979	3	0.801579714	3	Yes
TMGVMFTPLTVK	74.89938	O00303	EIF3F_HUMAN	662.8543595	2	0.773267865	2	Yes
TMGVMFTPLTVK	74.89938	O00303	EIF3F_HUMAN	442.238848	3	0.773267865	2	
VIGLSSDLQQVGGASAR	49.32953	O00303	EIF3F_HUMAN	829.4475745	2	0.852725506	2	Yes
VIGLSSDLQQVGGASAR	49.32953	O00303	EIF3F_HUMAN	553.3009913	3	0.852725506	2	
VIGTLLGTVDK	46.76376	O00303	EIF3F_HUMAN	558.3377135	2	0.817748845	2	Yes
VIGTLLGTVDK	46.76376	O00303	EIF3F_HUMAN	372.561084	3	0.817748845	2	
VSPNELILGWYATGHDITEHSVLIHEYYSR	91.77936	O00303	EIF3F_HUMAN	1750.368222	2	0.764630795	5	
VSPNELILGWYATGHDITEHSVLIHEYYSR	91.77936	O00303	EIF3F_HUMAN	1167.24809	3	0.764630795	5	
YAYYDTER	-6.18449	O00303	EIF3F_HUMAN	540.7358075	2	0.753014743	2	Yes
YAYYDTER	-6.18449	O00303	EIF3F_HUMAN	360.82648	3	0.753014743	2	
EAFGEQPLQLTTKPDLLAWDPAR	93.40605	O00257	CBX4_HUMAN	1298.674478	2	0.728629768	3	
EAFGEQPLQLTTKPDLLAWDPAR	93.40605	O00257	CBX4_HUMAN	866.118927	3	0.728629768	3	Yes
EAPSPTCPDLGAK	8.0284	O00257	CBX4_HUMAN	671.819559	2	0.731141031		
EAPSPTCPDLGAK	8.0284	O00257	CBX4_HUMAN	448.2156477	3	0.731141031		
GPKPKPLVVQVPTFAR	33.53885	O00257	CBX4_HUMAN	867.5254315	2	0.63365221		
GPKPKPLVVQVPTFAR	33.53885	O00257	CBX4_HUMAN	578.6862293	3	0.63365221		
GYLGAVKPLAGAAGAPGK	28.82417	O00257	CBX4_HUMAN	799.4572105	2	0.820383072		
GYLGAVKPLAGAAGAPGK	28.82417	O00257	CBX4_HUMAN	533.3074153	3	0.820383072		
LLIAFQNR	40.241	O00257	CBX4_HUMAN	487.7932755	2	0.770136535		
LLIAFQNR	40.241	O00257	CBX4_HUMAN	325.5314587	3	0.770136535		
MELPAVGEHVFAVESIEK	75.26815	O00257	CBX4_HUMAN	993.0066075	2	0.799815655		
MELPAVGEHVFAVESIEK	75.26815	O00257	CBX4_HUMAN	662.3403467	3	0.799815655		
MYDLQYQGGHK	-3.57769	O00257	CBX4_HUMAN	670.309153	2	0.707778811		
MYDLQYQGGHK	-3.57769	O00257	CBX4_HUMAN	447.2087103	3	0.707778811		
TCLGGSPAAERPADLPPAAALPQPEVILLDSDLDE	137.6159	O00257	CBX4_HUMAN	2339.700203	2	0.691839814		
TCLGGSPAAERPADLPPAAALPQPEVILLDSDLDE	137.6159	O00257	CBX4_HUMAN	1560.136077	3	0.691839814		
SNVLTGLQDSSTDNR	26.48349	O00257	CBX4_HUMAN	803.887546	2	0.908794761		
SNVLTGLQDSSTDNR	26.48349	O00257	CBX4_HUMAN	536.2609723	3	0.908794761		
YNTWEPEENILDPR	63.47552	O00257	CBX4_HUMAN	888.415935	2	0.82497406		
YNTWEPEENILDPR	63.47552	O00257	CBX4_HUMAN	592.6132317	3	0.82497406		
YYYQLNSK	3.807949	O00257	CBX4_HUMAN	539.7643675	2	0.646438956		
YYYQLNSK	3.807949	O00257	CBX4_HUMAN	360.1788533	3	0.646438956		
AHIQSLFSFITGWSPVGTK	115.4104	O00255	MEN1_HUMAN	1038.549832	2	0.661538482		
AHIQSLFSFITGWSPVGTK	115.4104	O00255	MEN1_HUMAN	692.702496	3	0.661538482		
DVHLALSEDHAWVVFGPNGEQTAEVTWHGK	71.67854	O00255	MEN1_HUMAN	1665.302896	2	0.744966567		
DVHLALSEDHAWVVFGPNGEQTAEVTWHGK	71.67854	O00255	MEN1_HUMAN	1110.537872	3	0.744966567		
EAEAAEAEEPWGEEAR	26.53697	O00255	MEN1_HUMAN	887.382294	2	0.818301558		
EAEAAEAEEPWGEEAR	26.53697	O00255	MEN1_HUMAN	591.9241377	3	0.818301558		
EFFEVANDVIPNLLK	125.4628	O00255	MEN1_HUMAN	874.4674435	2	0.788266838		
EFFEVANDVIPNLLK	125.4628	O00255	MEN1_HUMAN	583.3142373	3	0.788266838		
GAVDLSLYPR	46.42823	O00255	MEN1_HUMAN	545.7987505	2	0.83793354		
GAVDLSLYPR	46.42823	O00255	MEN1_HUMAN	364.2017753	3	0.83793354		
GLGTGQGAVSGPPR	0.070744	O00255	MEN1_HUMAN	627.3340255	2	0.824696422	2	Yes
GLGTGQGAVSGPPR	0.070744	O00255	MEN1_HUMAN	418.5586253	3	0.824696422	2	
KPPGTVAGTAR	-40.04176	O00255	MEN1_HUMAN	527.8043725	2	0.645490408		
KPPGTVAGTAR	-40.04176	O00255	MEN1_HUMAN	352.2055233	3	0.645490408		
LFAAELGR	30.56034	O00255	MEN1_HUMAN	438.7510765	2	0.730215609		
LFAAELGR	30.56034	O00255	MEN1_HUMAN	292.8366593	3	0.730215609		

LLWLLYDLGHLER	104.5246	O00255	MEN1 HUMAN	820.9621275	2	0.776776314		
LLWLLYDLGHLER	104.5246	O00255	MEN1 HUMAN	547.6440267	3	0.776776314		
LQLTAQSQVQMK	21.48289	O00255	MEN1 HUMAN	687.874669	2	0.810990214		
LQLTAQSQVQMK	21.48289	O00255	MEN1 HUMAN	458.9190543	3	0.810990214		
VSDVIWNSLSR	52.53644	O00255	MEN1 HUMAN	638.3387745	2	0.780786753		
VSDVIWNSLSR	52.53644	O00255	MEN1 HUMAN	425.8951247	3	0.780786753		
VSTPSDYTLSFLK	71.91136	O00255	MEN1 HUMAN	729.3803015	2	0.843376756		
VSTPSDYTLSFLK	71.91136	O00255	MEN1 HUMAN	486.589476	3	0.843376756		
INEELESQYQQSMDSK	24.55459	O00193	SMAP HUMAN	964.931289	2	0.643852055	2	Yes
INEELESQYQQSMDSK	24.55459	O00193	SMAP HUMAN	643.6234677	3	0.643852055	2	
LVIGDHK	-23.34815	O00193	SMAP HUMAN	391.2325195	2	0.612765491		
LVIGDHK	-23.34815	O00193	SMAP HUMAN	261.1576213	3	0.612765491		
SASPDDDLGSSNWEAADLGNEER	54.7215	O00193	SMAP HUMAN	1218.015659	2	0.846751273	3	
SASPDDDLGSSNWEAADLGNEER	54.7215	O00193	SMAP HUMAN	812.346381	3	0.846751273	3	Yes
AASAFFTYVSLSQEGR	75.42777	O00154	BACH HUMAN	867.428846	2	0.63932085	3	
AASAFFTYVSLSQEGR	75.42777	O00154	BACH HUMAN	578.621839	3	0.63932085	3	Yes
ATLWYVPLSLK	93.57278	O00154	BACH HUMAN	645.877001	2	0.801930308	2	Yes
ATLWYVPLSLK	93.57278	O00154	BACH HUMAN	430.920609	3	0.801930308	2	
HSVEVQVNVMSENILTGAK	58.41211	O00154	BACH HUMAN	1028.031145	2	0.762577057		
HSVEVQVNVMSENILTGAK	58.41211	O00154	BACH HUMAN	685.6900383	3	0.762577057		
LMDEVAGIVAAR	54.62701	O00154	BACH HUMAN	622.8375545	2	0.855746627	2	Yes
LMDEVAGIVAAR	54.62701	O00154	BACH HUMAN	415.560978	3	0.855746627	2	
LVAGQGCVGPR	-9.042347	O00154	BACH HUMAN	557.2958575	2	0.737955511		
LVAGQGCVGPR	-9.042347	O00154	BACH HUMAN	371.8665133	3	0.737955511		
MIEEAGAIISTR	37.08602	O00154	BACH HUMAN	645.840294	2	0.800258934		
MIEEAGAIISTR	37.08602	O00154	BACH HUMAN	430.8961377	3	0.800258934		
PDDANVAGNVHGGTILK	12.84274	O00154	BACH HUMAN	839.4319245	2	0.898166656		
PDDANVAGNVHGGTILK	12.84274	O00154	BACH HUMAN	559.9572247	3	0.898166656		
SLPVPQLVPETEDEK	63.79314	O00154	BACH HUMAN	840.9387165	2	0.772798061		
SLPVPQLVPETEDEK	63.79314	O00154	BACH HUMAN	560.9617527	3	0.772798061		
TNIVTASVDAINFHDK	45.16656	O00154	BACH HUMAN	872.947407	2	0.734524488	3	
TNIVTASVDAINFHDK	45.16656	O00154	BACH HUMAN	582.3008797	3	0.734524488	3	Yes
VLEVPPVVYSR	54.4728	O00154	BACH HUMAN	629.3642575	2	0.811767101	2	Yes
VLEVPPVVYSR	54.4728	O00154	BACH HUMAN	419.9121133	3	0.811767101	2	
ALGVSNFNHFQIER	40.70911	C9JRZ8	AK1BF HUMAN	816.4186195	2	0.825110078		
ALGVSNFNHFQIER	40.70911	C9JRZ8	AK1BF HUMAN	544.6150213	3	0.825110078		
EFSHLEDFPFDAEY	93.7397	C9JRZ8	AK1BF HUMAN	873.370662	2	0.776011586		
EFSHLEDFPFDAEY	93.7397	C9JRZ8	AK1BF HUMAN	582.5830497	3	0.776011586		
GTFLDAWEAMEELVDEGLVK	178.0528	C9JRZ8	AK1BF HUMAN	1126.543752	2	0.789137185		
GTFLDAWEAMEELVDEGLVK	178.0528	C9JRZ8	AK1BF HUMAN	751.3651093	3	0.789137185		
HIDCAYFYENQHEVGEAIQEK	32.74348	C9JRZ8	AK1BF HUMAN	1290.577169	2	0.700894535		
HIDCAYFYENQHEVGEAIQEK	32.74348	C9JRZ8	AK1BF HUMAN	860.7207207	3	0.700894535		
MPIVGLGTWR	67.64674	C9JRZ8	AK1BF HUMAN	565.3135185	2	0.760208309		
MPIVGLGTWR	67.64674	C9JRZ8	AK1BF HUMAN	377.2116207	3	0.760208309		
SMTPAHIVENIQVFDFK	80.81712	C9JRZ8	AK1BF HUMAN	988.5014925	2	0.76369679		
SMTPAHIVENIQVFDFK	80.81712	C9JRZ8	AK1BF HUMAN	659.3369367	3	0.76369679		
TGDDFFPK	29.59806	C9JRZ8	AK1BF HUMAN	463.7168995	2	0.711132467		
TGDDFFPK	29.59806	C9JRZ8	AK1BF HUMAN	309.4805413	3	0.711132467		
VWPTFFERPLVR	72.24324	C9JRZ8	AK1BF HUMAN	773.930635	2	0.813397408		
VWPTFFERPLVR	72.24324	C9JRZ8	AK1BF HUMAN	516.2896983	3	0.813397408		
LLHLHFK	-5.787056	A8MT69	CENPX HUMAN	454.279804	2	0.610617697		
LLHLHFK	-5.787056	A8MT69	CENPX HUMAN	303.1891443	3	0.610617697		
VSGDALQLMVELLK	136.5652	A8MT69	CENPX HUMAN	758.4267335	2	0.718228459		
VSGDALQLMVELLK	136.5652	A8MT69	CENPX HUMAN	505.953764	3	0.718228459		
AAAAASAAGPGGLVAGK	15.00149	A6NIH7	U119B HUMAN	670.370608	2	0.760997117	2	Yes
AAAAASAAGPGGLVAGK	15.00149	A6NIH7	U119B HUMAN	447.2496803	3	0.760997117	2	
LMIENPYETR	33.89469	A6NIH7	U119B HUMAN	633.3139075	2	0.825487494	2	Yes
LMIENPYETR	33.89469	A6NIH7	U119B HUMAN	422.5452133	3	0.825487494	2	
NTCEHIYEFPQLSEDVIR	65.5034	A6NIH7	U119B HUMAN	1125.528963	2	0.772752106	3	
NTCEHIYEFPQLSEDVIR	65.5034	A6NIH7	U119B HUMAN	750.6885833	3	0.772752106	3	Yes
SDSFYFVDNK	36.91319	A6NIH7	U119B HUMAN	611.2753045	2	0.845254719	2	Yes
SDSFYFVDNK	36.91319	A6NIH7	U119B HUMAN	407.8528113	3	0.845254719	2	
TVGATVEFTVGDKPVSNFR	48.36893	A6NIH7	U119B HUMAN	1012.526554	2	0.75323087		
TVGATVEFTVGDKPVSNFR	48.36893	A6NIH7	U119B HUMAN	675.353644	3	0.75323087		
VTENYLCKPEDNIYSIDFTR	65.95027	A6NIH7	U119B HUMAN	1239.086837	2	0.776233196		
VTENYLCKPEDNIYSIDFTR	65.95027	A6NIH7	U119B HUMAN	826.393833	3	0.776233196		
AFLHWYLR	47.98441	A6NHL2	TBAL3 HUMAN	553.3012635	2	0.799389482		
AFLHWYLR	47.98441	A6NHL2	TBAL3 HUMAN	369.2034507	3	0.799389482		
ALFVDLEPTVIDGIR	110.2134	A6NHL2	TBAL3 HUMAN	829.462162	2	0.85783112		
ALFVDLEPTVIDGIR	110.2134	A6NHL2	TBAL3 HUMAN	553.3107163	3	0.85783112		
AYHEQFSVSDITTACFESSNQLVK	66.70274	A6NHL2	TBAL3 HUMAN	1381.142877	2	0.616380632		
AYHEQFSVSDITTACFESSNQLVK	66.70274	A6NHL2	TBAL3 HUMAN	921.0978593	3	0.616380632		
EGMEEAEFLEAR	48.23723	A6NHL2	TBAL3 HUMAN	705.814473	2	0.793287456		
EGMEEAEFLEAR	48.23723	A6NHL2	TBAL3 HUMAN	470.8789237	3	0.793287456		
FEGPLNVDLIEFQTNLVPYPR	139.8271	A6NHL2	TBAL3 HUMAN	1231.142277	2	0.62709713		
FEGPLNVDLIEFQTNLVPYPR	139.8271	A6NHL2	TBAL3 HUMAN	821.0974597	3	0.62709713		
HSVQFVDWCPTGFK	55.65228	A6NHL2	TBAL3 HUMAN	854.401582	2	0.819517672		
HSVQFVDWCPTGFK	55.65228	A6NHL2	TBAL3 HUMAN	569.9369963	3	0.819517672		
IHFPMTAFAPIVSADK	76.32637	A6NHL2	TBAL3 HUMAN	872.9587325	2	0.785218716		
IHFPMTAFAPIVSADK	76.32637	A6NHL2	TBAL3 HUMAN	582.30843	3	0.785218716		
LAEQCGGLQGFLIFR	94.83508	A6NHL2	TBAL3 HUMAN	854.946157	2	0.696191788		
LAEQCGGLQGFLIFR	94.83508	A6NHL2	TBAL3 HUMAN	570.3000463	3	0.696191788		
LEFSVYPAPR	48.76846	A6NHL2	TBAL3 HUMAN	589.8144005	2	0.813448608		
LEFSVYPAPR	48.76846	A6NHL2	TBAL3 HUMAN	393.545542	3	0.813448608		
LGVECPSHASINR	-8.894169	A6NHL2	TBAL3 HUMAN	720.3571735	2	0.690181911		
LGVECPSHASINR	-8.894169	A6NHL2	TBAL3 HUMAN	480.5740573	3	0.690181911		
LVVQVVSSITASLR	90.8223	A6NHL2	TBAL3 HUMAN	736.4463145	2	0.68826139		
LVVQVVSSITASLR	90.8223	A6NHL2	TBAL3 HUMAN	491.3001513	3	0.68826139		
MEHTNASFDTFFCETR	46.08266	A6NHL2	TBAL3 HUMAN	996.9149245	2	0.70631963		
MEHTNASFDTFFCETR	46.08266	A6NHL2	TBAL3 HUMAN	664.9458913	3	0.70631963		
SFGGGTGSGFTSLLMER	76.85861	A6NHL2	TBAL3 HUMAN	852.407061	2	0.791698575		
SFGGGTGSGFTSLLMER	76.85861	A6NHL2	TBAL3 HUMAN	568.6073157	3	0.791698575		
SLFHPEQLLSGK	42.80641	A6NHL2	TBAL3 HUMAN	678.3700755	2	0.8167063		
SLFHPEQLLSGK	42.80641	A6NHL2	TBAL3 HUMAN	452.5826587	3	0.8167063		
VGINNRPPTVMPGGDLAK	28.39874	A6NHL2	TBAL3 HUMAN	918.494002	2	0.829196215		
VGINNRPPTVMPGGDLAK	28.39874	A6NHL2	TBAL3 HUMAN	612.6652763	3	0.829196215		
YSVGSEVIDLVLER	100.6213	A6NHL2	TBAL3 HUMAN	789.9228645	2	0.834285617		
YSVGSEVIDLVLER	100.6213	A6NHL2	TBAL3 HUMAN	526.9511847	3	0.834285617		
ELAALYSPGK	27.11893	A5PLL7	PDES1 HUMAN	524.7878515	2	0.645781577	2	Yes
ELAALYSPGK	27.11893	A5PLL7	PDES1 HUMAN	350.1945093	3	0.645781577	2	
LEDLIQGLTGEKPR	48.17281	A5PLL7	PDES1 HUMAN	784.9363115	2	0.817624509		

LEDLIQGLTGEKPR	48.17281	A5PLL7	PDES1_HUMAN	523.626816	3	0.817624509		
WVTLLQDWHVILPR	112.5407	A5PLL7	PDES1_HUMAN	888.501956	2	0.691075087	3	
WVTLLQDWHVILPR	112.5407	A5PLL7	PDES1_HUMAN	592.670579	3	0.691075087	3	Yes
APGDHQFYK	-26.73749	A2RUS2	DEND3_HUMAN	531.7543435	2	0.747036338		
APGDHQFYK	-26.73749	A2RUS2	DEND3_HUMAN	354.8388373	3	0.747036338		
DLAQPPQR	-8.250999	A2RUS2	DEND3_HUMAN	462.7490655	2	0.682859719		
DLAQPPQR	-8.250999	A2RUS2	DEND3_HUMAN	308.8353187	3	0.682859719		
ELVAHMDTVR	0.483871	A2RUS2	DEND3_HUMAN	585.800972	2	0.611954629		
ELVAHMDTVR	0.483871	A2RUS2	DEND3_HUMAN	390.869923	3	0.611954629		
ETEAEAQEVSLPWLVMEHLDK	101.6031	A2RUS2	DEND3_HUMAN	1227.597047	2	0.616855443		
ETEAEAQEVSLPWLVMEHLDK	101.6031	A2RUS2	DEND3_HUMAN	818.7339727	3	0.616855443		
FPYYNSLK	28.02027	A2RUS2	DEND3_HUMAN	516.2640075	2	0.67563045		
FPYYNSLK	28.02027	A2RUS2	DEND3_HUMAN	344.5119467	3	0.67563045		
GSSAVLNVTPK	12.59301	A2RUS2	DEND3_HUMAN	536.804037	2	0.772335231		
GSSAVLNVTPK	12.59301	A2RUS2	DEND3_HUMAN	358.2052997	3	0.772335231		
HMQLGDFMK	28.36111	A2RUS2	DEND3_HUMAN	553.7602615	2	0.709465802		
HMQLGDFMK	28.36111	A2RUS2	DEND3_HUMAN	369.5094493	3	0.709465802		
IFPTDLVK	50.35492	A2RUS2	DEND3_HUMAN	466.77676	2	0.685804307		
IFPTDLVK	50.35492	A2RUS2	DEND3_HUMAN	311.5204483	3	0.685804307		
IPEIHFPLESK	52.88927	A2RUS2	DEND3_HUMAN	655.361719	2	0.827700019		
IPEIHFPLESK	52.88927	A2RUS2	DEND3_HUMAN	437.2437543	3	0.827700019		
IYVIDAER	22.31445	A2RUS2	DEND3_HUMAN	489.766919	2	0.684844851		
IYVIDAER	22.31445	A2RUS2	DEND3_HUMAN	326.847221	3	0.684844851		
LFEALTVGQEK	49.53243	A2RUS2	DEND3_HUMAN	617.8380775	2	0.829230785		
LFEALTVGQEK	49.53243	A2RUS2	DEND3_HUMAN	412.2279933	3	0.829230785		
LISEILDKPHEASK	6.566494	A2RUS2	DEND3_HUMAN	790.438686	2	0.738875985		
LISEILDKPHEASK	6.566494	A2RUS2	DEND3_HUMAN	527.2950657	3	0.738875985		
LWCALSEGK	29.17743	A2RUS2	DEND3_HUMAN	532.2662335	2	0.708031833		
LWCALSEGK	29.17743	A2RUS2	DEND3_HUMAN	355.1800973	3	0.708031833		
LWCCTGNSIMVMK	53.68347	A2RUS2	DEND3_HUMAN	800.3616995	2	0.610092223		
LWCCTGNSIMVMK	53.68347	A2RUS2	DEND3_HUMAN	533.910408	3	0.610092223		
MDAFAQMDLDTQSEEDR	57.58052	A2RUS2	DEND3_HUMAN	1001.412412	2	0.768458247		
MDAFAQMDLDTQSEEDR	57.58052	A2RUS2	DEND3_HUMAN	667.944216	3	0.768458247		
MSNEMPMTLPETTLETLK	84.28766	A2RUS2	DEND3_HUMAN	1033.496902	2	0.806996703		
MSNEMPMTLPETTLETLK	84.28766	A2RUS2	DEND3_HUMAN	689.333876	3	0.806996703		
MVVSMPNLQDIAMPELAPR	117.3699	A2RUS2	DEND3_HUMAN	1056.536695	2	0.740864217		
MVVSMPNLQDIAMPELAPR	117.3699	A2RUS2	DEND3_HUMAN	704.6937383	3	0.740864217		
SLQIVLPAR	45.56216	A2RUS2	DEND3_HUMAN	498.814208	2	0.60660249	2	Yes
SLQIVLPAR	45.56216	A2RUS2	DEND3_HUMAN	332.878747	3	0.60660249	2	
STDDNVDIPDVPLLAAQTFIQR	143.9776	A2RUS2	DEND3_HUMAN	1214.621911	2	0.760883451		
STDDNVDIPDVPLLAAQTFIQR	143.9776	A2RUS2	DEND3_HUMAN	810.083882	3	0.760883451		
TVESMSAPEWEGAEQAPELMR	69.64426	A2RUS2	DEND3_HUMAN	1174.53085	2	0.806263685		
TVESMSAPEWEGAEQAPELMR	69.64426	A2RUS2	DEND3_HUMAN	783.356508	3	0.806263685		
VFNSEEFLK	38.71767	A2RUS2	DEND3_HUMAN	556.7853125	2	0.78457433		
VFNSEEFLK	38.71767	A2RUS2	DEND3_HUMAN	371.52615	3	0.78457433		
VLQILTCILTEQR	94.83859	A2RUS2	DEND3_HUMAN	793.950908	2	0.607041001		
VLQILTCILTEQR	94.83859	A2RUS2	DEND3_HUMAN	529.636547	3	0.607041001		
VPLEDCSEINCMIR	51.0062	A2RUS2	DEND3_HUMAN	868.3947815	2	0.6922791		
VPLEDCSEINCMIR	51.0062	A2RUS2	DEND3_HUMAN	579.265796	3	0.6922791		
VQSLQLHHELHAAHLLSSTDLK	25.91086	A2RUS2	DEND3_HUMAN	1239.167154	2	0.771523714		
VQSLQLHHELHAAHLLSSTDLK	25.91086	A2RUS2	DEND3_HUMAN	826.4473773	3	0.771523714		
VTVFNASSWTIHQHSFK	39.40266	A2RUS2	DEND3_HUMAN	995.003048	2	0.748249114		
VTVFNASSWTIHQHSFK	39.40266	A2RUS2	DEND3_HUMAN	663.671307	3	0.748249114		
AASMHCQELMATGYPK	13.66473	A2RRP1	NBAS_HUMAN	897.9027705	2	0.782931387		
AASMHCQELMATGYPK	13.66473	A2RRP1	NBAS_HUMAN	598.9377887	3	0.782931387		
AEEIEHYAR	-22.19432	A2RRP1	NBAS_HUMAN	559.2678145	2	0.655108809		
AEEIEHYAR	-22.19432	A2RRP1	NBAS_HUMAN	373.1811513	3	0.655108809		
AQDLHLFETLK	45.45329	A2RRP1	NBAS_HUMAN	657.8568015	2	0.864830256		
AQDLHLFETLK	45.45329	A2RRP1	NBAS_HUMAN	438.9071427	3	0.864830256		
ASAQWSAELLVINYR	87.62874	A2RRP1	NBAS_HUMAN	860.9550305	2	0.710192621		
ASAQWSAELLVINYR	87.62874	A2RRP1	NBAS_HUMAN	574.305962	3	0.710192621		
AVLQAVSDGQWWK	62.12821	A2RRP1	NBAS_HUMAN	744.3862575	2	0.893984675		
AVLQAVSDGQWWK	62.12821	A2RRP1	NBAS_HUMAN	496.5934467	3	0.893984675		
AVQEDEVGVPGSNSADLLR	51.92115	A2RRP1	NBAS_HUMAN	978.4876245	2	0.759696603		
AVQEDEVGVPGSNSADLLR	51.92115	A2RRP1	NBAS_HUMAN	652.6610247	3	0.759696603		
DDFTSIIGK	53.19097	A2RRP1	NBAS_HUMAN	498.2563885	2	0.604309022		
DDFTSIIGK	53.19097	A2RRP1	NBAS_HUMAN	332.5068673	3	0.604309022		
DGQMLSPSSLYTIWLQK	119.1691	A2RRP1	NBAS_HUMAN	984.001321	2	0.718879879		
DGQMLSPSSLYTIWLQK	119.1691	A2RRP1	NBAS_HUMAN	656.3368223	3	0.718879879		
DIVQSAIMK	39.0686	A2RRP1	NBAS_HUMAN	502.776434	2	0.745854318		
DIVQSAIMK	39.0686	A2RRP1	NBAS_HUMAN	335.520231	3	0.745854318		
EAGHEAEAGSLLLAVR	43.85126	A2RRP1	NBAS_HUMAN	811.9290175	2	0.791990817		
EAGHEAEAGSLLLAVR	43.85126	A2RRP1	NBAS_HUMAN	541.6219533	3	0.791990817		
ECISQSPTCYK	-2.290596	A2RRP1	NBAS_HUMAN	686.797765	2	0.728728473		
ECISQSPTCYK	-2.290596	A2RRP1	NBAS_HUMAN	458.2011183	3	0.728728473		
EGLGNEVLK	11.14033	A2RRP1	NBAS_HUMAN	479.7643805	2	0.744911313		
EGLGNEVLK	11.14033	A2RRP1	NBAS_HUMAN	320.178862	3	0.744911313		
ELLQYGLK	38.09834	A2RRP1	NBAS_HUMAN	482.2796625	2	0.77627039		
ELLQYGLK	38.09834	A2RRP1	NBAS_HUMAN	321.8557167	3	0.77627039		
EYFNSSTNLTDSCMDLAR	59.27241	A2RRP1	NBAS_HUMAN	1062.454606	2	0.652761042		
EYFNSSTNLTDSCMDLAR	59.27241	A2RRP1	NBAS_HUMAN	708.639012	3	0.652761042		
GQVLILLVEQALR	117.5519	A2RRP1	NBAS_HUMAN	726.4514005	2	0.624981642		
GQVLILLVEQALR	117.5519	A2RRP1	NBAS_HUMAN	484.6368753	3	0.624981642		
GTDLEALLAIGK	90.87758	A2RRP1	NBAS_HUMAN	600.8459025	2	0.757925034		
GTDLEALLAIGK	90.87758	A2RRP1	NBAS_HUMAN	400.8998767	3	0.757925034		
HEHEAWPEDLISLTK	57.10568	A2RRP1	NBAS_HUMAN	902.947407	2	0.841747344		
HEHEAWPEDLISLTK	57.10568	A2RRP1	NBAS_HUMAN	602.3008797	3	0.841747344		
IFQHSKPDLQQK	-25.96646	A2RRP1	NBAS_HUMAN	734.899532	2	0.85688746		
IFQHSKPDLQQK	-25.96646	A2RRP1	NBAS_HUMAN	490.268963	3	0.85688746		
IHVLQILGQSFHLTEEDSK	71.7958	A2RRP1	NBAS_HUMAN	1097.579317	2	0.667880356		
IHVLQILGQSFHLTEEDSK	71.7958	A2RRP1	NBAS_HUMAN	732.0554863	3	0.667880356		
IISALSGGSADLGGPR	41.60596	A2RRP1	NBAS_HUMAN	735.8997285	2	0.752419114		
IISALSGGSADLGGPR	41.60596	A2RRP1	NBAS_HUMAN	490.9357607	3	0.752419114		
ILPLQVR	33.53222	A2RRP1	NBAS_HUMAN	419.779637	2	0.631242633		
ILPLQVR	33.53222	A2RRP1	NBAS_HUMAN	280.189033	3	0.631242633		
IVDHLLASLQQGR	41.32832	A2RRP1	NBAS_HUMAN	725.4128065	2	0.806127429		
IVDHLLASLQQGR	41.32832	A2RRP1	NBAS_HUMAN	483.9444793	3	0.806127429		
LATVMLTR	22.6077	A2RRP1	NBAS_HUMAN	452.7684125	2	0.688361228		
LATVMLTR	22.6077	A2RRP1	NBAS_HUMAN	302.18155	3	0.688361228		
LATYEEILGVPHASEQR	48.836	A2RRP1	NBAS_HUMAN	956.992342	2	0.823212862	3	
LATYEEILGVPHASEQR	48.836	A2RRP1	NBAS_HUMAN	638.3308363	3	0.823212862	3	Yes

LFWTGDPHLIK	54.02683	A2RRP1	NBAS HUMAN	663.8644295	2	0.804431796		
LFWTGDPHLIK	54.02683	A2RRP1	NBAS HUMAN	442.912228	3	0.804431796		
LHDMVDQLEQILSVSELLEK	146.6643	A2RRP1	NBAS HUMAN	1170.112332	2	0.652837455		
LHDMVDQLEQILSVSELLEK	146.6643	A2RRP1	NBAS HUMAN	780.4108297	3	0.652837455		
LHPGDLITVVDAVTFSPK	102.5534	A2RRP1	NBAS HUMAN	955.025658	2	0.696472526		
LHPGDLITVVDAVTFSPK	102.5534	A2RRP1	NBAS HUMAN	637.0197137	3	0.696472526		
LLAAVQDQCVEIR	42.34247	A2RRP1	NBAS HUMAN	757.9039575	2	0.787119031		
LLAAVQDQCVEIR	42.34247	A2RRP1	NBAS HUMAN	505.6052467	3	0.787119031		
LLDFTQAQILQGLR	104.8499	A2RRP1	NBAS HUMAN	808.462497	2	0.761116624		
LLDFTQAQILQGLR	104.8499	A2RRP1	NBAS HUMAN	539.3109397	3	0.761116624		
LLGLAELLR	91.48814	A2RRP1	NBAS HUMAN	499.3244085	2	0.768995464		
LLGLAELLR	91.48814	A2RRP1	NBAS HUMAN	333.2188807	3	0.768995464		
LLLVGGCETAEVGMSK	53.41306	A2RRP1	NBAS HUMAN	832.4238655	2	0.768668234		
LLLVGGCETAEVGMSK	53.41306	A2RRP1	NBAS HUMAN	555.2851853	3	0.768668234		
LLMNSCSEDK	-5.927055	A2RRP1	NBAS HUMAN	598.7684795	2	0.758133471		
LLMNSCSEDK	-5.927055	A2RRP1	NBAS HUMAN	399.514928	3	0.758133471		
LSIWAIPSLK	92.90823	A2RRP1	NBAS HUMAN	564.3453405	2	0.766695261		
LSIWAIPSLK	92.90823	A2RRP1	NBAS HUMAN	376.5661687	3	0.766695261		
MVVEPNLQDESEFLYAAQPELLR	115.7077	A2RRP1	NBAS HUMAN	1346.170906	2	0.757001579		
MVVEPNLQDESEFLYAAQPELLR	115.7077	A2RRP1	NBAS HUMAN	897.783212	3	0.757001579		
SAVNVASIQNYLSK	57.58788	A2RRP1	NBAS HUMAN	747.402099	2	0.846073031		
SAVNVASIQNYLSK	57.58788	A2RRP1	NBAS HUMAN	498.6040077	3	0.846073031		
SEYVITNNPWVR	51.28356	A2RRP1	NBAS HUMAN	739.3758845	2	0.789136171		
SEYVITNNPWVR	51.28356	A2RRP1	NBAS HUMAN	493.253198	3	0.789136171		
SIDLVLAASR	48.62569	A2RRP1	NBAS HUMAN	522.8065795	2	0.754040241		
SIDLVLAASR	48.62569	A2RRP1	NBAS HUMAN	348.8736613	3	0.754040241		
SLAHLETLSHSFILSLK	68.28387	A2RRP1	NBAS HUMAN	948.533649	2	0.676731825		
SLAHLETLSHSFILSLK	68.28387	A2RRP1	NBAS HUMAN	632.6917077	3	0.676731825		
SLTYLRPLQGQK	18.4128	A2RRP1	NBAS HUMAN	702.404446	2	0.699974		
SLTYLRPLQGQK	18.4128	A2RRP1	NBAS HUMAN	468.6055723	3	0.699974		
STTPEELYQR	5.002159	A2RRP1	NBAS HUMAN	612.299312	2	0.799497724		
STTPEELYQR	5.002159	A2RRP1	NBAS HUMAN	408.535483	3	0.799497724		
SWDVCSQLGQSEGYQDLATR	61.61032	A2RRP1	NBAS HUMAN	1150.516584	2	0.732131004		
SWDVCSQLGQSEGYQDLATR	61.61032	A2RRP1	NBAS HUMAN	767.3469977	3	0.732131004		
SWVLHECLER	22.58152	A2RRP1	NBAS HUMAN	664.824978	2	0.806489825		
SWVLHECLER	22.58152	A2RRP1	NBAS HUMAN	443.5525937	3	0.806489825		
TDPEAFHQHMVK	-17.37468	A2RRP1	NBAS HUMAN	720.340993	2	0.743712664		
TDPEAFHQHMVK	-17.37468	A2RRP1	NBAS HUMAN	480.5632703	3	0.743712664		
TYAQESNVQALEILFTYHGSDLLPHR	106.0157	A2RRP1	NBAS HUMAN	1501.75451	2	0.611794651		
TYAQESNVQALEILFTYHGSDLLPHR	106.0157	A2RRP1	NBAS HUMAN	1001.505615	3	0.611794651		
VLEGVVAAVHASVDK	35.60741	A2RRP1	NBAS HUMAN	747.420297	2	0.726280689		
VLEGVVAAVHASVDK	35.60741	A2RRP1	NBAS HUMAN	498.6161397	3	0.726280689		
VMDWYQTR	26.60851	A2RRP1	NBAS HUMAN	549.756029	2	0.754342556		
VMDWYQTR	26.60851	A2RRP1	NBAS HUMAN	366.839961	3	0.754342556		
VTYADTLNHLEK	21.49508	A2RRP1	NBAS HUMAN	702.3624435	2	0.894131422		
VTYADTLNHLEK	21.49508	A2RRP1	NBAS HUMAN	468.5775707	3	0.894131422		
VVASGLNYK	-0.201828	A2RRP1	NBAS HUMAN	475.7694615	2	0.698731661		
VVASGLNYK	-0.201828	A2RRP1	NBAS HUMAN	317.5155827	3	0.698731661		
YDAEFFK	30.33983	A2RRP1	NBAS HUMAN	460.213988	2	0.745776296		
YDAEFFK	30.33983	A2RRP1	NBAS HUMAN	307.145267	3	0.745776296		
YIYPTIGGFDHER	40.2813	A2RRP1	NBAS HUMAN	784.3811635	2	0.833408356		
YIYPTIGGFDHER	40.2813	A2RRP1	NBAS HUMAN	523.2567173	3	0.833408356		
YSHLYDLSR	4.644947	A2RRP1	NBAS HUMAN	577.2860025	2	0.827682137		
YSHLYDLSR	4.644947	A2RRP1	NBAS HUMAN	385.1932767	3	0.827682137		
YVTSAYQWMVPFLHR	84.62685	A2RRP1	NBAS HUMAN	949.4750725	2	0.777259827		
YVTSAYQWMVPFLHR	84.62685	A2RRP1	NBAS HUMAN	633.3193233	3	0.777259827		
AGGQQVTLTTHAPFGLGAHFTVPLK	67.70389	A0PJW6	TM223 HUMAN	1274.687724	2	0.823993802		
AGGQQVTLTTHAPFGLGAHFTVPLK	67.70389	A0PJW6	TM223 HUMAN	850.1277577	3	0.823993802		
DVLLFEHDR	31.54849	A0PJW6	TM223 HUMAN	572.2938365	2	0.777826428		
DVLLFEHDR	31.54849	A0PJW6	TM223 HUMAN	381.865166	3	0.777826428		
FYFLLDK	66.51573	A0PJW6	TM223 HUMAN	473.2581985	2	0.71937412	2	Yes
FYFLLDK	66.51573	A0PJW6	TM223 HUMAN	315.8414073	3	0.71937412	2	
GEVPAMLPLK	51.686	A0PJW6	TM223 HUMAN	527.802452	2	0.771172047	2	Yes
GEVPAMLPLK	51.686	A0PJW6	TM223 HUMAN	352.204243	3	0.771172047	2	
LFDNTVGAYR	30.49033	A0PJW6	TM223 HUMAN	578.2938325	2	0.809381247		
LFDNTVGAYR	30.49033	A0PJW6	TM223 HUMAN	385.8651633	3	0.809381247		

Our DeepPRM method could also predicted the imformation about FragmentMz, FragmentType,FragmentCharge and FragmentLossType. These data are not shown.

SourceDataForFigure4C, D: The peak area of 113 peptides/100 proteins in 212 samples

No.	Uniprot Accession	Protein Name	Peptide Sequence	FC [(R&UR) *vs.* NC]	*P* [(R&UR) *vs.* NC]	FC (R *vs.* UR)	*P*(R *vs.* UR)
1	P02750	A2GL HUMAN	DLLLPQPDLR	1.0024	0.865	1.5909	0
2	P02750	A2GL HUMAN	ENQLEVLEVSWLHGLK	0.95648	0.351	1.6358	0.002
3	P08311	CATG HUMAN	IFGSYDPR	0.14827	P<0.001	1.175	0.378
4	P08311	CATG HUMAN	VSSFLPWIR	0.14114	P<0.001	1.1136	0.319
5	P01024	CO3 HUMAN	EYVLPSFEVIVEPTEK	0.83978	0.028	1.0455	0.686
6	P01024	CO3 HUMAN	NTLIIYLDK	1.2323	0.083	0.82029	0.604
7	P02671	FIBA HUMAN	GSESGIFTNTK	4.0802	0.001	0.44824	0.49
8	P02671	FIBA HUMAN	VQHIQLLQK	14.234	0.001	0.54964	0
9	P02675	FIBB HUMAN	AHYGGFTVQNEANK	16.295	0.001	0.52765	0
10	P02675	FIBB HUMAN	DNENVVNEYSSELEK	12.347	0.003	0.59745	0
11	P02679	FIBG HUMAN	EGFGHLSPTGTTEFWLGNE[illegible]	10.375	0.004	0.67997	0
12	P02751	FINC HUMAN	DLQFVEVTDVK	2.027	0.214	2.0213	0.025
13	P02751	FINC HUMAN	NLQPASEYTVSLVAIK	1.9946	0.063	1.2662	0.128
14	P06312	KV401 HUMAN	NYLAWYQQKPGQPPK	1.4134	0.145	0.47192	0
15	P06312	KV401 HUMAN	SSQSVLYSSNNK	0.93161	0.277	0.96043	0.699
16	P14780	MMP9 HUMAN	LGLGADVAQVTGALR	0.33527	P<0.001	1.7225	0.056
17	P14780	MMP9 HUMAN	SLGPALLLLQK	0.25354	P<0.001	1.3397	0.246
18	O00151	PDLI1 HUMAN	DFEQPLAISR	0.2667	P<0.001	0.48524	0.878
19	P05164	PERM HUMAN	VVLEGGIDPILR	0.28711	P<0.001	1.0787	0.281
20	P24158	PRTN3 HUMAN	LFPDFFTR	0.38298	P<0.001	1.2892	0.812
21	P24158	PRTN3 HUMAN	LVNVVLGAHNVR	0.24659	P<0.001	0.98278	0.078
22	P19320	VCAM1 HUMAN	LTAFPSESVK	0.87909	0.048	0.78111	0.17
23	P19320	VCAM1 HUMAN	SLEVTFTPVIEDIGK	0.78881	0.003	0.97241	0.582
24	A0A075B6I0	LV861 HUMAN	TLIYSTNTR	1.1471	0.647	0.79599	0.991
25	A0A075B6K4	LV310 HUMAN	SGQAPVLVIYEDSK	1.3022	0.577	0.83105	0.732
26	A0A0A0MT36	KVD21 HUMAN	YASQSISGVPSR	0.99917	0.919	0.78631	0.155
27	A0A0B4J1V6	HV373 HUMAN	ANSYATAYAASVK	1.2615	0.174	1.3836	0.162
28	A0A0C4DH25	KVD20 HUMAN	LLIYDASSR	1.1615	0.02	0.90233	0.065
29	O43866	CD5L HUMAN	LVGGLHR	1.1783	0.035	0.89866	0.302
30	O75015	FCG3B HUMAN	AVVFLEPQWYSVLEK	0.76328	0.134	0.98751	0.463
31	O95497	VNN1 HUMAN	FGQTPVQER	1.1924	0.349	1.3514	0.374
32	P00450	CERU HUMAN	ALYLQYTDETFR	1.1906	0.005	0.83951	0.342
33	P00488	F13A HUMAN	GTYIPVPIVSELQSGK	1.7195	0.003	0.9882	0.246
34	P00736	C1R HUMAN	TLDEFTIIQNLQPQYQFR	1.2195	0.099	1.0036	0.682
35	P00738	HPT HUMAN	TEGDGVYTLNNEK	1.147	0.257	1.4975	0.022
36	P00739	HPTR HUMAN	VGYVSGWGQSDNFK	1.1038	0.123	0.85675	0.119
37	P00740	FA9 HUMAN	IIPHHNYNAAINK	1.0126	0.891	1.2493	0.065
38	P00751	CFAB HUMAN	DISEVVTPR	0.84463	0.003	0.96119	0.823
39	P00915	CAH1 HUMAN	VLDALQAIK	0.7596	0.123	2.0904	0.246
40	P01009	A1AT HUMAN	LSITGTYDLK	0.50726	0.001	0.68686	0.626
41	P01011	AACT HUMAN	EQLSLLDR	1.2087	0.038	1.237	0.011
42	P01019	ANGT HUMAN	VLSALQAVQGLLVAQGR	1.0977	0.242	1.0459	0.435
43	P01023	A2MG HUMAN	HYDGSYSTFGER	1.1462	0.093	0.94537	0.696
44	P01023	A2MG HUMAN	VSNQTLSLFFTVLQDVPVR	1.4268	0.408	0.89654	0.347
45	P01031	CO5 HUMAN	LQGTLPVEAR	0.98618	0.886	1.0049	0.635
46	P01344	IGF2 HUMAN	FFQYDTWK	1.5397	0.313	0.41213	0.072
47	P01591	IGJ HUMAN	SSEDPNEDIVER	1.11	0.96	0.8583	0.708
48	P01619	KV320 HUMAN	LLIYGASSR	1.3052	P<0.001	0.83685	0.09
49	P01780	HV307 HUMAN	GLEWVANIK	0.9092	0.479	0.9739	0.981
50	P01871	IGHM HUMAN	QIQVSWLR	1.2079	0.022	0.94097	0.836
51	P02042	HBD HUMAN	VNVDAVGGEALGR	0.93042	0.094	1.6795	0.787
52	P02649	APOE HUMAN	SELEEQLTPVAEETR	1.2875	0.113	0.81854	0.713
53	P02745	C1QA HUMAN	GHIYQGSEADSVFSGFLIFPS	0.98353	0.171	1.2092	0.341
54	P02746	C1QB HUMAN	LEQGENVFLQATDK	1.0535	0.835	0.829	0.089
55	P02747	C1QC HUMAN	FNAVLTNPQGDYDTSTGK	1.2226	0.024	0.84703	0.26
56	P02763	A1AG1 HUMAN	YVGGQEHFAHLLILR	1.0942	0.105	1.1246	0.253
57	P02765	FETUA HUMAN	HTLNQIDEVK	0.95887	0.996	0.88263	0.046
58	P02775	CXCL7 HUMAN	NIQSLEVIGK	0.59141	0.001	0.91149	0.339
59	P02787	TRFE HUMAN	EFQLFSSPHGK	0.9642	0.446	0.86771	0.291
60	P02788	TRFL HUMAN	YLGPQYVAGITNLK	0.39949	P<0.001	1.0171	0.675
61	P04003	C4BPA HUMAN	GYILVGQAK	0.92353	0.52	0.89753	0.229
62	P04070	PROC HUMAN	YLDWIHGHIR	1.2192	P<0.001	0.91317	0.31
63	P04430	KV116 HUMAN	SLIYAASSLQSGVPSK	1.0505	0.612	0.81109	0.186
64	P05062	ALDOB HUMAN	ALQASALAAWGGK	1.2394	0.782	1.6294	0.017
65	P05090	APOD HUMAN	NILTSNNIDVK	0.9785	0.5	0.90836	0.62
66	P05109	S10A8 HUMAN	ALNSIIDVYHK	0.15789	0.006	0.43059	0.096
67	P05154	IPSP HUMAN	GFQQLLQELNQPR	1.135	0.283	0.78746	0.229
68	P05160	F13B HUMAN	GDTYPAELYITGSILR	0.94352	0.537	1.2009	0.408
69	P05362	ICAM1 HUMAN	VELAPLPSWQPVGK	1.113	0.349	0.95552	0.535
70	P05556	ITB1 HUMAN	GEVFNELVGK	0.70437	0.045	0.92334	0.696
71	P06681	CO2 HUMAN	NQGILEFYGDDIALLK	0.84683	0.015	1.1151	0.057
72	P06702	S10A9 HUMAN	LGHPDTLNQGEFK	0.12779	0.043	1.0443	0.038
73	P06702	S10A9 HUMAN	NIETIINTFHQYSVK	0.24398	0.073	0.78937	0.21
74	P06727	APOA4 HUMAN	SELTQQLNALFQDK	1.0735	0.479	0.3891	0
75	P07225	PROS HUMAN	NNLELSTPLK	1.1937	0.017	0.86618	0.911
76	P07996	TSP1 HUMAN	GFLLLASLR	0.64801	0.025	1.1133	0.557
77	P08253	MMP2 HUMAN	AFQVWSDVTPLR	1.1618	0.145	0.71678	0.002
78	P08519	APOA HUMAN	TPENYPNAGLTR	1.2595	0.614	1.295	0.362
79	P08571	CD14 HUMAN	AFPALTSLDLSDNPGLGER	0.98848	0.227	1.2152	0.002
80	P08697	A2AP HUMAN	FDPSLTQR	1.2057	0.146	1.2083	0.113
81	P08709	FA7 HUMAN	FSLVSGWGQLLDR	1.244	0.187	0.83284	0.376
82	P0C0L4	CO4A HUMAN	ANSFLGEK	1.0608	0.056	1.0229	0.95
83	P0DJI8	SAA1 HUMAN	FGHGAEDSLADQAANEWG	1.9191	0.16	7	0
84	P0DOX7	IGK HUMAN	GTVAAPSVFIFPPSDEQLK	1.0368	0.331	0.97027	0.656
85	P10451	OSTP HUMAN	AIPVAQDLNAPSDWDSR	1.512	0.023	1.5063	0.055
86	P12111	CO6A3 HUMAN	VVESLDVGQDR	1.0282	0.306	0.89993	0.719
87	P13598	ICAM2 HUMAN	ILLDEQAQWK	0.98091	0.804	0.95066	0.656
88	P19320	VCAM1 HUMAN	NTVISVNPSTK	0.87038	0.13	0.95424	0.933
89	P19827	ITIH1 HUMAN	FAHYVVTSQVVNTANEAR	1.1399	0.426	0.8095	0.375
90	P20742	PZP HUMAN	SLFTDLVAEK	0.74988	0.958	1.2203	0.757
91	P20851	C4BPB HUMAN	ALLAFQESK	0.96009	0.742	0.91058	0.114
92	P24592	IBP6 HUMAN	HLDSVLQQLQTEVYR	0.90064	0.67	0.95102	0.541
93	P25774	CATS HUMAN	GPVSVGVDAR	1.3179	0.017	0.89044	0.015
94	P32942	ICAM3 HUMAN	TFVLPVTPPR	1.1021	0.711	1.0377	0.298
95	P35542	SAA4 HUMAN	GPGGVWAAK	1.1333	0.027	1.1895	0.023
96	P41222	PTGDS HUMAN	AQGFTEDTIVFLPQTDK	0.86706	0.655	0.65002	0
97	P48740	MASP1 HUMAN	TGVITSPDFPNPYPK	1.0131	0.876	0.88392	0.412
98	P49747	COMP HUMAN	NALWHTGDTESQVR	1.0525	0.959	0.592	0
99	P49913	CAMP HUMAN	SSDANLYR	1.1652	0.065	0.79844	0.4
100	P81605	DCD HUMAN	DAVEDLESVGK	0.95009	0.517	1.0812	0.888
101	Q08830	FGL1 HUMAN	IDLADFEK	1.1711	0.731	1.5542	0
102	Q13508	NAR3 HUMAN	TSQGTSFTFGGLNQAR	1.0335	0.473	0.61423	0.047
103	Q14515	SPRL1 HUMAN	AQSIAYHLK	0.59716	0.465	0.58297	0.021
104	Q14624	ITIH4 HUMAN	NGIDIYSLTVDSR	1.4746	0.002	1.5032	0.133
105	Q14766	LTBP1 HUMAN	EAQPGQSQVSYQGLPVQK	0.68601	0.007	0.82306	0.795
106	Q15485	FCN2 HUMAN	VDGSVDFYR	1.3544	0.145	1.0241	0.246
107	Q86VB7	C163A HUMAN	EAEFGQGTGPIWLNEVK	0.84577	0.137	0.86672	0.827
108	Q8IZF2	AGRF5 HUMAN	FSNVPSSPESPIGGTITYK	1.142	0.061	1.2504	0.027
109	Q96IY4	CBPB2 HUMAN	DTGTYGFLLPER	1.1278	0.447	0.8069	0.135
110	Q99784	NOE1 HUMAN	LTGISDPVTVK	0.9124	0.01	0.92324	0.703
111	Q99969	RARR2 HUMAN	GLQVALEEFHK	1.0115	0.873	0.72024	0.033
112	Q9Y490	TLN1 HUMAN	GLAGAVSELLR	0.99189	0.004	0.49998	0.787
113	Q9Y6Z7	COL10 HUMAN	DEAANTLIADYVAK	0.90544	0.226	0.83312	0.122

No.	N_B1_R1	N_B1_R2	N_B1_R3	N_B1_R4	N_B1_R5	N_B1_R6	N_B1_R7	N_B1_R8	N_B1_R9	N_B1_R10
1	431328524	280618666	111702262	243305092	229303750	234765386	429962831	650918277	52789840.7	404455622
2	65117375.7	48034227.1	33002021.1	37022853.7	45347406.8	60752746.9	111770116	191528460	13448499.2	105066185
3	3149058.17	1584811.62	318525.943	290153.419	41130.8569	602567.621	560434.966	2174676.35	224727.258	399480.695
4	3441337.8	1763102.97	1175538.1	1461935.22	966388.139	1168131.99	1672690.27	3232871.06	1581833.84	1904210.91
5	284634362	351104407	260647070	224289622	242541940	284494920	155970696	683379045	106682159	215670164
6	1205438322	1372386169	125903812	197817531	719872715	942062084	151567290	1317615216	70155362.5	289789519
7	95412782.2	82276031.7	43186148.4	75857193.7	86766717.5	91773511.4	45297340.3	122448033	44221162.5	86921405.4
8	1771498.94	1054710.92	880527.689	1129327.77	69124.3575	1084580.33	3767290.38	4651893.43	1260708.82	3104154.6
9	733021.3	343979.031	783620.419	1375310.99	402762.377	554200.556	1664862.07	4610259.16	978571.66	640834.129
10	443517.139	981567.858	1520334.37	2645959.03	862995.292	970429.64	9200443.58	17721688.1	2893005.36	4096955.1
11	610771.658	255956.068	540141.128	803152.858	437115.262	411977.248	2048228.55	3405720.86	492671.664	966359.946
12	2154215.01	6551069.76	137743.204	204158.967	1214056.04	1880266.61	535079.565	2857750.91	489508.901	1658884.84
13	12334297.4	46322829.4	353162.894	1064189.44	8203578.5	23204867.1	2074550.82	31749563.1	1703126.54	13144461.8
14	519534.783	462554.722	123076.381	338965.99	490819.727	462318.615	655782.519	3125977.48	430420.793	276415.201
15	30809515	9069356.09	2895969.49	6442678.02	10227793.3	10178879.2	23673159	40190573	10563962.7	21290130.2
16	8531580.71	6859371.99	847461.001	2227939.12	3351148.98	5068016.97	2602159.55	5925741.35	688971.709	4245748.61
17	19880047.3	8635330.99	5513136.47	4488872.97	4980853.11	4086266.23	5336520.23	8150639.84	5449665.87	7262650.91
18	2540731.7	2085719.71	315079.411	713954.278	355373.344	262846.537	150321.639	1326191.13	434366.293	1172243.68
19	4390405.71	3357230.38	82983.4519	474621.891	452144.046	731058.369	181386.831	1851486.65	63341.9593	1369241.44
20	5106351.12	3458745.92	283496.387	847240.819	1284056.21	1380270.87	1519338.52	4531997.05	287911.635	2004692.77
21	14700213.9	9129733.64	847527.877	1725477.47	5790307.84	2248793.2	1585064.79	7906385.95	734113.94	1813896.32
22	3976048.34	1879771.2	1541323.14	1145939.5	2865704.56	3128282.23	4890567.77	9046482.08	2282428.11	5048857.09
23	437464.074	164257.58	372751.978	410985.051	566988.693	421989.692	749344.776	1191840.34	906680.102	562526.093
24	107826491	74968296	15483008.6	36432771.6	34154321.2	24557191.8	80867796.2	114351195	18105505.3	70145086.1
25	14588274.7	17582129.4	5383817.43	8908270.21	2083089.6	2034183.95	6337629.91	10130641.9	4576807.2	17153823.2
26	7153363.44	4489326.06	472595.938	1259648	4522470.51	4978579.01	13855310	34758447.5	683361.808	6577167.49
27	281289.646	187306.276	40558.1536	276239.642	141997.841	60016.6031	122382.004	568338.105	99501.419	80839.1536
28	63672500.1	70444689.6	4151281.32	2605368.17	35443241.9	40688554.4	12201244.2	88787790.1	5524028.4	72392464.7
29	260674745	144657089	43073696	64600246.6	101033850	54994062.8	234187371	586287390	117411611	118818382
30	299501.418	133878.623	1030.72852	1057.4203	21742.3301	31986.9885	457708.653	615871.141	311037.055	268947.592
31	465764.661	18490.8388	1347756.6	43252.6591	2514.23878	375252.648	1134375.06	1819638.81	973931.07	1710177.39
32	625508428	268154184	94466474.7	208093971	261100362	251682829	192750528	947144038	165254288	453730459
33	2885167.21	1368955.32	131416.631	464601.46	2833696.87	3683129.87	3706154.65	13194525.5	644740.529	3318117.28
34	28417497.5	17415471.6	23972997.6	20368645.1	39094407.5	37481354.6	38694891.6	85347465.8	49499006.8	31097038.4
35	3304156840	3763964410	692405403	491574287	427788958	1233216452	465133193	1874011797	661445446	2142480754
36	88391134.8	56752562.7	26030443.4	26729235.4	52504093.4	84910428.8	22960995.9	349952535	79165527	153423738
37	2117015.71	1012676.17	715018.271	1371949.41	2245770.63	816364.57	595721.028	1186261.18	608877.074	783472.894
38	1448699745	762917168	852282448	581386332	977929396	47640024.3	830796442	515516294	1093543293	571062835
39	4778085.85	3213560.3		402797.731		3448700.86	1570032.91	1188673.99	1230265.7	28905588.8
40	87840263.9	45348984.8	120553319	101260205	56873293.7	54945466.2	63445569.8	44258948.9	30816090.5	42508113.6
41	17525254.2	24460189.4	4894109.03	2786502.86	18863054.2	33303370.9	5645539.38	43070342.1	4887229.81	22719620.2
42	128813739	167829735	21707586.2	46391685.4	112930540	300164548	89294406.6	310881069	33718243.7	431489915
43	2530040606	1153265454	265138518	252540382	3175220417	2530349359	2026840524	4000258536	1553521757	3758076642
44	20578931.9	19112378.6	2176883.67	2378926.44	45161724.8	70161187.3	21158693.7	67262775.8	7328046.11	13167950.8
45	581623538	318439134	110179070	199821063	456211520	363392980	178900116	59474392.6	176325069	141273017
46	47027.6014	22825.4663	10212.3402	17713.2997	87570.133	11989.6453	34569.4036	81652.3539	26526.558	61248.6479
47	775312314	532481443	281881604	709346320	103633679	54869427.5	978389598	1201216078	940588316	581165900
48	549859776	372502896	32418632	86268387.9	110432329	65264553.8	226712755	490404085	49202420.1	291928957
49	311926544	275796198	24582186.3	47333432.6	274091459	557125552	170292823	345594557	66616489.3	115899179
50	2676522984	1101385593	484831531	598528854	824041335	180484732	1417948659	4184775779	509104712	708270777
51	16509438.1	22027347.8	792189.038	4776350.18	1936865.23	9154720.95	2350560.47	7614876.75	5608466.31	61938006.9
52	307171947	695041033	107506048	193469417	869527555	941845497	798293885	375783666	19986001.9	166333268
53	12266280.1	5050059.94	6049015.11	3642247.63	8870229.22	17211123.8	17086013.6	21388364.3	17121481.3	6392328.42
54	181193013	232539163	22946182.1	155396329	156898556	128394003	160310579	246093216	88573758	540020970
55	155030566	257172286	14087998.9	75086899.2	377711530	289552254	117978943	735336377	24462991.7	76621103.6
56	1901905265	1823146357	398670215	989502629	2389312676	1972609491	1691087125	5062375126	724042686	600589183
57	861410285	469202191	315654023	175731615	249700632	301290268	459822257	856681399	570998473	555023093
58	255427696	150033012	78883445.1	35841276.5	49643980.5	65053704	89754167.4	80758662.5	151245022	202205625
59	4282181134	2403001862	1327411427	1425531906	3688893468	3148817300	2041643654	5071881784	385888316	6160314023
60	262828.684	264724.401	91383.9713	206337.885	243250.717	235673.512	361210.642	371540.135	172034.624	75511.0345
61	706152594	341992844	217630327	145545369	385749361	374262741	361146336	871023838	442573705	842433006
62	9003428.74	5481178.27	421191.646	290579.38	3312602.41	12451261.6	1817991.8	19057651.2	1463823.86	7256010.26
63	2146136.7	1397867.27	668256.29	350452.472	798131.134	2179438.99	1300947.12	1433584.74	716081.568	471338.343
64	640002.37	348534.908	301952.691	443244.481	285289.35	1195207.99	158847.084	319883.565	754757.68	1143274.65
65	23121343.5	15673765.5	10608336.4	6154108.05	5492779.31	11113265.6	9793246.56	22053663.9	21585757.5	13326503.1
66	74261.265	45499.9894	8940.91488	40656.7161	19462.5046	36464.3227	52383.9654	98873.4037	113624.985	224333.237
67	343555	246677.494	516998.3	49530.597	277090.351	319916.22	191849.567	871689.024	25934.5953	395583.389
68	13759211.9	7037777.22	1110576.26	1449349.48	9316065.74	8198801.35	2376558.56	18862659.2	2158337.35	9397671.9
69	624769.587	639867.553	91232.7298	176318.387	621430.923	773887.41	144377.765	1115407.79	251727.197	1047982.28
70	58860.2428	272911.436		174222.499	94905.7337	269848.945	100080.641	2026205.01	42849.2022	
71	7963155.39	4816676.59	5726097.89	5221690.08	6629349.44	7054100.31	9739004.88	15780809.9	12413122.4	9903085.82
72	94580.3036	89968.9948	68484.3455	204730.781	42877.4415	55180.4549	59092.8939	135689.809	392385.588	281836.839
73	632389.915	382231.154	266113.03	121764.482	141328.231	426403.267	231260.106	1319884.88	2128558.44	1189862.51
74	34624809.4	26041335.6	27311945.8	27450226.4	84369688	196215256	17640638.4	49003666.9	103224259	179153851
75	130975598	104224006	15812984.4	17290564.8	18707604.3	13216026.6	13615083.2	76012982.8	15903260.6	66769530.8
76	12697931	5862700.91	2015055.86	1562076.57	7851287.75	6848339.02	4420143.58	12333312.9	3707009.42	5786885.09
77	371172.922	215544.499	38299.6063	42899.2782	300973.814	207777.454	428815.16	508258.41	99649.0085	334275.812
78	6850298.13	4996345.6	4946305.71	857899.425	1817729.15	4557966.78	854837.312	1162901.12	2953005.2	3693479.12
79	24467021.9	11196300.7	7657021.64	10387341.9	11635484.8	7785535.56	30256241.6	46022048.8	16766428.3	10435754.3
80	4791279.1	5348860.32	1239750.14	4929718.84	3572005.57	5755538.06	5524867.7	8154892.52	19986.4586	8855648.54
81	635581.51	409728.569	8687.4326	38000.794	624621.919	1026958.18	238370.907	968989.942	33408.1521	513066.829
82	31533027.6	39847681.8	105197.3	332530.995	38674816.4	53619534.4	54018236	239400564	7249478.3	221318613
83	38921809.9	46729796	1158419865	1043877496	15160006.2	5844925.46	111834054	457394989	562474519	615866598
84	452083813	307135515	42884372.2	67689148.9	270618715	266342530	339479024	650248446	116321280	148780239
85	327170.89	188950.241	51520.3042	22548.6315	112664.189	1208645.62	457262.305	69140.5417		83469.9946
86	168729.571	271014.269	168915.73	192407.079	223695.569	241404.808	184142.467	68884.0711	138883.214	224807.263
87	1662891.93	735012.242	491332.77	409544.207	346132.846	953877.038	526668.664	675388.67	1554238.88	889632.831
88	1187907.58	1053508.03	1401962.41	1659017.83	1418136.79	830013.56	1717630.81	3946275.95	1894488.55	1401933.4
89	820005130	408957080	119027708	596071293	671819870	806284307	502349479	1071692433	234638912	420923653
90	1786852.79	1165259.53	32584.1862	43001.5149	14714663.9	15625866.3	3923686.97	9475571.5	3656836.83	15336974.1
91	70688962.3	24962109.1	16952355.5	14969573.9	80085279.8	39987212.5	23899471.9	68503574.1	76520949.8	22137363.2
92	580996.238	260746.086	100196.193	254134.865	649232.657	892221.659	381777.187	217352.67	259747.766	652581.378
93	863984.661	364504.898	94051.8942	1028989.05	537520.782	348478.747	485557.315	641851.307	595776.969	398065.001
94	1384466.01	763372.228	301226.298	230136.769	752361.505	655410.846	737820.958	1301055.99	511513.194	787268.067
95	556956497	250062490	73216248.9	179262295	388476721	241213591	325143633	432450176	137912368	897473597
96	12275680.9	5951310.09	1906755.49	1705236.94	2764829.06	4258224.95	4825789.89	10542345.5	5156793.06	2883106.36
97	18440392.3	12401401.9	8073025.04	10057127.8	16271788.7	13162149.1	14466997.7	34743523.3	14882174.8	13993468.5
98	2795119.52	1745762.95	485140.399	875869.999	2056941.65	1202120.75	1562872.54	3235707.38	768291.172	1789256.05
99	6210208.83	4877471.04	552438.323	676555.346	1739186.07	1164737.85	1111539.11	3842407.4	2324858.52	5893231.3
100	143301.086	131358.017	91288.0096	40419.4604	256762.948	78263.4592	101739.312	363269.95	34798.7249	569531.237
101	375004.633	710555.088	135116.598	366380.011	460574.203	416680.165	1468624.29	3631507.84	503058.557	911606.284
102	109130.459	144647.95	6587.69757	34493.878	117171.951	28006.0701	101661.523	106376.441	64146.5639	163345.334
103	367085.426	218122.066	40944.135	64239.4468	9202.65804	241749.889	156447.408	237347.825	157230.613	459390.807
104	21013567.4	15303622.9	6794722.35	8484454.24	31191762.6	20878091.2	16594560.1	7759191.37	4073259.75	21960781.5
105	528782.234	268146.218	167336.209	245438.355	214219.751	123430.875	84511.8445	217773.508	104712.069	138741.546
106	5755659.62	4197238.61	2502867.46	6747457.64	1303473.76	2296278.01	806385.907	2134807.13	2995852.66	2196880.41
107	405499.079	216907.771	153204.522	213623.105	564050.681	849370.896	951966.265	1491142.25	550936.506	364121.253
108	149967.068	124832.787	67045.4049	19577.4489	254535.071	109098.23	43459.5388	211737.769	177217.079	26534.3125
109	275568829	170888601	38484105.6	60592115.7	127094734	56546536.1	37486302.6	63806057.5	20280070.3	56895456.7
110	4903677.46	1788014.04	1293767.25	1521401.93		436606.831	701484.16	1376252.18	70376.2804	174415.274
111	709876.566	472238.476	235267.764	491911.234	371154.982	535786.682	347294.62	395995.743	768171.901	211649.593
112	829440.662	426363.659	112156.771	187370.76	87435.7247	152061.715	186875.122	584226.691	70433.6681	237840.88
113	552060.259	321146.329	154409.335	211479.744	335746.669	214109.459	134618.862	408719.148	165443.05	67996.6817

No.	N_B2_R1	N_B2_R2	N_B2_R3	N_B2_R4	N_B2_R5	N_B2_R6	N_B2_R7	N_B2_R8	N_B2_R9	N_B2_R10	N_B3_R1	N_B3_R2	N_B3_R3	N_B3_R4
1	172714355	133721570	107014833	87822535.6	89193154.5	125854990	170178919			65425529.7	194290957	116258210	119834857	118608986
2	50979468.7	48072278.3	36057841.3	33219788.8	38804803.3		32689263.5	266831.711	295272.406	8961781.4	56670972.3	29209521.9	7336545.39	35577086.7
3	548063.233	886482.211	514788.566	69808.7634	49477.5463	155860.104	682339.231	1068677.49	241205.921	198198.739	800444.274	264654.028	210127.495	231536.273
4	670889.376	2279328.99	1342068.59	139579.05	192293.231	232173.79	774454.58	16253.7089	31511.3496	211249.7	1031464.44	351827.357	284778.141	204659.922
5	486347154	418692356	158633967	248590433	274995823	263558726	507186847	25235.3635	31293.5685	51962811.6	418573165	270121077	45979758.3	648711034
6	250372983	759347301	305451412	429866901	519130127		362588721	142770.468	136071.076	764920577	645106641	358610865	274610260	765206015
7	75263637.9	69264293.2	28537470.9	25093495.7	36397515.3	28541298.9	68145989.7	94284943	111067196	50869776.2	67997168.3	36923809.8	36810612.4	34555518.5
8	259975.06	1236272.86	311680.092	526324.64	326133.848	245864.248	202770.368	1126100.84	637034.49	344548.486	207512.158	225725.134	605429.934	271358.562
9	266644.818	724734.559	224122.959	484040.044	165979.363	169007.976	88215.7649	765791.999	540276.486	186037.975	92089.7833	190873.019	369998.982	252883.742
10	1175719.07	2068247.74	532893.091	974297.085	363191.698	454476.896	602297.529			826317.89	640122.067	796038.455	1280366.16	1151524.81
11	104314.431	588977.043	123328.681	137150.358	112823.048	135043.139	87096.6346		13518.0706	112390.105	155932.412	204270.999	189119.307	241345.902
12	4205815.43	2775340.89	1497612.96	2011663.41	1949970.28	2971131.89	5883154.54			2540878.98	4430888.28	2602562.09	1332245.76	4846587.38
13	12466703.4	20978012.9	10503258.9	13859066.9	18901544.3		15249769.5	22822.2771	2122.46363	15273127.4	26204743.5	16738088.9	10821529	32073468.3
14							211259.729			593997.042	531857.547	380126.786	594492.467	354047.734
15	20941470.3	10330178.8	11513594	8271725.93	5287501.07	12292823.2	9893564.72	16803480.2	15834276.6	9423143.53	7913975.16	6714574.93	12726630	5217752.13
16	990844.964	2796477.42	2742853.79	238149.792	336957.238	910649.371	955272.713	269635.364	137200.481	513294.556	9997181.1	2502223.05	1322112.55	1684637.62
17	994865.999	3623885.44	3278861.65	568698.237	343943.963	1048397.47	1138839.72			1439723.04	19527139.7	4618071.75	3205588.04	2432314.98
18	894140.2	905692.317	159886.004	219878.299	522167.212	237273.455	614709.886			528010.007	631748.108	199211.751	499898.585	225370.753
19	1046685.09	2009455.41	813541.841	272343.597	147252.532	425203.204	1406232.5	184449.544	69362.2318	426953.566	3387901.99	314019.345	380861.88	447943.945
20	299725.224	1843651.59	816788.489	104261.588		95115.2554	506503.542			413896.03	1450824.13	645596.584	405230.805	179560.497
21	95264.2761	2500713.2	927535.409	227648.044	173671.849	380999.644	478935.806	2265926.19	112736.657	1067246.7	1989186.38	816981.219	586182.714	721594.531
22	2652260.39	1980074.02	1286742	1247424.65	1391490.86	1251243.29	1116390.74	4260546.34	3319718.29	1362906.03	2048968	2118189.81	1459160.36	2230299.49
23	670880.316	539958.945	174341.942	336844.749	417718.343		262872.195				647281.826	446607.128	112088.657	720582.524
24	7266654.22	25965935.9	26983099.4	30415559	61677658	306851.678	40346427.1	167823619	4769692.68	98069.5117	14482570.2	39785886.5	34100.9758	18870340.3
25	19415666.1	13679300.7	1937977.46	10001210.6	4496108.1	14496048.1	4358393.91	440161.91	588616.041	6837238.19	7097602.14	4298886.28	9323752.45	6290699.8
26	12310731.8	2217211.55	4448533.03	1083516.03	4788384.39	11219926.5	2065778.59	6719923.55	10898142.7	8299850.5	5673056.87	6767387.44	4751464.6	312375.473
27	128258.206	49430.2554	82228.8576	67985.4559	156162.242	19543.6045	73808.0541	228523.43	87876.2532	306712.289	102547.825	301275.967	59528.9321	142862.044
28	46409671.8	26656611.9	22057526.1	29802281.8	19900702.2	18818800.7	31518033.7	27994330.2	27336351.9	30197451.3	24865852.4	19736104.9	27550014.9	27400161.9
29	73927382.1	171706359	114444271	66834305.7	71774841.1	185020484	79701635.7	334933921	142211918	102577531	20794328.2	62405968.8	81555580.3	70776849
30	686378.292	334145.196	193707.533	69566.3907	16573.2393	274009.485	9216.93058	268958.605		80592.1784	297717.315	407322.227	101873.364	970113.39
31	1314252.47	1311089.37	7059.27398	682524.048	278460.446	303897.646	279733.586	1278593.75	874330.599	191989.402	504031.38	149529.988	581272.655	923191.004
32	214069232	295048282	206072439	210449162	259084259	219017451	187488078	36863.7599	71181.5728	246296428	196053696	103034949	125694915	133789390
33	2859316.23	4794743.35	4962647.01	2758400.65	2050809.16		1669719.83			187688.205	2647896.16	2061414.59	3054674.53	1731294.45
34	41691405.2	27479175.5	17286443.7	17599172.5	27999200.9	12257665.1	17417485.6	52990229.1	31669441.2	22829446.2	45810490.2	23432990	10766971.5	57311043.5
35	1235760615	887492122	592660841	168375941	289188920	336463000	1445473896	189123710	1648364052	619119521	1230474630	1042417946	609932762	1280123856
36	24602564.6	76247907.5	83214337.7	20306648.3	37214054.1	18584054.4	57524843.2	63673409.9		19090075.9	76551356.2	63077341.8	12544477.7	68034888.8
37	337884.936	677431.714	372755.901	426606.732	423735.029	495015.105	385853.896	2041437.93	647828.442	437558.572	1263917.34	529081.22	682036.202	514389.883
38	1445035838	859618700	347357059	468790940	468152827	500127506	976103836	1472790419	1336310974	448836140	788094063	643239612	506913480	658484232
39	3581215.75	5865652.28	1417282.91	807194.975	1419118.42	1909318.1	3040532.21			1895122.57	7655489.6	5607771.26	2477196.51	1207230.28
40	38122432.9	60570261	31019364.3	32026048.1	32406626.6	43899497.1	34039285.4			41877315.8	37590636.4	33464943.2	32401153.3	26459948.5
41	13745900.1	18344424.5	10188256.7	9304394.41	12155554.9	14773176.4	15575423.8	29688663.3	27465533.3	13054522.6	14030127.8	11924080	14344279.3	12365947.2
42	97258840.9	154563248	87974277.7	86058927.2	154006110	120610966	104959308		102410363	99082822.1	206066504	130683920	66148873	257019341
43			1235213104	1598464297	1698690785	1546017531	2201789780	2725006936	2864542388	1653511530	1608099113	1286081019	1709129175	2066056812
44	20333870.5	16113731.9	10859235.9	21014497.5	33974657	19832103.7	11782214.8	23455948.4	15624868.6		59633858.7	52469450.6	395027.072	85214913.9
45	185401641	167558532	108804980	121094218	119589054	100051007	174599111	347946586	277054607	157353502	223863962	173767657	179548388	183830430
46	56338.3592	89801.2924	31705.7091	18609.4617	73706.6034	48093.7543	5291.46392	147906.85	36377.035	65783.9725	29347.2191	10618.6378	24525.485	14002.0175
47	682437696	757419181	483250655	239000155	213807512	574435829	654335583	1048819305	888135643	363070399	299046691	628190949	339586104	501597164
48	97018249.3	148124678	129029139	122235448	84564680.4	131487096	78275004.8	184471563	86627465.9	172215207	124330296	88057564.3	101314170	88688616.3
49	152740576	157441838	145316361	82605196.8	93239192.6		80428628.3		1648588.28	140710397	172041585	160800397	73984580.9	89952014.7
50	382393006	1271684861	644191567	424744949	495853478	1132443920	380517878			808492360	199509483	620626111	518159758	498661312
51	10187676.3	16491832.1	4227610.04	2073994.11	4152631.6	4736301.51	8538628.36	12760237.5	13631970.7	7324867.79	25488186	10986217.5	7565023.33	7615629.41
52	852802284	489023820	257220322	418492635	498299052	363527267	409763599	1122693676		399139462	488506147	326386419	469225745	583398537
53	12877646	8984916.81	5198007.19	6497034.16	16796410.3	10415892.5	14168775.2	13779922.1	14491630.2	4674845.9	17553467.7	9745961.19	312078.91	19261135.9
54	297742759	197149944	133794694	154095921	145811367	144392873	242611604			154311158	179640750	124019814	151092323	173111762
55	355572267	221772454	124983997	157692720	129555304	186219727	282722849	426676324	426003215	216587556	230606358	150913585	198648015	247154509
56	370392658	946459918	476372041	763161318	799685772	880604689	450258065		684214539	1108163951	1231581466	809261943	597017930	1023344195
57	826081277	861974366	335018371	399919869	525991724	449563503	794681473	1210794559	960976295	624839551	697031710	421421419	466313263	696027312
58	124204507	155228620	59348735.7	56038969.7	86799911.3	62481496.4	114938178	151304379	120546643	117494192	158047044	96887051.5	77148316.6	92220822.8
59	3099971540	3507616235	2187535425	1839515695	2464974164	2120774204	2605275656	5234004899	3789583068	3374199226	2642718031	2708515700	1815820114	2800817246
60	197786.616	562551.194	267550.501	51696.5419	68131.8959	128768.59	259769.092	4237.20701	8015.07682	165872.708	346835.214	13449.9857	18772.2529	29696.5892
61	216544033	409145322	202113155	243102623	271811307	263699315	308810265	865809581	335063124	329405703	317697486	197476150	356957749	298533277
62	2002954.86	3742611.91	2605708.74	2878682.6	4079507.82	2955030.01	1890269.26	11141734.9	3313474.66	2837125.11	2540206.23	3758400.96	2037140.68	2522887.37
63	895830.557	1451255.16	1911108.93	1174192.86	998301.47	1257698.59	708493.242			1613822.74	208658.532	328812.136	973491.017	522191.469
64	211340.53	610684.02	507693.199	534169.122	911108.561	330297.96	264865.014			989460.279	599271.385	419380.97	198944.118	1685310.82
65	14423452.3	18094725.3	14477411.8	9671159.67	12427386.6	17055353.4	18159562.4		21030534.8	16575827.6	19600029.3	13771062.6	17475018.7	11888053.5
66	43142.8243	132084.07	53287.762	7060.2977	9538.93353	29638.9505	52197.6311	89338.6415	62147.3185	12493.111	73683.1997	44295.2635	22758.6637	138703.872
67	300300.951	145776.184	72617.3535	313388.069	467698.606		284949.794		502.848739	111135.775	847356.49	571973.75	398248.216	777182.217
68	5693921.57	9910452.11	4583374.77	6234801	8070004.43		6560693.38			1232173.3	12929406.3	7259957.86	2300075.7	9109515.04
69	1237588.78	713909.783	577825.472	594288.196	592562.019	377811.119	660746.834			300741.432	1168405.05	659242.143	451265.666	821689.523
70	258433.026	233830.46	145073.868	169183.368	190614.957	147173.022	225108.041	337603.764	407541.421		287785.613	135487.806	248891.547	300151.192
71	10862338.3	7848820.12	3374683.22	4026022.12	9679010.73	6421940.98	8877405.69	2652203.35		5154835.92	11762607.3	6874260.32	2022991.07	9442065.03
72	90685.8331	168630.504	75404.5198	10006.2574	15829.3878	56674.153	130804.569	63976.1656	151851.859	29362.6312	172467.996	24579.2975	21947.1765	20472.4143
73	177824.119	521430.174	256623.667	51386.3748	82686.9695	185697.816	263647.237	6711.03283	12774.8349	131725.001	310981.018	910450.341	113819.273	2684099.56
74	222529611	228392425	132845991	170533491			189467571	123022.308	35912.9022	107193785	102738905	175328281	109456673	122240790
75	27779446.7	22397081.9	12128706.2	19260921.9	15491935.9	15279188.1	21167009.5	52474160.7	40445674.6	29186532.2	27853386.2	14933930	18047918.7	15295468
76	3013841.83	25800994.9	12226578.1	16401653.1	28161963.3		4397143.74	78917.9086	89860.4773	12801985.2	8854545.03	6605043.12	3396875.74	10633547.8
77	291232.059	344743.087	234590.765	177214.207	215544.499	179452.609	237127.197			82575.2018	189497.795	220984.614	300333.142	169650.942
78	485735.191	199597.798	567160.707	5881421.8	208673.851	242559.841	3254369.65	8724787.63	552693.248	1232792.94	1118749.9		1547949.87	1026102.42
79	17719705.8	15096238.9	8779254.97	7511727.04	12472316.2		14694648.8	131843.723	48155.9467	2478918.12	13431093.2	9565236.87	10661934.2	8293880.39
80	5442412.88	5548352.27	2851560.69	2895933.65	4160707.82	3646611.71	6417193.12	9925116.65	8106450.16	4087374.82	4774219.74	4028415.01	4372083.83	4666072.5
81	509948.503	705765.271	293138.42	495656.796	883724.939	635751.112	779724.74	1204.39687	604.106159	442177.121	440990.347	443367.089	188617.103	500274.421
82	93768511.5	50843629.3	31562134.5	43205458.5	36904721.7	21830966.2	32317530.8	96515018.5	107673240	35634827.5	99578352.1	39211474.7	42648783.4	45221865.7
83	44447733.4	19798679	5951228.64	38519900.6	40933726.3	26696399.2	29445309.1	2829962.41		14787531.2	27552886.5	37824379.3	13349895.6	13855457.8
84	303064067	203202134	170079227				261557146	54082.5797	210292.079	99588066.1	150822460	106318911	86706857.1	94048540.8
85	112842.238	81471.188	63122.5752	115123.912	81948.4485	110086.549	86653.853	311743.969		63984.4328	40725.2804	134847.18	146666.466	106541.158
86	246301.776	296704.982	160457.641	261047.066	207828.196	223469.992	192026.982	355087.719	375273.882	284122.76	414265.72	396495.513	407710.666	509141.996
87	944139.635	663389.029	415425.82	520048.131	592494.297	414221.91	800892.168	1558629.48	1112401.49	791368.763	833116.532	432510.873	504622.934	1014956.22
88	1512308.13	1411488.28	722212.029	784718.164	979862.091	776284.954	479424.646	2473898.89	1850097.61	845154.12	1165798.56	1370860.75	990797.197	1490282.99
89	134877484	796329070	366131916	342219808	563375851	438571120	218358354	668373644	284440467	683879312	635609244	431443998	337262250	550145490
90	3730740.99	2584.16022	2729161.91	2017309.27	3595834.85	2973221.05	2316306.22	7138.21235		2322997.82	2121562.88	6663.43781	397611.601	2179906.91
91	42109353.3	37622320.6	19916128.2	24015714.5	26791697.6	28234559.5	37657977.1	70925212.5	52509627.3	38587075.9	32419066.3	18284481.2	31162385.8	22961411.8
92	272724.182	27229.6781	152367.039		415030.108	405441.701	248339.597	536180.932	705871.078	429237.498	679551.006	337001.749	260246.447	164914.149
93	598973.174	754426.565	403940.476	459127.896	587450.973	293821.723	488542.172	1528794.87	1041419.14	701241.454	746398.056	612384.52	510979.816	542020.189
94	580736.793	636477.885	469613.278	412307.873	428429.871	455941.592	228605.113		11773.7077	478558.537	771858.702	571724.962	380575.812	530236.227
95	219499931	182669017	128629027	121942733	172424744	190708479	232032478	355692055	291441459	169905922	155219999	119256023	169731536	127600213
96	4049182.25	4339126.23	1883012.62	3124487.21	2953823.97	2652423.2	3914964.92	7844895.21	8857283.38	451234.957	2996111.53	2960020	2574818.52	2116159.87
97	17032790.4	18684062	14967064.9	7955626.58	19726847	13319864.8	19856966.3	1279606.11	78561.2787	10930404.2	16994113.5	9791905.97	11259046.7	21481318.3
98	1623045.87	1986684.86	1697939.88	1352550.35	997653.083	1237134.56	1140662.74	1934916.69	2484434.21	1512389.11	1826758.17	1280084.89	1780981.42	1765522.82
99	3864508.19	2665953.78	2013158.36	2018771.56	1793957.77	1167505.84	1961819.55	6087189.86	3674723.3	1310422.12	4103818.54	2298975.37	1935589.05	1467416.85
100	95954.611	146732.449	68152.9543	31580.1789	51822.7353	76030.9541	110580.529	153036.884	101359.027	65184.2857	196908.752	137413.425	73051.2775	146183.077
101	60023.3752		1828674.44	14626.9304	34698.5261	71780.6492	2671621.04	111970.228	84960.4627	1642429.92	1289119.11	26175.1888	72791.1581	63450.2078
102	14509.6667	150055.978	51891.569	49982.5607	63475.0971	53759.71	17485.1337	187168.859	60351.095	72586.8207	63321.1342	207005.99	145399.135	109449.169
103	284217.235	329145.334	11397.0341	2992337.39	91231.2338	284613.17	268757.082	3975354.6	7842465.84	957263.173	398599.477	80511.9696	155341.534	109963.806
104	13507442	18735219.3	8640991.25	11233796.3	12724191.6	20030165	19730787.8	34195.1881	25750.2724	10325997.3	18558787.2	11892604.9	10756475.3	14143256.2
105	163933.317	232669.054	15350.8957	70849.5651	156283.421	69016.0215	132035.067	339619.398	223370.273	174023.796	166531.338	122486.998	90018.3698	255869.777
106	2868090.14	3147550.25	2076949.54	1562151.66	1980766.72	3114913.54	2682773.19	7450165.86	2951987.53	1621029.97	4832848.12	3346206.67	1403342.15	4557450.32
107	560376.539	286888.744	167922.182	146313.087	113532.051	166821.456	202050.93	138451.014	189679.657	58565.8842	488645.271	300653.64	308851.523	328295.147
108	123518.988	72943.4003	45683.6509	30291.5583	123736.861	59367.0137	66692.1494		16177.9782	90673.4101	87299.0027	86174.0222	86271.5639	97814.4177
109	60290091.4	71688734.4	42268725.7	28191988	60631064.1	53863551.3	44063502.3	177193.166	13461.0293	10204889.2	42062833.1	37547508.2	42810843.3	34873508.3
110	2613472.02	1597578.12	985947.825	840171.024	1235160.42	883996.84	1872058.17	2230968.61	2955372.94	831012.625	1798400.87	1351035.18	1284936.21	1369621.87
111	275966.549	645347.78	314396.633	405792.711	544299.028	436638.429	272564.189	1092413	404635.072	222484.775	718701.527	488851.237	434790.374	631730.365
112	63660.2232	152554.974	32911.7905	34954.2392	98991.3144	5462.14266	63140.4914	20332.6682	17058.2043	93771.356	198979.309	62048.3942	149325.313	190634.628
113	331622.947	297959.782	142981.783	118816.714	234657.394	170178.973	226988.778	1368.52349		143795.634	257773.557	198724.716	147370.115	200280.525

N_B3_R5	N_B3_R6	N_B3_R7	N_B3_R8	N_B3_R9	N_B3_R10	N_B4_R1	N_B4_R2	N_B4_R3	N_B4_R4	N_B4_R5	N_B4_R6	N_B4_R7	N_B4_R8	N_B4_R9	N_B4_R10	N_B5_R1	N_B5_R2	N_B5_R3
97772181.7	151698598	149331434	101013132	252545620	124360710	245649156	190099592	161932980	154509996	150143160	165407156	179227789	213565397	479380621	185402897	4814421.05	57304862.5	66453037.3
6216656.07	1801816.18	10158016.1	39997086.2	57670.3374	68247562.9	25602869.8	66059608.2	37244585.3	50644943.1	46377553.8	40608942.5	55949415.6	61933965.2	111720060	45677891.2	22614951	95652402	44307301.8
50869.0474	161713.417	147328.764	99171.7219	170071.847	163982.55	1522332.8	1283172.24	655828.567	428240.668	695107.221	623000.606	773808.867	897903.214	556569.026	114381.626	420641.801	540507.246	591497.702
30975.6174	89878.8517	175958.578	142422.701	273057.351	95193.3569	2260433.55	2079214.29	1174014.1	867926.521	755164.485	964710	715014.566	993543.37	4438333.29	1084008.87	3238437.45	4174527.49	1739354.95
79177581.2	27276445.5	84867749	368455297	3275193.4	703623181	108523876	420595347	184198764	298528573	368128784	340718932	501648150	521842406	419458044	226520475	289956343	601707828	307685930
278155191	172482572	283601402	427820512	72273197.3	720872890	480918770	541783837	389651232	444454680	561646634	462545760	623978228	573840937	58602249.7	18166493.5	4642276.47	16501401.1	16569288.6
34443893.5	48990566.8	50200411.7	28967025.9	49918027.7	33041297.7	60201342.4	63683363.3	35119215	32141894.2	42040084.7	38330879.9	82501680.7	80319160.3	91407321.9	31099840.8	35816350.5	43563381.3	64863418.6
264261.571	678683.366	532515.575	450675.173	485273.937	386866.879	538582.891	658219.837	1872350.08	1173131.45	926438.904	1030080.49	4238879.45	7029087.21	7495400.39	2945513.77	1809780.86	2259502.11	1820840.85
163557.204	434435.586	319492.718	398605.074	247571.884	126942.001	345984.727	534655.102	1214497.35	845036.151	559422.291	550696.879	3006095.72	3630611.76	4214893.17	2205397.36	1735972.95	1865781.44	1327605.06
459995.594	1880771.34	1105932.95	1448236.36	938541.26	677071.08	1430070.2	1615846.53	4098928.55	2222273.4	1712744.19	1852163.96	9094150.78	11328251.5	15132990	5121007.44	7436740.12	5824018.74	4362568.93
84359.3601	158517.563	175785.273	295970.844	12407.5272	150741.669	292933.735	506140.163	720601.759	495004.247	392596.558	502164.24	2825919.64	3324191.84	3158630.79	1106436.64	1135103.16	893387.933	582274.655
1906991.46	2744603.6	2407423.84	3165370.81	511547.29	5045826.85	975676.929	1366340.82	958440.823	1068217.26	1868448.21	2555391.99	967261.084	1207108.77	1003449.27	636948.662	14405.5194	13491.0893	
13774473.2	16059101.4	12073341.9	17828959.9	3513677.45	26802648.3	6689038.05	10063397.1	7924595.19	6828960.32	14013361.6	19413055.4	6125986.86	5719838.47	140157.894	139799.765		3565.03894	
516103.705	371669.757	620871.945	316426.804	939350.047	542916.186	680436.629	402773.51	240875.307	419656.779	166825.733	244644.117	145089.163	97497.2466	445864.603	31922.1923	453712.964	172110.745	83545.3282
13437187.4	13524151	16097317.4	19157586.7	10217489.4	23002598	8234436.49	7306953.06	13258262.7	12017444.1	11455367	11885544.2	5680288.13	6601956.41	12898588.8	7475923.27	6581335.04	9253949.12	8813061.45
125626.608	597236.394	377033.654	614879.413	522348.557	2054946.37	5455025.86	5465363.27	2621290.11	2784994.99	1790596.2	1564929.98	3982839.76	5264851.32	3879732.99	1113912.57	1263371.36	3541406.23	5388572.08
366421.674	2229405.81	1045234.8	884845.786	3673779.16	3272032.79	5801010.58	4514865.31	2601048.49	2355800.56	1301240.72	1238077.52	3385413.79	3797749.89	7200865.69	2339963.98	25810183.7	22366078.7	17409017.6
233330.961	235461.94	383991.15	79454.2745	212369.109	119612.563	454393.274	436040.939	625466.134	553183.936	100762.743	128760.062	1062090.6	1299963.29	2163306.62	678066.24	526052.322	826812.942	561470.983
121108.894	310505.434	310801.066	173194.914	305490.434	331038.725	1787587.75	1794064.9	734770.895	583704.43	994281.368	954805.38	1232783.73	1169966.96	203127.382				
88334.7012	144809.808		179198.046	285467.121	90806.3495	1568464.09	1354459.59	402808.287	321418.942	268033.344	428870.051	377421.482	446630.244	459067.883	32071.7108			
258545.577	459575.625	434459.622	268448.731	1117630.3	594956.106	11475160.1	11227757.1	3815545.83	3872287.56	3503340.39	2902053.7	4978549.7	5014144.11	1787520.96	427622.811	154163.129	545997.517	539741.768
1066361.99	1384909.39	1537482.76	1216384.12	2346441.8	1868805.05	1983645.77	1948520.82	2226578.47	2260424.02	2291073.4	2030447.13	2019868.73	2219223.04	3432852.75	1084008.87	2064014.02	2100972.33	1504077.95
42994.8696		95424.0404	372111.445		671128.328	167225.951	660596.082	587007.051	659051.897	360343.583	519992.557	623029.933	579412.208	895791.754	263152.499	375848.741	407704.406	228821.083
39637852.9	44886152.1	25239482.8	44619747.7	44464236.2	35377411.2	33791175	35049612.2	913909.254	963303.062	54607623.3	56852519.5	25888458.4	30084864.7	59211631.8	29380378.4	8573914.17		
7412672.51	8040813.03	22719878	5017306.88	7965652.81	17545532.9	14877343.2	11144588.6	6993480.56	7258154.75	7685105.43	6388479.98	5130276.92	6778380	10765751.2	6675999.48	4080735.1	9130007.37	8003432.92
227583.922	4108367.78	4622010.53	5797225.62	1186888.37	17615099.8	4763056.4	4229715.92	3906633.13	2928059.8	294725.462	296148.142	4826822.47	5784836.63	525084.282	154751.612			278186.129
165450.151	236809.355	279381.463	170556.821	204435.519	92271.0362	272174.652	162772.776	150800.087	166908.946	152367.503	55961.1037	678979.349	820833.962	999386.719	586859.976		343257.537	276977.19
29384446.2	24789226.2	20728885	13415764.3	33749242.5	38852158.4	22143022.5	19247583.7	26010484.9	22604240.2	30028631.9	27534843.9	12991644	13278196.4	17265827.5	4717307.58	477097.087	1777288.24	2712702.29
95531134.8	113091404	123806756	53215960.1	115895131	137772760	76347296.4	100871596	112341005	101099133	110327418	115884055	68656571	79390615.1	160470632	79244786.6	32167901.2	63126159.8	17448561
57818.1783		8761.32812	900384.311		339176.213	159152.974	583368.122	655828.567	829775.905	293613.29	351614.014		13742.469	557584.663	164470.312	367694.986	385119.622	130741.367
1463923.97	844731.866	440850.859	343869.365	738456.865	1141140.69	2006711.41	1984164.49	828894.439	841221.09	1857326.49	1624357.7	218107.891	280420.652	39711.4032	30127.9708	185265.165	558104.045	975078.894
108784570	73486963.9	127202695	170618075	39817721	178998081	282554193	352872367	263141092	319511412	231331683	207997023	204831759	202422855	197033560	80739971.3	18278729.3	58914605.2	61604782.3
1274627.3	2676451.85	2515597.23	1855605.17	2111498.8	3080374.66	2710213.69	2411888.57	4341828.02	2832683.26	1668257.33	2763389.01	1906073.31	3073484.63	2925034.3	1226051.42	832712.825	749070.37	959210.439
13869737.3	3362738.02	8582389.31	35978499.1	304420.281	68167790.1	9572244.1	31247620.4	26314109.2	30615869.6	37146529.9	34765216.6	43906066.8	43084497.6	24070594.8	11737199.5	2408077.47	6132760.81	4674594.7
1168946248	1085398487	448322466	446408716	837466994	817721481	1055253416	931488000	705420542	785902696	931999761	865663799	2181078914	2182081234	881572837	425380034	1017366284	884072148	532211799
70330703.7	53953227.4	40327954.8	9921539.71	53804782.3	101058484	104141403	85188379.6	48883518.2	47116011.1	53829103.2	45561252.6	87432815.6	86540413.2	88360411.1	29156100.7	25405673.2	43150842.7	61641337.8
622947.529	320487.322	428475.748	256679.962	214930.8	230822.181	149926.715	320793.061	325890.121	260377.956	1037656.06	1188554.42	677082.759	1439245.07	1381266.2	653395.693	349264.393	638445.259	817897.713
543427431	676697783	658028720	443613965	666427688	829811217	855735557	733071551	594091619	507403197	679536819	645781232	895190650	903474485	1696113639	569665353	1072309212	1212687736	665859232
1040173.19	8314005.7	1865936.72	1275788.6	4612424.43	2014621.53	9779834.94	9659435.51	2155732.79	1754928.35	9775987.95	9132059.76	8259651.02	9378306.58	11781388.2	3311834.01	14652267.7	49561102.6	26349698.7
29634052.8	21109125	31763730.5	26573230.9	23835138.2	33185326.5	68850960.6	81267575.5	44329153.2	52170967.8	48824331.2	37736602.7	56992540.3	49677168.5	218361936	177926974	1147322725	949901806	181122064
11845191.5	13860665.5	15844338.7	10482904.9	14515337.8	11777686.7	12340121.9	13188159.2	14573968.2	14592610.7	12567538.5	14955976.4	24371186.1	17456649.9	1492986.26	376786.533	54668.9272	356434.767	301132.615
44080780.2	25741613.5	41102514.5	127389753	3494920.93	248017518	118788089	260198816	122461816	136388453	174610934	138664682	169744837	172709408	65407017	18689808.2	2819015.89	7334704.79	35273813.9
1704229805	1726255697	1554063149	1395537020	3133315941	1597828651	1314741961	1165548123	1882470888	1974294394	1557040174	1317314477	1137954216	1281392384	2203932094	941966331	28974193.3	83726063	118447092
2456392.73		8218991.21	61437570.1	170374.924	85998827	3436781.62	20316893.9	25403236.2	48069776.5	30251066.2	22582533.9	22474595.8	20149431	7911811.53	2571717.6	275207.275	531449.629	981352.278
138617508	158888341	132100575	89961993.8	102049488	159660396	189138317	201980816	164969223	143064811	174610934	169369004	213366416	221922304	201096108	63022033.1	33145374.9	76698787	152120798
13197.0974	8921.16224	6298.75925	7867.56547	9750.92019	7405.42787	70350.2278	23643.6367	16699.3385	7000.63809	89196.1586	175311.776	32621.3542	73540.7803	34023.8365	42687.5218	20908.9779	16701.7942	8343.74896
645783758	460925197	692193586	458166931	500046985	913389006	527050067	470496490	579922483	549368875	551637090	588334435	483630542	507914228	698758194	277356753	107052641		
83659263.3	151834154	102794171	82949774.6	170670588	149900855	110484456	105267649	110316842	105867960	137909273	136683758	80320601.7	78090651.8	105626239	34688284	2219158.45	6657260.21	7931830.57
127356123	107368969	166843948	108153102	118494737	133084286	98605647.1	101465657	120437654	123989503	145694473	127769600	139399391	153209959	139142257	53677129	47201806.6	82923027.2	44276279.9
696416766	535645120	765882110	397143419	404047911	756471662	841896168	664160449	675058109	540784986	836353008	823073932	502596445	566412576	1320327982	458274096	168482348	330674212	106337885
5347777.07	44272418.5	9441105.66	6660138.37	16357021.8	10441819	30100671.2	29227812.2	5698016.72	5255247.4	28249157.4	25553919.9	15646870.5	19499449.3	18789282.8	9942977.94	8279541.31	45961065.2	45896180
479162443	594252225	742551154	257583694	765134677	764627473	446320297	345743633	556644617	521709678	510486743	466507608	571821994	600768748	340238365	117371995	19720586.4	67999165.2	82737948.2
1787653.13	340035.064	2950596.5	14897637.1	13259.5441	52603577.8	1972112.94	13188159.2	8268702.77	12780456.5	13568492.9	12083636.6	12612325.9	10678269.9	12797025.1	6892801.25	5179614.88	8314849.44	2549455.97
186637521	117224899	122682838	124733006	157598566	195418297	196058012	163960898	181162521	153556231	152367503	151540688	123278373	114211060	176720822	79244786.6	148693255	180855416	117097529
216868347	149123373	127803276	123363339	291703230	226298875	232963049	198416449	221645766	188845551	199078708	219882567	165951657	181066315	138126620	45827409.6	5337313.74	31877529.3	33287773.9
620869405	449061168	613146930	669951064	496730388	1198989819	1499267149	1390103265	1153772480	1192206760	1043216917	965700462	2000902830	1188537864	765790230	283337492	652460.556	7677328.22	10610365.8
646771961	649894178	408047107	368567039	885464235	478528566	461312969	755645878	440255288	408211595	734033225	667571396	401128861	340776091	487505717	359591909	530160353	523422372	267654319
82317799.8	105827454	61002504.7	46529822	126536823	73469159.7	82113708.5	65584359.2	45341234.3	43682455.7	50603805.7	45165067.8	97674403.5	90997430.2	105626239	36706783.2	156500423	167101166	86483644.5
2052915762	2608868857	2681289552	2872324584	2636380117	2371918838	3655905278	3897041633	2165853602	1878917853	2958376331	2614819713	1991419878	2479215700	2630499596	1293334725	3665252140	3289633693	1113816836
		25387.5861		44773.4276	15888.9262	462466.251	156832.163	56777.751	63616.1527		29119.5832	42673.2831	82083.3962	60938.2146	48144.9458	444524.023	733501.411	108399.84
231611371	328505480	227481741	191212477	259750814	325010482	414028390	344555510	321841797	253701598	180171792	189178244	356558988	333347729	999386719	354358763	505038286	587495199	414186094
2742681.72	3620349.92	2639016.71	1920647.8	3510255.92	3401984.48	5235902.2	4241597.14	3582767.17	3519394.35	5438518.89	4169845.07	3489726.26	3602755.4	1675800.9	361087.094	39629.4517	496741.697	697416.275
1242007.96	1283604.35	1618217.3	1727546.02	1241414.19	2023255.27	918012.808	657031.714	3329746.89	2641930.18	547188.404	109941.283	571821.994	352847.178	1970335.6	518081.482	909513.733	1009363.75	340547.673
351977.235	182248.656	227729.815	24857.7962	70517.9225	698976.797	630845.485	640398	33702.3014	39295.1348	207976.08	60021.998	518717.463	474486.6	1147669.71	346135.247	250613.8	277425.972	467359.72
12671704.4	12030134.3	15003778.4	15047868.3	18654630	14040560.9	12686106.6	11287163.3	23581490.2	20219826.6	14791881.7	13866468.2	21146982.5	22470794	24679976.9	8522552.52	13947639.4	12425487.9	17574416.8
30392.1613	21358.822	17509.7726	9567.67665	20105.5824	122961.746	73118.1056	45386.2776			45376.5994	48037.4076	68466.912	39648.8803	46516.1705	20932.5851	27502.3746	224529.988	60697.1987
217476.15	53966.3656	142106.955	393210.933	15121.7468	771405.829	160306.257	335050.531	86735.3522	76491.9857	423737.362	280300.75	269315.831	233064.847	832822.266	221287.329	7079.9029	11796.9463	
2205652.59	212497.872	2143775.64	9025790.26		18408638.6	2444958.74	8768343.67	4564485.86	5360161.59	5983482.96	5922962.83	5300970.06	5023429.56	517974.824	154751.612		93291.945	285610.572
324310.121	424772.017	450869.521	209491.399	472450.071	648873.707	789998.459	598813.714	583970.808	635207.762	345885.353	624981.53	376473.186	326847.912			24620.5133		
242152.151	275812.448	206556.566	148961.655	262457.309	112036.077	221430.225	200792.694	430134.477	304251.165	163489.218	163426.232	220952.777	168995.227	177736.459	69301.8087	57184.0456	70117.205	72990.0053
1469055.87	469563.828	2049455.15	7320527.47		15338612.9	3102329.72	10455477.6	6699977.03	7992554.12	10120761.1	8369404.01	9331224.57	9656870.14	18586155.4	9045867.15	5682783.17	11416567.2	6275754.92
	20949.4741	25699.5905	22984.0101	33758.4701	43693.5669	37597.007		18622.2927	10109.9133	16126.4875	215920.719	64673.7313	104925.608	118829.518	53153.8144	224062.023	338336.383	109661.589
714630.619	112390.248	84092.1816	1297761.46	44298.6892	151759.085	8292.10062				231331.683	305062.3	184917.56	171780.863	133048.435	33641.6547	244279.195	1437007.46	522154.483
99292554.9	35879905.6	80764740.8	208072657	13092188.7	150701810	83151662.6	94336922.5	78132662.7	80020917.7	184620478	155502536	129916440	129996329	158439358	59358830.7	51973638.1	68133561	75668981.7
18457174.8	22290271.5	20339335.8	13571265	17858675.6	21859249.8	23411633.2	22455514.3	19128333.2	18979931.6	10365438.9	9815478.54	18491756	20335140	15539244.7	8373034.06	2023814.4	5978717.29	7590397
1974422.54	1796989.49	1663479.97	5499736.05	1140747.92	7863960.53	3505978.56	6154474.29	2590927.67	3872287.56	3102958.63	2000733.27	2797470.78	1207108.77	3595354.66	1771793.81	2318777	4322744.37	2123580.49
132336.387	99118.7422	209582.721	292651.634	219627.195	77097.9922	191444.882	180594.612	352204.231	408211.595	264696.83	364490.021	156468.705	146710.143	260003.049	117371.995	78447.2937	84175.0013	66063.2238
72229.8658	1129959.88	11639623.4	1967023.28	2034212.41	265999.101	1499267.15	576239.388	595103.7	587519.491	445980.793	883492.115	2057800.54	3138482.8	19398665	6399390.32	765752.312	2245672.91	517249.874
7269269.33	8767793.36	8900850.71	8233646.91	6369599.03	9833778.65	16953251.6	13188159.2	12549805.9	10777549.1	9931691.97	12975052.4	12327837.3	12721069.3	37984820.4	10391533.3	4774559.13	7734704.53	6957163.77
4862604.19	6567718.64	3481960.48	4660318.22	4839904.22	4453028.36	4878384.65	4360409.39	3532163.12	3118812.88	5216084.58	4734408.42	3470760.36	3027057.37	3788325.67	1674606.81	246629.606	1154040.29	1522067.3
87876.6637	25068.8932	117454.914	785870.453		854062.587	343678.162	1163171.88	445315.694	461622.457	690658.534	840902.249	439060.668	283206.288	118829.518	7625.44173			4773.64797
50904210.9	53092595.2	33499762.6	50843629.3	70120864	39950665.8	60316670.7	49544706.1	45746066.7	49500424.7	96202839.3	85476871.7	23802209	26370683.8	19500228.7	5203242.59	26584686.2	44539157.3	36254547.9
50590394.7	10331261.5	17623488.6	13287935.3	5465099.05	12274616	365590528	304159347	41596534.1	35670826.2	13457275.8	11885544.2	262677765	299920102	478364984	104662926	437202252	1065825281	1217837916
122944545	120046411	115610731	91239451.1	250346617	120458585	81883052	55010069.4	143715519	129712095	169050076	184225934	151727229	158781230	198049197	64666736.2	102132927	112649542	96152968
162641.662	27234.6302	104708.366	39703.1984	361620.059	50633.9178	106217.311	139010.327	178126.278	206967.093	120114.528	75770.344	374576.596	547841.671	308753.62	45378.8542	28831.0267	55254.1874	61372.0166
326747.34	452955.542	287655.023	336071.411	198764.064	176262.219	54896.2433	48237.7714	305648.499	170724.008	191293.507	250586.889	24371.1861	22099.3759			6900.27717		
349988.91	595370.158	404577.437	568024.859	227276.953	480748.544	1395471.73	1306934.69	1204376.54	1468798.73	438195.592	623991.068	513027.692	253492.841	1980491.97	707222.341	677917.715	881264.548	533139.988
867300.938	916596.745	835827.24	681221.985	1877151.66	1143137.29	1349340.43	1065745.84	953380.418	1087292.56	1223388.71	1287600.62	1365545.06	1374246.9	2041430.19	665357.171	970275.561	891120.086	801774.387
359707404	485373773	405197787	278249702	391577403	348068789	485531900	452674653	342083419	337632954	490467655	414013121	437164078	452201515	202111745	60928774.6	16308406.2	54658370.6	75169824.1
4898436.54	7975620.4	6911829.82	836501.171	805613.069	71023.2725	19375.1447	11097.0637	2935035.26	2765919.68	18350830.6	23176811.1	1555204.1	1884946.77	5271155.56	1540040.19			8903.90215
22162917	30138395.5	23370967.6	18327294.6	25073971.6	27418058.7	43594075.6	37782293.9	34208341.9	31188128.8	22465865.4	20799702.3	30535104.8	28970610.4	106641875	31324118.5	67468562.4	57269520	32463238.7
105577.739	82534.6978	85599.3011	222785.549		200316.809	186831.752	349308	249984.037	269915.61	558310.12	542773.183	294919.801	320348.096	370707.472	90458.6715	23873.7493	91007.1728	302228.51
546136.273	218104.131	398939.087	167397.225	418679.911	714919.742	1372406.08	1283172.24	564741.266	515987.086	764061.857	696294.795	377421.482	384417.715	551490.842	122605.142	60091.4285	146320.383	143193
238562.323	350710.432	291259.638	252350.589	539835.247	398651.308	584714.188	458615.265	435194.883	416795.483	636162.128	671533.245	567080.518	572912.392	636804.342	79992.3789			52035.0288
121477808	107876778	136959442	96358073.7	176530075	114965958	276787781	263763184	192295413	186938020	219097796	202054251	552856090	664838367	556569026	159984758	5260046.76	24618263.7	60808365.8
2782987.37	1682059.34	3515665.9	3621693.74	1040126.97	4431317.45	3263789.25	3718823.27	2530202.81	3042511.65	6228160.7	5328685.63	3594038.73	3231337.32	2914877.93	1113912.57	1880847.55	2214882.88	2376218.34
12318329.9	11088191.1	11073985.2	5837700.66	12312707.7	12128083.3	16376610.4	12831722.4	13055846.5	10777549.1	15014316	11588405.5	12991644	14299596.2	28437833.5	10316774.1	6984444.77	9059869.13	7986016.75
1095784.54	2034999.48	2029558.88	924142.216	1398281.43	1162205.29	1081778.91	1118023.22	2864189.58	2908984.49	2769307.17	2674247.43	1081056.51	915545.573	1025793.28	299036.931	33891.8501	327460.872	478243.205
767464.053	4199719.57	3121731.91	1199968.17	2266949.72	3477974.26	3379117.5	3481198.78	1072805.99	1440185.77	2102004.24	2218634.91	1232783.73	1559955.95	2163306.62	624239.593	401166.751	738010.493	1183284.25
93538.2945	179694.344	34737.3171	99291.7325	8974.36321	101347.133	147620.15	63445.7388	124485.978	128758.33	140133.616	21294.9333	63630.6066	50327.1502	139142.257	31623.1554	83053.7611	189854.762	144854.094
864247.871	100579.828	1127049.78	75622.7909	1411657.05	712926.587	472845.793	560793.796	1163893.29	673358.378	209088.252	234739.497	549062.909	713122.718	2082055.66	716941.041	1058136.49	692568.646	360755.245
27102.5537	35200.9434	17170.0299	36097.6638	257452.249	50820.889	84189.6168	38732.7918	75906.0842	41870.3014	23911.6884	33081.4312	25888.4584	14856.7233	55250.6479	42911.7995		3022.95307	118110.249
61905.3939	150661.738	103201.619	169417.953	353428.032	54431.9507	226043.355	292278.122	275286.065	267054.314	181283.963	270396.129	155520.41	194065.948	568756.669	113634.034	35061.3524	51682.5197	108761.724
11185474.6	12400823.8	8937499.59	16266233.8	5543916.81	20642454.8	10829321.9	12237661.2	9736220.4	11540561.4	9308875.9	10399851.1	11853689.8	7409790.74	7881342.42	3513683.93	452795.422	1219375.05	1109231.97
146807.296	345510.224	47441.0199	111693.82	346497.122	72658.5459	168379.234	135445.959	84306.3575	105867.96	175723.105	93598.6602	132761.325	194994.493	375785.657	124847.919	627239.28	478570.583	125579.569
2771617.52	1214604.56	1732093.21	856478.81	1407487.31	7153125.05	4716925.11	3813873.06	2226578.47	2098283.9	2279951.68	2079970.23	2683675.36	2265650.3	217346.299	205587.89	217702.27	379536.828	230885.679
134854.106	84575.283	168636.436	48570.035	58895.6751	171189.592	273327.934	247129.469	357264.636	394858.879	309183.692	346661.704	350869.217	345418.817	341254.002	119614.772	492944.033	448364.737	170729.277
19752.6379	136279.937	23768.2227	25615.4182	109885.186	69910.7093	132627.479	154455.918	100499.655	73058.4302	97315.0109	75176.0668	71122.1385	81061.9965	253909.227	40968.0595	72054.3042	52714.1978	8554.61067
28679353.9	35599198.2	29927182.7	26555436.4	53739824.3	30373178.8	84766258	66059608.2	39673580	41488795.2	73625756.8	70718987.7	55095950	60726856.5	35242600.8	11139125.7	471451.968	4427189.3	9213561.5
1431613.49	1747596.23	1378824	1121177.49	1727534.11	1181830.82	1799120.58	1722777.55	1558604.93	1564175.27	1390214.44	1148935.93	1602618.85	1337105.1	2051586.56	614520.892	2147236.47	1872389.06	1465097.91
478247.606	350491.619	184720.271	199009.556	331551.599	536452.132	1044873.87	686734.776	241887.388	188845.551	616143.04	596258.132	505441.331	360275.54	794228.063	225772.883	314327.665	317402.141	383098.225
28750.2195	9840.46751	63620.913	23499.8977	60530.0829	50139.2692	9906.69601	51089.2653	165981.304	39962.7706	44709.2964	51107.8398	110950.536	181994.86	811493.891	94944.2255	186258.409	558706.603	255047.572
174758.059	268094.34	195853.795	130408.232	241890.596	200842.382	215663.813	99208.2245	139667.195	220319.809	468224.224	258510.585	132761.325	174566.499	296565.978	199607.151	265836.831	191590.066	39476.9223

N_B5_R4	N_B5_R5	N_B5_R6	N_B5_R7	N_B5_R8	N_B5_R9	N_B5_R10	N_B6_R1	N_B6_R2	N_B6_R3	N_B6_R4	N_B6_R5	N_B6_R6	N_B6_R7	N_B6_R8	N_B6_R9	N_B6_R10	N_B7_R1
27731439.3	20224554.8	6930676.15	2513218.35	25650029.9	5086636.33	2720973.16	586328931	261490009	151458797	139852329	768637279	643037956	184503334	175059499	183535402	197224317	183277669
9288440.91	8832669.9	18617339.9	28317738.4	26249087.2	11385145.9	17257204.2	188264631	37608317.7	14402164.8	32016369	133288585	165238118	30998160.5	44121865	40417051.8	34468442.3	33576151.7
502250.592	178173.62	90154.9524	200901.588	50302.5824	74257.4419		1038134.3	696672.604	181663.036	138911.688	1661315.17	1566917.16	1024042.8	1117117.41	483501.656	364051.188	358291.582
1797275.7	1234018.04	632370.488	389238.938	285081.222	258463.43	50172.4112	3137210.06	1853535.82	103360.638	46455.6102	3781648.95	3802533.51	2487285.88	1909828.95	1408211.28	1333673.77	875049.922
101071318	109695170	261901545	247012073	294372596	207911439	202805084	840594311	253358069	125824344	156979103	263431561	331892896	303744052	435102402	295949715	150576284	449373508
5777579.46	3071436.68	7241763.38	12070998.1	9641880.95	6319971.67	4896127.22	1358956018	718177179	148734676	324955381	600595425	453961269	553821449	792253433	380858600	419691662	527202272
46247156.4	17541356.5	20680562.4	32155874.7	25816383.5	16488430.5	15902200.7	64448964.8	50052508.1	26912596.9	27298572.2	182974201	159528830	34476392.5	35377500.7	41538915.3	40756036.6	100111264
2641265.14	1992315	550602.442	274188.205	309698.572	305758.51	168194.739	2421683.34	1001578.74	452405.517	505401.425	42832969.3	27170901.5	1005802.11	1229377.38	1479490.1	785521.15	13868659.1
2142940.61	1996151.94	454275.08	261941.538	156150.819	233549.451	118033.765	895479.966	818561.366	232402.758	280651.515	23837575.4	18192208.1	574413.082	839689.67	720140.829	368111.923	6593396.52
8353348.22	6497078.56	1434099.5	851800.353	395190.838	523120.941	314445.915	3606264.35	2213889.07	1358964.47	1333958.53	68427527.3	49743514.3	1877178.49	2534481.97	2978670.91	1220232.55	23645208.7
595592.528	653473.068	284947.405	228724.159	96929.2997	133716.205	61634.3916	1271060.02	502510.619	249590.596	417912.424	21089446.2	16755591	420037.104	518505.333	585035.387	218875.17	4767045.17
		58723.9466	72721.8896	80283.4375	15217.0842	14846.6688	7825173.93	1638758.17	1299489.29	2133157.91	10107762.4	4790303.74	1743470.77	2102137.29	5398317.55	1808818.15	743163.582
		3716.6024	4736.27084	4410.41466			18636121.2	6343249.72	7560927.32	13386410.1	26900719.1	17085530.2	12477217.5	15129614.1	30412582.9	13964325.8	5448575.75
150379.285	409789.335	388707.448	537726.973	395923.202	180909.156	302952.106	414275.855	258061.527	183951.432	197460.369	214032.144	137417.367	532198.643	563109.099	386789.974	439527.824	525009.274
13372207.9	16187226.1	8588362.37	10858708	8540664.1	5832463.99	4499841.05	10737294.5	7716446.34	12888417.4	11725498.2	14521400	12170734.1	7523579.9	9695875.65	9474391.5	12885326.5	11115511.2
2654257.73	33887.4452	331200.664	513147.494	514602.216	286520.595	477957.428	5190660.63	2186734.38	614879.413	1119995.35	5676912.14	6742488.27	6612517.34	6916463.33	3172375.77	2974953.72	2696052.45
24280112.1	3917317.45	4035104.53	7809258.62	2754995.22	1676642.72	4882485.21	4967577.19	2432366.7	1936453.1	1549438.21	7924074.25	6870225.75	9385702.6	8121006.27	3911356.26	4260238.85	5626845.77
750313.78	291942.09	180140.649	613172.141	157629.321	482955.248	632765.421	235838.565	166041.068	183083.282	140333.21	219274.249	160755.886	247691.711	244889.067	301733.502	311166.936	428546.811
			37855.9267				2048569.18	871151.029	282506.669	239439.094	1938866.1	2165282.83	1365002.53	1576134.46	747604.137	602610.753	465773.262
							328663.1	268033.344	5104.67352		639829.978	431551.493	381708.717	273546.708	445991.43	351322.402	645182.074
22776.4588	58144.1322	57866.1236	376234.849	148068.117	89699.5171	51093.7354	2032965	1189919.28	584994.78	544652.591	3333449.1	3079934.06	2485521	1964555.25	1631371.21	1580616.05	774575.027
2447762.16	5127129.4	3531264.01	3683870.2	2497308.09	1672463.24	2552203.24	2796591.79	1488195.95	1752047.68	1558818.34	3543936.58	3117399.97	2034532.74	2088838.66	2361926.77	2368739.33	1174500.25
53935.7828	340685.765	662649.044	740888.474	619823.365	300660.478	504135.403	1038799.29	299260.283	375644.275	410797.481	394146.222	421517.195	425327.085	487243.677	579538.753	521564.364	255015.344
4801066.3	19971171		14542202.2		2381611.02		105678983	50347599.3	115323583	135658532	416783.639	312225.562	25661221.9	30269053.8		424307.678	57776684.9
10687915.7	9562495.23	9489416.53	16571269.8	7539910.27	11863255.9	7600515.6	35745976.9	10381310.3	7979070.83	7837906.35	11587994	10327900.2	2523393.67	2970675.22	11615138.6	9262392.76	10501305.4
			53950.7549		62058.9444	40423.3509	9762448.77	5057773.58	3296305.91	3790087.54	6622288.57	5444298.22	3923934.87	5414605.94	3683614.89	4187150.29	5476018.29
463578.696	28474.2679	184223.594	40936.8862	214322.181	250826.375		501885.173	109241.639	88505.6579	146362.192	327543.102	309785.774	280637.113	238341.195	45191.3724	37949.6116	343165.527
726619.008	1165428.4	2647577.21	1366953.3	7951387.17	966941.334	476069.765	62047070.4	25345391.6	10967344.2	9345396.7	35823580.1	22985096.3	28144787	24015689.6	13319532.6	10749430.4	26321907.1
16643639.2	90990041.4	67749212.9	74039276.2	131075480	31263564.9	58937808.5	92397430.3	79485110.4	33359078.1	37168540.4	153735409	179428355	76354508.9	84127628.1	134385636	114671233	177478700
21665.1221		757286.725	67958.8231	373566.467	276670.542	184610.45	414427.466	120007.138	97227.6767	144331.142	149634.583	229413.231	228136.984	257262.357	290632.603	164032.356	619661.446
691768.283	58362.1403	504337.169	1152197.62	162991.428	87058.9404	753075.331											677741.112
13776313.2	14031788.1	40301156.1	40443934.7	30284202.1	24780598.8	23562427.1	378260682	155936805	21567328.1	35281006.5	231319229	224381989	67411176	69662498.3	267069624	157074487	336081027
1230591.81	3812409.69	7426626.88	2154902.33	2272024.15	1297654.36	1753385.54	2183619.68	2050656.59	2458052.72	2935724.65	3435887.1	1837293.26	4004899.13	5010746.01	5393187.7	2565307.3	1111076.77
515826.776	1003860.8	7888632.4	9187550.71	7807806.25	4560364.95	8527419.73	59994168.9	19874941.6	7617425.35	30024674.8	42022007.7	44011355.9	20689163.6	22166160.2	28819250.5	20969523.9	22783895.9
666610447	268405443	43283840.8	325097923	131100095	139900269	435331717	3739382948	2113256308	1703251248	2001810962	4623096307	4262625709	93021140.2	102488892	1110211385	1304962367	1561885254
56422671.6	33732190.7	27634768.7	34113741.7	35550198.8	21515501.3	40006022.6	57160697.5	41672394	38852740.9	38619440.8	59947023.8	61932545.6	24426884.6	30072593.3	55141979	53729738.8	67333079.2
735586.186	1128801.53	766465.749	944771.776	976125.644	350135.016	16891.9648	1439563.07	851887.407	212259.036	307085.664		1044395.43	713600.86	758592.101	444393.548	578250.834	1581397.86
1027476153	829914259	801378542	1999604979	907752898	464969456	767245418	1215895046	848511875	466483538	541351481	1025647536	1109200827	770607958	703194072	618559188	622974360	1134566150
31627148	2610388.28	2709047.46	1355520.23	1618391.99	4265195.97	2080789.7	3734778.7	1786350.99	58347090.7	46423391	4192850.71	2708768.75	919439.553	825860.468	2476322.09	2184431.73	413695.126
345847518	511238351	721024106	487711369	167333847	322246822		49687618.4	27371686	10451466.4	10148144.8	102713825	49837014.8	35099794.8	54874992.4	37522294.1	35877787.1	69766626.8
133521.724	41799.5747	65060.111	291696.454	34909.3029	69703.4306	84590.2914	33464993.8	19700155.5	12240586.4	13515737.3	93803087.1	69752448.4	18162264.5	17231090.1	12123501.1	12416184.2	14862369.2
1579260.34	1091944.3	4421252.15	4853601.43	11277660	2798273.45	3992301.08	312288554	82452890.7	86024655.1	67545780.3	169214127	263749978	149331177	208124118	154108471	97355854.5	204634489
65106450.3	90373139.4	383123175	59892521.1	311635438	133848527	49219338.2	1338853034	1553630239	1162130563	1107464681	2123340214	2352513556	2359788359	3240587954	1948408742	2254553628	2338775603
18923.2453	11402.4222	6603979.34	976879.685	2962719.3	583757.873	408438.748	92550291.9	3152935.04	2020967.78	20450375.7	47020019.3	31679696.4	9845652.33	58207584	33562388.4	13987959.9	23983646.2
78415004.5	39697780.6	47354466.1	84753614.3	44178625.2	32768285.3	32455679.8	361728042	191039245	123141370	109431647	357414548	315109007	212936279	224642076	155375618	150882387	174806956
29012.233	30479.1516	38290.2861	9688.26735	22425.255	11539.4223	21439.0312	84718.8789	47962.5654	31764.0517	26813.4608	43523.7792	22877.7641	81940.6091	109297.911	57521.336	90549.3101	35682.5902
49159929.2							452939042	349771830	469931145	543714714	1521147921	1647542371	517633463	629259255	501460562	554698325	606514983
4052478.96	7359333.88	5639875.63	6552498.7	13992698.4	5572752.62	2580722.31	251612529	122804820	58144664.8	54403683.1	187352054	147865807	121289363	131854752	111021047	99895861.1	174347240
40739339.5	112893229	184994557	289912182	150615038	90497064.4	82456527.5	425606243	130383026	116644652	154412777	245051700	197798425	117223803	128736911	107126485	63429620.1	290485930
87977080	444436318	368577893	621997807	518295945	173652362	291888310	588371487	377640003	240780092	352343759	1033980701	929998802	411874503	477840771	722629456	634819913	1403067771
30708703.9	2618107.13	4881175.34	4539762.89	5015624.91	6663290.7	3375263.05	11206764.3	5620885.24	23383592.2	25019491.2	10129808.5	8428923.28	2846629.55	3513957.67	8959003.47	8522824.85	2551939.19
47796901	61037448.3	53872542.3	41425951.8	365674373	44261471.5	40001585	533911973	270880743	495672460	365977544	800269489	626978185	729349646	754501252	539624212	495063077	327311370
13969.7825	339147.994	12934565.7	21379681.7	15386708.3	5449453.63	10543537.2	39258811.7	2739902.79	2519026.12	12567675	23376556	19961151.9	5190943.99	11770930.4	11401904.8	3931586.45	11118706.6
115821034	150484327	171276826	245612919	195464775	115638188	115366484	323009569	152724426	119335195	136603624	224596061	256911619	155132379	189804980	172821060	139066142	156009466
15182120.7	19500078	22928759.3	15039900.7	40712188.2	15972961.8	7883409.6	357983880	166081081	148011574	174369110	240119927	279873665	192279284	266146389	237451765	196275262	197075117
2380435.13	705516.889	1100272.73	4214788.95	4644015.06	1202889.66	1920101.35	2977116844	1415462373	840606283	764191199	4052962107	2767735264	1202390796	1183653734	936381715	866187154	914496303
411881367	672347169	795419470	855931461	665860956	529138399	860611738	459060435	362997690	307623940	290786072	423836996	485402185	530005749	626892485	559035322	590731113	1148244243
132632659	50130845.7	86999634.3	357417436	98761387.4	86451912.7	220832916	109098270	67619891.7	53835401.2	60954288.1	112070615	72654171.1	75666604.5	73572379.4	63442878.2	63104532.5	390454787
1807595242	4481279076	4481102352	6897479872	3555320033	3148241404	5044167850	2571557753	1572237871	3516430500	3266867946	2554540959	2385173774	2125208251	2190190845	2708158768	2794958303	3203287388
164764.429	76980.7792	27751.7023	32979.9043		21591.3367	73835.762	255682.247	84759.8235	85431.1287	46979.2118	648687.293	480796.765	141404.862	396316.358	101217.763	100869.58	99062.398
480199294	443249154	381089679	729986422	380958223	278838074	342335730	606697222	435957740	111640579	111079593	628689988	577404171	389393623	393282146	299832003	278621740	331257344
174706.166	204607.476	319166.087	322942.541	576047.827	289081.111	288111.236	3490284.25	3007627.37	1510770.68	1560239.96	3618824.19	3232892.95	3485367.7	3552914.6	3500420.28	3001452.5	5562647.98
281999.836	1518168.19	1682970.76	1009924.39	1086041.46	616245.468	751470.62	1576295.13	707217.791	981438.945	975331.509	1882153.91	1626068.8	962233.091	1329212.53	493927.681	299118.913	2000069.54
388815.686	156837.41	727361.902	1232941.49	4282867.74	1242034.77	2045391.11	179087.453	48275.148	2179781.41	3043493.76	514992.241	474920.781	166385.814	168047.359	493662.961	251758.638	184441.499
21354360.9	16692438.4	24173149.9	29545962.6	11726676.9	17935556.4	15372786.6	12922443.4	10688541.2	15441015.7	15046665.3	20752042.5	17926255.3	24318523.2	26205505.7	15565114.9	15541557.5	22351410.9
187525.342	75339.2088	48798.3058	240969.338	112030.543	66145.1789	158859.527	498353.115	183490.645	69734.6383	59147.2095	1112451.36	370734.338	45612.9843	57200.8485	93779.1733	26259.6084	56144.6038
4919.32121	5863.48875			6599.84939	7696.16605		670529.341	144862.699	171001.119	184019.796	657986.503	447548.265	323022.813	306846.255	302829.55	231173.882	480785.234
51881.1064		129327.619	94989.382	189208.602	43614.5857	82846.2364	23829839.6	3540573.29	1762396.14	3023089.47	7179725.27	6926683.7	5259230.99	6418288.52	4586638.29	2404040.18	4334093.67
				9779.44574			842039.165	620208.973	867650.955	878506.824	997318.238	1074456.46	640095.735	606593.419	512643.297	571316.594	503757.068
142177.278	264802.833	353923.271	309324.848	270615.178	173643.678	230164.295	197931.725	84391.1552	129418.221	148505.859	329166.884	278460.898	303841.626	363349.494	280846.058	221219.052	
918819.346	1042270.69	10823705.6	18467035.2	12500454.8	6571532.26	10802922.7	20935718.6	4693732.82	3015692.92	6893337.45	17074056.6	23913058.8	6778203.44	7875012.15	8776436.99	5851992.9	5651992.54
215298.918	62540.0724				17558.9499	26687.2148	675169.883	242166.708	161926.765	185381.916	1597116.87	803309.955	76177.5734	196770.712	113711.744	53153.8144	74154.3674
78173.4152	79488.1565	98898.9692			67168.6542		2553290.72	784332.972	719171.189	737829.528	5961924.22	3279845.46	281312.398	972588.39	449943.466	180495.129	124673.613
49974375.9	13769892	104145113	106453264	119442811	57060797.3	83370595.4	56461256.2	26078125.5	10062502.9	11046262.2	78824024.6	90223391.1	90449849.7	94621845.4	79806735	80940846.5	136748118
3346601.4	2392787.13	1391844.4	3152915.39	3633971.64	2132749.19	1688633.38	31650841.7	26029432.4	16343656.6	16402037.8	38779446	34828986	20494565	30899607.6	13178877.8	14983230.5	20907635.8
342513.475	759092.804	6161647.61	15788927	6632765.25	5674389.25	8019874.77	9324311.01	990581.713	2616595.98	84625.0157	6503984.28	586170.743	7761817.17	64114.7901	7895842.7	11311671.9	9639966.8
	215544.499	43213.0276	321202.457	32087.4358	47525.1708	62332.1221	132075.517	58476.3166	6720.28263		48292.2824	44095.108	289763.792	215544.499	245054.593	283703.838	380752.323
202079.76	169982.404	101947.806	1155515	11073513.6	200548.725	42123.2439	2567931.28	1847243.01	889167.581	877871.811	660481.516	717163.481	1487965.49	1464901.07	5504508.09	5918420.19	1400263.53
3837512.91	8432153.77	5771939.77	5910691.84	7183731.61	2746523.48	4943299.76	25218178.9	11645513.2	6417559.04	8336799.18	33186465.5	35117680.1	14636268.1	13282760.8	9363257.39	8168665.72	7407099.04
721004.772	837585.106	1171834.79	727034.3	883049.292	611407.88	459356.494	11976073.3	4788372.8	5475394.21	4765622.77	15785422.2	13076895.2	4398269.11	3914842.88	4851576.37	4547698.13	4914423.75
				4475.02014			956355.242	184424.092	1814.7672	13507.241	506391.618	464848.796	383622.2	361983.514	425833.083	263874.601	767353.66
51075078.8	15776784.3	15958519.2	133205852	5093468.3	10999602.7	22286623.6	102837094	33132847.8	19939516.3	22185908	222119517	215784516	101975771	97907982.1	15786407.7	17863352.2	77951240.2
482775874	306433810	7407281.32	7644903.61	19564620.2	3974832.35	11837947.6	1485167267	1006737617	50820510	57311719	735803284	784230084	9117335.8	9597022.14	19065320.8	18831348.6	10596760.4
123482835	209098446	120055257	315287995	253041709	132148633	137104708	365927709	132924459	104983804	94217600.7	281649682	251705777	137060528	122729425	92758815.6	89812289	138767335
50988.9929	54800.4192	18046.005	34011.1715	30852.2673	11834.4432	14984.5484	311596.83	92786.6441	195982.602	132265.002	193543.89	220870.033	181865.931	204562.438	173970.751	149529.8	159731.158
	5417.68799		8245.85719	2964.21355		6573.88615	468241.315	195696.292	97907.6108	79093.7562	443615.744	364956.662	432218.407	430937.985	245142.37	164924.327	227280.97
506297.192	528587.662	799795.387	1347747.69	678031.666	569156.434	896452.687	1008378.78	688268.874	377780.593	431769.61	1250430.29	1126560.38	836338.804	812536.706	634009.281	560988.491	814043.318
1285679.43	2189437.97	1852349.14	1628157.2	1304680.47	702822.522	1063542.2	2042157.52	1246153.47	1679256.01	1699641.47	2919487.87	2665348.44	1073836.01	1407243.01	1074190.7	1355996.88	513367.632
40286888.9	33028787.2	51442675	105519079	24514177.7	31518388.8	37242176.9	477549124	267583406	236463188	235899908	525670124	453705075	490386670	465917827	484072070	373847653	450136814
				22394.7743			7795055.91	3903795.96	1640048.8	1530888.88	6519016.87	5900743.71	6752891.28	6599297.22	2377833.88	2262889.07	86407471.1
45863842.7	41230008.9	35464341.8	72203444.6	30986181.7	24377667.1	29874527.9	56831664.1	29324535.9	6665835.93	6942263.02	56052016.1	43578367.6	34133378.4	33863839.3	19100835.3	18003119.8	37140754.9
67034.0619	8963.72693	329757.105	380129.56	287261.437	266756.825	292503.299	291501.818	66197.7305	106825.285	80013.4027	385080.216	161035.892	180210.919	93464.8633	235109.713	185932.902	281573.738
37949.1327	97892.0691	147580.338	118267.143	220511.928	99285.7264	80327.0701	635199.96	398791.441	301131.891	363811.721	323024.215	323081.246	522532.991	526591.528	460348.665	458537.04	564703.584
19957.7844	26345.2019			18843.7886	9918.04683		937065.269	610860.153	301982.828	263213.028	1109867.95	789894.203	564386.161	598615.522	470400.132	413095.963	434911.628
15976864	6354198.38	5067328.25	5605937.45	13022184.3	7112092.62	4901322.15	377390887	239852840	274620702	277416222	437930976	410606822	214743343	145317723	187949432	201724567	250298002
1923433.12	1304498.65	2796768.97	3712571.05	2625152.79	2506813.33	2416710.63	3088602.21	1531414.2	1145916.4	1723852.97	3494626.92	2871379.83	3803976.61	4573909.89	4033542.22	3315751.12	3105825.87
8160326.82	7765708.57	11752210.2	19318675.6	14476931.8	7930521.17	12572673.3	21541380	10196636	8519433.6	7619801.23	18709846.1	17373914	16529452.3	18176854.1	12024213.1	10901130.6	14495707.5
210545.218	238754.793		729599.346		536515.138		1186140.41	577942.7	239776.737	273404.684	1218316.38	908555.853	1614139.5	1827067.81	877550.92	754361.222	1204189.16
549949.976	208204.6	413513.945	1228554.48	641876.871	405709.001	202438.824	3106170.49	1877829.04	1061906.98	923679.023	3829668.24	2958791.76	2207135.12	2365403.2	2715771.9	2827042.77	3889615.81
229306.41	286954.19	141231.129	73503.6693	70155.9364	49247.9456	104575.026	188261.717	44763.8709	70900.1604	133213.993	93811.2345	89856.8062	149018.857	138845.727	39990.1889	102352.163	71687.9396
586883.169	1932657.19	223586.412	460574.203	13822.6827	10460.9824	11912.3342	4822669.54	2239420.55	151941.706	121398.57	6697213.59	3664403.1	2329649.54	2824733.93	460574.203	92583.7276	783612.836
52975.716		29009.2994	193356.923	9162.77683	30938.586	12732.7294	61901.9718	17605.2076	8479.90228	8278.83399	134448.293	118309.316	194620.78	34814.4848	195196.627	11822.4343	195934.524
90054.0374	129164.498	256998.617	146153.524	182394.602	137090.966	107197.212	61139.4995	186853.236	87794.1066	11506.6991	325131.148	345558.56	84979.4322	414479.305	797397.175	602662.386	121656.963
498102.962	327321.093	508573.172	511129.934	531943.852	368040.675	102926.008	32830740.1	8006456.89	6111571.32	9521264.2	20646379.5	19547566.9	13618174.2	14871703.7	11923264.3	9797312.21	15319215.8
167086.729	92127.893	133108.433	1001292.48	135092.756	162855.407	407499.248	209653.073	158117.972	121761.261	116399.227	134096.924	100164.991	85796.7216	89840.751	275542.637	222314.386	1244580.33
365781.047	123349.676	149261.78	531170.521	332943.841	70515.4794	475634.059	3575104.6	2256081.56	1722554.22	1644682.09	3444673.57	3041491.82	3198522.96	3948344.55	873193.604	811423.46	3030874.93
270028.448	981860.126	428626.004	397420.336	745584.89	350485.076	507257.224	280792.883	116829.098	441239.01	469384.07	524937.808	516450.132	335783.392	340811.306	263672.284	282494.908	280459.506
75575.6241	67794.6151	43162.1892	217278.549	88375.0707	50079.4616	77397.0946	108293.521	63898.5115	69025.9363	43723.5451	141366.778	109223.187	111477.822	115636.707	96398.7838	77396.6763	109436.81
2583087.3	2688060.85	1906914.96	1304528.24	3960515.7	1453942.75	1124376.82	50750436.2	21983745	44892640.3	42158336.2	53075253.6	50202026.2	69449612.4	75033028.2	36044015.7	49455540	39487928.6
2206596.77	2095297.5	1742062.14	3134040.7	2174964.1	1328249.37	1779639.71	2257171.82	1222594.64	1349559.35	1371119.64	2324614.24	2323103.97	1833499.6	1717275.96	1280319.2	1511330.56	1112073.08
236327.068	231381.04	466691.256	914986.575	765996.491	349830.53	681186.298	993060.252	376242.219	306053.239	563312.072	1089521.75	740489.562	259977.966	301786.272	383777.916	278212.793	544209.206
105248.152	14565.763	91130.9494	85117.516	74192.6378	255147.373	155593.092	234545.325	109202.944	33744.7113	84877.1146	125957.665	148911.975	207280.606	201381.425	468679.152	198736.496	77061.6013
197368.369	321926.943	379115.876	707008.427	670041.859	173859.29	382072.751	231368.774	138962.904	130342.836	133730.044	245916.863	237751.255	148441.575	200045.061	127791.53	191422.16	213277.518

N_B7_R2	N_B7_R3	N_B7_R4	N_B7_R5	N_B7_R6	N_B7_R7	N_B7_R8	N_B7_R9	N_B7_R10	N_B7_R11	N_B7_R12	N_B7_R13	N_B7_R14	N_B7_R15	N_B7_R16	N_B7_R17	N_B7_R18	N_B7_R19	N_B7_R20	N_B7_R21	N_B7_R22	N_B7_R23	N_B7_R24	N_B7_R25	N_B7_R26	N_B7_R27	N_B7_R28	N_B7_R29	N_B7_R30	N_B7_R31	N_B7_R32	N_B7_R33	N_B7_R34	N_B7_R35	N_B7_R36	N_B7_R37	N_B7_R38
376895662	155468296	370094490	353537848	210428142	259370294	96270763.7	199330006	234087653	95379827.5	153443403	300746969	248384771	45085417.5		372684977	194445082	199752825	162547375	129359774	164436828	118149724	108370954	100591791	137753680	127147306	196060436	443177892	211837317	171936203	157745669	94166947.8	159702288	137019263	94824806.1	144795448	297815442
64903774.2	33919675.1	71890836.4	51362272.4	30770069.2	47064585.5	13844219.6	32867208.2	44668551.6	16699252.4	29201954.6	32234431.1	57696815.3	9593910.48	52787087.8	110802328	46826096.7	38275607.8	36310104.6	31498644.6	42522793.1	24440415.4	24287430.9	16807203.1	32645671.6	19317609	35004764.7	72478676.4	44954321.1	49587546.7	30389120.8	29541287.2	42928413.4	22927362.3	22935078	32161711.3	45058818.4
38004.2745	178762.782	23831.9029	67298.7932	480878.215	299834.476	4786.09203	275817.235	149445.178	3083.72237	1645738.79	161512.492	244132.824	91789.034	23833.2008	30455.4798	73605.1309	200707.422	97822.4854	485675.224	67956.5161	80757.8068	40088.0645	68985.6357	1640086.17	14938.63	89571.0464	716.007938	209975.788	2017290.38	1709642.08	98017.885	36517.0411	2483.36638	1266639.62	32525.0633	48347.8347
102049.241	403235.718		110924.529	434247.217	885022.199	71195.2821	440664.257	327362.817	3840.36471	618493.307	422623.028	329050.581	186643.373	18611.051	53937.3111	145115.665	366863.738	94083.8124	1031322.71	83047.8819	86774.2353	48908.9247	126247.549	2866248.48	40416.7851	232961.234		28085.987	605328.014	824991.164	59792.7265	44028.5608		2724887.73	47364.5341	130798.138
687525575	655974805	907558819	334357625	474539435	233419488	175297946	339257877	636108952	362123909	214986837	137193504	245831937	347403019		226990785	355295513	197268674	233708826	89831329.5	436356770	216413121	89680137.8	232701052	142410430	77779307.5	125153528	178687344	213071325	195253121	150103997	142299482	281872364	165284963	92295383.1	111119638	144043822
428556963	726513856	798556873	353299909	528598698	278081994	250308183	545088765	640469358	539353915	246973104	257599101	763079766	384739322	1208775382	547948843	527741766	365352174	413686904	304453908	746761900	534576161	494514859	477710242	545007068	293431456	358734600	371460523	485168672	315452412	304759294	623880189	678343652	343327343	444570445	421406762	240439742
34979009	61446642.5	41610214.4	22745817.9	65773279.6	44295226.5	286531258	74035802.2	47975737.2	10643989.6	18048348.5	47879994.8	90819994	7695082.67	18096242.5	24212302.7	47388853.2	32039132.6	84570411.4	39239042.8	22365257.6	19637105.9	14913979	19631676.1	13183514.1	21057685.9	53546780.2	15881542.8	50675682.9	29234668.7	22207957.5	47942066.3	54833856.6	8914769.68	11805231.3	38941232.1	31367839
736484.435	1149761.8	1817909.34	1174671.07	1575006.33	1669178.44	152128920	37440246.9	1133929.89	317869.63	36025354.3	1301105.15	38388161.9	81019.3269	480677.6	710110.016	1456626.76	1912891.77	4195539.03	868350.717	439358.714	353729.424	285022.671	362485.621	20126155.4	443032.485	10698990.6	1163450.77	917247.01	56630298.2	45347161.9	800471.855	2019872.14	81135.6949	13451481.1	1406786.36	924870.715
481349.098	680007.003	1026009.02	724369.368	969509.475	902979.003	108585222	19036832.3	577097.319	153227.548	19866048	827783.192	16794609.8	25309.9639	640607.771	393246.609	809583.359	1100073.15	2139433.79	390310.778	211417.694	166267.797	138416.528	197074.898	9912500.82	254656.409	6393598.36	533959.564	648720.144	27455298.2	28748669.3	464110.45	1217431.33	26520.5432	8686415.88	984054.845	581901.683
1382136.11	2363627.46	5450476.84	2452239.38	2439903.4	2994914.48	259838889	38965680.3	1855949.33	530055.844	115817125	2632188.99	37940702.7	137072.062	1421544.27	1323600.88	2425234.19	4031307.12	6999431.64	2086871.28	1172227.26	878365.987	584741.73	724151.368	48589885.4	939770.185	18165943.9	2953239.44	3268978.8	186626579	145067228	2123472.68	4690069.69	187342.124	37185337.8	3057288.38	1680423.98
344561.441	499075.983	1154224.35	511449.255	542574.016	559083.109	46758638.2	12668484.9	469568.553	97774.0501	27131400.5	416991.351	14470662.1	30858.3141	362790.572	305201.933	649152.892	803718.706	1500418.81	447913.106	257154.849	186969.521	122268.853	154971.394	9589349.02	170530.707	5262173.45	587477.156	740146.997	52948739.3	33695470.4	500288.337	1091290.31	45837.5628	6070713.69	640242.711	349232.624
122414.046	1763708.91	2067261.6	1271798.91	1061197.44	403805.406	121907.969	1883207.09	1976629.97	1923670.15	3315230.46	411360.201	2648621.12	2255315.62	7735411.1	184460.156	1788059.77	588972.626	923676.206	3281262.4	16362416.7	6249476.67	2535849.1	3120793.64	9309178.19	1476766.37	924566.931	510569.838	1743043.15	6328719.85	9657882.42	11806430.2	2391397.81	1046517.86	8167205.74	2726490.28	811496.323
1334742.09	17292838.9	8084566.34	9580849	10972697.7	4528995.19	1764167.88	13625281.5	13673713.6	9901452.89	20809550.3	4247454.18	16238553.3	14147305.6	26720735.3	2142044.5	11826389.9	3806620.86	7604402.47	23696857.8	45673209.7	20038268.3	12680609.6	26501218.2	58724734	11974945	5927714.28	3698421.16	10954777.2	34512094.5	58285230.8	48042924.7	24060886.2	9447272.88	52063951.1	17979676.8	5423658.23
208715.195	501663.81	312513.452	199290.165	485063.431	124669.357	231955.442	670446.25	433776.813	403776.234	317406.293	227182.201	567713.412	323620.677	932565.684	314731.838	405968.42	395857.599	285396.191	465277.637	431989.81	339335.797	1139861.81	448925.135	335473.533	304525.337	392481.491	249392.569	1240691.79	394044.232	341980.55	637501.724	538859.967	392116.064	290885.533	289187.726	231399.548
9291079.96	9546570.56	26716150.2	7412196.28	13033955.9	6433751.39	11104467.9	21105901.8	15422517	21708600.4	12651180.1	16166557	16166071.8	17291052.9	14238534	10112245.3	16834019	13908169	7895046.62	6151825.53	11540607.9	8122558.88	10279484.8	9319697.74	11057084	8403941.45	12713784.4	7312324.12	5960825.53	6306846.61	17379916.4	7514221.28	6480997.87	9818983.47	7144687.1	5678352.87	10127693.6
374696.579	1627175.12	567832.796	1201967.43	3282073.01	2582647.65	157129.511	6133806.14	1655243.41	37461.6845	91551.3921	3488848.69	6901472.94	465088.282	188603.006	297366.955	335210.682	947080.994	2612771.14	4078636.61	708718.49	614879.413	620671.25	465855.999	353623.056	105085.625	2620859.2		2633106.81	192503.12	113840.145	1024563.8	309953.853	75532.091	92487.3483	206581.13	1072263.68
669934.685	3394841.39	956777.911	3027405.58	7378016.75	5936906.24	460761.62	14079410.8	3126445.57	58931.8832	162310.184	7963484.08	13539645.3	1164578.89	185812.278	649366.987	592844.465	2279512.5	7987004.11	9025763.56	1324544.5	1238077.52	1240700.92	1034722.25	575160.05	225043.202	5485322.69		5510956.3	291197.365	169472.721	1713133.21	508952.404	105959.713	163842.776	448788.394	2943443.04
10674.5265	453253.949	78179.0202	1904.72498	1327470.82	351145.602	905.91612	148571.835	1618664.73	5039.45623	16366.0503	99686.0292	179657.733	271010.722	13430.2077	3479.2187	566476.379	33347.8017	124453.405	380223.107	821939.298	724385.744	810404.836	620591.206	21433.3648	118482.387	8756.01534		851677.621	40671.6768	19607.3979	859457.291	341566.408	309773.014		202808.192	14337.8607
65373.7456	331374.049	54837.363	293970.56	955499.372	649164.341		470286.416	379227.862	4591.47842	4860479.44	447368.583	479084.56	125814.313	33578.1915	54677.9077	121130.2	518130.931	234693.151	1306318.76	179331.189	151712.479	123751.421	127456.503	3592348.47	35678.3914	174651.9	18455.023	429699.863	7172168.82	5388669.15	184449.544	71947.8905	10756.808	2863337.58	51004.5785	109359.653
101638.432	640545.404		178835.526	1178330.33	3287503.11	187951.321	1548933.95	500691.293		35730.7621	2090923.42	1608436.57	277422.39	18499.6892	94418.5989	400778.136	650033.937	706738.501	2754426.54	112290.99	161954.825	193128.169	186660.9	360698.907	50809.2685	956309.464		332012.115	31474.9698	44064.3144	152405.476	81174.4665	246054.488	268033.344	113300.534	598407.082
235271.726	568179.249	191914.636	664287.125	2345292.97	2435075.38	63351.4946	1906006.02	1039275.26	48542.3758	421848.011	1985236.82	1822728.73	359108	63841.3284	169998.026	491144.622	1472088.31	1195235.57	1488656.41	209050.223	177638.469	173294.046	259060.448	325519.898	83955.5962	520217.096	36509.8358	1775120.25	372099.376	447229.357	250301.607	139039.231	161375.779	217246.191	114718.912	358298.665
3639119.68	2322189.64	3745312.69	3025116.8	1021925.37	2054636.05	627879.169	1126341.14	706837.309	733922.6	2562992.88	2608615.03	1446828.32	888103.587	1997118.64	3874845.58	2986179.72	2894410.31	2342282.62	1935375.79	1470617.9	1338236.7	1837422.12	1064018.76	2485995.21	2066913.81	1032318.57	2238556.13	1470823.4	3845837.7	2767666.38	957254.226	3054997.98	1594403.6	1699697	2668639.73	2615859.42
846641.451	635312.123	1696091.73	859267.535	214842.814	408792.645	134572.712	219897.092	176913.973	154985.87	832439.33	406090.107	298370.126	188459.559	529247.389	841355.689	753868.809	543474.36	392111.293	357962.941	390014.831	222067.259	289583.416	158042.286	550168.664	293944.143	146734.13	405885.965	382045.776	1239756.79	629115.507	324563.891	630542.574	201202.712	362435.437	491320.785	353950.372
48309676.3	6262609.07	47035306.3	44222444.8	17223417.1	7757623.75	25681129.6	28315577.5	31871292	305412.971	16638495	53274515.1	44936147.2	30407947.5	554378.168	51127094.4	924294.348	30311258.1	5530482.5	26165282.8	121325435	99283635.7	39669047.3	72327936.2	9201900.53	23002094.8	25388647	18170303.1	72407794.9	19137993.1	17255896	68082.9887	36200152.9	38104050.9	6062642.14	26777521.7	37240145.8
11520656.6	6591810.18	9101083.88	9282404.37	4940250.5	2944128.57	4028204.33	7302219.43	8426752.42	3136754.88	4452379.34	3899053.91	15326604.5	11942932.6	5229125.98	8509642.37	29758193.2	8021622.63	5612334.11	6665093.46	34533546.5	23076295.7	5446745.92	13981801.5	5341667.13	11272312.5	8352909.82	5089093.87	7984709.32	5125331.99	4096426.47	15531126.6	21148380.7	11987007.4	3610382.37	14021137.4	3602329.54
2824036.47	7704314.65	5007222.71	2585442.56	16336005.7	2349264.45	3319836.52	6285600.97	1945421.9	12751854.4	1139064.5	6644496.47	9691478.05	7631431.86	21360876.4	1875074.88	585159.828	7489069.03	1941889.03	2645941.68	21180017.5	10062967.6	7157879.39	8156537.87	956391.253	3585502.44	6010449.09	4192224.17	4299077.62	1334799.92	1390124.99	14457687.4	2550494.73	4330025.57	679884.839	2468372.63	4605810.53
329258.309	80622.2287	37008.1221	248161.188	310257.535	26442.7537	183055.27	37707.6995	157787.587	418056.989	90212.1019	124763.024	117188.391	656886.07	469904.538	330827.054	406538.903	265713.536	98339.5219	135534.957	386655.306	245383.199	163697.673	130633.174	168020.016	164973.07	43675.1624	117477.537	86611.3538	40291.9114	105604.213	144061.036	6094.9267	198339.961	2767.20012	37450.5281	148700.106
11903055	30197980.3	24035626.8	8306314.86	24790555	9223437.29	17807801.6	22719871.1	44276342.7	32684532.5	5520078.07	16785879.8	31661239.8	29430908.5	77525834.6	11140856.1	35315175.3	25300517.5	28096221.4	26241148.6	68300208.7	48174348.6	45122811.4	33225108.3	21177970.9	15172290.2	27455466.6	15831580.2	9776951.2	6057590.62	7209905.04	64517222.6	26907322.4	32659986.9	12967108.2	25263966.7	23185196.5
118909091	222969027	17311972.8	59040612.4	45965610.8	29111945.1	134084921	217013113	110212850	145116361	32324952.7	23608565.1	237715477	126840648	221059691	89819935.8	81799853.2	35267764	30921378.7	100111878	146577341	169308769	200217499	136166876	252559296	73373978.4	175692291	8782574.78	23318733.2	46183187.6	31963460.8	185339801	86290614.1	101866343	198192244	97806877.7	22723566.7
69956.2358	404819.32	59132.1411	66792.552	437901.67	210396.966	428025.962	1056102.85	965043.532	154256.049	120909.135	1588298.02	1627571.62	530294.414	149332.742	77651.5566	204522.446	68679.5402	104685.251	230050.036	1527918.88	1151713.4	206713.605	1177480.42	197684.038	215245.045	878315.943	107416.113	117973.189	106373.573	111942.594	208595.443	415024.031	137561.641	206128.758	343945.302	1414225.14
415475.895	77706.4399	768661.691			238707.927	330493.708	495862.307	1597374.21	267948.513	1238426.35	290121.517	659086.028	1485159.18	909885.3	435452.16	203672.569	340769.801	440560.039	862091.092	1448928.43	1142619.8	824027.825	1028254.27	1667755.44	1092241.9	329550.186	662808.537	1167845.49	1494129.57	1258792.66	803544.377	1868502.35	258203.607	1127357.18	1326861.04	249250.037
570173162	477921992	57868219.2	251186535	217348834	247694127	608694801	228219039	236069016	457670217	26389253.3	192118766	313012540	140938660	442661462	799900163	355095145	209893386	169868085	208629322	250051570	196769216	311384346	210630846	165002803	194620072	215712671	143011035	51739632.4	24001340.8	38386949.9	333201835	275712118	260394501	132726966	253246221	175518582
2667956.07	1923679.53	906600.843	1386464.25	1222617.54	989785.939	7728111.05	5722501.24	2738482.97	2511462.34	2898060.61	710162.237	6432944.78	1533901.58	8752841.65	3065957.66	5476324.39	2258172.9	1906464.59	6734548.64	4358961.73	3012826.35	2145476.51	3446173.74	229281.204	1884024.74	3191049.98	4335776.58	4296248.9	4961504.23	3624126.44	3495510.68	4115868.64	3779899.03	163377.911	2821089.83	832757.363
28961631.6	47267390	26247743.7	47112215.6	20837353	20203131.9	16122225.3	26965775.2	25094648.6	16001830.9	27605689	37473750.5	40569645	15546795.3	37117359.1	30356792.4	39203254.7	26359382.6	17503542.7	16495195.2	33793521.2	24219117.1	23345145.7	22565080.5	27466520.3	16043229	22742381.1	28694574.9	26786101	37686026.8	27088159.8	26282458.1	34075831.1	19905633.8	24378111.7	27455153.9	31089961.3
912808670	47897319.3	5559625853	657841441	1069526505	1440772898	358683216	951071810	3394600590	1222671339	45881931.6	1452969863	1328386256	677648649	3020494344	601244673	547844442	1542906415	2631821849	205098149	1527476207	822031845	277848884	718618328	1557628259	614753701	513843300	1945452123	1678627458	30531932.2	55229245.8	733167140	804682469	417870338	1221181078	688873667	1135529905
36781909	107696137	37621167.5	21817526.2	20361423.2	28434650	54894789.7	139164749	77688140.1	103368091	43758888.2	26763625.7	211949160	59463019.5	133472780	32204935.4	80331824.7	23397608.9	29638066.3	92022801.2	80138306.1	65084394.5	54543645.7	72567307.3	46519615.4	33471294.8	124252790	82324590.2	36933803.4	59888652.1	56877710	62093923.2	59316394.6	81378722.5	36966270.3	43185941.6	25488206
616471.721	988211.181	603679.755	595992.289	1668979.85	1145107.78	1054062.35	635026.004	1442202.69	463740.286	453008.9	664273.216	951901.703	940716.9	956386.4	655853.056	1106476.54	927654.397	930972.34	987146.206	774811.978	354569.206	663460.206	234518.646	614952.125	210094.112	600565.89	769246.607	310647.182	697663.046	265866.34	853385.506	614750.52	607840.489	509605.003	515708.274	564898.775
857252924	1133771970	1130677561	604506406	984160925	548584833	552131338	719087360	962191215	583979463	631785566	517497421	986480916	469754206		1028202681	663509876	598326901	938229140	514145629	813739772	662197264	662444054	539290535	919089240	621628778	770069741	1173088125	1366533816	1117459057	656225965	580952181	1222805469	446049450	413080832	567494344	583456599
4658282.11	9626076.66	25324814.7	626806.41	3205665.16	2251824.61	1507670.64	4825337.49	1531043.22	1395398.28	3784975.34	2568615.99	7319005.59	2133075.39	6139893.57	1308067.82	1149120.27	1256228.2	853595.792	5316654.85	1562305.31	5401529.53	2424589.7	3796933.49	17419571.7	190932.638	4276439.61	356827.904	1543487.43	3142121.39	2219023	17128659.5	4651066	1076233.28	3445344.67	3876442.23	694333.706
89376463.4	90935945.7	18090295.7	70579966.8	35671134	84431530.3	74372718.7	40211767.8	46923193.9	26971717.8	11897577.8	37038326.5	61599481.3	29676441.3	90172248.6	124064949	64228136.1	38445701.1	38103107.5	87499803.1	21787718.3	23711564.3	41097603.2	28245656.9	59555949.5	20069667.8	25984346.7	30337547.5	38020175.2	17234679.3	16657729.1	50718258.2	67521413.2	28303073.8	46308938.5	39121999.1	48195677.6
36118769.3	29050757.8	44560096.2	45358165.3	22209489	25542988.6	2783973.48	12660058.9	25790162.3	11429121.6	5983437.62	33862193.3	20725698.3	5868291.79	35125796.3	34786745.3	17892591.3	42546666.5	31393027	13854756	17005789.3	12749339.9	5023954.3	10362935.9	2982143.84	12486201.4	9158287.22	40334181.3	30258601.9	4675219.37	6123895.45	9713101.47	24635552.3	7537696	2329170.24	14684518.9	32709184.5
139438689	154148775	160279731	97232753.8	96296986.6	250300275	136859975	117985094	108668772	102023596	114639510	95714071.5	203916140	84086305.5	191631002	169471869	236598102	199279842	115719375	100428174	211504249	144374369	140675633	84890843.7	270659877	61840230.2	123295279	215065932	95800791.7	177888106	116808788	431204766	117813883	76704643.5	143669067	77189114.4	98560321.8
2199401588	1694460857	1862194910	1708591679	2349266451	642526764	1490694302	1512361252	3086644950	1190852733	1619098787	1002921235	2188978126	2506809671		1969792585	4526507023	1555701835	2037763346	1151699860	2242893015	1616529111	1364461457	1604254473	2285062117	1717373462	996329608	1428685176	2257778597	2500087927	1731287730	2582869009	3134966157	1489954229	1736542091	1933235755	926547004
26523247.4	27653843.3	14614123.4	23694752.3	15793233	6581281.87	17603090.1	16024842.1	27189481.3	17398286.7	12443914.8	9956699.59	28645499.3	20052714	32858453	35559935.6	73893634.1	11371769.1	24368075.7	10636098.1	60227234.1	20380666.7	13663931.3	25718502.2	34550533.7	10889463.3	14173914.5	17414738.9	24982721.1	19613920.5	11037683.8	39748812.2	48162102.9	11377458	25265441.3	43403476.2	4462146.43
193008957	268969458	254363663	134492338	230037013	85404591.6	101756096	179599789	228264639	171949787	124703266	167636047	222795399	183863056	242400232	222130332	284191790	172746308	159724600	99522582.8	264632784	201499908	151136731	208585695	185917340	131540353	115469285	153269320	167340254	133414963	117664944	162882142	151577156	121997629	118868491	108418566	154711565
70280.8883	63477.7339	93744.9069	96993.5231	96587.1054	138960.749	13161.9199	36658.1674	65116.2246	62356.2282	21631.7375	72453.734	24730.0326	31999.0096	32857.593	75092.1154	108818.905	88871.0014	41397.4639	18638.0322	54244.6008	24619.1768	24761.8978	42685.3537	14211.3814	39703.3774	23255.615	43752.2135	17241.2358	4716.21247	23772.5846	10452.3931	19104.5794	22188.6576	12310.7005	21747.4894	33779.2127
438309680	492357863	265007416	527320376	546356092	124336928	282660156	862368782	593122635	493367868	886931818	398335972	1036891096	460522043	896911641	334562815	394593407	590925848	455992199	312832224	1003648337	759312239	409683324	664614602	778154752	311037450	542280137	204693412	554457013	897502992	699557505	620989250	555461890	223274199	554014759	351135596	355373938
84148808.1	175082500	55741709.7	41004546.3	115856069	55852547.2	128286687	141251170	159977784	122300406	30041306.8	100407340	207635480	187761956	276761798	92599489.3	158347784	137886612	107142413	114639010	265526117	176825292	200989992	135714987	122942879	105000961	138817212	52680035.5	44555852.6	36086271.9	44651861.3	160085233	136198336	169030229	89676099.1	130519535	99265190.9
124951168	154275882	94285932.1	92972800.7	123093281	49105965.7	116185623	167222552	110703649	190612620	59866739	72730158.2	309037234	127852036	478076329	122598327	125426050	132502445	88616109.4	122467033	283197510	169586622	135941086	112031515	74599959.9	72871075.1	211842582	124234384	147572646	77555653.3	66375507.8	383985396	149658200	145510809	57818844.3	120423478	59940177.5
775967441	1670448862	365436976	567682652	381872369	308512322	1169895518	1716742249	843128362	1554092915	580388227	346216907	2229113098	778554685	945701602	641571175	750063783	334263521	170817369	577352434	1091602240	853714488	932098928	661291967	1647598258	351373642	800798526	79360456.9	170102904	518866674	526919505	791313718	502402242	642455744	959769531	299769009	185895073
8099641	26444616	116934406	1262334.96	28062748.5	14140713.2	8493475.54	34562721.7	8164330.17	5445999.47	17128313.9	11000528.1	46706244.9	8199797.59	23173763.9	5117157.49	3870526.98	5439850.69	5877959.68	18649398.4	8031769.05	8963029.74	5369107.76	7324109.47	54297064.4	2078752.15	32695082.9	2829225.42	10773183	6803939.67	8822428.85	63206306.3	30061043.8	2553937.92	21559315.5	20111315.3	6683352.83
168810033	365968736	734013076	263951496	800535472	524332138	220521041	299061930	234791931	403748668	744900464	702067243	455529136	315404257	785408223	207315647	624524160	458704761	579326405	464127492	1271321335	1196170240	537525734	1034389192	1303096676	266071934	255152393	700998677	563450844	1224246908	798415912	430915800	562102157	208147766	872708337	358123425	683547739
13237834.3	25333947.1	14540152	14784672.7	9569166.84	4198289.39	3679349.6	8483700.52	10574491.5	7691744.44	5618854.85	14627495.1	15645856	7411123.25	14749704.5	12668541.7	14244978	7638129.94	8523212.5	3335656.86	17851092.1	8134347.57	7017551.51	8490641.18	8211591.04	2737321.37	5570353.51	6458023.06	12706766.7	4969418.72	3659035.27	9180293.22	11823905.7	3888382.3	6165815.78	9240715.5	6030689.61
175125056	283905249	220800849	178352260	185706214	68410146.2	66912910.7	143182304	163697541	108864738	91974001.8	177713813	208298034	155335992	316319692	164913148	232400126	153293085	131397668	107745487	216583977	170376060	200736929	119333333	138725771	81376387.2	99525787	81077514.9	182516111	150293776	88909427.5	166161208	188032386	119809112	68940976.1	106000640	162223160
200300606	324980432	250379013	249658245	266748498	66018010	66942785.3	206089281	221616366	138966084	110287636	248428889	301213786	214373611	305440697	213364256	356803420	250410698	220998047	132640104	274143721	208409062	241362107	176473827	166928112	121124815	131013582	104689305	261062448	153782178	120117131	179113861	238746842	161896206	106575898	144856092	199048650
2267979782	1582533053	1713908160	1471121054	1442698572	1634549168	640783585	1062672933	1743883830	958097393	400467032	1318677424	1050395186	375361337	1767168064	2480806247	1971545359	1071114283	1162385296	502087056	1072509557	828943972	687224267	982783185	611580831	645648920	496742287	776908777	606005833	390516945	350820295	474043645	971183351	709047191	495830023	888150805	1582353233
602451077	860372114	451110286	305514424	527686110	222972294	513667511	591542039	379276633	589323350	598348561	169917241	844457775	609687515	2624574967	550500061	639336087	262415837	209861905	497754108	741156900	564738780	771726161	334287124	784154595	242912634	512609720	228485541	316290936	893046661	524862927	796443827	614088839	394485888	622212329	493932430	93515597.7
2088966.11	75489491.9	64956456.4	79319.8964	449182120	61634106.7	23574046.7	85648637.3	533876651	4922857.06	4644359.09	30118167.3	110203054	255629090	11163936.2	2390350.51	196413962	15236977.8	13468246.9	135157199	309420643	240329647	578651177	221666102	3796342.98	39352383.9	85130314.3	242236.303	234713397	6599210.01	4943166.19	366435863	71401181.4	79804847.1	1731278.45	52360889.8	37673925.7
3076655058	3885914836	3528173027	1420653564	1708317955	1106392821	2347957778	4829209631	2025879130	3156075890	2813444757	1423861731	6630580451	2208287441	8768168673	2597900441	2195255192	1524482125	1163918215	2495901506	4032319905	3382993028	2613032850	3655274050	3802906776	1767056385	3545842116	1292387845	1772656356	3436688207	2543616695	3704064180	4430941030	2194794007	2624843804	3172062112	1509562526
	59795.95	12499.3514	18645.3789	283245.674	88139.1943	99909.7821	386391.758	239893.29			313636.032	540593.745	19488.2026	97531.6463		4527.06235	92064.4321	57132.8608	341604.994	54213.5802	64083.2952	24561.0532	52968.1649	27651.6546	8960.6173	233034.504	67755.6579	537502.086	13674.8948	5860.52039	97127.723	19105.8023	16079.0098	4216.08891	12569.3725	86753.6832
457252050	441458544	336159966	257771102	488358138	163956743	279447265	380942758	647215325	417559833	99811739.5	312726179	480840964	361710070	623318946	573380102	822662116	269826918	417200374	263307982	706860785	615068052	302981050	556432696	357317279	196693430	316170156	221267719	315760644	100152531	121943266	374880992	313787864	264340766	252696809	227671216	340212979
1940811.55	1867625.84	2834633.54	2287485.72	4190866.59	3531531.08	4861254.56	5331923.89	5011984.08	3835454.88	1705003.6	1182022.4	7405269.86	3907141.74	4508875.27	3497576.81	6247700.42	2248088.28	2708434.59	4092541.75	9325667.21	5413268.41	5317353.34	4421528.04	5216043.14	2737472.18	5254407.82	2081122.79	2956218.92	2842988.72	2204527.69	4929474.37	3085189.62	2934659.46	3640736.28	1919996.58	2545116.76
716183.9	1186684.95	333818.693	659386.274	1227144.01	352393.727	1005465.89	1372383.81	730638.672	663793.363	924551.336	438212.337	1818494.65	362359.932	773836.89	700997.607	1444535.8	1294687.73	415135.455	996335.109	2077717.69	1436372.76	944501.796	1265628.37	1312619.17	964220.55	974242.227	842659.381	617830.537	1058883.13	1066507.39	1662125.55	945105.28	1654805.91	873978.313	1024880.13	519420.384
204928.159	255783.798	748325.344	127736.711	159498.02	729169.112	137212.47	284039.628	94790.5429	452489.331	1267549.4	213516.188	346031.98	606845.763	587945.962	245048.473	554523.542	1365626.9	403726.985	2231035.3	2722120.38	1940468.16	813016.723	1882094.29	1240301.58	280918.027	245373.472	3609621.27	95321.1182	1642893.84	1800391.87	263486.969	506229.922	211998.234	960319.335	365624.691	250875.36
15939588.8	14862555.5	38291491.8	10097282.5	15446272.5	6679084.72	12190760.7	16879962.8	17512698.6	16898248.4	25796065.5	13697029.2	18459770.3	21441413.9	18727181.7	14431916.3	48101091	7405296.59	9960165.91	11002593.8	20682607.4	16871808.1	13478583.6	17592466.8	16610976.5	10071327.8	10804395.1	15162872.6	12589097.9	22258658.7	26039737.8	12634823.4	15482205.1	8058538.7	12592039.1	10639081.5	11284648.6
23186.937	68766.7055	90184.7253	37002.7956	150457.727	34778.2692	40690892.9	95224.5678	39001.4359	19559.4667	330071.84	204344.269	70646.7873	38253.5837	2268.50298	34584.6862	31198.5765	58302.3009	224429.629	233811.729	57909.8412	47416.3559	48928.385	32590.3086	44360.8711	12526.3125	35791.8256	35517.0542	154360.952	108637.356	333645.901	36764.5423	19266.9631	13884.4648	27541.5137	17064.0278	42006.6839
309673.315	456596.629	264580.275	263348.661	325501.908	59784.8079	125002.117	486435.996	287441.823	381558.437	164639.15	262720.212	689753.53	536191.091	1179251.2	285152.167	662417.015	333128.482	84623.6403	425935.857	1014210.09	653368.928	878969.97	392986.962	403060.776	144642.748	276667.492	23774.7953	185306.93	176480.784	152076.735	719778.34	267060.786	247178.035	328214.387	187388.684	210026.453
6795755.62	5655780.07	6471097.19	2980329.65	3455886.68	2488509.66	2006220.9	4524240.5	7589771.89	6361509.38	7527484.11	3243509.96	8420333.46	6643382.91	10679520.5	6720934.03	20590946.6	3071877.36	3292058.92	12767228.7	13046506.6	6647677.43	6659774.23	4209771.74	4463224.82	2418053.76	4397198.43	3277019.15	4639558.68	8071988.27	7121909.5	6817652.49	4527871.23	3393814.5	3567020.7	4211919.79	2882091.91
833681.467	613827.001	2302213.55	930103.875	615401.329	1136714.31	188662.806	479339.856	533485.344	316465.813	955784.277	1029673.53	623767.414	358076.835	612192.305	727820.011	680068.14	867626.894	932190.403	639021.868	569178.456	538594.887	369557.812	391703.112	636444.145	770971.769	425339.588	1517353.4	2242984.69	1059455.97	832241.643	396212.091	1784305.73	416162.957	425718.025	1120143.27	1084787.69
		4536.31721	157883.812	101976.62	44890.0966	71810.0105	188742.979	98750.0097	179734.812	1387482.65	62581.0748	233555.226	166283.279	624067.685	173310.742	440439.002	109874.156	59994.3147	245360.382	269173.098	216175.453	235364.487	196388.25	872574.909	149300.024	126959.971	102090.163	105503.581	1646928.24	1374874.9	308509.649	324680.153	149182.999	724681.393	205474.76	63717.0143
10807267.7	8495080.31	10378163.9	8772565.88	6457862.74	5185076.56	3456725.62	6386219.02	9355540.94	6194271.3	4671261.97	5362765.97	10745125.8	5512744.72	10754710.3	9655186.23	8454914.81	5214908.12	4948695.21	3755515.33	12600630.5	7989156.11	5229592.78	5473356.29	7220784.01	4498584.34	5655452.45	6612656.99	7706696.92	7063355.09	4887284.22	6725515.85	11000524.8	4404720.75	6705654.45	10339495.5	5086651.43
6090.75976	34385.0286	137663.093	14919.8479	210495.118	26142.4906	37235148.1	72052.3297	46532.3888	6020.84999	409447.51	214958.441	108311.197	47364.4382	23252.4196	5101.76299	57188.5544	87827.0453	407875.236	447003.277	96023.144	49403.7315	16567.4002	22746.8425	125059.107	11778.1071	57318.0818	3264.77314	109097.407	785137.844	752502.979	33571.5546	14050.9482	5229.5364	75235.4967	36413.9669	43504.8745
17730.8622	82554.7658	448010.593	31826.4841	444078.834	31870.0578	46110115.4	268287.116	131933.504	29370.8911	1096291.25	425276.116	339792.632	115433.767		11143.5046	163068.271	238751.792	718745.237	1445186.34	362426.993	202615.434	65883.3371	68210.2881	375834.393	18335.1663	193915.206		370020.494	2001937.08	2170361.07	136870.313	44052.9681	30022.9797	263096.835	122835.994	124430.66
82172955.4	80973993.5	173252765	66802970.6	35556938.9	13824654.7	105617400	132869135	136552686	99030125.5	44882706.8	158453204	186397518	100511451	203274963	73096891.4	405504446	14385772.2	33274124.9	74932422.5	201253778	136130581	161217741	159796072	93040339.4	39162860.7	80756212.5	15089971.3	9779739.94	70041773.6	42301368.6	102036680	58666814.9	52107295.4	59186305.4	47403811.6	121260634
27602834.4	24371850.2	40342446.2	24897039.7	31052598	8893115.17	9135567.54	19732618.8	35447720.4	20647901.1	20781119.5	15785327.9	21638127.8	22932742.6	78737372.1	20717673.4	35487663.6	15109696.8	21755481.3	9559646.38	22635233.5	15323264.1	10069563.2	10351768.6	11294666.4	9127708.34	8053373.81	10874581.7	17062385.3	15460539	15027927.2	20027505.3	19225435.9	11588501.3	17172843.6	14916782.7	11040243
357929.9	2933848.6	183644.752	111633.247	16208301.1	7955858.14	1694693.9	5970750.1	29102155.3	155425.5	1119144.81	2233649.44	6232579.04	20632917.3	263727.485	594349.164	11350509.4	1458973.65	1223612.89	9624388.31	23815861.2	13113269.3	32290403.5	13374187.8	91786.2478	2624247.99	3658886.83	1013202.66	2958719.86	1936084.13	838113.635	13064319	5114545.95	6442423.96	49808.361	3597066.01	1092504.72
410936.468	498971.392		33922.9536	51772.128	101501.632	983050.984	82464.5763	131759.71	173708.61	14497.825	213871.379	159643.39	215544.499	436496.634	507687.331	1048035.77	205208.011	264185.736	334473.49	450282.269	372289.347	531475.465	383071.978	157444.471	276673.34	165819.21	40854.0716	8493.95292	16207.7239	5332.77923	170339.091	327538.885	408312.76	126783.282	303111.6	327341.807
63349.7368	106288.247	650757.897	831038.02	1261037.48	148588.79	7934229.19	992778.038	1406241.43	129046.867	2669658.96	576552.598	1108931.06	155221.864	123889.245	331705.962	1806707.12	595056.288	1697928.66	2339179.08	7555621.88	8039663.19	90177.7695	6628034.79	5179756	288582.267	1617078.57	337759.21	100043.337	4004340.86	2053879.46	1151387.48	404885.082	2796847.39	3350162.31	216822.612	1163126.55
11810238	12466424.1	16691992.4	7394633.96	10028330.3	15514437.4	5424745.68	10100542	11304898.2	5156441.25	10294929.7	18139322	13777397.1	5270007.16	11260955.9	10504055.1	18548822.6	16748222.6	8896648.92	15279675.6	12451170.8	9809676.05	10463874.7	7497560.81	12542836.6	8879678.67	10915314.6	11123150.5	13301743.9	15050112	12628270.5	6658044.15	11425685.6	8472337.29	9138283.89	5802146.32	17120021.9
6598048.43	4625276.96	14062358.8	4707727.71	7426167.71	8193170.99	3080554.86	5374225.19	4455751.69	6559111.01	3624035.82	6977829.25	7003536.82	5861497.33	5799551.32	6896075.25	7238109.3	4073789.91	3880697.88	3974949.93	16414032.7	9311392.19	4322039.7	8587742.03	2861025.93	4873039.25	3201155.38	6001833.79	6536818.67	1855102	3953252.3	4378081.31	4152998.52	3147176.38	2022902.79	3506078.65	4174445.47
318517.805	760532.133	102593.743	75597.3787	557125.587	304920.519	984260.634	575090.752	428307.814	566257.969	3886.10115	259057.42	899357.57	573833.232	498285.354	525586.999	892109.161	126777.862	193215.018	2180409.95	1084903.72	581564.086	608238.253	619497.217	456495.538	213472.409	521710.407	61049.0482	60112.1272	1233.74339	7552.85891	742481.623	284937.793	424352.492	332765.732	199165.327	351382.369
95002693.5	36938001.9	54594833.1	44088461.8	81135150.1	30836917.2	47207125.8	49500779	72839458.2	58479039.4	23295773.3	42942106.5	77997261	52249449.1	93957413.8	107685332	45792386	23550113.3	41855082.2	61892858	125975232	103151886	29064891.6	98828309.1	46051777.4	89363277.1	47650272.8	54286040	22872631	34212538.7	27685655.3	82914148.7	138330399	52222908.2	41409122.6	100810920	35218805.4
58072803.1	9158558.73	46375000.3	80435483.2	117529818	97577098.2	4039869.2	99424078.6	55450417.5	2715763.76	51259293.8	62984946.6	126175897	1131371.27	4763814.32	53463586.1	5428212.84	63089630	35569361.5	19912716.1	1951897.86	1516985.87	1764866.71	1179629.45	2627478.53	35846306.9	56097851.5	52861303	56360004	67693604	48779875.9	2212276.18	66306598	7744612.63	2310469.51	47602574.8	41392904
52130290.9	177829024	151740076	41115545.8	112786108	32953068.8	52628109.8	182256749	350532581	143598702	122237722	122002413	211427041			48686330.1	145014007	169411158	275810894	152510867	199544765	152586364	179934285	145651189	151399804	238510159	139574455	134447550	176768832	154085295	125334676	48353162.5	324661128	213946938	104759828	199964887	138524737
110775.078	119576.104	141352.903	160789.525	149150.794	491674.696	136179.666	57604.9796	139916.187	15160.9166	260426.327	765000.627	103384.907	133428.936	21831.2956	179680.372	1281929.29	1991345.62	429669.454	895870.251	172811.119	144326.443	91912.5808	106643.556	143479.969	110591.22	89801.1672	275936.215	224298.982	18484.749	317792.779	36827.2551	132029.864	115162.666	97384.0859	72919.9136	1075055.05
251604.709	223636.674	414872.916	159918.275	102437.514	130467.941	42699.5216	106504.627	152929.239	189594.934	284329.65	273617.928	159359.778	216637.452	320386.362	189435.709	610969.708	730682.792	203261.668	150128.83	584695.81	378308.322	216217.438	283153.568	214429.345	134901.503	88752.5017	159861.915	85922.4481	203620.825	313343.191	193007.298	187277.398	133715.783	151418.972	146737.852	255629.617
1182160.15	2385300.15	386573.75	667191.328	748060.336	718526.256	684266.371	816191.008	598021.231	325512.899	415059.064	521049.845	1178858.26	504142.555	391236.175	1258863.47	1567888.13	503484.136	526411.845	1204727.64	1480516.59	898559.895	1356279.84	902267.065	929043.109	753889.621	572700.825	268438.639	352714.062	431399.95	514150.113	509898.077	1467881.43	974819.322	713838.215	761574.033	368979.345
1540844.75	1203832.09	4289854.98	1910551.48	599830.468	972912.486	259780.446	633274.565	474766.16	413470.926	1695172.55	1331126.17	832560.251	446629.008	1381019.32	2081230.95	1998109.63	1601929.35	1254558.18	1176845.3	891272.871	752903.086	1006588.27	570364.567	1665876.83	1001992.57	707658.707	1425259	1383050.44	4031801.12	2189755.22	873662.751	2816458.31	887621.721	1474630.67	1687541.84	1754202.51
336860905	446709691	498668307	229420471	332817054	157277776	227829873	427893351	298923103	382085094	396292108	146898698	582653123	431941642	897880035	400776120	660703622	151076554	213608061	526841321	573369684	478466019	586973516	403654600	602756318	195132702	397342390	233081508	342283690	791285420	360957898	624774705	309908389	301162963	377898188	219961074	158048978
52353.8415	1804459.95	7569078.66	72912.2075	212646.959	2037372.87	22940015.3	119957.979	2088245.21	5191265.51	484448.681	3272196.95	130521.061	1301805.47	10840686.7	45839.1675	29925179.1	1859427.81	6909571.61	3677107.51	40334841.1	34409024.8	2576696.58	23693816.1	642227.139	422724.887	145332.962	15799212	5463799.71	482489.831	568425.532	17424141.9	536052.438	9453577.42	482535.383	508266.321	3074524.89
51490588.5	56554996.6	24391874.9	23489118.3	60088097.3	16461906.1	29083870.7	39657284.8	65728755.6	42672574.4	6812374.19	28669999.3	56110224.7	37538771.2	38306191.7	56514037.1	102280543	27842236.9	42124932.2	22547990	57927633.2	46999503.7	32076139.8	44354846.1	26289717.5	19383935.7	36396053.7	15428498.1	19812727.7	5133819.53	10391246.5	26354525.5	29330864.9	27416649.4	21979297.5	20300478.8	31300872.2
218228.841	218277.299	191647.593	216121.754	135203.287	159166.657	152840.862	71478.8283	139333.413	101381.63	346433.339	350725.516	170100.509	310581.468	215934.414	258995.951	1506601.7	497111.087	274399.001	599699.465	521573.709	359532.394	403538.964	268114.577	292617.145	140289.968	107388.697	190293.36	261502.982	481380.252	567508.505	141542.941	266743.918	286872.077	177445.353	234947.549	348462.823
455314.41	795595.95	1262346.35	210442.835	878992.169	308202.717	338324.401	793528.686	675193.473	454804.804	618579.418	293240.949	845277.086	680781.425	582929.282	514882.426	589080.882	363488.804	555251.644	1711882.43	815886.002	719050.896	480363.502	640466.037	679025.868	264588.84	479049.104	288859.739	844292.938	630326.379	576882.306	433640.679	453021.759	251288.374	475832.882	341731.094	179832.79
572424.584	467406.851	618533.767	532154.83	488867.766	299300.503	136195.656	487048.611	437207.019	240411.666	211945.494	495666.499	617374.393	508610.244	909486.103	760602.068	605992.159	1169271.13	478433.541	577801.152	751460.66	576611.343	587485.916	412728.285	358223.626	321300.299	481226.384	101211.75	412113.561	446979.33	284959.172	488812.316	558740.253	303902.34	306719.223	328343.191	508424.978
364361802	357156869	223854610	188780394	417550384	95619388.6	238482558	272192462	264870240	199837726	210532855	356511410	394452092	181612819	152476282	437088721	294438378	85869945.1	234131364	173178968	135146943	129887753	209096657	131958709	215570999	155825710	259227892	251406215	194044411	237711405	178622211	131910705	184942721	198367918	157809166	161426632	341936375
2586667.36	4343541.58	4683549.25	2052610.71	2223498.64	1684205.11	2038949.17	1911161.27	3161945.98	1652153.39	2012932.11	3345881.7	3065681.03	2943468.5	5242459.28	2916538.01	40633734.8	6116311.81	5510690.41	8006644.15	5319103.66	3598798.93	3654424.37	3012959.05	3549285.5	1489387.57	1373187.05	951339.843	2873614.46	3888270.97	1911890.28	3077109.99	3466388.71	1798517.06	2700671.25	2556780.94	2585630.39
15939764.9	19507319.6	15459342.2	13563518.8	8356892.41	8008311.06	6108993.14	10642052.3	10019094.3	10618013.8	20091388.4	7070680.25	13727251.3	10232714.8	25923933.4	11363717.8	22959935.2	15246073	7023966.88	9456674.63	17580410.5	13140905.8	18134079.4	10385252	18485527.5	6789588.73	10021347	9518104.03	9087855.44	28538021.3	19803145.7	18241727.9	13158223.6	13621046.4	13572508.9	8034284.06	6147326.34
1177348.68	1157237.34	1553818.37	691414.247	1005491.09	520557.091	686203.721	1399915.41	2888967.06	1594479.71	3473515.31	1676641.21	1446496.95	1200055.89	2318924.87	1136676	1346776.41	2057669.78	879361.198	1211595.87	2489069.88	1528843.92	981099.583	852432.825	1338791.32	1015149.04	606717.597	399784.961	406368.577	6283148.29	2434317.07	499092.405	1958946.09	319891.932	906625.953	1631675.02	1812397.98
724088.756	3967983.26	2404816.16	655981.253	4398432.77	812287.399	1848958.26	7755762.24	2045251.13	277704.557	557036.417	2721774.94	7695759.15	2171043.39		851640.239	1979071.29	65922.795	5871709.87	5613502.95	5012037.02	3729668.55	2179058.16	2882169.73	833723.436	615154.211	2900480.31	110755.006	4485477.39	375101.997	598995.599	2661760.44	713320.369	258645.548	794927.982	697968.204	2572340
63677.6442	96358.2717	160518.746	42196.346	79283.0846	88052.3124	125784.969	128110.941	95367.3175	228439.237	79184.9202	127392.491	100422.368	62748.4358	69656.6691	64510.1071	558099.674	201515.269	178581.98	1273118.13	103736.115	37237.7676	155371.671	120461.069	118312.4	106176.903	116143.179	54178.6248	37546.0712	261457.402	297821.938	54585.8456	64789.9012	96385.906	70642.8204	85138.3546	47690.9844
500747.608	23442.0814	128804.187	442263.338	345038.602	1301444.03	951059.292	286712.644	302100.841	26041.6679	707676.385	672185.124	379462.937	17700.1572	1132878.45	576901.52	479643.185	548197.091	888750.116	586410.616	73097.0044	46955.9962	37083.0723	601349.704	261080.011	319332.137	508382.463	2298185.05	307589.938	979352.559	809589.969	326933.405	1051187.46	69928.2112	442044.308	764949.857	705352.85
73209.2883	50337.977	57395.7816	51283.1021	25760.5198	37799.2597	91988.9973	1991.22368	14875.7206	141062.591	27590.7389	30471.9029	28304.7476	78799.0358	383397.498	113497.993	1382894.96	66105.5244	77749.0172	162590.043	81335.7242	79882.5656	171174.714	69527.291	83151.4062		14410.0033	56929.2249	17767.6483	68458.483	40462.1792	46781.9621	11776.9592	30973.003	45848.2682		30788.6932
128727.791	180613.849	6400.5225	26549.9971	46078.1081	411417.945	1271319.25	210189.242	98925.6696	241795.971	50793.1343	503613.44	195127.752	280727.602	167740.012	373901.481	1280934	177412.197	390112.64	1069932.8	217868.232	122809.656	162619.476	267367.482	165141.527	234065.339	190679.323	110001.207	69661.9606	51679.2865	38793.8053	76312.6528	219521.457	168077.704	99295.3834	103905.71	383779.846
16621166.9	13239416.6	30355554.4	8014644.78	12731178.5	17812956.2	12712038.6	8024892.92	14805867.2	13871578.3	17434866.6	5969504.99	11978658.2	7250781.37	9004031.68	12474623.3	24002799.9	9938230.76	9365414.35	11699917	30945861.2	19508890.6	12303791.3	10854414.9	16961740.2	5078568.77	11780309.7	14732966.1	14035091.3	6730700.11	20732843.3	20384167	7683280.45	6813962.62	11411400.1	5748068.89	6423343.83
	134096.924	192088.466	45129.1587	2333729.46	29003.3242	103969.151	173811.4	1872014.88		457176.034	97474.6996	195561.102	674830.102		17370.4174	545695.551	183624.44	66345.3302	120899.555	464778.009	387825.054	513639.482	275466.556		17291.1438	26246.4473	16357.302	483187.984	71574.6313	83162.9127	827951.46		146507.394	79856.589	68038.8073	109900.805
4700683.82	5642527.97	8483160.58	4678065.03	2863507.34	1609096.32	600110.346	1070928.54	3703191.09	1251644.27	4944413.82	359545.563	1515233.36	2396498.71	1252590.63	2971688.14	2867264.39	2472174.89	2053659.57	1024944.07	3806811.76	2404317.3	2176937.98	1622195.97	2317313.69	685602.871	789029.318	2404399.36	2340775.93	3638636.81	4197177.61	849885.86	974683.489	1181791.39	1777174.63	753352.289	278820.738
358278.307	146948.504	1134432.68	673099.284	209339.081	603334.318	137577.192	198828.888	403067.825	120133.311	673665.577	900152.424	251910.418	162926.559	165166.724	264971.995	283867.308	1175155.04	484515.062	395184.47	348666.282	279415.569	180644.518	233577.872	280551.484	175929.592	124233.739	305021.299	222882.678	603895.832	553869.193	135209.521	330026.505	159142.803	175022.898	172452.918	652443.372
63542.7582	133739.335	143915.449	71459.9842	72414.7266	44752.2246	61193.0427	122578.004	73219.2321	29173.7901	206026.79	82388.8566	190272.944	68817.6512	19354.5511	75135.1454	138158.822	132071.111	92481.1861	180478.094	92856.4318	69691.6667	64575.7133	60819.6121	174021.852	97607.098	131429.398	41525.7383	101600.203	283418.034	205721.203	88900.9717	181883.036	78692.9012	163011.085	122152.454	79719.3178
23442376.7	23149509.5	81409411.3	45159112.5	96647150.9	50055107.8	56863046.9	26701725.5	73037453.4	53587450.8	25998335.2	45381074.4	30349406.7	54741534.9	100495491	20512290.2	66467152.9	76919698	59109807.8	15923754.6	47827194.1	32973502	21885304.9	41055407.4	23709194.3	32156144.6	36359329.9	48436555.1	43151501.9	33763468.5	29172186.9	59947839.9	22938382	18013065.1	22048446.5	19969954	45526336.4
2996000.68	1978508.4	1130696.91	3402501.7	1311320.66	481084.553	524055.869	1007941.04	1897309.31	1245431.21	1505287.12	1074113.92	1321714.16	1092104.28	2525379.38	3065172.5	2322575.62	1578933.71	1364809.91	868154.76	2217088.21	1641745.04	1418039.19	1293863.28	1613837.04	1626428.45	847614.952	735348.874	1355798.58	1993986.12	1484610.63	988367.245	2744738.16	1901085.9	1123731.79	1816708.82	1178657.18
822905.155	608602.14	607327.961	401603.128	626062.359	414413.724	218574.239	441968.797	1005078.35	440739.202	166078.635	744166.837	496083.298	395588.306	430790.862	729552.239	1978422.21	931363.413	153188.731	336369.546	958195.883	684884.016	796959.824	554169.521	289667.824	283223.744	237574.799	186798.888	442964.986	200645.415	111691.004	626935.529	533951.923	366295.258	198048.463	382562.16	603182.334
5500.20224	140236.318	9921.68727	5332.24301	424176.367	126792.922	5167.01941	81215.9595	602768.297	4546.06797	17930.0958	84628.6997	93612.8349	75677.8543	5123.37753	3661.54073	228436.526	5018.11906	32275.6336	169643.163	204845.491	262613.664	220591.888	260083.009	23736.7966	36679.0735	10605.0853	1219.57091	387794.54	26160.1662	16988.3398	138392.934	156809.497	92840.4208	7870.19208	158779.739	13998.7689
196938.454	136210.09	206972.881	278405.045	175584.374	109527.707	132910.218	139182.847	166038.711	149554.218	393716.677	248546.334	232935.273	168820.471	555948.462	244555.466	314327.227	316100.958	173645.57	132674.086	222193.336	190253.469	198174.474	182396.657	332445.602	126477.611	135989.283	148629.34	227276.29	598884.187	381329.64	235033.661	229590.165	117565.437	279442.28	171379.058	282109.981

N_B7_R39	N_B7_R40	UR_B1_R1	UR_B1_R2	UR_B1_R3	UR_B1_R4	UR_B1_R5	UR_B1_R6	UR_B1_R7	UR_B1_R8	UR_B1_R9	UR_B1_R10	UR_B2_R1	UR_B2_R2	UR_B2_R3	UR_B2_R4	UR_B2_R5	UR_B2_R6	UR_B2_R7	UR_B2_R8	UR_B2_R9	UR_B2_R10	UR_B3_R1	UR_B3_R2	UR_B3_R3	UR_B3_R4	UR_B3_R5	UR_B3_R6	UR_B3_R7	UR_B3_R8	UR_B3_R9	UR_B3_R10	UR_B4_R1	UR_B4_R2	UR_B4_R3	UR_B4_R4	UR_B4_R5
123700642	158841347	244893123	217455667	158798939	263344335	125692607	8789579.85	66311826.5	7953296.84	140877251	104665286	93268680.3	270060403	127058812	100712070	172844594	104743964	144826550			191888211	110037163	154647557	112570287	105117824	132771795	136975477	198093375	461772177	248986807	211332592	125630372	150242887	48362082.7	54210507.4	119281425
9700536.11	23497049.1	53155313	52044046.8	33438993.5	69963858.1	28374734.7	4406423.63	5501039.55	3575061.62	15325844.2	27218403.9	26019063.5	100332023	37287830	26494702.3	32673937.7	19168595.1	47986682.9	29390.9402	17561.4243	8327747.06	3487178.77	18109231.9	40540078.4	51700123.6	32968893.9	29861147.5	6959458.51	33472593	3832962.45	52562156.8	28762029.1	6353127.81	12140688.8	19187911.5	24357467.5
14144.311	331189.405	26327.0282	63031.5169	7140.80598	28878.9144	14337.3424		101029.801	6903.99557	371082.924		7525.48905	60661.2451	31933.4246	59802.807	6154.40312		97588.9582	90673.2586	159138.301	109659.268	74019.1809	133044.173	18114.7415	66206.5091	9896.75565	32846.1659	12454.3036	256433.91	23591.9048	328353.815	90356.1857	131069.033	8859.69275	74050.4353	49817.5365
15958.9695	755467.679	38115.7978	81193.6321	6857.46054	95604.7693	42456.7491	52854.0868	250591.505	99873.4653	25718.391	44370.5747	14034.9298	357200.362	217314.524	82306.2312	55841.1911	12122.4455	503698.057		18224.3087	37580.6371	79126.1729	204089.328		130496.707	13453.6049	99330.3971	40478.9782	628822.116	77751.4435	937410.145	234709.011	253266.581	8237.60787	72001.2409	148350.008
320431667	277429850	617451036	454329593	303625125	797830530	268100801	20915227.4	83194218.4	28799306.2	272715664	267214086	253403100	417937551	72337826.6	215821697	127217666	217460288	459761271	32439.6887	12329.9265	248291413	15375880.8	149517232	315365131	699360436	243913328	333679069	41755176.5	27221840.6	11967383.2	700238218	165517337	40064769.9	118396800	148100871	183432780
397989692	385392290	2082510633	1692672119	1136041159	1992556684	932932118	12383395	79072242.4	20710214.5	819990470	1373198080	489418014	734015546	273240238	349984464	316013847	141206352	715240349	62262.9631		1671020500	274304750	445335729	822576293	763726242	491056035	673734170	259858479	352888659	97208403.9	865482114	487055115	260421005	364220665	371649355	476123336
14164087.2	56578458.8	75444187.1	12581174.9	12275705	28811788.8	16125506.7	15492157.5	13662648	7622735.44	26937572.4	24575594.7	13316175.5	1971334108	29723664.2	9979126.58	20175608.1	6575491.29	212746.326	3159958854	77643042.8	30790483.8	13585475.1	33918071.8	15081354.4	13465883.1	894354140	23760182.7	36324006.6	51422425.1	1114033132	113100809	34867176.8	50653316.3	338133234	10245972.2	10725304.6
107837.309	15069029.8	1212310.53	1033718.65	446625.734	565181.16	98334.0248	205069.493	912376.196	180269.415	2210874.62	11045270.9	428323.395	1229523682	1180301.57	1599218.16	2147997.95	648846.969		1495144197	520047.279	2201749.64	1167352.3	19843636.2	686096.631	750696.81	861091034	1630105.42	1309262.49	2672784.47	1111588162	1319996.42	1276654.22	878563.17	255857492	301790.453	577362.193
48856.6933	7957790.68	569088.264	483439.239	206018.928	376292.109	148437.954	141205.944	430750.833	197073.163	748369.2	2942514.89	260880.17	881579049	648502.634	1026597.41	1537389.02	541626.654		1078654221	716596.624	2360186.83	633404.659	12527182.4	292776.478	452103.161	701524116	1324741.46	833686.841	1184151.34	746732450	705990.732	633578.659	693979.051	169568298	163004.103	574355.099
224145.931	19899441.2	1288503.36	1429743.2	553972.894	2800928.18	272524.654	1186397.29	1877325.57	903475.333	3503290.85	3213643.77	774370.31	2554954528	1561636.36	2540621.48	3771449.22	1649166.01	2702037.83		1650937.31	5664282.81	2742876.12	43563036.3	1658482.77	1963513.41	2251687944	4596649.2	2403456.44	5764410.77	2669497331	2293201.99	2577729.02	2504048.12	276928109	596129.291	1573712.92
28815.9039	3964907.34	325145.552	254350.835	108668.522	561601.315	67120.0152	375639.942	248550.129	183837.509	993728.863	268727.666	123528.98	604661926	350319.445	492094.147	762426.258	291840.046	550766.709	731707.999	62638.1153	1313544	295186.641	8343285.93	465443.924	518102.63	504351709	1073046.13	413849.94	1066120.55	162507811	738175.452	565743.685	82418.9553	103346359	122951.666	316747.314
4805657.86	2694595.65	15642743	14046030.2	4757894.16	14632761.5	2875909.28	13148.7637	466165.42	118850.309	23514466.5	8820704.66	3330034.43	534115.267	578949.199	1483014.27	2396671.63	1767486.42	1233354.13	1336.75354		5957612.29	771516.615	758663.221	1179050.41	1577401.58	175039.252	4068738.1	2139260.8	1993917.73	73486.3182	2938769.61	2035049.23	1303535.91	243817.139	968710.098	908142.617
21612277.5	16060964.7	87943121.6	87260964.8	38735547.3	151057954	23840753.6	54040.5255	3221471.53	58040.3078	213994126	157237350	15603464.1	4734839.84	6237323.52	16303230.9	14290883.2	9732392.38	17767581.3			38729993.2	8715619.15	5506928.14	8167578.48	12543265.7	2064218.21	29403172.6	14266455.6	14480005.4	941324.151	20432801.6	20621832.2	12276990.2	1896355.53	10059681.8	9071402.52
232597.572	402984.757	387963.504	714406.264	577295.763	1533361	339554.535	21576.1734	352869.08	38061.5507	1751021.66	446565.066							392298.735			532492.867	711358.553	678493.689	567417.269	1553481.08	386969.07	370169.91	350026.542	507258.481	489647.836	844815.269	2007915.24	2003238.5	961221.479	429399.38	867045.655
8161779.59	10330830.6	6090656.82	14954780.4	4899172.34	38780488	10916479.8	922254.495	8145525.35	1228907.42	17704299	13351373.7	4510549.28	9532572.9	5525323.95	10077993.3	7556646.2	8760340.57	4168.58397	18864648.7	19386429	13989910.8	13438752.5	14325212.8	8256471.97	8614416.65	8314627.61	10745447.2	6508526.81	16301289.1	10269071	15635516.8	24827600.6	11447077.1	7334581.43	4647945.56	14233581.8
79594.5871	1432152.69	235327.831	526317.41	55531.3764	614879.413	96904.4319	107670.729	1318268.07	32764.087	775450.127	127342.596	72135.6736	1258445.27	868697.576	622028.901	285921.779	45716.6355	1243051.28	357891.814	564021.644	607812.099	551053.975	219223.218	198677.153	853941.617	28602.9599	288779.881	242496.166	2995639.07		2648994.41	1040588.5	527996.433	23880.0326	54117.3622	48915.408
200858.851	3352764.58	325669.634	645748.296	162866.611	598144.635	117365.094	289454.675	2404102.13	561588.077	1046858.88	186411.494		1467108.61	1345962.83	883622.074	284003.709		2108425.19				1828481.96	887578.357	199395.099	1235192.97	27820.9897	379626.324	470583.462	7783883.92	65004.6838	4389040.34	1245450.13	1659826.18	6963.33723	82992.3747	40295.0697
4326.88518	184442.783	156866.861	78838.7909	51612.9055		97177.9434	59917.7287	275939.527	170157.54	146388.543		142153.659	187272.256	119938.358	34799.3375		133913.623	69584.6299		166944.438	42649.472		106266.192		43034.6344			35325.2649	561455.613		4835356.98	67834.9742	171706.157			
20551.6894	184739.533	110093.829	152465.271	39151.7363	202430.023	43856.7239	787588.906	114557.211	806100.945	242756.32	107030.926	48643.0191	1158679.97	84173.4192	135248.431	53471.0389	60435.5801	305850.154		63495.8966	467331.978	94037.796	230481.498	21179.0675	43492.4103	384797.199	56635.3232		401229.567	182319.026	1149136.67	105008.54	38347.7084	17960.1926	123883.118	180425.685
32113.9466	1005197.65					47121.9773		36013.5232		172866.143	147203.111		494266.59	164091.872	143530.655	98198.7555	20429.0983	212293.007			122712.752	293719.852	757379.675		142492.043		184789.213	147425.314	1512186.74	137333.646	4658497.66		62958.9242			
41395.0598	563616.57	212052.875	319435.125	160150.288	545040.46	225028.748	60577.077	123630.366	4326.04775	330620.317	175200.365	74415.805	331218.35	139646.206	240045.551	70277.0347	4386375.8	349584.969	115703.373	835980.416	517584.574	340199.874	321724.29	97156.498	217938.21	115586.849	185370.245	148683.963	997229.518	138040.718	2025720.43	834370.183	1403697.83	191642.278	274778.345	295697.651
574425.706	822411.96	1589042.55	4102873.43	1629513.56	2746971.64	4234567.17	190257.757	806292.748	1251438.34	3551685.23	915518.414	1021407.55	2674615.45	854549.454	1366173.93	1428907.11	1373987.04	2408986.5	3719027.1	4792907.44	6779170.59	2392618.83	2224410.07	1550463.73	2584630.22	1249787.71	1404770.49	2249300.39	2433339.66	1253035.05	1881652.99	3717356.59	3534285.06	966238.292	1182944.06	2285392.02
73262.9487	141062.792	196184.092	352532.117	287600.826	633225.418	749389.285	212100.074	572460.37	270156.538	203924.181	203740.271	263890.208	527667.847	73692.6741	255807.09	127947.522	197933.555	685475.625				53312.2938	219849.283	415103.853	712949.138	175184.114	326921.592	23639.6336	15523.8611		484347.083	625438.462	34913.5852	144484.231	218891.224	517220.298
1611849.2	22271564	37941746.5	28531007.7	11382075.7	11388011.8	342194.412	5064332.44	77639987.5	6681178.88	53150927.4	157803.355	7819311.62	59955137.8	30713464.3	16206231.9	19423495.4	20955.8597	1353747.47	157786749	113473898	115192027	7539432.2	20036806.6	9275446.35	19938130.8	33964196.5	22407999.2	60455556.6	61066821.9	75443648.7	43917608.3	151950.342	77267770.6	19465236.6	18722185.5	27063852.8
4227581	12821796.3	35489270.4	69406090.4	3160676.69	22584016.6	8441007.31	375451.907	3737664.2	349562.907	45859421.3	20259129.3	4568953.32	21765053.3	8003144.69	8287097.88	13269894.7	6402478.99	20423151.5	20024.6995		39106036.4	9100442.9	24859357.4	1507097.2	7719185.75	19841525.4	11742852.3	2193479.84	14911526.1	1068401.08	23558004	22385541.5	12906579.5	7254312.41	5542139.5	9793105.26
1813133.29	4519435.64	1869802.47	5289188.15	8021440.07	12064433.5	5980800.86	1122746.49	4146527.95	1163719.42	22587127.8	8351440.43	4233725.38	4396864.3	2490035.66	3342687.44	4214072.59	3297621.23	5200444.71	4490606.82	2911549.56	9697839.62	4406563.72	4683374.97	4972600.61	5061061.11	3641921.37	1831915.93	3849290.64	1481073.23	5459816.1	7996768.82	7434713.18	14451934.9	2046859.93	2785041.53	5913953.02
381606.947	93477.8122	37264.6909	150860.684	24136.1921	132474.66	37511.0115	958597.025	64562.1323	803033.352	237187.436	29762.5571	27494.0053	220465.339	46714.1353	209844.24	91052.5868	134805.486	136439.478	441898.808	251844.058	186645.449	195060.669	118844.286	48628.1773	395802.911	23072.2571	149948.094	218676.627	52028.9058	154186.794	84157.809	1763709.33	123771.521	17659.1837	111774.242	259612.514
23390442.7	21494262.7	24941410.1	76660278.6	23433584.1	53682861.5	41769571.2	1823911.76	27284655.4	3325005.67	25111160.7	48056672.9	21215467.4	24988805.3	17635753.7	20539165.7	25747235.4	23064736.5	32762014.7		30971378.8	57483189.6	25187463.8	34594505.6	21854668.7	29801798.2	36290482.2	17627709.3	25605739.1	23924790.5	24174053.4	30310478.4	42736033.8	46789927.8	21873307.1	24124607.2	27364562.3
52005938	171839598	98871317	99214716.6	73083204.1	458397089	99795031.7	47841231.6	139041861	47196667.6	119173653	156211116	42184970.7	224258535	48854709.8	75158043.8	93837936.3	82239671		125350397	201624345	287831778	198850898	107969864	52878518.1	87805683.4	81422972.9	137249337	99433035.4	203668208	137690647	223527016	246919306	131212122	83981459	70138336.9	239565216
145054.59	765632.437	4749.53562	412214.827	2125.02701	259789.768	248593.625	80514.9892	399970.934	212722.766	374738.306	332876.901	211800.068	238436.198	1886.66426	193506.575	124239.56	142448.54	633595.688		960595.496	754335.106	3773.39211	3439.01728	91936.8709	2283868.35	132452.632	398785.747	49913.768	316268.897	3302.73527	703527.877	60373.1271		36422.0665	445234.064	209494.268
555454.758	642677.653	957897.595	2018156.21	791478.737	1599486	1916.52647	168452.644	137512.969	1219100.69	126388.885	417272.631	817986.641	1280282.68	98454.2846	1196474.55	605393.568	876160.973	2053.40616	1331462.07	1632029.97	779609.194	565466.416	705462.504	254945.448	95990.3715	1302575.04	1012838.95	186836.714	1259044.64	1364264.36	1099228.99	44906.7529	505102.278	1033463.59	175112.979	559319.625
178220438	193046504	302254609	445686586	140078706	849239470	212340930	15752594.1	131974346	99730645.6	324897053	341192869	169015747	513015870	169075142	232322328	155956990	176359677	332531999	152202.408		537200015	154640803	267536974	225044378	367117675	225638147	425513526	212044785	242915610	81826372.1	424565121	230638912	142086845	231776787	225411388	292690556
1747629.91	3028606.62	1745705.15	4983730.92	4875922.53	5916035.05	1934149.62	80317.766	1219470.96	161377.269	4636317.95	10533035.3	2931366.4	16959927.6	2883603.16	3678722.77	3994715.67	3215337.8	1617319.73	57865.8183		837503.575	3502642.47	9689006.53	4124054.92	1931073.34	18040154	6673340.05	3179840.77	6476251.25	16138781.9	1060003.12	5576034.88	3748917.76	12140688.8	3055162.62	1904493.35
8285793.03	16550426.6	23924004.1	33993065.1	26282530.3	42580931.4	25458819.4	16110076.3	56216065.6	24967830.6	27229000.2	10024024.8	13738391.2	44220928.2	9301079.16	25211432.1	12691701.2	23544276.7	50617469.4	46316708.1	112538923	36286514.6	3031283.26	28334815.8	54013727.3	64825623.2	26346319.9	26295489.1	7538056.57	7049452.75	1916674.73	68643001.1	34460166.9	3877697.38	17057166.1	21889122.4	30772603
1843911484	1856002760	5410708910	2795541910	2231459441	1547065942	254876152	1104149513	427759123	214093870	3092818959	1798498045	1165353087	1539878533	637594171	929382460	1234942020	958109521	906790099	1463273995	45616137.3	3228686872	24072324	645794681	629063409	902654278	1166358854	44228731.7	381166483	1573275592	828014366	1412164883	949689639	9644162.48	729444692	464794556	762799703
55382770.3	55416330.6	106132195	57151197.4	92196363.7	210216516	30054297.3	3420876.48	35016099.8	10793413.7	68651806	59252250.4	48461357.2	84161035.9	55694290.4	56142265.6	50434158.4	43881331.6	112500394			396441207	82385542.6	51068897.2	76864369.9	56085265.6	58782029.4	15974111.8	63680928.9	83252959.8	72524046.4	98888552.6	130921500	58523181.8	57793692.2	51136715.7	121286155
767693.543	1164438.96	278938.501	1186074.27	717245.54	1977435.96	796772.002	212239.48	206266.896	199924.191	440460.403	250666.06	545360.878	1036372.98	413740.79	657472.044	629797.209	467630.105	1153.81733	1089391.51	1412472.24	1158194.01	534352.062	841562.509	646181.014	1090503.39	1073534.62	512971.633	488435.792	1311543.2	782253.216	287554.443	907631.955	141800.668	209702.807	617552.687	979310.526
839221633	584926205	609097126	731501230	448066563	873306177	330710681	345482740	339418341	322313986	1021228273	309894407	607195925	1444756203	540091841	662444054	566926074	594707477	878998230		806089746	1311256801	614950465	707097750	623747070	682085430	721950383	434302455	559244123	1135320447	570875276	1017348769	789599100	1214821060	573923472	546762334	686619969
1934074.8	383823.113	5101219.17	1827535.5	2386719.3	5090943.41	4049979.94	414583.127	892817.634		2287554.61	1922616.26	2099596.85	5666632.65	509400.818	2182872.4	3734546.55	1589786.33	2337053.02		4641767.28	5173394.73	1521677.34	6054513.73	2204003.89	1713229.15	1616307.9	2182840.87	1237966.89	2499668.7	778927.792	17709802	1831544.3	2532665.81	986305.546	1201573.1	673589.226
26673861.6	31478397.7	24243282.8	30229484	12318832.7	53394344	17709647.9	7290643.61	18664824.9	33994894.6	25992822	63685011.7	36406571.8	65977781.9	17894720.8	31702931.8	24789730.5	28066395.7	48240406.6			113419770	38769134.3	69749976.4	38795622.1	70640878.8	49694808.7	54169664.6	38094719.8	50126712.9	18133483.1	69182628.7	48027161.8	16312084.9	33411978.3	22634284	33980170.8
8624348.59	11653735.9	28158994	42887146.4	39281490.9	129135594	46679246.9	968187.348	2175031.01	1123367.3	16070035.6	21453205.5	11077894	27033869.2	9394251.07	12378698.8	11266591.6	9116801.69	11151706.8		10138240.2	42319583.1	14104830.5	13923007.1	12041443.2	15511064	14376838.7	14372936.7	15080099	20455170.4	15035595.1	25494936.2	13268521	27616073.6	11337998.7	10059681.8	16037838.7
52216196.1	105601412	223472788	273075200	187063094	452219681	129751058	1735930.18	48953760.3	5694953.81	252833296	302921899	114937822	154358721	48984576.6	86294686.9	82182942.9	61767309.9	125096735			370329898	39427735.7	86614524.6	231433002	224433577	114192898	169363650	32825594	97389370.7	14133816.2	168621577	154663741	24038862	114383349	105254078	96427505.2
980730390	2239856886	1929395061	3359994041	1494865609	4202177379	1383771201	242737064	1544763367	333945877	1997189619	2122263547			1206871159	1392189959	3216474821	1373950954	286636357	5063869617	3539147692	4042197666	2310698967	2555141871	1415400971	1381819851	1367495394	2308179954	1782662912	2054362731	991394060	1742371329	5087623068	2132018115	1785985628	1294718303	1413334536
761215.59	14381752.1	50207971.5	34917759.7	40845673.1	41820797.4	34076500.2	328747.921	4605220.28	1501763.28	24374668.1	25598995	10204258	31042447.2	2619414.78	3831751.45	3425001.17	7087905.27	23401764	1929826.48	48635782.6	25717060.2	110114.365	4401950.06	19832103.7	208713439	31194063.4	6859219.68	56777.6625	97522.8874	129377.586	20937027.4	44635413	3734608.91	19364900.4	27943560.5	10123885.7
103672151	142816004	272046182	342937497	166489839	435162636	196384480	17462690.2	260550441	25981826.7	163714129	50527774.6	117162094	275868800	109575561	139482573	145554983	127447077	186701722	325113177	208583970	424770861	150601960	210559565	149469630	171144711	115667701	145187808	105979660	259110889	119125842	196143572	206218322	214632696	44248295.6	95008105.7	79487538.1
25013.8068	22285.1896	26296.9393	62174.0703	11533.8839	223529.05	17612.912	15302.8757	58367.6171	8021.4051	48290.0896	32106.5308	43055.4729	35006.4279	53718.2241	27179.6689	21061.1156	19114.176	64995.1588	1887158.85	17552.9786	205087.275	171504.086	160974.172	107928.664	127128.497	36133.3347	112553.562	54771.7121	135608.58	68181.7329	11990.2128	165517.337	87140.8746	43746.6143	17418.1527	9251.8282
242102016	526428530	917980804	663929616	355209249	1394152792	247669048	210611361	649562010	125135826	346544981	632867838	258464197	687428553	165736493	347803515	429599185	415362192	444305249	762161790	582008047	984102462	883101584	924814270	384360286	484393174	454435751	691261023	441536405	475696234	454130124	706096348	1082646189	446436008	636131960	306447714	793873016
108285180	162410239	206665590	425047258	119207957	587705165	119479885	9474078.05	129094898	47438691.2	271248843	129399611	62693009.4	120050360	117399616	109053899	95842341.8	63423815.5	248798821	179024619	186902685	275503165	159532681	245558991	108163639	210919301	159643271	111650611	156454104	158751104	138838001	186130889	303900685	193169427	115386712	136923446	166392577
81037689.8	94953720.7	160610844	312443219	89569298.4	170135983	173798283	6286707.72	73309644.3	9348311.01	183552447	130208259	71175262.1	151446721	76771513.6	63483001.5	83409916.4	96067759.3	151547871	2739632.32		251241123	129338853	341294551	116040044	154814238	115196957	127446597	105064404	186111390	105164894	112046991	293047089	58952447.2	72442787.8	62873011.1	147347643
222479664	580056384	769960216	746090509	630166268	3633400661	654083070	392191333	513055302	281457283	592166062	587029025	218328915	1856297017	468496130	678175536	607332113	527112867	974494329	1269412.91		2223118526	1213971033	903839184	717980985	637075145	713919181	938880939	536313643	1174467377	548476493	1187622346	1817977309	558045010	432449329	474109077	1453429132
4857597.89	3158317.04	28909017.3	12105223.6	17186301.7	12154073.3	11756594.9	525343.925	604565.092	501097.664	23745129.3	9145884.11	6876632.82	11995275.5	1341280.72	4867859.36	9793081.77	4189054.01	6520525.61	50186271.2	15501451.3	14742792.9	5456332.45	16471713.5	7039748.4	4558523.68	8395377.18	5037476.33	2667540.28	9111034.97	3346343.56	30684588.6	7678919.08	16168996.4	2287666.98	2887501.25	1453429.13
297511585	410552465	1872946224	1047653619	1521151223	4948363188	1337615659	1151818.04	75555635.9	58531375.5	2518799222	2834426955	528135528	787502621	257001429	555917373	578504394	357963862	513362108			1479817684	479732409	569582506	665475855	441680485	376983796	714578047	533584745	473532265	304332628	353556167	470774721	585231818	411378712	357677574	620463885
713050.911	4613762.8	7882773.42	9628401.52	7513584.51	9914243.37	8837584.87	2122964.53	17598503.7	12087614.6	14774223.8	2930125.3	5138622.8	4383680.24	947190.732	2191597.74	1016119.99	9481964.02	12687794.5	1954932.2	11518845.1	5815010.59	69209.4729	3784995.92	11033033.2	32478868.3	8628505.12	7547892.85	542219.359	227333.281	74442.0676	26330851.3	8425103.8	1347893.33	5378024.14	9146858.81	6074331.41
56716298.5	97116546	273059914	415651171	344421488	232766989	119713537	1826977.99	199306674	15005093.9	164294423	77742862.9	163433786	177847258	104709030	148897929	102102303	145427329	217414997		296352760	595511402	157783794	209214048	212903124	145488132	162252087	135623953	101638003	147842272	90937413.2	230781139	218428617	171706157	158531309	163935555	175413861
82143716.5	119294496	422925906	458199161	415452240	530950507	428750503	987360.737	97599738.5	2444369.82	394505537	284087206	219816247	196434708	116195564	167632177	130773670	202965991	211287826	452386959	315053392	773216884	254769726	297330074	286192065	204048010	165410840	143594021	143889198	200704574	139553990	237324984	328321275	402078584	198665817	138786351	182430415
883309833	1097646927	1301627364	2223107750	945791960	1857157360	635132227	139757690	793753149	330221585	2808836984	1148247964	786980265	1512181672	946856715	1023333558	810418610	430750550	982712825		1809265137	3533601006	893818220	906929646	1068232614	733884319	1096384370	1239647897	869244581	1739003277	793963373	2047956983	965970033	910042632	522751973	591472031	1052483165
377497320	380379169	515424362	618671619	505780434	526061377	282927465	313296695	526483958	534324256	529422832	619608443	530429829	573364345	279357280	351027988	390589204	506477649	21035390	1001180362	1186968709	1238852484	792515794	591726624	531482499	545894458	594574865	539544554	568424524	562676445	374263870	446083759	852007276	922920593	598004177	578431702	591395302
2154738.07	218596470	109508380	62712965.8	54247648.6	163670658	27437397	63839002.3	61680191.3	49676885.2	134488217	42099291.4	54864176.3	50115237.6	65541126.6	32424600.2	43409213.3	13286169.1	74553447.2	53693682.3		148354745	62203527.1	36742979.8	61992728.9	37667599.1	29821698.3	64361835.3	99883032	136867824	29131840.9	129791107	200791524	165982618	19164227.8	52068167.7	61044023.6
2060838942	1703390479	2040439752	3298992119	2735817959	4154277235	1508970547	2282155216	4470543313	2481305151	1849435226	4457320154	1712451913	2605635675	1832476138	1986058990	1955803887	2639001860	3364097460	6637161116	5578656177	6728097783	2887405151	2215722035	2398256350	2650421329	3230286459	2271440107	3023127672	3380305028	1770546893	2374668483	6023745712	4264036230	2026792679	1760444312	2596125140
98651.3344	286334.05	99791.3696	16000.2594	7858.16644	53247.9816	48760.0473	143029.69	154698.762	17885.5485	8014.18639		14470.5228	196112.222	40114.2641	57798.2783	19624.5219	10102.5682	139446.75	2242.45568	1535.55544	139330.767	67709.1635	65893.8982	77565.0214	253691.861		102001.877	38789.4121	79159.2934		100258.022	114912.446	68253.1974	178598.563	97802.4618	92017.0996
320833165	615566904	434804474	453651868	248709510	517412052	85115979.4	34684430.2	331574154	71103982.3	128965378	291878592	389991819	540177718	260591338	388443214	342427640	184434994	352013956	565611592	542353290	1066179159	312810457	602396284	380595184	375829332	223647117	515773213	348998721	453453341	338975950	435754111	503335509	715442320	307028990	269189633	322761504
3146256.86	6511094.79	3881351.19	7248244.32	5355428.75	5504120.97	2563088.72	492582.084	410796.253	963859.305	11087216.2	2710128.77	2915698.21	6762210.53	3594380.79	3779223.59	2619674.66	2727947.23	4121659.04	6226108.63	7752206.88	8068061.41	5358337.92	3898171.77	4541116.9	3551417.29	5186840.37	4042343.35	4710733.64	5633902.75	2502471.81	3775494.9	3513851.67	7183040.9	3842879.19	3278711.1	4209932.66
973321.149	827722.305	885348.958	1938444.39	1384219.69	2278495.39	261313.209	78595.3685	1060029.77	347102.89	1990415.66	704729.497	367456.545	717035.08	591326.091	1522906.66	580218.264	530028.634	2144446.7	45975.0158		1925516	1348006.8	2466190.86	682503.684	1811301.92	416068.563	684629.612	749147.877	1741384.58	2409239.17	2160929.28	4409273.33	784124.783	484624.19	275709.797	1132672.36
117951.15	127605.084	384203.792	1353973.02	1294900.53	1462568.44	90041.1762		1381699.11	351755.603	194125.86	307197.946	317030.323	631007.041	384028.228	455494.028	216793.994	189732.413	384379.437			1116744.1	909656.593	544475.035	1536911.74	822895.116	510751.367	945923.002	122363.541	1220052.22	10596.6195	2809829.97	389372.752	766954.168	733458.143	237520.264	315744.949
9716525.9	16441743.3	20250056.5	16920448.7	19293172.7	22159159	7823422.49	7885782.39	6675491.69	2276874.65	25783305.5	4950405.47	16627298.2	30475235.8	15622676.5	16225555.9	10196695.3	9347985.82	23572457.4		14331088.6	84044878.9	23040190.4	22348414.5	14240428.5	15564321.6	13717772.4	16675768.8	17176725.1	18751266.4	12191447.3	13265911.7	13838334.7	22321800.4	9562046.65	11643150.2	7708186.23
35149.1158	96613.4294	81951.0466	10873.121	79522.491	2455357.4	38719.3131	95658.7152	48603.363	29985.9161	123568.847	27306.9538	9133.15465	133547.68	32480.0265	38301.0879	30144.3847	27972.0656	687483.668	71368.0535	262779.8	13394.9984	42601.6829	48288.3919	148200.052	43741.3023	88770.3555	72433.5063	16765.7588	63842.7997	69642.6892	70880.7292	77738.8805	41066.3892	20970.2807	34091.1438	36786.7925
208861.861	308395.267	423083.429	357003.495	378411.395	1125726.63	341548.061	200000.004	87044.7148	14672.698	471962.332	650235.688	380202.616	652733.571	418676.225	694357.744	309616.692	548536.4	512549.804	3781.65824		267164.4	86509.879	325024.906	938007.911	625640.908	428513.997	756514.077	186413.136	272735.092	56121.7491	580909.89	606444.67	66106.8704	180605.288	180701.691	320756.774
2254565.09	7101206.94	10818505.2	8881659.92	8013684.35	14331458.2	6371995.75	503861.46	4243052.23	1589285.75	3525328.25	6124031.57	5162518.37	5266162.78	1709149.06	3644107.76	2086923.78	3191368.34	6425020.06			1738750.73	544900.308	3662364.95	5485236.5	8673528.19	4583374.77	6479780.92	607009.455	720896.822	150768.881	6636479.78	3920861.51	386338.853	2076960.82	3129678.78	2435746.75
372146.295	380023.28	1046720.03	1535163.77	1048468.41	1571300.7	836779.475	19939.868	88707.8577	67804.4465	1189766.7	657911.753	539121.772	1093617.7	462352.729	966224.365	608728.231	537443.037	873276.963			149508.425	846394.002	1079434.07	778649.485	389574.705	597882.904	646267.895	614285.688	877935.825	462632.077	823183.995	1191182.15	1422299.33	744495.133	300859.001	842988.897
80466.6446	159373.354	187604.126	439886.229	173310.742	60536.2257	133697.464			23250.507	571593.781	131845.366	100110.782	152162.807	90754.7001	133149.095	153441.613	142619.124	210870.71	461822.007	256382.997		237895.206	158878.745	217768.728	171705.811	124682.324	152532.811	226310.575	183888.263	135678.466	125752.558	554890.089	668223.127	196659.092	164867.007	116274.331
2978310.3	4645579.04	6712760.89	7149892.94	4073991.69	10625929.6	5222937.66	5777891.46	5575430.56	5509213.13	7324983.89	3459574.75	4605779.74	9233393.37	1781593.1	6216594.32	3805951.82	8127762.23	11510914.5	2570872.05	782064.595	17178659.7	513812.081	4650651.94	8033735.2	9798650.92	5560492.67	7141749.64	1050242.44	1613840.06	378931.028	9745056.41	4341438.35	1171894.52	2558574.92	3465001.5	3989412.38
35602.3712	107495.683	248419.156	39223.5971	38295.644	50837.6422	21229.1398	31250.1799	20479.0879	18828.2532	86420.2111	35099.1923	11737.0403	105319.476	35558.9	30935.6778	33262.231	39027.014	918066.241	103918.024	86039.818	49852.1431	43892.1897		18968.0301	62678.8125		93321.7424	48668.9891	58051.9143	330252.201	151815.884	66478.2747	31479.4621			9502.41943
90805.9409	331009.389	670682.078	106483.891	118158.783	150100.626	229619.77	63301.6712	154264.286	119332.105	331850.675	88595.818	61730.9403	554796.895	130577.351	228394.589	132947.329	85141.4075	3671030.94	19868.0574	3949.06266	257582.645	388680.946	127086.666	887219.206	1019785	35852.9684	38213.5526			155865.869	497842.066	189937.928	45072.8662	218733.071		
66423501	113564608	165189178	291889022	439020487	75108532.4	236541519	47392617.5	350719778	137404621	477312394	187959989	117049872	92143432.1	140616378	84435616.7	51941802.5		210209921	250437.551	237644.681	393909592	61035251.6	84046399	344624824	123799552	114500642	159395792	58710509.6	32716165	25748749.7	82385856.5	213001819	42354185.4	172578387	98733913.8	169399671
19324462.7	24005690.5	115933421	111734440	76259811.7	195949922	50425998.9	91983.4502	30144918.2	7927556.18	19859369.1	13462738.1	23098064.2	24774687	13298928.6	14188068.6	14951729.7	15164831.5	17852145.8		24492237.9	38725544.1	16677431.3	25473139.1	22229456.2	15256166.2	17675725.1	23259169.8	17484209.4	24893588.7	20447042.8	25000622.2	29576048.8	36201381.4	18963555.3	15927829.5	21250136.3
844248.469	7896035.32	8985151.62	2074300.14	2935161.52	1621315.25	2957396.4	2179595.12	3634831.39	1144673.61	3321761.43	1577532.57	2946257.98	1906551.41	5917069.39	2420793.85	1334432.63	22206.8809	13408234.7	265352.808	174478.834	1664571.21	966209.5	877530.115	6262227.38	3556254.13	395695.693	4483334.04	2201643.2	2555417.62	558176.618	12425948.2	10243081.1	6338818.96	1404707.8	6492220.56	3989412.38
134920.209	268720.093	200342.714	458635.468	40750.3169	388041.179	231253.828	51138.8263	435154.822		220038.569	336038.16	131051.727	488365.079	159323.647	248538.398	178807.721	233203.858	364090.15	6371.16327		166533.493	387790.886	442921.116	119537.495	490271.087	227145.352	332016.515	573407.33	211537.697	161337.6	274551.073	590164.276	83992.9284	142477.505	329734.014	314742.585
977480.394	9958648.6	9275016.28	1097951.77	837960.022	3593196.46	89922.4061	591562.341	733900.59	162520.343	282728.102	517336.948	1866617.86	4485024.01	576777.034	842095.988	1394736.92	54658.4336	1144345.48	5434024.53	4203106.68	1436654.38	221303.314	3173686.02	188428.61	2332972.11	7816991.88	4590200.43	656538.045	618593.012	32354902.4	46302.989	1370266.48	6410363.19	133447.241	44523.4064	2275368.37
8106566.06	9181522.44	9351770.51	11926274.9	6538077.29	23043745.5	7090156.49	7482177.24	13416785.7	10267733.1	10015206.1	5688485.92	7284281.11	16096816.4	5705452.3	10537089.6	9224407.08	9718041.23	12241605.7	35220.9799	9535.71702	8715863	9647497.01	12503438.4	10744882.3	6646218.82	9901960.73	11422717.4	10414620.7	13402304.5	7508233.36	12441281.6	17637093.3	3605829.3	5006779.94	5532824.98	11527196.6
3769093.61	2178207.57	4381439.39	7119218.65	4544620.34	24830152.1	6531835.8	211677.619	2881167.58	394605.86	5965890.82	15188276.4	3448132.05	5865131.35	2456637.45	4630119.61	4220738.17	4847887.11	5371463.32	14374996.3	8068117.26	13978654.2	5159008.55	4139592.15	3567690.62	4611003.75	6509249.63	4968221.48	5726884.88	7096963.75	5914839.15	2716104.05	4599211.25	5966788.95	1896355.53	2049194.44	1904493.35
211924.036	626995.22	620349.194	880968.122	305391.361	1138928.18	748440.99	11924.1549	339264.918	10158.8975	792471.602	745264.153	454502.033	642539.562	194635.53	418838.635	238256.599	376816.192	779158.139	812.994539		166547.068	74029.1912	417956.323	712266.098	1709773.94	463148.496	1214892.47	142846.296	120609.695	10647.4864	1061743.88	519615.903	58093.9164	302012.176	475971.981	663565.576
24013841	34148137.1	76767474.2	117576328	80189030.1	146601494	63119030.6	2498288.32	45667554.1	7499444.27	14704487	137129067	68269500	94016626.3	29694765.7	17692016	39617912	59631596.7		153759487	121027521	123391258	37729709.1	58632127.2	42011626.9	49562990.2		24710462.8	63404715.7	132083043	45990559.5	40362486.7	18451113	96155447.9	58195037.3	37071790.3	53325813.7
33536737.8	5833729.24	24928536.2	3209393.78	2553864.08	3414876.55	1049980.68	101747135	17773919.9	21314458.9	11477261	1903995.99	13693604.8	465594350	18757952.5	16841722.2	28623697.9	88931575.2	36926490.8			37450986.2	8638797.57	76683916.6	35473709.9	20186591.5	39228211.3	26286750.5	21163952.1	147329519	163405486	301871290	22385541.5	9214897.09	4304426.04	6184841.39	6876223.34
58825800.1	200877185	220820806	340348515	175557812	368998496	87819567.2	27554164.6	148215660	36082379.7	369709411	113515818	149424235	241315507	139824142	187198047			236976713	10091.5542	35218.4604	33807451.8	167647152	321824603	125912559	93332109.9	184948974	103051131	131891468	230538194	171154904	273167422	188581228	70685701.3	148497681	99665365.8	90012369.7
99246.0838	389171.569	140632.711	319623.129	113708.361	1133763.22	15924.9954	63434.8452	617483.489	66379.6777	393364.983	388865.873	187091.003	344939.925	72280.7406	58336.6318	95915.6101	214549.415	141463.947	46310.5159		342344.751	257539.697	176093.553	62947.3928	198829.244	153875.058	156382.19	82070.9098	170470.612	757871.783	27122.6256	3364614.72	141943.756	58997.7275	59240.3483	172406.766
144946.213	146964.452	401755.811	816578.062	322417.942	612795.162	778018.211	69158.4654	29271.7539	93978.0634	706563.311	134151.731	159152.287	246306.643	133698.844	177473.553	136886.922	188831.903	266650.008	603970.968	345440.166	595962.935	205345.265	148189.419	159234.775	304387.884	103856.418	105719.246	184103.732	143328.001	150231.93	77029.6309	419220.141	294762.236	104349.722	53465.3458	84599.5992
185718.776	186462.377	567715.902	1822605.69	404321.436	534851.296	262434.931	741647.908	448712.78	1042861.81	562557.415	286347.438	603825.867	717485.386	387531.851	680788.061	614538.728	371381.858	790329.105			2596466.81	913072.241	454806.893	1032488.77	1185425.89	707650.953	690249.173	724579.401	773728.792	365791.635	909617.922	1709441.35	655345.166	524758.699	728395.477	1212861.55
501513.502	630427.461	1076157.31	1188737.21	967651.502	1873166.23	910130.319	237388.577	1605230.18	541339.226	1497494.36	715736.254	731937.978	1469375.59	551264.823	638742.464	744531.719	744860.133	1571422.83	1897178.76	2141171.48	3646958.09	1359687.47	1299098.34	1159089.31	1409299.67	957419.362	877634.032	1193663.77	1313829.02	1186870.34	1048421.67	1831544.3	1645517.34	539809.139	552351.046	1012388.57
308036740	412376238	309621631	726959893	367851333	1228493209	626919560	122636169	231154765	139880949	1172881946	414810289	333015372	210822804	283379776	328702871	315284988	166107021	518907103	397910709	896021099	2384833469	555791232	350085159	415174254	395826642	369242819	515226434	295103008	494117238	207363581	514078072	793669199	639605435	330106332	293407385	509201379
4327468.13	10286014.6	1814207.18	16030309.2	6644037.73	15433933.4	8295855.04	410207.077	1819986.59	232396.292	9794814.65	8796865.81	3729090.26	1066995.57	889794.075	1039938.8	884386.985	9771247.35	2270201.46	26826.1608	5249.23024	16829784.2	730461.134	761030.078	4769222.13	7845820.54	379898.918	6608085.1	1524787.94	5517038.05	210615.939	2468350.48	894064.96	2332341.96	3622139.39	1313347.34	2856740.02
31211291.4	54789069.4	30627742.7	31897246	13976217.3	9870722.38	40110879.8	19620398.8	46769455.5	36452886	83792488.9	19841853.5	32132914.1	43354264.4	26827670.8	23114207.7	25638378.5	22914222.2	28490810.3		49179880.1	93714197	42392768.4	49529419.9	34057087.3	39469724.8	27649449.6	48579248.5	38664826.7	42484264.2	25873498.8	46645324.1	50333550.9	72259674.4	28997182.4	28316141.3	38490812.9
113328.171	1018928.95	241342.045	352672.632	209345.403	748128.127	248676.237	84204.0614	404555.233	274583.677	549960.32	100115.462		17770.1595	8457.06722	8659.81282	322893.001	418281.187	26383.9376	581292.687	83825.8672		394584.214	400502.301	559540.042	288510.607	434843.017	556862.019	205691.512	126370.463	131442.568	270581.161	312040.881	44357.4239	137460.692	178838.787	117276.696
465183.616	369467.671	547588.034	698955.566	716981.573	1533097.4	425968.273	65557.7884	179536.041	92211.7767	531866.53	621192.465	546714.122	829722.023	358994.781	467319.305	389070.168	356645.565	115694.967	1344860.67	1530899.21	2725598.8	914270.204	807768.966	927087.841	670692.623	527451.411	710715.773	971810.905	668127.797	339656.67	432635.392	678349.742	1302105.02	533788.963	535584.91	1132672.36
96790.9171	399687.259	559159.302	864949.326	481272.92	478387.28	286297.73	100437.685	279640.395	89732.8264	608045.857	281599.154	323330.071	667941.975	366352.482	443496.052	432879.526	265694.561	741542.4	12800.8138	279225.976	1716811.19	733818.949	598249.352	461236.722	486090.579	247577.188	472164.333	572963.305	520373.112	627641.581	545737.122	1075862.69	1179048.94	168564.936	252423.497	377891.574
303304995	370699776	255808475	115460022	242443505	223193963	120005052	9865333.46	203342319	30079599.6	124956340	719787457	99895321	303419740	145467726	140856862	202219711	142119041	81221.3542	504170237	343310972	465351926	281412028	323975405	278291302	173128648	191146722	229366239	190127518	364043347	259470730	297511521	204861622	362013814	157527946	125746022	213503728
1369849.5	7865096.97	6200184.39	10312629.7	4956447.04	2941844.49	2830218	649685.343	4063933.52	2768429.49	3286664.94	3967621.3	3001915.22	2978011.09	1449602.22	3330260.5	2042653.42	3042124.4	4516321.07	11232324.7	4283048.38	4969297.36	2650440.32	3484597.47	3012770.77	3618396.04	2850952.14	4015584.63	2896656.49	3452166.71	1797414.66	3918159.93	6783497.42	1702752.72	2117095.32	3455686.98	2666290.68
9296299.85	5314404.17	15172299.6	16746637.2	12041343.8	35324396.1	11499243.4	5122218.99	10094335.1	5919981.98	12868365.3	10659147.5	12012222	20665969.2	5661772.81	14138395.8	11041882.7	11778993.2	19741727.8	63616.9102		34745272.5	15977693.4	21407331.7	14779726.3	9444223.75	11549925.7	15125620.8	11649609.1	14132814.2	9384413.37	13003295.8	20757502.1	31622550.6	14047078	7731051.74	9141568.06
727670.44	760934.901	1236847.71	1989764.98	1455938.72	2575829.91	242735.551	211145.426	2419647.67	56109.618	3837708.28	746235.184	707351.166	1193181.4	946104.264	1010053.08	1073183.64	1259787.33	1452519.1	2601983.98	1883262.82	2917413.03	1317687.5	1590925.81	1025470.68	1504678.96	1425847.53	1181462.79	1774175	863266.572	1934504.84	1131847.15	3717356.59	5237037.79	1675615.73	1816331.43	1764162.26
785441.43	2591291.35	3206346.67	2291771.45	4090614.12	8320602.54	3710543.39	328116.672	1825248.41	495431.024	4301444.09	4980672.02	1001180.31	5123391.34	1453106.76	2133525.67	1632435.03	3295589.67	91748.3487	6521172.99	15161928.7	4239673.18	2494656.43	1604898.53	2489715.47	2672584.95	1056661.79	2307371.8	1372315.6	2832381.44	1569717.58	4204070.38	3351047.73	5723538.56	1204035.26	1248145.7	914156.806
65333.9336	249482.508	86463.0972	176079.296	209768.328	26734.9884	126604.956	54372.2422	179382.963	7859.54267	47964.6429	12259.5835	99136.0099	145602.014	41734.9151	187092.776	48995.7846	42295.7556	42363.5772	117757.76	114735.857	285739.058	376723.268	140641.646	45684.2105	110015.019	262621.678	79662.728	69296.5569	48923.6722	119403.442	218864.1	54946.3291	427834.508	43846.9505	54769.3786	31374.022
288567.205	248775.573	884114.778	965095.849	185747.955	117410.05	1290210.2		141158.022	155077.064	227911.266	1025301.96	143509.914	11930280.7	132641.793	445911.092	731123.006	276727.143	135821.333		110459.16	270099.56	71599.5563	792230.447	98518.0748	111740.311	466062.544	455150.493	116709.951	850047.304	1467494.57	67259.4834	139740.047	142229.933	282948.285	97802.4618	29369.2921
26060.0532	122413.589	65855.2282	63740.4976	76326.8883	34034.8194	92755.0307	22958.1623	101044.862	304710.426	28181.1296	13368.2147	44278.3549	77338.8767	63604.6939	56189.4679	6015.11085	27357.1668	57327.2175	66693.9115	89045.8922	257792.899	224098.285	170585.283	56723.3782	78744.5843	36811.5025	73744.4312	143994.956	16185.5122	252527.202	24362.08	211645.12	214632.696	26589.1119	163935.555	59139.5302
113531.202	113225.501	67645.5224	200949.668	42962.5303	40196.3107	45743.8136	75838.7724	236331.313	266605.454	245346.061	268286.151	48383.7633	349406.478	131259.889	19911.5474	400927.959	214790.34	757.594003	22059.264	2690144.69	125194.403	346095.027	261655.417	114976.428	409784.764	57168.5725	125821.659	479196.071	200460.074	360798.07	135888.025	421933.54	722596.744	207696.082	132266.186	300709.476
11650578.9	14080148	23642676.7	23837661.6	20337782.3	5487457.72	4127492.54	748375.445	555528.799	1006264.52	40393407	25390683.1	19992536.9	21218926.3	7586812.34	13071619.3	14807660.7	10440275.3	24067637.1	35335.5057		31780095.3	12341828.9	10787746.8	18583129.5	14884321.3	14578169	17348842.2	12277373.8	9870298.83	5239589.7	16965350.1	19536472.6	12606093.7	12542033.9	11363714.6	13732399.4
74439.2636	85665.6634	226763.023	114510.958	138758.532	129607.789	2520.93982	532177.642	30271.6906	113790.218	181870.653	69689.7344	47607.5516	104824.684	136190.979	68415.707	85257.7746	8982.89373	183026.856	93951.4929	204965.67	450665.956	60393.5629	46983.9926	242646.291	57577.2804	26778.8087	163021.639	223153.677	195479.029	18364.0581	301657.191	978180.328	605264.203	23679.36	112705.694	155366.562
1900692.31	2338653.2	3722094.24	5968789.22	4075213.18	22275001.3	1824946.21	594659.805	1578279.95	405094.452	3047259.77	790443.352	3400653.59	1123458.82	1786522.92	2503875.41	1056549.84	1427470.94	2298415.63	5599615.91	389277.408	27511861.4	2669058.75	1383169.8	3355871.01	1263596.9	2555574.82	2486408.69	1019371.82	3167585.8	950083.467	1733617.8	4368572.34	2117709.27	2558574.92	1825645.95	1333145.34
128950.042	111764.432	266625.196	379128.068	246031.755	250053.455	215681.835	277683.063	260461.394	142516.965	448833.585	118630.462	241867.58	479643.526	269496.537	246564.957	321294.442	198145.612	459747.484	318854.385	131028.25	2916512.69	505932.183	571457.274	453174.162	174364.579	230481.895	313185.632	278324.897	277347.814	219857.483	204025.667	198078.125	208909.158	368234.116	208645.252	174411.496
29534.6955	66747.1818	88886.4227	188966.234	87712.6341	263932.818	65046.9009	23813.1045	72074.1747	129221.16	49059.443	11722.9838	57869.7787	56674.2111	14809.9312	191389.053	135979.524	61234.9034	102239.191			172033.853	64322.6993	113484.145	86064.5329	73908.775	42203.5404	94387.3494	109799.629	98105.0632	89065.132	121554.002	234709.011	314794.621	64014.5411	49925.8281	66657.2671
67677396.7	86963267.3	172064305	276099980	130690180	258005454	117277070	2212350.58	30742238.8	7653935.79	54018025	28632058.5	36696046.1	56899566	31544726.1	39947107.2	43720022	56841892.8	34842843.3	92870.3965	1765083.71	117474399	54743369.7	49200039.4	43796229.5	27477953.1	49544513.5	54150429.8	44776112.3	66027957.1	56837054.8	38206742.4	65799925	81274247.6	47860401.4	34370579.4	42700745.5
979164.477	1108639.18	1733534.86	3448601.69	1890534.06	663577.209	1042976.66	352827.033	1898346.72	870681.319	3963368.8	243718.238	1202983.25	1771425.75	435740.837	930223.122	1289231.27	912276.009	1569740.67	4069464.83	2970494.03	2045645.21	1738038.53	1382584.82	1595065.55	1260196.15	855630	1269270.68	1295727.58	1568101.39	745275.61	831630.026	3120408.81	2031856.19	939147.499	872770.54	1152719.66
466650.621	963490.757	828011.072	831532.977	562009.225	1638816.87	437723.515	8142.33868	237084.337	347267.541	1317369.2	621373.664	489168.454	700201.256	345415.589	469158.791	342741.729	234859.956	845587.563		1011412.76	1432373.6	408159.703	456382.074	470054.312	406274.844	485531.266	690849.987	358748.308	763088.599	219460.792	725219.094	1356699.48	447866.893	253850.766	290613.029	584378.748
3279.83759	30387.5554	45285.2181	9338.99623	6117.77277	36087.7255	19884.5635	34683.322	9911.47658	36132.365	27503.7285	73521.4096	51653.7943	35761.6161	53738.4256	5844.30096	74731.6322	9259.67099	41561.0581	241178.203	180185.924	202399.097	7665.36139	389458.197	18650.8217	74251.616	26348.7632	16810.6013	35501.828	276021.263	25527.5524	5335952.94	211645.12	102594.429	27492.1383	11177.4242	15436.4198
138980.994	161823.319	277149.84	776777.025	324777.151	546078.002	155732.513	111681.381	52356.7044	101355.353	77097.5383	263524.003	150007.296	197555.497	89448.6229	124403.001	184548.447	200205.002	240368.451			319212.702	399022.229	334948.471	311884.923	236930.329	115471.716	164029.406	317370.497	222054.227	196393.158	179405.166	449067.529	109748.852	352180.312	133197.638	229541.566

UR_B4_R6	UR_B4_R7	UR_B4_R8	UR_B5_R1	UR_B5_R2	UR_B5_R3	UR_B5_R4	UR_B5_R5	UR_B5_R6	UR_B5_R7	UR_B5_R8	UR_B5_R9	UR_B5_R10	UR_B6_R1	UR_B6_R2	UR_B6_R3	UR_B6_R4	UR_B6_R5	UR_B6_R6	UR_B6_R7	UR_B6_R8	UR_B6_R9	R_B1_R1	R_B1_R2	R_B1_R3	R_B1_R4	R_B1_R5	R_B1_R6	R_B1_R7	R_B1_R8	R_B1_R9	R_B1_R10	R_B2_R1	R_B2_R2	R_B2_R3	R_B2_R4	R_B2_R5
68676901.2	124705083	114470771	20891516.8	163559695	7448178.74	127492882	92216522.4	653811832	236011321	201012646	178246391	175544157	80631730.8	58168262.4	189638959	247433141	69004041.7	133502102	76908408.6	172116376	117487911	400774535	19236102.1	75883672.9	78419627.1	167623235	166384292	95655467.1	49599161.8	36606774.9	248017174	99626406.9	259735262	199608334	156606676	263918666
17271423.1	29629927.6	5943674.66	45461175.5	28689949.7	10096536.7	38189006.9	17663019.8	207039013	40937190.6	24816694.1	34401084.1	32079057.9	13624358.8	11506773.5	71479872.5	59887206.9	15462539.5	27370736.1	12443324.2	57526149.8	16435728.8	85274874.4	4318228.77	13830043.2	29666883.6	34394640.5	26798098	13580752.1	14684999.2	14631036.9	68018591	30288602	95833345.8	83941992.1	52846383	58809226.2
11241.7547	27135.826	46844.9618	17921.5863	102148.292		125071.048	144724.881	106004.118	31957.1145	83076.2558	14321.0581	67777.7577	90938.2894	20644.9357	34895.3698	90673.2586	8213.54923	64388.559	16875.9481	12186.5587	7411.82891	223935.645	7900.89673	5591.83022		12548.8905	89884.9219	90673.2586	36250.2125	39639.0806	104462.485	27693.5752	149329.383	28970.6324	14894.1146	42438.8586
47215.3696	30827.0964	60933.6721	341863.929	102769.337	65629.1655		461653.433	30729.0159	70922.9572	59088.2199		24726.9793	271584.681	117277.08		176922.925	18751.3759	25170.1966	52315.6352	26257.4316	26184.4199	333140.998	215850.871	27222.3436	142948.084	48343.8176	49914.1106	57227.2126	147316.115	75774.3886	177579.359	125159.045	1251682.8	204511.856	106890.661	150871.899
145120833	227462071	188436500	232561262	246274605	104758270	382450294	85983077.6	447212535	221624715	139650750	232486780	215340132	179622965	114826292	98218568.3	303596542	131526514	207822842	192119590	415592415	247926627	450309493	32533155	42786037.3	123824425	82588153.6	177965747	229724478	33008444.3	99522086.3	83396172.6	143629416	427698256	322747223	208721794	236938968
379153725	354162435	228060998	7376030.45	641825408	9667301.06	919712030	272905020	1084069440	596380596	466801434	734490023	442454512	407096748	395917574	1184626443	561101906	330457244	510855172	357705089	995333912	315375390	1582374852	16053477.4	39502852.7	78099845.6	486177579	496394021	391881008	36755923.4	129371586	286050463	342114854	443672238	635581712	443215701	331405769
35667021.6	13966969.2	13032057	28459741.3	92530439	9043917.22	12926812.6	37370615	5110024605	2122397817	15064166.9	34852017.5	22138875.2	40246174.5	9358521.24	104872837	46666998.4	29761727.1	28574059.8	11411332.4	29342570.7	18429252.9	80425857.8	9336505.93	16267732.4	13980367.9	34716223	32281654.3	35931360.9	18890942	44769406.7	31846441.3	1799396729	138775177	25797743	9588829.88	9713139.65
162494.454	1197168.79	3724702.79	2902257.2	851485.14	365379.212	216648.355	332855.88	995940459	386695732	618564.054	386757.713	390232.121	361856.307	121496.989	1708229.55	1141430.81	399555.801	1336850.06	426131.859	460642.269	5890260.91	10448725.8	470909.158	22690966.5	987864.716	15291303.5	20111328.9	7973035.08	2752541.19	1514203.41	7155988.95	1161467421	11220788.4	3447822.51	1714492.93	10158638.7
151252.699	354162.435	2624022.3	3554270.71	840496.024	301831.914	58389.6001	229717.669	559731795	284698788	378884.286	230228.953	286453.649	294918.55	89960.1496	687354.426	563673.617	261123.874	814337.089	185354.78	218119.009	4118170.33	7274901.23	306426.503	10500180.6	1034114.74	8712279.15	7513877.77	2259441.03	2539528.91	925617.022	1840762.53	792469812	7991941.01	3007164.27	1128550.6	6781464.6
421054.811	1576272.24	7898483.22	9217531.63	2158956.23	607409.634	202256.682	700324.176	2194070667	1108138634	1272071.86	733403.604	855993.073	733673.821	212601.143	3083573.95	2216774.54	571253.82	2546775.87	650870.905	625183.738	12700577.5	19249899.7	1402394.8	35150085.6	2439477.5	35068474.8	29030068.3	16363666.4	7734193.03	1084859.47	4471039.81	2300212541	20510265.9	6583912.25	3063285.14	15290160.7
46091.1941	363141.2	1373649.26	1519637.7	269057.544	114234.022	15135.1869	111684.387	481518685	243976321	384267.962	223480.739	231701.818	184021.362	39119.2139	1346095.01	661922.977	79064.8071	505916.366	209963.678	255038.657	2558153.04	4423903.8	239623.263	6101312.52	505571.007	6832266.78	3878597.61	955410.074	1173502.32	634165.18	586312.559	450101583	3691055.53	1639289.35	703519.028	3765378.19
636692.105	2783417.44	7352545.69	45965.9877	281275.796	25909.3259	2346348.46	201525.442	2470486.66	802310.712	3438569.63	2635397.2	3826694.34	723346.154	781083.714	15898627	14427006.1	3307489.62	4532395.57	701397.023	1626199.87	1274327.33	5309771.25	31214.2828	165457.763	342777.729	396239.317	6929368.61	7444989.38	318903.091	2022257.94	5474573.65	837552.887	970038.317	1883395	2135458.21	993091.13
3628020.82	18256824.1	45436090.8		446129.182	72343.0409	11043334.3	169857.083	17567107.6	5903429.54	17966111	14951492.9	14770047.8	7650141.45	6677617.09	64944737.2	33479602.4	10609995	17547886.1	8653001.04	17103580.3	6837120.17	30127911.4		407725.09	657315.624	2955361.64	83785988.4	74168040.2	858418.161	11265865	41132709.2	9065062.69	5284087.87	17164463.9	21430480.7	10922129.5
122637.324	382096.373	487821.595	515012.547	627527.158	505935.954	1389142.37	1874295.45	355453.783	756841.018	301429.467	749911.213	279144.146	3696706.4	1464627.18	4929417.82	344733.83	369000.601	304625.456	1706729.28	1441418.49	115909.509	396682.409	30023.3297	94618.4076	165939.049	142963.863	254180.009	666307.467	116944.463	82496.5943	704225.139					
6724613.24	14765081.8	14881200.3	7188859.44	19685732.7	8210985.64	10755444.8	14918768.9	7441200.98	17123915.5	8796729.22	8330983.1	7238110.96	8463363.98	6781372.88	18825515.3	15982383.2	11814404	14677290.6	6040079.91	8217054.13	6956000.05	20932239.4	1958026.49	2521124.66	8337202.7	4440605.1	17274967.8	15688019.2	4535705.96	4725177.6	12737000.4	10975347.2	10753946.3	6984932.54	8066232.29	5521529.22
105263.703	287320.51	171706.157	194038.321	299321.782		1503767.9	614879.413	112787.858	614964.719	319664.787	1408019.27	444269.288	465065.346	268279.416	592744.589	1474602.14	914357.092	146163.013	89950.0178	396527.738	26611.0722	3158386.51	7409.36012		141420.809	287860.747	432683.368	500298.377	181300.196	144556.912	2606311.38	67345.5785	4463280.44	487237.668	34374.7246	626994.489
137966.989	183565.882	66921.374	6062558.13	883860.756	988501.661	845693.713	2886723.91	80802.5873	834440.008	351582.583	1454021.2	345328.836	1130196.14	646749.453	609456.809	2307456.32	1356256.56	95166.0419	221414.781	613312.204	43785.1137	3621924.33	439322.829	274253.375	1480406.28	245202.568	190869.114	797552.718	1464224.03	410694.588	3213163.49		6978972.94	484773.671		740529.963
55493.3889	43098.0765	79425.1044	27046.9239	58910.148	6508.75718	63295.7016	270746.692		37927.179		298548.79	6432.16314	11153.6727		21299.0403	1486.03982	7841.08099	2718.15293		11656.6772		417128.382	52914.1736	62011.1925		238421.057	102569.832	52401.0841	52020.6962	37328.147		58843.6507	377541.46	221270.829	61169.8153	61406.7033
14205.49		187555.956				186780.939		2322367.14	1057790.35	103939.343	47048.9544	22559.8062	33358.8107	29756.7773	66548.6513	268084.505	69455.0404	60418.9007	41155.729	44385.2454	12618.1279	404428.427	49804.8071	18156.8975	1561974.31	187563.97	62510.9139	147339.062	58053.5714	12714.2179	334742.381	1651331.03	444082.435	342780.632	76437.5551	147807.276
		36982.8646				78047.9382	374469.238	250919.502	409082.71	105037.723	268033.344	64365.006	68884.6775	10743.4865			6777.28697					10813.8129				51097.9402	152262.328	112814.465	42494.5095		253413.718	309295.902	1113137.05	239692.451	428479.06	463741.927
101277.99	175584.756	268566.04	16451.8495	169885.768	26395.3539	506445.601	425611.304	702819.528	583378.046	357273.461	313363.559	290251.988	327365.197	159168.538	214945.53	393040.916	112043.292	90618.4291	123585.021	123224.374	26241.2067	660563.813	35444.6904	46182.9171	25923.7578	389171.235	414657.616	212732.141	69123.1725	50853.5812	1190429.6	170885.82	609093.961	307214.161	281494.781	368059.065
1512526.99	2593865.72	1276789.37	4777797.97	2533329.76	3484246.59	1734499.14	2156369.08	2569026.84	1491515.16	846303.017	781252.215	671370.015	1504509.43	1678370.17	6101159.06	2041978.18	1398050.41	1339018.72	2202525.02	3533195.24	1218821.75	2524554.54	905149.029	1323189.77	2834354.12	134937.817	881511.297	2056194.43	1517052.04	798983.93	2463948.27	2261614.03	2672819.87	2452099.34	1947074.84	1382707.36
384263.614	524758.988	253596.786	1132271.78	368411.311	639544.744	471395	523263.11	685139.054	217514.542	137692.35	177305.043	117654.717	352715.44	284353.498	1771343.95	368611.84	217733.317	324702.424	289981.866	793893.874	156829.299	182345.565	541834.095	403596.367	1098643.56	371472.013	236305.749	179872.199	250380.589	323832.381	78856.1962	370377.805	720638.534	479122.687	373853.2	256256.569
12672523.4	422999.64	13032057	6736046.09	27771931	191697.544	19441.6129	77957100.8	77608492.8	70659322.6	35556737.1	51873497	17315726.7	22994600.2	12240883	327166944	105479522	15186.621	149148037	40766042.1	121454958	13086953.6	48184406.4	7532114.22	15878288.7	9543583.84	47688.5895	30908373.7	14756451.8	17405107.8	76296072.5	41934843.9	57641244.5	33573028.3	53914044.3	40462935.6	18742721.6
12774721.2	10175934.7	29674346.1	3933013.58	6709713.01	3531543.58	7065840.19	7393427.97	11198553.4	3274010.65	8668871.98	4915985.79	4709948.44	2194093.88	2763163.17	18966941.6	4207114.24	5538020.24	67684002.9	8027291.19	21894618	7763598.23	40569262.7	515368.499	2048963.05	4235302.55	7587995.44	12627523.5	29487626.4	1860804.38	1080765.51	19954096.3	4158411	4512451.19	4001010.63	2381654.2	10016806.7
6264723.28	4778698.76	2668049.52	18308.7142	21226460	139446.136	4314523.84	5521634.43	2998196.78	1481825.96	9398747.07	2000958.08	12672690.5	1828452.18	638913.712	8535446.62	15738042.8	8782715.07	4445772.11	2590008.32	3126460.54	10984380	6547641.97	725692.524	1168271.67	1263642.07	2132980.3	2046105.77	18390288.5	2170178.13	2185928.21	2162293.39	4791271.6	2153358.08	3033423.16	2455343.31	579314.188
142054.9	74324.2292	123276.215	15579.5628		150383.236	134204.254	168911.373	83243.4968	364902.945	43347.8887	54470.0204	17462.9295	160943.562	49892.004	115524.803	1637072.07	28009.9151	815390.892	75023.7076	34776.2558	43017.6063	27316.0598	3347654.42	810900.435	44112.2276	66685.5359	224515.481	170481.762	310376.569	239062.968	303712.725	228567.625	284350.199	47640.1696	162622.382	21104.6363
12876919	28233230.7	42090022.1	2365368.39	32330269.1	1649636.67	54640917.1	40602179	30399564.3	29080000.7	49117685.8	27862497	34518272	22583965.5	13853617	75081147.4	39325950.1	29173119.2	32053672.8	26423759.6	50853518.2	24710912.5	22517177.4	4076512.03	8461985.49	13995727.7	24561051.3	56050970.5	65349350.1	10116371.8	10479164.3	75174202.7	18677338.6	31456856.3	22732390.5	16728035.2	20002347.2
137966989	155631943	86909731.7	28996352.6	168626353	126187075	103736981	390783989	146938240	165763256	137449511	78456915.5	74955700.5	126456242	132850771	412827275	187838519	68886492.5	155526324	65167729.1	128291050	35658059.4	136394448	74727432.8	100936774	185393406	69099749.6	91400498.5	95033005.8	117684782	131016142	77134925.8	177816772	173554829	75135136.8	51737215.9	148172830
	18655.8804		167953.532	304744.836	229111.435	265766.683		9865.43448	123191.922	3336.95251	7017.18062	4599.61366	192374.982	139142.933	591282.867	390412.492	104046.753	150029.1	736784.999	1051729.73	81431.3598	195995.555	110262.306	224225.532	317184.452	206314.769	135796.169	536565.533	187237.28	1929.11646	11370.416	329490.799	1052468.23	207213.571	2612.01023	349667.08
518142.692	370124.685	807459.21	71264.0191	3448813.33	1332836.95	804199.352	1324046.58	971116.98	1620984	166336.559	623818.252	593640.051										763505.011	625551.317	1007925.5	2093985.52	462965.262	660541.871	1571286.22	826699.447	682318.546	499026.066	581999.64	1749236.13	1450.94182	468572.069	216118.739
265714201	282332307	155856358	64377474	724802589	62505442.4	276058008	559248585	598017155	320121972	230806887	387780104	232605111	170730423	230336515	430656225	272583586	226386115	172292233	208951252	503172817	190538483	281903701	13433427.6	87412426.5	193553547	332673928	301347155	220800095	63778466.2	79899928.7	63003316.3	229943730	371846127	344762279	188335933	244388726
2197252.05	6275159.76	8444420.74	1252009.1	2562637.58	1487792.57	4371342.48	3298504.58	15112895.6	12572567.6	3432572.22	2973865.04	3187062.89	6093935.27	2234563.55	9945073.31	2547016.71	2740694.81	3654338.07	4320541.62	2536773.13	4200288.73	3020276.68	21400.0212	258904.02	759026.494	2074052.22	3892154.07	9202136.46	567878.916	4208667.61	3088882.96	22283495.6	1893240.08	5226448.14	2111260.05	1912942.51
26775815.6	26138185.3	25095515.2	8665293.98	40017942.9	11769330.1	43633271.7	40965032.3	66793342.5	34724940.2	26450484.3	26191935.1	19688285.2	27188736.5	25480748.9	92846984	86608791.4	19316764.2	22869665.9	20848295.2	48049268.3	16269180.5	41133072.2	24709472.4	13933722.8	40008943.9	26359310.1	22712050.5	28671872.1	18457688	25253179.7	9183786.49	22698430.6	58544237.7	24466100.4	28597269.9	29598899.3
242208714	351169513	1391260143	571307617	8382643.57	51703736.4	1154044697	166773848	1826037034	1019788622	1481814866	1074314246	1442145094	954690708	618940272	909348532	2527765182	787981551	870922632	495315994	646073617	613902632	2287740806	684729711	453029105	786258187	444609198	736598061	2049224202	643054209	320285680	1805909468	665917287	437840782	888827084	1045599005	934111231
91262608.3	49882033	55474296.9	59654433.6	98406519.3	28452452.1	56747210.7	144820510	55863253.1	66396003.8	69068749.3	40960842.9	48041234.4	41978733.1	60056328.4	89635062.7	64021613.1	86185166.6	41694456.6	76229683.3	88522212.9	24816554.1	153882123	25978609	29046736.7	61690909.9	49541261.3	47433255.1	89920476.2	15873050.7	13970023	55538531.3	28580701.1	70249674.1	52053460.7	76172267.3	58362127.3
674505.28	766188.027	66216.9385	899045.233	905878.017	557569.69	592326.925	870436.156	526261.227	491318.971	530424.018	663866.587	391572.781	124567.409	377853.763	1200089.96	791186.602	401326.89	721292.317	676311.895	1066357.85	519138.606	1204097.44	596957.178	1140157.05	1189805.69	616374.243	361493.657	122307.943	184947.5	144049.331	438857.387	1006025.29	1148294.17	670323.322	734627.736	874158.867
505878960	559676411	615500532	942367610	787641295	514948841	414941092	454042698	798133630	531960296	392642831	541794761	562388254	549961017	504760549	1149920555	894462190	490110468	624055494	843722721	1343696184	747006569	866004118	766745500	516467744	781273105	720463299	886847723	361176679	759477171	267981003	339052061	524215766	888614380	568874814	585374471	578872695
18089005.2	6464711.48	3407706.81	370322.973	1361120.8	643829.196	3205921.91	2175864.6	2304312.03	2496410.98	2148647.36	1830301.81	1682369.86	77365.7228	723611.574	4165255.42	3260059.31	11478137	2204782.15	637207.663	2501860.82	979132.182	27766876.9			848102.338	747379.819	9603438.7				16917975.8	2082480.15	12614057.4	4210405.06	2336610.95	781479.186
39857130.2	40105154.6	26768549.6	869653175	58428004.4	170075386	25573523.6	53803306.6	38782376	18771050.1	15625598.4	23698905	23872477.9	40603576.9	40084774.1	111414261	33055218.1	23724916.5	25555916.4	63685723.5	74417487.5	29799207.8	37005547.5	14533103.2	40862806.5	92095093.3	48279335.3	27147465.3	27359138	40644519.1	31560046.6	21563149.6	26013509	49778657.1	48459239.8	36776757.2	39453743.4
4977031.38	12470508.3	19371976.7	621166.939	3382866.98	52027.3986	20263105.8	871826.478	38315782	25473153.2	16854720.1	12948701.7	19802671.1	10805789.1	7120956.97	51231686.7	20533973.5	9657037.51	11215723.9	11199107.8	16837305.9	11418893.9	21698058.4	1091790.61	3642732.87	4694661.91	6386974.59	17113768.7	8349235.84	1363325.79	1789589.16	13580974.4	9875426.79	11996906.7	16938048.3	17593171.1	16024123.7
74604371.8	99764066.1	75990981.2	7603754.04	225153070	5896605.02	336171202	52662170.3	286346835	180869150	103045779	146087954	151751816	77580274.1	81132254.3	391692106	208084755	131754969	113924414	89254509	233177449	83538984	64646984.8	5218508.92	15449591.8	34179815.4	84184312.2	143652661	176349367	11332854.4	18801265.3	205405411	135270627	145864564	153458958	115422574	216642169
1696482976	2464172432	2122111991	88552578.9	2883686314	197856702	1787774140	1920404173	2714267940	2109863342	1676586062	3212519720	1929428562	1402310691	1051114667	3064130835	1370418205	1264846588	1999636247	1778949945	2985886949	1684902497	2533361684	586253304	710567687	1301261898	1291156467	3349792321	2224962731	234081627	1072285424	1949130377			2136446136	1611917978	1764510194
19519774	29230871.4	2491940.64	2731298.67	27592616.8	1755283.13	59337292.3	85384893.1	230321433	18763475.5	20961593.1	64556185.1	16728351.6	13298324.1	9732593.68	343322591	50399726.4	19232523.9	14581069.6	12088118.9	56866983.9	9106400.27	30413248.7	334352.294	1588883.77	6907852.59	9077555.29	41703593.4	36288504.2	2553439.63	5833995.18	3785420.19	6984487.06	31673910.1	40626683.2	9132930.46	20541870
33214275.1	54171887.9	103904239	68243509.9	254009423	54469593.4	193457895	170472545	233282612	222407364	221627739	165931488	164265018	161618109	115200764	340718939	231174902	56489909.9	118882225	192574884	164762067	99108637.2	207794899	63445539.4	149261139	135895584	128809091	107203799	210719355	129451398	42079723	101345141	162669956	193888034	192287056	150810323	142248149
17884.6097	83602.2874	30378.7816	33725.5255	103326.378	39947.1972	66942.1644	156760.334	93618.1179	66124.9143	59875.2647	28668.9638	13670.0497	1613.13898	8479.85613	5026.20581	16985.6562	53790.586	75104.5538	10033.3663	34942.1299	52482.4589	56597.5467	52060.5406	51209.8488	82801.543	45335.9033	29814.5904	22452.6291	66583.3657	21351.9793	27841.0314	15971.2244	25293.0773	25701.7298	21942.0102	28418.5537
436384476	768183309	493104861	115910761	695760411	336633386	409659106	1642868533	539207574	890700635	689109644	332647216	358130248	536280033	513515370	2748253529	1239056004	257660986	1070312319	302168150	688174475	127880956	674183169	521326131	375196491	452635767	272932869	416159550	599684705	616729716	345040767	255324638	632081508	674567161	368215283	161728668	438152077
111395569	141664974	255357874	9768466.02	179229172	9547942.19	261138302	165648228	274327757	232215088	164250682	155957768	145347081	103074266	94265359.2	413067112	259837102	92809301.1	168730527	178410404	173268602	104282548	267791383	30391367.5	64290816.6	74207910.7	90315900.3	169871602	391656558	59266973.8	122173183	161311345	95513629.7	169808570	137405662	78224487.2	112692900
50179104.9	121712161	220136099	68423583.9	121401756	68431498.3	143534345	150143387	168118152	100887440	101364919	124306524	75340522.5	85943722.8	59737276.8	303653992	386156879	61874520.2	244981799	97727189.2	146470043	85765330	376257900	3707635.76	17252408.6	58355820.9	47723613.6	135249957	180148220	6277243.11	65978057.5	100483985	130713373	135755475	116281389	52928765	132650817
774659094	1057499100	409453143	177285645	1186961243	583425586	842643440	2255350251	1152756700	1252215185	934896620	469491638	386546773	670225273	659360251	3178904139	1261030461	300472518	1010209187	356631999	649288213	159496206	1316801982	496100275	859984419	1170385817	830735973	380834908	738092997	542622434	433105360	552838255	1619680672	1707282836	667702262	421465947	1161954419
33520868.4	24841252.5	9069607.26	433396.119	7027702.61	1020644.95	19653961.2	7845143.2	9151953.97	8411671.33	7974253.07	6619439.32	9460813.49	306715.026	1179443.44	21560111	11551776	32718838.9	8262595.11	2659597.52	5740587.43	7157386.94	71997967.1	262648.723	285981.636	1882023.68	3117650.53	11064295.3	9149515.66	1152248.78	354444.346	27889516.4	7344978.82	43170907.3	8156436.53	5214539.82	2953460.35
330098796	513784940	487821595	20688306.3	554974560	58571480.6	671833704	258118765	672118491	745573946	515875508	471735829	639191927	217783018	324401945	1452204581	566764487	187927629	539567190	331855283	491567583	249094036	865233771	8760556.76	47966326.2	302971302	166210667	647598558	788852356	86487727.1	173906516	1679342024	444203688	606592415	604070062	597111862	741900346
6561096.81	10774519.1	2879380.17	11707514.8	5687371	8378345.23	15732771.7	22261655	36567474.1	4964418.74	3984231.08	5412916.66	2895251.48	3326618.04	4192524.48	42211066.9	22849740	3248950.65	8567982.38	4689200.71	21654652.1	4168684.76	19032641.9	2259013.27	3353648.3	10027998	3098022.14	3335602.09	5380197.85	3426671.55	8990369.86	1128879.5	2110782.14	9380913.53	7753514.1	2017067.8	5237538.31
126725234	149646099	193719767	61895509	252548364	153948159	180318658	136738721	202982651	216044676	154675012	117344105	115422584	153494284	119011215	647396006	278457237	93873330.5	215403836	145926375	225943930	149357816	341034709	1109086.86	22248281.2	40315144.8	81022836.9	153233565	207486000	16073994.3	119574089	138486085	145893210	151805350	126346801	118945168	161635045
147164788	239433759	302907272	2523962.65	303977783	12673409.3	243748714	111637906	318782180	336707186	223023460	154597791	187421876	193244748	137964787	618708690	275664422	118060675	244404939	198595873	301331324	200757270	341039307	1767285.71	12335890.6	18036581.6	75273069	356859117	545466377	8639411.26	23747782.3	378274489	182380462	160054627	112481957	104983575	157167044
736845919	648466429	695630072	10111632.9	886253427	12317412.1	1433576664	659219018	2430381665	1321962579	1024927063	1242239082	962740948	208297319	501064726	2120225863	1612043896	456459156	1137666667	462345597	1252463926	889713531	1837060284	240604096	440168195	654465339	2859481788	338771826	983819145	691717658	387314248	668220330	843709524	995410111	1762509279	736327440	1676720811
859483243	575638661	354859391	438013635	634105802	550003168	583738378	622728586	535256579	500212245	331354930	361247679	510546774	522565796	546972102	890651549	480173893	448895539	598794645	503405386	964724973	648549950	555096349	735164801	446212849	547412316	503350071	462815624	586775841	467670609	648217034	732896424	522012661	643089300	291789304	321651428	435455058
77976898.2	34618130.9	32932360.4	63650305.9	98790297.5	55933458.3	62898883.3	54445297.1	35324640.3	7657928.32	43694681.6	62871364.9	67757005.8	75994076.5	41272970.4	131914235	132745557	61861127.7	62587256.4	34655365.5	115519772	30576359.5	128199211	70198236.7	52657734.8	55127253.6	46557449.1	53176366.5	43126087.7	22751940.9	142150349	32288288.3	12816104.5	117567202	54506910.8	25691051.5	20615905.9
1992856508	5886079898	2624022295	1815062555	3761435587	2907307451	2962189437	2079809825	2520362235	2345345812	1881751398	2213516542	1840440385	3144821736	2827065788	6588948415	3860183139	1187010073	1943167391	3164555128	3869515628	1519656302	3472157856	3472663613	2461637583	2199058257	2275630908	3138758610	3584863815	1652442034	2986980751	1207344352	2056651323	3173368937	2252318829	2194086188	2441452533
81758.2157	22546.6789	36894.8101					127197.319		15891.2302	44087.7569			2221.13701	5432.84077	84770.8563	46084.3544	42195.5983	8170.97987	62731.7371	69215.4566		24176.1857	10266.7485	9319.01606	52973.9054	40604.7879	111338.741	15999.0023	57501.607	10010.8671		93921.2662	138934.217	75382.3016	17840.1729	63729.4143
281043867	354162435	340770681	433461149	483760611	524846861	276090517	257777167	334401387	313943476	273059811	228027448	224899207	317208402	310058850	672489944	394600896	192729490	328842031	338629028	532264018	280036271	329978854	147432625	352745575	470974479	168631959	402582545	287321396	249922730	335838774	254272846	332341727	295140791	313832185	293742577	296588751
2902416.66	4908392.05	2632827.74	176195.125	7989833.86	313171.824	8947086.71	2800300.82	5715770.61	5350950.46	4344167.13	4525511.05	3694474.32	5550037.56	4630449.95	8836615.65	3952135.08	3623001.12	3387821.46	5168762.69	7764903	3286434.23	5469104.63	257509.904	603011.114	1077392.24	5934074.87	8795598.32	11779367.4	2636438.23	114101.333	10787021.6	3157282.13	4117925.46	4163050.97	3577076.01	3370044.84
1113955.69	1107381.13	2271804.54	929789.937	1254991.97	1194602.92	871790.427	2623503.9	512900.047	1198298.6	404752.952	2019261.59	1421671.69	811850.573	507026.189	3015505.91	2928777.56	281554.694	1463928.49	586260.153	1218131.84	496019.645	2601912.09	143766.891	345200.864	1062127.46	290883.843	2508712.14	3001690.55	447484.487	457699.85	1252250.64	828012.09	968760.163	642990.999	528913.715	709910.717
556977.845	379103.451	518640.648	1890673.3	365037.134	195524.421	940448.852	173718.083	436204.047	424934.963	514521.716	193771.861	379646.602	855691.003	330704.779	1930794.8	276125.631	99463.7719	329719.614	1833154.67	906515.882	432401.568	669604.752	710444.336	279364.596	50539.0431	127418.563	1648765.91	152391.159	374999.233	1124225.97	1591441.26	874582.582	581821.211	330006.612	296979.273	161611.874
12059336.8	25439836.8	15849799.1	20819763.9	16158696.5	20913662.3	15235355.1	17512815.1	11349440.1	12758775.9	10383069.6	6853996.87	14872639.9	15832074.6	12548499.5	33321319.3	11977439.1	14706043.4	15168034.7	21499449.1	18194956	12946921.1	21213333.7	16005741.2	14942794.9	23468549.4	17246950.8	849162.268	21799451.4	4731840	15728514.9	8623920.87	12351692.3	11146074.1	20539937.2	14023277.8	13703527.8
23607.6848	95174.919	67097.4829	36456.6316		10045.0493	26197.8399	186941.748	84970.413	27982.4118	17850.7956	25736.0675	39677.1541	29233.7028	20013.8741	46381.4454	168962.288	35295.0547	39950.8962	9632.57904	18214.9397	6016.82315	60779.8517	4471.11689	5375.42018	18563.5381	13725.3854	27674.4476	115568.155	56312.4199	8092.77211	40955.0225	49642.7783	66140.8382	38928.8093	23233.6836	87153.0581
162494.454	316252.089	458763.63	11459.97	116229.681	23130.5715	685552.744	56111.7025	346752.066	525909.126	295714.809	299767.53	403829.944	246225.088	292724.728	1612639.95	983904.058	287491.306	480623.922	180173.048	451003.04	252435.823	437181.937	3134.53405	10790.5311	16617.6568	268823.424	393812.282	332353.58	32478.4377	103255.708	190062.639	706251.599	200333.795	222103.882	295189.534	390540.981
2248350.93	6125513.66	3346068.7	50466.8012	4605236.98	91367.0746	10682176.4	5272170.02	13192349.7	5415060.22	4363712.84	4883058.48	4561949.94	3309574.95	2688536.15	10752788	6897443.03	4580113.56	7398107.61	3581974.3	6476324.96	3267583.08	12936999.9	2201360.98	1118176.25	3277866.3	2809224.94	6750700.59	4950993.55	1143514.74	3503752.46	3995313.88	3349246.67	5644850.22	5207077.71	2832351.75	2875669.62
513032.804	662433.399	924571.614		475143.413	8944.75528	895694.281	116402.337	1472309.94	496477.104	755689.862	581291.956	533490.814	563752.237	410579.761	1310844.26	886220.538	370350.116	613404.295	677306.319	803550.725	495170.974	1003071.56	27280.9365	25653.9465	33071.5749	216754.245	1130530.82	740997.311	101526.616	109343.573	635423.245	887128.261	768939.283	540725.72	651435.514	645058.603
129791.167	438961.891	120634.582	56255.1365	185856.682	89385.8827	221925.741	87275.2801	223469.366	149571.421	130030.759	136884.675	161001.892	29875.9438	66633.2202	395829.782	234656.393	111626.352	175834.257	120469.159	354560.839	137990.59	302258.489					755352.951	92787.9493		80741.7218	535752.872	177832.058	164194.101	147954.179	155074.568	110829.836
5130328.04	6155442.88	4323472.98	11423681.7	6196913.16	6696768.24	6002623.62	6248485.52	11993455	4821972.72	3141119.83	4202828.41	3637264.72	3195166.08	2842092.88	17681902.7	18614959.8	4738601.46	5994348.09	5672113.84	13465306.1	4567162.91	7741808.89	7235742.38	4479959.06	6865226.43	5849172.4	5144889.21	7124042.74	5487750.36	6221021.58	2188505.5	5500929.17	11533445.7	8931023.22	5617355.31	8134459.12
	150643.74	13472.3292	35561.1446		16286.4929	25879.4375	129688.495				17613.5812		5166.76761	9420.08402	9903.69182	150072.347	49113.9055	32631.8708		11881.4912	9045.62351	95956.1729	45249.275	41661.2962	68563.8752	68874.3211	25260.9721	228118.378	51201.6074	24145.3106	50816.3437	48405.2661	147928.749	29472.2552	23697.7638	80346.919
177824.119			80309.2053		49825.5334	200583.963	1103188.18	146497.994			565693.446	151491.893	45294.1762	38308.8353	111310.777	694768.836	128136.239	175192.636	11296.417	33494.4174	34574.1939	564502.868	98617.2608	68571.6261	133641.614	201446.329	377159.207	34792.6777	103946.876	156971.922	356905.692	211662.697	336734.67	178424.199	133360.63	363729.215
169648298	465898189	117112404	30256947.8	80754851.1	92432408.2	96923477.6	129464826	84634259	107333579	92466790.4	56945827.6	139885693	136749518	71195566	267345175	118861373	82206607	187888729	138990368	153717585	98743993.5	207414440	80745961.7	46191094.7	82405017.1	41886776.1	62399258	31415768.2	49318028.5	216026645	64058984	51099876.9	47988051.9	49517554.2	46600276.9	32932569.5
14920874.4	19354228.8	19195867.8	2053742.92	23101606.6	3725853.08	25292536.3	13167644.8	28190550	27451563.5	19723282.3	15991722.8	23637795	17778596.1	16199337.2	50475287.5	24476128.8	16890919.1	24652261.7	15379499.6	21637003.6	18855172.3	115627534	3367250.42	10364805.9	22825048	3552443.72	15029253.4	50057524.3	14988339.3	1414110.18	84661277.4	16010372.7	18701698.2	19274885.7	13428724.8	16808473.3
4619339.19	997640.661	117992.949	3335244.86	11826021.5	1689945.47	1819144.4	3116779.71	3181449.36	103547.115	1166229.99	6058374.52	4960737.56	2499630.67	1736809.6	6871509	2169353.16	6622281.69	7210070.17	11263555.8	121393.298	8088253.22	8994267.4	462683.501	626977.12	1719663.55	3510082.64	2491414.03	1812648.4	1374479.49	5964625.19	2623758.39	380221.676	29032714.5	4639370.1	660760.136	921824.015
328054.841	534735.394	224538.821	96281.906	715210.461	93540.1533	246387.937	809854.006	373771.205	305477.574	322907.338	378579.995	322186.231	258426.362	343313.003	757989.191	147968.274	249192.996	127719.565	334904.419	515250.349	158060.373	252512.083	41452.4667	99939.6113	191465.644	283727.377	141305.067	302185.374	93767.1339	127409.348	180761.272	263118.917	244232.867	158082.781	281285.736	114132.667
733779.986	931796.377	1840337.78	358827.443	784330.637	4671980.1	202372.948	3185650.8	4471016.05	4740066.26	5781513.64	14196.7379	1341804.37	1562109.42	3082009.48	2665331.59	344560.117	4619609.02	1435960.3	737085.56	438388.954	108329.258	956637.509	14460147.6	13885186.9	4656714.85	465551.772	12855319.9	532296.538	9083081.05	66551.1514	629924.725	5294250.03	464809.42	17328647.5	2128501.47	982061.153
6796151.68	9976406.61	8550086.07	5873161.77	18941930.9	8350616.44	11517269.5	11862879.5	18907085.4	16687688	7686647.49	9955056.19	11709001.6	10744854.3	7821061.96	24116873.6	16019048.2	6592904.77	14258190.9	10670695.2	13841579.4	9393749.57	15330439.4	11364536.7	9873748.69	16351965.9	11878292.4	12659233.6	10763132.2	9957021.24	9068225.53	10652574.2	13740790	16068825.6	18614053.7	10707710.5	15663542.2
1400109.44	3621435.6	6586472.07	280148.409	3464401.96	807109.284	8469582	945639.464	7770816.63	8959536.07	7033300.63	4546933.67	7973794.74	1309249.07	2556210.24	6285641.26	10622213.3	3840156.09	4017354.84	1795467.28	3242695	2760084.76	4050239.06	315260.71	336326.797	1145494.45	2220253.07	6863751.22	14762301.9	666689.468	1469091.2	6273221.87	5174926.95	4463457.77	3939095.14	4503252.98	4702803.89
616252.551	1107381.13	351337.213		806222.909	14190.8062	1110163.56	462750.981	1088423.69	434504.914	416984.939	556932.664	331622.414	440781.95	443576.707	2495322.36	723003.815	371533.604	532352.082	498146.331	972227.983	343530.526	846268.748	34707.6129	22784.0861	106808.039	477195.443	652819.084	508256.131	10656.7445	120625.754	233798.453	450549.216	226111.145	395048.424	306553.981	596253.865
47726358.4	52176606.6	61726162.1	14945771.2	106153030	13529204.9	62129068.9	39157348.9	104263988	31768000.3	26676178.1	117624039	50613228.6	25087638.8	44715384.3	80533659.6	125499314	40886099.6	75079797	52898349	73338166.8	90275559	48211433.9	4538828.86	14507449.8	58319972.3	74416671	105466106	55832778	21081575.1	22957779.5	60907588.3	29168901.6	54028157.5	51885489.5	38390751.3	42377831.1
3985713.02	7941219.66	22894154.3	672335621	14906504.8	29279337.9	38559959.6	38479883.2	1399438067	16771319.8	13173861.1	35131965.8	6169565.36	43267626.5	5171411.19	20712207.5	101767348	3433812.88	6539408.63	9132917.62	22677539.6	47512482.6	157562748	148202789	77925979.8	92129433.4	38122141.4	10457947.9	980962.623	157027145	11268940.9	128452429	109590538	1356662470	502780767	1399341854	911055928
85743928.7	139669692	269446585	79730426.3	147040659	46456534.1	124653145	178543069	184580994	131075300	142885331	149969142	291408243	141445717	88679872	408901872	365758905	155901231	203722123	239038156	181700670	166053000	332789216	23594653.6	69634237.1	44450138.5	131434716	178659377	295567306	95759533.1	60881597.2	144259397	96933634.5	482023100			246573000
31476.9131	115726.317	63399.1964	16323.1356	108988.598	80461.7181	291990.248	127877.323	548896.885	315370.143	316763.025	117337.377	177317.449	85259.7023	38337.6414	249329.899	41906.3101	118578.989	316286.652	80791.154	233654.609	79379.196	187136.508	32601.8715	44775.8633	87090.7199	1737128.75	4905301.43	109058.575	795056.503	2006636.52	50149.5745	194374.906	166154.145	145195.433	139806.359	154511.13
29024.1666	138672.052	243910.797	4646.13238	10921.1081		225803.404	8989.10338	217646.469	234448.224	190660.618	151513.512	287780.473	111315.996	74456.7774	438450.498	351221.684	130820.894	209560.419	328802.971	224852.89	83859.7785	329124.861	312331.283	190186.381	32466.8735	171236.514	142170.775	80361.191	106163.052	223302.5	292649.403	167723.748	132840.589	115844.739	123983.164	112349.052
739911.852	954742.112	321398.704	587924.711	762084.053	843632.5	1188396.99	1420902.81	883836.387	809794.826	493055.79	629941.372	347037.82	709687.018	639341.763	1853857.23	1093592.54	613120.433	740491.946	601207.486	1633270.22	452678.778	949649.151	1135449.58	710497.18	1057936.33	227010.623	1012920.05	274719.729	700077.909	699565.998	698226.452	875962.478	1069695.02	718304.964	558570.946	572340.596
687790.99	1237074.42	1241567.6	2109537.02	1393620.19	1834788.2	1072376.77	1218289.06	1668163.58	1228768.93	694623.937	493661.403	599910.407	914155.233	856106.331	2321173.41	1916555.01	1153880.08	1110386.11	668244.925	1252867.72	611528.035	913730.613	1715145.76	1406686.84	2030574.68	864936.696	1300760.66	937222.343	621514.93	242961.788	1191789.17	1298488.95	1579585.21	1321141.16	1082593.89	795340.31
362802082	335207262	263282774	46380687.7	975582853	50176073.8	637984243	566582229	524904750	380970692	426936237	376965393	402976632	334328341	329425696	991772028	450688356	313074563	436522823	405024095	719937995	339474289	534610789	102435065	136981880	273596479	372765306	369901898	406200107	124685480	163130909	320230848	305214185	421377543	394709478	333685850	383617105
1103735.91	2204785.86	2307026.31		2101857.4		14984304.9	4899046.78	14277550.8	584158.941	2663820.6	1647423.96	956417.812	1335969.32	2634139.66	3936194.01	8264256.85	53041.5887	2067984.78	383275.611	6393048.88	226267.253	326952.977	1119041.34	67792.0109	6101925.1	239935.719	465456.77	4480916.59	1255109.63	2956491.17	1507344.23	6601086.06	15605285.6	13542.03	1001338.65	8254729.9
29637353.2	29929219.8	39536443.3	46391646.4	49423865.2	45077163.7	29883995.2	39065141.8	40852620	30546986	25650869.8	25307364.3	26848174.6	25333443.9	25382276.4	64586490.9	38963366.3	23925182.7	31213481.2	31298676.2	46444114.4	33195473.2	25360783.2	24929580.5	41638822.1	48812424.9	17901460.7	24881936.8	39754605	72275054.7	21604435.1	34465542.3	28721986.6	38042169.5	27166807.2	21577577	27421683.7
536538.291	475874.595	348695.58	46303.4837	341514.97	287557.58	123798.576	566160.034	192663.322	243325.151	215474.881	97816.4572	258863.368	175298.746	190549.294	497101.869	441996.504	313918.952	290415.073	320686.821	290265.048	288070.674	238367.572	79021.5074	103654.132	142149.719	660931.279	269401.021	172536.292	93274.2185	260942.757	263681.273	19292.3573			18264.6337	207444.536
929999.704	609558.444	455241.452	79214.161	1541940.31	329774.275	731802.151	747032.036	1233437.17	1119862.21	611629.882	358540.687	574783.543	542905.266	629898.483	1178911.55	793959.506	654286.95	815075.443	899862.045	841080.139	429034.905	725167.778	6413680.57	138621.599	170451.44	1178988.73	716147.885	918379.393	269599.924	321346.112	253158.41	323094.412	539684.699	356771.687	529733.961	286678.931
321922.974	324233.215	382156.267	7342.41134	587306.128	14562.595	850561.104	527629.712	938871.01	515086.224	390318.396	479827.931	406046.62	489444.682	303101.801	1030189.96	589463.647	217575.749	569717.986	424836.363	765403.095	316751.202	711686.777	407345.902	258944.878	564649.171	268660.446	444385.52	506898.46	241105.931	420933.518	424378.274	684099.832	586795.413	788091.255	551904.193	628855.077
212571361	130690927	207808477	6758959.83	487108497	14027598.5	292819925	417056117	509314924	261406840	212032654	328272411	214046172	155702275	130166981	265640633	211344284	160618414	161159849	312758572	337250564	169447387	132634857	57184784.5	190500170	256839471	474657222	272710179	54409477.4	82308213.5	161303392	334807591	229555019	437932980	187886019	292041045	340801833
3433845.06	10475226.9	3980060.66	3003563.14	3254837.11	2988641.03	3287927.28	4050525.19	3090072.78	3659207.61	2033663.57	2967418.96	3298274.73	2335063.47	1861026.83	12534887.9	5428130.68	4509510.13	6017343.76	3833235.07	3945743.14	2379446.53	7109642.4	2107899.5	1458552.27	3014622.32	2236987.98	2236832.95	3591184.96	2090139.82	2385362.25	2868593.81	2611623.64	2672657.03	2836967.22	1642390.94	1728812.55
9657689.23	18556116.3	14000655.9	11504476.1	20849703.5	10884866	10776566.9	11928625.1	15897146.7	19430075.8	11137349	10293742.5	12030406.3	8758290.3	11620697	44093492.2	10693100.2	6883882.88	12054471.3	18804968.3	22161832.3	12814589.9	14119409.9	13204343.7	9822015.72	10176253.8	11009023.2	16115783.2	11664621.3	8543187.85	11868419	8700660.19	15207066.9	14910122.9	11689414.3	11867112	12220046.8
2473186.03	4279878.43	1963614	17657.9468	1759954.91	692842.78	1790036.05	1949926.21	1617202.55	1872900.1	1826824.92	859150.666	1654771.39	1399085.28	1245035.02	5475157.73	1438311.8	1159131.44	3136770.72	2927661.23	2379159.98	949250.332	869977.418	418050.572	569945.286	530543.822	56317.8358	240898.037	1487774.97		892243.022	2115833.73	930069.007	661564.688	672489.111	1211127.08	423615.992
1400109.44	2444219.62	1990030.33	153992.147	2078708.21	1004417.03	5226470.72	1512697.83	3457205.77	3716017.12	1690189.99	3247140.64	2512755.53	1691735.38	1967181.53	5114026.77	3812812.65	2009861.24	2857722.87	1223211.96	2081264.17	1743087.39	3348101.84	1092731.68	424192.641	908340.831	393250.031	1671922.48	2676365.5	1279292.26	1526644.09	2698028.94	2657857.37	4194281.48	2645092.11	1974215.53	3052200.36
40674.7123	96372.0878	235985.898	88134.3225	96372.0878	76751.2605	65477.905	9504.67331	303464.832	195835.714	46204.4508	128645.252	60708.8434	58045.8469	81903.0576	183816.766	39761.7583	138355.189	381404.572	110073.319	145294.191	129794.981	275260.664	106193.198	76423.2282	77479.2133	72642.0247	149190.828	76827.7869	184215.622	135434.469	41507.142	152758.996	96791.381	874757.956	35862.9245	58655.2795
9402.19481	166605.99	286176.928	4318089.44	130308.489	48863.5199	21149.7268	102328.385	6060593.14	969325.707	264468.624		190030.606	483429.212		1499250.86	421786.852	90290.0498	180828.277	324654.885	440398.241	871474.368	538389.429	30009.4757	178759.459	152427.819	247968.844	988907.363	1071642.99	2096309.37	483320.242	403504.046	8241907.39	2236173.48	464950.128	1688872.2	1832119.01
287175.733	275348.822	173467.246	23868.4224	19085.1142	70899.2453	30155.2825	203667.983	103324.773	46014.9133	27097.2622	22531.6748	89612.8055	98463.0904	52785.0287	415744.171	263782.165	294091.289	218889.437		12183.2672	8205.50306	23419.9841	25736.4084	10489.8306	32479.7736	44933.9794	101948.916	51262.5016	305511.973	30731.0296	6340.16046	66560.3477	6901.39819	60523.0194	56997.313	15899.3641
368933.948	465898.189	125037.304	76263.9914	776704.926	441061.156	171348.52	824456.719	252055.455	158620.328	204181.349	164538.078	123008.602	213347.584	168847.245	118178.162	161606.697	166975.518	465365.86	241750.734	449175.366	133494.644	127725.433	23903.9842	120856.443	172088.608	231021.154	287457.674	163830.462	207713.478	135753.296	224022.993	239197.305	56350.3116	43602.7546	6877.91257	101847.921
3004614.43	5886079.9	49398540.5	881227.851	4896249.96	885360.995	23185291.2	2468305.27	21173706.2	17602227.8	13959922.9	20848137.2	18464653.3	4164919.65	6937901.85	21459400.2	14070221.8	12285440.8	16754384.1	10574063.6	19420795.5	13772814.2	14653211	2322279.66	5226747.5	12430505.2	11234048.8	12347459.7	17024253.2	3604636.56	4256571.35	15025944.1	11346501.7	6650062.89	19818168.4	9112294.47	7504684.57
153296.654	102756.988	43058.6209	186491.666	161511.322	129596.052	25005.5427	76681.5991	84268.9559	40820.4327	35431.9871	72857.3116	140011.488	300775.964	87321.9295	936607.99	860179.061	119249.162	167349.91	28240.8513	363341.805	42082.9275	153685.446	142741.163	85496.6659	80395.9452	38898.7048	26174.7544	6237.36745	305220.164	14216.1055	139385.726	36891.4669	213489.054	81441.7109	11612.2377	20706.1343
472153.696	674405.087	2201360.99	525241.051	958832.898	419805.593	5830875.63	703591.787	2926264.23	4533837.75	6375620.66	3500311.74	3431475.27	1130522.61	357371.565	5509919.83	3330445.81	3646744.24	3097325.54	1622516.09	2270311.3	2060319.07	4574833.78	1473824.26	2010318.18	1780339.03	1281572.19	511194.471	3422999.38	1072850.53	2360108.77	2444391.79	845064.37	550470.878	2536959.66	1291439.61	1489974.74
206439.495	341193.106	399767.155	526140.695	375488.687	513614.125	160048.63	243117.586	420115.778	439262.335	92433.816	56372.1389	88837.1912	113061.694	157608.055	350081.871	254143.31	80264.0528	314553.509	223430.431	295149.763	103124.558	205291.346	381642.369	360452.242	191418.089	409022.145	320914.1	279108.546	198354.334	209121.791	265363.17	646822.91	804129.229	588616.265	605739.327	535182.546
51303.2804	207509.257	82507.0098	24655.6735	108452.103	96942.8281	111464.325	172063.705	103987.949	167922.304	88616.6085	64880.4642	46921.2719	80946.4999	23641.736	139724.608	148281.869	31577.3013	85288.0812	107351.825	141261.191	63497.8547	166259.916	103338.995	90673.4101	83247.5623	92983.9573	152595.641	98842.5703	83854.9346		53373.1788	259559.568	264098.746	130299.788	129240.963	59280.4742
40981305.6	55768112.9	39184225.6	1287035.21	56297136.4	4284550.5	61392327.9	31682391.1	68492512.1	55605777.4	45857261.3	38851789.3	57379357.1	23415547.6	20909817.5	57935558.7	38022174.9	42022590.3	85808747.3	28491492.1	29878620	59061295.2	218673570	17338067.1	39659933.8	25980927.5	27769766.9	44669829.7	28480743.6	9772663.01	14075554.6	31988717.6	41912843.6	41485316.5	54626431.7	32886673.5	54434738.2
945329.369	1656083.5	715882.593	1382935.51	1673294.41	1879066.58	1201287.62	1776689.07	1278747.74	1136064.53	863690.312	709477.044	936189.98	1269044.66	681454.096	4604196.32	1473396.9	822890.268	1571410.58	821730.7	2455976.82	770327.937	2504008.8	784908.92	1283053.62	2253192.51	1360296.78	2964637.84	3532485.6	106584.173	2858222.46	635230.368	1435279.59	1808997.67	1087567.75	1073760.85	921217.033
438428.432	480862.798	385678.445	179573.754	453789.019	590338.751	487992.108	559214.89	895670.309	715962.379	339505.055	455519.806	375043.338	625878.915	481832.854	2215044.01	1254846.25	325465.389	543678.575	188900.562	531911.616	343399.428	734555.657	103764.118	279257.014	250141.146	371152.077	172536.813	768476.115	392822.407	662424.329	546977.58	619918.103	478943.071	338050.738	211741.731	350109.342
227901.026	80808.8935	156736.902	48520.4975	219620.994	17140.4427	51521.6437	1132302.96	32249.0995	37948.0311	6701.54071	37611.1003	9461.94487	14696.6589	5790.25709	192764.172	41275.8905	20721.4807	44373.5716	30601.024	62555.1084	28394.5767	599485.495	9767.82081	11588.4844	15327.9677	34074.9089	61295.798	114458.103	10137.7724	444395.217	31759.5897	10875.6461	324088.941	261439.8	100210.802	74134.6104
438428.432	283329.948	42354.1854	63935.878	157603.227	168487.59	125011.33	249244.143	231516.345	213160.41	149882.632	133547.406	208108.028	209869.449	198227.684	611926.716	304664.162	110289.145	186107.636	272069.675	253785.785	152212.99	449856.265	43038.1609	159993.512	221808.275	184548.051	262992.017	240996.832	81514.509	131587.288	145134.554	206106.416	262141.903	133042.691	112789.018	197461.595

R_B2_R6	R_B2_R7	R_B2_R8	R_B2_R9	R_B2_R10	R_B3_R1	R_B3_R2	R_B3_R3	R_B3_R4	R_B3_R5	R_B3_R6
447971872	168713427	358037844	196017524	348770868	449497534	226083638	325616077	331548545	175426025	283797955
100534689	32475009	73572.6034	88554943.3	115171457	88457693.7	58893226.3	151715872	53558202.3	52903809.4	4351179.34
15560.6233	92492.2861	80254.524	73472.2175	279407.123	111657.37	54887.0556	60926.5857	154917.894	84426.0501	78050.858
128181.822	118420.529		111975.465	380553.503	383291.657	208281.997	183753.049	145115.665	58818.8004	405425.063
98378472.9	352461161	135899543	185830530	272593025	279089913	311419401	597162658	211242315	607632276	35169238.8
315886154	569935703	498540387	552826279	731616233	586970537	544864816	761387901	551488727	896090643	160260773
15239485.1	617696080	25533773.1	118803470	49038471.3	32979097.5	38400861.5	24882638.7	22213122.2	25806609.5	28344880.1
7390677.09	461502397	907709.971	54871599.1	24432800.2	1712511.2	1146679	2663622.32	3057001.94	11513669.5	5735622.16
5169132.78	368943757	721418.167	56493508.5	25828493.1	1263469.15	640002.368	1391249.77	1923586.69	5923777.66	4691022.81
11261002.3	959817809	1632381.69	90508384	48090664.4	4184215.5	2685614.52	5486518.62	7162257.68	25004781.4	14689550.8
2544816.32	218617394	298909.042	16394844.2	13867708.2	1043078.32	687102.712	1624097.73	1327974.44	7332003.64	2417861.03
2222865.48	358885.84	690291.168	6165547.82	2432229.21	951781.137	2742130.71	945668.279	1451913.42	8341004.56	1832424.83
11163501.3	1730431.77		47280321.5	11752911.4	7338409.82	20209999.5	4670182.89	5887106.95	37421413.3	9726920.48
	153631.032				338718.906	282780.878	342784.402	385162.12	395806.819	1725148.74
6291280.84	13151073.3	12511388	16657336.2	18886553.4	10101228.6	5365311.94	7729237.3	9953708.85	12486329.2	8342853.72
525809.559	655527.82	745529.631	828391.79	2471305.83	4068080.16	2825564.99	2757578.06	692708.723	962437.28	436954.503
578712.816	1593069.64		2415559.04	5612366.23	7837371.01	5005212	4334389.31	1141002	860576.21	1240968.8
47324.0776	55979.3368	35867.2563	499858.702	266034.998	19959.9794	15026.4013	22633.3338		14232.1056	163377.107
97459.2111	137410.005	166194.172	50027.6991	845367.87	515756.676	154028.294	146282.713	237565.403	101339.936	74766.6423
671789.934	14411.1304		129438.502		1019648.33	768107.889	75992.1983	326574.235	64610.2208	2280605.51
397678.74	264666.565	511933.841	428041.019	518306.428	939007.359	378212.712	207502.626	519826.523	252671.303	496580.678
1601257.43	1952035.64	2635021.15	2460908.76	3092919.46	1952071.47	2289862.48	2008845.27	2404605.31	1980037.68	2314944.32
233979.397	561310.765		505204.111	787080.042	408650.371	402194.033	522568.513	438586.578	557018.366	48307.8442
18264010.6	22527890.4	8300552.47	2064315.95	86658147.9	13474144.4	27759737.9	32177261.6	15235396.1	14669067.2	29449669
4825714.68	12434346.6	13027780.3	9974388.83	14276297.1	3707071.97	7694960.23	15575427.6	9768313.69	16927882.5	8472626.7
3061391.2	1253005.47	8894468.05	9126415.16	8226684.61	8803209.11	4224406.41	3891167.06	2715100.83	1553612.54	4647840.02
169933.135	277548.11	386235.31	137177.15	199147.989	50210.7592	61600.8277	120500.9	667129.226	94096.3529	601148.788
18060900.2	25026141.4	27950826.9	30730653.2	17629390.7	14956903.5	33228670.1	18093623.7	12712354.6	32045607.8	25800535.4
51096405.8	85428303.8	100985300	79607047.1	270202278	67982878.1	51733229.4	175073688	58717606.8	102561798	109589848
205919.833	693152.191	270016.125	4369.00689	191167.896	334738.085	242727.929	637013.542	171021.82	185877.326	76106.8829
763275.126	401888.47	470407.213	828417.831	1195490.09	130887.974	538971.252	793100.904	599365.718	1608537.78	323716.711
162631316	193885902		415693494	255558788	296246002	340663960	309458047	235762344	221108468	222631975
2231174.92	8180697.97		11603014.9	1597916.63	2448579.65	3927496.79	1202274.38	1122796.38	2621303.21	7740872.92
16656770.8	11645904.8	43926300.8	46537435.4	16832807.9	27413126	38096121.9	73537342.7	31761117.9	73581124.5	8416070.3
1426124757	800401972	978728519	1225045910	2823150881	2265347710	999367950	1902822054	466120003	1549681461	60846635.5
43346959.5	49788059.3	60210162.6	83792441.3	128838653	73617924.2	49078286.8	69246776.7	38346623.2	52709882.8	8773876.17
443727.93	889464.173	1505163.41	1345430.95	1476186.59	2206292.72	2419870.68	1672031.54	1201195.3	1615181.11	1385171.91
525827083	800015908	929997117	730346656	923287471	722452991	704710814	806776164	966084873	819830582	558904070
1523332.8	3849767.6	1574038.61	7289916.77	6498393.13	2812067.03	3230749.13	3367167.79	607658.336	1847196.09	2717928.27
37299416.2	46090455.2	70033506.9	64015274.4	64125680.2	72557583.1	59431406.1	55948920.8	59983343.9	45077281	78305228.2
20211245.5	15301131.2	22706774.9	19651989.8	24818981.9	35038477.6	20400642.1	23563539.7	26168739.8	29294991.3	42988672.6
87619570.7	164189551	220513282	196792893	211674336	204558845	180743645	167135878	130115749	219833611	41786327.8
949072821	2711858578	1673140404	2145649069	1791443938	1906715282	1855679372	1779385870	1736334432	1681800016	2850259449
3634152.81	11650944.3	28613276.1	30817095.9	27106437.6	53754362.7	92339480.6	119850860	41945613.9	20932684.3	18765587.8
99471156.6	146678960	214394088	179060575	204216691	217407196	165480755	241712671	246243852	164712441	179371143
35318.4761	12168.3205	10582.8707	16703.8453	60534.1732	89764.4348	74953.2581	62433.1337	132994.949	31118.9691	14495.5755
212469089	629769678	435303912	592449712	789804506	837129073	474849914	826217839	637244186	808826117	571626019
101423435	134192404	159090765	208489068	151174486	107418530	126855120	96183507.2	113768671	192620288	142130516
74938404.1	129780272		134557499	275273509	153848094	143631286	209335032	106918794	404262458	94044029.1
474700358	291685404	642013307	508470447	820806692	646989747	467332464	1355033368	437358833	745404125	571978336
6096254.54	13044603.1	8932803.2	30480962.9	21093055.5	11832708.9	8117445.52	8281828.89	2668528.28	8295610.64	13169601.4
462408382	479006667	364501931	493312234	407051019	413259824	554841654	528046403	498165605	698979996	462735951
1167189.12	12506436.1	13827607.7	21836461	23619024.8	8519158.24	25307567.1	33864342.4	12858398.9	30807601.2	1234464.42
85692714.3	200067181	191159006	262937610	267441402	84877278.8	168768673	193131701	172478173	293202868	138445896
97478288.4	292520492	195838626	258054409	324354451	98633977.4	181701171	262565493	208680042	316016153	147406697
1123250480	984937145	1926844698	1241643354	2022284134	1856198442	1251783288	1521745710	1986334476	1668250328	735423751
249275628	512463260	526553677	749731615	775038101	337874986	499758406	324584831	521113822	574738940	457747463
20608959.4	63273479.2	74788522.8	98127922.8	112475393	57133858.7	121403357	60667778.9	50814476.1	116387702	85004063.4
1045587565	2518084762	3073037557	3955180509	5299256344	2166695622	2655317357	1629994205	2311835634	2510706824	2602046928
34267.1749	54578.242	42309.5616	107265.828	265794.596	24879.1434	42712.7186	133309.94	128944.922	31524.5689	224056.619
215634587	422551527	365935439	423723565	486023282	462476026	361485638	599923185	509773232	436667305	287618865
1136703.97	3429879.94	5100207.4	6249780.69	5711358.8	4141068.84	3815746.51	3370325.28	3098895.15	5189182.43	3115984.04
794862.926	1285830.1	1542813.44	1806755.51	1574696.52	617780.576	747539.215	1006865.87	463096.715	500676.463	806338.495
81831.8212	224719.465	121387.739	512434.748	998437.592	270502.108	162412.045	287664.177	1644048.49	429546.326	599115.101
14991660.5	22780356.7	6535139.51	9234955.58	17438272.3	18085273.4	23695522.7	19506667.7	23436944.1	13937557.8	20080679.9
129848.251	35768.9849	43192.6541	20868.6812	62933.9909	36977.4629	23859.3462	80585.0615	59388.394	17858.7725	200193.588
140010.135	622984.032		289524.837	93844.737	180676.248	419423.003	223071.479	204527.889	780049.292	26650.4133
1556101.75	3607633.9	79672.0749	4617465.03	7223727.37	5743684.61	7826197.13	6678555.6	2995083.6	12644546.6	569862.535
561384.968	893278.459		630073.899	777779.85	788238.457	1051091.63	502384.578	803136.003	647959.628	752030.017
102347.025	183074.828	184062.25	296964.335	177538.807	236682.791	174930.674	154968.048	139229.504	234759.751	417234.897
4443973.72	6398929.71	13577258.5	9884415.42	8874469.11	7588325.13	7277814.19	13428333.6	6421940.98	14564489.4	747242.237
88684.1068	48391.0881	38065.1697	36794.9417	112154.776	64898.5146	23075.7772	137508.609	86899.2896	70437.1571	359048.25
384052.355	136099.284		131896.433	407205.78	164347.527	94242.54	403159.334			435205.963
27574005.4	105617143		23079090	78804094.9	41766499.5	83092583.8	74687946.9	40070885.5	46991218.7	16137323
16227335.7	20921115.5	23804012.8	23910637.8	22224984.9	27501485.9	20128823.7	26207062	17506485.2	34240008.3	16015655.4
1287874.93	12722326.8	169379.054	10720773.4	19491064.1	5204970.53	16463526.2	5642319.9	1210799.17	7743679.6	1972878.49
125726.211	99712.9291	255471.902	229669.264	134937.341	240576.432	481073.229	136452.951	98788.9071	179861.362	397780.238
579046.501	679149.729	789460.891	13876095.6	1115755.13	1065835.38	772059.561	22700092.3	3654228.9	121387.475	6767235.79
14836032.2	15050762.3	2689.29943	18078722	25039628.9	13946906.6	14404165.6	17047140.6	16176597.8	10786006.3	13295375.8
3475254.92	3145844.82	5357534.41	4028004.25	6055120.71	3345931.83	5226051.4	3367486.25	4300635.94	7449198.22	4140214.41
208961.621	570865.488	433919.849	483210.535	1073942.45	770705.426	778507.602	592187.271	221194.914	941687.842	46181.7121
15009194.6	50936140.2	53762952.8	65128978.9	5695.11846	49073663.2	61172263.8	36530532.5	58585118.6	74994279.4	67541222
5179376500	34687036.4	464876025	524881249	563086669	2400794734	112713698	1364826340	1387802226	49081016.7	1880795442
	147576873	105753.679	155119891	118512142	192925831	162208311	222403471	146208737	294033441	172083076
378598.39	191983.795	163493.35	175145.699	132645.677	272130.921	127338.895	348434.434	118274.75	92176.8583	412546.86
117862.673	190562.834	147766.661	286545.774	135638.894	180987.515	179092.562	152055.668	140296.876	337670.906	390545.668
503468.724	500851.086	1018702.6	1119803.85	840541.186	833239.347	796137.399	720607.569	678910.649	776203.757	780130.319
925918.037	1091055.98	1745730.82	1608750.43	2057893.22	1244449.2	1354787.68	1120057.77	1388483.21	1281951.67	1410891.37
236316779	624083900	787876552	633415064	901162714	385726004	411113335	403319455	386685509	609239838	238514448
5556825.45	366768.874		18319275.9	3184731.33	2671991.54	709452.829	4712756.63	5367412.27	5278912.68	38754.4726
22681026.8	31256049.7	40322049.4	46053988.4	32720073.1	44438381.9	39020171.3	41981136	31349995.1	43003360.9	26993195
5798.82793		366159.278	532530.997	375609.463	575670.361	633703.145	211266.039	243335.559	325130.665	110680.429
262849.203	555206.134	315546.691	614721.181	808258.071	338673.154	894547.676	404069.689	445369.88	543322.274	513883.036
479827.931	535692.998	731423.668	986619.166	619724.597	489463.407	421243.03	433263.235	473645.969	767919.511	714761.733
206764429	163132955	317532991	286816611	325767878	368412210	337366850	398668856	481785514	165074242	386973156
1619610.78	3080291.89		1255213.52	2002811.08	3199167.13	4669854.16	3130702.93	1951907.63	4572612.5	1580308.31
8095661.89	15839252.1	12147276.3	10523069.9	22169216.6	9038335.69	15313983.1	12454110.9	13614278.2	17844439	6451701.43
794657.312	1093626.72	596499.883	1491882.67	834758.598	1437838.19	687157.218	461074.763	469267.724	683548.217	1186726.81
1879076.43	2072078.02	1525532.03	1811343.37	1609476.42	2508258.81	1760209.23	2161126.19	1461224.78	1782447.21	1718373.65
83832.0806	23566.8415	87826.8125	335594.397	174196.629	93037.7521	132465.887	20942.3242	114398.979	221433.739	41659.0969
2779069.61	1007096.08	456239.462	2880592.61	1568492.28	1025999.53	225479.615	1408391.4	1951318.9	407522.592	3139580.72
45706.333	42468.0413	58679.0081	60887.4983	15165.5645	270126.67	110858.987	51848.8153	46352.0951	57604.0684	45327.989
131102.155	456690.986	37918.7153	8836.54259	514359.056	155389.415	255502.942	123936.099	166413.038	49832.4103	483932.945
13919855.3	25604181.5		10697909.8	12062468.6	16583179	22706810.7	13410965.8	10544440.3	23861898.6	10470561.3
52432.5509	457265.139	141684.587	406551.71	263000.972	153455.986	225453.445	153069.315	98274.8436	225819.641	236243.683
3455687.98	2201819.57	983248.9	2291126.96	7469886.67	4194689.95	2618762.95	1716471.59	2472574.42	9353798.59	905629.16
436902.585	272097.962		328643.248	261172.947	383850.839	266518.641	424823.201	419821.054	266947.483	368846.473
80393.8259	53048.1336	272013.724	176112.001	136278.099	349709.456	129575.402	110118.878	115688.907	137613.187	66286.7812
26775621.1	38229117.7	88975770.1	65150680.9	125507446	33407762.2	50971248.3	42118002	43528490.9	50802672.7	34007021.4
873061.713	1066039.89	1877227.86	1849918.45	2056449.98	1674005.97	1609078.77	1256337.74	1135206.11	1570061.91	1549945.66
138510.03	454653.589	483757.461	598119.519	632439.094	456234.827	642787.734	430540.591	520415.013	689589.57	94322.779
12480.4954	11085.3884	8809.41034	354885.411	39962.3937	34583.2796	18238.6593	111617.756	69912.7963	37453.497	281912.487
162761.986	268128.772	293016.41	229999.319	241520.313	314451.975	281489.598	118615.942	183194.827	162539.976	79250.7079

R_B3_R7	R_B3_R8	R_B3_R9	R_B3_R10	R_B4_R1	R_B4_R2	R_B4_R3	R_B4_R4	R_B4_R5	R_B4_R6	R_B4_R7	R_B4_R8	R_B4_R9	R_B4_R10
156590080	168731308	315202525	468345231	172142504	207756610	275889701	74919864.2	190093683	252971903	411673388	51315259.8	60167075.2	163164101
2702476.22	56100715.7	58088394.1	1705565.2	46242201.9	69791292.2	23701433.4	24666696.4	61264478.5	22551758.8	131070899	2397008.11	11696816	52424915.2
8886.59667	48027.7039	33881.3781	97382.7369		15550.6444	70351.8738	11706.2288	106235.213	90991.4442	47167.0631	14946.8609		11295.9762
20573.8494	241215.586	71902.4684	75078.0044	14513.9758	55609.1045	38624.5582	13127.6994	84944.7202	145115.665	10522.5933	5524.13938	27937.7188	
23991874.3	348661561	191578276	26344545.6	325158062	625757932	238268378	184791183	489898578	92168057.7	592588151	31271312.7	115285165	497216046
147075113	562439026	373847174	161873108	428668587	801169201	446439698	285130287	823377212	261796504	658123601	95053769.9	164092023	481768557
28366821.1	13121008.4	14591134	28989480.3	22839822.4	29857237.3	46650440.4	7583963.93	28242490.1	29905593.2	20583669.4	730123160	15231105.7	16509503.7
1604440.28	1028615.4	11214007.1	6869300.66	30378088.9	955431.593	5204283	729966.98	630024.779	20198531.8	17445352.1	695683388	613451.717	12744178.3
1520653.97	830658.761	8152567.12	4299658.09	18114341.9	464031.23	2746356.57	525107.977	544211.059	12550544	10614896.8	360928807	366892.934	8293370.56
2041610.98	2794702.35	22306636	21061250.2	74145039.1	2226852.28	10107595.4	1020114.22	1422987	41083421.5	34983007.7	950537699	1363226.04	26936558.6
262875.598	585490.113	5209086.59	893610.429	18114341.9	536186.22	2006470.55	193152.775	410602.356	7098901.46	11630234.7	170132472	295365.642	7800981.85
1644463.78	3469209.88	4321868.62	1236858.07	35553615.1	16172670.2	18309043.8	12960467.6	14229870	11962237.3	30275531.7	2155929.71	7354688.63	17474971.7
12396978.6	17093349.9	27480301.3	7047905.48	69869604.4	87954444.9	55930366.7	41724344	76906473.1	43632750.7	117225382	12329438.3	37025892.4	78106365.3
158264.141	260779.059	388821.743	382341.833	1800183.04	1189313.29	255824.996	226599.143	1052575.88	283367.752	168915.314	148091.018	153994.052	861197.501
6897617.44	8361273.38	10689836.8	10189144.2	15189044.4	6357103.44	11913418.9	10368374.1	6843372.6	8363760.98	13568607.2	8954340.64	6311231.66	10040867.7
27353.9753	602338.076	453674.994	668740.505	132763.499	2139768.67	716059.179	401356.415	1966111.81	148057.199	294448.007	15566.7768	247400.281	425771.41
239324.369	1214944.19	961320.593	2894658.96	50517.6367	1592385.99	477790.801	234124.576	1260049.56	173550.492	163377.107		159043.038	159302.228
11727.7246				18451.8762	34957.8487			69411.3507	52751.5053	163377.107			251021.693
25945.7207	184449.544	159781.256	66531.6206	54005.4913	237613.847	64708.6754	43647.5102	291114.898	181394.582	172607.452	210082.607	110236.18	35432.6775
54519.5387	1762204.67	251664.267			12440.5155	22321.9849	7174.24592		18139.4582	30183.2283			
100830.663	384597.776	140213.251	201142.351	129388.156	440394.25	240776.467	164723.362	300891.145	228459.122	495669.528	140514.269	134639.609	189231.738
958769.248	1319809.77	1921345.57	2571819.36	837085.115	2102447.13	1780742.62	1697403.17	2085599.27	1304079.96	1532237.28	1136512.47	564644.859	1062014.86
	370423.883	295663.355		235148.91	850931.263	213187.496	384633.231	538779.811	72263.6793	309216.559	65573.3253	117809.658	234608.736
13975879.4	36261054.3	186731.957	38944.9631	13276349.9	50010872.5	37746727.3	59450919	46708733.6	43632750.7	19568331.5	19492910.8	9340622.86	13709646.3
3999264.26	5987209.69	7524434.53	10191730.5	14626487.2	3906321.88	28466801	15803408.9	3465136.29	8334345.64	21875917.7	2493439.47	2070083.98	5261800.88
1309364.88	1583137.92	691697.037	7071209.25	5029261.38	2052685.06	15173933.6	4406558.98	2313711.69	4735869.35	1098411.06	1990618.8	4401032.21	4460462.39
210645.486	100120.322	25935.5994	192582.278	590685.061	66681.1633	2595871.28	339480.635	101347.089	105895.215	129224.83	79900.2704	29789.0134	13130.3655
12941690.8	12666270.9	26919097.7	17950444	62218826.4	27244729	45772609.5	21322059.6	25961365.9	39416552.3	44582566.5	17908681.3	13295661.4	15640582.4
33571811	93656787.6	141263848	124519809	84721114.5	79121678.8	102455403	187299660	101238465	62654669	95995588.3	93676179	56885234.7	168956909
19828.3658	550166	209494.268		228398.224	831026.438	284668.01	41139.0326	752770.986	54320.3233	164300.142	39605.7375	3559.53466	
244626.118	1262742.28	1022060.37	1897917	141764.415	2313935.89	1341827.18	96158.3078	2118186.76	645176.404	1901451.08	819666.567	337440.519	1033050.81
143222421	321616969	279046282	49682357.9	187894105	255030569	193122791	94485989.4	271562405	133349530	280602489	45460498.7	72957838	111028826
2344061.75	4346702.16	3884217.95	4229518.53	8978412.93	2886199.61	3874996.26	986667.854	2661311.57	3098415.56	2529114.54	8816581.56	1438960.82	1901972.06
3541240.35	27160764.9	24389633.9	2002171.3	26327677	33216176.5	17431212.9	9699446.7	32696113.5	9805112.52	42644194	2782733.55	10939468.2	37846347.6
1129277286	961770874	762944540	3818236579	2632767701	4553228688	4865691094	1505086557	4768635830	3421984269	3867514568	867882247	838131564	5116980669
36372152.4	31072529	40366581.8	56199323.4	56818277.3	64317465.3	13292867.4	5669159.37	47577733.3	55202783.5	117225382	12053920.1	12370014.1	116821634
1110769.37	89400.3833	111341.864	1401674.88	865212.975	745186.881	800080.134	410554.166	1140562.1	585365.217	1698383.49	172887.654	512472.011	182473.462
625872193	432026789	421599611	1096765627	588434832	1105961831	806350354	394667142	988487154	567716015	807655190	263808651	232253325	722170102
4842277.54	4390911.23	793241.736	3491141.87	2115215.08	442882.353	39376984.6	995029.446	240061.166	1657064.02	5252066.32	1267383.6	942477.261	1148906.98
35906495.4	50033668.8	53954643.3	24551940.8	27002745.7	43915019.9	23074411.4	15719792.9	42363735.2	23924474.5	30367835.2	9987533.79	15736004.3	23171233.2
20829705.3	12783961.4	21989441.6	55715362.3	27340280	48144795.1	45270991.9	14967249.7	41494735.5	41867830.5	41536552.6	10538570.1	10434569.7	33115554.1
37329211.3	141308098	105285880	20985186.9	153015559	245078156	132928674	115389969	219422423	94717386.9	245527178	40707810.2	40644331.9	323431797
1388489104	1707288975	1810718145	3479992163	1372639571	1990482486	3410999942	1145538102	1900936834	1921802054	2639878679	1246719736	917232334	1766806533
88627.1454	57518430.7	18610149.5	19982.2171	15751601.6	31474504.3	10559051.3	11371765.1	29980489.5	3304322.92	41905766.4	1012529.29	3130370.9	41128939
106411276	168810541	96352923	307570843	140639300	294840218	169295953	68230590.6	278079903	133349530	165223176	35679603.5	45188418.7	167991441
8294.05257	68670.4387	34808.2311	31886.2402	34653.5236	26000.6775	66715.1459		28894.2399	14707.6688	93226.4848	7370.11114		25874.5438
117089719	331873908	666379003	553425160	415167214	665567581	526698521	620430125	645232274	437308018	422749802	345775308	242351296	733755719
58040445.4	110596980	183637003	80398432	342034778	164214805	210679408	115389969	167282441	182375093	175376556	72323520.6	77333625.3	91912558.4
45847382.2	88819971.4	137008282	54723873.5	153015559	191583939	142961027	79518739.8	194438682	84814223.3	142147313	37883748.9	25833974.9	49142323.8
207409142	787690982	946549859	356880676	610937120	604609055	678437856	1128814918	539866061	385340922	700583188	620604686	346696992	730859314
9554725.91	8418723.54	3091817.46	10227788.3	7560768.78	1281373.1	68972425.3	2650624.66	1238324.57	5000607.38	9968772.63	2624310.6	4443107.09	2877094.79
436041088	390082155	571088800	696307659	559181858	498864673	861528294	400520256	450793592	633410269	642432014	151534996	249083276	585073639
362254.167	8177413.44	11407354.8	424926.494	8202083.99	14182187.7	2495547.75	5418311.61	11948745.8	1608038.45	18829903.9	398812.556	2659132.27	16992237.7
111885133	92088051.6	133495827	161388030	139514186	210244713	147977203	111209173	207473677	135310553	155069797	70257134.3	95089223.7	189231738
119354054	95360996.4	140540414	296938832	200270364	299816425	224473893	121243084	318271139	206887874	234450764	86099429.3	104345697	287709479
909262242	1099918596	1389454097	825701344	814582827	1139551223	1555014680	586983757	457311090	1098172602	1089180714	309269150	434212738	963537115
485155509	504612490	426755275	628266452	551306057	491400364	474028668	540158842	513796070	490255626	539052150	372638330	547814908	309915244
36211833.2	61035157	30689605.3	36670066	67731887	164214805	121266064	14883633.7	171627440	31180257.8	73012029.2	6020072.09	32565955.4	84381907.6
1939705913	1601490658	3390578914	3145676544	2981553166	3446022805	1956308790	1672318397	3910498630	2088488966	4827470452	1343151096	1783974816	3823253481
	15629.3162		344439.871		43666.2095			51379.607	172569.98	167992.28	17151.0063	6530.02102	91912.5584
295805048	317605938	244488406	650185604	289154401	419245374	443931610	199842048	410602356	317685646	370136836	126049564	135481106	334051946
3781438.96	2829330.81	2223551.56	3952943.24	2621516.56	3769476.21	4426775.66	2249268.24	3899636.13	3588671.18	4135194.57	1088296.79	1287491.26	3272936.69
273790.903	968760.163	621597.644		1777680.76	2512984.14	1404529.39	953221.486	2704761.55	249049.858	1504546.24	590986.482	984552.139	562867.874
57041.3146	213011.69	57127.7554	189131.084	186768.991	923086.253	106719.153	420588.077	775582.228	150998.733	191068.142	303758.786	60924.423	29736416
10997764.8	10489875.4	14999813.4	19916748.5	8224586.28	20526850.6	22949007	16137872.5	20421492.8	9334467.12	18922207.3	4924887.35	10602869.2	15833676
22769.1551	38984.8549	7869.35048	29854.5783	118137.012	10860.5701	54174.705	7617.4103	20095.618	6696.89185	76888.7741	13569.2701	12033.415	116821.634
41847.122	598671.077	184021.054	35385.8576	711072.302	374459.518	583130.505	430621.987	400826.11	256893.948	400596.974	143269.45	111077.677	535834.768
181162.121	8173338.67	3081788.39	174714.066	16989227.5	7862405.82	4602341.83	5042039.97	7277872.45	2402252.57	9414951.93	737011.114	2617057.39	6449326.58
444276.935	268366.213	314089.082	610806.712	649191.01	1380897.22	707280.87	301017.311	1336087.03	478489.491	664584.842	387103.034	286109.169	463424.664
112989.475	72634.1589	83470.5691	157494.62	167642.046	237613.847	205663.232	33864.4475	97871.0907	278465.196	272295.178	125360.769	70769.9443	133234.591
550440.198	8054619.09	5299788.76	261073.047	9833499.88	9442351.29	5630657.99	4741022.66	7940484.72	3167051.34	13753214.1	991865.425	2128988.81	8496118.85
46271.9955	70618.0389		70777.7843	363411.952		29093.823			26866.0083	75227.312		192702.94	
	192405.8	43835.4445		589559.947			7575.60234			122763.589		356794.963	
11139840.2	84841951	41766088.9	12794418.7	41741744.3	50757303.4	46901249.2	45403444.5	47903608.2	19708276.2	35998345.6	12673836	74977432.1	23267780
16865185.5	13977910.1	15604346.2	33139272.8	20702105	33838202.3	33733786.2	14047474.5	29545989.6	20786838.5	26029573	11916161	15483555	30991524.4
1082164.06	5278577.38	1020821.58	1170149.1	3611617.23	9628959.03	3987860.23	792678.92	8451022.04	577521.127	1836838.66	396057.375	1009797.07	2490907.57
159020.657	336364.841	205665.159	70305.2754	158641.131	223929.28	245792.643	64049.7946	207473.677	147076.688	275987.316	71634.7251	160726.033	82354.4247
349969.921	3522067.68	993578.139	5225239.06	1912694.48	834758.593	1555014.68	277604.854	678906.012	3147441.12	2547575.23	1976842.9	565486.357	739548.527
10765725.9	12248919.8	12491287.7	10670667.4	11251144	10748605.4	17682021.8	6764527.92	10666971.3	9089339.3	13384000.3	3850366.48	4703971.33	10523601.8
5179548.77	4994859.65	3998939.65	4666567.97	12938815.6	9044254.8	20817132	9783062.62	5409523.11	9569789.82	15230069.3	6309366.18	3652099.39	6082448.72
23229.6479	1290722.98	349632.188		679569.099	817341.871	239522.422	426441.191	567022.301	146096.177	809501.259	78522.6795	239826.803	320535.393
47013696	82779184.8	61193280.6	110609764	58393437.5	66681163.3	175566174	33529983.9	75277098.6	131388508	105225933	23419044.8	28610916.9	49431964.2
161489405	27664035.7	772410333	399310647	76057733.6	789972737	803842266	1429832.23	726700995	435346996	497515597	9918654.25	6588925.85	41708219.8
87387243.5	104853429	312978404	237754664	190144334		238268378	67645279.2	191179933	206887874	191991177	32648903.6	41233380.2	194059078
348611.206	121417.081	121876.491	316589.084	151890.444	139333.774	569336.02	107864.537	256354.91	200024.295	333215.456	63782.4572	52593.5972	588935.511
73383.0285	205549.233	34516.3819	260350.454	414042.1	379435.724	300970.583	279277.172	459483.589	250030.369	198452.418	107452.088	185129.462	364946.923
384202.801	823269.814	588681.208	264927.984	514177.282	2201971.25	524190.432	690667.498	1944386.82	303958.488	1061489.68	307891.559	252449.266	414185.794
761020.697	776932.831	1240082.19	1715528.28	686319.785	1405778.26	1098542.63	1204069.25	1379537.02	1176613.5	1467624.86	799002.704	445152.206	838026.268
223458522	359120213	263571801	366256144	303780889	469007436	445185654	291819560	423637352	365730697	443979596	85410633.8	143896082	467286537
8597075.05	6005915.29	3799237.28	14172.8032	18901922	972848.315	2056632.32	8779671.58	925484.676	22845912.2	17537655.6	4256755.78	50826.4523	3128116.48
22090995.3	27744255	26807429.8	65013396.5	30940646.1	54116242.6	58940072.5	22492682.4	52031356.8	38730194.4	45413297.6	15153499.6	17082400.4	27226199
46518.5281	271413.689	399150.257	63258.7387	1053107.08	522501.653	228236.036	175593.432	250923.662	152959.755	324908.145	34439.7717	106028.692	151578.484
286392.9	405170.697	248377.193	769929.103	433169.045	990265.037	354894.479	339480.635	860309.699	288270.308	450440.838	190107.54	307988.105	511698.067
201809.513	425092.823	344438.366	602498.469	568182.773	854663.418	630784.181	205695.163	819032.213	502021.761	556589.805	306513.968	216264.872	309915.244
322784070	197870350	289746110	489479454	183393648	313500992	408818375	131276994	323702387	324549224	330446352	141203064	66899055.6	345637562
1119708	4281180.72	2213352.26	1204791.34	1923945.63	3968524.46	2382683.78	2157290.73	3215298.87	1333495.3	2473732.47	833442.475	1800804.77	2355742.04
9148578.39	6584122.91	6551456.33	18773858.9	10632331.1	11631882	11637529.2	4515259.67	10036946.5	15884282.3	17445352.1	4931775.31	5680108.49	10813242.2
408311.815	562417.494	580705.74	1217714.38	1462648.72	1226634.83	879084.912	1036837.41	1002608.4	586345.729	1061489.68	874770.201	1178096.58	1071669.54
645612.273	1790582.05	1829086.18	3295336.48	1350137.28	3856559.82	4602341.83	3695823.66	3475998.78	2510108.8	2769103.51	1033193.15	1497865.65	1419238.03
28993.1037	52383.6801	33486.107	80095.3062	58505.9489	159238.599	130420.586		152074.947	11373.9305	179991.728	347841.694	17671.4486	565764.278
332321.48	308368.066	838090.137	698149.567	342034.778	476471.745	569336.02	642170.264	448621.093	1068757.26	1163023.47	1370702.91	30714.6607	353361.307
12708.0104	28219.0757	38404.6795	65437.2055	94172.0755	141821.877	106468.344	100339.104	48338.1081	26277.7015	42828.8009	36643.9171	9508.92237	82933.7056
226606.316	198798.908	295607.454	172171.236	113636.555	103131.874	82766.9104	36372.9251	135781.202	76774.031	128301.796	50419.8258	27937.7188	29832.9628
11328040	18619375.3	12377013.4	6158337.16	82808420	65437111.7	70602682.6	49333392.7	56159105.3	50888534	63873987.6	11985040.6	19101994.5	63817438.2
88250.223	51679.1966	82027.2003	54647.1991	28690.4173	631978.189	344862.127	59116.4553	527917.315	78342.849	234450.764	19768.429	47292.1626	172818.781
2040241.19	1509993.19	790015.908	2508289.26	3802886.68	5448945.81	4715205.8	1923166.16	5767985.48	4922166.48	3682907.67	1102072.69	1405300.92	7308593.14
117487.712	178193.319	177277.883	290779.737	239649.368	422977.528	270873.525	137130.109	441017.346	189238.672	174453.521	231435.266	39466.2353	359154.115
77346.7004	88424.1077	92310.7294	127241.18	93271.984	118433.708	158009.556	85288.2382	191179.933	146096.177	219682.212	82655.4521	50153.2543	88147.233
29498235.1	29276562.1	35680254.2	88853847.9	46017179.1	43915019.9	57184410.8	18144654.6	34434112.9	40789268.1	33321545.6	19079633.5	21458187.6	31570805.3
976554.414	659888.688	1239743.66	2270866.43	1383890.71	2637389.29	904165.794	953221.486	2389749.16	742247.018	1504546.24	678463.503	636172.151	838991.736
13184.5075	453077.831	98027.1041	310580.02	554681.4	461543.127	754934.546	94485.9894	647404.773	143154.643	563974.081	381592.67	210374.389	261641.842
52921.3517	21704.7109	52451.2978	143237.12	83933.5344	38192.3827	160517.644	13211.3153	54312.481	46280.1311	74396.581	30513.6377	6084.02732	6903.09656
70725.0461	35850.452	61436.6131	222154.823	113636.555	282399.703	42888.3081	60036.2304	195524.932	181394.582	452286.907	34715.2899	35090.448	284813.075

R_B5_R1	R_B5_R2	R_B5_R3	R_B5_R4	R_B5_R5	R_B5_R6	R_B5_R7	R_B5_R8	R_B5_R9	R_B5_R10	R_B6_R1	R_B6_R2
534197122	330948714	354251937	419504503	161541319	186270252	226122682	366076808	247476144	545668367	138162252	171282430
148088340	88545966.8	67871883.6	25062454.3	30995120.9	44595126.7	65582178.6	51810363	71326710.2	107686454	23982257.6	58424507.4
295445.019	90405.3173	79843.9171	49062.558	48498.8545	90941.994	443912.643	64529.7506	37987.981	332228.349	33479.4751	55837.9121
79131.2841	48016.7988	25717.8225	11327.6921			376103.115	48573.8623	10163.8401	204910.89	119026.725	399122.601
354718290	444924184	496753756	118515282	248085025	191785148	204072128	136872660	219492542	180401096	92935201.9	291448793
806698467	1112205569	1028934826	430606435	462103707	417787841	568144311	576998562	598140649	561301537	314887484	506691469
68786393.2	25475098.5	26745030.4	49427307.7	37668087	1095553315	24774398.6	151420211	104825919	18724878	6906707.64	32506217.4
13005058	3255341.75	7189933.9	1651913.04	913607.091	236772867	545154.819	8934298.96	3420710.02	925212.498	1243207.02	1705516.59
8859027.59	2229960.24	4908417.28	697727.734	613392.155	157705862	267442.825	5491097.33	1914073.7	653889.215	891407.89	1291568.71
25680042.3	7914624.3	15093460.7	2959594.82	2162537.21	649876082	973938.367	17292645.3	7865387	2180959.86	2834320.73	3288310.89
6584654.06	1737721.36	3585462.21	811348.096	442991.382	177562854	240272.43	4734923.62	1782436.47	520963.716	661680.124	1210039.45
2148923.54	6794656.84	4070479.26	1678513.78	4779961.81	1949970.28	4345495.5	15268245.9	80319189.6	2963511.71	384269.969	7315913.01
13144461.8	38178241.6	21260203.4	7617579.9	20124228.3	7635720.99	17367337.7	97004091.4	232242600	16580499.8	5534698.59	30467689
306881.152	472170.892	322162.167	381865.128	383648.261	199721.309	414935.576	480621.849	162057.731	922459.831	431442.252	397885.953
15505415.5	9995280.58	10415662.8	10538244.8	34284864.7	13218827.8	16411580.3	5624528.41	12457083.7	21225895.3	4380710.78	12780476.6
2569757.62	1356158.4	452155.049	824494.469	180232.009	1044527.55	1073198.33	714864.529	310440.405	1763388.55	499655.996	502329.39
1902653.67	1034660.53	369229.25	1054204.67	89588.839	502663.36	799918.154	903767.354	140287.454	1605862.15	807381.83	768583.957
130205.268		17150.818	213374.413		12022.4505	163377.107	7282.89116	17555.4305			
247086.956	173479.951	184449.544	33274.967	20815.6502	802862.413	604698.785	66841.1678	192090.938	374264.123	151547.339	109268.351
335360.244						1395279.36		219360.492	1188360.34	50431.6301	101891.191
997751.263	389642.908	486641.73	360138.468	290882.378	340448.758	1246938.12	573052.831	436302.457	1815120.57	198864.059	481782.898
1665032.08	2179879.06	1008277.27	858803.42	1411799.99	1121854.49	1235563.27	1872649.62	1229310.29	2564815.32	1899785.14	2203983.31
509137.272	543029.333	223785.064	56886.5127	206303.722	319860.195	387443.735	335764.431	252518.071	410457.607	300895.691	469034.549
	17768515.5	44085297.7	38162541.9	79525376.7	72816529	191099584	25228401.6	942220.334	62636684.9	22899041.8	173686.199
26297057.3	7269704.69	27565669.9	8002856.47	3576333.54	14721019	22713667.5	6701722.42	32106043.2	12571638	3151407.55	21867703.2
12698298.5	9120631.34		5472316.15	5223762.46	1049744.76	7081506.53	8449530.31	1961778.24	6895813.46	3318293.96	8221241.28
107074.438	34928.9229	146362.192	139897.524	185614.733	83088.3526	665873.98	20630.8497	331809.024	206047.162	497005.774	152333.959
21685281.5	39823945	42552802.9	34354512.7	35567275.7	16084797.8	50590347.5	22533782.1	27794169.3	36860696.1	14887180.4	50064204.7
192517238	108400733	98306609.7	93282617	324598496	190339870	231986601	152368280	49439414.2	485982016	89486423.2	110371369
391015.371	274224.076	164336.68	191556.779	116902.865	15288.7444	252222.431	275623.072	410774.662	558526.824	522321.317	397911.843
505491.296	3695257.08	4095605.77	1520745.93	1706676.85	588487.404	339917.168	772141.072	214301.727	1492446.94	721771.175	
550809276	525804517	330634485	172707077	228815512	130289085	360688630	204902618	244612347	247819472	352228654	363893125
2765449.76	1824576.49	2956368.78	1869971.91	3098132.41	8719298.08	2035351.46	16125618.9	36739204.4	3745706.16	2784896.5	6725989.14
55756438.9	86562051.5	61454299.2	13724119.6	32538934	40970397.3	46886971.2	30292577.3	56062267.9	42700721	29871018	77841620.5
2012378844	2335326185	2914636034	1984067683	1039663827	981986137	279368781	2002135310	2410724595	41479099.3	582864610	984273785
88466104.4	156121194	140414677	63401559.7	51350939.5	29521693.4	71261978	143841197	130120897	44186022.9	30537056.3	66867544.7
637403.656	1726024.19		532796.225	884748.392	117471.498	684099.036	880549.124	813386.148	539805.86	981780.484	651650.294
659046391	1009405038	856853896	531702964	347250979	337298345	424076317	516580157	466565904	691956481	529331604	587228981
2369362.6	2046248	4639260.69	32445621.1	2663395.02	1717815.63	89725523.7	2180141.02	7178023.18	1401210.36	1165004.24	2118971.15
25498145.3	43492698.3	23551013.8	20319747.2	13576288.8	7846796.88	13315852.9	18081457.7	11919121.1	21984326	66918869.6	65550695.3
51721329.3	48128448.9	46854619.4	29373614	19468539.3	17036781.2	16934995.6	28779818.6	20374946.7	31845413	16119053.8	17399895.2
273451318	284090284	320388068	117165697	124157543	174386926	174826454	157191000	186418344	168034125	208993958	127689971
4177541644	3377992085	2006743353	1868925311	3207657342	1458912372	2203286810	2745226616	1297088870	1792711734	1374663070	1892623940
147940218	128807742	37194485.3	4506571.22	23594822.8	41654737.4	45855107.2	33862961.5	47162132.6	35078761.2	16419205.6	68254982.4
264369144	257381948	281176708	177958372	189803437	132447818	163588605	282239760	217579422	268207404	124463517	203673919
6549.26454	62830.8331	32909.8985	42222.2827	76822.7186	5234.74382	51904.8802	74574.8918	24257.7906	49302.8038	5640.63314	15066.4291
	641890457		514823055	941047221	965395142	1054887662	503402909	321108014	1284116921	376965645	674703850
132039705	216595067	120506084	153684368	206217882	93516056.8	215091899	118908375	130880816	197552353	81995262.7	301793540
148989077	126079975	116491318	91648332.8	237838514	192817726	168251747	141781348	199485301	157152468	96483530.6	234044347
1772088335	930977245	662672895	712234186	2023665144	1193609906	1720608570	698322932	448682630	2970904759	907410006	841170276
9815521.18	17975844.7	25879493.2	115374251	10707655.9	8897875.3	235289331	7988647.33	29435877.2	7386322.98	2426656.48	6396014.2
911290222	848491926	1084151910	1289090192	718317664	337063914	688459187	795497989	704981705	571992387	429092433	1014536910
29932004.7	35993890.6	17310046.9	592089.06	6282724.11	15205392	15769399.3	6882523.6	19168850.1	6523681.63	5634654	36157348
168962208	249106637	175658063	151476216	195666740	130599487	169208252	142515064	131537314	161111845	84813140.1	243226561
256705862	486314783	315191135	266024800	353692155	212726316	248643008	290641993	259753901	314445491	127398097	210150780
3106701086	1895706123	3356684457	1133231508	966499077	1135479272	1138911081	1482467864	1249294833	1490731017	993706115	1092382235
381861977	642385281	416517279	593742748	551296892	433756313	359258888	506167522	300225959	512448997	391760154	634014038
70227282.3	67022237.6	47192418.8	62408628.6	58275716.4	23380304	55619061.9	73130002.7	18976925.8	28131955.4	47784034.9	103411721
1501758480	3574244923	3328246673	2617046902	2304261108	2394567731	1725231741	2316139038	2593915756	3075714011	1495384598	3211778342
	17757.4057	58806.271			11201.9782		74137.6205	146281.62	26348.0051	25853.4544	3630.67032
242616785	323883269	294222199	245505454	237800649	115541758	251087411	332999389	344921788	477010310	282287555	427007262
5341398.2	7303209.76	6757666.33	4447189.26	4124266.68	2682557.96	3995230.7	6061083.78	3480163.13	4958279.15	4771886.01	5279082.23
633130.891	1179812.24	233668.607	919791.785	1025202.71	263111.073	1051452.71	653066.475	380385.031	1507961.16	648711.468	1335724.39
528180.068	438388.867	306134.56	57022.0031	325577.51	4546577.09	358769.081	231922.978	353194.496	271838.846	341378.397	618329.621
26848503.2	18766668.3	14227218.3	9805095.51	9656540.78	9310072.23	10982180.7	24160184.9	13075007.9	18492182.6	11894679.8	16380343
30663.8988	72494.5499	43167.732	13042.0712	12653.9046	217485.719	36657.8579	46648.6319	32348.422	19687.1363	14632.4515	80891.6372
403961.294	338864.229	318207.024	127257.625	389020.42	537452.117	630016.34	345437.549	552392.115	211280.473	199775.437	468505.176
12675056	8838652.03	9098513.63	1908646.05	5511188	8750629.52	7217489.09	4604900.21	13987053.3	4503925.09	2466520.63	14515037.5
1312749.6	931912.367	784839.743	612586.009	838159.65	698043.819	630073.899	1121069.32	562248.858	1287922.44	497760.621	1083935.8
157966.249	291948.872	157427.794	156884.881	177227.357	172978.444	192587.637	241257.701	199620.167	196952.146	52826.6447	207235.899
9759103.28	10954261.4	9594764.01	1337586.36	5095912.18	7201759.44	7377751.04	4234959.41	7389754.4	4366704.2	5233710.95	8612456.85
42848.1224		61286.2438	58658.3476		53153.8144	120515.269	24452.9143		41693.1613	35338.5722	122957.967
	165011.564	742725.185	273451.481		56623.976	1436203.46	65770.5199	191631.524	444156.524	130670.143	508682.789
82395946.1	47913840.6	33801621	23961825	74198379.9	94018263.3	94821775.7	49230808.8	97780621	48099567.8	17758089.7	60100531.1
20783280	39838701.9	34292618.9	24297540.5	25308174.7	18836683.1	20162985.6	30770454.3	21870011.4	20331401.7	12191211.1	28621781.5
11265709	3926861.78	666765.294	939200.985	1737945.44	206136.265	3673477.87	2785065.65	756139.894	1080305.22	575459.199	
667363.89	202220.818	161595.57	142001.764	254417.697	130802.16	317010.161	467314.501	167745.315	271016.62	454928.349	484574.311
1378587.34	238731.338	2655859.96	2491256.93	1171142.46	658340.472	1104969.94	2193084.66	477487.729	3777485.35	1789426.34	667544.701
20321277.9	16793558.9	14989401.1	16010032.1	11983348	9657299.62	14272713.3	13525405.2	15137872.8	17639973.4	9341016.38	15701150.1
11134711.2	5657836.08	9564082.01	4788841.46	8808579.47	11878102.5	13326375.5	6048532.25	16272326.7	14633164.4	2646619.28	5983572.09
831870.881	496603.773	592538.635	170601.796	442177.121	267419.535	748297.173	370198.737	567274.067	417711.86	468128.315	1211377.44
77147983.4	133143602	128324908	79043500.1	115083587	41969184.3	54524381.2	88643344.1	126845623	123421979	36272358.4	48315115
2076651700	146092818	677774342	304225153	9554992.72	27084828.8	6641223.31	61723124.3	245691532	1469850231	152325663	272814425
109001992	251516207	144439448	195902495	240474918	150205898	228489631	180946688	208682348	161165638	105194736	247352438
377816.271	673213.158	341504.822	460177.531	154660.41	285073.229	182937.271	467712.712	257082.328	378121.869	233580.531	27324.6709
288134.764	191293.507	205253.014	143946.13	181373.638	275841.782	306416.981	212141.362	247145.92	306662.452	100132.178	186989.776
721802.749	875534.635	455018.207	565848.6	641628.907	720402.171	1040152.02	537405.38	512269.552	975568.332	472970.666	1074225.46
1286293.84	1549378.22	615063.652	687549.903	1021846.41	948894.124	1162880.13	1500916.33	927092.1	1715314.96	571652.299	1227791.48
434723164	750753752	617086861	481050962	456911652	321842013	397451328	491511097	477397999	504488433	368892445	312056060
4046368.66	212262.223	77911.2317	8399664.95	2560009.2	26166072.9	8502562.85	3605385.47	1907624.65	722715.505	1319224.03	1078821.52
21251825.3	37641885.7	37465768.8	28015310.2	22537935.6	14234950.2	27829067.7	31528268	23729225.8	25184296.7	25733614.7	45167296.8
279972.688	379064.007	261636.916	47988.2232	208966.91	298272.183	214300.733	315997.044	405205.952	216628.513	224429.712	667629.96
327305.618	882966.599	497386.046	639092.109	726108.041	615694.298	940342.761	633312.774	678086.934	911857.304	411911.625	1324270.01
469553.747	652205.666	252827.266	485492.228	508852.281	378102.372	737721.081	584651.35	605539.767	766717.196	434684.383	607261.286
334921695	413041212	374700833	432929395	203157775	178316724	249993837	328652550	237927189	399913158	311957065	364755923
3477861.23	4074783.48	3647357.07	995974.256	3170117.89	3453459.7	3895276.04	2353685.19	2806009.81	2059086.34	1467521.13	2678466.97
13094260.9	19824975.7	17160993.8	14659035.2	17494468.3	10152355.6	13211075.2	14596084.9	21344792.8	12412676.7	10742745.9	22420192
3074283.26	1202120.75	1112926.63	1141612.42	2828327.21	2163597.69	2181741.83	790297.34	1219287.03	2142059.26	713626.669	1734840.56
4004675.36	2221963.99	3059267.99	3630154.68	3086358.09	2012332.54	2839144.48	3087242.65	3461532.56	4993024.87	556403.425	2889274.02
99768.0061	47438.8653	344008.717	74420.4105	162497.18	89818.0423		165166.9	197487.746	82030.585	63330.6972	99002.8471
553786.256	1198744.83	831889.5	412105.021	219024.427	610803.919	159573.948	2134007.11	4945572.44	1366505.58	417097.532	1575513.82
68673.5367	77810.9131	103789.701	27975.806	61679.1542	68986.2188	21542.2999	119318.169	120996.92	203554.404	46741.7994	149300.573
249004.382	254772.631	97248.3066	229140.935	200117.23	61114.7951	148038.072	293279.492	177079.716	242157.546	553724.278	94259.1829
25151647.5	21892678	28383362.9	10994699.8	18941139.9	21542358.3	23057114.1	14886276.9	23420741.1	17741488	9229575.09	14304933.7
210347.715	291789.294	55554.614	53173.8093	137262.857	25998.1186	135896.186	283494.269	37913.0713	38074.7211	114648.463	348841.843
8665552.72	10999916.8	12805532.4	3115688.63	3997721.8	6006863.02	2191790.19	4130852.51	2301610.9	2540447.42	883339.229	2236022.08
204375.092	189608.493	107607.03	93535.7284	274183.337	285618.564	187002.777	98113.3204	99097.0754	180069.014	103721.395	287278.64
58267.8002	200359.375	111506.391	145285.467	62796.2416	99568.3796	104206.234	156588.879	92777.9504	169470.784	49988.6491	161488.016
60739790.9	55426393.2	63120718.9	48667939.6	48736111.4	39348208.6	45023713.9	54780673.5	53707929.2	49107104.6	20564836.6	30918325.6
1338028.65	1511502.07	1426295.55	1156572.94	1135343.86	835335.96	1053435.28	1245256.75	592155.374	1270115.05	594080.339	1266295.91
614054.246	699161.721	559575.359	251191.959	313461.777	519735.783	470857.577	390404.779	379464.462	413425.169	159070.67	843338.669
228886.314	34506.4389	25867.3516	63299.4334	53876.2621	34044.5105	354892.675	16420.0659	28209.8695	20776.2703	29103.4937	141801.269
103939.468	260073.567	94717.9592	150329.603	236002.487	159630.988	197742.803	384891.25	220546.347	139041.004	299801.935	200484.656

R_B6_R3	R_B6_R4	R_B6_R5
154257790	74268951.1	119894336
36915490.9	11929336.9	28640320.5
20425.9146	8479.17976	
64307.3867	64997.0107	
171158489	112237475	367202927
397607788	155191929	609415302
16037697.5	992749935	10125660.5
1810191.56	462694948	2029656.95
1123633.49	331691599	1323972.72
3280969.61	859110612	3949478.32
774083.999	221198067	1022609.11
2550230.87	433439.968	1743644.92
12906886.6	3564438.61	12428720.1
248803.41	88226.4532	524927.238
6864389.42	3687456.74	19109319.1
160187.679		45047.7875
367811.273	8143.21864	42797.7564
40027.353	224495.094	23962.0502
6653.6999		
255983.307	192848.513	54546.4846
1540896.21	986336.17	1309248.47
288035.3	144182.936	276298.107
70290.4973	26469632.2	187636017
5994940.1	4931788.96	15405084.2
2407885.46	1449855.65	5708567.07
17330.4347		161209.939
19149573.1	13340535.4	25636651.2
45153974.4	34683063.7	224066621
151097.676	106676.371	309919.382
357958582	167498742	178261587
2387060.83	13281698.5	1933340.31
56319889.9	23473201.7	18712364.9
889816862	350780007	1132390585
65861274.5	11575404.4	94311119.5
	298397.798	538722.147
748853986	388954586	601510936
4304767.96	1001502.88	5441263.76
41151296.6	29979849.7	34374195.9
13556342.7	4518129.62	16099234.6
105179308	26323823.2	108101997
1072187660	1248508527	1850717968
32123481.8	10108067.1	27682306.3
203437442	93911856.8	165430912
17040.0834	6915.43633	56305.3373
302647408	149547669	847404251
128430908	49391213	135287763
92815650.3	49551219.6	158840953
403727854	149074259	903652044
13917260.4	2514862.53	17759277.2
336064398	296932957	476009681
24715441.9	5818428.39	8130814.94
222040275	89069607	135680388
231412971	96621124	189876622
894213003	474467478	761117761
759633955	447714784	532409403
43286977.8	4510614.9	39258884.5
2639800404	1563216030	1851938788
4975.07673	5279.63369	
328085757	241302875	410394467
4628024.65	2878278.7	4227962.49
263559.706	384020.968	1584823.21
207987.049	203305.3	702865.625
7424800.13	11039949.4	14155825.8
36123.9452	40853.5666	10153.9839
425947.581	225233.911	103775.622
5750960.51	2679434	4074797.9
742062.543	571379.532	588612.589
106742.454	92404.7041	170823.444
10441107.1	6084403.67	5202141.44
44818.9412	33455.1898	5044.42407
128933.014	123839.91	5313.6326
27612580.2	88950611.8	41008215.3
19169078.1	13918649.2	19381277.3
759940.453	2946257.98	8122268.81
115272.9	105600.37	20577.943
568135.082	820788.731	843373.633
9640122.69	5919867.44	7737396.81
6402079.49	4674825.11	4657830.82
693990.124	264432.823	215379.891
48868720.6	60722355.9	21833103.8
34293139.3	4503452.55	44754696.2
100936188	120617794	258047739
53828.9218	43960.546	97878.9973
143803.16	65939.8823	176690.353
1057954.95	580025.721	423913.083
972778.414	665744.529	620439.337
368767459	173924096	430128116
1489840.15	4494866.04	1153715.72
31777600	20041897.4	30696854
413947.295	371350.689	120013.131
571519.895	488178.232	595760.114
251770.137	250876.09	416371.266
187829494	176048263	191393959
2484358.34	2091677.22	2548530.38
13708454.6	8680769.92	12387896.7
608385.424	948635.799	1659294.73
1315297.02	1238290.42	1953344.01
41904.5672	41620.6807	74336.1383
138162.763	808554.114	332843.934
15562.9209	13759.6735	
55502.4702	26533.969	136855.153
10508149.6	3198066.63	12493256.4
161674.442		86842.2301
2824323.28	504746.237	2171351.66
128304.343	170556.868	212259.579
84386.1031	25337.0098	158196.613
30358996.7	24722318.7	51140787.8
856199.759	591770.418	1540342.92
737913.138	429007.454	121390.609
24456.9373	7491.47008	21883.566
167029.692	174303.706	196885.59

SourceDataForFigure4E, F														
Accession	P02750	P08311	P08311	P02671	P02675	P02675	P02679	P06312	P14780	P14780	O00151	P05164	P24158	P24158
ID	A2GL_HUMAN	CATG_HUMAN	CATG_HUMAN	FIBA_HU	FIBB_HU	FIBB_HU	FIBG_HU	KV401_H	MMP9_H	MMP9_HUMAN	PDLI1_HUMAN	PERM_HUMAN	PRTN3_HUMAN	PRTN3_HUMAN
Peptide	DLLLPQPDLR	IFGSYDPR	VSSFLPWIR	VQHIQLL	AHYGGF	DNENVV	EGFGHLS	NYLAWY	LGLGAD	SLGPALLLLQK	DFEQPLAISR	VVLEGGIDPILR	LFPDFFTR	LVNVVLGAHNVR
N_B3_R1	-1.353933091	-0.167701069	-0.540201385	-4.91309	-4.76868	-2.11711	-5.36507	-3.54987	-2.17785	-0.052479585	-2.881334203	0.152859114	-3.097130601	-1.851999828
N_B3_R2	-2.112710192	-2.16589926	-2.178450393	-4.69944	-3.87528	-1.44037	-4.87098	-4.0187	-4.16123	-1.968768879	-4.904639261	-3.177390803	-4.245378948	-3.317749447
N_B3_R3	-2.287336038	-2.510948591	-2.327897781	-3.1959	-3.03236	-1.4316	-4.96157	-3.41753	-5.13772	-2.960444837	-3.643765777	-3.292719529	-4.92194058	-3.997261315
N_B3_R4	-2.250388537	-2.082625865	-3.042945019	-4.83565	-3.40832	-1.01537	-4.86677	-4.33406	-4.58715	-2.807485942	-4.76245579	-3.086099239	-5.995955566	-3.505045687
N_B3_R5	-2.029742763	-3.98769313	-4.890790215	-4.365	-3.64817	-2.44957	-5.58638	-3.18335	-7.79485	-5.034126559	-4.243121957	-4.419420303	-6.443549744	-4.61456151
N_B3_R6	-2.250601151	-2.864666753	-4.298213757	-3.48386	-2.88888	-1.24739	-5.19109	-4.01148	-6.21568	-3.136573828	-4.915457255	-3.69093592	-6.460778607	-4.439059119
N_B3_R7	-1.696726974	-2.540927353	-2.700451949	-3.35438	-2.74469	-0.74261	-4.75734	-2.96564	-6.50695	-3.798157611	-3.670065723	-2.96379295	#NUM!	-4.017877086
N_B3_R8	-2.248595255	-2.893620676	-2.96585337	-3.66059	-2.7631	-0.85356	-4.05429	-4.07226	-5.895	-3.953451524	-5.942557013	-3.945784295	-5.680107988	-4.757141914
N_B3_R9	-1.70642037	-2.783199899	-2.773883869	-3.8196	-3.61185	-1.82252	-8.93965	-2.78816	-6.36159	-2.417148815	-5.074458499	-3.581879005	-5.487537756	-2.731551641
N_B3_R10	-1.880062122	-2.2289851	-3.460555525	-3.93471	-4.33418	-1.57763	-5.12429	-3.12428	-4.29825	-2.206963156	-5.257720229	-2.729243203	-6.814584486	-3.346214091
UR_B3_R1	-2.959243147	-4.612545592	-4.974874774	-2.99735	-2.45042	-0.96123	-5.07394	-3.37041	-7.19268	-4.10436836	#NUM!	-6.063099825	-6.036654043	-5.121689643
UR_B3_R2	-1.983459272	-3.199625227	-3.227487761	1.117624	1.779696	3.437384	0.189526	-3.5207	-7.81746	-4.80713207	-5.956481256	-4.155266842	-4.364315606	-4.769350208
UR_B3_R3	-2.083930163	-6.040947221	#NUM!	-3.57639	-3.19295	-1.03258	-3.58481	-3.50938	-7.68729	-6.359328488	#NUM!	-7.078178006	#NUM!	-6.288543268
UR_B3_R4	-1.935497532	-3.902495944	-3.208799096	-2.79571	-2.55205	-0.5505	-3.50017	-2.01481	-5.54605	-3.485824634	-7.064014993	-5.569671679	-6.262603713	-5.103667904
UR_B3_R5	-1.849123618	-6.373099631	-6.449179665	7.441846	7.911869	9.534388	6.706577	-3.64702	-10.314	-9.06004616	#NUM!	-2.820452721	#NUM!	-5.83985686
UR_B3_R6	-1.93785695	-4.95759813	-4.029311138	-1.89188	-1.03433	0.707522	-2.61067	-3.80711	-7.32318	-5.71717877	#NUM!	-5.820858774	-6.059169279	-5.060355606
UR_B3_R7	-1.840095634	-6.447272238	-5.142838462	-2.41607	-1.77058	-0.51969	-3.89058	-3.98395	-7.53513	-5.263753909	-7.504150021	#NUM!	-6.452494914	-5.610153079
UR_B3_R8	-0.227592307	-1.754743014	-0.933885786	-1.36719	-1.15086	1.15991	-2.42197	-3.43577	-3.84135	-1.251028013	-3.332328749	-2.860929153	-2.995113219	-2.944306729
UR_B3_R9	-1.396311443	-5.007675254	-4.189065823	7.628255	8.001062	9.545977	5.214744	-3.2901	#NUM!	-7.986600128	#NUM!	-4.045514231	-6.400552324	-5.29178953
UR_B3_R10	-1.331680592	-1.177084845	-0.243999501	-2.18499	-2.08576	0.294659	-2.84452	-2.50967	-4.05624	-1.646967017	0.003848071	-1.264151101	-1.399192201	-1.772925538
R_B3_R1	-0.416686676	-3.399723823	-2.344298908	-2.09256	-1.43307	0.235328	-2.58385	-4.53095	-3.70903	-1.275417981	-8.386751782	-3.036351151	-3.81852856	-3.599728628
R_B3_R2	-1.298490685	-4.369177115	-3.129883222	-2.6918	-2.16474	-0.54641	-3.42464	-4.5731	-4.14201	-1.914224633	-8.64767551	-4.398434513	-4.169867533	-4.492680024
R_B3_R3	-0.646613768	-3.651729381	-3.105433223	-1.51304	-1.01601	0.517747	-2.00165	-4.15377	-3.91383	-1.680452907	-7.99651161	-4.151352633	-7.289930456	-5.139408425
R_B3_R4	-0.613179335	-2.847921655	-3.453553233	-1.06472	-0.43543	1.244542	-2.12143	-4.05808	-6.24286	-4.05192828	#NUM!	-3.835282121	-5.222288929	-3.75506235
R_B3_R5	-1.74644432	-3.628968858	-4.541133954	0.687712	1.141027	3.101354	0.373008	-3.74011	-5.58713	-4.055200462	-8.599131535	-4.784395103	-7.543479386	-4.876449151
R_B3_R6	-0.968464445	-3.682680349	-1.64782367	-0.03964	0.743183	2.244801	-1.04546	-1.58384	-6.32249	-3.705214024	-4.870904654	-5.26662605	-2.219415824	-3.692674317
R_B3_R7	-1.622241425	-6.313154826	-5.878461356	-1.89411	-0.94301	-0.41299	-3.87449	-4.75572	-10.0213	-5.801125528	-8.724426943	-6.419515948	-7.427866394	-5.601199085
R_B3_R8	-1.18692978	-3.954317505	-2.168386794	-1.88387	-1.16027	0.563426	-2.68481	-3.79555	-5.62807	-3.209213851	#NUM!	-3.374037352	-2.346304934	-3.505690758
R_B3_R9	-0.528943383	-4.627118481	-3.720153218	1.010083	1.45408	3.315428	0.310035	-3.6735	-6.02414	-3.593969785	#NUM!	-3.597146308	-5.268804764	#DIV/0!
R_B3_R10	-0.445888881	-3.148705901	-4.167586102	0.262969	0.695721	2.42039	-2.5896	-3.95125	-5.93772	-2.350698634	#NUM!	-5.828959471	#NUM!	-5.184338057
N_B4_R1	-1.075862015	0.517699274	0.78181983	-3.39129	-2.94486	-1.05143	-4.08479	-3.04217	-2.66505	-1.487064857	-3.668543069	-0.588061739	-2.670760373	0.722538524
N_B4_R2	-1.153805336	0.162430465	0.618321098	-3.15947	-2.19465	-0.94596	-3.51796	-3.7112	-2.81183	-1.839535328	-3.580368972	-0.692332598	-2.864077512	0.46000423
N_B4_R3	-1.469234794	-0.900941329	-0.186413124	-1.50479	-1.13203	0.330861	-2.77693	-4.39001	-3.81291	-2.67385555	-3.02774499	-1.9627606	-4.639499785	-0.98654372
N_B4_R4	-1.548893246	-1.146530407	-0.805088315	-2.21736	-1.51945	-0.37899	-3.69035	-3.83289	-3.75049	-2.881355504	-3.229481846	-2.269111396	-5.105232404	-0.92712112
N_B4_R5	-1.701261532	-0.80240004	-0.880444615	-2.72042	-2.1804	-0.73884	-3.75625	-5.05311	-4.46674	-3.517652566	-5.578712164	-1.285981398	-5.403392196	-1.020464103
N_B4_R6	-1.480481852	-0.594867496	-0.492182216	-2.42109	-2.2198	-0.68192	-3.22787	-4.44234	-4.48167	-3.59454855	-5.230297619	-1.163926261	-4.680048841	-1.136160024
N_B4_R7	-1.634974269	-0.349942471	-1.181359897	-0.48589	0.046244	1.458304	-1.37142	-5.47605	-3.71621	-1.918963428	-2.164188632	-1.532221039	-5.126172642	-0.598637438
N_B4_R8	-1.316901278	0.244946738	-0.543935232	0.16327	0.486903	2.263981	-0.67858	-6.02342	-2.9949	-1.754495519	-2.082605278	-1.126007885	-4.778357225	-0.678071905
N_B4_R9	-0.142496513	-0.359348796	1.913508474	0.38537	0.44904	2.065363	-0.80404	-3.79888	-3.53892	-1.012157614	-1.290429942	-3.786596362	-4.789461512	-1.790866799
N_B4_R10	-1.062611977	-2.611434712	0.249171752	-0.96666	-0.47842	1.116179	-1.76209	-6.95016	-4.56628	-2.289297091	-2.713715213	#NUM!	-8.091315167	-3.827392675
UR_B4_R1	-2.208931135	-3.901116943	-2.925999419	-2.40874	-1.97137	-0.44539	-3.37351	-1.79968	-5.51	-3.900169628	-6.466039743	-4.921233585	#NUM!	-3.413805404
UR_B4_R2	-2.180602554	-3.488068149	-2.970042503	-2.87323	-1.80259	-0.58287	-6.06684	-1.79003	-6.50056	-4.03068616	-5.429030064	-6.630878336	-7.901278321	-2.286193883
UR_B4_R3	-3.505912008	-6.183903685	-7.736663537	5.36691	5.823825	6.204214	3.802176	-2.40471	-10.955	-11.55800434	#NUM!	-7.63872915	#NUM!	-5.244424859
UR_B4_R4	-3.327716992	-3.708235831	-4.479984138	-4.31919	-3.8532	-1.6343	-5.72315	-3.62023	-9.63236	-7.834323844	#NUM!	-4.450975551	#NUM!	-4.99513187
UR_B4_R5	-2.263589959	-4.079208964	-3.307822328	-3.39365	-2.51726	-1.17217	-4.18765	-2.57973	-9.76372	-8.645828677	#NUM!	-4.003552267	#NUM!	-4.446839382
UR_B4_R6	-2.800715046	-6.613638801	-4.867211871	-5.2131	-4.06987	-1.78781	-6.54112	-5.26847	-8.45716	-6.983289222	-6.710394207	-7.587393054	#NUM!	-6.056715629
UR_B4_R7	-1.99988458	-5.344687537	-5.338869774	-2.43545	-3.22098	-0.55519	-4.16735	-4.13547	-7.23608	-6.690984845	-6.988895038	#NUM!	#NUM!	-5.460884015
UR_B4_R8	-2.232755202	-4.120500996	-5.464870314	-0.72589	-0.4424	1.164026	-2.39724	-3.72308	-8.14967	-8.230020436	-6.309962784	-4.134112935	-8.511564423	-4.945963771
R_B4_R1	-1.673645099	#NUM!	-6.85508576	2.57102	2.802721	4.305255	1.35453	-1.69966	-8.56538	-8.66300729	-8.297978719	-6.096275911	#NUM!	-6.011305053

R_B4_R2	-1.462694997	-6.168224826	-4.979583445	-2.87815	-2.63451	-0.88713	-3.67144	-2.55854	-4.6651	-3.500443565	-7.425428721	-3.771631196	-10.09618805	-4.222959956
R_B4_R3	-1.11545704	-3.867121238	-5.314060858	-0.08265	0.151857	1.068059	-1.87652	-4.40406	-6.28854	-5.248624877	#NUM!	-5.907309923	-9.448777552	-5.052001911
R_B4_R4	-2.315149562	-5.954246172	-6.016701846	-2.87088	-2.37519	-0.97019	-4.0433	-4.31197	-6.09113	-5.978669606	#NUM!	-5.574038852	-10.19048087	-5.144435679
R_B4_R5	-1.472591746	-3.390911835	-4.3395311	-3.02915	-2.47661	-0.86883	-3.84383	-2.44561	-4.7149	-3.568178909	-6.343357773	-3.169266906	#NUM!	-4.51365492
R_B4_R6	-1.126609238	-3.337668036	-3.398154363	2.019716	2.063032	3.812066	0.474983	-4.11906	-8.05575	-6.420662048	-6.502510199	-4.050666409	-9.325822011	-4.71088032
R_B4_R7	-0.226563823	-3.862995757	-7.055662993	1.450689	1.755272	3.645046	0.784612	-5.36486	-7.20621	-6.459061079	-5.208241248	-4.090273788	-8.597910173	-4.097337719
R_B4_R8	-3.28822972	-6.04157084	-7.762926672	6.773121	6.793139	8.398704	5.229885	-5.26957	-11.1396	#NUM!	#NUM!	-3.92325831	#NUM!	-5.676579376
R_B4_R9	-3.05393765	#NUM!	-5.406115339	-3.39104	-3.4083	-1.21562	-4.1464	-5.32108	-7.241	-6.78717704	#NUM!	-4.848570383	#NUM!	-5.670125098
R_B4_R10	-1.709238423	-6.603721437	#NUM!	1.068901	1.246828	3.456627	0.447711	-2.94778	-6.41108	-6.435790204	-4.61069552	-6.421227886	#NUM!	-5.597330497
N_B5_R1	-6.612762077	-1.311306773	0.991869019	-1.71208	-0.61691	0.986094	-2.48251	-3.79293	-5.19158	0.34907485	-3.505369878	#NUM!	#NUM!	-5.661272161
N_B5_R2	-3.353946962	-1.385789938	1.146668711	-1.73883	-1.37223	0.857317	-2.49902	-5.23896	-3.18887	-0.598915685	-3.977575754	#NUM!	#NUM!	-4.686278277
N_B5_R3	-3.034240041	-1.288126679	0.162996365	-2.00749	-1.81128	0.804867	-2.94465	-6.18853	-2.38702	-0.822089387	-4.495651539	#NUM!	#NUM!	-4.898913063
N_B5_R4	-4.682595024	-1.886099132	-0.014654105	-1.81026	-1.40748	1.671901	-2.99121	-5.4884	-3.6075	-0.701540398	-4.329668521	#NUM!	#NUM!	-9.49846819
N_B5_R5	-5.38248462	-2.815980545	-0.809345932	-2.21867	-1.22721	0.518774	-3.74331	-4.2648	-10.7255	-2.818208868	-4.890079956	#NUM!	#NUM!	-7.579584217
N_B5_R6	-5.483546148	-3.51314768	-1.404793874	-3.47179	-2.72488	-1.05323	-4.38288	-3.9985	-6.90282	-1.975312804	-4.697167576	#NUM!	#NUM!	-7.049000379
N_B5_R7	-7.387778318	-2.834236163	-2.004321433	-4.53786	-3.82059	-2.22012	-5.21218	-3.73482	-6.98945	-1.827026124	-3.54370064	-6.765953277	#NUM!	-4.084914055
N_B5_R8	-4.097560416	-3.652453358	-2.311425202	-4.2357	-4.16078	-2.90465	-5.97461	-3.82389	-6.38342	-2.554575407	-4.806853808	#NUM!	#NUM!	-5.626276996
N_B5_R9	-5.943583961	-3.208371778	-1.7372931	-4.03736	-3.39463	-2.006	-5.28351	-4.69848	-6.86358	-2.864012557	-3.034441104	#NUM!	#NUM!	-6.327280297
N_B5_R10	-6.304923637	#NUM!	-4.966538251	-5.07486	-4.64968	-2.84733	-6.08785	-3.9681	-6.14689	-1.353522872	-2.752741208	#NUM!	#NUM!	-7.067009846
UR_B5_R1	-4.821743669	-6.350659399	-2.376962538	-1.50758	-0.84001	1.655904	-1.81495	-3.6065	-7.32091	-2.377656282	-8.964613473	#NUM!	#NUM!	-9.568264245
UR_B5_R2	-2.246515782	-4.302250009	-4.565839982	-3.28719	-2.98749	-0.56012	-4.6791	-3.62321	-7.2626	-5.679112181	-8.091583953	#NUM!	#NUM!	-5.746327602
UR_B5_R3	-6.619653836	#NUM!	-4.759454852	-4.40511	-3.73864	-2.76804	-6.13507	-3.86274	#NUM!	-4.435783955	-10.23805475	#NUM!	#NUM!	-8.666334907
UR_B5_R4	-2.088688008	-3.289509537	#NUM!	-5.1138	-5.6896	-4.13601	-8.9663	-2.33192	-5.20514	-4.269893282	-6.695578539	-4.277409279	-7.347453706	-3.973434654
UR_B5_R5	-2.802880459	-2.764675449	-1.898299671	-4.42247	-3.69505	-2.33895	-6.03416	-1.66194	-6.39816	-2.763739064	-4.614789498	#NUM!	-5.269727349	-3.813308817
UR_B5_R6	0.686321505	-2.874798152	-4.827169907	7.715075	8.588326	10.09378	6.727665	-3.65594	-8.20259	-6.668465768	#NUM!	0.289627955	-5.235817296	-2.885348233
UR_B5_R7	-1.472158894	-5.009484615	-4.604942833	5.973424	6.6624	8.339901	5.179002	-2.82609	-6.25154	-4.653968852	-7.175984893	-1.434655686	-5.037346696	-3.651104381
UR_B5_R8	-1.426658702	-3.350396303	-4.581203153	-3.11596	-2.68675	-1.17914	-3.69623	-4.08032	-6.99179	-5.568092176	#NUM!	-4.464342388	-6.555822466	-4.078702167
UR_B5_R9	-1.695124046	-6.046003152	#NUM!	-3.80018	-3.49227	-2.04434	-4.68503	-2.73966	-5.03865	-3.508169206	-4.465092025	-6.272245523	-5.543803522	#DIV/0!
UR_B5_R10	-1.609197657	-3.76328342	-5.843524242	-3.87206	-3.19522	-1.69681	-4.64935	-4.21611	-6.56903	-5.795198583	-9.674414744	-6.968787969	-7.532578649	-4.564785867
R_B5_R1	0.026747957	-1.749072905	-4.167294561	0.972814	1.672672	2.971428	-0.20114	-4.30458	-4.34758	-2.31260612	-5.446078005	-3.642269838	-5.110788591	-3.79401704
R_B5_R2	-0.352385551	-3.287593233	-4.597839645	-0.71151	-0.0771	1.581742	-1.72454	-3.42364	-4.85916	-3.367437423	#NUM!	-3.724012314	#NUM!	-3.94531823
R_B5_R3	-0.587570454	-3.596848414	-5.729800473	0.009597	0.617847	2.12451	-1.14437	-4.31384	-6.8413	-5.297376557	-8.385870705	-4.132611568	#NUM!	-3.968517531
R_B5_R4	-0.622854115	-4.377218483	-6.976202891	-2.02564	-2.06422	-0.24469	-3.04662	-3.93535	-5.77703	-4.127311719	-4.968844454	-6.655256288	#NUM!	-4.373224434
R_B5_R5	-1.579401864	-4.041006769	#NUM!	-2.55231	-2.01027	-0.50494	-3.4171	-3.66482	-7.62886	-7.194412554	#NUM!	-6.643094155	#NUM!	-4.290431829
R_B5_R6	-1.164403919	-2.831489003	#NUM!	5.702713	6.460731	8.079799	5.063212	-4.40445	-5.14889	-4.48786908	-8.454278985	-1.348207296	#NUM!	-4.209404546
R_B5_R7	-0.988561916	-1.153140782	-1.667553455	-3.44676	-3.14225	-1.49617	-4.3905	-3.49584	-5.30099	-4.149765587	-4.893132741	-1.84236335	-2.909331103	-2.161473267
R_B5_R8	-0.383890281	-3.889124043	-4.643874525	0.69762	1.247392	2.734791	-0.08858	-3.44233	-5.88207	-3.950844948	-9.523928712	-5.250662317	#NUM!	-3.445815489
R_B5_R9	-0.504042376	-4.094188135	-6.467009447	-0.17852	0.265337	2.025235	-0.85661	-4.68136	-6.66495	-6.031126197	-7.936076651	-3.18333021	-5.335704818	-3.447616743
R_B5_R10	0.070165446	-1.513320139	-2.560548713	-2.30376	-1.76201	-0.25738	-3.40344	-2.37873	-4.61057	-3.08237557	#NUM!	-2.87452256	-3.284133208	-1.979903037
N_B6_R1	-0.061695299	-0.007699627	0.820758019	-1.33412	-1.39165	0.147236	-1.97845	-4.29575	-3.17887	-1.844145898	-4.409309665	-0.574232334	-5.408491152	-2.345355406
N_B6_R2	-0.989000993	-0.459504758	0.323944892	-2.17936	-1.4375	-0.25051	-3.20849	-4.36792	-4.52151	-2.645499241	-5.405394615	-2.179908145	-5.679049523	-2.563475031
N_B6_R3	-2.007571059	-2.655201838	-4.038389092	-3.99069	-3.62112	-1.52837	-4.41466	-5.07041	-5.64781	-3.376510966	-5.369125606	-3.64252309	-11.37287749	-3.732905541
N_B6_R4	-1.764969115	-2.569316082	-4.738236818	-3.52122	-3.1559	-0.90748	-3.62273	-4.96115	-4.99744	-3.254478623	-5.206354976	-3.437808278	#NUM!	-3.729553971
N_B6_R5	0.37240729	1.029015985	1.204673001	2.523557	2.992788	4.629726	1.598269	-5.0156	-2.66232	-0.684625845	-4.628645528	-0.443592707	-4.373410272	-1.357363599
N_B6_R6	0.290599209	1.02428823	1.413106964	2.130046	2.677179	3.901099	1.368272	-5.30861	-2.87019	-0.932099334	-4.292414008	-0.258653898	-4.723877956	-1.491757143
N_B6_R7	-1.741746832	-0.340469245	0.646427838	-2.67356	-2.39121	-0.73148	-3.78496	-3.62538	-2.90496	-0.953014061	-4.564565172	-1.246005679	-5.074146436	-1.913893621
N_B6_R8	-1.477138126	0.09772133	0.718790274	-2.22839	-1.62771	-0.1132	-3.29904	-3.05063	-2.52053	-0.766724664	-4.542637513	-0.703326936	-5.31827648	-1.982611099
N_B6_R9	-1.78565077	-1.496907587	-0.053929159	-2.21841	-1.98569	-0.25881	-3.47867	-4.15488	-4.08581	-2.243400798	-4.228968047	-2.25468372	-4.991862275	-2.487638779
N_B6_R10	-1.506082212	-1.662483814	-0.113005621	-2.97731	-2.72381	-0.99367	-4.22399	-3.52405	-3.97339	-1.94876045	-4.196631181	-2.264881435	-5.158562698	-2.414666456
UR_B6_R1	-2.587046426	-3.541071786	-2.600924564	-3.38059	-3.25522	-1.44767	-5.05979	-0.724	-6.12644	-3.999465928	-9.236621779	-6.401937017	-7.422156789	-4.317553624
UR_B6_R2	-2.809517534	-5.447271093	-3.519224342	-5.08655	-4.86387	-3.1515	-7.12139	-1.8252	-6.62298	-4.443361371	#NUM!	-6.374373801	-9.654036978	-5.194800814
UR_B6_R3	-1.093424483	-4.859919221	#NUM!	-1.88428	-1.97473	0.058137	-2.40718	-0.56922	-6.3653	-4.920773562	-8.297340296	-5.350833809	#NUM!	-4.972065605
UR_B6_R4	-1.016280202	-3.401629218	-3.073537482	-2.3814	-2.44379	-0.43731	-3.3867	-4.31319	-4.70871	-2.830319848	-11.72038356	-3.377486564	#NUM!	-4.223984048
UR_B6_R5	-2.94390737	-6.83591913	-6.242892924	-3.84079	-3.48454	-2.21652	-6.06387	-3.78043	-5.56072	-3.502775957	-9.007573342	-5.50265504	-10.80590389	-6.21220595

UR_B6_R6	-1.905707072	-3.86853219	-5.595349816	-1.84528	-1.82014	-0.24913	-3.3108	-4.1781	-8.13065	-7.294367062	-10.44066626	-5.412735443	#NUM!	-6.32568308
UR_B6_R7	-2.942232252	-6.112155797	-4.465783687	-3.59067	-3.76536	-2.07237	-4.80866	-1.80082	-9.16828	-6.358485253	#NUM!	-6.137176746	#NUM!	-5.981985228
UR_B6_R8	-1.744655667	-6.599895572	-5.219069189	-3.79077	-3.51331	-2.11967	-4.57493	-2.1215	-7.07404	-4.875081987	-9.181109025	-6.082921942	#NUM!	-5.863181937
UR_B6_R9	-2.359145831	-7.227611758	-5.951433932	-0.17394	0.370079	2.006524	-1.15635	-5.60563	-10.9271	-8.724681285	#NUM!	-8.094921538	#NUM!	-8.116506507
R_B6_R1	-2.031108222	-4.770733919	-2.835903247	-2.01563	-1.32163	-0.09961	-3.17359	-3.74973	-6.82492	-4.50796025	#NUM!	-4.550070546	-8.082053199	-4.920545192
R_B6_R2	-1.617933136	-3.879920311	-1.52695034	-1.97942	-1.38892	-0.05928	-2.53783	-3.78017	-6.36319	-4.3265617	#NUM!	-4.893610139	-6.826922856	-4.033069553
R_B6_R3	-1.554570846	-5.736331552	-4.027253543	-1.57438	-1.45545	0.141938	-2.86316	-4.04462	-7.71627	-5.075296835	#NUM!	-5.735950692	-10.40225801	-4.801379296
R_B6_R4	-3.170865623	-6.601609984	-4.590811133	5.985132	6.894619	8.033893	5.184371	-5.80015	#NUM!	-11.06222925	#NUM!	-3.968566226	#NUM!	-5.083051954
R_B6_R5	-1.74631647	#NUM!	#NUM!	-1.06534	-0.89721	0.863097	-1.87369	-3.08874	-9.64074	-8.197660425	#NUM!	-6.287483974	#NUM!	-6.925357178
N_B7_R1	-1.527488969	-1.689889707	-1.153335604	0.809148	1.238228	3.314172	-0.23577	-3.50003	-3.77919	-1.985832437	-3.812737566	-2.51140516	-4.125748952	-3.318469818
N_B7_R2	-0.502752491	-4.716542722	-4.277229473	-3.02278	-2.54227	-0.99687	-4.30308	-4.76038	-7.04915	-0.692056038	-9.183315909	-5.762631308	-6.780922361	-5.023985309
N_B7_R3	-1.624064187	-2.69121977	-2.047390605	-2.57969	-2.03564	-0.41729	-3.70737	-3.75409	-4.93344	-2.501000855	-3.9194323	-3.467709026	-4.316731556	-3.913869366
N_B7_R4	-0.393116361	-4.815062773	#NUM!	-1.79537	-1.48157	0.907257	-2.58354	-4.00182	-6.4175	-4.240229421	-5.964207344	-5.930621791	#NUM!	-5.41217387
N_B7_R5	-0.505714905	-3.661918712	-3.85552038	-2.40766	-2.16089	-0.23501	-3.46184	-4.74956	-5.02497	-0.664962729	-11.29624675	-3.346164145	-5.818107426	-3.62244569
N_B7_R6	-1.327924478	-0.996764535	-1.796410707	-2.08317	-1.64032	0.031064	-3.37576	-3.50119	-3.66281	-0.660805104	-1.881489545	-1.761487355	-3.018054904	-1.78438867
N_B7_R7	-0.891976426	-1.475614297	-0.65692436	-1.80819	-1.73405	0.185641	-3.02622	-5.36041	-3.61826	-0.458897357	-3.802266121	-2.050485383	-1.608673924	-1.592797405
N_B7_R8	-2.800451754	-7.648053777	-4.650412129	4.400394	4.810204	6.268927	3.082768	-4.64176	-7.82197	-5.409530411	-12.6450592	#NUM!	-6.02649078	-7.082577462
N_B7_R9	-1.406573704	-1.960110653	-1.785268216	2.543996	2.785912	3.99266	1.102518	-3.15633	-2.46573	-0.199739073	-5.104282126	-2.555413381	-2.794044694	-2.027599563
N_B7_R10	-0.816619019	-1.832875465	-1.757348323	-2.42166	-2.26607	-0.12094	-3.30077	-3.50035	-4.18975	1.821945785	-1.38204313	-2.371430146	-4.091091326	-2.725804276
N_B7_R11	-2.418443278	-8.080092851	-8.46281733	-4.49963	-4.52924	-2.34745	-5.80521	-3.97715	-10.0677	-7.854709897	-10.08813	-9.335671888	#NUM!	-7.603660851
N_B7_R12	-1.305771335	0.847244269	-0.667318364	2.605236	3.020858	5.603953	2.468168	-4.05548	-8.54169	-6.595827135	-8.140679499	0.955803067	-8.009259336	-4.162985546
N_B7_R13	-0.864807812	-2.4014823	-1.862082699	-2.34438	-2.03581	-0.26125	-3.71168	-4.54011	-3.48097	-0.276539953	-5.678951001	-2.790243772	-2.344460861	-2.052741893
N_B7_R14	-0.939737304	-2.070886078	-1.427784889	2.223714	2.403065	3.668494	1.265613	-3.56495	-2.47362	1.33243753	-4.935363262	-2.457792891	-2.711943614	-2.155087662
N_B7_R15	-3.827366842	-3.859347676	-3.375304891	-6.6527	-6.98006	-4.58588	-7.74505	-4.53061	-6.60198	-4.763200431	-4.531912457	-4.939500113	-5.499843586	-4.813115156
N_B7_R16	#DIV/0!	-5.493444754	-6.599048293	-3.25305	-2.90784	-0.61949	-3.73818	-2.04361	-7.77012	0.675873442	-8.78430784	-6.092576081	-8.915186931	-6.580982787
N_B7_R17	-0.57408102	-5.014524859	-4.812779779	-3.09475	-2.75174	-1.26648	-4.43745	-4.48598	-7.26819	-2.161672513	-10.76598187	-6.161245408	-7.102042125	-5.834806109
N_B7_R18	-1.4928386	-4.009412024	-3.365320802	-2.23744	-1.53905	-0.11726	-3.22708	-4.21161	-7.03891	-3.626271929	-3.227718752	-4.757289177	-4.821844399	-4.195994001
N_B7_R19	-1.605200806	-1.954892944	-2.077744212	-1.5711	-1.38663	0.559195	-2.82286	-3.78082	-5.39476	-2.974158463	-7.306485329	-2.39813842	-4.182465864	-2.391976399
N_B7_R20	-1.515597598	-3.042698172	-3.89814568	-0.60704	-0.50426	1.420338	-1.98055	-4.42079	-4.05194	-1.43163415	-5.412252822	-3.809040145	-4.127131944	-2.884216455
N_B7_R21	-1.821220172	-1.070562104	-0.150776243	-2.66147	-2.57937	-0.50013	-3.73477	-3.77104	-3.21514	-1.323770117	-3.858923225	-1.136495669	-2.190870384	-1.764081292
N_B7_R22	-2.016942753	-3.923346553	-4.221642573	-3.60872	-3.70396	-1.59792	-4.8188	-4.07985	-6.17748	-1.795426119	-2.788026867	-4.166382	-6.926091825	-4.977586927
N_B7_R23	-2.382438637	-3.892099445	-3.934957572	-3.96147	-3.87022	-2.00309	-5.31727	-4.39206	-6.3578	-3.735640337	-2.985268959	-4.617572018	-6.284383733	-4.888936074
N_B7_R24	-2.10009502	-4.861897643	-4.666020404	-4.49436	-4.06327	-2.54034	-5.77535	-2.59378	-6.11212	-3.727003593	-2.949834055	-4.715159672	-6.002593782	-5.081391706
N_B7_R25	-2.851735324	-4.112863004	-3.517323896	-4.0827	-3.95012	-2.33166	-5.5784	-4.09424	-6.79141	#DIV/0!	-3.35710196	-4.834594903	-6.288342243	-4.897750362
N_B7_R26	-1.956542201	0.654111876	1.248232627	1.826624	1.972632	4.01632	0.512616	-4.13361	-7.13354	-4.972853468	-7.975231325	0.435567829	-5.301197894	-4.031790827
N_B7_R27	-2.465851141	-6.042950038	-5.402271938	-3.69807	-3.67595	-1.91553	-5.19311	-4.34058	-8.82359	-6.587787117	-5.655044416	-6.634228788	-7.937986316	-6.4386857
N_B7_R28	-1.766473811	-3.663128915	-2.526475594	1.061893	1.045833	2.29045	-0.40579	-3.98168	-4.21005	-1.912088504	-9.527761905	-4.43601282	-3.896010418	-3.469899286
N_B7_R29	-0.433466237	-10.12573552	#NUM!	-2.40771	-2.35723	-0.41394	-3.23542	-4.43119	#NUM!	#NUM!	#NUM!	-7.303282015	#NUM!	-7.422551355
N_B7_R30	-1.013348431	-1.85724538	-5.166416734	-2.36901	-1.97905	0.407538	-2.57861	-1.92873	-3.65835	-1.40541076	-2.558907482	-2.234865692	-4.859957324	-1.791445883
N_B7_R31	-1.147379493	1.108410806	-1.112933641	3.111524	3.671293	6.139588	3.189156	-3.84201	-7.88728	-6.138973285	-7.149957312	1.40404687	-8.670313422	-3.780172898
N_B7_R32	-1.507513403	0.942939922	0.021562735	3.02259	3.289838	5.739368	2.712988	-3.94661	-8.08144	-6.662912767	-8.029444505	1.47176214	-7.813850081	-4.088010245
N_B7_R33	-2.193404812	-3.481879327	-4.613248316	-2.59449	-2.53473	-0.6516	-3.63545	-3.27332	-5.71275	-3.493132554	-3.173117839	-3.918659168	-6.444573839	-4.534206426
N_B7_R34	-1.566925191	-4.811333426	-4.924051761	-1.40305	-1.01467	0.463427	-2.45541	-3.37005	-7.11838	-4.715052428	-4.245711438	-5.122441065	-7.248075195	-4.975323876
N_B7_R35	-2.100045027	-8.765798439	#NUM!	-6.09437	-6.67418	-4.38312	-6.86196	-3.99734	-9.27552	-7.813125657	-4.418305518	-8.339558082	-5.625208635	-5.139567247
N_B7_R36	-2.478148799	0.493061975	1.192235541	1.385385	1.640422	3.537238	0.457183	-4.25515	-9.01898	-7.146926833	#NUM!	-0.007456454	-5.562014348	-4.563469695
N_B7_R37	-1.6782863	-5.026731704	-4.965350751	-1.9217	-1.70673	0.007373	-2.97495	-4.27403	-7.50804	-3.73337373	-4.936773038	-5.649677187	-6.539458515	-5.476550252
N_B7_R38	-0.615323432	-3.705923094	-3.21991689	-2.46926	-2.43537	-0.55143	-3.49808	-4.40628	-4.92819	0.29644091	-8.600310925	-4.484399083	-4.036686547	-3.985311309
N_B7_R39	-2.350623526	-6.123041408	-6.514273799	-5.51611	-5.84056	-3.80544	-7.33846	-4.28885	-9.18955	-7.286045615	-10.4851415	-7.52734123	-8.638410717	-6.725375732
N_B7_R40	-1.81701607	-1.506285823	-0.767152763	1.191377	1.104012	3.027005	-0.09826	-3.7393	-4.56007	-0.904856406	-4.71628879	-3.709968249	-3.396195025	-3.269946044
