# Supplementary material for: Circulating proteomic panels for risk stratification of intracranial aneurysm and its rupture
Source: EMBO Mol Med. 2022 Jan 3;14(2):e14713. doi: 10.15252/emmm.202114713 (PMC8819334; doi:10.15252/emmm.202114713)
Supplement: Supplementary file 23 — Source Data for Figure 5 [file EMMM-14-e14713-s001.pdf]

ourceDataForFigure5B, C, D: The original dataset and predicted value based on LR model (P6: IA vs. NC) of training, internal validation and external validation se

Training	grade	PRTN3_HUMAN	KV320_HUMAN	PDLI1_HUMAN	CATG_HUMAN	PERM_HUMAN	IGHM_HUMAN	X0	X1	obs	pred
N_B1_R1	0	7.167323654	8.740251951	6.404958807	6.498180684	6.642504655	9.427570977	0.889761055	0.110238945	0	0
N_B1_R2	0	6.960458107	8.571129654	6.319255945	6.199977648	6.525981145	9.041939391	0.88729981	0.11270019	0	0
N_B1_R6	0	6.351949519	7.814677373	5.41970226	5.780005791	5.863952053	8.256440469	0.950492128	0.049507872	0	0
N_B1_R7	0	6.200047018	8.355475955	5.177021504	5.748525224	5.258605754	9.151660506	0.725886527	0.274113473	0	0
N_B1_R8	0	6.897978011	8.690554079	6.12260612	6.337394632	6.267520584	9.621672193	0.843048271	0.156951729	0	0
N_B1_R9	0	5.865763471	7.691986465	5.637856117	5.351655752	4.801691495	8.706807117	0.936615758	0.063384242	0	0
N_B1_R10	0	6.258612459	8.465277176	6.0690179	5.601495797	6.136480035	8.850199323	0.75421372	0.24578628	0	0
N_B2_R2	0	6.398063887	8.17062742	5.956980683	5.947670025	6.303078373	9.104379501	0.918730981	0.081269019	0	0
N_B2_R3	0	5.967330498	8.1106878	5.203810448	5.711628892	5.910379894	8.809015035	0.835196504	0.164803496	0	0
N_B2_R4	0	5.357263923	8.087197169	5.342182368	4.843909947	5.43511717	8.628128223	0.57724866	0.42275134	0	0
N_B2_R5	0	5.239729429	7.927189012	5.717809598	4.694408158	5.168062772	8.695353364	0.694802581	0.305197419	0	0
N_B2_R6	0	5.58092457	8.118883134	5.375249155	5.192734961	5.628596529	9.054016704	0.682096622	0.317903378	0	0
N_B2_R9	0	5.052065152	7.93765561	2.258452118	5.382387966	4.841123061	5.404632907	0.475946934	0.524053066	0	1
N_B3_R1	0	6.298675477	8.094576967	5.80054395	5.903331102	6.529930838	8.299963543	0.930115045	0.069884955	0	0
N_B3_R2	0	5.912212073	7.944766668	5.299314953	5.422678509	5.496956403	8.792830043	0.847958625	0.152041375	0	0
N_B3_R3	0	5.768033007	8.005670189	5.698881907	5.322482884	5.580767507	8.714463681	0.844225627	0.155774373	0	0
N_B3_R4	0	5.858293233	7.947867879	5.352897556	5.364619038	5.651223671	8.697805675	0.839172094	0.160827906	0	0
N_B3_R5	0	5.412537113	7.922514036	5.367972369	4.706453609	5.08317604	8.842869218	0.637298802	0.362701198	0	0
N_B3_R6	0	5.662356986	8.181369475	5.371920718	5.208746055	5.492069206	8.728877152	0.669664446	0.330335554	0	0
N_B3_R7	0	5.637949421	8.01196849	5.584321215	5.168287546	5.4924825	8.884161925	0.779549303	0.220450697	0	0
N_B3_R8	0	5.428861356	7.91881521	4.900117268	4.996387855	5.238535134	8.59894737	0.675926085	0.324073915	0	0
N_B3_R9	0	6.048298169	8.232158684	5.327091345	5.230632428	5.484997615	8.606432866	0.653780182	0.346219818	0	0
N_B3_R10	0	5.774484926	8.175804109	5.077776797	5.214797636	5.5198788	8.878792664	0.614488482	0.385511518	0	0
N_B4_R1	0	7.059758753	8.043301182	5.657431895	6.182509604	6.252267371	8.925258533	0.95609579	0.04390421	0	0
N_B4_R2	0	7.05029301	8.022294924	5.639527266	6.108284957	6.253838149	8.82227301	0.953464368	0.046535632	0	0
N_B4_R3	0	6.581556675	8.042641823	5.7962038	5.81679033	5.866151945	8.829341158	0.928610022	0.071389978	0	0
N_B4_R4	0	6.5879676	8.024764546	5.74286956	5.631687908	5.766192989	8.733024626	0.90753953	0.09246047	0	0
N_B4_R5	0	6.544482336	8.139593468	5.003299981	5.8420518	5.997509301	8.922389623	0.836772926	0.163227074	0	0
N_B4_R7	0	6.697102847	7.904826953	6.026161566	5.888633702	6.090886895	8.701219413	0.964401885	0.035598115	0	0
N_B4_R8	0	6.700196812	7.892599048	6.113931088	5.953229526	6.068173597	8.753132887	0.970984842	0.029015158	0	0
N_B4_R9	0	6.252251143	8.023771815	6.335118079	5.745519034	5.307768471	9.120681827	0.947513809	0.052486191	0	0
N_B4_R10	0	5.631060864	7.540182816	5.831272122	5.058356267	2.962995565	8.66112531	0.950545635	0.049454365	0	0
N_B5_R4	0	4.357486223	6.607720769	5.875242923	5.700920457	2.962995565	7.944369543	0.998913772	0.001086228	0	0
N_B5_R5	0	4.764505897	6.866838506	5.465296713	5.250843404	2.962995565	8.647809541	0.992134756	0.007865244	0	0
N_B5_R8	0	5.170461553	7.145901474	5.197637006	4.701590285	2.962995565	8.714577811	0.947066787	0.052933213	0	0
N_B5_R9	0	4.952790107	6.746069764	5.68390689	4.870739986	2.962995565	8.239680696	0.992007896	0.007992104	0	0
N_B5_R10	0	4.708367659	6.411741276	5.801242738	2.15648235	2.962995565	8.465216702	0.74520329	0.25479671	0	0
N_B6_R1	0	6.308129902	8.400732263	5.372614824	6.01625354	6.311450635	8.769651618	0.81180177	0.18819823	0	0

N_B6_R3	0	5.767151991	7.764509872	5.26264869	5.259266567	5.451028705	8.381620575	0.881568384	0.118431616	0	0
N_B6_R4	0	5.736119574	7.735628302	5.147160459	5.14273879	5.379195061	8.546966584	0.852977384	0.147022616	0	0
N_B6_R5	0	6.522893828	8.272658458	5.340987633	6.22045203	6.287547818	9.014512433	0.894145779	0.105854221	0	0
N_B6_R6	0	6.488541419	8.169867758	5.206166885	6.195046036	6.335514631	8.968482389	0.906958411	0.093041589	0	0
N_B6_R7	0	6.395417438	8.083822716	5.393911473	6.010318109	6.135133457	8.614764908	0.92059728	0.07940272	0	0
N_B6_R8	0	6.293264247	8.120095787	5.388969396	6.048098821	6.197593264	8.679283202	0.91480382	0.08519618	0	0
N_B6_R10	0	6.198826387	7.999547495	5.492993444	5.561162453	5.780036877	8.802650541	0.876939933	0.123060067	0	0
N_B7_R3	0	5.754485368	8.243242739	5.656341596	5.252277104	5.520318495	9.222833185	0.67539992	0.32460008	0	0
N_B7_R6	0	6.370197103	8.063918787	6.123024984	5.682035103	5.980230406	8.581918235	0.928173051	0.071826949	0	0
N_B7_R7	0	6.38651241	7.747042984	5.545487233	5.476881569	5.812354656	8.489272515	0.935953246	0.064046754	0	0
N_B7_R8	0	4.801756868	8.10818159	2.957101368	3.679981525	2.962995565	9.068147077	0.025160243	0.974839757	0	1
N_B7_R10	0	6.016730589	8.204059677	6.209156903	5.174481908	5.578900237	8.925893699	0.779134997	0.220865003	0	0
N_B7_R11	0	4.686121033	8.087427898	3.70238411	3.489076426	3.661953068	9.191476981	0.031388641	0.968611359	0	1
N_B7_R13	0	6.29781232	8.001765461	4.998634298	5.208206119	5.650665482	8.539348272	0.734909376	0.265090624	0	0
N_B7_R14	0	6.260722038	8.317301567	5.254445916	5.387626175	5.680412175	9.348132104	0.607532963	0.392467037	0	0
N_B7_R16	0	4.805101918	8.442106143	4.128082788	4.377182392	4.526057311	8.975754125	0.070271004	0.929728996	0	1
N_B7_R17	0	5.230443879	7.966608591	3.541482636	4.483665458	4.737811892	8.807244844	0.191784223	0.808215777	0	1
N_B7_R18	0	5.691209393	8.19961199	5.753181805	4.866908092	5.083252436	8.875098196	0.595210599	0.404789401	0	0
N_B7_R21	0	6.172794472	8.059332428	5.580038507	5.686345949	6.116049165	8.761441001	0.886160365	0.113839635	0	0
N_B7_R22	0	5.320250635	8.424107244	5.914839745	4.832231109	5.253655828	9.038064418	0.427897518	0.572102482	0	1
N_B7_R23	0	5.249537022	8.247544383	5.859969895	4.907184518	5.181021306	8.931312651	0.581648594	0.418351406	0	0
N_B7_R24	0	5.238783642	8.303174432	5.908702024	4.603015095	5.092550197	8.969462009	0.420147155	0.579852845	0	1
N_B7_R25	0	5.413401112	8.13262781	5.792805617	4.838758673	5.105362	8.820393247	0.631872152	0.368127848	0	0
N_B7_R26	0	5.51257754	8.089703378	4.33109038	6.214866665	6.555378458	9.216851324	0.835382959	0.164617041	0	0
N_B7_R27	0	4.924049652	8.021193273	5.073653797	4.17431082	4.552405274	8.54576918	0.304343264	0.695656736	0	1
N_B7_R28	0	5.71618462	8.142443316	3.942306657	4.952167649	5.242173314	8.903523265	0.306478664	0.693521336	0	1
N B7 R29	0	4.562409888	7.721646059	2.258452118	2.854939256	4.266114623	7.899604159	0.01154688	0.98845312	0	1
N B7 R30	0	6.249227778	7.648904759	5.930275236	5.32216922	5.633165216	8.230711727	0.955895725	0.044104275	0	0
N B7 R31	0	5.570658941	7.557342017	4.609292084	6.304768418	6.855650504	8.715055777	0.97447397	0.02552603	0	0
N B7 R32	0	5.650530304	7.649839567	4.292419991	6.2329052	6.73148152	8.721744275	0.952956291	0.047043709	0	0
N B7 R33	0	5.398463638	8.204351274	5.9342243	4.991305328	5.265877586	8.898348694	0.663498433	0.336501567	0	0
N B7 R34	0	5.143137358	8.134171801	5.533475153	4.562495588	4.857018067	8.701051569	0.465387676	0.534612324	0	1
N B7 R36	0	5.336952171	7.952676708	2.258452118	6.102653068	6.456872554	8.982166959	0.539014864	0.460985136	0	0
N B7 R37	0	5.059635019	8.115675519	5.307085494	4.512218161	4.707609167	8.476786733	0.424959322	0.575040678	0	1
N B7 R38	0	5.55424519	7.996796982	4.156484411	4.684377033	5.038857126	8.26926788	0.368896046	0.631103954	0	1
N B7 R39	0	4.61694852	8.034569024	3.636175957	4.150581852	4.312847554	8.347290321	0.113043727	0.886956273	0	1
N B7 R40	0	5.750983753	8.210613406	5.265861667	5.520076435	5.266559841	8.763470211	0.750882606	0.249117394	0	0
UR B1 R1	1	5.326444166	8.315268173	5.195531206	4.420401854	5.041762975	8.886468286	0.226220335	0.773779665	1	1
UR B1 R2	1	5.50438267	8.628437219	4.896739957	4.799557762	5.18317093	8.872791515	0.152864534	0.847135466	1	1
UR B1 R3	1	5.204527724	8.076305246	4.712758312	3.853747449	4.592751035	8.799455152	0.133266674	0.866733326	1	1

UR_B1_R4	1	5.736428743	8.769159507	2.258452118	4.460580877	5.306274924	9.560313291	0.006473489	0.993526511	1	1
UR_B1_R5	1	5.352238005	8.077294797	4.987567705	4.156468709	4.642036193	8.815632908	0.247527208	0.752472792	1	1
UR_B1_R6	1	4.782308317	6.976536958	4.777555345	2.15648235	5.89629959	8.593497992	0.149992446	0.850007554	1	1
UR_B1_R7	1	5.092125156	8.110909078	5.440813915	5.004449498	5.059022431	8.71016418	0.644087835	0.355912165	1	0
UR_B1_R8	1	3.636091896	7.676132699	5.230851198	3.839100735	5.906389431	8.449412491	0.364561985	0.635438015	1	1
UR_B1_R9	1	5.519329538	8.433367895	5.16550709	5.569470971	5.385170546	8.772443514	0.603470422	0.396529578	1	0
UR_B1_R10	1	5.243535008	8.111932971	2.258452118	2.15648235	5.029509285	8.768659575	0.000915072	0.999084928	1	1
UR_B2_R1	1	4.871665186	7.797219117	5.152758042	3.876534922	4.687020527	8.339111256	0.35317889	0.64682111	1	1
UR_B2_R2	1	5.520114389	8.079363466	5.272473442	4.782911324	6.063963501	9.268647467	0.494053142	0.505946858	1	1
UR_B2_R5	1	4.84681343	7.981557417	2.258452118	3.789186229	4.728118626	8.783426245	0.022066833	0.977933167	1	1
UR_B2_R6	1	6.642105837	7.802252365	5.126824761	2.15648235	4.781292698	8.721903618	0.028560153	0.971439847	1	1
UR_B2_R7	1	5.543552751	8.395848318	4.842513324	4.989400683	5.485508703	8.988779316	0.308545499	0.691454501	1	1
UR_B2_R8	1	5.06334602	8.252912759	2.258452118	4.957479225	2.962995565	6.103602911	0.152893206	0.847106794	1	1
UR_B2_R9	1	5.922196104	8.27161554	5.222571956	5.201774719	4.802745663	5.404632907	0.764741938	0.235258062	1	0
UR_B2_R10	1	5.713981324	8.440126593	4.629913665	5.040045343	5.6696255	9.346962618	0.249008806	0.750991194	1	1
UR_B3_R1	1	5.531734149	8.202849664	2.258452118	4.869344277	4.973302443	9.084208324	0.07160753	0.92839247	1	1
UR_B3_R2	1	5.507483852	8.390155841	5.02639512	5.12399586	5.362636068	8.956091165	0.404241268	0.595758732	1	1
UR_B3_R3	1	4.987471853	8.034081288	2.258452118	4.258032175	4.325906859	8.856112942	0.043774005	0.956225995	1	1
UR_B3_R4	1	5.33833338	8.324116324	4.633818124	4.820900692	4.638413482	8.804190662	0.277142415	0.722857585	1	1
UR_B3_R5	1	5.062908424	8.203150617	2.258452118	3.99549296	5.585231902	8.853649051	0.015471857	0.984528143	1	1
UR_B3_R6	1	5.268040023	8.047861105	2.258452118	4.516484693	4.753087387	8.972610522	0.062298311	0.937701689	1	1
UR_B3_R7	1	5.172264129	8.194386959	4.548085436	4.095319517	2.962995565	8.729418845	0.148553099	0.851446901	1	1
UR_B3_R9	1	5.140007212	8.142508351	2.258452118	4.372763026	5.260831993	8.739158019	0.037308274	0.962691726	1	1
UR_B4_R2	1	6.147273629	8.28593839	5.234785868	5.117500096	4.583739424	8.746669229	0.569668346	0.430331654	1	0
UR_B4_R3	1	5.282491325	8.062155798	2.258452118	3.947418801	4.254311023	8.635935228	0.026254576	0.973745424	1	1
UR_B4_R4	1	5.438982504	8.136477822	2.258452118	4.869527618	5.093012129	8.67587827	0.091171788	0.908828212	1	1
UR_B4_R6	1	5.005515073	8.046867916	4.744241251	4.05083419	4.152456273	8.889110624	0.195311038	0.804688962	1	1
UR_B4_R8	1	5.429051096	8.407149255	4.899957796	4.670662894	5.27313086	8.61220421	0.222282534	0.777717466	1	1
UR_B5_R1	1	4.216214769	6.98982637	4.432117895	4.253376481	2.962995565	8.248673572	0.871691322	0.128308678	1	0
UR_B5_R3	1	4.421527506	6.979909781	3.813498329	2.15648235	2.962995565	8.765985471	0.089783765	0.910216235	1	1
UR_B5_R4	1	5.704532803	8.416870576	4.801374221	5.09715679	5.271332555	8.925643844	0.341239364	0.658760636	1	1
UR_B5_R6	1	5.84684382	8.438269753	2.258452118	5.025322738	6.365930877	9.061737655	0.04416216	0.95583784	1	1
UR_B5_R7	1	5.765950082	8.365890434	4.578950548	4.50456757	6.024399602	9.097678966	0.137266951	0.862733049	1	1
UR_B5_R8	1	5.553000757	8.215507182	2.258452118	4.919476917	5.016779968	8.97076359	0.076747547	0.923252453	1	1
UR_B5_R9	1	5.496048491	8.193007011	5.475015315	4.15597516	4.672549981	8.671627861	0.269387286	0.730612714	1	1
UR_B5_R10	1	5.462775204	8.162406314	3.808357316	4.8310872	4.353335386	8.587202052	0.257250445	0.742749555	1	1
UR_B6_R1	1	5.515032507	8.013150252	4.047417984	4.958746782	4.523210568	8.8262208	0.431665603	0.568334397	1	1
UR_B6_R3	1	5.332328418	8.616020618	4.328360059	4.542767814	4.823139261	9.502277432	0.061727302	0.938272698	1	1
UR_B6_R5	1	5.049385863	7.967591502	3.894376118	3.914531027	4.841703771	8.477804757	0.109253091	0.890746909	1	1
UR_B6_R8	1	5.090696623	8.238719872	4.06657485	4.08588116	4.647238631	8.812437519	0.076298532	0.923701468	1	1

UR_B6_R9	1	4.418983819	8.018211634	2.258452118	3.869925586	4.100994995	8.202750357	0.026386833	0.973613167	1	1
R_B1_R1	1	5.819914778	8.427796598	5.620269741	5.350123229	5.606841675	9.119520472	0.589524568	0.410475432	1	0
R_B1_R2	1	4.549551196	7.482750242	4.723572021	3.897676561	4.697271267	8.695569468	0.466445707	0.533554293	1	1
R_B1_R7	1	5.327833112	8.592905403	4.719340276	4.957479225	5.168317901	8.868111085	0.182552829	0.817447171	1	1
R_B1_R8	1	4.839623665	7.772812752	4.716176164	4.559310565	4.763828946	8.734497745	0.547877295	0.452122705	1	0
R_B1_R9	1	4.706321546	8.08697589	4.572036439	4.59812358	4.10428972	8.636593558	0.333464067	0.666535933	1	1
R_B1_R10	1	6.075703719	8.207664912	2.258452118	5.018960354	5.524710702	8.742598088	0.097112862	0.902887138	1	1
R_B2_R2	1	5.784684293	8.229959605	5.576964651	5.174145273	5.647463596	9.232305474	0.635866918	0.364133082	1	0
R_B2_R4	1	5.449470348	7.893342725	4.786537172	4.17301474	4.883306789	8.62476249	0.334798554	0.665201446	1	1
R_B2_R5	1	5.565917519	8.051896554	4.788215785	4.627763701	5.169695814	9.065189092	0.384892119	0.615107881	1	1
R_B2_R10	1	5.714586595	8.1794785	5.424938774	5.446237474	5.927045737	8.914240889	0.747982033	0.252017967	1	0
R_B3_R2	1	5.577736122	8.103308001	4.176855031	4.739469937	5.187600506	8.669625951	0.302108427	0.697891573	1	1
R_B3_R3	1	5.317023596	7.983100609	4.354748551	4.784806844	5.165193007	9.13194999	0.401637535	0.598362465	1	1
R_B3_R6	1	5.695989818	8.152687331	5.213191202	4.892377683	4.873707879	8.75737958	0.542212957	0.457787043	1	0
R_B3_R7	1	5.003592622	7.763730736	4.06921384	3.948735608	4.414065754	8.316827895	0.226521508	0.773478492	1	1
R_B3_R8	1	5.585006768	8.043743269	2.258452118	4.681491829	5.265877586	8.896355874	0.082384579	0.917615421	1	1
R_B3_R9	1	5.146789061	8.263960196	2.258452118	4.529961076	5.203525831	8.976143495	0.033063909	0.966936091	1	1
R_B3_R10	1	5.303503523	7.905247579	2.258452118	4.988481977	4.823028104	8.552523033	0.200617573	0.799382427	1	1
R_B4_R1	1	5.111894525	8.534070268	4.266040564	2.15648235	4.732437925	8.785996514	0.001432282	0.998567718	1	1
R_B4_R2	1	5.64384164	8.215412309	4.543544707	4.191748436	5.375871745	8.781474647	0.135745254	0.864254746	1	1
R_B4_R3	1	5.381614037	8.32362209	2.258452118	4.847275672	4.810962512	8.831510073	0.052509647	0.947490353	1	1
R_B4_R4	1	5.216755198	8.062168058	2.258452118	4.068417088	4.639959481	9.05262274	0.028014378	0.971985622	1	1
R_B4_R6	1	5.358808503	8.260965526	4.722234861	4.959000559	5.258624311	8.585845132	0.383571451	0.616428549	1	1
R_B4_R7	1	5.695192221	8.243971536	5.213191202	4.67363884	5.237059542	8.845459711	0.369444268	0.630555732	1	1
R_B4_R8	1	5.147720428	7.859279559	2.258452118	4.174550043	5.322390099	8.792815051	0.057626386	0.942373614	1	1
R_B4_R9	1	5.129172841	7.88836837	2.258452118	2.15648235	5.042324155	8.539950074	0.001812934	0.998187066	1	1
R B4 R10	1	5.276993978	7.963374855	5.399711255	4.052923854	4.54940398	8.863833786	0.346922377	0.653077623	1	1
R B5 R1	1	5.999022286	8.120704546	5.114628558	5.470476673	5.392849819	9.248485367	0.742047683	0.257952317	1	0
R B5 R3	1	5.687209347	8.081008973	4.234284877	4.902241837	5.265877586	8.821299207	0.384216606	0.615783394	1	1
R B5 R4	1	5.556469513	8.186629695	5.32914234	4.690750192	4.522117643	8.852622815	0.453547711	0.546452289	1	1
R B5 R5	1	5.463717413	8.314326323	2.258452118	4.685731486	4.318390006	9.306138651	0.039335927	0.960664073	1	1
R B5 R6	1	5.532051754	7.970886186	4.079993074	4.958764474	5.904641127	9.076862414	0.419159418	0.580840582	1	1
R B5 R7	1	6.095844903	8.332624055	5.213191202	5.647297514	5.781539096	9.235682082	0.691443128	0.308556872	1	0
R B5 R8	1	5.758194663	8.075212445	3.862304027	4.809759989	4.825044031	8.844056304	0.294412335	0.705587665	1	1
R B5 R9	1	5.639787659	8.116875995	4.24441152	4.57964622	5.283506877	8.651939257	0.250131865	0.749868135	1	1
R B5 R10	1	6.258905477	8.295682206	2.258452118	5.521436688	5.573178197	9.472888729	0.146789899	0.853210101	1	1
R B6 R1	1	5.298556299	7.913788762	2.258452118	4.52477865	5.180548317	8.957803564	0.087317142	0.912682858	1	1
R B6 R2	1	5.682851379	8.479709939	2.258452118	4.746929174	5.038494388	8.924883918	0.028502099	0.971497901	1	1
R B6 R3	1	5.408211646	8.108669554	2.258452118	4.310181537	4.602356878	8.606088713	0.041879969	0.958120031	1	1
R B6 R4	1	5.285216294	7.693649692	2.258452118	3.928353995	5.351206854	8.17340266	0.069894007	0.930105993	1	1

R	B6	R5	1	4.73676677	8.131258516	2.258452118	2.15648235	4.379523992	8.956001235	0.0008353	0.9991647	1	1

SourceDataForFigure5B, C, D: The original dataset and predicted value based on LR model (P6: IA vs. NC) of training, internal validation and external validation set.

Internal validation	grade	PRTN3_HUMAN	KV320_HUMAN	PDLI1_HUMAN	CATG_HUMAN	PERM_HUMAN	IGHM_HUMAN	X0	X1	obs	pred
N_B1_R3	0	5.928153992	7.510794685	5.498420025	5.50314481	4.918991498	8.685590856	0.966632087	0.033367913	0	0
N_B1_R4	0	6.236909294	7.935851682	5.8536704	5.462627693	5.676347765	8.777085092	0.908507489	0.091492511	0	0
N_B1_R5	0	6.762701653	8.043096233	5.550684849	4.614167764	5.655276816	8.915948997	0.571687078	0.428312922	0	0
N_B2_R1	0	4.978930073	7.986853434	5.951405621	5.738830668	6.019816038	8.58250994	0.928862134	0.071137866	0	0
N_B2_R7	0	5.680277307	7.893623103	5.788670197	5.834000341	6.148057132	8.580375066	0.949839368	0.050160632	0	0
N_B2_R8	0	6.355245759	8.265929429	2.258452118	6.028846661	5.265877586	5.404632907	0.547685544	0.452314456	0	0
N_B2_R10	0	6.028264822	8.236071497	5.722642153	5.297100888	5.630380646	8.907675921	0.727110279	0.272889721	0	0
N_B4_R6	0	6.462705444	8.13571691	5.109781176	5.794488469	5.979914857	8.915438847	0.838132297	0.161867703	0	0
N_B5_R1	0	5.187980518	6.346188313	5.721028942	5.623912429	2.962995565	8.226554406	0.999359655	0.000640345	0	0
N_B5_R2	0	5.737190668	6.823295532	5.917407266	5.732801521	2.962995565	8.519400327	0.998207458	0.001792542	0	0
N_B5_R3	0	5.732186028	6.899373429	5.749327316	5.771953062	2.962995565	8.026688018	0.997859329	0.002140671	0	0
N_B5_R6	0	4.762424394	6.751269527	5.255611724	4.95498959	2.962995565	8.566529284	0.989027502	0.010972498	0	0
N_B5_R7	0	5.57545902	6.816406943	5.787582415	5.302983371	4.57813389	8.793788853	0.994696977	0.005303023	0	0
N_B6_R2	0	6.0755175	8.089215414	5.22021552	5.843028732	5.940093454	8.577077992	0.879830844	0.120169156	0	0
N_B6_R9	0	6.212552793	8.04540532	5.479623534	5.684397966	5.873671696	8.85891566	0.882229852	0.117770148	0	0
N_B7_R1	0	5.889063491	8.241415076	5.631998268	5.554236604	5.668174554	9.147078649	0.779408724	0.220591276	0	0
N_B7_R2	0	5.371569739	7.925047969	4.028348718	4.579832454	4.815403371	8.889843499	0.318922995	0.681077005	0	1
N_B7_R4	0	5.283108098	7.746180285	4.893090225	4.37715874	4.739076566	8.562812488	0.548026492	0.451973508	0	0
N_B7_R5	0	5.822355835	7.612832011	3.279835304	4.828007279	5.46830384	8.754105622	0.493853368	0.506146632	0	1
N_B7_R9	0	6.280124269	8.149992054	5.171936488	5.4406214	5.672362434	9.234705095	0.726024543	0.273975457	0	0
N_B7_R12	0	5.625156006	7.47771882	4.213943923	6.216360906	6.686679111	8.763718594	0.967855596	0.032144404	0	0
N_B7_R15	0	5.555225081	8.2736076	5.432986474	4.9627908	5.099730051	8.891289123	0.513104717	0.486895283	0	0
N_B7_R19	0	6.167933863	8.139522099	4.52306722	5.302563434	5.714439519	8.524088983	0.592442961	0.407557039	0	0
N_B7_R20	0	6.07745351	8.029961424	5.095006784	4.990438694	5.370500416	8.23253203	0.67082204	0.32917796	0	0
N_B7_R35	0	5.207838352	8.227964379	5.491043581	3.395042578	4.031683511	8.807843217	0.082367966	0.917632034	0	1
UR_B2_R3	1	5.14502914	8.069666678	5.078958101	4.504245507	4.92517497	8.670706008	0.394370767	0.605629233	1	1
UR_B2_R4	1	5.38029366	8.037641198	4.541570985	4.776721573	5.131132237	8.831342119	0.416812375	0.583187625	1	1
UR_B3_R8	1	5.998795125	8.200716754	5.749315428	5.408975455	5.603392929	9.069840958	0.779253451	0.220746549	1	0
UR_B3_R10	1	6.306579507	8.269818452	6.684428542	5.516342067	6.060371682	9.074678361	0.881706913	0.118293087	1	0
UR_B4_R1	1	5.921358776	8.482731678	4.831453667	4.955957891	5.021224622	9.259588458	0.250762831	0.749237169	1	1
UR_B4_R5	1	5.470847875	8.221133947	2.258452118	4.697382252	5.256298364	9.162393861	0.048808677	0.951191323	1	1
UR_B4_R7	1	5.24448681	8.151262486	4.634457894	4.43354306	2.962995565	9.024280006	0.265101753	0.734898247	1	1
UR_B5_R2	1	5.230156999	8.253408699	4.770190117	5.009231111	2.962995565	9.074436538	0.447001908	0.552998092	1	1
UR_B5_R5	1	5.629013155	8.219186795	5.43256316	5.160543203	2.962995565	9.353213996	0.663833816	0.336166184	1	0
UR_B6_R2	1	5.201857229	7.974352127	2.258452118	4.31481356	4.473585907	8.819122762	0.057671413	0.942328587	1	1
UR_B6_R4	1	5.594437764	8.414701163	3.17203542	4.957479225	5.428271714	9.100725577	0.090226939	0.909773061	1	1
UR_B6_R6	1	4.957216531	8.227193662	3.434275374	4.808808708	4.781172822	9.004411314	0.142252867	0.857747133	1	1
UR_B6_R7	1	5.091965836	8.251420177	2.258452118	4.227268219	4.614430305	8.552220308	0.023794518	0.976205482	1	1
R_B1_R3	1	4.664481366	7.808148942	4.792470086	3.747554328	4.259041675	8.934490583	0.218204433	0.781795567	1	1
R_B1_R4	1	4.413697972	7.870450204	2.258452118	2.15648235	6.193673886	9.06832905	0.001376849	0.998623151	1	1

R_B1_R5	1	5.590140733	7.955764215	5.377344609	4.098605398	5.273149417	8.919463017	0.354165629	0.645834371	1	1
R_B1_R6	1	5.617689646	8.230120784	5.011019644	4.953686847	4.795955851	8.58073675	0.481152992	0.518847008	1	1
R_B2_R1	1	5.232706028	7.980065349	4.769699612	4.442379041	6.217834142	9.2094294	0.313868165	0.686131835	1	1
R_B2_R3	1	5.487441231	8.138004629	5.344924163	4.461957989	5.535016275	8.824582848	0.368356043	0.631643957	1	1
R_B2_R6	1	5.599532375	8.006138315	4.675082163	4.192027035	4.988822893	8.676419559	0.254082801	0.745917199	1	1
R_B2_R7	1	5.422699081	8.127727932	4.748027752	4.966105515	5.138018355	8.464914698	0.498219593	0.501780407	1	1
R_B2_R8	1	5.709213839	8.20164497	4.554698165	4.904469525	5.220615792	8.80754403	0.367808461	0.632191539	1	1
R_B2_R9	1	5.631485389	8.319083287	5.698847257	4.86612315	4.699210534	8.706265716	0.516399008	0.483600992	1	0
R_B3_R1	1	5.972668996	8.031079204	4.300160115	5.047887394	5.712444859	8.810897398	0.492668163	0.507331837	1	1
R_B3_R4	1	5.715858435	8.056022685	2.258452118	5.190101585	5.375783195	8.640837902	0.182960885	0.817039115	1	1
R_B3_R5	1	5.40255592	8.284702028	4.153269211	4.926476473	5.005780627	8.872391792	0.24896509	0.75103491	1	1
R_B4_R5	1	5.478409407	8.223450358	4.841430498	5.026268493	5.464064431	8.732286026	0.452354889	0.547645111	1	1
R_B5_R2	1	5.590666776	8.33564856	2.258452118	4.956193976	5.239249292	8.968939066	0.05789988	0.94210012	1	1

SourceDataForFigure5B, C, D: The original dataset and predicted value based on LR model (P6: IA vs. NC) of training, internal validation and external validation set.

External validation	grade	CATG_HUMAN	PDLI1_HUMAN	PERM_HUMAN	PRTN3_HUMAN	KV320_HUMAN	IGHM_HUMAN	X0	X1	obs	pred
NC_B8_R1	0	5.643452676	6.485721426	6.198657087	6.485721426	8.546542663	9.037426498	0.782594	0.217406	0	0
NC_B8_R2	0	6.294466226	5.826074803	6.617000341	6.636487896	8.720159303	8.796574333	0.801861	0.198139	0	0
NC_B8_R3	0	5.093421685	6.621176282	6.117271296	6.348304863	8.544068044	8.997386384	0.608859	0.391141	0	0
NC_B8_R4	0	6.217483944	6.57634135	6.515873844	6.501059262	8.544068044	9.021189299	0.911801	0.088199	0	0
NC_B8_R5	0	5.414973348	6.648360011	6.155336037	6.33243846	8.338456494	8.893761762	0.837317	0.162683	0	0
NC_B8_R6	0	5.668385917	6.056904851	5.833784375	6.123851641	7.995196292	8.62838893	0.934301	0.065699	0	0
NC_B8_R7	0	5.683947131	6.10720997	5.879669206	6.46834733	8.181843588	8.658964843	0.901365	0.098635	0	0
NC_B8_R8	0	5.212187604	6.033423755	5.36361198	6.139879086	8.33243846	9.120573931	0.690421	0.309579	0	0
NC_B8_R9	0	2.795184599	4.511883361	5.652246341	6.012837225	8.017033339	8.804139432	0.023478	0.976522	0	1
NC_B8_R10	0	5.625312451	6.071882007	6.079181246	6.828659897	8.392696953	9.158362492	0.798661	0.201339	0	0
NC_B8_R11	0	2.795184599	6.096910013	5.608526034	5.982271233	8.385606274	8.810904281	0.029726	0.970274	0	1
NC_B8_R12	0	5.46686762	5.606381365	6.075546961	6.481442629	8.161368002	8.784617293	0.807164	0.192836	0	0
NC_B8_R13	0	5.824776462	5.819543936	6.06069784	6.552668216	8.494154594	9.068185862	0.770916	0.229084	0	0
NC_B8_R14	0	4.919078092	6.285557309	5.848189117	6.397940009	8.281033367	9.167317335	0.64962	0.35038	0	0
NC_B8_R15	0	5.311753861	6.012837225	5.906873535	6.269512944	8.376576957	8.787460475	0.703951	0.296049	0	0
NC_B8_R16	0	5.674861141	6.416640507	5.751279104	6.744292983	8.096910013	8.686636269	0.939228	0.060772	0	0
NC_B8_R17	0	2.795184599	6.624282096	3.982271233	4.356025857	7.592176757	8.5132176	0.329872	0.670128	0	1
NC_B8_R18	0	4.64738297	5.959994838	5.33243846	6.127104798	8.184691431	8.481442629	0.582575	0.417425	0	0
NC_B8_R19	0	5.079181246	6.264817823	5.876794976	6.123851641	8.416640507	8.976349979	0.621361	0.378639	0	0
NC_B8_R20	0	5.26245109	6.161368002	5.658011397	6.220108088	8.419955748	9.049218023	0.676411	0.323589	0	0
R_B8_R1	1	2.795184599	3.812913357	5.484299839	6.017033339	8.201397124	8.783903579	0.008034	0.991966	1	1
R_B8_R2	1	5.260071388	3.812913357	6.025305865	6.428134794	7.84509804	8.692846919	0.628259	0.371741	1	0
R_B8_R3	1	2.795184599	3.812913357	5.252853031	5.781036939	8.198657087	8.71432976	0.008196	0.991804	1	1
R_B8_R4	1	4.943494516	4.571708832	5.843855423	6.255272505	8.127104798	8.930439595	0.432297	0.567703	1	1
R_B8_R5	1	4.789580712	3.812913357	4.681241237	5.812913357	8.173186268	8.657055853	0.237061	0.762939	1	1
R_B8_R6	1	4.506505032	3.812913357	5.28780173	5.792391689	8.170261715	8.730782276	0.149721	0.850279	1	1
UR_B8_R1	1	4.967547976	3.812913357	5.193124598	6.086359831	8.292256071	9.326335861	0.201266	0.798734	1	1
UR_B8_R2	1	2.795184599	5.33243846	5.871572936	5.909556029	8.28780173	9.315970345	0.018223	0.981777	1	1
UR_B8_R3	1	2.795184599	3.812913357	5.859738566	5.660865478	8.206825876	8.859738566	0.007168	0.992832	1	1
UR_B8_R4	1	4.527629901	3.812913357	5.204119983	5.64246452	8.372912003	9.283301229	0.081461	0.918539	1	1
UR_B8_R5	1	2.795184599	3.812913357	5.103803721	5.503790683	8.267171728	9.225309282	0.005886	0.994114	1	1
UR_B8_R6	1	2.795184599	3.812913357	5.08278537	5.979092901	8.456366033	8.859138297	0.003936	0.996064	1	1

SourceDataForFigure5E, F, G: The original dataset and predicted value based on LR model (P8: R vs. UR) of training, internal validation and external validation dataset.

Training	grade	ITIH4_HUMAN	APOA4_HUMAN	FIBG_HUMAN	FINC_HUMAN	A2GL_HUMAN	COMP_HUMAN	IGHM_HUMAN	KV320_HUMAN	X0	X1	obs	pred
UR_B1_R1	0	7.373696643	8.217981593	5.512077817	7.194312909	7.72554668	6.09231623	8.886468286	8.315268173	0.630484	0.369516	0	0
UR_B1_R2	0	7.37726365	8.465217762	5.405433169	7.147553598	7.716371058	6.298801782	8.872791515	8.628437219	0.955024	0.044976	0	0
UR_B1_R3	0	7.308303594	8.642484787	5.036103763	6.677414777	7.524253197	6.163143095	8.799455152	8.076305246	0.966037	0.033963	0	0
UR_B1_R4	0	6.739371187	7.875689276	5.749428116	7.165326294	7.844873751	6.410917181	9.560313291	8.769159507	0.709418	0.290582	0	0
UR_B1_R5	0	6.615686297	8.373907381	4.826852049	6.458775182	7.452931809	5.385133387	8.815632908	8.077294797	0.950808	0.049192	0	0
UR_B1_R6	0	5.87411953	7.675710696	5.574771764	4.118884984	6.644086248	5.324581678	8.593497992	6.976536958	0.830315	0.169685	0	0
UR_B1_R7	0	5.744706578	8.544960258	5.395413993	5.668540055	6.740444767	6.383752131	8.71016418	8.110909078	0.999794	0.000206	0	0
UR_B1_R9	0	7.606310486	8.678802711	5.997267904	7.37133513	7.185424405	6.584071959	8.772443514	8.433367895	0.967564	0.032436	0	0
UR_B1_R10	0	7.404674385	8.274065411	5.429312381	6.945503281	7.434862655	5.872875721	8.768659575	8.111932971	0.651923	0.348077	0	0
UR_B2_R2	0	7.326723404	7.964464384	8.781512623	5.727634992	8.001439567	6.076706474	9.268647467	8.079363466	0.25863	0.74137	0	1
UR_B2_R3	0	6.880059342	8.148035906	5.544464244	5.762640458	7.57156711	5.975939	8.670706008	8.069666678	0.939745	0.060255	0	0
UR_B2_R4	0	7.116329392	7.92652568	5.6920482	6.17114533	7.423159043	6.004344198	8.831342119	8.037641198	0.584869	0.415131	0	0
UR_B2_R7	0	7.381433454	8.322653209	5.740967682	6.091087793	7.68112073	6.162121852	8.988779316	8.395848318	0.939253	0.060747	0	0
UR_B2_R8	0	4.54821132	5.398699448	5.864337803	3.126057487	4.468213492	6.415304618	6.103602911	8.252912759	0.974659	0.025341	0	0
UR_B2_R10	0	7.502155195	8.595396556	6.118444624	6.775072237	6.920527526	6.464997918	9.346962618	8.440126593	0.966109	0.033891	0	0
UR_B3_R1	0	7.09137952	7.785580739	5.470096699	5.887345283	6.542474212	6.119812425	9.084208324	8.202849664	0.743204	0.256796	0	0
UR_B3_R2	0	7.032930745	7.924519111	6.921337127	5.880049031	7.257900029	6.201649927	8.956091165	8.390155841	0.848117	0.151883	0	0
UR_B3_R3	0	7.269118854	8.537346558	5.667867366	6.071532374	7.607884584	6.010923246	8.856112942	8.034081288	0.957202	0.042798	0	0
UR_B3_R5	0	7.16370298	8.05880792	8.702733497	5.24313545	7.518104377	6.154073086	8.853649051	8.203150617	0.854044	0.145956	0	0
UR_B3_R8	0	6.994330301	7.51476239	6.027806316	6.299707236	7.524689356	5.936144924	9.069840958	8.200716754	0.148185	0.851815	0	1
UR_B3_R9	0	6.719297279	7.410756146	8.210874242	4.866206491	6.583534565	6.28656982	8.739158019	8.142508351	0.706061	0.293939	0	0
UR_B3_R10	0	7.229562825	7.915852661	5.868159599	6.46816554	7.720673178	6.053787782	9.074678361	8.269818452	0.450503	0.549497	0	1
UR_B4_R1	0	7.290846152	8.328383312	5.752619715	6.308574919	7.458819521	6.570234223	9.259588458	8.482731678	0.960935	0.039065	0	0
UR_B4_R3	0	7.098367971	8.236986405	8.014295182	5.387064231	7.084243328	6.224174429	8.635935228	8.062155798	0.95595	0.04405	0	0
UR_B4_R4	0	7.055520318	7.994466353	5.089734419	5.986193827	7.283027708	6.259195099	8.67587827	8.136477822	0.895508	0.104492	0	0
UR_B4_R5	0	7.137746426	8.228912563	5.500712941	5.958154056	7.386632132	6.246538526	9.162393861	8.221133947	0.932843	0.067157	0	0
UR_B4_R6	0	6.477788748	8.229549506	4.663617965	5.803929465	7.237328123	6.393256784	8.889110624	8.046867916	0.990439	0.009561	0	0
UR_B4_R8	0	7.693714118	8.068602898	6.137875855	6.866437732	6.77405503	6.29305612	8.61220421	8.407149255	0.696825	0.303175	0	0
UR_B5_R1	0	5.945088215	7.480825116	6.181740058	4.662436602	7.657640661	4.246940239	8.248673572	6.98982637	0.117846	0.882154	0	1
UR_B5_R2	0	6.689863581	7.90716862	5.429845173	5.449132362	7.457729787	6.245501542	9.074436538	8.253408699	0.944759	0.055241	0	0
UR_B5_R3	0	5.947120385	7.965824268	5.057795469	4.41345613	7.00417243	5.840634696	8.765985471	6.979909781	0.943758	0.056242	0	0
UR_B5_R5	0	6.39239887	8.112151791	5.047992465	5.304329883	7.247064955	6.290018177	9.353213996	8.219186795	0.990078	0.009922	0	0
UR_B5_R6	0	7.325796882	7.927546196	8.682613144	6.392782513	8.316052189	6.208764417	9.061737655	8.438269753	0.287759	0.712241	0	1
UR_B5_R7	0	7.245567637	8.030735611	8.387347678	5.90434259	7.612118035	6.272514612	9.097678966	8.365890434	0.706155	0.293845	0	0
UR_B5_R8	0	7.14488302	7.965985783	5.584634177	6.536377823	7.394743927	6.261696928	8.97076359	8.215507182	0.661996	0.338004	0	0
UR_B5_R9	0	7.319067257	7.755461909	5.349240099	6.42084608	7.536572129	5.934069331	8.671627861	8.193007011	0.330021	0.669979	0	1
UR_B5_R10	0	7.266341157	8.145773298	5.364929442	6.582823774	7.506221605	6.218738003	8.587202052	8.162406314	0.820882	0.179118	0	0
UR_B6_R1	0	6.619606627	8.135925804	5.26486824	5.859346177	7.134316074	6.145844189	8.8262208	8.013150252	0.965928	0.034072	0	0
UR_B6_R2	0	6.841228152	7.852452947	4.592390126	5.892697583	7.060953563	6.095181568	8.819122762	7.974352127	0.817279	0.182721	0	0
UR_B6_R4	0	7.148300945	8.075040742	5.820807457	7.159176215	7.777334058	6.157853043	9.100725577	8.414701163	0.575003	0.424997	0	0
UR_B6_R5	0	7.089390745	7.914906723	4.897983218	6.51949849	7.189280822	6.064132687	8.477804757	7.967591502	0.640699	0.359301	0	0
UR_B6_R6	0	7.224128468	8.273900729	5.704078728	6.656327806	7.437286477	6.496482775	9.004411314	8.227193662	0.890598	0.109402	0	0
UR_B6_R7	0	7.02424192	8.142984705	5.322144171	5.845963918	7.094936417	6.466520822	8.552220308	8.251420177	0.980985	0.019015	0	0
R_B1_R1	1	7.165932805	8.316838988	6.645805674	6.725075812	7.930821088	5.93950798	9.119520472	8.427796598	0.814459	0.185541	1	0
R_B1_R2	1	6.365914519	7.907120811	5.379528978	4.494353373	6.635305646	5.621228822	8.695569468	7.482750242	0.950231	0.049769	1	0
R_B1_R3	1	6.71823152	7.664558255	6.785423271	5.218687148	7.140823536	5.755833166	8.934490583	7.808148942	0.485407	0.514593	1	1
R_B1_R5	1	7.050536306	7.622076935	6.834564816	5.597957566	7.536490774	4.75064596	8.919463017	7.955764215	0.073854	0.926146	1	1
R_B1_R6	1	7.091577617	7.795179425	6.588674726	6.840693664	7.428103972	5.381833262	8.58073675	8.230120784	0.201771	0.798229	1	1

R_B1_R7	1	7.23106807	7.497147684	5.980189816	6.871864083	7.13292382	6.172537248	8.868111085	8.592905403	0.231465	0.768535	1	1
R_B1_R8	1	6.556861483	7.693005707	6.069483951	5.503658729	7.166873926	3.547971079	8.734497745	7.772812752	0.101487	0.898513	1	1
R_B1_R9	1	6.629059917	8.334507321	5.802202392	6.30583655	7.165275107	5.95048316	8.636593558	8.08697589	0.976439	0.023561	1	0
R_B2_R1	1	7.054861984	7.708419853	8.65331054	5.92301224	7.481279228	5.968515172	9.2094294	7.980065349	0.120405	0.879595	1	1
R_B2_R2	1	6.822825752	7.68113312	6.567150579	5.98678889	7.981516651	5.820572316	9.232305474	8.229959605	0.300894	0.699106	1	1
R_B2_R3	1	7.297063514	7.694759186	6.214655617	6.274941412	7.923979272	5.827685256	8.824582848	8.138004629	0.142849	0.857151	1	1
R_B2_R4	1	6.959627746	7.668388498	5.847275848	6.329491078	7.723015268	6.083189713	8.62476249	7.893342725	0.233508	0.766492	1	1
R_B2_R5	1	6.875332443	7.517625616	6.575808603	5.996989103	7.769445465	5.626972347	9.065189092	8.051896554	0.100195	0.899805	1	1
R_B2_R6	1	7.143634721	7.440499857	6.405656442	6.346913181	8.00231594	5.900179884	8.676419559	8.006138315	0.043447	0.956553	1	1
R_B2_R7	1	7.408310897	8.023734417	8.339684713	5.554956323	7.51154928	6.038869112	8.464914698	8.127727932	0.706332	0.293668	1	0
R_B2_R8	1	3.711812238	3.856280712	5.475539053	5.839032317	4.866716126	5.775610363	8.80754403	8.20164497	2.65E-05	0.999974	1	1
R_B2_R9	1	7.029298931	7.36321868	7.214707294	6.789971671	7.947212809	6.173734668	8.706265716	8.319083287	0.051953	0.948047	1	1
R_B2_R10	1	7.081436198	7.896548786	7.142004695	6.3860045	8.061344863	5.921560901	8.914240889	8.1794785	0.32144	0.67856	1	1
R_B3_R1	1	7.219667788	7.620828078	6.018316917	5.978537093	7.946735612	6.157710013	8.810897398	8.031079204	0.190697	0.809303	1	1
R_B3_R2	1	7.356156139	7.919562264	5.837021663	6.438088152	7.770065347	5.837056113	8.669625951	8.103308001	0.337938	0.662062	1	1
R_B3_R3	1	7.127460054	7.873250521	6.21061216	5.975738822	8.181031019	5.663771352	9.13194999	7.983100609	0.214961	0.785039	1	1
R_B3_R4	1	7.023023534	7.60282894	6.123189714	6.161940719	7.728825991	5.671420685	8.640837902	8.056022685	0.17552	0.82448	1	1
R_B3_R5	1	7.377704996	7.672016708	6.865222672	6.921218359	7.723486945	5.834769155	8.872391792	8.284702028	0.063623	0.936377	1	1
R_B3_R6	1	7.019969963	7.207831492	6.383431335	6.263026169	6.638606983	6.074350754	8.75737958	8.152687331	0.081833	0.918167	1	1
R_B3_R8	1	7.269965107	7.928610647	5.767519566	6.540230575	7.748968402	5.750058821	8.896355874	8.043743269	0.249968	0.750032	1	1
R_B3_R9	1	7.09261586	7.620823809	6.716761577	6.635671561	7.76408937	5.763956119	8.976143495	8.263960196	0.10076	0.89924	1	1
R_B3_R10	1	6.789463462	7.10702056	5.951148229	6.092319866	6.231868326	6.085545434	8.552523033	7.905247579	0.097187	0.902813	1	1
R_B4_R1	1	7.918074498	7.620570594	7.25802256	7.550883767	7.665038506	6.165140036	8.785996514	8.534070268	0.02059	0.97941	1	1
R_B4_R4	1	7.693140983	7.657088801	5.285900952	7.11262067	7.392110988	6.015710657	9.05262274	8.062168058	0.026528	0.973472	1	1
R_B4_R5	1	7.74942018	7.680368227	5.613421437	7.153200933	7.787208741	6.001131338	8.732286026	8.223450358	0.039117	0.960883	1	1
R_B4_R6	1	7.70661994	7.294648639	6.851191148	7.077812412	7.353180418	5.768153766	8.585845132	8.260965526	0.006279	0.993721	1	1
R_B4_R10	1	7.804939366	7.366754949	6.892149267	7.242416482	7.719537736	6.030060885	8.863833786	7.963374855	0.001832	0.998168	1	1
R_B5_R1	1	7.400566437	7.915905845	6.818532963	6.332220964	8.170520866	6.48774388	9.248485367	8.120704546	0.242562	0.757438	1	1
R_B5_R2	1	7.34029889	7.680460984	6.239980139	6.832167528	7.947168784	6.079948093	8.968939066	8.33564856	0.112023	0.887977	1	1
R_B5_R5	1	7.277406113	7.870394423	5.646395277	6.679424426	7.491293335	6.451529651	9.306138651	8.314326323	0.429287	0.570713	1	1
R_B5_R6	1	7.333293245	7.973212225	8.249352117	6.290027992	7.649287402	6.335176508	9.076862414	7.970886186	0.237112	0.762888	1	1
R_B5_R7	1	7.362804949	7.976908084	5.38070394	6.638039305	7.816785839	6.338803359	9.235682082	8.332624055	0.501184	0.498816	1	0
R_B5_R8	1	7.172786093	7.692236971	6.675312977	7.183789146	7.714416635	5.89779052	8.844056304	8.075212445	0.04991	0.95009	1	1
R_B6_R1	1	6.965181707	7.249396244	5.820648089	5.584636446	7.379890064	5.853471072	8.957803564	7.913788762	0.065615	0.934385	1	1
R_B6_R2	1	7.15548585	7.77887831	6.082799531	6.864268533	7.76659506	6.239259566	8.924883918	8.479709939	0.404451	0.595549	1	1
R_B6_R3	1	7.021526247	7.44110699	5.888788091	6.406579499	7.567208648	5.7841788	8.606088713	8.108669554	0.100504	0.899496	1	1
R_B6_R5	1	7.096675652	7.612870869	6.009709657	6.24145805	7.456977873	6.219923535	8.956001235	8.131258516	0.256164	0.743836	1	1

SourceDataForFigure5E, F, G: The original dataset and predicted value based on LR model (P8: R vs. UR) of training, internal validation and external validation dataset.

Internal validation	grade	ITIH4_HUMAN	APOA4_HUMAN	FIBG_HUMAN	FINC_HUMAN	A2GL_HUMAN	COMP_HUMAN	IGHM_HUMAN	KV320_HUMAN	X0	X1	obs	pred
UR_B1_R8	0	6.002712161	8.138001339	5.264434126	5.075000315	6.553283531	4.749037315	8.449412491	7.676132699	0.982403	0.017597	0	0
UR_B2_R1	0	7.300867907	8.068370945	5.091768858	6.522448724	7.415291662	5.849635074	8.339111256	7.797219117	0.52686	0.47314	0	0
UR_B2_R5	0	7.170486454	7.715517017	5.882197845	6.379608535	7.514201477	6.030674043	8.783426245	7.981557417	0.208126	0.791874	0	1
UR_B2_R6	0	7.018711951	3.856280712	5.465144886	6.247356085	7.282590283	6.100297235	8.721903618	7.802252365	1.25E-09	1	0	1
UR_B2_R9	0	3.711812238	5.375928099	4.796838685	2.427234893	4.244559771	6.274910932	5.404632907	8.27161554	0.999475	0.000525	0	0
UR_B3_R4	0	7.172729037	8.092719072	5.714415797	6.197942272	7.713491581	6.177443847	8.804190662	8.324116324	0.868481	0.131519	0	0
UR_B3_R6	0	7.239270497	8.202476852	6.030618394	6.609459736	7.475106493	6.072420047	8.972610522	8.047861105	0.658404	0.341596	0	0
UR_B3_R7	0	7.089105479	7.76871585	5.616842897	6.330263733	6.84257545	6.248996455	8.729418845	8.194386959	0.660422	0.339578	0	0
UR_B4_R2	0	7.100580531	7.626896333	4.916027107	6.115122999	6.802987592	6.719085707	8.746669229	8.28593839	0.781807	0.218193	0	0
UR_B4_R7	0	6.769826153	8.668291022	5.560075525	6.444578345	7.471730591	6.631431433	9.024280006	8.151262486	0.99505	0.00495	0	0
UR_B5_R4	0	7.365212556	7.986428988	4.179987837	6.37039251	7.581938365	6.252861776	8.925643844	8.416870576	0.818883	0.181117	0	0
UR_B6_R3	0	7.33161758	8.42707235	6.129075715	7.201359619	7.854183769	6.738396635	9.502277432	8.616020618	0.905108	0.094892	0	0
UR_B6_R8	0	7.288267015	8.186723553	5.406606013	6.211173921	7.759865308	6.376423647	8.812437519	8.238719872	0.895283	0.104717	0	0
UR_B6_R9	0	7.13902269	7.994510687	6.407926523	6.105280999	7.215788968	5.977380758	8.202750357	8.018211634	0.788203	0.211797	0	0
R_B1_R4	1	7.09448878	7.915953654	5.70378216	5.535012597	7.472271928	5.724721262	9.06832905	7.870450204	0.522134	0.477866	1	0
R_B1_R10	1	7.176841768	7.806580046	5.768129197	6.738350303	7.832627631	6.325481536	8.742598088	8.207664912	0.372583	0.627417	1	1
R_B3_R7	1	7.054154772	7.04687896	5.419750273	6.216024312	6.431761882	5.610991947	8.316827895	7.763730736	0.019687	0.980313	1	1
R_B4_R2	1	7.815824122	7.705498541	5.729315648	7.20878173	7.843801239	6.088715293	8.781474647	8.215412309	0.03383	0.96617	1	1
R_B4_R3	1	7.848821203	7.67118441	6.302432791	7.262665664	7.374774612	5.944030826	8.831510073	8.32362209	0.030544	0.969456	1	1
R_B4_R7	1	7.80532403	7.556282542	7.06558848	7.481091779	8.11750628	6.025915776	8.845459711	8.243971536	0.005634	0.994366	1	1
R_B4_R8	1	7.078639508	7.102908083	8.230787213	6.333634597	6.379669504	5.941893981	8.792815051	7.859279559	0.009343	0.990657	1	1
R_B4_R9	1	7.281078715	7.874930562	5.470359975	6.866564291	7.068067659	6.071180894	8.539950074	7.88836837	0.283922	0.716078	1	1
R_B5_R3	1	7.45306385	7.528937527	6.554545149	6.609645546	7.831689902	6.046466535	8.821299207	8.081008973	0.032548	0.967452	1	1
R_B5_R4	1	7.041183377	7.379519892	5.909207222	6.22492491	7.399023598	6.057518686	8.852622815	8.186629695	0.12574	0.87426	1	1
R_B5_R9	1	7.369600633	7.990252791	6.25101406	7.904819318	7.853252193	6.086105955	8.651939257	8.116875995	0.096192	0.903808	1	1
R_B5_R10	1	7.248990042	7.682141174	5.716807477	6.471806648	8.032161077	6.330831481	9.472888729	8.295682206	0.156597	0.843403	1	1
R_B6_R4	1	6.504887508	7.949148939	8.344781328	5.636928956	7.076616305	5.97709951	8.17340266	7.693649692	0.836644	0.163356	1	0

urceDataForFigure5E, F, G: The original dataset and predicted value based on LR model (P8: R vs. UR) of training, internal validation and external validation datas

External validation	grade	KV320_HUMAN	ITIH4_HUMAN	FIBG_HUMAN	IGHM_HUMAN	FINC_HUMAN	APOA4_HUMAN	A2GL_HUMAN	COMP_HUMAN
R_B8_R1	1	8.201397124	7.201397124	6.890421019	8.783903579	6.413299764	7.629409599	7.45484486	5.893761762
R_B8_R2	1	7.84509804	7.556302501	5.72427587	8.692846919	6.635483747	8.008600172	7.432969291	6.155336037
R_B8_R3	1	8.198657087	7.654176542	6.802773725	8.71432976	6.663700925	7.987666265	7.610660163	6.133538908
R_B8_R4	1	8.127104798	7.702430536	6.969881644	8.930439595	7.068185862	7.710117365	7.485721426	6.004321374
R_B8_R5	1	8.173186268	7.57634135	6.544068044	8.657055853	6.685741739	7.895974732	7.494154594	6.170261715
R_B8_R6	1	8.170261715	7.575187845	6.740362689	8.730782276	6.699837726	7.935507266	7.587710965	6.113943352
UR_B8_R1	0	8.292256071	7.547774705	5.550228353	9.326335861	6.766412847	7.989004616	7.330413773	5.683047038
UR_B8_R2	0	8.28780173	7.572871602	8.962369336	9.315970345	5.945468585	8.383815366	7.195899652	5.999130541
UR_B8_R3	0	8.206825876	7.88422877	8.953276337	8.859738566	6.307496038	8.460897843	7.240549248	5.633468456
UR_B8_R4	0	8.372912003	7.534026106	6.100370545	9.283301229	6.852479994	8.36361198	7.309630167	5.996511672
UR_B8_R5	0	8.267171728	7.608526034	6.378397901	9.225309282	7.440909082	8.450249108	7.439332694	6.212187604
UR_B8_R6	0	8.456366033	7.691081492	6.572871602	8.859138297	6.654176542	8.344392274	7.550228353	6.173186268
