# Supplementary material for: Circulating proteomic panels for risk stratification of intracranial aneurysm and its rupture
Source: EMBO Mol Med. 2022 Jan 3;14(2):e14713. doi: 10.15252/emmm.202114713 (PMC8819334; doi:10.15252/emmm.202114713)
Supplement: Supplementary file 24 [file EMMM-14-e14713-s015.pdf]

EMBO Molecular Medicine

Circulating proteomic panels for risk stratification of intracranial aneurysm and its rupture

Yueting Xiong, Yongtao Zheng, Yan Yan, Jun Yao, Hebin Liu, Fenglin Shen, Siyuan Kong, Shuang Yang, Guoquan Yan, Huanhuan Zhao, Xinwen Zhou, Jia Hu, Bin Zhou, Tao Jin, Huali Shen, Bing Leng, Pengyuan Yang, Xiaohui Liu
DOI: 10.15252/emmm.202114713

Corresponding authors: Xiaohui Liu (liuxiaohui@fudan.edu.cn), Bing Leng (lengbing99999@126.com)

Review Timeline:

Submission Date:	16th Jun 21
Editorial Decision:	5th Aug 21
Revision Received:	29th Oct 21
Editorial Decision:	7th Dec 21
Revision Received:	9th Dec 21
Accepted:	13th Dec 21

Editor: Zeljko Durdevic

Transaction Report:

(Note: With the exception of the correction of typographical or spelling errors that could be a source of ambiguity, letters and reports are not edited. Depending on transfer agreements, referee reports obtained elsewhere may or may not be included in this compilation. Referee reports are anonymous unless the Referee chooses to sign their reports.)

5th Aug 2021

Dear Dr. Liu,

Thank you for the submission of your manuscript to EMBO Molecular Medicine. We have now received feedback from the three reviewers who agreed to evaluate your manuscript. As you will see from the reports below, while referee #2 has no major concerns and supports publication of the manuscript, referees #1 and #3 recognize potential interest of the study but also raise serious critique particularly regarding statistical analyses.

Further consideration of a revision that addresses reviewers' concerns in full will entail a second round of review. EMBO Molecular Medicine encourages a single round of revision only and therefore, acceptance or rejection of the manuscript will depend on the completeness of your responses included in the next, final version of the manuscript. For this reason, and to save you from any frustrations in the end, I would strongly advise against returning an incomplete revision.

We would welcome the submission of a revised version within three months for further consideration. However, we realize that the current situation is exceptional on the account of the COVID-19/SARS-CoV-2 pandemic. Please let us know if you require longer to complete the revision.

I look forward to seeing a revised form of your manuscript as soon as possible. Use this link to login to the manuscript system and submit your revision: Link Not Available

Yours sincerely,

Zeljko Durdevic

Zeljko Durdevic
Editor
EMBO Molecular Medicine

***** Reviewer's comments *****

Referee #1 (Comments on Novelty/Model System for Author):

The technical quality of the wet lab work is high, but the statistics are quite low - the machine learning lacks some rigor. Additionally, many other works have published similar results, in both proteomics and transcriptomics, so the results are not novel (these are even included in their candidate markers).

Referee #1 (Remarks for Author):

It appears that the machine learning model is overfit - A/S/S in training was 1/1/1. I suggest implementing a more rigorous machine learning strategy. This is needed given that the number of samples in the training dataset is low, but the number of queried proteins are high. Nested cross-validation or Mote-Carlo CV could help make a more generalized model.

What is the rationale of including tissue proteins in a serum panel as a biomarker? Since you have serum protein data, does it make sense to add the proteins that can only be derived from tissue to a serum panel? I would remove these, as they could introduce noise into a biomarker panel.

Why was random forest chosen instead of simpler methods or other linear discriminants. These could be more easily generalized to give a useful equation.

Several of these serum proteins have been demonstrated to be differentially expressed in blood tissue FN1 (https://www.ncbi.nlm.nih.gov/pmc/articles/PMC7647097/) , PTGDS (https://www.ncbi.nlm.nih.gov/pmc/articles/PMC5771622/), PRTN3 (https://www.mdpi.com/2075-4418/11/6/1092). THe authors should mentions these and how they may be related to the observations in their work.

There seems to be a disconnect of semantics. The authors say they have a rupture predictor, but in reality is is a rupture classifier. They did not perform experiments to develop panels in patients where some of the unruptured IAs progress to rupture. Therefore it cannot be called a risk predictor, only a rupture stratifier/classifier.

Referee #2 (Remarks for Author):

Well done study and needs to be validated in larger cohort form multi-centers

Referee #3 (Remarks for Author):

This manuscript by Xiong et al. titled "Circulating proteomic panels for risk stratification of intracranial aneurysm (IA) and its rupture" describes a research strategy of identifying and validating the protein biomarkers of IA and its rupture by developing mass spectrometry-based targeted assays driven by machine learning analysis. The authors carried out discovery proteomics analysis of five paired IA tissues and 60 serum samples by label-free and TMT methods to identify altered proteins. By combining the altered proteins in this study with the published candidate biomarkers and applying a proposed deep learning-based method - DeepPRM they identified 113 peptides corresponding to 100 serum proteins to assay in 212 patient serum samples using PRM assay. Further machine learning analysis of the resulting data identified smaller subset of protein biomarkers of IA and rupture with accuracies as high as 100%. I feel that the manuscript is suitable for publication after the following issues are addressed:

Major comments
1. Prosit (Gessulat et al. 2019) is also a tool to predict the retention time and MS/MS spectra from peptide sequence alone. Prosit is probably the best tool to compare against instead of Picky (Zauber et al. 2018). How do the DeepPRM results compare to Prosit predictions?
2. In Table S7, it is not clear which peptide charge state(s) were identified by DeepPRM and DDA in the HeLa data. Table S7 currently shows identification of peptides in both charge states of 2 and 3 with equal "Detectability" score in the DDA mode. Criteria of the peptide charge state picked up for PRM analysis of 367 peptides in Table S8 needs to be described. Also, what is the collision energy used in the PRM analysis?
3. Provide a summary table with details of the studies in the literature reporting biomarkers of IA that were used to build PCBB and SPCBB
4. Random forest (RF) modeling to model 113 features and 160 training set with 1,000 trees could overfit the model and in P28-based data which might also be reflective of less performance in the test data set as shown in Tables 1 and 2 with . Can a simple logistic regression modeling or RF with fewer trees improve the results?

Minor comments
1. Language improvements throughout the manuscript and supplementary information (examples: line 332: construct; line 464: "Peak area"; lines 64-67 delete "of" etc.)
2. Provide reference(s) for the sentence described in lines 64-67
3. In page 37 and Table 1 correct accuracy (%) in last row to 100% for the training data set. While it is correct in page 38 and Table 1

Response Letter

Point-by-point responses to the reviewers' comments

To Reviewer 1:

We deeply appreciate your professional review work on our article. As you mentioned, there are several problems that must be addressed. According to your suggestions, we made extensive corrections to our previous draft, and the detailed responses are listed below.

Referee #1 (Comments on Novelty/Model System for Author):

The technical quality of the wet lab work is high, but the statistics are quite low - the machine learning lacks some rigor. Additionally, many other works have published similar results, in both proteomics and transcriptomics, so the results are not novel (these are even included in their candidate markers).

Response: Thank you for your positive comments on our wet lab work. We performed all the experiments under strict quality controls to ensure reliable results generated for further candidate biomarker selection and determination using machine learning strategies.

We also thank your critique on the statistical analysis. We have changed the machine learning strategies using a classical logistic regression (LR) for the model training. As described above, we take two steps for the biomarker candidate section, including differential feature reservation (DFR), candidate feature selection, and final model construction (CFS & FMC). For each step, we set strict criteria and a series of evaluation to make the statistical analysis rigorous. As for DFR, three standards must be meet [fold change (FC) >1.2, *P*-value < 0.05, and variable importance in projection (VIP) > 1.0] for the selection of highly ranked proteins with altered abundance.

Afterwards, we manually check the MS raw data and calculate the area under the curve (AUC) of each peptide to select the best peptide of each protein, and ensure one protein corresponding to one peptide, which resulted in 27 peptides. In the CFS & FMC step, the dataset of cohort I was randomly separated to training set (75% of cohort I) and internal validation set (25% of cohort I). Logistic regression was used for model building, and recursive feature elimination (RFE) with cross-validation (10-fold CV, repeated 10 times) was performed to select optimal biomarker combination on the training dataset based on the highest average accuracy (Ac). The performance of newly developed panels was evaluated by six parameters as mentioned above in the training set, internal validation set, and a newly collected external validation set (cohort II). Finally, we also checked the learning curves of each model to make sure that the model was not overfitted. To summarize, we used a simpler method for machine learning with a more rigid control of each step of the statistical analysis. Detailed results are illustrated in the answer of the following question.

Furthermore, we wish to summarize and explain the novelty of our work other than similar proteomics and transcriptomics-based biomarker discovery studies:

1) We constructed a pipeline to support a comprehensive serum protein biomarker survey for IA, which fully integrated the results of current proteome research and previously reported studies. The newly developed DeepPRM method in the current work enabled us to conduct such an extensive biomarker survey study of a specific disease, which has not been reported to date.

   There are two main reasons that require a thorough survey of biomarkers is necessary, rather than employing regular serum proteomic-based biomarker

discovery strategies.

One reason is that the identification of serum proteins is limited by the high dynamic range of protein abundance (up to 12 orders), which hinders the deep mining of serum biomarker discovery. For example, scientists could detect about 500 proteins (Niu *et al*, 2020) in serum without high abundant protein (HAP) depletion, and about 1400 proteins (Shen *et al*, 2021) could be identified when the HAP was removed combined with fractionation using two-dimensional liquid chromatography. These two main strategies either sacrifice the depth or the throughput of serum biomarker discovery. Some low abundance proteins and tissue leakage proteins lack in the detection using the direct serum proteome analysis.

The other reason is that in most reported biomarker discovery studies, scientists conducted the omics-based analysis of available specimen to discover the altered molecules (Demichev *et al*, 2021; Lee *et al*, 2021), followed by interpreting their correlation (Ben-Hamo *et al*, 2020; Haider *et al*, 2018; Li *et al*, 2020), and/or selecting a part of the significantly changed protein expression or metabolites for further verification and validation (Carnielli *et al*, 2018; Shen *et al*, 2020; Shu *et al*, 2020). Different scientists have investigated the diseases separately using various strategies and perspectives, thus generating diverse results.

Considering that each technology has its advantages and disadvantages, which result in different biomarker findings, and to avoid the limitations of the direct comparative serum proteomic research, we proposed a comprehensive serum protein biomarker discovery strategy not only based on our current proteomic analysis, but also make full use of the valuable results of previously conducted studies. The targeted proteomics strategy was used to connect the collected candidate biomarkers and the serum protein detection and quantitation.

In the current study, by analyzing the quantitative results of 113 peptides in 212 individual serum samples (cohort I), 27 targeted proteins showed significant change of their abundance among unruptured, and ruptured IA groups and

healthy controls. Among them, five proteins originated from tissue results, 18 are from direct serum comparative proteome analysis results, and 11 are derived from the literature. These results clearly demonstrate the newly proposed extensive serum biomarker survey strategy benefit the serum proteomic signature discovery of IA. We regard it has the potential to be widely used in the comprehensive biomarker discovery studies in other diseases. Therefore, we assume that it will meet the interest and demands of readers working on biomarker related research.

2) In the technical aspect, as described in the discussion section, we proposed a highly-efficient and time-saving PRM assay approach (DeepPRM) to facilitate highly efficient PRM method design for large scale candidate proteins, especially for the proteins lacking information of peptide detection in serum, such as those collected from tissue and the literature. Without prior knowledge of the retention time (RT) and detectability, scientists continuously face difficulties regarding peptide selection or the decision to synthesize hundreds and thousands of peptides, which is costly (Kusebauch *et al*, 2016). DeepPRM, precisely predicting the detectability and RT of each peptide, perform the extensive validation of candidate biomarkers possible in a cost-effective way. Furthermore, the algorithm used by DeepPRM was based on the deep learning of peptide spectrums, thus showing the potential to be widely used without the restriction of actual specimens from humans and mouse models. To this extent, we regard the method to have the potential to aid scientists in validating their interesting putative proteins more flexibly. We hope that DeepPRM will rise the interest of readers conducting targeted proteomics research and eventually contribute to their work.

3) We obtain several notable findings on IA among the numerous results reported in this study. During our collection of the IA related literature, little systematic proteome-based IA analysis was found. We made an in-depth analysis of the tissue and serum proteome of IA, which resulted in 5915 and 1557 proteins detected separately. Through the bioinformation interpretation of the dysregulate

proteins, we noticed that the immune system and related pathways were distinctively enhanced in the serum collected from ruptured IA patients, and the key energy metabolic pathways, such as the TCA cycle, were markedly down-regulated, indicating different energy mediation of IA patients.

4) Last but not least, regarding the novelty of the protein biomarkers, we wish to explain that we aimed to discover new protein biomarker combinations related to IA in the study. Two proteomic panels were discovered to discriminate the IA group from NC group, and classify IA rupture with high accuracy. According to the bioinformatic analysis (KEGG & Reactome database) of these signatures, we noticed that the IA formation and the IA rupture are complex pathological process. Proteins involved in immune systems, platelet degranulation, complement, and coagulation cascades, neutrophil degranulation, integrin cell surface interactions, foal adhesion, degradation of the extracellular matrix and metabolism together contributed to the highly accurate classification of IA and IA rupture. Among these proteins, CTSG, PRTN3, MPO, and LRG1 have a strong relationship with neutrophil degranulation, while PDLIM1 is putatively related with vascular endothelial cell function, which was seldomly reported in relation to IA. The biomarker combinations and these proteins are novel findings of our study.

   In our future study, we aim to determine the effect of PDLIM1 on endothelial cells (ECs) function and vascular remodeling. For this purpose, we knocked down PDLIM1 in human umbilical vein endothelial cells (HUVECs) using short interfering RNA (siRNA) and performed a cell viability and migration ability test.

   We added **SI Fig 1** and **SI Fig 2** for the reviewer. PDLIM1 expression was efficiently reduced in siRNA-transfected HUVECs. PDLIM1 knockdown suppressed ECs proliferation, as shown in the result of CCK-8 proliferation assay (**SI Fig 1**). Furthermore, migration ability of HUVECs decreased significantly at 24 and 48 h after siRNA transfection compared to the control groups (**SI Fig 2**). These results primarily demonstrate that PDLIM1 can mediate the migration

dysfunction of vascular endothelial cells.

To summarize, the current omics-based IA biomarker discovery study has brought a new conception of a comprehensive protein candidate biomarker discovery pipeline for IA. It takes full advantage of data from the present and previous studies using our newly developed DeepPRM method, reached proteome based novel findings of the IA, and discovered new protein biomarker panels for classifying the IA and IA rupture which included proteins revealed to function in the migration dysfunction of vascular endothelial cells which was not reported before. We have described these novelties in the discussion section.

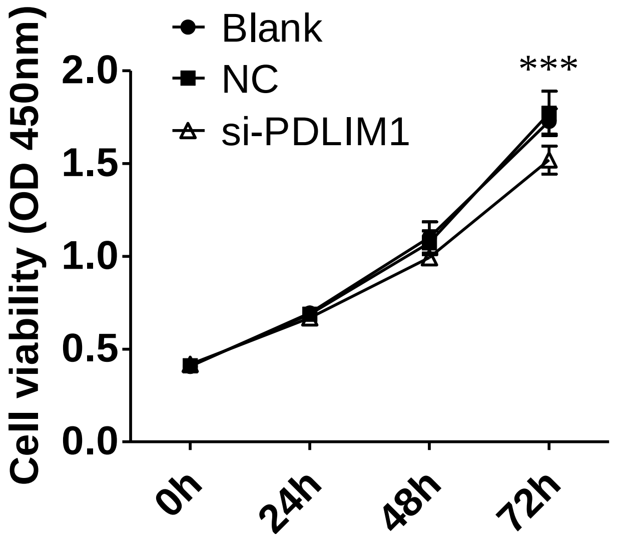

SI Figure 1 for reviewer. Cell viability detected by CCK-8 proliferation assay. Cell activity gradually decreases with time after silencing PDLIM1.

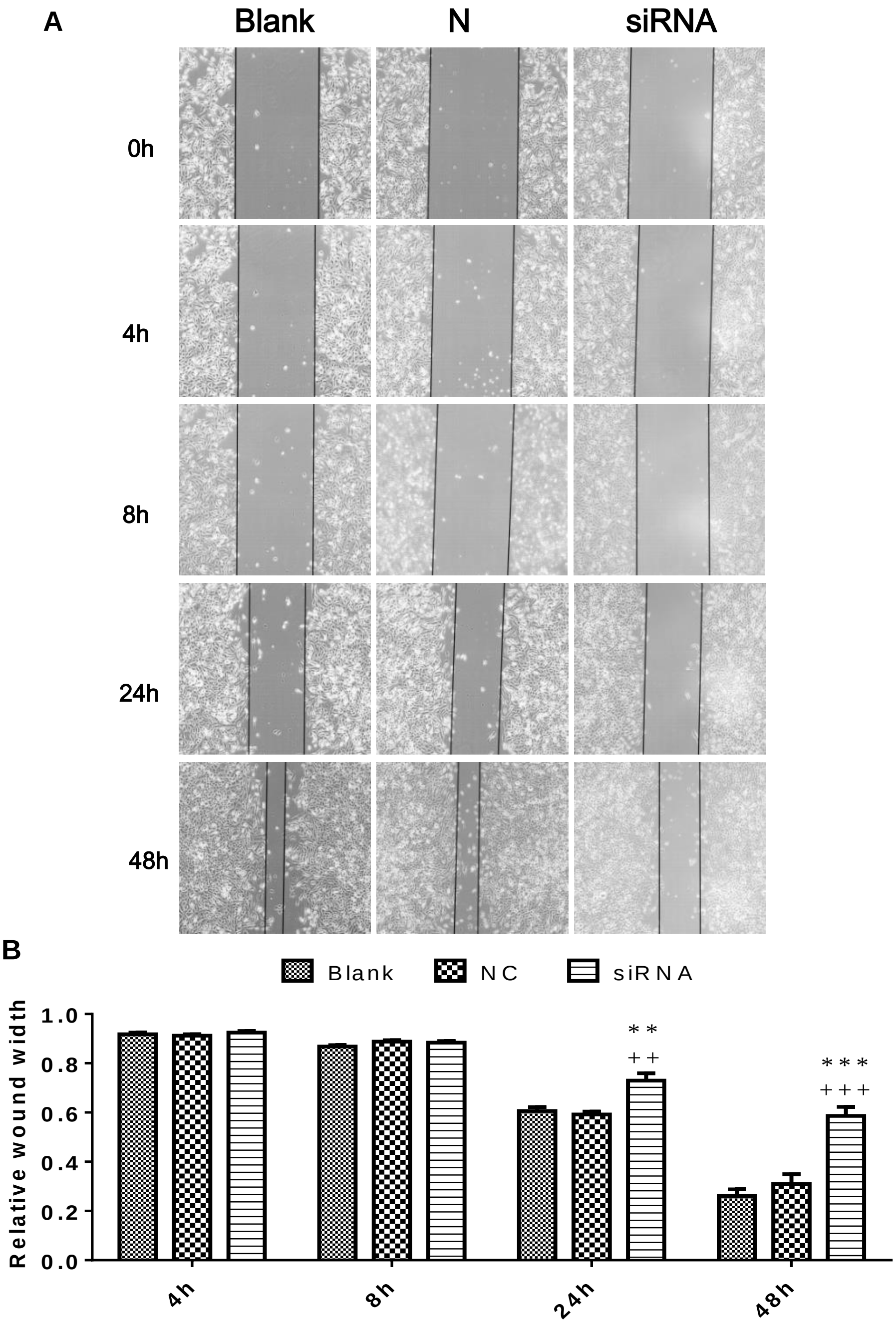

SI Figure 2 for reviewer. Comparison of migration ability of vascular endothelial cells after silencing PDLIM1 by siRNA. (A) Migration ability of different groups at different time periods after wound healing assay. (B) Difference comparison of relative wound width in each group. siRNA vs. Blank：*p＜0.05，**p＜0.01，***p＜0.001；siRNA vs. NC：#p ＜0.05，##p＜0.01，###p＜0.001. Blank: positive control; NC: negative control.

Referee #1 (Remarks for Author):

It appears that the machine learning model is overfit - A/S/S in training was 1/1/1. I suggest implementing a more rigorous machine learning strategy. This is needed given that the number of samples in the training dataset is low, but the number of queried proteins are high. Nested cross-validation or Mote-Carlo CV could help make a more generalized model.

Response: Thank you for your constructive correction and suggestion. We made a corresponding change in the machine learning strategy in this study. To avoid overfitting, we first reduced the candidate features by preselect differential proteins followed by RFE with CV (10-fold, repeated 10 times) to select the optimal combinations for further model training. Binary logistic regression was used to construct a more generalized model in the revised manuscript. The models consisting of six and eight proteins to classify IA and IA rupture were further evaluated on the internal and external validation set. The learning curve was checked to evaluate the overfit states of the model based on the accuracy of training set and internal validation set.

We wish to introduce the whole altered statistical analysis procedure in this part. In brief, based on the serum proteomic data of PRM cohort I (n = 212), we developed a computational pipeline including differential feature reservation (DFR), candidate feature selection, and final model construction (CFS & FMC) for identifying potential biomarker combinations to classify IA cases and IA rupture. In the DFR step, we selected the highly ranked different expressed proteins (DEPs) based on the criteria of fold change (FC > 1.2), the P-value of Whitney U test (P < 0.05), and the variable importance in projection (VIP >1.0). Then, the combined dataset (cohort I) was randomly divided into a training set and an internal validation set with a ratio of three to one. In the following CFS & FMC step, RFE with CV (10-fold, repeated 10 times) was performed to remove features of low importance that could improve accuracy, reduce both model complexity and over fitting, which finally helped select the optimal combinations of the features with the highest average accuracy. Classical binary logistic regression was used for model training and parameter optimization of the candidate biomarkers in the training set (**Fig 5A**).

We then developed two new proteomic panels in this study, one is P6 (CTSG, PDLIM1, PRTN3, MPO, IGHM and IGKV3D-20), for optimal discrimination of the IA group from the NC group; and the other is P8 (ITIH4, APOA4, FGG, FN1, LRG1, COMP, IGHM, and IGKV3D-20) for classifying ruptured and unruptured IA with the highest average accuracy (**Dataset EV 16**). Then, to evaluate the accuracy of these two LR models on the entire training set, true positive (TP), true negative (TN), false positive (FP), and false negative (FN) numbers were counted. Six measurements including sensitivity (Sn), specificity (Sp), accuracy (Ac), positive predictive value (PPV), negative predictive value (NPV), and Mathew correlation coefficient (MCC) were calculated and evaluated on the internal validation set (**Table 1**). The new models generated by LR are shown in **Figure 5 B, E,** which are much simpler and more general than the previous version.

To assess the transferability of the proteomic predictors, we further evaluated our model (P6 & P8) in the independent cohort II (n = 32). These two proteomic models demonstrated a comparably high patient classification performance when applied to this external validation cohort. More specifically, the AUC values of P6 model were calculated as 0.929 (95% CI = 0.780–0.990) and Ac of 87.50% for distinguishing IA cases from NC. Similarly, the P8 model yield an excellent classification Ac of 91.67% and a great AUC value of 0.905, further demonstrating the robustness of the model (**Figure 5D, G; Table 1**).

Finally, we plotted and checked the learning curves of both P6 and P8 models (**Fig S6C, E**). It is evident that the curves of the training and internal validation sets getting flattened, and the accuracy became stable and larger than 0.8, which indicates that the model fits well (Emmert-Streib & Dehmer, 2019).

We changed the machine learning strategies using a classical logistic regression (LR) for model training. This resulted in a new **Fig 5**, a new **Table 1**, and an additional supplemental figure (**Appendix Figure S6**).

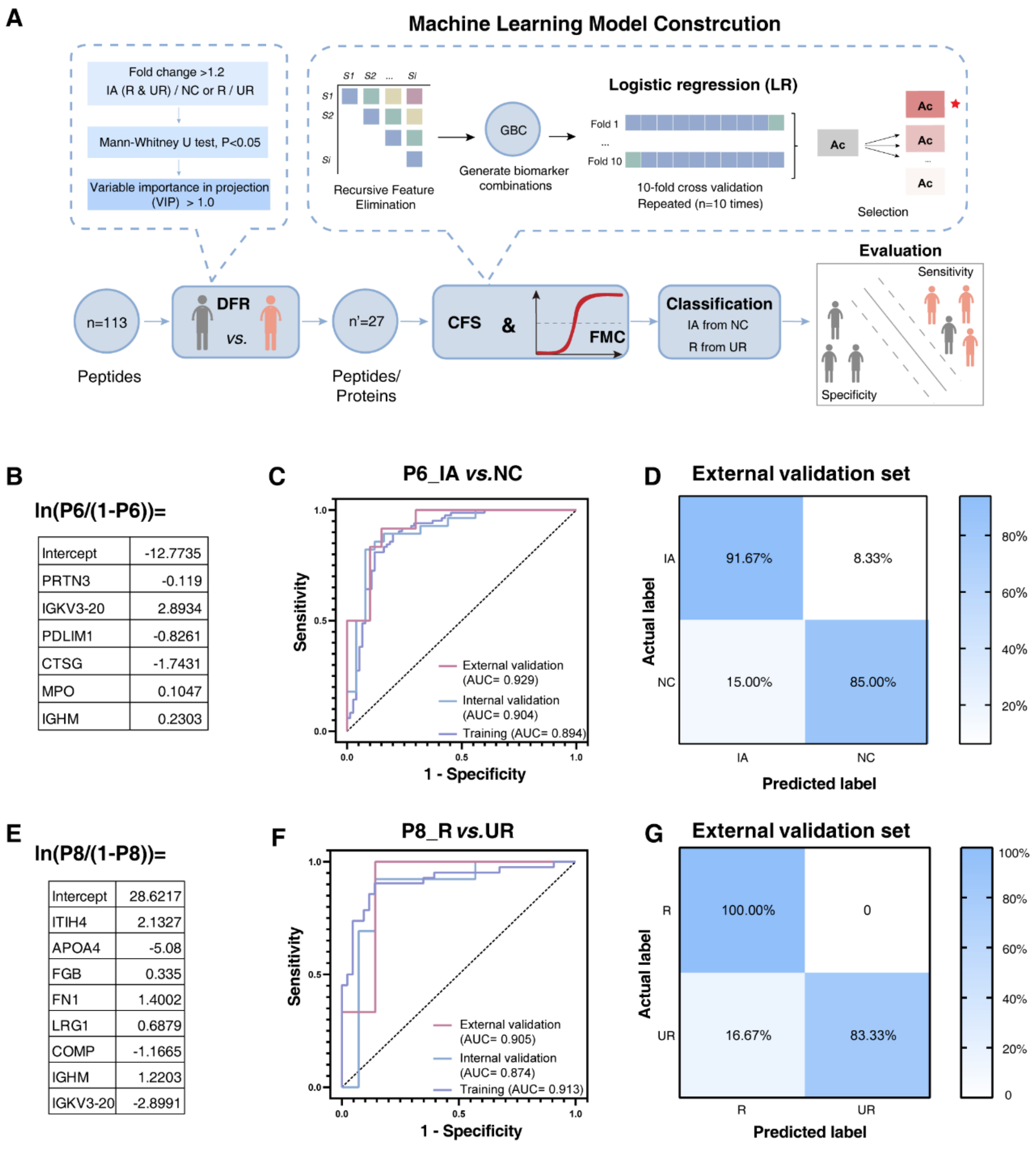

Figure 5. Identification of potential biomarker combinations for classification of IA different outcomes from healthy controls using machine-learning method.

A Workflow of data processing and machine-learning model construction.

B, E Logistic regression (LR) model for the classification of IA and NC (B) or R and UR (E).

C, F Receiver operating characteristic (ROC) curve of the LR-based model in IA *vs.* NC (C) or R *vs.* UR (F) in training, internal validation and external validation set.

D, G Confusion matrix showing the model performance for classifying IA and NC (D) or R and UR (G) in the external validation set.

Table 1 Performance of circulating protein panel P6 in differential diagnosis of IA from healthy controls and P8 in classification of IA rupture.

Classification	Dataset	AUC (95% CI)	Sn	Sp	Ac	PPV	NPV	MCC
P6 (IA *vs*. NC)	Training	0.894 0.836 to 0.937)	85.71%	81.33%	83.65%	83.72%	83.56%	0.672
	Internal validation	0.904 (0.792 to 0.968)	85.71%	88.00%	86.79%	88.89%	84.62%	0.736
	External validation	0.929 (0.780 to 0.990)	91.67%	85.00%	87.50%	78.57%	94.44%	0.748
P8 (R *vs*. UR)	Training	0.913 (0.832 to 0.963)	88.10%	86.05%	87.06%	86.05%	88.10%	0.741
	Internal validation	0.874 (0.689 to 0.969)	84.62%	85.71%	85.19%	84.62%	85.71%	0.703
	External validation	0.905 (0.616 to 0.996)	100.00%	83.33%	91.67%	85.71%	100.00%	0.845

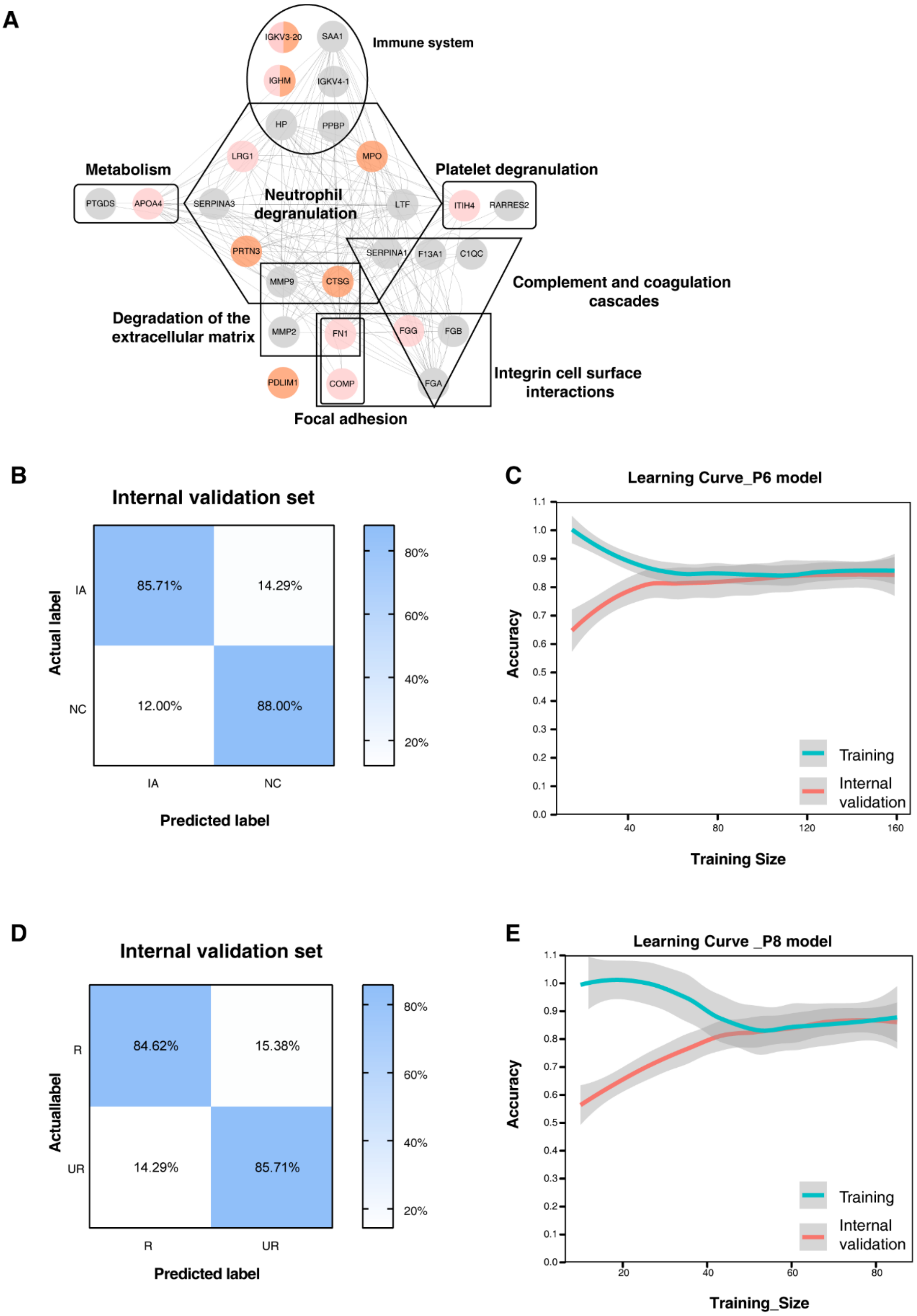

Appendix Figure S6: Machine learning strategy for the classification of IA cases and healthy controls.

A) A serum protein regulatory network based on 27 highly ranked proteins associated with IA.

B, D) Confusion matrix showing the model performance for classifying IA and NC (**B**) or R and UR (**D**) in the internal validation set.

C, E) The learning curve showing the fitting situation of P6 model (**C**) and P8 model (**E**) based on the accuracy of the training set and the internal validation set.

-These new analyses resulted in the following paragraph, which we included in the revised Abstract section:

"A total of 113 potential markers were further quantitated in serum cohort I (n=212) & II (n = 32). Combined with a machine-learning-based pipeline, we built two sets of biomarker combinations (P6 & P8) to accurately distinguish IA from healthy controls (accuracy: 87.50%) or classify IA rupture patients (accuracy: 91.67%) upon evaluation in the external validation set (n = 32)."

New Introduction section:

"To identify potential biomarkers for the accurate classification of different samples, we developed a machine-learning-based pipeline, resulting in a six-protein based biomarker combinations to accurately distinguish IA, as well as an eight-protein panel to classify IA rupture. Both proteomic models trained on the training set (75% of cohort I) demonstrated a comparably high patient stratification performance when applied to an internal validation set (25% of cohort I) and external validation cohort II (n = 32) (**Dataset EV2**). Some identified biomarkers were further examined for their serum levels via the enzyme-linked immunosorbent assay (ELISA), whose findings were in accordance with the proteomic results."

New Results section:

"**Machine-learning-based selection of biomarker combinations for classification of IA cases.** We investigated the possibility of discriminating patients with ruptured or unruptured IA from healthy controls based on the molecular signatures of serum proteins. On the basis of the serum proteomic data of PRM cohort I (n = 212), we developed a computational pipeline including differential feature reservation (DFR), candidate feature selection, and final

model construction (CFS & FMC) for identifying potential biomarker combinations to classify IA cases (**Fig 5A**). In the DFR step, 32 peptides corresponding to 27 proteins were identified as highly ranked DEPs (fold change (FC) >1.2, P-value < 0.05 and VIP > 1.0). For proteins associated with multiple peptides, we selected the best peptide the basis of area under the curve (AUC) and PRM raw data, to ensure that each peptide corresponded to a single protein (Kim et al, 2021) which resulted in remaining 27 peptides (**Fig S6A**). Subsequently, the combined dataset (cohort I) was randomly divided into a training and an internal validation set with a ratio of three to one (**Dataset EV14 & EV15**). In the CFS & FMC step, logistic regression was used for model building, and recursive feature elimination (RFE) with cross-validation (10-fold CV, repeated 10 times) was performed to select the optimal biomarker combination on the training set (75% of cohort I), based on the highest average accuracy (Ac) (**Fig 5A**).

We sought to utilize the above machine-learning strategy to classify different clinical outcomes of IA (e.g., IA *vs.* NC, or R *vs.* UR) based on the significant features with more economical combinations of the molecules. For the classification of IA (R & UR) patients and healthy controls, we identified a compact biomarker combination containing six proteins (dubbed P6) (**Fig 5B**), including CTSG, PDLIM1, myeloblastin (PRTN3), myeloperoxidase (MPO), immunoglobulin heavy constant mu (IGHM), and immunoglobulin kappa variable 3–20 (IGKV3-20). This model reached an AUC of 0.894 (95% CI = 0.836–0.937) based on the receiver operating characteristic curve (ROC) analysis and an Ac of 83.65% in the training set (**Fig 5C; Table 1; Dataset EV16**).

We then tested the P6 model on the internal validation set (25% of cohort I), resulting in a promising AUC of 0.904 (95% CI = 0.792–0.968) with high sensitivity and specificity for distinguishing different IA groups (R & UR) from healthy controls (**Fig 5B; Table 1**). To evaluate the reliability of the machine-learning strategy, confusion matrices were compiled, and the results demonstrated that different samples could be correctly classified with a high Ac

of 86.79% (**Fig S6B; Table 1**).

To validate the Ac of the machine-learning-based classification of IA cases, we further collected 32 serum samples of a new cohort (II) as the external validation set. The AUC values were calculated as 0.929 (95% CI = 0.780–0.990) for distinguishing IA cases from NC (**Fig 5C; Table 1**). Accordingly, the corresponding confusion matrix results also demonstrated that P6 exhibited a promising Ac of 87.50% on the independent cohort (**Fig 5D; Table 1; Dataset EV16**).

Finally, we plotted the learning curves of P6 model (**Fig S6C**). The curves of the training and internal validation sets are getting flattened, and the accuracy becomes stable and larger than 0.8, indicating a good fit of the model (Emmert-Streib & Dehmer, 2019).

Biomarker combinations for classification of ruptured and unruptured IA. The potential IA rupture is a complex and challenging condition that confuses clinicians regarding the choice of treatments. To classify UR and R outcomes, we identified an eight-protein combination (P8) containing inter-alpha-trypsin inhibitor heavy chain H4 (ITIH4), apolipoprotein A-IV (APOA4), FGG, fibronectin (FN1), LRG1, cartilage oligomeric matrix protein (COMP), IGHM, and IGKV3D-20, with an AUC of 0.913 (95% CI = 0.832–0.963) and an Ac of 87.06% in the training set, and an AUC of 0.874 (95% CI = 0.689–0.969) and an Ac of 85.19% in the internal validation set to distinguish ruptured from unruptured IA patients (**Fig 5E, F; Table 1; Fig S6D; Dataset EV16**). It also yielded an excellent classification Ac of 91.67% and a high AUC value of 0.905 in external validation set, which further demonstrates the robustness of the model (**Fig 5F, G; Table 1**). The learning curves of the P8 model also illustrated that the model fits well (**Fig S6E**).

We further validated two low abundant proteins PRTN3 (tissue original) and CTSG (serum original) using ELISA in an additional cohort (III) that included 40 R and 40 UR patients plus 40 healthy controls, showing a high concordance with the PRM results (**Appendix Fig S7A-D; Dataset EV17**).

In summary, these data strongly supported the potential clinical value of serum proteomics-derived panels for the identification of IA and determination of its rupture.

New Discussion section:

"Herein, we performed a systematic proteomic analysis of the diseased organs and serum samples, developed a comprehensive biomarker discovery strategy, and identified six- and eight-protein based biomarker combinations with potential clinical utility for classifying IA and its rupture."

"Accordingly, two biomarker combinations were identified as biomarkers using the LR machine-learning strategy. These biomarkers could classify the different outcomes of IA cases with high Ac, AUC, sensitivity, and specificity. Further, the alternations of these proteins provide valuable insight into the pathogenesis of IA. Moreover, the Ac of these biomarkers to distinguish IA formation and rupture were further validated via proteomics and ELISA using the serum samples from 25% of cohort I and two additional cohorts (II & III) of IA and NC, respectively."

New Methods section:

"**Machine learning strategy.** For the identification of different types of biomarker combinations, we first classified the proteomic datasets of R, UR, and NC into different groups: (*i*) For the classification of IA and NC, the R and UR groups were combined and set as the IA group. (*ii*) For the classification of the R and UR groups.

In the DFR step, we compared the PRM quantitation results of peptides in UR, R, and NC groups, and reserved high potential DEPs as a candidate reservoir (FC > 1.2, $P < 0.05$, and VIP > 1). To avoid over-fitting, the number of proteins in a combination must be significantly smaller than the sample size (Shu *et al.*, 2020). Thus, in the CFS & FMC step, we used binary logistic regression to construct the models, and RFE with CV (10-fold, repeated 10 times) was further implemented to remove features of low importance and select the best subsets of

biomarker combinations based on the highest average Ac.

The combined dataset (cohort I) was randomly divided into a training set and internal validation set with a ratio of three (training set) to one (the internal validation set). The newly enrolled cohort II (n = 32) was set as external validation set. The internal and external validation sets were only used to evaluate the performance, but not for model training. To evaluate the accuracy of the LR model, true positive (TP), true negative (TN), false positive (FP) and false negative (FN) numbers were counted. Then, six measurements including sensitivity (Sn), specificity (Sp), accuracy (Ac), positive predictive value (PPV), negative predictive value (NPV), and Mathew correlation coefficient (MCC) were calculated, as shown in **Table 1**.

To evaluate whether the model was overfitting, we plotted a learning curve of the P6/P8 model based on the accuracy of the training and internal validation sets using R package (version 4.6-14) (**Fig S6C, E**). The ROC curve was illustrated using MedCalc (version 19.5.6) and GraphPad Prism (version 8.0.2).

What is the rationale of including tissue proteins in a serum panel as a biomarker? Since you have serum protein data, does it make sense to add the proteins that can only be derived from tissue to a serum panel? I would remove these, as they could introduce noise into a biomarker panel.

Response: Thank you for your comments. There are several reasons for including the tissue proteins in our candidate biomarker list. In our observation and other scientist's reports, they contributed significantly in the classification of diseases rather than introducing noise into a biomarker panel.

As we have described in the manuscript, tissue leakage proteins or secreted proteins are reported to be among the main biomarkers in serum or plasma, which is the main reason we initially took the proteins derived from tissue into account (Geyer *et al*, 2017; Robinson *et al*, 2019). It has been reported that there are more than 100 FDA-cleared or FDA-approved clinical plasma or serum proteins, which can be categorized as abundant, functional plasma/serum proteins (50%), followed by tissue

leakage markers without a dedicated function in the circulation (25%), and signal molecules include receptor ligands, immunoglobulins, and aberrant secretions (Anderson, 2010; Geyer et al, 2017). Moreover, the secretome from tissue is reported as a valuable reservoir of potential biomarkers for cancer and other diseases (Makridakis & Vlahou, 2010; Xue *et al*, 2008).

A specific example stems from the study of Herrington et al., who described a proteomic architecture of human coronary arteries and aortas, which identified hundreds of tissue proteins. Among these proteins, a subset of tissue protein biomarkers indicative of early atherosclerosis was shown to predict anatomically defined coronary atherosclerosis when measured in plasma samples in a separate clinical cohort (area under the curve =0 .92 [0.83–0.96]), thereby validating the value of human tissue proteomics to discover relevant plasma biomarkers for clinical applications (Herrington *et al*, 2018). Meanwhile, we previously utilized dysregulated proteins identified in primary tissue and potentially secreted into the blood as biomarker candidates, followed by validation in plasma samples using the targeted multiple reaction monitoring (MRM) method. We finally achieved 88.6% accurate classification of dilated cardiomyopathy, ischemic cardiomyopathy, and healthy controls with machine learning based on 14 peptides (Xie *et al*, 2020).

Besides, the high dynamic range of serum protein concentration limited the depth of protein identification; therefore, we assume that adding the proteins derived from tissue may benefit the serum biomarker panel discovery. In the current study, there are proteins derived from the diseased tissue (such as PRTN3, MPO, PDLIM1, RARRES2, and COMP) that contributed to markedly discriminate IA from NC, and R from UR group. Without these proteins, the accuracy of the classifying panels decreases dramatically. Therefore, we would like to introduce the tissue leakage proteins or secreted proteins into our candidate biomarker discovery study.

Why was random forest chosen instead of simpler methods or other linear discriminants. These could be more easily generalized to give a useful equation.

Response: Thank you for your helpful suggestions. We chose the random forest

method aimed to construct a three-phenotype classification model to discriminate the NC, UR, and R groups directly. Meanwhile, we agree with your comments that the method is not good enough to generate a simple and useful equation. We thus studied the generalized linear models for machine learning and chose the classical binary logistic regression for the model training and optimization. We finally obtained a more easily generalized model to classify the IA groups from the NC groups (Equation: ln(P6/(1-P6))= –12.7735 + (–0.119*PRTN3) + 2.8934*IGKV3-20 + (–0.8261*PDLIM1) + (–1.7431*CTSG) + 0.1047*MPO + 0.2303*IGHM), as well as ruptured IA and un ruptured IA (Equation: ln(P8/(1-P8))= 28.6217 + 2.1327*ITIH4 + (–5.08*APOA4) + 0.335*FGB + 1.4002*FN1 + 0.6879*LRG1 + (–1.1665*COMP) + 1.2203*IGHM+ (–2.8991* IGKV3-20)). Thanks again for such constructive advice that helped we make further modification of the statistical analysis.

Several of these serum proteins have been demonstrated to be differentially expressed in blood tissue FN1 (https://www.ncbi.nlm.nih.gov/pmc/articles/PMC7647097/) , PTGDS (https://www.ncbi.nlm.nih.gov/pmc/articles/PMC5771622/), PRTN3 (https://www.mdpi.com/2075-4418/11/6/1092). THe authors should mention these and how they may be related to the observations in their work.

Response: Thank you for your serious checking of our manuscript. With the new machine learning strategy, PTGDS is no longer included in our newly developed panels, so we have omitted it from this discussion. Meanwhile for FN1 and PRTN3, we noticed that the related studies focused on transcriptome and genome analysis of the whole BLOOD and the peripheral blood mononuclear cells (PBMCs), and we have verified and quantify the relative abundance of these two proteins in SERUM samples. We noticed that the transcription level and genome analysis revealed that FN1 and PRTN3 are upregulated in IA blood samples, while the protein abundance were decreased in IA serum samples in this study. For one reason, the differences may be caused by different types of specimen analyzed in different studies, as the reported sample included BLOOD and PBMCs, and the proteome data were generated in SERUM samples. The other reason may be on account of the imperfect correlations of

the abundant of mRNA level and protein level due to technical (for example, measurement error) or biological (for example, post-transcriptional regulation) reasons (Buccitelli & Selbach, 2020). For example, the correlation between mRNA and protein in Brain is about 0.56 (**SI Fig 3a for reviewer**) for across-gene correlation, while the within-gene correlation various from 0.074 to 0.91(**SI Fig 3c for reviewer**) (Buccitelli & Selbach, 2020; Wang *et al*, 2019). Therefore, we quantitated these proteins in serum by mass spectrometry to offer proteomic evidence of the protein abundance level for further references.

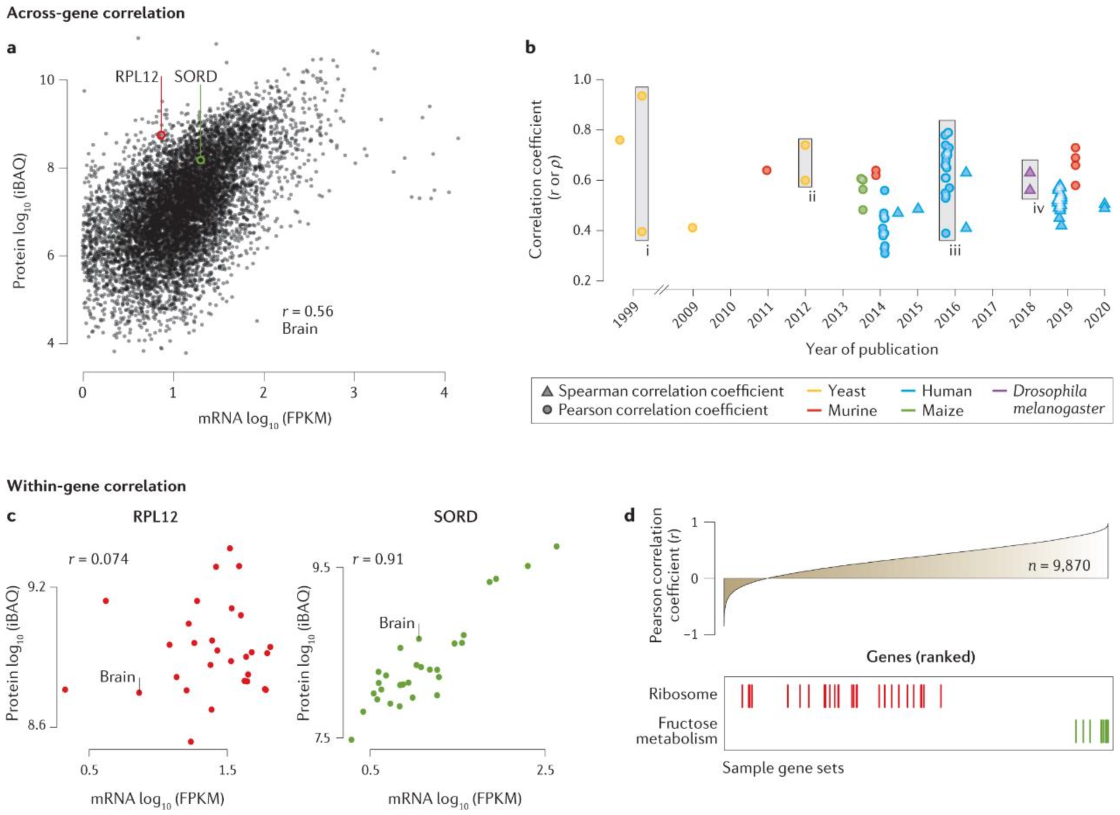

SI Figure 3 for reviewer. Across-gene and within-gene correlations between mRNA and protein levels (Buccitelli & Selbach, 2020).

a Example of an across-gene correlation analysis comparing estimates of absolute mRNA abundance (expressed in fragments per kilobase of transcript per million mapped reads (FPKM)) to protein abundance (expressed as intensity-based absolute quantification (iBAQ)) in brain tissue from ref (Wang *et al.*, 2019). c | Example of a within-gene correlation analysis comparing absolute estimates of mRNA and protein abundance across 29 tissues from ref (Wang *et al.*, 2019). for two genes: the genes encoding 60S ribosomal protein L12 (RPL12) and sorbitol dehydrogenase

(SORD).

We have rephrased the statement of candidate biomarker proteins and added a statement about PRTN3 in the Discussion section:

"Notably, some of these proteins were reported to be strongly relevant to cardiovascular or cerebrovascular disease. For instance, the markedly increased levels of two acute-phase proteins (APPs) including FGG and ITIH4 are usually reported to occur in response to inflammation or tissue injury (Gabay & Kushner, 1999). FN1 and COMP are mapped in the focal adhesion pathway, which is known to maintain cerebrovascular integrity (Liu *et al*, 2012; Xu *et al*, 2019). The four regulators LRG1, MPO, CTSG, and PRTN3 are all involved in the neutrophil degranulation pathway. Notably, MPO, CTSG, and PRTN3 are all neutrophil-related proteins, of which MPO has been revealed to be correlated with the severity and outcome of vascular disease (Gounis *et al*, 2014; Schindhelm *et al*, 2009). Further, CTSG has been reported to induce early atherosclerotic lesion formation (Ortega-Gomez *et al*, 2016), while PRTN3 is possibly involved in neuroinflammation caused by excess neutrophils entering the brain (Kwon *et al*, 2013)."

There seems to be a disconnect of semantics. The authors say they have a rupture predictor, but in reality is a rupture classifier. They did not perform experiments to develop panels in patients where some of the unruptured IAs progress to rupture. Therefore it cannot be called a risk predictor, only a rupture stratifier/classifier.

Response: Thank you for your comments and correction. As you have mentioned, the biomarker panel in our study is a rupture classifier, more accurately than a rupture predictor. We have followed your suggestions, and made corrections in the revised manuscript.

We agree with the Reviewer and we rephrased the statement in Introduction selection:

"In this regard, it is of immense value to develop biological signatures that assist in the early diagnosis of IA and the prediction or classification of IA rupture (Baker *et al*,

1995; Mack *et al*, 2002)."

"Therefore, we hypothesize that a system-wide analysis of IA, linking changes in circulation levels and dysregulation in the diseased organs, as well as the previously reported proteins, would provide informative knowledge for deciphering the molecular mechanisms underlying these events and contribute to a comprehensive serum biomarker survey for the classification of IA and IA rupture"

"To identify potential biomarkers for the accurate classification of different samples, we developed a machine-learning-based pipeline, resulting in a six-protein based biomarker combinations to accurately distinguish IA, as well as an eight-protein panel to classify IA rupture"

New Result section:

"We sought to utilize the above machine-learning strategy to classify different clinical outcomes of IA (e.g., IA *vs.* NC, or R *vs.* UR) based on the significant features with more economical combinations of the molecules."

"In summary, these data strongly support the potential clinical value of serum proteomics-derived panels for the identification of IA and determination of its rupture."

New Method section:

"(*ii*) For the classification of the R and UR groups."

Referee #2 (Remarks for Author):

Well done study and needs to be validated in larger cohort form multi-centers

Response: Thank you for your positive comments and helpful suggestion. We keep collecting more IA related serum samples after finishing the work reported in our previous version of manuscript. However, the number of samples collected from ruptured IA patients are far lower than the unruptured IA patients and healthy individuals. During the submission and revision of the manuscript, we assembled 32 serum samples, including 6 ruptured IA, 6 unruptured IA, and 20 healthy controls. We utilized these samples for further validation of our biomarker panel in the revised version of manuscript, which could accurately distinguish IA from healthy controls (AUC = 0.929, accuracy = 87.50%) or classify IA rupture patients (AUC = 0.905, accuracy = 91.67%). We would like to further validate our results in a larger cohort from multi-centers, which we think requires more effort and time to organize and initiate. Nevertheless, it is highly important advice, and we will take it into account in our future work.

Referee #3 (Remarks for Author):

This manuscript by Xiong et al. titled "Circulating proteomic panels for risk stratification of intracranial aneurysm (IA) and its rupture" describes a research strategy of identifying and validating the protein biomarkers of IA and its rupture by developing mass spectrometry-based targeted assays driven by machine learning analysis. The authors carried out discovery proteomics analysis of five paired IA tissues and 60 serum samples by label-free and TMT methods to identify altered proteins. By combining the altered proteins in this study with the published candidate biomarkers and applying a proposed deep learning-based method - DeepPRM they identified 113 peptides corresponding to 100 serum proteins to assay in 212 patient serum samples using PRM assay. Further machine learning analysis of the resulting data identified smaller subset of protein biomarkers of IA and rupture with accuracies as high as 100%. I feel that the manuscript is suitable for publication after the following issues are addressed:

Response: We thank the reviewer for the positive and supportive evaluation of our work. We also thank the reviewer for the useful comments and are pleased to provide the point-by-point response to each comment below.

Major comments

1. Prosit (Gessulat et al. 2019) is also a tool to predict the retention time and MS/MS spectra from peptide sequence alone. Prosit is probably the best tool to compare against instead of Picky (Zauber et al. 2018). How do the DeepPRM results compare to Prosit predictions?

Response: Thank you for your professional comments and suggestions. DeepPRM used in our work was derived from our previously reported DeepDIA for the prediction of peptide detectability and retention time using similar deep learning model constructed based on instrument-specific raw data (Yang *et al*, 2020). Prosit is a deep learning model for MS/MS and retention time (RT) prediction by taking collision energy (CE) into consideration, and DeepDIA aims at training instrument-specific models for more accurate MS/MS spectrum and RT prediction. In

addition, DeepDIA can select a list of target peptides to be included in in-silico spectral libraries from protein sequence databases, e.g., SwissProt, by predicting the MS detectability of candidate proteotypic peptides.

There are two reasons for the comparison of DeepPRM with Picky rather than Prosit in our manuscript. The first one is that Picky is more suitable for the PRM method design than Prosit. For PRM acquisition, the key parameters needed are the targeted peptide list and their corresponding precursor m/z and RT information. Users submitted the list of candidate proteins to Picky and DeepPRM, both methods offered suggesting targeted peptides and their related m/z and RT information. In contrast, although Prosit can make precise predictions of the MS/MS spectrum and RT, it could not support the predictions detectability of peptides of candidate proteins. Grossegesse *et al* (2021) evaluated the application of spectral library prediction by Prosit for parallel reaction monitoring (PRM) of viral peptides. Without prior knowledge of the peptides' detectability by nLC-MS/MS, they ordered a total of 1569 crude synthetic viral peptides in six pools from JPT (Berlin, Germany) for further analysis. When testing the Prosit-based PRM in a potential diagnostic PRM application example, they must identify the potential targeted peptides using DDA acquisition method before PRM method development (Grossegesse *et al.*, 2021). In our current work, the precise prediction of the detectivity of peptides of DeepPRM facilitated the highly efficient PRM assay development and made the large-scale biomarker validation more cost-effective.

The other reason is that we have compared the DeepDIA with Prosit to generate in silico spectral libraries for DIA analysis using the MS/MS and RT information in our previously reported work (Yang *et al.*, 2020). Because DeepPRM and DeepDIA are generated by similar instrument specific model, we think that we can answer the question of the comparison of DeepPRM and Prosit with our former results. We previously benchmark the performance of DeepDIA on datasets of HeLa cells and mixed proteome samples, and the results indicate that the instrument-specific libraries by DeepDIA outperform Prosit in terms of detectable peptides and proteins as well as reproducibility among technical replicates (Yang *et*

al., 2020). We show the detailed comparison results between the instrument specific model for DeepDIA and Prosit in the following **SI Fig 4 for the reviewer**.

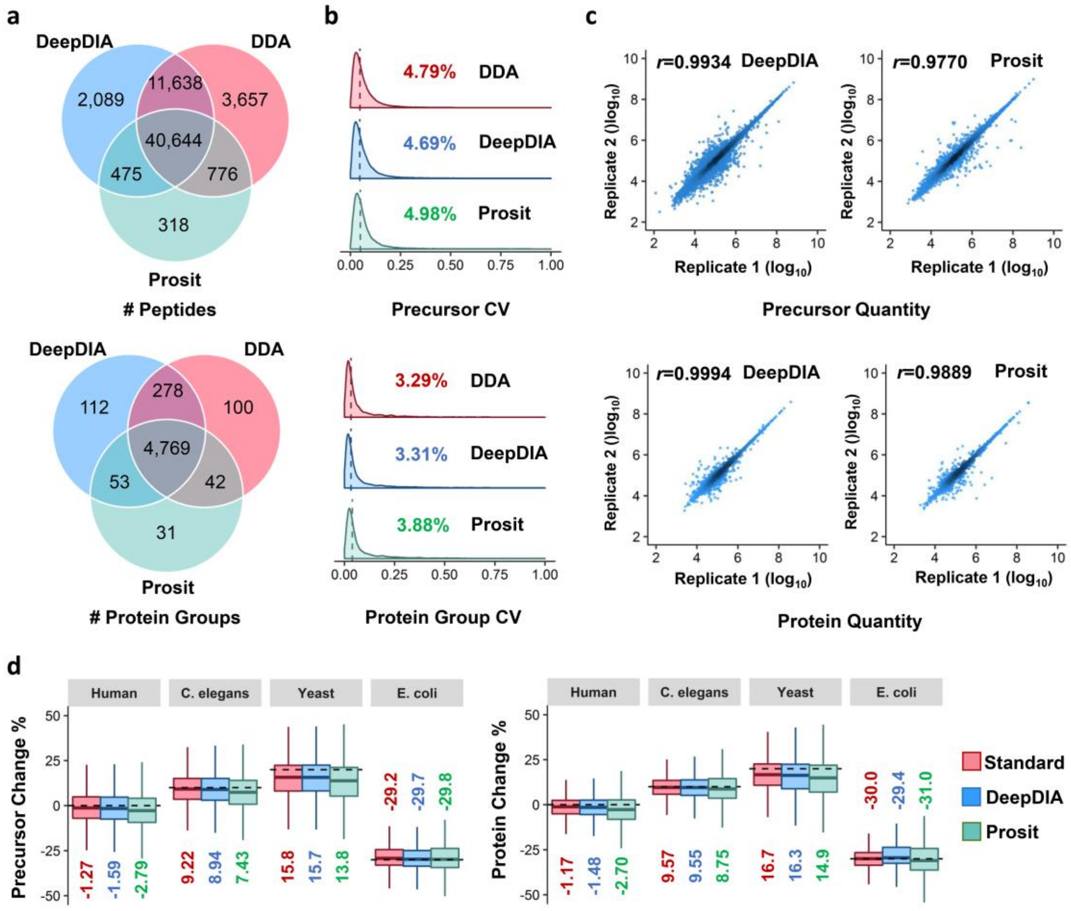

SI Figure 4 for reviewer. Performance comparison of predicted libraries and DDA libraries for DIA analysis on data of HeLa cells (HeLa1). (a-c) and mixed proteome samples (Mix) (d). (a) The numbers of peptides and protein groups detected using HeLaDDA (DDA), HeLaPredicted (DeepDIA), and HeLaProsit (Prosit) libraries. The Prosit library was built with the optimal normalized collision energy (CE = 30). Among the 3,657 peptides only detected using HeLaDDA, 1,886 with 1+ or > 3+ charge state were absent in the predicted libraries (HeLaPredicted and HeLaProsit). Among the peptides detected using HeLaDDA and HeLaPredicted, 674 (639 using HeLaDDA and 376 using HeLaPredicted) with length > 30 amino acids were absent in the HeLaProsit library. (b) The distributions of coefficient of variation (CV) of peptide precursor and protein group quantification results detected in three technical replicates using the three libraries. The medians are indicated. (c) Correlation between replicates of precursor and protein group quantification results. (d) Box plot visualization of percent change of identified precursors and

proteins of two mixed proteome samples. Percent changes were calculated based on the mean quantities in three replicates of each sample. Only overlapping identifications by the three libraries (Standard: MixStandard, DeepDIA: MixPredicted, Prosit: MixProsit) are shown. The medians are indicated. The boxes indicate the interquartile ranges (IQR), and whiskers indicate 1.5× IQR values; no outliers are shown. Details of the spectral libraries are described in Supplementary Table 2. The dashed lines indicate theoretical fold changes of the organisms. Source data are provided as a Source Data file

2. In Table S7, it is not clear which peptide charge state(s) were identified by DeepPRM and DDA in the HeLa data. Table S7 currently shows identification of peptides in both charge states of 2 and 3 with equal "Detectability" score in the DDA mode. Criteria of the peptide charge state picked up for PRM analysis of 367 peptides in Table S8 needs to be described. Also, what is the collision energy used in the PRM analysis?

Response: Thank you for raising such an important issue. We are sorry for missing the information and have updated the charge states of identified peptides in the Table S7 **(current: Dataset EV10)**. As for most peptides, 2+ and 3+ are the main charge states in the electrospray ion source coupled mass spectrometry systems (ESI-MS). Hence, we previously marked "YES" for all these identified peptides mainly considering their m/z, while ignoring their charge state. As you mentioned, this does not mean that the peptides in different charge state have an equal "Detectablity" score. Therefore, for a strict comparation, we re-checked the suggested m/z of peptides from DeepPRM and Picky, and the DDA identification results with the same m/z and charge state of each peptide. The results including the number of the suggested proteins and proteins corresponding to the detected peptides from DDA validation were illustrated in the main **Fig 4 (4B)**. According to the **Fig 4B**, the detection rate of DeepPRM (85.23%) was still significantly higher than that obtained by Picky (30.70%).

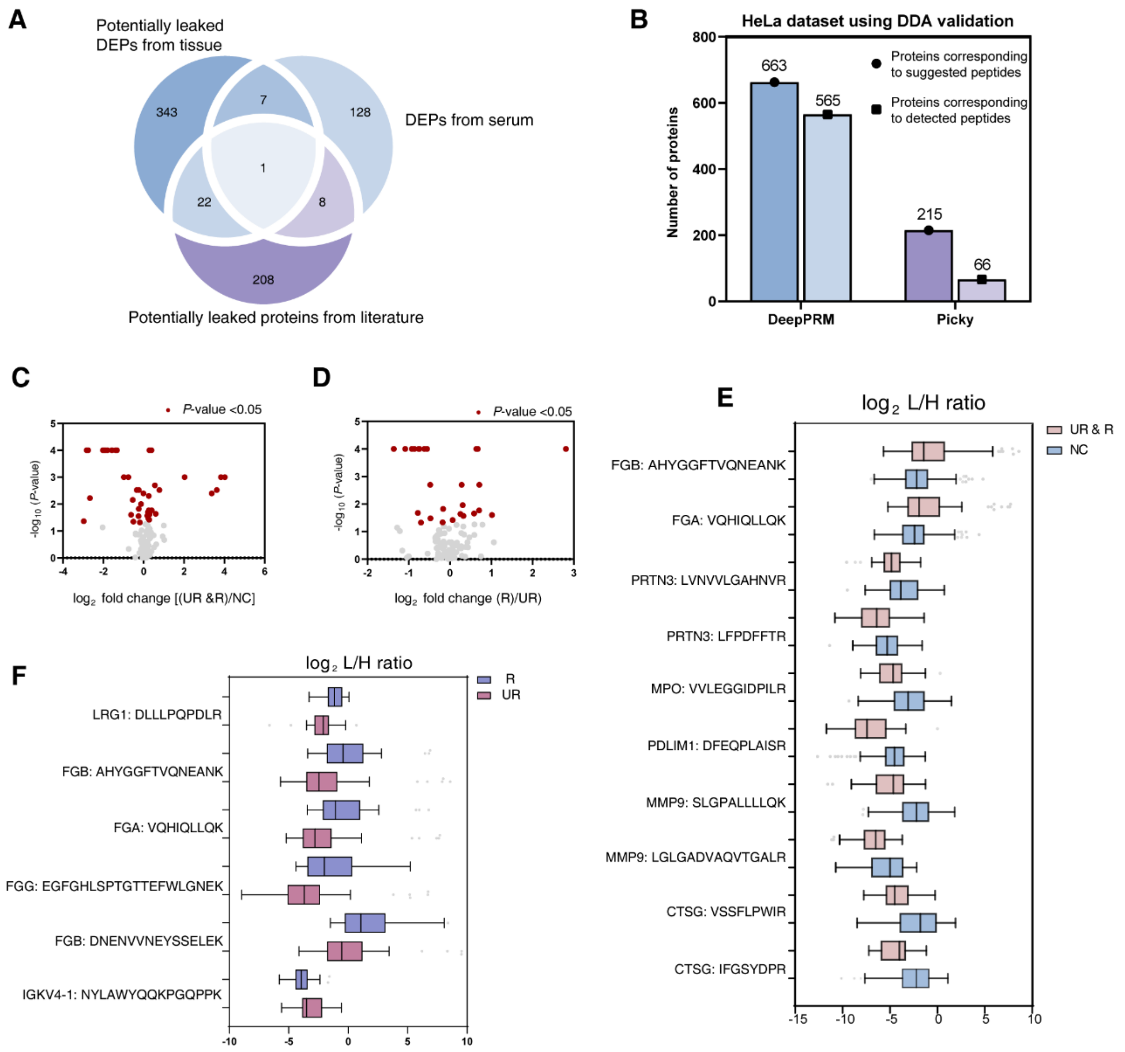

Figure 4. Targeted proteomics of IA candidate biomarker bank.

A Overlap of serum candidate proteins related to IA.

B Histogram showing the number of proteins corresponding to suggested peptides by DeepPRM and Picky methods, and proteins corresponding to detected peptides from DDA validation.

C, D Volcano analyses showing log2 fold change of (UR & R)/NC (C), R/ UR (D) of peptides according to the *P*-value. Proteins meeting the indicated statistical cut-off criteria (Mann–Whitney U test, *P*-value < 0.05) are colored in red.

E, F Ten peptide relative quantification (log2 L/H ratio) between (R & UR) and NC serum

samples (E), and six peptide relative quantification (log2 L/H ratio) between R and UR serum samples (F). For each protein, 1-2 proteotypic peptides were monitored. The light peptide (corresponding to the endogenous peptide present in serum) and the heavy peptide (which corresponds to the synthetic peptide spiked-in serum) were monitored, and the light/heavy ratio for each of the 12 peptides was obtained by Skyline. Box plots represent the median and interquartile range, whiskers represent the 1–99 percentile, and outliers are represented by empty circles.

-These new analyses resulted in the following paragraph, which we included in the revised Results section:

"Consequently, DeepPRM achieves more targeted peptides with corresponding RT information, and the detection rate (detected peptides/suggested peptides) of DeepPRM (85.23%) is far larger than that obtained by Picky (30.70%) (**Fig 4B, Appendix Fig S2B-D, Dataset EV8-10**)."

Further, this important issue provides some inspiration for us to further optimize DeepPRM method. For peptides with m/z greater than 1500, we must add the peptides in charge state of 4, 5 or 6 for multiple choice.

As for the criteria of the peptide charge state picked up for PRM analysis of 367 peptides in Table S8 (**current Dataset EV11**), we first verified the m/z of the peptides with different charge states and chose those m/z values within the optimal MS scan range of 350-1250. Then, when two charge states could be detected simultaneously, we manually checked the MS signals regarding the signal to noise ratio, transitions, peak shape, and peak area to select the better charge state for further validation in the large cohort of serum samples. For instance, the serum peptide (ITYGETGGNSPVQEFTVPGSK) could be detected in both the 2+ and 3+ charge state, peptide with charge 2 was selected in the final list due to the better mass

spectrum signal response (**Appendix Fig S3A**). Another serum peptide (LVPFATELHER) of apolipoprotein A-IV with charge 3 was enrolled for the same reason (**Appendix Fig S3B**).

In summary, the criteria of the peptide charge state picked up for PRM analysis was based on the information of mass-to-charge ratio of each peptide in different charge state and their mass spectrum signal response. We add the brief description of criteria of the charge state selection in the revised Method selection and **Appendix Figure S3** as follows:

"**Charge state selection**: the peptide charge state picked up for PRM analysis were selected based on the following criteria: (1) The m/z of targeted peptide was selected within the optimal MS scan range of 350-1250; (2) When two charge states of a peptide could be detected simultaneously, we selected the better peptide charge state based on the manually check the mass spectrum signal response such the signal to noise ratio, the transitions, the peak shape, and the peak area (**Appendix Fig S3**)."

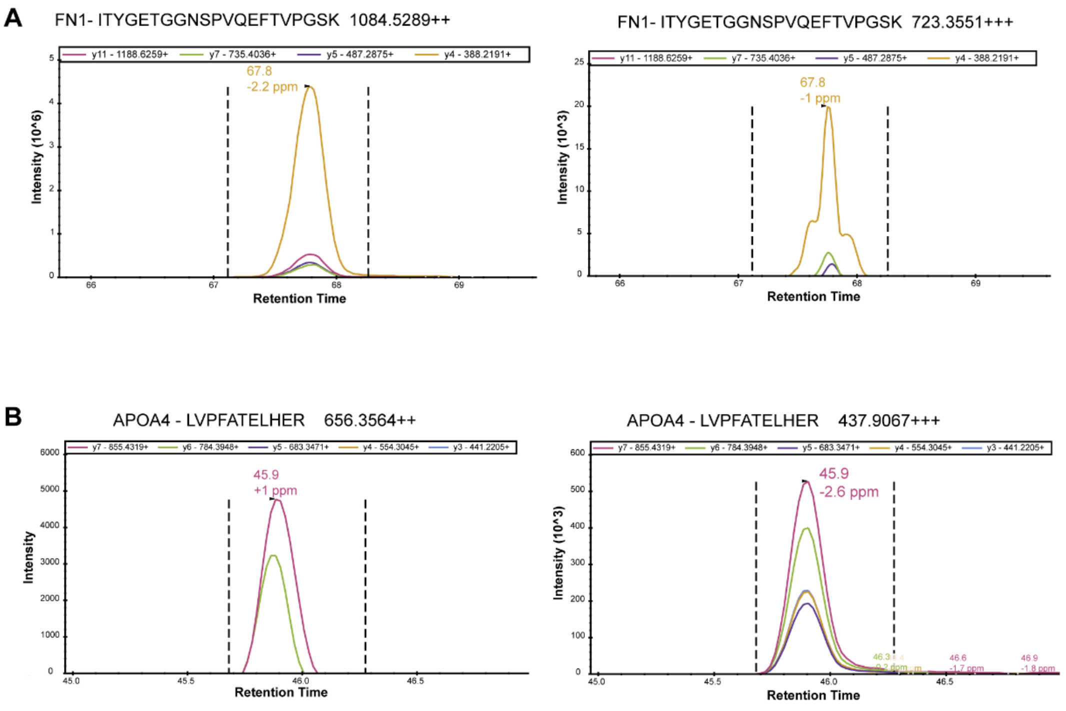

Appendix Figure S3. The comparation of same peptide with charge state of 2 and 3.

A) Skyline display of ion pattern and chromatograph of serum peptide (ITYGETGGNSPVQEFTVPGSK) of fibronectin protein with charge state of 2 and 3.

B) Skyline display of ion pattern and chromatograph of serum peptide (LVPFATELHER) of apolipoprotein A-IV protein with charge state of 2 and 3.

As for the last question, the collision energy used in the PRM analysis was 30, which meets the criteria of quantification due to the good mass spectrum signal response, signal to noise ratio, and the transition signal.

3. Provide a summary table with details of the studies in the literature reporting biomarkers of IA that were used to build PCBB and SPCBB

Response: Thank you for your advice. We have added the list of PCBB **(Dataset EV6)** and SPCBB **(Dataset EV7)** in the revised version of manuscript, which are shown in SI Table 1 for the reviewer [Position and content of tables in the revised manuscript] in the bottom summary. We also updated the exact position of each table in the revised manuscript.

4. Random forest (RF) modeling to model 113 features and 160 training set with 1,000 trees could overfit the model and in P28-based data which might also be reflective of less performance in the test data set as shown in Tables 1 and 2 with. Can a simple logistic regression modeling or RF with fewer trees improve the results?

Response: Thank you very much for your suggestions. In agreement with your comments, to avoid overfitting, we first reduced the candidate features by preselected the significantly changed proteins followed by a recursive feature elimination (RFE) to weight the importance of features. Then, the cross-validation (10-fold CV, 10 times repeated) was used to select the optimal candidate biomarker combinations. A simple binary logistic regression (LR) was used for model training and parameter optimization as you suggested in the comments. Consequently, two proteomic panels, one with six and the other with eight proteins, were discovered to discriminate the IA group from NC group, as well as IA ruptured group and unruptured groups with highest accuracy. We also obtain a simper equation of both models, as described above (**Fig 5B, E**). Therefore, we choose the simple LR to help us.

Minor comments

1. Language improvements throughout the manuscript and supplementary information (examples: line 332: construct; line 464: "Peak area"; lines 64-67 delete "of" etc.)

Response: Thank you for your comment. We have done our best to improve the incorrect words or sentences, and made them clearer in the revised version of the manuscript. The examples you listed have been corrected carefully:

The word “of” has been removed. (Lines 80-83)

“For example, multiplex quantitative proteomics revealed characteristic protein changes in the sera of severe COVID-19 patients, which might be used in the selection of potential blood biomarkers for severity evaluation (Demichev *et al.*, 2021; Geyer *et al*, 2021; Messner *et al*, 2020).”

The word “constructed” has been corrected to “construct”. (Line 350-352)

“In this study, we used DeepPRM and our rapid serum sample preparation (RSP) method (Shen *et al.*, 2021), to construct a highly efficient PRM assay developing strategy and validate the candidate peptides in serum samples.”

The word “Peak aera” has been corrected to “peak area”. (Line 488-492)

“Acquired DeepPRM raw data were analyzed using the open-source Skyline-daily software for transition identification and peak area integration. The peak area of targeted peptides was exported from Skyline into an Excel report spreadsheet and transformed to log10 format, which was more closely conformed to normal distribution and better suitable for the statistical modeling assumptions for downstream analysis.”

2. Provide reference(s) for the sentence described in lines 64-67

Response: We are sorry for the missed citations. The references have been added at these positions as follows:

“For example, multiplex quantitative proteomics revealed characteristic protein changes in the sera of severe COVID-19 patients, which might be used in the selection of potential blood biomarkers for severity evaluation (Demichev *et al.*, 2021;

Geyer *et al.*, 2021; Messner *et al.*, 2020). ” (Lines 80-83)

3. In page 37 and Table 1 correct accuracy (%) in last row to 100% for the training data set. While it is correct in page 38 and Table 1

Response: Thank you for your suggestion. We have corrected all the statements of accuracy (%) in our revised version of the manuscript.

Table 1. Position and content of figures in the revised manuscript

Current Figure	Original Figure	Content
Figure 1	Figure 1	Framework for biomarker discovery in intracranial aneurysms disease
Figure 2	Figure 2	IA tissue proteome remodeling due to IA formation and rupture
Figure 3	Figure 3	Remodeled serum proteome due to IA formation and rupture
Figure 4	Figure 4	Targeted proteomics of IA candidate biomarker bank
Figure 5	Figure 5	Identification of potential biomarker combinations for classification of IA different outcomes from healthy controls using machine-learning method
Appendix Figure S1	Figure S1	IA tissue proteome remodeling due to IA formation and rupture
Appendix Figure S2	Figure S2	Developed DeepPRM method for IA candidate proteins biomarker bank
Appendix Figure S3	New	The comparation of same peptide with charge state of 2 and 3
Appendix Figure S4	Figure S3	SPCBB quantification in large-scale IA serum samples by DeepPRM method
Appendix Figure S5	Figure S4	Skyline display of ion pattern and chromatograph of 8 serum peptides
Appendix Figure S6	New	Machine learning strategy for the classification of IA cases and healthy controls
Appendix Figure S7	Figure S6	Serological Validation of IA Biomarkers
SI Figure 1	Only for reviewer	The cell viability detected by CCK-8 proliferation assay
SI Figure 2	Only for reviewer	The comparison of migration ability of vascular endothelial cells after silencing PDLIM1 by siRNA
SI Figure 3	Only for reviewer	Across-gene and within-gene correlations between mRNA and protein levels
SI Figure 4	Only for reviewer	Performance comparison of predicted libraries and DDA libraries for DIA analysis on data of HeLa cells (HeLa1)

Table 2. Position and content of tables in the revised manuscript

Current Table	Original Table	Content
Table 1	New	Performance of circulating protein panel P6 in differential diagnosis of IA from healthy controls and P8 in classification of IA rupture.
Dataset EV1	Table S10	Patient characteristics in the cohort I (n=212) for the DeepPRM validation
Dataset EV2	New	Patient characteristics in the cohort II (n=32) for the DeepPRM validation
Dataset EV3	Table S2	Differentially expressed proteins in IA vs. STA
Dataset EV4	Table S3	Differentially expressed proteins in (R & UR) vs. NC or R vs. (UR & N)
Dataset EV5	Table S4	A list of 446 IA-related proteins from data mining
Dataset EV6	New	The list of IA-related protein candidate biomarker bank (PCBB)
Dataset EV7	New	The list of IA-related Serum Protein Candidate Biomarker Bank (SPCBB)
Dataset EV8	Table S5	The number of suggested peptides by Picky method and peptides detected by DDA analysis
Dataset EV9	Table S6	HeLa DDA dataset from public resource
Dataset EV10	Table S7	The number of suggested peptides of HeLa dataset by Picky/DeepPRM method and peptides detected by DDA analysis
Dataset EV11	Table S8	The spectral library for further DeepPRM analysis
Dataset EV12	Table S9	The peak area of 113 peptides/100 proteins in cohort I of 212 serum samples
Dataset EV13	New	The variable importance in projection (VIP) of 113 peptides.
Dataset EV14	New	The original dataset and predicted value based on LR model (P6: IA vs. NC) of training, internal validation and external validation set
Dataset EV15	New	The original dataset and predicted value based on LR model (P8: R vs. UR) of training, internal validation and external validation set
Dataset EV16	New	The resampling performance of different subject size in IA *vs.* NC or R *vs.* UR.
Dataset EV17	Table S13	The serum levels of 2 candidate proteins from the samples of cohort III were detected via ELISA
Dataset EV18	Table S14	The concentration of 16 heavy peptides and 11 iRT peptides skiked in 212 serum samples and their calibration curve tests

References

Baker CJ, Fiore A, Connolly ES, Jr., Baker KZ, Solomon RA (1995) Serum elastase and alpha-1-antitrypsin levels in patients with ruptured and unruptured cerebral aneurysms. *Neurosurgery* 37: 56-61

Ben-Hamo R, Berger AJ, Gavert N, Miller M, Pines G, Oren R, Pikarsky E, Benes CH, Neuman T, Zwang Y *et al* (2020) Predicting and affecting response to cancer therapy based on pathway-level biomarkers. *Nat Commun* 11

Buccitelli C, Selbach M (2020) mRNAs, proteins and the emerging principles of gene expression control. *Nat Rev Genet* 21: 630-644

Carnielli CM, Macedo CCS, De Rossi T, Granato DC, Rivera C, Domingues RR, Pauletti BA, Yokoo S, Heberle H, Busso-Lopes AF *et al* (2018) Combining discovery and targeted proteomics reveals a prognostic signature in oral cancer. *Nat Commun* 9

Demichev V, Tober-Lau P, Lemke O, Nazarenko T, Thibeault C, Whitwell H, Röhl A, Freiwald A, Szyrwiel L, Ludwig D *et al* (2021) A time-resolved proteomic and prognostic map of COVID-19. *Cell Syst* 12: 780-794.e787

Emmert-Streib F, Dehmer M (2019) Evaluation of Regression Models: Model Assessment, Model Selection and Generalization Error. *Mach Learn Know Extr* 1

Gabay C, Kushner I (1999) Acute-phase proteins and other systemic responses to inflammation. *N Engl J Med* 340: 448-454

Geyer PE, Arend FM, Doll S, Louiset ML, Virreira Winter S, Müller-Reif JB, Torun FM, Weigand M, Eichhorn P, Bruegel M *et al* (2021) High-resolution serum proteome trajectories in COVID-19 reveal patient-specific seroconversion. *EMBO Mol Med* 13: e14167

Geyer PE, Holdt LM, Teupser D, Mann M (2017) Revisiting biomarker discovery by plasma proteomics. *Mol Syst Biol* 13

Gounis MJ, Vedantham S, Weaver JP, Puri AS, Brooks CS, Wakhloo AK, Bogdanov AA, Jr. (2014) Myeloperoxidase in human intracranial aneurysms: preliminary evidence. *Stroke* 45: 1474-1477

Grossegesse M, Nitsche A, Schaade L, Doellinger J (2021) Application of spectral library prediction for parallel reaction monitoring of viral peptides. *Proteomics*: e2000226

Haider S, Yao CQ, Sabine VS, Grzadkowski M, Stimper V, Starmans MHW, Wang JX, Nguyen F, Moon NC, Lin XH *et al* (2018) Pathway-based subnetworks enable cross-disease biomarker discovery. *Nat Commun* 9

Herrington DM, Mao CH, Parker SJ, Fu ZM, Yu GQ, Chen LL, Venkatraman V, Fu Y, Wang YZ, Howard TD *et al* (2018) Proteomic Architecture of Human Coronary and Aortic Atherosclerosis. *Circulation* 137: 2741-2756

Kusebauch U, Campbell DS, Deutsch EW, Chu CS, Spicer DA, Brusniak MY, Slagel J, Sun Z, Stevens J, Grimes B *et al* (2016) Human SRMAtlas: A Resource of Targeted Assays to Quantify the Complete Human Proteome. *Cell* 166: 766-778

Kwon KJ, Cho KS, Kim JN, Kim MK, Lee EJ, Kim SY, Jeon SJ, Kim KC, Han JE, Kang YS *et al* (2013) Proteinase 3 induces oxidative stress-mediated neuronal death in rat primary cortical neuron. *Neurosci Lett* 548: 67-72

Lee JW, Su YP, Baloni P, Chen D, Pavlovitch-Bedzyk AJ, Yuan D, Duvvuri VR, Ng RH, Choi J, Xie JY *et al* (2021) Integrated analysis of plasma and single immune cells uncovers metabolic changes in individuals with COVID-19. *Nat Biotechnol*

Li MB, Parker BL, Pearson E, Hunter B, Cao J, Koay YC, Guneratne O, James DE, Yang J, Lal S *et al* (2020) Core functional nodes and sex-specific pathways in human ischaemic and dilated cardiomyopathy. *Nat Commun* **11**

Liu J, Zeng L, Kennedy RM, Gruenig NM, Childs SJ (2012) βPix plays a dual role in cerebral vascular stability and angiogenesis, and interacts with integrin αvβ8. *Dev Biol* **363**: 95-105

Mack WJ, Mocco J, Hoh DJ, Huang J, Choudhri TF, Kreiter KT, Lozier A, Opperman M, Poisik A, Yorgason J *et al* (2002) Outcome prediction with serum intercellular adhesion molecule-1 levels after aneurysmal subarachnoid hemorrhage. *J Neurosurg* **96**: 71-75

Makridakis M, Vlahou A (2010) Secretome proteomics for discovery of cancer biomarkers. *J Proteomics* **73**: 2291-2305

Messner CB, Demichev V, Wendisch D, Michalick L, White M, Freiwald A, Textoris-Taube K, Vernardis SI, Egger A-S, Kreidl M *et al* (2020) Ultra-High-Throughput Clinical Proteomics Reveals Classifiers of COVID-19 Infection. *Cell Syst* **11**: 11-24.e14

Niu L, Thiele M, Geyer PE, Rasmussen DN, Webel HE, Santos A, Gupta R, Meier F, Strauss M, Kjaergaard M *et al* (2020) A paired liver biopsy and plasma proteomics study reveals circulating biomarkers for alcohol-related liver disease. *bioRxiv*: 2020.2010.2016.337592

Ortega-Gomez A, Salvermoser M, Rossaint J, Pick R, Brauner J, Lemnitzer P, Tilgner J, de Jong RJ, Megens RT, Jamasbi J *et al* (2016) Cathepsin G Controls Arterial But Not Venular Myeloid Cell Recruitment. *Circulation* **134**: 1176-1188

Robinson JL, Feizi A, Uhlén M, Nielsen J (2019) A Systematic Investigation of the Malignant Functions and Diagnostic Potential of the Cancer Secretome. *Cell Rep* **26**: 2622-2635.e2625

Schindhelm RK, van der Zwan LP, Teerlink T, Scheffer PG (2009) Myeloperoxidase: a useful

biomarker for cardiovascular disease risk stratification? *Clin Chem* **55**: 1462-1470

Shen B, Yi X, Sun YT, Bi XJ, Du JP, Zhang C, Quan S, Zhang FF, Sun R, Qian LJ *et al* (2020) Proteomic and Metabolomic Characterization of COVID-19 patient sera. *Cell* **182**: 59-72

Shen F, Xiong Y, Zhang L, Li H, Zhao H, Liu X, Yang P (2021) Rapid Sample Preparation Workflow for Serum Sample Analysis with Different Mass Spectrometry Acquisition Strategies. *Anal Chem* **93**: 1578-1585

Shu T, Ning WS, Wu D, Xu JQ, Han QQ, Huang MH, Zou XJ, Yang QY, Yuan Y, Bie YY *et al* (2020) Plasma Proteomics Identify Biomarkers and Pathogenesis of COVID-19. *Immunity* **53**: 1108-1122

Wang D, Eraslan B, Wieland T, Hallström B, Hopf T, Zolg DP, Zecha J, Asplund A, Li L-h, Meng C *et al* (2019) A deep proteome and transcriptome abundance atlas of 29 healthy human tissues. *Mol Syst Biol* **15**: e8503

Xie J, Zhang L, Chen Z, Hu A, Liu S, Lu D, Xia Y, Qian J, Yang P, Shen H (2020) Protein-protein correlations based variable dimension expansion algorithm for high efficient serum biomarker discovery. *Anal Chim Acta* **1119**: 25-34

Xu Z, Rui Y-N, Hagan JP, Kim DH (2019) Intracranial Aneurysms: Pathology, Genetics, and Molecular Mechanisms. *Neuromolecular Med* **21**: 325-343

Xue H, Lu B, Lai M (2008) The cancer secretome: a reservoir of biomarkers. *J Transl Med* **6**

Yang Y, Liu XH, Shen CP, Lin Y, Yang PY, Qiao L (2020) In silico spectral libraries by deep learning facilitate data-independent acquisition proteomics. *Nat Commun* **11**: 146

7th Dec 2021

Dear Dr. Liu,

Thank you for the submission of your revised manuscript to EMBO Molecular Medicine. I am pleased to inform you that we will be able to accept your manuscript pending the following final amendments:

1) Please address minor issues raised by referees #3.
2) In the main manuscript file, please do the following:
- In M&M, include a statement that in addition to the WMA Declaration of Helsinki that the experiments also conformed to the principles set out in the Department of Health and Human Services Belmont Report.
- Please be aware that all deposited data sets should be made freely available prior the publication of the manuscript. Use the following format to report the accession number of your data:

The datasets produced in this study are available in the following databases:
[data type]: [full name of the resource] [accession number/identifier] ([doi or URL or identifiers.org/DATABASE:ACCESSION])

Please check "Author Guidelines" for more information.
https://www.embopress.org/page/journal/17574684/authorguide#availabilityofpublishedmaterial
3) Synopsis: Please check your synopsis text and image, revise them if necessary and submit their final versions with your revised manuscript. Please be aware that in the proof stage minor corrections only are allowed (e.g., typos).
4) For more information: There is space at the end of each article to list relevant web links for further consultation by our readers. Could you identify some relevant ones and provide such information as well? Some examples are patient associations, relevant databases, OMIM/proteins/genes links, author's websites, etc...
5) As part of the EMBO Publications transparent editorial process initiative (see our Editorial at http://embomolmed.embopress.org/content/2/9/329), EMBO Molecular Medicine will publish online a Review Process File (RPF) to accompany accepted manuscripts. This file will be published in conjunction with your paper and will include the anonymous referee reports, your point-by-point response and all pertinent correspondence relating to the manuscript. Let us know whether you agree with the publication of the RPF and as here, if you want to remove or not any figures from it prior to publication. Please note that the Authors checklist will be published at the end of the RPF.
6) Please provide a point-by-point letter INCLUDING my comments as well as the reviewer's reports and your detailed responses (as Word file).

I look forward to reading a new revised version of your manuscript as soon as possible.

Yours sincerely,

Zeljko Durdevic

Zeljko Durdevic
Editor
EMBO Molecular Medicine

***** Reviewer's comments *****

Referee #3 (Remarks for Author):

The authors have addressed my previous concerns. I have one more comment - it would be great if the authors could comment on two of the biomarker candidates being derived from immunoglobulins, one of the most abundant components of blood present at mg/ml concentration: immunoglobulin heavy constant mu
273 (IGHM) and immunoglobulin kappa variable 3-20 (IGKV3-20).

Response Letter

Point-by-point responses to the editor and reviewers' comments

To Reviewer 3:

Referee #3 (Remarks for Author):

The authors have addressed my previous concerns. I have one more comment - it would be great if the authors could comment on two of the biomarker candidates being derived from immunoglobulins, one of the most abundant components of blood present at mg/ml concentration: immunoglobulin heavy constant mu 273 (IGHM) and immunoglobulin kappa variable 3-20 (IGKV3-20).

Response: Thank you for your constructive suggestion. We have added the comment of IGHM and IFKV3-20 in the sixth paragraph of the **Discussion** part as following:

"For instance, two of the biomarker candidates derived from immunoglobulins, IGHM and IGKV3-20 are important mediators of the inflammatory responses which are reported to be the shared pathological mechanisms in a variety of vascular diseases, including atherosclerosis, abdominal aortic aneurysm, and arteritis (Chalouhi *et al*, 2012; Chyatte *et al*, 1999) ."

Reference:

Chalouhi N, Ali MS, Jabbour PM, Tjoumakaris SI, Gonzalez LF, Rosenwasser RH, Koch WJ, Dumont AS (2012) Biology of intracranial aneurysms: role of inflammation. ***Journal of Cerebral Blood Flow and Metabolism*** **32**: 1659-1676

Chyatte D, Bruno G, Desai S, Todor DR (1999) Inflammation and Intracranial Aneurysms. ***Neurosurgery*** **45**: 1137-1147

2nd Revision - Editorial Decision 13th Dec 2021

We are pleased to inform you that your manuscript is accepted for publication and is now being sent to our publisher to be included in the next available issue of EMBO Molecular Medicine.

EMBO PRESS

YOU MUST COMPLETE ALL CELLS WITH A PINK BACKGROUND ↓

PLEASE NOTE THAT THIS CHECKLIST WILL BE PUBLISHED ALONGSIDE YOUR PAPER

Corresponding Author Name: Xiaohui Liu
Journal Submitted to: EMBO Molecular Medicine
Manuscript Number: EMM-2021-14713

USEFUL LINKS FOR COMPLETING THIS FORM

http://www.antibodypedia.com
http://1degreebio.org
http://www.equator-network.org/reporting-guidelines/improving-bioscience-research-repor
http://grants.nih.gov/grants/olaw/olaw.htm
http://www.mrc.ac.uk/Ourresearch/Ethicsresearchguidance/Useofanimals/index.htm
http://ClinicalTrials.gov
http://www.consort-statement.org
http://www.consort-statement.org/checklists/view/32-consort/66-title
http://www.equator-network.org/reporting-guidelines/reporting-recommendations-for-tumo
http://datadryad.org
http://figshare.com
http://www.ncbi.nlm.nih.gov/gap
http://www.ebi.ac.uk/ega
http://biomodels.net/
http://biomodels.net/miriam/
http://jjj.biochem.sun.ac.za
https://osp.od.nih.gov/biosafety-biosecurity-and-emerging-biotechnology/
http://www.selectagents.gov/

Reporting Checklist For Life Sciences Articles (Rev. June 2017)

This checklist is used to ensure good reporting standards and to improve the reproducibility of published results. These guidelines are consistent with the Principles and Guidelines for Reporting Preclinical Research issued by the NIH in 2014. Please follow the journal's authorship guidelines in preparing your manuscript.

A- Figures
1. Data
The data shown in figures should satisfy the following conditions:

- ➔ the data were obtained and processed according to the field's best practice and are presented to reflect the results of the experiments in an accurate and unbiased manner.
- ➔ figure panels include only data points, measurements or observations that can be compared to each other in a scientifically meaningful way.
- ➔ graphs include clearly labeled error bars for independent experiments and sample sizes. Unless justified, error bars should not be shown for technical replicates.
- ➔ if n< 5, the individual data points from each experiment should be plotted and any statistical test employed should be justified
- ➔ Source Data should be included to report the data underlying graphs. Please follow the guidelines set out in the author ship guidelines on Data Presentation.

2. Captions

Each figure caption should contain the following information, for each panel where they are relevant:

- ➔ a specification of the experimental system investigated (eg cell line, species name).
- ➔ the assay(s) and method(s) used to carry out the reported observations and measurements
- ➔ an explicit mention of the biological and chemical entity(ies) that are being measured.
- ➔ an explicit mention of the biological and chemical entity(ies) that are altered/varied/perturbed in a controlled manner.

- ➔ the exact sample size (n) for each experimental group/condition, given as a number, not a range;
- ➔ a description of the sample collection allowing the reader to understand whether the samples represent technical or biological replicates (including how many animals, litters, cultures, etc.).
- ➔ a statement of how many times the experiment shown was independently replicated in the laboratory.
- ➔ definitions of statistical methods and measures:
  - common tests, such as t-test (please specify whether paired vs. unpaired), simple χ2 tests, Wilcoxon and Mann-Whitney tests, can be unambiguously identified by name only, but more complex techniques should be described in the methods section;
  - are tests one-sided or two-sided?
  - are there adjustments for multiple comparisons?
  - exact statistical test results, e.g., P values = x but not P values < x;
  - definition of 'center values' as median or average;
  - definition of error bars as s.d. or s.e.m.

Any descriptions too long for the figure legend should be included in the methods section and/or with the source data.

In the pink boxes below, please ensure that the answers to the following questions are reported in the manuscript itself. Every question should be answered. If the question is not relevant to your research, please write NA (non applicable). We encourage you to include a specific subsection in the methods section for statistics, reagents, animal models and human subjects.

B- Statistics and general methods

Please fill out these boxes ↓ (Do not worry if you cannot see all your text once you press return)

1.a. How was the sample size chosen to ensure adequate power to detect a pre-specified effect size?	Three independent patient cohorts were used in the present study. The selection of the cohorts and size was based on availability and no power calculation has been performed.
1.b. For animal studies, include a statement about sample size estimate even if no statistical methods were used.	No animal models have been used.
2. Describe inclusion/exclusion criteria if samples or animals were excluded from the analysis. Were the criteria pre-established?	Samples were not excluded within this study.
3. Were any steps taken to minimize the effects of subjective bias when allocating animals/samples to treatment (e.g. randomization procedure)? If yes, please describe.	Samples were randomized for the proteomics sample preparation and data acquisition.
For animal studies, include a statement about randomization even if no randomization was used.	No animal models have been used.
4.a. Were any steps taken to minimize the effects of subjective bias during group allocation or/and when assessing results (e.g. blinding of the investigator)? If yes please describe.	No.
4.b. For animal studies, include a statement about blinding even if no blinding was done	No animal models have been used.
5. For every figure, are statistical tests justified as appropriate?	Yes.
Do the data meet the assumptions of the tests (e.g., normal distribution)? Describe any methods used to assess it.	Quantitative proteomics data are log10 or log2 transformed to meet the assumption of normal distribution.
Is there an estimate of variation within each group of data?	The variation of each protein and each group e.g. UR,R patients and healthy controls has been determined.

Is the variance similar between the groups that are being statistically compared?	Yes.

C- Reagents

6. To show that antibodies were profiled for use in the system under study (assay and species), provide a citation, catalog number and/or clone number, supplementary information or reference to an antibody validation profile. e.g., Antibodypedia (see link list at top right), 1DegreeBio (see link list at top right).	The antibodies used are described in the Supplementary Information part of the manuscript.
7. Identify the source of cell lines and report if they were recently authenticated (e.g., by STR profiling) and tested for mycoplasma contamination.	NA

* for all hyperlinks, please see the table at the top right of the document

D- Animal Models

8. Report species, strain, gender, age of animals and genetic modification status where applicable. Please detail housing and husbandry conditions and the source of animals.	NA
9. For experiments involving live vertebrates, include a statement of compliance with ethical regulations and identify the committee(s) approving the experiments.	NA
10. We recommend consulting the ARRIVE guidelines (see link list at top right) (PLoS Biol. 8(6), e1000412, 2010) to ensure that other relevant aspects of animal studies are adequately reported. See author guidelines, under 'Reporting Guidelines'. See also: NIH (see link list at top right) and MRC (see link list at top right) recommendations. Please confirm compliance.	NA

E- Human Subjects

11. Identify the committee(s) approving the study protocol.	The Research Ethics Committee from the Huashan Hospital, Fudan University.(Page 24)
12. Include a statement confirming that informed consent was obtained from all subjects and that the experiments conformed to the principles set out in the WMA Declaration of Helsinki and the Department of Health and Human Services Belmont Report.	The study was conducted in accordance with the guidelines of the Declaration of Helsinki and the Department of Health and Human Services Belmont Report, with the approval of the Research Ethics Committee from the Huashan Hospital, Fudan University. Written informed consent was obtained from all patients or their legal representatives prior to their participation in the study.(Page 26)
13. For publication of patient photos, include a statement confirming that consent to publish was obtained.	NA
14. Report any restrictions on the availability (and/or on the use) of human data or samples.	NA
15. Report the clinical trial registration number (at ClinicalTrials.gov or equivalent), where applicable.	NA
16. For phase II and III randomized controlled trials, please refer to the CONSORT flow diagram (see link list at top right) and submit the CONSORT checklist (see link list at top right) with your submission. See author guidelines, under 'Reporting Guidelines'. Please confirm you have submitted this list.	NA
17. For tumor marker prognostic studies, we recommend that you follow the REMARK reporting guidelines (see link list at top right). See author guidelines, under 'Reporting Guidelines'. Please confirm you have followed these guidelines.	NA

F- Data Accessibility

18: Provide a "Data Availability" section at the end of the Materials & Methods, listing the accession codes for data generated in this study and deposited in a public database (e.g. RNA-Seq data: Gene Expression Omnibus GSE39462, Proteomics data: PRIDE PXD000208 etc.) Please refer to our author guidelines for 'Data Deposition'.<br><br>Data deposition in a public repository is mandatory for:<br>a. Protein, DNA and RNA sequences<br>b. Macromolecular structures<br>c. Crystallographic data for small molecules<br>d. Functional genomics data<br>e. Proteomics and molecular interactions	The mass spectrometry proteomics data have been deposited to the ProteomeXchange Consortium (http://proteomecentral.proteomexchange.org) via the iProX partner repository with the dataset identifier PXD024615
19. Deposition is strongly recommended for any datasets that are central and integral to the study; please consider the journal's data policy. If no structured public repository exists for a given data type, we encourage the provision of datasets in the manuscript as a Supplementary Document (see author guidelines under 'Expanded View' or in unstructured repositories such as Dryad (see link list at top right) or Figshare (see link list at top right).	NA
20. Access to human clinical and genomic datasets should be provided with as few restrictions as possible while respecting ethical obligations to the patients and relevant medical and legal issues. If practically possible and compatible with the individual consent agreement used in the study, such data should be deposited in one of the major public access-controlled repositories such as dbGAP (see link list at top right) or EGA (see link list at top right).	NA
21. Computational models that are central and integral to a study should be shared without restrictions and provided in a machine-readable form. The relevant accession numbers or links should be provided. When possible, standardized format (SBML, CellML) should be used instead of scripts (e.g. MATLAB). Authors are strongly encouraged to follow the MIRIAM guidelines (see link list at top right) and deposit their model in a public database such as Biomodels (see link list at top right) or JWS Online (see link list at top right). If computer source code is provided with the paper, it should be deposited in a public repository or included in supplementary information.	NA

G- Dual use research of concern

22. Could your study fall under dual use research restrictions? Please check biosecurity documents (see link list at top right) and list of select agents and toxins (APHIS/CDC) (see link list at top right). According to our biosecurity guidelines, provide a statement only if it could.	No.
